# Top-Down Sequencing of Intact Proteoforms Using the timsOmni Mass Spectrometer: Accurate Determination of Co-Occurring Histone Modifications

**DOI:** 10.1016/j.mcpro.2026.101640

**Published:** 2026-08-17

**Authors:** Francis Berthias, Nurgül Bilgin, Athanasios Smyrnakis, Elisa Le Boiteux, Mariangela Kosmopoulou, Christian Albers, Detlev Suckau, Jasmin Mecinović, Dimitris Papanastasiou, Ole N. Jensen

**Affiliations:** 1 Department of Biochemistry and Molecular Biology, University of Southern Denmark, Odense, Denmark; 2 Department of Physics, Chemistry and Pharmacy, University of Southern Denmark, Odense, Denmark; 3 Fasmatech Science & Technology SA, Chalandri, Greece; 4 Bruker Daltonics GmbH & Co. KG, Bremen, Germany

**Keywords:** histones, proteoforms, top-down proteomics, Omnitrap, positional isomers, multimodal mass spectrometry

## Abstract

Deep characterization of intact proteoforms remains an analytical challenge in functional proteomics, particularly for heterogeneous multi-site post-translational modifications at distinct amino acid residues. Histones are among the most dynamically and diversely post-translationally modified proteins in eukaryotic cells, carrying multiple, co-occurring, and reversible modifications that can give rise to isomeric proteoform species. Tandem mass spectrometry with multimodal fragmentation capabilities is a promising approach for deep characterization of intact proteoforms, such as modified histones. We applied the novel timsOmni mass spectrometer, which incorporates the Omnitrap platform enabling multimodal MS^n^ workflows using controlled *in vitro* acetylation of recombinant histones H3.1 and H4 by GCN5, PCAF and p300, followed by analysis of endogenous H4 proteoforms. Complementary MS^2^ electron- and collision-based dissociation (ECD, EID, _R_CID and ECciD), together with MS^3^ strategies, produced complete or near-complete backbone fragmentation of intact protein ions (>92% amino acid sequence coverage). For monoacetylated species generated by the more site-selective lysine acetyltransferases, the dominant proteoform matched the known catalytic preferences of the enzymes (H3.1K14ac for GCN5 and PCAF, and H4K8ac for PCAF), while minor positional isomers were also identified and their relative abundance estimated. In contrast, the broader substrate specificity of p300 produced a wide distribution of H4 proteoforms bearing up to seven acetylated lysine residues. Species carrying six and seven acetylations were characterized by multimodal MS^2^/MS^3^ experiments, enabling localization of individual acetylation sites and discrimination of positional isomers. Finally, top-down sequencing of endogenous intact H4 proteoforms from human liver extracts yielded amino acid sequence coverages of 92 to 93% for the most abundant species and confident localization of multiple, distant PTMs (acetylation and methylation). These results demonstrate that multimodal MS^n^ fragmentation of intact proteins supports residue-level assignment of combinatorial histone marks and coexisting positional isomers in controlled and endogenous proteoform mixtures.

## Highlights

• Novel timsOmni mass spectrometer platform for proteoform analysis.
• Effective top-down sequencing of intact proteins by multi-modal mass spectrometry.
• Accurate localization of co-occurring site-specific post-translational modifications.

## Graphical Abstract

**Figure:**
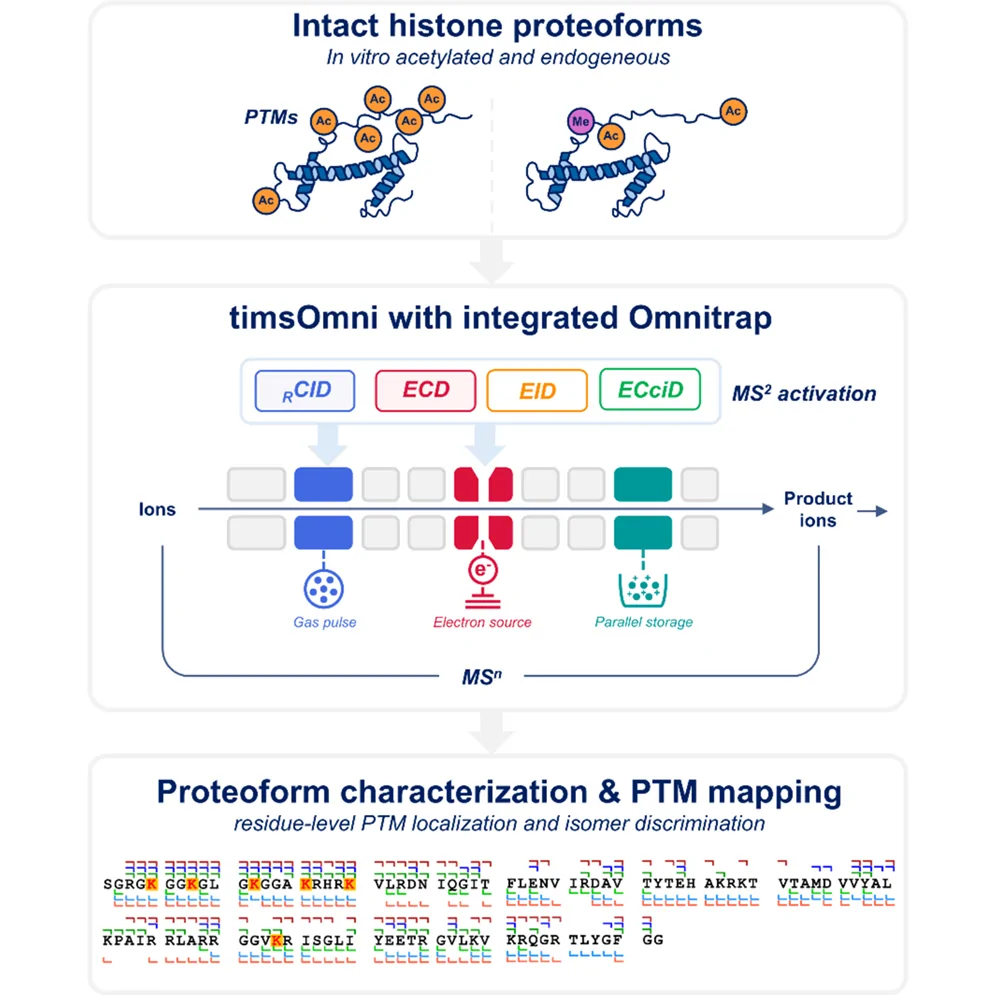

## In Brief

We present the timsOmni platform and demonstrate its abilities and versatility for efficient and accurate top-down proteoform analysis and amino acid sequencing, *viz*. determination of multi-site, co-occurring PTMs in histone proteins. Using *in vitro* modified histones and endogenous histones, we show how multi-modal MS/MS and MS/MS/MS can determine the exact location and types of PTMs, including determination of up to seven acetylation sites in histone H4, and multiple acetylation and methylation sites in endogenous histones extracted from hepatocytes.

The diversity of the eukaryote proteome arises from alternative splicing, sequence variants, and post-translational modifications (PTMs) of proteins that extend beyond the information encoded in the 20,000 human genes, thus generating millions of distinct proteoforms (1, 2, 3). PTMs modulate protein function by introducing chemical modifications at specific amino acid residues within proteins. Many modifications are reversible and dynamically alter or modulate protein structure, interactions, localization, and activity (4, 5, 6). The actual site of location, the chemical nature, and the combination of PTMs in a protein dictate the biological outcome. To date, more than 500 discrete PTMs have been described (7, 8, 9).

Histones are among the most densely post-translationally modified proteins in cells. These small basic proteins package the DNA into nucleosomes and modulate chromatin structure and gene accessibility *via* site-specific and combinatorial patterns of PTMs, often referred to as the “histone code” (10, 11). More than 75 distinct types of histone modification have been reported across over 200 residue sites, dominated by lysine acetylation, lysine/arginine methylation, and serine/threonine phosphorylation (12). The combinatorial distribution of different co-occurring PTMs across individual histones provides a vast proteoform space, exceeding that observed for many other cellular proteins.

The biological function of histone PTMs depends not only on their presence but also on their precise residue localization. Among them, lysine acetylation (Kac) represents a central regulator, as it modulates histone-DNA interactions through neutralization of the ε-amino group of lysine side chains, weakening histone-DNA electrostatic interactions and promoting recruitment of acetyl-lysine reader proteins (13). Importantly, the same modifications on a different residue can have distinct regulatory consequences. For example, acetylation of histone H3 at K9 is associated with active promoters and early transcriptional steps, while K27ac marks active enhancers and correlates with transcriptional output and elongation (14, 15, 16, 17). Similarly, acetylation of histone H4 at K16 alters higher-order chromatin folding, in contrast to acetylation of other H4 lysine residues (K5, K8 and K12) (18). Defects in histone PTMs deposition and removal are implicated in numerous diseases, including cancer (19, 20, 21) and neurodegenerative disorders (22). Consequently, interpretation of histone PTM function requires analytical strategies capable of determining both PTM identity and its precise site of occurrence.

Antibody-based assays and bottom-up proteomics by mass spectrometry (MS) are the standard approaches for identifying individual histone PTMs (23, 24, 25). However, bottom-up workflows rely on proteolytic digestion into short peptides, which inherently lose the link between distantly located PTM sites, while antibody assays are often limited by epitope occlusion (26, 27). Although partial PTM connectivity can be retained using middle-down proteomics strategies (28, 29, 30) or alternative proteases designed to detect local PTM crosstalk (31), preservation of the complete combinatorial pattern of histone PTMs generally requires analysis of intact histone proteoforms.

Top-down mass spectrometry analysis aims to address this limitation by directly analyzing intact proteins and preserving full proteoform information (PTM identification and localization, protein sequence variants, truncations). The theoretical search space increases rapidly when different PTM types and potential modification sites are combined. For histone H4 alone, an early top-down study generated a database of nearly 50,000 candidate modified sequences (32). Only a fraction of these theoretical combinations is expected to occur in biological samples, and intact protein measurements are needed to determine which PTMs coexist on individual histone proteins. Nevertheless, unambiguous localization of PTMs at the protein level remains analytically challenging. First, the combinatorial space of positional PTM isomers increases rapidly with the number of potential modification sites, complicating proteoform identification when sequence coverage is incomplete, in contrast to peptide-level analyses where the number of potential modification sites is inherently limited. Second, co-isolation of positional PTM isomers can generate chimeric mass spectra, complicating unambiguous proteoform assignment. Finally, many PTMs introduce only small mass differences relative to the intact protein mass. For example, acetylation and trimethylation differ by only 36.4 mDa, while citrullination of arginine produces a +0.98 Da mass shift, requiring high-quality fragment ion information for confident PTM assignments. Separation strategies applied prior to MS analysis can mitigate these challenges by reducing spectral complexity. Liquid chromatography (LC) can separate distinct proteoform families at intact protein level (33) and separation by degree of acetylation or methylation has been demonstrated for histone tails in middle-down workflows (∼5 kDa) (34, 35). However, PTM positional isomers often exhibit similar retention behavior. Gas-phase separation such as ion mobility spectrometry (IMS) provides an additional dimension of separation based on ion structure (36, 37, 38) and has demonstrated high isomeric separation capabilities for large molecular ions (39, 40, 41, 42), including targeted separation of positional isomers of acetylated histones (43, 44).

Even with effective separation, unambiguous PTM site assignment depends on the informative content of tandem mass spectra and the extent of backbone fragmentation. Collision-based dissociation (*e.g*., CID or HCD) typically yields limited sequence coverage and is biased toward preferential cleavage pathways. Electron-based dissociation methods (ECD/ETD) generate more extensive backbone fragmentation and are particularly suited for large, highly charged protein ions while preserving labile PTMs (45, 46, 47). Combining complementary ion activation methods into multimodal workflows is a powerful strategy to reach near complete amino acid sequence coverage (48). MS^n^ (n ≥ 2) and hybrid workflows can effectively reduce the theoretical proteoform search, enabling targeted interrogation of informative fragment ions (49, 50). Furthermore, recent computational evidence suggests that the addition of internal fragment ions may be critical for expanding effective sequence coverage and achieving unambiguous characterization of complex histone PTM patterns (51). Altogether, this underscores that top-down proteomics remains an actively developing analytical workflow, in which fragmentation strategies and data analysis approaches are still being refined to fully exploit the complexity of the recorded information.

In this study, we leverage the timsOmni platform (Bruker Daltonics), which integrates the Omnitrap segmented linear ion trap enabling multiple complementary activation methods within a time-of-flight mass spectrometer. The system supports resonance collision-induced dissociation (_R_CID), electron-capture dissociation (ECD), electron-induced dissociation (EID), as well as hybrid activation strategies, such as electron-capture collision-induced dissociation (ECciD). Using *in vitro* enzymatically acetylated histones H3.1 and H4, we exploit the timsOmni platform to localize acetylation sites, resolve positional isomers and compare complementary fragmentation pathways, including MS^3^ analysis. We demonstrate that multimodal MS^n^ analysis enables complete or near complete sequence coverage, allowing confident, site-resolved mapping of PTMs in intact histone proteoforms isolated from human cells.

## Experimental Procedures

### Histone Proteins and *In Vitro* Enzymatic Acetylation

Recombinant unmodified histone H4 (*Xenopus laevis* sequence, monoisotopic neutral mass of 11,229.4 Da) was expressed and purified in-house as descrived previously (52). The *X*. *laevis* H4 amino acid sequence is identical to the canonical human histone H4 sequence. Recombinant human PCAF and GCN5 were expressed and purified in-house as described previously (53). Human p300 was purchased from Active Motif and used without further purification. Recombinant human histone H3.1 (monoisotopic neutral mass of 15,263.4 Da) was purchased from Sigma-Aldrich.

**Fig. 1:**
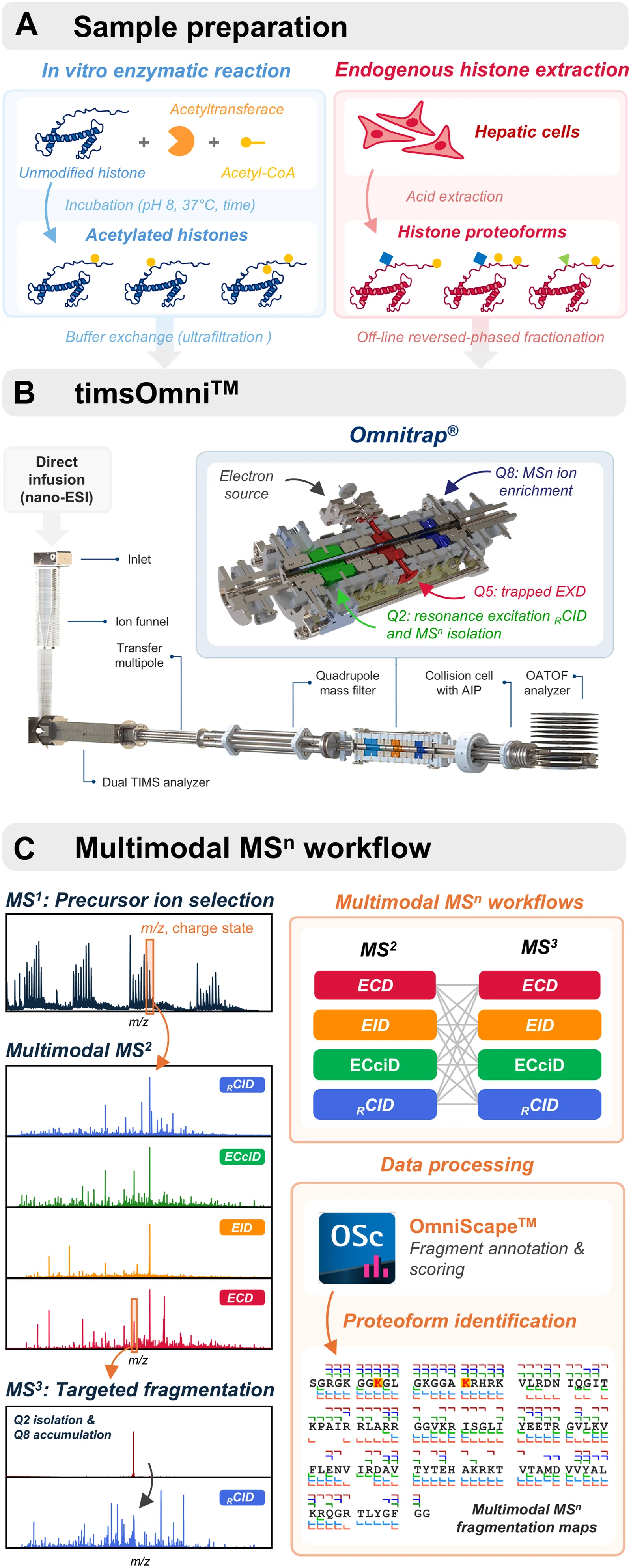
**Overview of sample preparation and multimodal MS^n^ workflow for top-down analysis of histone proteoforms.** (*A*) Histone H4 and H3.1 proteoforms were generated by *in vitro* enzymatic acetylation reactions using p300, PCAF, and GCN5 while endogenous H4 was isolated from hepatic cells by acid extraction followed by reversed-phase micropurification. (*B*) Schematic of the timsOmni prototype integrating an Omnitrap platform, which enables multiple ion activation modes. (*C*) Multimodal MS^n^ workflow. Precursor ions of specific m/z and charge state are isolated and subjected to complementary MS^2^ fragmentation (_R_CID, ECD, EID, and ECciD). Targeted MS^3^ experiments can be performed on selected product ions to refine PTM localization. Fragment ions are annotated and scored using OmniScape, yielding multimodal fragmentation maps for proteoform identification.

Enzymatic acetylation reactions were performed *in vitro* in a total volume of 30 μl using a reaction buffer composed of 50 mM HEPES, 0.1 mM EDTA, and 1 mM DTT at pH 8.0. Acetyl-coenzyme A (acetyl-CoA, Sigma-Aldrich) was used as co-substrate at a final concentration of 100 μM. Histone proteins were added to a final concentration of 20 μM in a total reaction volume of 30 μl, corresponding to 600 pmol of starting histone material per reaction (approximately 6.7 μg of H4 and 9.2 μg of H3.1). Recombinant lysine acetyltransferases (p300, PCAF, or GCN5) were diluted in reaction buffer and added to initiate the reaction, yielding a final enzyme concentration of 100 nM (enzyme-to-substrate molar ratio ∼1:200). Reactions were carried out at 37 °C with agitation (750 rpm) using a thermomixer (Eppendorf) for 10 min, 30 min and 60 min for GCN5, PCAF and p300 reactions, respectively. The *in vitro* enzymatic acetylation workflow is summarized in Figure 1*A* (left).

Following incubation, enzyme reactions were quenched by addition of 10% (v/v) trifluoroacetic acid (TFA) or formic acid (FA). Samples were subsequently prepared for mass spectrometry by buffer exchange using centrifugal ultrafiltration units equipped with polyethersulfone (PES) membranes (Vivaspin, Sartorius). A 3 kDa MWCO filter (VS0191) was used for histone H4, and a 5 kDa MWCO filter (VS0111) for histone H3.1. Membranes were pre-conditioned with Milli-Q water prior to sample loading. Quenched reaction mixtures were diluted up to ten-fold with Milli-Q water, centrifuged (5000–8000*g*), and washed at least twice to remove non-volatile buffer components while restoring the retentate volume to approximately the initial volume. Protein concentration after buffer exchange was determined using a NanoPhotometer N60 (Implen).

### Endogenous Histone Preparation

The endogenous histone preparation workflow is summarized in Figure 1*A* (right). Histones were extracted from frozen cell pellets (human hepatocellular carcinoma cell line HepG2/C3A) by acid extraction (adapted from Garcia *et al*., 2008) (54). Briefly, cell pellets were resuspended in nucleus isolation buffer (15 mM Tris-HCl pH 7.5, 60 mM KCl, 11 mM CaCl_2_, 5 mM NaCl, 5 mM MgCl_2_, 250 mM sucrose, 1 mM dithiothreitol, 10 mM sodium butyrate and 0.1% Igepal) supplemented with protease inhibitors (cOmplete Protease Inhibitor Cocktail, Roche) and phosphatase inhibitors (PhosSTOP, Roche). Nuclei were isolated by centrifugation (1000*g*, 5 min) and washed twice with nucleus isolation buffer without Igepal. Histones were extracted by resuspending the pellet in 0.2 M H_2_SO_4_ for 1 h under gentle agitation, and the supernatant was collected after centrifugation (20,000*g*, 5 min). Histones were precipitated by addition of trichloroacetic acid to a final concentration of 20% and incubated overnight. Precipitated proteins were recovered by centrifugation (20,000*g*, 15 min) and washed once with 0.1% HCl in acetone and twice with pure acetone. Pellets were air-dried and resuspended in ultrapure H_2_O. All steps were performed at 4 °C. Histone purity was assessed by SDS-PAGE (Supplemental Fig. S1), and protein concentration was determined using a NanoPhotometer N60 (Implen).

Acid-extracted histones were subjected to off-line reversed-phase micropurification. Microcolumns were prepared by packing a small C18 disk and reversed-phase resin (POROS R1 Applied Biosystems) into 10-μl pipette tips. The packed material was equilibrated with 100% acetonitrile followed by 0.1% formic acid in Milli-Q water. Histone samples, acidified in 0.1% formic acid, were loaded onto the microcolumn and washed twice with 0.1% formic acid. Proteins were then eluted stepwise using mixtures of water and acetonitrile, both containing 0.1% formic acid, with the acetonitrile content increased in 10% (v/v) increments. Fractions were collected separately and the fraction eluting at 25% acetonitrile, corresponding to the analytical LC retention window of H4, was selected for subsequent analysis. The selected histone fraction was recovered in a final volume of approximately 20 μl, fourfold before direct-infusion MS analysis.

### timsOmni Prototype Instrument

All experiments were performed on a prototype timsOmni platform (Bruker Daltonics), generated through extensive modification of a timsTOF Ultra system, and recently described for top-down analysis of intact proteins and oligonucleotides (55, 56). The prototype retains the timsTOF Ultra front-end, a reconfigured quadrupole mass filter, an upgraded collision cell, a high-resolution orthogonal time-of-flight (oTOF) mass analyzer and integrates the latest version of the Omnitrap platform (50). Samples were introduced by direct infusion using a nano-electrospray ionization (nESI) source (NEOS, Bruker) with an ESI voltage of 1.0 to 1.4 kV (Fig. 1*B*). Samples were analyzed at a final concentration of 1 to 5 μM in water/methanol/formic acid (49:50:1). Direct infusion was performed at an estimated flow rate of approximately 1 to 5 μl/h, and individual MS/MS acquisitions were typically 2 min. Electrosprayed ions were introduced into the mass spectrometer through a 1.0 mm inner diameter resistive glass capillary into differentially pumped regions comprising RF ion funnels and quadrupole ion guides for efficient transmission and collisional cooling. The instrument also incorporates a trapped ion mobility spectrometry (TIMS) analyzer, which was not employed in the present study and was set in transparent mode.

Precursor ions were selected using the quadrupole mass filter upstream of the Omnitrap platform. Selected ions were transferred into the Omnitrap through an RF hexapole and DC ion optics. The Omnitrap is a linear ion trap composed of nine hyperbolic quadrupole segments (Q1-Q9), with spatially separated regions for ion accumulation, isolation and ion enrichment, as illustrated in Figure 1*B*. It provides radial confinement through variable-frequency rectangular waveforms and enables dynamic axial ion manipulation *via* independently applied DC potentials across each segment. A Q10 transfer section guides processed ions from the Omnitrap toward the collision cell and TOF analyzer.

Resonance CID (_R_CID) was performed in Q2 by dipolar excitation of trapped ions in the presence of a pulsed N_2_ buffer gas. Electron-based dissociation (EXD, where X = C for ECD or X = I for EID) was performed in Q5, which is equipped with an axial electron source enabling interaction of the trapped ion cloud with a pulsed electron beam of controlled kinetic energy. The reaction time was defined by the electron irradiation period while ions remained radially and axially confined. The electron kinetic energy was adjusted according to the selected dissociation mode, typically ∼0 eV for ECD and ∼35 eV for EID. Collisionally activated ECD (ECciD) was performed by combining ECD with supplemental _R_CID.

The multimodal MS^2^ and targeted MS^3^ workflows used in this study are summarized in Figure 1*C*. Modular multimodal MS^3^ workflows were performed by combining isolation, activation, transfer and re-isolation steps. In the present experiments, MS^2^ product ions generated by _R_CID or ECD were isolated and subjected to a second activation step by ECD or _R_CID, enabling sequential MS^3^ combinations. Product ions could optionally be accumulated in parallel in downstream segments (Q8) to enhance ion statistics prior to ejection (ion enrichment workflow).

Precursor ions were accumulated for 30 ms to 1 s prior to activation, depending on precursor abundance. For electron-based dissociation experiments, irradiation times were adjusted according to precursor intensity and desired fragmentation efficiency. For _R_CID experiments, excitation amplitude and duration were optimized independently. Acquisition parameters for each MS^2^/MS^3^ spectrum (accumulation times, irradiation times, electron energy, excitation settings and isolation settings) are indicated directly in the corresponding figures and compiled in the supplementary tables.

### Data Analysis

Top-down MS^2^ and MS^3^ spectra were exported from Compass DataAnalysis (Bruker Daltonics) as simple ASCII (.xy) files containing m/z and intensity values. Spectra were recalibrated in OmniScape (hereafter abbreviated as OSc) software version 2025b (Bruker Daltonics), when necessary, using the Calibration workflow. Recalibration was performed based on matched theoretical fragment ions of the most probable proteoform using a quadratic fit function. For each calibration, more than 10 calibration points were selected across the full *m/z* range.

Proteoform analysis was performed using the “Confirmation” workflow in OSc, a workflow for fragment identification for proteins with known sequence and user-defined PTMs. For *in vitro* reactions, variable acetylation was set on all possible lysine residues of histone H4 and H3.1. Regarding endogenous histone, acetylation, mono-, di-, and trimethylation were set as variable PTMs on all possible lysine residues of histone H4. The software operates directly on profile *m/z* spectra, using the OmniWave algorithm, calculating theoretical isotopic distributions for all possible charge states and selecting the optimal set of isotopic distributions that best explain the mass spectrum. Thus, for each candidate proteoform, theoretical fragment ions and their isotopic distributions are assigned with high confidence, even in highly congested top-down mass spectra. Fragment ion types follow the classical a, b, c and x, y, z nomenclature (57) with numbering from the N- and C-termini, respectively.

Fragment matches were accepted based on agreement between experimental and theoretical isotopic envelopes using the following criteria: mass tolerance 5 ppm; score threshold 3; a minimum intensity within mass tolerance (ei score) of 60 to 80%; a correlation threshold of 20 to 50%; and a stringency of peak acceptance set to “High” (0.35).

Proteoform ranking relied primarily on the MS score, which quantifies the agreement between experimental and theoretical fragment ion isotopic distributions for a given proteoform hypothesis. It is derived from fragment-level matches satisfying mass accuracy and isotopic quality criteria. In addition, sequence-level validation is assessed using the Sequence Validation Percentage (SVP). SVP estimates the fraction of a protein sequence for which unexpected sequence variants or post-translational modifications can be excluded based on fragment constraints, while tolerating terminal and internal gaps where sequence length and composition remain mass constrained (58). SVP thus reflects experimentally constrained backbone coverage but is intrinsically insensitive to positional PTM isomerism when multiple proteoforms satisfy the same constraints. SVP parameters were set to a terminal gap size of two residues and an internal gap size of a single residue. Accepted fragment ion assignments were displayed along the protein sequence as fragmentation maps. When several MS^2^ or MS^3^ datasets were considered together, the corresponding fragment assignments were summarized in a combined fragmentation map to show the complementary sequence coverage and the fragment ions supporting PTM localization (Fig. 1*C*).

Reports generated by OSc for proteoform identification are provided in Supporting Information.

Deconvoluted spectra were generated using UNIDEC (59). The following parameters were applied: *m/z* range 500 to 1400; charge state range 1 to 50, mass range 5000 to 25,000 Da; mass sampling interval 0.2 Da; peak full width at half maximum 0.01 Th; and Gaussian peak shape function.

**Fig. 2:**
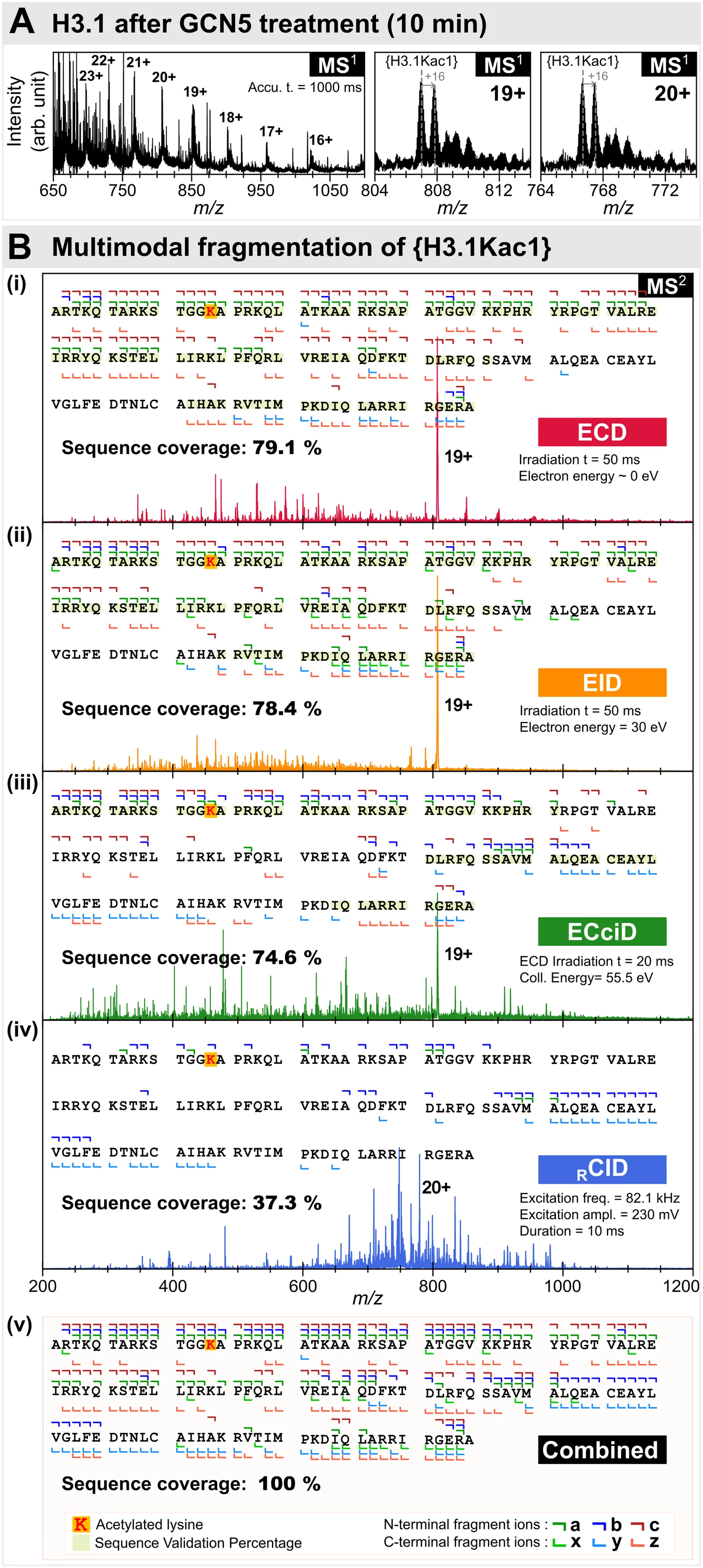
**Multimodal fragmentation analysis of monoacetylated H3.1 produced by GCN5.** (*A*) MS^1^ spectrum of histone H3.1 after a 10 min *in vitro* reaction with GCN5. (*B*) Multimodal MS^2^ fragmentation of the identified proteoform [H3.1K14ac]. (*i–iv*) Representative spectra obtained by ECD, EID, ECciD (19^+^ precursor) and _R_CID (20^+^ precursor), with corresponding fragment assignments along the H3.1 sequence. (*v*) Combined fragmentation map summarizing fragment ions identified across all datasets.

**Fig. 3:**
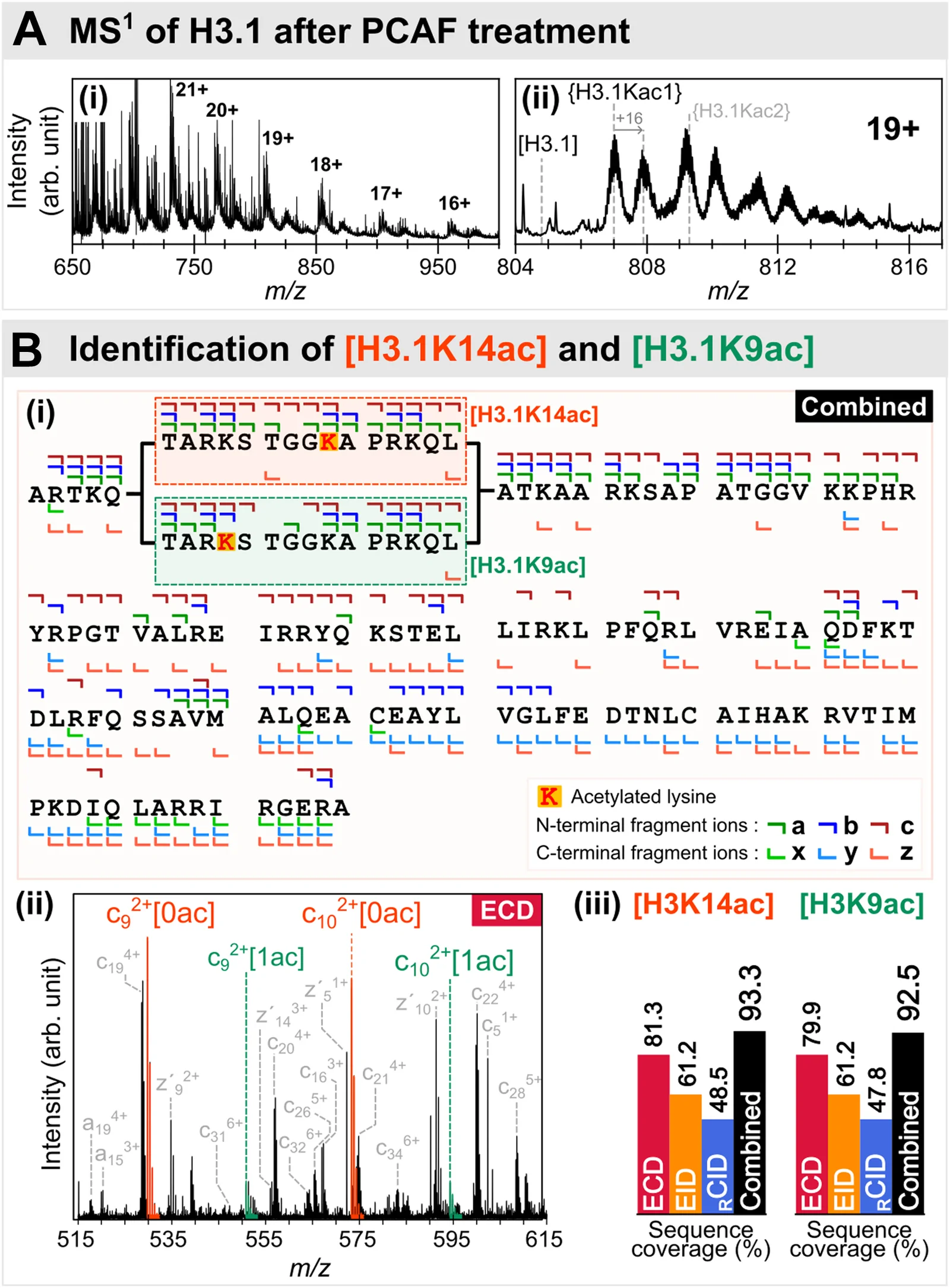
**Identification of monoacetylation sites in histone H3.1 after PCAF treatment using multimodal MS^2^ analysis.** (*A*) (*i*) MS^1^ spectrum of H3.1 after a 30 min *in vitro* acetylation reaction with PCAF. (*ii*) Zoomed view of the charge-state 19^+^ region showing the monoacetylated population. (*B*) Characterization of the monoacetylated species {H3.1Kac1}. (*i*) Combined fragmentation map summarizing fragment ions identified using ECD, EID, and _R_CID. (*ii*) Zoomed ECD spectrum showing diagnostic unmodified ([0ac]) and monoacetylated ([1ac]) of c_9_^2+^ and c_10_^2+^ ions, supporting the dominant [H3.1K14ac] and minor [H3.1K9ac] proteoforms. (*iii*) Sequence coverage obtained for each individual fragmentation method (*colored bars*) and for the combined dataset (*black bars*) for both identified proteoforms.

**Fig. 4:**
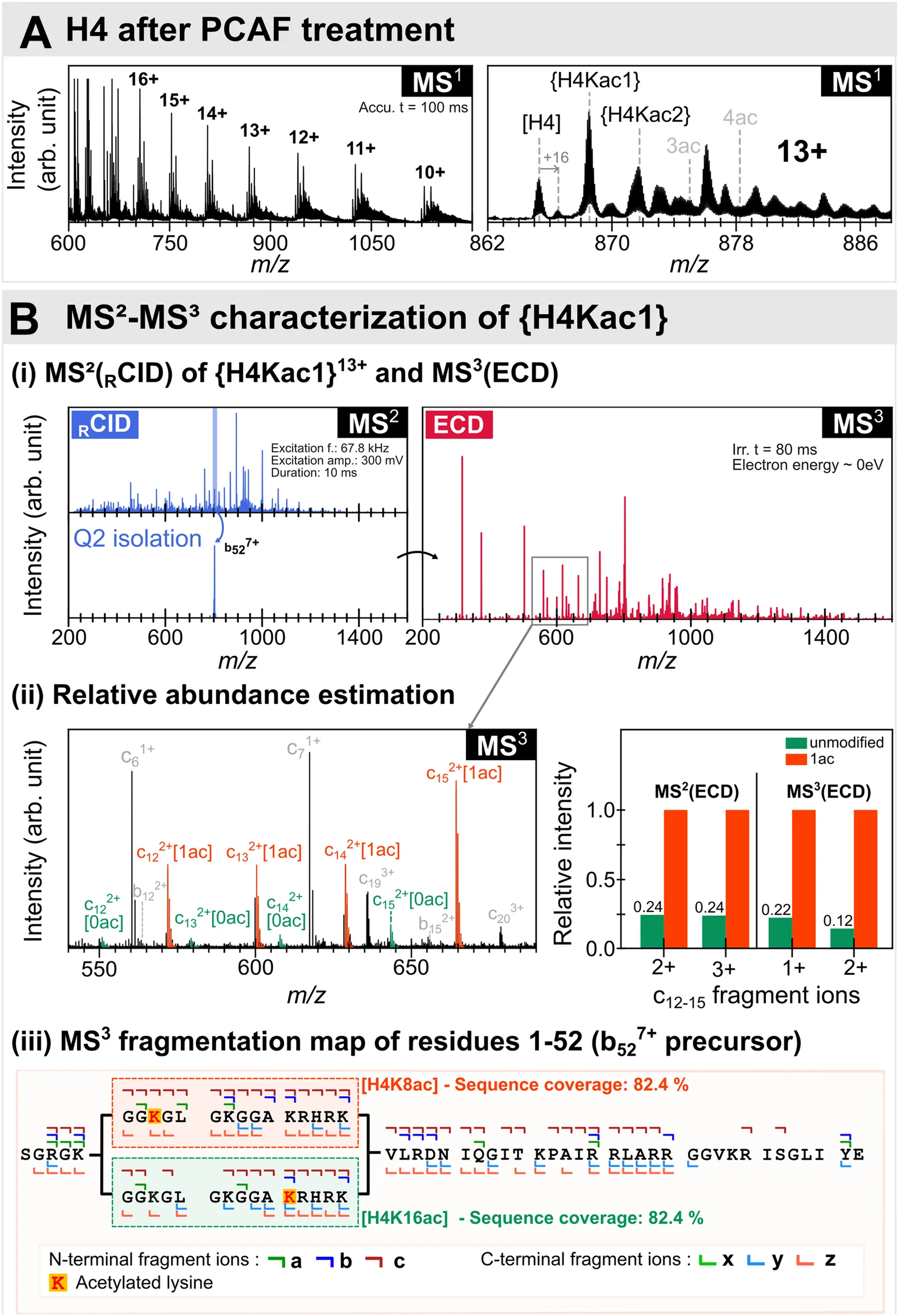
**Localization of monoacetylation sites in histone H4 after PCAF treatment using targeted MS^3^ analysis.** (*A*) MS^1^ spectrum of H4 after *in vitro* acetylation by PCAF for 30 min, showing the charge-state distribution and a zoomed view of the 13^+^ precursor region containing a dominant {H4Kac1} population. (*B*) (*i*) targeted MS^2^(_R_CID)→MS^3^(ECD) workflow applied to {H4Kac1}^13+^. The N-terminal b_52_^7+^ fragment (residue 1–52) bearing one acetylation was isolated in Q2 and accumulated (left spectra) before MS^3^ analysis by ECD (*right spectrum*). (*ii*) *Left*: zoomed view of diagnostic unmodified ([0ac]) and monoacetylated ([1ac]) c-type fragment ions (c_12_-_15_^2+^). *Right*: histograms of the mean relative intensities of matched c_12_-c_15_ ion pairs detected in direct MS^2^(ECD) and targeted MS^2^(_R_CID)→ MS^3^(ECD), shown separately for each detected fragment charge state. (*iii*) Fragmentation map and PTM localization within the H4 N-terminal tail for the main ([H4K8ac]) and secondary ([H4K16ac]) proteoforms.

**Fig. 5:**
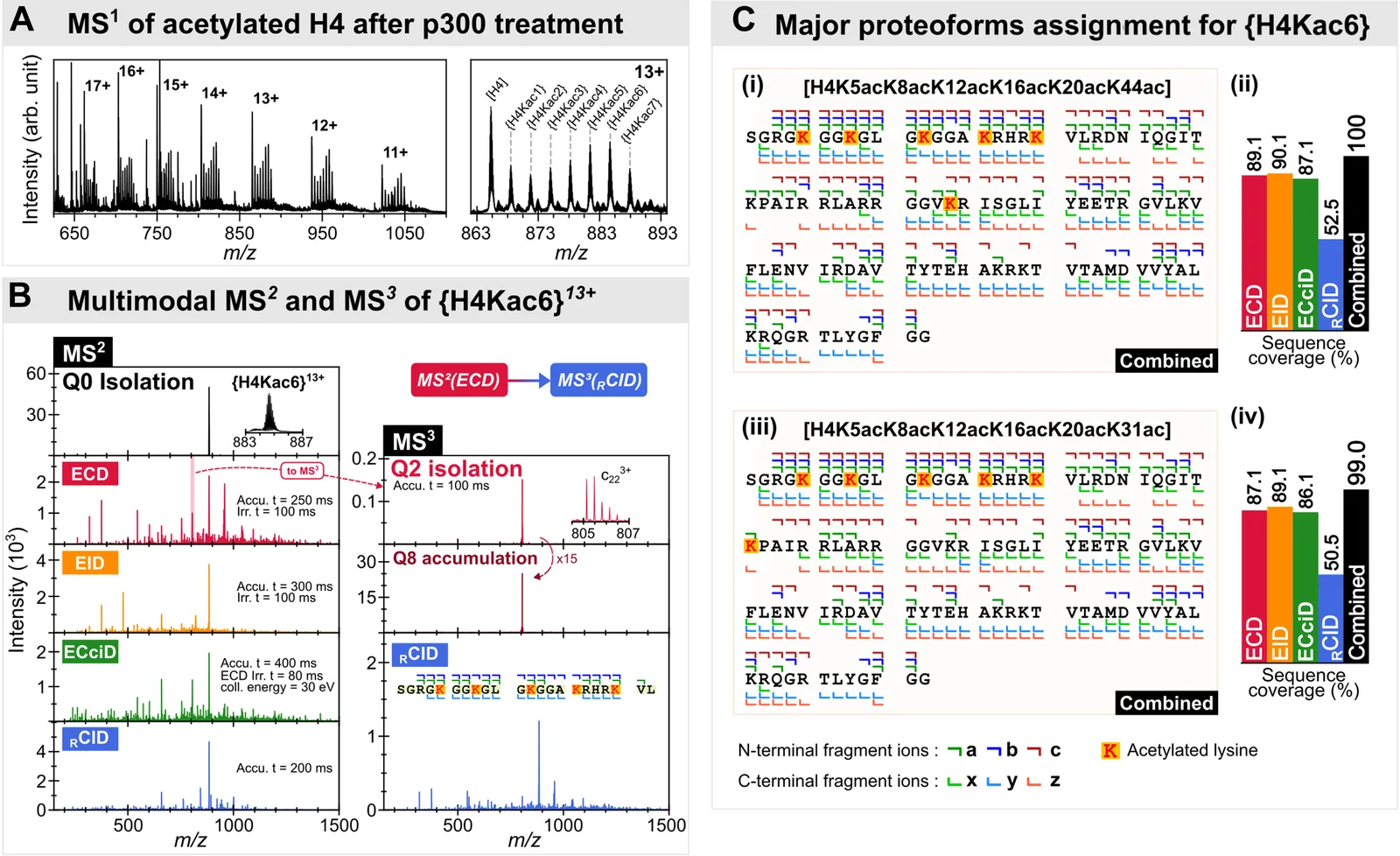
**Proteoform characterization of hexa-acetylated histone H4 ({H4Kac6}) generated by p300 using a multimodal MS^n^ workflow.** (*A*) MS^1^ spectrum of H4 after 1 h *in vitro* acetylation by p300, showing the charge-state distribution (11^+^–17^+^) and zoomed view of the 13^+^ region. (*B*) Multimodal MS^2^ and targeted MS^3^ workflow applied to the {H4Kac6}^13+^ precursor. *Left panels*: MS^2^ spectra acquired using ECD, EID, ECciD, and _R_CID. *Right panels*: targeted MS^3^ experiment based on ECD followed by _R_CID after isolation of the c_22_^3+^ fragment ion in Q2 and accumulation in Q8. (*C*) Proteoform assignment for {H4Kac6}. (*i*, *iii*) Combined fragmentation maps showing identified fragment ions (a, b, c and x, y, z series) and localized acetylated lysine residues for the two main proteoforms. (*ii*, *iv*) Sequence coverage obtained for each fragmentation method (ECD, EID, ECciD, _R_CID) and for the combined dataset.

**Fig. 6:**
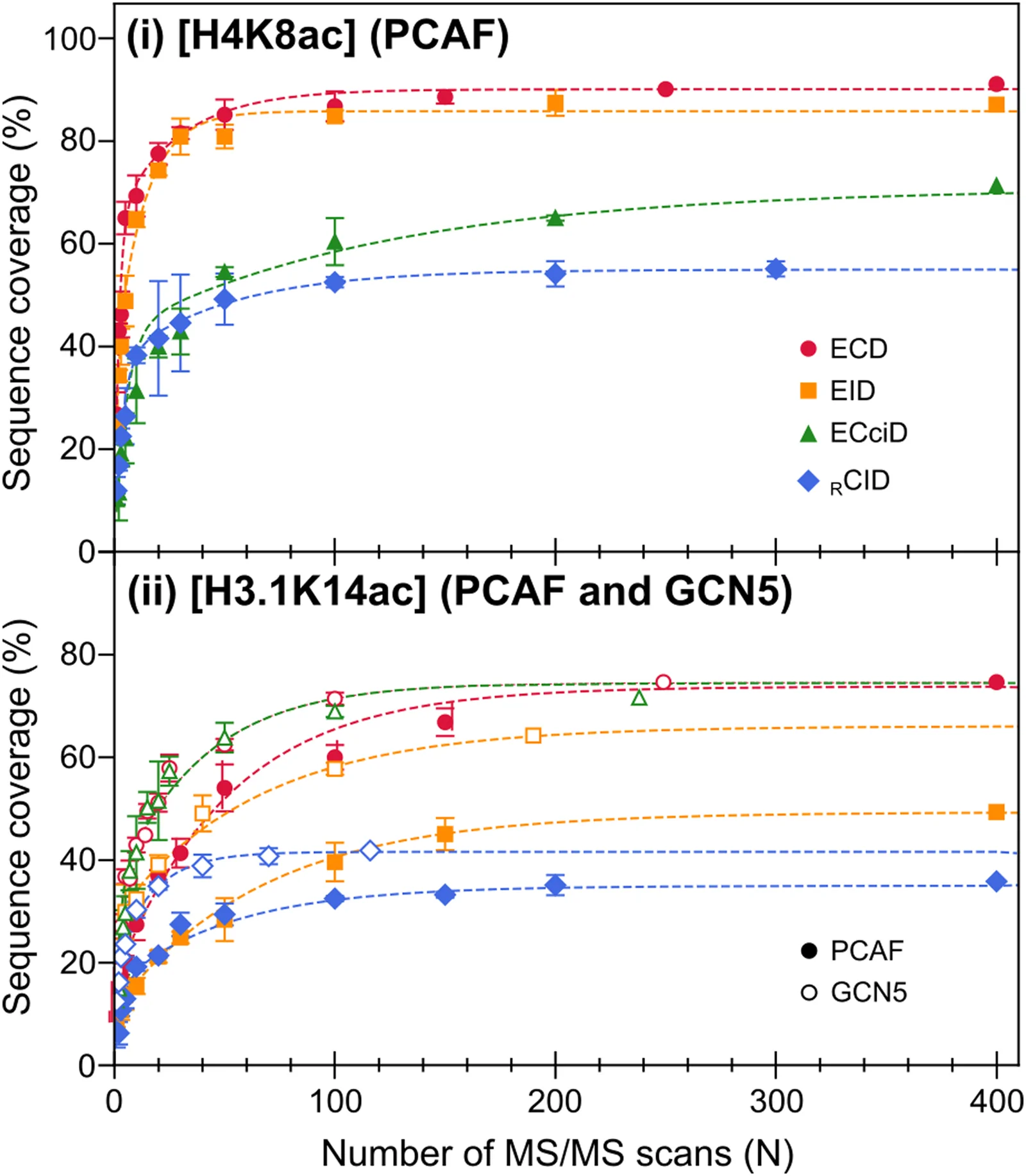
**Sequence coverage as a function of the number of accumulated MS/MS scans (N) for (i) [H4K8ac] generated by PCAF and (ii) [H3.1K14ac] generated by PCAF and GCN5.** Experimental values are shown as mean ± standard deviation (n = 3). Fragmentation methods are indicated by color and symbol: ECD, *red circles*; EID, *orange squares*; ECciD, *green triangles*; and _R_CID, *blue diamonds*. In panel (*ii*), filled symbols correspond to PCAF-treated samples and open symbols correspond to GCN5-treated samples. Dotted lines represent fits of the data using a bi-exponential model.

**Fig. 7:**
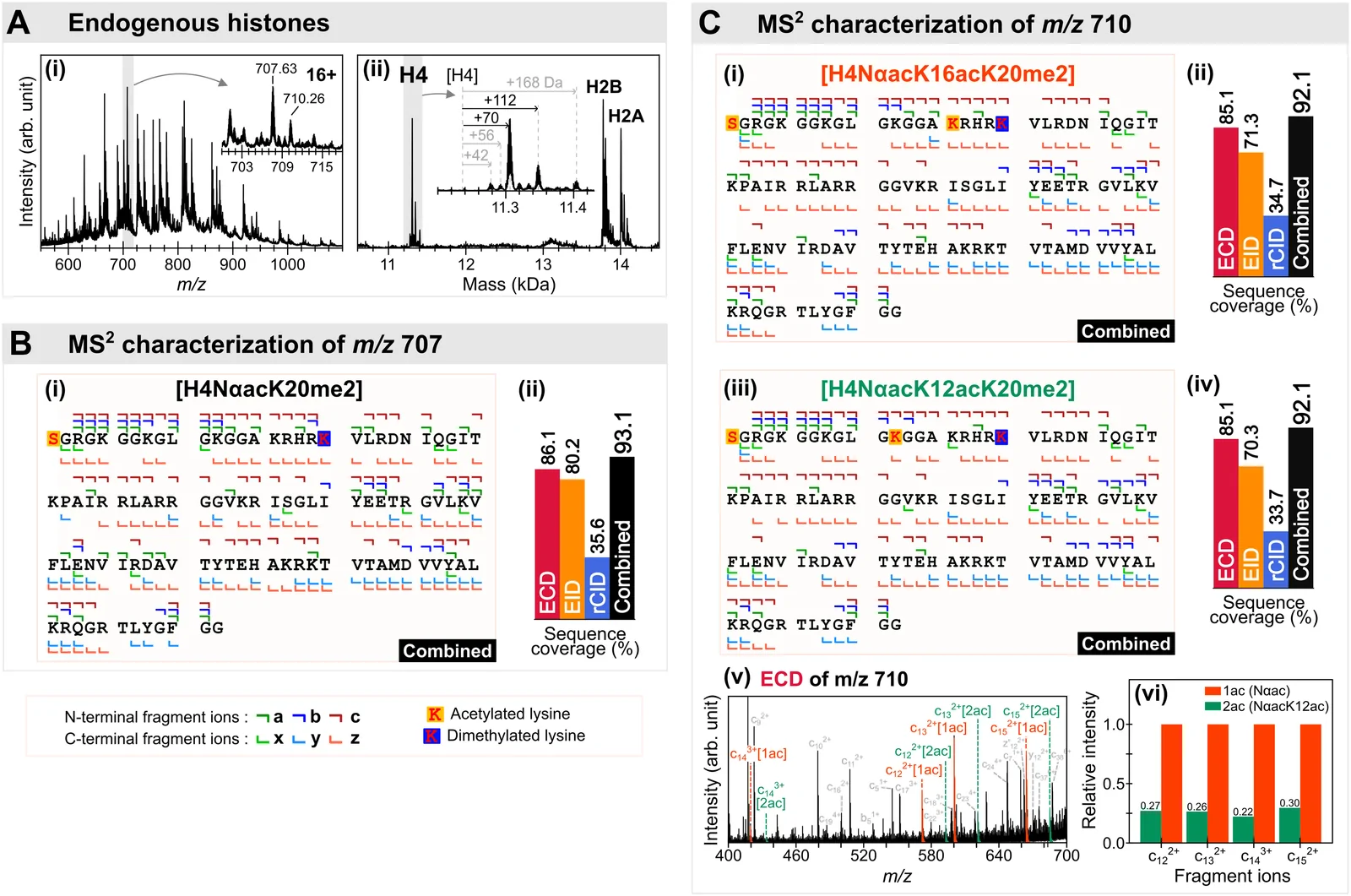
**Characterization of endogenous histone H4 proteoforms using multimodal MS^2^ analysis.** (*A*) MS^1^ spectrum (*i*) and deconvoluted mass spectrum (*ii*) of an offline histone fraction extracted from liver cells. (*B*) Multimodal MS^2^ analysis of the precursor ion at *m/z* 707 (16^+^). (*i*) Combined fragmentation map showing identified a-, b-, c-, x-, y- and z-type fragment ions and annotated PTMs. (*ii*) Sequence coverage obtained for each fragmentation method (ECD, EID and _R_CID) and for the combined dataset. (*C*) Multimodal MS^2^ analysis of the precursor ion at m/z 710 (16^+^). (*i*, *iii*) Combined fragmentation maps corresponding to two candidate proteoforms, showing identified fragment ions and PTM annotations. (*ii*, *iv*) Sequence coverage obtained for each fragmentation method (ECD, EID and _R_CID) and for the combined dataset. (*v*) Zoomed view of the ECD spectrum highlighting selected diagnostic fragment ions. (*vi*) Relative intensities of the corresponding diagnostic fragment ions.

### Estimation of Positional Isomer Abundance

Relative proteoform abundances were estimated from matched diagnostic fragment ion intensities, assuming comparable ionization, fragmentation and detection efficiencies for the corresponding positional isomers and fragment ion pairs. Intensity ratios were calculated between the different forms (for instance, unmodified and singly acetylated) of fragments produced by the same backbone cleavage and detected at the same charge state. When several charge states were detected for the same matched fragment, ratios were evaluated separately for each charge state and, where indicated, after summing their intensities. This approach is supported by Top-down studies showing that precursor ion ratios of intact acetylated histone proteoforms remained close to the expected mixture compositions and that matched ECD/ETD fragment ion ratios reproduced known positional-isomer proportions (60, 61, 62). In addition, matched fragment ions have the same sequence length and charge state and differ only by number of PTMs, substantial differences in ion transmission and MCP detection response are therefore not expected over the small corresponding m/z interval (63, 64). Because standards of known positional isomer composition were not available in the present study, the estimated proportion should be interpreted as rough estimates rather than absolute quantification.

### Proteoform Nomenclatures

Proteoforms are described in this work using bracket-based notation introduced by Kelleher and co-workers (65). Square brackets ‘[ ]’ indicate fully defined proteoforms in which all PTMs are chemically identified and site-localized. For example, [H3.1K14ac] denotes histone variant H3.1 acetylated exclusively at K14. Curly braces ‘{ }’ denote partially defined proteoforms, where the presence of at least one or more PTMs is established but their localization remains unresolved. For example, {H4Kac2} indicates a histone H4 carrying two lysine acetylations without specification of the modified lysine residues.

## Experimental Design and Statistical Rationale

This study was designed as an analytical evaluation of multimodal top-down MS^n^ workflows on the timsOmni platform for characterization of intact histone proteoforms. Recombinant histones H3.1 and H4 were subjected to independent *in vitro* acetylation reactions with GCN5, PCAF, or p300, and a liver cell fraction containing endogenous histone H4 was also analyzed. The aim was to assess how complementary ion activation methods (_R_CID, ECD, EID, and ECciD) and targeted MS^3^ experiments improve sequence coverage, PTM localization, and discrimination of positional isomers. The *in vitro* systems were selected to provide controlled proteoform mixtures spanning increasing levels of complexity, whereas the endogenous H4 experiment demonstrates the application to complex protein samples of biological origin.

No formal statistical hypothesis testing was applied. Proteoform assignments were based on analytical criteria implemented in OSc, including fragment-isotope matching, mass accuracy, MS score, and SVP. Supplemental Tables S1–S6 summarize the precursor m/z selected and the MS score, SVP, SVP range, and sequence coverage (%) for the top-ranked candidate proteoforms identified by OSc. OSc reports are also provided in the Supporting Information for every experiment presented.

## Results and Discussion

### Predominant Formation of the [H3.1K14ac] proteoform by GCN5

Figure 2 presents the results of a 10-min *in vitro* reaction of histone variant H3.1 performed in the presence of the histone acetyltransferase GCN5 (KAT2A). The MS^1^ signal of H3.1 was dominated by a single monoacetylated species {H3.1Kac} (15,315 Da), together with its oxidized form (15,331 Da) (Fig. 2*A*). MS/MS spectra were acquired for 19+ and 20+ precursor ions using _R_CID, ECD, EID, and ECciD (Fig. 2*B*).

Across fragmentation modes, the acetylation was consistently localized to K14 (Supplemental Table S1), and electron-based methods (ECD, EID and ECciD) provided individual sequence coverages ranging from 66.4 to 79.1% (Fig. 2*B*(*i-iii*)). The identification of [H3.1K14ac] was based on the multiple diagnostic a-, b- and c-type fragment ions carrying a +42 Da mass shift in the 14 to 17 residues region. Sparse, low intensity signals potentially consistent with minor [H3.1K9ac] and [H3.1K18ac] populations were examined across activation methods and precursor charge states (Supplemental Fig. S2). Candidate [H3.1K18ac] signals overlapped with fragments assigned to the dominant [H3.1K14ac] proteoform or occurred at low intensity in congested spectral regions (Supplemental Fig. S2, *A–C*). One c’_9_^3+^[1ac] ion potentially supporting [H3.1K9ac] was detected only by ECD of the 19+ precursor (Supplemental Fig. S2*D*), with no supporting evidence under the other conditions. Neither alternative proteoform was therefore retained as a confident assignment, although a very low-abundance [H3.1K9ac] population cannot be formally excluded. _R_CID alone provided lower sequence coverages, with 41.8% and 37.3% for the charge states 19+ and 20+, respectively. Nevertheless, both datasets remained consistent with acetylation at K14, which gave the highest MS score, although SVP was limited to the residues 1 to 6 and 132 to 135 (Fig. 2*B*(*iv*)). The lower sequence coverage likely resulted from the preferential cleavage of labile bonds during resonance activation. When all the different fragmentation data were combined, the sequence coverage reaches 100% (Fig. 2*B*(*v*)), with the best continuous SVP across residues 1 to 86 and 112 to 135 obtained for ECD, covering all possible lysine residues.

To confirm K14 localization, an MS^3^ experiment was performed in which ECD of {H3.1Kac1}^19+^ produced a charge-reduced radical intermediate (18+•), followed by _R_CID (Supplemental Fig. S3*A*). This increased MS^3^ sequence coverage to 56%, representing a ∼35 to 50% gain relative to direct MS^2^(_R_CID) (Supplemental Fig. S3*B*(*i*)). The SVP score also increased from 6.7% to 17.8%. Fragments spanning residues 1 to 17 localized the acetylation to K14 while excluding modification at K9 and K18, confirming that [H3.1K14ac] was the predominant proteoform.

Overall, application of multimodal MS^n^ workflows confirmed that GCN5 predominantly acetylated H3.1K14, while no other major acetylation sites were detected, in agreement with the previously reported preference of GCN5 for H3K14 (66).

### Distinct Monoacetylated Proteoform Mixtures of Histones H3.1 and H4 Generated by PCAF

PCAF (KAT2B) displays a well-established preference for histone H3.1, whereas histone H4 is a comparatively weaker substrate (67, 68, 69). To evaluate enzyme specificity at the intact proteoform level, separate 30 min *in vitro* acetylation reactions were performed with recombinant H3.1 and H4.

The MS^1^ spectrum of H3.1 after PCAF treatment is presented in Figure 3*A*(*i*). A zoomed view of the region of the charge state 19+ (Fig. 3*A*(*ii*)) showed only acetylated species, with no detectable unmodified H3.1 ([H3.1]). The monoacetylated precursor {H3.1Kac1}^19+^ was selected for ECD, EID and _R_CID characterization. Across all activation modes, [H3.1K14ac] was identified as the top-ranked proteoform, followed by [H3.1K9ac] and [H3.1K18ac] (Supplemental Table S2). In ECD spectra, [H3.1K14ac] gave the highest MS score (28.7) and the largest SVP (67.4%), with N-terminal sequence validation extending from residues 1 to 64. By contrast, although [H3.1K18ac] ranked second (MS score 27.1), its SVP dropped to 29.6%, restricted to residues 1 to 13 in the N-terminal region. Moreover, no unmodified N-terminal fragments were detected in region 14 to 17, supporting localization of the acetylation at or before K14, thus excluding K18. No fragment ions provided evidence for acetylation at K4, while [H3.1K9ac], ranked third (MS score 26.7), was supported by SVP validation up to residue 10 and by two low-intensity acetylated c_9_^2+^ and c_10_^2^+ fragments.

Based on the relative intensities of the acetylated and unmodified forms of c_9_^2+^ and c_10_^2+^ fragment ions, the relative abundance of [H3.1K14ac] *versus* [H3.1K9ac] was estimated to be approximately 7.1 and 9.5 (Fig. 3*B*(*ii*)). This suggests that [H3.1K14ac] was the major monoacetylated proteoform, accounting for roughly 90% of the population and [H3.1K9ac] represented a minor fraction of about 10%.

Among the different MS^2^ techniques tested, ECD provided the highest sequence coverage (81.3%) for [H3.1K14ac], while EID and _R_CID offered complementary coverage (61.2% and 48.5%, respectively) (Fig. 3*B*(*iii*)). Combined, all fragmentation modes increase the total sequence coverage to 93.3% for the [H3.1K14ac] and 92.5% for [H3.1K9ac]. The MS^1^ spectrum also suggests the presence of a diacetylated H3.1 species (Fig. 3*A*(*ii*)), but the spectral quality in this region did not allow acquisition of MS/MS data sufficient for confident localization of a second acetylation site.

The activity of PCAF toward histone H4 was examined under identical reaction conditions (Fig. 4). The total MS^1^ spectrum showed a dominant monoacetylated species, {H4Kac1}, and a lower-abundance doubly acetylated species, {H4Kac2}, together with residual unmodified H4 (Fig. 4*A* right panel). No clear signals were detected at the expected positions for {H4Kac3} or {H4Kac4} (light grey annotation). Instead, several low-intensity peaks appear consistent with adducted and oxidized forms of the protein.

The monoacetylated precursor {H4Kac1}^13+^ was first analyzed by MS^2^ using ECD, EID, ECciD and _R_CID. Across all activation modes, [H4K8ac] was consistently ranked as the top candidate proteoform, followed by [H4K12ac], while [H4K16ac] and [H4K5ac] ranked third and fourth depending on the fragmentation method (Supplemental Table S3). However, N-terminal c-type ions spanning residues 5 to 8 were detected only in their unmodified forms, thus excluding [H4K5ac] despite its comparable MS score.

To refine localization within the N-terminal tail, the abundant _R_CID fragment b_52_^7+^ was mass-selected and subjected to MS^3^(ECD) analysis (Fig. 4*B*). In this experiment, [H4K8ac] remained the top-ranked proteoform (MS score 28.1, SVP 82.4%, supported by SVP across N-terminal residues 1-40) with [H4K12ac] and [H4K16ac] ranked second and third, respectively (MS score 26.1 and 25.0). The c_8_-c_11_ ions were only detected unmodified and therefore did not provide evidence for [H4K12ac]. By contrast, low-intensity diagnostic fragments (acetylated and unmodified c_12-15_^2+^ and y_39_^3+^), supported the presence of [H4K16ac]. Intensity ratios of matched c_12_-c_15_ fragment ion from MS^2^(ECD) were similar for charge states 2+ and 3+, with mean [H4K16ac]/[H4K8ac] ratios of 0.24 ± 0.02 and 0.24 ± 0.05 (mean ± standard deviation), respectively. In contrast, MS^3^(ECD) yielded ratios of 0.22 ± 0.03 for charge state 1+ and 0.12 ± 0.01 for the charge state 2+ (Fig. 4*B*(*ii*) and Supplemental Fig. S4). This systematic charge state dependence in the MS^3^ experiment may result from altered charge partitioning following _R_CID formation of the b_52_^7+^ intermediate. Nevertheless, all estimates consistently identified [H4K8ac] as the dominant proteoform, representing approximately 81 to 89% of the selected monoacetylated population.

Taken together, the data indicated that {H4Kac1} predominantly corresponded to [H4K8ac] under the present *in vitro* conditions, with a minor contribution from [H4K16ac]. Combined MS^2^ and targeted MS^3^ data yielded 99% sequence coverage for the dominant assignment.

A lower-abundance doubly acetylated population, {H4Kac2}, was also detected (Fig. 4*A*), although partially overlapped with isobaric/isomeric background signals. MS^2^ (ECD and _R_CID) produced several closely ranked candidate proteoforms (Supplemental Table S4). Targeted MS^3^(ECD) experiments (Supplemental Fig. S5*B*(*i*)) were performed on the doubly acetylated _R_CID b_52_^7+^ fragment and consistently supported acetylation at K8 within the {H4Kac2} population. Site-specific fragments further supported [H4K8acK16ac] and [H4K8acK44ac] (Supplemental Fig. S5*B*(*ii-iii*)), whereas intermediate lysines (K20 and K31) remained plausible but could not be uniquely assigned. A detailed description of the MS^3^ analysis is provided in the Supporting Information.

The multimodal MS^n^ workflow demonstrates that PCAF acetylates both histones H3.1 and H4 *in vitro*, with distinct levels of site-specificity. On monoacetylated H3.1, PCAF showed a predominant preference for K14, with a minor contribution from K9, whereas the monoacetylated H4 population was assigned mainly to [H4K8ac] with minor [H4K16ac]. These results are consistent with previous studies reporting H3K14 as the primary PCAF target with minor acetylation at H3K9, and H4K8 as the preferred site on H4 with minor acetylation at H4K16 (69, 70, 71). In contrast, the doubly acetylated H4 population showed greater positional heterogeneity but K8 remains an anchor site, while the second acetylation was distributed across multiple possible lysine residues with K16 and K44 directly supported.

### Multimodal MS^n^ Resolves Highly Acetylated H4 Positional Isomers Generated by p300

The histone lysine acetyltransferase p300 (KAT3B) is a transcriptional co-activator with broad substrate specificity, acetylating all four core histones (H2A, H2B, H3, and H4), as well as numerous non-histone proteins *in vivo* (72). In contrast to the more site-selective acetyltransferases GCN5 and PCAF, p300 can modify multiple lysine residues within the histone tails. On histone H4, reported sites include the N-terminal tail K5, K8, K12, and K16 with a preference for K5 and K8, together with additional lysine residues at lower abundance (69, 73, 74). Consistent with this broad specificity, incubation of H4 with p300 for 1h produced a wide distribution of acetylated species (Fig. 5*A*), ranging from unmodified H4 (11,236 Da) to proteoforms carrying up to seven acetylations. Subsequent analysis focused on {H4Kac6} and {H4Kac7}, as these highly acetylated species provide an informative model for localization of multiple co-occurring acetylation sites on intact histones.

Figure 5*B* summarizes the multimodal MS^n^ workflow applied to {H4Kac6}^13+^. ECD of the intact precursor generated a relatively abundant c_22_^3+^ fragment corresponding to the H4 N-terminal tail, which was isolated in Q2 for 100 ms and accumulated in Q8, resulting in a ∼15-fold signal enhancement. Subsequent _R_CID on this fragment yielded 95.2% sequence coverage of the tail region and showed that major {H4Kac6} population carried five N-terminal acetylation sites at K5, K8, K12, K16 and K20, thereby constraining the remaining acetylation to the downstream sequence.

Multimodal MS^2^ analysis provides complementary information to localize the sixth site. [H4K5acK8acK12acK16acK20acK44ac] was the top-scoring proteoform (Supplemental Table S5), while [H4K5acK8acK12acK16acK20acK31ac] was ranked second, in the EXD datasets. ECD, EID and ECciD provided sequence coverage of 89.1%, 90.1%, and 87.1% for [H4K5acK8acK12acK16acK20acK44ac], with an MS score of 54.7, 35.3 and 40.8, and N-terminal sequence validation extending to residue 41, 46 and 30, respectively. In the ECD spectrum, a continuous c-ion series spanning residues 33 to 41 was observed with either five or six acetylations, consistent with coexisting [H4K5acK8acK12acK16acK20acK44ac] and [H4K5acK8acK12acK16acK20acK31ac]. _R_CID data supports the presence of the [H4K5acK8acK12acK16acK20acK44ac], yielding 52.5% sequence coverage through diagnostic b- and y-type fragments (b_24_^4+^, y_63_^7+^, y_49_^5+^ and y_47_^4+^). In addition, two low-intensity c_22_^3+^ and c_23_^3+^ ions carrying four and five acetylations suggested a third minor configuration, [H4K5acK8acK12acK16acK31acK44ac], although this proteoform is not supported by ECciD, suggesting that its abundance is near the detection limit. No consistent fragment evidence supports acetylation beyond K44.

The relative abundances of the two principal proteoforms, [H4K5acK8acK12acK16acK20acK44ac] and [H4K5acK8acK12acK16acK20acK31ac], were estimated from ECD data, which provide the largest number of diagnostic fragments, using shared c_34_ and c_36_ to c_41_ ions observed with five or six acetylations together with complementary z_59_ to z_67_ bearing one and no acetylation at identical charge states. The average abundance ratio between these two proteoforms is 1.8 ± 0.5, indicating that {H4Kac6} was dominated by [H4K5acK8acK12acK16acK20acK44ac] (∼70%), followed by [H4K5acK8acK12acK16acK20acK31ac] (∼25%). The possible configuration [H4K5acK8acK12acK16acK31acK44ac] accounted for only a minor fraction (∼5%). ECciD data yields abundance ratios of 1.9 ± 0.6 between the two main proteoforms, estimated based on c_34_^5+^, c_36_^5+^-c_41_^5+^, z’_59_^6+^-z’_64_^6+^ and z_66_^6+^, which is consistent with ECD-derived estimates. Integration of the multimodal MS^n^ workflow yielded overall in-sequence coverages of 100% and 99% for [H4K5acK8acK12acK16acK20acK44ac] and [H4K5acK8acK12acK16acK20acK31ac], respectively (Fig. 5*C*).

The most heavily modified species, {H4Kac7}^13+^, was characterized by ECD and _R_CID (Supplemental Fig. S6). Both methods identified [H4K5acK8acK12acK16acK20acK31acK44ac] as the top-scoring proteoform. and restricted the hepta-acetylated population to two positional isomers differing by acetylation at either K44 or K59, with the K44ac configuration dominating (∼75–80%). Combined ECD and _R_CID sequence coverages lead to 95.0% and 93.1% sequence coverage for [H4K5acK8acK12acK16acK20acK31acK44ac] and [H4K5acK8acK12acK16acK20acK31acK59ac], respectively. Detailed analysis is provided in the Supporting Information.

### Evolution of Sequence Coverage During Data Acquisition

To evaluate how backbone information accumulates with increasing data acquisition, sequence coverage was analyzed as a function of the number of accumulated scans, N (Fig. 6). For each value of N, at least three subsets were evaluated when possible, and sequence coverage is reported as mean ± standard deviation. The analysis was restricted to datasets dominated by a single proteoform to minimize bias from coexisting positional isomers.

Sequence coverage was expressed as a function of the number of accumulated MS/MS scans rather than acquisition time because scan duration depends on experimental settings, in particular precursor accumulation, and varied from 30 to 1000 ms per scan. Under these conditions, N provides a more comparable metric for direct comparison across different experiments.

For all fragmentation methods, sequence coverage increased rapidly at low scan numbers and then more gradually as additional scans were accumulated. This behavior reflects the early detection of the most abundant fragment ions arising from the most favored backbone cleavages, while less probable cleavages required accumulating higher number of scans to be detected. The dependence of sequence coverage on N was described empirically using a bi-exponential saturation function (dotted lines in Fig. 6):

(1) $$SC\left(N\right)=C_{\infty }\left[1-\left(ae^{-k_{1}N}+\left(1-a\right)e^{-k_{2}N}\right)\right]$$

Where SC(N) is the sequence coverage obtained from N spectra, $C_{\infty }$ is the asymptotic plateau, k1 and k2 describe a fast and a slow accumulation regime, and *a* is the fractional contribution of the fast component. Parameters were estimated by nonlinear least-squares minimization.

The bi-exponential model provided excellent agreement with the data (R^2^ = 0.9980–0.9999, mean 0.9995), whereas a single-exponential model resulted in lower goodness-of-fit (R^2^ = 0.775–0.968, mean 0.918), and systematically overestimated sequence coverage at low scan numbers while underestimating the plateau level (Supplemental Fig. S7). Based on the bi-exponential fits, electron-based fragmentation reached high sequence coverage after relatively few scans, typically 2 to 4 ECD and EID scans for H4 to reach 50% sequence coverage, 5 to 20 scans for H3.1, depending on the activation method and precursor. _R_CID showed a similar behavior but reached lower plateau values. The faster convergence observed for H4 likely reflects a combination of higher precursor signal intensity and the shorter H4 sequence, which reduced the number of backbone cleavages required to reach a given sequence coverage. The present experiments were performed in direct infusion, and compatibility with online LC-MS^2^(EXD) therefore remains to be demonstrated. Nevertheless, the rapid accumulation of sequence coverage within the first few scans suggests that informative fragmentation could be achieved on chromatographic timescales.

### Multimodal MS^2^ Identifies Endogenous H4 Proteoforms and Positional Isomers

Figure 7*A* shows the MS^1^ (i) and deconvoluted (ii) spectra of a histone fraction extracted from human liver cells after off-line reversed-phase fractionation. Histone H4 was a major component, together with signals in the mass range expected for H2B (13,700–13,900 Da) and H2A (13,950–14,100 Da). The deconvoluted spectrum reveals two major H4 species at 11,306 and 11,348 Da, corresponding to +70 Da and +112 Da relative to unmodified H4 (11,236 Da). Minor neighboring peaks differing by +14 Da were also observed, consistent with methylation. Precursor ions at *m/z* 707 and 710 (16+) were mass-selected for multimodal MS^2^ analysis by ECD, EID, and _R_CID.

For the *m/z* 707 precursor, ECD and EID consistently supported a doubly modified proteoform carrying one +42 Da modification and one +28 Da modification. In both datasets, the two top-ranked proteoforms consistently identified by OSc were [H4NαacK20me2] and [H4K5acK20me2] (Supplemental Table S6). These assignments differ only in the position of the acetylation, located either at the protein N terminus or at K5, while both place a dimethylation at K20 (K20me2). N-terminal fragment ions up to residue four were consistently detected only in their acetylated form, supporting N-terminal acetylation (Nαac). The alternative K5ac assignment lacked consistent corroboration across the different MS/MS experiments and was supported only by a single low-intensity ion (a_3_^1+^) in EID dataset. No fragment ions supported lysine modifications at K8, K12, or K16. For the additional +28 Da shift, fragment ions do not support the presence of two monomethylated lysine residues. Fragment ions spanning the K20-K31 region carried both modifications, supporting dimethylation at K20 rather than two monomethylation events on two different lysine residues. _R_CID also ranked [H4NαacK20me2] as the top proteoform, although with lower sequence coverage (33.7%). Taken together, [H4NαacK20me2] remains as the only proteoform identified for the *m/z* 707 precursor, with an overall sequence coverage of 93.1% (Fig. 7*B*(*ii*)).

For the *m/z* 710 precursor, Osc and analysis of fragment ion indicated a modification pattern consistent with the *m/z* 707 species carrying one additional acetylation. ECD fragment-ion analysis localized the second at K16, thus supporting the presence of [H4NαacK16acK20me2] with a sequence coverage of 85.1% and 71.3% using ECD and EID, respectively (Fig. 7*C*(*ii*)). In addition, ECD revealed lower intensity diagnostic c-type fragment ions between K12 and K16 carrying two acetyl groups (c_12_-c_15_), consistent with a minor [H4NαacK12acK20me2] positional isomer (Fig. 7*C*(*v*)). EID does not provide clear evidence for this [H4NαacK12acK20me2] isomer, likely reflecting its lower sequence coverage and reduced fragment ion signal relative to ECD under the selected conditions. No additional proteoform candidates are supported by the observed fragment ion series. Based on the relative intensities of c_12_-c_15_ ions, [H4NαacK16acK20me2] was estimated to be ∼3.8-fold more abundant than [H4NαacK12acK20me2] (Fig. 7*C*(*vi*)).

Because a +42 Da mass shift is compatible with both acetylation and trimethylation events, an important ambiguity could arise from these near-isobaric PTMs. This possibility was considered for the endogenous H4 proteoforms. Indeed, these two modifications differ in mass by only 36.4 mDa, corresponding to a relative mass difference of ∼3.2 ppm for intact H4, which can be insufficient to exclude both possibilities based on precursor mass alone. However, at the fragment level, the same absolute mass difference corresponds to a larger relative difference, reaching ∼7.4 ppm for 5 kDa fragments and increasing further for smaller fragments. This exceeds both the 5 ppm mass tolerance applied for analysis and the experimental fragment mass error. Indeed, as an example for the ECD datasets of the *m/z* 707 and 710 precursor, fragment mass errors were centered around zero, with mean errors (μ) of 0.7 and 0.1 ppm and standard deviations (σ) of 1.6 and 1.3 ppm, respectively (Supplemental Fig. S10). These values support confident distinction between acetylation and trimethylation at the fragment level and explain why OSc favored acetylated rather than trimethylated candidates for the endogenous H4 proteoforms identified.

Overall, the endogenous H4 proteoforms identified here, [H4NαacK20me2], [H4NαacK16acK20me2] and [H4NαacK12acK20me2], are consistent with the known H4 PTM landscape, dominated by N-terminal acetylation, acetylation within the K5-K16 region, and prevalent dimethylation at K20 (75, 76, 77, 78, 79).

## Conclusion

The timsOmni platform enables multimodal MS^n^ workflows for detailed top-down characterization of intact proteoforms. Integration of the Omnitrap enabled flexible ion trapping, isolation, and complementary ion activation by _R_CID, ECD, EID, and hybrid workflows, producing dense and complementary fragment ion series for unambiguous proteoform assignment and PTM localization. In monoacetylated H3.1, the dominant proteoform generated by GCN5 and PCAF was consistently identified as [H3.1K14ac], in agreement with known enzyme specificity. For H4, multimodal MS^2^ and targeted MS^3^ experiments identified two monoacetylated proteoforms, [H4K8ac] and [H4K16ac], after PCAF treatment, and multiple co-occurring acetylations and resolved site ambiguity in highly modified species generated by p300 {H4Kac6} and {H4Kac7}. The p300 reaction generates a broad distribution of highly acetylated proteoforms yielding a more extreme combinatorial scenario than typically encountered *in vivo*, providing a particularly demanding analytical case evaluating top-down proteoform analysis. Across these experiments, complementary fragmentation approaches yielded complete or near-complete backbone sequence coverage and enabled discrimination of positional acetylation isomers in intact proteins. Targeted MS^3^ workflows (ECD→_R_CID, _R_CID→ECD, and charge reduction→_R_CID) were employed to resolve residual site ambiguities and to enhance local diagnostic coverage, particularly in highly modified regions. Analysis of endogenous H4 fraction further showed that the workflow remains effective in complex biological samples and supports confident assignment of co-occurring acetylation and methylation. Together, these results highlight the capability of the timsOmni platform to support rapid multimodal MS^n^ analysis of intact proteins and residue-level mapping of combinatorial PTMs. Future integration with online LC separation, automated precursor selection and higher-throughput data analysis will extend these workflows toward large-scale top-down proteomics and systematic characterization of proteoform complexity.

## Data Availability

The mass spectrometry data supporting this study have been deposited in MassIVE repository under dataset identifier MSV000101646. The deposited files include Bruker raw mass spectrometry files, exported ascii profile spectra, recalibrated spectra, and associated OSc analysis files. These materials are available from the corresponding author upon reasonable request.

## Supplemental Data

The supporting information document contains Supplemental Figs. S1–S10 and Supplemental Tables S1–S6, including SDS-PAGE, MS^3^ workflows, additional fragmentation maps, sequence-coverage analyses, and OmniScape ranking tables for the proteoform assignments discussed in the main text. OmniScape PDF reports are provided separately as five supplemental PDF files (Supplemental Data S1–S5) corresponding to the H3.1 GCN5, H3.1 PCAF, H4 PCAF, H4 p300, and endogenous H4 datasets.

## Conflict of Interest

The authors declare the following conflict of interest(s): A. Smyrnakis, M. Kosmopoulou, D. Suckau, C. Albers and D. Papanastasiou are employees of Bruker, which develops and manufactures the timsOmni platform and the OmniScape software used in this study. Other authors declare no conflict of interest.
