# Supplementary material for: Top-Down Sequencing of Intact Proteoforms Using the timsOmni Mass Spectrometer: Accurate Determination of Co-Occurring Histone Modifications
Source: Mol Cell Proteomics. 2026 Aug 17;25(9):101640. doi: 10.1016/j.mcpro.2026.101640 (PMC13595144; doi:10.1016/j.mcpro.2026.101640)

Supplemental Data S1. Endogenous H4 - OmniScape reports

OmniScape reports for endogenous histone H4 proteoforms.

**Histone**	H4
**Enzyme**	Not applicable
**Reaction/source**	Endogenous sample
**Nb of Osc reports**	6

Included reports

#	Experiment	Precursor	Charge	Fragmentation / workflow	File
1	MS2	m/z 707	16+	ECD	MS2_mz-707_16+_ECD.pdf
2	MS2	m/z 707	16+	EID	MS2_mz-707_16+_EID.pdf
3	MS2	m/z 707	16+	RCID	MS2_mz-707_16+_RCID.pdf
4	MS2	m/z 710	16+	ECD	MS2_mz-710_16+_ECD.pdf
5	MS2	m/z 710	16+	EID	MS2_mz-710_16+_EID.pdf
6	MS2	m/z 710	16+	RCID	MS2_mz-710_16+_RCID.pdf

Supplemental Data S1

Endogenous H4

**Report**	1 of 6
**Experiment**	MS2
**Histone/sample**	H4
**Enzyme**	Not applicable
**Reaction/source**	Endogenous sample
**Precursor**	m/z 707
**Charge state**	16+
**Fragmentation / workflow**	ECD
**Original file**	MS2_mz-707_16+_ECD.pdf

Project Information

Project Directory: C:/Users/LAB-ADMIN
Processing date: Tue Apr 28 14:58:29 2026

Sample Info & Protocols

Name: Top-Down Protein Sequencing ExD

Dataset

Imported spectrum file name:	250430 Liver-Histone-25 mz707z16 MS2 ECD_001-311_calibrated.txt
Dataset name:	Information is only available from imported JPEAKS files
Date acquired:	Information is only available from imported JPEAKS files
Date imported:	Information is only available from imported JPEAKS files
Instrument Name:	Information is only available from imported JPEAKS files
Instrument Serial Number:	Information is only available from imported JPEAKS files

Workflow Result Info

Analysis: 250430 Liver-Histone-25 mz707z16 MS2 ECD_001-311_calibrated
Location: C:/Users/LAB-ADMIN
Method: -

Method Information

Name: Unnamed

Spectral Parameters

Mass Error [ppm]: 6
Intensity Deviation [au]: 0.15
Min/Max Charges: 1-16
Experiment type: ExD

a ions	b ions	c ions	x ions	y ions	z ions	satellite	internal	precursor
a	b	c		y	z+1			M
a+1		c+1			z+2			M+-2H

Noise threshold calculation: Simple moving average noise threshold (SMA)
Window size [datapoints]: 600
Noise curve offset: 3.1327336
SVP parameters: terminal gap: 2, internal gap: 1

Proteoforms

(top 10)

Index	Parent Sequence	Chain	Var. Mods	Mono Mass [Da]	Chemical Composition
1	H4Kac_me1-3	H4Kac_me1-3_Nterm[Acetyl]_K20[Dim]	Dim: K20, Ace: N-term	11299.3831	C498H843N163O135S
2	H4Kac_me1-3	H4Kac_me1-3K5[Ace]K20[Dim]	Ace: K5, Dim: K20	11299.3831	C498H843N163O135S
3	H4Kac_me1-3	H4Kac_me1-3_Nterm[Acetyl]_K16[Dim]	Dim: K16, Ace: N-term	11299.3831	C498H843N163O135S
4	H4Kac_me1-3	H4Kac_me1-3_Nterm[Acetyl]_K16[Met]K20[Met]	Met: K16, K20, Ace: N-term	11299.3831	C498H843N163O135S
5	H4Kac_me1-3	H4Kac_me1-3K8[Ace]K20[Dim]	Ace: K8, Dim: K20	11299.3831	C498H843N163O135S
6	H4Kac_me1-3	H4Kac_me1-3K5[Ace]K16[Dim]	Ace: K5, Dim: K16	11299.3831	C498H843N163O135S
7	H4Kac_me1-3	H4Kac_me1-3K5[Ace]K16[Met]K20[Met]	Ace: K5, Met: K16, K20	11299.3831	C498H843N163O135S
8	H4Kac_me1-3	H4Kac_me1-3_Nterm[Acetyl]_K12[Met]K20[Met]	Met: K12, K20, Ace: N-term	11299.3831	C498H843N163O135S
9	H4Kac_me1-3	H4Kac_me1-3_Nterm[Acetyl]_K12[Met]K16[Met]	Met: K12, K16, Ace: N-term	11299.3831	C498H843N163O135S
10	H4Kac_me1-3	H4Kac_me1-3K12[Ace]K20[Dim]	Ace: K12, Dim: K20	11299.3831	C498H843N163O135S

Results

Proteoforms Table (top 10)

Index	Protein	SC [%]	SVP [%]	IC [%]	MS Score [a.u.]	Var. Mods	Mono Mass [Da]	Chemical Formula
1	H4Kac_me1-3	86.1	18.6	45.4	39.1	Dim: K20, Ace: N-term	11299.3831	C498H843N163O135S
2	H4Kac_me1-3	85.1	0.0	44.7	38.0	Ace: K5, Dim: K20	11299.3831	C498H843N163O135S
3	H4Kac_me1-3	82.2	14.7	44.7	36.7	Dim: K16, Ace: N-term	11299.3831	C498H843N163O135S
4	H4Kac_me1-3	82.2	14.7	44.7	36.7	Met: K16, K20, Ace: N-term	11299.3831	C498H843N163O135S
5	H4Kac_me1-3	82.2	0.0	44.0	36.1	Ace: K8, Dim: K20	11299.3831	C498H843N163O135S
6	H4Kac_me1-3	81.2	0.0	43.9	35.7	Ace: K5, Dim: K16	11299.3831	C498H843N163O135S
7	H4Kac_me1-3	81.2	0.0	43.9	35.7	Ace: K5, Met: K16, K20	11299.3831	C498H843N163O135S
8	H4Kac_me1-3	81.2	10.8	43.6	35.4	Met: K12, K20, Ace: N-term	11299.3831	C498H843N163O135S
9	H4Kac_me1-3	81.2	10.8	43.6	35.4	Met: K12, K16, Ace: N-term	11299.3831	C498H843N163O135S
10	H4Kac_me1-3	79.2	0.0	43.5	34.5	Ace: K12, Dim: K20	11299.3831	C498H843N163O135S

Sequence

Name: H4Kac_me1-3

```
                 10                    20                    30                    40                    50                    60
S G R G K G G K G L   G K G G A K R H R K   V L R D N I Q G I T   K P A I R R L A R R   G G V K R I S G L I   Y E E T R G V L K V
                 70                    80                    90                   100                   110                   120
F L E N V I R D A V   T Y T E H A K R K T   V T A M D V V Y A L   K R Q G R T L Y G F   G G
```

Modifications

Modification	Position	Type
Acetyl	N-term	variable
Acetyl	5	variable
Trimethyl	5	variable
Methyl	5	variable
Dimethyl	5	variable
Acetyl	8	variable
Methyl	8	variable
Dimethyl	8	variable
Trimethyl	8	variable
Methyl	12	variable
Acetyl	12	variable
Dimethyl	12	variable
Trimethyl	12	variable

Modification	Position	Type
Dimethyl	16	variable
Methyl	16	variable
Acetyl	16	variable
Dimethyl	20	variable
Methyl	20	variable
Dimethyl	31	variable
Methyl	31	variable

Sequence Map

Proteoform:

Index	Protein	SC [%]	SVP [%]	IC [%]	MS Score [a.u.]	Var. Mods	Mono Mass [Da]	Chemical Formula
1	H4Kac_me1-3	86.1	18.6	45.4	39.1	Dim: K20, Ace: N-term	11299.3831	C498H843N163O135S

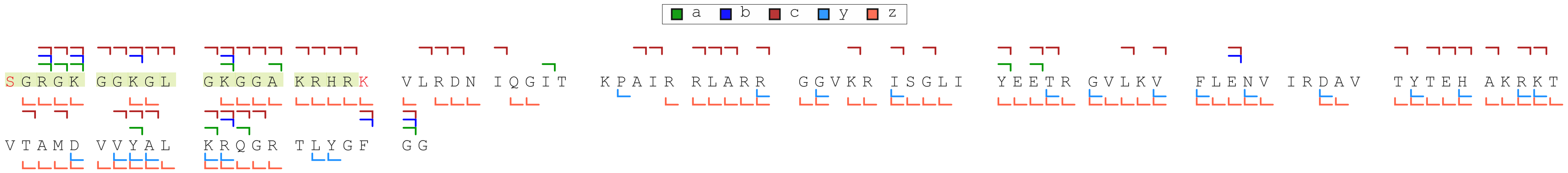

Proteoform:

Index	Protein	SC [%]	SVP [%]	IC [%]	MS Score [a.u.]	Var. Mods	Mono Mass [Da]	Chemical Formula
2	H4Kac_me1-3	85.1	0.0	44.7	38.0	Ace: K5, Dim: K20	11299.3831	C498H843N163O135S

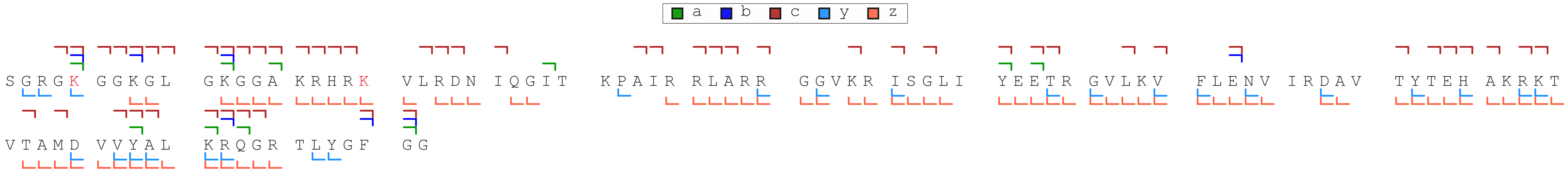

Proteoform:

Index	Protein	SC [%]	SVP [%]	IC [%]	MS Score [a.u.]	Var. Mods	Mono Mass [Da]	Chemical Formula
3	H4Kac_me1-3	82.2	14.7	44.7	36.7	Dim: K16, Ace: N-term	11299.3831	C498H843N163O135S

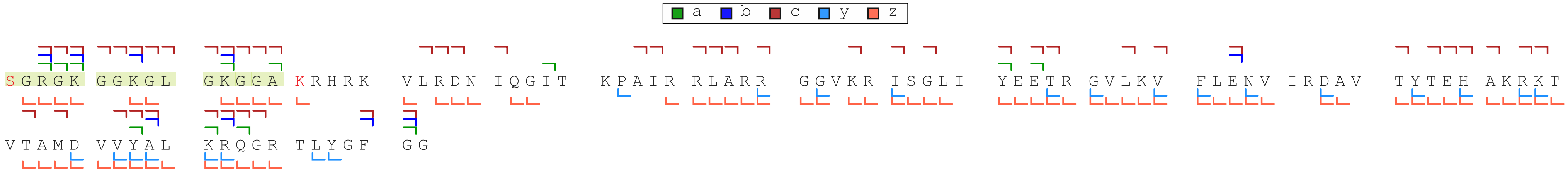

Proteoform:

Index	Protein	SC [%]	SVP [%]	IC [%]	MS Score [a.u.]	Var. Mods	Mono Mass [Da]	Chemical Formula
4	H4Kac_me1-3	82.2	14.7	44.7	36.7	Met: K16, K20, Ace: N-term	11299.3831	C498H843N163O135S

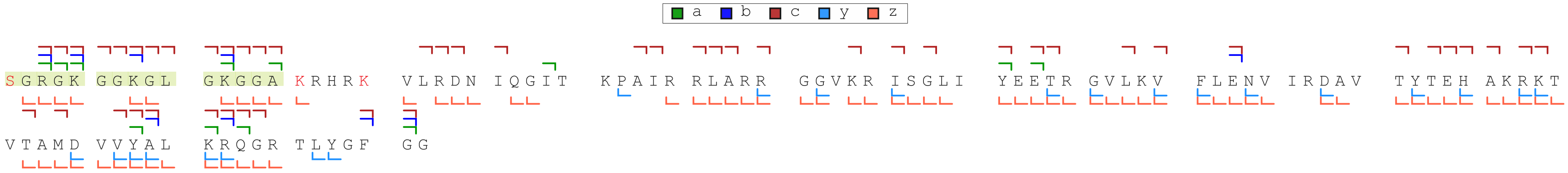

Proteoform:

Index	Protein	SC [%]	SVP [%]	IC [%]	MS Score [a.u.]	Var. Mods	Mono Mass [Da]	Chemical Formula
5	H4Kac_me1-3	82.2	0.0	44.0	36.1	Ace: K8, Dim: K20	11299.3831	C498H843N163O135S

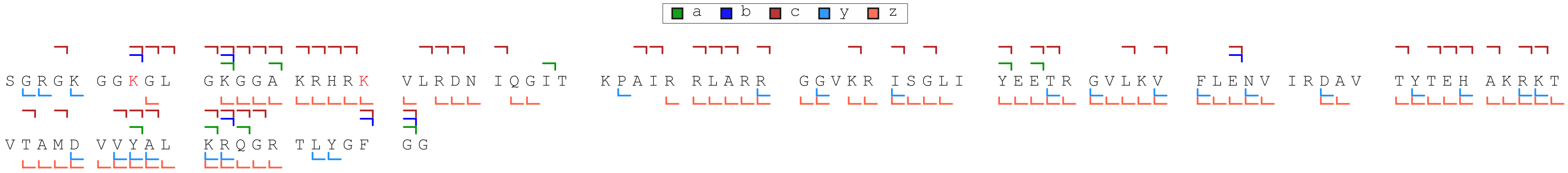

Proteoform:

Index	Protein	SC [%]	SVP [%]	IC [%]	MS Score [a.u.]	Var. Mods	Mono Mass [Da]	Chemical Formula
6	H4Kac_me1-3	81.2	0.0	43.9	35.7	Ace: K5, Dim: K16	11299.3831	C498H843N163O135S

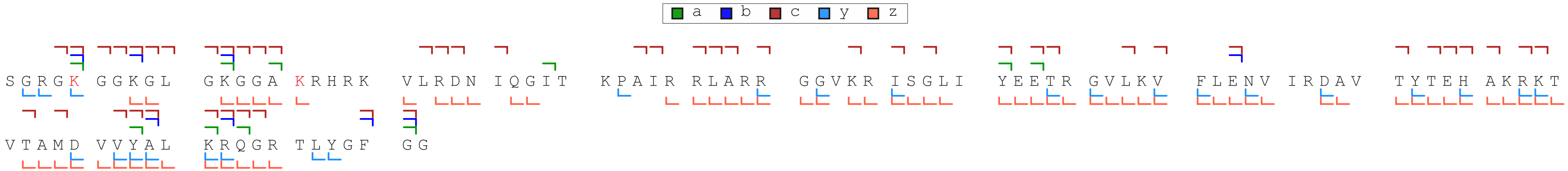

Proteoform:

Index	Protein	SC [%]	SVP [%]	IC [%]	MS Score [a.u.]	Var. Mods	Mono Mass [Da]	Chemical Formula
7	H4Kac_me1-3	81.2	0.0	43.9	35.7	Ace: K5, Met: K16, K20	11299.3831	C498H843N163O135S

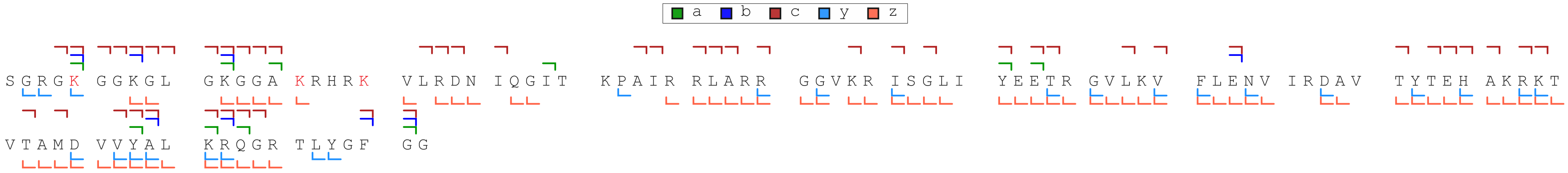

Proteoform:

Index	Protein	SC [%]	SVP [%]	IC [%]	MS Score [a.u.]	Var. Mods	Mono Mass [Da]	Chemical Formula
8	H4Kac_me1-3	81.2	10.8	43.6	35.4	Met: K12, K20, Ace: N-term	11299.3831	C498H843N163O135S

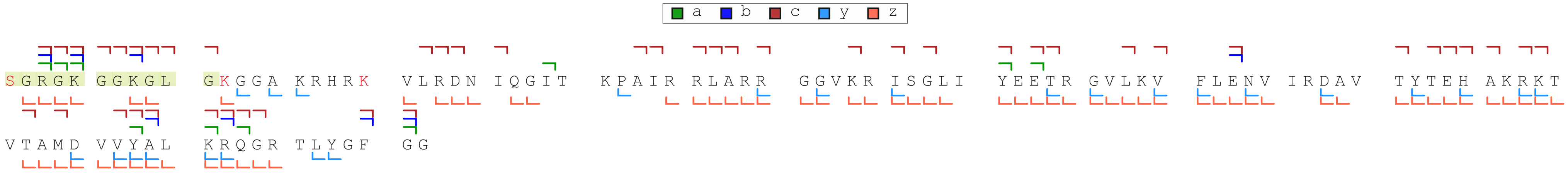

Proteoform:

Index	Protein	SC [%]	SVP [%]	IC [%]	MS Score [a.u.]	Var. Mods	Mono Mass [Da]	Chemical Formula
9	H4Kac_me1-3	81.2	10.8	43.6	35.4	Met: K12, K16, Ace: N-term	11299.3831	C498H843N163O135S

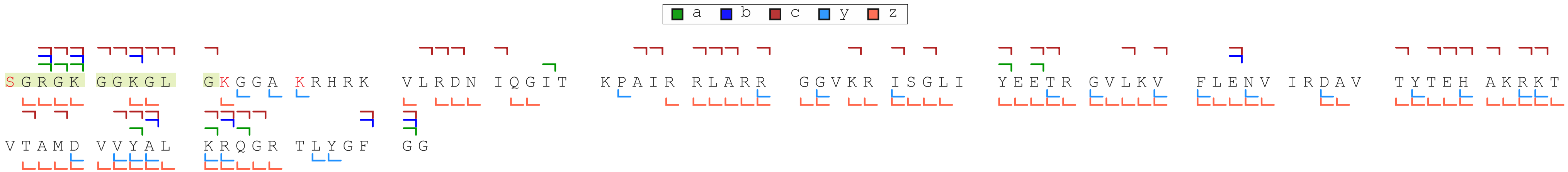

Proteoform:	Index	Protein	SC [%]	SVP [%]	IC [%]	MS Score [a.u.]	Var. Mods	Mono Mass [Da]	Chemical Formula
	10	H4Kac_me1-3	79.2	0.0	43.5	34.5	Ace: K12, Dim: K20	11299.3831	C498H843N163O135S

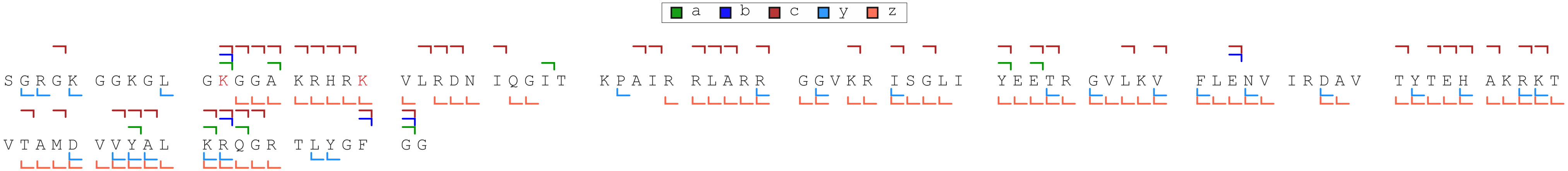

Error Plot

Proteoform:	Index	Protein	SC [%]	SVP [%]	IC [%]	MS Score [a.u.]	Var. Mods	Mono Mass [Da]	Chemical Formula
	1	H4Kac_me1-3	86.1	18.6	45.4	39.1	Dim: K20, Ace: N-term	11299.3831	C498H843N163O135S

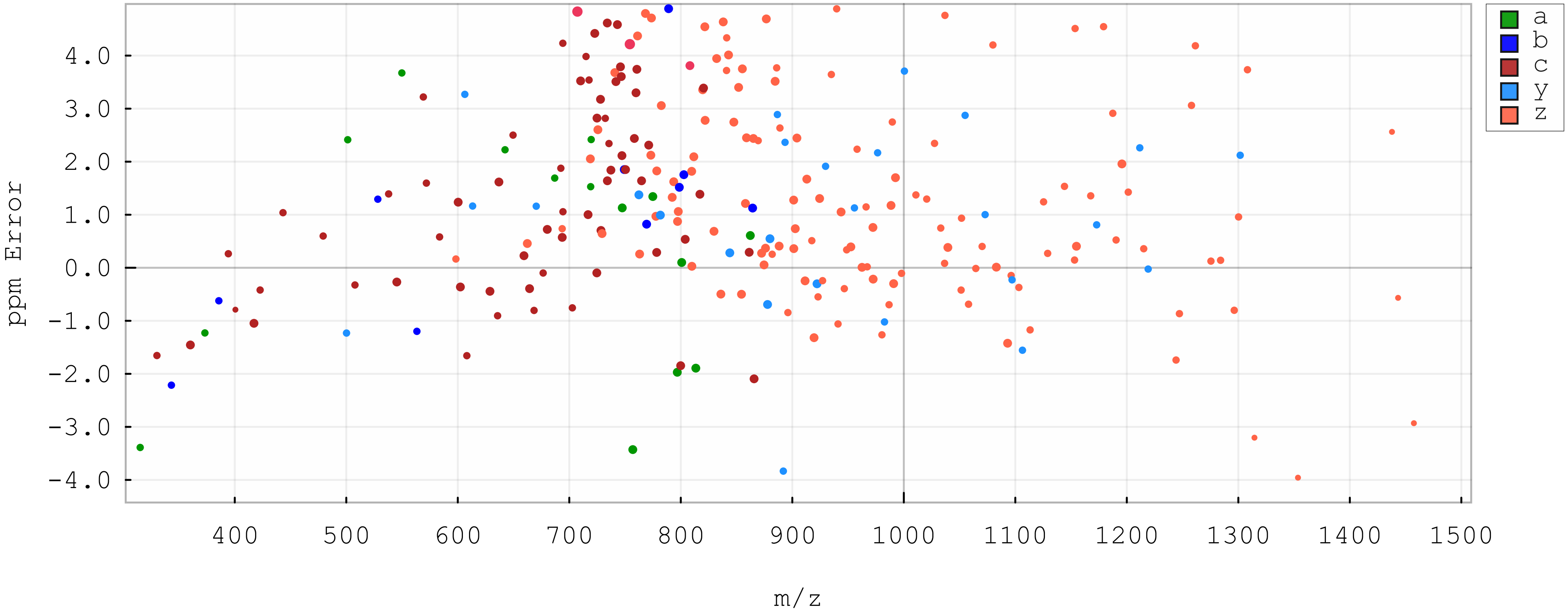

Proteoform:

Index	Protein	SC [%]	SVP [%]	IC [%]	MS Score [a.u.]	Var. Mods	Mono Mass [Da]	Chemical Formula
2	H4Kac_me1-3	85.1	0.0	44.7	38.0	Ace: K5, Dim: K20	11299.3831	C498H843N163O135S

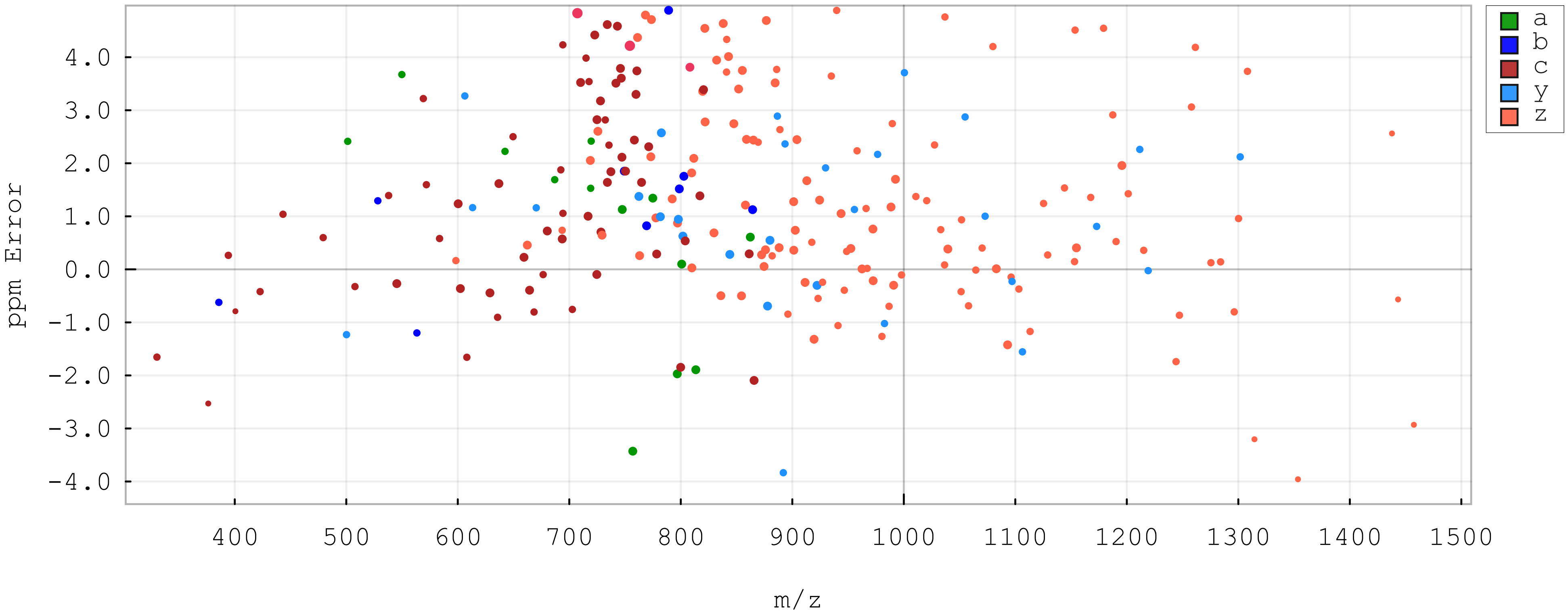

Proteoform:

Index	Protein	SC [%]	SVP [%]	IC [%]	MS Score [a.u.]	Var. Mods	Mono Mass [Da]	Chemical Formula
3	H4Kac_me1-3	82.2	14.7	44.7	36.7	Dim: K16, Ace: N-term	11299.3831	C498H843N163O135S

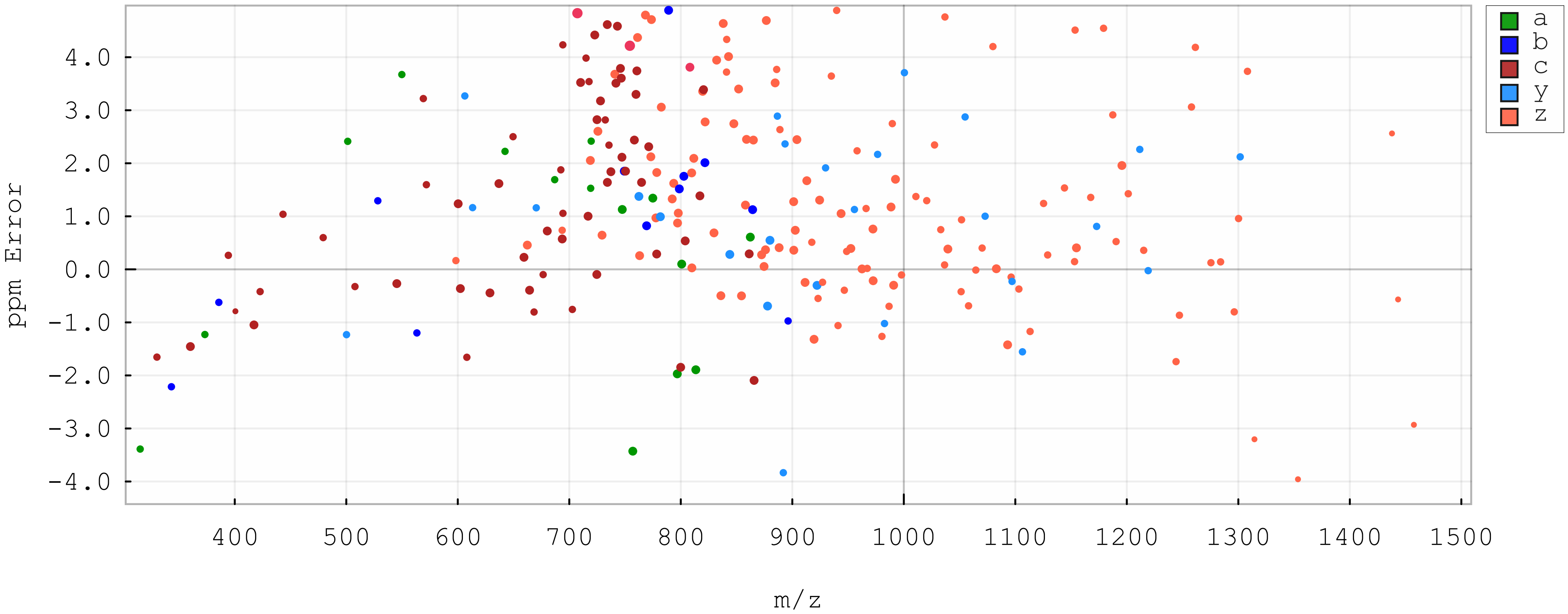

Proteoform:

Index	Protein	SC [%]	SVP [%]	IC [%]	MS Score [a.u.]	Var. Mods	Mono Mass [Da]	Chemical Formula
4	H4Kac_me1-3	82.2	14.7	44.7	36.7	Met: K16, K20, Ace: N-term	11299.3831	C498H843N163O135S

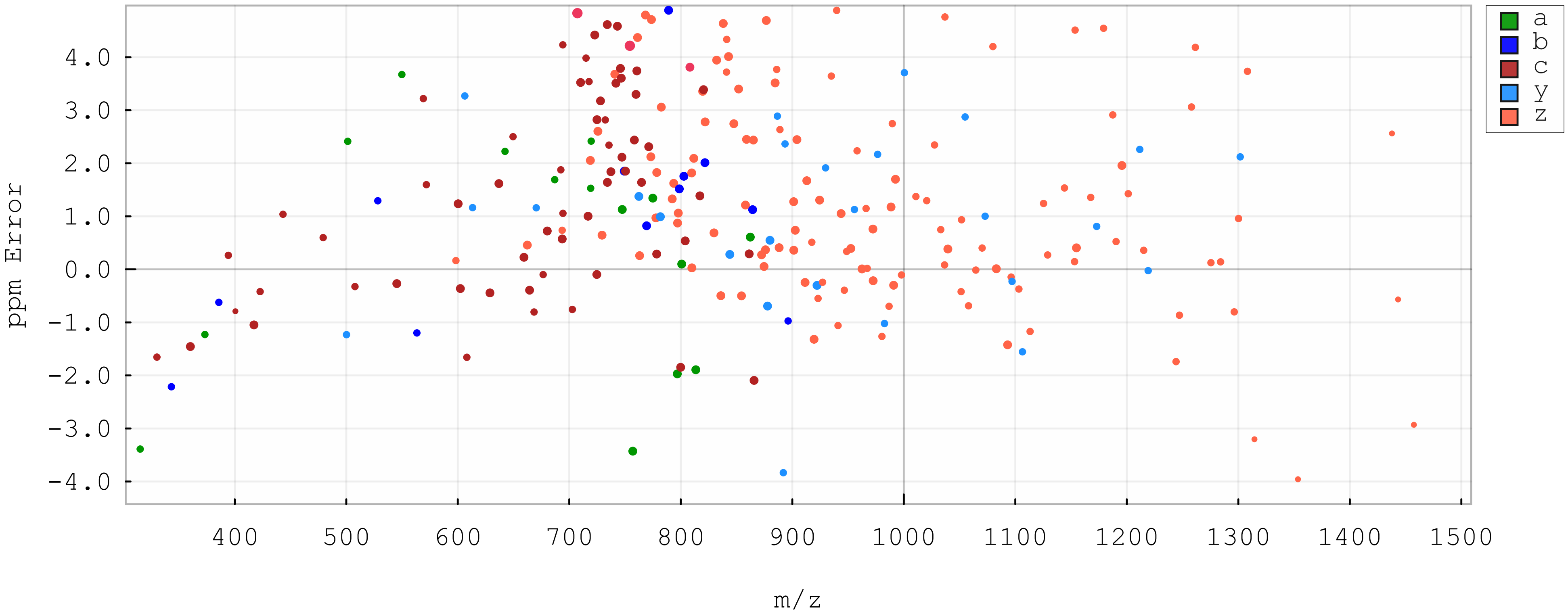

Proteoform:

Index	Protein	SC [%]	SVP [%]	IC [%]	MS Score [a.u.]	Var. Mods	Mono Mass [Da]	Chemical Formula
5	H4Kac_me1-3	82.2	0.0	44.0	36.1	Ace: K8, Dim: K20	11299.3831	C498H843N163O135S

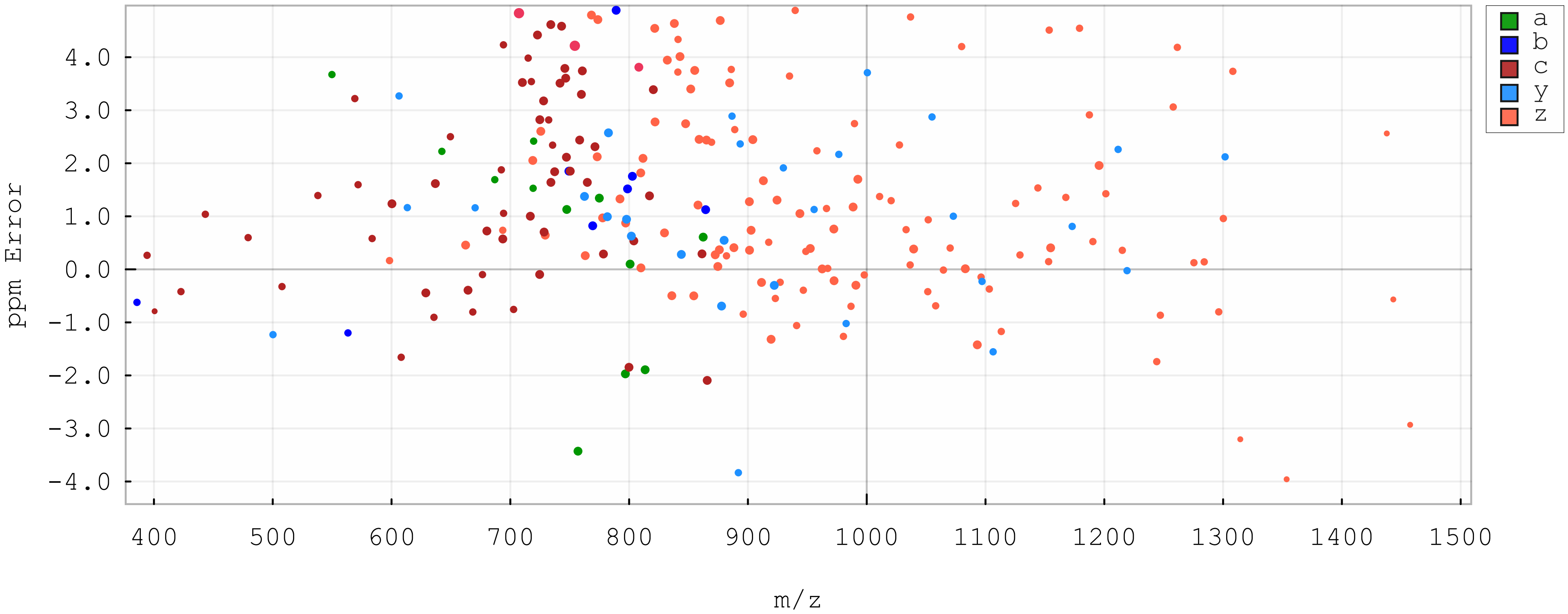

Proteoform:

Index	Protein	SC [%]	SVP [%]	IC [%]	MS Score [a.u.]	Var. Mods	Mono Mass [Da]	Chemical Formula
6	H4Kac_me1-3	81.2	0.0	43.9	35.7	Ace: K5, Dim: K16	11299.3831	C498H843N163O135S

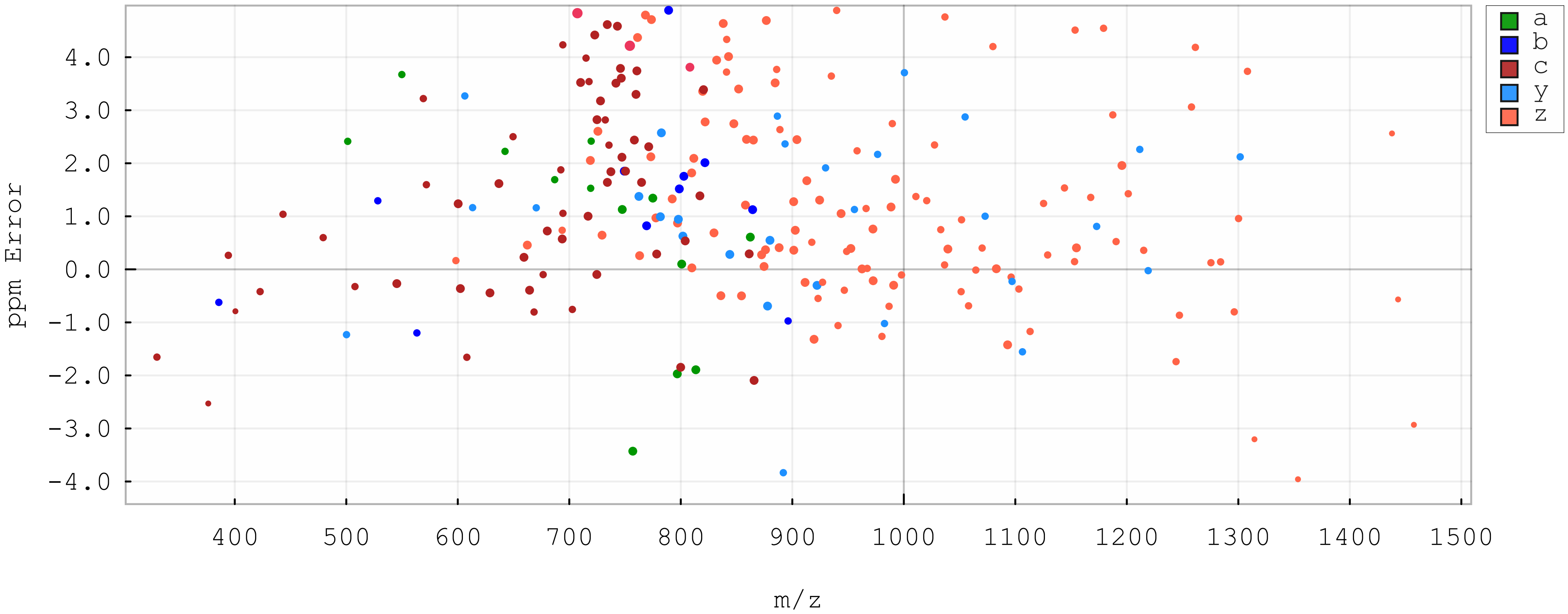

Proteoform:

Index	Protein	SC [%]	SVP [%]	IC [%]	MS Score [a.u.]	Var. Mods	Mono Mass [Da]	Chemical Formula
7	H4Kac_me1-3	81.2	0.0	43.9	35.7	Ace: K5, Met: K16, K20	11299.3831	C498H843N163O135S

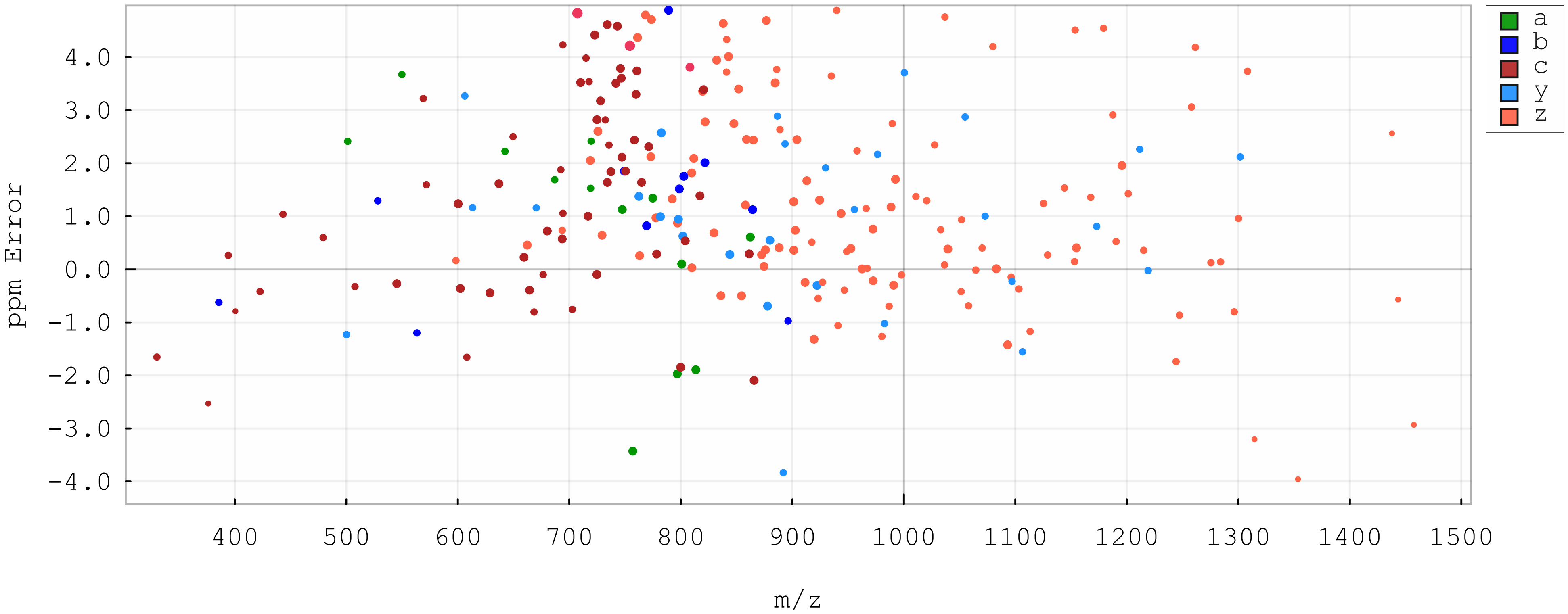

Proteoform:

Index	Protein	SC [%]	SVP [%]	IC [%]	MS Score [a.u.]	Var. Mods	Mono Mass [Da]	Chemical Formula
8	H4Kac_me1-3	81.2	10.8	43.6	35.4	Met: K12, K20, Ace: N-term	11299.3831	C498H843N163O135S

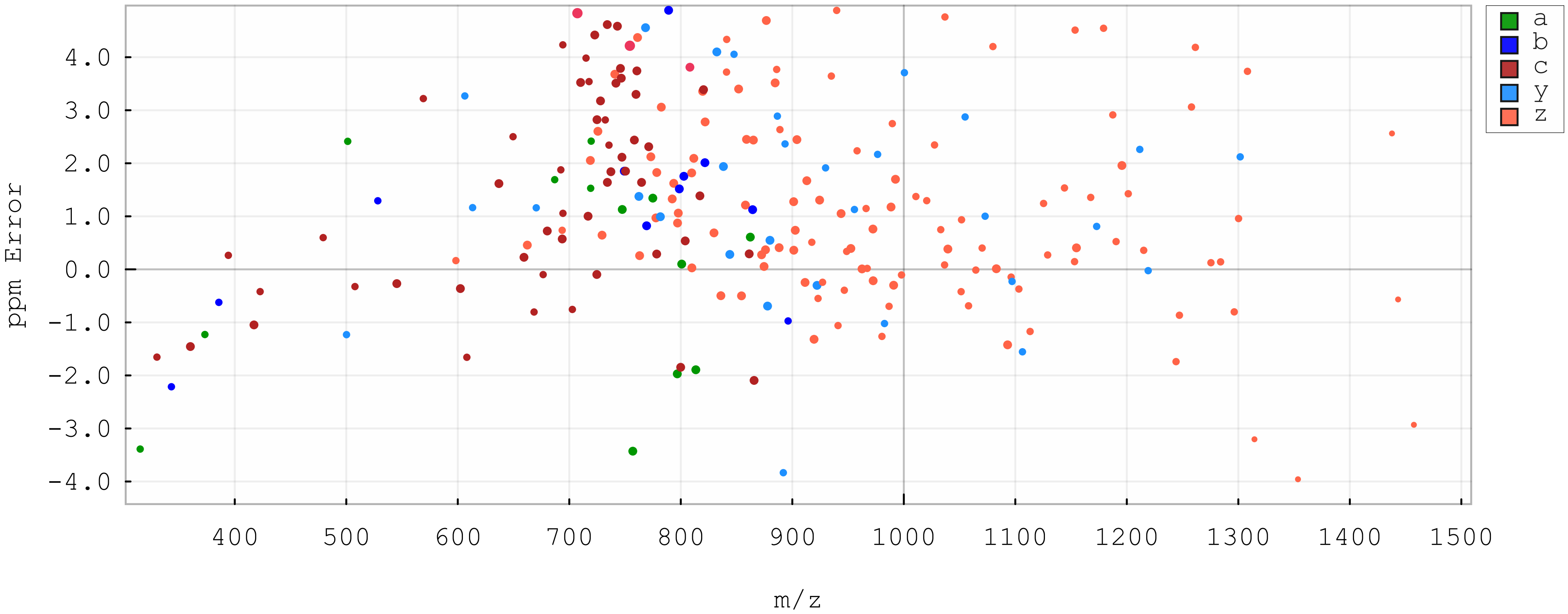

Proteoform:

Index	Protein	SC [%]	SVP [%]	IC [%]	MS Score [a.u.]	Var. Mods	Mono Mass [Da]	Chemical Formula
9	H4Kac_me1-3	81.2	10.8	43.6	35.4	Met: K12, K16, Ace: N-term	11299.3831	C498H843N163O135S

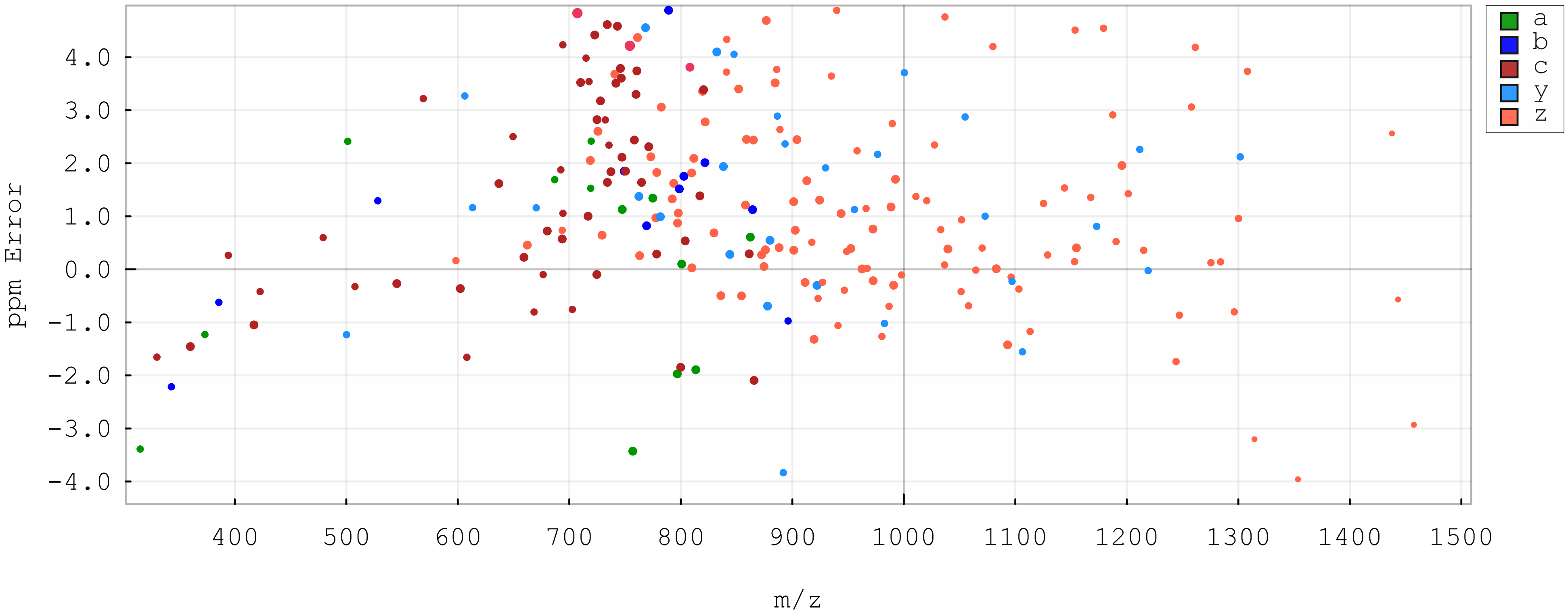

Proteoform:

Index	Protein	SC [%]	SVP [%]	IC [%]	MS Score [a.u.]	Var. Mods	Mono Mass [Da]	Chemical Formula
10	H4Kac_me1-3	79.2	0.0	43.5	34.5	Ace: K12, Dim: K20	11299.3831	C498H843N163O135S

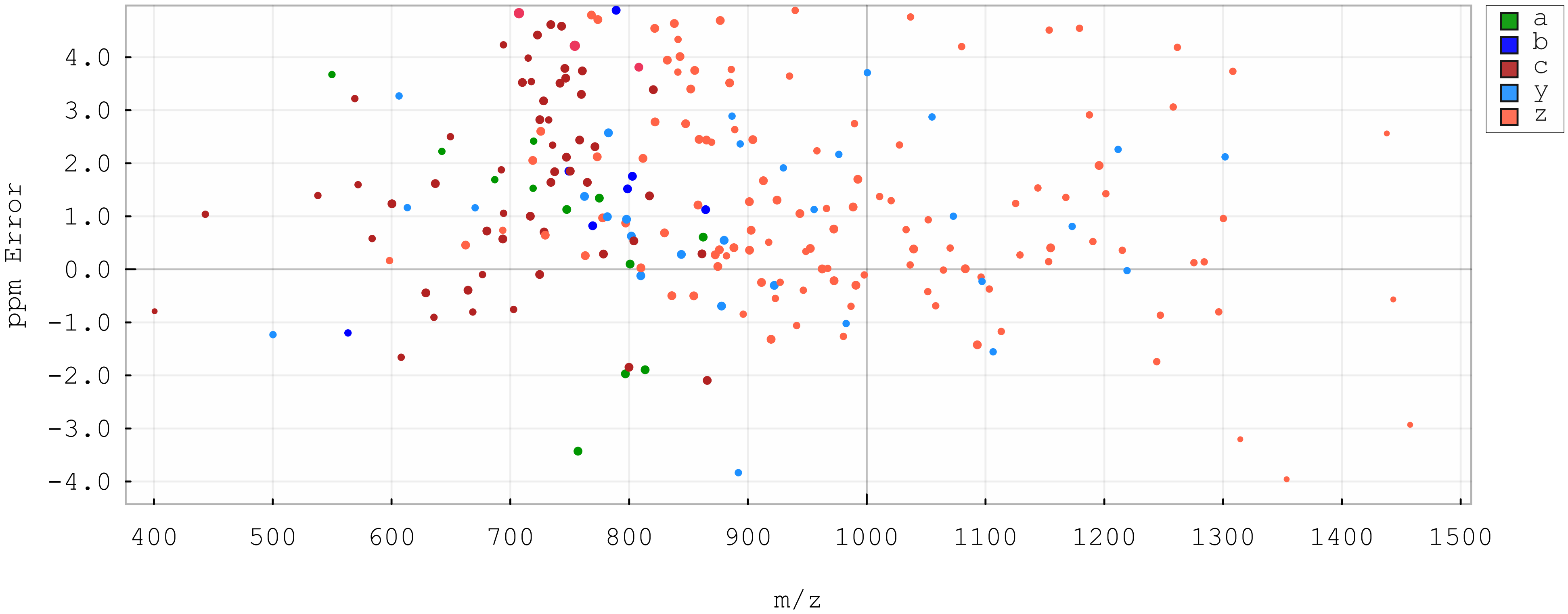

Primary Fragment Plots

Proteoform:

Index	Protein	SC [%]	SVP [%]	IC [%]	MS Score [a.u.]	Var. Mods	Mono Mass [Da]	Chemical Formula
1	H4Kac_me1-3	86.1	18.6	45.4	39.1	Dim: K20, Ace: N-term	11299.3831	C498H843N163O135S

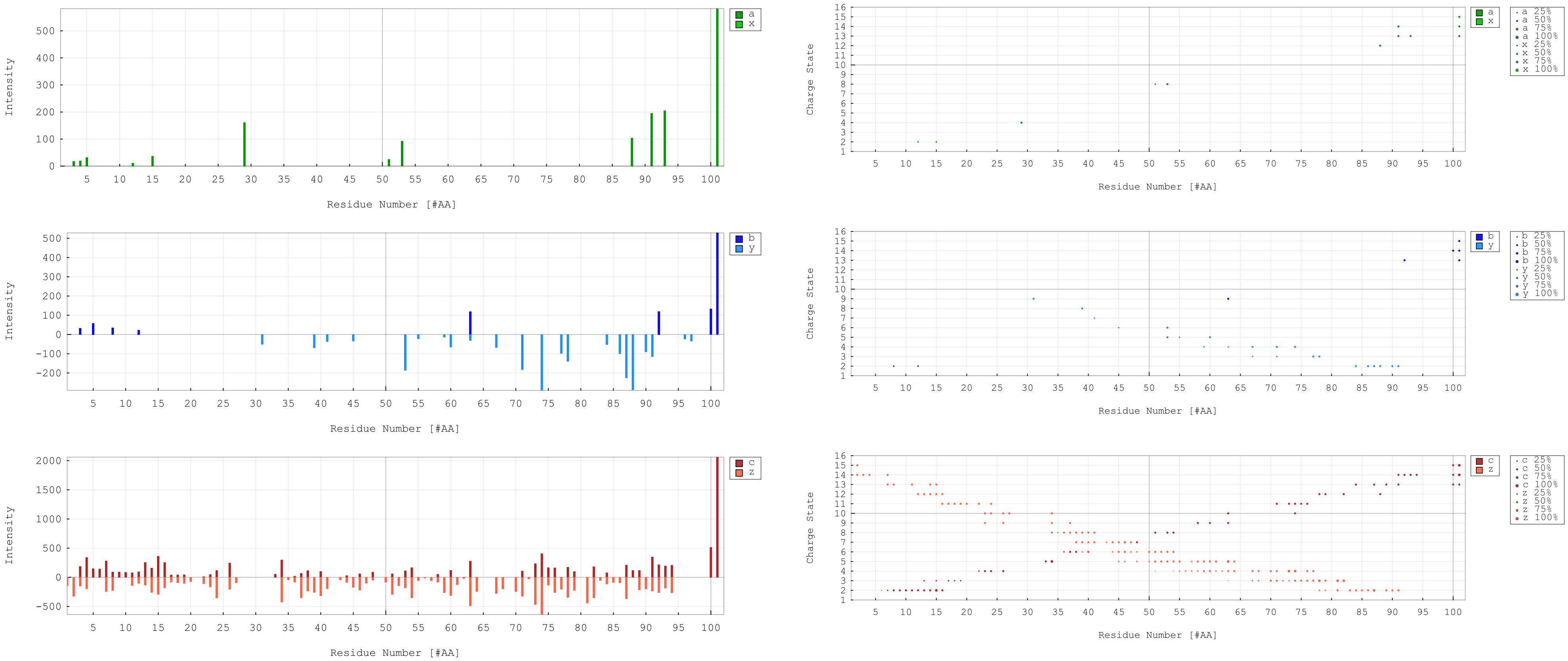

Proteoform:

Index	Protein	SC [%]	SVP [%]	IC [%]	MS Score [a.u.]	Var. Mods	Mono Mass [Da]	Chemical Formula
2	H4Kac_me1-3	85.1	0.0	44.7	38.0	Ace: K5, Dim: K20	11299.3831	C498H843N163O135S

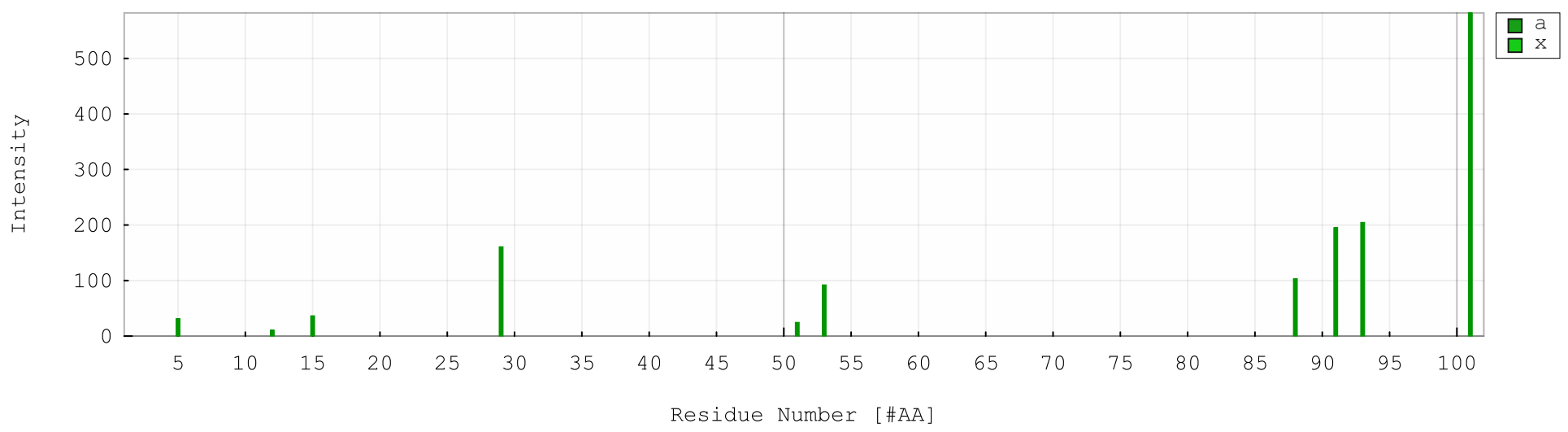

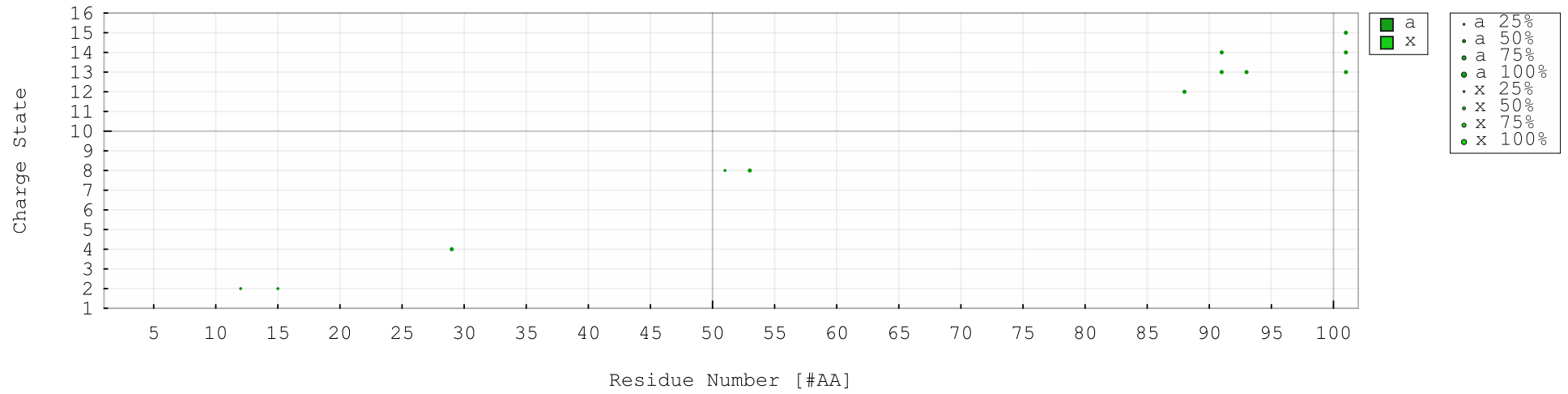

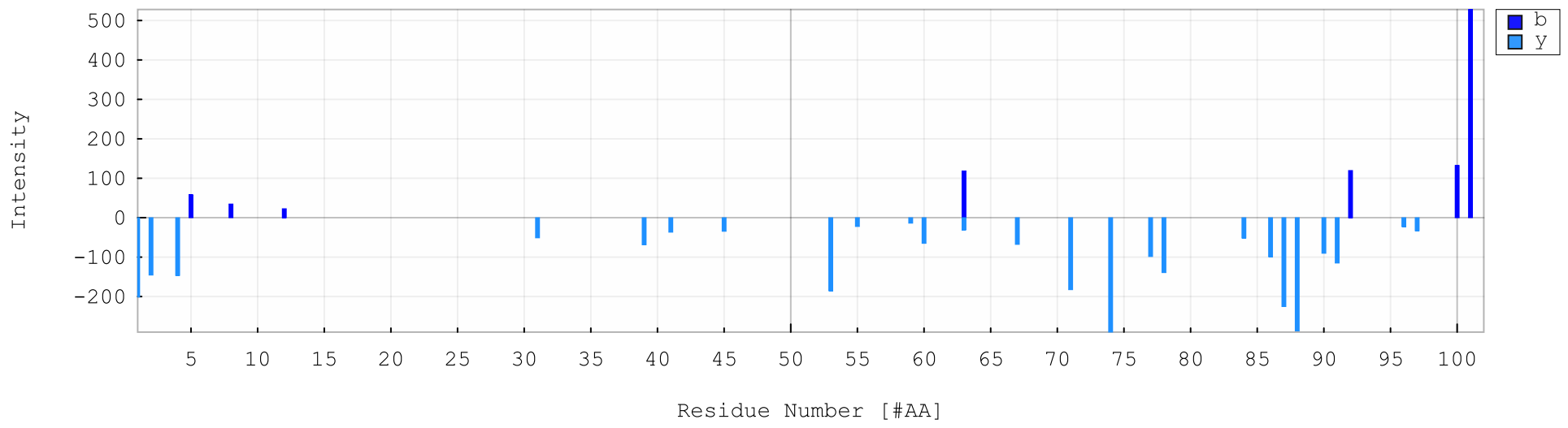

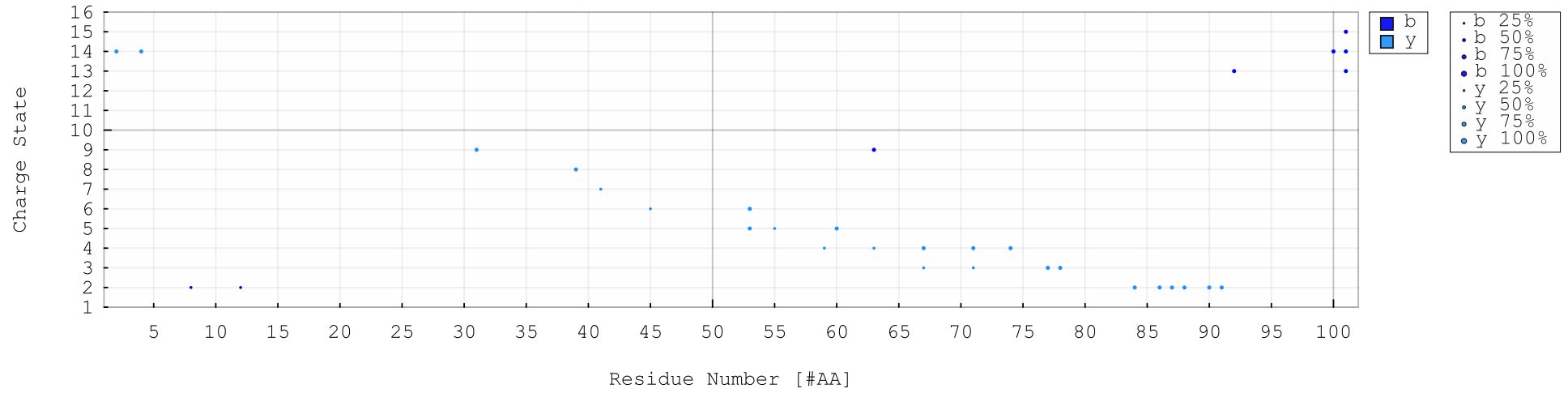

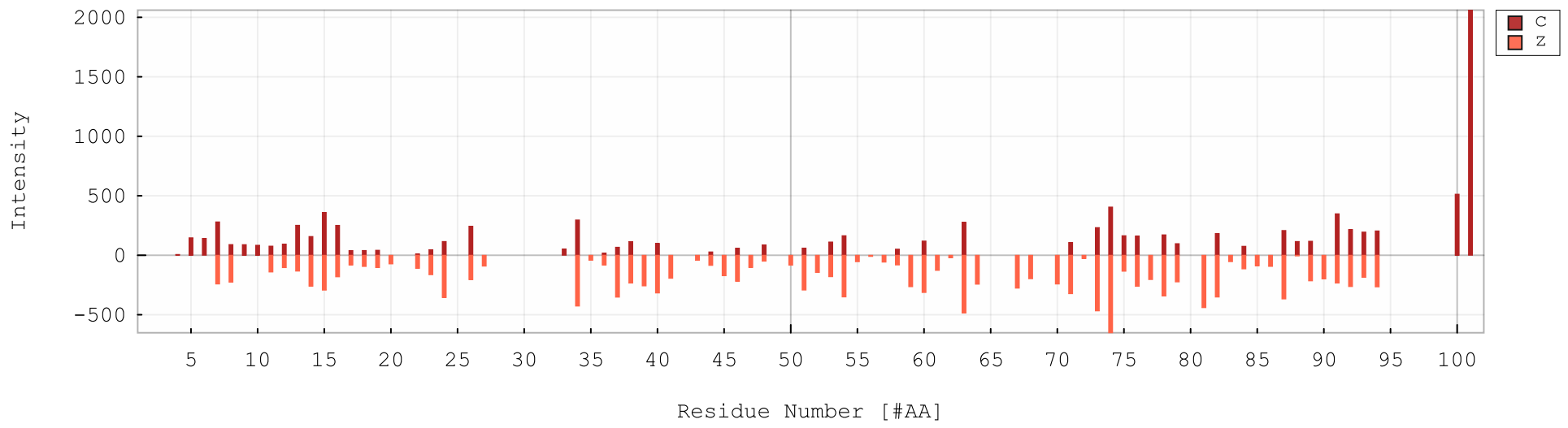

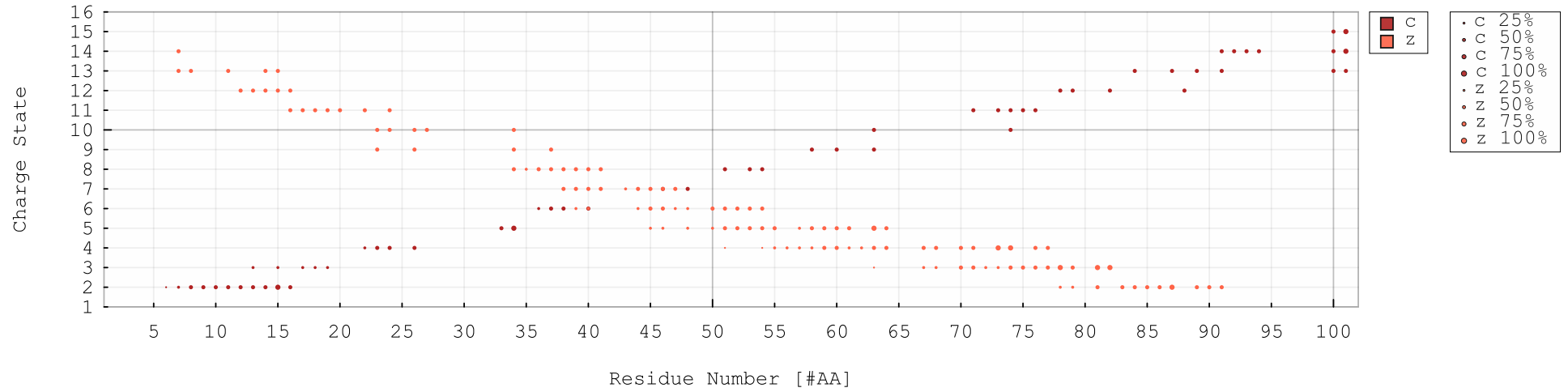

Proteoform:

Index	Protein	SC [%]	SVP [%]	IC [%]	MS Score [a.u.]	Var. Mods	Mono Mass [Da]	Chemical Formula
3	H4Kac_me1-3	82.2	14.7	44.7	36.7	Dim: K16, Ace: N-term	11299.3831	C498H843N163O135S

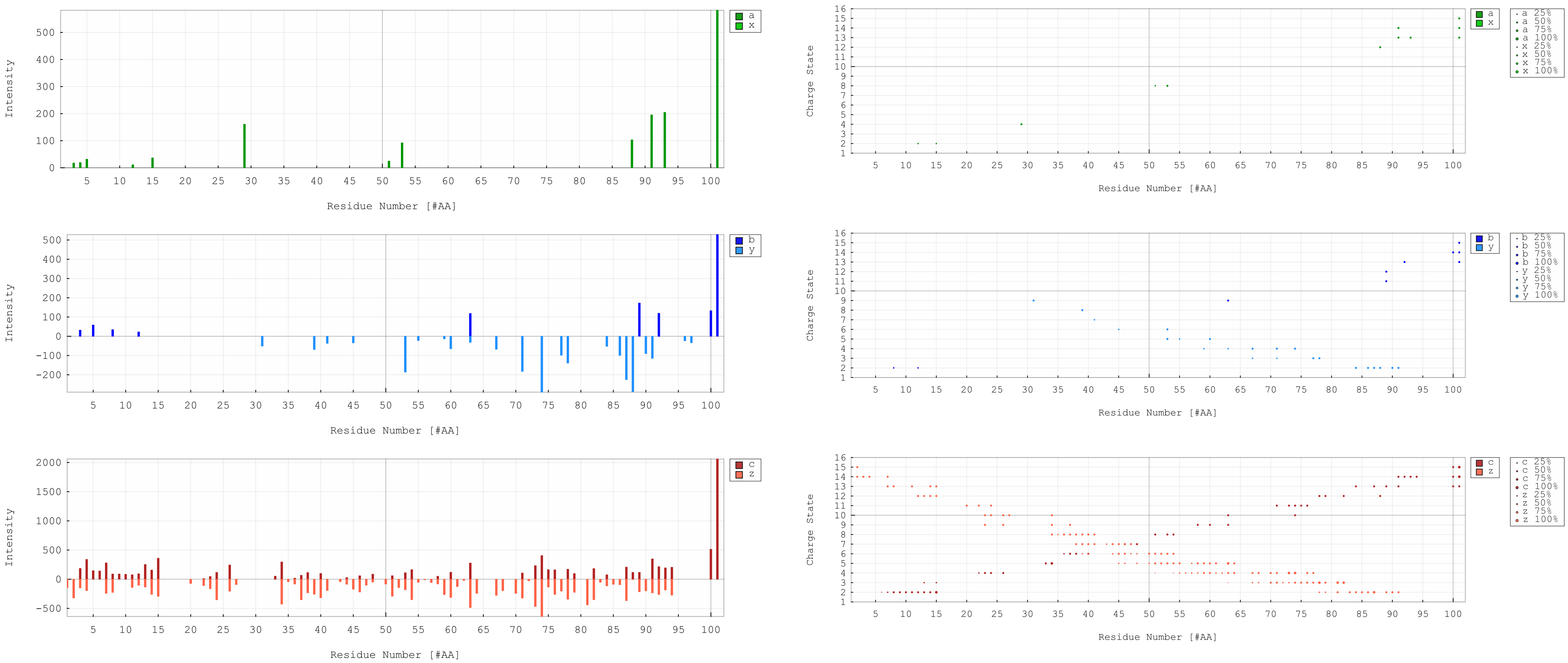

Proteoform:

Index	Protein	SC [%]	SVP [%]	IC [%]	MS Score [a.u.]	Var. Mods	Mono Mass [Da]	Chemical Formula
4	H4Kac_me1-3	82.2	14.7	44.7	36.7	Met: K16, K20, Ace: N-term	11299.3831	C498H843N163O135S

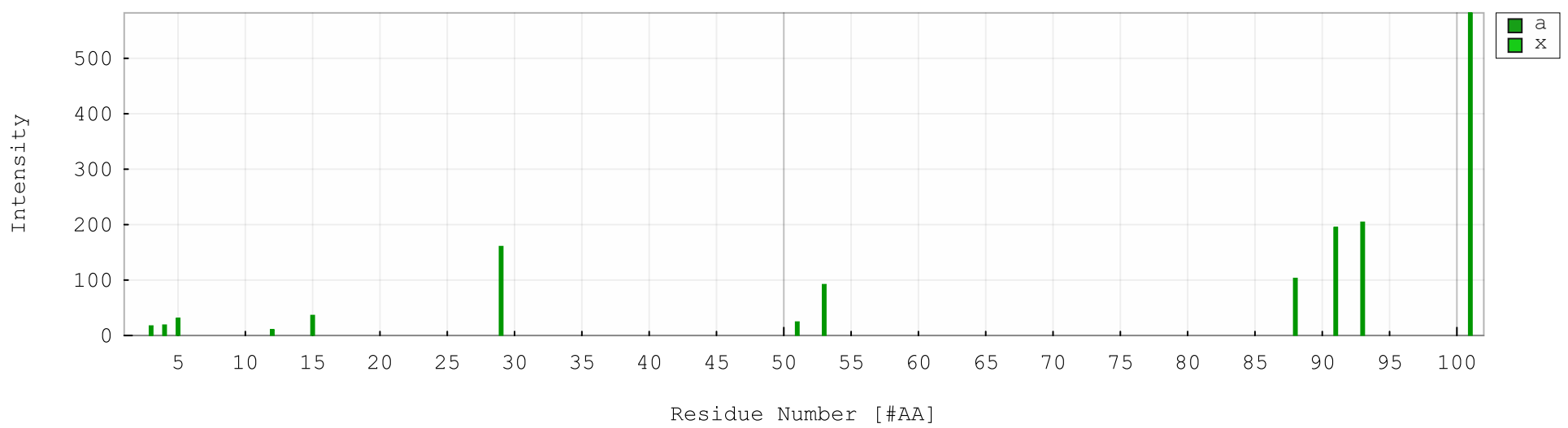

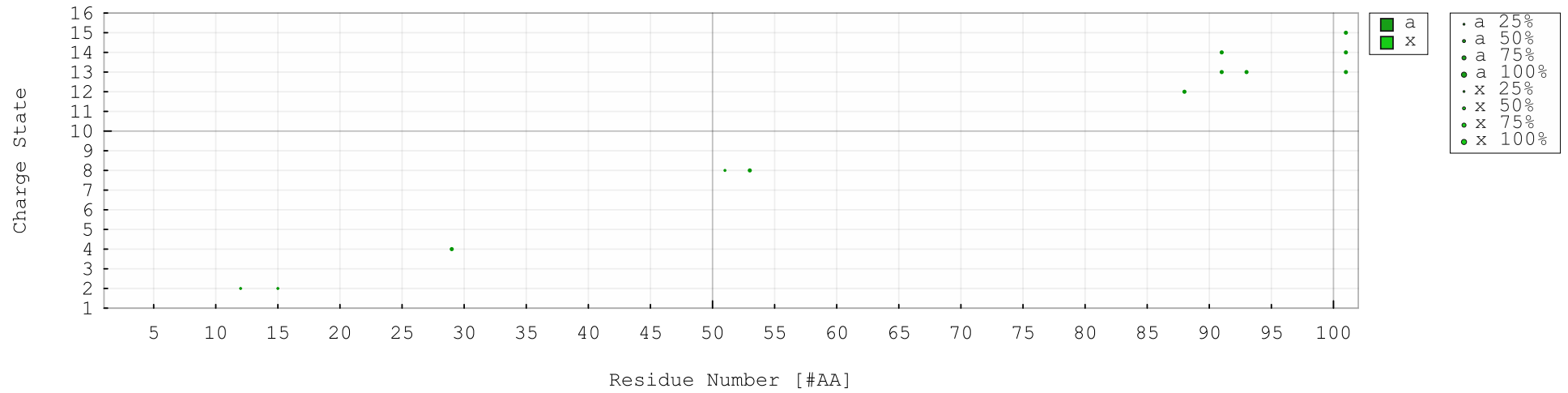

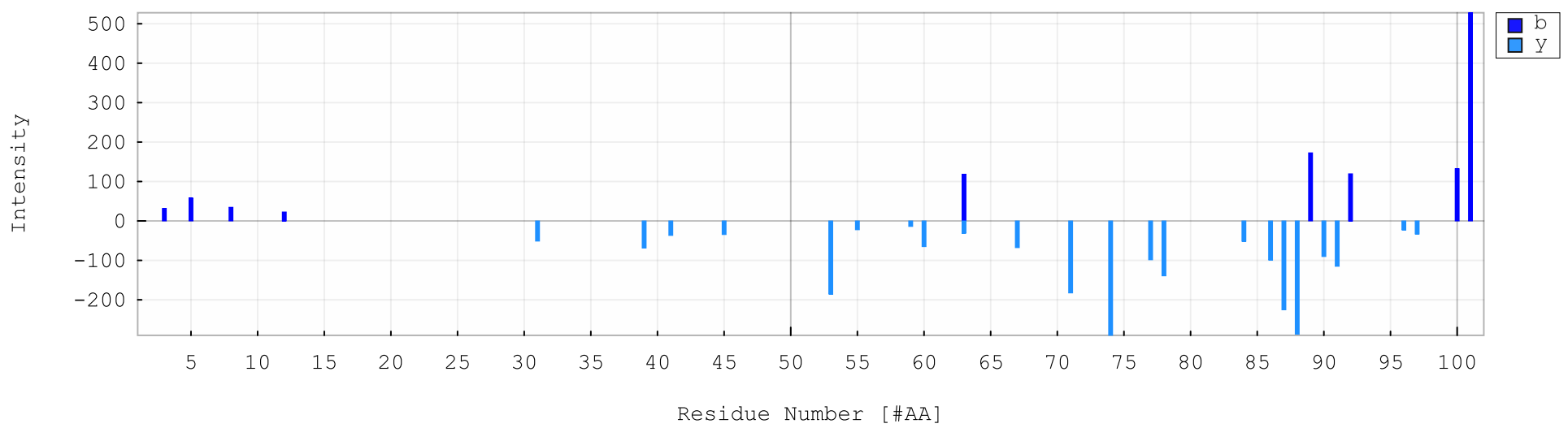

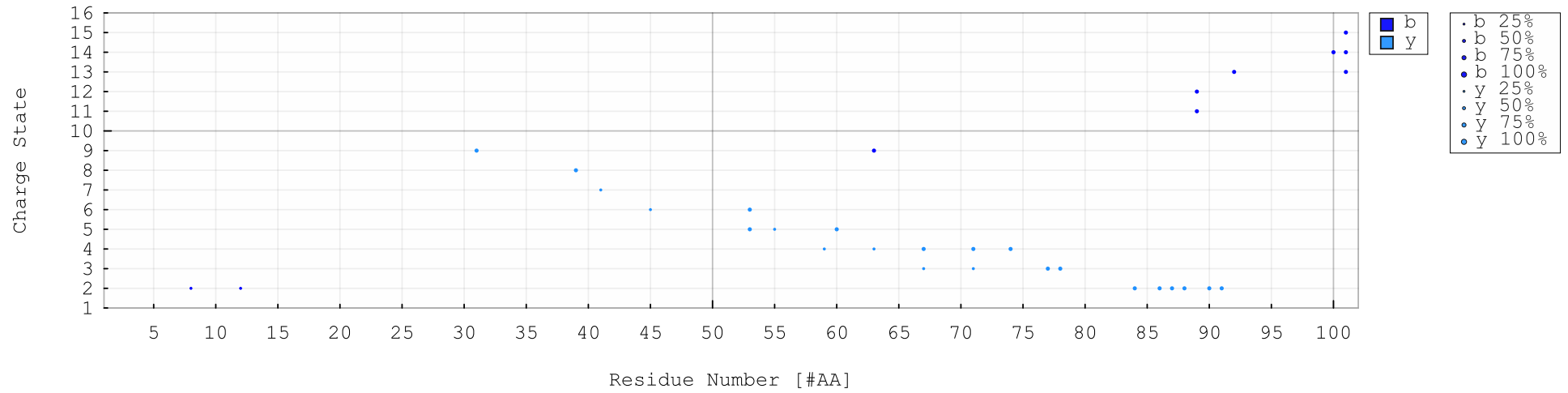

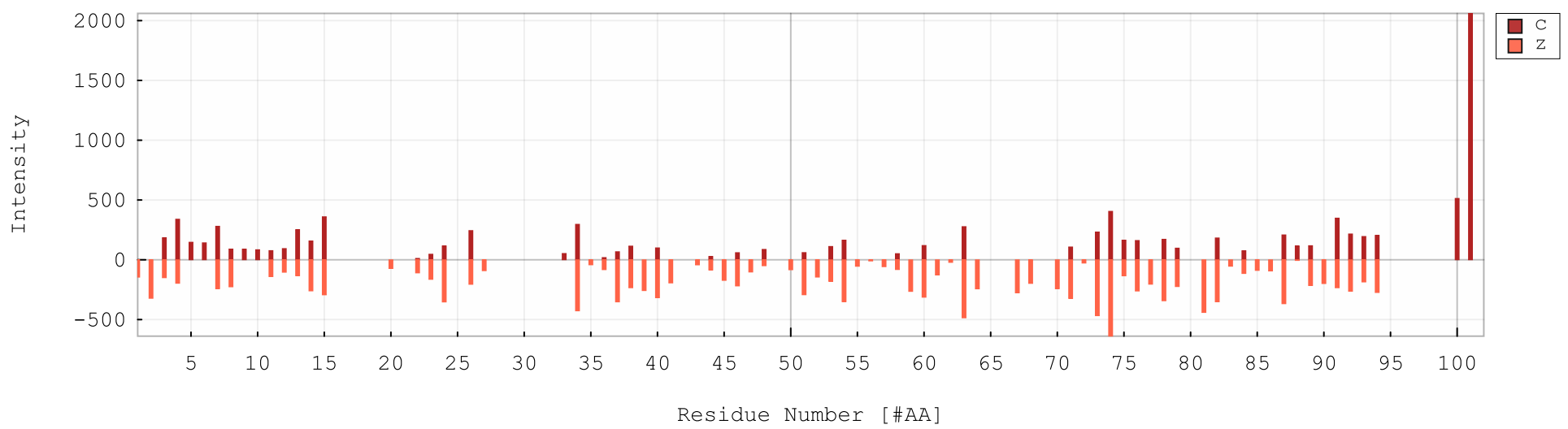

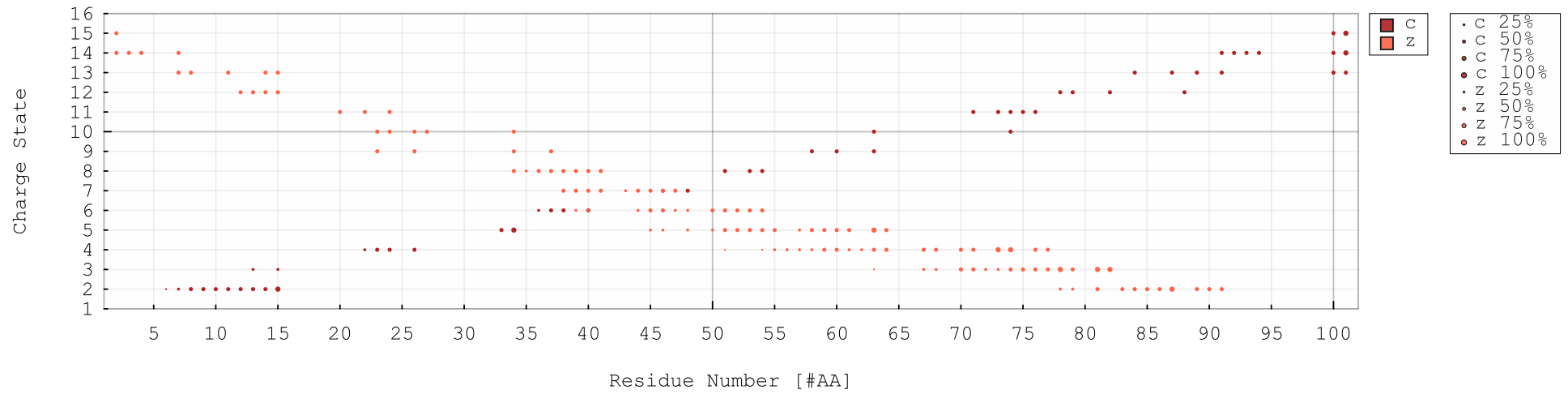

Proteoform:

Index	Protein	SC [%]	SVP [%]	IC [%]	MS Score [a.u.]	Var. Mods	Mono Mass [Da]	Chemical Formula
5	H4Kac_me1-3	82.2	0.0	44.0	36.1	Ace: K8, Dim: K20	11299.3831	C498H843N163O135S

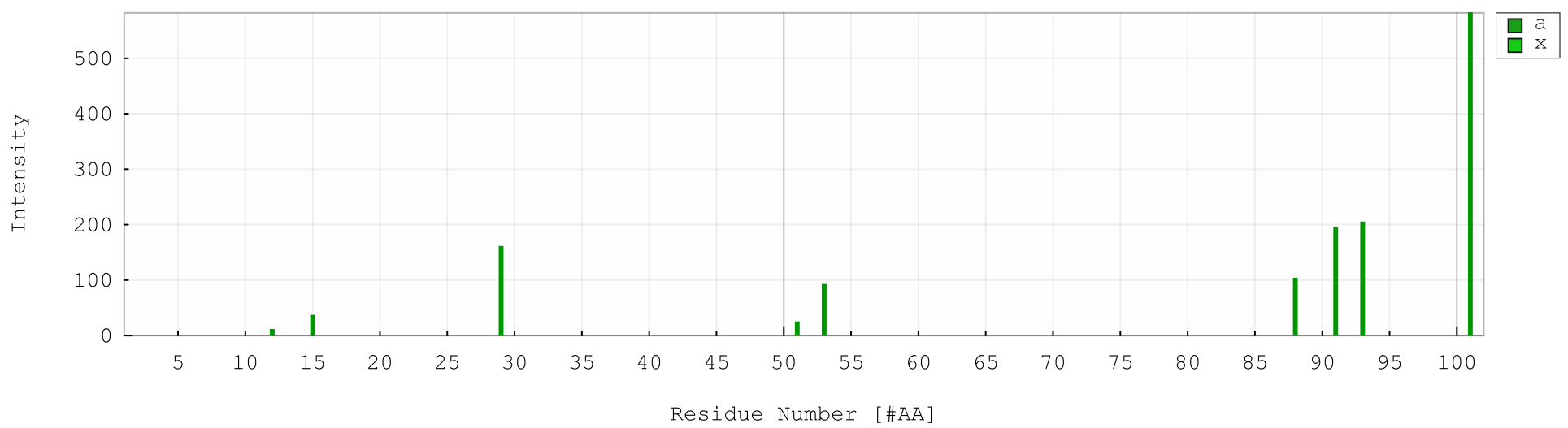

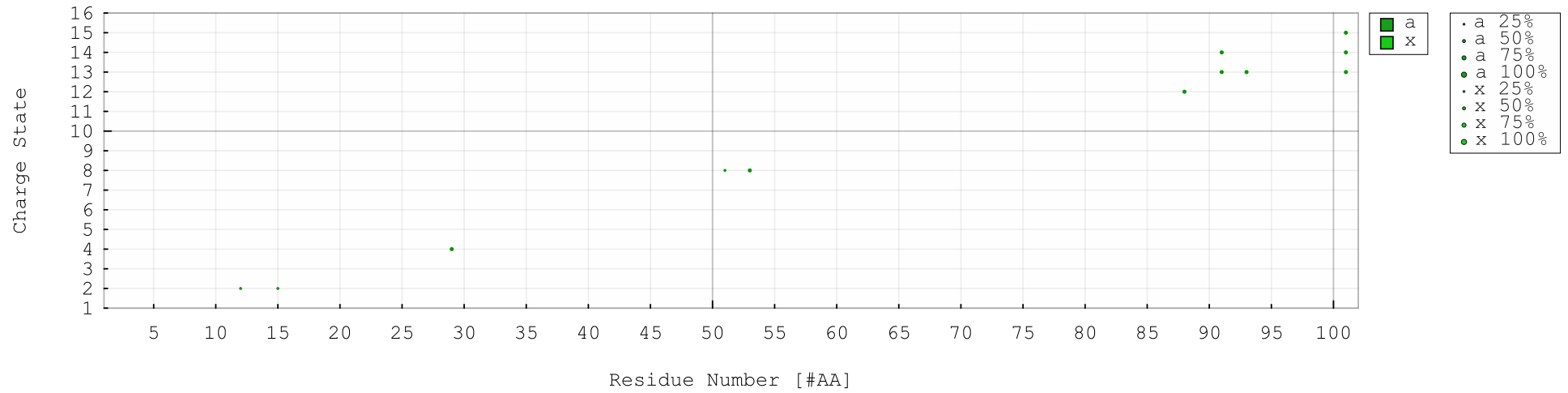

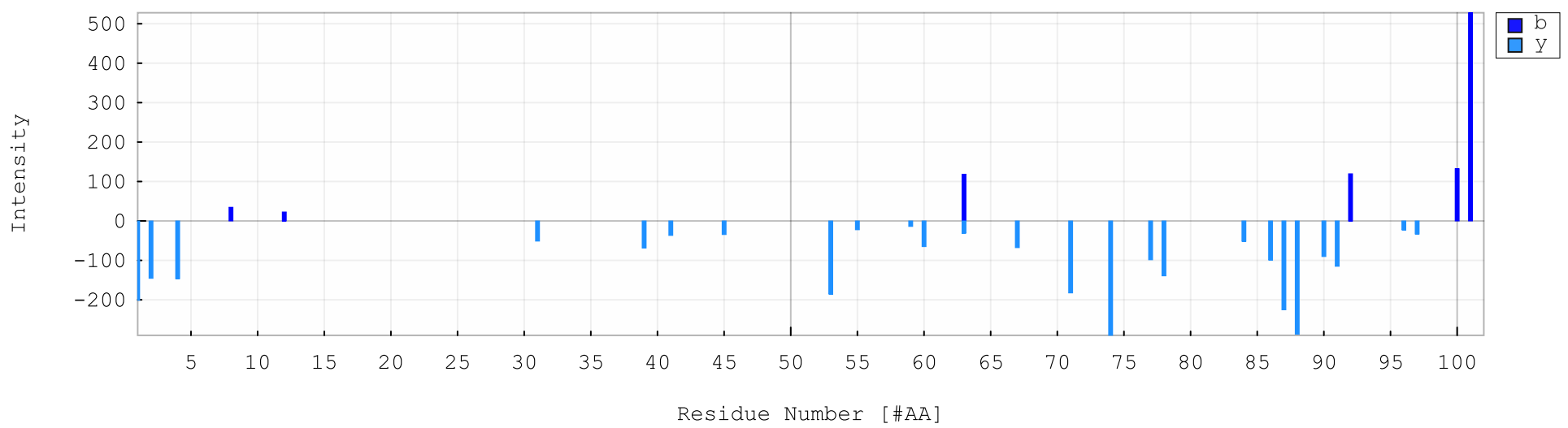

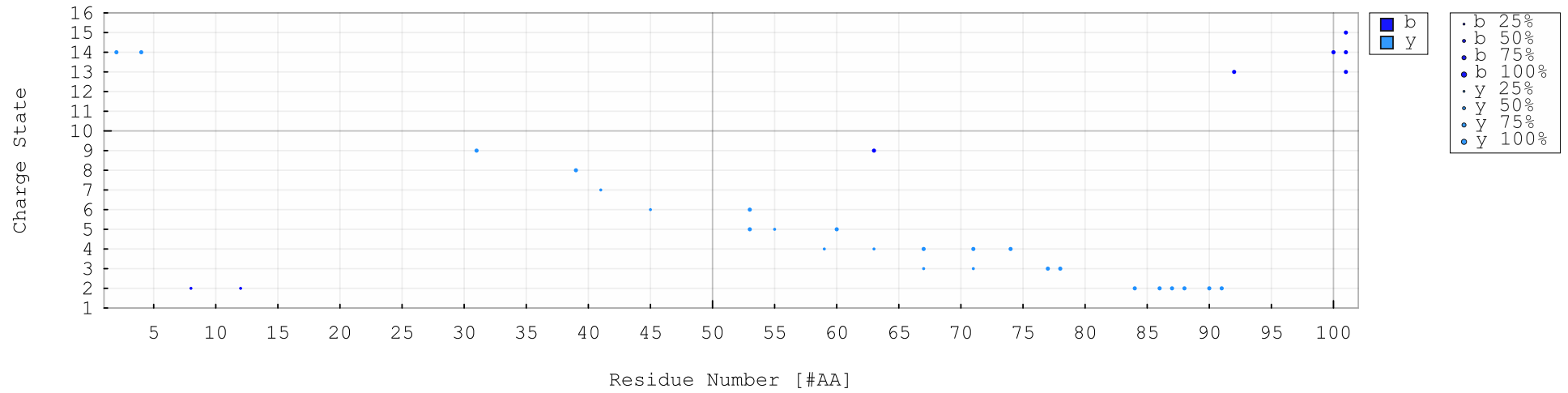

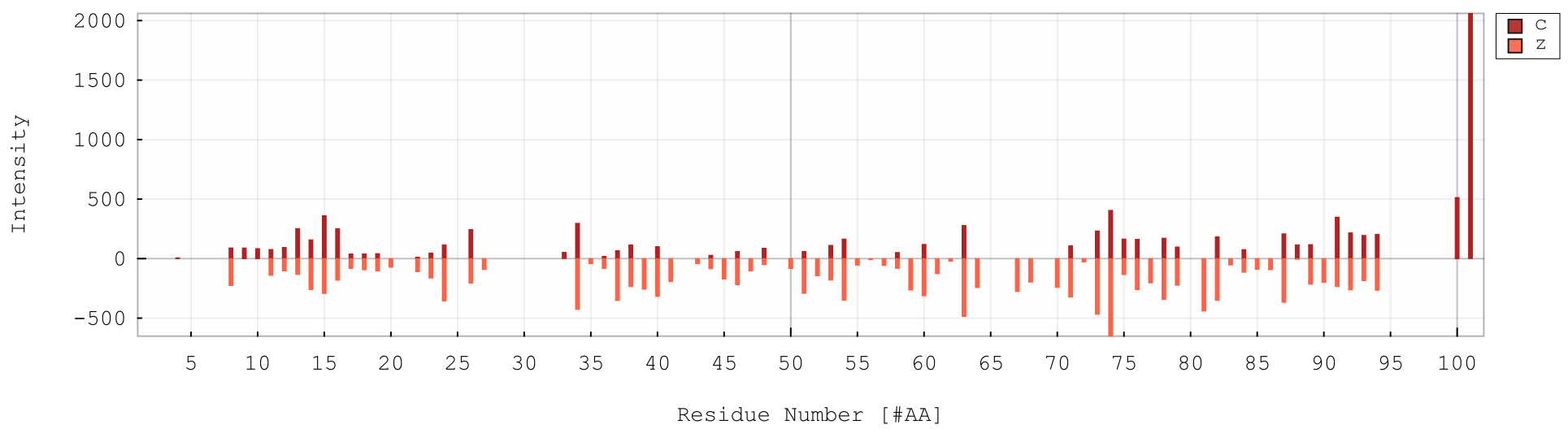

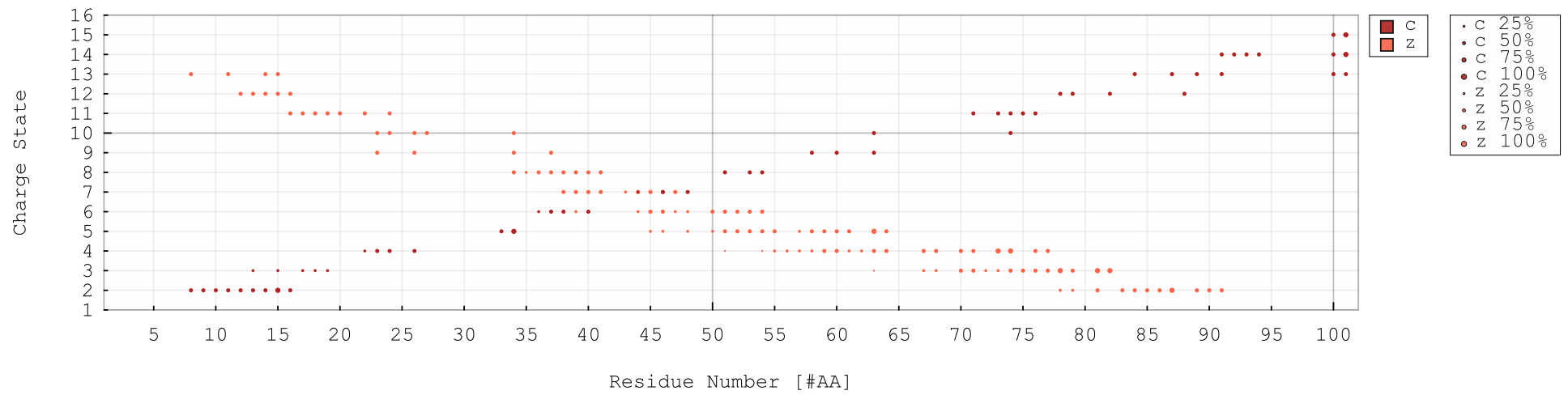

Proteoform:

Index	Protein	SC [%]	SVP [%]	IC [%]	MS Score [a.u.]	Var. Mods	Mono Mass [Da]	Chemical Formula
6	H4Kac_me1-3	81.2	0.0	43.9	35.7	Ace: K5, Dim: K16	11299.3831	C498H843N163O135S

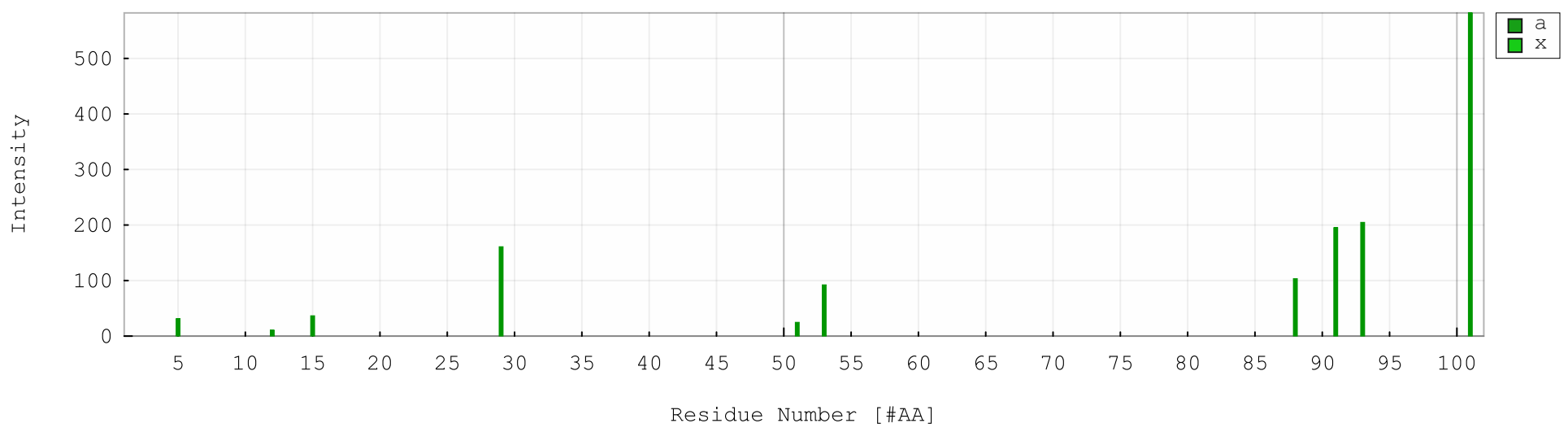

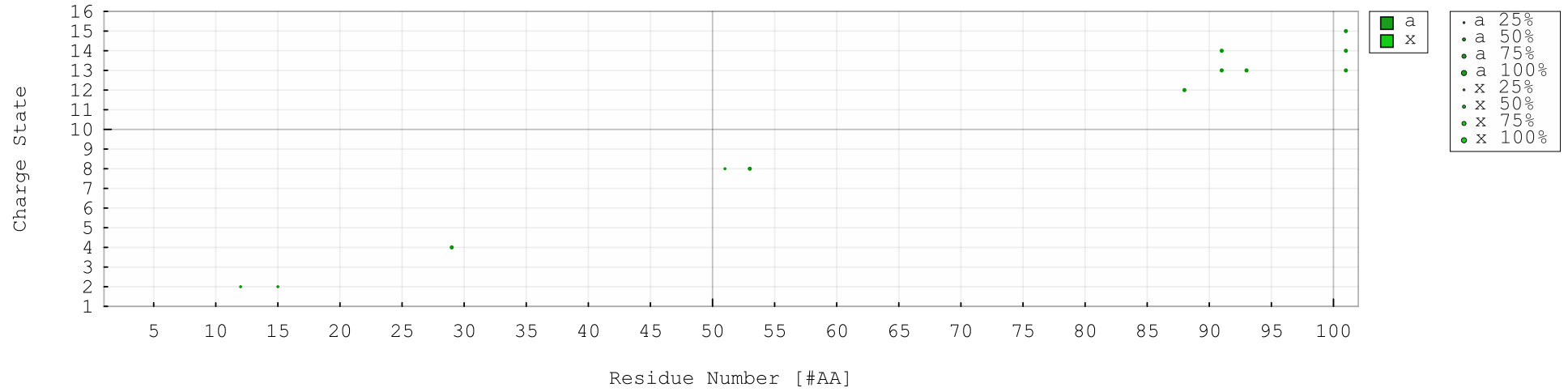

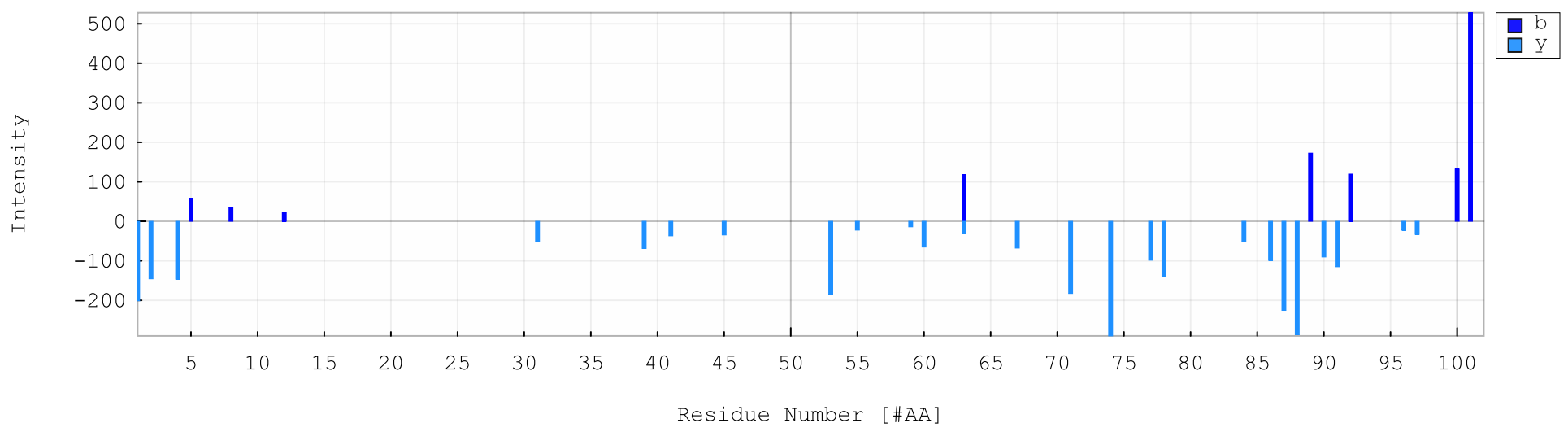

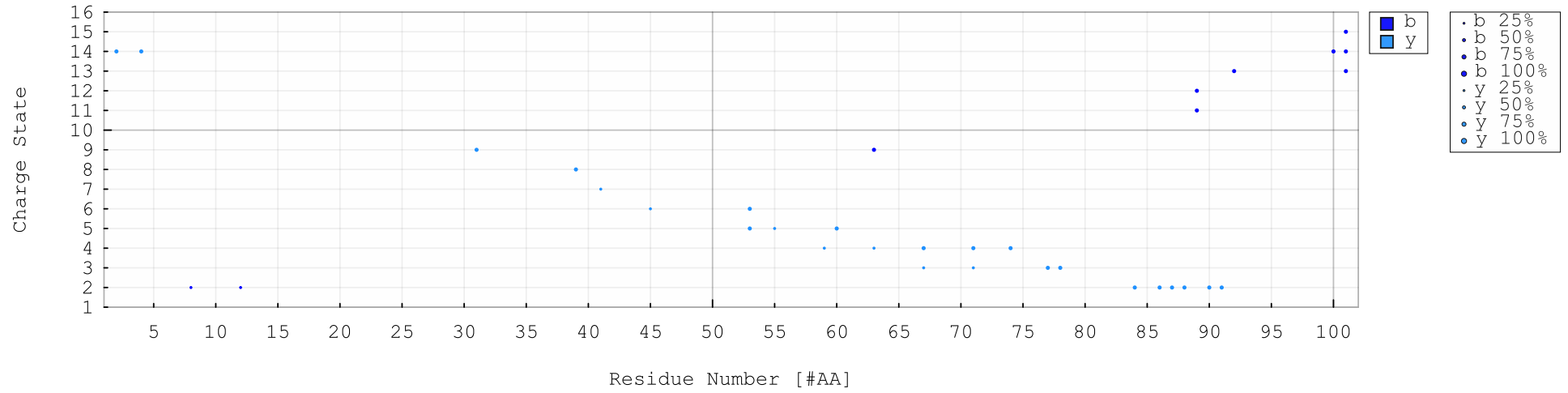

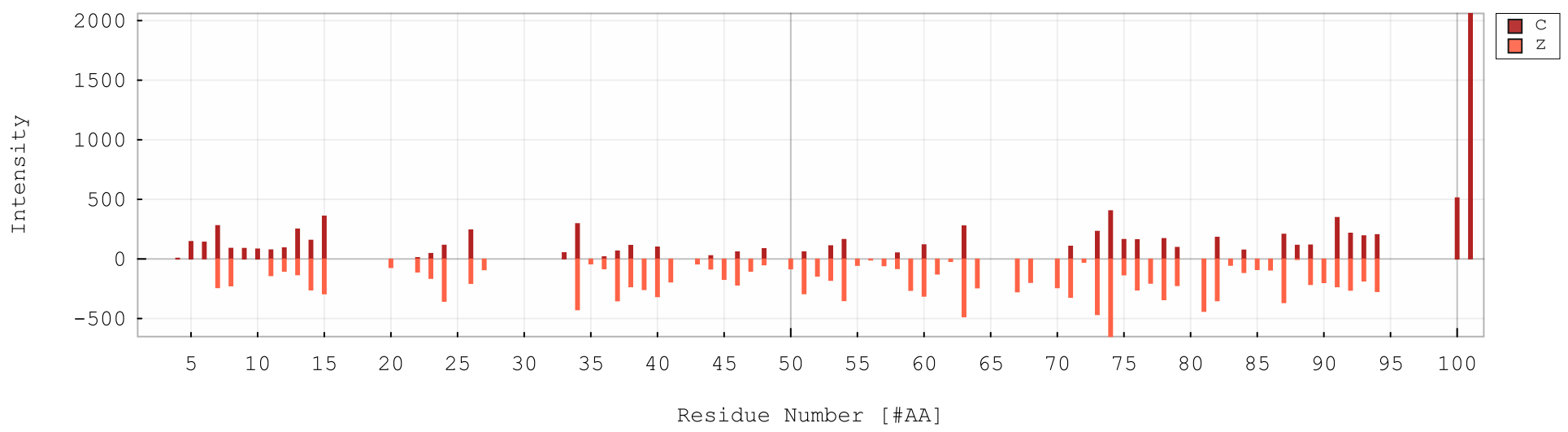

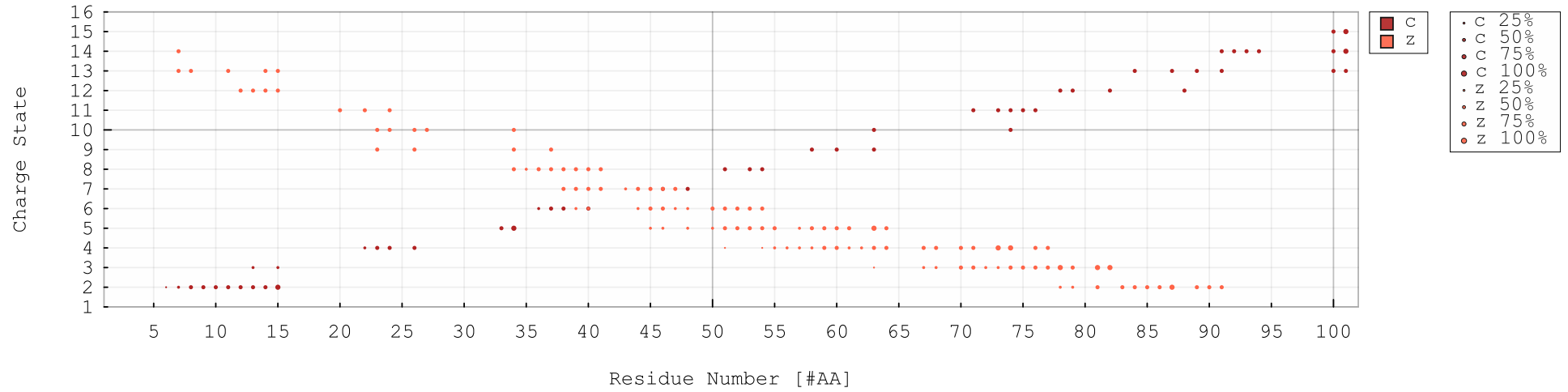

Proteoform:

Index	Protein	SC [%]	SVP [%]	IC [%]	MS Score [a.u.]	Var. Mods	Mono Mass [Da]	Chemical Formula
7	H4Kac_me1-3	81.2	0.0	43.9	35.7	Ace: K5, Met: K16, K20	11299.3831	C498H843N163O135S

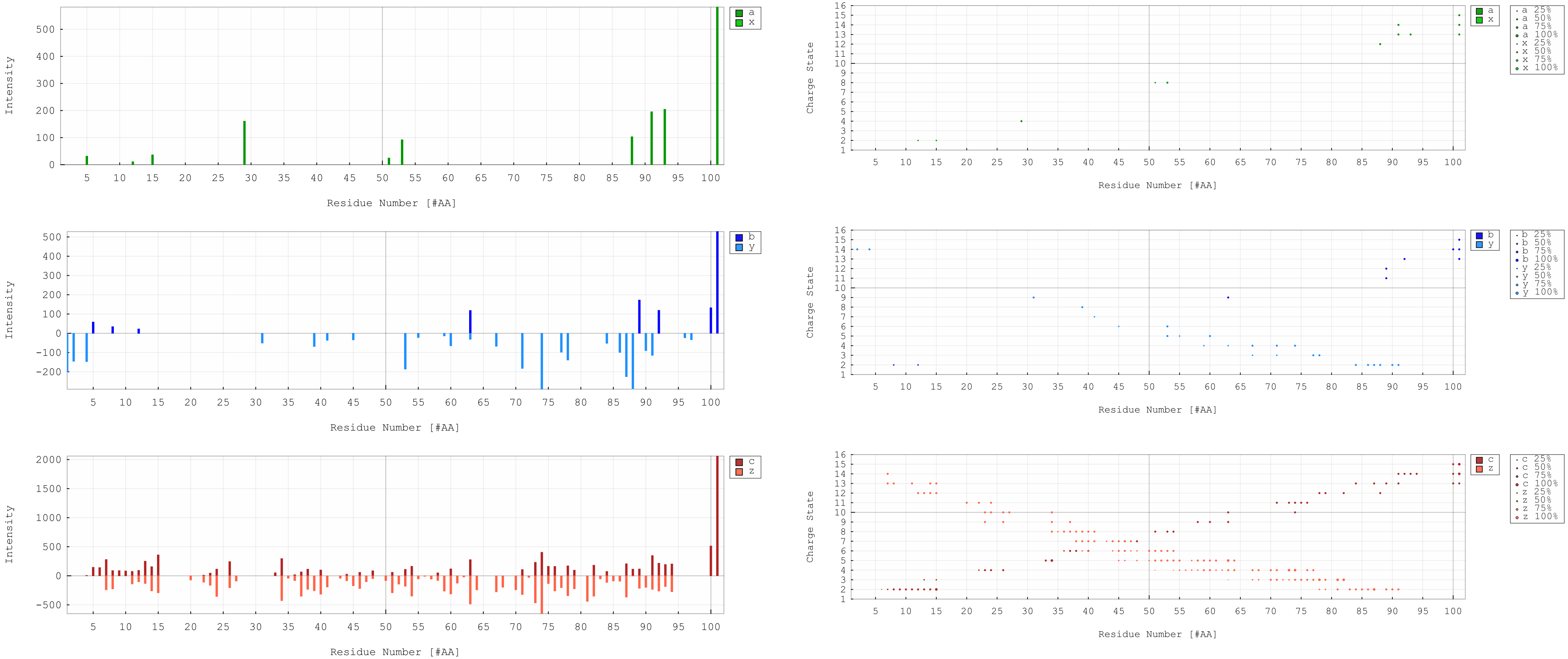

Top-Down Result OmniScape 2025
Sequence Confirmation
250430 Liver-Histone-25 mz707z16 MS2 ECD_001-311_calibrated

Proteoform:

Index	Protein	SC [%]	SVP [%]	IC [%]	MS Score [a.u.]	Var. Mods	Mono Mass [Da]	Chemical Formula
8	H4Kac_me1-3	81.2	10.8	43.6	35.4	Met: K12, K20, Ace: N-term	11299.3831	C498H843N163O135S

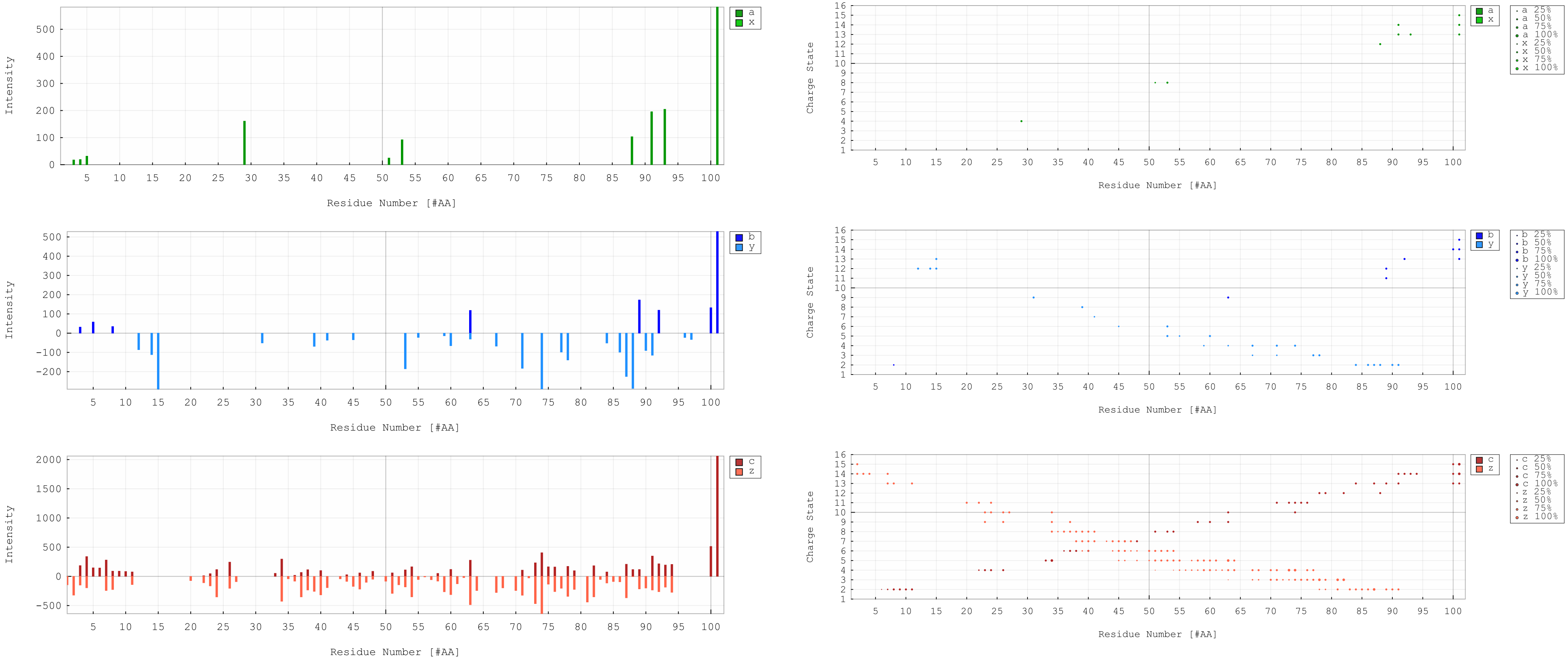

Proteoform:

Index	Protein	SC [%]	SVP [%]	IC [%]	MS Score [a.u.]	Var. Mods	Mono Mass [Da]	Chemical Formula
9	H4Kac_me1-3	81.2	10.8	43.6	35.4	Met: K12, K16, Ace: N-term	11299.3831	C498H843N163O135S

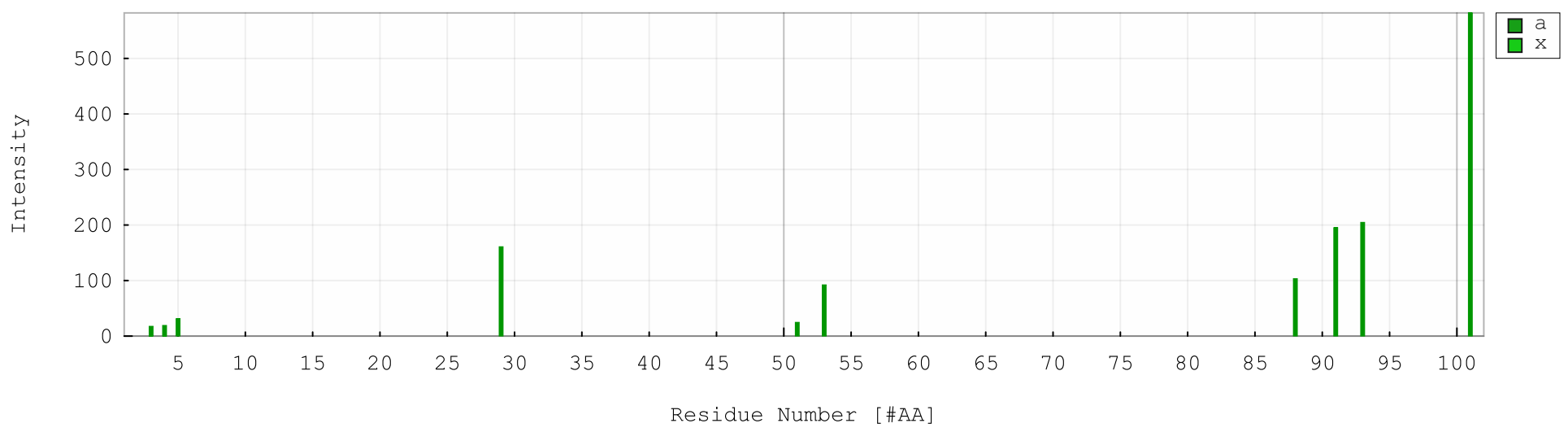

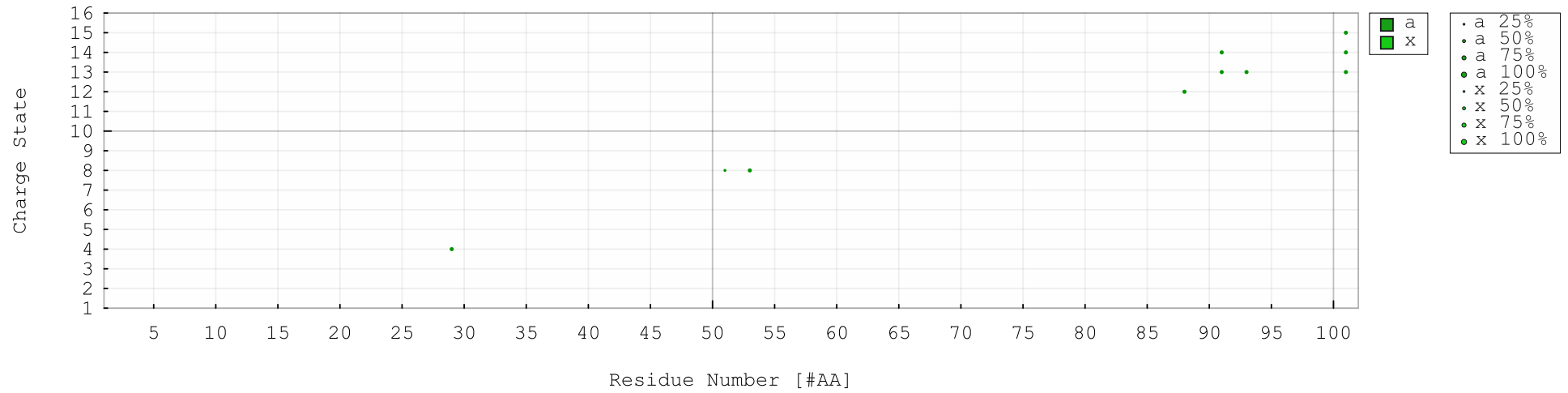

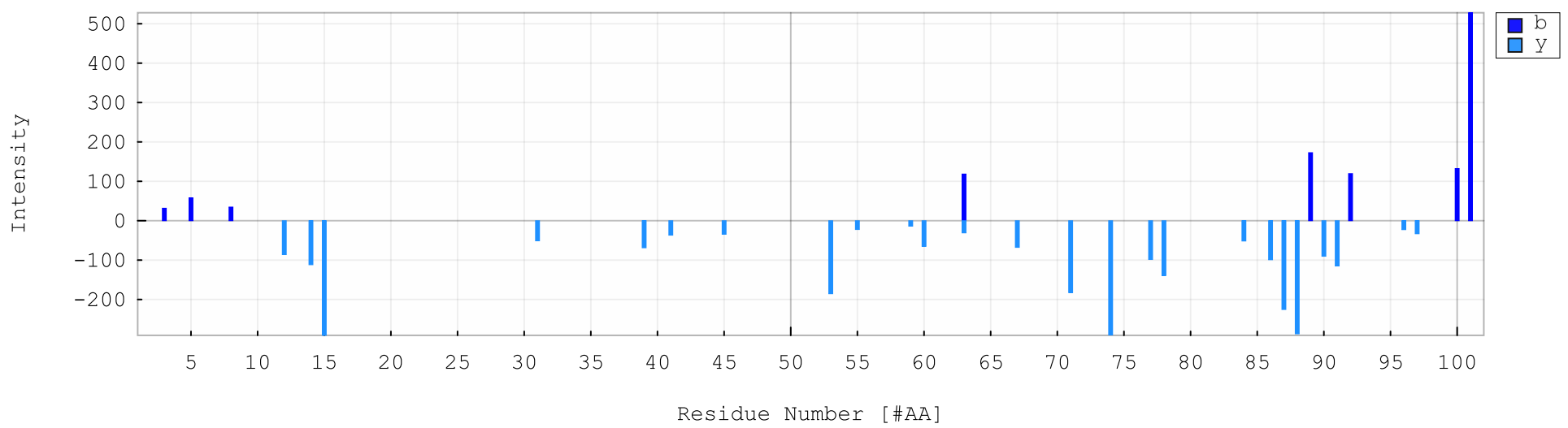

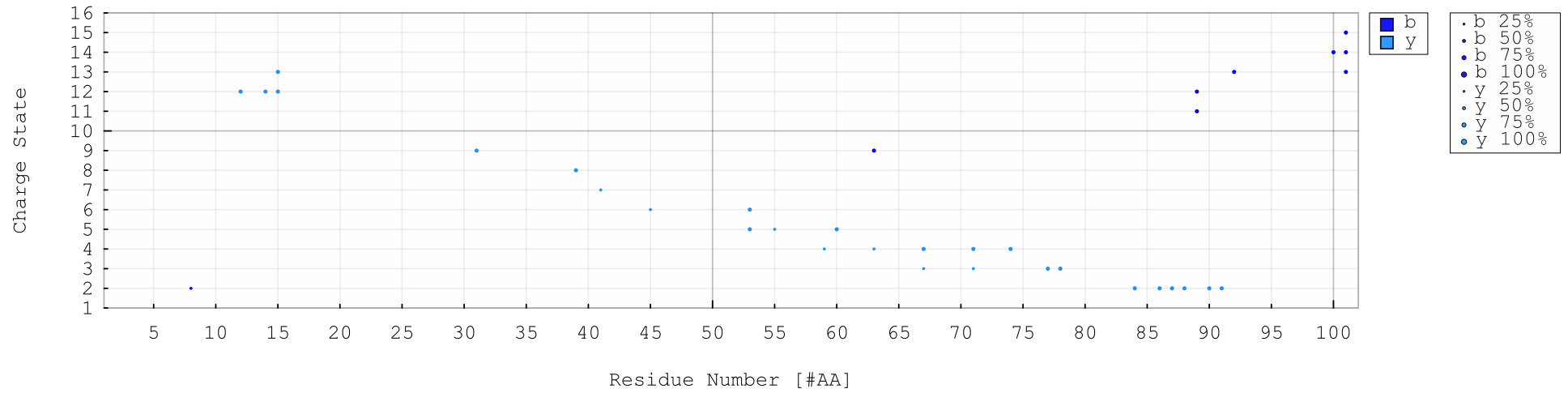

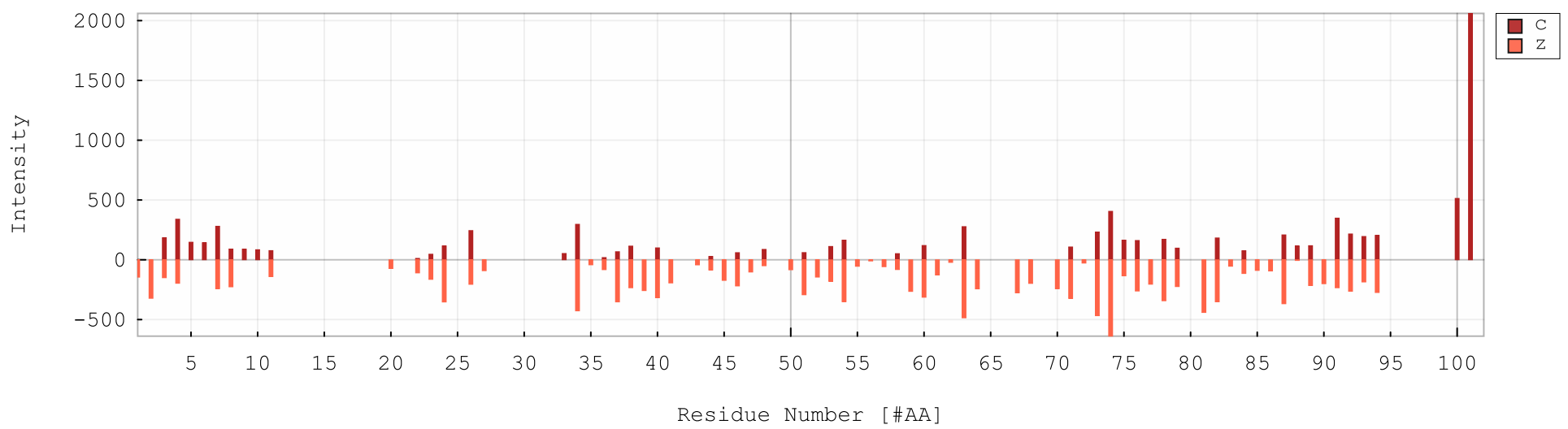

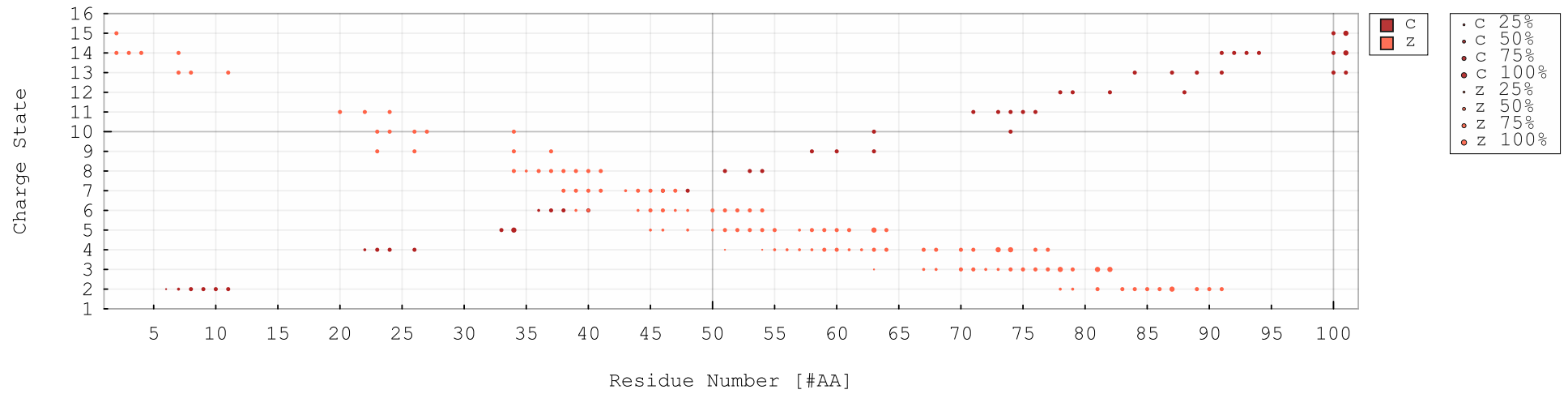

Proteoform:

Index	Protein	SC [%]	SVP [%]	IC [%]	MS Score [a.u.]	Var. Mods	Mono Mass [Da]	Chemical Formula
10	H4Kac_me1-3	79.2	0.0	43.5	34.5	Ace: K12, Dim: K20	11299.3831	C498H843N163O135S

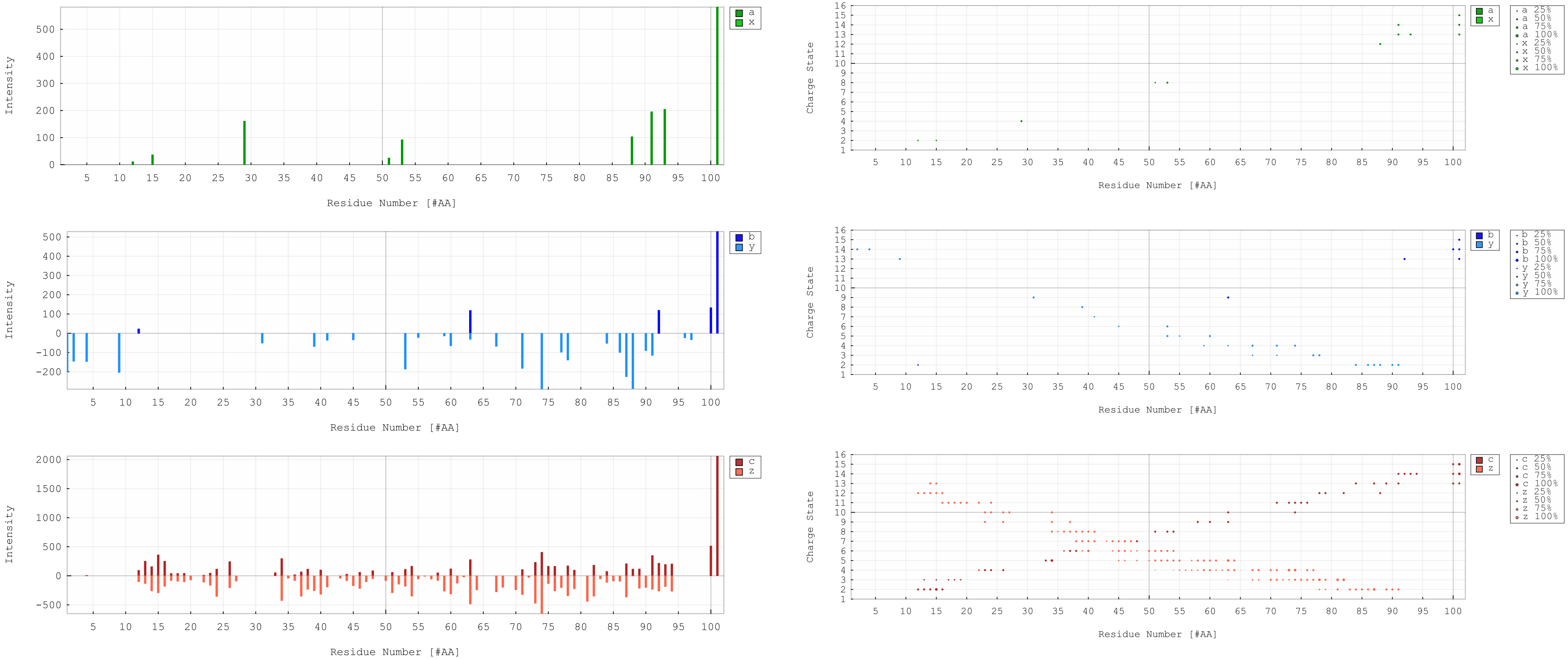

Supplemental Data S1

Endogenous H4

**Report**	2 of 6
**Experiment**	MS2
**Histone/sample**	H4
**Enzyme**	Not applicable
**Reaction/source**	Endogenous sample
**Precursor**	m/z 707
**Charge state**	16+
**Fragmentation / workflow**	EID
**Original file**	MS2_mz-707_16+_EID.pdf

Project Information

Project Directory: C:/Users/LAB-ADMIN
Processing date: Tue Apr 28 14:59:13 2026

Sample Info & Protocols

Name: Top-Down Protein Sequencing EID

Dataset

Imported spectrum file name:	250430 Liver-Histone-25 mz707z16 MS2 EID_003-314_calibrated.txt
Dataset name:	Information is only available from imported JPEAKS files
Date acquired:	Information is only available from imported JPEAKS files
Date imported:	Information is only available from imported JPEAKS files
Instrument Name:	Information is only available from imported JPEAKS files
Instrument Serial Number:	Information is only available from imported JPEAKS files

Workflow Result Info

Analysis: 250430 Liver-Histone-25 mz707z16 MS2 EID_003-314_calibrated
Location: C:/Users/LAB-ADMIN
Method: -

Method Information

Name: Unnamed

Spectral Parameters

Mass Error [ppm]: 6
Intensity Deviation [au]: 0.15
Min/Max Charges: 1-16
Experiment type: EID

a ions	b ions	c ions	x ions	y ions	z ions	satellite	internal	precursor
a	b	c	x	y	z			M
a+1			x+1	y-1	z+1			M+-1H

Noise threshold calculation: Simple moving average noise threshold (SMA)
Window size [datapoints]: 600
Noise curve offset: 4.20458
SVP parameters: terminal gap: 2, internal gap: 1

Proteoforms

(top 10)

Index	Parent Sequence	Chain	Var. Mods	Mono Mass [Da]	Chemical Composition
1	H4Kac_me1-3	H4Kac_me1-3_Nterm[Acetyl]_K20[Dim]	Dim: K20, Ace: N-term	11299.3831	C498H843N163O135S
2	H4Kac_me1-3	H4Kac_me1-3K5[Ace]K20[Dim]	Ace: K5, Dim: K20	11299.3831	C498H843N163O135S
3	H4Kac_me1-3	H4Kac_me1-3K8[Ace]K20[Dim]	Ace: K8, Dim: K20	11299.3831	C498H843N163O135S
4	H4Kac_me1-3	H4Kac_me1-3_Nterm[Acetyl]_K16[Met]K20[Met]	Met: K16, K20, Ace: N-term	11299.3831	C498H843N163O135S
5	H4Kac_me1-3	H4Kac_me1-3_Nterm[Acetyl]_K16[Dim]	Dim: K16, Ace: N-term	11299.3831	C498H843N163O135S
6	H4Kac_me1-3	H4Kac_me1-3K5[Ace]K16[Dim]	Ace: K5, Dim: K16	11299.3831	C498H843N163O135S
7	H4Kac_me1-3	H4Kac_me1-3K5[Ace]K16[Met]K20[Met]	Ace: K5, Met: K16, K20	11299.3831	C498H843N163O135S
8	H4Kac_me1-3	H4Kac_me1-3K12[Ace]K20[Dim]	Ace: K12, Dim: K20	11299.3831	C498H843N163O135S
9	H4Kac_me1-3	H4Kac_me1-3K8[Ace]K16[Met]K20[Met]	Ace: K8, Met: K16, K20	11299.3831	C498H843N163O135S
10	H4Kac_me1-3	H4Kac_me1-3K8[Ace]K16[Dim]	Ace: K8, Dim: K16	11299.3831	C498H843N163O135S

Results

Proteoforms Table (top 10)

Index	Protein	SC [%]	SVP [%]	IC [%]	MS Score [a.u.]	Var. Mods	Mono Mass [Da]	Chemical Formula
1	H4Kac_me1-3	80.2	30.4	35.3	28.3	Dim: K20, Ace: N-term	11299.3831	C498H843N163O135S
2	H4Kac_me1-3	79.2	30.4	35.7	28.3	Ace: K5, Dim: K20	11299.3831	C498H843N163O135S
3	H4Kac_me1-3	78.2	7.8	35.6	27.9	Ace: K8, Dim: K20	11299.3831	C498H843N163O135S
4	H4Kac_me1-3	76.2	19.6	34.9	26.6	Met: K16, K20, Ace: N-term	11299.3831	C498H843N163O135S
5	H4Kac_me1-3	76.2	19.6	34.9	26.6	Dim: K16, Ace: N-term	11299.3831	C498H843N163O135S
6	H4Kac_me1-3	75.2	19.6	35.3	26.6	Ace: K5, Dim: K16	11299.3831	C498H843N163O135S
7	H4Kac_me1-3	75.2	19.6	35.3	26.6	Ace: K5, Met: K16, K20	11299.3831	C498H843N163O135S
8	H4Kac_me1-3	75.2	7.8	35.3	26.5	Ace: K12, Dim: K20	11299.3831	C498H843N163O135S
9	H4Kac_me1-3	74.3	7.8	35.2	26.1	Ace: K8, Met: K16, K20	11299.3831	C498H843N163O135S
10	H4Kac_me1-3	74.3	7.8	35.2	26.1	Ace: K8, Dim: K16	11299.3831	C498H843N163O135S

Sequence

Name: H4Kac_me1-3

```
                  10                    20                    30                    40                    50                    60
S G R G K G G K G L   G K G G A K R H R K   V L R D N I Q G I T   K P A I R R L A R R   G G V K R I S G L I   Y E E T R G V L K V
                  70                    80                    90                   100                   110                   120
F L E N V I R D A V   T Y T E H A K R K T   V T A M D V V Y A L   K R Q G R T L Y G F   G G
```

Modifications

Modification	Position	Type
Acetyl	N-term	variable
Acetyl	5	variable
Methyl	5	variable
Dimethyl	5	variable
Acetyl	8	variable
Methyl	8	variable
Dimethyl	8	variable
Methyl	12	variable
Dimethyl	12	variable
Acetyl	12	variable
Methyl	16	variable
Dimethyl	16	variable
Acetyl	16	variable

Modification	Position	Type
Dimethyl	20	variable
Methyl	20	variable
Acetyl	20	variable
Methyl	31	variable
Dimethyl	31	variable
Acetyl	31	variable
Dimethyl	44	variable
Methyl	44	variable

Sequence Map

Proteoform:

Index	Protein	SC [%]	SVP [%]	IC [%]	MS Score [a.u.]	Var. Mods	Mono Mass [Da]	Chemical Formula
1	H4Kac_me1-3	80.2	30.4	35.3	28.3	Dim: K20, Ace: N-term	11299.3831	C498H843N163O135S

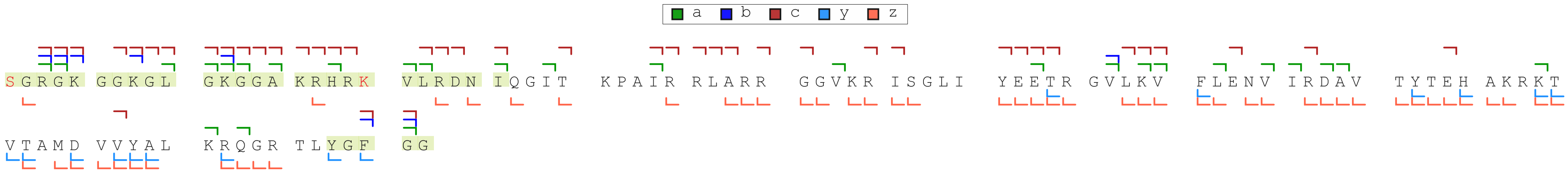

Proteoform:

Index	Protein	SC [%]	SVP [%]	IC [%]	MS Score [a.u.]	Var. Mods	Mono Mass [Da]	Chemical Formula
2	H4Kac_me1-3	79.2	30.4	35.7	28.3	Ace: K5, Dim: K20	11299.3831	C498H843N163O135S

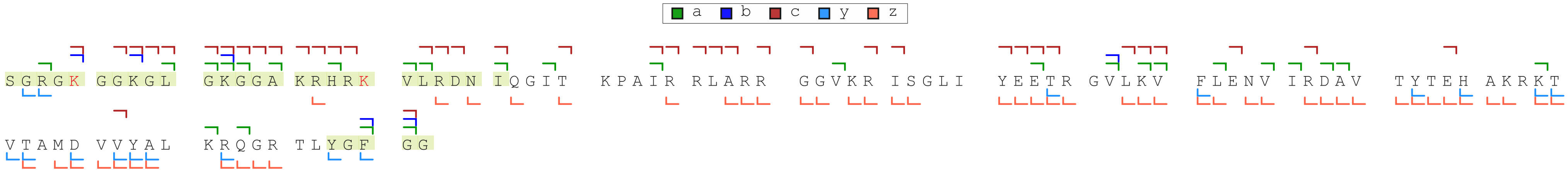

Proteoform:	Index	Protein	SC [%]	SVP [%]	IC [%]	MS Score [a.u.]	Var. Mods	Mono Mass [Da]	Chemical Formula
	3	H4Kac_me1-3	78.2	7.8	35.6	27.9	Ace: K8, Dim: K20	11299.3831	C498H843N163O135S

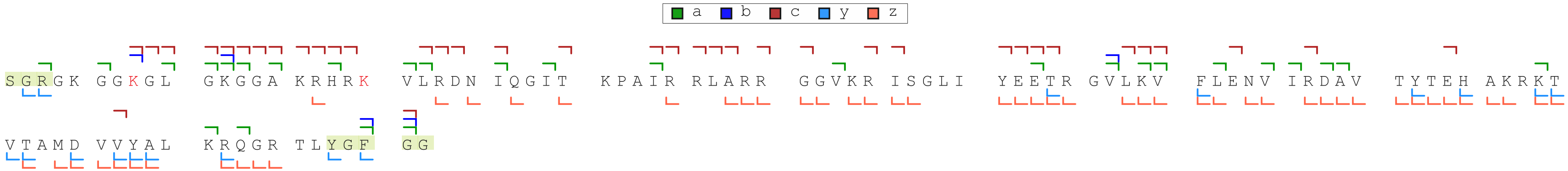

Proteoform:

Index	Protein	SC [%]	SVP [%]	IC [%]	MS Score [a.u.]	Var. Mods	Mono Mass [Da]	Chemical Formula
4	H4Kac_me1-3	76.2	19.6	34.9	26.6	Met: K16, K20, Ace: N-term	11299.3831	C498H843N163O135S

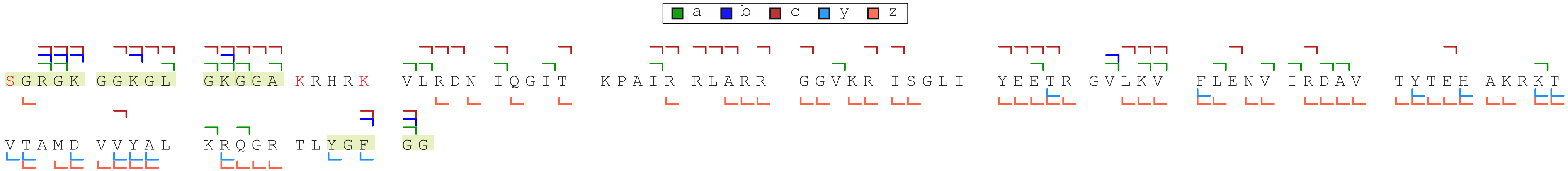

Proteoform:

Index	Protein	SC [%]	SVP [%]	IC [%]	MS Score [a.u.]	Var. Mods	Mono Mass [Da]	Chemical Formula
5	H4Kac_me1-3	76.2	19.6	34.9	26.6	Dim: K16, Ace: N-term	11299.3831	C498H843N163O135S

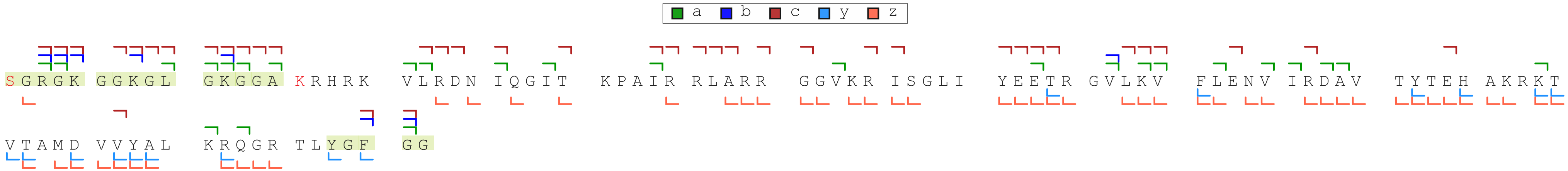

Proteoform:

Index	Protein	SC [%]	SVP [%]	IC [%]	MS Score [a.u.]	Var. Mods	Mono Mass [Da]	Chemical Formula
6	H4Kac_me1-3	75.2	19.6	35.3	26.6	Ace: K5, Dim: K16	11299.3831	C498H843N163O135S

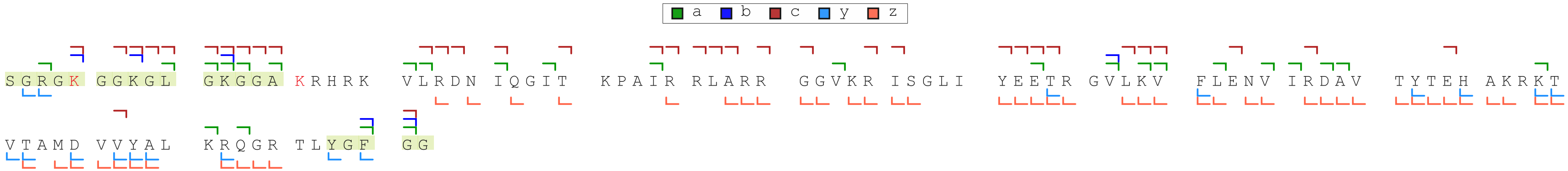

Proteoform:	Index	Protein	SC [%]	SVP [%]	IC [%]	MS Score [a.u.]	Var. Mods	Mono Mass [Da]	Chemical Formula
	7	H4Kac_me1-3	75.2	19.6	35.3	26.6	Ace: K5, Met: K16, K20	11299.3831	C498H843N163O135S

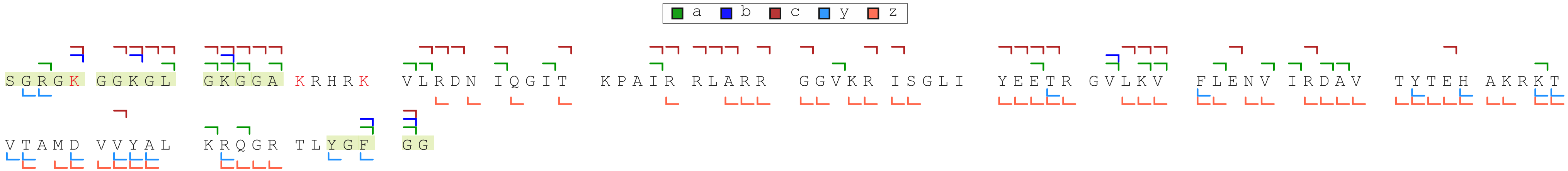

Proteoform:

Index	Protein	SC [%]	SVP [%]	IC [%]	MS Score [a.u.]	Var. Mods	Mono Mass [Da]	Chemical Formula
8	H4Kac_me1-3	75.2	7.8	35.3	26.5	Ace: K12, Dim: K20	11299.3831	C498H843N163O135S

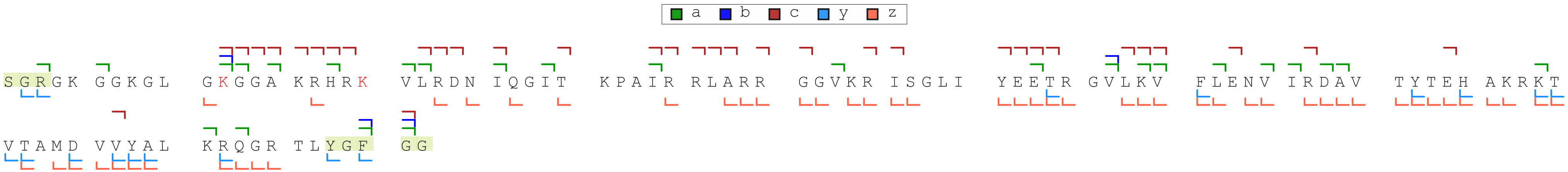

Proteoform:

Index	Protein	SC [%]	SVP [%]	IC [%]	MS Score [a.u.]	Var. Mods	Mono Mass [Da]	Chemical Formula
9	H4Kac_me1-3	74.3	7.8	35.2	26.1	Ace: K8, Met: K16, K20	11299.3831	C498H843N163O135S

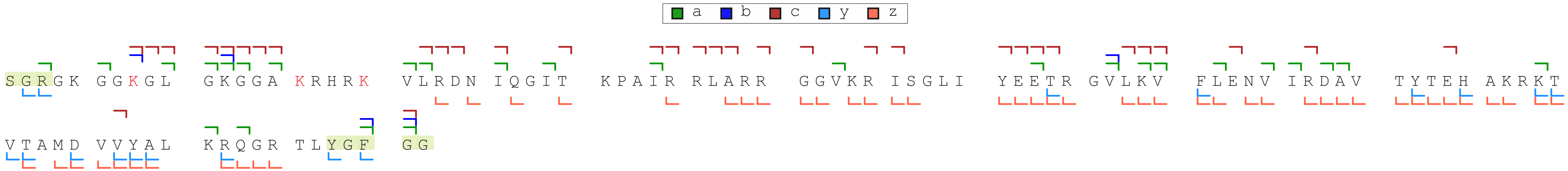

Proteoform:	Index	Protein	SC [%]	SVP [%]	IC [%]	MS Score [a.u.]	Var. Mods	Mono Mass [Da]	Chemical Formula
	10	H4Kac_me1-3	74.3	7.8	35.2	26.1	Ace: K8, Dim: K16	11299.3831	C498H843N163O135S

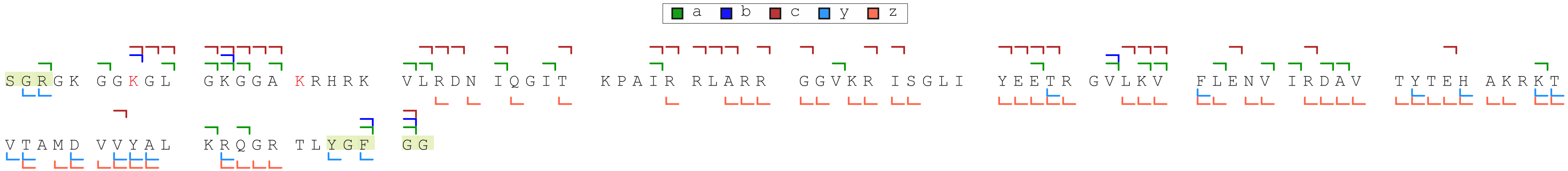

Error Plot

Proteoform:

Index	Protein	SC [%]	SVP [%]	IC [%]	MS Score [a.u.]	Var. Mods	Mono Mass [Da]	Chemical Formula
1	H4Kac_me1-3	80.2	30.4	35.3	28.3	Dim: K20, Ace: N-term	11299.3831	C498H843N163O135S

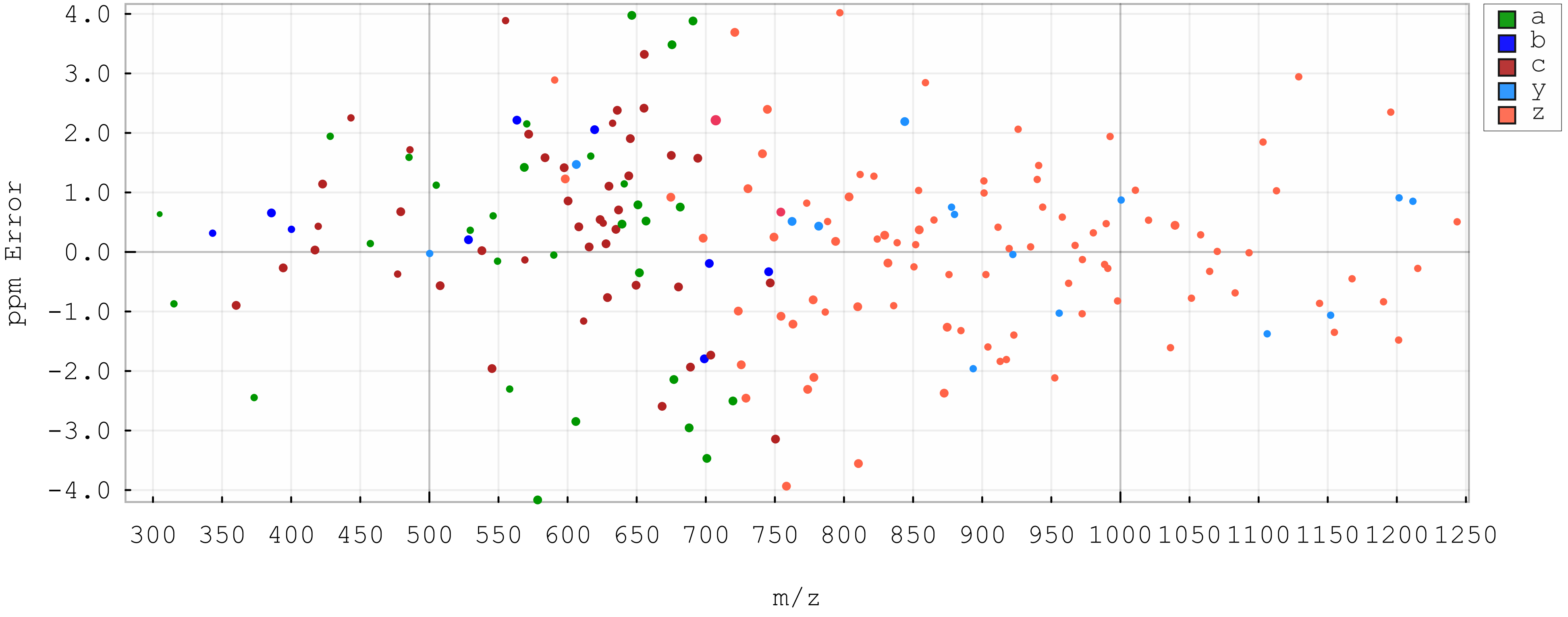

Proteoform:

Index	Protein	SC [%]	SVP [%]	IC [%]	MS Score [a.u.]	Var. Mods	Mono Mass [Da]	Chemical Formula
2	H4Kac_me1-3	79.2	30.4	35.7	28.3	Ace: K5, Dim: K20	11299.3831	C498H843N163O135S

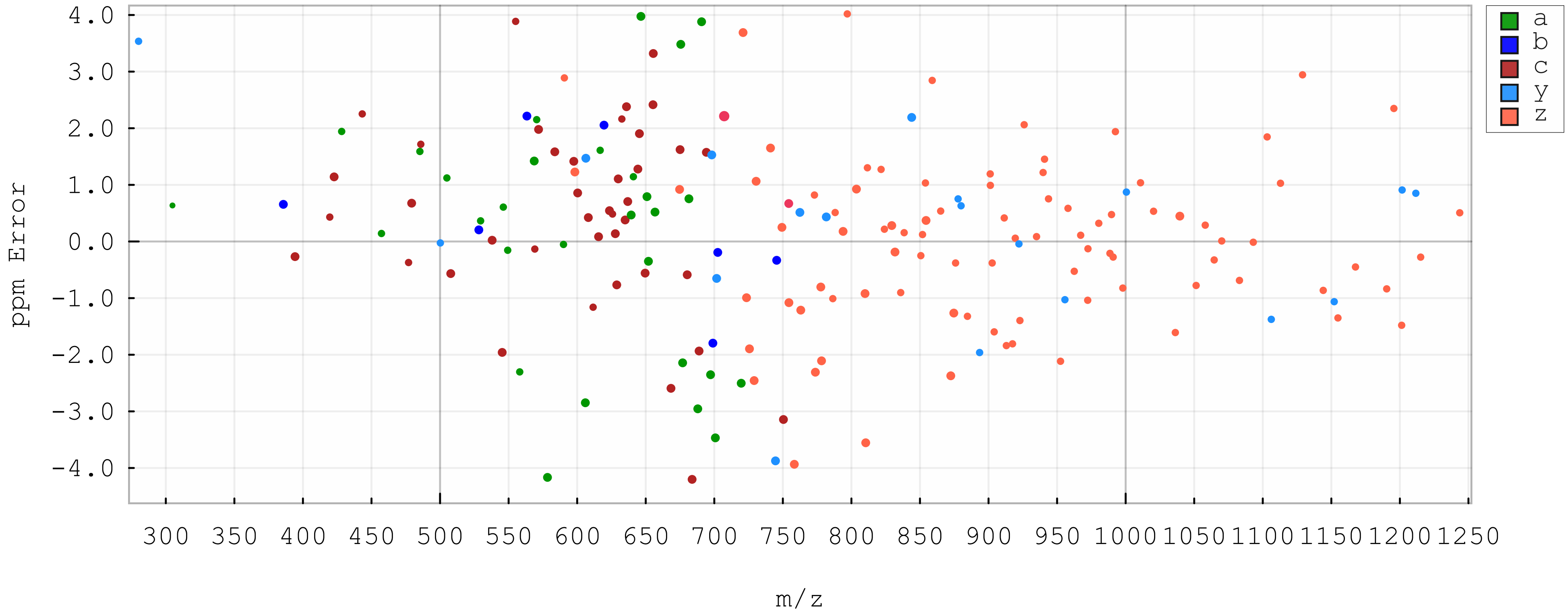

Proteoform:

Index	Protein	SC [%]	SVP [%]	IC [%]	MS Score [a.u.]	Var. Mods	Mono Mass [Da]	Chemical Formula
3	H4Kac_me1-3	78.2	7.8	35.6	27.9	Ace: K8, Dim: K20	11299.3831	C498H843N163O135S

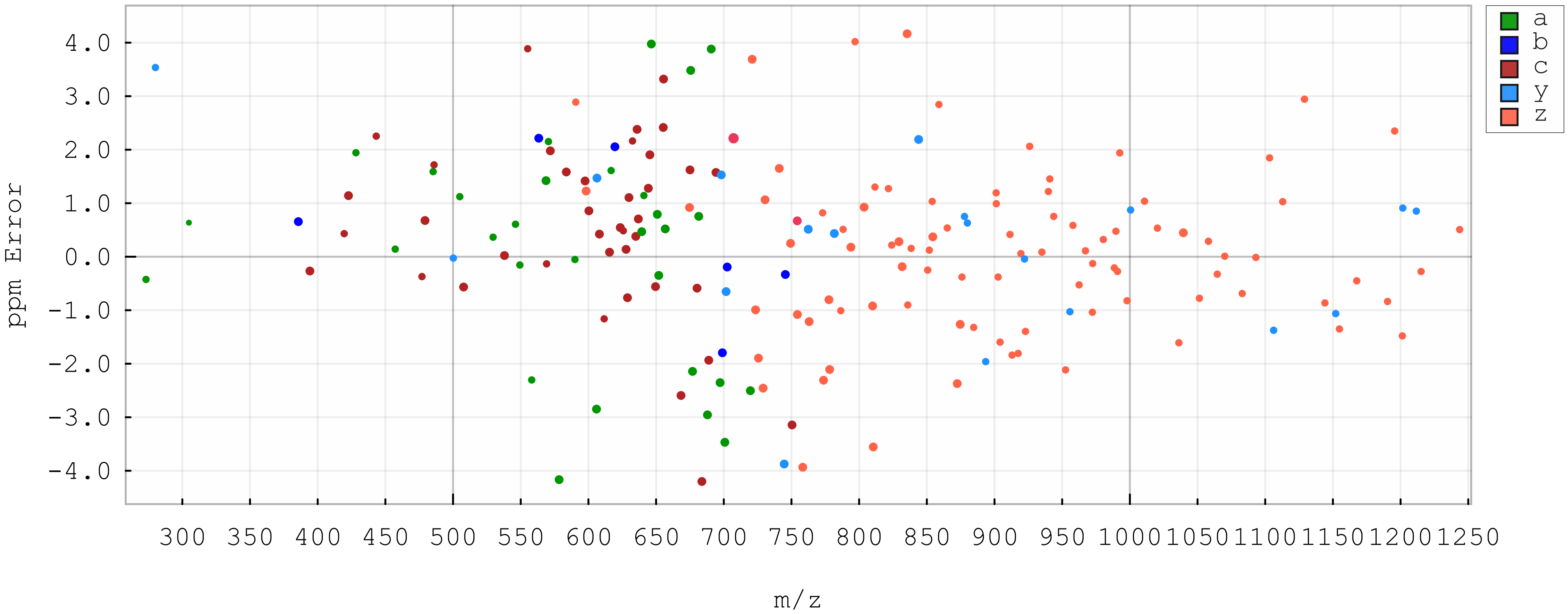

Proteoform:

Index	Protein	SC [%]	SVP [%]	IC [%]	MS Score [a.u.]	Var. Mods	Mono Mass [Da]	Chemical Formula
4	H4Kac_me1-3	76.2	19.6	34.9	26.6	Met: K16, K20, Ace: N-term	11299.3831	C498H843N163O135S

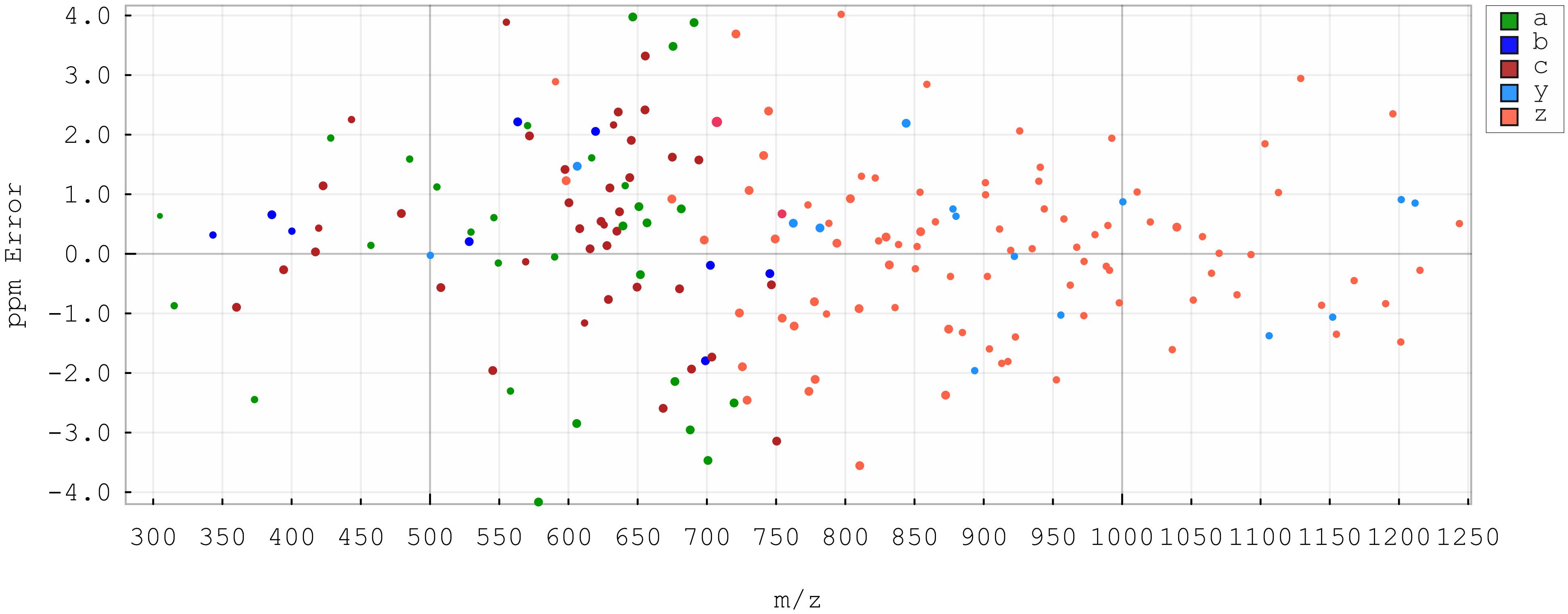

Proteoform:

Index	Protein	SC [%]	SVP [%]	IC [%]	MS Score [a.u.]	Var. Mods	Mono Mass [Da]	Chemical Formula
5	H4Kac_me1-3	76.2	19.6	34.9	26.6	Dim: K16, Ace: N-term	11299.3831	C498H843N163O135S

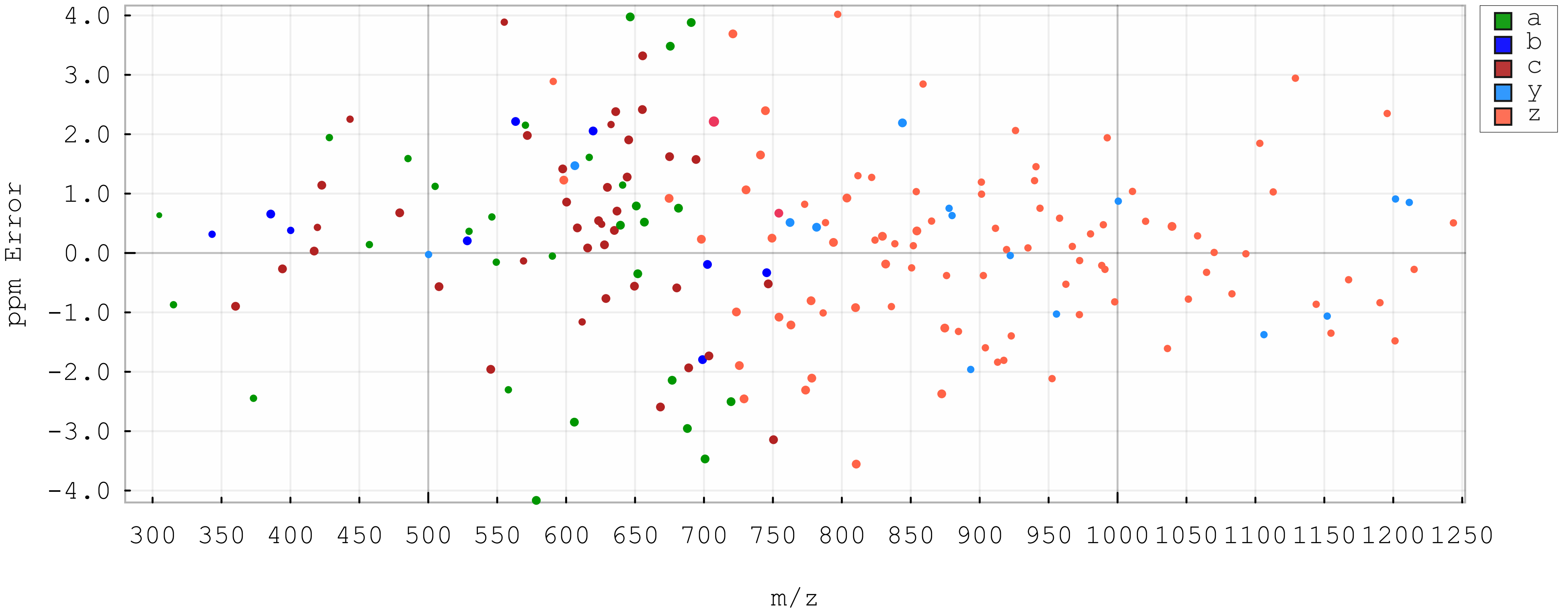

Proteoform:

Index	Protein	SC [%]	SVP [%]	IC [%]	MS Score [a.u.]	Var. Mods	Mono Mass [Da]	Chemical Formula
6	H4Kac_me1-3	75.2	19.6	35.3	26.6	Ace: K5, Dim: K16	11299.3831	C498H843N163O135S

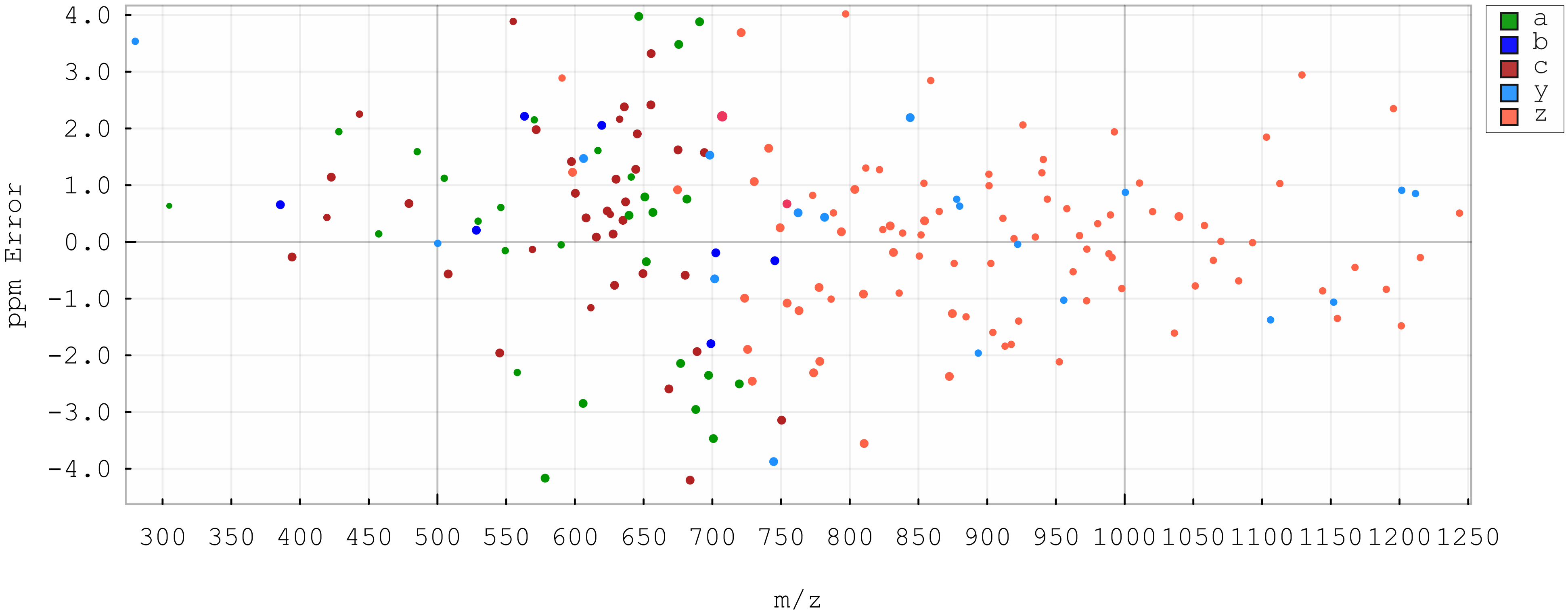

Proteoform:	Index	Protein	SC [%]	SVP [%]	IC [%]	MS Score [a.u.]	Var. Mods	Mono Mass [Da]	Chemical Formula
	7	H4Kac_me1-3	75.2	19.6	35.3	26.6	Ace: K5, Met: K16, K20	11299.3831	C498H843N163O135S

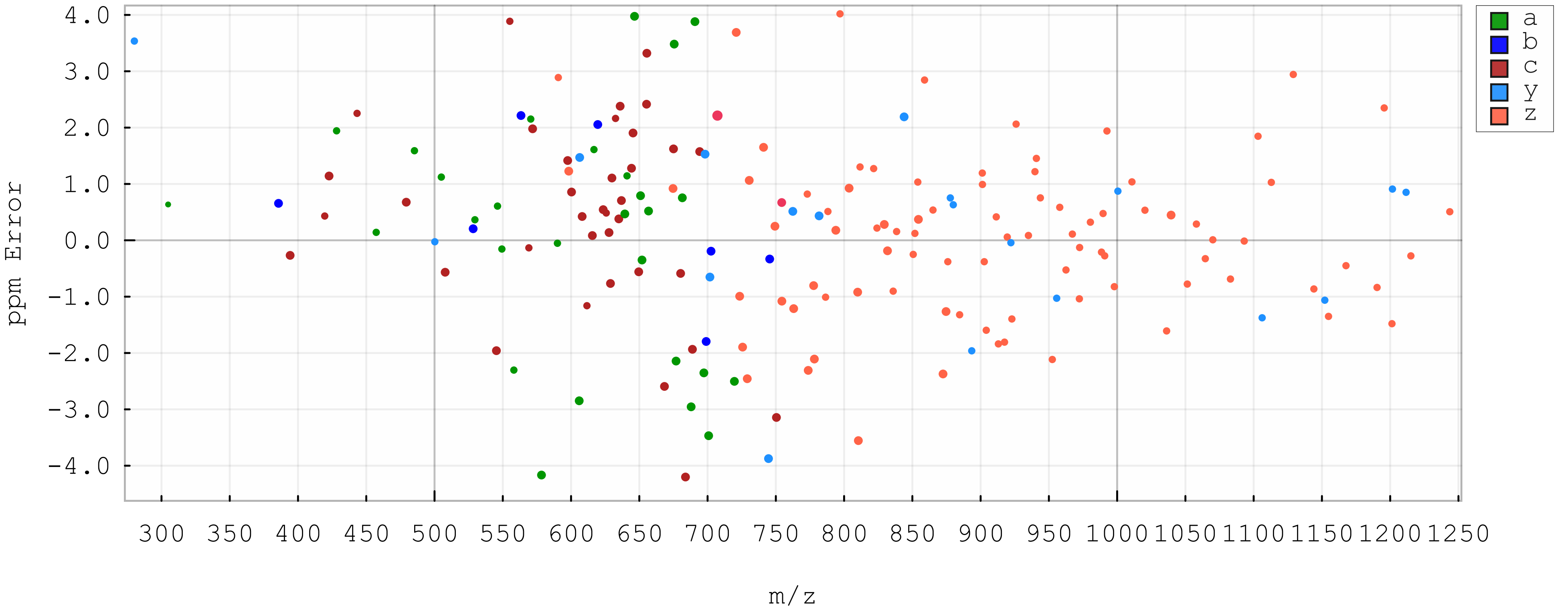

Proteoform:

Index	Protein	SC [%]	SVP [%]	IC [%]	MS Score [a.u.]	Var. Mods	Mono Mass [Da]	Chemical Formula
8	H4Kac_me1-3	75.2	7.8	35.3	26.5	Ace: K12, Dim: K20	11299.3831	C498H843N163O135S

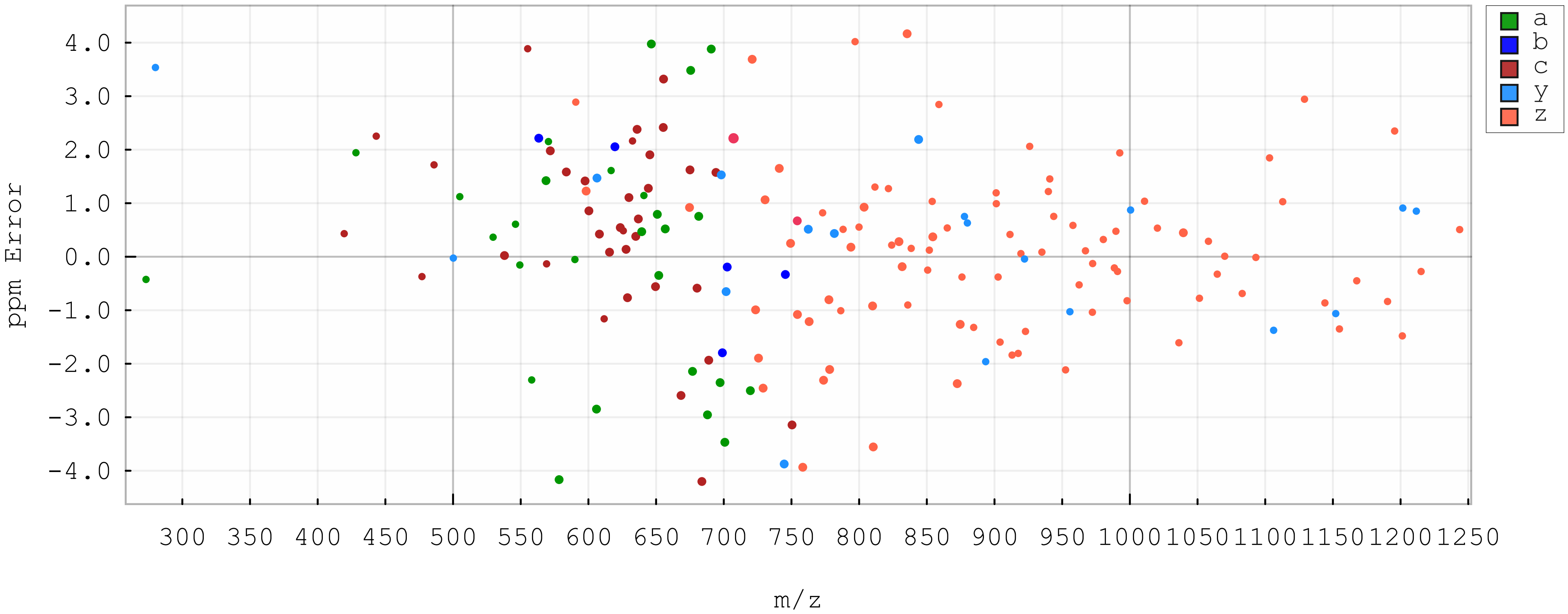

Proteoform:

Index	Protein	SC [%]	SVP [%]	IC [%]	MS Score [a.u.]	Var. Mods	Mono Mass [Da]	Chemical Formula
9	H4Kac_me1-3	74.3	7.8	35.2	26.1	Ace: K8, Met: K16, K20	11299.3831	C498H843N163O135S

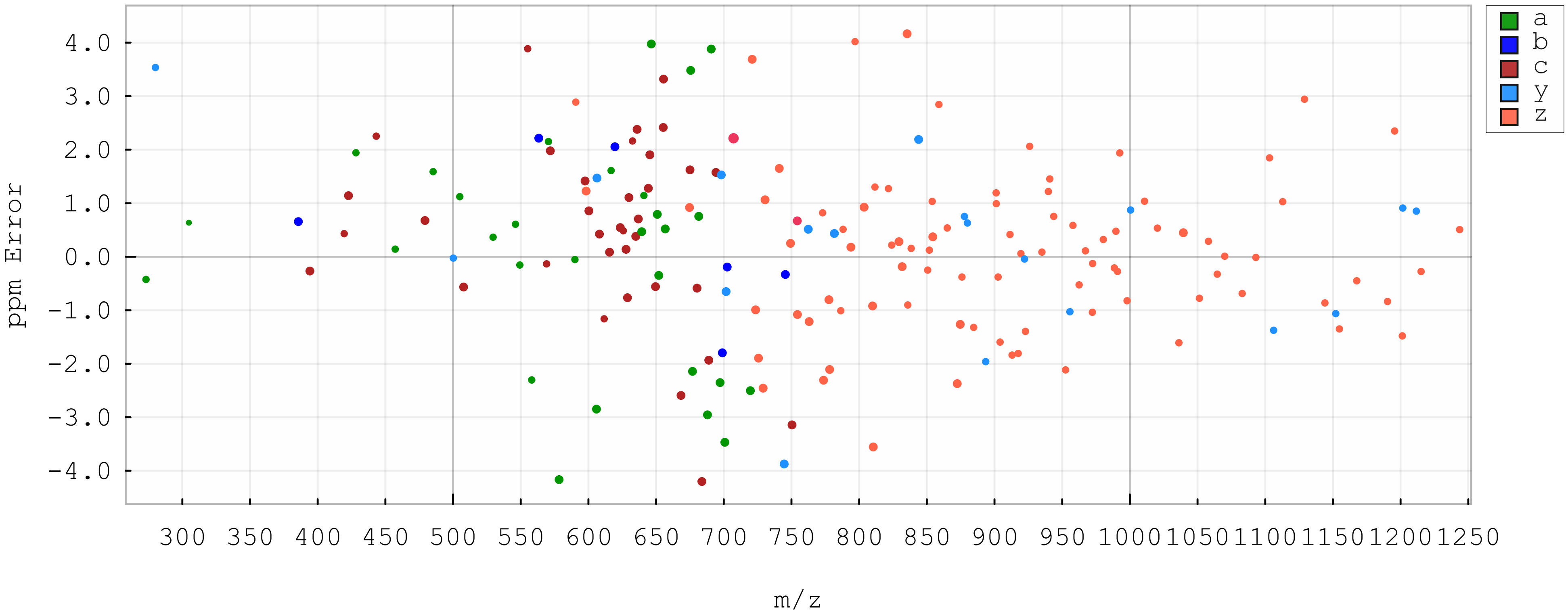

Proteoform:	Index	Protein	SC [%]	SVP [%]	IC [%]	MS Score [a.u.]	Var. Mods	Mono Mass [Da]	Chemical Formula
	10	H4Kac_me1-3	74.3	7.8	35.2	26.1	Ace: K8, Dim: K16	11299.3831	C498H843N163O135S

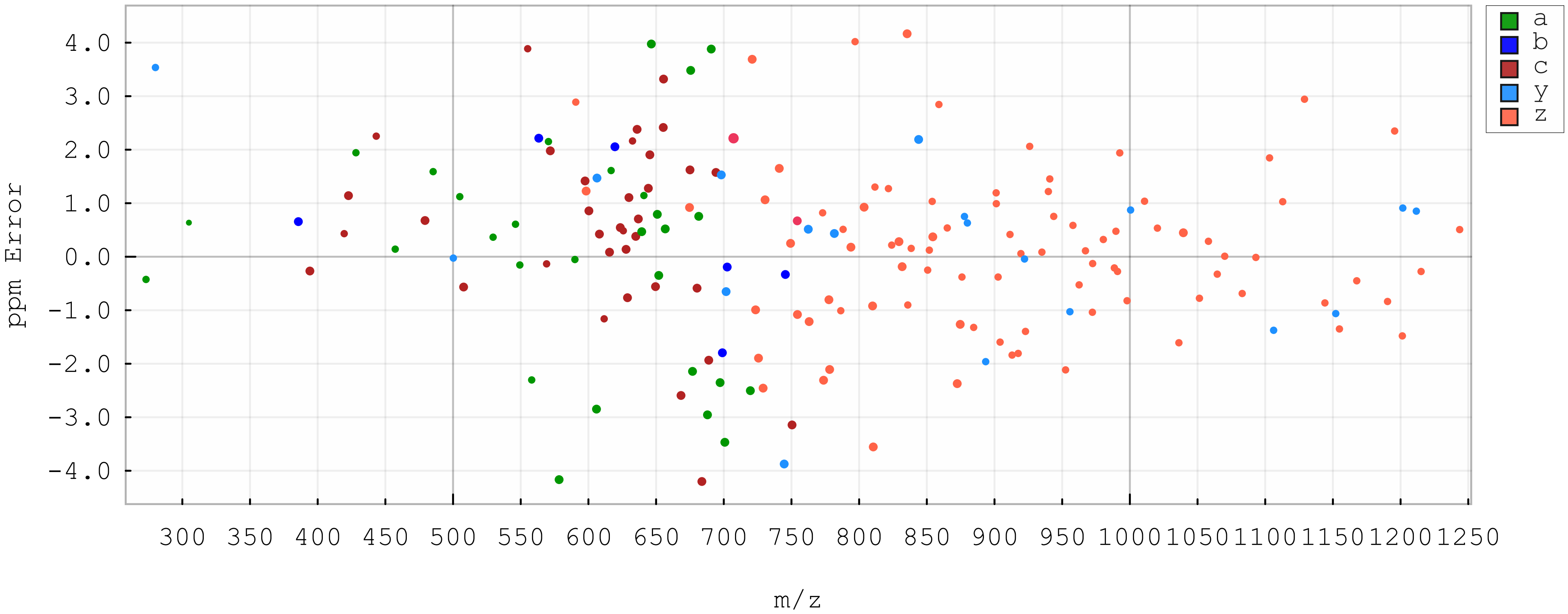

Primary Fragment Plots

Proteoform:

Index	Protein	SC [%]	SVP [%]	IC [%]	MS Score [a.u.]	Var. Mods	Mono Mass [Da]	Chemical Formula
1	H4Kac_me1-3	80.2	30.4	35.3	28.3	Dim: K20, Ace: N-term	11299.3831	C498H843N163O135S

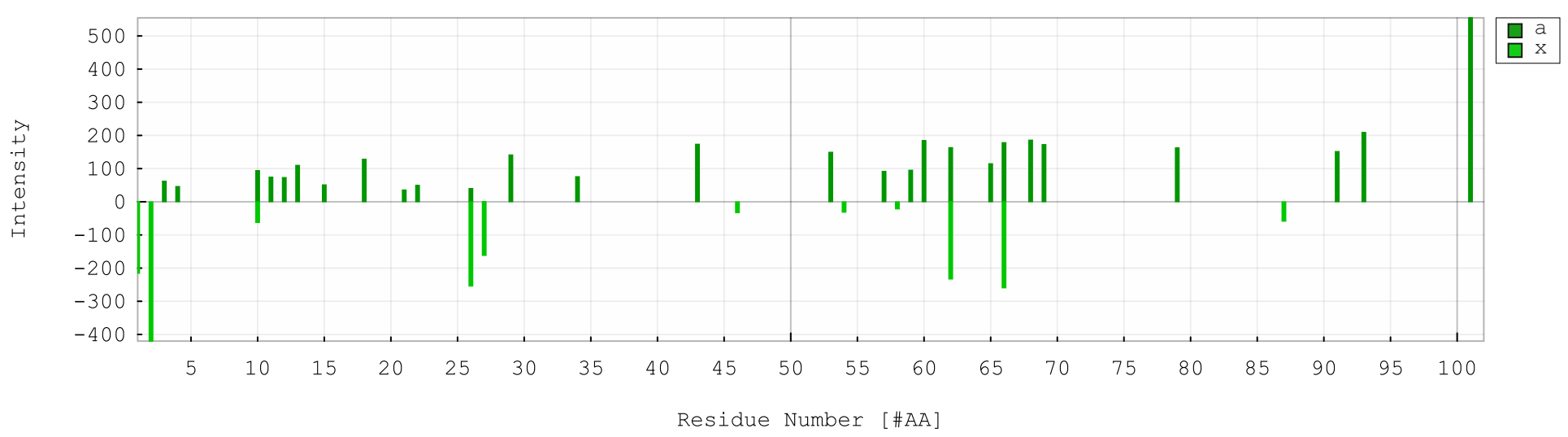

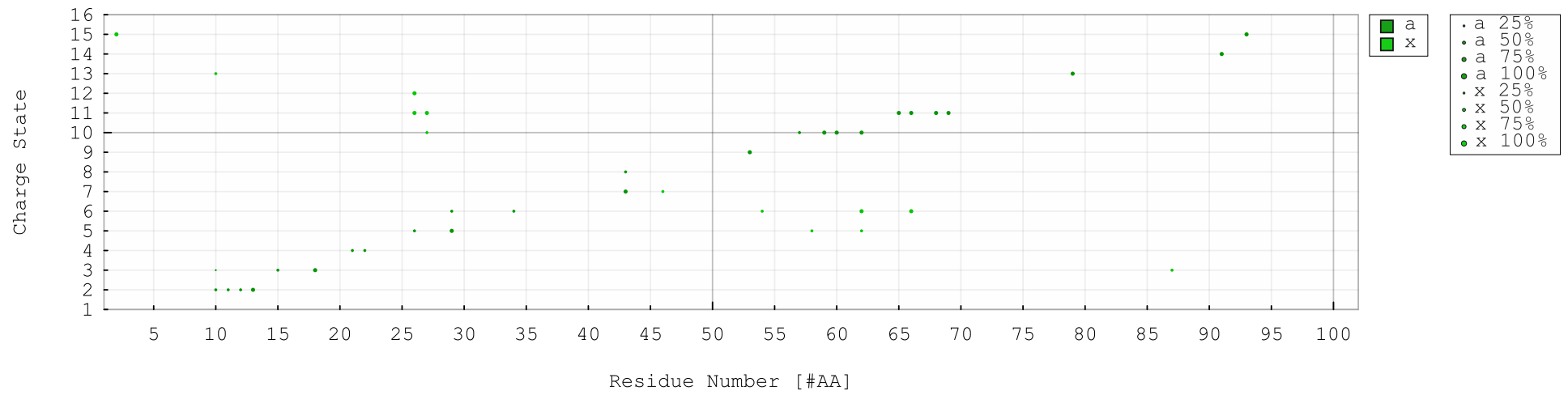

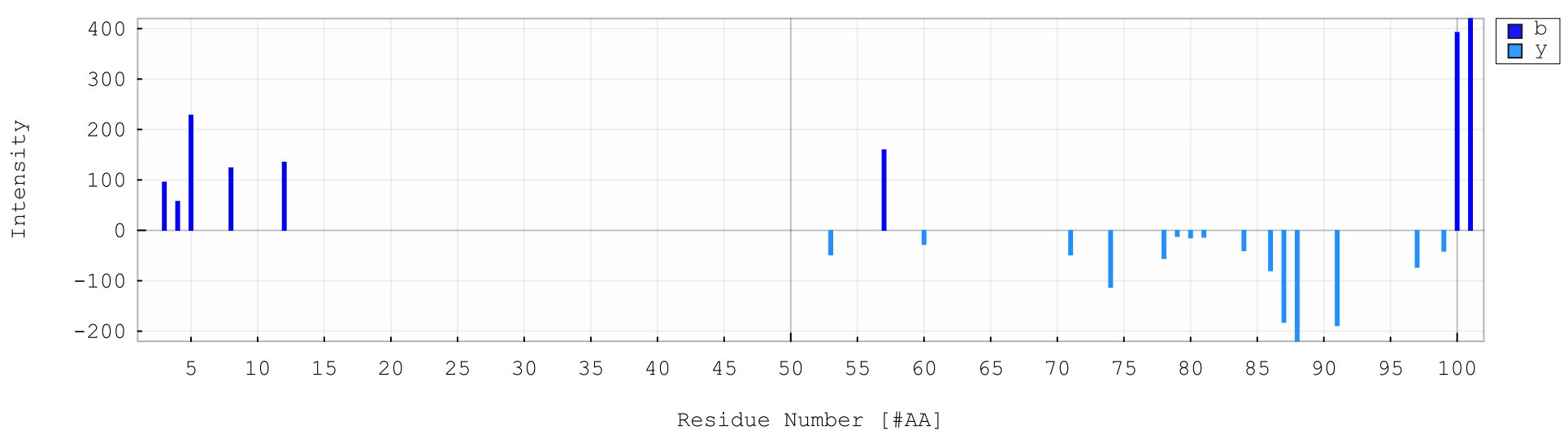

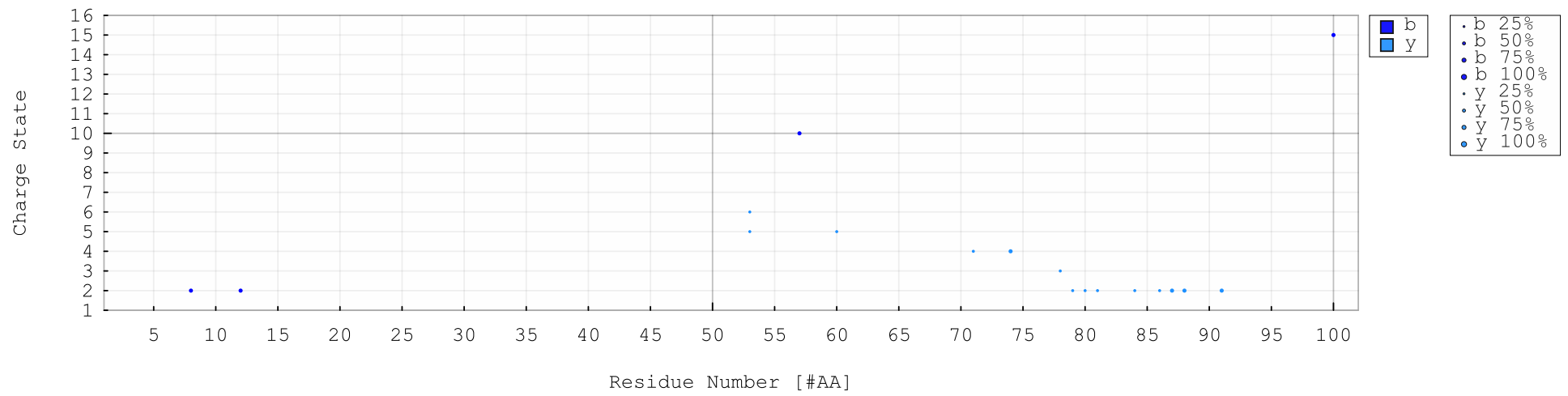

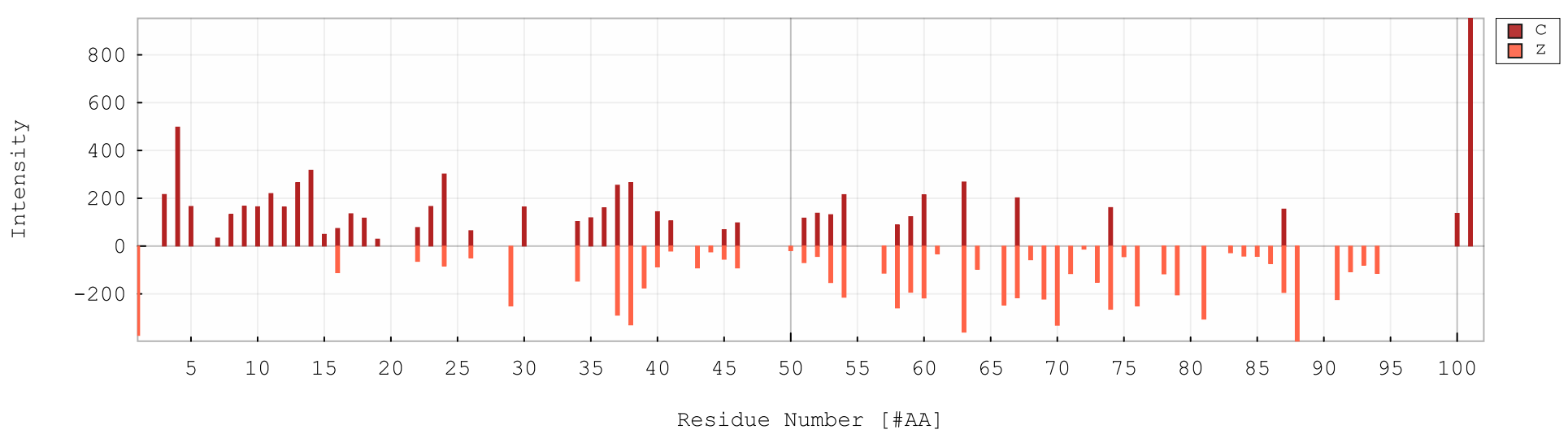

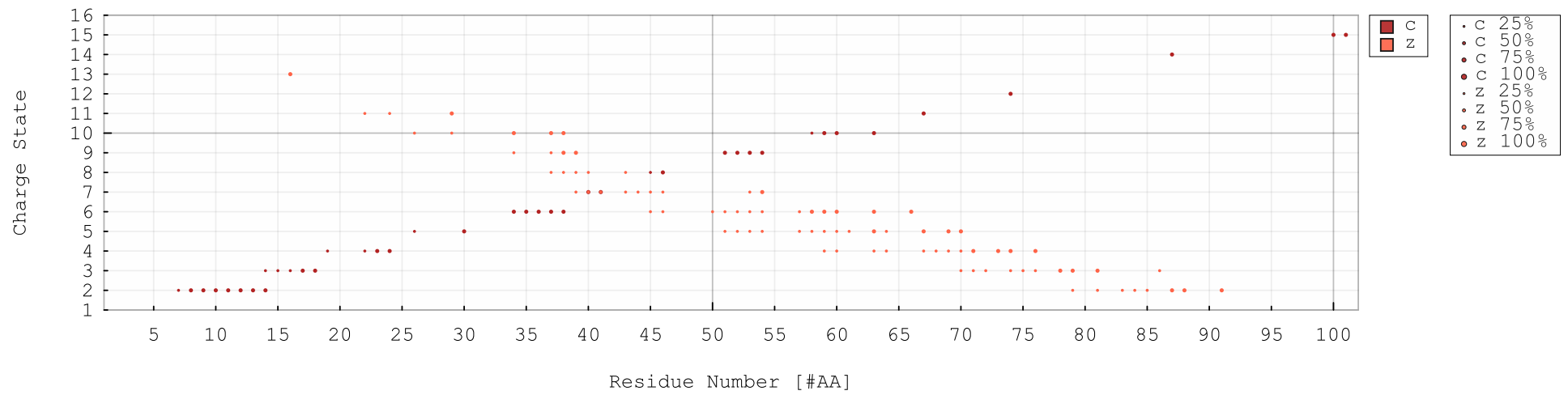

Proteoform:

Index	Protein	SC [%]	SVP [%]	IC [%]	MS Score [a.u.]	Var. Mods	Mono Mass [Da]	Chemical Formula
2	H4Kac_me1-3	79.2	30.4	35.7	28.3	Ace: K5, Dim: K20	11299.3831	C498H843N163O135S

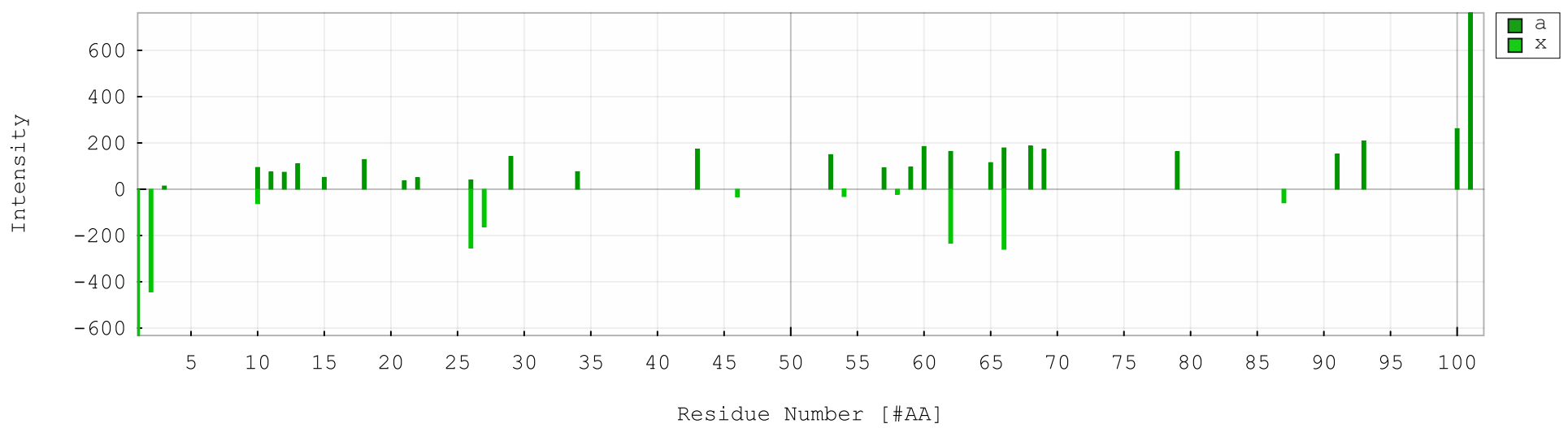

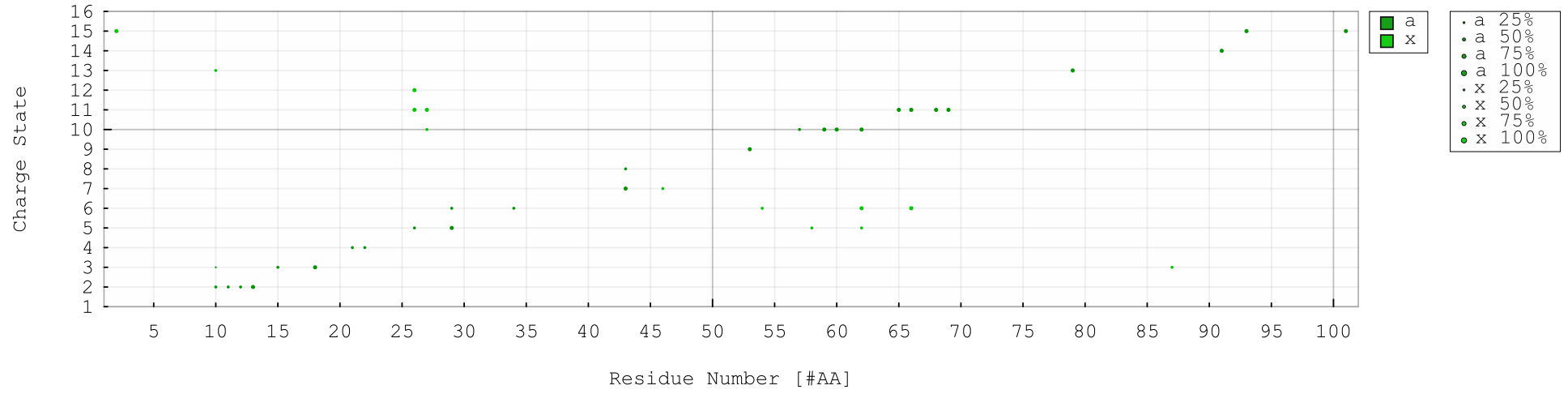

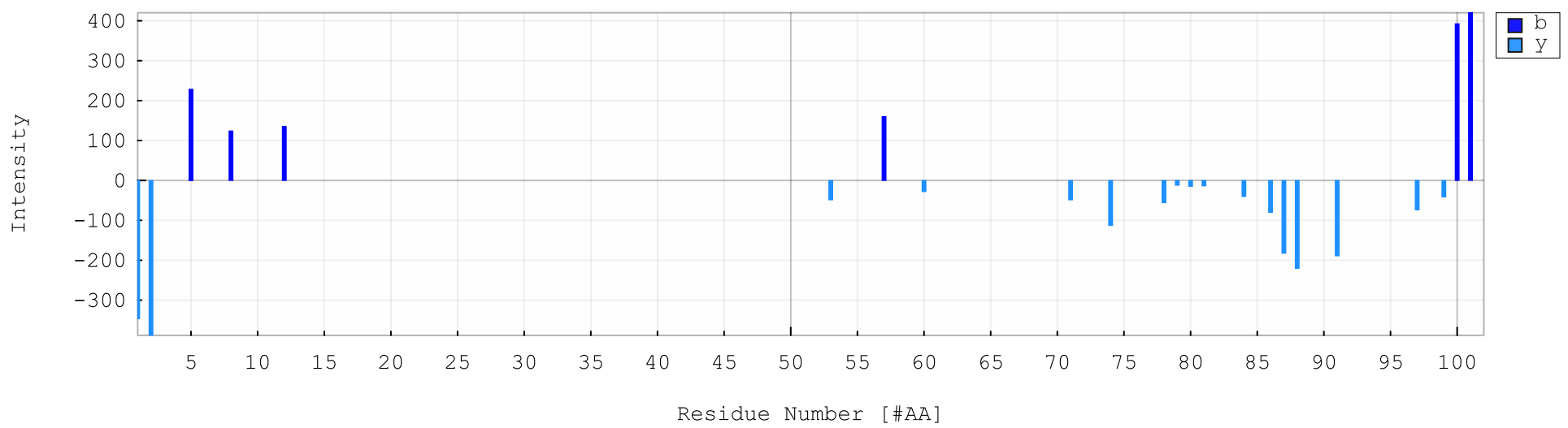

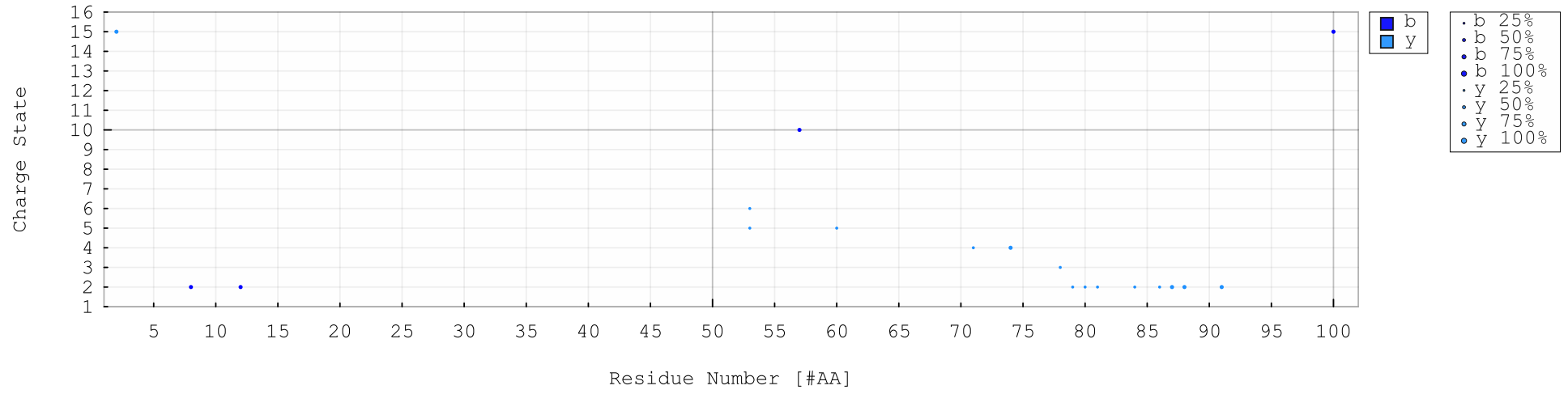

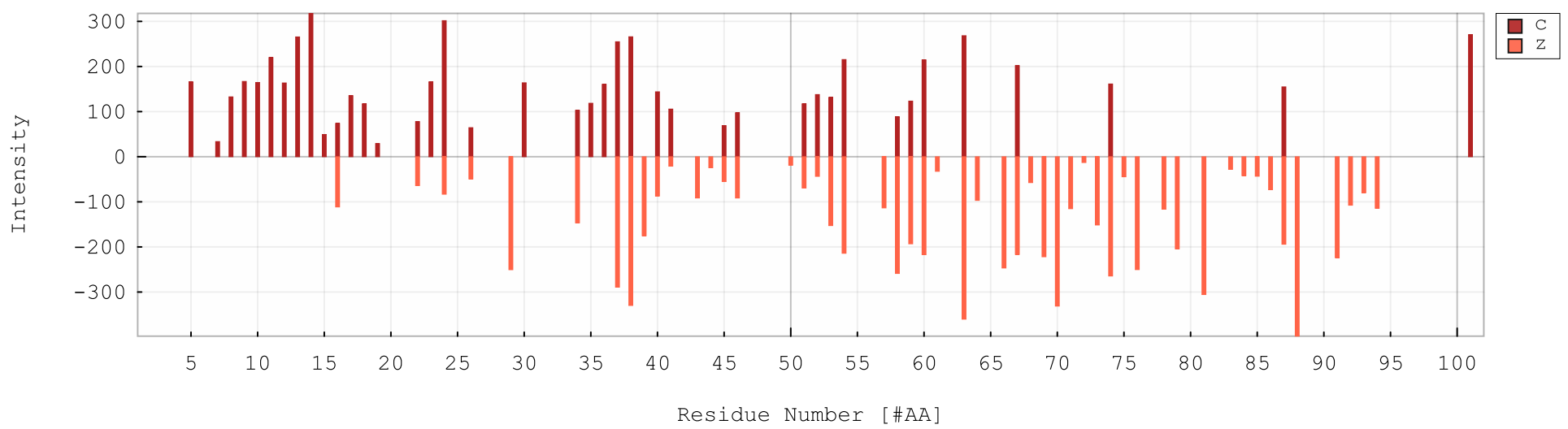

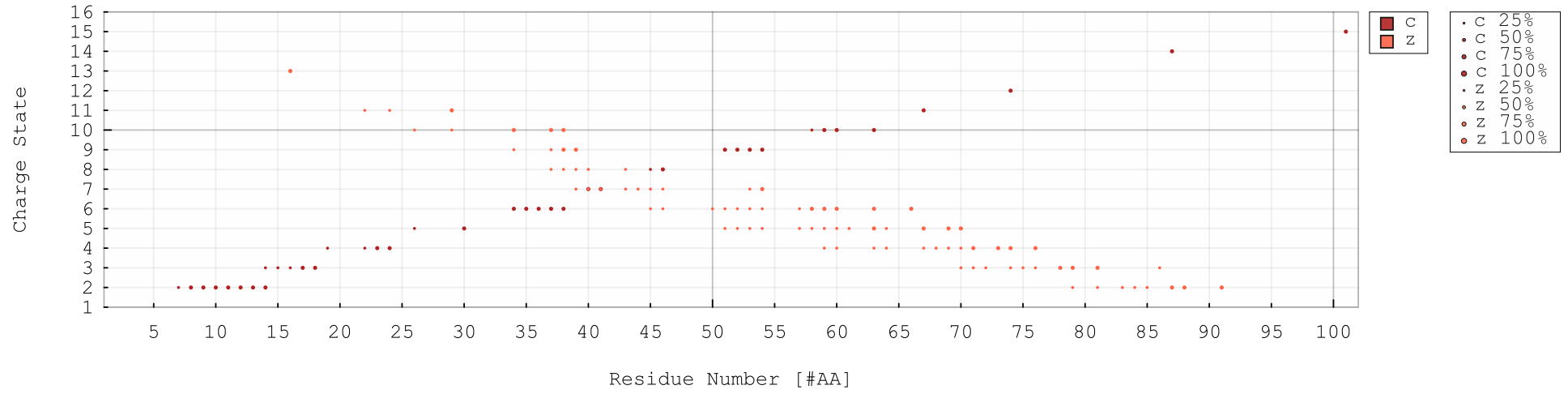

Top-Down Result OmniScape 2025
Sequence Confirmation
250430 Liver-Histone-25 mz707z16 MS2 EID_003-314_calibrated

Proteoform:

Index	Protein	SC [%]	SVP [%]	IC [%]	MS Score [a.u.]	Var. Mods	Mono Mass [Da]	Chemical Formula
3	H4Kac_me1-3	78.2	7.8	35.6	27.9	Ace: K8, Dim: K20	11299.3831	C498H843N163O135S

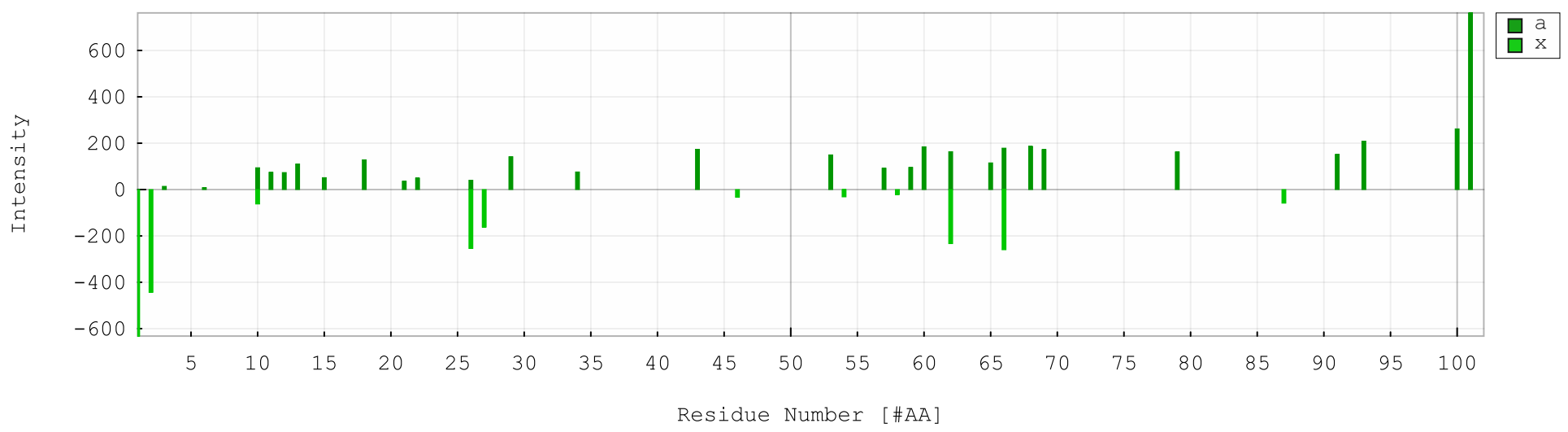

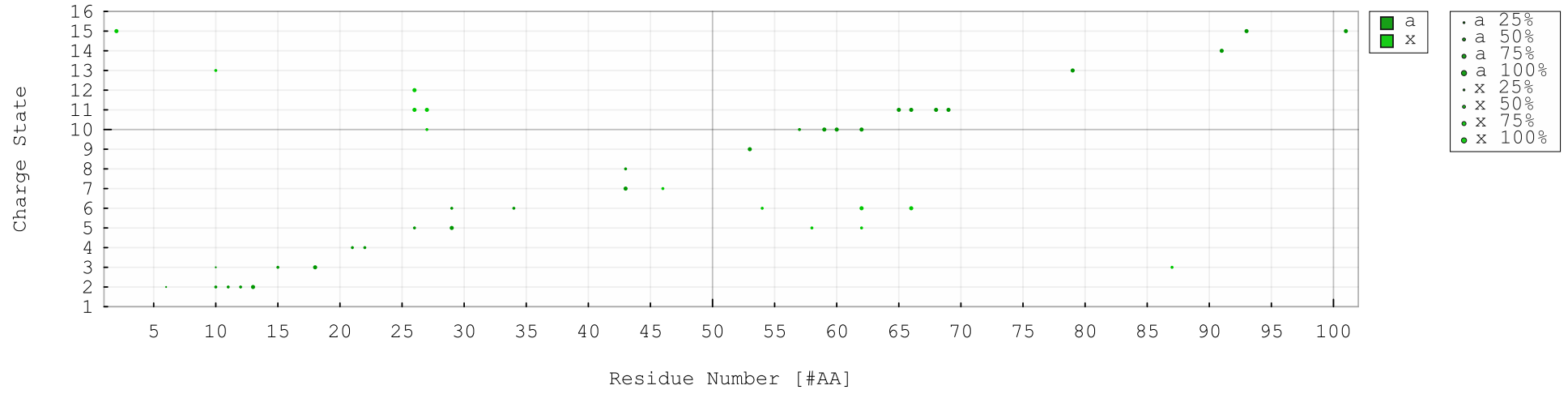

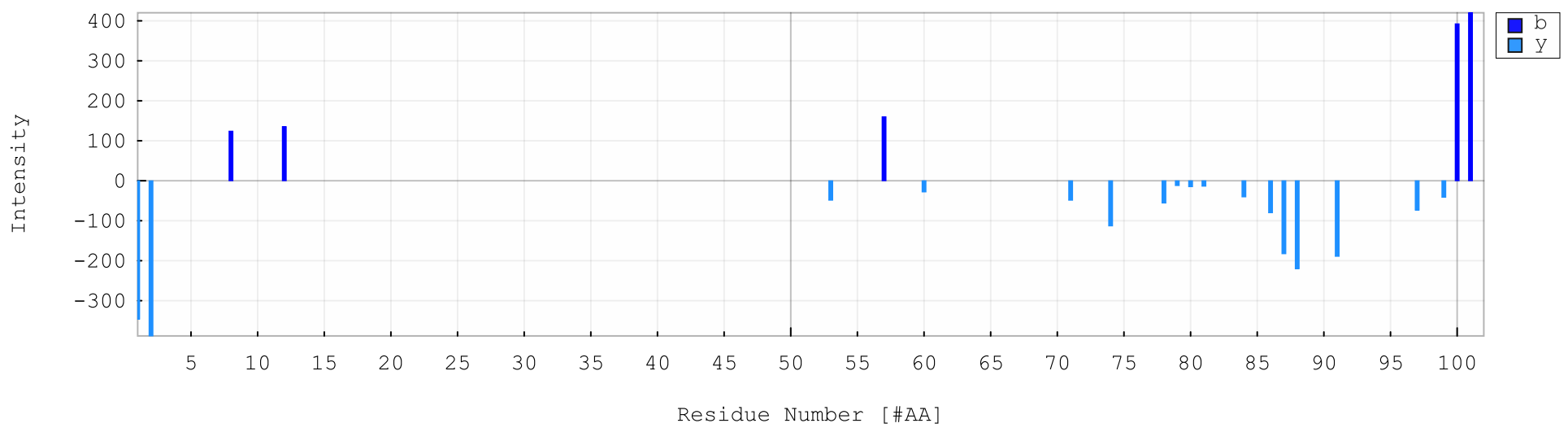

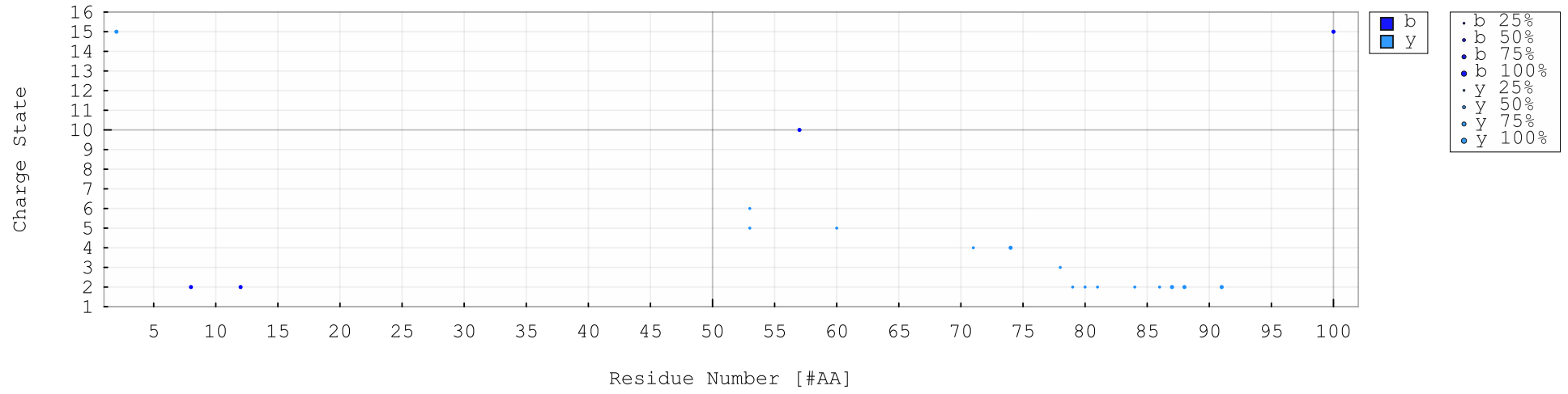

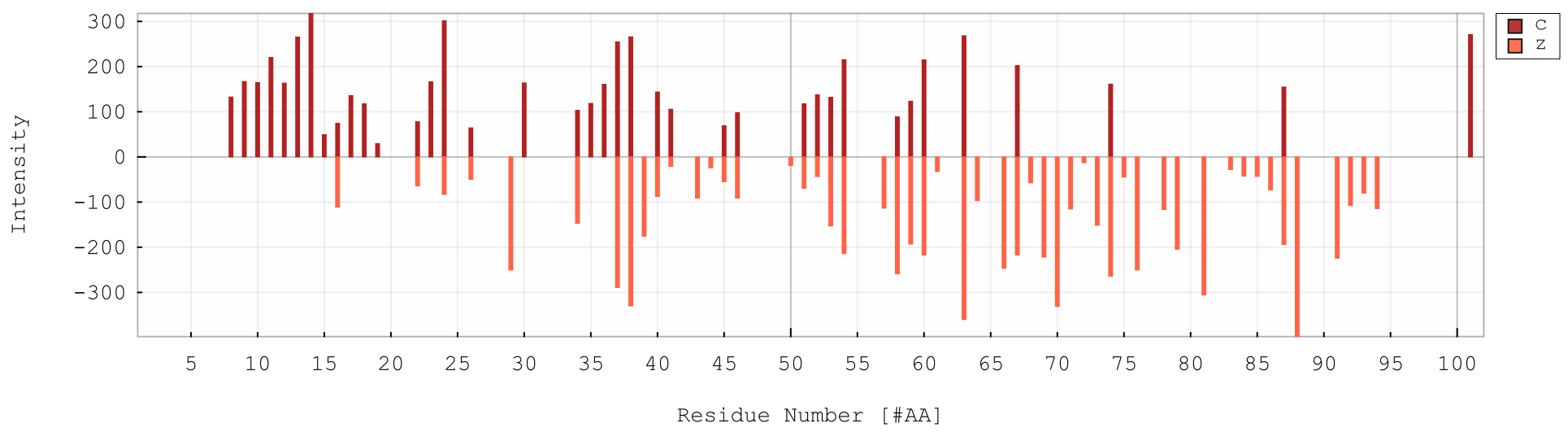

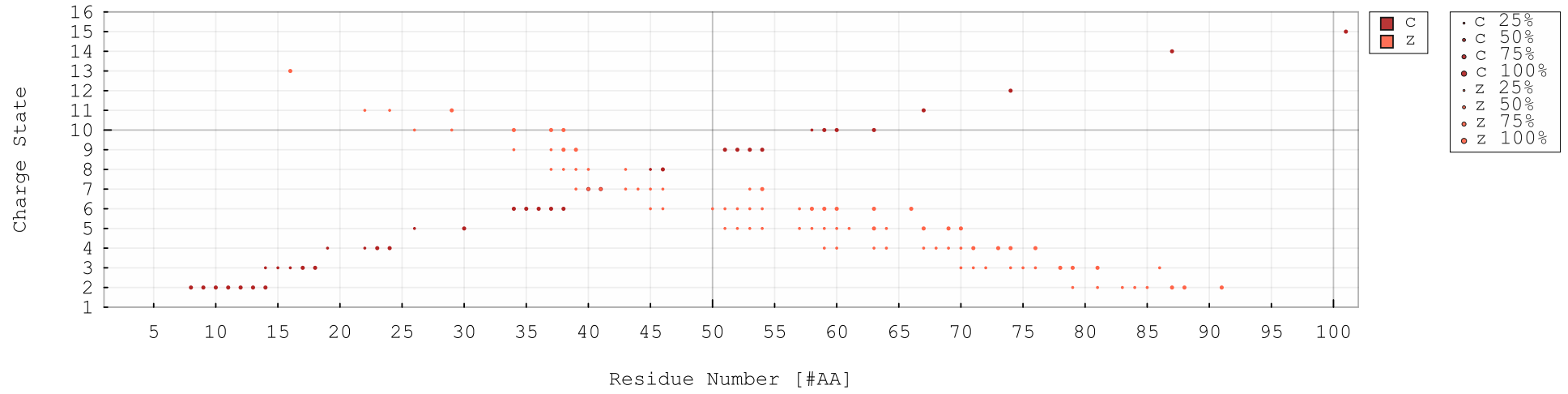

Proteoform:

Index	Protein	SC [%]	SVP [%]	IC [%]	MS Score [a.u.]	Var. Mods	Mono Mass [Da]	Chemical Formula
4	H4Kac_me1-3	76.2	19.6	34.9	26.6	Met: K16, K20, Ace: N-term	11299.3831	C498H843N163O135S

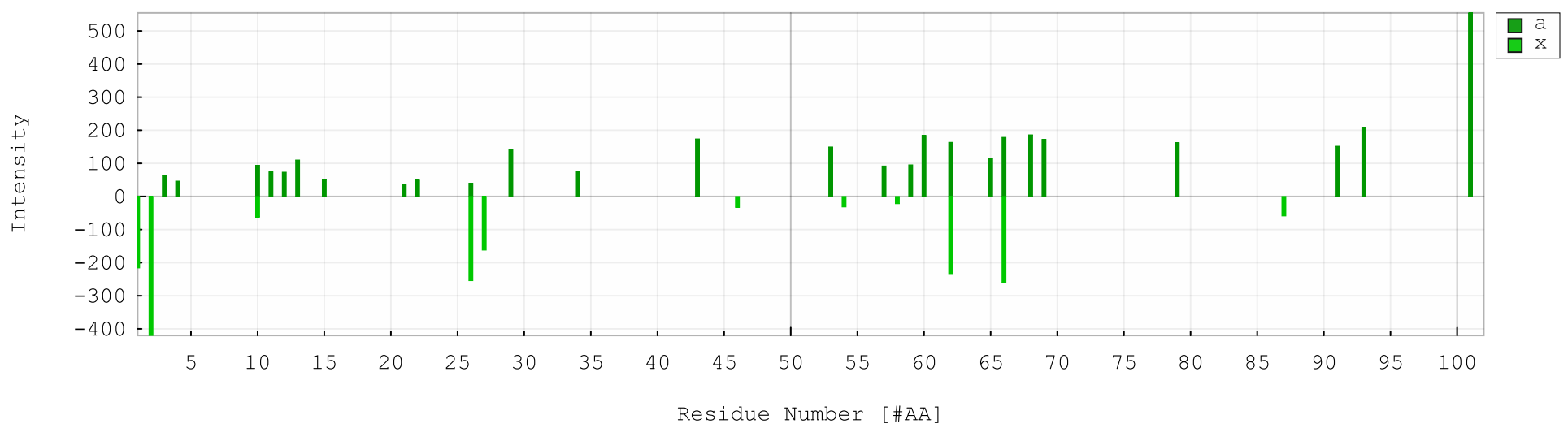

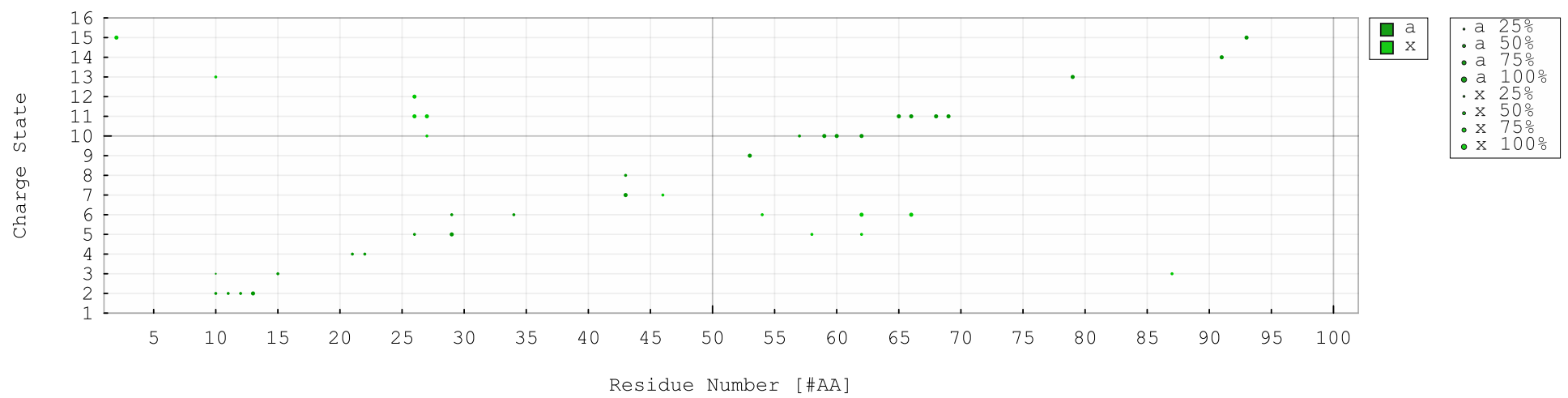

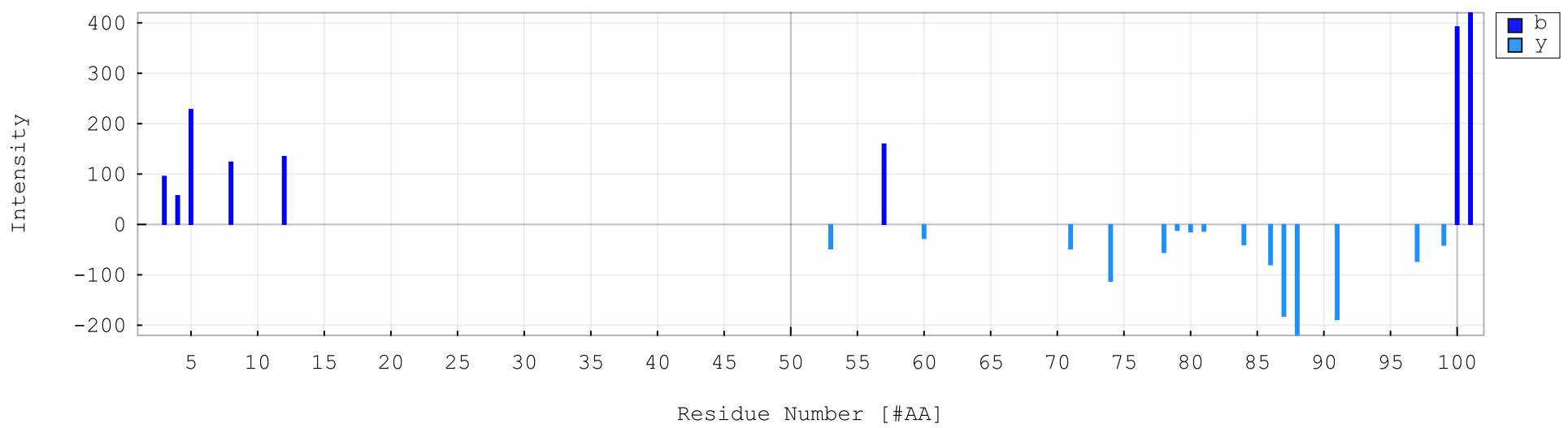

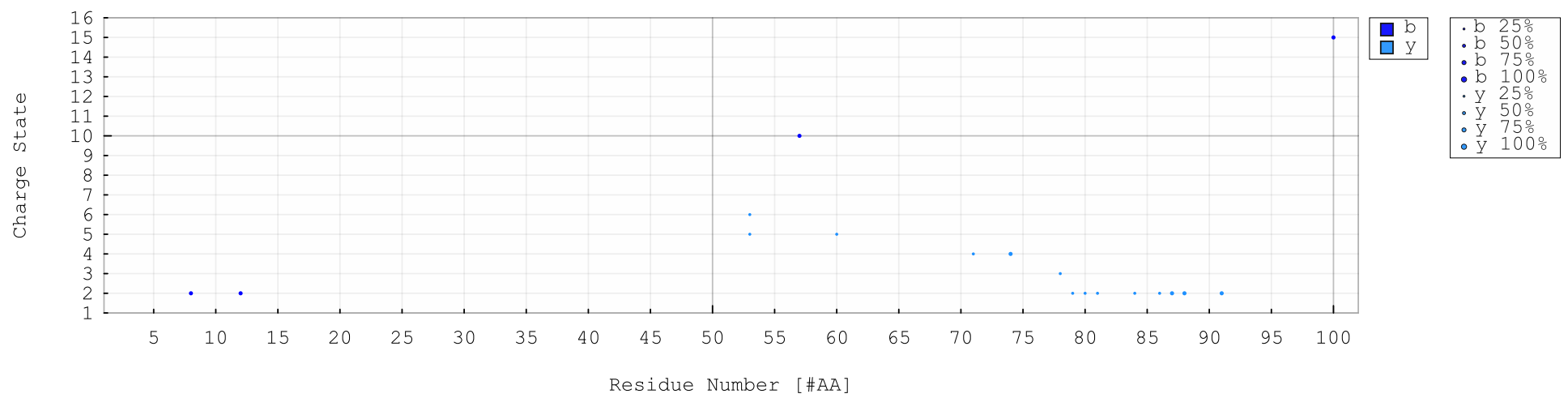

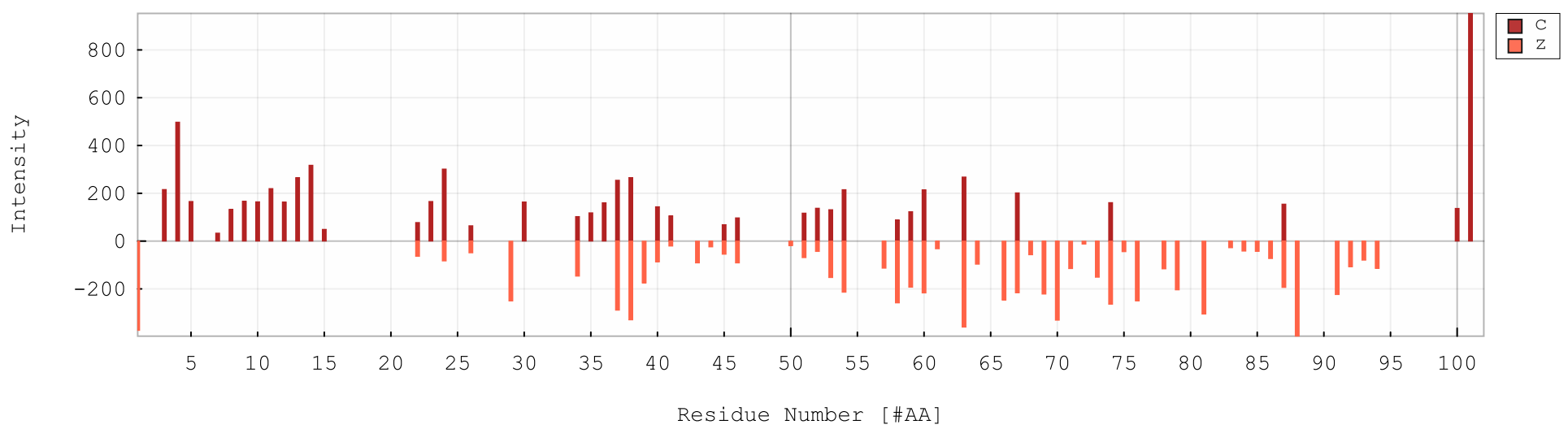

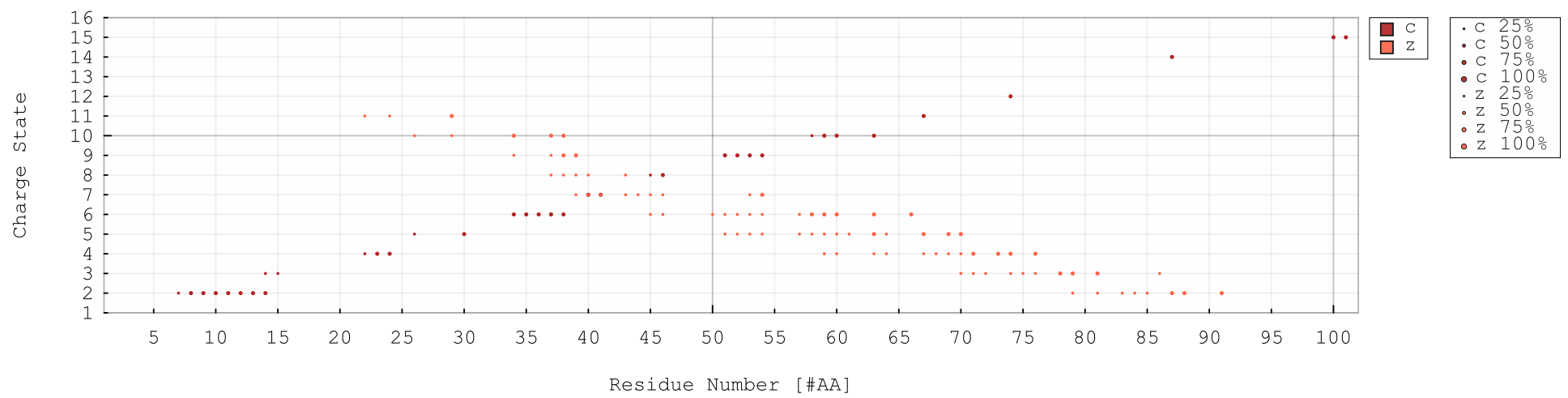

Proteoform:

Index	Protein	SC [%]	SVP [%]	IC [%]	MS Score [a.u.]	Var. Mods	Mono Mass [Da]	Chemical Formula
5	H4Kac_me1-3	76.2	19.6	34.9	26.6	Dim: K16, Ace: N-term	11299.3831	C498H843N163O135S

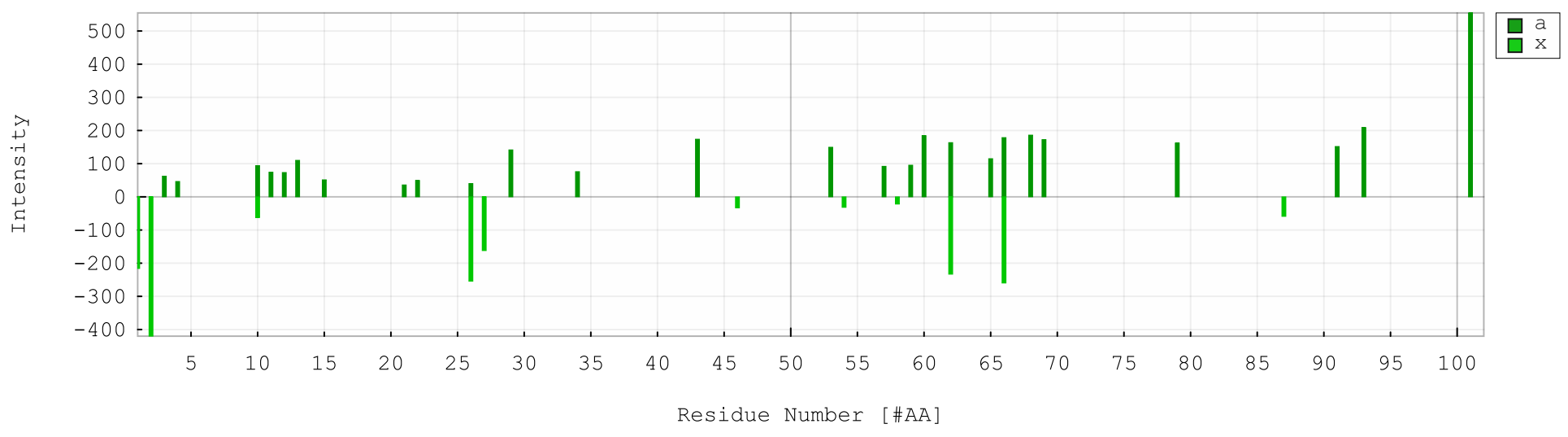

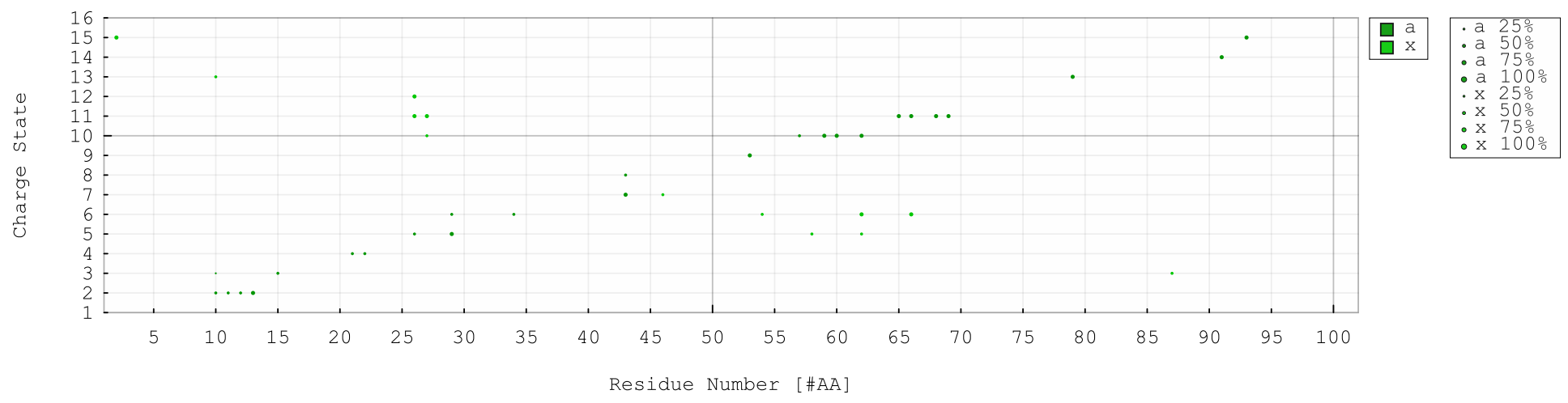

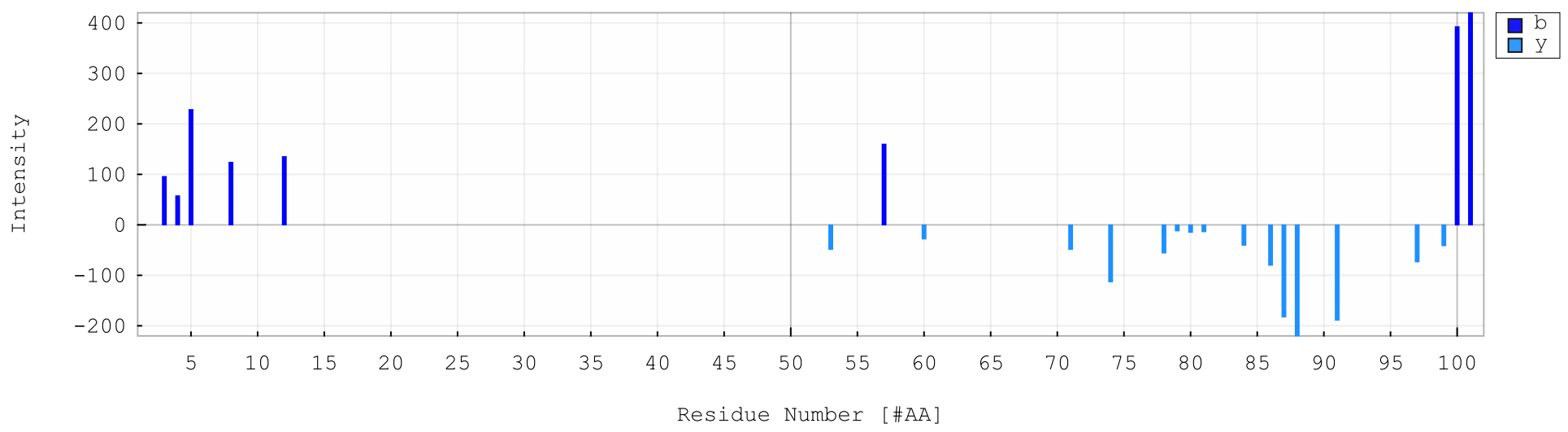

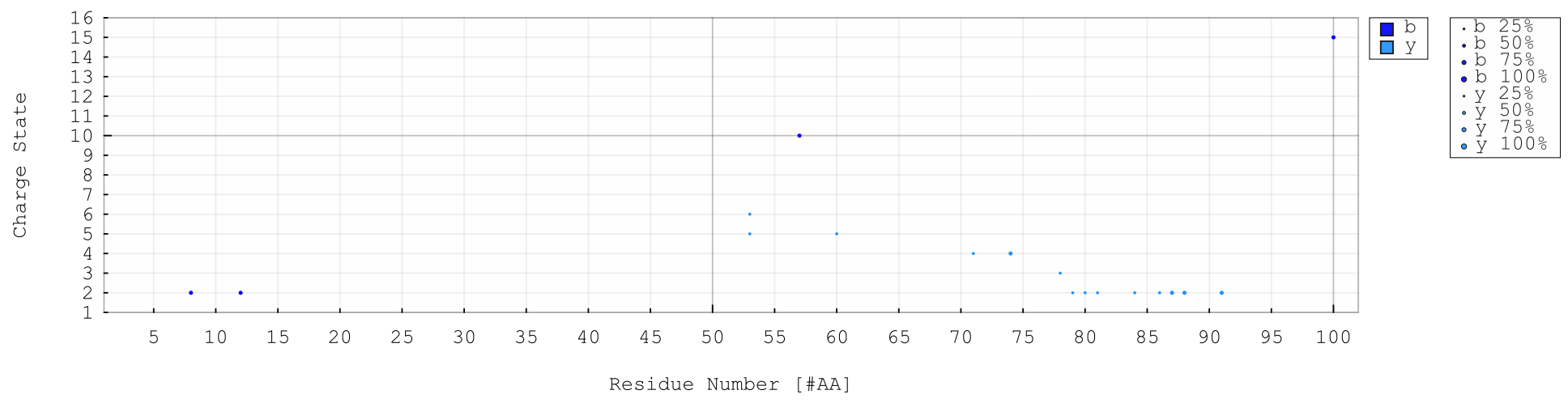

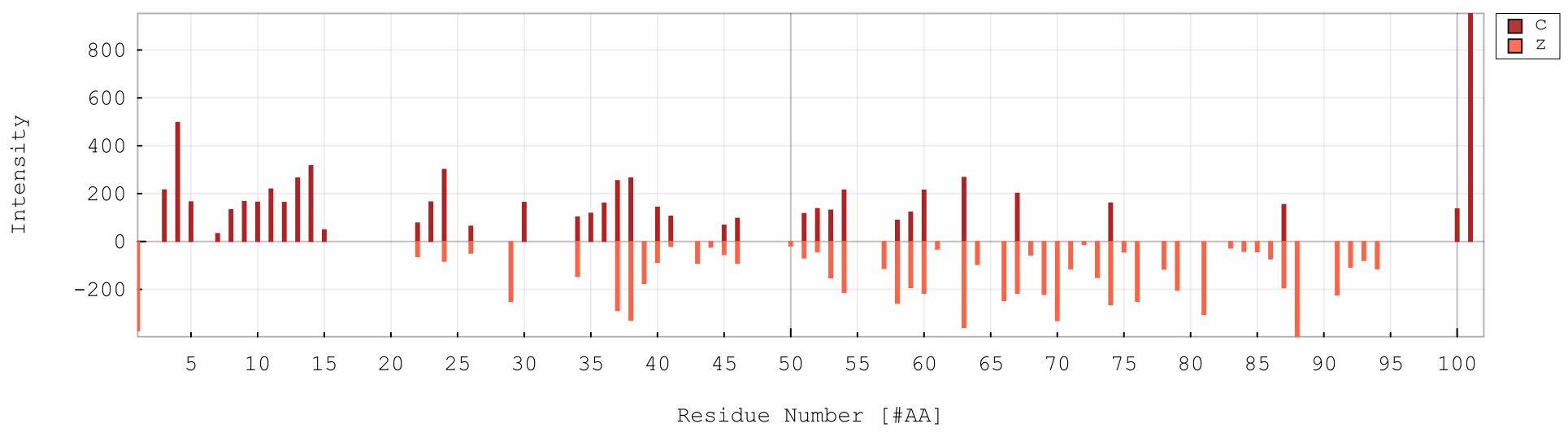

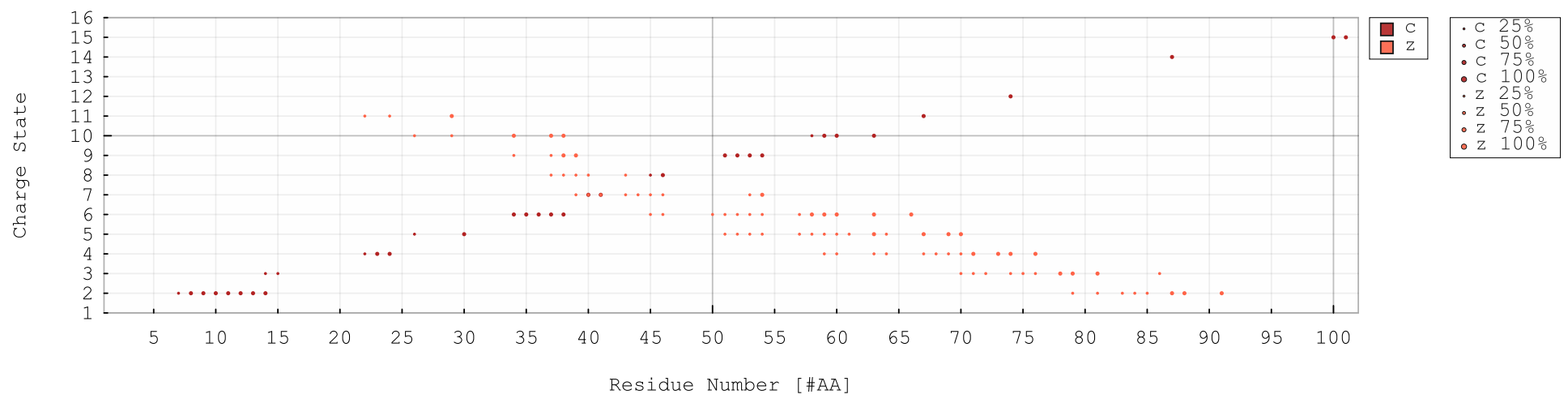

Proteoform:

Index	Protein	SC [%]	SVP [%]	IC [%]	MS Score [a.u.]	Var. Mods	Mono Mass [Da]	Chemical Formula
6	H4Kac_me1-3	75.2	19.6	35.3	26.6	Ace: K5, Dim: K16	11299.3831	C498H843N163O135S

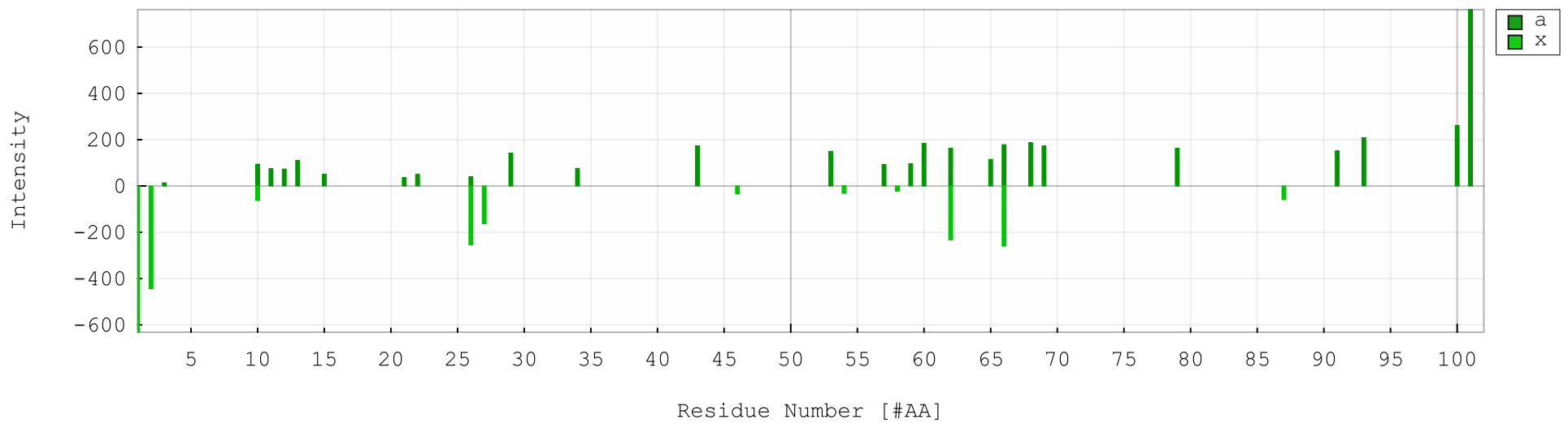

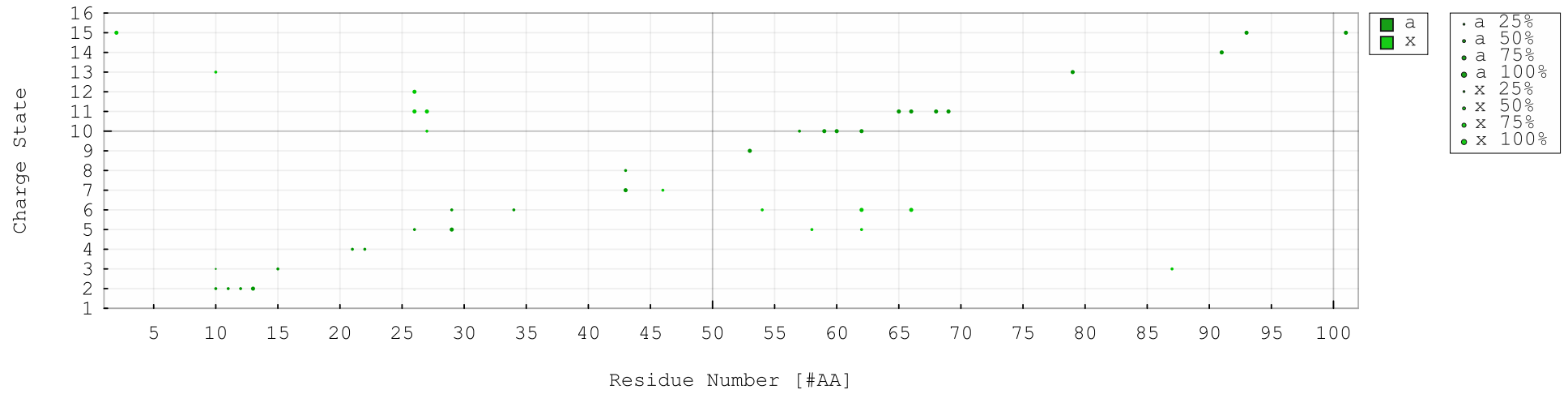

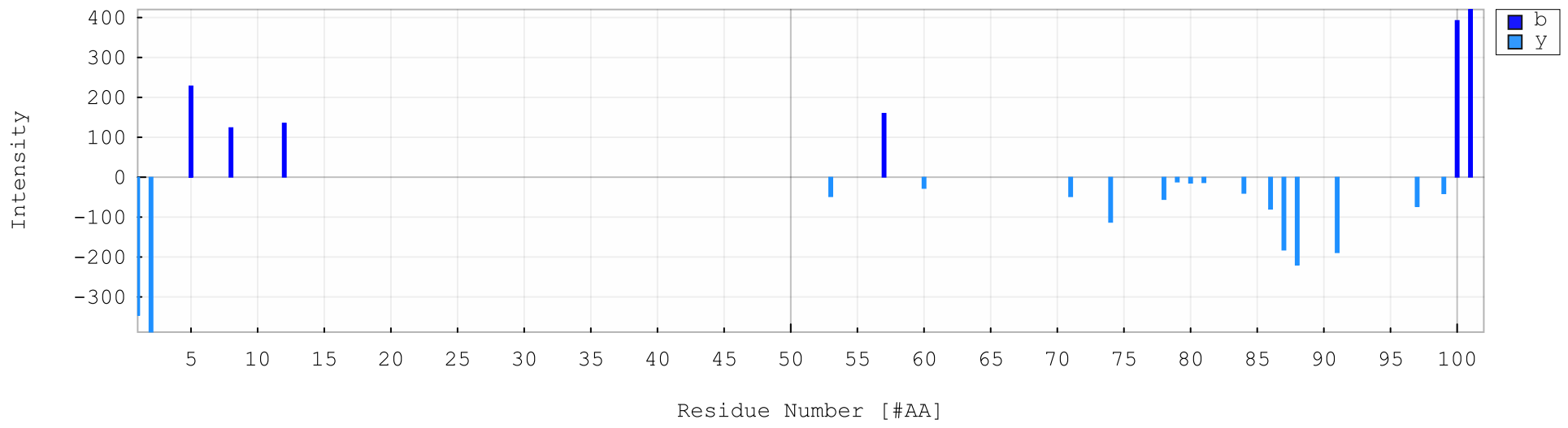

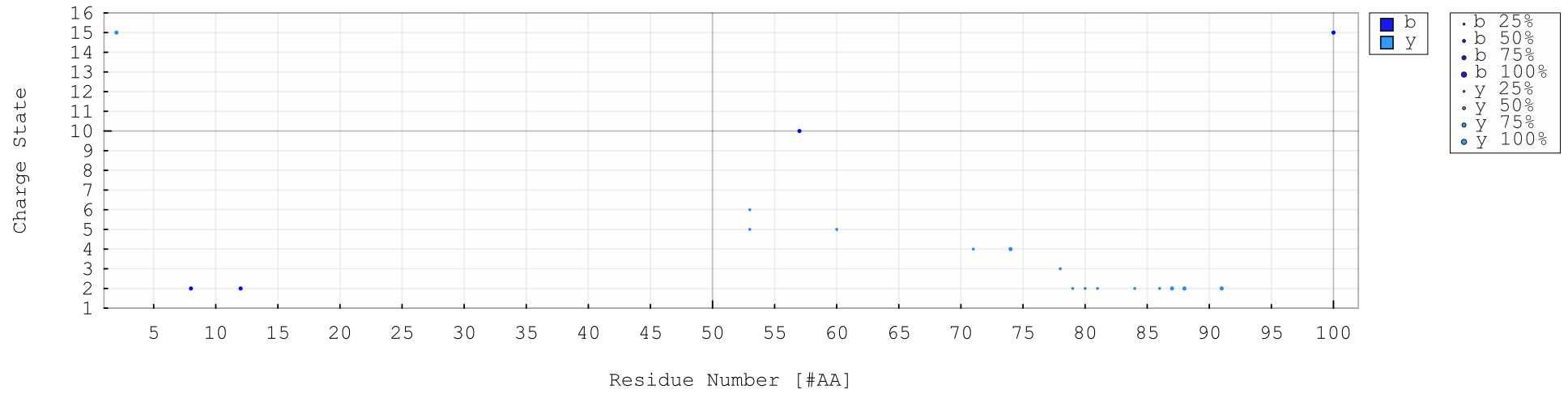

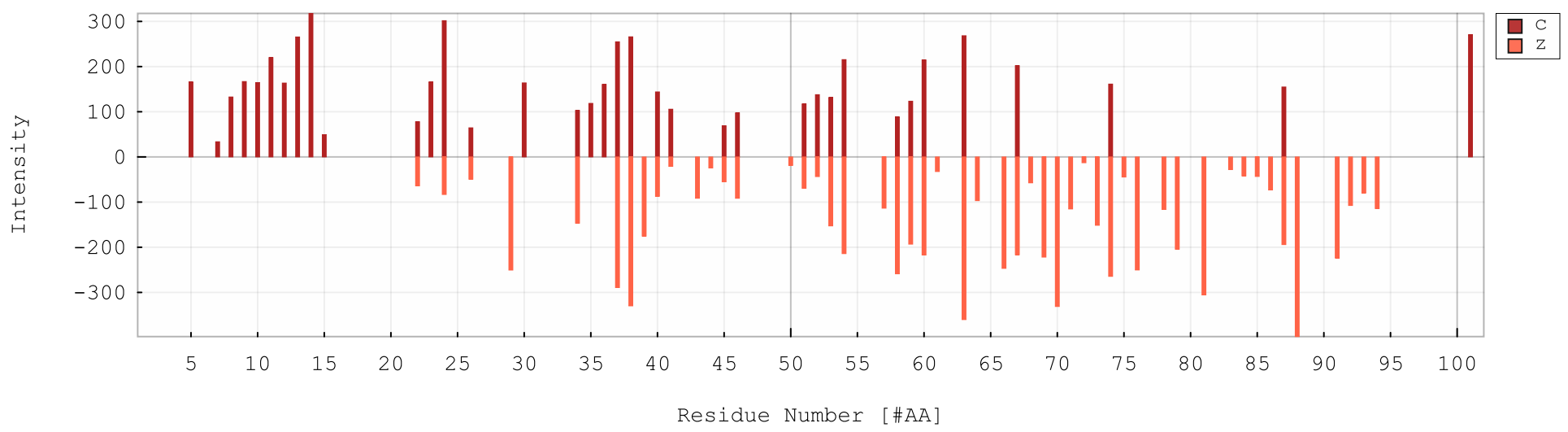

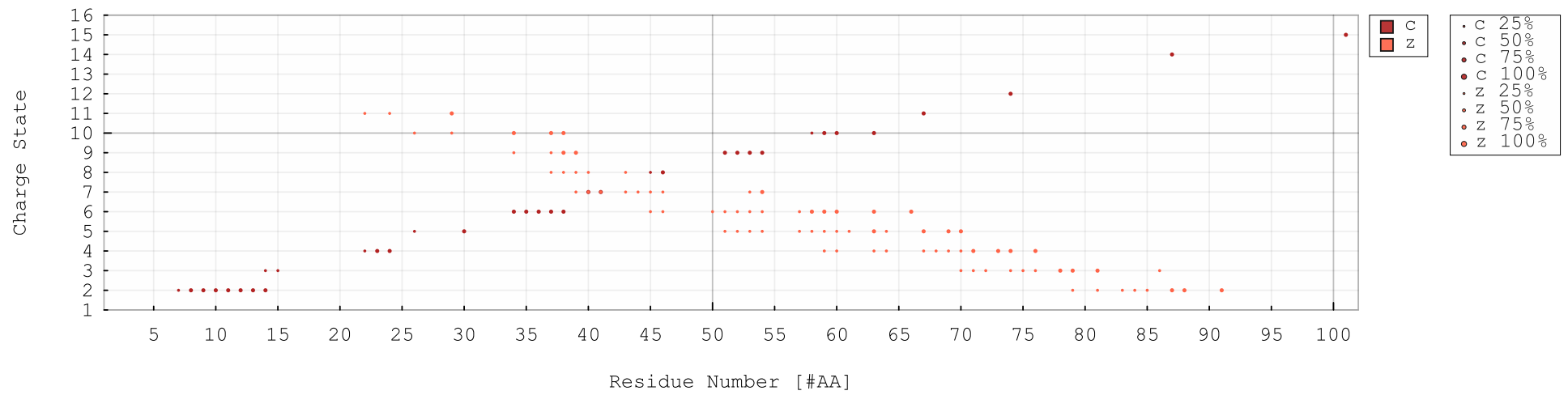

Proteoform:

Index	Protein	SC [%]	SVP [%]	IC [%]	MS Score [a.u.]	Var. Mods	Mono Mass [Da]	Chemical Formula
7	H4Kac_me1-3	75.2	19.6	35.3	26.6	Ace: K5, Met: K16, K20	11299.3831	C498H843N163O135S

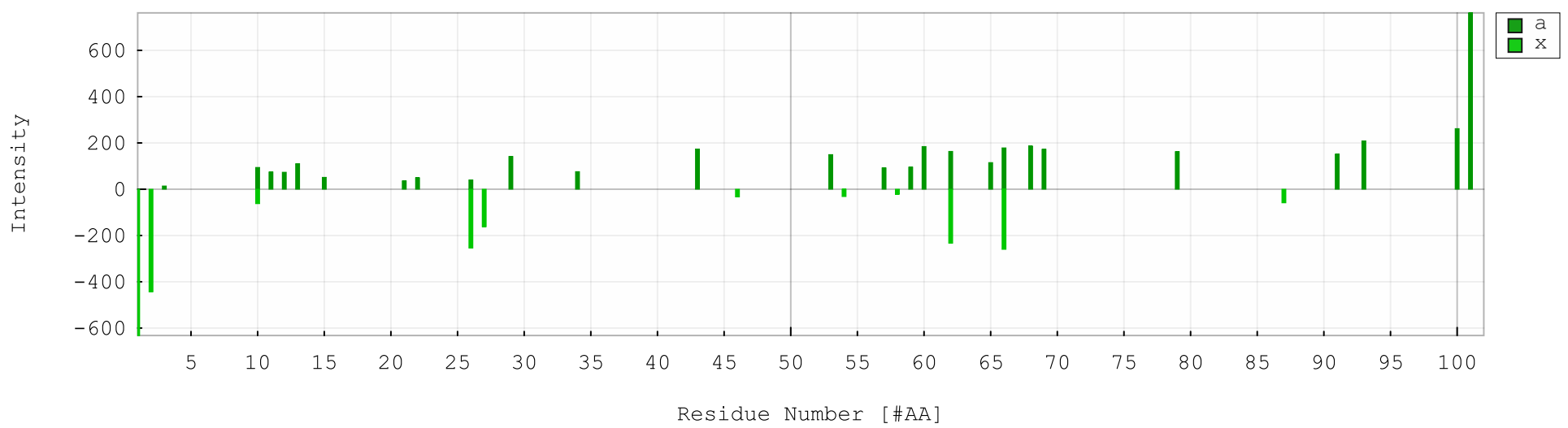

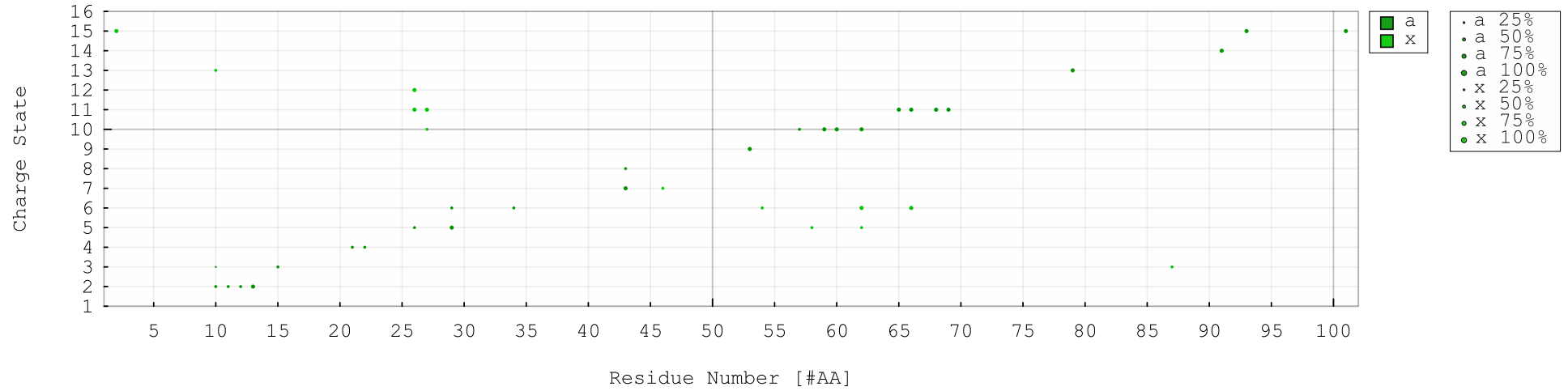

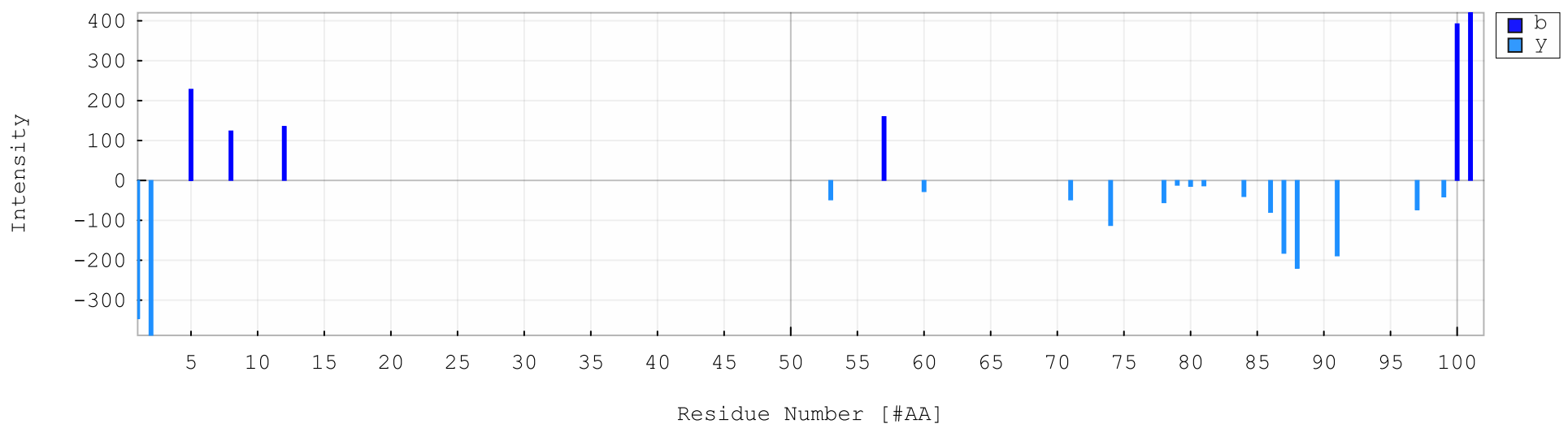

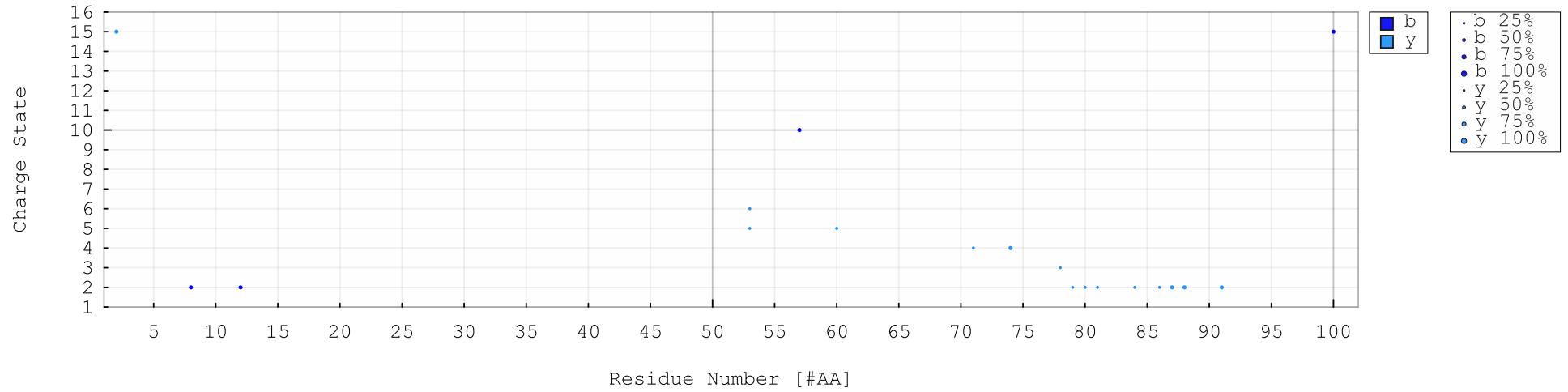

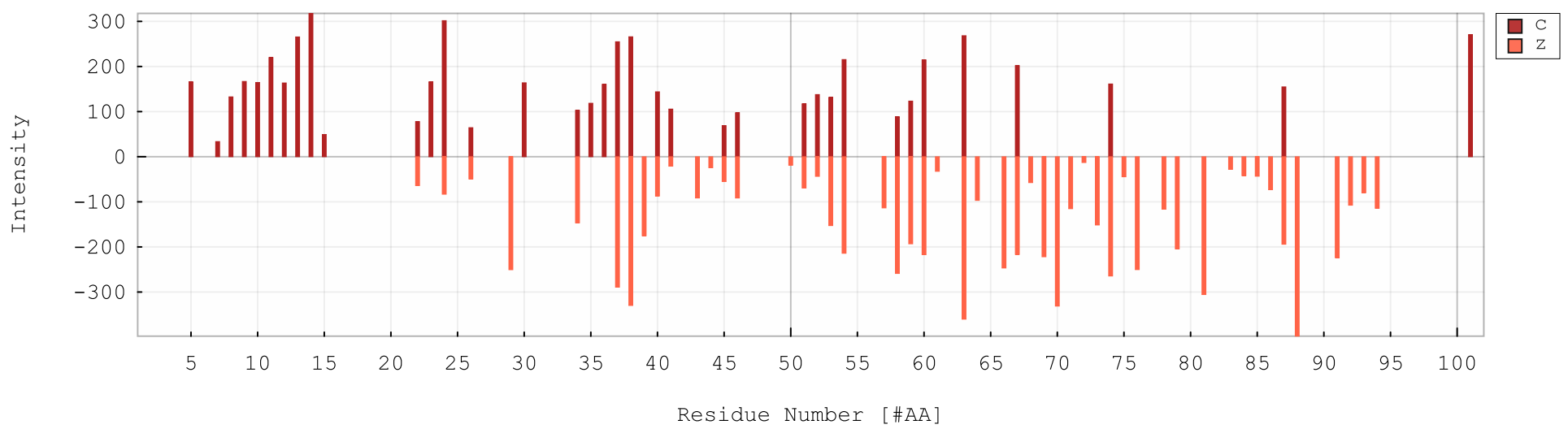

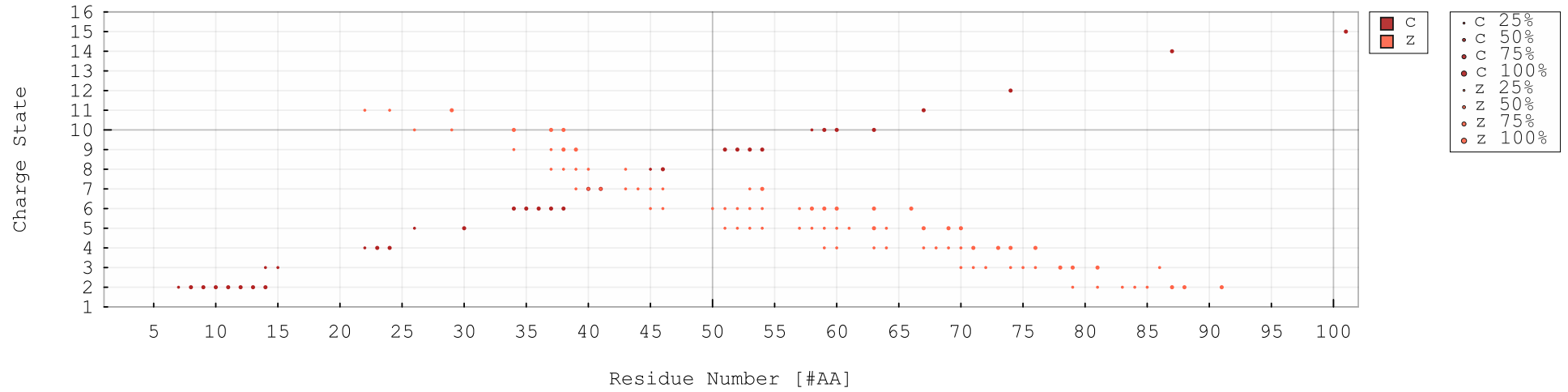

Proteoform:

Index	Protein	SC [%]	SVP [%]	IC [%]	MS Score [a.u.]	Var. Mods	Mono Mass [Da]	Chemical Formula
8	H4Kac_me1-3	75.2	7.8	35.3	26.5	Ace: K12, Dim: K20	11299.3831	C498H843N163O135S

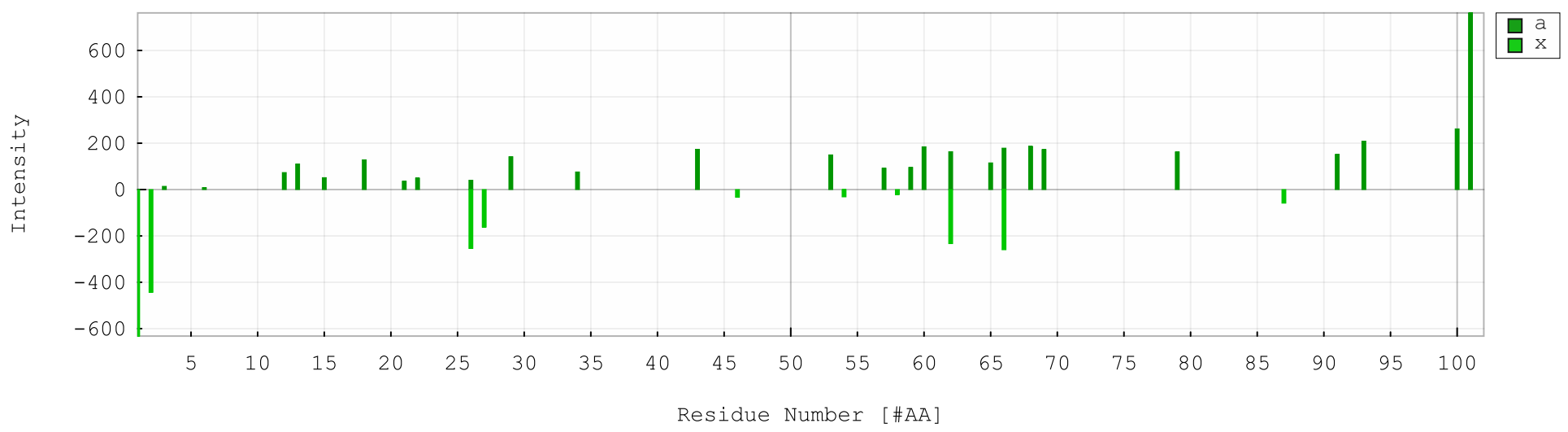

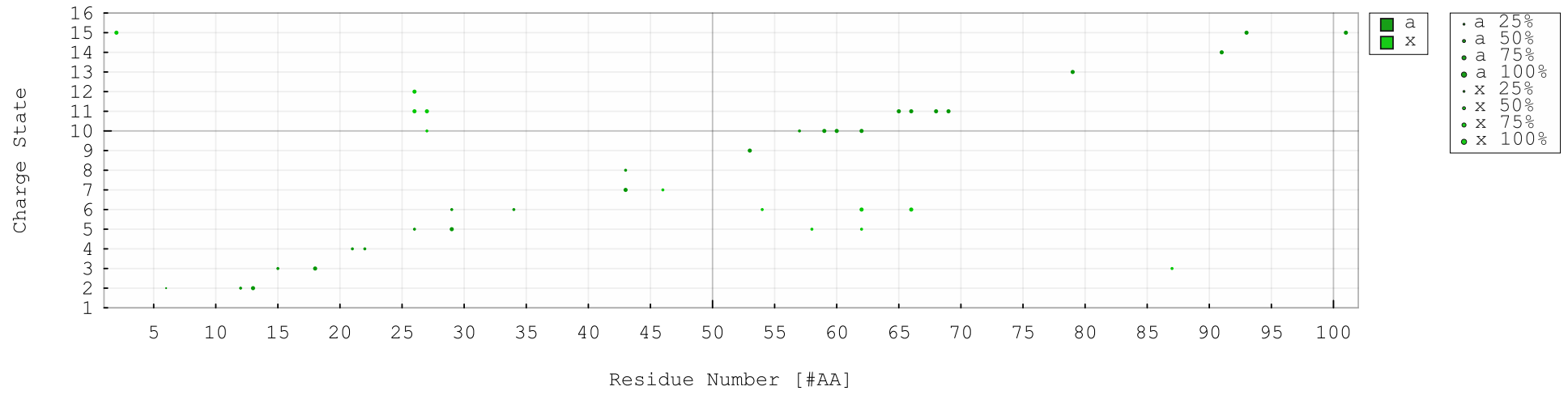

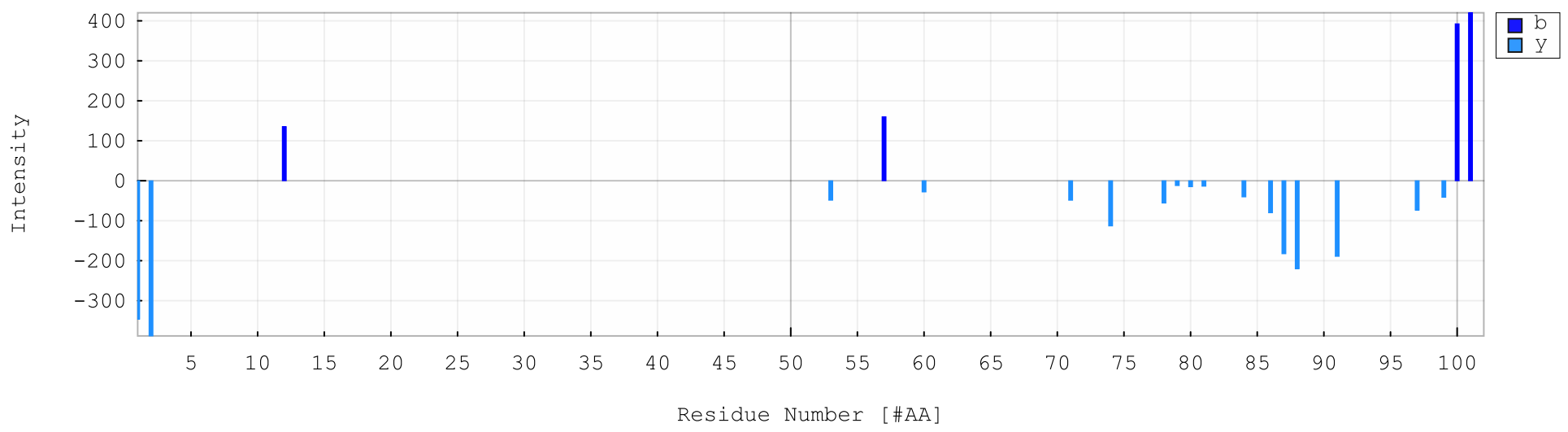

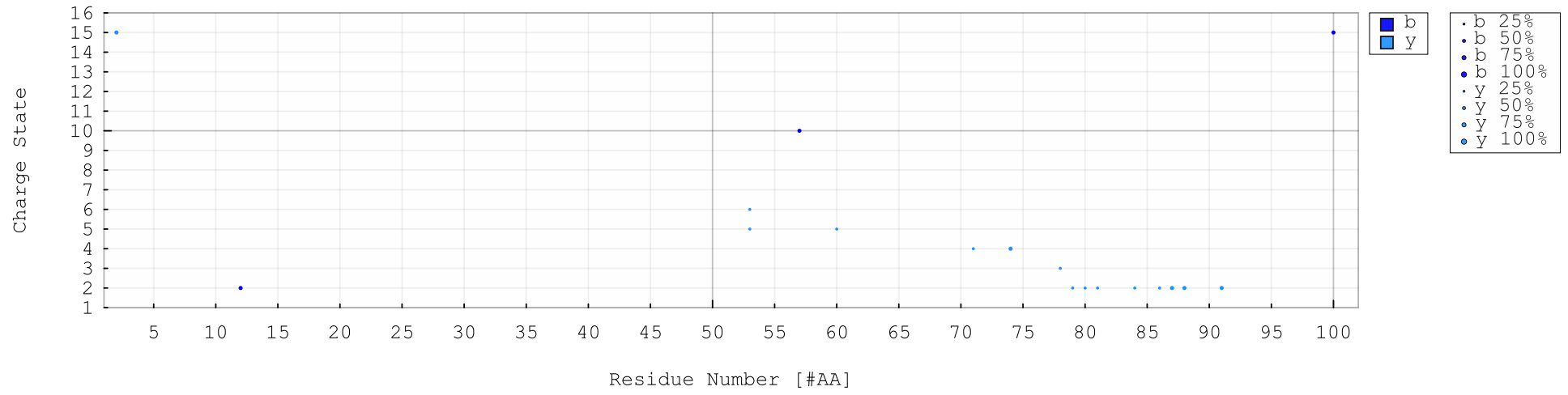

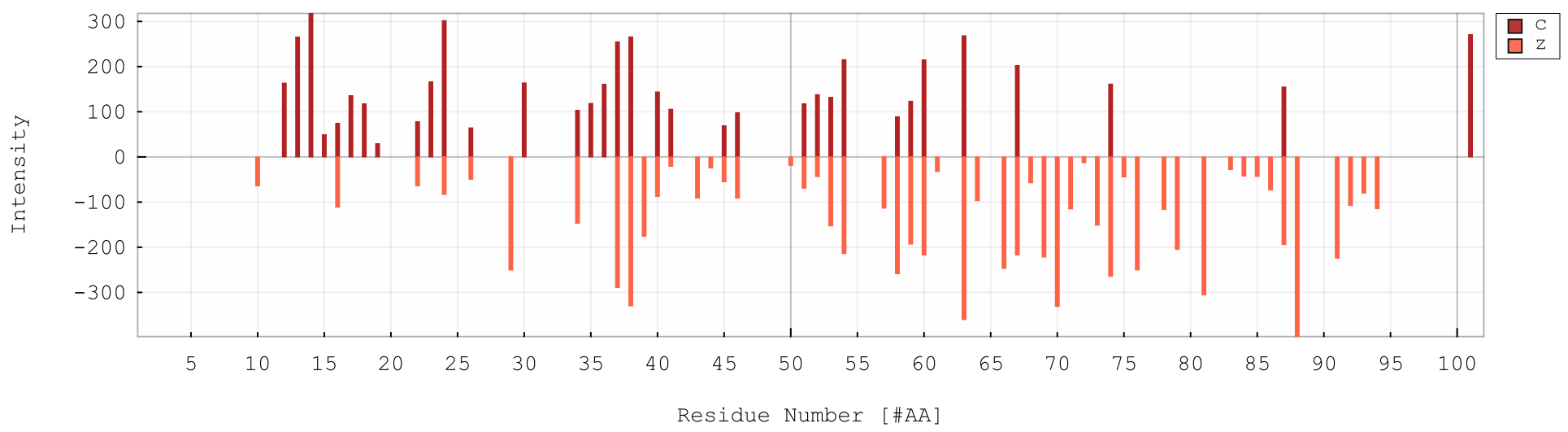

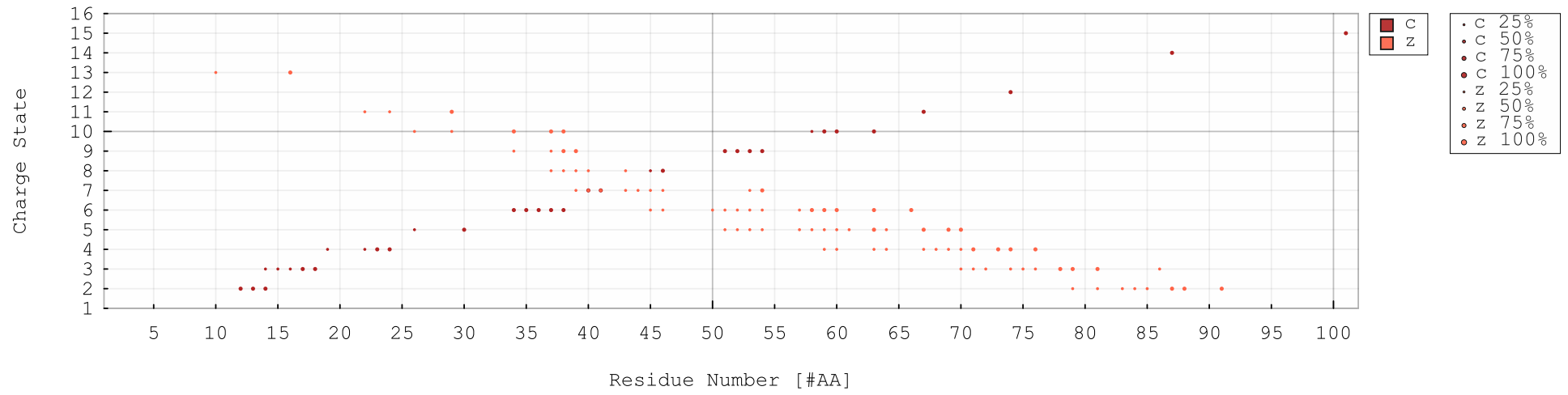

Proteoform:

Index	Protein	SC [%]	SVP [%]	IC [%]	MS Score [a.u.]	Var. Mods	Mono Mass [Da]	Chemical Formula
9	H4Kac_me1-3	74.3	7.8	35.2	26.1	Ace: K8, Met: K16, K20	11299.3831	C498H843N163O135S

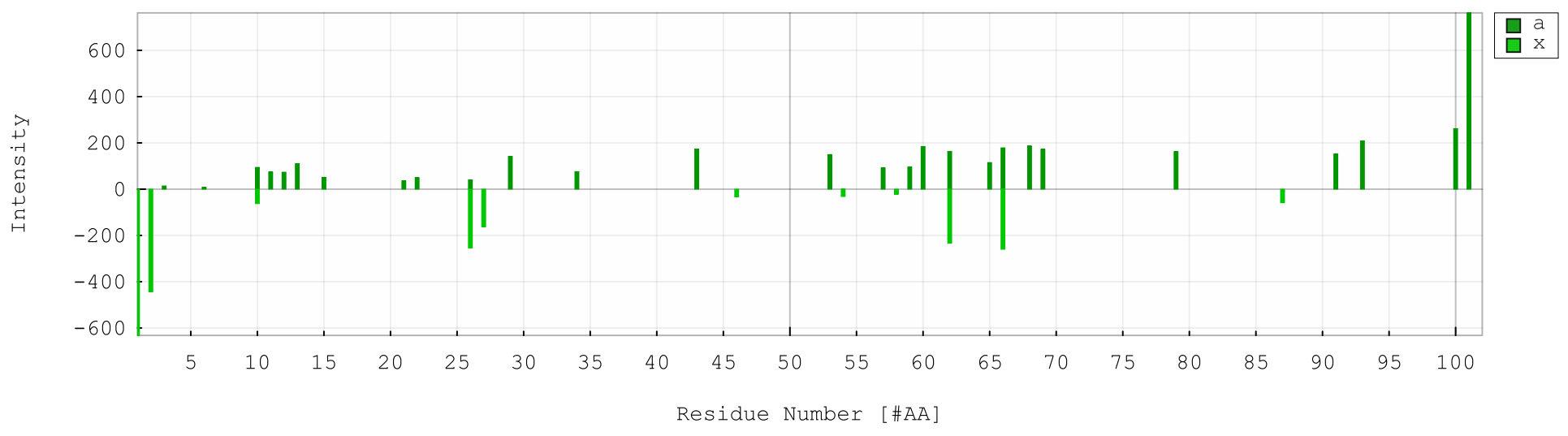

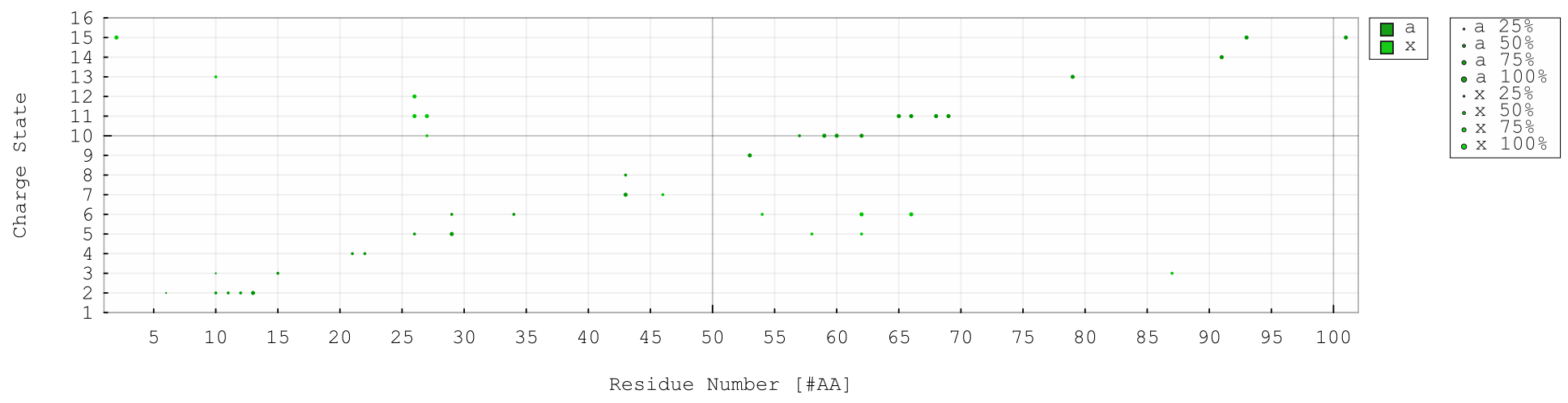

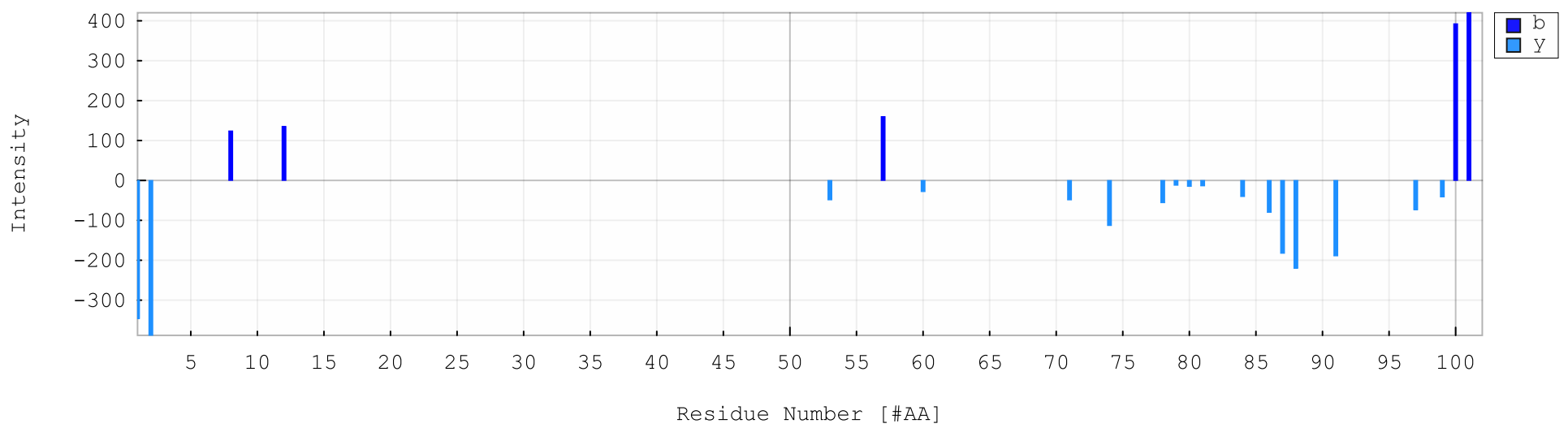

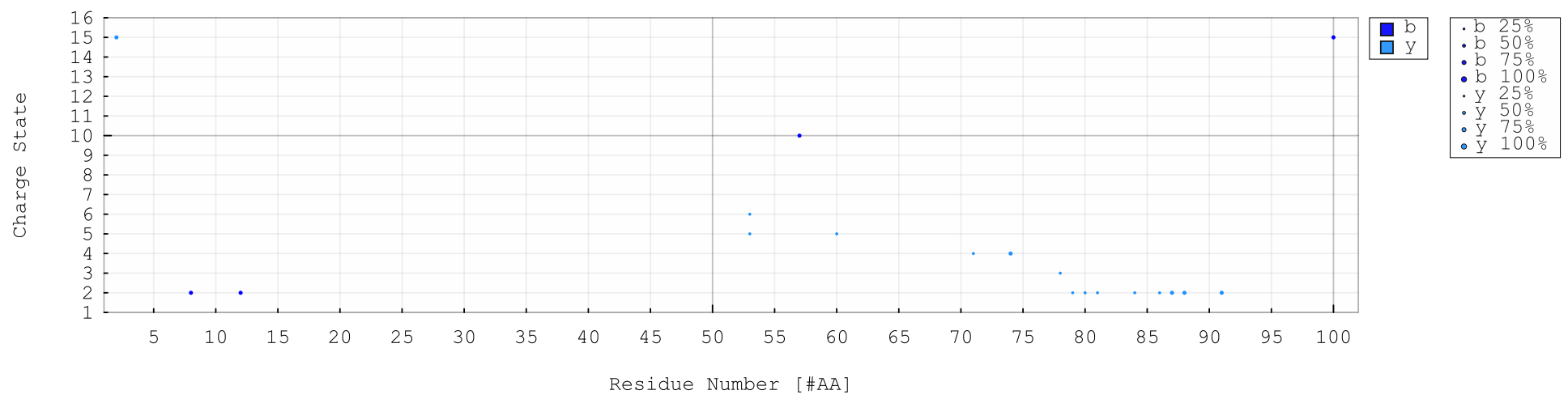

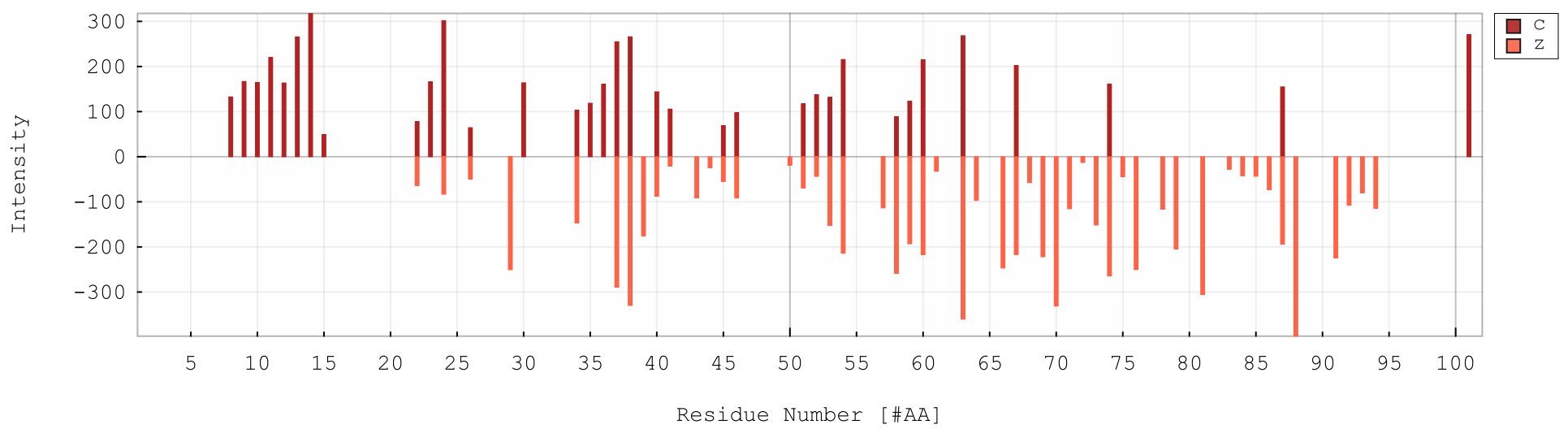

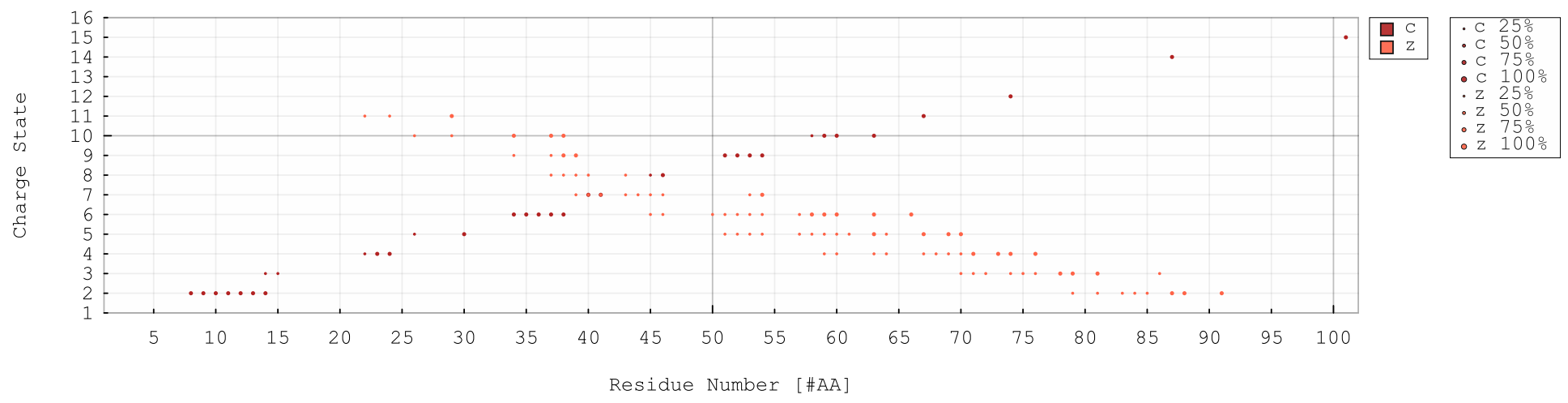

Proteoform:

Index	Protein	SC [%]	SVP [%]	IC [%]	MS Score [a.u.]	Var. Mods	Mono Mass [Da]	Chemical Formula
10	H4Kac_me1-3	74.3	7.8	35.2	26.1	Ace: K8, Dim: K16	11299.3831	C498H843N163O135S

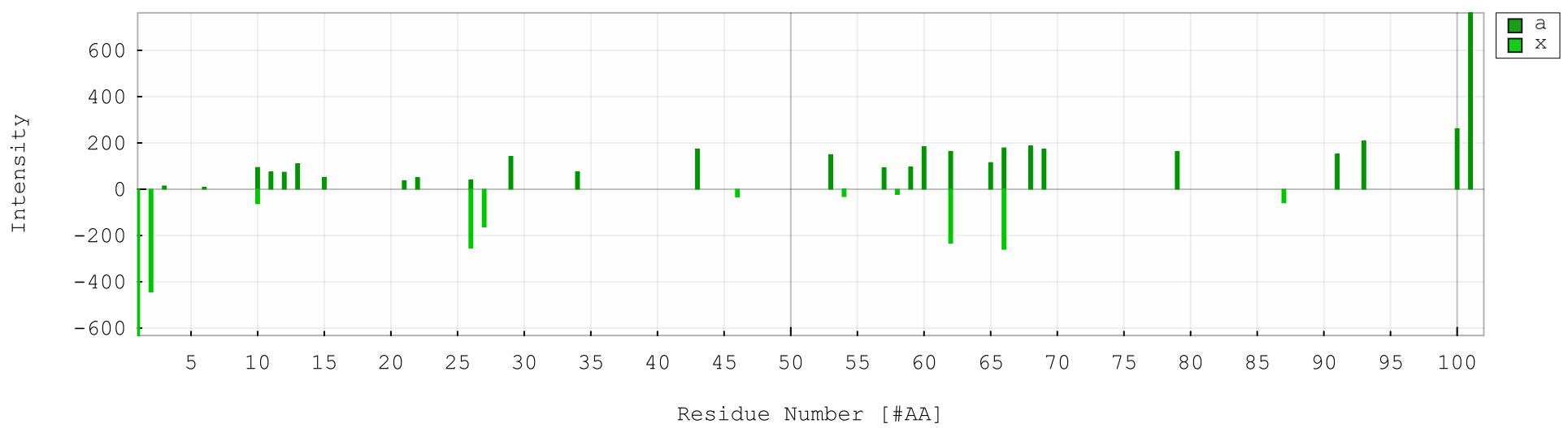

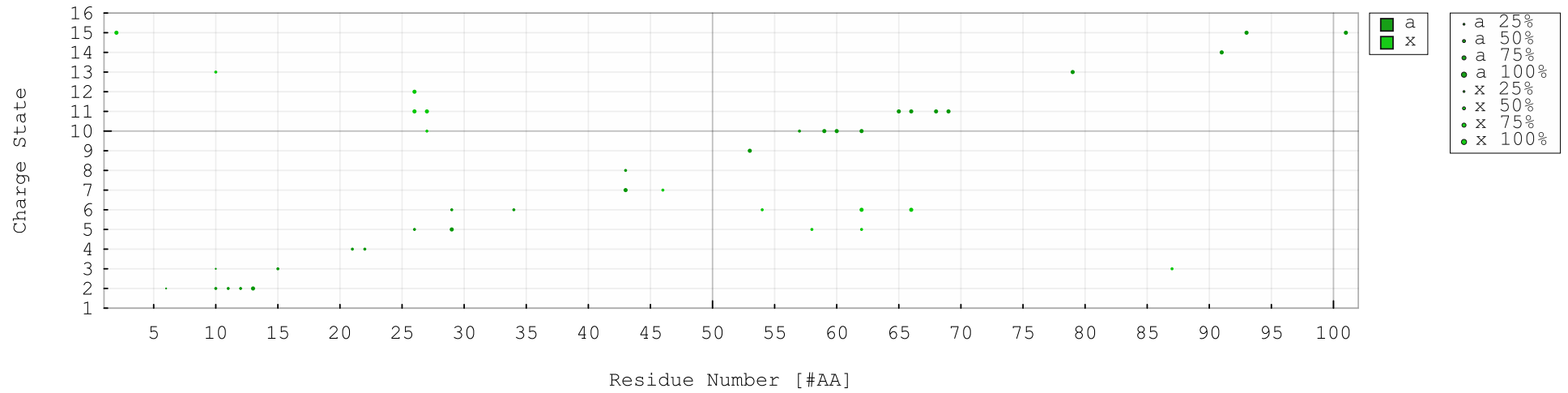

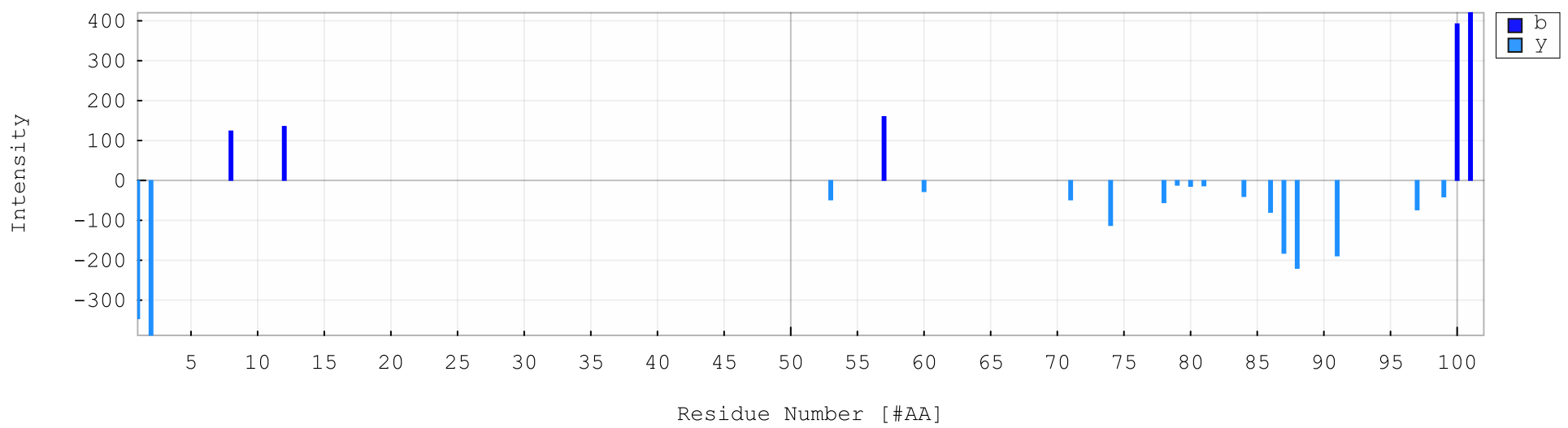

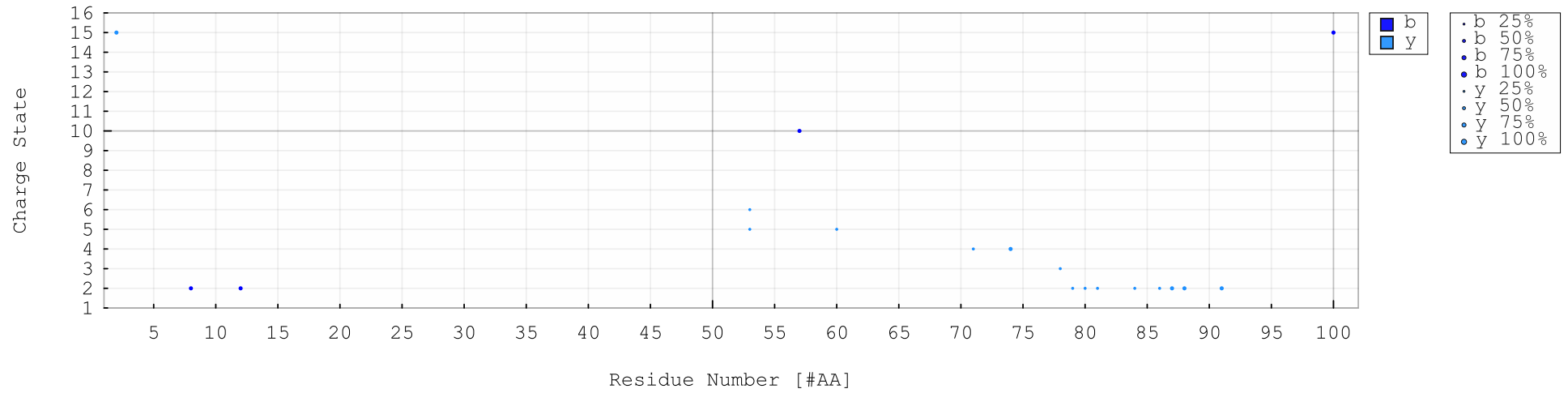

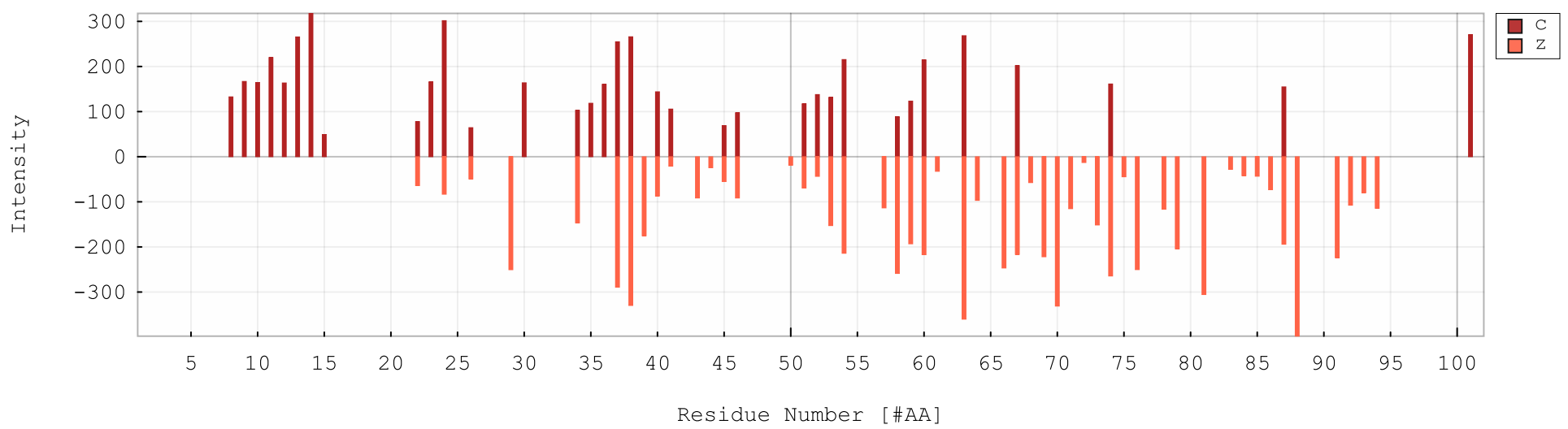

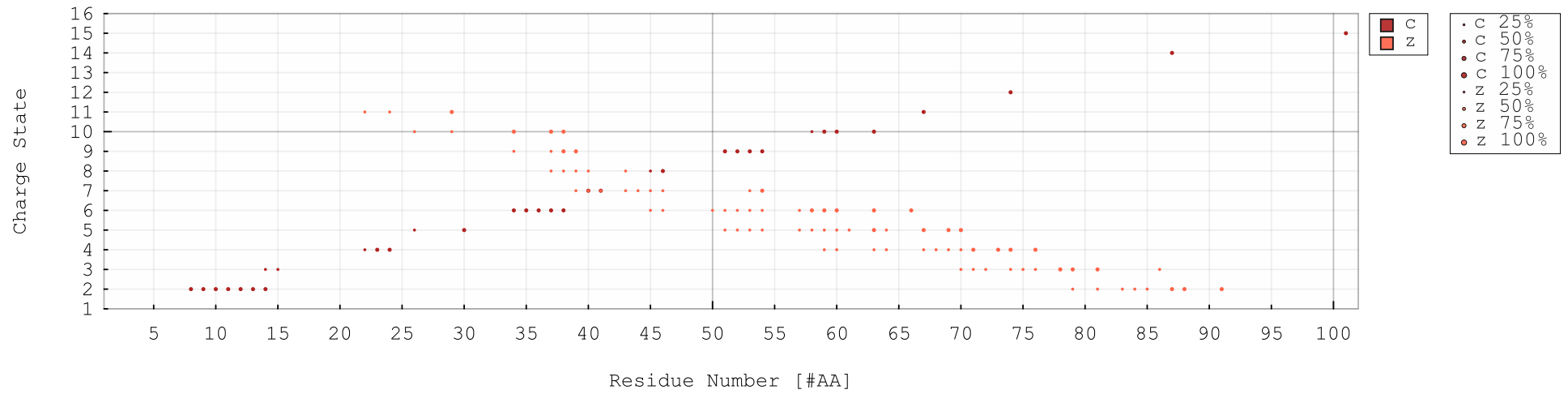

Supplemental Data S1

Endogenous H4

**Report**	3 of 6
**Experiment**	MS2
**Histone/sample**	H4
**Enzyme**	Not applicable
**Reaction/source**	Endogenous sample
**Precursor**	m/z 707
**Charge state**	16+
**Fragmentation / workflow**	RCID
**Original file**	MS2_mz-707_16+_RCID.pdf

Project Information

Project Directory: C:/Users/LAB-ADMIN
Processing date: Tue Apr 28 14:59:36 2026

Sample Info & Protocols

Name: Top-Down Protein Sequencing CID

Dataset

Imported spectrum file name:	250430 Liver-Histone-25 mz707z16 MS2 shCID_0001-1096_calibrated.txt
Dataset name:	Information is only available from imported JPEAKS files
Date acquired:	Information is only available from imported JPEAKS files
Date imported:	Information is only available from imported JPEAKS files
Instrument Name:	Information is only available from imported JPEAKS files
Instrument Serial Number:	Information is only available from imported JPEAKS files

Workflow Result Info

Analysis: 250430 Liver-Histone-25 mz707z16 MS2 shCID_0001-1096_calibrated
Location: C:/Users/LAB-ADMIN
Method: -

Method Information

Name: Unnamed

Spectral Parameters

Mass Error [ppm]: 6
Intensity Deviation [au]: 0.15
Min/Max Charges: 1-16
Experiment type: CID

a ions	b ions	c ions	x ions	y ions	z ions	satellite	internal	precursor
a	b			y				M

Noise threshold calculation: Simple moving average noise threshold (SMA)
Window size [datapoints]: 600
Noise curve offset: 1.233225
SVP parameters: terminal gap: 2, internal gap: 1

Proteoforms

(top 10)

Index	Parent Sequence	Chain	Var. Mods	Mono Mass [Da]	Chemical Composition
1	H4Kac_me1-3	H4Kac_me1-3_Nterm[Acetyl]_K20[Dim]	Dim: K20, Ace: N-term	11299.3831	C498H843N163O135S
2	H4Kac_me1-3	H4Kac_me1-3_Nterm[Acetyl]_K16[Dim]	Dim: K16, Ace: N-term	11299.3831	C498H843N163O135S
3	H4Kac_me1-3	H4Kac_me1-3_Nterm[Acetyl]_K16[Met]K20[Met]	Met: K16, K20, Ace: N-term	11299.3831	C498H843N163O135S
4	H4Kac_me1-3	H4Kac_me1-3_Nterm[Acetyl]_K12[Dim]	Dim: K12, Ace: N-term	11299.3831	C498H843N163O135S
5	H4Kac_me1-3	H4Kac_me1-3_Nterm[Acetyl]_K12[Met]K20[Met]	Met: K12, K20, Ace: N-term	11299.3831	C498H843N163O135S
6	H4Kac_me1-3	H4Kac_me1-3_Nterm[Acetyl]_K12[Met]K16[Met]	Met: K12, K16, Ace: N-term	11299.3831	C498H843N163O135S
7	H4Kac_me1-3	H4Kac_me1-3K5[Ace]K20[Dim]	Ace: K5, Dim: K20	11299.3831	C498H843N163O135S
8	H4Kac_me1-3	H4Kac_me1-3K5[Ace]K16[Met]K20[Met]	Ace: K5, Met: K16, K20	11299.3831	C498H843N163O135S
9	H4Kac_me1-3	H4Kac_me1-3K5[Ace]K16[Dim]	Ace: K5, Dim: K16	11299.3831	C498H843N163O135S
10	H4Kac_me1-3	H4Kac_me1-3_Nterm[Acetyl]_K8[Met]K20[Met]	Met: K8, K20, Ace: N-term	11299.3831	C498H843N163O135S

Results

Proteoforms Table (top 10)

Index	Protein	SC [%]	SVP [%]	IC [%]	MS Score [a.u.]	Var. Mods	Mono Mass [Da]	Chemical Formula
1	H4Kac_me1-3	35.6	11.8	26.9	9.6	Dim: K20, Ace: N-term	11299.3831	C498H843N163O135S
2	H4Kac_me1-3	34.7	11.8	26.9	9.3	Dim: K16, Ace: N-term	11299.3831	C498H843N163O135S
3	H4Kac_me1-3	34.7	11.8	26.9	9.3	Met: K16, K20, Ace: N-term	11299.3831	C498H843N163O135S
4	H4Kac_me1-3	33.7	10.8	26.8	9.0	Dim: K12, Ace: N-term	11299.3831	C498H843N163O135S
5	H4Kac_me1-3	33.7	10.8	26.8	9.0	Met: K12, K20, Ace: N-term	11299.3831	C498H843N163O135S
6	H4Kac_me1-3	33.7	10.8	26.8	9.0	Met: K12, K16, Ace: N-term	11299.3831	C498H843N163O135S
7	H4Kac_me1-3	33.7	0.0	26.7	9.0	Ace: K5, Dim: K20	11299.3831	C498H843N163O135S
8	H4Kac_me1-3	32.7	0.0	26.6	8.7	Ace: K5, Met: K16, K20	11299.3831	C498H843N163O135S
9	H4Kac_me1-3	32.7	0.0	26.6	8.7	Ace: K5, Dim: K16	11299.3831	C498H843N163O135S
10	H4Kac_me1-3	31.7	8.8	26.6	8.4	Met: K8, K20, Ace: N-term	11299.3831	C498H843N163O135S

Sequence

Name: H4Kac_me1-3

```
                 10                  20                  30                  40                  50                  60
S G R G K G G K G L  G K G G A K R H R K  V L R D N I Q G I T  K P A I R R L A R R  G G V K R I S G L I  Y E E T R G V L K V
                 70                  80                  90                 100                 110                 120
F L E N V I R D A V  T Y T E H A K R K T  V T A M D V V Y A L  K R Q G R T L Y G F  G G
```

Modifications

Modification	Position	Type
Acetyl	N-term	variable
Acetyl	5	variable
Methyl	8	variable
Acetyl	8	variable
Dimethyl	12	variable
Methyl	12	variable
Acetyl	12	variable
Dimethyl	16	variable
Methyl	16	variable
Acetyl	16	variable
Trimethyl	16	variable
Dimethyl	20	variable
Methyl	20	variable

Modification	Position	Type
Acetyl	20	variable
Trimethyl	20	variable

Sequence Map

Proteoform:	Index	Protein	SC [%]	SVP [%]	IC [%]	MS Score [a.u.]	Var. Mods	Mono Mass [Da]	Chemical Formula
	1	H4Kac_me1-3	35.6	11.8	26.9	9.6	Dim: K20, Ace: N-term	11299.3831	C498H843N163O135S

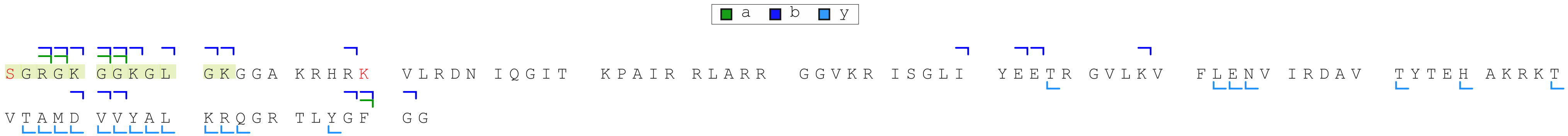

Proteoform:	Index	Protein	SC [%]	SVP [%]	IC [%]	MS Score [a.u.]	Var. Mods	Mono Mass [Da]	Chemical Formula
	2	H4Kac_me1-3	34.7	11.8	26.9	9.3	Dim: K16, Ace: N-term	11299.3831	C498H843N163O135S

a b y

SGRGK GGKGL GKGGA KRHRK VLRDN IQGIT KPAIR RLARR GGVKR ISGLI YEETR GVLKV FLENV IRDAV TYTEH AKRKT
VTAMD VVYAL KRQGR TLYGF GG

Proteoform:

Index	Protein	SC [%]	SVP [%]	IC [%]	MS Score [a.u.]	Var. Mods	Mono Mass [Da]	Chemical Formula
3	H4Kac_me1-3	34.7	11.8	26.9	9.3	Met: K16, K20, Ace: N-term	11299.3831	C498H843N163O135S

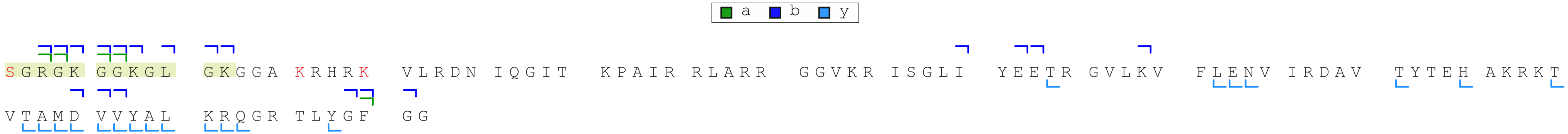

Proteoform:

Index	Protein	SC [%]	SVP [%]	IC [%]	MS Score [a.u.]	Var. Mods	Mono Mass [Da]	Chemical Formula
4	H4Kac_me1-3	33.7	10.8	26.8	9.0	Dim: K12, Ace: N-term	11299.3831	C498H843N163O135S

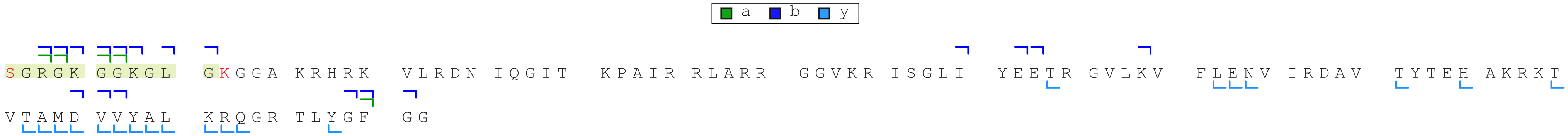

Proteoform:

Index	Protein	SC [%]	SVP [%]	IC [%]	MS Score [a.u.]	Var. Mods	Mono Mass [Da]	Chemical Formula
5	H4Kac_me1-3	33.7	10.8	26.8	9.0	Met: K12, K20, Ace: N-term	11299.3831	C498H843N163O135S

a b y

SGRGK GGKGL GKGGA KRHRK VLRDN IQGIT KPAIR RLARR GGVKR ISGLI YEETR GVLKV FLENV IRDAV TYTEH AKRKT
VTAMD VVYAL KRQGR TLYGF GG

Proteoform:	Index	Protein	SC [%]	SVP [%]	IC [%]	MS Score [a.u.]	Var. Mods	Mono Mass [Da]	Chemical Formula
	6	H4Kac_me1-3	33.7	10.8	26.8	9.0	Met: K12, K16, Ace: N-term	11299.3831	C498H843N163O135S

a b y

SGRGK GGKGL GKGGA KRHRK VLRDN IQGIT KPAIR RLARR GGVKR ISGLI YEETR GVLKV FLENV IRDAV TYTEH AKRKT
VTAMD VVYAL KRQGR TLYGF GG

Proteoform:	Index	Protein	SC [%]	SVP [%]	IC [%]	MS Score [a.u.]	Var. Mods	Mono Mass [Da]	Chemical Formula
	7	H4Kac_me1-3	33.7	0.0	26.7	9.0	Ace: K5, Dim: K20	11299.3831	C498H843N163O135S

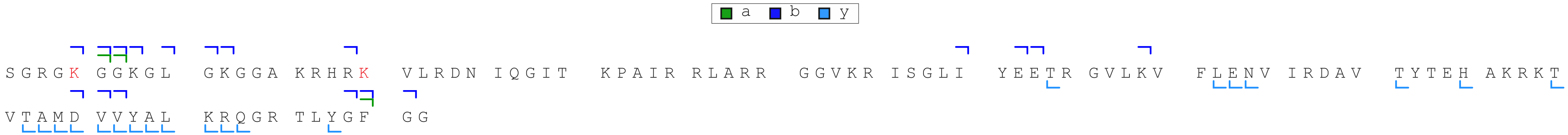

Proteoform:

Index	Protein	SC [%]	SVP [%]	IC [%]	MS Score [a.u.]	Var. Mods	Mono Mass [Da]	Chemical Formula
8	H4Kac_me1-3	32.7	0.0	26.6	8.7	Ace: K5, Met: K16, K20	11299.3831	C498H843N163O135S

a b y

SGRGK GGKGL GKGGA KRHRK VLRDN IQGIT KPAIR RLARR GGVKR ISGLI YEETR GVLKV FLENV IRDAV TYTEH AKRKT
VTAMD VVYAL KRQGR TLYGF GG

Proteoform:

Index	Protein	SC [%]	SVP [%]	IC [%]	MS Score [a.u.]	Var. Mods	Mono Mass [Da]	Chemical Formula
9	H4Kac_me1-3	32.7	0.0	26.6	8.7	Ace: K5, Dim: K16	11299.3831	C498H843N163O135S

a b y

S G R G K G G K G L G K G G A K R H R K V L R D N I Q G I T K P A I R R L A R R G G V K R I S G L I Y E E T R G V L K V F L E N V I R D A V T Y T E H A K R K T
V T A M D V V Y A L K R Q G R T L Y G F G G

Proteoform:

Index	Protein	SC [%]	SVP [%]	IC [%]	MS Score [a.u.]	Var. Mods	Mono Mass [Da]	Chemical Formula
10	H4Kac_me1-3	31.7	8.8	26.6	8.4	Met: K8, K20, Ace: N-term	11299.3831	C498H843N163O135S

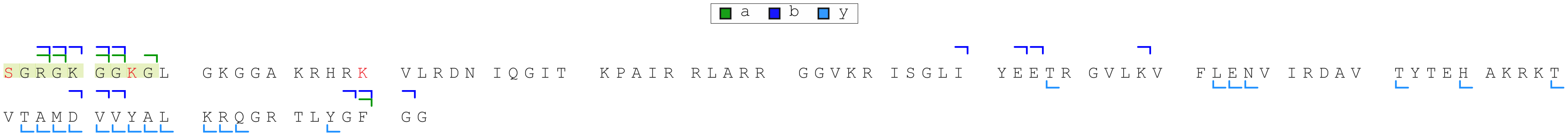

Error Plot

Proteoform:

Index	Protein	SC [%]	SVP [%]	IC [%]	MS Score [a.u.]	Var. Mods	Mono Mass [Da]	Chemical Formula
1	H4Kac_me1-3	35.6	11.8	26.9	9.6	Dim: K20, Ace: N-term	11299.3831	C498H843N163O135S

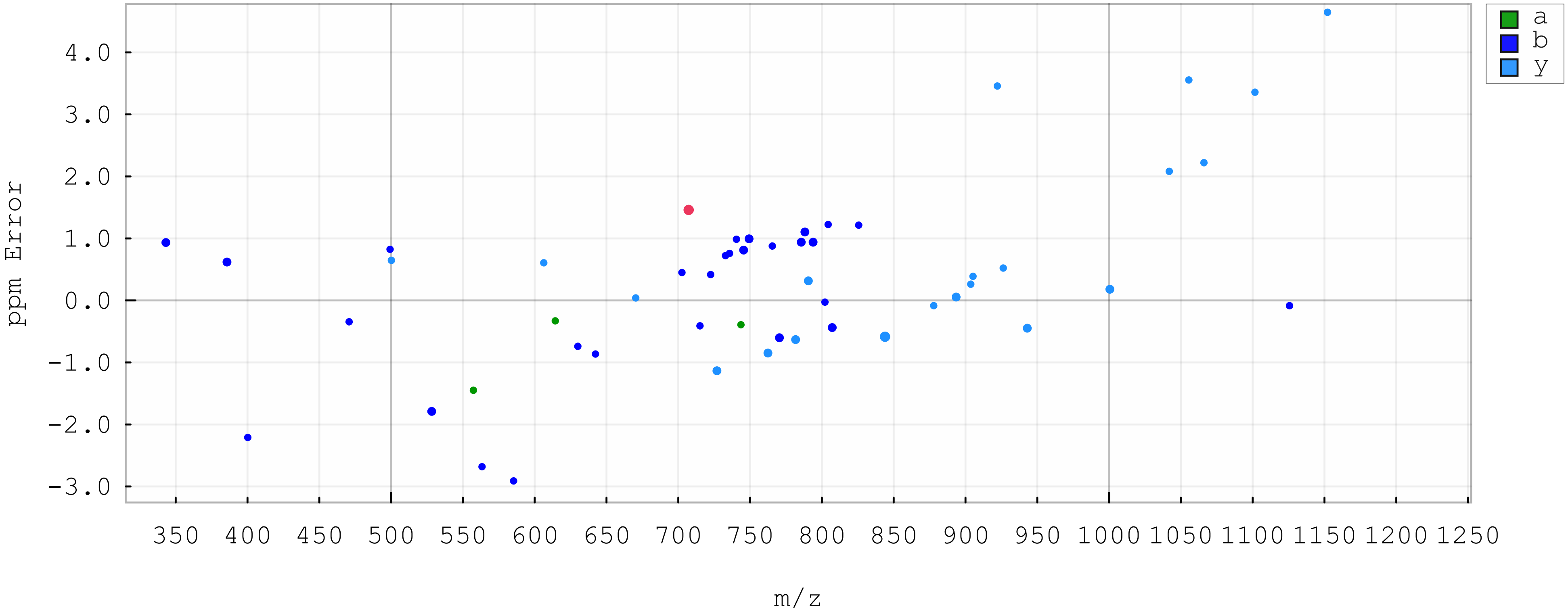

Proteoform:

Index	Protein	SC [%]	SVP [%]	IC [%]	MS Score [a.u.]	Var. Mods	Mono Mass [Da]	Chemical Formula
2	H4Kac_me1-3	34.7	11.8	26.9	9.3	Dim: K16, Ace: N-term	11299.3831	C498H843N163O135S

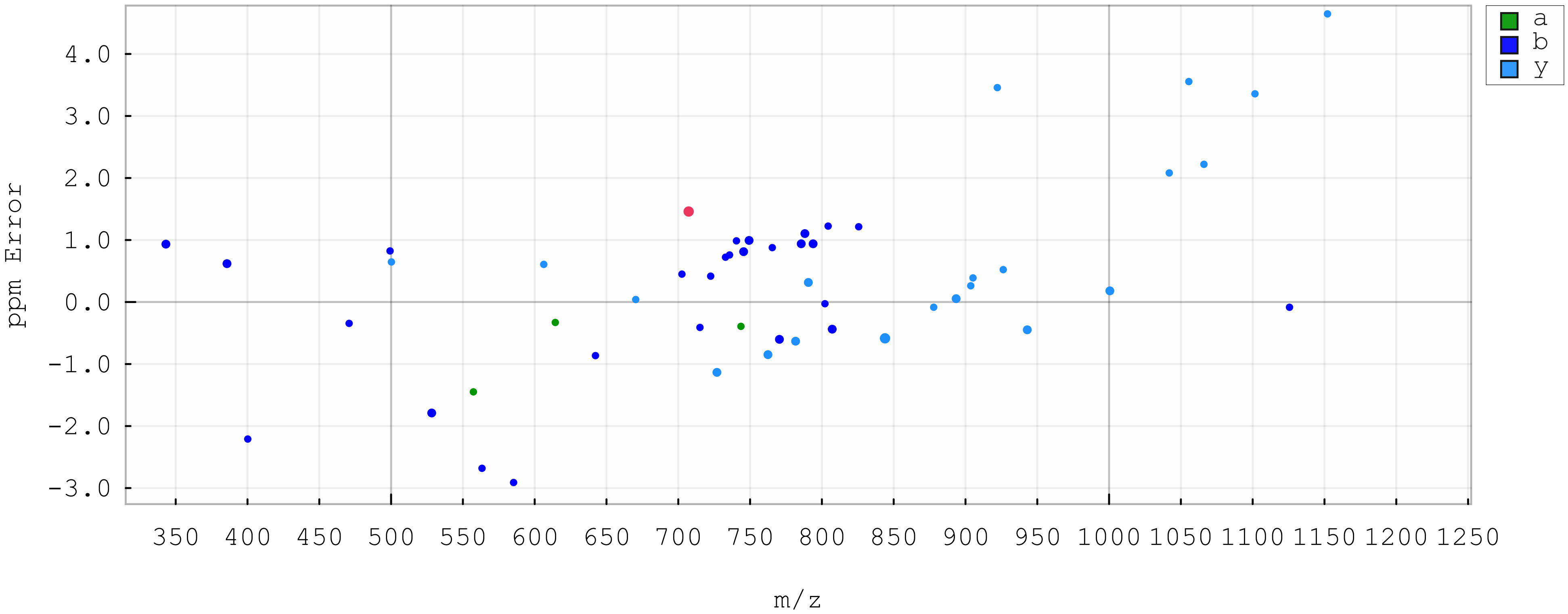

Proteoform:

Index	Protein	SC [%]	SVP [%]	IC [%]	MS Score [a.u.]	Var. Mods	Mono Mass [Da]	Chemical Formula
3	H4Kac_me1-3	34.7	11.8	26.9	9.3	Met: K16, K20, Ace: N-term	11299.3831	C498H843N163O135S

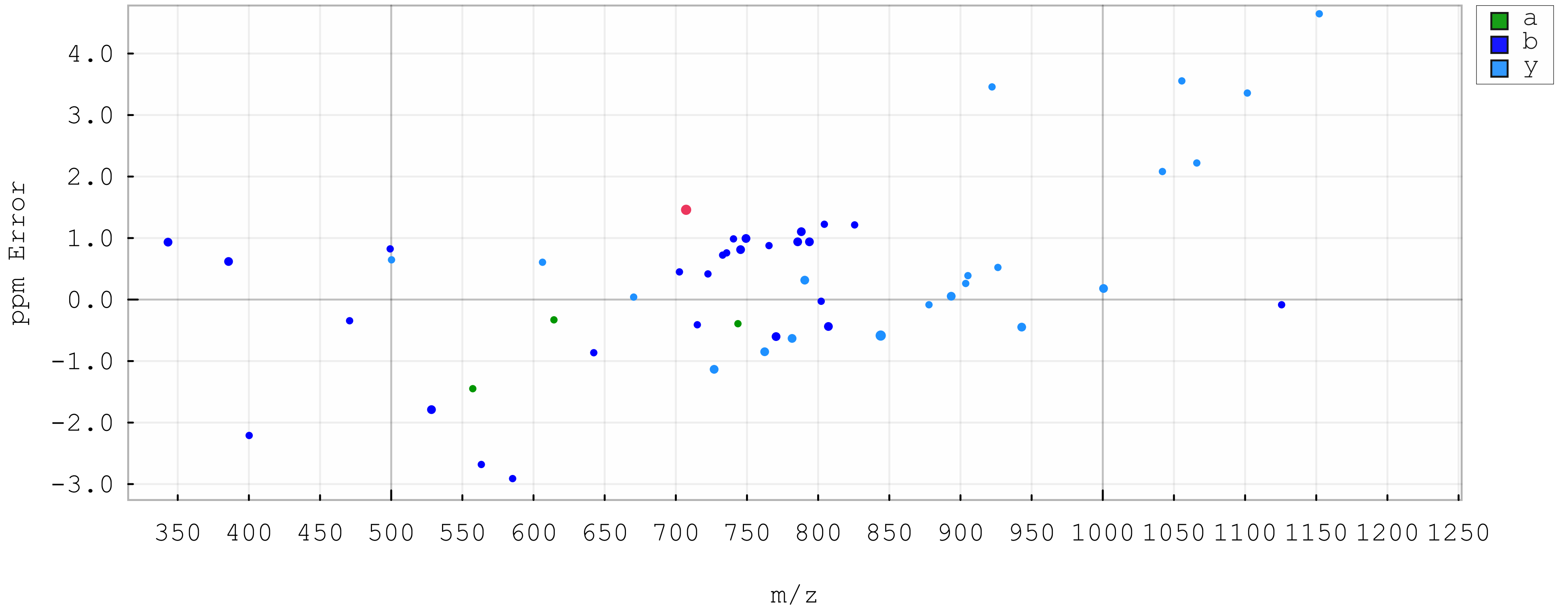

Proteoform:

Index	Protein	SC [%]	SVP [%]	IC [%]	MS Score [a.u.]	Var. Mods	Mono Mass [Da]	Chemical Formula
4	H4Kac_me1-3	33.7	10.8	26.8	9.0	Dim: K12, Ace: N-term	11299.3831	C498H843N163O135S

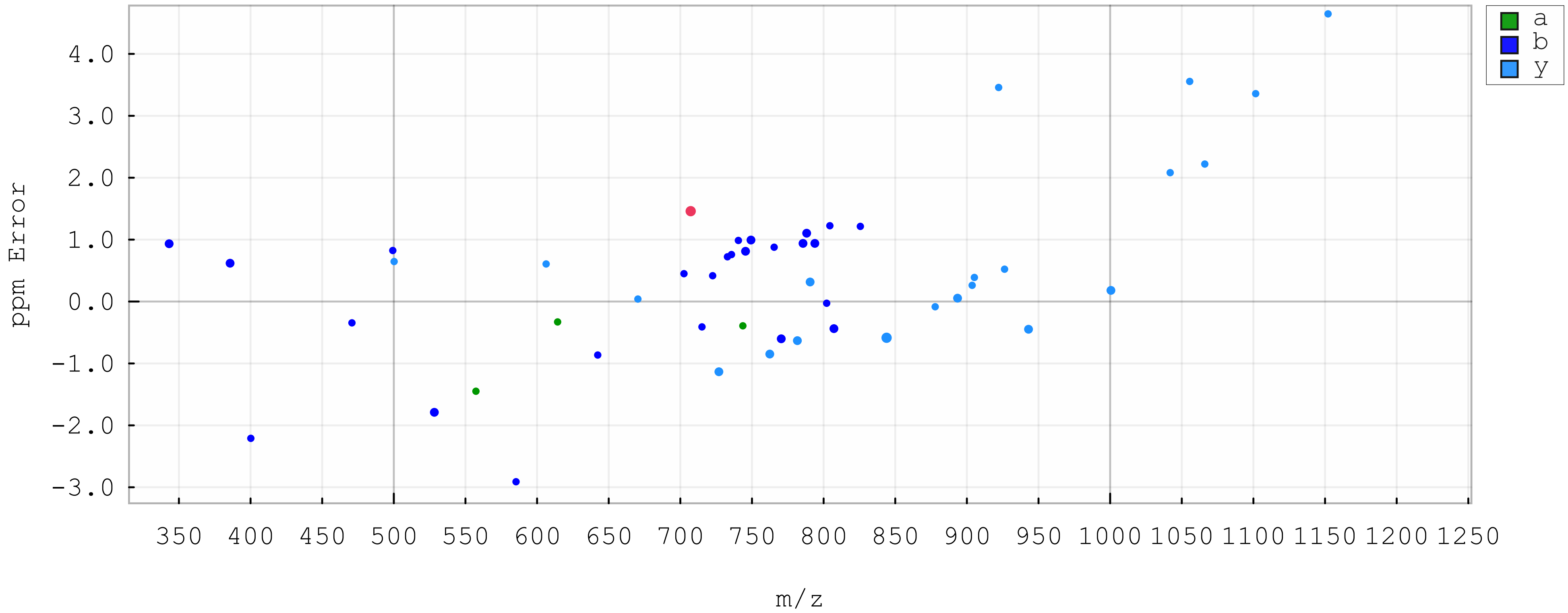

Proteoform:

Index	Protein	SC [%]	SVP [%]	IC [%]	MS Score [a.u.]	Var. Mods	Mono Mass [Da]	Chemical Formula
5	H4Kac_me1-3	33.7	10.8	26.8	9.0	Met: K12, K20, Ace: N-term	11299.3831	C498H843N163O135S

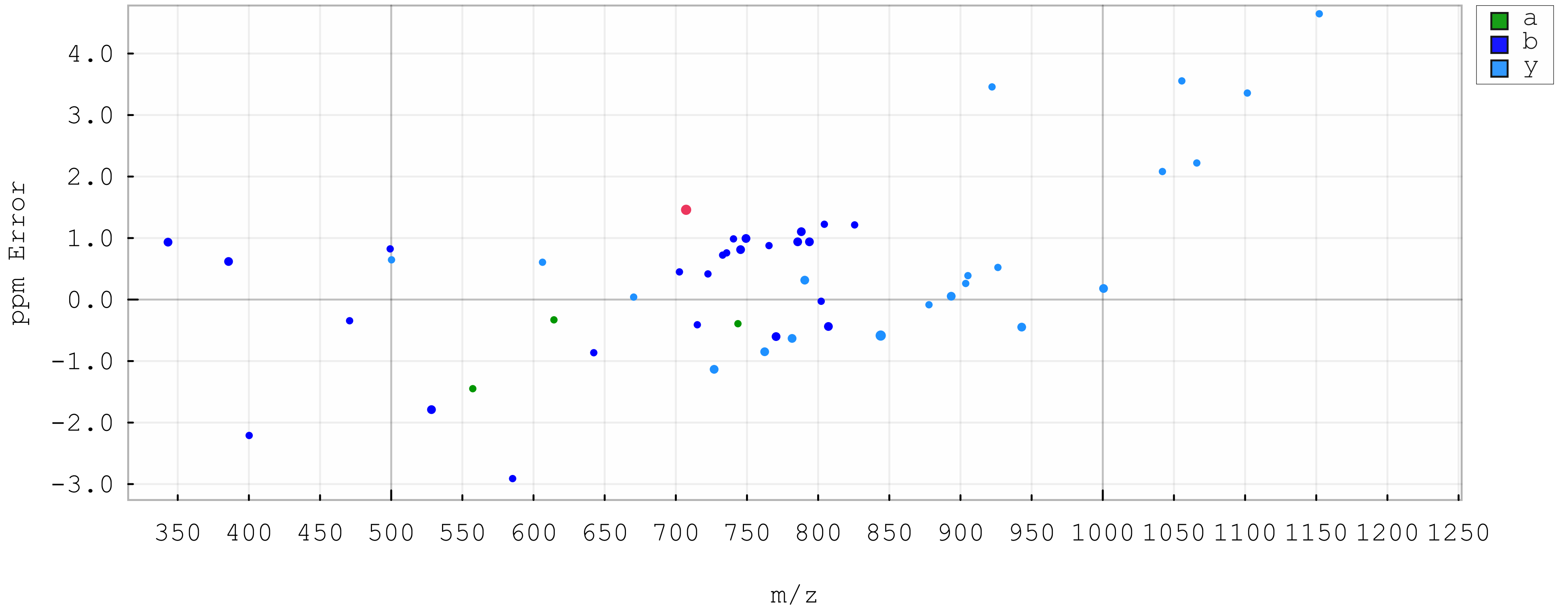

Proteoform:

Index	Protein	SC [%]	SVP [%]	IC [%]	MS Score [a.u.]	Var. Mods	Mono Mass [Da]	Chemical Formula
6	H4Kac_me1-3	33.7	10.8	26.8	9.0	Met: K12, K16, Ace: N-term	11299.3831	C498H843N163O135S

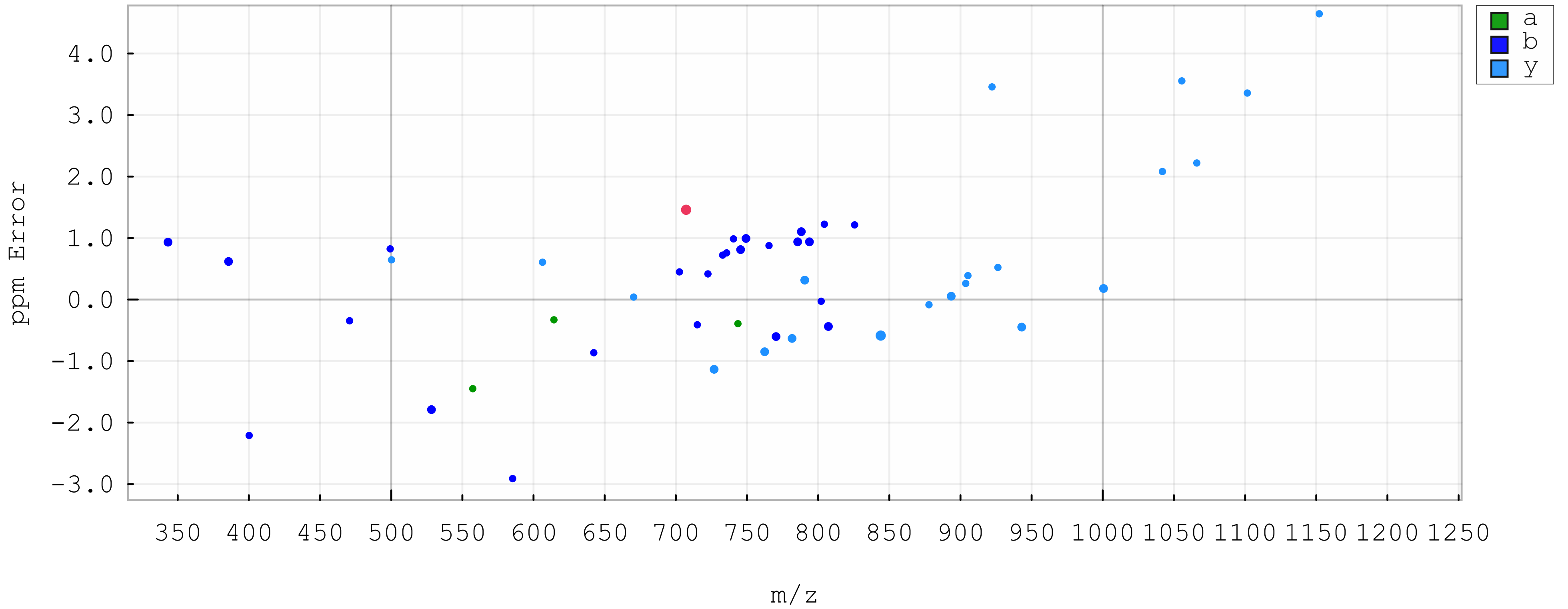

Proteoform:

Index	Protein	SC [%]	SVP [%]	IC [%]	MS Score [a.u.]	Var. Mods	Mono Mass [Da]	Chemical Formula
7	H4Kac_me1-3	33.7	0.0	26.7	9.0	Ace: K5, Dim: K20	11299.3831	C498H843N163O135S

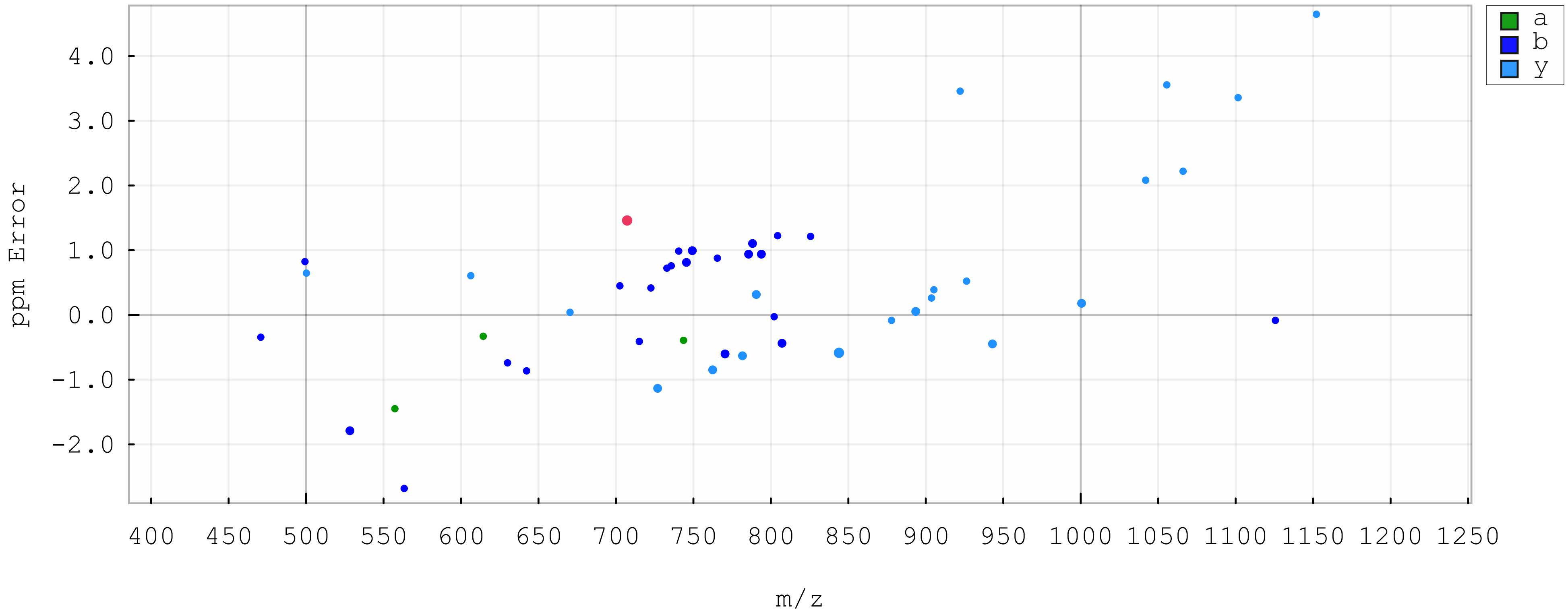

Proteoform:

Index	Protein	SC [%]	SVP [%]	IC [%]	MS Score [a.u.]	Var. Mods	Mono Mass [Da]	Chemical Formula
8	H4Kac_me1-3	32.7	0.0	26.6	8.7	Ace: K5, Met: K16, K20	11299.3831	C498H843N163O135S

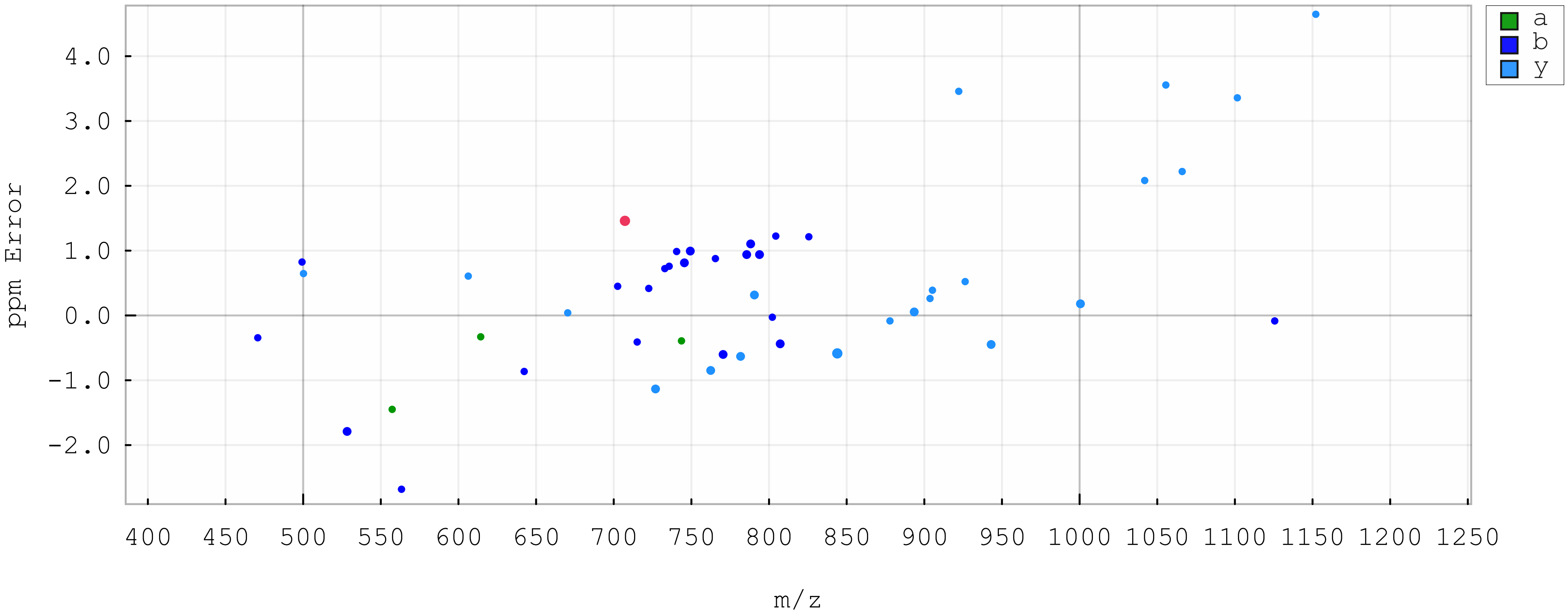

Proteoform:

Index	Protein	SC [%]	SVP [%]	IC [%]	MS Score [a.u.]	Var. Mods	Mono Mass [Da]	Chemical Formula
9	H4Kac_me1-3	32.7	0.0	26.6	8.7	Ace: K5, Dim: K16	11299.3831	C498H843N163O135S

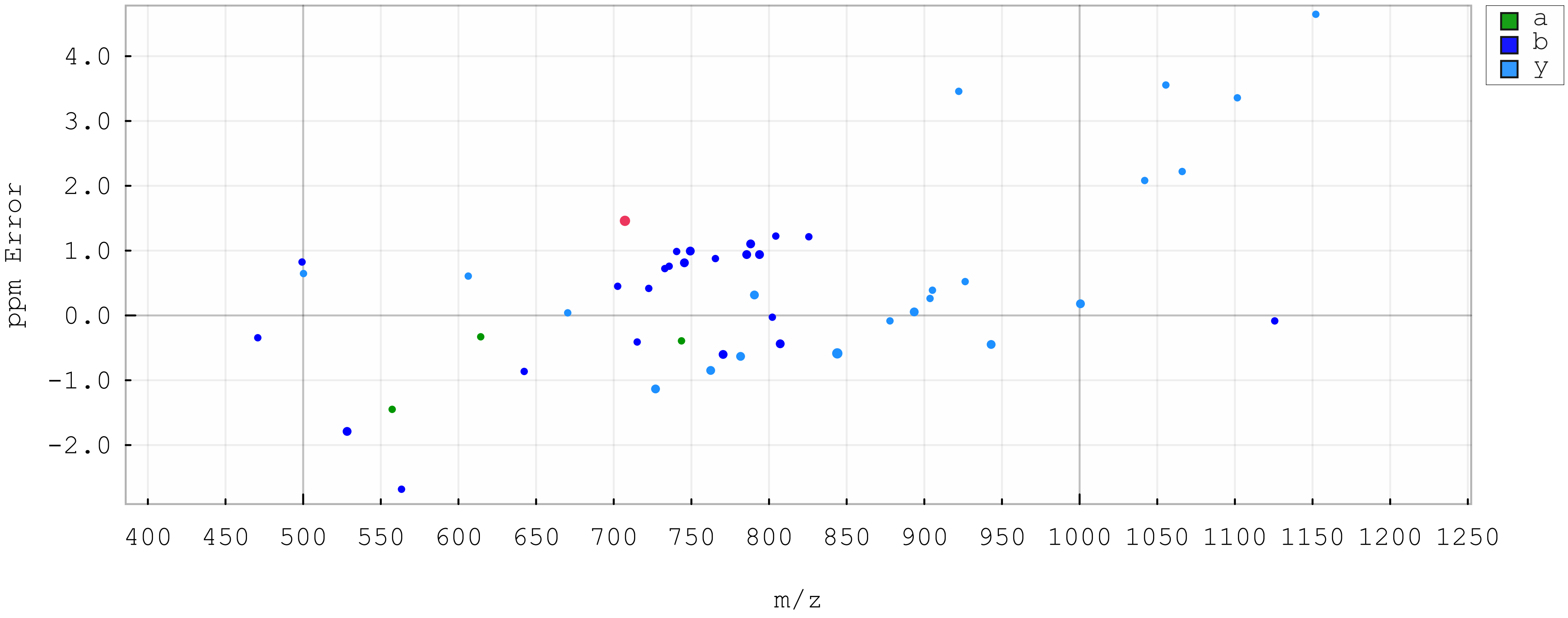

Proteoform:

Index	Protein	SC [%]	SVP [%]	IC [%]	MS Score [a.u.]	Var. Mods	Mono Mass [Da]	Chemical Formula
10	H4Kac_me1-3	31.7	8.8	26.6	8.4	Met: K8, K20, Ace: N-term	11299.3831	C498H843N163O135S

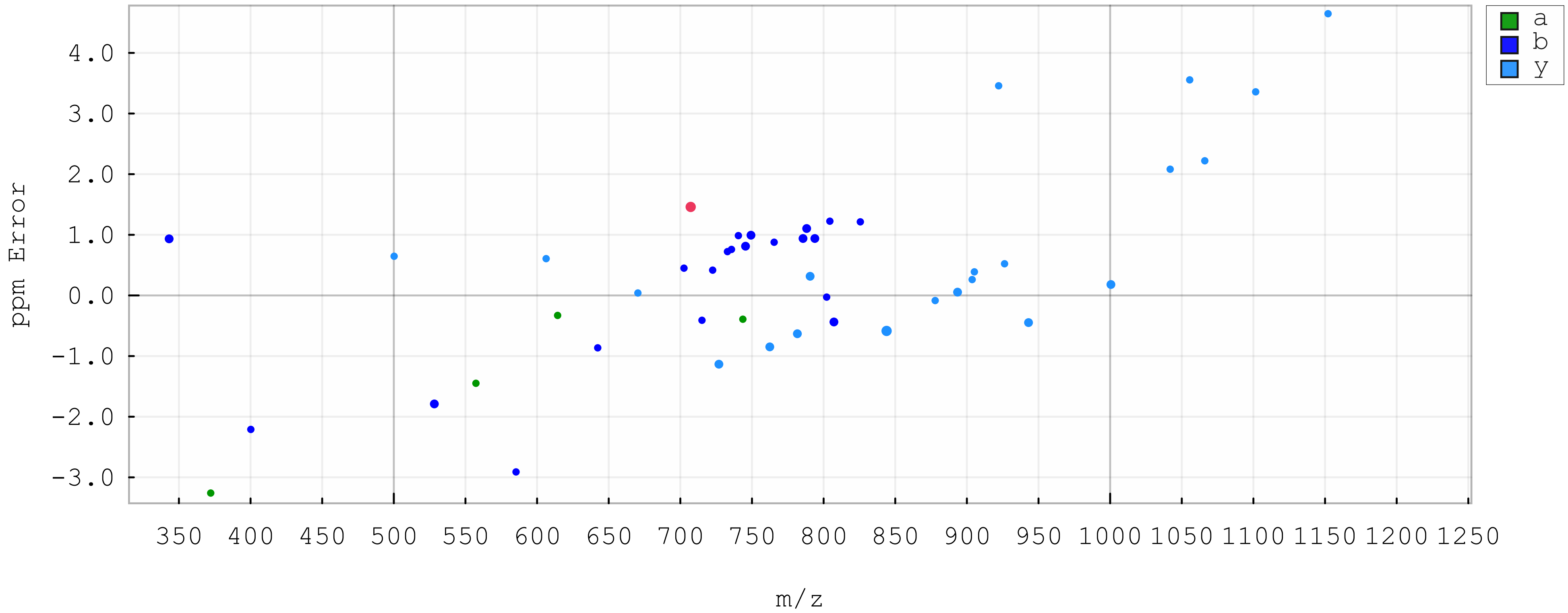

Primary Fragment Plots

Proteoform:

Index	Protein	SC [%]	SVP [%]	IC [%]	MS Score [a.u.]	Var. Mods	Mono Mass [Da]	Chemical Formula
1	H4Kac_me1-3	35.6	11.8	26.9	9.6	Dim: K20, Ace: N-term	11299.3831	$C_{498}H_{843}N_{163}O_{135}S$

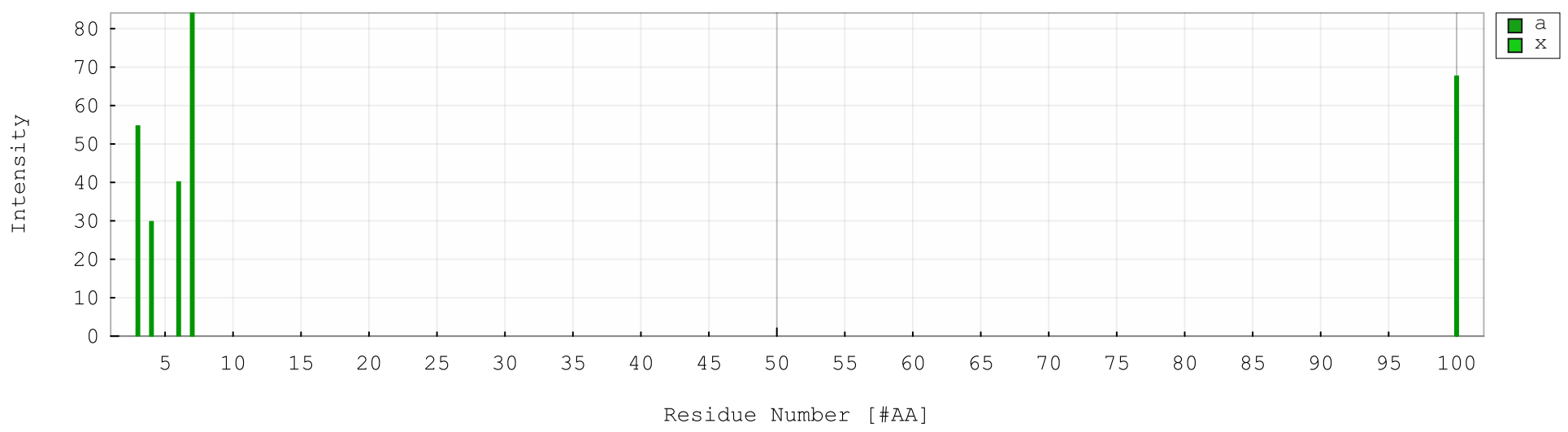

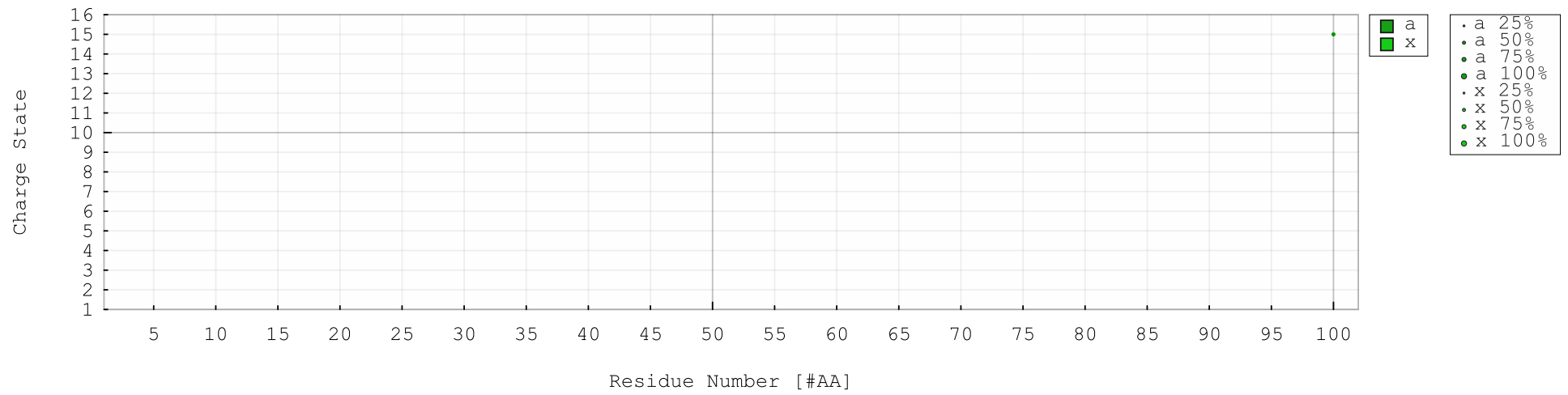

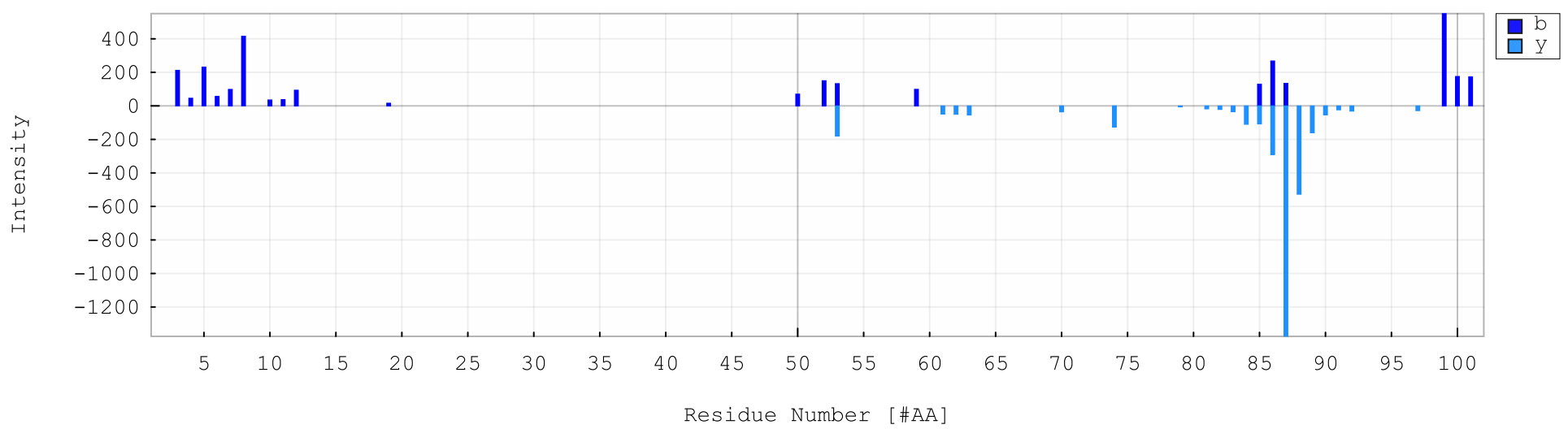

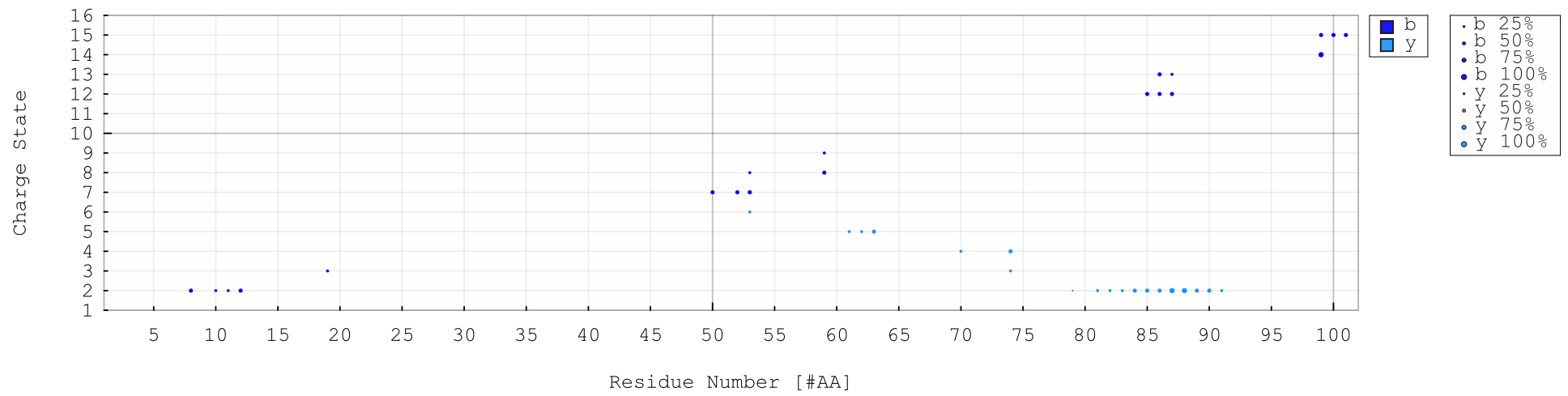

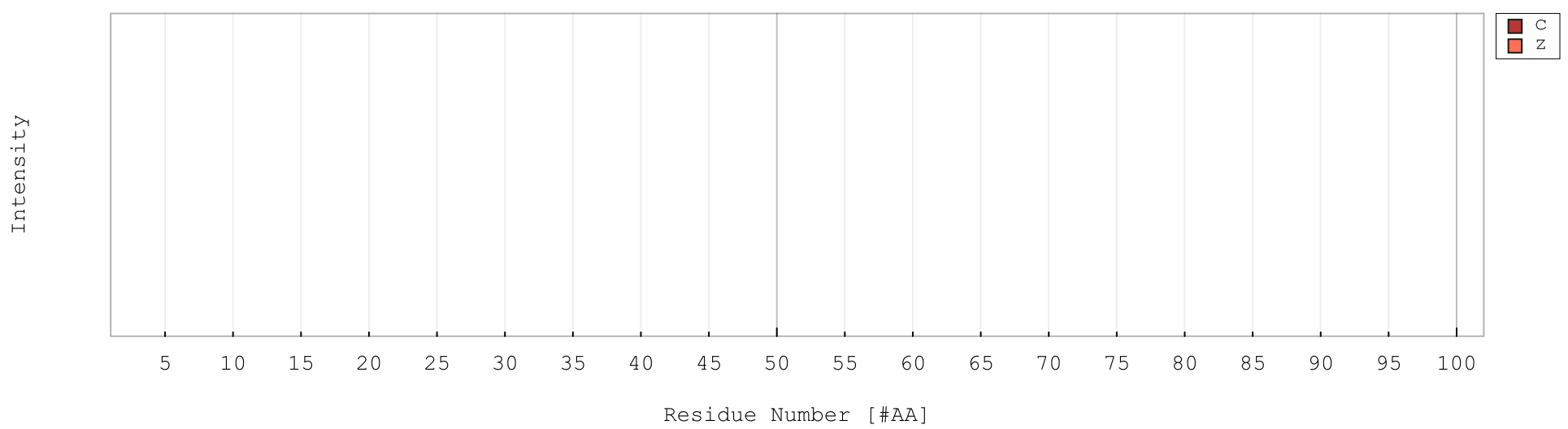

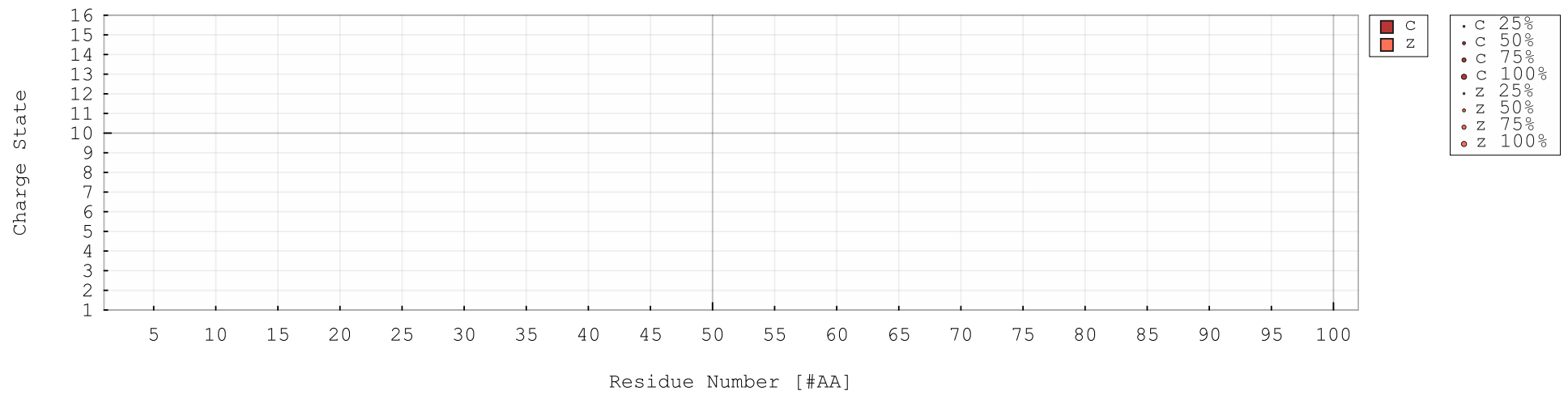

Proteoform:	Index	Protein	SC [%]	SVP [%]	IC [%]	MS Score [a.u.]	Var. Mods	Mono Mass [Da]	Chemical Formula
	2	H4Kac_me1-3	34.7	11.8	26.9	9.3	Dim: K16, Ace: N-term	11299.3831	C498H843N163O135S

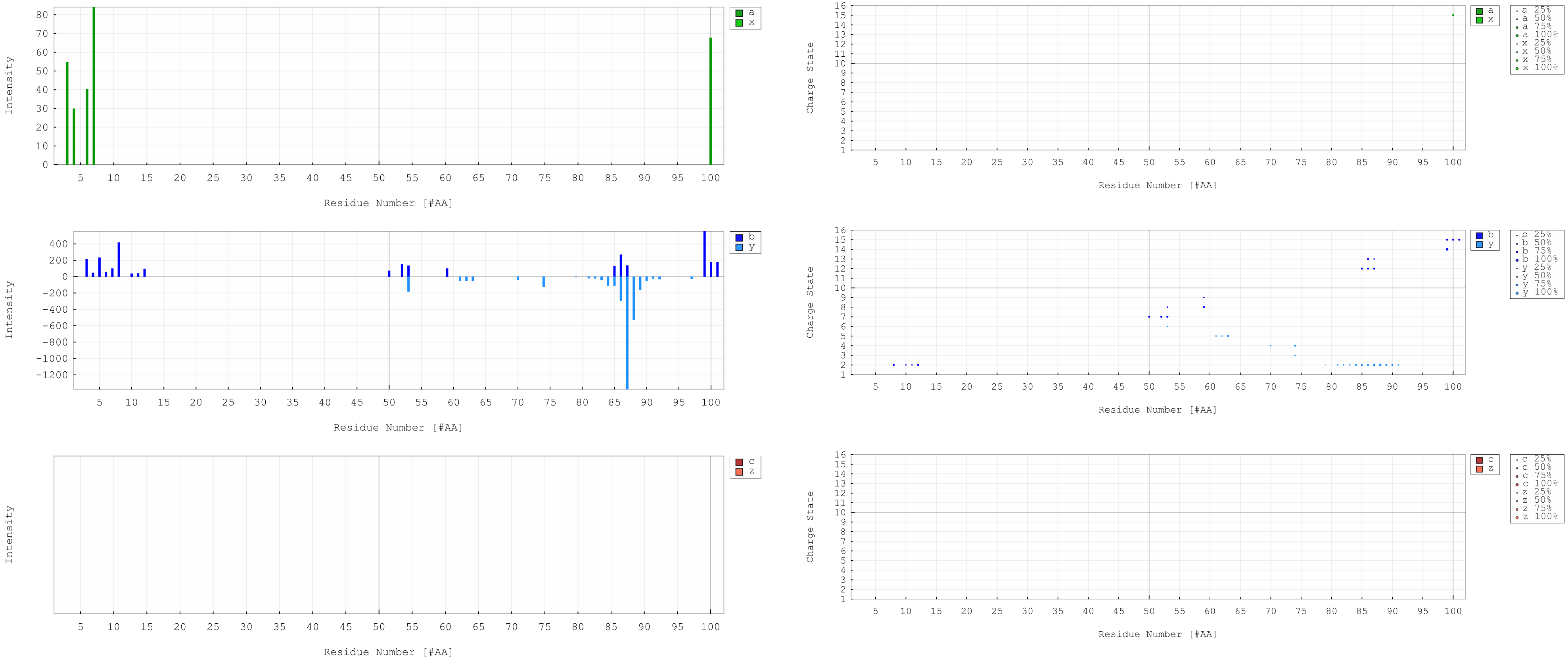

Proteoform:

Index	Protein	SC [%]	SVP [%]	IC [%]	MS Score [a.u.]	Var. Mods	Mono Mass [Da]	Chemical Formula
3	H4Kac_me1-3	34.7	11.8	26.9	9.3	Met: K16, K20, Ace: N-term	11299.3831	C498H843N163O135S

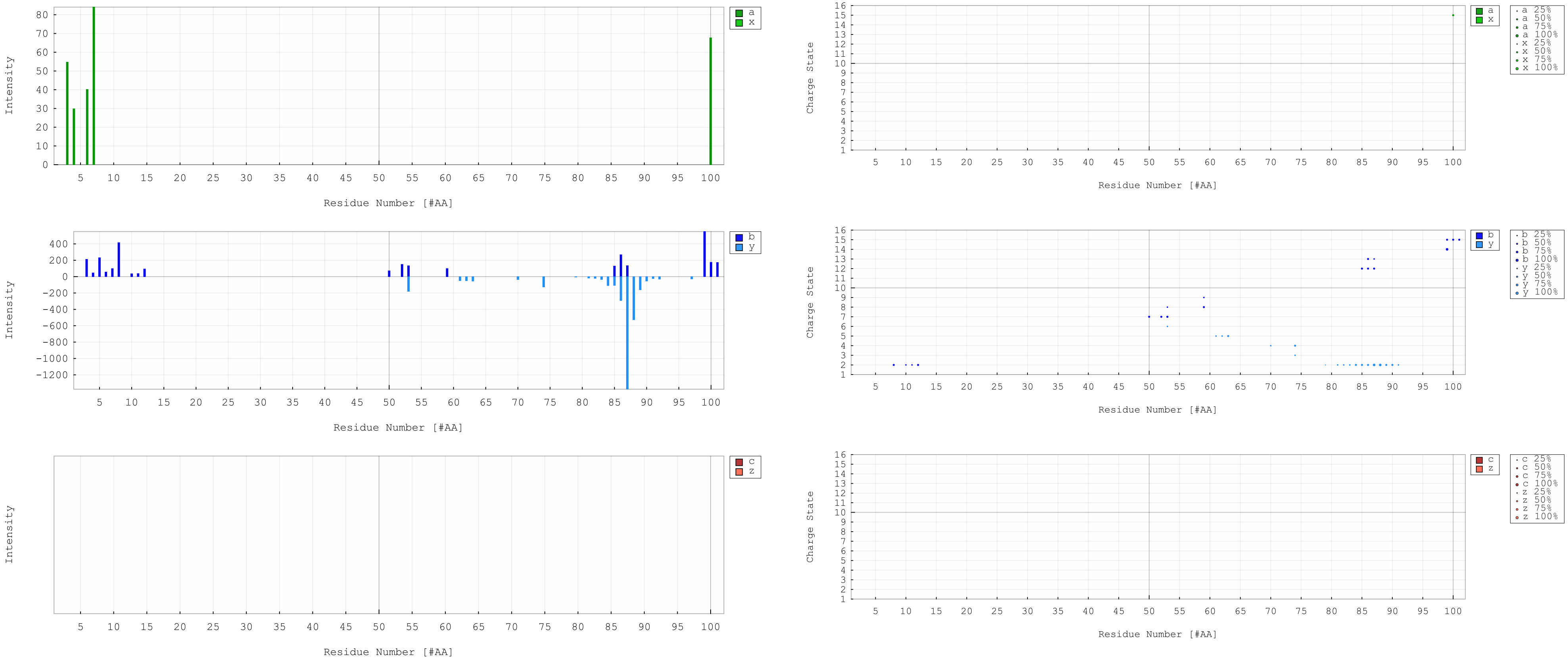

Proteoform:	Index	Protein	SC [%]	SVP [%]	IC [%]	MS Score [a.u.]	Var. Mods	Mono Mass [Da]	Chemical Formula
	4	H4Kac_me1-3	33.7	10.8	26.8	9.0	Dim: K12, Ace: N-term	11299.3831	C498H843N163O135S

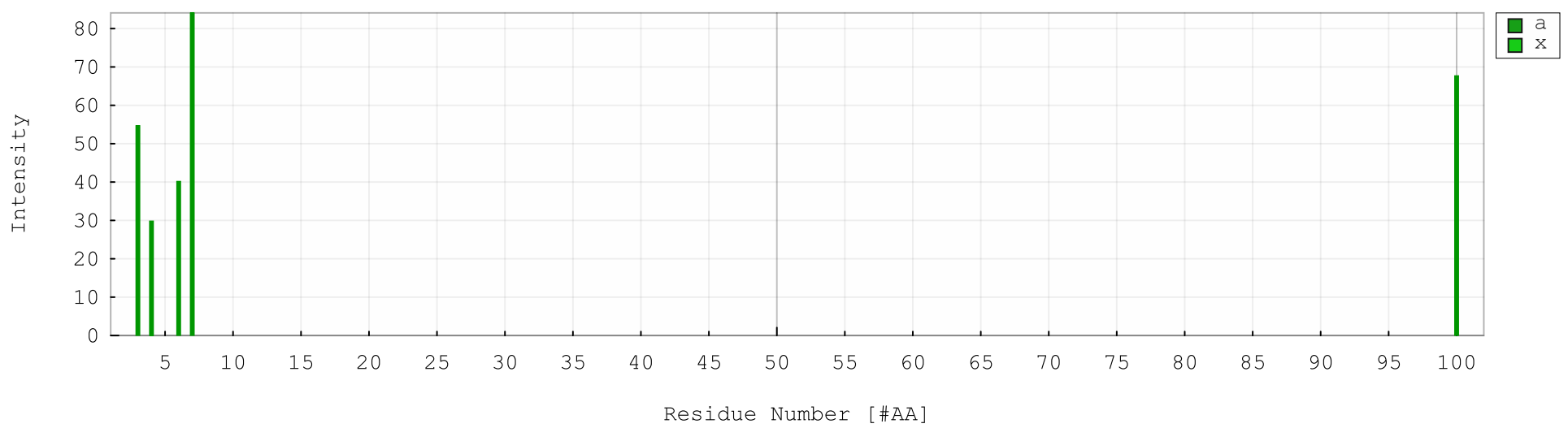

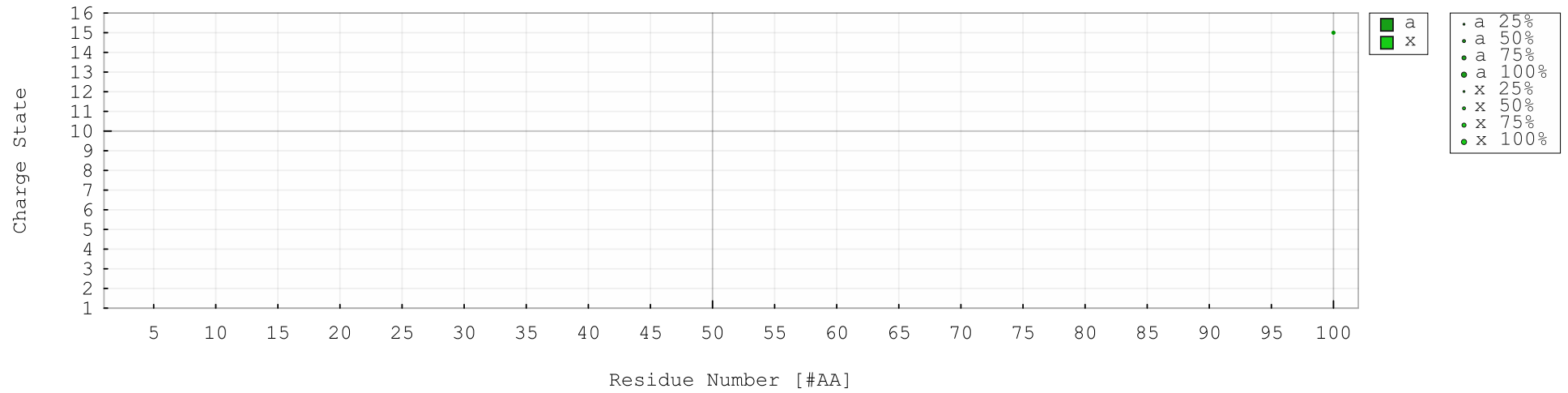

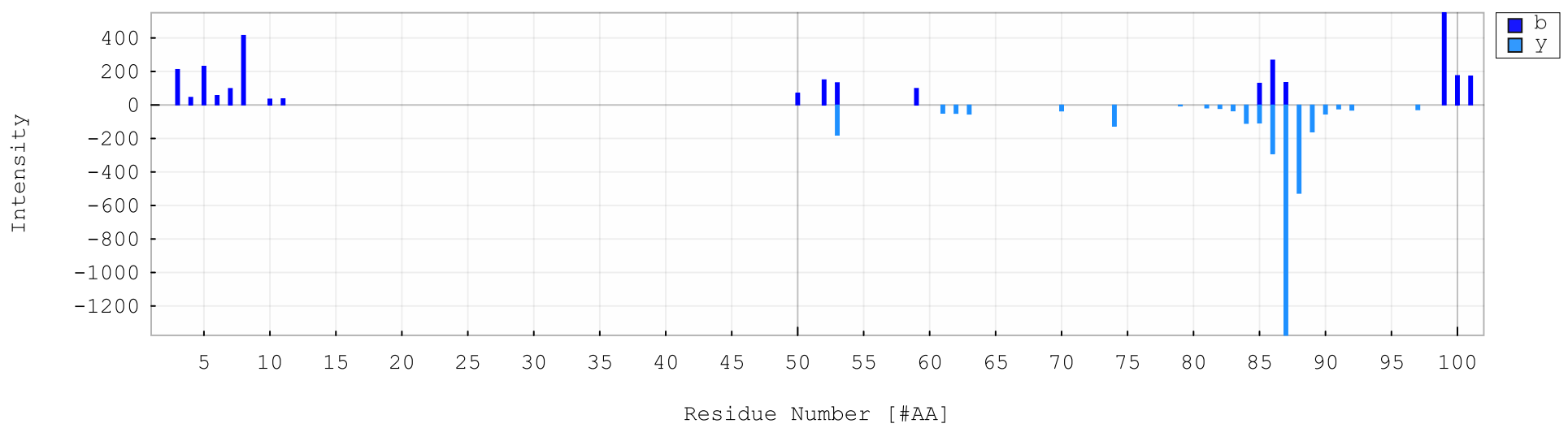

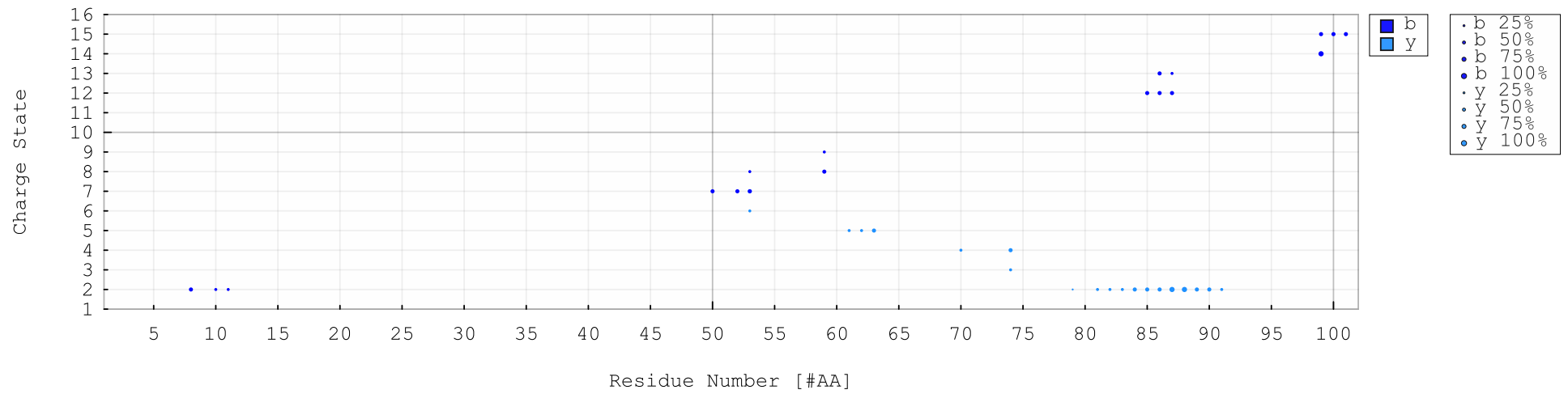

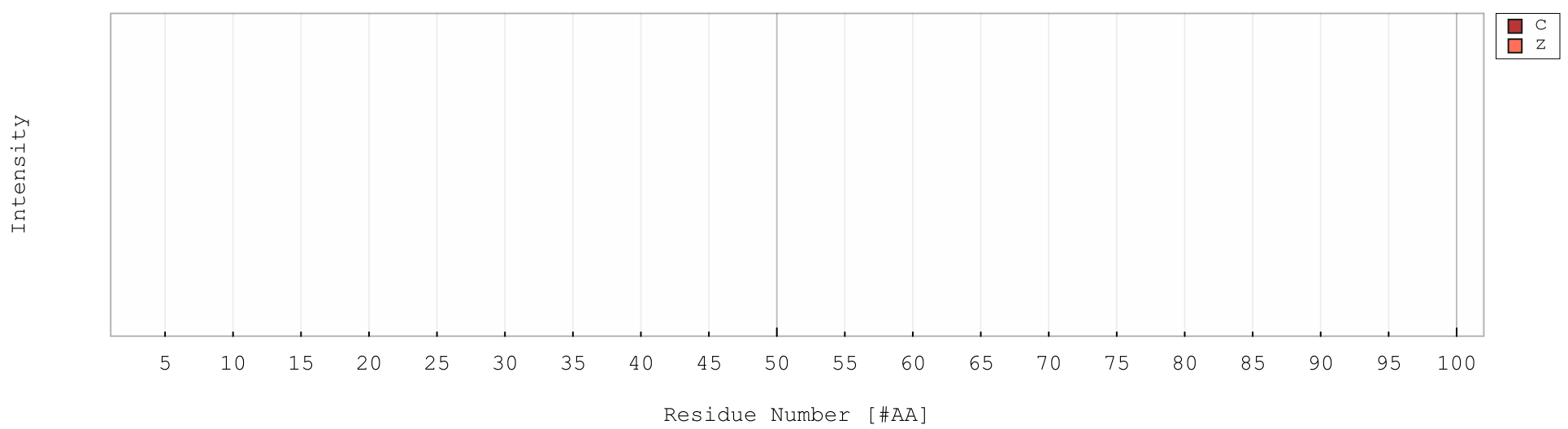

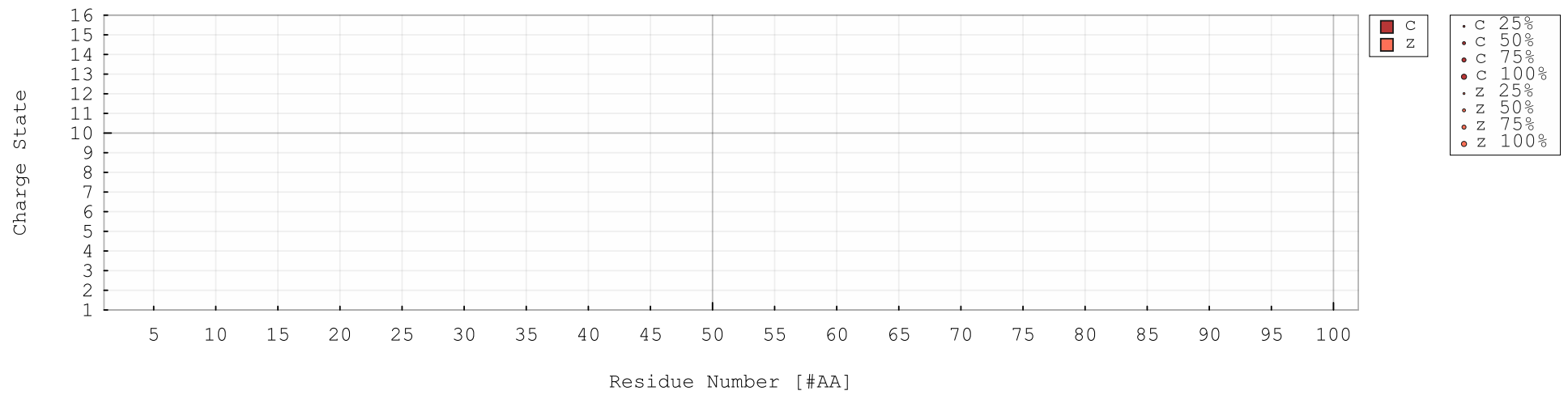

Proteoform:

Index	Protein	SC [%]	SVP [%]	IC [%]	MS Score [a.u.]	Var. Mods	Mono Mass [Da]	Chemical Formula
5	H4Kac_me1-3	33.7	10.8	26.8	9.0	Met: K12, K20, Ace: N-term	11299.3831	C498H843N163O135S

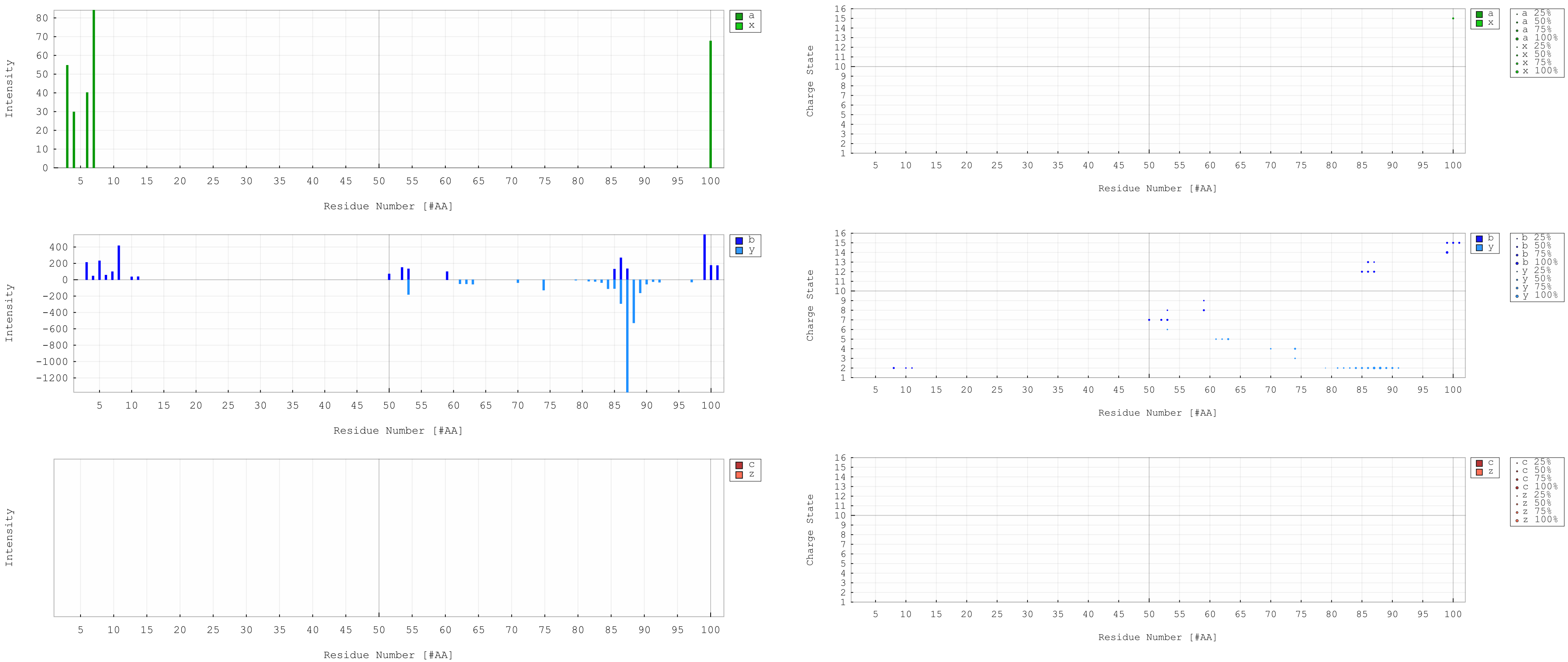

Proteoform:

Index	Protein	SC [%]	SVP [%]	IC [%]	MS Score [a.u.]	Var. Mods	Mono Mass [Da]	Chemical Formula
6	H4Kac_me1-3	33.7	10.8	26.8	9.0	Met: K12, K16, Ace: N-term	11299.3831	C498H843N163O135S

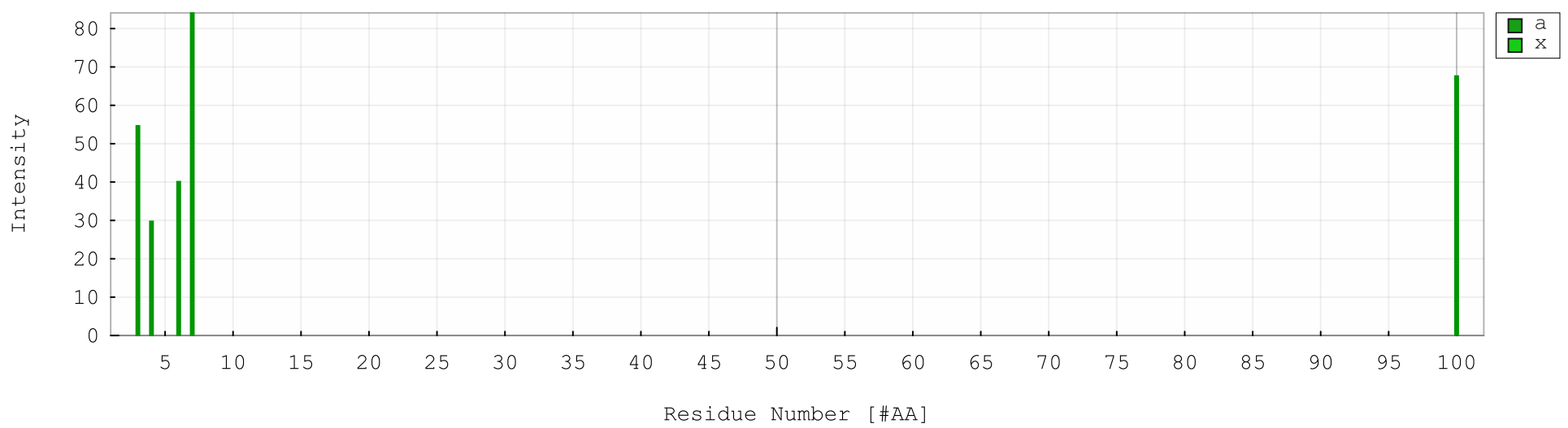

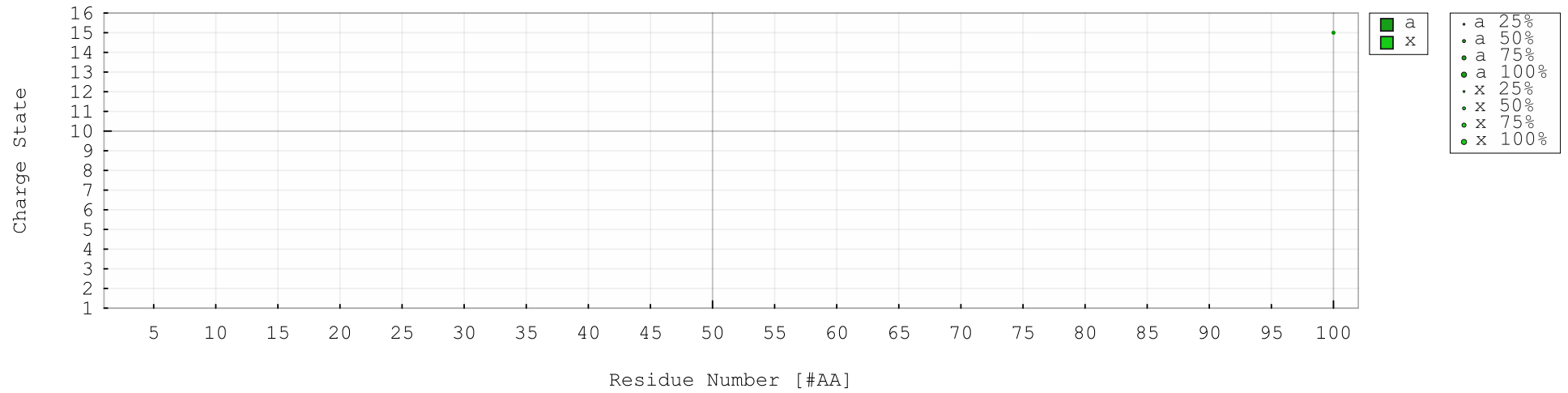

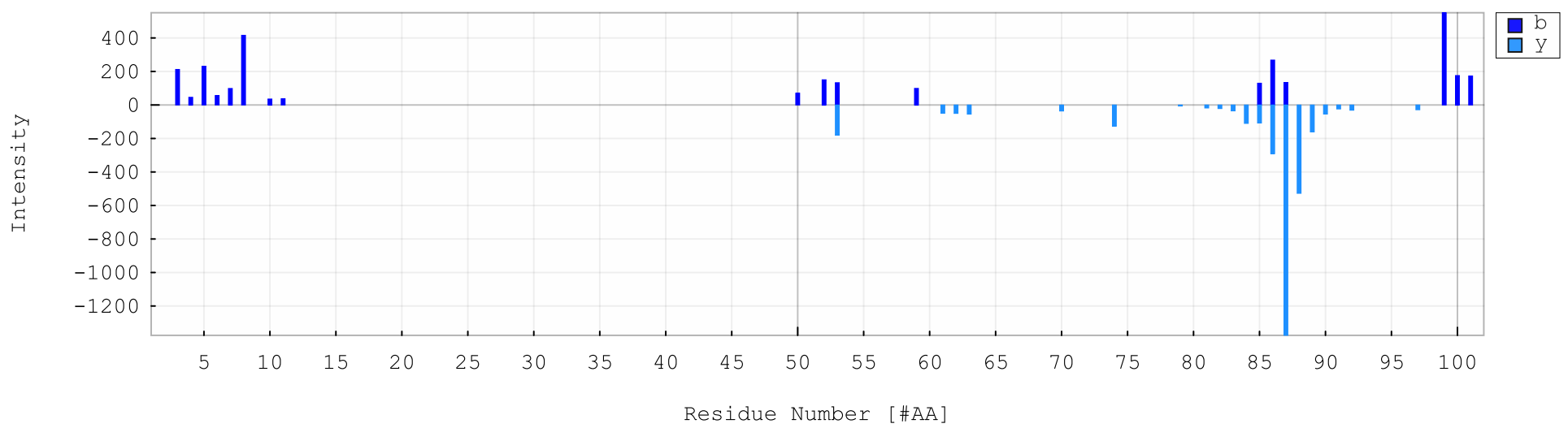

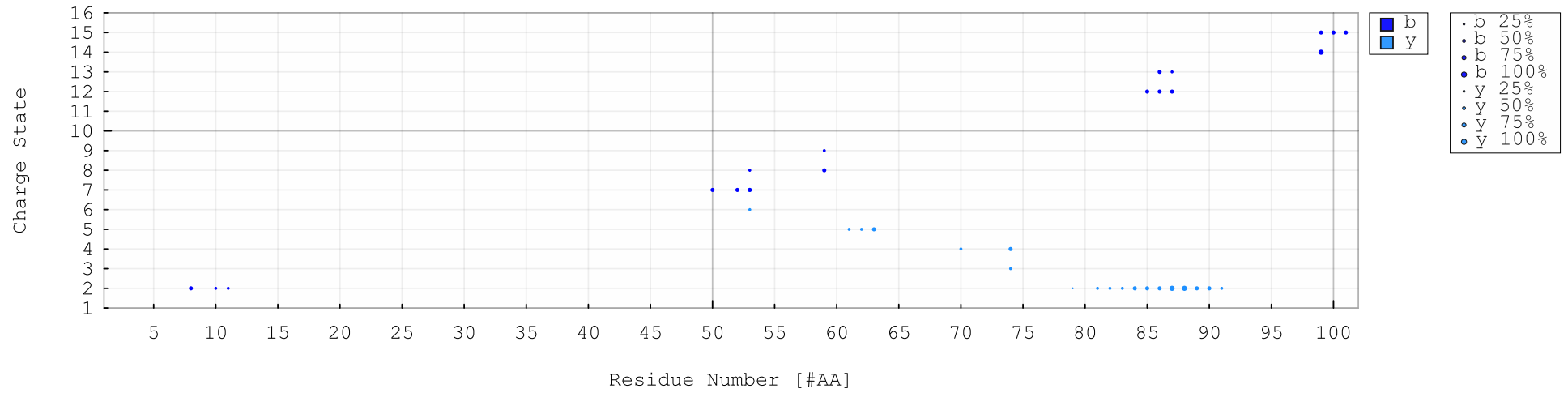

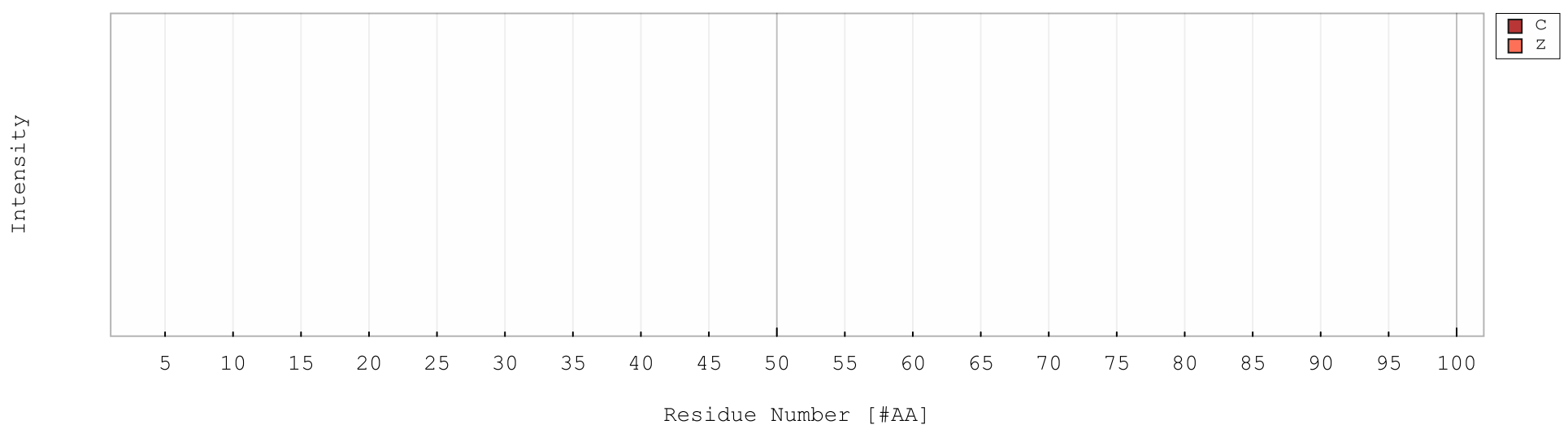

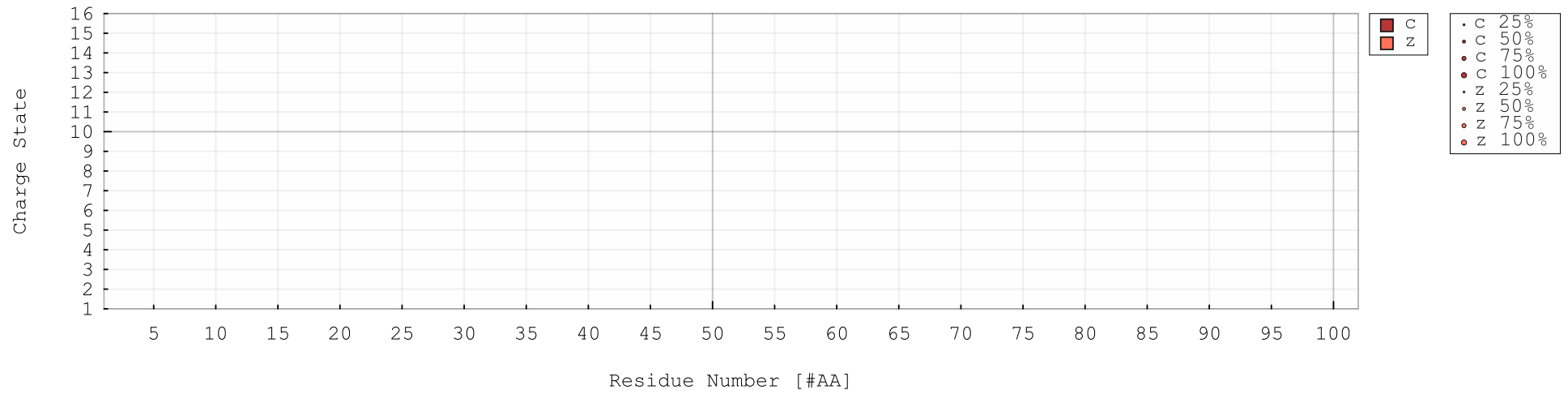

Proteoform:

Index	Protein	SC [%]	SVP [%]	IC [%]	MS Score [a.u.]	Var. Mods	Mono Mass [Da]	Chemical Formula
7	H4Kac_me1-3	33.7	0.0	26.7	9.0	Ace: K5, Dim: K20	11299.3831	C498H843N163O135S

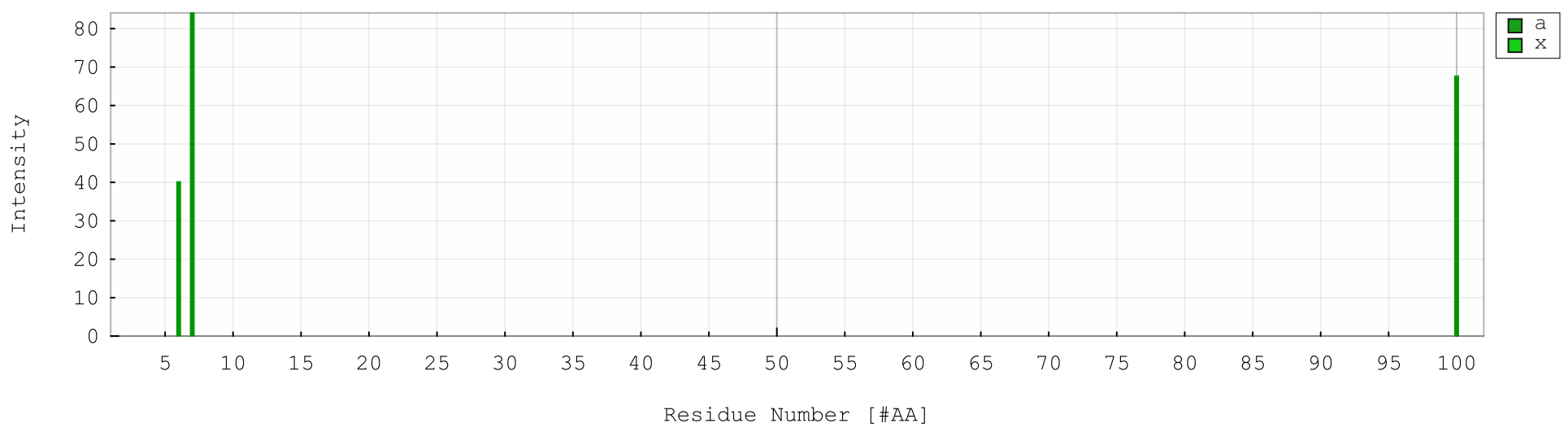

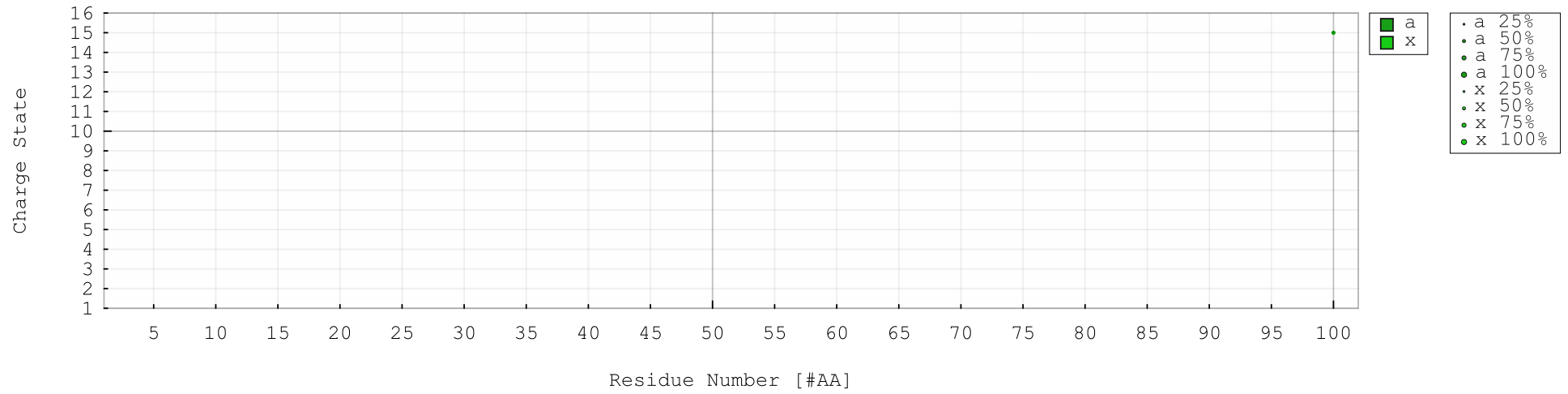

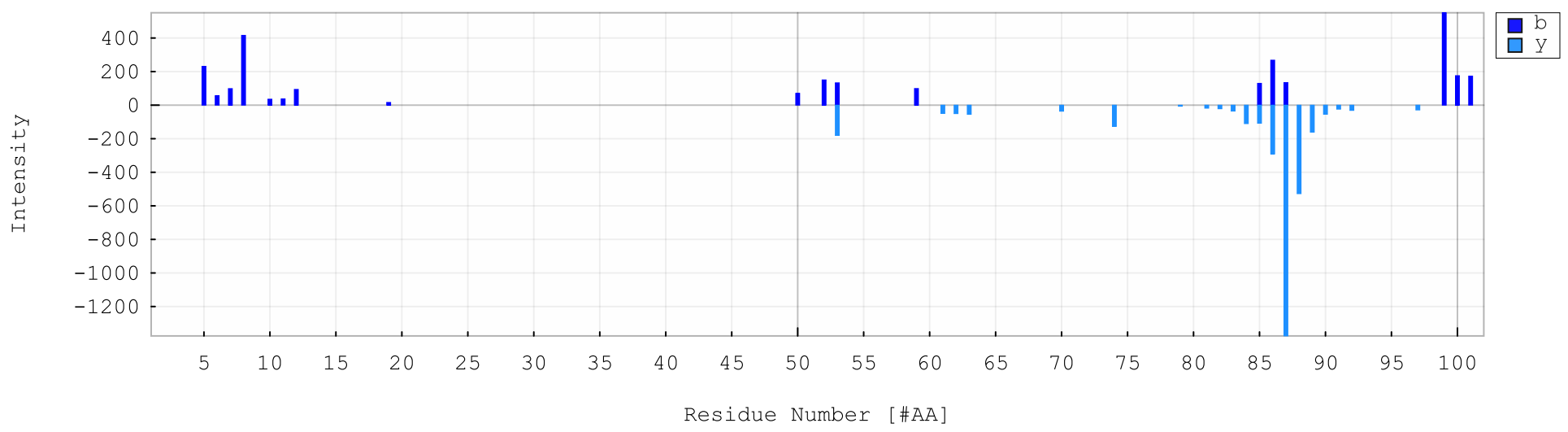

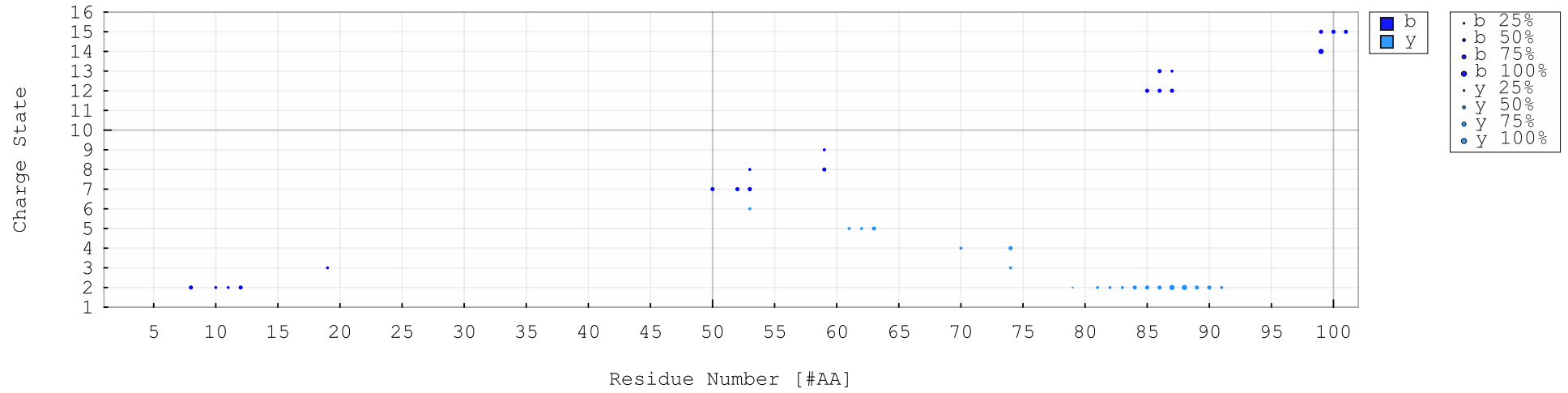

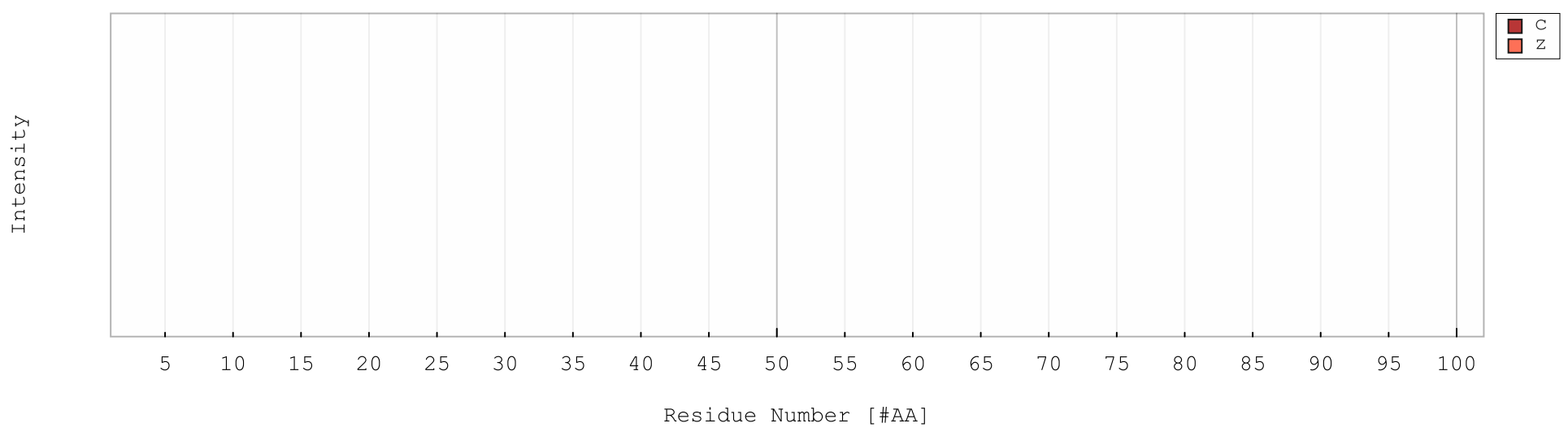

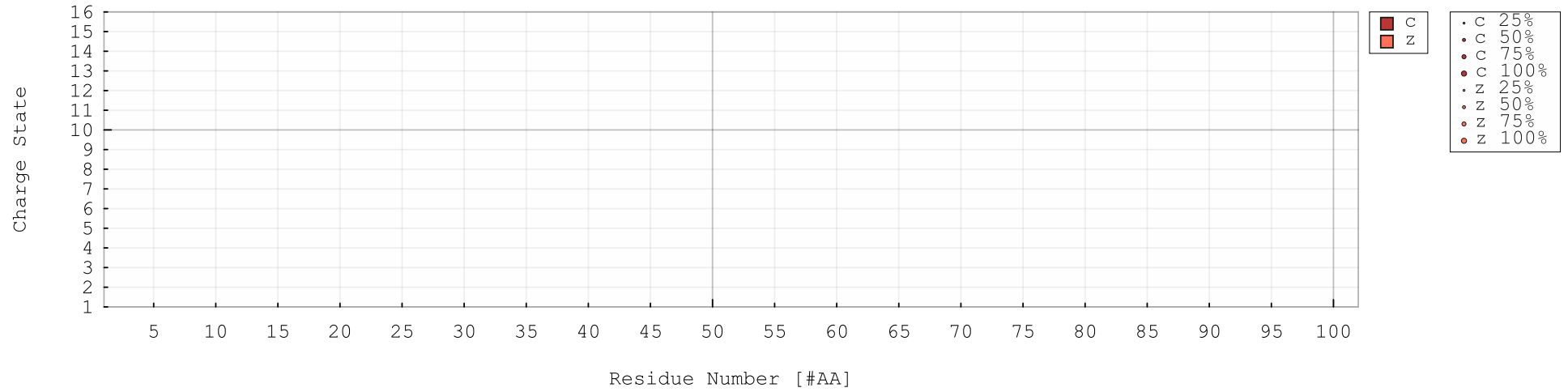

Proteoform:

Index	Protein	SC [%]	SVP [%]	IC [%]	MS Score [a.u.]	Var. Mods	Mono Mass [Da]	Chemical Formula
8	H4Kac_me1-3	32.7	0.0	26.6	8.7	Ace: K5, Met: K16, K20	11299.3831	C498H843N163O135S

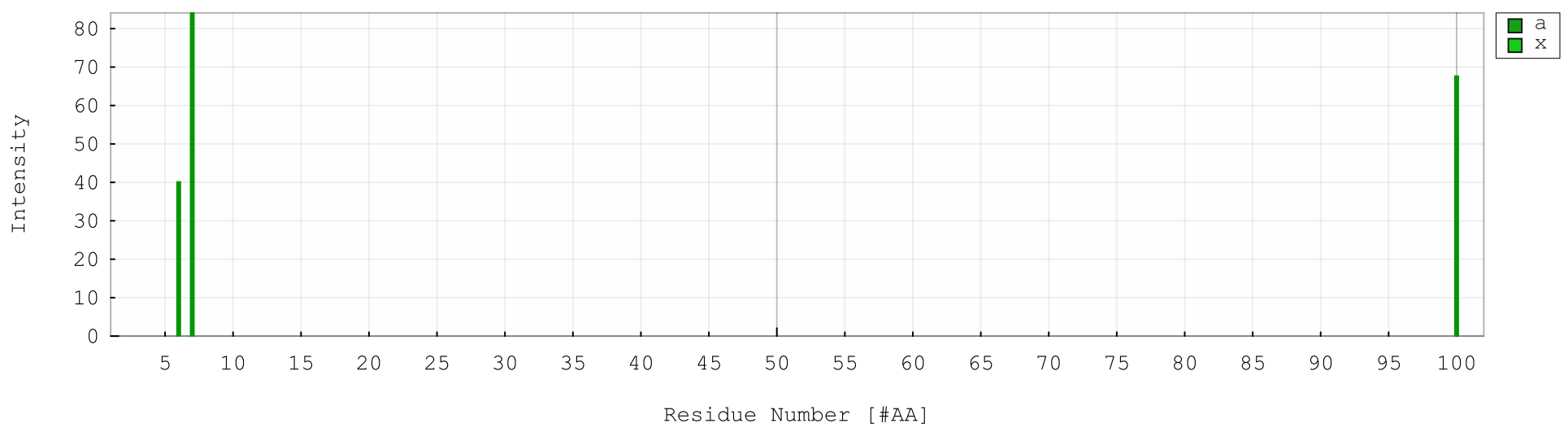

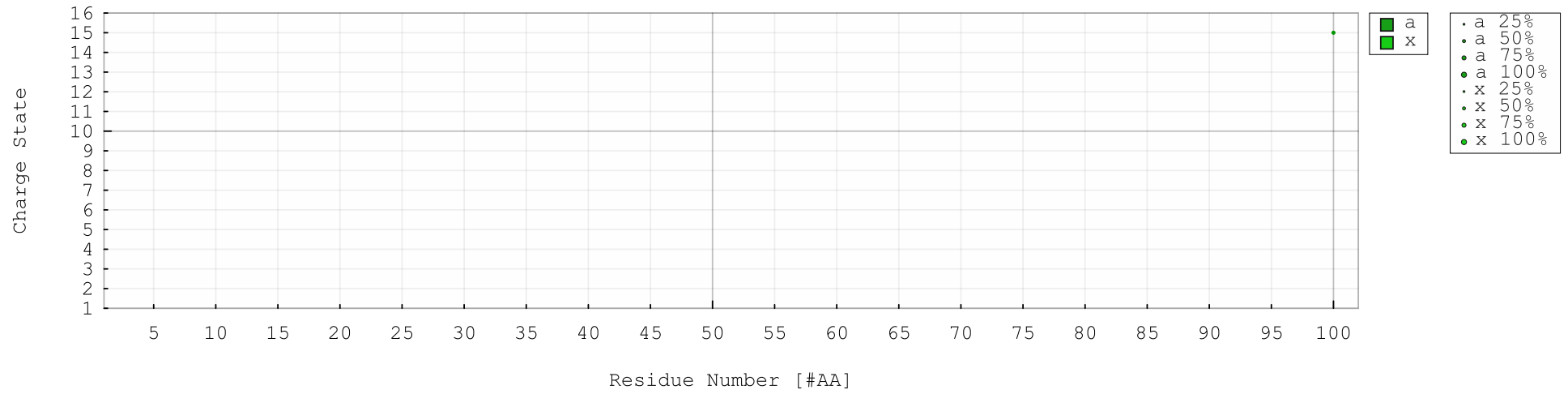

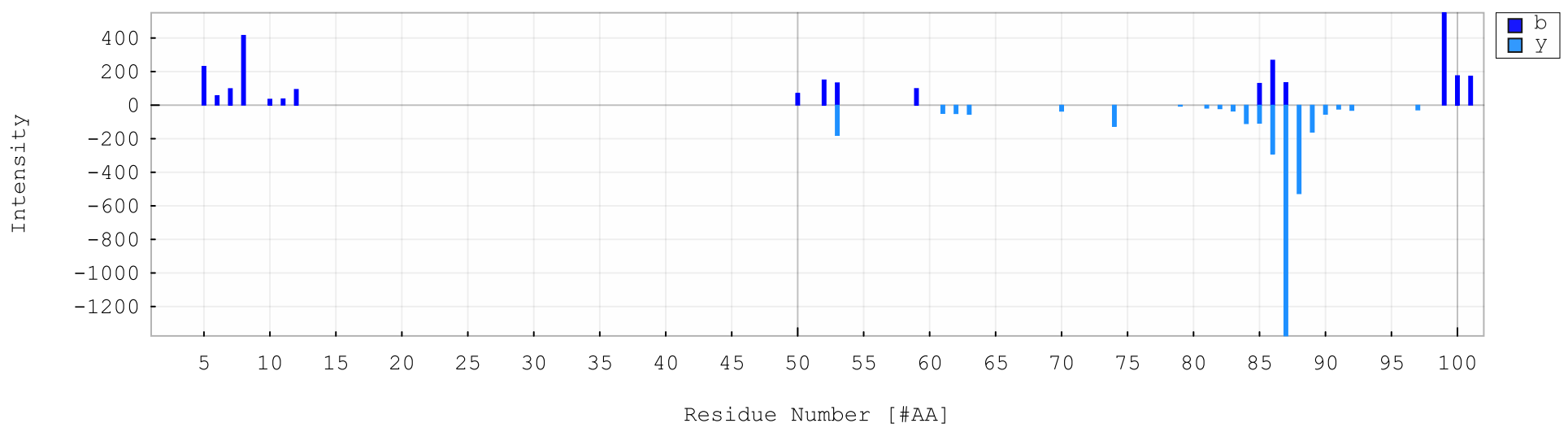

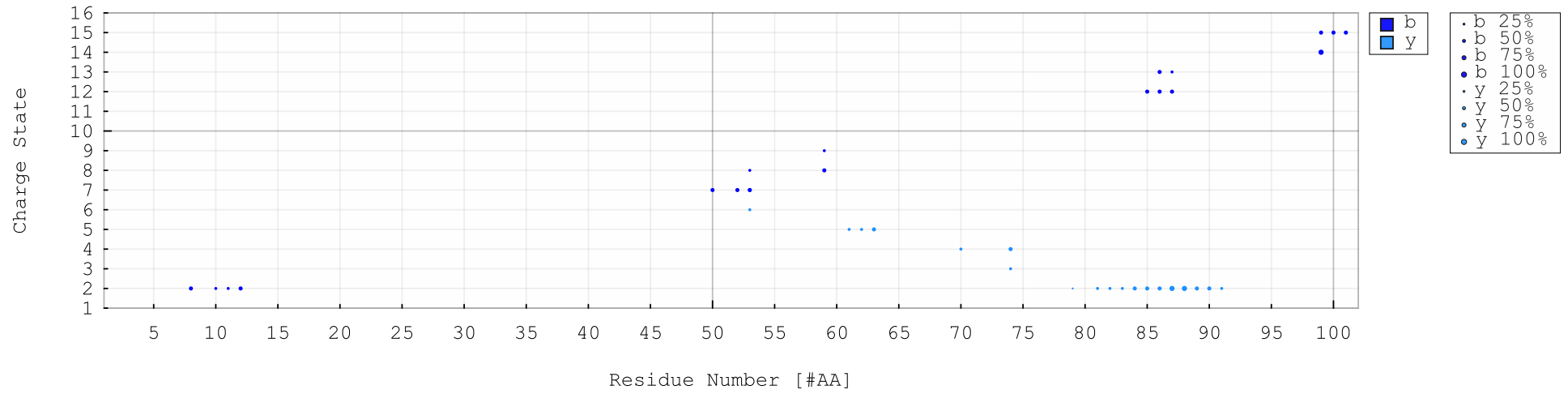

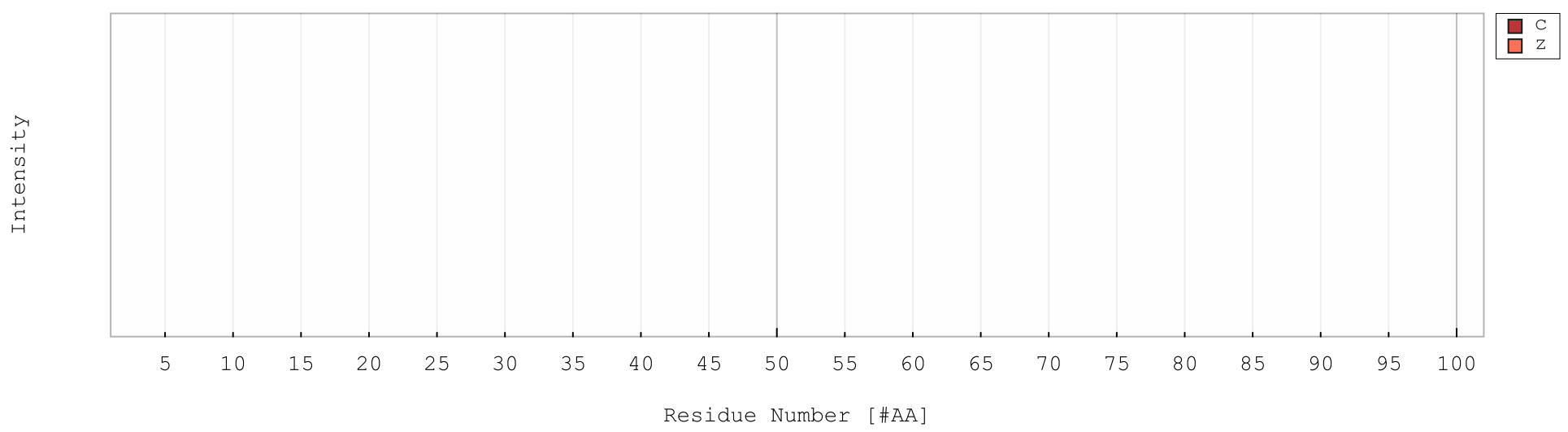

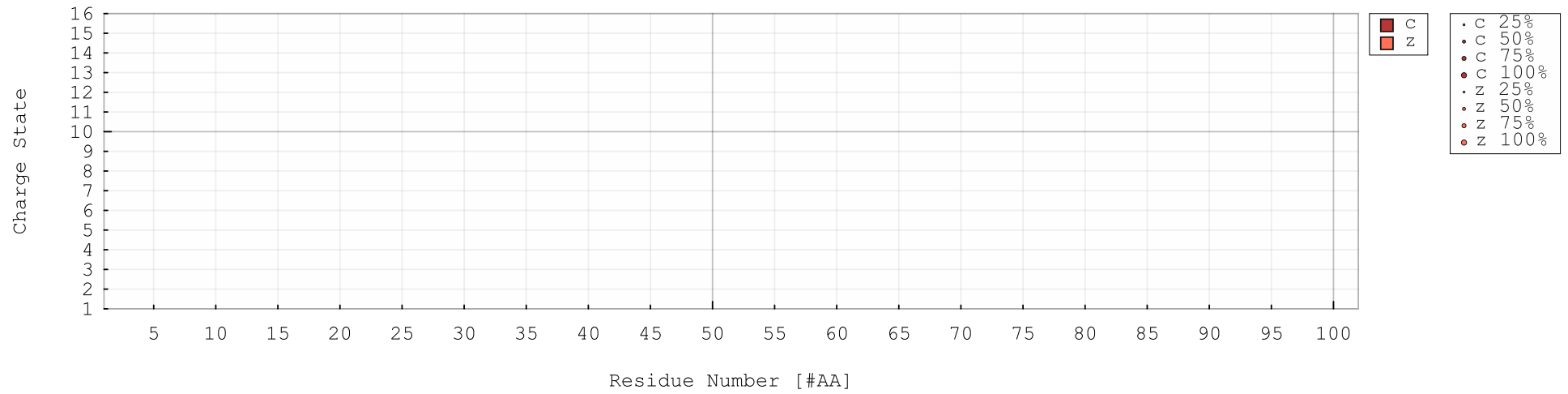

Proteoform:

Index	Protein	SC [%]	SVP [%]	IC [%]	MS Score [a.u.]	Var. Mods	Mono Mass [Da]	Chemical Formula
9	H4Kac_me1-3	32.7	0.0	26.6	8.7	Ace: K5, Dim: K16	11299.3831	C498H843N163O135S

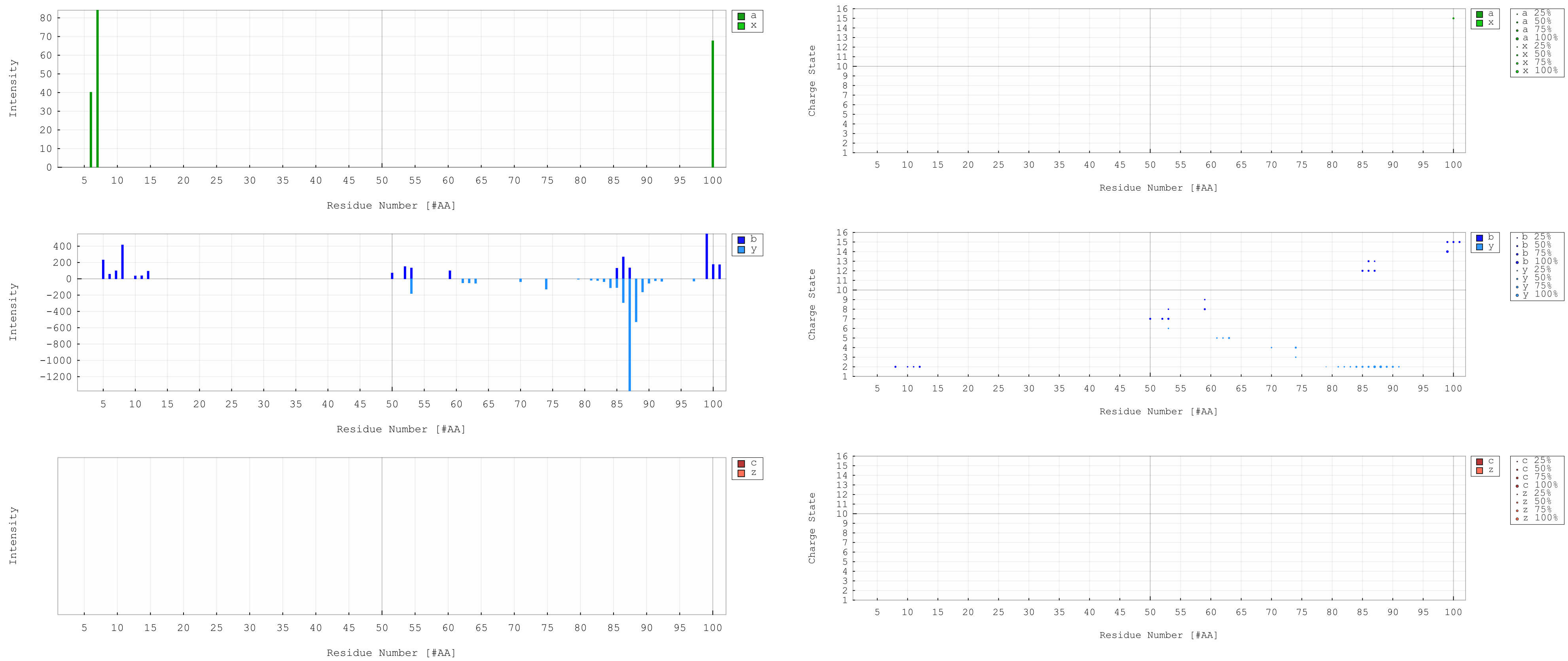

Proteoform:

Index	Protein	SC [%]	SVP [%]	IC [%]	MS Score [a.u.]	Var. Mods	Mono Mass [Da]	Chemical Formula
10	H4Kac_me1-3	31.7	8.8	26.6	8.4	Met: K8, K20, Ace: N-term	11299.3831	C498H843N163O135S

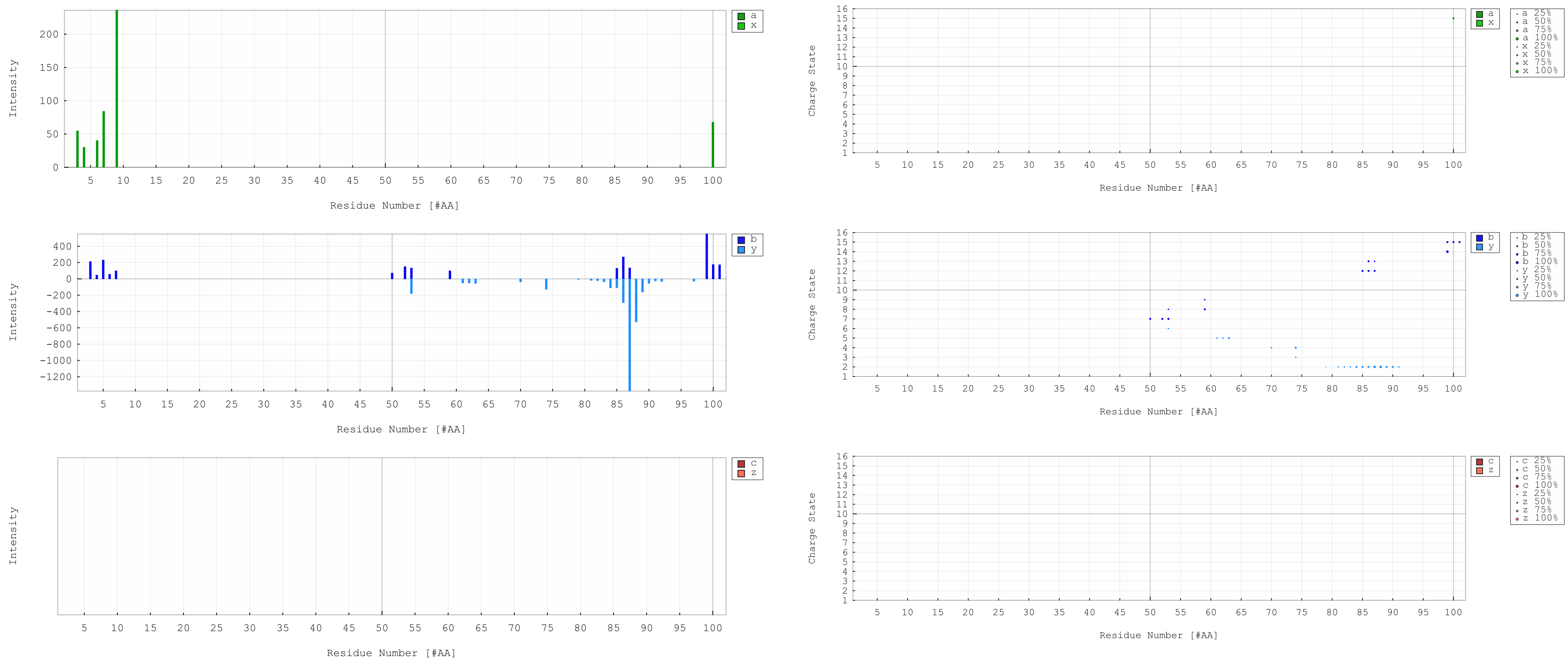

Supplemental Data S1

Endogenous H4

**Report**	4 of 6
**Experiment**	MS2
**Histone/sample**	H4
**Enzyme**	Not applicable
**Reaction/source**	Endogenous sample
**Precursor**	m/z 710
**Charge state**	16+
**Fragmentation / workflow**	ECD
**Original file**	MS2_mz-710_16+_ECD.pdf

Project Information

Project Directory: C:/Users/LAB-ADMIN
Processing date: Tue Apr 28 15:00:11 2026

Sample Info & Protocols

Name: Top-Down Protein Sequencing ExD

Dataset

Imported spectrum file name:	250430 Liver-Histone-25 mz710z16 MS2 ECD_001-478_calibrated.txt
Dataset name:	Information is only available from imported JPEAKS files
Date acquired:	Information is only available from imported JPEAKS files
Date imported:	Information is only available from imported JPEAKS files
Instrument Name:	Information is only available from imported JPEAKS files
Instrument Serial Number:	Information is only available from imported JPEAKS files

Workflow Result Info

Analysis: 250430 Liver-Histone-25 mz710z16 MS2 ECD_001-478_calibrated
Location: C:/Users/LAB-ADMIN
Method: -

Method Information

Name: Unnamed

Spectral Parameters

Mass Error [ppm]: 6
Intensity Deviation [au]: 0.15
Min/Max Charges: 1-16
Experiment type: ExD

a ions	b ions	c ions	x ions	y ions	z ions	satellite	internal	precursor
a	b	c		y	z+1			M
a+1		c+1			z+2			M+-2H

Noise threshold calculation: Simple moving average noise threshold (SMA)
Window size [datapoints]: 600
Noise curve offset: 2.9595578
SVP parameters: terminal gap: 2, internal gap: 1

Proteoforms

(top 10)

Index	Parent Sequence	Chain	Var. Mods	Mono Mass [Da]	Chemical Composition
1	H4Kac_me1-3	H4Kac_me1-3_Nter-m[Acetyl]_K16[Ace]K20[Dim]	Ace: K16, N-term, Dim: K20	11341.3937	C500H845N163O136S
2	H4Kac_me1-3	H4Kac_me1-3K5[Ace]K16[Ace]K20[Dim]	Ace: K5, K16, Dim: K20	11341.3937	C500H845N163O136S
3	H4Kac_me1-3	H4Kac_me1-3_Nter-m[Acetyl]_K12[Ace]K20[Dim]	Ace: K12, N-term, Dim: K20	11341.3937	C500H845N163O136S
4	H4Kac_me1-3	H4Kac_me1-3K5[Ace]K12[Ace]K20[Dim]	Ace: K5, K12, Dim: K20	11341.3937	C500H845N163O136S
5	H4Kac_me1-3	H4Kac_me1-3K8[Ace]K16[Ace]K20[Dim]	Ace: K8, K16, Dim: K20	11341.3937	C500H845N163O136S
6	H4Kac_me1-3	H4Kac_me1-3_Nter-m[Acetyl]_K12[Ace]K16[Met]K20[Met]	Ace: K12, N-term, Met: K16, K20	11341.3937	C500H845N163O136S
7	H4Kac_me1-3	H4Kac_me1-3K5[Ace]K12[Ace]K16[Met]K20[Met]	Ace: K5, K12, Met: K16, K20	11341.3937	C500H845N163O136S
8	H4Kac_me1-3	H4Kac_me1-3_Nter-m[Acetyl]_K16[Dim]K20[Ace]	Dim: K16, Ace: K20, N-term	11341.3937	C500H845N163O136S
9	H4Kac_me1-3	H4Kac_me1-3K5[Ace]K16[Dim]K20[Ace]	Ace: K5, K20, Dim: K16	11341.3937	C500H845N163O136S
10	H4Kac_me1-3	H4Kac_me1-3K8[Ace]K12[Ace]K20[Dim]	Ace: K8, K12, Dim: K20	11341.3937	C500H845N163O136S

Results

Proteoforms Table (top 10)

Index	Protein	SC [%]	SVP [%]	IC [%]	MS Score [a.u.]	Var. Mods	Mono Mass [Da]	Chemical Formula
1	H4Kac_me1-3	85.1	23.5	41.6	35.4	Ace: K16, N-term, Dim: K20	11341.3937	C500H845N163O136S
2	H4Kac_me1-3	84.2	4.9	41.9	35.2	Ace: K5, K16, Dim: K20	11341.3937	C500H845N163O136S
3	H4Kac_me1-3	85.1	23.5	40.0	34.1	Ace: K12, N-term, Dim: K20	11341.3937	C500H845N163O136S
4	H4Kac_me1-3	84.2	4.9	40.3	33.9	Ace: K5, K12, Dim: K20	11341.3937	C500H845N163O136S
5	H4Kac_me1-3	81.2	4.9	41.4	33.6	Ace: K8, K16, Dim: K20	11341.3937	C500H845N163O136S
6	H4Kac_me1-3	84.2	19.6	39.4	33.1	Ace: K12, N-term, Met: K16, K20	11341.3937	C500H845N163O136S
7	H4Kac_me1-3	83.2	4.9	39.6	33.0	Ace: K5, K12, Met: K16, K20	11341.3937	C500H845N163O136S
8	H4Kac_me1-3	81.2	19.6	40.4	32.8	Dim: K16, Ace: K20, N-term	11341.3937	C500H845N163O136S
9	H4Kac_me1-3	80.2	4.9	40.7	32.6	Ace: K5, K20, Dim: K16	11341.3937	C500H845N163O136S
10	H4Kac_me1-3	81.2	4.9	39.8	32.4	Ace: K8, K12, Dim: K20	11341.3937	C500H845N163O136S

Sequence

Name: H4Kac_me1-3

```
         10         20         30         40         50         60
SGRGKGGKGL GKGGAKRHRK VLRDNIQGIT KPAIRRLARR GGVKRISGLI YEE RGVLKV
         70         80         90        100        110        120
FLENVIRDAV TYTEHAKRKT VTAMDVVYAL KRQGRTLYGF GG
```

Modifications

Modification	Position	Type
Acetyl	N-term	variable
Acetyl	5	variable
Acetyl	8	variable
Acetyl	12	variable
Methyl	12	variable
Dimethyl	12	variable
Acetyl	16	variable
Dimethyl	16	variable
Trimethyl	16	variable
Methyl	16	variable
Dimethyl	20	variable
Acetyl	20	variable
Methyl	20	variable

Modification	Position	Type
Trimethyl	20	variable
Dimethyl	31	variable
Methyl	31	variable
Acetyl	31	variable

Sequence Map

Proteoform:	Index	Protein	SC [%]	SVP [%]	IC [%]	MS Score [a.u.]	Var. Mods	Mono Mass [Da]	Chemical Formula
	1	H4Kac_me1-3	85.1	23.5	41.6	35.4	Ace: K16, N-term, Dim: K20	11341.3937	C500H845N163O136S

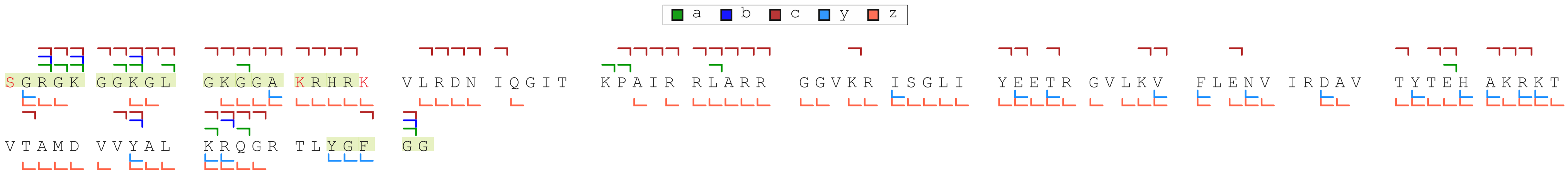

Proteoform:

Index	Protein	SC [%]	SVP [%]	IC [%]	MS Score [a.u.]	Var. Mods	Mono Mass [Da]	Chemical Formula
2	H4Kac_me1-3	84.2	4.9	41.9	35.2	Ace: K5, K16, Dim: K20	11341.3937	C500H845N163O136S

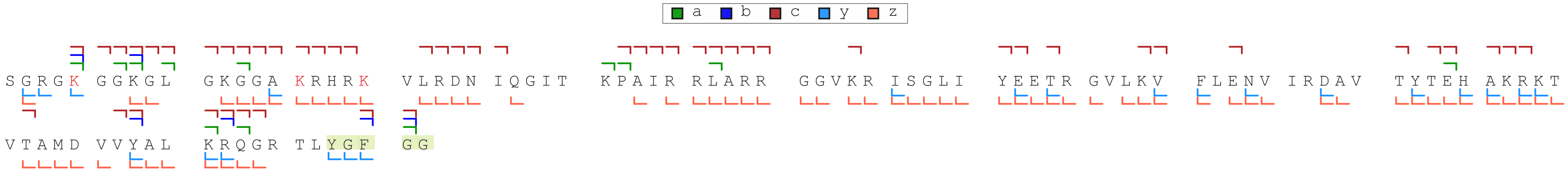

Proteoform:

Index	Protein	SC [%]	SVP [%]	IC [%]	MS Score [a.u.]	Var. Mods	Mono Mass [Da]	Chemical Formula
3	H4Kac_me1-3	85.1	23.5	40.0	34.1	Ace: K12, N-term, K20 Dim:	11341.3937	C500H845N163O136S

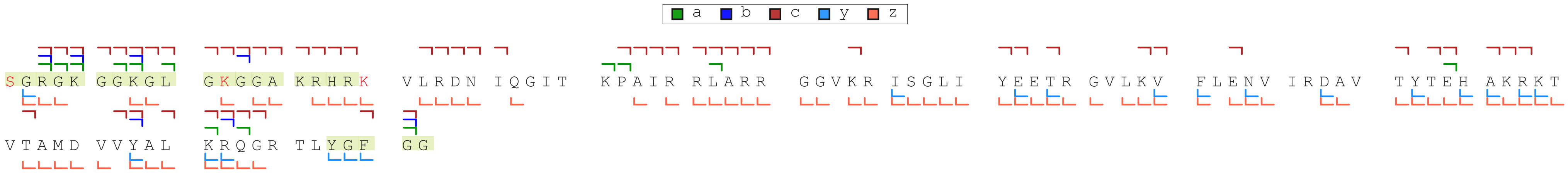

Proteoform:

Index	Protein	SC [%]	SVP [%]	IC [%]	MS Score [a.u.]	Var. Mods	Mono Mass [Da]	Chemical Formula
4	H4Kac_me1-3	84.2	4.9	40.3	33.9	Ace: K5, K12, Dim: K20	11341.3937	C500H845N163O136S

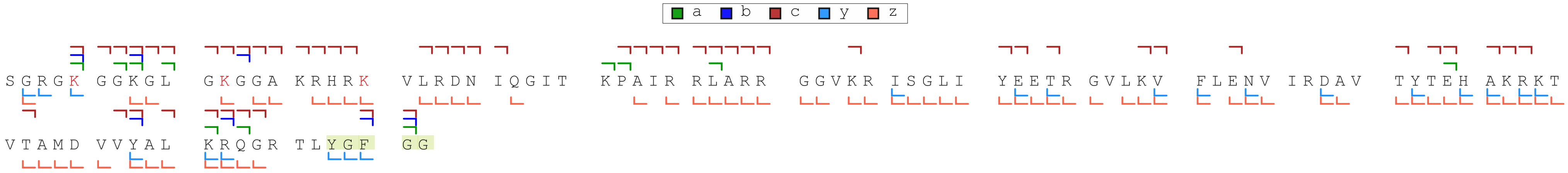

Proteoform:

Index	Protein	SC [%]	SVP [%]	IC [%]	MS Score [a.u.]	Var. Mods	Mono Mass [Da]	Chemical Formula
5	H4Kac_me1-3	81.2	4.9	41.4	33.6	Ace: K8, K16, Dim: K20	11341.3937	C500H845N163O136S

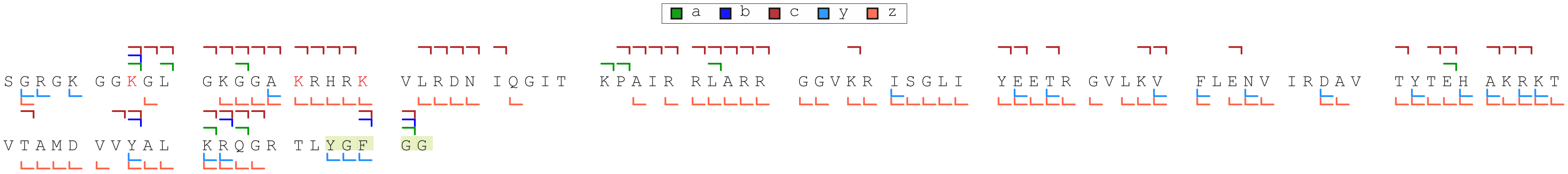

Top-Down Result OmniScape 2025
Sequence Confirmation
250430 Liver-Histone-25 mz710z16 MS2 ECD_001-478_calibrated

Proteoform:

Index	Protein	SC [%]	SVP [%]	IC [%]	MS Score [a.u.]	Var. Mods	Mono Mass [Da]	Chemical Formula
6	H4Kac_me1-3	84.2	19.6	39.4	33.1	Ace: K12, N-term, Met: K16, K20	11341.3937	C500H845N163O136S

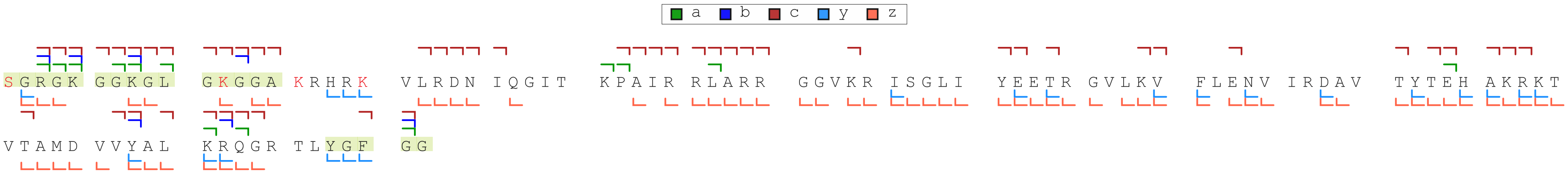

Proteoform:

Index	Protein	SC [%]	SVP [%]	IC [%]	MS Score [a.u.]	Var. Mods	Mono Mass [Da]	Chemical Formula
7	H4Kac_me1-3	83.2	4.9	39.6	33.0	Ace: K5, K12, Met: K16, K20	11341.3937	C500H845N163O136S

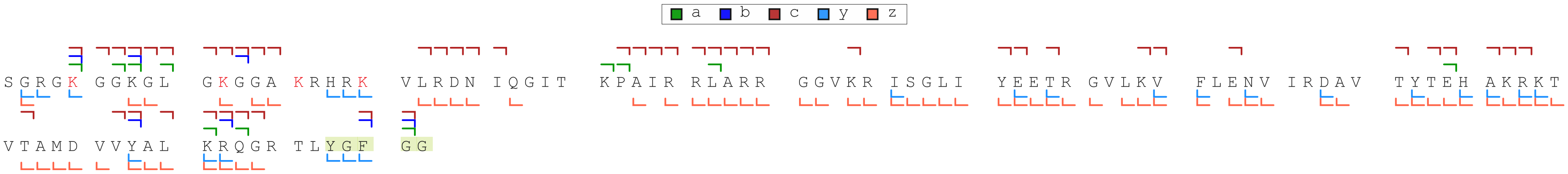

Proteoform:

Index	Protein	SC [%]	SVP [%]	IC [%]	MS Score [a.u.]	Var. Mods	Mono Mass [Da]	Chemical Formula
8	H4Kac_me1-3	81.2	19.6	40.4	32.8	Dim: K16, Ace: K20, N-term	11341.3937	C500H845N163O136S

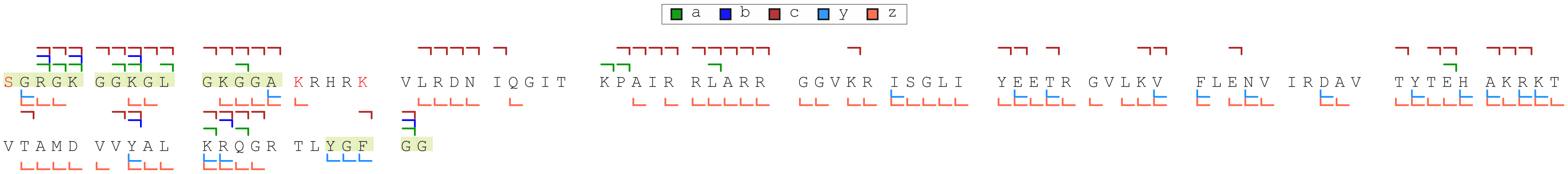

Proteoform:

Index	Protein	SC [%]	SVP [%]	IC [%]	MS Score [a.u.]	Var. Mods	Mono Mass [Da]	Chemical Formula
9	H4Kac_me1-3	80.2	4.9	40.7	32.6	Ace: K5, K20, Dim: K16	11341.3937	C500H845N163O136S

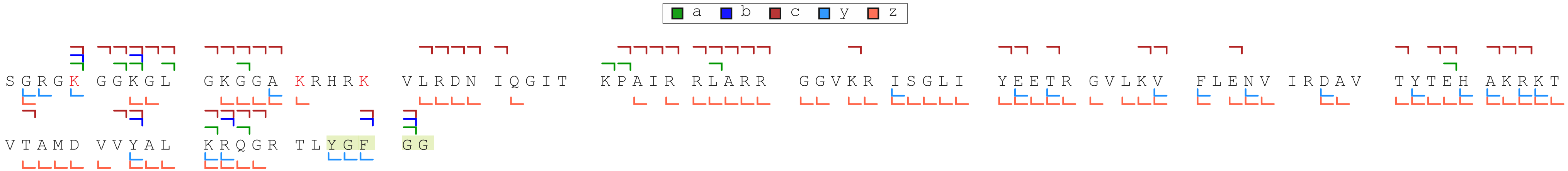

Proteoform:

Index	Protein	SC [%]	SVP [%]	IC [%]	MS Score [a.u.]	Var. Mods	Mono Mass [Da]	Chemical Formula
10	H4Kac_me1-3	81.2	4.9	39.8	32.4	Ace: K8, K12, Dim: K20	11341.3937	C500H845N163O136S

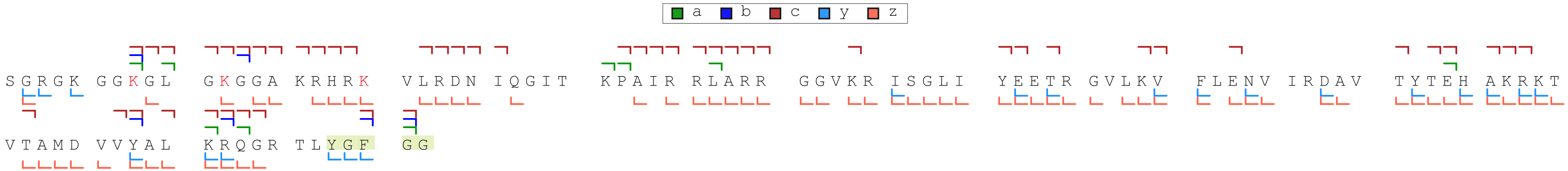

Error Plot

	Index	Protein	SC [%]	SVP [%]	IC [%]	MS Score [a.u.]	Var. Mods	Mono Mass [Da]	Chemical Formula
Proteoform:	1	H4Kac_me1-3	85.1	23.5	41.6	35.4	Ace: K16, N-term, Dim: K20	11341.3937	C500H845N163O136S

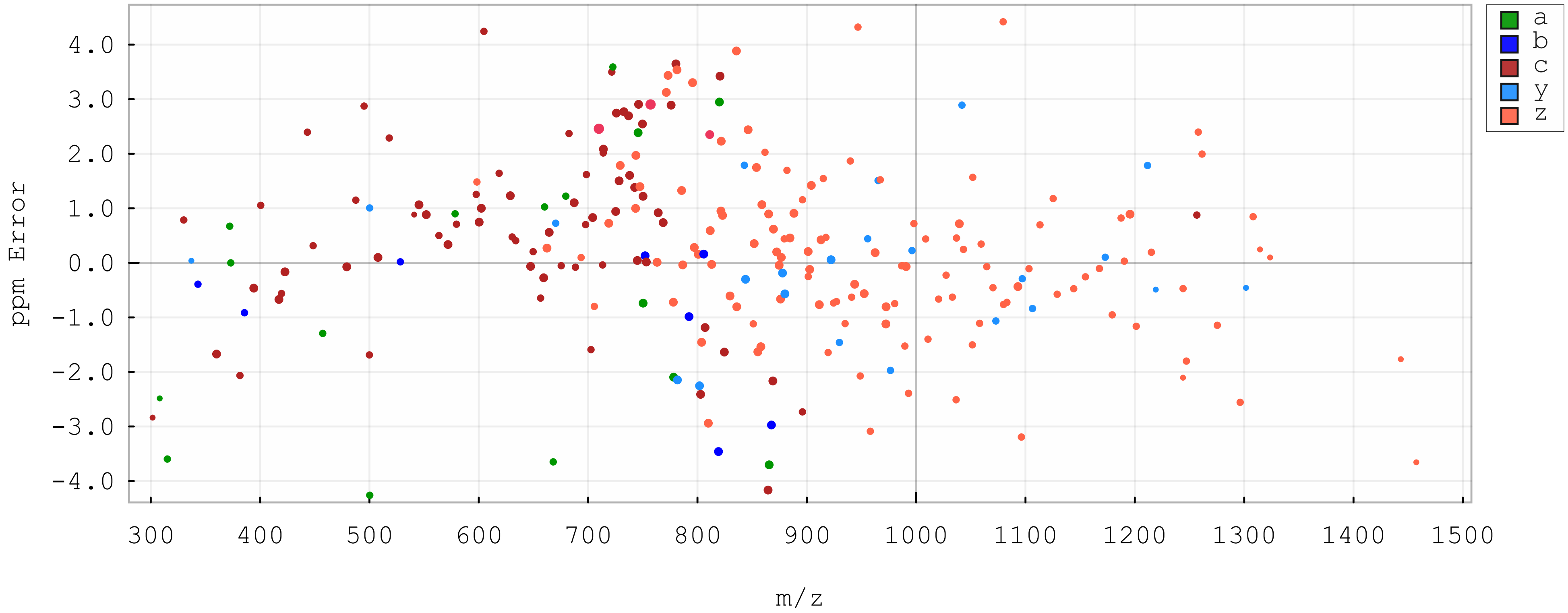

Proteoform:

Index	Protein	SC [%]	SVP [%]	IC [%]	MS Score [a.u.]	Var. Mods	Mono Mass [Da]	Chemical Formula
2	H4Kac_me1-3	84.2	4.9	41.9	35.2	Ace: K5, K16, Dim: K20	11341.3937	C500H845N163O136S

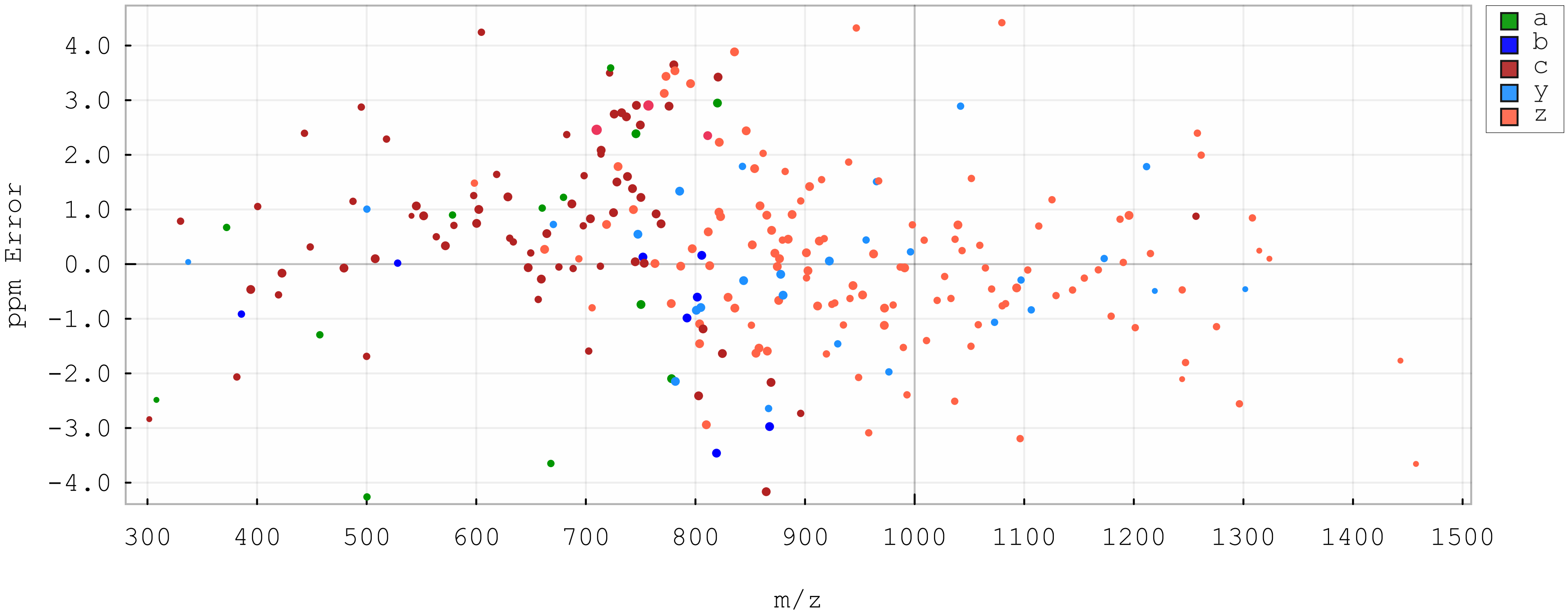

Proteoform:

Index	Protein	SC [%]	SVP [%]	IC [%]	MS Score [a.u.]	Var. Mods	Mono Mass [Da]	Chemical Formula
3	H4Kac_me1-3	85.1	23.5	40.0	34.1	Ace: K12, N-term, Dim: K20	11341.3937	C500H845N163O136S

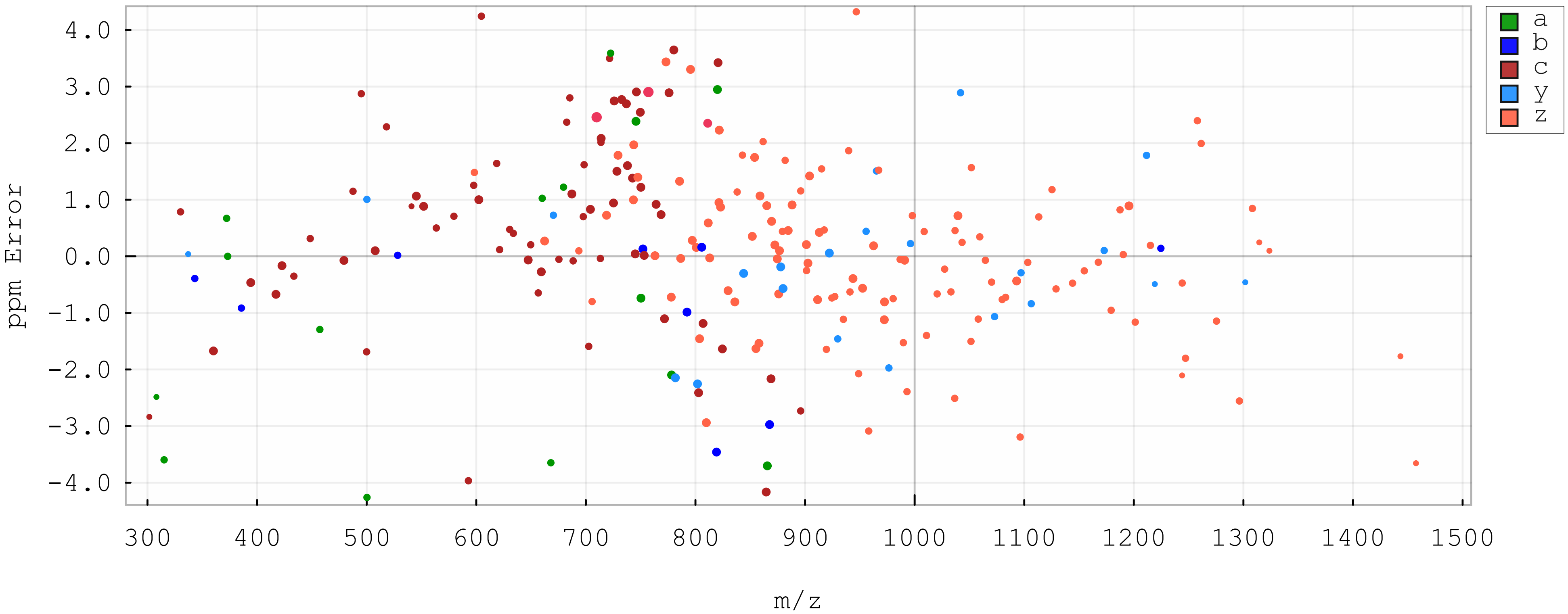

Proteoform:

Index	Protein	SC [%]	SVP [%]	IC [%]	MS Score [a.u.]	Var. Mods	Mono Mass [Da]	Chemical Formula
4	H4Kac_me1-3	84.2	4.9	40.3	33.9	Ace: K5, K12, Dim: K20	11341.3937	C500H845N163O136S

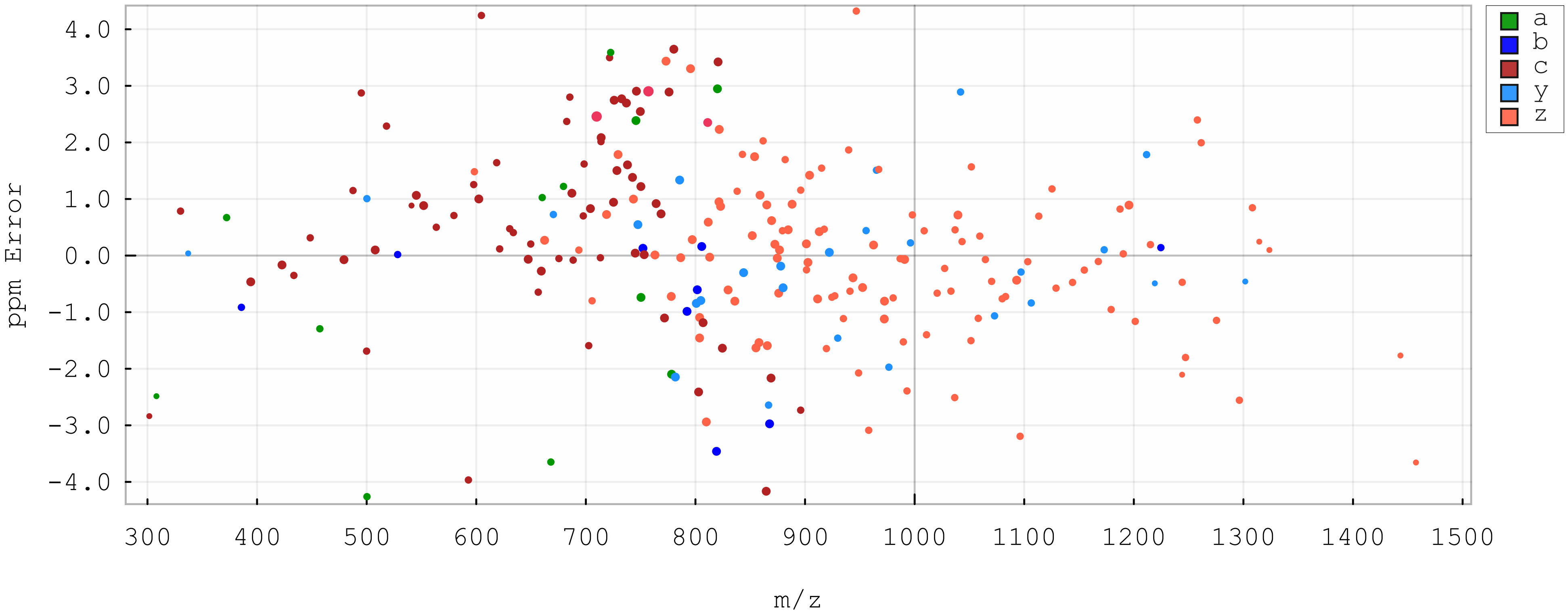

Proteoform:

Index	Protein	SC [%]	SVP [%]	IC [%]	MS Score [a.u.]	Var. Mods	Mono Mass [Da]	Chemical Formula
5	H4Kac_me1-3	81.2	4.9	41.4	33.6	Ace: K8, K16, Dim: K20	11341.3937	C500H845N163O136S

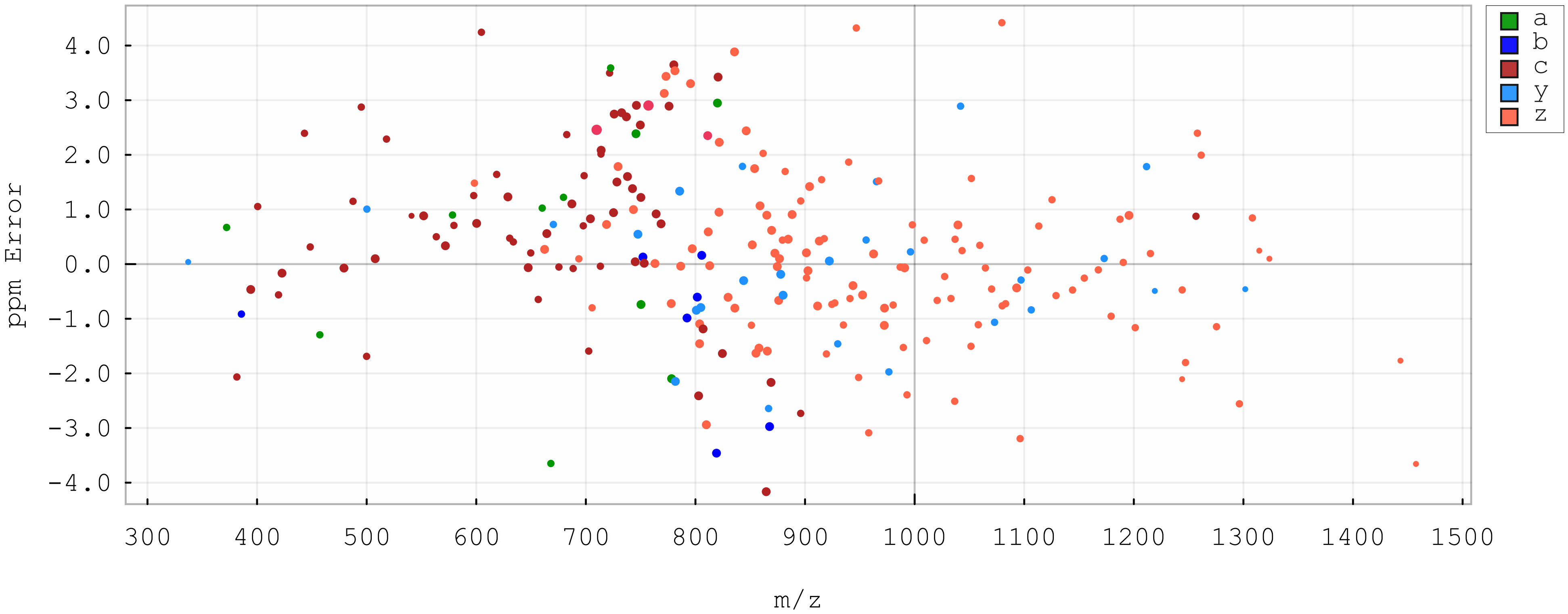

Proteoform:

Index	Protein	SC [%]	SVP [%]	IC [%]	MS Score [a.u.]	Var. Mods	Mono Mass [Da]	Chemical Formula
6	H4Kac_me1-3	84.2	19.6	39.4	33.1	Ace: K12, N-term, Met: K16, K20	11341.3937	C500H845N163O136S

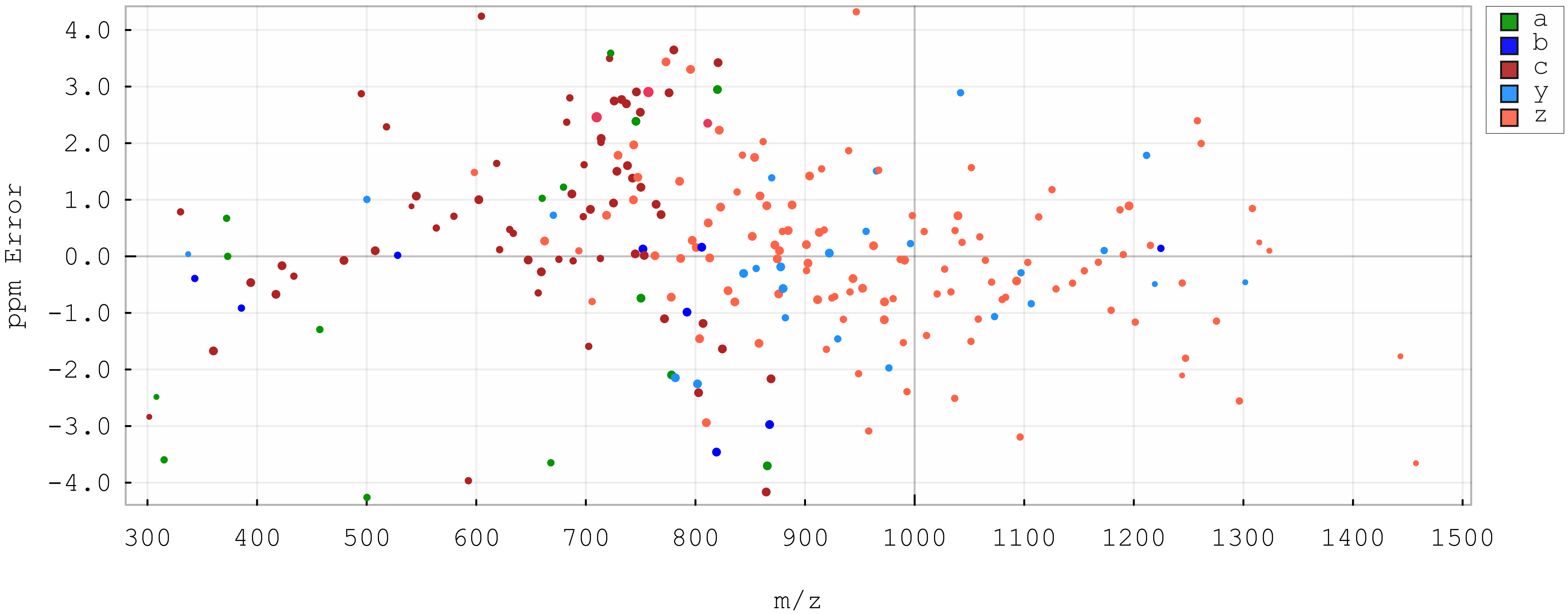

Proteoform:

Index	Protein	SC [%]	SVP [%]	IC [%]	MS Score [a.u.]	Var. Mods	Mono Mass [Da]	Chemical Formula
7	H4Kac_me1-3	83.2	4.9	39.6	33.0	Ace: K5, K12, Met: K16, K20	11341.3937	C500H845N163O136S

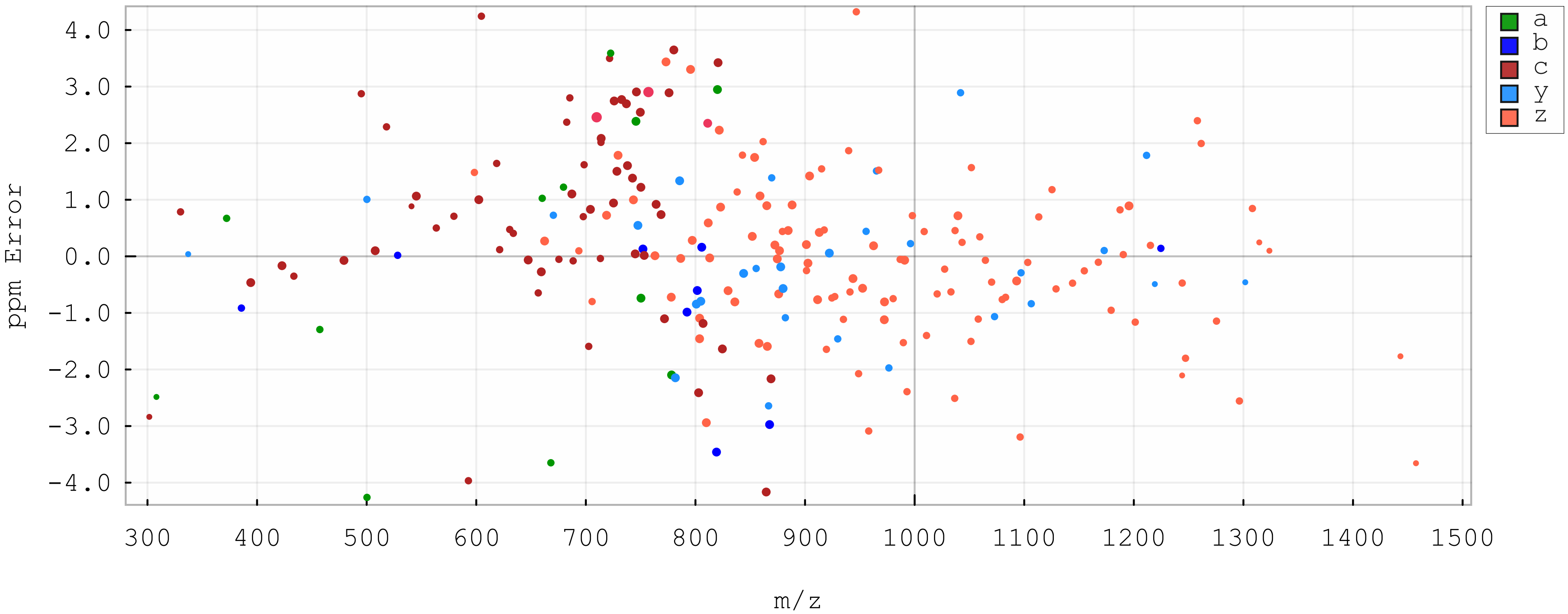

Proteoform:

Index	Protein	SC [%]	SVP [%]	IC [%]	MS Score [a.u.]	Var. Mods	Mono Mass [Da]	Chemical Formula
8	H4Kac_me1-3	81.2	19.6	40.4	32.8	Dim: K16, Ace: K20, N-term	11341.3937	C500H845N163O136S

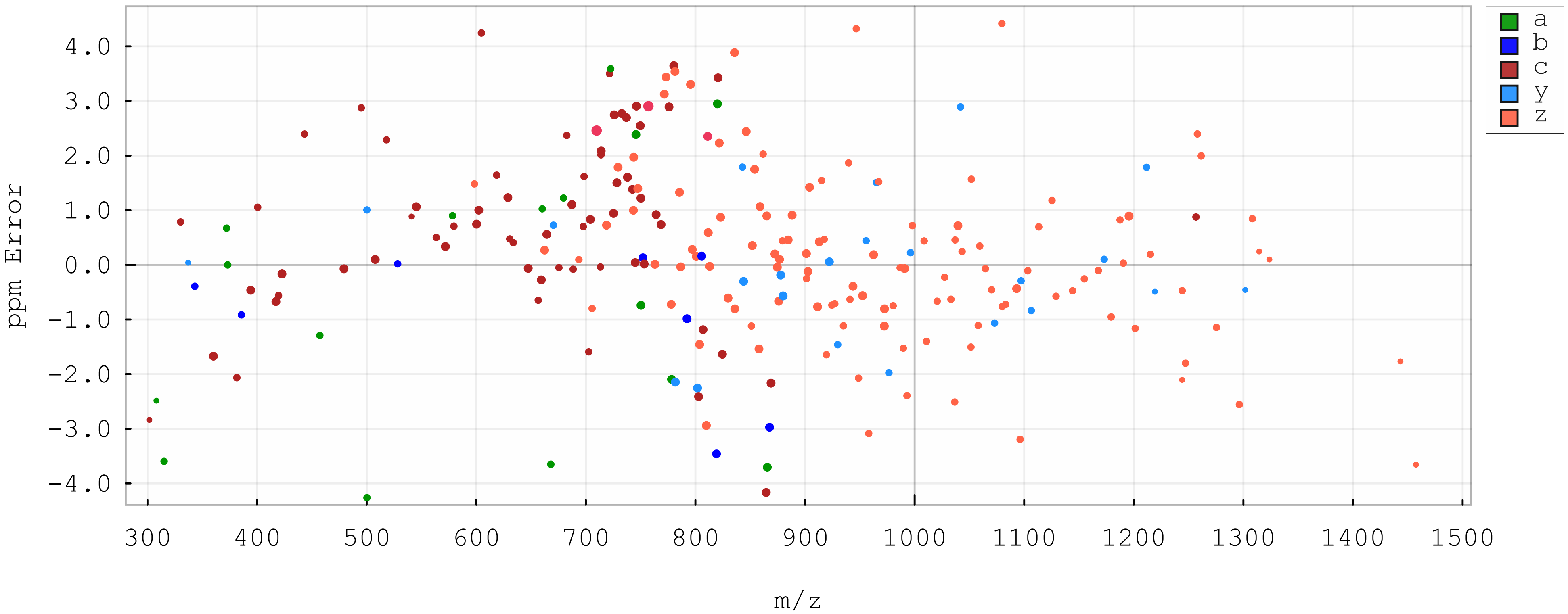

Proteoform:

Index	Protein	SC [%]	SVP [%]	IC [%]	MS Score [a.u.]	Var. Mods	Mono Mass [Da]	Chemical Formula
9	H4Kac_me1-3	80.2	4.9	40.7	32.6	Ace: K5, K20, Dim: K16	11341.3937	C500H845N163O136S

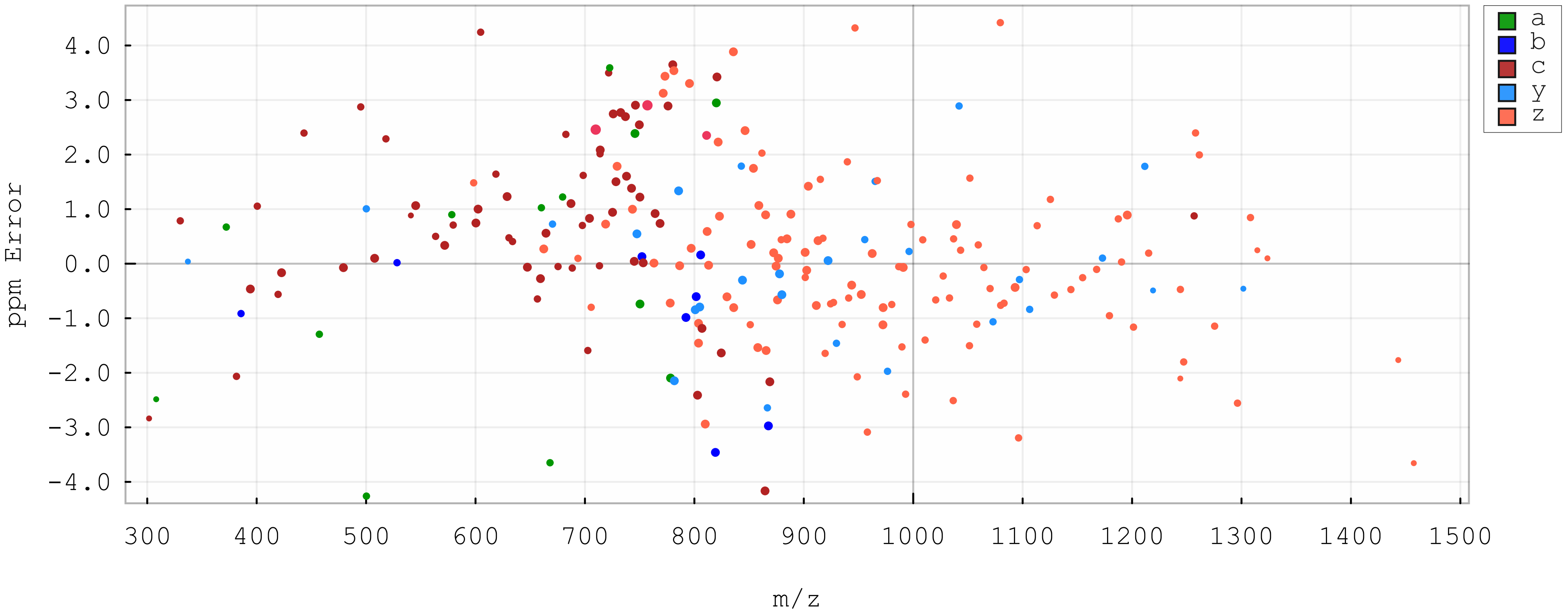

Proteoform:

Index	Protein	SC [%]	SVP [%]	IC [%]	MS Score [a.u.]	Var. Mods	Mono Mass [Da]	Chemical Formula
10	H4Kac_me1-3	81.2	4.9	39.8	32.4	Ace: K8, K12, Dim: K20	11341.3937	C500H845N163O136S

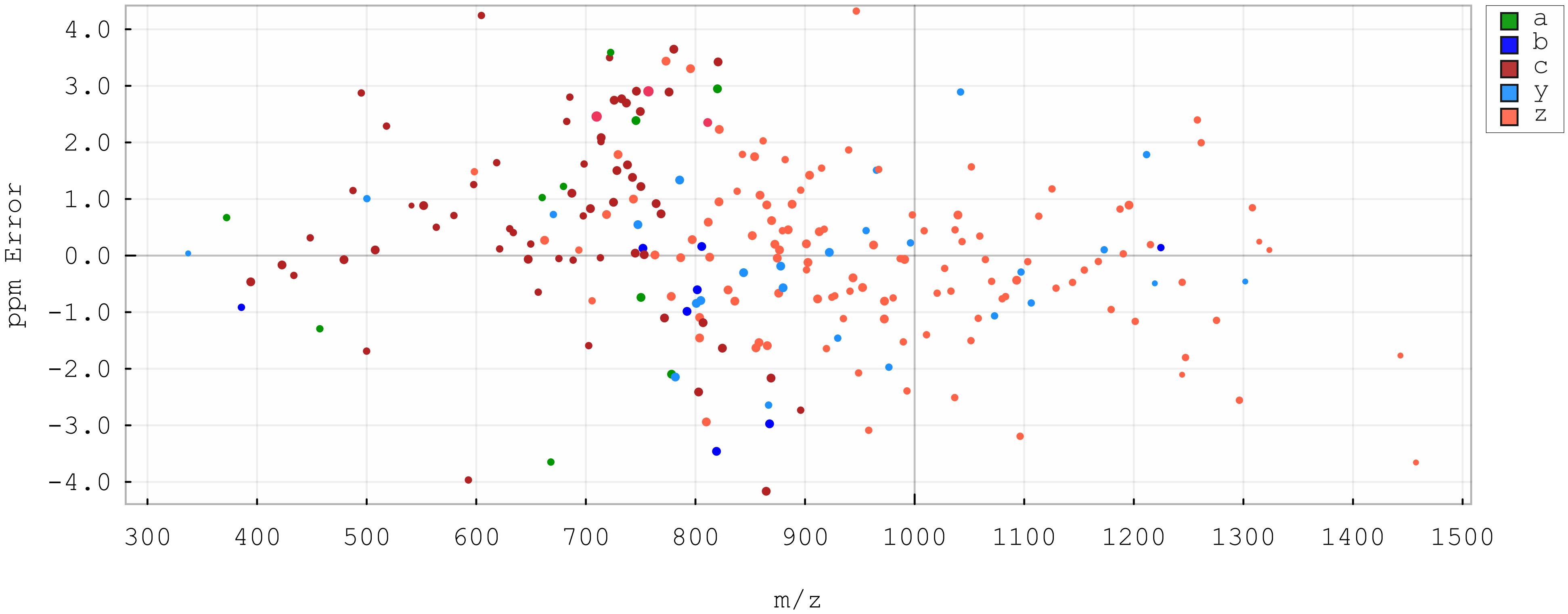

Primary Fragment Plots

Proteoform:

Index	Protein	SC [%]	SVP [%]	IC [%]	MS Score [a.u.]	Var. Mods	Mono Mass [Da]	Chemical Formula
1	H4Kac_me1-3	85.1	23.5	41.6	35.4	Ace: K16, N-term, Dim: K20	11341.3937	C500H845N163O136S

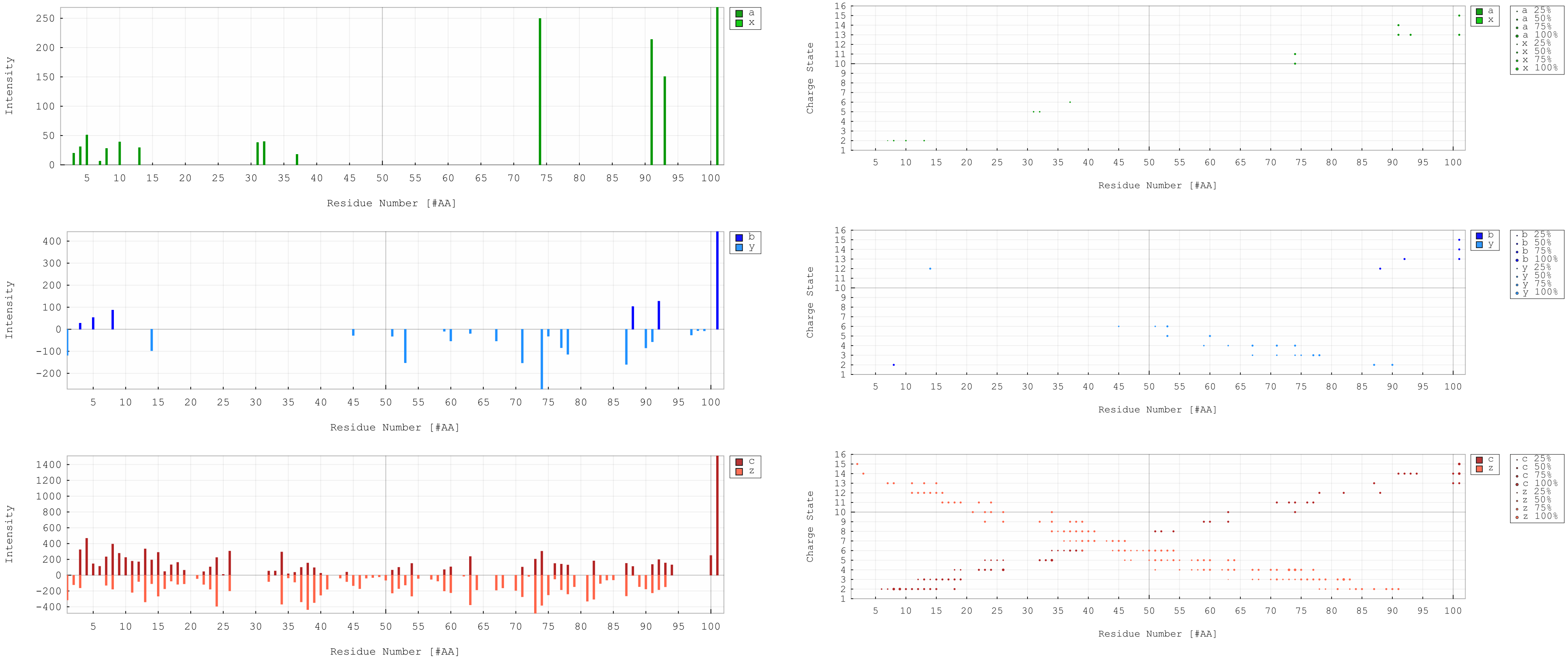

Proteoform:

Index	Protein	SC [%]	SVP [%]	IC [%]	MS Score [a.u.]	Var. Mods	Mono Mass [Da]	Chemical Formula
2	H4Kac_me1-3	84.2	4.9	41.9	35.2	Ace: K5, K16, Dim: K20	11341.3937	C500H845N163O136S

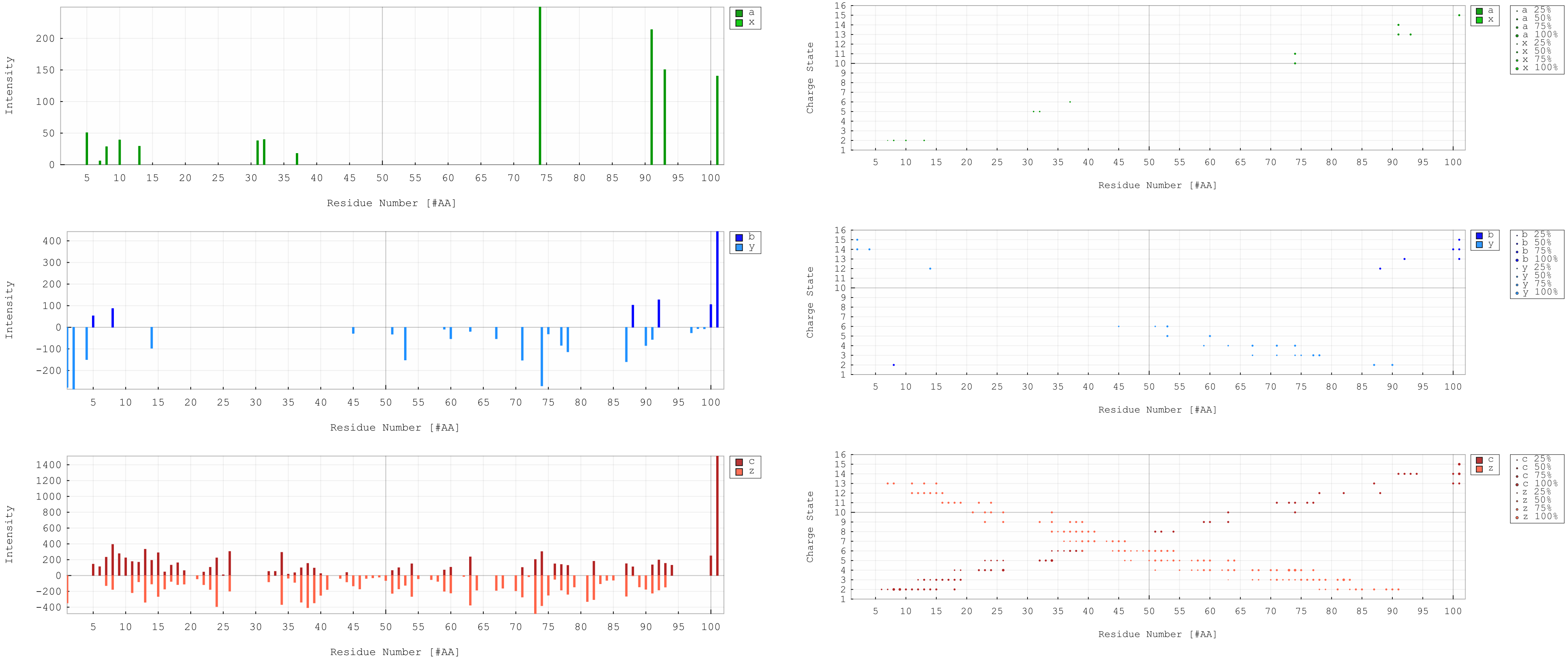

Proteoform:

Index	Protein	SC [%]	SVP [%]	IC [%]	MS Score [a.u.]	Var. Mods	Mono Mass [Da]	Chemical Formula
3	H4Kac_me1-3	85.1	23.5	40.0	34.1	Ace: K12, N-term, Dim:<br>K20	11341.3937	C500H845N163O136S

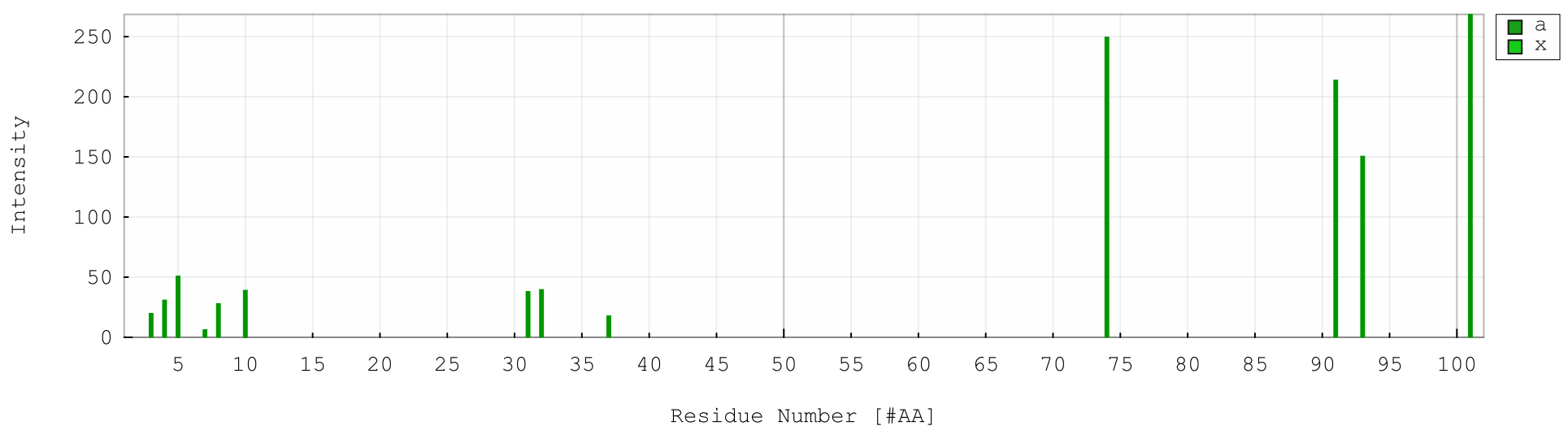

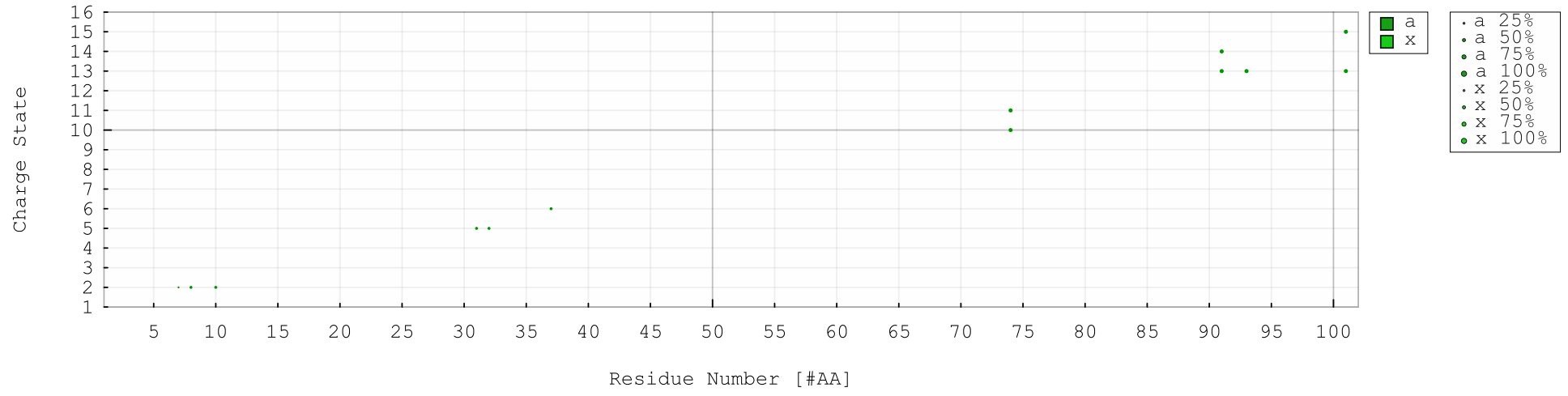

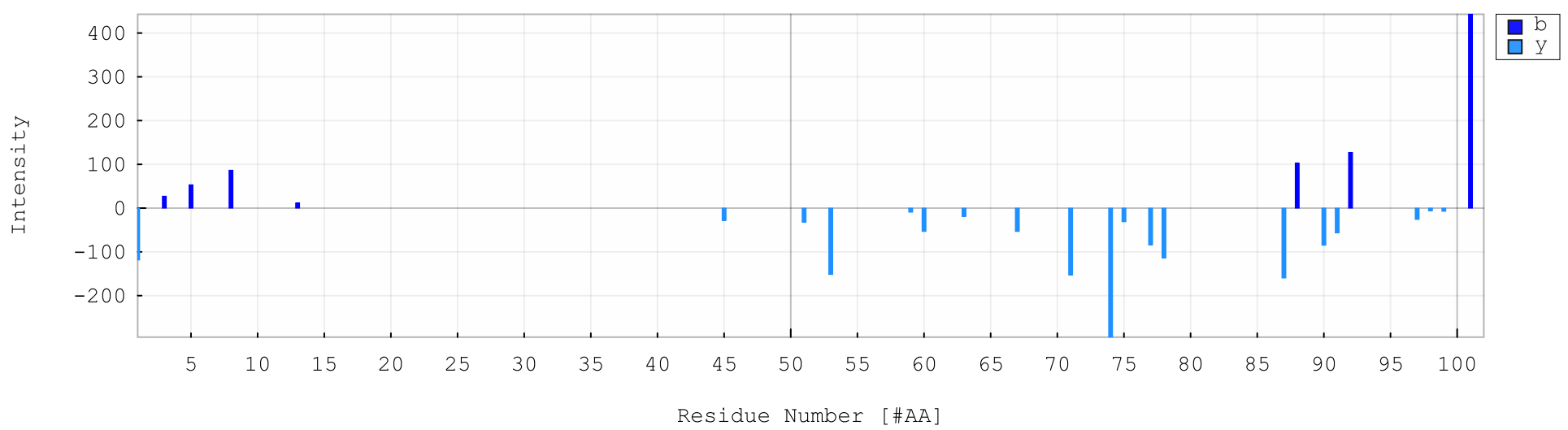

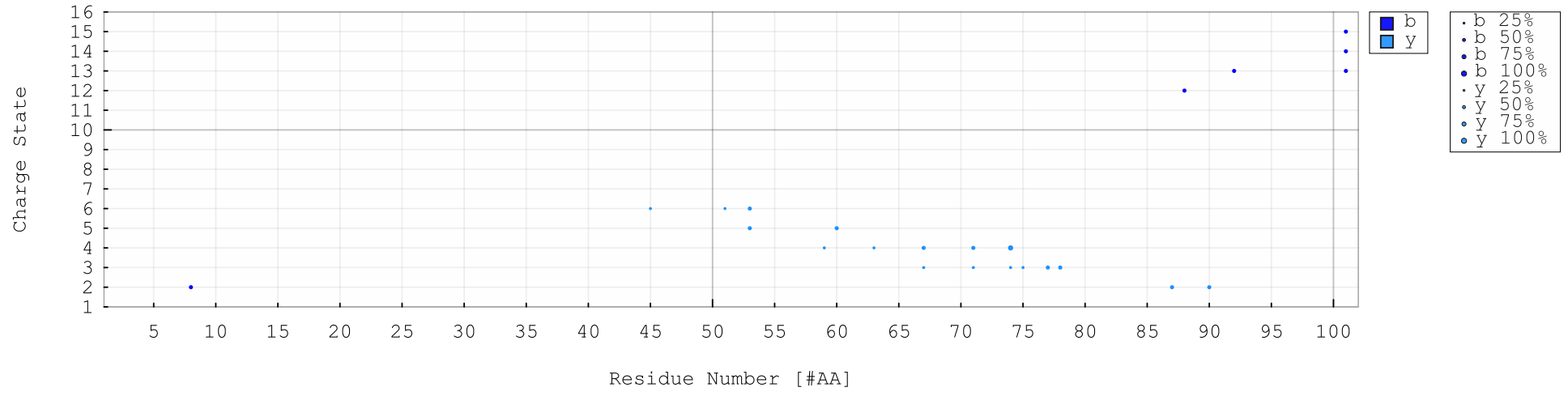

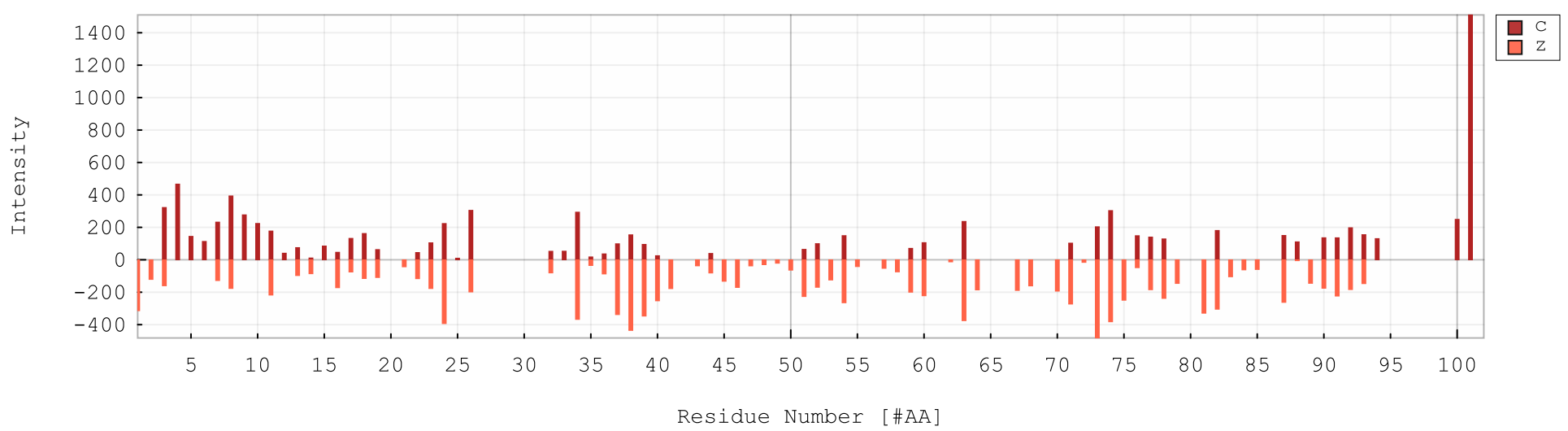

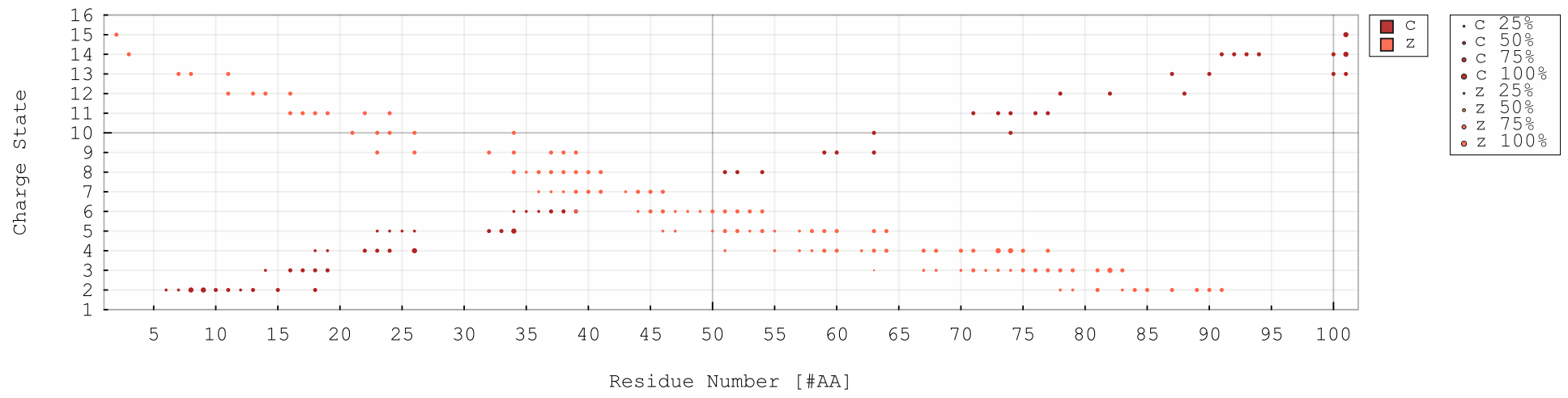

Proteoform:

Index	Protein	SC [%]	SVP [%]	IC [%]	MS Score [a.u.]	Var. Mods	Mono Mass [Da]	Chemical Formula
4	H4Kac_me1-3	84.2	4.9	40.3	33.9	Ace: K5, K12, Dim: K20	11341.3937	C500H845N163O136S

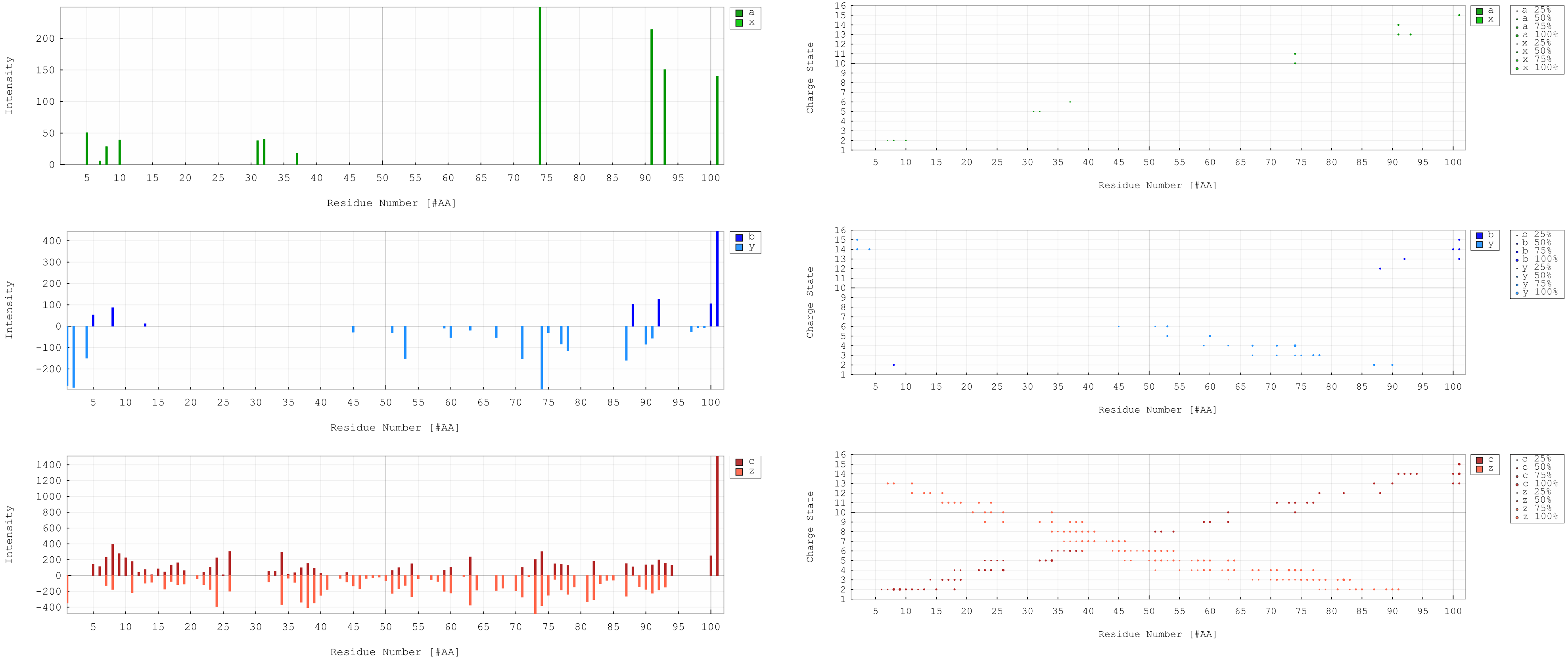

Proteoform:

Index	Protein	SC [%]	SVP [%]	IC [%]	MS Score [a.u.]	Var. Mods	Mono Mass [Da]	Chemical Formula
5	H4Kac_me1-3	81.2	4.9	41.4	33.6	Ace: K8, K16, Dim: K20	11341.3937	C500H845N163O136S

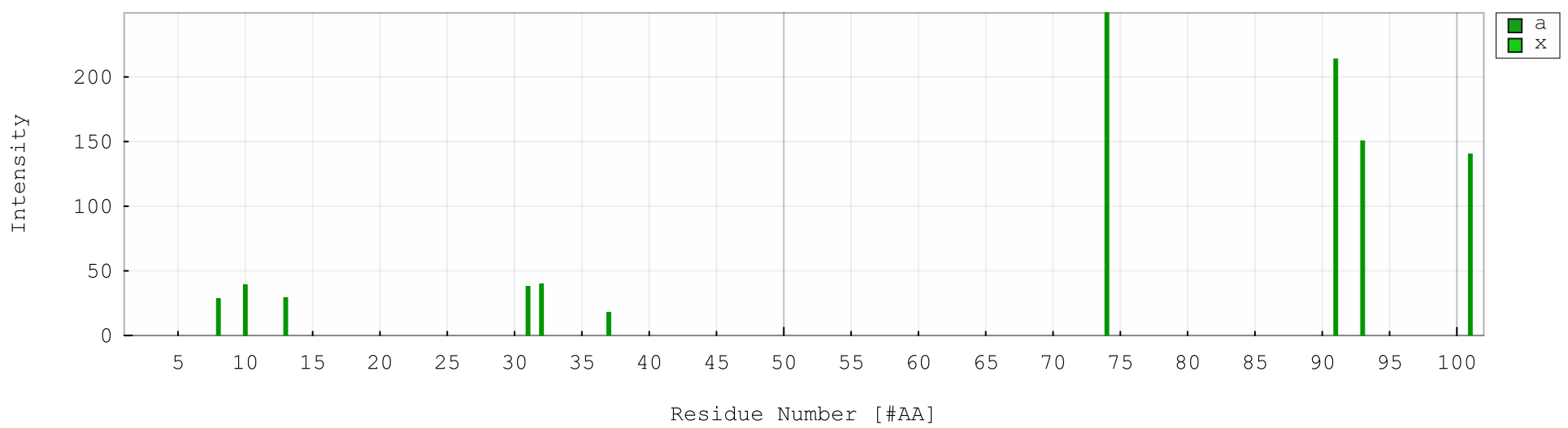

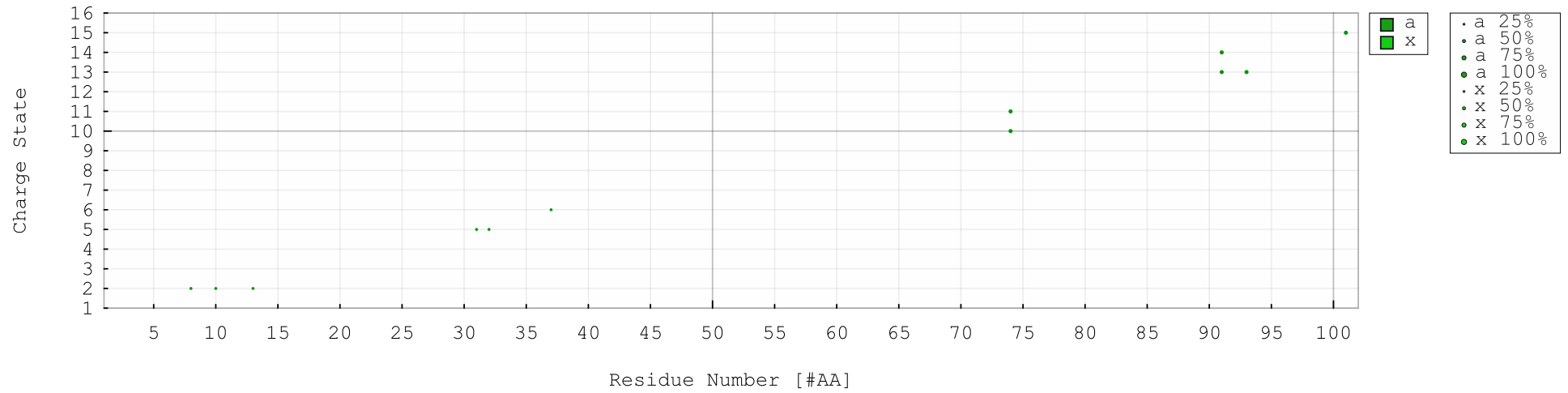

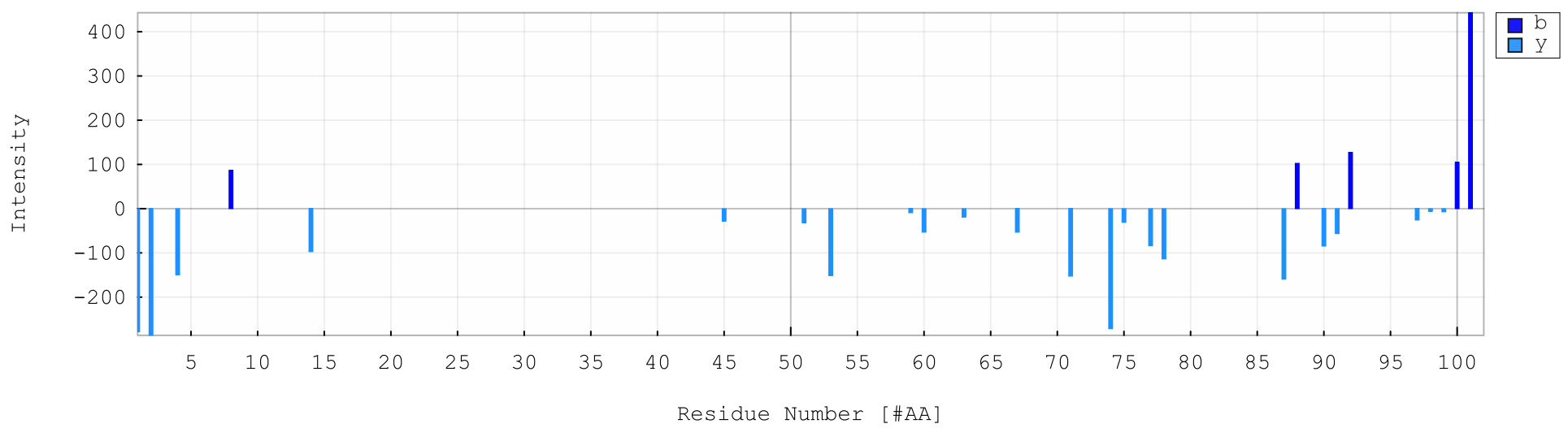

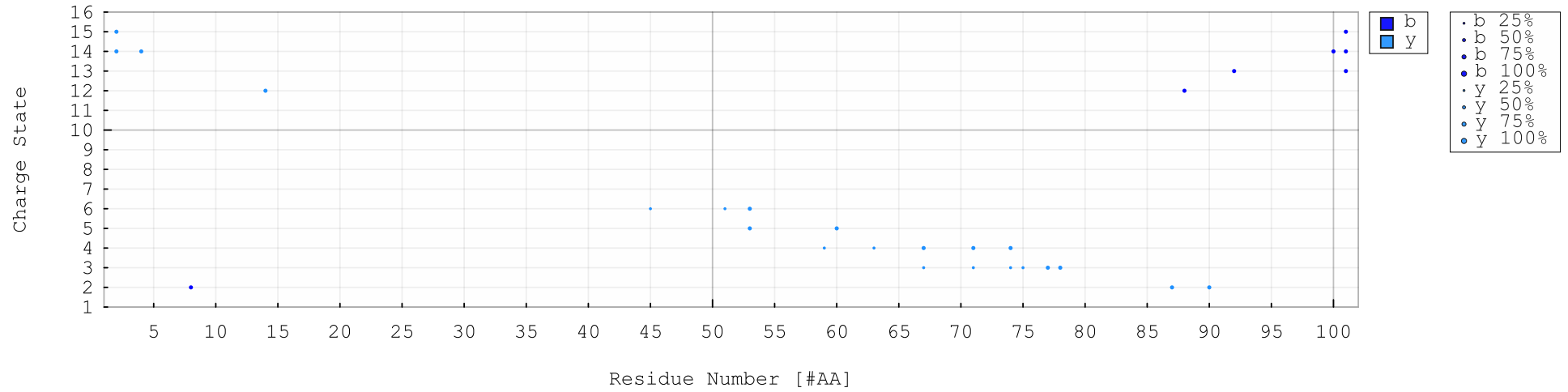

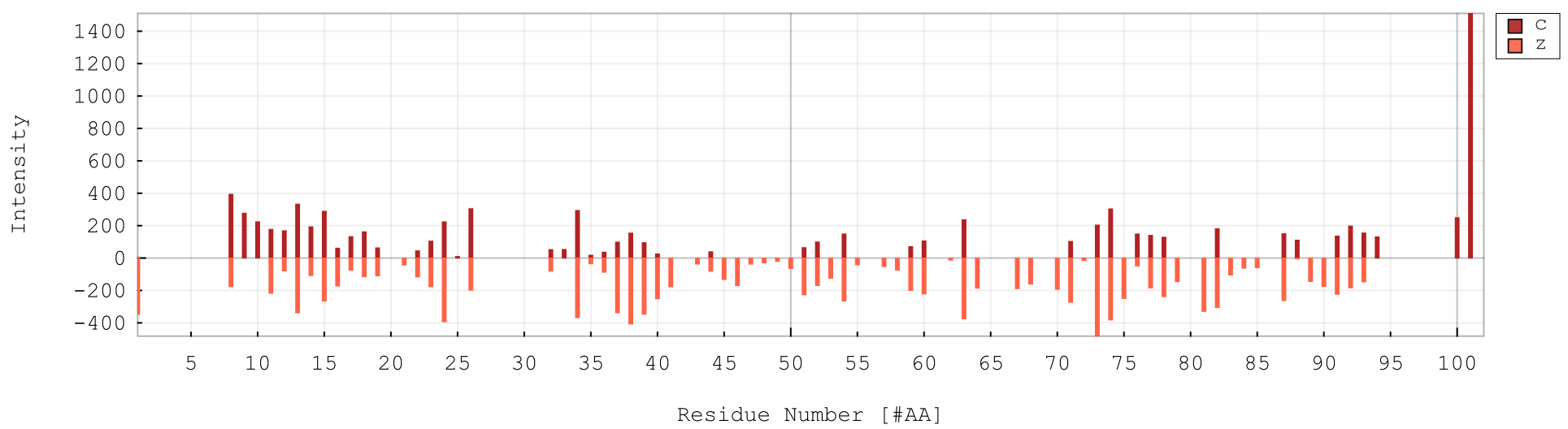

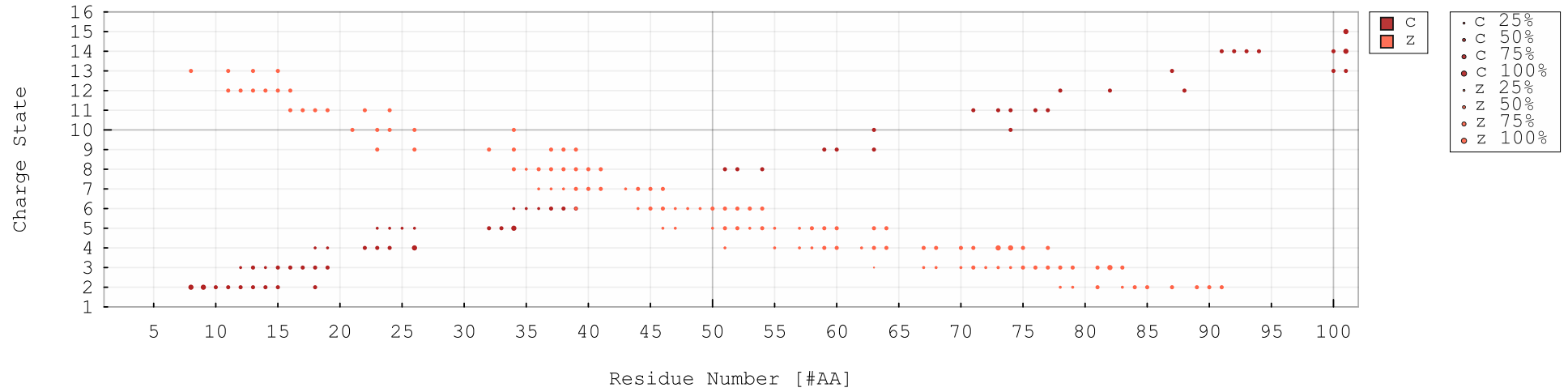

Proteoform:

Index	Protein	SC [%]	SVP [%]	IC [%]	MS Score [a.u.]	Var. Mods	Mono Mass [Da]	Chemical Formula
6	H4Kac_me1-3	84.2	19.6	39.4	33.1	Ace: K12, K16, K20 N-term, Met:	11341.3937	C500H845N163O136S

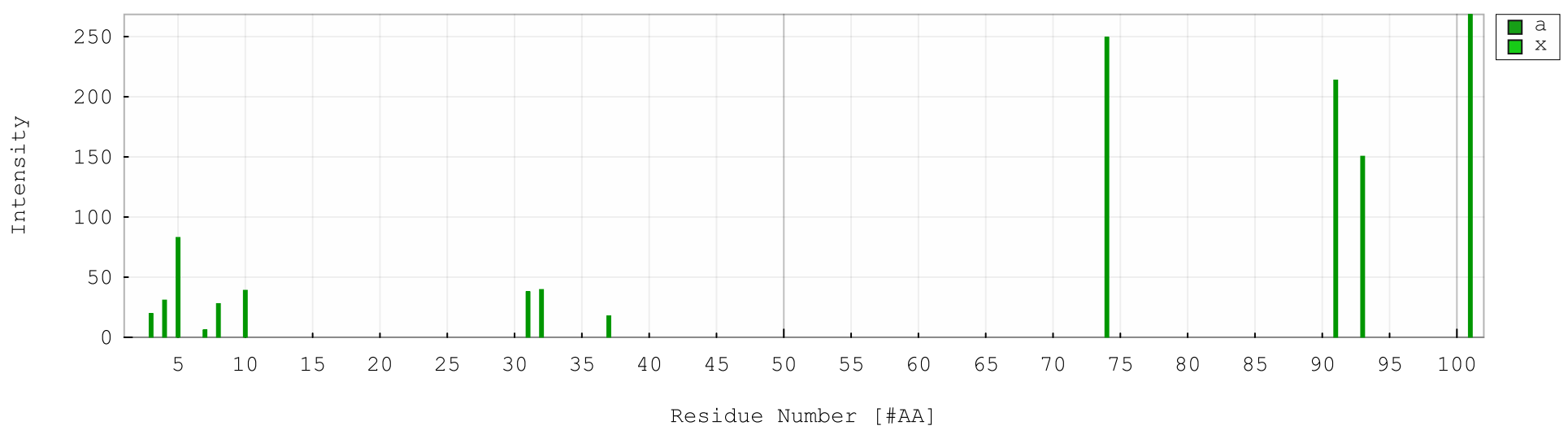

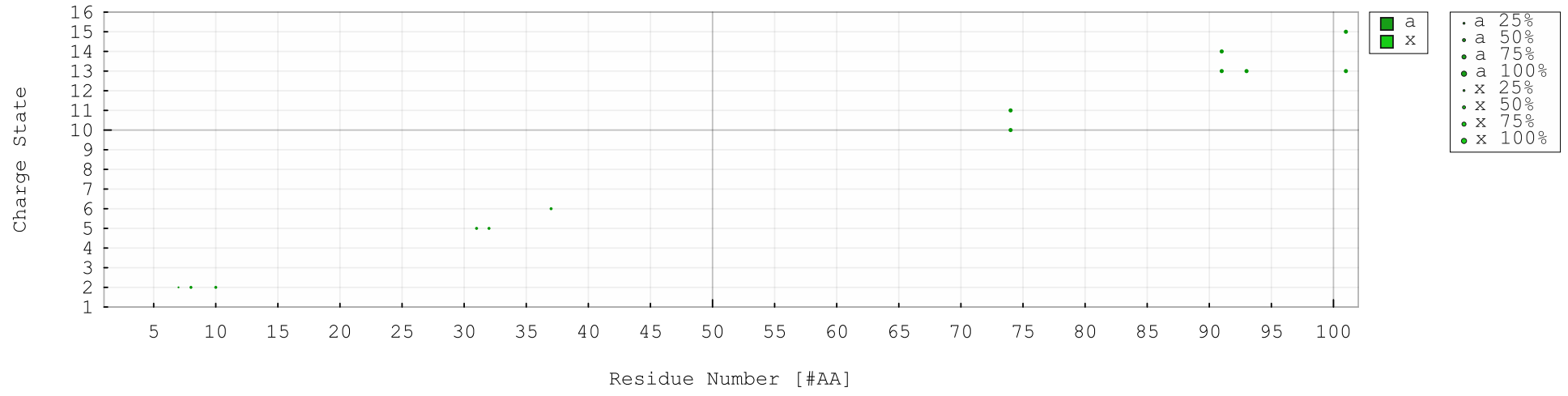

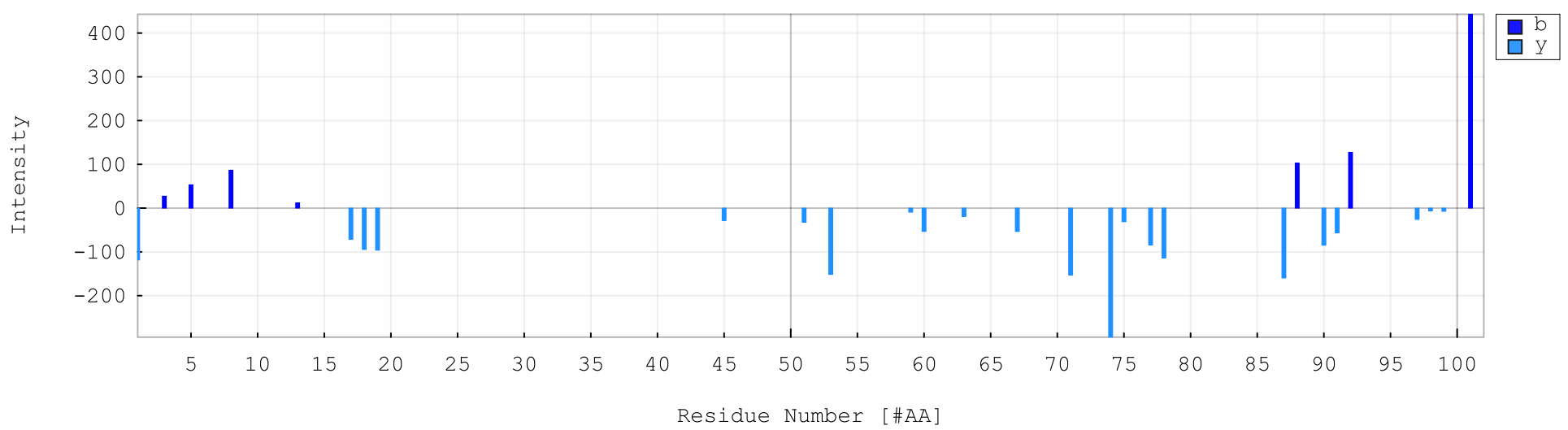

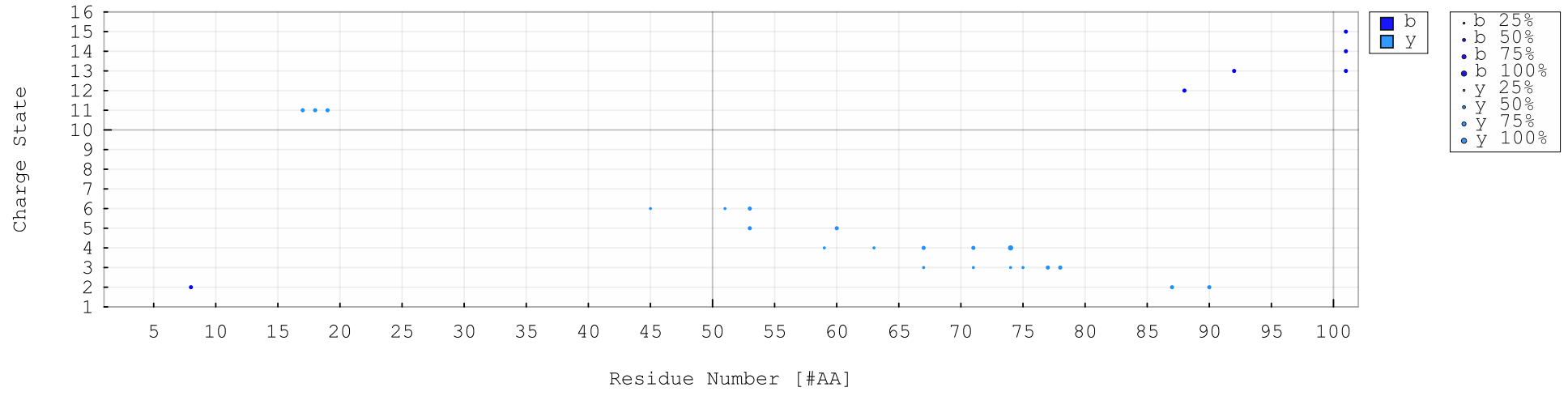

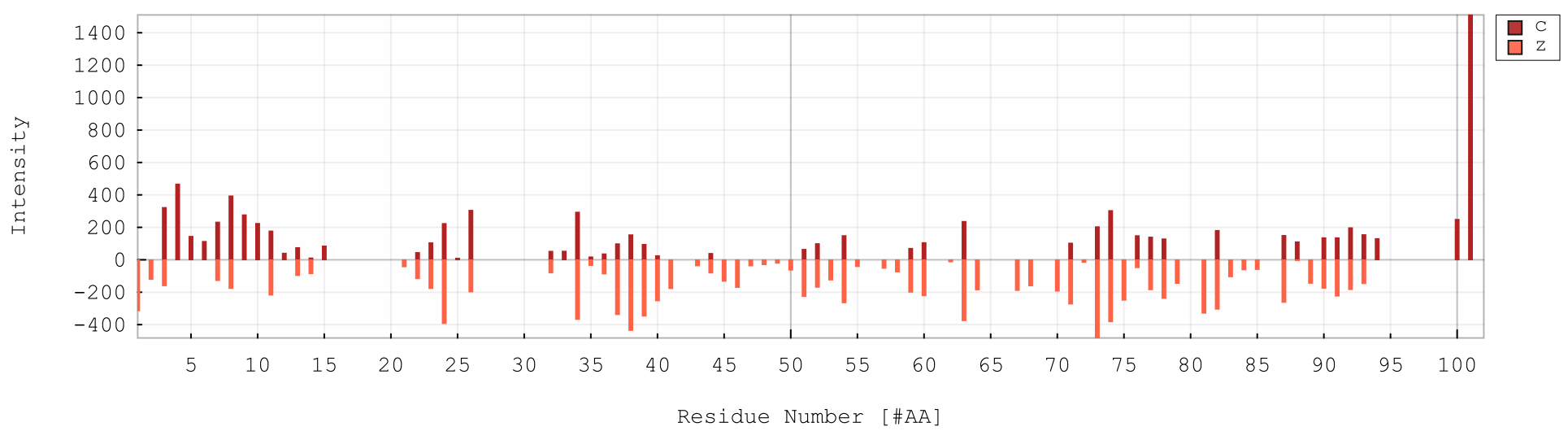

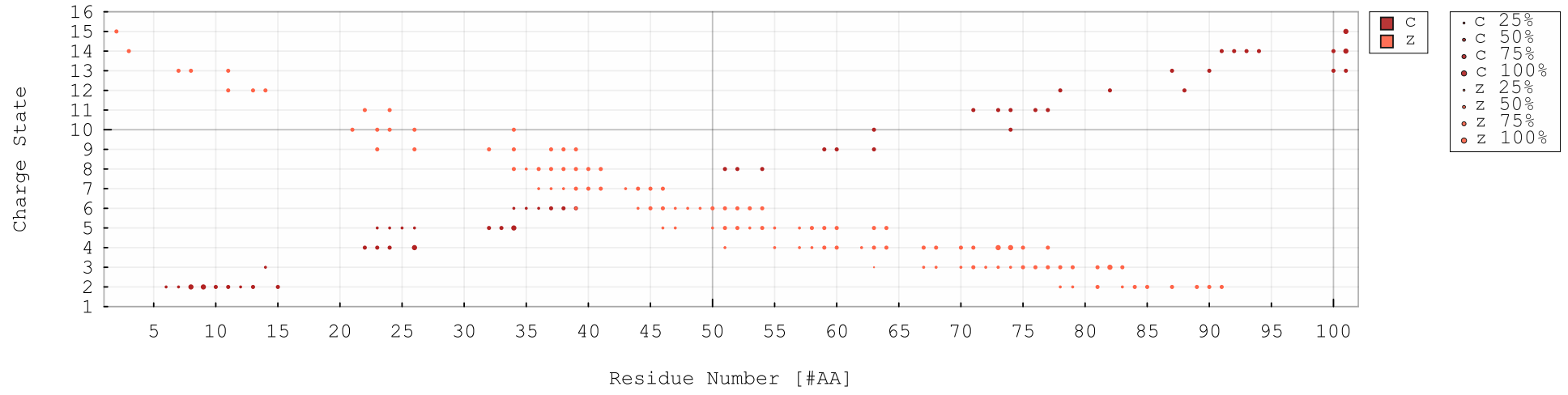

Proteoform:

Index	Protein	SC [%]	SVP [%]	IC [%]	MS Score [a.u.]	Var. Mods	Mono Mass [Da]	Chemical Formula
7	H4Kac_me1-3	83.2	4.9	39.6	33.0	Ace: K5, K12, Met: K16, K20	11341.3937	$C_{500}H_{845}N_{163}O_{136}S$

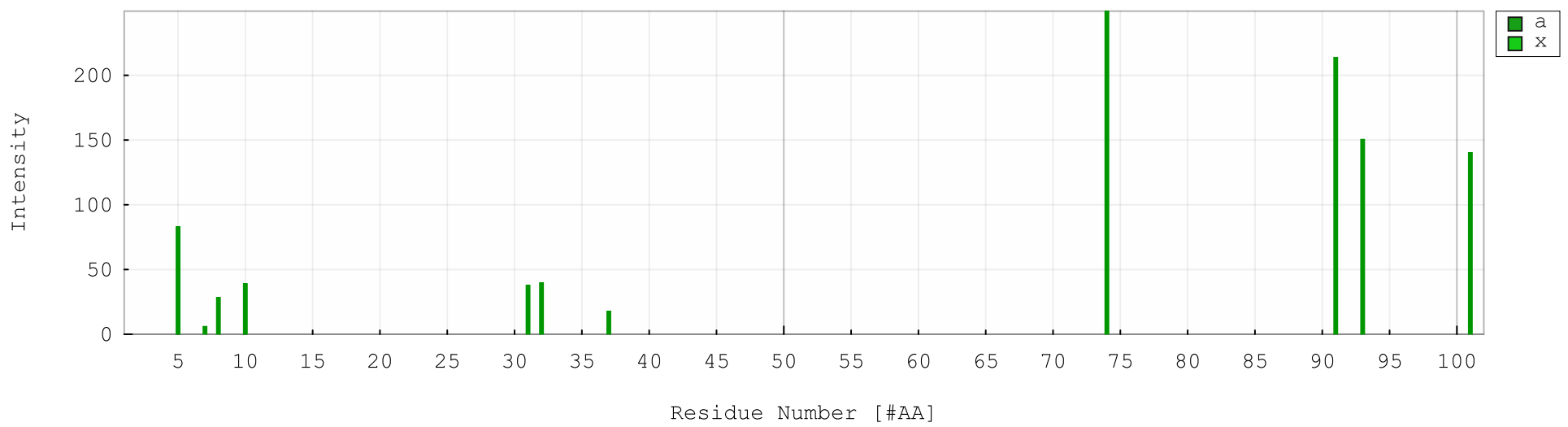

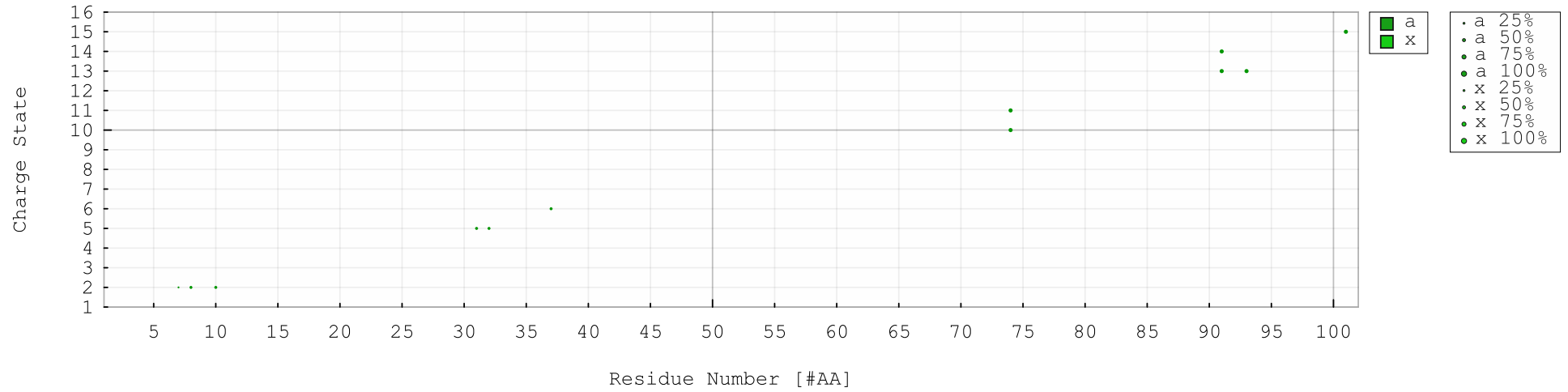

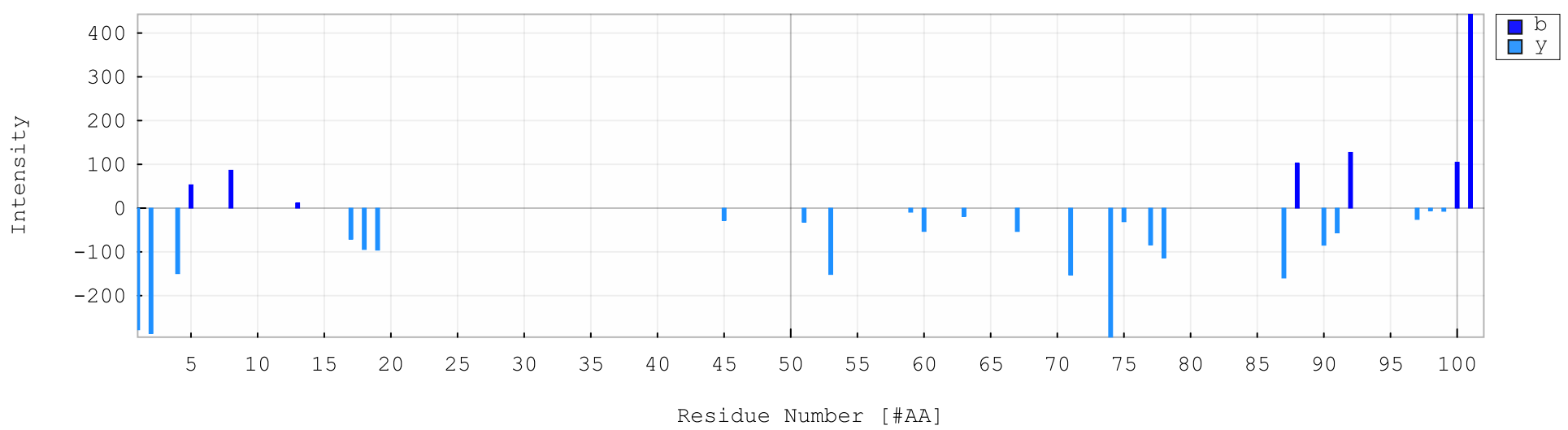

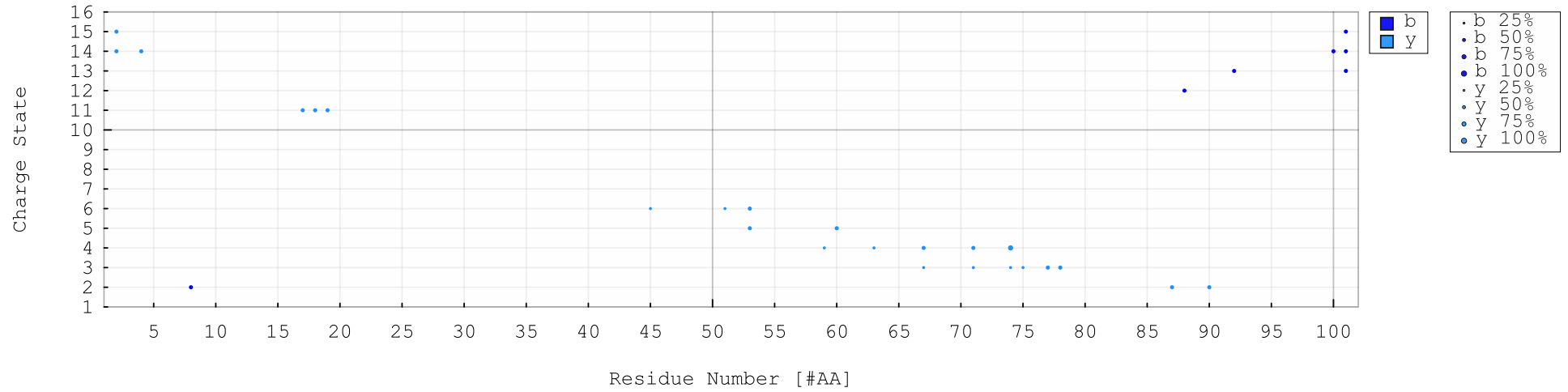

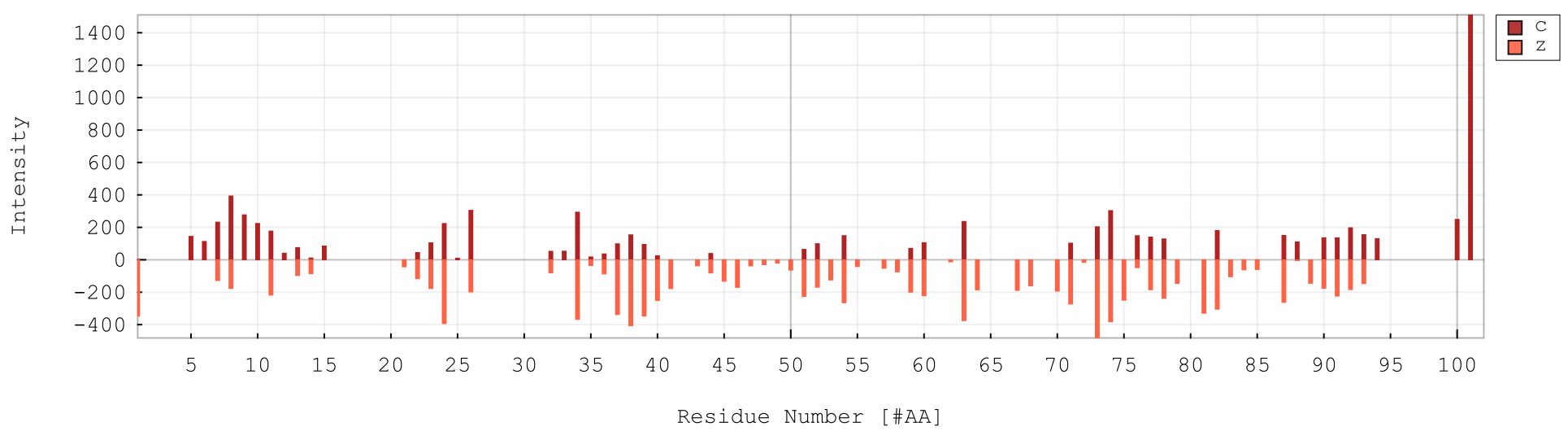

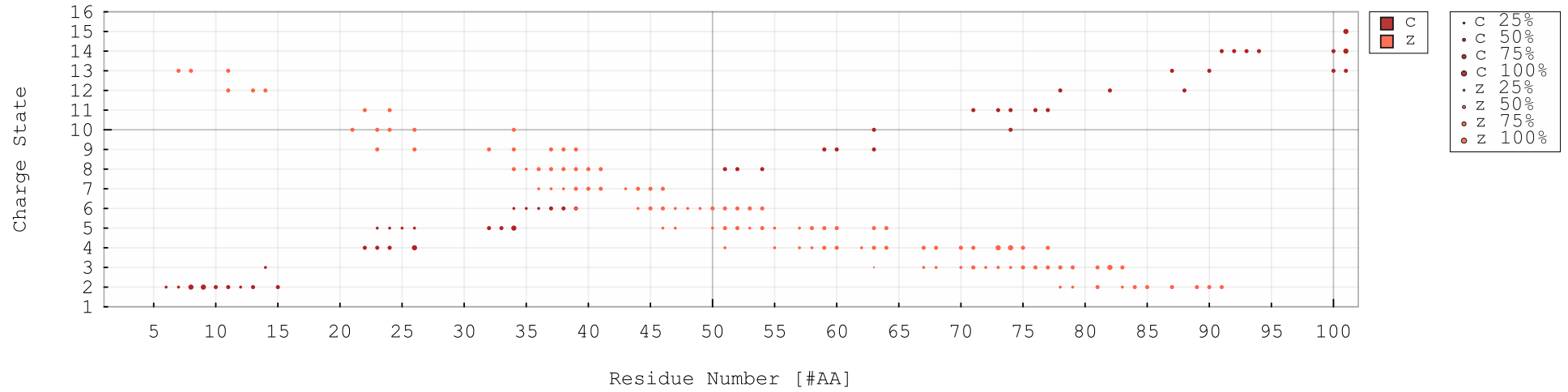

Proteoform:

Index	Protein	SC [%]	SVP [%]	IC [%]	MS Score [a.u.]	Var. Mods	Mono Mass [Da]	Chemical Formula
8	H4Kac_me1-3	81.2	19.6	40.4	32.8	Dim: K16, Ace: K20, N-term	11341.3937	C500H845N163O136S

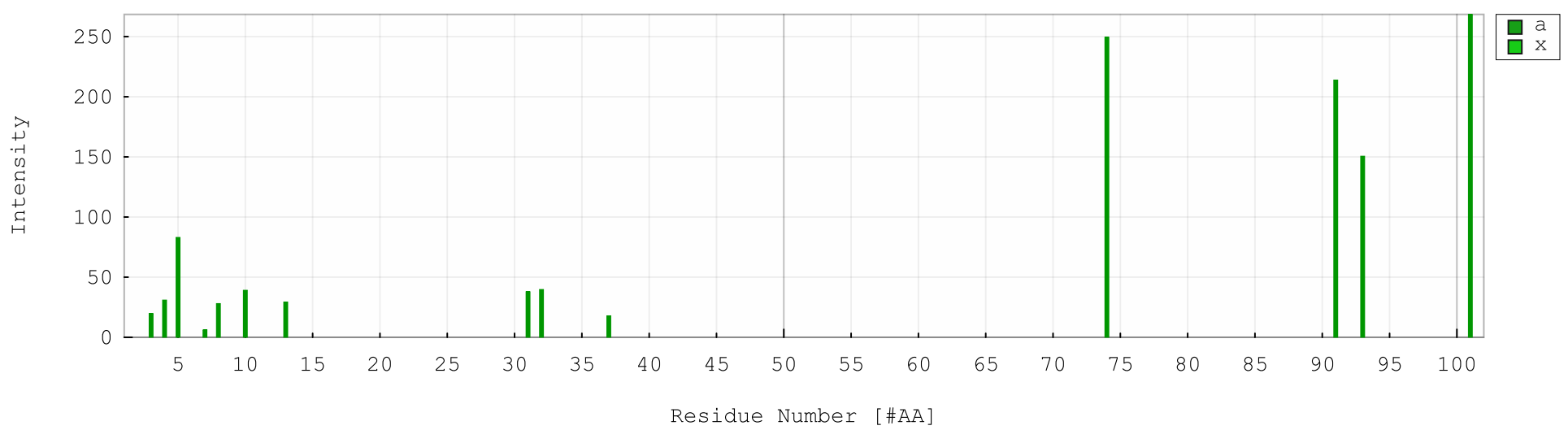

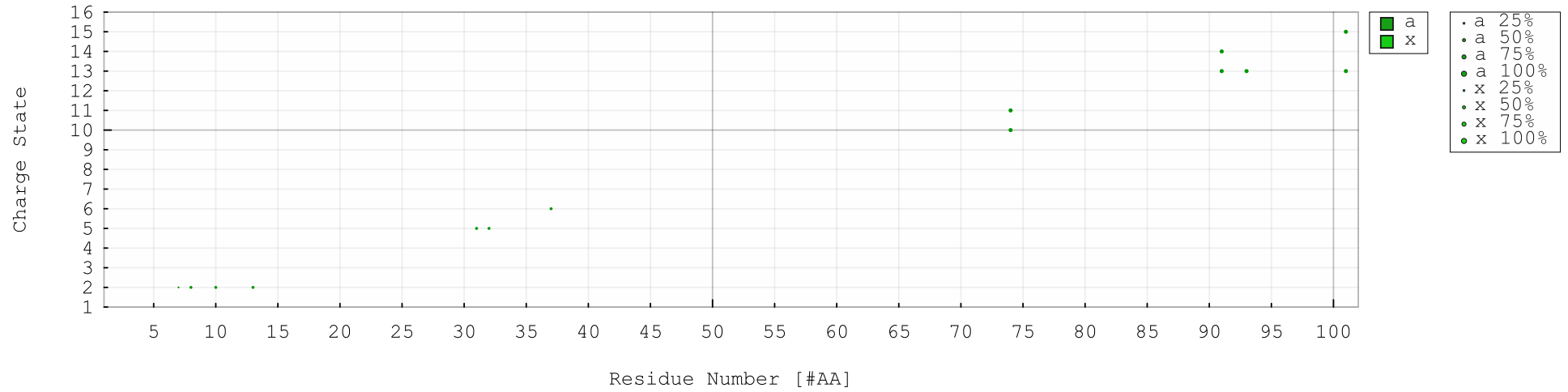

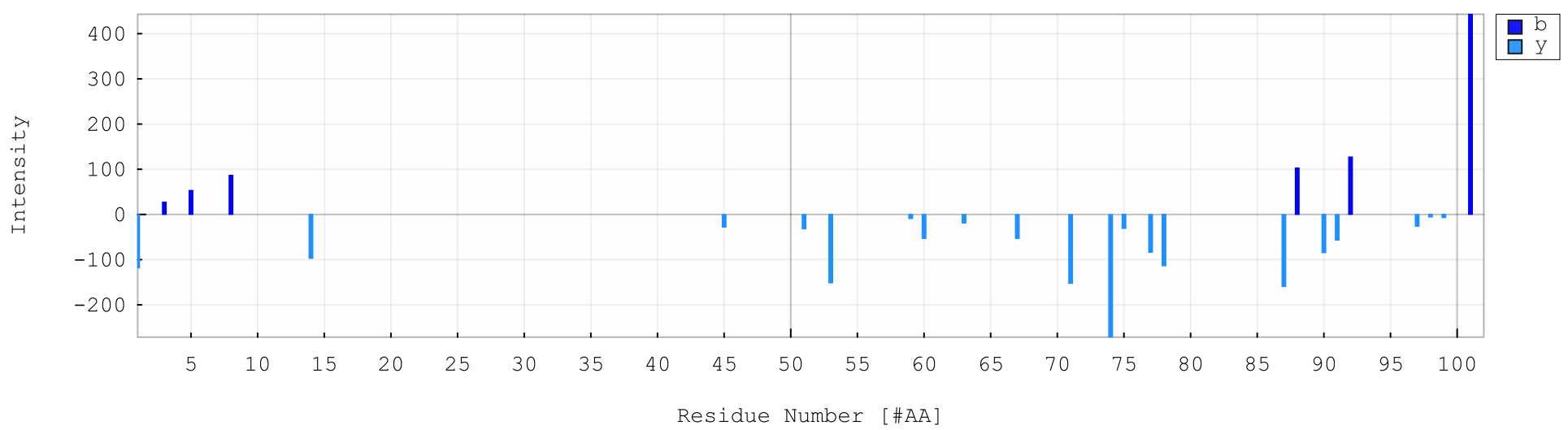

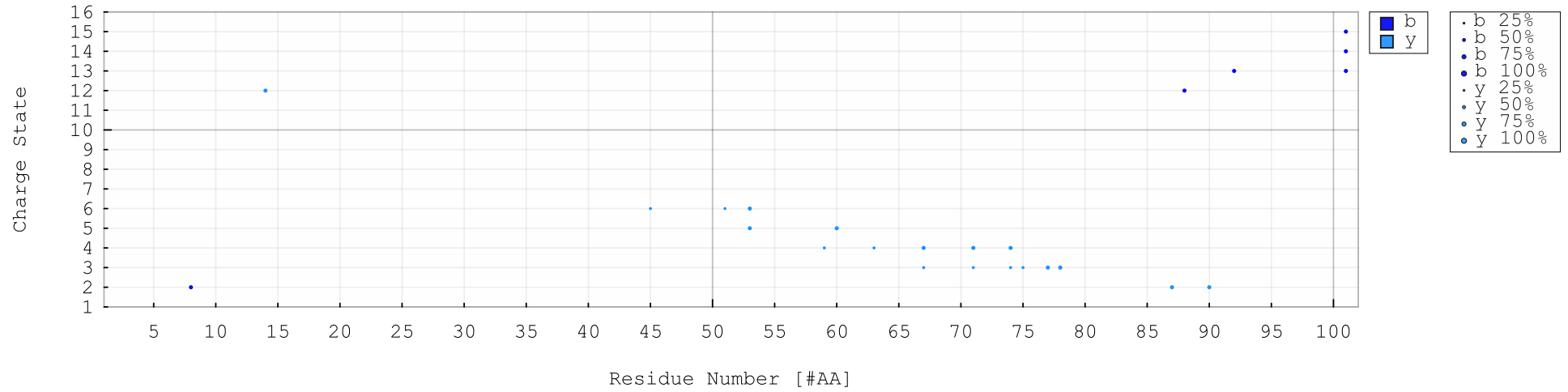

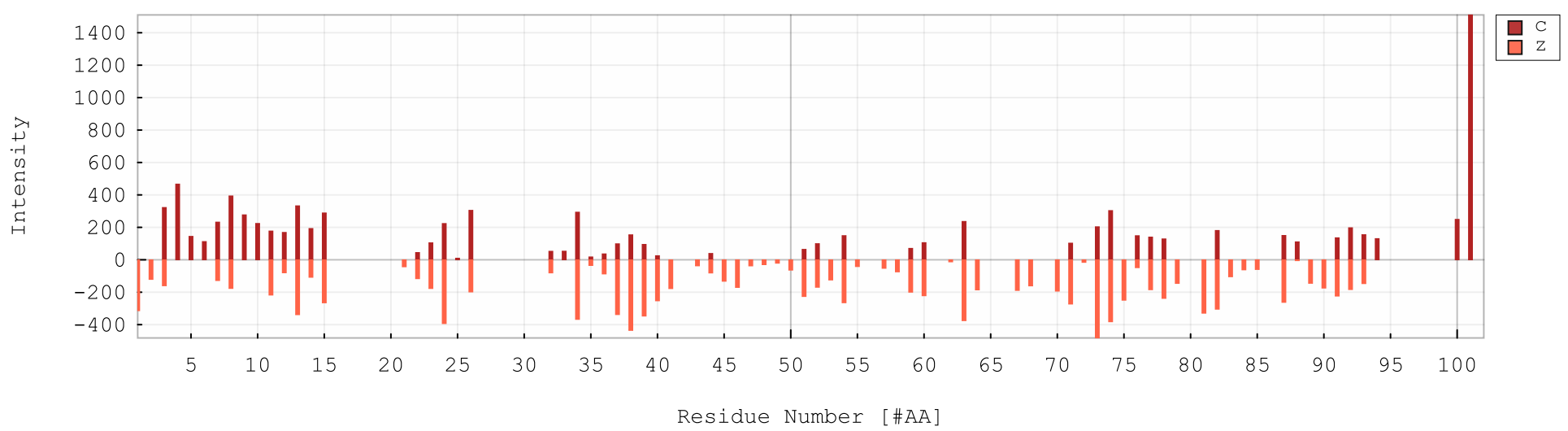

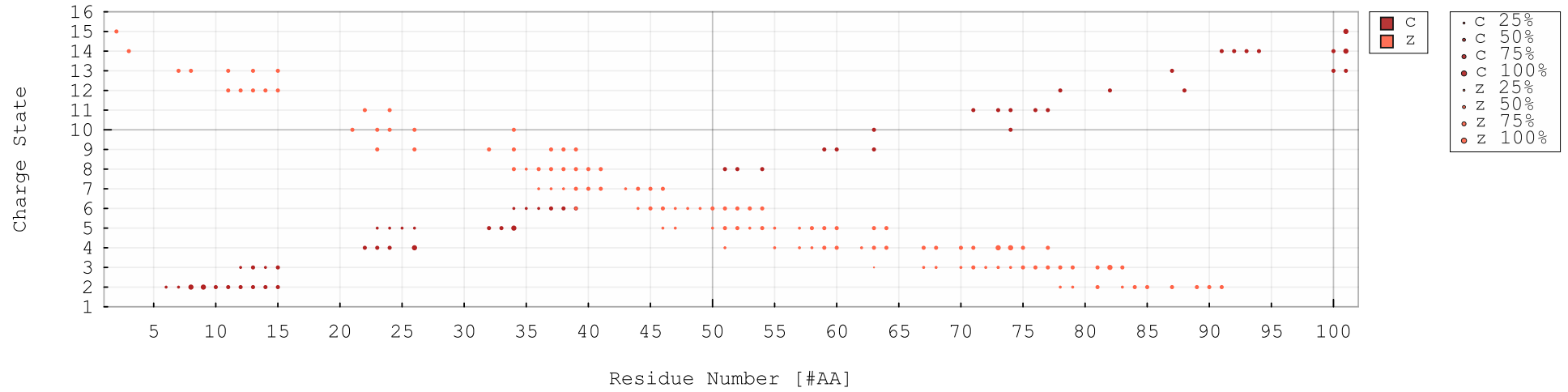

Proteoform:

Index	Protein	SC [%]	SVP [%]	IC [%]	MS Score [a.u.]	Var. Mods	Mono Mass [Da]	Chemical Formula
9	H4Kac_me1-3	80.2	4.9	40.7	32.6	Ace: K5, K20, Dim: K16	11341.3937	C500H845N163O136S

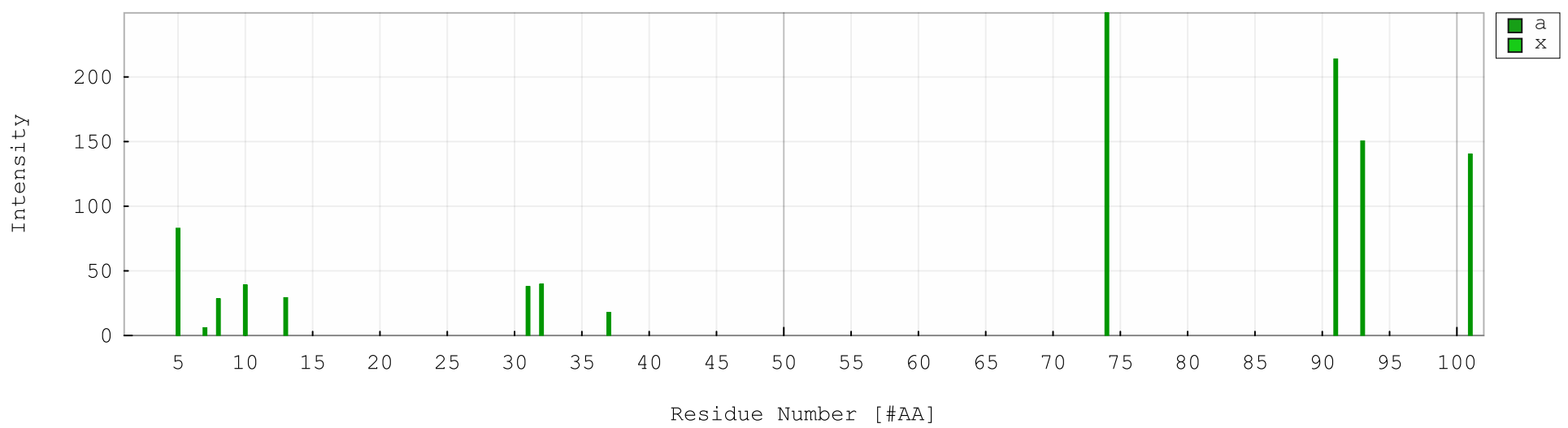

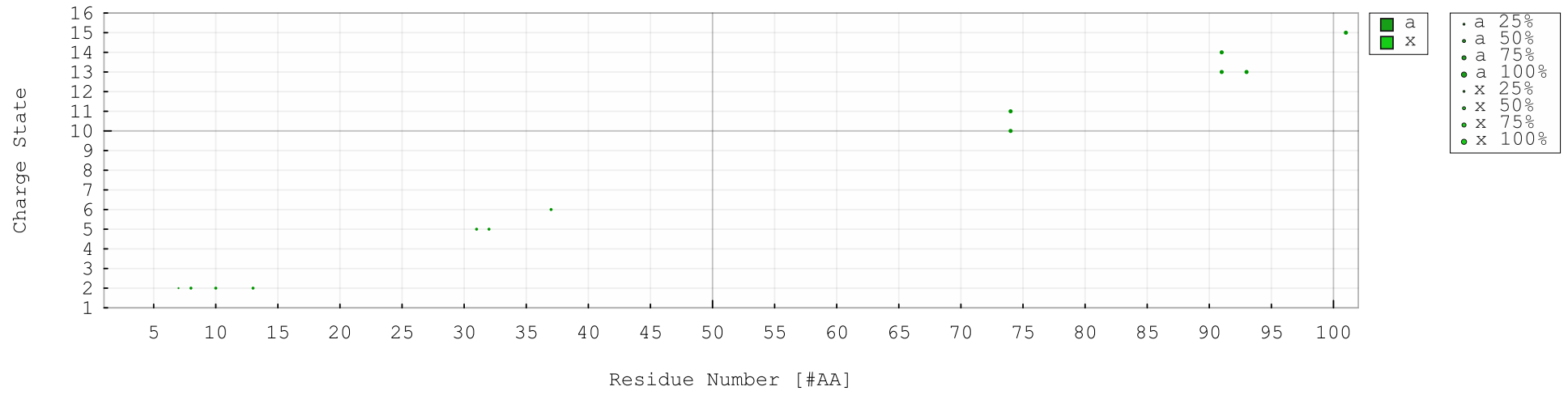

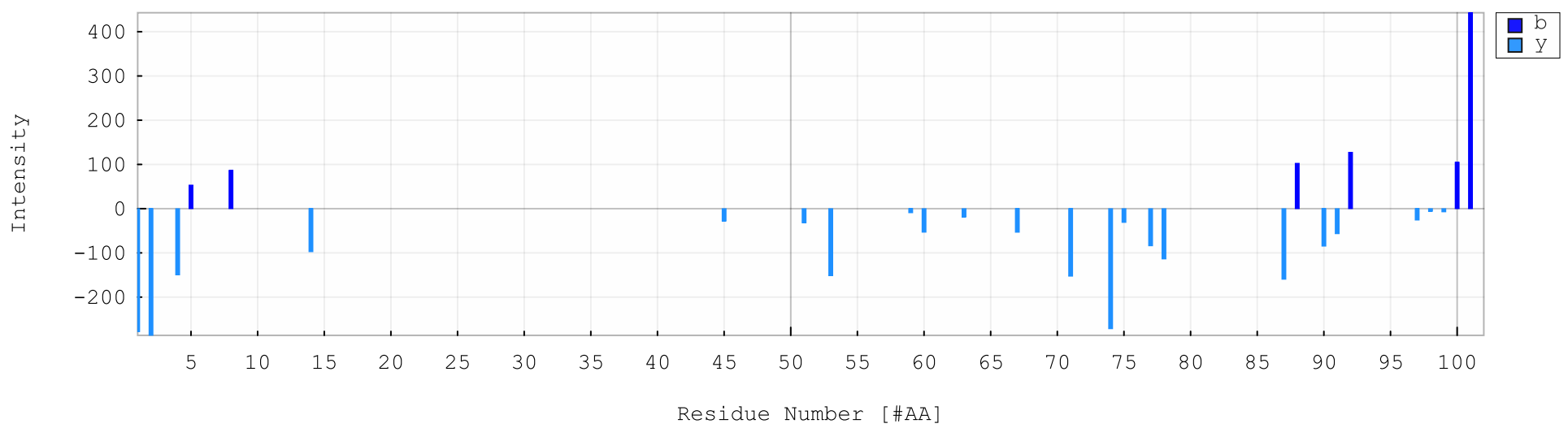

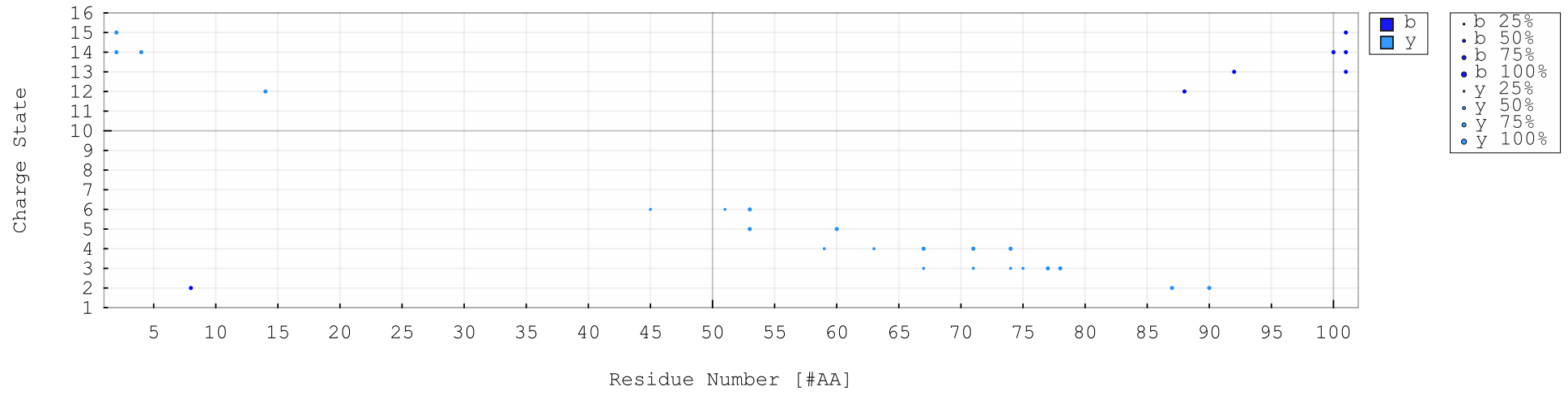

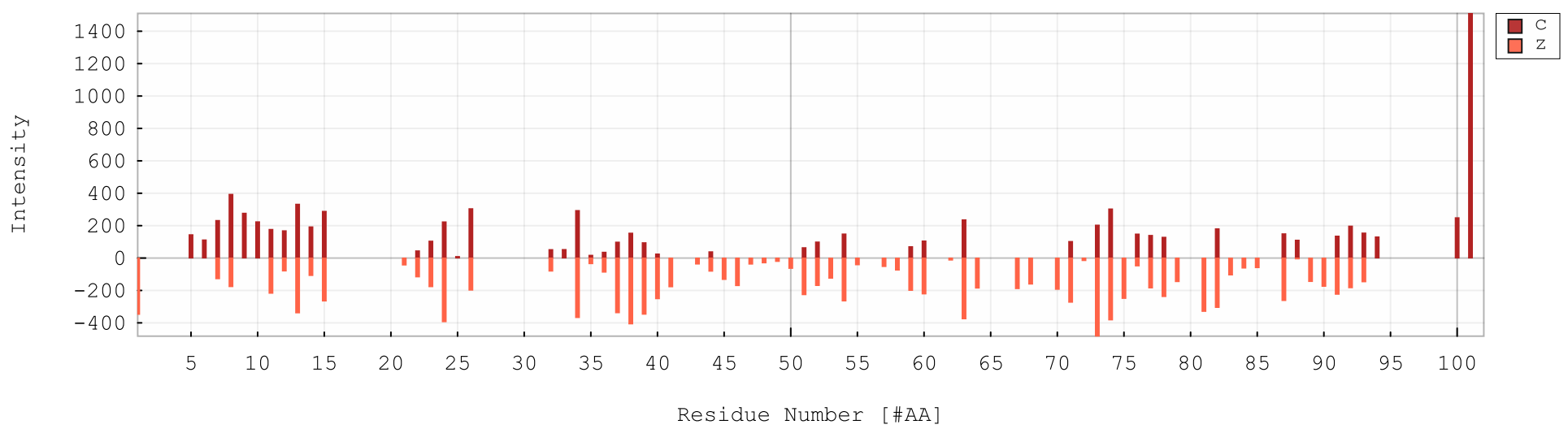

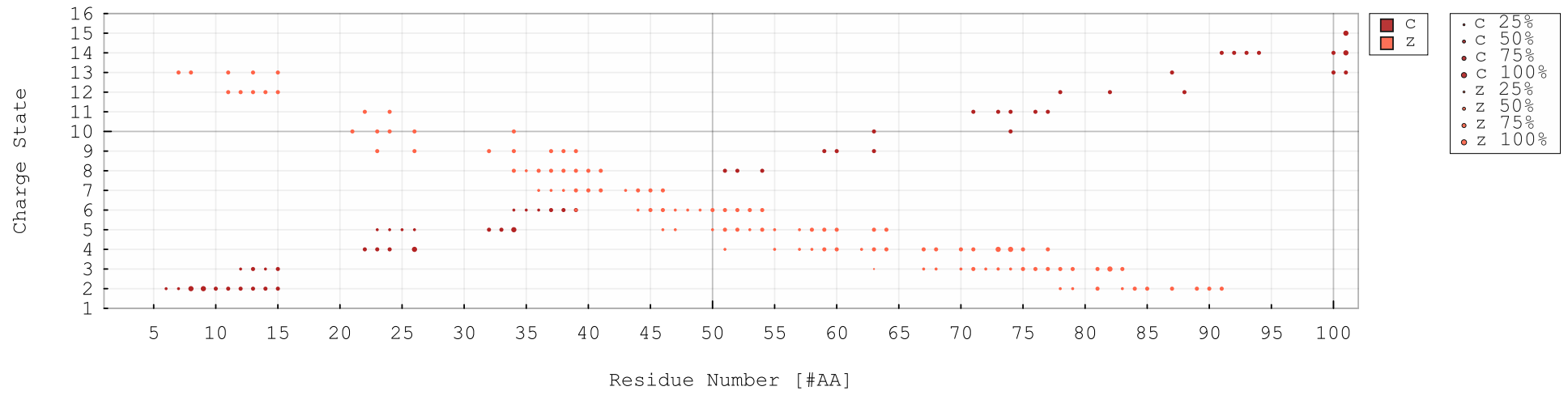

Top-Down Result OmniScape 2025
Sequence Confirmation
250430 Liver-Histone-25 mz710z16 MS2 ECD_001-478_calibrated

Proteoform:

Index	Protein	SC [%]	SVP [%]	IC [%]	MS Score [a.u.]	Var. Mods	Mono Mass [Da]	Chemical Formula
10	H4Kac_me1-3	81.2	4.9	39.8	32.4	Ace: K8, K12, Dim: K20	11341.3937	C500H845N163O136S

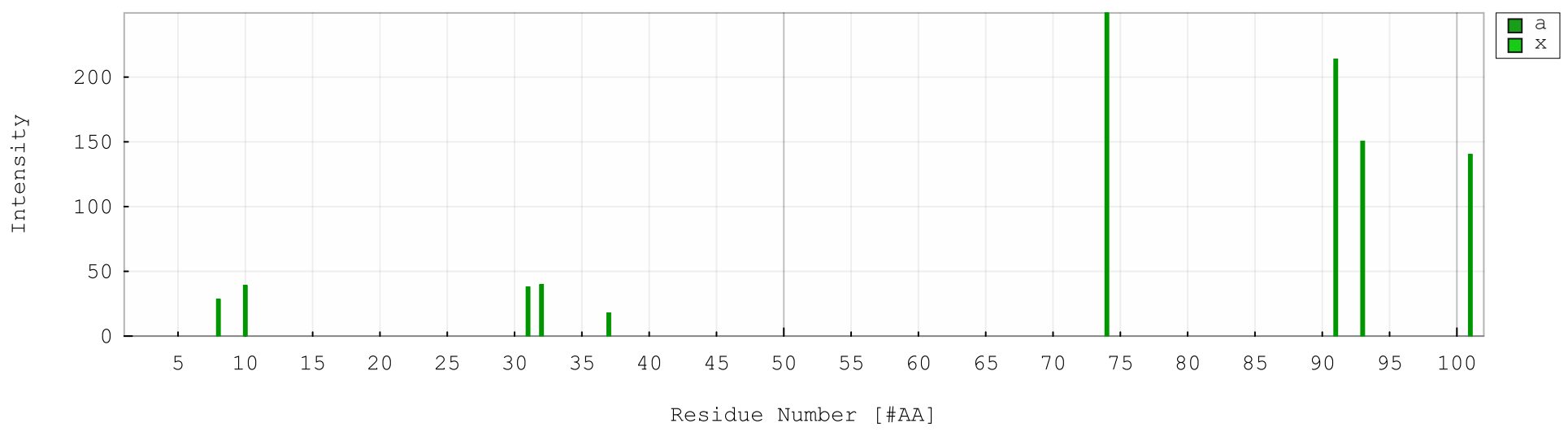

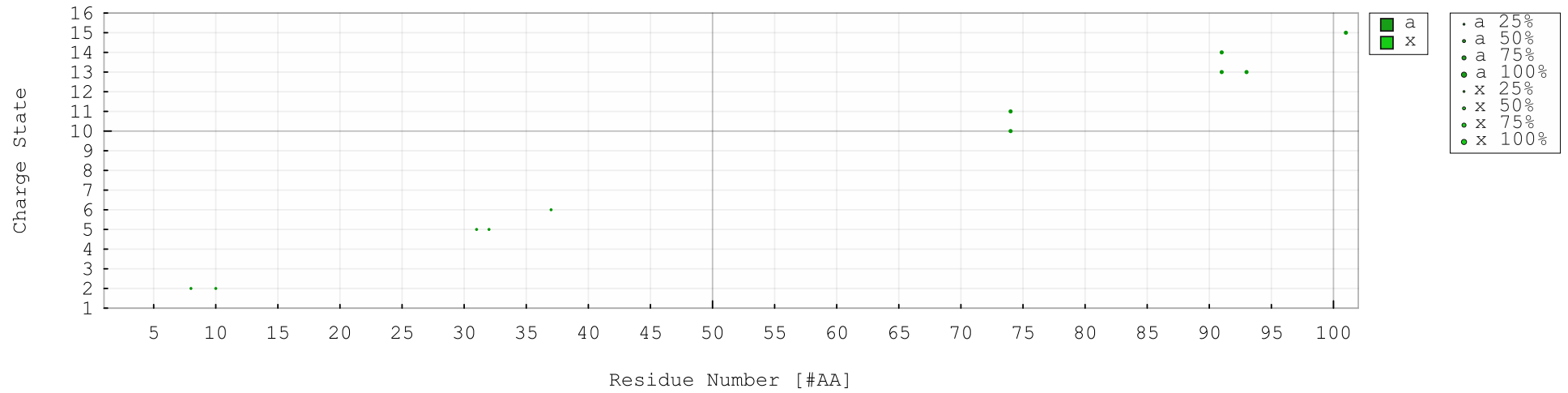

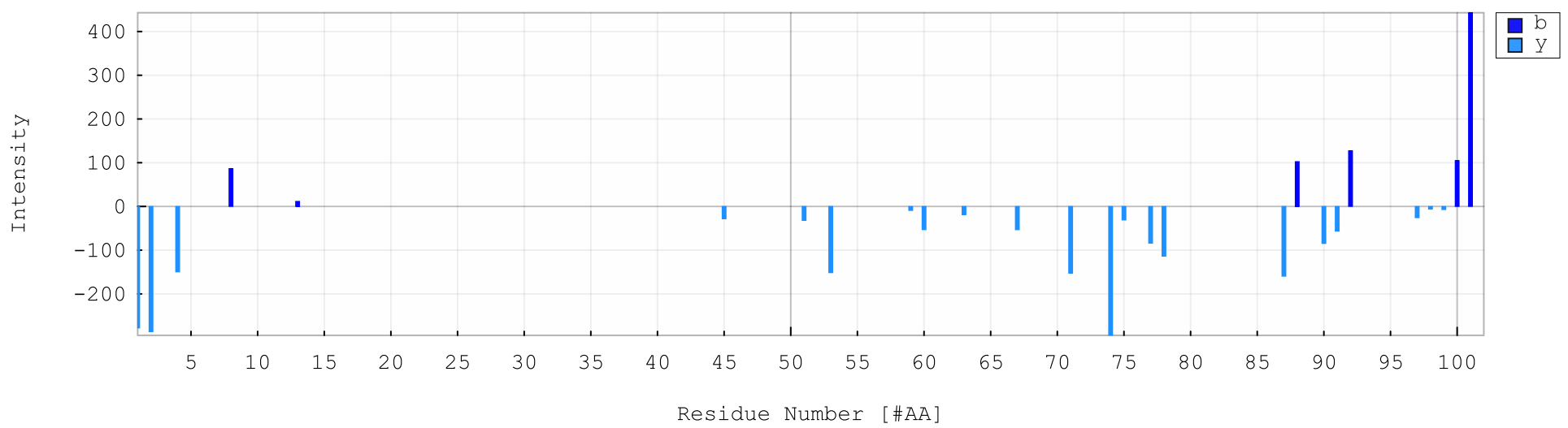

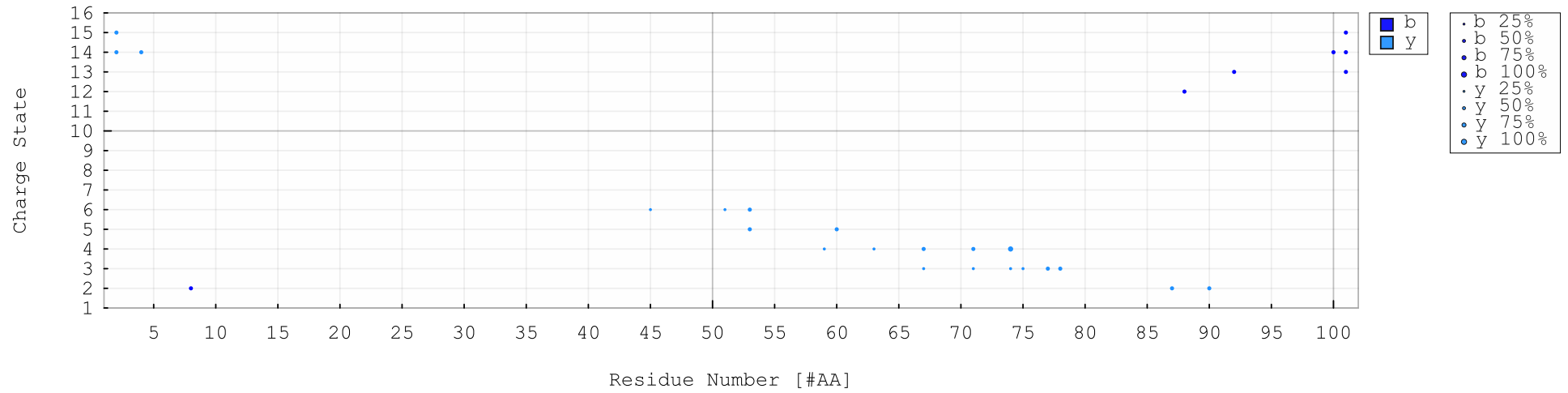

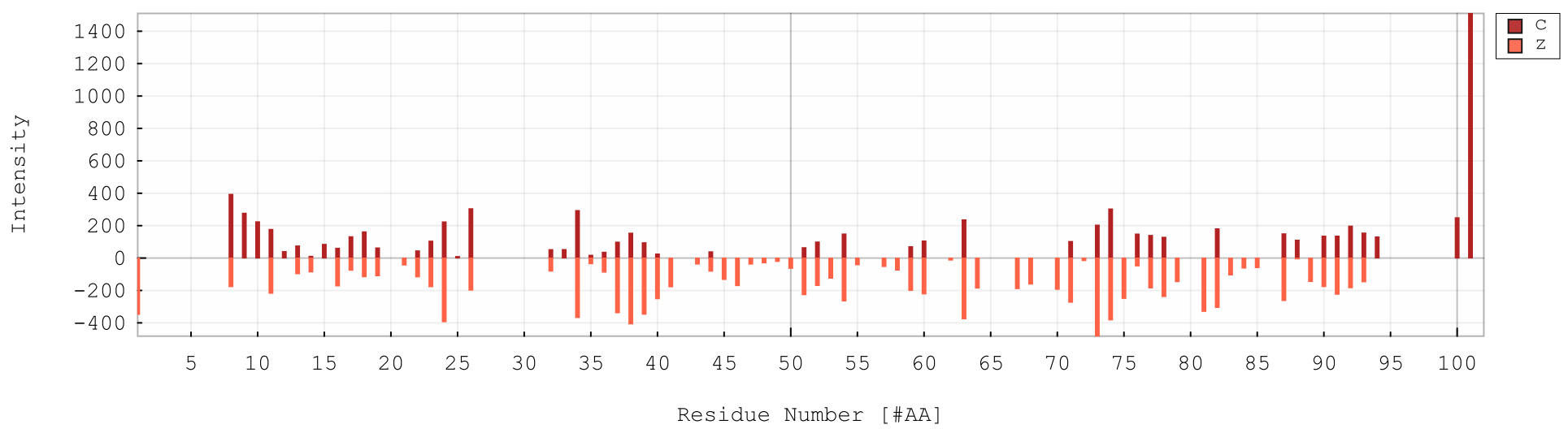

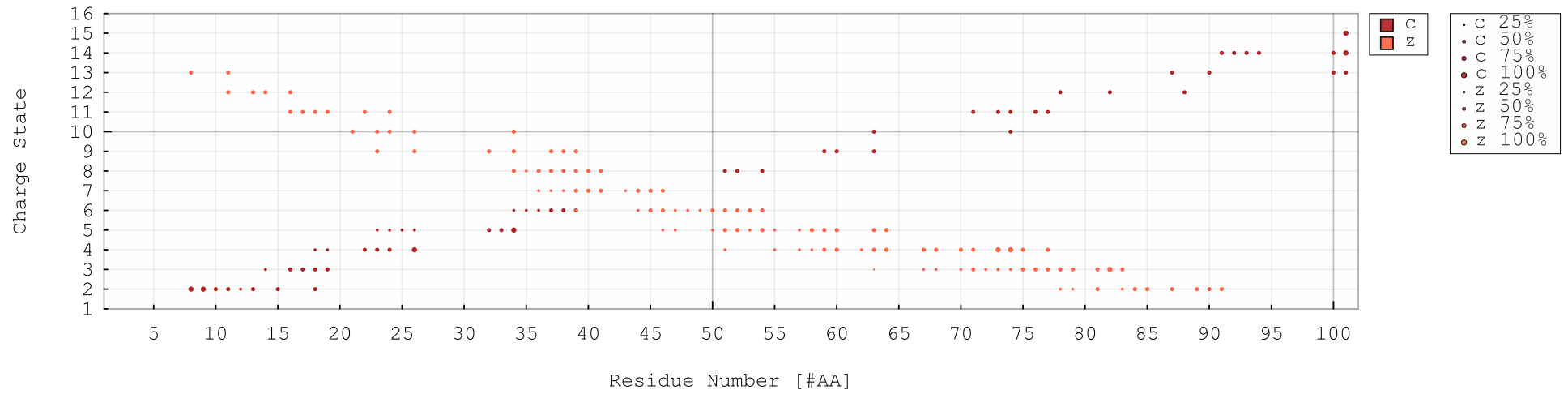

Supplemental Data S1

Endogenous H4

**Report**	5 of 6
**Experiment**	MS2
**Histone/sample**	H4
**Enzyme**	Not applicable
**Reaction/source**	Endogenous sample
**Precursor**	m/z 710
**Charge state**	16+
**Fragmentation / workflow**	EID
**Original file**	MS2_mz-710_16+_EID.pdf

Project Information

Project Directory: C:/Users/LAB-ADMIN
Processing date: Tue Apr 28 15:00:30 2026

Sample Info & Protocols

Name: Top-Down Protein Sequencing EID

Dataset

Imported spectrum file name:	250430 Liver-Histone-25 mz710z16 MS2 EID_001-356_calibrated.txt
Dataset name:	Information is only available from imported JPEAKS files
Date acquired:	Information is only available from imported JPEAKS files
Date imported:	Information is only available from imported JPEAKS files
Instrument Name:	Information is only available from imported JPEAKS files
Instrument Serial Number:	Information is only available from imported JPEAKS files

Workflow Result Info

Analysis: 250430 Liver-Histone-25 mz710z16 MS2 EID_001-356_calibrated
Location: C:/Users/LAB-ADMIN
Method: -

Method Information

Name: Unnamed

Spectral Parameters

Mass Error [ppm]: 6
Intensity Deviation [au]: 0.15
Min/Max Charges: 1-16
Experiment type: EID

a ions	b ions	c ions	x ions	y ions	z ions	satellite	internal	precursor
a	b	c	x	y	z			M
a+1			x+1	y-1	z+1			M+-1H

Noise threshold calculation: Simple moving average noise threshold (SMA)
Window size [datapoints]: 600
Noise curve offset: 3.6058304
SVP parameters: terminal gap: 2, internal gap: 1

Proteoforms

(top 10)

Index	Parent Sequence	Chain	Var. Mods	Mono Mass [Da]	Chemical Composition
1	H4Kac_me1-3	H4Kac_me1-3K5[Ace]K16[Ace]K20[Dim]	Ace: K5, K16, Dim: K20	11341.3937	C500H845N163O136S
2	H4Kac_me1-3	H4Kac_me1-3_Nterm[Acetyl]_K16[Ace]K20[Dim]	Ace: K16, N-term, Dim: K20	11341.3937	C500H845N163O136S
3	H4Kac_me1-3	H4Kac_me1-3K8[Ace]K16[Ace]K20[Dim]	Ace: K8, K16, Dim: K20	11341.3937	C500H845N163O136S
4	H4Kac_me1-3	H4Kac_me1-3K5[Ace]K16[Dim]K20[Ace]	Ace: K5, K20, Dim: K16	11341.3937	C500H845N163O136S
5	H4Kac_me1-3	H4Kac_me1-3_Nterm[Acetyl]_K16[Dim]K20[Ace]	Dim: K16, Ace: K20, N-term	11341.3937	C500H845N163O136S
6	H4Kac_me1-3	H4Kac_me1-3K5[Ace]K12[Ace]K20[Dim]	Ace: K5, K12, Dim: K20	11341.3937	C500H845N163O136S
7	H4Kac_me1-3	H4Kac_me1-3_Nterm[Acetyl]_K12[Ace]K20[Dim]	Ace: K12, N-term, Dim: K20	11341.3937	C500H845N163O136S
8	H4Kac_me1-3	H4Kac_me1-3K5[Ace]K16[Ace]K31[Dim]	Ace: K5, K16, Dim: K31	11341.3937	C500H845N163O136S
9	H4Kac_me1-3	H4Kac_me1-3K8[Ace]K12[Ace]K20[Dim]	Ace: K8, K12, Dim: K20	11341.3937	C500H845N163O136S
10	H4Kac_me1-3	H4Kac_me1-3_Nterm[Acetyl]_K16[Ace]K31[Dim]	Ace: K16, N-term, Dim: K31	11341.3937	C500H845N163O136S

Results

Proteoforms Table (top 10)

Index	Protein	SC [%]	SVP [%]	IC [%]	MS Score [a.u.]	Var. Mods	Mono Mass [Da]	Chemical Formula
1	H4Kac_me1-3	71.3	4.9	23.4	16.6	Ace: K5, K16, Dim: K20	11341.3937	C500H845N163O136S
2	H4Kac_me1-3	73.3	23.5	22.5	16.5	Ace: K16, N-term, Dim: K20	11341.3937	C500H845N163O136S
3	H4Kac_me1-3	69.3	4.9	23.2	16.1	Ace: K8, K16, Dim: K20	11341.3937	C500H845N163O136S
4	H4Kac_me1-3	68.3	4.9	23.2	15.8	Ace: K5, K20, Dim: K16	11341.3937	C500H845N163O136S
5	H4Kac_me1-3	70.3	19.6	22.3	15.7	Dim: K16, Ace: K20, N-term	11341.3937	C500H845N163O136S
6	H4Kac_me1-3	68.3	4.9	22.9	15.7	Ace: K5, K12, Dim: K20	11341.3937	C500H845N163O136S
7	H4Kac_me1-3	70.3	15.7	22.1	15.5	Ace: K12, N-term, Dim: K20	11341.3937	C500H845N163O136S
8	H4Kac_me1-3	67.3	4.9	22.7	15.2	Ace: K5, K16, Dim: K31	11341.3937	C500H845N163O136S
9	H4Kac_me1-3	66.3	4.9	22.8	15.1	Ace: K8, K12, Dim: K20	11341.3937	C500H845N163O136S
10	H4Kac_me1-3	69.3	23.5	21.8	15.1	Ace: K16, N-term, Dim: K31	11341.3937	C500H845N163O136S

Sequence

Name: H4Kac_me1-3

```
                 10                    20                    30                    40                    50                    60
S G R G K G G K G L   G K G G A K R H R K   V L R D N I Q G I T   K P A I R R L A R R   G G V K R I S G L I   Y E E T R G V L K V
                 70                    80                    90                   100                   110                   120
F L E N V I R D A V   T Y T E H A K R K T   V T A M D V V Y A L   K R Q G R T L Y G F   G G
```

Modifications

Modification	Position	Type
Acetyl	N-term	variable
Acetyl	5	variable
Acetyl	8	variable
Acetyl	12	variable
Dimethyl	12	variable
Methyl	12	variable
Acetyl	16	variable
Dimethyl	16	variable
Methyl	16	variable
Trimethyl	16	variable
Dimethyl	20	variable
Acetyl	20	variable
Methyl	20	variable

Modification	Position	Type
Trimethyl	20	variable
Dimethyl	31	variable
Methyl	31	variable
Acetyl	31	variable

Sequence Map

Proteoform:	Index	Protein	SC [%]	SVP [%]	IC [%]	MS Score [a.u.]	Var. Mods	Mono Mass [Da]	Chemical Formula
	1	H4Kac_me1-3	71.3	4.9	23.4	16.6	Ace: K5, K16, Dim: K20	11341.3937	C500H845N163O136S

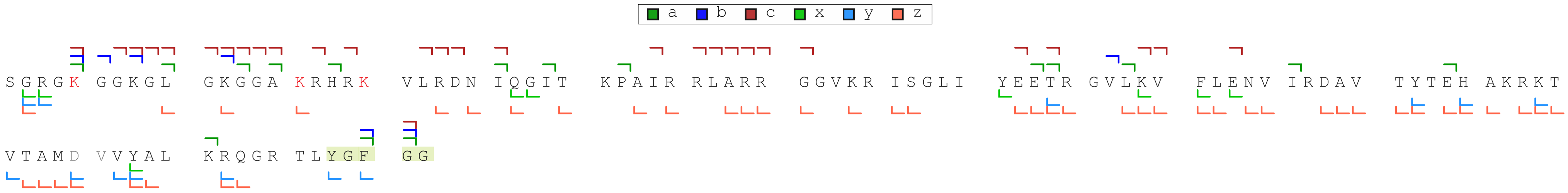

Proteoform:

Index	Protein	SC [%]	SVP [%]	IC [%]	MS Score [a.u.]	Var. Mods	Mono Mass [Da]	Chemical Formula
2	H4Kac_me1-3	73.3	23.5	22.5	16.5	Ace: K16, N-term, Dim: K20	11341.3937	C500H845N163O136S

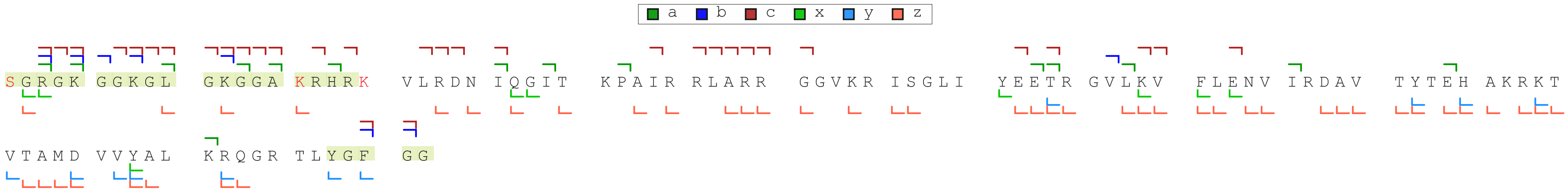

Proteoform:

Index	Protein	SC [%]	SVP [%]	IC [%]	MS Score [a.u.]	Var. Mods	Mono Mass [Da]	Chemical Formula
3	H4Kac_me1-3	69.3	4.9	23.2	16.1	Ace: K8, K16, Dim: K20	11341.3937	C500H845N163O136S

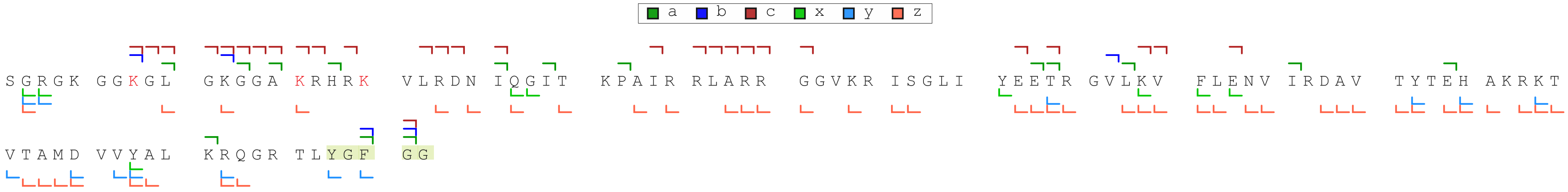

Proteoform:

Index	Protein	SC [%]	SVP [%]	IC [%]	MS Score [a.u.]	Var. Mods	Mono Mass [Da]	Chemical Formula
4	H4Kac_me1-3	68.3	4.9	23.2	15.8	Ace: K5, K20, Dim: K16	11341.3937	C500H845N163O136S

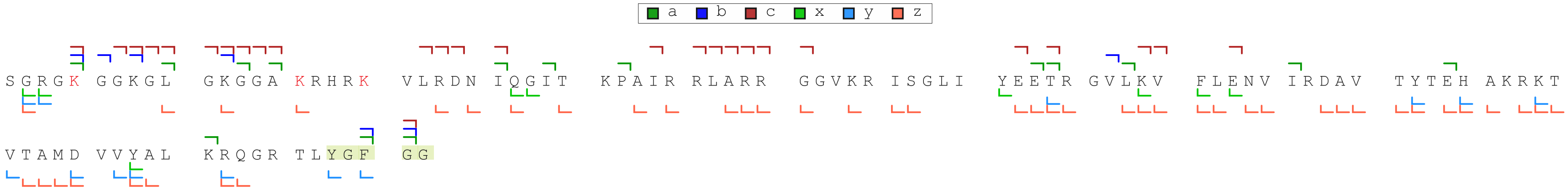

Proteoform:

Index	Protein	SC [%]	SVP [%]	IC [%]	MS Score [a.u.]	Var. Mods	Mono Mass [Da]	Chemical Formula
5	H4Kac_me1-3	70.3	19.6	22.3	15.7	Dim: K16, Ace: K20, N-term	11341.3937	C500H845N163O136S

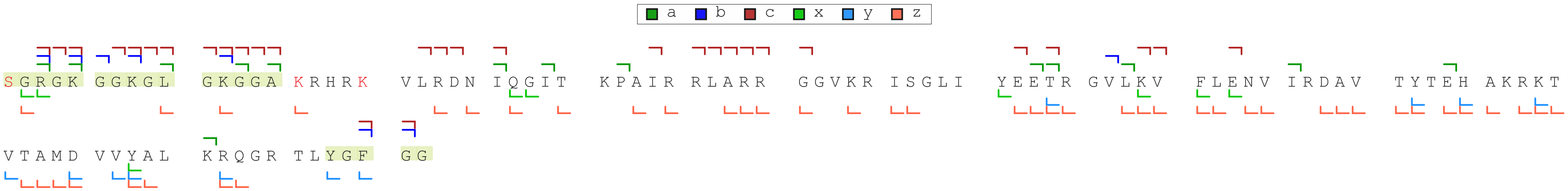

Proteoform:	Index	Protein	SC [%]	SVP [%]	IC [%]	MS Score [a.u.]	Var. Mods	Mono Mass [Da]	Chemical Formula
	6	H4Kac_me1-3	68.3	4.9	22.9	15.7	Ace: K5, K12, Dim: K20	11341.3937	C500H845N163O136S

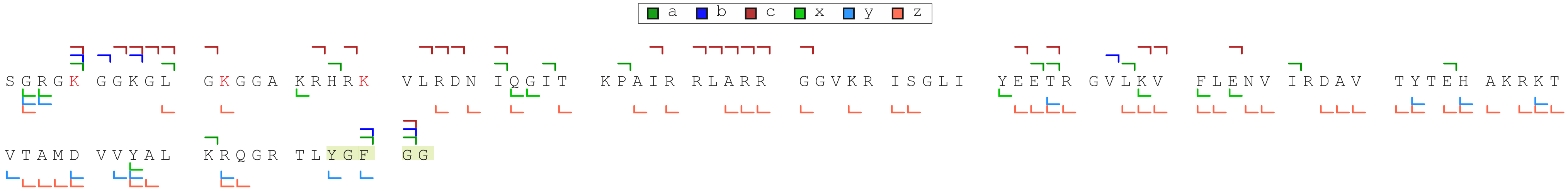

Proteoform:

Index	Protein	SC [%]	SVP [%]	IC [%]	MS Score [a.u.]	Var. Mods	Mono Mass [Da]	Chemical Formula
7	H4Kac_me1-3	70.3	15.7	22.1	15.5	Ace: K12, N-term, K20 Dim:	11341.3937	C500H845N163O136S

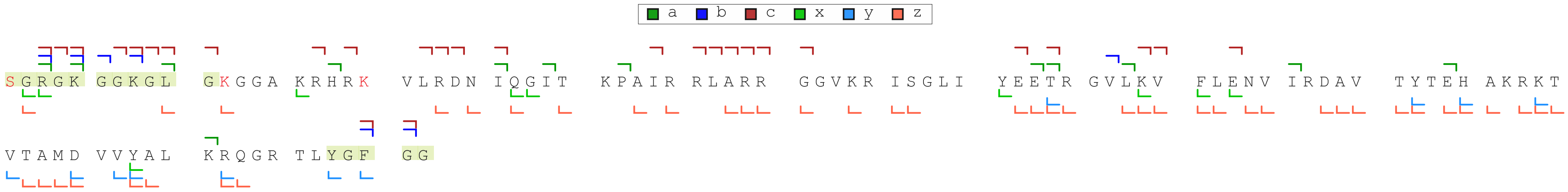

Proteoform:	Index	Protein	SC [%]	SVP [%]	IC [%]	MS Score [a.u.]	Var. Mods	Mono Mass [Da]	Chemical Formula
	8	H4Kac_me1-3	67.3	4.9	22.7	15.2	Ace: K5, K16, Dim: K31	11341.3937	C500H845N163O136S

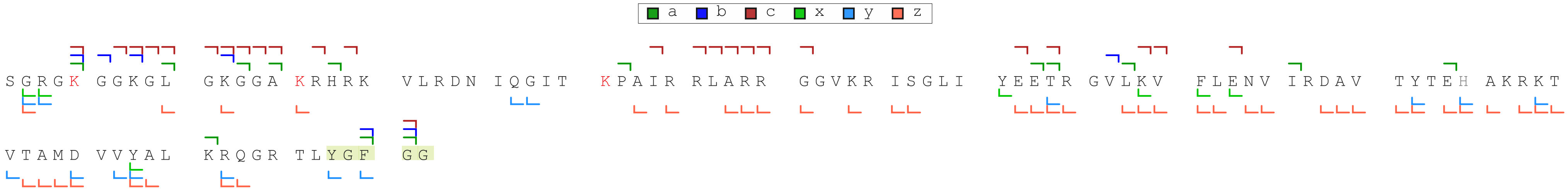

Proteoform:

Index	Protein	SC [%]	SVP [%]	IC [%]	MS Score [a.u.]	Var. Mods	Mono Mass [Da]	Chemical Formula
9	H4Kac_me1-3	66.3	4.9	22.8	15.1	Ace: K8, K12, Dim: K20	11341.3937	C500H845N163O136S

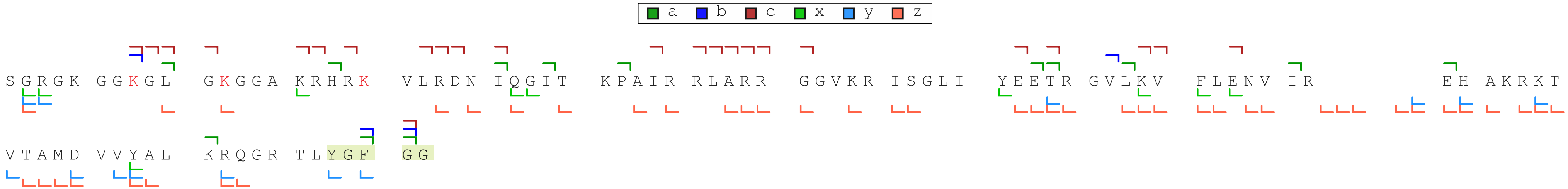

Proteoform:

Index	Protein	SC [%]	SVP [%]	IC [%]	MS Score [a.u.]	Var. Mods	Mono Mass [Da]	Chemical Formula
10	H4Kac_me1-3	69.3	23.5	21.8	15.1	Ace: K16, N-term, Dim: K31	11341.3937	C500H845N163O136S

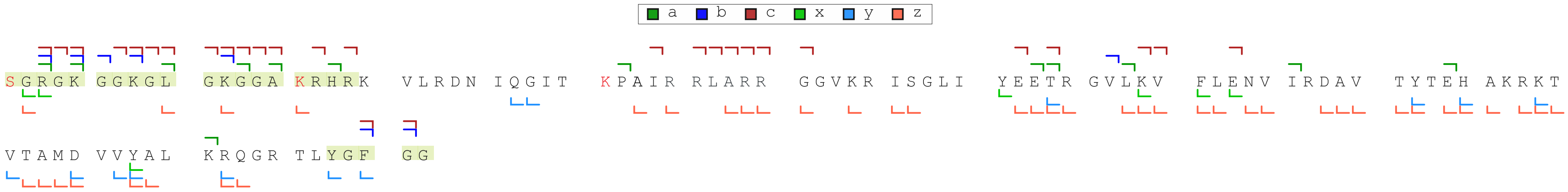

Error Plot

Proteoform:

Index	Protein	SC [%]	SVP [%]	IC [%]	MS Score [a.u.]	Var. Mods	Mono Mass [Da]	Chemical Formula
1	H4Kac_me1-3	71.3	4.9	23.4	16.6	Ace: K5, K16, Dim: K20	11341.3937	C500H845N163O136S

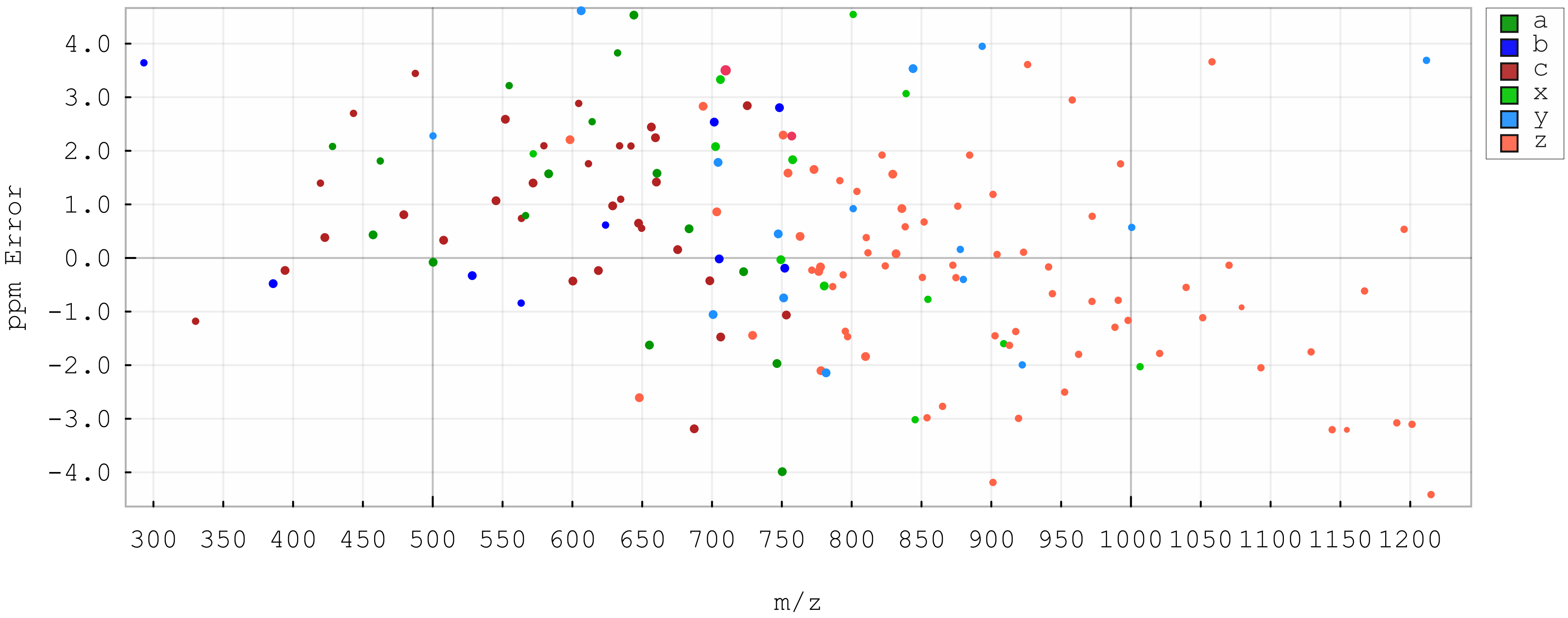

Proteoform:

Index	Protein	SC [%]	SVP [%]	IC [%]	MS Score [a.u.]	Var. Mods	Mono Mass [Da]	Chemical Formula
2	H4Kac_me1-3	73.3	23.5	22.5	16.5	Ace: K16, N-term, Dim: K20	11341.3937	C500H845N163O136S

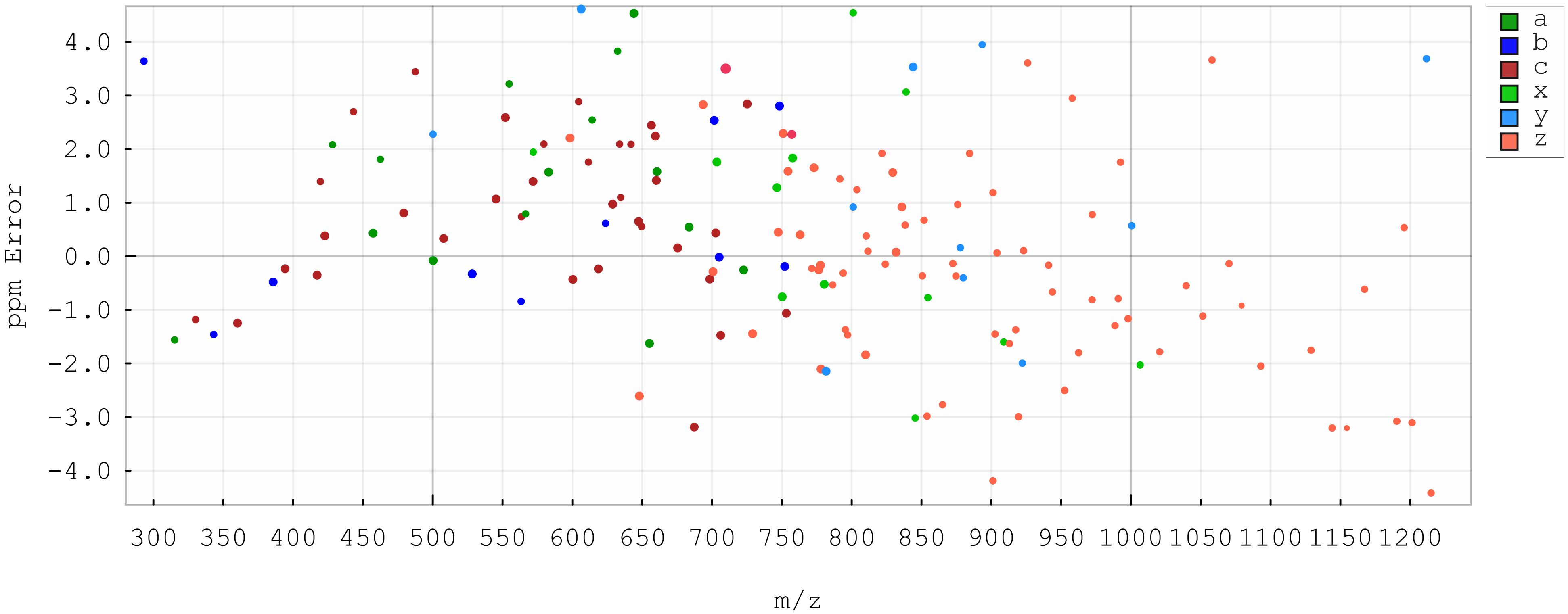

Proteoform:	Index	Protein	SC [%]	SVP [%]	IC [%]	MS Score [a.u.]	Var. Mods	Mono Mass [Da]	Chemical Formula
	3	H4Kac_me1-3	69.3	4.9	23.2	16.1	Ace: K8, K16, Dim: K20	11341.3937	C500H845N163O136S

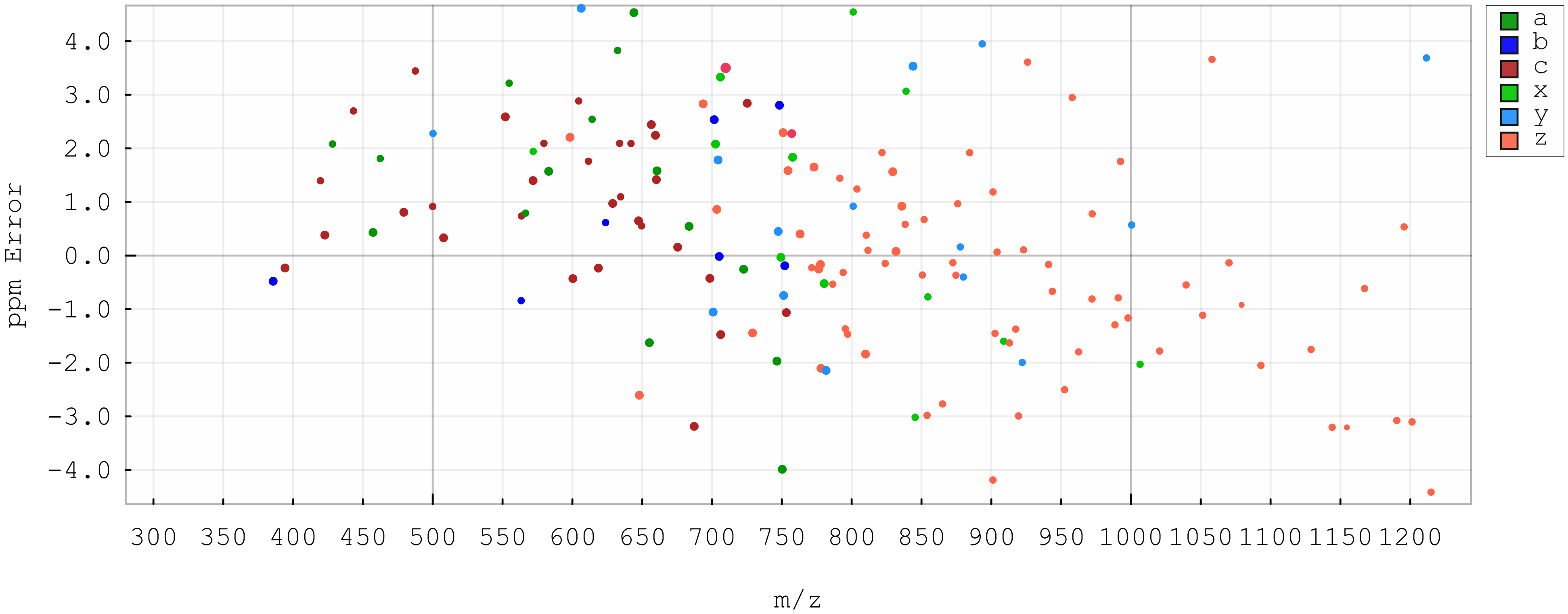

Proteoform:

Index	Protein	SC [%]	SVP [%]	IC [%]	MS Score [a.u.]	Var. Mods	Mono Mass [Da]	Chemical Formula
4	H4Kac_me1-3	68.3	4.9	23.2	15.8	Ace: K5, K20, Dim: K16	11341.3937	C500H845N163O136S

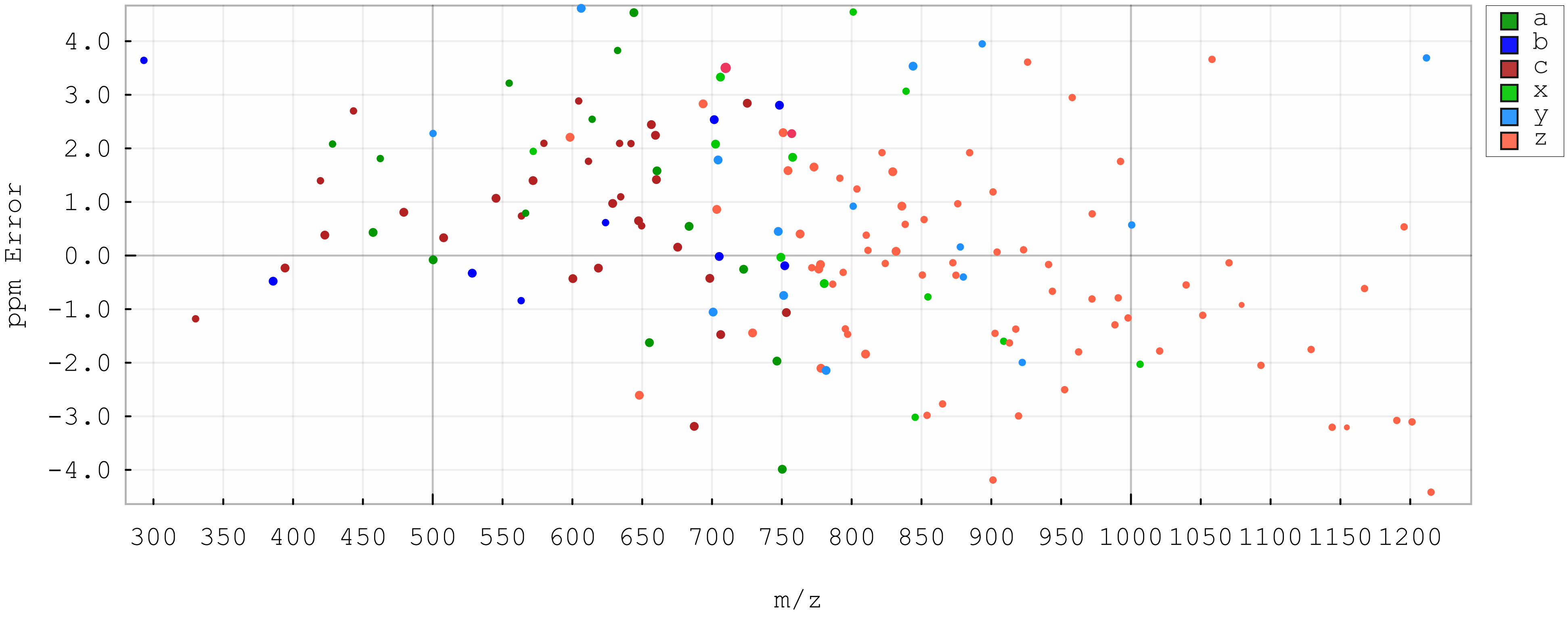

Proteoform:

Index	Protein	SC [%]	SVP [%]	IC [%]	MS Score [a.u.]	Var. Mods	Mono Mass [Da]	Chemical Formula
5	H4Kac_me1-3	70.3	19.6	22.3	15.7	Dim: K16, Ace: K20, N-term	11341.3937	C500H845N163O136S

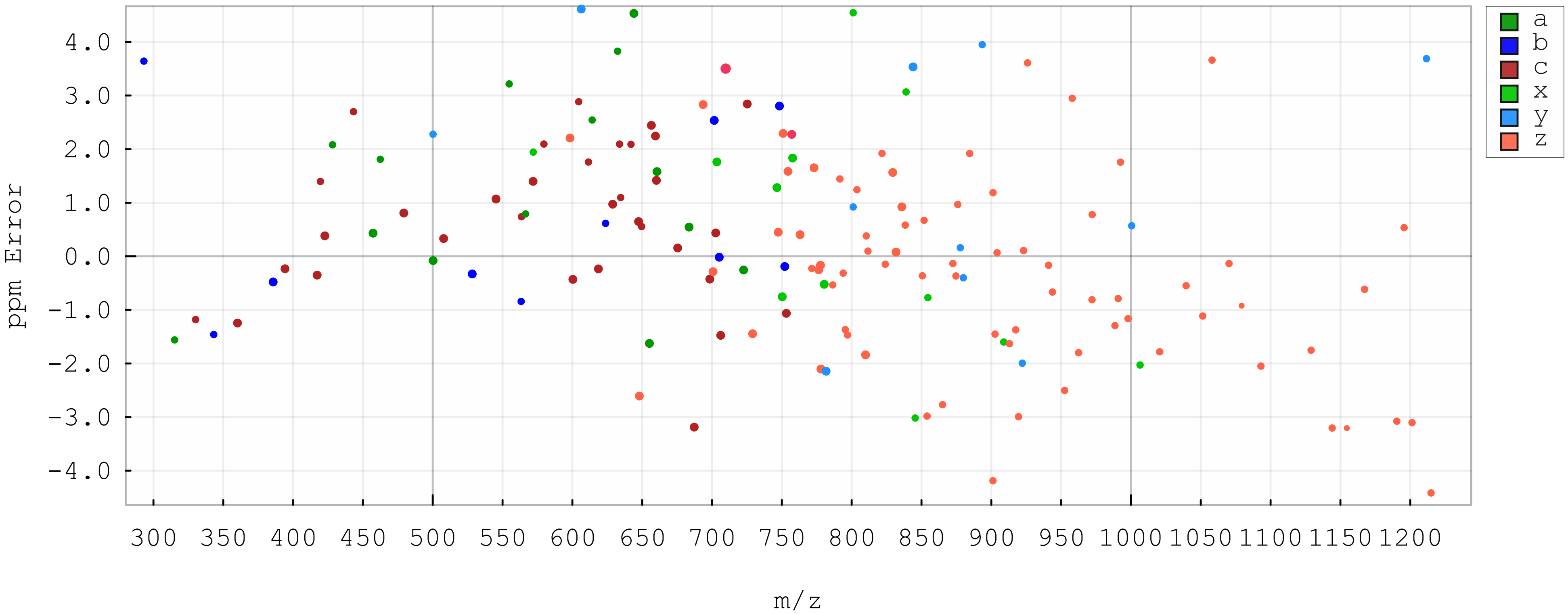

Proteoform:

Index	Protein	SC [%]	SVP [%]	IC [%]	MS Score [a.u.]	Var. Mods	Mono Mass [Da]	Chemical Formula
6	H4Kac_me1-3	68.3	4.9	22.9	15.7	Ace: K5, K12, Dim: K20	11341.3937	C500H845N163O136S

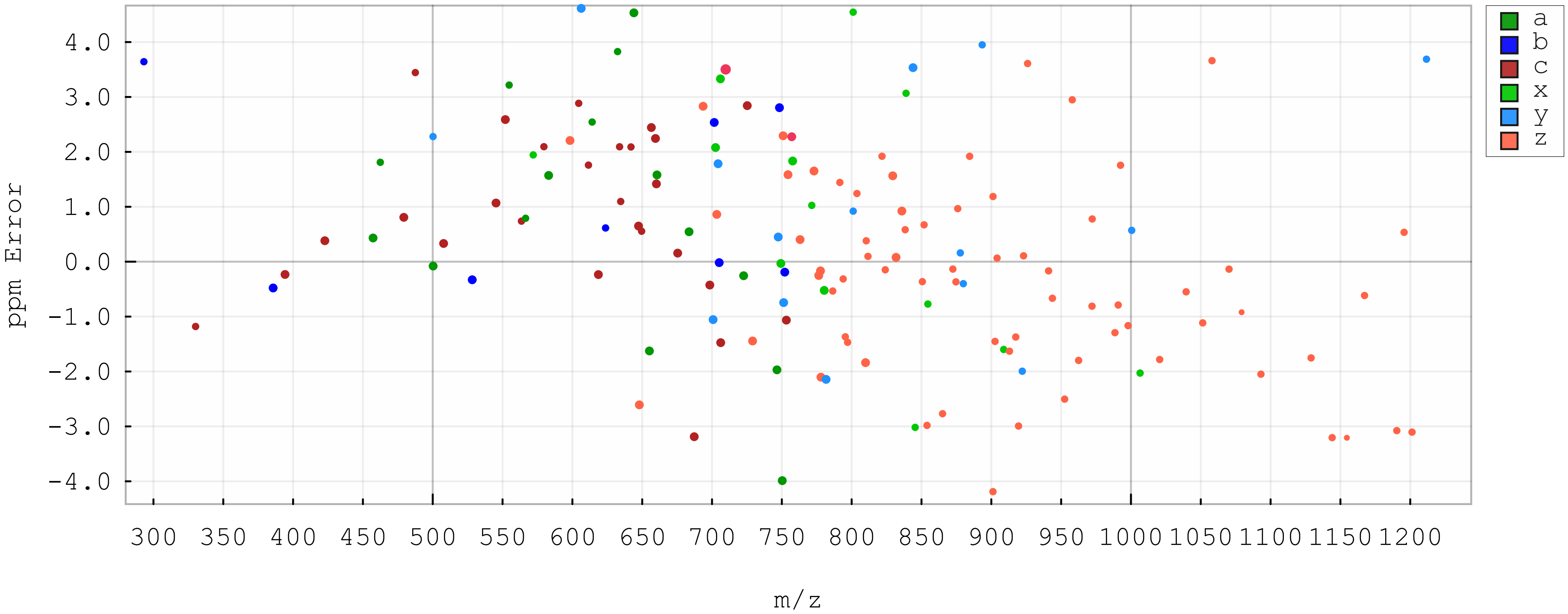

Proteoform:

Index	Protein	SC [%]	SVP [%]	IC [%]	MS Score [a.u.]	Var. Mods	Mono Mass [Da]	Chemical Formula
7	H4Kac_me1-3	70.3	15.7	22.1	15.5	Ace: K12, N-term, Dim: K20	11341.3937	C500H845N163O136S

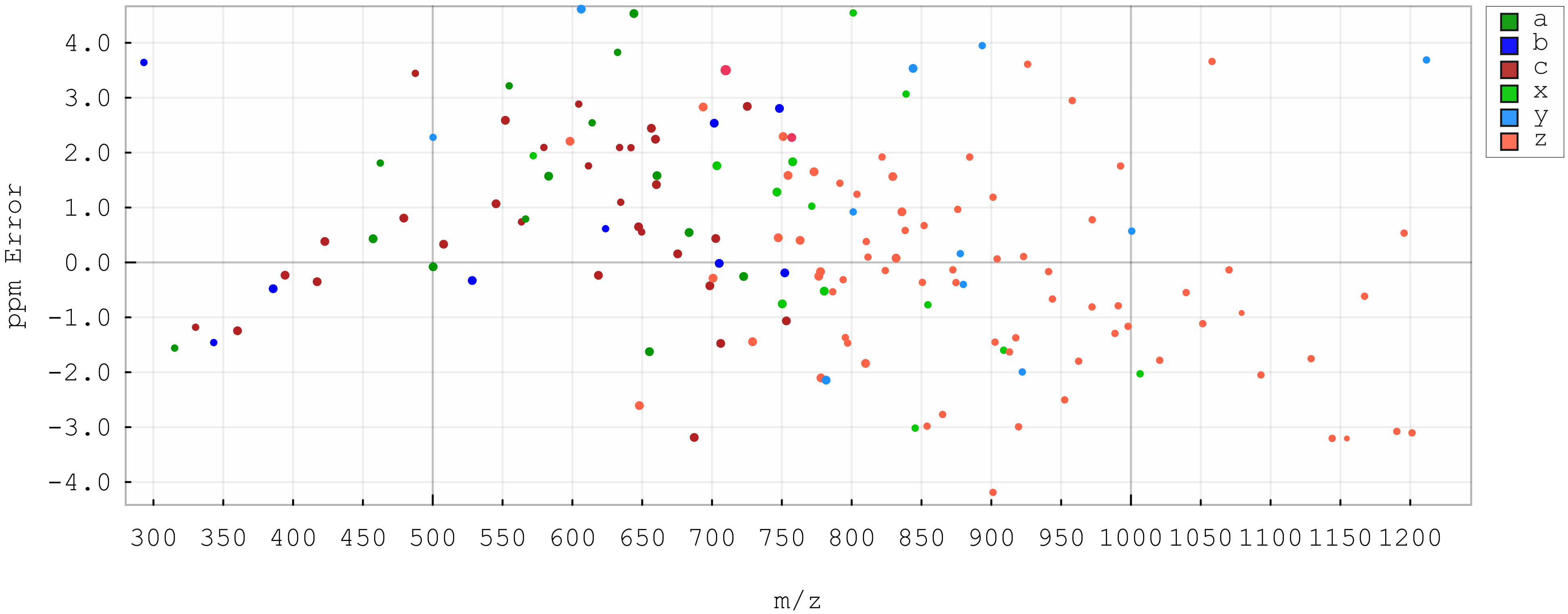

Proteoform:

Index	Protein	SC [%]	SVP [%]	IC [%]	MS Score [a.u.]	Var. Mods	Mono Mass [Da]	Chemical Formula
8	H4Kac_me1-3	67.3	4.9	22.7	15.2	Ace: K5, K16, Dim: K31	11341.3937	C500H845N163O136S

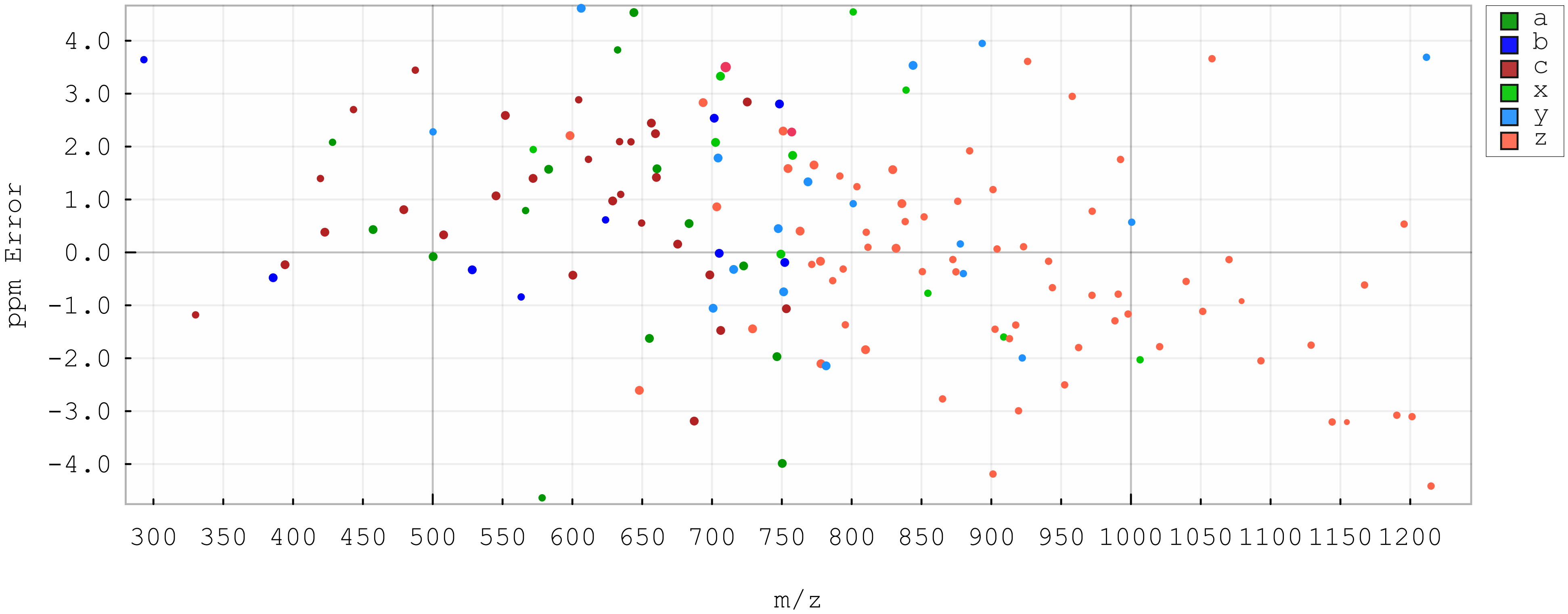

Proteoform:

Index	Protein	SC [%]	SVP [%]	IC [%]	MS Score [a.u.]	Var. Mods	Mono Mass [Da]	Chemical Formula
9	H4Kac_me1-3	66.3	4.9	22.8	15.1	Ace: K8, K12, Dim: K20	11341.3937	C500H845N163O136S

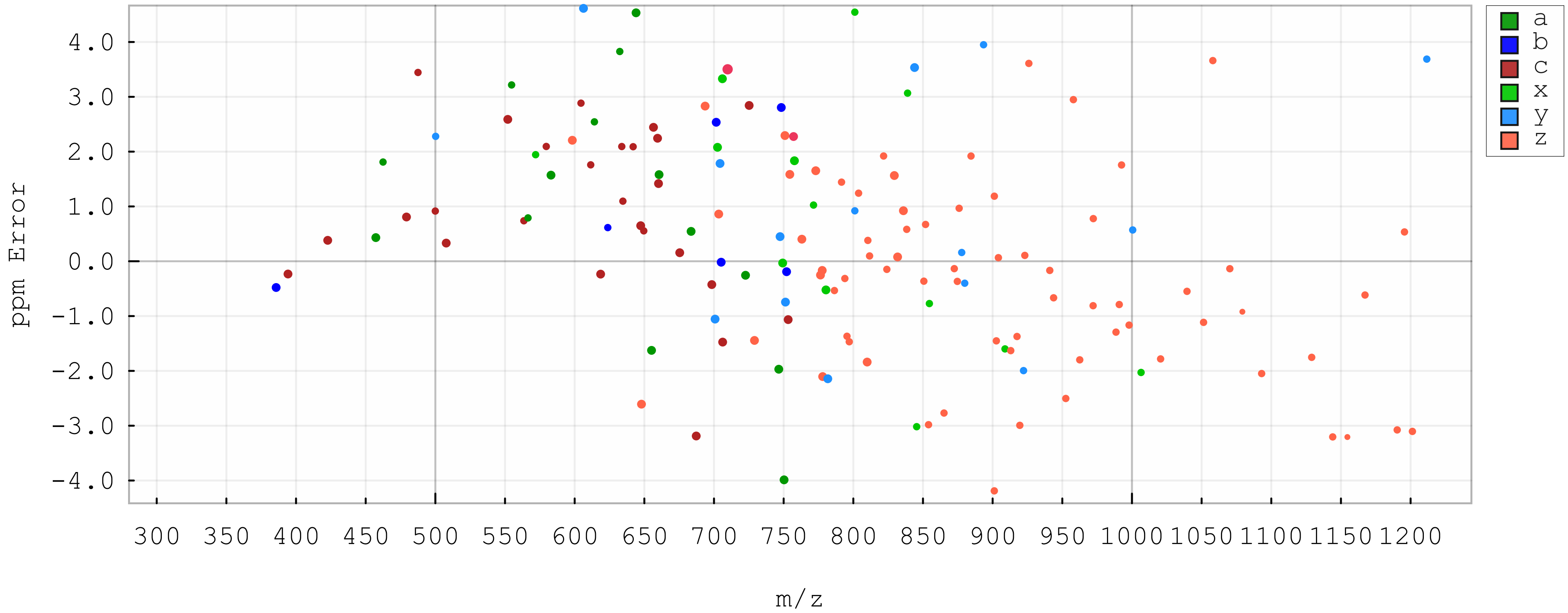

Proteoform:

Index	Protein	SC [%]	SVP [%]	IC [%]	MS Score [a.u.]	Var. Mods	Mono Mass [Da]	Chemical Formula
10	H4Kac_me1-3	69.3	23.5	21.8	15.1	Ace: K16, N-term, Dim: K31	11341.3937	C500H845N163O136S

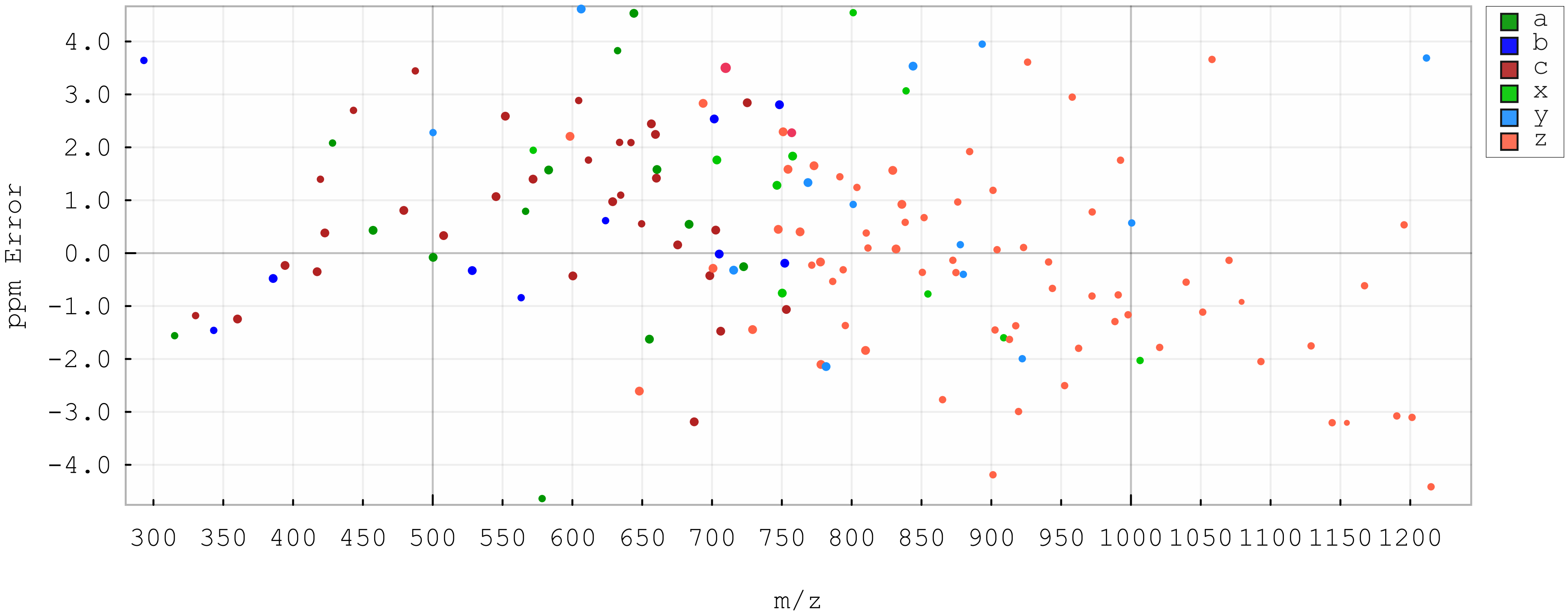

Primary Fragment Plots

Proteoform:

Index	Protein	SC [%]	SVP [%]	IC [%]	MS Score [a.u.]	Var. Mods	Mono Mass [Da]	Chemical Formula
1	H4Kac_me1-3	71.3	4.9	23.4	16.6	Ace: K5, K16, Dim: K20	11341.3937	C500H845N163O136S

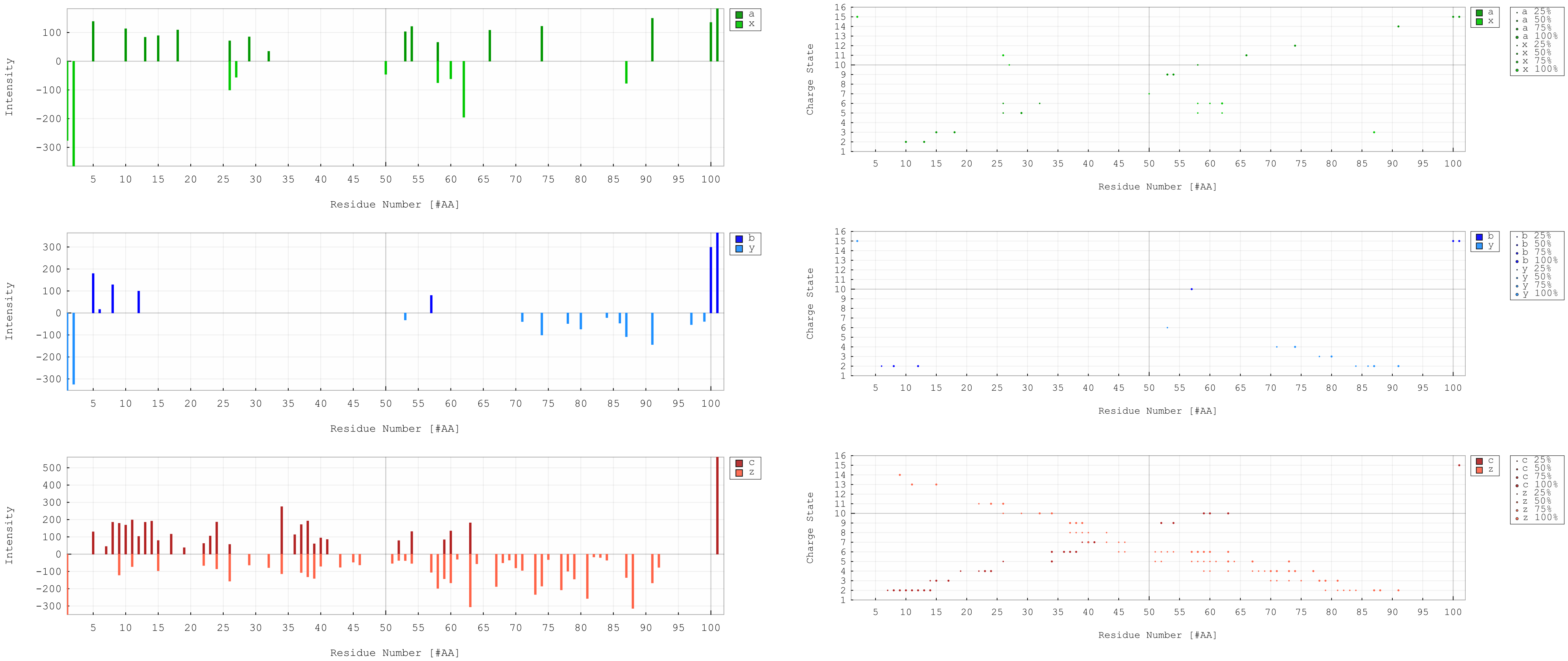

Proteoform:

Index	Protein	SC [%]	SVP [%]	IC [%]	MS Score [a.u.]	Var. Mods	Mono Mass [Da]	Chemical Formula
2	H4Kac_me1-3	73.3	23.5	22.5	16.5	Ace: K16, N-term, Dim: K20	11341.3937	C500H845N163O136S

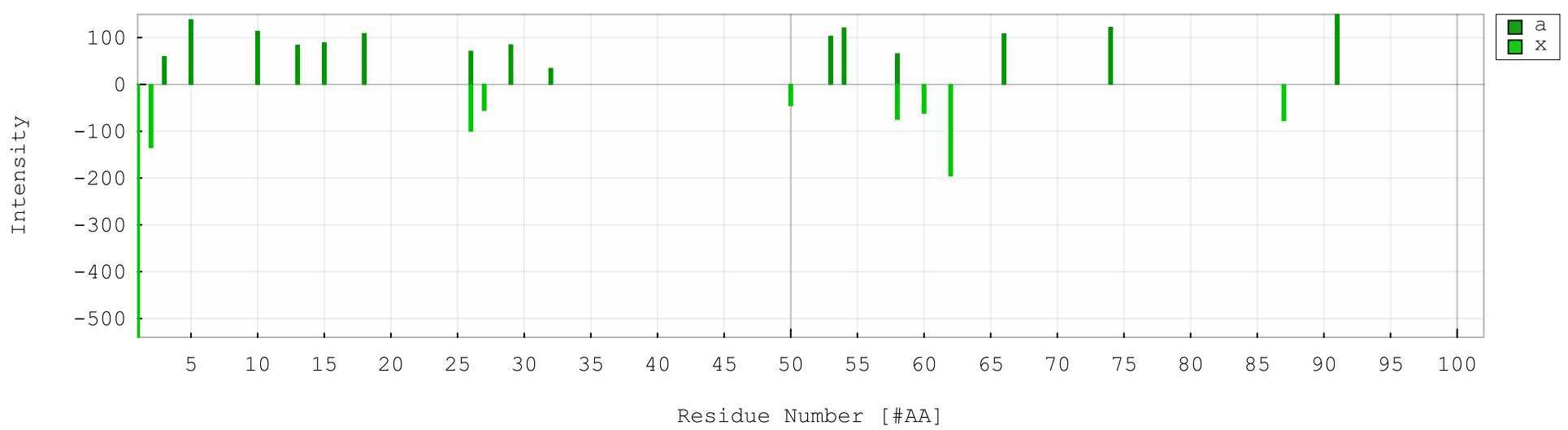

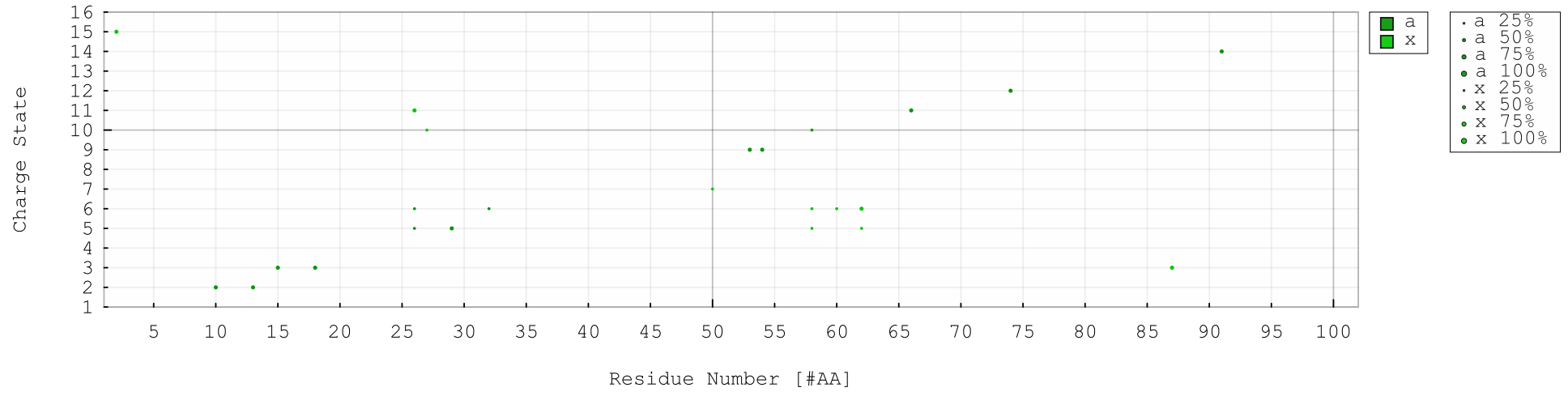

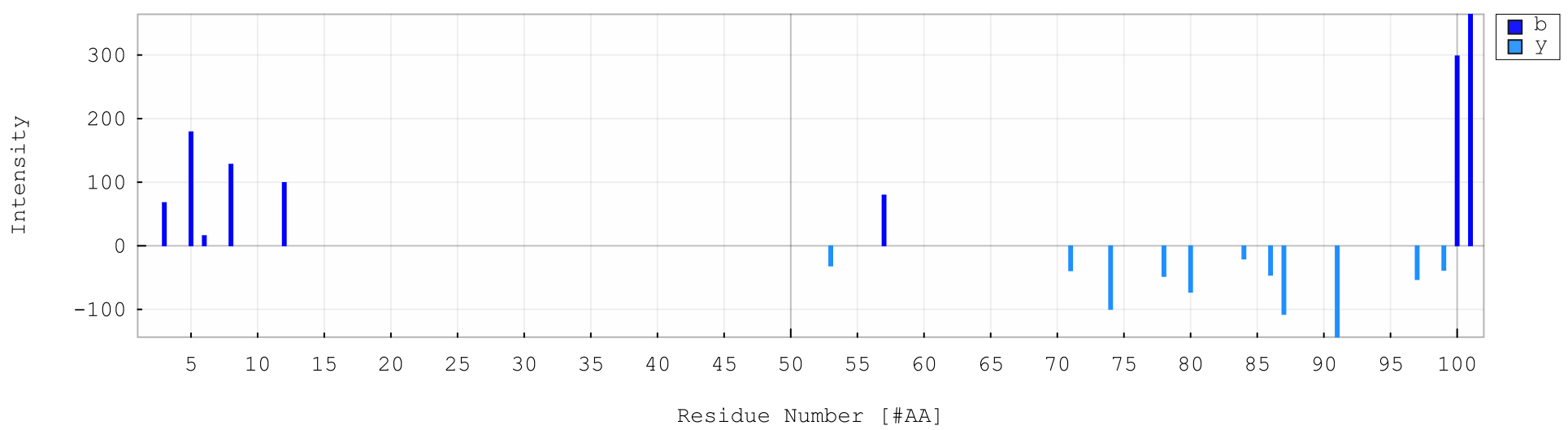

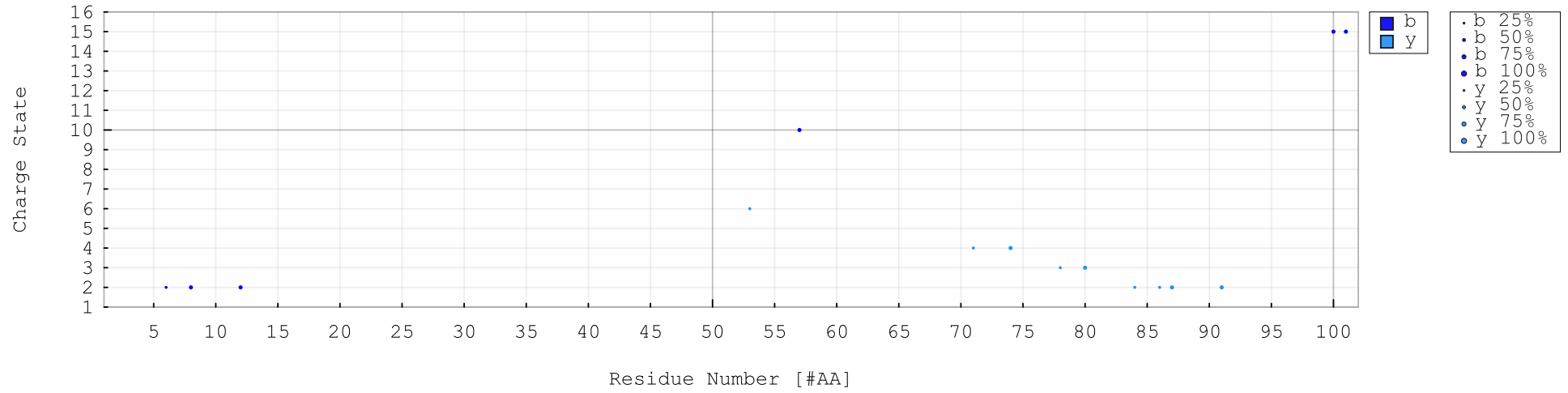

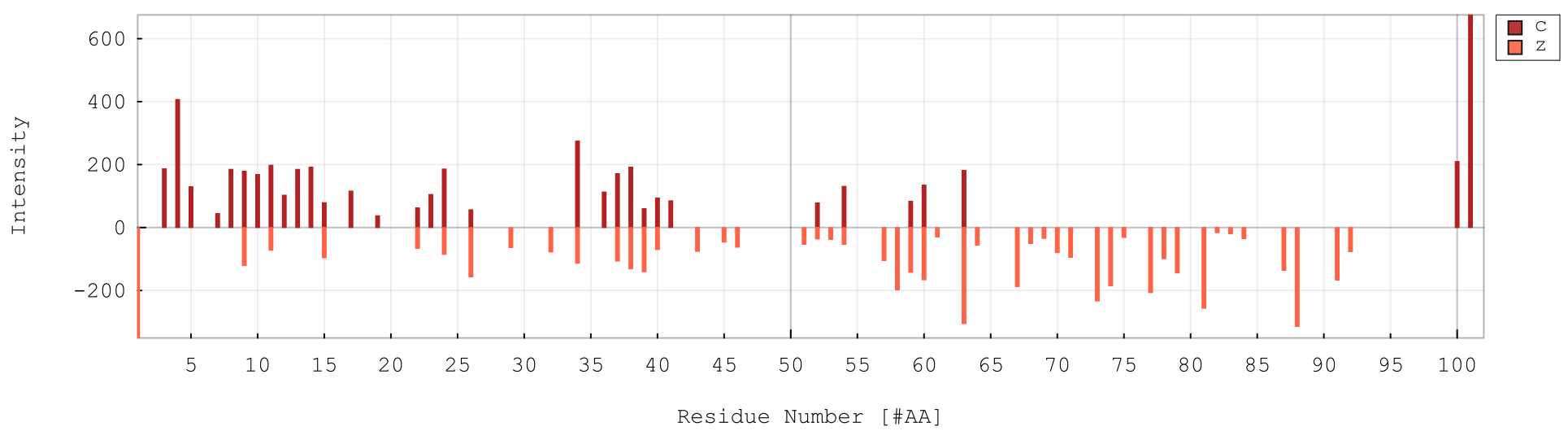

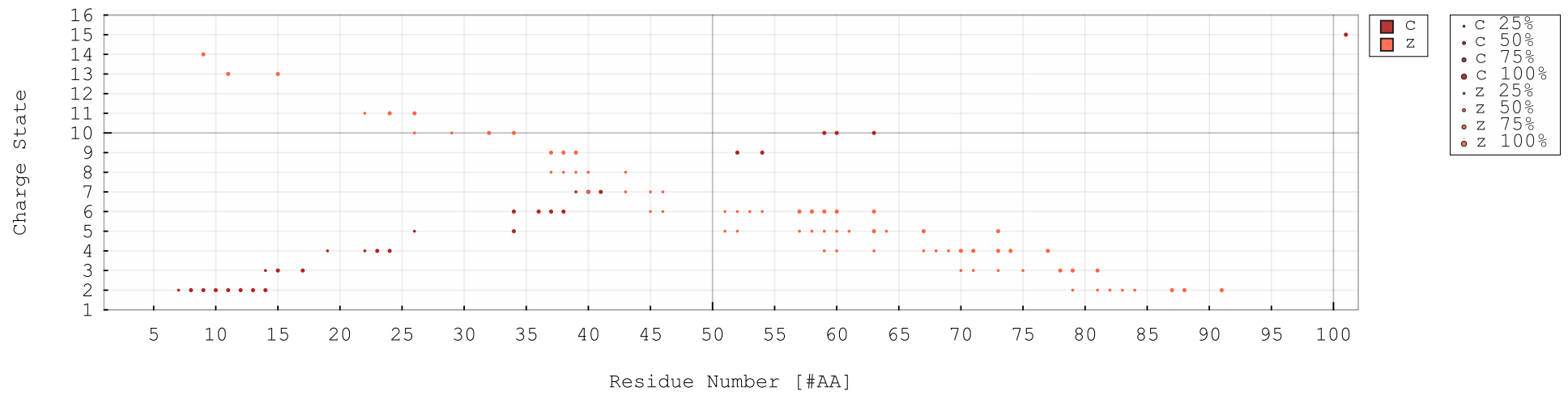

Proteoform:

Index	Protein	SC [%]	SVP [%]	IC [%]	MS Score [a.u.]	Var. Mods	Mono Mass [Da]	Chemical Formula
3	H4Kac_me1-3	69.3	4.9	23.2	16.1	Ace: K8, K16, Dim: K20	11341.3937	C500H845N163O136S

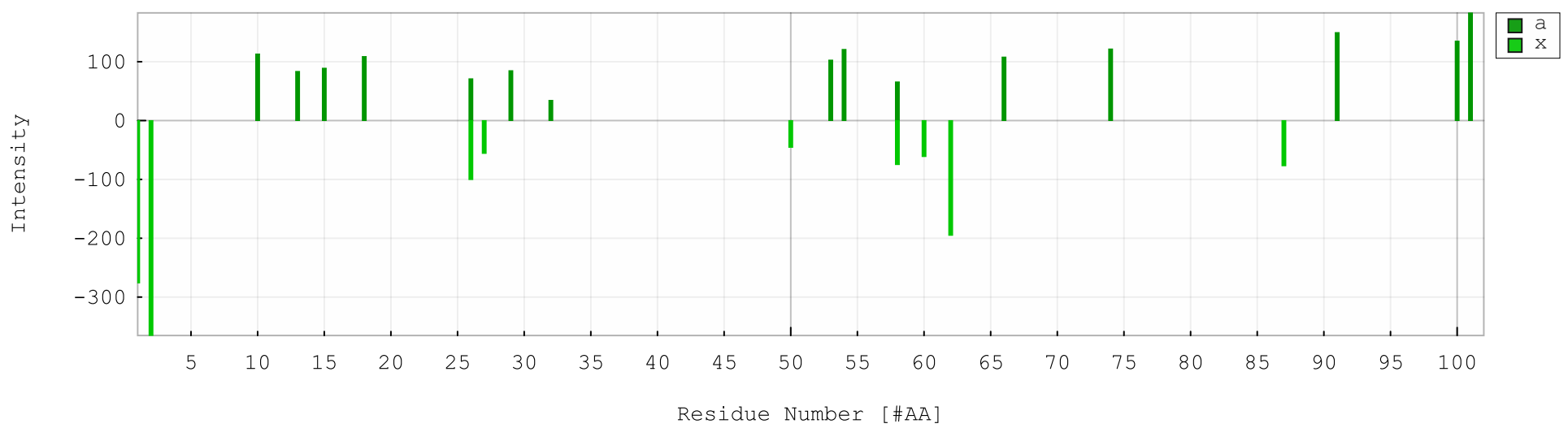

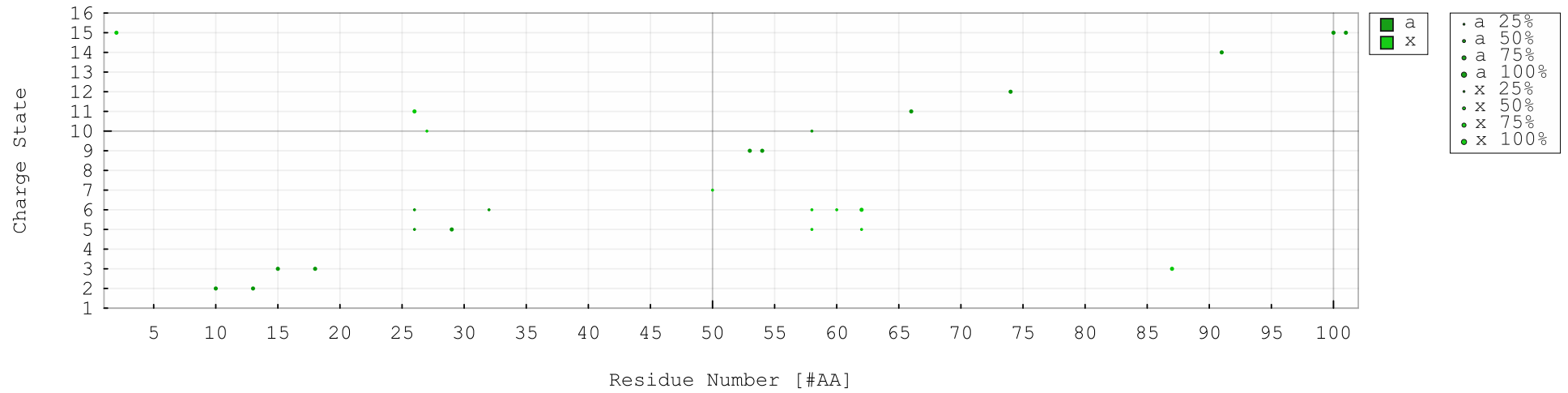

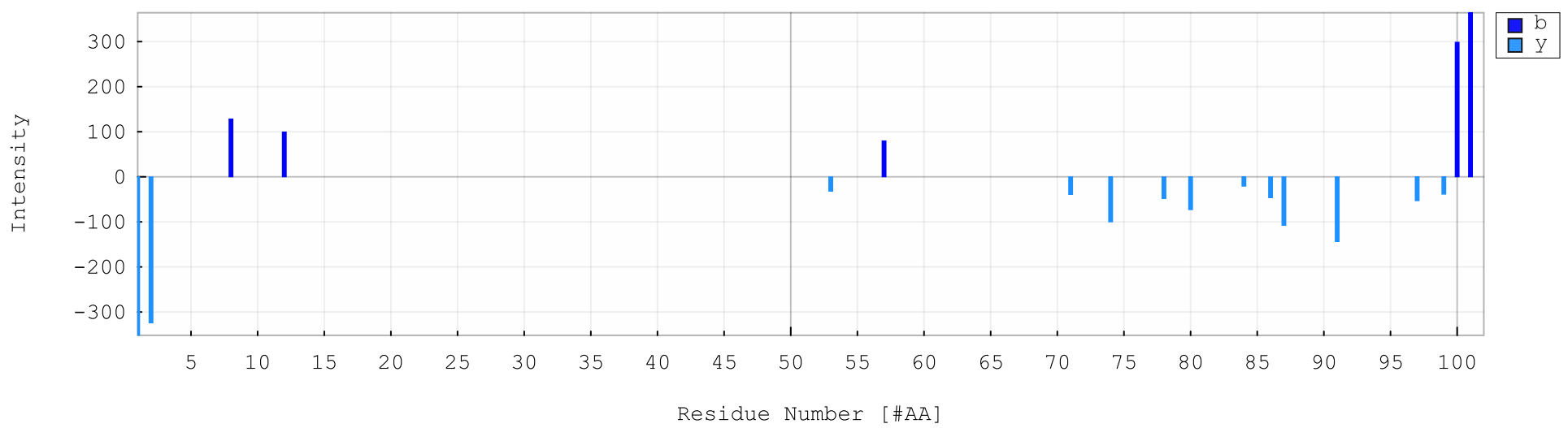

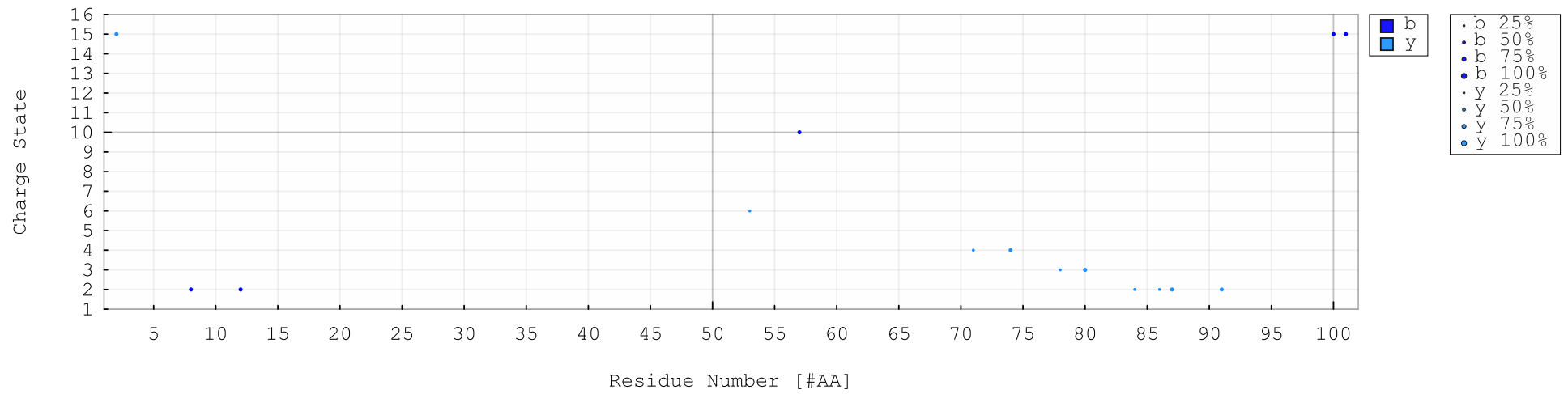

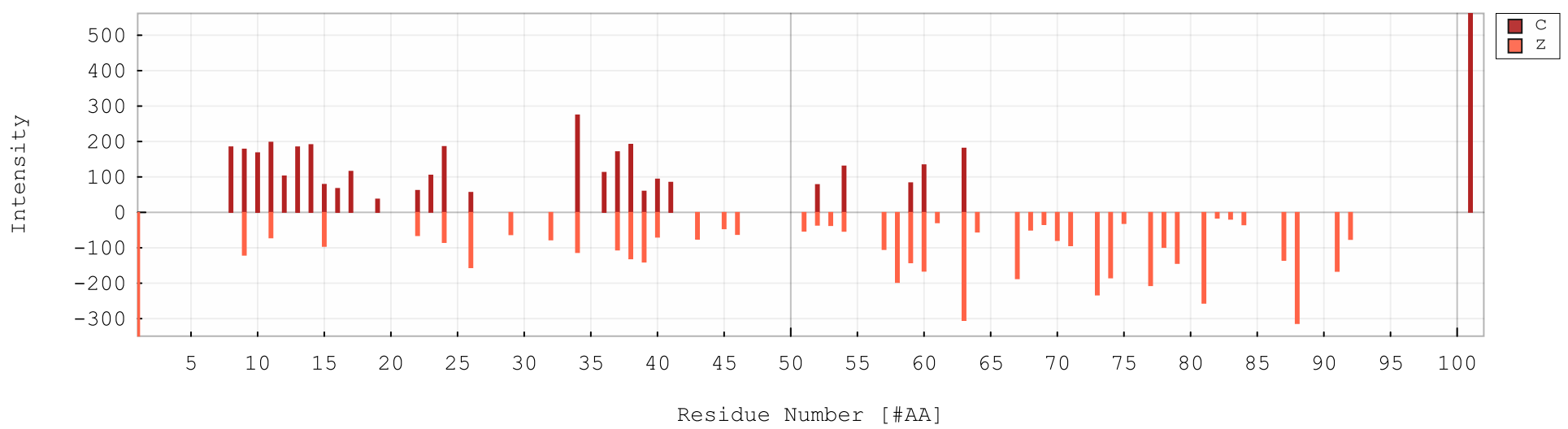

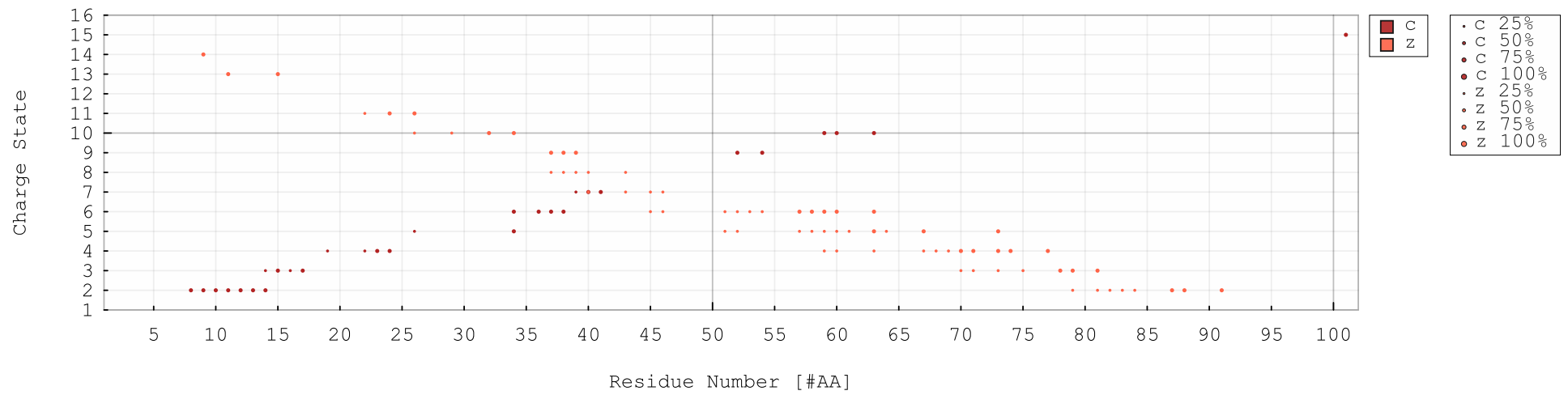

Proteoform:

Index	Protein	SC [%]	SVP [%]	IC [%]	MS Score [a.u.]	Var. Mods	Mono Mass [Da]	Chemical Formula
4	H4Kac_me1-3	68.3	4.9	23.2	15.8	Ace: K5, K20, Dim: K16	11341.3937	C500H845N163O136S

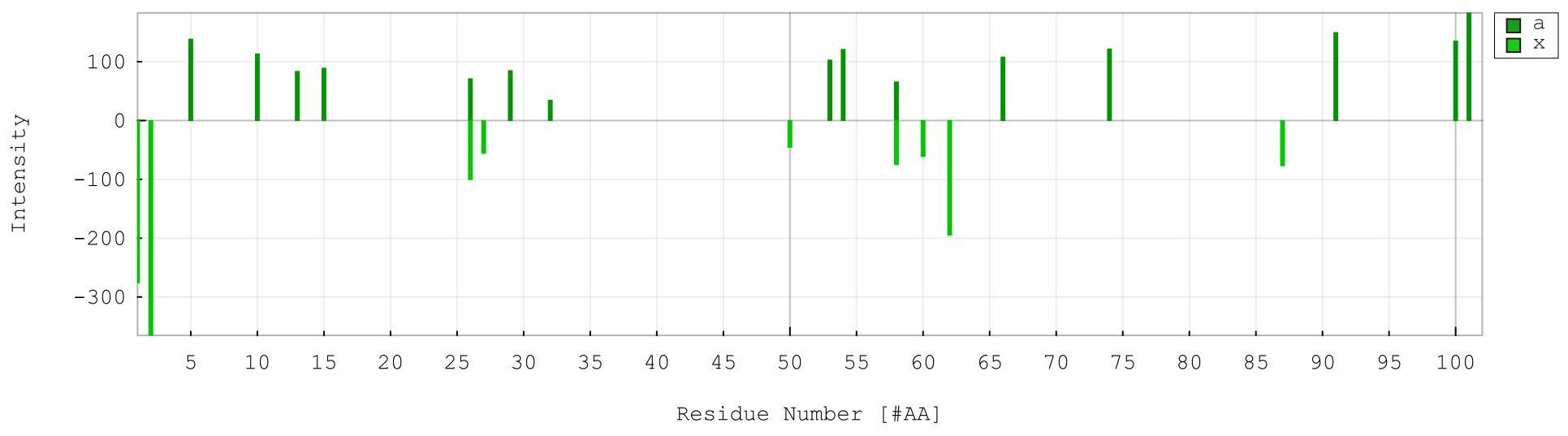

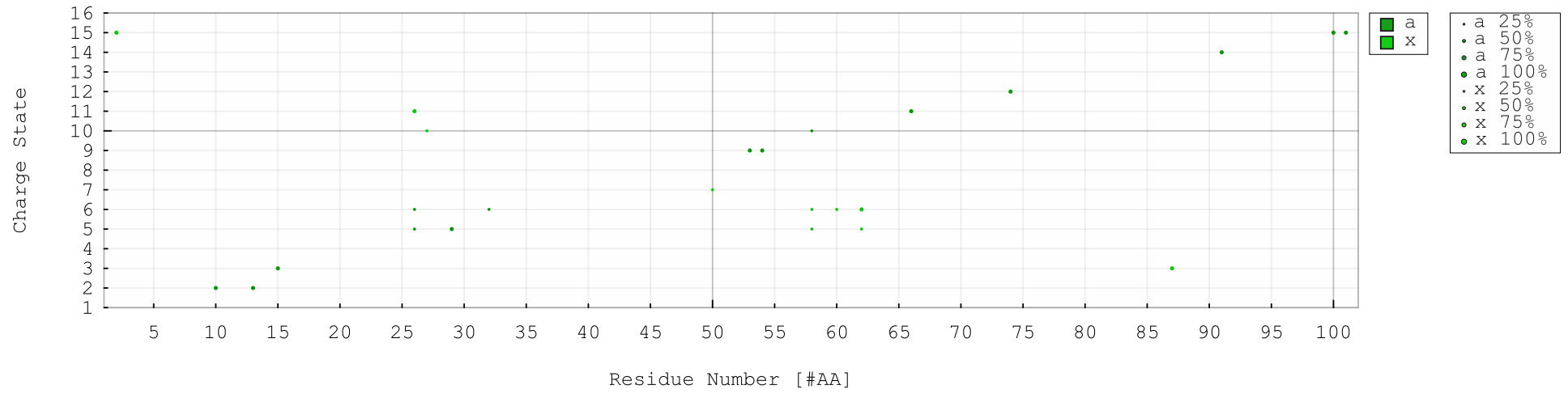

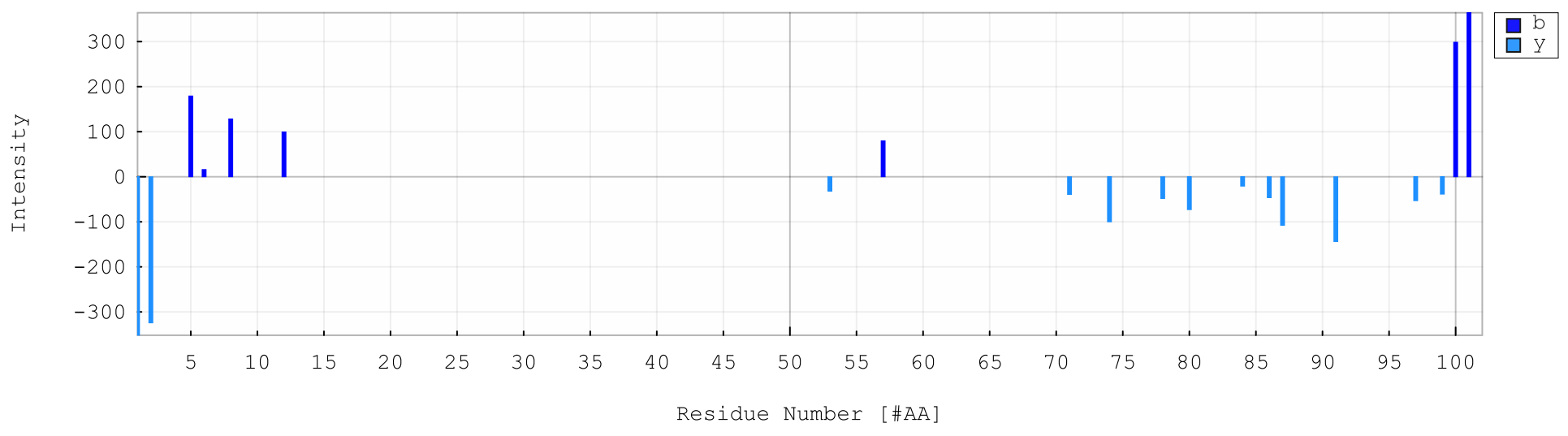

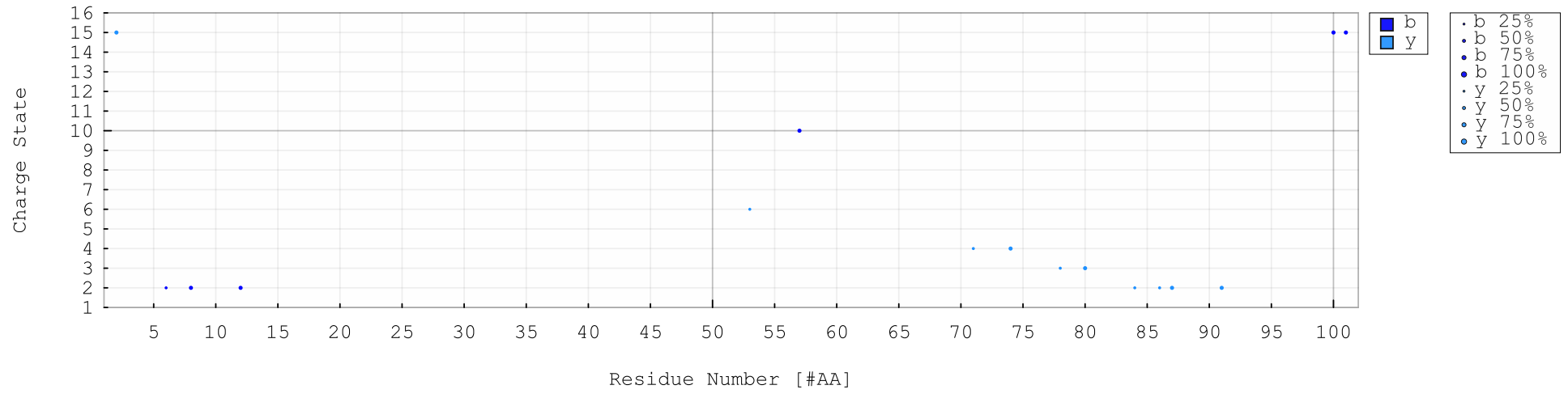

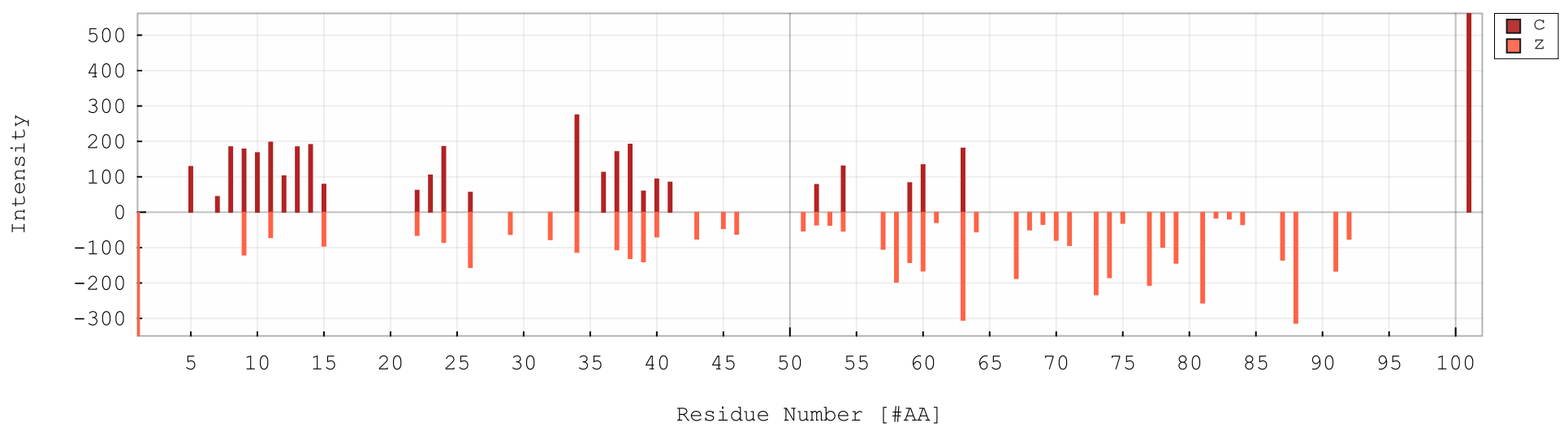

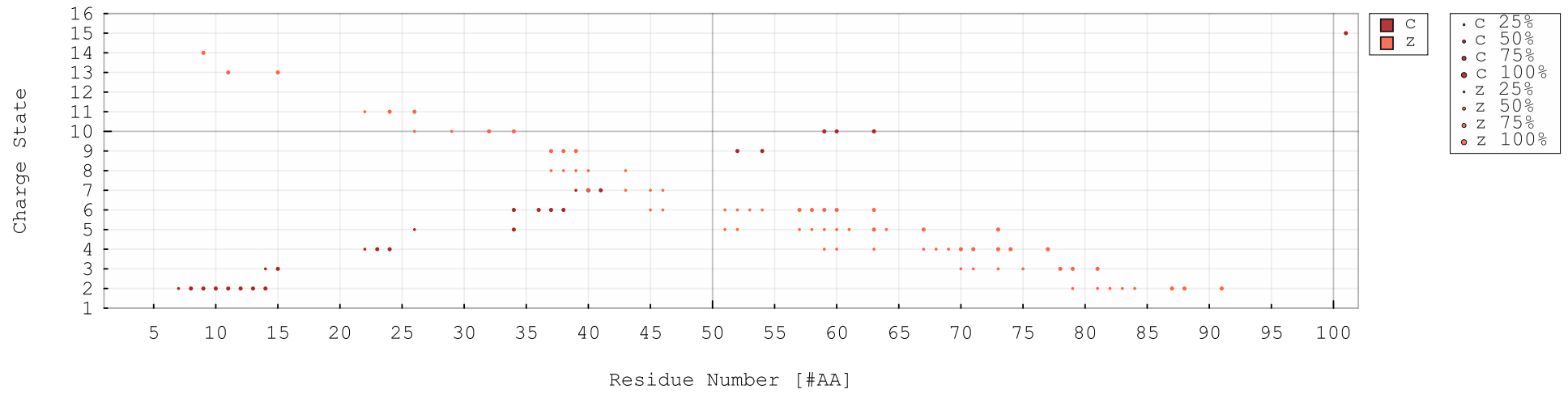

Proteoform:

Index	Protein	SC [%]	SVP [%]	IC [%]	MS Score [a.u.]	Var. Mods	Mono Mass [Da]	Chemical Formula
5	H4Kac_me1-3	70.3	19.6	22.3	15.7	Dim: K16, Ace: K20, N-term	11341.3937	C500H845N163O136S

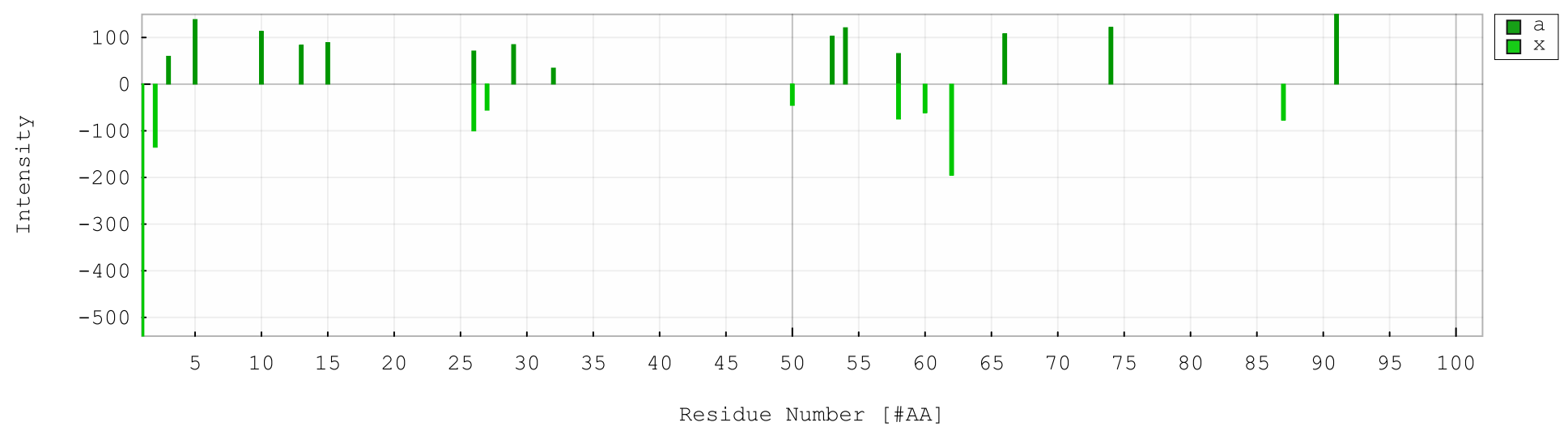

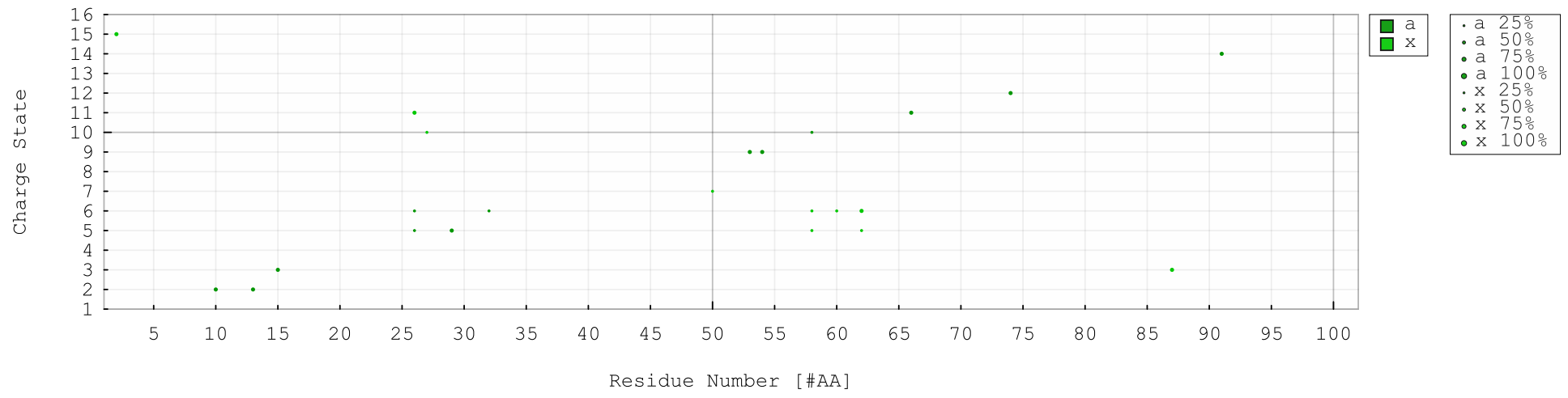

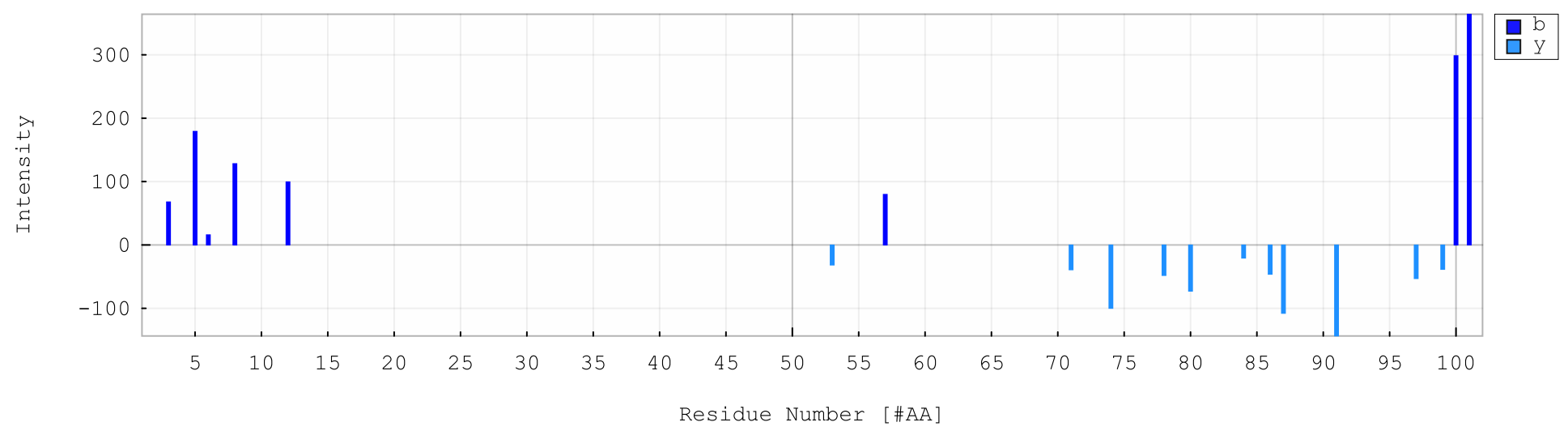

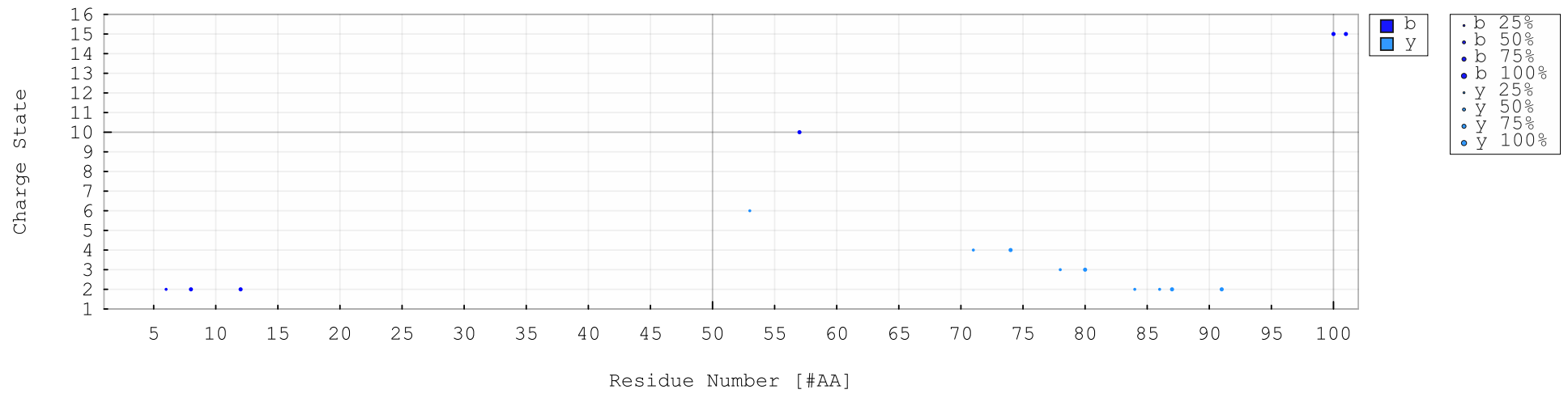

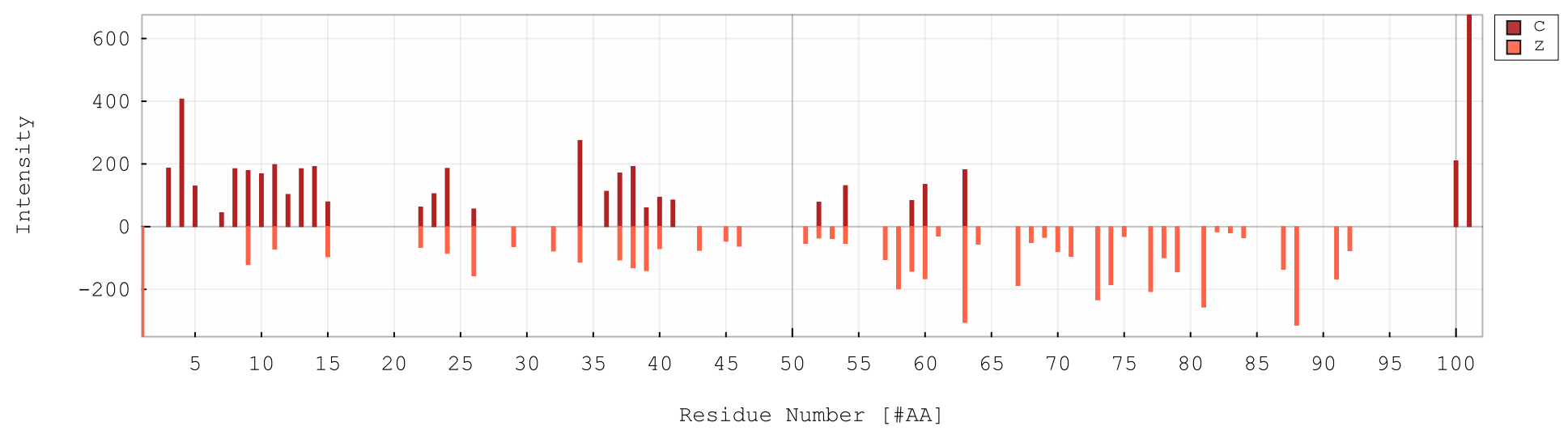

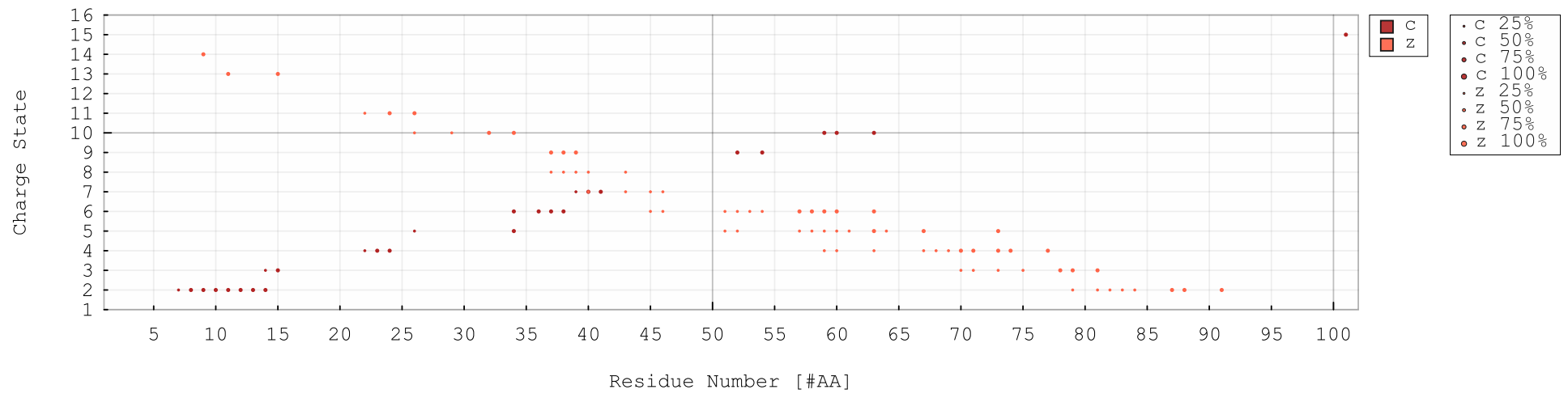

Proteoform:

Index	Protein	SC [%]	SVP [%]	IC [%]	MS Score [a.u.]	Var. Mods	Mono Mass [Da]	Chemical Formula
6	H4Kac_me1-3	68.3	4.9	22.9	15.7	Ace: K5, K12, Dim: K20	11341.3937	C500H845N163O136S

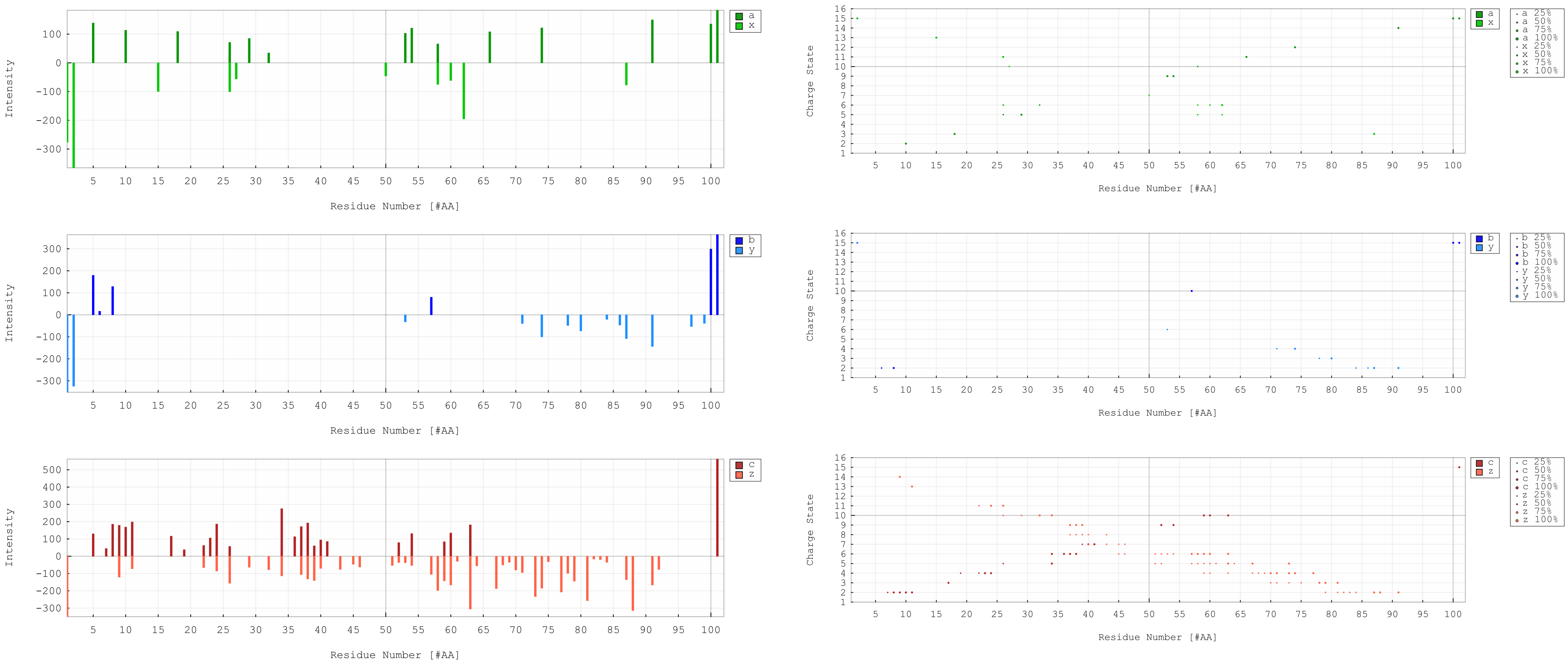

Proteoform:

Index	Protein	SC [%]	SVP [%]	IC [%]	MS Score [a.u.]	Var. Mods	Mono Mass [Da]	Chemical Formula
7	H4Kac_me1-3	70.3	15.7	22.1	15.5	Ace: K12, N-term, Dim: K20	11341.3937	C500H845N163O136S

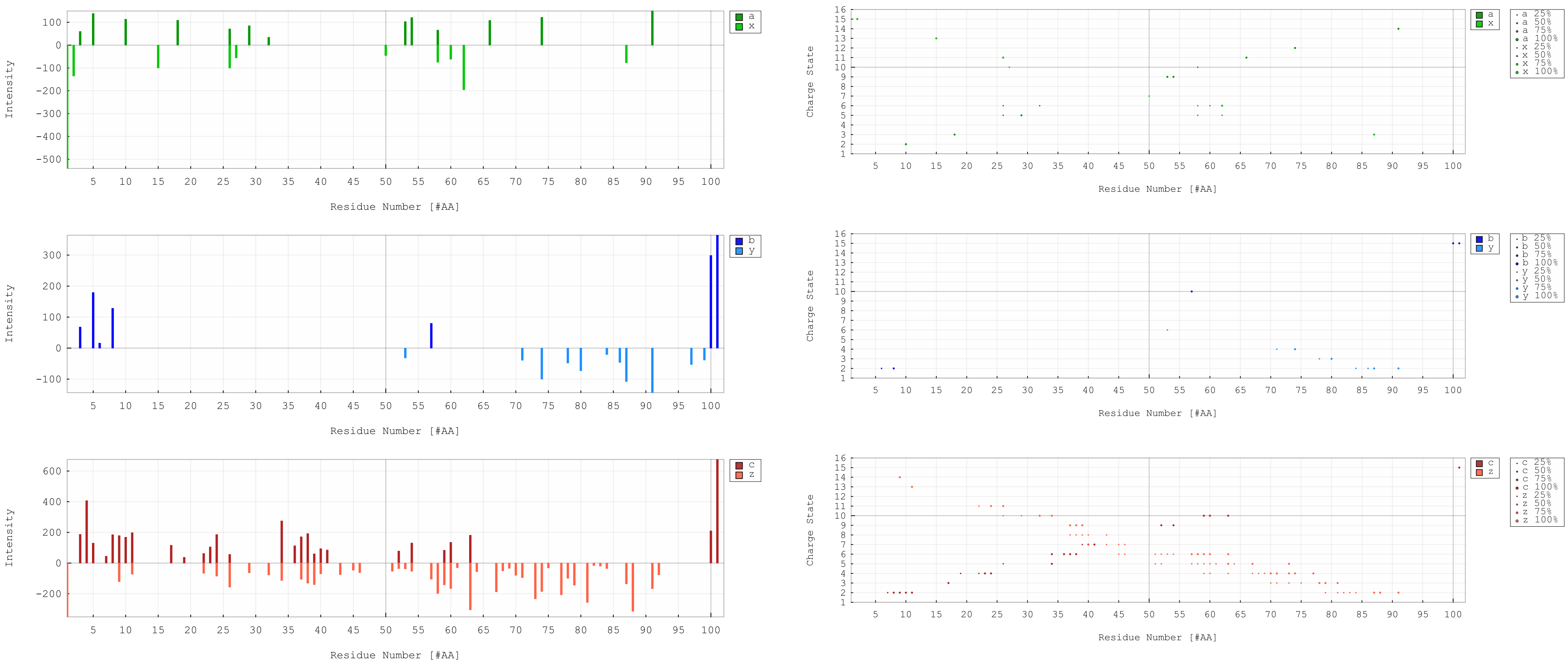

Proteoform:	Index	Protein	SC [%]	SVP [%]	IC [%]	MS Score [a.u.]	Var. Mods	Mono Mass [Da]	Chemical Formula
	8	H4Kac_me1-3	67.3	4.9	22.7	15.2	Ace: K5, K16, Dim: K31	11341.3937	C500H845N163O136S

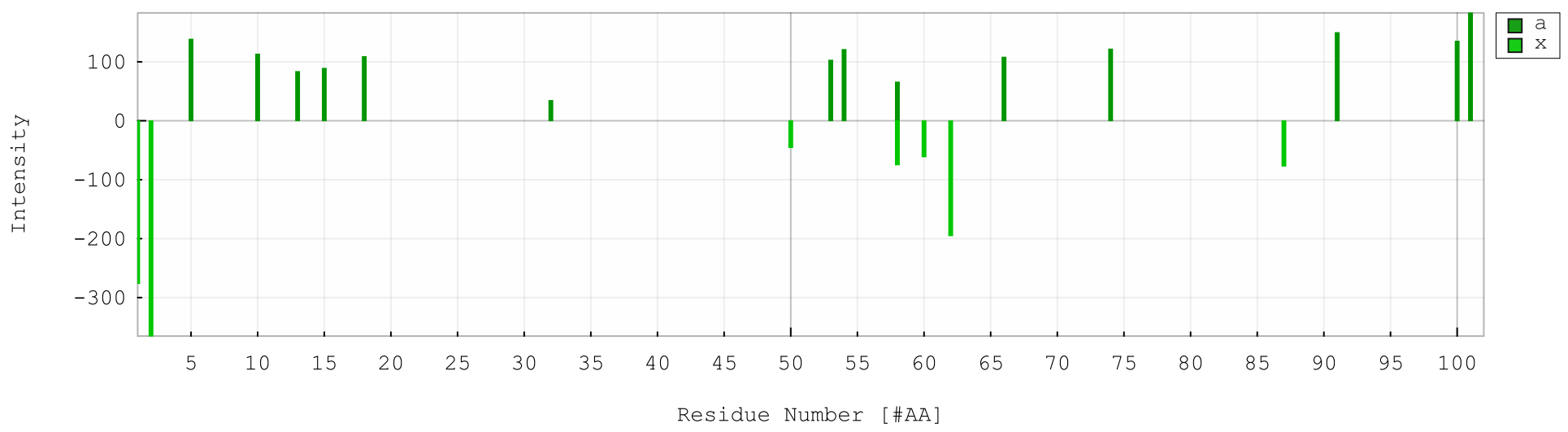

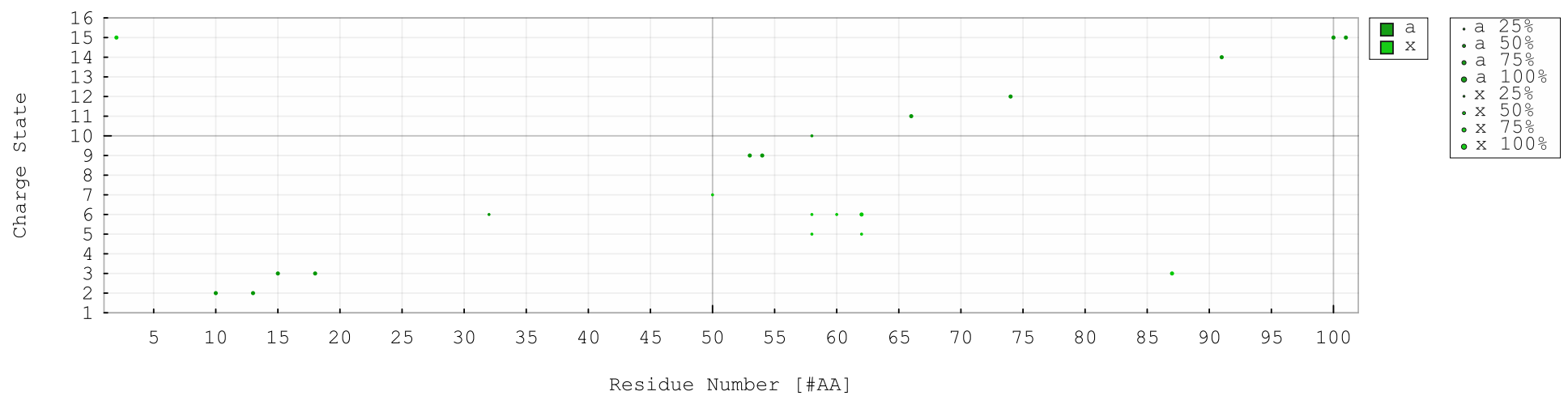

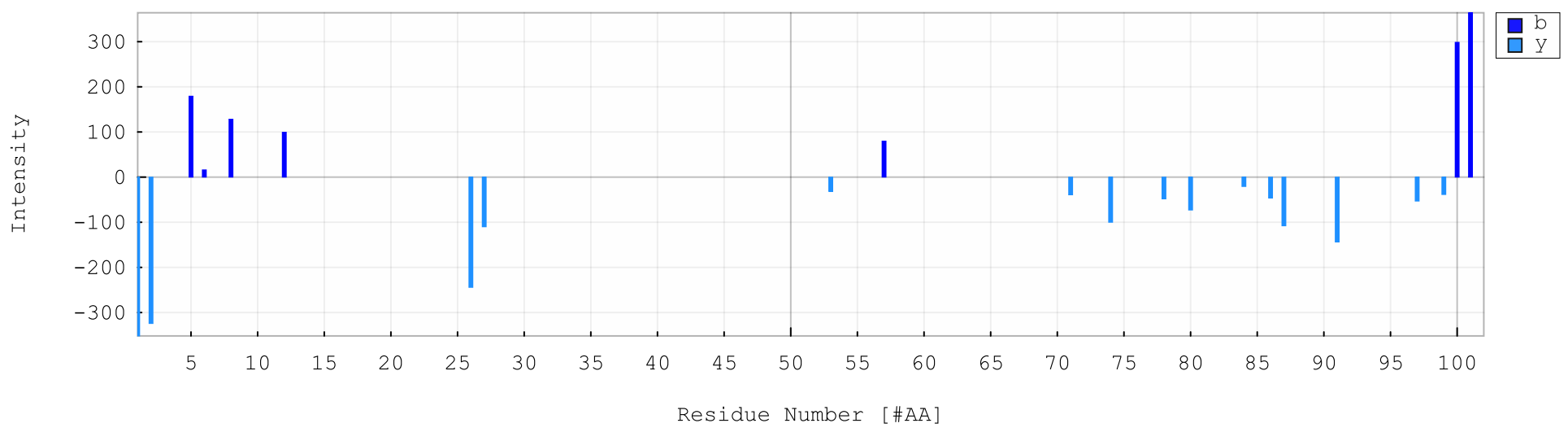

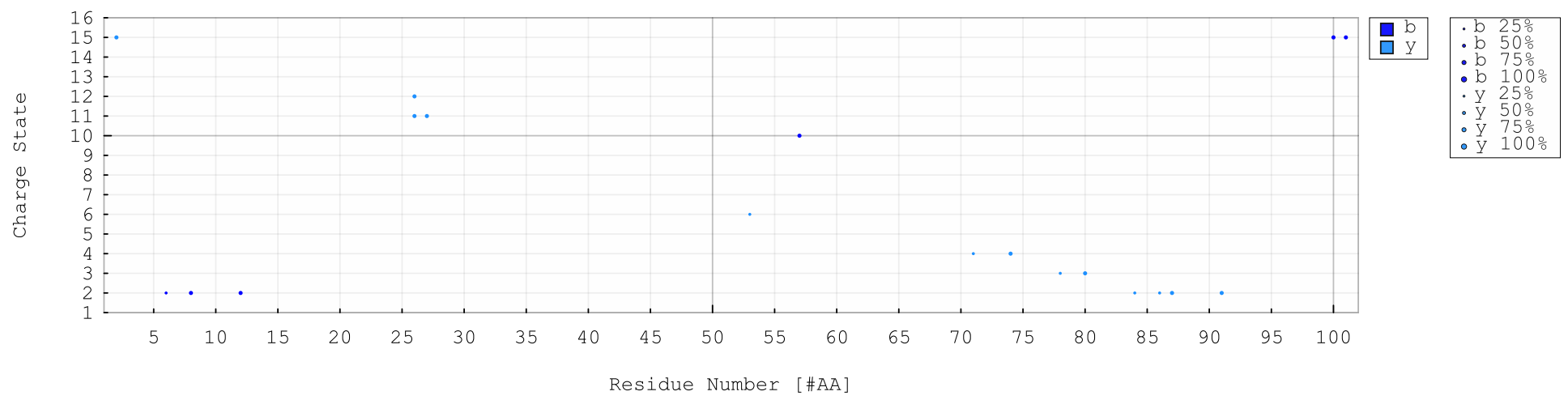

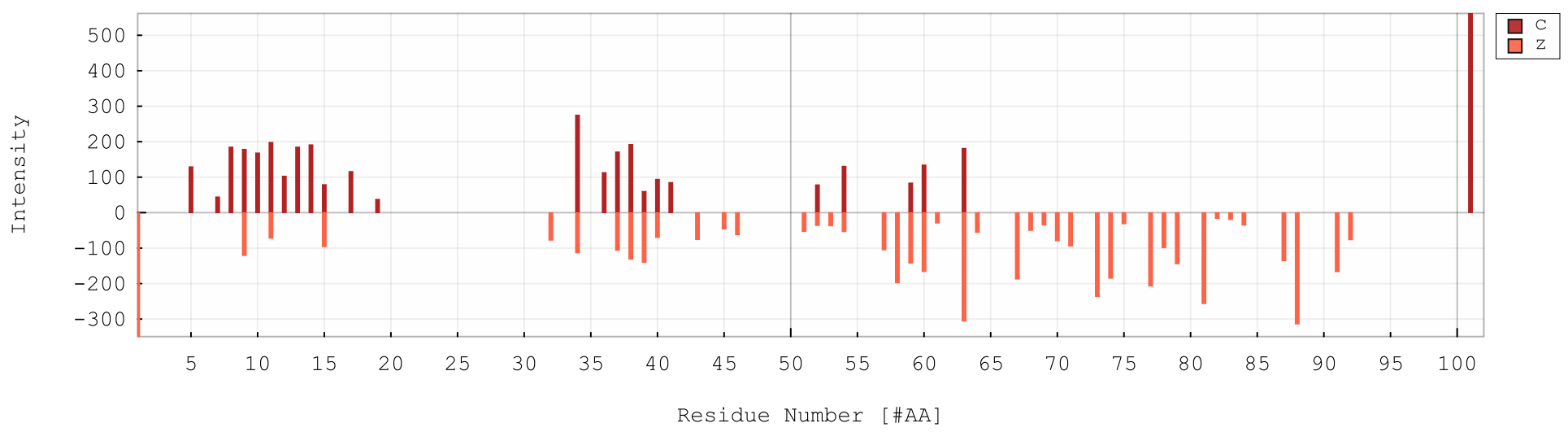

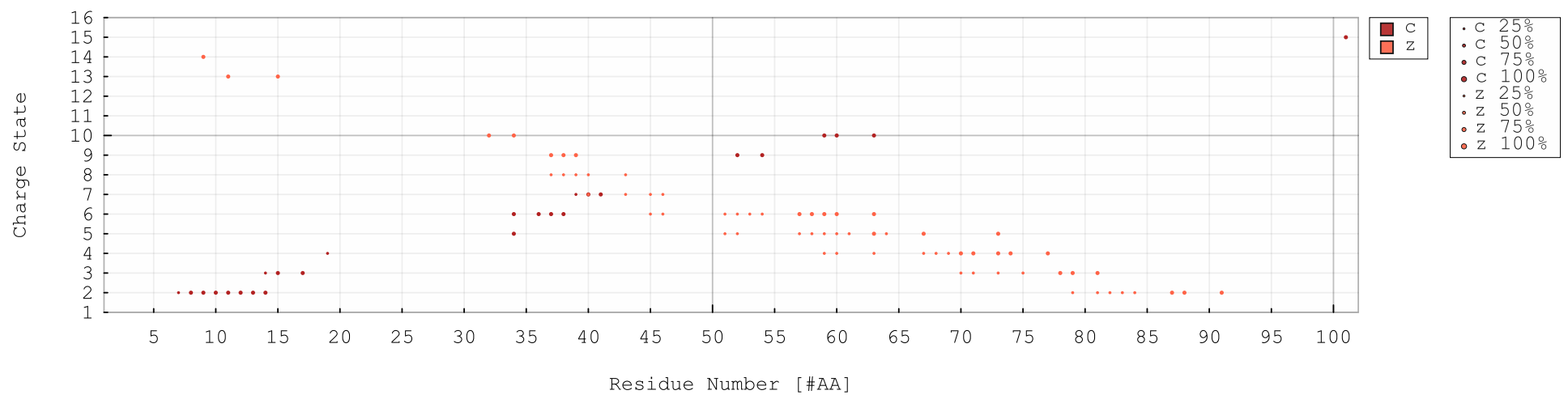

Proteoform:

Index	Protein	SC [%]	SVP [%]	IC [%]	MS Score [a.u.]	Var. Mods	Mono Mass [Da]	Chemical Formula
9	H4Kac_me1-3	66.3	4.9	22.8	15.1	Ace: K8, K12, Dim: K20	11341.3937	C500H845N163O136S

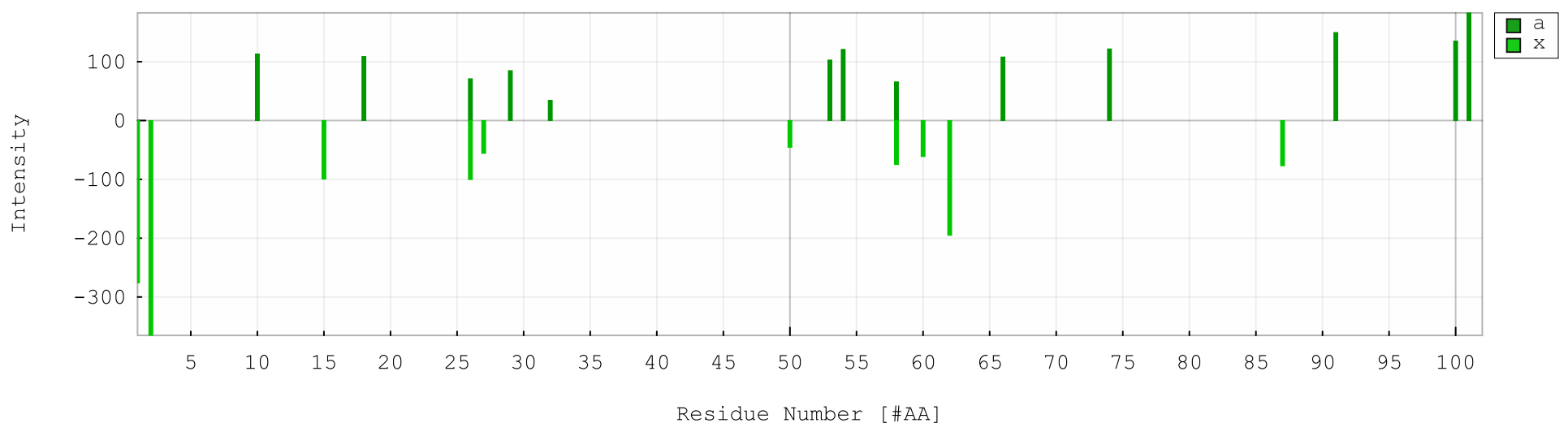

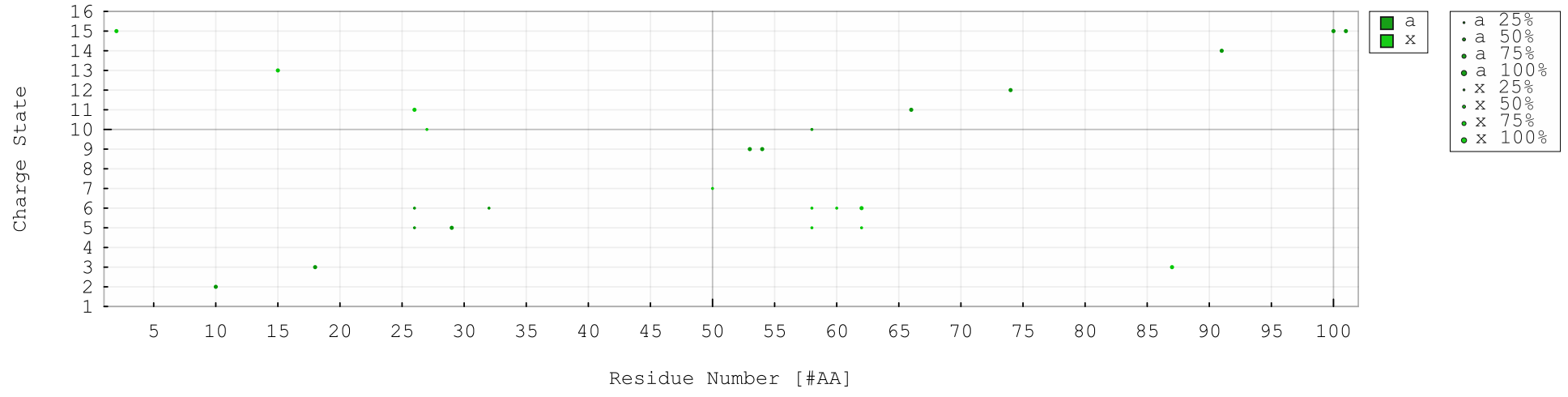

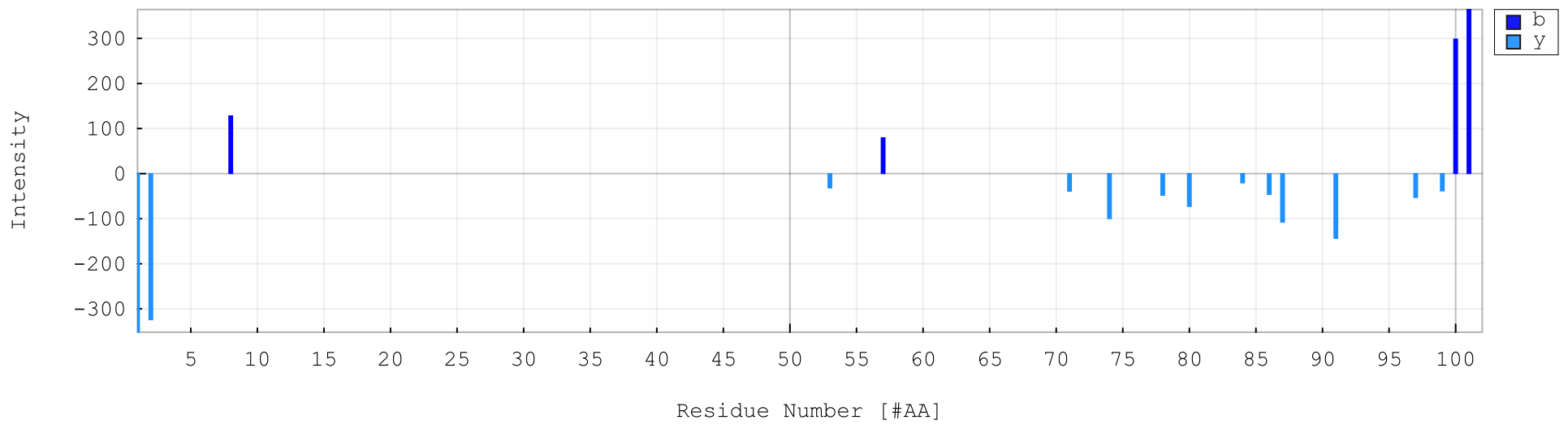

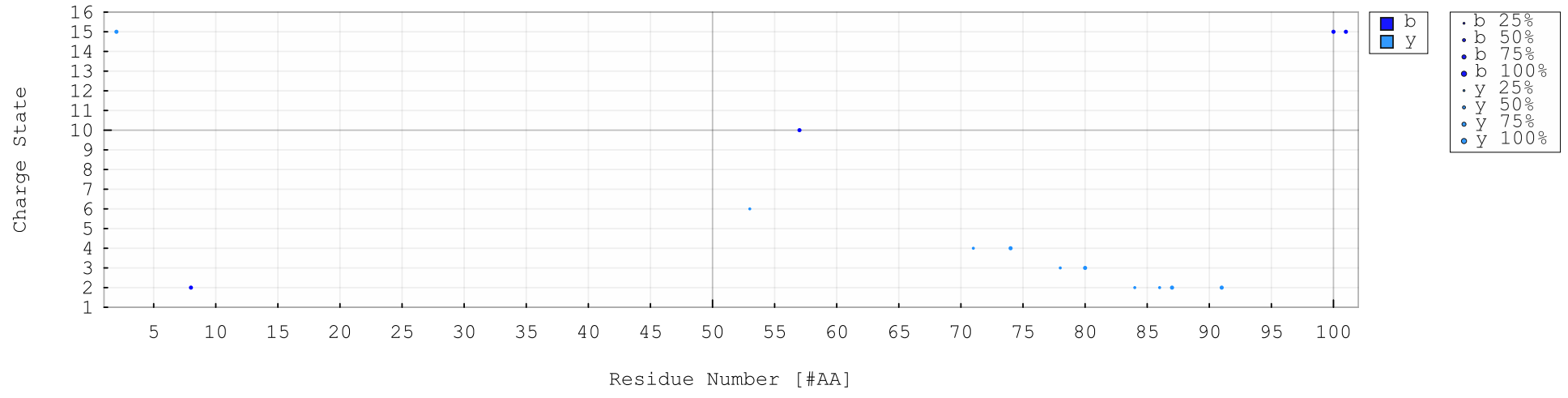

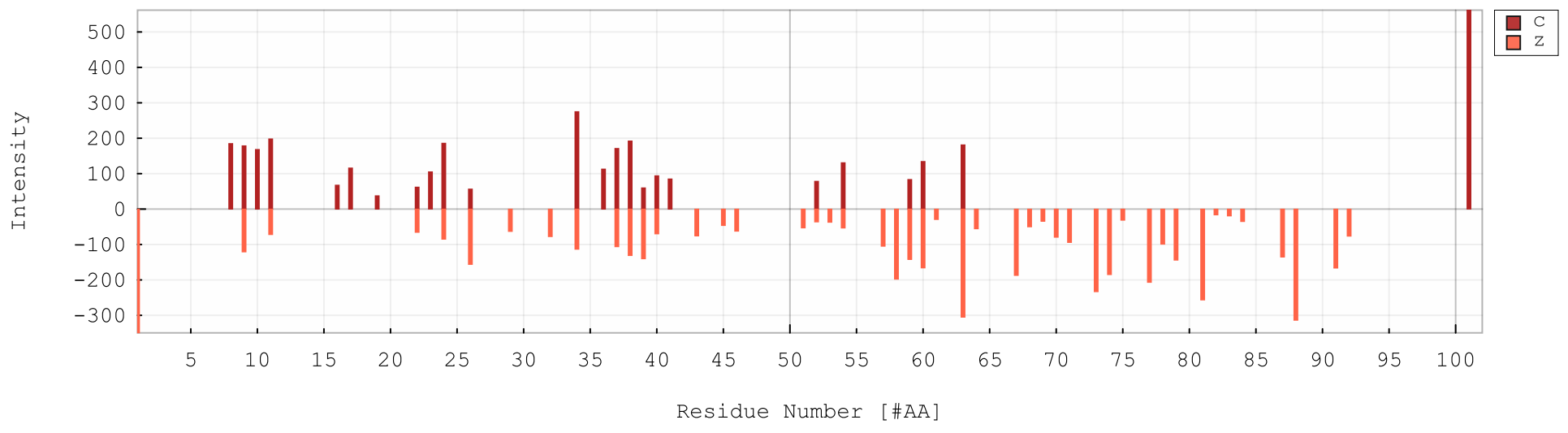

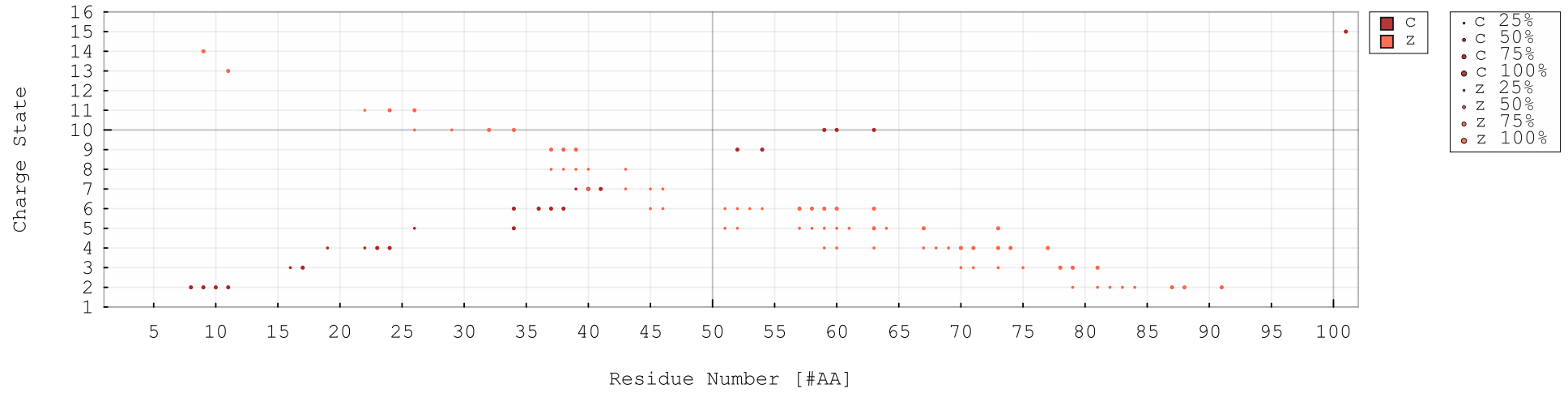

Proteoform:

Index	Protein	SC [%]	SVP [%]	IC [%]	MS Score [a.u.]	Var. Mods	Mono Mass [Da]	Chemical Formula
10	H4Kac_me1-3	69.3	23.5	21.8	15.1	Ace: K16, N-term, Dim: K31	11341.3937	C500H845N163O136S

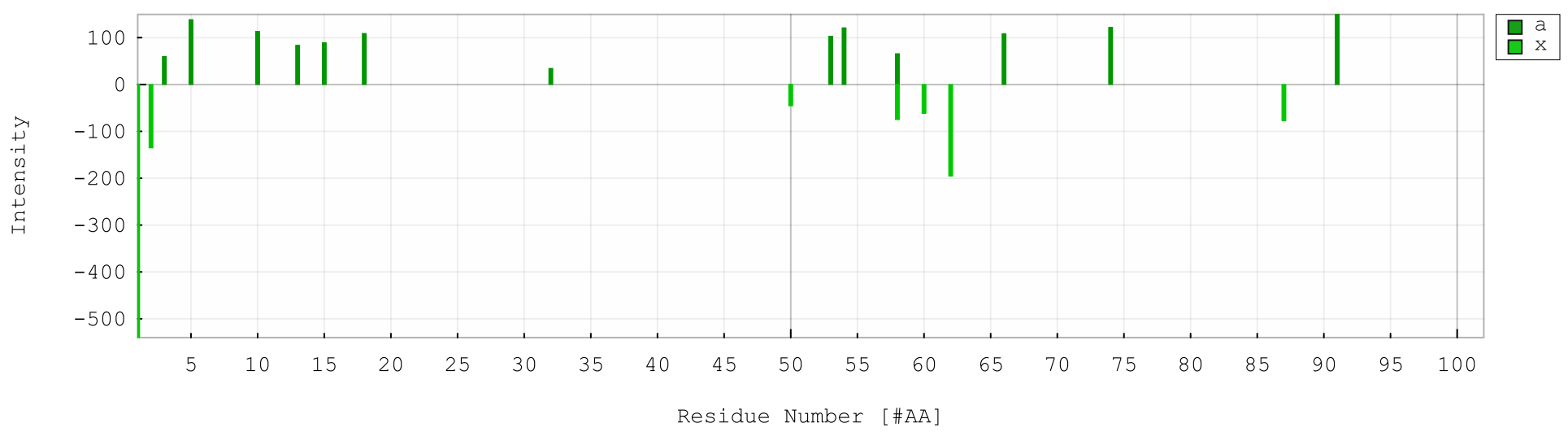

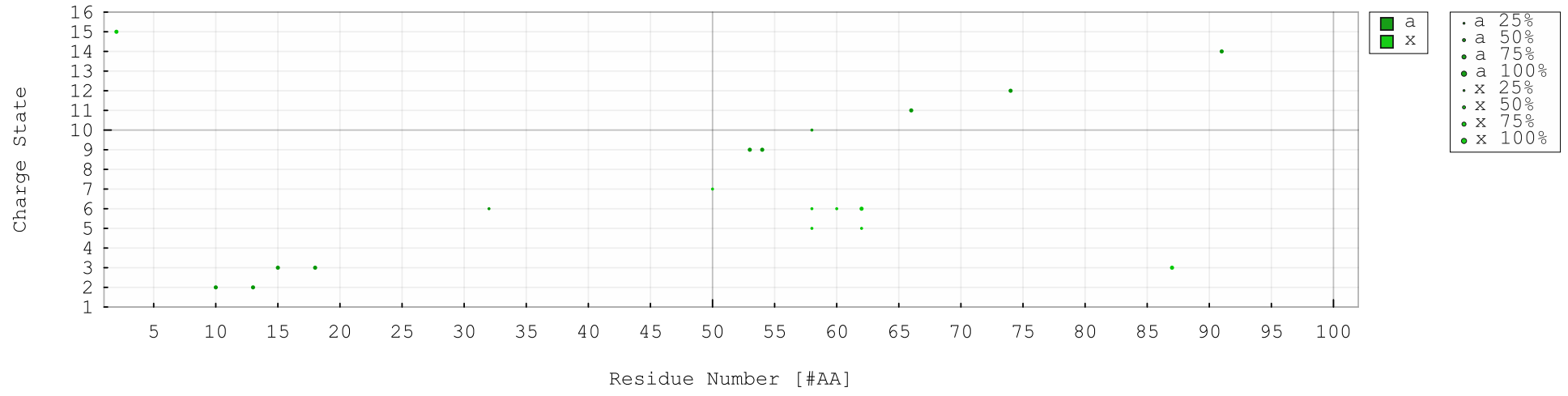

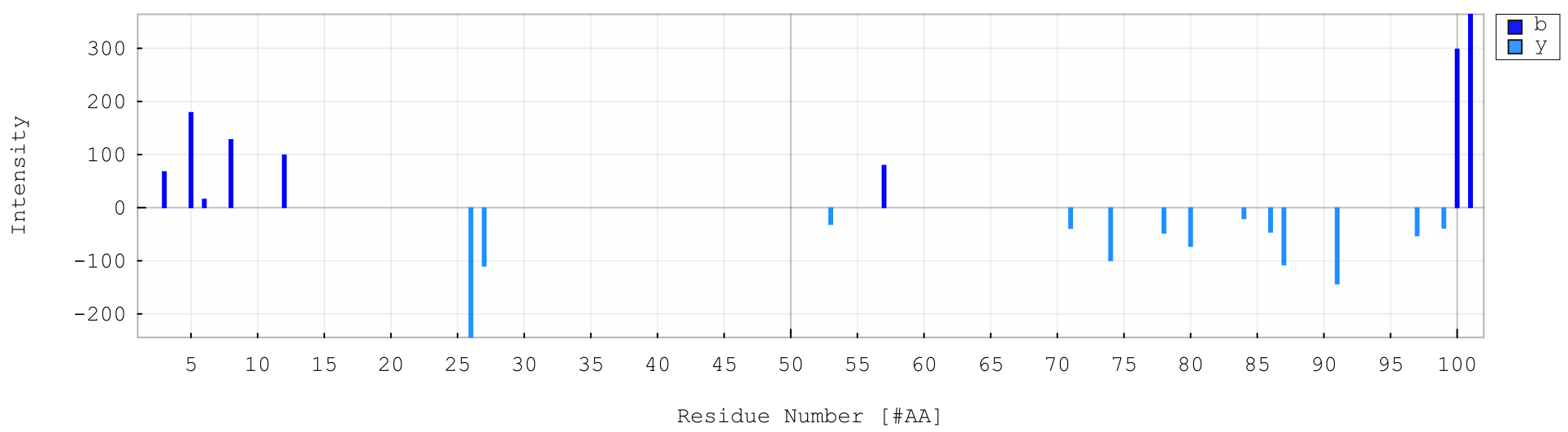

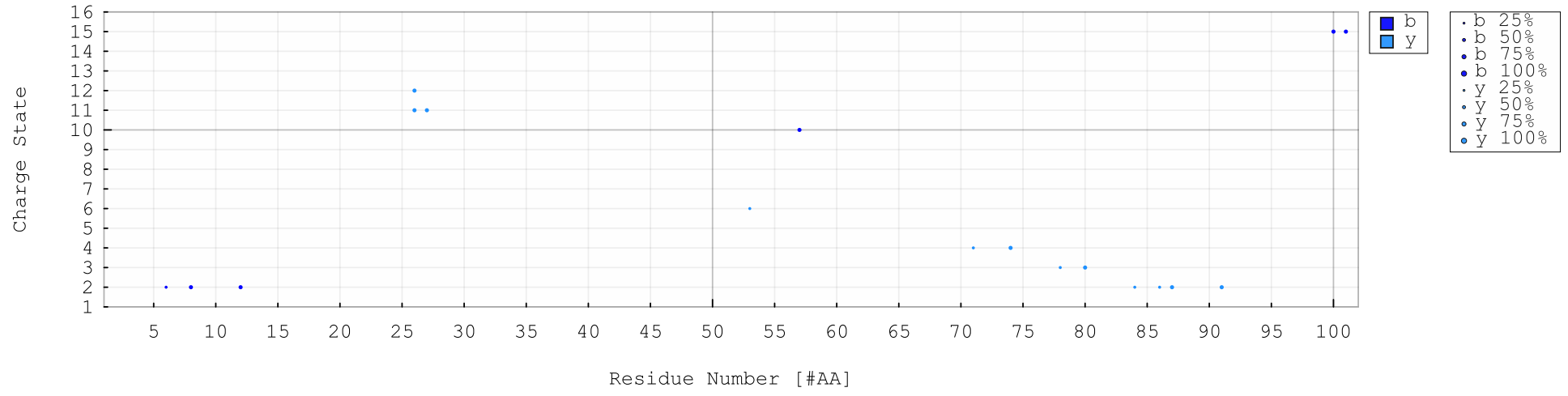

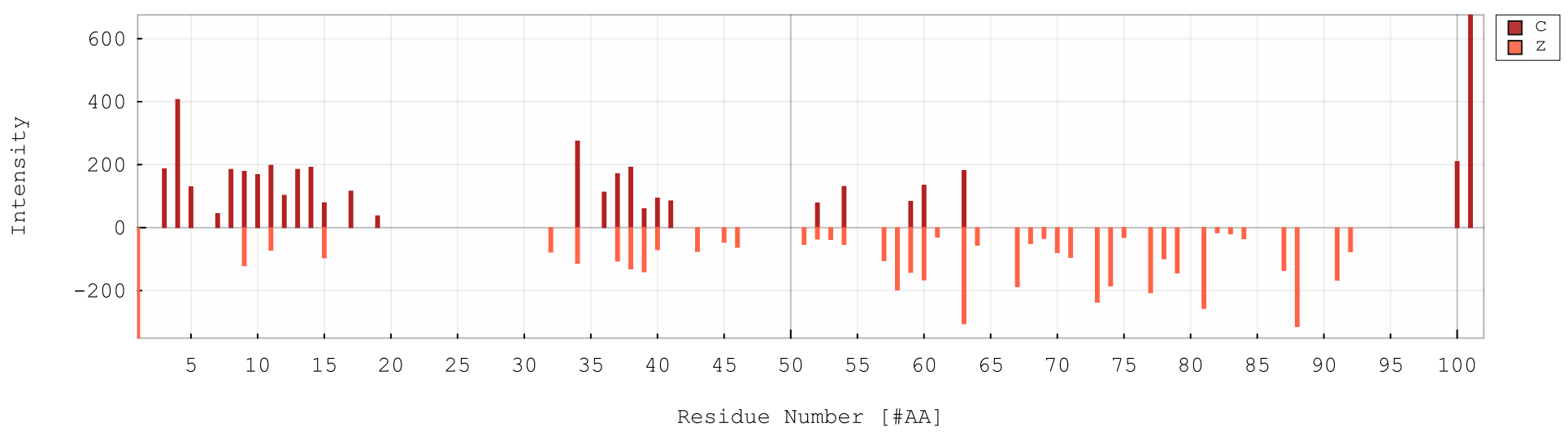

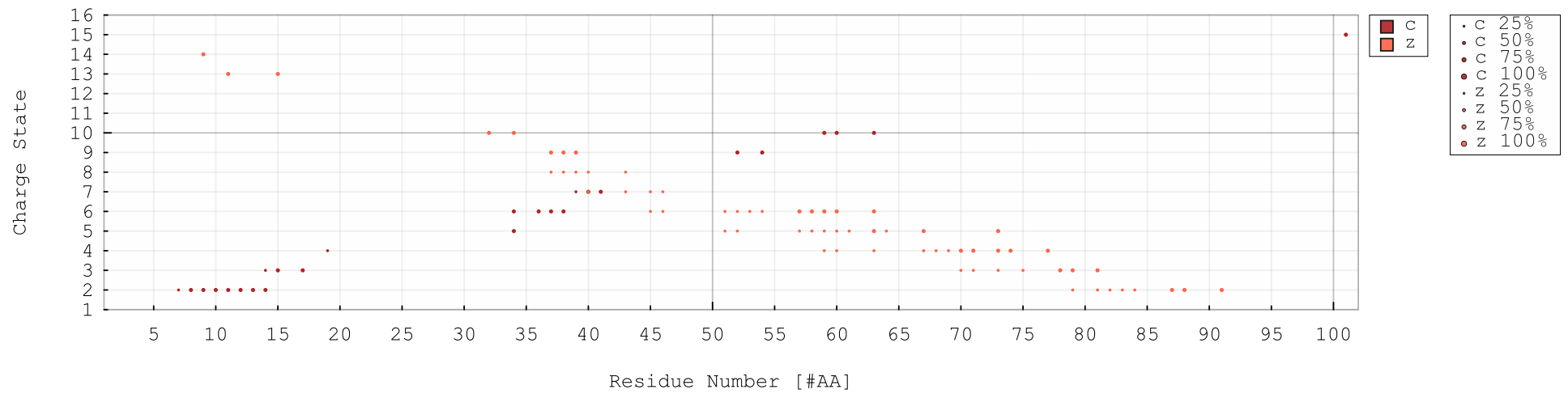

Supplemental Data S1

Endogenous H4

**Report**	6 of 6
**Experiment**	MS2
**Histone/sample**	H4
**Enzyme**	Not applicable
**Reaction/source**	Endogenous sample
**Precursor**	m/z 710
**Charge state**	16+
**Fragmentation / workflow**	RCID
**Original file**	MS2_mz-710_16+_RCID.pdf

Project Information

Project Directory: C:/Users/LAB-ADMIN
Processing date: Tue Apr 28 15:00:49 2026

Sample Info & Protocols

Name: Top-Down Protein Sequencing CID

Dataset

Imported spectrum file name:	250430 Liver-Histone-25 mz710z16 MS2 shCID_001-875_calibrated.txt
Dataset name:	Information is only available from imported JPEAKS files
Date acquired:	Information is only available from imported JPEAKS files
Date imported:	Information is only available from imported JPEAKS files
Instrument Name:	Information is only available from imported JPEAKS files
Instrument Serial Number:	Information is only available from imported JPEAKS files

Workflow Result Info

Analysis: 250430 Liver-Histone-25 mz710z16 MS2 shCID_001-875_calibrated
Location: C:/Users/LAB-ADMIN
Method: -

Method Information

Name: Unnamed

Spectral Parameters

Mass Error [ppm]: 6
Intensity Deviation [au]: 0.15
Min/Max Charges: 1-16
Experiment type: CID

a ions	b ions	c ions	x ions	y ions	z ions	satellite	internal	precursor
a	b			y				M

Noise threshold calculation: Simple moving average noise threshold (SMA)
Window size [datapoints]: 600
Noise curve offset: 1.3779447
SVP parameters: terminal gap: 2, internal gap: 1

Proteoforms

(top 10)

Index	Parent Sequence	Chain	Var. Mods	Mono Mass [Da]	Chemical Composition
1	H4_liver	H4_liver_Nterm[Acetyl]_K16[Dim]K20[Ace]	Dim: K16, Ace: K20, N-term	11341.3937	C500H845N163O136S
2	H4_liver	H4_liver_Nterm[Acetyl]_K16[Ace]K20[Dim]	Ace: K16, N-term, Dim: K20	11341.3937	C500H845N163O136S
3	H4_liver	H4_liver_Nterm[Acetyl]_K12[Dim]K16[Ace]	Dim: K12, Ace: K16, N-term	11341.3937	C500H845N163O136S
4	H4_liver	H4_liver_Nterm[Acetyl]_K12[Ace]K16[Dim]	Ace: K12, N-term, Dim: K16	11341.3937	C500H845N163O136S
5	H4_liver	H4_liver_Nterm[Acetyl]_K12[Ace]K20[Dim]	Ace: K12, N-term, Dim: K20	11341.3937	C500H845N163O136S
6	H4_liver	H4_liver_Nter-m[Acetyl]_K12[Met]K16[Ace]K20[Met]	Met: K12, K20, Ace: K16, N-term	11341.3937	C500H845N163O136S
7	H4_liver	H4_liver_Nter-m[Acetyl]_K12[Ace]K16[Met]K20[Met]	Ace: K12, N-term, Met: K16, K20	11341.3937	C500H845N163O136S
8	H4_liver	H4_liver_Nter-m[Acetyl]_K12[Met]K16[Met]K20[Ace]	Met: K12, K16, Ace: K20, N-term	11341.3937	C500H845N163O136S
9	H4_liver	H4_liver_Nterm[Acetyl]_K12[Dim]K20[Ace]	Dim: K12, Ace: K20, N-term	11341.3937	C500H845N163O136S
10	H4_liver	H4_liverK5[Ace]K16[Ace]K20[Dim]	Ace: K5, K16, Dim: K20	11341.3937	C500H845N163O136S

Results

Proteoforms Table (top 10)

Index	Protein	SC [%]	SVP [%]	IC [%]	MS Score [a.u.]	Var. Mods	Mono Mass [Da]	Chemical Formula
1	H4_liver	34.7	16.7	16.0	5.6	Dim: K16, Ace: K20, N-term	11341.3937	C500H845N163O136S
2	H4_liver	34.7	16.7	16.0	5.6	Ace: K16, N-term, Dim: K20	11341.3937	C500H845N163O136S
3	H4_liver	33.7	15.7	15.9	5.4	Dim: K12, Ace: K16, N-term	11341.3937	C500H845N163O136S
4	H4_liver	33.7	15.7	15.9	5.4	Ace: K12, N-term, Dim: K16	11341.3937	C500H845N163O136S
5	H4_liver	33.7	15.7	15.9	5.4	Ace: K12, N-term, Dim: K20	11341.3937	C500H845N163O136S
6	H4_liver	33.7	15.7	15.9	5.4	Met: K12, K20, Ace: K16, N-term	11341.3937	C500H845N163O136S
7	H4_liver	33.7	15.7	15.9	5.4	Ace: K12, N-term, Met: K16, K20	11341.3937	C500H845N163O136S
8	H4_liver	33.7	15.7	15.9	5.4	Met: K12, K16, Ace: K20, N-term	11341.3937	C500H845N163O136S
9	H4_liver	33.7	15.7	15.9	5.4	Dim: K12, Ace: K20, N-term	11341.3937	C500H845N163O136S
10	H4_liver	32.7	4.9	15.8	5.2	Ace: K5, K16, Dim: K20	11341.3937	C500H845N163O136S

Sequence

Name: H4_liver

```
                  10                    20                    30                    40                    50                    60
S G R G K G G K G L   G K G G A K R H R K   V L R D N I Q G I T   K P A I R R L A R R   G G V K R I S G L I   Y E E T R G V L K V
                  70                    80                    90                   100                   110                   120
F L E N V I R D A V   T Y T E H A K R K T   V T A M D V V Y A L   K R Q G R T L Y G F   G G
```

Modifications

Modification	Position	Type
Acetyl	N-term	variable
Acetyl	5	variable
Acetyl	8	variable
Dimethyl	8	variable
Methyl	8	variable
Dimethyl	12	variable
Acetyl	12	variable
Methyl	12	variable
Dimethyl	16	variable
Acetyl	16	variable
Methyl	16	variable
Acetyl	20	variable
Dimethyl	20	variable

Modification	Position	Type
Methyl	20	variable

Sequence Map

Proteoform:

Index	Protein	SC [%]	SVP [%]	IC [%]	MS Score [a.u.]	Var. Mods	Mono Mass [Da]	Chemical Formula
1	H4_liver	34.7	16.7	16.0	5.6	Dim: K16, Ace: K20, N-term	11341.3937	C500H845N163O136S

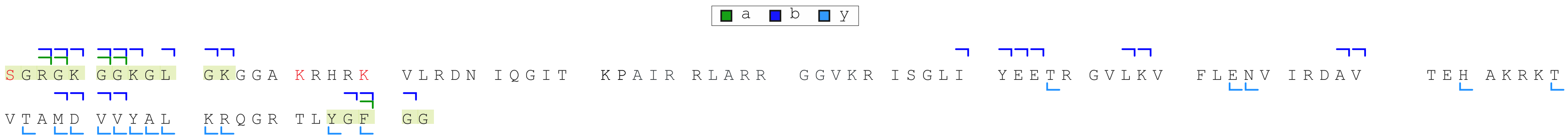

Proteoform:

Index	Protein	SC [%]	SVP [%]	IC [%]	MS Score [a.u.]	Var. Mods	Mono Mass [Da]	Chemical Formula
2	H4_liver	34.7	16.7	16.0	5.6	Ace: K16, N-term, Dim: K20	11341.3937	C500H845N163O136S

a b y

S G R G K G G K G L G K G G A K R H R K V L R D N I Q G I T K P A I R R L A R R G G V K R I S G L I Y E E T R G V L K V F L E N V I R D A V T Y T E H A K R K T
V T A M D V V Y A L K R Y G F G G

Proteoform:

Index	Protein	SC [%]	SVP [%]	IC [%]	MS Score [a.u.]	Var. Mods	Mono Mass [Da]	Chemical Formula
3	H4_liver	33.7	15.7	15.9	5.4	Dim: K12, Ace: K16, N-term	11341.3937	C500H845N163O136S

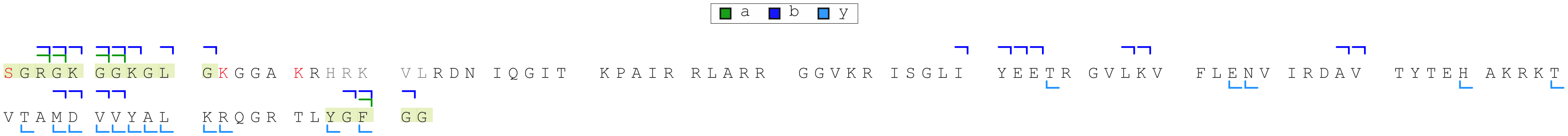

Proteoform:	Index	Protein	SC [%]	SVP [%]	IC [%]	MS Score [a.u.]	Var. Mods	Mono Mass [Da]	Chemical Formula
	4	H4_liver	33.7	15.7	15.9	5.4	Ace: K12, N-term, Dim: K16	11341.3937	C500H845N163O136S

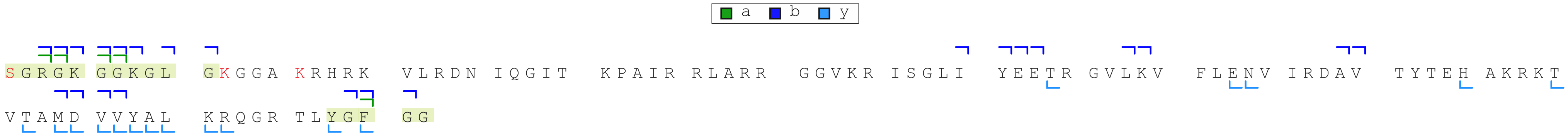

Proteoform:	Index	Protein	SC [%]	SVP [%]	IC [%]	MS Score [a.u.]	Var. Mods	Mono Mass [Da]	Chemical Formula
	5	H4_liver	33.7	15.7	15.9	5.4	Ace: K12, N-term, Dim: K20	11341.3937	C500H845N163O136S

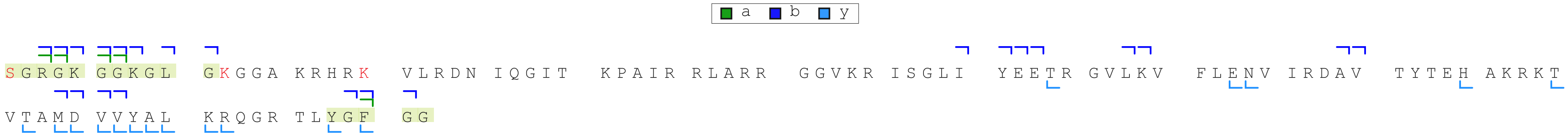

Proteoform:

Index	Protein	SC [%]	SVP [%]	IC [%]	MS Score [a.u.]	Var. Mods	Mono Mass [Da]	Chemical Formula
6	H4_liver	33.7	15.7	15.9	5.4	Met: K12, K20, Ace: K16, N-term	11341.3937	C500H845N163O136S

a b y

SGRGK GGKGL GKGGA KRHRK VLRDN IQGIT KPAIR RLARR GGVKR ISGLI YEETR GVLKV FLENV IRDAV TYTEH AKRKT
VTAMD VVYAL KRQGR TLYGF GG

Proteoform:

Index	Protein	SC [%]	SVP [%]	IC [%]	MS Score [a.u.]	Var. Mods	Mono Mass [Da]	Chemical Formula
7	H4_liver	33.7	15.7	15.9	5.4	Ace: K12, N-term, Met: K16, K20	11341.3937	C500H845N163O136S

a b y

SGRGK GGKGL GKGGA KRHRK VLRDN IQGIT KPAIR RLARR GGVKR ISGLI YEETR GVLKV FLENV IRDAV TYTEH AKRKT
VTAMD VVYAL KRQGR TLYGF GG

Proteoform:

Index	Protein	SC [%]	SVP [%]	IC [%]	MS Score [a.u.]	Var. Mods	Mono Mass [Da]	Chemical Formula
8	H4_liver	33.7	15.7	15.9	5.4	Met: K12, K16, Ace: K20, N-term	11341.3937	C500H845N163O136S

a b y

SGRGK GGKGL GKGGA KRHRK VLRDN IQGIT KPAIR RLARR GGVKR ISGLI YEETR GVLKV FLENV IRDAV TYTEH AKRKT
VTAMD VVYAL KRQGR TLYGF GG

Proteoform:

Index	Protein	SC [%]	SVP [%]	IC [%]	MS Score [a.u.]	Var. Mods	Mono Mass [Da]	Chemical Formula
9	H4_liver	33.7	15.7	15.9	5.4	Dim: K12, Ace: K20, N-term	11341.3937	C500H845N163O136S

a b y

SGRGK GGKGL GKGGA KRHRK VLRDN IQGIT KPAIR RLARR GGVKR ISGLI YEETR GVLKV FLENV IRDAV TYTEH AKRKT
VTAMD VVYAL KRQGR TLYGF GG

Proteoform:

Index	Protein	SC [%]	SVP [%]	IC [%]	MS Score [a.u.]	Var. Mods	Mono Mass [Da]	Chemical Formula
10	H4_liver	32.7	4.9	15.8	5.2	Ace: K5, K16, Dim: K20	11341.3937	C500H845N163O136S

a b y

S G R G K G G K G L G K G G A K R H R K V L R D N I Q G I T K P A I R R L A R R G G V K R I S G L I Y E E T R G V L K V F L E N V I R D A V T Y T E H A K R K T
V T A M D V V Y A L K R Q G R T L Y G F G G

Error Plot

Proteoform:

Index	Protein	SC [%]	SVP [%]	IC [%]	MS Score [a.u.]	Var. Mods	Mono Mass [Da]	Chemical Formula
1	H4_liver	34.7	16.7	16.0	5.6	Dim: K16, Ace: K20, N-term	11341.3937	C500H845N163O136S

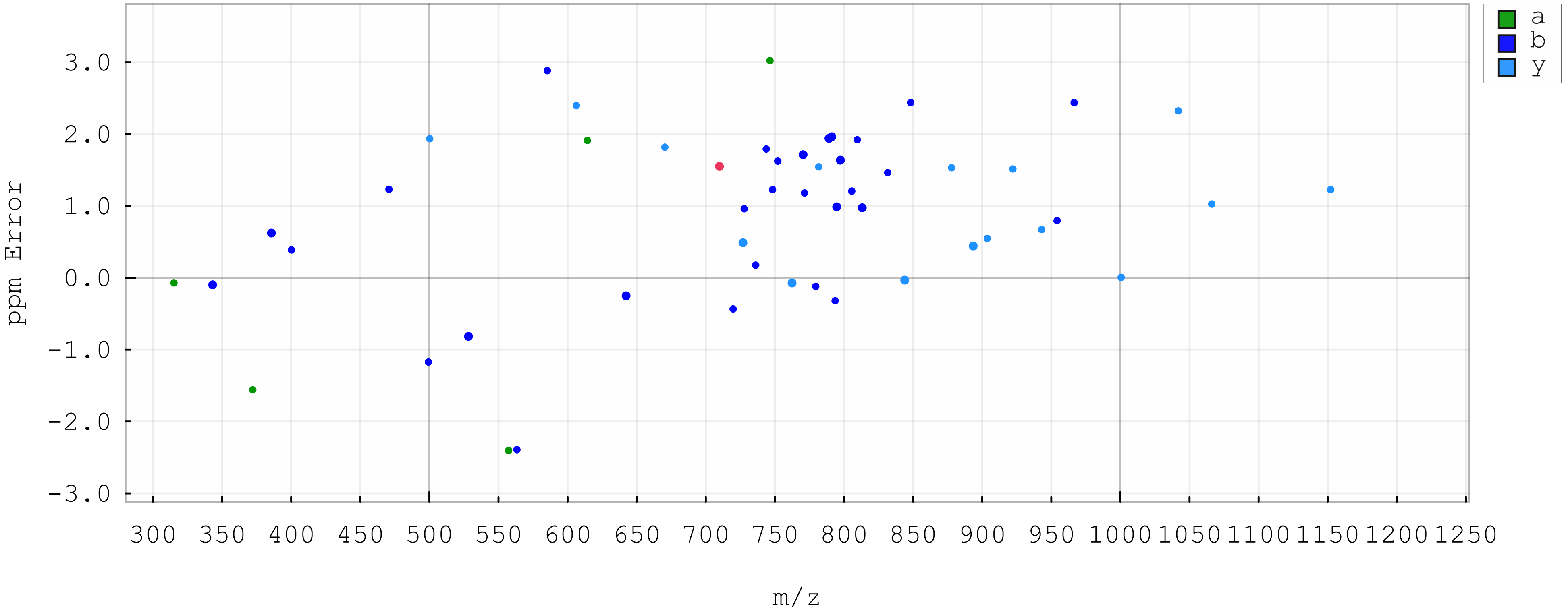

Proteoform:

Index	Protein	SC [%]	SVP [%]	IC [%]	MS Score [a.u.]	Var. Mods	Mono Mass [Da]	Chemical Formula
2	H4_liver	34.7	16.7	16.0	5.6	Ace: K16, N-term, Dim: K20	11341.3937	C500H845N163O136S

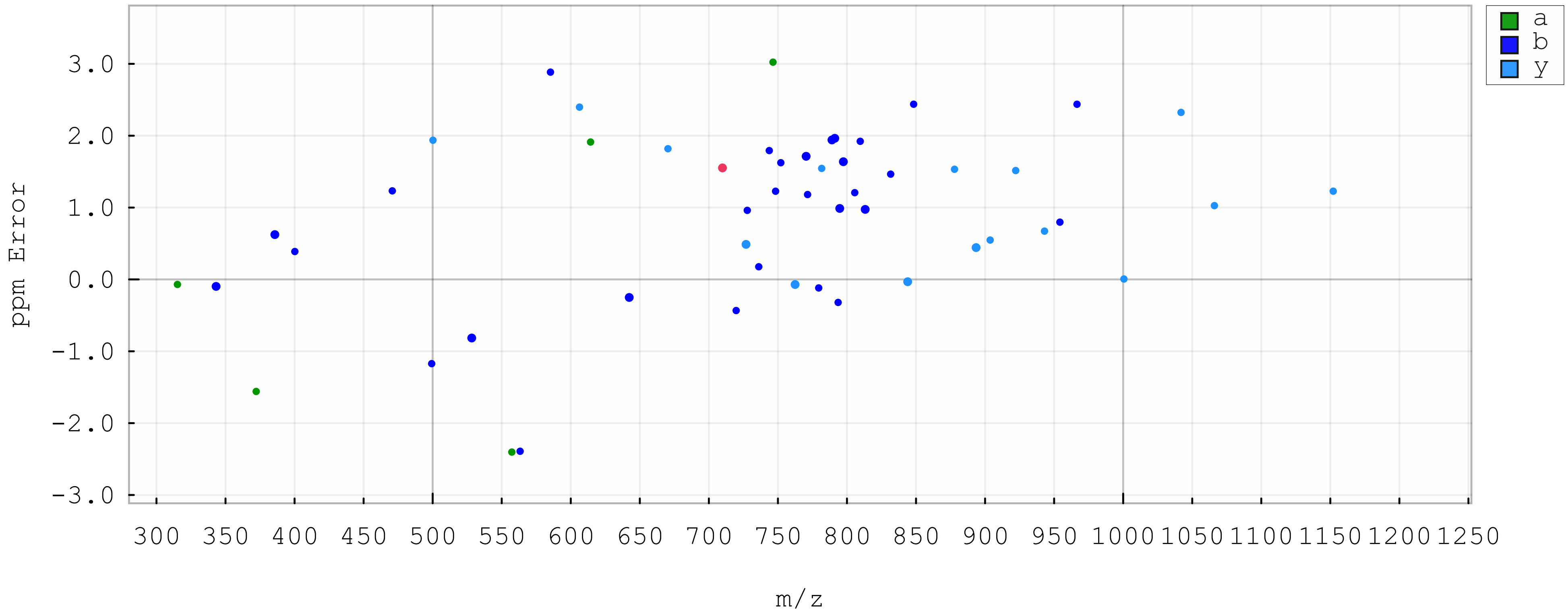

Proteoform:	Index	Protein	SC [%]	SVP [%]	IC [%]	MS Score [a.u.]	Var. Mods	Mono Mass [Da]	Chemical Formula
	3	H4_liver	33.7	15.7	15.9	5.4	Dim: K12, Ace: K16, N-term	11341.3937	C500H845N163O136S

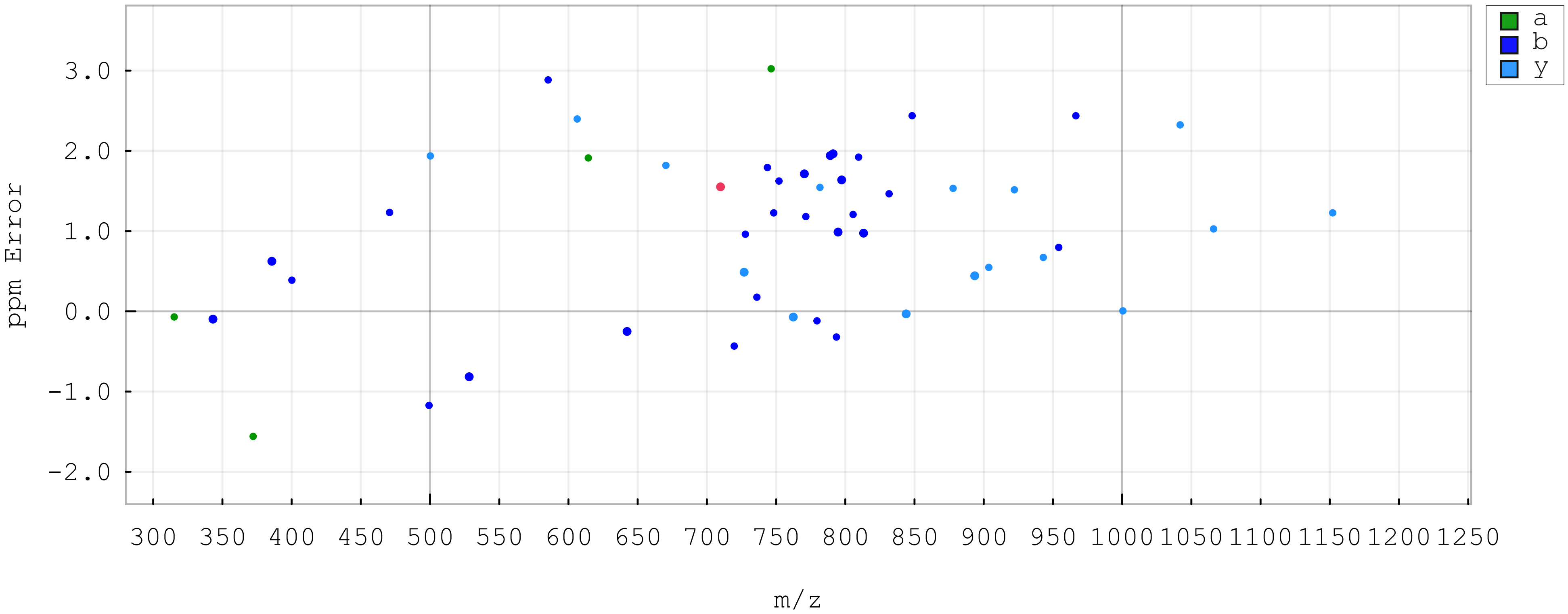

Proteoform:

Index	Protein	SC [%]	SVP [%]	IC [%]	MS Score [a.u.]	Var. Mods	Mono Mass [Da]	Chemical Formula
4	H4_liver	33.7	15.7	15.9	5.4	Ace: K12, N-term, Dim: K16	11341.3937	C500H845N163O136S

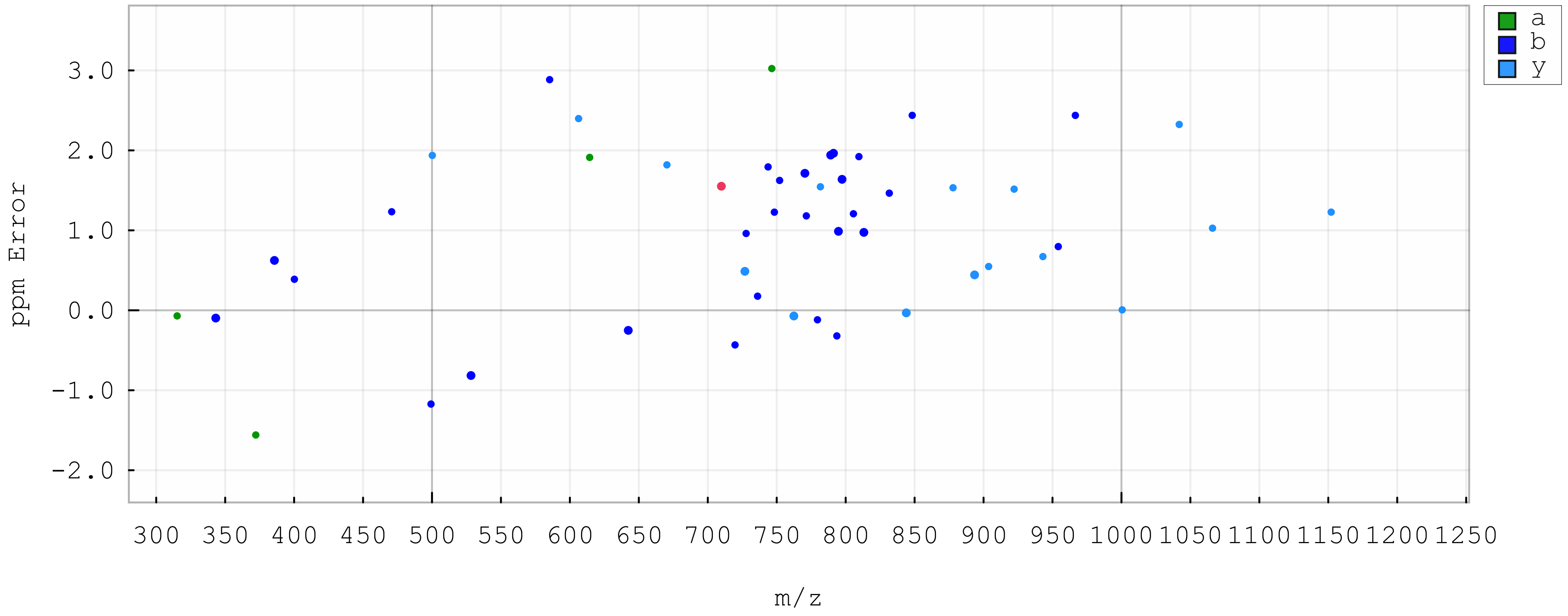

Proteoform:

Index	Protein	SC [%]	SVP [%]	IC [%]	MS Score [a.u.]	Var. Mods	Mono Mass [Da]	Chemical Formula
5	H4_liver	33.7	15.7	15.9	5.4	Ace: K12, N-term, Dim: K20	11341.3937	C500H845N163O136S

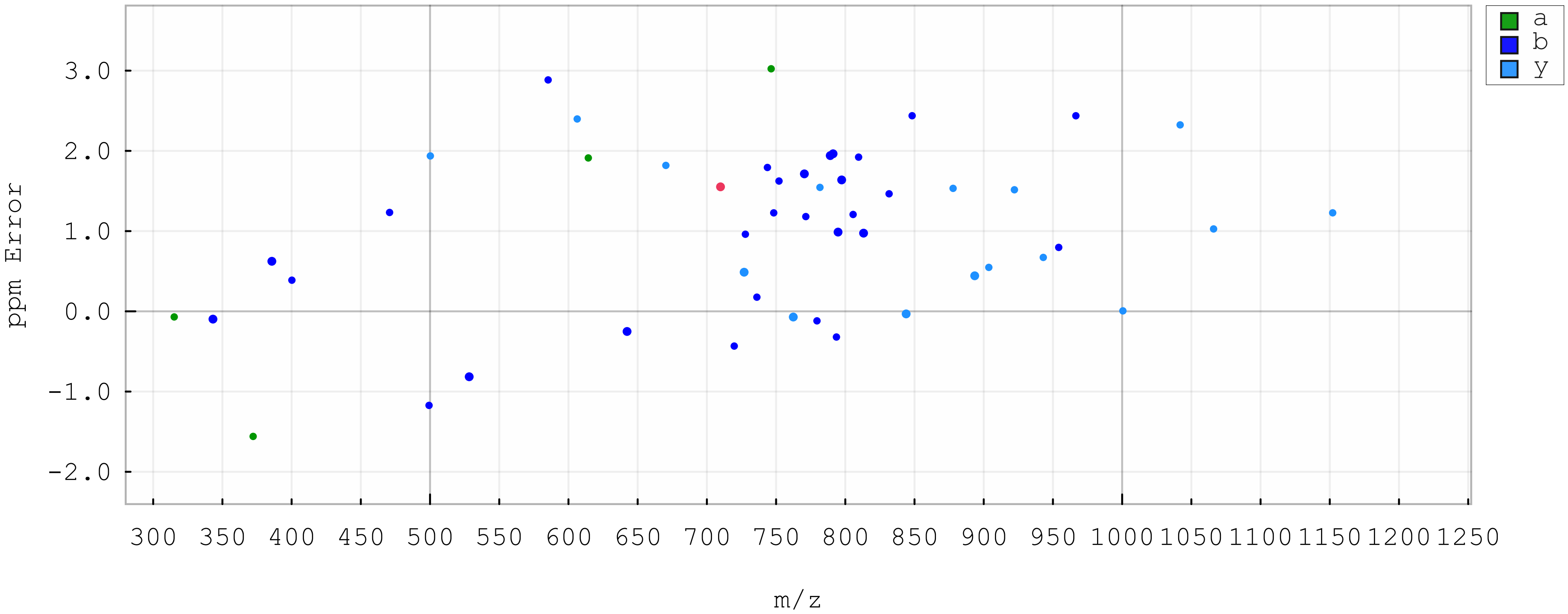

Proteoform:

Index	Protein	SC [%]	SVP [%]	IC [%]	MS Score [a.u.]	Var. Mods	Mono Mass [Da]	Chemical Formula
6	H4_liver	33.7	15.7	15.9	5.4	Met: K12, K20, Ace: K16, N-term	11341.3937	C500H845N163O136S

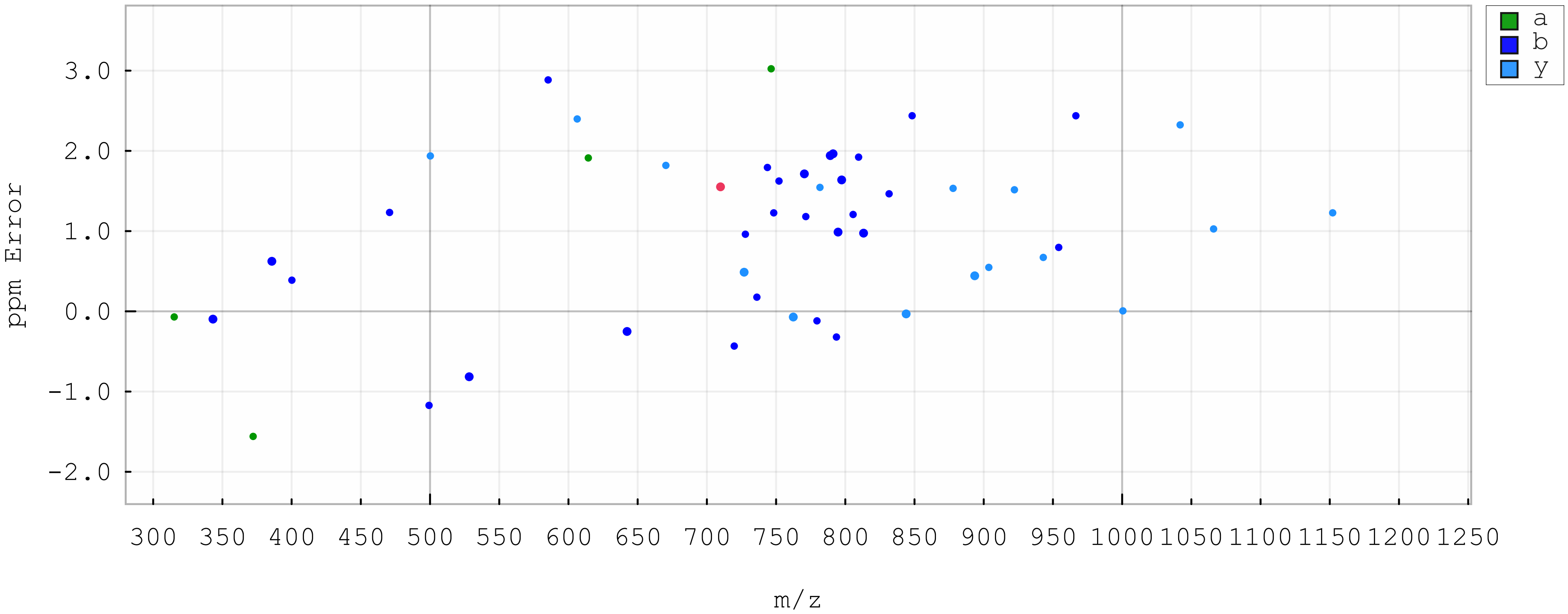

Proteoform:

Index	Protein	SC [%]	SVP [%]	IC [%]	MS Score [a.u.]	Var. Mods	Mono Mass [Da]	Chemical Formula
7	H4_liver	33.7	15.7	15.9	5.4	Ace: K12, N-term, Met: K16, K20	11341.3937	C500H845N163O136S

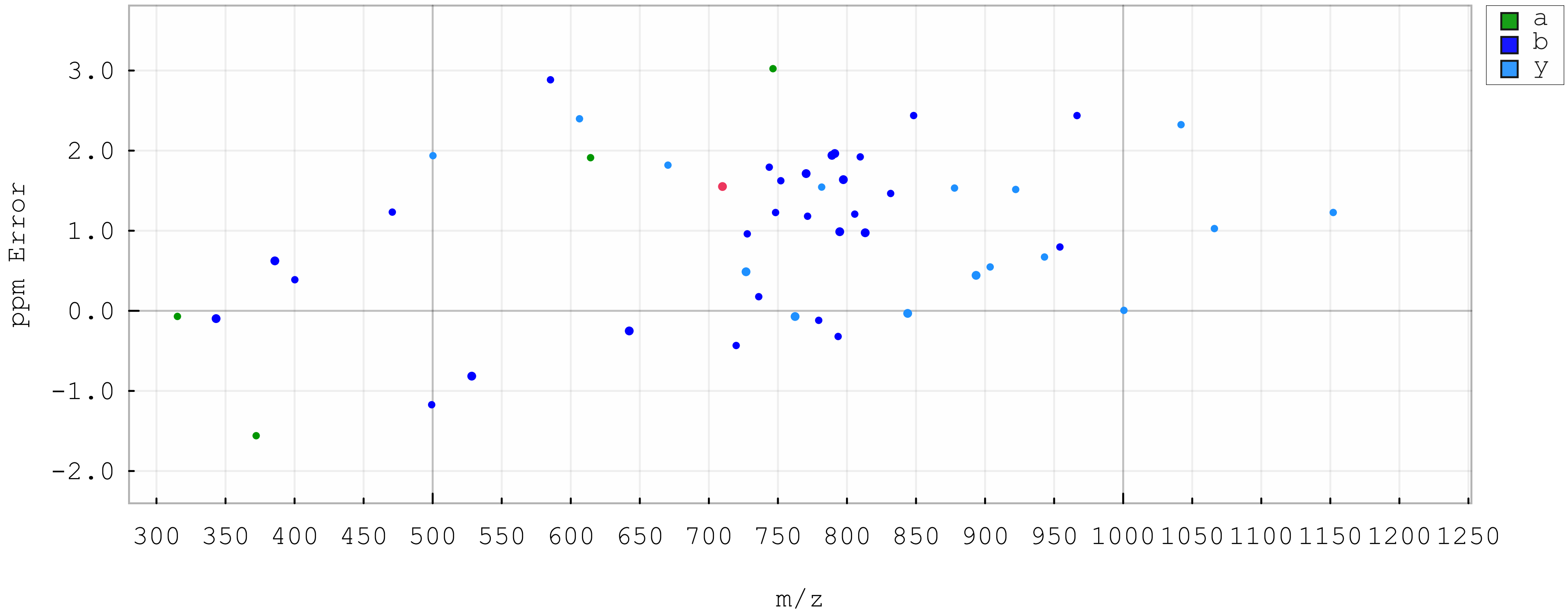

Proteoform:

Index	Protein	SC [%]	SVP [%]	IC [%]	MS Score [a.u.]	Var. Mods	Mono Mass [Da]	Chemical Formula
8	H4_liver	33.7	15.7	15.9	5.4	Met: K12, K16, Ace: K20, N-term	11341.3937	C500H845N163O136S

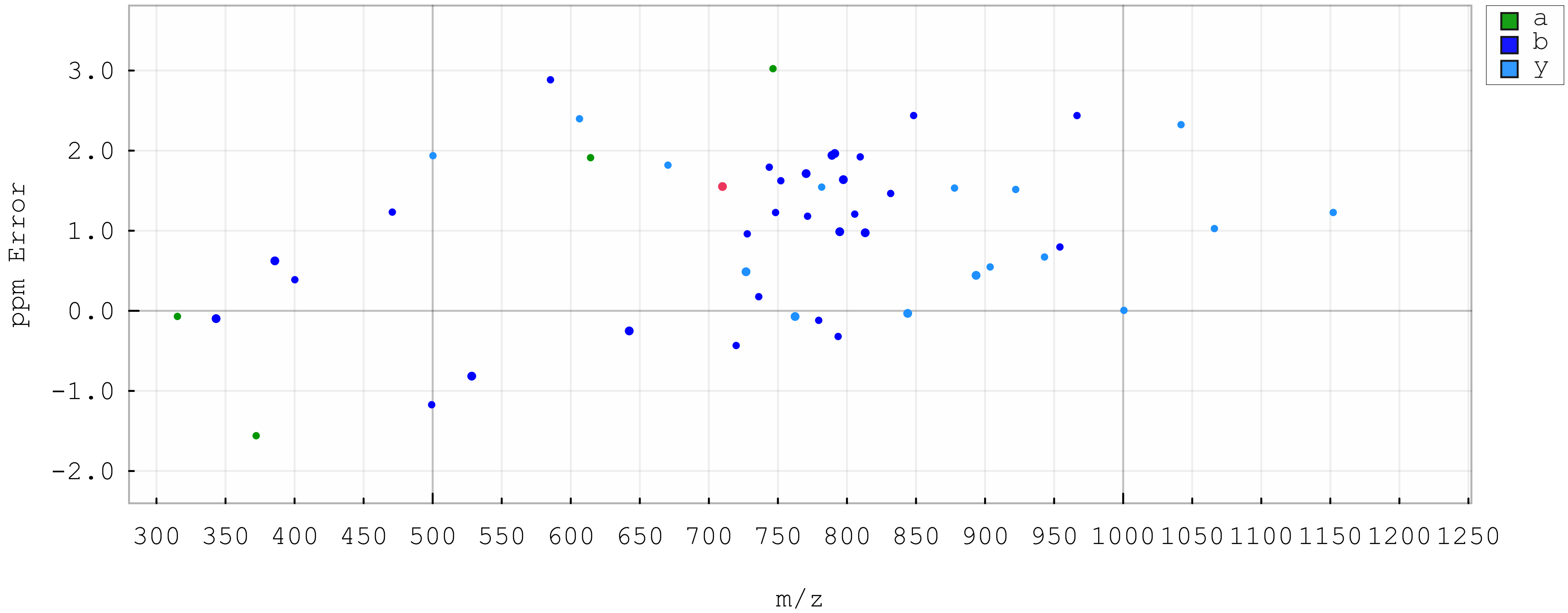

Proteoform:

Index	Protein	SC [%]	SVP [%]	IC [%]	MS Score [a.u.]	Var. Mods	Mono Mass [Da]	Chemical Formula
9	H4_liver	33.7	15.7	15.9	5.4	Dim: K12, Ace: K20, N-term	11341.3937	C500H845N163O136S

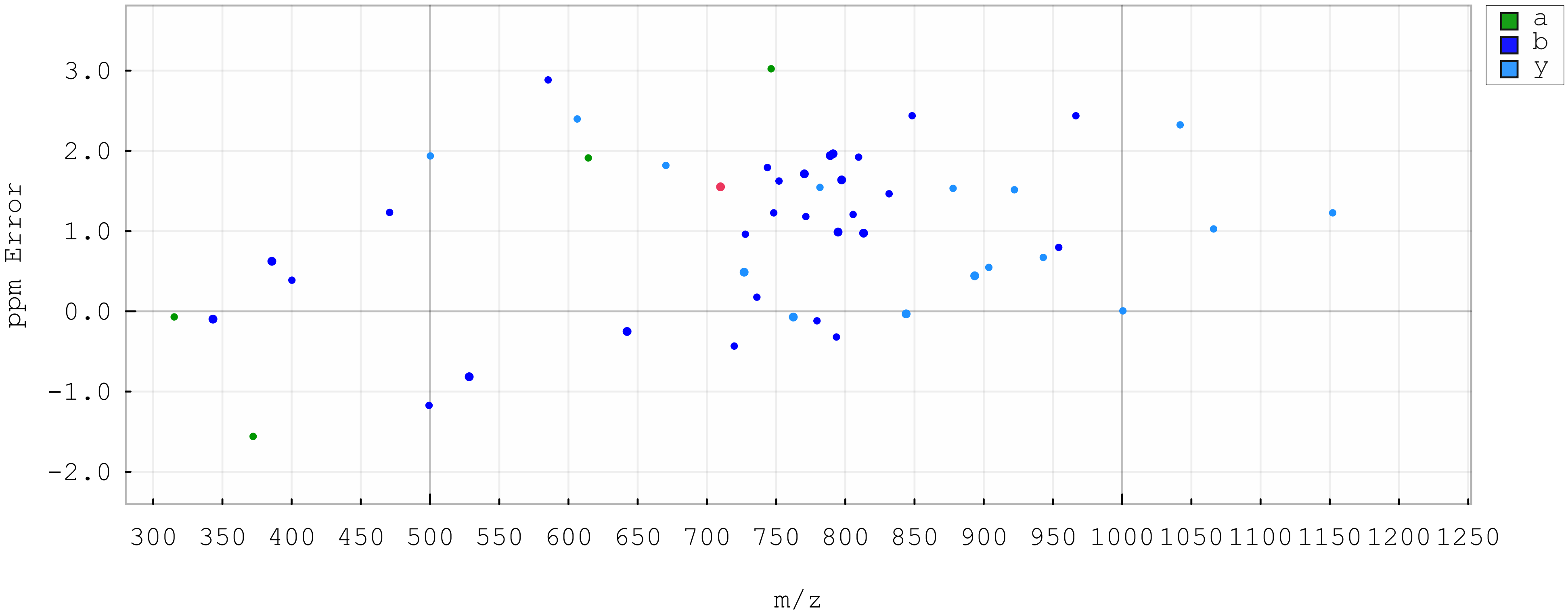

Proteoform:

Index	Protein	SC [%]	SVP [%]	IC [%]	MS Score [a.u.]	Var. Mods	Mono Mass [Da]	Chemical Formula
10	H4_liver	32.7	4.9	15.8	5.2	Ace: K5, K16, Dim: K20	11341.3937	C500H845N163O136S

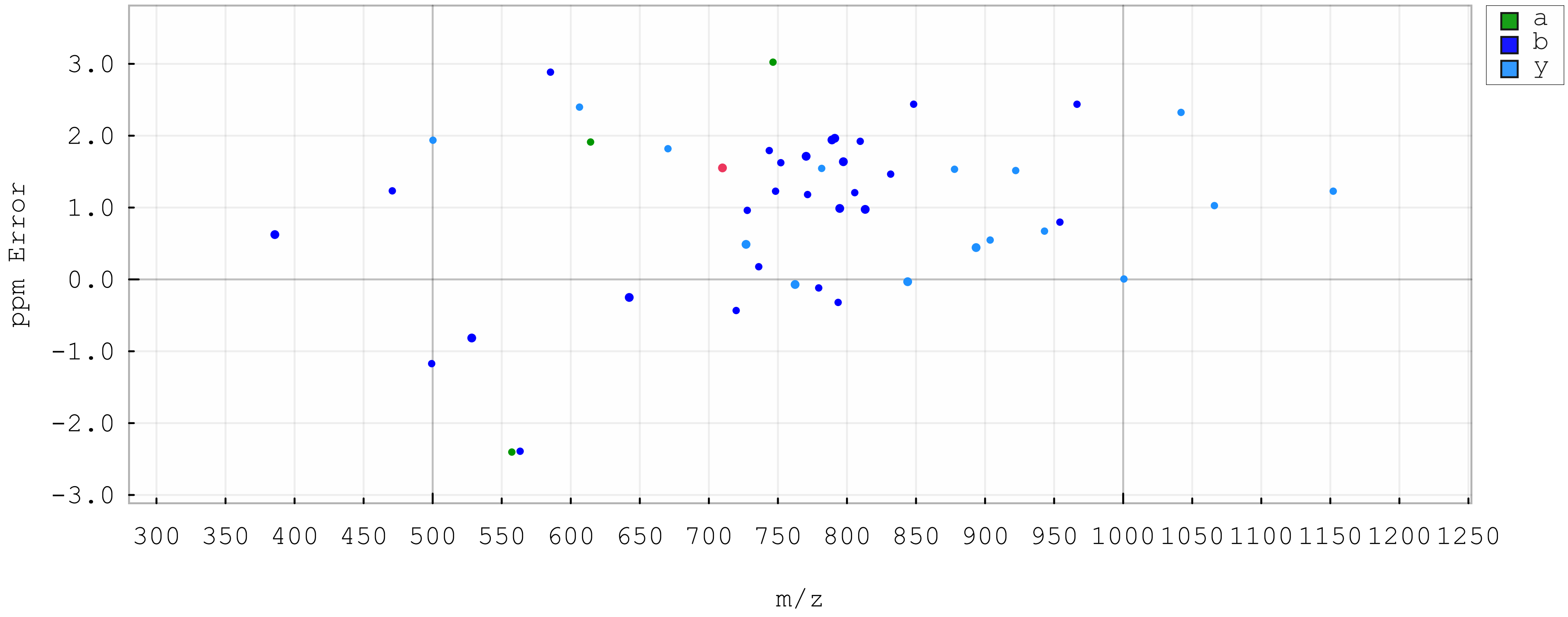

Primary Fragment Plots

Proteoform:	Index	Protein	SC [%]	SVP [%]	IC [%]	MS Score [a.u.]	Var. Mods	Mono Mass [Da]	Chemical Formula
	1	H4_liver	34.7	16.7	16.0	5.6	Dim: K16, Ace: K20, N-term	11341.3937	C500H845N163O136S

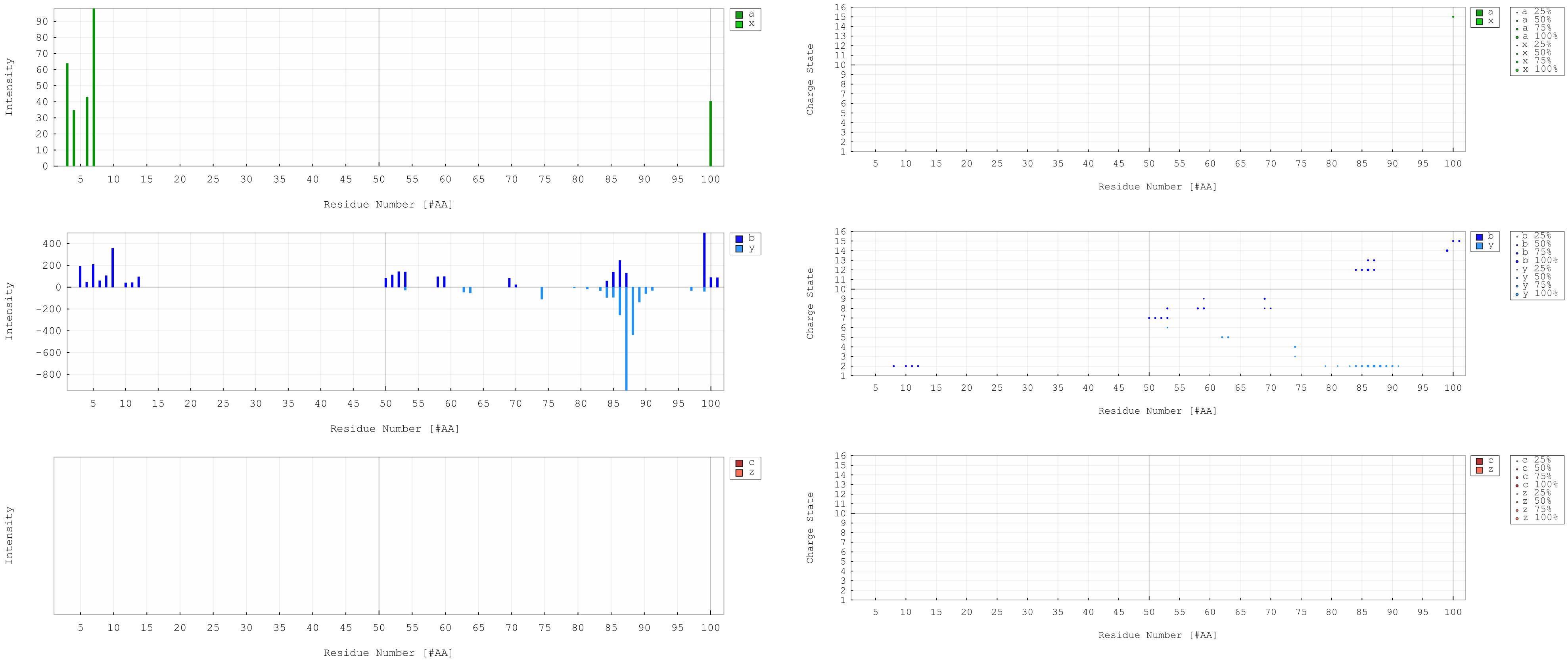

Proteoform:

Index	Protein	SC [%]	SVP [%]	IC [%]	MS Score [a.u.]	Var. Mods	Mono Mass [Da]	Chemical Formula
2	H4_liver	34.7	16.7	16.0	5.6	Ace: K16, N-term, Dim: K20	11341.3937	C500H845N163O136S

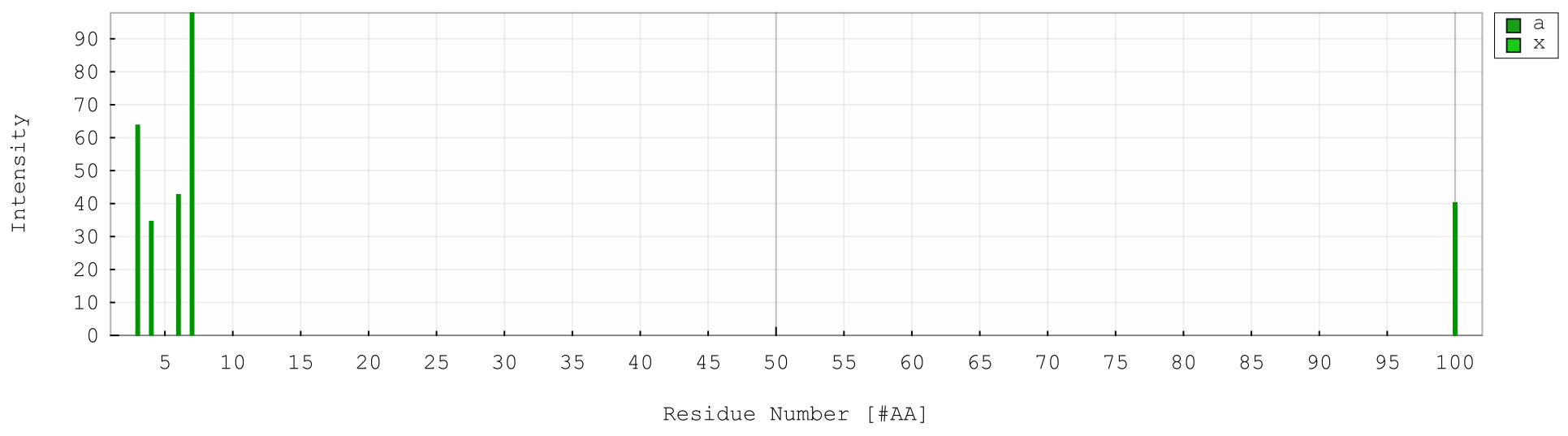
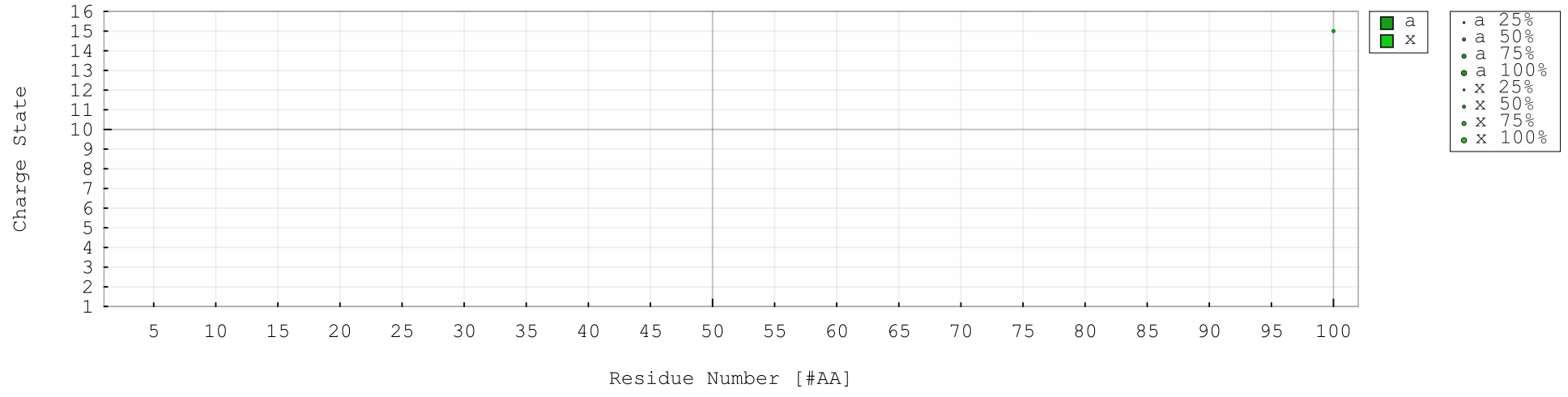
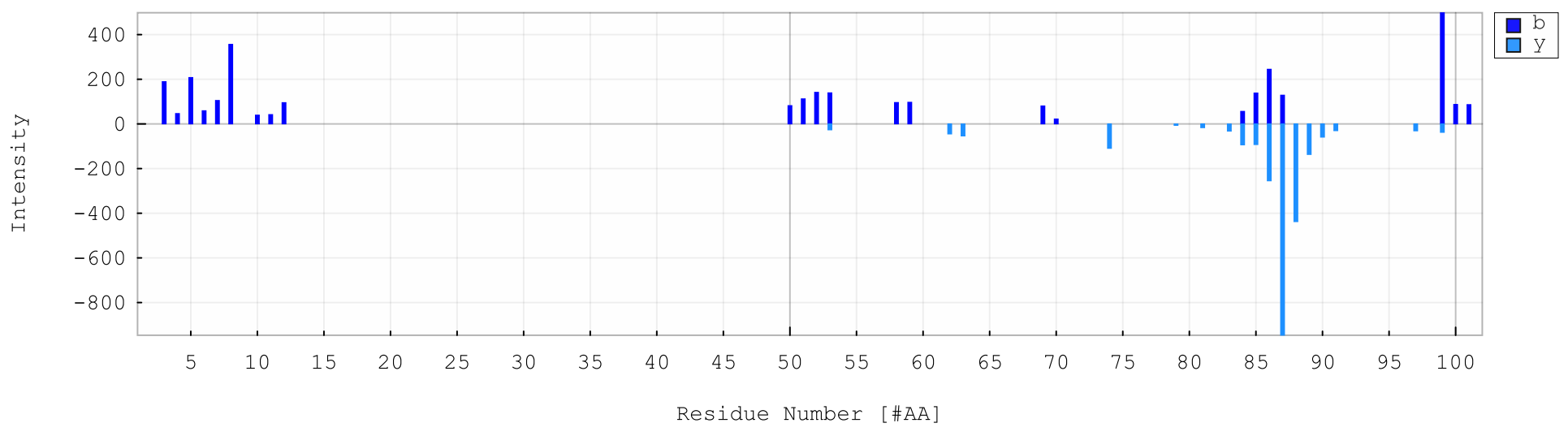
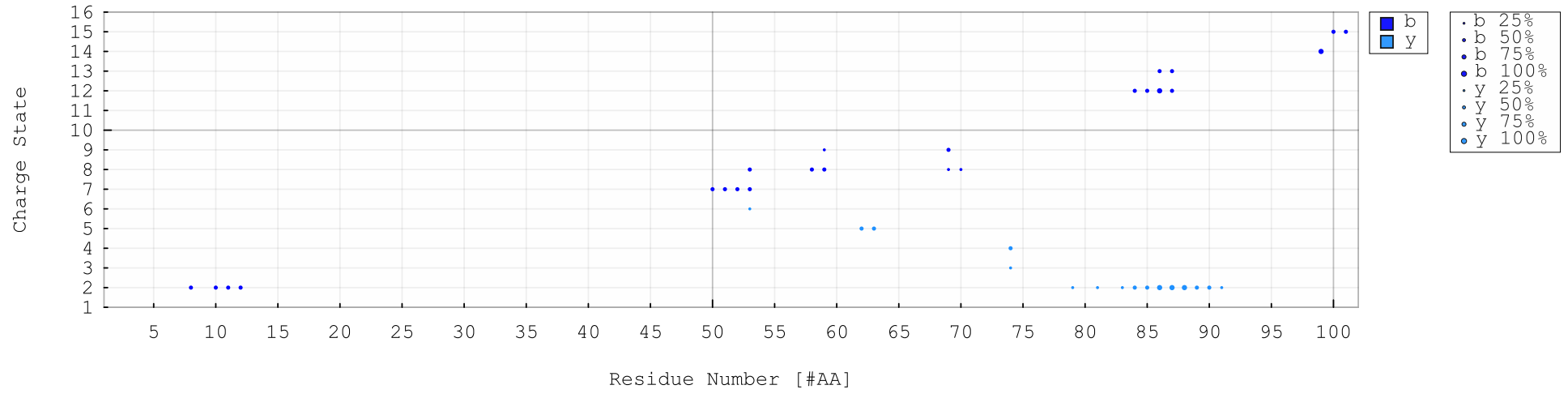
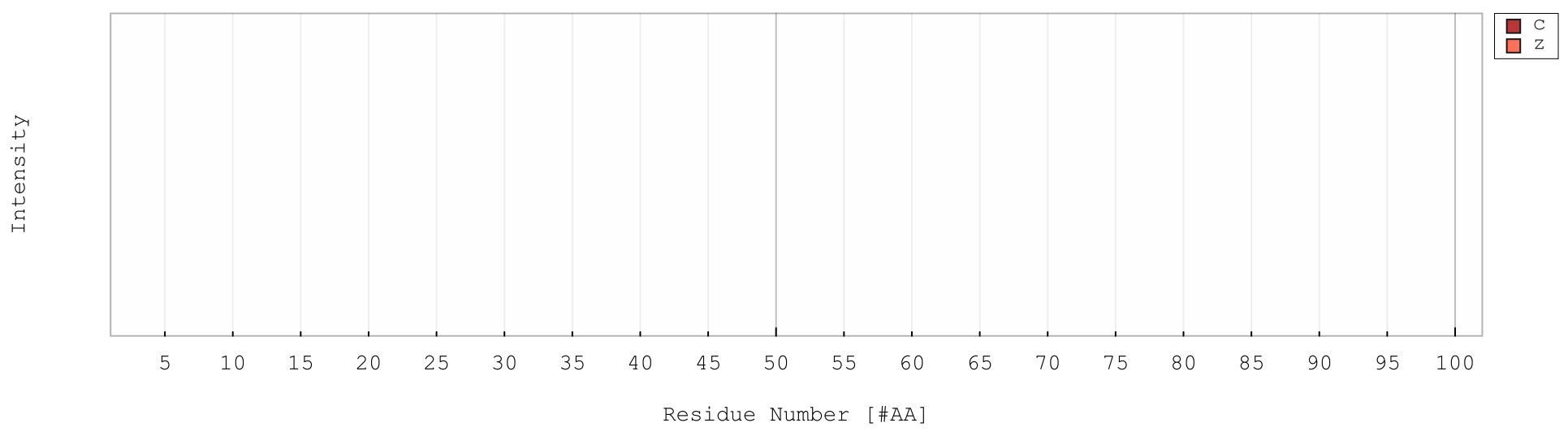
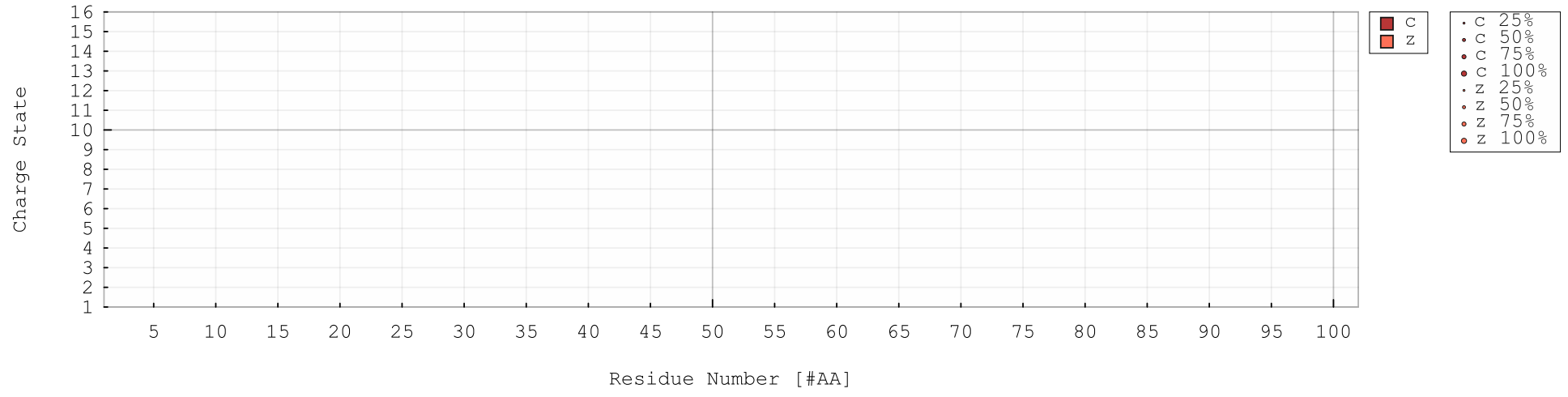

Proteoform:	Index	Protein	SC [%]	SVP [%]	IC [%]	MS Score [a.u.]	Var. Mods	Mono Mass [Da]	Chemical Formula
	3	H4_liver	33.7	15.7	15.9	5.4	Dim: K12, Ace: K16, N-term	11341.3937	C500H845N163O136S

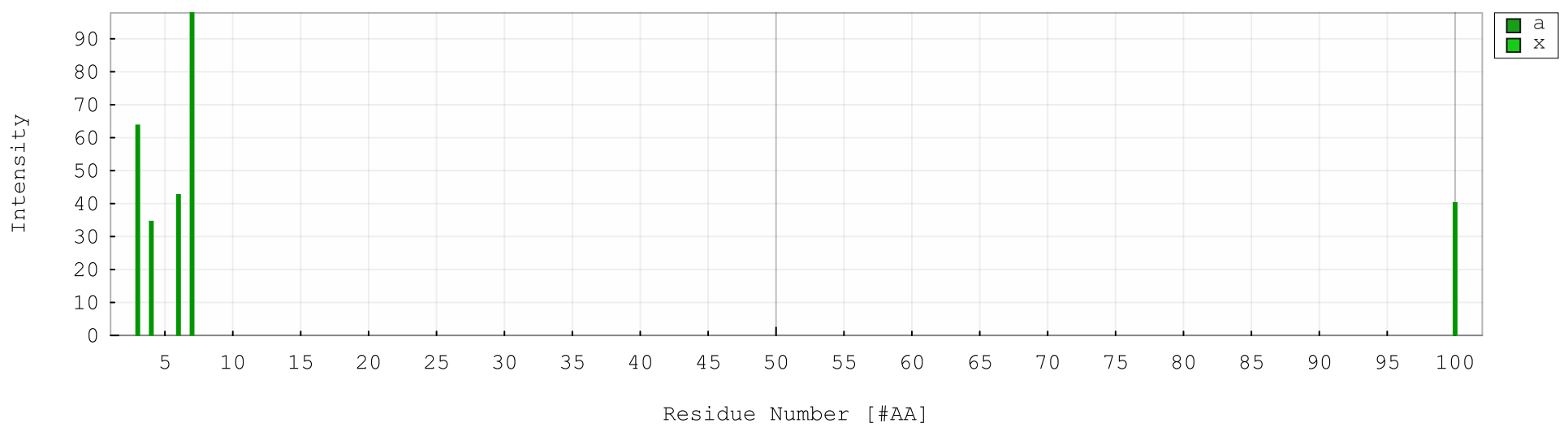

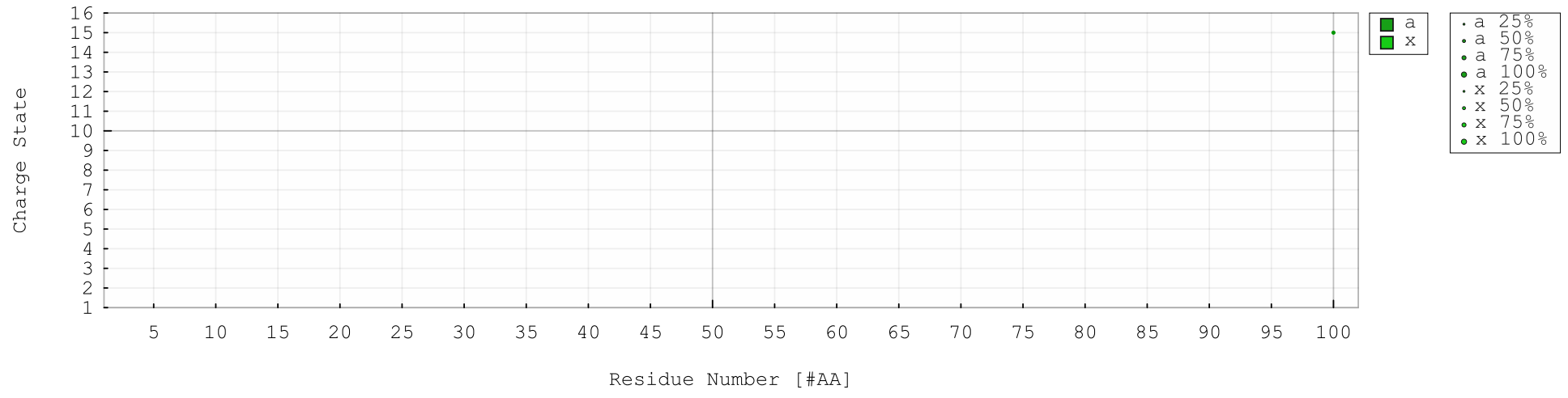

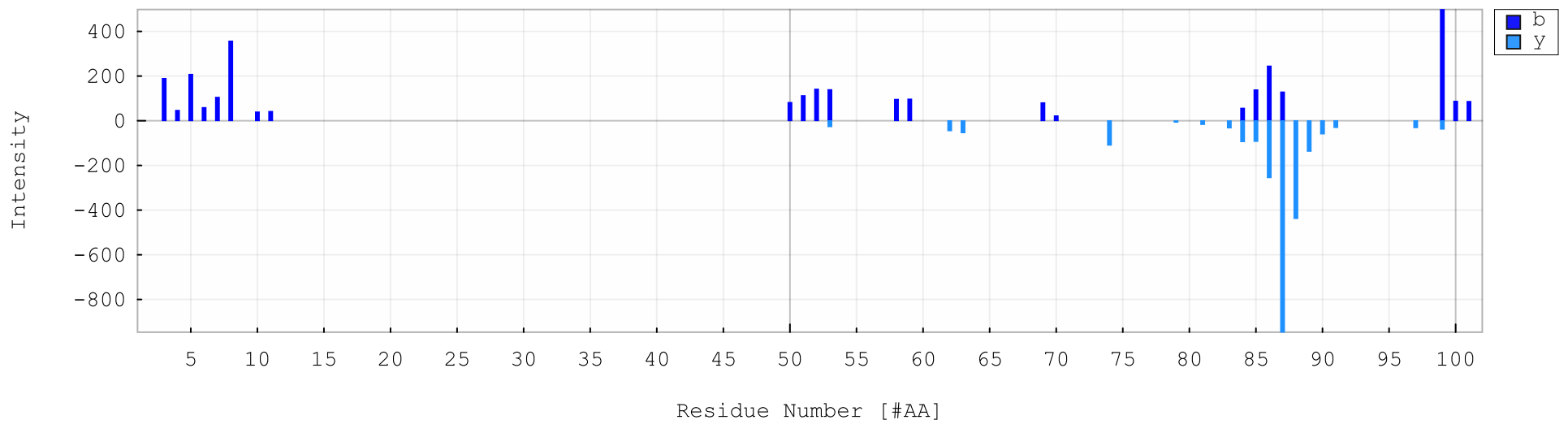

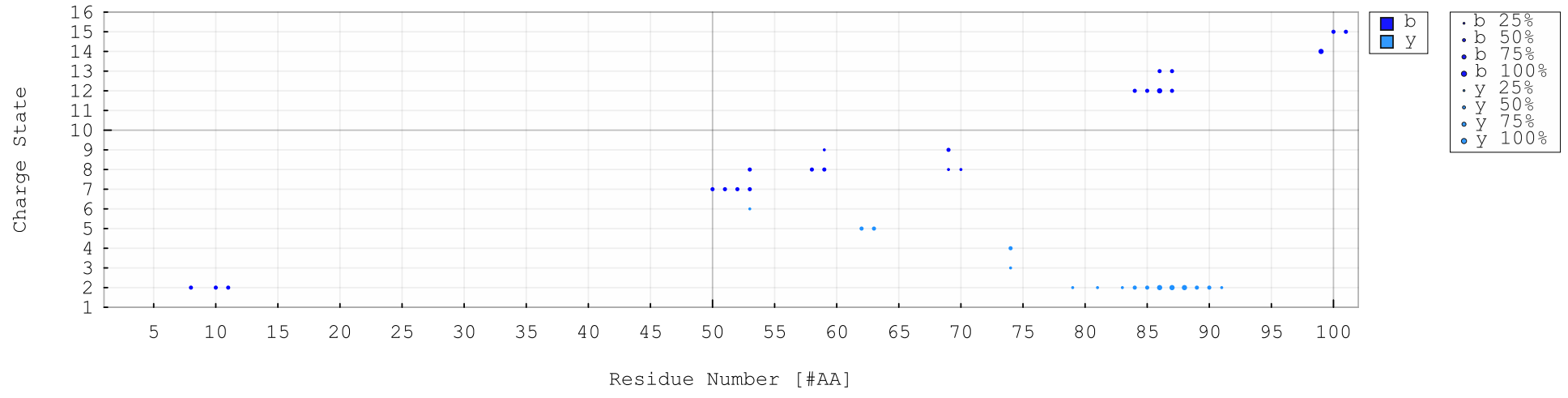

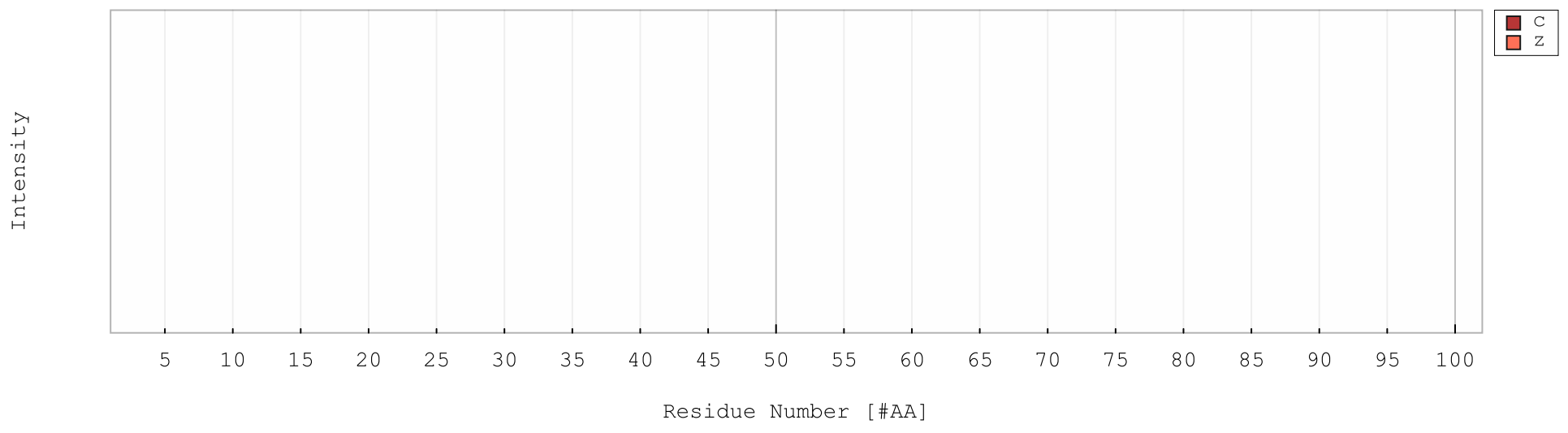

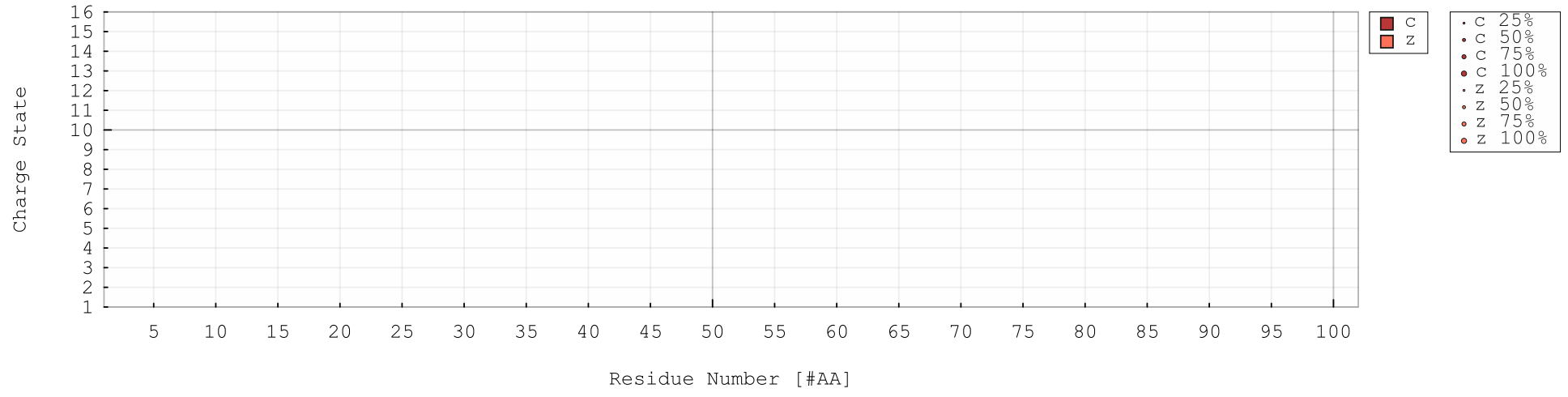

Proteoform:	Index	Protein	SC [%]	SVP [%]	IC [%]	MS Score [a.u.]	Var. Mods	Mono Mass [Da]	Chemical Formula
	4	H4_liver	33.7	15.7	15.9	5.4	Ace: K12, N-term, Dim: K16	11341.3937	C500H845N163O136S

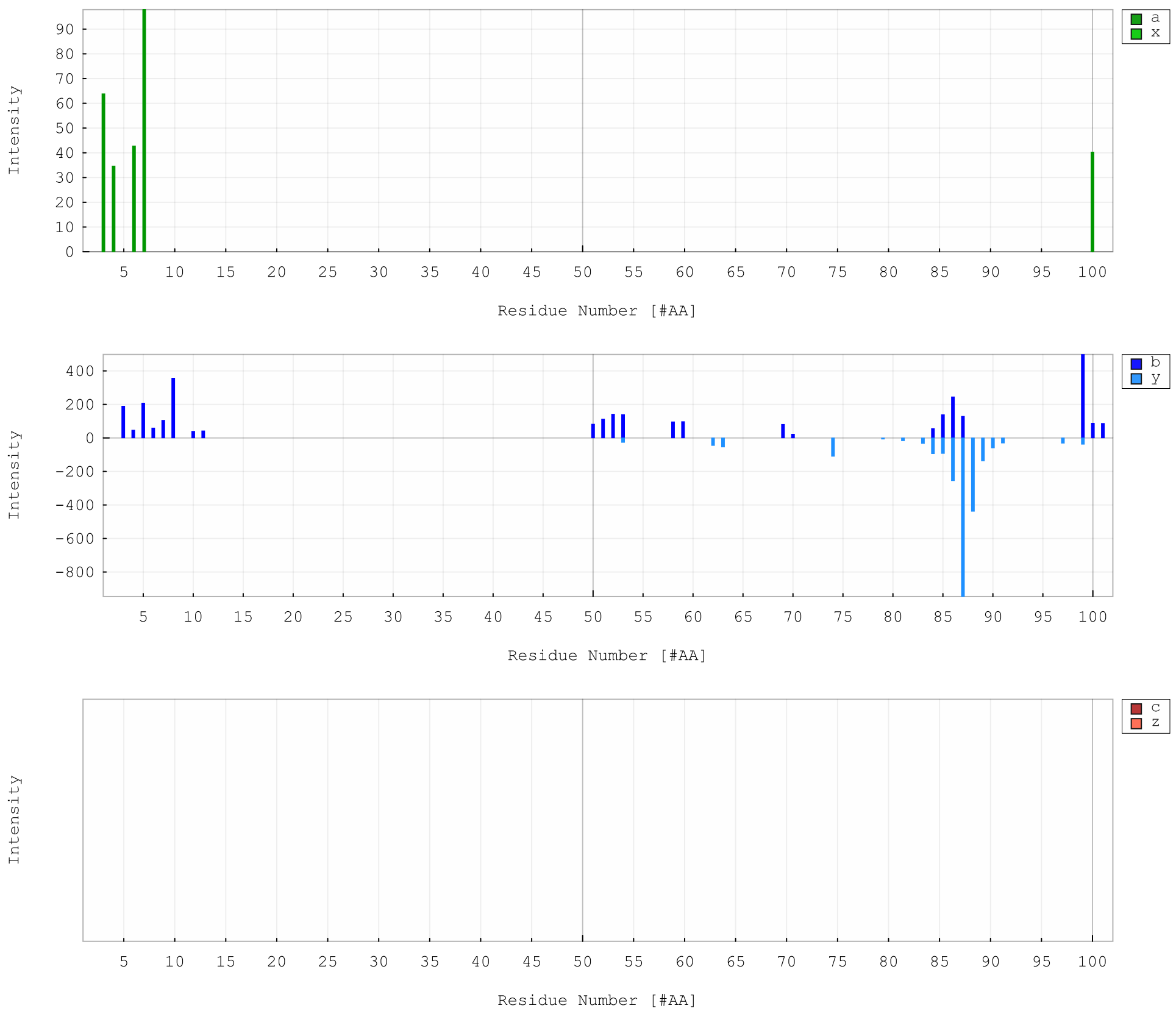

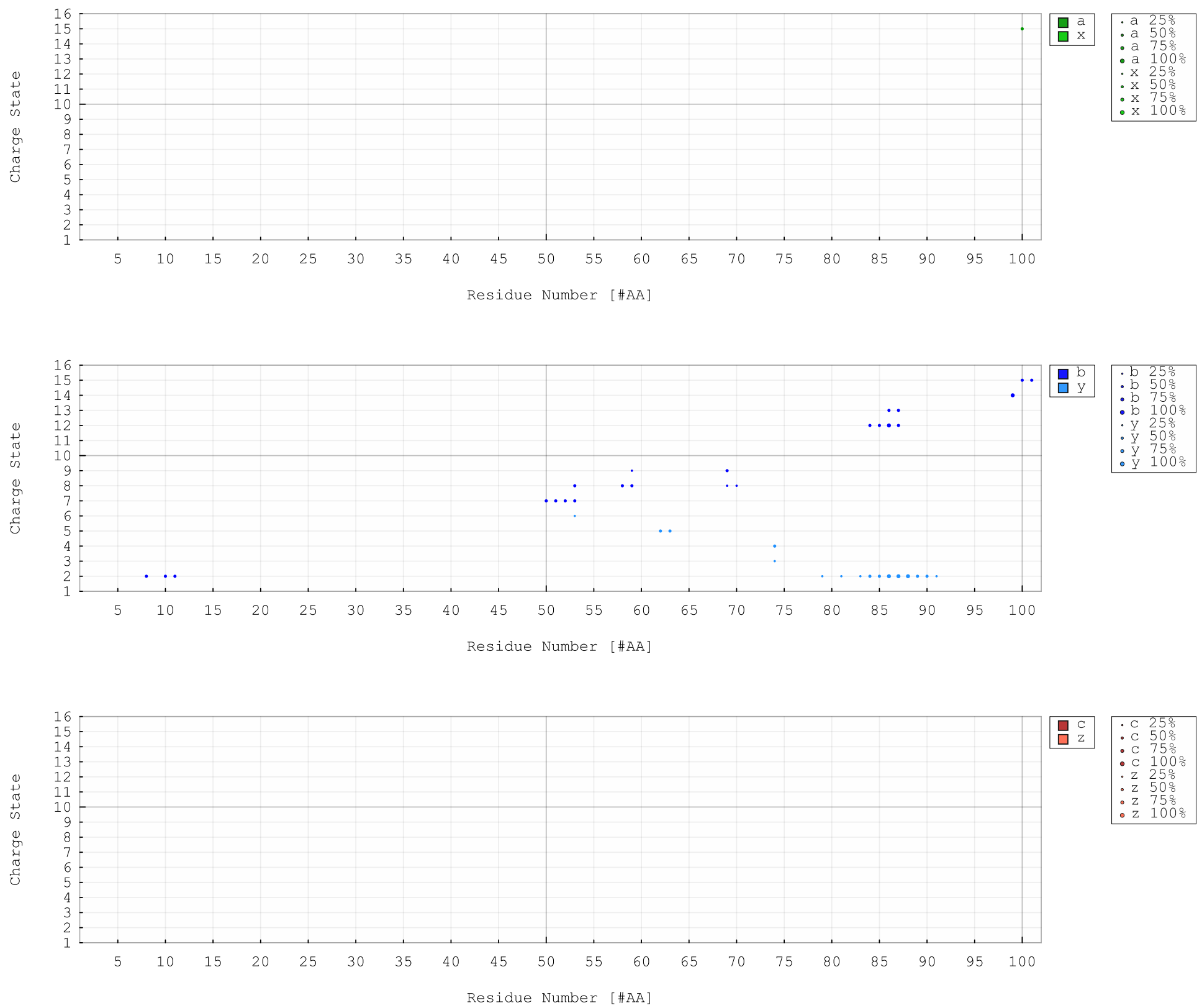

Proteoform:

Index	Protein	SC [%]	SVP [%]	IC [%]	MS Score [a.u.]	Var. Mods	Mono Mass [Da]	Chemical Formula
5	H4_liver	33.7	15.7	15.9	5.4	Ace: K12, N-term, Dim: K20	11341.3937	C500H845N163O136S

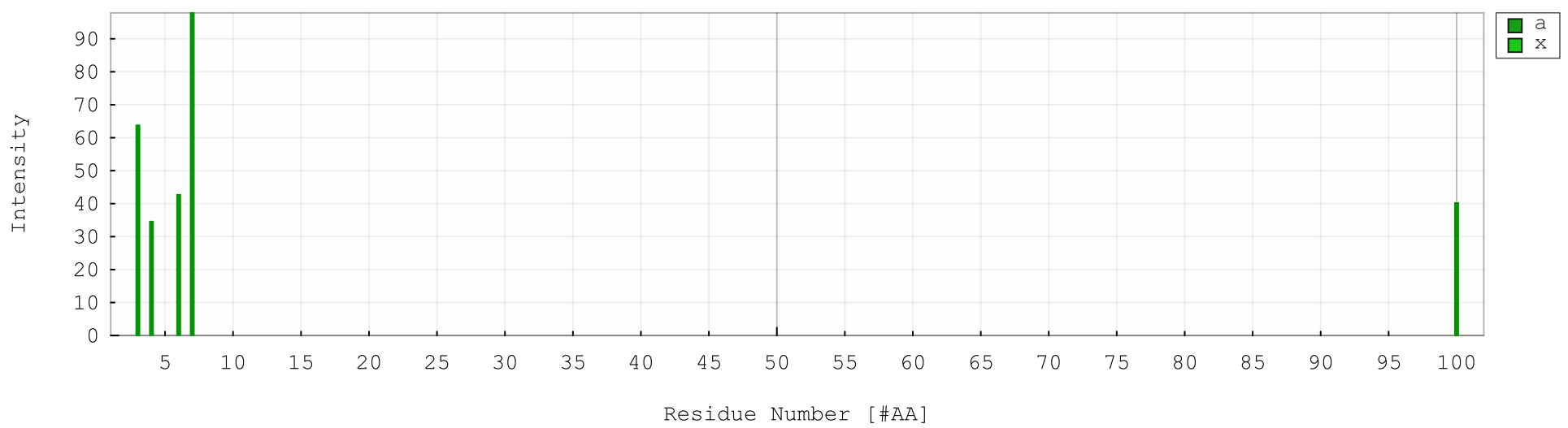

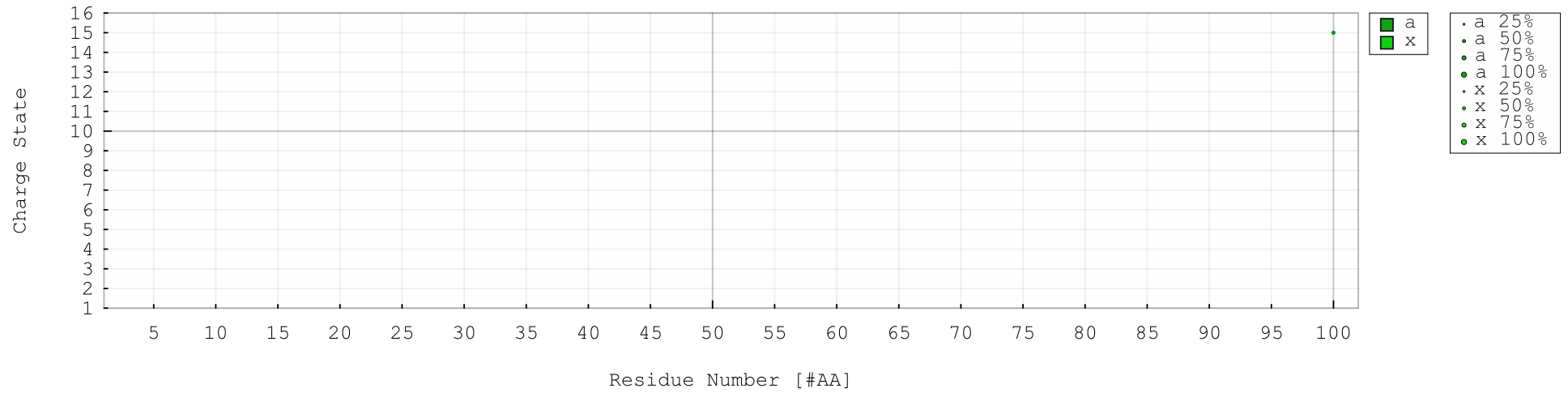

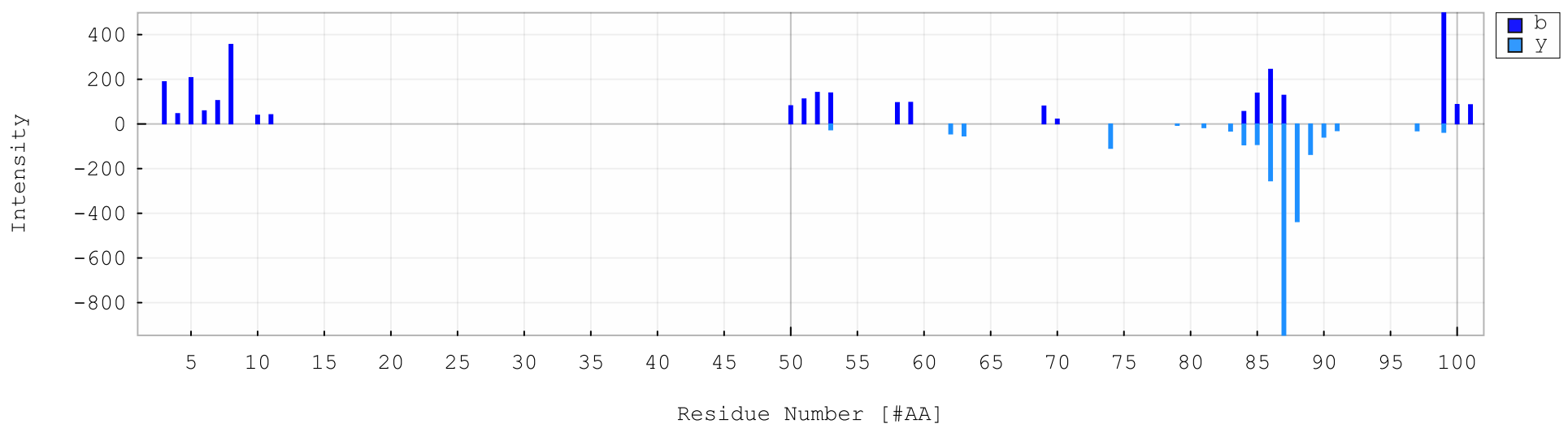

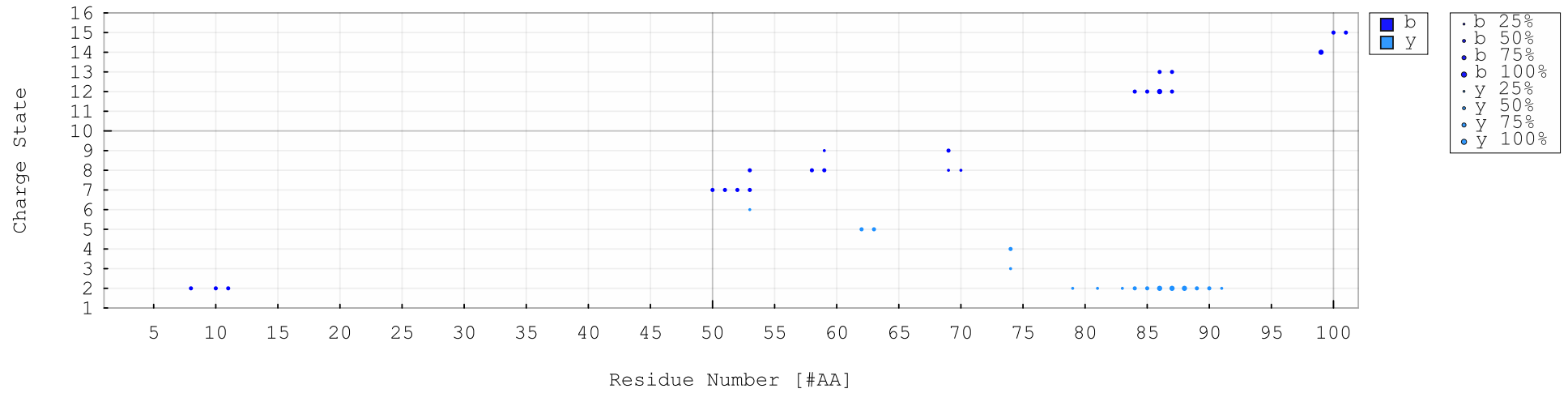

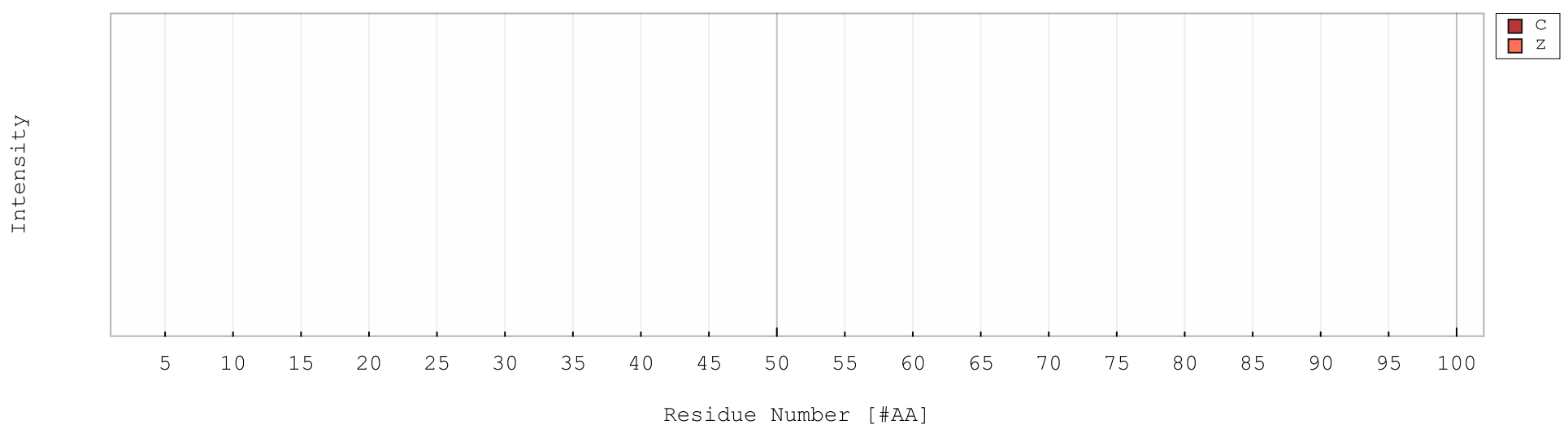

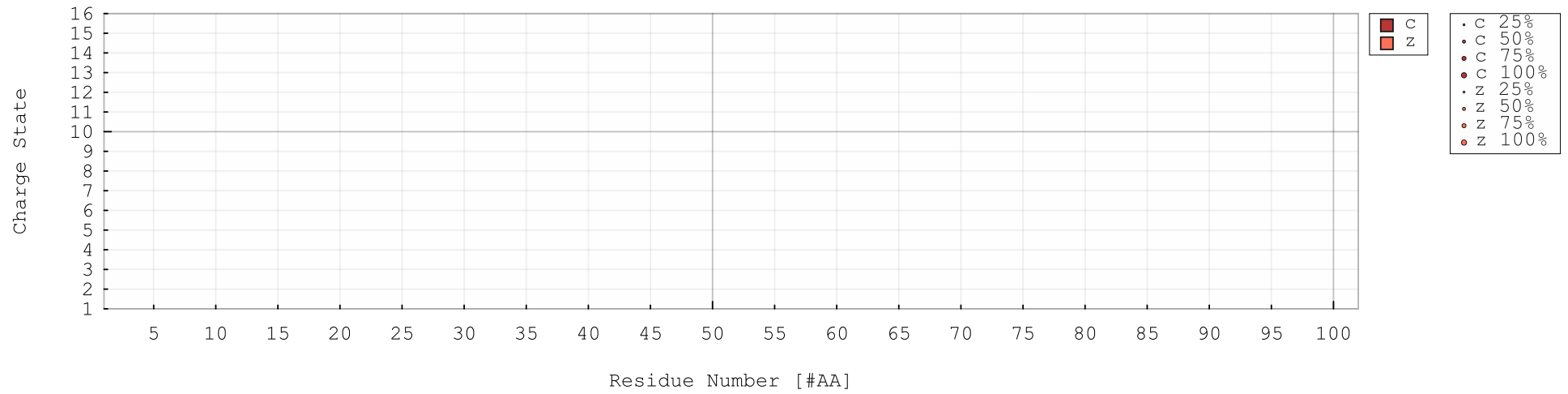

Proteoform:

Index	Protein	SC [%]	SVP [%]	IC [%]	MS Score [a.u.]	Var. Mods	Mono Mass [Da]	Chemical Formula
6	H4_liver	33.7	15.7	15.9	5.4	Met: K12, K20, Ace: K16, N-term	11341.3937	C500H845N163O136S

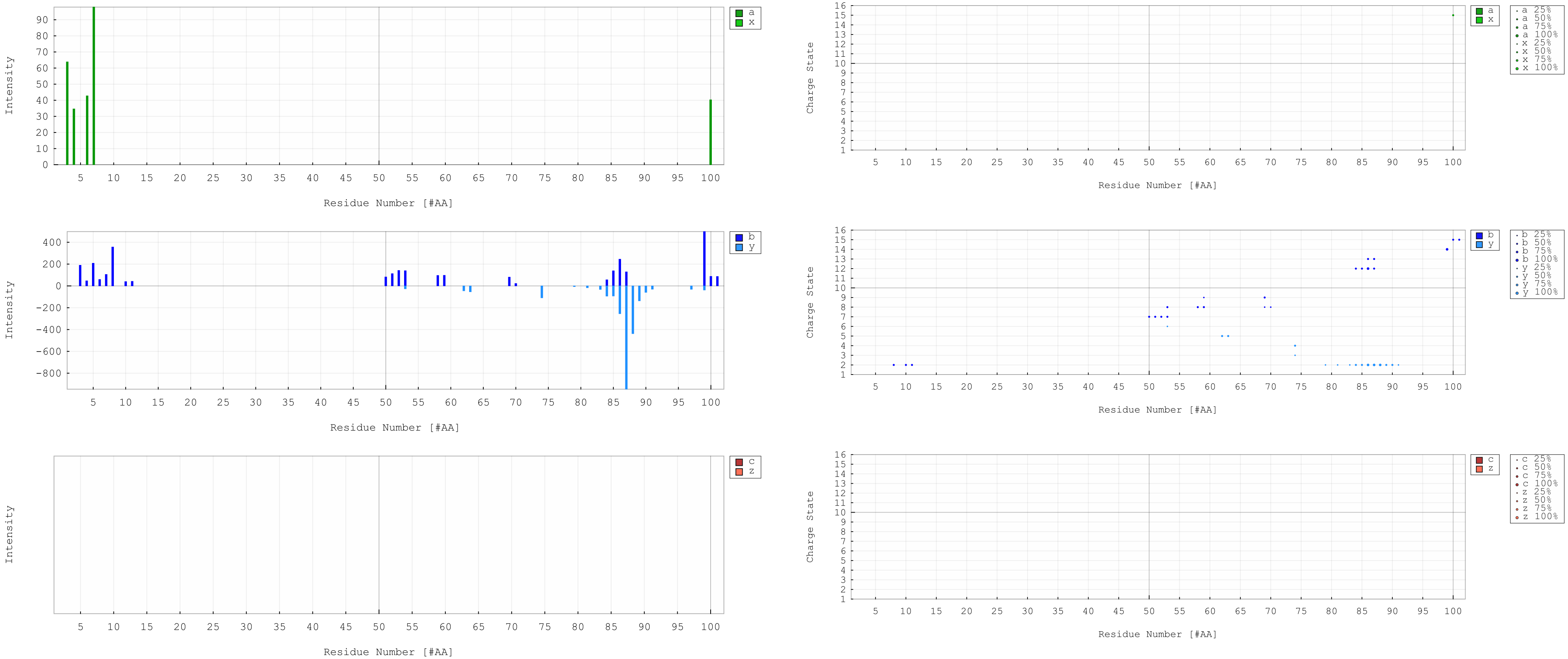

Proteoform:

Index	Protein	SC [%]	SVP [%]	IC [%]	MS Score [a.u.]	Var. Mods	Mono Mass [Da]	Chemical Formula
7	H4_liver	33.7	15.7	15.9	5.4	Ace: K12, N-term, Met: K16, K20	11341.3937	$C_{500}H_{845}N_{163}O_{136}S$

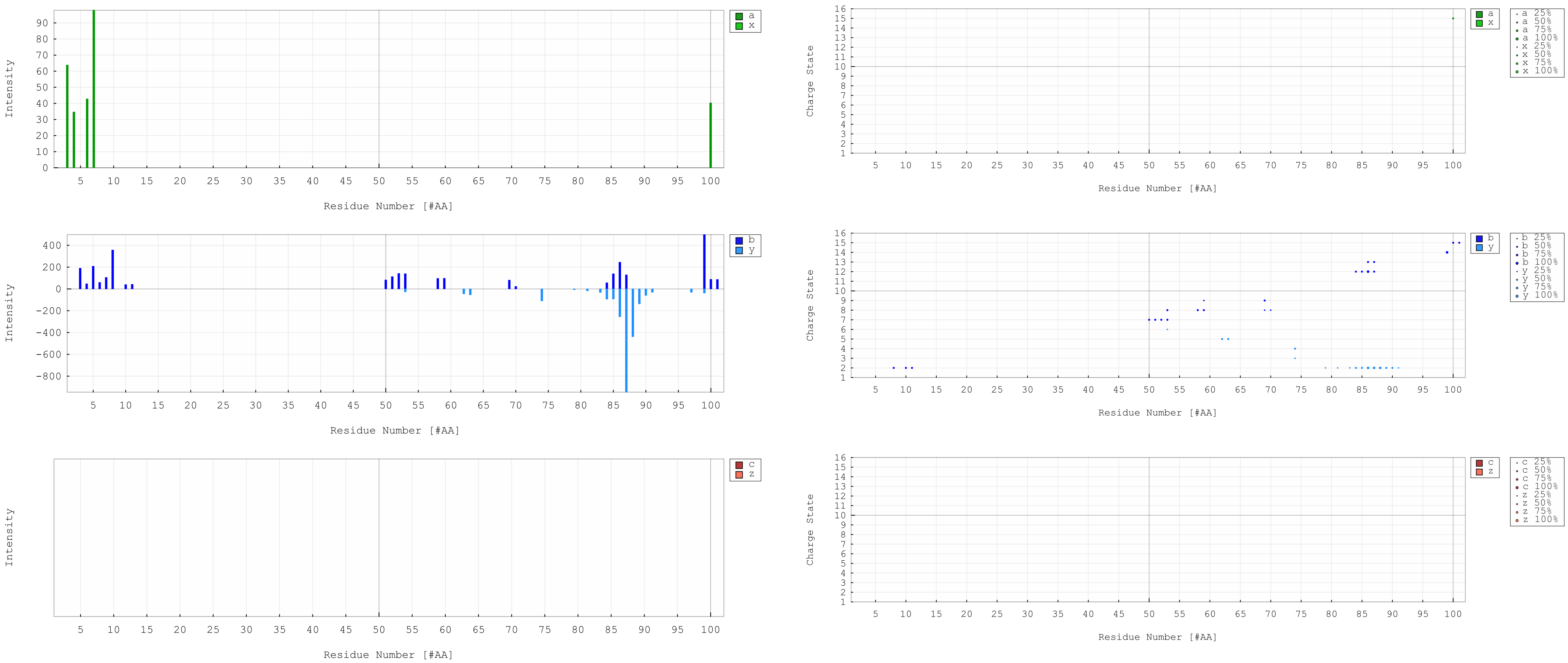

Proteoform:	Index	Protein	SC [%]	SVP [%]	IC [%]	MS Score [a.u.]	Var. Mods	Mono Mass [Da]	Chemical Formula
	8	H4_liver	33.7	15.7	15.9	5.4	Met: K12, K16, Ace: K20, N-term	11341.3937	C500H845N163O136S

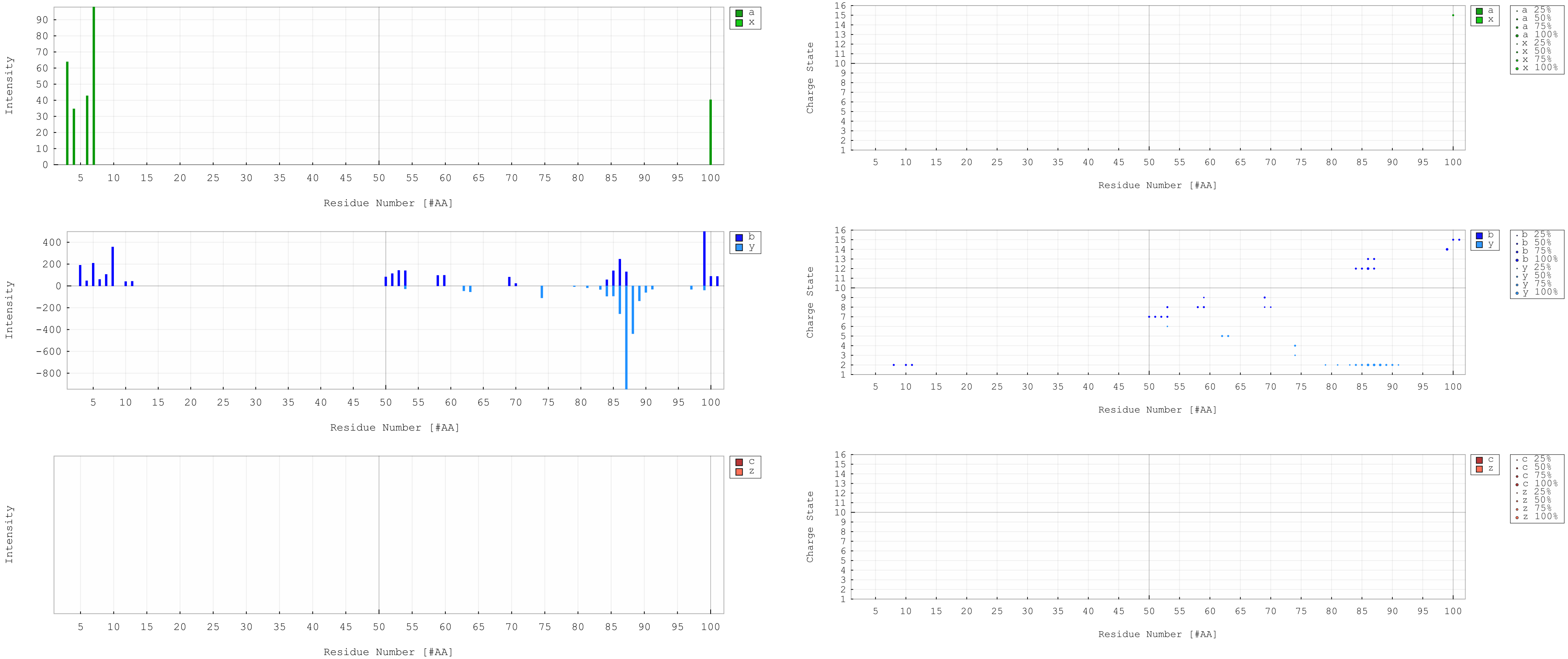

Proteoform:

Index	Protein	SC [%]	SVP [%]	IC [%]	MS Score [a.u.]	Var. Mods	Mono Mass [Da]	Chemical Formula
9	H4_liver	33.7	15.7	15.9	5.4	Dim: K12, Ace: K20, N-term	11341.3937	C500H845N163O136S

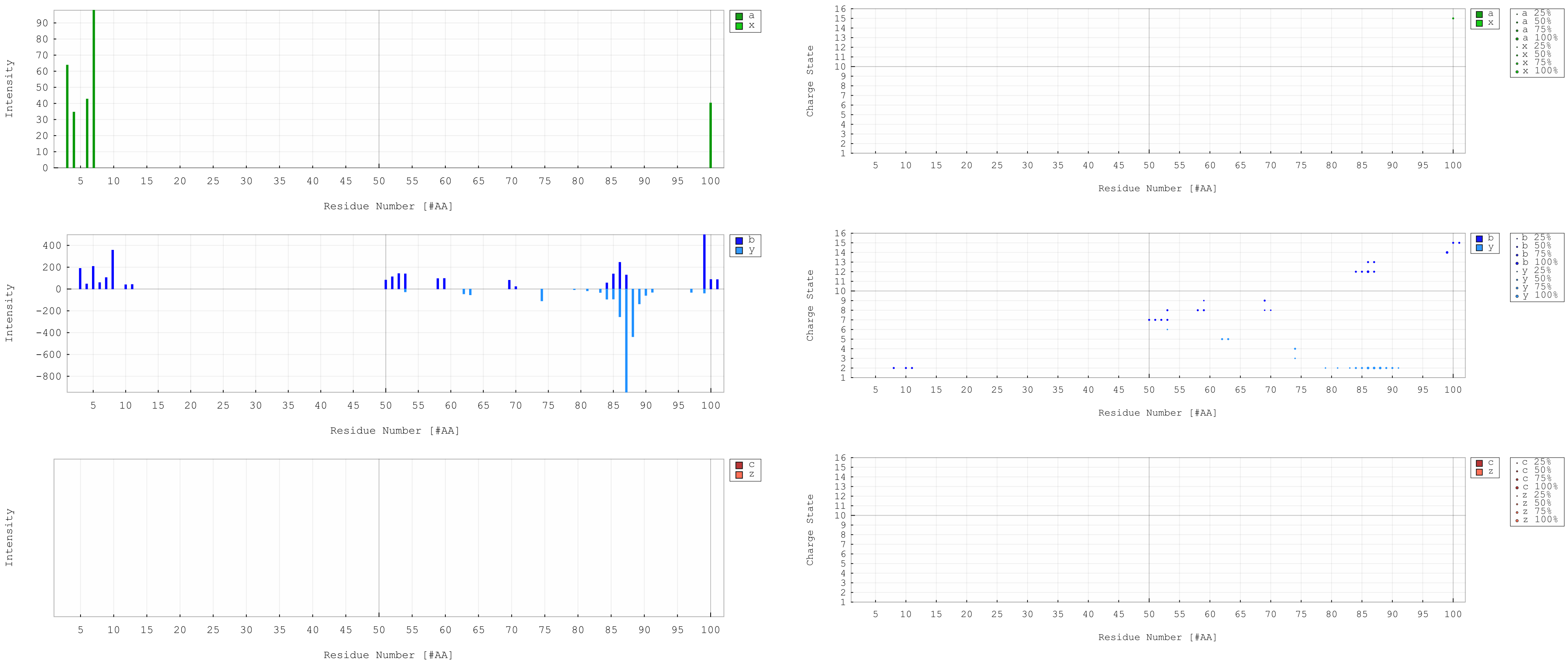

Proteoform:

Index	Protein	SC [%]	SVP [%]	IC [%]	MS Score [a.u.]	Var. Mods	Mono Mass [Da]	Chemical Formula
10	H4_liver	32.7	4.9	15.8	5.2	Ace: K5, K16, Dim: K20	11341.3937	C500H845N163O136S

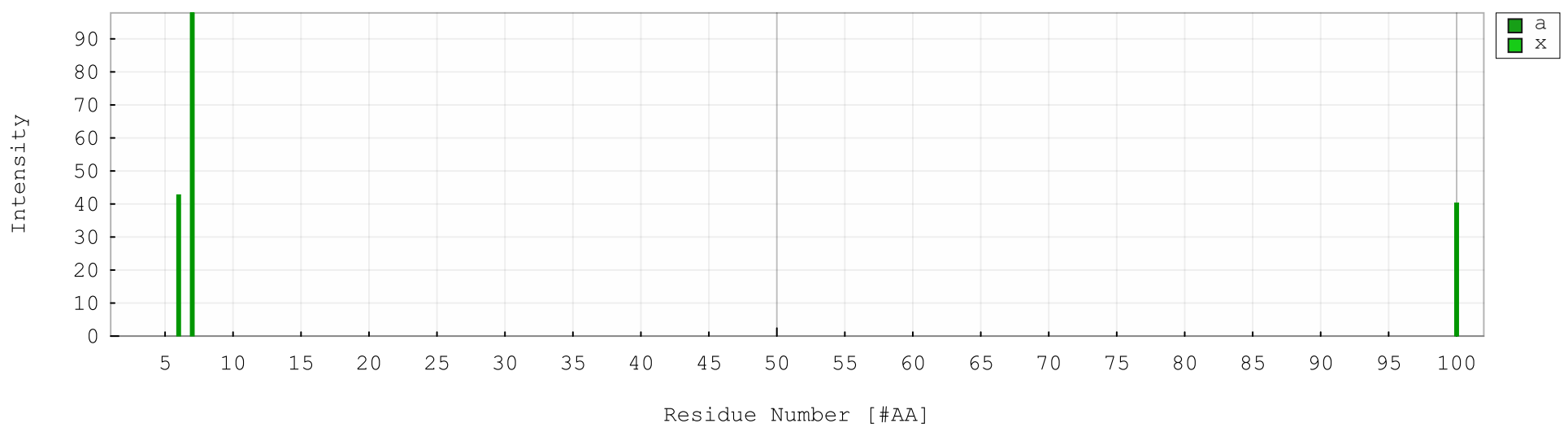

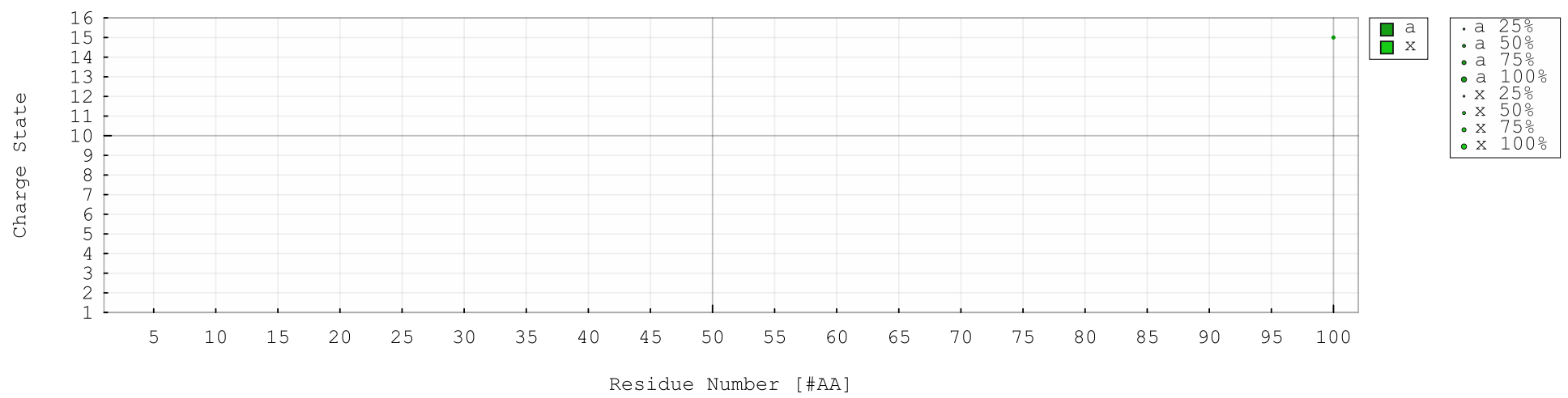

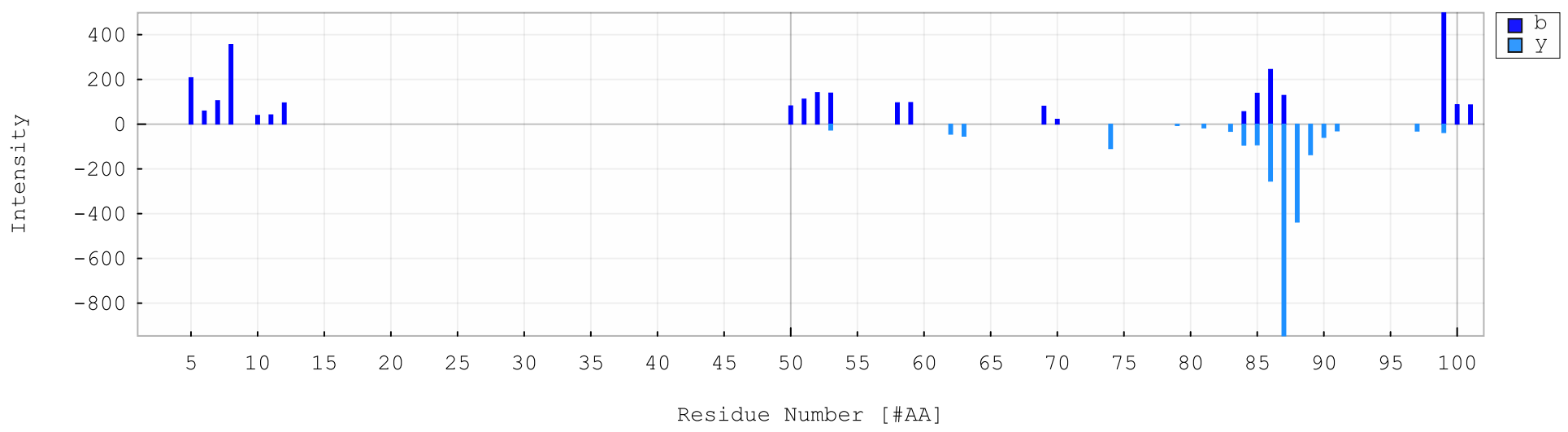

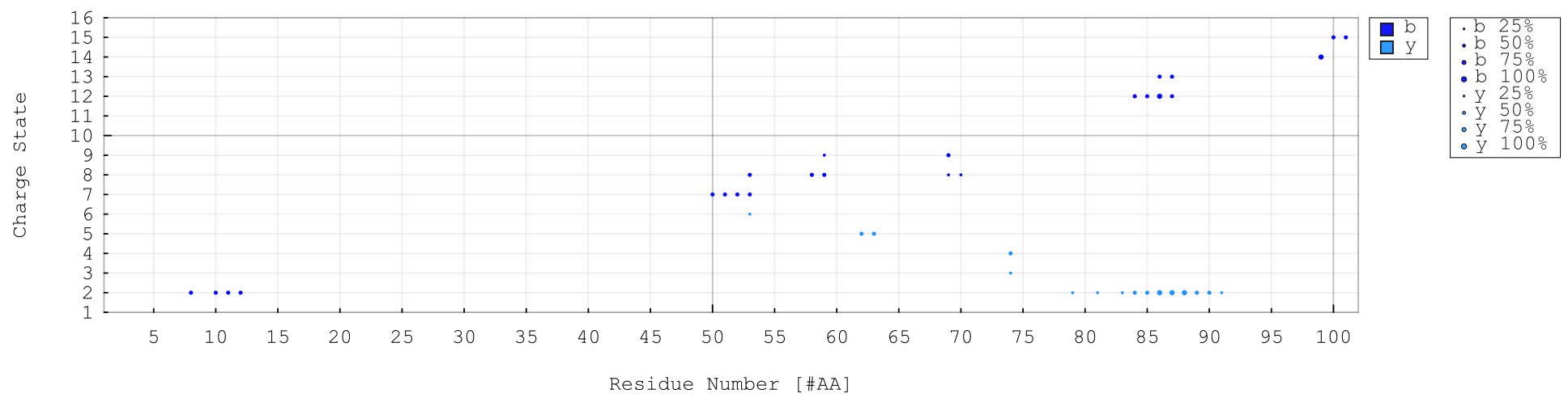

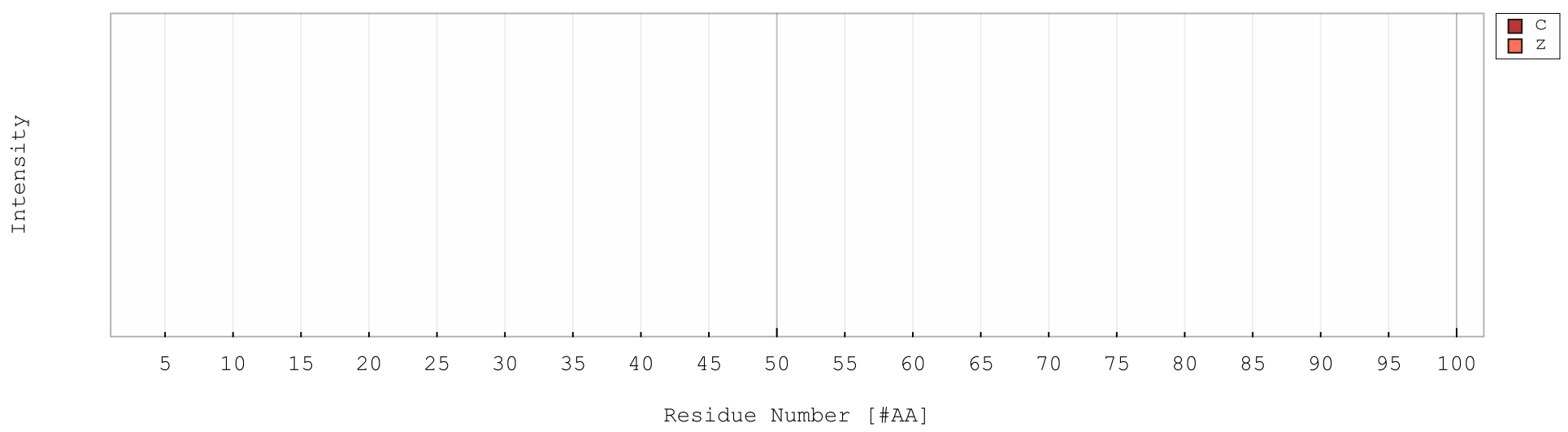

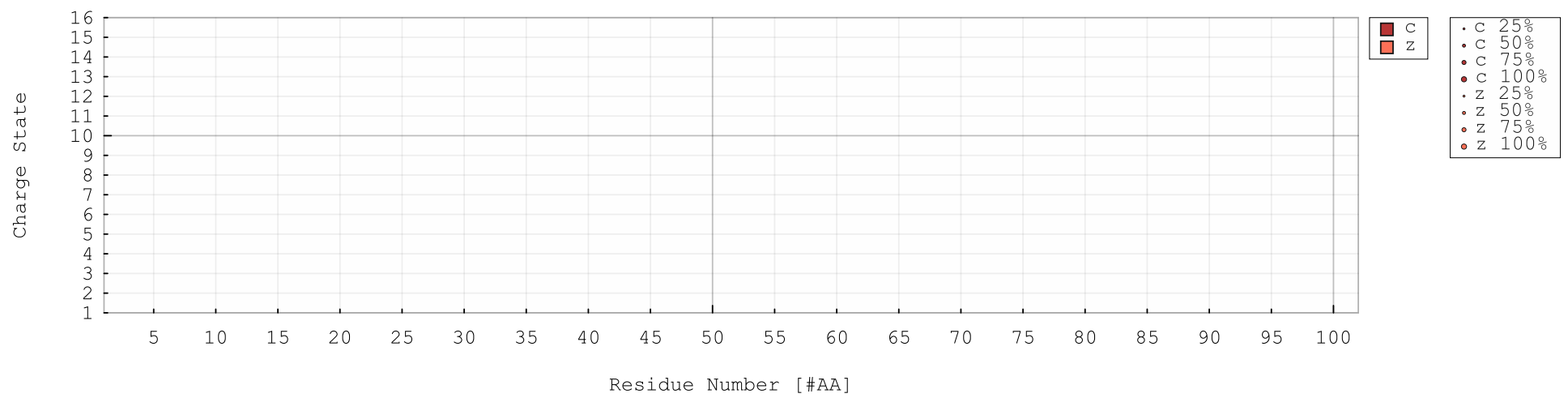

Supplement: Supplementary file 1 [file mmc1.pdf]
