# Supplementary material for: Top-Down Sequencing of Intact Proteoforms Using the timsOmni Mass Spectrometer: Accurate Determination of Co-Occurring Histone Modifications
Source: Mol Cell Proteomics. 2026 Aug 17;25(9):101640. doi: 10.1016/j.mcpro.2026.101640 (PMC13595144; doi:10.1016/j.mcpro.2026.101640)

Supplemental Data S2. H3.1 - GCN5, 10 min - OmniScape reports

OmniScape reports for monoacetylated H3.1 generated by GCN5.

**Histone**	H3.1
**Enzyme**	GCN5
**Reaction/source**	10 min
**Nb of Osc reports**	7

Included reports

#	Experiment	Precursor	Charge	Fragmentation / workflow	File
1	MS2	H3.1Kac1	19+	ECciD	MS2_H3.1Kac1_19+_ECciD.pdf
2	MS2	H3.1Kac1	19+	ECD	MS2_H3.1Kac1_19+_ECD.pdf
3	MS2	H3.1Kac1	19+	EID	MS2_H3.1Kac1_19+_EID.pdf
4	MS2	H3.1Kac1	19+	RCID	MS2_H3.1Kac1_19+_RCID.pdf
5	MS2	H3.1Kac1	20+	ECD	MS2_H3.1Kac1_20+_ECD.pdf
6	MS2	H3.1Kac1	20+	RCID	MS2_H3.1Kac1_20+_RCID.pdf
7	MS3	H3.1Kac1	19+	MS2 ECD -> MS3 RCID	MS3_H3.1Kac1_19+_MS2-ECD_MS3-RCID.pdf

Supplemental Data S2

H3.1 - GCN5, 10 min

**Report**	1 of 7
**Experiment**	MS2
**Histone/sample**	H3.1
**Enzyme**	GCN5
**Reaction/source**	10 min
**Precursor**	H3.1Kac1
**Charge state**	19+
**Fragmentation / workflow**	ECciD
**Original file**	MS2_H3.1Kac1_19+_ECciD.pdf

Project Information

Project Directory: C:/Users/LAB-ADMIN
Processing date: Wed Dec 10 11:26:45 2025

Sample Info & Protocols

Name: Top-Down Protein Sequencing ExD

Dataset

Imported spectrum file name:	241210 H31-GNC5_10 z19 MS2 ECD 30ms + ccCID 31eV_001-242_calibrated.txt
Dataset name:	Information is only available from imported JPEAKS files
Date acquired:	Information is only available from imported JPEAKS files
Date imported:	Information is only available from imported JPEAKS files
Instrument Name:	Information is only available from imported JPEAKS files
Instrument Serial Number:	Information is only available from imported JPEAKS files

Workflow Result Info

Analysis: 241210 H31-GNC5_10 z19 MS2 ECD 30ms + ccCID 31eV_001-242_calibrated
Location: C:/Users/LAB-ADMIN
Method: -

Method Information

Name: Unnamed

Spectral Parameters

Mass Error [ppm]: 6
Intensity Deviation [au]: 0.15
Min/Max Charges: 1-19
Experiment type: ExD

a ions	b ions	c ions	x ions	y ions	z ions	satellite	internal	precursor
a	b	c		y	z+1			M
a+1		c+1			z+2			M+-2H

Noise threshold calculation: Simple moving average noise threshold (SMA)
Window size [datapoints]: 600
Noise curve offset: 5.50429
SVP parameters: terminal gap: 2, internal gap: 1

Proteoforms

(top 10)

Index	Parent Sequence	Chain	Var. Mods	Mono Mass [Da]	Chemical Composition
1	H31Kac1	H31Kac1K14[Ace]	Ace: K14	15305.4534	C671H1131N215O186S4
2	H31Kac1	H31Kac1K18[Ace]	Ace: K18	15305.4534	C671H1131N215O186S4
3	H31Kac1	H31Kac1K9[Ace]	Ace: K9	15305.4534	C671H1131N215O186S4
4	H31Kac1	H31Kac1K4[Ace]	Ace: K4	15305.4534	C671H1131N215O186S4
5	H31Kac1	H31Kac1K23[Ace]	Ace: K23	15305.4534	C671H1131N215O186S4
6	H31Kac1	H31Kac1K27[Ace]	Ace: K27	15305.4534	C671H1131N215O186S4
7	H31Kac1	H31Kac1K36[Ace]	Ace: K36	15305.4534	C671H1131N215O186S4
8	H31Kac1	H31Kac1K37[Ace]	Ace: K37	15305.4534	C671H1131N215O186S4
9	H31Kac1	H31Kac1K56[Ace]	Ace: K56	15305.4534	C671H1131N215O186S4
10	H31Kac1	H31Kac1K64[Ace]	Ace: K64	15305.4534	C671H1131N215O186S4

Results

Proteoforms Table (top 10)

Index	Protein	SC [%]	SVP [%]	IC [%]	MS Score [a.u.]	Var. Mods	Mono Mass [Da]	Chemical Formula
1	H31Kac1	74.6	39.3	24.2	18.1	Ace: K14	15305.4534	C671H1131N215O186S4
2	H31Kac1	72.4	18.5	23.9	17.3	Ace: K18	15305.4534	C671H1131N215O186S4
3	H31Kac1	70.9	14.8	23.4	16.6	Ace: K9	15305.4534	C671H1131N215O186S4
4	H31Kac1	68.7	12.6	22.6	15.5	Ace: K4	15305.4534	C671H1131N215O186S4
5	H31Kac1	68.7	18.5	22.5	15.4	Ace: K23	15305.4534	C671H1131N215O186S4
6	H31Kac1	65.7	18.5	22.0	14.4	Ace: K27	15305.4534	C671H1131N215O186S4
7	H31Kac1	59.7	18.5	20.0	12.0	Ace: K36	15305.4534	C671H1131N215O186S4
8	H31Kac1	59.0	18.5	19.8	11.7	Ace: K37	15305.4534	C671H1131N215O186S4
9	H31Kac1	50.7	18.5	17.8	9.0	Ace: K56	15305.4534	C671H1131N215O186S4
10	H31Kac1	48.5	18.5	17.2	8.4	Ace: K64	15305.4534	C671H1131N215O186S4

Sequence

Name: H31Kac1

```
         10         20         30         40         50         60
ARTKQTARKS TGGKAPRKQL ATKAARKSAP ATGGVKKPHR YRPGTVALRE IRRYQKSTEL
         70         80         90        100        110        120
LIRKLPFQRL VREIAQDFKT DLRFQSSAVM ALQEACEAYL VGLFEDTNLC AIHAKRVTIM
        130        140        150        160        170        180
PKDIQLARRI RGERA
```

Modifications

Modification	Position	Type
Acetyl	4	variable
Acetyl	9	variable
Acetyl	14	variable
Acetyl	18	variable
Acetyl	23	variable
Acetyl	27	variable
Acetyl	36	variable
Acetyl	37	variable
Acetyl	56	variable
Acetyl	64	variable
Acetyl	79	variable

Modification	Position	Type
Acetyl	115	variable
Acetyl	122	variable

Sequence Map

Proteoform:

Index	Protein	SC [%]	SVP [%]	IC [%]	MS Score [a.u.]	Var. Mods	Mono Mass [Da]	Chemical Formula
1	H31Kac1	74.6	39.3	24.2	18.1	Ace: K14	15305.4534	C671H1131N215O186S4

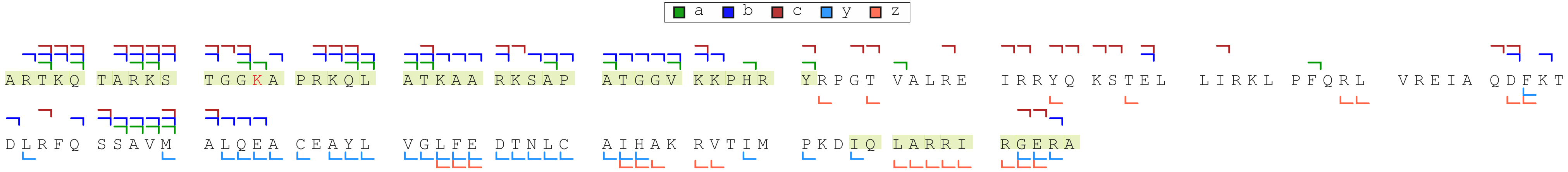

Proteoform:

Index	Protein	SC [%]	SVP [%]	IC [%]	MS Score [a.u.]	Var. Mods	Mono Mass [Da]	Chemical Formula
2	H31Kac1	72.4	18.5	23.9	17.3	Ace: K18	15305.4534	C671H1131N215O186S4

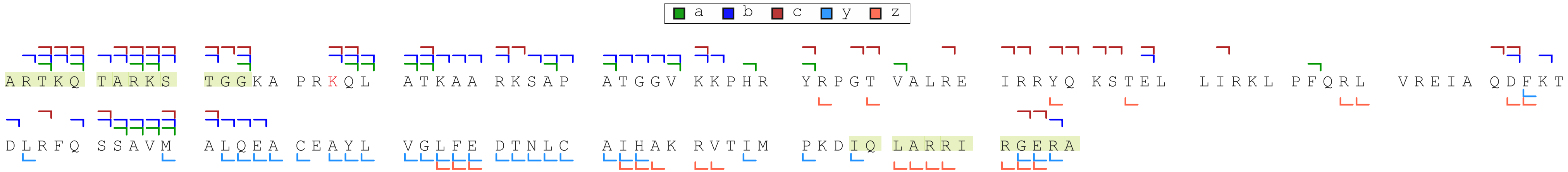

Proteoform:

Index	Protein	SC [%]	SVP [%]	IC [%]	MS Score [a.u.]	Var. Mods	Mono Mass [Da]	Chemical Formula
3	H31Kac1	70.9	14.8	23.4	16.6	Ace: K9	15305.4534	C671H1131N215O186S4

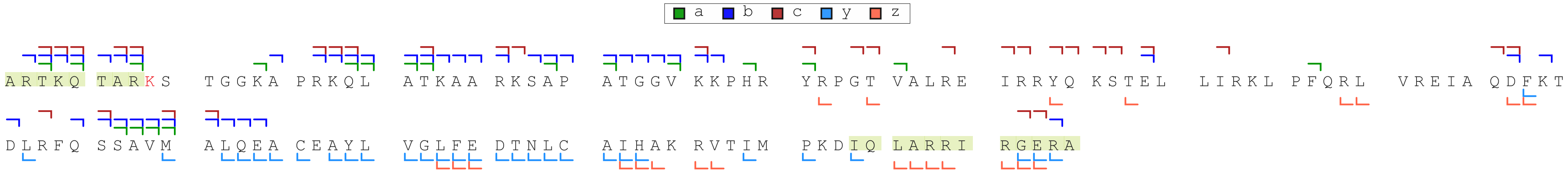

Proteoform:

Index	Protein	SC [%]	SVP [%]	IC [%]	MS Score [a.u.]	Var. Mods	Mono Mass [Da]	Chemical Formula
4	H31Kac1	68.7	12.6	22.6	15.5	Ace: K4	15305.4534	C671H1131N215O186S4

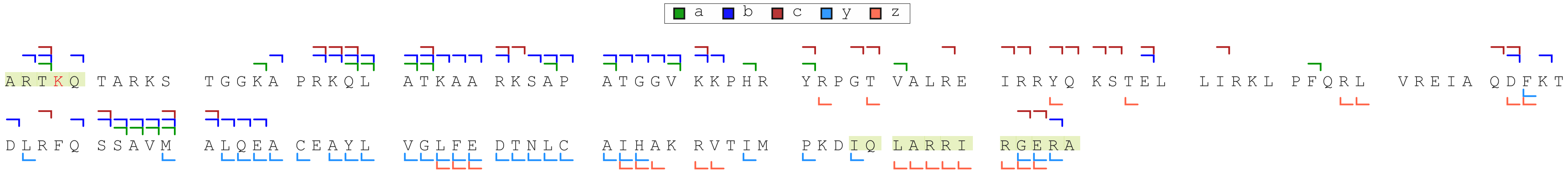

Proteoform:

Index	Protein	SC [%]	SVP [%]	IC [%]	MS Score [a.u.]	Var. Mods	Mono Mass [Da]	Chemical Formula
5	H31Kac1	68.7	18.5	22.5	15.4	Ace: K23	15305.4534	C671H1131N215O186S4

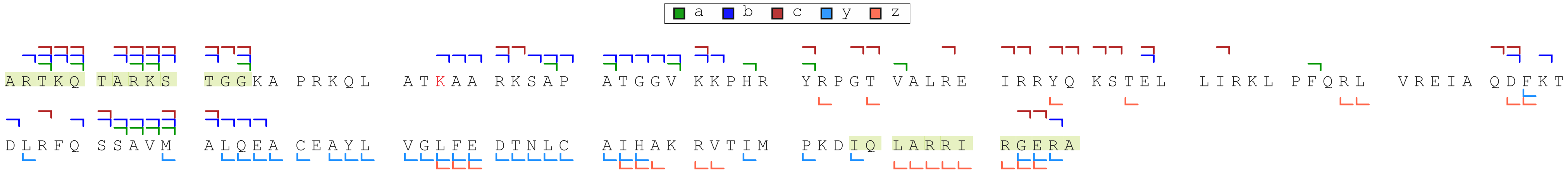

Proteoform:

Index	Protein	SC [%]	SVP [%]	IC [%]	MS Score [a.u.]	Var. Mods	Mono Mass [Da]	Chemical Formula
6	H31Kac1	65.7	18.5	22.0	14.4	Ace: K27	15305.4534	C671H1131N215O186S4

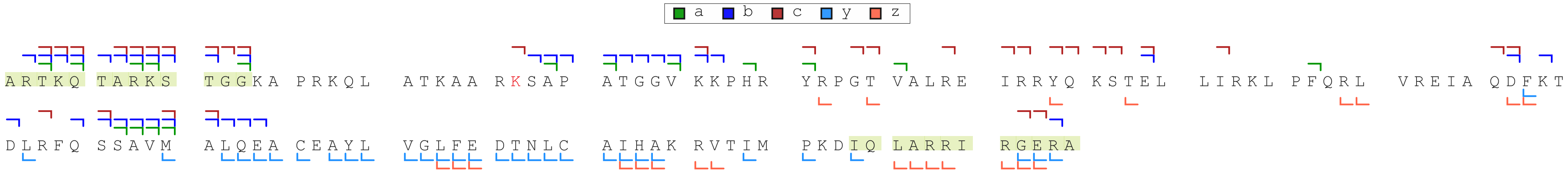

Proteoform:

Index	Protein	SC [%]	SVP [%]	IC [%]	MS Score [a.u.]	Var. Mods	Mono Mass [Da]	Chemical Formula
7	H31Kac1	59.7	18.5	20.0	12.0	Ace: K36	15305.4534	C671H1131N215O186S4

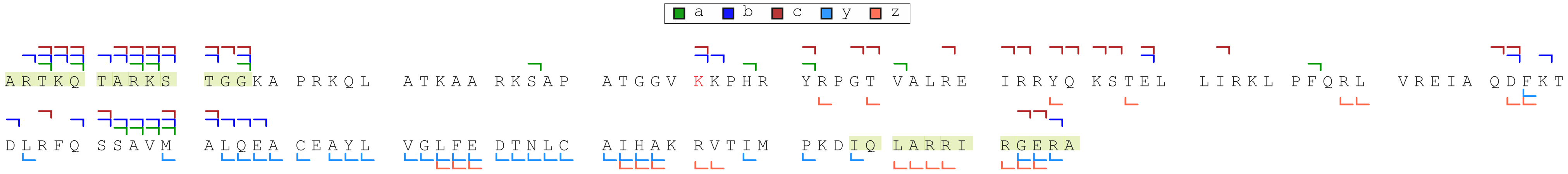

Proteoform:

Index	Protein	SC [%]	SVP [%]	IC [%]	MS Score [a.u.]	Var. Mods	Mono Mass [Da]	Chemical Formula
8	H31Kac1	59.0	18.5	19.8	11.7	Ace: K37	15305.4534	C671H1131N215O186S4

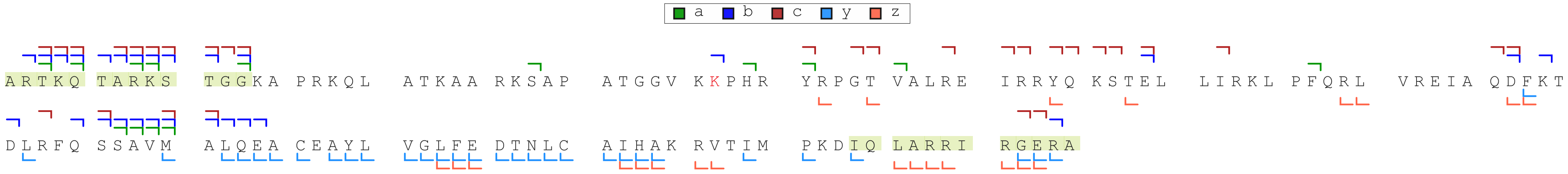

Proteoform:

Index	Protein	SC [%]	SVP [%]	IC [%]	MS Score [a.u.]	Var. Mods	Mono Mass [Da]	Chemical Formula
9	H31Kac1	50.7	18.5	17.8	9.0	Ace: K56	15305.4534	C671H1131N215O186S4

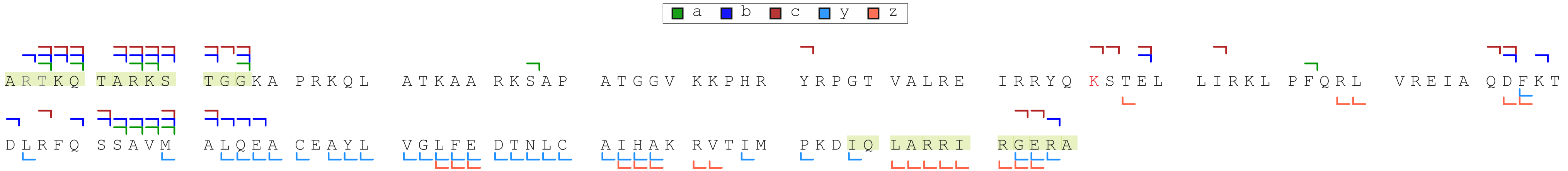

Proteoform:

Index	Protein	SC [%]	SVP [%]	IC [%]	MS Score [a.u.]	Var. Mods	Mono Mass [Da]	Chemical Formula
10	H31Kac1	48.5	18.5	17.2	8.4	Ace: K64	15305.4534	C671H1131N215O186S4

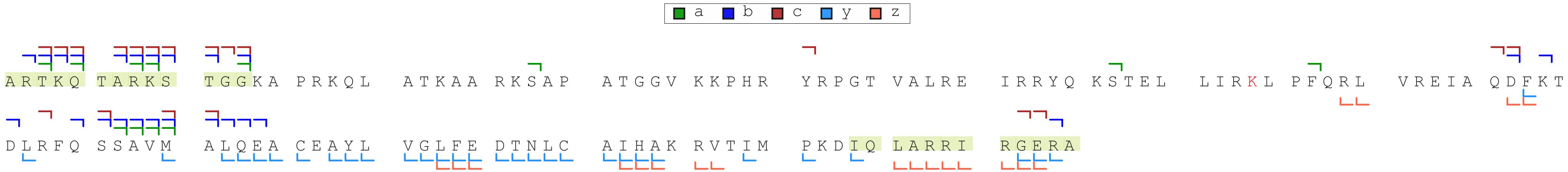

Error Plot

Proteoform:

Index	Protein	SC [%]	SVP [%]	IC [%]	MS Score [a.u.]	Var. Mods	Mono Mass [Da]	Chemical Formula
1	H31Kac1	74.6	39.3	24.2	18.1	Ace: K14	15305.4534	C671H1131N215O186S4

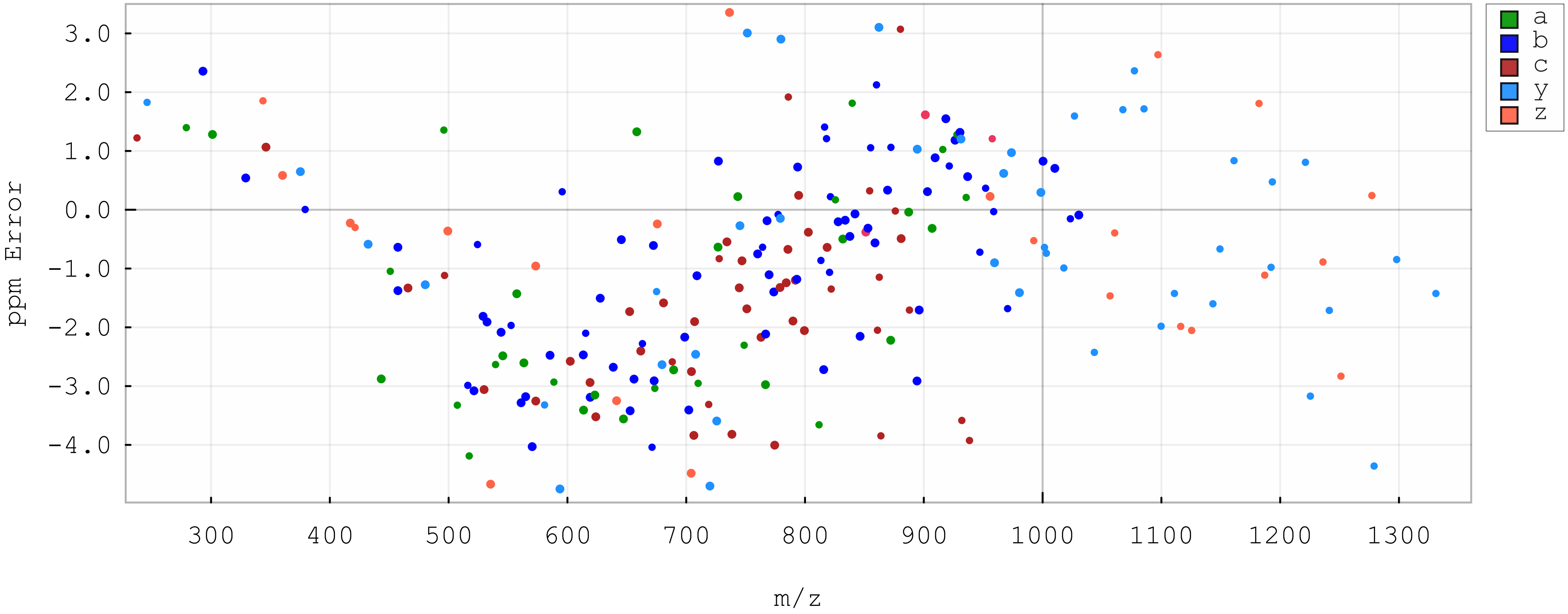

Proteoform:

Index	Protein	SC [%]	SVP [%]	IC [%]	MS Score [a.u.]	Var. Mods	Mono Mass [Da]	Chemical Formula
2	H31Kac1	72.4	18.5	23.9	17.3	Ace: K18	15305.4534	C671H1131N215O186S4

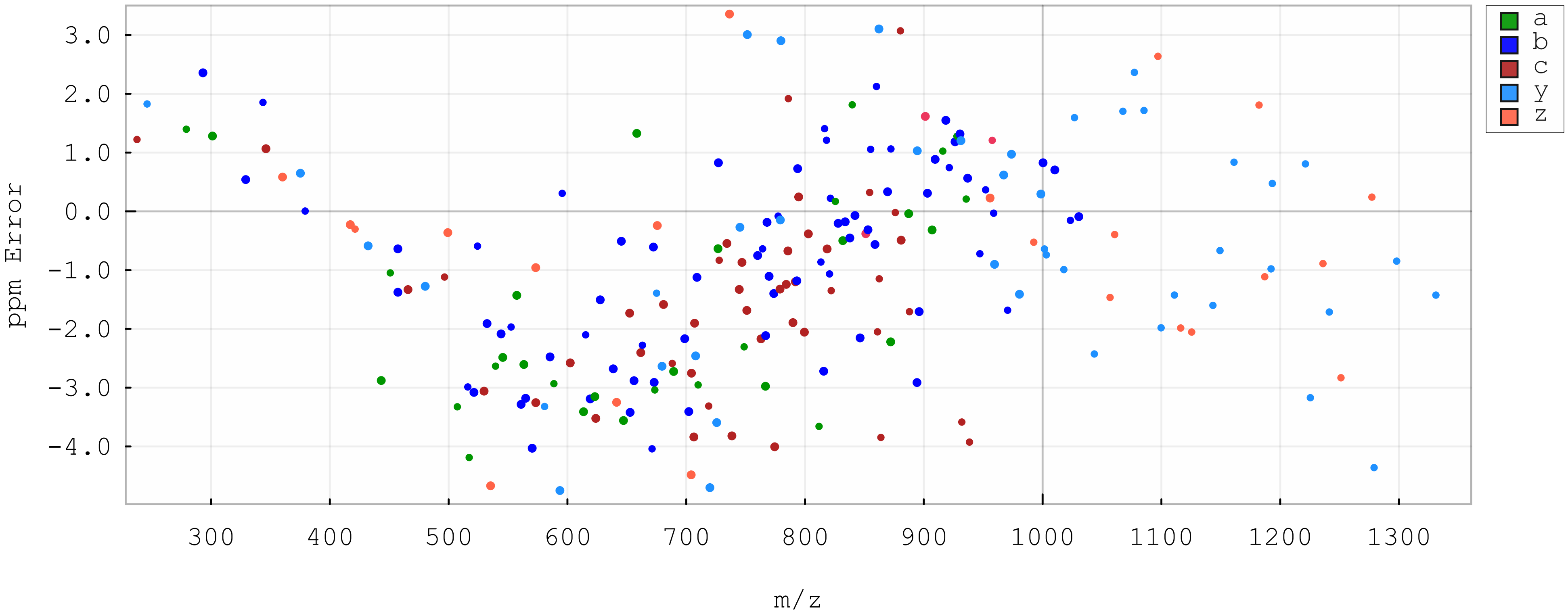

Proteoform:	Index	Protein	SC [%]	SVP [%]	IC [%]	MS Score [a.u.]	Var. Mods	Mono Mass [Da]	Chemical Formula
	3	H31Kac1	70.9	14.8	23.4	16.6	Ace: K9	15305.4534	C671H1131N215O186S4

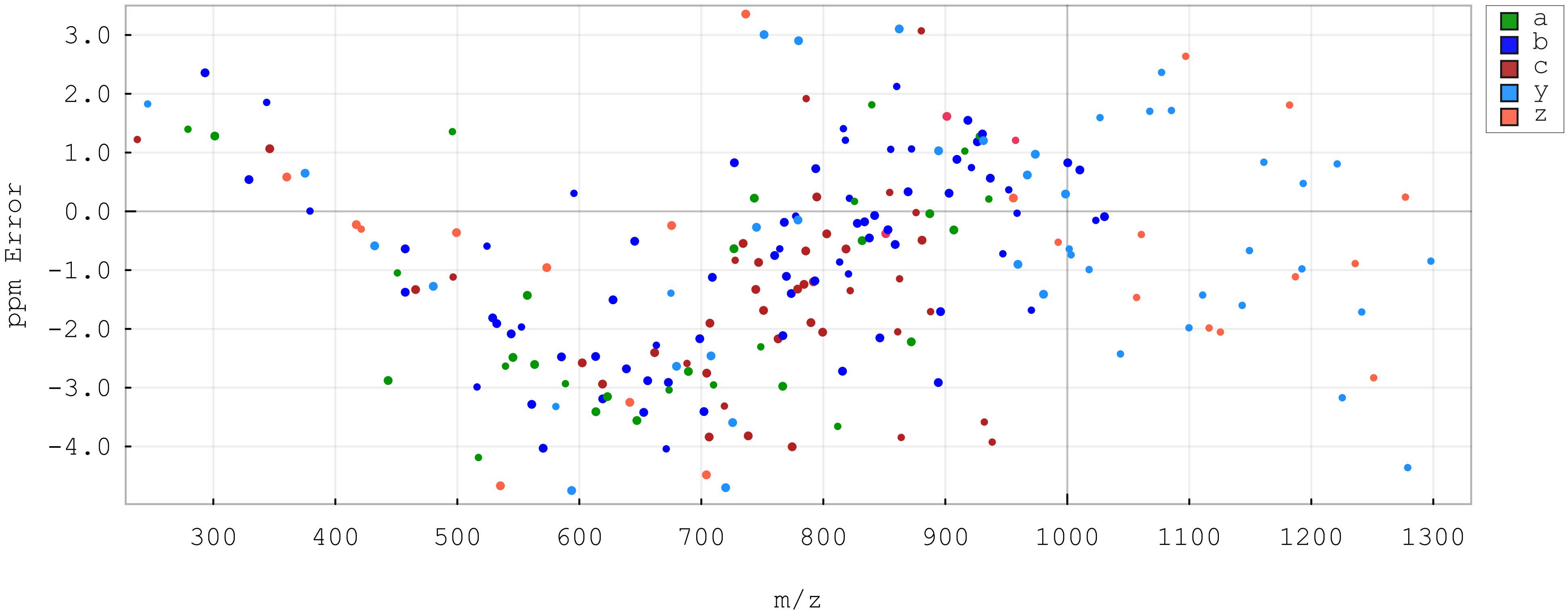

Proteoform:

Index	Protein	SC [%]	SVP [%]	IC [%]	MS Score [a.u.]	Var. Mods	Mono Mass [Da]	Chemical Formula
4	H31Kac1	68.7	12.6	22.6	15.5	Ace: K4	15305.4534	C671H1131N215O186S4

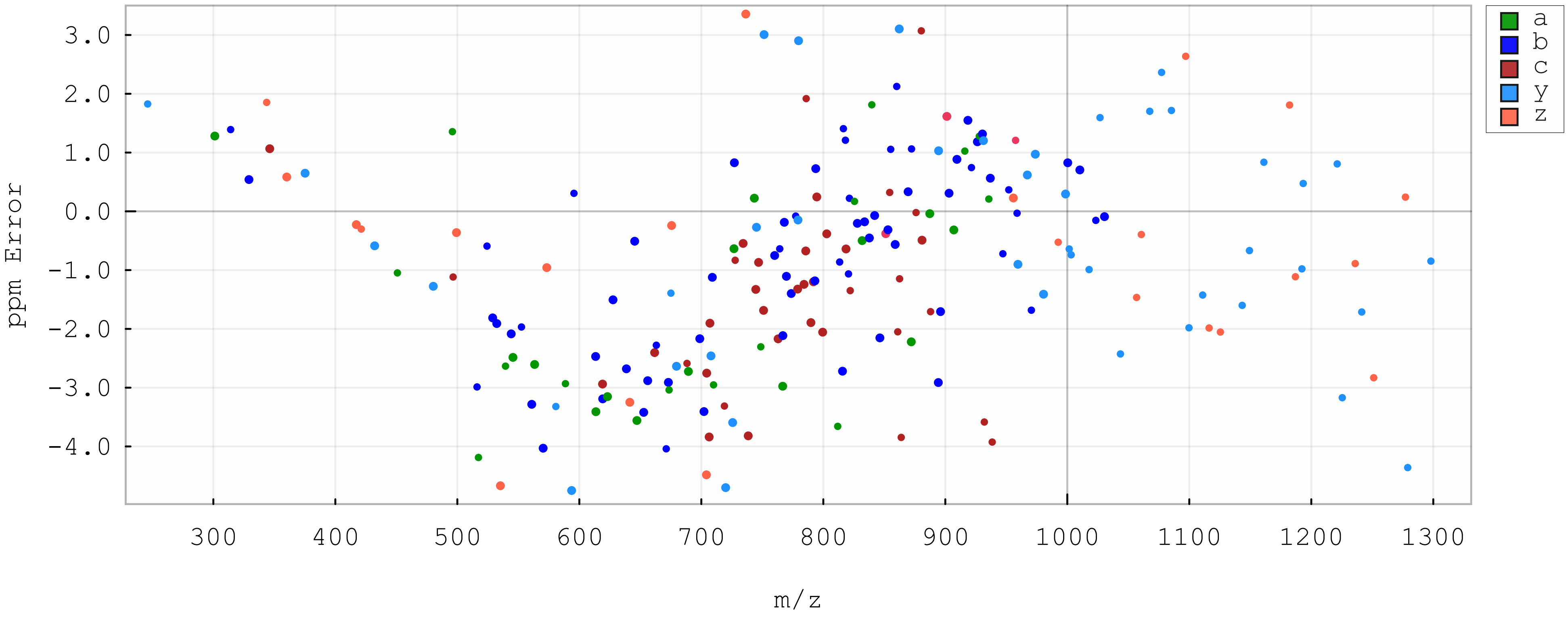

Proteoform:

Index	Protein	SC [%]	SVP [%]	IC [%]	MS Score [a.u.]	Var. Mods	Mono Mass [Da]	Chemical Formula
5	H31Kac1	68.7	18.5	22.5	15.4	Ace: K23	15305.4534	C671H1131N215O186S4

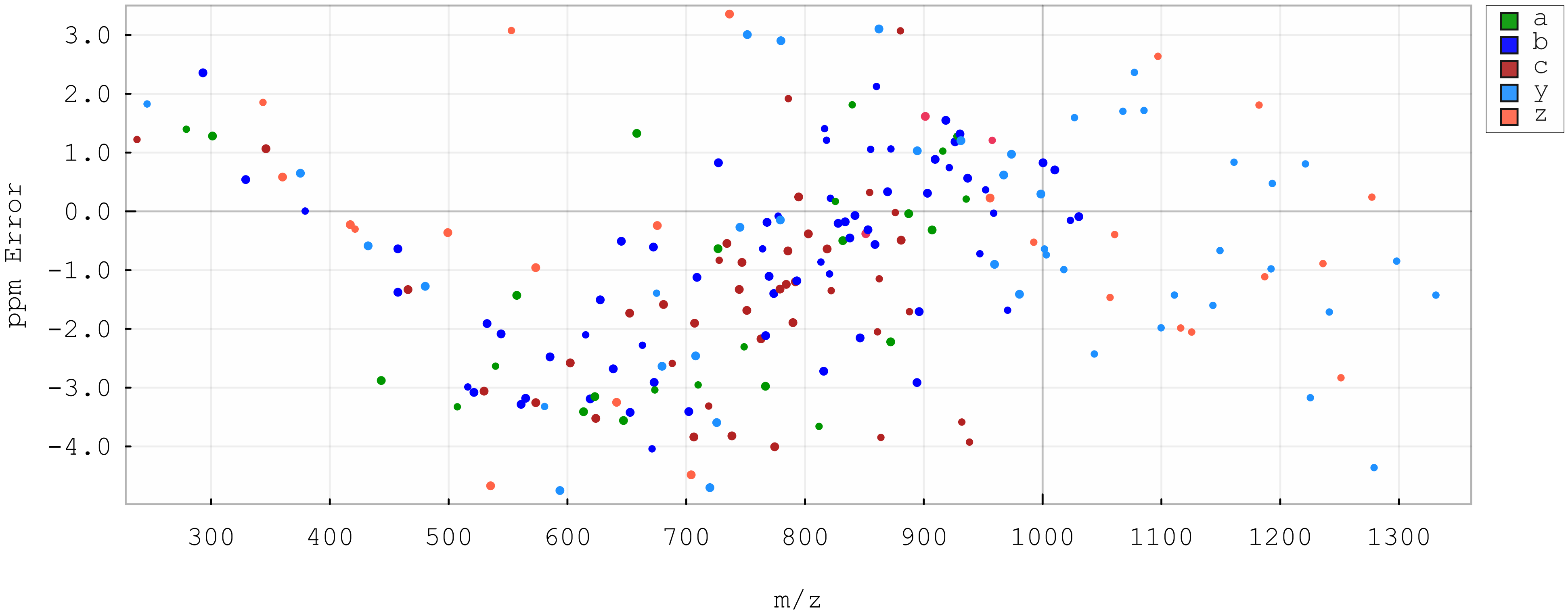

Proteoform:

Index	Protein	SC [%]	SVP [%]	IC [%]	MS Score [a.u.]	Var. Mods	Mono Mass [Da]	Chemical Formula
6	H31Kac1	65.7	18.5	22.0	14.4	Ace: K27	15305.4534	C671H1131N215O186S4

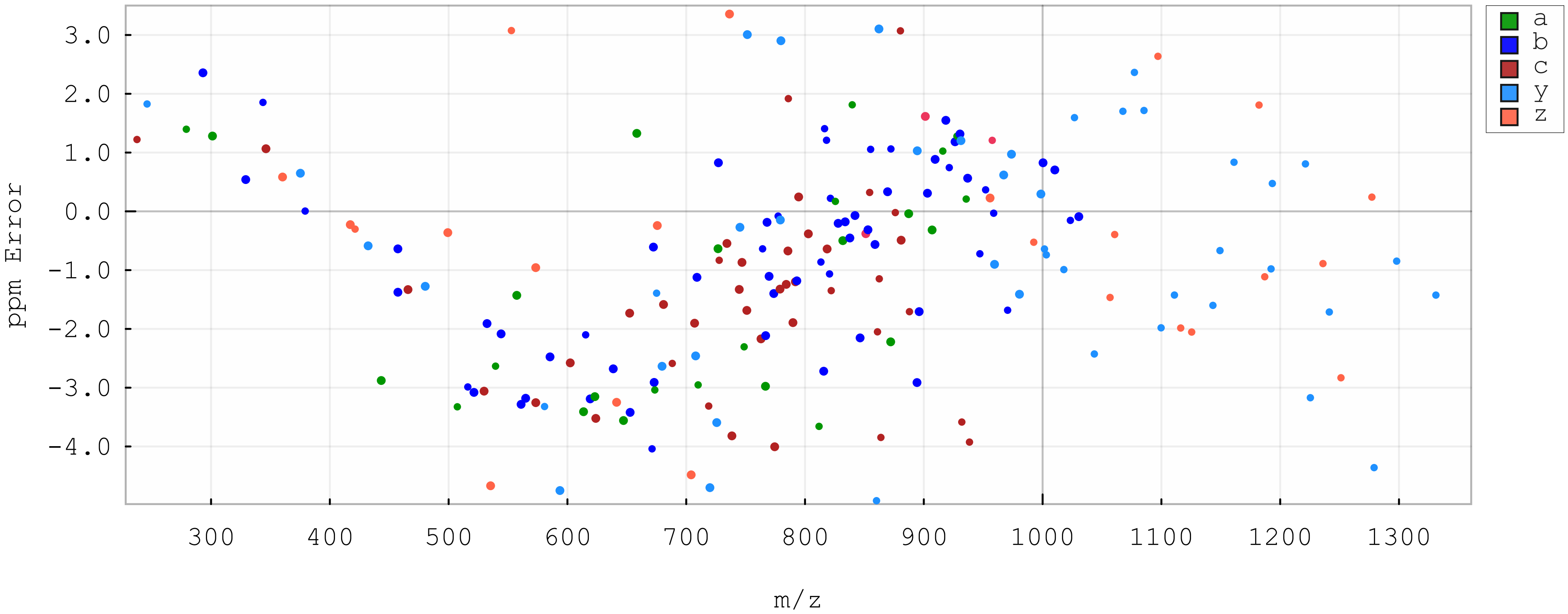

Proteoform:

Index	Protein	SC [%]	SVP [%]	IC [%]	MS Score [a.u.]	Var. Mods	Mono Mass [Da]	Chemical Formula
7	H31Kac1	59.7	18.5	20.0	12.0	Ace: K36	15305.4534	C671H1131N215O186S4

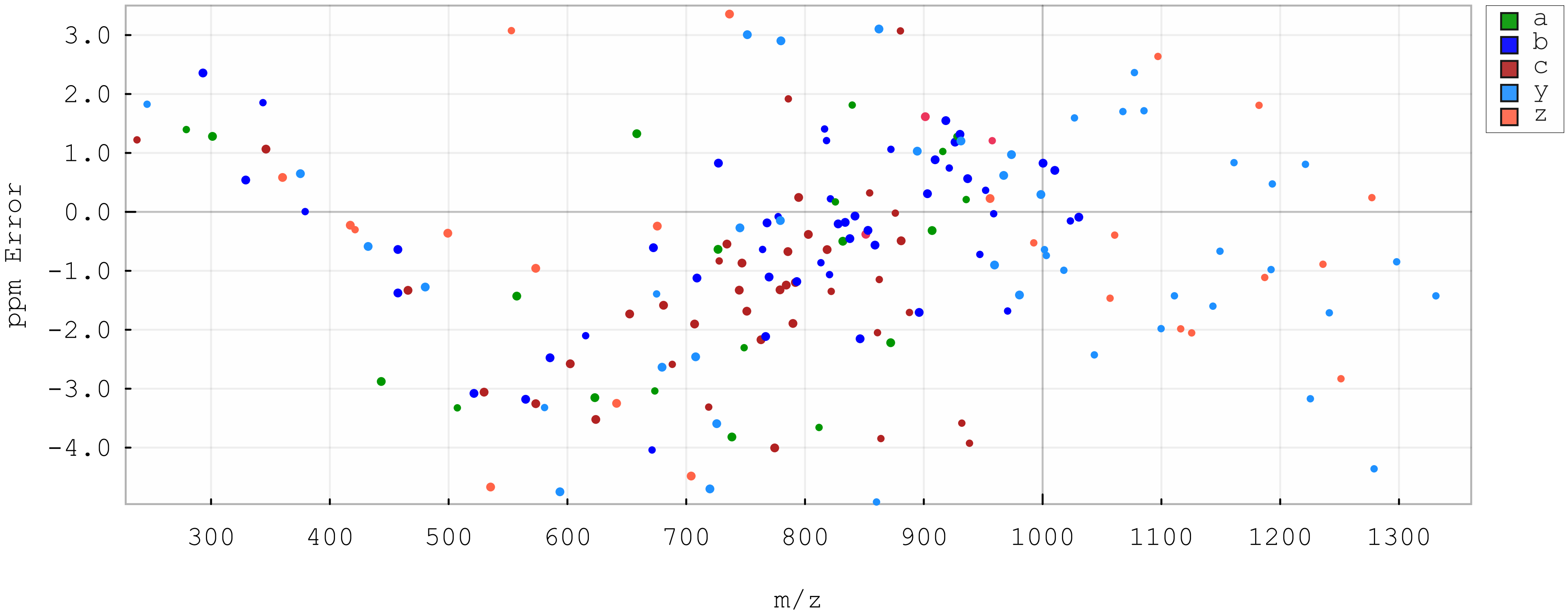

Proteoform:

Index	Protein	SC [%]	SVP [%]	IC [%]	MS Score [a.u.]	Var. Mods	Mono Mass [Da]	Chemical Formula
8	H31Kac1	59.0	18.5	19.8	11.7	Ace: K37	15305.4534	C671H1131N215O186S4

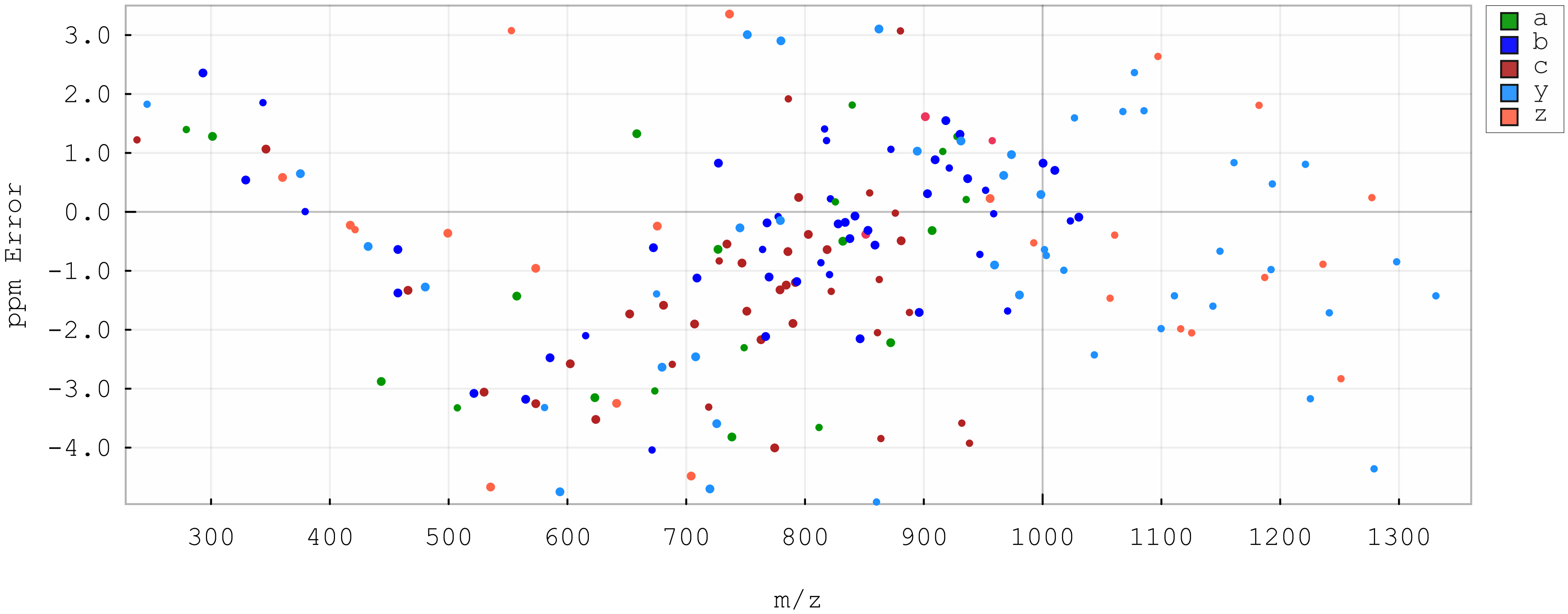

Proteoform:

Index	Protein	SC [%]	SVP [%]	IC [%]	MS Score [a.u.]	Var. Mods	Mono Mass [Da]	Chemical Formula
9	H31Kac1	50.7	18.5	17.8	9.0	Ace: K56	15305.4534	C671H1131N215O186S4

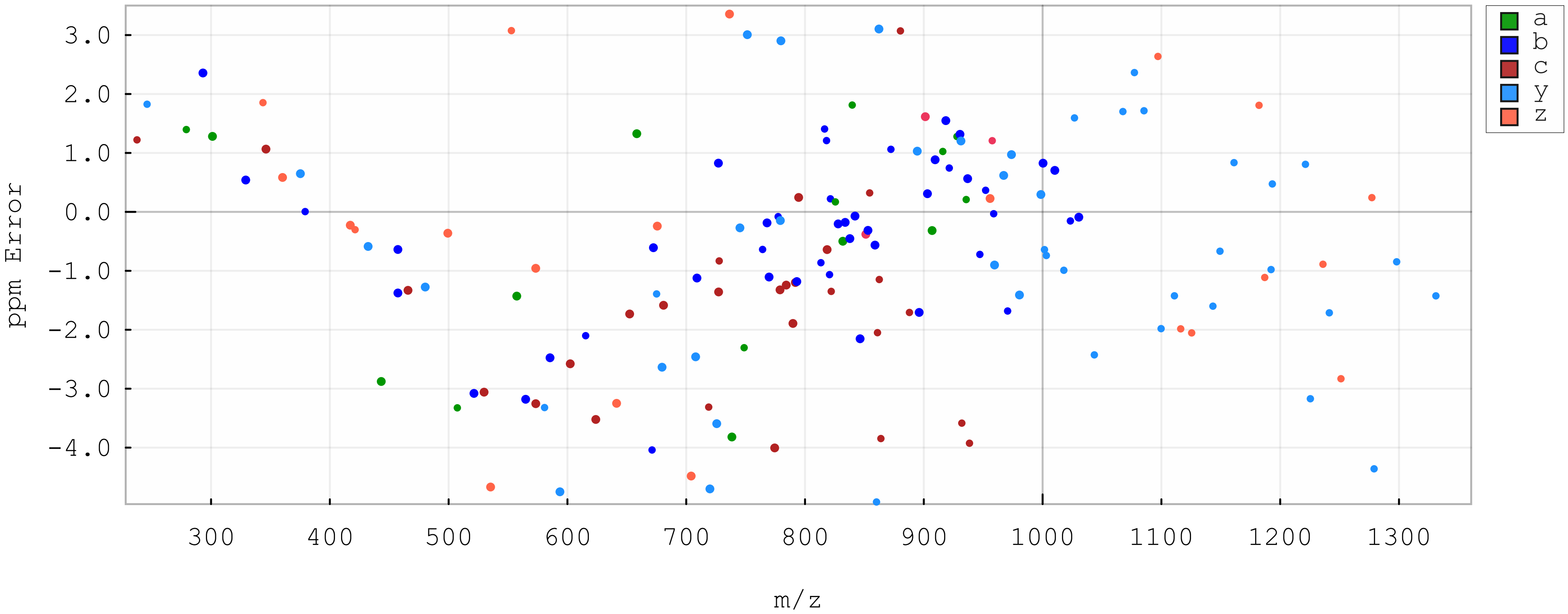

Proteoform:

Index	Protein	SC [%]	SVP [%]	IC [%]	MS Score [a.u.]	Var. Mods	Mono Mass [Da]	Chemical Formula
10	H31Kac1	48.5	18.5	17.2	8.4	Ace: K64	15305.4534	C671H1131N215O186S4

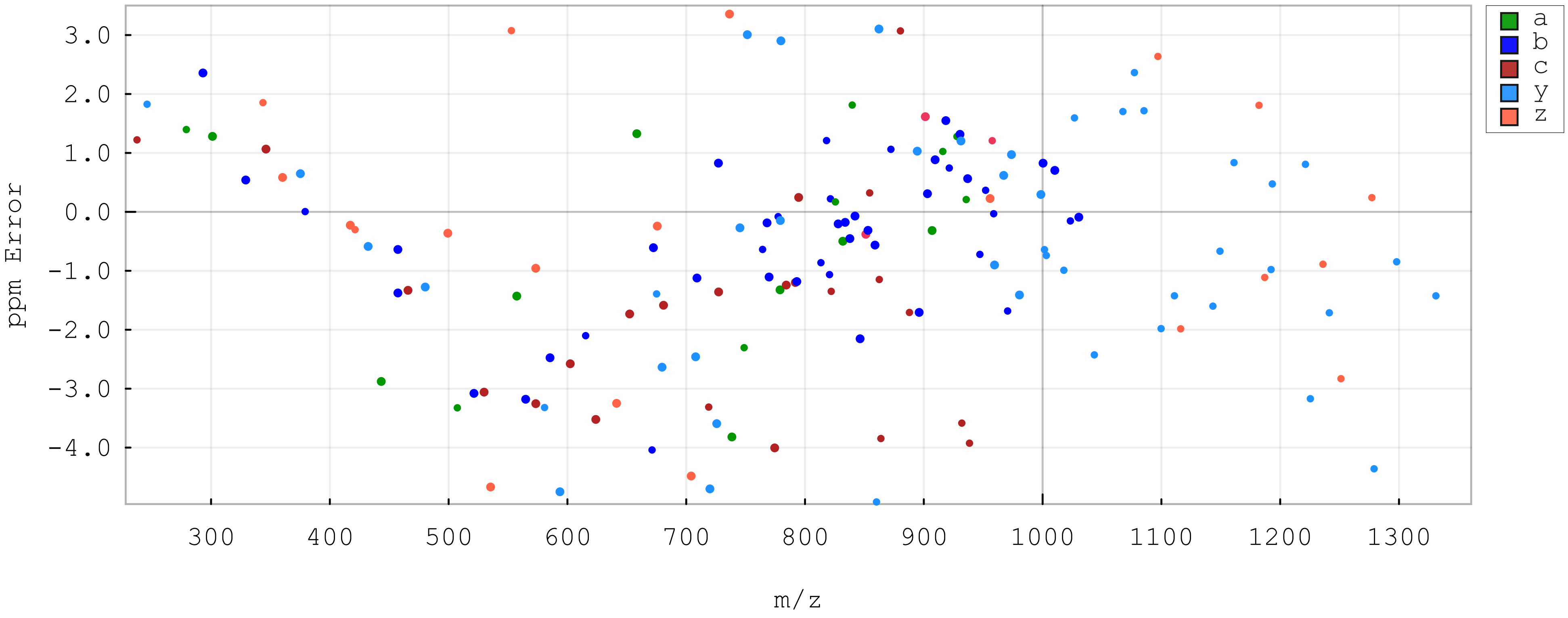

Primary Fragment Plots

Proteoform:

Index	Protein	SC [%]	SVP [%]	IC [%]	MS Score [a.u.]	Var. Mods	Mono Mass [Da]	Chemical Formula
1	H31Kac1	74.6	39.3	24.2	18.1	Ace: K14	15305.4534	C671H1131N215O186S4

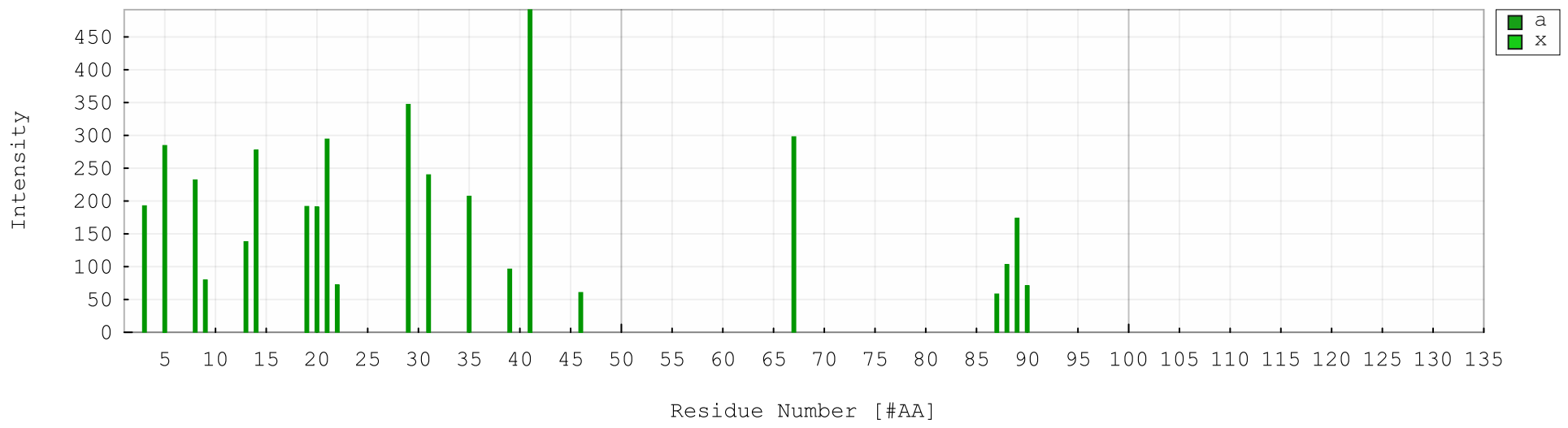

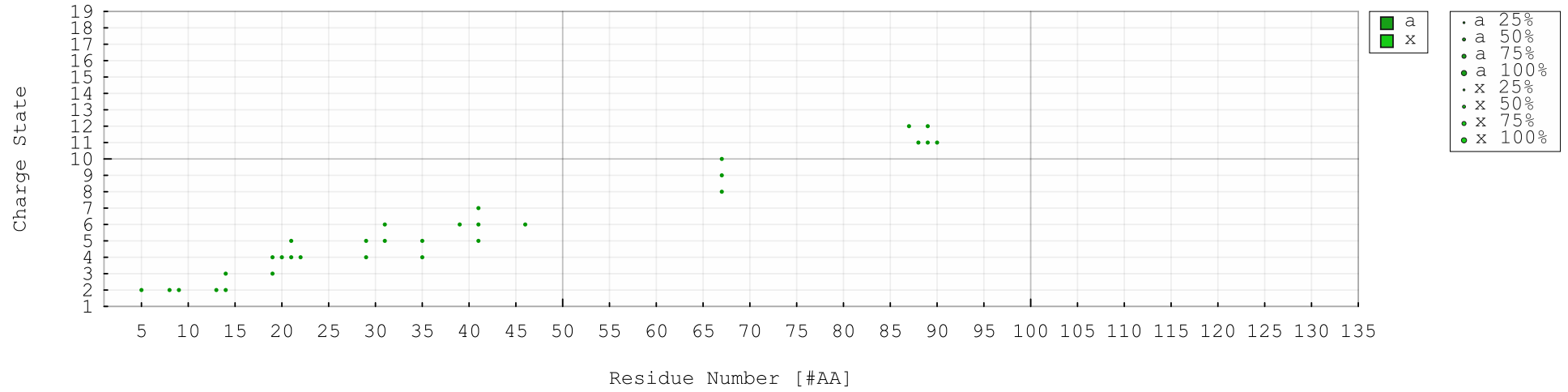

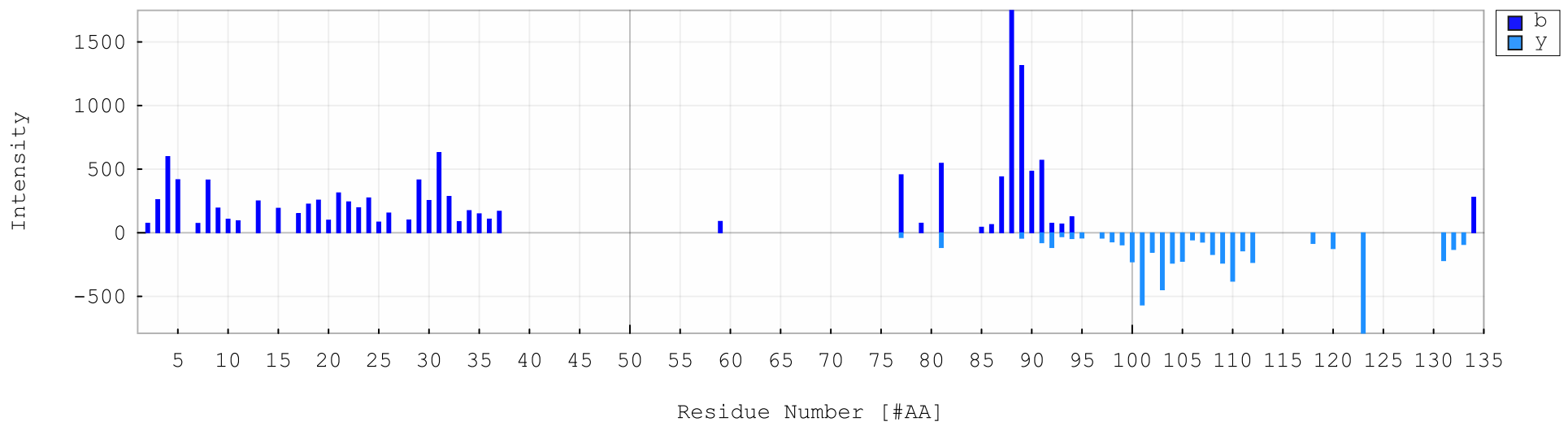

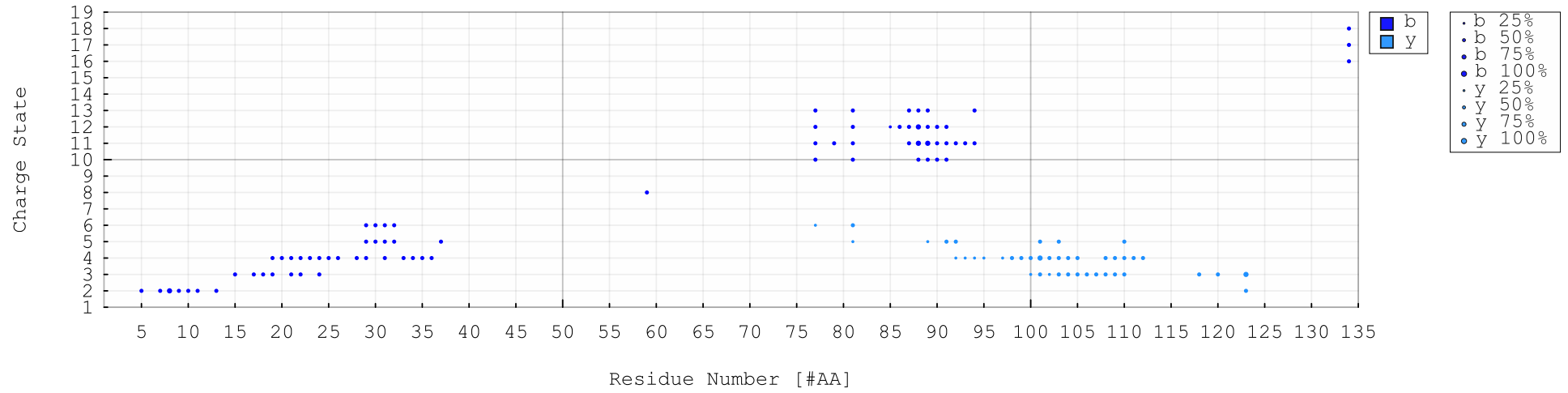

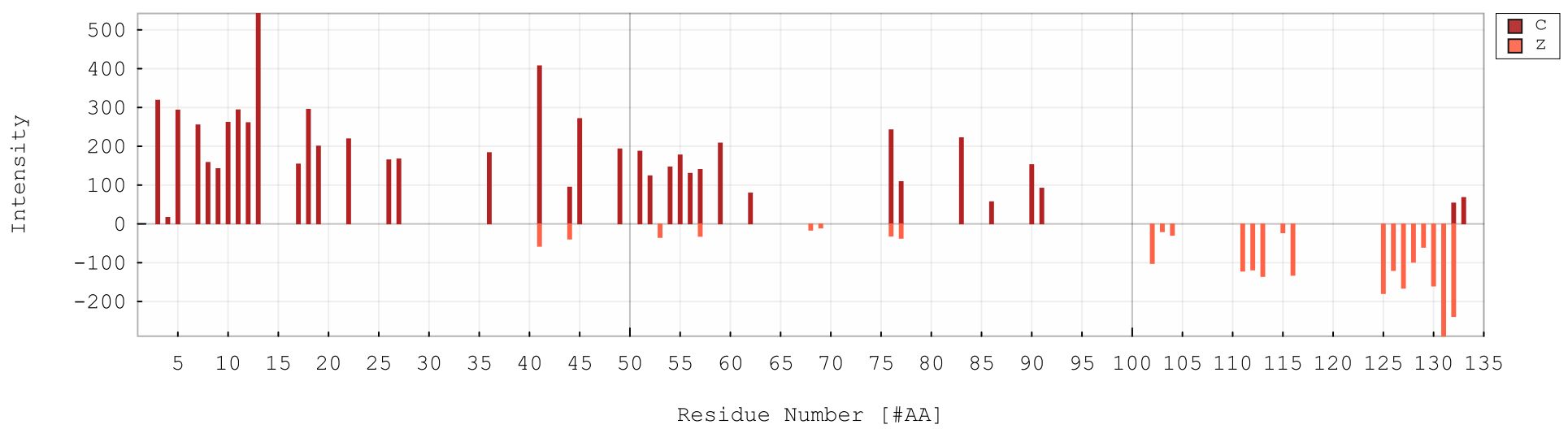

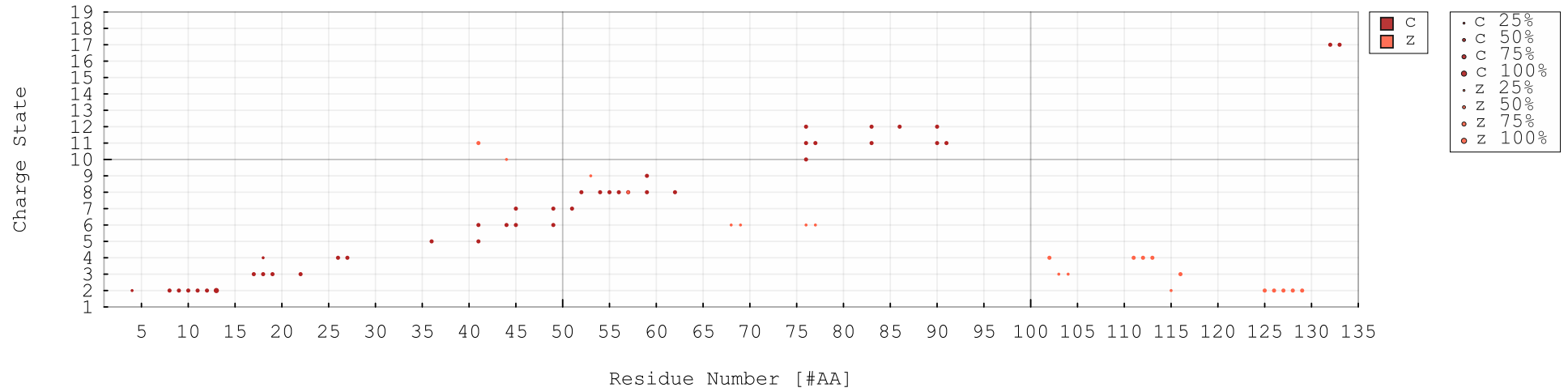

Proteoform:

Index	Protein	SC [%]	SVP [%]	IC [%]	MS Score [a.u.]	Var. Mods	Mono Mass [Da]	Chemical Formula
2	H31Kac1	72.4	18.5	23.9	17.3	Ace: K18	15305.4534	C671H1131N215O186S4

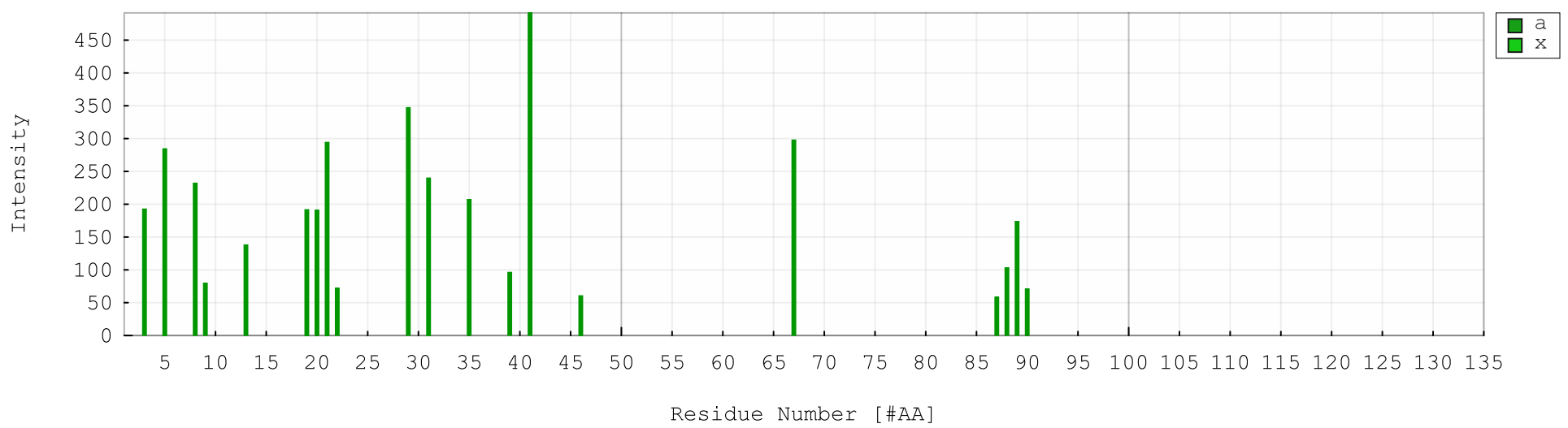

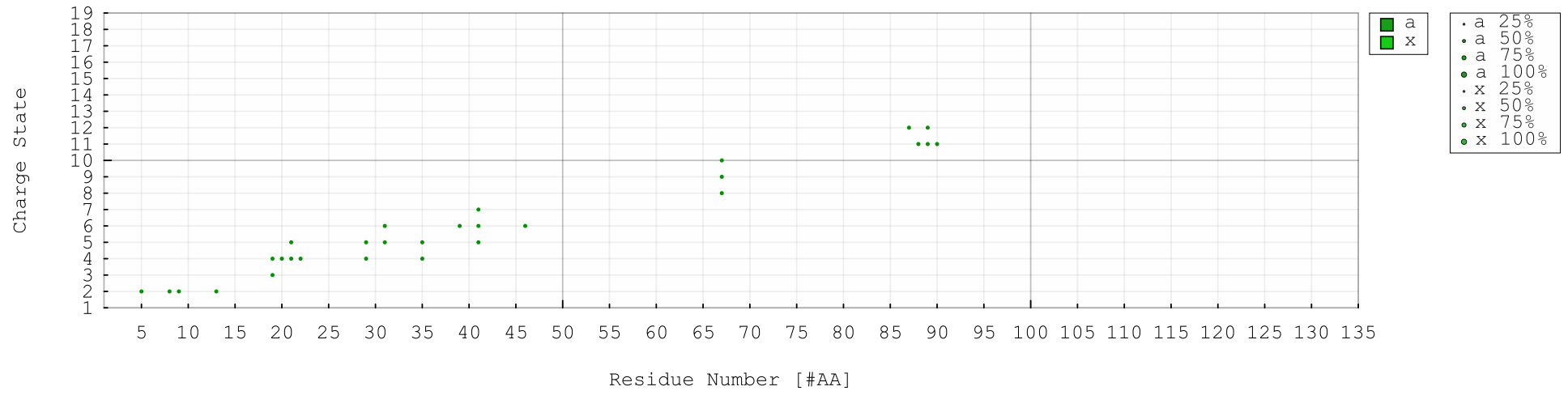

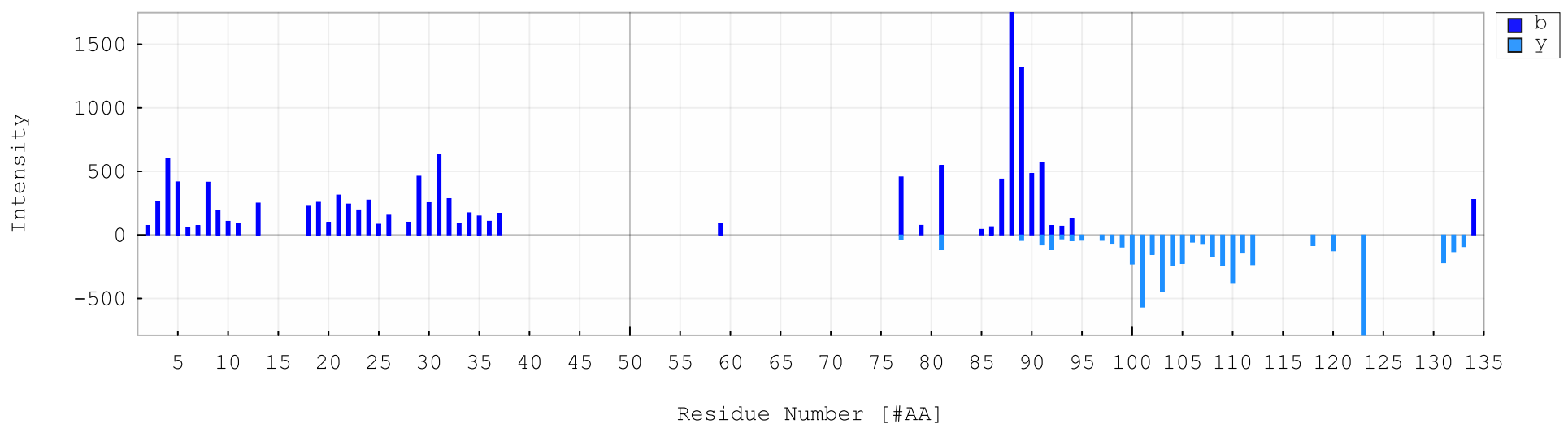

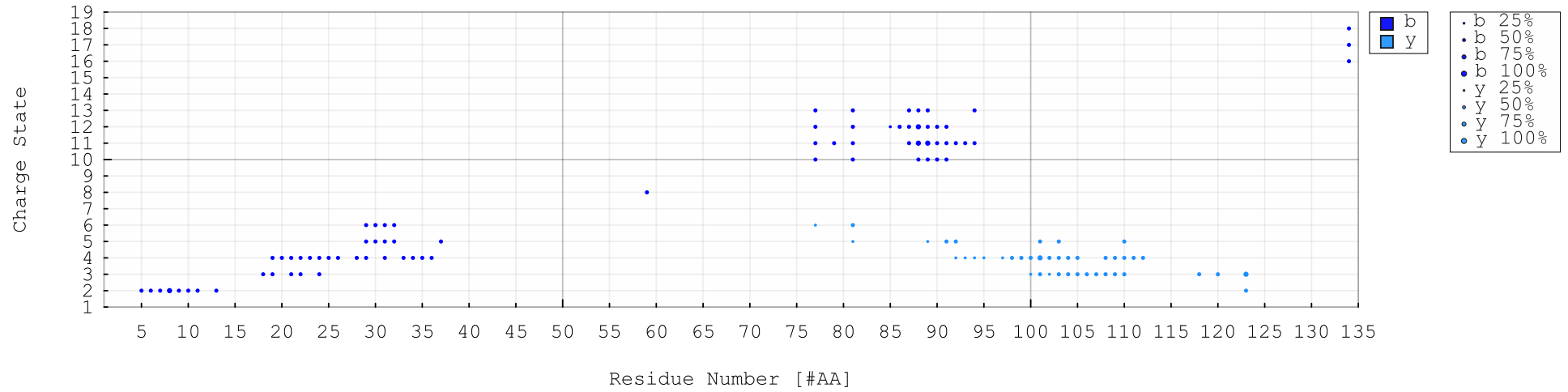

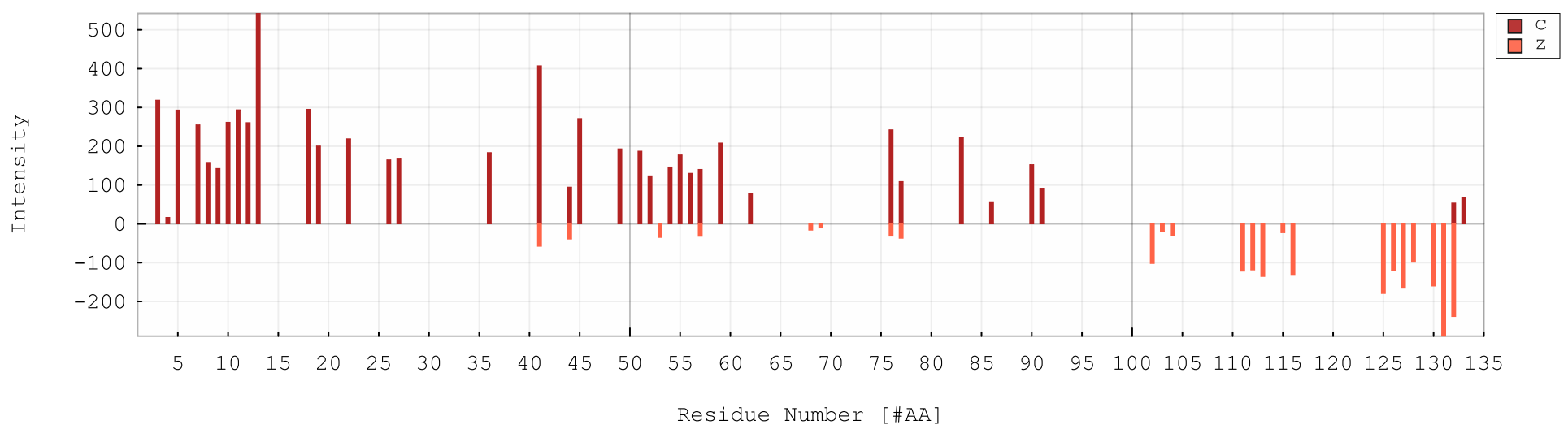

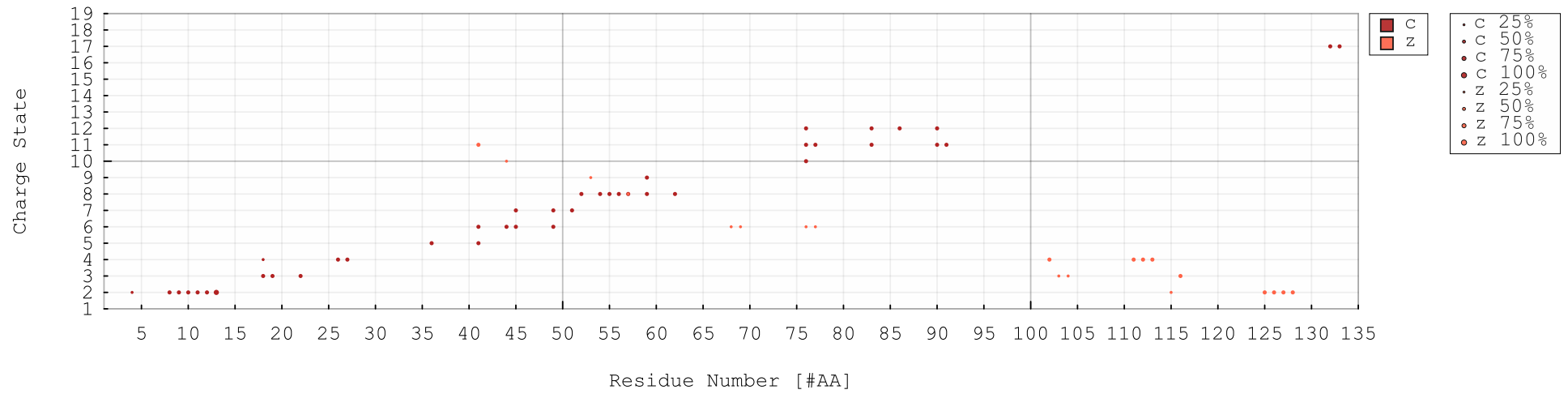

Top-Down Result OmniScape 2025
Sequence Confirmation
241210 H31-GNC5_10 z19 MS2 ECD 30ms + ccCID 31eV_001-242_calibrated

Proteoform:

Index	Protein	SC [%]	SVP [%]	IC [%]	MS Score [a.u.]	Var. Mods	Mono Mass [Da]	Chemical Formula
3	H31Kac1	70.9	14.8	23.4	16.6	Ace: K9	15305.4534	C671H1131N215O186S4

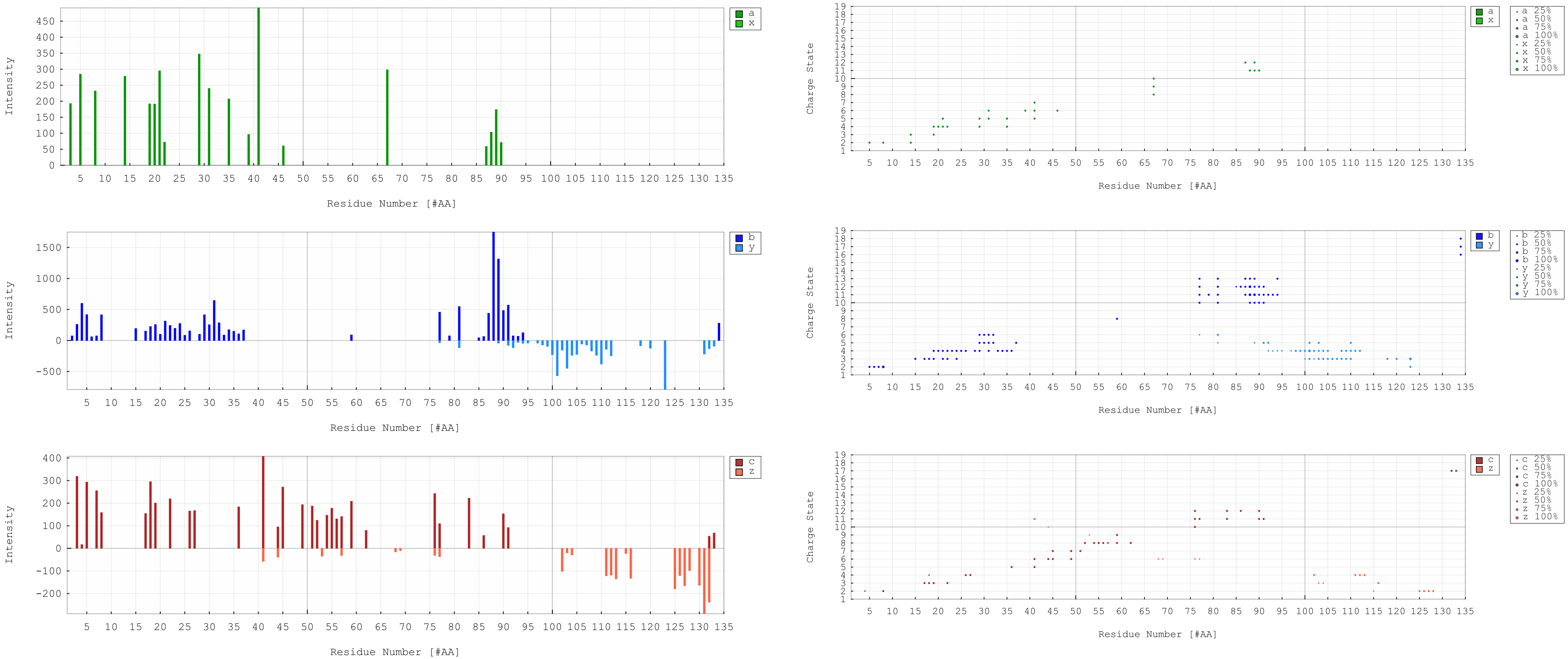

Proteoform:

Index	Protein	SC [%]	SVP [%]	IC [%]	MS Score [a.u.]	Var. Mods	Mono Mass [Da]	Chemical Formula
4	H31Kac1	68.7	12.6	22.6	15.5	Ace: K4	15305.4534	C671H1131N215O186S4

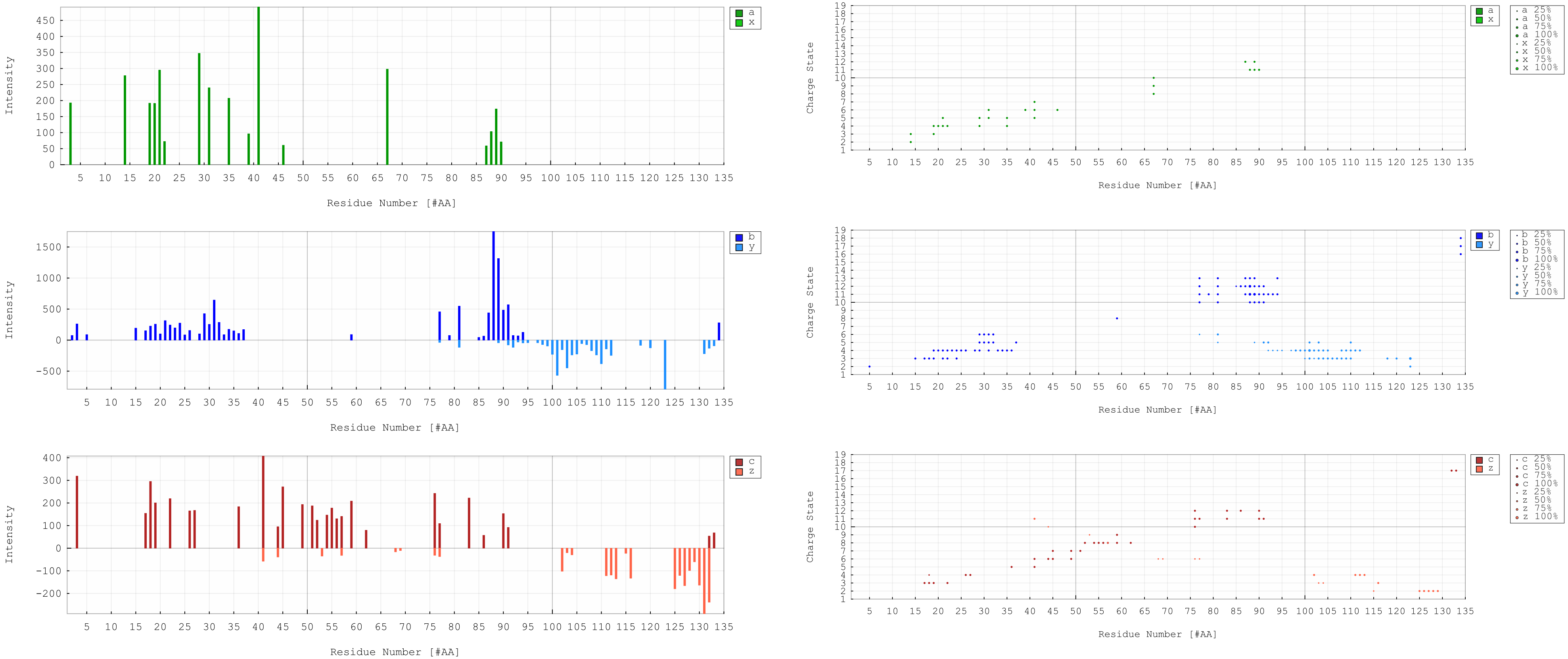

Top-Down Result OmniScape 2025
Sequence Confirmation
241210 H31-GNC5_10 z19 MS2 ECD 30ms + ccCID 31eV_001-242_calibrated

Proteoform:

Index	Protein	SC [%]	SVP [%]	IC [%]	MS Score [a.u.]	Var. Mods	Mono Mass [Da]	Chemical Formula
5	H31Kac1	68.7	18.5	22.5	15.4	Ace: K23	15305.4534	C671H1131N215O186S4

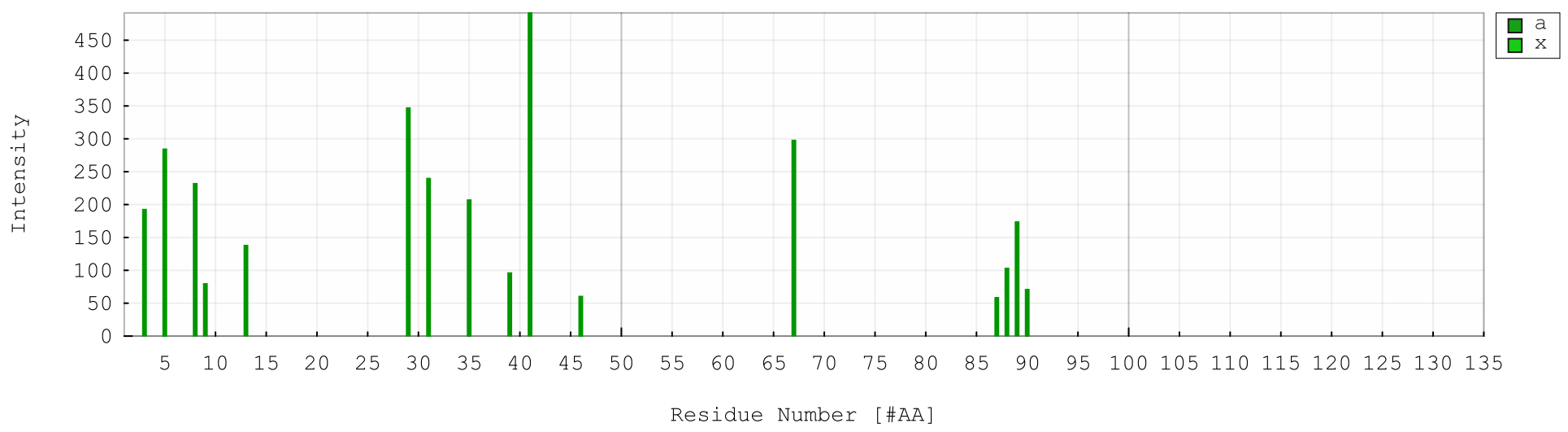

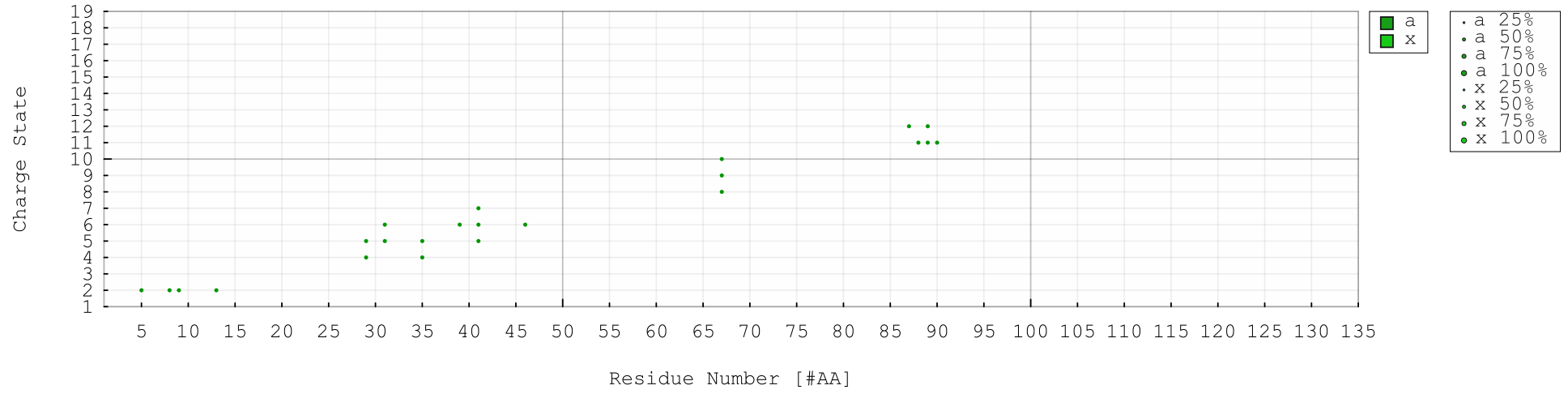

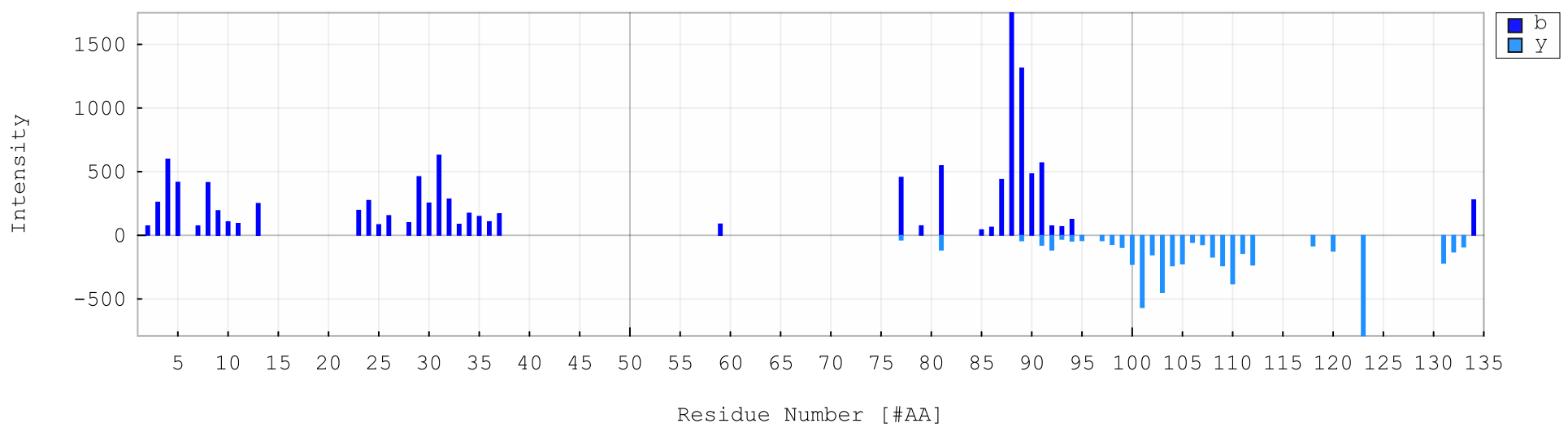

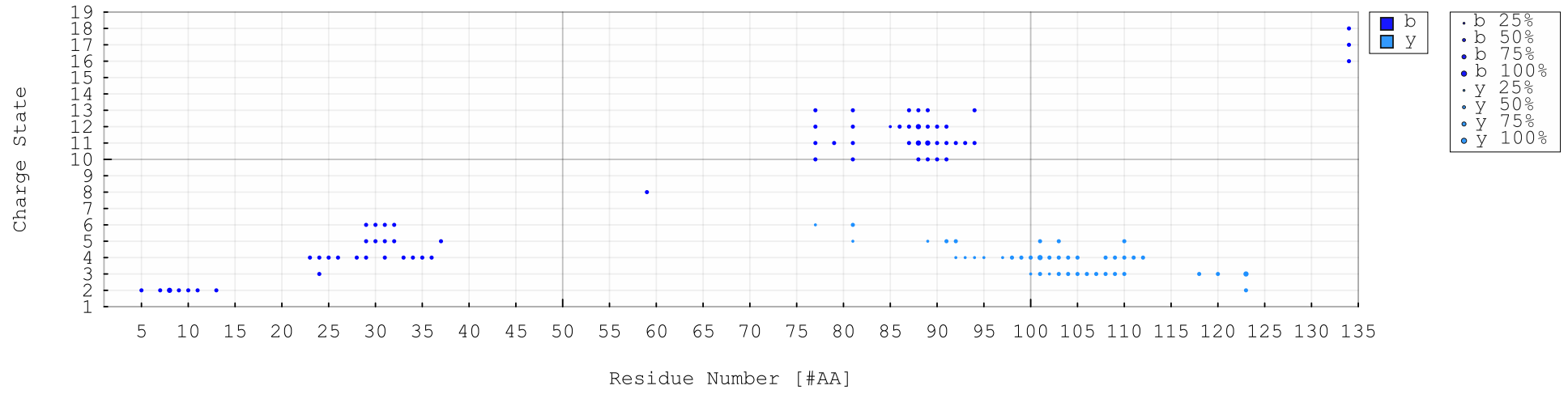

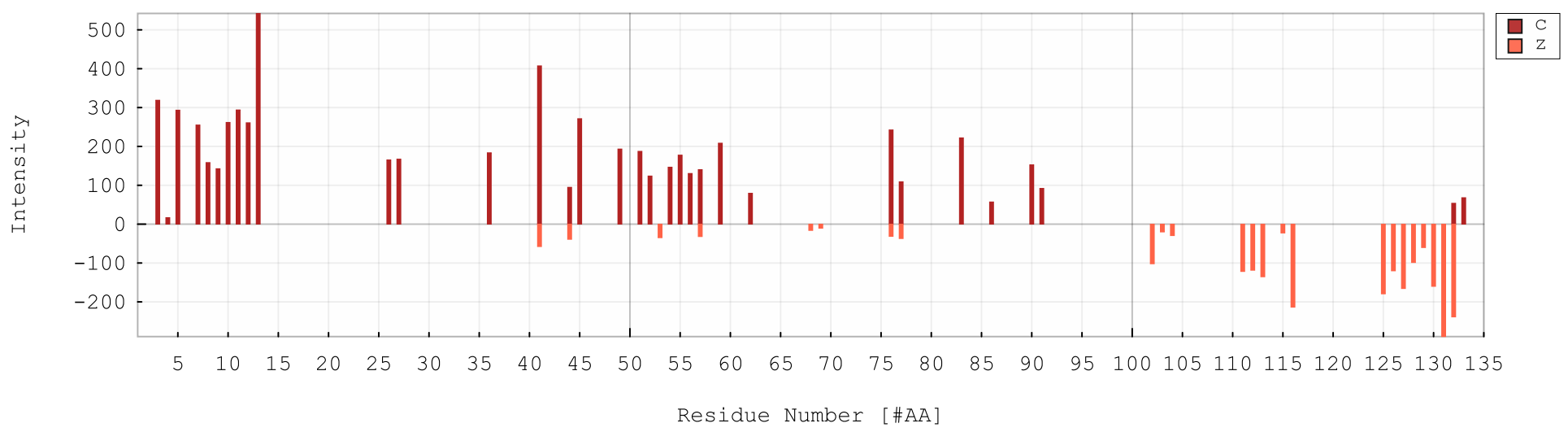

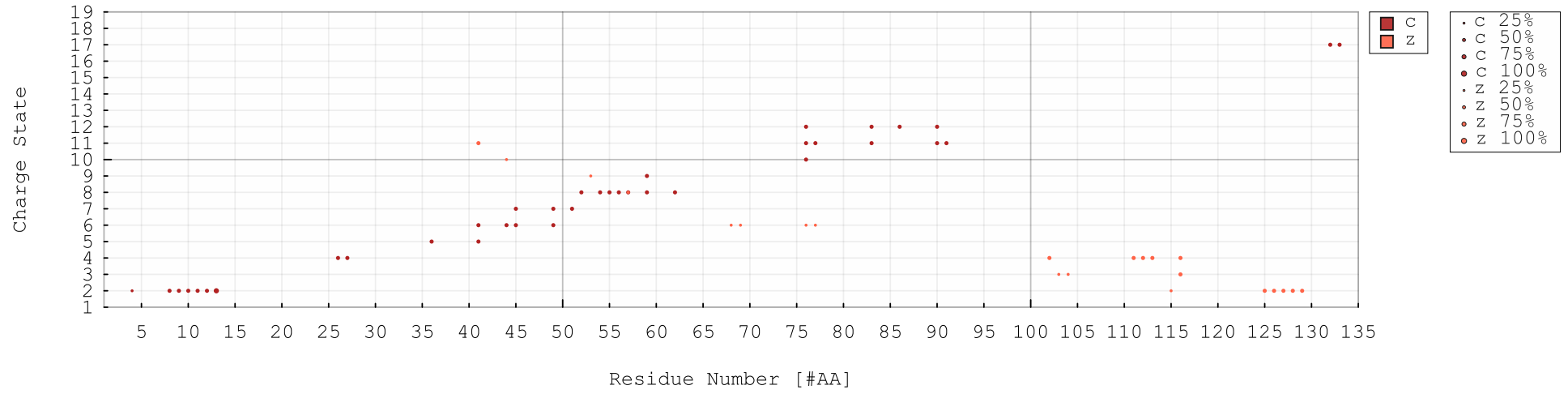

Top-Down Result OmniScape 2025
Sequence Confirmation
241210 H31-GNC5_10 z19 MS2 ECD 30ms + ccCID 31eV_001-242_calibrated

Proteoform:

Index	Protein	SC [%]	SVP [%]	IC [%]	MS Score [a.u.]	Var. Mods	Mono Mass [Da]	Chemical Formula
6	H31Kac1	65.7	18.5	22.0	14.4	Ace: K27	15305.4534	C671H1131N215O186S4

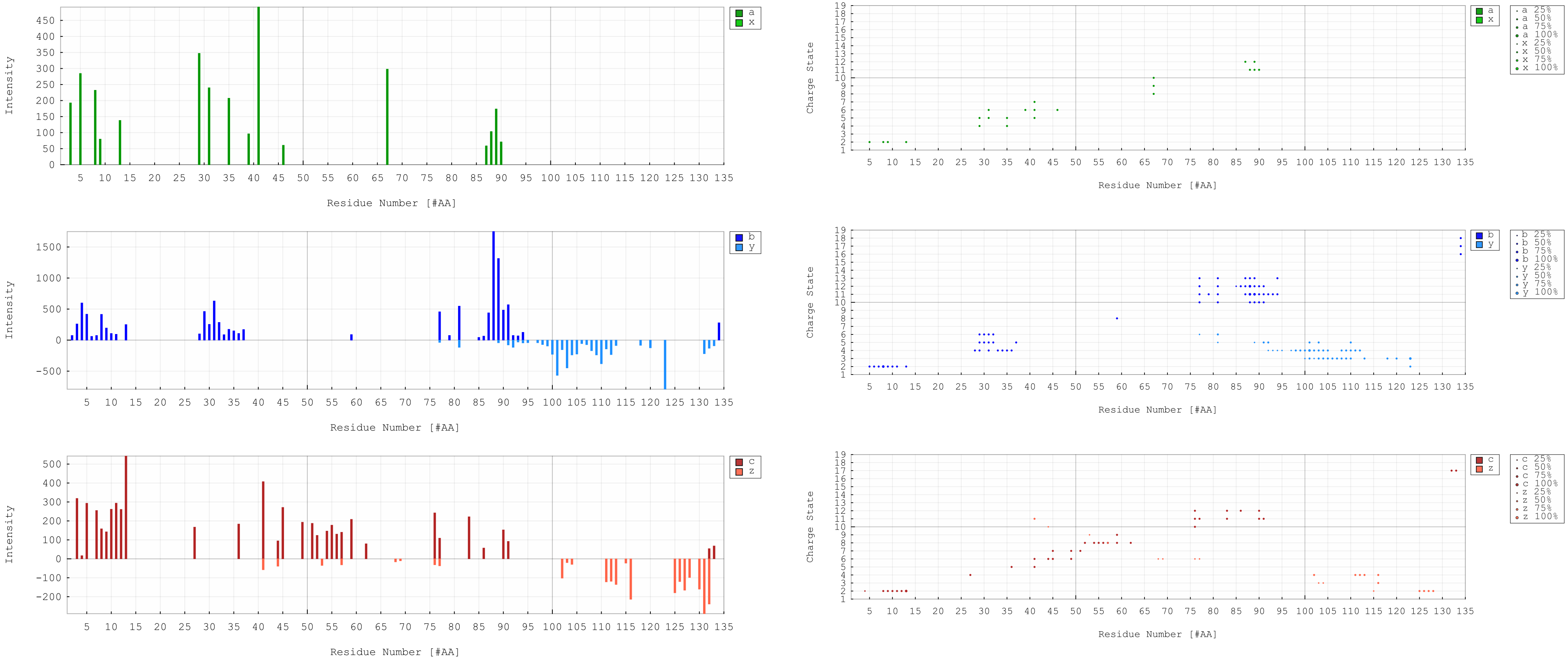

Proteoform:

Index	Protein	SC [%]	SVP [%]	IC [%]	MS Score [a.u.]	Var. Mods	Mono Mass [Da]	Chemical Formula
7	H31Kac1	59.7	18.5	20.0	12.0	Ace: K36	15305.4534	C671H1131N215O186S4

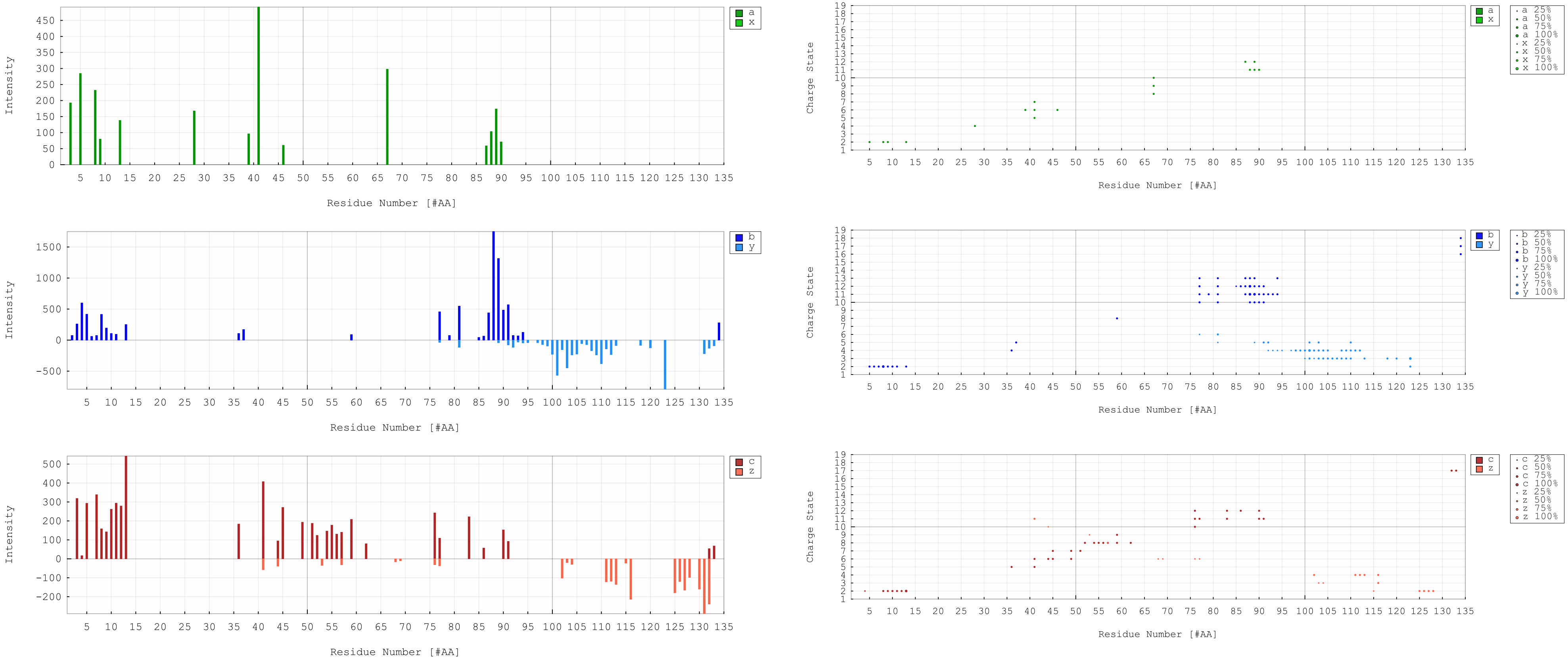

Top-Down Result OmniScape 2025
Sequence Confirmation
241210 H31-GNC5_10 z19 MS2 ECD 30ms + ccCID 31eV_001-242_calibrated

Proteoform:

Index	Protein	SC [%]	SVP [%]	IC [%]	MS Score [a.u.]	Var. Mods	Mono Mass [Da]	Chemical Formula
8	H31Kac1	59.0	18.5	19.8	11.7	Ace: K37	15305.4534	C671H1131N215O186S4

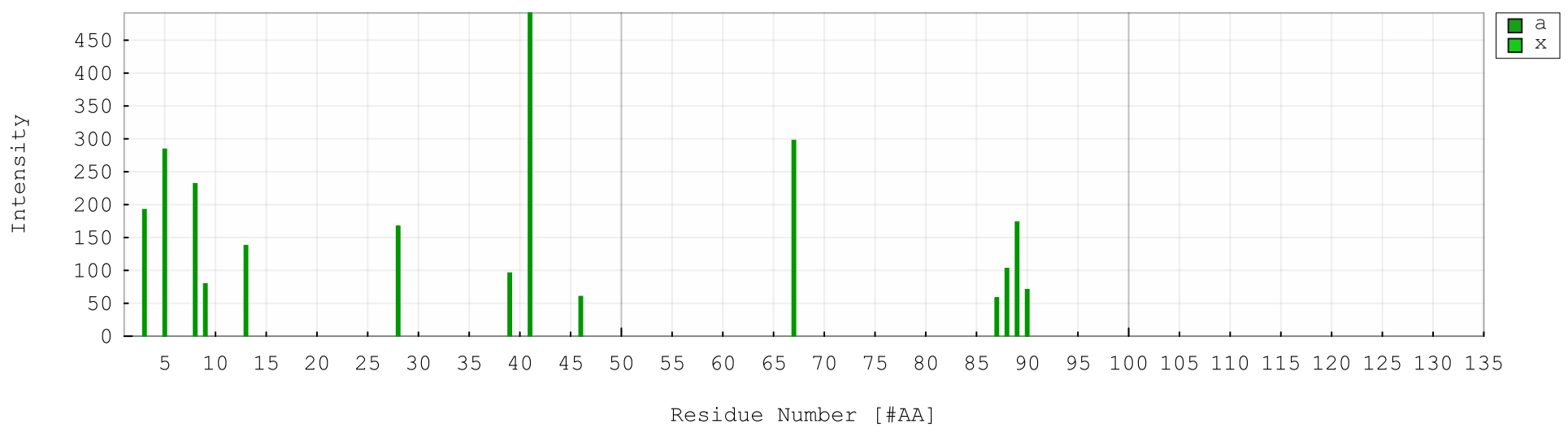

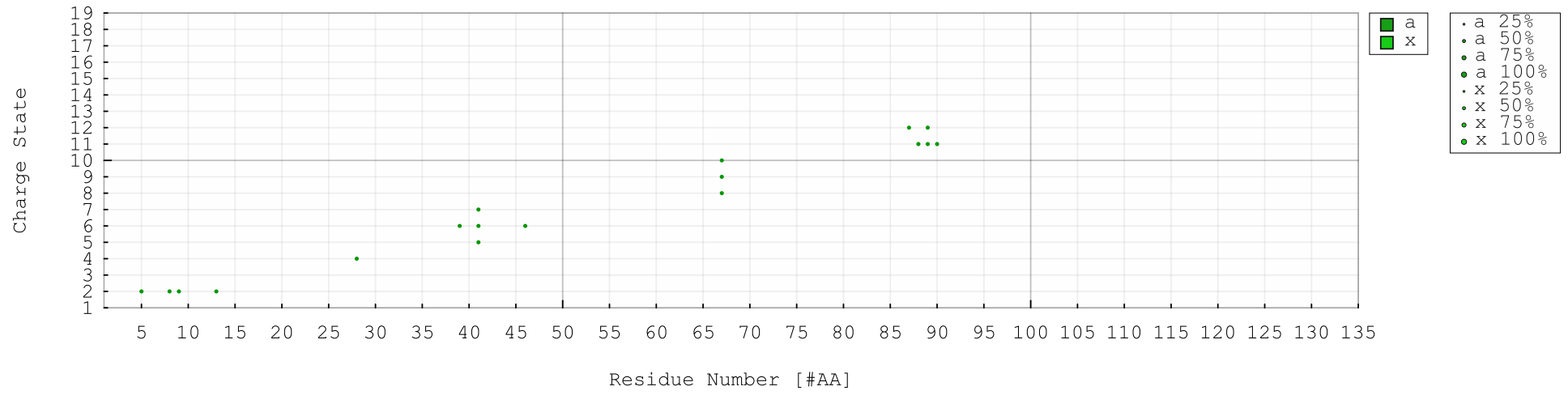

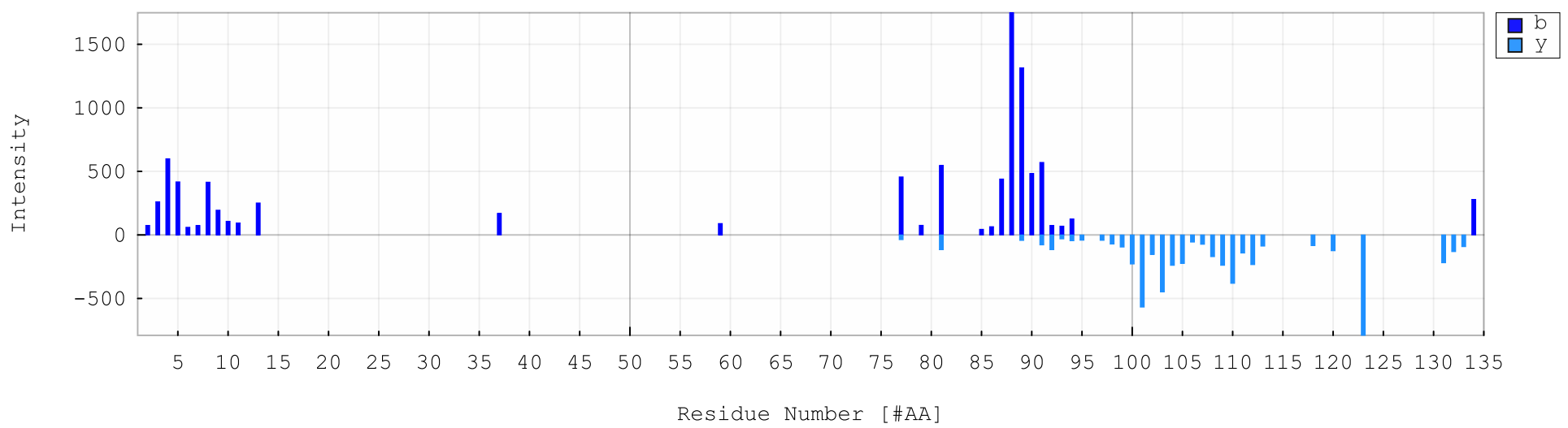

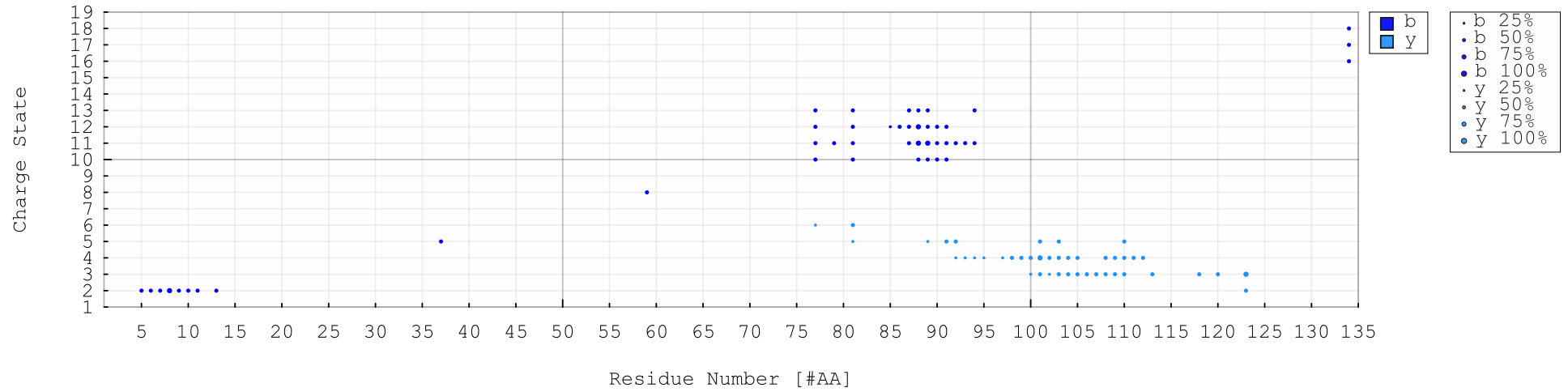

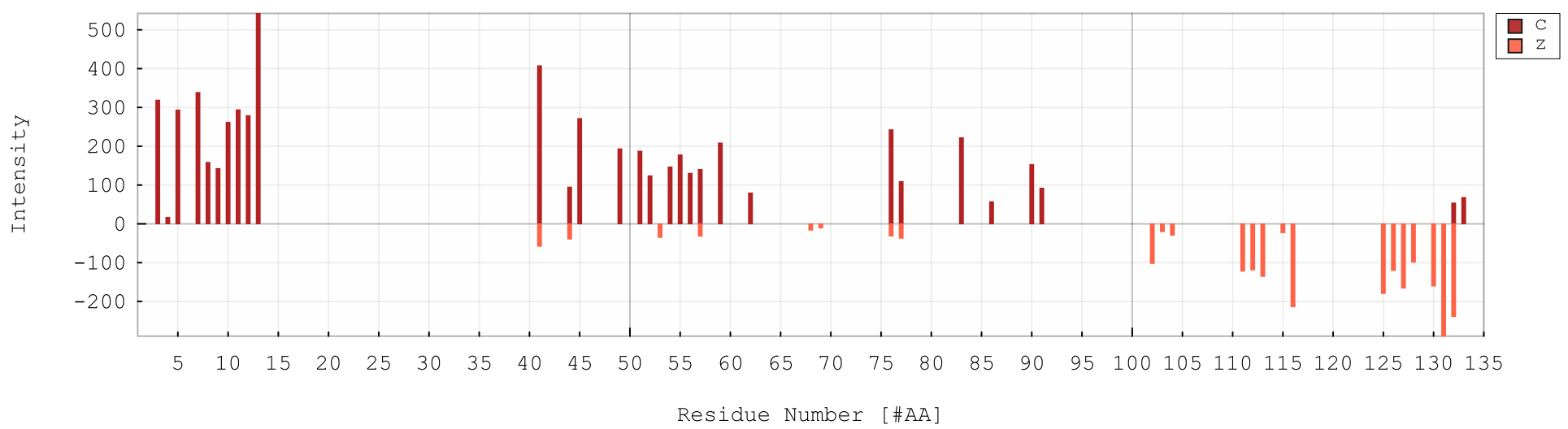

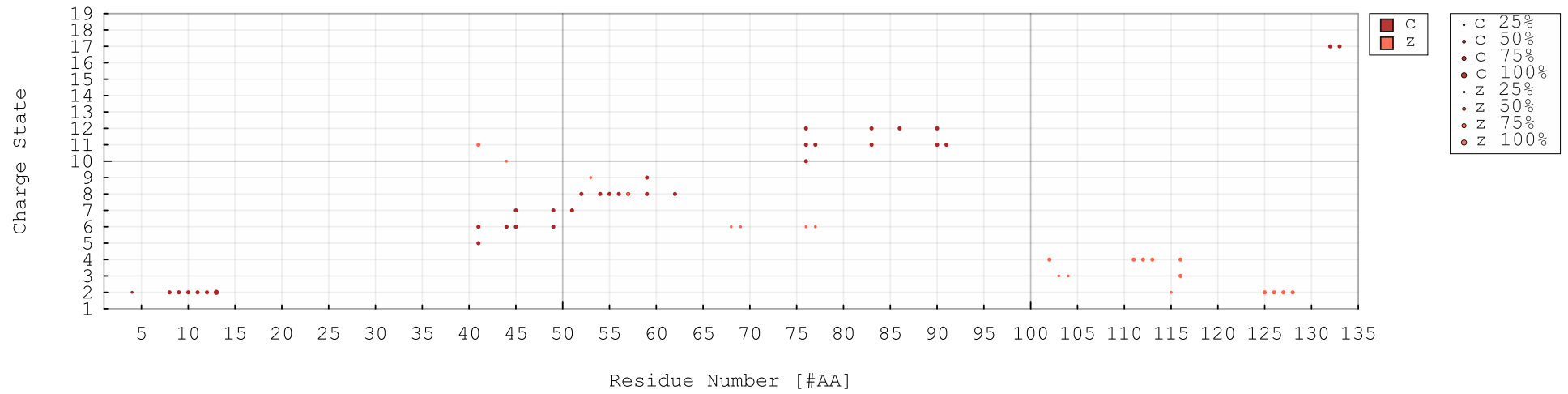

Top-Down Result OmniScape 2025
Sequence Confirmation
241210 H31-GNC5_10 z19 MS2 ECD 30ms + ccCID 31eV_001-242_calibrated

Proteoform:	Index	Protein	SC [%]	SVP [%]	IC [%]	MS Score [a.u.]	Var. Mods	Mono Mass [Da]	Chemical Formula
	9	H31Kac1	50.7	18.5	17.8	9.0	Ace: K56	15305.4534	C671H1131N215O186S4

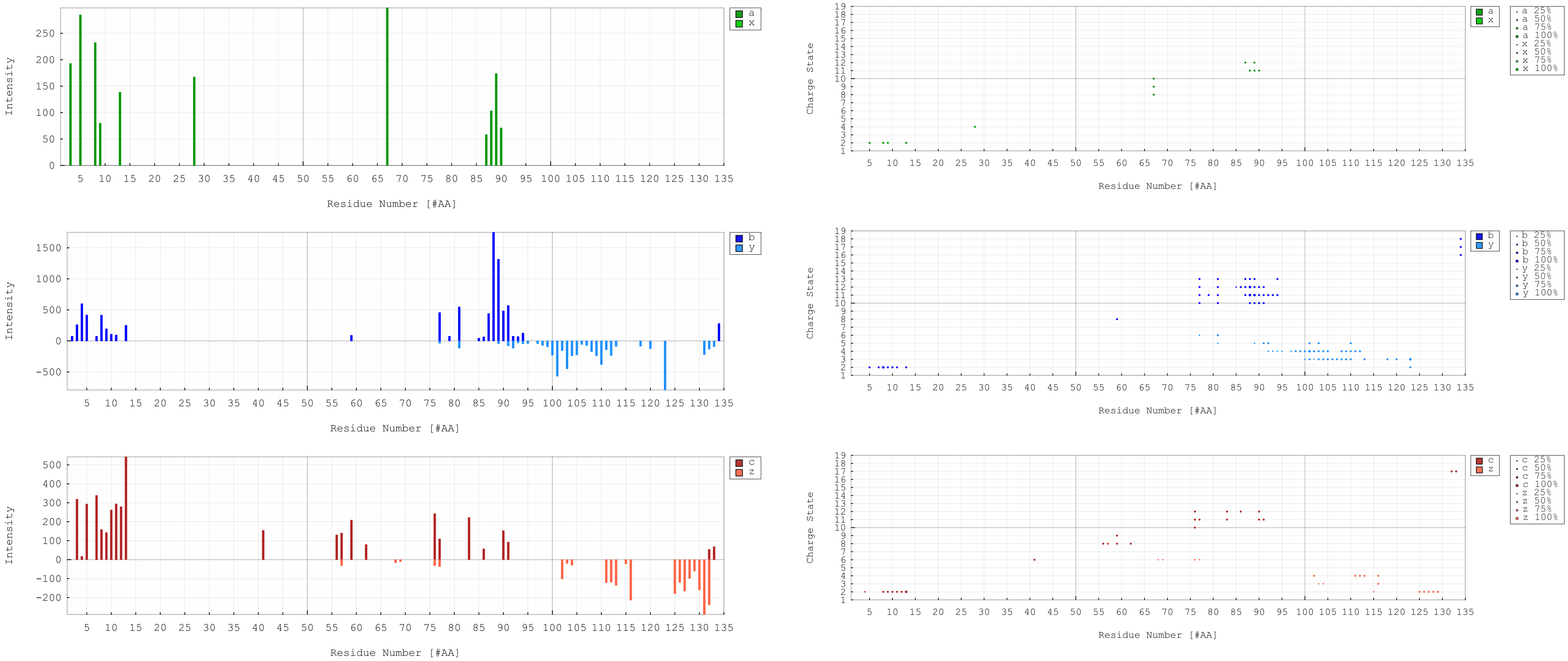

241210 H31-GNC5_10 z19 MS2 ECD 30ms + ccCID 31eV_001-242_calibrated

Proteoform:

Index	Protein	SC [%]	SVP [%]	IC [%]	MS Score [a.u.]	Var. Mods	Mono Mass [Da]	Chemical Formula
10	H31Kac1	48.5	18.5	17.2	8.4	Ace: K64	15305.4534	C671H1131N215O186S4

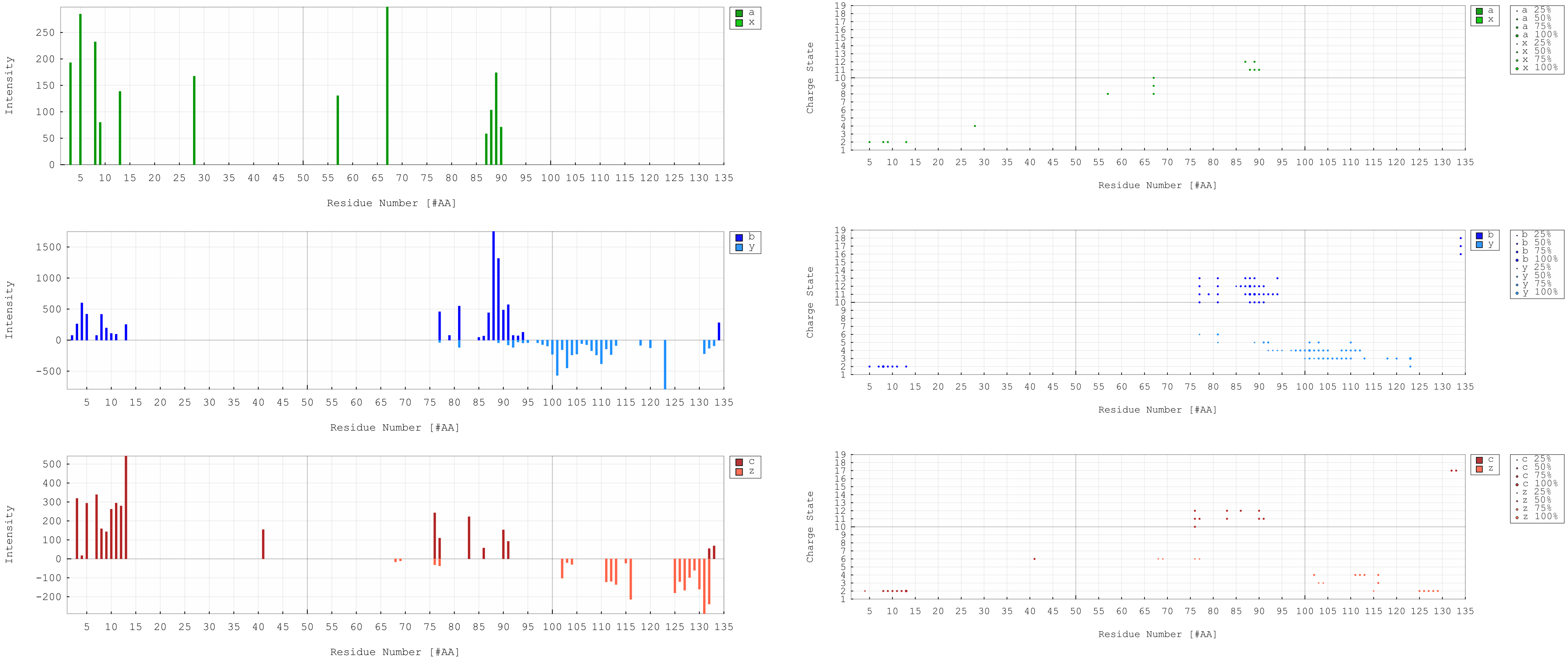

Supplemental Data S2

H3.1 - GCN5, 10 min

**Report**	2 of 7
**Experiment**	MS2
**Histone/sample**	H3.1
**Enzyme**	GCN5
**Reaction/source**	10 min
**Precursor**	H3.1Kac1
**Charge state**	19+
**Fragmentation / workflow**	ECD
**Original file**	MS2_H3.1Kac1_19+_ECD.pdf

Project Information

Project Directory: C:/Users/LAB-ADMIN
Processing date: Tue Feb 10 11:56:26 2026

Sample Info & Protocols

Name: Top-Down Protein Sequencing ExD

Dataset

Imported spectrum file name:	241210 H31-GNC5_10 z19 isCID 40eV MS2 ECD 50ms_001-247_calibrated.txt
Dataset name:	Information is only available from imported JPEAKS files
Date acquired:	Information is only available from imported JPEAKS files
Date imported:	Information is only available from imported JPEAKS files
Instrument Name:	Information is only available from imported JPEAKS files
Instrument Serial Number:	Information is only available from imported JPEAKS files

Workflow Result Info

Analysis: 241210 H31-GNC5_10 z19 isCID 40eV MS2 ECD 50ms_001-247_calibrated
Location: C:/Users/LAB-ADMIN
Method: -

Method Information

Name: Unnamed

Spectral Parameters

Mass Error [ppm]: 6
Intensity Deviation [au]: 0.15
Min/Max Charges: 1-19
Experiment type: ExD

a ions	b ions	c ions	x ions	y ions	z ions	satellite	internal	precursor
a	b	c		y	z+1			M
a+1		c+1			z+2			M+-2H

Noise threshold calculation: Simple moving average noise threshold (SMA)
Window size [datapoints]: 600
Noise curve offset: 3.3279638
SVP parameters: terminal gap: 2, internal gap: 1

Proteoforms

(top 10)

Index	Parent Sequence	Chain	Var. Mods	Mono Mass [Da]	Chemical Composition
1	H31Kac1	H31Kac1K14[Ace]	Ace: K14	15305.4534	C671H1131N215O186S4
2	H31Kac1	H31Kac1K18[Ace]	Ace: K18	15305.4534	C671H1131N215O186S4
3	H31Kac1	H31Kac1K9[Ace]	Ace: K9	15305.4534	C671H1131N215O186S4
4	H31Kac1	H31Kac1K23[Ace]	Ace: K23	15305.4534	C671H1131N215O186S4
5	H31Kac1	H31Kac1K4[Ace]	Ace: K4	15305.4534	C671H1131N215O186S4
6	H31Kac1	H31Kac1K27[Ace]	Ace: K27	15305.4534	C671H1131N215O186S4
7	H31Kac1	H31Kac1K36[Ace]	Ace: K36	15305.4534	C671H1131N215O186S4
8	H31Kac1	H31Kac1K37[Ace]	Ace: K37	15305.4534	C671H1131N215O186S4
9	H31Kac1	H31Kac1K56[Ace]	Ace: K56	15305.4534	C671H1131N215O186S4
10	H31Kac1	H31Kac1K64[Ace]	Ace: K64	15305.4534	C671H1131N215O186S4

Results

Proteoforms Table (top 10)

Index	Protein	SC [%]	SVP [%]	IC [%]	MS Score [a.u.]	Var. Mods	Mono Mass [Da]	Chemical Formula
1	H31Kac1	79.1	81.5	46.2	36.5	Ace: K14	15305.4534	C671H1131N215O186S4
2	H31Kac1	77.6	27.4	45.0	34.9	Ace: K18	15305.4534	C671H1131N215O186S4
3	H31Kac1	76.1	24.4	44.0	33.5	Ace: K9	15305.4534	C671H1131N215O186S4
4	H31Kac1	76.1	27.4	40.8	31.1	Ace: K23	15305.4534	C671H1131N215O186S4
5	H31Kac1	72.4	20.0	42.2	30.5	Ace: K4	15305.4534	C671H1131N215O186S4
6	H31Kac1	74.6	27.4	39.2	29.3	Ace: K27	15305.4534	C671H1131N215O186S4
7	H31Kac1	72.4	27.4	35.9	26.0	Ace: K36	15305.4534	C671H1131N215O186S4
8	H31Kac1	71.6	27.4	34.8	24.9	Ace: K37	15305.4534	C671H1131N215O186S4
9	H31Kac1	61.9	27.4	26.7	16.5	Ace: K56	15305.4534	C671H1131N215O186S4
10	H31Kac1	58.2	27.4	24.9	14.5	Ace: K64	15305.4534	C671H1131N215O186S4

Sequence

Name: H31Kac1

```
                 10                   20                   30                   40                   50                   60
A R T K Q T A R K S  T G G K A P R K Q L  A T K A A R K S A P  A T G G V K K P H R  Y R P G T V A L R E  I R R Y Q K S T E L
                 70                   80                   90                  100                  110                  120
L I R K L P F Q R L  V R E I A Q D F K T  D L R F Q S S A V M  A L Q E A C E A Y L  V G L F E D T N L C  A I H A K R V T I M
                130                  140                  150                  160                  170                  180
P K D I Q L A R R I  R G E R A
```

Modifications

Modification	Position	Type
Acetyl	4	variable
Acetyl	9	variable
Acetyl	14	variable
Acetyl	18	variable
Acetyl	23	variable
Acetyl	27	variable
Acetyl	36	variable
Acetyl	37	variable
Acetyl	56	variable
Acetyl	64	variable
Acetyl	79	variable

Modification	Position	Type
Acetyl	115	variable
Acetyl	122	variable

Sequence Map

Proteoform:

Index	Protein	SC [%]	SVP [%]	IC [%]	MS Score [a.u.]	Var. Mods	Mono Mass [Da]	Chemical Formula
1	H31Kac1	79.1	81.5	46.2	36.5	Ace: K14	15305.4534	C671H1131N215O186S4

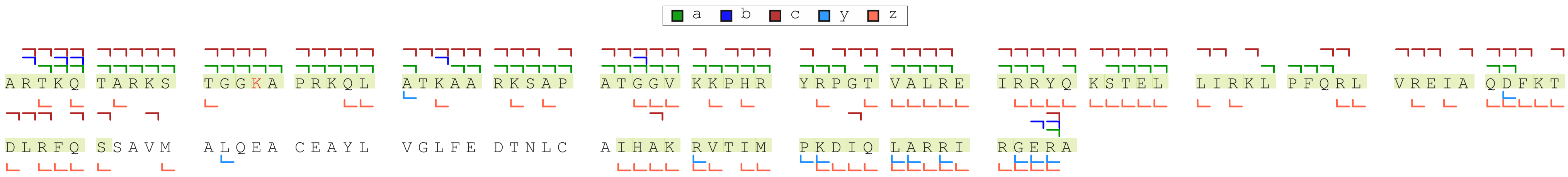

Top-Down Result OmniScape 2025
Sequence Confirmation
241210 H31-GNC5_10 z19 isCID 40eV MS2 ECD 50ms_001-247_calibrated

Proteoform:

Index	Protein	SC [%]	SVP [%]	IC [%]	MS Score [a.u.]	Var. Mods	Mono Mass [Da]	Chemical Formula
2	H31Kac1	77.6	27.4	45.0	34.9	Ace: K18	15305.4534	C671H1131N215O186S4

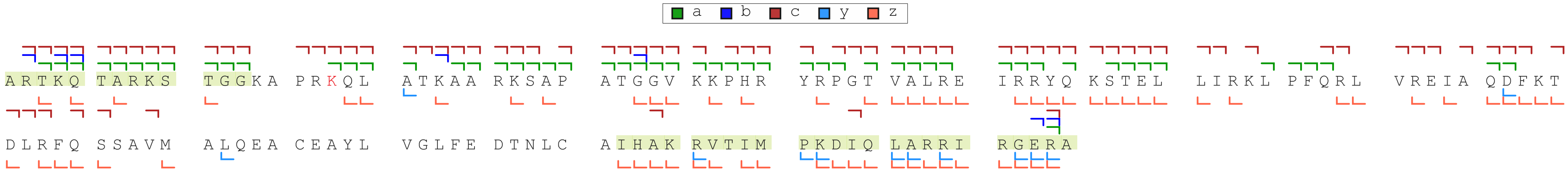

Proteoform:

Index	Protein	SC [%]	SVP [%]	IC [%]	MS Score [a.u.]	Var. Mods	Mono Mass [Da]	Chemical Formula
3	H31Kac1	76.1	24.4	44.0	33.5	Ace: K9	15305.4534	C671H1131N215O186S4

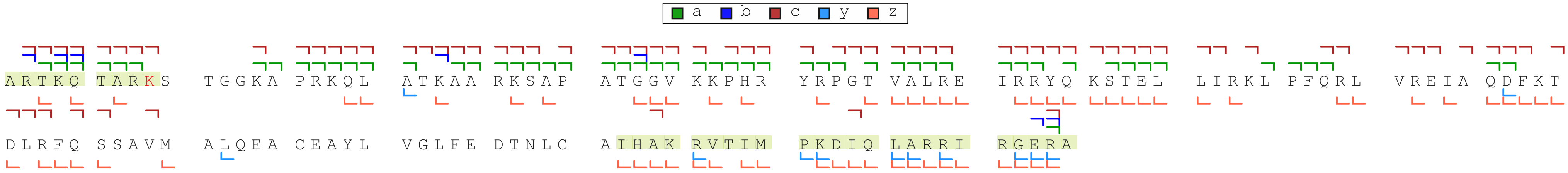

Proteoform:

Index	Protein	SC [%]	SVP [%]	IC [%]	MS Score [a.u.]	Var. Mods	Mono Mass [Da]	Chemical Formula
4	H31Kac1	76.1	27.4	40.8	31.1	Ace: K23	15305.4534	C671H1131N215O186S4

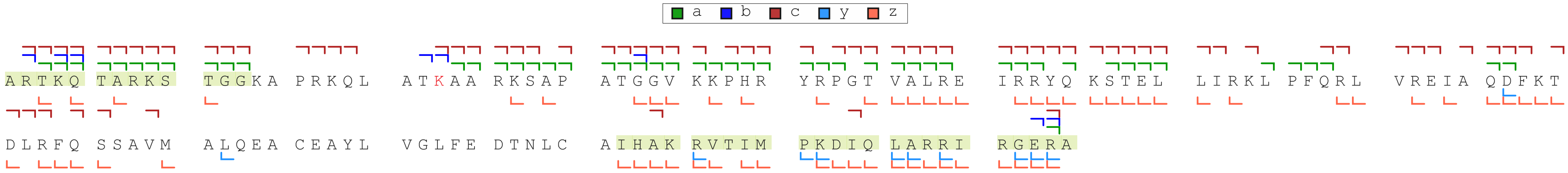

Proteoform:

Index	Protein	SC [%]	SVP [%]	IC [%]	MS Score [a.u.]	Var. Mods	Mono Mass [Da]	Chemical Formula
5	H31Kac1	72.4	20.0	42.2	30.5	Ace: K4	15305.4534	C671H1131N215O186S4

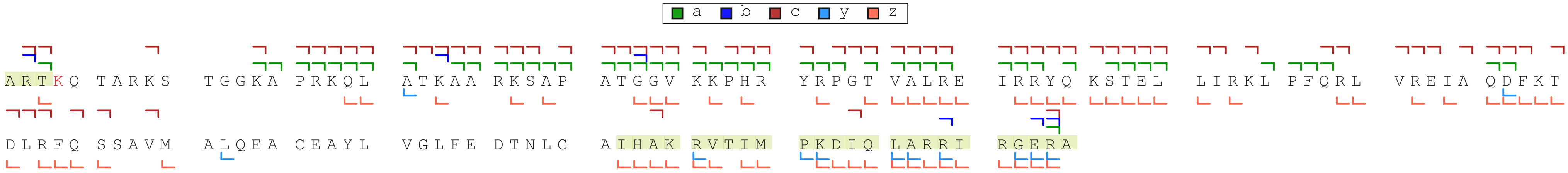

Proteoform:

Index	Protein	SC [%]	SVP [%]	IC [%]	MS Score [a.u.]	Var. Mods	Mono Mass [Da]	Chemical Formula
6	H31Kac1	74.6	27.4	39.2	29.3	Ace: K27	15305.4534	C671H1131N215O186S4

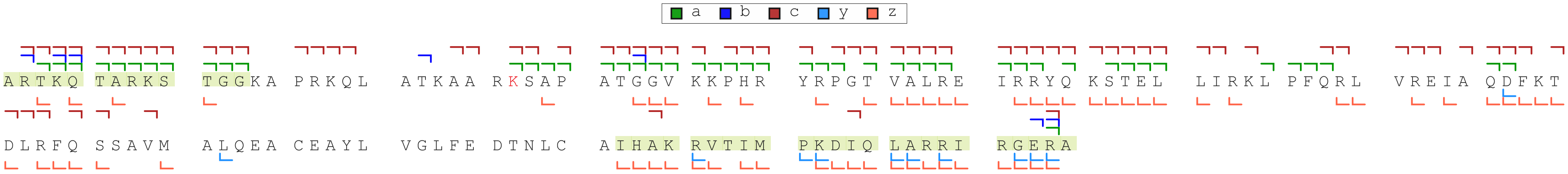

Proteoform:

Index	Protein	SC [%]	SVP [%]	IC [%]	MS Score [a.u.]	Var. Mods	Mono Mass [Da]	Chemical Formula
7	H31Kac1	72.4	27.4	35.9	26.0	Ace: K36	15305.4534	C671H1131N215O186S4

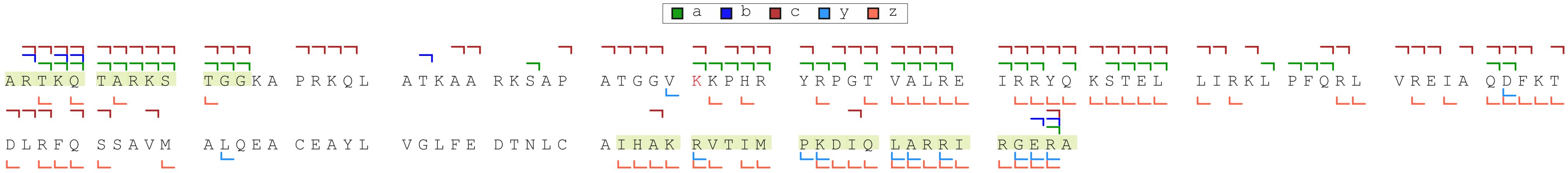

Proteoform:

Index	Protein	SC [%]	SVP [%]	IC [%]	MS Score [a.u.]	Var. Mods	Mono Mass [Da]	Chemical Formula
8	H31Kac1	71.6	27.4	34.8	24.9	Ace: K37	15305.4534	C671H1131N215O186S4

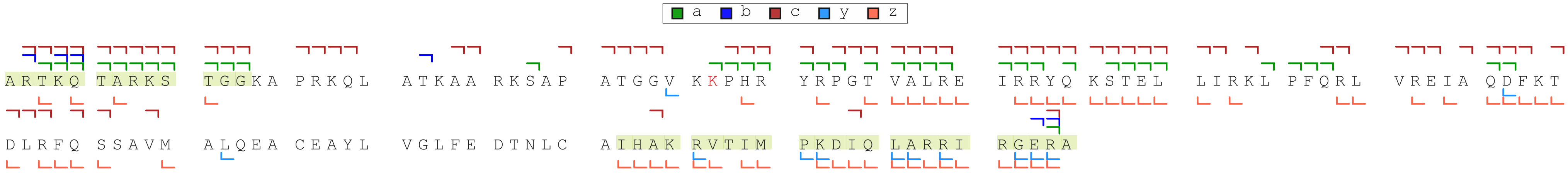

Top-Down Result OmniScape 2025
Sequence Confirmation
241210 H31-GNC5_10 z19 isCID 40eV MS2 ECD 50ms_001-247_calibrated

Proteoform:

Index	Protein	SC [%]	SVP [%]	IC [%]	MS Score [a.u.]	Var. Mods	Mono Mass [Da]	Chemical Formula
9	H31Kac1	61.9	27.4	26.7	16.5	Ace: K56	15305.4534	C671H1131N215O186S4

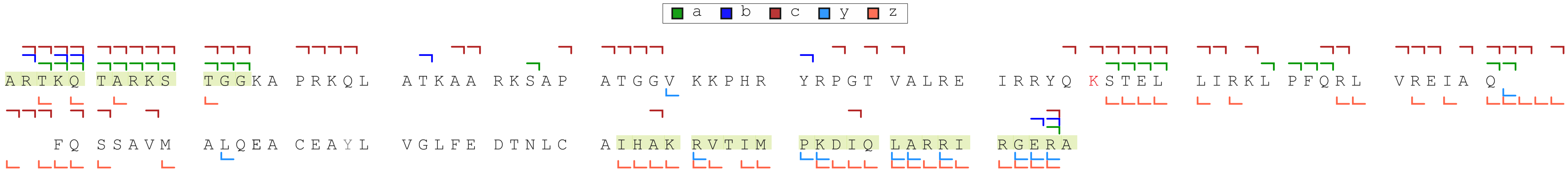

Proteoform:

Index	Protein	SC [%]	SVP [%]	IC [%]	MS Score [a.u.]	Var. Mods	Mono Mass [Da]	Chemical Formula
10	H31Kac1	58.2	27.4	24.9	14.5	Ace: K64	15305.4534	C671H1131N215O186S4

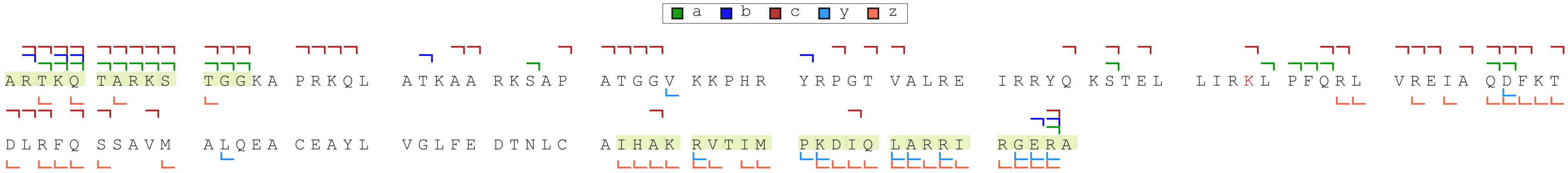

Error Plot

Proteoform:

Index	Protein	SC [%]	SVP [%]	IC [%]	MS Score [a.u.]	Var. Mods	Mono Mass [Da]	Chemical Formula
1	H31Kac1	79.1	81.5	46.2	36.5	Ace: K14	15305.4534	C671H1131N215O186S4

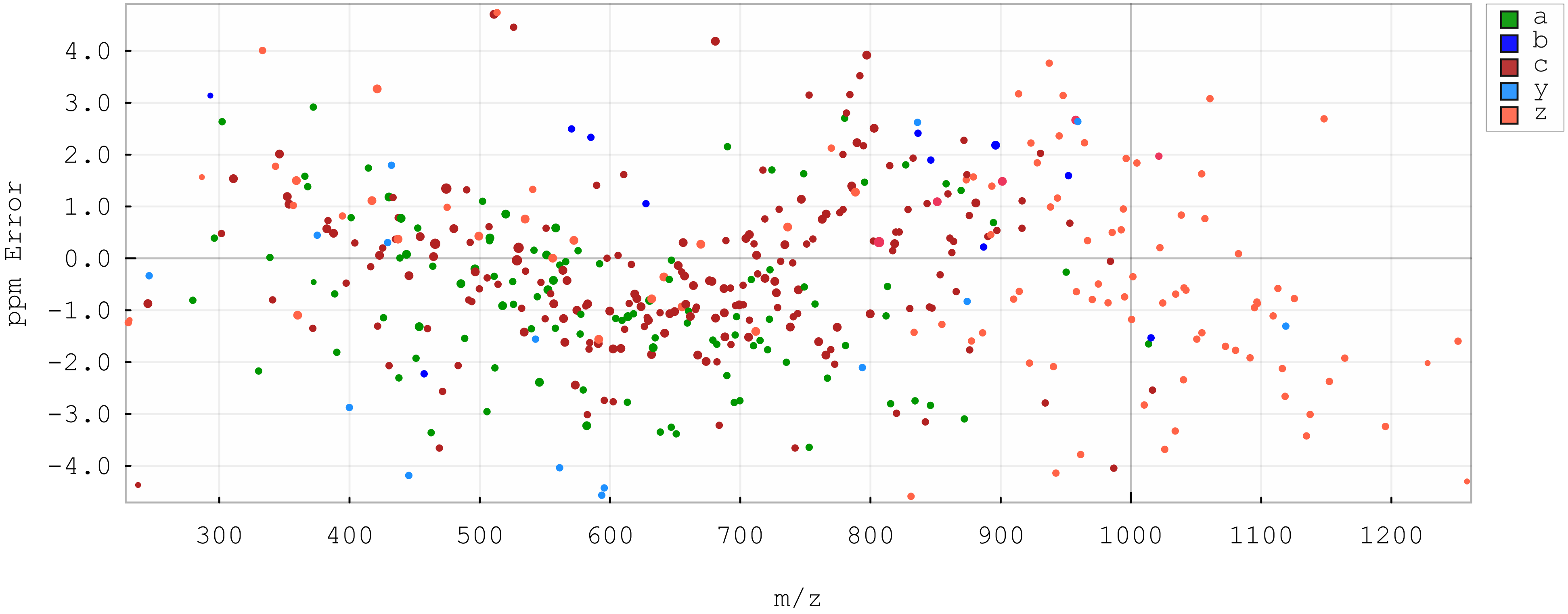

Proteoform:

Index	Protein	SC [%]	SVP [%]	IC [%]	MS Score [a.u.]	Var. Mods	Mono Mass [Da]	Chemical Formula
2	H31Kac1	77.6	27.4	45.0	34.9	Ace: K18	15305.4534	C671H1131N215O186S4

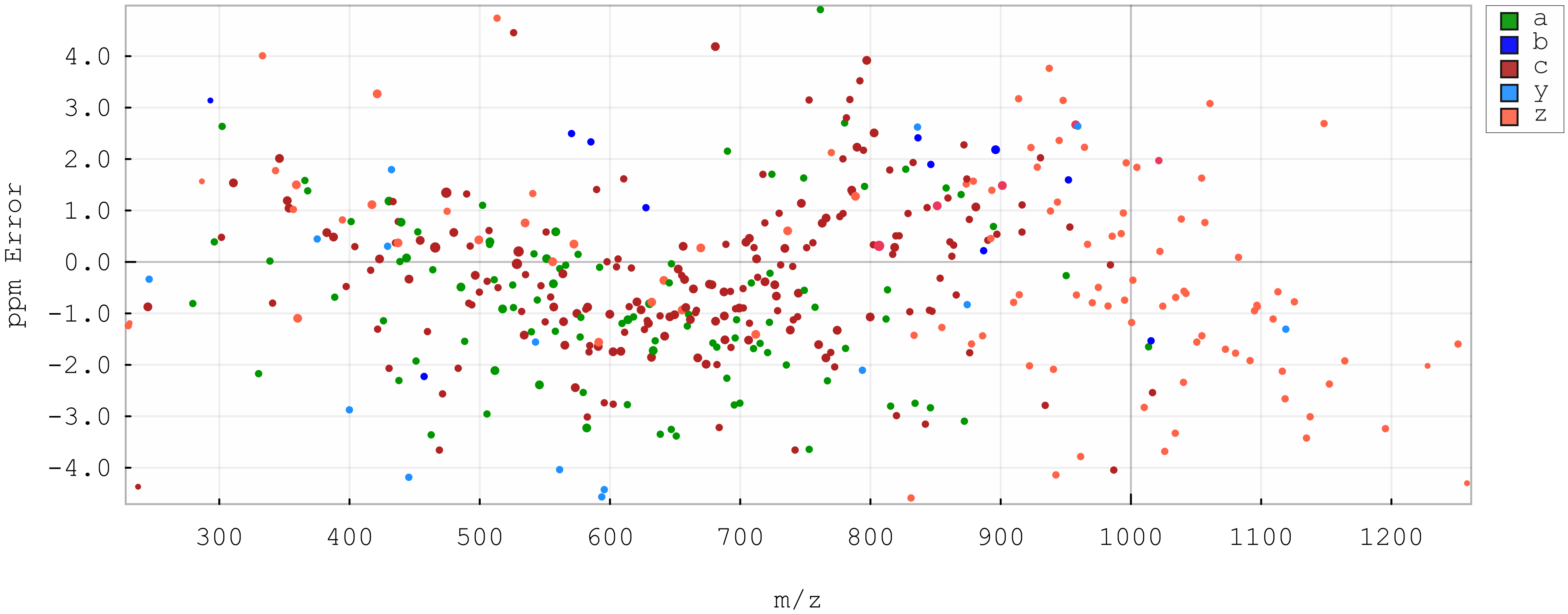

Proteoform:

Index	Protein	SC [%]	SVP [%]	IC [%]	MS Score [a.u.]	Var. Mods	Mono Mass [Da]	Chemical Formula
3	H31Kac1	76.1	24.4	44.0	33.5	Ace: K9	15305.4534	C671H1131N215O186S4

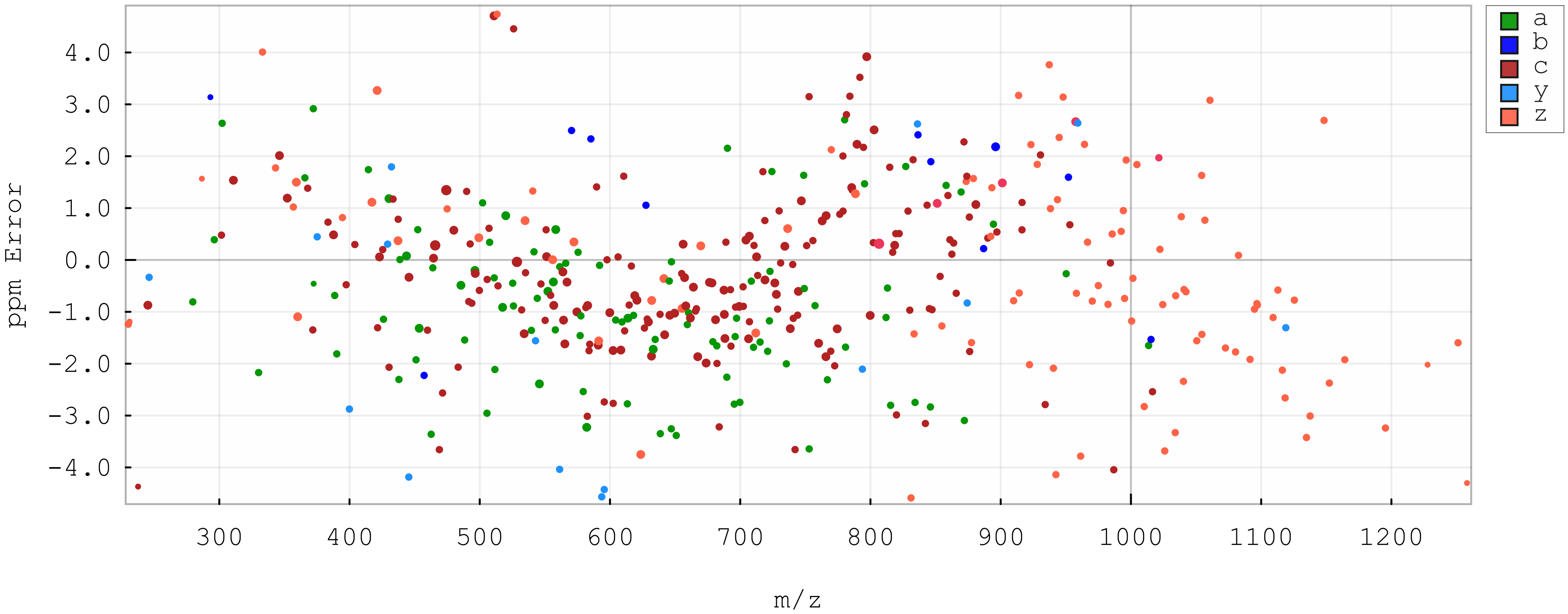

Proteoform:

Index	Protein	SC [%]	SVP [%]	IC [%]	MS Score [a.u.]	Var. Mods	Mono Mass [Da]	Chemical Formula
4	H31Kac1	76.1	27.4	40.8	31.1	Ace: K23	15305.4534	C671H1131N215O186S4

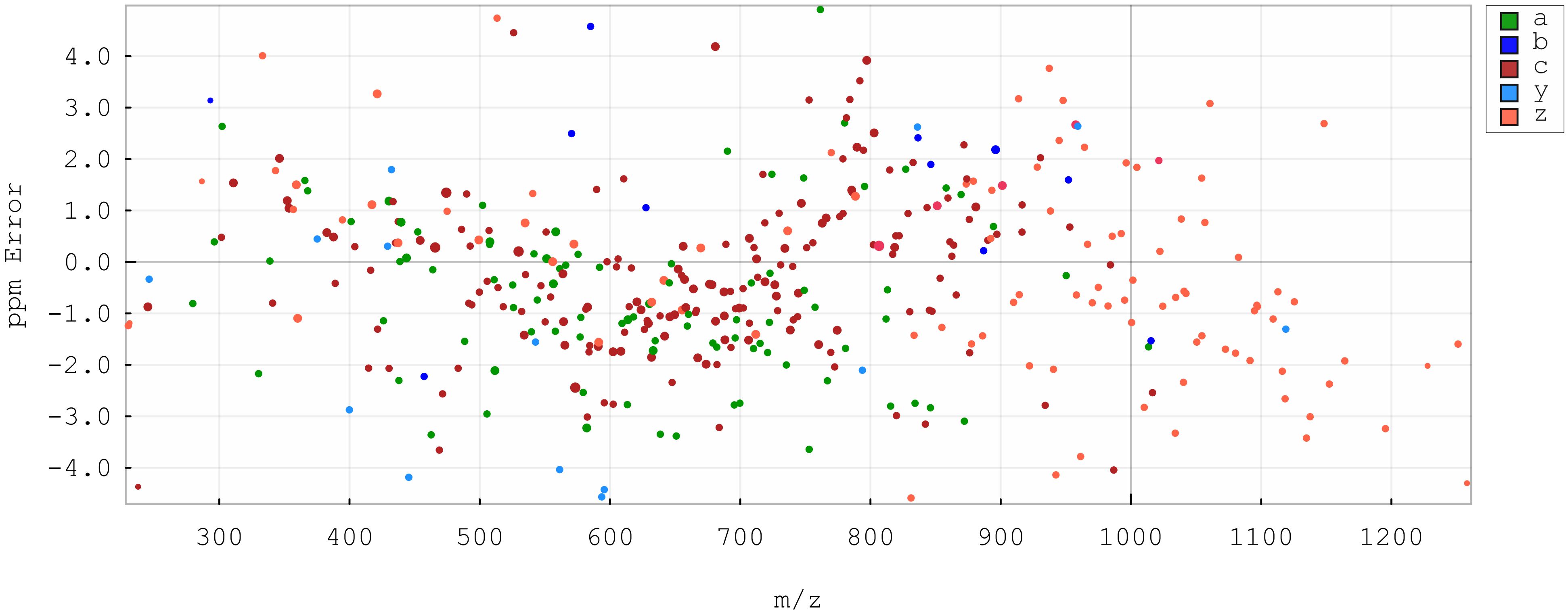

Proteoform:

Index	Protein	SC [%]	SVP [%]	IC [%]	MS Score [a.u.]	Var. Mods	Mono Mass [Da]	Chemical Formula
5	H31Kac1	72.4	20.0	42.2	30.5	Ace: K4	15305.4534	C671H1131N215O186S4

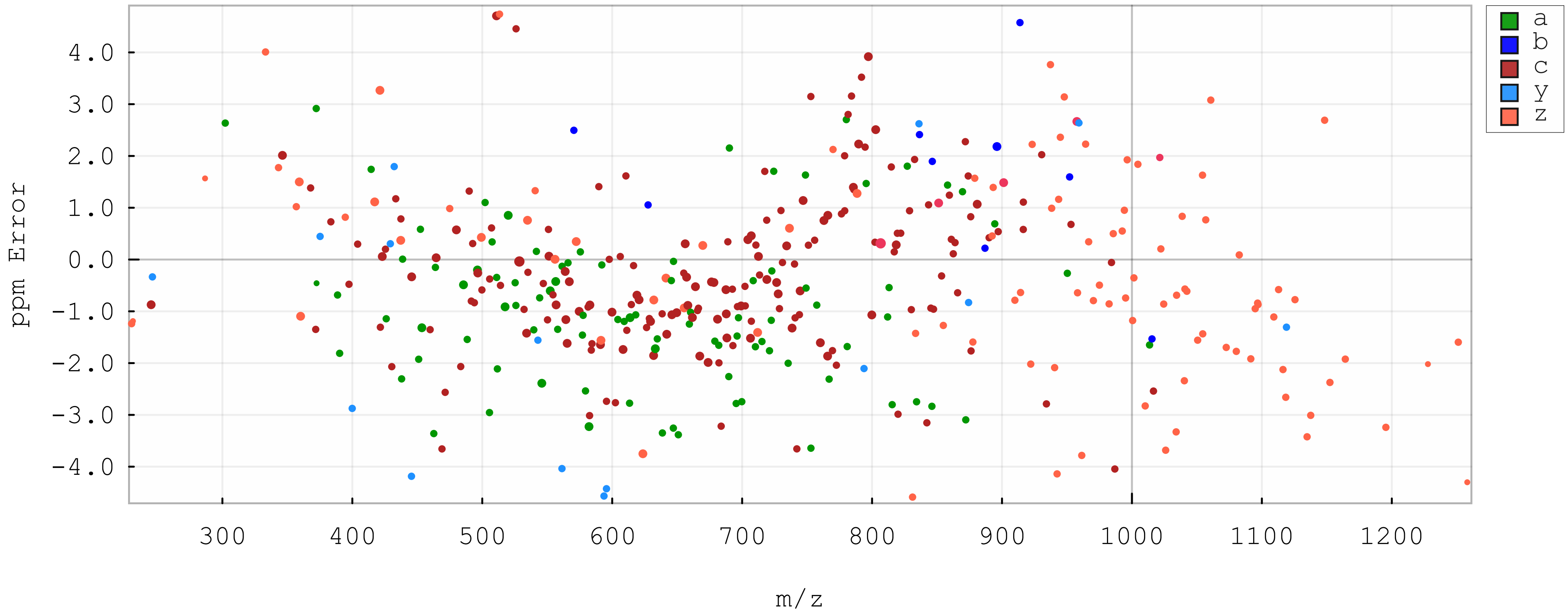

Proteoform:

Index	Protein	SC [%]	SVP [%]	IC [%]	MS Score [a.u.]	Var. Mods	Mono Mass [Da]	Chemical Formula
6	H31Kac1	74.6	27.4	39.2	29.3	Ace: K27	15305.4534	C671H1131N215O186S4

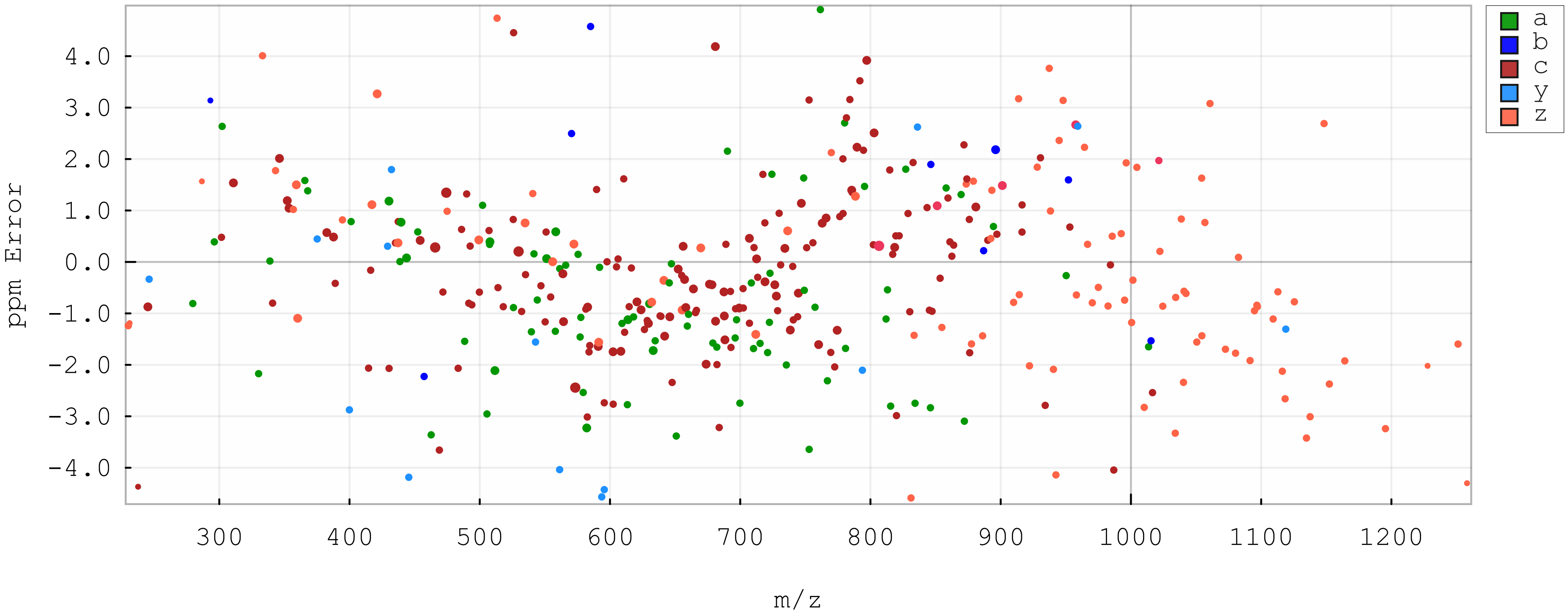

Proteoform:

Index	Protein	SC [%]	SVP [%]	IC [%]	MS Score [a.u.]	Var. Mods	Mono Mass [Da]	Chemical Formula
7	H31Kac1	72.4	27.4	35.9	26.0	Ace: K36	15305.4534	C671H1131N215O186S4

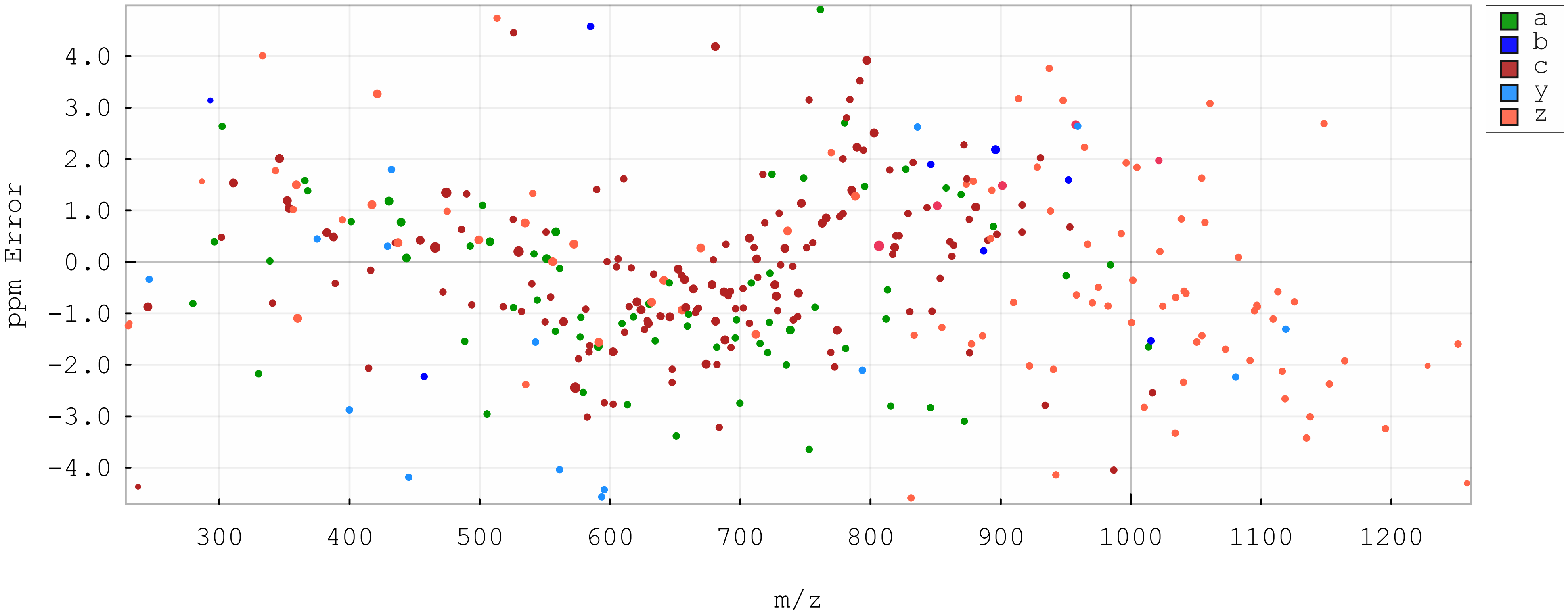

Proteoform:

Index	Protein	SC [%]	SVP [%]	IC [%]	MS Score [a.u.]	Var. Mods	Mono Mass [Da]	Chemical Formula
8	H31Kac1	71.6	27.4	34.8	24.9	Ace: K37	15305.4534	C671H1131N215O186S4

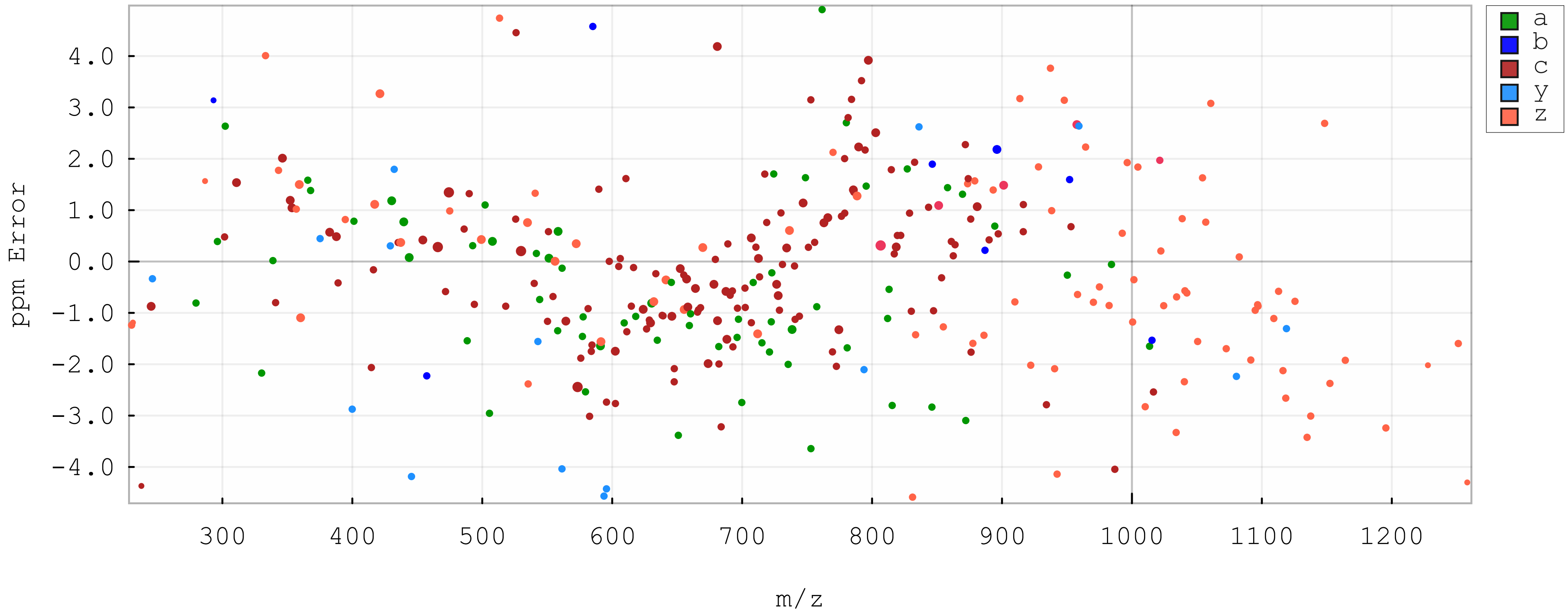

Proteoform:

Index	Protein	SC [%]	SVP [%]	IC [%]	MS Score [a.u.]	Var. Mods	Mono Mass [Da]	Chemical Formula
9	H31Kac1	61.9	27.4	26.7	16.5	Ace: K56	15305.4534	C671H1131N215O186S4

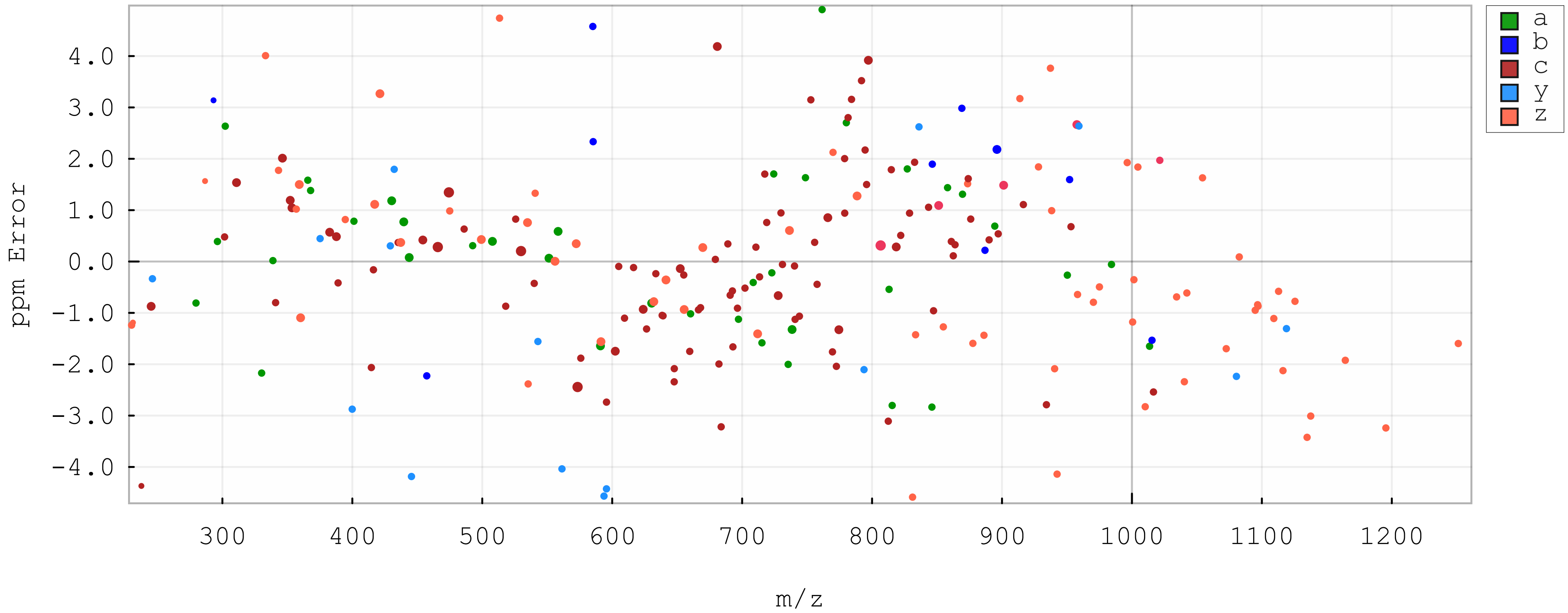

Proteoform:

Index	Protein	SC [%]	SVP [%]	IC [%]	MS Score [a.u.]	Var. Mods	Mono Mass [Da]	Chemical Formula
10	H31Kac1	58.2	27.4	24.9	14.5	Ace: K64	15305.4534	C671H1131N215O186S4

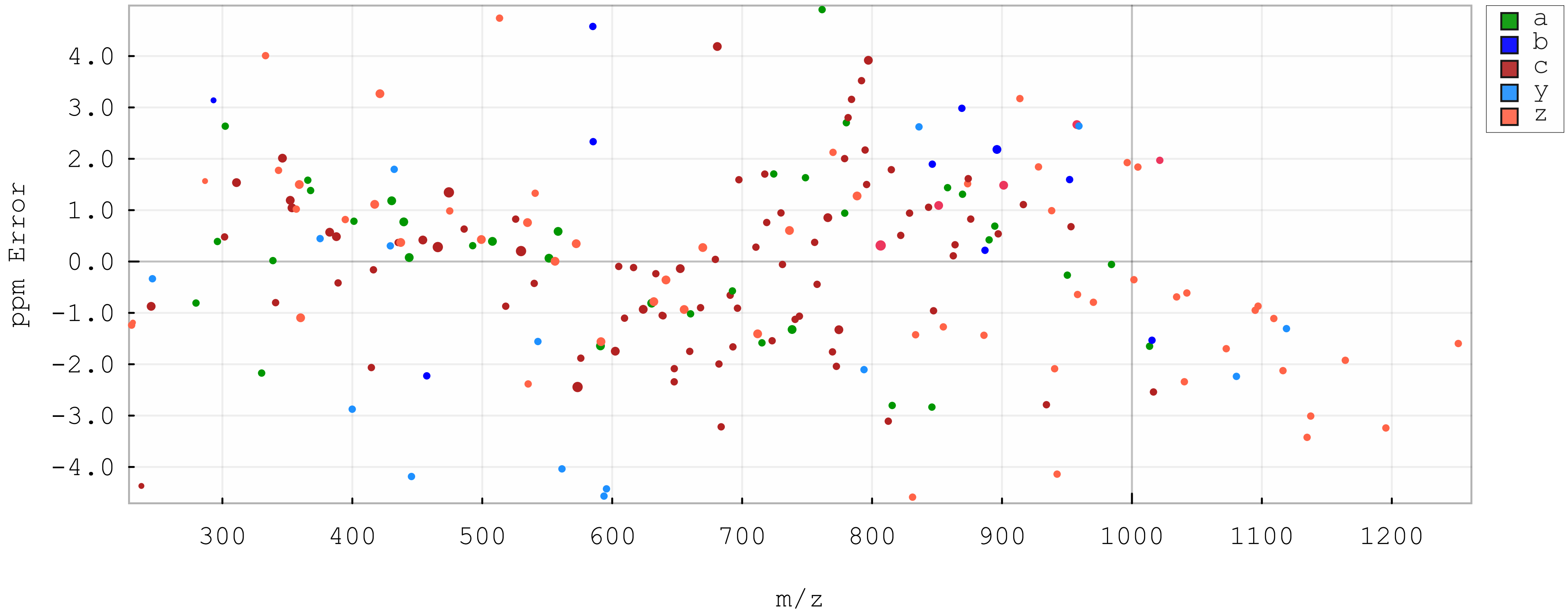

Primary Fragment Plots

Proteoform:

Index	Protein	SC [%]	SVP [%]	IC [%]	MS Score [a.u.]	Var. Mods	Mono Mass [Da]	Chemical Formula
1	H31Kac1	79.1	81.5	46.2	36.5	Ace: K14	15305.4534	C671H1131N215O186S4

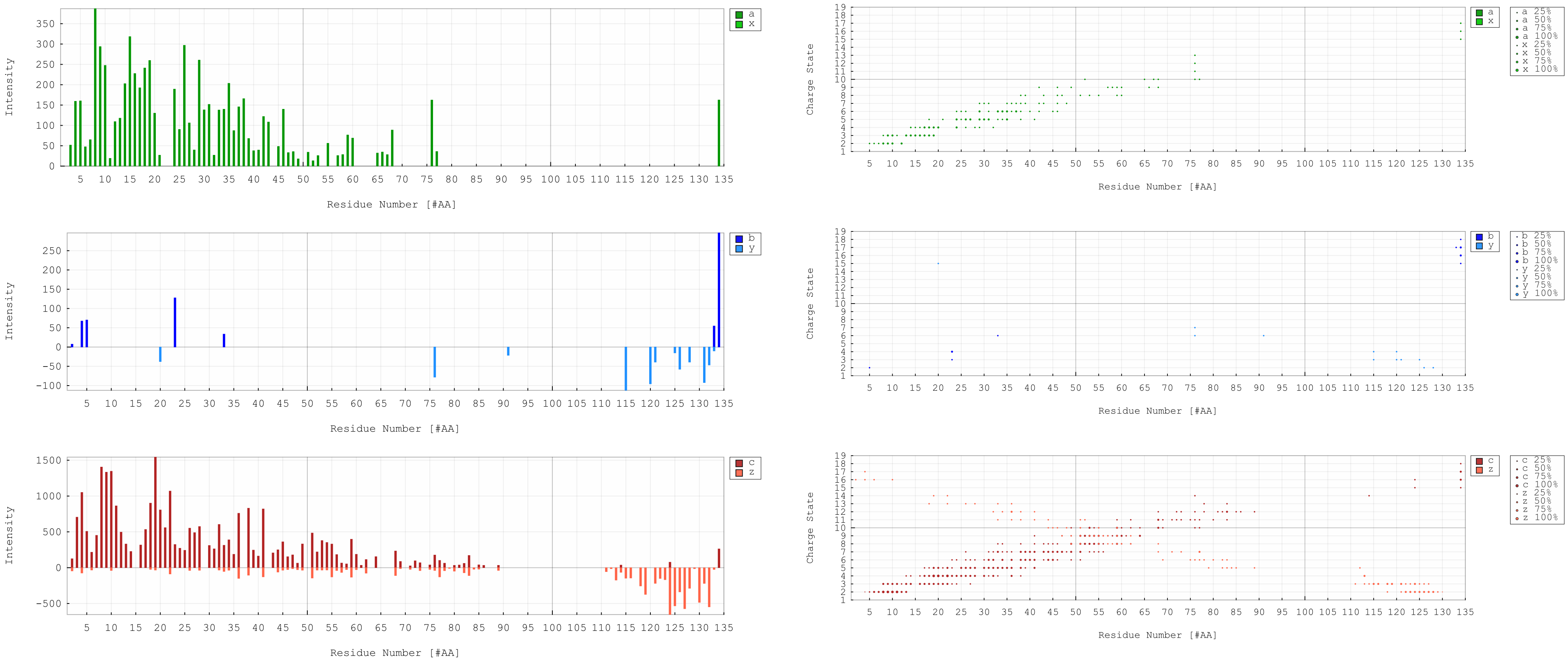

Top-Down Result OmniScape 2025
Sequence Confirmation
241210 H31-GNC5_10 z19 isCID 40eV MS2 ECD 50ms_001-247_calibrated

Proteoform:

Index	Protein	SC [%]	SVP [%]	IC [%]	MS Score [a.u.]	Var. Mods	Mono Mass [Da]	Chemical Formula
2	H31Kac1	77.6	27.4	45.0	34.9	Ace: K18	15305.4534	C671H1131N215O186S4

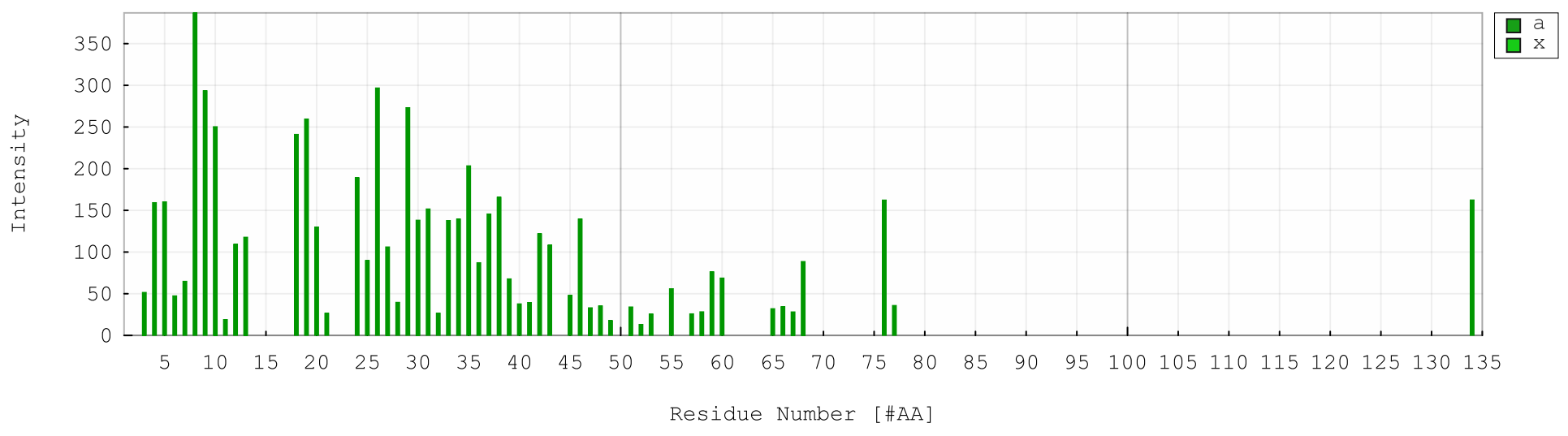

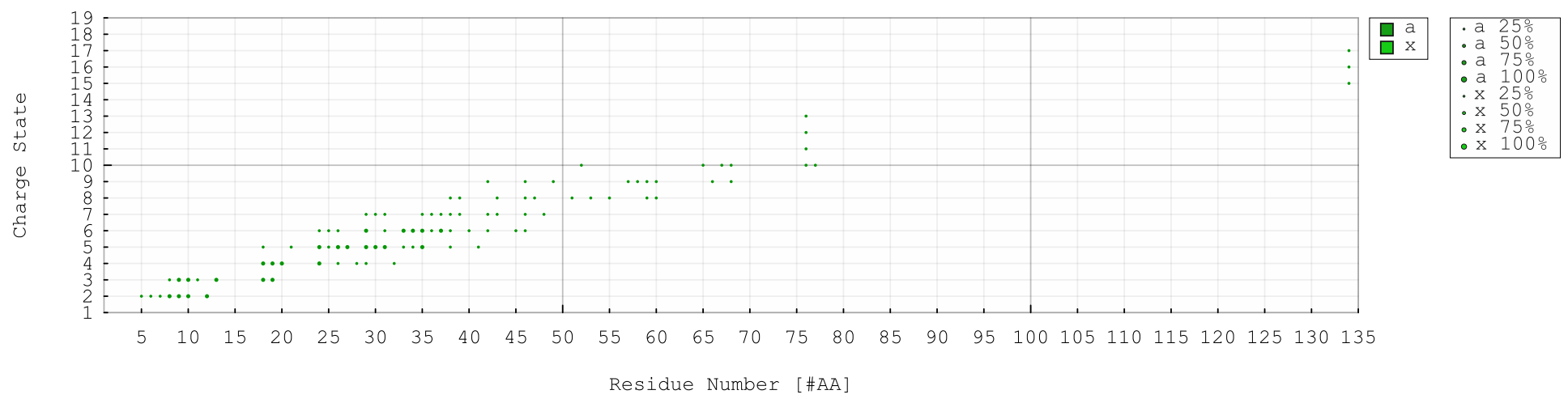

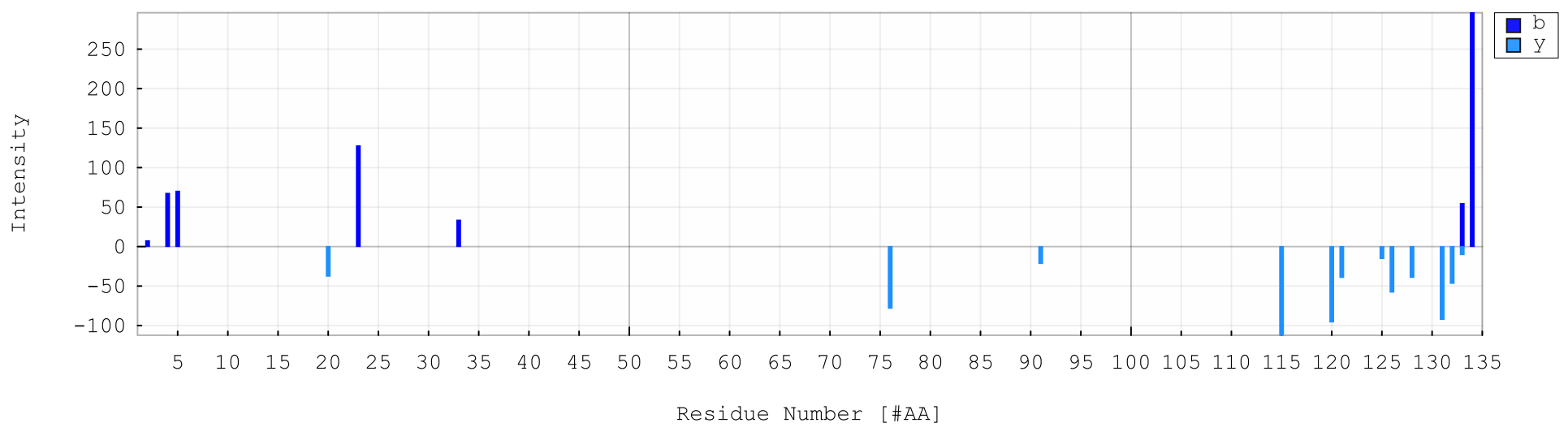

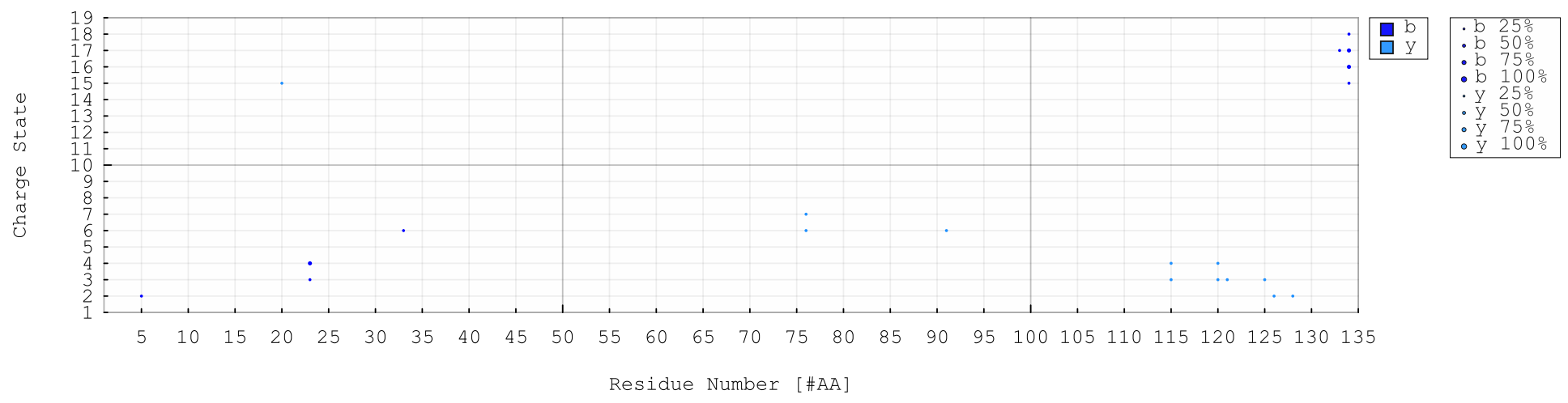

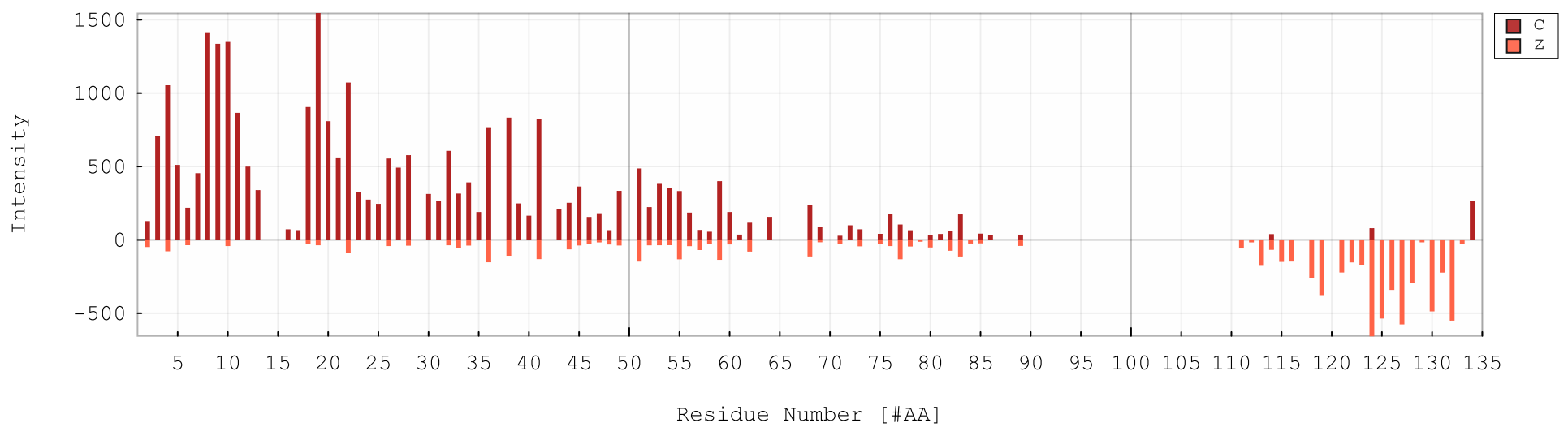

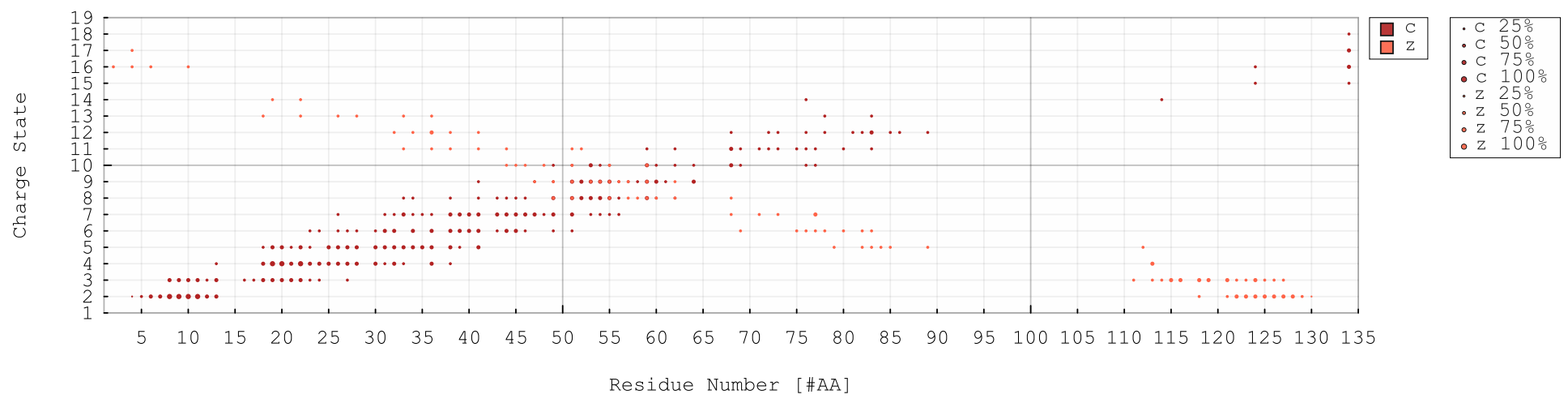

Top-Down Result OmniScape 2025
Sequence Confirmation
241210 H31-GNC5_10 z19 isCID 40eV MS2 ECD 50ms_001-247_calibrated

Proteoform:

Index	Protein	SC [%]	SVP [%]	IC [%]	MS Score [a.u.]	Var. Mods	Mono Mass [Da]	Chemical Formula
3	H31Kac1	76.1	24.4	44.0	33.5	Ace: K9	15305.4534	$C_{671}H_{1131}N_{215}O_{186}S_4$

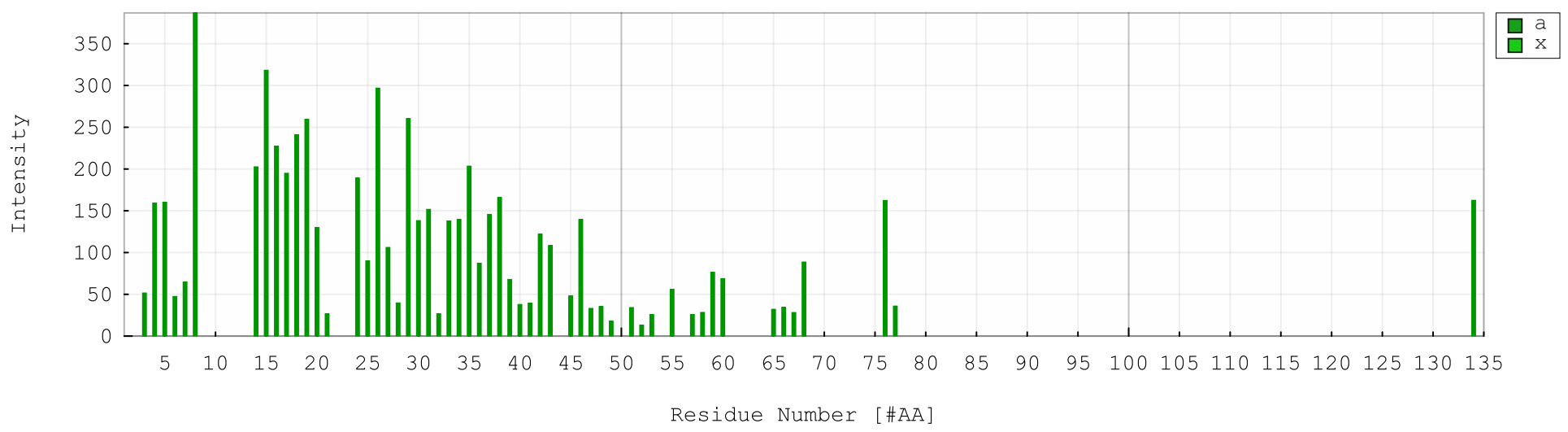

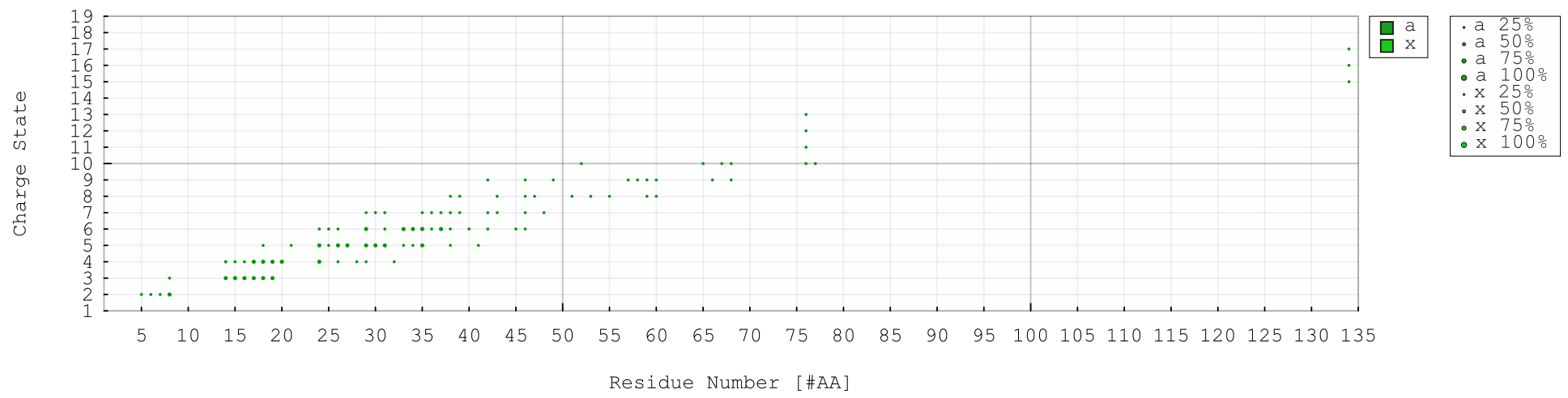

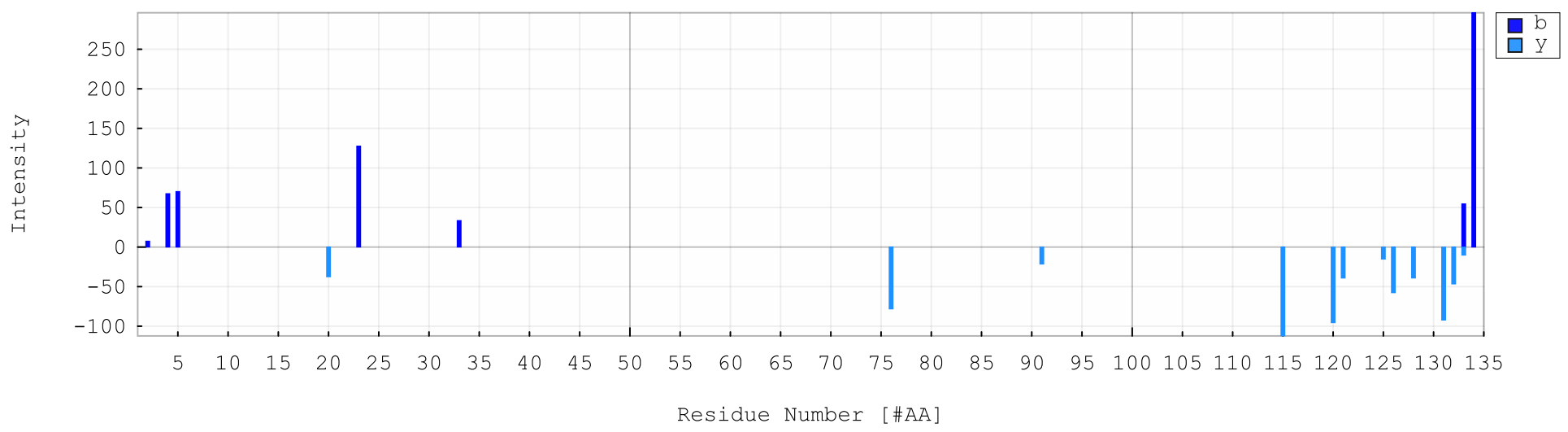

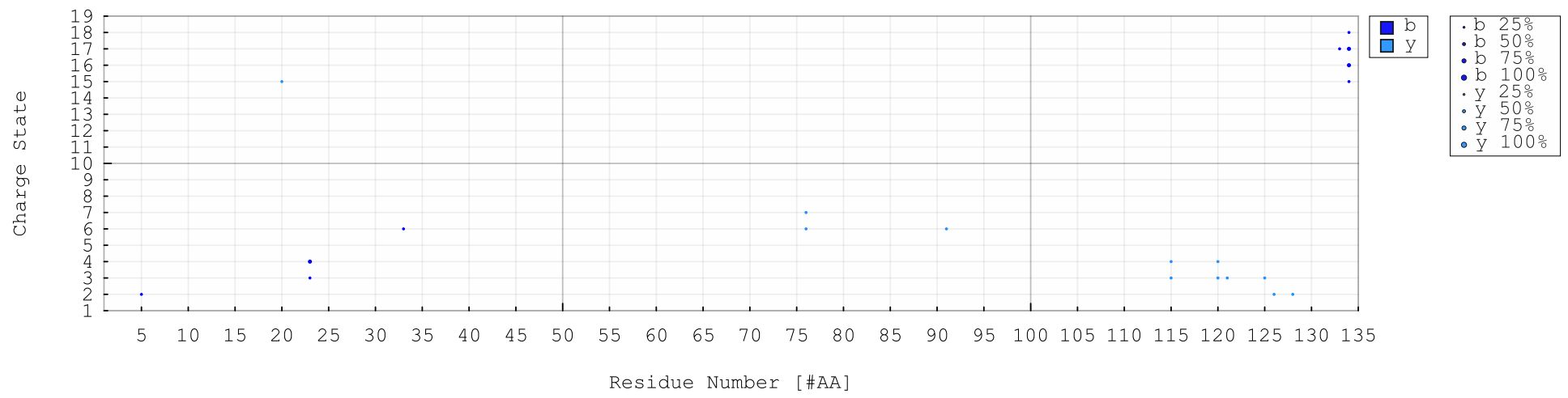

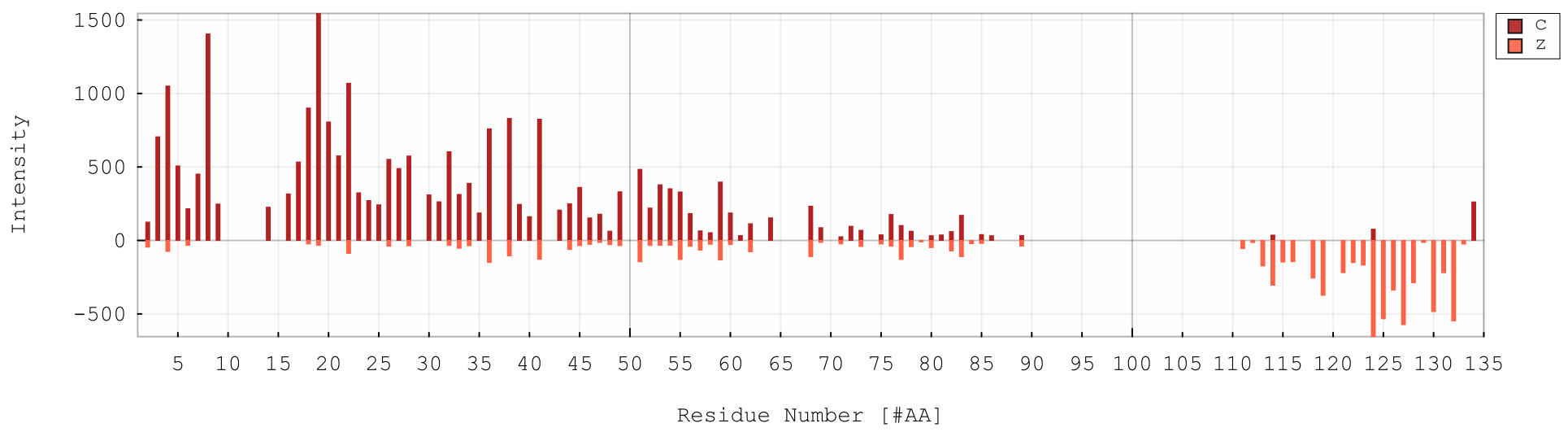

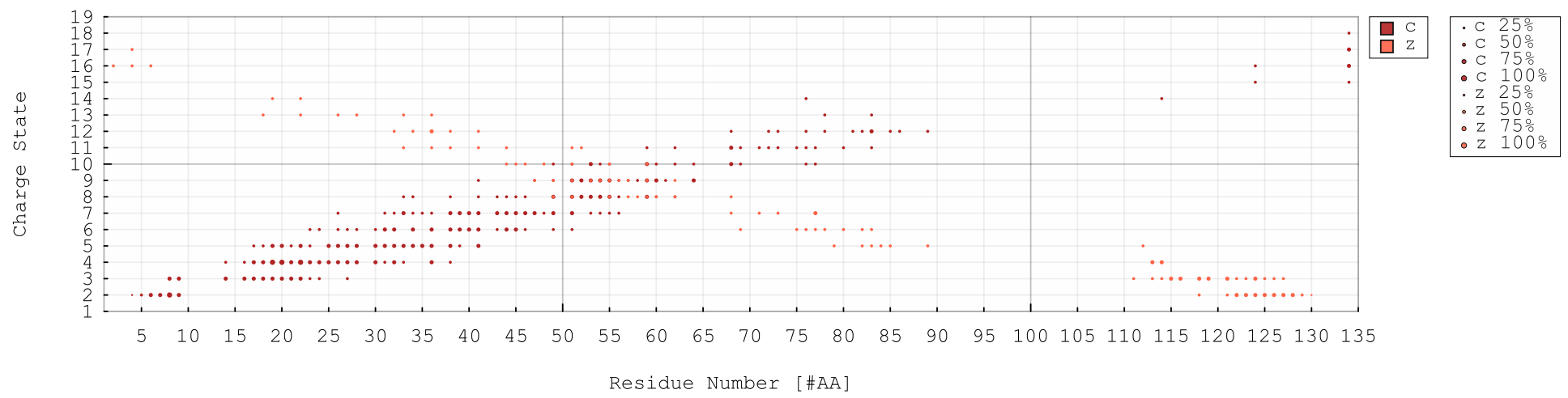

Proteoform:

Index	Protein	SC [%]	SVP [%]	IC [%]	MS Score [a.u.]	Var. Mods	Mono Mass [Da]	Chemical Formula
4	H31Kac1	76.1	27.4	40.8	31.1	Ace: K23	15305.4534	C671H1131N215O186S4

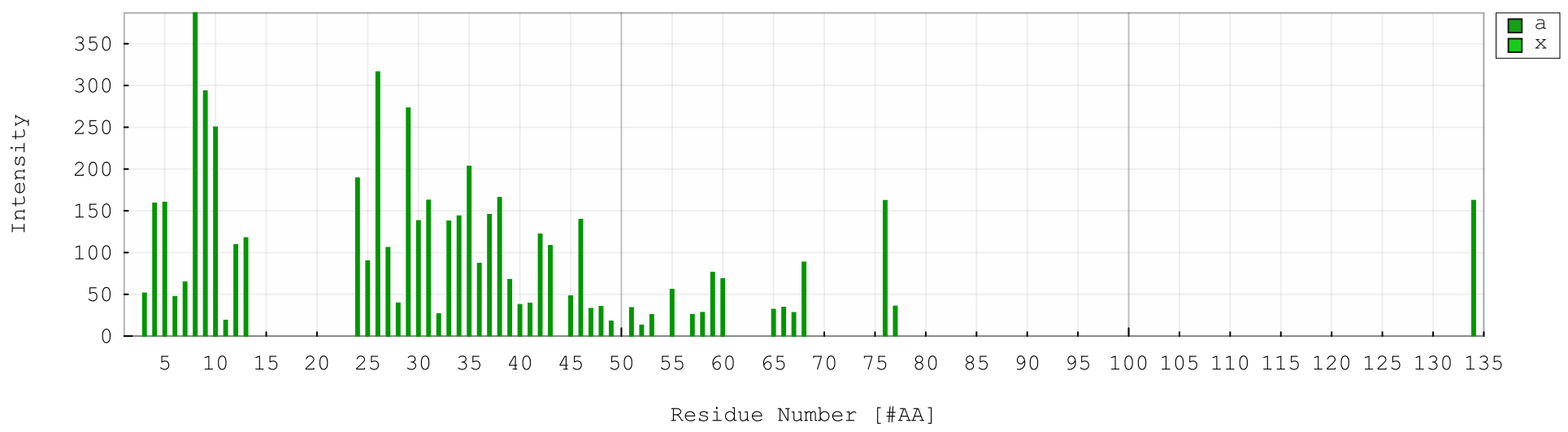

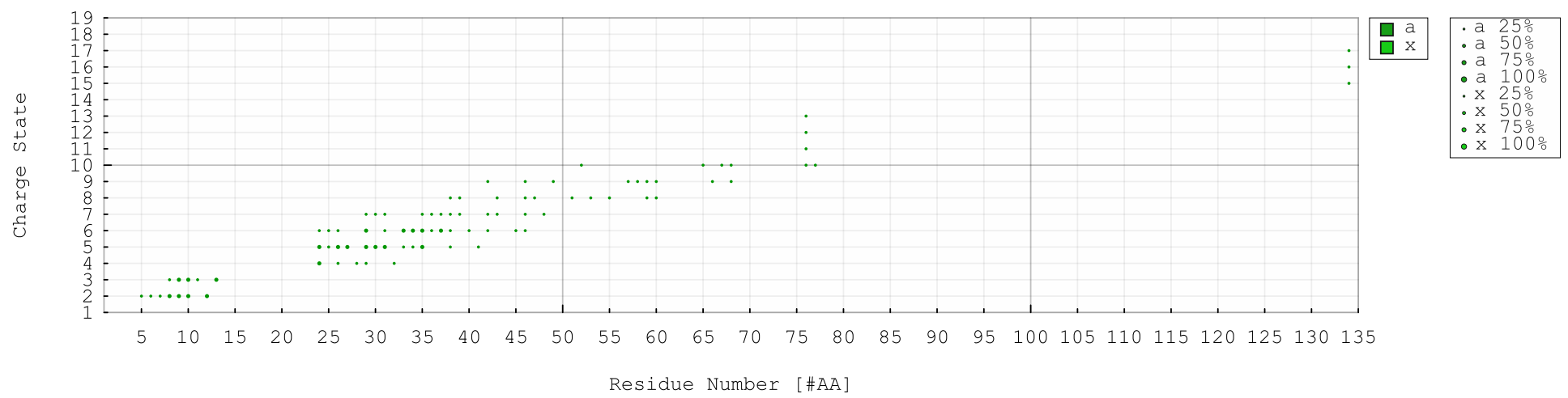

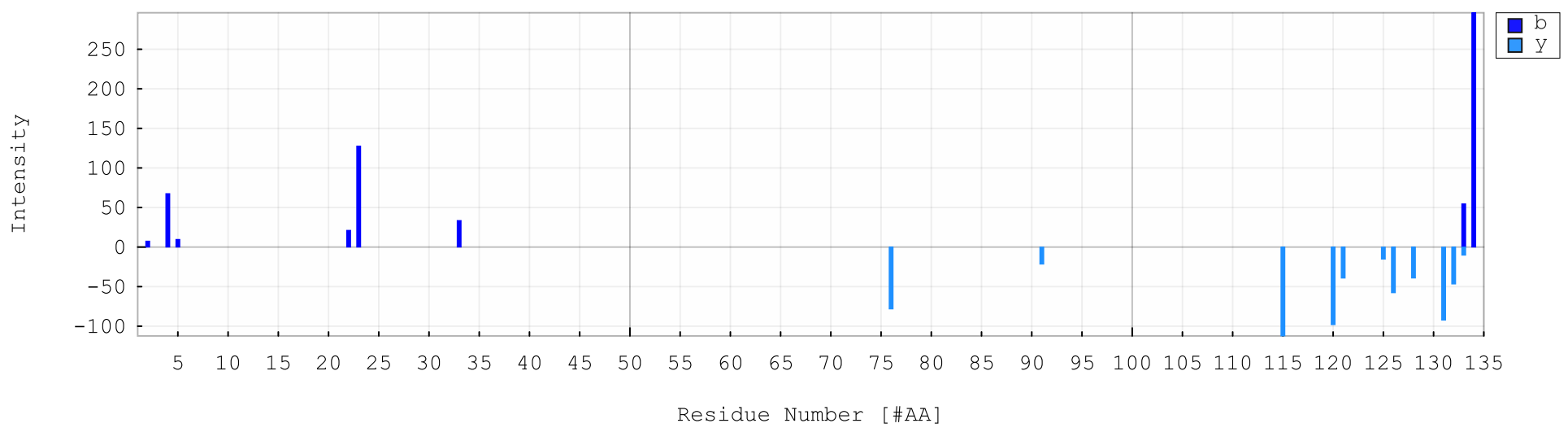

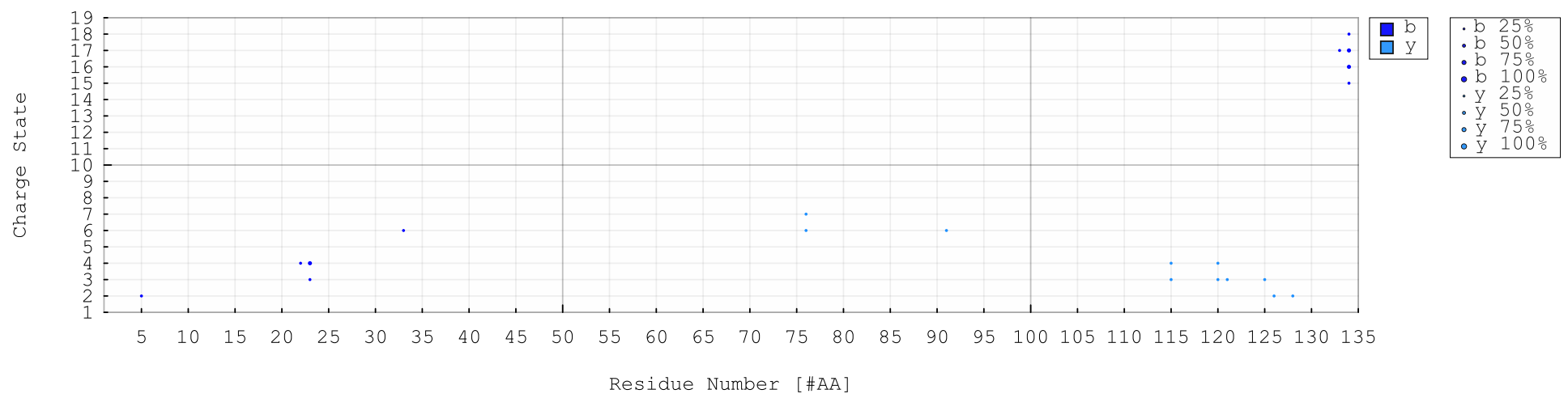

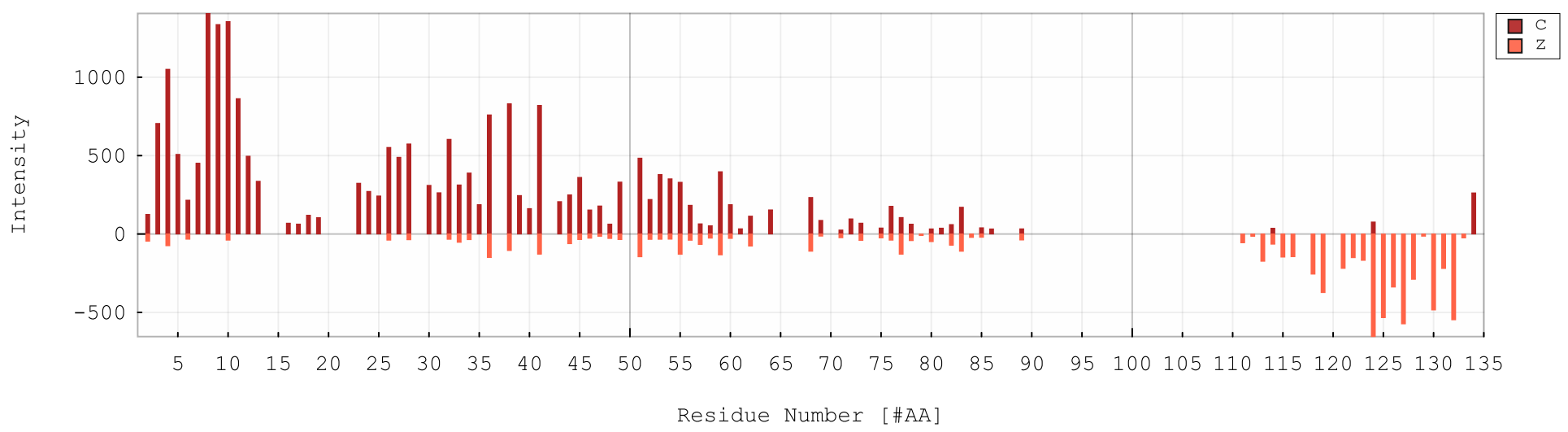

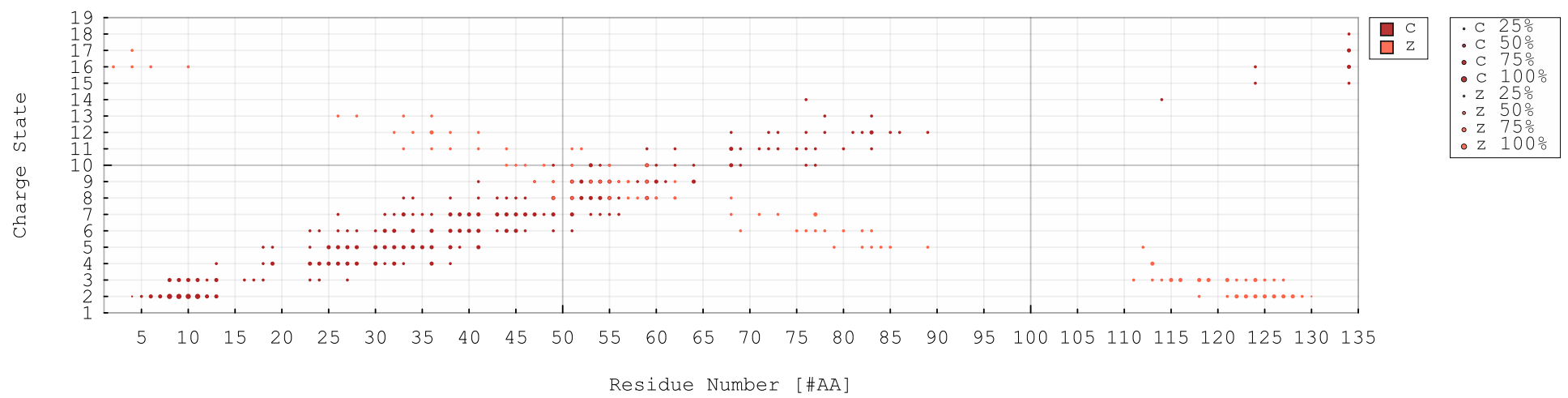

241210 H31-GNC5_10 z19 isCID 40eV MS2 ECD 50ms_001-247_calibrated

Proteoform:

Index	Protein	SC [%]	SVP [%]	IC [%]	MS Score [a.u.]	Var. Mods	Mono Mass [Da]	Chemical Formula
5	H31Kac1	72.4	20.0	42.2	30.5	Ace: K4	15305.4534	C671H1131N215O186S4

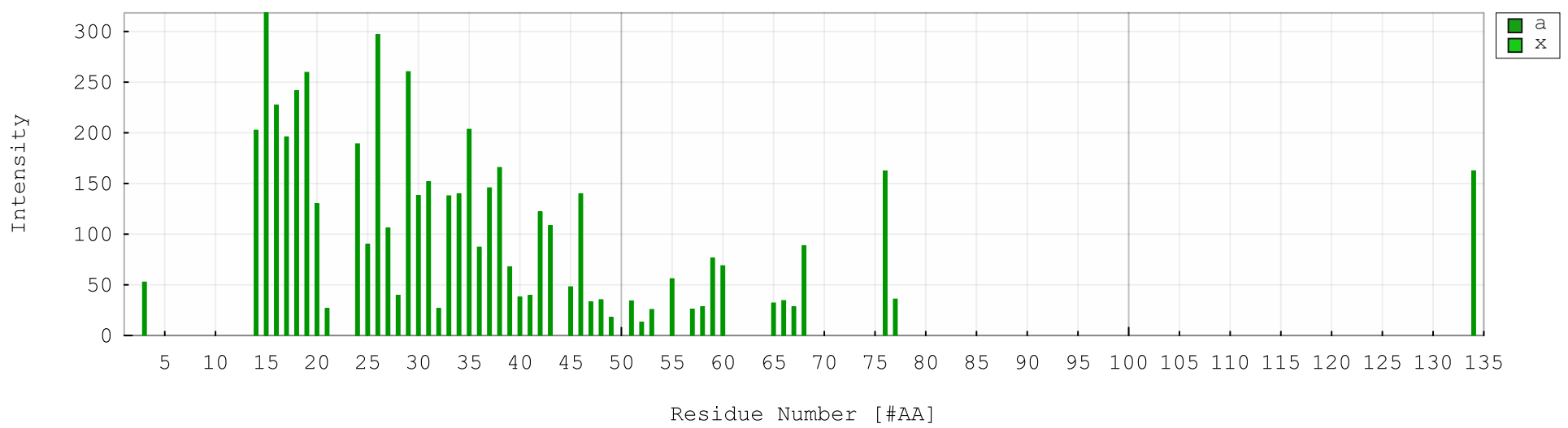

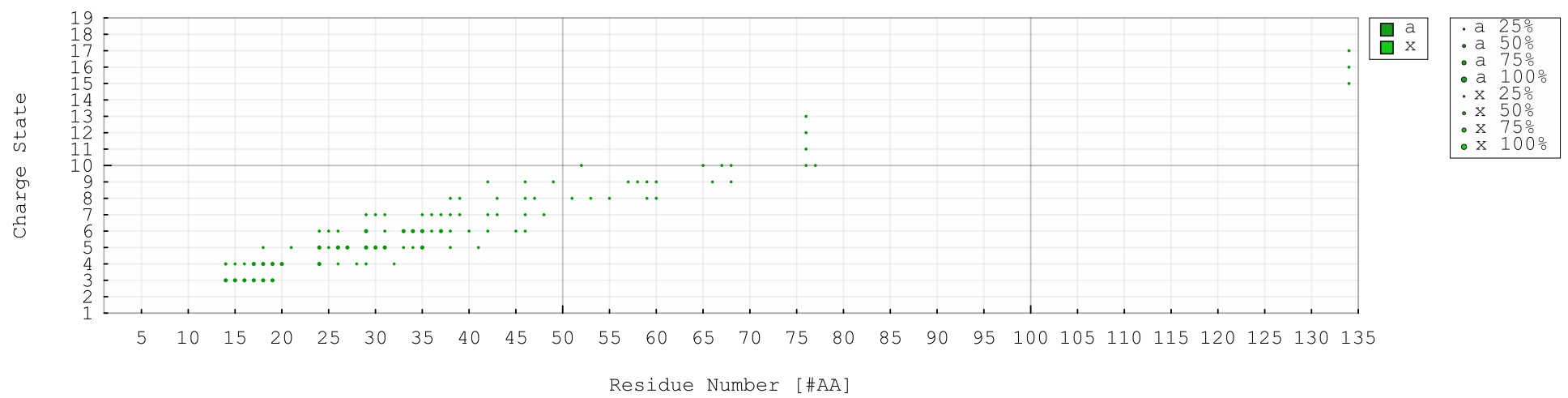

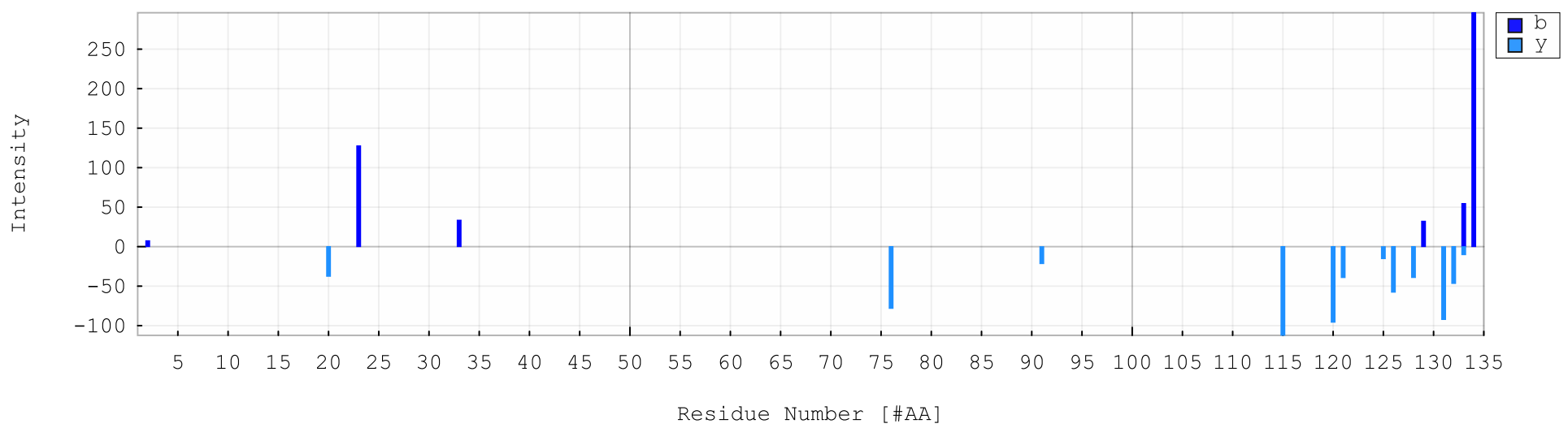

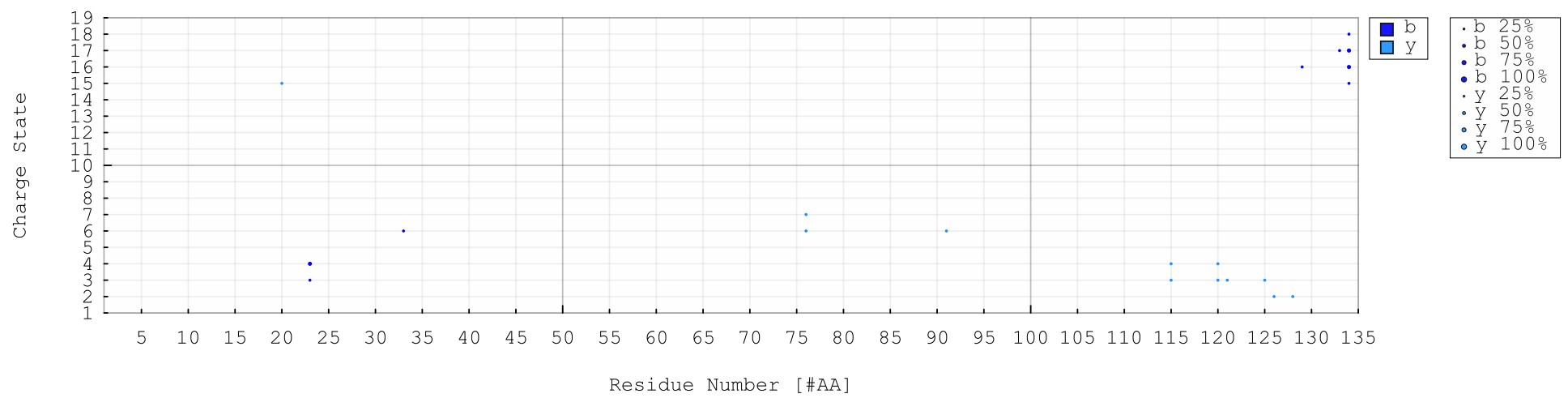

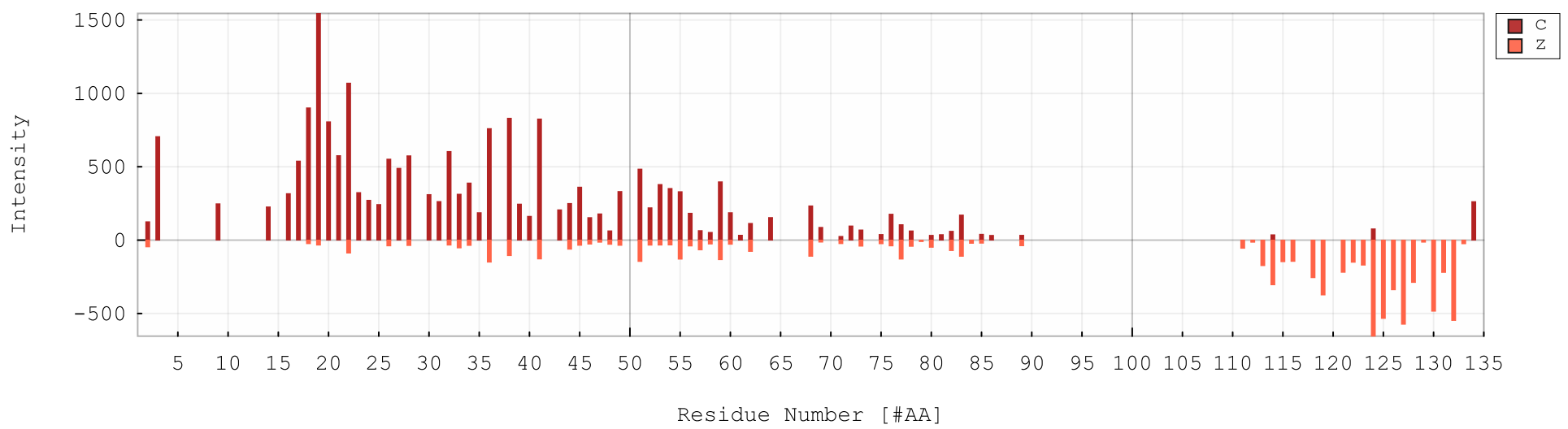

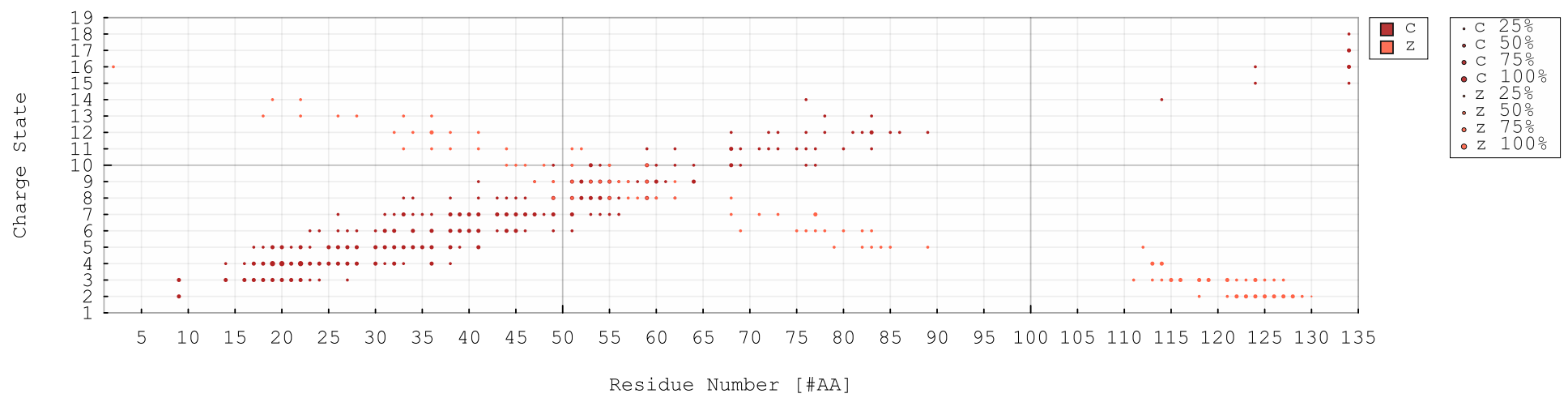

Proteoform:	Index	Protein	SC [%]	SVP [%]	IC [%]	MS Score [a.u.]	Var. Mods	Mono Mass [Da]	Chemical Formula
	6	H31Kac1	74.6	27.4	39.2	29.3	Ace: K27	15305.4534	C671H1131N215O186S4

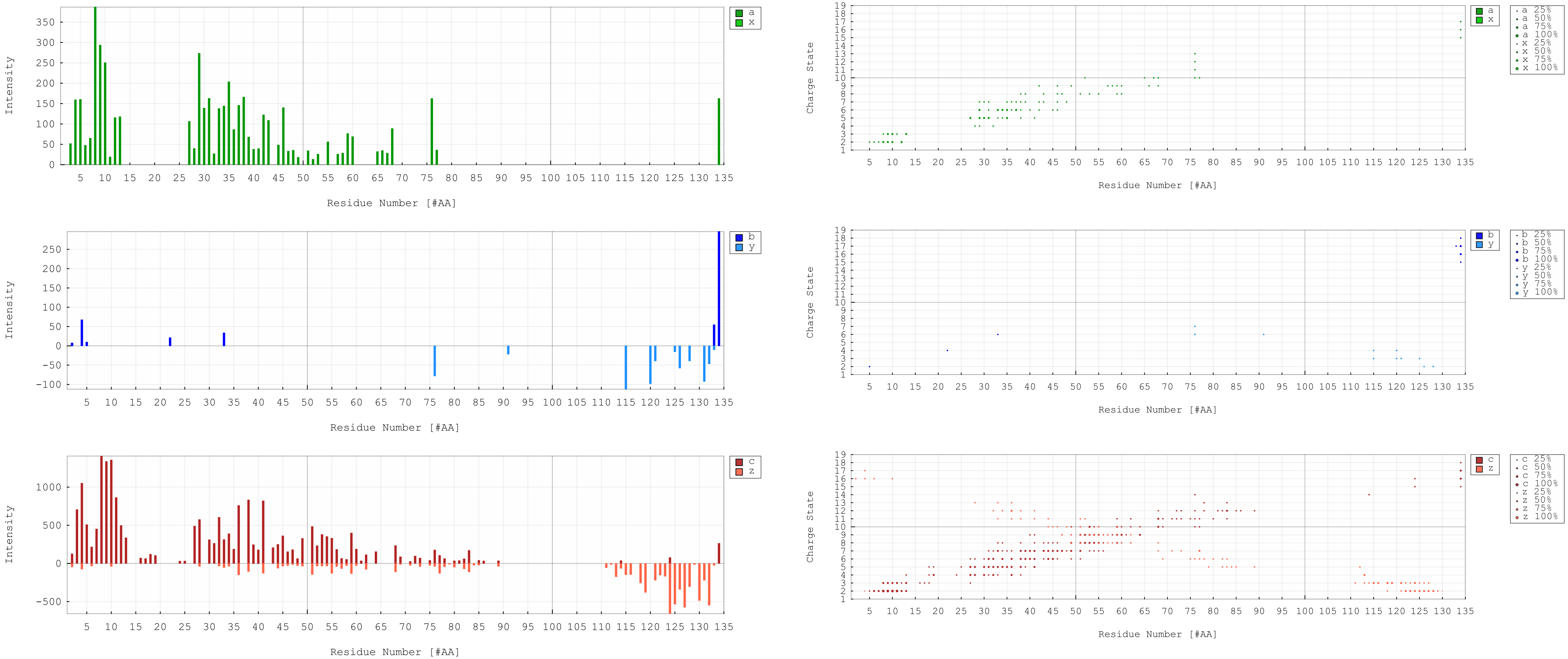

Top-Down Result OmniScape 2025
Sequence Confirmation
241210 H31-GNC5_10 z19 isCID 40eV MS2 ECD 50ms_001-247_calibrated

Proteoform:

Index	Protein	SC [%]	SVP [%]	IC [%]	MS Score [a.u.]	Var. Mods	Mono Mass [Da]	Chemical Formula
7	H31Kac1	72.4	27.4	35.9	26.0	Ace: K36	15305.4534	C671H1131N215O186S4

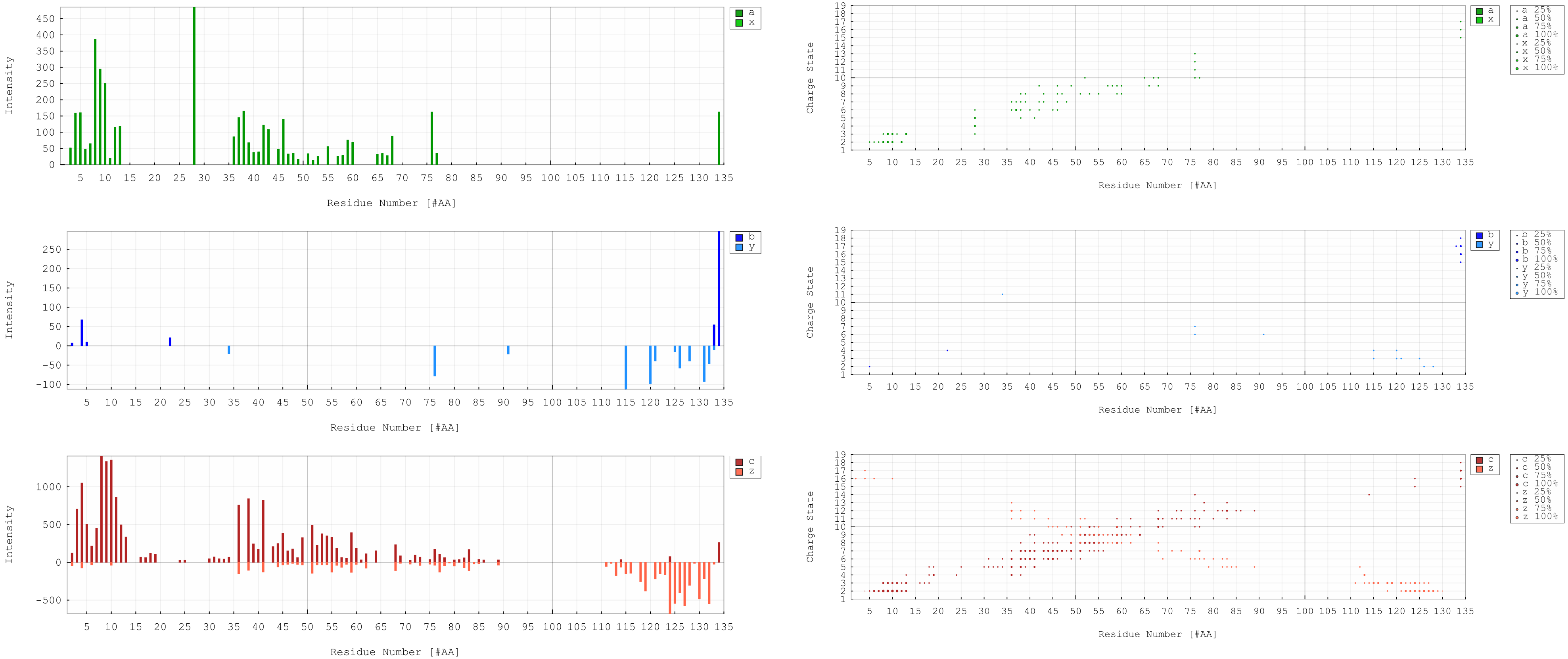

Proteoform:

Index	Protein	SC [%]	SVP [%]	IC [%]	MS Score [a.u.]	Var. Mods	Mono Mass [Da]	Chemical Formula
8	H31Kac1	71.6	27.4	34.8	24.9	Ace: K37	15305.4534	C671H1131N215O186S4

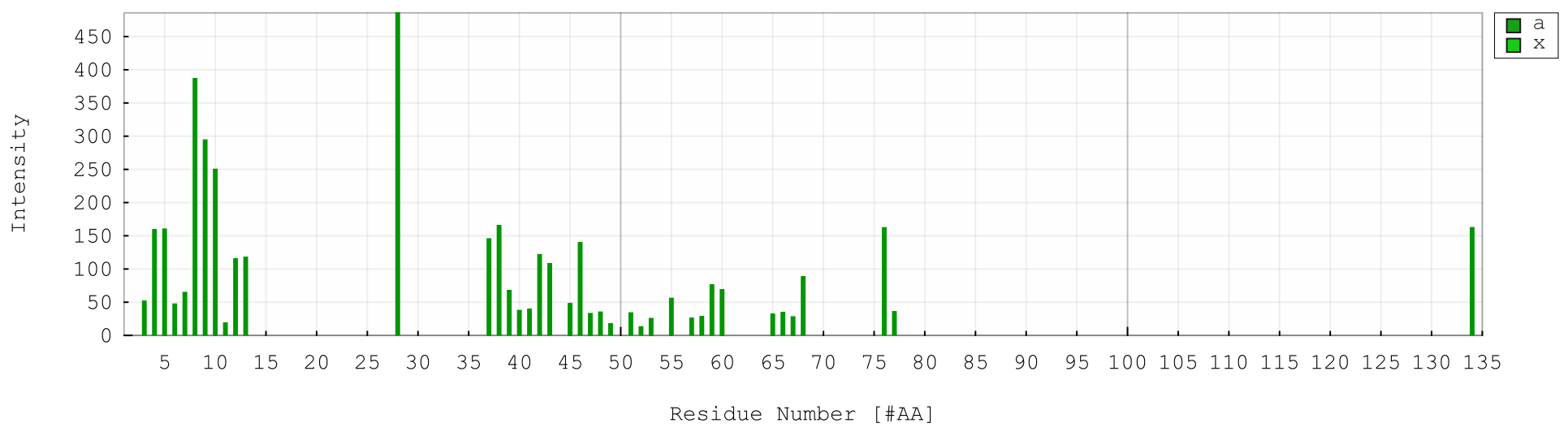

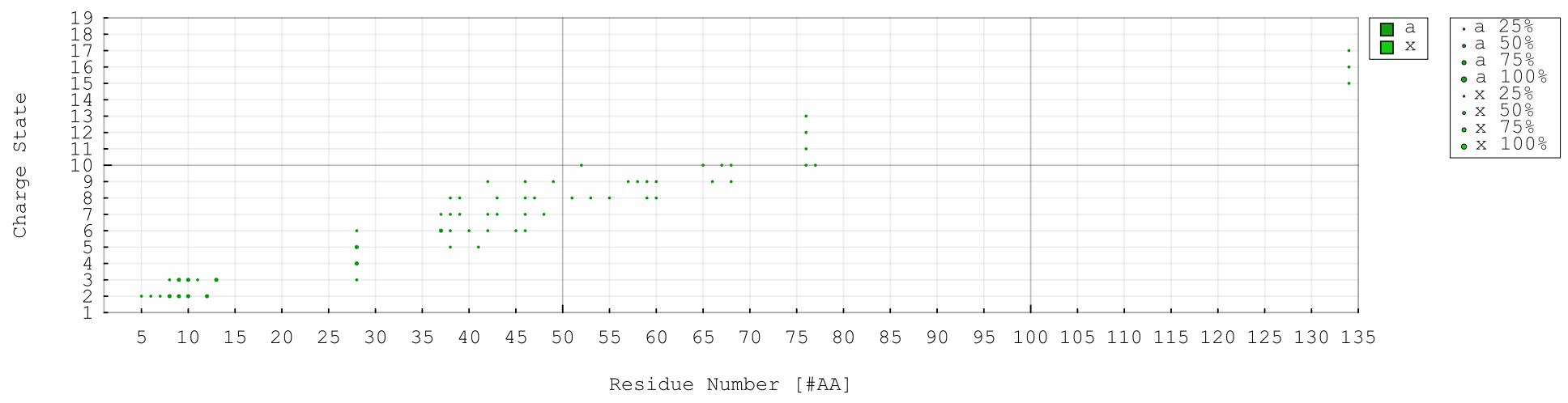

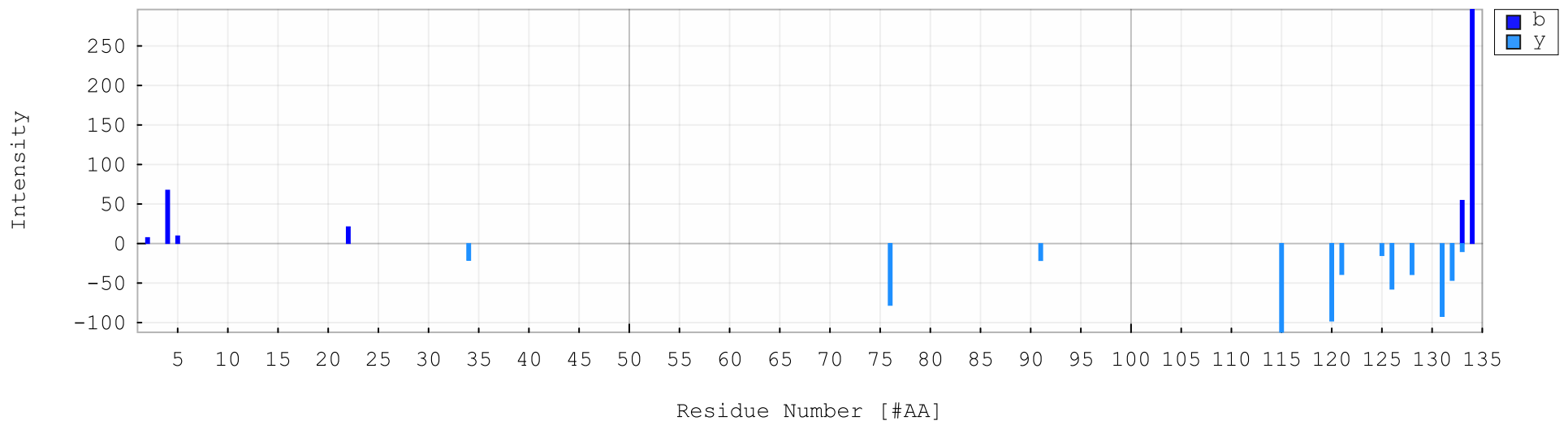

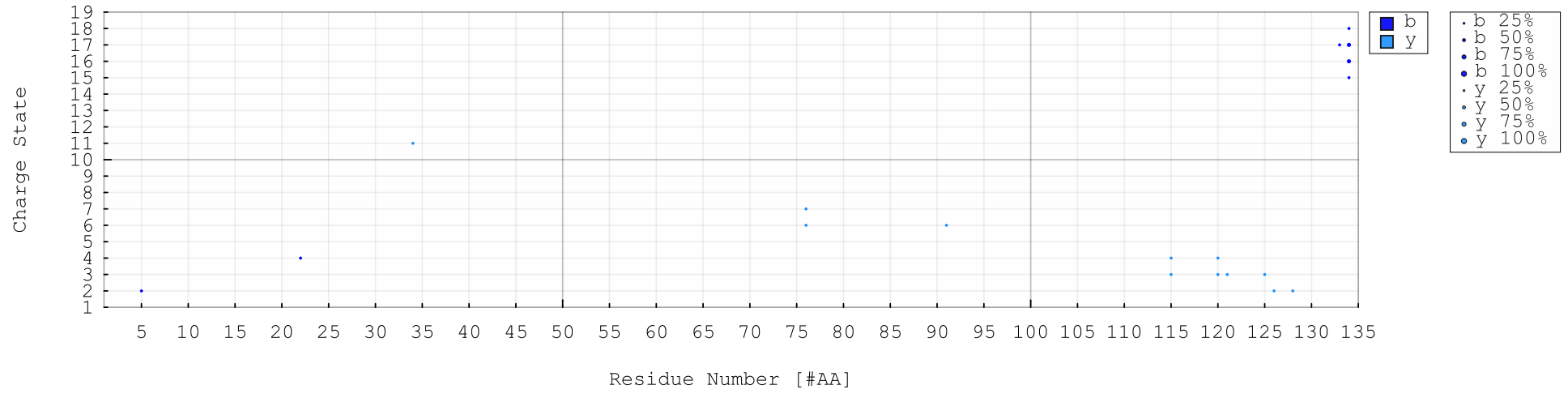

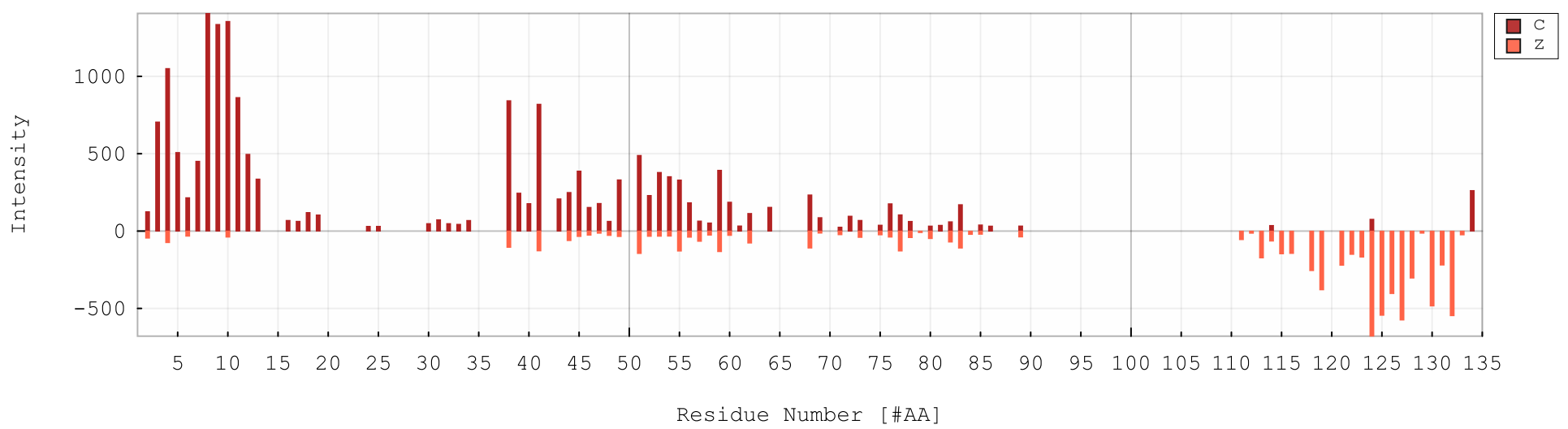

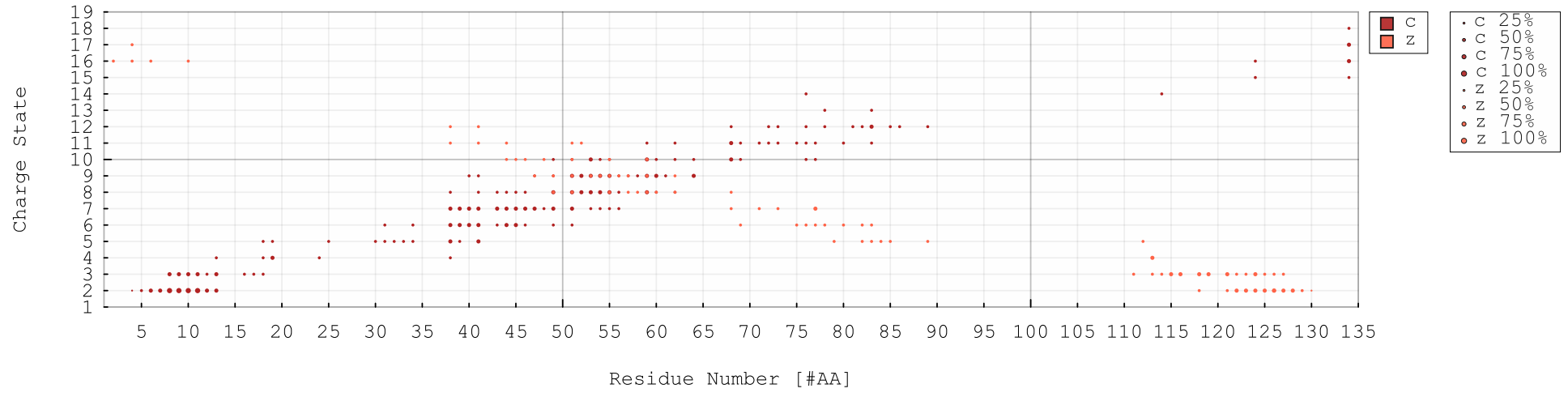

Proteoform:

Index	Protein	SC [%]	SVP [%]	IC [%]	MS Score [a.u.]	Var. Mods	Mono Mass [Da]	Chemical Formula
9	H31Kac1	61.9	27.4	26.7	16.5	Ace: K56	15305.4534	C671H1131N215O186S4

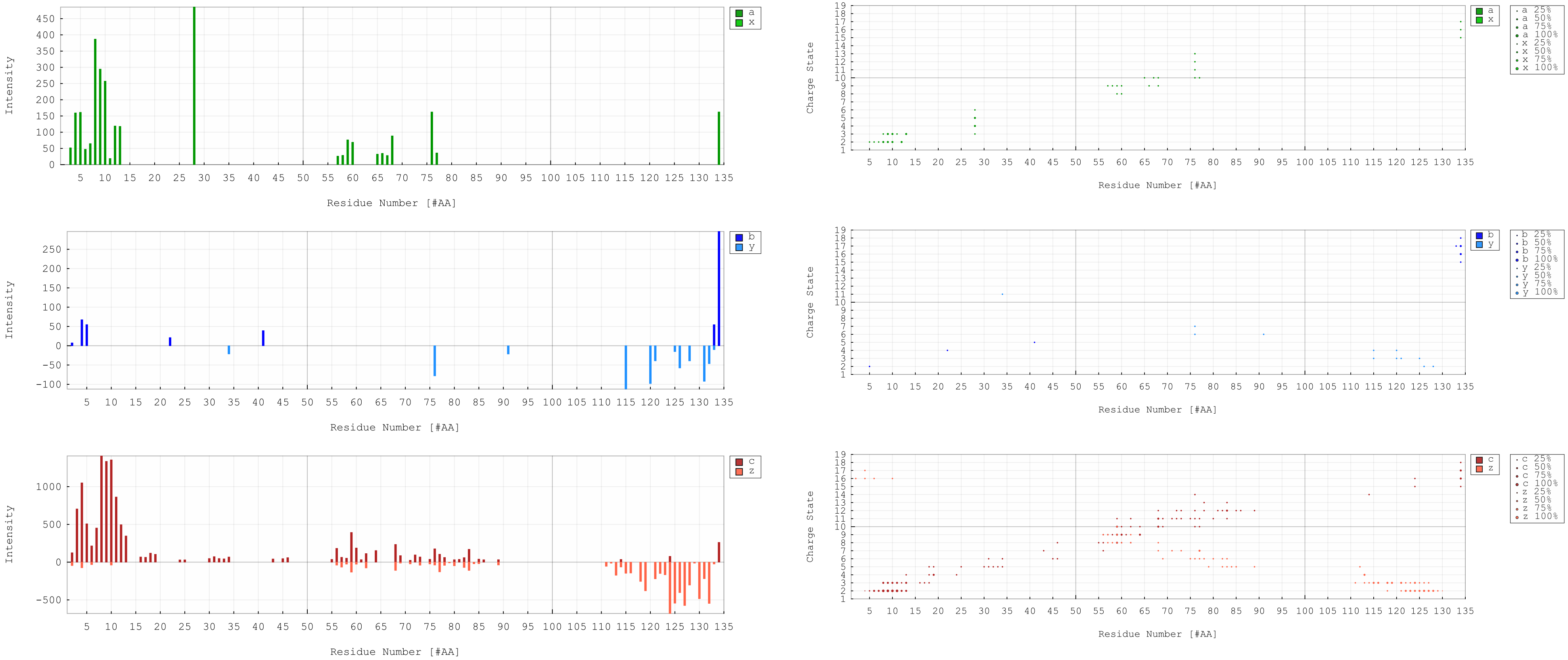

Proteoform:

Index	Protein	SC [%]	SVP [%]	IC [%]	MS Score [a.u.]	Var. Mods	Mono Mass [Da]	Chemical Formula
10	H31Kac1	58.2	27.4	24.9	14.5	Ace: K64	15305.4534	C671H1131N215O186S4

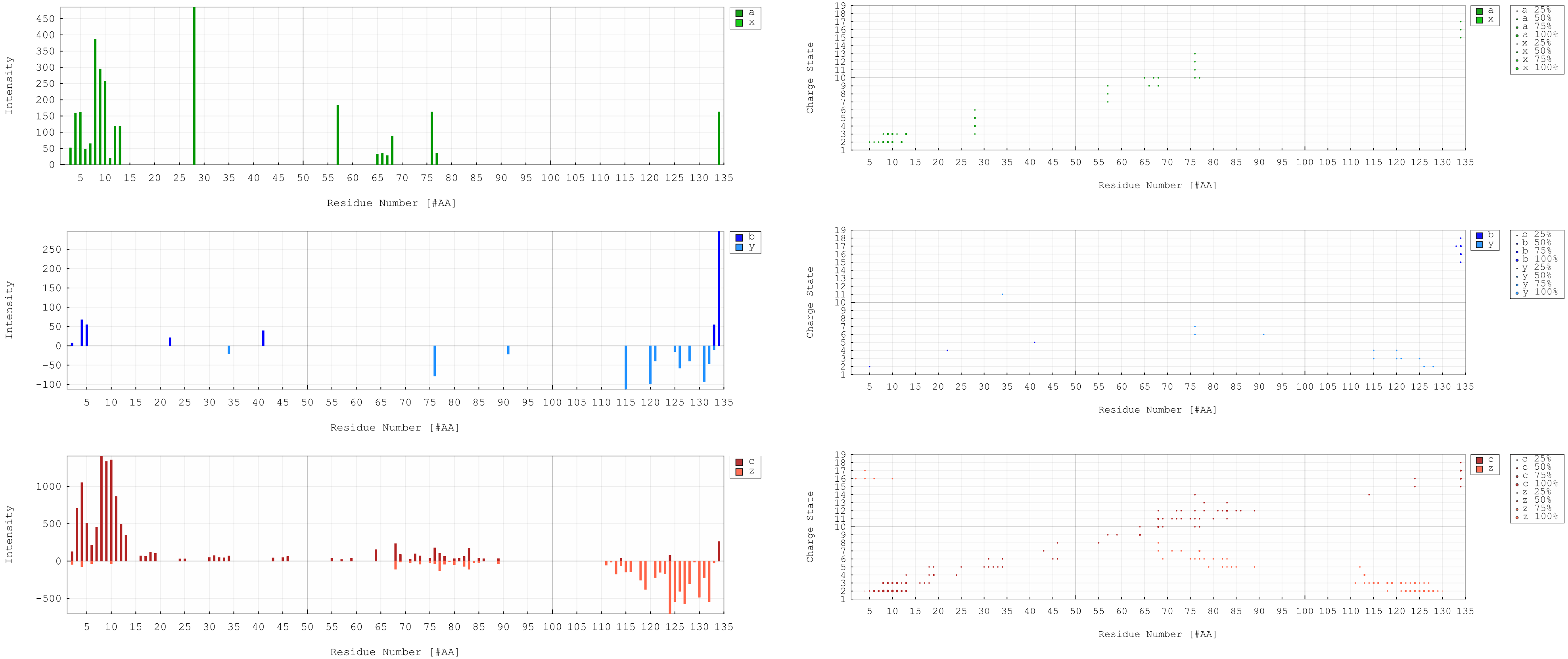

Supplemental Data S2

H3.1 - GCN5, 10 min

**Report**	3 of 7
**Experiment**	MS2
**Histone/sample**	H3.1
**Enzyme**	GCN5
**Reaction/source**	10 min
**Precursor**	H3.1Kac1
**Charge state**	19+
**Fragmentation / workflow**	EID
**Original file**	MS2_H3.1Kac1_19+_EID.pdf

Project Information

Project Directory: C:/Users/LAB-ADMIN
Processing date: Wed Dec 10 11:31:30 2025

Sample Info & Protocols

Name: Top-Down Protein Sequencing EID

Dataset

Imported spectrum file name:	241210 H31-GNC5_10 z19 MS2 EID 50ms_001-189_calibrated.txt
Dataset name:	Information is only available from imported JPEAKS files
Date acquired:	Information is only available from imported JPEAKS files
Date imported:	Information is only available from imported JPEAKS files
Instrument Name:	Information is only available from imported JPEAKS files
Instrument Serial Number:	Information is only available from imported JPEAKS files

Workflow Result Info

Analysis: 241210 H31-GNC5_10 z19 MS2 EID 50ms_001-189_calibrated
Location: C:/Users/LAB-ADMIN
Method: -

Method Information

Name: Unnamed

Spectral Parameters

Mass Error [ppm]: 6
Intensity Deviation [au]: 0.15
Min/Max Charges: 1-19
Experiment type: EID

a ions	b ions	c ions	x ions	y ions	z ions	satellite	internal	precursor
a	b	c	x	y	z			M
a+1			x+1	y-1	z+1			M+-1H

Noise threshold calculation: Simple moving average noise threshold (SMA)
Window size [datapoints]: 600
Noise curve offset: 3.7230983
SVP parameters: terminal gap: 2, internal gap: 1

Proteoforms

(top 10)

Index	Parent Sequence	Chain	Var. Mods	Mono Mass [Da]	Chemical Composition
1	H31Kac1	H31Kac1K14[Ace]	Ace: K14	15305.4534	C671H1131N215O186S4
2	H31Kac1	H31Kac1K18[Ace]	Ace: K18	15305.4534	C671H1131N215O186S4
3	H31Kac1	H31Kac1K9[Ace]	Ace: K9	15305.4534	C671H1131N215O186S4
4	H31Kac1	H31Kac1K4[Ace]	Ace: K4	15305.4534	C671H1131N215O186S4
5	H31Kac1	H31Kac1K23[Ace]	Ace: K23	15305.4534	C671H1131N215O186S4
6	H31Kac1	H31Kac1K27[Ace]	Ace: K27	15305.4534	C671H1131N215O186S4
7	H31Kac1	H31Kac1K36[Ace]	Ace: K36	15305.4534	C671H1131N215O186S4
8	H31Kac1	H31Kac1K37[Ace]	Ace: K37	15305.4534	C671H1131N215O186S4
9	H31Kac1	H31Kac1K56[Ace]	Ace: K56	15305.4534	C671H1131N215O186S4
10	H31Kac1	H31Kac1K64[Ace]	Ace: K64	15305.4534	C671H1131N215O186S4

Results

Proteoforms Table (top 10)

Index	Protein	SC [%]	SVP [%]	IC [%]	MS Score [a.u.]	Var. Mods	Mono Mass [Da]	Chemical Formula
1	H31Kac1	78.4	62.2	34.0	26.6	Ace: K14	15305.4534	C671H1131N215O186S4
2	H31Kac1	76.9	62.2	32.5	25.0	Ace: K18	15305.4534	C671H1131N215O186S4
3	H31Kac1	75.4	22.2	32.6	24.6	Ace: K9	15305.4534	C671H1131N215O186S4
4	H31Kac1	72.4	17.8	31.7	22.9	Ace: K4	15305.4534	C671H1131N215O186S4
5	H31Kac1	74.6	29.6	29.9	22.3	Ace: K23	15305.4534	C671H1131N215O186S4
6	H31Kac1	73.1	29.6	28.5	20.9	Ace: K27	15305.4534	C671H1131N215O186S4
7	H31Kac1	69.4	29.6	25.5	17.7	Ace: K36	15305.4534	C671H1131N215O186S4
8	H31Kac1	68.7	29.6	25.0	17.1	Ace: K37	15305.4534	C671H1131N215O186S4
9	H31Kac1	59.7	32.6	20.3	12.1	Ace: K56	15305.4534	C671H1131N215O186S4
10	H31Kac1	57.5	32.6	18.9	10.9	Ace: K64	15305.4534	C671H1131N215O186S4

Sequence

Name: H31Kac1

```
                  10         20         30         40         50         60
ARTKQTARKS TGGKAPRKQL ATKAARKSAP ATGGVKKPHR YRPGTVALRE IRRYQKSTEL
                  70         80         90        100        110        120
LIRKLPFQRL VREIAQDFKT DLRFQSSAVM ALQEACEAYL VGLFEDTNLC AIHAKRVTIM
                 130        140        150        160        170        180
PKDIQLARRI RGERA
```

Modifications

Modification	Position	Type
Acetyl	4	variable
Acetyl	9	variable
Acetyl	14	variable
Acetyl	18	variable
Acetyl	23	variable
Acetyl	27	variable
Acetyl	36	variable
Acetyl	37	variable
Acetyl	56	variable
Acetyl	64	variable
Acetyl	79	variable

Modification	Position	Type
Acetyl	115	variable
Acetyl	122	variable

Sequence Map

Proteoform:

Index	Protein	SC [%]	SVP [%]	IC [%]	MS Score [a.u.]	Var. Mods	Mono Mass [Da]	Chemical Formula
1	H31Kac1	78.4	62.2	34.0	26.6	Ace: K14	15305.4534	C671H1131N215O186S4

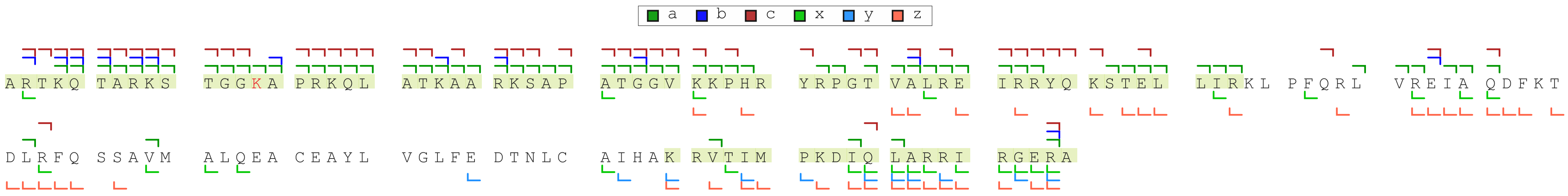

Proteoform:

Index	Protein	SC [%]	SVP [%]	IC [%]	MS Score [a.u.]	Var. Mods	Mono Mass [Da]	Chemical Formula
2	H31Kac1	76.9	62.2	32.5	25.0	Ace: K18	15305.4534	C671H1131N215O186S4

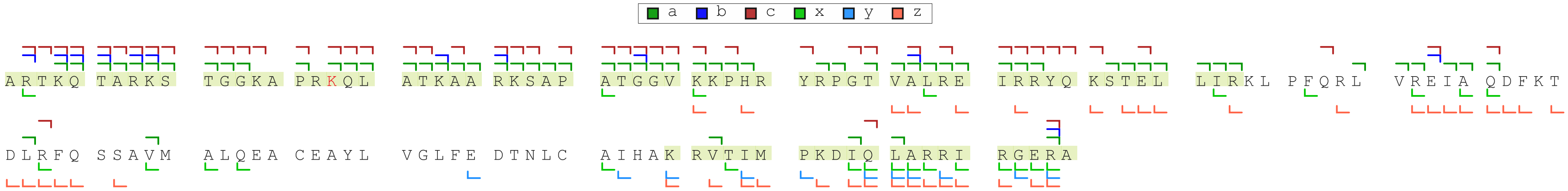

Proteoform:	Index	Protein	SC [%]	SVP [%]	IC [%]	MS Score [a.u.]	Var. Mods	Mono Mass [Da]	Chemical Formula
	3	H31Kac1	75.4	22.2	32.6	24.6	Ace: K9	15305.4534	C671H1131N215O186S4

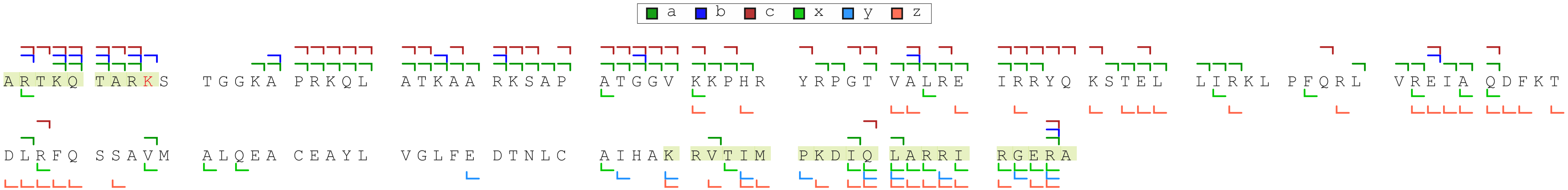

Proteoform:

Index	Protein	SC [%]	SVP [%]	IC [%]	MS Score [a.u.]	Var. Mods	Mono Mass [Da]	Chemical Formula
4	H31Kac1	72.4	17.8	31.7	22.9	Ace: K4	15305.4534	C671H1131N215O186S4

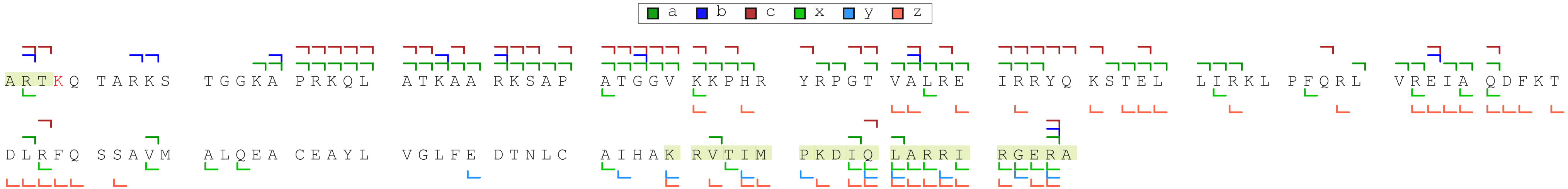

Proteoform:

Index	Protein	SC [%]	SVP [%]	IC [%]	MS Score [a.u.]	Var. Mods	Mono Mass [Da]	Chemical Formula
5	H31Kac1	74.6	29.6	29.9	22.3	Ace: K23	15305.4534	C671H1131N215O186S4

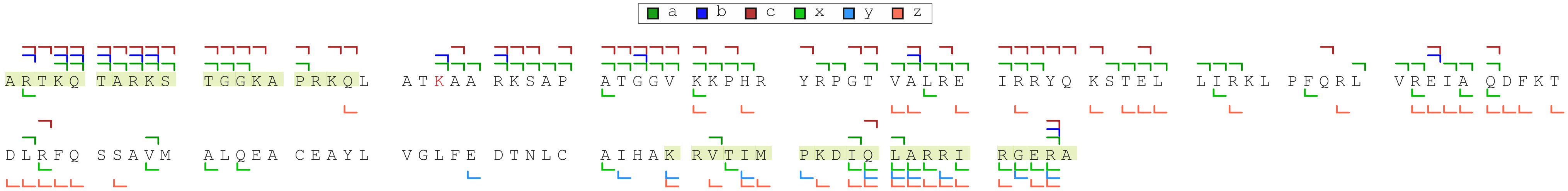

Proteoform:

Index	Protein	SC [%]	SVP [%]	IC [%]	MS Score [a.u.]	Var. Mods	Mono Mass [Da]	Chemical Formula
6	H31Kac1	73.1	29.6	28.5	20.9	Ace: K27	15305.4534	C671H1131N215O186S4

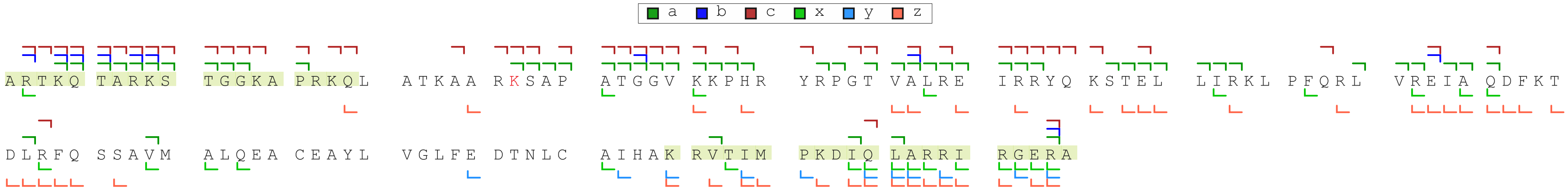

Proteoform:

Index	Protein	SC [%]	SVP [%]	IC [%]	MS Score [a.u.]	Var. Mods	Mono Mass [Da]	Chemical Formula
7	H31Kac1	69.4	29.6	25.5	17.7	Ace: K36	15305.4534	C671H1131N215O186S4

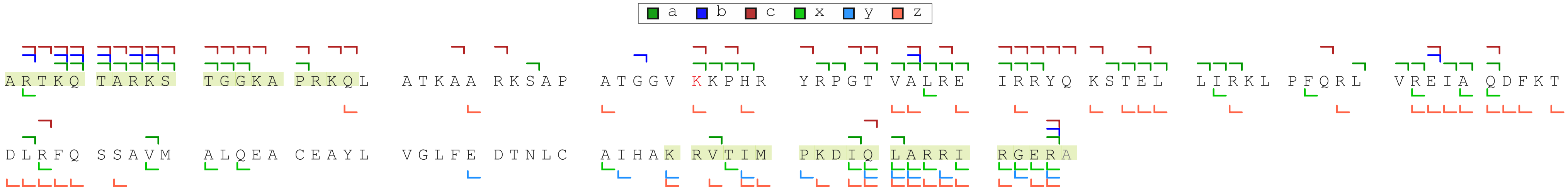

Top-Down Result OmniScape 2025
Sequence Confirmation
241210 H31-GNC5_10 z19 MS2 EID 50ms_001-189_calibrated

Proteoform:

Index	Protein	SC [%]	SVP [%]	IC [%]	MS Score [a.u.]	Var. Mods	Mono Mass [Da]	Chemical Formula
8	H31Kac1	68.7	29.6	25.0	17.1	Ace: K37	15305.4534	C671H1131N215O186S4

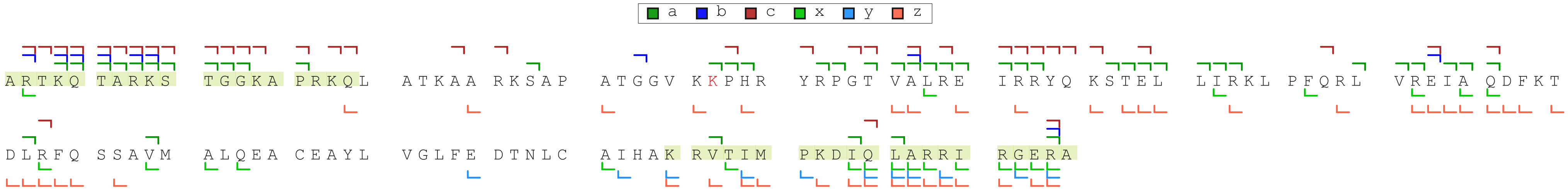

Proteoform:	Index	Protein	SC [%]	SVP [%]	IC [%]	MS Score [a.u.]	Var. Mods	Mono Mass [Da]	Chemical Formula
	9	H31Kac1	59.7	32.6	20.3	12.1	Ace: K56	15305.4534	C671H1131N215O186S4

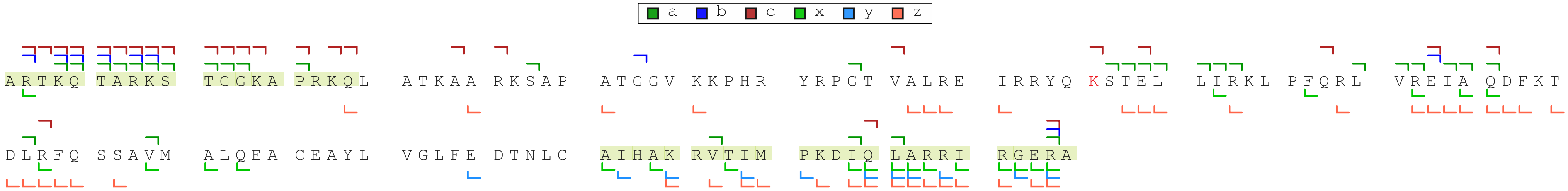

Proteoform:	Index	Protein	SC [%]	SVP [%]	IC [%]	MS Score [a.u.]	Var. Mods	Mono Mass [Da]	Chemical Formula
	10	H31Kac1	57.5	32.6	18.9	10.9	Ace: K64	15305.4534	C671H1131N215O186S4

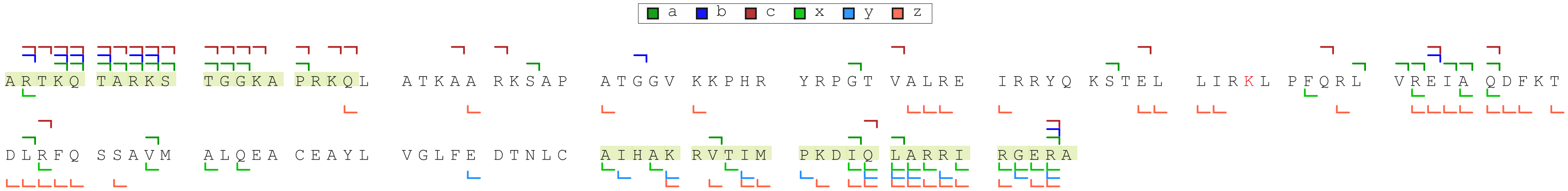

Error Plot

Proteoform:

Index	Protein	SC [%]	SVP [%]	IC [%]	MS Score [a.u.]	Var. Mods	Mono Mass [Da]	Chemical Formula
1	H31Kac1	78.4	62.2	34.0	26.6	Ace: K14	15305.4534	C671H1131N215O186S4

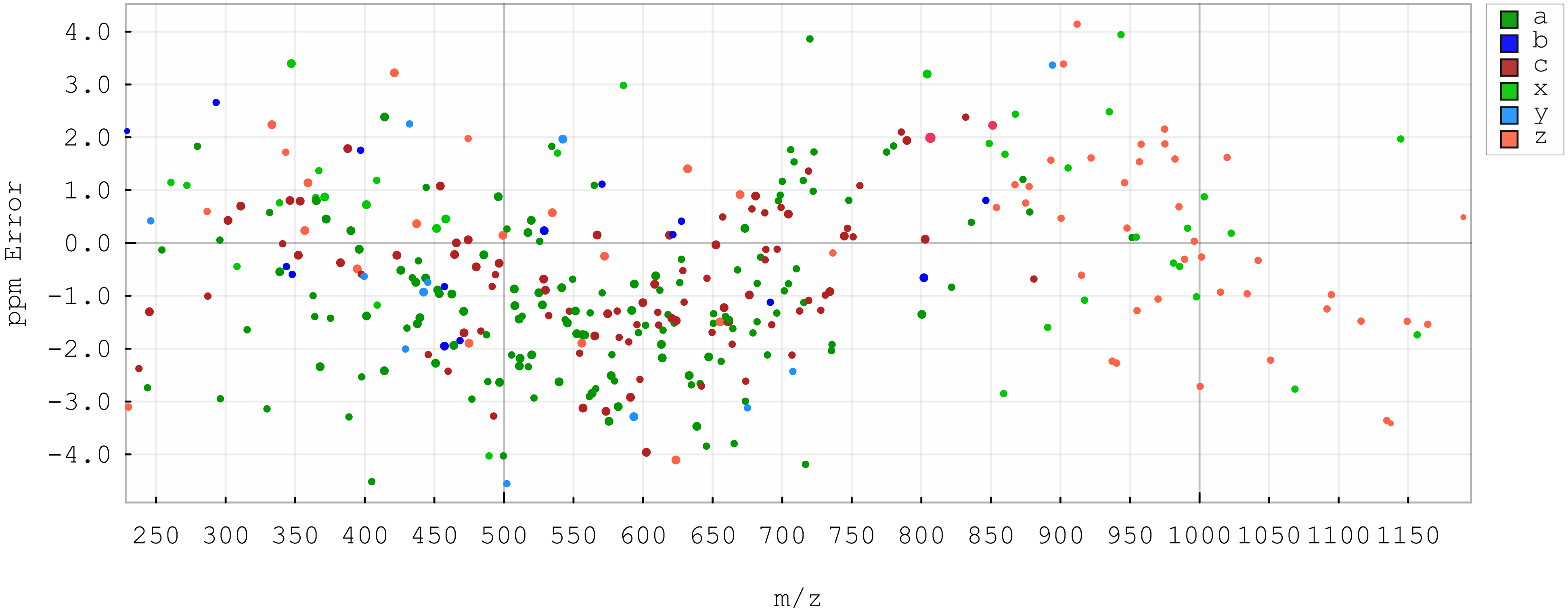

Proteoform:	Index	Protein	SC [%]	SVP [%]	IC [%]	MS Score [a.u.]	Var. Mods	Mono Mass [Da]	Chemical Formula
	2	H31Kac1	76.9	62.2	32.5	25.0	Ace: K18	15305.4534	C671H1131N215O186S4

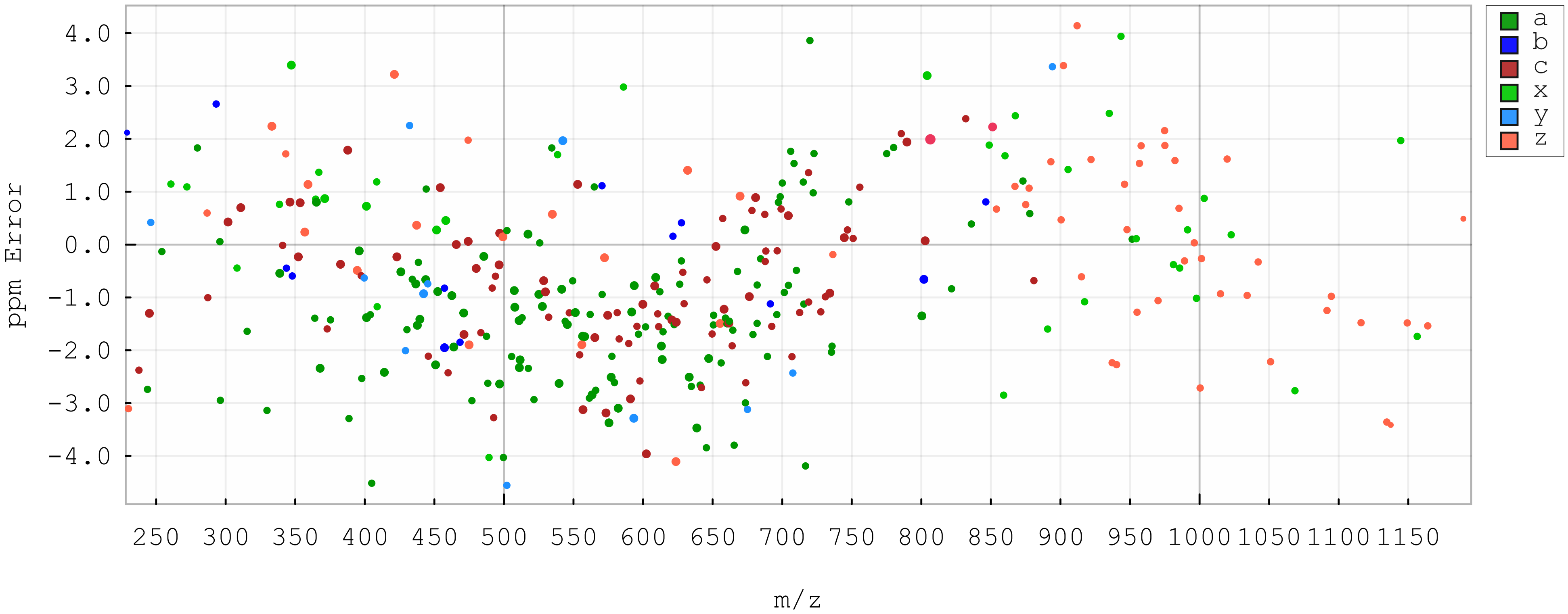

Proteoform:	Index	Protein	SC [%]	SVP [%]	IC [%]	MS Score [a.u.]	Var. Mods	Mono Mass [Da]	Chemical Formula
	3	H31Kac1	75.4	22.2	32.6	24.6	Ace: K9	15305.4534	C671H1131N215O186S4

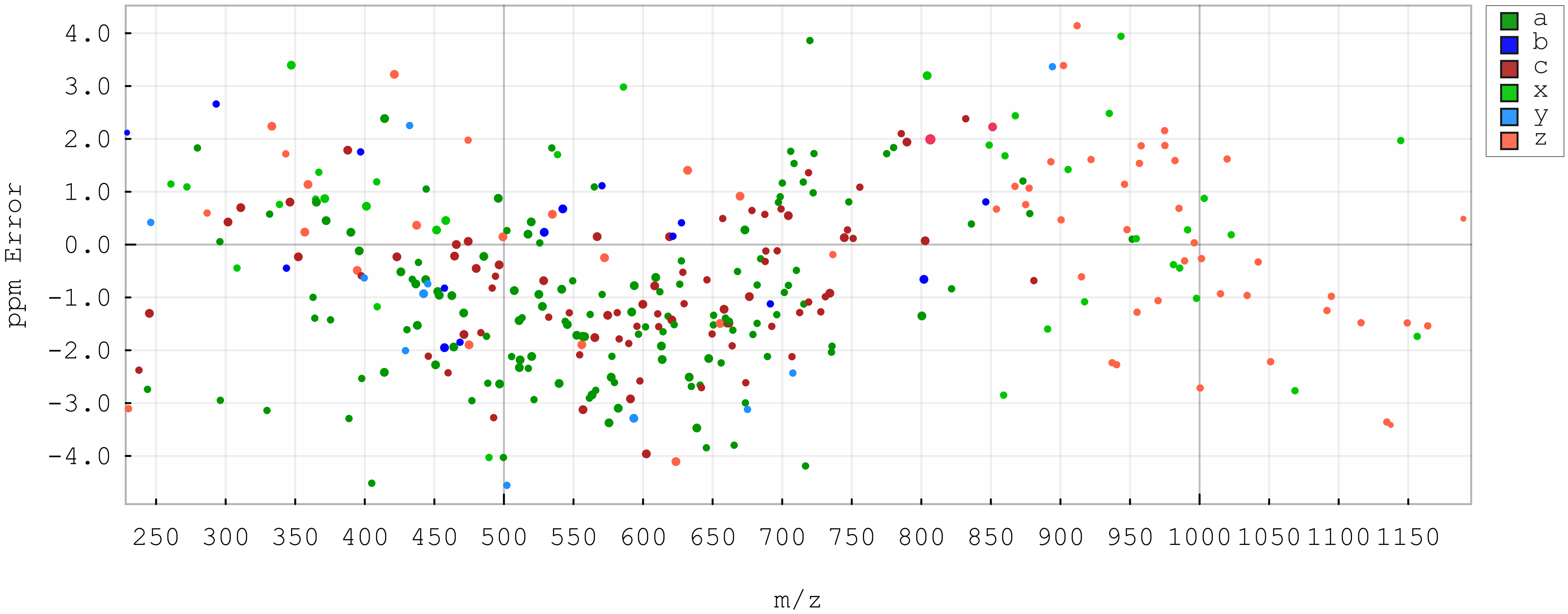

Proteoform:

Index	Protein	SC [%]	SVP [%]	IC [%]	MS Score [a.u.]	Var. Mods	Mono Mass [Da]	Chemical Formula
4	H31Kac1	72.4	17.8	31.7	22.9	Ace: K4	15305.4534	C671H1131N215O186S4

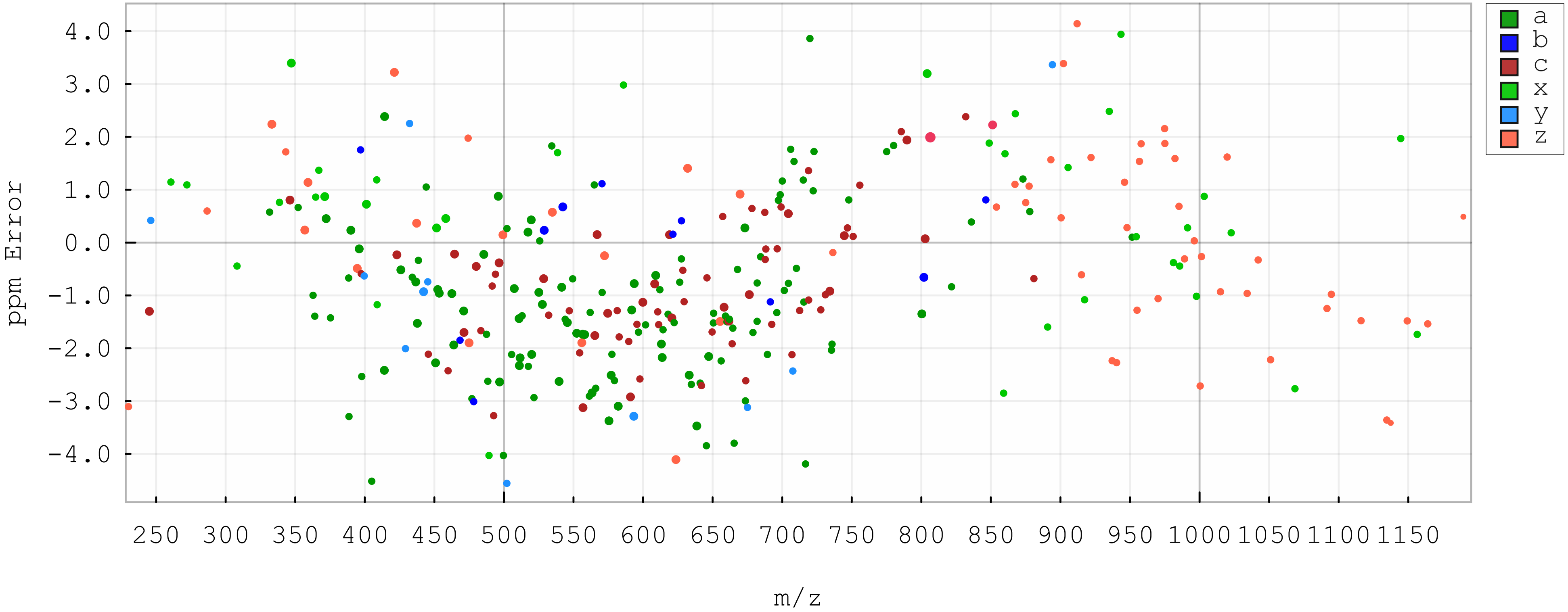

Proteoform:

Index	Protein	SC [%]	SVP [%]	IC [%]	MS Score [a.u.]	Var. Mods	Mono Mass [Da]	Chemical Formula
5	H31Kac1	74.6	29.6	29.9	22.3	Ace: K23	15305.4534	C671H1131N215O186S4

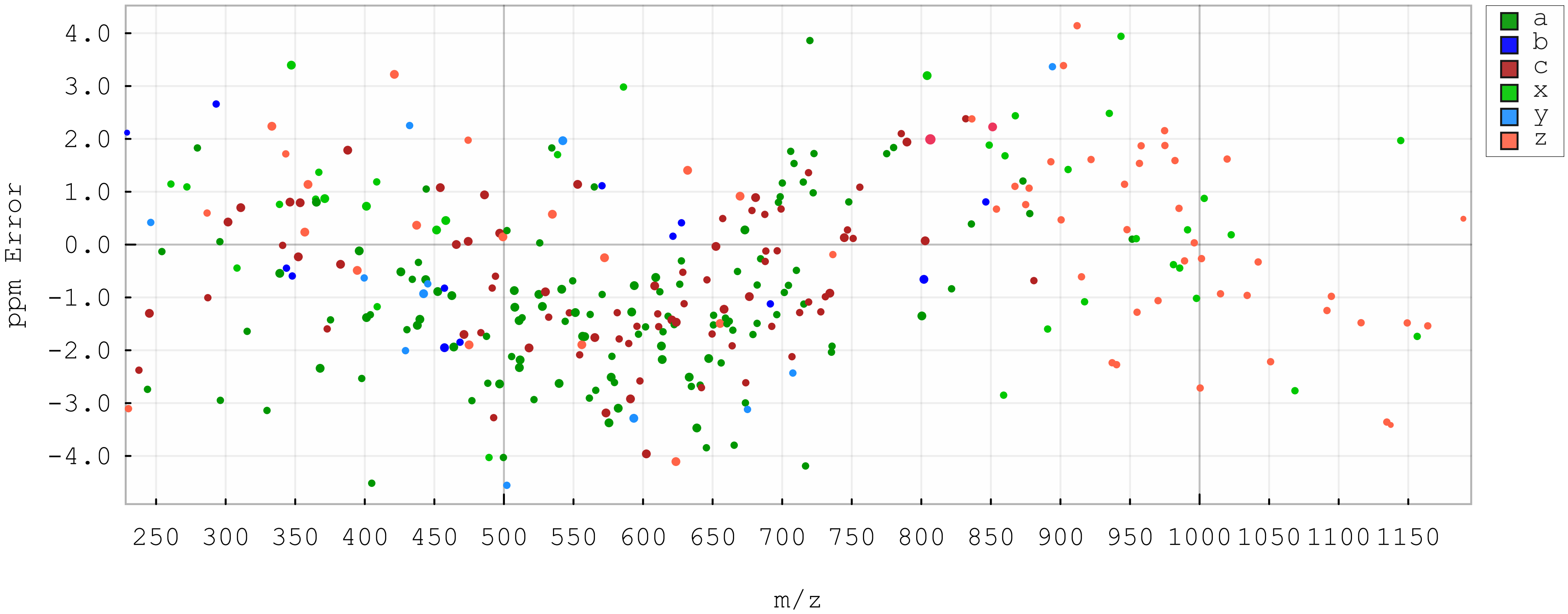

Proteoform:

Index	Protein	SC [%]	SVP [%]	IC [%]	MS Score [a.u.]	Var. Mods	Mono Mass [Da]	Chemical Formula
6	H31Kac1	73.1	29.6	28.5	20.9	Ace: K27	15305.4534	C671H1131N215O186S4

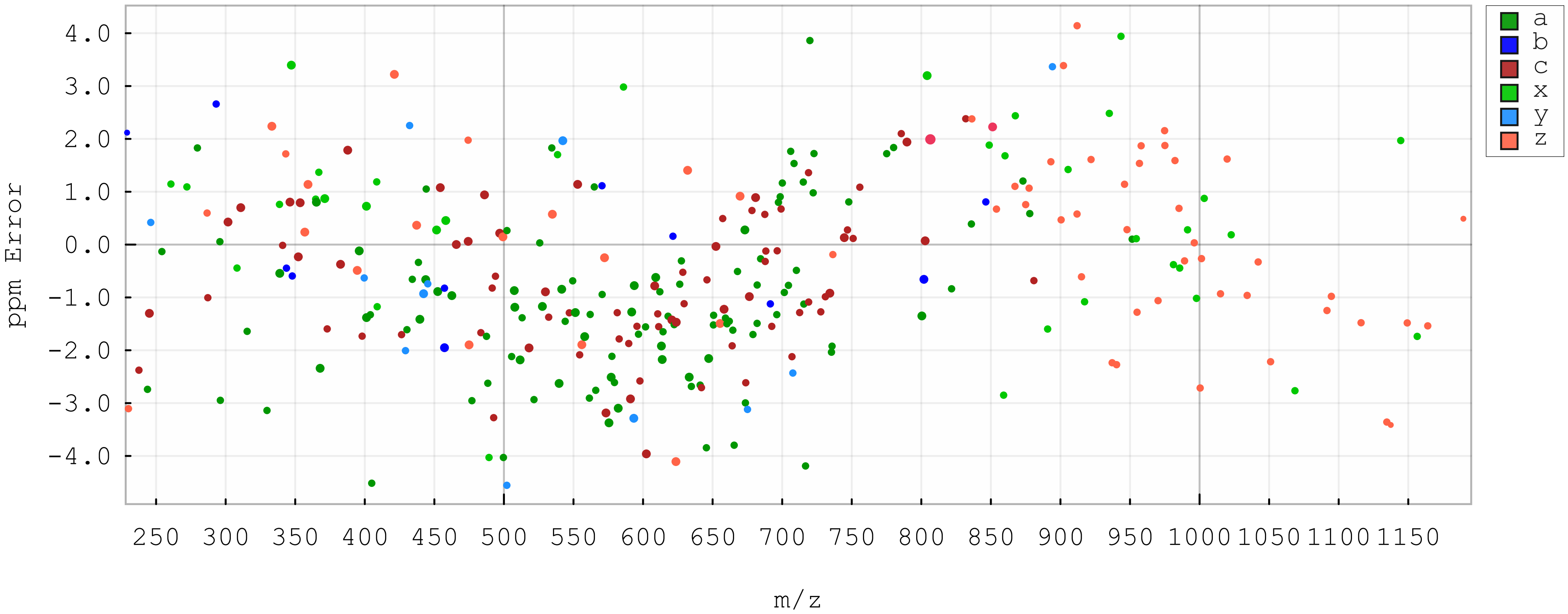

Proteoform:	Index	Protein	SC [%]	SVP [%]	IC [%]	MS Score [a.u.]	Var. Mods	Mono Mass [Da]	Chemical Formula
	7	H31Kac1	69.4	29.6	25.5	17.7	Ace: K36	15305.4534	C671H1131N215O186S4

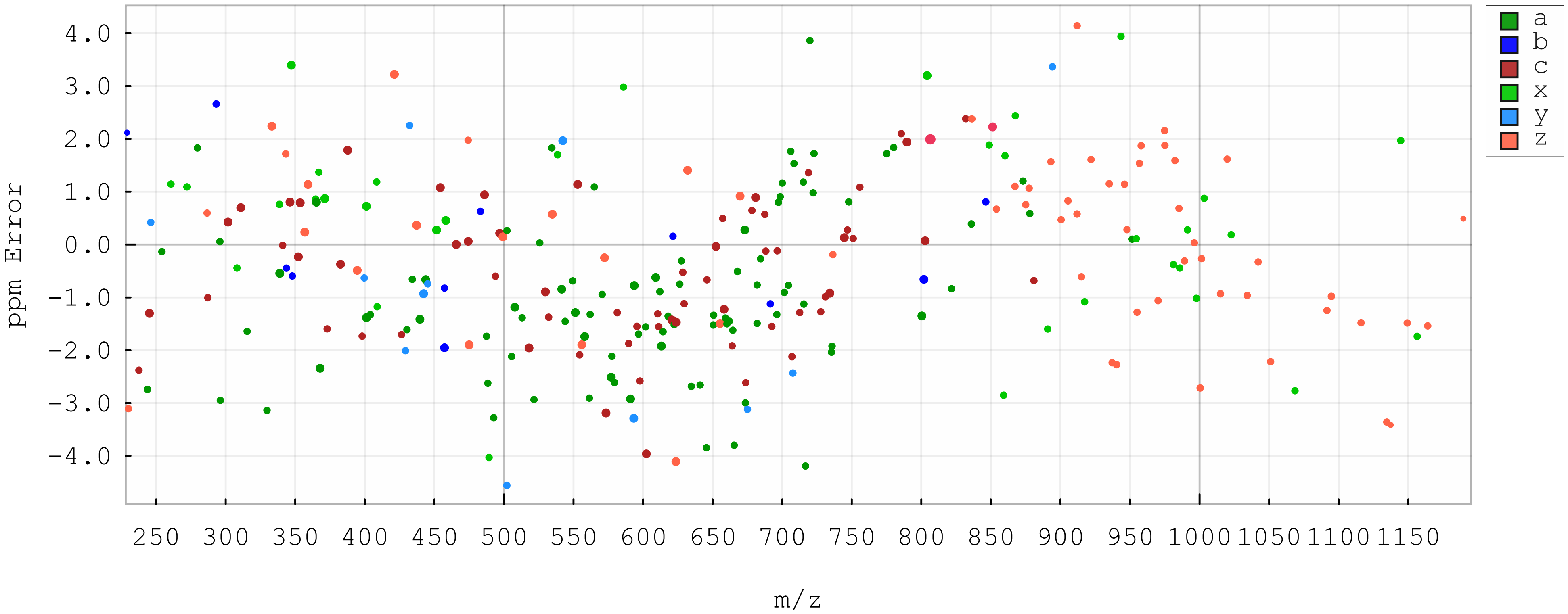

Proteoform:

Index	Protein	SC [%]	SVP [%]	IC [%]	MS Score [a.u.]	Var. Mods	Mono Mass [Da]	Chemical Formula
8	H31Kac1	68.7	29.6	25.0	17.1	Ace: K37	15305.4534	C671H1131N215O186S4

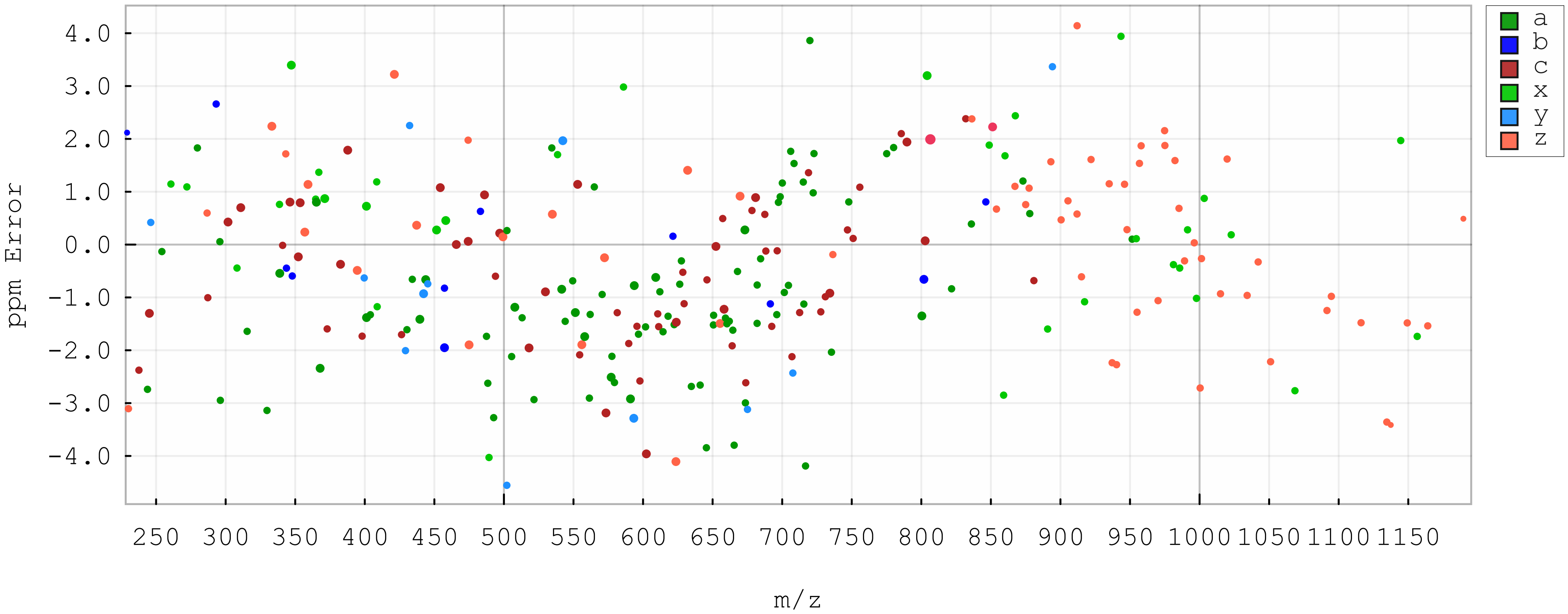

Proteoform:

Index	Protein	SC [%]	SVP [%]	IC [%]	MS Score [a.u.]	Var. Mods	Mono Mass [Da]	Chemical Formula
9	H31Kac1	59.7	32.6	20.3	12.1	Ace: K56	15305.4534	C671H1131N215O186S4

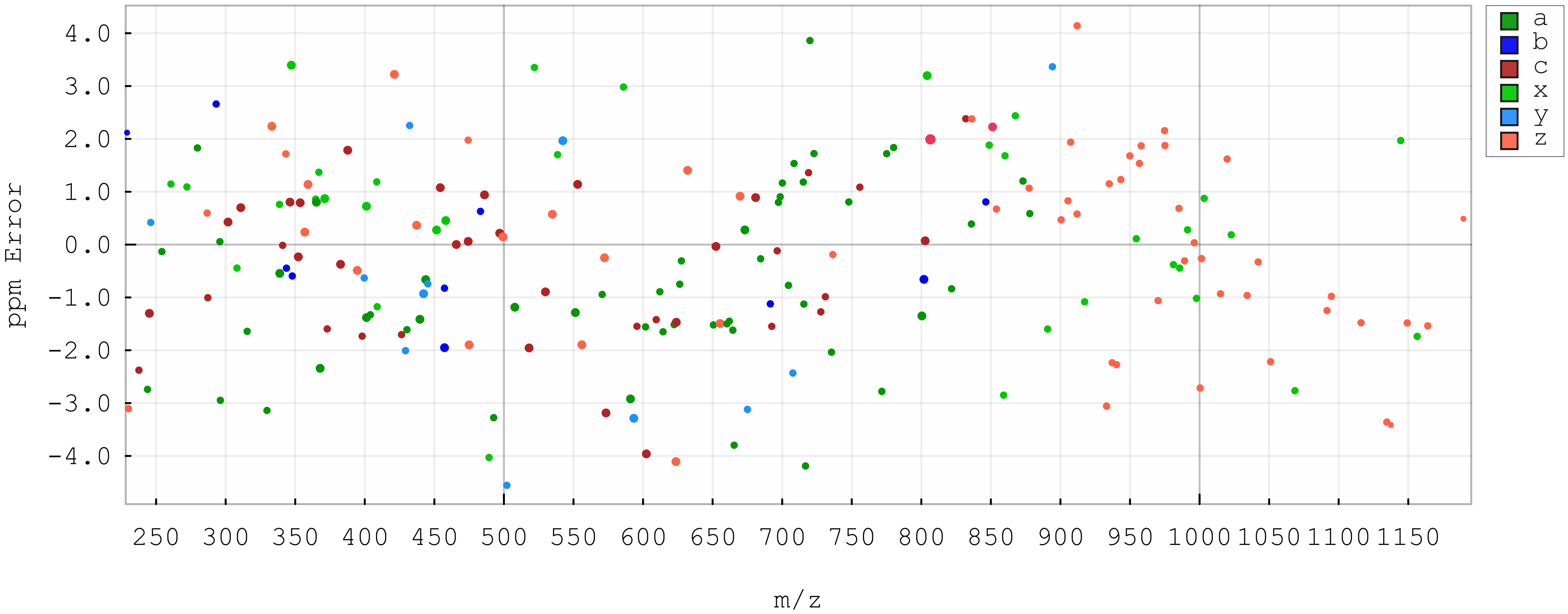

Proteoform:

Index	Protein	SC [%]	SVP [%]	IC [%]	MS Score [a.u.]	Var. Mods	Mono Mass [Da]	Chemical Formula
10	H31Kac1	57.5	32.6	18.9	10.9	Ace: K64	15305.4534	C671H1131N215O186S4

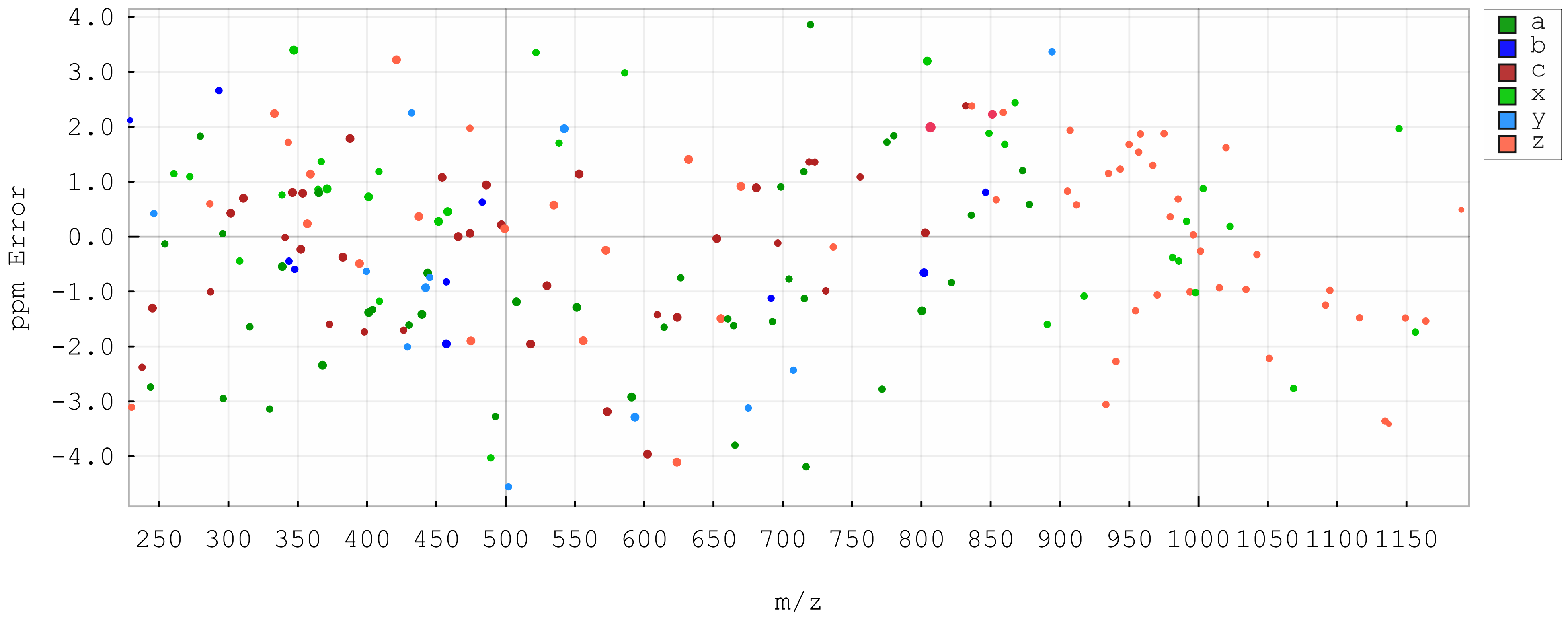

Primary Fragment Plots

Proteoform:

Index	Protein	SC [%]	SVP [%]	IC [%]	MS Score [a.u.]	Var. Mods	Mono Mass [Da]	Chemical Formula
1	H31Kac1	78.4	62.2	34.0	26.6	Ace: K14	15305.4534	$C_{671}H_{1131}N_{215}O_{186}S_4$

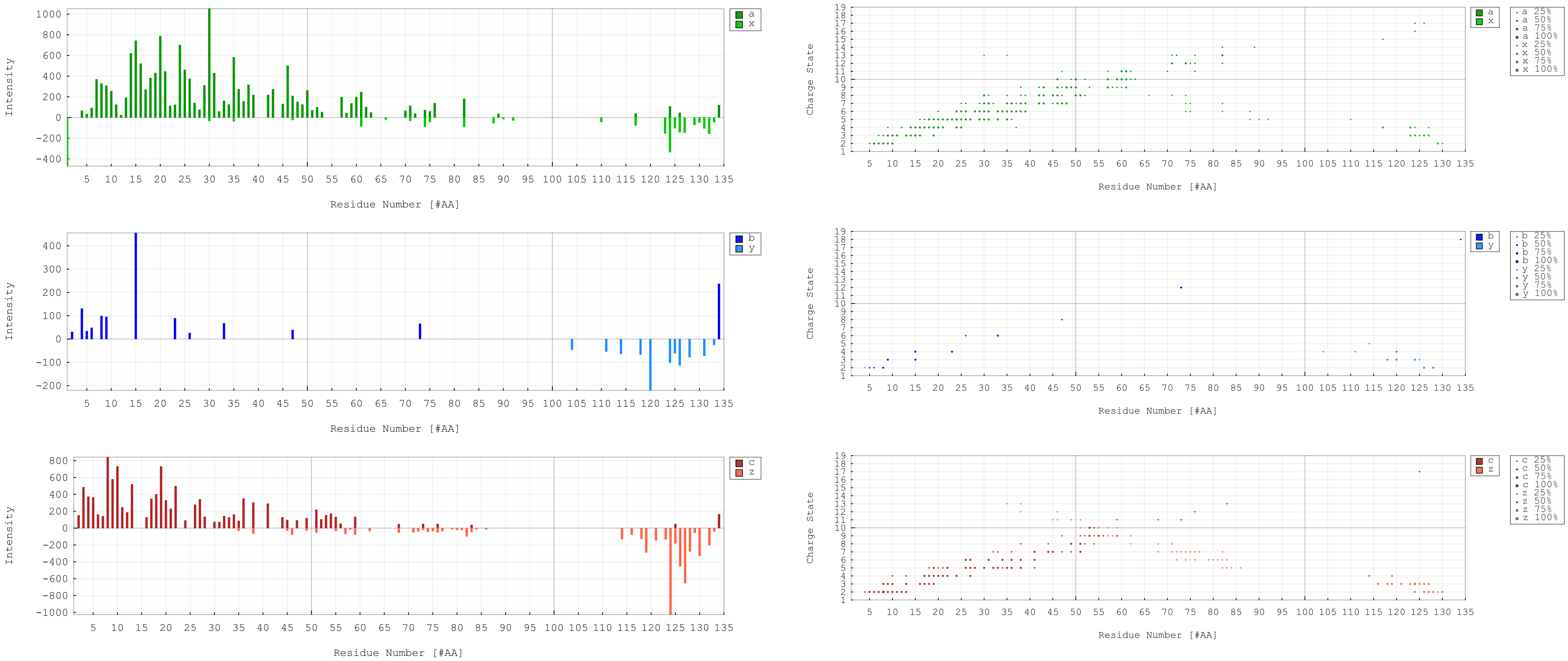

Proteoform:

Index	Protein	SC [%]	SVP [%]	IC [%]	MS Score [a.u.]	Var. Mods	Mono Mass [Da]	Chemical Formula
2	H31Kac1	76.9	62.2	32.5	25.0	Ace: K18	15305.4534	C671H1131N215O186S4

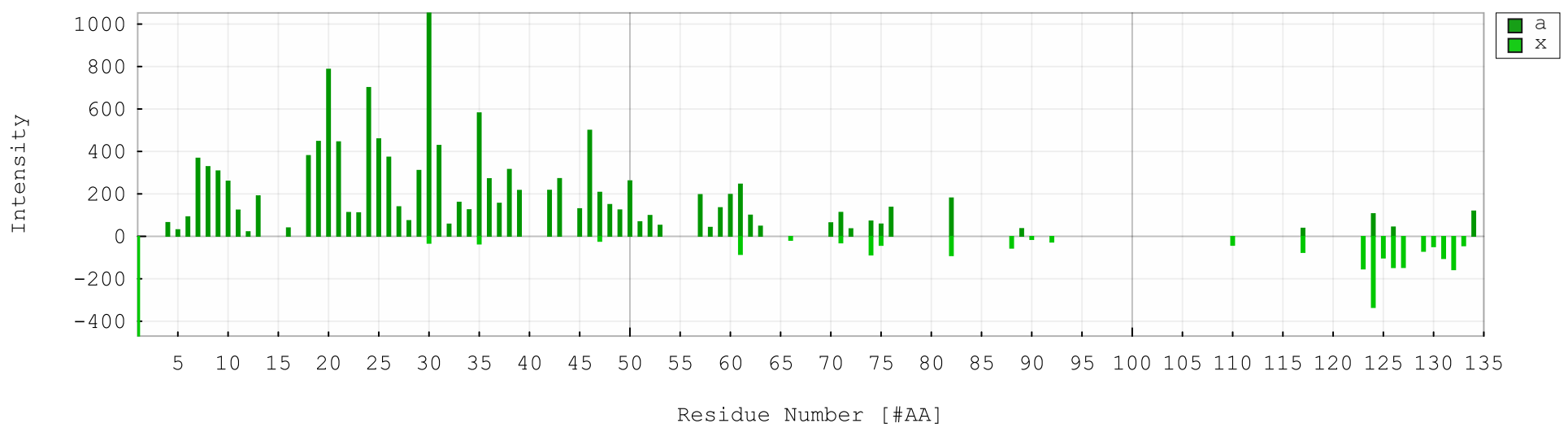

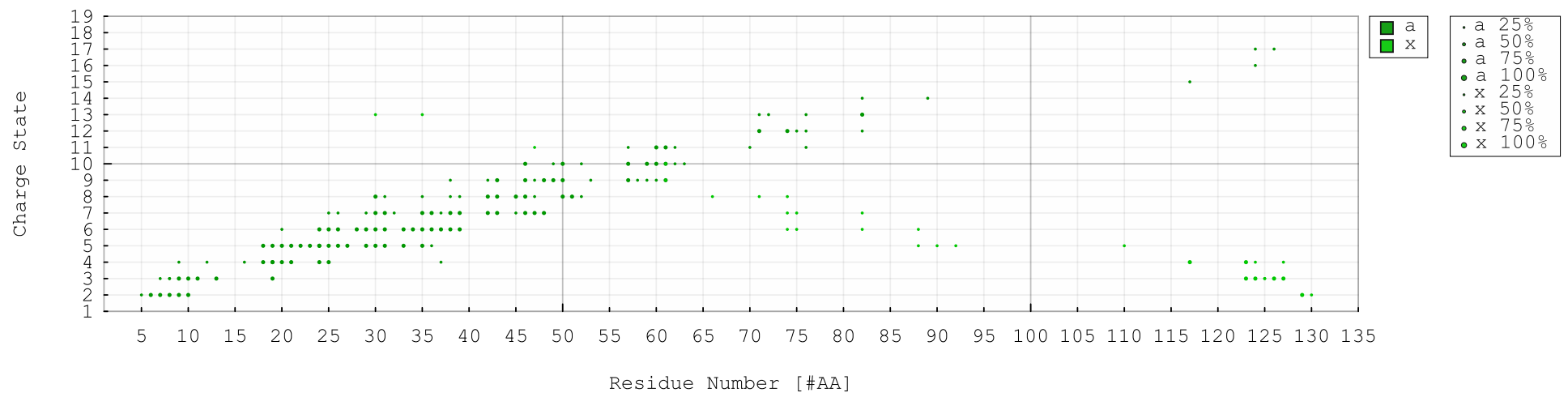

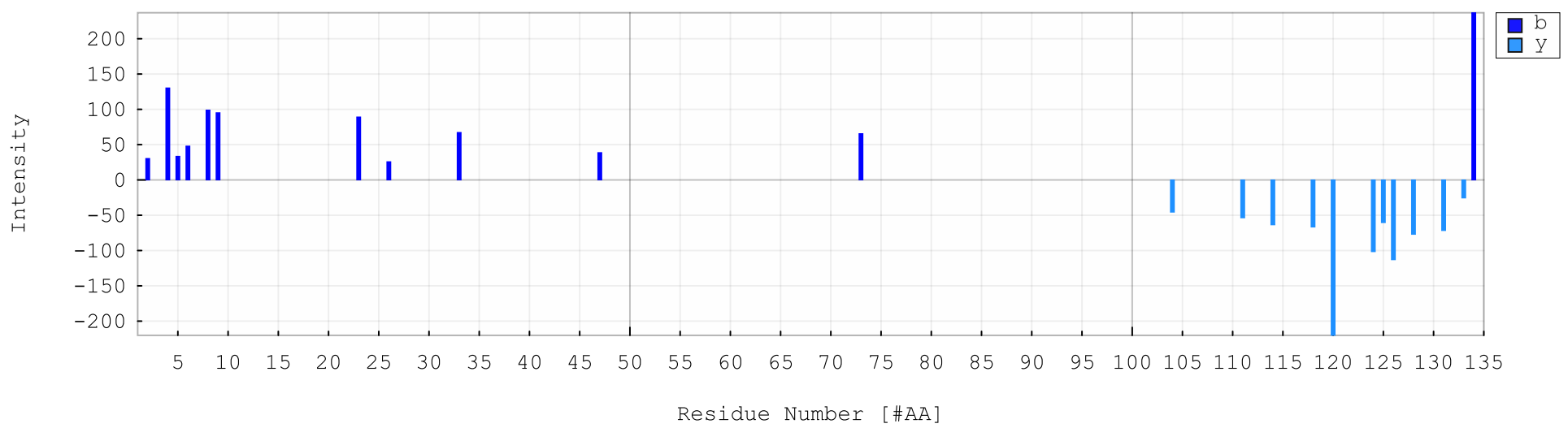

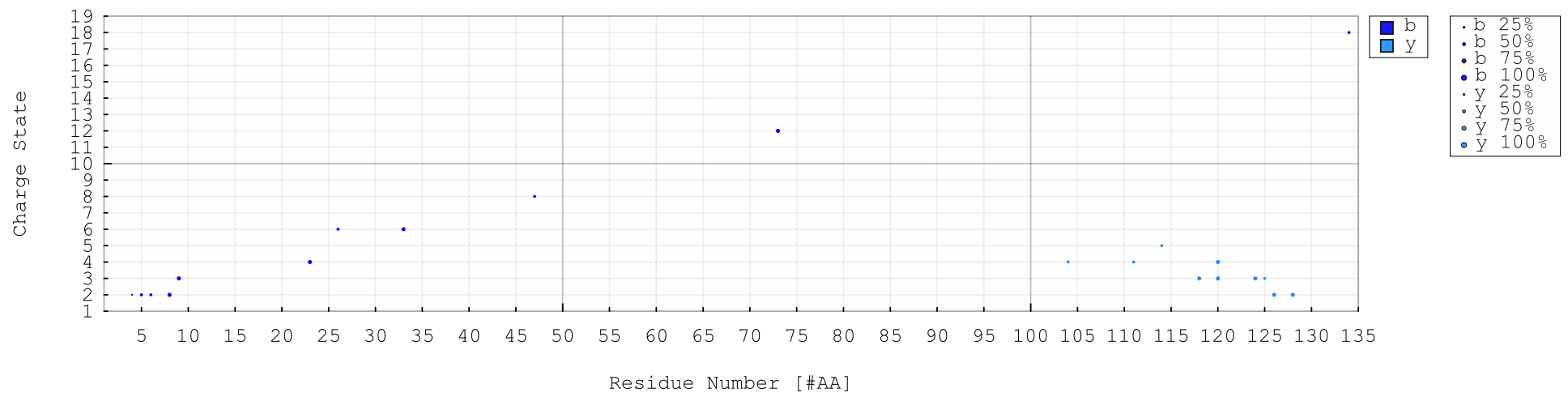

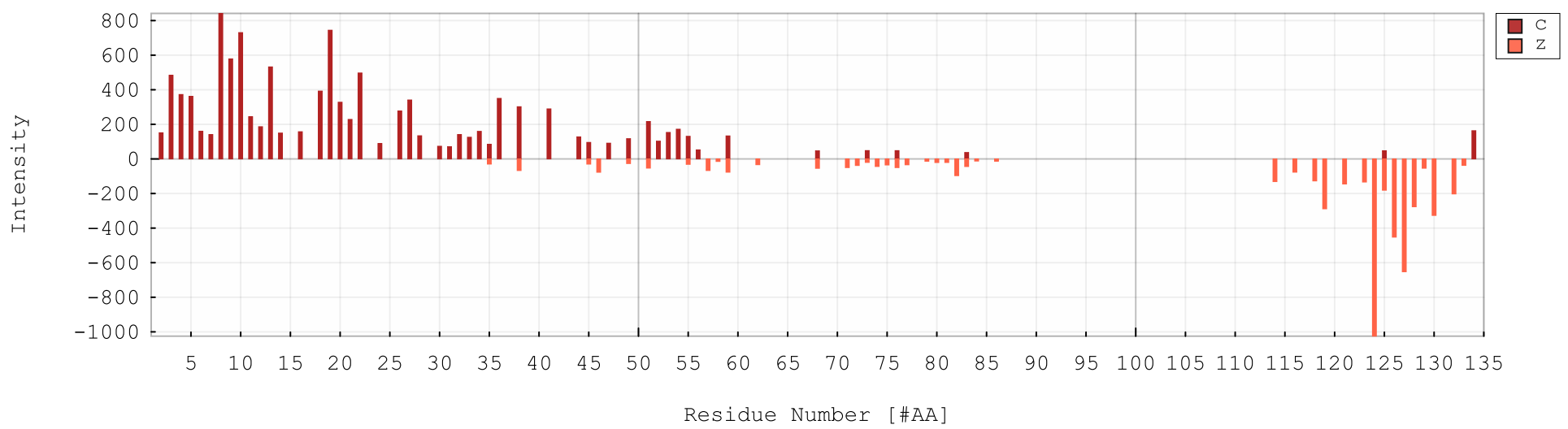

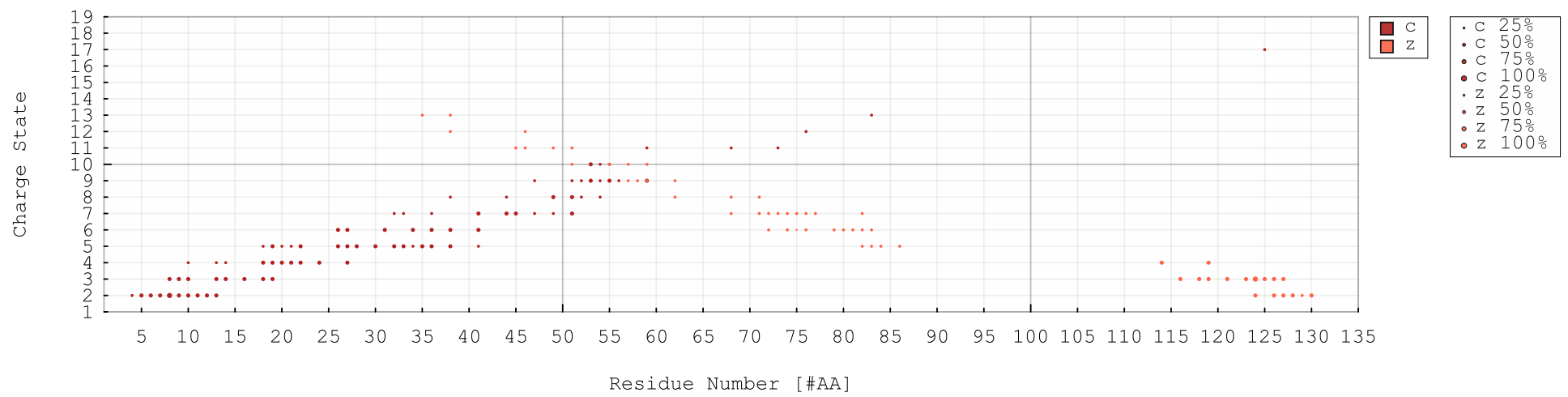

Proteoform:

Index	Protein	SC [%]	SVP [%]	IC [%]	MS Score [a.u.]	Var. Mods	Mono Mass [Da]	Chemical Formula
3	H31Kac1	75.4	22.2	32.6	24.6	Ace: K9	15305.4534	C671H1131N215O186S4

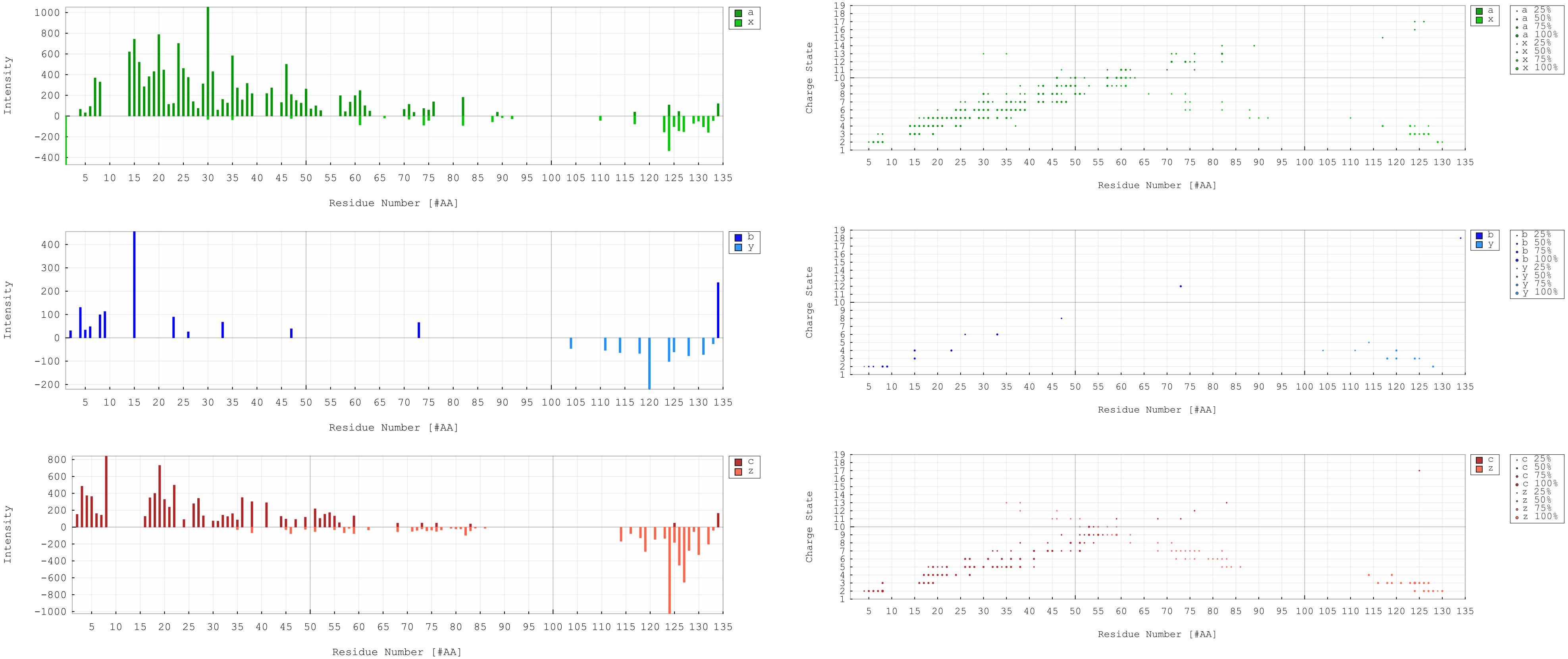

Proteoform:

Index	Protein	SC [%]	SVP [%]	IC [%]	MS Score [a.u.]	Var. Mods	Mono Mass [Da]	Chemical Formula
4	H31Kac1	72.4	17.8	31.7	22.9	Ace: K4	15305.4534	C671H1131N215O186S4

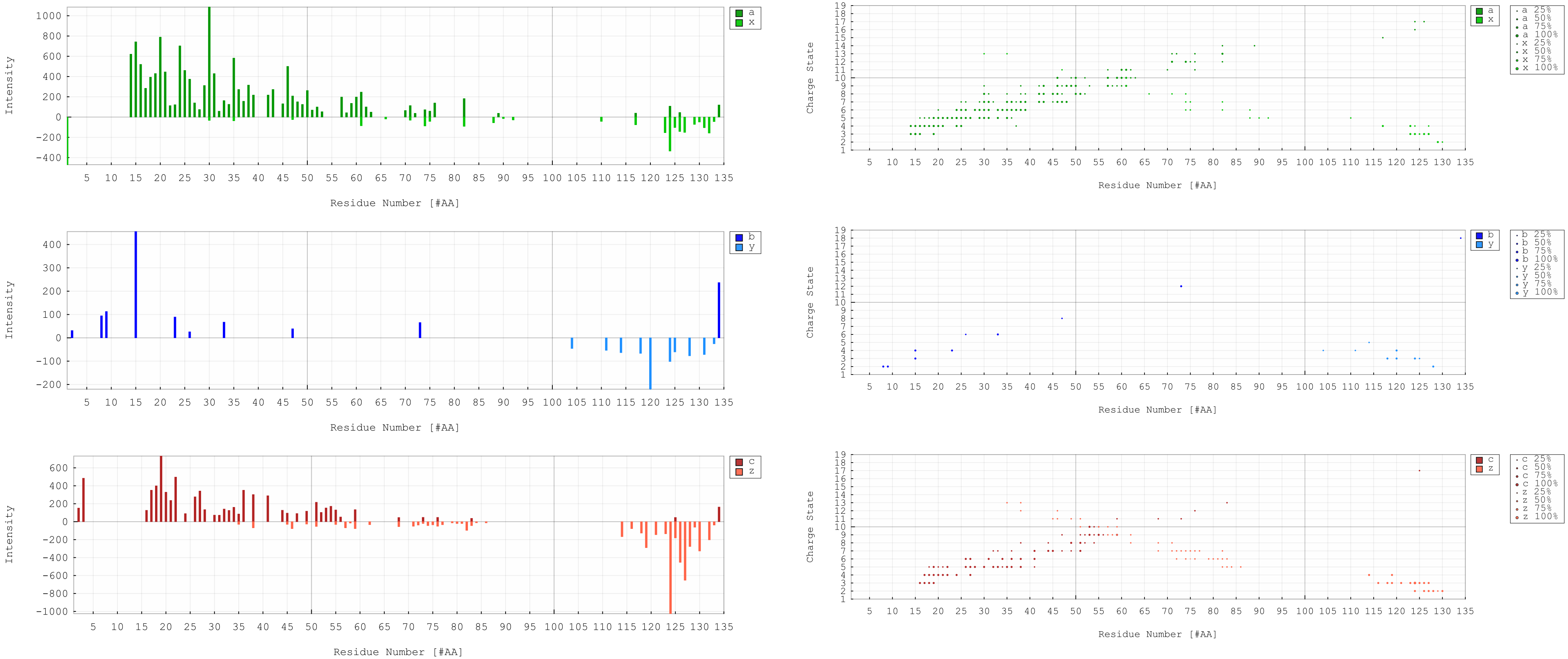

Proteoform:

Index	Protein	SC [%]	SVP [%]	IC [%]	MS Score [a.u.]	Var. Mods	Mono Mass [Da]	Chemical Formula
5	H31Kac1	74.6	29.6	29.9	22.3	Ace: K23	15305.4534	C671H1131N215O186S4

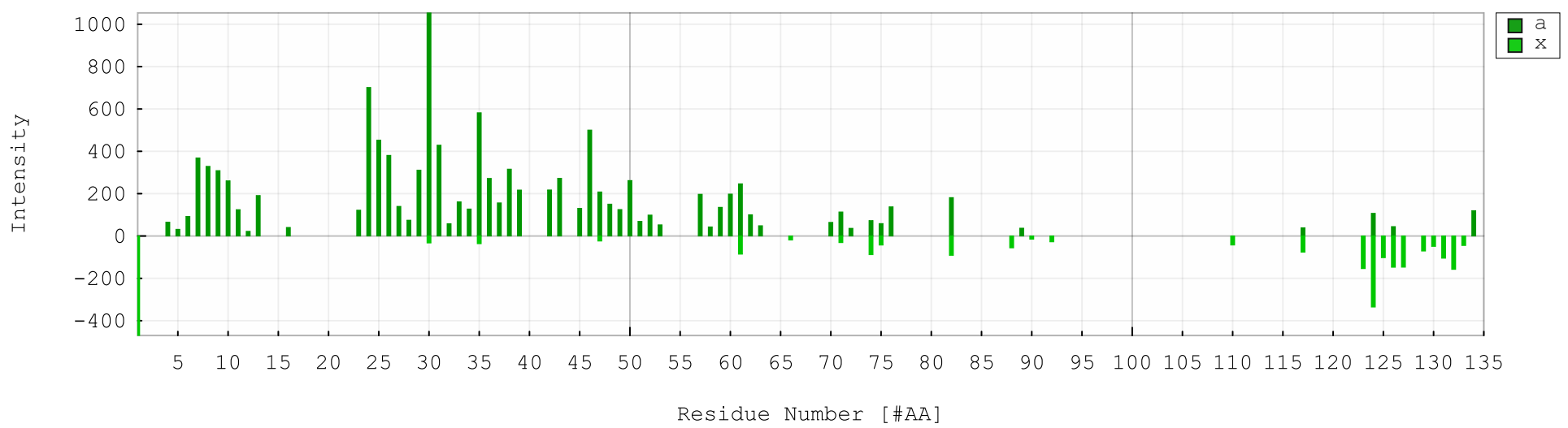

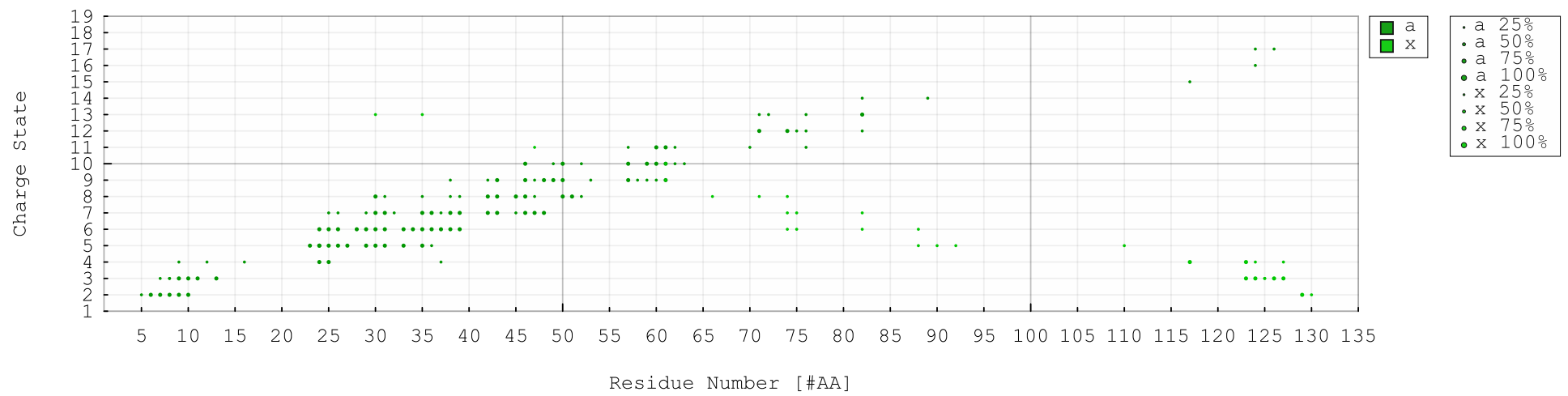

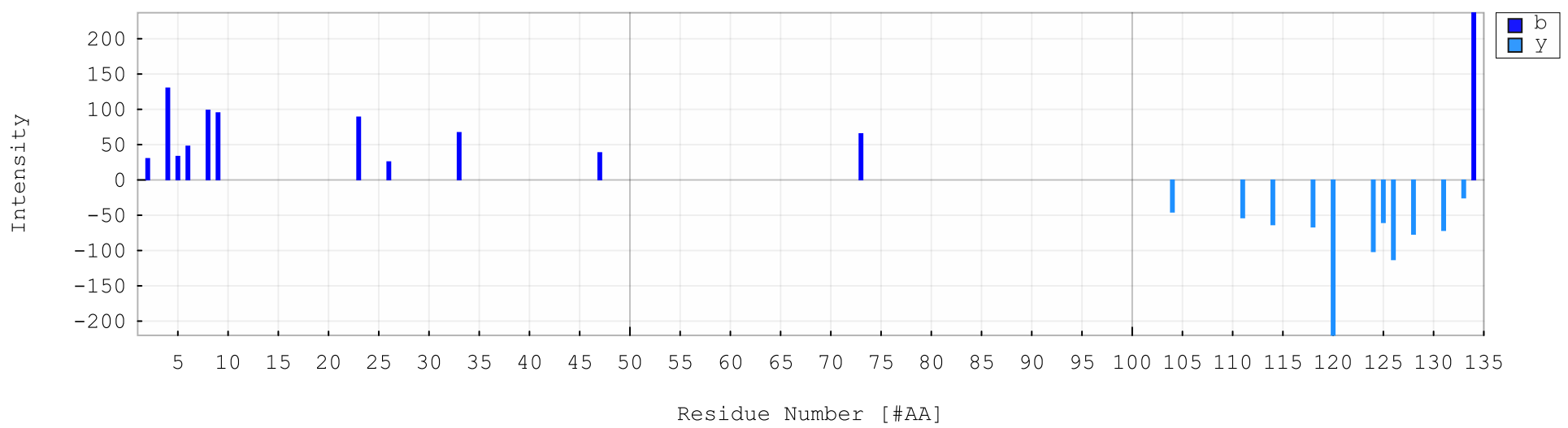

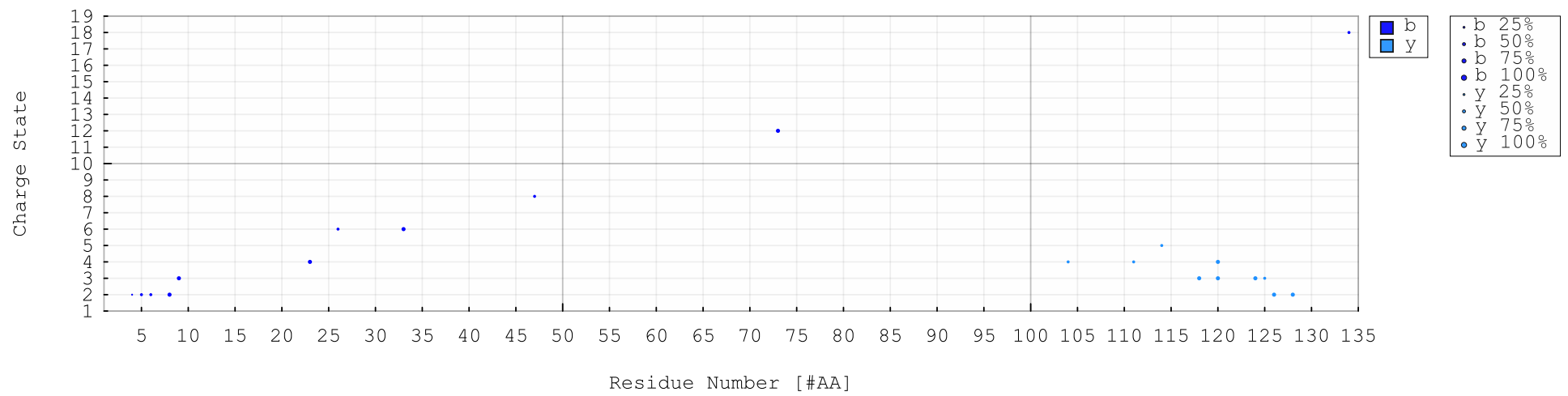

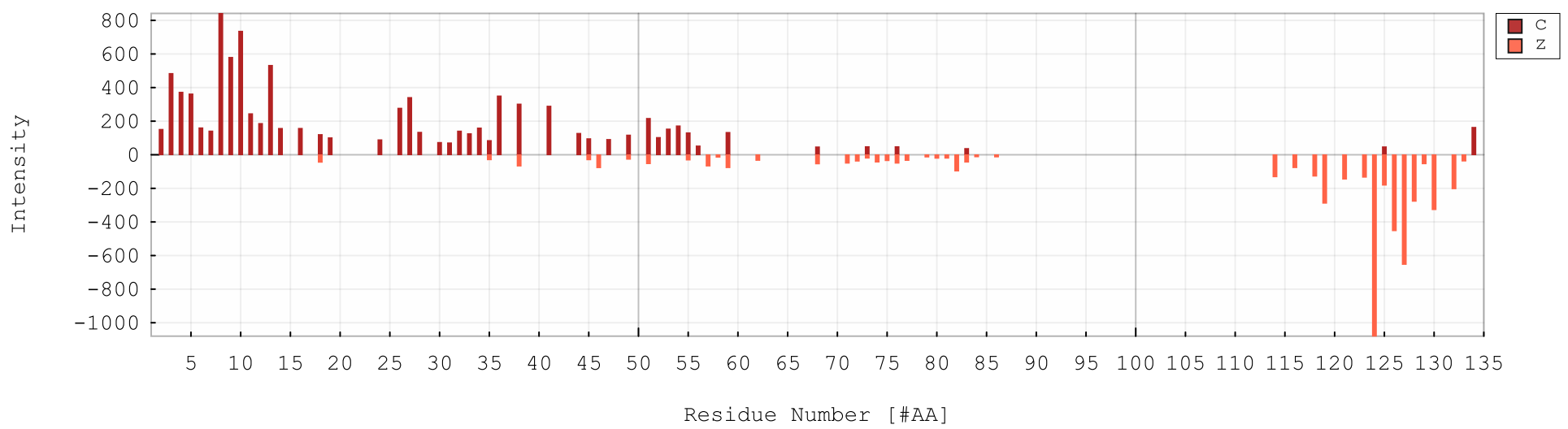

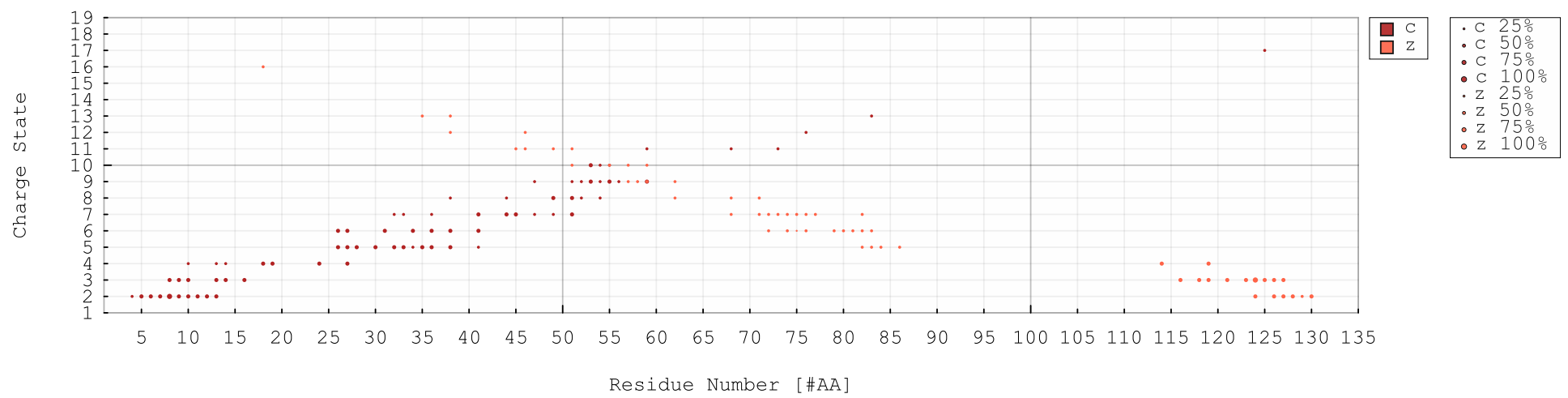

Proteoform:

Index	Protein	SC [%]	SVP [%]	IC [%]	MS Score [a.u.]	Var. Mods	Mono Mass [Da]	Chemical Formula
6	H31Kac1	73.1	29.6	28.5	20.9	Ace: K27	15305.4534	$C_{671}H_{1131}N_{215}O_{186}S_4$

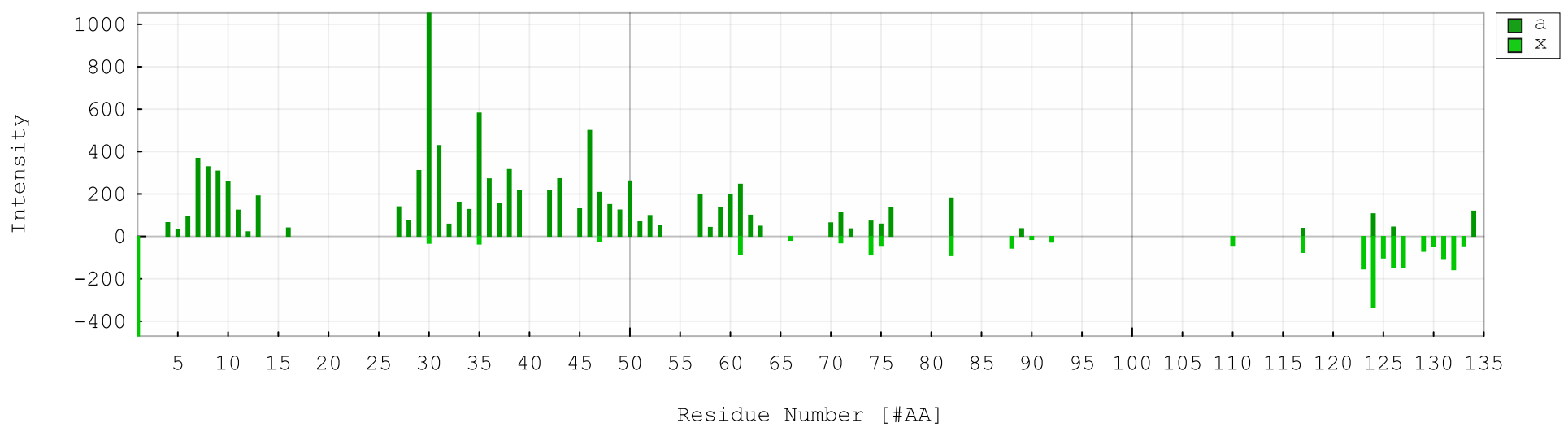

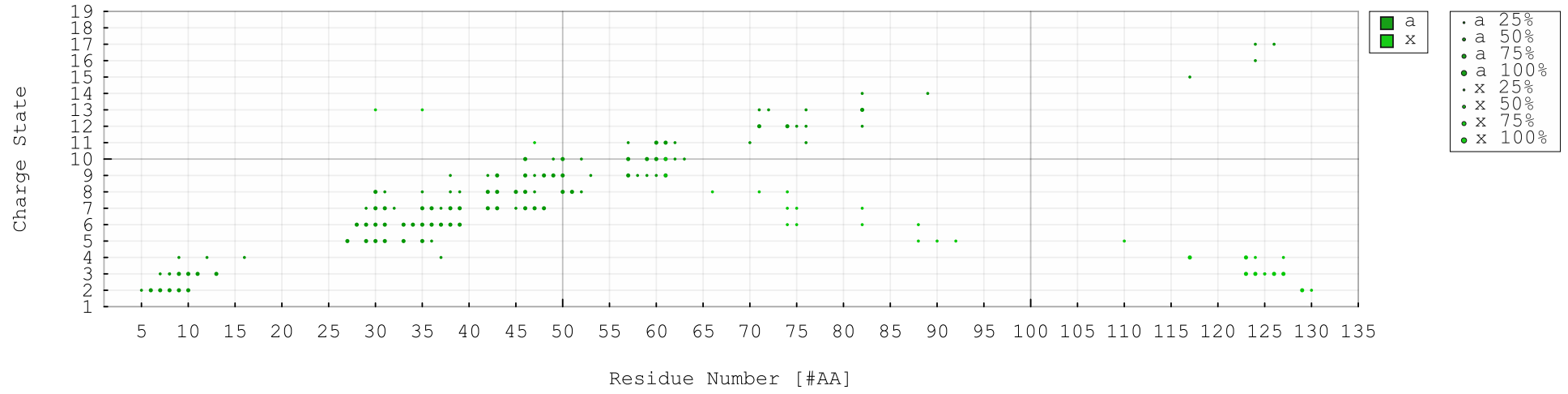

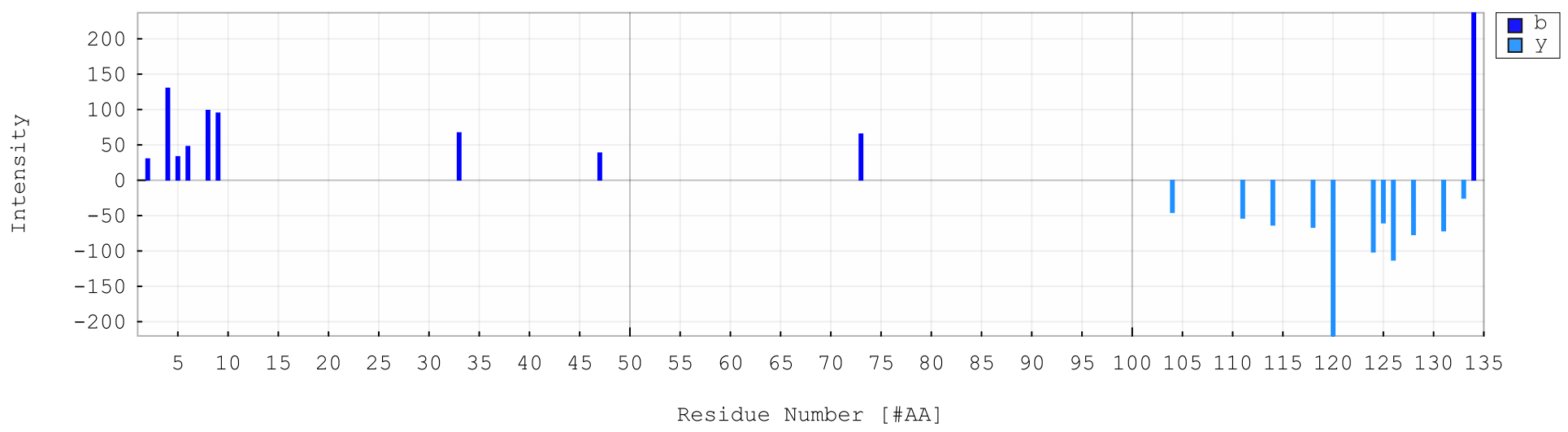

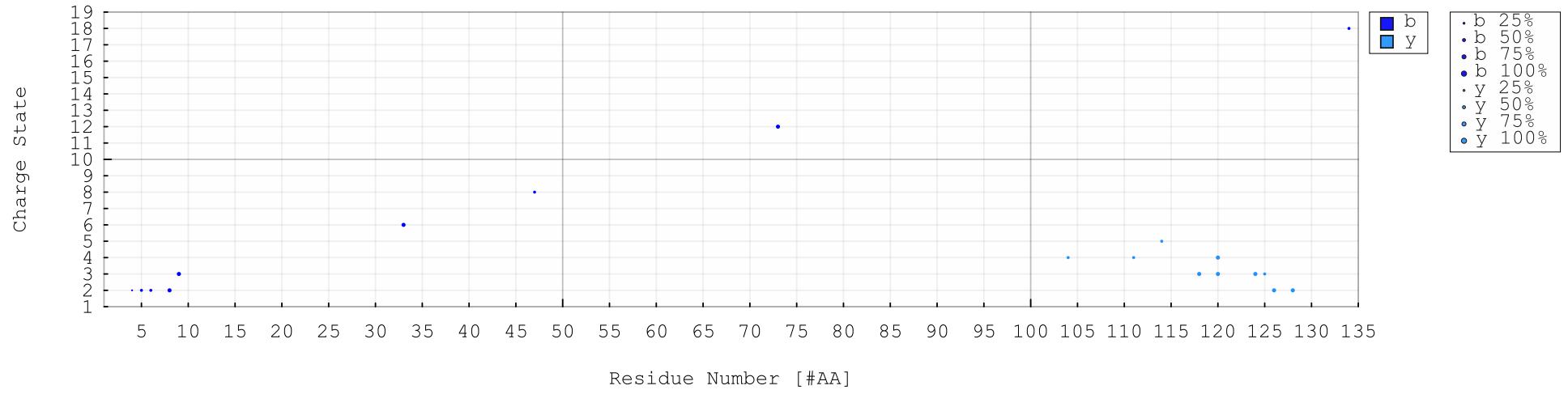

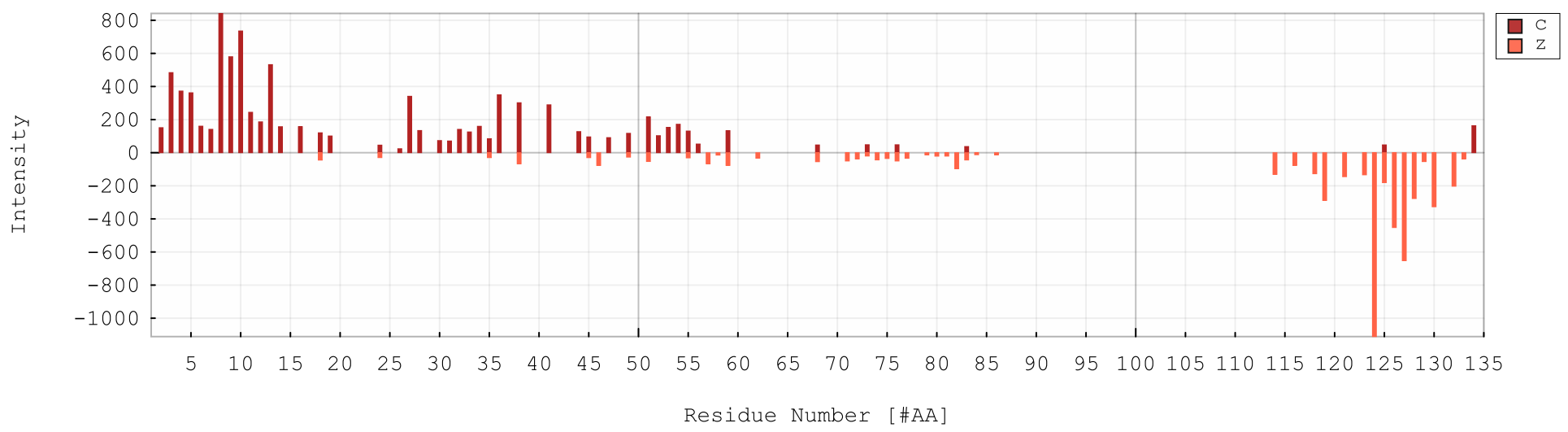

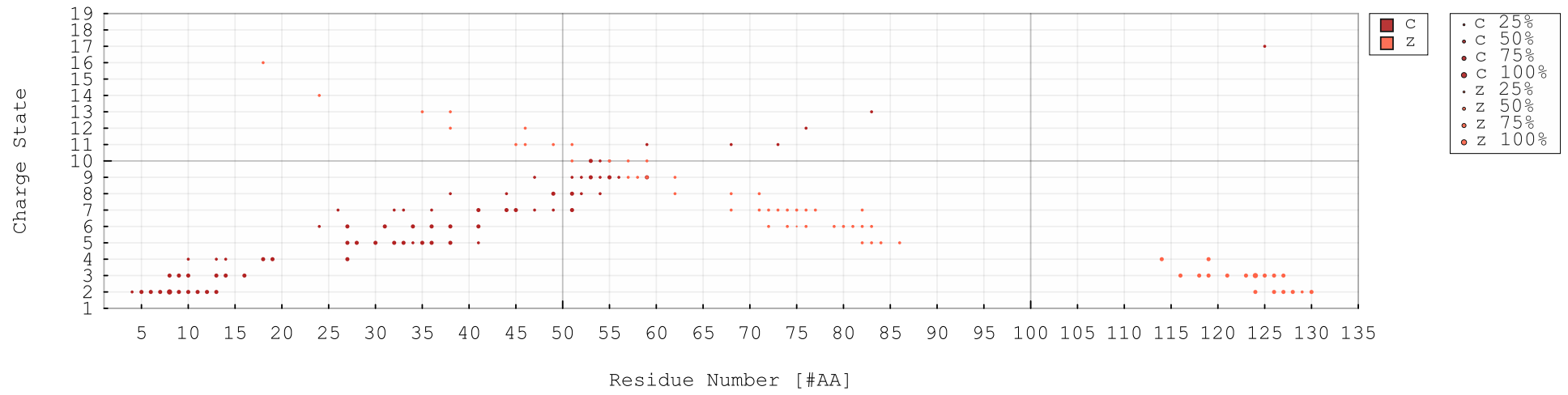

Proteoform:

Index	Protein	SC [%]	SVP [%]	IC [%]	MS Score [a.u.]	Var. Mods	Mono Mass [Da]	Chemical Formula
7	H31Kac1	69.4	29.6	25.5	17.7	Ace: K36	15305.4534	C671H1131N215O186S4

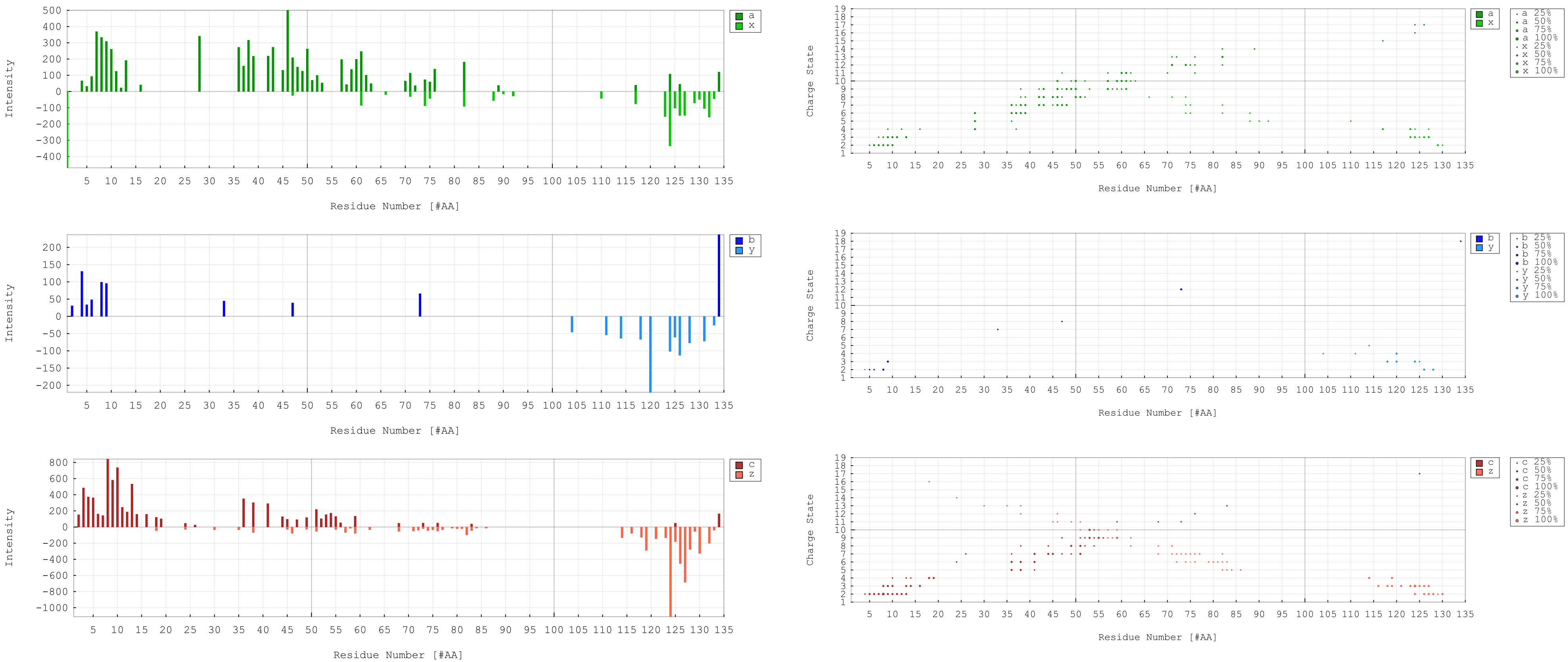

Proteoform:

Index	Protein	SC [%]	SVP [%]	IC [%]	MS Score [a.u.]	Var. Mods	Mono Mass [Da]	Chemical Formula
8	H31Kac1	68.7	29.6	25.0	17.1	Ace: K37	15305.4534	C671H1131N215O186S4

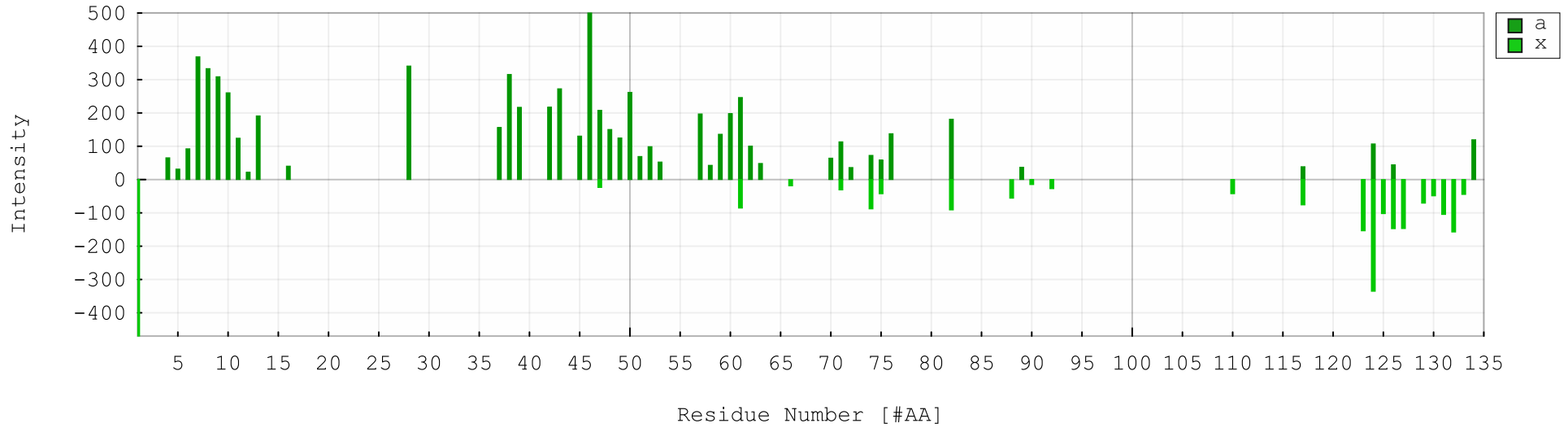

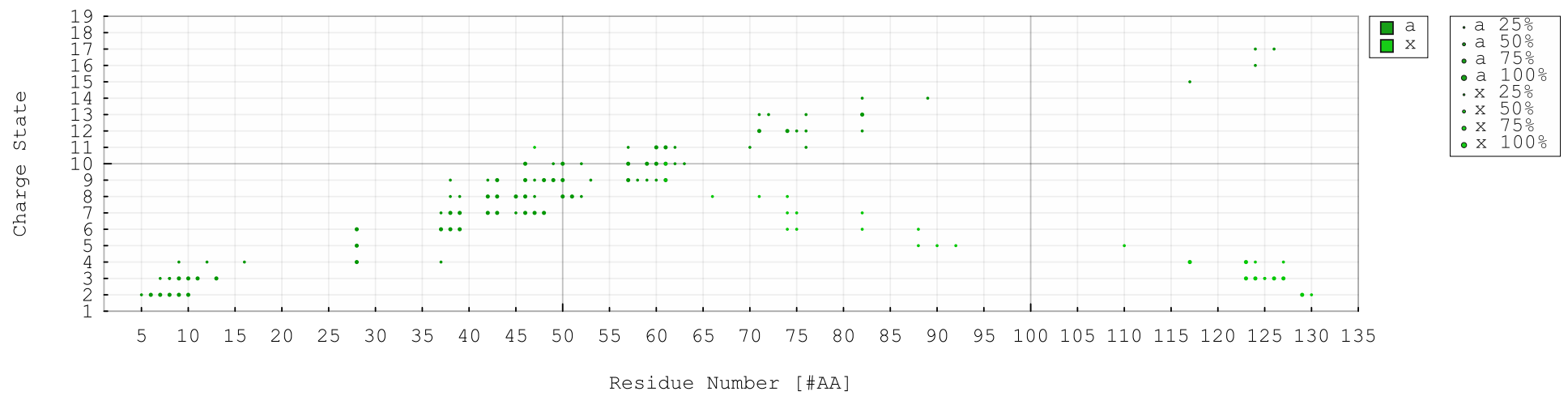

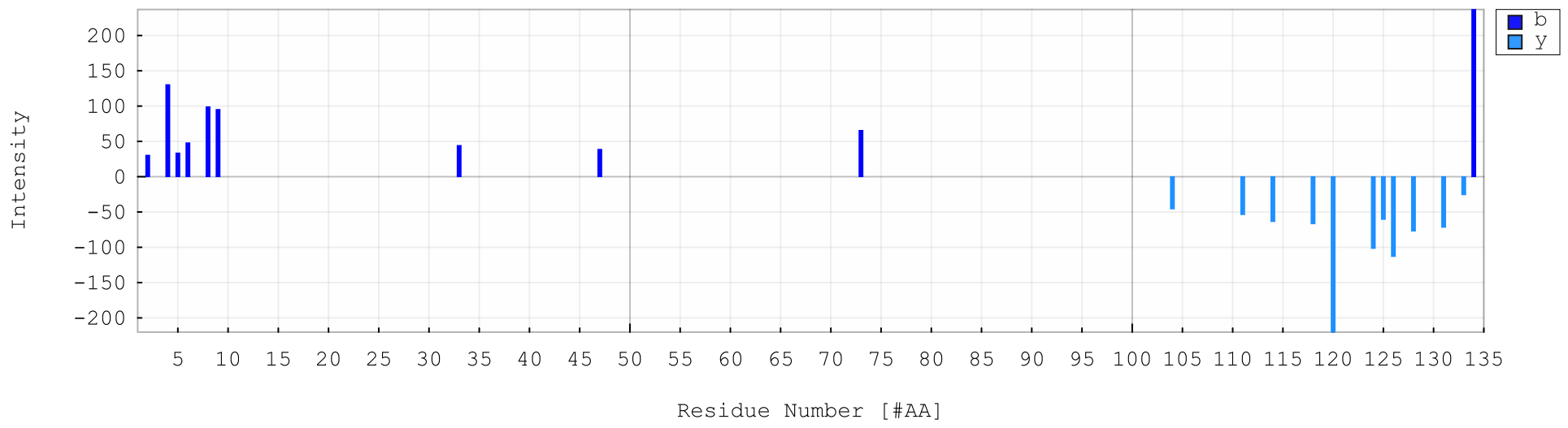

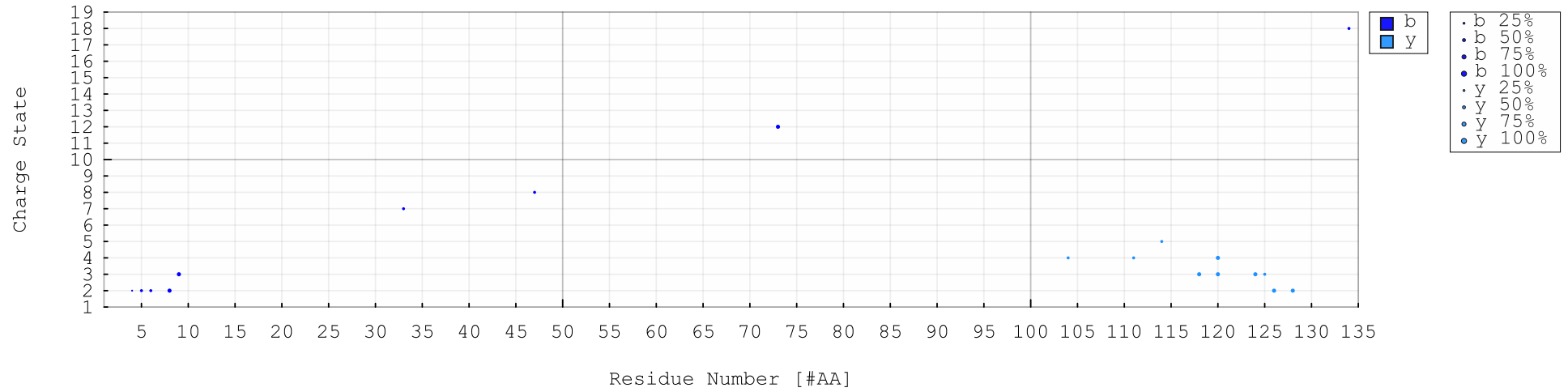

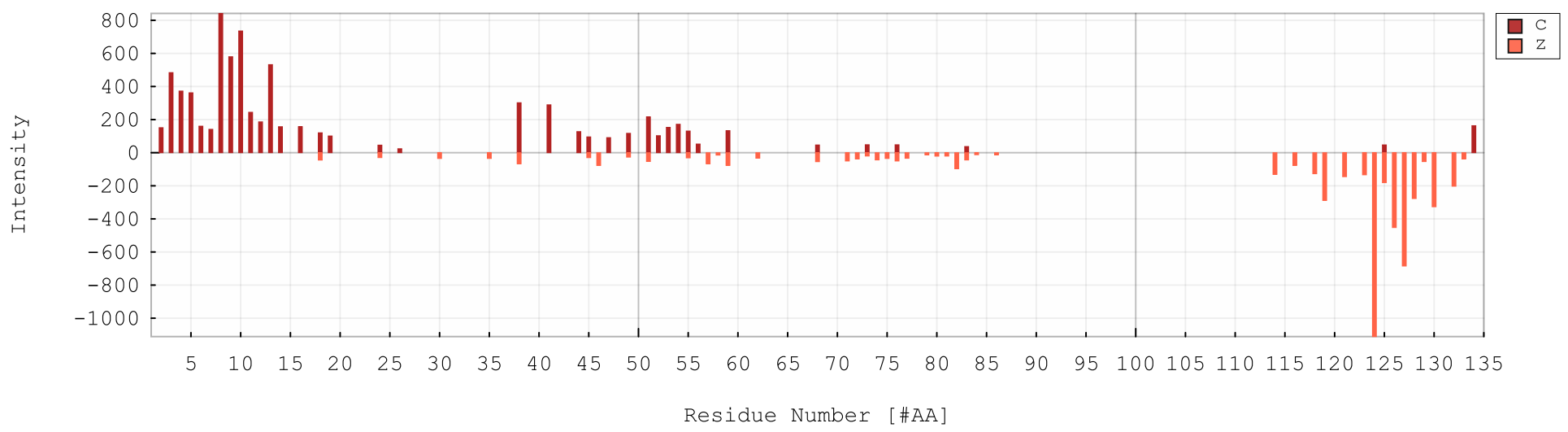

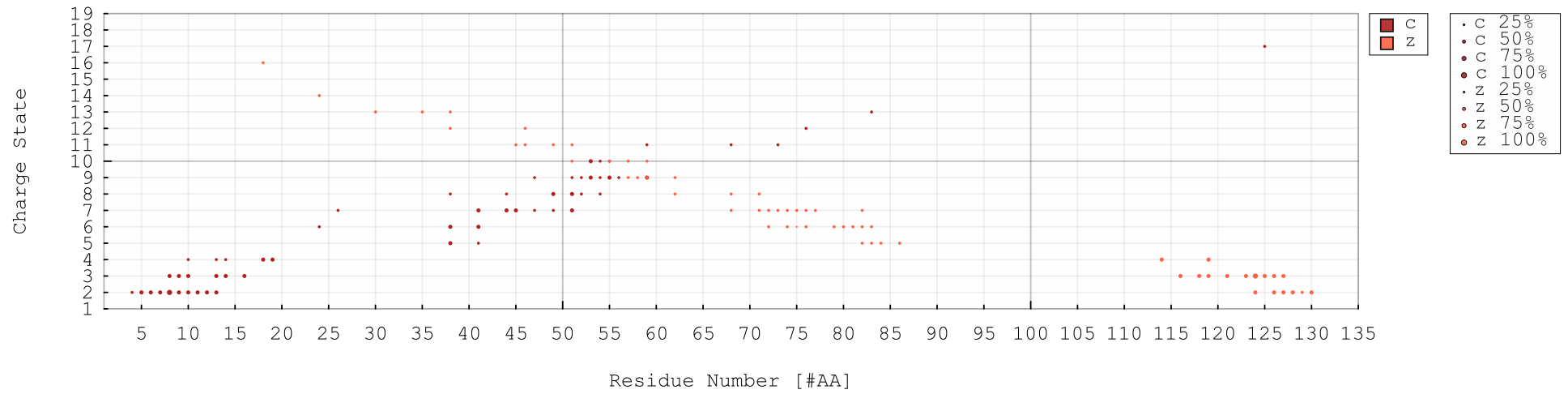

Proteoform:

Index	Protein	SC [%]	SVP [%]	IC [%]	MS Score [a.u.]	Var. Mods	Mono Mass [Da]	Chemical Formula
9	H31Kac1	59.7	32.6	20.3	12.1	Ace: K56	15305.4534	C671H1131N215O186S4

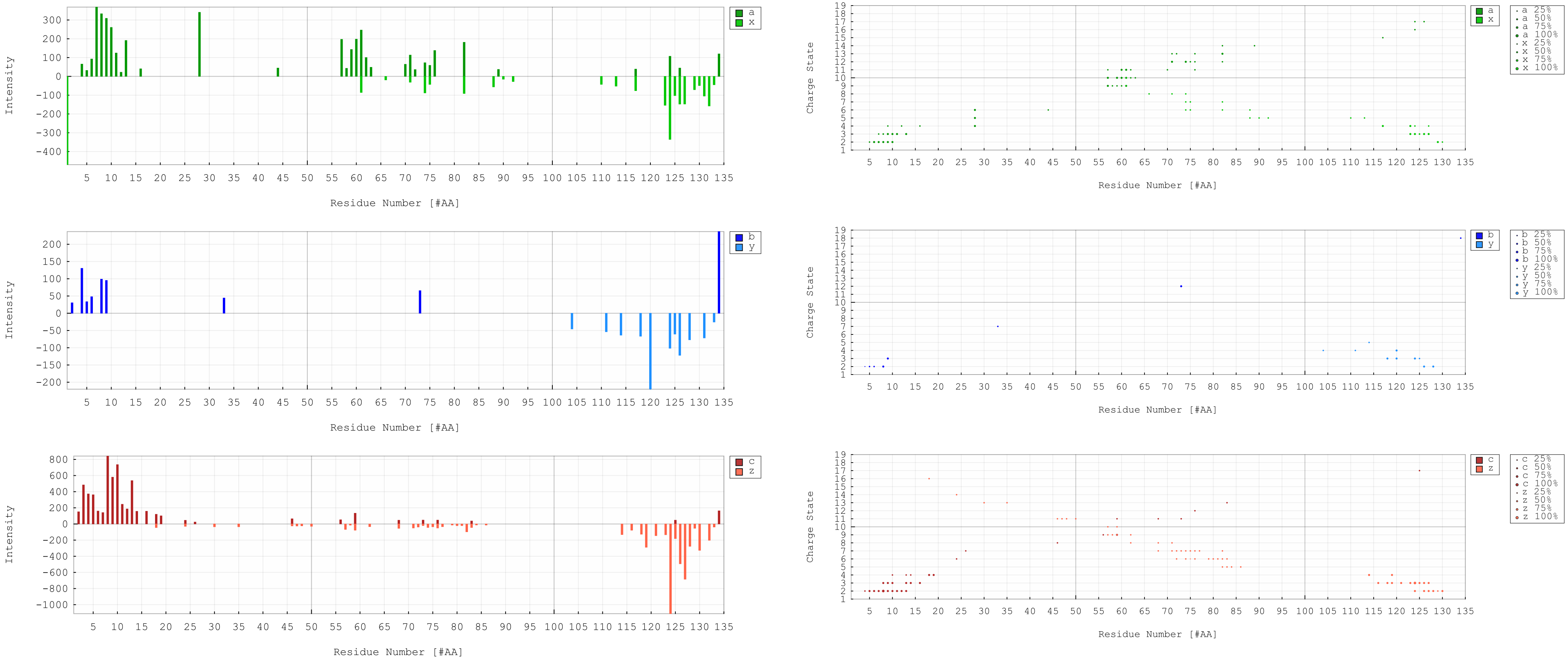

Proteoform:

Index	Protein	SC [%]	SVP [%]	IC [%]	MS Score [a.u.]	Var. Mods	Mono Mass [Da]	Chemical Formula
10	H31Kac1	57.5	32.6	18.9	10.9	Ace: K64	15305.4534	C671H1131N215O186S4

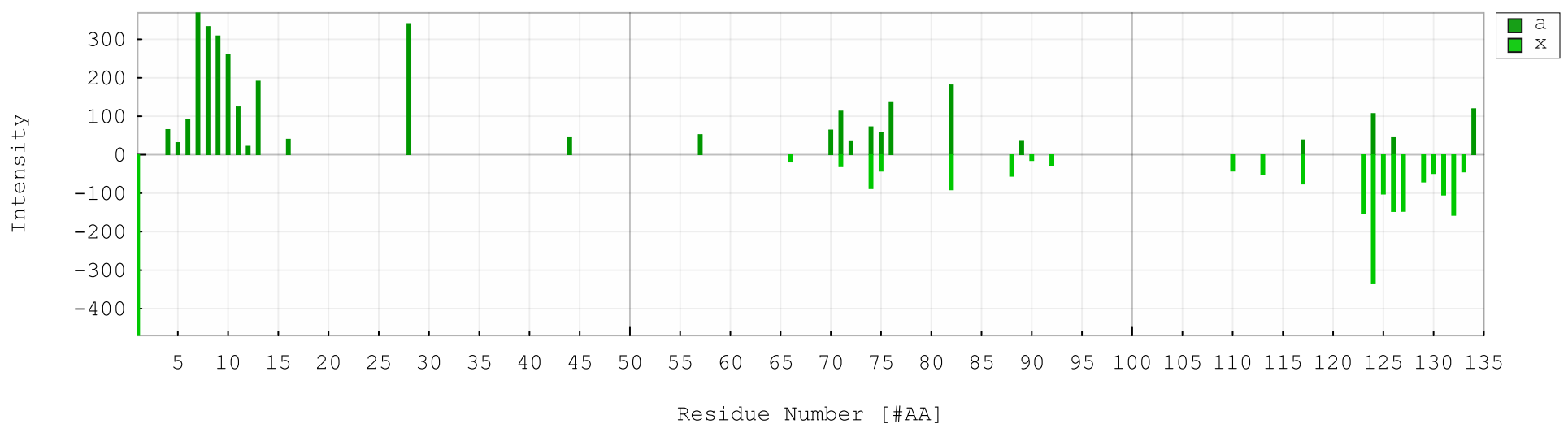

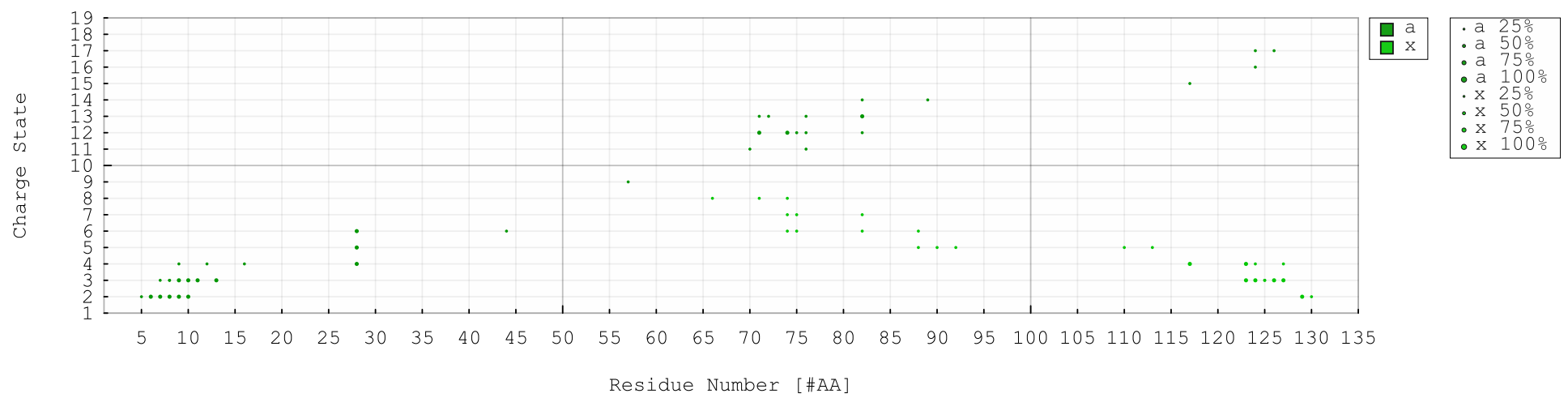

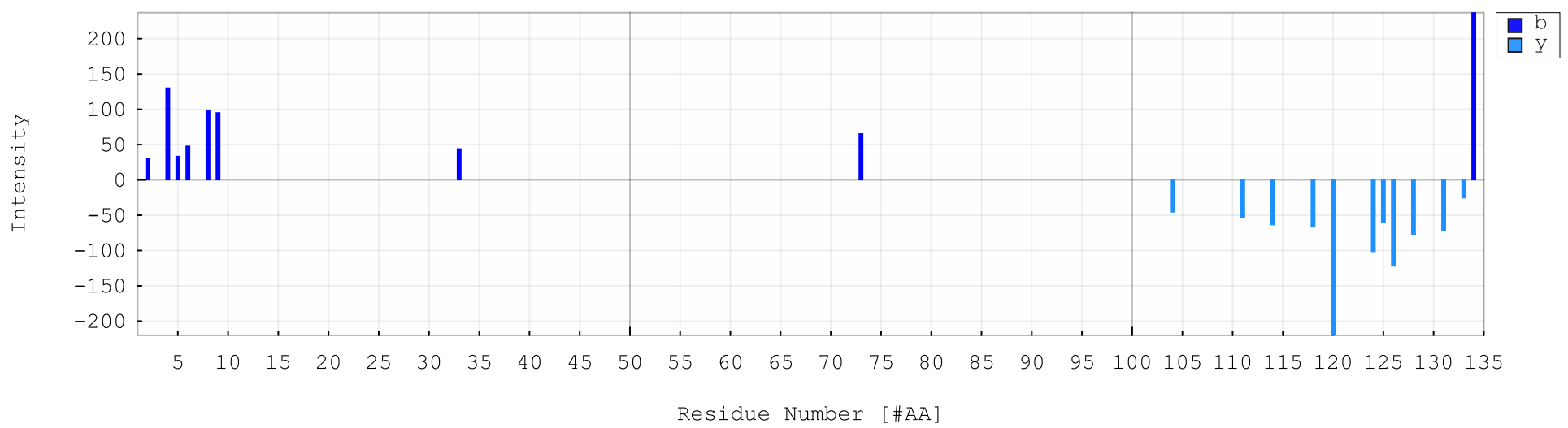

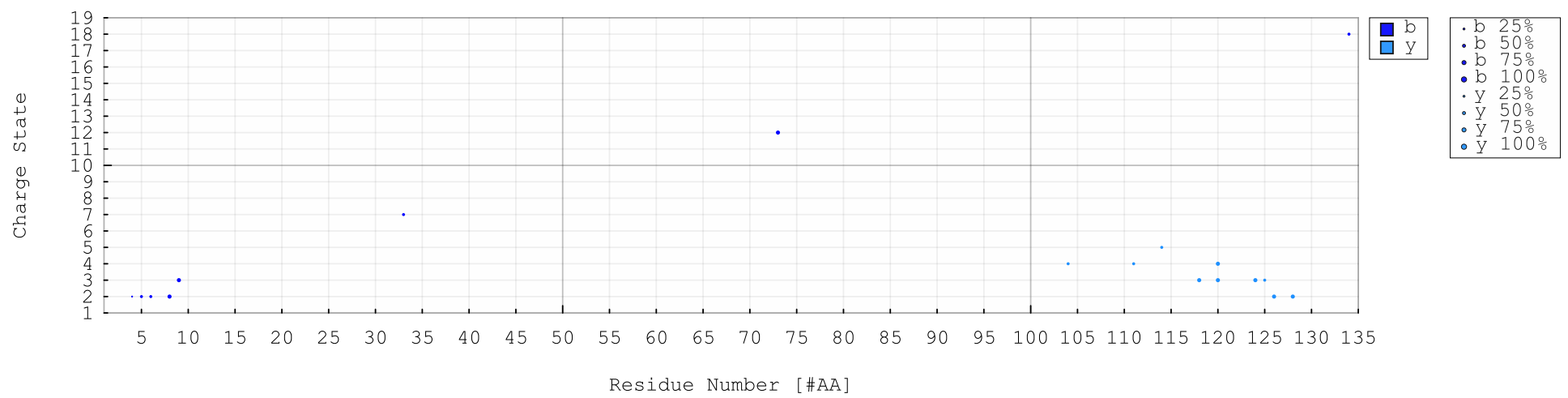

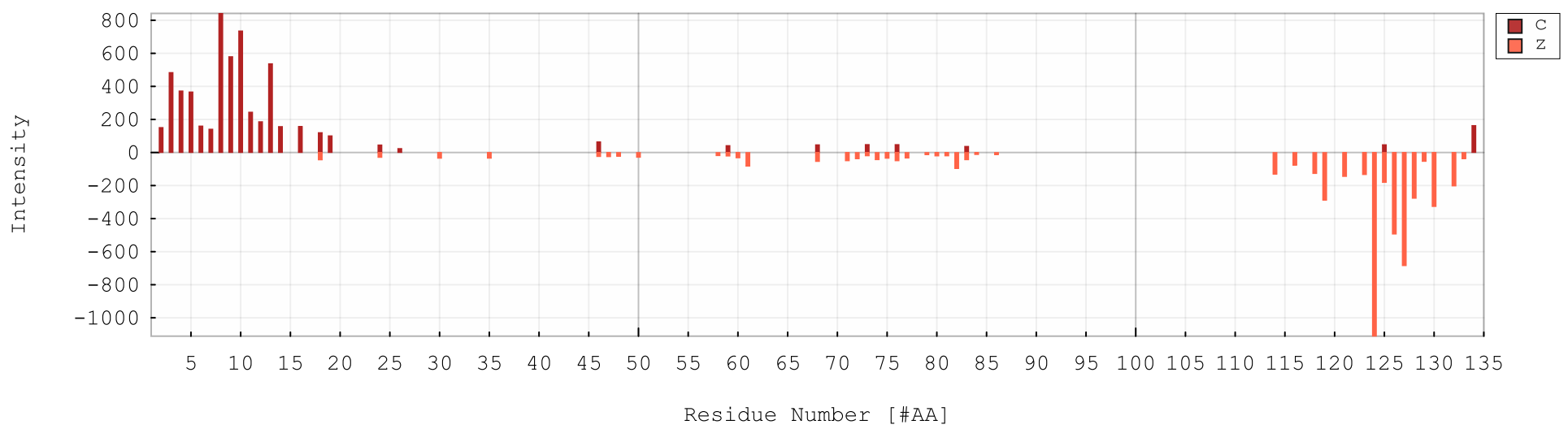

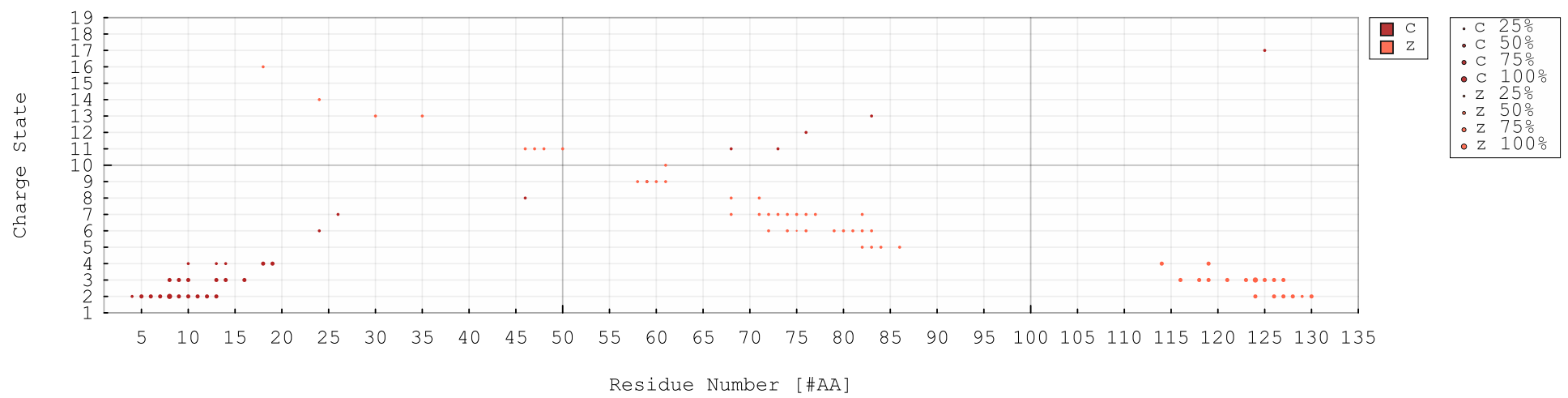

Supplemental Data S2

H3.1 - GCN5, 10 min

**Report**	4 of 7
**Experiment**	MS2
**Histone/sample**	H3.1
**Enzyme**	GCN5
**Reaction/source**	10 min
**Precursor**	H3.1Kac1
**Charge state**	19+
**Fragmentation / workflow**	RCID
**Original file**	MS2_H3.1Kac1_19+_RCID.pdf

Project Information

Project Directory: C:/Users/LAB-ADMIN
Processing date: Wed Dec 10 11:35:05 2025

Sample Info & Protocols

Name: Top-Down Protein Sequencing CID

Dataset

Imported spectrum file name:	241210 H31-GNC5_10 z19-2 MS2 shCID Q2_013-128_calibrated.txt
Dataset name:	Information is only available from imported JPEAKS files
Date acquired:	Information is only available from imported JPEAKS files
Date imported:	Information is only available from imported JPEAKS files
Instrument Name:	Information is only available from imported JPEAKS files
Instrument Serial Number:	Information is only available from imported JPEAKS files

Workflow Result Info

Analysis: 241210 H31-GNC5_10 z19-2 MS2 shCID Q2_013-128_calibrated
Location: C:/Users/LAB-ADMIN
Method: -

Method Information

Name: Unnamed

Spectral Parameters

Mass Error [ppm]: 6
Intensity Deviation [au]: 0.15
Min/Max Charges: 1-19
Experiment type: CID

a ions	b ions	c ions	x ions	y ions	z ions	satellite	internal	precursor
a	b			y				M

Noise threshold calculation: Simple moving average noise threshold (SMA)
Window size [datapoints]: 600
Noise curve offset: 7.1159825
SVP parameters: terminal gap: 2, internal gap: 1

Proteoforms

(top 10)

Index	Parent Sequence	Chain	Var. Mods	Mono Mass [Da]	Chemical Composition
1	H31Kac1	H31Kac1K14[Ace]	Ace: K14	15305.4534	C671H1131N215O186S4
2	H31Kac1	H31Kac1K9[Ace]	Ace: K9	15305.4534	C671H1131N215O186S4
3	H31Kac1	H31Kac1K18[Ace]	Ace: K18	15305.4534	C671H1131N215O186S4
4	H31Kac1	H31Kac1K23[Ace]	Ace: K23	15305.4534	C671H1131N215O186S4
5	H31Kac1	H31Kac1K4[Ace]	Ace: K4	15305.4534	C671H1131N215O186S4
6	H31Kac1	H31Kac1K27[Ace]	Ace: K27	15305.4534	C671H1131N215O186S4
7	H31Kac1	H31Kac1K36[Ace]	Ace: K36	15305.4534	C671H1131N215O186S4
8	H31Kac1	H31Kac1K37[Ace]	Ace: K37	15305.4534	C671H1131N215O186S4
9	H31Kac1	H31Kac1K56[Ace]	Ace: K56	15305.4534	C671H1131N215O186S4
10	H31Kac1	H31Kac1K64[Ace]	Ace: K64	15305.4534	C671H1131N215O186S4

Results

Proteoforms Table (top 10)

Index	Protein	SC [%]	SVP [%]	IC [%]	MS Score [a.u.]	Var. Mods	Mono Mass [Da]	Chemical Formula
1	H31Kac1	41.8	6.7	23.3	9.7	Ace: K14	15305.4534	C671H1131N215O186S4
2	H31Kac1	41.0	6.7	23.2	9.5	Ace: K9	15305.4534	C671H1131N215O186S4
3	H31Kac1	40.3	6.7	23.1	9.3	Ace: K18	15305.4534	C671H1131N215O186S4
4	H31Kac1	38.8	6.7	23.0	8.9	Ace: K23	15305.4534	C671H1131N215O186S4
5	H31Kac1	38.8	5.2	23.0	8.9	Ace: K4	15305.4534	C671H1131N215O186S4
6	H31Kac1	35.8	6.7	22.6	8.1	Ace: K27	15305.4534	C671H1131N215O186S4
7	H31Kac1	32.8	6.7	21.7	7.1	Ace: K36	15305.4534	C671H1131N215O186S4
8	H31Kac1	32.8	6.7	21.7	7.1	Ace: K37	15305.4534	C671H1131N215O186S4
9	H31Kac1	31.3	6.7	21.5	6.8	Ace: K56	15305.4534	C671H1131N215O186S4
10	H31Kac1	30.6	6.7	21.3	6.5	Ace: K64	15305.4534	C671H1131N215O186S4

Sequence

Name: H31Kac1

```
                 10                   20                   30                   40                   50                   60
A R T K Q T A R K S  T G G K A P R K Q L  A T K A A R K S A P  A T G G V K K P H R  Y R P G T V A L R E  I R R Y Q K S T E L
                 70                   80                   90                  100                  110                  120
L I R K L P F Q R L  V R E I A Q D F K T  D L R F Q S S A V M  A L Q E A C E A Y L  V G L F E D T N L C  A I H A K R V T I M
                130                  140                  150                  160                  170                  180
P K D I Q L A R R I  R G E R A
```

Modifications

Modification	Position	Type
Acetyl	4	variable
Acetyl	9	variable
Acetyl	14	variable
Acetyl	18	variable
Acetyl	23	variable
Acetyl	27	variable
Acetyl	36	variable
Acetyl	37	variable
Acetyl	56	variable
Acetyl	64	variable
Acetyl	79	variable

Modification	Position	Type
Acetyl	115	variable
Acetyl	122	variable

Sequence Map

Proteoform:

Index	Protein	SC [%]	SVP [%]	IC [%]	MS Score [a.u.]	Var. Mods	Mono Mass [Da]	Chemical Formula
1	H31Kac1	41.8	6.7	23.3	9.7	Ace: K14	15305.4534	C671H1131N215O186S4

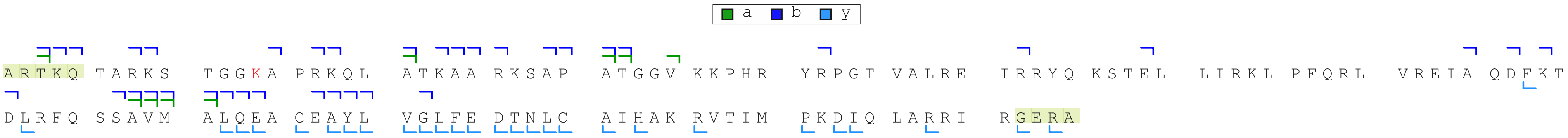

Proteoform:

Index	Protein	SC [%]	SVP [%]	IC [%]	MS Score [a.u.]	Var. Mods	Mono Mass [Da]	Chemical Formula
2	H31Kac1	41.0	6.7	23.2	9.5	Ace: K9	15305.4534	C671H1131N215O186S4

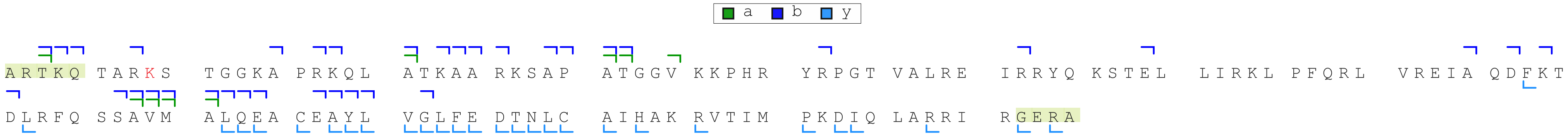

Proteoform:

Index	Protein	SC [%]	SVP [%]	IC [%]	MS Score [a.u.]	Var. Mods	Mono Mass [Da]	Chemical Formula
3	H31Kac1	40.3	6.7	23.1	9.3	Ace: K18	15305.4534	C671H1131N215O186S4

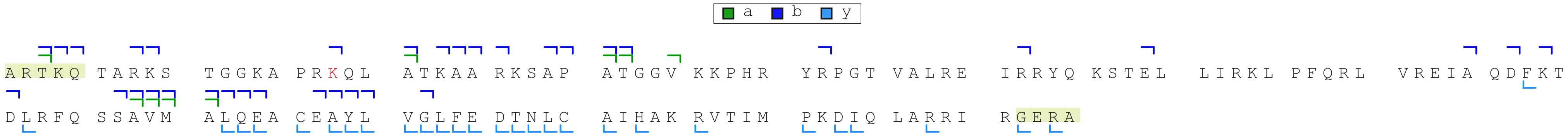

Proteoform:

Index	Protein	SC [%]	SVP [%]	IC [%]	MS Score [a.u.]	Var. Mods	Mono Mass [Da]	Chemical Formula
4	H31Kac1	38.8	6.7	23.0	8.9	Ace: K23	15305.4534	C671H1131N215O186S4

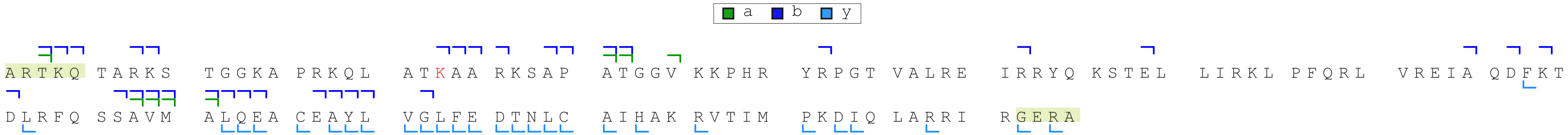

Proteoform:

Index	Protein	SC [%]	SVP [%]	IC [%]	MS Score [a.u.]	Var. Mods	Mono Mass [Da]	Chemical Formula
5	H31Kac1	38.8	5.2	23.0	8.9	Ace: K4	15305.4534	C671H1131N215O186S4

a b y

ARTKQ TARKS TGGKA PRKQL ATKAA RKSAP ATGGV KKPHR YRPGT VALRE IRRYQ KSTEL LIRKL PFQRL VREIA QDFKT
DLRFQ SSAVM ALQEA CEAYL VGLFE DTNLC AIHAK RVTIM PKDIQ LARRI RGERA

Proteoform:	Index	Protein	SC [%]	SVP [%]	IC [%]	MS Score [a.u.]	Var. Mods	Mono Mass [Da]	Chemical Formula
	6	H31Kac1	35.8	6.7	22.6	8.1	Ace: K27	15305.4534	C671H1131N215O186S4

a b y

ARTKQ TARKS TGGKA PRKQL ATKAA RKSAP ATGGV KKPHR YRPGT VALRE IRRYQ KSTEL LIRKL PFQRL VREIA QDFKT
DLRFQ SSAVM ALQEA CEAYL VGLFE DTNLC AIHAK RVTIM PKDIQ LARRI RGERA

Proteoform:

Index	Protein	SC [%]	SVP [%]	IC [%]	MS Score [a.u.]	Var. Mods	Mono Mass [Da]	Chemical Formula
7	H31Kac1	32.8	6.7	21.7	7.1	Ace: K36	15305.4534	C671H1131N215O186S4

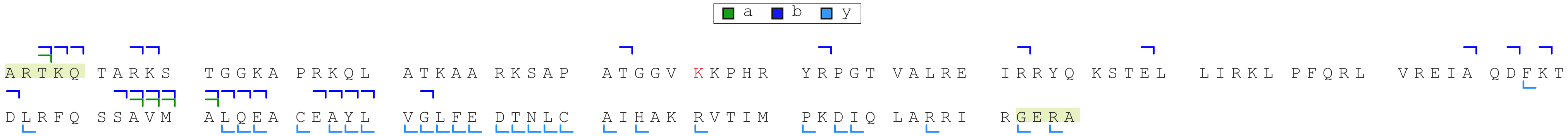

Proteoform:

Index	Protein	SC [%]	SVP [%]	IC [%]	MS Score [a.u.]	Var. Mods	Mono Mass [Da]	Chemical Formula
8	H31Kac1	32.8	6.7	21.7	7.1	Ace: K37	15305.4534	C671H1131N215O186S4

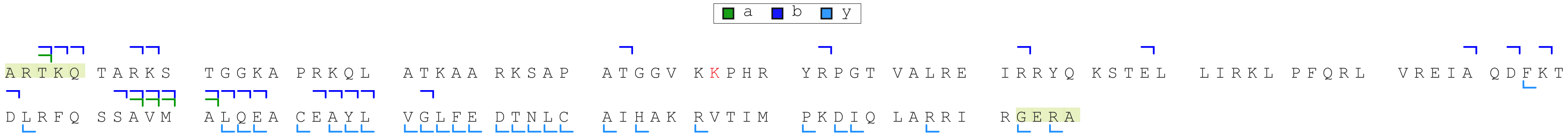

Proteoform:

Index	Protein	SC [%]	SVP [%]	IC [%]	MS Score [a.u.]	Var. Mods	Mono Mass [Da]	Chemical Formula
9	H31Kac1	31.3	6.7	21.5	6.8	Ace: K56	15305.4534	C671H1131N215O186S4

a b y

ARTKQ TARKS TGGKA PRKQL ATKAA RKSAP ATGGV KKPHR YRPGT VALRE IRRYQ KSTEL LIRKL PFQRL VREIA QDFKT
DLRFQ SSAVM ALQEA CEAYL VGLFE DTNLC AIHAK RVTIM PKDIQ LARRI RGERA

Proteoform:

Index	Protein	SC [%]	SVP [%]	IC [%]	MS Score [a.u.]	Var. Mods	Mono Mass [Da]	Chemical Formula
10	H31Kac1	30.6	6.7	21.3	6.5	Ace: K64	15305.4534	C671H1131N215O186S4

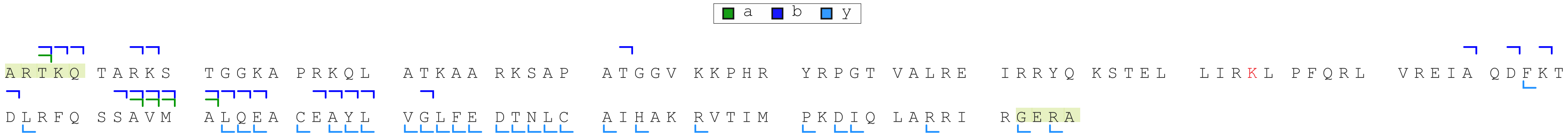

Error Plot

Proteoform:

Index	Protein	SC [%]	SVP [%]	IC [%]	MS Score [a.u.]	Var. Mods	Mono Mass [Da]	Chemical Formula
1	H31Kac1	41.8	6.7	23.3	9.7	Ace: K14	15305.4534	C671H1131N215O186S4

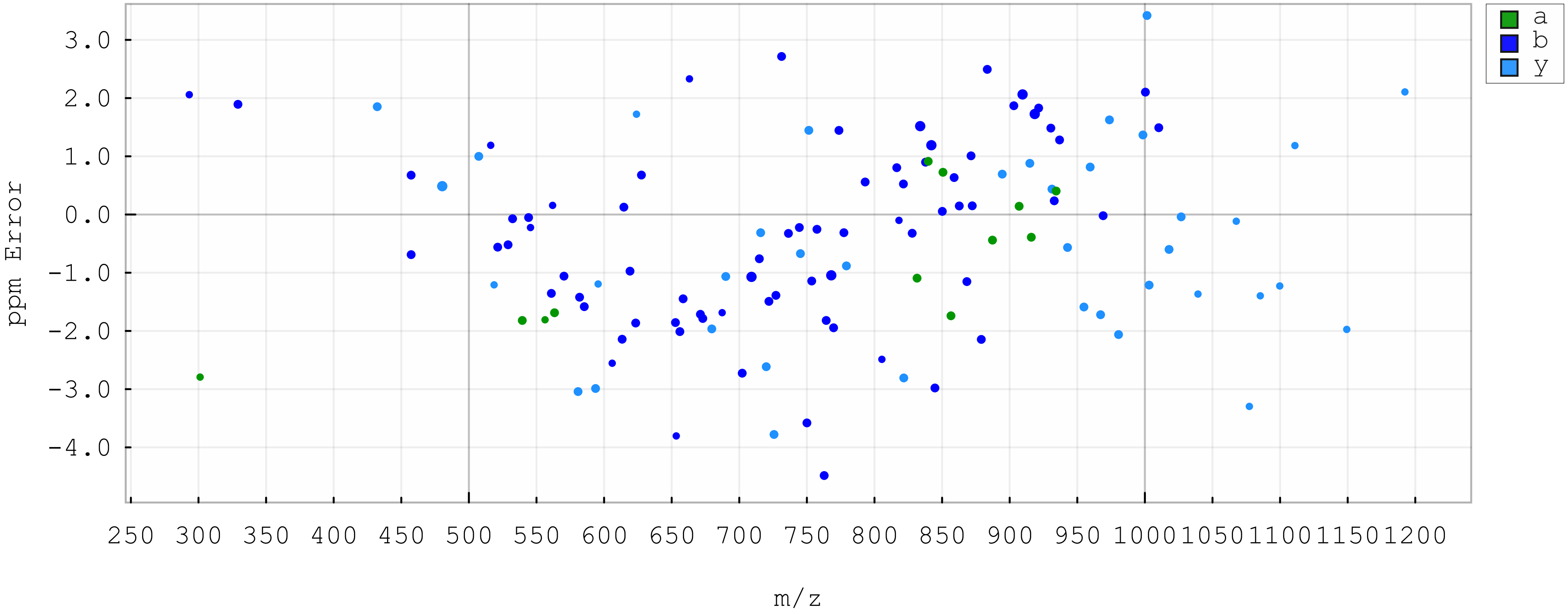

Proteoform:

Index	Protein	SC [%]	SVP [%]	IC [%]	MS Score [a.u.]	Var. Mods	Mono Mass [Da]	Chemical Formula
2	H31Kac1	41.0	6.7	23.2	9.5	Ace: K9	15305.4534	C671H1131N215O186S4

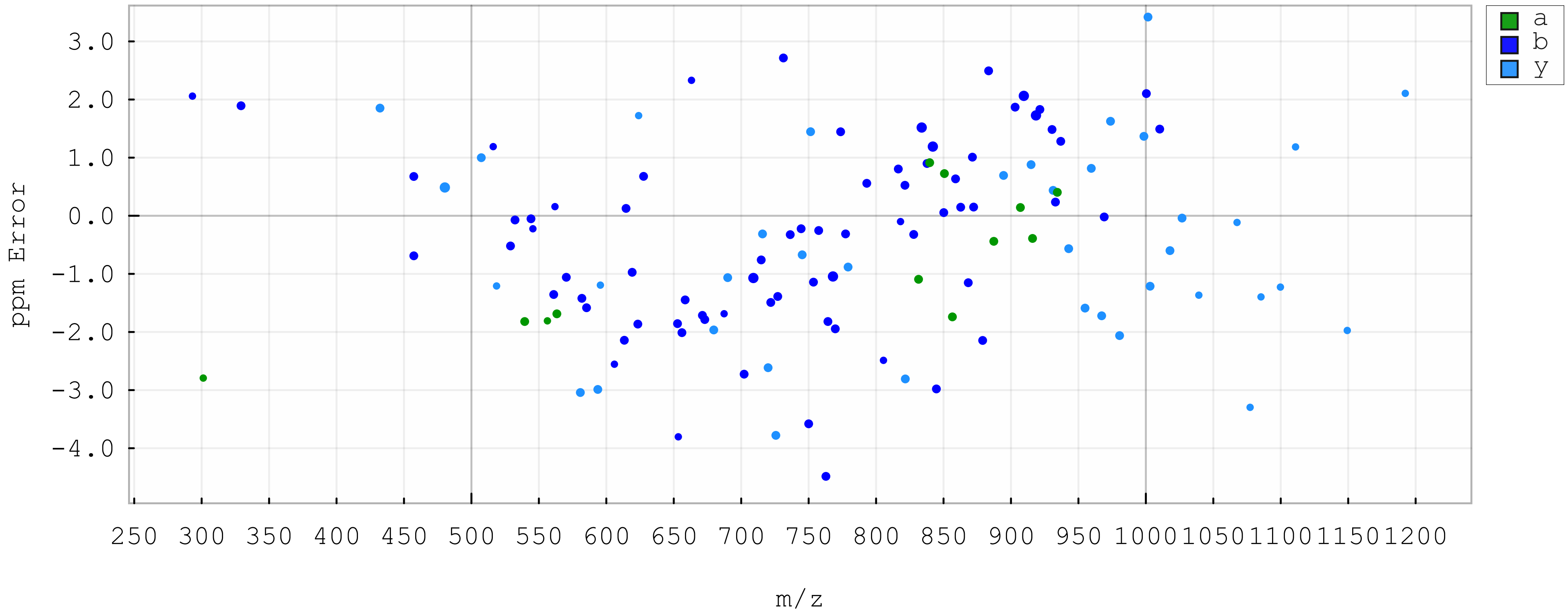

Proteoform:

Index	Protein	SC [%]	SVP [%]	IC [%]	MS Score [a.u.]	Var. Mods	Mono Mass [Da]	Chemical Formula
3	H31Kac1	40.3	6.7	23.1	9.3	Ace: K18	15305.4534	C671H1131N215O186S4

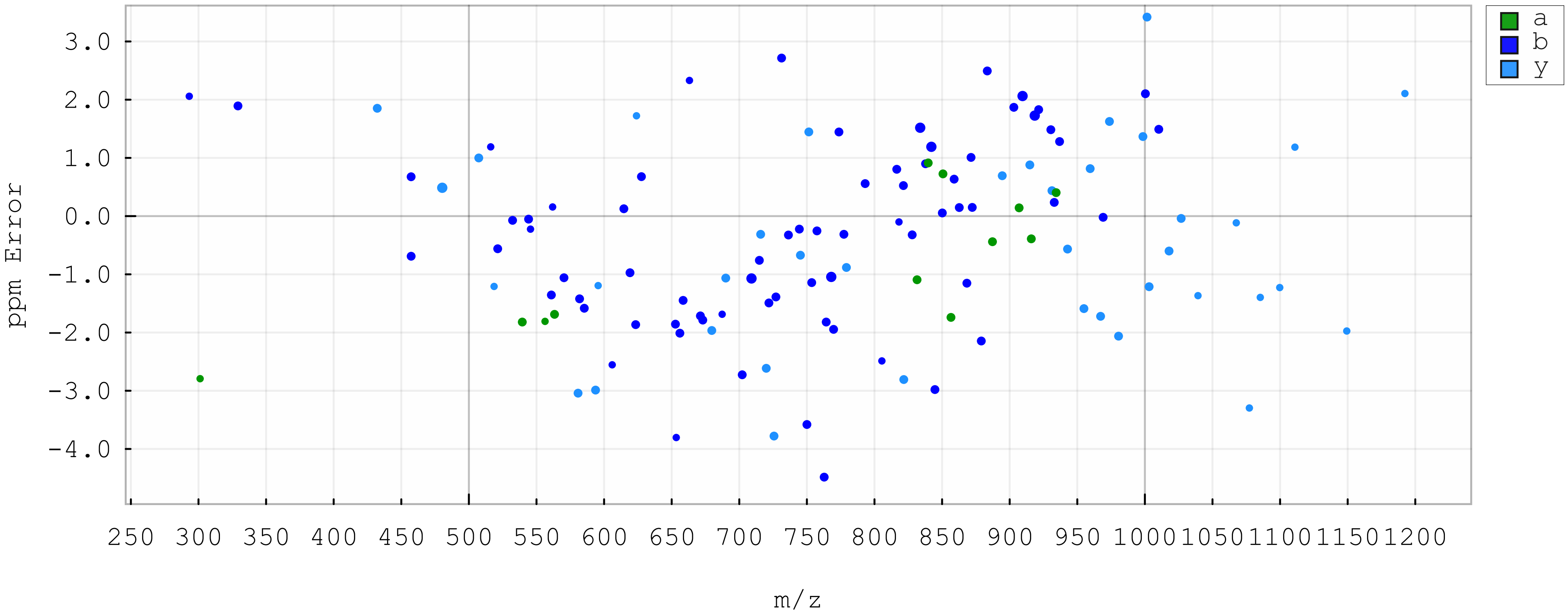

Proteoform:

Index	Protein	SC [%]	SVP [%]	IC [%]	MS Score [a.u.]	Var. Mods	Mono Mass [Da]	Chemical Formula
4	H31Kac1	38.8	6.7	23.0	8.9	Ace: K23	15305.4534	C671H1131N215O186S4

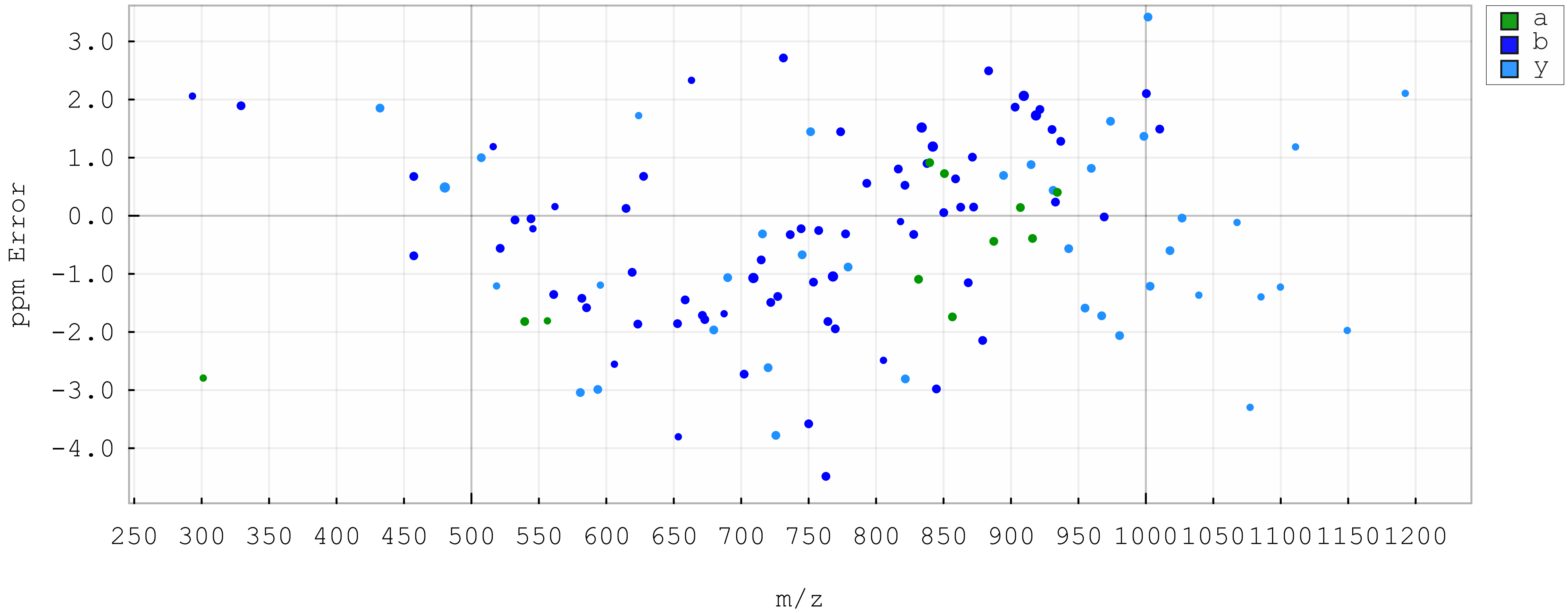

Proteoform:

Index	Protein	SC [%]	SVP [%]	IC [%]	MS Score [a.u.]	Var. Mods	Mono Mass [Da]	Chemical Formula
5	H31Kac1	38.8	5.2	23.0	8.9	Ace: K4	15305.4534	C671H1131N215O186S4

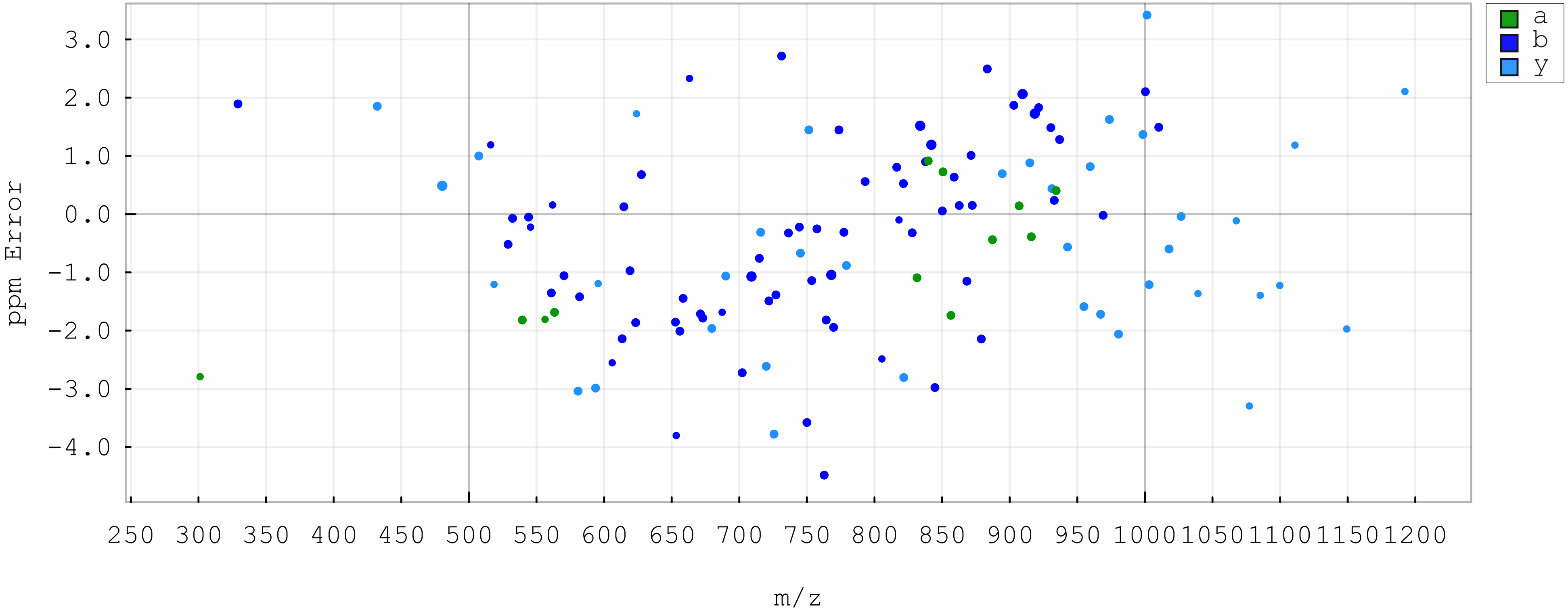

Proteoform:

Index	Protein	SC [%]	SVP [%]	IC [%]	MS Score [a.u.]	Var. Mods	Mono Mass [Da]	Chemical Formula
6	H31Kac1	35.8	6.7	22.6	8.1	Ace: K27	15305.4534	C671H1131N215O186S4

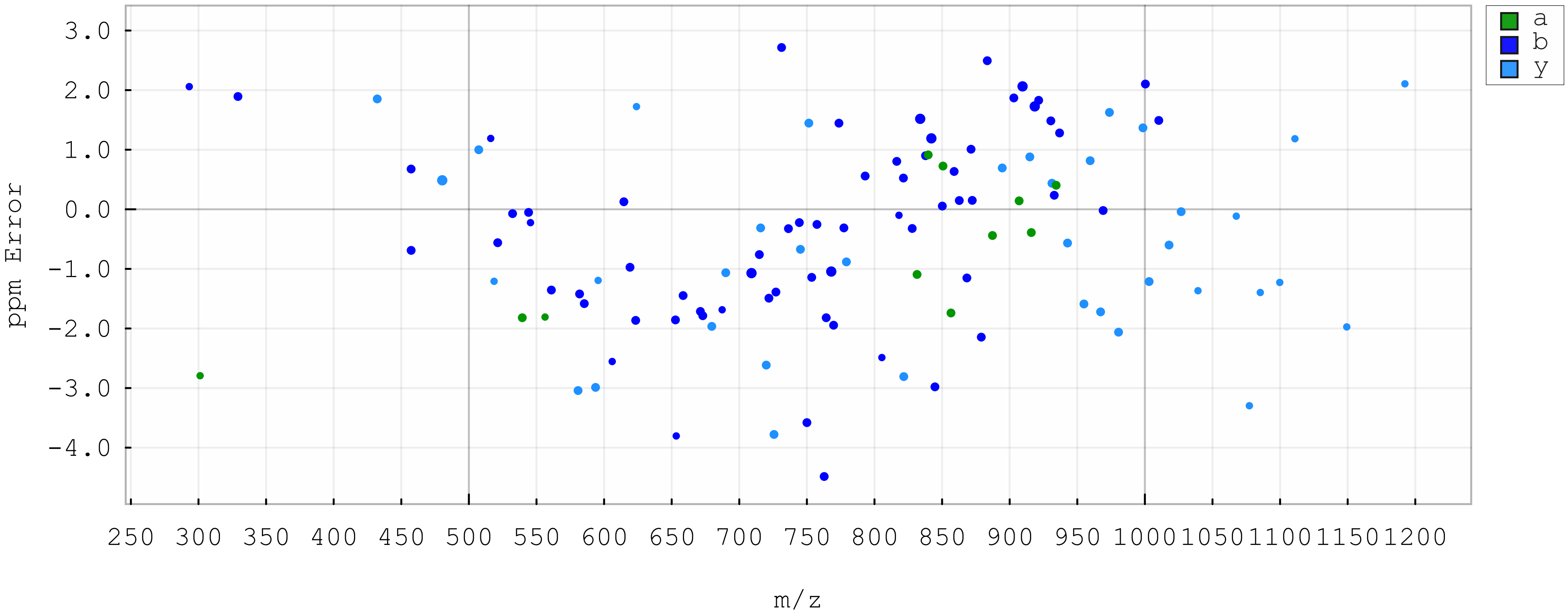

Proteoform:

Index	Protein	SC [%]	SVP [%]	IC [%]	MS Score [a.u.]	Var. Mods	Mono Mass [Da]	Chemical Formula
7	H31Kac1	32.8	6.7	21.7	7.1	Ace: K36	15305.4534	C671H1131N215O186S4

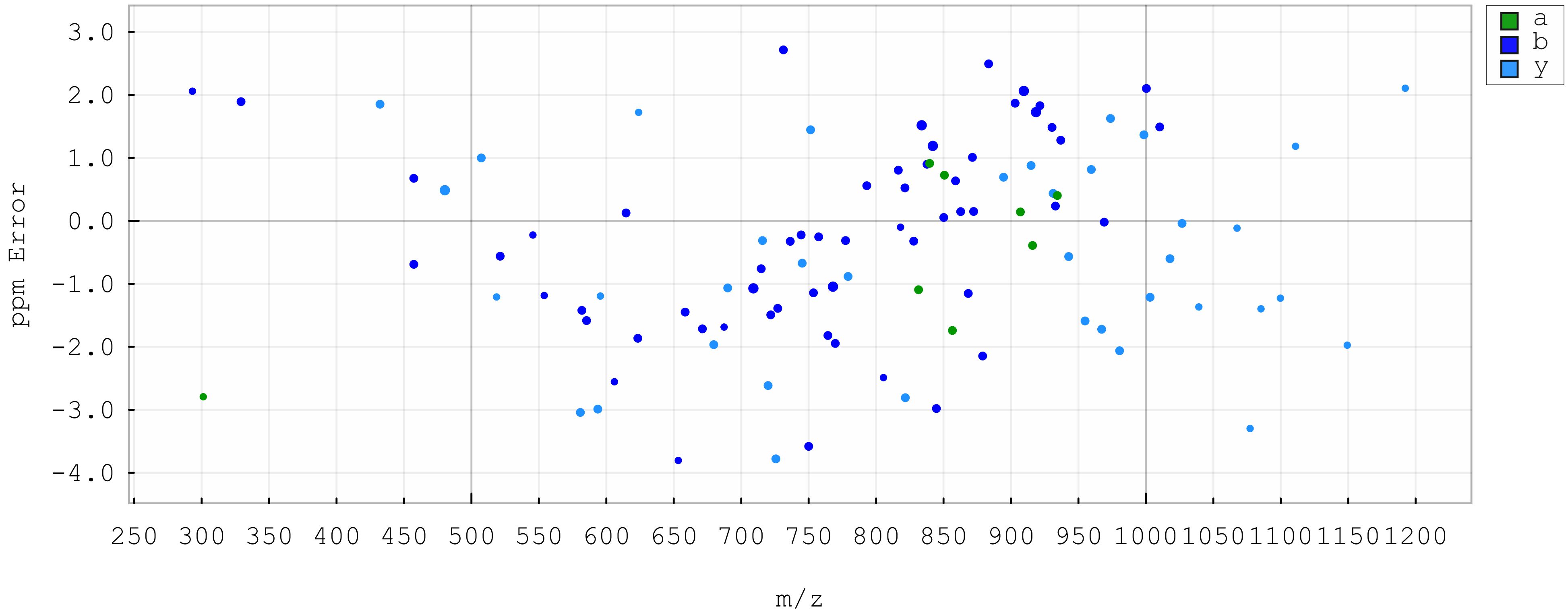

Proteoform:

Index	Protein	SC [%]	SVP [%]	IC [%]	MS Score [a.u.]	Var. Mods	Mono Mass [Da]	Chemical Formula
8	H31Kac1	32.8	6.7	21.7	7.1	Ace: K37	15305.4534	C671H1131N215O186S4

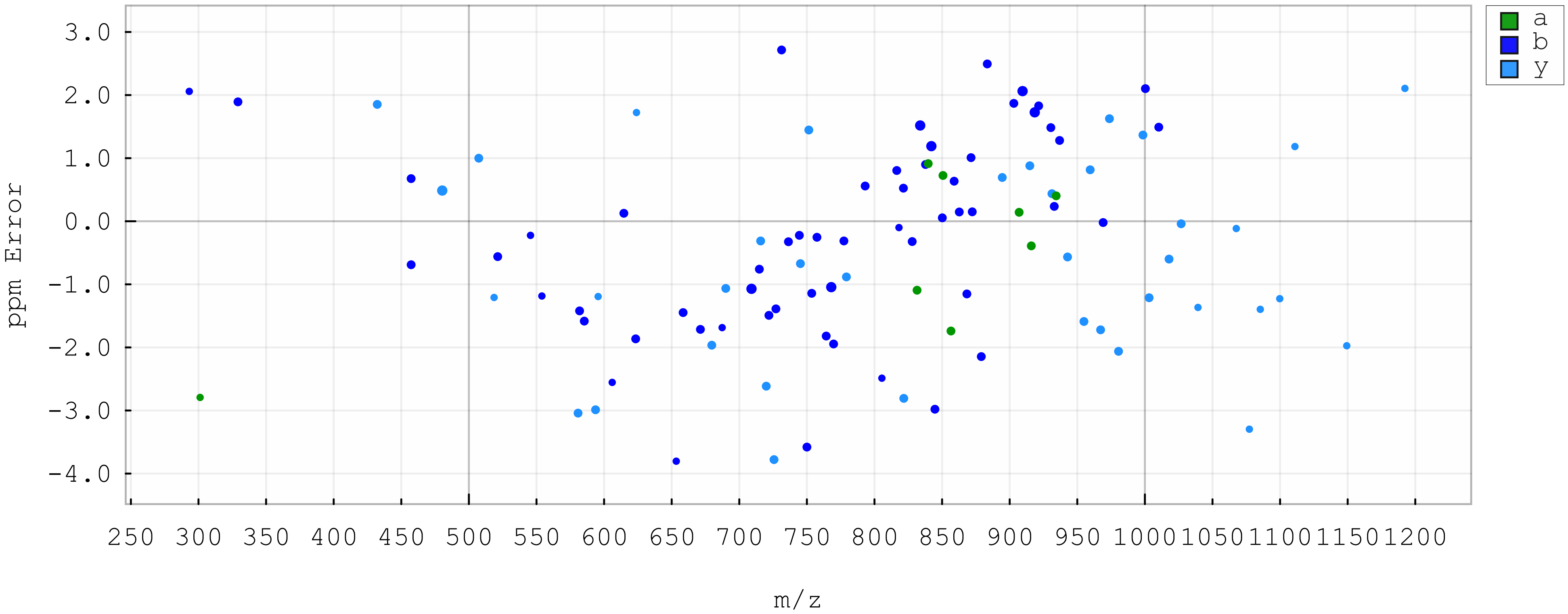

Proteoform:

Index	Protein	SC [%]	SVP [%]	IC [%]	MS Score [a.u.]	Var. Mods	Mono Mass [Da]	Chemical Formula
9	H31Kac1	31.3	6.7	21.5	6.8	Ace: K56	15305.4534	C671H1131N215O186S4

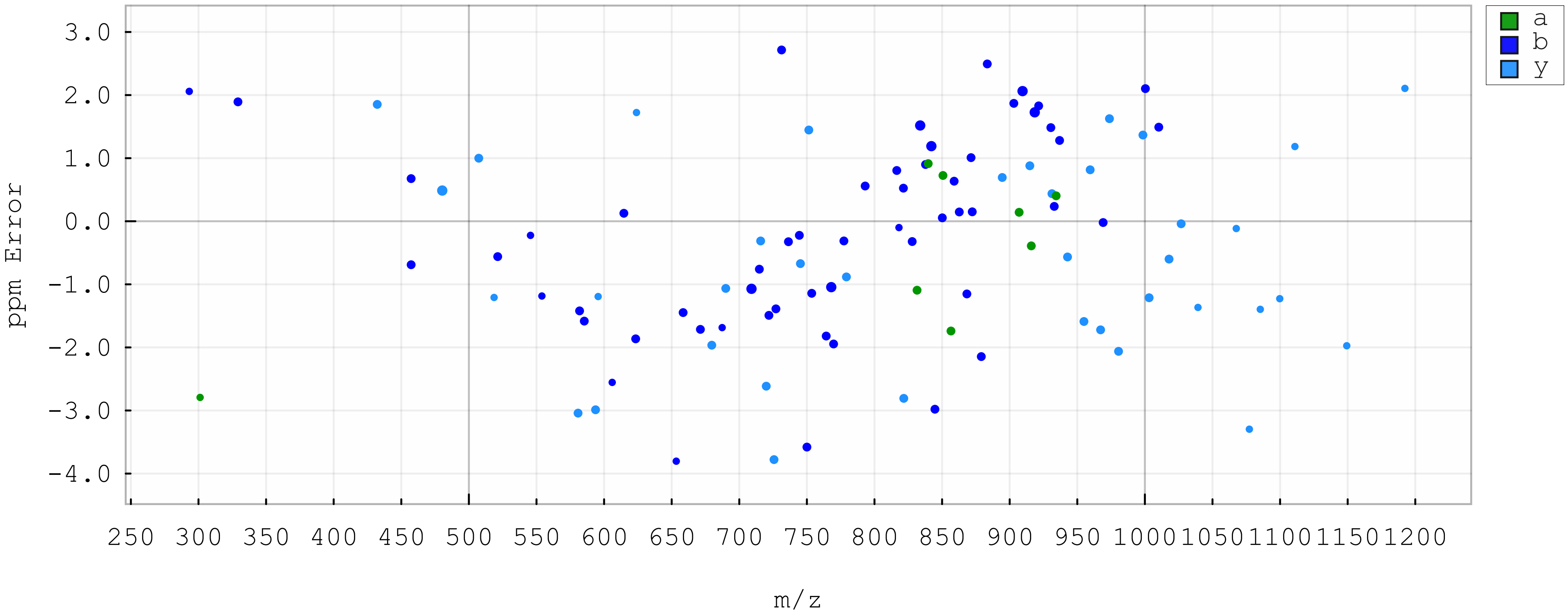

Proteoform:

Index	Protein	SC [%]	SVP [%]	IC [%]	MS Score [a.u.]	Var. Mods	Mono Mass [Da]	Chemical Formula
10	H31Kac1	30.6	6.7	21.3	6.5	Ace: K64	15305.4534	C671H1131N215O186S4

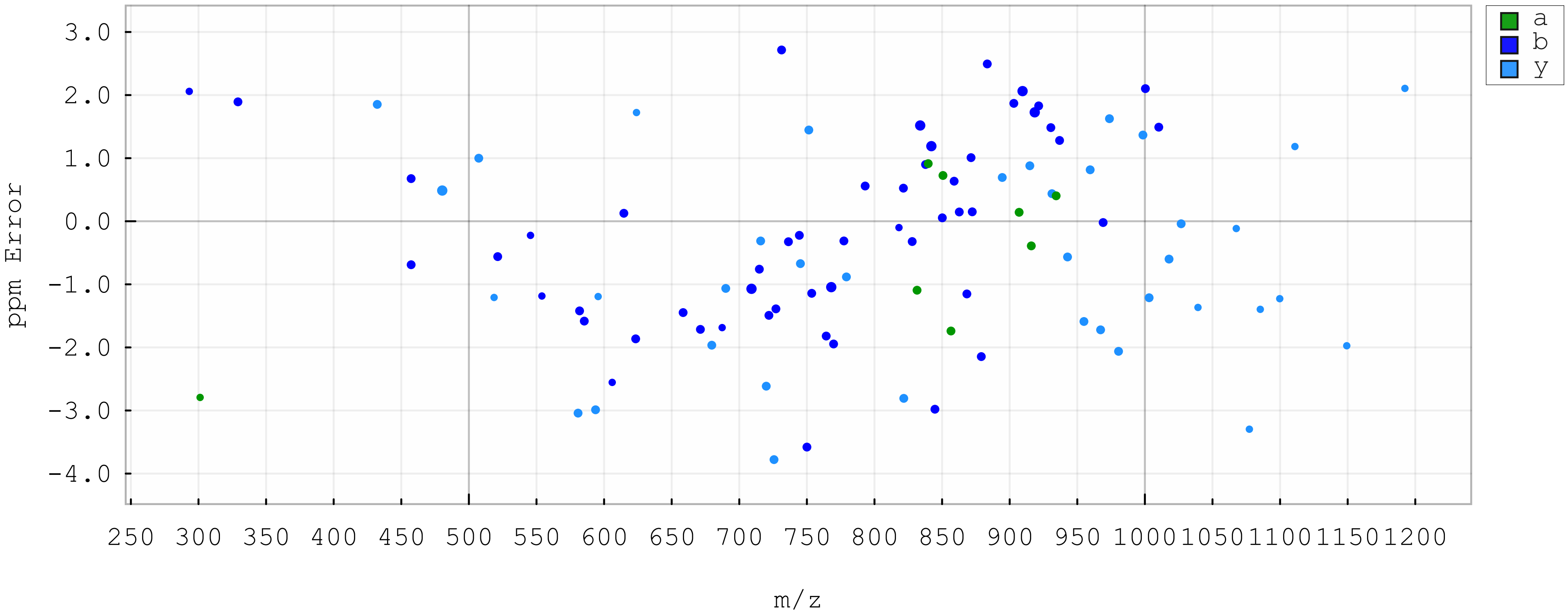

Primary Fragment Plots

Proteoform:

Index	Protein	SC [%]	SVP [%]	IC [%]	MS Score [a.u.]	Var. Mods	Mono Mass [Da]	Chemical Formula
1	H31Kac1	41.8	6.7	23.3	9.7	Ace: K14	15305.4534	C671H1131N215O186S4

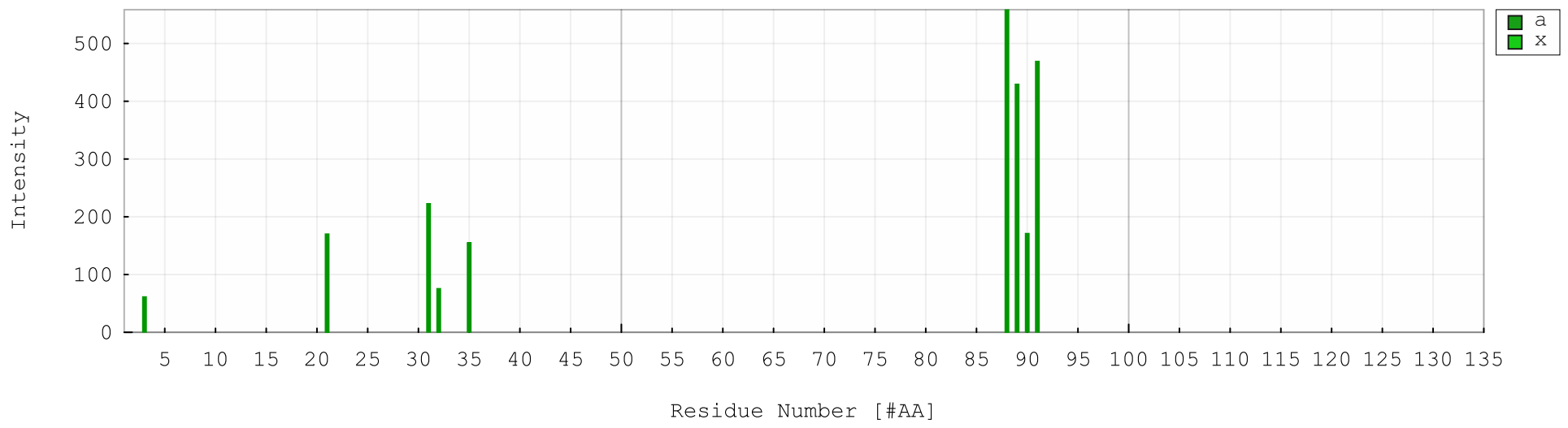

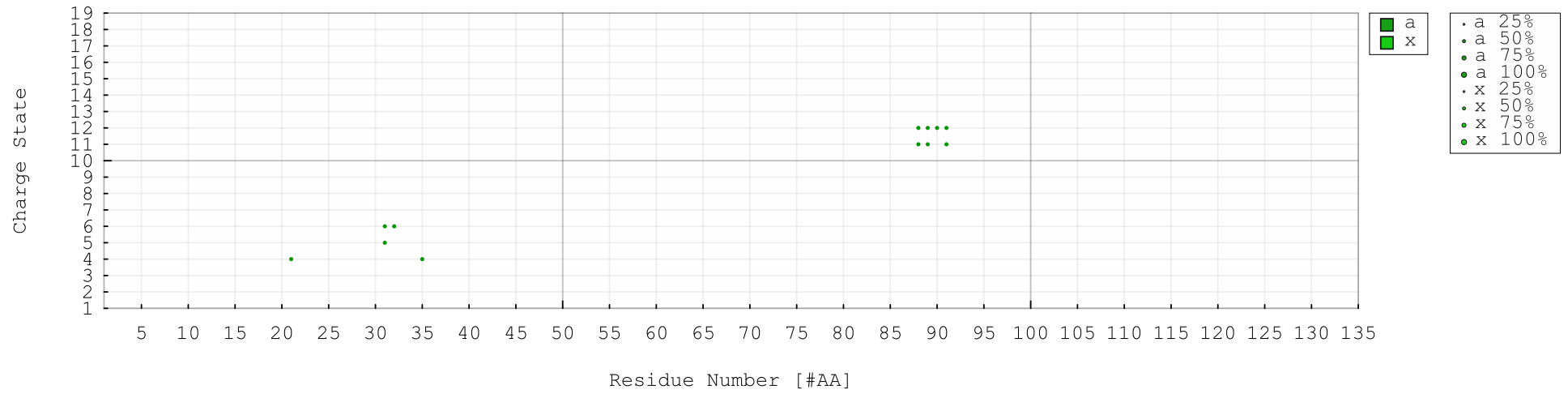

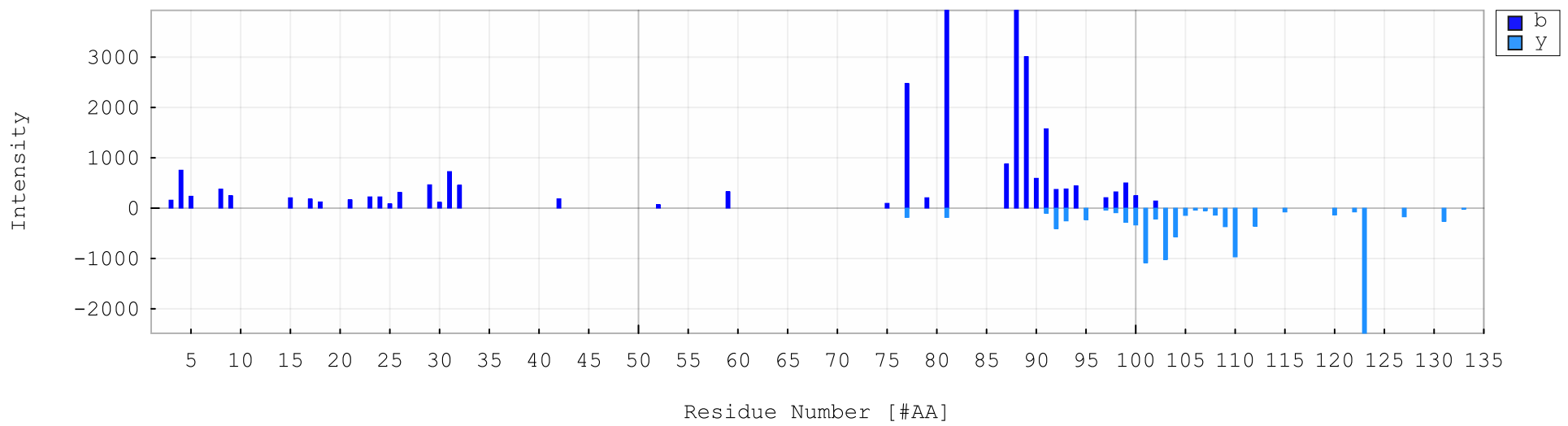

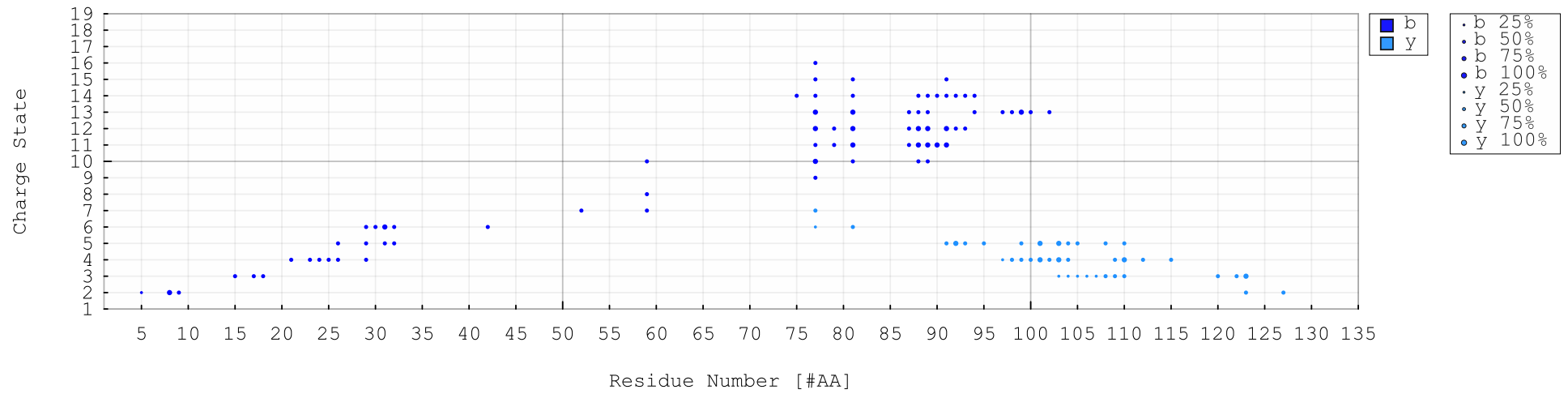

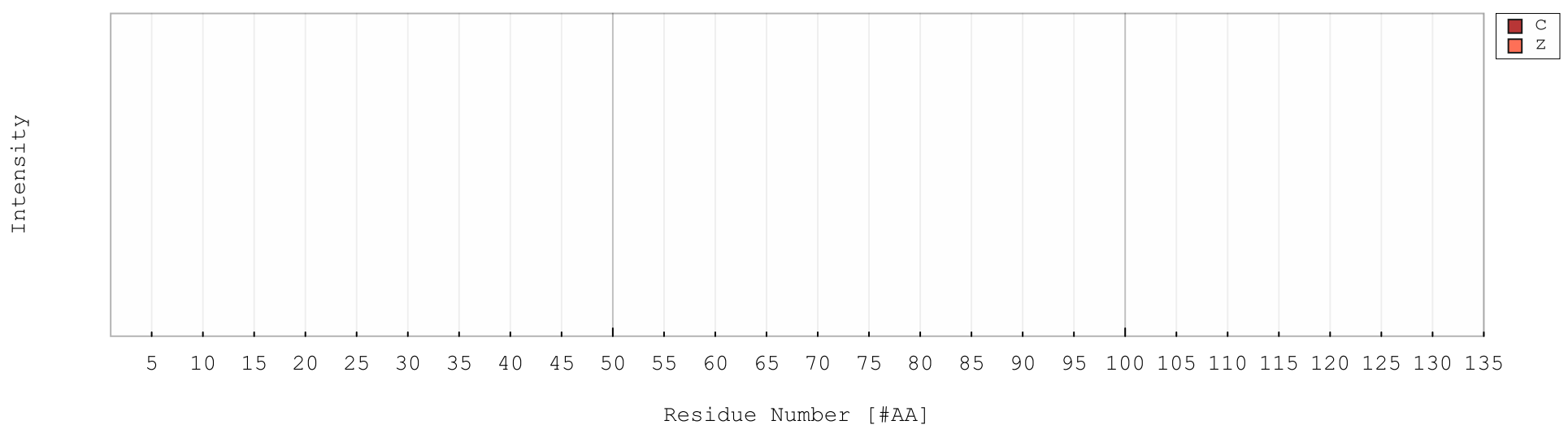

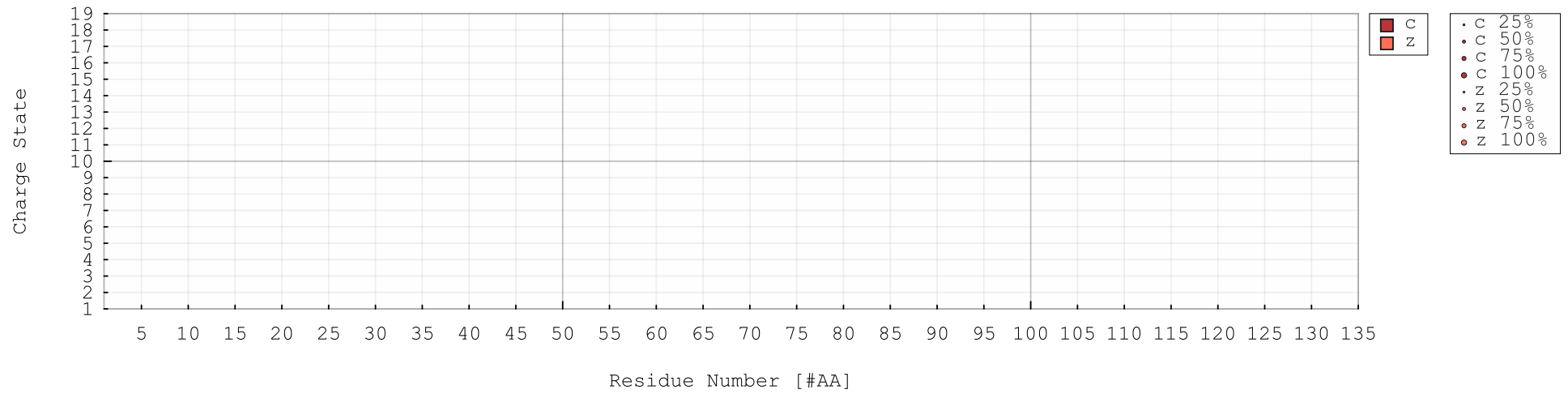

Proteoform:

Index	Protein	SC [%]	SVP [%]	IC [%]	MS Score [a.u.]	Var. Mods	Mono Mass [Da]	Chemical Formula
2	H31Kac1	41.0	6.7	23.2	9.5	Ace: K9	15305.4534	C671H1131N215O186S4

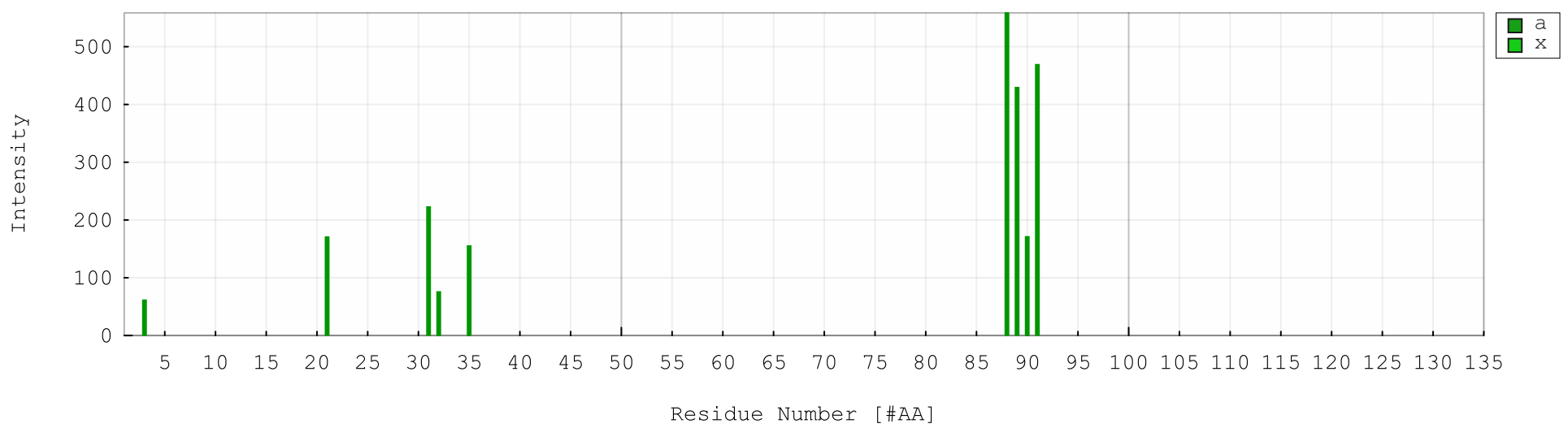

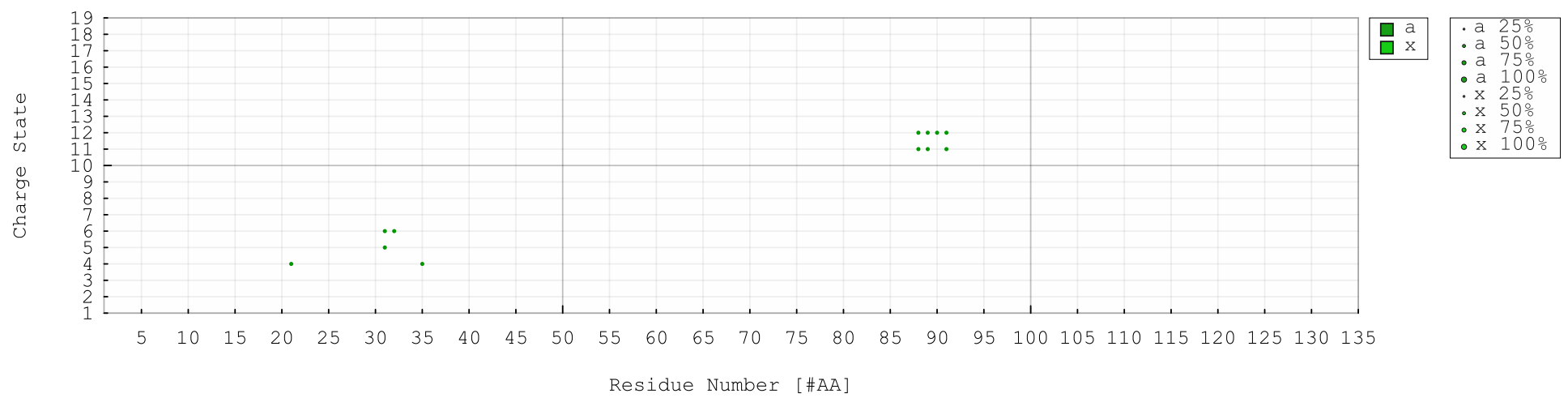

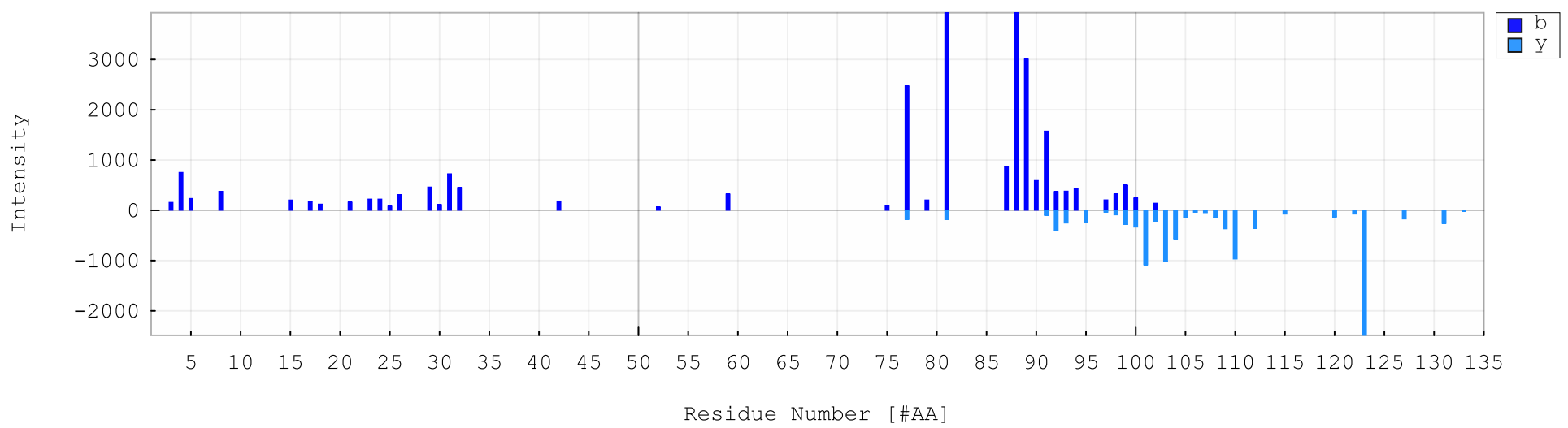

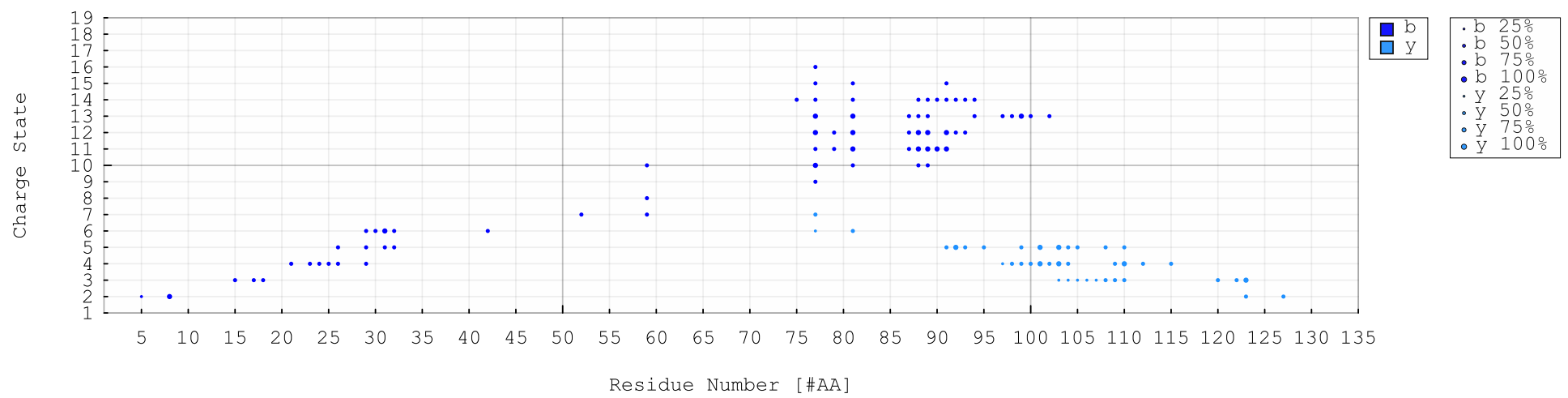

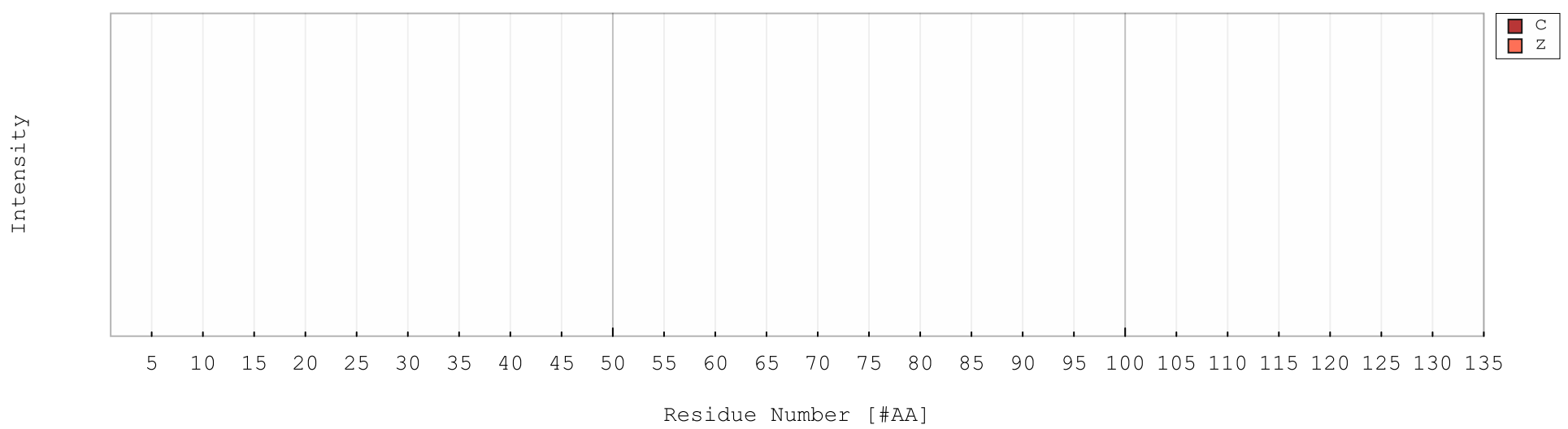

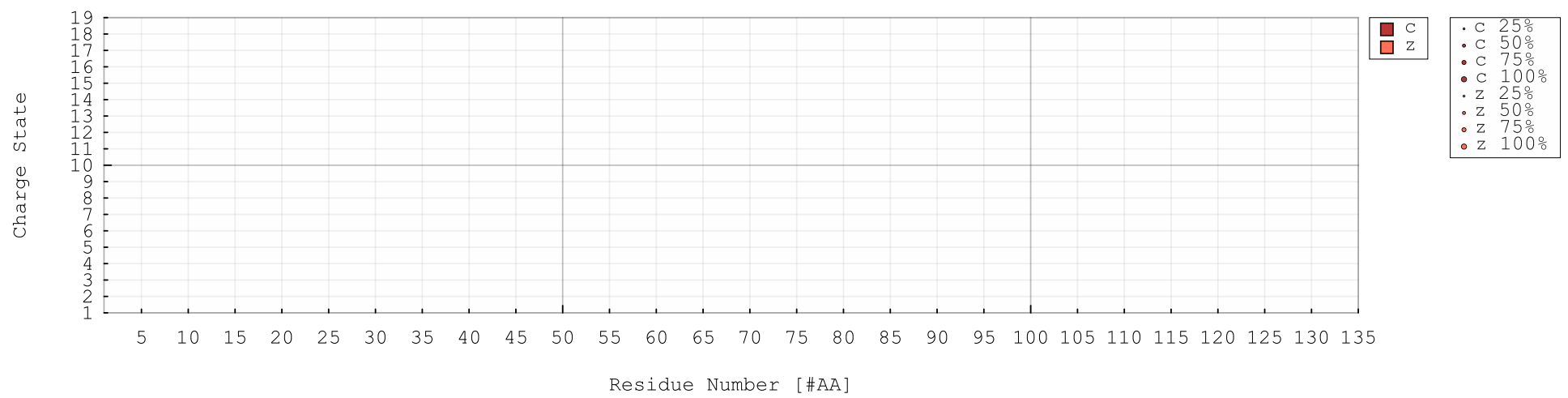

Proteoform:

Index	Protein	SC [%]	SVP [%]	IC [%]	MS Score [a.u.]	Var. Mods	Mono Mass [Da]	Chemical Formula
3	H31Kac1	40.3	6.7	23.1	9.3	Ace: K18	15305.4534	C671H1131N215O186S4

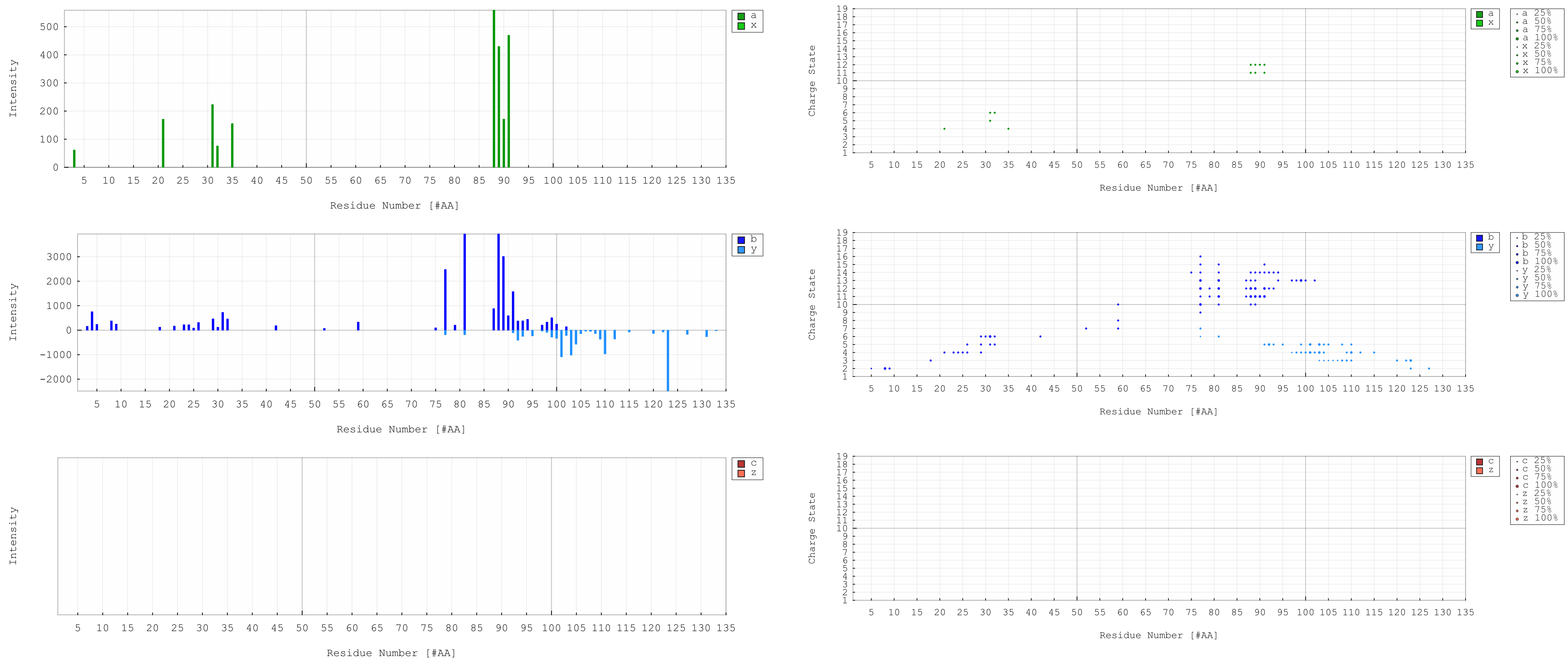

Proteoform:	Index	Protein	SC [%]	SVP [%]	IC [%]	MS Score [a.u.]	Var. Mods	Mono Mass [Da]	Chemical Formula
	4	H31Kac1	38.8	6.7	23.0	8.9	Ace: K23	15305.4534	C671H1131N215O186S4

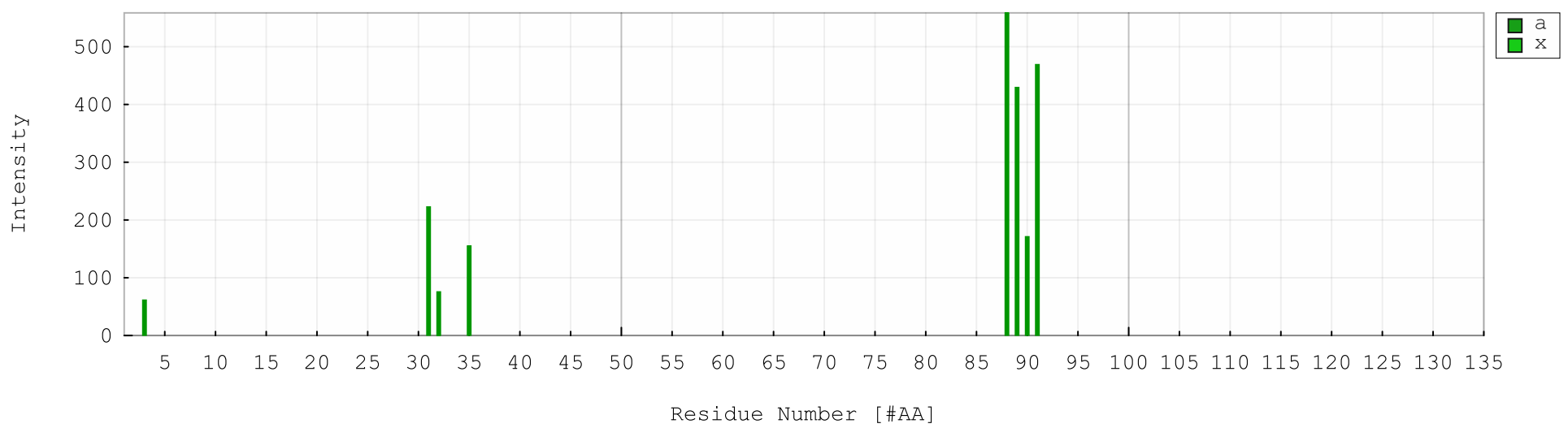

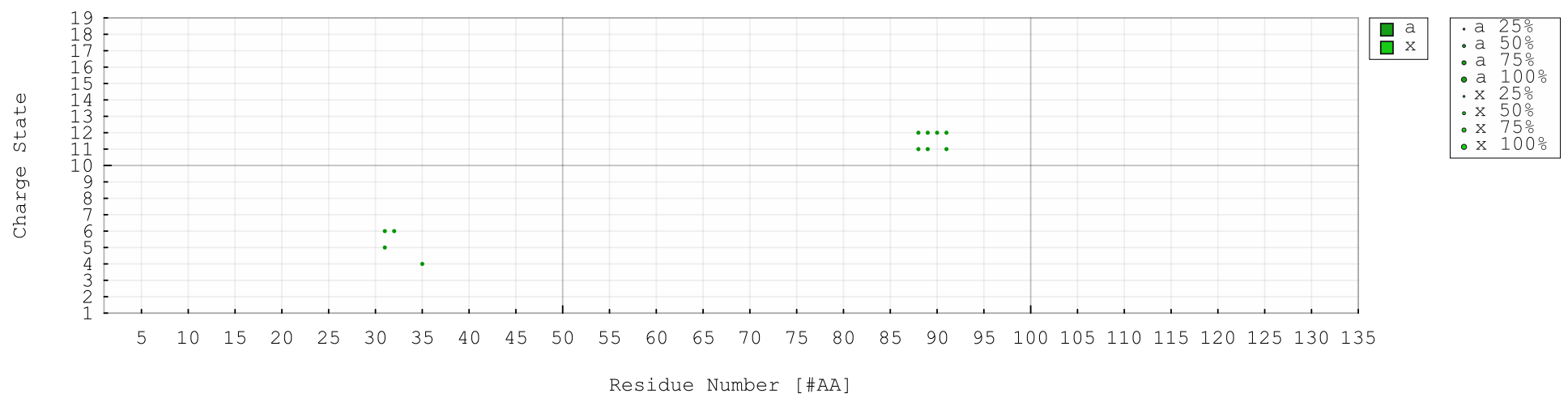

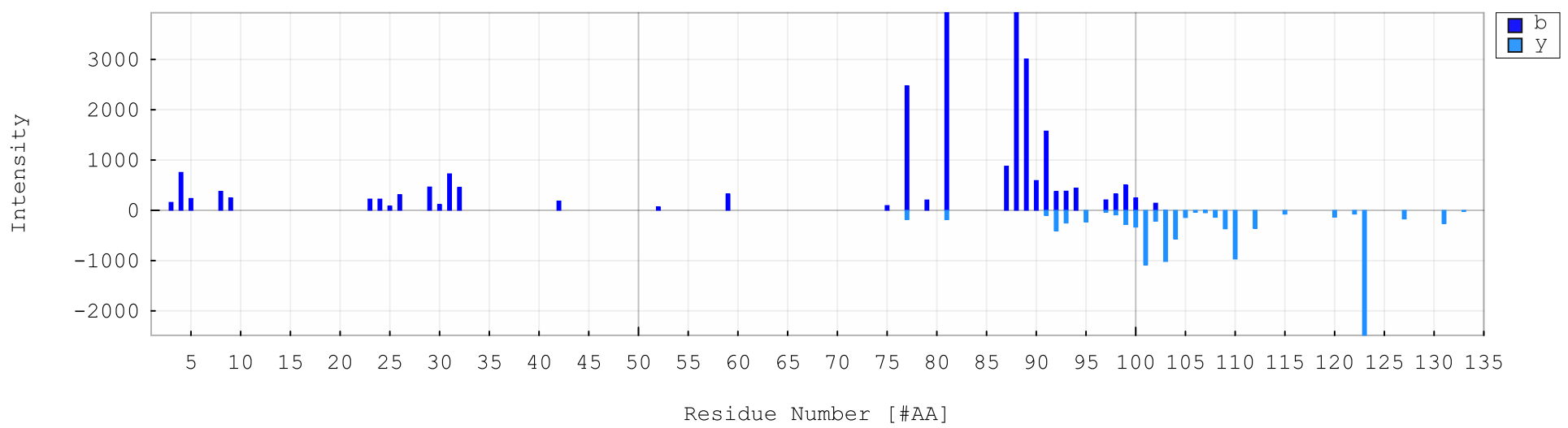

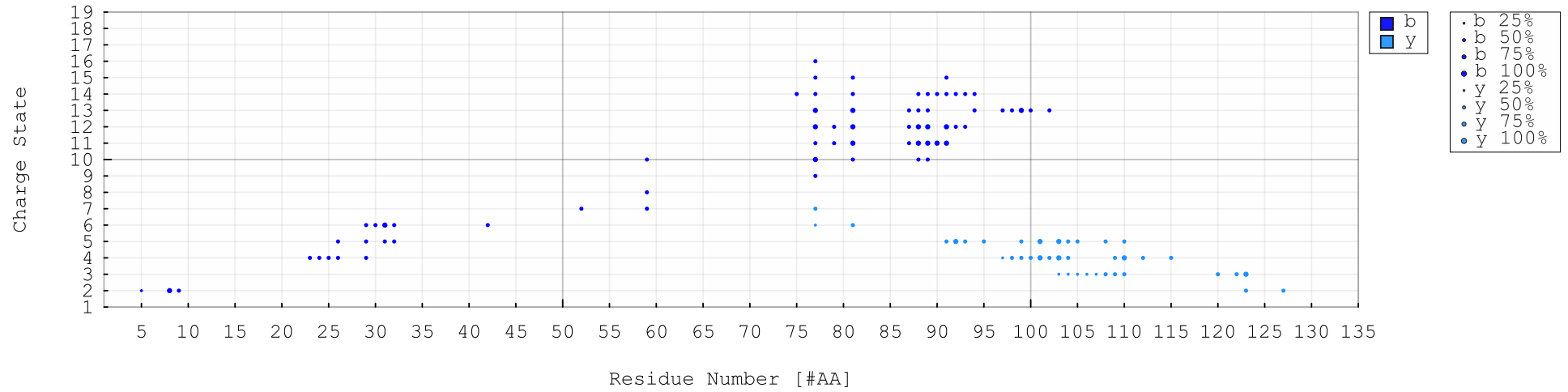

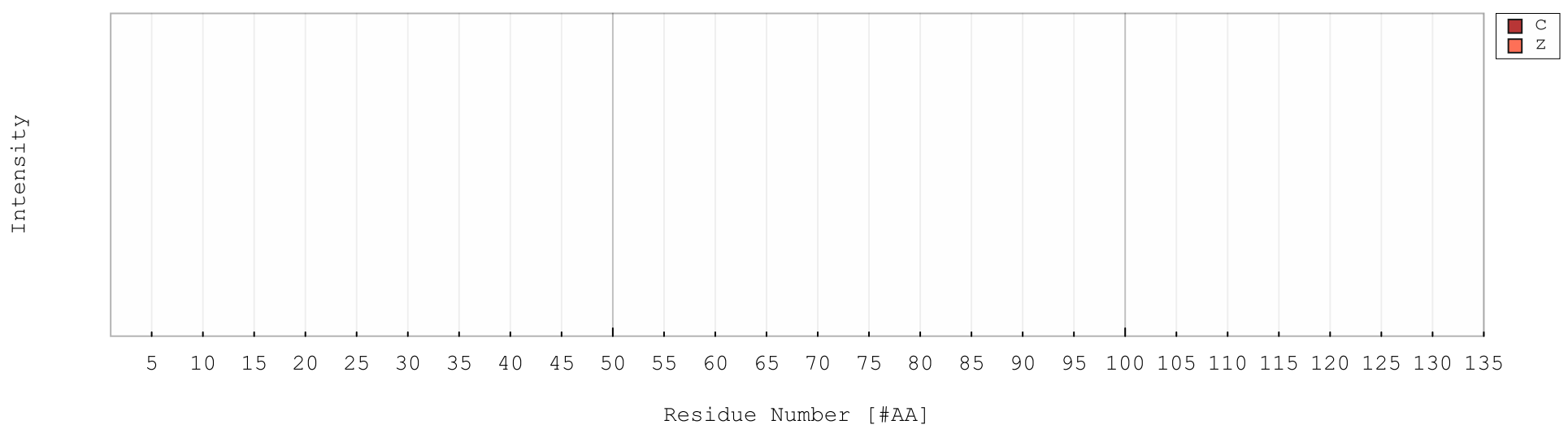

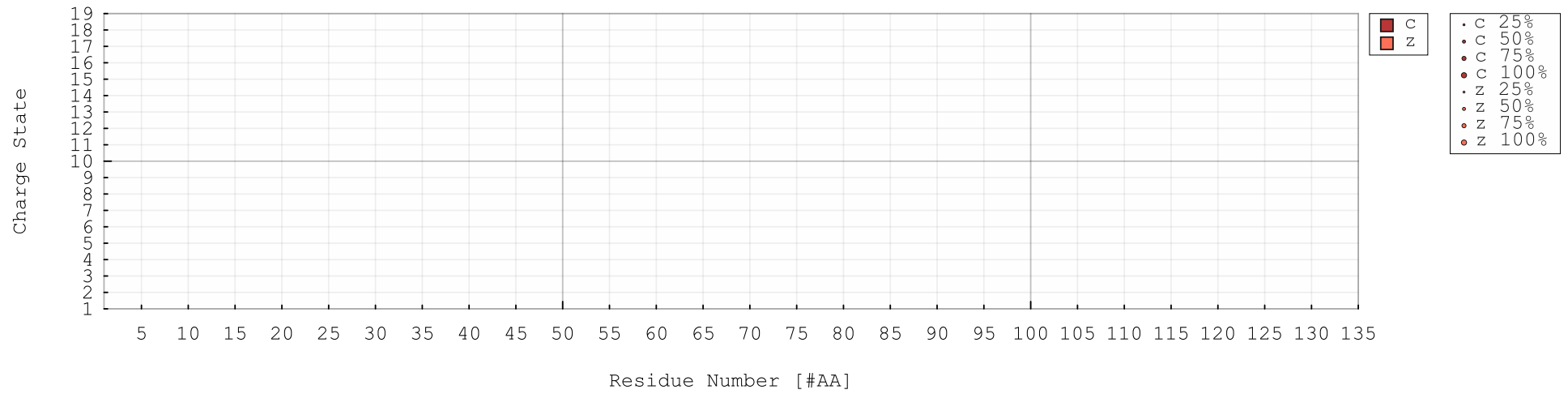

Proteoform:

Index	Protein	SC [%]	SVP [%]	IC [%]	MS Score [a.u.]	Var. Mods	Mono Mass [Da]	Chemical Formula
5	H31Kac1	38.8	5.2	23.0	8.9	Ace: K4	15305.4534	C671H1131N215O186S4

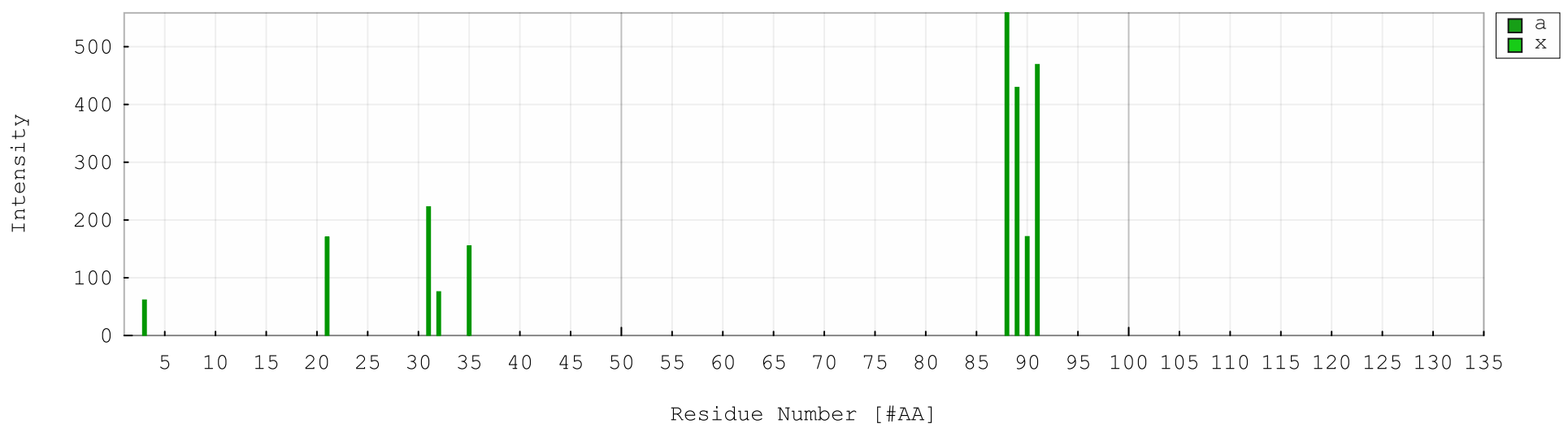

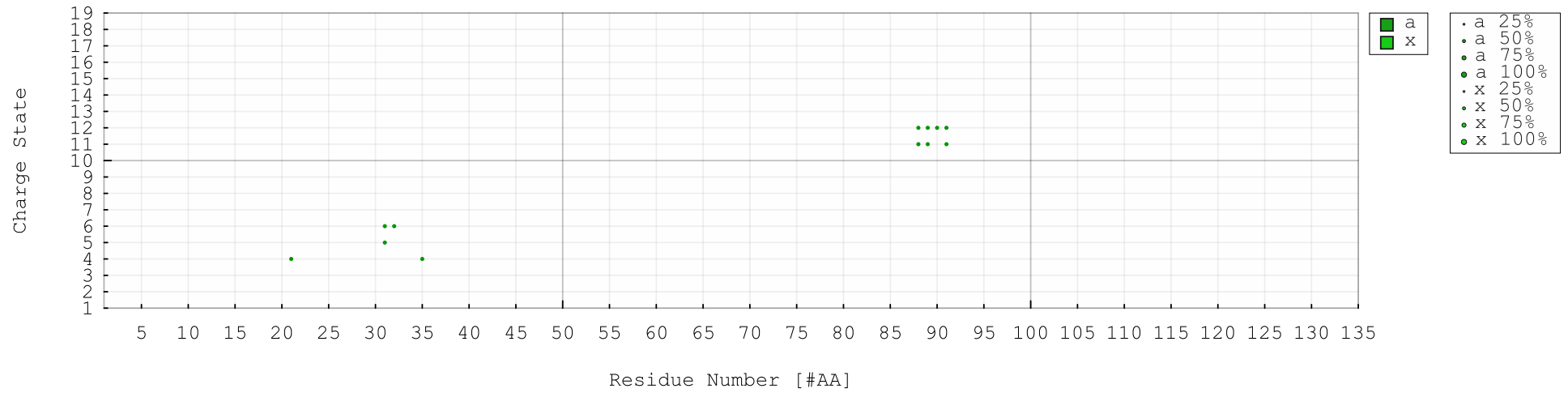

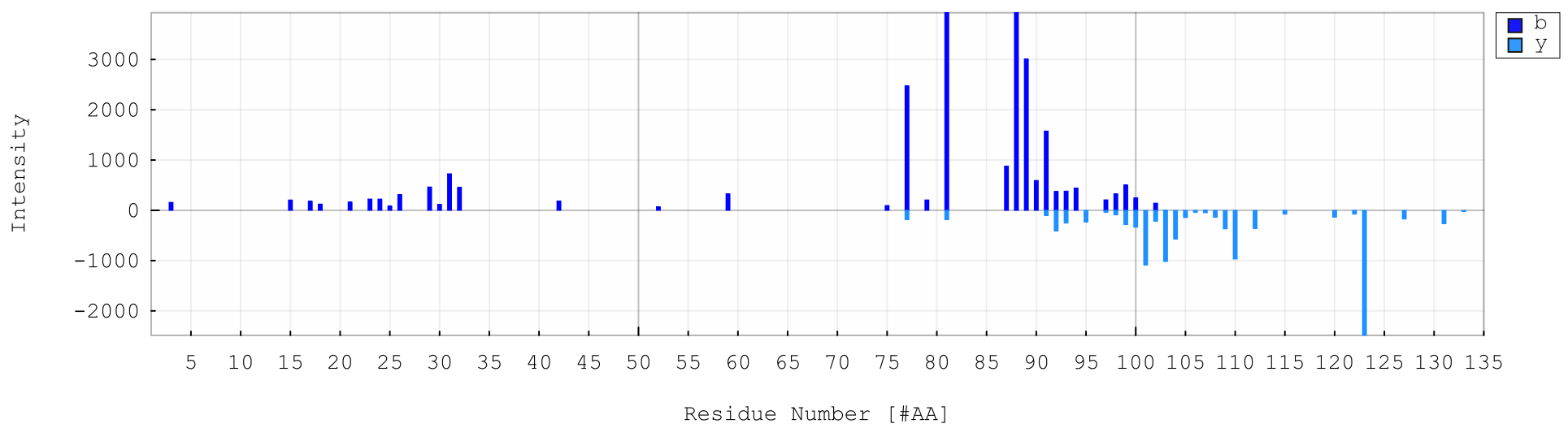

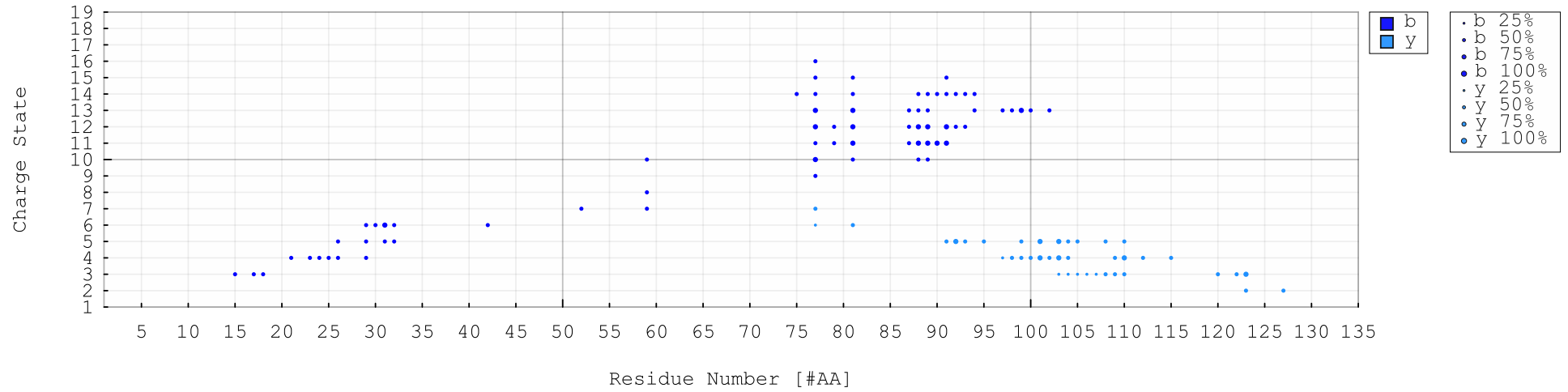

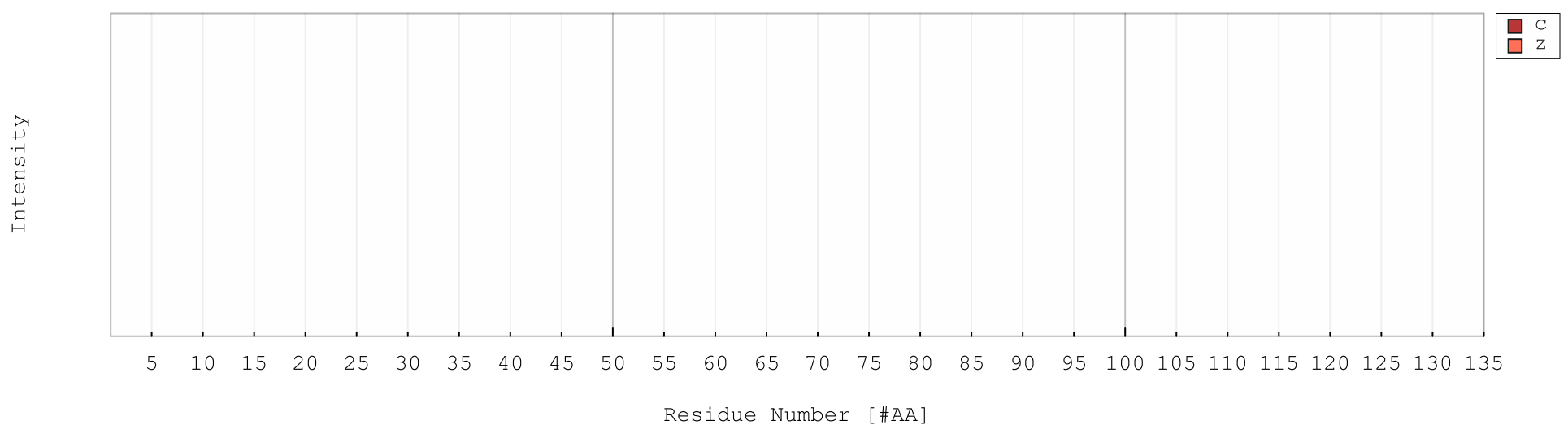

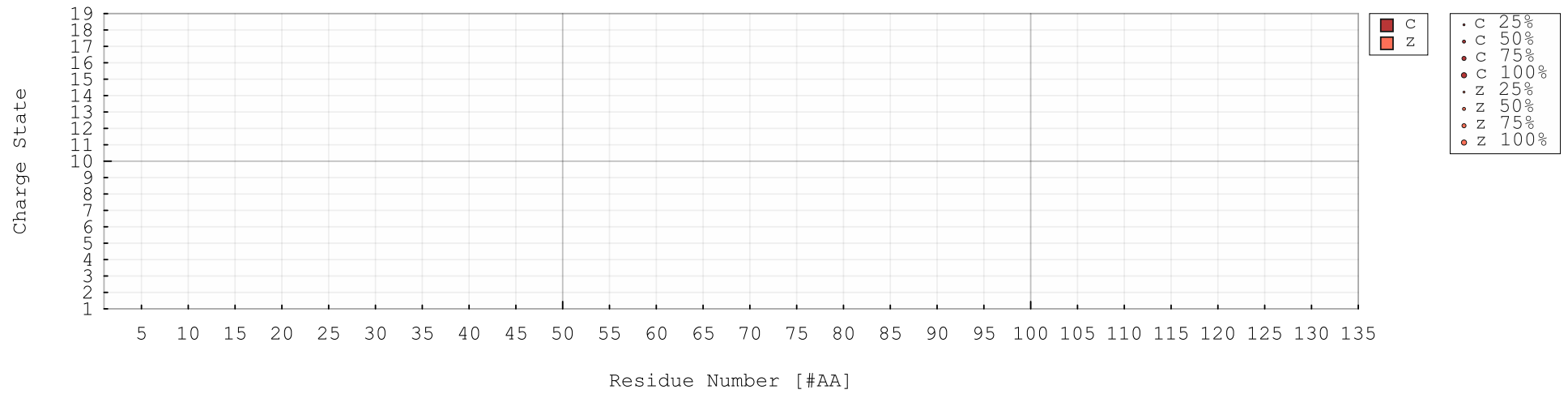

Proteoform:

Index	Protein	SC [%]	SVP [%]	IC [%]	MS Score [a.u.]	Var. Mods	Mono Mass [Da]	Chemical Formula
6	H31Kac1	35.8	6.7	22.6	8.1	Ace: K27	15305.4534	C671H1131N215O186S4

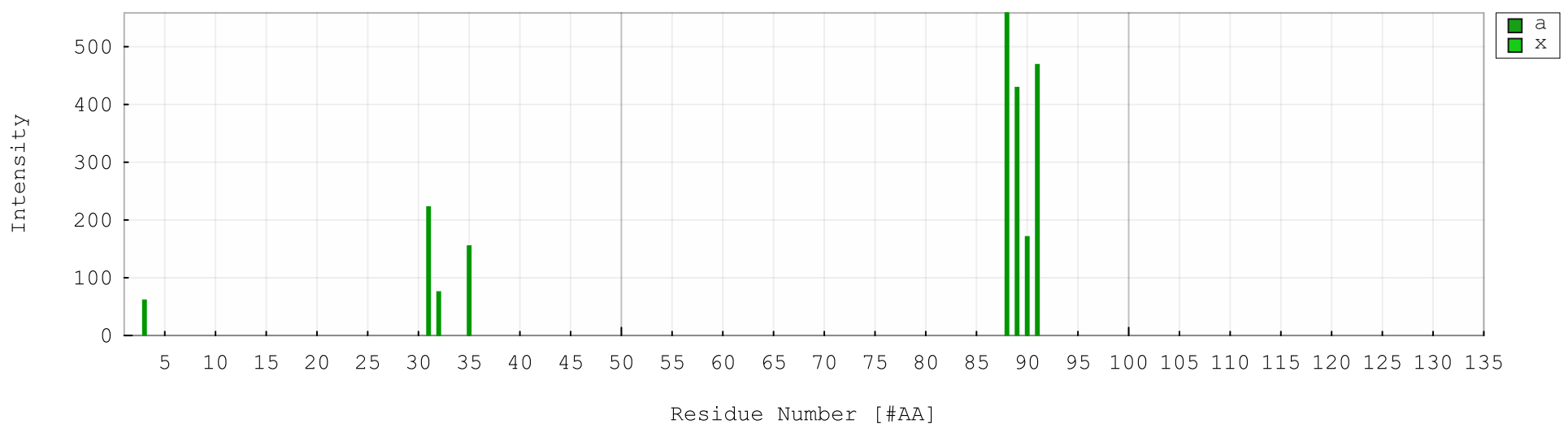

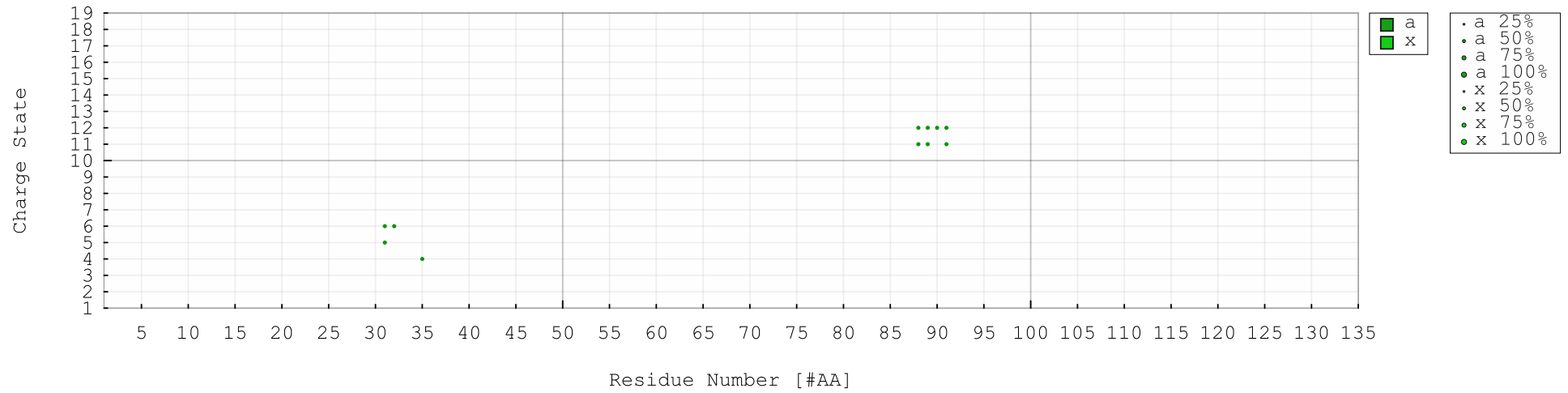

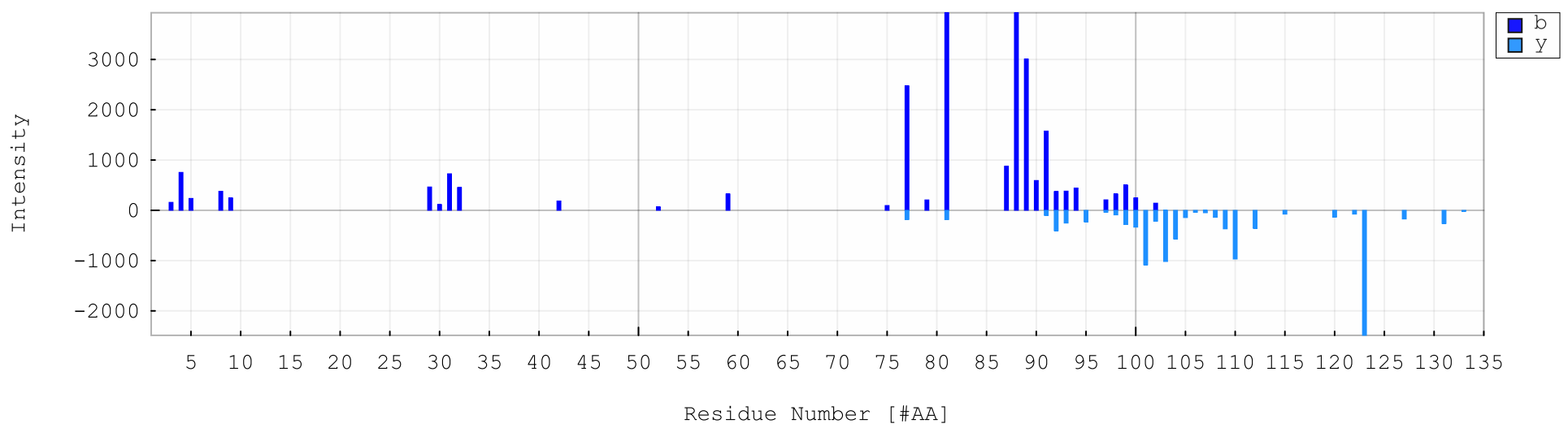

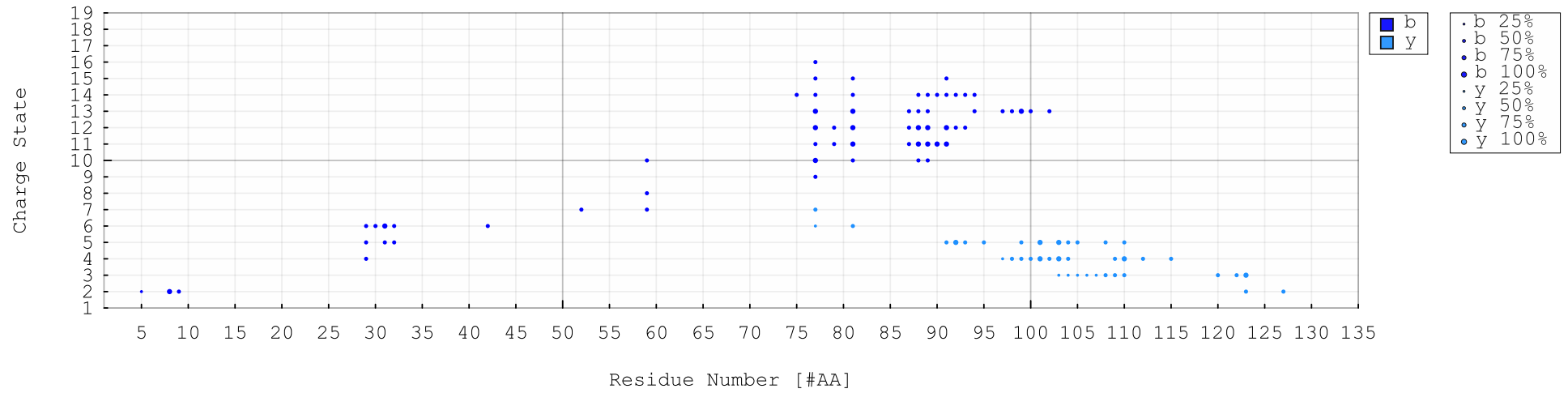

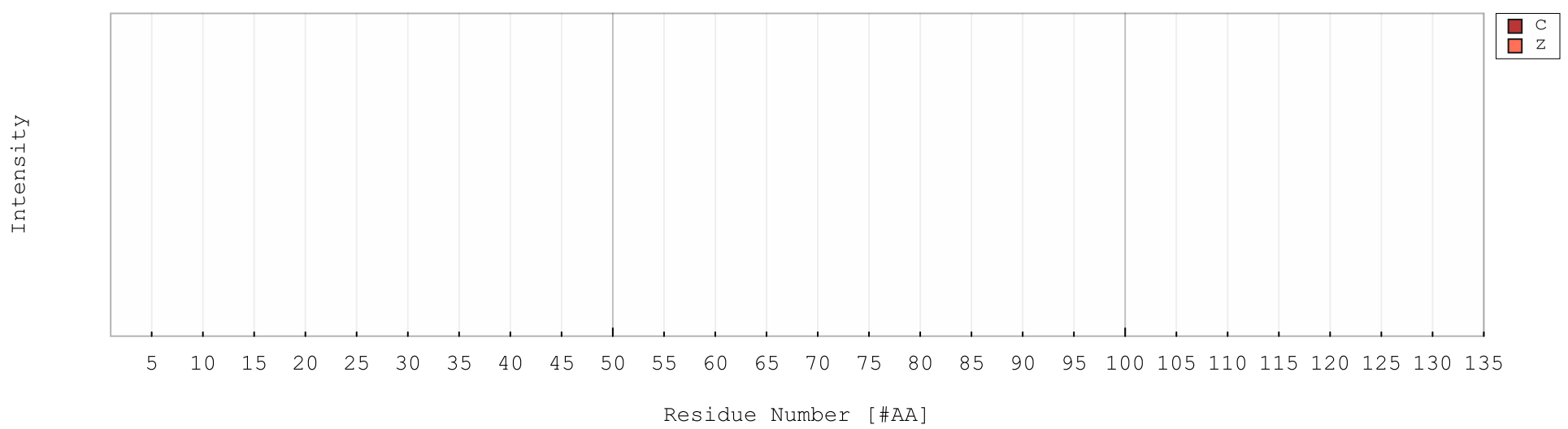

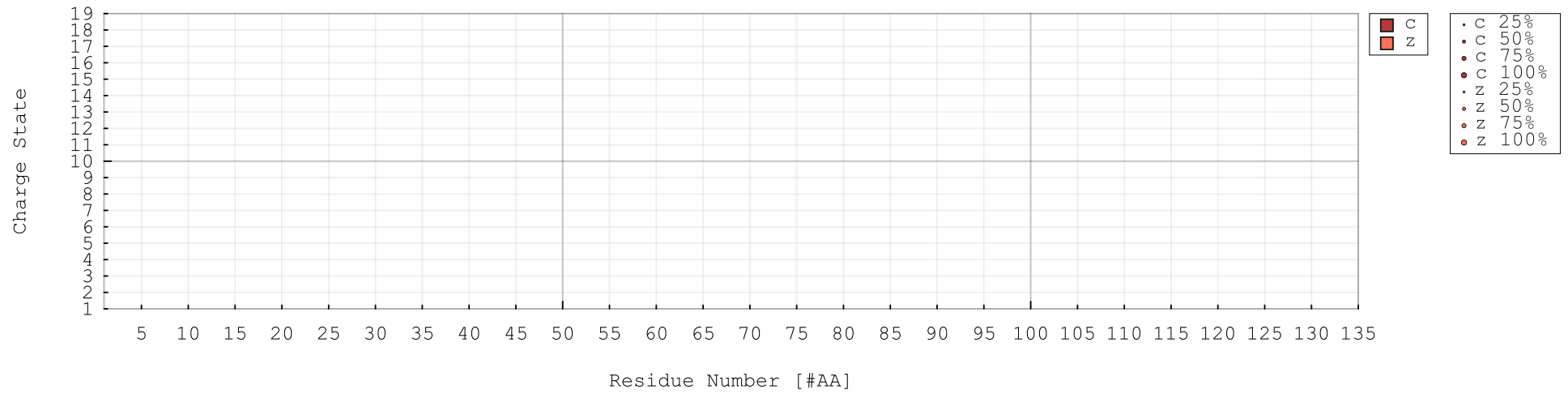

Proteoform:

Index	Protein	SC [%]	SVP [%]	IC [%]	MS Score [a.u.]	Var. Mods	Mono Mass [Da]	Chemical Formula
7	H31Kac1	32.8	6.7	21.7	7.1	Ace: K36	15305.4534	C671H1131N215O186S4

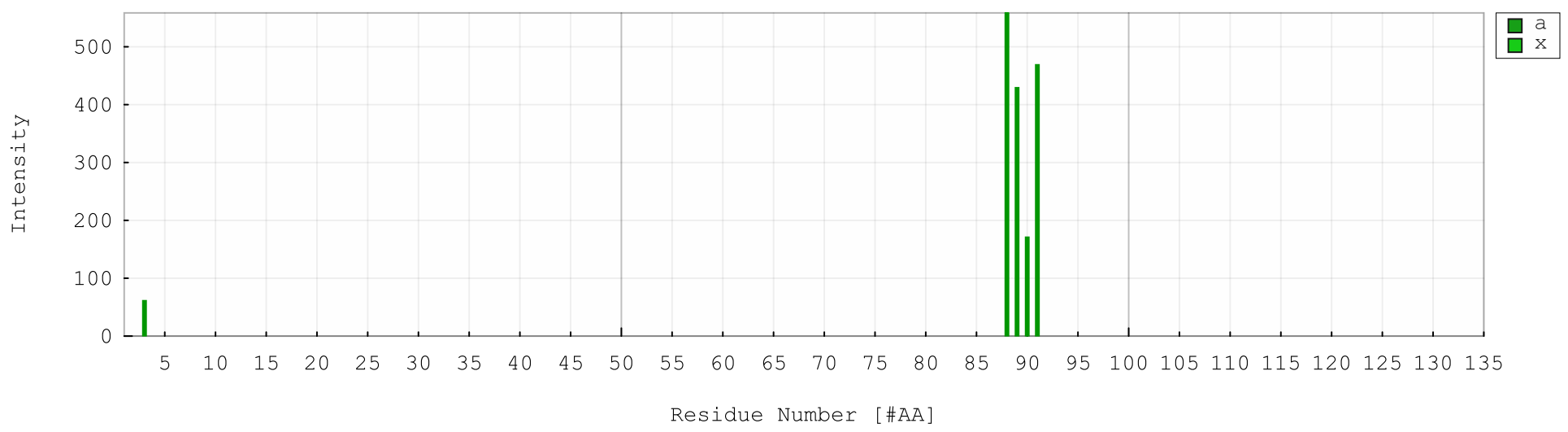

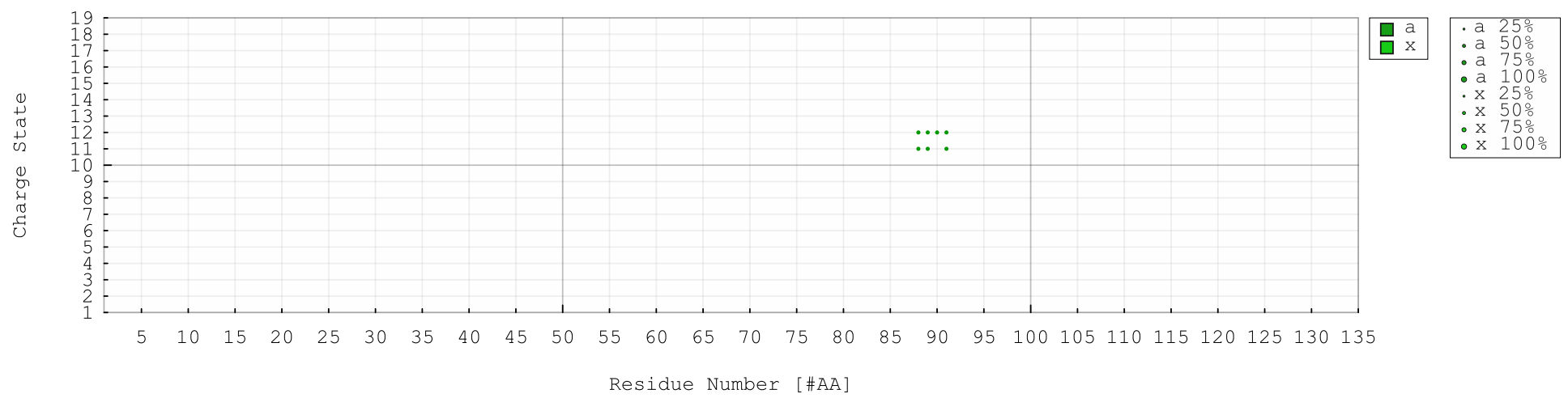

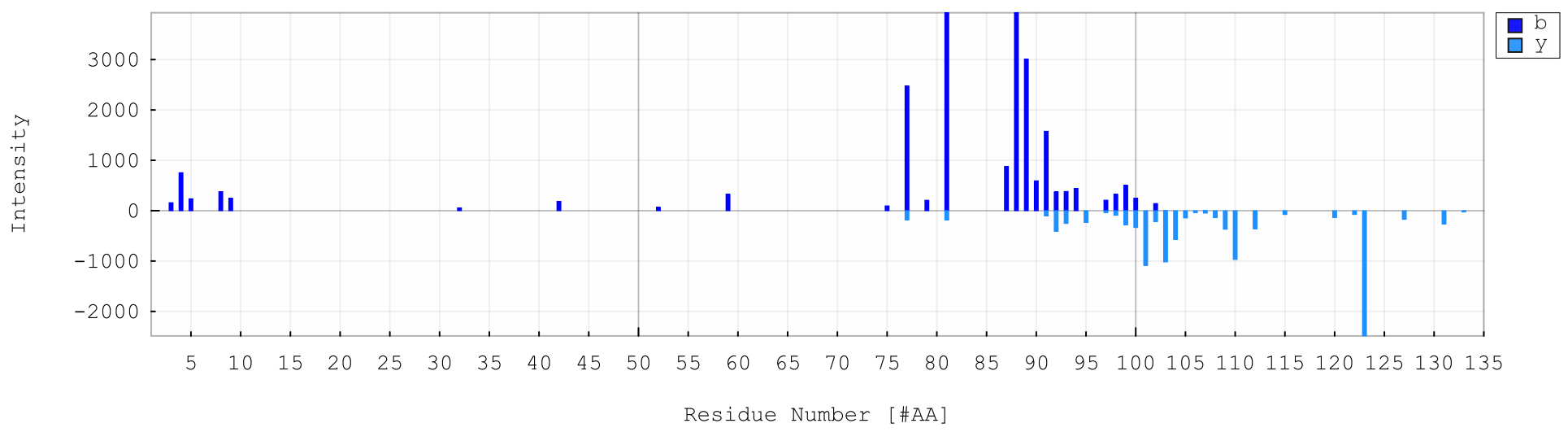

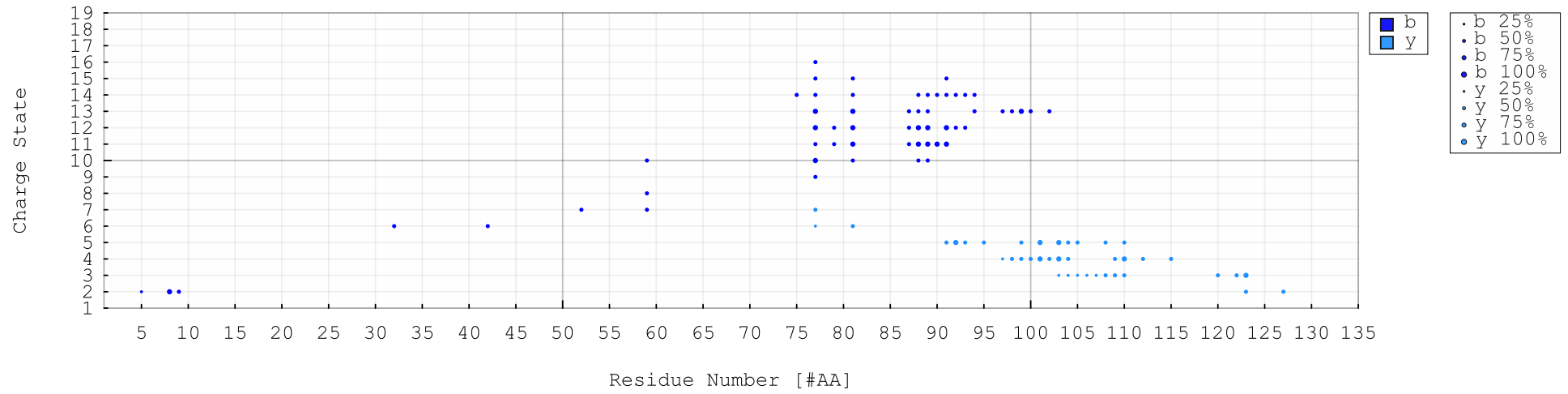

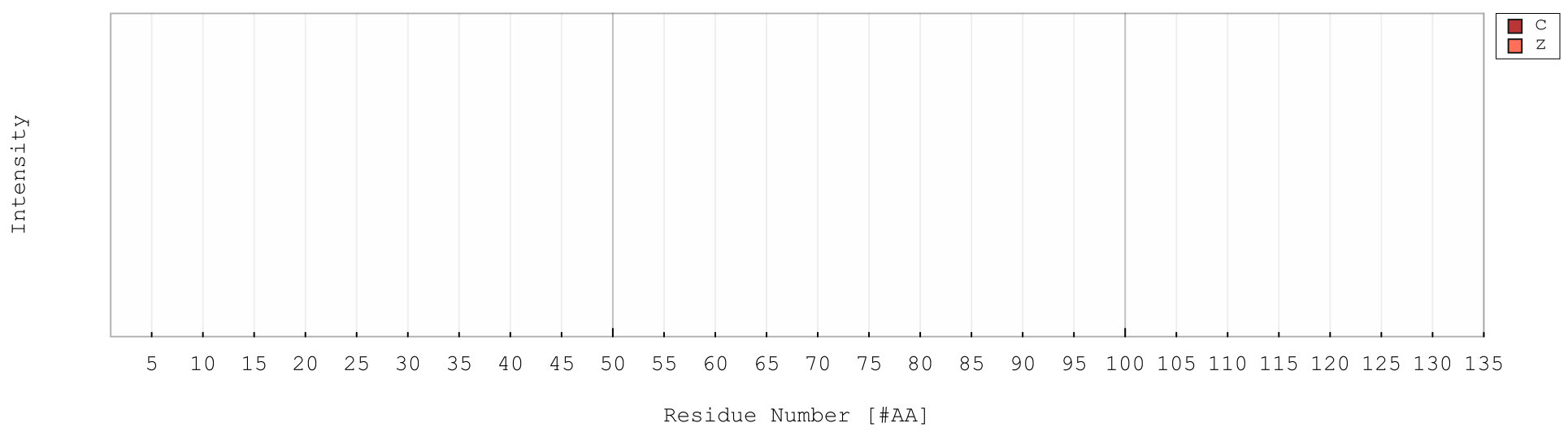

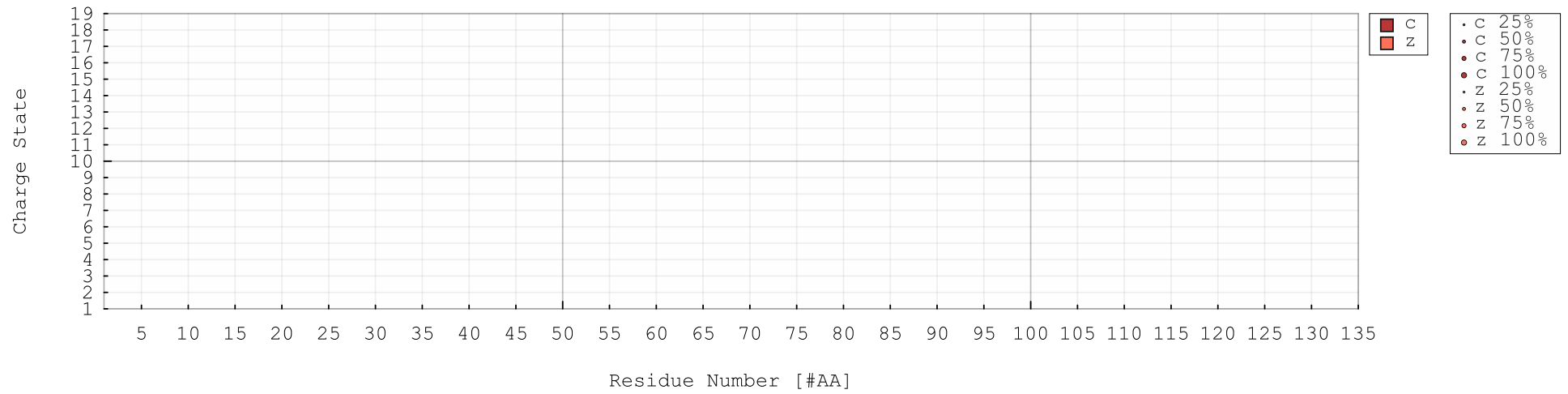

Proteoform:

Index	Protein	SC [%]	SVP [%]	IC [%]	MS Score [a.u.]	Var. Mods	Mono Mass [Da]	Chemical Formula
8	H31Kac1	32.8	6.7	21.7	7.1	Ace: K37	15305.4534	C671H1131N215O186S4

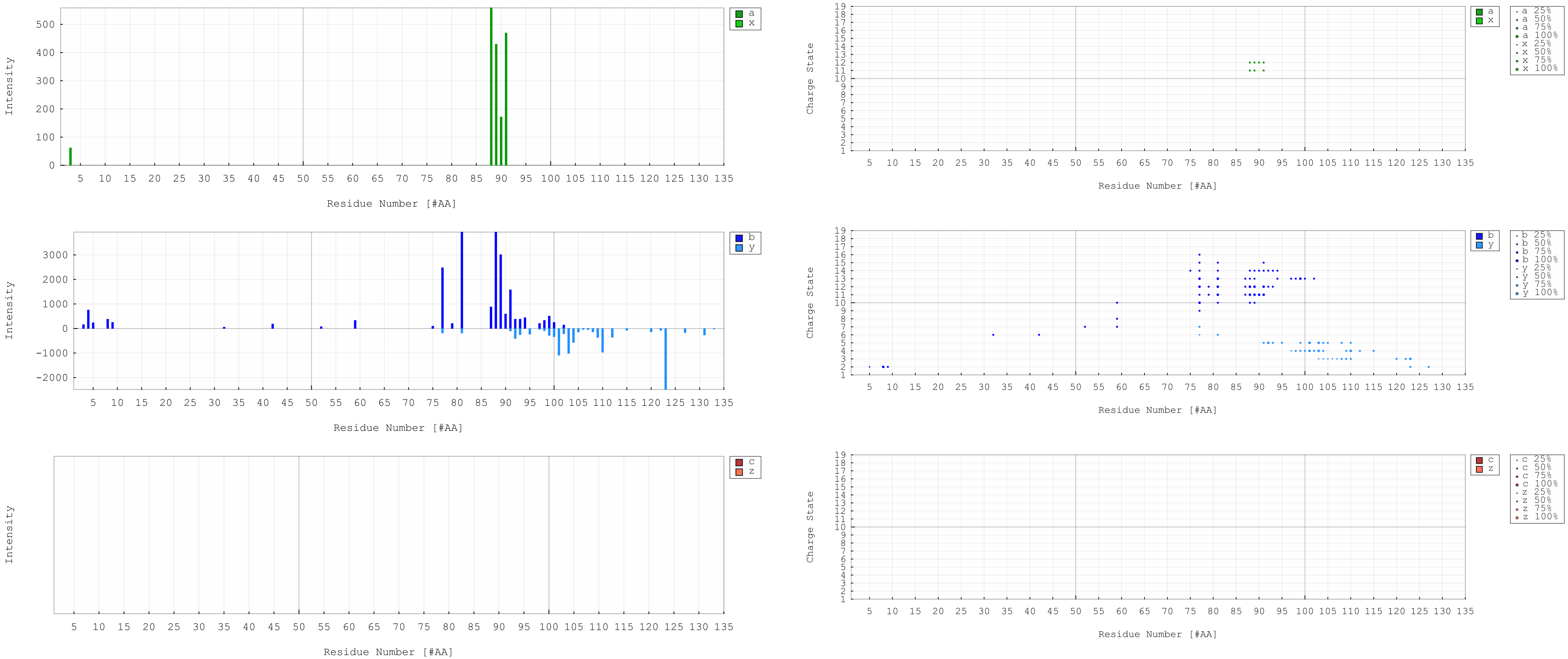

Proteoform:

Index	Protein	SC [%]	SVP [%]	IC [%]	MS Score [a.u.]	Var. Mods	Mono Mass [Da]	Chemical Formula
9	H31Kac1	31.3	6.7	21.5	6.8	Ace: K56	15305.4534	C671H1131N215O186S4

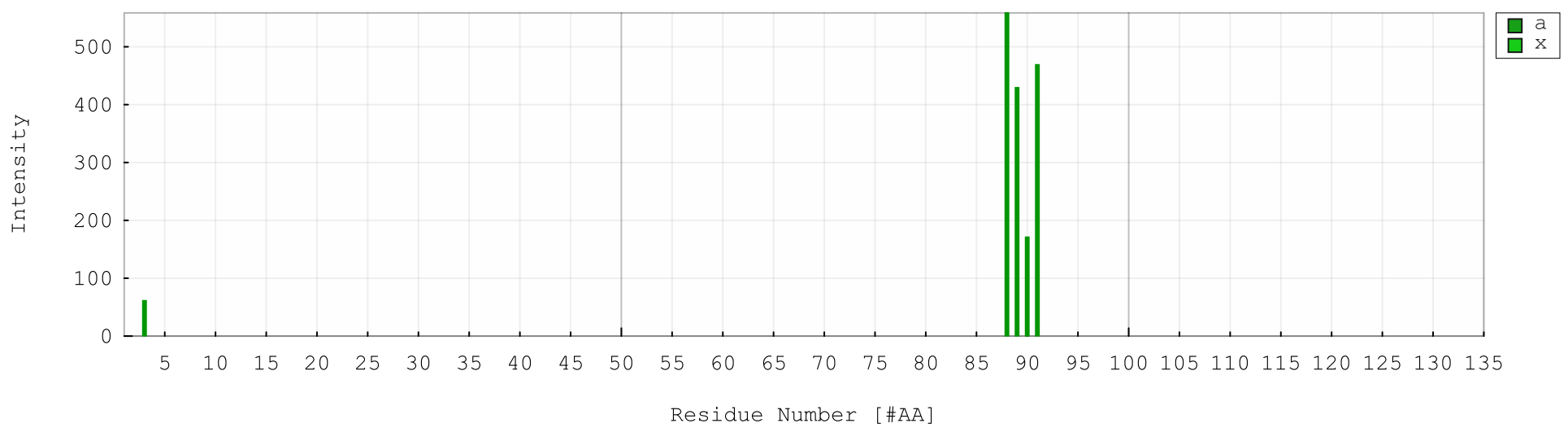

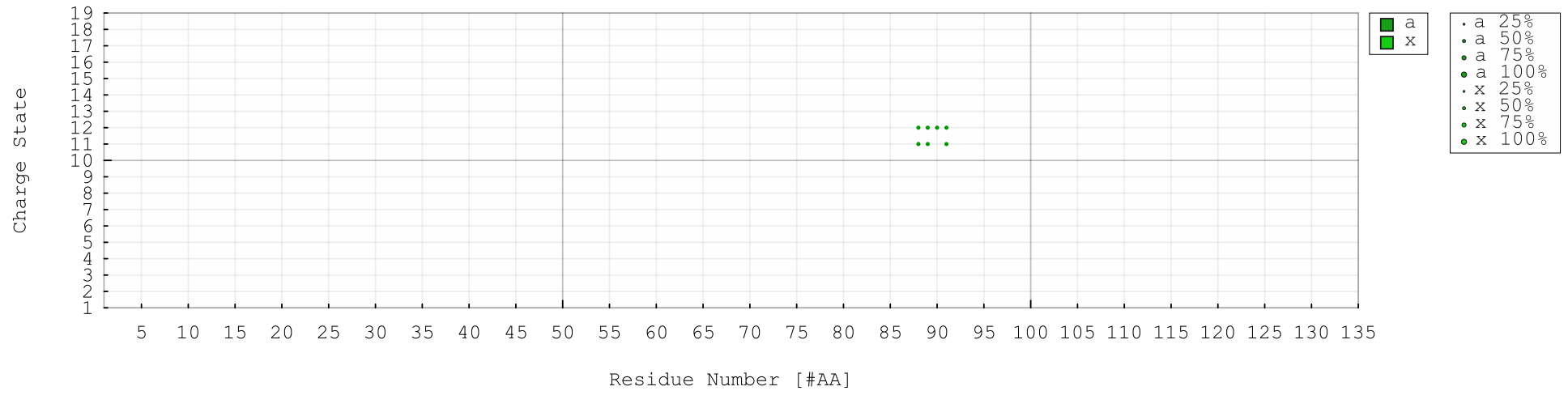

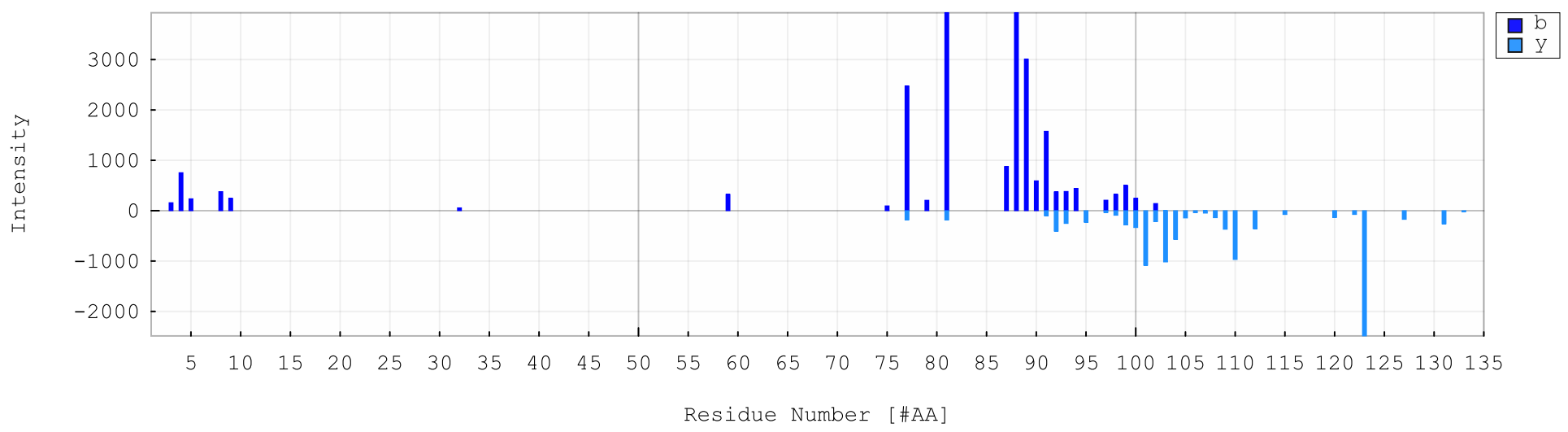

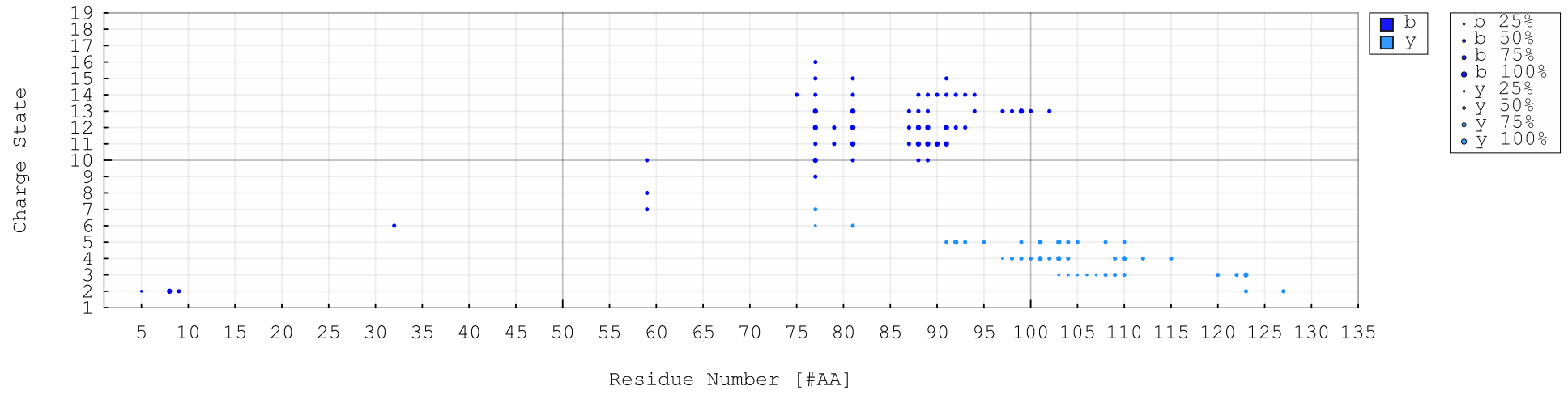

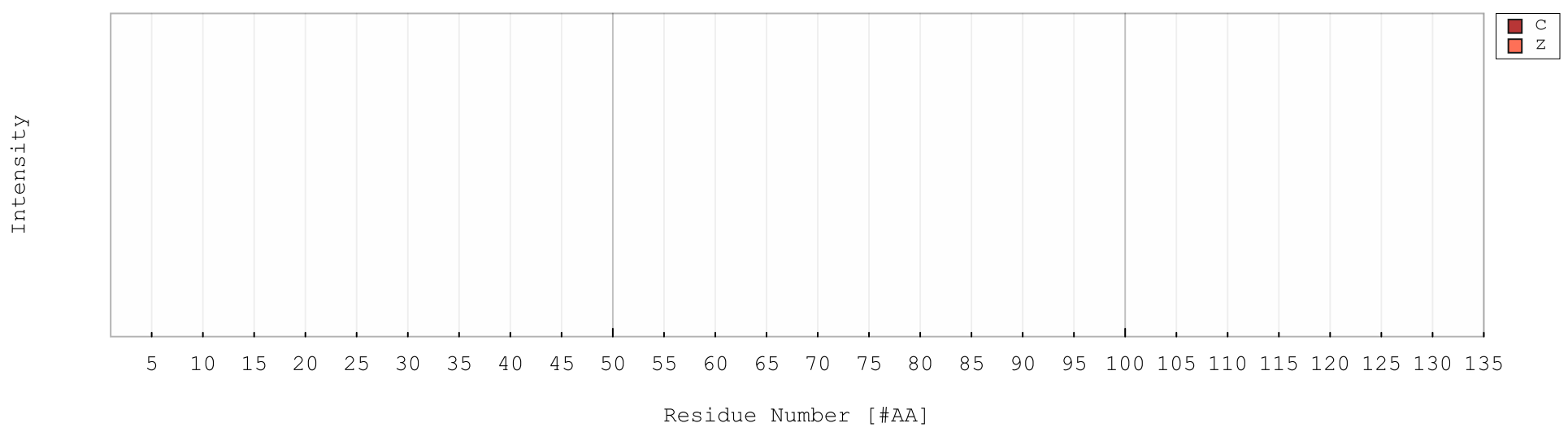

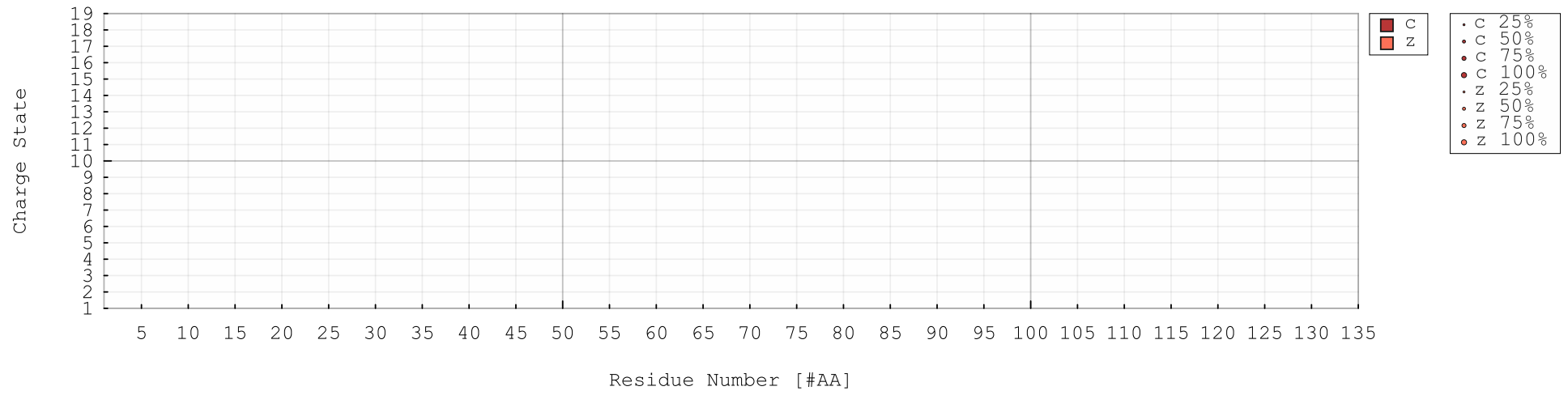

Proteoform:

Index	Protein	SC [%]	SVP [%]	IC [%]	MS Score [a.u.]	Var. Mods	Mono Mass [Da]	Chemical Formula
10	H31Kac1	30.6	6.7	21.3	6.5	Ace: K64	15305.4534	C671H1131N215O186S4

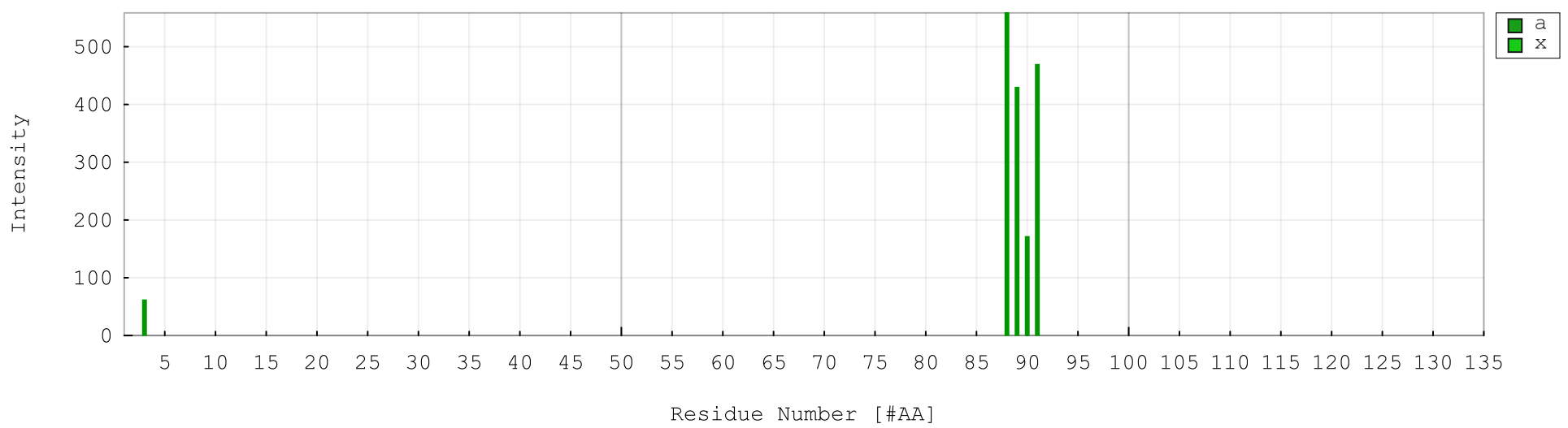

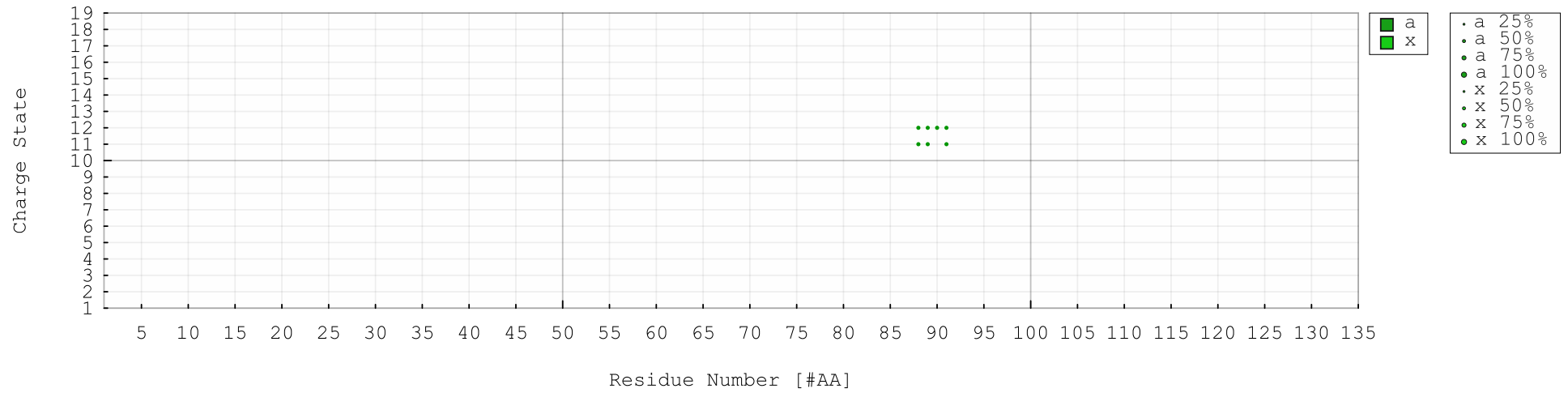

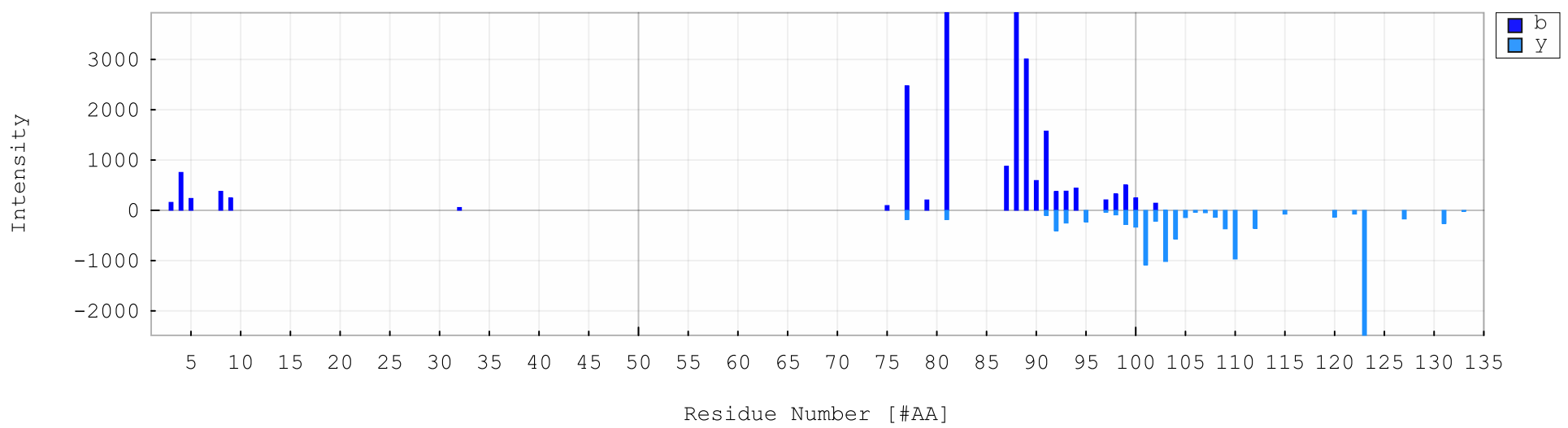

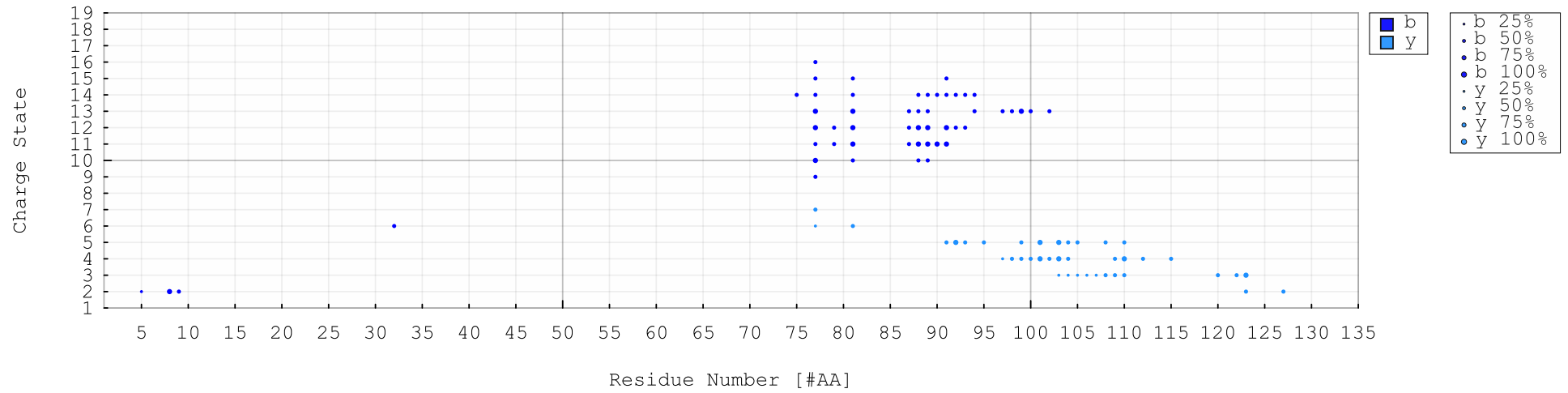

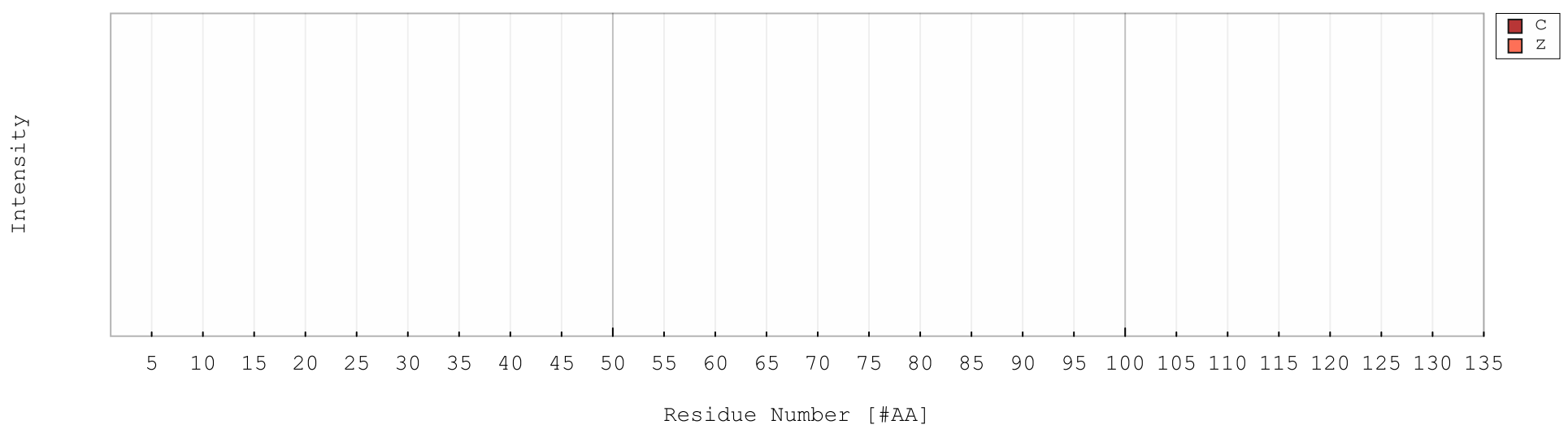

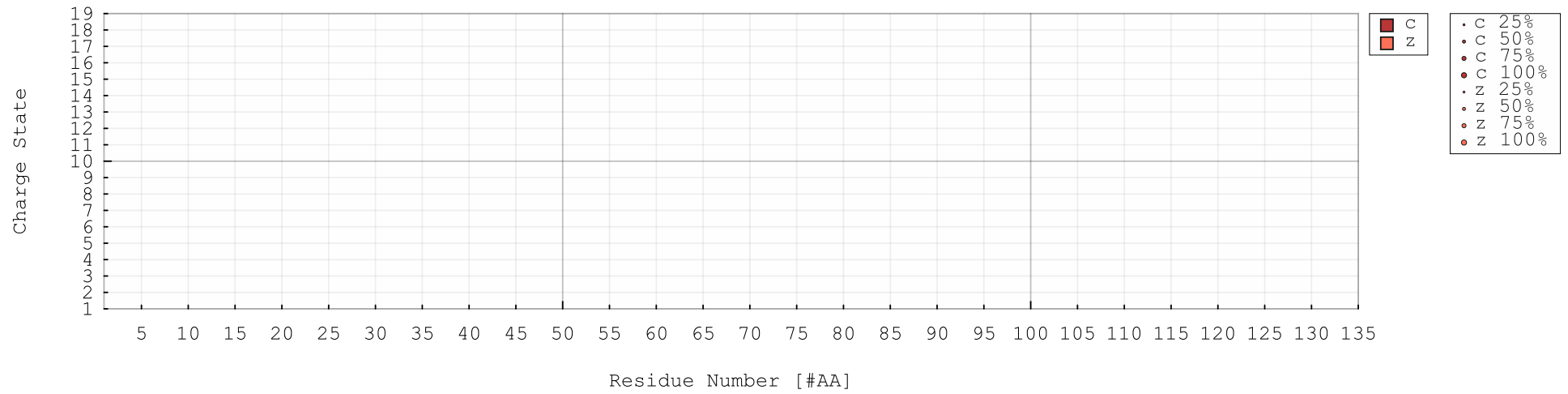

Supplemental Data S2

H3.1 - GCN5, 10 min

**Report**	5 of 7
**Experiment**	MS2
**Histone/sample**	H3.1
**Enzyme**	GCN5
**Reaction/source**	10 min
**Precursor**	H3.1Kac1
**Charge state**	20+
**Fragmentation / workflow**	ECD
**Original file**	MS2_H3.1Kac1_20+_ECD.pdf

Project Information

Project Directory: C:/Users/LAB-ADMIN
Processing date: Wed Dec 10 11:22:24 2025

Sample Info & Protocols

Name: Top-Down Protein Sequencing ExD

Dataset

Imported spectrum file name:	241209 H31-GNC5_10 z20 MS2 ECD 40ms_001-169_calibrated.txt
Dataset name:	Information is only available from imported JPEAKS files
Date acquired:	Information is only available from imported JPEAKS files
Date imported:	Information is only available from imported JPEAKS files
Instrument Name:	Information is only available from imported JPEAKS files
Instrument Serial Number:	Information is only available from imported JPEAKS files

Workflow Result Info

Analysis: 241209 H31-GNC5_10 z20 MS2 ECD 40ms_001-169_calibrated
Location: C:/Users/LAB-ADMIN
Method: -

Method Information

Name: Unnamed

Spectral Parameters

Mass Error [ppm]: 6
Intensity Deviation [au]: 0.15
Min/Max Charges: 1-20
Experiment type: ExD

a ions	b ions	c ions	x ions	y ions	z ions	satellite	internal	precursor
a	b	c		y	z+1			M
a+1		c+1			z+2			M+-2H

Noise threshold calculation: Simple moving average noise threshold (SMA)
Window size [datapoints]: 600
Noise curve offset: 0.9915274
SVP parameters: terminal gap: 2, internal gap: 1

Proteoforms
(top 10)

Index	Parent Sequence	Chain	Var. Mods	Mono Mass [Da]	Chemical Composition
1	H31Kac1	H31Kac1K14[Ace]	Ace: K14	15305.4534	C671H1131N215O186S4
2	H31Kac1	H31Kac1K18[Ace]	Ace: K18	15305.4534	C671H1131N215O186S4
3	H31Kac1	H31Kac1K9[Ace]	Ace: K9	15305.4534	C671H1131N215O186S4
4	H31Kac1	H31Kac1K4[Ace]	Ace: K4	15305.4534	C671H1131N215O186S4
5	H31Kac1	H31Kac1K23[Ace]	Ace: K23	15305.4534	C671H1131N215O186S4
6	H31Kac1	H31Kac1K27[Ace]	Ace: K27	15305.4534	C671H1131N215O186S4
7	H31Kac1	H31Kac1K36[Ace]	Ace: K36	15305.4534	C671H1131N215O186S4
8	H31Kac1	H31Kac1K37[Ace]	Ace: K37	15305.4534	C671H1131N215O186S4
9	H31Kac1	H31Kac1K56[Ace]	Ace: K56	15305.4534	C671H1131N215O186S4
10	H31Kac1	H31Kac1K64[Ace]	Ace: K64	15305.4534	C671H1131N215O186S4

Results

Proteoforms Table (top 10)

Index	Protein	SC [%]	SVP [%]	IC [%]	MS Score [a.u.]	Var. Mods	Mono Mass [Da]	Chemical Formula
1	H31Kac1	68.7	64.4	30.3	20.8	Ace: K14	15305.4534	C671H1131N215O186S4
2	H31Kac1	67.2	26.7	28.8	19.3	Ace: K18	15305.4534	C671H1131N215O186S4
3	H31Kac1	65.7	23.7	29.0	19.0	Ace: K9	15305.4534	C671H1131N215O186S4
4	H31Kac1	61.9	19.3	28.1	17.4	Ace: K4	15305.4534	C671H1131N215O186S4
5	H31Kac1	65.7	26.7	25.6	16.8	Ace: K23	15305.4534	C671H1131N215O186S4
6	H31Kac1	64.2	26.7	24.4	15.7	Ace: K27	15305.4534	C671H1131N215O186S4
7	H31Kac1	59.0	26.7	21.7	12.8	Ace: K36	15305.4534	C671H1131N215O186S4
8	H31Kac1	58.2	26.7	20.8	12.1	Ace: K37	15305.4534	C671H1131N215O186S4
9	H31Kac1	47.0	26.7	13.3	6.3	Ace: K56	15305.4534	C671H1131N215O186S4
10	H31Kac1	44.8	26.7	12.4	5.5	Ace: K64	15305.4534	C671H1131N215O186S4

Sequence

Name: H31Kac1

```
                   10                   20                   30                   40                   50                   60
A R T K Q T A R K S  T G G K A P R K Q L  A T K A A R K S A P  A T G G V K K P H R  Y R P G T V A L R E  I R R Y Q K S T E L
                   70                   80                   90                  100                  110                  120
L I R K L P F Q R L  V R E I A Q D F K T  D L R F Q S S A V M  A L Q E A C E A Y L  V G L F E D T N L C  A I H A K R V T I M
                  130                  140                  150                  160                  170                  180
P K D I Q L A R R I  R G E R A
```

Modifications

Modification	Position	Type
Acetyl	4	variable
Acetyl	9	variable
Acetyl	14	variable
Acetyl	18	variable
Acetyl	23	variable
Acetyl	27	variable
Acetyl	36	variable
Acetyl	37	variable
Acetyl	56	variable
Acetyl	64	variable
Acetyl	79	variable

Modification	Position	Type
Acetyl	115	variable
Acetyl	122	variable

Sequence Map

Proteoform:

Index	Protein	SC [%]	SVP [%]	IC [%]	MS Score [a.u.]	Var. Mods	Mono Mass [Da]	Chemical Formula
1	H31Kac1	68.7	64.4	30.3	20.8	Ace: K14	15305.4534	C671H1131N215O186S4

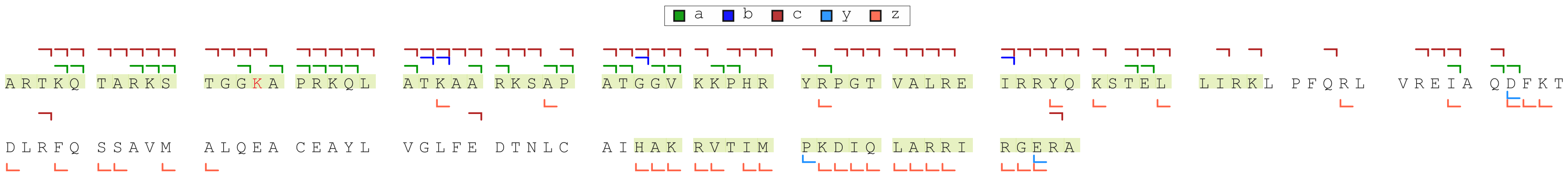

Proteoform:

Index	Protein	SC [%]	SVP [%]	IC [%]	MS Score [a.u.]	Var. Mods	Mono Mass [Da]	Chemical Formula
2	H31Kac1	67.2	26.7	28.8	19.3	Ace: K18	15305.4534	C671H1131N215O186S4

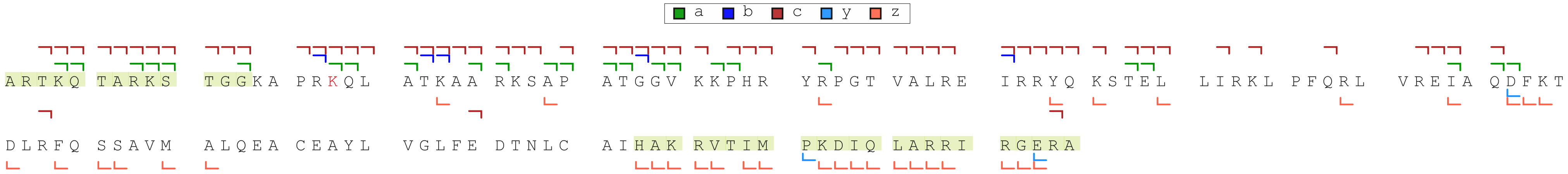

Proteoform:

Index	Protein	SC [%]	SVP [%]	IC [%]	MS Score [a.u.]	Var. Mods	Mono Mass [Da]	Chemical Formula
3	H31Kac1	65.7	23.7	29.0	19.0	Ace: K9	15305.4534	C671H1131N215O186S4

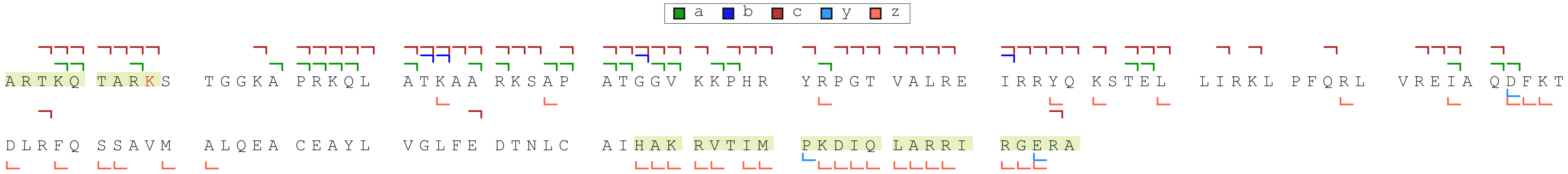

Proteoform:

Index	Protein	SC [%]	SVP [%]	IC [%]	MS Score [a.u.]	Var. Mods	Mono Mass [Da]	Chemical Formula
4	H31Kac1	61.9	19.3	28.1	17.4	Ace: K4	15305.4534	C671H1131N215O186S4

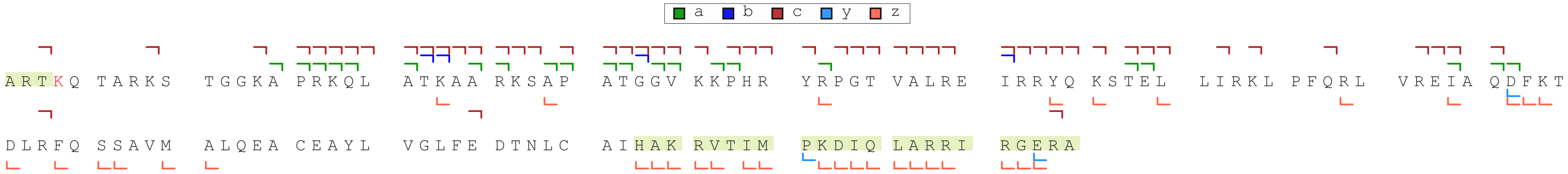

Proteoform:

Index	Protein	SC [%]	SVP [%]	IC [%]	MS Score [a.u.]	Var. Mods	Mono Mass [Da]	Chemical Formula
5	H31Kac1	65.7	26.7	25.6	16.8	Ace: K23	15305.4534	C671H1131N215O186S4

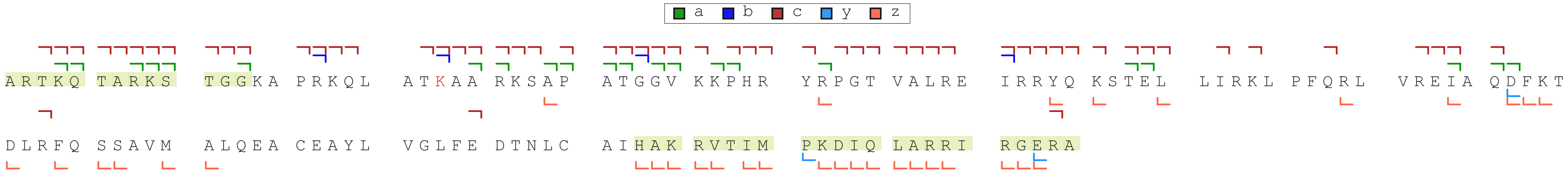

Proteoform:

Index	Protein	SC [%]	SVP [%]	IC [%]	MS Score [a.u.]	Var. Mods	Mono Mass [Da]	Chemical Formula
6	H31Kac1	64.2	26.7	24.4	15.7	Ace: K27	15305.4534	C671H1131N215O186S4

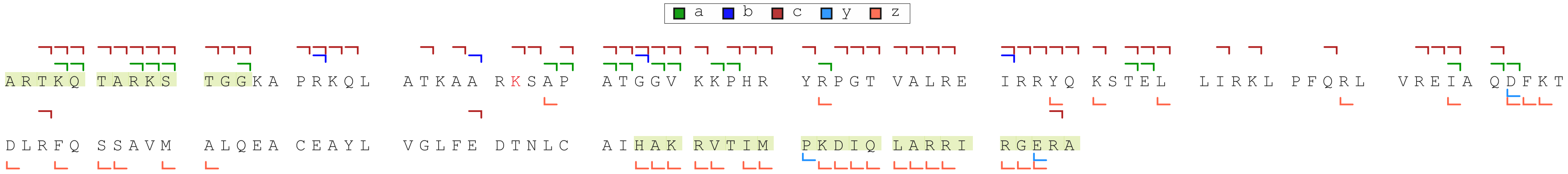

Proteoform:

Index	Protein	SC [%]	SVP [%]	IC [%]	MS Score [a.u.]	Var. Mods	Mono Mass [Da]	Chemical Formula
7	H31Kac1	59.0	26.7	21.7	12.8	Ace: K36	15305.4534	C671H1131N215O186S4

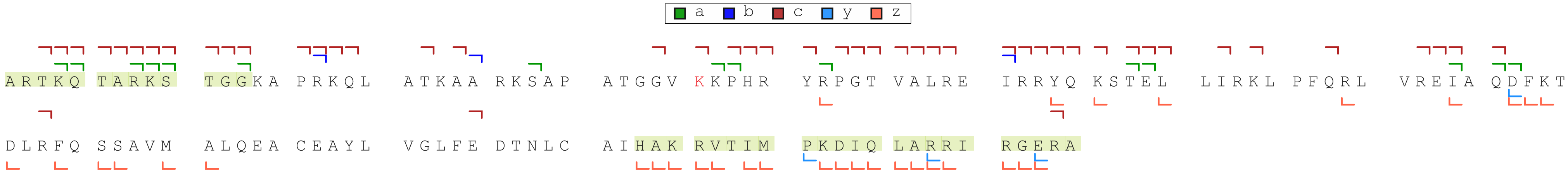

Proteoform:

Index	Protein	SC [%]	SVP [%]	IC [%]	MS Score [a.u.]	Var. Mods	Mono Mass [Da]	Chemical Formula
8	H31Kac1	58.2	26.7	20.8	12.1	Ace: K37	15305.4534	C671H1131N215O186S4

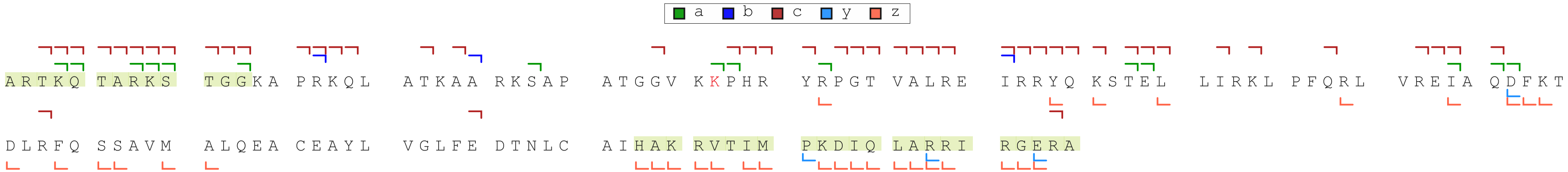

Proteoform:

Index	Protein	SC [%]	SVP [%]	IC [%]	MS Score [a.u.]	Var. Mods	Mono Mass [Da]	Chemical Formula
9	H31Kac1	47.0	26.7	13.3	6.3	Ace: K56	15305.4534	C671H1131N215O186S4

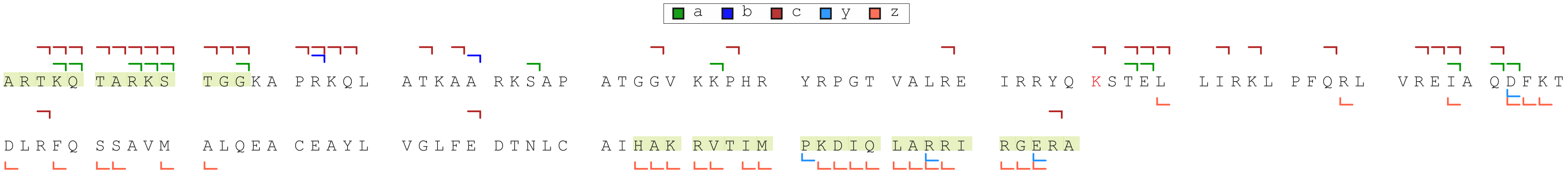

Top-Down Result OmniScape 2025

Sequence Confirmation

241209 H31-GNC5_10 z20 MS2 ECD 40ms_001-169_calibrated

Proteoform:

Index	Protein	SC [%]	SVP [%]	IC [%]	MS Score [a.u.]	Var. Mods	Mono Mass [Da]	Chemical Formula
10	H31Kac1	44.8	26.7	12.4	5.5	Ace: K64	15305.4534	C671H1131N215O186S4

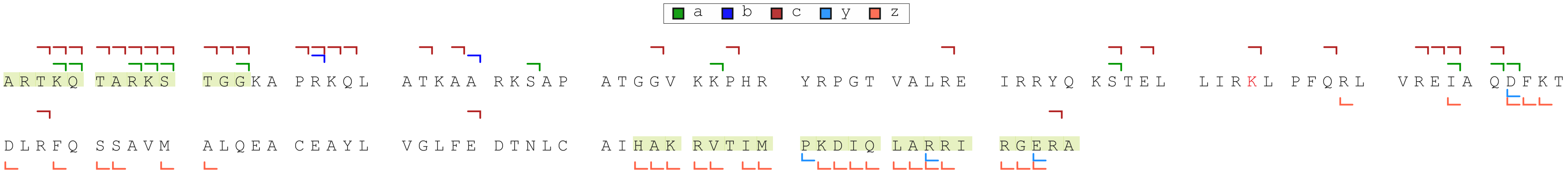

Error Plot

Proteoform:

Index	Protein	SC [%]	SVP [%]	IC [%]	MS Score [a.u.]	Var. Mods	Mono Mass [Da]	Chemical Formula
1	H31Kac1	68.7	64.4	30.3	20.8	Ace: K14	15305.4534	C671H1131N215O186S4

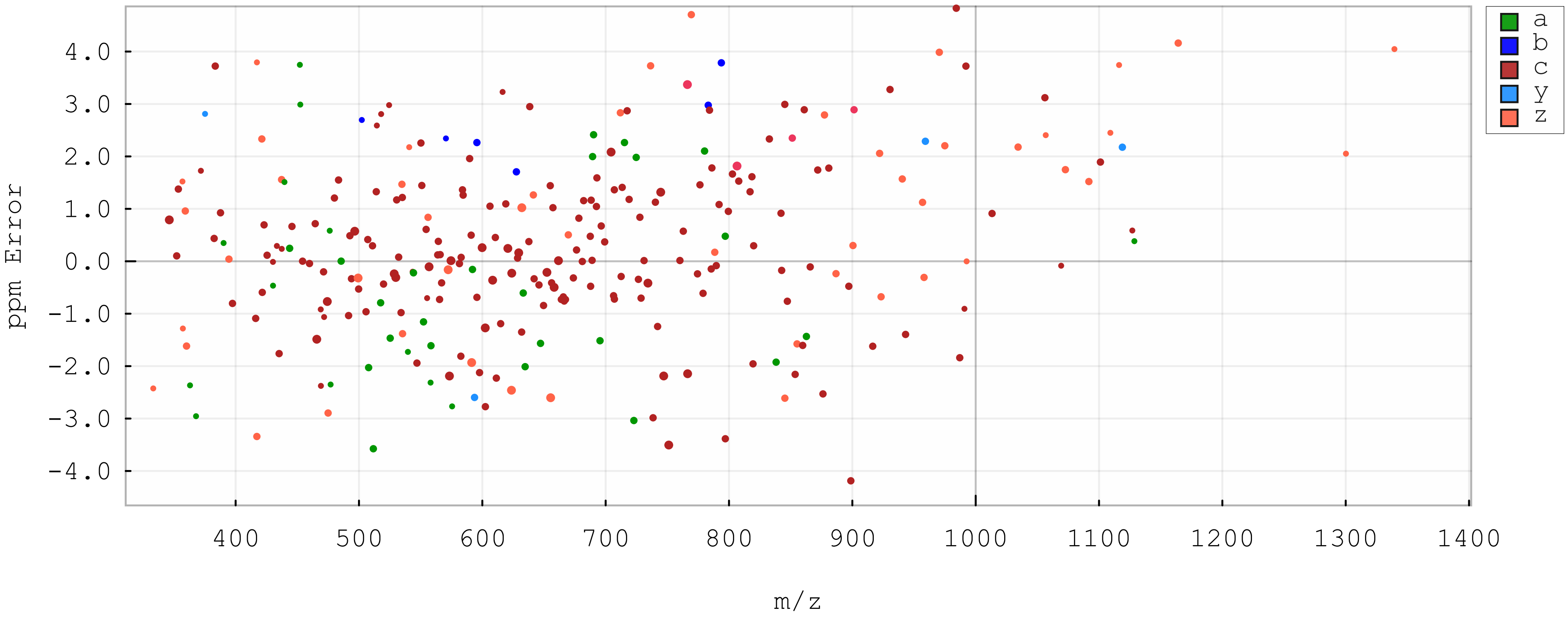

Proteoform:

Index	Protein	SC [%]	SVP [%]	IC [%]	MS Score [a.u.]	Var. Mods	Mono Mass [Da]	Chemical Formula
2	H31Kac1	67.2	26.7	28.8	19.3	Ace: K18	15305.4534	C671H1131N215O186S4

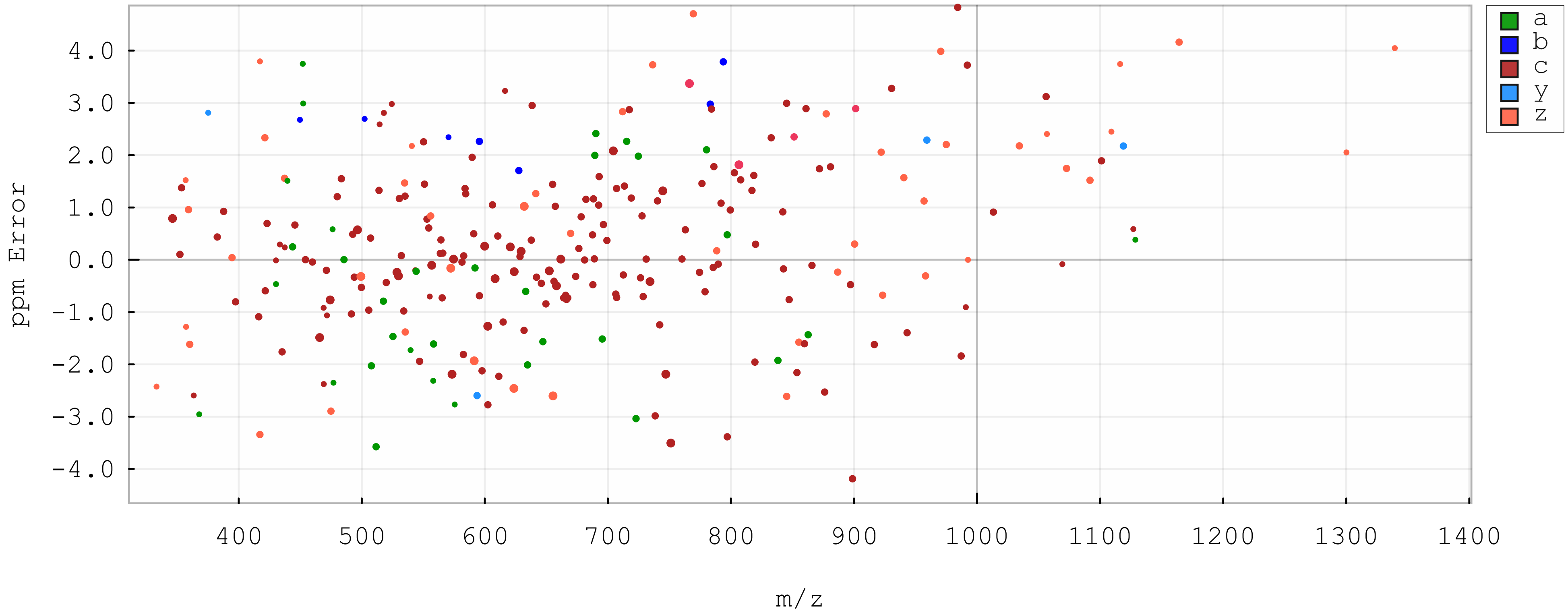

Proteoform:	Index	Protein	SC [%]	SVP [%]	IC [%]	MS Score [a.u.]	Var. Mods	Mono Mass [Da]	Chemical Formula
	3	H31Kac1	65.7	23.7	29.0	19.0	Ace: K9	15305.4534	C671H1131N215O186S4

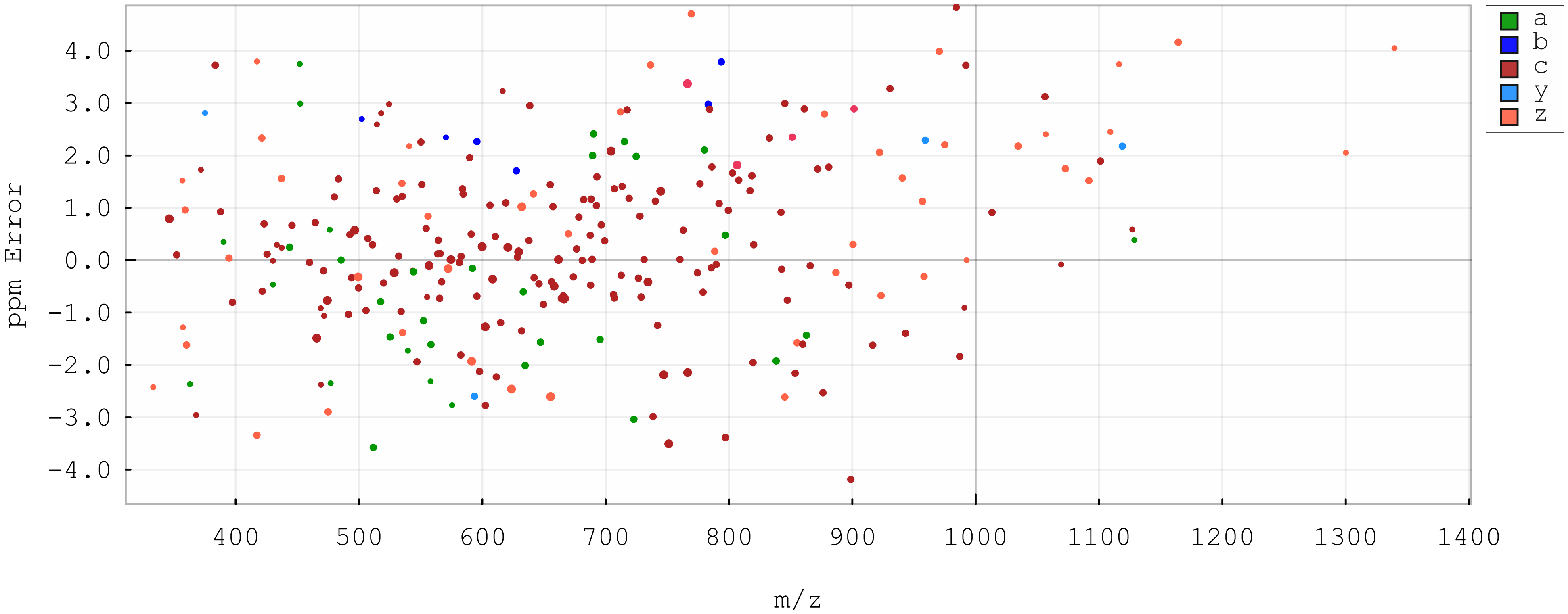

Proteoform:

Index	Protein	SC [%]	SVP [%]	IC [%]	MS Score [a.u.]	Var. Mods	Mono Mass [Da]	Chemical Formula
4	H31Kac1	61.9	19.3	28.1	17.4	Ace: K4	15305.4534	C671H1131N215O186S4

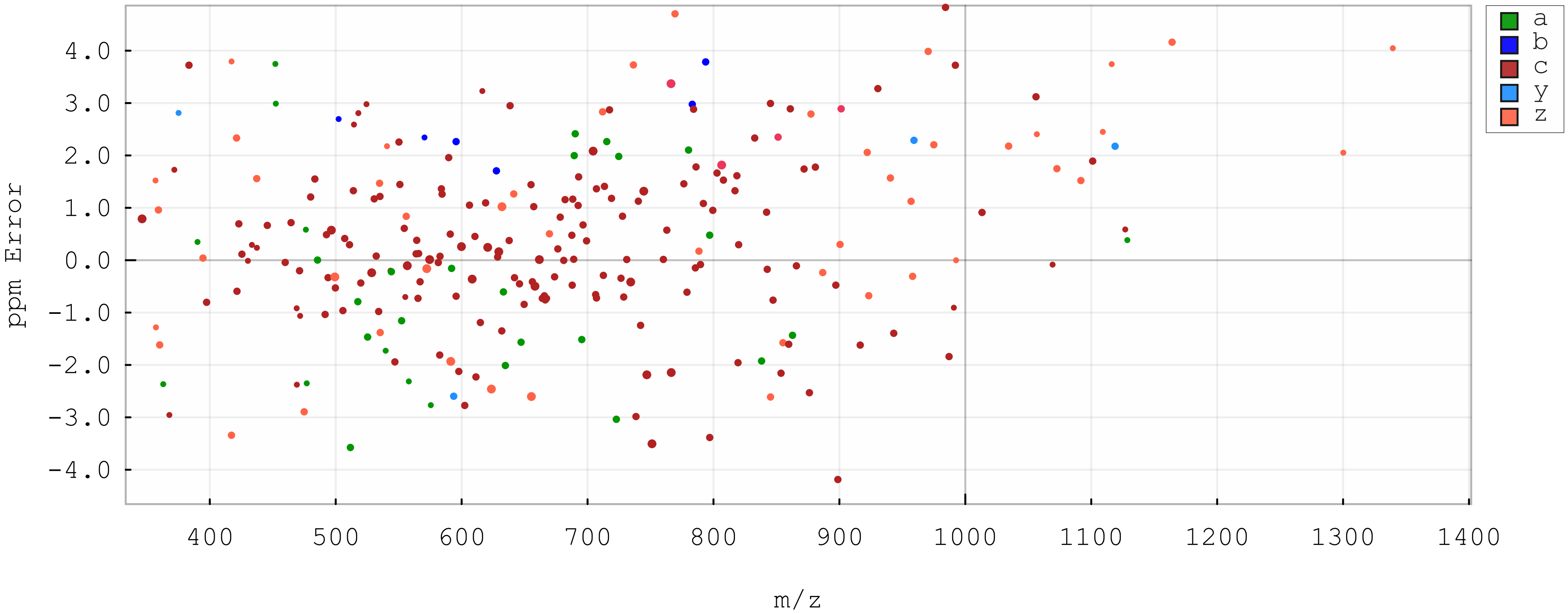

Proteoform:

Index	Protein	SC [%]	SVP [%]	IC [%]	MS Score [a.u.]	Var. Mods	Mono Mass [Da]	Chemical Formula
5	H31Kac1	65.7	26.7	25.6	16.8	Ace: K23	15305.4534	C671H1131N215O186S4

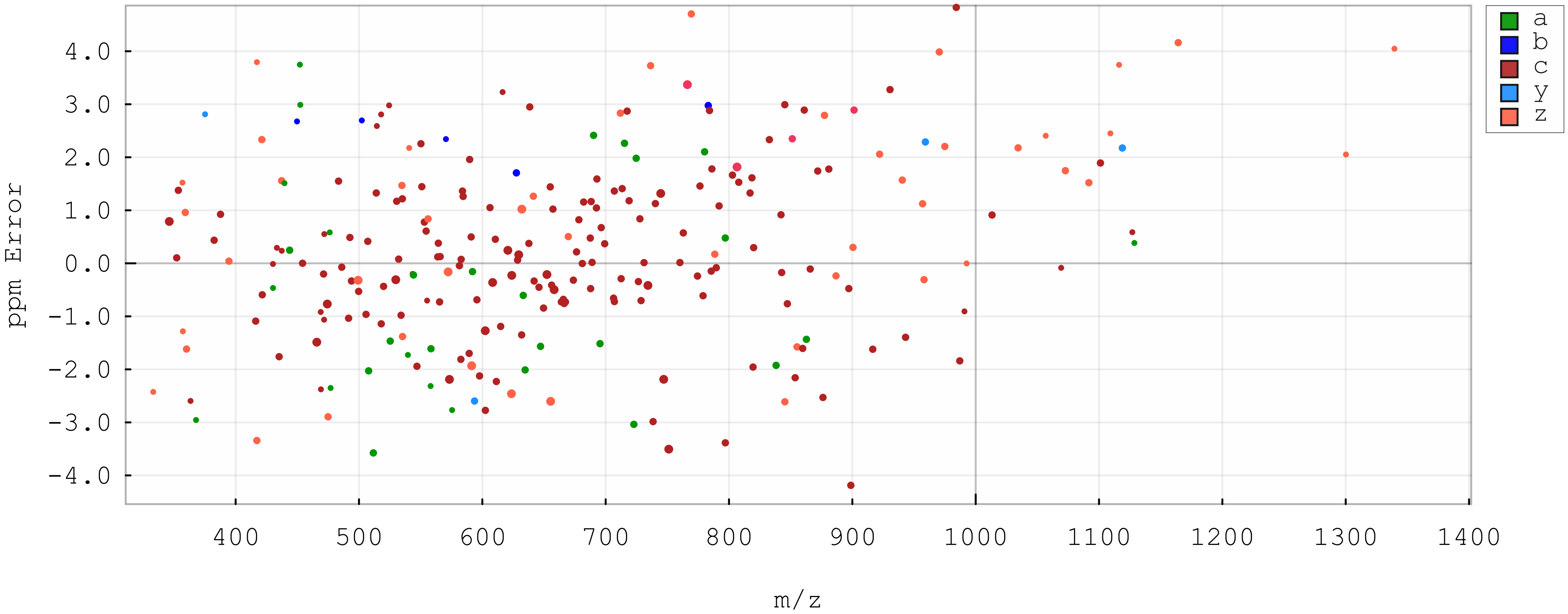

Proteoform:

Index	Protein	SC [%]	SVP [%]	IC [%]	MS Score [a.u.]	Var. Mods	Mono Mass [Da]	Chemical Formula
6	H31Kac1	64.2	26.7	24.4	15.7	Ace: K27	15305.4534	C671H1131N215O186S4

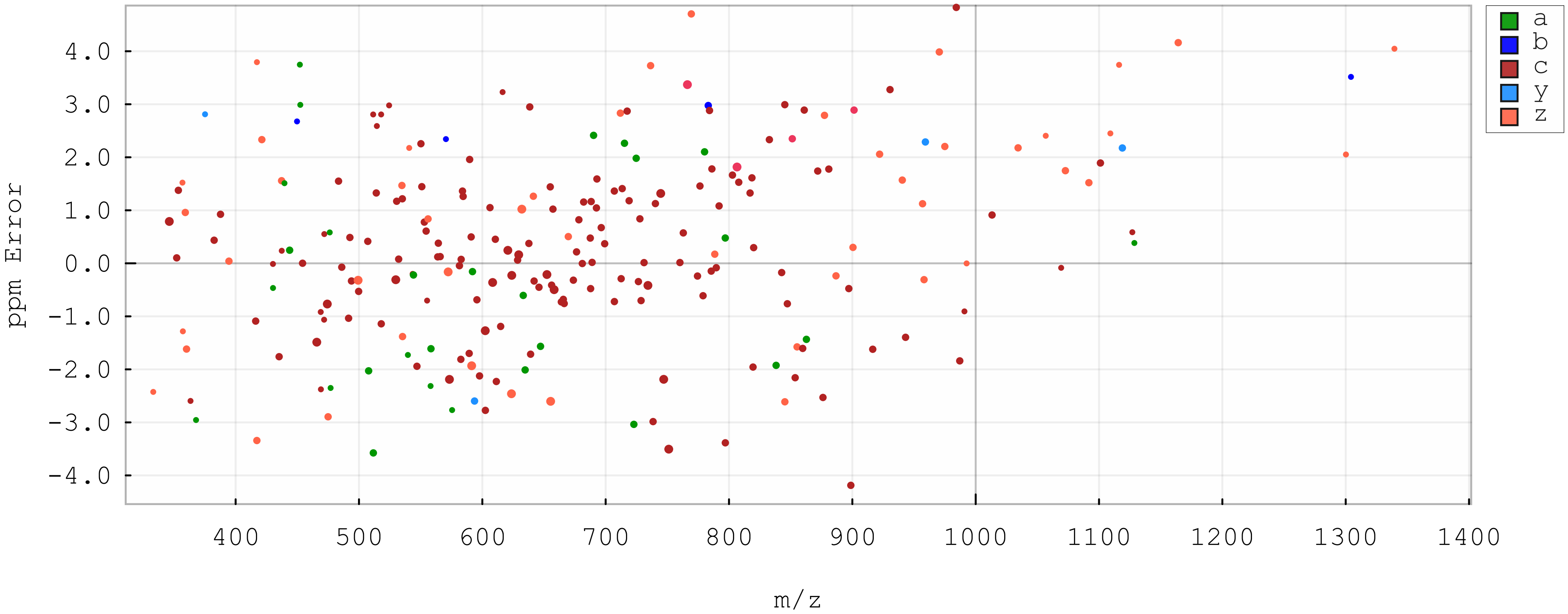

Proteoform:

Index	Protein	SC [%]	SVP [%]	IC [%]	MS Score [a.u.]	Var. Mods	Mono Mass [Da]	Chemical Formula
7	H31Kac1	59.0	26.7	21.7	12.8	Ace: K36	15305.4534	$C_{671}H_{1131}N_{215}O_{186}S_{4}$

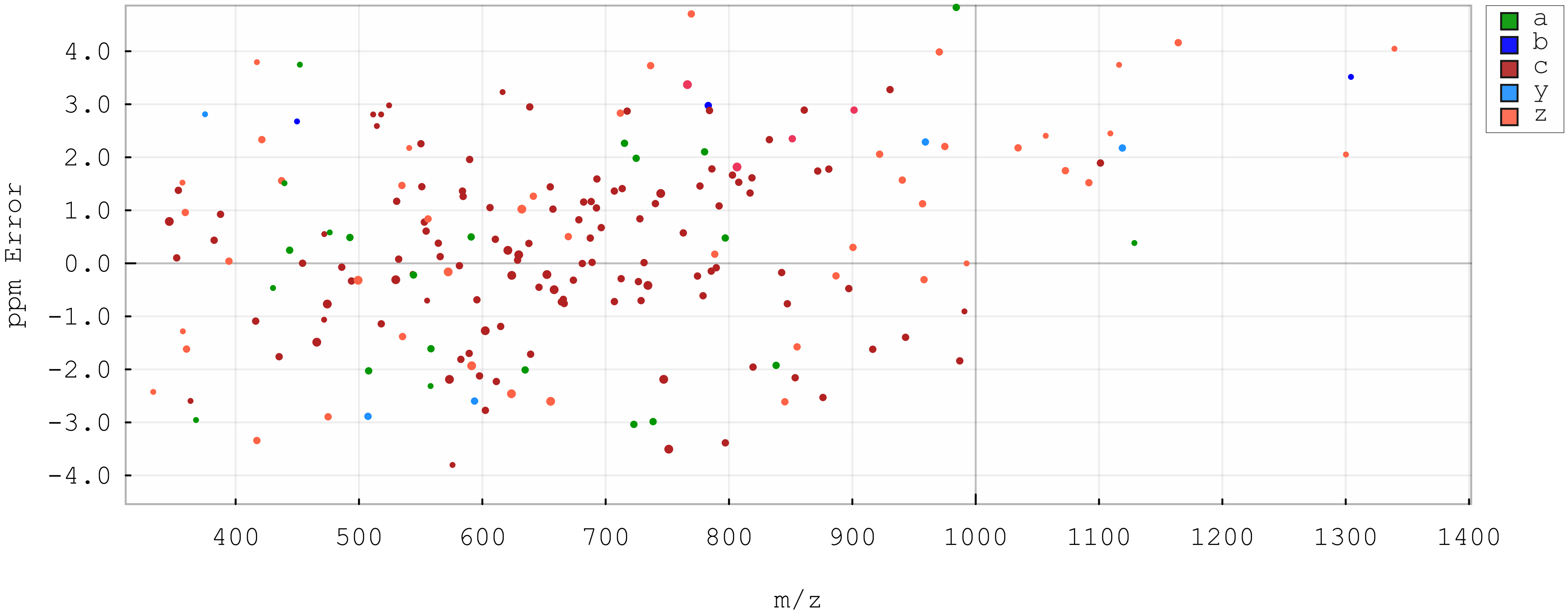

I

Proteoform:

Index	Protein	SC [%]	SVP [%]	IC [%]	MS Score [a.u.]	Var. Mods	Mono Mass [Da]	Chemical Formula
8	H31Kac1	58.2	26.7	20.8	12.1	Ace: K37	15305.4534	C671H1131N215O186S4

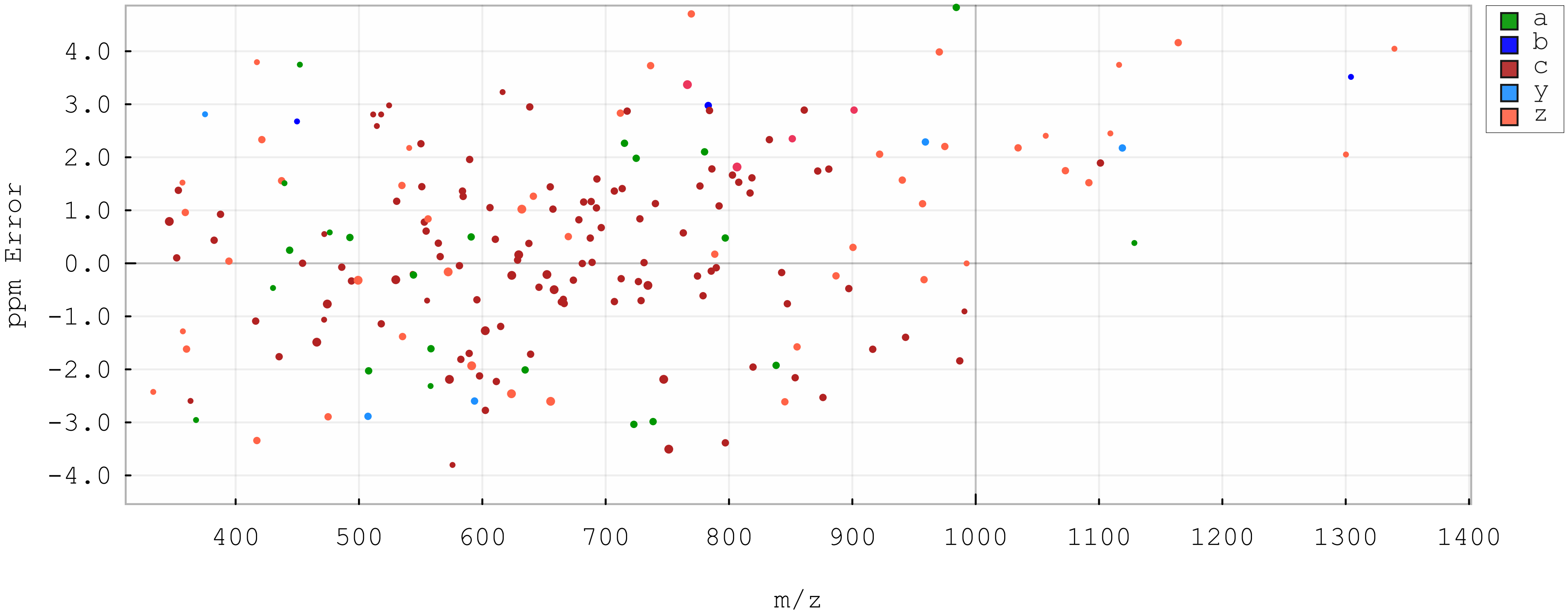

Proteoform:

Index	Protein	SC [%]	SVP [%]	IC [%]	MS Score [a.u.]	Var. Mods	Mono Mass [Da]	Chemical Formula
9	H31Kac1	47.0	26.7	13.3	6.3	Ace: K56	15305.4534	C671H1131N215O186S4

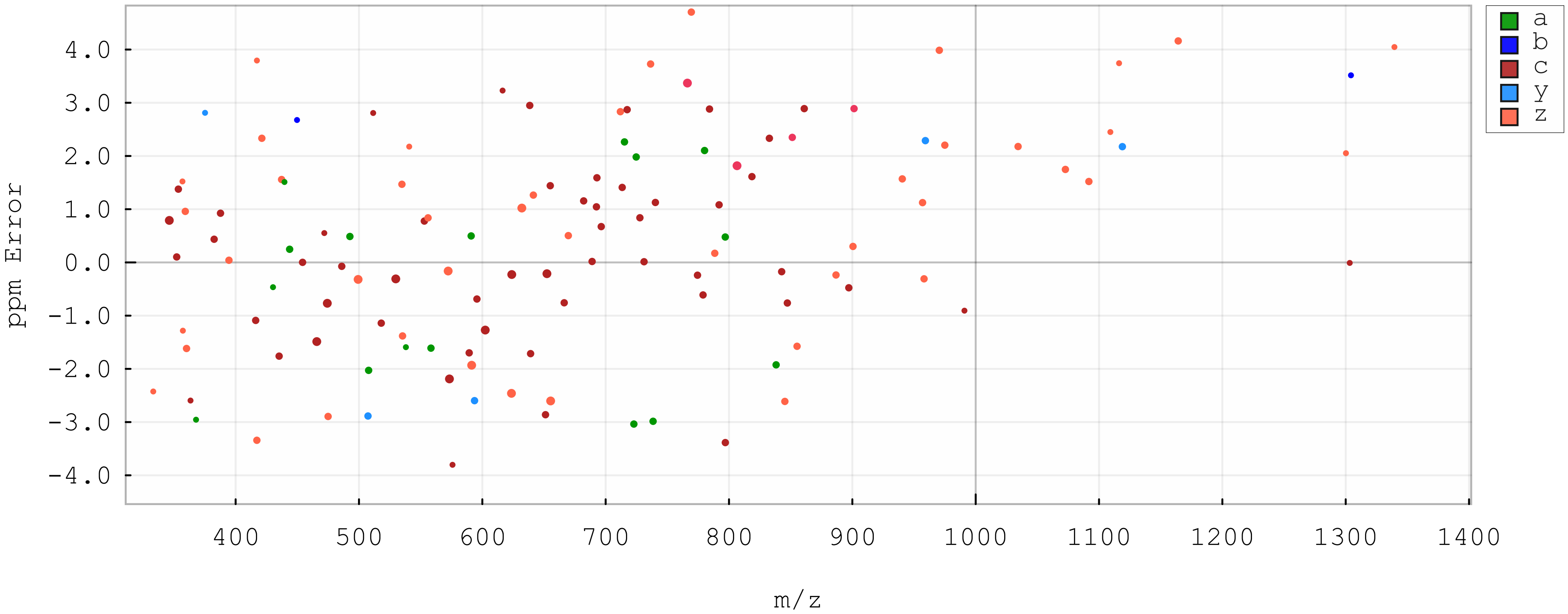

Proteoform:

Index	Protein	SC [%]	SVP [%]	IC [%]	MS Score [a.u.]	Var. Mods	Mono Mass [Da]	Chemical Formula
10	H31Kac1	44.8	26.7	12.4	5.5	Ace: K64	15305.4534	C671H1131N215O186S4

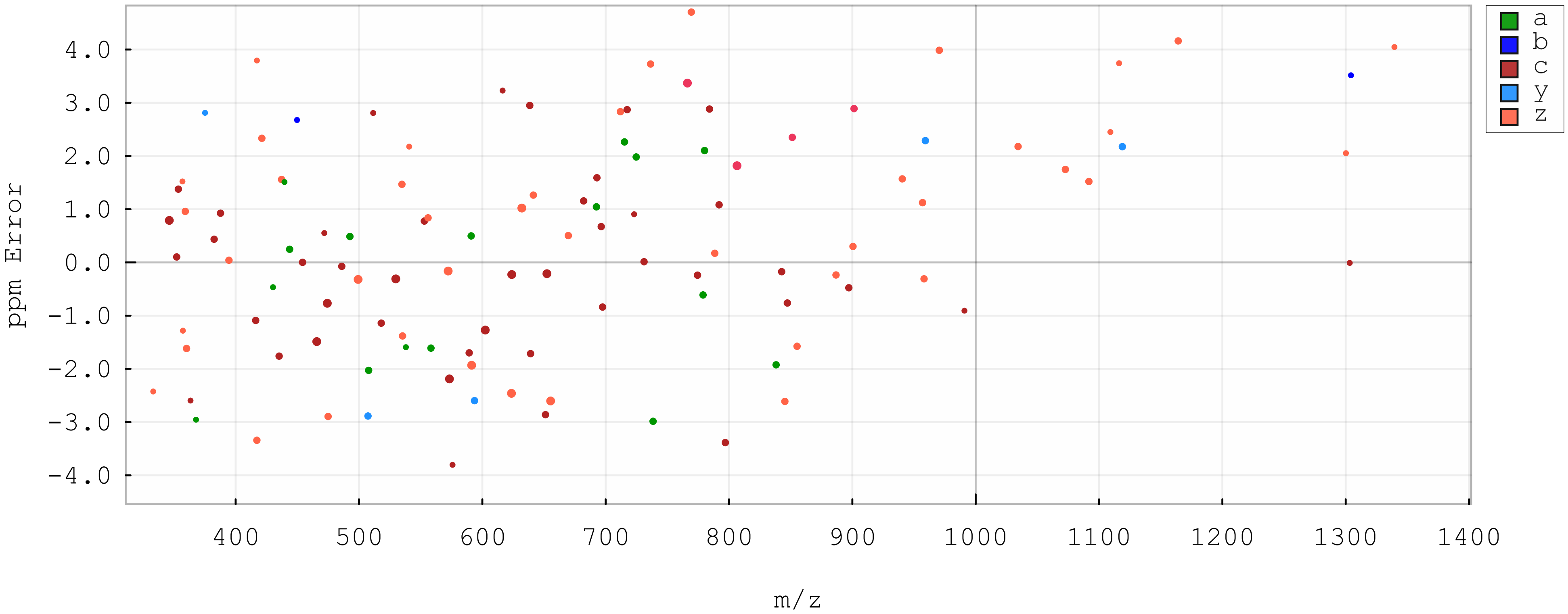

Primary Fragment Plots

Proteoform:

Index	Protein	SC [%]	SVP [%]	IC [%]	MS Score [a.u.]	Var. Mods	Mono Mass [Da]	Chemical Formula
1	H31Kac1	68.7	64.4	30.3	20.8	Ace: K14	15305.4534	C671H1131N215O186S4

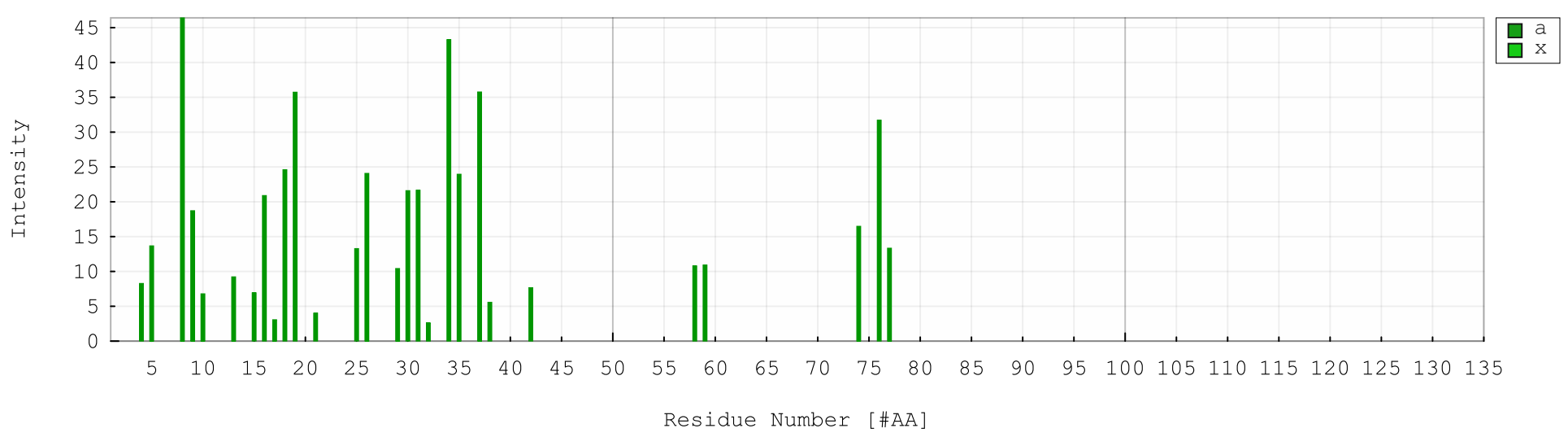

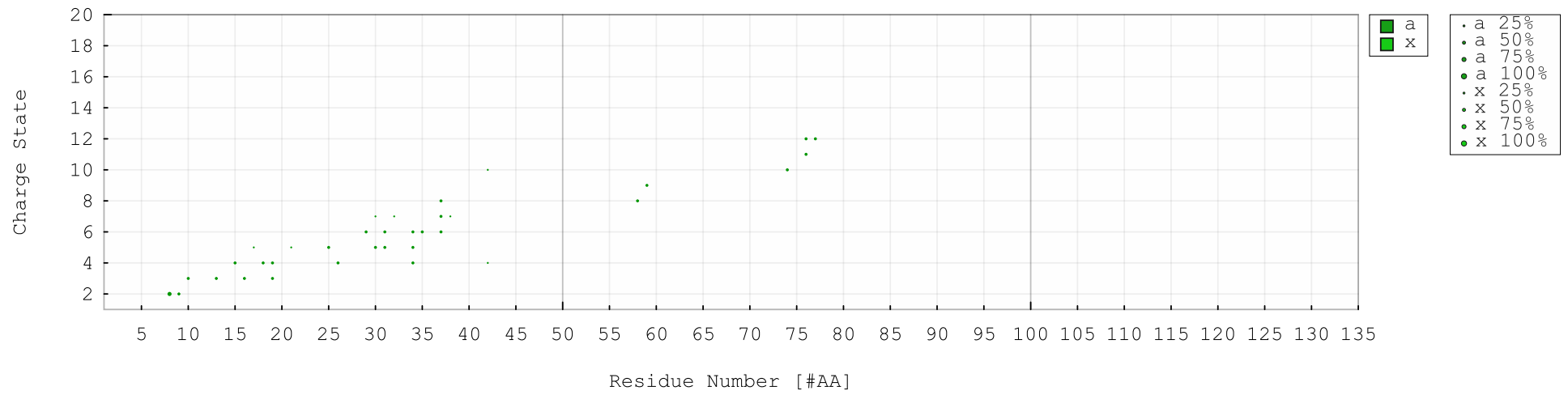

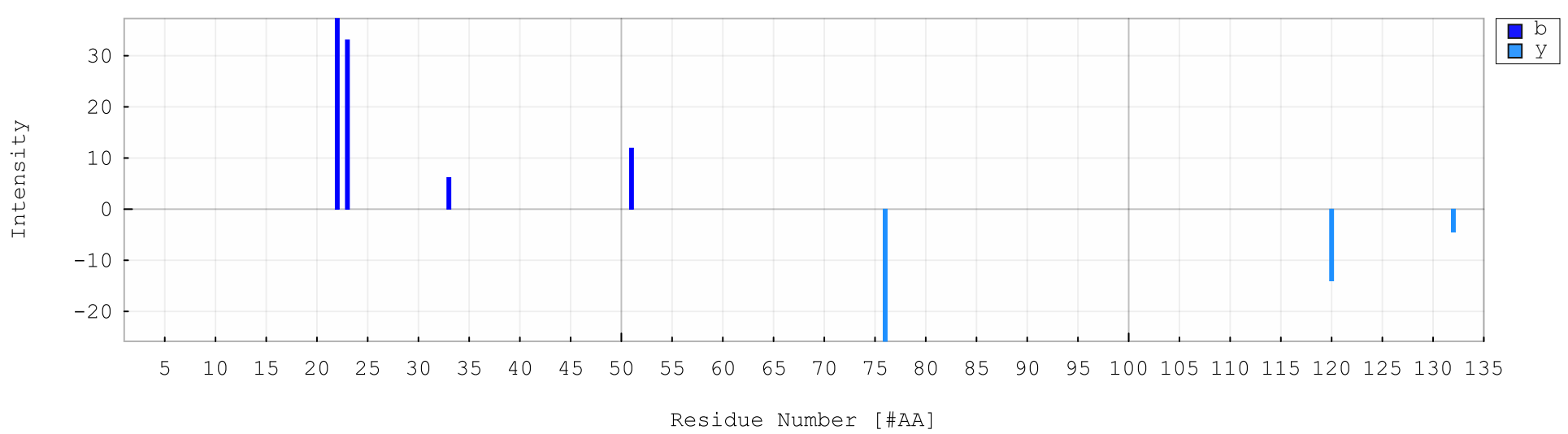

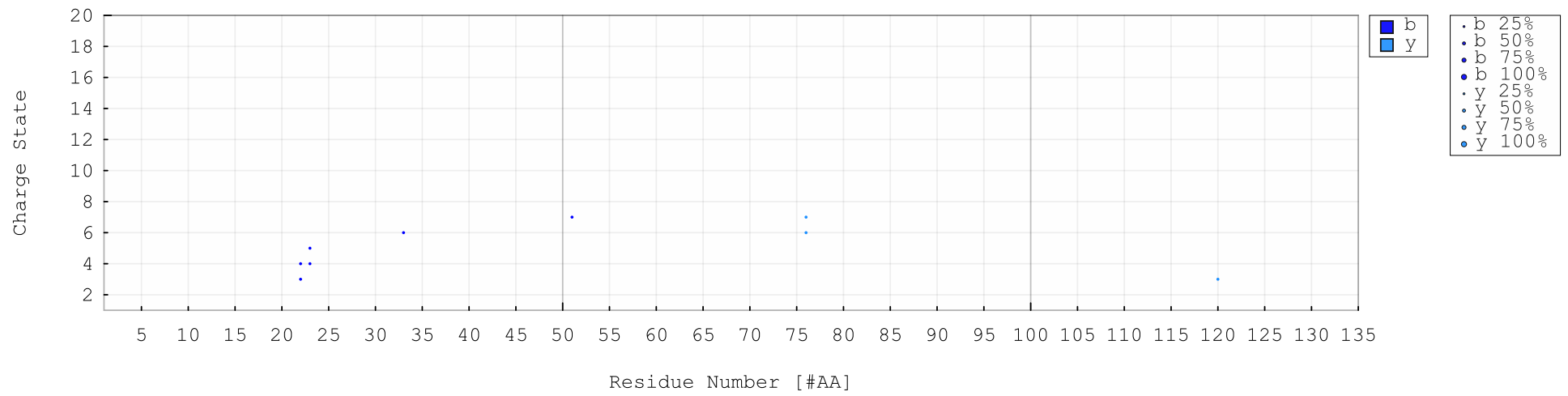

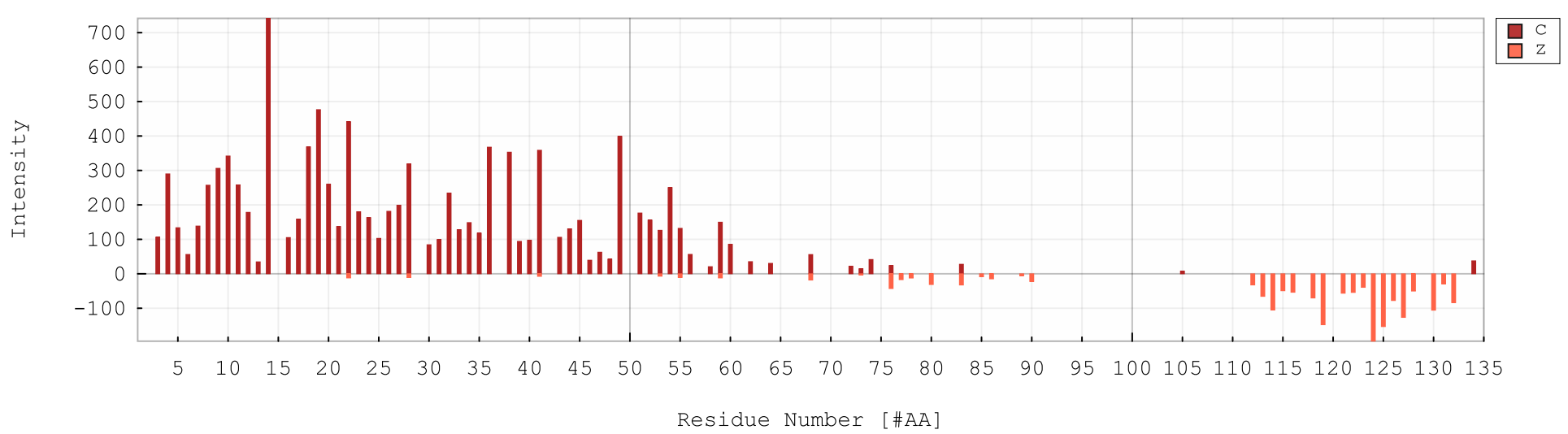

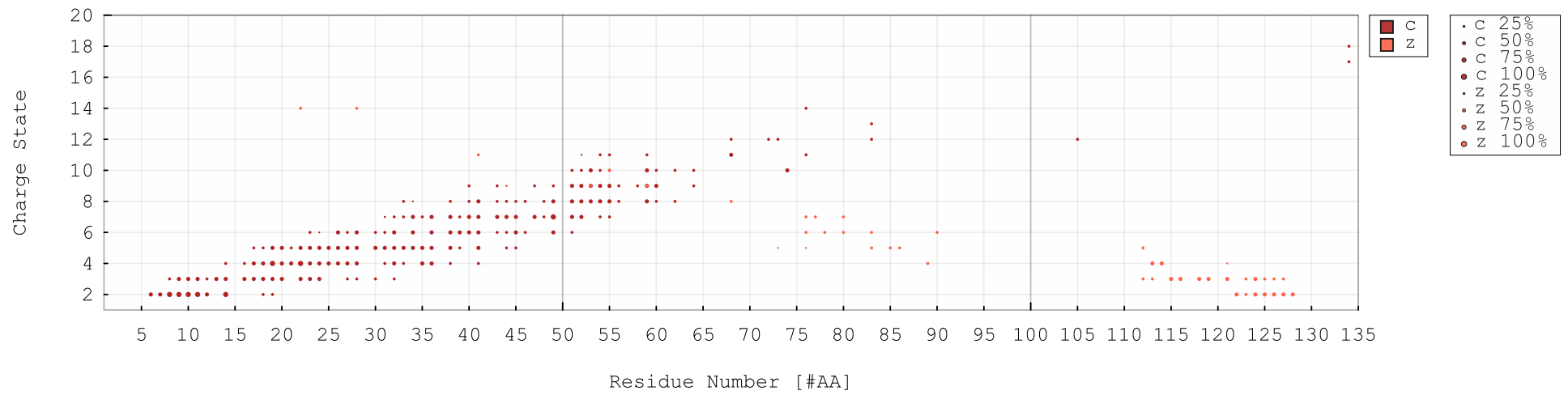

Top-Down Result OmniScape 2025
Sequence Confirmation
241209 H31-GNC5_10 z20 MS2 ECD 40ms_001-169_calibrated

Proteoform:	Index	Protein	SC [%]	SVP [%]	IC [%]	MS Score [a.u.]	Var. Mods	Mono Mass [Da]	Chemical Formula
	2	H31Kac1	67.2	26.7	28.8	19.3	Ace: K18	15305.4534	C671H1131N215O186S4

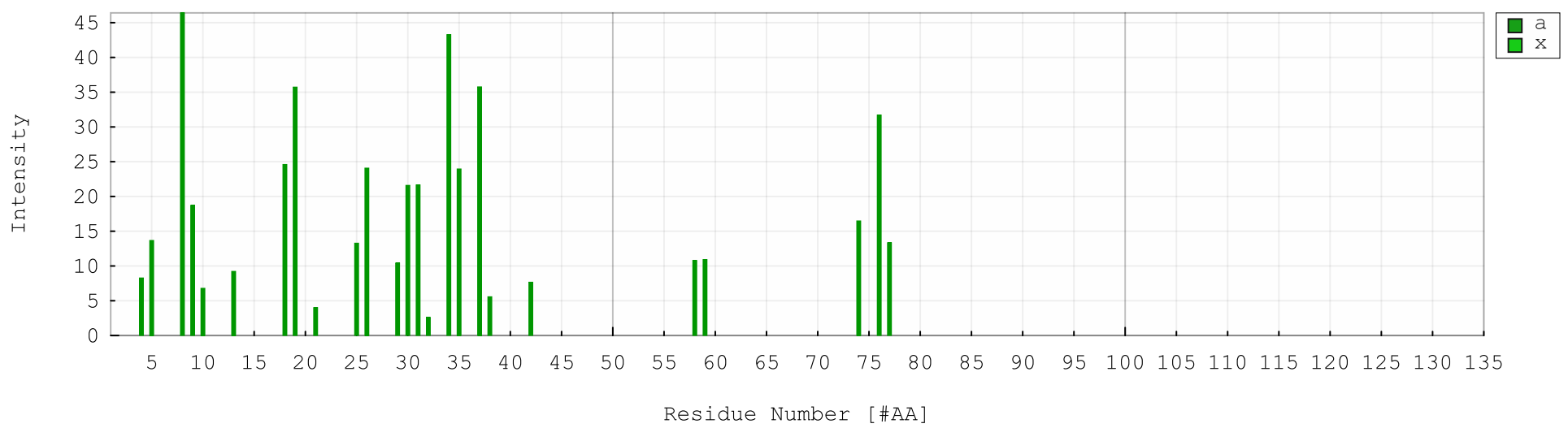

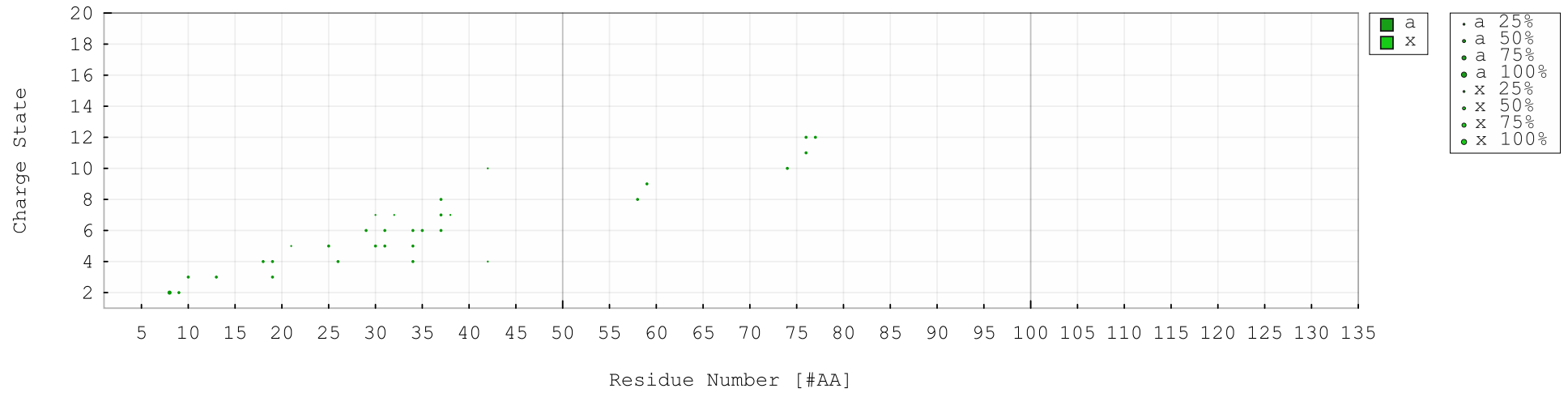

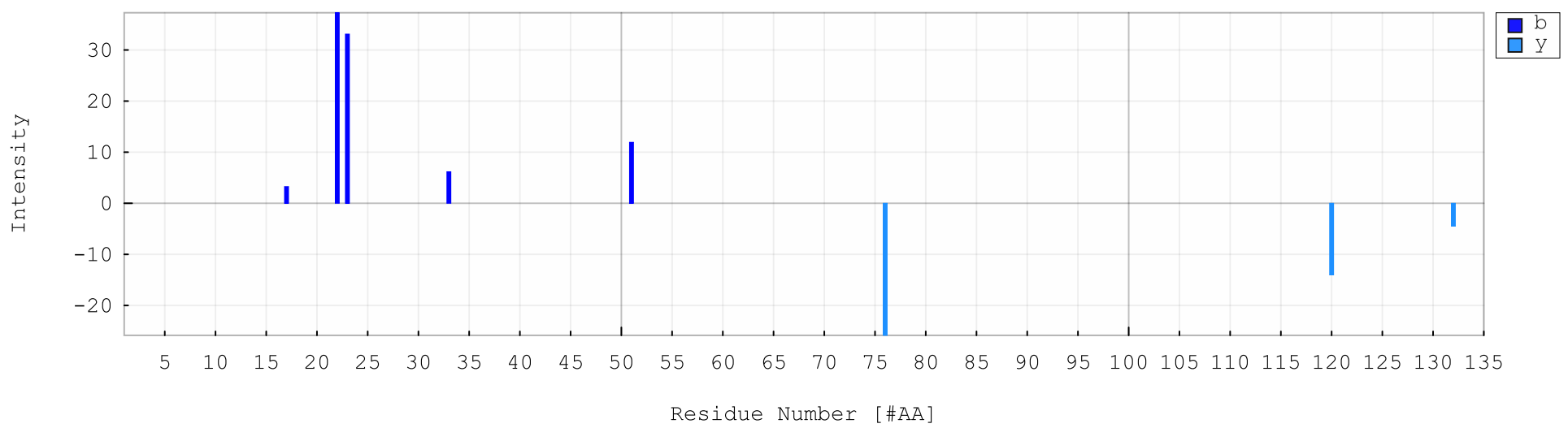

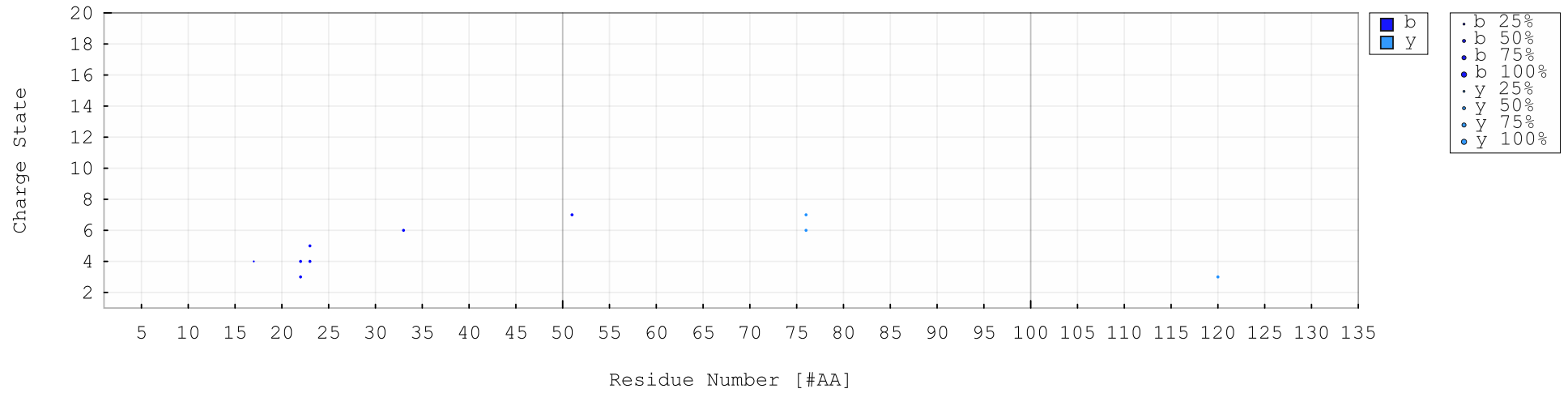

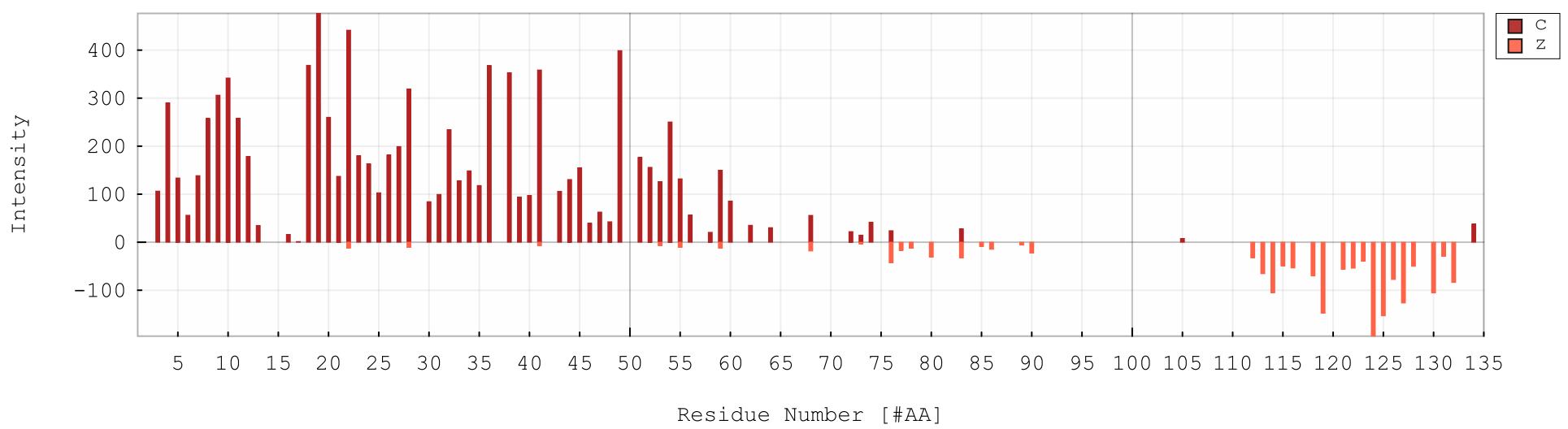

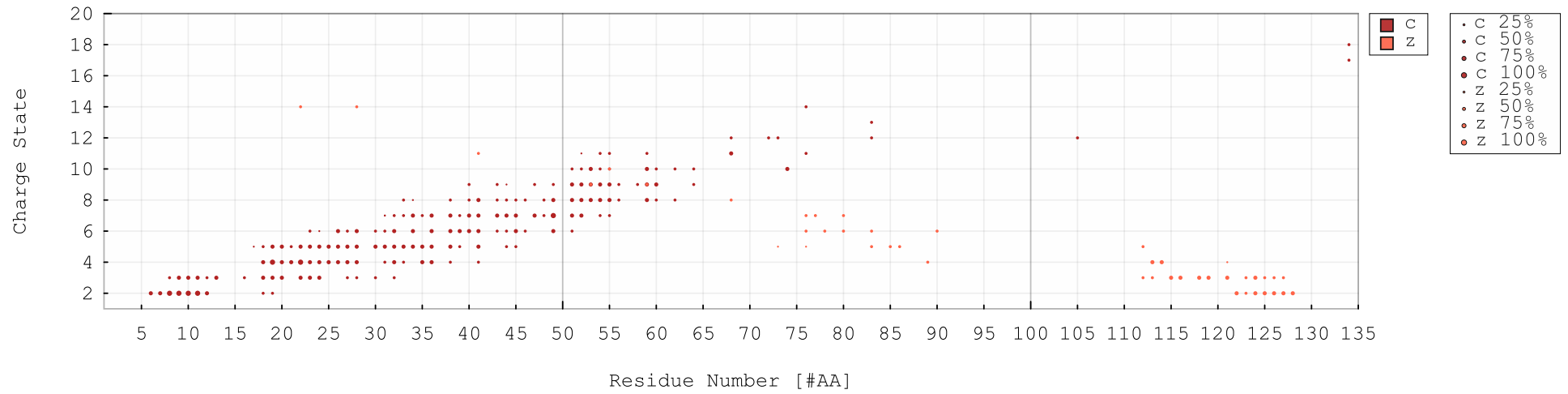

Proteoform:

Index	Protein	SC [%]	SVP [%]	IC [%]	MS Score [a.u.]	Var. Mods	Mono Mass [Da]	Chemical Formula
3	H31Kac1	65.7	23.7	29.0	19.0	Ace: K9	15305.4534	C671H1131N215O186S4

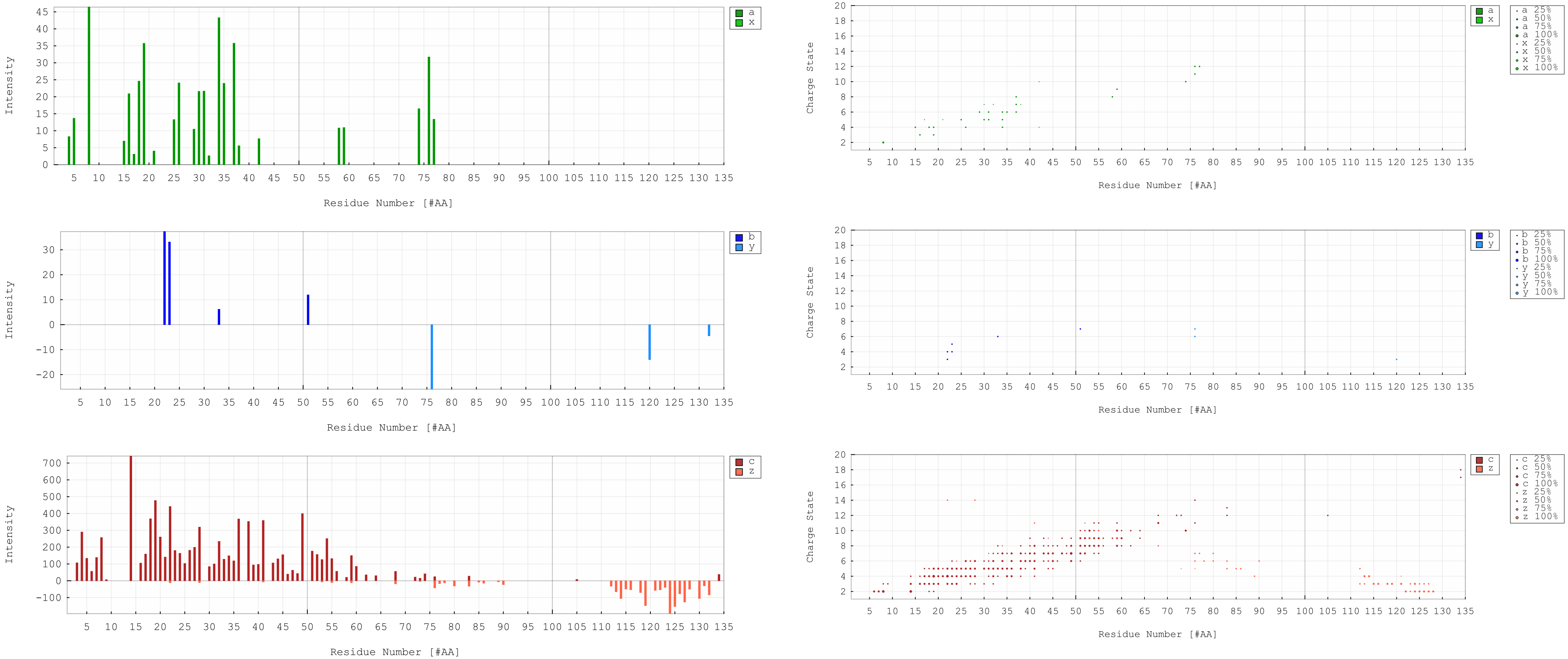

Proteoform:

Index	Protein	SC [%]	SVP [%]	IC [%]	MS Score [a.u.]	Var. Mods	Mono Mass [Da]	Chemical Formula
4	H31Kac1	61.9	19.3	28.1	17.4	Ace: K4	15305.4534	C671H1131N215O186S4

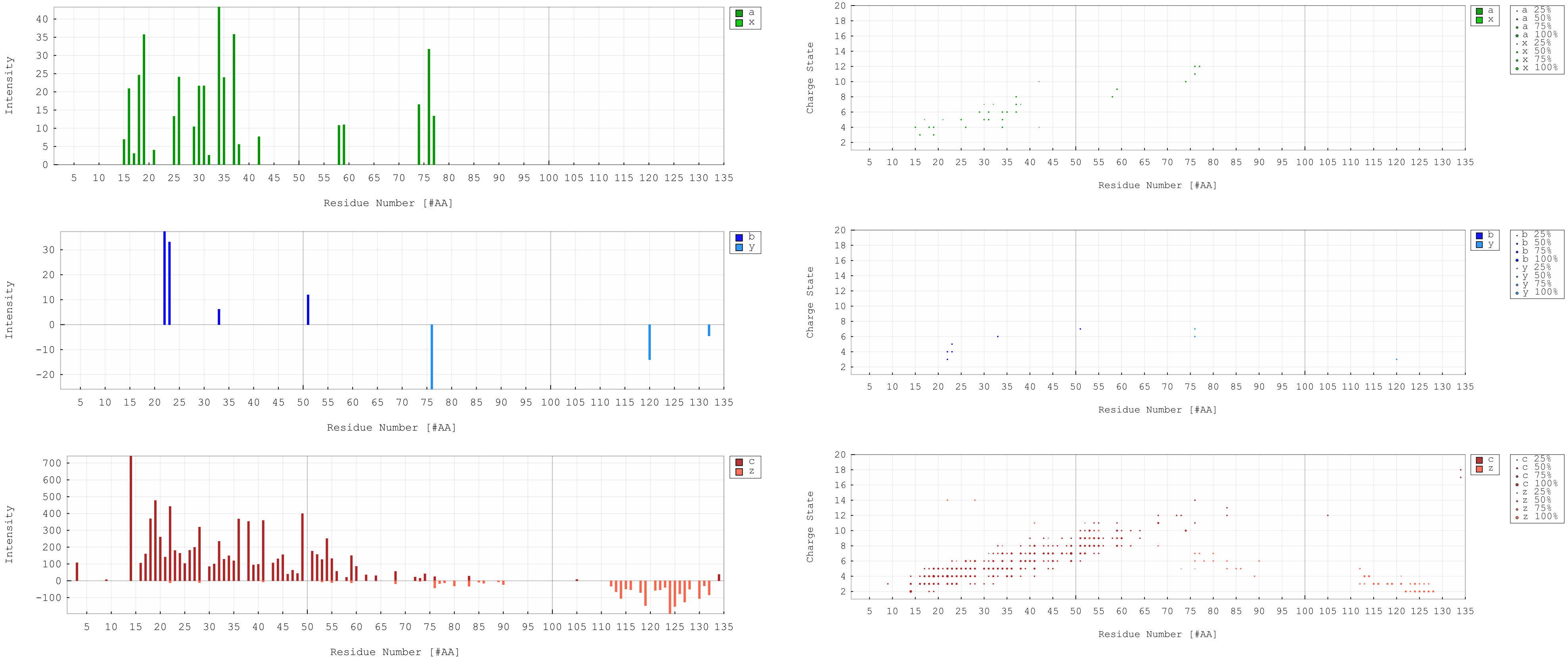

Top-Down Result OmniScape 2025
Sequence Confirmation
241209 H31-GNC5_10 z20 MS2 ECD 40ms_001-169_calibrated

Proteoform:

Index	Protein	SC [%]	SVP [%]	IC [%]	MS Score [a.u.]	Var. Mods	Mono Mass [Da]	Chemical Formula
5	H31Kac1	65.7	26.7	25.6	16.8	Ace: K23	15305.4534	C671H1131N215O186S4

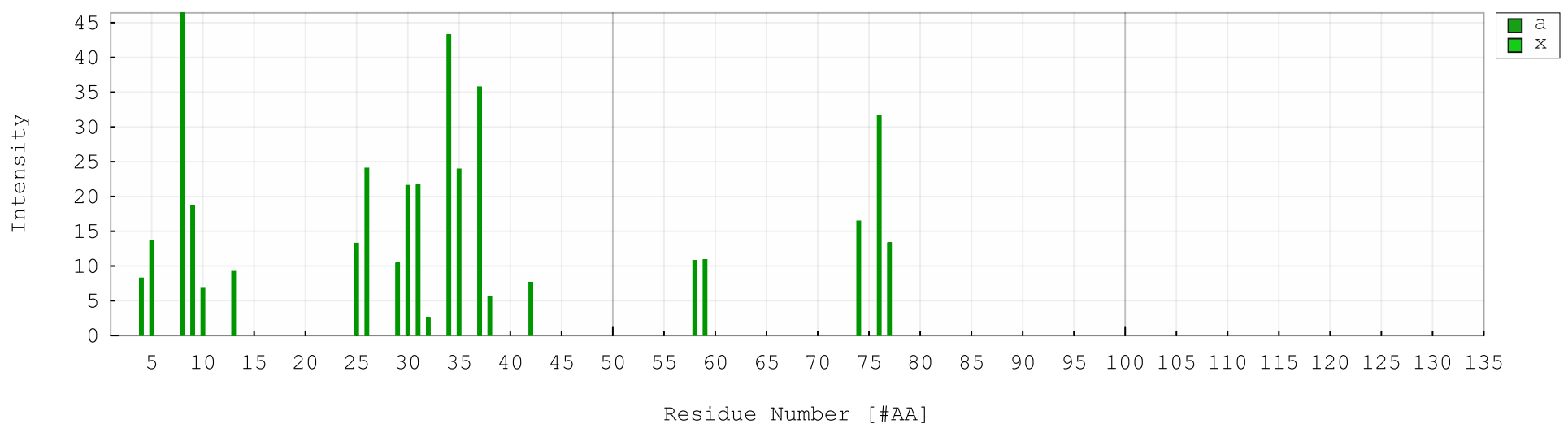

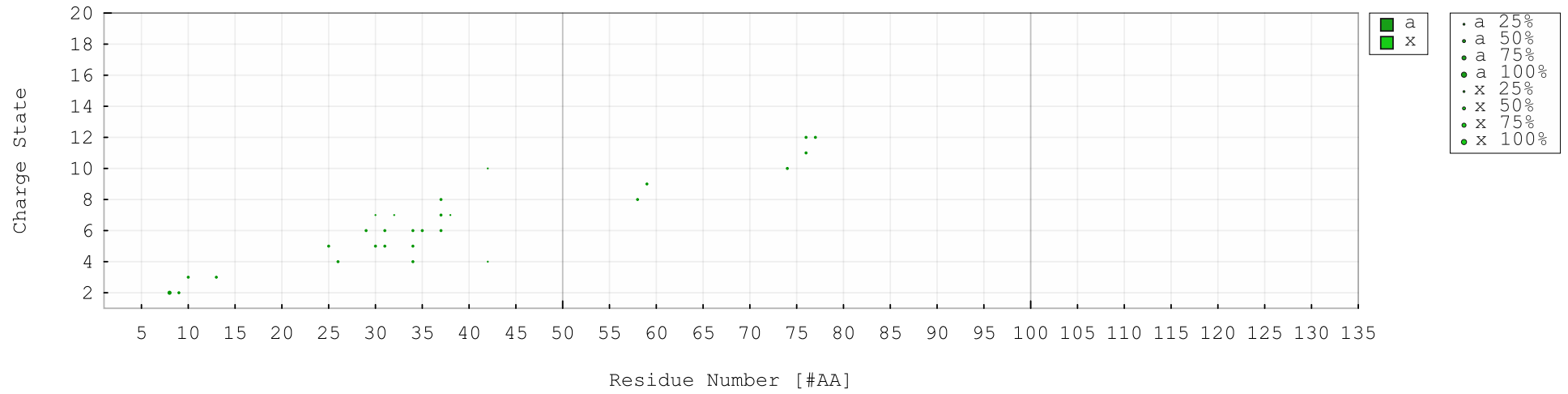

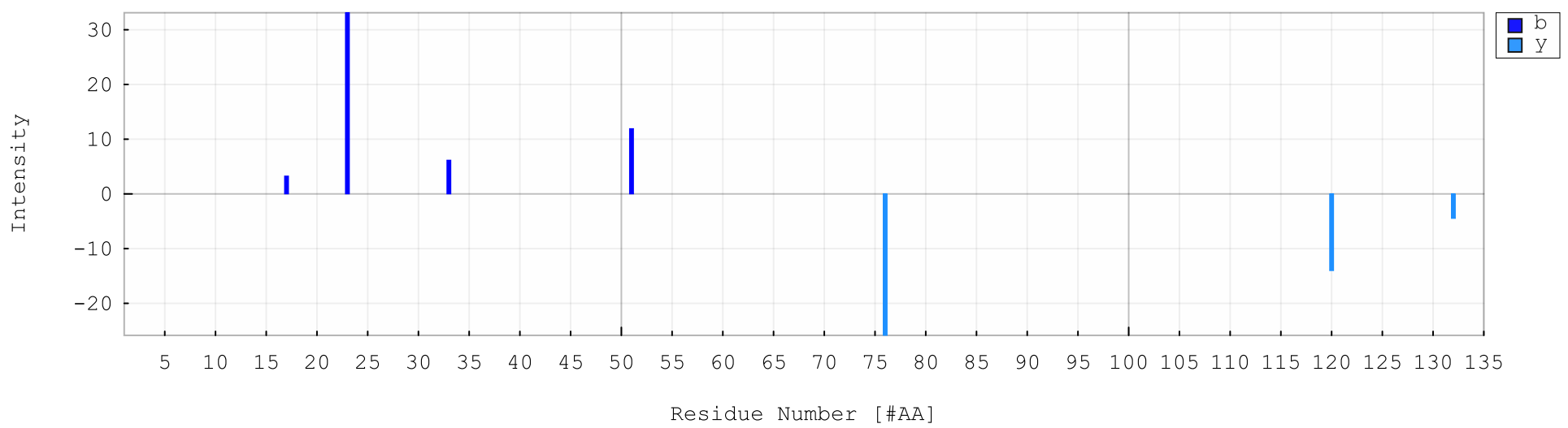

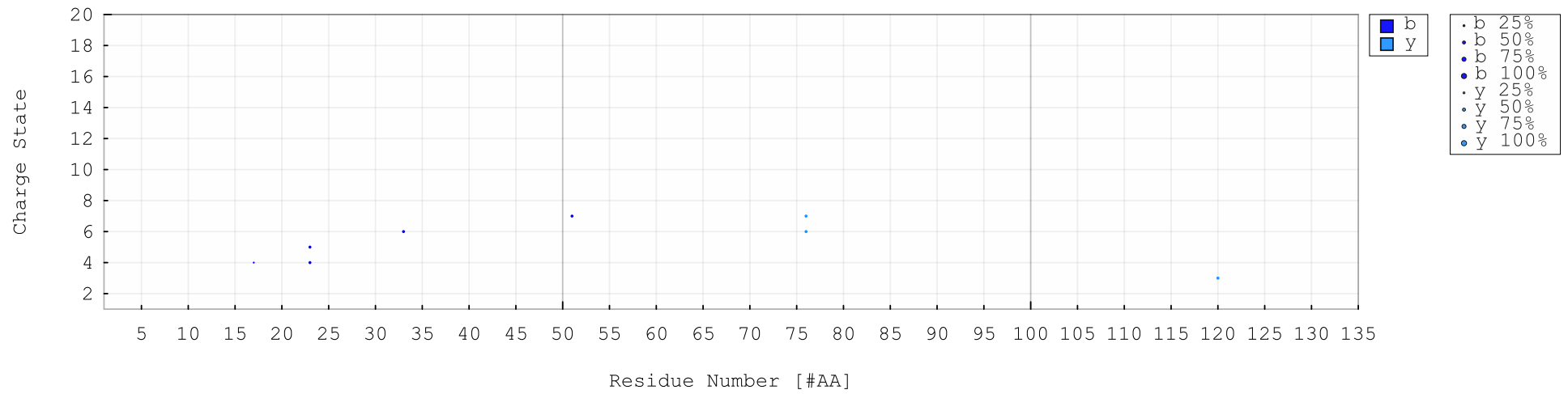

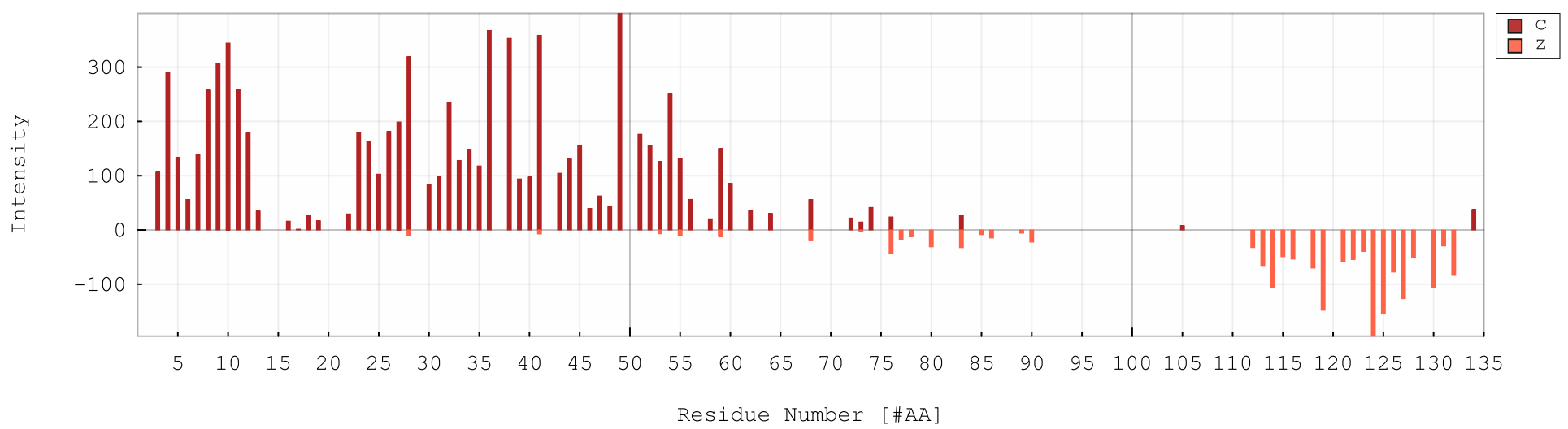

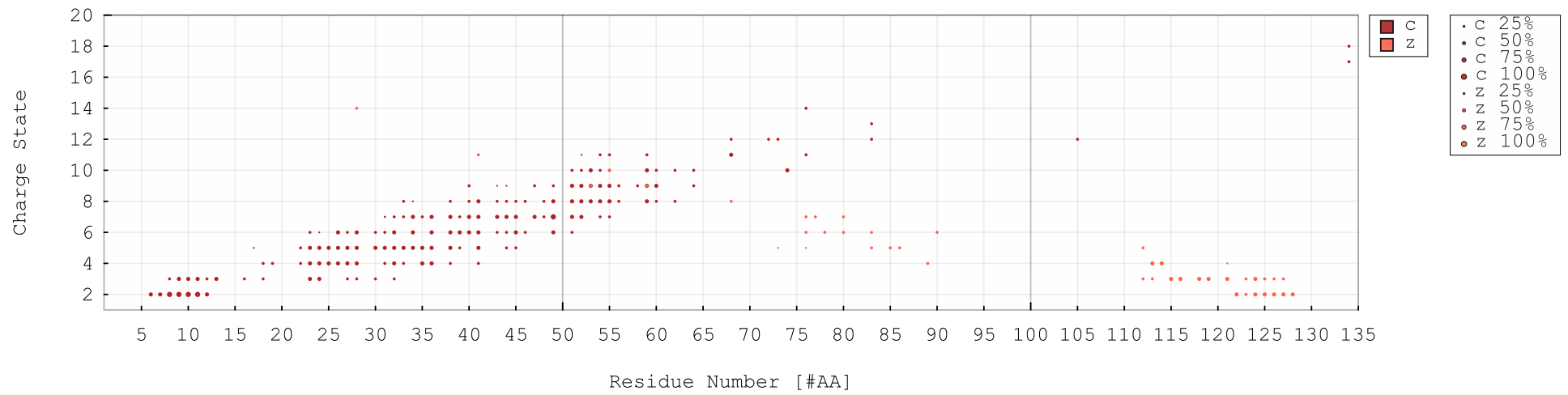

Top-Down Result OmniScape 2025
Sequence Confirmation
241209 H31-GNC5_10 z20 MS2 ECD 40ms_001-169_calibrated

Proteoform:

Index	Protein	SC [%]	SVP [%]	IC [%]	MS Score [a.u.]	Var. Mods	Mono Mass [Da]	Chemical Formula
6	H31Kac1	64.2	26.7	24.4	15.7	Ace: K27	15305.4534	C671H1131N215O186S4

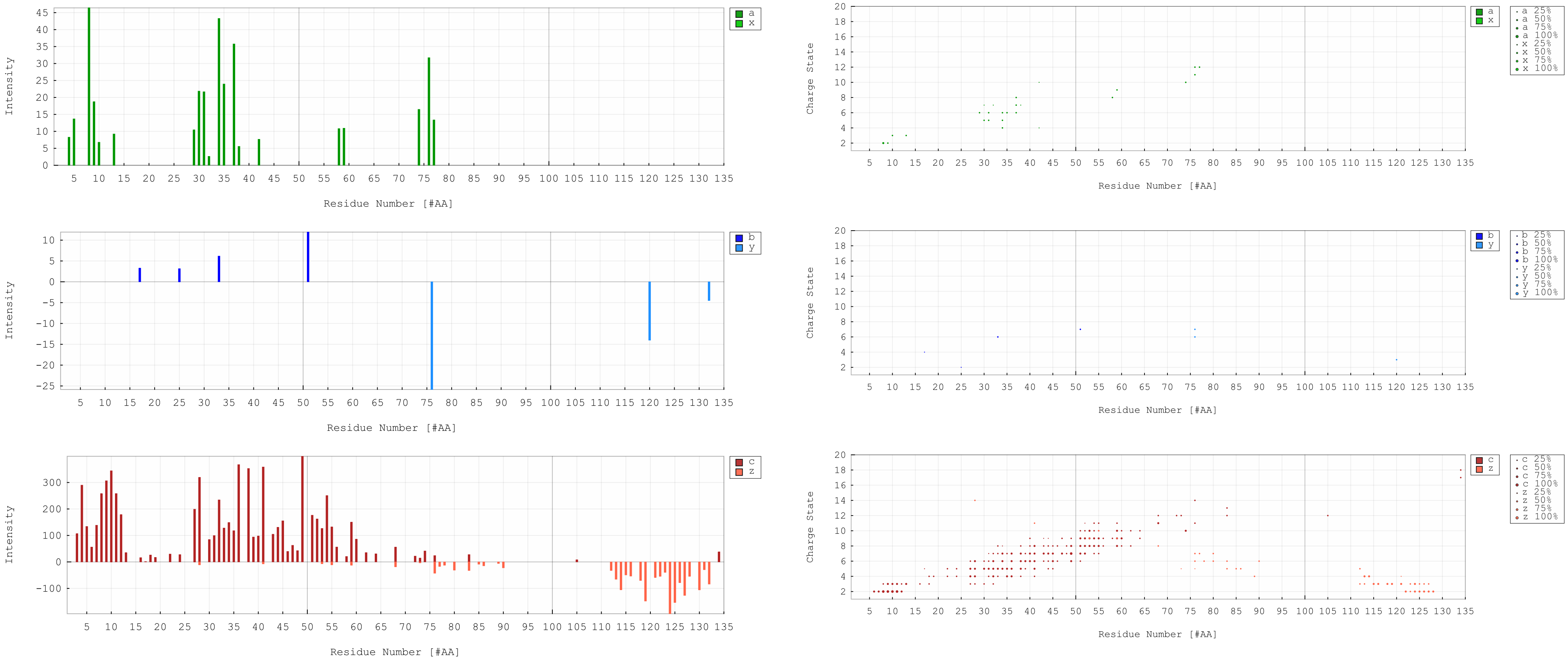

Proteoform:

Index	Protein	SC [%]	SVP [%]	IC [%]	MS Score [a.u.]	Var. Mods	Mono Mass [Da]	Chemical Formula
7	H31Kac1	59.0	26.7	21.7	12.8	Ace: K36	15305.4534	C671H1131N215O186S4

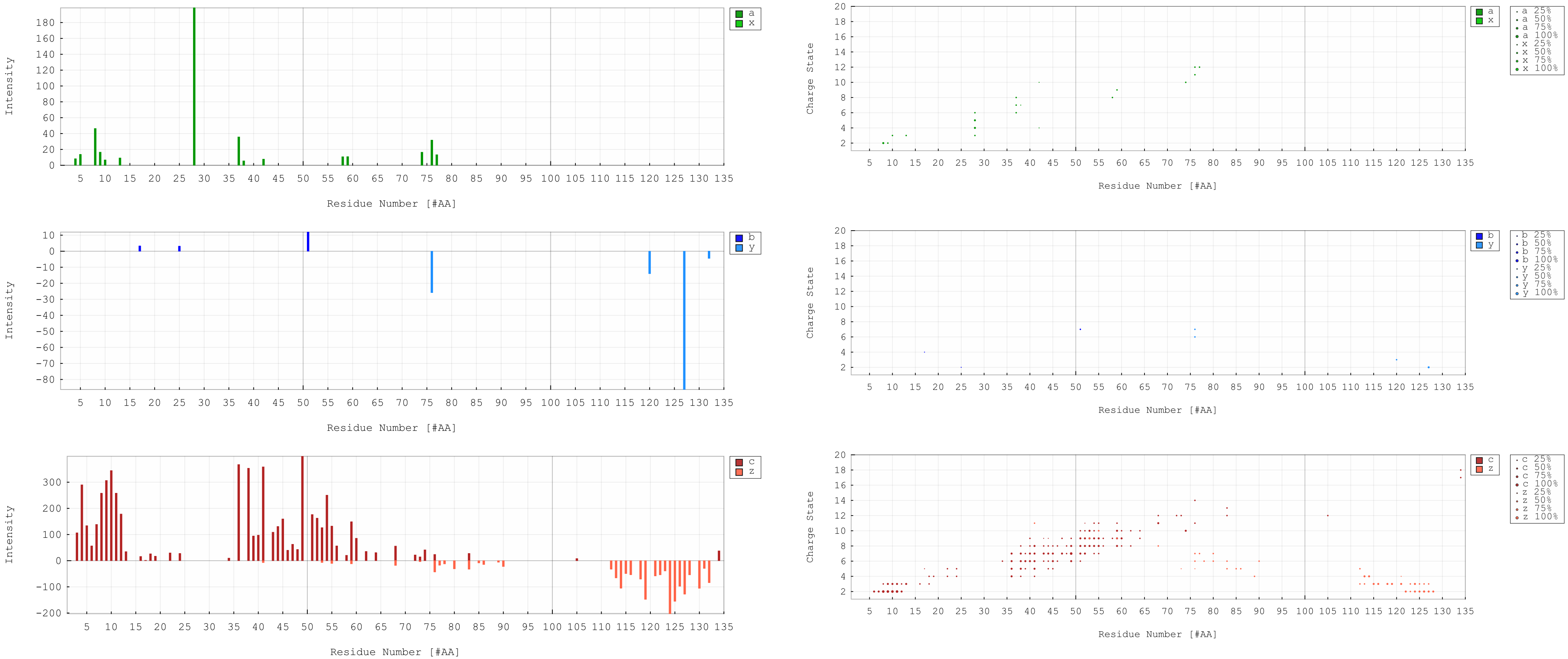

Top-Down Result OmniScape 2025
Sequence Confirmation
241209 H31-GNC5_10 z20 MS2 ECD 40ms_001-169_calibrated

Proteoform:

Index	Protein	SC [%]	SVP [%]	IC [%]	MS Score [a.u.]	Var. Mods	Mono Mass [Da]	Chemical Formula
8	H31Kac1	58.2	26.7	20.8	12.1	Ace: K37	15305.4534	C671H1131N215O186S4

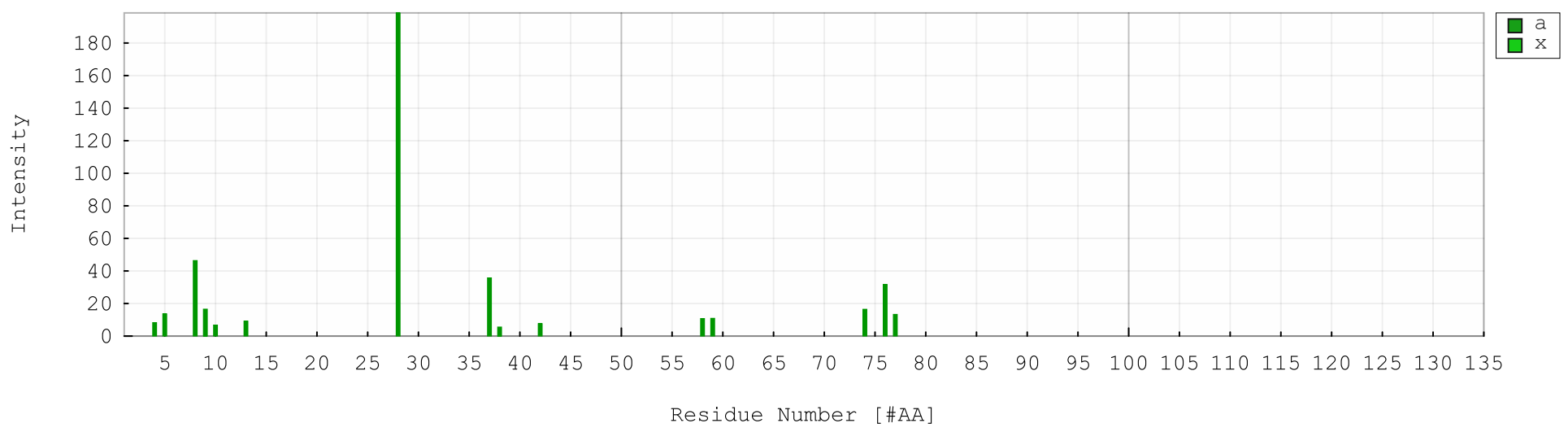

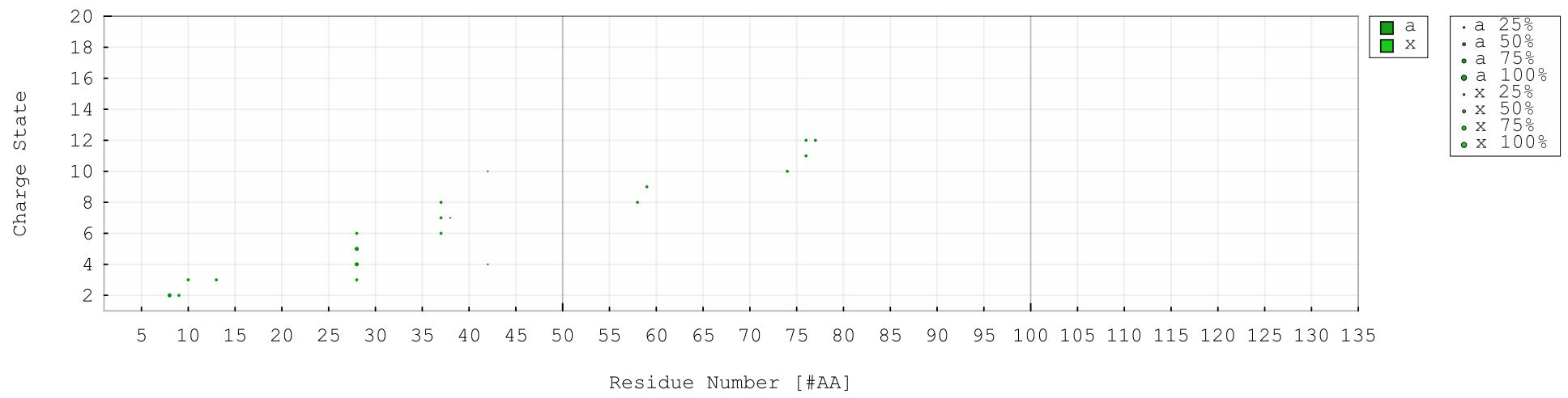

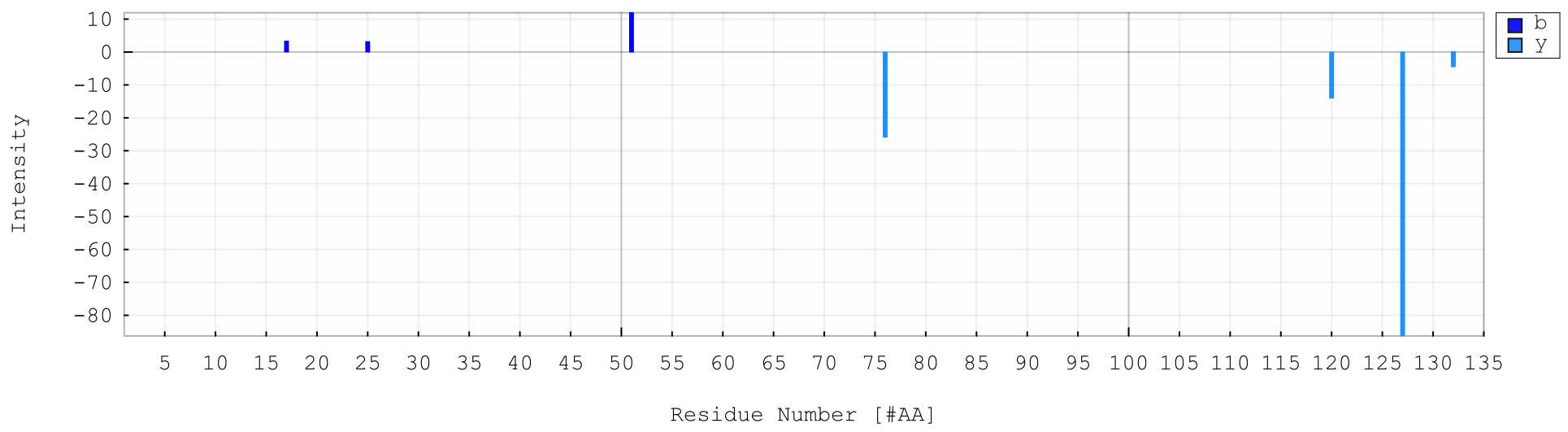

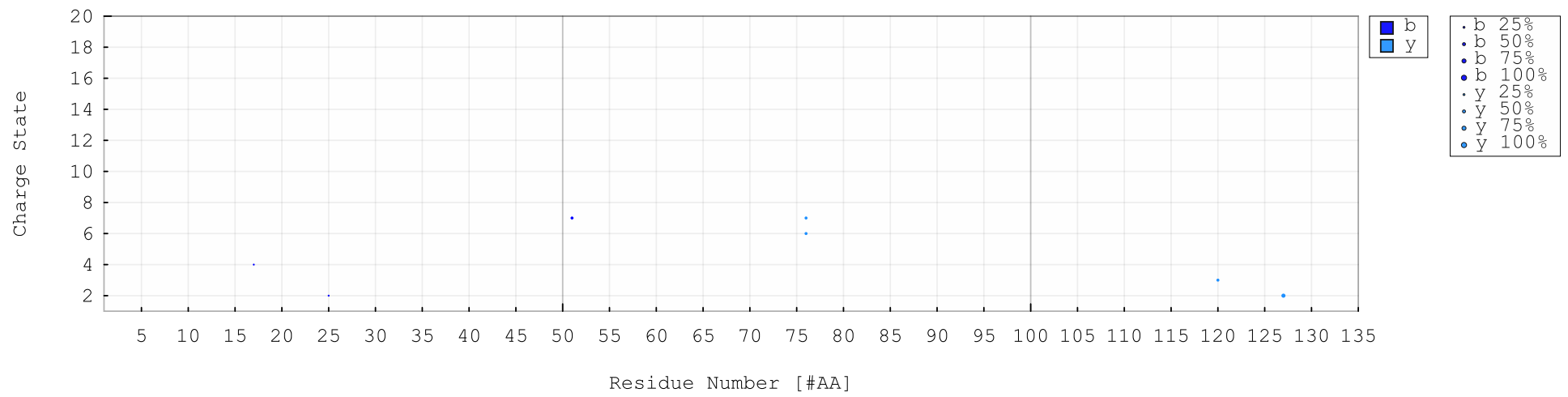

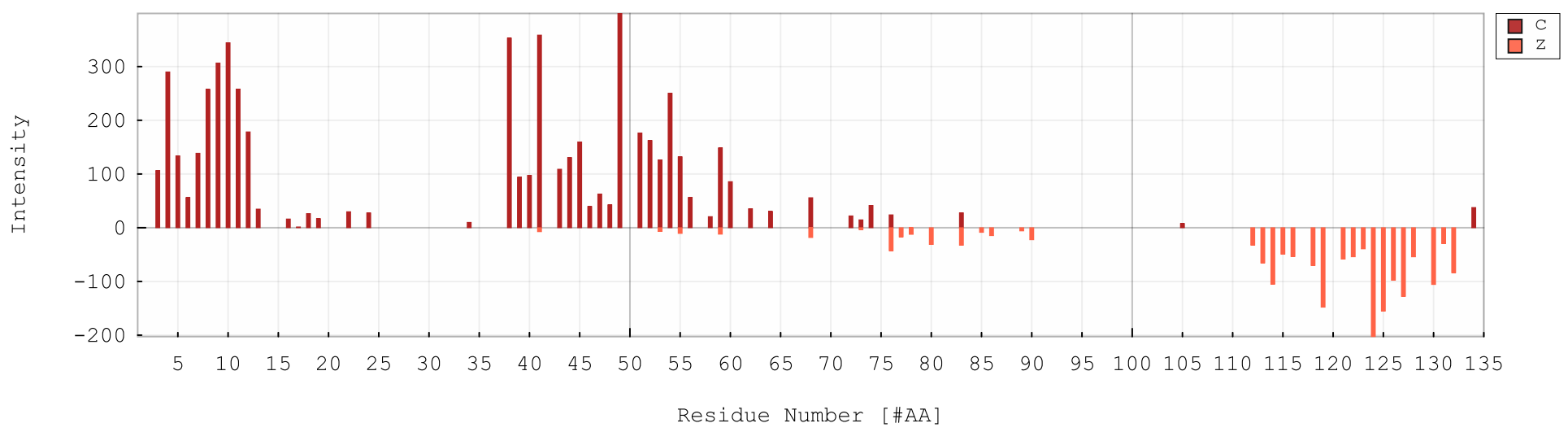

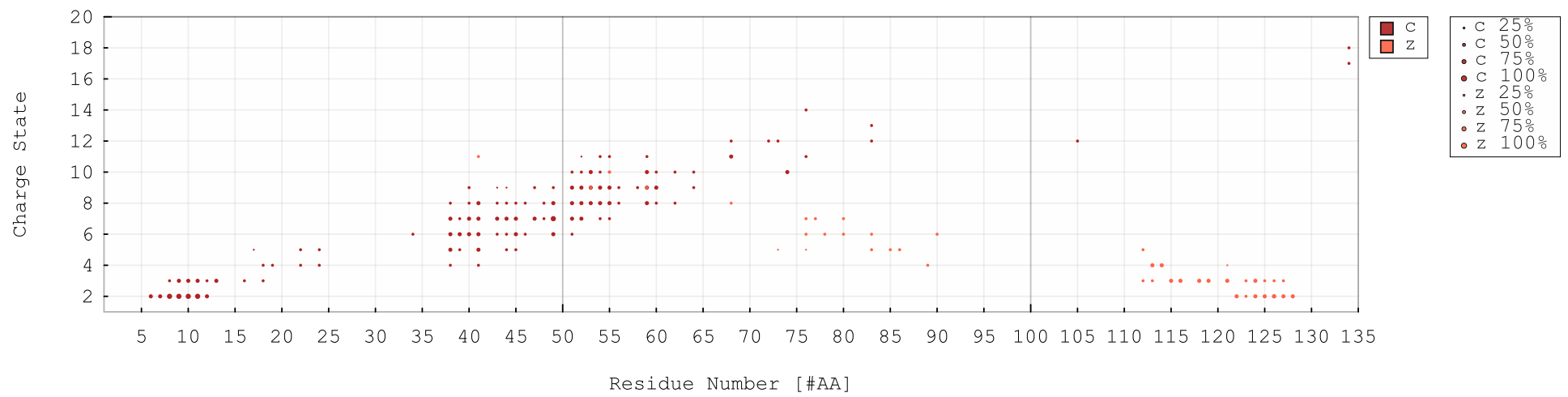

Top-Down Result OmniScape 2025
Sequence Confirmation
241209 H31-GNC5_10 z20 MS2 ECD 40ms_001-169_calibrated

Proteoform:	Index	Protein	SC [%]	SVP [%]	IC [%]	MS Score [a.u.]	Var. Mods	Mono Mass [Da]	Chemical Formula
	9	H31Kac1	47.0	26.7	13.3	6.3	Ace: K56	15305.4534	C671H1131N215O186S4

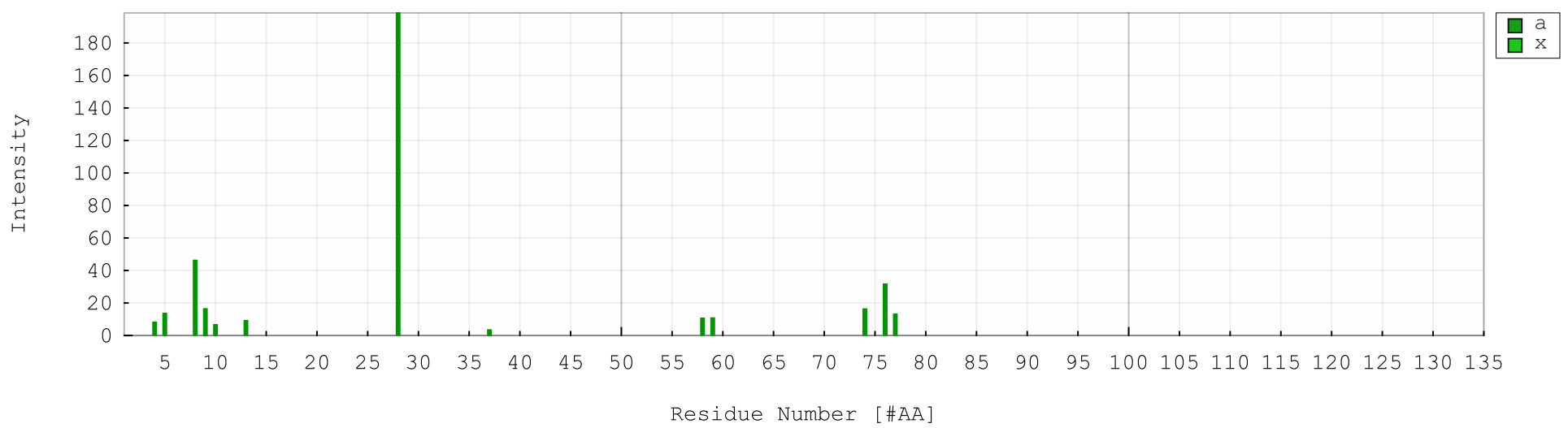

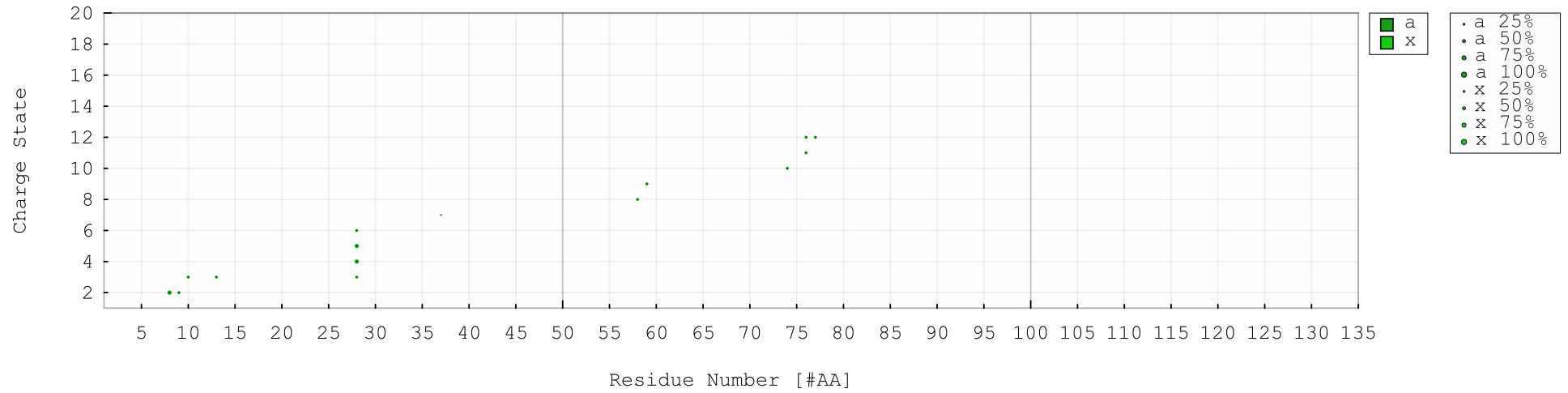

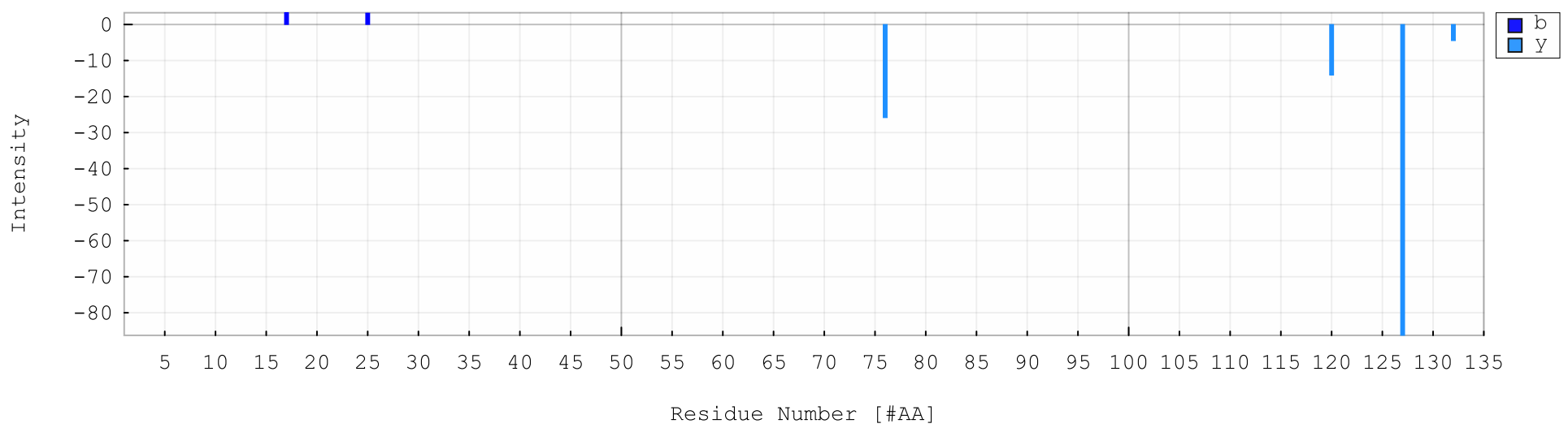

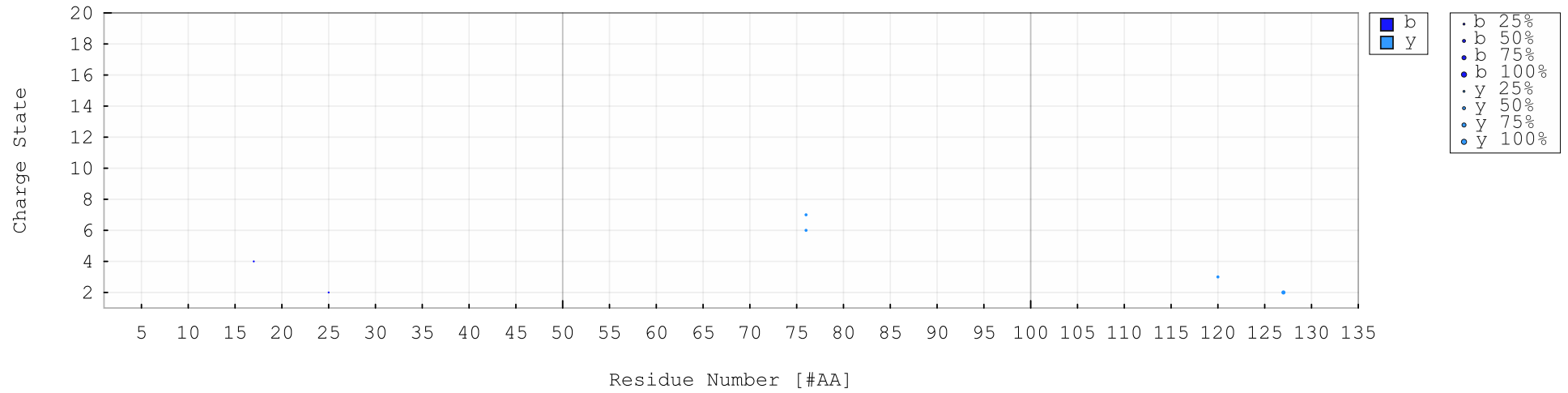

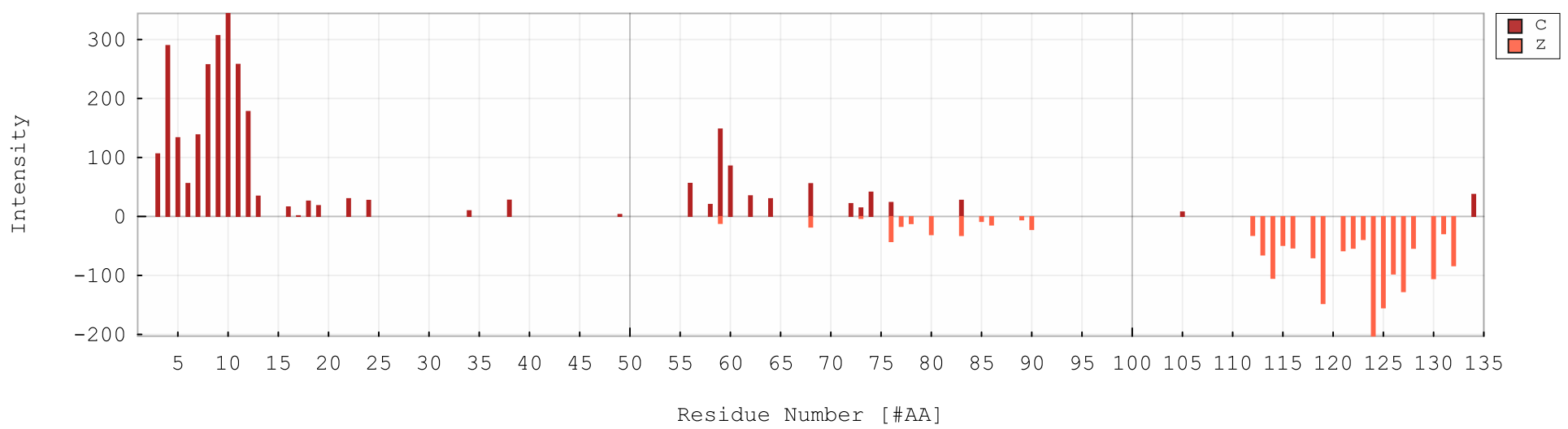

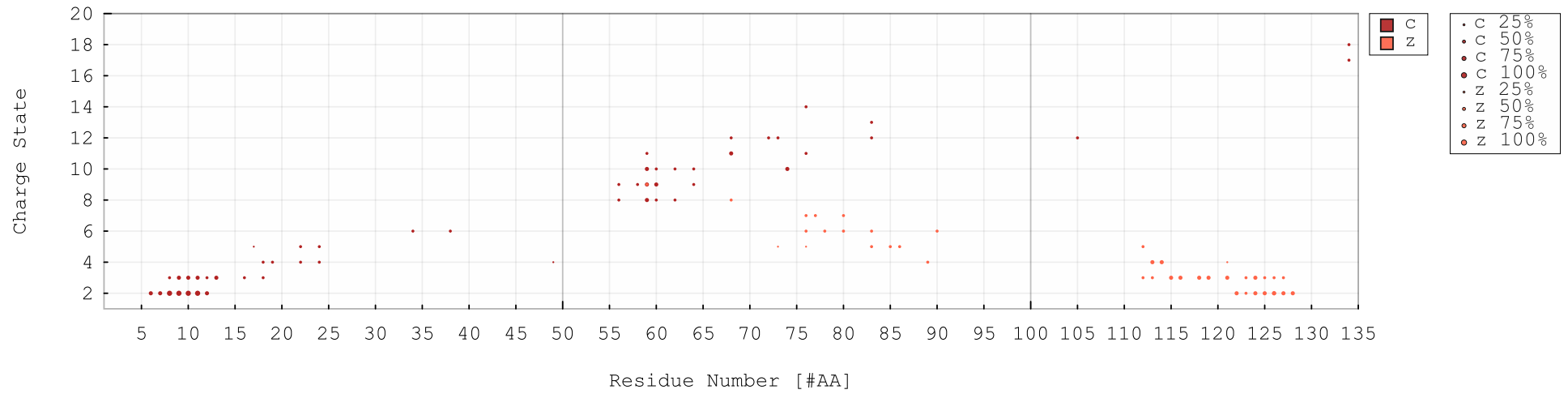

Top-Down Result OmniScape 2025
Sequence Confirmation
241209 H31-GNC5_10 z20 MS2 ECD 40ms_001-169_calibrated

Proteoform:

Index	Protein	SC [%]	SVP [%]	IC [%]	MS Score [a.u.]	Var. Mods	Mono Mass [Da]	Chemical Formula
10	H31Kac1	44.8	26.7	12.4	5.5	Ace: K64	15305.4534	C671H1131N215O186S4

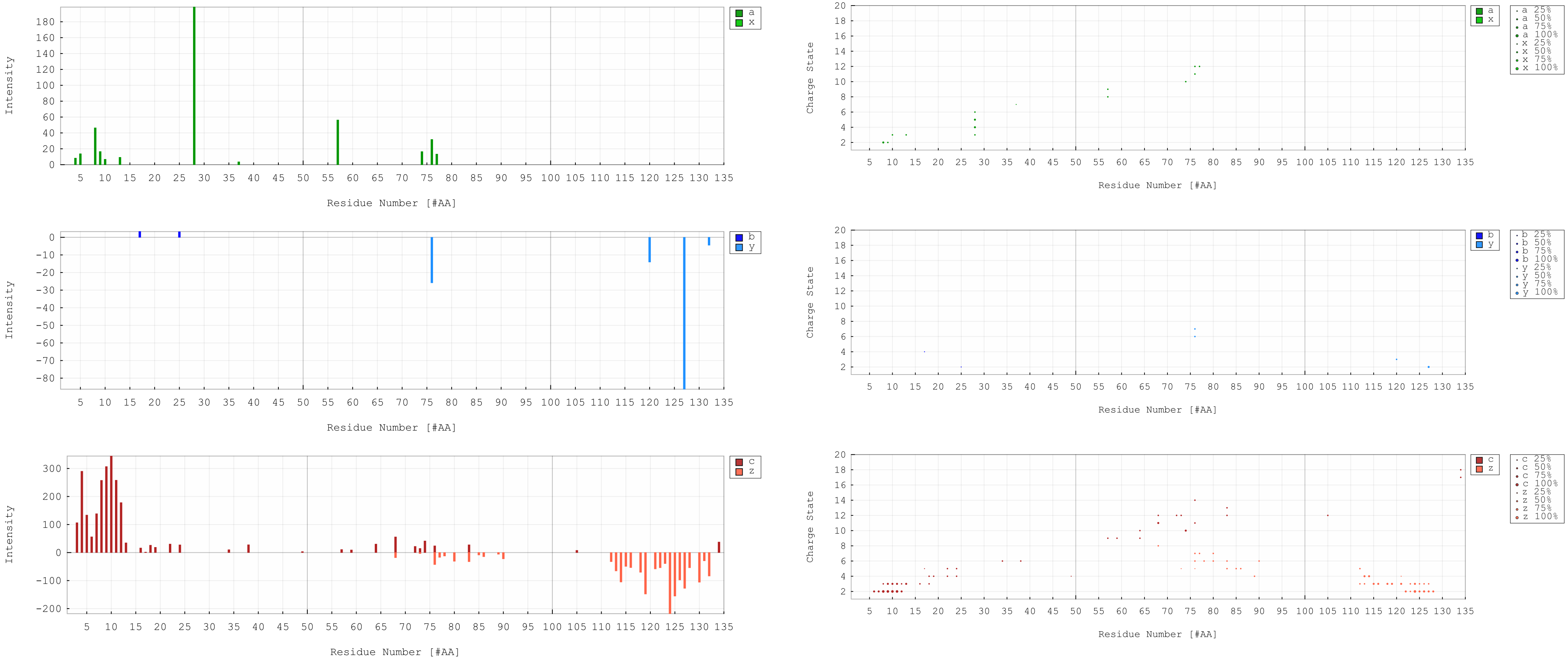

Supplemental Data S2

H3.1 - GCN5, 10 min

**Report**	6 of 7
**Experiment**	MS2
**Histone/sample**	H3.1
**Enzyme**	GCN5
**Reaction/source**	10 min
**Precursor**	H3.1Kac1
**Charge state**	20+
**Fragmentation / workflow**	RCID
**Original file**	MS2_H3.1Kac1_20+_RCID.pdf

Project Information

Project Directory: C:/Users/LAB-ADMIN
Processing date: Wed Dec 10 11:13:46 2025

Sample Info & Protocols

Name: Top-Down Protein Sequencing CID

Dataset

Imported spectrum file name:	241209 H31-GNC5_10 MS2 shCID Q2_001-197_calibrated.txt
Dataset name:	Information is only available from imported JPEAKS files
Date acquired:	Information is only available from imported JPEAKS files
Date imported:	Information is only available from imported JPEAKS files
Instrument Name:	Information is only available from imported JPEAKS files
Instrument Serial Number:	Information is only available from imported JPEAKS files

Workflow Result Info

Analysis: 241209 H31-GNC5_10 MS2 shCID Q2
Location: C:/Users/LAB-ADMIN
Method: -

Method Information

Name: Unnamed

Spectral Parameters

Mass Error [ppm]: 6
Intensity Deviation [au]: 0.15
Min/Max Charges: 1-20
Experiment type: CID

a ions	b ions	c ions	x ions	y ions	z ions	satellite	internal	precursor
a	b			y				M

Noise threshold calculation: Simple moving average noise threshold (SMA)
Window size [datapoints]: 600
Noise curve offset: 0.7346993
SVP parameters: terminal gap: 2, internal gap: 1

Proteoforms
(top 10)

Index	Parent Sequence	Chain	Var. Mods	Mono Mass [Da]	Chemical Composition
1	H31Kac1	H31Kac1K14[Ace]	Ace: K14	15305.4534	C671H1131N215O186S4
2	H31Kac1	H31Kac1K18[Ace]	Ace: K18	15305.4534	C671H1131N215O186S4
3	H31Kac1	H31Kac1K9[Ace]	Ace: K9	15305.4534	C671H1131N215O186S4
4	H31Kac1	H31Kac1K23[Ace]	Ace: K23	15305.4534	C671H1131N215O186S4
5	H31Kac1	H31Kac1K27[Ace]	Ace: K27	15305.4534	C671H1131N215O186S4
6	H31Kac1	H31Kac1K4[Ace]	Ace: K4	15305.4534	C671H1131N215O186S4
7	H31Kac1	H31Kac1K36[Ace]	Ace: K36	15305.4534	C671H1131N215O186S4
8	H31Kac1	H31Kac1K37[Ace]	Ace: K37	15305.4534	C671H1131N215O186S4
9	H31Kac1	H31Kac1K56[Ace]	Ace: K56	15305.4534	C671H1131N215O186S4
10	H31Kac1	H31Kac1K64[Ace]	Ace: K64	15305.4534	C671H1131N215O186S4

Results

Proteoforms Table (top 10)

Index	Protein	SC [%]	SVP [%]	IC [%]	MS Score [a.u.]	Var. Mods	Mono Mass [Da]	Chemical Formula
1	H31Kac1	37.3	0.0	37.7	14.1	Ace: K14	15305.4534	C671H1131N215O186S4
2	H31Kac1	35.8	0.0	37.6	13.5	Ace: K18	15305.4534	C671H1131N215O186S4
3	H31Kac1	35.1	0.0	37.6	13.2	Ace: K9	15305.4534	C671H1131N215O186S4
4	H31Kac1	35.1	0.0	37.6	13.2	Ace: K23	15305.4534	C671H1131N215O186S4
5	H31Kac1	33.6	0.0	37.5	12.6	Ace: K27	15305.4534	C671H1131N215O186S4
6	H31Kac1	32.8	0.0	37.5	12.3	Ace: K4	15305.4534	C671H1131N215O186S4
7	H31Kac1	30.6	0.0	37.0	11.3	Ace: K36	15305.4534	C671H1131N215O186S4
8	H31Kac1	29.9	0.0	37.0	11.1	Ace: K37	15305.4534	C671H1131N215O186S4
9	H31Kac1	29.9	0.0	37.0	11.1	Ace: K56	15305.4534	C671H1131N215O186S4
10	H31Kac1	29.1	0.0	36.9	10.7	Ace: K64	15305.4534	C671H1131N215O186S4

Sequence

Name: H31Kac1

```
                 10                    20                    30                    40                    50                    60
A R T K Q T A R K S   T G G K A P R K Q L   A T K A A R K S A P   A T G G V K K P H R   Y R P G T V A L R E   I R R Y Q K S T E L
                 70                    80                    90                   100                   110                   120
L I R K L P F Q R L   V R E I A Q D F K T   D L R F Q S S A V M   A L Q E A C E A Y L   V G L F E D T N L C   A I H A K R V T I M
                130                   140                   150                   160                   170                   180
P K D I Q L A R R I   R G E R A
```

Modifications

Modification	Position	Type
Acetyl	4	variable
Acetyl	9	variable
Acetyl	14	variable
Acetyl	18	variable
Acetyl	23	variable
Acetyl	27	variable
Acetyl	36	variable
Acetyl	37	variable
Acetyl	56	variable
Acetyl	64	variable
Acetyl	79	variable

Modification	Position	Type
Acetyl	115	variable
Acetyl	122	variable

Sequence Map

Proteoform:

Index	Protein	SC [%]	SVP [%]	IC [%]	MS Score [a.u.]	Var. Mods	Mono Mass [Da]	Chemical Formula
1	H31Kac1	37.3	0.0	37.7	14.1	Ace: K14	15305.4534	C671H1131N215O186S4

a b y

ARTKQ TARKS TGGKA PRKQL ATKAA RKSAP ATGGV KKPHR YRPGT VALRE IRRYQ KSTEL LIRKL PFQRL VREIA QDFKT
DLRFQ SSAVM ALQEA CEAYL VGLFE DTNLC AIHAK RVTIM PKDIQ LARRI RGERA

Proteoform:	Index	Protein	SC [%]	SVP [%]	IC [%]	MS Score [a.u.]	Var. Mods	Mono Mass [Da]	Chemical Formula
	2	H31Kac1	35.8	0.0	37.6	13.5	Ace: K18	15305.4534	C671H1131N215O186S4

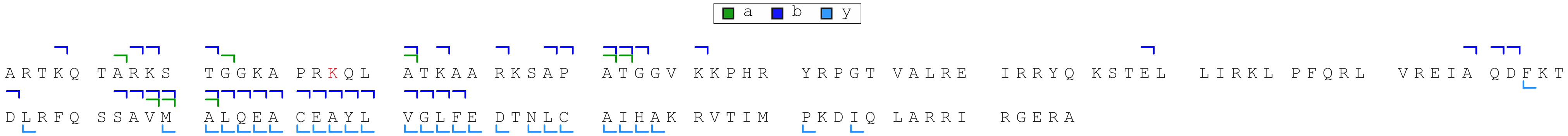

Proteoform:

Index	Protein	SC [%]	SVP [%]	IC [%]	MS Score [a.u.]	Var. Mods	Mono Mass [Da]	Chemical Formula
3	H31Kac1	35.1	0.0	37.6	13.2	Ace: K9	15305.4534	C671H1131N215O186S4

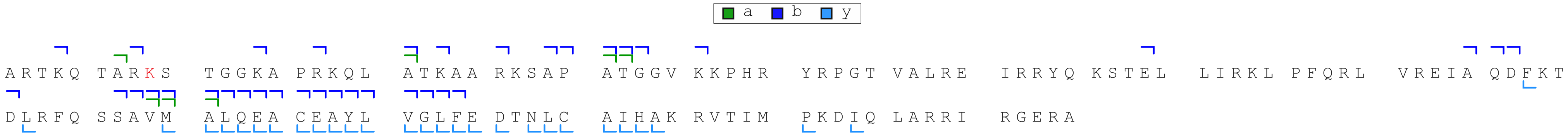

Proteoform:	Index	Protein	SC [%]	SVP [%]	IC [%]	MS Score [a.u.]	Var. Mods	Mono Mass [Da]	Chemical Formula
	4	H31Kac1	35.1	0.0	37.6	13.2	Ace: K23	15305.4534	C671H1131N215O186S4

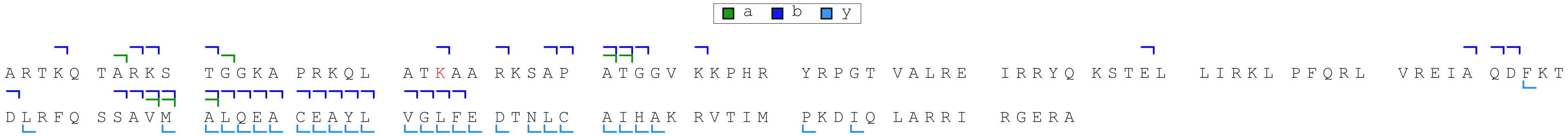

Proteoform:

Index	Protein	SC [%]	SVP [%]	IC [%]	MS Score [a.u.]	Var. Mods	Mono Mass [Da]	Chemical Formula
5	H31Kac1	33.6	0.0	37.5	12.6	Ace: K27	15305.4534	C671H1131N215O186S4

a b y

ARTKQ TARKS TGGKA PRKQL ATKAA RKSAP ATGGV KKPHR YRPGT VALRE IRRYQ KSTEL LIRKL PFQRL VREIA QDFKT
DLRFQ SSAVM AL EA CEAYL VGLFE DTNLC AIHAK RVTIM PKDIQ LARRI RGERA

Proteoform:

Index	Protein	SC [%]	SVP [%]	IC [%]	MS Score [a.u.]	Var. Mods	Mono Mass [Da]	Chemical Formula
6	H31Kac1	32.8	0.0	37.5	12.3	Ace: K4	15305.4534	C671H1131N215O186S4

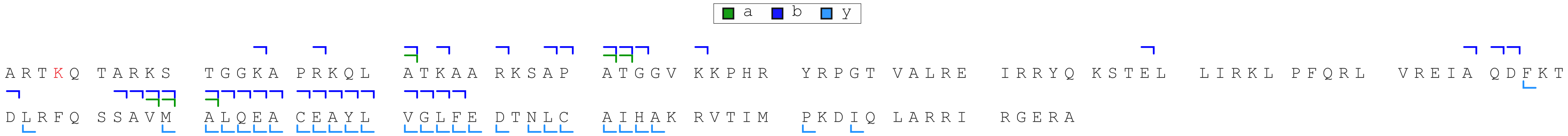

Top-Down Result OmniScape 2025
Sequence Confirmation
241209 H31-GNC5_10 MS2 shCID Q2

Proteoform:

Index	Protein	SC [%]	SVP [%]	IC [%]	MS Score [a.u.]	Var. Mods	Mono Mass [Da]	Chemical Formula
7	H31Kac1	30.6	0.0	37.0	11.3	Ace: K36	15305.4534	C671H1131N215O186S4

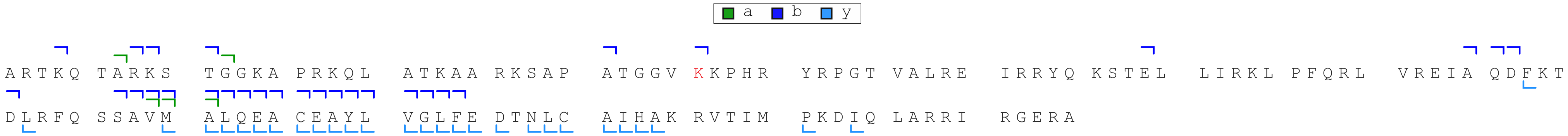

Proteoform:	Index	Protein	SC [%]	SVP [%]	IC [%]	MS Score [a.u.]	Var. Mods	Mono Mass [Da]	Chemical Formula
	8	H31Kac1	29.9	0.0	37.0	11.1	Ace: K37	15305.4534	C671H1131N215O186S4

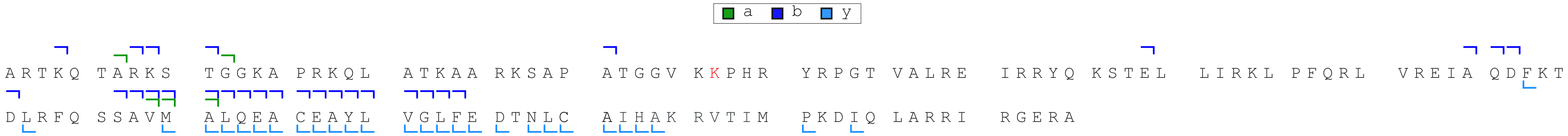

Proteoform:	Index	Protein	SC [%]	SVP [%]	IC [%]	MS Score [a.u.]	Var. Mods	Mono Mass [Da]	Chemical Formula
	9	H31Kac1	29.9	0.0	37.0	11.1	Ace: K56	15305.4534	C671H1131N215O186S4

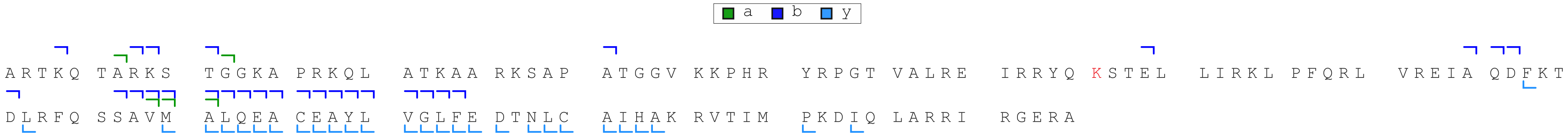

Proteoform:

Index	Protein	SC [%]	SVP [%]	IC [%]	MS Score [a.u.]	Var. Mods	Mono Mass [Da]	Chemical Formula
10	H31Kac1	29.1	0.0	36.9	10.7	Ace: K64	15305.4534	C671H1131N215O186S4

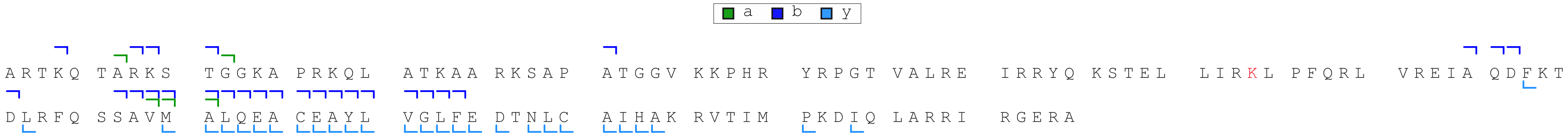

Error Plot

Proteoform:

Index	Protein	SC [%]	SVP [%]	IC [%]	MS Score [a.u.]	Var. Mods	Mono Mass [Da]	Chemical Formula
1	H31Kac1	37.3	0.0	37.7	14.1	Ace: K14	15305.4534	C671H1131N215O186S4

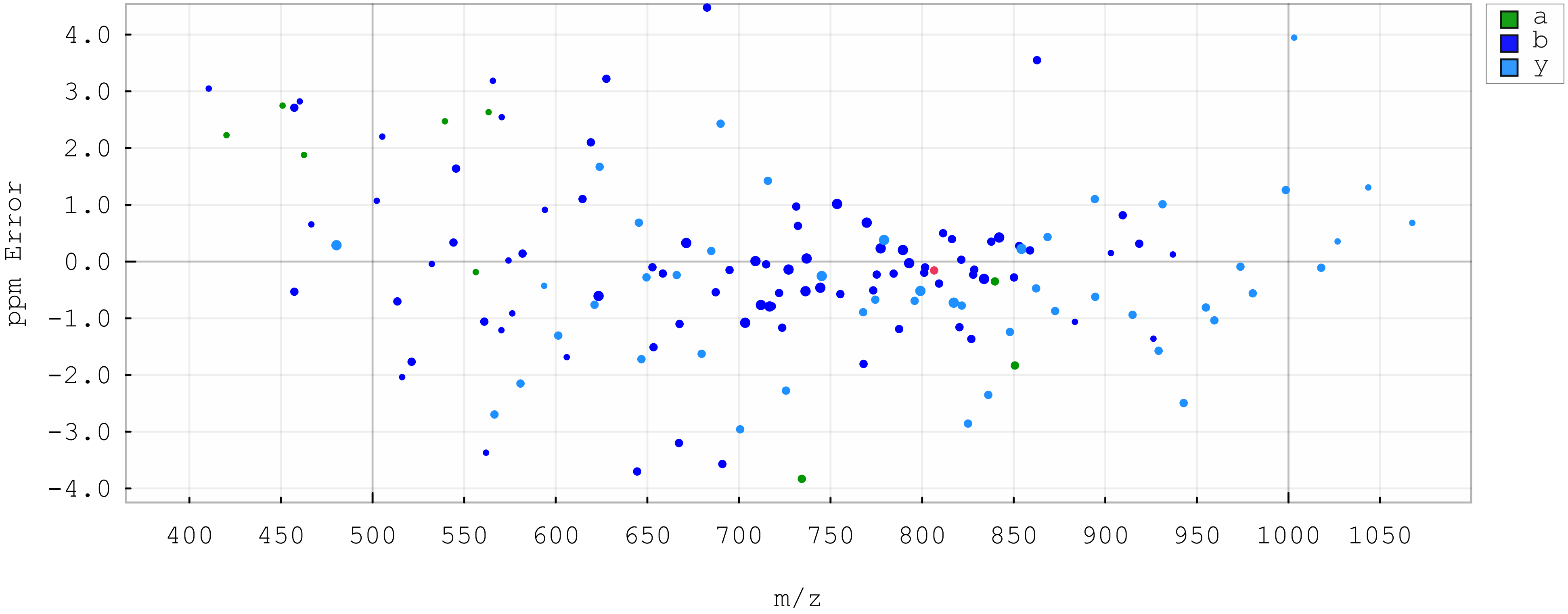

Proteoform:

Index	Protein	SC [%]	SVP [%]	IC [%]	MS Score [a.u.]	Var. Mods	Mono Mass [Da]	Chemical Formula
2	H31Kac1	35.8	0.0	37.6	13.5	Ace: K18	15305.4534	C671H1131N215O186S4

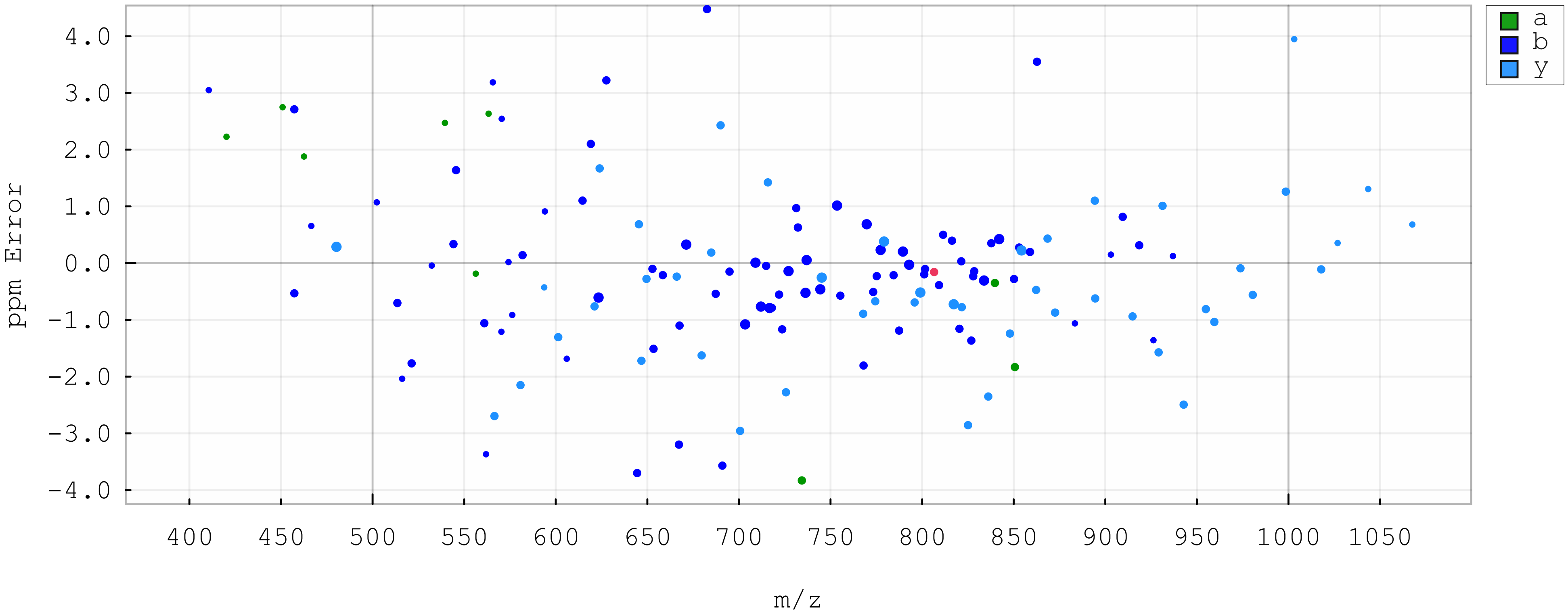

Proteoform:

Index	Protein	SC [%]	SVP [%]	IC [%]	MS Score [a.u.]	Var. Mods	Mono Mass [Da]	Chemical Formula
3	H31Kac1	35.1	0.0	37.6	13.2	Ace: K9	15305.4534	C671H1131N215O186S4

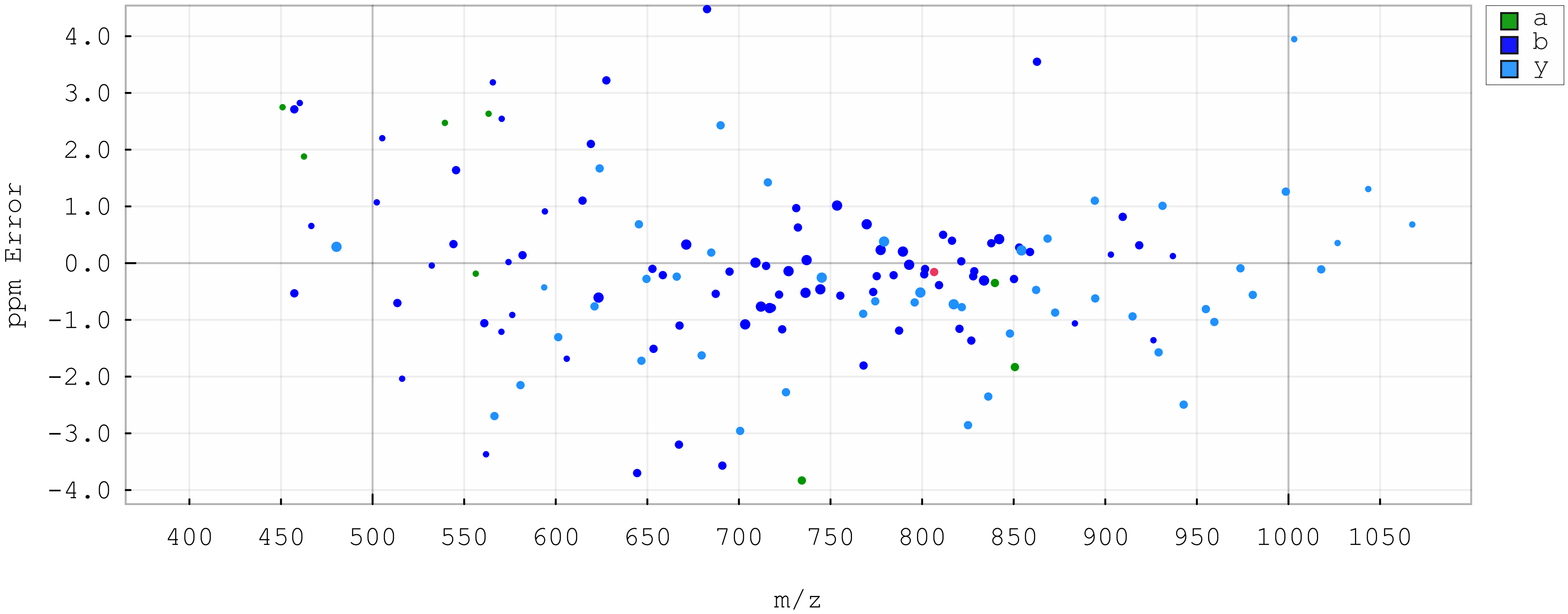

Proteoform:

Index	Protein	SC [%]	SVP [%]	IC [%]	MS Score [a.u.]	Var. Mods	Mono Mass [Da]	Chemical Formula
4	H31Kac1	35.1	0.0	37.6	13.2	Ace: K23	15305.4534	C671H1131N215O186S4

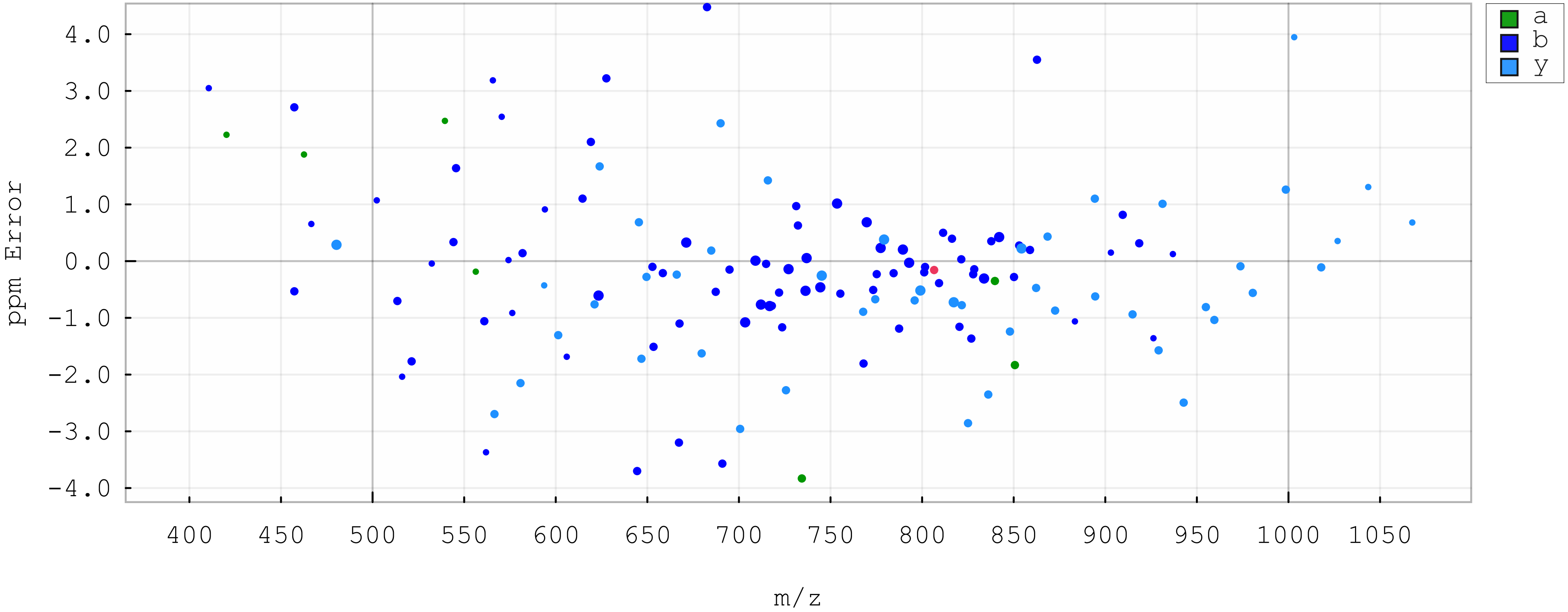

Proteoform:

Index	Protein	SC [%]	SVP [%]	IC [%]	MS Score [a.u.]	Var. Mods	Mono Mass [Da]	Chemical Formula
5	H31Kac1	33.6	0.0	37.5	12.6	Ace: K27	15305.4534	C671H1131N215O186S4

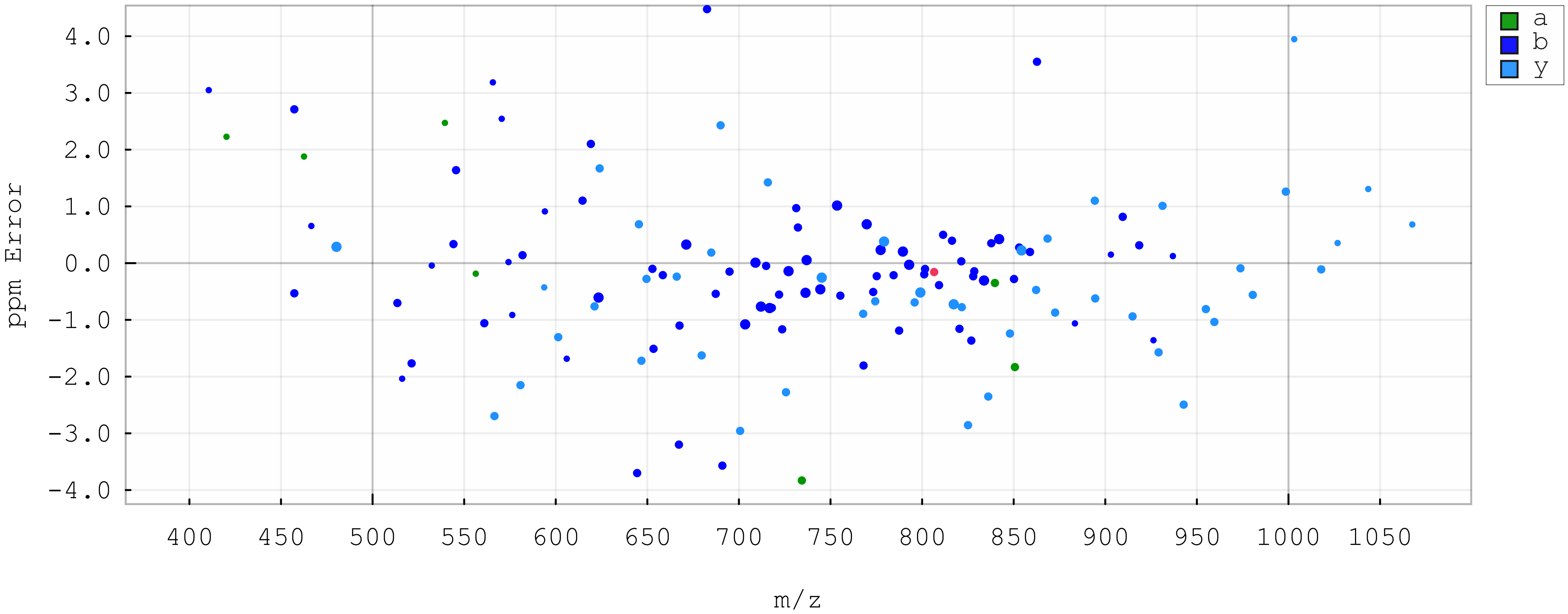

Proteoform:

Index	Protein	SC [%]	SVP [%]	IC [%]	MS Score [a.u.]	Var. Mods	Mono Mass [Da]	Chemical Formula
6	H31Kac1	32.8	0.0	37.5	12.3	Ace: K4	15305.4534	C671H1131N215O186S4

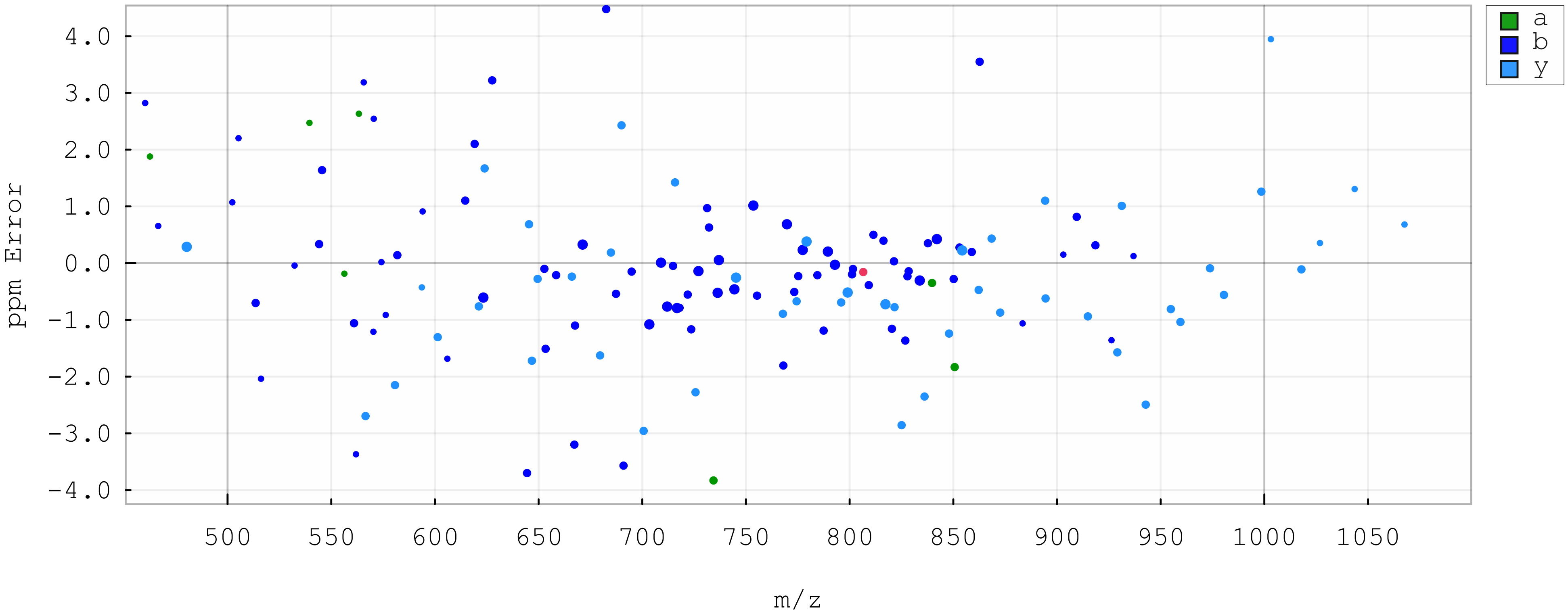

Proteoform:

Index	Protein	SC [%]	SVP [%]	IC [%]	MS Score [a.u.]	Var. Mods	Mono Mass [Da]	Chemical Formula
7	H31Kac1	30.6	0.0	37.0	11.3	Ace: K36	15305.4534	C671H1131N215O186S4

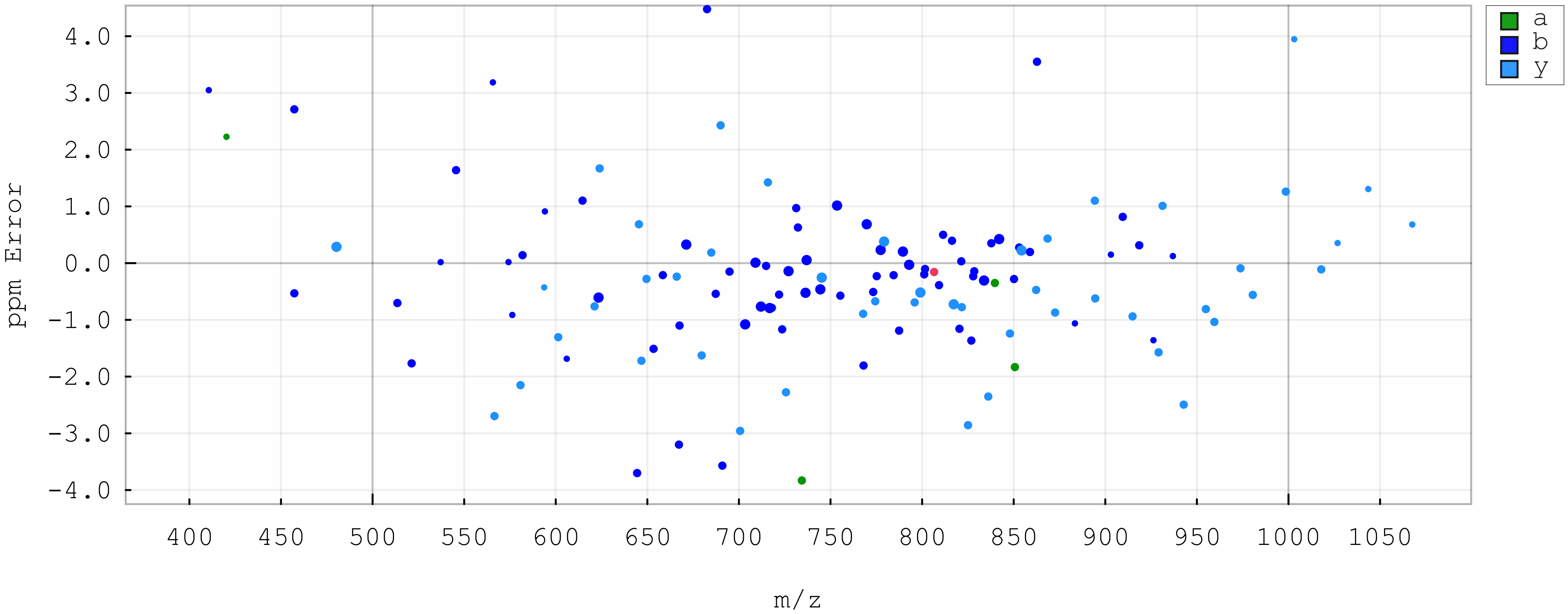

Proteoform:

Index	Protein	SC [%]	SVP [%]	IC [%]	MS Score [a.u.]	Var. Mods	Mono Mass [Da]	Chemical Formula
8	H31Kac1	29.9	0.0	37.0	11.1	Ace: K37	15305.4534	C671H1131N215O186S4

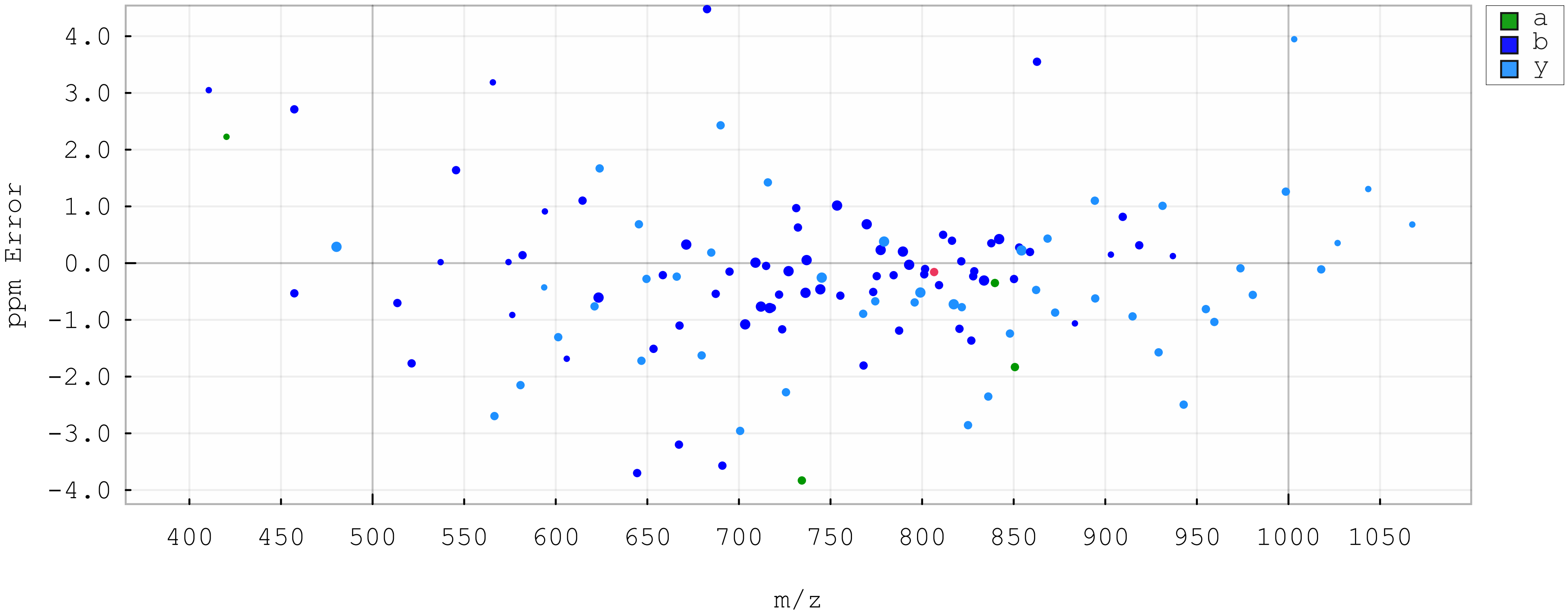

Proteoform:	Index	Protein	SC [%]	SVP [%]	IC [%]	MS Score [a.u.]	Var. Mods	Mono Mass [Da]	Chemical Formula
	9	H31Kac1	29.9	0.0	37.0	11.1	Ace: K56	15305.4534	C671H1131N215O186S4

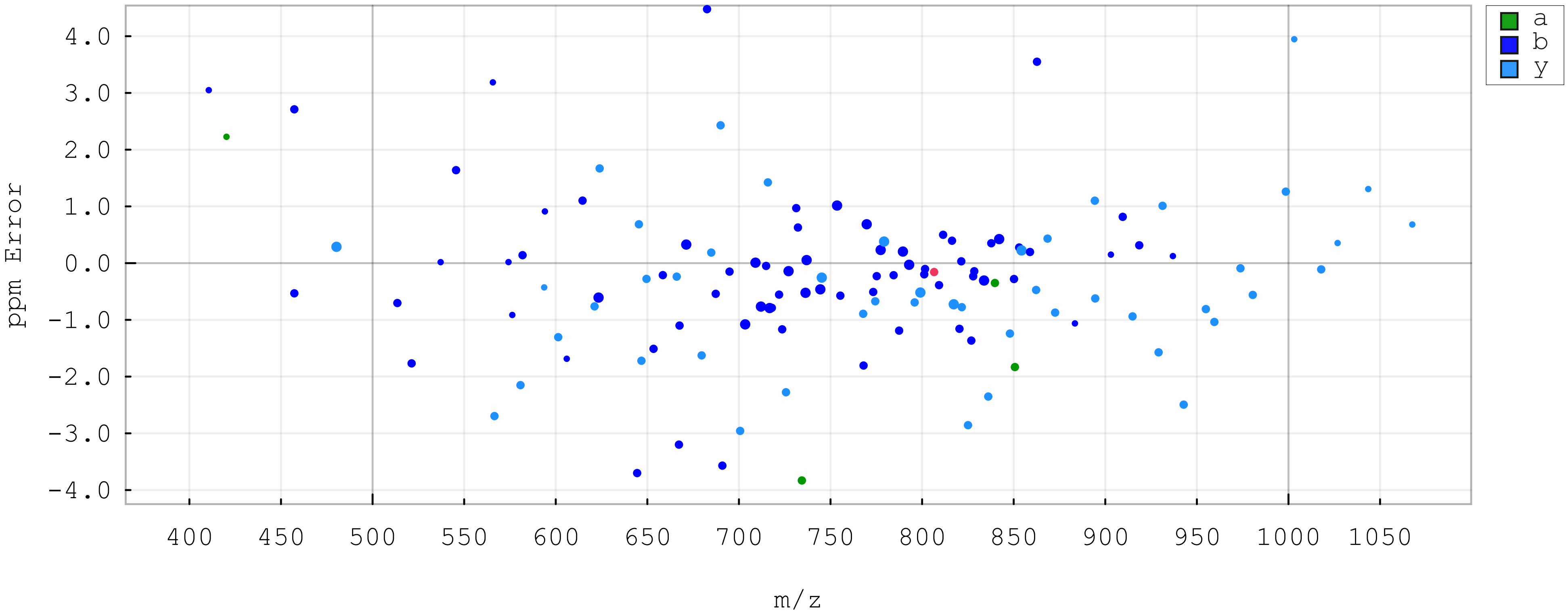

Proteoform:

Index	Protein	SC [%]	SVP [%]	IC [%]	MS Score [a.u.]	Var. Mods	Mono Mass [Da]	Chemical Formula
10	H31Kac1	29.1	0.0	36.9	10.7	Ace: K64	15305.4534	C671H1131N215O186S4

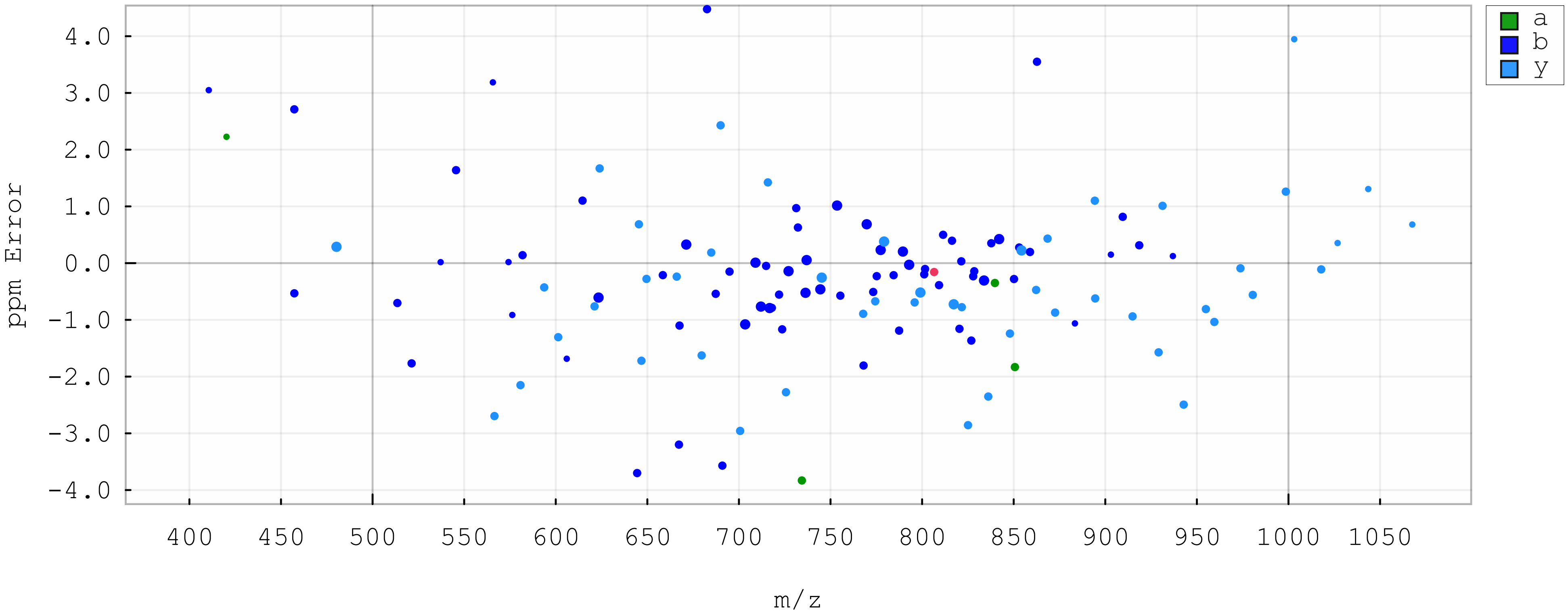

Primary Fragment Plots

Proteoform:

Index	Protein	SC [%]	SVP [%]	IC [%]	MS Score [a.u.]	Var. Mods	Mono Mass [Da]	Chemical Formula
1	H31Kac1	37.3	0.0	37.7	14.1	Ace: K14	15305.4534	C671H1131N215O186S4

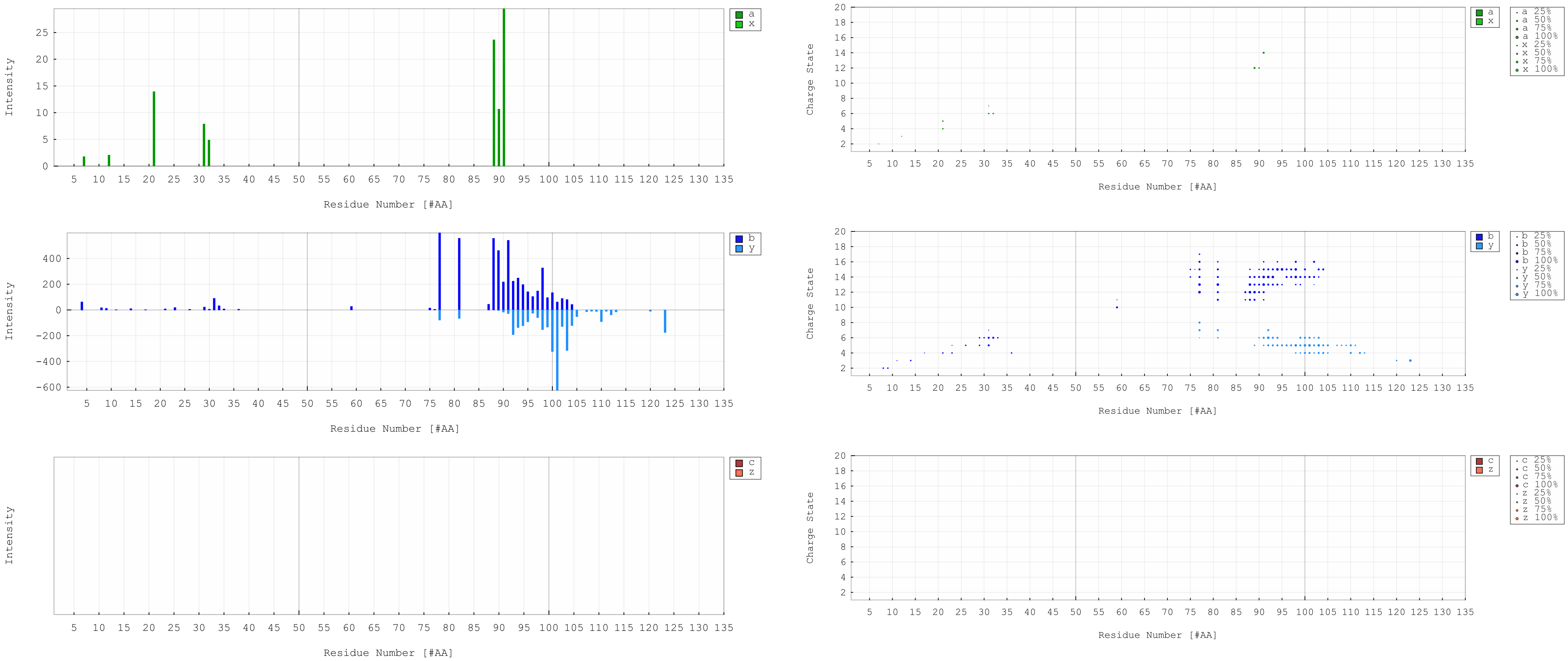

Proteoform:

Index	Protein	SC [%]	SVP [%]	IC [%]	MS Score [a.u.]	Var. Mods	Mono Mass [Da]	Chemical Formula
2	H31Kac1	35.8	0.0	37.6	13.5	Ace: K18	15305.4534	C671H1131N215O186S4

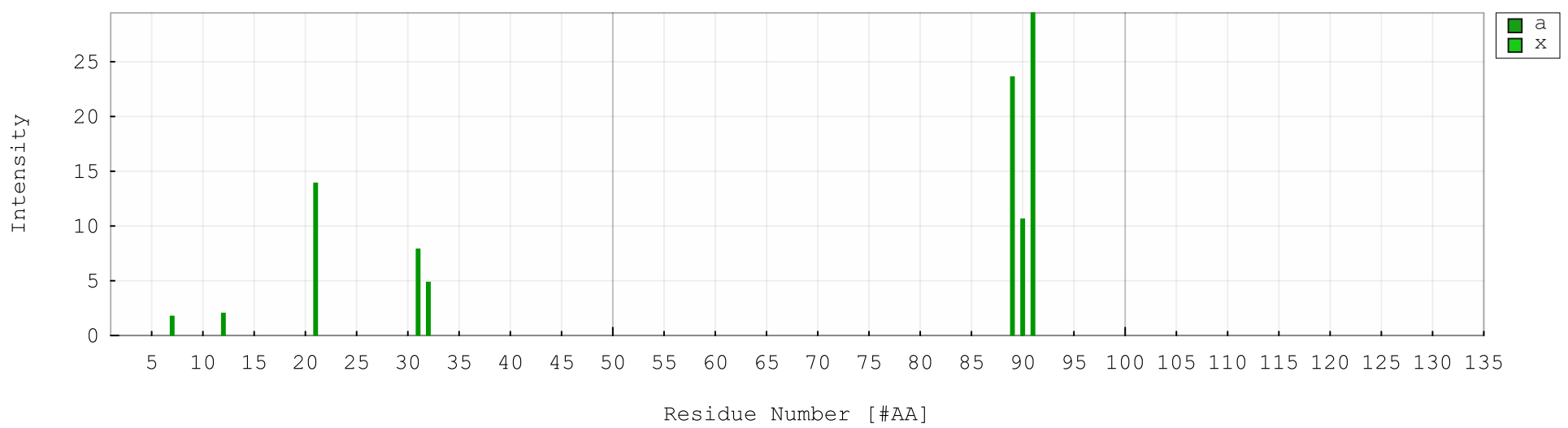

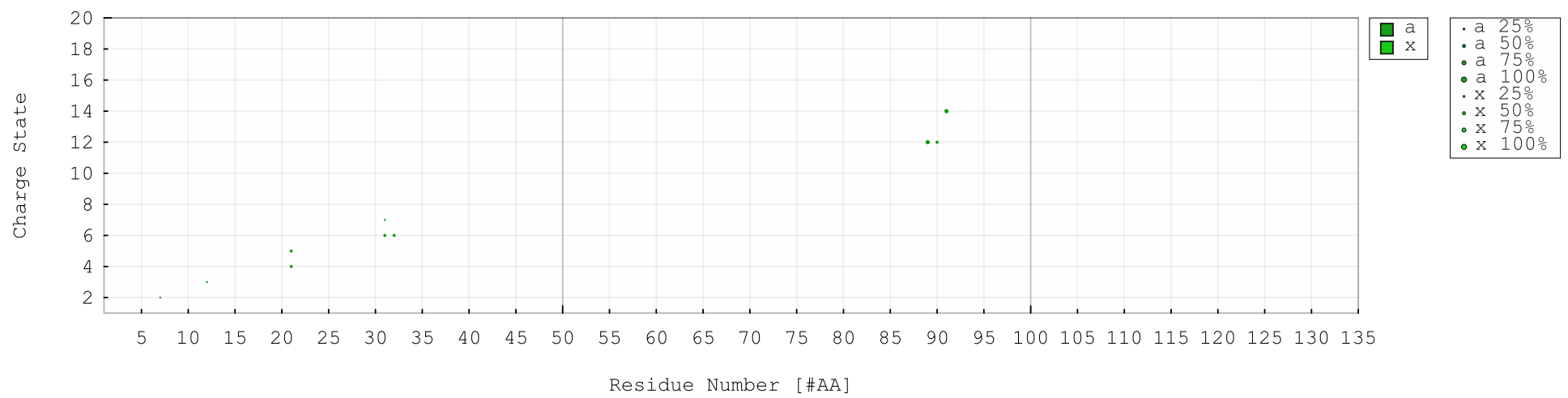

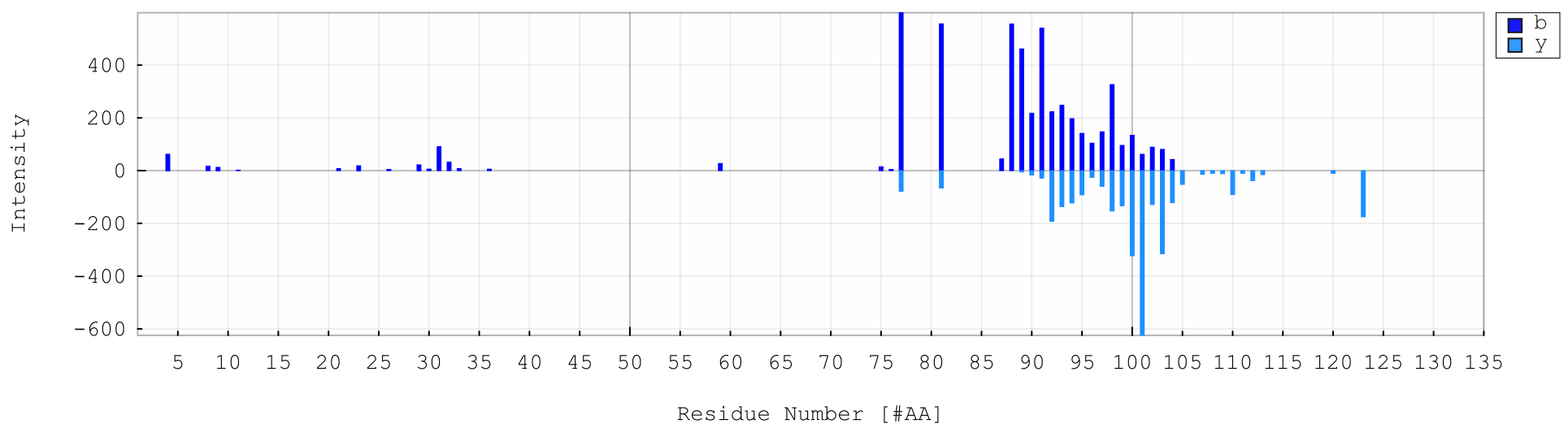

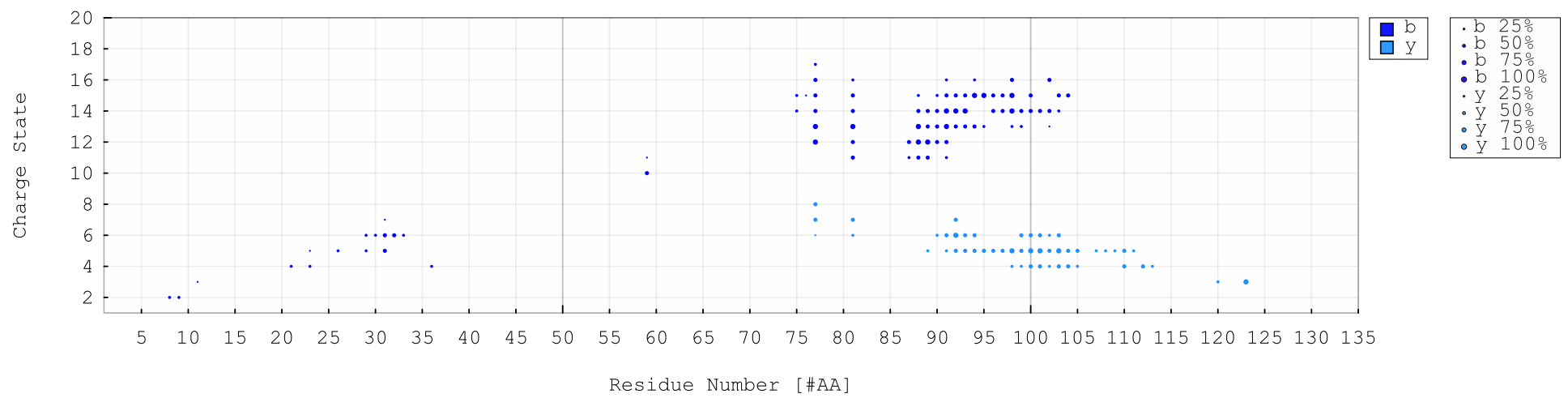

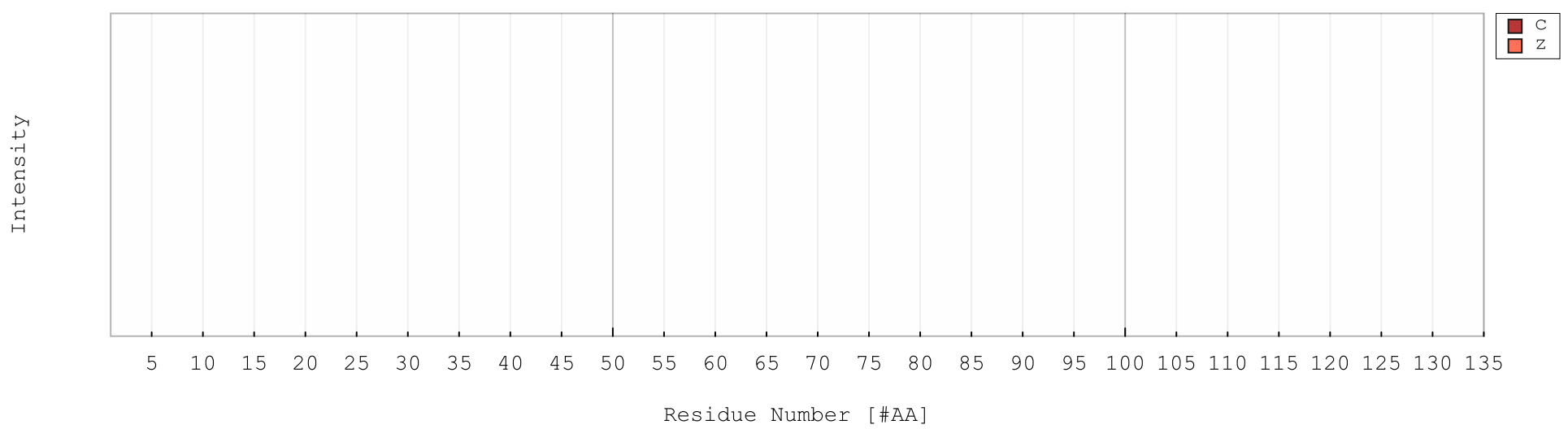

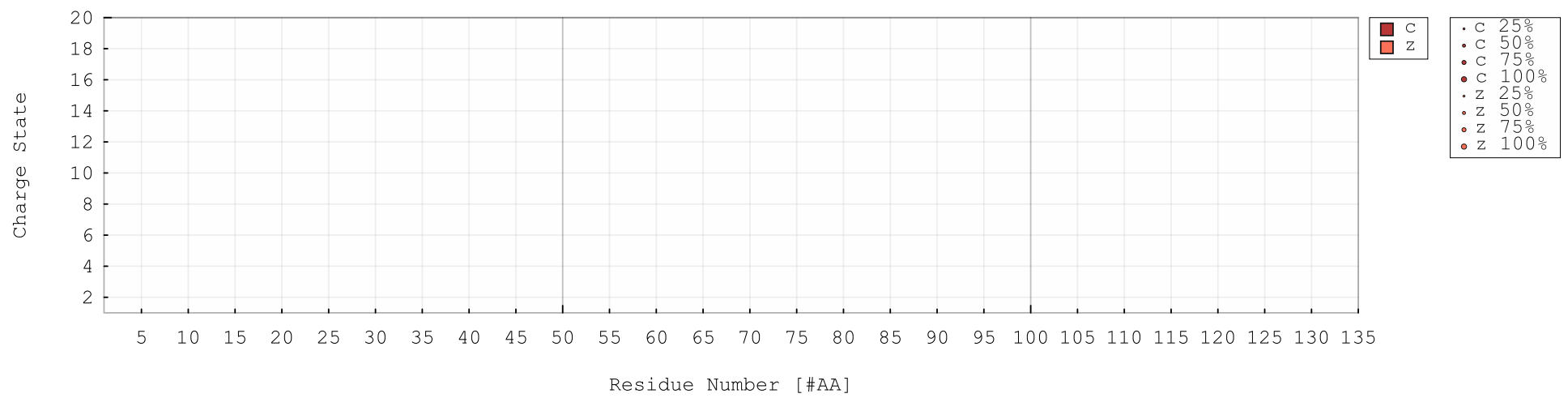

Proteoform:

Index	Protein	SC [%]	SVP [%]	IC [%]	MS Score [a.u.]	Var. Mods	Mono Mass [Da]	Chemical Formula
3	H31Kac1	35.1	0.0	37.6	13.2	Ace: K9	15305.4534	C671H1131N215O186S4

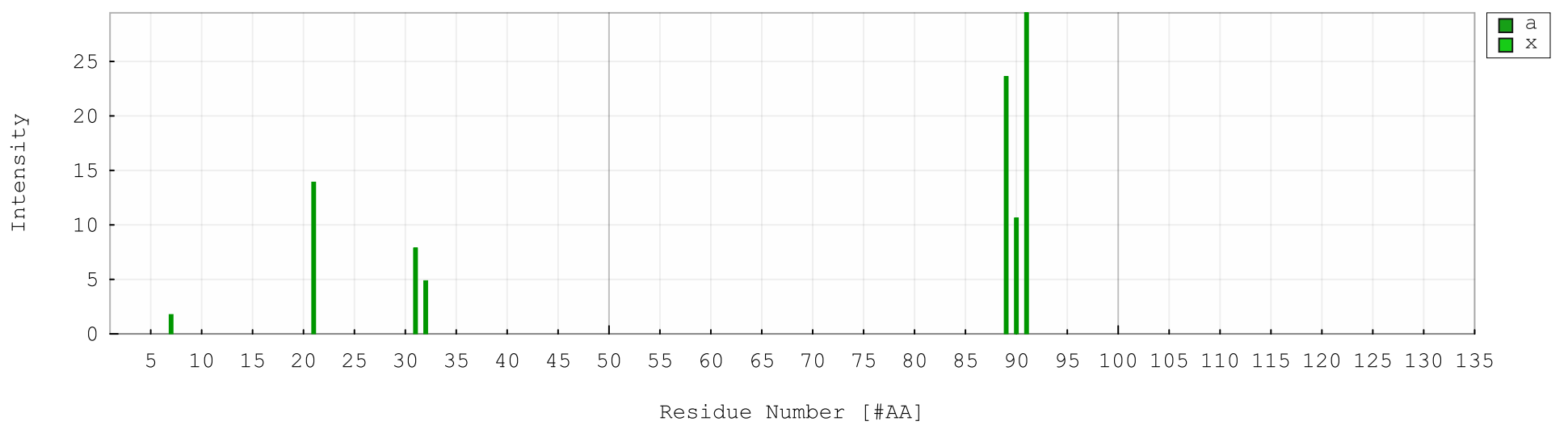

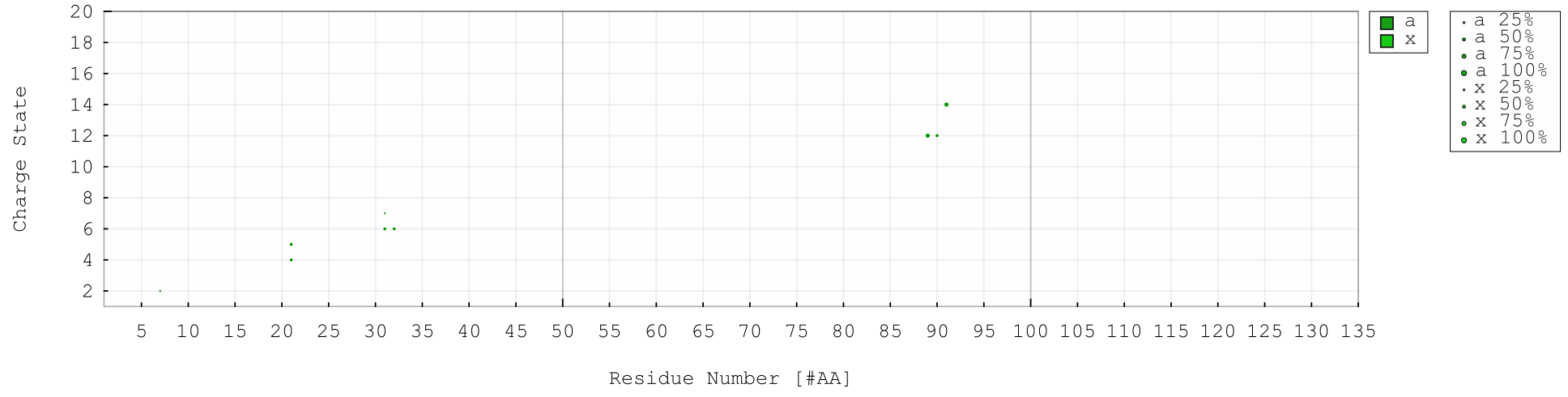

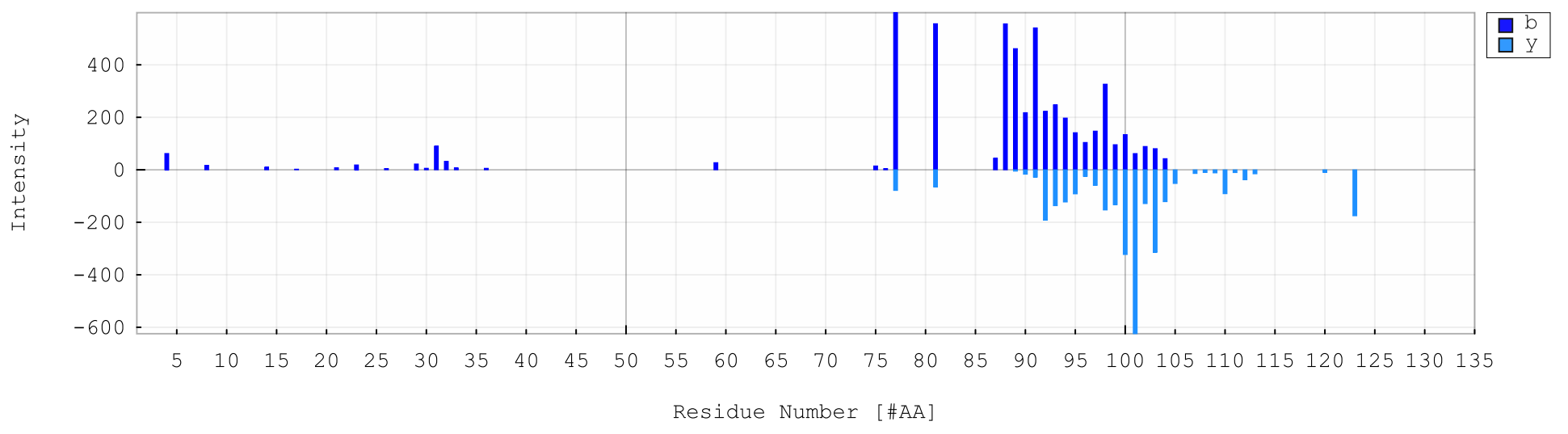

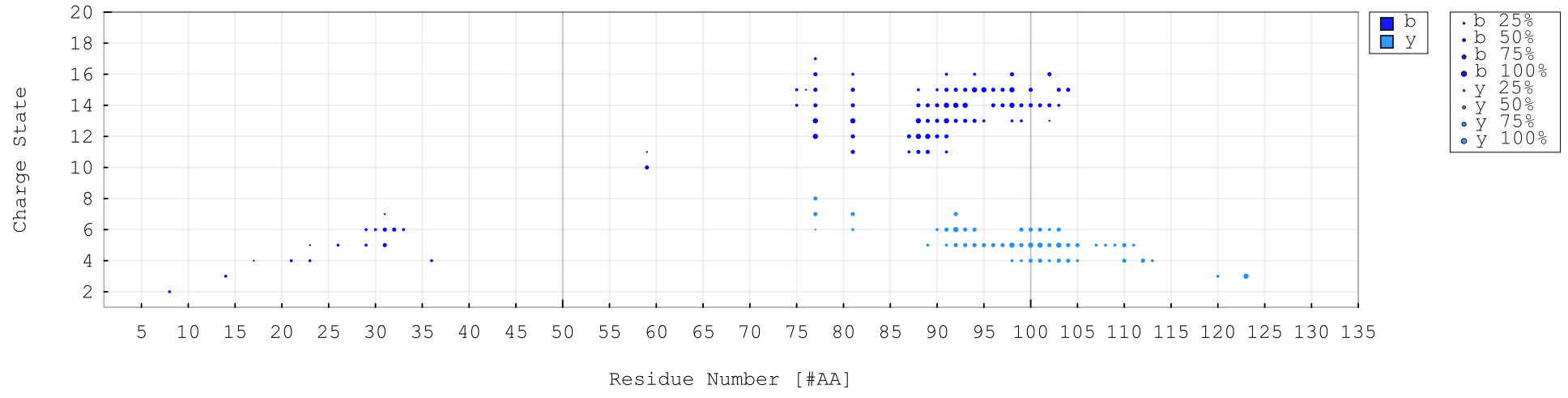

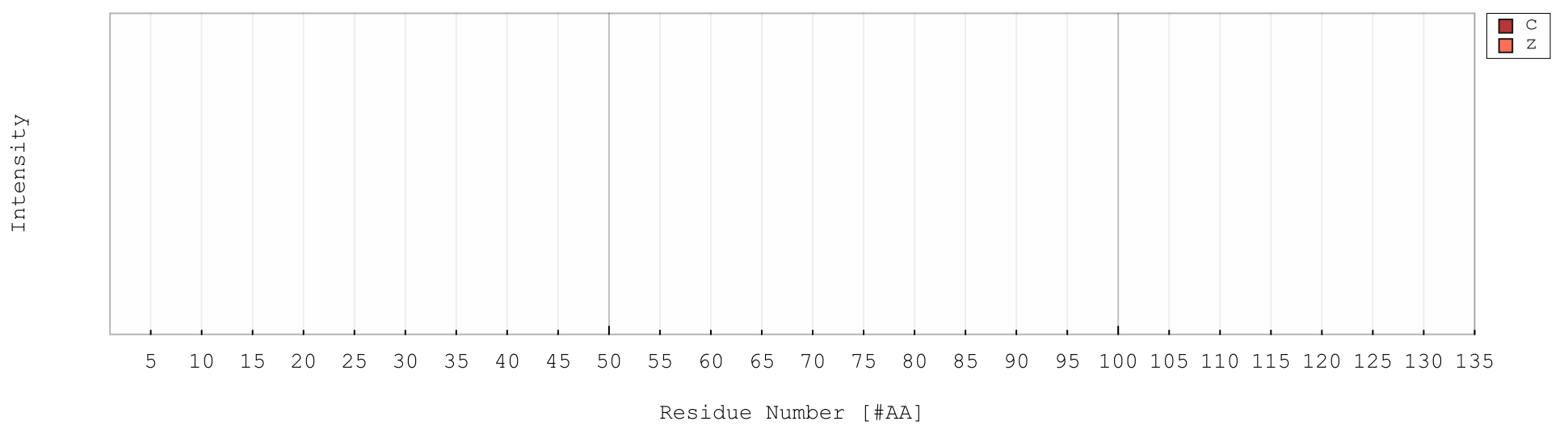

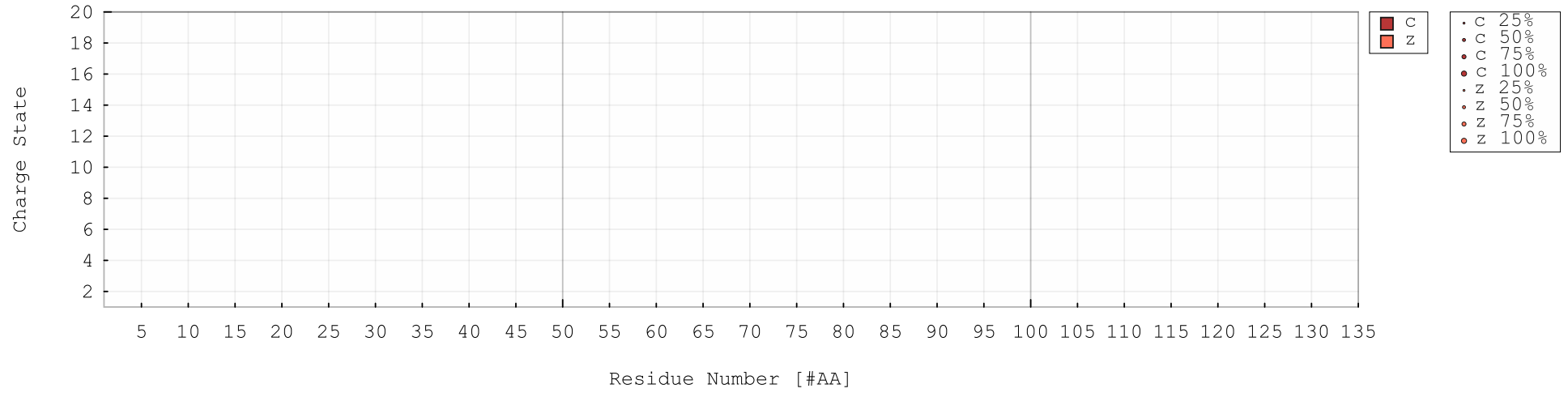

Proteoform:

Index	Protein	SC [%]	SVP [%]	IC [%]	MS Score [a.u.]	Var. Mods	Mono Mass [Da]	Chemical Formula
4	H31Kac1	35.1	0.0	37.6	13.2	Ace: K23	15305.4534	C671H1131N215O186S4

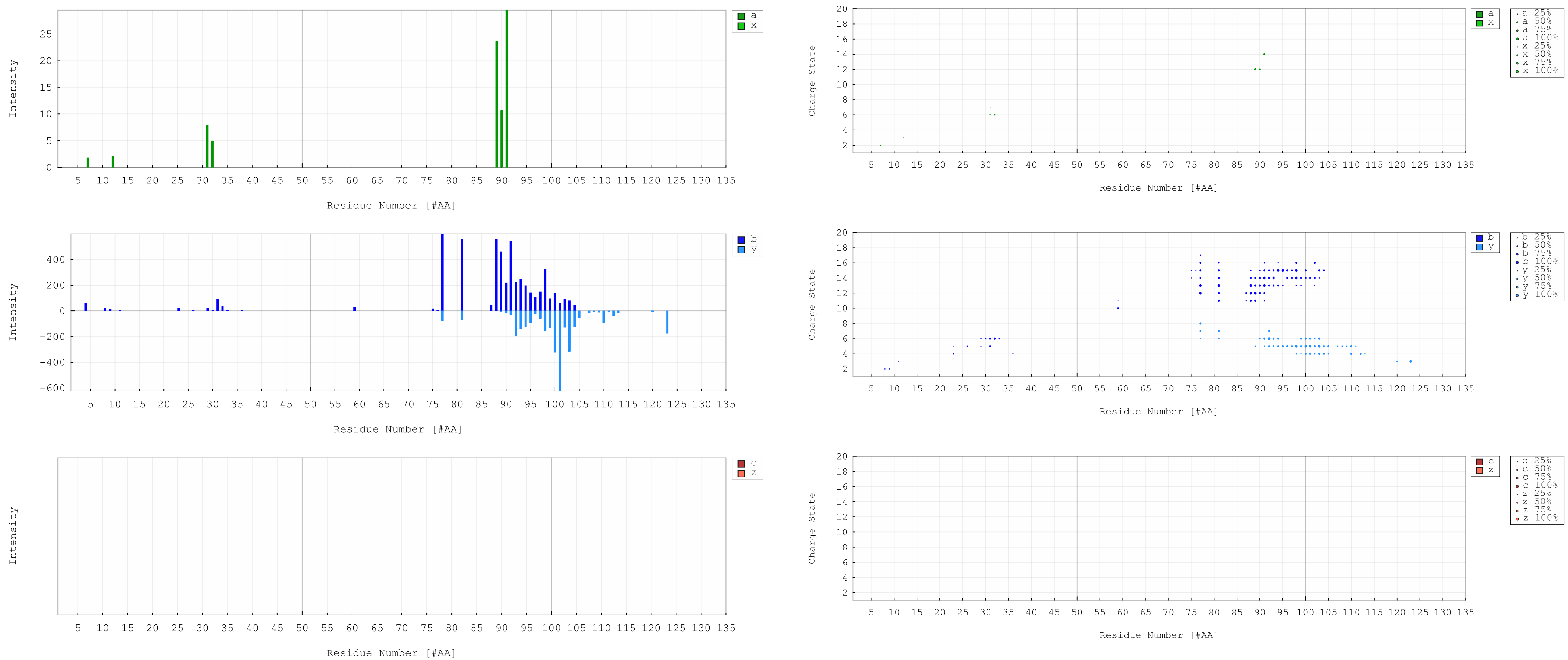

Proteoform:

Index	Protein	SC [%]	SVP [%]	IC [%]	MS Score [a.u.]	Var. Mods	Mono Mass [Da]	Chemical Formula
5	H31Kac1	33.6	0.0	37.5	12.6	Ace: K27	15305.4534	C671H1131N215O186S4

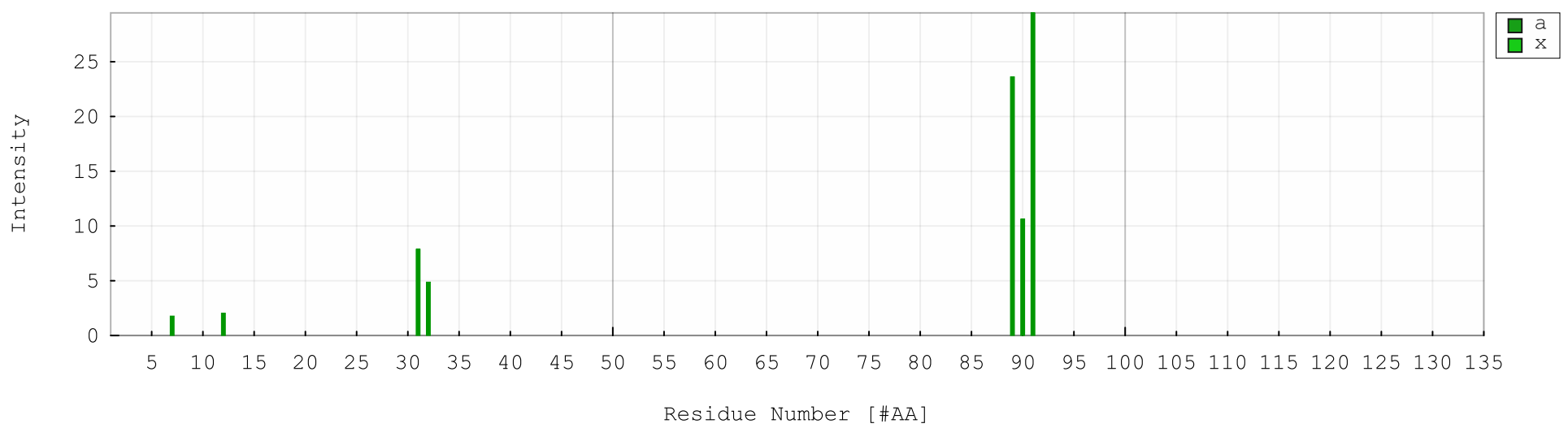

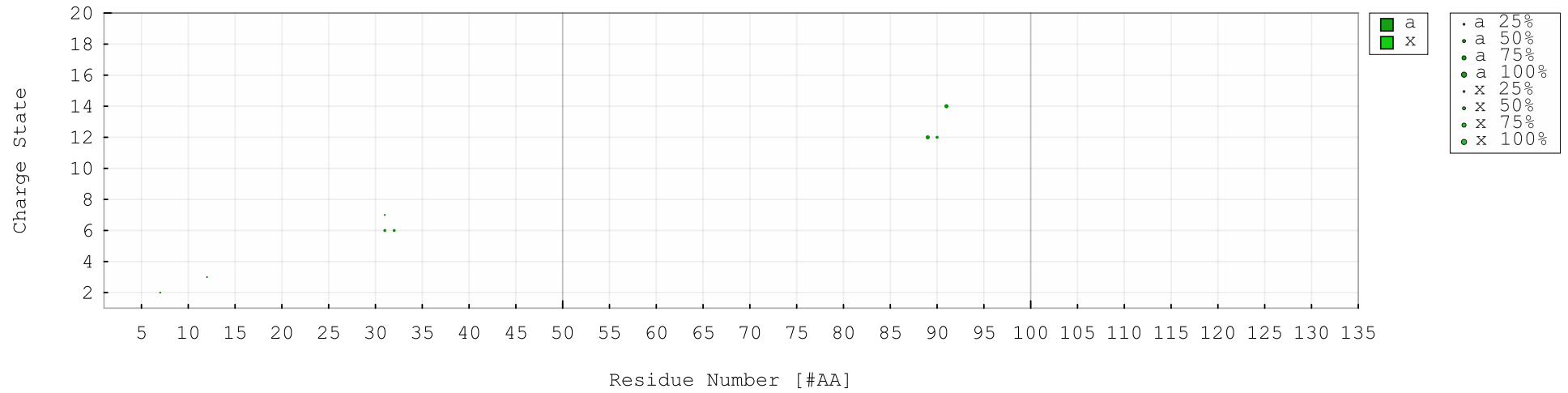

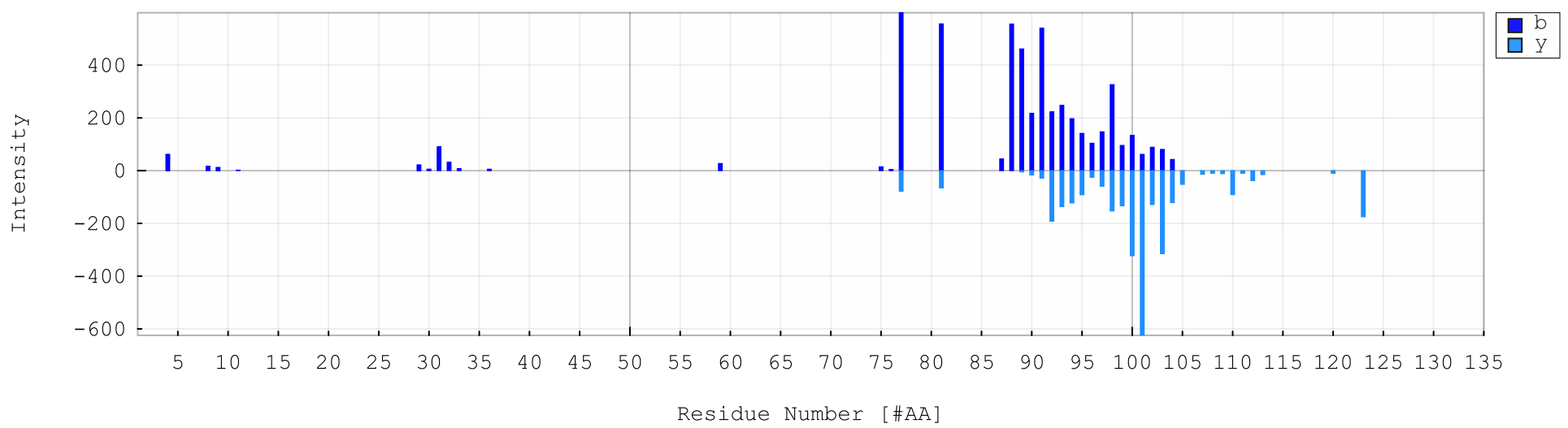

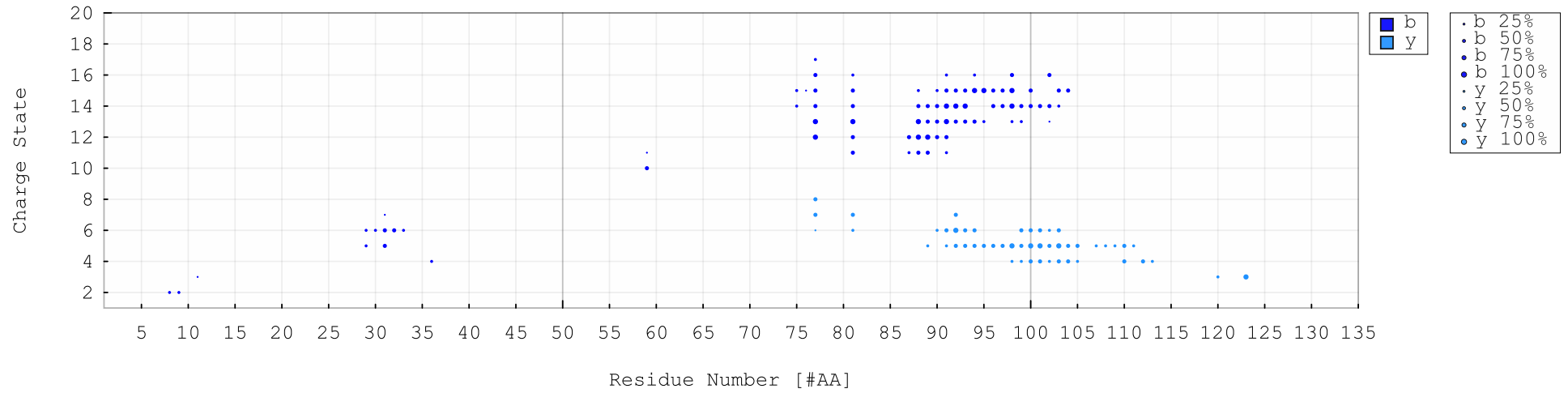

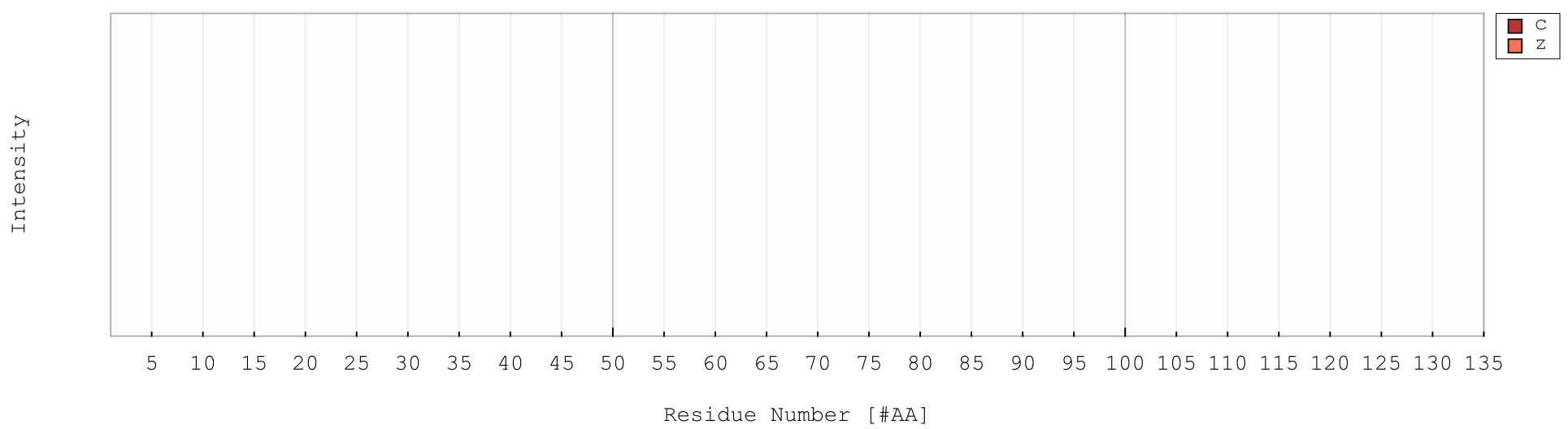

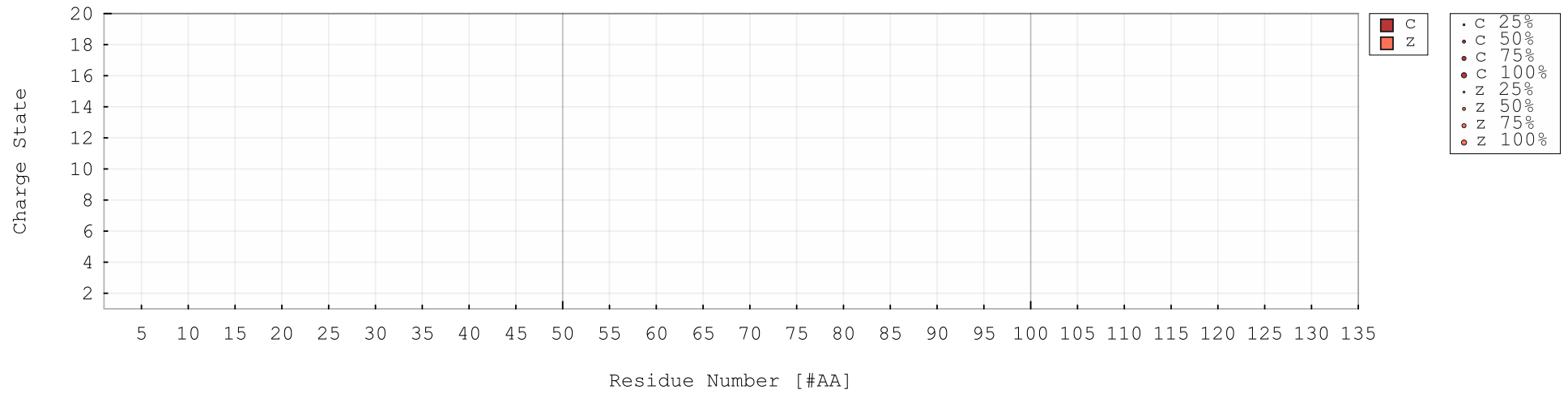

Top-Down Result OmniScape 2025
Sequence Confirmation
241209 H31-GNC5_10 MS2 shCID Q2

Proteoform:

Index	Protein	SC [%]	SVP [%]	IC [%]	MS Score [a.u.]	Var. Mods	Mono Mass [Da]	Chemical Formula
6	H31Kac1	32.8	0.0	37.5	12.3	Ace: K4	15305.4534	C671H1131N215O186S4

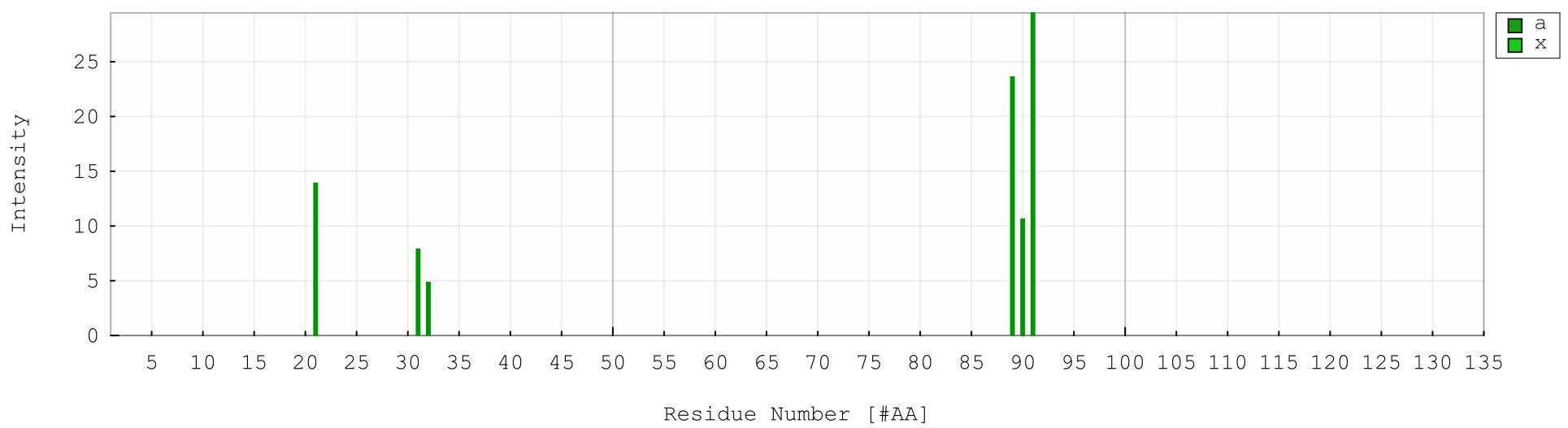

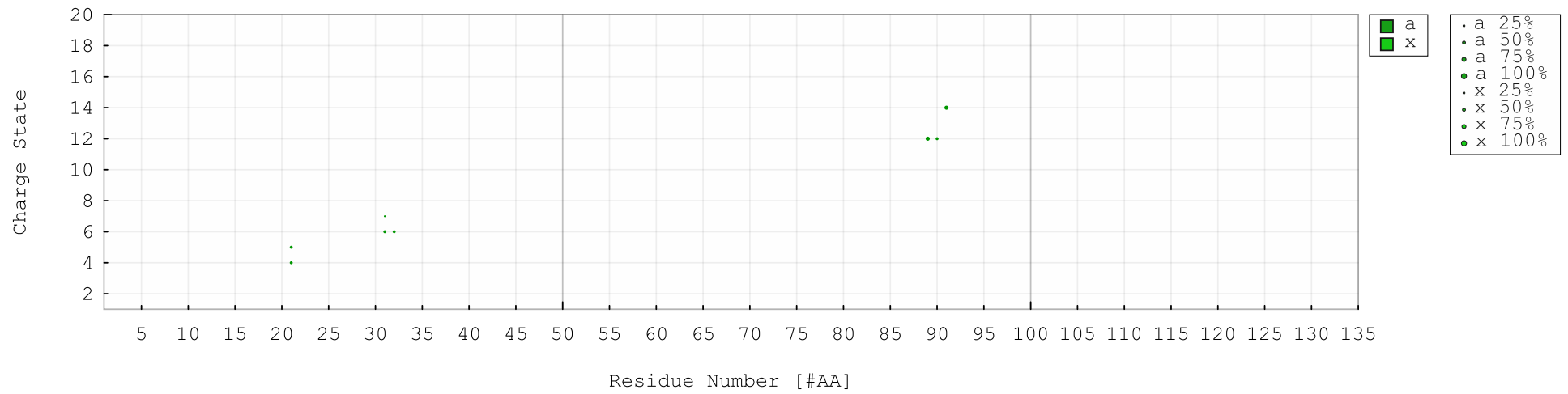

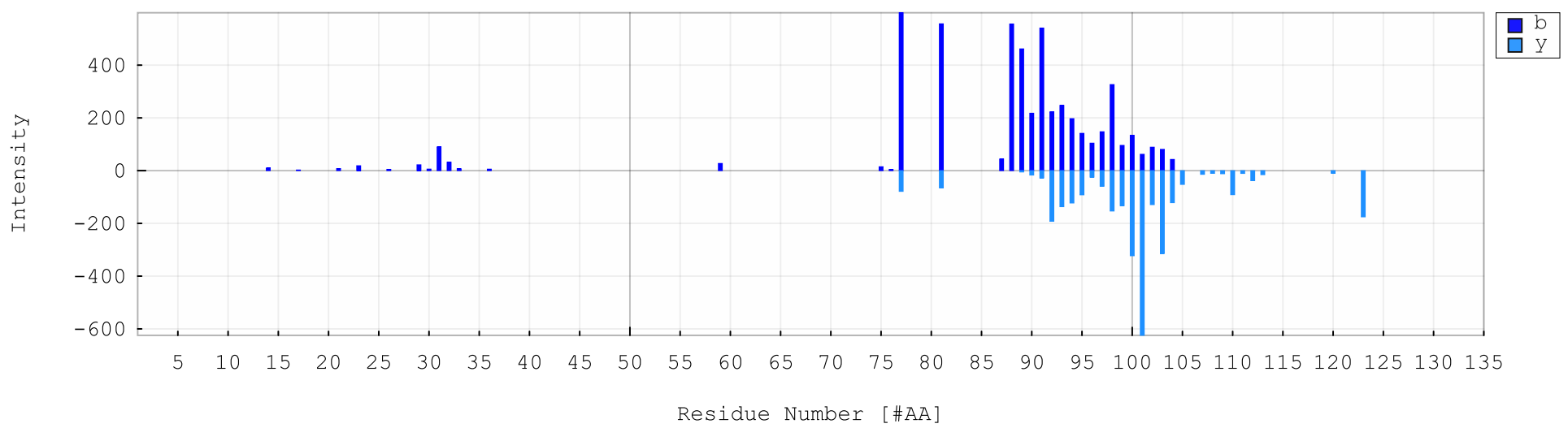

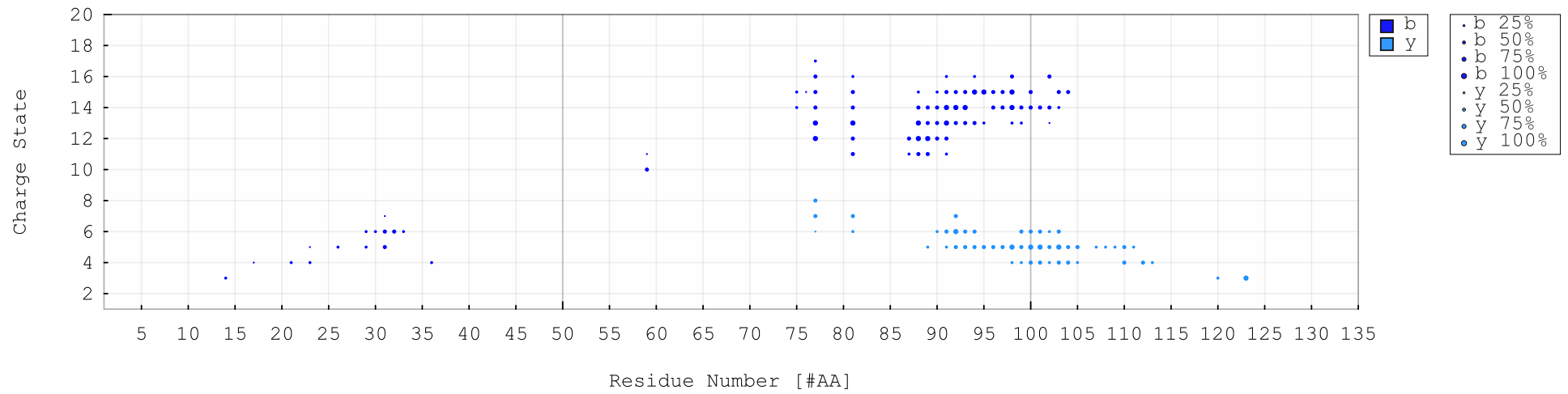

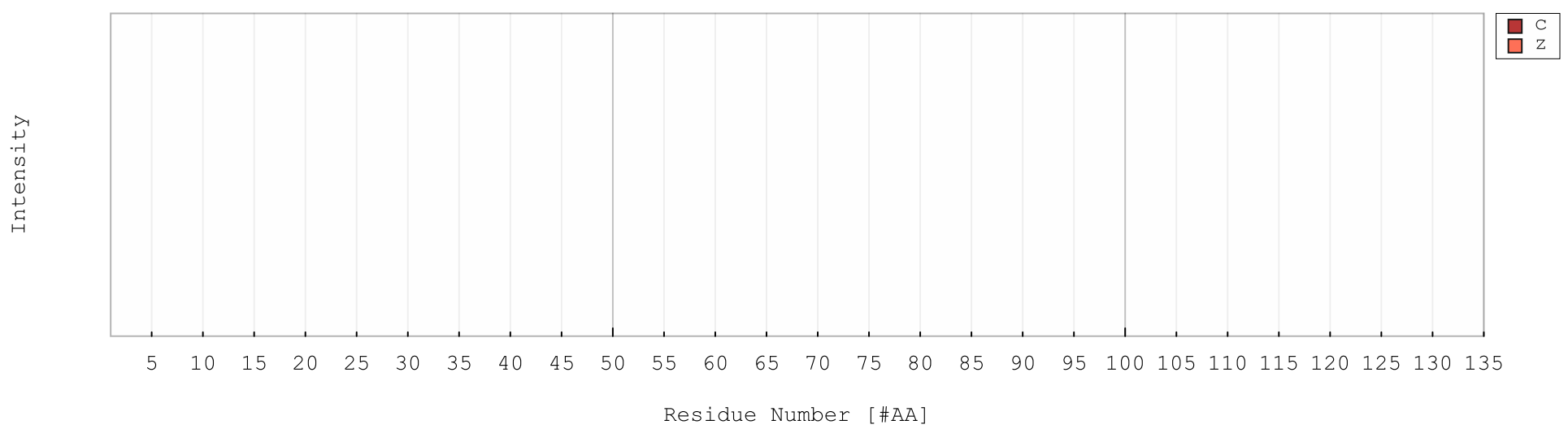

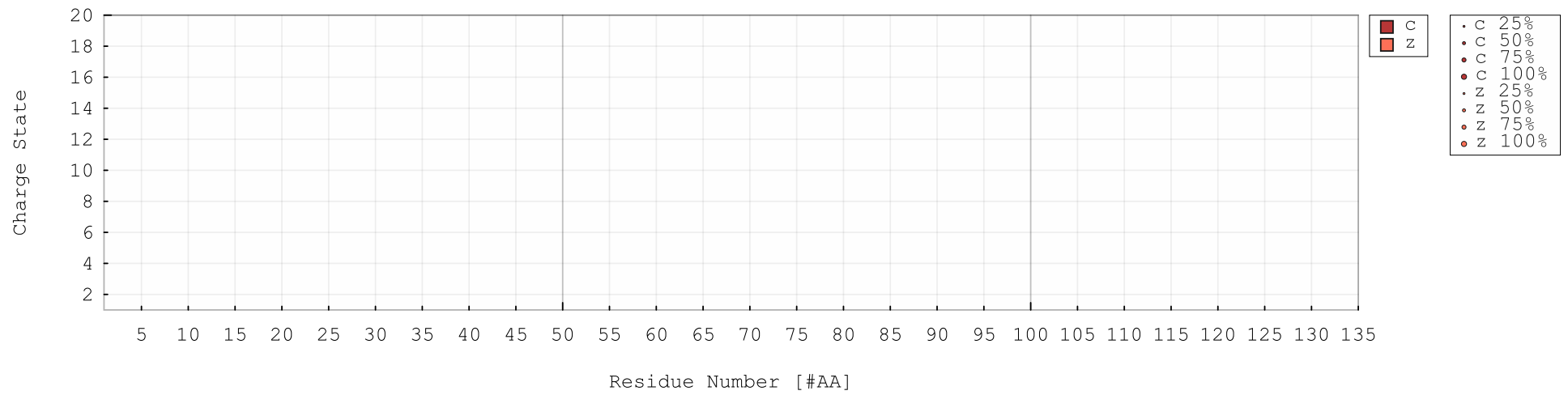

Proteoform:

Index	Protein	SC [%]	SVP [%]	IC [%]	MS Score [a.u.]	Var. Mods	Mono Mass [Da]	Chemical Formula
7	H31Kac1	30.6	0.0	37.0	11.3	Ace: K36	15305.4534	C671H1131N215O186S4

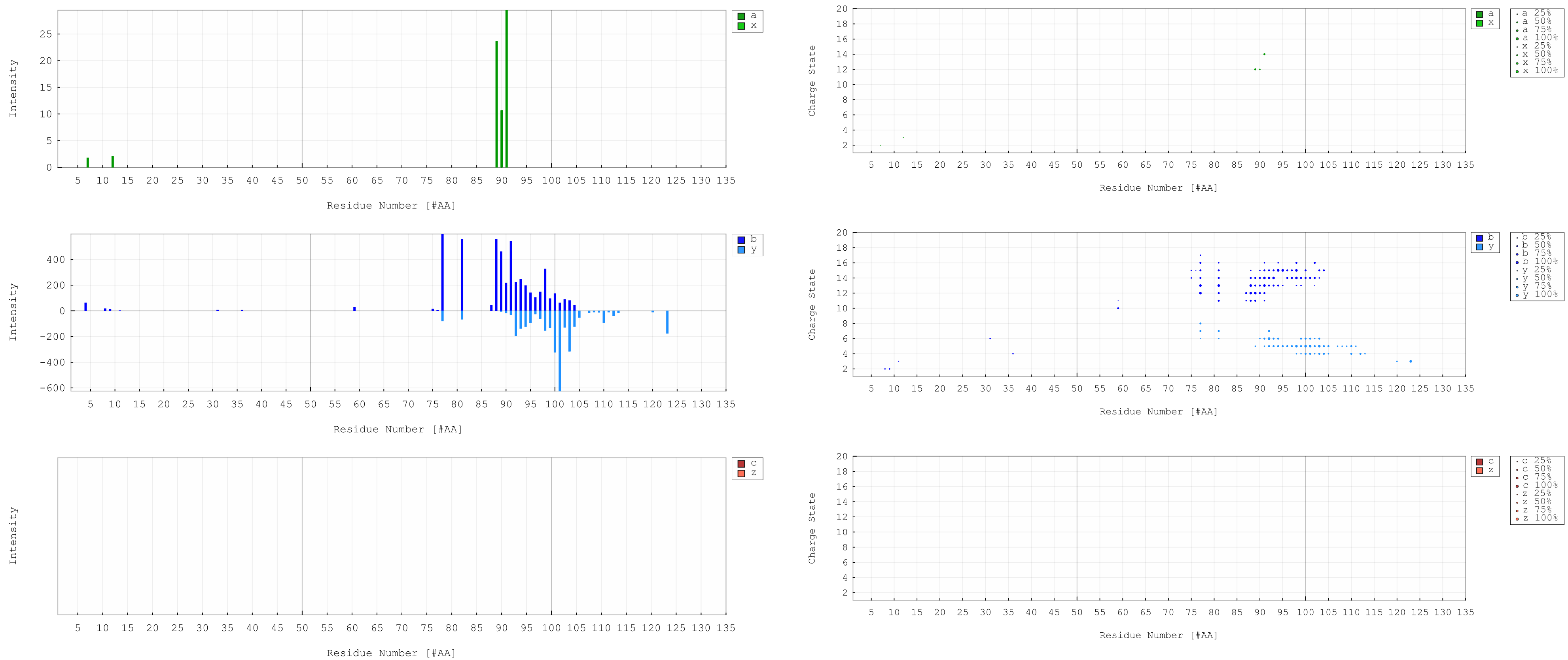

Top-Down Result OmniScape 2025
Sequence Confirmation
241209 H31-GNC5_10 MS2 shCID Q2

Proteoform:	Index	Protein	SC [%]	SVP [%]	IC [%]	MS Score [a.u.]	Var. Mods	Mono Mass [Da]	Chemical Formula
	8	H31Kac1	29.9	0.0	37.0	11.1	Ace: K37	15305.4534	C671H1131N215O186S4

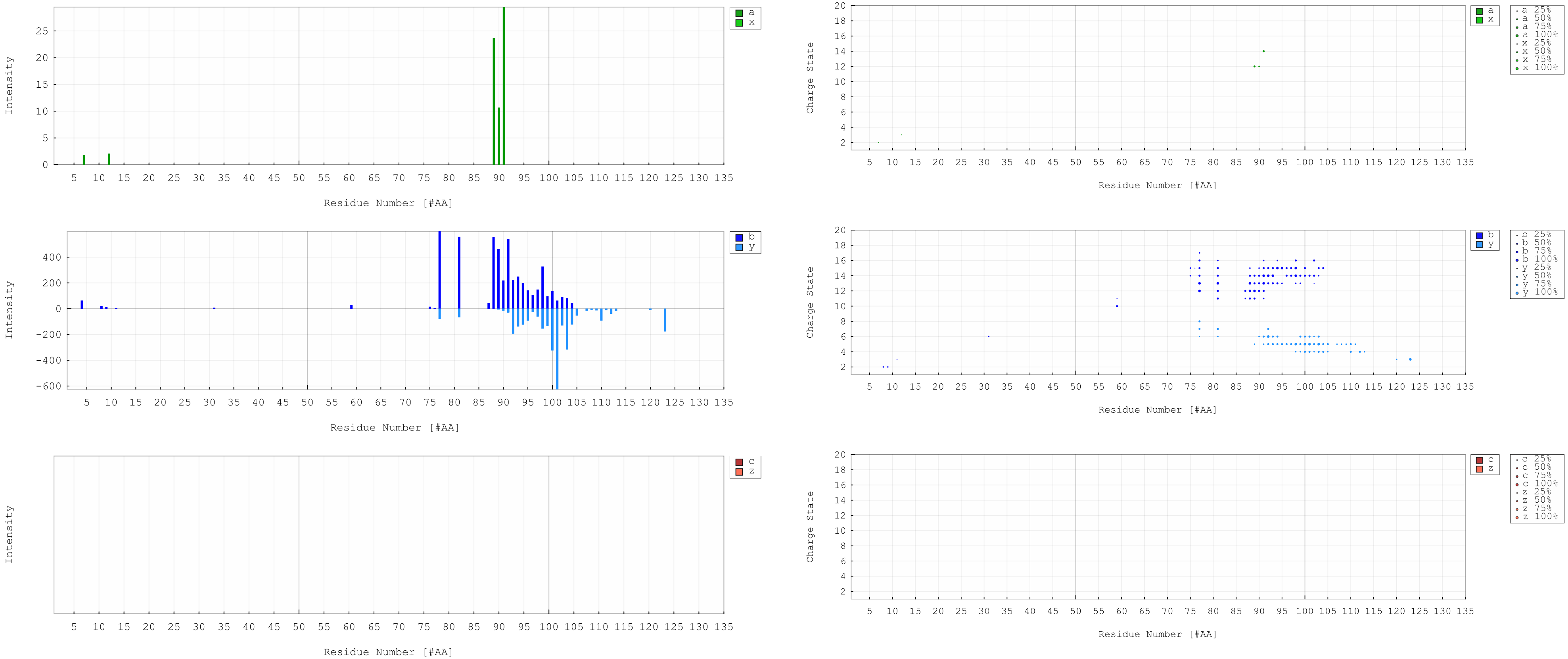

Proteoform:

Index	Protein	SC [%]	SVP [%]	IC [%]	MS Score [a.u.]	Var. Mods	Mono Mass [Da]	Chemical Formula
9	H31Kac1	29.9	0.0	37.0	11.1	Ace: K56	15305.4534	C671H1131N215O186S4

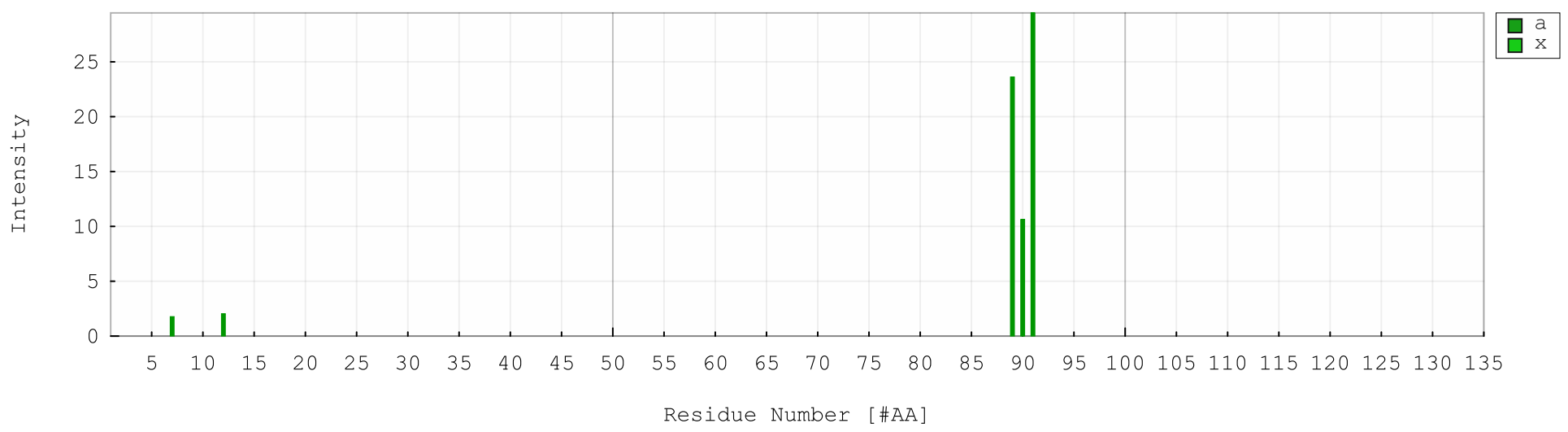

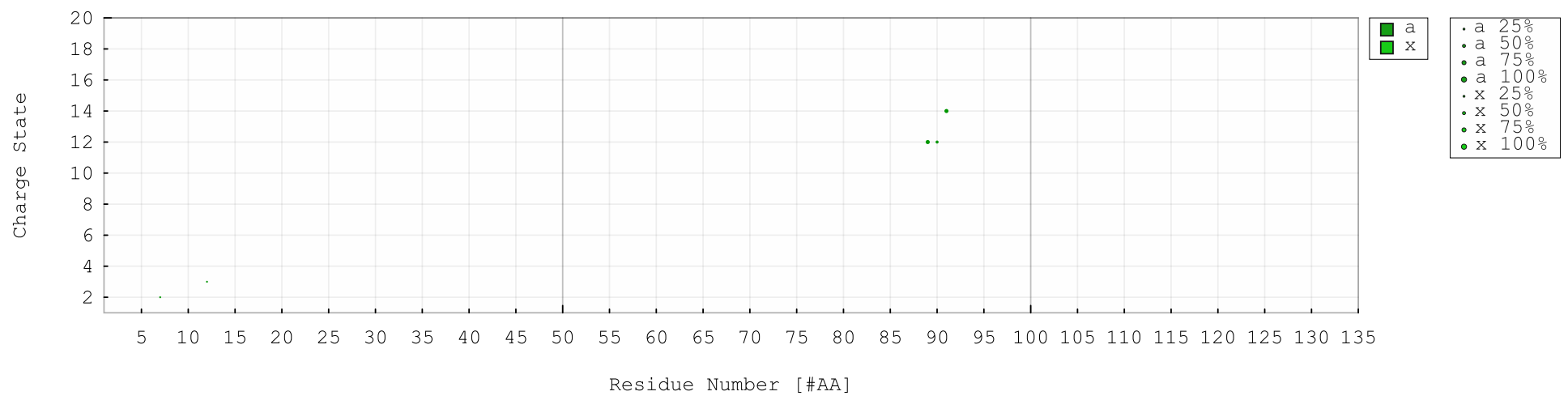

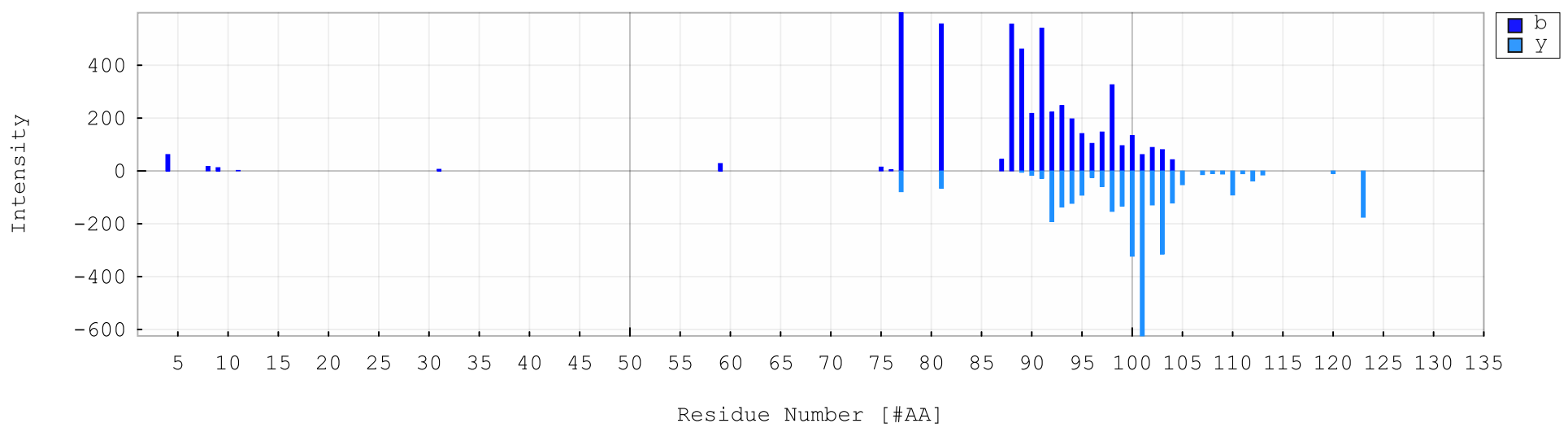

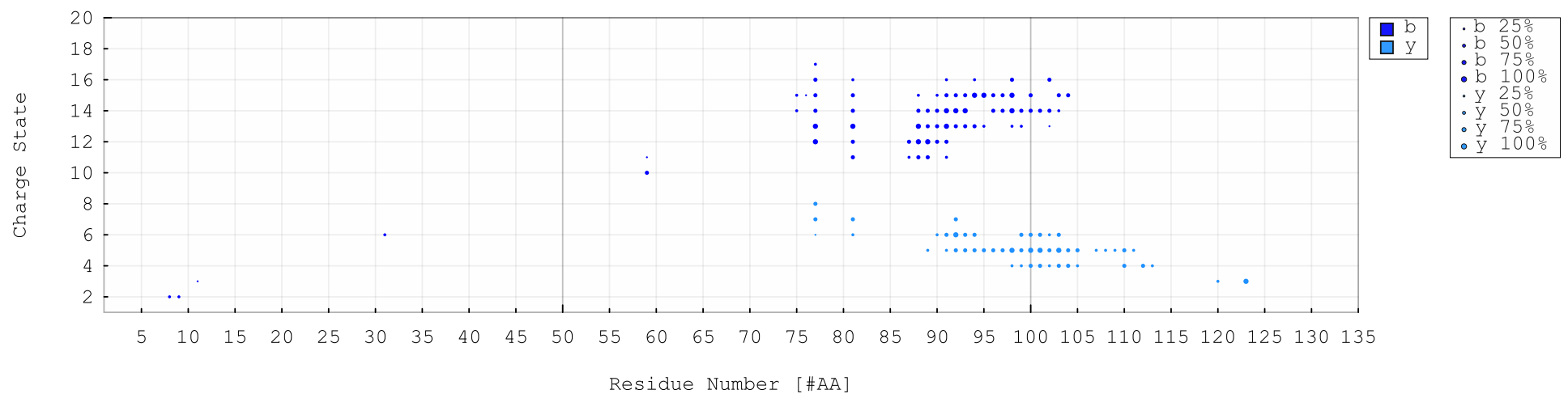

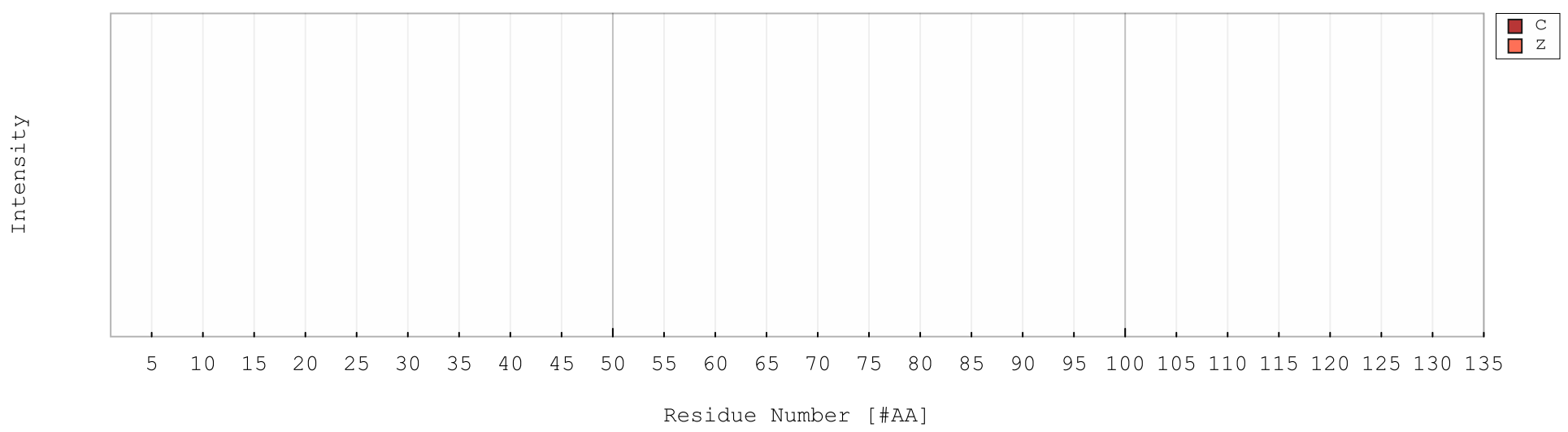

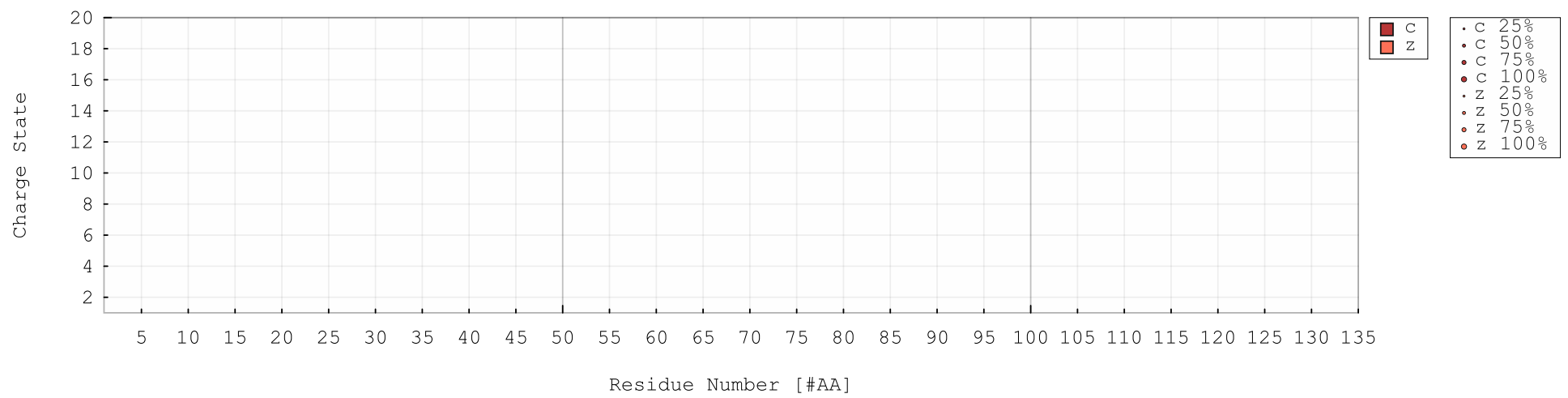

Proteoform:

Index	Protein	SC [%]	SVP [%]	IC [%]	MS Score [a.u.]	Var. Mods	Mono Mass [Da]	Chemical Formula
10	H31Kac1	29.1	0.0	36.9	10.7	Ace: K64	15305.4534	C671H1131N215O186S4

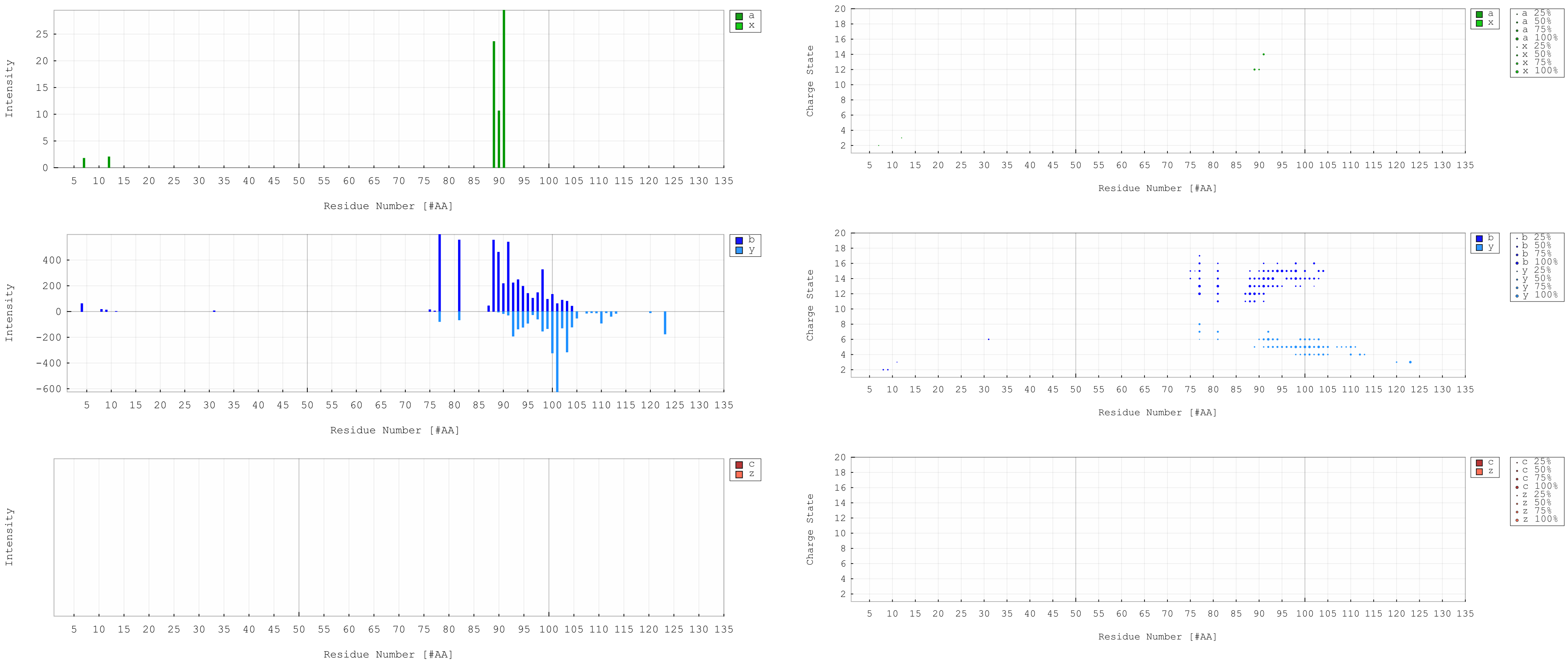

Supplemental Data S2

H3.1 - GCN5, 10 min

**Report**	7 of 7
**Experiment**	MS3
**Histone/sample**	H3.1
**Enzyme**	GCN5
**Reaction/source**	10 min
**Precursor**	H3.1Kac1
**Charge state**	19+
**Fragmentation / workflow**	MS2 ECD -> MS3 RCID
**Original file**	MS3_H3.1Kac1_19+_MS2-ECD_MS3-RCID.pdf

Project Information

Project Directory: C:/Users/LAB-ADMIN
Processing date: Wed Jan 21 15:49:25 2026

Sample Info & Protocols

Name: Top-Down Protein Sequencing CID

Dataset

Imported spectrum file name:	241210 H31-GNC5_10 z19 MS2 ECD 20ms MS3 shCID z18d_001-366_calibrated.txt
Dataset name:	Information is only available from imported JPEAKS files
Date acquired:	Information is only available from imported JPEAKS files
Date imported:	Information is only available from imported JPEAKS files
Instrument Name:	Information is only available from imported JPEAKS files
Instrument Serial Number:	Information is only available from imported JPEAKS files

Workflow Result Info

Analysis: 241210 H31-GNC5_10 z19 MS2 ECD 20ms MS3 shCID z18d_001-366_calibrated
Location: C:/Users/LAB-ADMIN
Method: -

Method Information

Name: Unnamed

Spectral Parameters

Mass Error [ppm]:	6
Intensity Deviation [au]:	0.15
Min/Max Charges:	1-19
Experiment type:	CID

a ions	b ions	c ions	x ions	y ions	z ions	satellite	internal	precursor
a	b			y				M

Noise threshold calculation:	Simple moving average noise threshold (SMA)
Window size [datapoints]:	600
Noise curve offset:	0.17585362
SVP parameters:	terminal gap: 2, internal gap: 1

Proteoforms

(top 10)

Index	Parent Sequence	Chain	Var. Mods	Mono Mass [Da]	Chemical Composition
1	H31Kac1	H31Kac1K14[Ace]	Ace: K14	15305.4534	C671H1131N215O186S4
2	H31Kac1	H31Kac1K9[Ace]	Ace: K9	15305.4534	C671H1131N215O186S4
3	H31Kac1	H31Kac1K18[Ace]	Ace: K18	15305.4534	C671H1131N215O186S4
4	H31Kac1	H31Kac1K4[Ace]	Ace: K4	15305.4534	C671H1131N215O186S4
5	H31Kac1	H31Kac1K23[Ace]	Ace: K23	15305.4534	C671H1131N215O186S4
6	H31Kac1	H31Kac1K27[Ace]	Ace: K27	15305.4534	C671H1131N215O186S4
7	H31Kac1	H31Kac1K36[Ace]	Ace: K36	15305.4534	C671H1131N215O186S4
8	H31Kac1	H31Kac1K37[Ace]	Ace: K37	15305.4534	C671H1131N215O186S4
9	H31Kac1	H31Kac1K56[Ace]	Ace: K56	15305.4534	C671H1131N215O186S4
10	H31Kac1	H31Kac1K64[Ace]	Ace: K64	15305.4534	C671H1131N215O186S4

Results

Proteoforms Table (top 10)

Index	Protein	SC [%]	SVP [%]	IC [%]	MS Score [a.u.]	Var. Mods	Mono Mass [Da]	Chemical Formula
1	H31Kac1	56.0	17.8	28.5	16.0	Ace: K14	15305.4534	C671H1131N215O186S4
2	H31Kac1	53.7	11.1	28.4	15.3	Ace: K9	15305.4534	C671H1131N215O186S4
3	H31Kac1	53.7	14.8	28.2	15.2	Ace: K18	15305.4534	C671H1131N215O186S4
4	H31Kac1	51.5	7.4	28.3	14.6	Ace: K4	15305.4534	C671H1131N215O186S4
5	H31Kac1	51.5	14.8	27.9	14.4	Ace: K23	15305.4534	C671H1131N215O186S4
6	H31Kac1	50.7	14.8	27.8	14.1	Ace: K27	15305.4534	C671H1131N215O186S4
7	H31Kac1	47.0	14.8	26.3	12.4	Ace: K36	15305.4534	C671H1131N215O186S4
8	H31Kac1	47.0	14.8	26.3	12.4	Ace: K37	15305.4534	C671H1131N215O186S4
9	H31Kac1	41.8	14.8	24.5	10.2	Ace: K56	15305.4534	C671H1131N215O186S4
10	H31Kac1	41.0	14.8	24.4	10.0	Ace: K64	15305.4534	C671H1131N215O186S4

Sequence

Name: H31Kac1

```
                 10                   20                   30                   40                   50                   60
A R T K Q T A R K S  T G G K A P R K Q L  A T K A A R K S A P  A T G G V K K P H R  Y R P G T V A L R E  I R R Y Q K S T E L
                 70                   80                   90                  100                  110                  120
L I R K L P F Q R L  V R E I A Q D F K T  D L R F Q S S A V M  A L Q E A C E A Y L  V G L F E D T N L C  A I H A K R V T I M
                130                  140                  150                  160                  170                  180
P K D I Q L A R R I  R G E R A
```

Modifications

Modification	Position	Type
Acetyl	4	variable
Acetyl	9	variable
Acetyl	14	variable
Acetyl	18	variable
Acetyl	23	variable
Acetyl	27	variable
Acetyl	36	variable
Acetyl	37	variable
Acetyl	56	variable
Acetyl	64	variable
Acetyl	79	variable

Modification	Position	Type
Acetyl	115	variable
Acetyl	122	variable

Sequence Map

Proteoform:

Index	Protein	SC [%]	SVP [%]	IC [%]	MS Score [a.u.]	Var. Mods	Mono Mass [Da]	Chemical Formula
1	H31Kac1	56.0	17.8	28.5	16.0	Ace: K14	15305.4534	C671H1131N215O186S4

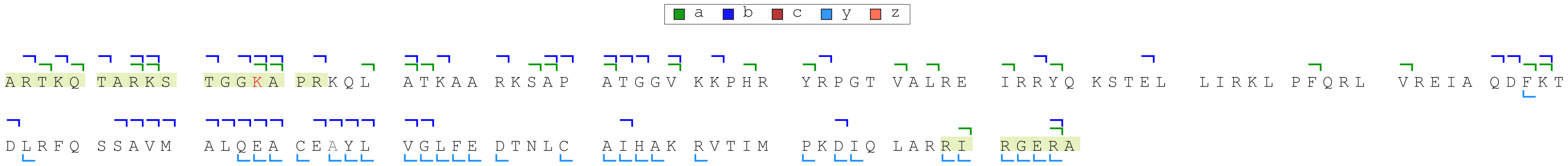

Proteoform:	Index	Protein	SC [%]	SVP [%]	IC [%]	MS Score [a.u.]	Var. Mods	Mono Mass [Da]	Chemical Formula
	2	H31Kac1	53.7	11.1	28.4	15.3	Ace: K9	15305.4534	C671H1131N215O186S4

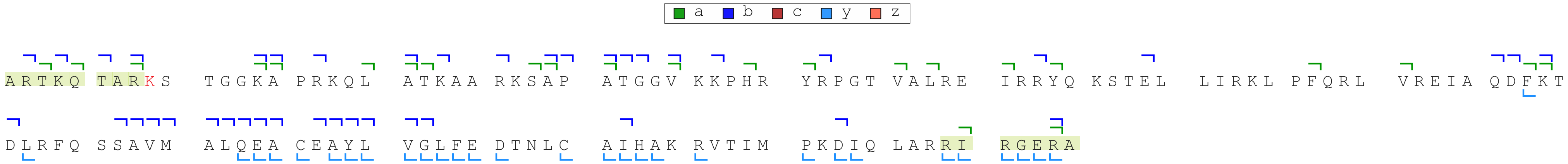

Proteoform:	Index	Protein	SC [%]	SVP [%]	IC [%]	MS Score [a.u.]	Var. Mods	Mono Mass [Da]	Chemical Formula
	3	H31Kac1	53.7	14.8	28.2	15.2	Ace: K18	15305.4534	C671H1131N215O186S4

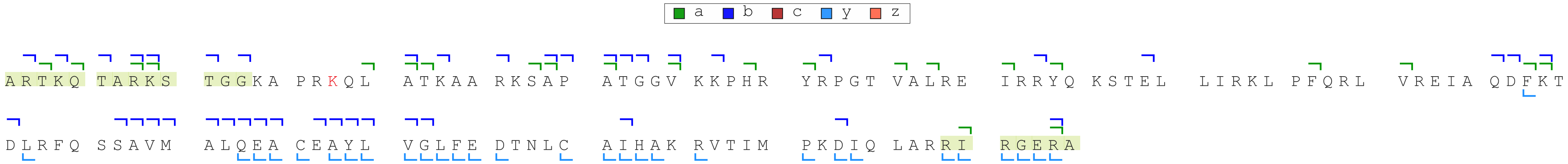

Proteoform:	Index	Protein	SC [%]	SVP [%]	IC [%]	MS Score [a.u.]	Var. Mods	Mono Mass [Da]	Chemical Formula
	4	H31Kac1	51.5	7.4	28.3	14.6	Ace: K4	15305.4534	C671H1131N215O186S4

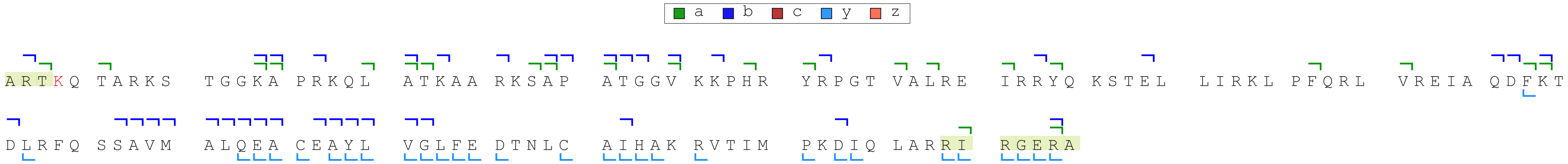

Proteoform:

Index	Protein	SC [%]	SVP [%]	IC [%]	MS Score [a.u.]	Var. Mods	Mono Mass [Da]	Chemical Formula
5	H31Kac1	51.5	14.8	27.9	14.4	Ace: K23	15305.4534	C671H1131N215O186S4

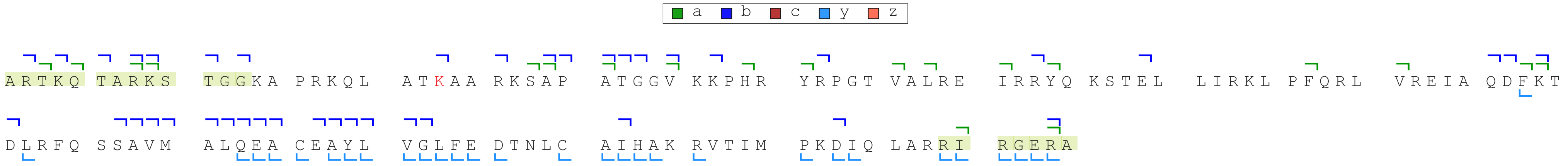

Top-Down Result OmniScape 2025
Sequence Confirmation
241210_H31-GNC5_10_z19_MS2_ECD_20ms_MS3_shCID_z18d_001-366_calibrated

Proteoform:

Index	Protein	SC [%]	SVP [%]	IC [%]	MS Score [a.u.]	Var. Mods	Mono Mass [Da]	Chemical Formula
6	H31Kac1	50.7	14.8	27.8	14.1	Ace: K27	15305.4534	C671H1131N215O186S4

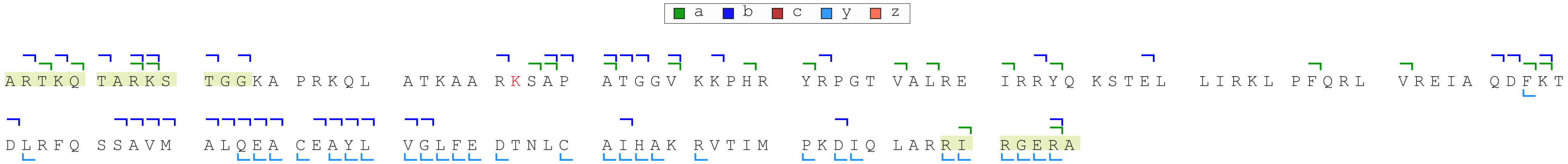

Proteoform:

Index	Protein	SC [%]	SVP [%]	IC [%]	MS Score [a.u.]	Var. Mods	Mono Mass [Da]	Chemical Formula
7	H31Kac1	47.0	14.8	26.3	12.4	Ace: K36	15305.4534	C671H1131N215O186S4

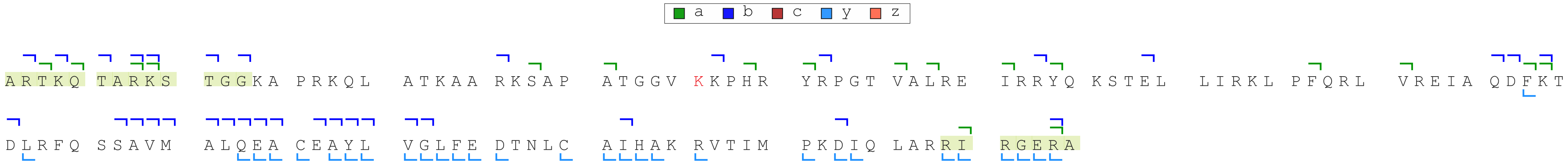

Proteoform:	Index	Protein	SC [%]	SVP [%]	IC [%]	MS Score [a.u.]	Var. Mods	Mono Mass [Da]	Chemical Formula
	8	H31Kac1	47.0	14.8	26.3	12.4	Ace: K37	15305.4534	C671H1131N215O186S4

a b c y z

ARTKQ TARKS TGGKA PRKQL ATKAA RKSAP ATGGV KKPHR YRPGT VALRE IRRYQ KSTEL LIRKL PFQRL VREIA QDFKT
DLRFQ SSAVM ALQEA CEAYL VGLFE DTNLC AIHAK RVTIM PKDIQ LARRI RGERA

Top-Down Result OmniScape 2025
Sequence Confirmation
241210 H31-GNC5_10 z19 MS2 ECD 20ms MS3 shCID z18d_001-366_calibrated

Proteoform:

Index	Protein	SC [%]	SVP [%]	IC [%]	MS Score [a.u.]	Var. Mods	Mono Mass [Da]	Chemical Formula
9	H31Kac1	41.8	14.8	24.5	10.2	Ace: K56	15305.4534	C671H1131N215O186S4

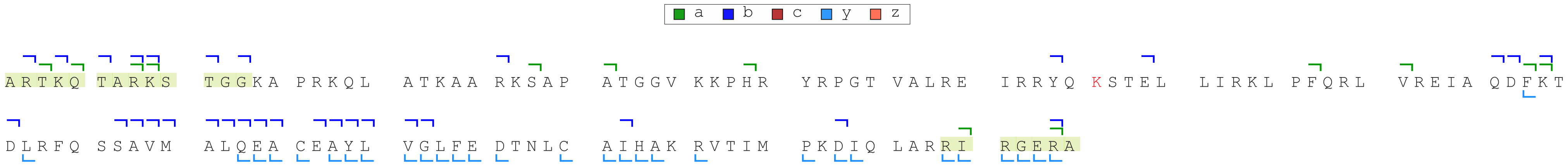

Proteoform:

Index	Protein	SC [%]	SVP [%]	IC [%]	MS Score [a.u.]	Var. Mods	Mono Mass [Da]	Chemical Formula
10	H31Kac1	41.0	14.8	24.4	10.0	Ace: K64	15305.4534	C671H1131N215O186S4

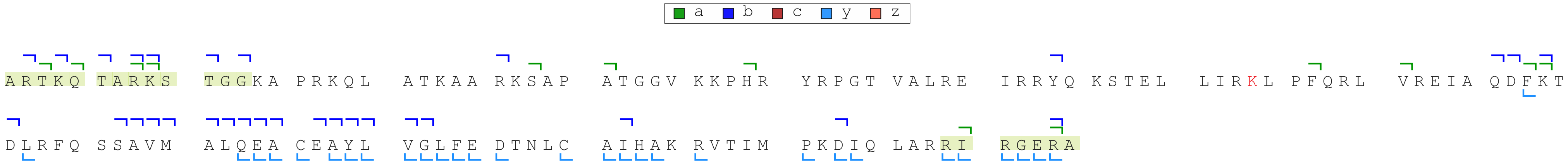

Error Plot

Proteoform:

Index	Protein	SC [%]	SVP [%]	IC [%]	MS Score [a.u.]	Var. Mods	Mono Mass [Da]	Chemical Formula
1	H31Kac1	56.0	17.8	28.5	16.0	Ace: K14	15305.4534	C671H1131N215O186S4

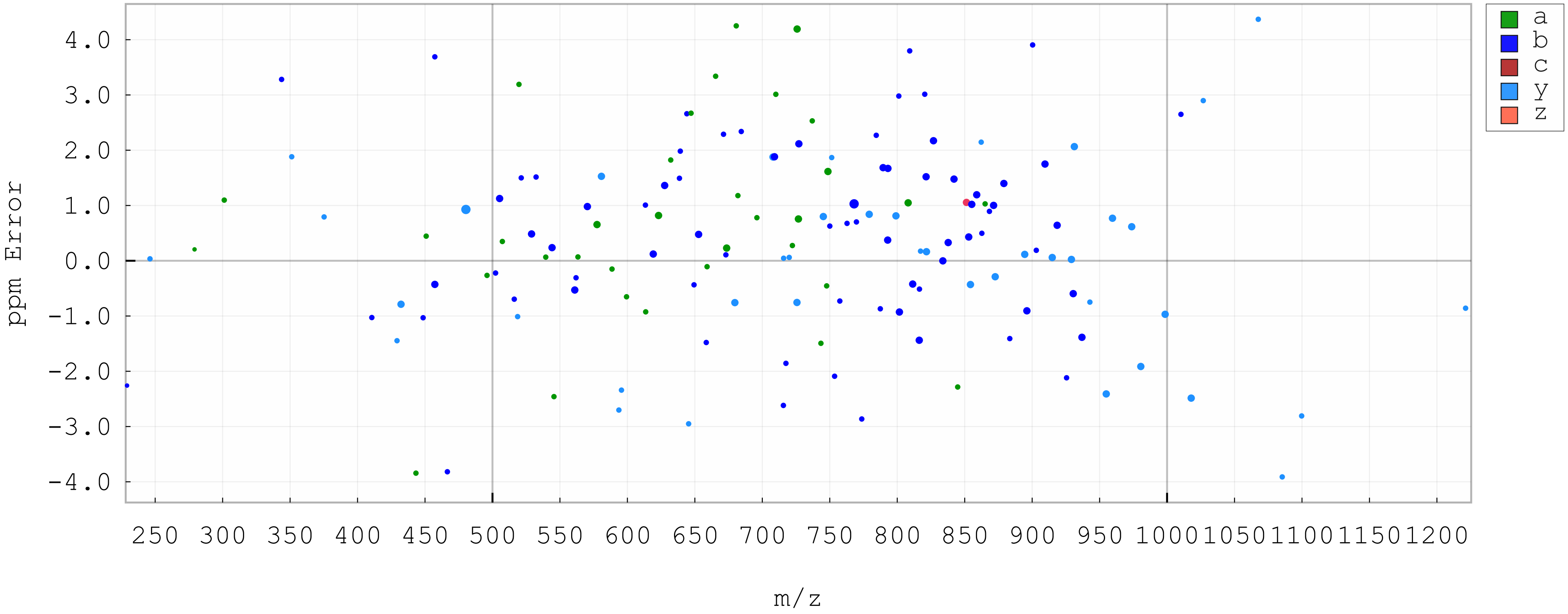

Proteoform:	Index	Protein	SC [%]	SVP [%]	IC [%]	MS Score [a.u.]	Var. Mods	Mono Mass [Da]	Chemical Formula
	2	H31Kac1	53.7	11.1	28.4	15.3	Ace: K9	15305.4534	C671H1131N215O186S4

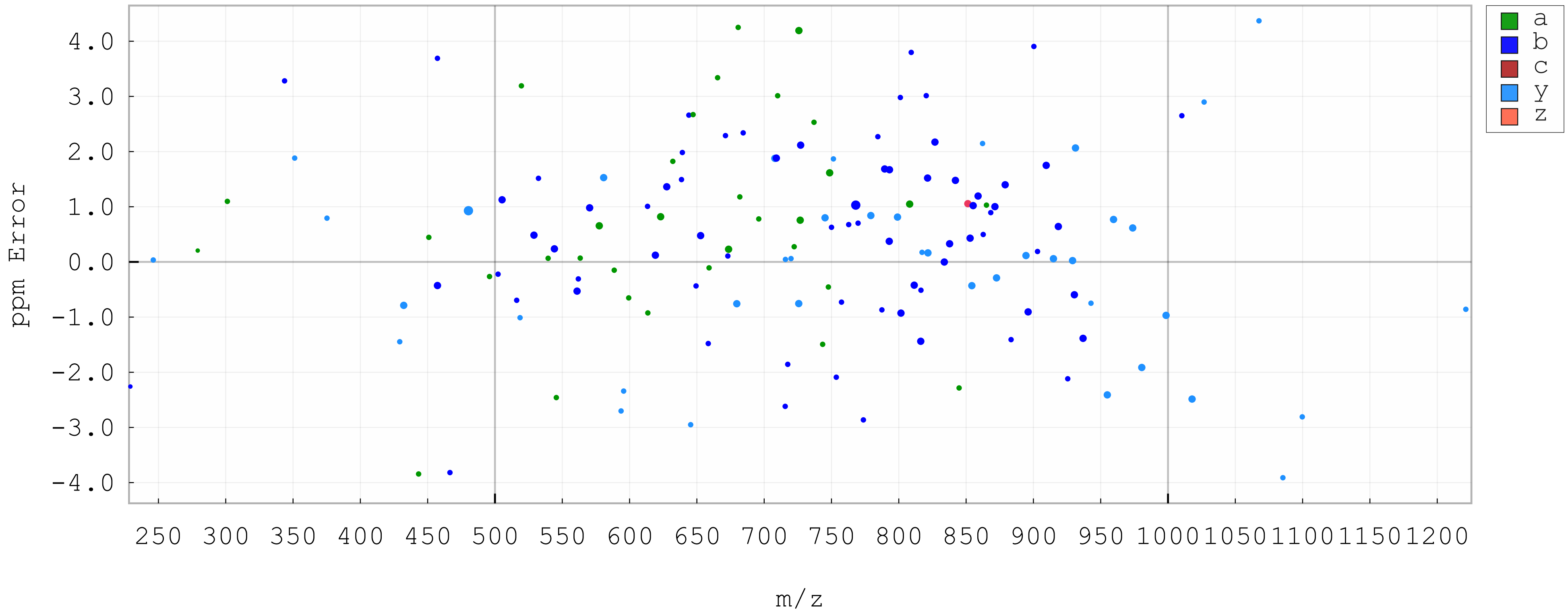

Proteoform:

Index	Protein	SC [%]	SVP [%]	IC [%]	MS Score [a.u.]	Var. Mods	Mono Mass [Da]	Chemical Formula
3	H31Kac1	53.7	14.8	28.2	15.2	Ace: K18	15305.4534	C671H1131N215O186S4

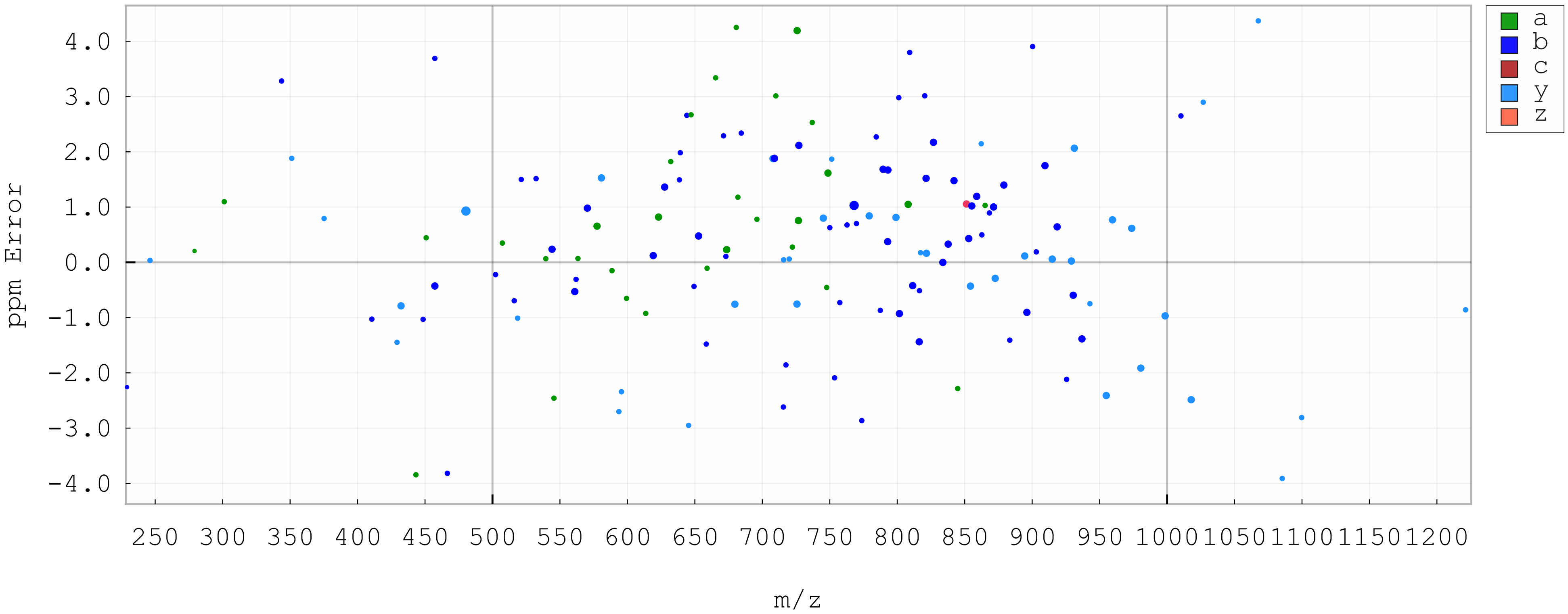

Proteoform:

Index	Protein	SC [%]	SVP [%]	IC [%]	MS Score [a.u.]	Var. Mods	Mono Mass [Da]	Chemical Formula
4	H31Kac1	51.5	7.4	28.3	14.6	Ace: K4	15305.4534	C671H1131N215O186S4

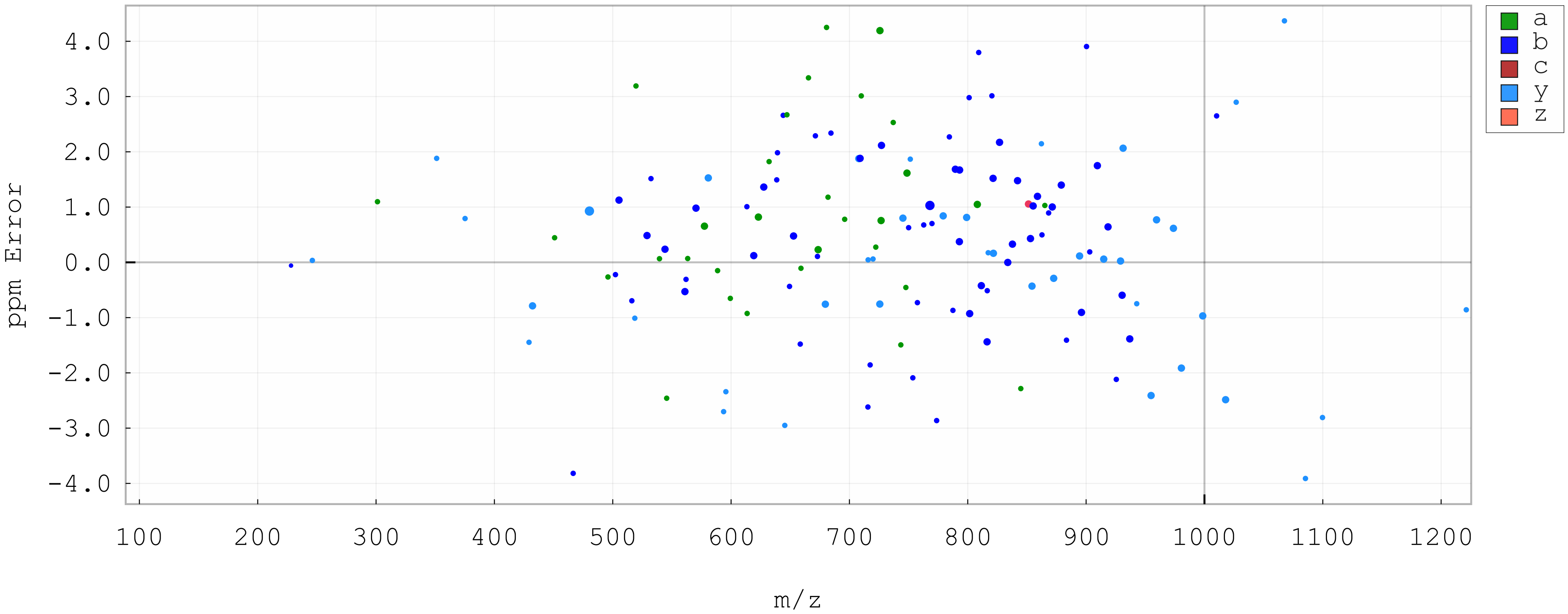

Proteoform:

Index	Protein	SC [%]	SVP [%]	IC [%]	MS Score [a.u.]	Var. Mods	Mono Mass [Da]	Chemical Formula
5	H31Kac1	51.5	14.8	27.9	14.4	Ace: K23	15305.4534	$C_{671}H_{1131}N_{215}O_{186}S_4$

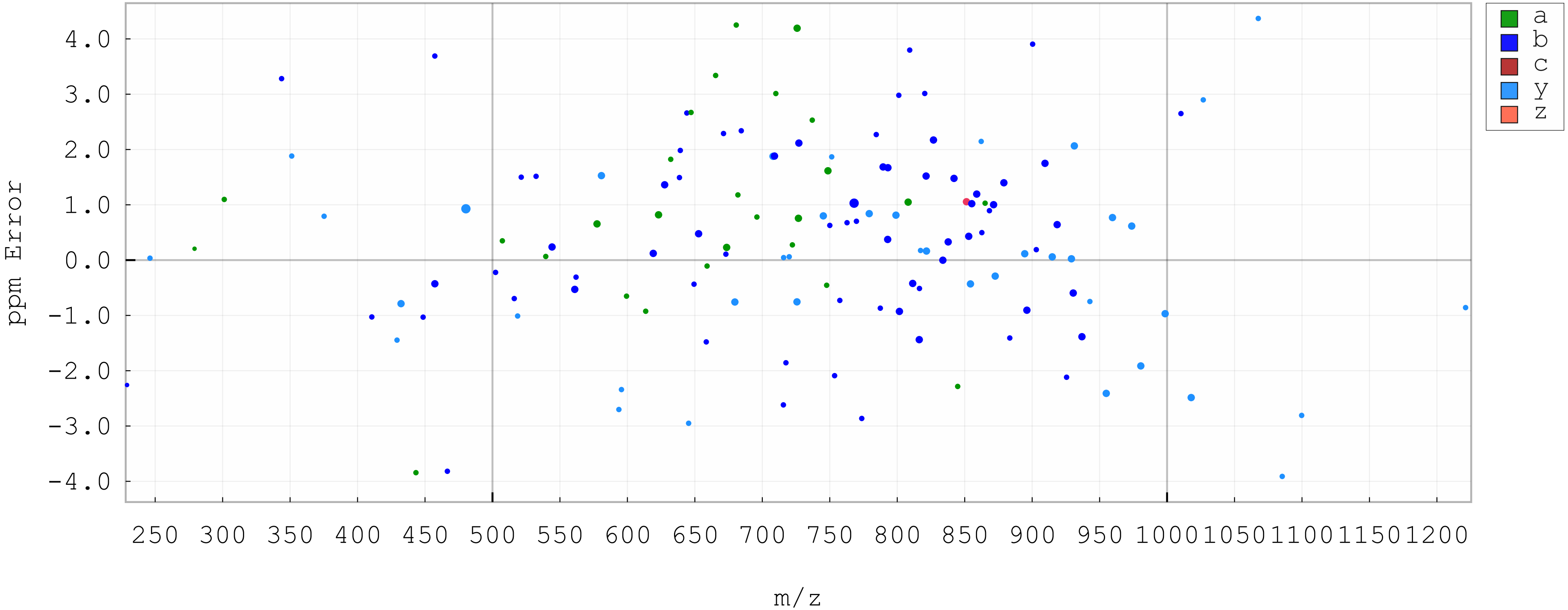

Proteoform:	Index	Protein	SC [%]	SVP [%]	IC [%]	MS Score [a.u.]	Var. Mods	Mono Mass [Da]	Chemical Formula
	6	H31Kac1	50.7	14.8	27.8	14.1	Ace: K27	15305.4534	C671H1131N215O186S4

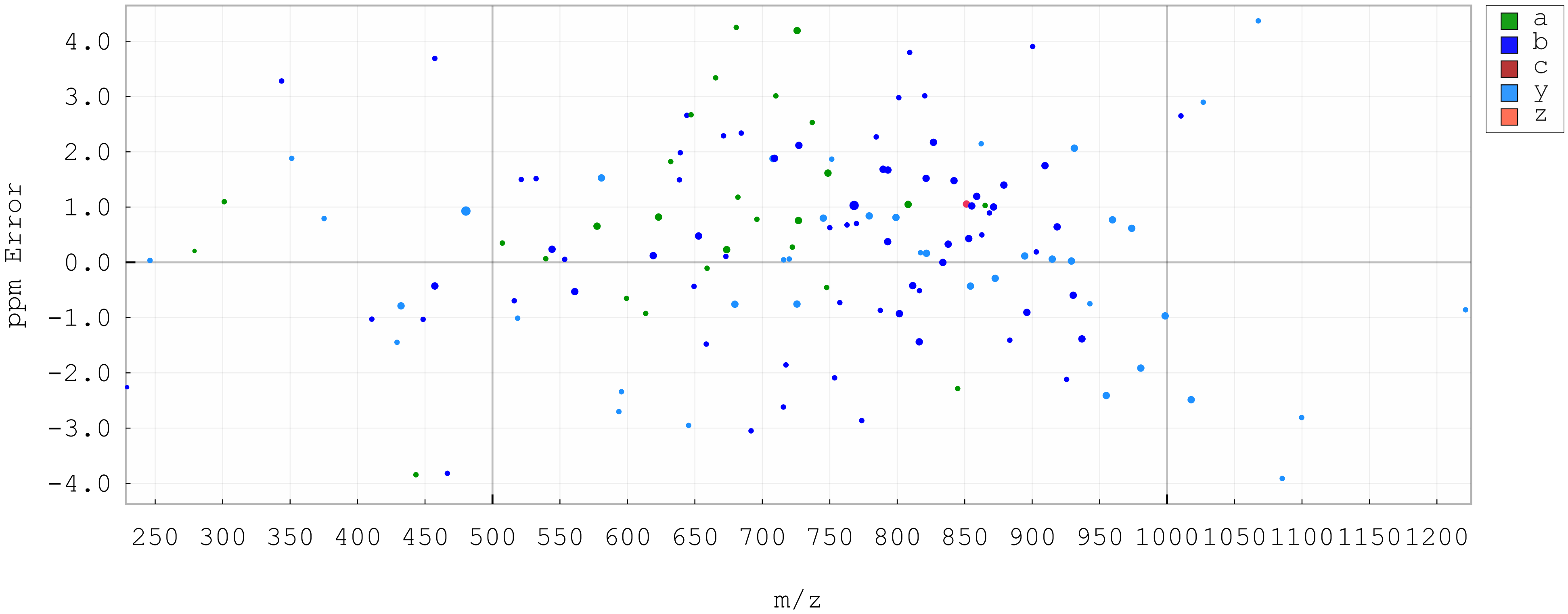

Proteoform:	Index	Protein	SC [%]	SVP [%]	IC [%]	MS Score [a.u.]	Var. Mods	Mono Mass [Da]	Chemical Formula
	7	H31Kac1	47.0	14.8	26.3	12.4	Ace: K36	15305.4534	C671H1131N215O186S4

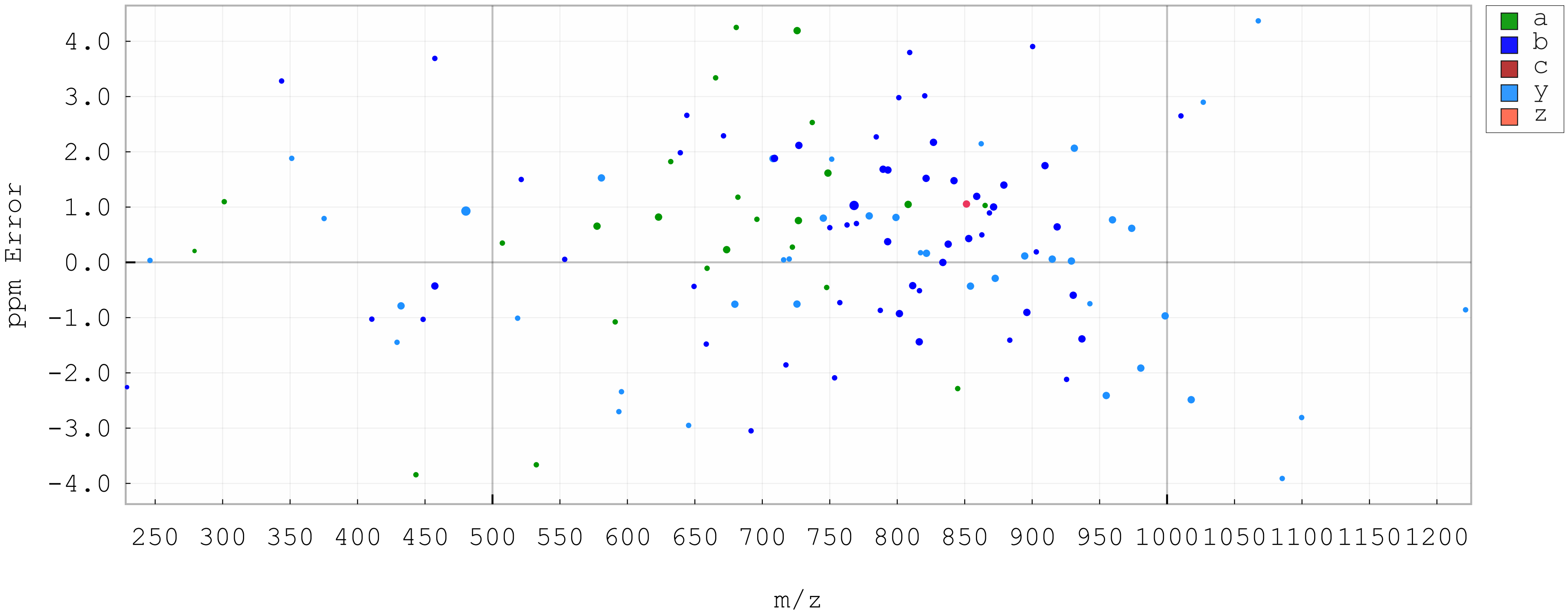

Proteoform:

Index	Protein	SC [%]	SVP [%]	IC [%]	MS Score [a.u.]	Var. Mods	Mono Mass [Da]	Chemical Formula
8	H31Kac1	47.0	14.8	26.3	12.4	Ace: K37	15305.4534	C671H1131N215O186S4

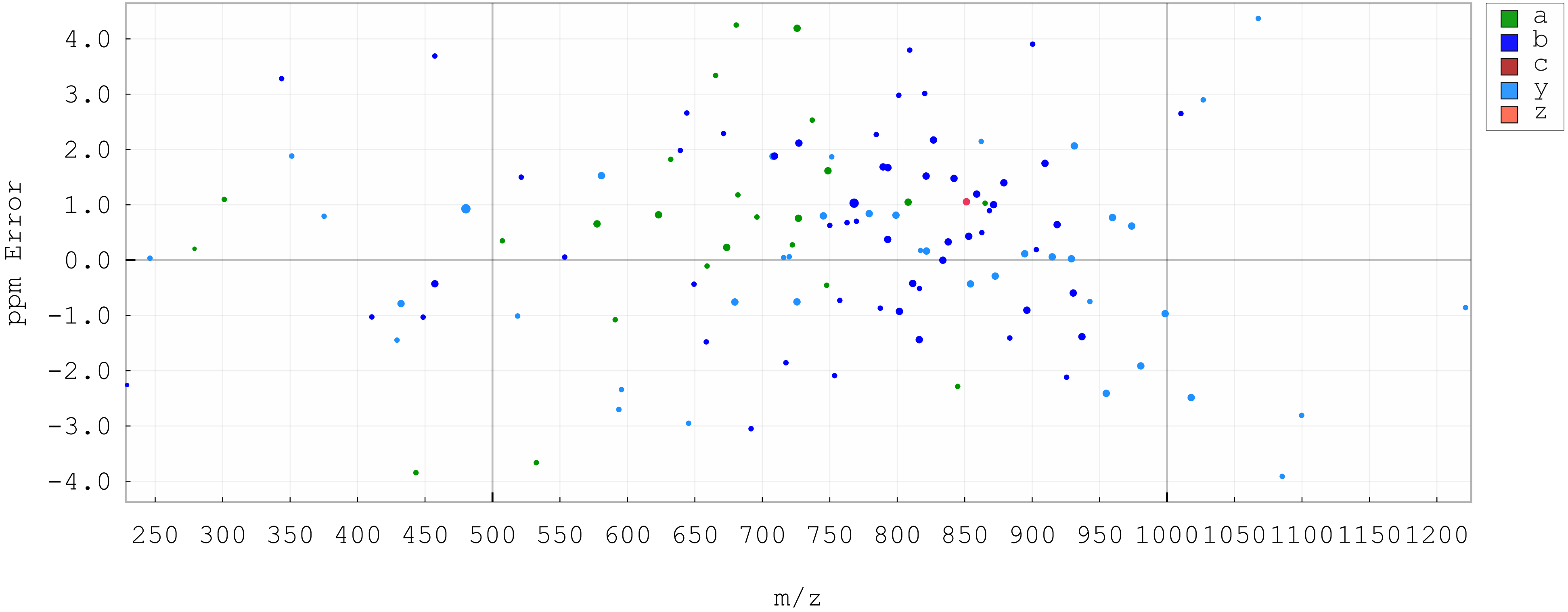

Proteoform:

Index	Protein	SC [%]	SVP [%]	IC [%]	MS Score [a.u.]	Var. Mods	Mono Mass [Da]	Chemical Formula
9	H31Kac1	41.8	14.8	24.5	10.2	Ace: K56	15305.4534	$C_{671}H_{1131}N_{215}O_{186}S_4$

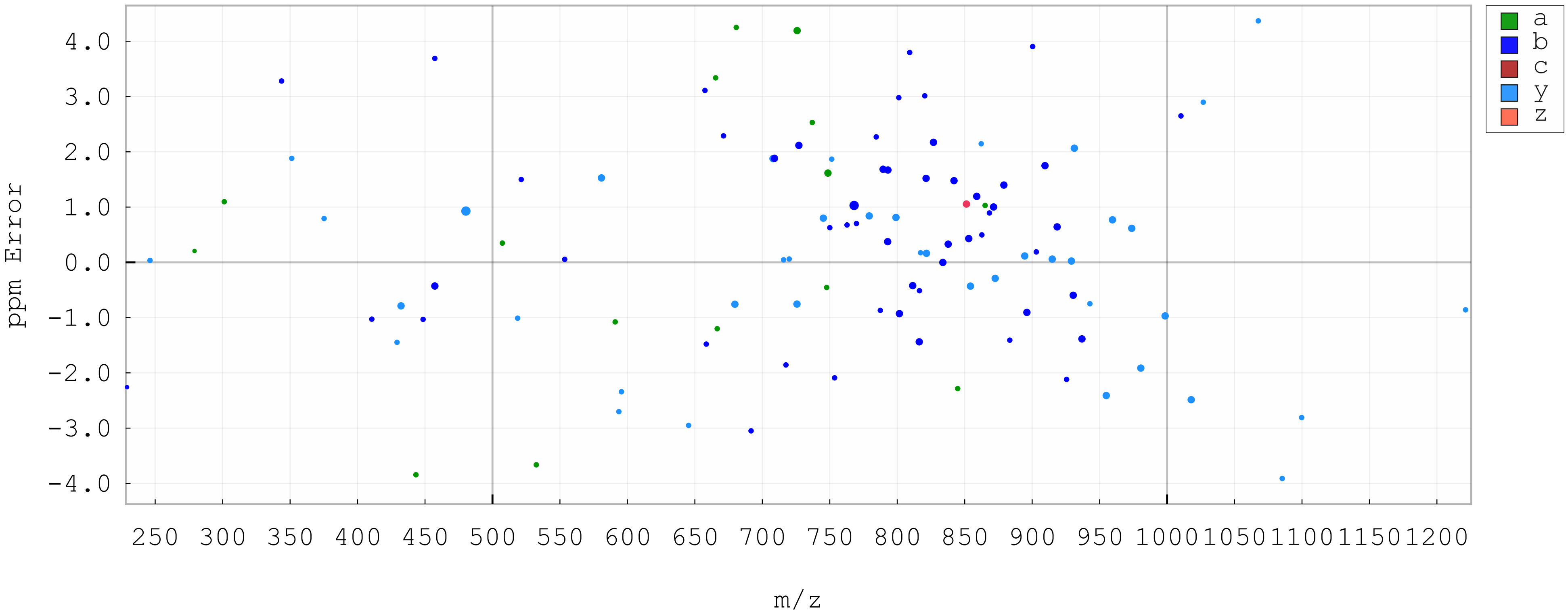

Proteoform:	Index	Protein	SC [%]	SVP [%]	IC [%]	MS Score [a.u.]	Var. Mods	Mono Mass [Da]	Chemical Formula
	10	H31Kac1	41.0	14.8	24.4	10.0	Ace: K64	15305.4534	C671H1131N215O186S4

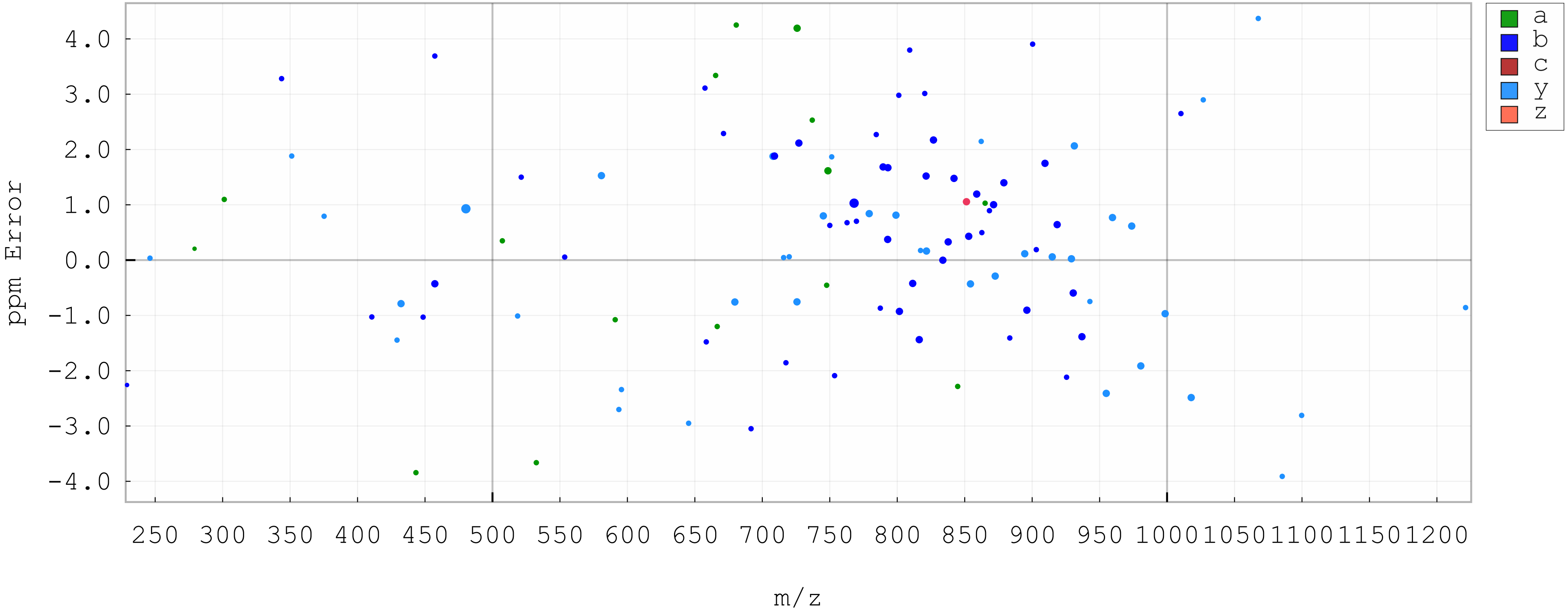

Primary Fragment Plots

Proteoform:

Index	Protein	SC [%]	SVP [%]	IC [%]	MS Score [a.u.]	Var. Mods	Mono Mass [Da]	Chemical Formula
1	H31Kac1	56.0	17.8	28.5	16.0	Ace: K14	15305.4534	C671H1131N215O186S4

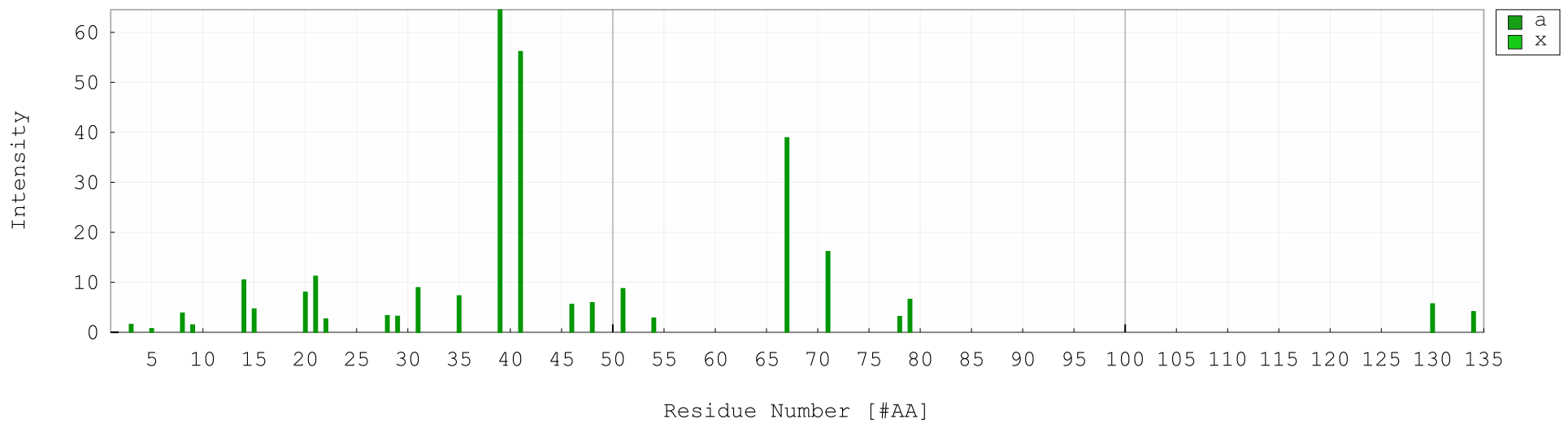

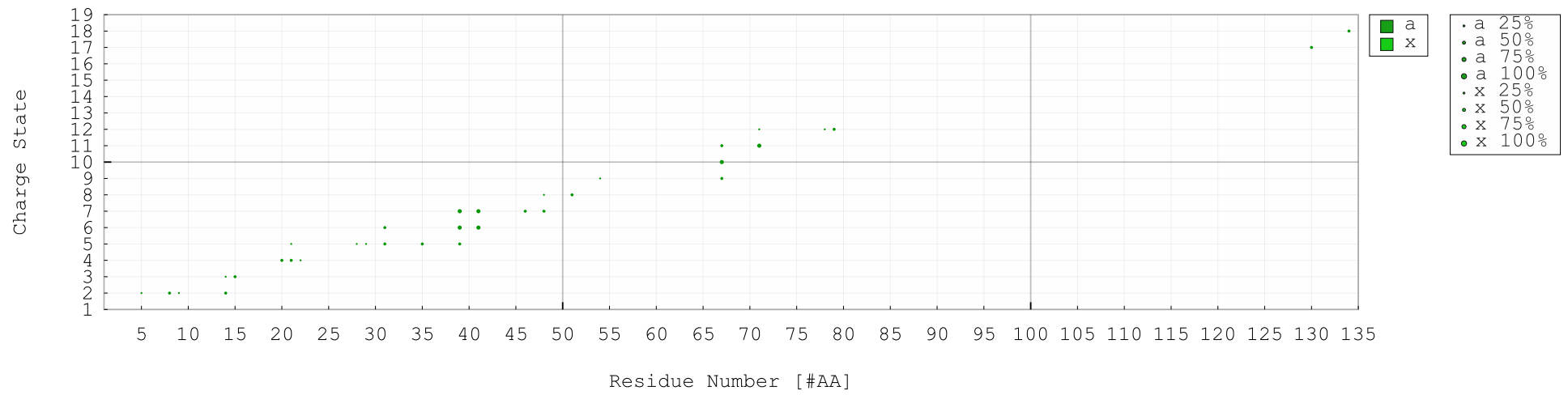

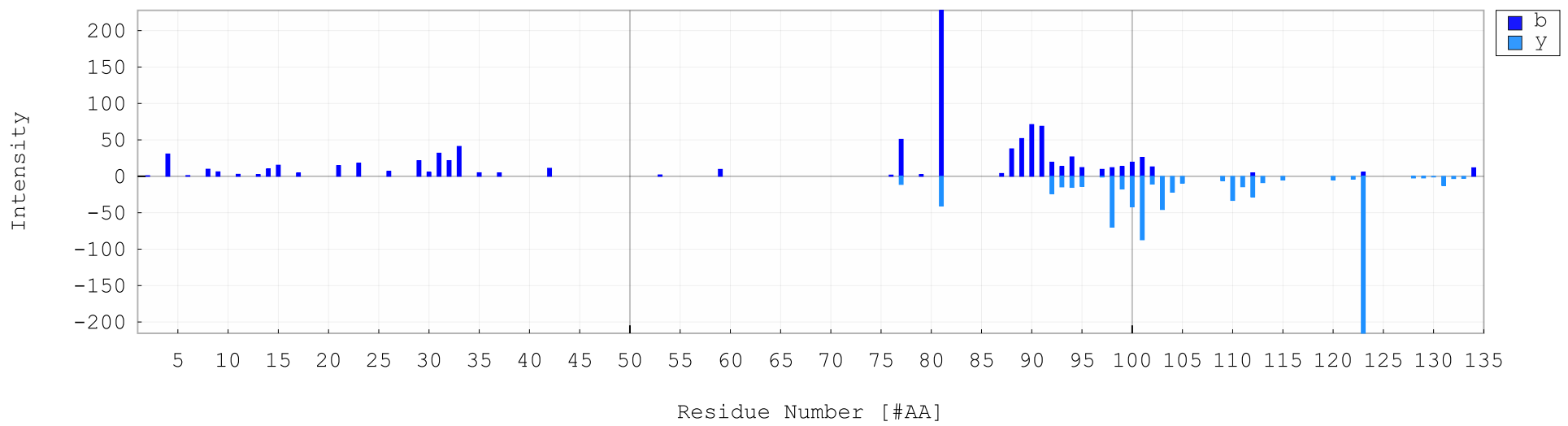

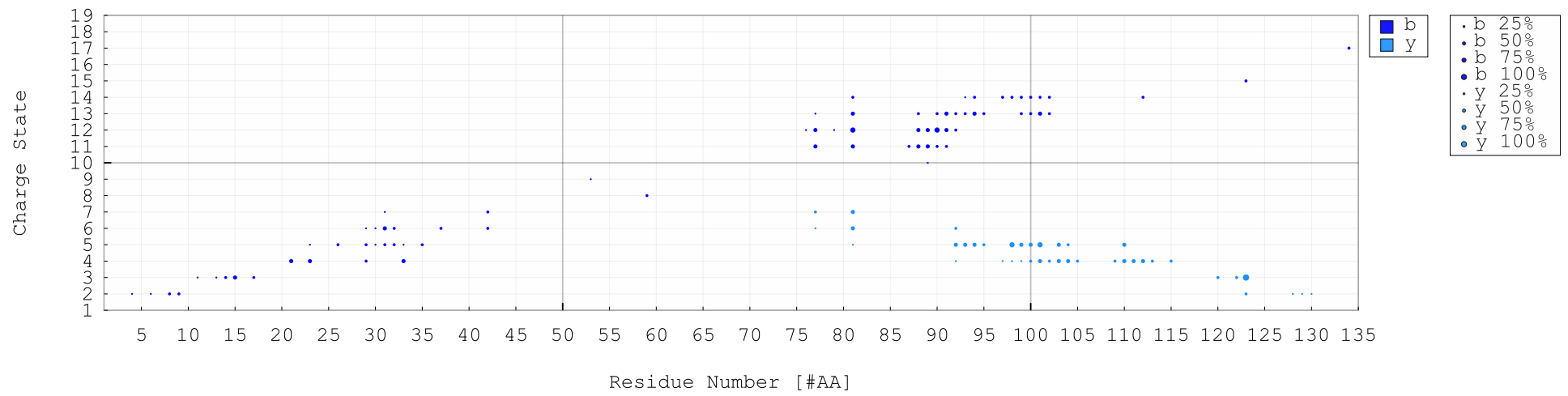

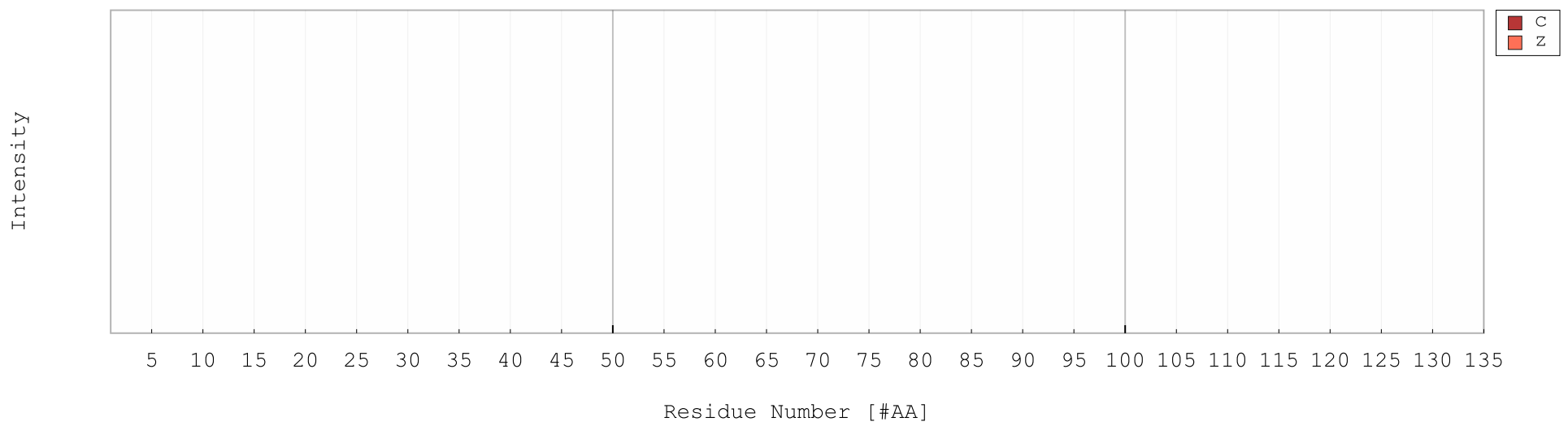

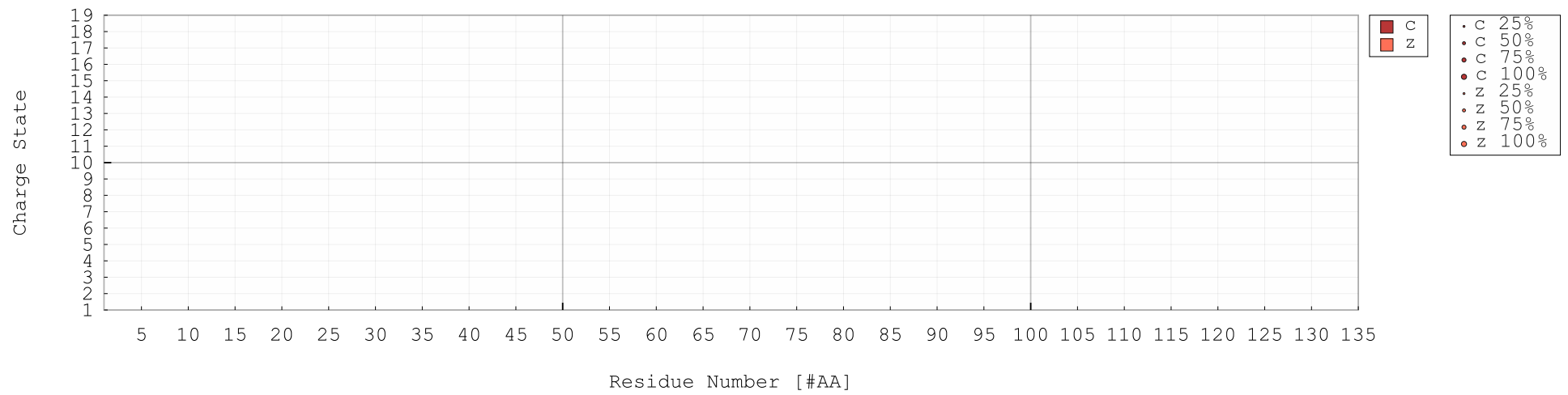

Proteoform:

Index	Protein	SC [%]	SVP [%]	IC [%]	MS Score [a.u.]	Var. Mods	Mono Mass [Da]	Chemical Formula
2	H31Kac1	53.7	11.1	28.4	15.3	Ace: K9	15305.4534	C671H1131N215O186S4

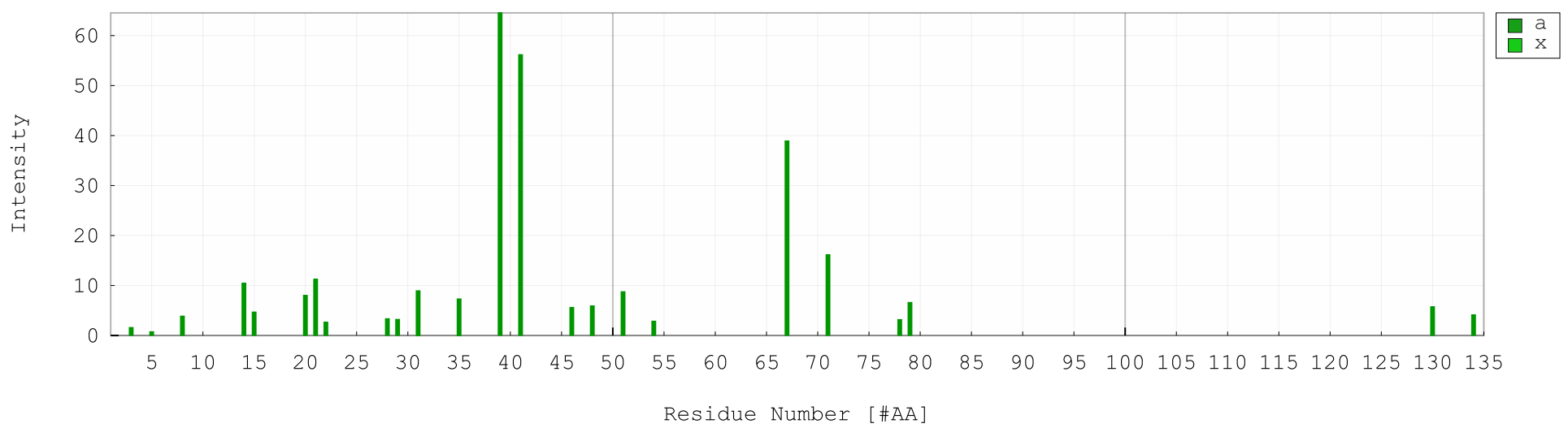

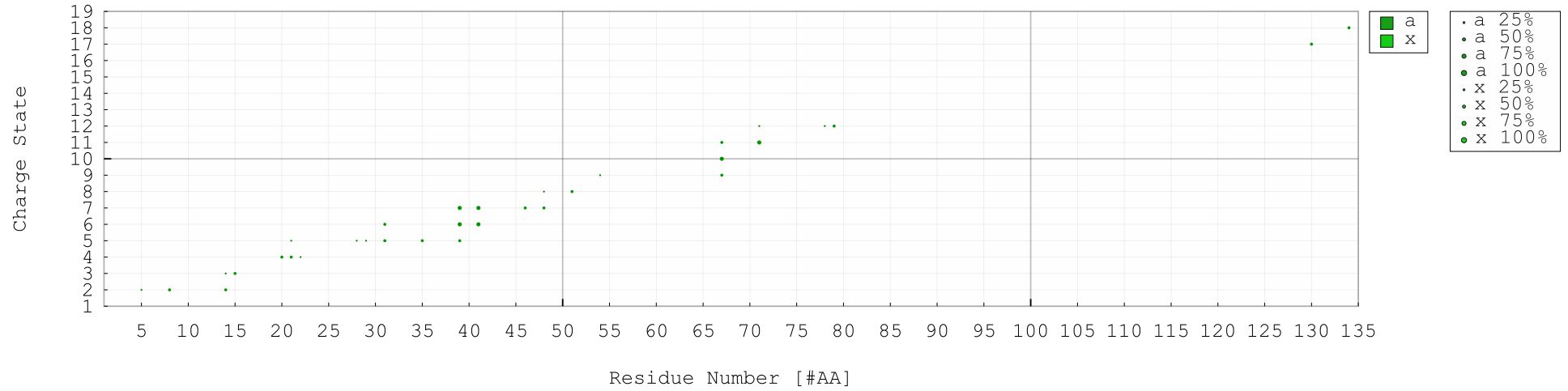

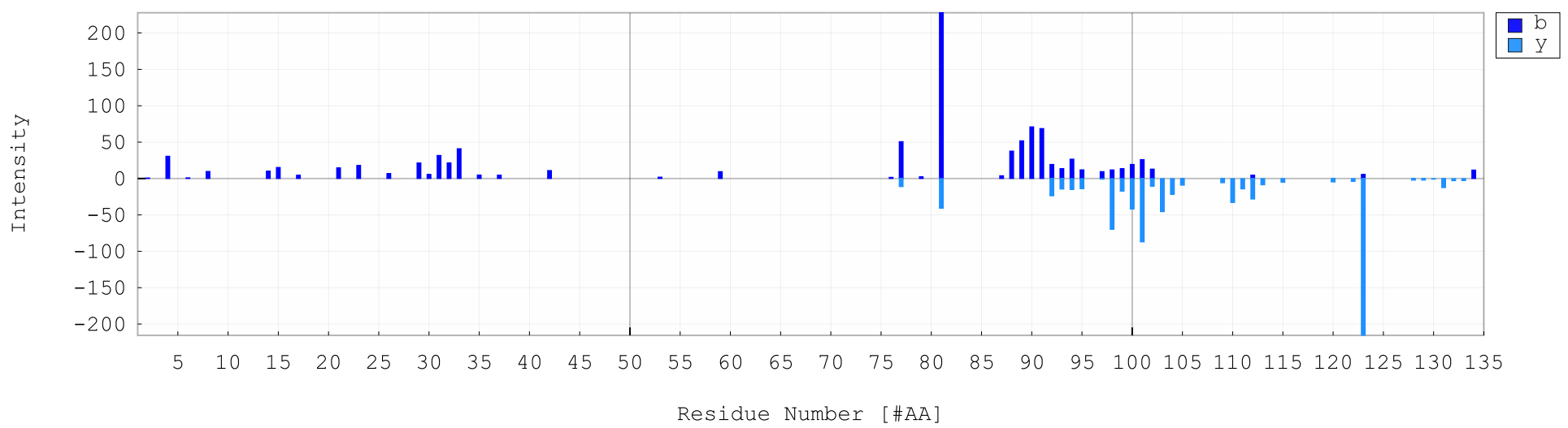

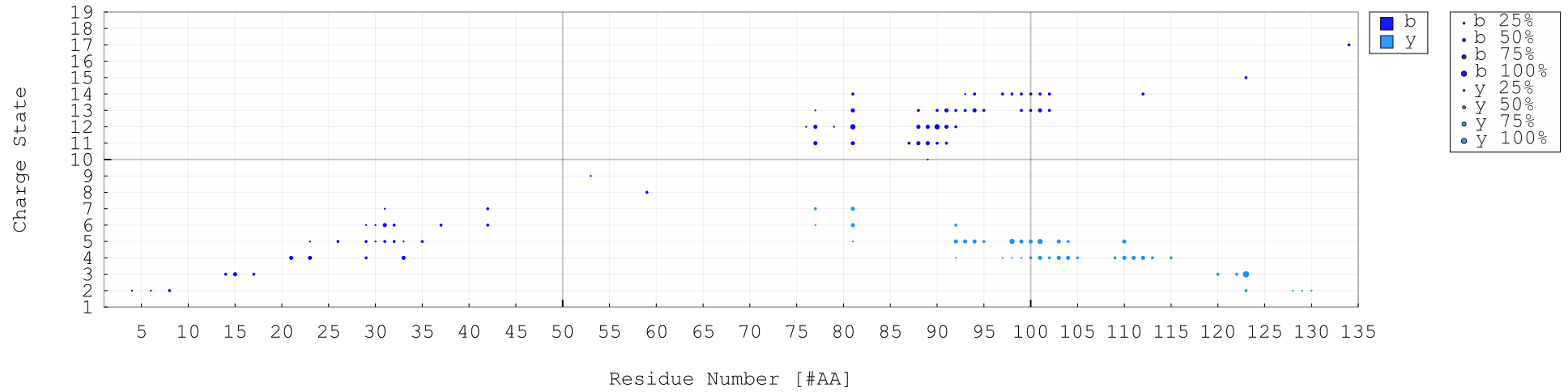

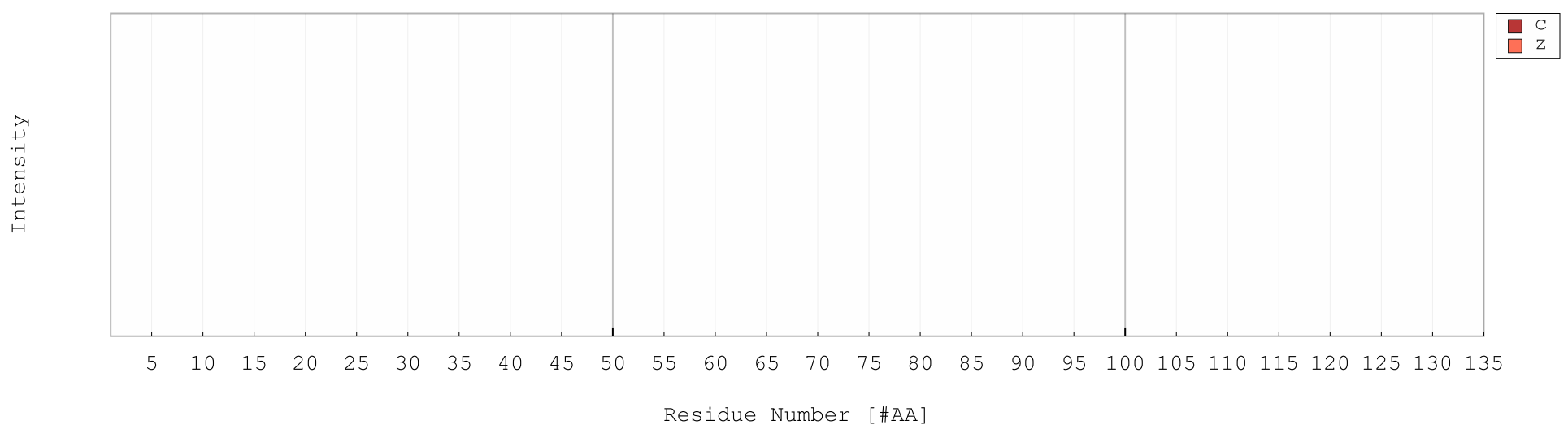

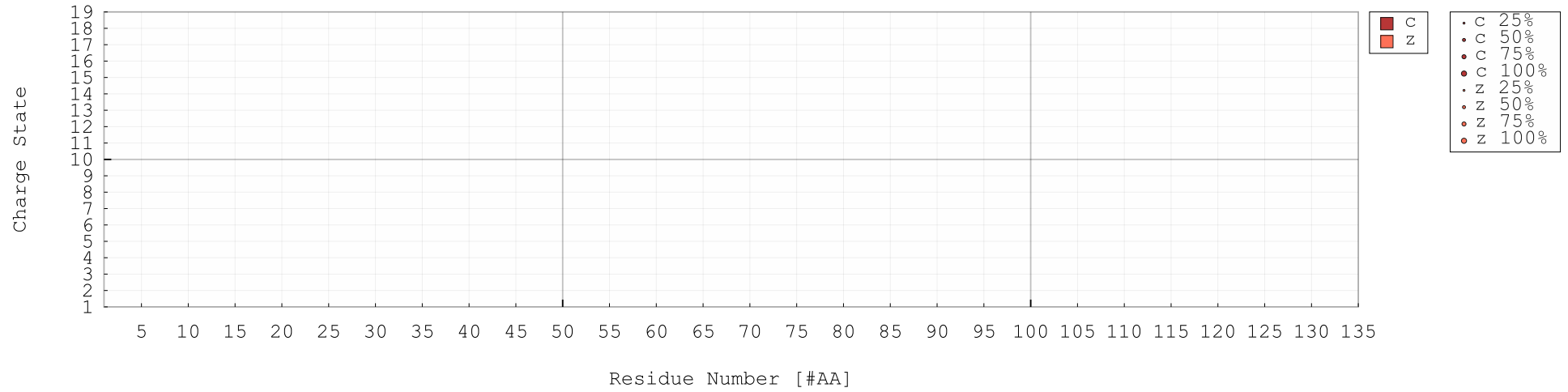

Proteoform:

Index	Protein	SC [%]	SVP [%]	IC [%]	MS Score [a.u.]	Var. Mods	Mono Mass [Da]	Chemical Formula
3	H31Kac1	53.7	14.8	28.2	15.2	Ace: K18	15305.4534	C671H1131N215O186S4

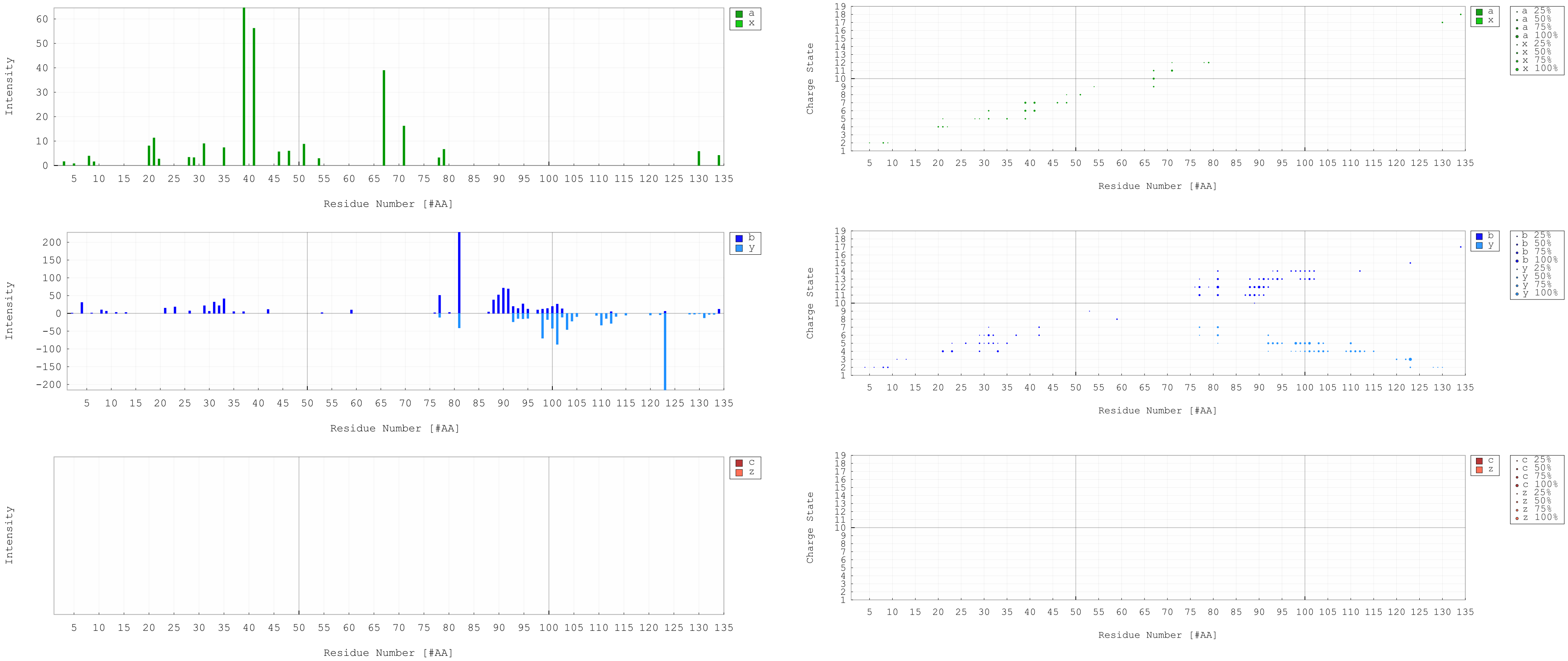

Top-Down Result OmniScape 2025
Sequence Confirmation
241210 H31-GNC5_10 z19 MS2 ECD 20ms MS3 shCID z18d_001-366_cali-
brated

Proteoform:

Index	Protein	SC [%]	SVP [%]	IC [%]	MS Score [a.u.]	Var. Mods	Mono Mass [Da]	Chemical Formula
4	H31Kac1	51.5	7.4	28.3	14.6	Ace: K4	15305.4534	C671H1131N215O186S4

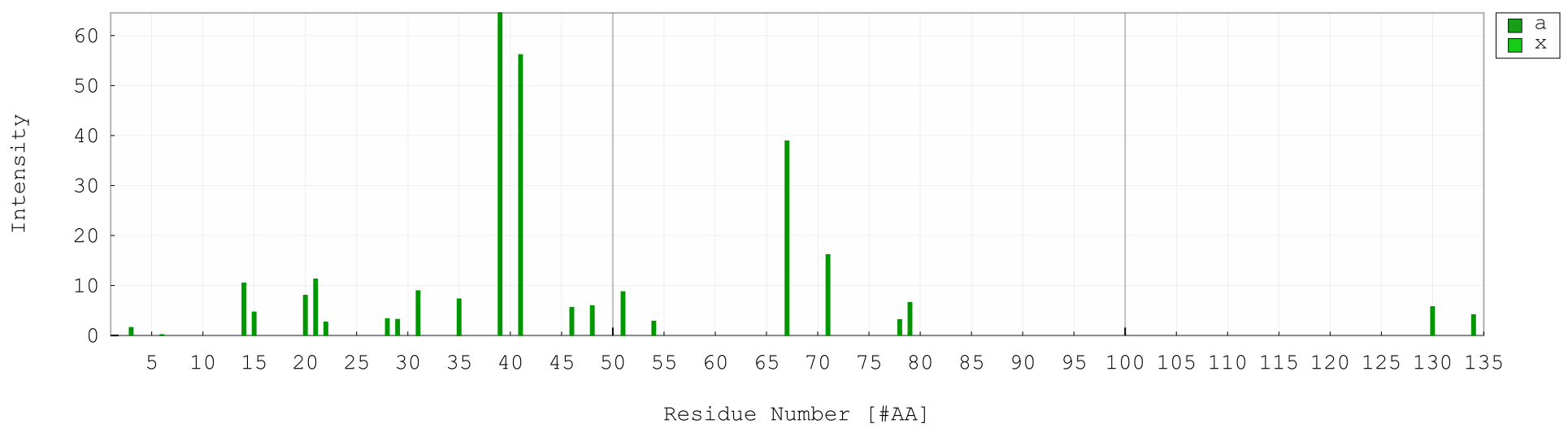

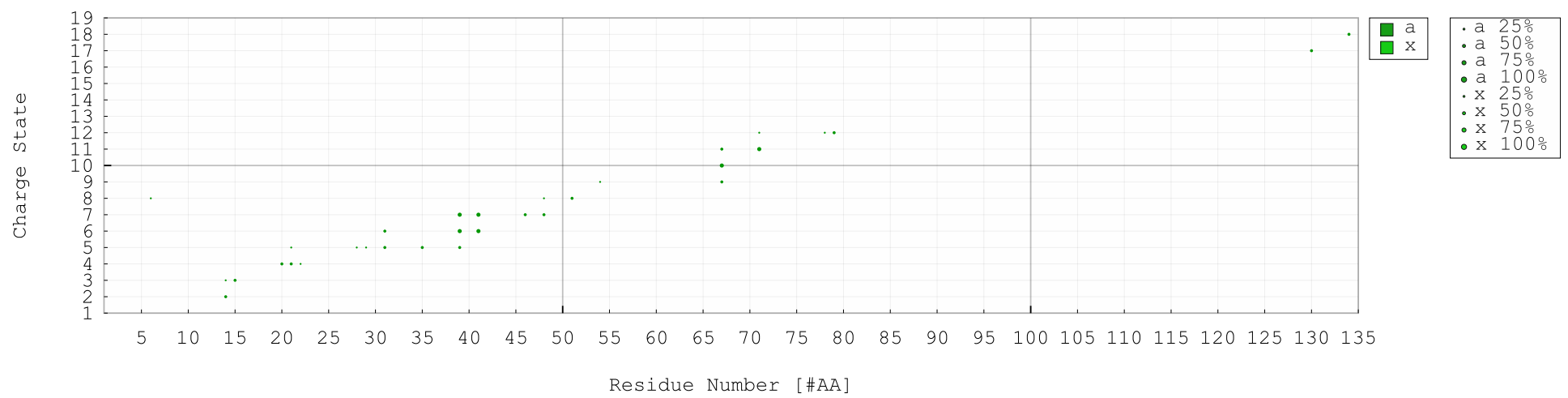

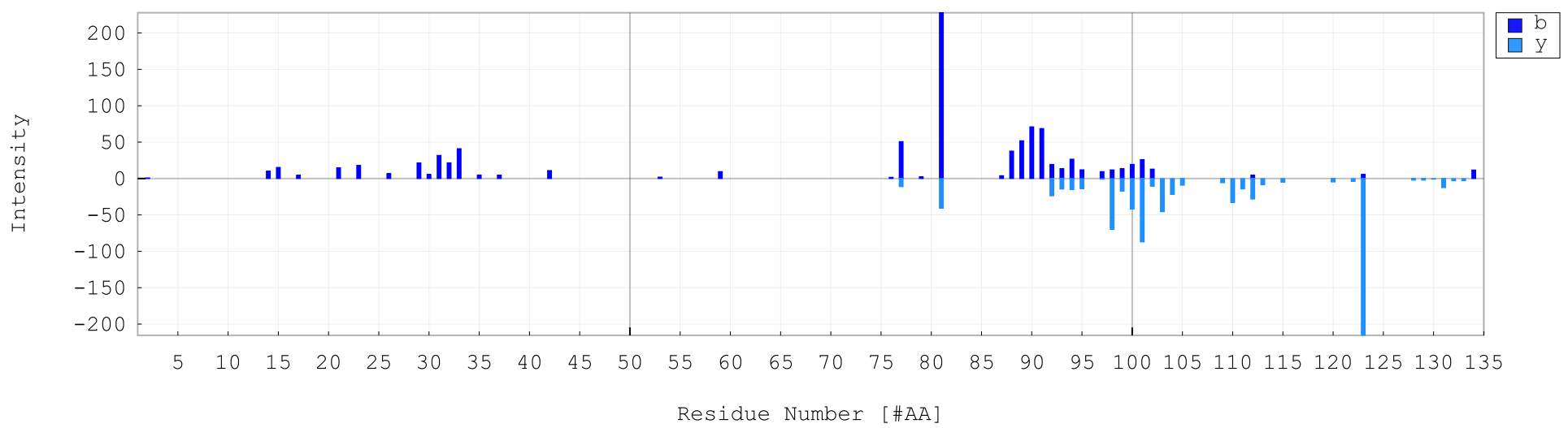

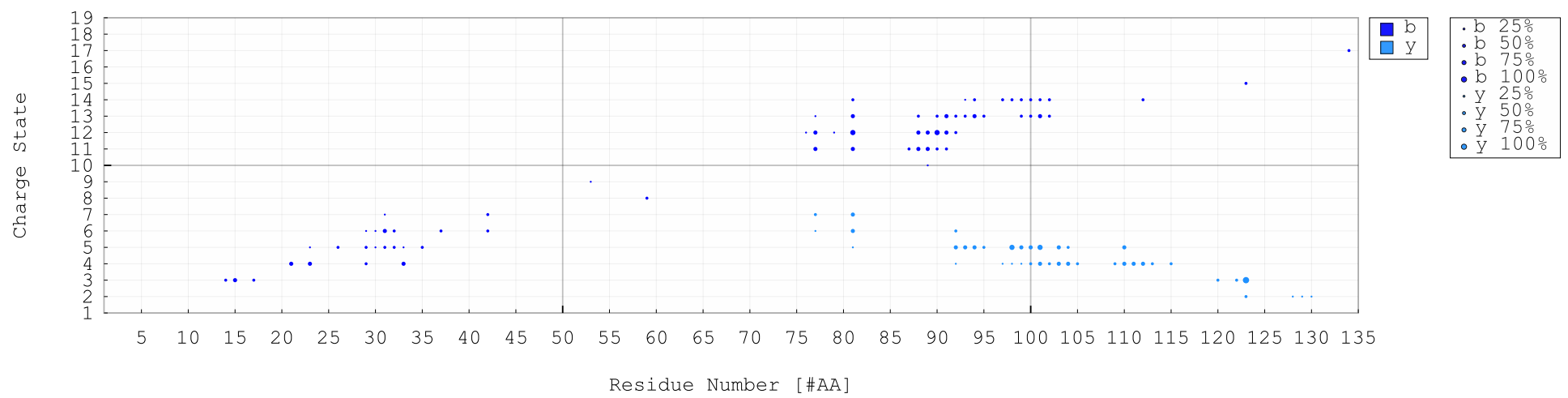

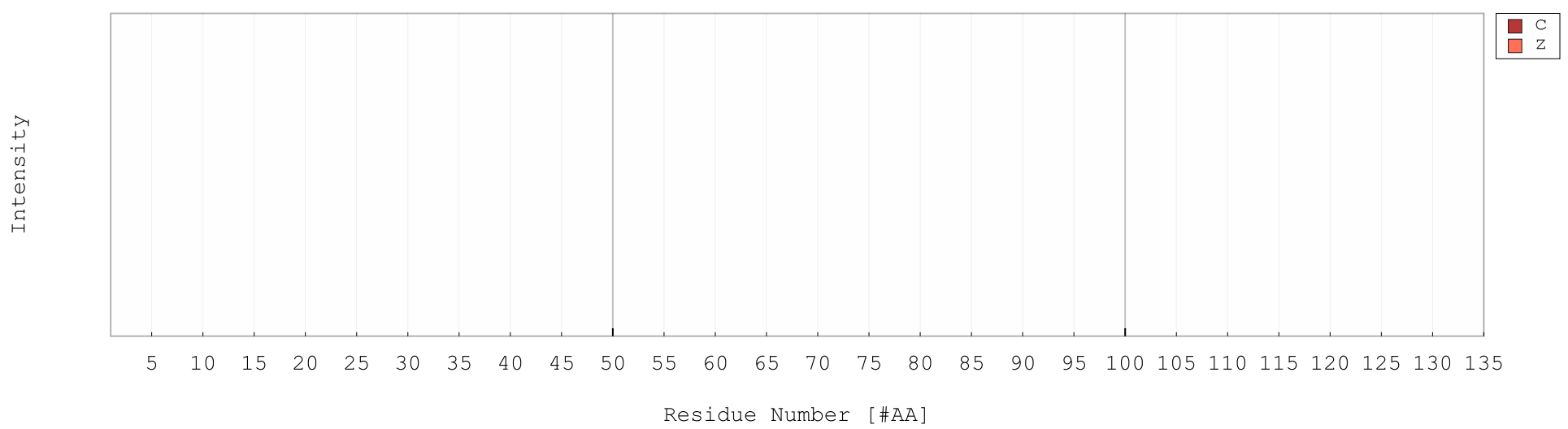

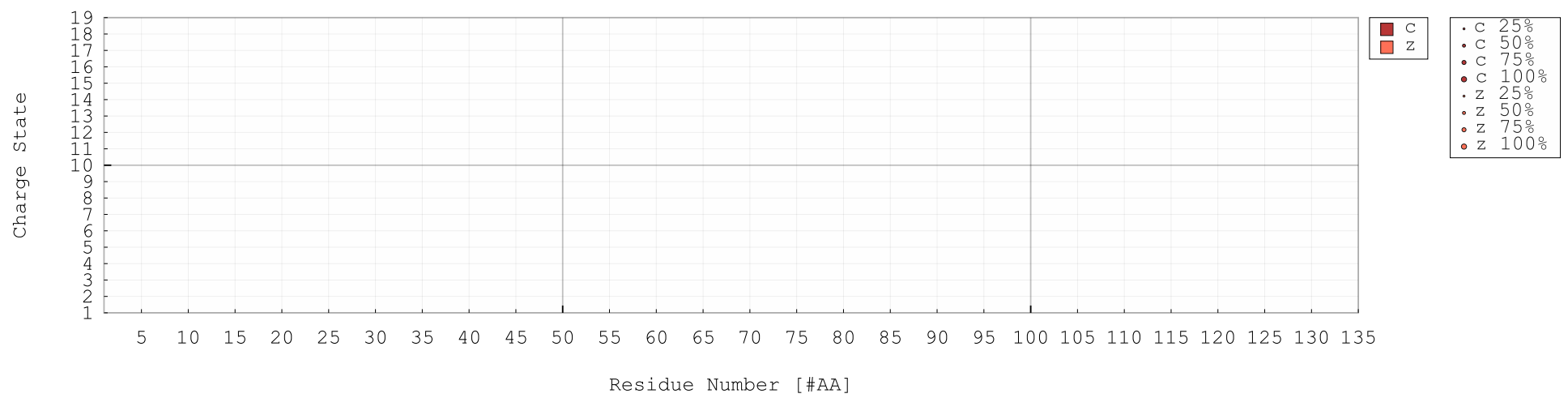

Proteoform:

Index	Protein	SC [%]	SVP [%]	IC [%]	MS Score [a.u.]	Var. Mods	Mono Mass [Da]	Chemical Formula
5	H31Kac1	51.5	14.8	27.9	14.4	Ace: K23	15305.4534	C671H1131N215O186S4

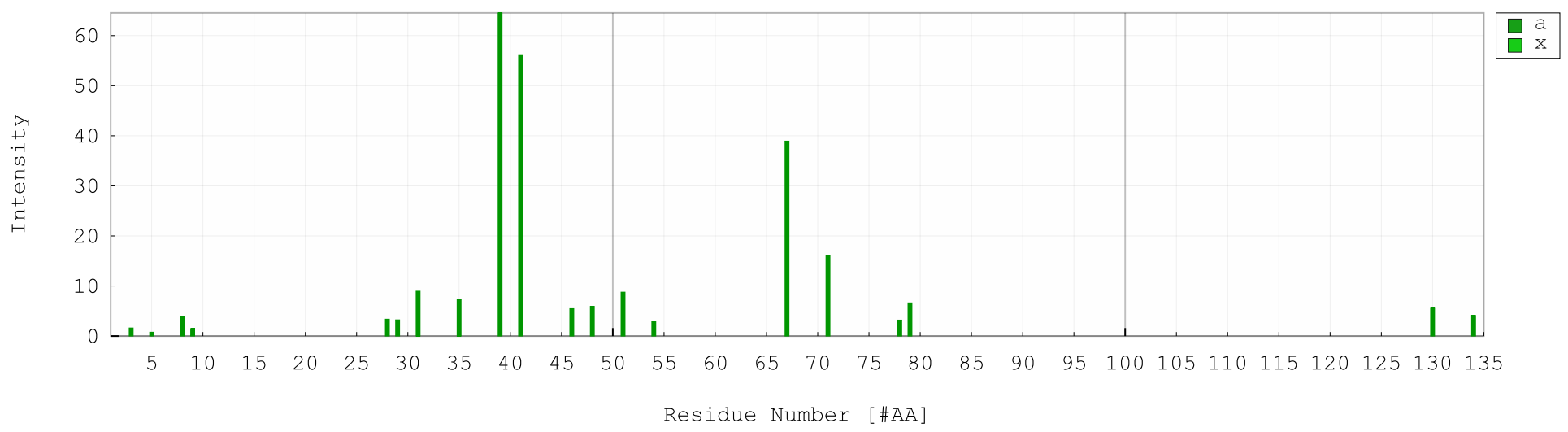

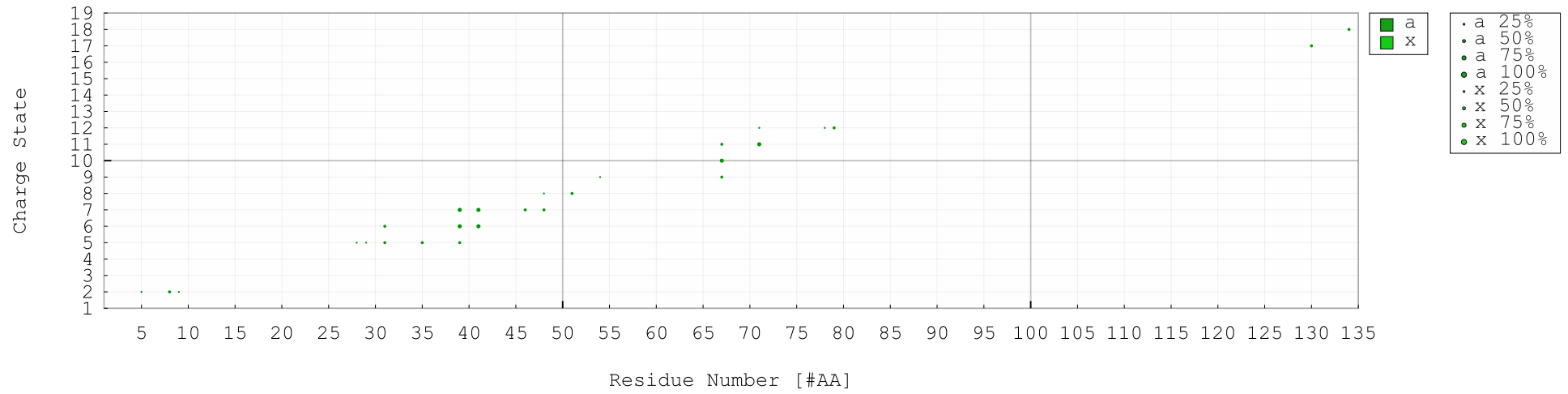

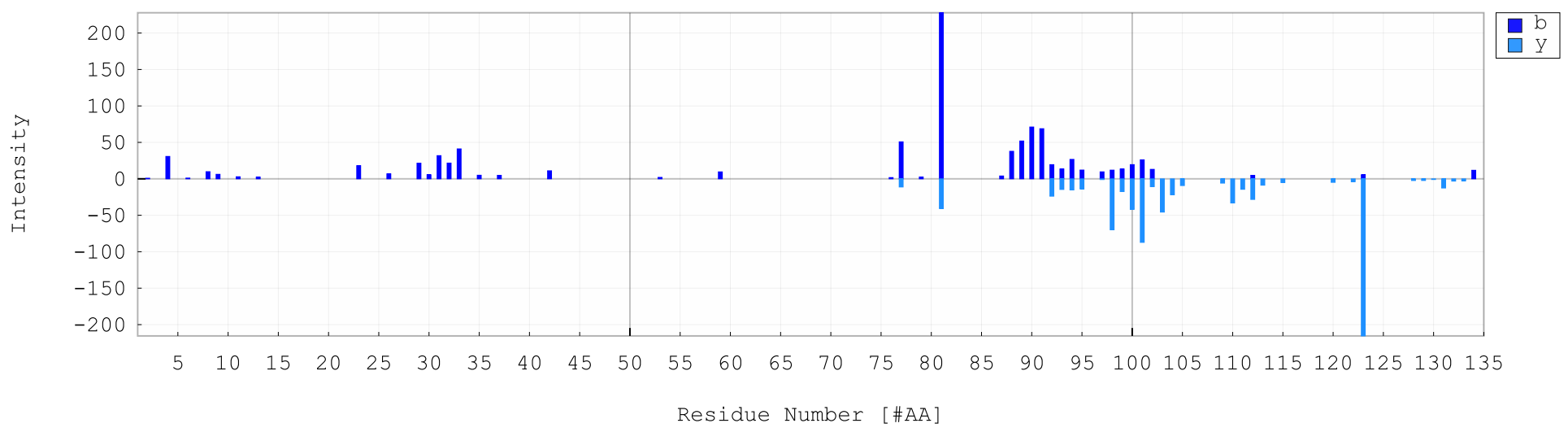

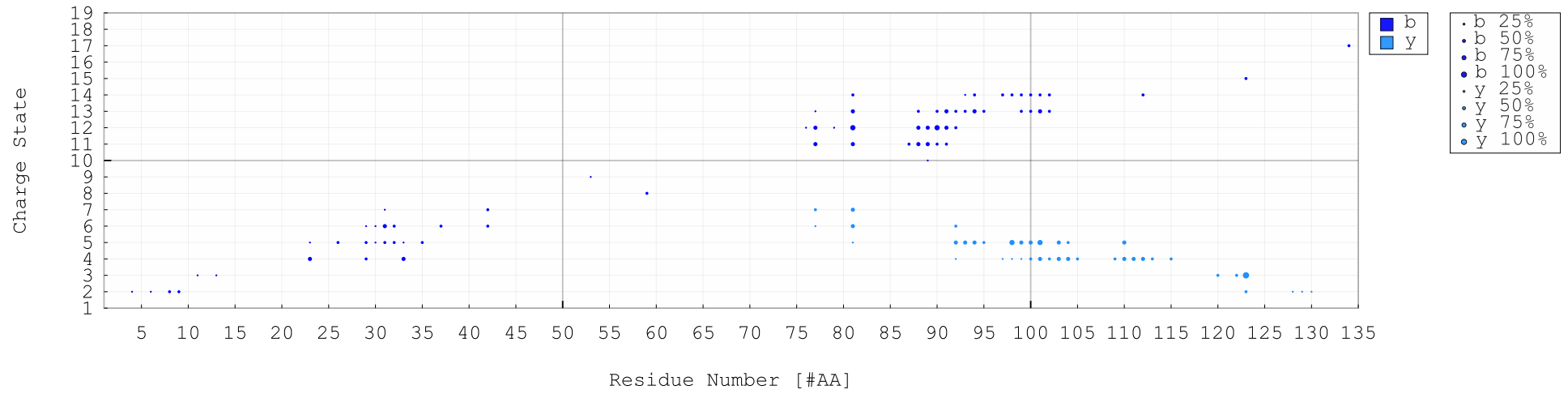

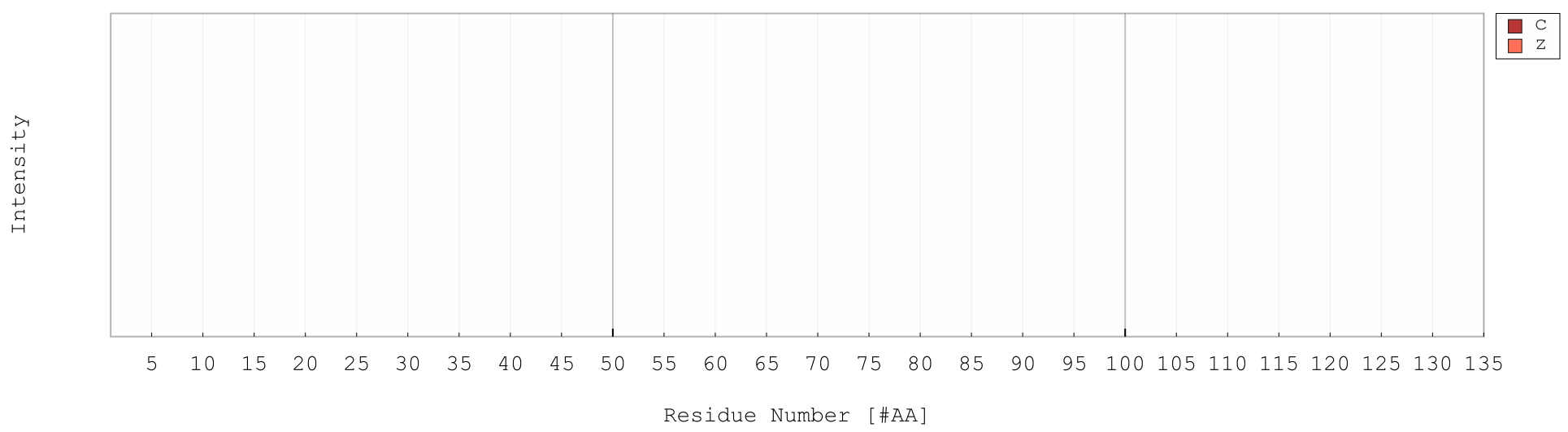

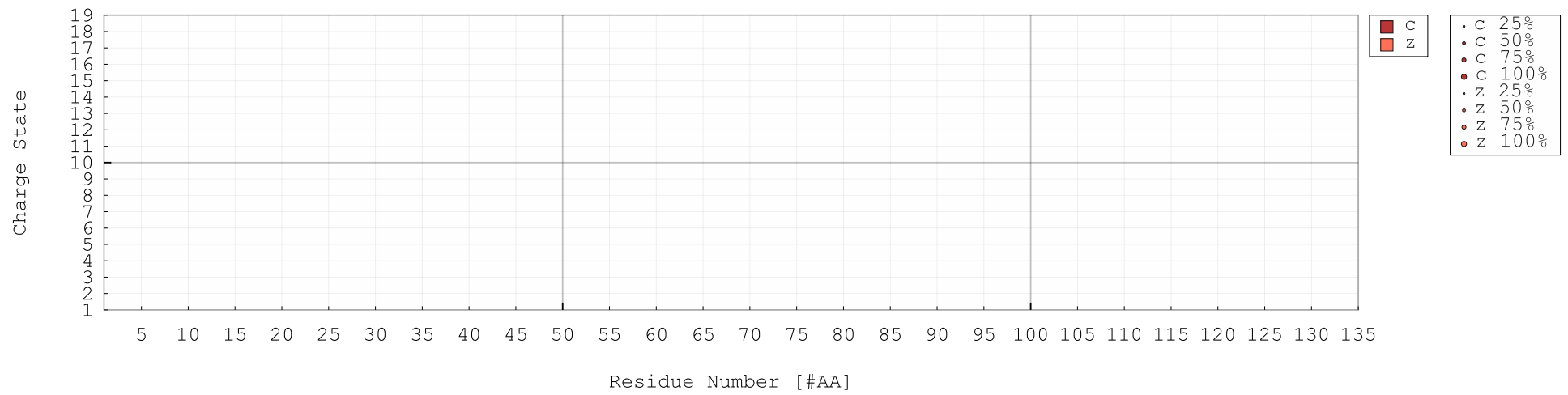

Top-Down Result OmniScape 2025
Sequence Confirmation
241210 H31-GNC5_10 z19 MS2 ECD 20ms MS3 shCID z18d_001-366_cali-brated

Proteoform:

Index	Protein	SC [%]	SVP [%]	IC [%]	MS Score [a.u.]	Var. Mods	Mono Mass [Da]	Chemical Formula
6	H31Kac1	50.7	14.8	27.8	14.1	Ace: K27	15305.4534	C671H1131N215O186S4

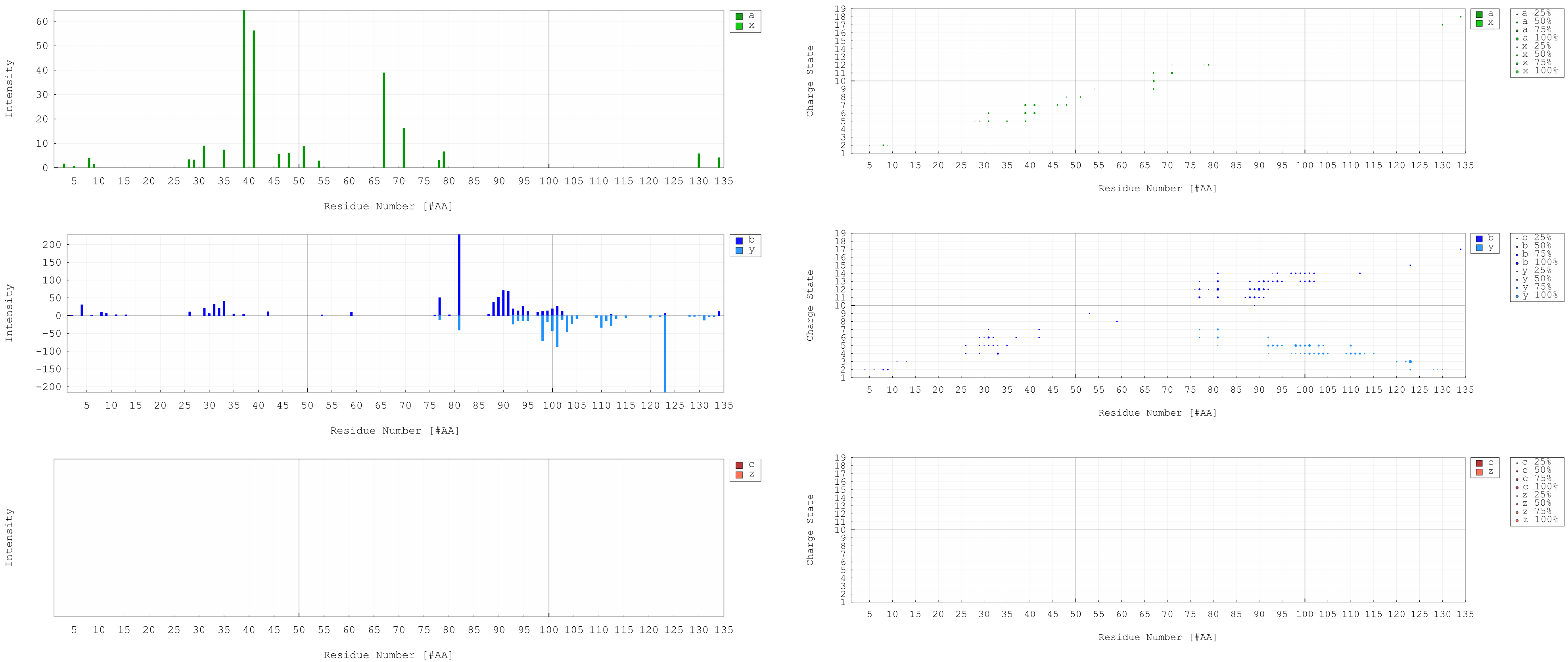

Proteoform:

Index	Protein	SC [%]	SVP [%]	IC [%]	MS Score [a.u.]	Var. Mods	Mono Mass [Da]	Chemical Formula
7	H31Kac1	47.0	14.8	26.3	12.4	Ace: K36	15305.4534	C671H1131N215O186S4

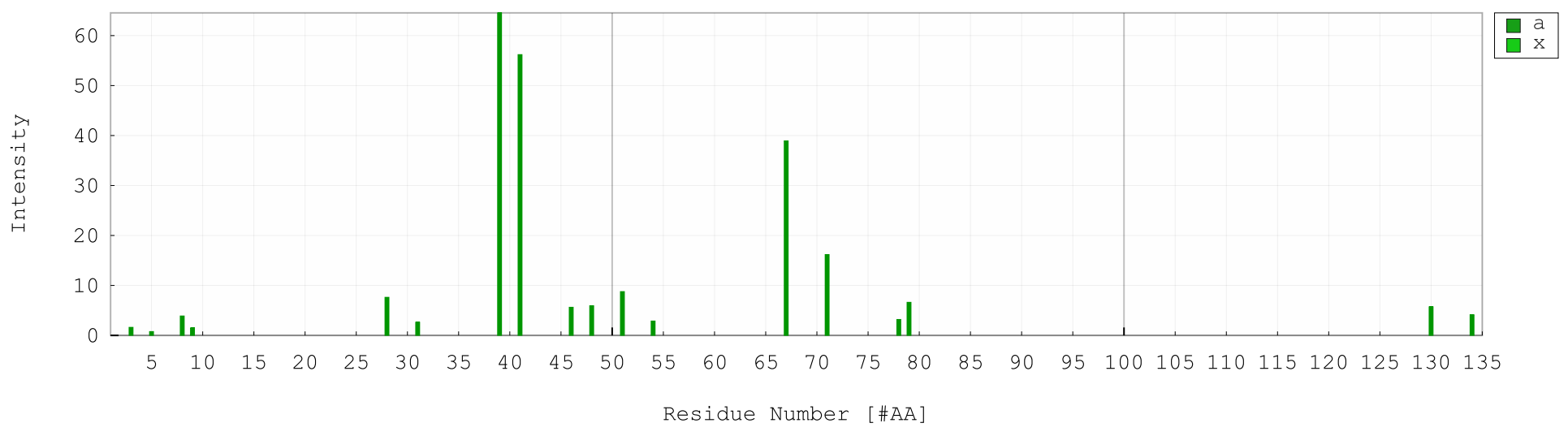

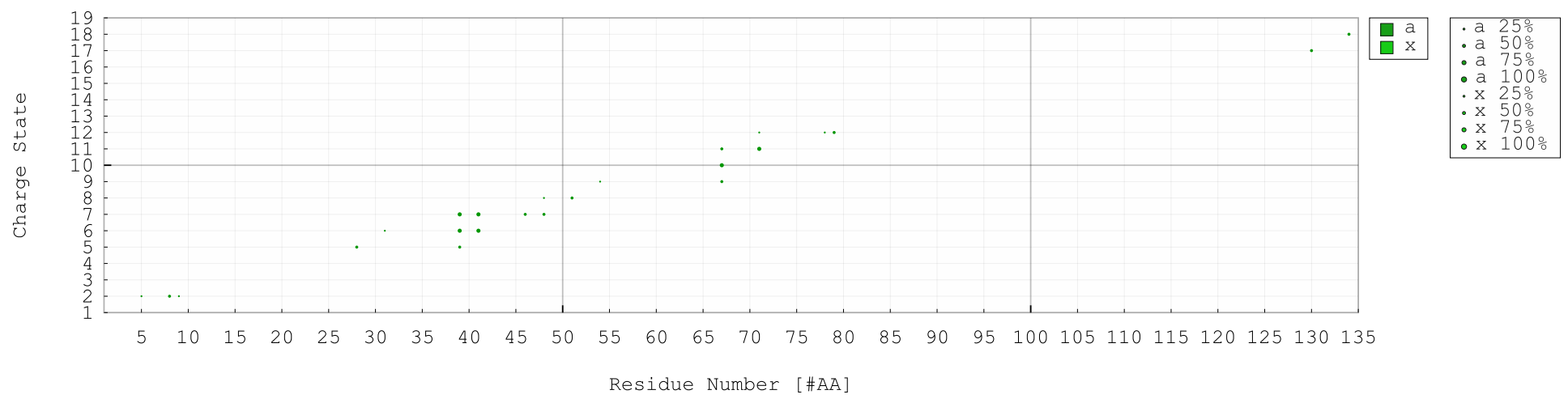

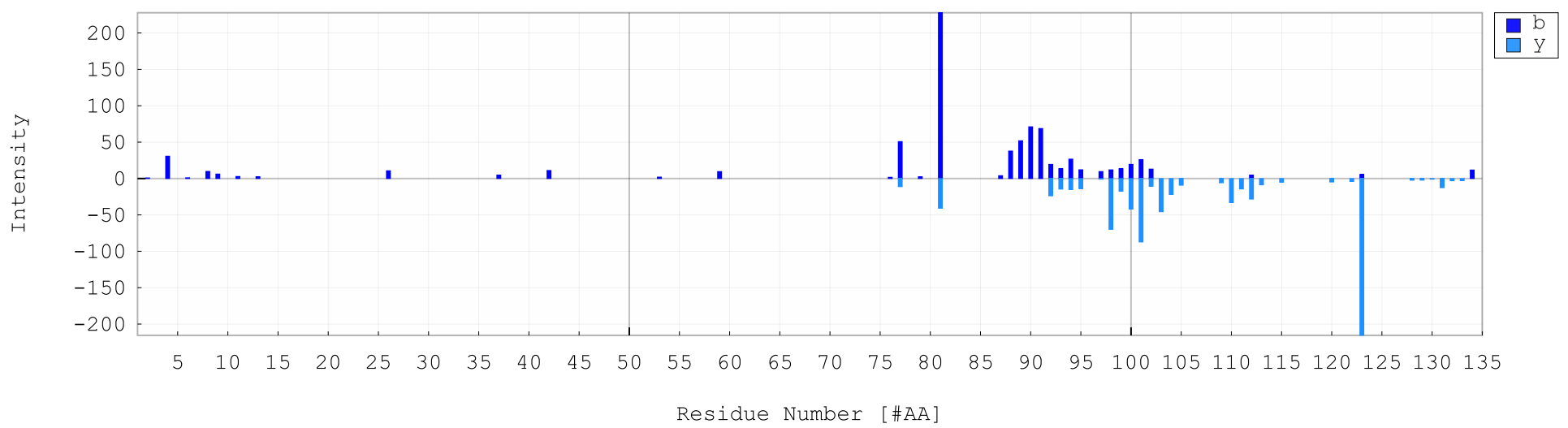

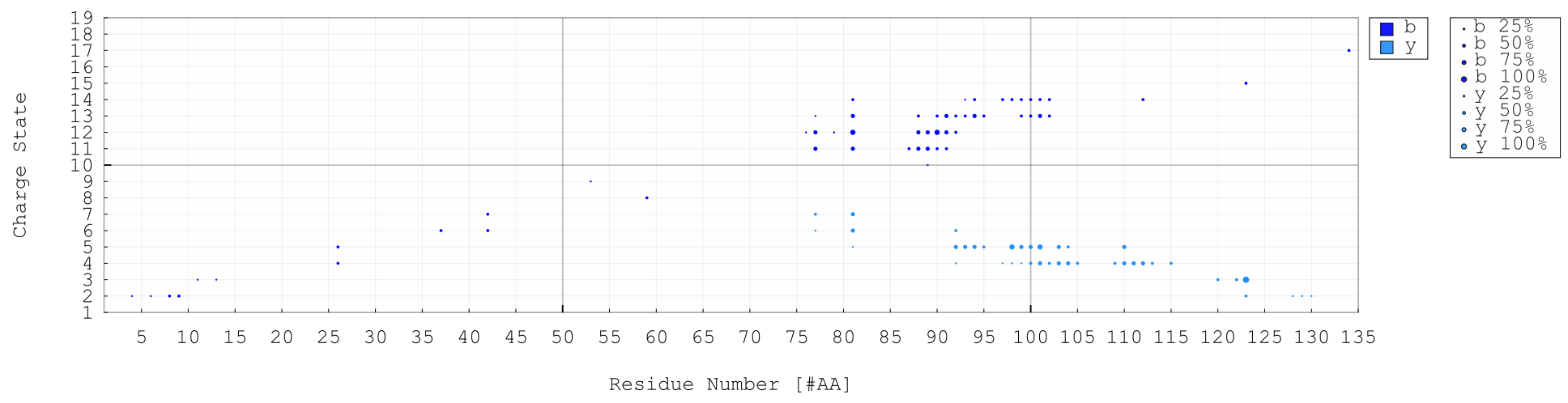

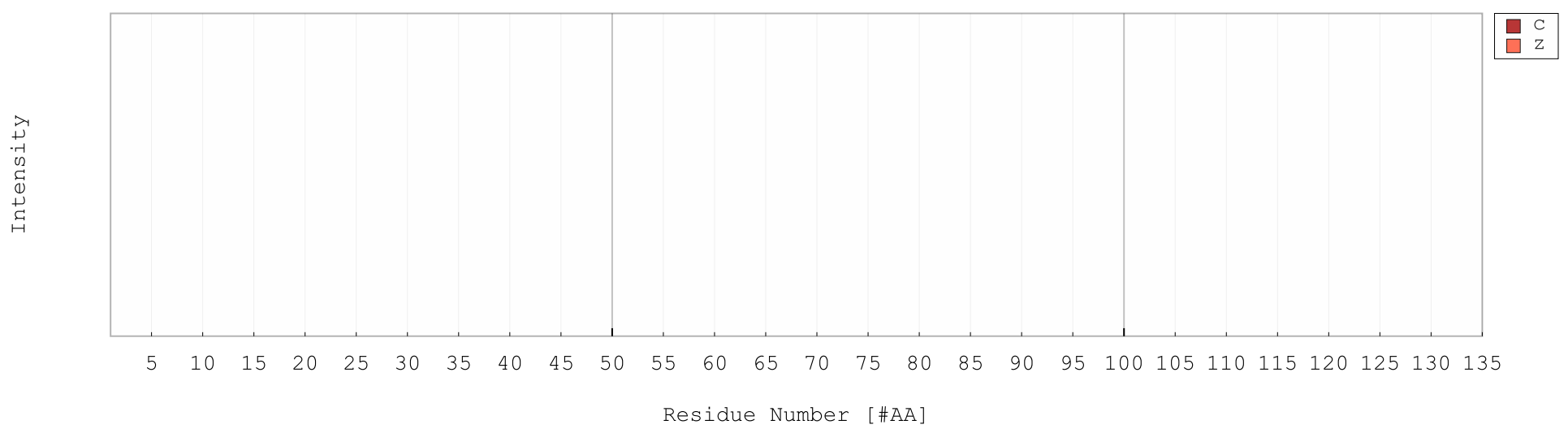

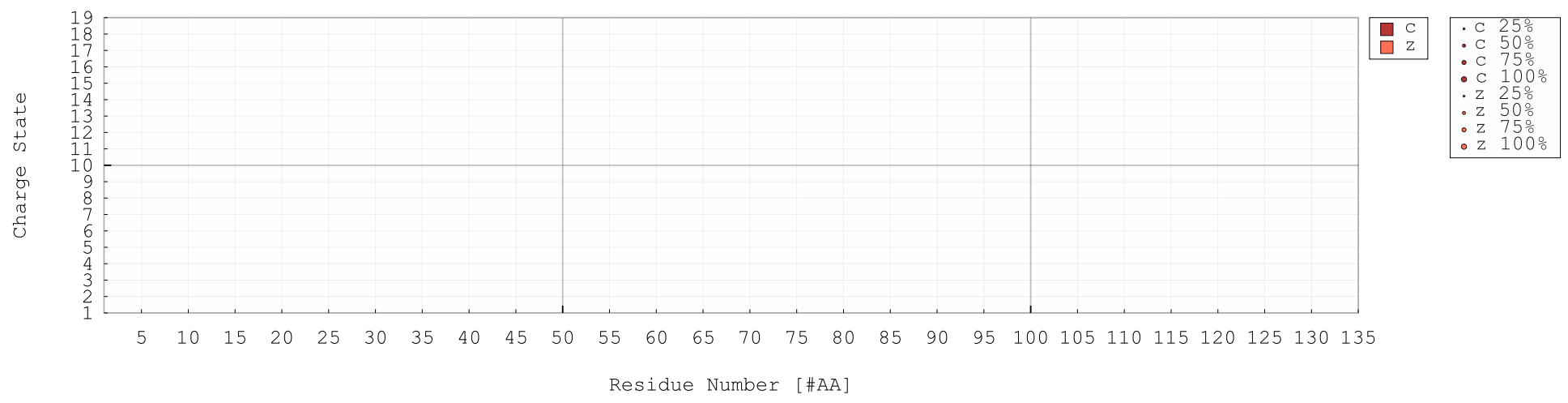

Proteoform:

Index	Protein	SC [%]	SVP [%]	IC [%]	MS Score [a.u.]	Var. Mods	Mono Mass [Da]	Chemical Formula
8	H31Kac1	47.0	14.8	26.3	12.4	Ace: K37	15305.4534	C671H1131N215O186S4

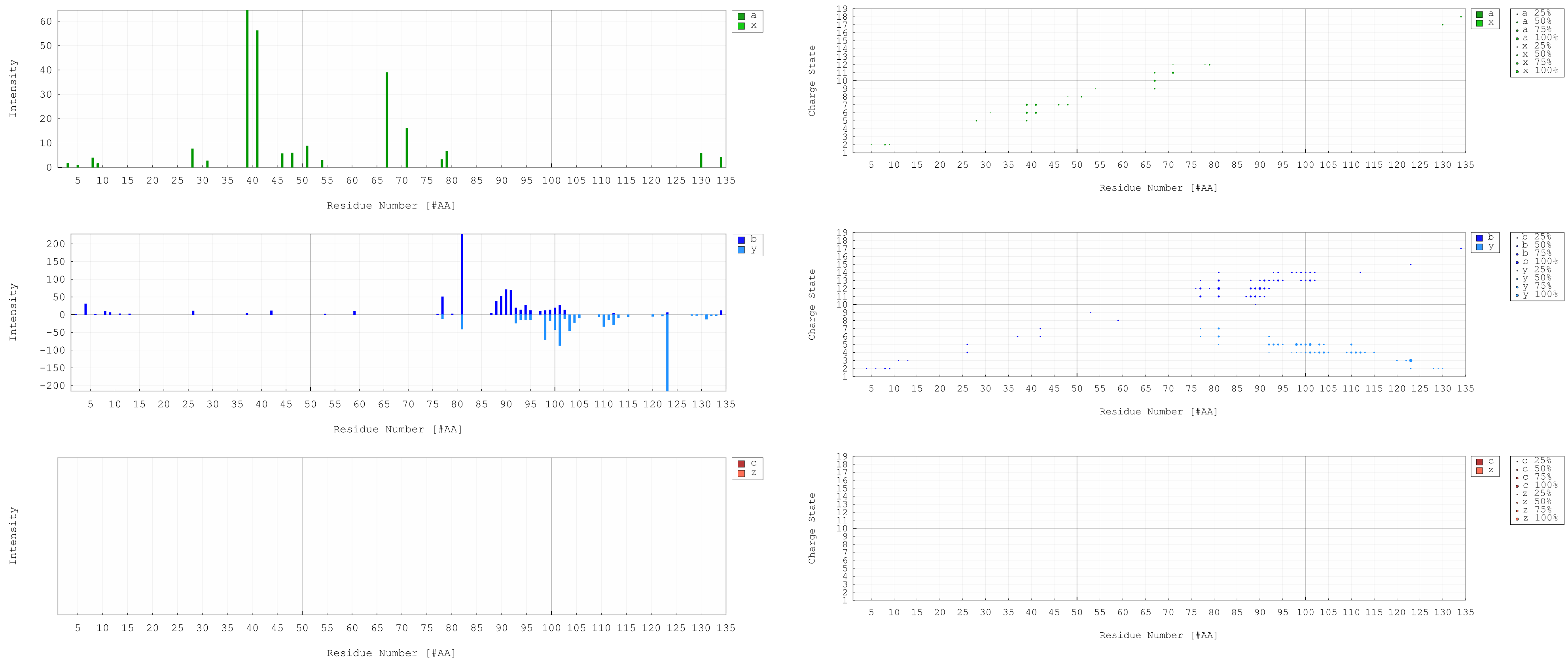

Top-Down Result OmniScape 2025
Sequence Confirmation
241210 H31-GNC5_10 z19 MS2 ECD 20ms MS3 shCID z18d_001-366_calibrated

Proteoform:

Index	Protein	SC [%]	SVP [%]	IC [%]	MS Score [a.u.]	Var. Mods	Mono Mass [Da]	Chemical Formula
9	H31Kac1	41.8	14.8	24.5	10.2	Ace: K56	15305.4534	C671H1131N215O186S4

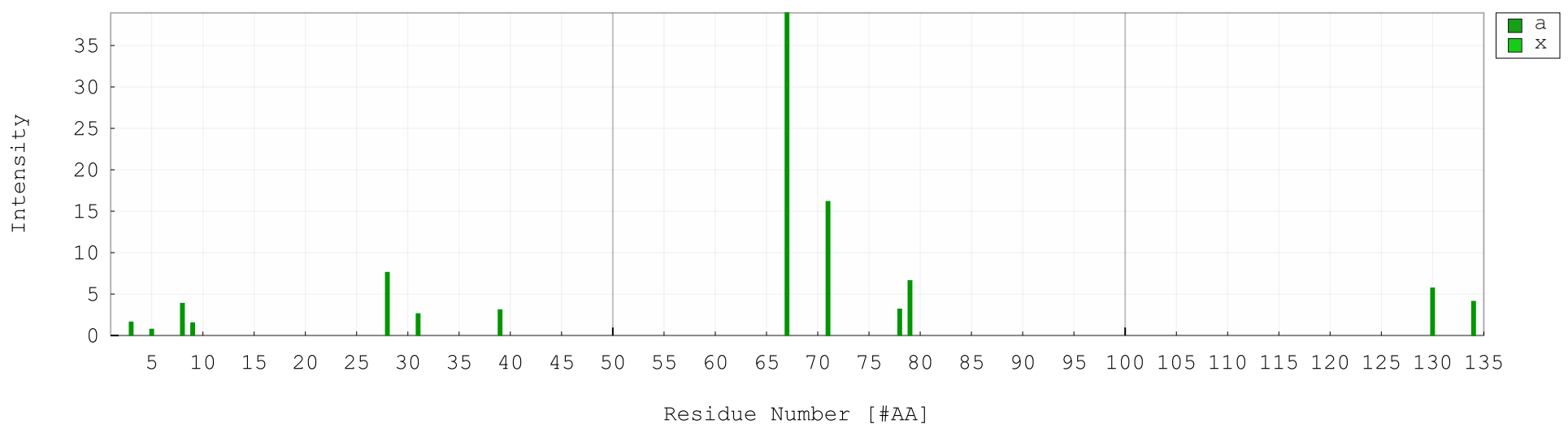

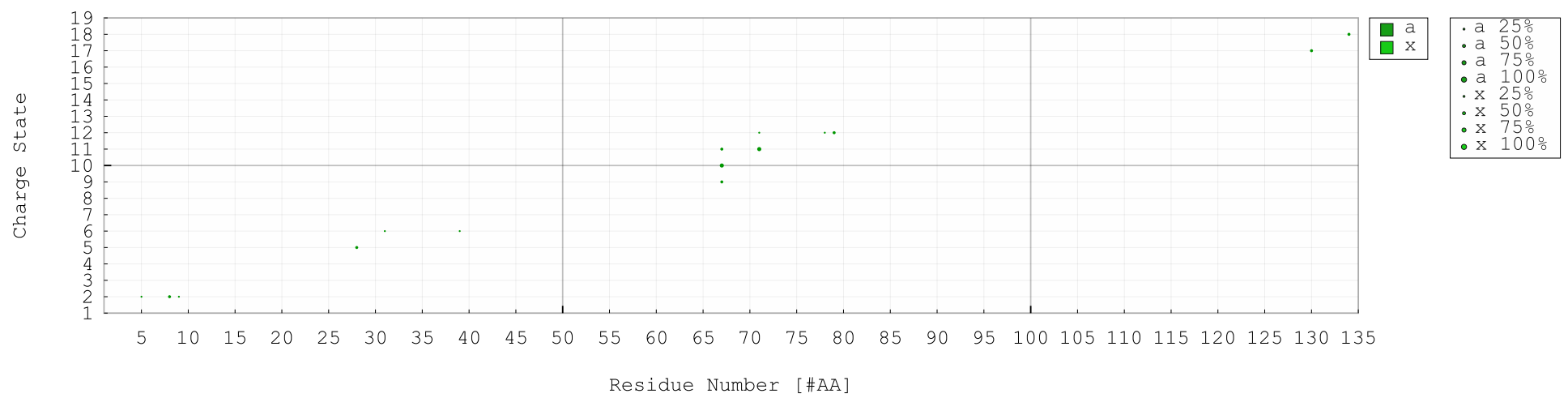

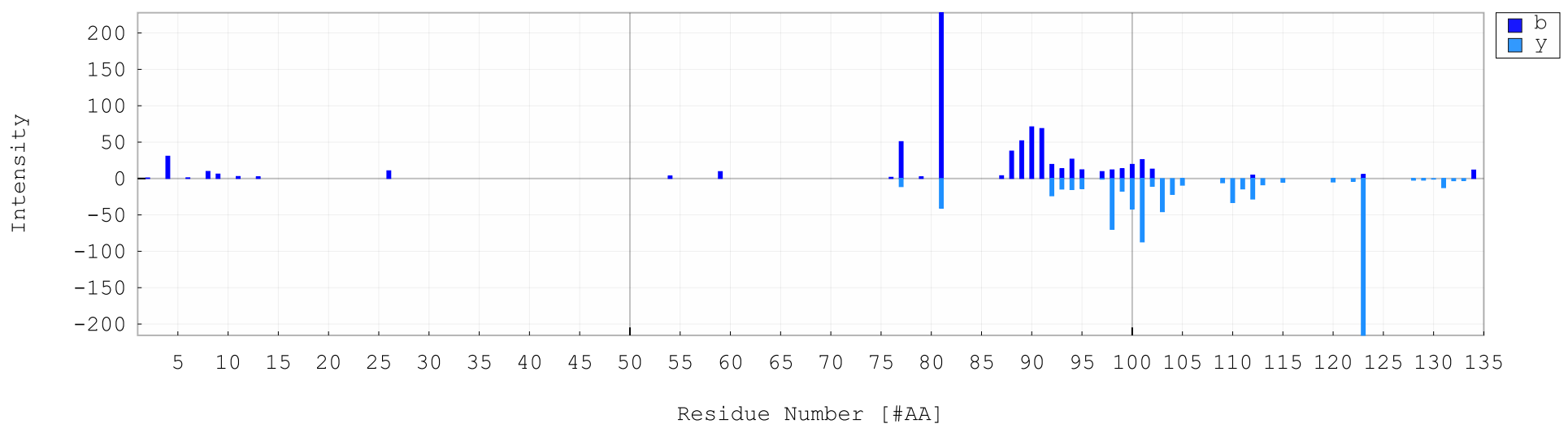

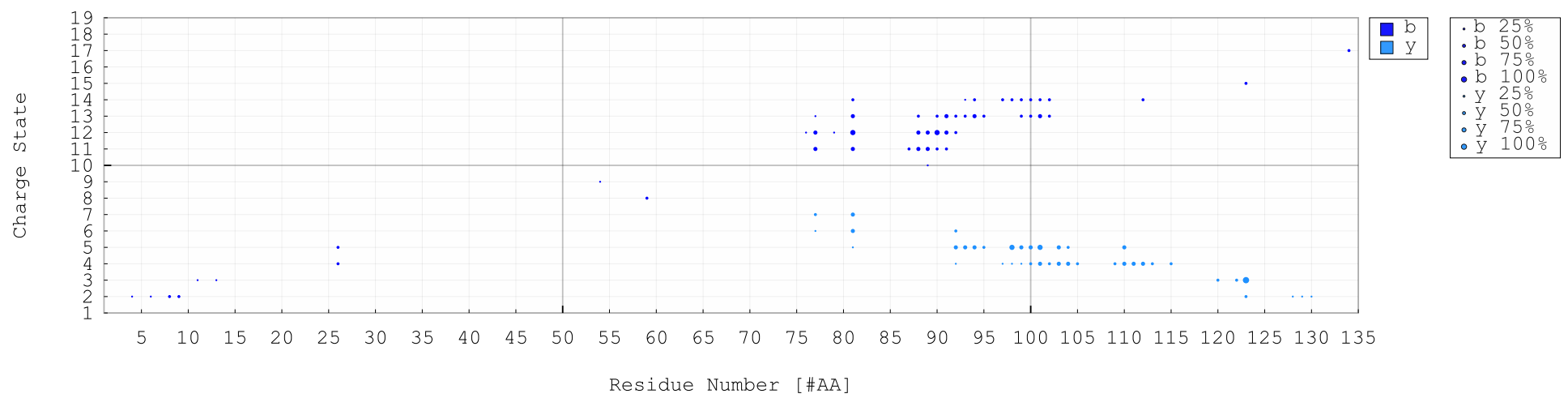

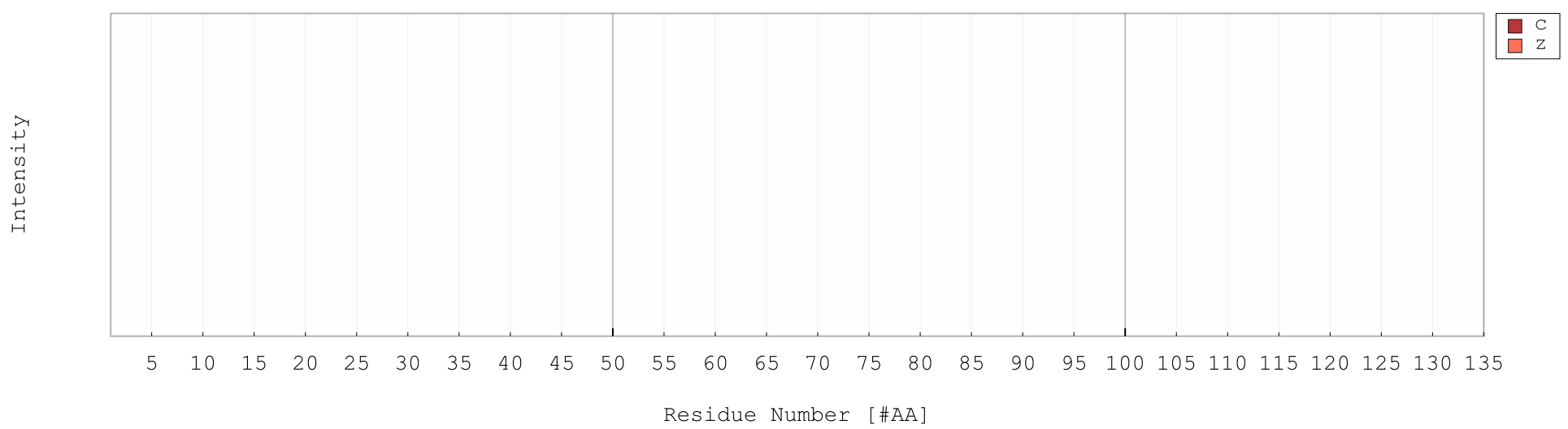

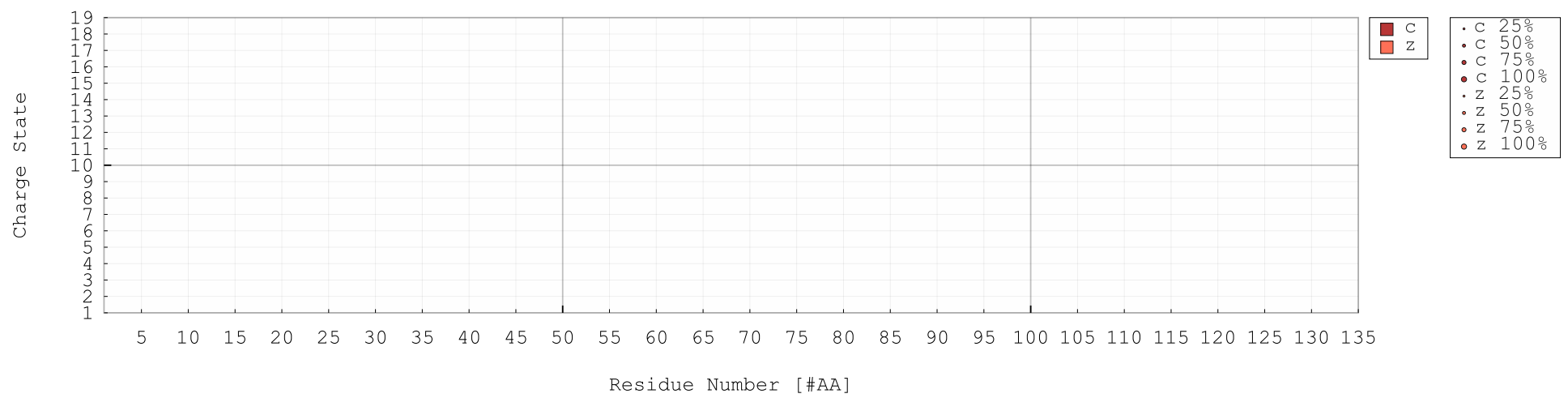

Top-Down Result OmniScape 2025
Sequence Confirmation
241210_H31-GNC5_10_z19_MS2_ECD_20ms_MS3_shCID_z18d_001-366_calibrated

Proteoform:

Index	Protein	SC [%]	SVP [%]	IC [%]	MS Score [a.u.]	Var. Mods	Mono Mass [Da]	Chemical Formula
10	H31Kac1	41.0	14.8	24.4	10.0	Ace: K64	15305.4534	C671H1131N215O186S4

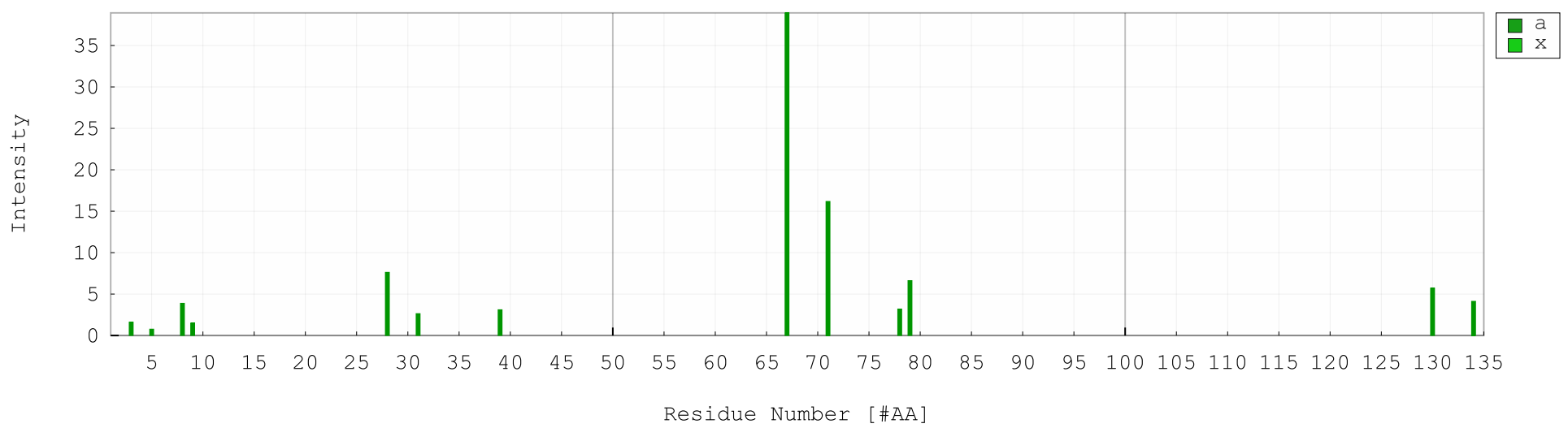

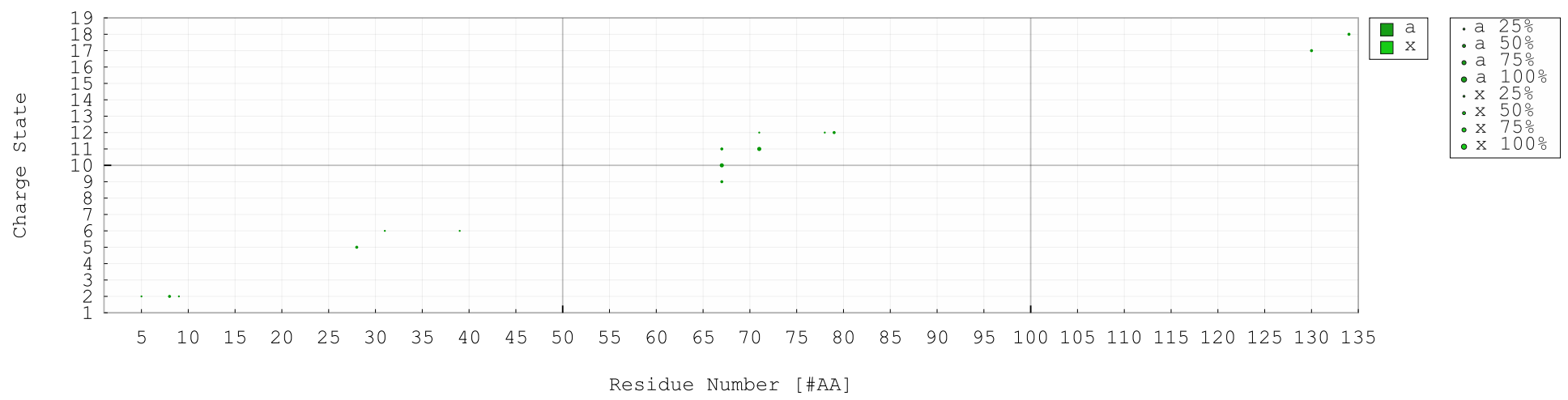

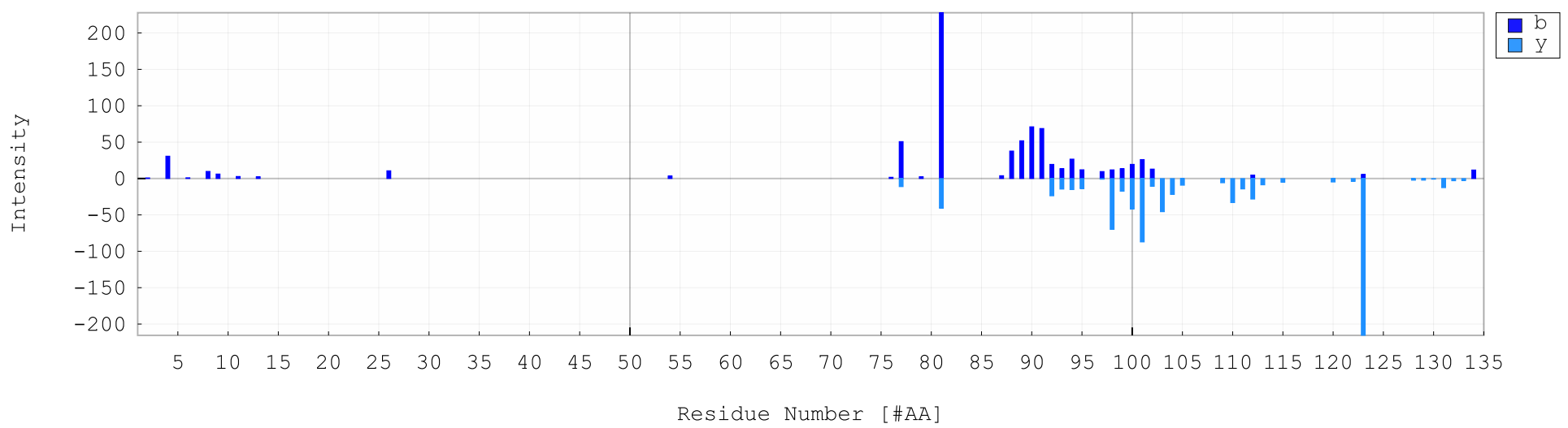

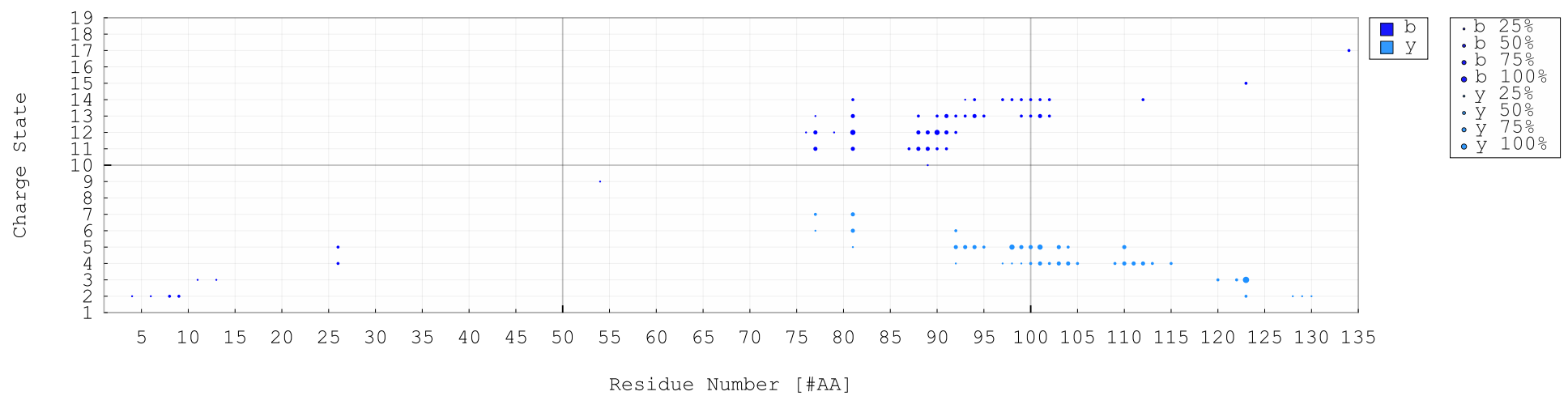

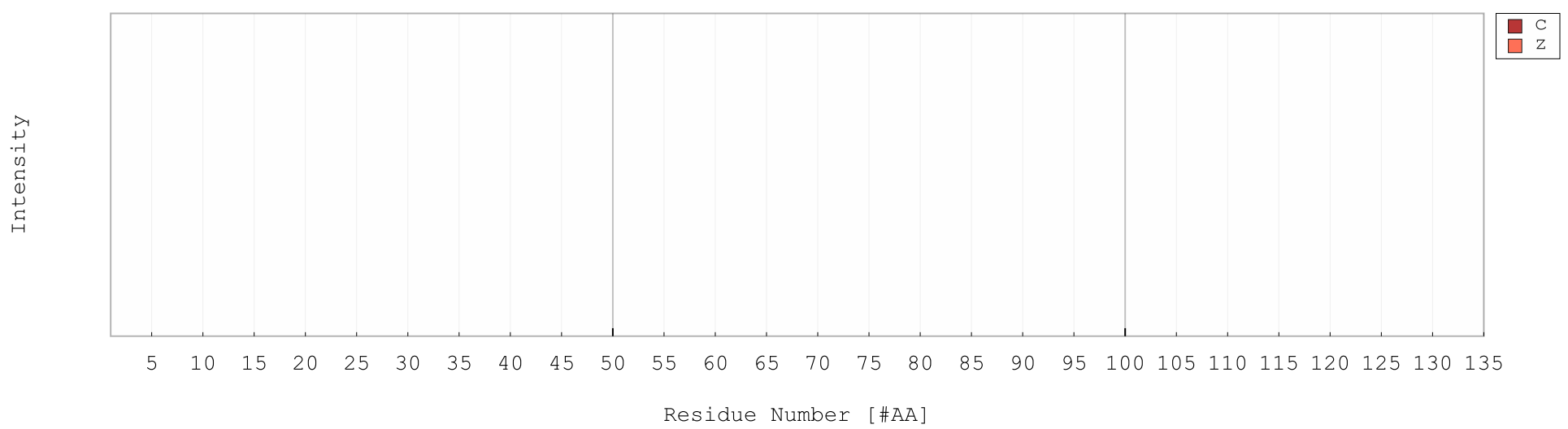

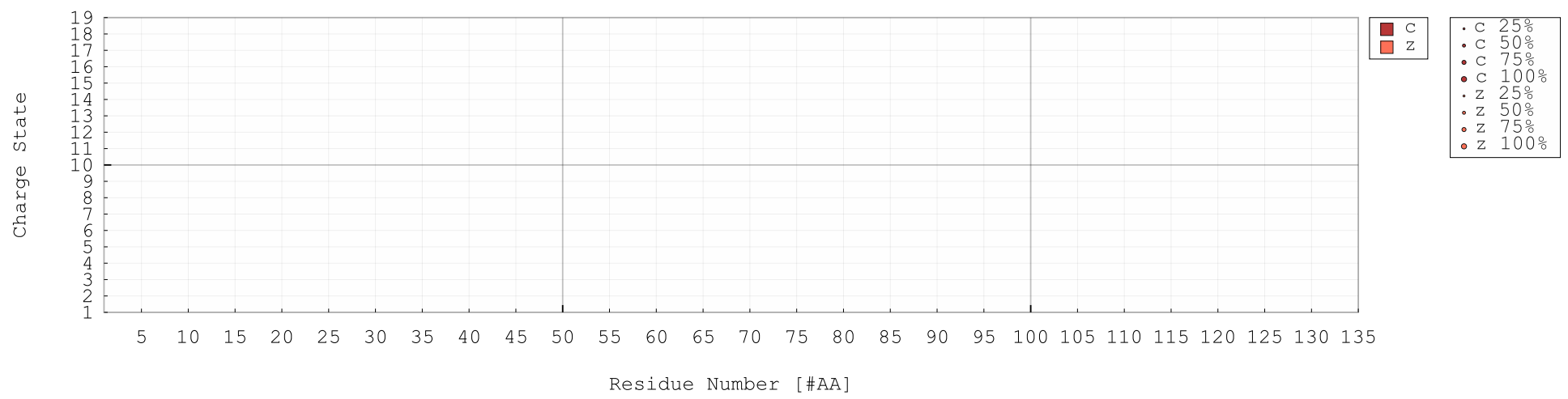

Supplement: Supplementary file 2 [file mmc2.pdf]
