# Supplementary material for: Top-Down Sequencing of Intact Proteoforms Using the timsOmni Mass Spectrometer: Accurate Determination of Co-Occurring Histone Modifications
Source: Mol Cell Proteomics. 2026 Aug 17;25(9):101640. doi: 10.1016/j.mcpro.2026.101640 (PMC13595144; doi:10.1016/j.mcpro.2026.101640)

Supplemental Data S3. H3.1 - PCAF, 30 min - OmniScape reports

OmniScape reports for monoacetylated H3.1 generated by PCAF.

**Histone**	H3.1
**Enzyme**	PCAF
**Reaction/source**	30 min
**Nb of Osc reports**	3

Included reports

#	Experiment	Precursor	Charge	Fragmentation / workflow	File
1	MS2	H3.1Kac1	19+	ECD	MS2_H3.1Kac1_19+_ECD.pdf
2	MS2	H3.1Kac1	19+	EID	MS2_H3.1Kac1_19+_EID.pdf
3	MS2	H3.1Kac1	19+	RCID	MS2_H3.1Kac1_19+_RCID.pdf

Supplemental Data S3

H3.1 - PCAF, 30 min

**Report**	1 of 3
**Experiment**	MS2
**Histone/sample**	H3.1
**Enzyme**	PCAF
**Reaction/source**	30 min
**Precursor**	H3.1Kac1
**Charge state**	19+
**Fragmentation / workflow**	ECD
**Original file**	MS2_H3.1Kac1_19+_ECD.pdf

Project Information

Project Directory: C:/Users/LAB-ADMIN
Processing date: Tue Apr 28 13:05:22 2026

Sample Info & Protocols

Name: Top-Down Protein Sequencing ExD

Dataset

Imported spectrum file name:	250428 H31-PCAF100-30min z19ac1 MS2 ECD_calibrated.txt
Dataset name:	Information is only available from imported JPEAKS files
Date acquired:	Information is only available from imported JPEAKS files
Date imported:	Information is only available from imported JPEAKS files
Instrument Name:	Information is only available from imported JPEAKS files
Instrument Serial Number:	Information is only available from imported JPEAKS files

Workflow Result Info

Analysis: 250428 H31-PCAF100-30min z19ac1 MS2 ECD_calibrated2
Location: C:/Users/LAB-ADMIN
Method: -

Method Information

Name: Unnamed

Spectral Parameters

Mass Error [ppm]: 6
Intensity Deviation [au]: 0.15
Min/Max Charges: 1-19
Experiment type: ExD

a ions	b ions	c ions	x ions	y ions	z ions	satellite	internal	precursor
a	b	c		y	z+1			M
a+1		c+1			z+2			M+-2H

Noise threshold calculation: Simple moving average noise threshold (SMA)
Window size [datapoints]: 600
Noise curve offset: 1.7267058
SVP parameters: terminal gap: 2, internal gap: 1

Proteoforms

(top 10)

Index	Parent Sequence	Chain	Var. Mods	Mono Mass [Da]	Chemical Composition
1	H31Kac1	H31Kac1K14[Ace]	Ace: K14	15305.4534	C671H1131N215O186S4
2	H31Kac1	H31Kac1K18[Ace]	Ace: K18	15305.4534	C671H1131N215O186S4
3	H31Kac1	H31Kac1K9[Ace]	Ace: K9	15305.4534	C671H1131N215O186S4
4	H31Kac1	H31Kac1K23[Ace]	Ace: K23	15305.4534	C671H1131N215O186S4
5	H31Kac1	H31Kac1K4[Ace]	Ace: K4	15305.4534	C671H1131N215O186S4
6	H31Kac1	H31Kac1K27[Ace]	Ace: K27	15305.4534	C671H1131N215O186S4
7	H31Kac1	H31Kac1K36[Ace]	Ace: K36	15305.4534	C671H1131N215O186S4
8	H31Kac1	H31Kac1K37[Ace]	Ace: K37	15305.4534	C671H1131N215O186S4
9	H31Kac1	H31Kac1K56[Ace]	Ace: K56	15305.4534	C671H1131N215O186S4
10	H31Kac1	H31Kac1K64[Ace]	Ace: K64	15305.4534	C671H1131N215O186S4

Results

Proteoforms Table (top 10)

Index	Protein	SC [%]	SVP [%]	IC [%]	MS Score [a.u.]	Var. Mods	Mono Mass [Da]	Chemical Formula
1	H31Kac1	81.3	67.4	35.3	28.7	Ace: K14	15305.4534	C671H1131N215O186S4
2	H31Kac1	78.4	29.6	34.6	27.1	Ace: K18	15305.4534	C671H1131N215O186S4
3	H31Kac1	79.9	27.4	33.4	26.7	Ace: K9	15305.4534	C671H1131N215O186S4
4	H31Kac1	76.9	29.6	31.4	24.1	Ace: K23	15305.4534	C671H1131N215O186S4
5	H31Kac1	76.1	22.2	31.7	24.1	Ace: K4	15305.4534	C671H1131N215O186S4
6	H31Kac1	73.9	29.6	30.2	22.3	Ace: K27	15305.4534	C671H1131N215O186S4
7	H31Kac1	67.9	29.6	28.4	19.3	Ace: K36	15305.4534	C671H1131N215O186S4
8	H31Kac1	67.2	29.6	27.5	18.5	Ace: K37	15305.4534	C671H1131N215O186S4
9	H31Kac1	58.2	29.6	22.0	12.8	Ace: K56	15305.4534	C671H1131N215O186S4
10	H31Kac1	54.5	29.6	20.7	11.3	Ace: K64	15305.4534	C671H1131N215O186S4

Sequence

Name: H31Kac1

```
                 10                   20                   30                   40                   50                   60
A R T K Q T A R K S  T G G K A P R K Q L  A T K A A R K S A P  A T G G V K K P H R  Y R P G T V A L R E  I R R Y Q K S T E L
                 70                   80                   90                  100                  110                  120
L I R K L P F Q R L  V R E I A Q D F K T  D L R F Q S S A V M  A L Q E A C E A Y L  V G L F E D T N L C  A I H A K R V T I M
                130                  140                  150                  160                  170                  180
P K D I Q L A R R I  R G E R A
```

Modifications

Modification	Position	Type
Acetyl	4	variable
Acetyl	9	variable
Acetyl	14	variable
Acetyl	18	variable
Acetyl	23	variable
Acetyl	27	variable
Acetyl	36	variable
Acetyl	37	variable
Acetyl	56	variable
Acetyl	64	variable
Acetyl	79	variable

Modification	Position	Type
Acetyl	115	variable
Acetyl	122	variable

Sequence Map

Proteoform:

Index	Protein	SC [%]	SVP [%]	IC [%]	MS Score [a.u.]	Var. Mods	Mono Mass [Da]	Chemical Formula
1	H31Kac1	81.3	67.4	35.3	28.7	Ace: K14	15305.4534	C671H1131N215O186S4

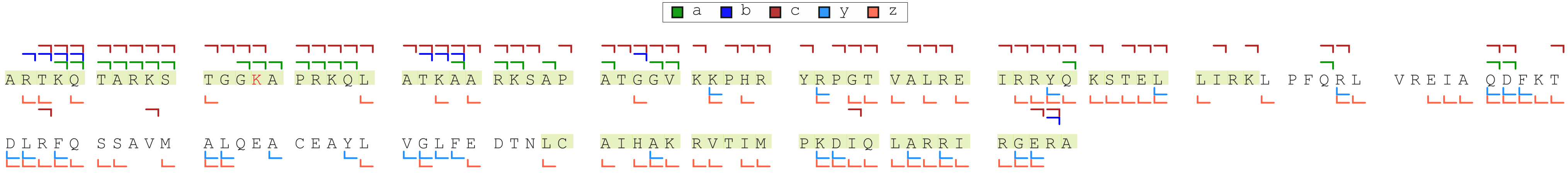

Proteoform:

Index	Protein	SC [%]	SVP [%]	IC [%]	MS Score [a.u.]	Var. Mods	Mono Mass [Da]	Chemical Formula
2	H31Kac1	78.4	29.6	34.6	27.1	Ace: K18	15305.4534	C671H1131N215O186S4

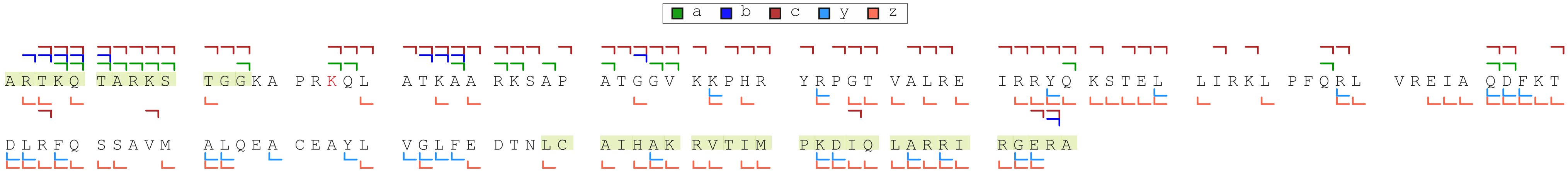

Proteoform:

Index	Protein	SC [%]	SVP [%]	IC [%]	MS Score [a.u.]	Var. Mods	Mono Mass [Da]	Chemical Formula
3	H31Kac1	79.9	27.4	33.4	26.7	Ace: K9	15305.4534	C671H1131N215O186S4

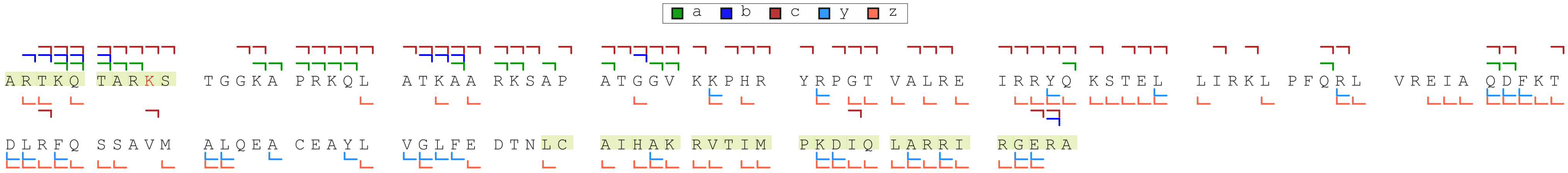

Top-Down Result OmniScape 2025
Sequence Confirmation
250428 H31-PCAF100-30min z19ac1 MS2 ECD_calibrated2

Proteoform:	Index	Protein	SC [%]	SVP [%]	IC [%]	MS Score [a.u.]	Var. Mods	Mono Mass [Da]	Chemical Formula
	4	H31Kac1	76.9	29.6	31.4	24.1	Ace: K23	15305.4534	C671H1131N215O186S4

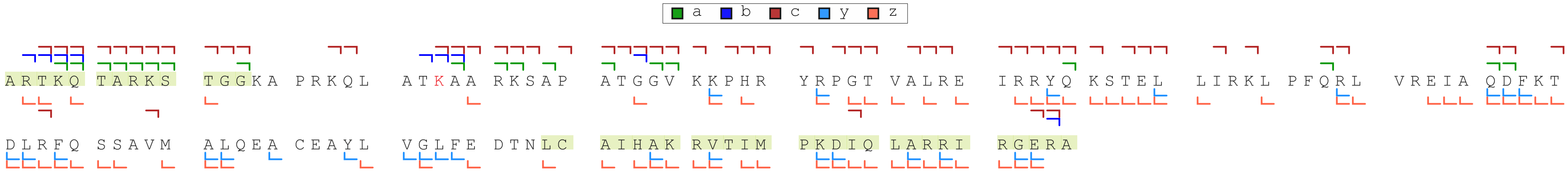

Proteoform:

Index	Protein	SC [%]	SVP [%]	IC [%]	MS Score [a.u.]	Var. Mods	Mono Mass [Da]	Chemical Formula
5	H31Kac1	76.1	22.2	31.7	24.1	Ace: K4	15305.4534	C671H1131N215O186S4

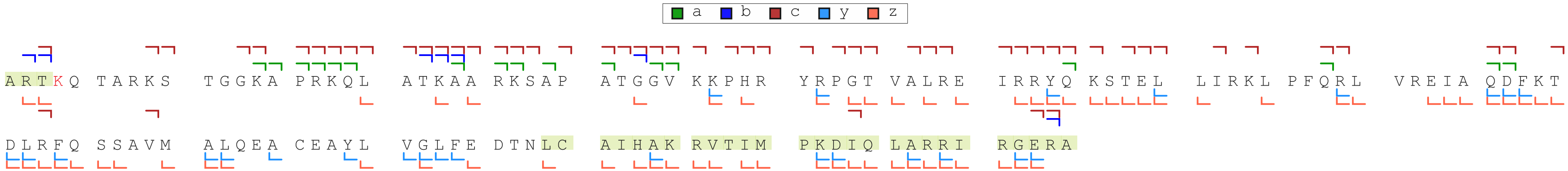

Proteoform:

Index	Protein	SC [%]	SVP [%]	IC [%]	MS Score [a.u.]	Var. Mods	Mono Mass [Da]	Chemical Formula
6	H31Kac1	73.9	29.6	30.2	22.3	Ace: K27	15305.4534	C671H1131N215O186S4

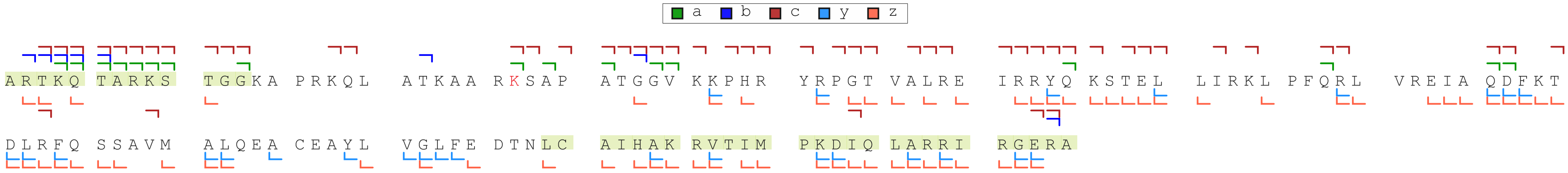

Proteoform:

Index	Protein	SC [%]	SVP [%]	IC [%]	MS Score [a.u.]	Var. Mods	Mono Mass [Da]	Chemical Formula
7	H31Kac1	67.9	29.6	28.4	19.3	Ace: K36	15305.4534	C671H1131N215O186S4

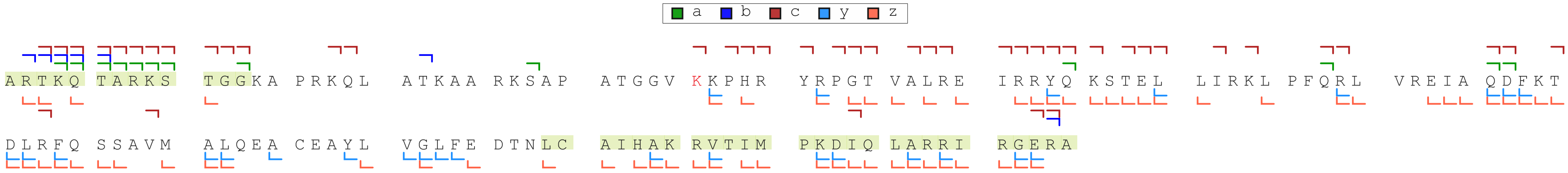

Proteoform:

Index	Protein	SC [%]	SVP [%]	IC [%]	MS Score [a.u.]	Var. Mods	Mono Mass [Da]	Chemical Formula
8	H31Kac1	67.2	29.6	27.5	18.5	Ace: K37	15305.4534	C671H1131N215O186S4

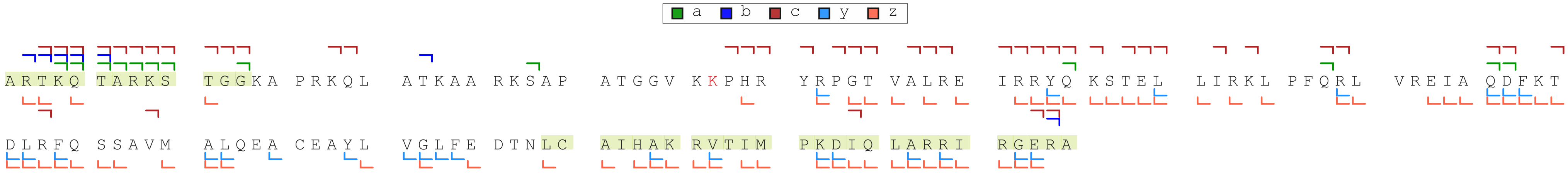

Proteoform:

Index	Protein	SC [%]	SVP [%]	IC [%]	MS Score [a.u.]	Var. Mods	Mono Mass [Da]	Chemical Formula
9	H31Kac1	58.2	29.6	22.0	12.8	Ace: K56	15305.4534	C671H1131N215O186S4

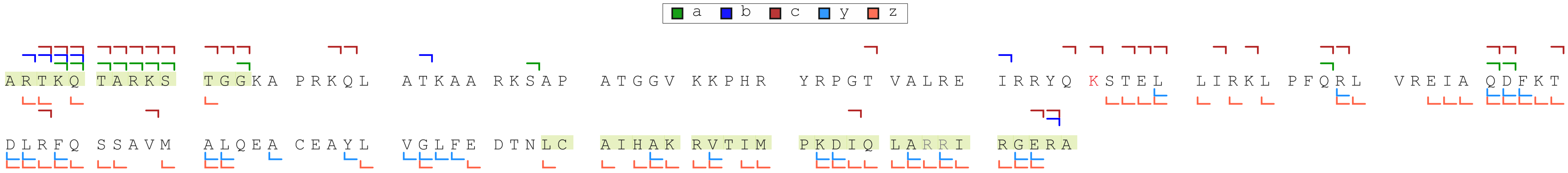

Proteoform:

Index	Protein	SC [%]	SVP [%]	IC [%]	MS Score [a.u.]	Var. Mods	Mono Mass [Da]	Chemical Formula
10	H31Kac1	54.5	29.6	20.7	11.3	Ace: K64	15305.4534	C671H1131N215O186S4

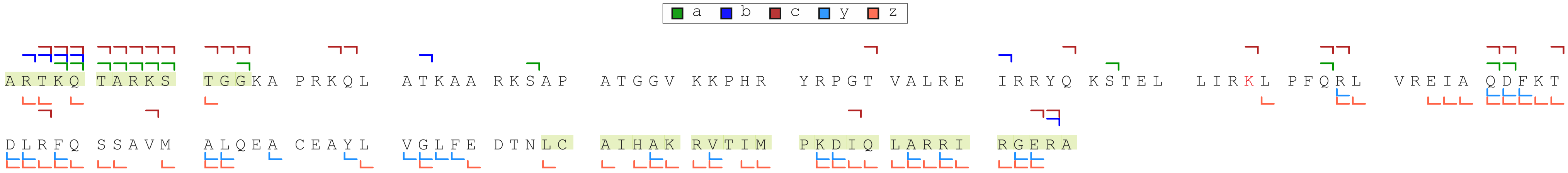

Error Plot

Proteoform:

Index	Protein	SC [%]	SVP [%]	IC [%]	MS Score [a.u.]	Var. Mods	Mono Mass [Da]	Chemical Formula
1	H31Kac1	81.3	67.4	35.3	28.7	Ace: K14	15305.4534	C671H1131N215O186S4

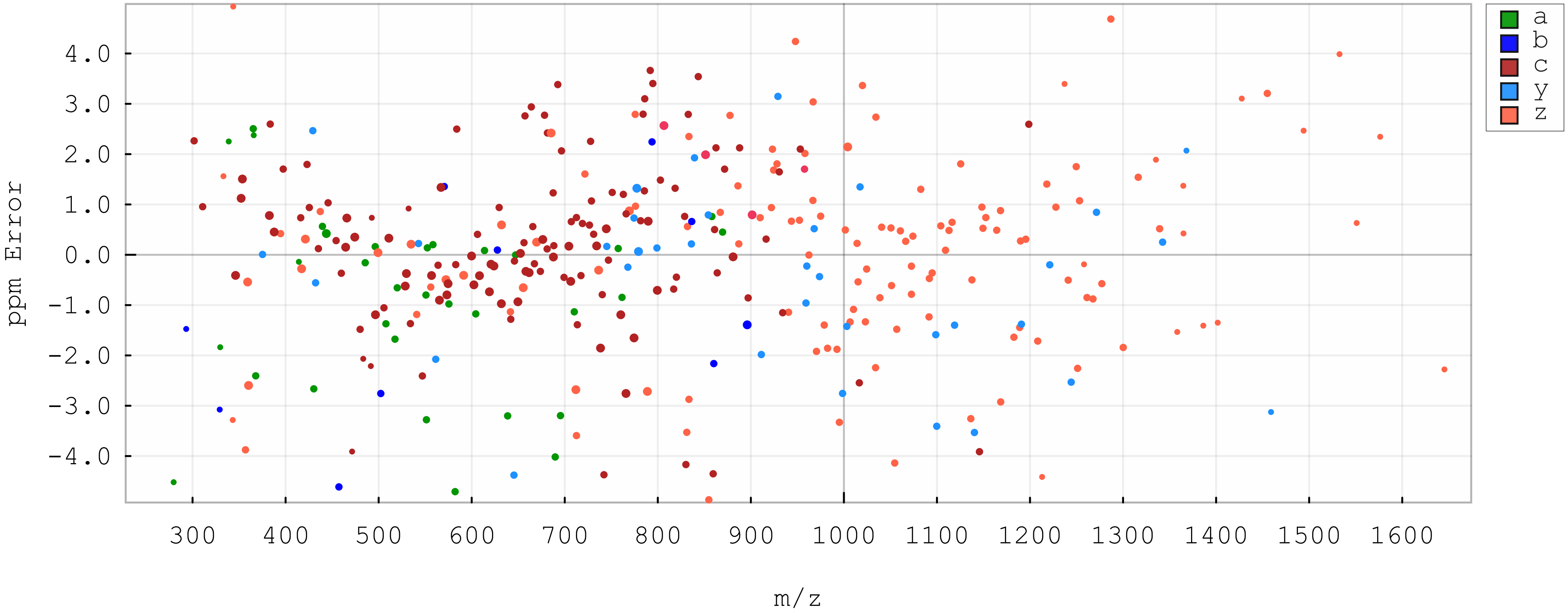

Proteoform:

Index	Protein	SC [%]	SVP [%]	IC [%]	MS Score [a.u.]	Var. Mods	Mono Mass [Da]	Chemical Formula
2	H31Kac1	78.4	29.6	34.6	27.1	Ace: K18	15305.4534	C671H1131N215O186S4

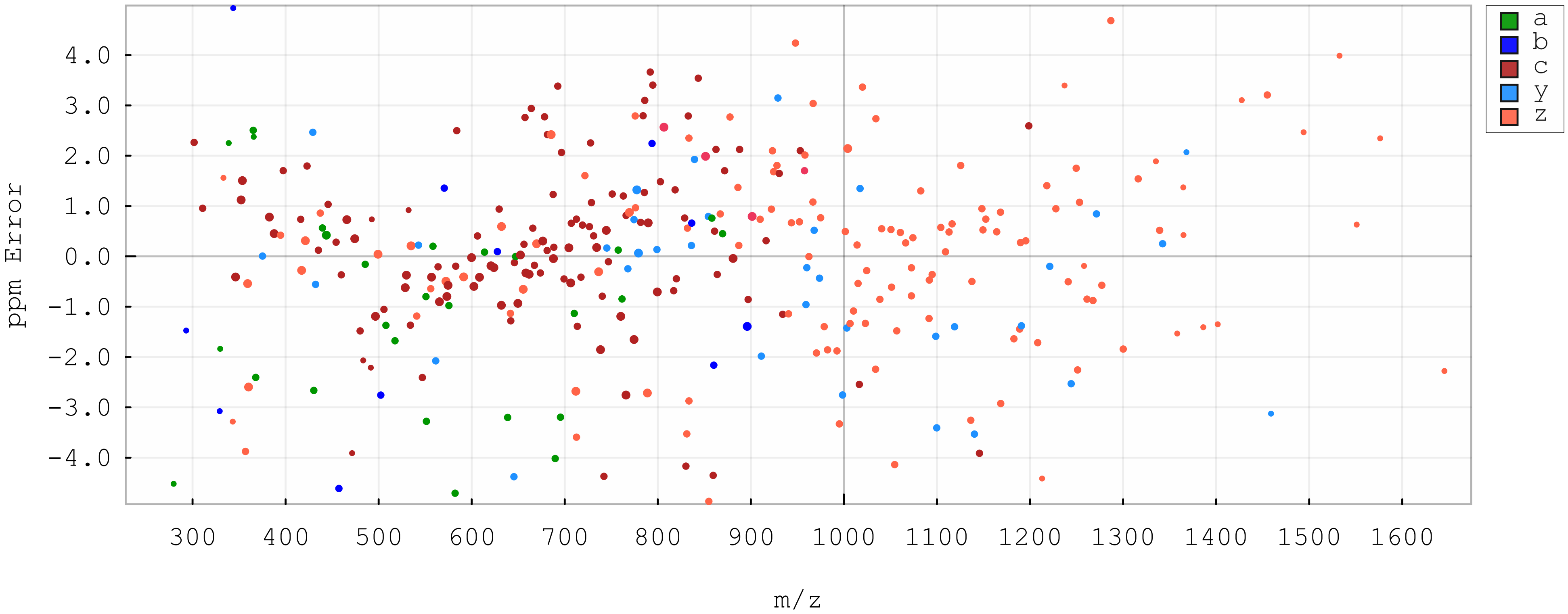

Proteoform:

Index	Protein	SC [%]	SVP [%]	IC [%]	MS Score [a.u.]	Var. Mods	Mono Mass [Da]	Chemical Formula
3	H31Kac1	79.9	27.4	33.4	26.7	Ace: K9	15305.4534	C671H1131N215O186S4

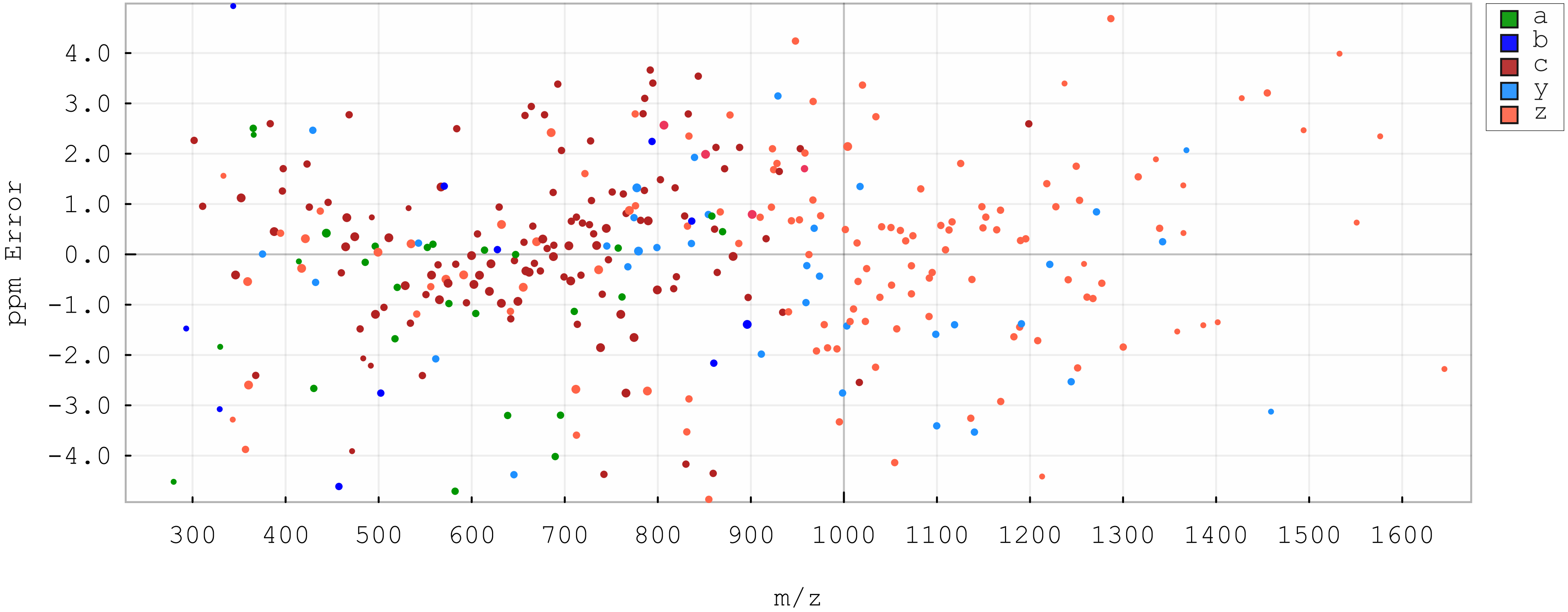

Proteoform:

Index	Protein	SC [%]	SVP [%]	IC [%]	MS Score [a.u.]	Var. Mods	Mono Mass [Da]	Chemical Formula
4	H31Kac1	76.9	29.6	31.4	24.1	Ace: K23	15305.4534	C671H1131N215O186S4

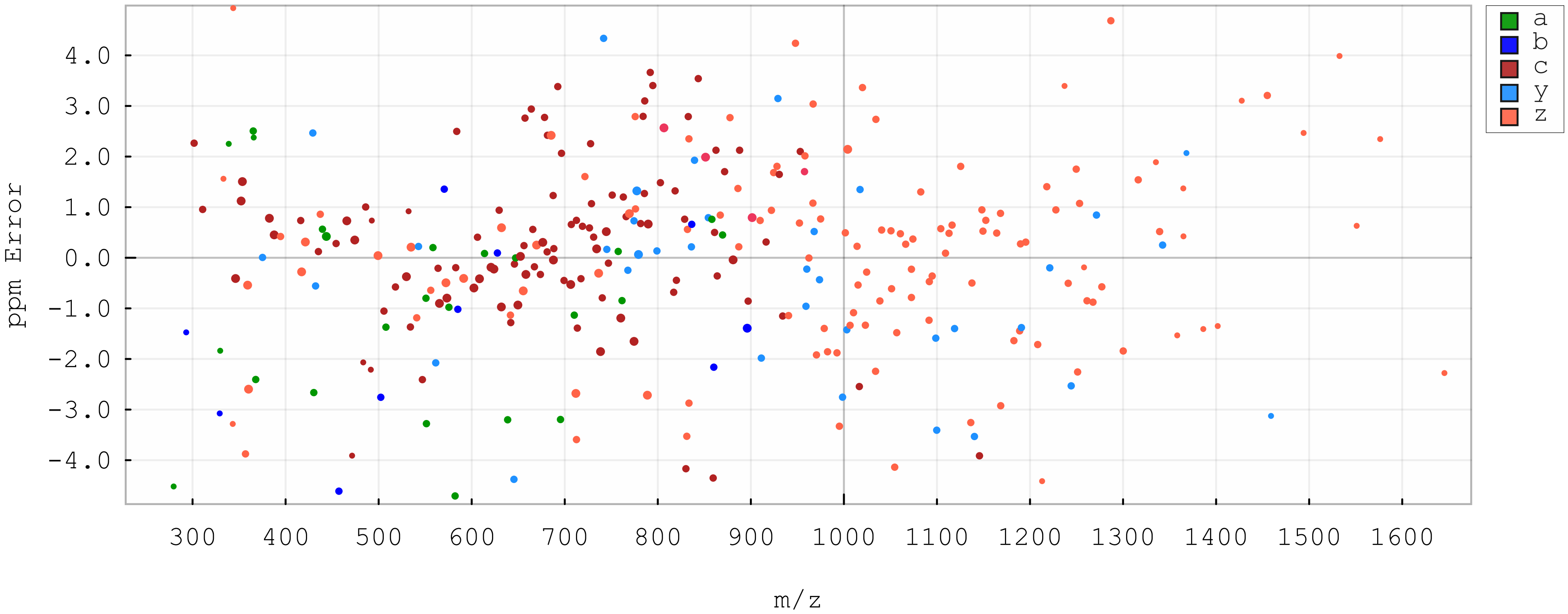

Proteoform:

Index	Protein	SC [%]	SVP [%]	IC [%]	MS Score [a.u.]	Var. Mods	Mono Mass [Da]	Chemical Formula
5	H31Kac1	76.1	22.2	31.7	24.1	Ace: K4	15305.4534	C671H1131N215O186S4

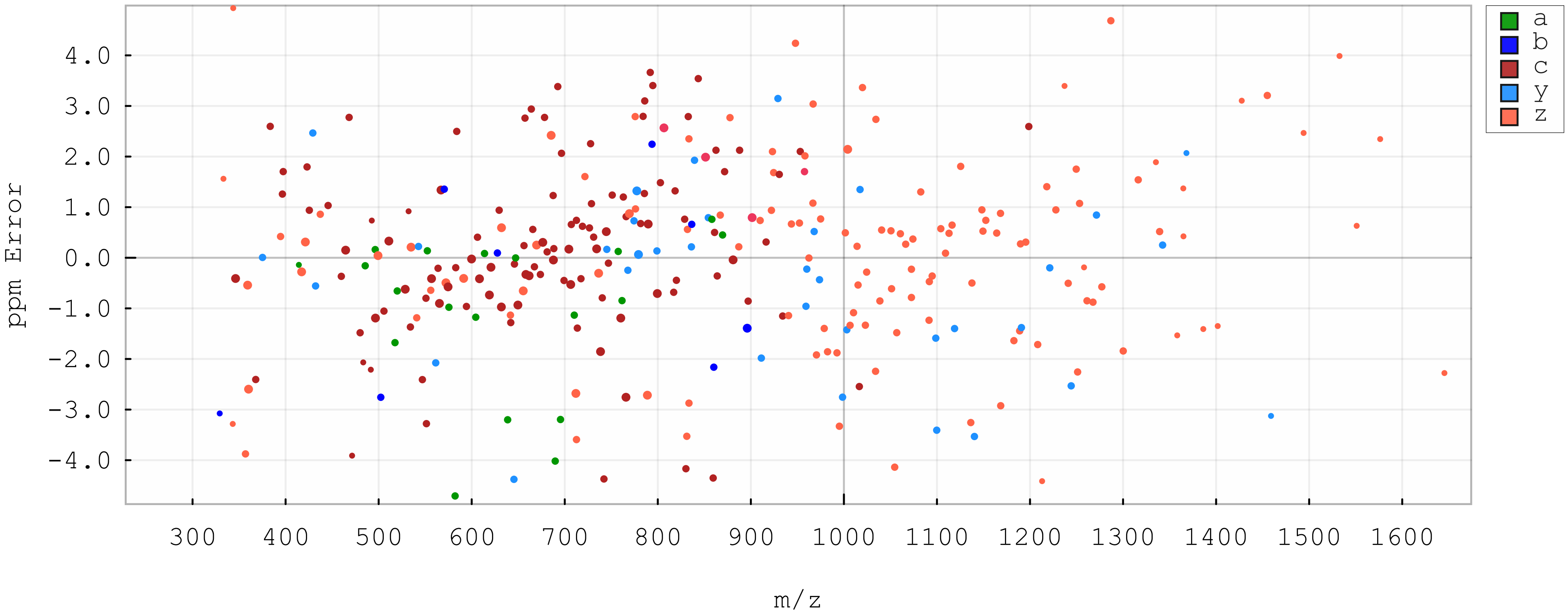

Proteoform:

Index	Protein	SC [%]	SVP [%]	IC [%]	MS Score [a.u.]	Var. Mods	Mono Mass [Da]	Chemical Formula
6	H31Kac1	73.9	29.6	30.2	22.3	Ace: K27	15305.4534	C671H1131N215O186S4

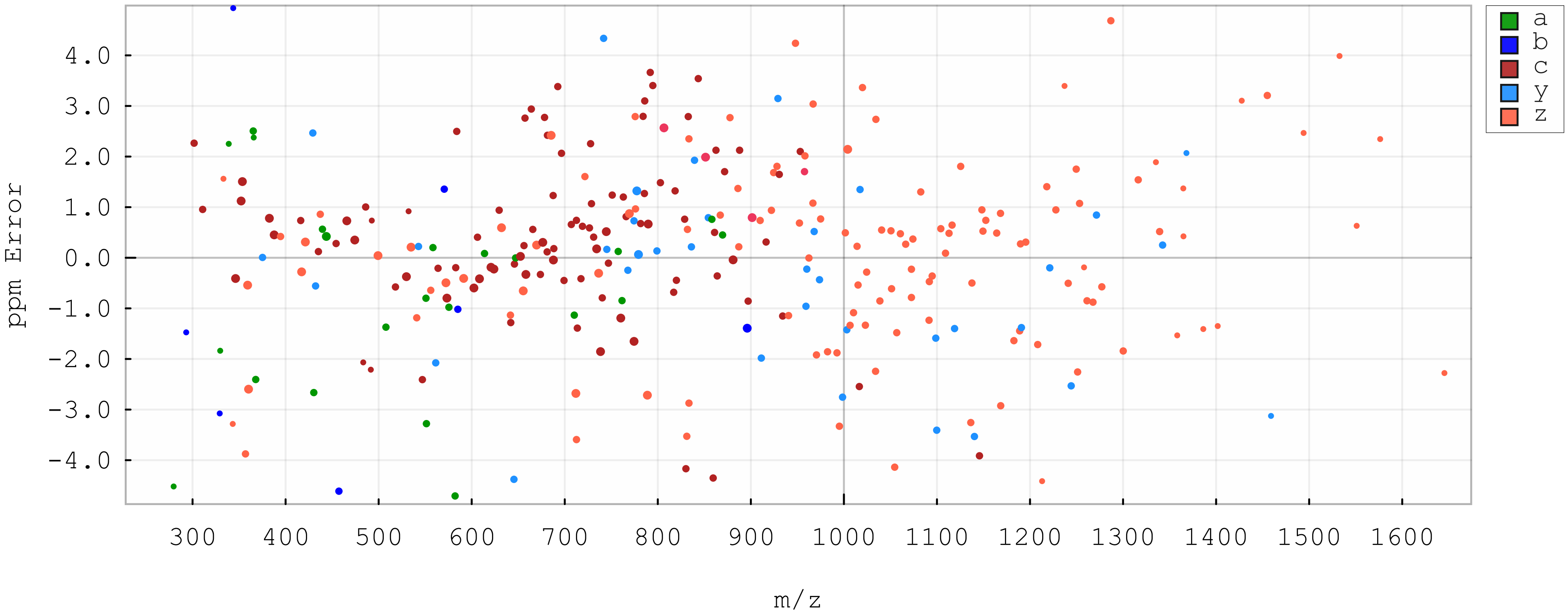

Proteoform:

Index	Protein	SC [%]	SVP [%]	IC [%]	MS Score [a.u.]	Var. Mods	Mono Mass [Da]	Chemical Formula
7	H31Kac1	67.9	29.6	28.4	19.3	Ace: K36	15305.4534	C671H1131N215O186S4

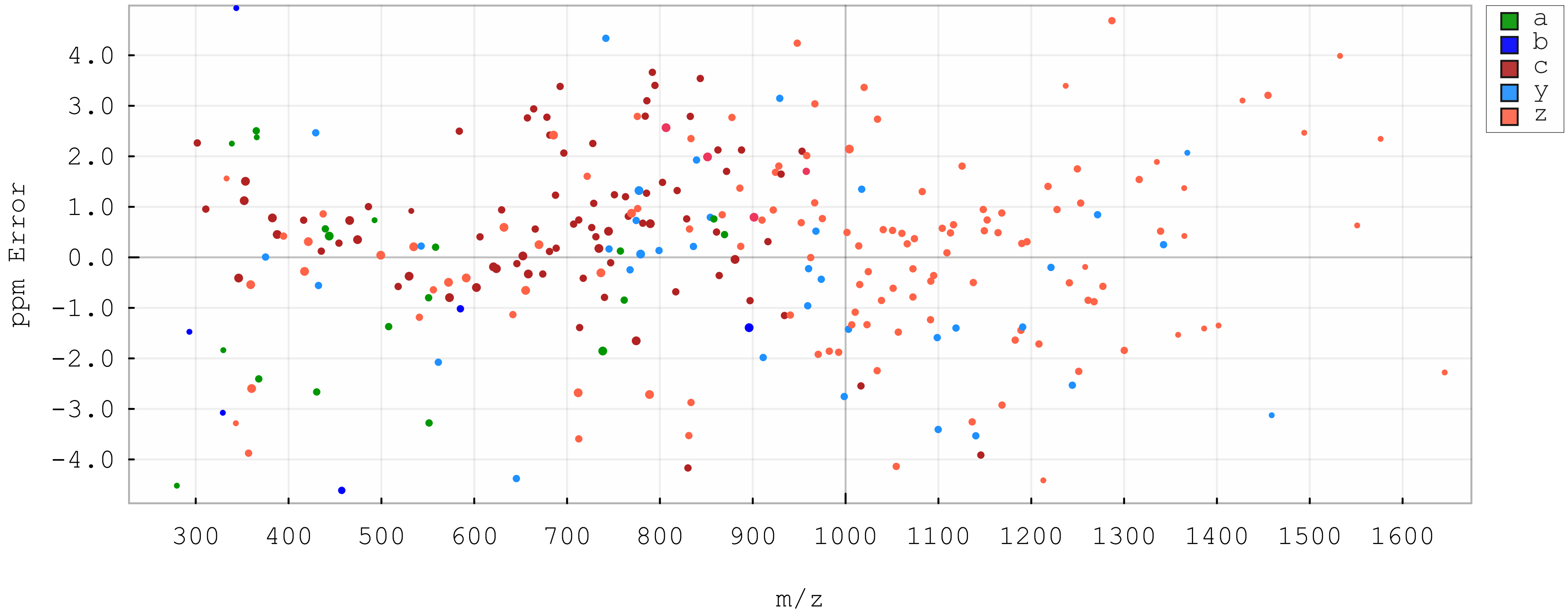

Proteoform:

Index	Protein	SC [%]	SVP [%]	IC [%]	MS Score [a.u.]	Var. Mods	Mono Mass [Da]	Chemical Formula
8	H31Kac1	67.2	29.6	27.5	18.5	Ace: K37	15305.4534	C671H1131N215O186S4

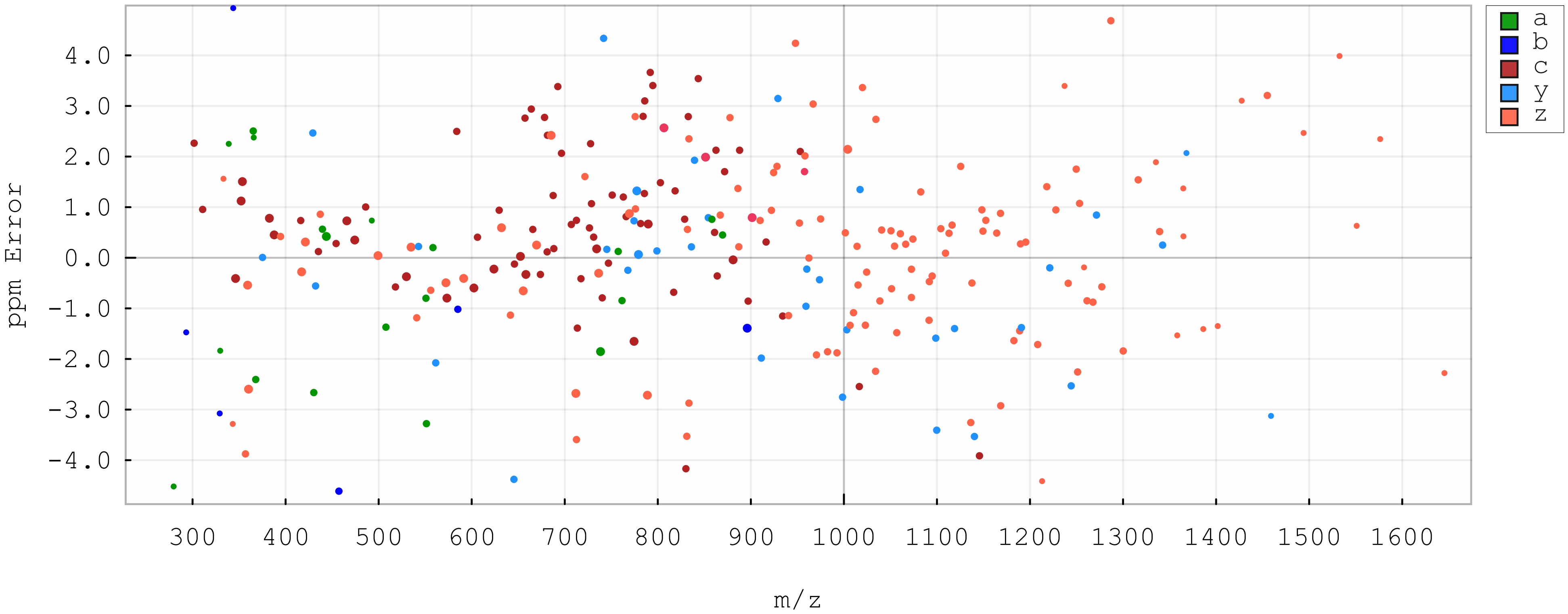

Proteoform:

Index	Protein	SC [%]	SVP [%]	IC [%]	MS Score [a.u.]	Var. Mods	Mono Mass [Da]	Chemical Formula
9	H31Kac1	58.2	29.6	22.0	12.8	Ace: K56	15305.4534	C671H1131N215O186S4

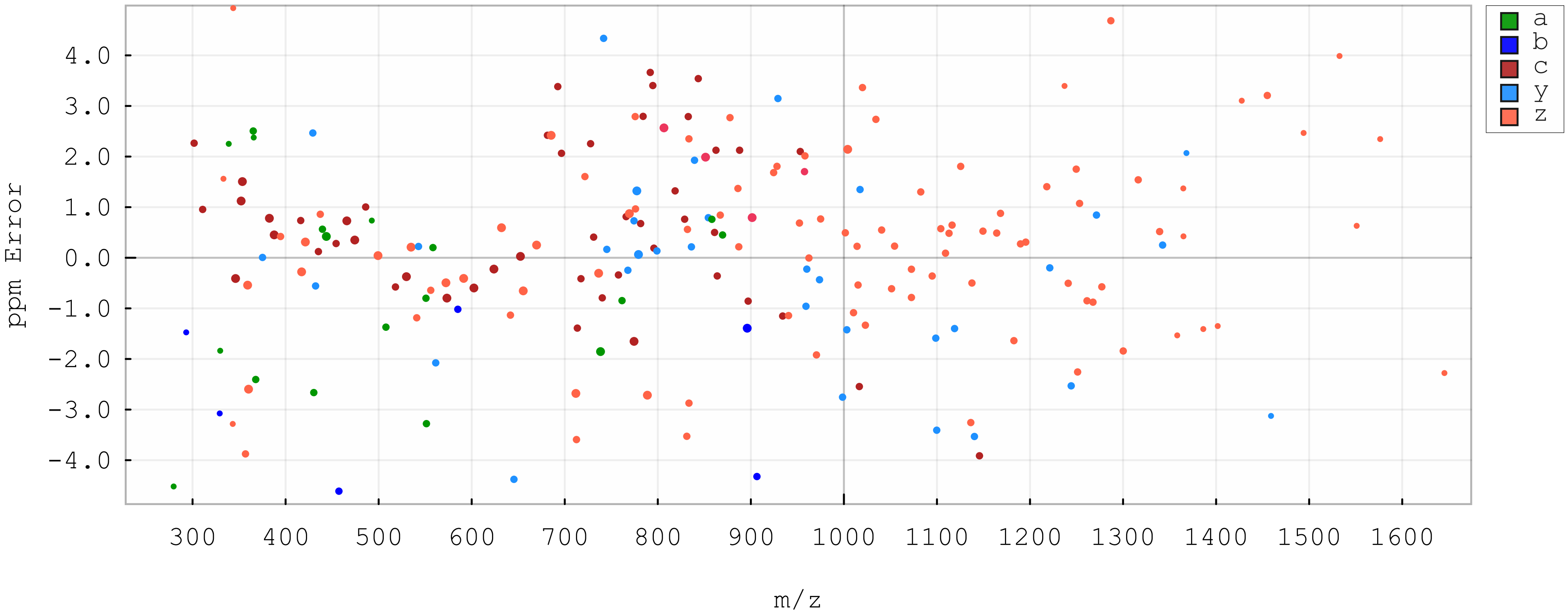

Proteoform:

Index	Protein	SC [%]	SVP [%]	IC [%]	MS Score [a.u.]	Var. Mods	Mono Mass [Da]	Chemical Formula
10	H31Kac1	54.5	29.6	20.7	11.3	Ace: K64	15305.4534	C671H1131N215O186S4

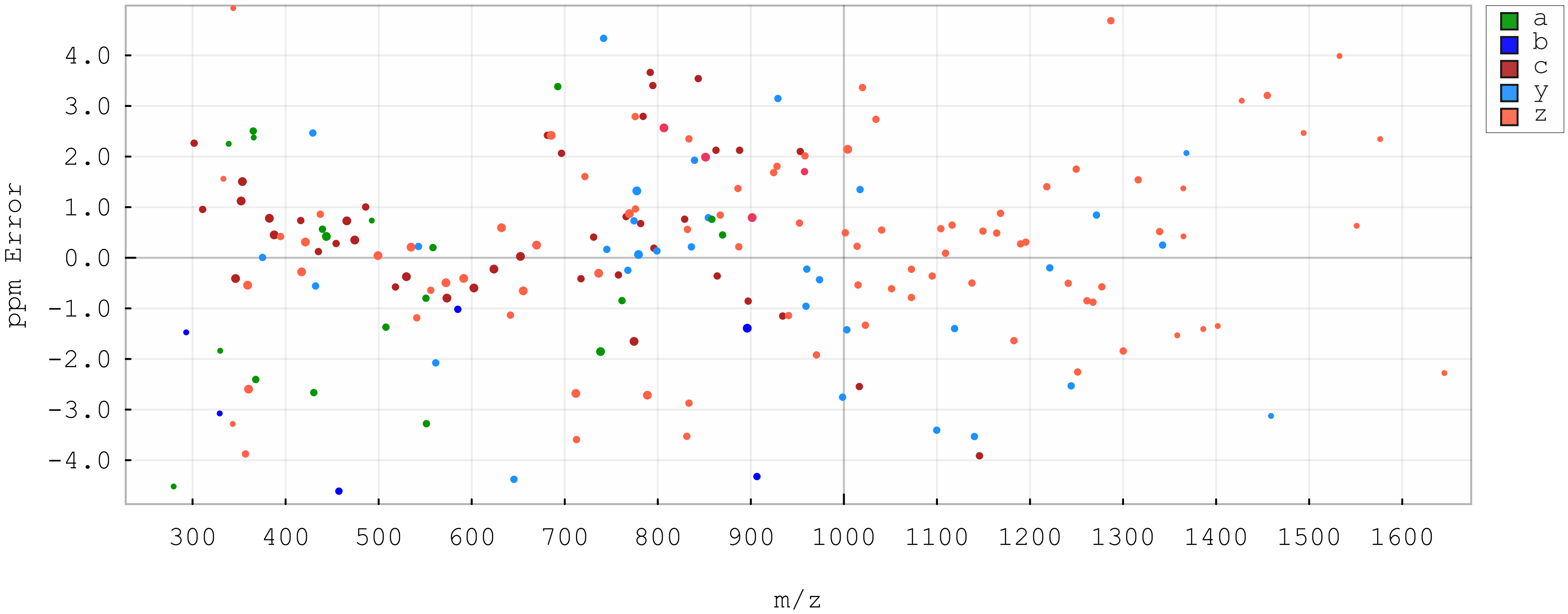

Primary Fragment Plots

Proteoform:

Index	Protein	SC [%]	SVP [%]	IC [%]	MS Score [a.u.]	Var. Mods	Mono Mass [Da]	Chemical Formula
1	H31Kac1	81.3	67.4	35.3	28.7	Ace: K14	15305.4534	C671H1131N215O186S4

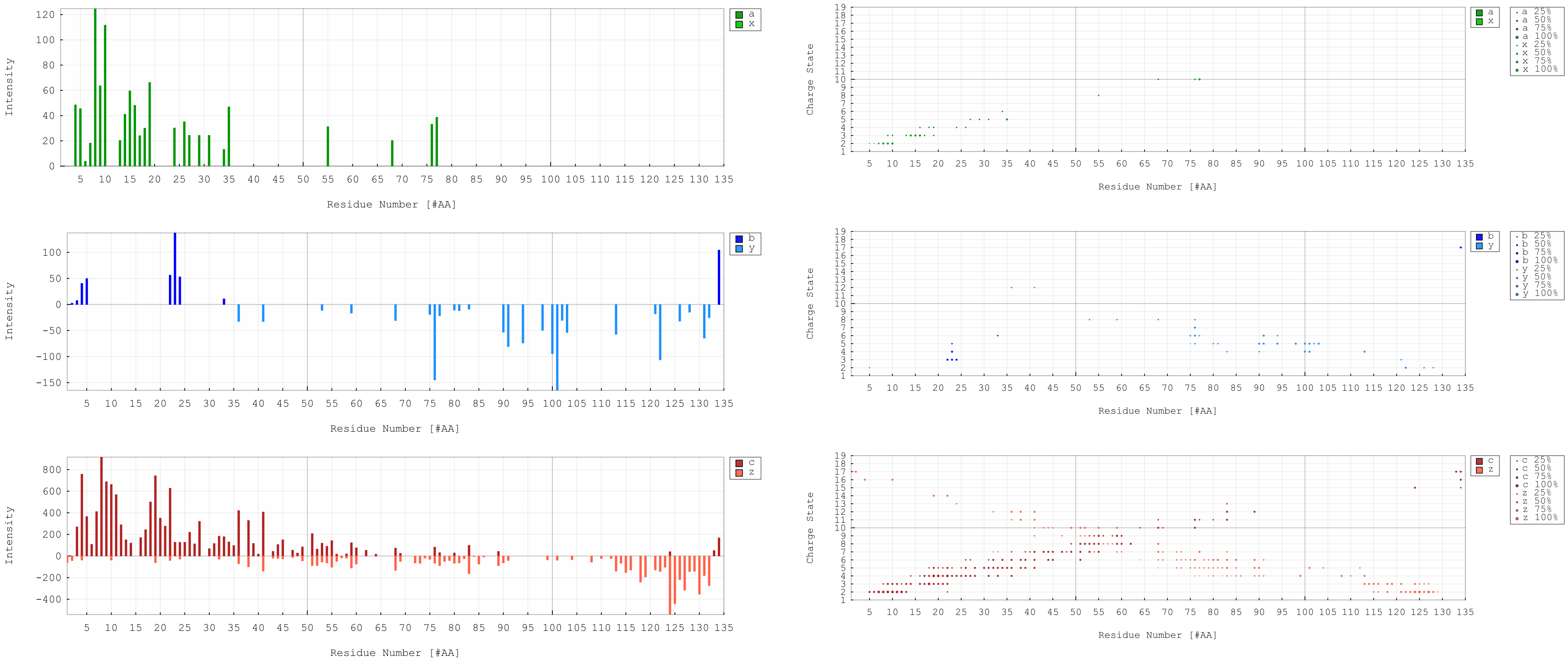

Proteoform:

Index	Protein	SC [%]	SVP [%]	IC [%]	MS Score [a.u.]	Var. Mods	Mono Mass [Da]	Chemical Formula
2	H31Kac1	78.4	29.6	34.6	27.1	Ace: K18	15305.4534	C671H1131N215O186S4

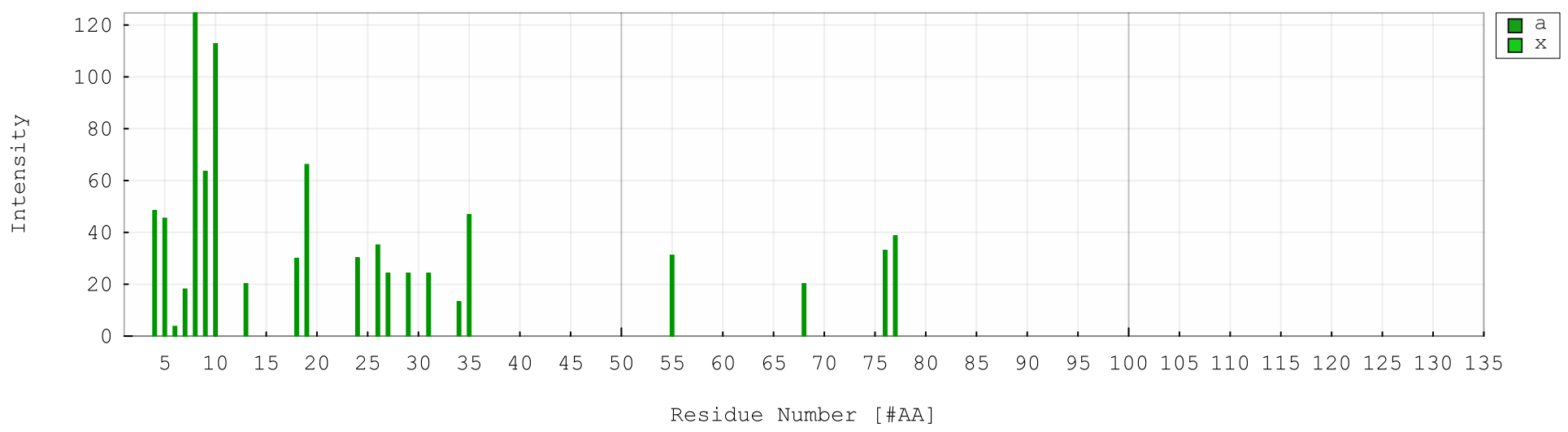

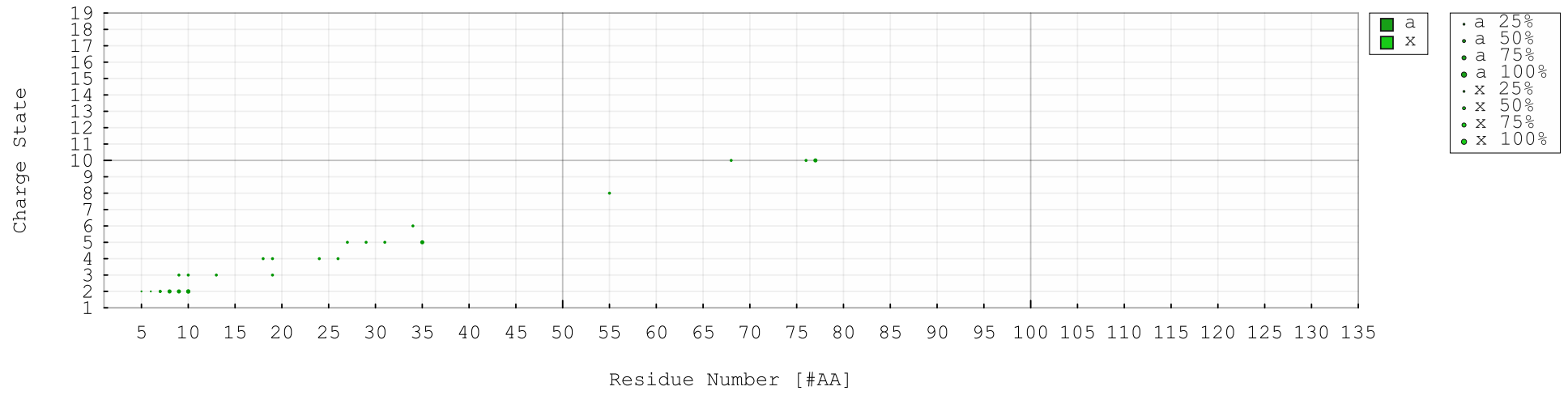

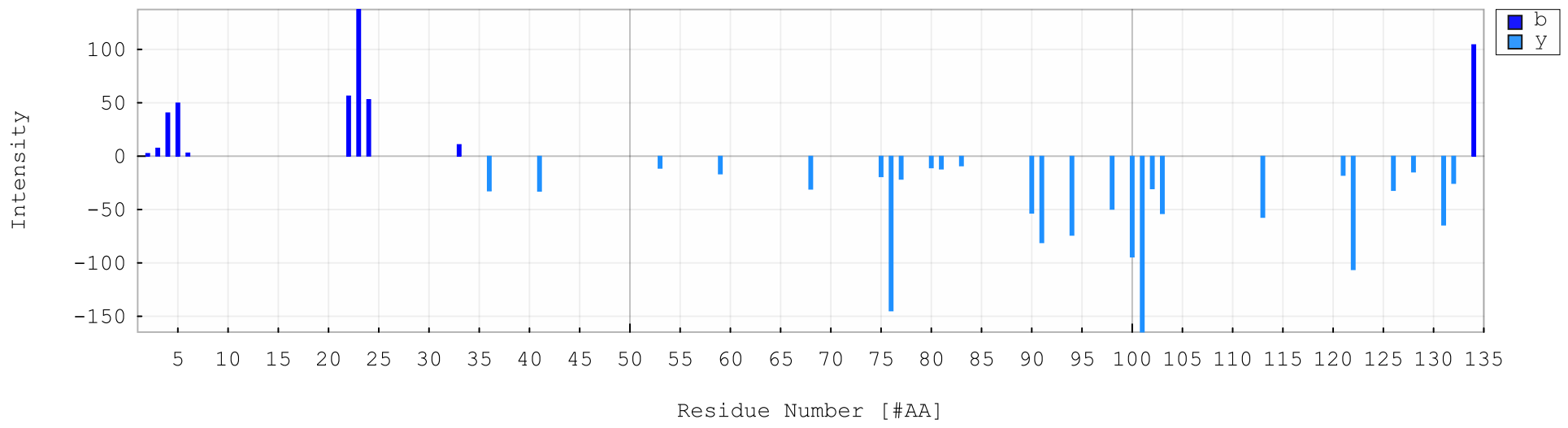

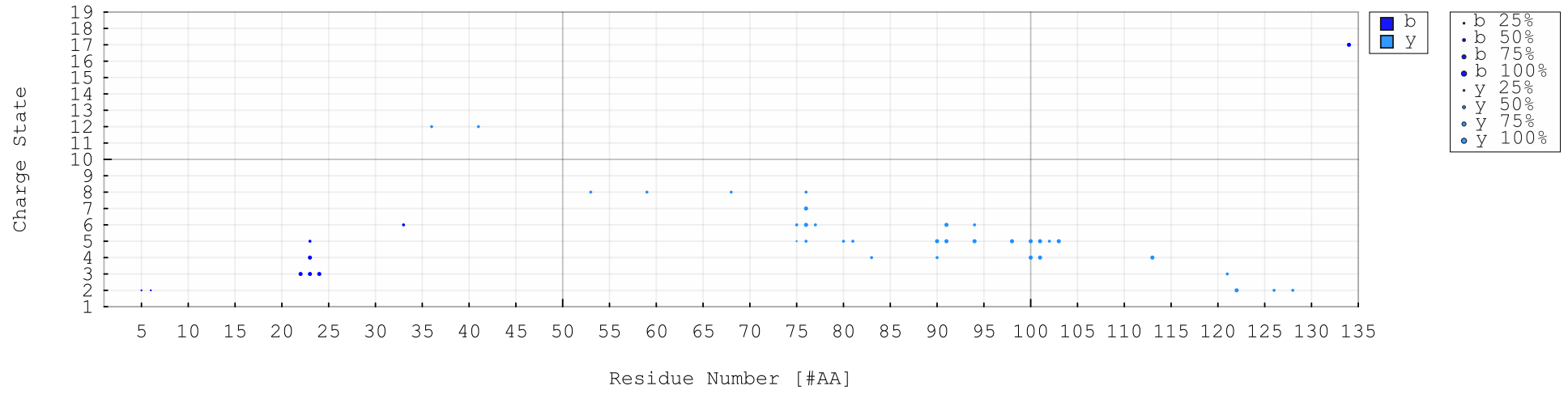

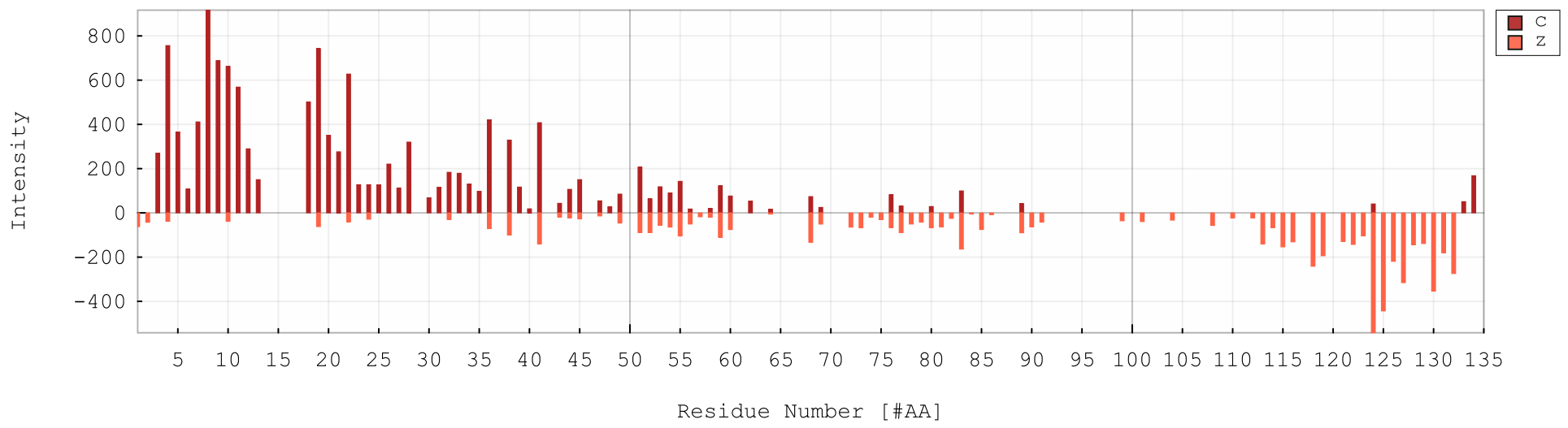

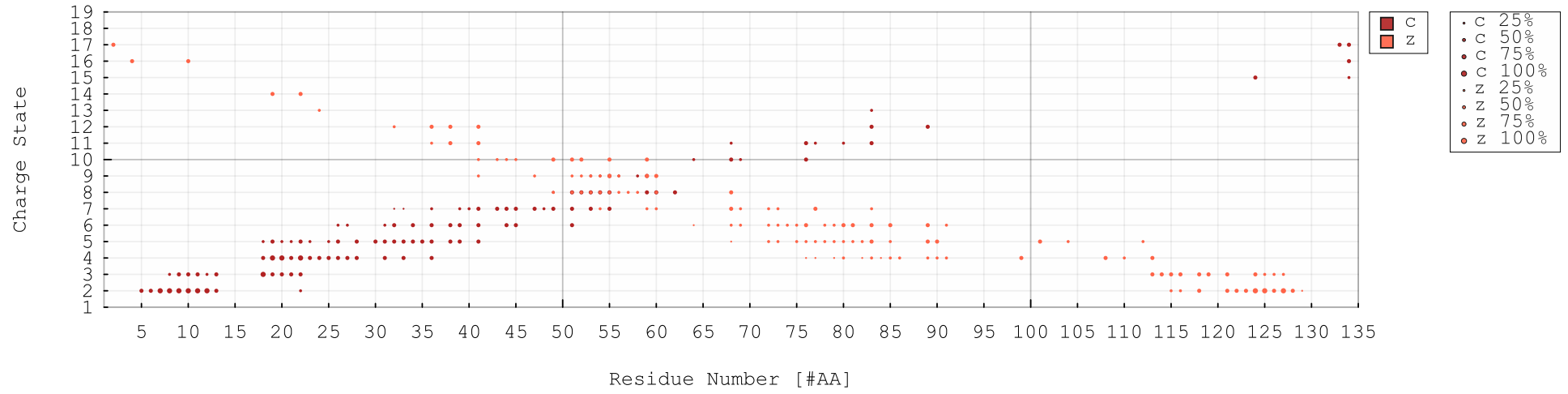

Proteoform:

Index	Protein	SC [%]	SVP [%]	IC [%]	MS Score [a.u.]	Var. Mods	Mono Mass [Da]	Chemical Formula
3	H31Kac1	79.9	27.4	33.4	26.7	Ace: K9	15305.4534	C671H1131N215O186S4

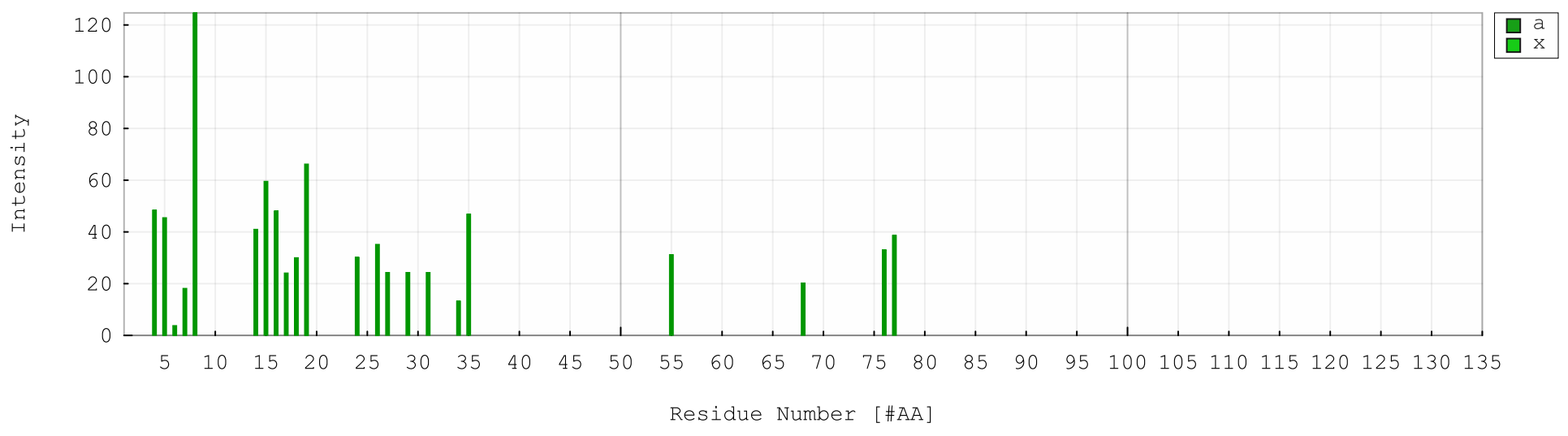

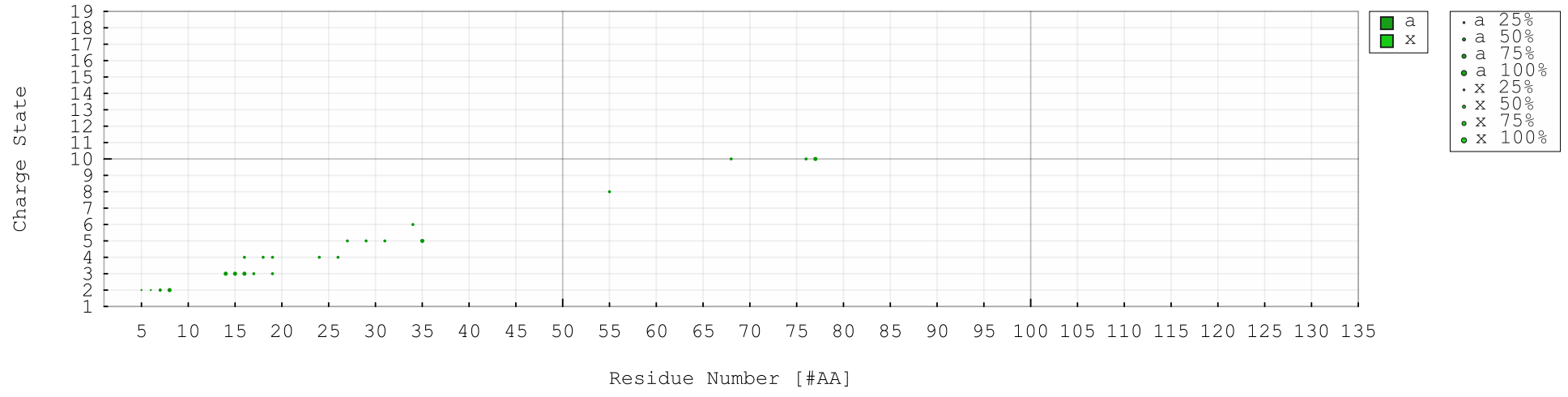

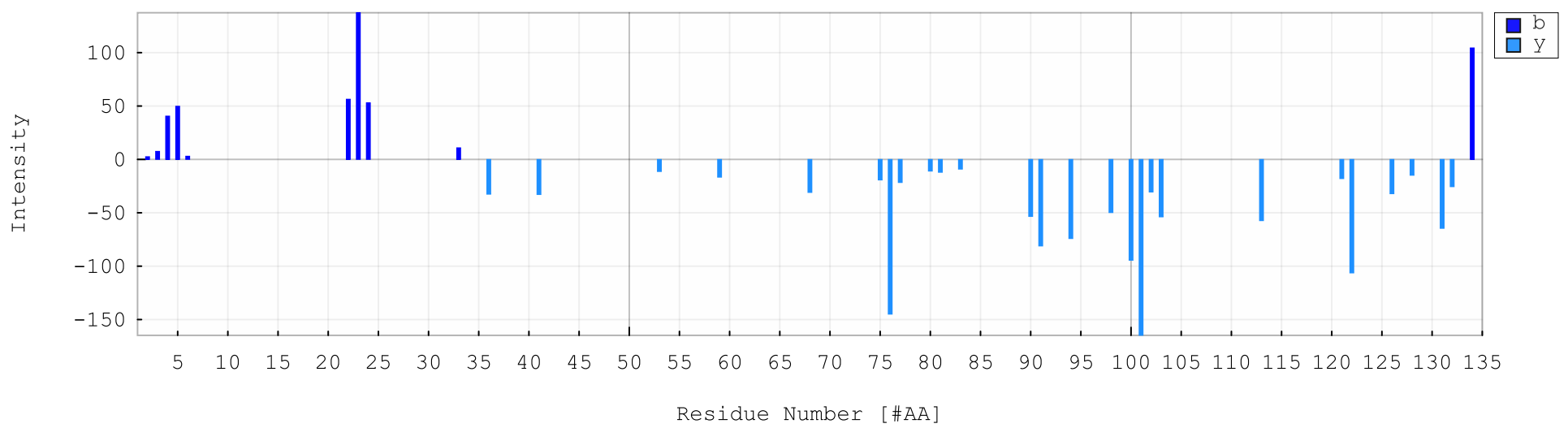

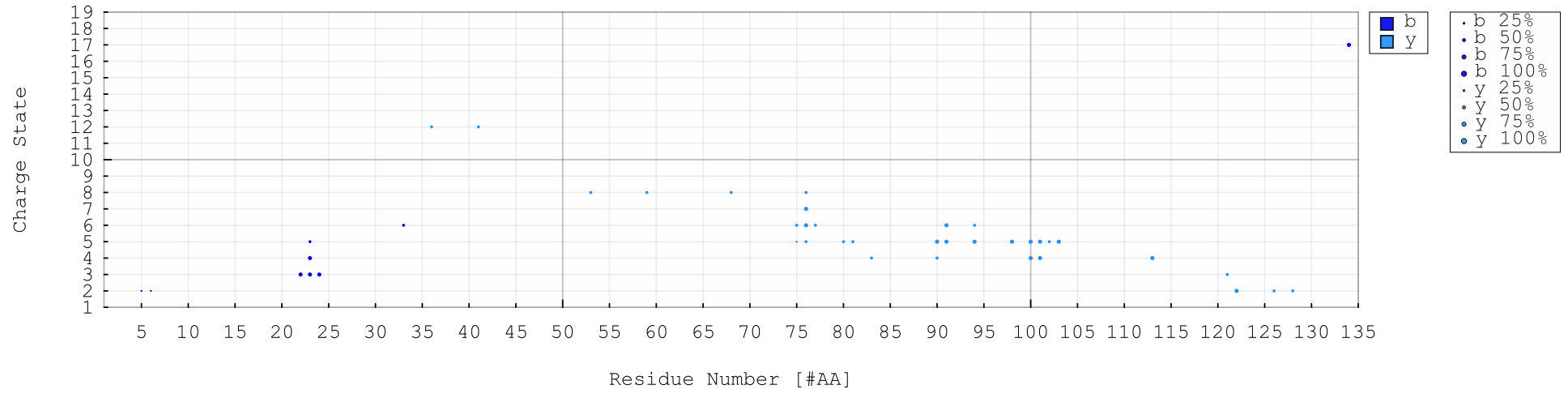

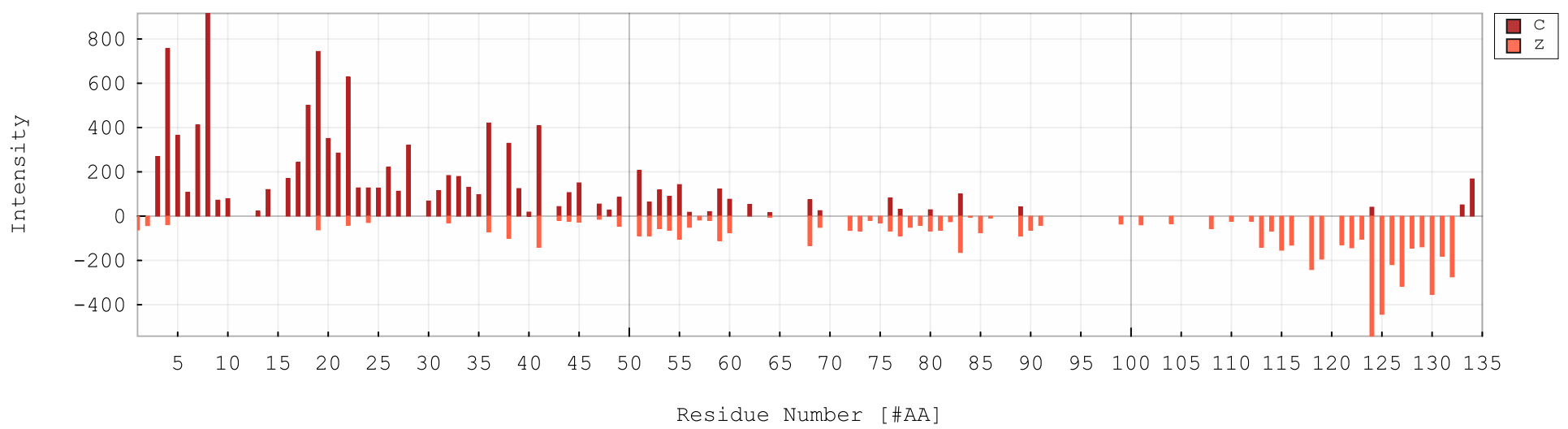

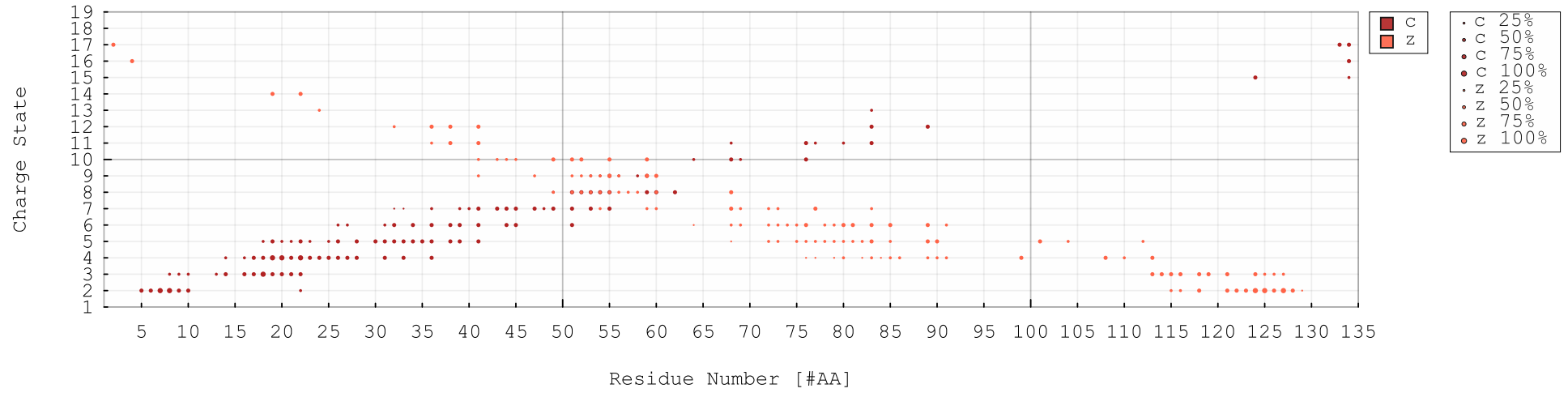

Proteoform:

Index	Protein	SC [%]	SVP [%]	IC [%]	MS Score [a.u.]	Var. Mods	Mono Mass [Da]	Chemical Formula
4	H31Kac1	76.9	29.6	31.4	24.1	Ace: K23	15305.4534	C671H1131N215O186S4

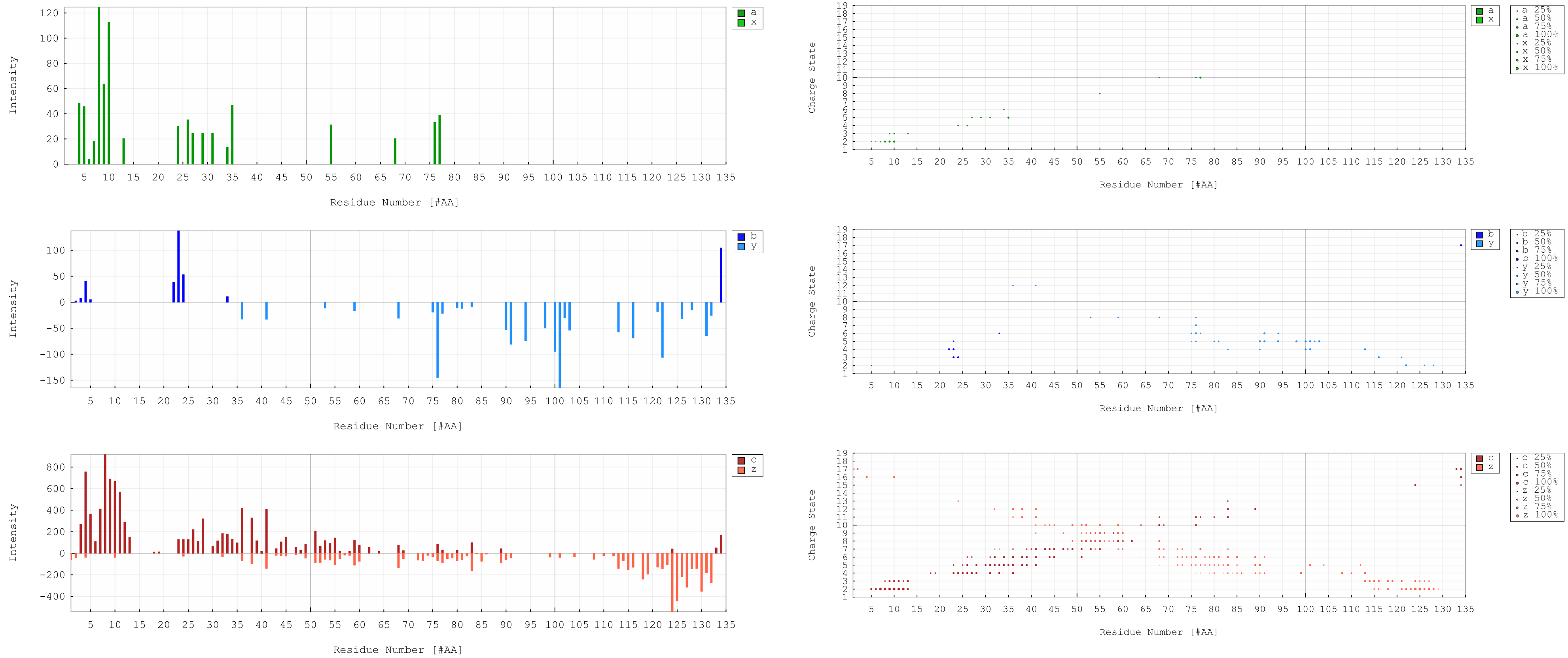

Proteoform:

Index	Protein	SC [%]	SVP [%]	IC [%]	MS Score [a.u.]	Var. Mods	Mono Mass [Da]	Chemical Formula
5	H31Kac1	76.1	22.2	31.7	24.1	Ace: K4	15305.4534	C671H1131N215O186S4

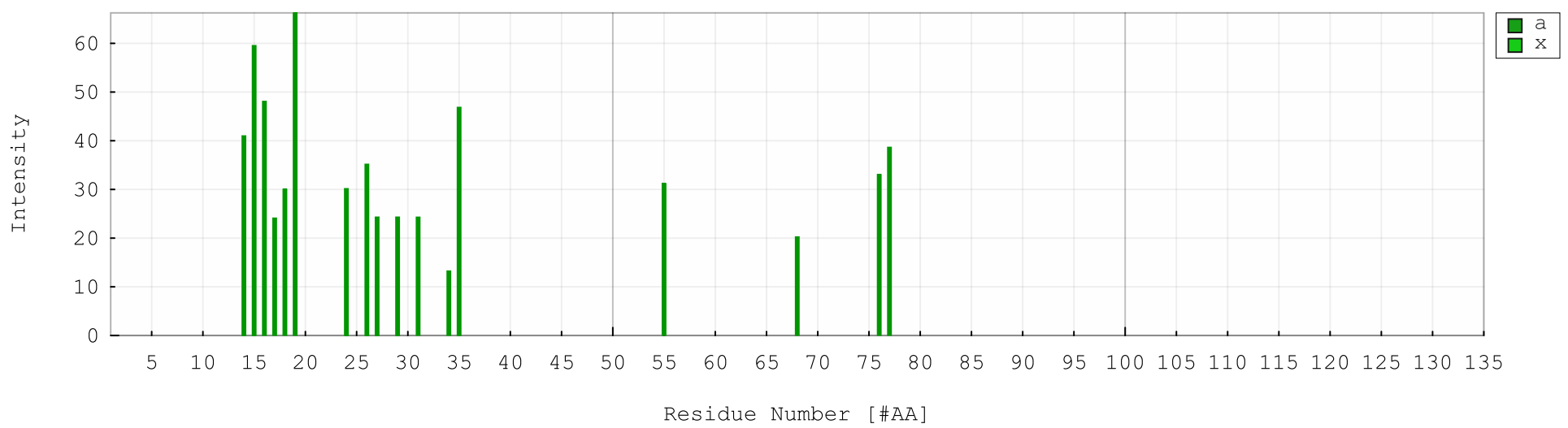

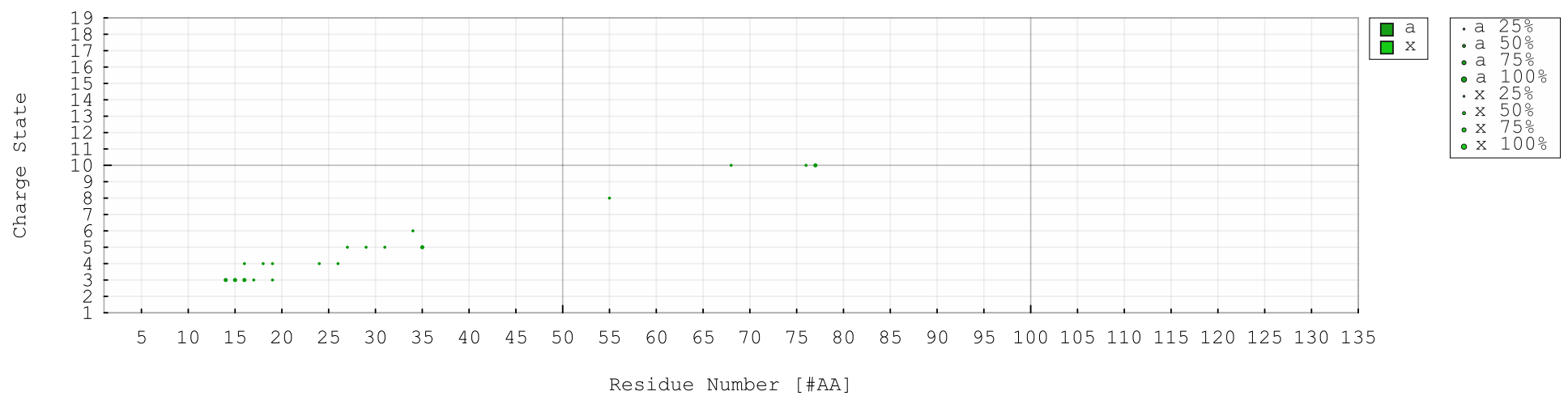

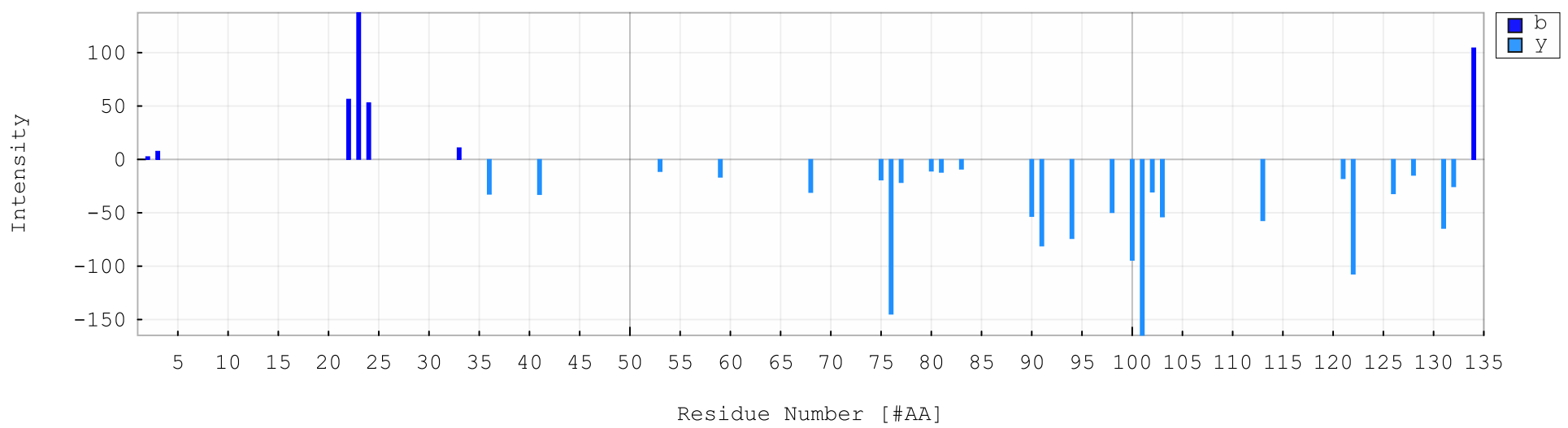

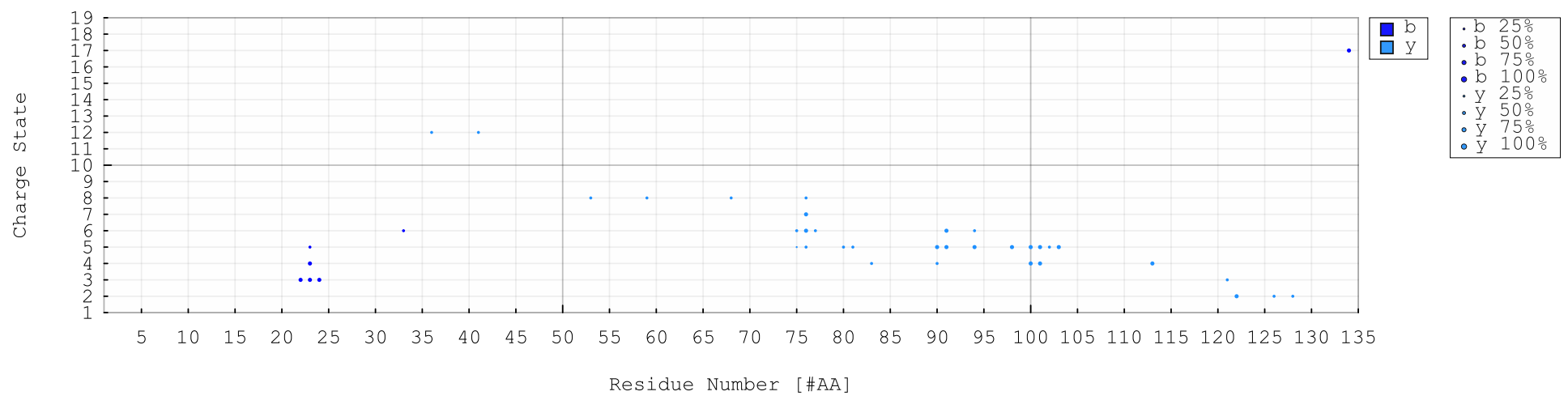

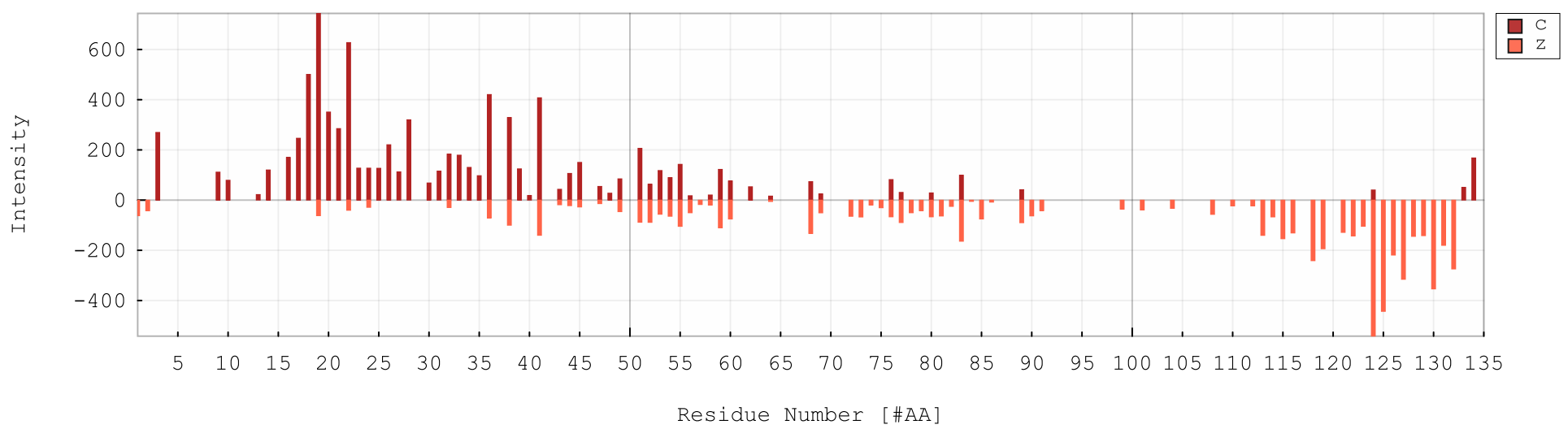

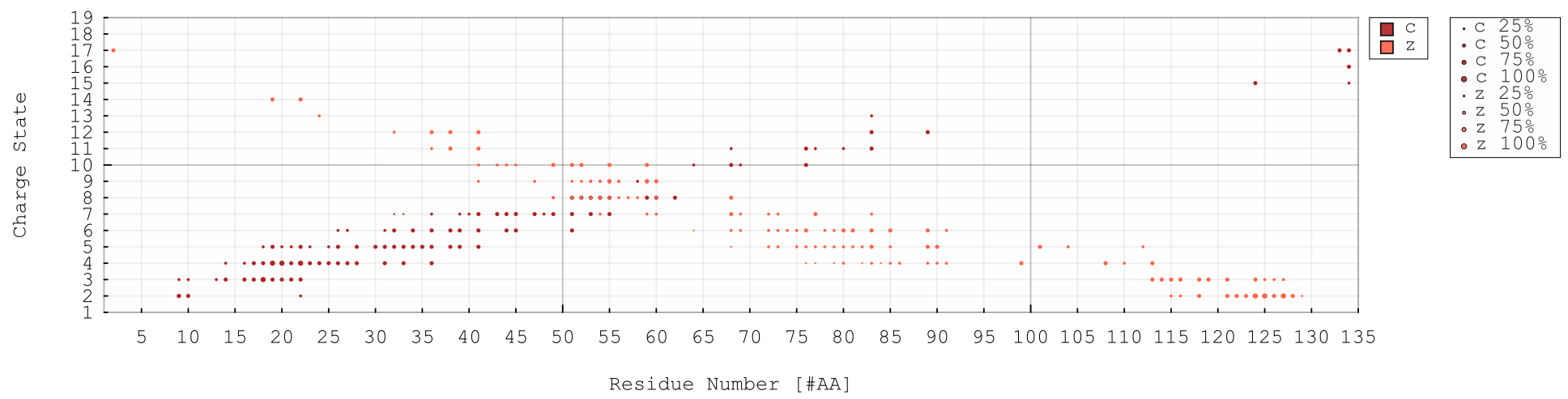

Proteoform:

Index	Protein	SC [%]	SVP [%]	IC [%]	MS Score [a.u.]	Var. Mods	Mono Mass [Da]	Chemical Formula
6	H31Kac1	73.9	29.6	30.2	22.3	Ace: K27	15305.4534	C671H1131N215O186S4

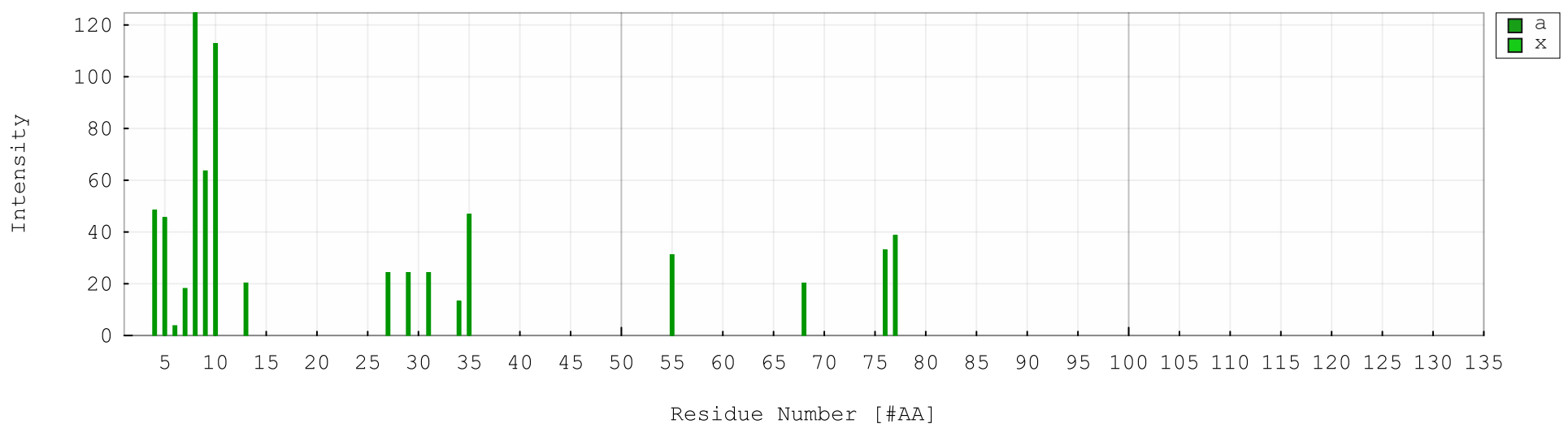

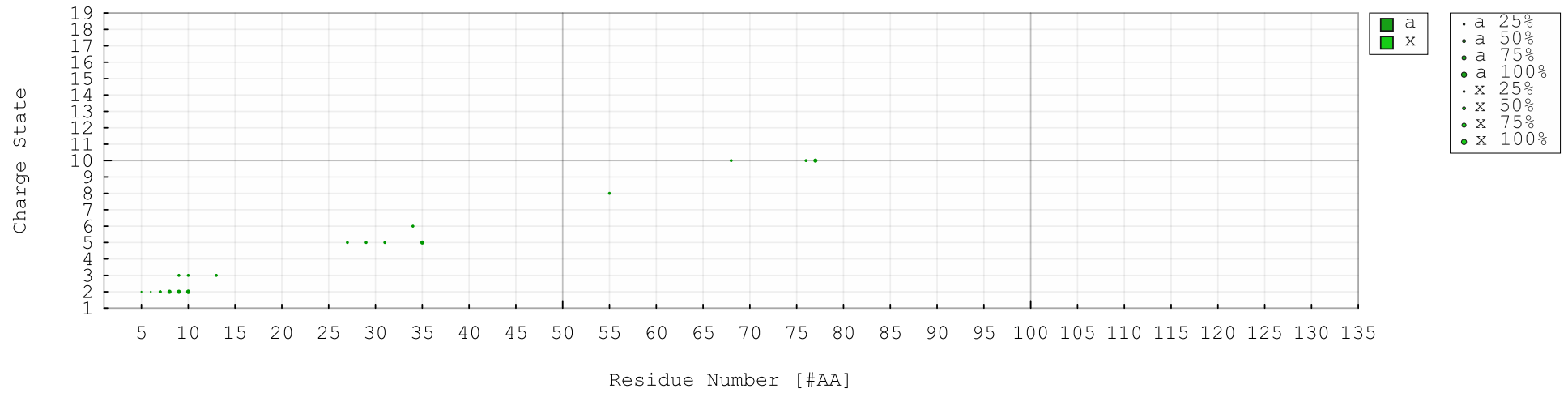

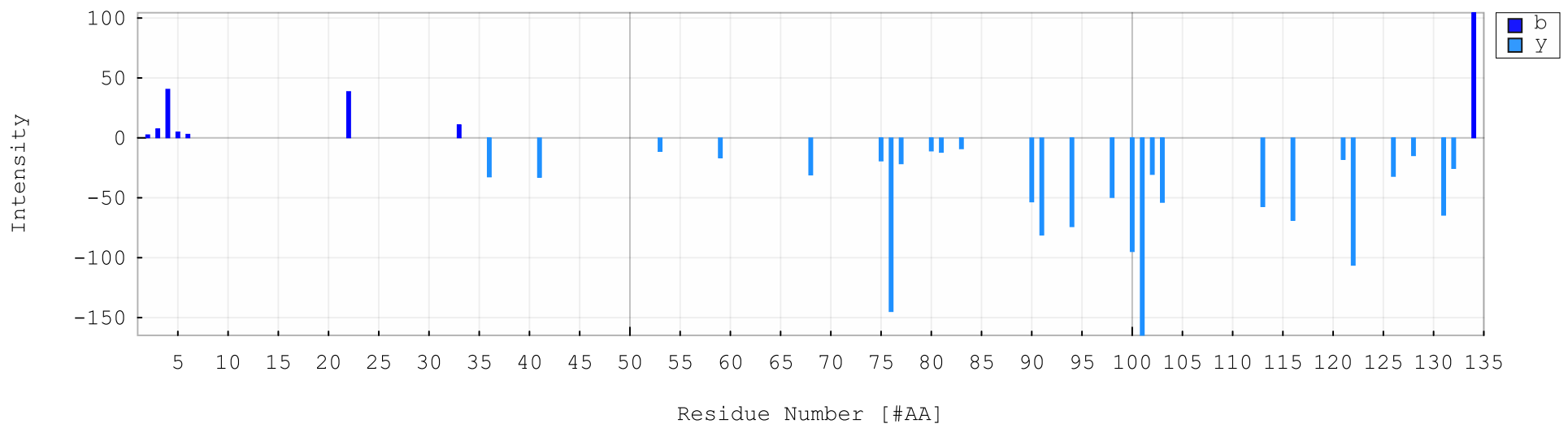

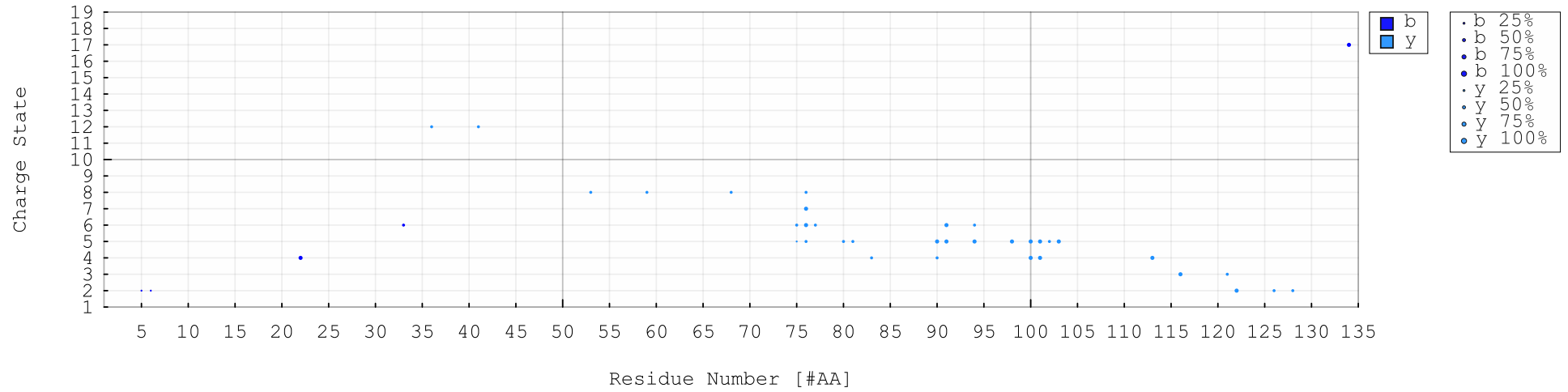

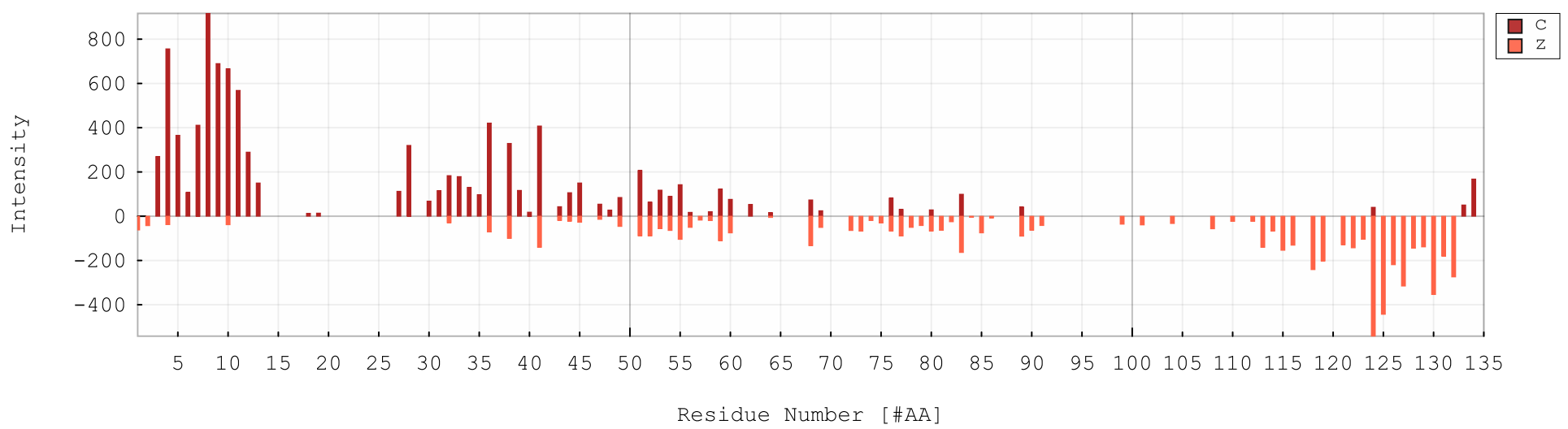

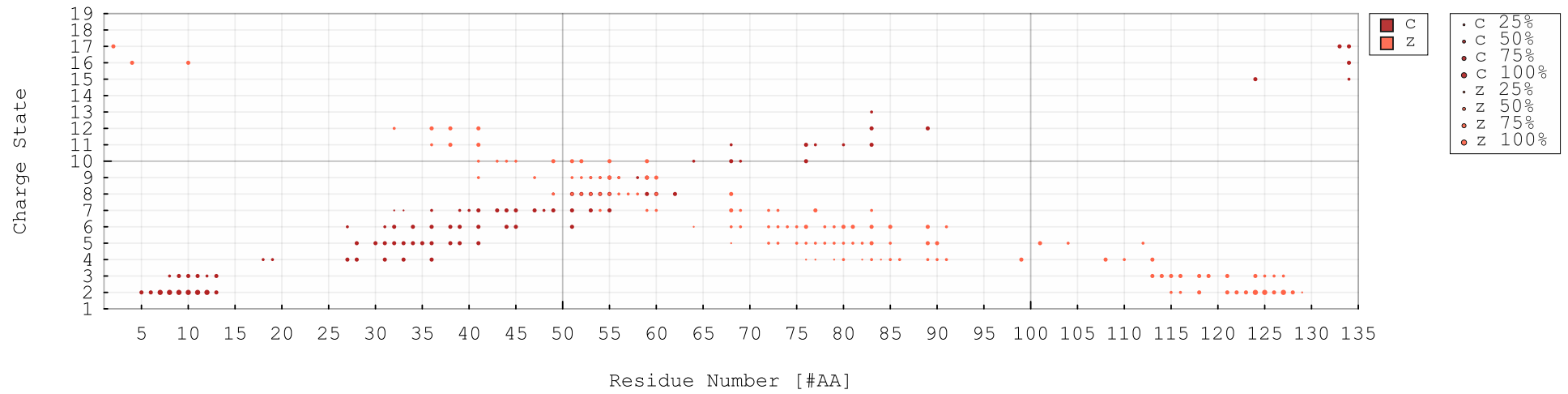

Proteoform:

Index	Protein	SC [%]	SVP [%]	IC [%]	MS Score [a.u.]	Var. Mods	Mono Mass [Da]	Chemical Formula
7	H31Kac1	67.9	29.6	28.4	19.3	Ace: K36	15305.4534	C671H1131N215O186S4

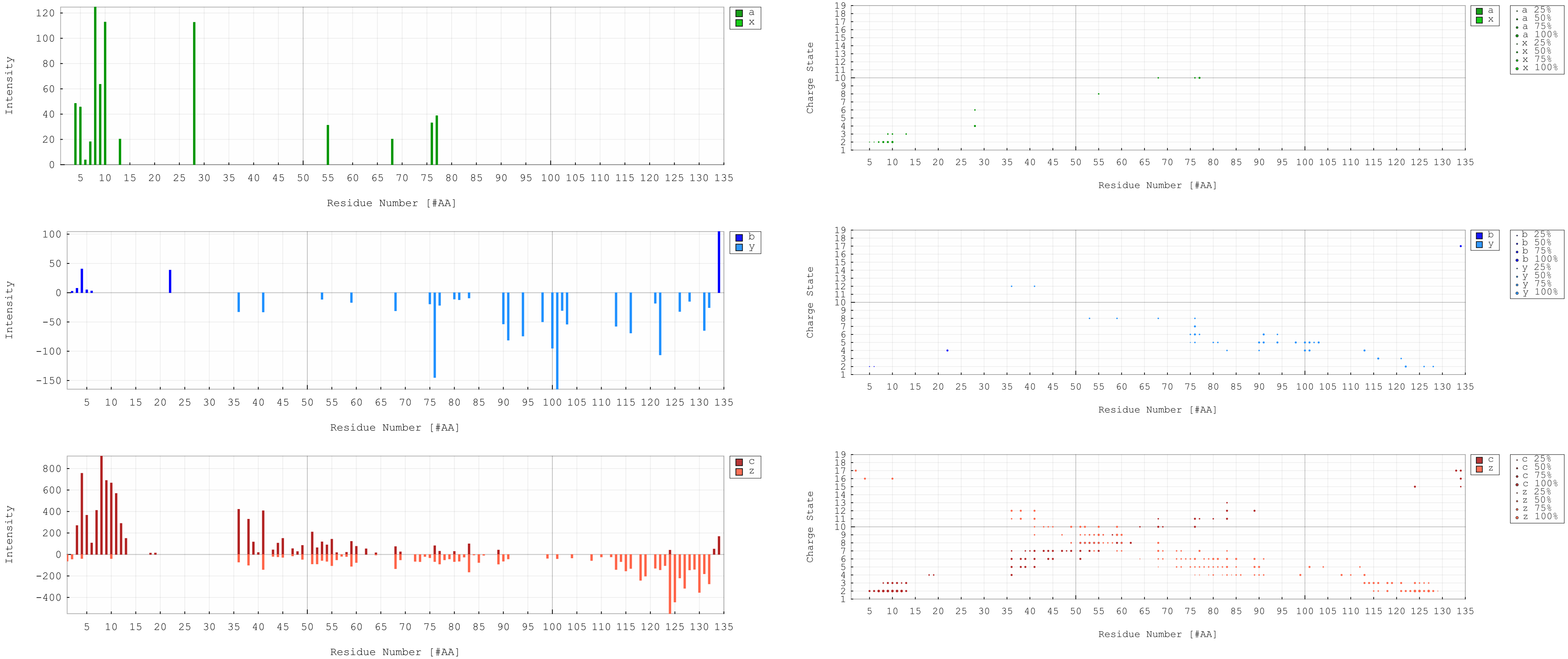

Proteoform:

Index	Protein	SC [%]	SVP [%]	IC [%]	MS Score [a.u.]	Var. Mods	Mono Mass [Da]	Chemical Formula
8	H31Kac1	67.2	29.6	27.5	18.5	Ace: K37	15305.4534	C671H1131N215O186S4

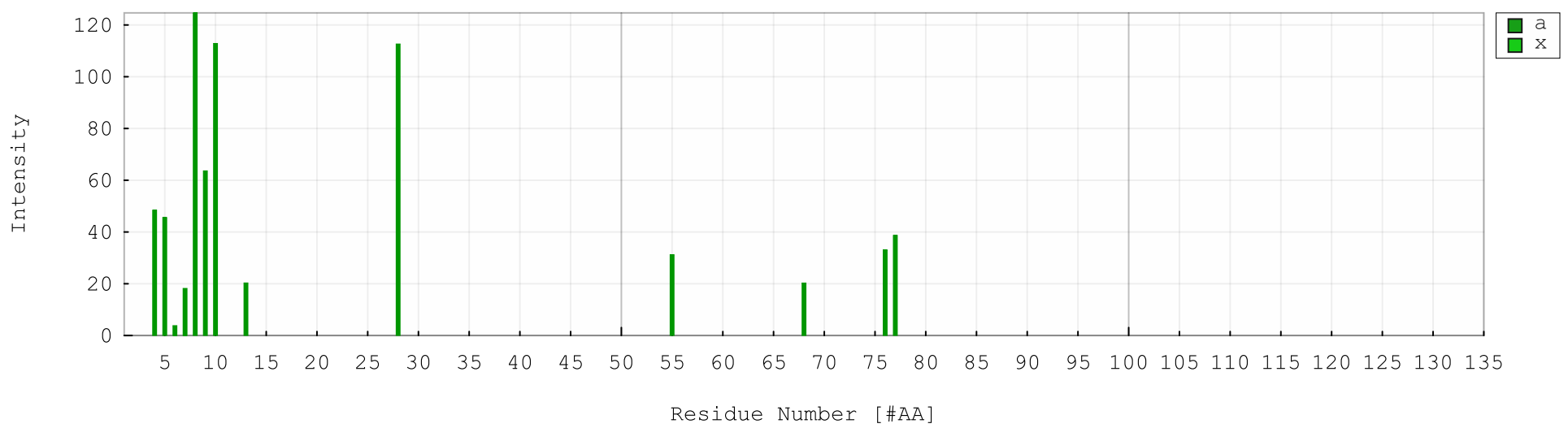
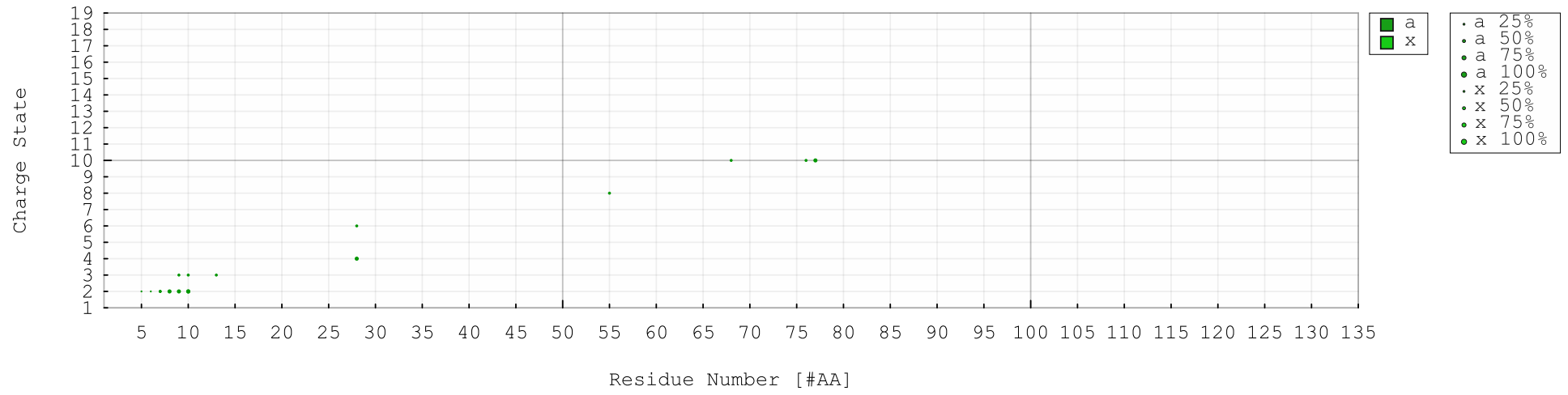
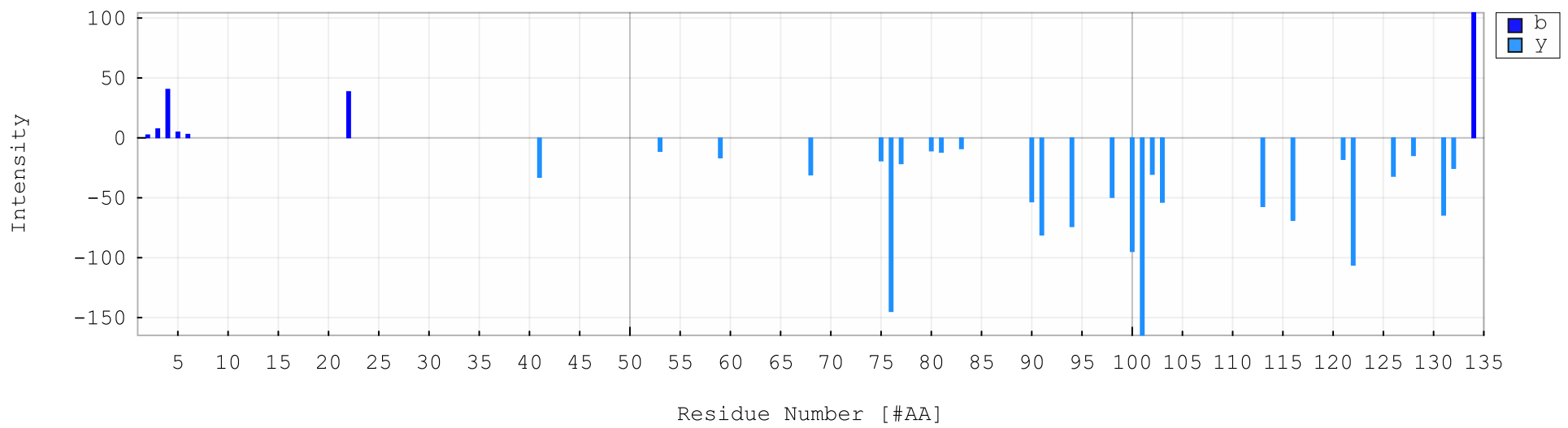
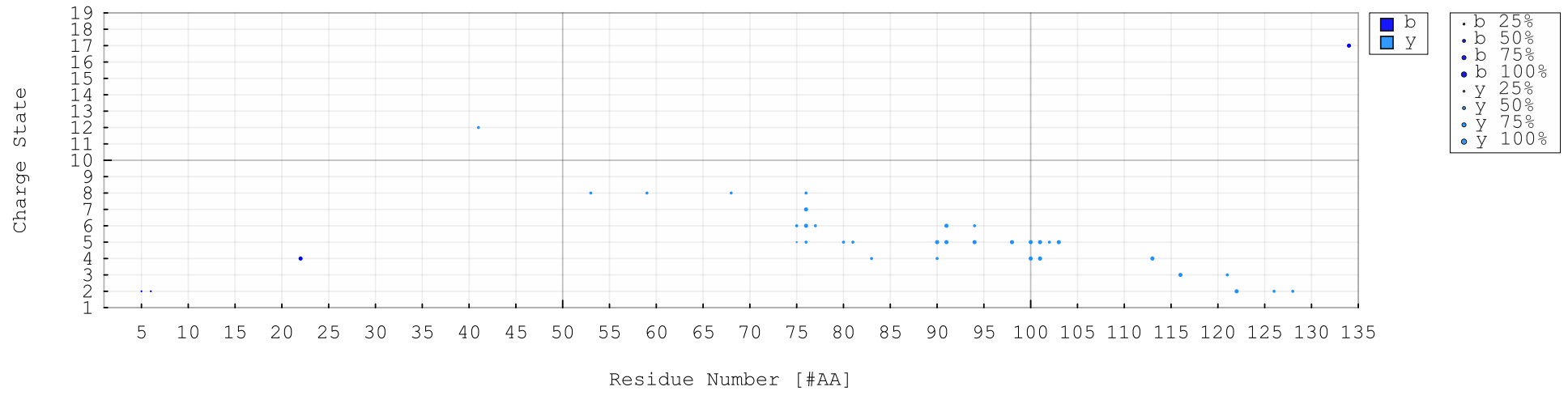
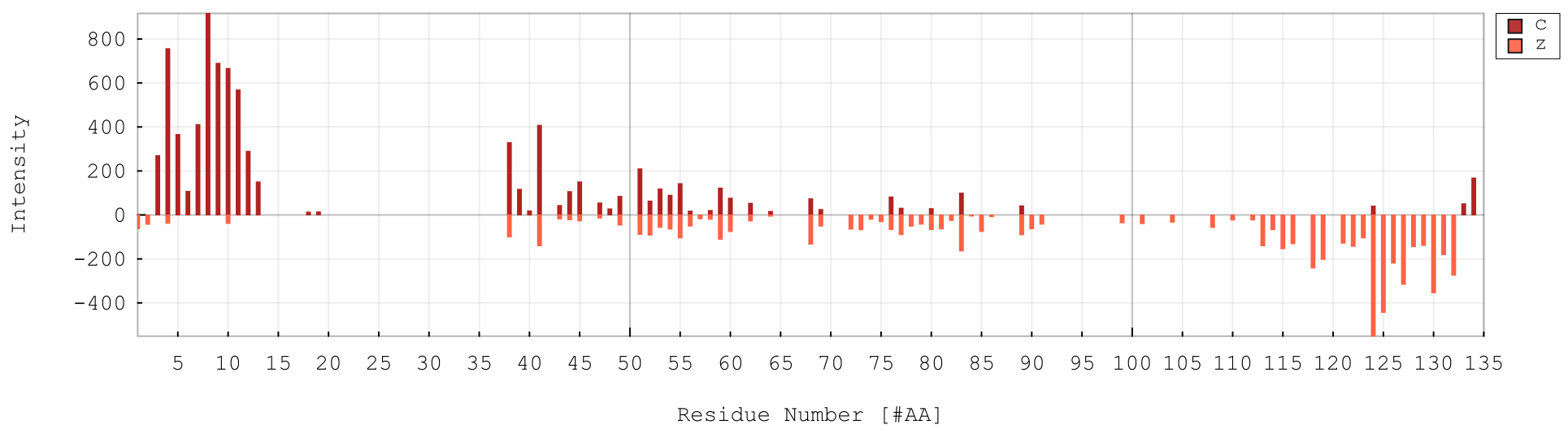
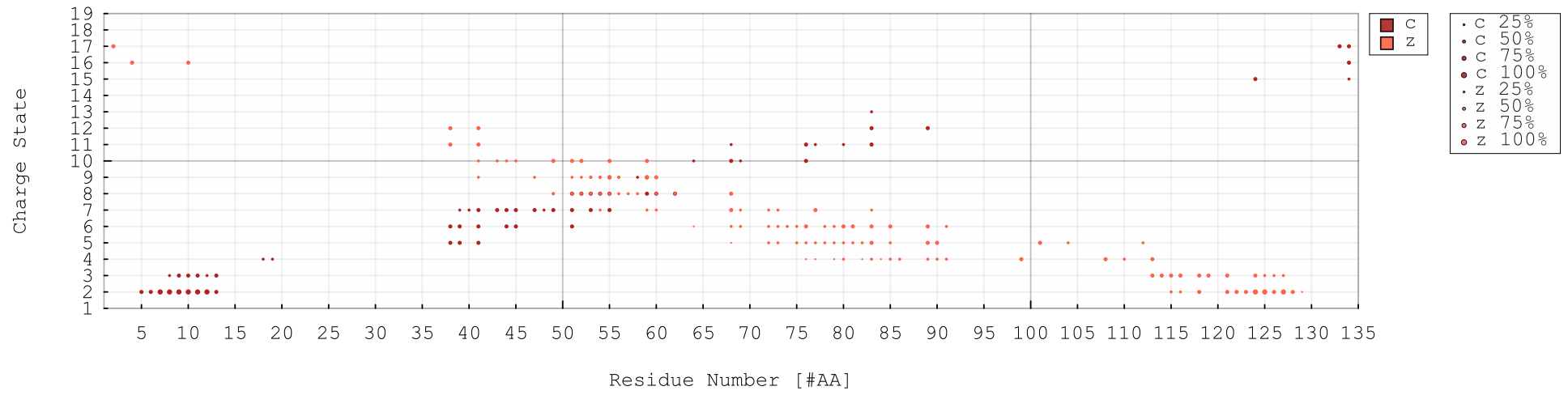

Proteoform:

Index	Protein	SC [%]	SVP [%]	IC [%]	MS Score [a.u.]	Var. Mods	Mono Mass [Da]	Chemical Formula
9	H31Kac1	58.2	29.6	22.0	12.8	Ace: K56	15305.4534	C671H1131N215O186S4

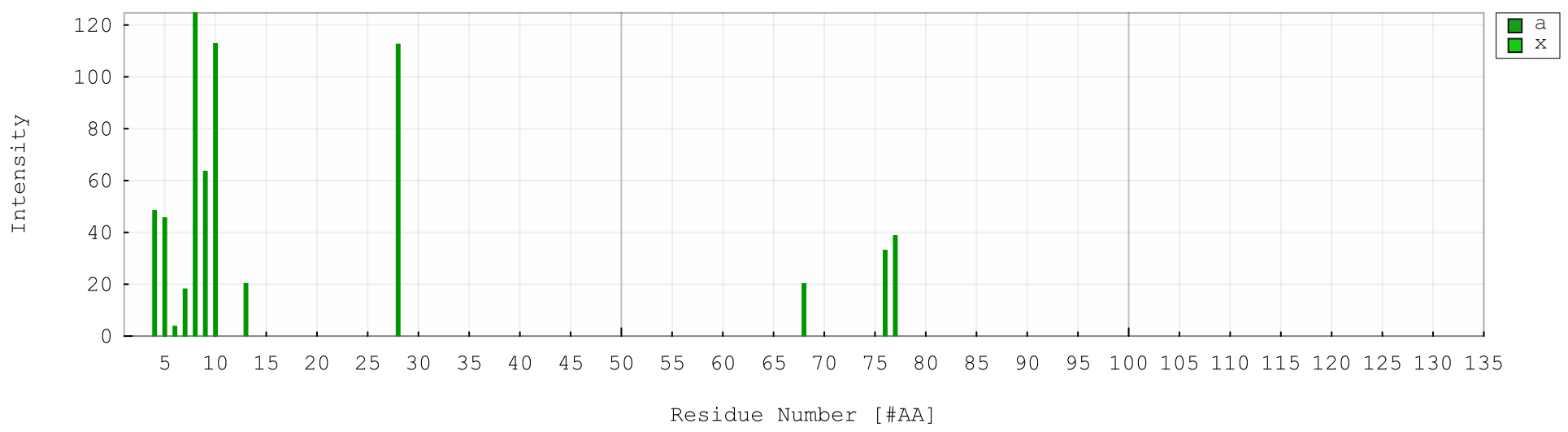

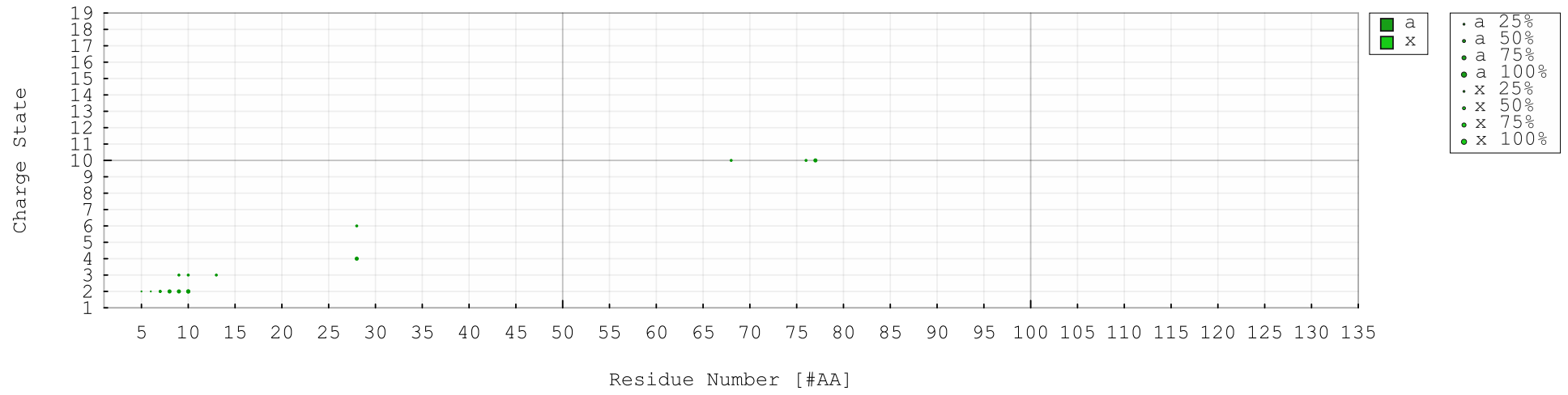

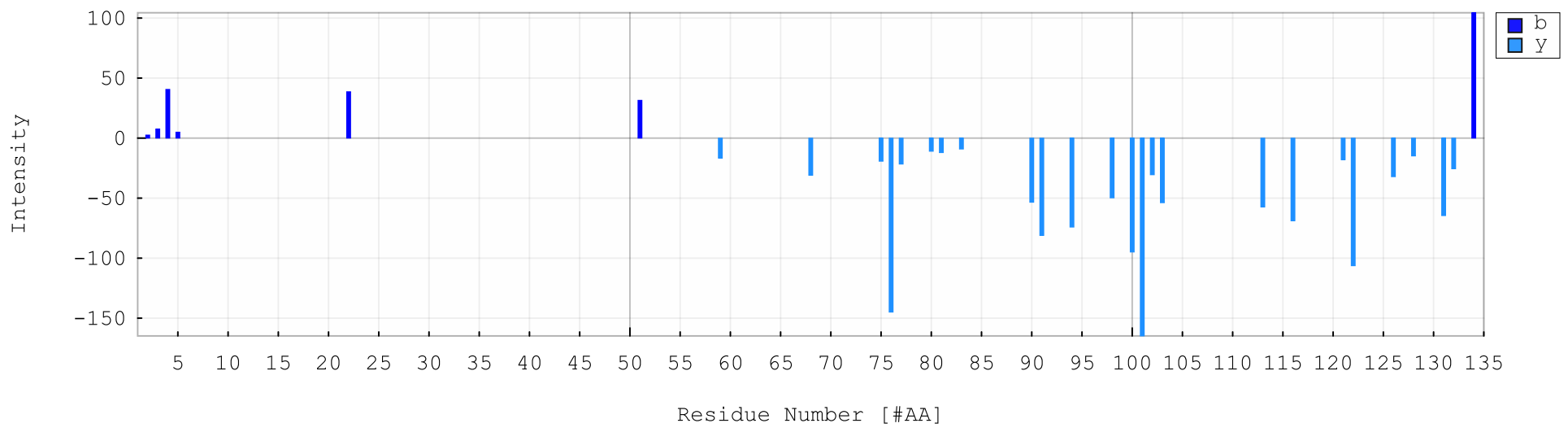

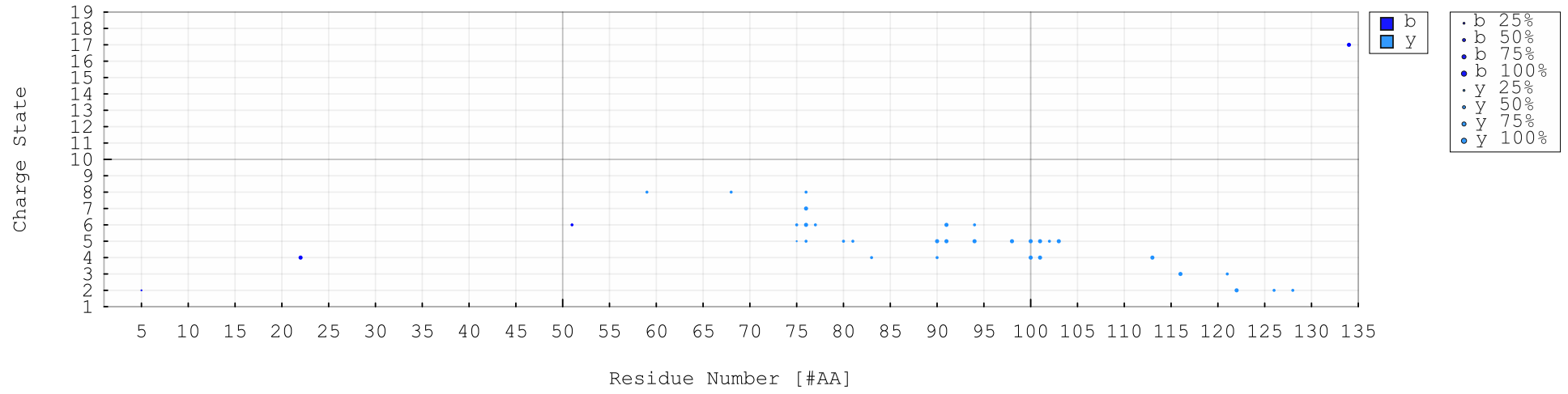

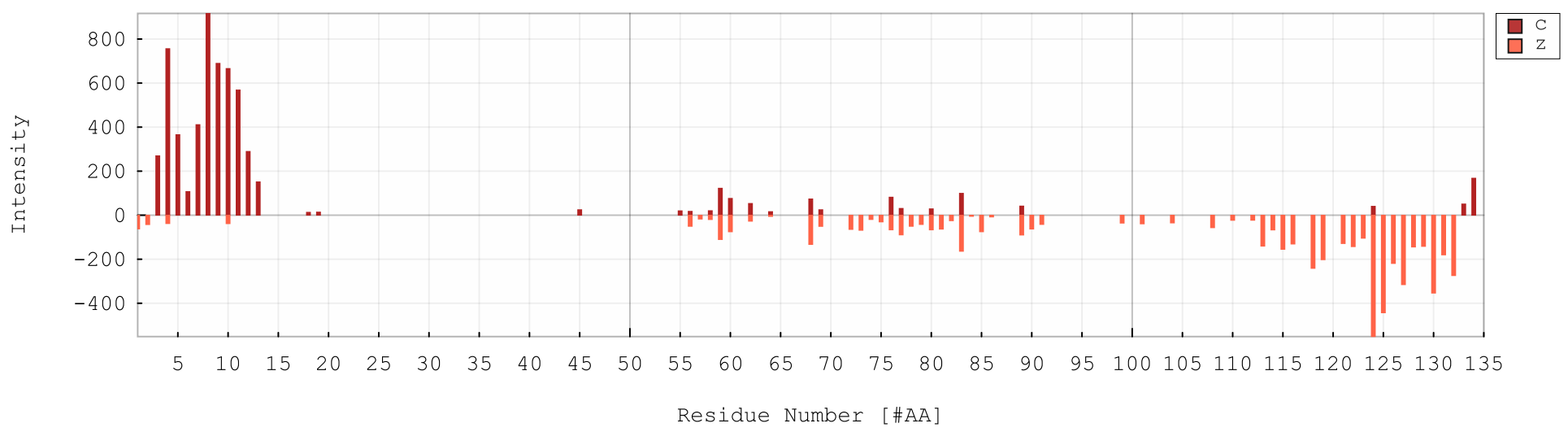

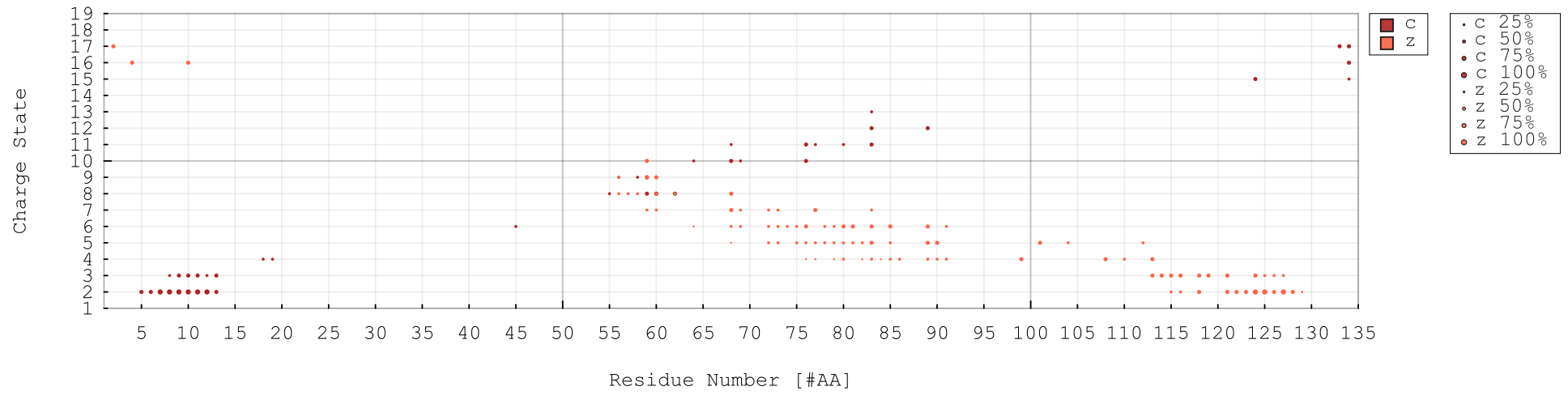

Proteoform:

Index	Protein	SC [%]	SVP [%]	IC [%]	MS Score [a.u.]	Var. Mods	Mono Mass [Da]	Chemical Formula
10	H31Kac1	54.5	29.6	20.7	11.3	Ace: K64	15305.4534	C671H1131N215O186S4

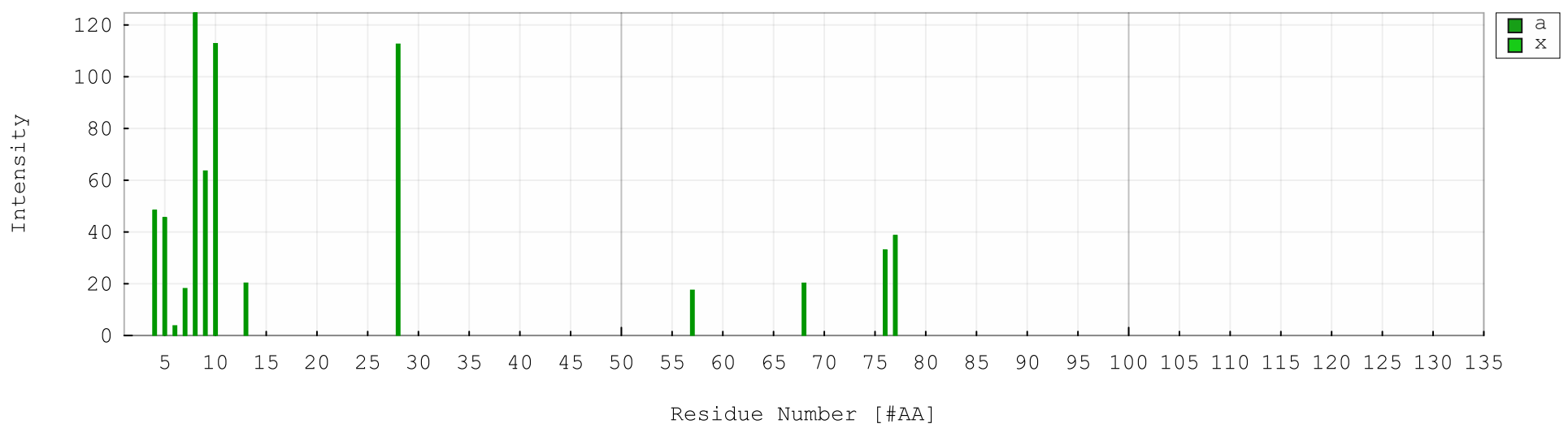

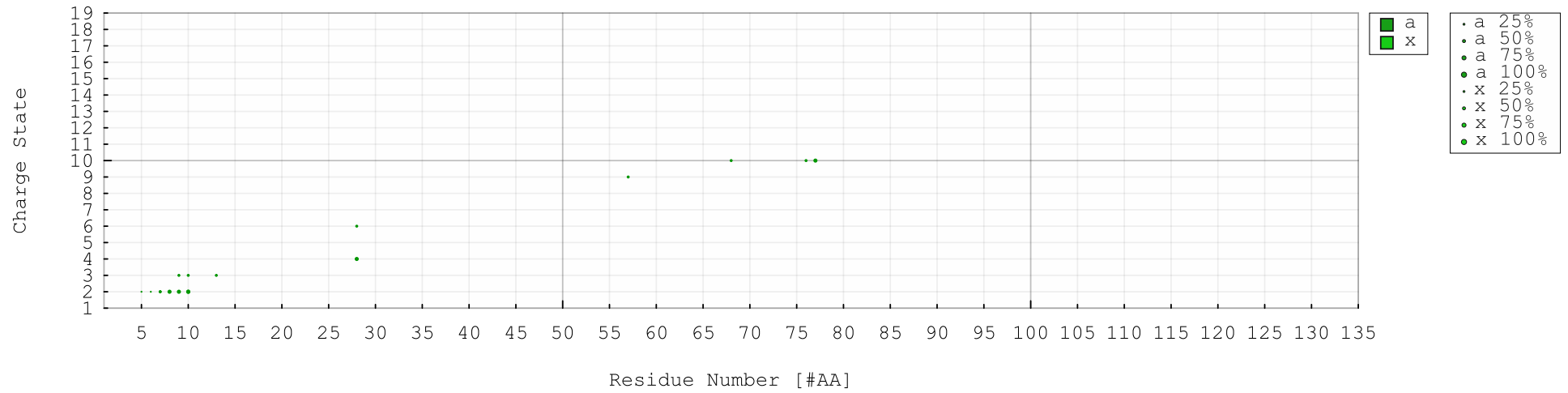

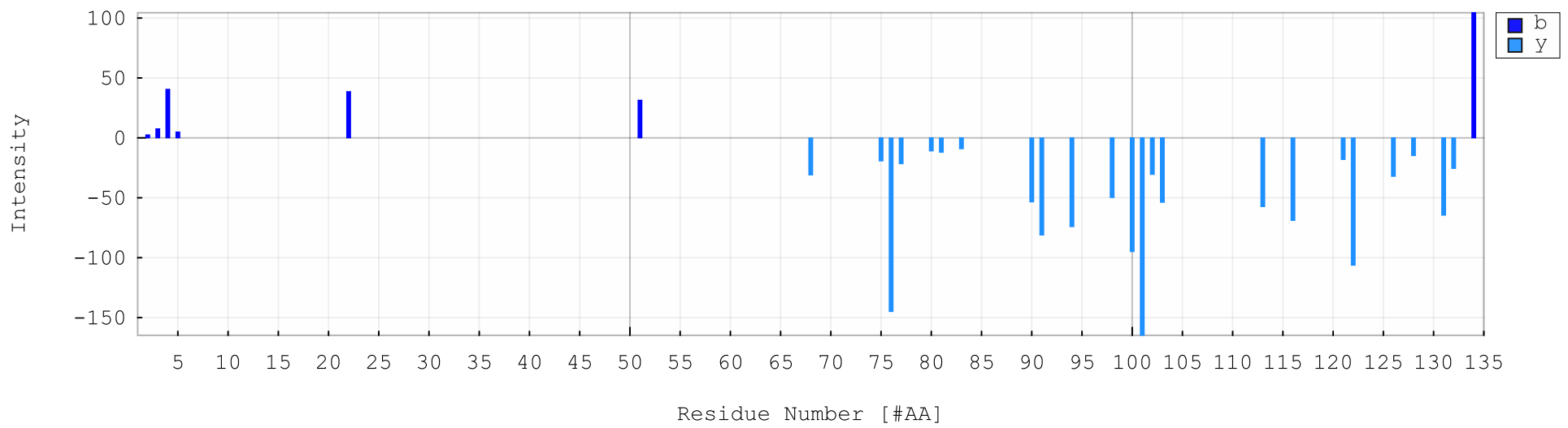

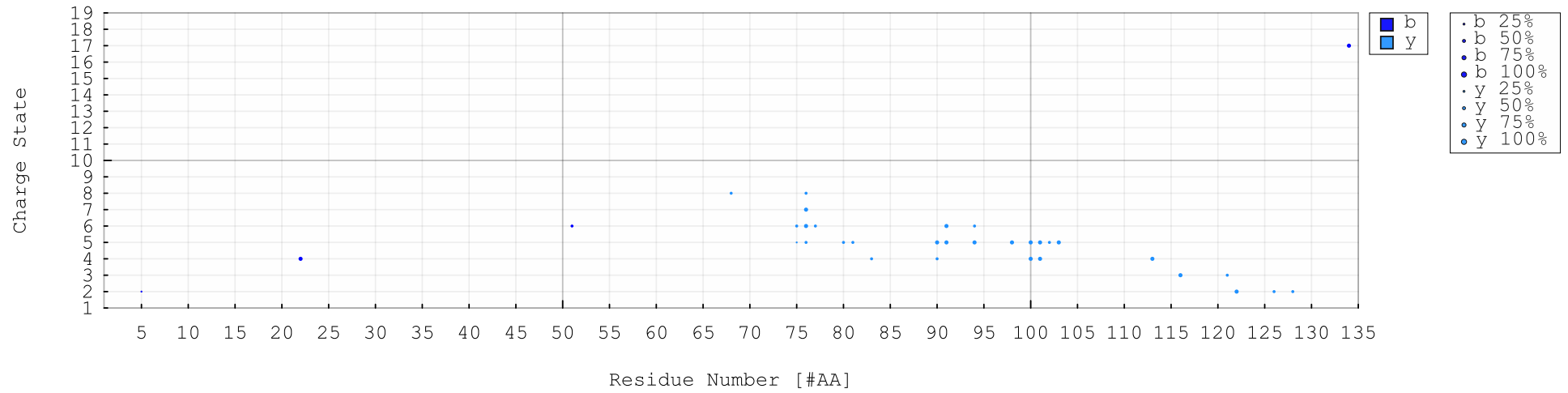

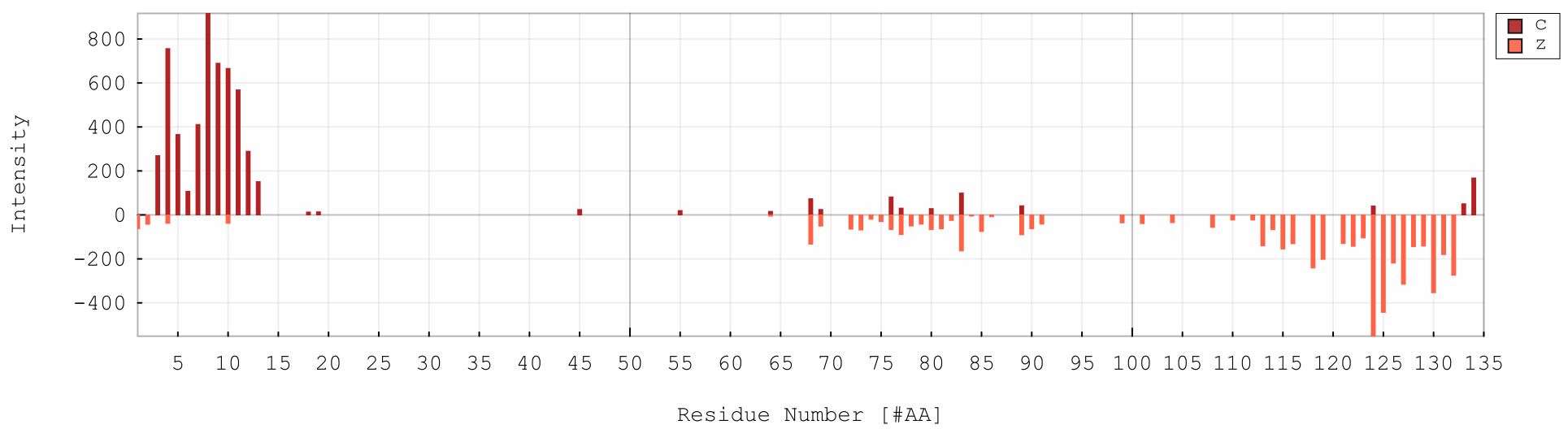

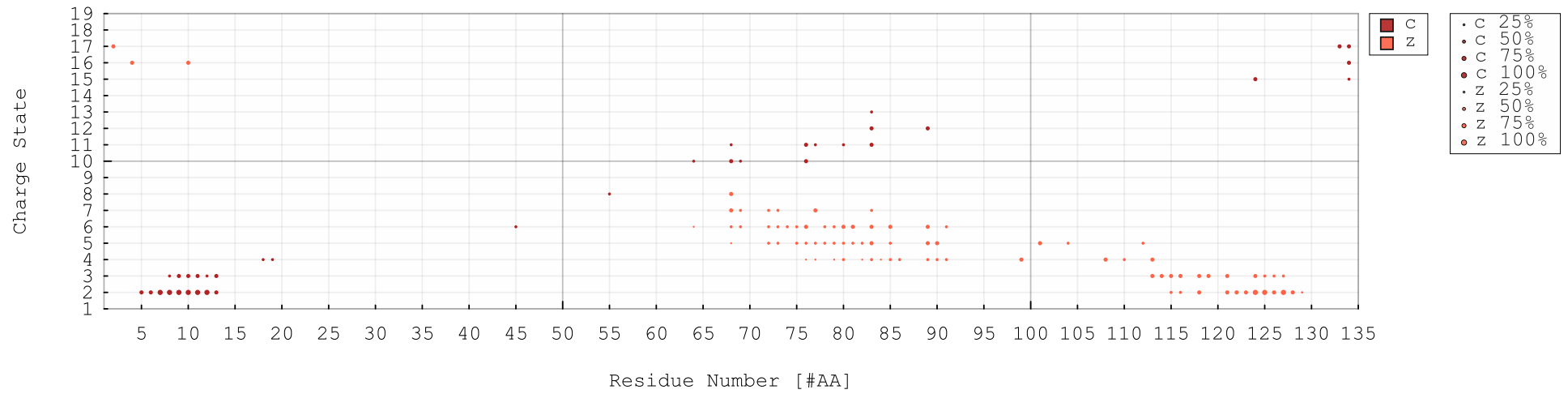

Supplemental Data S3

H3.1 - PCAF, 30 min

**Report**	2 of 3
**Experiment**	MS2
**Histone/sample**	H3.1
**Enzyme**	PCAF
**Reaction/source**	30 min
**Precursor**	H3.1Kac1
**Charge state**	19+
**Fragmentation / workflow**	EID
**Original file**	MS2_H3.1Kac1_19+_EID.pdf

Project Information

Project Directory: C:/Users/LAB-ADMIN
Processing date: Tue Apr 28 13:06:03 2026

Sample Info & Protocols

Name: Top-Down Protein Sequencing EID

Dataset

Imported spectrum file name:	250428 H31-PCAF100-30min z19ac1 MS2 EID_calibrated.txt
Dataset name:	Information is only available from imported JPEAKS files
Date acquired:	Information is only available from imported JPEAKS files
Date imported:	Information is only available from imported JPEAKS files
Instrument Name:	Information is only available from imported JPEAKS files
Instrument Serial Number:	Information is only available from imported JPEAKS files

Workflow Result Info

Analysis: 250428 H31-PCAF100-30min z19ac1 MS2 EID_calibrated2
Location: C:/Users/LAB-ADMIN
Method: -

Method Information

Name: Unnamed

Spectral Parameters

Mass Error [ppm]: 6
Intensity Deviation [au]: 0.15
Min/Max Charges: 1-19
Experiment type: EID

a ions	b ions	c ions	x ions	y ions	z ions	satellite	internal	precursor
a	b	c	x	y	z			M
a+1			x+1	y-1	z+1			M+-1H

Noise threshold calculation: Simple moving average noise threshold (SMA)
Window size [datapoints]: 600
Noise curve offset: 1.883298
SVP parameters: terminal gap: 2, internal gap: 1

Proteoforms

(top 10)

Index	Parent Sequence	Chain	Var. Mods	Mono Mass [Da]	Chemical Composition
1	H31Kac1	H31Kac1K14[Ace]	Ace: K14	15305.4534	C671H1131N215O186S4
2	H31Kac1	H31Kac1K9[Ace]	Ace: K9	15305.4534	C671H1131N215O186S4
3	H31Kac1	H31Kac1K18[Ace]	Ace: K18	15305.4534	C671H1131N215O186S4
4	H31Kac1	H31Kac1K4[Ace]	Ace: K4	15305.4534	C671H1131N215O186S4
5	H31Kac1	H31Kac1K23[Ace]	Ace: K23	15305.4534	C671H1131N215O186S4
6	H31Kac1	H31Kac1K27[Ace]	Ace: K27	15305.4534	C671H1131N215O186S4
7	H31Kac1	H31Kac1K36[Ace]	Ace: K36	15305.4534	C671H1131N215O186S4
8	H31Kac1	H31Kac1K37[Ace]	Ace: K37	15305.4534	C671H1131N215O186S4
9	H31Kac1	H31Kac1K56[Ace]	Ace: K56	15305.4534	C671H1131N215O186S4
10	H31Kac1	H31Kac1K64[Ace]	Ace: K64	15305.4534	C671H1131N215O186S4

Results

Proteoforms Table (top 10)

Index	Protein	SC [%]	SVP [%]	IC [%]	MS Score [a.u.]	Var. Mods	Mono Mass [Da]	Chemical Formula
1	H31Kac1	61.2	45.2	21.8	13.3	Ace: K14	15305.4534	C671H1131N215O186S4
2	H31Kac1	61.2	45.9	20.5	12.5	Ace: K9	15305.4534	C671H1131N215O186S4
3	H31Kac1	59.0	24.4	20.4	12.0	Ace: K18	15305.4534	C671H1131N215O186S4
4	H31Kac1	58.2	17.8	19.1	11.1	Ace: K4	15305.4534	C671H1131N215O186S4
5	H31Kac1	57.5	24.4	18.3	10.5	Ace: K23	15305.4534	C671H1131N215O186S4
6	H31Kac1	54.5	24.4	17.5	9.5	Ace: K27	15305.4534	C671H1131N215O186S4
7	H31Kac1	49.3	24.4	15.7	7.7	Ace: K36	15305.4534	C671H1131N215O186S4
8	H31Kac1	48.5	24.4	15.3	7.4	Ace: K37	15305.4534	C671H1131N215O186S4
9	H31Kac1	43.3	24.4	14.0	6.0	Ace: K56	15305.4534	C671H1131N215O186S4
10	H31Kac1	41.8	24.4	13.5	5.6	Ace: K64	15305.4534	C671H1131N215O186S4

Sequence

Name: H31Kac1

```
                 10                  20                  30                  40                  50                  60
A R T K Q T A R K S  T G G K A P R K Q L  A T K A A R K S A P  A T G G V K K P H R  Y R P G T V A L R E  I R R Y Q K S T E L
                 70                  80                  90                 100                 110                 120
L I R K L P F Q R L  V R E I A Q D F K T  D L R F Q S S A V M  A L Q E A C E A Y L  V G L F E D T N L C  A I H A K R V T I M
                130                 140                 150                 160                 170                 180
P K D I Q L A R R I  R G E R A
```

Modifications

Modification	Position	Type
Acetyl	4	variable
Acetyl	9	variable
Acetyl	14	variable
Acetyl	18	variable
Acetyl	23	variable
Acetyl	27	variable
Acetyl	36	variable
Acetyl	37	variable
Acetyl	56	variable
Acetyl	64	variable
Acetyl	79	variable

Modification	Position	Type
Acetyl	115	variable
Acetyl	122	variable

Sequence Map

Proteoform:

Index	Protein	SC [%]	SVP [%]	IC [%]	MS Score [a.u.]	Var. Mods	Mono Mass [Da]	Chemical Formula
1	H31Kac1	61.2	45.2	21.8	13.3	Ace: K14	15305.4534	C671H1131N215O186S4

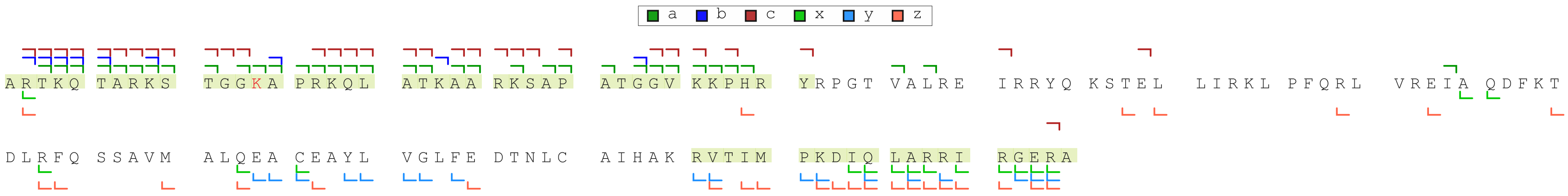

Proteoform:

Index	Protein	SC [%]	SVP [%]	IC [%]	MS Score [a.u.]	Var. Mods	Mono Mass [Da]	Chemical Formula
2	H31Kac1	61.2	45.9	20.5	12.5	Ace: K9	15305.4534	C671H1131N215O186S4

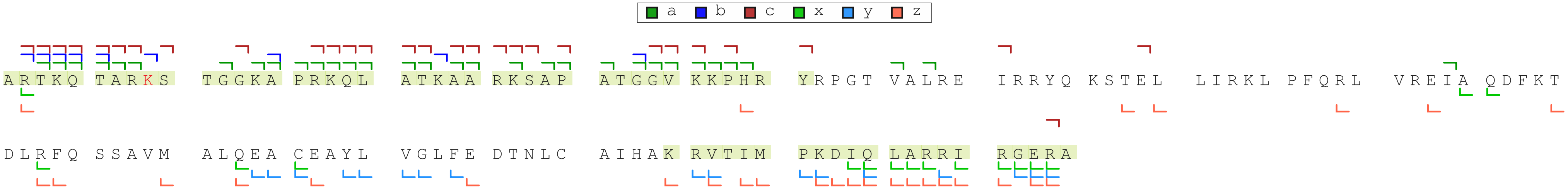

Proteoform:

Index	Protein	SC [%]	SVP [%]	IC [%]	MS Score [a.u.]	Var. Mods	Mono Mass [Da]	Chemical Formula
3	H31Kac1	59.0	24.4	20.4	12.0	Ace: K18	15305.4534	C671H1131N215O186S4

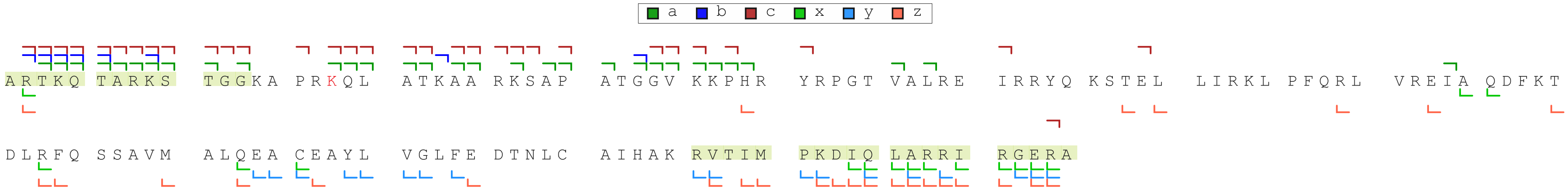

Proteoform:

Index	Protein	SC [%]	SVP [%]	IC [%]	MS Score [a.u.]	Var. Mods	Mono Mass [Da]	Chemical Formula
4	H31Kac1	58.2	17.8	19.1	11.1	Ace: K4	15305.4534	C671H1131N215O186S4

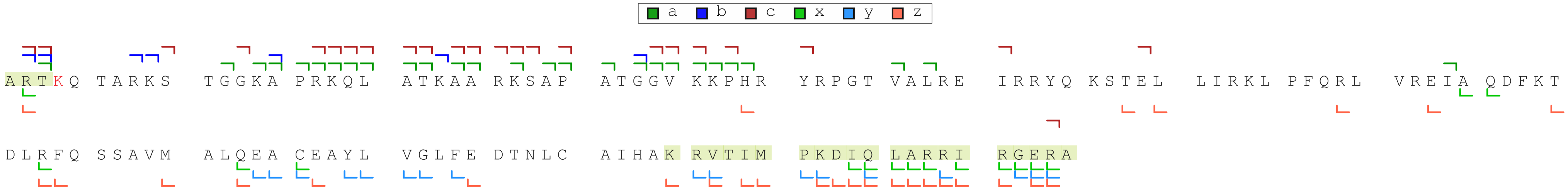

Proteoform:

Index	Protein	SC [%]	SVP [%]	IC [%]	MS Score [a.u.]	Var. Mods	Mono Mass [Da]	Chemical Formula
5	H31Kac1	57.5	24.4	18.3	10.5	Ace: K23	15305.4534	C671H1131N215O186S4

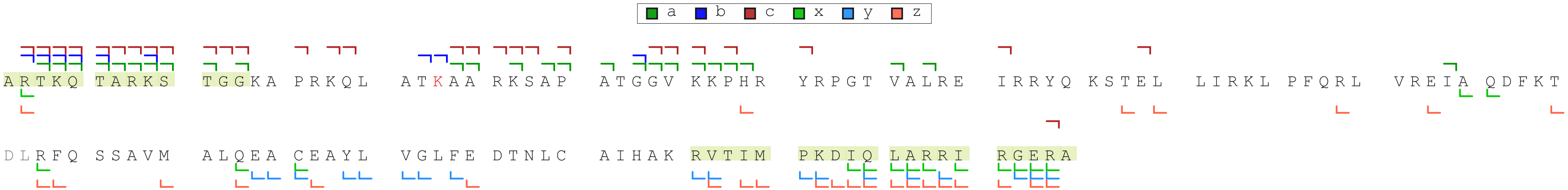

Proteoform:

Index	Protein	SC [%]	SVP [%]	IC [%]	MS Score [a.u.]	Var. Mods	Mono Mass [Da]	Chemical Formula
6	H31Kac1	54.5	24.4	17.5	9.5	Ace: K27	15305.4534	C671H1131N215O186S4

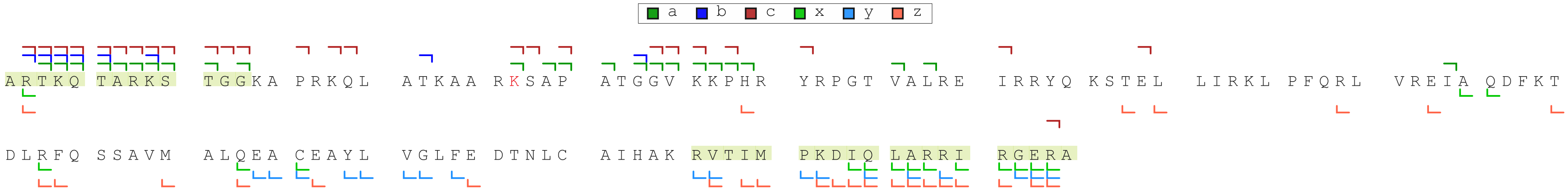

Proteoform:

Index	Protein	SC [%]	SVP [%]	IC [%]	MS Score [a.u.]	Var. Mods	Mono Mass [Da]	Chemical Formula
7	H31Kac1	49.3	24.4	15.7	7.7	Ace: K36	15305.4534	C671H1131N215O186S4

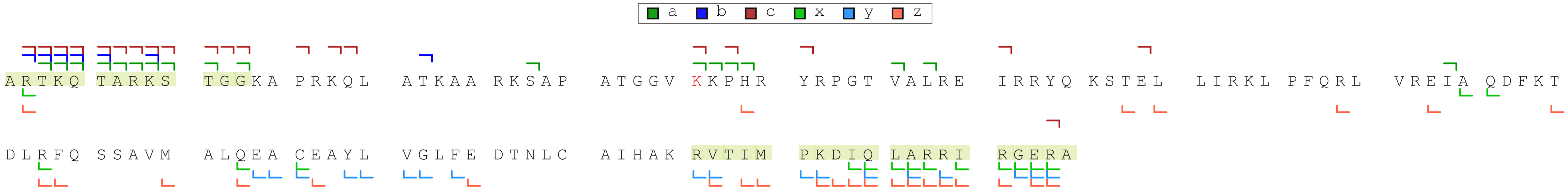

Proteoform:

Index	Protein	SC [%]	SVP [%]	IC [%]	MS Score [a.u.]	Var. Mods	Mono Mass [Da]	Chemical Formula
8	H31Kac1	48.5	24.4	15.3	7.4	Ace: K37	15305.4534	C671H1131N215O186S4

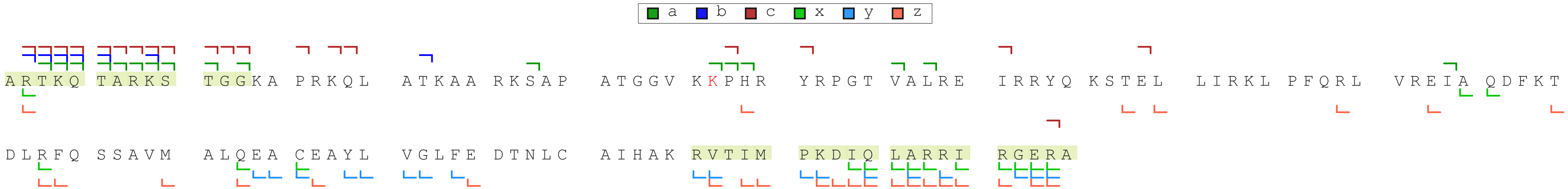

Proteoform:

Index	Protein	SC [%]	SVP [%]	IC [%]	MS Score [a.u.]	Var. Mods	Mono Mass [Da]	Chemical Formula
9	H31Kac1	43.3	24.4	14.0	6.0	Ace: K56	15305.4534	C671H1131N215O186S4

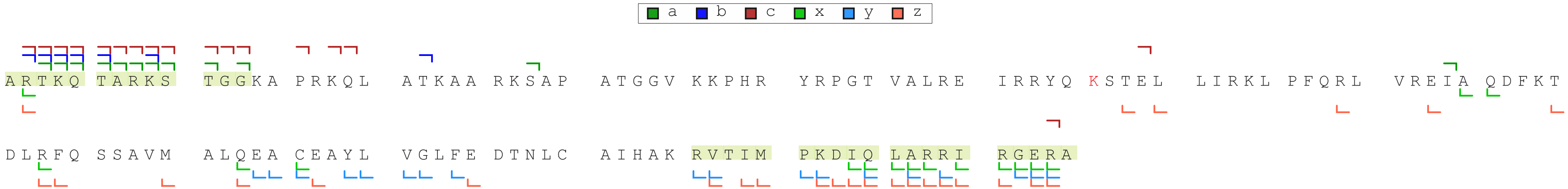

Proteoform:

Index	Protein	SC [%]	SVP [%]	IC [%]	MS Score [a.u.]	Var. Mods	Mono Mass [Da]	Chemical Formula
10	H31Kac1	41.8	24.4	13.5	5.6	Ace: K64	15305.4534	C671H1131N215O186S4

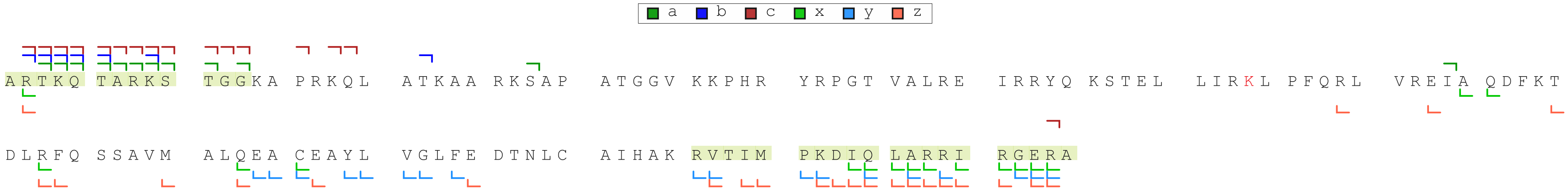

Error Plot

Proteoform:

Index	Protein	SC [%]	SVP [%]	IC [%]	MS Score [a.u.]	Var. Mods	Mono Mass [Da]	Chemical Formula
1	H31Kac1	61.2	45.2	21.8	13.3	Ace: K14	15305.4534	C671H1131N215O186S4

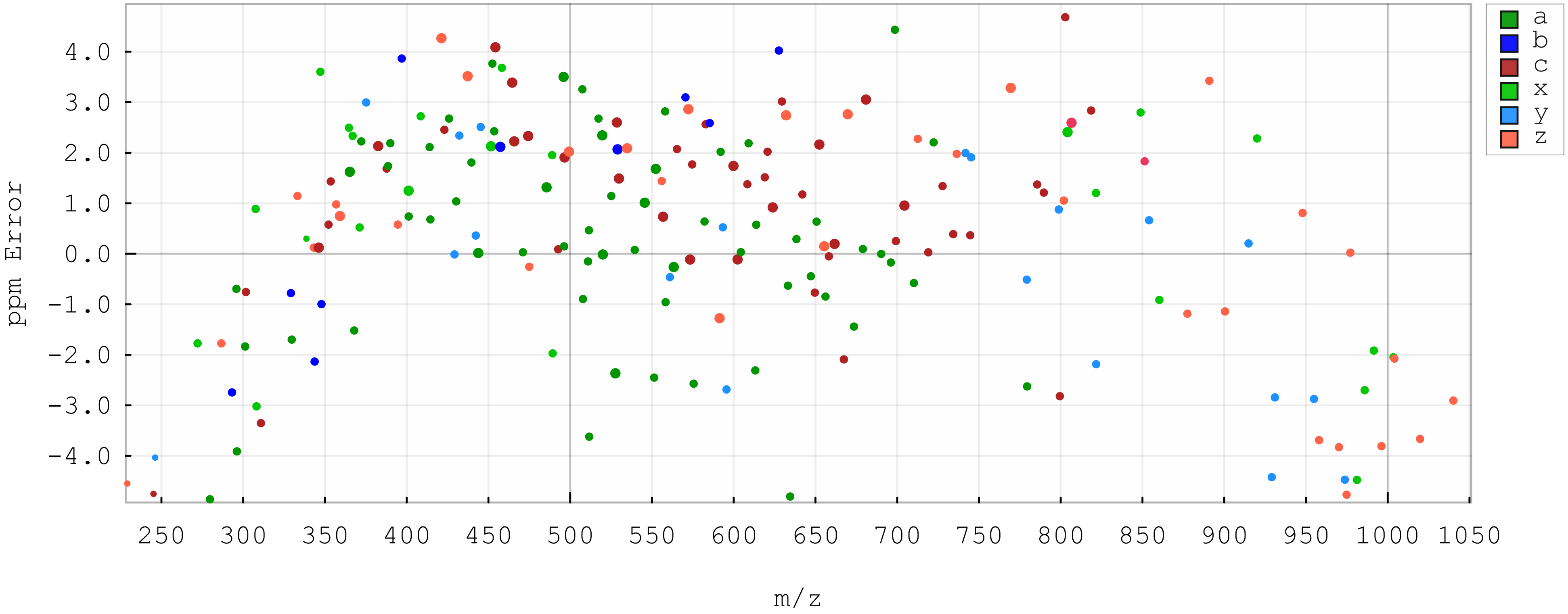

Proteoform:

Index	Protein	SC [%]	SVP [%]	IC [%]	MS Score [a.u.]	Var. Mods	Mono Mass [Da]	Chemical Formula
2	H31Kac1	61.2	45.9	20.5	12.5	Ace: K9	15305.4534	C671H1131N215O186S4

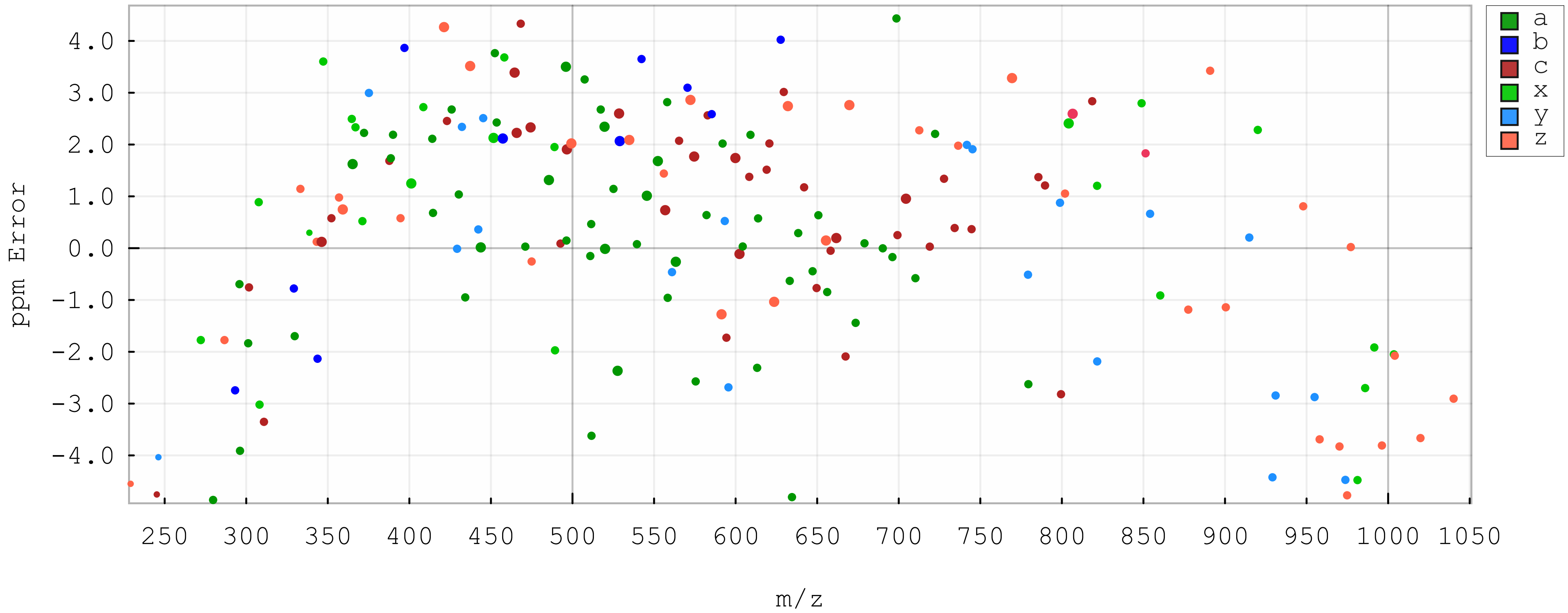

Proteoform:

Index	Protein	SC [%]	SVP [%]	IC [%]	MS Score [a.u.]	Var. Mods	Mono Mass [Da]	Chemical Formula
3	H31Kac1	59.0	24.4	20.4	12.0	Ace: K18	15305.4534	C671H1131N215O186S4

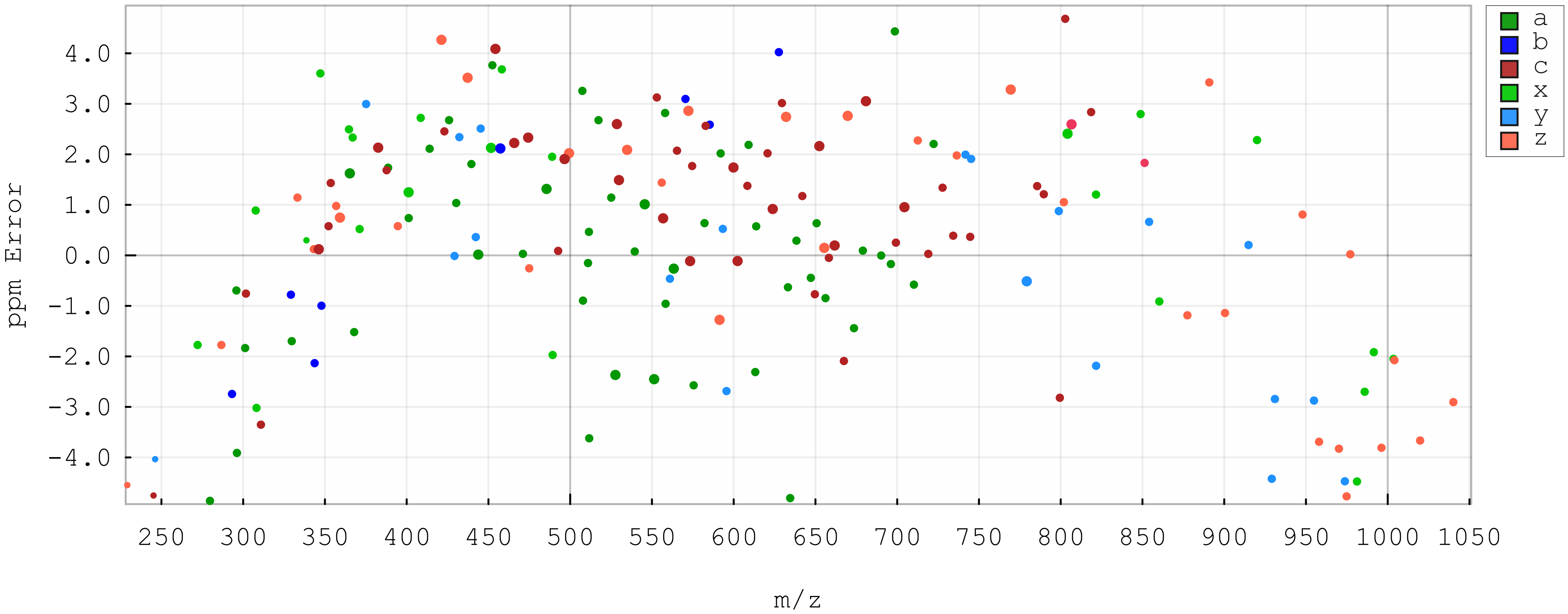

Proteoform:	Index	Protein	SC [%]	SVP [%]	IC [%]	MS Score [a.u.]	Var. Mods	Mono Mass [Da]	Chemical Formula
	4	H31Kac1	58.2	17.8	19.1	11.1	Ace: K4	15305.4534	C671H1131N215O186S4

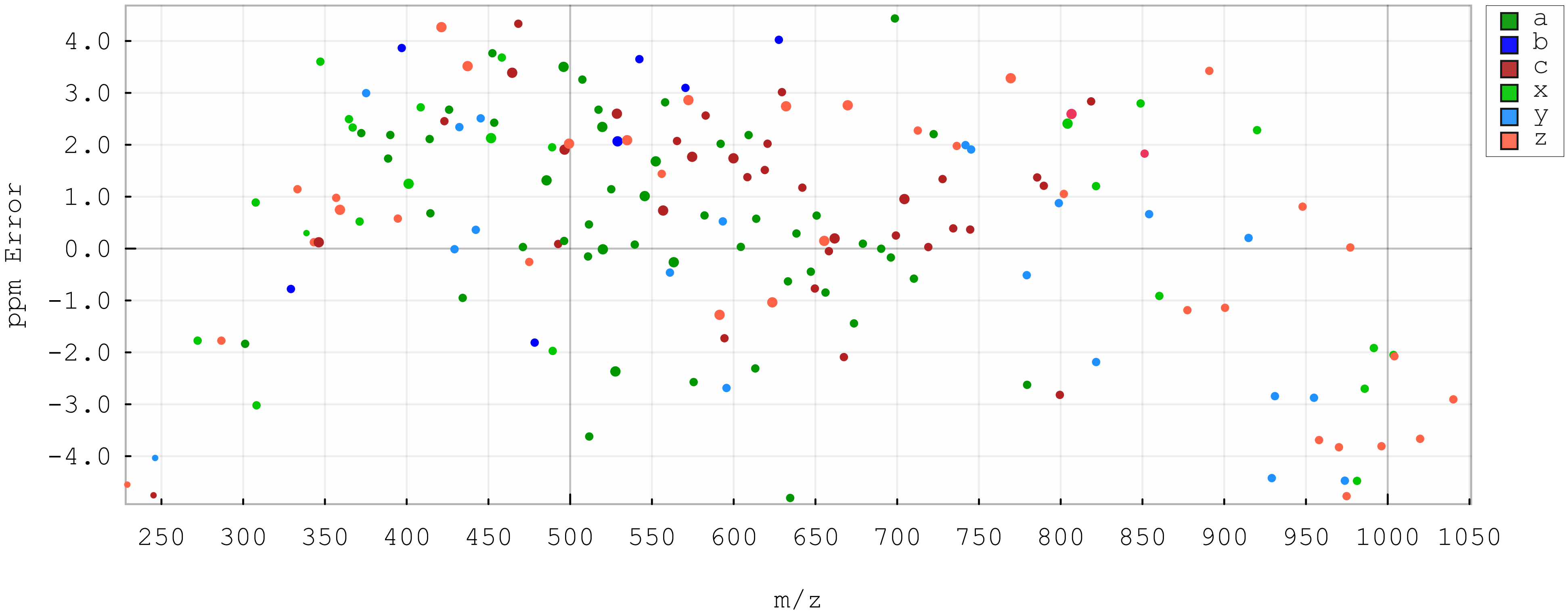

Proteoform:

Index	Protein	SC [%]	SVP [%]	IC [%]	MS Score [a.u.]	Var. Mods	Mono Mass [Da]	Chemical Formula
5	H31Kac1	57.5	24.4	18.3	10.5	Ace: K23	15305.4534	C671H1131N215O186S4

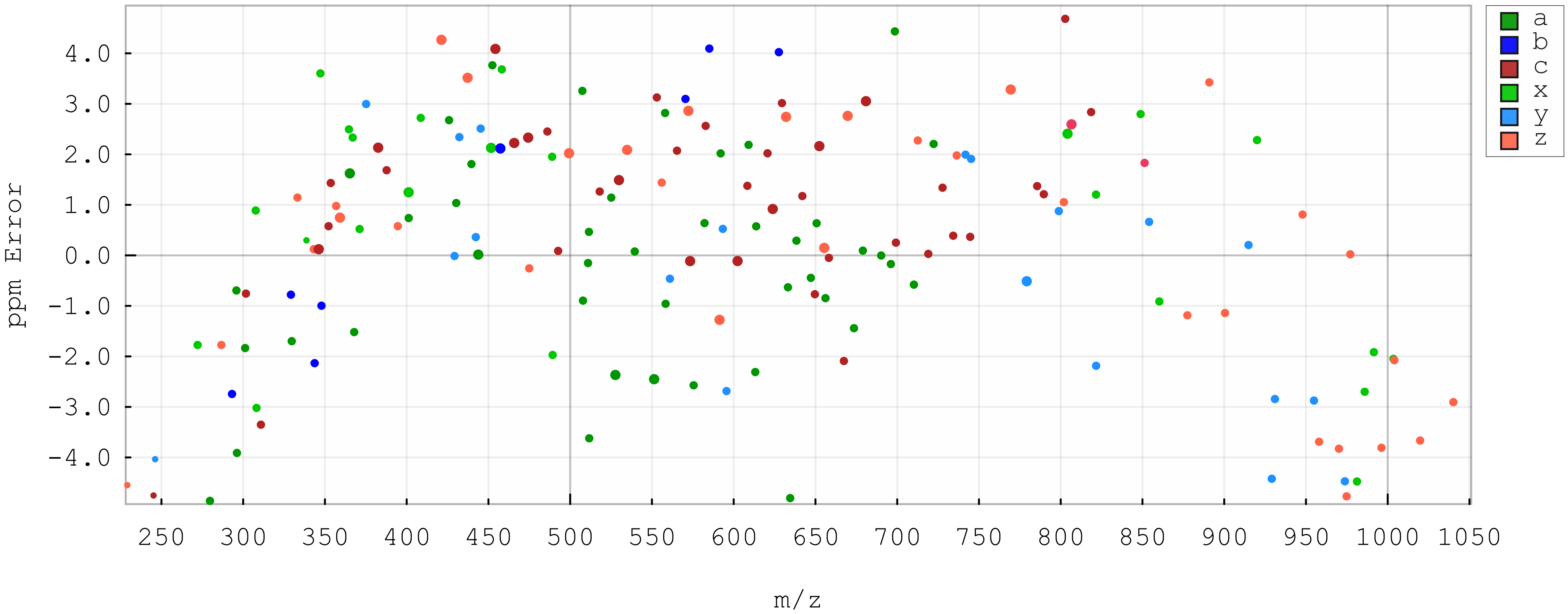

Proteoform:	Index	Protein	SC [%]	SVP [%]	IC [%]	MS Score [a.u.]	Var. Mods	Mono Mass [Da]	Chemical Formula
	6	H31Kac1	54.5	24.4	17.5	9.5	Ace: K27	15305.4534	C671H1131N215O186S4

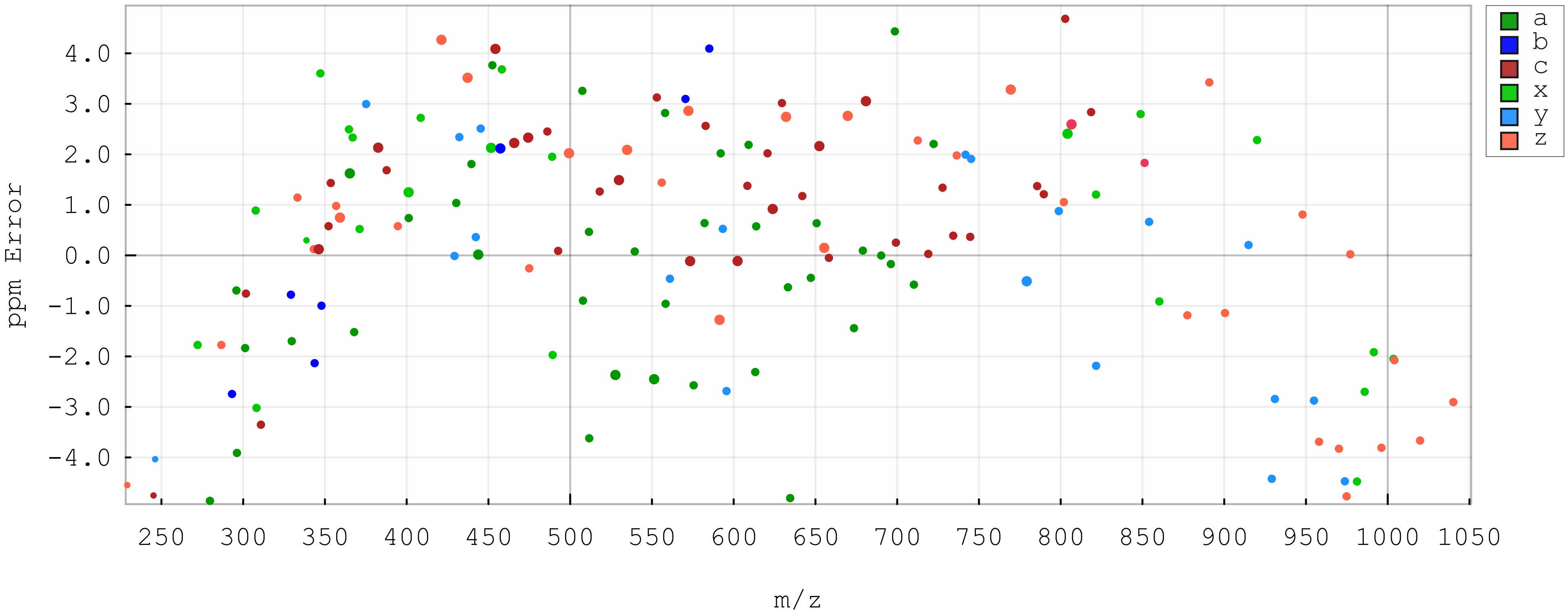

Proteoform:	Index	Protein	SC [%]	SVP [%]	IC [%]	MS Score [a.u.]	Var. Mods	Mono Mass [Da]	Chemical Formula
	7	H31Kac1	49.3	24.4	15.7	7.7	Ace: K36	15305.4534	C671H1131N215O186S4

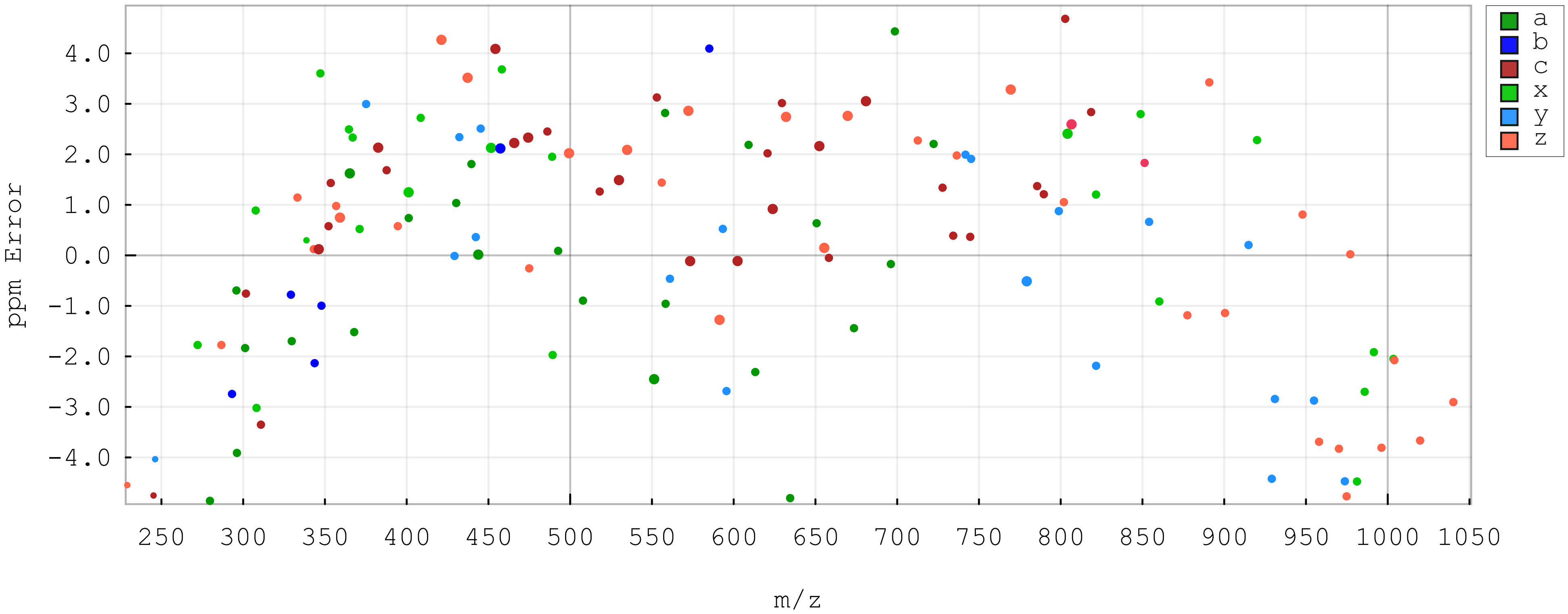

Proteoform:	Index	Protein	SC [%]	SVP [%]	IC [%]	MS Score [a.u.]	Var. Mods	Mono Mass [Da]	Chemical Formula
	8	H31Kac1	48.5	24.4	15.3	7.4	Ace: K37	15305.4534	C671H1131N215O186S4

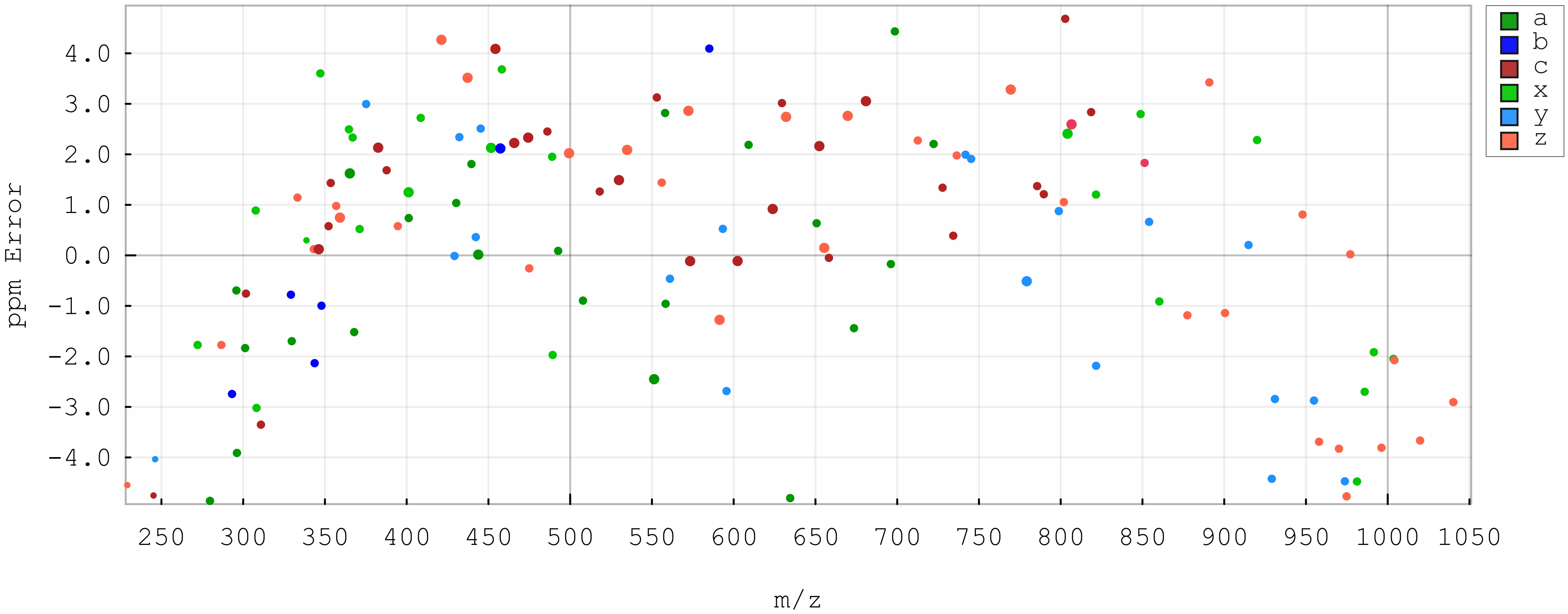

Proteoform:

Index	Protein	SC [%]	SVP [%]	IC [%]	MS Score [a.u.]	Var. Mods	Mono Mass [Da]	Chemical Formula
9	H31Kac1	43.3	24.4	14.0	6.0	Ace: K56	15305.4534	C671H1131N215O186S4

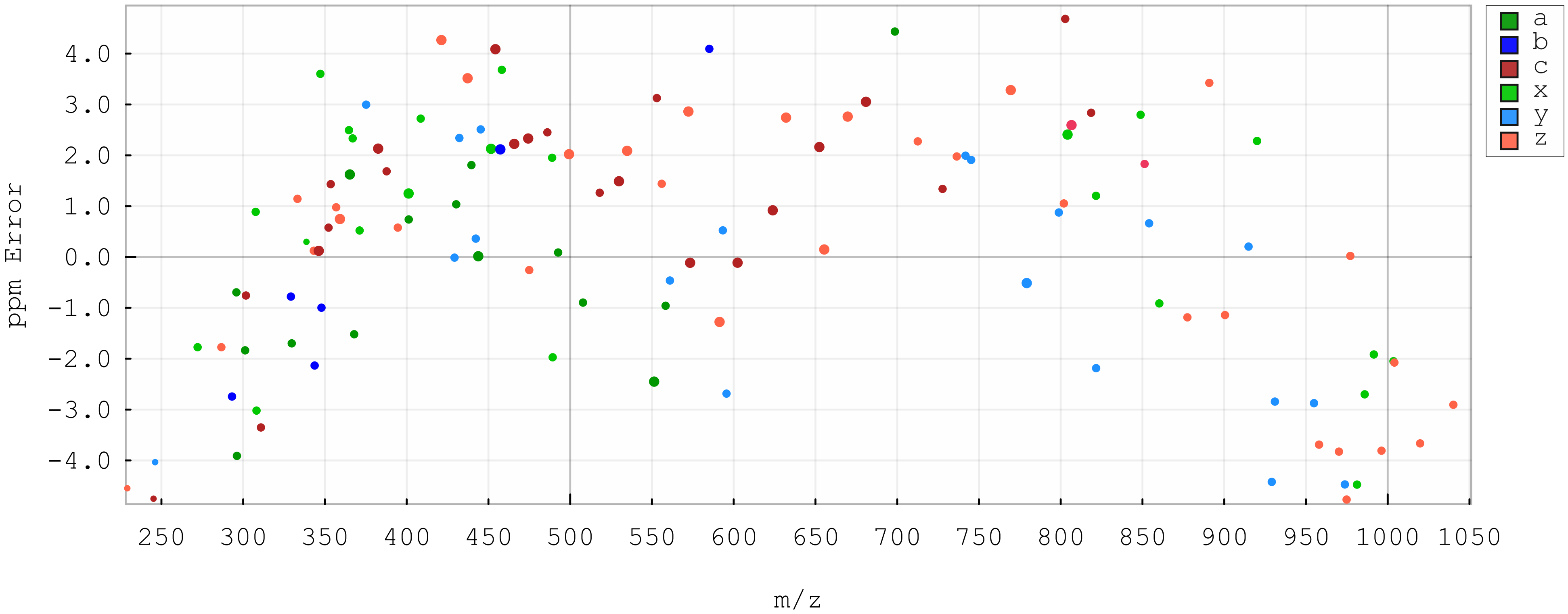

Proteoform:

Index	Protein	SC [%]	SVP [%]	IC [%]	MS Score [a.u.]	Var. Mods	Mono Mass [Da]	Chemical Formula
10	H31Kac1	41.8	24.4	13.5	5.6	Ace: K64	15305.4534	C671H1131N215O186S4

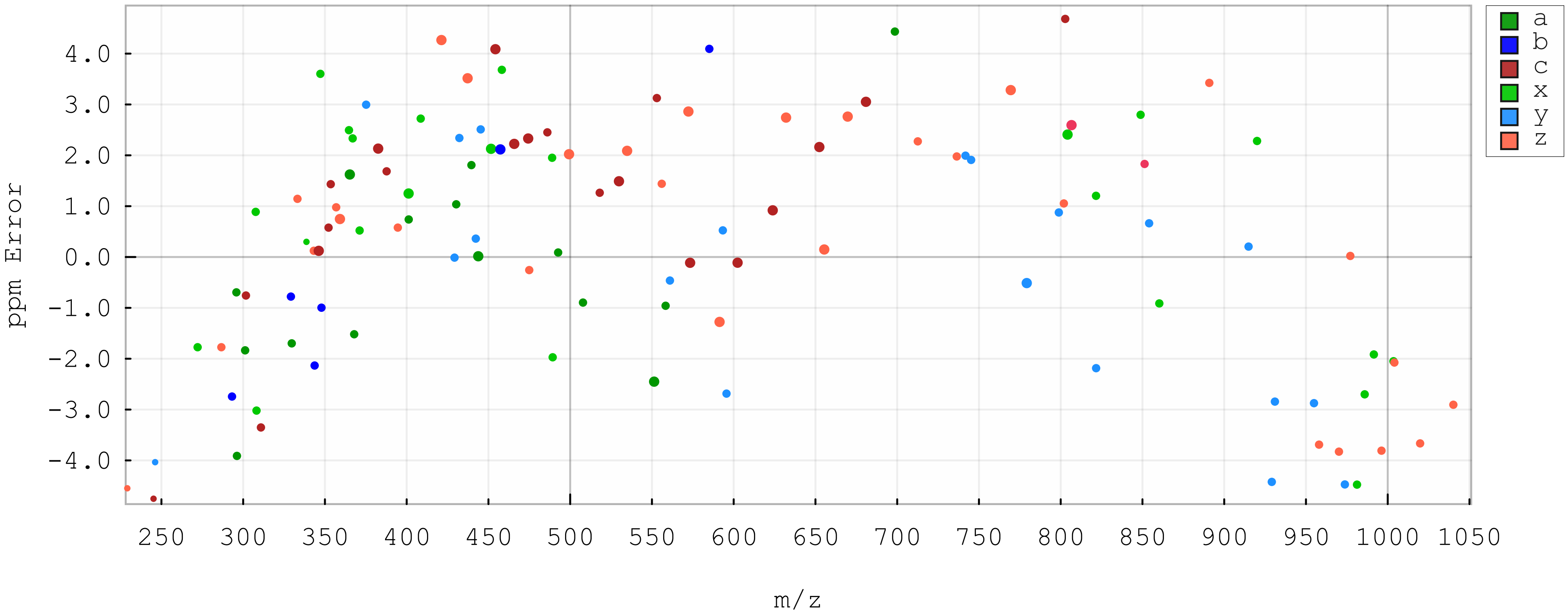

Primary Fragment Plots

Proteoform:

Index	Protein	SC [%]	SVP [%]	IC [%]	MS Score [a.u.]	Var. Mods	Mono Mass [Da]	Chemical Formula
1	H31Kac1	61.2	45.2	21.8	13.3	Ace: K14	15305.4534	C671H1131N215O186S4

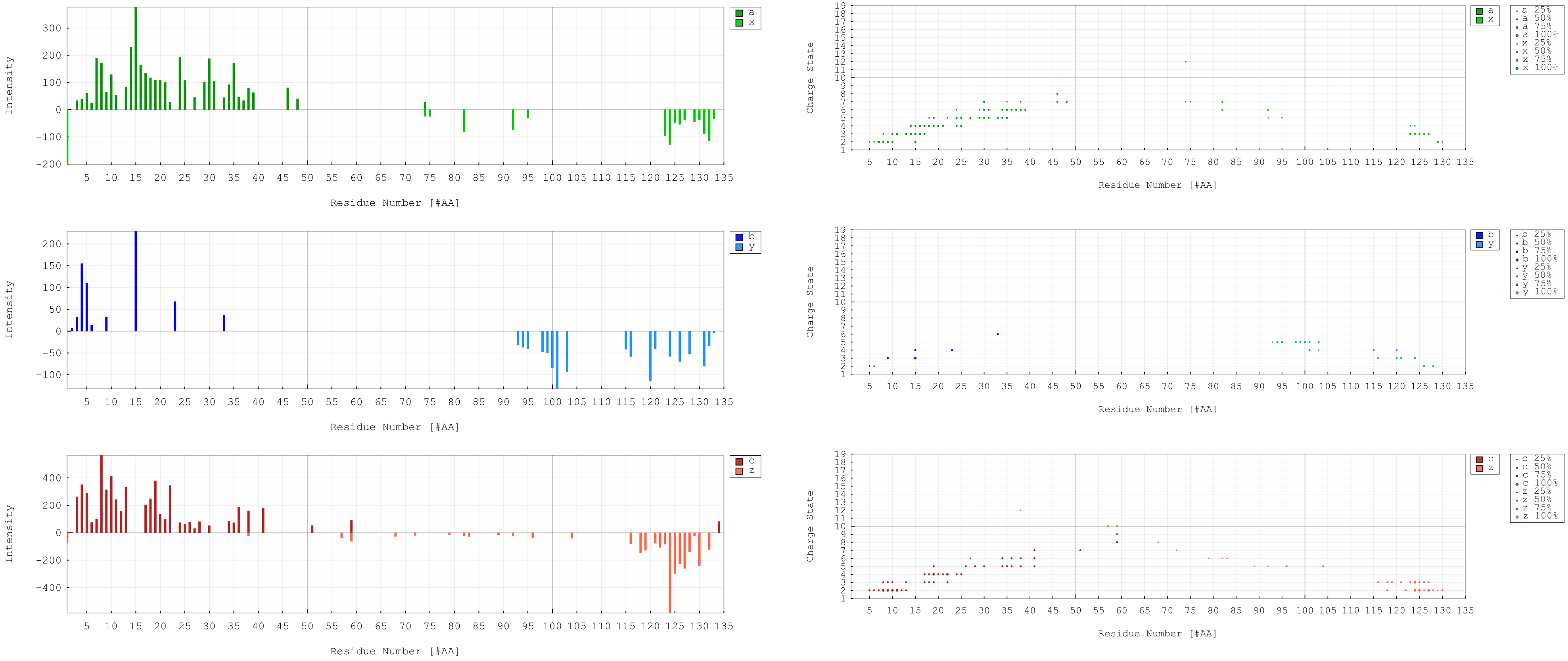

Proteoform:	Index	Protein	SC [%]	SVP [%]	IC [%]	MS Score [a.u.]	Var. Mods	Mono Mass [Da]	Chemical Formula
	2	H31Kac1	61.2	45.9	20.5	12.5	Ace: K9	15305.4534	C671H1131N215O186S4

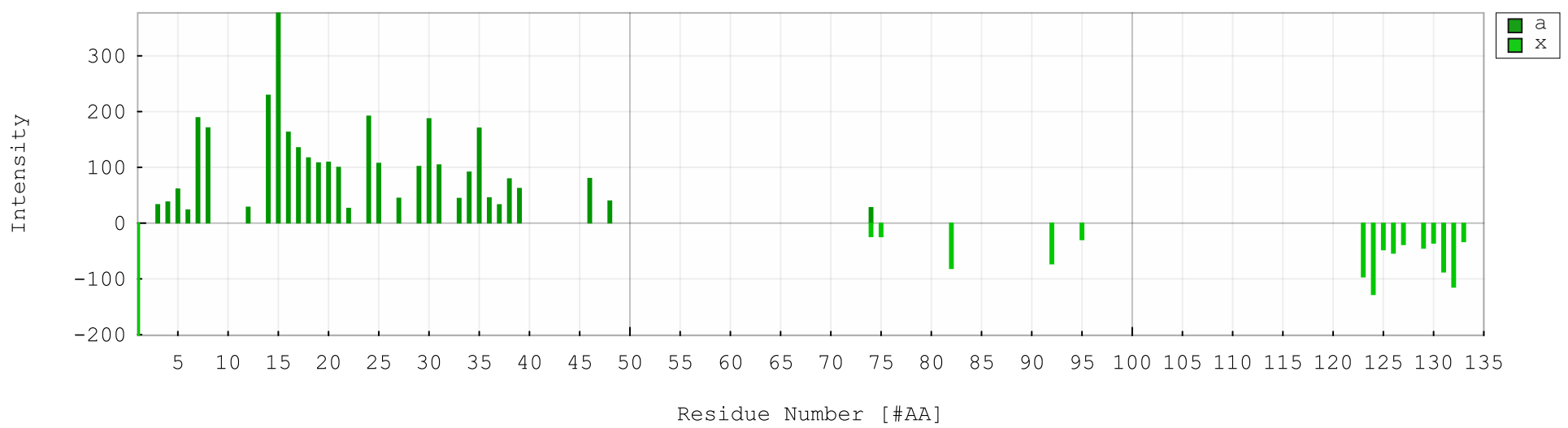

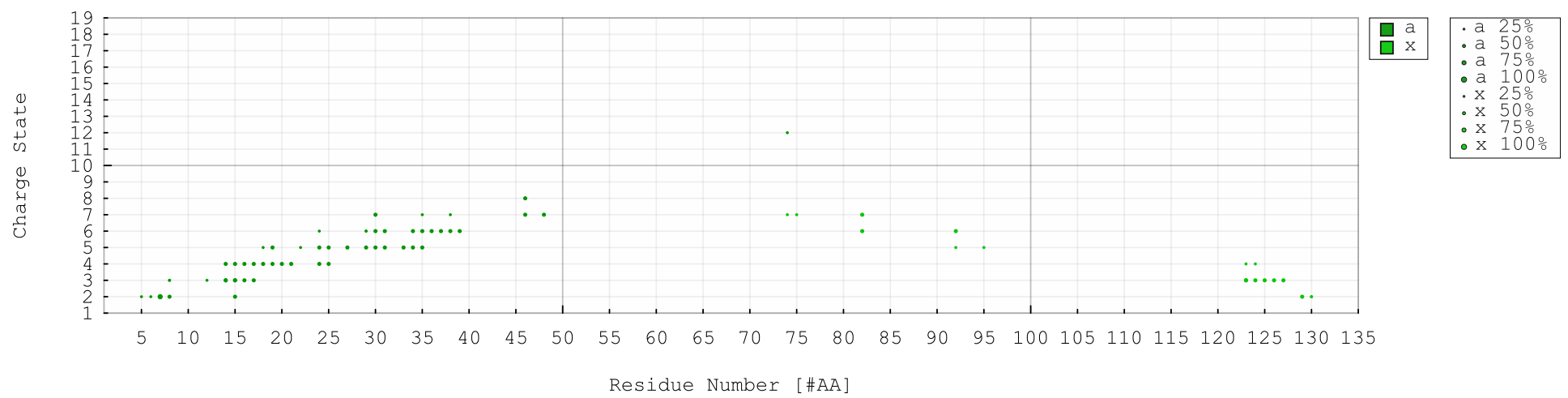

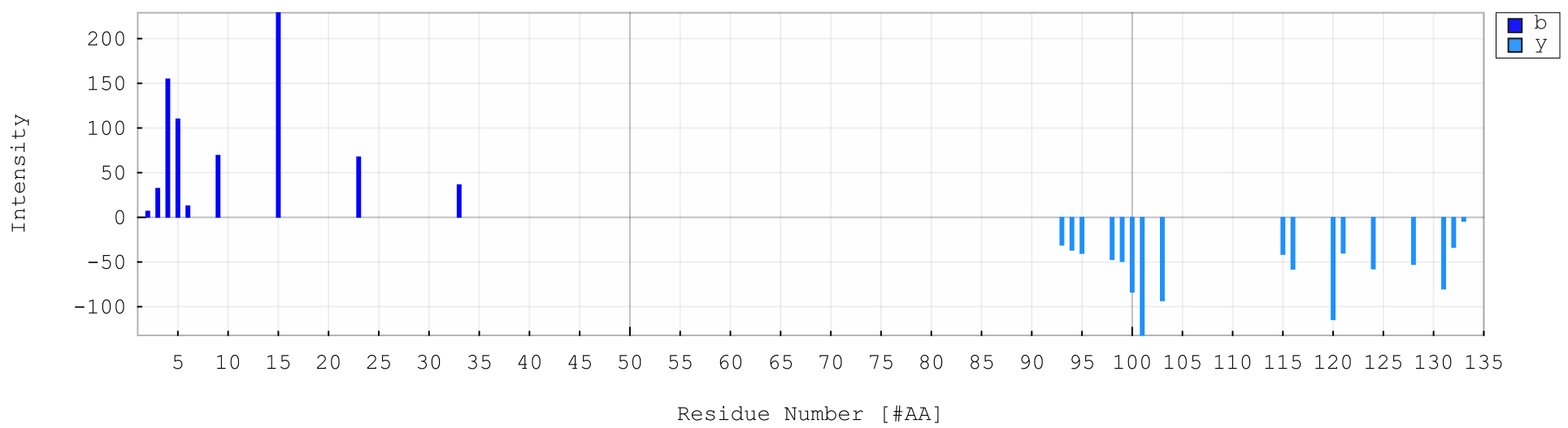

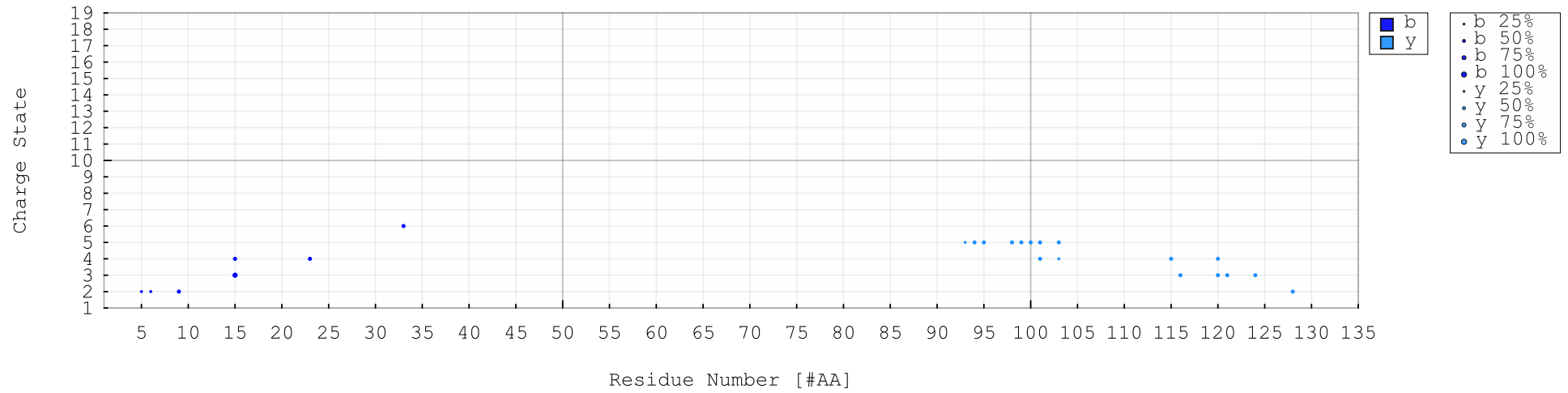

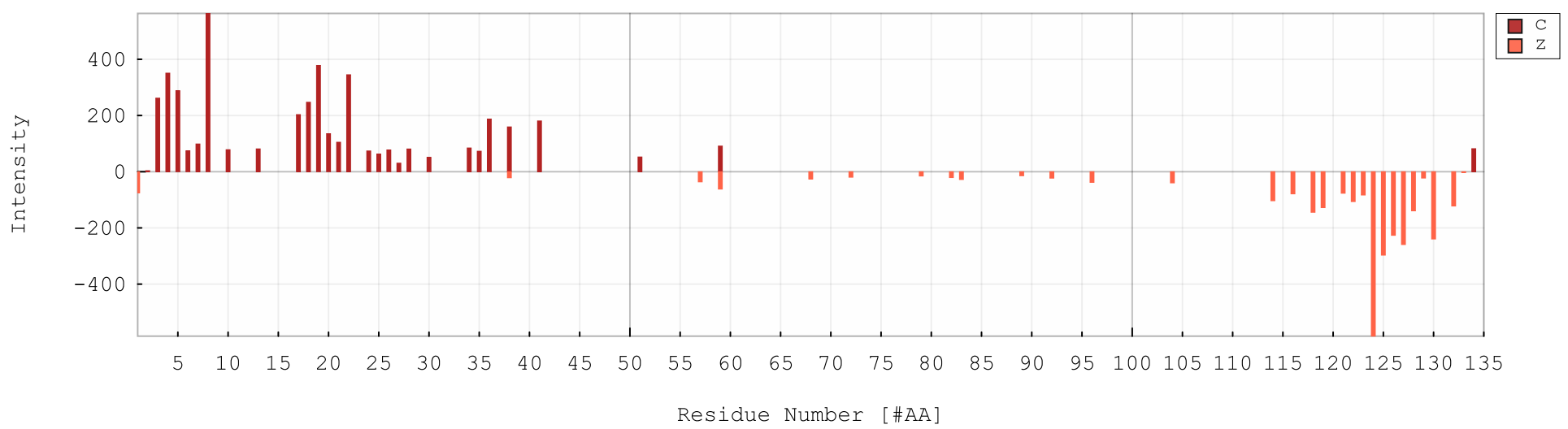

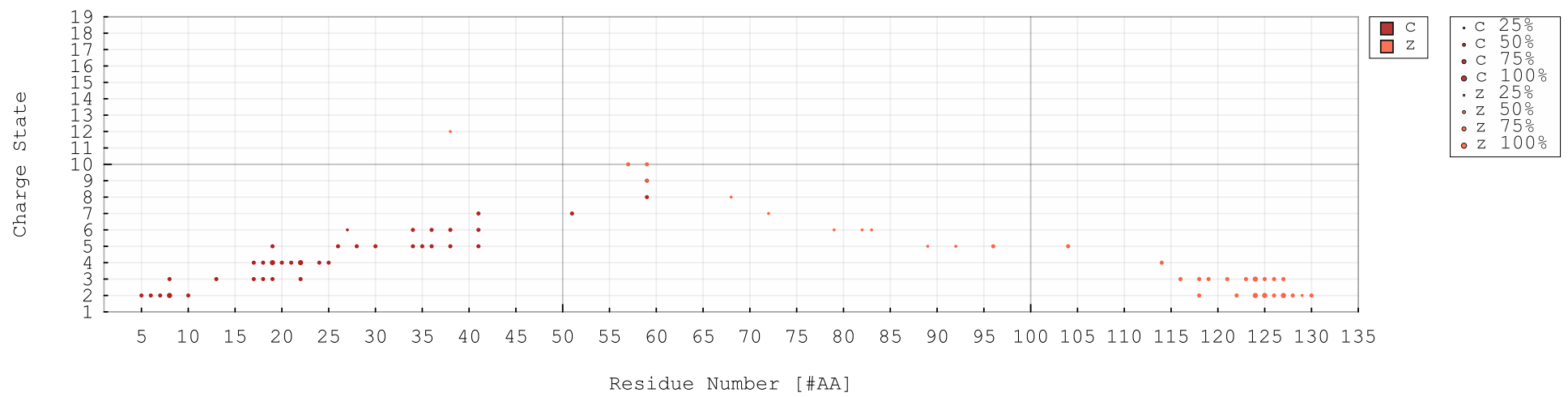

Proteoform:

Index	Protein	SC [%]	SVP [%]	IC [%]	MS Score [a.u.]	Var. Mods	Mono Mass [Da]	Chemical Formula
3	H31Kac1	59.0	24.4	20.4	12.0	Ace: K18	15305.4534	C671H1131N215O186S4

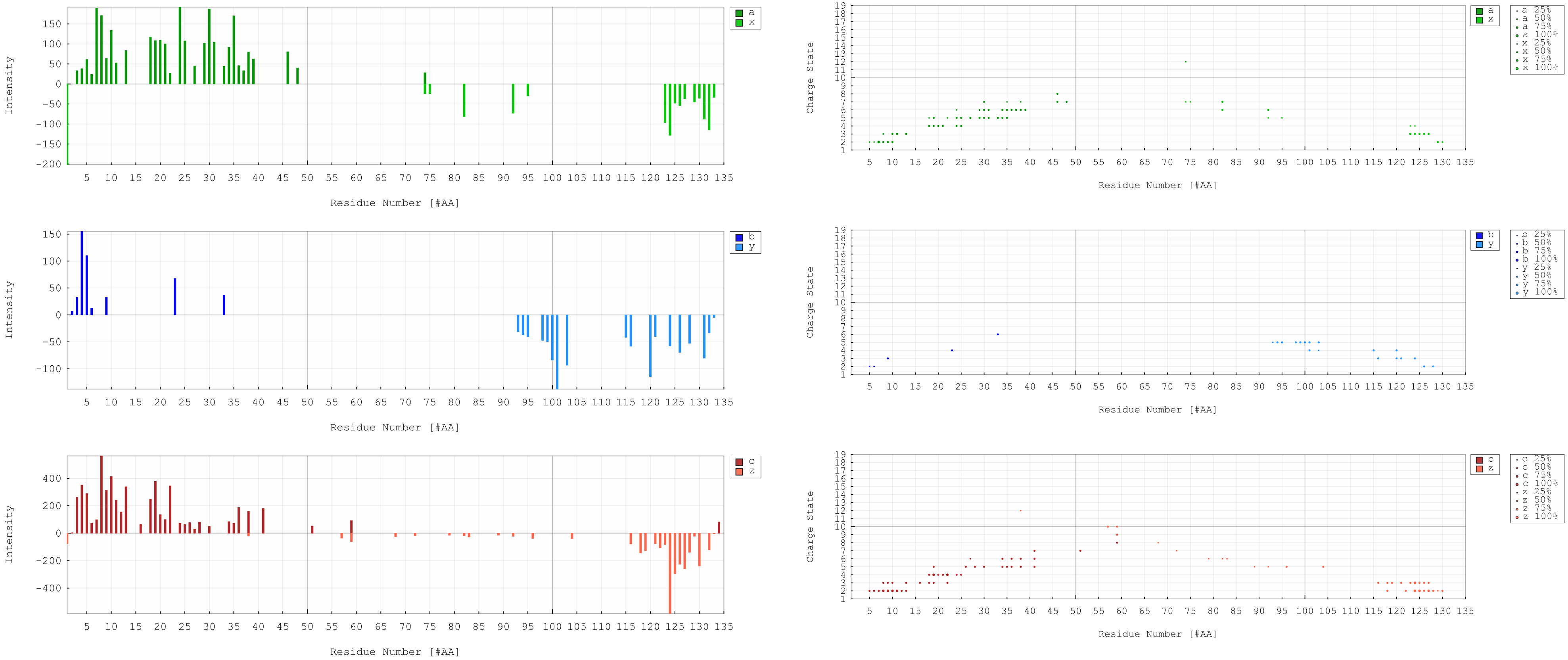

Proteoform:

Index	Protein	SC [%]	SVP [%]	IC [%]	MS Score [a.u.]	Var. Mods	Mono Mass [Da]	Chemical Formula
4	H31Kac1	58.2	17.8	19.1	11.1	Ace: K4	15305.4534	C671H1131N215O186S4

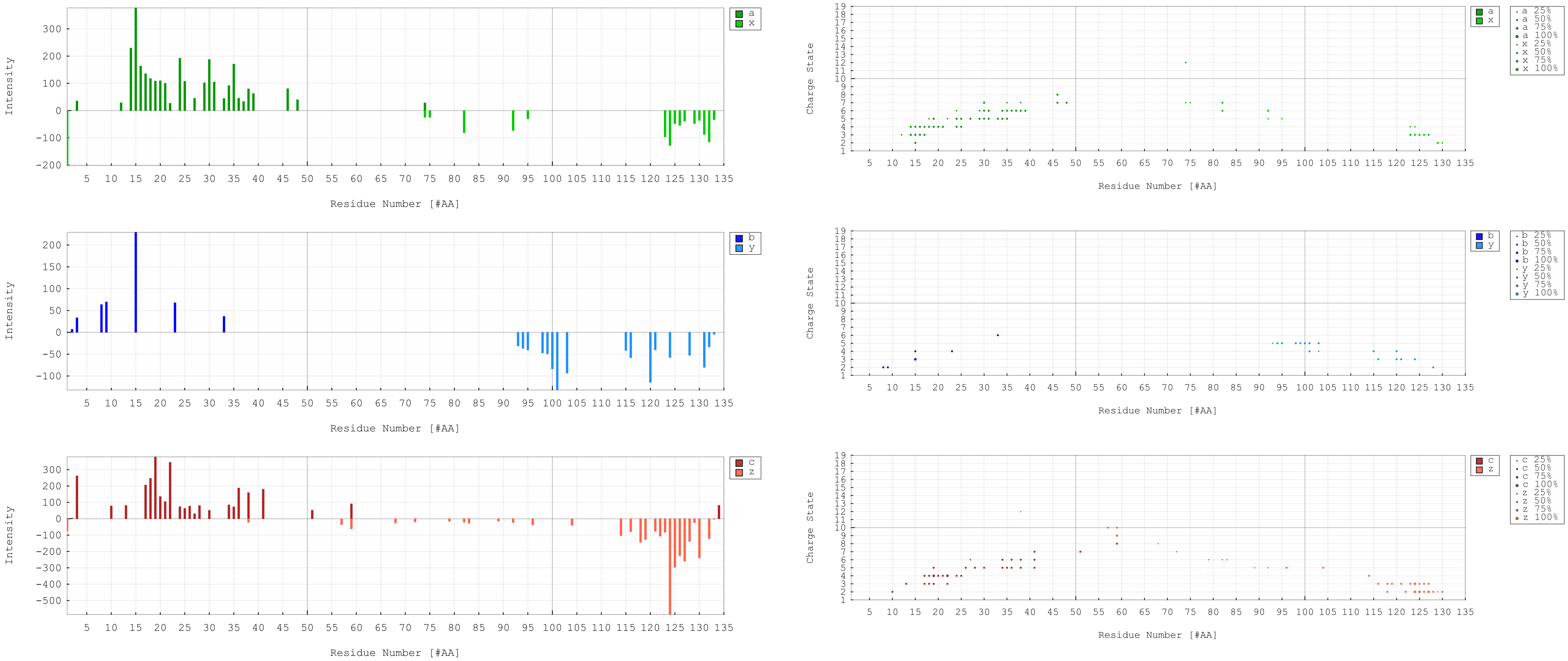

Proteoform:

Index	Protein	SC [%]	SVP [%]	IC [%]	MS Score [a.u.]	Var. Mods	Mono Mass [Da]	Chemical Formula
5	H31Kac1	57.5	24.4	18.3	10.5	Ace: K23	15305.4534	C671H1131N215O186S4

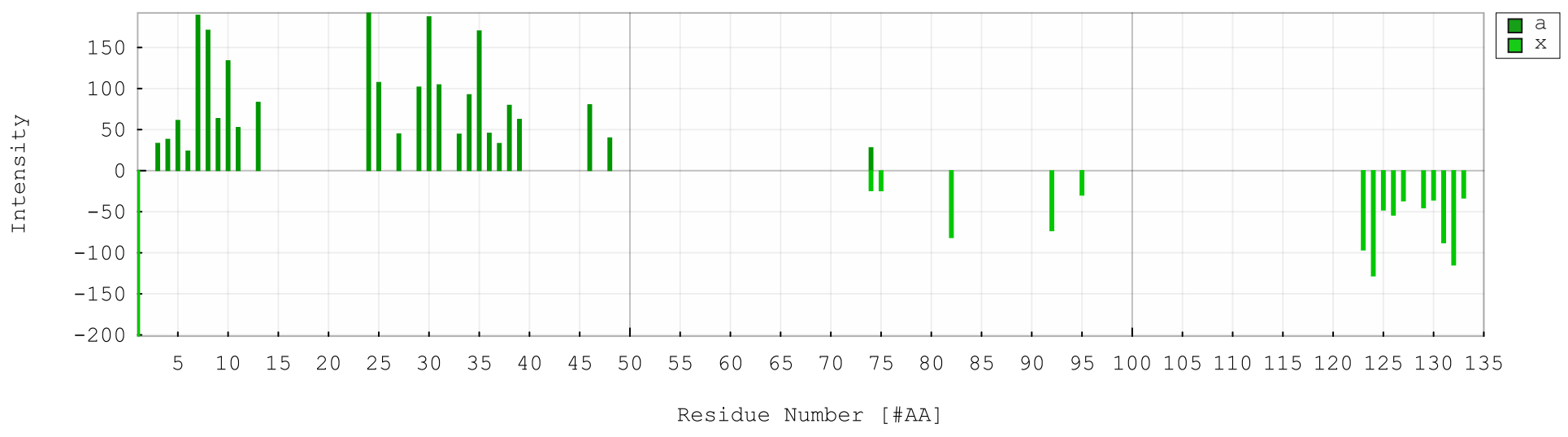

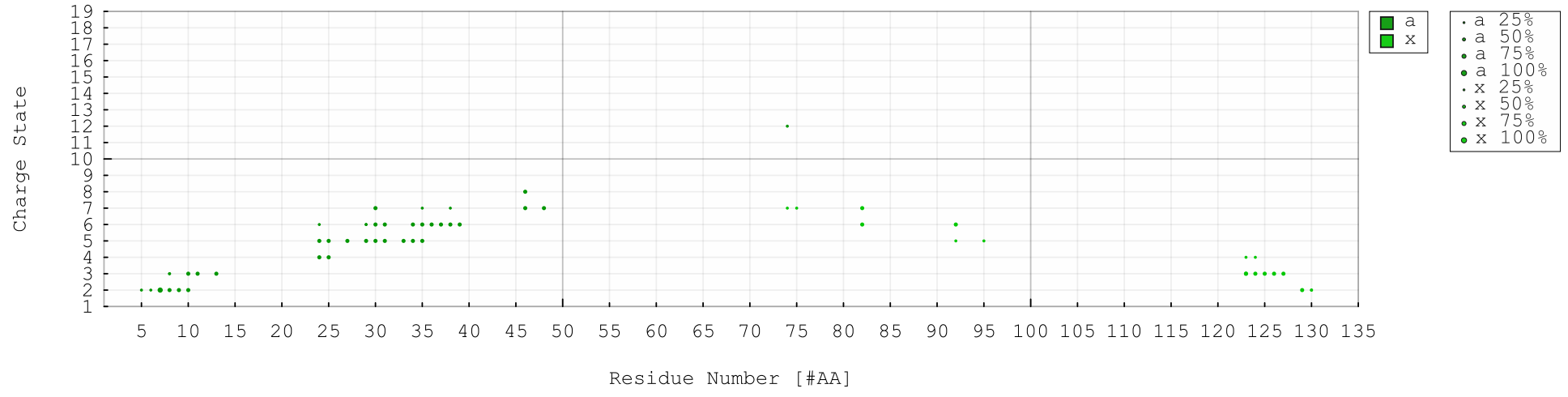

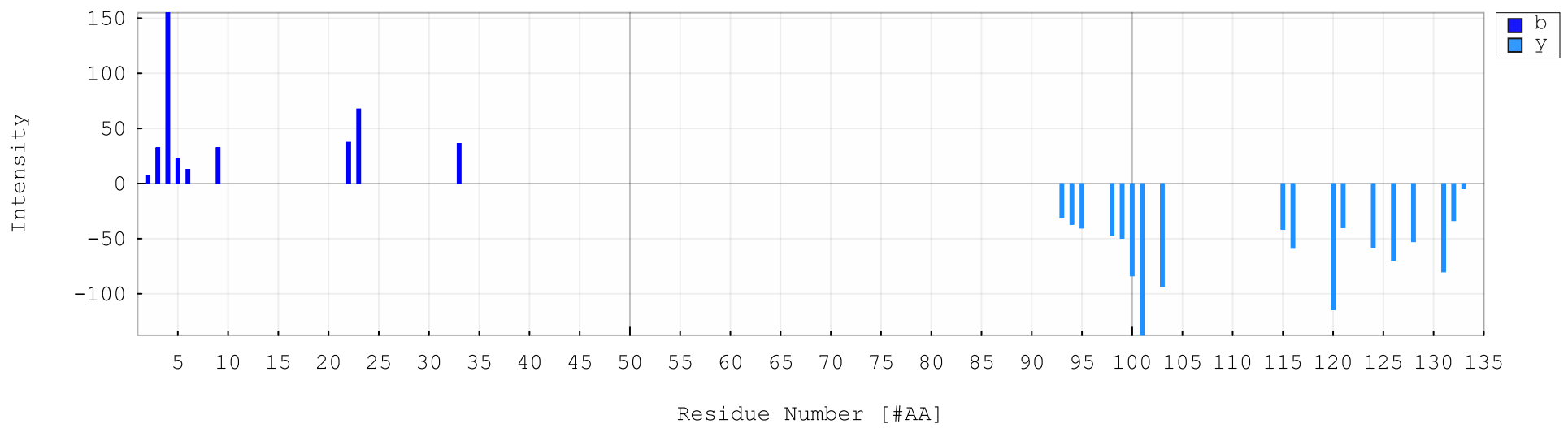

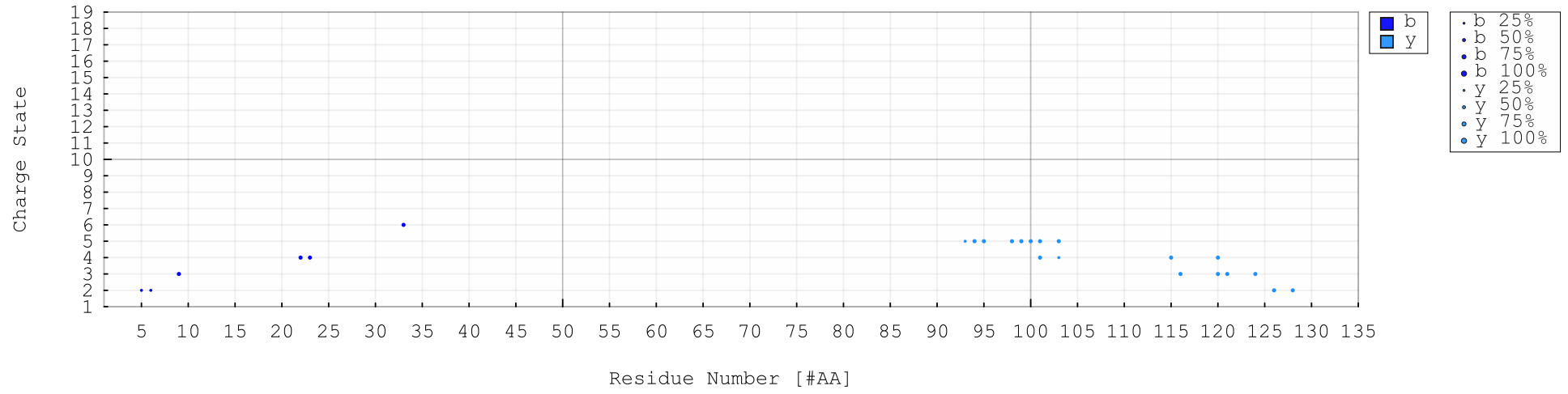

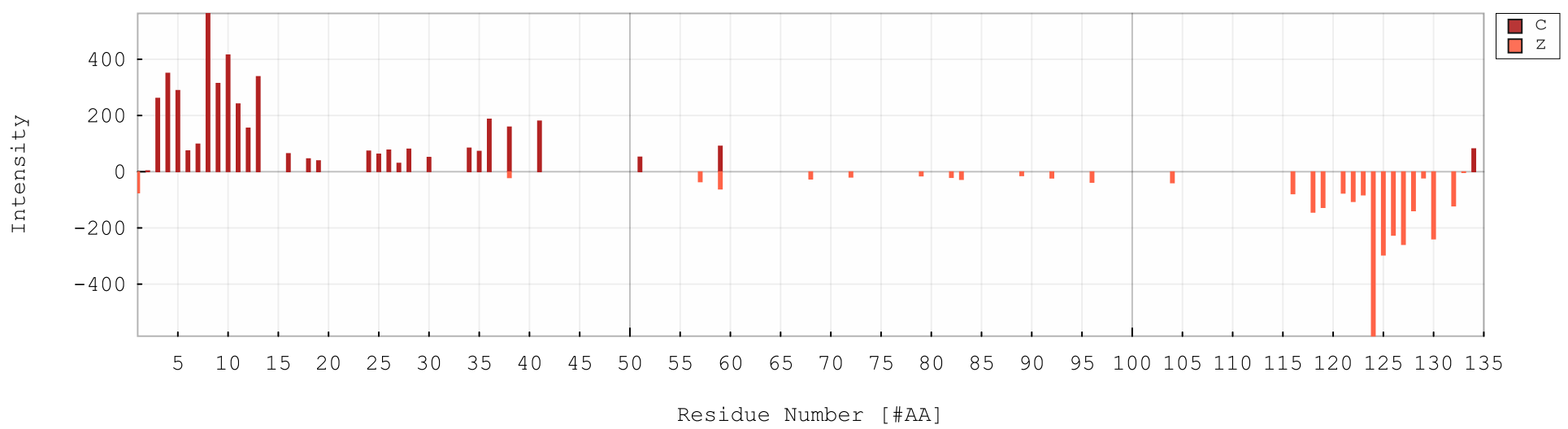

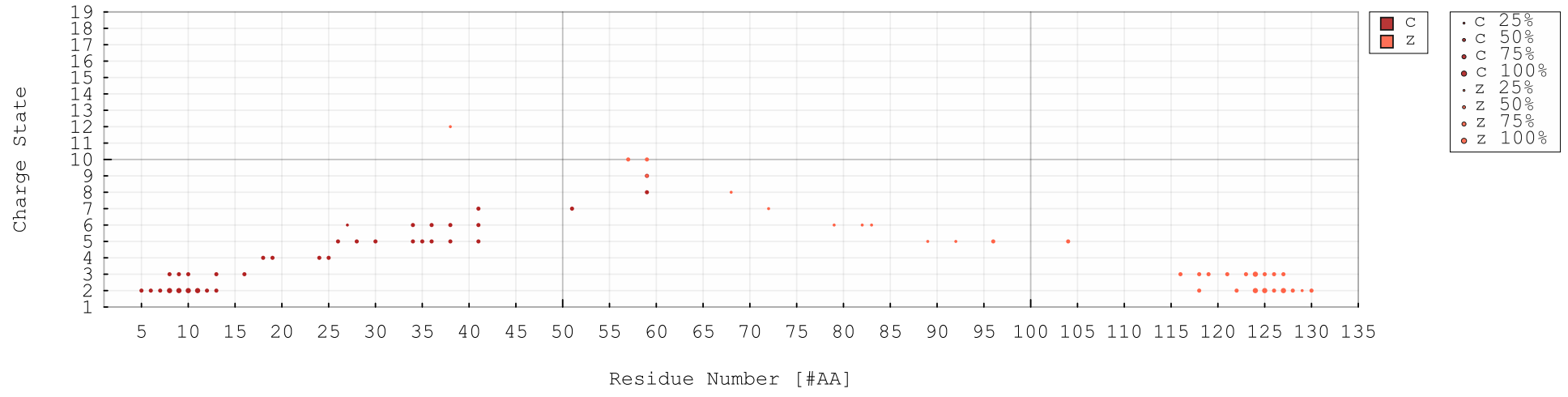

Proteoform:

Index	Protein	SC [%]	SVP [%]	IC [%]	MS Score [a.u.]	Var. Mods	Mono Mass [Da]	Chemical Formula
6	H31Kac1	54.5	24.4	17.5	9.5	Ace: K27	15305.4534	C671H1131N215O186S4

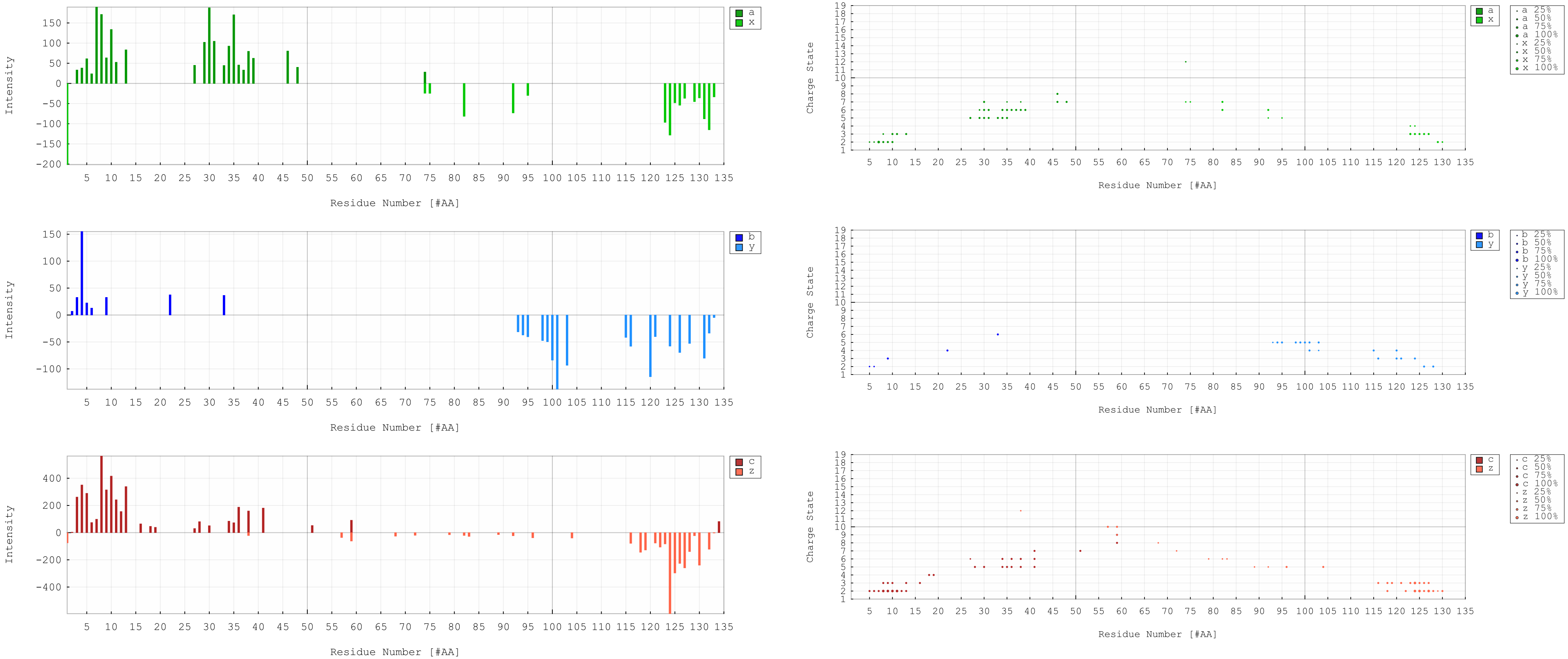

Proteoform:	Index	Protein	SC [%]	SVP [%]	IC [%]	MS Score [a.u.]	Var. Mods	Mono Mass [Da]	Chemical Formula
	7	H31Kac1	49.3	24.4	15.7	7.7	Ace: K36	15305.4534	C671H1131N215O186S4

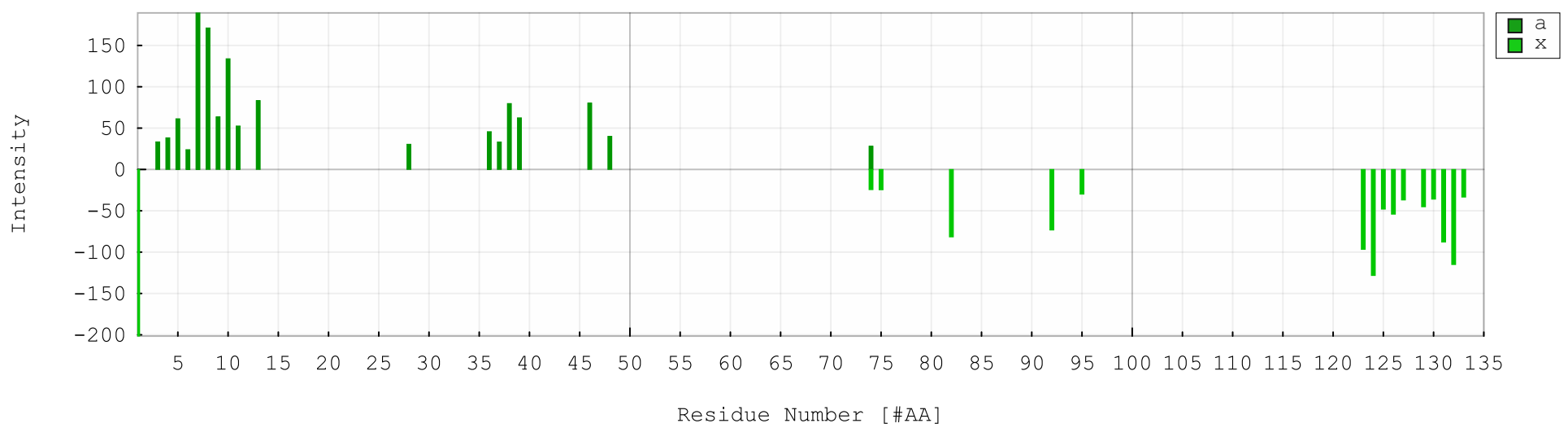

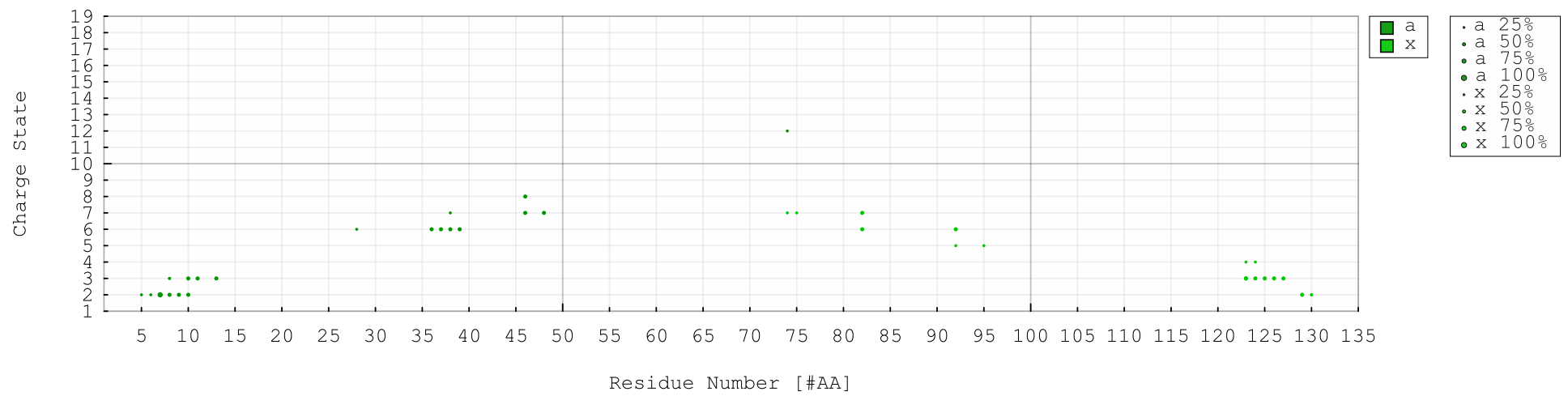

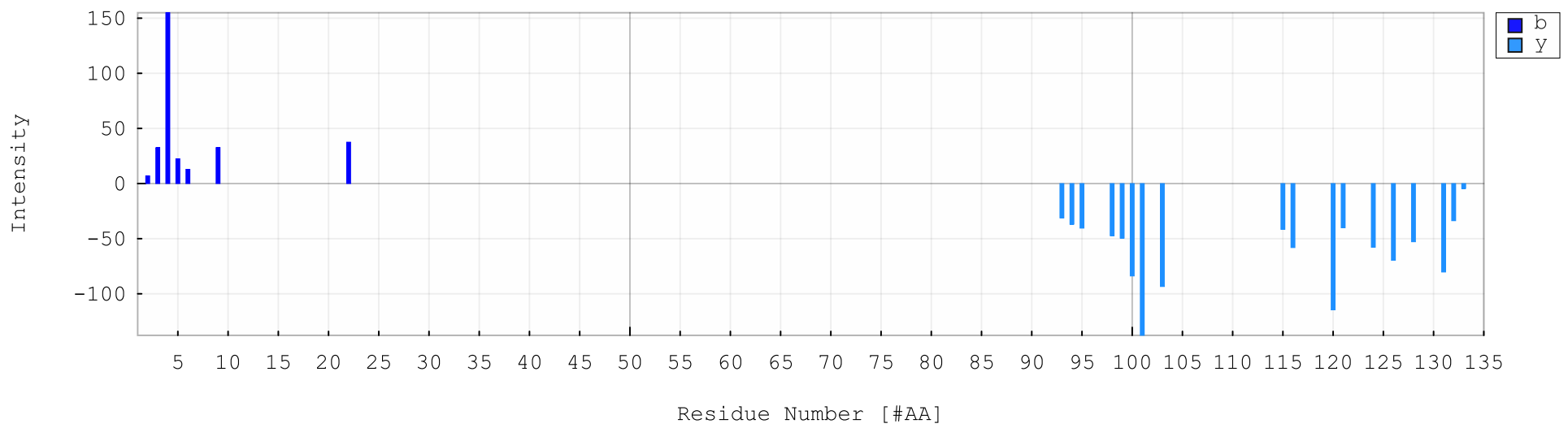

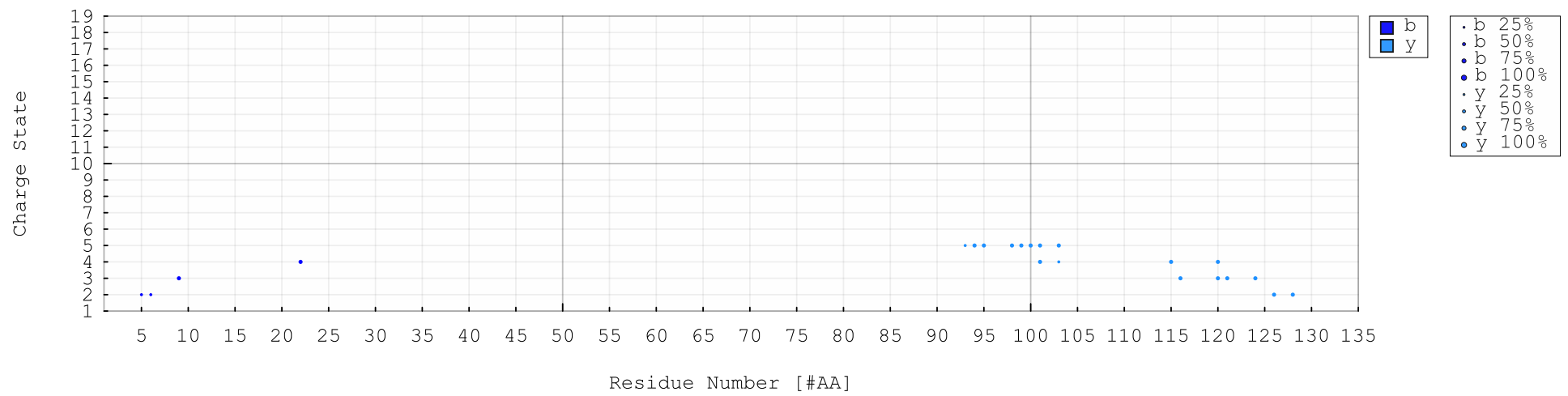

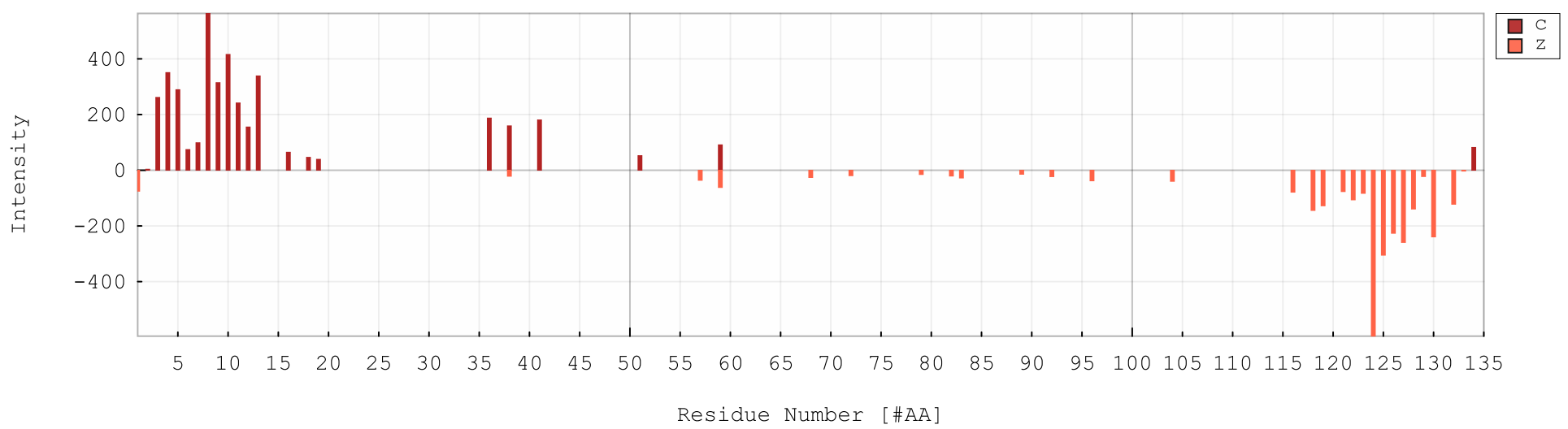

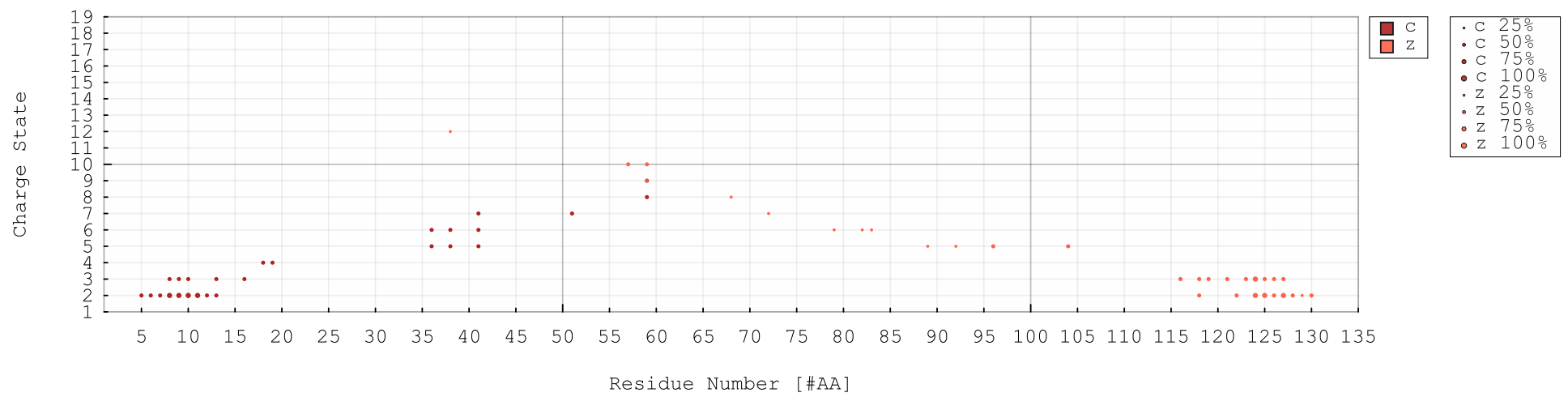

Proteoform:

Index	Protein	SC [%]	SVP [%]	IC [%]	MS Score [a.u.]	Var. Mods	Mono Mass [Da]	Chemical Formula
8	H31Kac1	48.5	24.4	15.3	7.4	Ace: K37	15305.4534	C671H1131N215O186S4

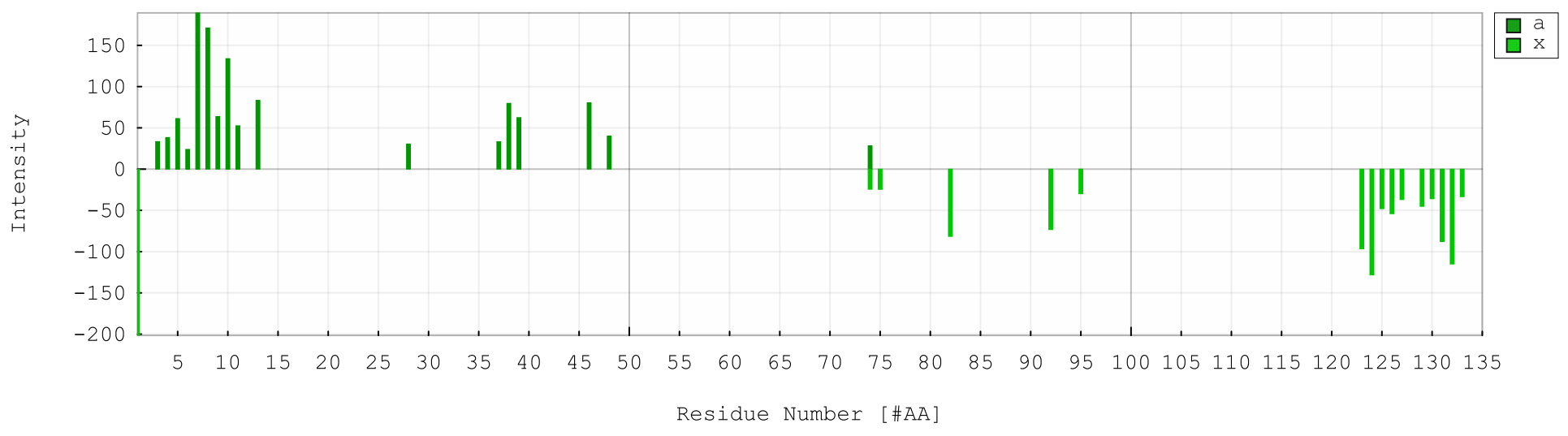

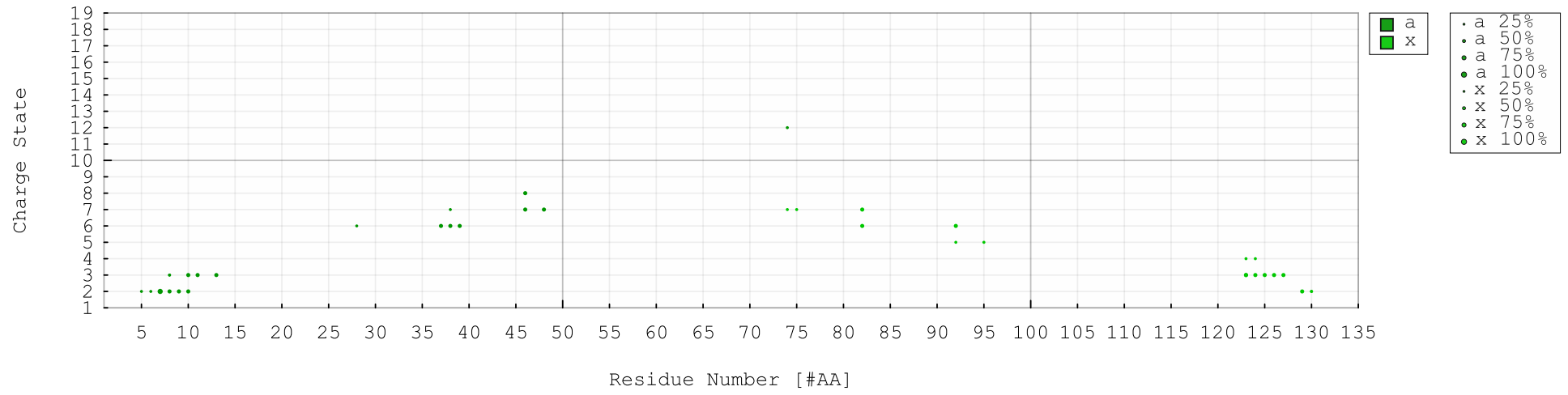

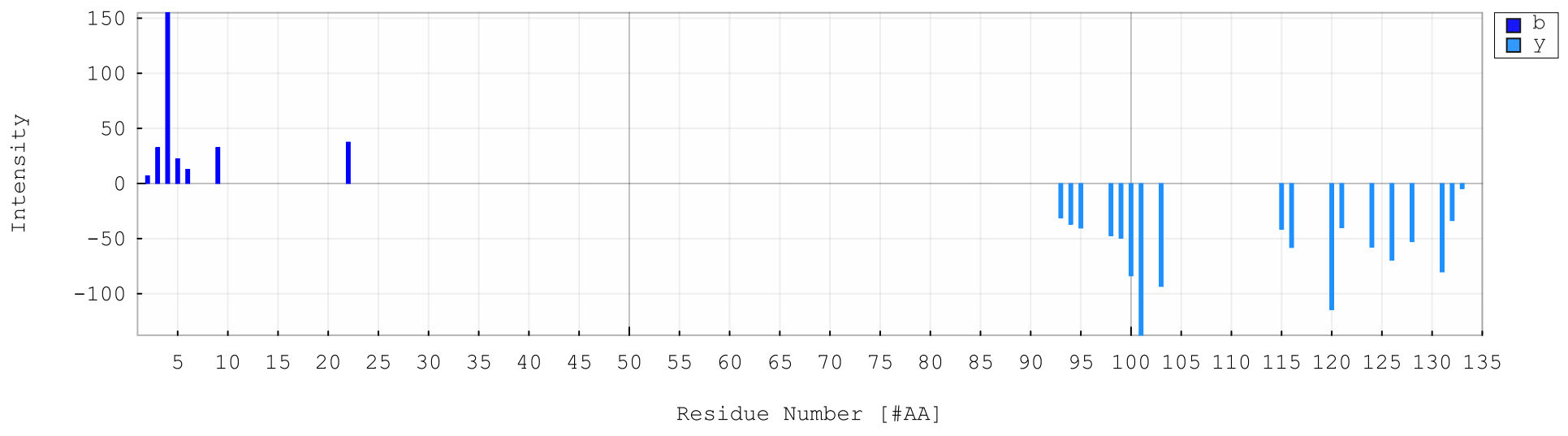

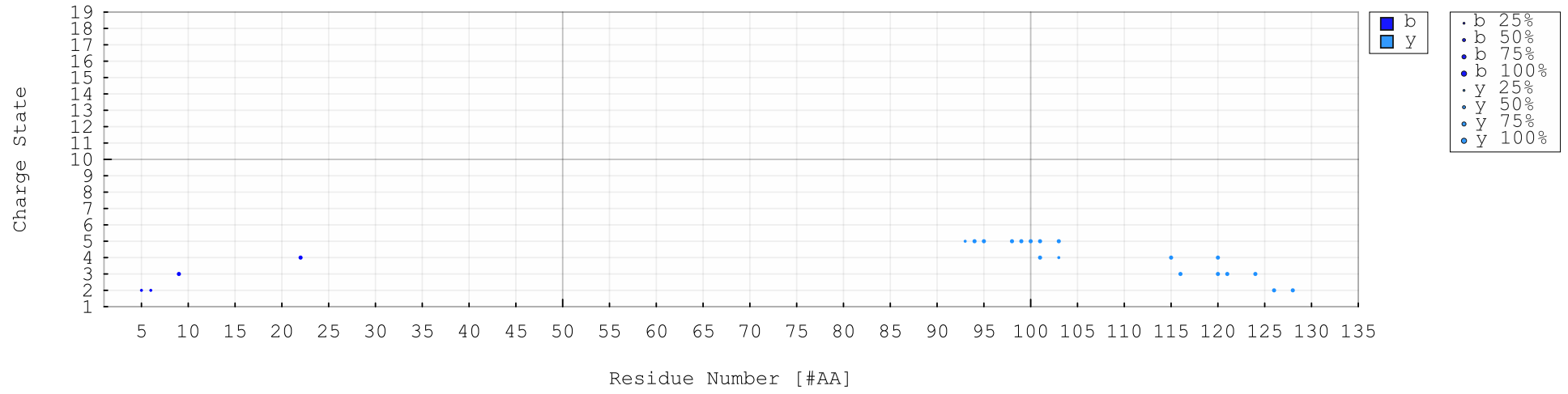

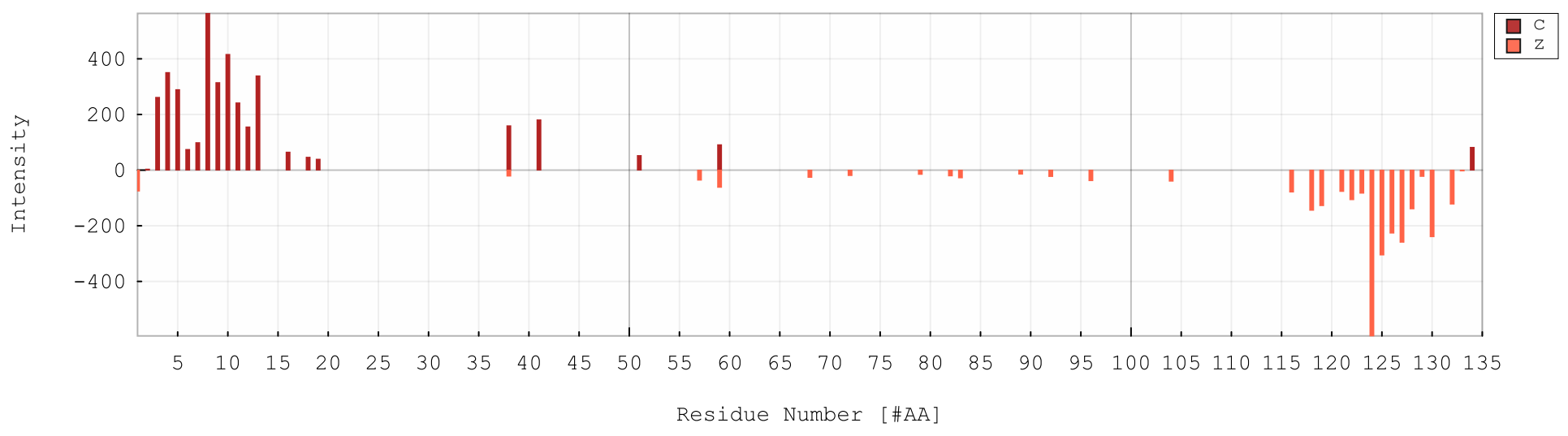

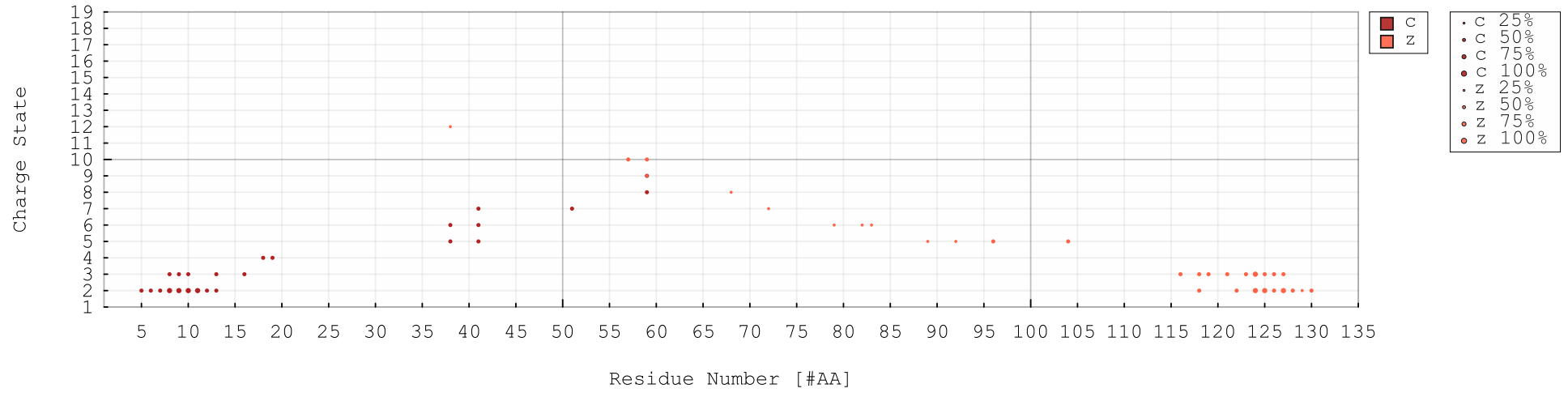

Proteoform:

Index	Protein	SC [%]	SVP [%]	IC [%]	MS Score [a.u.]	Var. Mods	Mono Mass [Da]	Chemical Formula
9	H31Kac1	43.3	24.4	14.0	6.0	Ace: K56	15305.4534	C671H1131N215O186S4

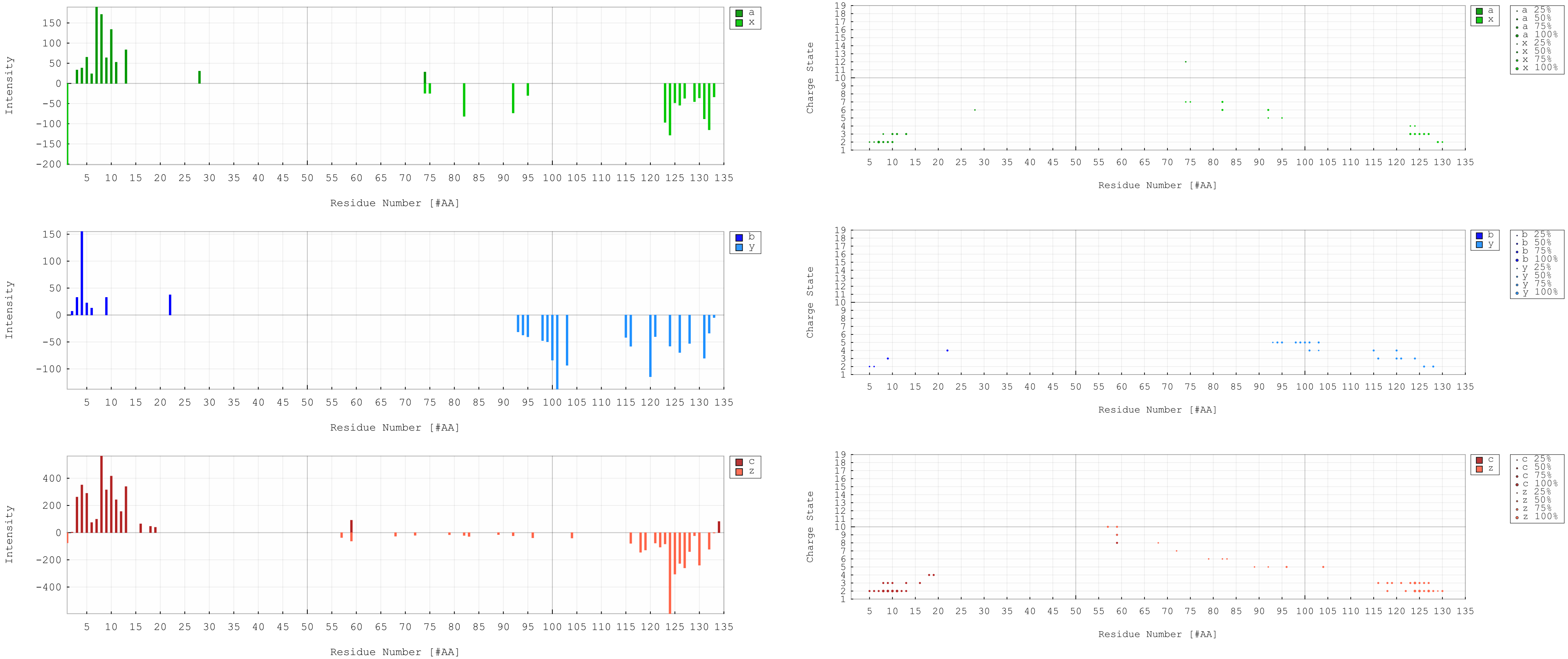

Proteoform:

Index	Protein	SC [%]	SVP [%]	IC [%]	MS Score [a.u.]	Var. Mods	Mono Mass [Da]	Chemical Formula
10	H31Kac1	41.8	24.4	13.5	5.6	Ace: K64	15305.4534	C671H1131N215O186S4

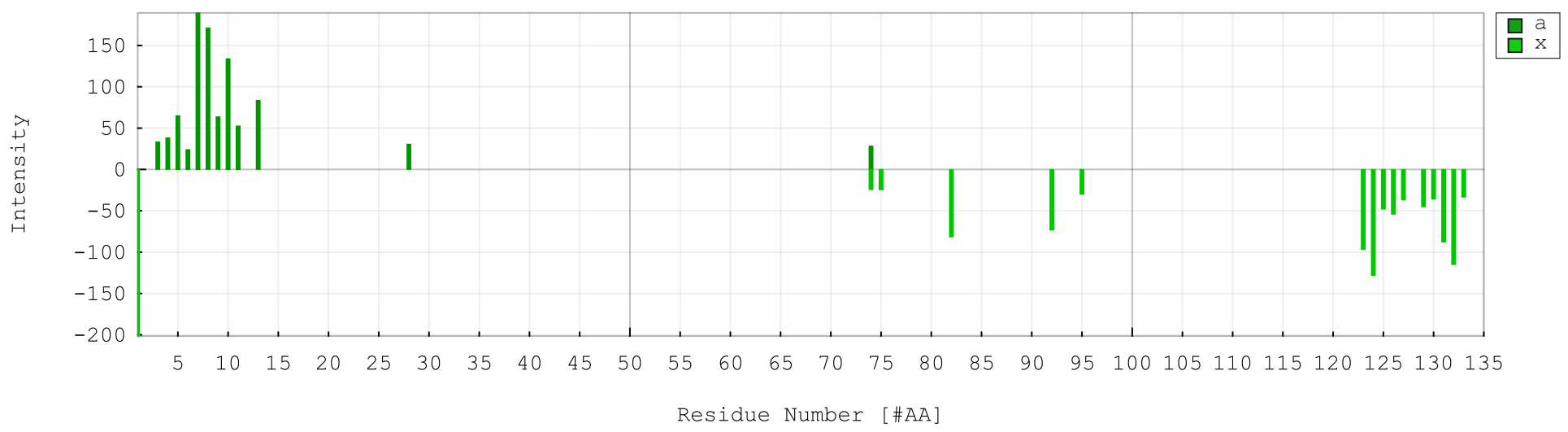

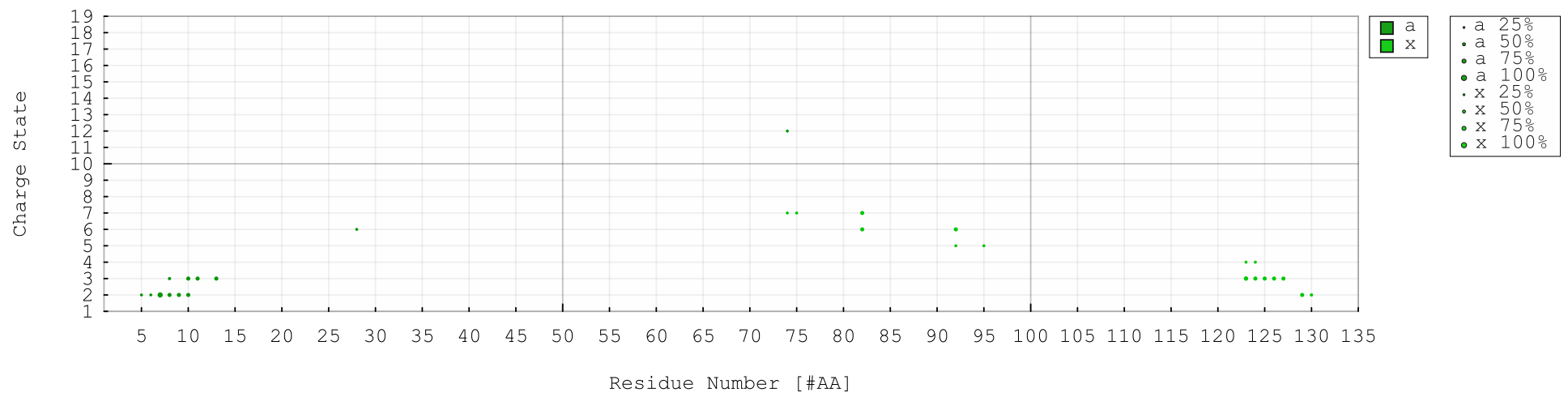

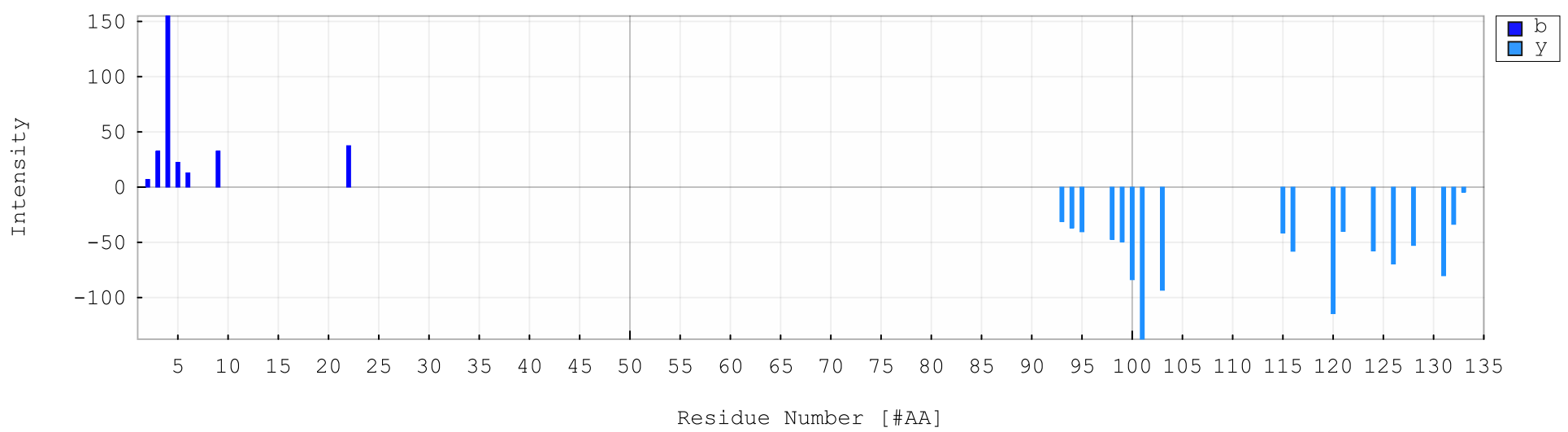

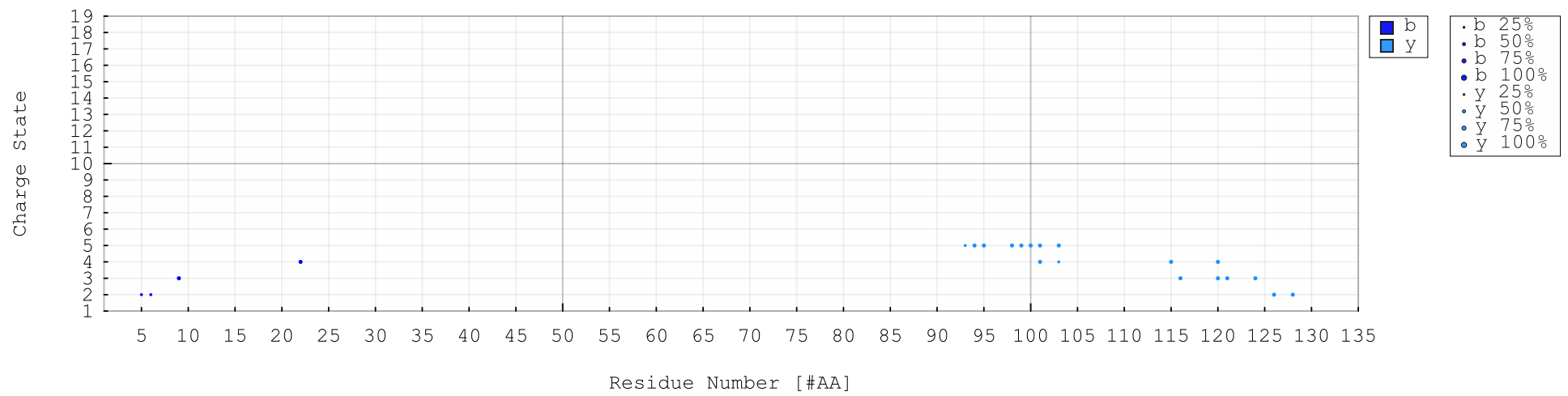

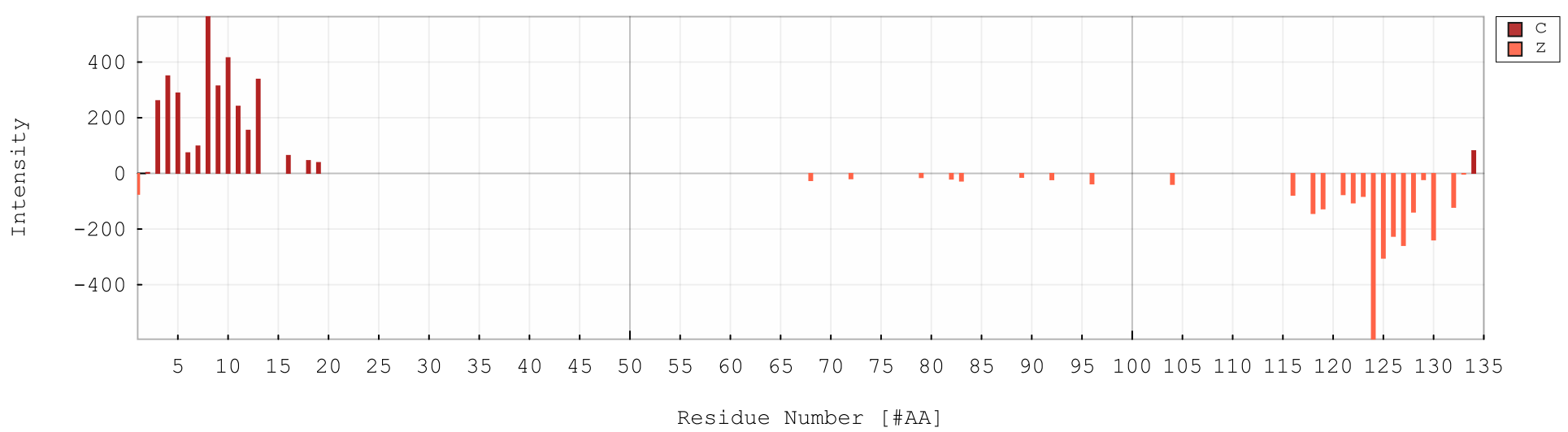

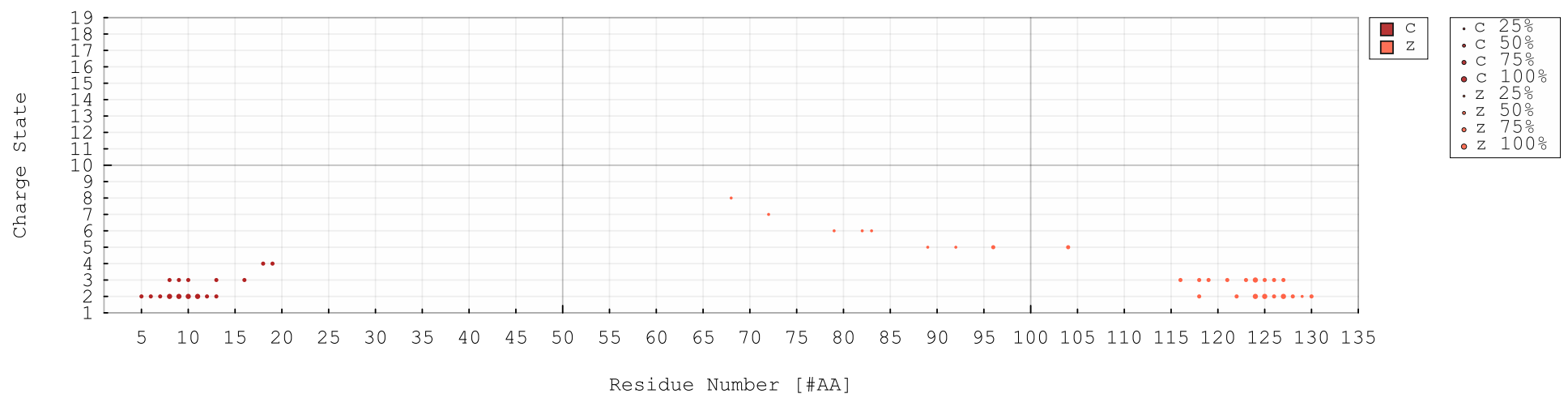

Supplemental Data S3

H3.1 - PCAF, 30 min

**Report**	3 of 3
**Experiment**	MS2
**Histone/sample**	H3.1
**Enzyme**	PCAF
**Reaction/source**	30 min
**Precursor**	H3.1Kac1
**Charge state**	19+
**Fragmentation / workflow**	RCID
**Original file**	MS2_H3.1Kac1_19+_RCID.pdf

Project Information

Project Directory: C:/Users/LAB-ADMIN
Processing date: Tue Apr 28 14:33:36 2026

Sample Info & Protocols

Name: Top-Down Protein Sequencing CID

Dataset

Imported spectrum file name:	250428 H31-PCAF100-30min z19ac1 MS2 shCID_calibrated.txt
Dataset name:	Information is only available from imported JPEAKS files
Date acquired:	Information is only available from imported JPEAKS files
Date imported:	Information is only available from imported JPEAKS files
Instrument Name:	Information is only available from imported JPEAKS files
Instrument Serial Number:	Information is only available from imported JPEAKS files

Workflow Result Info

Analysis: 250428 H31-PCAF100-30min z19ac1 MS2 shCID_calibrated2
Location: C:/Users/LAB-ADMIN
Method: -

Method Information

Name: Unnamed

Spectral Parameters

Mass Error [ppm]: 6
Intensity Deviation [au]: 0.15
Min/Max Charges: 1-19
Experiment type: CID

a ions	b ions	c ions	x ions	y ions	z ions	satellite	internal	precursor
a	b			y				M

Noise threshold calculation: Simple moving average noise threshold (SMA)
Window size [datapoints]: 600
Noise curve offset: 1.7049497
SVP parameters: terminal gap: 2, internal gap: 1

Proteoforms

(top 10)

Index	Parent Sequence	Chain	Var. Mods	Mono Mass [Da]	Chemical Composition
1	H31Kac1	H31Kac1K14[Ace]	Ace: K14	15305.4534	C671H1131N215O186S4
2	H31Kac1	H31Kac1K9[Ace]	Ace: K9	15305.4534	C671H1131N215O186S4
3	H31Kac1	H31Kac1K18[Ace]	Ace: K18	15305.4534	C671H1131N215O186S4
4	H31Kac1	H31Kac1K4[Ace]	Ace: K4	15305.4534	C671H1131N215O186S4
5	H31Kac1	H31Kac1K23[Ace]	Ace: K23	15305.4534	C671H1131N215O186S4
6	H31Kac1	H31Kac1K27[Ace]	Ace: K27	15305.4534	C671H1131N215O186S4
7	H31Kac1	H31Kac1K36[Ace]	Ace: K36	15305.4534	C671H1131N215O186S4
8	H31Kac1	H31Kac1K37[Ace]	Ace: K37	15305.4534	C671H1131N215O186S4
9	H31Kac1	H31Kac1K56[Ace]	Ace: K56	15305.4534	C671H1131N215O186S4
10	H31Kac1	H31Kac1K64[Ace]	Ace: K64	15305.4534	C671H1131N215O186S4

Results

Proteoforms Table (top 10)

Index	Protein	SC [%]	SVP [%]	IC [%]	MS Score [a.u.]	Var. Mods	Mono Mass [Da]	Chemical Formula
1	H31Kac1	48.5	8.9	48.5	23.5	Ace: K14	15305.4534	C671H1131N215O186S4
2	H31Kac1	47.8	8.9	48.5	23.2	Ace: K9	15305.4534	C671H1131N215O186S4
3	H31Kac1	46.3	8.9	48.4	22.4	Ace: K18	15305.4534	C671H1131N215O186S4
4	H31Kac1	45.5	7.4	48.2	21.9	Ace: K4	15305.4534	C671H1131N215O186S4
5	H31Kac1	44.0	8.9	48.2	21.2	Ace: K23	15305.4534	C671H1131N215O186S4
6	H31Kac1	42.5	8.9	48.0	20.4	Ace: K27	15305.4534	C671H1131N215O186S4
7	H31Kac1	38.1	8.9	47.6	18.1	Ace: K36	15305.4534	C671H1131N215O186S4
8	H31Kac1	38.1	8.9	47.6	18.1	Ace: K37	15305.4534	C671H1131N215O186S4
9	H31Kac1	36.6	8.9	47.3	17.3	Ace: K56	15305.4534	C671H1131N215O186S4
10	H31Kac1	35.8	8.9	47.0	16.8	Ace: K64	15305.4534	C671H1131N215O186S4

Sequence

Name: H31Kac1

```
                   10                   20                   30                   40                   50                   60
A R T K Q T A R K S  T G G K A P R K Q L  A T K A A R K S A P  A T G G V K K P H R  Y R P G T V A L R E  I R R Y Q K S T E L
                   70                   80                   90                  100                  110                  120
L I R K L P F Q R L  V R E I A Q D F K T  D L R F Q S S A V M  A L Q E A C E A Y L  V G L F E D T N L C  A I H A K R V T I M
                  130                  140                  150                  160                  170                  180
P K D I Q L A R R I  R G E R A
```

Modifications

Modification	Position	Type
Acetyl	4	variable
Acetyl	9	variable
Acetyl	14	variable
Acetyl	18	variable
Acetyl	23	variable
Acetyl	27	variable
Acetyl	36	variable
Acetyl	37	variable
Acetyl	56	variable
Acetyl	64	variable
Acetyl	79	variable

Modification	Position	Type
Acetyl	115	variable
Acetyl	122	variable

Sequence Map

Proteoform:

Index	Protein	SC [%]	SVP [%]	IC [%]	MS Score [a.u.]	Var. Mods	Mono Mass [Da]	Chemical Formula
1	H31Kac1	48.5	8.9	48.5	23.5	Ace: K14	15305.4534	C671H1131N215O186S4

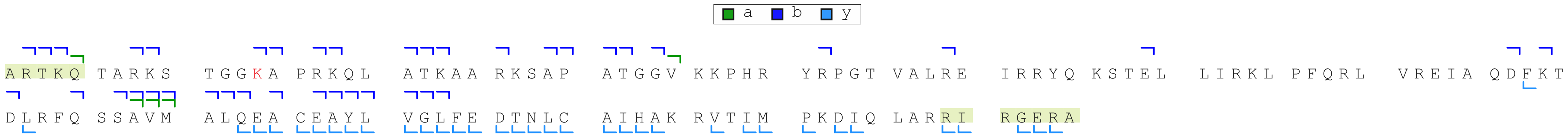

Proteoform:

Index	Protein	SC [%]	SVP [%]	IC [%]	MS Score [a.u.]	Var. Mods	Mono Mass [Da]	Chemical Formula
2	H31Kac1	47.8	8.9	48.5	23.2	Ace: K9	15305.4534	C671H1131N215O186S4

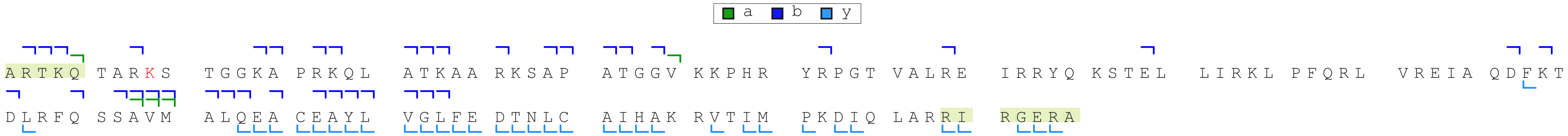

Top-Down Result OmniScape 2025
Sequence Confirmation
250428 H31-PCAF100-30min z19ac1 MS2 shCID_calibrated2

Proteoform:

Index	Protein	SC [%]	SVP [%]	IC [%]	MS Score [a.u.]	Var. Mods	Mono Mass [Da]	Chemical Formula
3	H31Kac1	46.3	8.9	48.4	22.4	Ace: K18	15305.4534	C671H1131N215O186S4

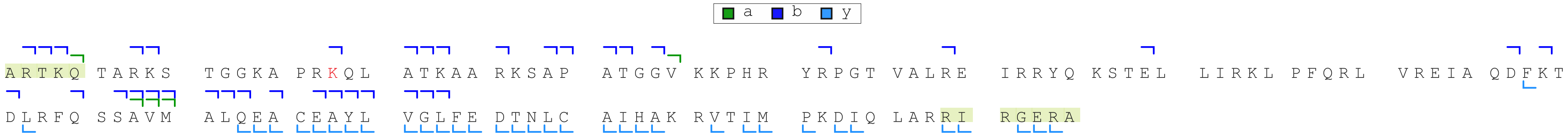

Proteoform:	Index	Protein	SC [%]	SVP [%]	IC [%]	MS Score [a.u.]	Var. Mods	Mono Mass [Da]	Chemical Formula
	4	H31Kac1	45.5	7.4	48.2	21.9	Ace: K4	15305.4534	C671H1131N215O186S4

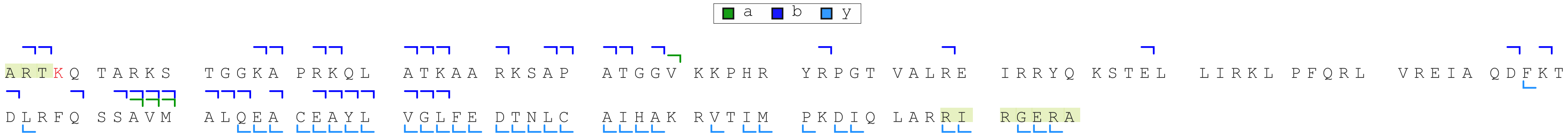

Top-Down Result OmniScape 2025
Sequence Confirmation
250428 H31-PCAF100-30min z19ac1 MS2 shCID_calibrated2

Proteoform:

Index	Protein	SC [%]	SVP [%]	IC [%]	MS Score [a.u.]	Var. Mods	Mono Mass [Da]	Chemical Formula
5	H31Kac1	44.0	8.9	48.2	21.2	Ace: K23	15305.4534	C671H1131N215O186S4

a b y

ARTKQ TARKS TGGKA PRKQL ATKAA RKSAP ATGGV KKPHR YRPGT VALRE IRRYQ KSTEL LIRKL PFQRL VREIA QDFKT
DLRFQ SSAVM ALQEA CEAYL VGLFE DTNLC AIHAK RVTIM PKDIQ LARRI RGERA

Proteoform:

Index	Protein	SC [%]	SVP [%]	IC [%]	MS Score [a.u.]	Var. Mods	Mono Mass [Da]	Chemical Formula
6	H31Kac1	42.5	8.9	48.0	20.4	Ace: K27	15305.4534	C671H1131N215O186S4

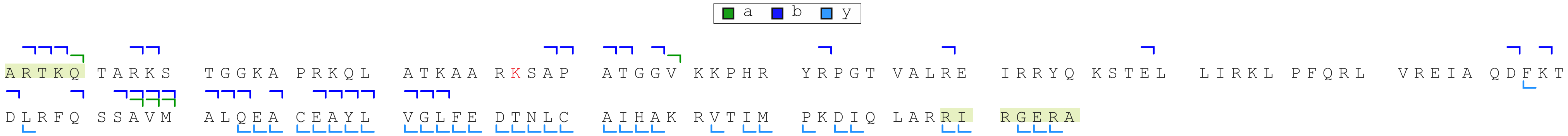

Proteoform:

Index	Protein	SC [%]	SVP [%]	IC [%]	MS Score [a.u.]	Var. Mods	Mono Mass [Da]	Chemical Formula
7	H31Kac1	38.1	8.9	47.6	18.1	Ace: K36	15305.4534	C671H1131N215O186S4

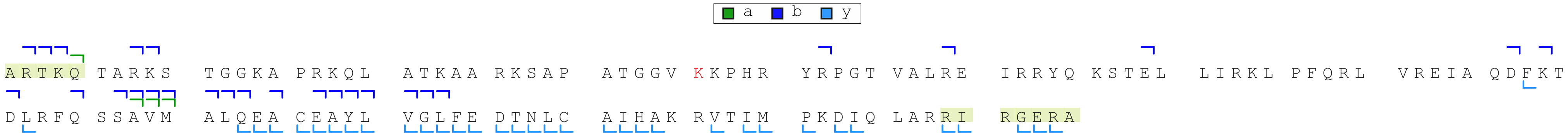

Proteoform:

Index	Protein	SC [%]	SVP [%]	IC [%]	MS Score [a.u.]	Var. Mods	Mono Mass [Da]	Chemical Formula
8	H31Kac1	38.1	8.9	47.6	18.1	Ace: K37	15305.4534	C671H1131N215O186S4

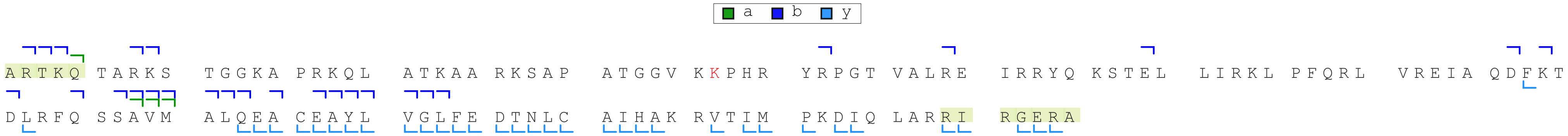

Proteoform:

Index	Protein	SC [%]	SVP [%]	IC [%]	MS Score [a.u.]	Var. Mods	Mono Mass [Da]	Chemical Formula
9	H31Kac1	36.6	8.9	47.3	17.3	Ace: K56	15305.4534	C671H1131N215O186S4

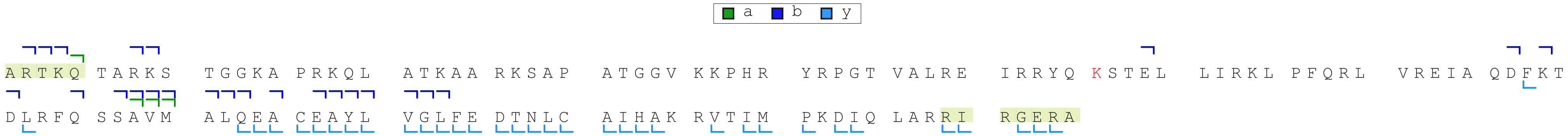

Proteoform:	Index	Protein	SC [%]	SVP [%]	IC [%]	MS Score [a.u.]	Var. Mods	Mono Mass [Da]	Chemical Formula
	10	H31Kac1	35.8	8.9	47.0	16.8	Ace: K64	15305.4534	C671H1131N215O186S4

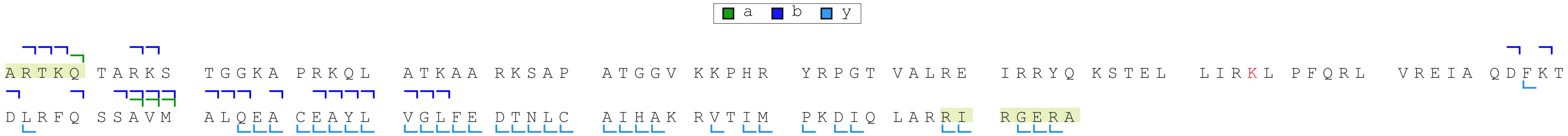

Error Plot

Proteoform:

Index	Protein	SC [%]	SVP [%]	IC [%]	MS Score [a.u.]	Var. Mods	Mono Mass [Da]	Chemical Formula
1	H31Kac1	48.5	8.9	48.5	23.5	Ace: K14	15305.4534	C671H1131N215O186S4

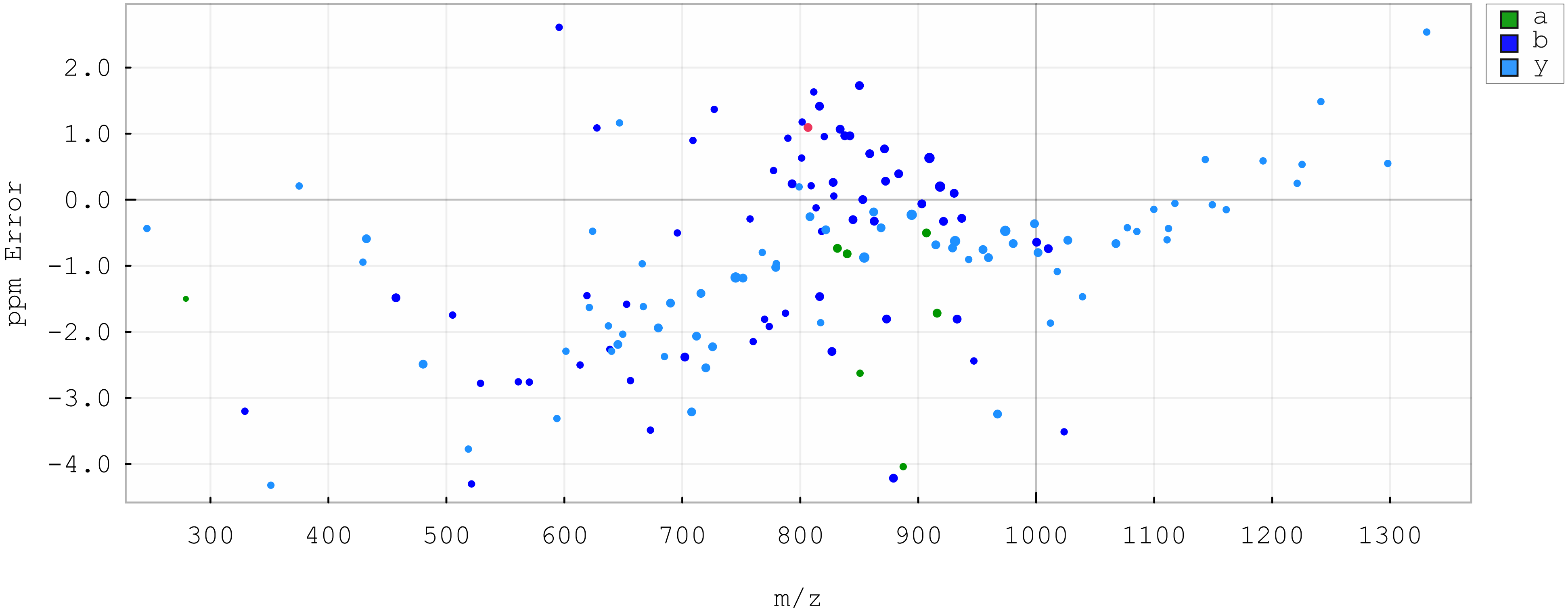

Proteoform:

Index	Protein	SC [%]	SVP [%]	IC [%]	MS Score [a.u.]	Var. Mods	Mono Mass [Da]	Chemical Formula
2	H31Kac1	47.8	8.9	48.5	23.2	Ace: K9	15305.4534	C671H1131N215O186S4

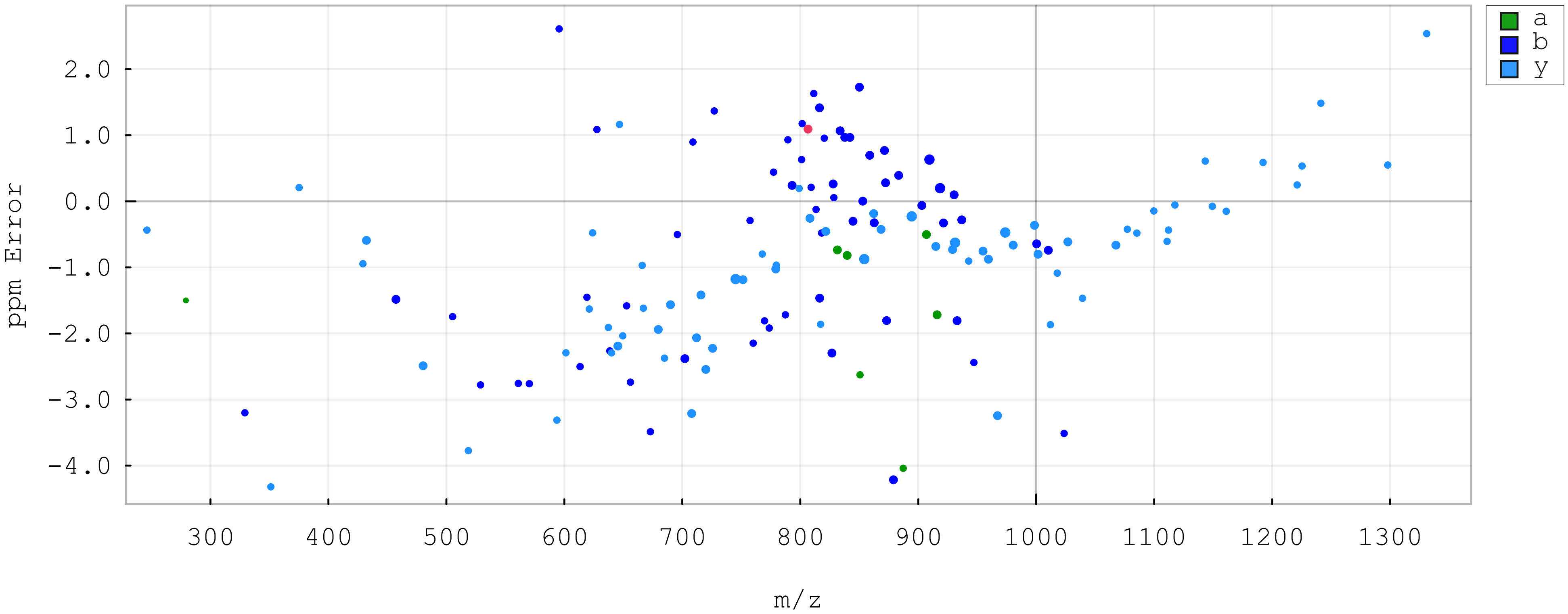

Proteoform:

Index	Protein	SC [%]	SVP [%]	IC [%]	MS Score [a.u.]	Var. Mods	Mono Mass [Da]	Chemical Formula
3	H31Kac1	46.3	8.9	48.4	22.4	Ace: K18	15305.4534	C671H1131N215O186S4

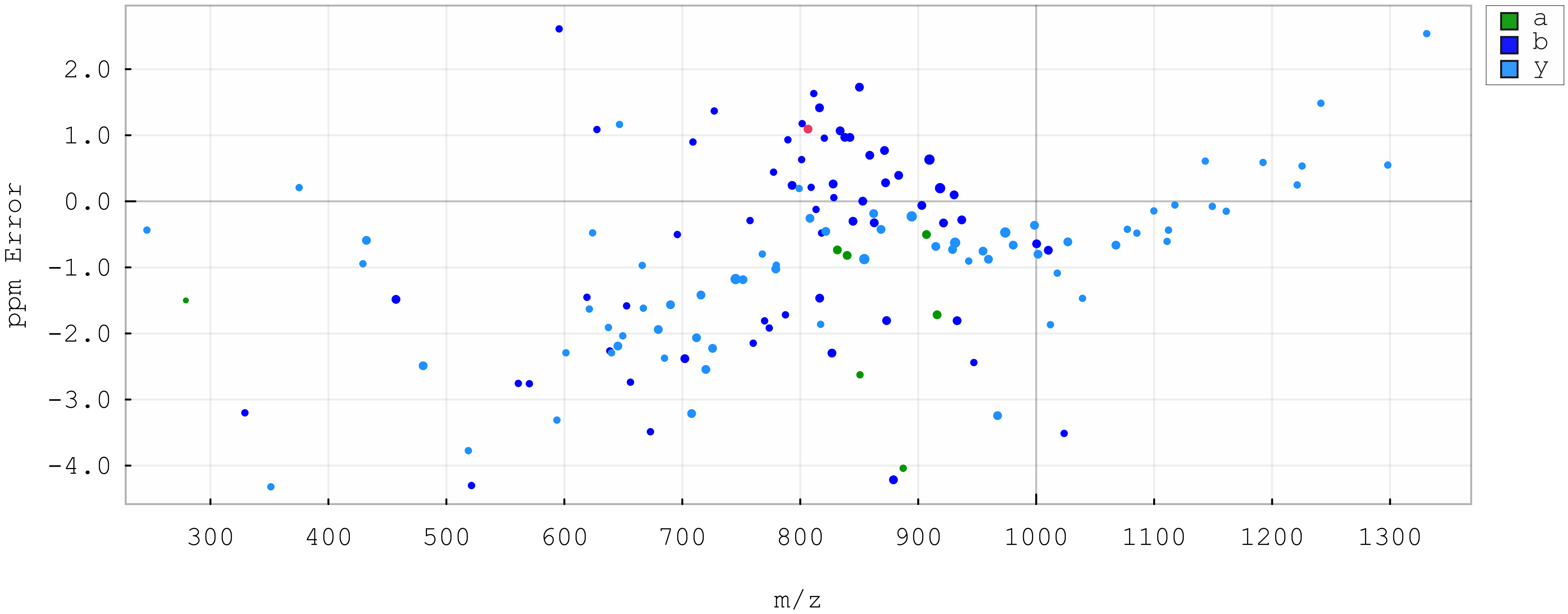

Proteoform:

Index	Protein	SC [%]	SVP [%]	IC [%]	MS Score [a.u.]	Var. Mods	Mono Mass [Da]	Chemical Formula
4	H31Kac1	45.5	7.4	48.2	21.9	Ace: K4	15305.4534	C671H1131N215O186S4

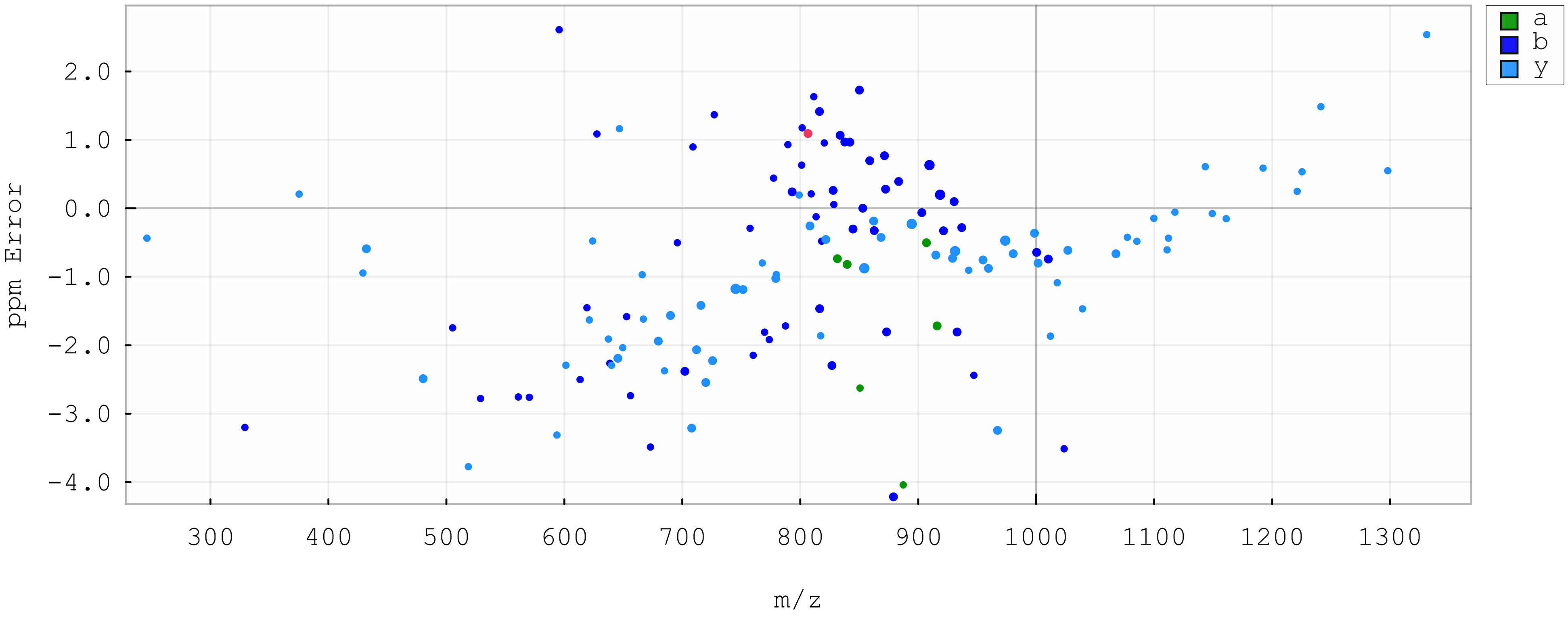

Proteoform:

Index	Protein	SC [%]	SVP [%]	IC [%]	MS Score [a.u.]	Var. Mods	Mono Mass [Da]	Chemical Formula
5	H31Kac1	44.0	8.9	48.2	21.2	Ace: K23	15305.4534	C671H1131N215O186S4

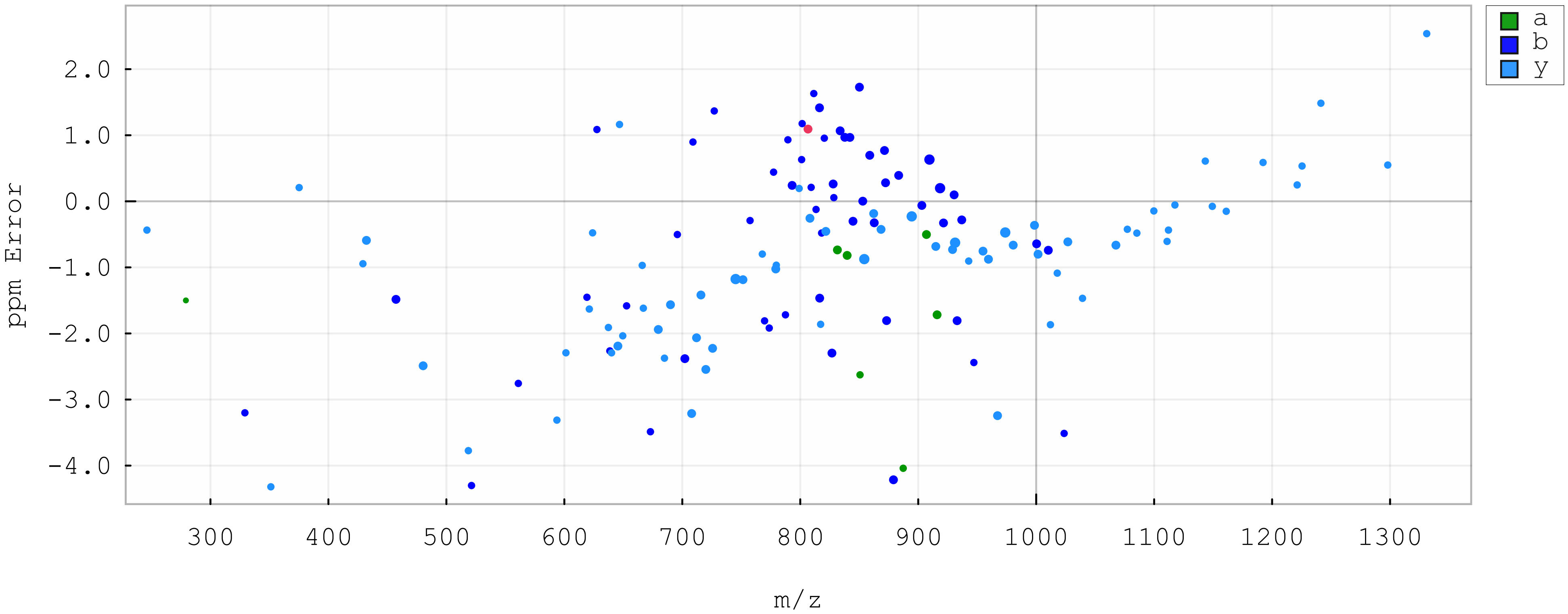

Proteoform:

Index	Protein	SC [%]	SVP [%]	IC [%]	MS Score [a.u.]	Var. Mods	Mono Mass [Da]	Chemical Formula
6	H31Kac1	42.5	8.9	48.0	20.4	Ace: K27	15305.4534	C671H1131N215O186S4

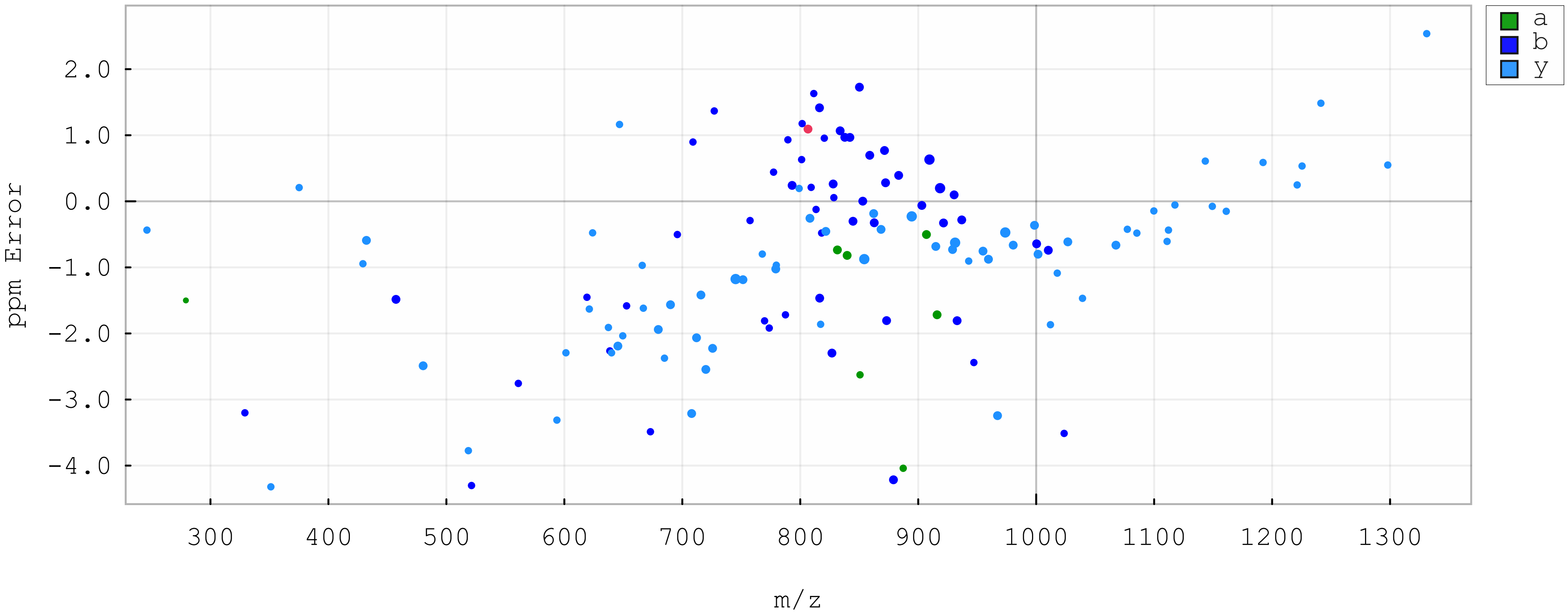

Proteoform:	Index	Protein	SC [%]	SVP [%]	IC [%]	MS Score [a.u.]	Var. Mods	Mono Mass [Da]	Chemical Formula
	7	H31Kac1	38.1	8.9	47.6	18.1	Ace: K36	15305.4534	C671H1131N215O186S4

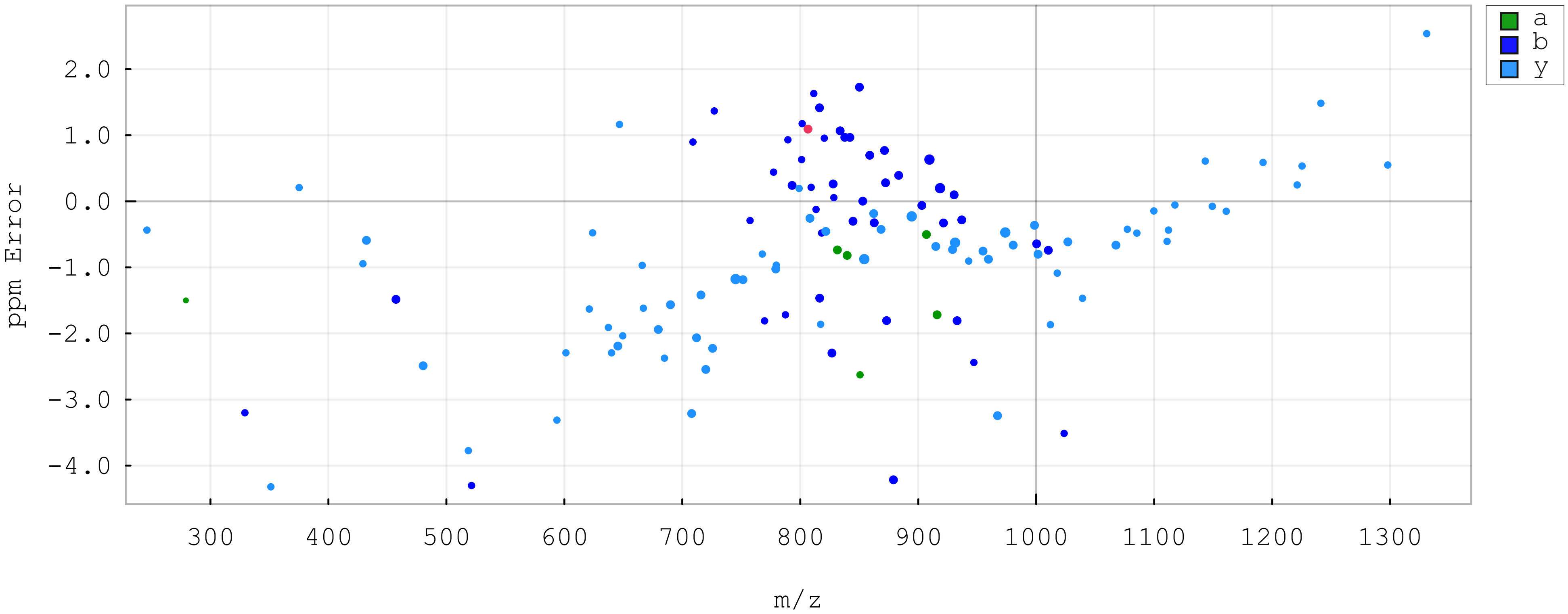

Proteoform:

Index	Protein	SC [%]	SVP [%]	IC [%]	MS Score [a.u.]	Var. Mods	Mono Mass [Da]	Chemical Formula
8	H31Kac1	38.1	8.9	47.6	18.1	Ace: K37	15305.4534	C671H1131N215O186S4

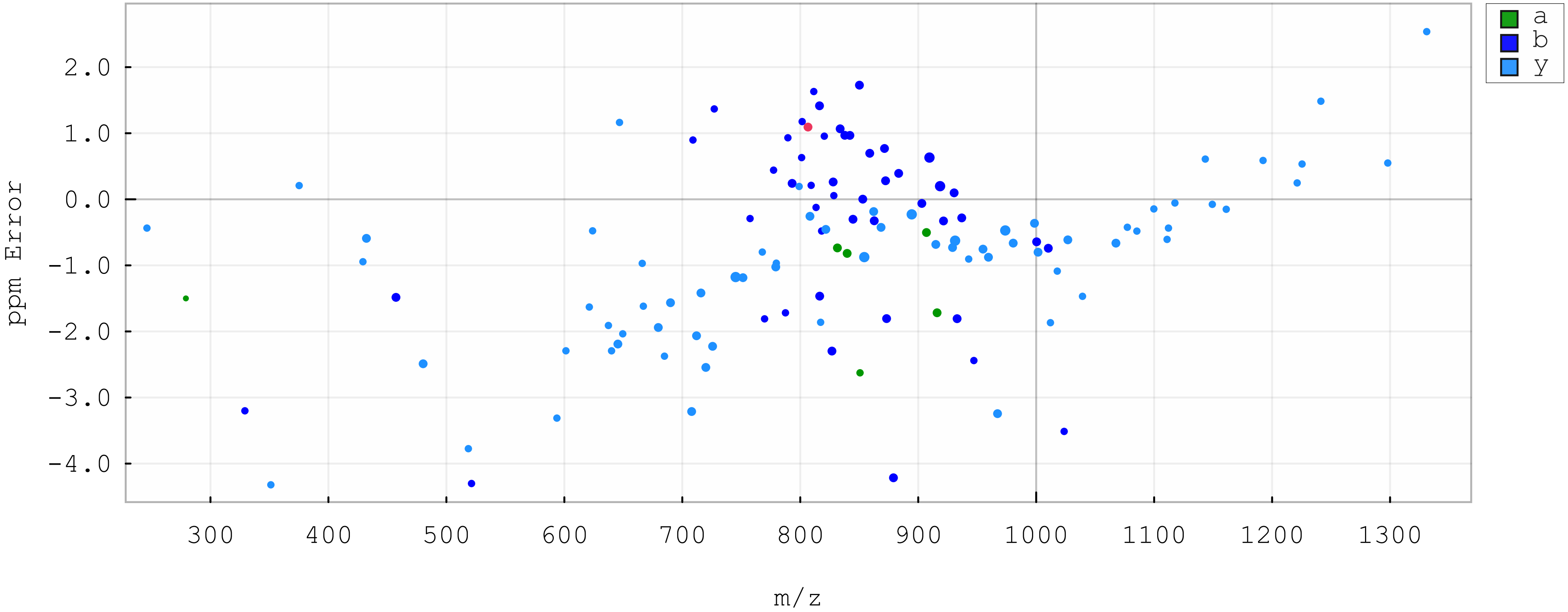

Proteoform:	Index	Protein	SC [%]	SVP [%]	IC [%]	MS Score [a.u.]	Var. Mods	Mono Mass [Da]	Chemical Formula
	9	H31Kac1	36.6	8.9	47.3	17.3	Ace: K56	15305.4534	C671H1131N215O186S4

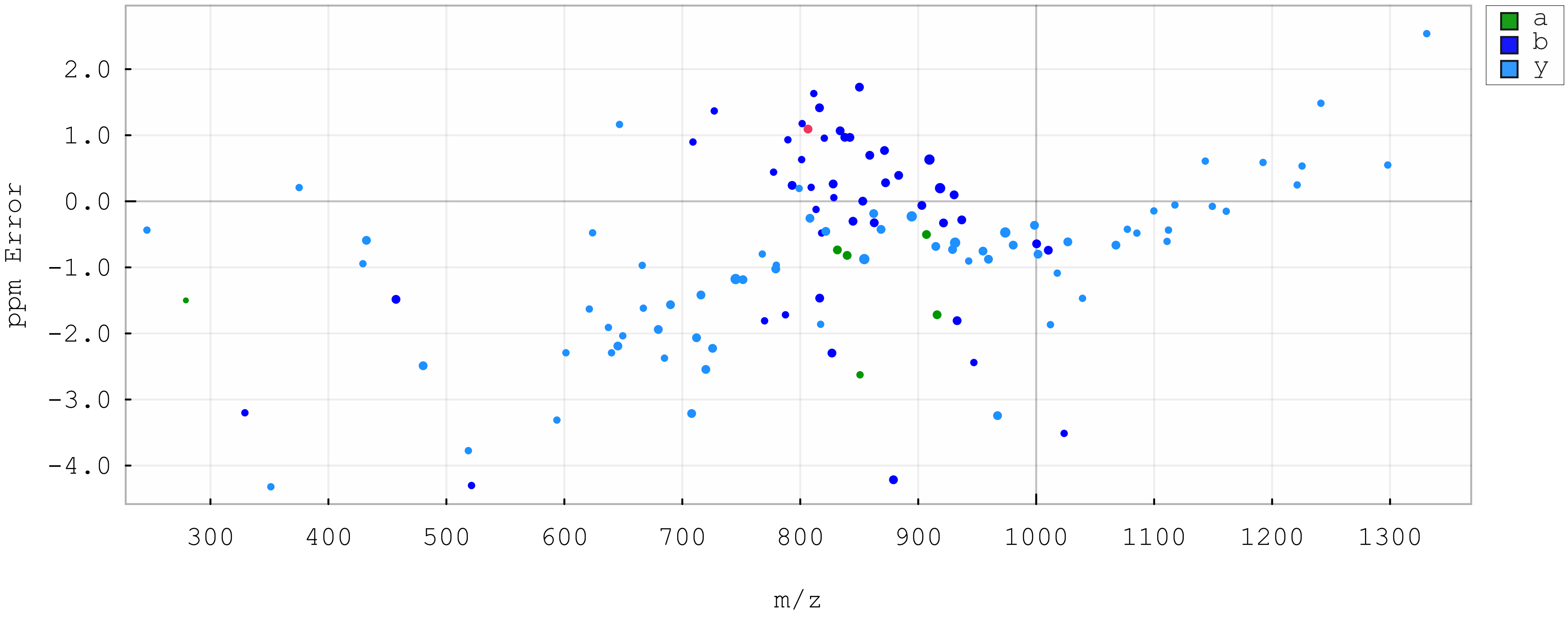

Proteoform:

Index	Protein	SC [%]	SVP [%]	IC [%]	MS Score [a.u.]	Var. Mods	Mono Mass [Da]	Chemical Formula
10	H31Kac1	35.8	8.9	47.0	16.8	Ace: K64	15305.4534	C671H1131N215O186S4

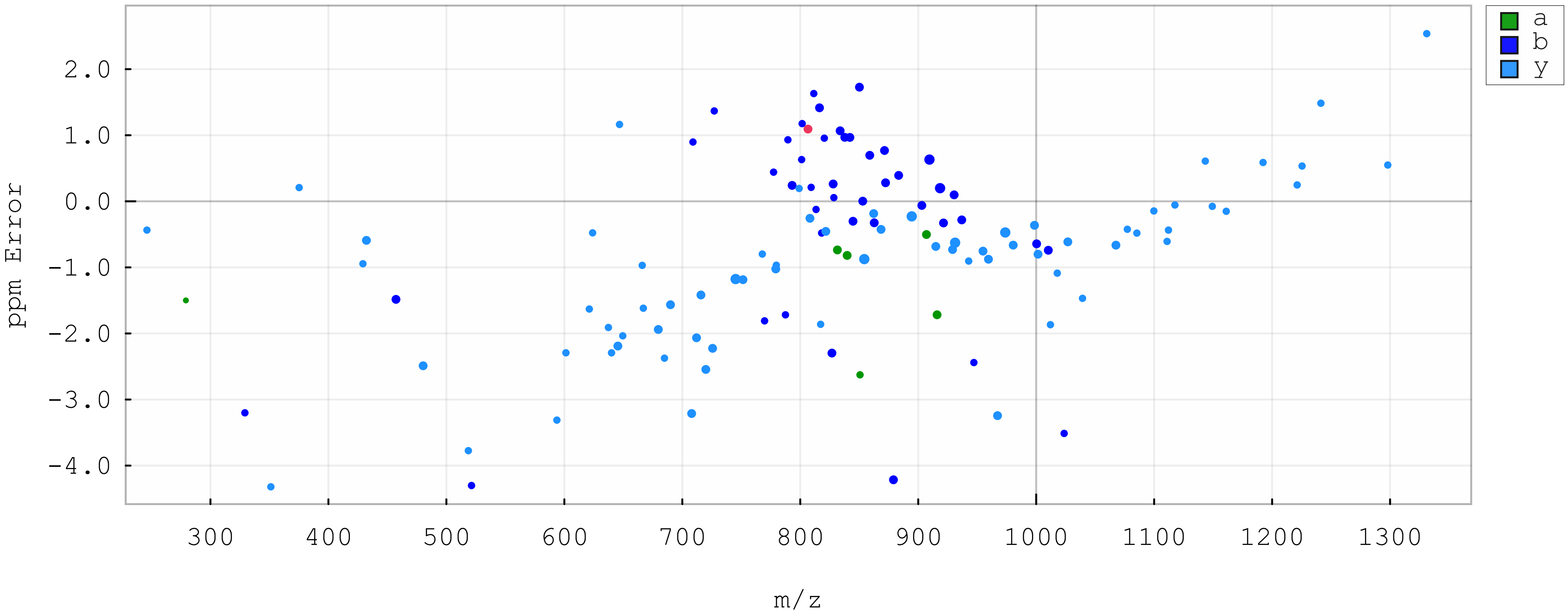

Primary Fragment Plots

Proteoform:

Index	Protein	SC [%]	SVP [%]	IC [%]	MS Score [a.u.]	Var. Mods	Mono Mass [Da]	Chemical Formula
1	H31Kac1	48.5	8.9	48.5	23.5	Ace: K14	15305.4534	C671H1131N215O186S4

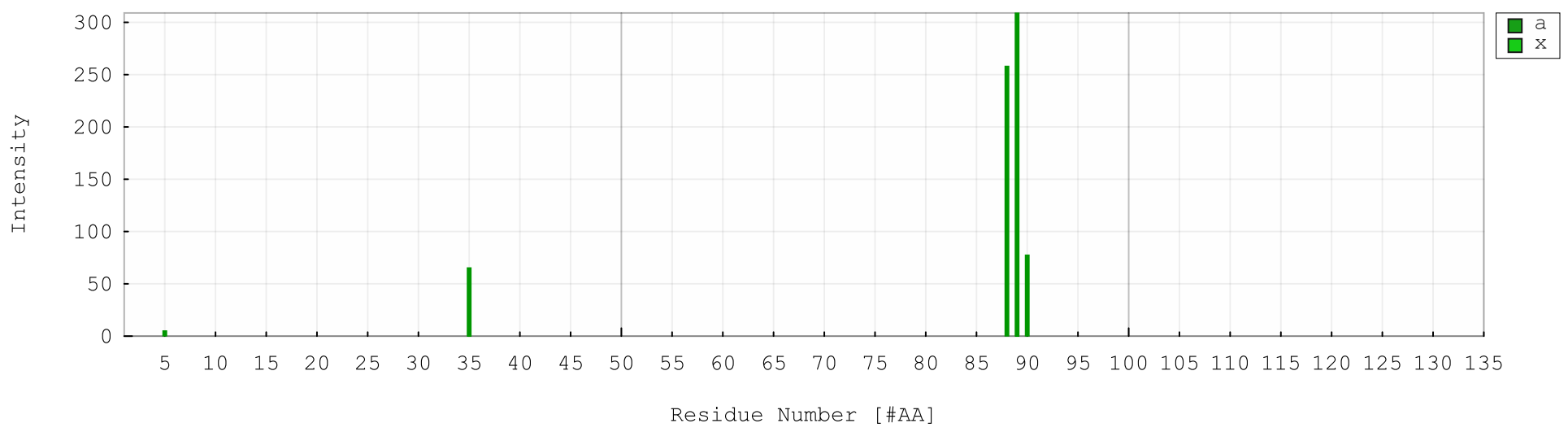

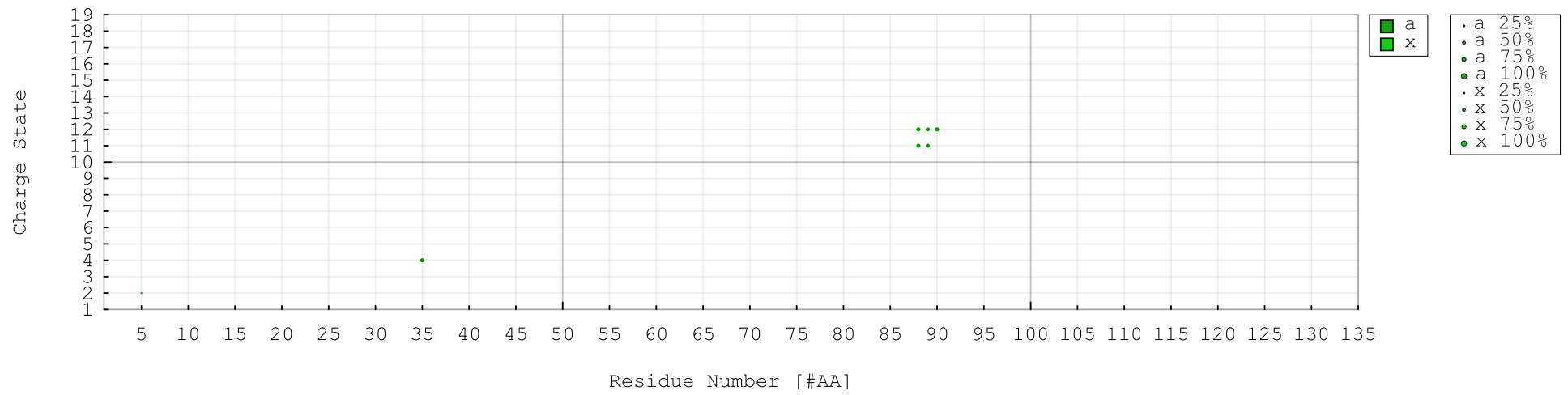

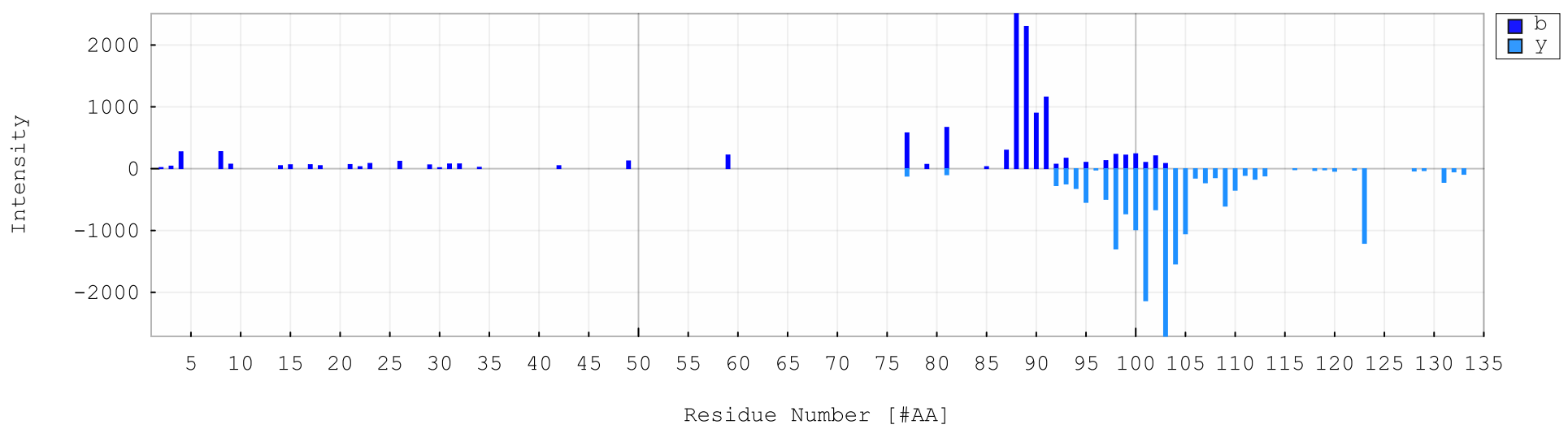

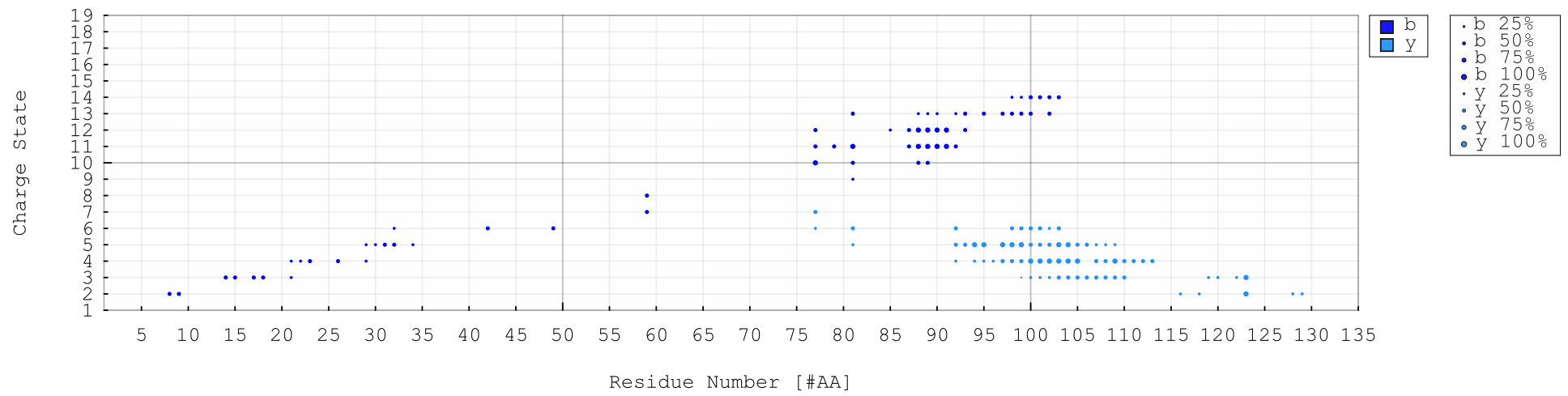

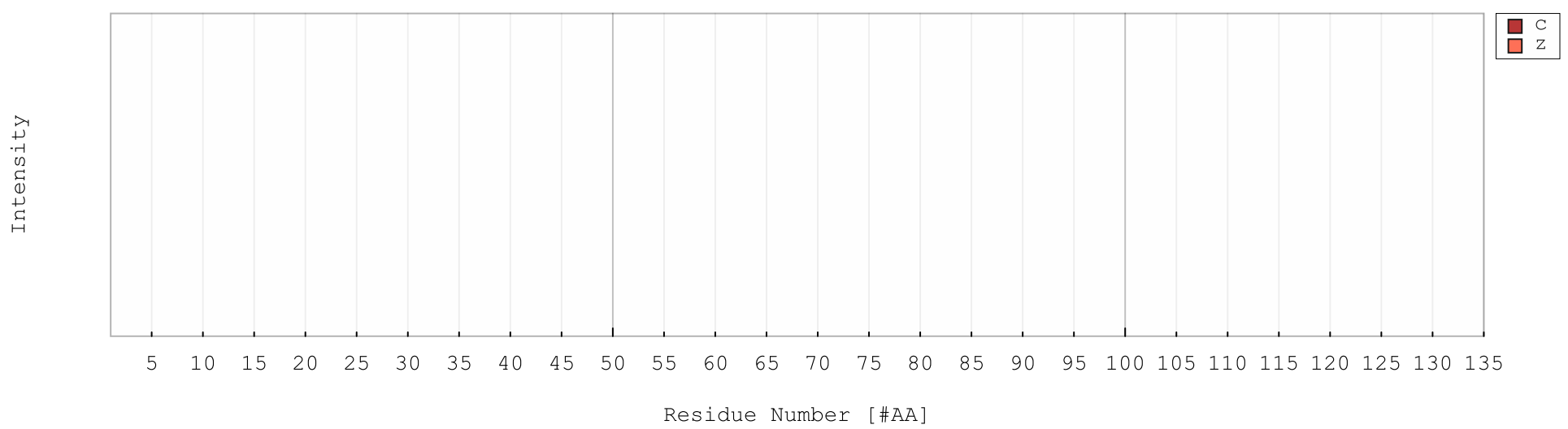

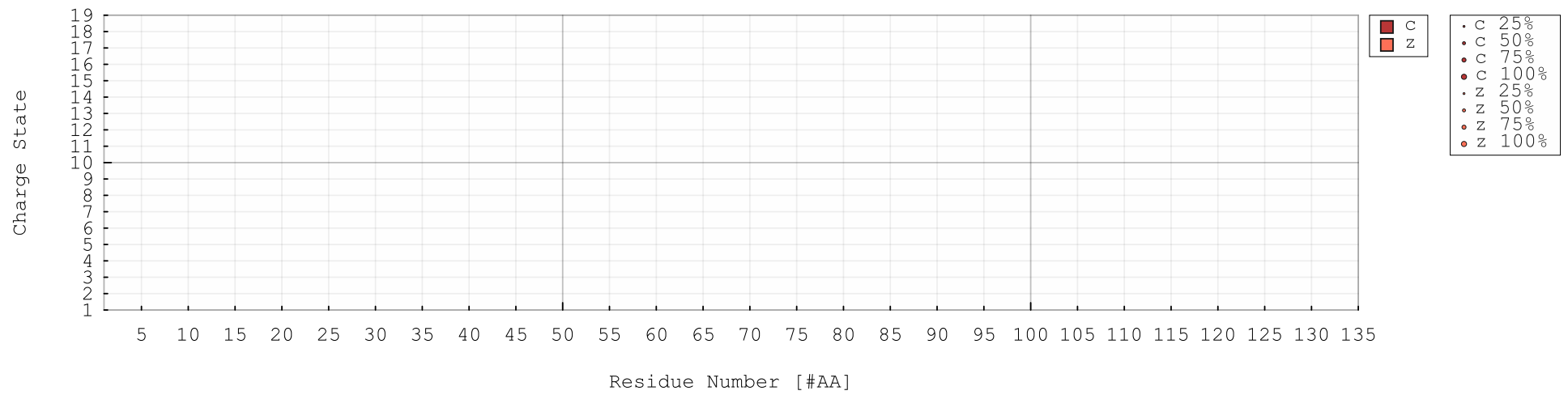

Proteoform:

Index	Protein	SC [%]	SVP [%]	IC [%]	MS Score [a.u.]	Var. Mods	Mono Mass [Da]	Chemical Formula
2	H31Kac1	47.8	8.9	48.5	23.2	Ace: K9	15305.4534	C671H1131N215O186S4

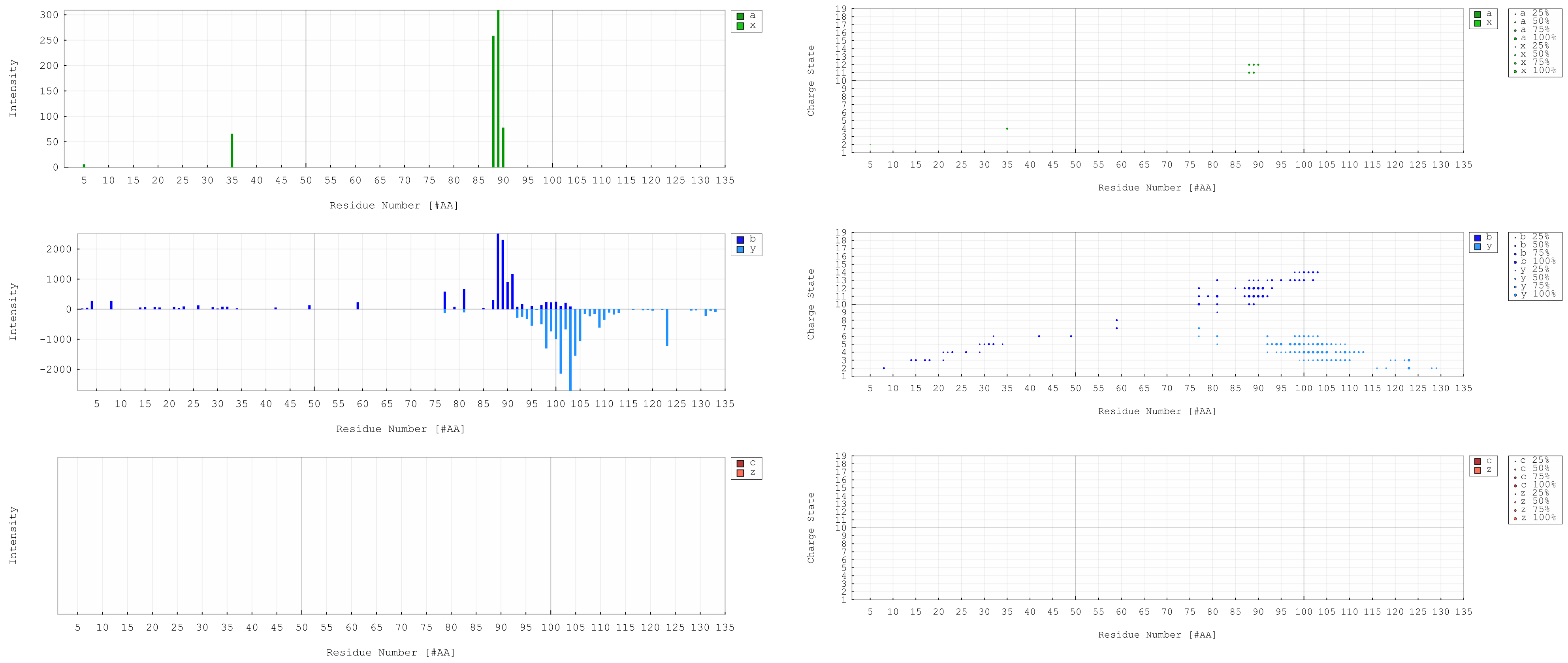

Proteoform:

Index	Protein	SC [%]	SVP [%]	IC [%]	MS Score [a.u.]	Var. Mods	Mono Mass [Da]	Chemical Formula
3	H31Kac1	46.3	8.9	48.4	22.4	Ace: K18	15305.4534	C671H1131N215O186S4

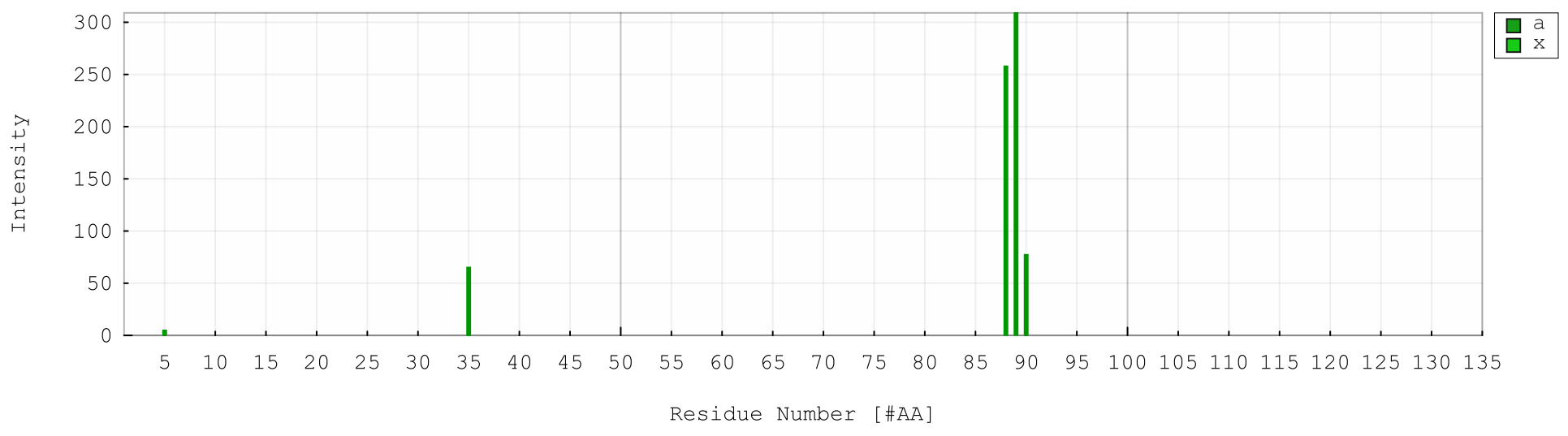
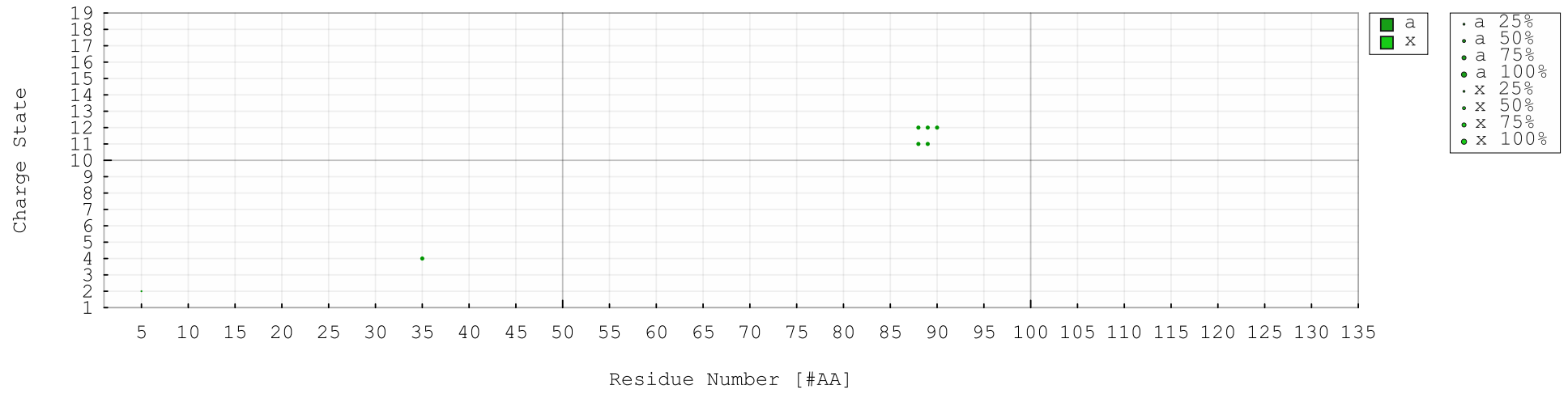
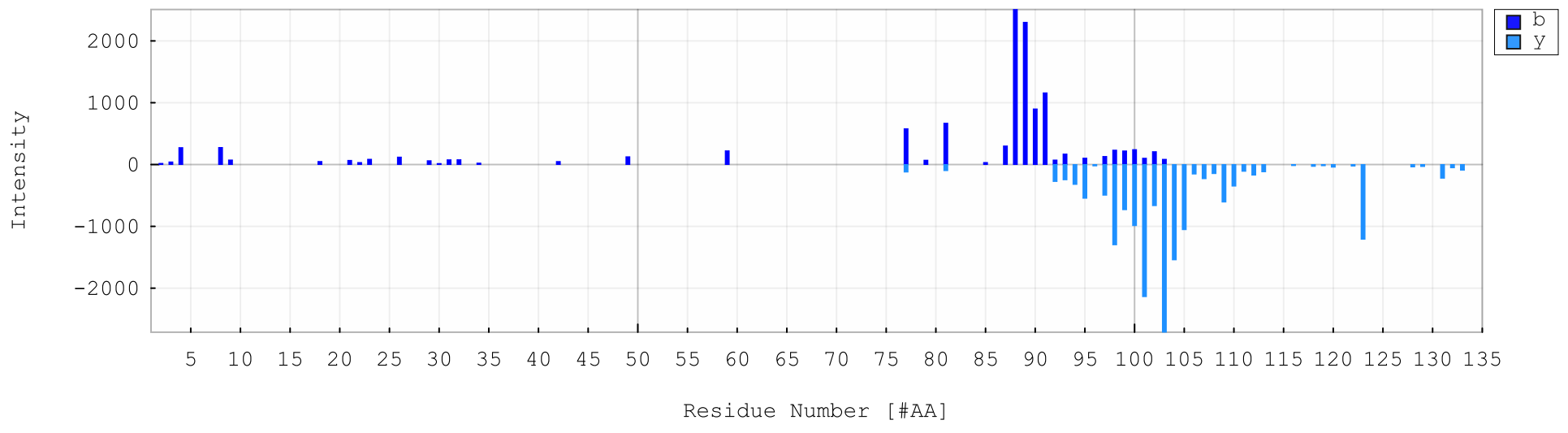
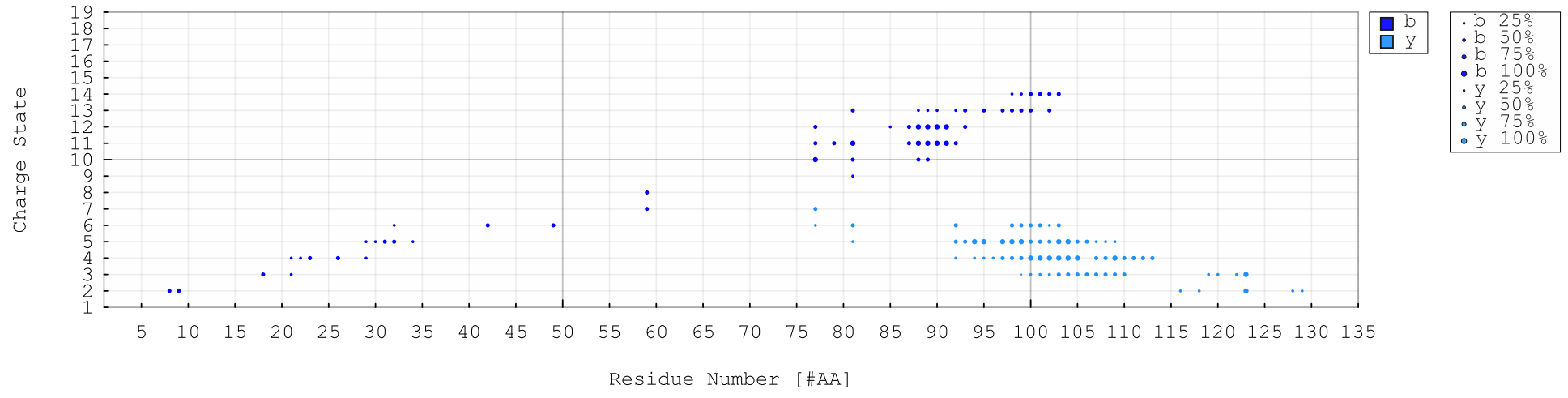
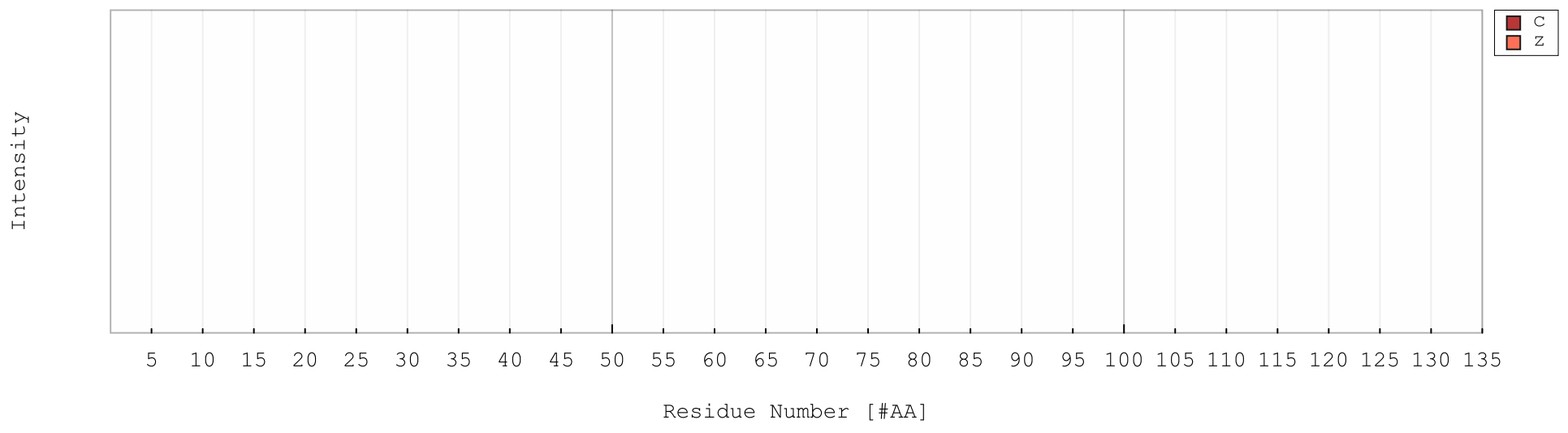
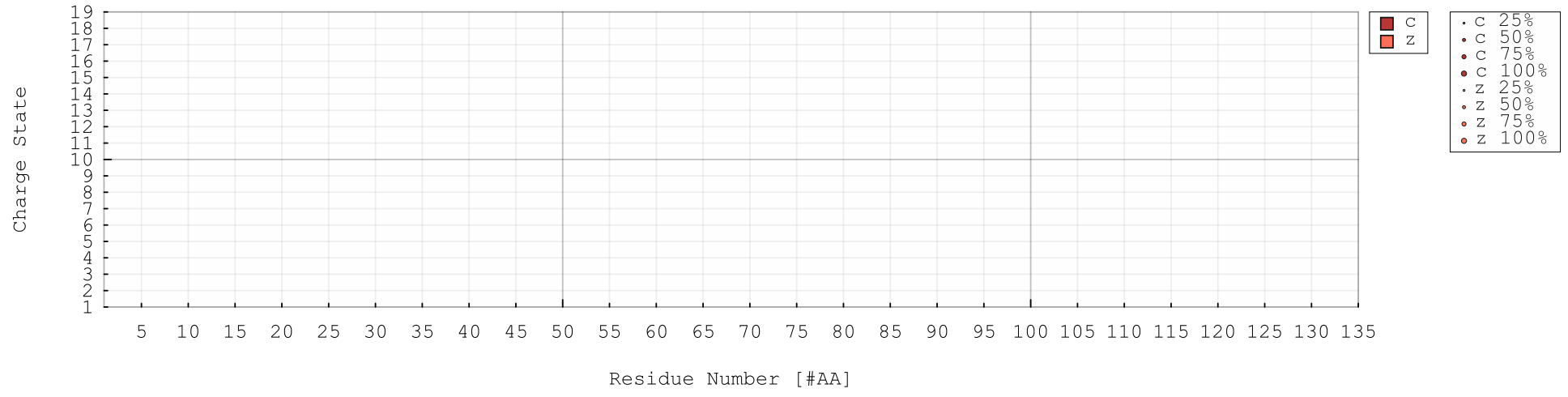

Top-Down Result OmniScape 2025
Sequence Confirmation
250428 H31-PCAF100-30min z19ac1 MS2 shCID_calibrated2

Proteoform:

Index	Protein	SC [%]	SVP [%]	IC [%]	MS Score [a.u.]	Var. Mods	Mono Mass [Da]	Chemical Formula
4	H31Kac1	45.5	7.4	48.2	21.9	Ace: K4	15305.4534	C671H1131N215O186S4

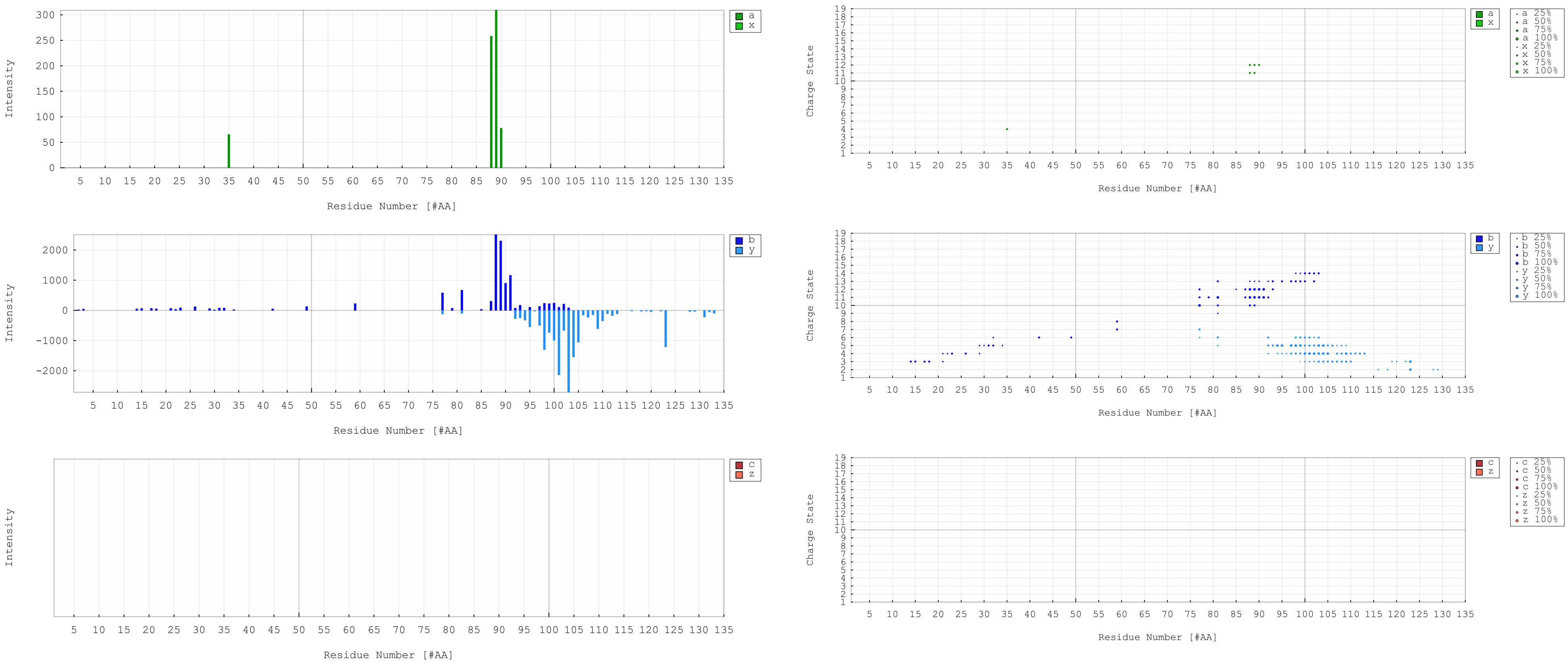

Proteoform:

Index	Protein	SC [%]	SVP [%]	IC [%]	MS Score [a.u.]	Var. Mods	Mono Mass [Da]	Chemical Formula
5	H31Kac1	44.0	8.9	48.2	21.2	Ace: K23	15305.4534	C671H1131N215O186S4

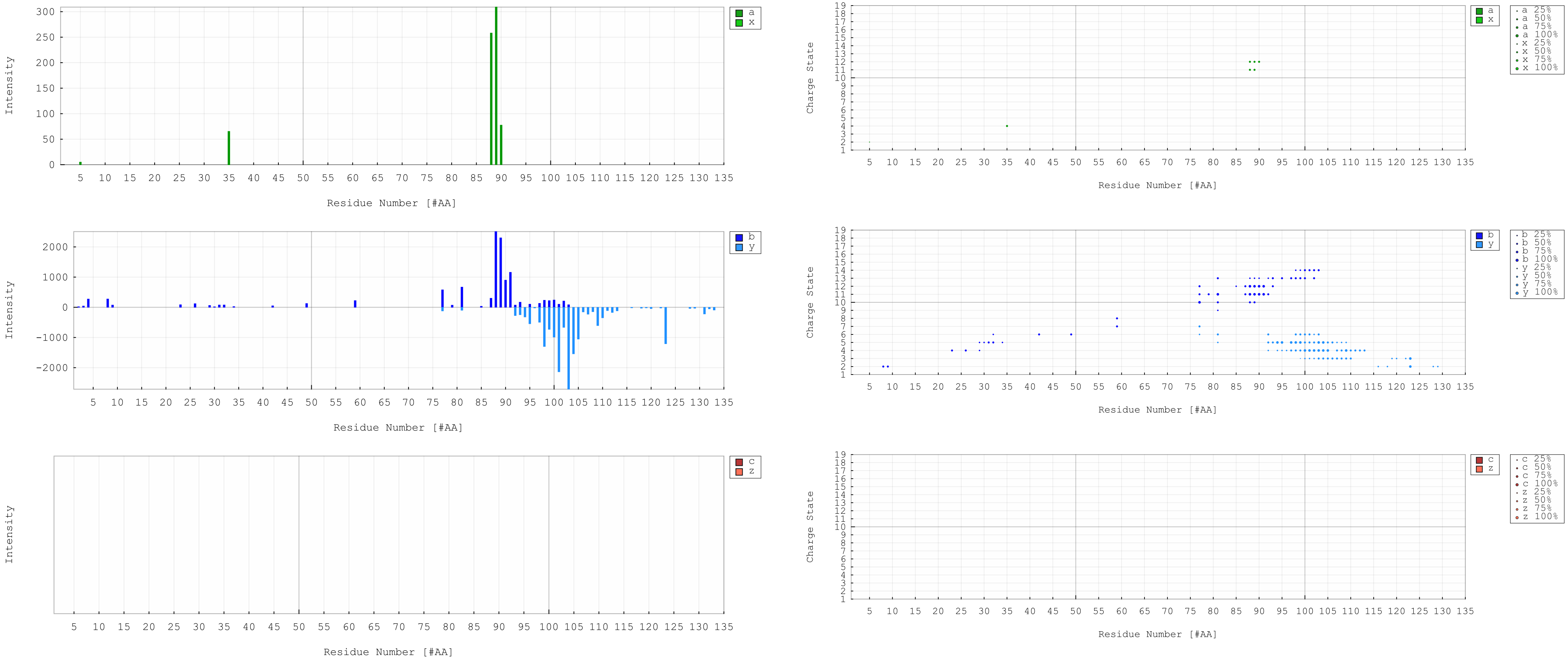

Proteoform:

Index	Protein	SC [%]	SVP [%]	IC [%]	MS Score [a.u.]	Var. Mods	Mono Mass [Da]	Chemical Formula
6	H31Kac1	42.5	8.9	48.0	20.4	Ace: K27	15305.4534	C671H1131N215O186S4

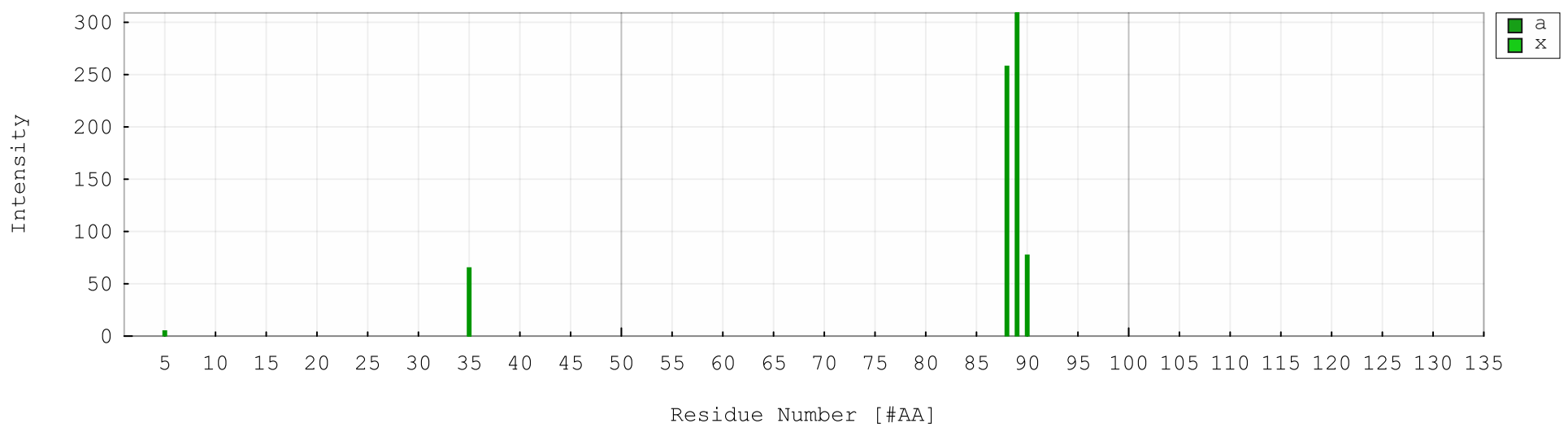

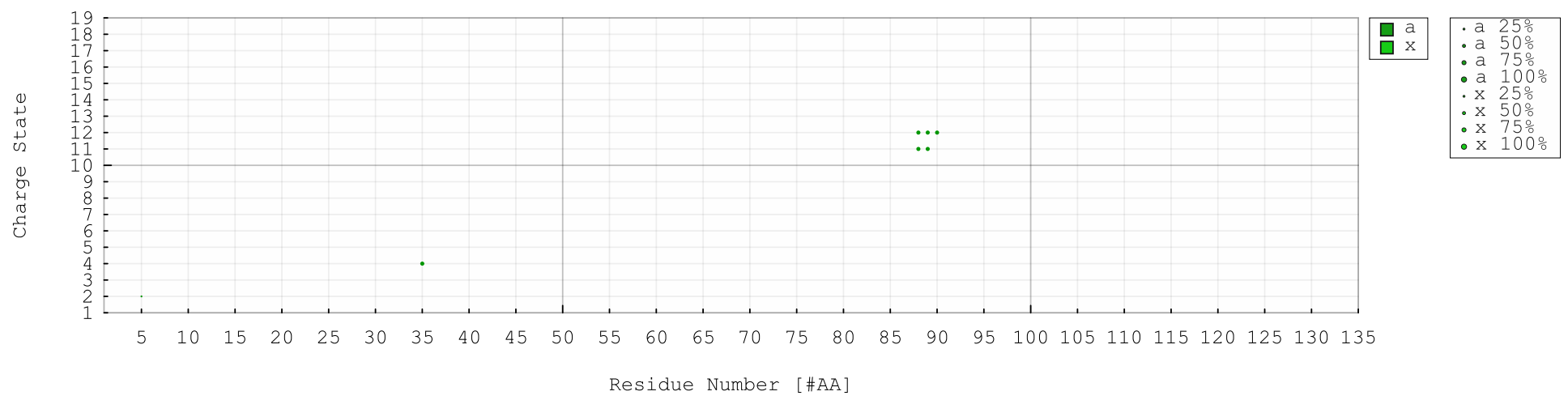

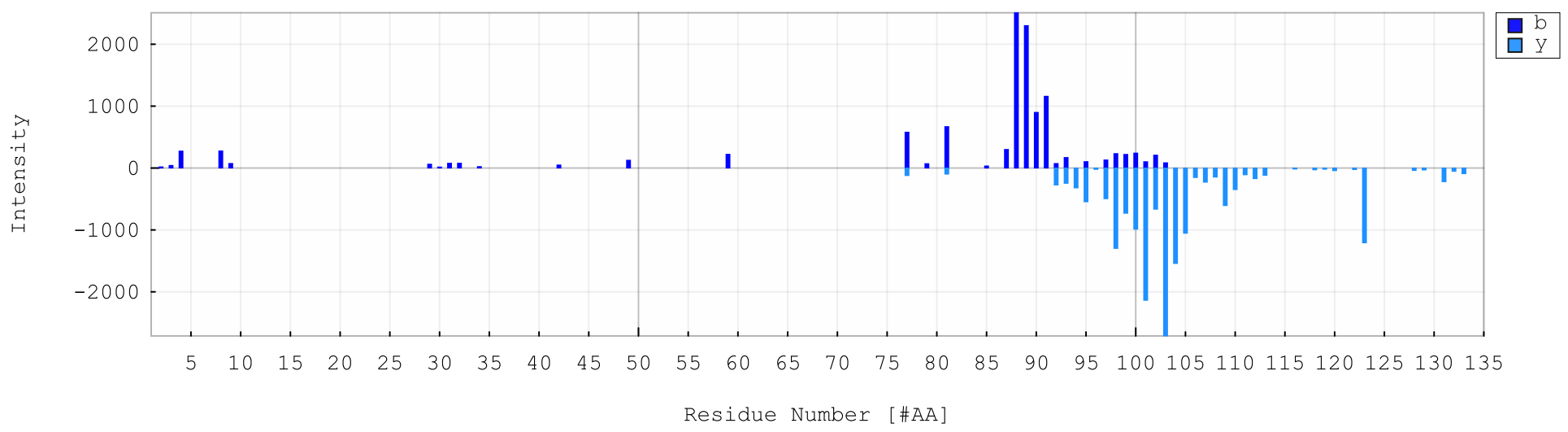

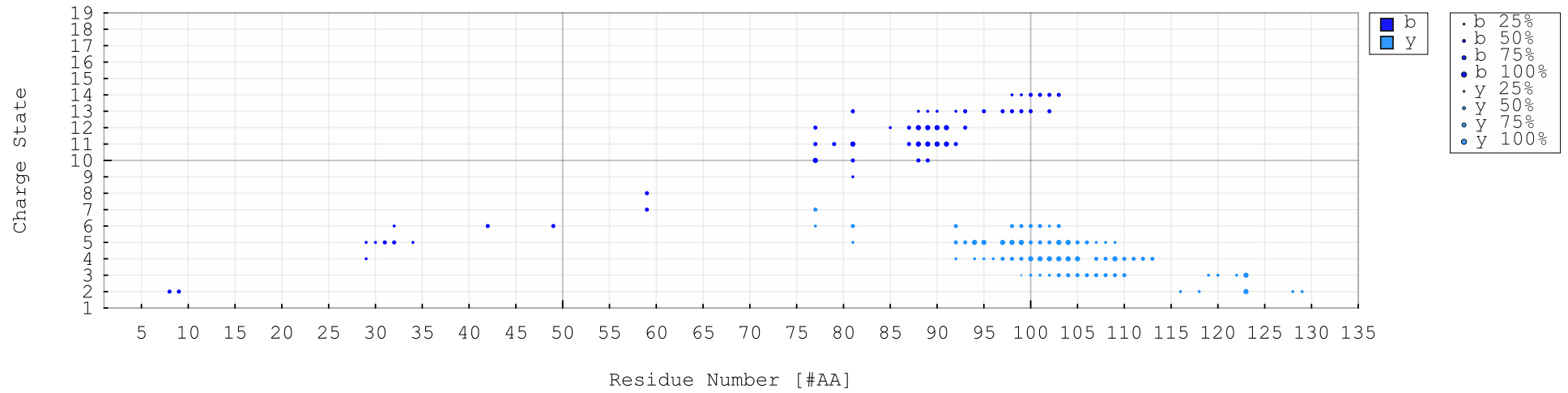

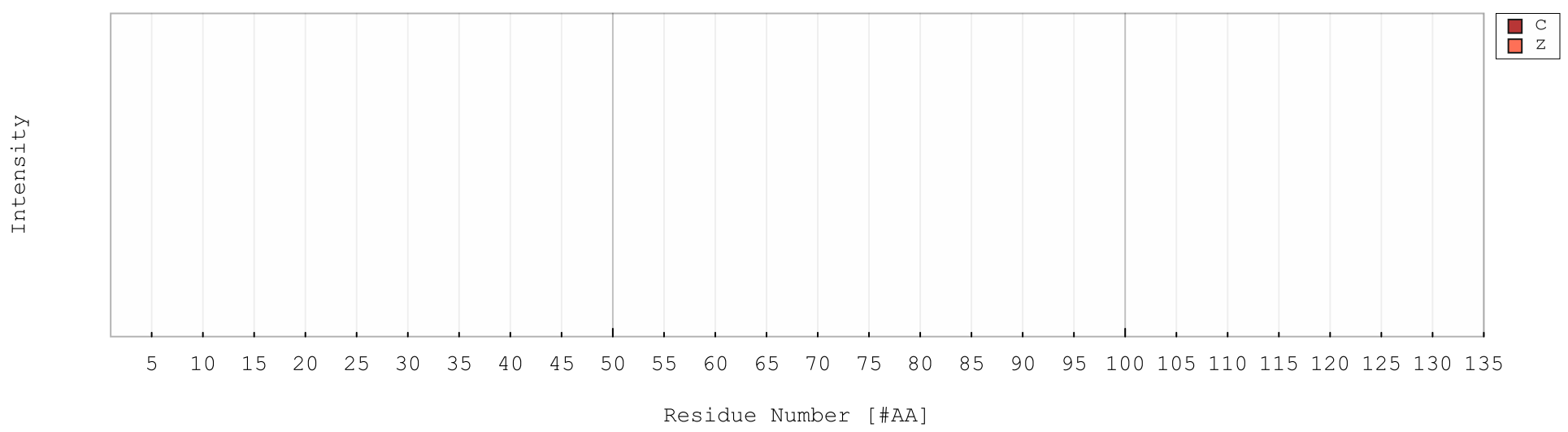

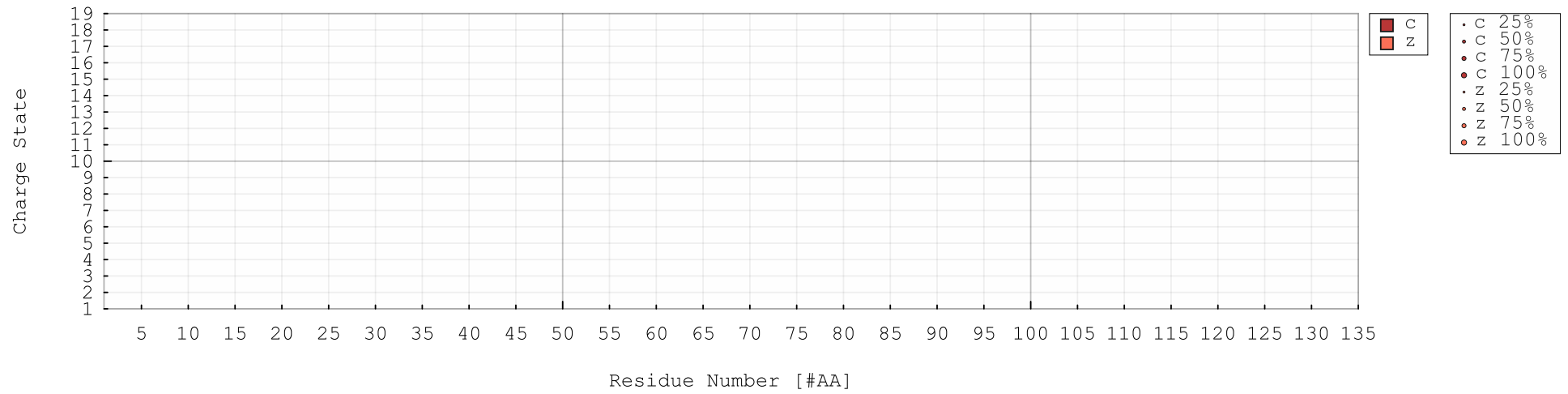

Proteoform:

Index	Protein	SC [%]	SVP [%]	IC [%]	MS Score [a.u.]	Var. Mods	Mono Mass [Da]	Chemical Formula
7	H31Kac1	38.1	8.9	47.6	18.1	Ace: K36	15305.4534	C671H1131N215O186S4

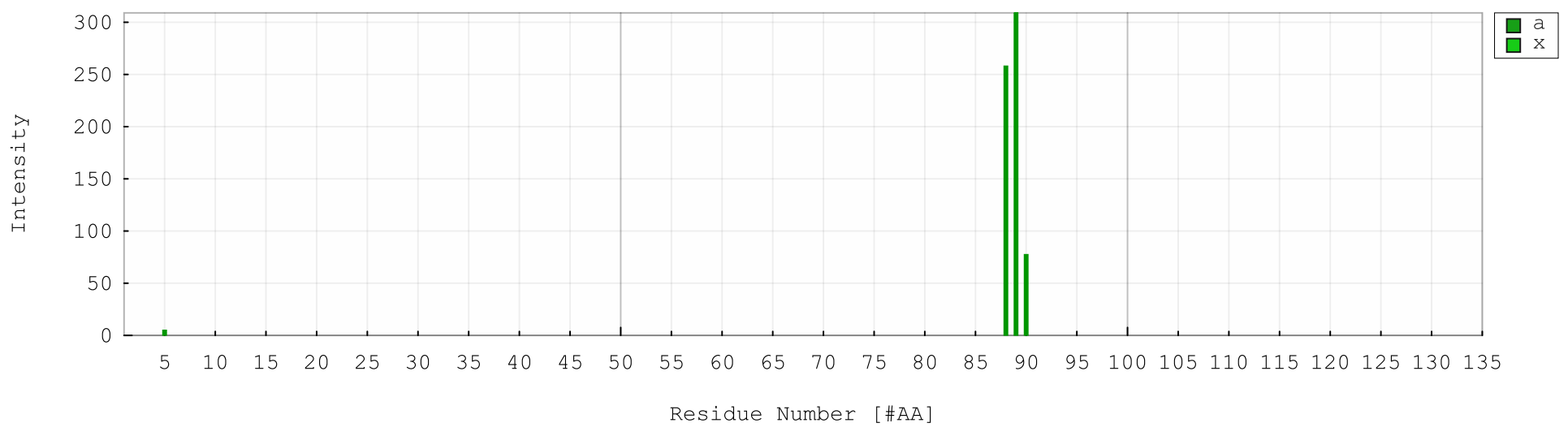
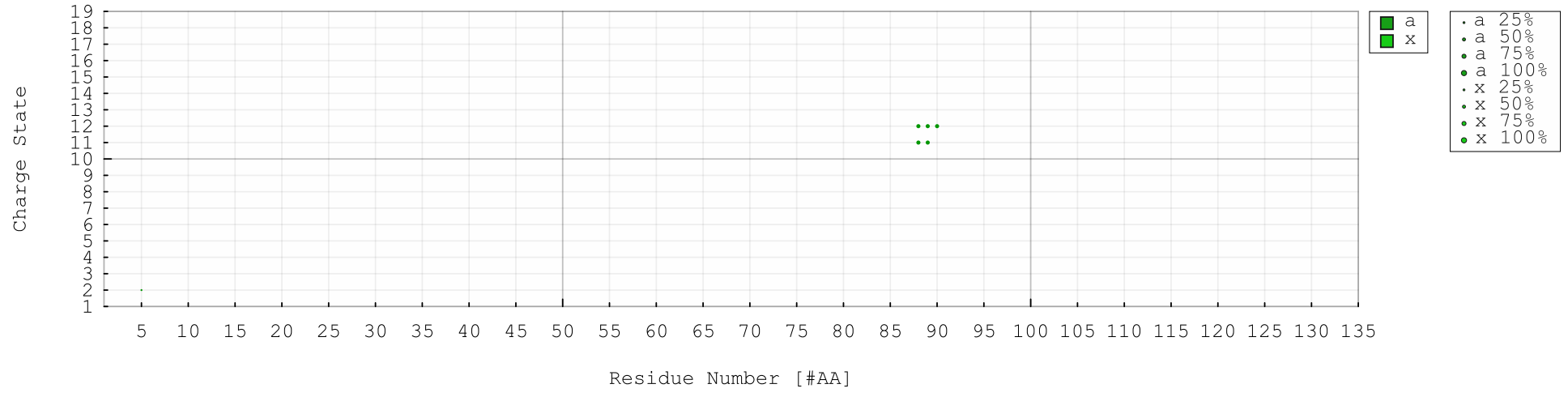
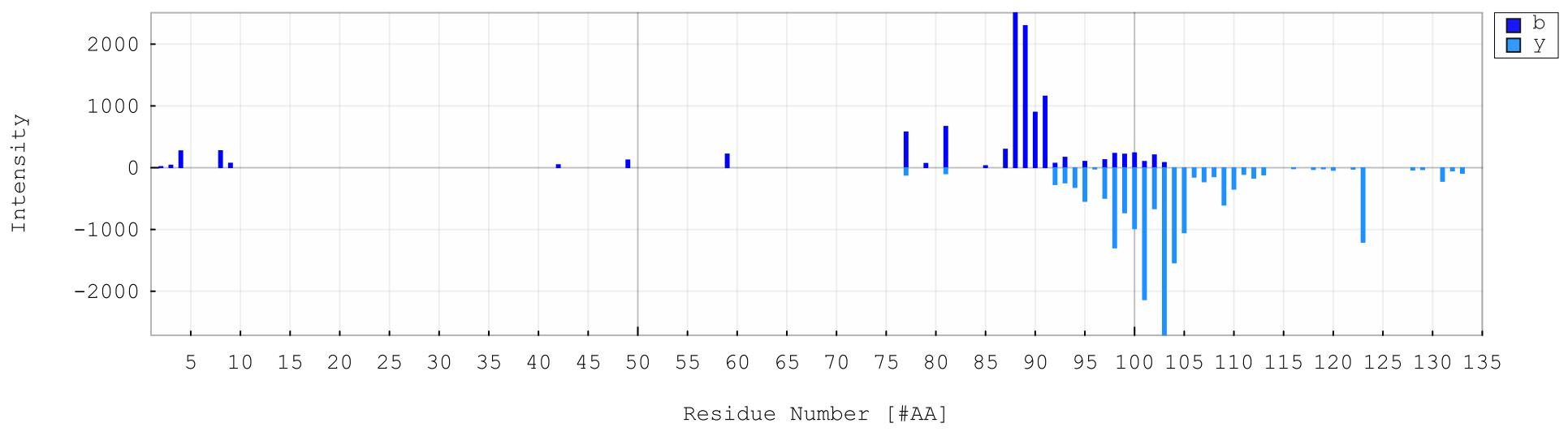
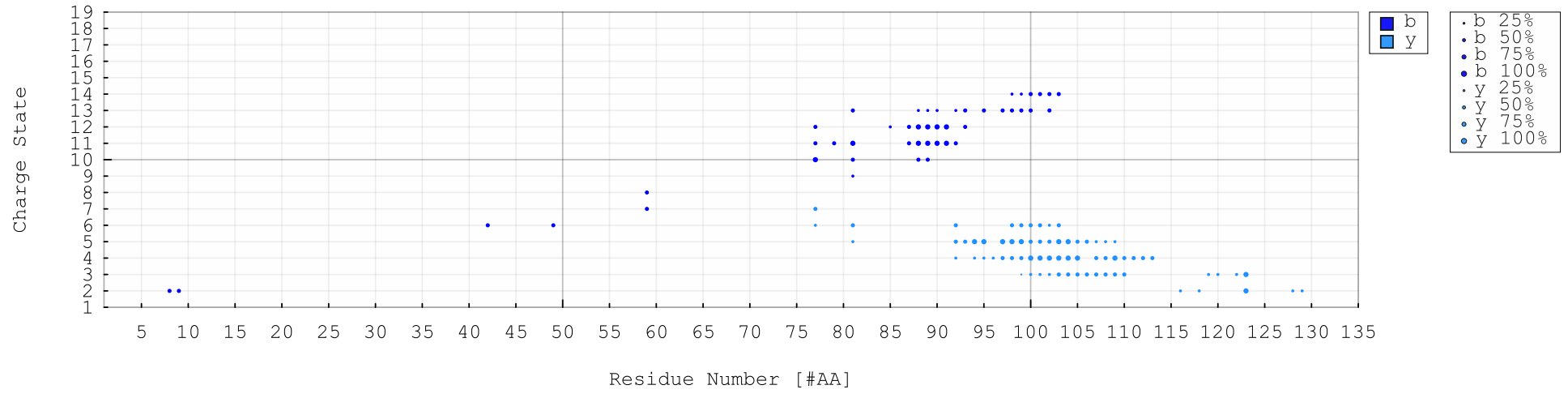
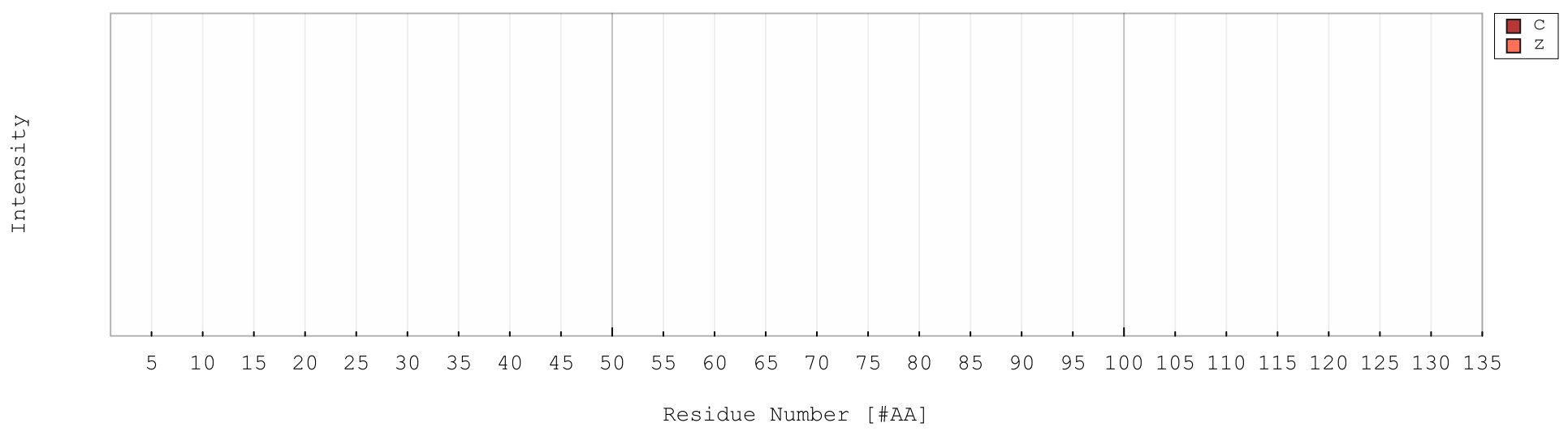
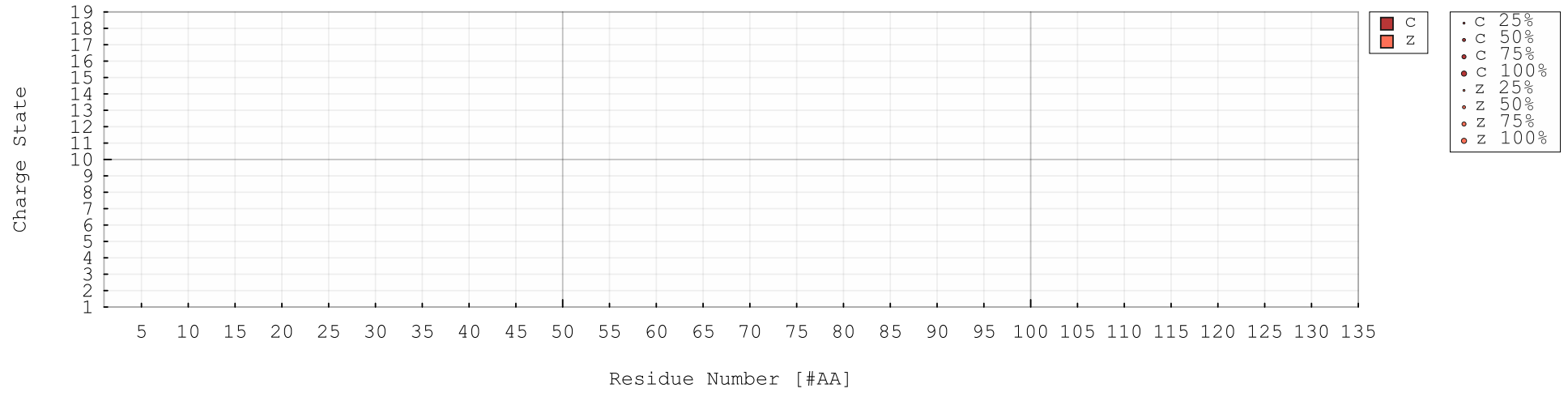

Proteoform:

Index	Protein	SC [%]	SVP [%]	IC [%]	MS Score [a.u.]	Var. Mods	Mono Mass [Da]	Chemical Formula
8	H31Kac1	38.1	8.9	47.6	18.1	Ace: K37	15305.4534	C671H1131N215O186S4

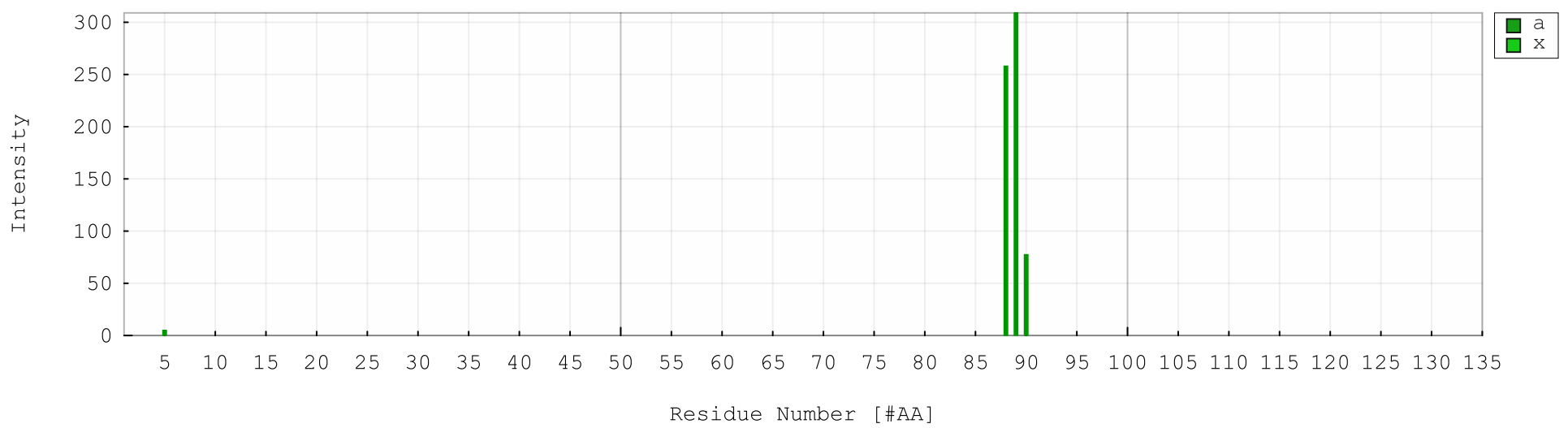
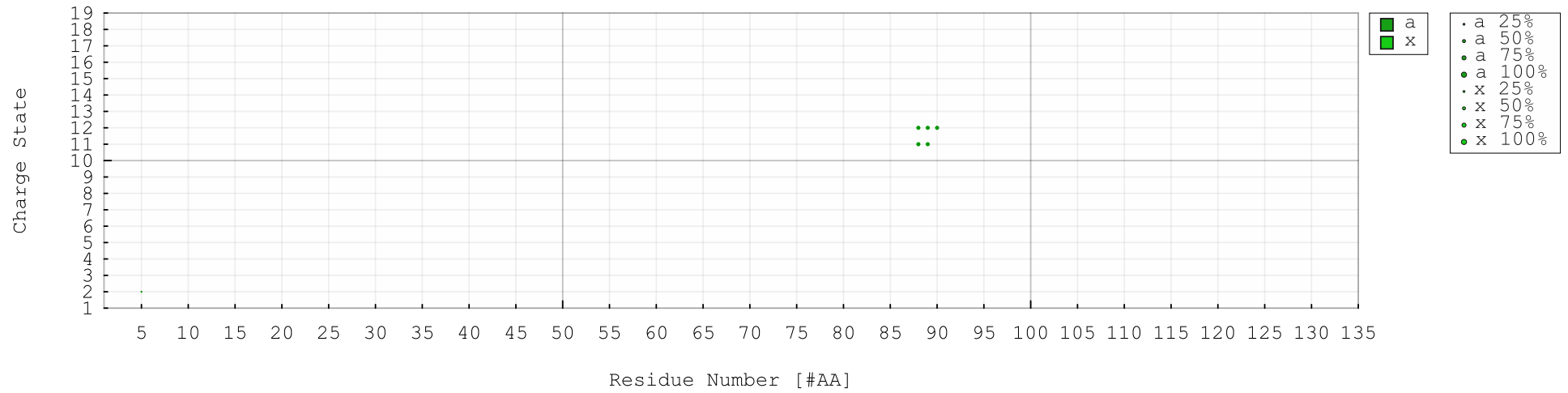
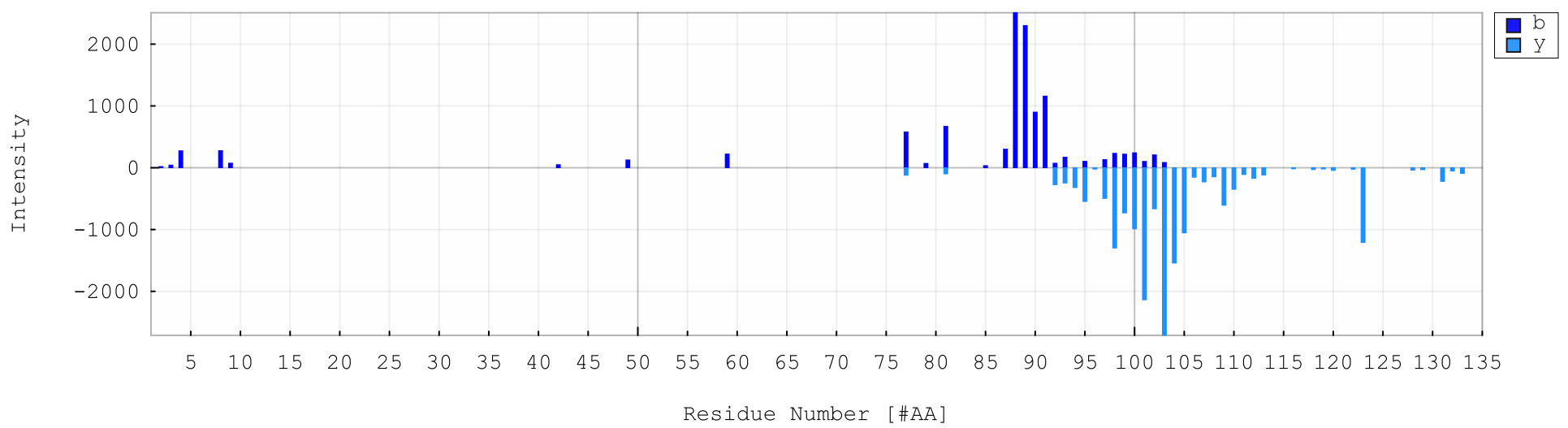
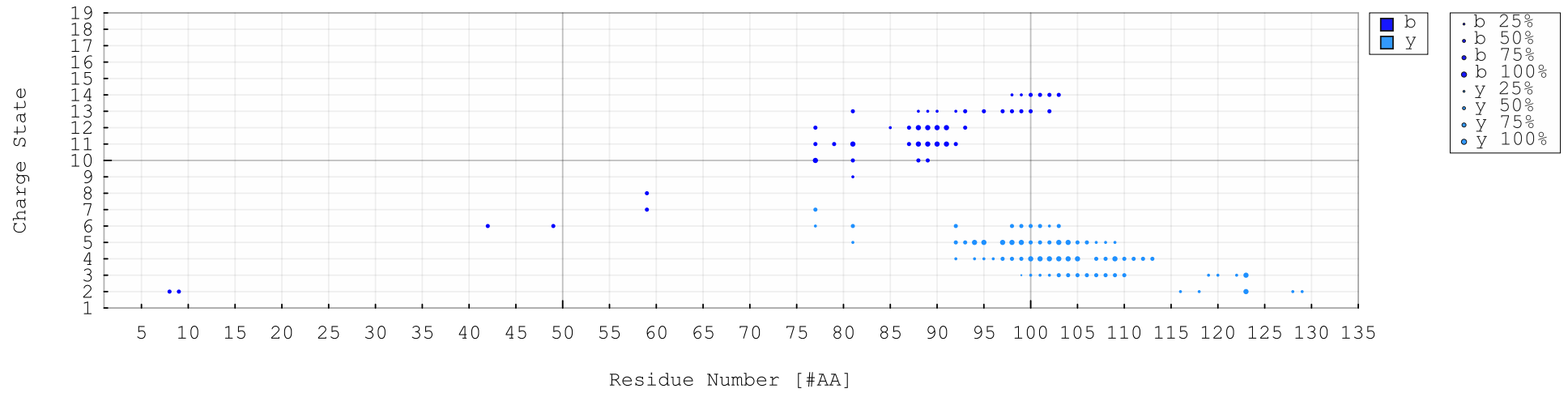
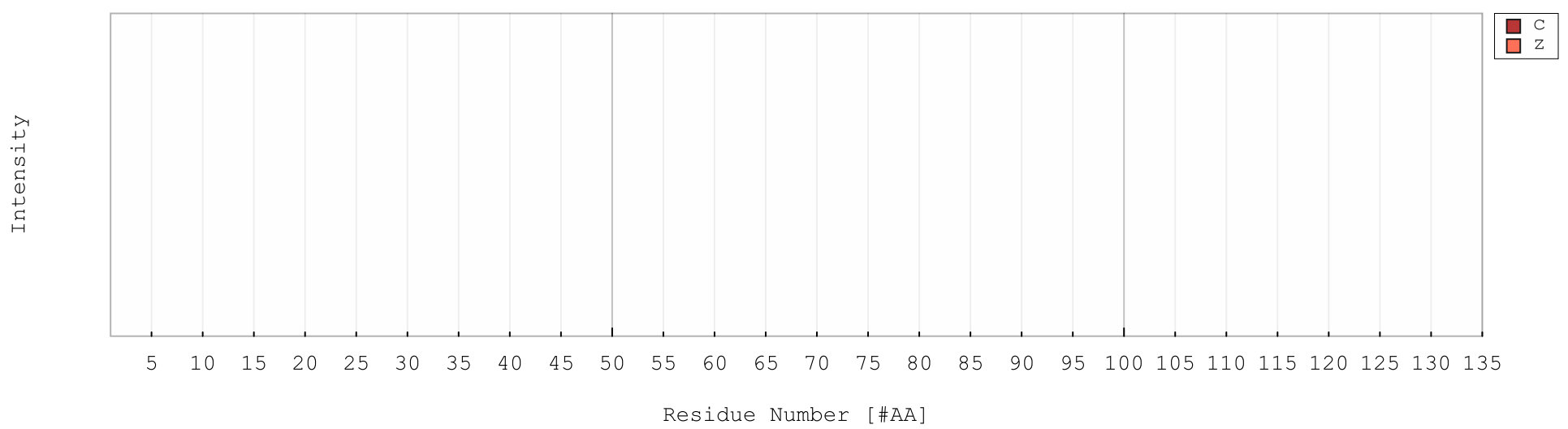
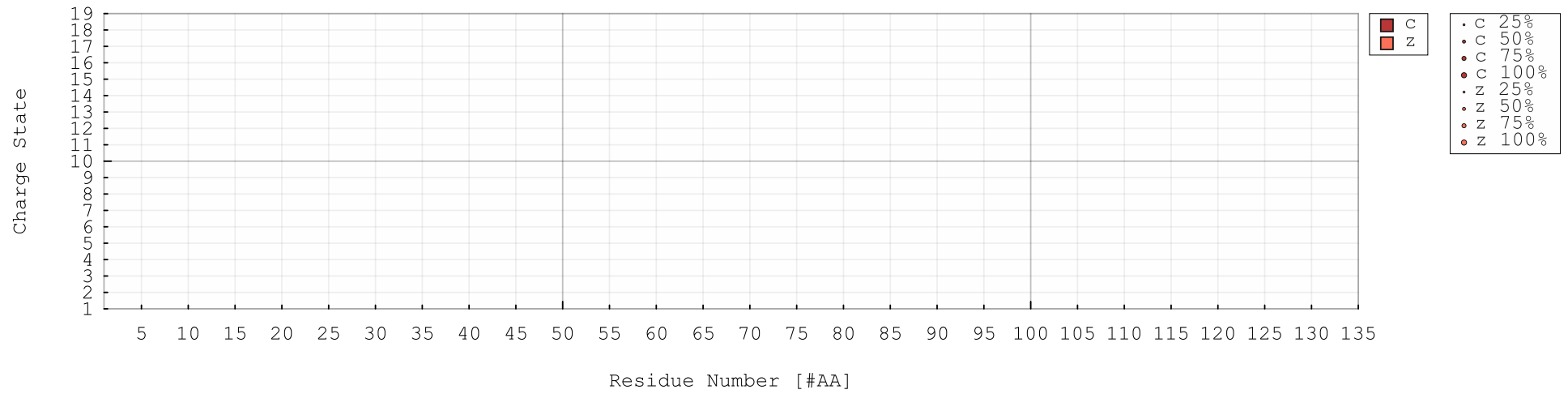

Top-Down Result OmniScape 2025
Sequence Confirmation
250428 H31-PCAF100-30min z19ac1 MS2 shCID_calibrated2

Proteoform:

Index	Protein	SC [%]	SVP [%]	IC [%]	MS Score [a.u.]	Var. Mods	Mono Mass [Da]	Chemical Formula
9	H31Kac1	36.6	8.9	47.3	17.3	Ace: K56	15305.4534	C671H1131N215O186S4

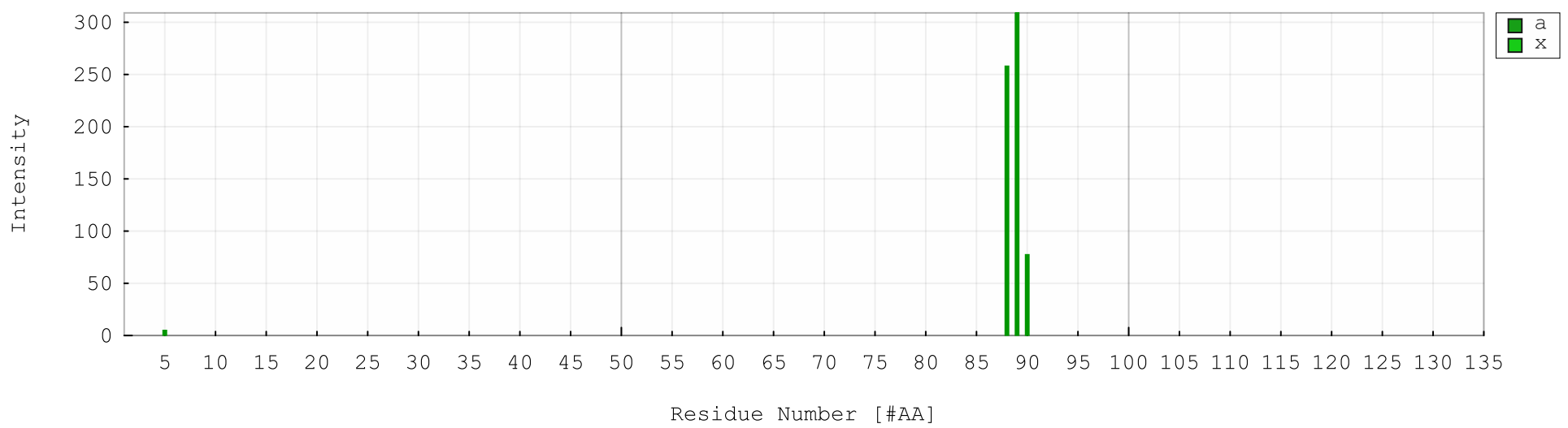

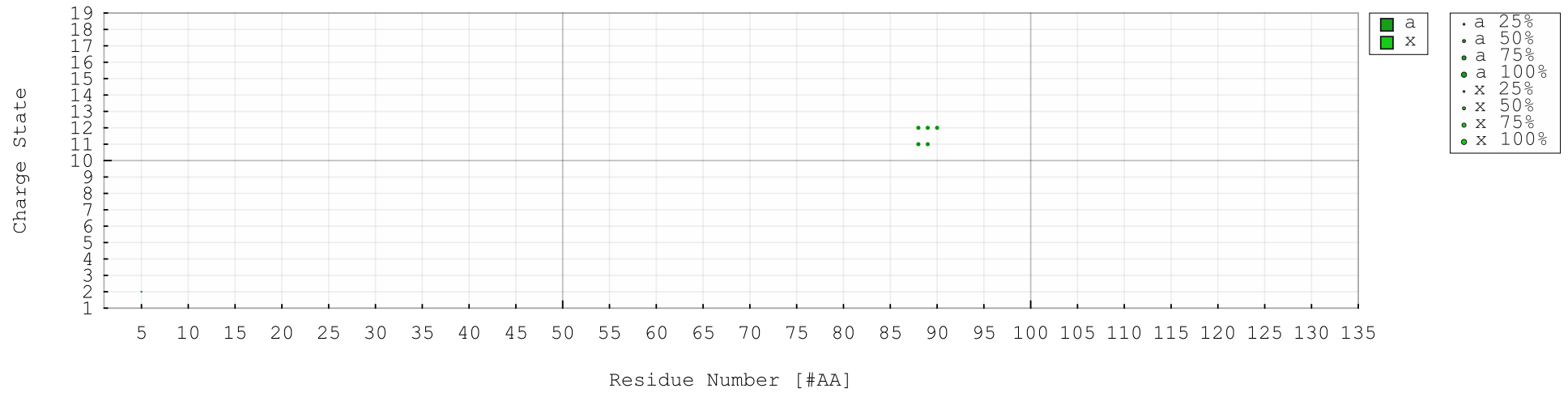

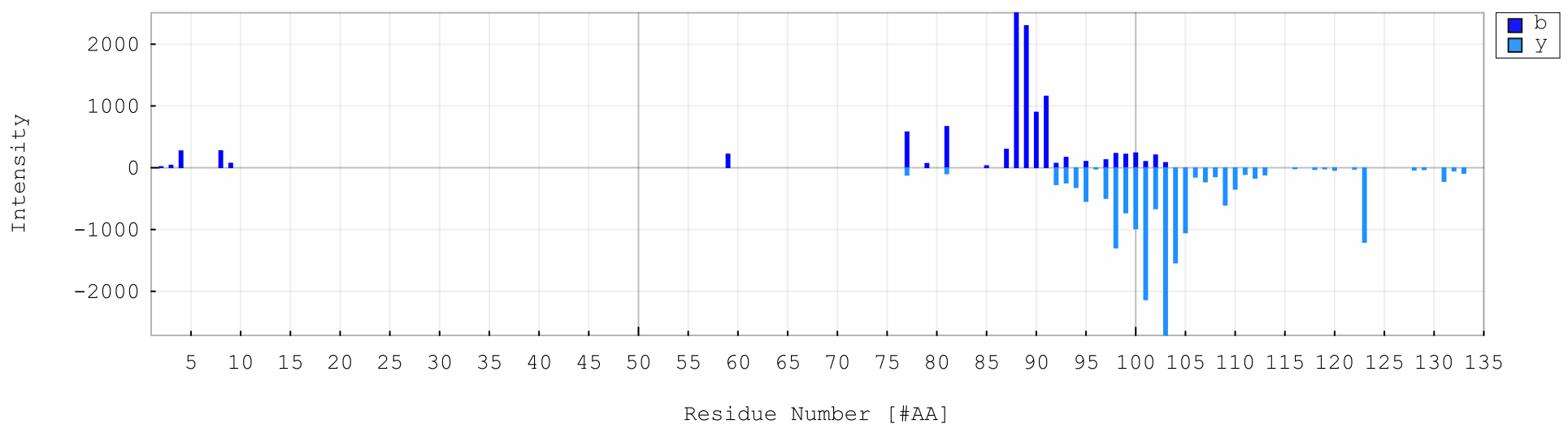

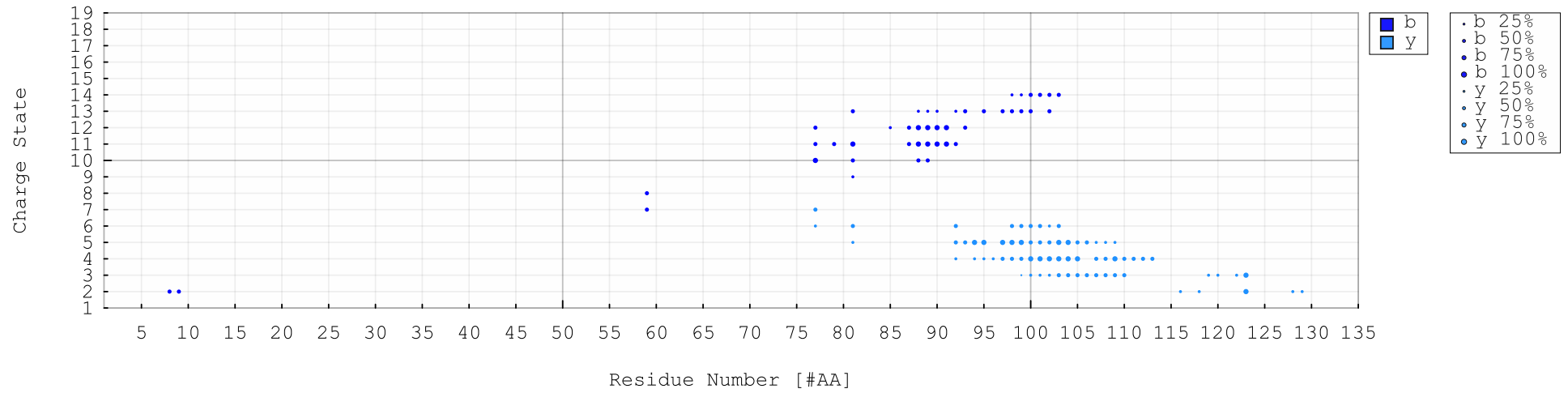

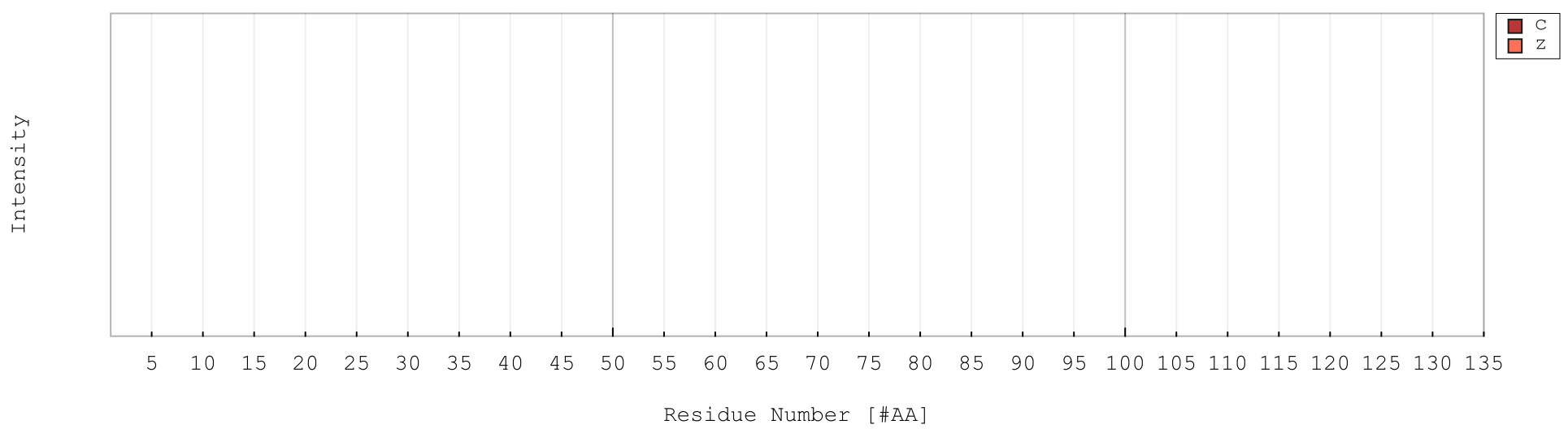

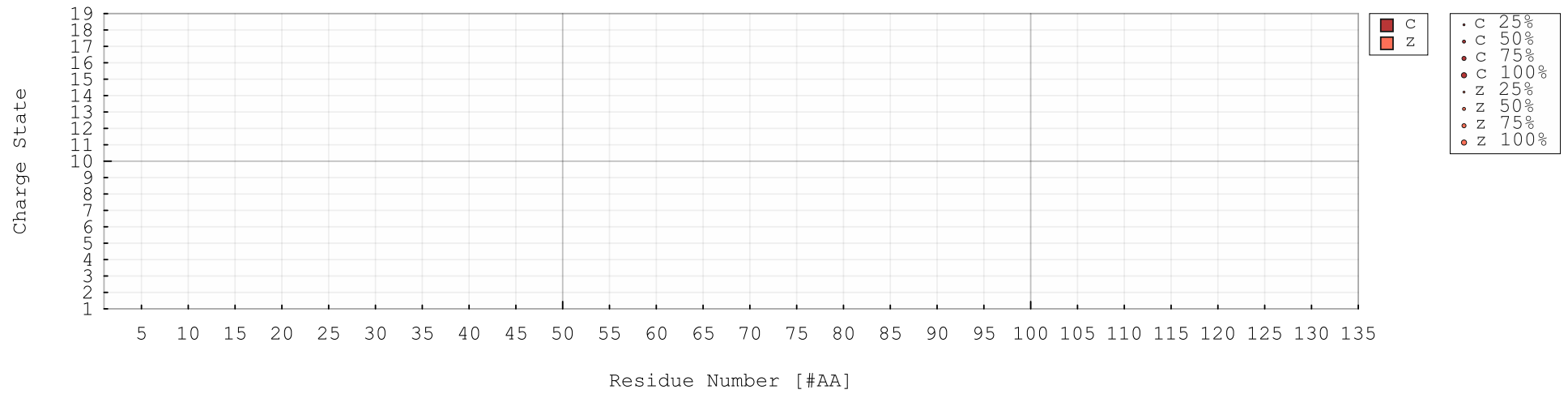

Top-Down Result OmniScape 2025
Sequence Confirmation
250428 H31-PCAF100-30min z19ac1 MS2 shCID_calibrated2

Proteoform:

Index	Protein	SC [%]	SVP [%]	IC [%]	MS Score [a.u.]	Var. Mods	Mono Mass [Da]	Chemical Formula
10	H31Kac1	35.8	8.9	47.0	16.8	Ace: K64	15305.4534	C671H1131N215O186S4

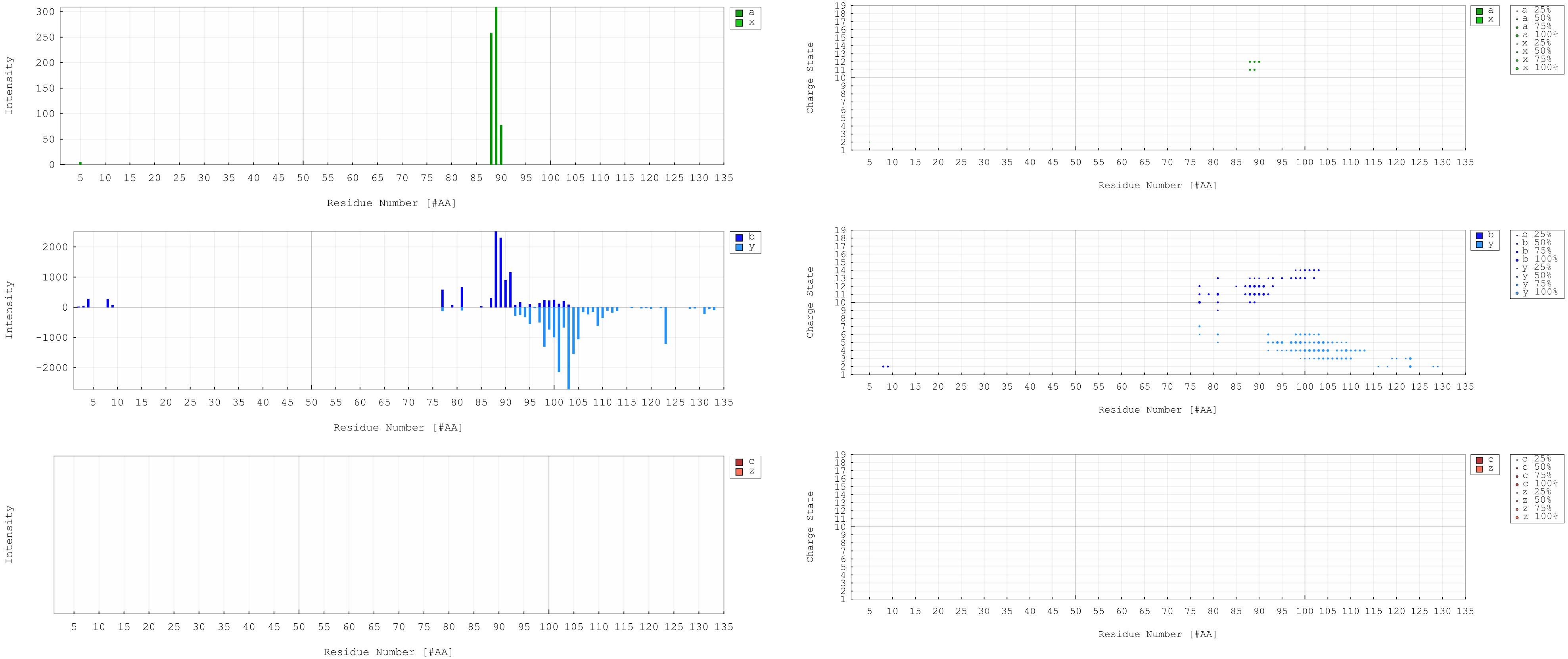

Supplement: Supplementary file 3 [file mmc3.pdf]
