# Supplementary material for: Top-Down Sequencing of Intact Proteoforms Using the timsOmni Mass Spectrometer: Accurate Determination of Co-Occurring Histone Modifications
Source: Mol Cell Proteomics. 2026 Aug 17;25(9):101640. doi: 10.1016/j.mcpro.2026.101640 (PMC13595144; doi:10.1016/j.mcpro.2026.101640)

Supplemental Data S4. H4 - p300, 60 min - OmniScape reports

OmniScape reports for highly acetylated H4 proteoforms generated by p300.

**Histone**	H4
**Enzyme**	p300
**Reaction/source**	60 min
**Nb of Osc reports**	7

Included reports

#	Experiment	Precursor	Charge	Fragmentation / workflow	File
1	MS2	H4Kac6	13+	ECciD	MS2_H4Kac6_13+_ECciD.pdf
2	MS2	H4Kac6	13+	ECD	MS2_H4Kac6_13+_ECD.pdf
3	MS2	H4Kac6	13+	EID	MS2_H4Kac6_13+_EID.pdf
4	MS2	H4Kac6	13+	RCID	MS2_H4Kac6_13+_RCID.pdf
5	MS2	H4Kac7	13+	ECD	MS2_H4Kac7_13+_ECD.pdf
6	MS2	H4Kac7	13+	RCID	MS2_H4Kac7_13+_RCID.pdf
7	MS3	H4Kac6	13+	MS2 ECD -> MS3 RCID	MS3_H4Kac6_13+_MS2-ECD_MS3-RCID.pdf

Supplemental Data S4

H4 - p300, 60 min

**Report**	1 of 7
**Experiment**	MS2
**Histone/sample**	H4
**Enzyme**	p300
**Reaction/source**	60 min
**Precursor**	H4Kac6
**Charge state**	13+
**Fragmentation / workflow**	ECciD
**Original file**	MS2_H4Kac6_13+_ECciD.pdf

Project Information

Project Directory: C:/Users/LAB-ADMIN
Processing date: Tue Apr 28 12:58:18 2026

Sample Info & Protocols

Name: Top-Down Protein Sequencing ExD

Dataset

Imported spectrum file name:	250428 H4_P300-50_60min z13ac6 MS2 CA-ECD_001-770_calibrated.txt
Dataset name:	Information is only available from imported JPEAKS files
Date acquired:	Information is only available from imported JPEAKS files
Date imported:	Information is only available from imported JPEAKS files
Instrument Name:	Information is only available from imported JPEAKS files
Instrument Serial Number:	Information is only available from imported JPEAKS files

Workflow Result Info

Analysis: 250428 H4_P300-50_60min z13ac6 MS2 CA-ECD_001-770_calibrated
Location: C:/Users/LAB-ADMIN
Method: -

Method Information

Name: Unnamed

Spectral Parameters

Mass Error [ppm]: 6
Intensity Deviation [au]: 0.15
Min/Max Charges: 1-13
Experiment type: ExD

a ions	b ions	c ions	x ions	y ions	z ions	satellite	internal	precursor
a	b	c		y	z+1			M
a+1		c+1			z+2			M+-2H

Noise threshold calculation: Simple moving average noise threshold (SMA)
Window size [datapoints]: 600
Noise curve offset: 2.211297
SVP parameters: terminal gap: 2, internal gap: 1

Proteoforms

(top 10)

Index	Parent Sequence	Chain	Var. Mods	Mono Mass [Da]	Chemical Composition
1	H4Kac	H4KacK5[Ace]K8[Ace]K12[Ace]K16[Ace]K20[Ace]K44[Ace]	Ace: K5, K8, K12, K16, K20, K44	11481.4046	C506H849N163O140S
2	H4Kac	H4KacK5[Ace]K8[Ace]K12[Ace]K16[Ace]K20[Ace]K31[Ace]	Ace: K5, K8, K12, K16, K20, K31	11481.4046	C506H849N163O140S
3	H4Kac	H4KacK5[Ace]K8[Ace]K12[Ace]K16[Ace]K31[Ace]K44[Ace]	Ace: K5, K8, K12, K16, K31, K44	11481.4046	C506H849N163O140S
4	H4Kac	H4KacK5[Ace]K8[Ace]K12[Ace]K20[Ace]K31[Ace]K44[Ace]	Ace: K5, K8, K12, K20, K31, K44	11481.4046	C506H849N163O140S
5	H4Kac	H4KacK5[Ace]K8[Ace]K12[Ace]K16[Ace]K20[Ace]K59[Ace]	Ace: K5, K8, K12, K16, K20, K59	11481.4046	C506H849N163O140S
6	H4Kac	H4KacK5[Ace]K8[Ace]K16[Ace]K20[Ace]K31[Ace]K44[Ace]	Ace: K5, K8, K16, K20, K31, K44	11481.4046	C506H849N163O140S
7	H4Kac	H4KacK5[Ace]K12[Ace]K16[Ace]K20[Ace]K31[Ace]K44[Ace]	Ace: K5, K12, K16, K20, K31, K44	11481.4046	C506H849N163O140S
8	H4Kac	H4KacK8[Ace]K12[Ace]K16[Ace]K20[Ace]K31[Ace]K44[Ace]	Ace: K8, K12, K16, K20, K31, K44	11481.4046	C506H849N163O140S
9	H4Kac	H4KacK5[Ace]K8[Ace]K12[Ace]K16[Ace]K31[Ace]K59[Ace]	Ace: K5, K8, K12, K16, K31, K59	11481.4046	C506H849N163O140S
10	H4Kac	H4KacK5[Ace]K8[Ace]K12[Ace]K20[Ace]K31[Ace]K59[Ace]	Ace: K5, K8, K12, K20, K31, K59	11481.4046	C506H849N163O140S

Results

Proteoforms Table (top 10)

Index	Protein	SC [%]	SVP [%]	IC [%]	MS Score [a.u.]	Var. Mods	Mono Mass [Da]	Chemical Formula
1	H4Kac	87.1	34.3	46.8	40.8	Ace: K5, K8, K12, K16, K20, K44	11481.4046	C506H849N163O140S
2	H4Kac	86.1	34.3	43.9	37.8	Ace: K5, K8, K12, K16, K20, K31	11481.4046	C506H849N163O140S
3	H4Kac	82.2	23.5	41.9	34.4	Ace: K5, K8, K12, K16, K31, K44	11481.4046	C506H849N163O140S
4	H4Kac	81.2	23.5	40.9	33.2	Ace: K5, K8, K12, K20, K31, K44	11481.4046	C506H849N163O140S
5	H4Kac	78.2	34.3	41.5	32.4	Ace: K5, K8, K12, K16, K20, K59	11481.4046	C506H849N163O140S
6	H4Kac	80.2	15.7	39.8	31.9	Ace: K5, K8, K16, K20, K31, K44	11481.4046	C506H849N163O140S
7	H4Kac	77.2	13.7	38.4	29.7	Ace: K5, K12, K16, K20, K31, K44	11481.4046	C506H849N163O140S
8	H4Kac	77.2	13.7	37.1	28.7	Ace: K8, K12, K16, K20, K31, K44	11481.4046	C506H849N163O140S
9	H4Kac	73.3	23.5	36.6	26.8	Ace: K5, K8, K12, K16, K31, K59	11481.4046	C506H849N163O140S
10	H4Kac	72.3	23.5	35.6	25.7	Ace: K5, K8, K12, K20, K31, K59	11481.4046	C506H849N163O140S

Sequence

Name: H4Kac

```
                   10                     20                     30                     40                     50                     60
S G R G K G G K G L    G K G G A K R H R K    V L R D N I Q G I T    K P A I R R L A R R    G G V K R I S G L I    Y E E T R G V L K V
                   70                     80                     90                    100                    110                    120
F L E N V I R D A V    T Y T E H A K R K T    V T A M D V V Y A L    K R Q G R T L Y G F    G G
```

Modifications

Modification	Position	Type
Acetyl	5	variable
Acetyl	8	variable
Acetyl	12	variable
Acetyl	16	variable
Acetyl	20	variable
Acetyl	31	variable
Acetyl	44	variable
Acetyl	59	variable
Acetyl	77	variable

Sequence Map

Proteoform:

Index	Protein	SC [%]	SVP [%]	IC [%]	MS Score [a.u.]	Var. Mods	Mono Mass [Da]	Chemical Formula
1	H4Kac	87.1	34.3	46.8	40.8	Ace: K5, K8, K12, K16, K20, K44	11481.4046	C506H849N163O140S

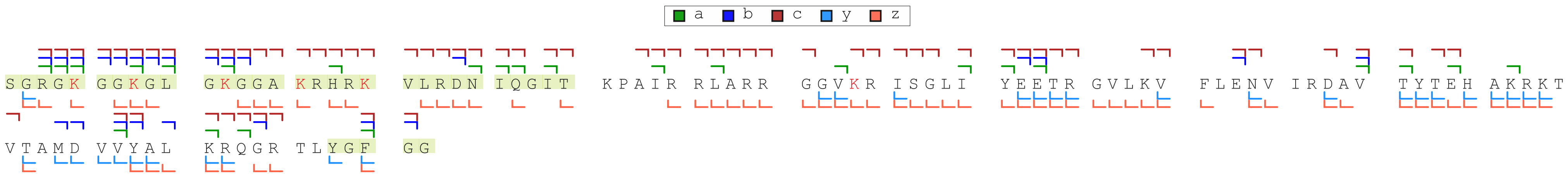

Proteoform:

Index	Protein	SC [%]	SVP [%]	IC [%]	MS Score [a.u.]	Var. Mods	Mono Mass [Da]	Chemical Formula
2	H4Kac	86.1	34.3	43.9	37.8	Ace: K5, K8, K12, K16, K20, K31	11481.4046	C506H849N163O140S

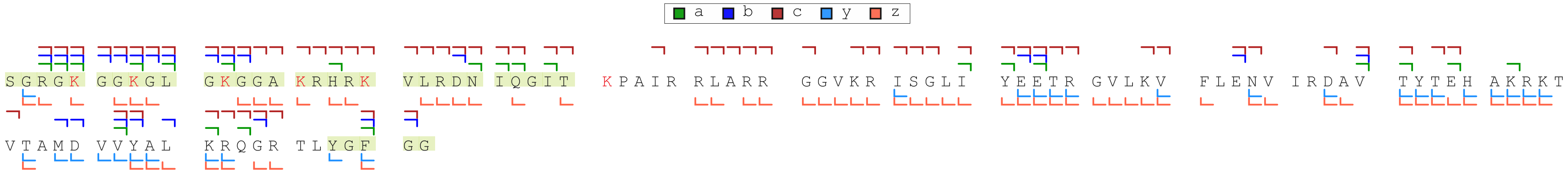

Proteoform:	Index	Protein	SC [%]	SVP [%]	IC [%]	MS Score [a.u.]	Var. Mods	Mono Mass [Da]	Chemical Formula
	3	H4Kac	82.2	23.5	41.9	34.4	Ace: K5, K8, K12, K16, K31, K44	11481.4046	C506H849N163O140S

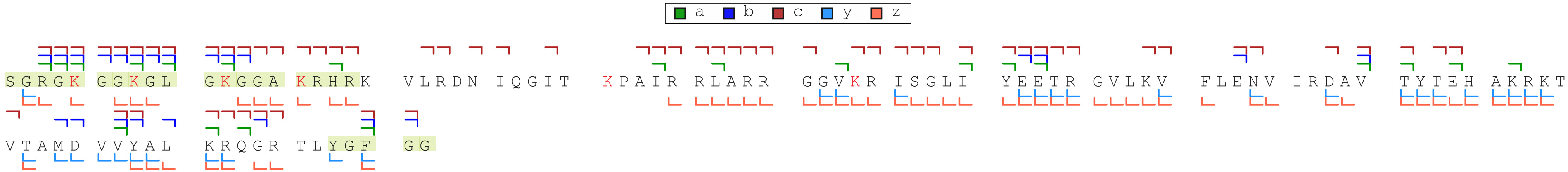

Proteoform:

Index	Protein	SC [%]	SVP [%]	IC [%]	MS Score [a.u.]	Var. Mods	Mono Mass [Da]	Chemical Formula
4	H4Kac	81.2	23.5	40.9	33.2	Ace: K5, K8, K12, K20, K31, K44	11481.4046	C506H849N163O140S

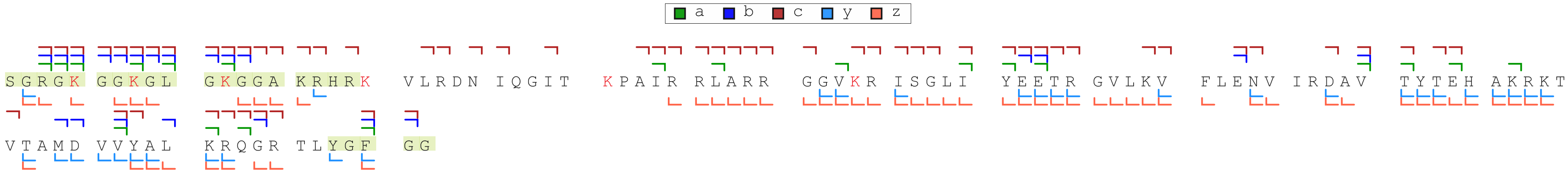

Proteoform:	Index	Protein	SC [%]	SVP [%]	IC [%]	MS Score [a.u.]	Var. Mods	Mono Mass [Da]	Chemical Formula
	5	H4Kac	78.2	34.3	41.5	32.4	Ace: K5, K8, K12, K16, K20, K59	11481.4046	C506H849N163O140S

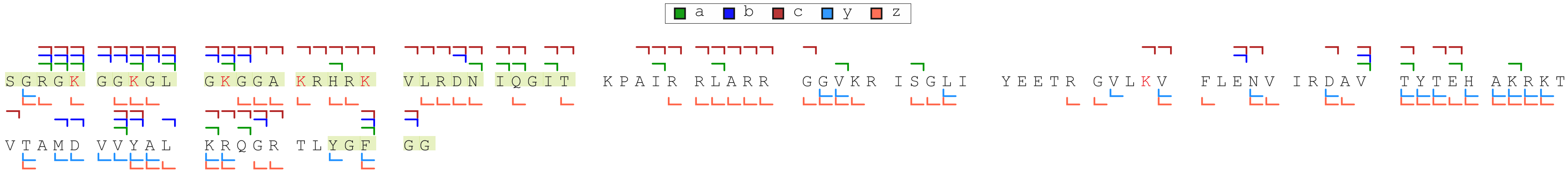

Proteoform:

Index	Protein	SC [%]	SVP [%]	IC [%]	MS Score [a.u.]	Var. Mods	Mono Mass [Da]	Chemical Formula
6	H4Kac	80.2	15.7	39.8	31.9	Ace: K5, K8, K16, K20, K31, K44	11481.4046	C506H849N163O140S

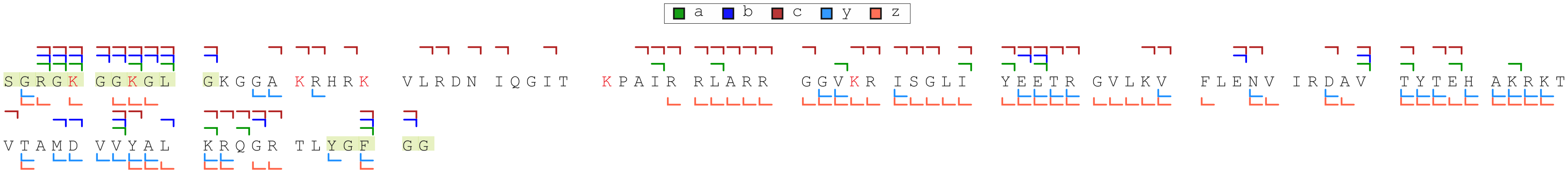

Proteoform:

Index	Protein	SC [%]	SVP [%]	IC [%]	MS Score [a.u.]	Var. Mods	Mono Mass [Da]	Chemical Formula
7	H4Kac	77.2	13.7	38.4	29.7	Ace: K5, K12, K16, K20, K31, K44	11481.4046	C506H849N163O140S

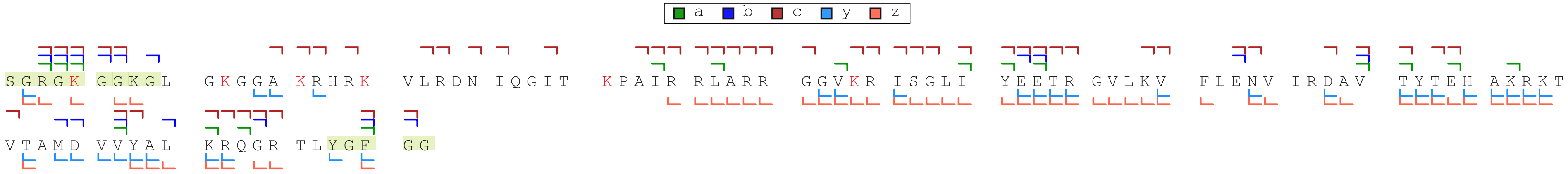

Proteoform:	Index	Protein	SC [%]	SVP [%]	IC [%]	MS Score [a.u.]	Var. Mods	Mono Mass [Da]	Chemical Formula
	8	H4Kac	77.2	13.7	37.1	28.7	Ace: K8, K12, K16, K20, K31, K44	11481.4046	C506H849N163O140S

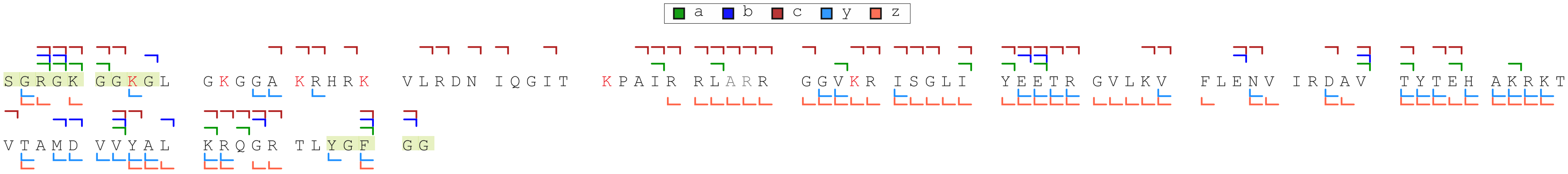

Proteoform:

Index	Protein	SC [%]	SVP [%]	IC [%]	MS Score [a.u.]	Var. Mods	Mono Mass [Da]	Chemical Formula
9	H4Kac	73.3	23.5	36.6	26.8	Ace: K5, K8, K12, K16, K31, K59	11481.4046	C506H849N163O140S

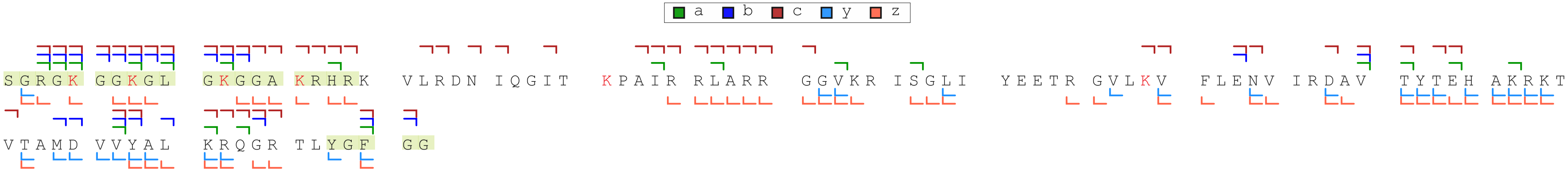

Top-Down Result OmniScape 2025
Sequence Confirmation
250428 H4_P300-50_60min z13ac6 MS2 CA-ECD_001-770_calibrated

Proteoform:

Index	Protein	SC [%]	SVP [%]	IC [%]	MS Score [a.u.]	Var. Mods	Mono Mass [Da]	Chemical Formula
10	H4Kac	72.3	23.5	35.6	25.7	Ace: K5, K8, K12, K20, K31, K59	11481.4046	C506H849N163O140S

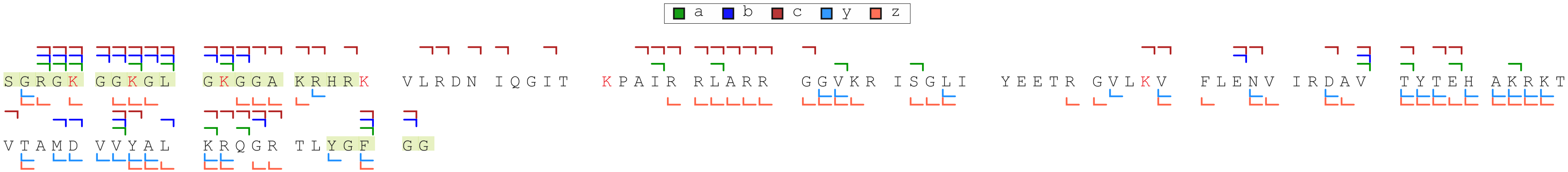

Error Plot

Proteoform:

Index	Protein	SC [%]	SVP [%]	IC [%]	MS Score [a.u.]	Var. Mods	Mono Mass [Da]	Chemical Formula
1	H4Kac	87.1	34.3	46.8	40.8	Ace: K5, K8, K12, K16, K20, K44	11481.4046	C506H849N163O140S

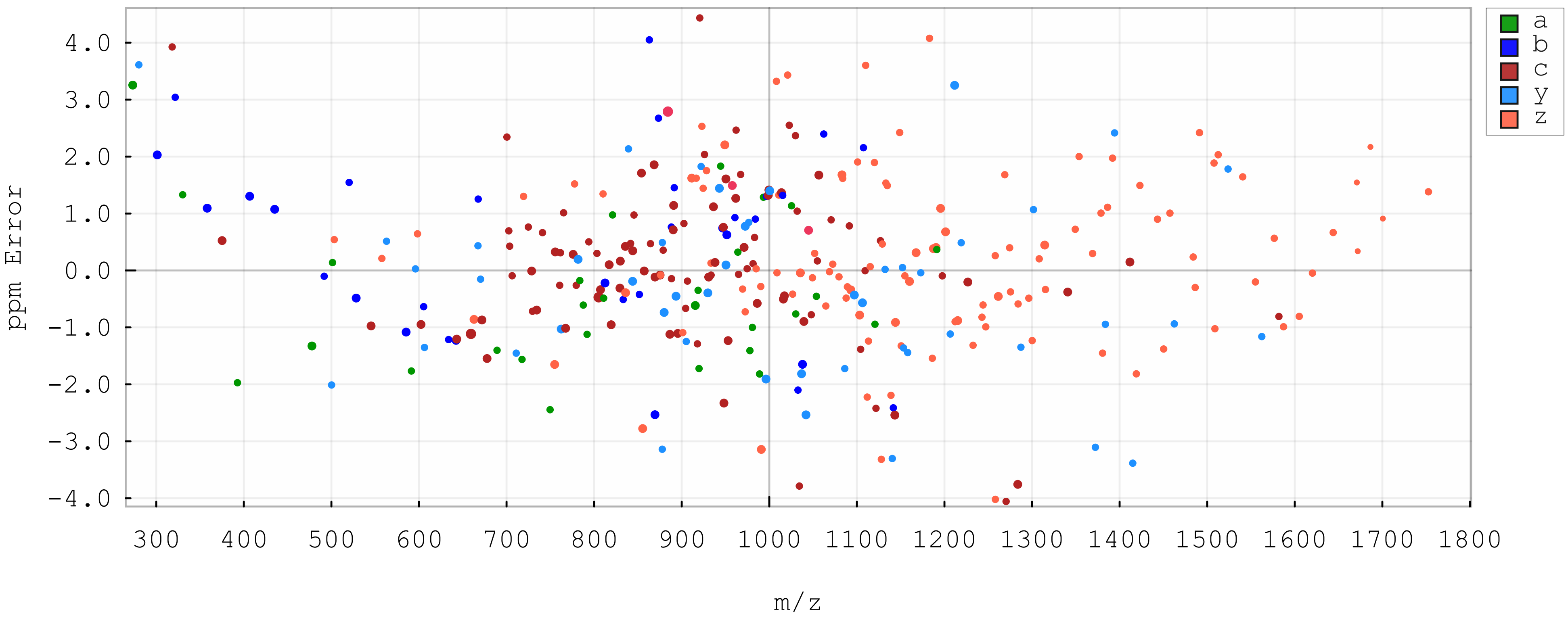

Proteoform:

Index	Protein	SC [%]	SVP [%]	IC [%]	MS Score [a.u.]	Var. Mods	Mono Mass [Da]	Chemical Formula
2	H4Kac	86.1	34.3	43.9	37.8	Ace: K5, K8, K12, K16, K20, K31	11481.4046	C506H849N163O140S

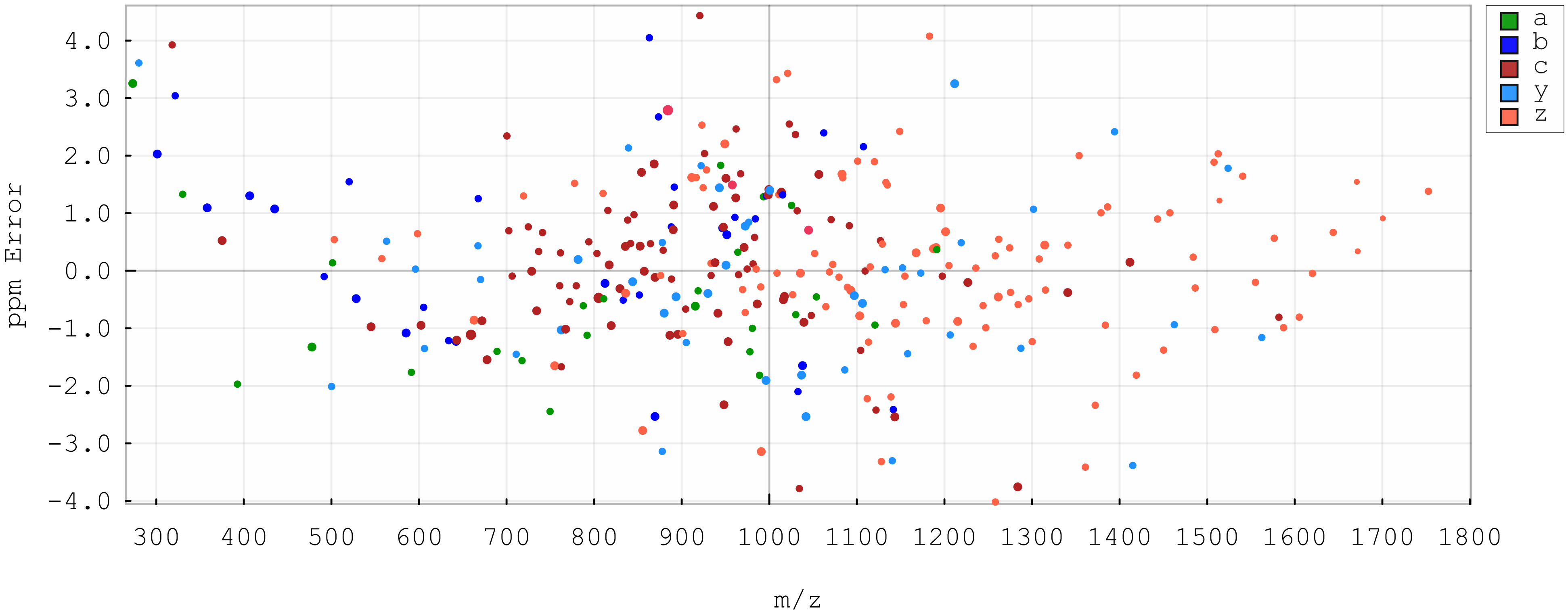

Proteoform:

Index	Protein	SC [%]	SVP [%]	IC [%]	MS Score [a.u.]	Var. Mods	Mono Mass [Da]	Chemical Formula
3	H4Kac	82.2	23.5	41.9	34.4	Ace: K5, K8, K12, K16, K31, K44	11481.4046	C506H849N163O140S

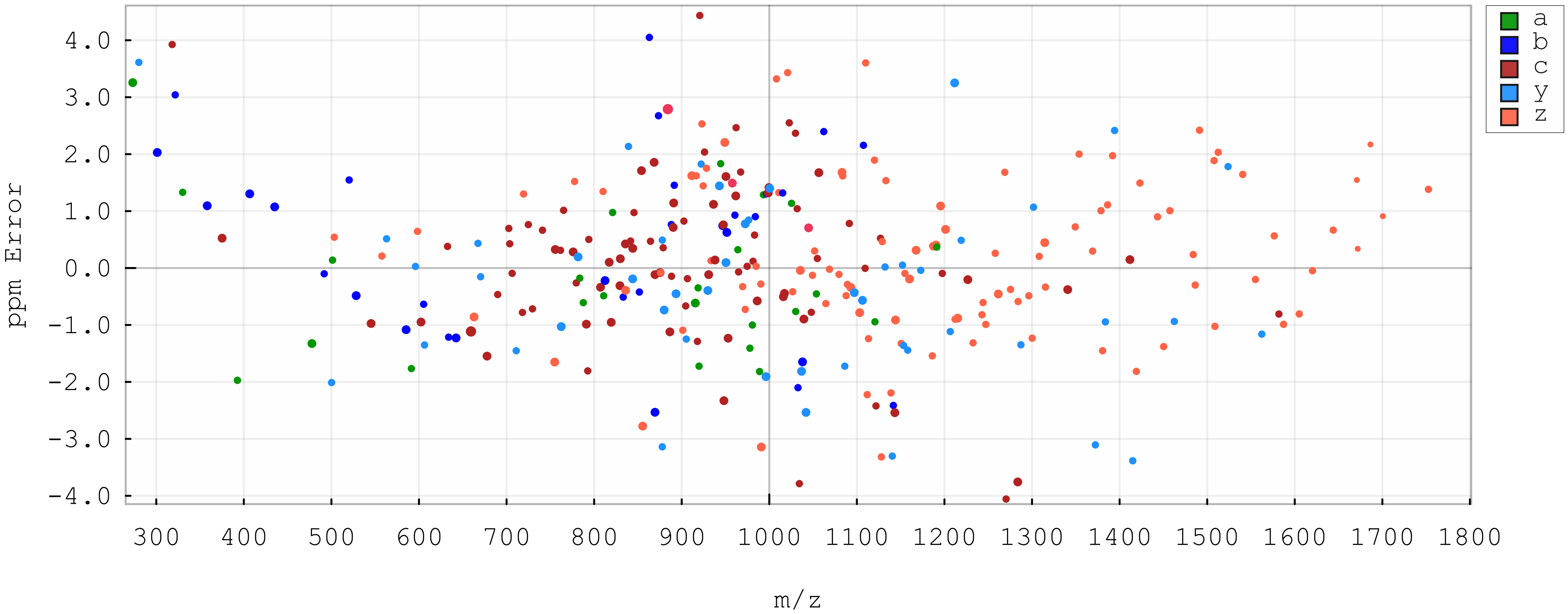

Proteoform:

Index	Protein	SC [%]	SVP [%]	IC [%]	MS Score [a.u.]	Var. Mods	Mono Mass [Da]	Chemical Formula
4	H4Kac	81.2	23.5	40.9	33.2	Ace: K5, K8, K12, K20, K31, K44	11481.4046	C506H849N163O140S

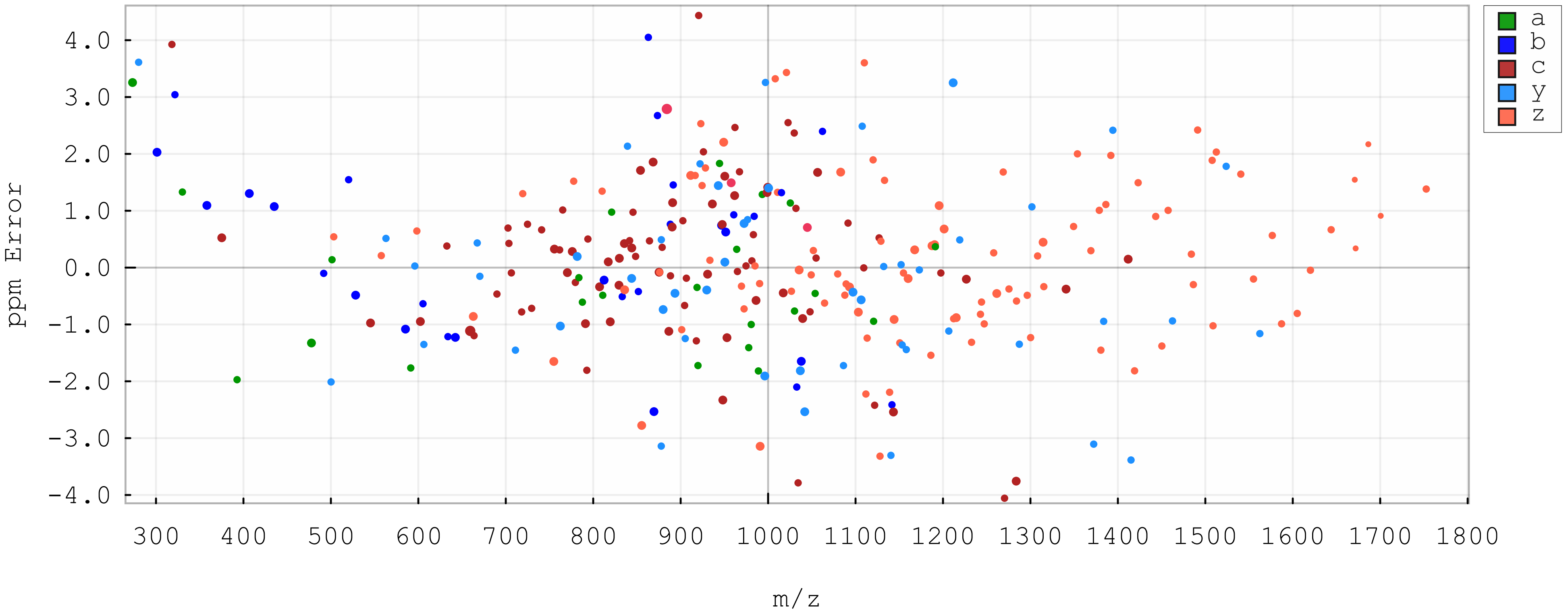

Proteoform:

Index	Protein	SC [%]	SVP [%]	IC [%]	MS Score [a.u.]	Var. Mods	Mono Mass [Da]	Chemical Formula
5	H4Kac	78.2	34.3	41.5	32.4	Ace: K5, K8, K12, K16, K20, K59	11481.4046	C506H849N163O140S

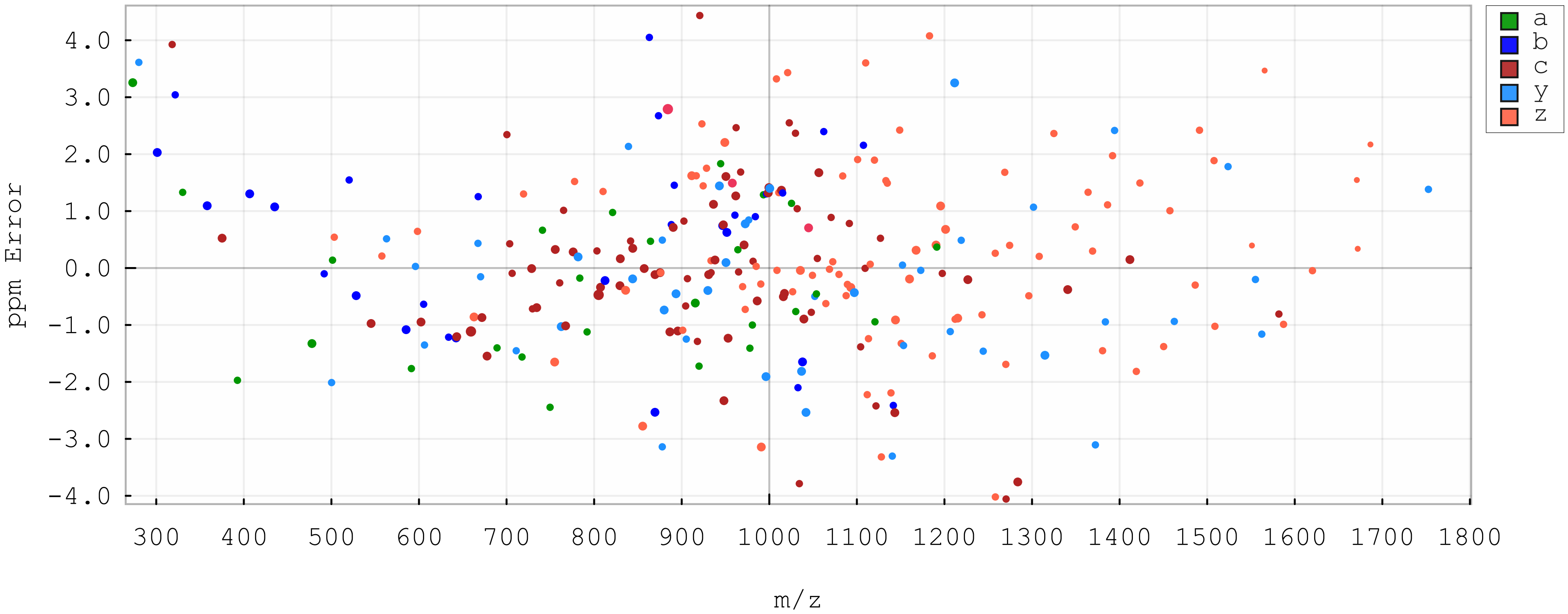

Proteoform:

Index	Protein	SC [%]	SVP [%]	IC [%]	MS Score [a.u.]	Var. Mods	Mono Mass [Da]	Chemical Formula
6	H4Kac	80.2	15.7	39.8	31.9	Ace: K5, K8, K16, K20, K31, K44	11481.4046	C506H849N163O140S

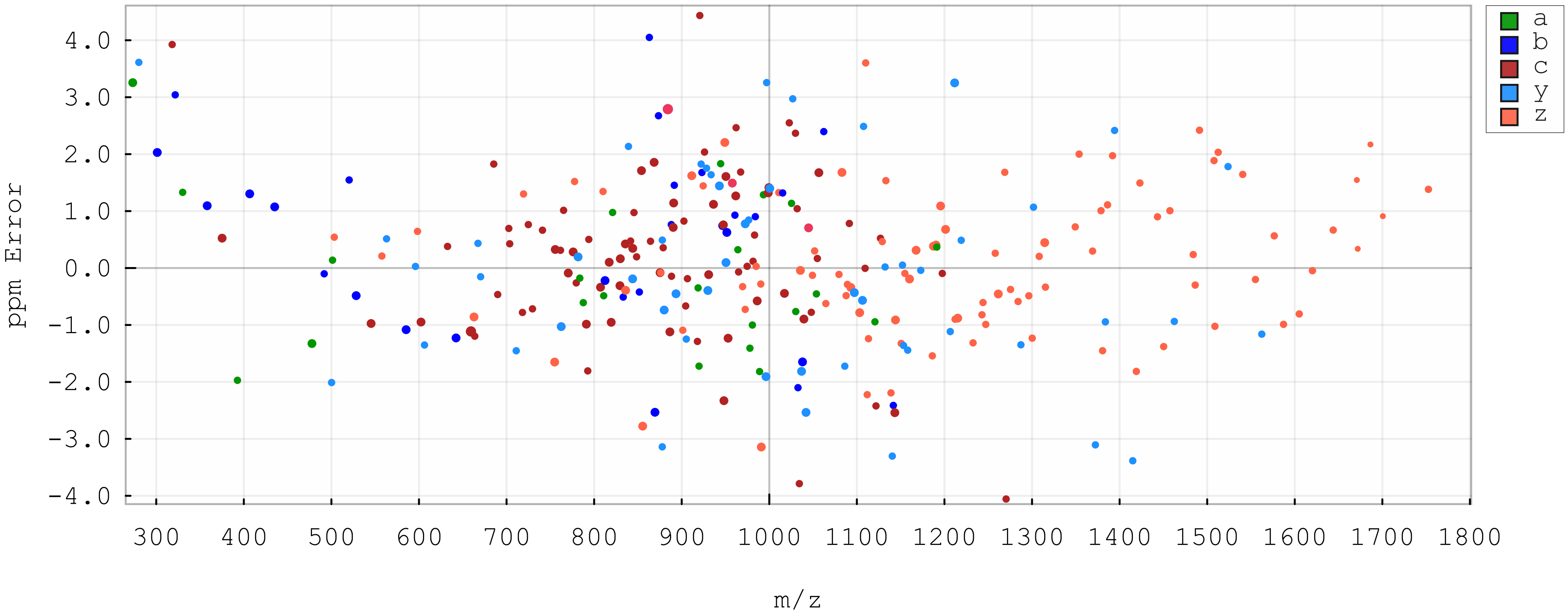

Proteoform:

Index	Protein	SC [%]	SVP [%]	IC [%]	MS Score [a.u.]	Var. Mods	Mono Mass [Da]	Chemical Formula
7	H4Kac	77.2	13.7	38.4	29.7	Ace: K5, K12, K16, K20, K31, K44	11481.4046	C506H849N163O140S

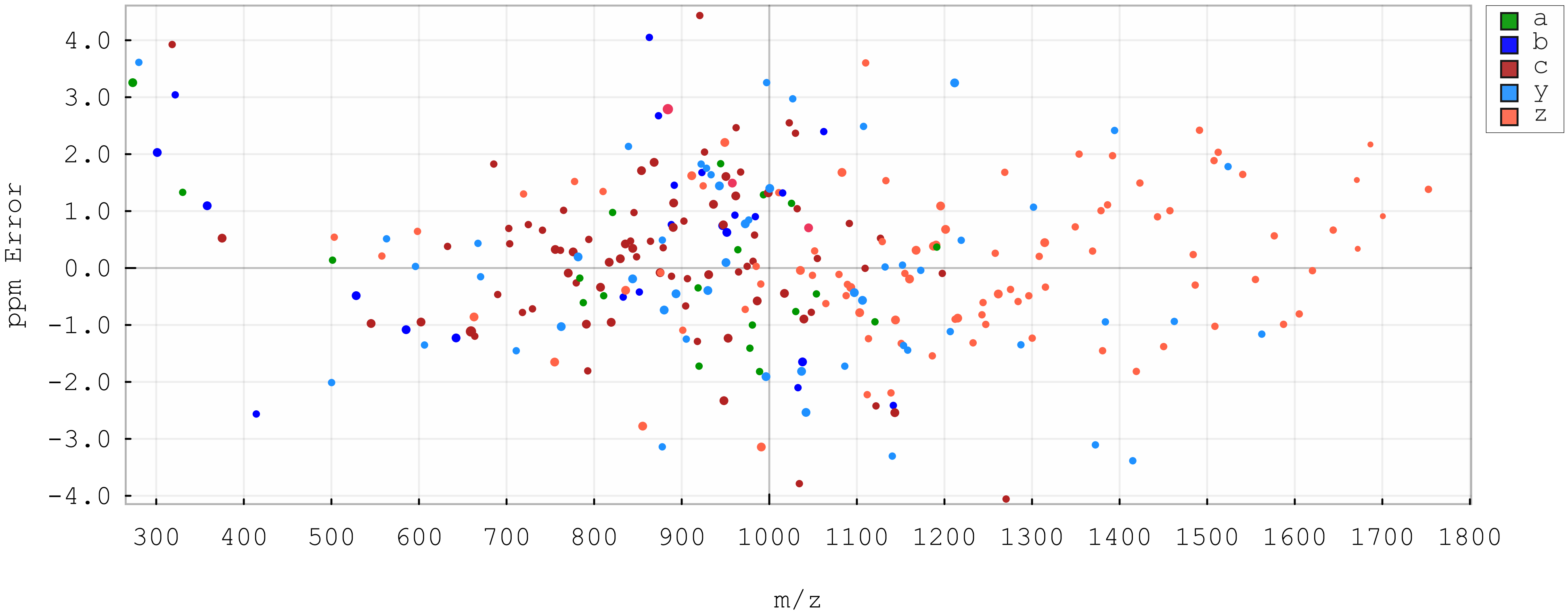

Proteoform:

Index	Protein	SC [%]	SVP [%]	IC [%]	MS Score [a.u.]	Var. Mods	Mono Mass [Da]	Chemical Formula
8	H4Kac	77.2	13.7	37.1	28.7	Ace: K8, K12, K16, K20, K31, K44	11481.4046	C506H849N163O140S

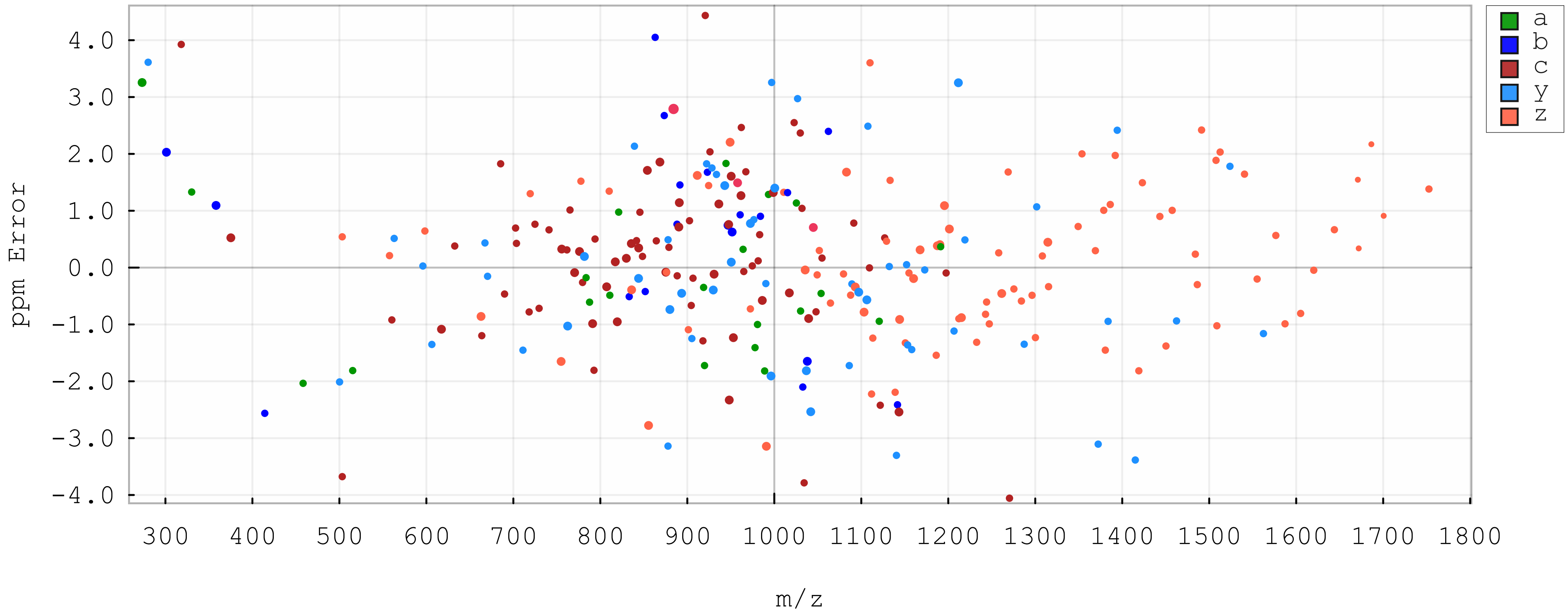

Proteoform:	Index	Protein	SC [%]	SVP [%]	IC [%]	MS Score [a.u.]	Var. Mods	Mono Mass [Da]	Chemical Formula
	9	H4Kac	73.3	23.5	36.6	26.8	Ace: K5, K8, K12, K16, K31, K59	11481.4046	C506H849N163O140S

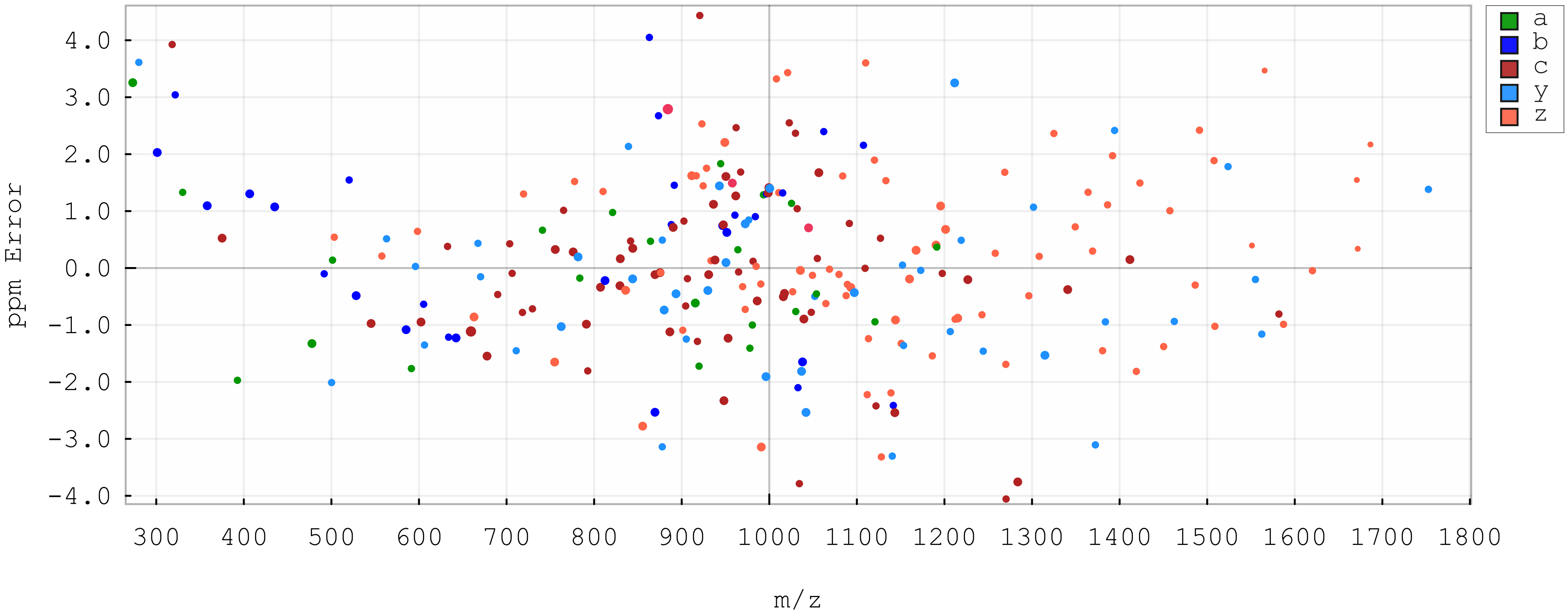

Proteoform:

Index	Protein	SC [%]	SVP [%]	IC [%]	MS Score [a.u.]	Var. Mods	Mono Mass [Da]	Chemical Formula
10	H4Kac	72.3	23.5	35.6	25.7	Ace: K5, K8, K12, K20, K31, K59	11481.4046	C506H849N163O140S

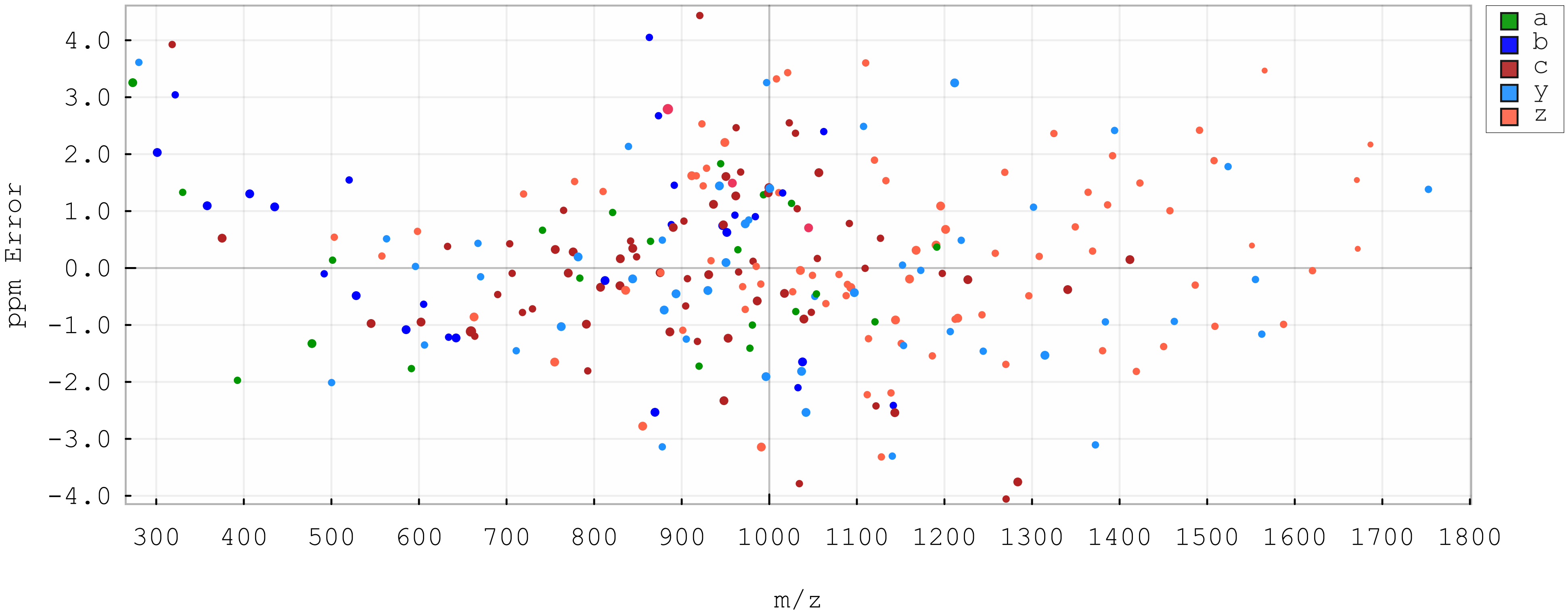

Primary Fragment Plots

Proteoform:

Index	Protein	SC [%]	SVP [%]	IC [%]	MS Score [a.u.]	Var. Mods	Mono Mass [Da]	Chemical Formula
1	H4Kac	87.1	34.3	46.8	40.8	Ace: K5, K8, K12, K16, K20, K44	11481.4046	$C_{506}H_{849}N_{163}O_{140}S$

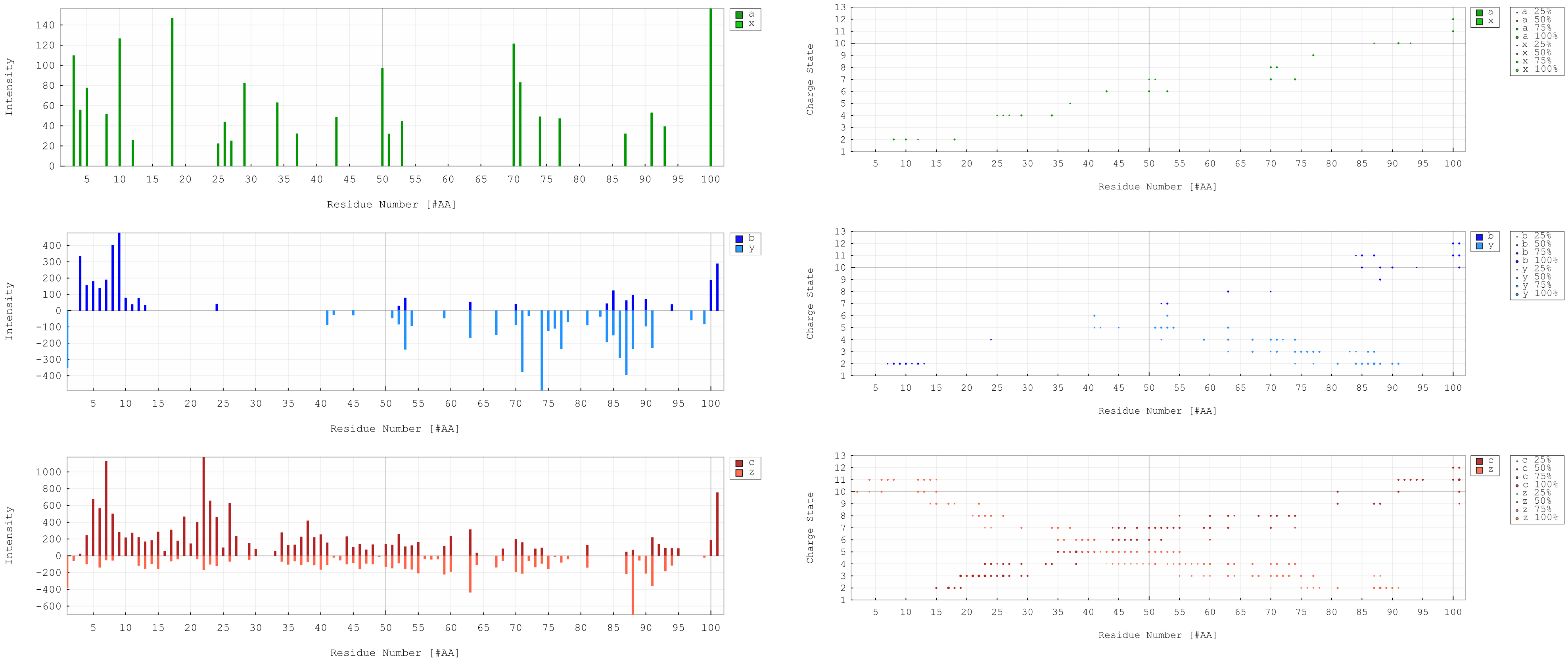

Proteoform:

Index	Protein	SC [%]	SVP [%]	IC [%]	MS Score [a.u.]	Var. Mods	Mono Mass [Da]	Chemical Formula
2	H4Kac	86.1	34.3	43.9	37.8	Ace: K5, K8, K12, K16, K20, K31	11481.4046	C506H849N163O140S

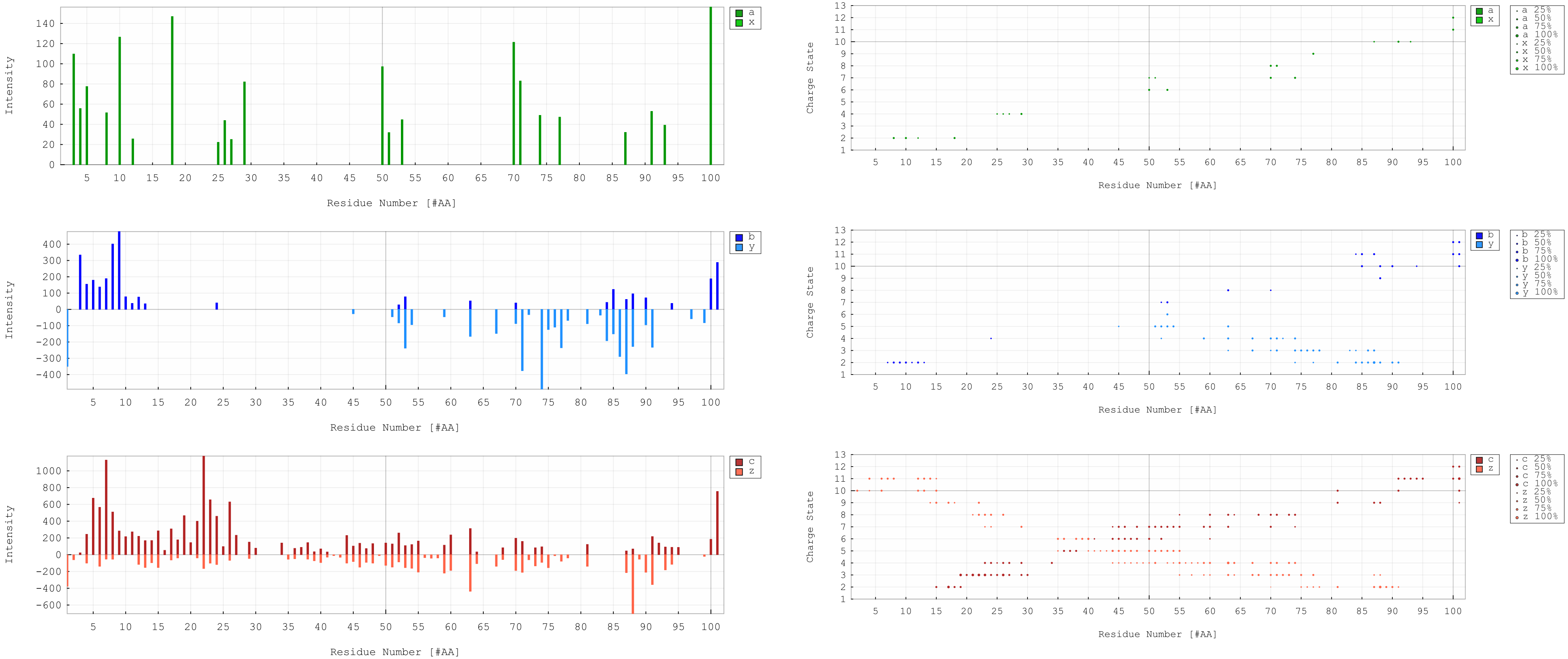

	Index	Protein	SC [%]	SVP [%]	IC [%]	MS Score [a.u.]	Var. Mods	Mono Mass [Da]	Chemical Formula
Proteoform:	3	H4Kac	82.2	23.5	41.9	34.4	Ace: K5, K8, K12, K16, K31, K44	11481.4046	C506H849N163O140S

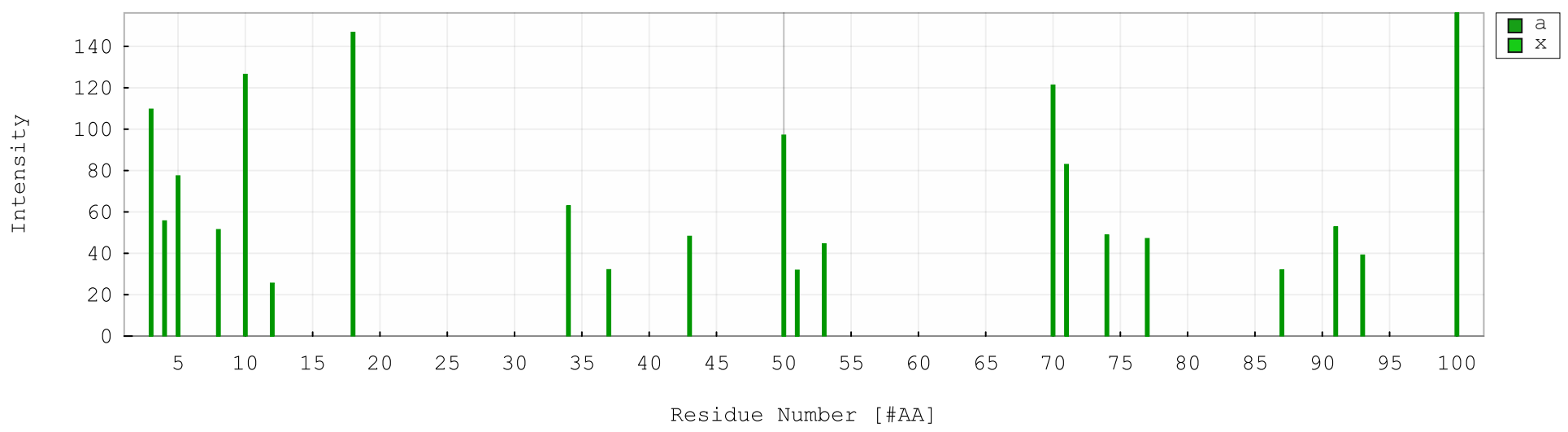

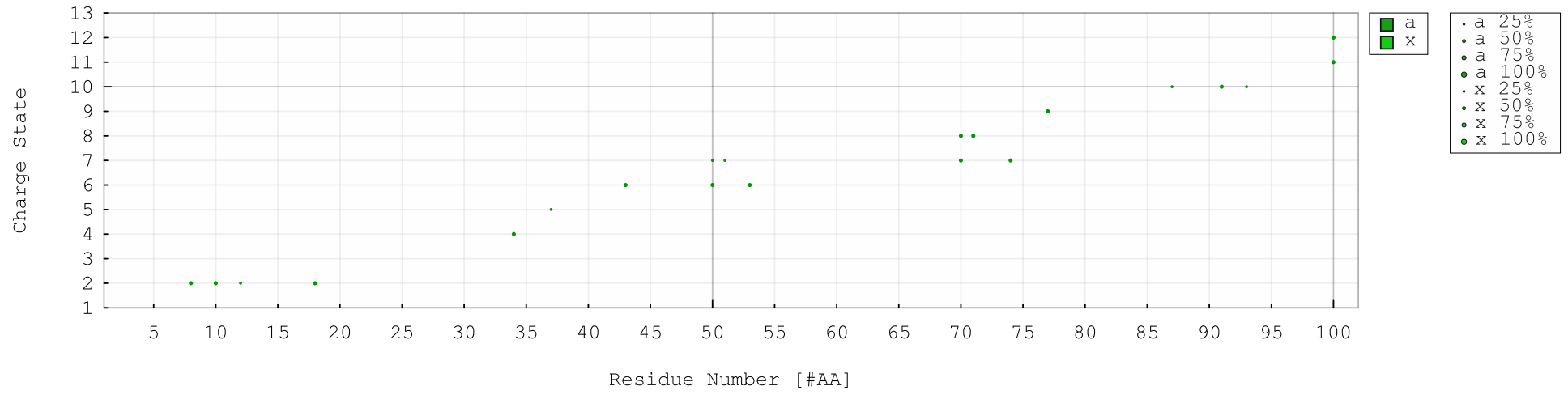

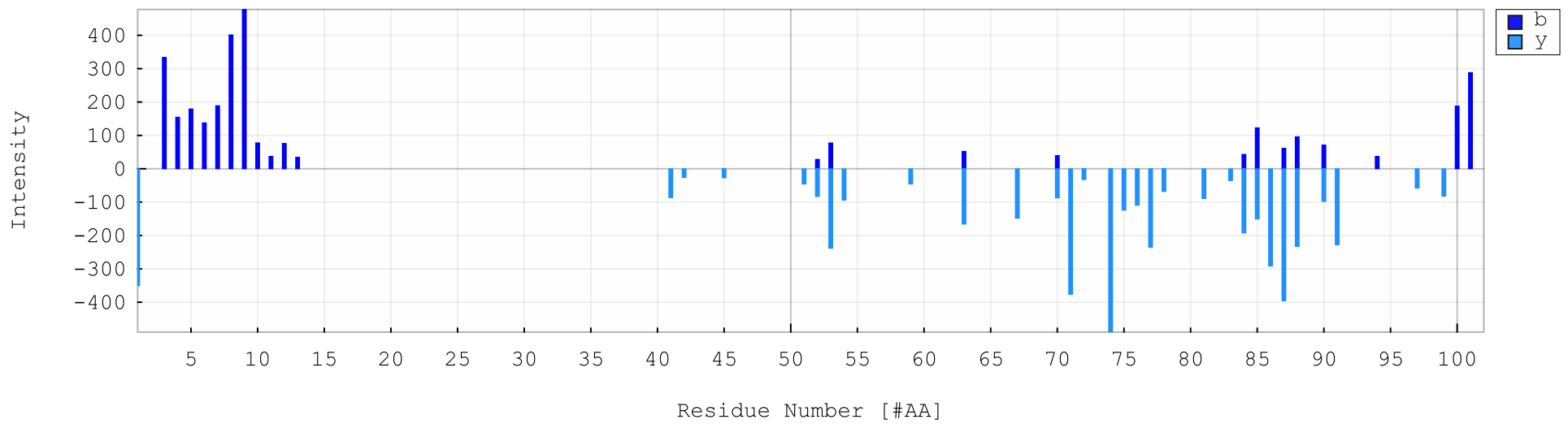

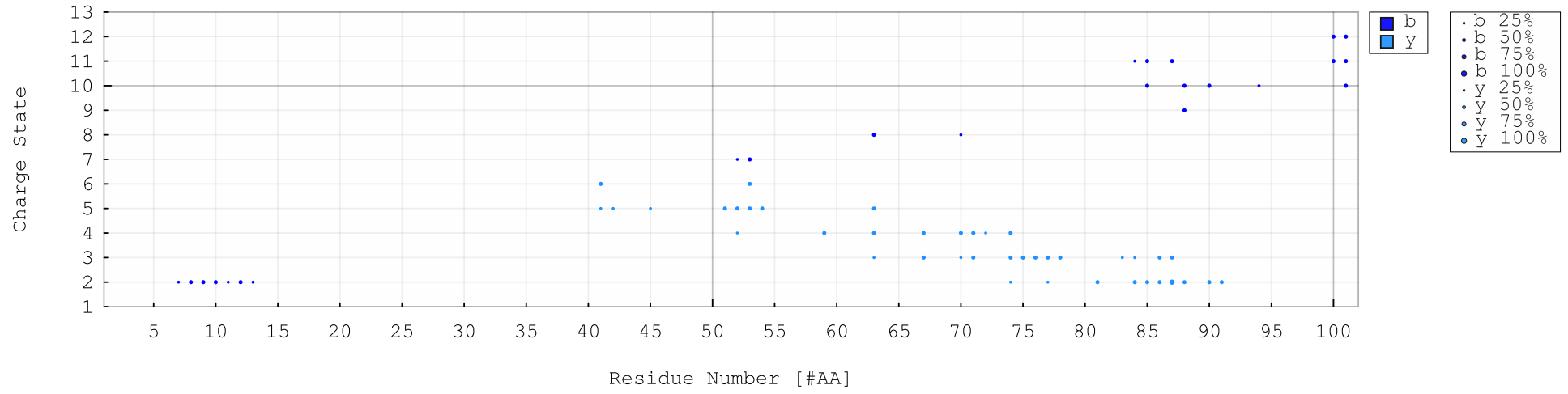

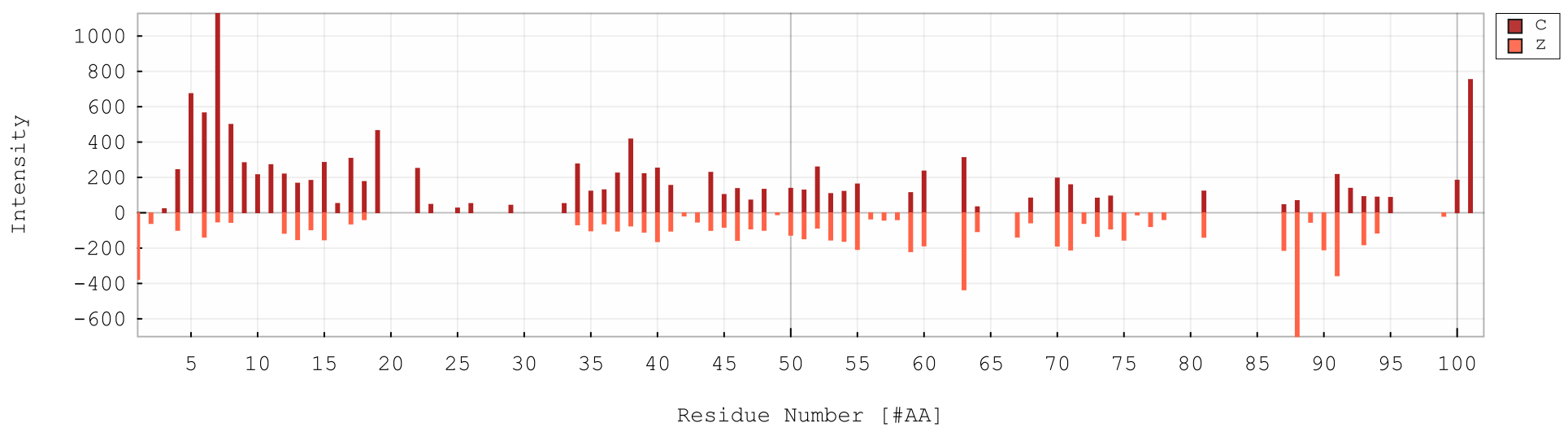

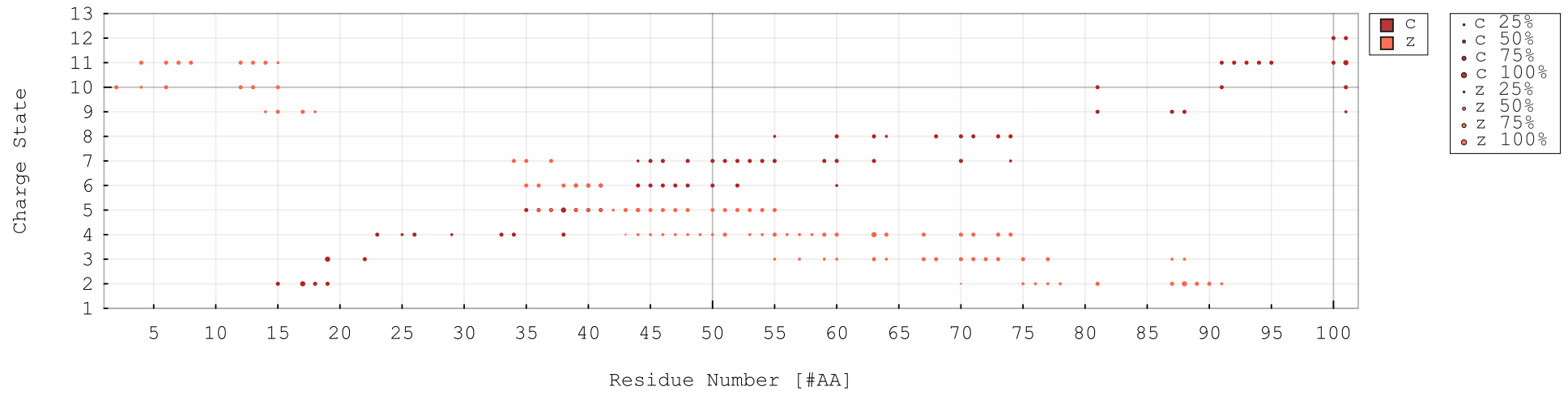

Proteoform:

Index	Protein	SC [%]	SVP [%]	IC [%]	MS Score [a.u.]	Var. Mods	Mono Mass [Da]	Chemical Formula
4	H4Kac	81.2	23.5	40.9	33.2	Ace: K5, K8, K12, K20, K31, K44	11481.4046	C506H849N163O140S

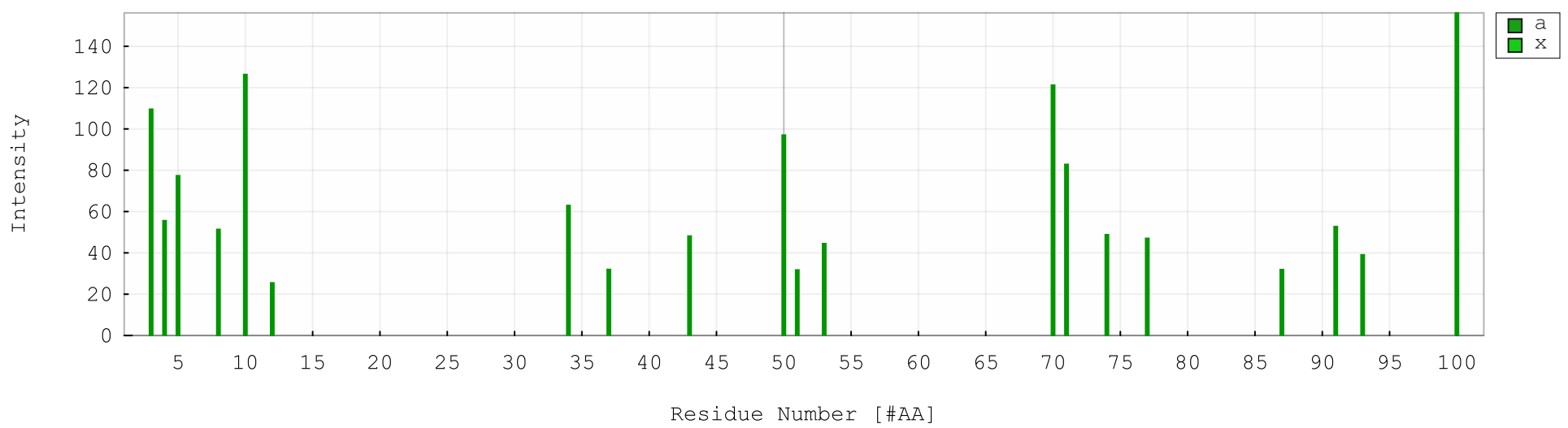

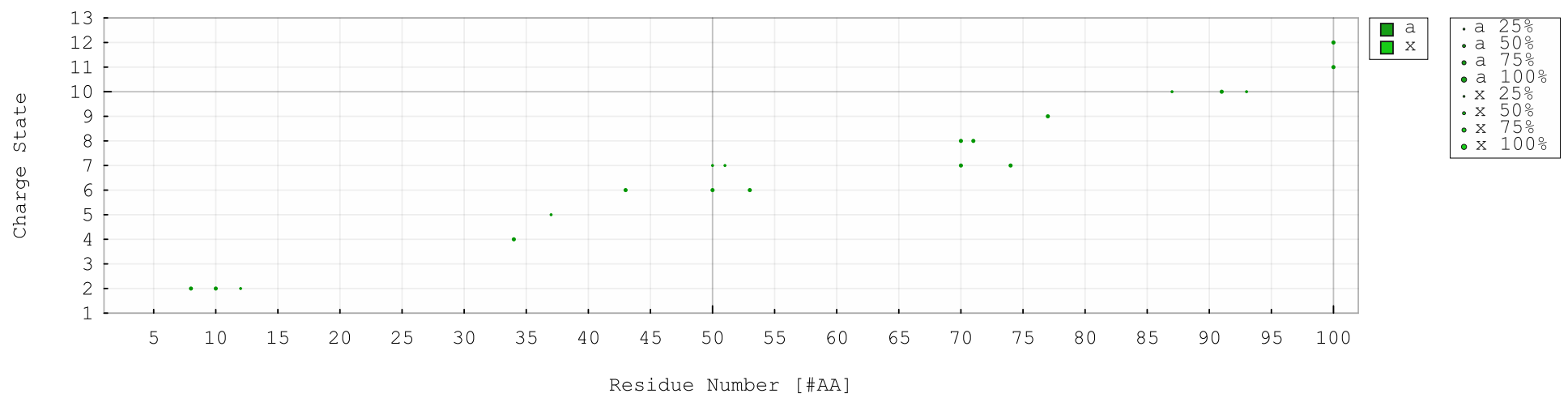

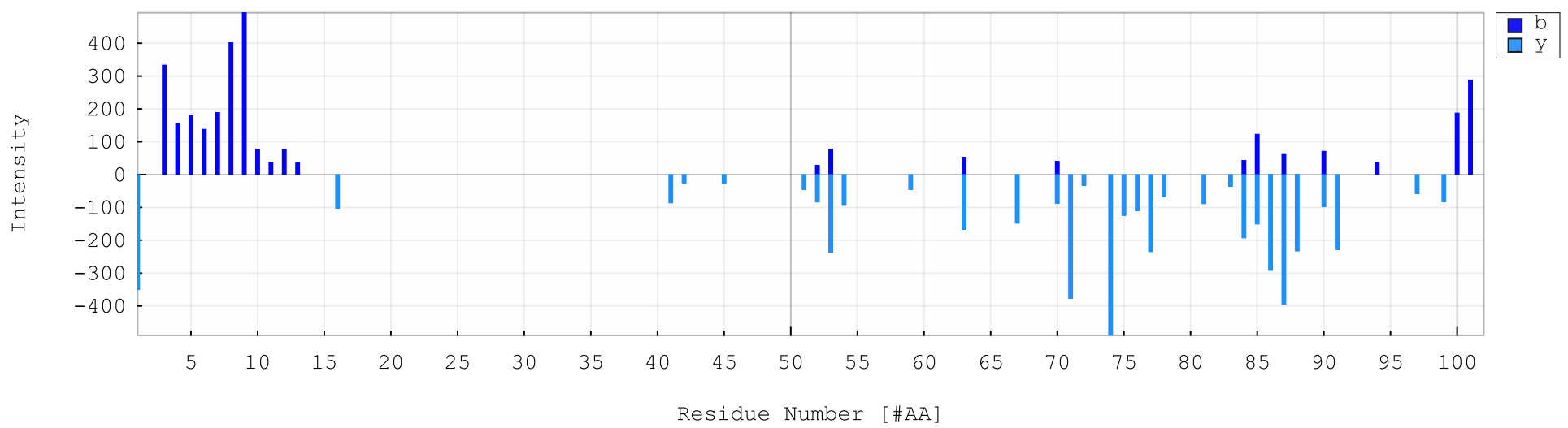

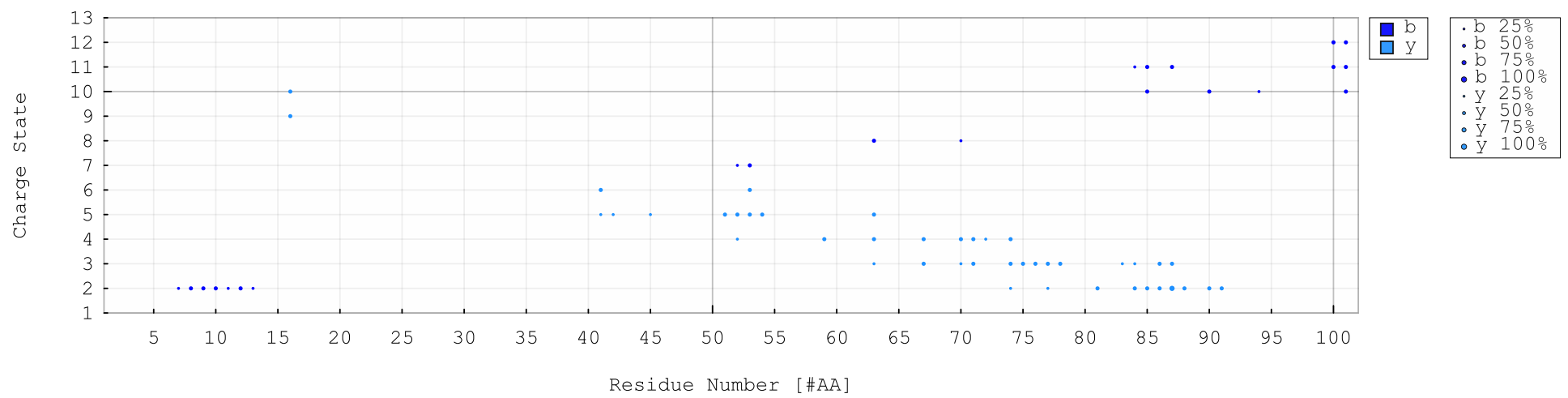

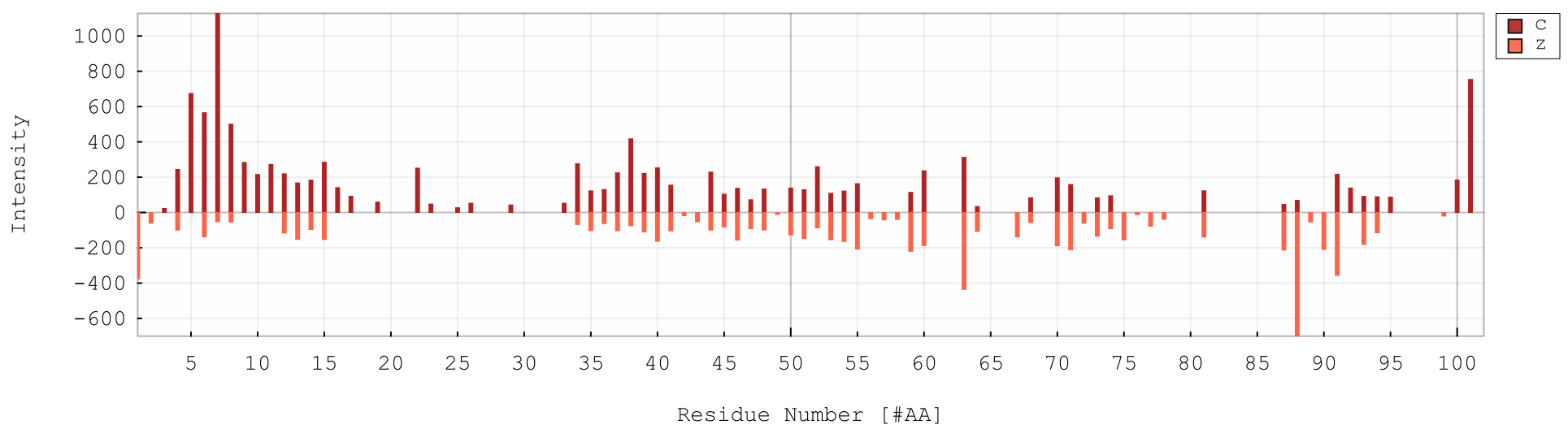

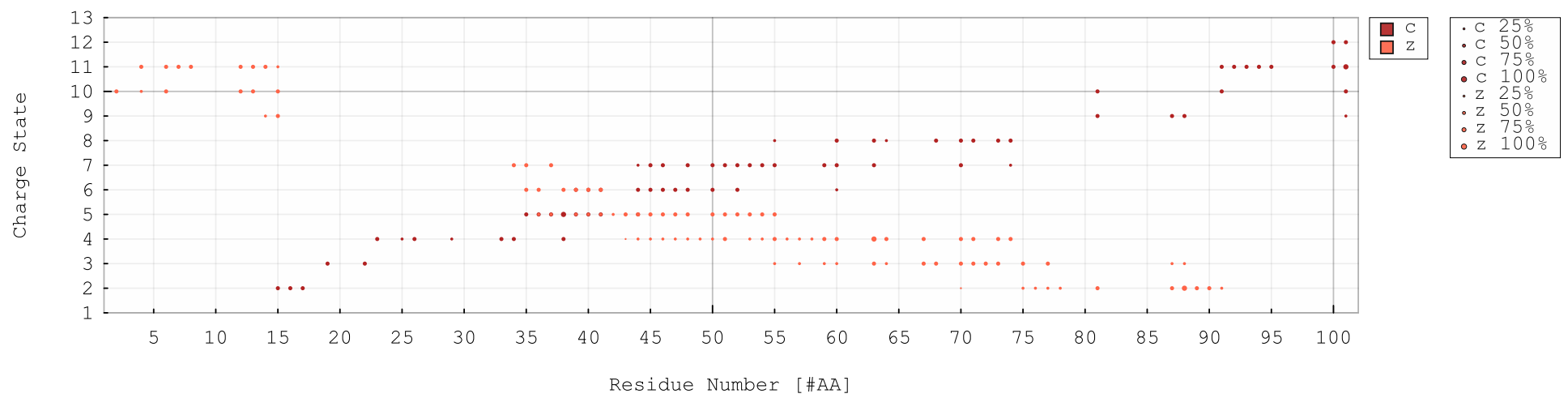

Proteoform:

Index	Protein	SC [%]	SVP [%]	IC [%]	MS Score [a.u.]	Var. Mods	Mono Mass [Da]	Chemical Formula
5	H4Kac	78.2	34.3	41.5	32.4	Ace: K5, K8, K12, K16, K20, K59	11481.4046	C506H849N163O140S

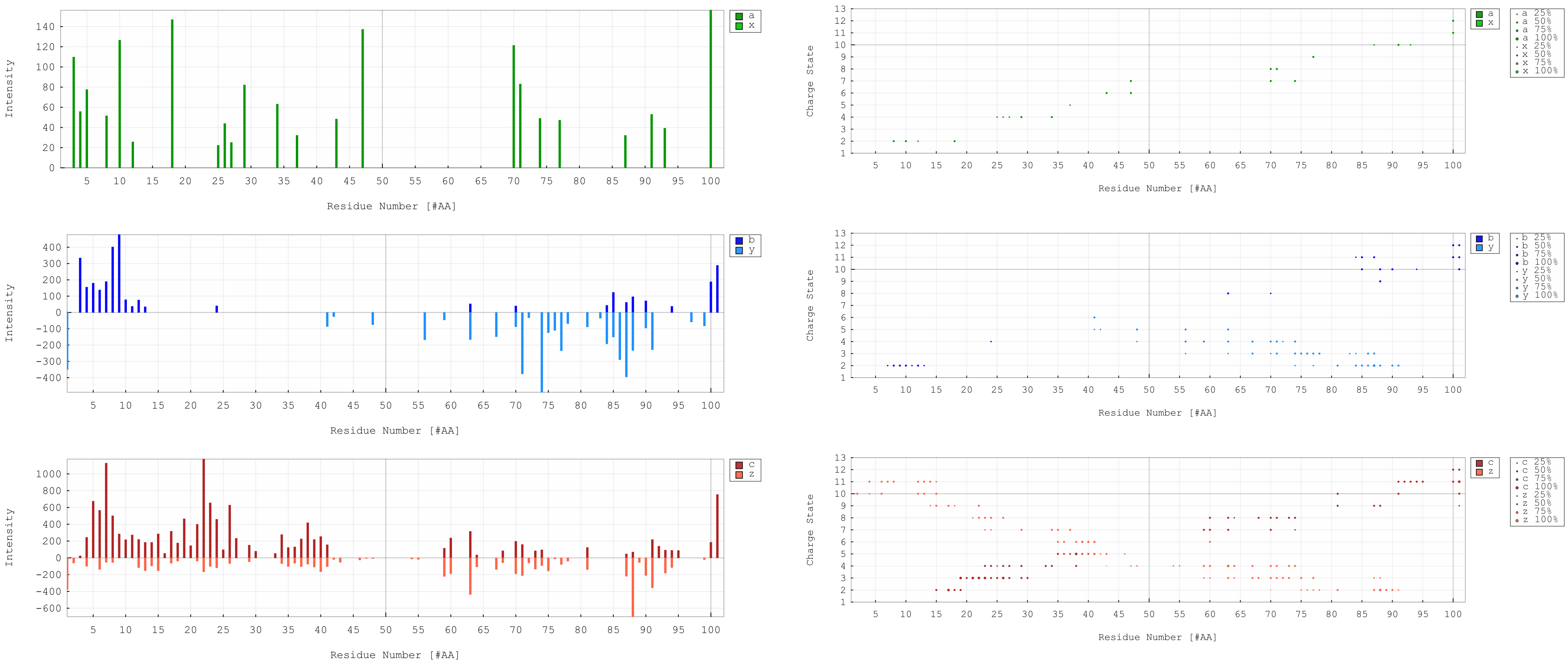

Proteoform:

Index	Protein	SC [%]	SVP [%]	IC [%]	MS Score [a.u.]	Var. Mods	Mono Mass [Da]	Chemical Formula
6	H4Kac	80.2	15.7	39.8	31.9	Ace: K5, K8, K16, K20, K31, K44	11481.4046	C506H849N163O140S

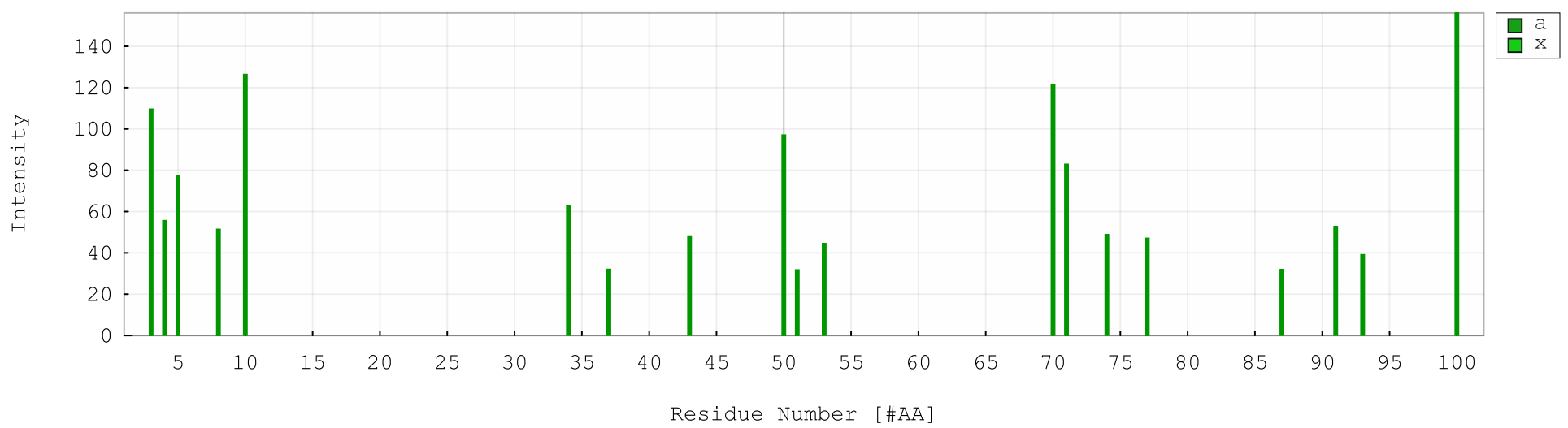

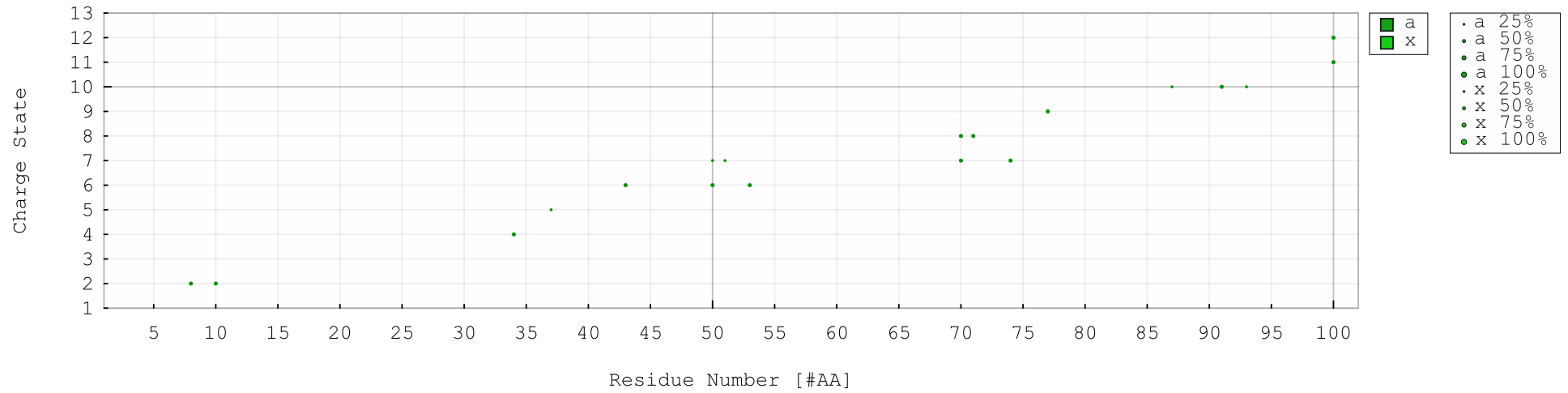

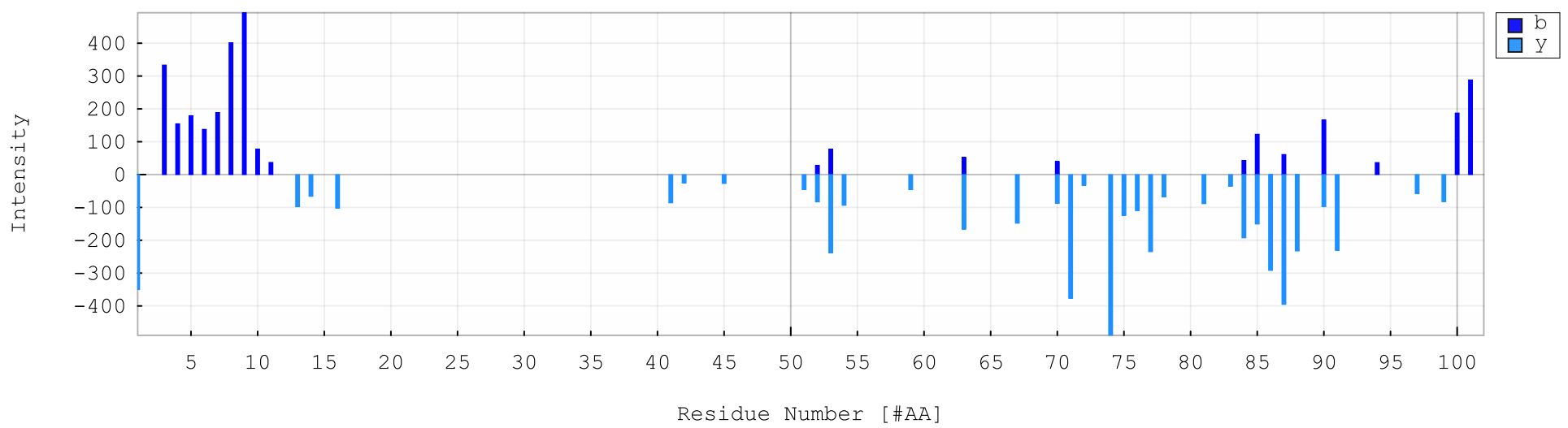

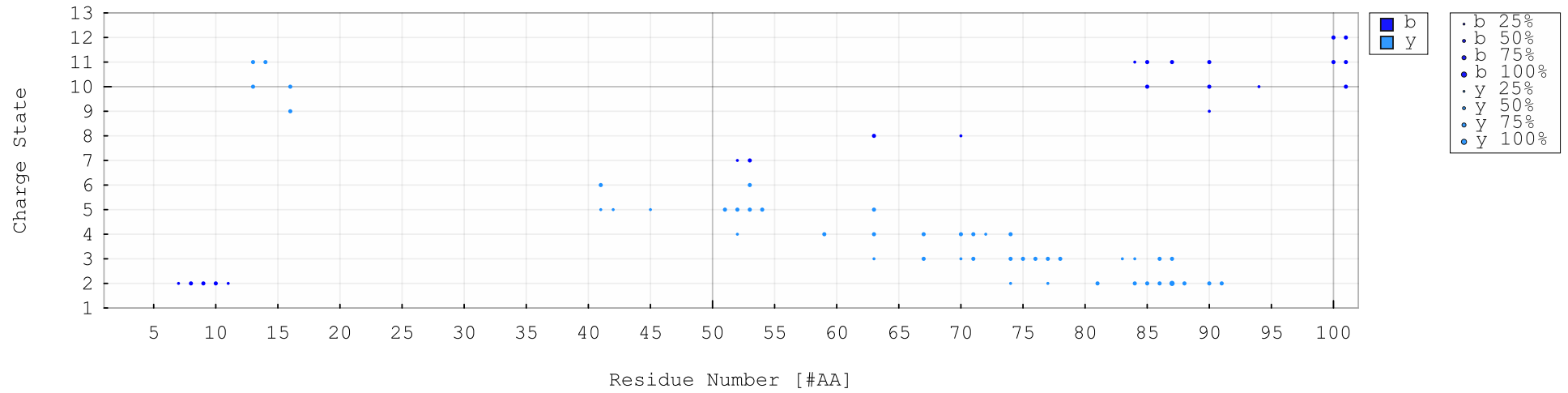

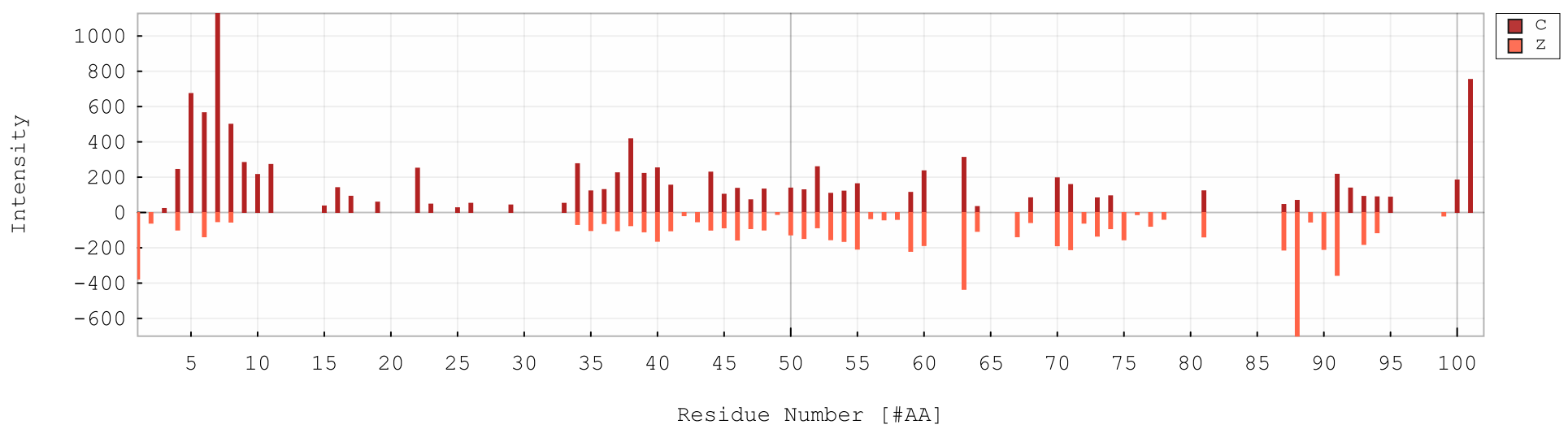

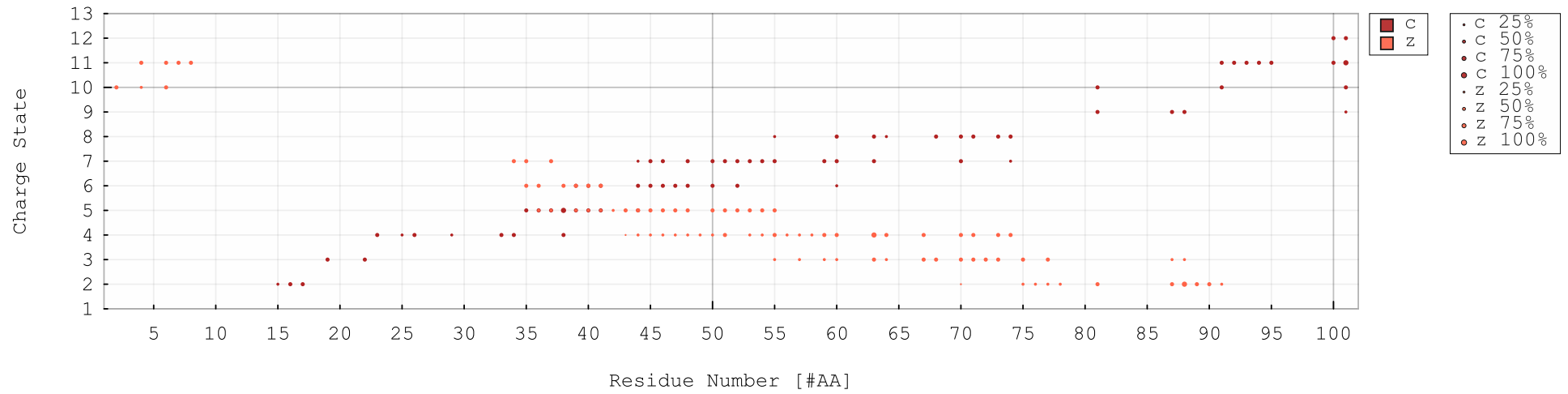

Proteoform:

Index	Protein	SC [%]	SVP [%]	IC [%]	MS Score [a.u.]	Var. Mods	Mono Mass [Da]	Chemical Formula
7	H4Kac	77.2	13.7	38.4	29.7	Ace: K5, K12, K16, K20, K31, K44	11481.4046	C506H849N163O140S

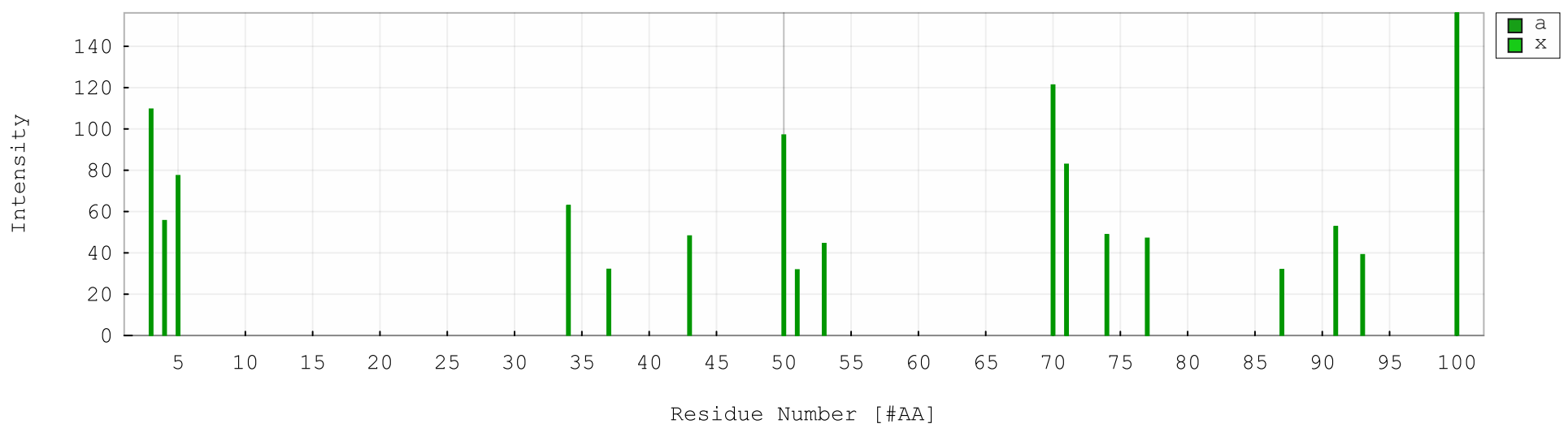

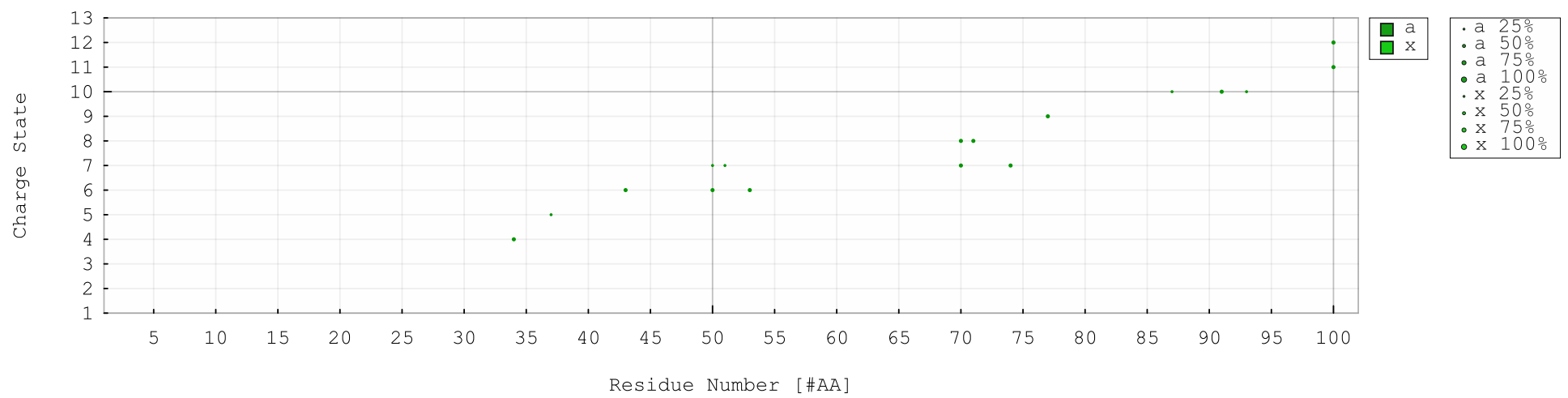

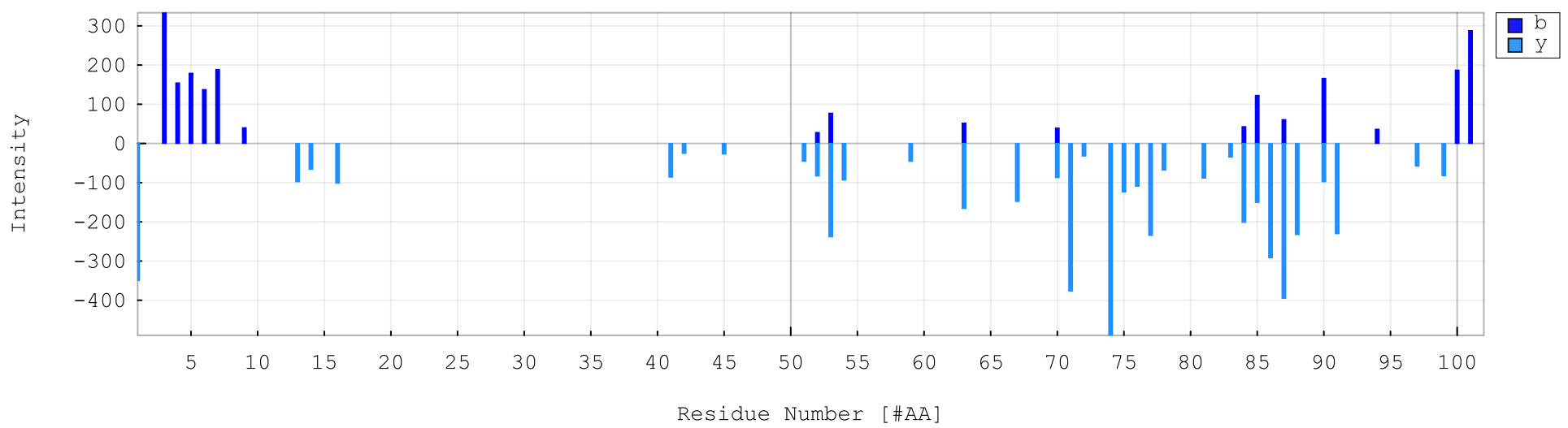

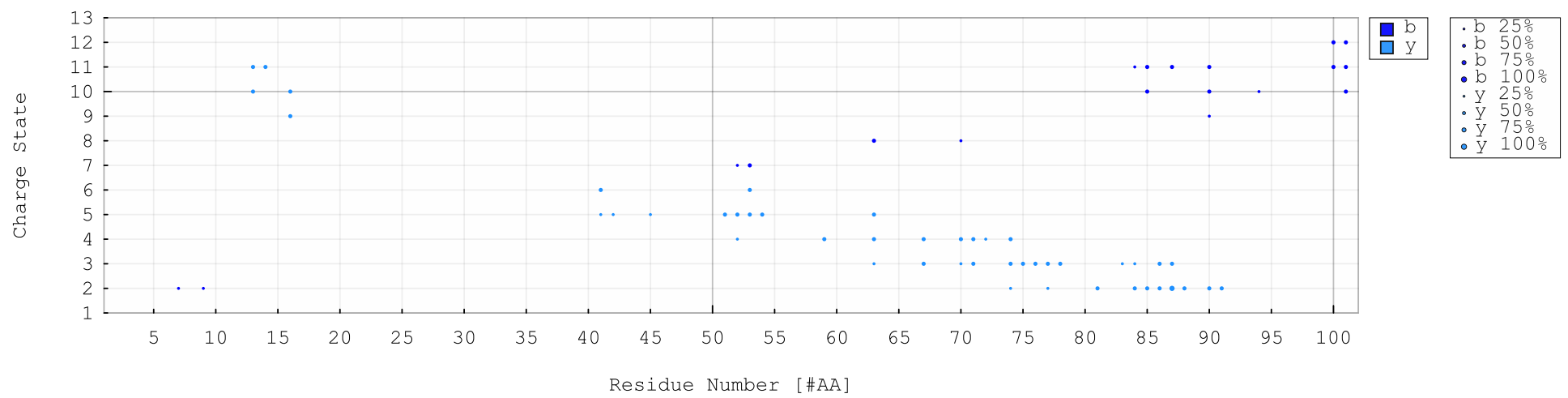

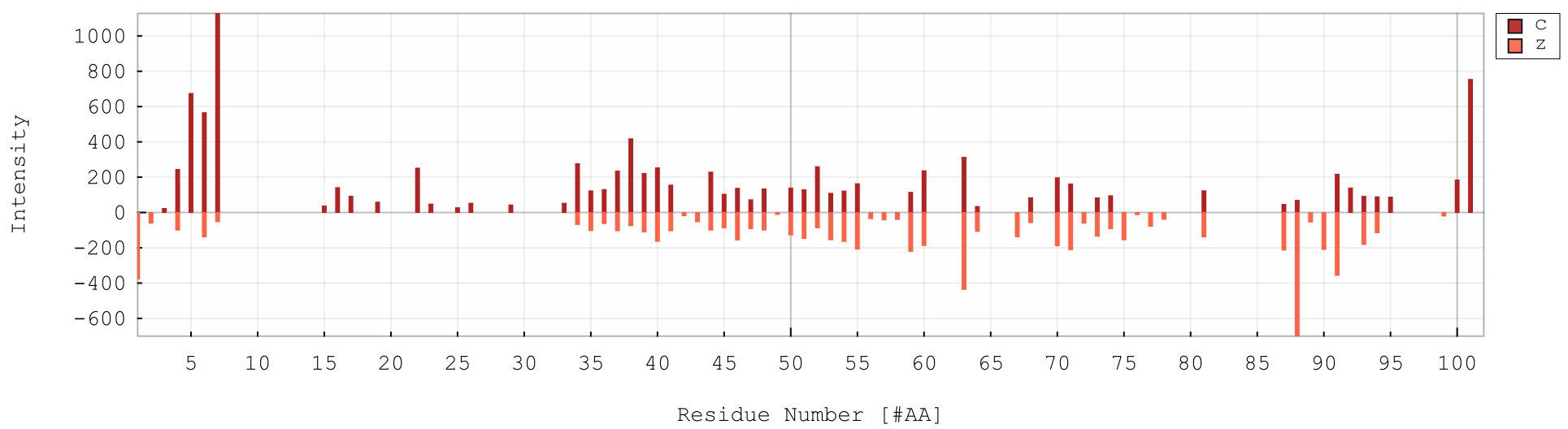

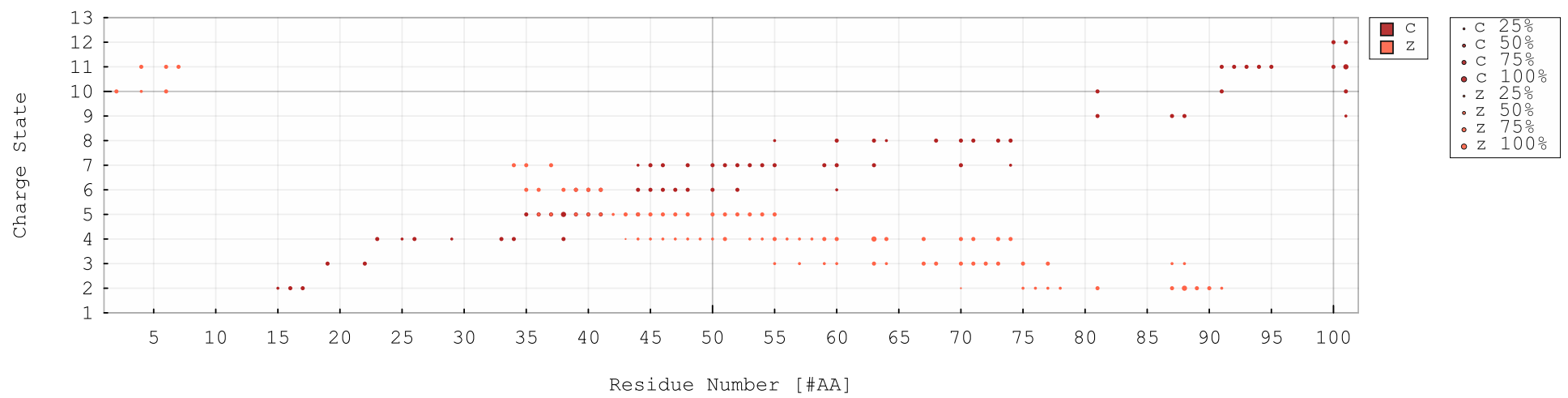

Proteoform:

Index	Protein	SC [%]	SVP [%]	IC [%]	MS Score [a.u.]	Var. Mods	Mono Mass [Da]	Chemical Formula
8	H4Kac	77.2	13.7	37.1	28.7	Ace: K8, K12, K16, K20, K31, K44	11481.4046	C506H849N163O140S

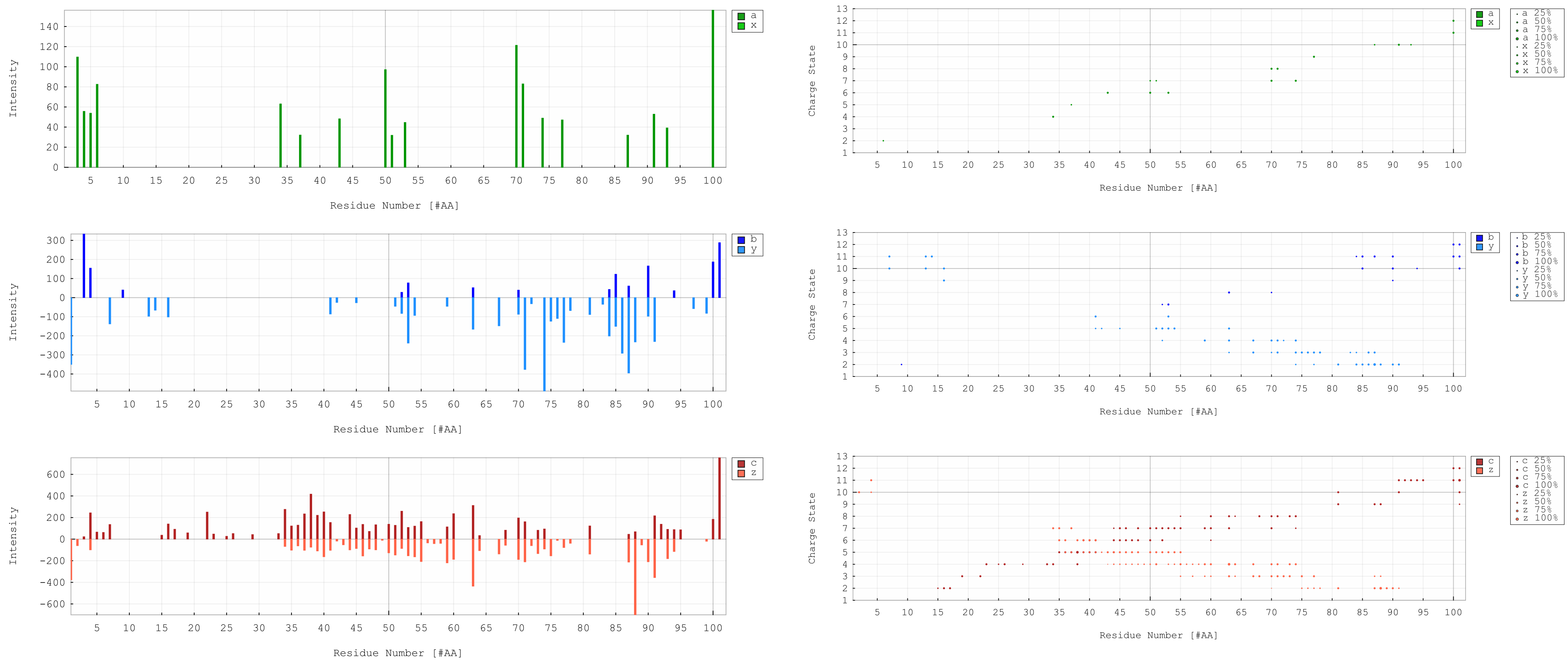

Proteoform:	Index	Protein	SC [%]	SVP [%]	IC [%]	MS Score [a.u.]	Var. Mods	Mono Mass [Da]	Chemical Formula
	9	H4Kac	73.3	23.5	36.6	26.8	Ace: K5, K8, K12, K16, K31, K59	11481.4046	C506H849N163O140S

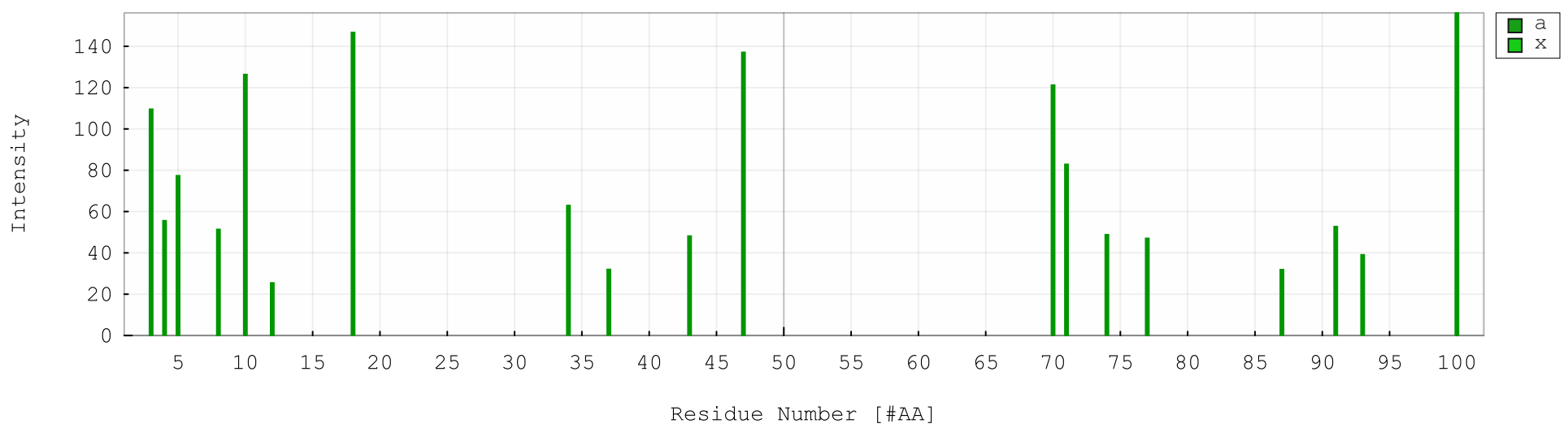

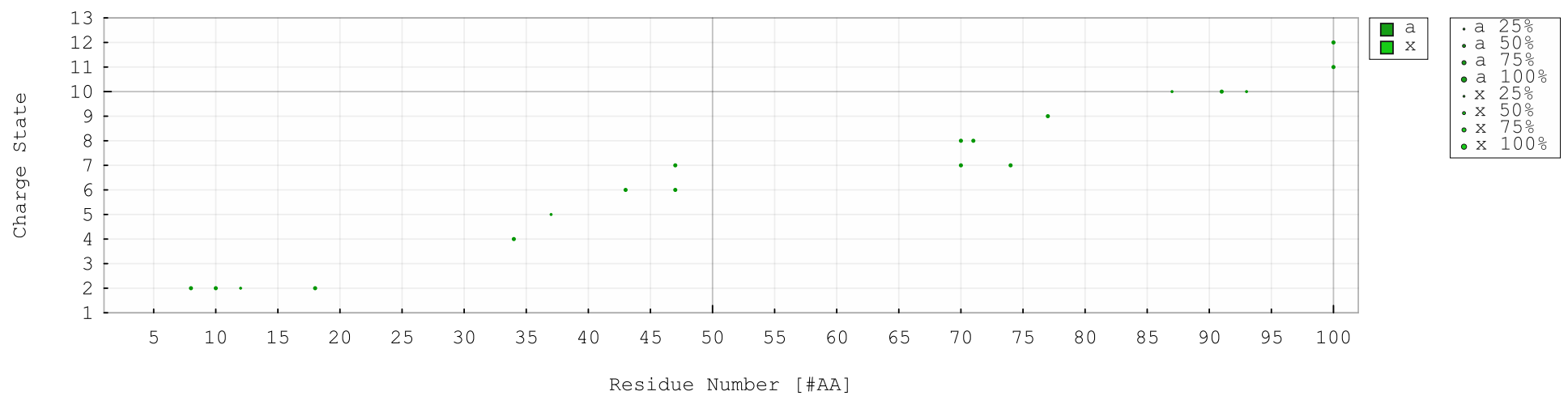

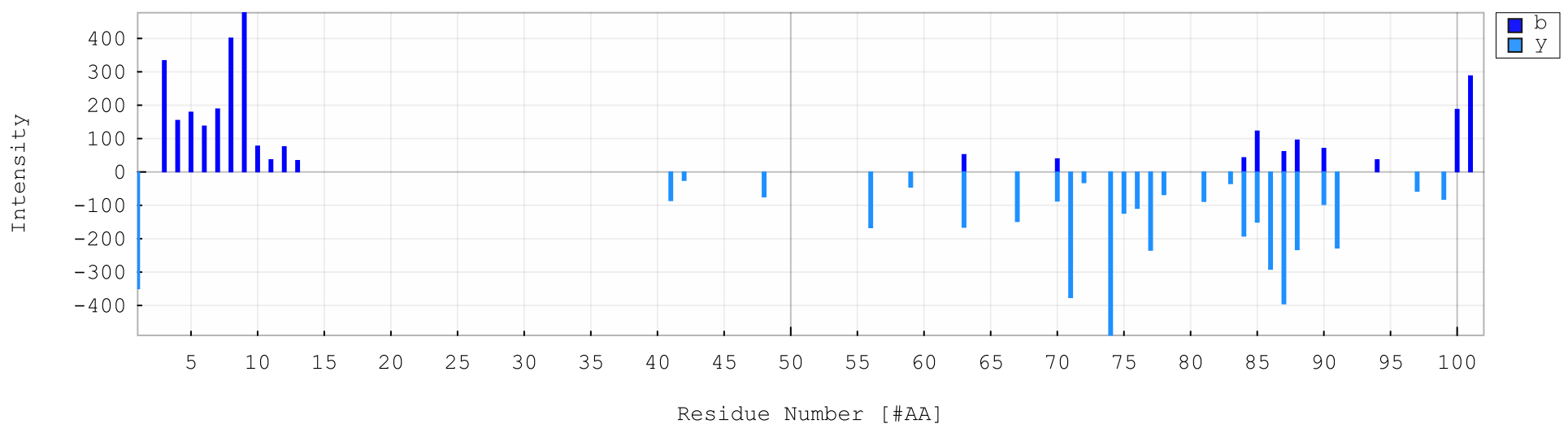

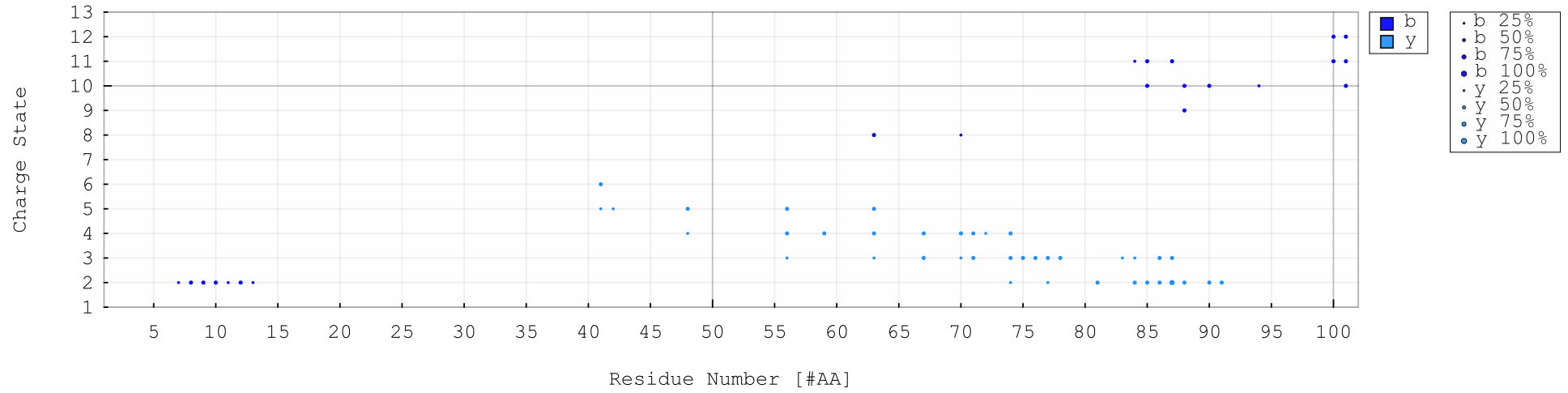

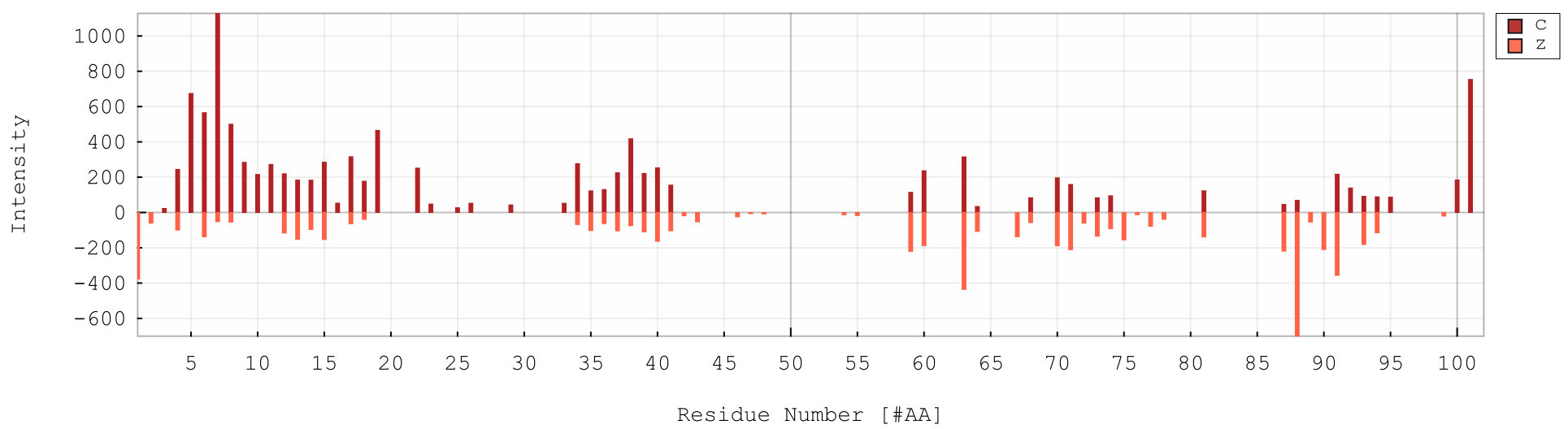

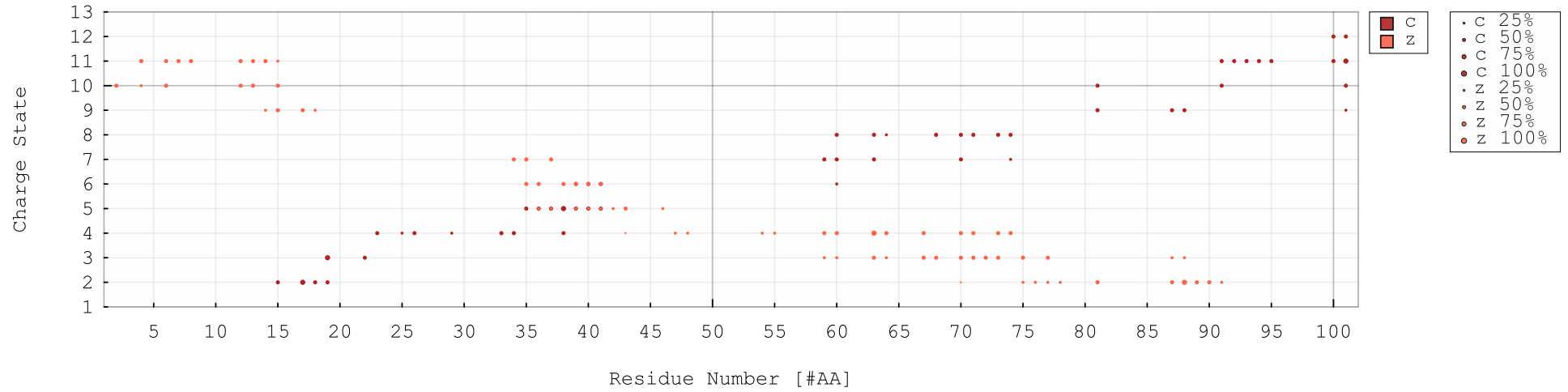

Proteoform:

Index	Protein	SC [%]	SVP [%]	IC [%]	MS Score [a.u.]	Var. Mods	Mono Mass [Da]	Chemical Formula
10	H4Kac	72.3	23.5	35.6	25.7	Ace: K5, K8, K12, K20, K31, K59	11481.4046	C506H849N163O140S

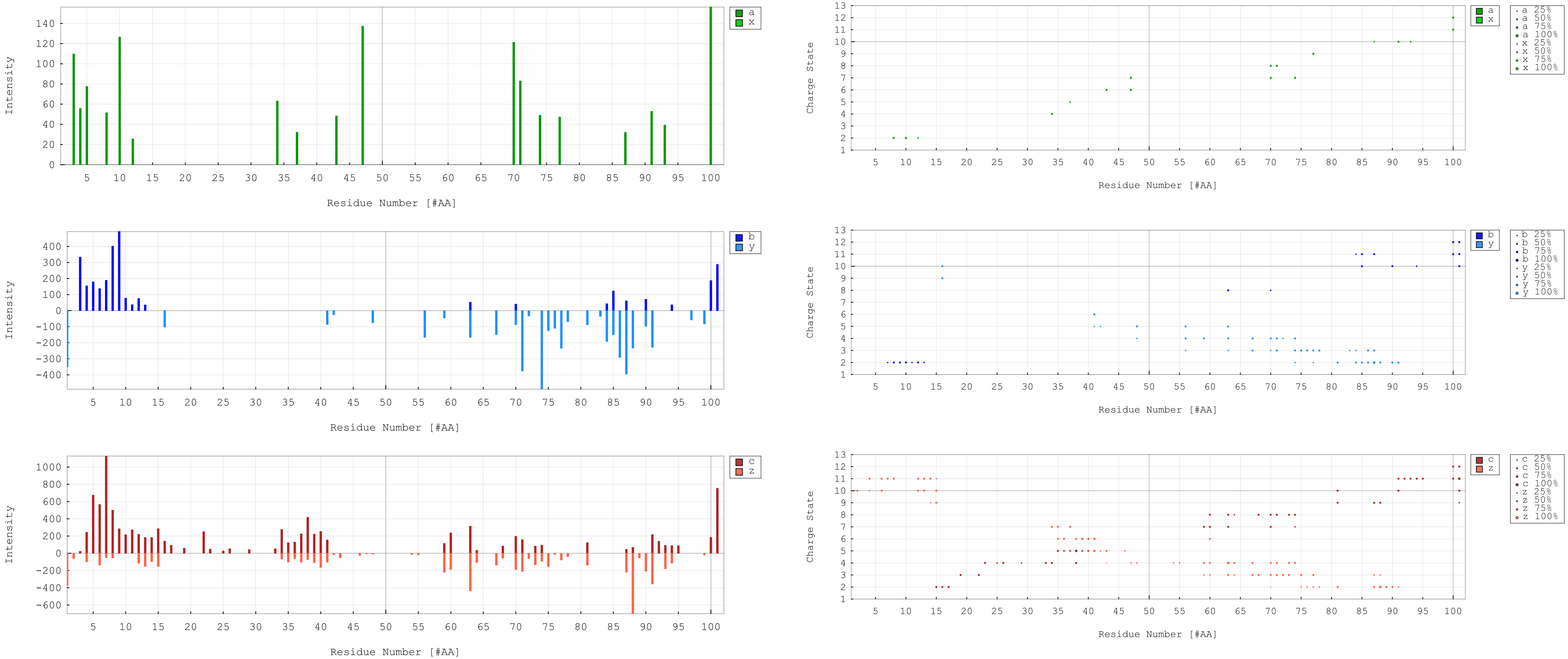

Supplemental Data S4

H4 - p300, 60 min

**Report**	2 of 7
**Experiment**	MS2
**Histone/sample**	H4
**Enzyme**	p300
**Reaction/source**	60 min
**Precursor**	H4Kac6
**Charge state**	13+
**Fragmentation / workflow**	ECD
**Original file**	MS2_H4Kac6_13+_ECD.pdf

Project Information

Project Directory: C:/Users/LAB-ADMIN
Processing date: Tue Apr 28 12:54:19 2026

Sample Info & Protocols

Name: Top-Down Protein Sequencing ExD

Dataset

Imported spectrum file name:	250428 H4_P300-50_60min z13ac6 MS2 ECD_061-736_calibrated.txt
Dataset name:	Information is only available from imported JPEAKS files
Date acquired:	Information is only available from imported JPEAKS files
Date imported:	Information is only available from imported JPEAKS files
Instrument Name:	Information is only available from imported JPEAKS files
Instrument Serial Number:	Information is only available from imported JPEAKS files

Workflow Result Info

Analysis: 250428 H4_P300-50_60min z13ac6 MS2 ECD_061-736_calibrated2
Location: C:/Users/LAB-ADMIN
Method: -

Method Information

Name: Unnamed

Spectral Parameters

Mass Error [ppm]: 6
Intensity Deviation [au]: 0.15
Min/Max Charges: 1-13
Experiment type: ExD

a ions	b ions	c ions	x ions	y ions	z ions	satellite	internal	precursor
a	b	c		y	z+1			M
a+1		c+1			z+2			M+-2H

Noise threshold calculation: Simple moving average noise threshold (SMA)
Window size [datapoints]: 600
Noise curve offset: 2.313631
SVP parameters: terminal gap: 2, internal gap: 1

Proteoforms

(top 10)

Index	Parent Sequence	Chain	Var. Mods	Mono Mass [Da]	Chemical Composition
1	H4Kac	H4KacK5[Ace]K8[Ace]K12[Ace]K16[Ace]K20[Ace]K44[Ace]	Ace: K5, K8, K12, K16, K20, K44	11481.4046	C506H849N163O140S
2	H4Kac	H4KacK5[Ace]K8[Ace]K12[Ace]K16[Ace]K20[Ace]K31[Ace]	Ace: K5, K8, K12, K16, K20, K31	11481.4046	C506H849N163O140S
3	H4Kac	H4KacK5[Ace]K8[Ace]K12[Ace]K16[Ace]K31[Ace]K44[Ace]	Ace: K5, K8, K12, K16, K31, K44	11481.4046	C506H849N163O140S
4	H4Kac	H4KacK5[Ace]K8[Ace]K12[Ace]K20[Ace]K31[Ace]K44[Ace]	Ace: K5, K8, K12, K20, K31, K44	11481.4046	C506H849N163O140S
5	H4Kac	H4KacK5[Ace]K8[Ace]K16[Ace]K20[Ace]K31[Ace]K44[Ace]	Ace: K5, K8, K16, K20, K31, K44	11481.4046	C506H849N163O140S
6	H4Kac	H4KacK5[Ace]K12[Ace]K16[Ace]K20[Ace]K31[Ace]K44[Ace]	Ace: K5, K12, K16, K20, K31, K44	11481.4046	C506H849N163O140S
7	H4Kac	H4KacK5[Ace]K8[Ace]K12[Ace]K16[Ace]K20[Ace]K59[Ace]	Ace: K5, K8, K12, K16, K20, K59	11481.4046	C506H849N163O140S
8	H4Kac	H4KacK8[Ace]K12[Ace]K16[Ace]K20[Ace]K31[Ace]K44[Ace]	Ace: K8, K12, K16, K20, K31, K44	11481.4046	C506H849N163O140S
9	H4Kac	H4KacK5[Ace]K8[Ace]K12[Ace]K16[Ace]K31[Ace]K59[Ace]	Ace: K5, K8, K12, K16, K31, K59	11481.4046	C506H849N163O140S
10	H4Kac	H4KacK5[Ace]K8[Ace]K12[Ace]K20[Ace]K31[Ace]K59[Ace]	Ace: K5, K8, K12, K20, K31, K59	11481.4046	C506H849N163O140S

Results

Proteoforms Table (top 10)

Index	Protein	SC [%]	SVP [%]	IC [%]	MS Score [a.u.]	Var. Mods	Mono Mass [Da]	Chemical Formula
1	H4Kac	89.1	54.9	58.9	52.5	Ace: K5, K8, K12, K16, K20, K44	11481.4046	C506H849N163O140S
2	H4Kac	87.1	43.1	56.4	49.2	Ace: K5, K8, K12, K16, K20, K31	11481.4046	C506H849N163O140S
3	H4Kac	87.1	41.2	53.9	46.9	Ace: K5, K8, K12, K16, K31, K44	11481.4046	C506H849N163O140S
4	H4Kac	87.1	41.2	52.4	45.6	Ace: K5, K8, K12, K20, K31, K44	11481.4046	C506H849N163O140S
5	H4Kac	87.1	41.2	51.0	44.5	Ace: K5, K8, K16, K20, K31, K44	11481.4046	C506H849N163O140S
6	H4Kac	87.1	41.2	50.0	43.6	Ace: K5, K12, K16, K20, K31, K44	11481.4046	C506H849N163O140S
7	H4Kac	81.2	54.9	53.2	43.2	Ace: K5, K8, K12, K16, K20, K59	11481.4046	C506H849N163O140S
8	H4Kac	87.1	41.2	48.8	42.5	Ace: K8, K12, K16, K20, K31, K44	11481.4046	C506H849N163O140S
9	H4Kac	79.2	41.2	48.1	38.1	Ace: K5, K8, K12, K16, K31, K59	11481.4046	C506H849N163O140S
10	H4Kac	79.2	41.2	46.6	36.9	Ace: K5, K8, K12, K20, K31, K59	11481.4046	C506H849N163O140S

Sequence

Name: H4Kac

```
                  10                    20                    30                    40                    50                    60
S G R G K G G K G L   G K G G A K R H R K   V L R D N I Q G I T   K P A I R R L A R R   G G V K R I S G L I   Y E E T R G V L K V
                  70                    80                    90                   100                   110                   120
F L E N V I R D A V   T Y T E H A K R K T   V T A M D V V Y A L   K R Q G R T L Y G F   G G
```

Modifications

Modification	Position	Type
Acetyl	5	variable
Acetyl	8	variable
Acetyl	12	variable
Acetyl	16	variable
Acetyl	20	variable
Acetyl	31	variable
Acetyl	44	variable
Acetyl	59	variable
Acetyl	77	variable

Sequence Map

Proteoform:

Index	Protein	SC [%]	SVP [%]	IC [%]	MS Score [a.u.]	Var. Mods	Mono Mass [Da]	Chemical Formula
1	H4Kac	89.1	54.9	58.9	52.5	Ace: K5, K8, K12, K16, K20, K44	11481.4046	C506H849N163O140S

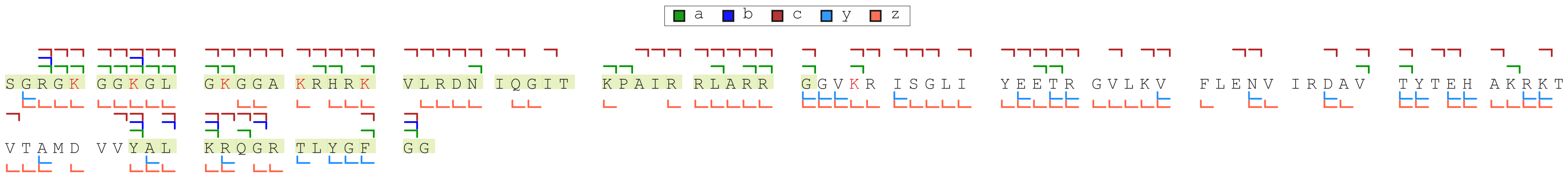

Proteoform:	Index	Protein	SC [%]	SVP [%]	IC [%]	MS Score [a.u.]	Var. Mods	Mono Mass [Da]	Chemical Formula
	2	H4Kac	87.1	43.1	56.4	49.2	Ace: K5, K8, K12, K16, K20, K31	11481.4046	C506H849N163O140S

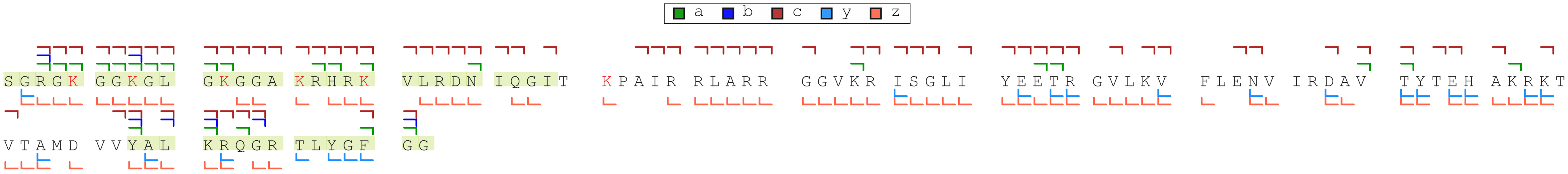

Top-Down Result OmniScape 2025
Sequence Confirmation
250428 H4_P300-50_60min z13ac6 MS2 ECD_061-736_calibrated2

Proteoform:

Index	Protein	SC [%]	SVP [%]	IC [%]	MS Score [a.u.]	Var. Mods	Mono Mass [Da]	Chemical Formula
3	H4Kac	87.1	41.2	53.9	46.9	Ace: K5, K8, K12, K16, K31, K44	11481.4046	C506H849N163O140S

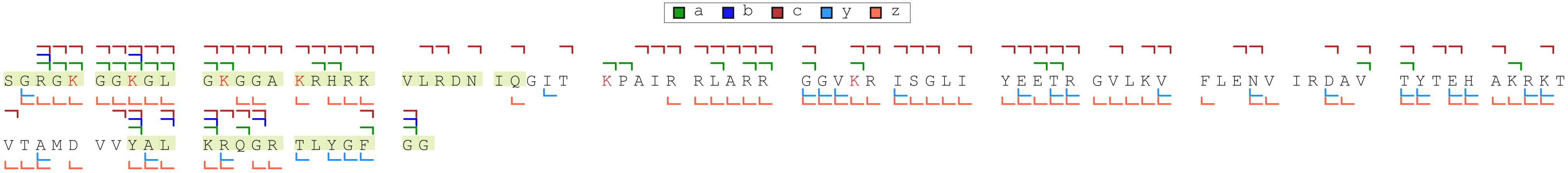

Proteoform:

Index	Protein	SC [%]	SVP [%]	IC [%]	MS Score [a.u.]	Var. Mods	Mono Mass [Da]	Chemical Formula
4	H4Kac	87.1	41.2	52.4	45.6	Ace: K5, K8, K12, K20, K31, K44	11481.4046	C506H849N163O140S

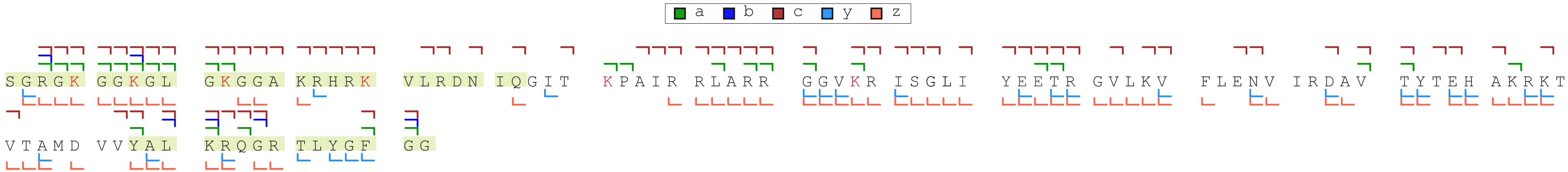

Proteoform:

Index	Protein	SC [%]	SVP [%]	IC [%]	MS Score [a.u.]	Var. Mods	Mono Mass [Da]	Chemical Formula
5	H4Kac	87.1	41.2	51.0	44.5	Ace: K5, K8, K16, K20, K31, K44	11481.4046	C506H849N163O140S

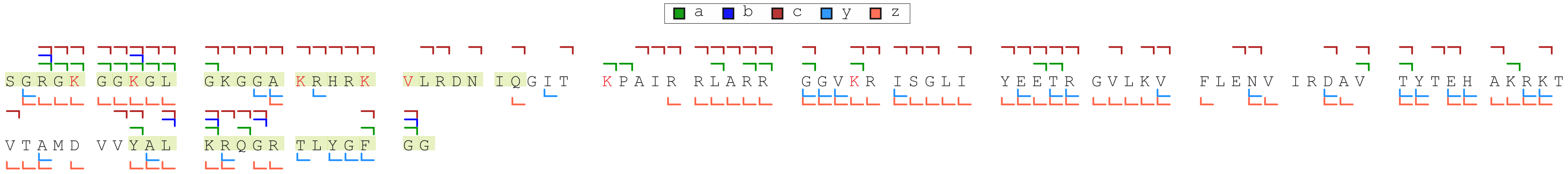

Proteoform:

Index	Protein	SC [%]	SVP [%]	IC [%]	MS Score [a.u.]	Var. Mods	Mono Mass [Da]	Chemical Formula
6	H4Kac	87.1	41.2	50.0	43.6	Ace: K5, K12, K16, K20, K31, K44	11481.4046	C506H849N163O140S

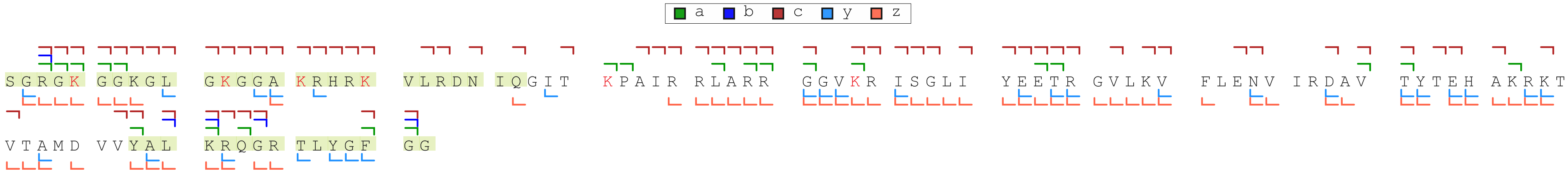

Proteoform:

Index	Protein	SC [%]	SVP [%]	IC [%]	MS Score [a.u.]	Var. Mods	Mono Mass [Da]	Chemical Formula
7	H4Kac	81.2	54.9	53.2	43.2	Ace: K5, K8, K12, K16, K20, K59	11481.4046	C506H849N163O140S

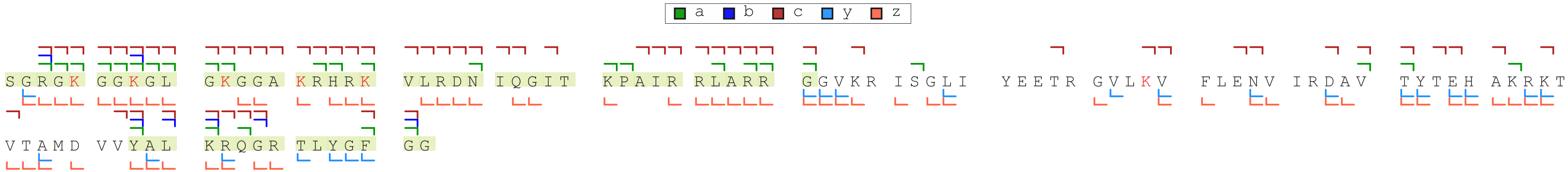

Proteoform:

Index	Protein	SC [%]	SVP [%]	IC [%]	MS Score [a.u.]	Var. Mods	Mono Mass [Da]	Chemical Formula
8	H4Kac	87.1	41.2	48.8	42.5	Ace: K8, K12, K16, K20, K31, K44	11481.4046	C506H849N163O140S

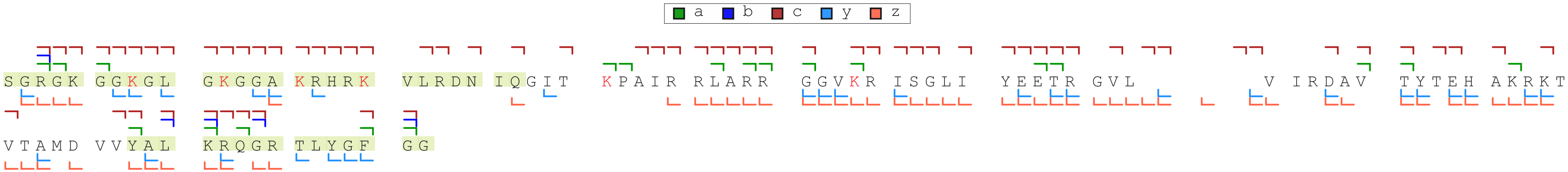

Proteoform:

Index	Protein	SC [%]	SVP [%]	IC [%]	MS Score [a.u.]	Var. Mods	Mono Mass [Da]	Chemical Formula
9	H4Kac	79.2	41.2	48.1	38.1	Ace: K5, K8, K12, K16, K31, K59	11481.4046	C506H849N163O140S

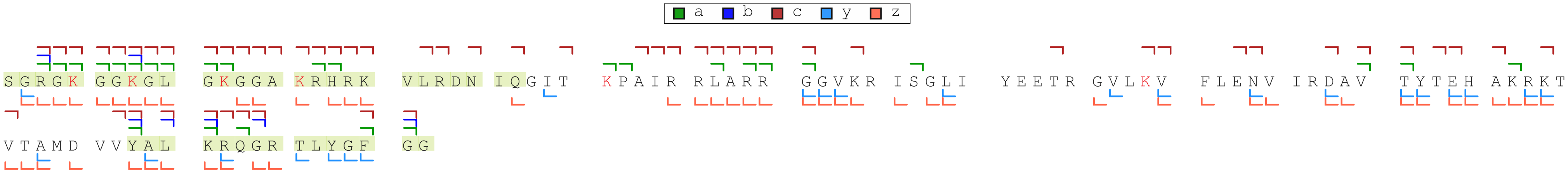

Proteoform:

Index	Protein	SC [%]	SVP [%]	IC [%]	MS Score [a.u.]	Var. Mods	Mono Mass [Da]	Chemical Formula
10	H4Kac	79.2	41.2	46.6	36.9	Ace: K5, K8, K12, K20, K31, K59	11481.4046	C506H849N163O140S

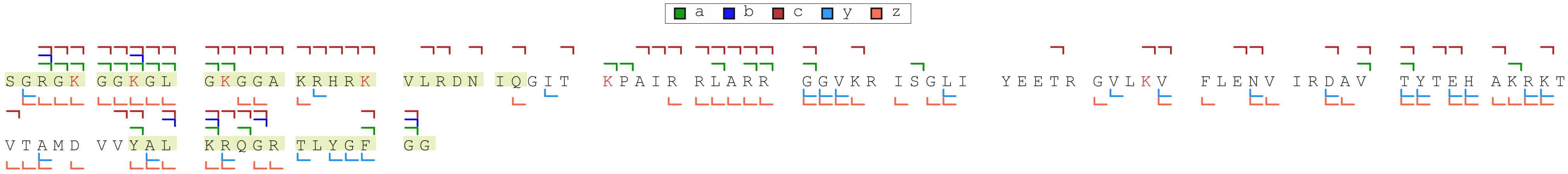

Error Plot

Proteoform:	Index	Protein	SC [%]	SVP [%]	IC [%]	MS Score [a.u.]	Var. Mods	Mono Mass [Da]	Chemical Formula
	1	H4Kac	89.1	54.9	58.9	52.5	Ace: K5, K8, K12, K16, K20, K44	11481.4046	C506H849N163O140S

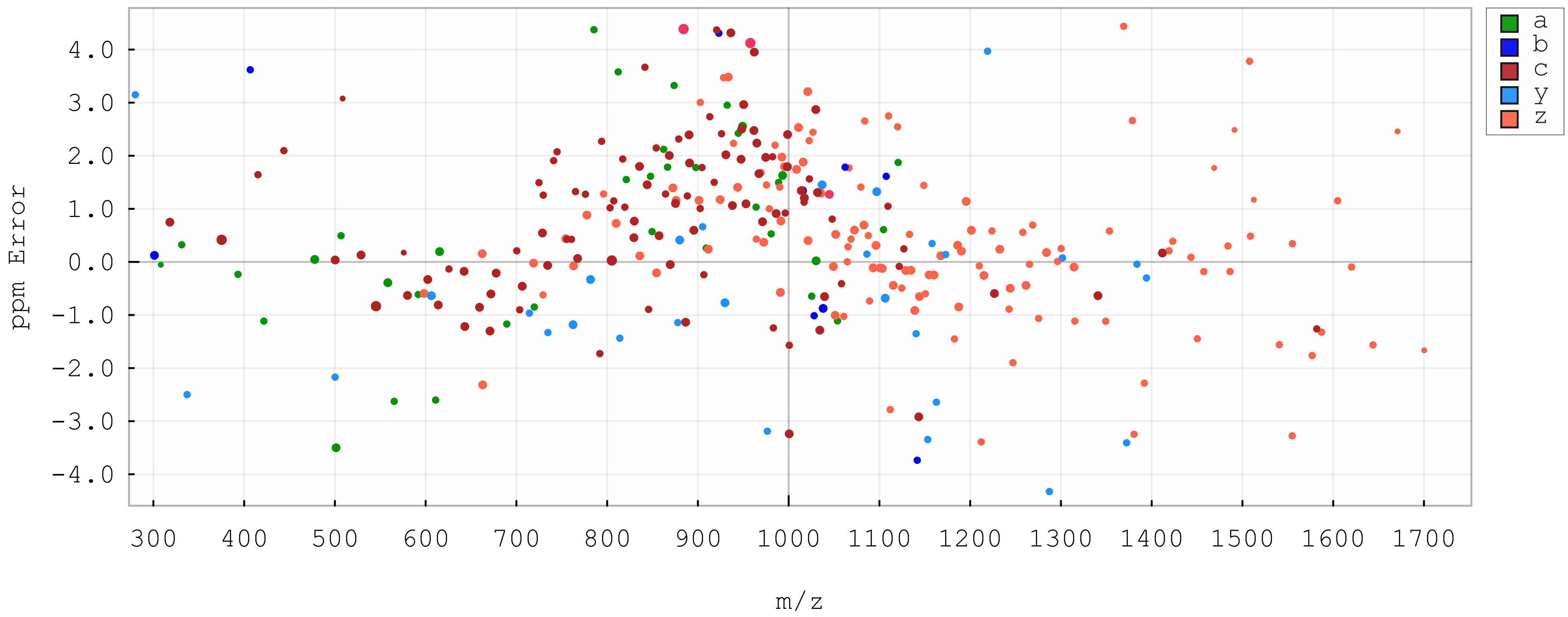

Proteoform:

Index	Protein	SC [%]	SVP [%]	IC [%]	MS Score [a.u.]	Var. Mods	Mono Mass [Da]	Chemical Formula
2	H4Kac	87.1	43.1	56.4	49.2	Ace: K5, K8, K12, K16, K20, K31	11481.4046	C506H849N163O140S

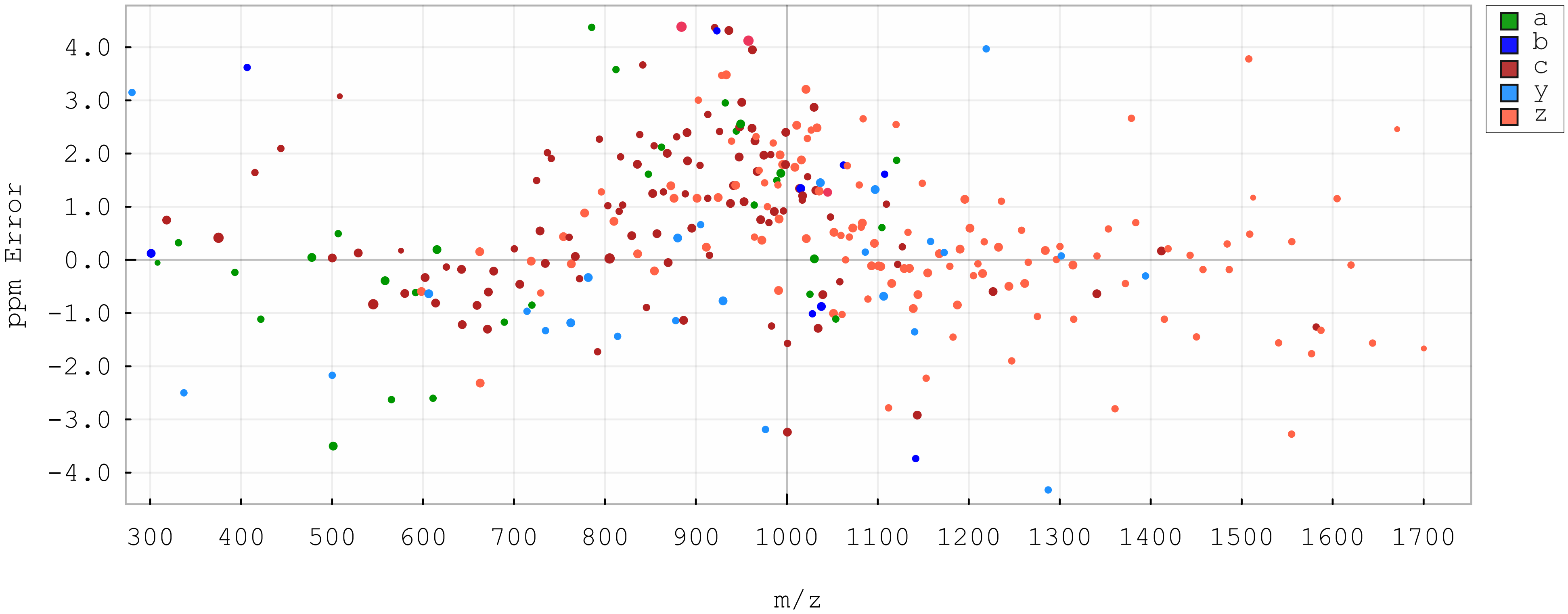

Proteoform:

Index	Protein	SC [%]	SVP [%]	IC [%]	MS Score [a.u.]	Var. Mods	Mono Mass [Da]	Chemical Formula
3	H4Kac	87.1	41.2	53.9	46.9	Ace: K5, K8, K12, K16, K31, K44	11481.4046	C506H849N163O140S

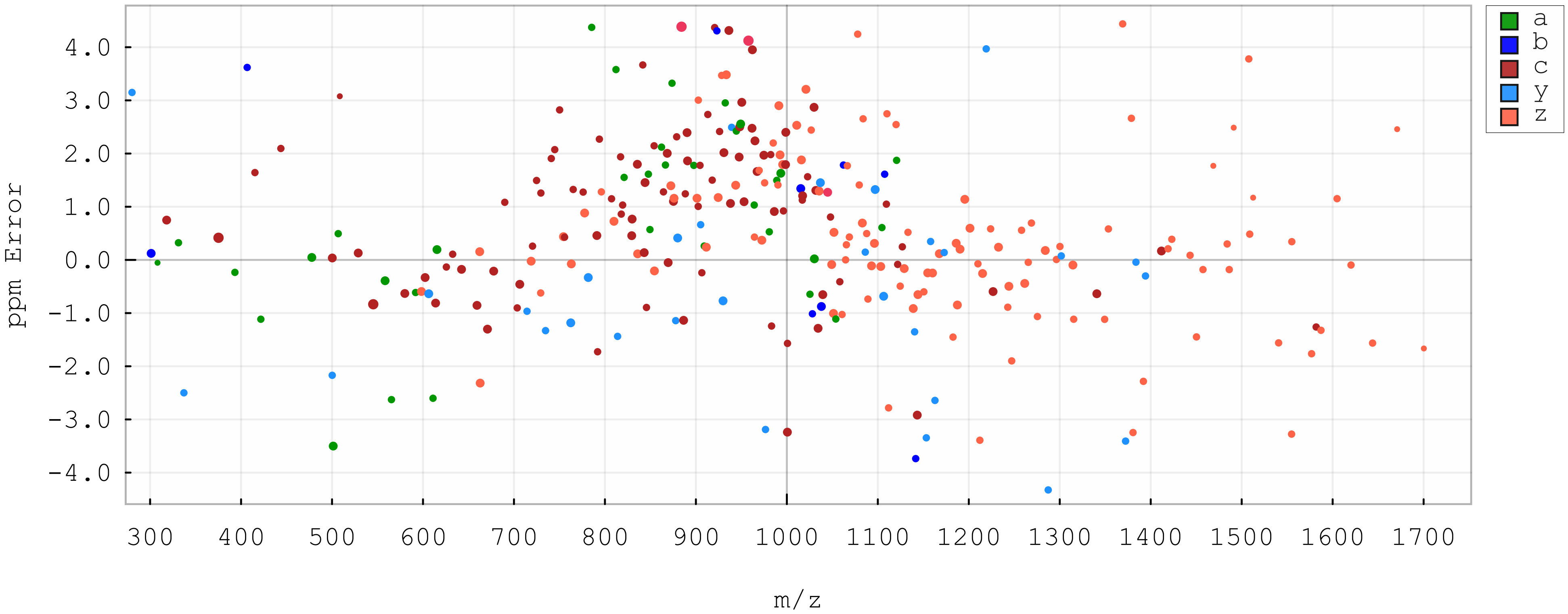

Proteoform:

Index	Protein	SC [%]	SVP [%]	IC [%]	MS Score [a.u.]	Var. Mods	Mono Mass [Da]	Chemical Formula
4	H4Kac	87.1	41.2	52.4	45.6	Ace: K5, K8, K12, K20, K31, K44	11481.4046	C506H849N163O140S

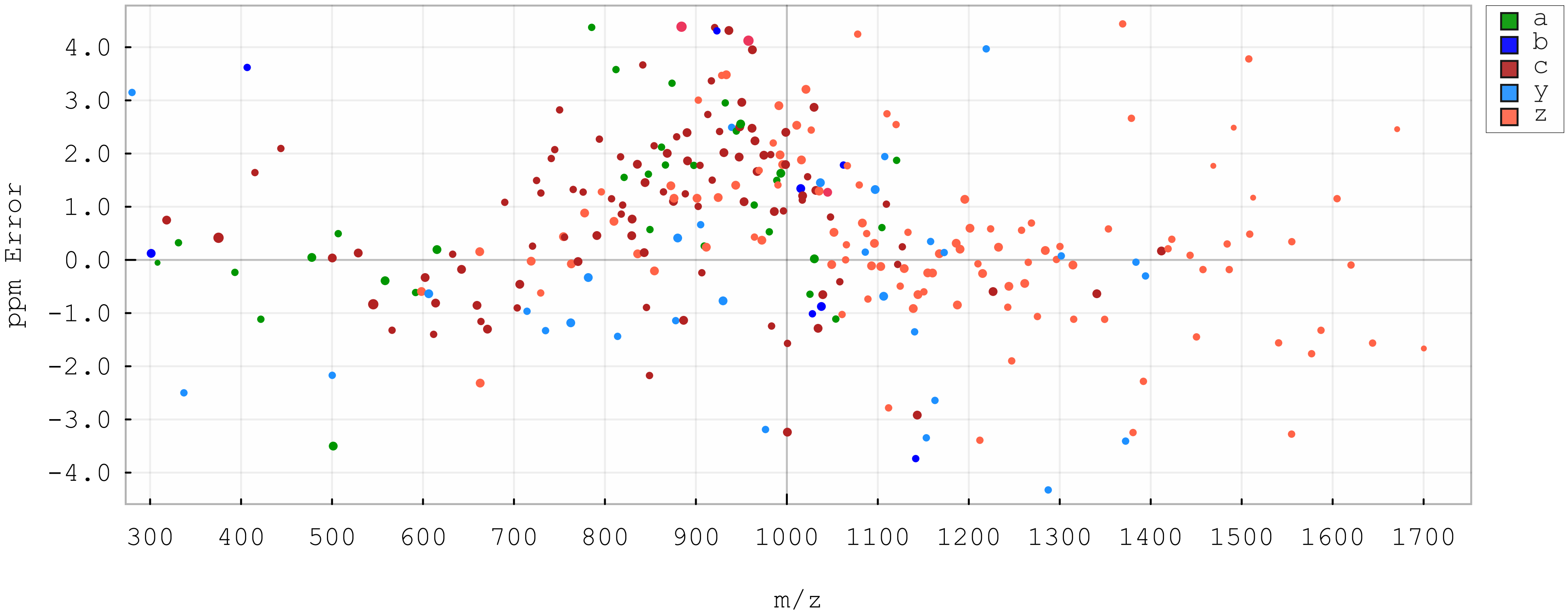

Proteoform:

Index	Protein	SC [%]	SVP [%]	IC [%]	MS Score [a.u.]	Var. Mods	Mono Mass [Da]	Chemical Formula
5	H4Kac	87.1	41.2	51.0	44.5	Ace: K5, K8, K16, K20, K31, K44	11481.4046	C506H849N163O140S

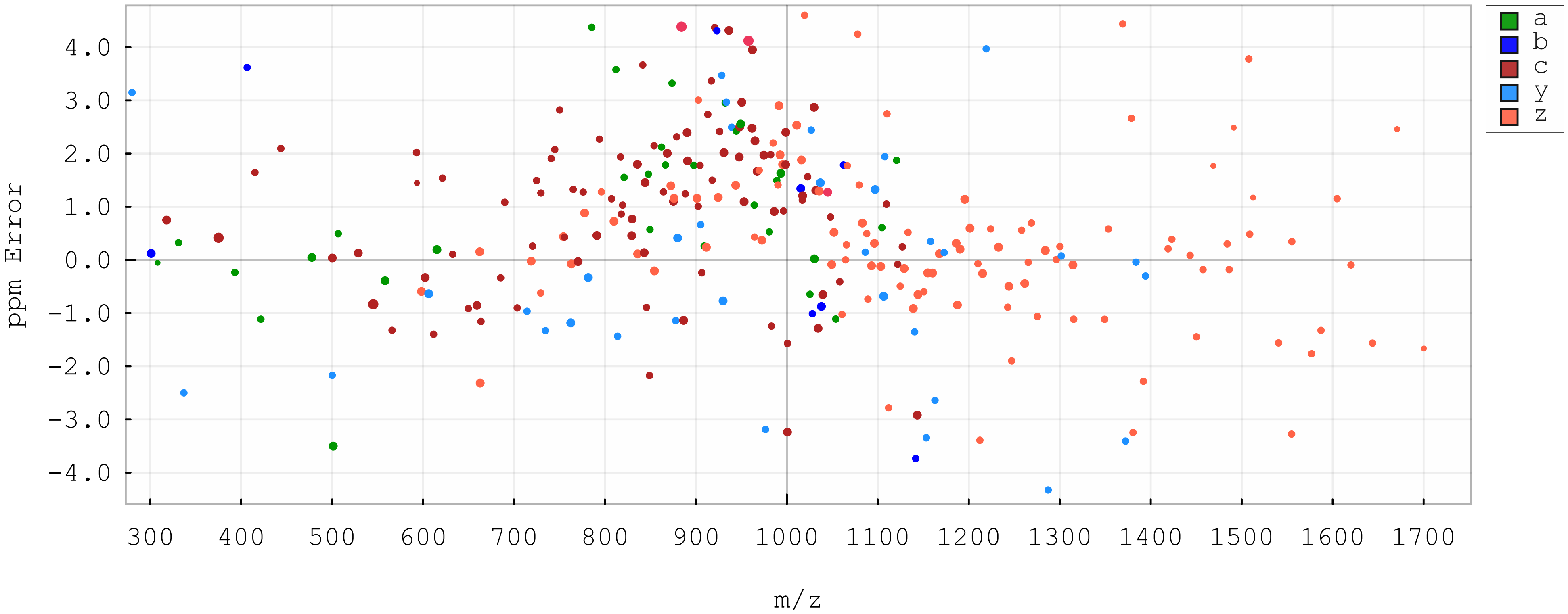

Proteoform:

Index	Protein	SC [%]	SVP [%]	IC [%]	MS Score [a.u.]	Var. Mods	Mono Mass [Da]	Chemical Formula
6	H4Kac	87.1	41.2	50.0	43.6	Ace: K5, K12, K16, K20, K31, K44	11481.4046	C506H849N163O140S

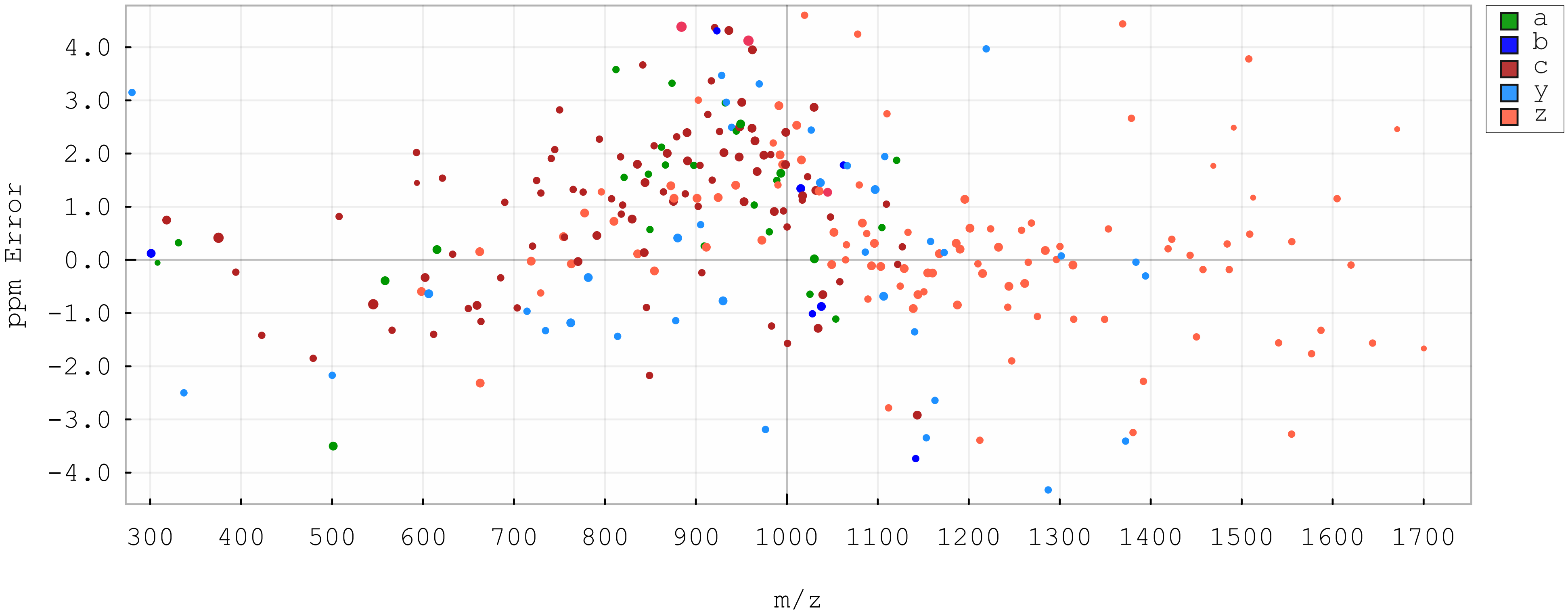

Proteoform:

Index	Protein	SC [%]	SVP [%]	IC [%]	MS Score [a.u.]	Var. Mods	Mono Mass [Da]	Chemical Formula
7	H4Kac	81.2	54.9	53.2	43.2	Ace: K5, K8, K12, K16, K20, K59	11481.4046	C506H849N163O140S

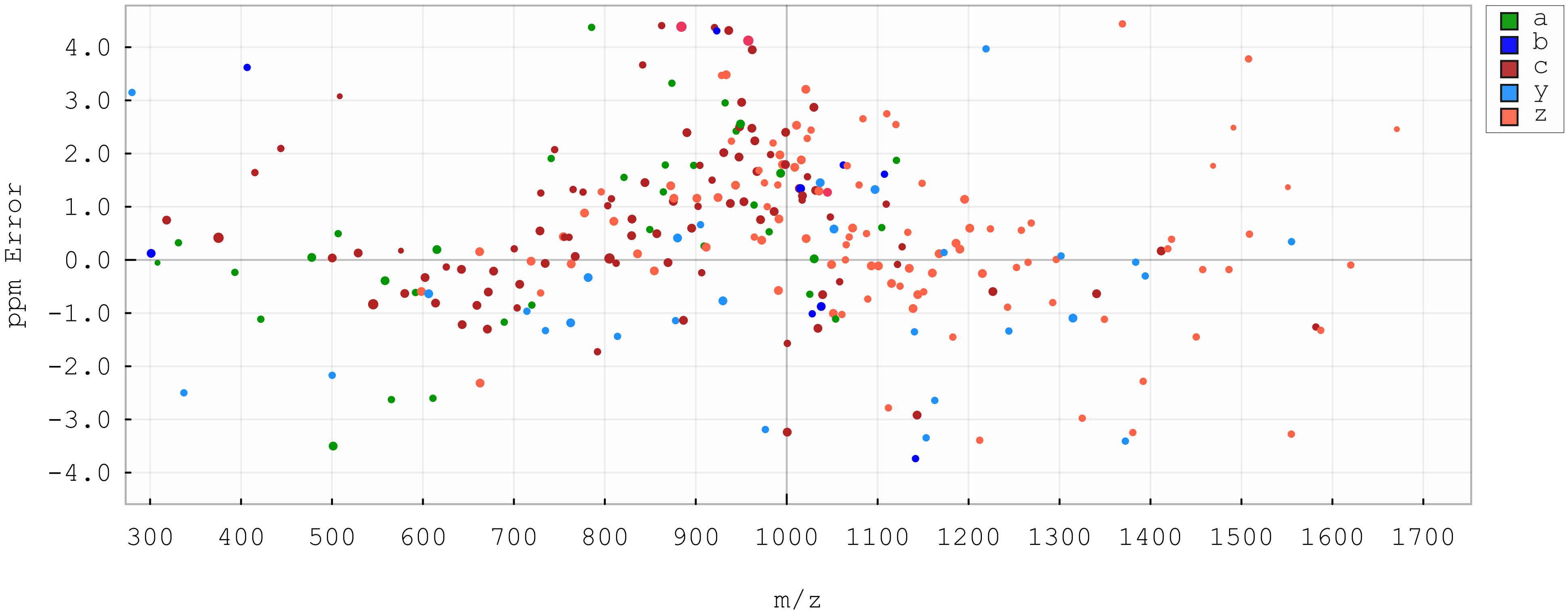

Proteoform:

Index	Protein	SC [%]	SVP [%]	IC [%]	MS Score [a.u.]	Var. Mods	Mono Mass [Da]	Chemical Formula
8	H4Kac	87.1	41.2	48.8	42.5	Ace: K8, K12, K16, K20, K31, K44	11481.4046	C506H849N163O140S

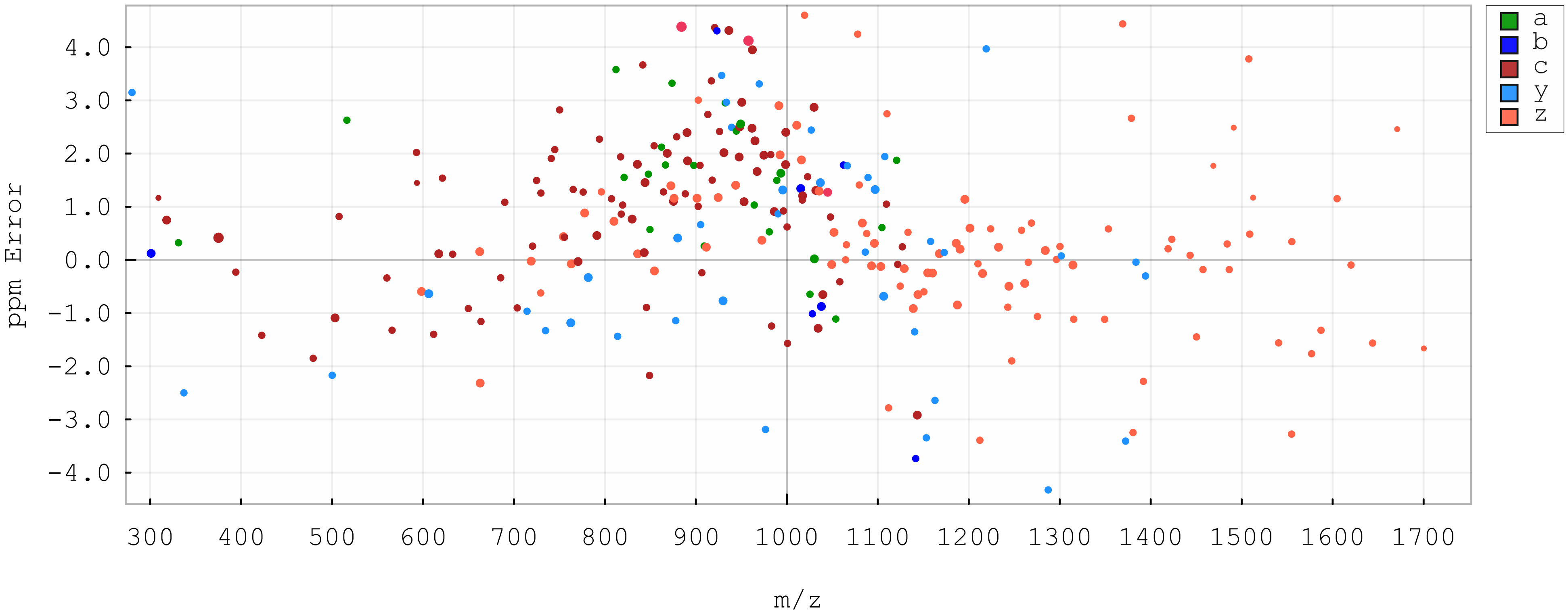

Proteoform:

Index	Protein	SC [%]	SVP [%]	IC [%]	MS Score [a.u.]	Var. Mods	Mono Mass [Da]	Chemical Formula
9	H4Kac	79.2	41.2	48.1	38.1	Ace: K5, K8, K12, K16, K31, K59	11481.4046	C506H849N163O140S

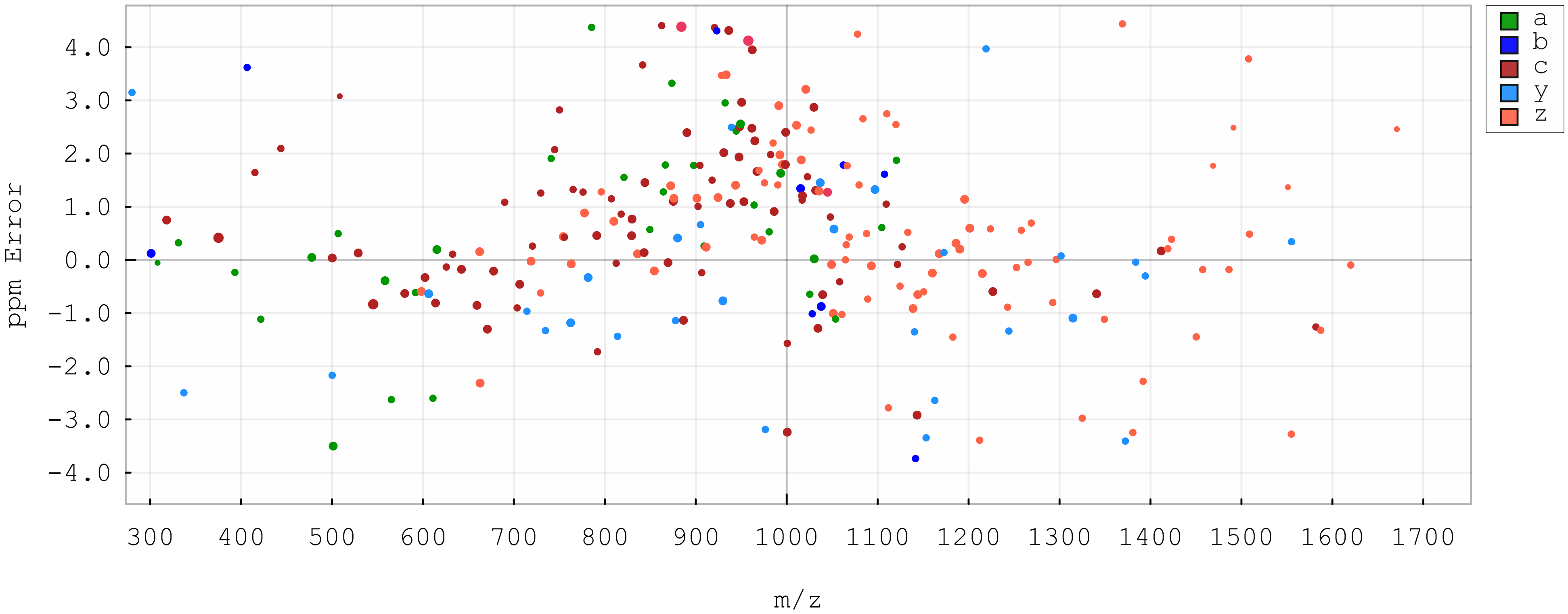

Proteoform:

Index	Protein	SC [%]	SVP [%]	IC [%]	MS Score [a.u.]	Var. Mods	Mono Mass [Da]	Chemical Formula
10	H4Kac	79.2	41.2	46.6	36.9	Ace: K5, K8, K12, K20, K31, K59	11481.4046	C506H849N163O140S

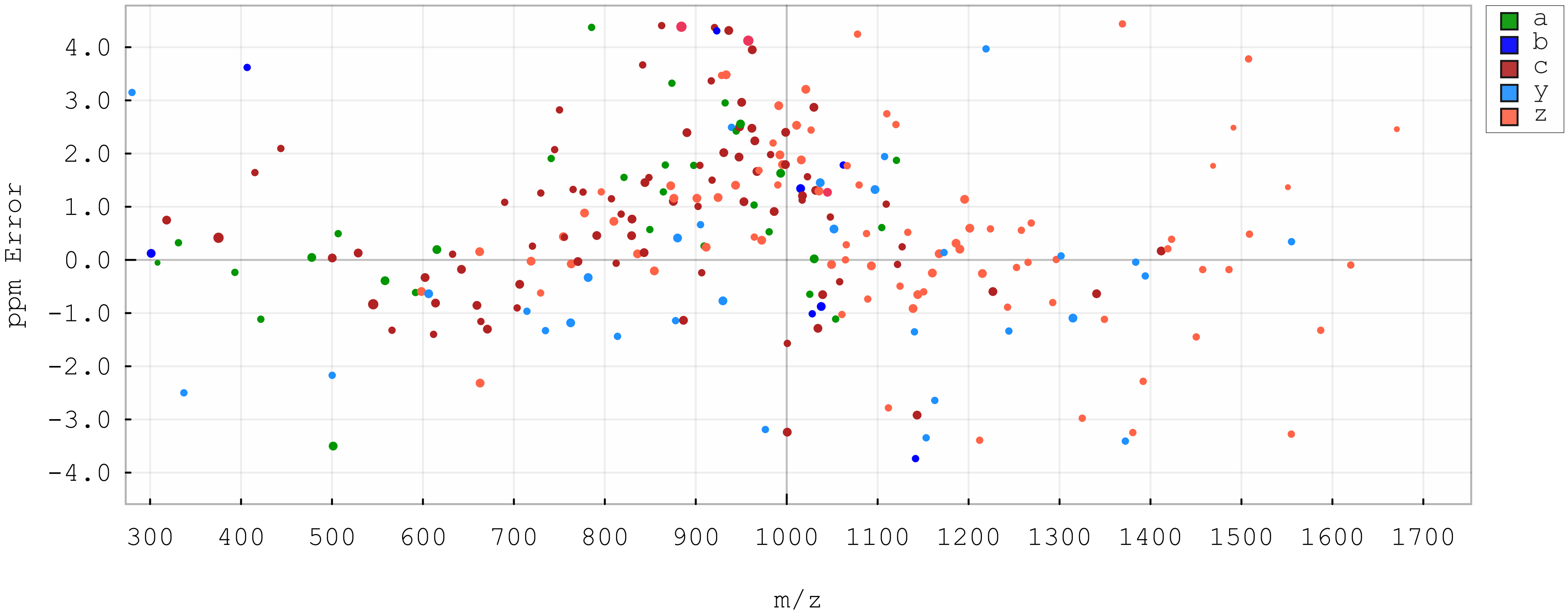

Primary Fragment Plots

Proteoform:

Index	Protein	SC [%]	SVP [%]	IC [%]	MS Score [a.u.]	Var. Mods	Mono Mass [Da]	Chemical Formula
1	H4Kac	89.1	54.9	58.9	52.5	Ace: K5, K8, K12, K16, K20, K44	11481.4046	C506H849N163O140S

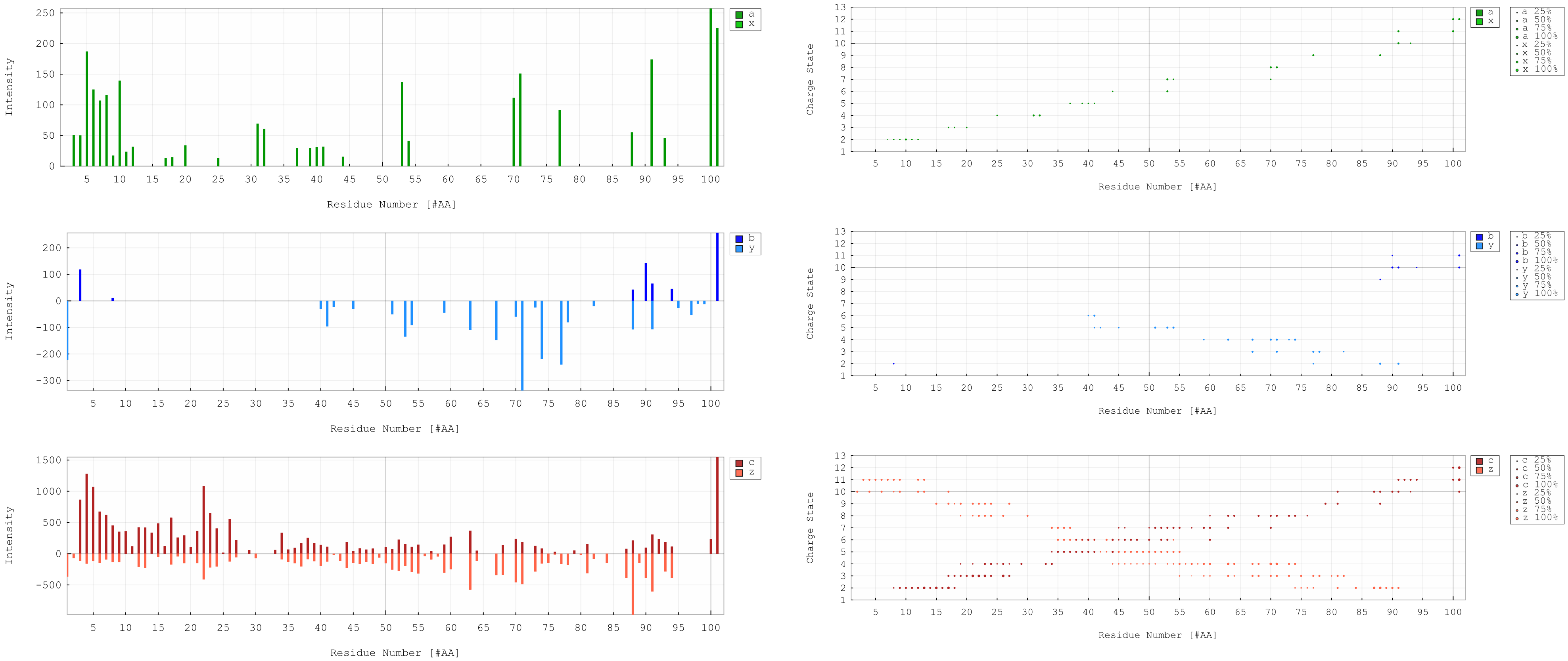

Top-Down Result OmniScape 2025
Sequence Confirmation
250428 H4_P300-50_60min z13ac6 MS2 ECD_061-736_calibrated2

Proteoform:	Index	Protein	SC [%]	SVP [%]	IC [%]	MS Score [a.u.]	Var. Mods	Mono Mass [Da]	Chemical Formula
	2	H4Kac	87.1	43.1	56.4	49.2	Ace: K5, K8, K12, K16, K20, K31	11481.4046	C506H849N163O140S

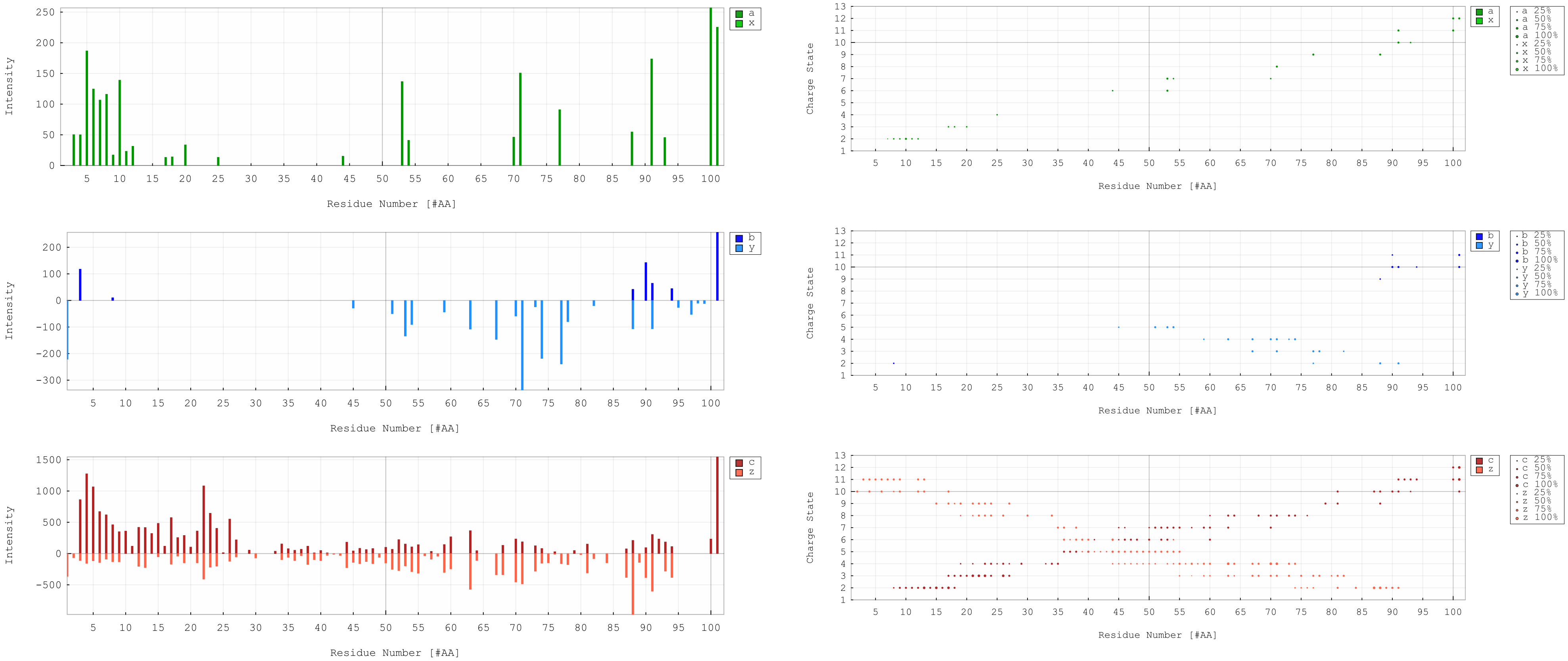

Proteoform:

Index	Protein	SC [%]	SVP [%]	IC [%]	MS Score [a.u.]	Var. Mods	Mono Mass [Da]	Chemical Formula
3	H4Kac	87.1	41.2	53.9	46.9	Ace: K5, K8, K12, K16, K31, K44	11481.4046	C506H849N163O140S

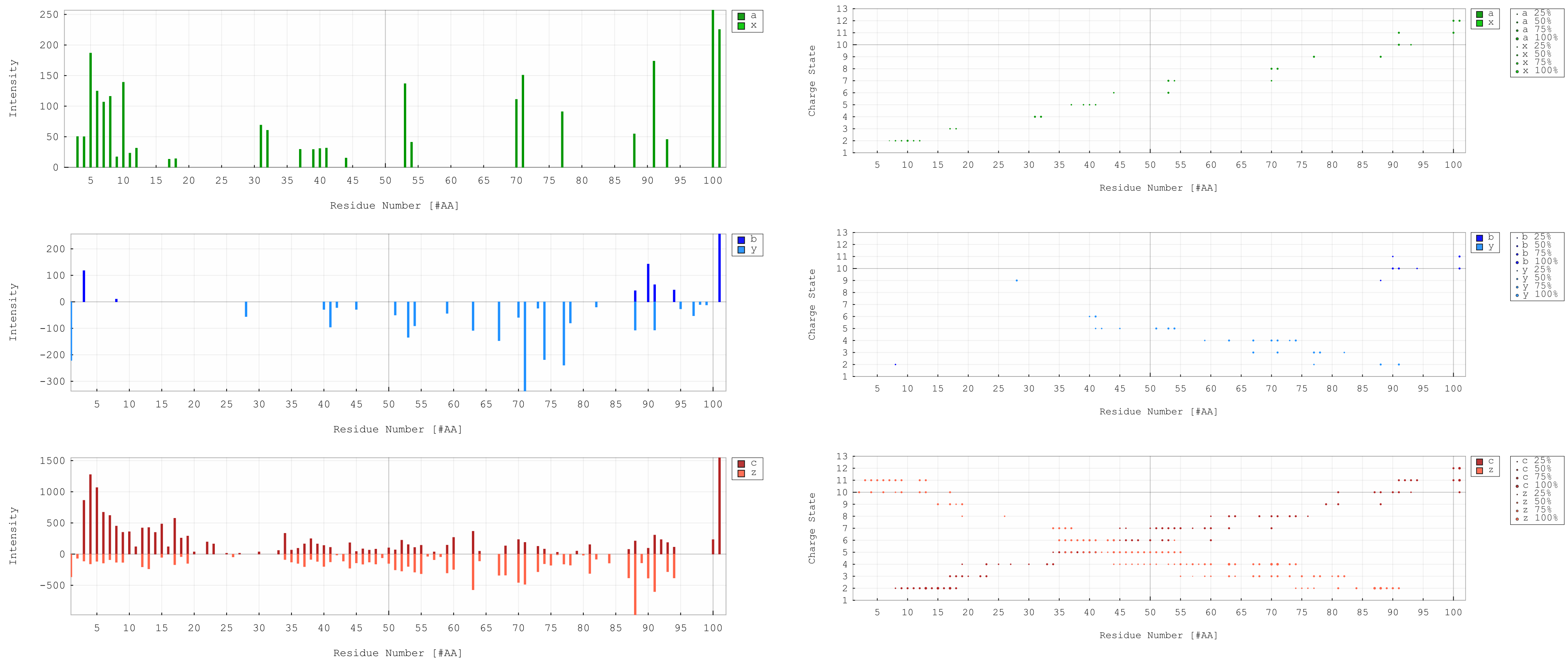

Proteoform:

Index	Protein	SC [%]	SVP [%]	IC [%]	MS Score [a.u.]	Var. Mods	Mono Mass [Da]	Chemical Formula
4	H4Kac	87.1	41.2	52.4	45.6	Ace: K5, K8, K12, K20, K31, K44	11481.4046	C506H849N163O140S

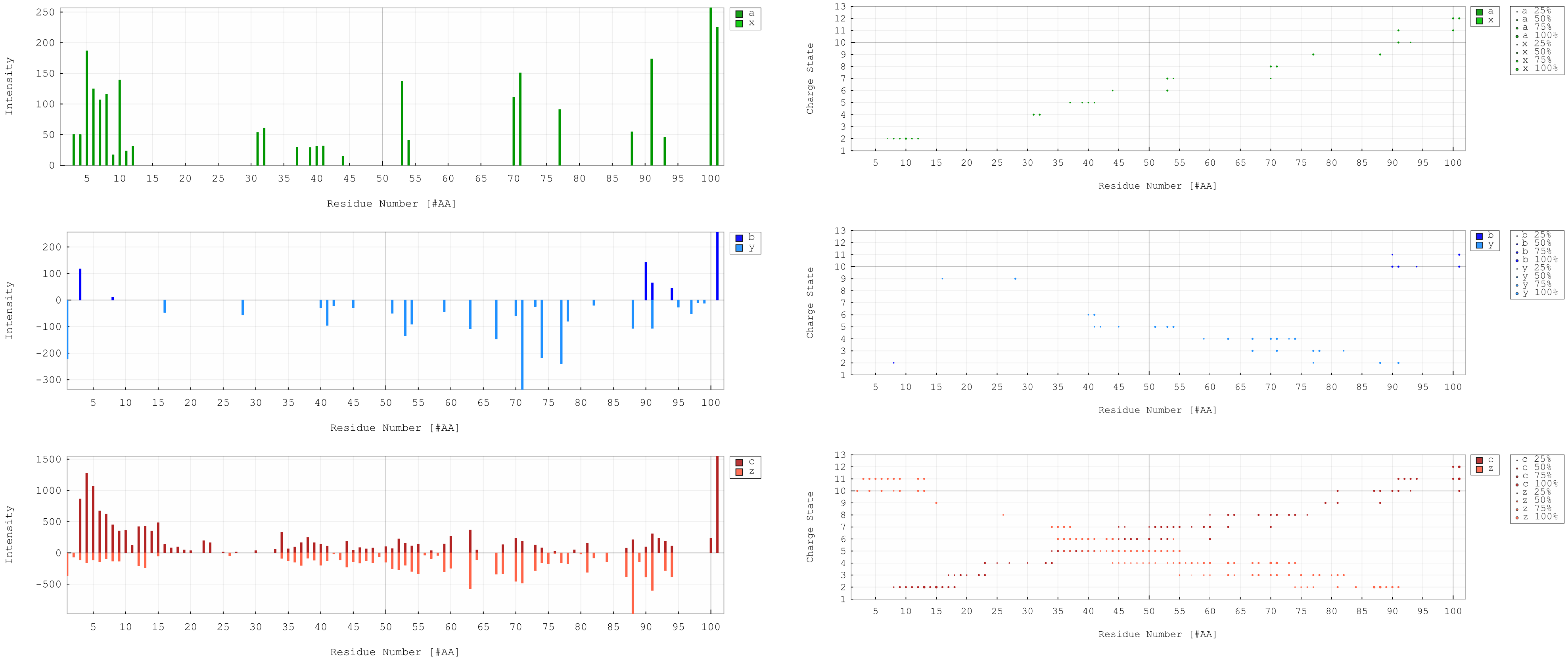

Top-Down Result OmniScape 2025

Sequence Confirmation

250428 H4_P300-50_60min z13ac6 MS2 ECD_061-736_calibrated2

Proteoform:

Index	Protein	SC [%]	SVP [%]	IC [%]	MS Score [a.u.]	Var. Mods	Mono Mass [Da]	Chemical Formula
5	H4Kac	87.1	41.2	51.0	44.5	Ace: K5, K8, K16, K20, K31, K44	11481.4046	C506H849N163O140S

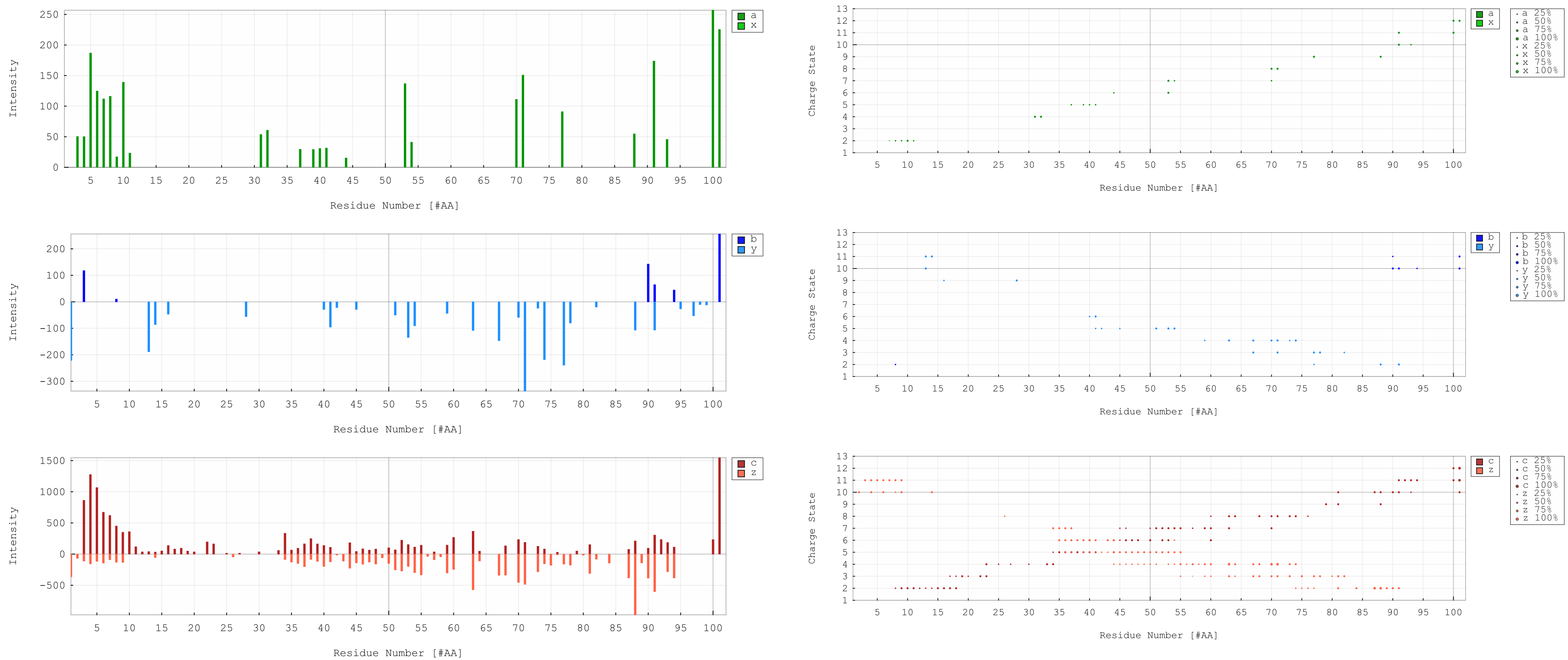

Proteoform:

Index	Protein	SC [%]	SVP [%]	IC [%]	MS Score [a.u.]	Var. Mods	Mono Mass [Da]	Chemical Formula
6	H4Kac	87.1	41.2	50.0	43.6	Ace: K5, K12, K16, K20, K31, K44	11481.4046	C506H849N163O140S

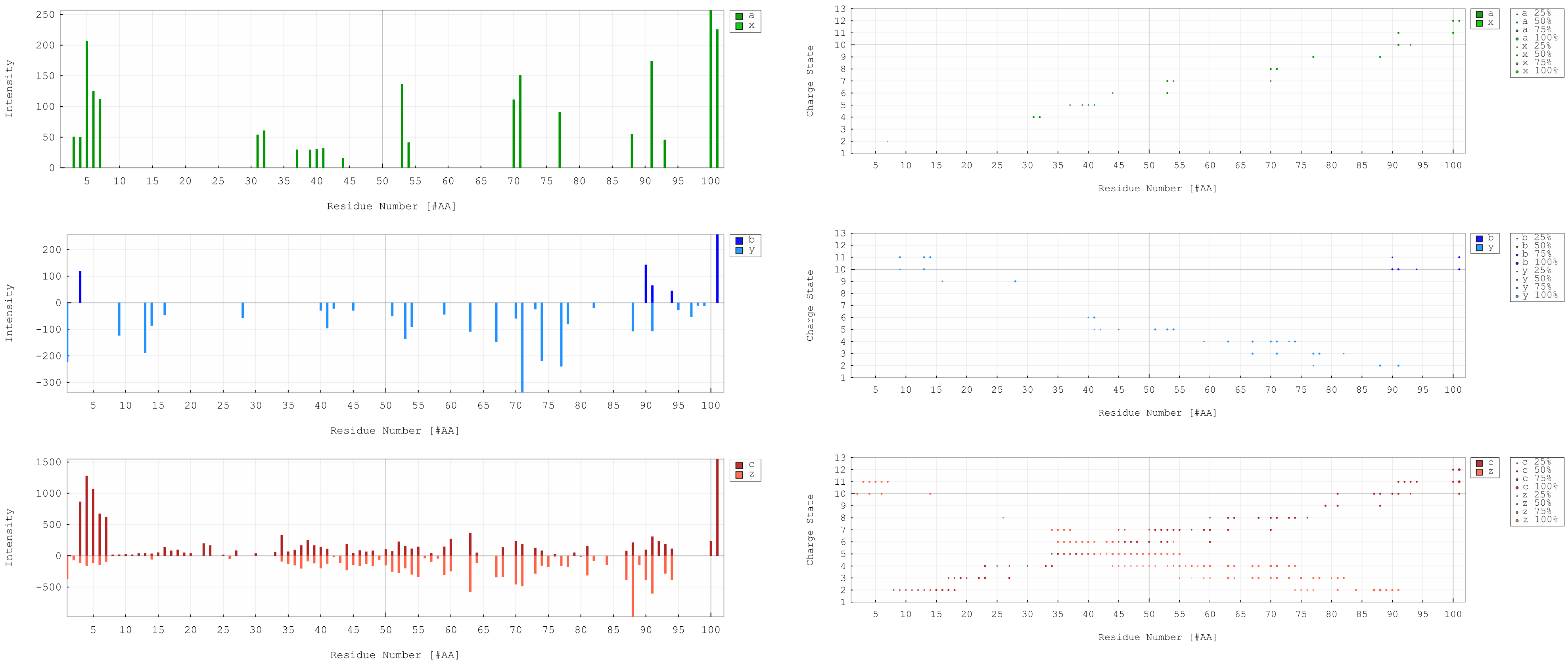

Proteoform:

Index	Protein	SC [%]	SVP [%]	IC [%]	MS Score [a.u.]	Var. Mods	Mono Mass [Da]	Chemical Formula
7	H4Kac	81.2	54.9	53.2	43.2	Ace: K5, K8, K12, K16, K20, K59	11481.4046	C506H849N163O140S

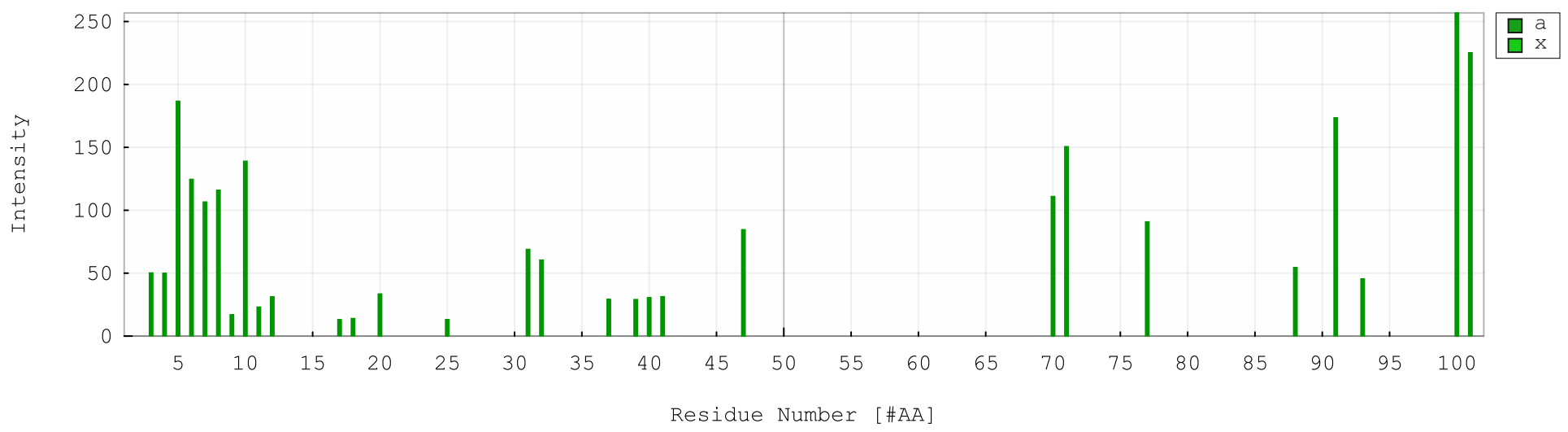

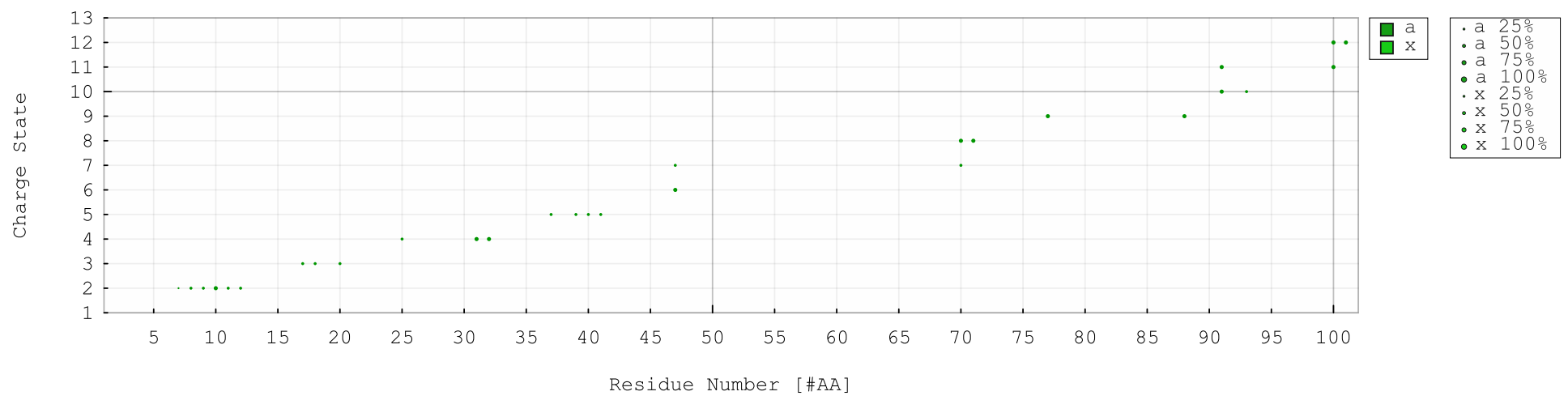

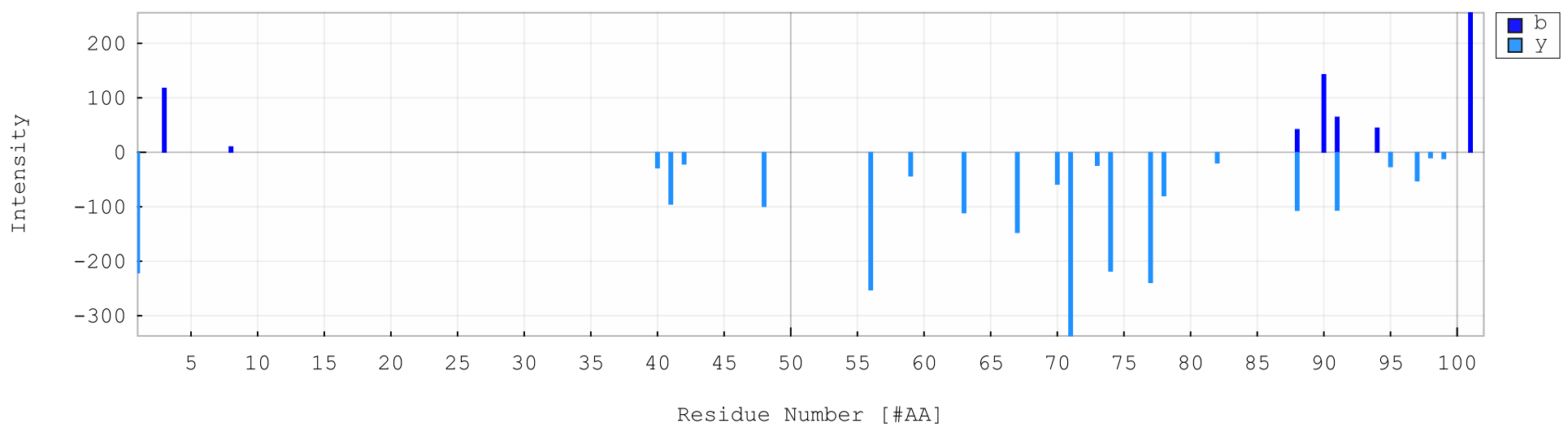

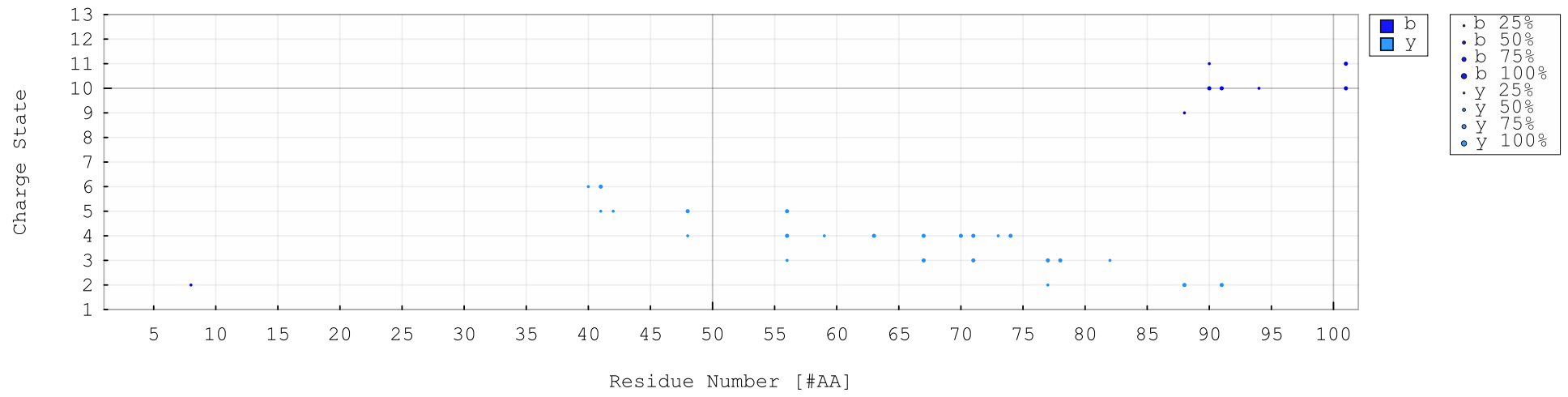

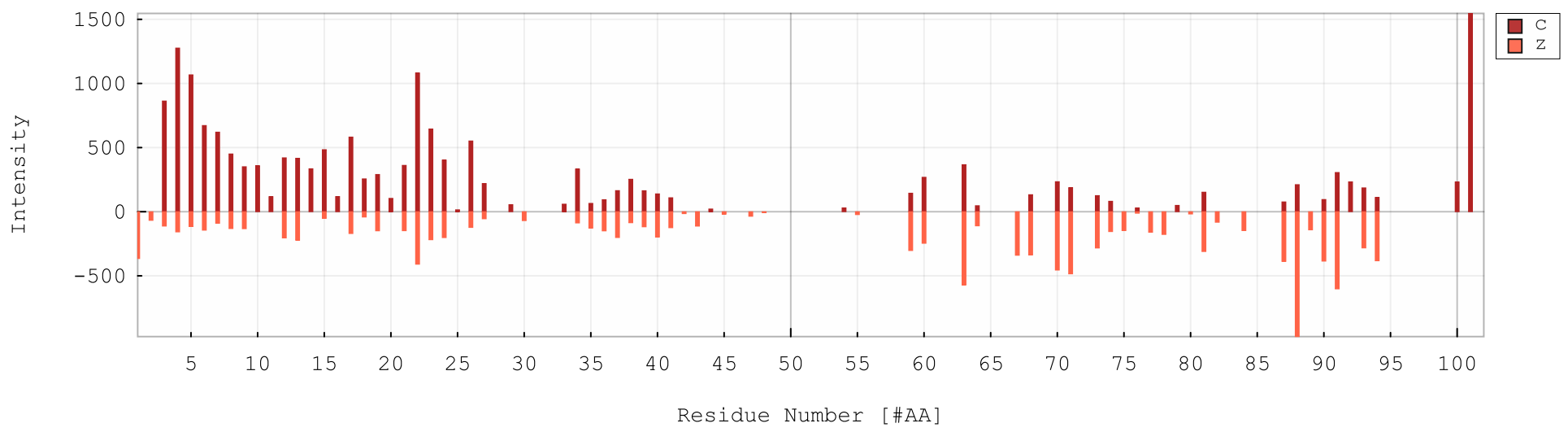

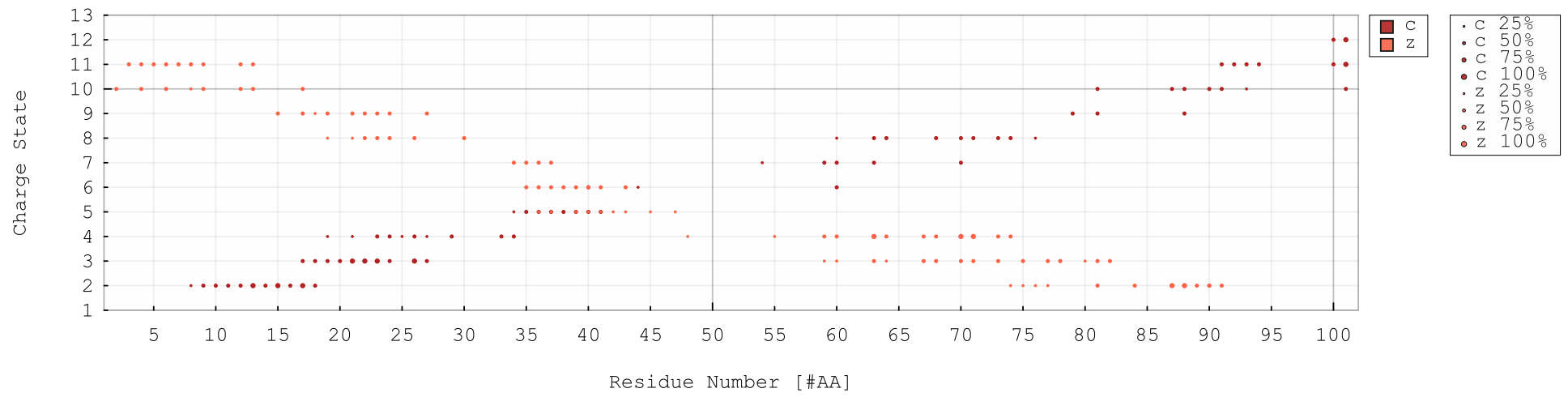

Proteoform:

Index	Protein	SC [%]	SVP [%]	IC [%]	MS Score [a.u.]	Var. Mods	Mono Mass [Da]	Chemical Formula
8	H4Kac	87.1	41.2	48.8	42.5	Ace: K8, K12, K16, K20, K31, K44	11481.4046	C506H849N163O140S

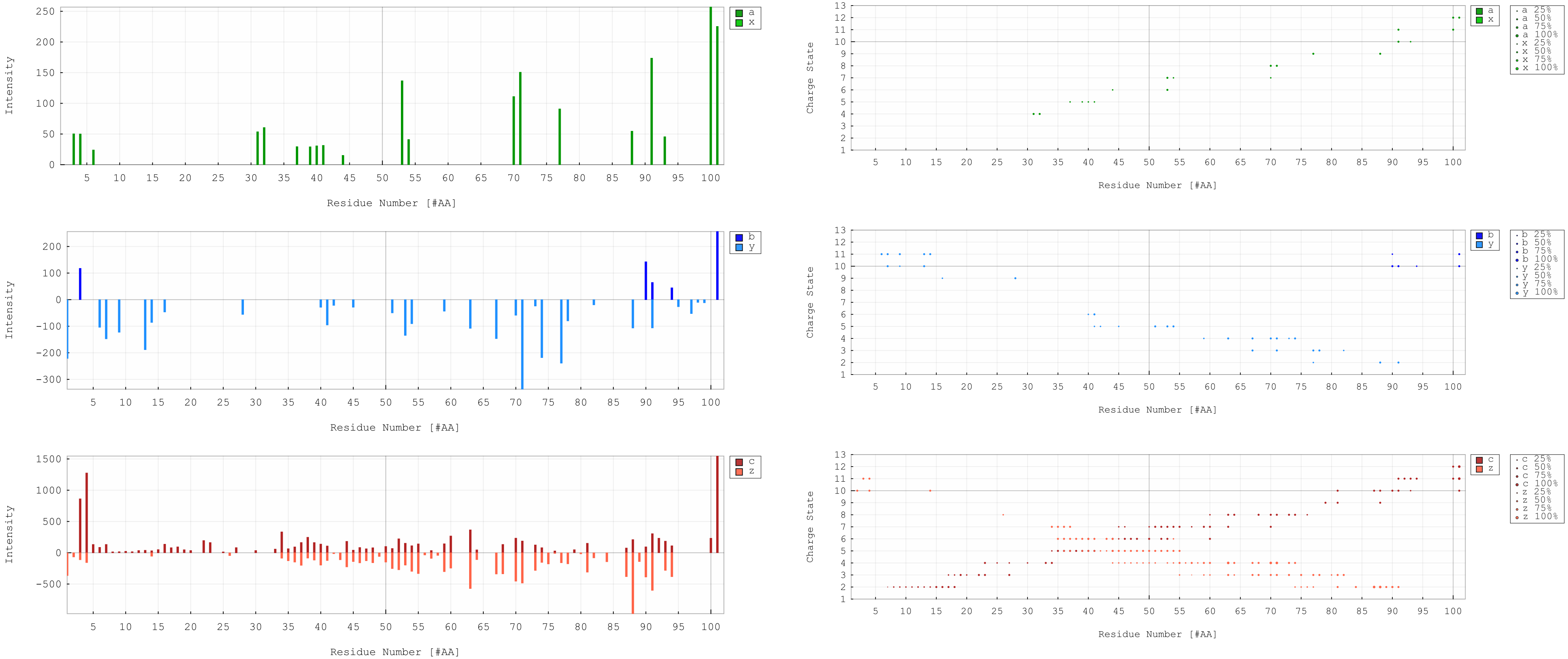

Top-Down Result OmniScape 2025
Sequence Confirmation
250428 H4_P300-50_60min z13ac6 MS2 ECD_061-736_calibrated2

Proteoform:

Index	Protein	SC [%]	SVP [%]	IC [%]	MS Score [a.u.]	Var. Mods	Mono Mass [Da]	Chemical Formula
9	H4Kac	79.2	41.2	48.1	38.1	Ace: K5, K8, K12, K16, K31, K59	11481.4046	C506H849N163O140S

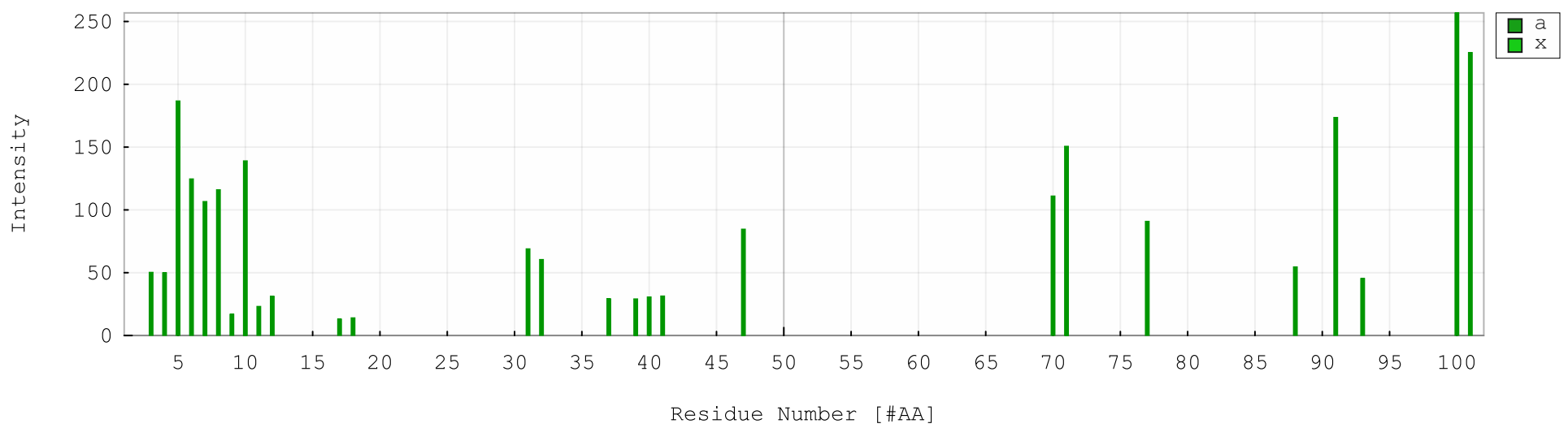

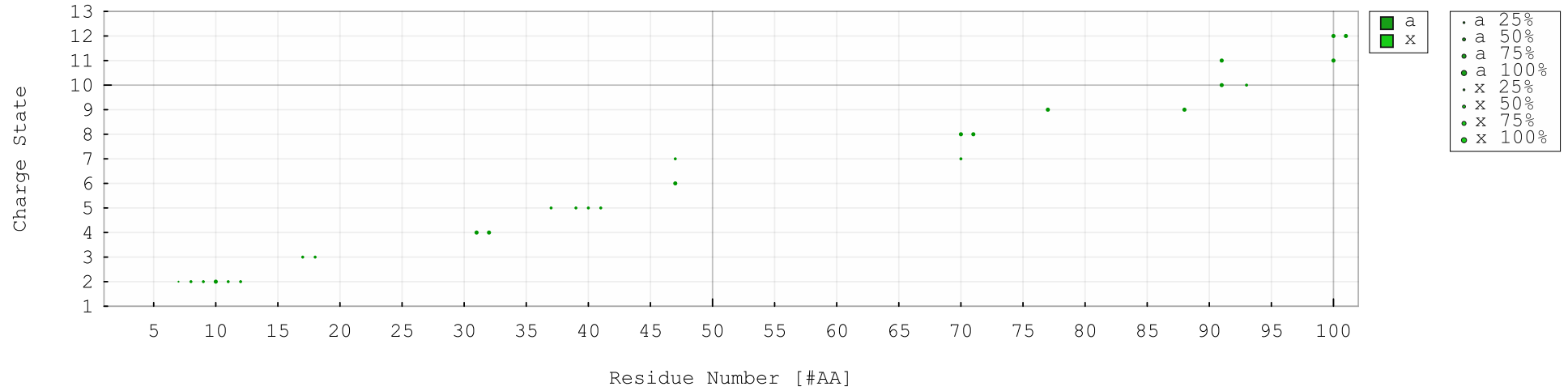

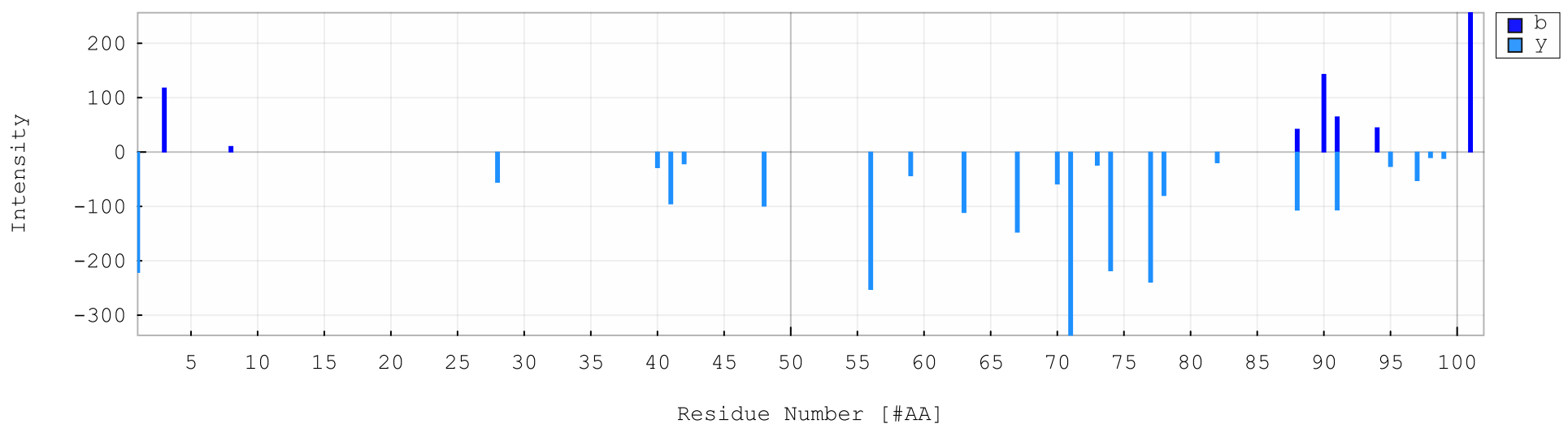

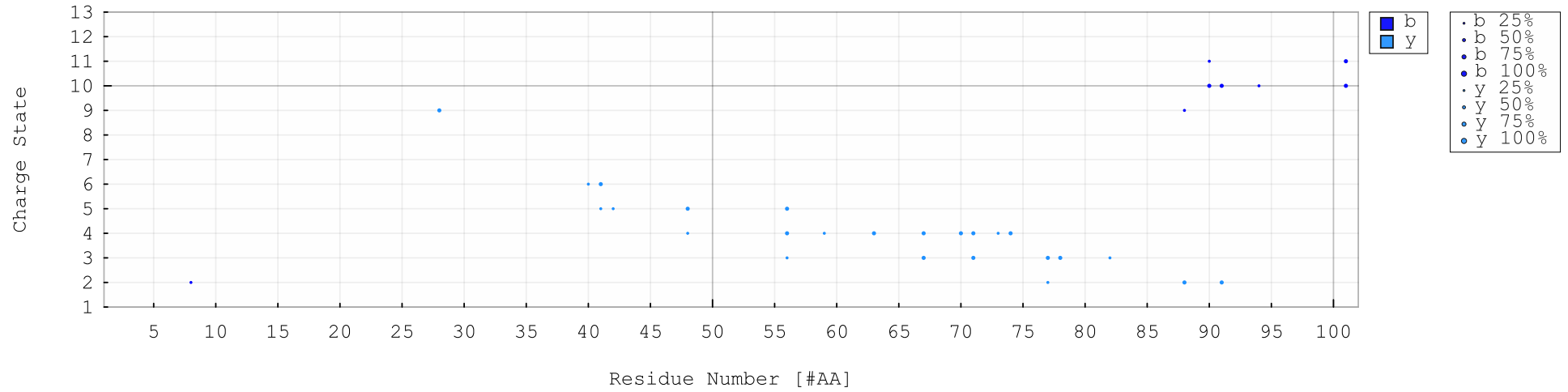

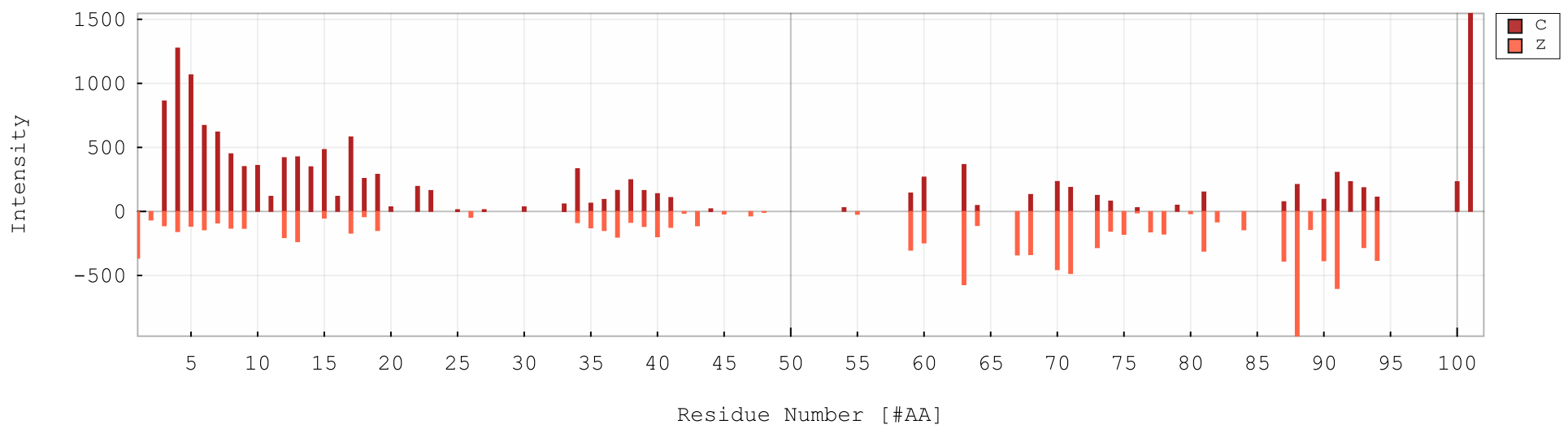

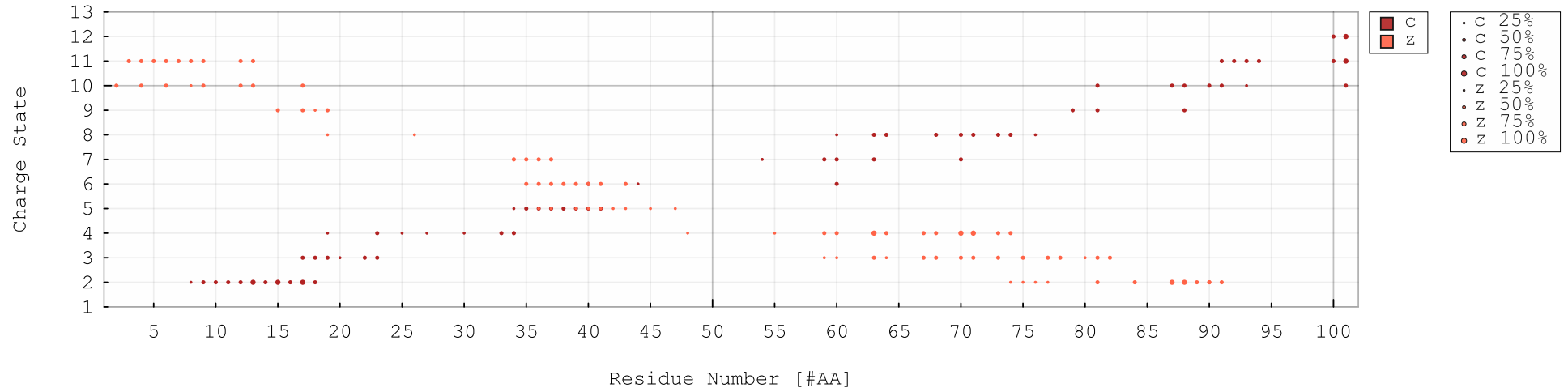

Proteoform:

Index	Protein	SC [%]	SVP [%]	IC [%]	MS Score [a.u.]	Var. Mods	Mono Mass [Da]	Chemical Formula
10	H4Kac	79.2	41.2	46.6	36.9	Ace: K5, K8, K12, K20, K31, K59	11481.4046	C506H849N163O140S

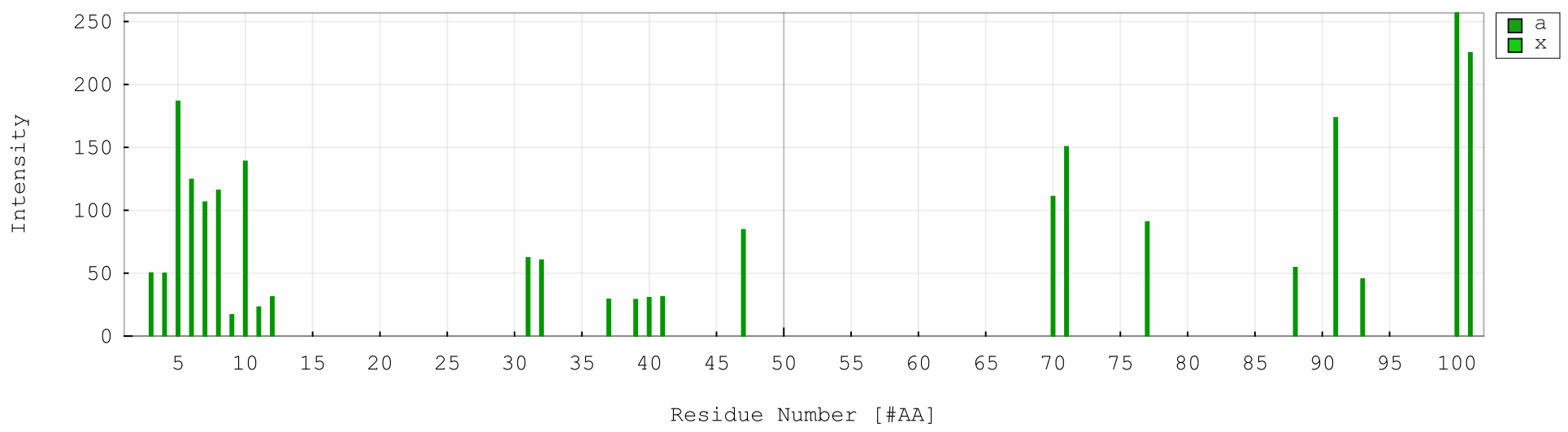

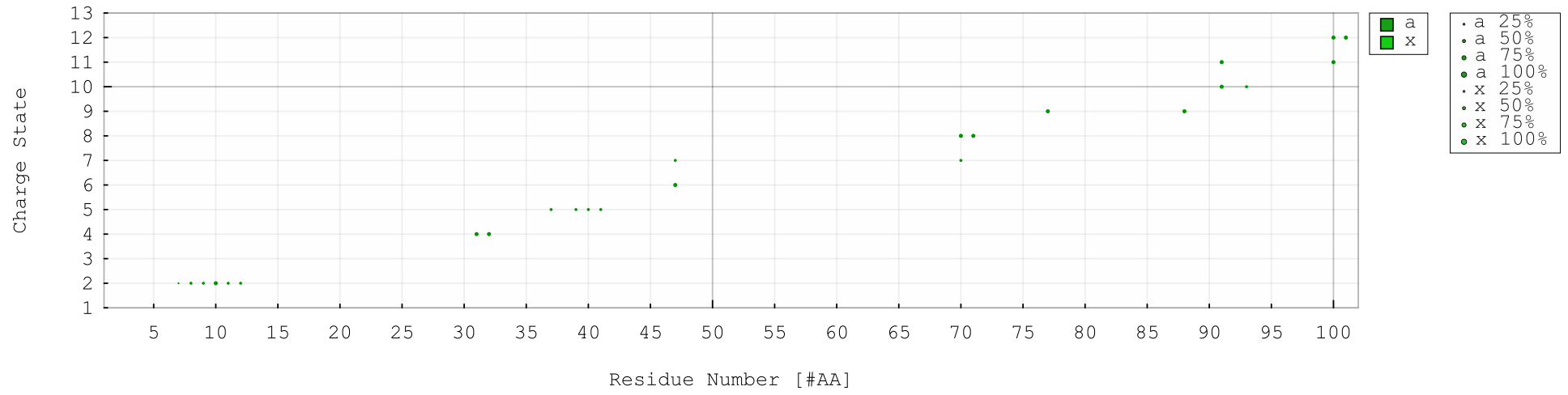

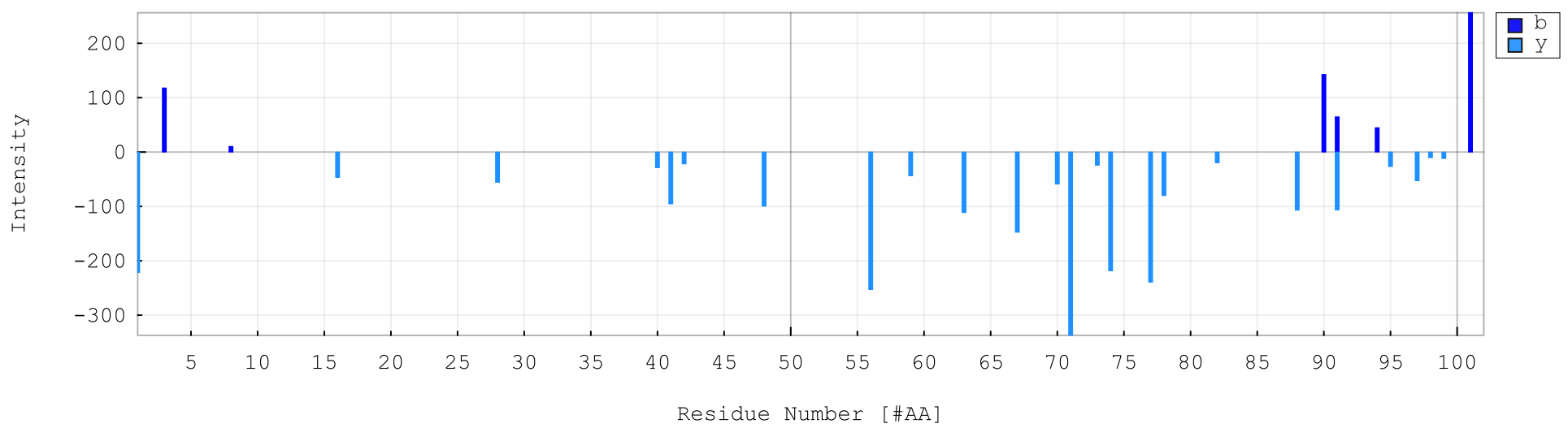

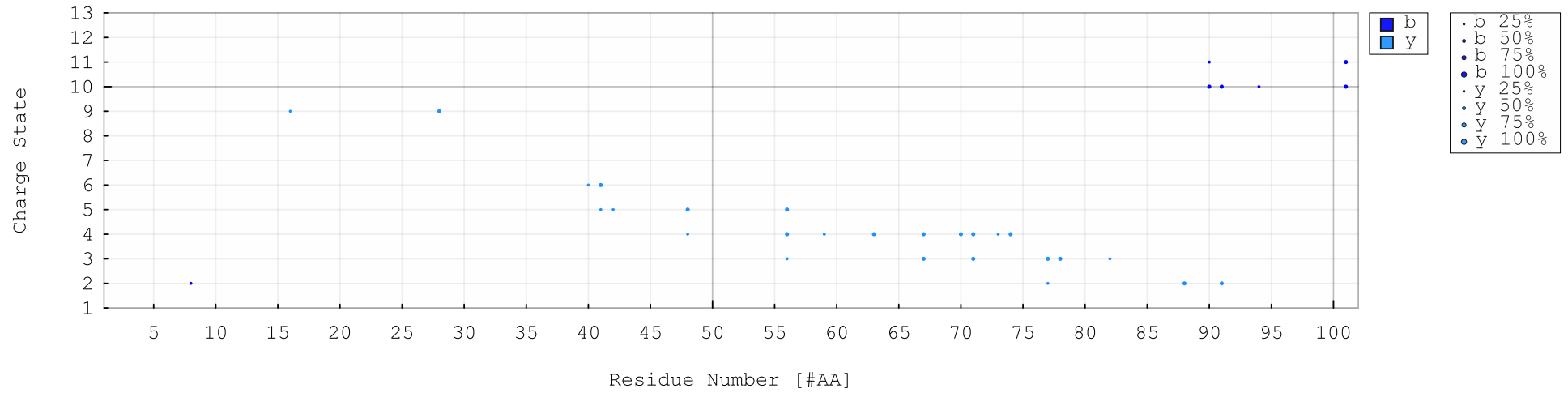

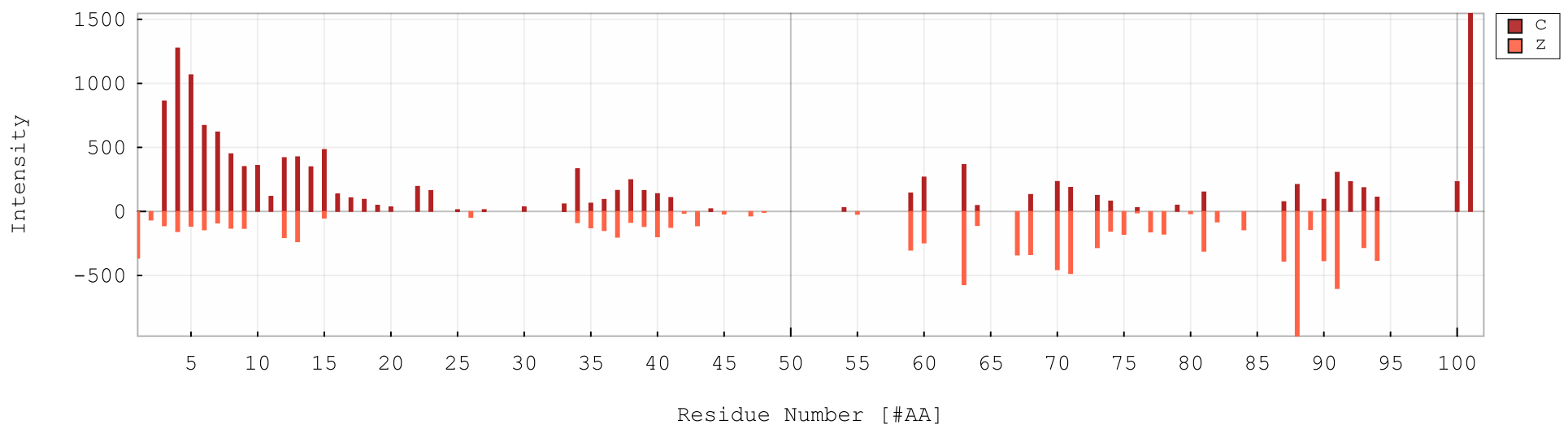

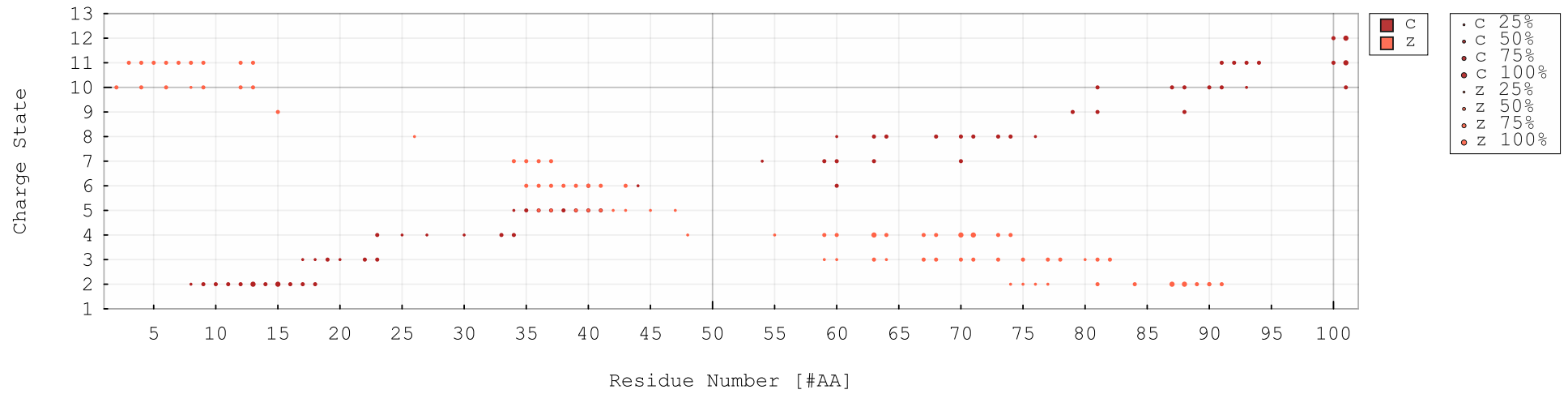

Supplemental Data S4

H4 - p300, 60 min

**Report**	3 of 7
**Experiment**	MS2
**Histone/sample**	H4
**Enzyme**	p300
**Reaction/source**	60 min
**Precursor**	H4Kac6
**Charge state**	13+
**Fragmentation / workflow**	EID
**Original file**	MS2_H4Kac6_13+_EID.pdf

Project Information

Project Directory: C:/Users/LAB-ADMIN
Processing date: Tue Apr 28 12:57:36 2026

Sample Info & Protocols

Name: Top-Down Protein Sequencing EID

Dataset

Imported spectrum file name:	250428 H4_P300-50_60min z13ac6 MS2 EID_001-598_calibrated.txt
Dataset name:	Information is only available from imported JPEAKS files
Date acquired:	Information is only available from imported JPEAKS files
Date imported:	Information is only available from imported JPEAKS files
Instrument Name:	Information is only available from imported JPEAKS files
Instrument Serial Number:	Information is only available from imported JPEAKS files

Workflow Result Info

Analysis: 250428 H4_P300-50_60min z13ac6 MS2 EID_001-598_calibrated
Location: C:/Users/LAB-ADMIN
Method: -

Method Information

Name: Unnamed

Spectral Parameters

Mass Error [ppm]: 6
Intensity Deviation [au]: 0.15
Min/Max Charges: 1-13
Experiment type: EID

a ions	b ions	c ions	x ions	y ions	z ions	satellite	internal	precursor
a	b	c	x	y	z			M
a+1			x+1	y-1	z+1			M+-1H

Noise threshold calculation: Simple moving average noise threshold (SMA)
Window size [datapoints]: 600
Noise curve offset: 2.591177
SVP parameters: terminal gap: 2, internal gap: 1

Proteoforms

(top 10)

Index	Parent Sequence	Chain	Var. Mods	Mono Mass [Da]	Chemical Composition
1	H4Kac	H4KacK5[Ace]K8[Ace]K12[Ace]K16[Ace]K20[Ace]K44[Ace]	Ace: K5, K8, K12, K16, K20, K44	11481.4046	C506H849N163O140S
2	H4Kac	H4KacK5[Ace]K8[Ace]K12[Ace]K16[Ace]K20[Ace]K31[Ace]	Ace: K5, K8, K12, K16, K20, K31	11481.4046	C506H849N163O140S
3	H4Kac	H4KacK5[Ace]K8[Ace]K12[Ace]K16[Ace]K20[Ace]K59[Ace]	Ace: K5, K8, K12, K16, K20, K59	11481.4046	C506H849N163O140S
4	H4Kac	H4KacK5[Ace]K8[Ace]K12[Ace]K16[Ace]K31[Ace]K44[Ace]	Ace: K5, K8, K12, K16, K31, K44	11481.4046	C506H849N163O140S
5	H4Kac	H4KacK5[Ace]K8[Ace]K12[Ace]K20[Ace]K31[Ace]K44[Ace]	Ace: K5, K8, K12, K20, K31, K44	11481.4046	C506H849N163O140S
6	H4Kac	H4KacK5[Ace]K8[Ace]K16[Ace]K20[Ace]K31[Ace]K44[Ace]	Ace: K5, K8, K16, K20, K31, K44	11481.4046	C506H849N163O140S
7	H4Kac	H4KacK5[Ace]K8[Ace]K12[Ace]K16[Ace]K31[Ace]K59[Ace]	Ace: K5, K8, K12, K16, K31, K59	11481.4046	C506H849N163O140S
8	H4Kac	H4KacK5[Ace]K8[Ace]K12[Ace]K20[Ace]K31[Ace]K59[Ace]	Ace: K5, K8, K12, K20, K31, K59	11481.4046	C506H849N163O140S
9	H4Kac	H4KacK5[Ace]K12[Ace]K16[Ace]K20[Ace]K31[Ace]K44[Ace]	Ace: K5, K12, K16, K20, K31, K44	11481.4046	C506H849N163O140S
10	H4Kac	H4KacK8[Ace]K12[Ace]K16[Ace]K20[Ace]K31[Ace]K44[Ace]	Ace: K8, K12, K16, K20, K31, K44	11481.4046	C506H849N163O140S

Results

Proteoforms Table (top 10)

Index	Protein	SC [%]	SVP [%]	IC [%]	MS Score [a.u.]	Var. Mods	Mono Mass [Da]	Chemical Formula
1	H4Kac	90.1	50.0	39.2	35.3	Ace: K5, K8, K12, K16, K20, K44	11481.4046	C506H849N163O140S
2	H4Kac	89.1	35.3	37.6	33.5	Ace: K5, K8, K12, K16, K20, K31	11481.4046	C506H849N163O140S
3	H4Kac	88.1	47.1	35.6	31.3	Ace: K5, K8, K12, K16, K20, K59	11481.4046	C506H849N163O140S
4	H4Kac	87.1	30.4	34.8	30.3	Ace: K5, K8, K12, K16, K31, K44	11481.4046	C506H849N163O140S
5	H4Kac	85.1	20.6	33.7	28.7	Ace: K5, K8, K12, K20, K31, K44	11481.4046	C506H849N163O140S
6	H4Kac	81.2	15.7	32.7	26.5	Ace: K5, K8, K16, K20, K31, K44	11481.4046	C506H849N163O140S
7	H4Kac	85.1	30.4	31.1	26.5	Ace: K5, K8, K12, K16, K31, K59	11481.4046	C506H849N163O140S
8	H4Kac	83.2	20.6	30.1	25.0	Ace: K5, K8, K12, K20, K31, K59	11481.4046	C506H849N163O140S
9	H4Kac	80.2	11.8	31.1	25.0	Ace: K5, K12, K16, K20, K31, K44	11481.4046	C506H849N163O140S
10	H4Kac	80.2	11.8	30.3	24.3	Ace: K8, K12, K16, K20, K31, K44	11481.4046	C506H849N163O140S

Sequence

Name: H4Kac

```
                  10                    20                    30                    40                    50                    60
S G R G K G G K G L   G K G G A K R H R K   V L R D N I Q G I T   K P A I R R L A R R   G G V K R I S G L I   Y E E T R G V L K V
                  70                    80                    90                   100                   110                   120
F L E N V I R D A V   T Y T E H A K R K T   V T A M D V V Y A L   K R Q G R T L Y G F   G G
```

Modifications

Modification	Position	Type
Acetyl	5	variable
Acetyl	8	variable
Acetyl	12	variable
Acetyl	16	variable
Acetyl	20	variable
Acetyl	31	variable
Acetyl	44	variable
Acetyl	59	variable
Acetyl	77	variable

Sequence Map

Proteoform:

Index	Protein	SC [%]	SVP [%]	IC [%]	MS Score [a.u.]	Var. Mods	Mono Mass [Da]	Chemical Formula
1	H4Kac	90.1	50.0	39.2	35.3	Ace: K5, K8, K12, K16, K20, K44	11481.4046	C506H849N163O140S

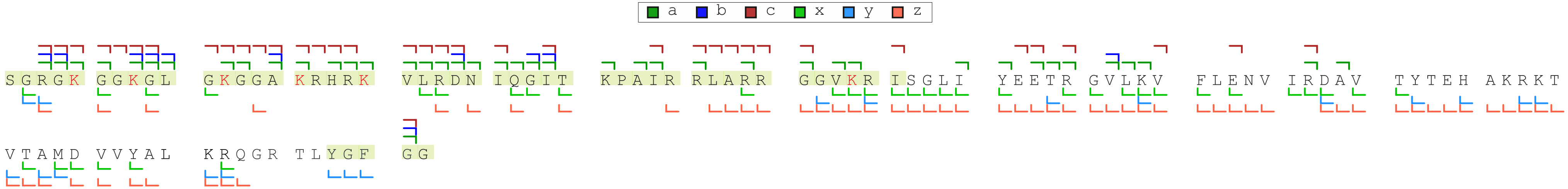

Proteoform:

Index	Protein	SC [%]	SVP [%]	IC [%]	MS Score [a.u.]	Var. Mods	Mono Mass [Da]	Chemical Formula
2	H4Kac	89.1	35.3	37.6	33.5	Ace: K5, K8, K12, K16, K20, K31	11481.4046	C506H849N163O140S

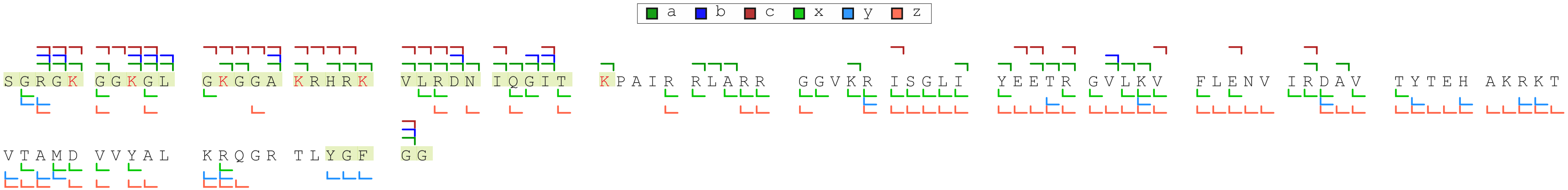

Proteoform:	Index	Protein	SC [%]	SVP [%]	IC [%]	MS Score [a.u.]	Var. Mods	Mono Mass [Da]	Chemical Formula
	3	H4Kac	88.1	47.1	35.6	31.3	Ace: K5, K8, K12, K16, K20, K59	11481.4046	C506H849N163O140S

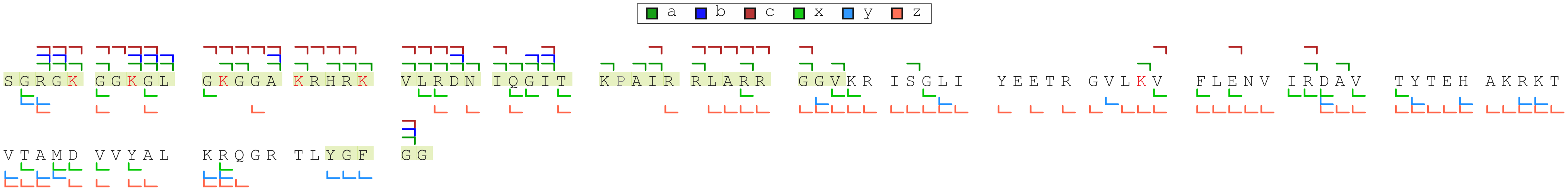

Proteoform:

Index	Protein	SC [%]	SVP [%]	IC [%]	MS Score [a.u.]	Var. Mods	Mono Mass [Da]	Chemical Formula
4	H4Kac	87.1	30.4	34.8	30.3	Ace: K5, K8, K12, K16, K31, K44	11481.4046	C506H849N163O140S

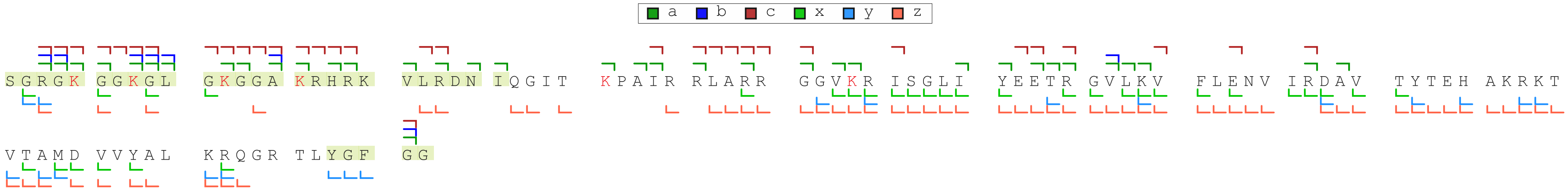

Proteoform:

Index	Protein	SC [%]	SVP [%]	IC [%]	MS Score [a.u.]	Var. Mods	Mono Mass [Da]	Chemical Formula
5	H4Kac	85.1	20.6	33.7	28.7	Ace: K5, K8, K12, K20, K31, K44	11481.4046	C506H849N163O140S

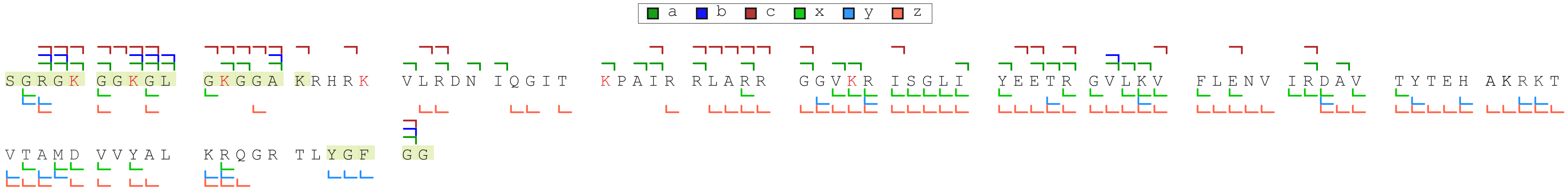

Proteoform:	Index	Protein	SC [%]	SVP [%]	IC [%]	MS Score [a.u.]	Var. Mods	Mono Mass [Da]	Chemical Formula
	6	H4Kac	81.2	15.7	32.7	26.5	Ace: K5, K8, K16, K20, K31, K44	11481.4046	C506H849N163O140S

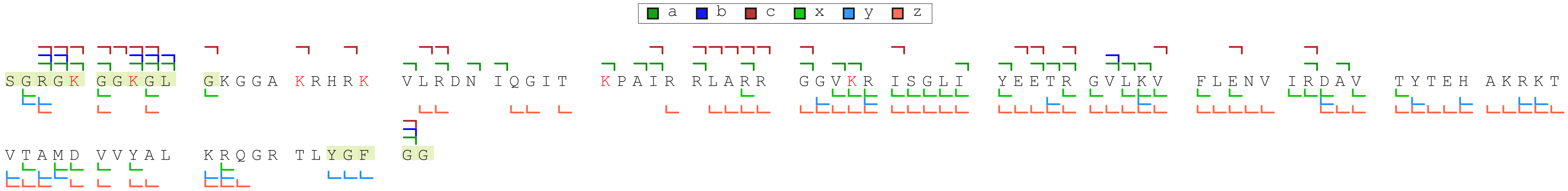

Proteoform:

Index	Protein	SC [%]	SVP [%]	IC [%]	MS Score [a.u.]	Var. Mods	Mono Mass [Da]	Chemical Formula
7	H4Kac	85.1	30.4	31.1	26.5	Ace: K5, K8, K12, K16, K31, K59	11481.4046	C506H849N163O140S

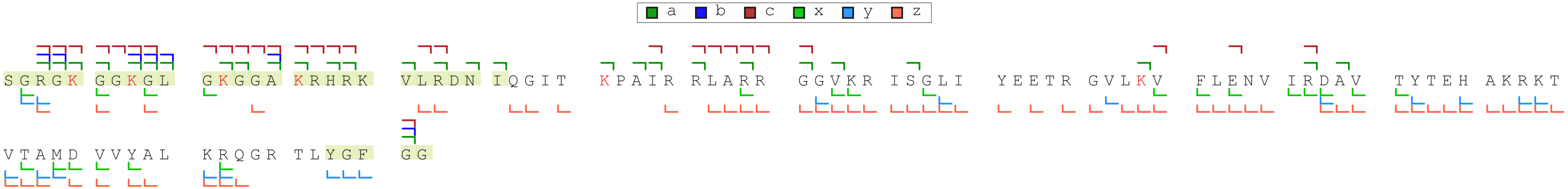

Proteoform:	Index	Protein	SC [%]	SVP [%]	IC [%]	MS Score [a.u.]	Var. Mods	Mono Mass [Da]	Chemical Formula
	8	H4Kac	83.2	20.6	30.1	25.0	Ace: K5, K8, K12, K20, K31, K59	11481.4046	C506H849N163O140S

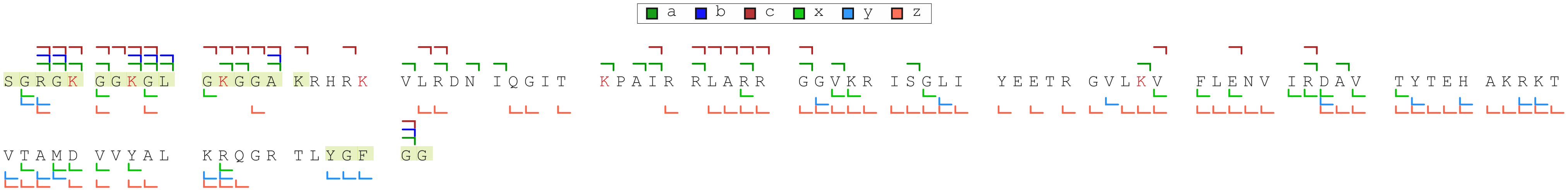

Proteoform:	Index	Protein	SC [%]	SVP [%]	IC [%]	MS Score [a.u.]	Var. Mods	Mono Mass [Da]	Chemical Formula
	9	H4Kac	80.2	11.8	31.1	25.0	Ace: K5, K12, K16, K20, K31, K44	11481.4046	C506H849N163O140S

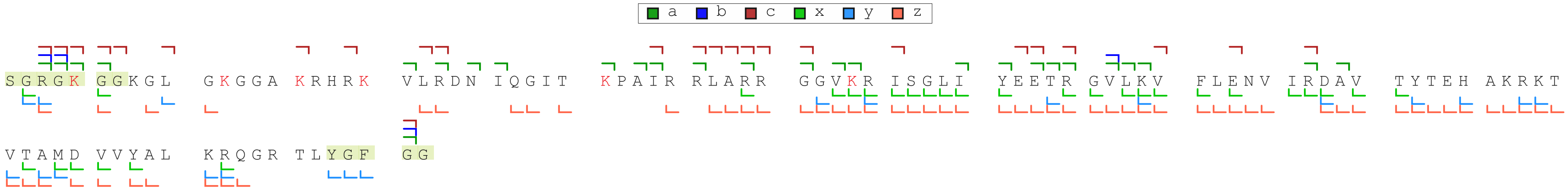

Proteoform:	Index	Protein	SC [%]	SVP [%]	IC [%]	MS Score [a.u.]	Var. Mods	Mono Mass [Da]	Chemical Formula
	10	H4Kac	80.2	11.8	30.3	24.3	Ace: K8, K12, K16, K20, K31, K44	11481.4046	C506H849N163O140S

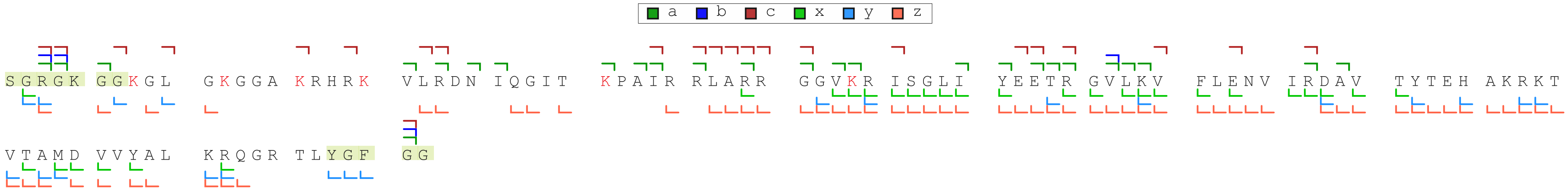

Error Plot

Proteoform:

Index	Protein	SC [%]	SVP [%]	IC [%]	MS Score [a.u.]	Var. Mods	Mono Mass [Da]	Chemical Formula
1	H4Kac	90.1	50.0	39.2	35.3	Ace: K5, K8, K12, K16, K20, K44	11481.4046	C506H849N163O140S

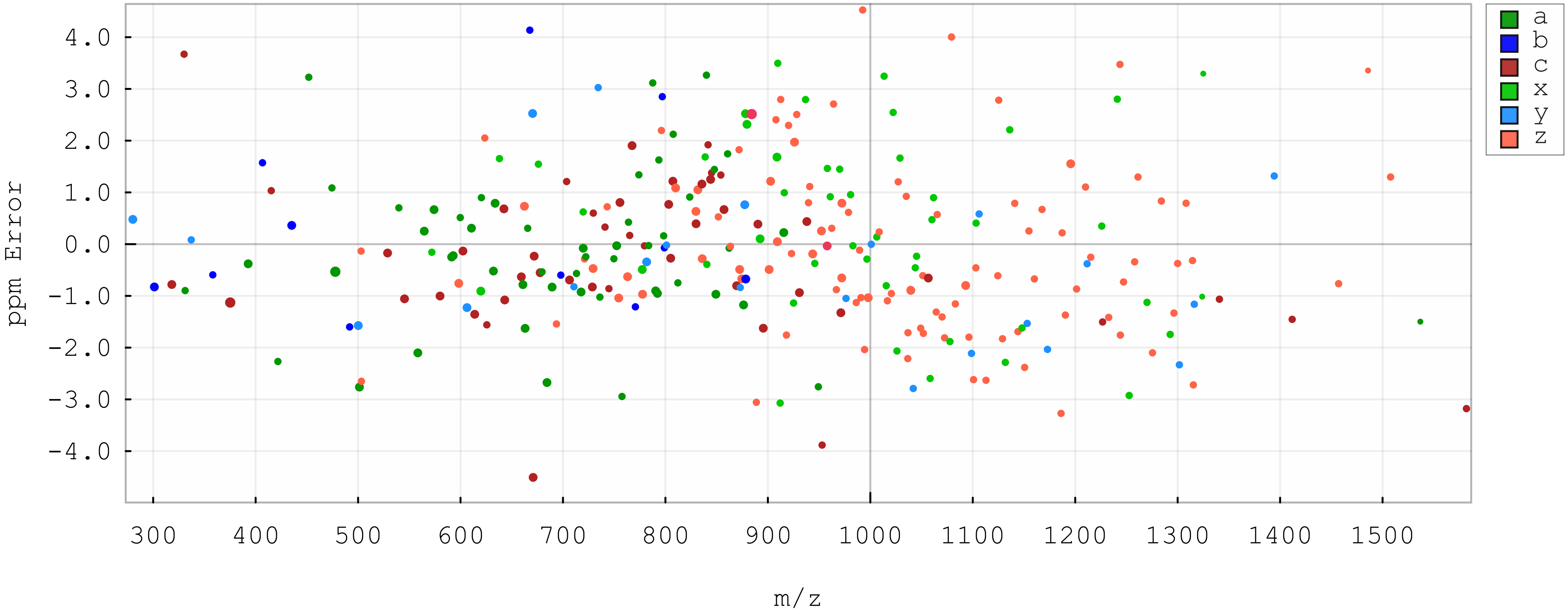

Proteoform:

Index	Protein	SC [%]	SVP [%]	IC [%]	MS Score [a.u.]	Var. Mods	Mono Mass [Da]	Chemical Formula
2	H4Kac	89.1	35.3	37.6	33.5	Ace: K5, K8, K12, K16, K20, K31	11481.4046	C506H849N163O140S

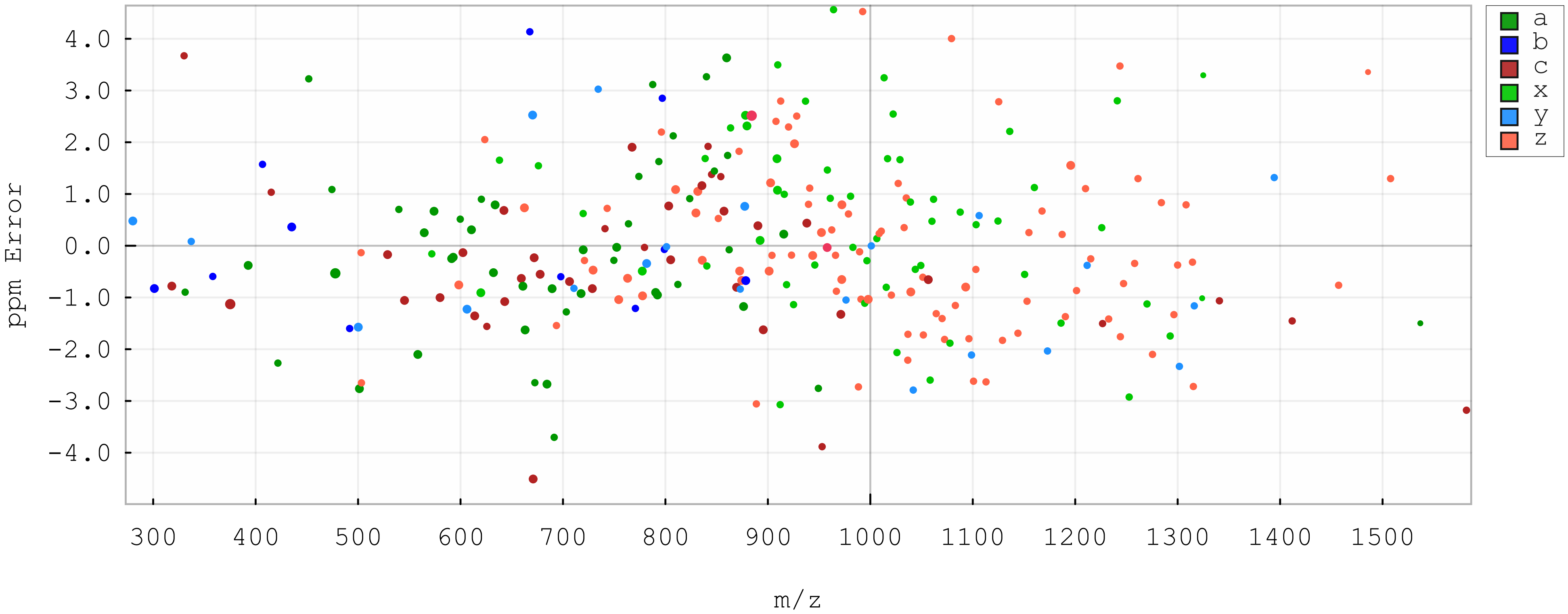

Proteoform:

Index	Protein	SC [%]	SVP [%]	IC [%]	MS Score [a.u.]	Var. Mods	Mono Mass [Da]	Chemical Formula
3	H4Kac	88.1	47.1	35.6	31.3	Ace: K5, K8, K12, K16, K20, K59	11481.4046	C506H849N163O140S

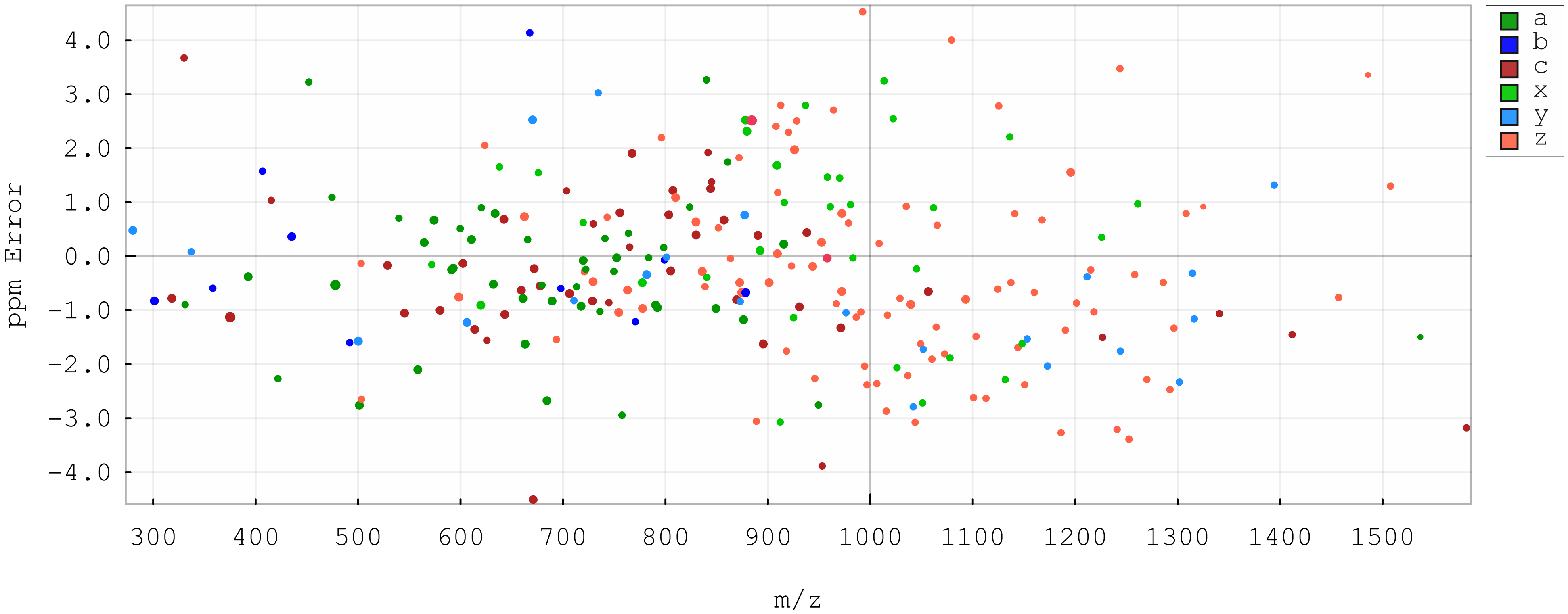

Proteoform:	Index	Protein	SC [%]	SVP [%]	IC [%]	MS Score [a.u.]	Var. Mods	Mono Mass [Da]	Chemical Formula
	4	H4Kac	87.1	30.4	34.8	30.3	Ace: K5, K8, K12, K16, K31, K44	11481.4046	C506H849N163O140S

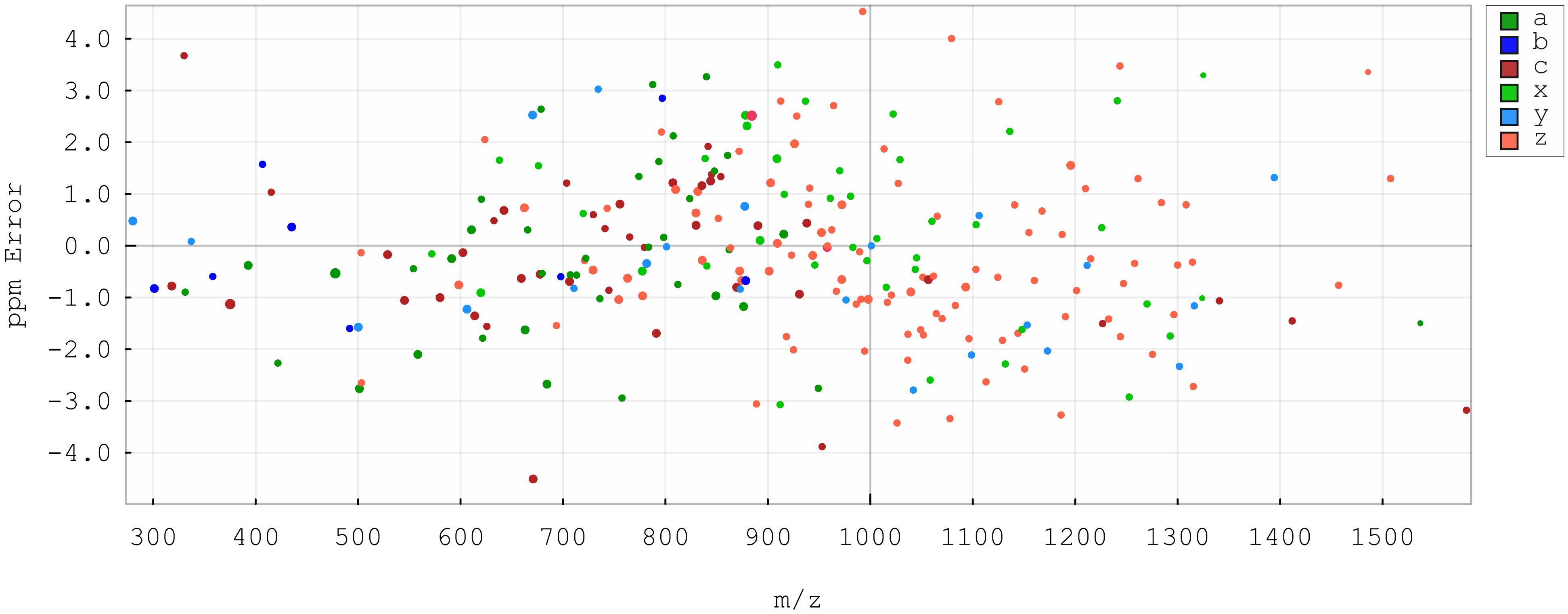

Proteoform:

Index	Protein	SC [%]	SVP [%]	IC [%]	MS Score [a.u.]	Var. Mods	Mono Mass [Da]	Chemical Formula
5	H4Kac	85.1	20.6	33.7	28.7	Ace: K5, K8, K12, K20, K31, K44	11481.4046	C506H849N163O140S

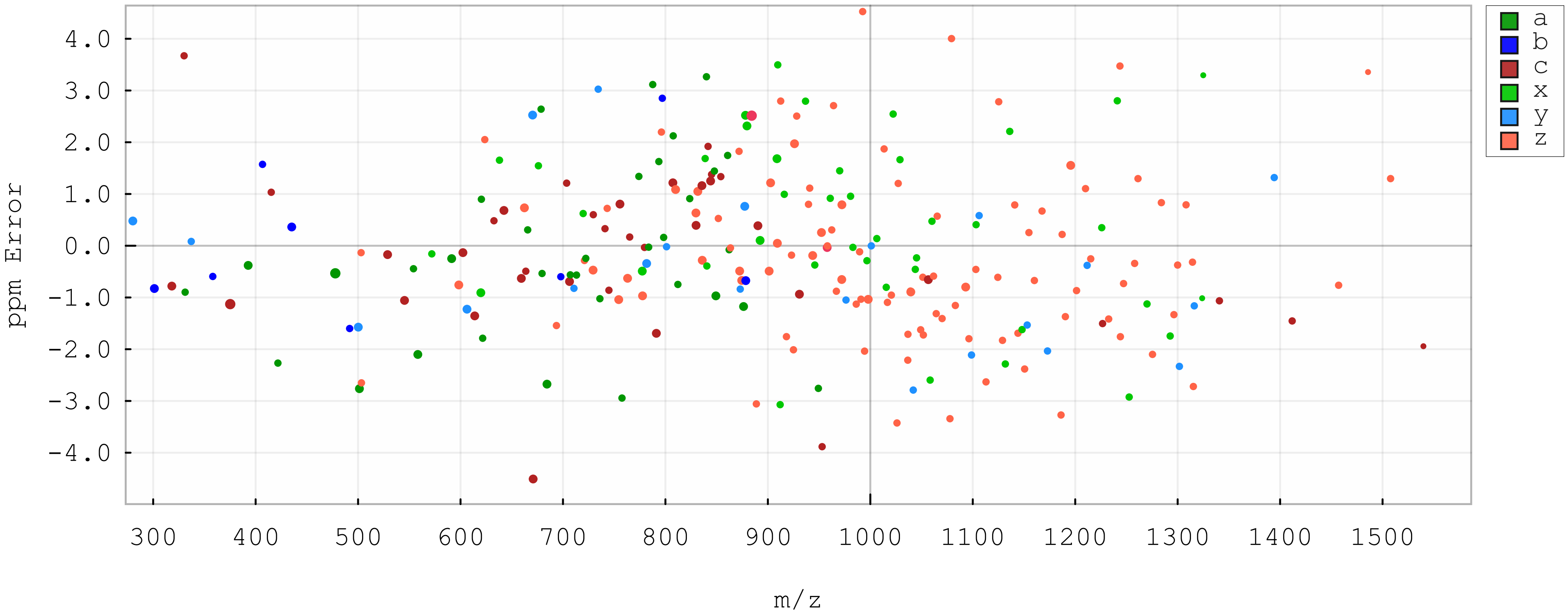

Proteoform:

Index	Protein	SC [%]	SVP [%]	IC [%]	MS Score [a.u.]	Var. Mods	Mono Mass [Da]	Chemical Formula
6	H4Kac	81.2	15.7	32.7	26.5	Ace: K5, K8, K16, K20, K31, K44	11481.4046	C506H849N163O140S

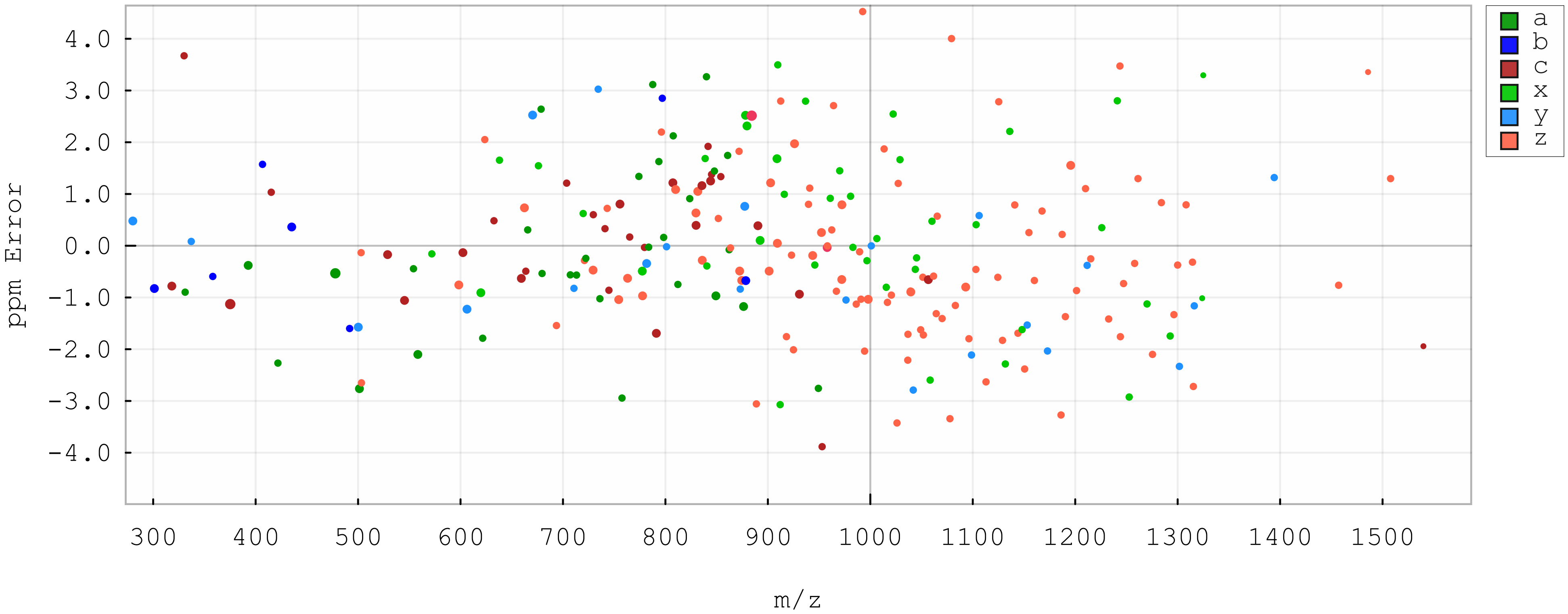

Proteoform:

Index	Protein	SC [%]	SVP [%]	IC [%]	MS Score [a.u.]	Var. Mods	Mono Mass [Da]	Chemical Formula
7	H4Kac	85.1	30.4	31.1	26.5	Ace: K5, K8, K12, K16, K31, K59	11481.4046	C506H849N163O140S

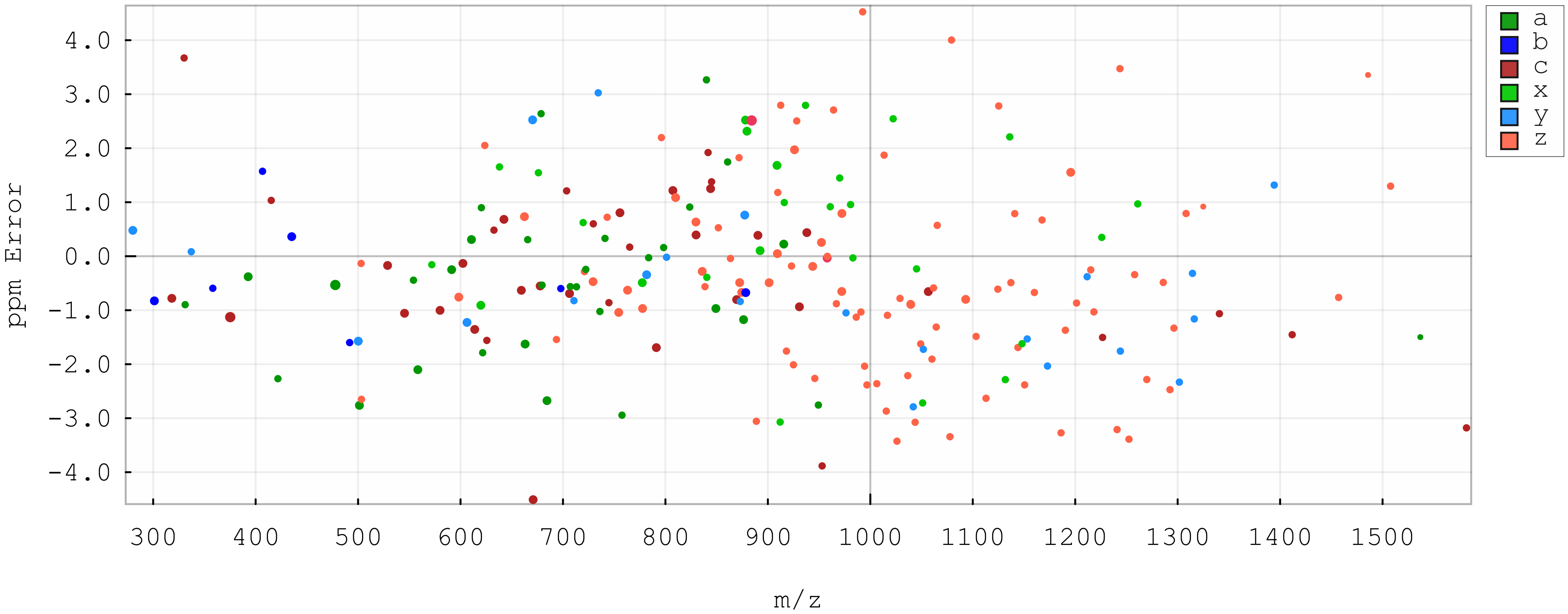

Proteoform:

Index	Protein	SC [%]	SVP [%]	IC [%]	MS Score [a.u.]	Var. Mods	Mono Mass [Da]	Chemical Formula
8	H4Kac	83.2	20.6	30.1	25.0	Ace: K5, K8, K12, K20, K31, K59	11481.4046	C506H849N163O140S

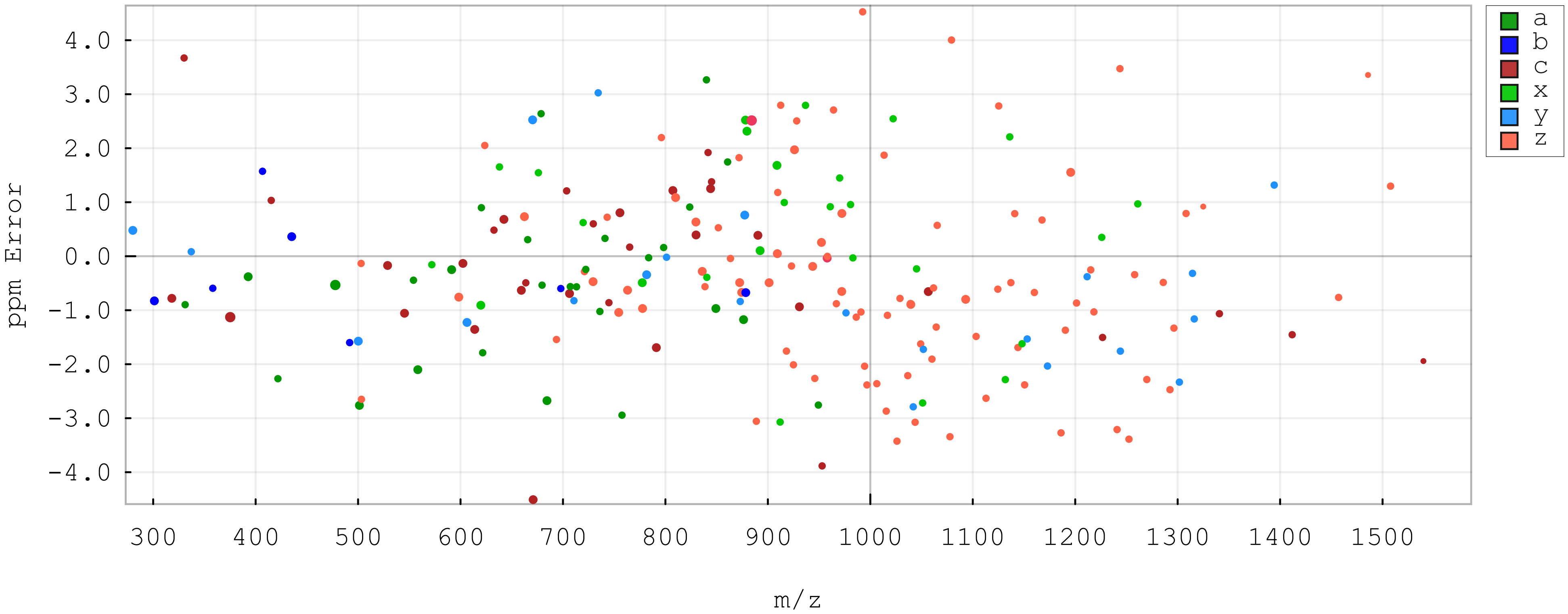

Proteoform:

Index	Protein	SC [%]	SVP [%]	IC [%]	MS Score [a.u.]	Var. Mods	Mono Mass [Da]	Chemical Formula
9	H4Kac	80.2	11.8	31.1	25.0	Ace: K5, K12, K16, K20, K31, K44	11481.4046	C506H849N163O140S

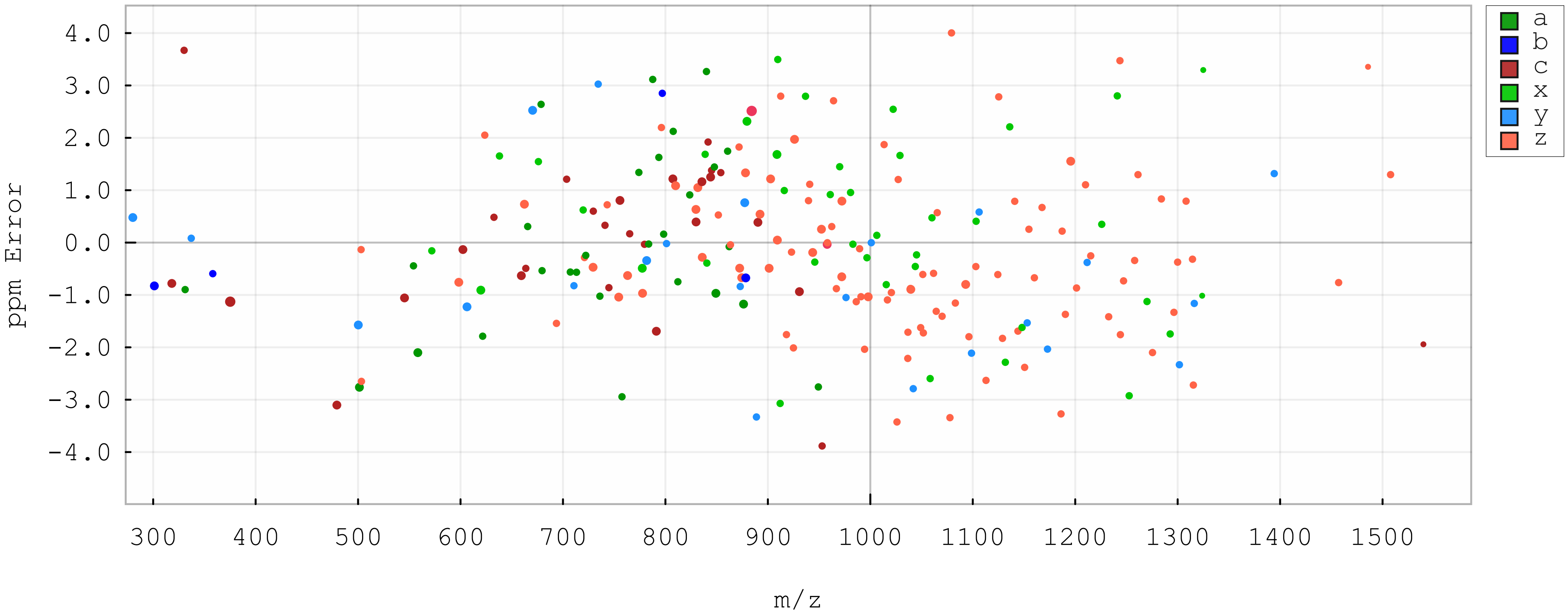

Proteoform:	Index	Protein	SC [%]	SVP [%]	IC [%]	MS Score [a.u.]	Var. Mods	Mono Mass [Da]	Chemical Formula
	10	H4Kac	80.2	11.8	30.3	24.3	Ace: K8, K12, K16, K20, K31, K44	11481.4046	C506H849N163O140S

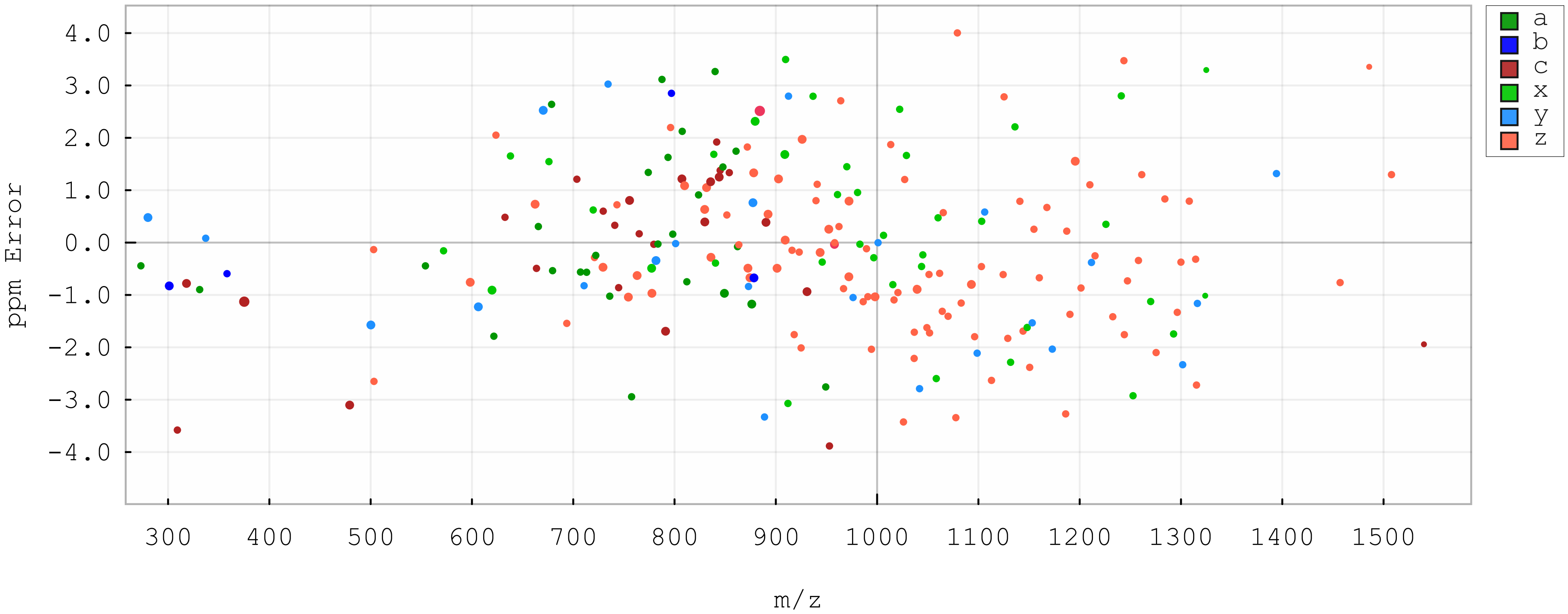

Primary Fragment Plots

Proteoform:

Index	Protein	SC [%]	SVP [%]	IC [%]	MS Score [a.u.]	Var. Mods	Mono Mass [Da]	Chemical Formula
1	H4Kac	90.1	50.0	39.2	35.3	Ace: K5, K8, K12, K16, K20, K44	11481.4046	C506H849N163O140S

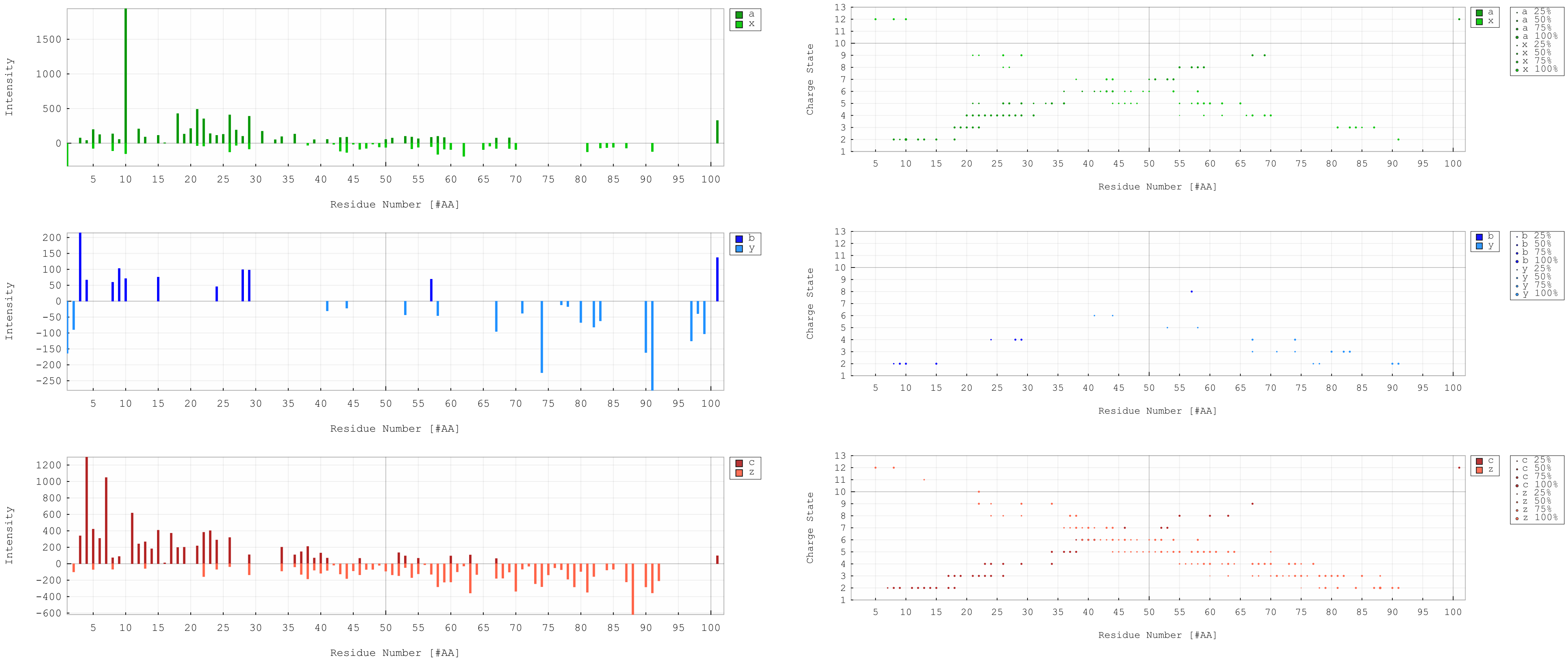

Proteoform:

Index	Protein	SC [%]	SVP [%]	IC [%]	MS Score [a.u.]	Var. Mods	Mono Mass [Da]	Chemical Formula
2	H4Kac	89.1	35.3	37.6	33.5	Ace: K5, K8, K12, K16, K20, K31	11481.4046	C506H849N163O140S

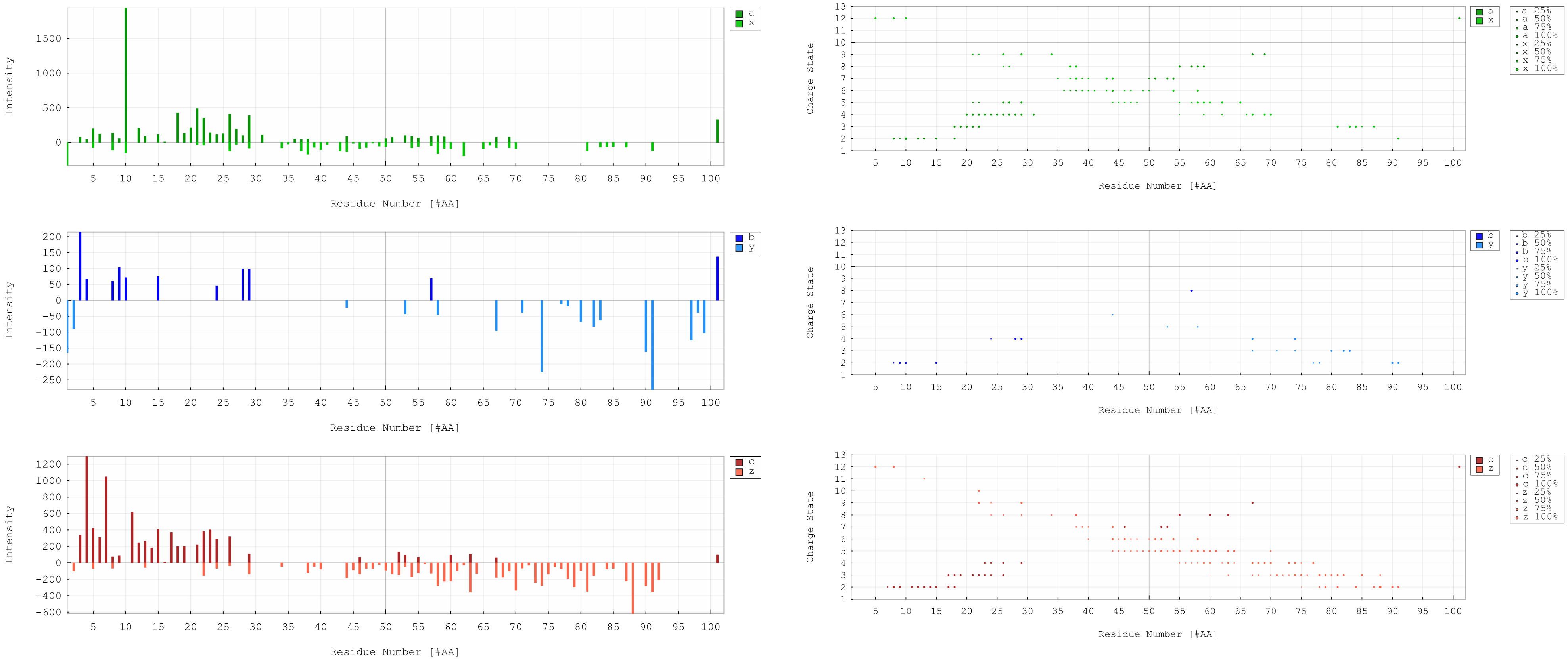

Proteoform:

Index	Protein	SC [%]	SVP [%]	IC [%]	MS Score [a.u.]	Var. Mods	Mono Mass [Da]	Chemical Formula
3	H4Kac	88.1	47.1	35.6	31.3	Ace: K5, K8, K12, K16, K20, K59	11481.4046	C506H849N163O140S

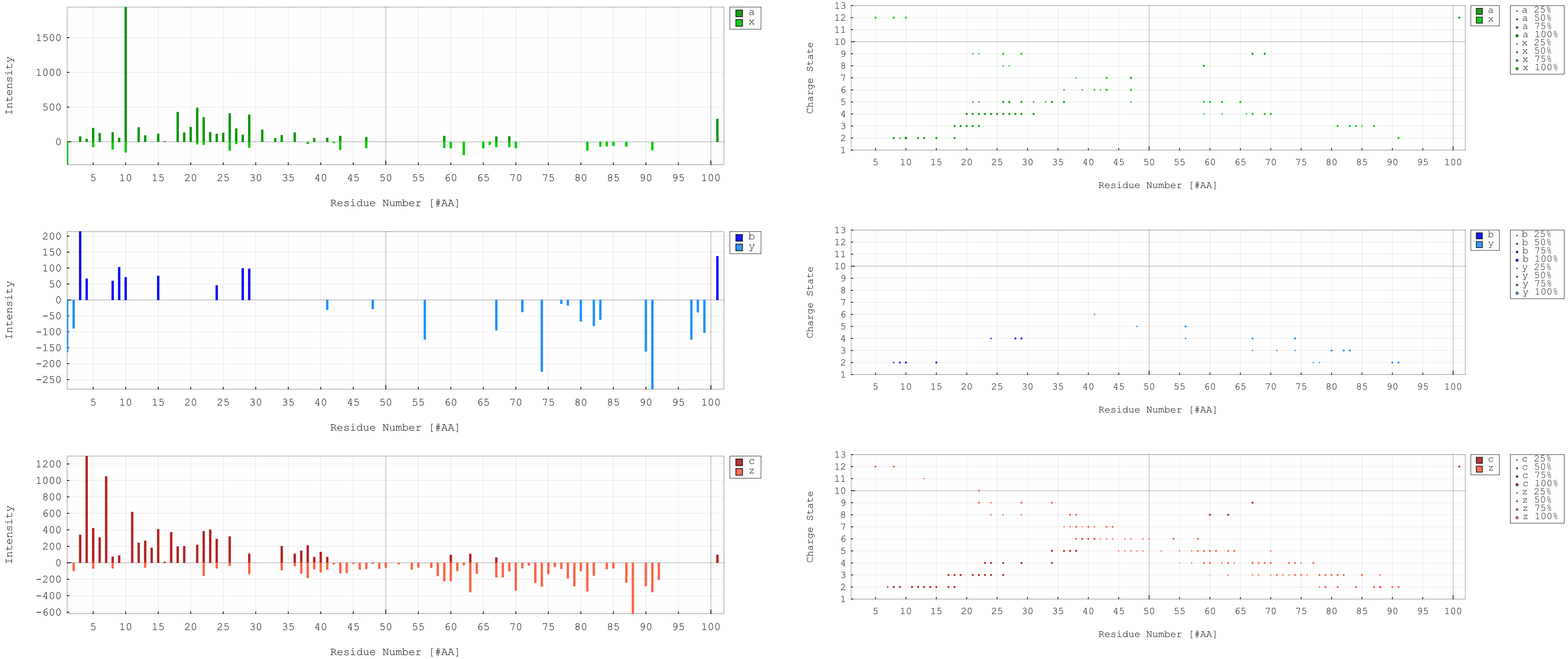

Proteoform:

Index	Protein	SC [%]	SVP [%]	IC [%]	MS Score [a.u.]	Var. Mods	Mono Mass [Da]	Chemical Formula
4	H4Kac	87.1	30.4	34.8	30.3	Ace: K5, K8, K12, K16, K31, K44	11481.4046	C506H849N163O140S

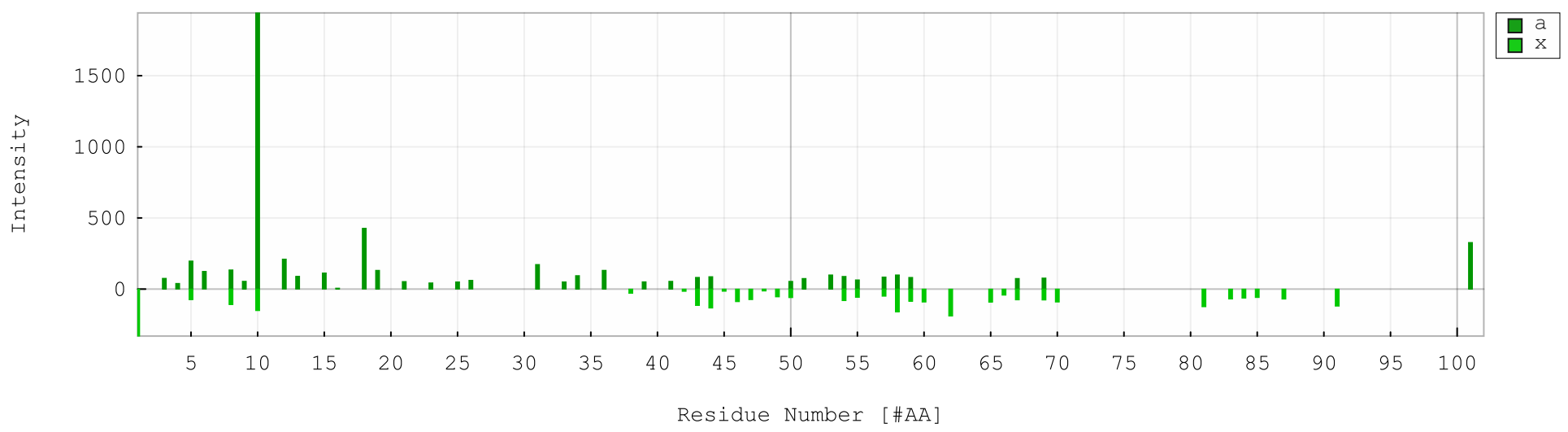

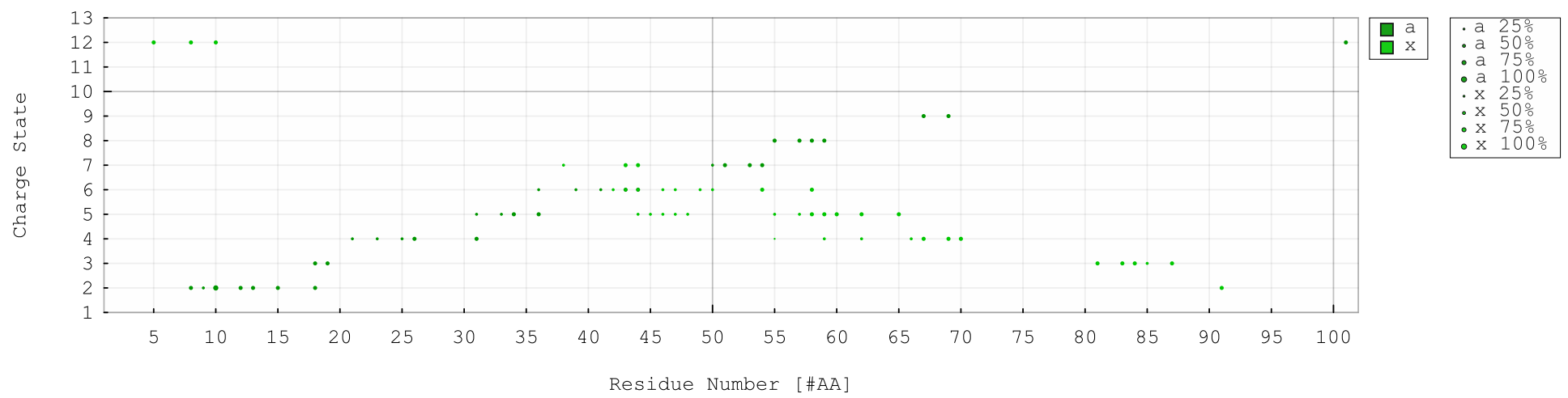

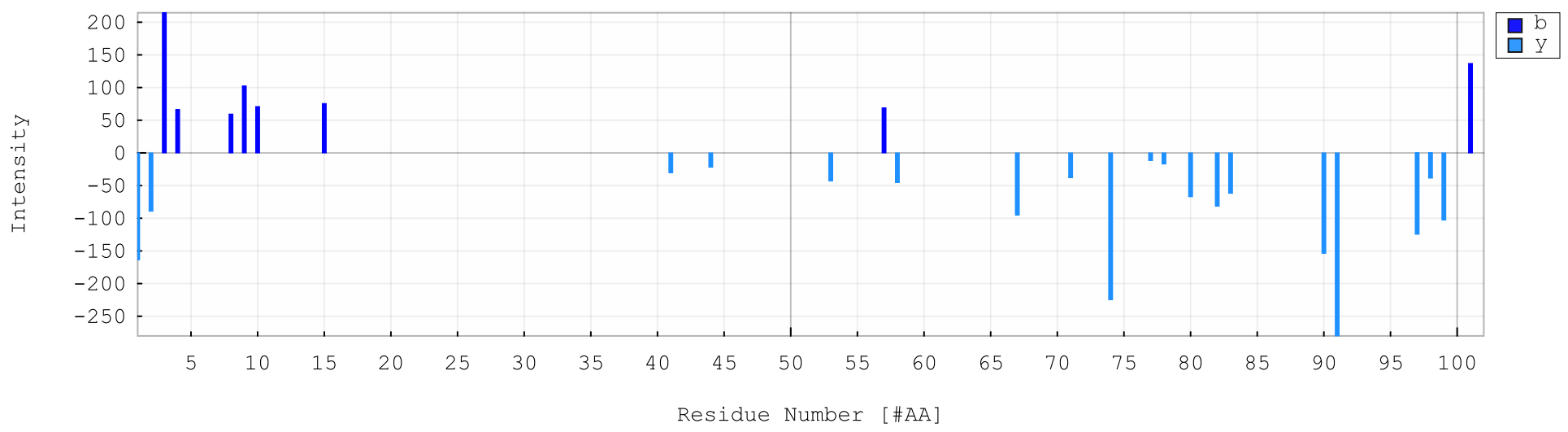

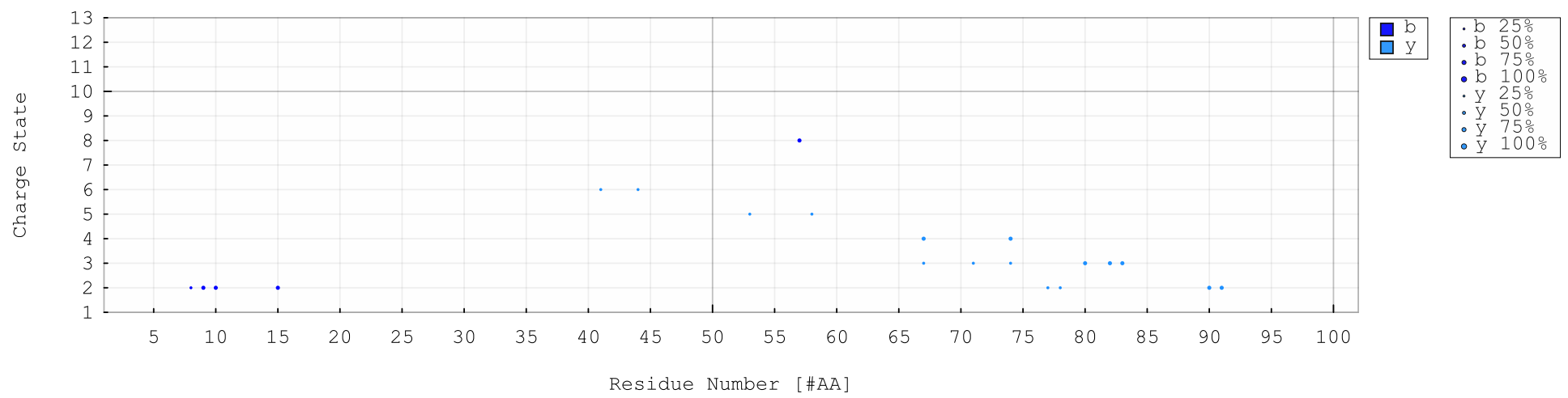

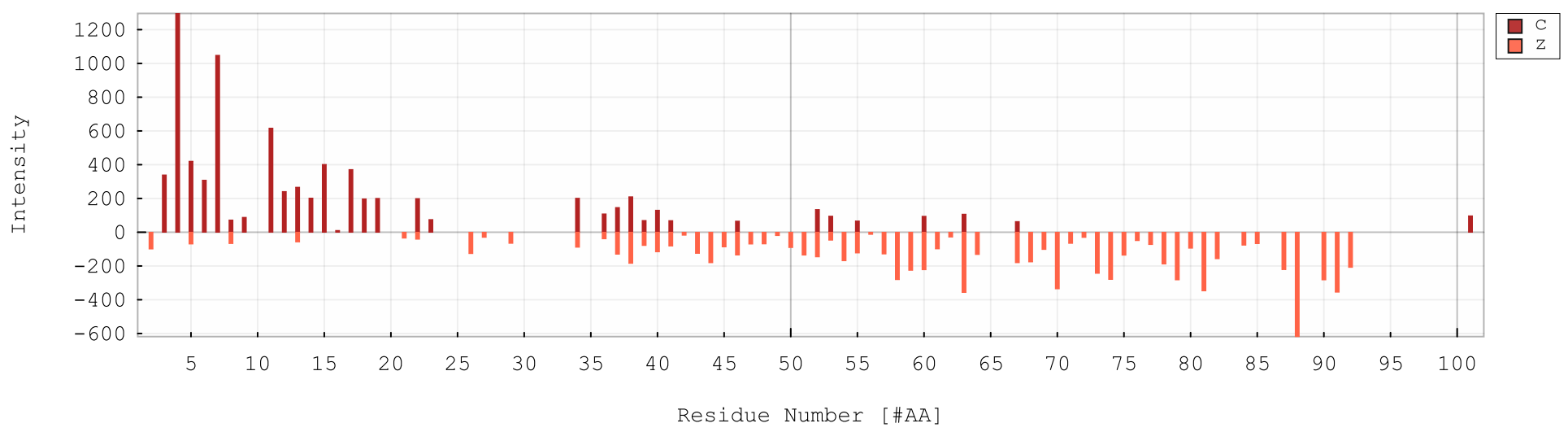

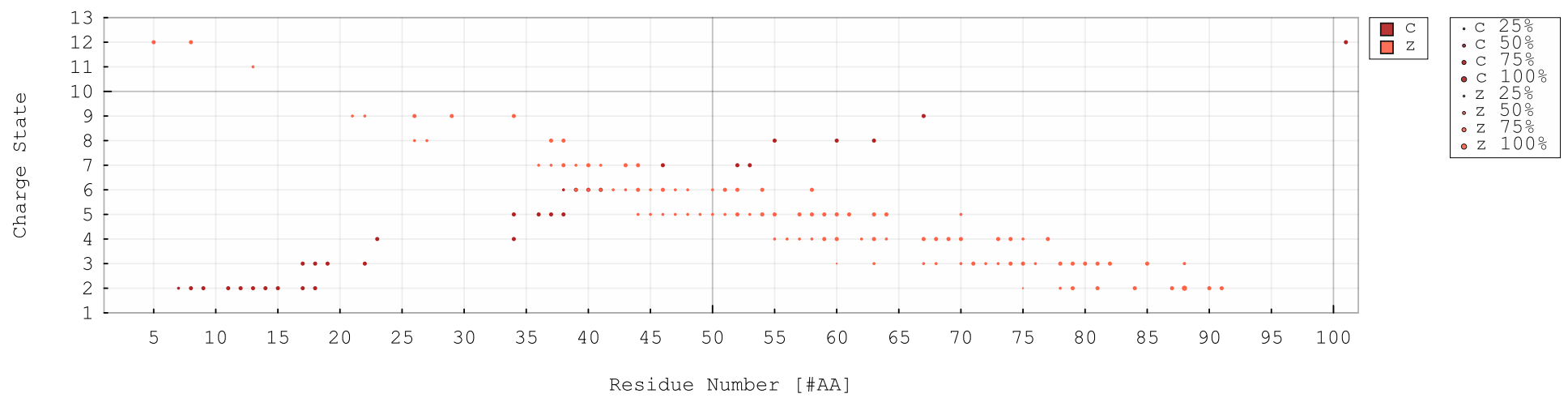

Proteoform:	Index	Protein	SC [%]	SVP [%]	IC [%]	MS Score [a.u.]	Var. Mods	Mono Mass [Da]	Chemical Formula
	5	H4Kac	85.1	20.6	33.7	28.7	Ace: K5, K8, K12, K20, K31, K44	11481.4046	C506H849N163O140S

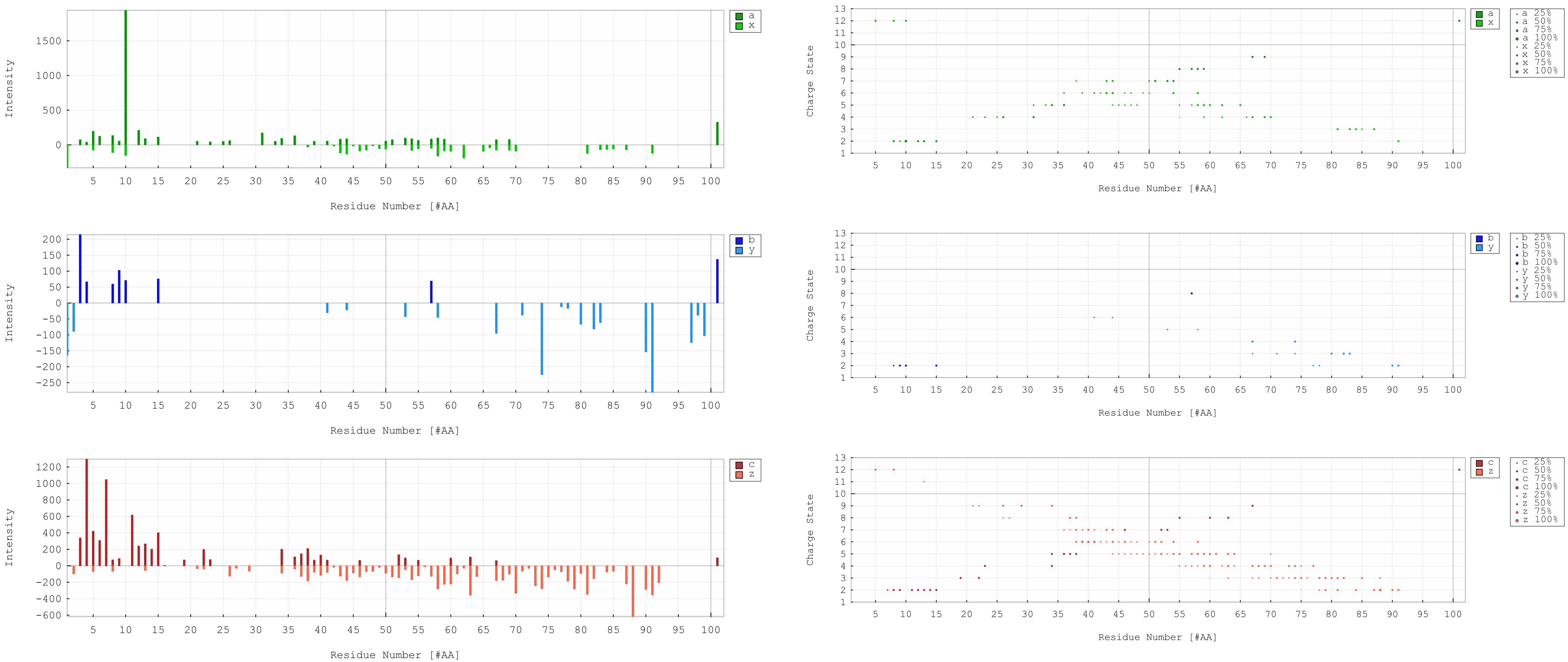

Proteoform:

Index	Protein	SC [%]	SVP [%]	IC [%]	MS Score [a.u.]	Var. Mods	Mono Mass [Da]	Chemical Formula
6	H4Kac	81.2	15.7	32.7	26.5	Ace: K5, K8, K16, K20, K31, K44	11481.4046	C506H849N163O140S

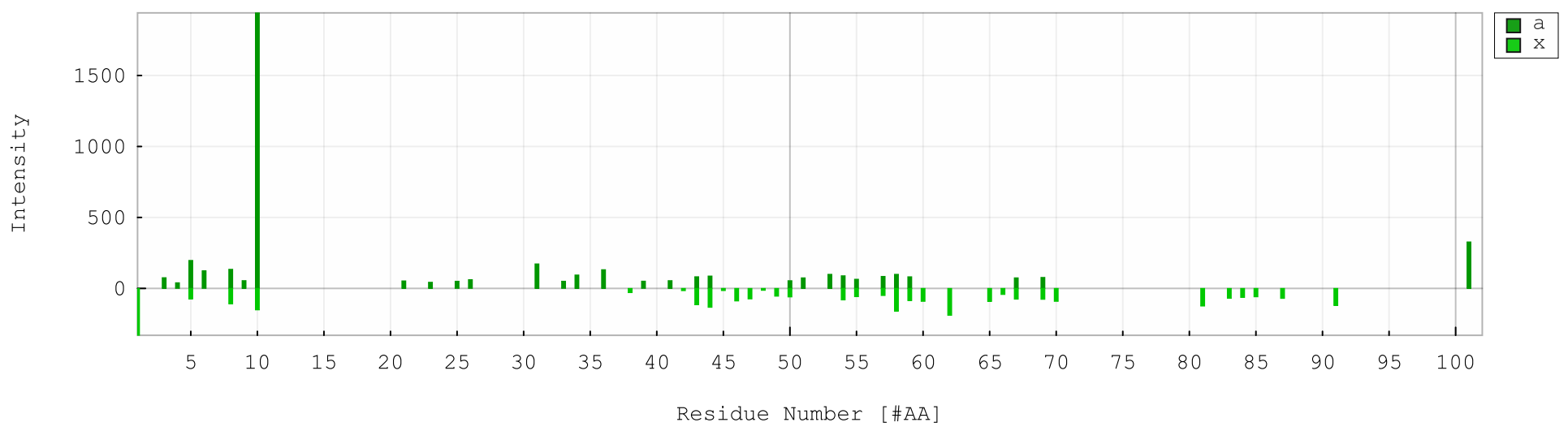

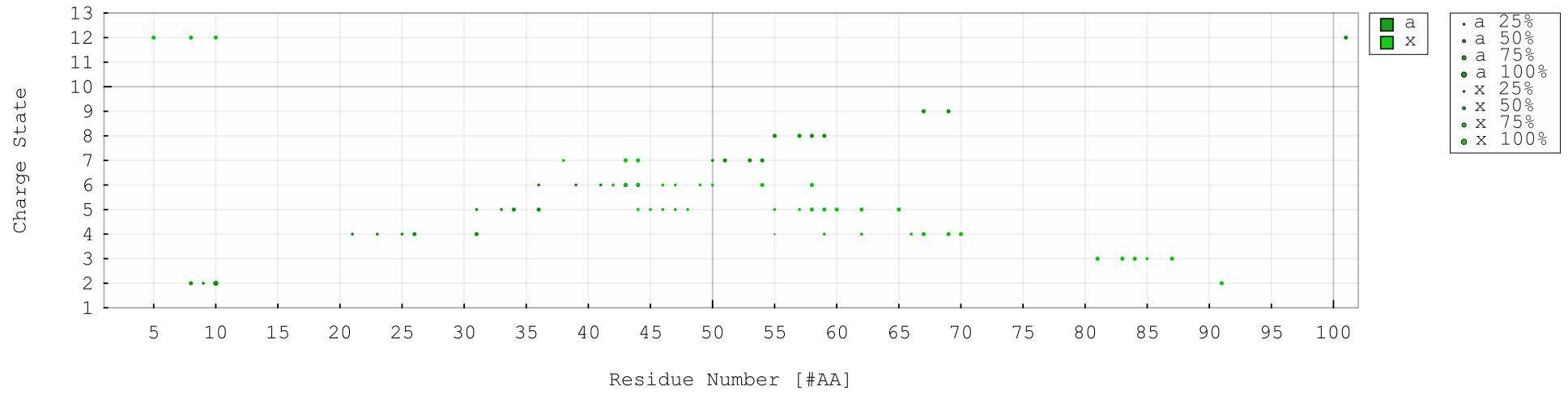

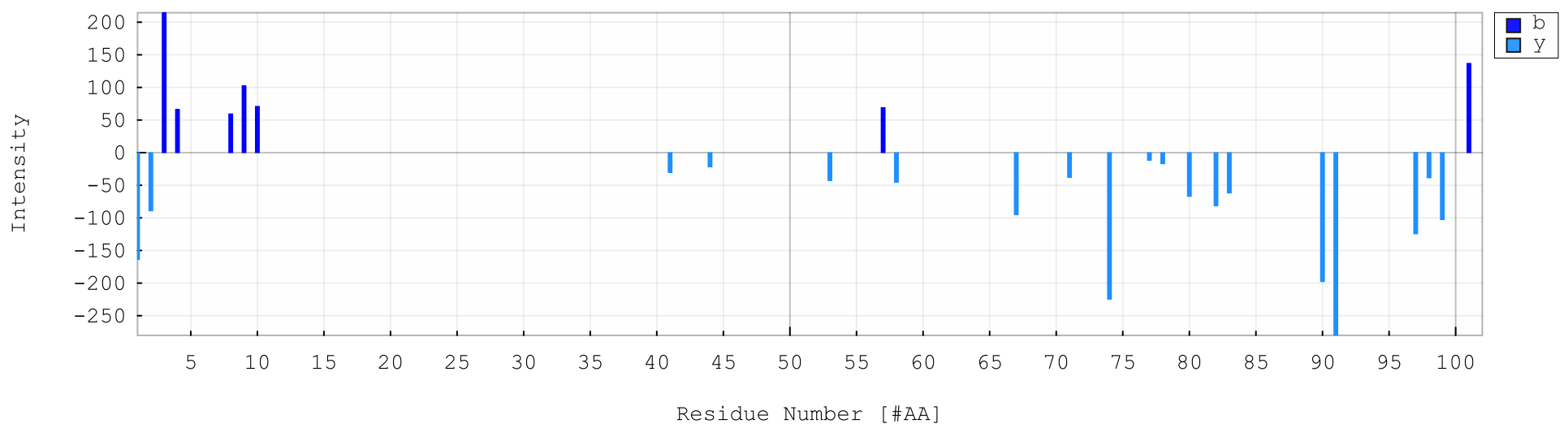

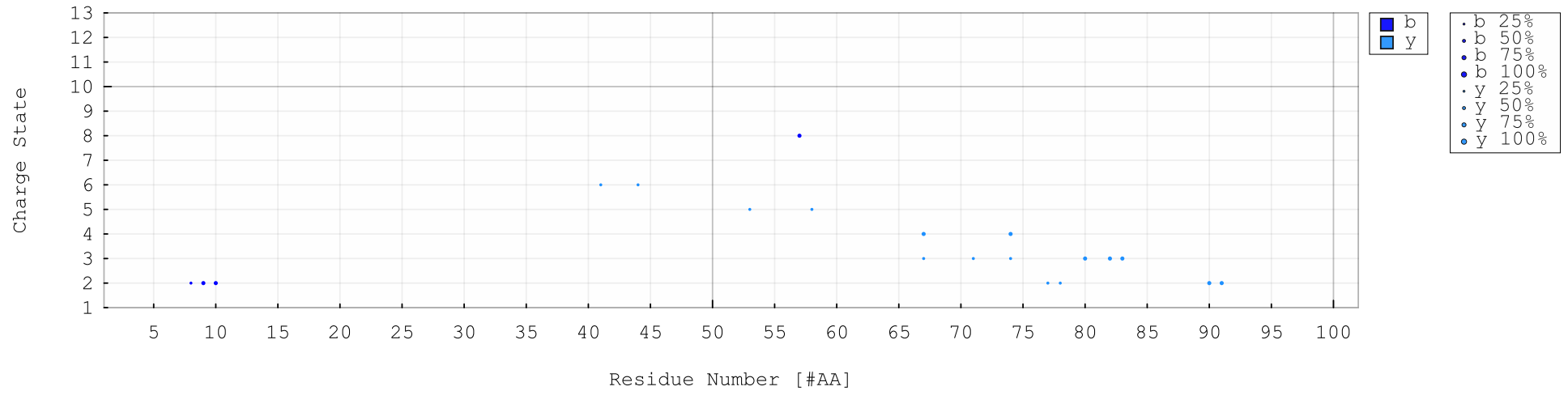

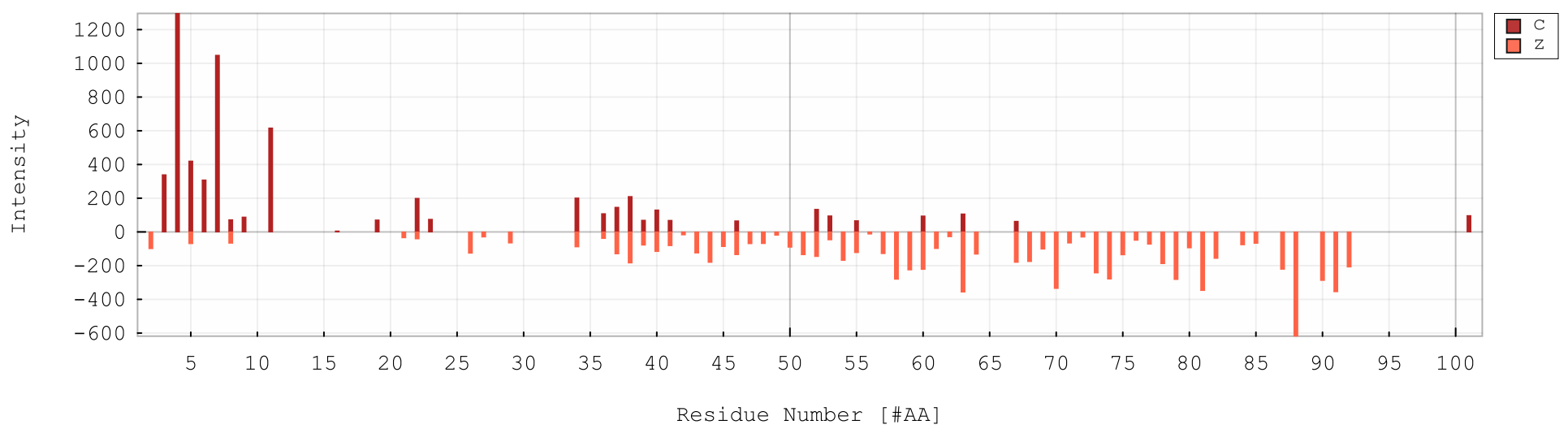

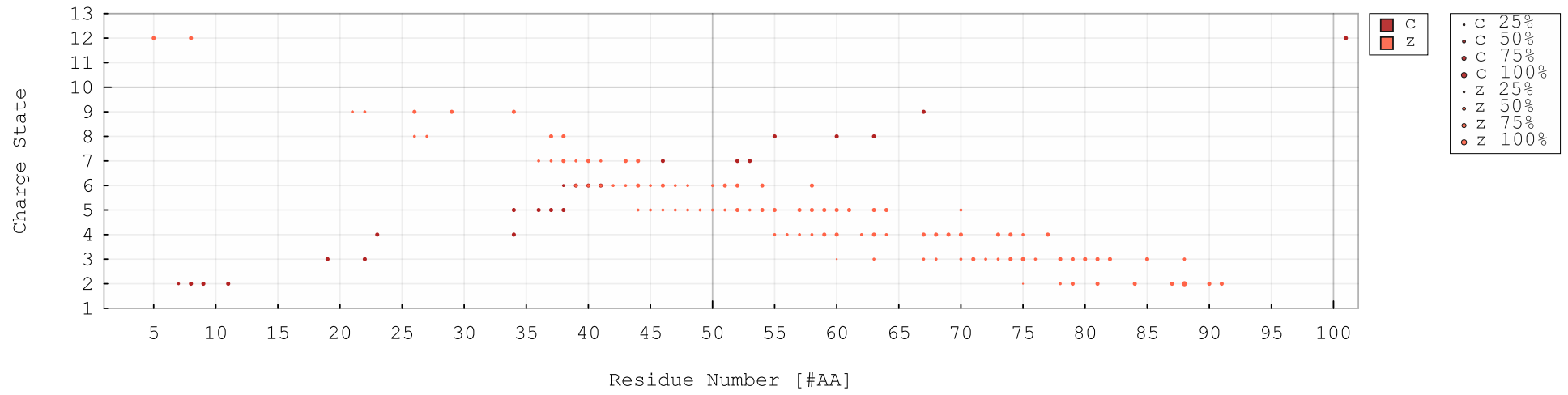

Proteoform:

Index	Protein	SC [%]	SVP [%]	IC [%]	MS Score [a.u.]	Var. Mods	Mono Mass [Da]	Chemical Formula
7	H4Kac	85.1	30.4	31.1	26.5	Ace: K5, K8, K12, K16, K31, K59	11481.4046	C506H849N163O140S

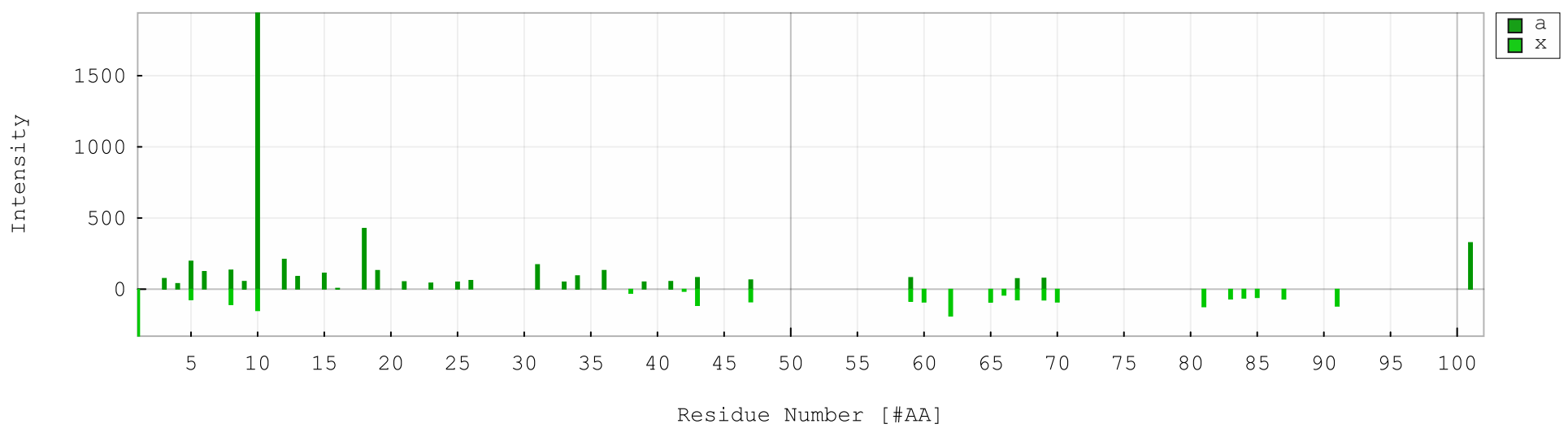

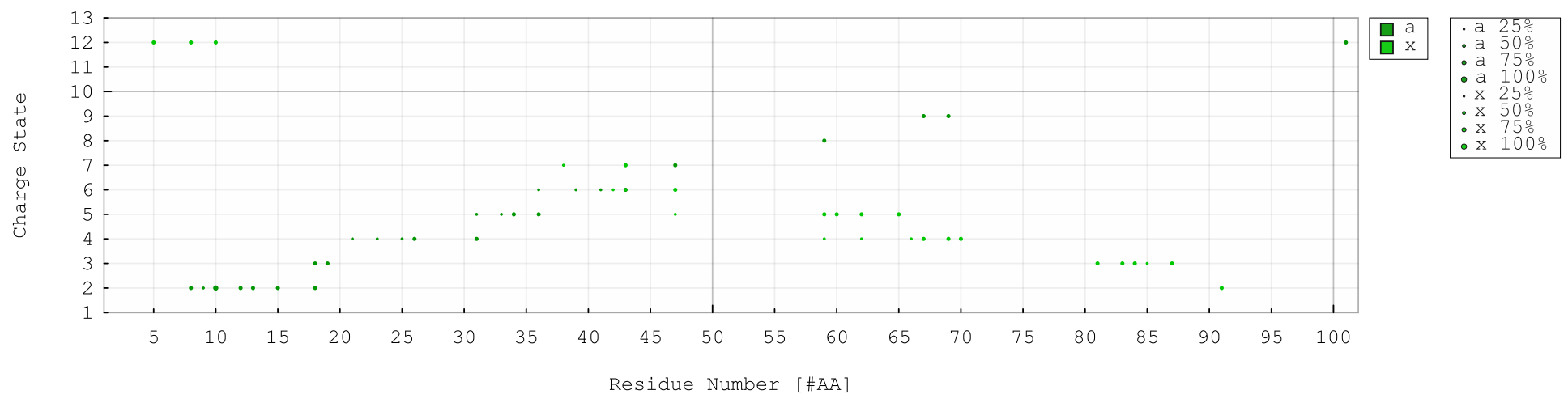

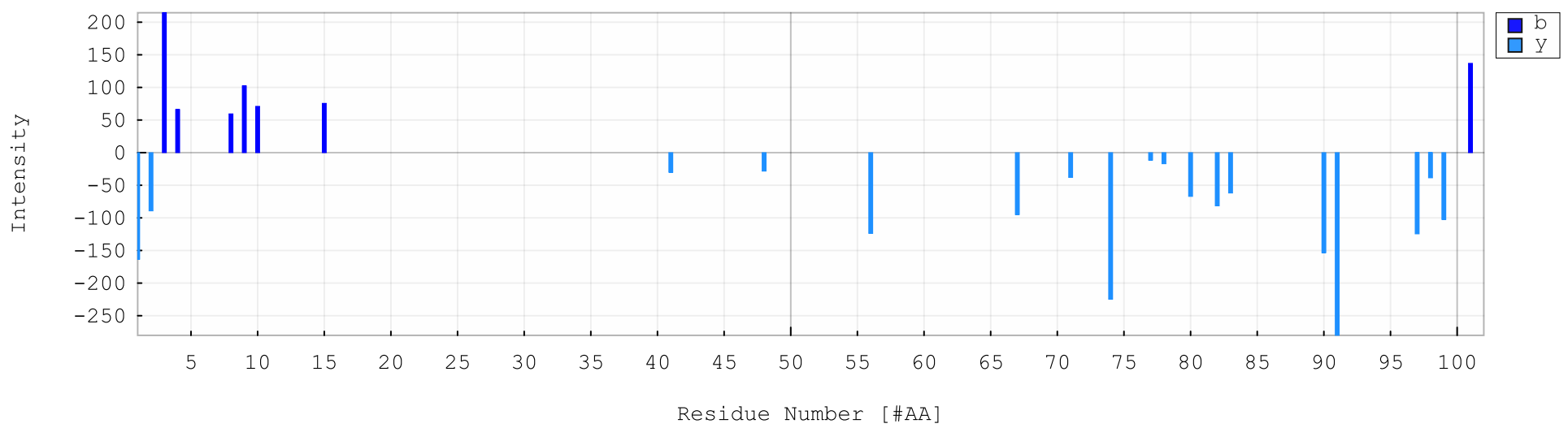

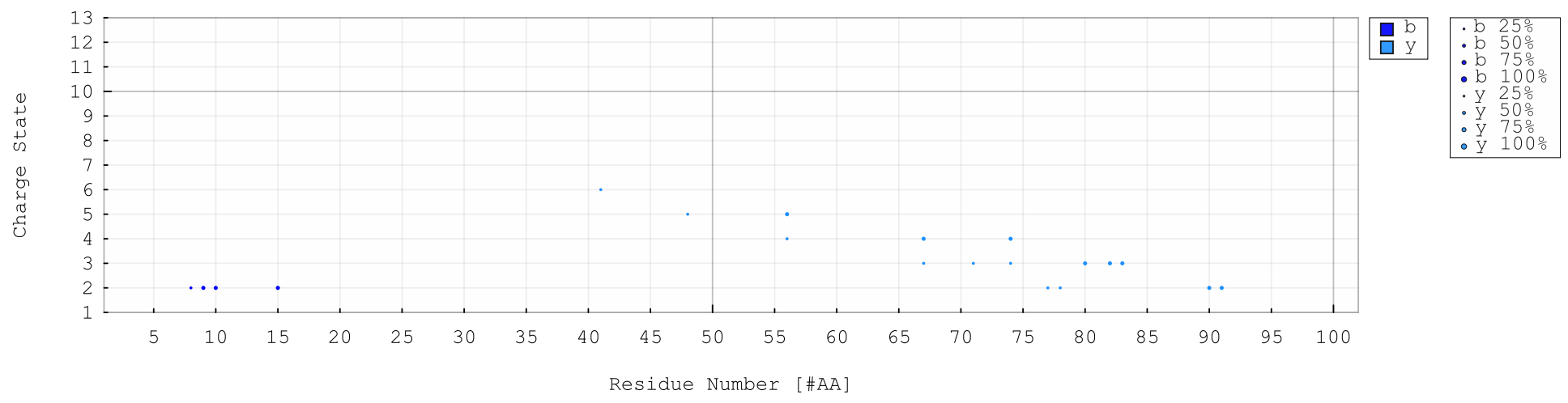

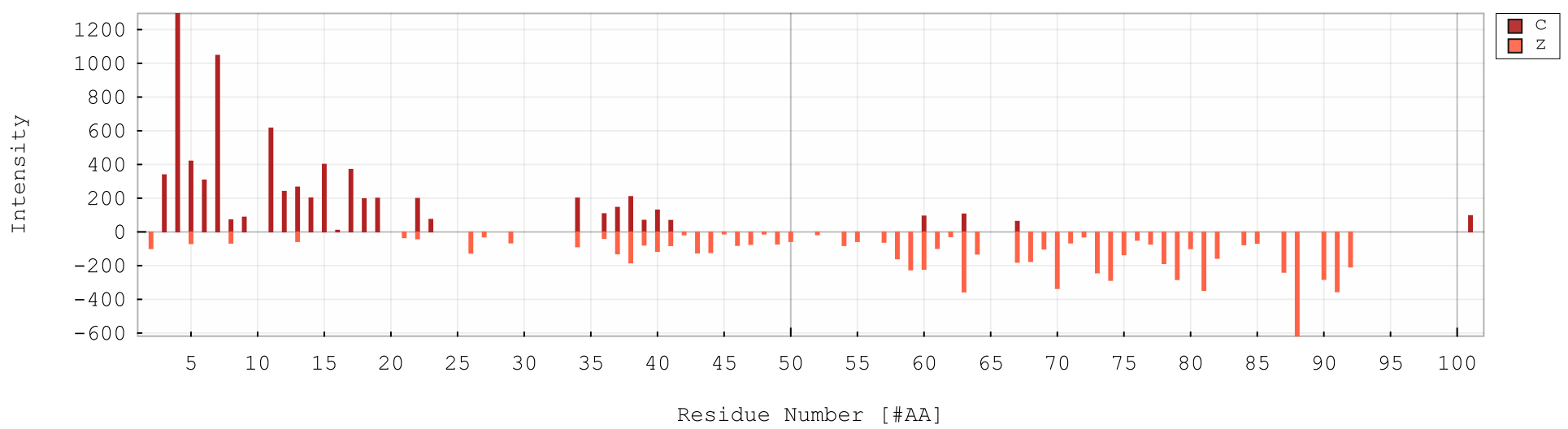

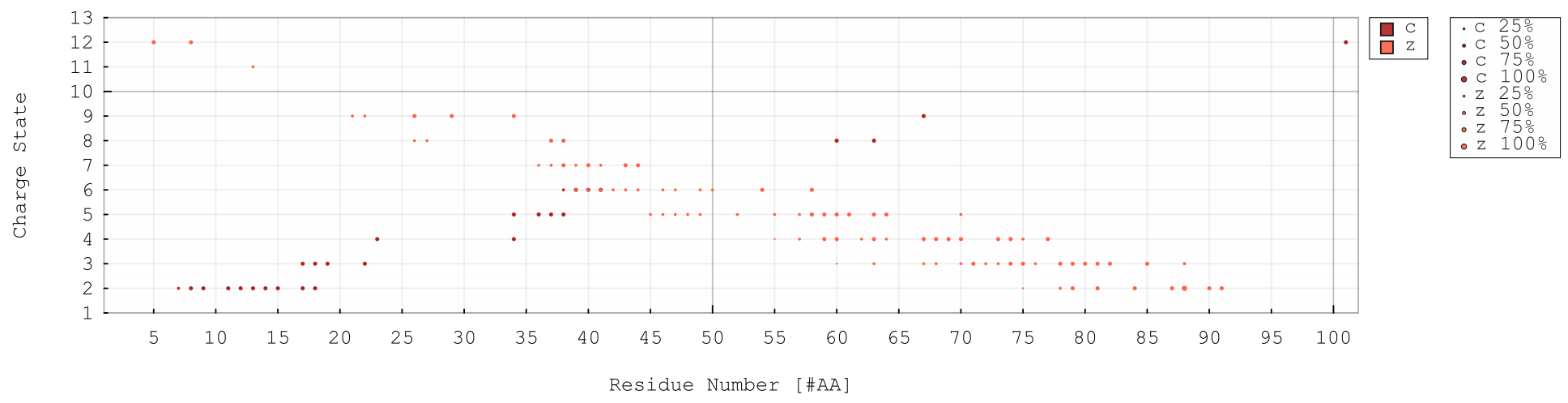

Proteoform:

Index	Protein	SC [%]	SVP [%]	IC [%]	MS Score [a.u.]	Var. Mods	Mono Mass [Da]	Chemical Formula
8	H4Kac	83.2	20.6	30.1	25.0	Ace: K5, K8, K12, K20, K31, K59	11481.4046	C506H849N163O140S

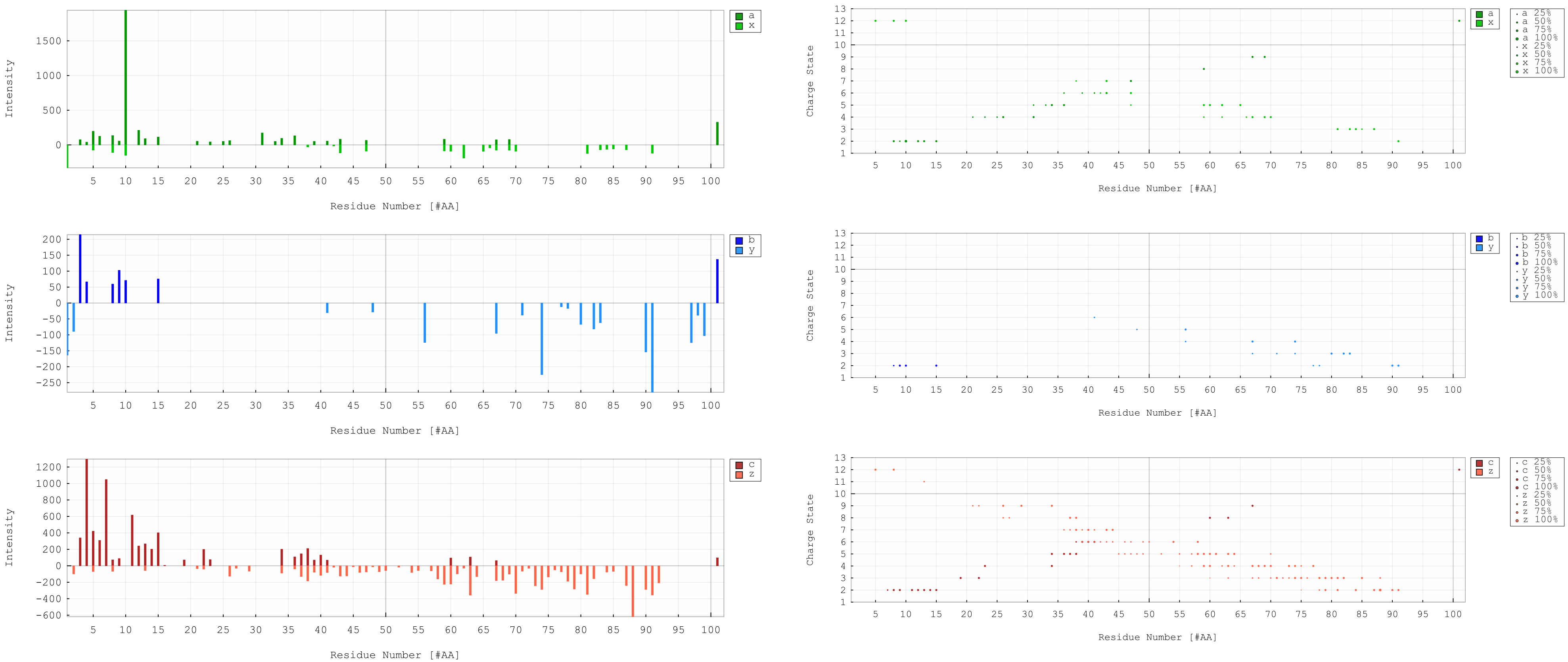

Proteoform:

Index	Protein	SC [%]	SVP [%]	IC [%]	MS Score [a.u.]	Var. Mods	Mono Mass [Da]	Chemical Formula
9	H4Kac	80.2	11.8	31.1	25.0	Ace: K5, K12, K16, K20, K31, K44	11481.4046	C506H849N163O140S

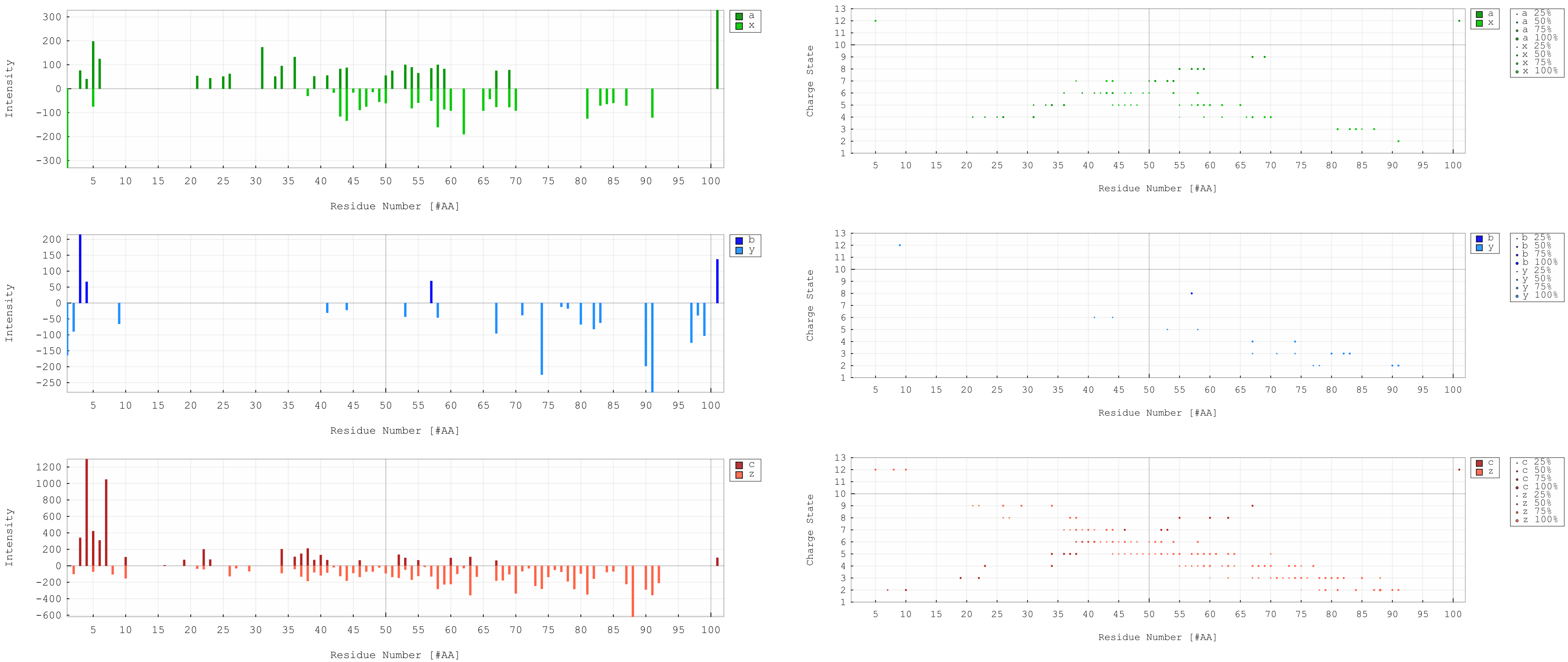

Proteoform:

Index	Protein	SC [%]	SVP [%]	IC [%]	MS Score [a.u.]	Var. Mods	Mono Mass [Da]	Chemical Formula
10	H4Kac	80.2	11.8	30.3	24.3	Ace: K8, K12, K16, K20, K31, K44	11481.4046	C506H849N163O140S

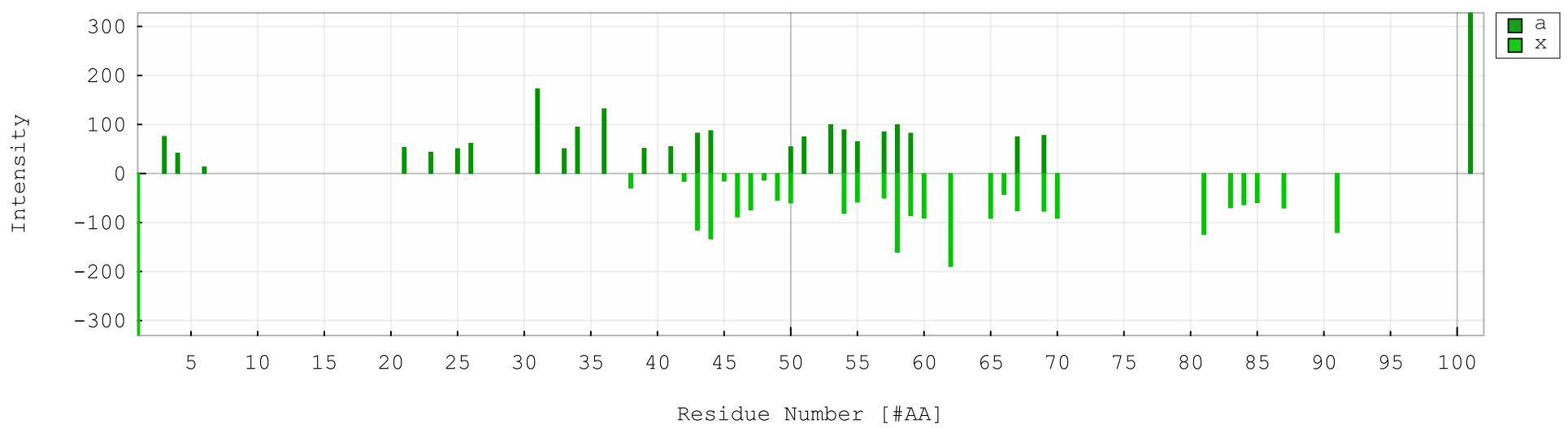

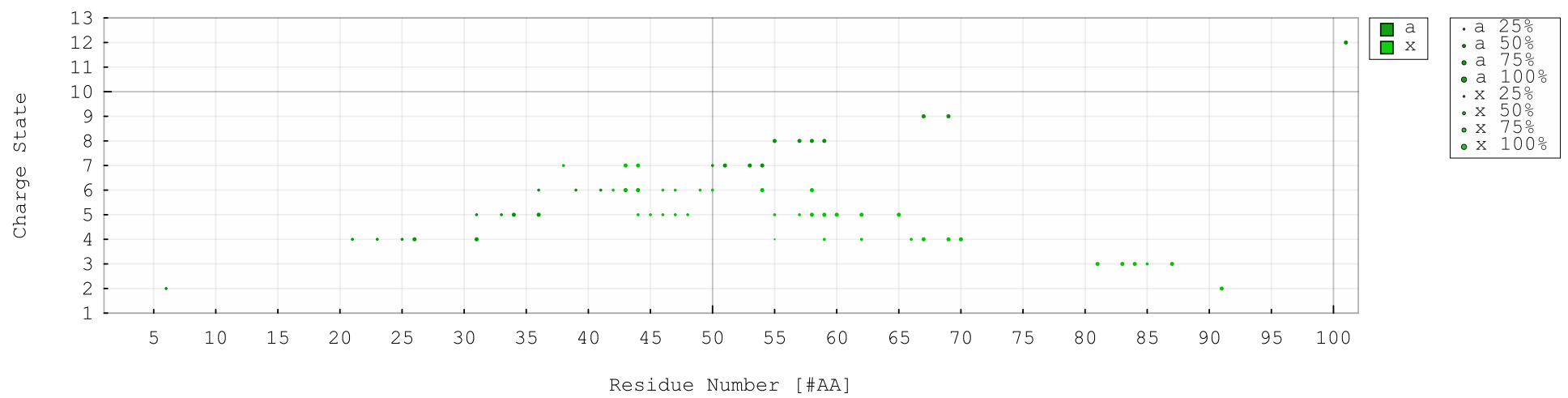

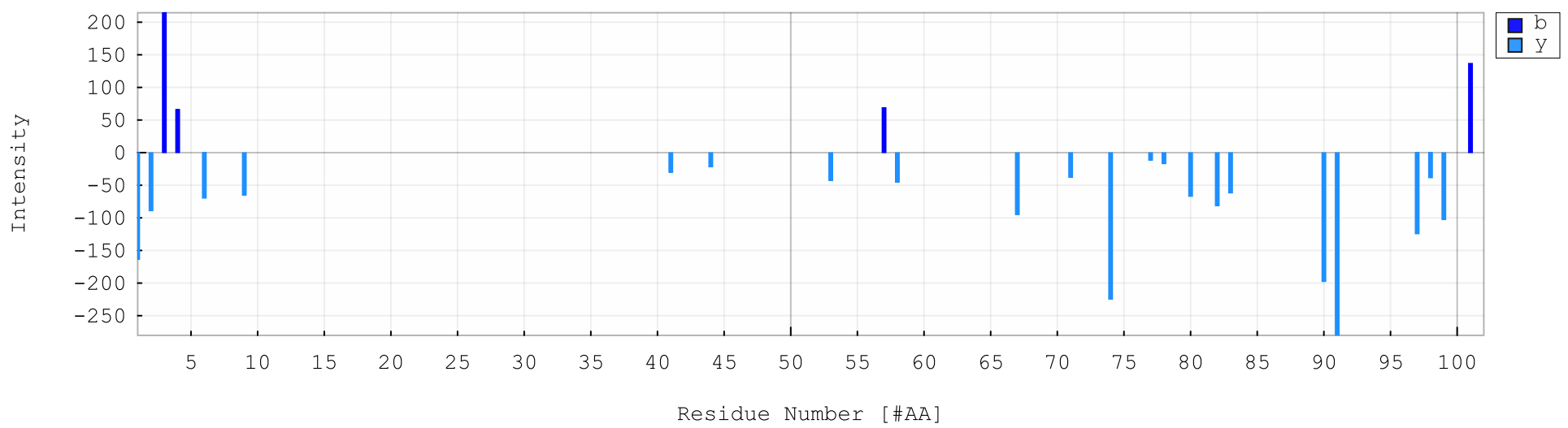

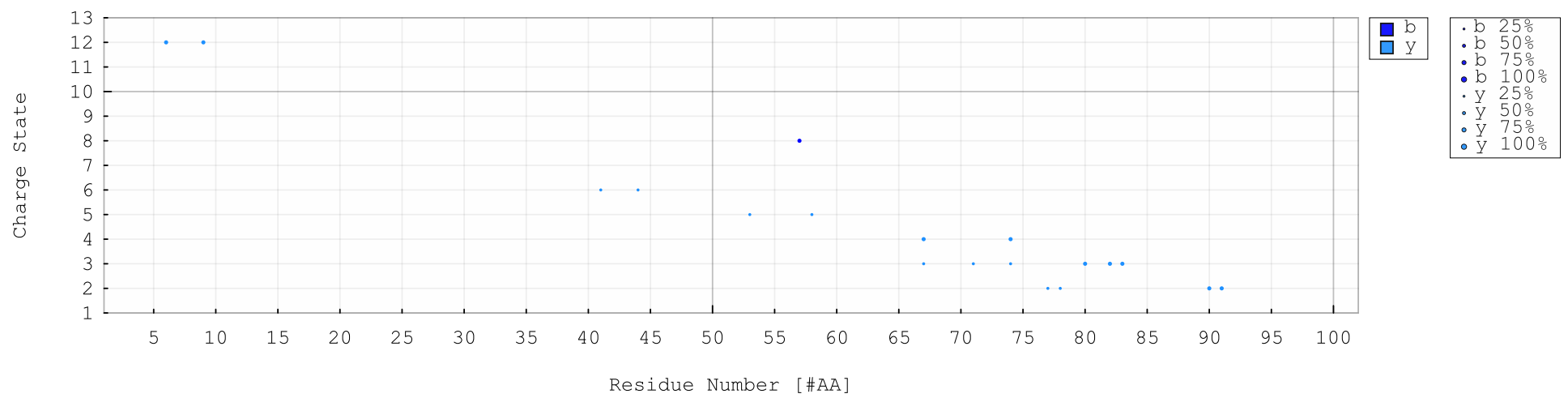

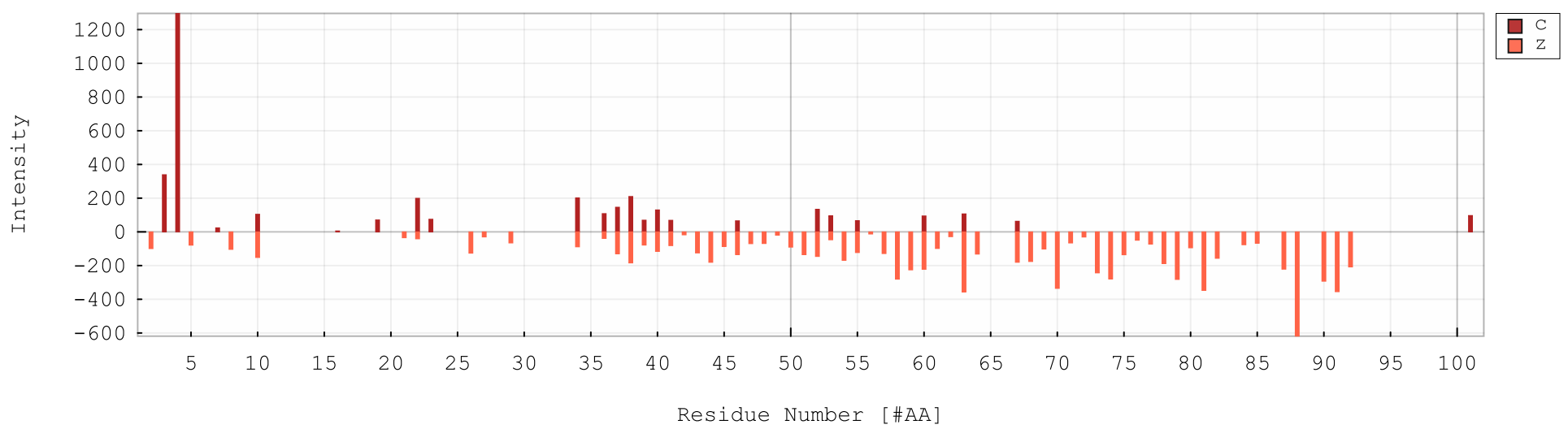

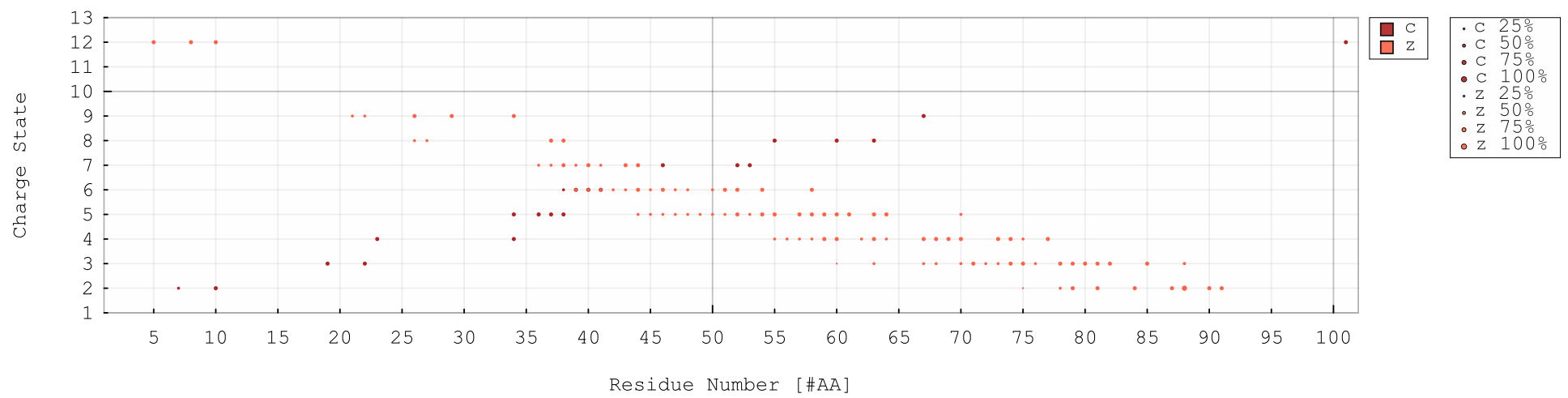

Supplemental Data S4

H4 - p300, 60 min

**Report**	4 of 7
**Experiment**	MS2
**Histone/sample**	H4
**Enzyme**	p300
**Reaction/source**	60 min
**Precursor**	H4Kac6
**Charge state**	13+
**Fragmentation / workflow**	RCID
**Original file**	MS2_H4Kac6_13+_RCID.pdf

Project Information

Project Directory: C:/Users/LAB-ADMIN
Processing date: Tue Apr 28 12:58:54 2026

Sample Info & Protocols

Name: Top-Down Protein Sequencing CID

Dataset

Imported spectrum file name:	250428 H4_P300-50_60min z13ac6 MS2 shCID_001-670_calibrated.txt
Dataset name:	Information is only available from imported JPEAKS files
Date acquired:	Information is only available from imported JPEAKS files
Date imported:	Information is only available from imported JPEAKS files
Instrument Name:	Information is only available from imported JPEAKS files
Instrument Serial Number:	Information is only available from imported JPEAKS files

Workflow Result Info

Analysis: 250428 H4_P300-50_60min z13ac6 MS2 shCID_001-670_calibrated
Location: C:/Users/LAB-ADMIN
Method: -

Method Information

Name: Unnamed

Spectral Parameters

Mass Error [ppm]: 6
Intensity Deviation [au]: 0.15
Min/Max Charges: 1-13
Experiment type: CID

a ions	b ions	c ions	x ions	y ions	z ions	satellite	internal	precursor
a	b			y				M

Noise threshold calculation: Simple moving average noise threshold (SMA)
Window size [datapoints]: 600
Noise curve offset: 1.2616721
SVP parameters: terminal gap: 2, internal gap: 1

Proteoforms

(top 10)

Index	Parent Sequence	Chain	Var. Mods	Mono Mass [Da]	Chemical Composition
1	H4Kac	H4KacK5[Ace]K8[Ace]K12[Ace]K16[Ace]K20[Ace]K44[Ace]	Ace: K5, K8, K12, K16, K20, K44	11481.4046	C506H849N163O140S
2	H4Kac	H4KacK5[Ace]K8[Ace]K12[Ace]K16[Ace]K31[Ace]K44[Ace]	Ace: K5, K8, K12, K16, K31, K44	11481.4046	C506H849N163O140S
3	H4Kac	H4KacK5[Ace]K8[Ace]K12[Ace]K16[Ace]K20[Ace]K31[Ace]	Ace: K5, K8, K12, K16, K20, K31	11481.4046	C506H849N163O140S
4	H4Kac	H4KacK5[Ace]K8[Ace]K12[Ace]K20[Ace]K31[Ace]K44[Ace]	Ace: K5, K8, K12, K20, K31, K44	11481.4046	C506H849N163O140S
5	H4Kac	H4KacK5[Ace]K8[Ace]K16[Ace]K20[Ace]K31[Ace]K44[Ace]	Ace: K5, K8, K16, K20, K31, K44	11481.4046	C506H849N163O140S
6	H4Kac	H4KacK5[Ace]K8[Ace]K12[Ace]K16[Ace]K20[Ace]K59[Ace]	Ace: K5, K8, K12, K16, K20, K59	11481.4046	C506H849N163O140S
7	H4Kac	H4KacK5[Ace]K8[Ace]K12[Ace]K16[Ace]K31[Ace]K59[Ace]	Ace: K5, K8, K12, K16, K31, K59	11481.4046	C506H849N163O140S
8	H4Kac	H4KacK5[Ace]K8[Ace]K12[Ace]K20[Ace]K31[Ace]K59[Ace]	Ace: K5, K8, K12, K20, K31, K59	11481.4046	C506H849N163O140S
9	H4Kac	H4KacK5[Ace]K8[Ace]K16[Ace]K20[Ace]K31[Ace]K59[Ace]	Ace: K5, K8, K16, K20, K31, K59	11481.4046	C506H849N163O140S
10	H4Kac	H4KacK5[Ace]K12[Ace]K16[Ace]K20[Ace]K31[Ace]K44[Ace]	Ace: K5, K12, K16, K20, K31, K44	11481.4046	C506H849N163O140S

Results

Proteoforms Table (top 10)

Index	Protein	SC [%]	SVP [%]	IC [%]	MS Score [a.u.]	Var. Mods	Mono Mass [Da]	Chemical Formula
1	H4Kac	52.5	16.7	39.3	20.6	Ace: K5, K8, K12, K16, K20, K44	11481.4046	C506H849N163O140S
2	H4Kac	51.5	16.7	39.1	20.2	Ace: K5, K8, K12, K16, K31, K44	11481.4046	C506H849N163O140S
3	H4Kac	50.5	16.7	39.1	19.7	Ace: K5, K8, K12, K16, K20, K31	11481.4046	C506H849N163O140S
4	H4Kac	50.5	16.7	39.1	19.7	Ace: K5, K8, K12, K20, K31, K44	11481.4046	C506H849N163O140S
5	H4Kac	50.5	16.7	39.1	19.7	Ace: K5, K8, K16, K20, K31, K44	11481.4046	C506H849N163O140S
6	H4Kac	51.5	16.7	36.4	18.8	Ace: K5, K8, K12, K16, K20, K59	11481.4046	C506H849N163O140S
7	H4Kac	50.5	16.7	36.3	18.3	Ace: K5, K8, K12, K16, K31, K59	11481.4046	C506H849N163O140S
8	H4Kac	49.5	16.7	36.2	17.9	Ace: K5, K8, K12, K20, K31, K59	11481.4046	C506H849N163O140S
9	H4Kac	49.5	16.7	36.2	17.9	Ace: K5, K8, K16, K20, K31, K59	11481.4046	C506H849N163O140S
10	H4Kac	47.5	12.7	36.2	17.2	Ace: K5, K12, K16, K20, K31, K44	11481.4046	C506H849N163O140S

Sequence

Name: H4Kac

```
                   10                    20                    30                    40                    50                    60
S G R G K G G K G L   G K G G A K R H R K   V L R D N I Q G I T   K P A I R R L A R R   G G V K R I S G L I   Y E E T R G V L K V
                   70                    80                    90                   100                   110                   120
F L E N V I R D A V   T Y T E H A K R K T   V T A M D V V Y A L   K R Q G R T L Y G F   G G
```

Modifications

Modification	Position	Type
Acetyl	5	variable
Acetyl	8	variable
Acetyl	12	variable
Acetyl	16	variable
Acetyl	20	variable
Acetyl	31	variable
Acetyl	44	variable
Acetyl	59	variable
Acetyl	77	variable

Sequence Map

Proteoform:	Index	Protein	SC [%]	SVP [%]	IC [%]	MS Score [a.u.]	Var. Mods	Mono Mass [Da]	Chemical Formula
	1	H4Kac	52.5	16.7	39.3	20.6	Ace: K5, K8, K12, K16, K20, K44	11481.4046	C506H849N163O140S

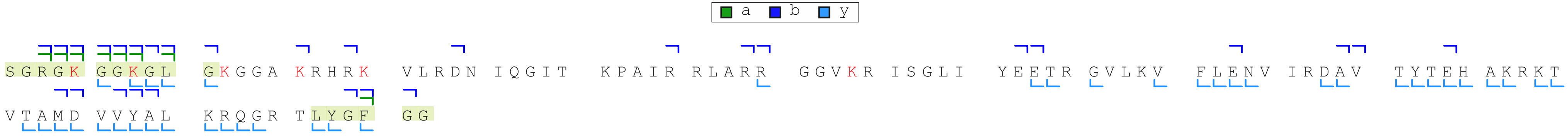

Proteoform:

Index	Protein	SC [%]	SVP [%]	IC [%]	MS Score [a.u.]	Var. Mods	Mono Mass [Da]	Chemical Formula
2	H4Kac	51.5	16.7	39.1	20.2	Ace: K5, K8, K12, K16, K31, K44	11481.4046	C506H849N163O140S

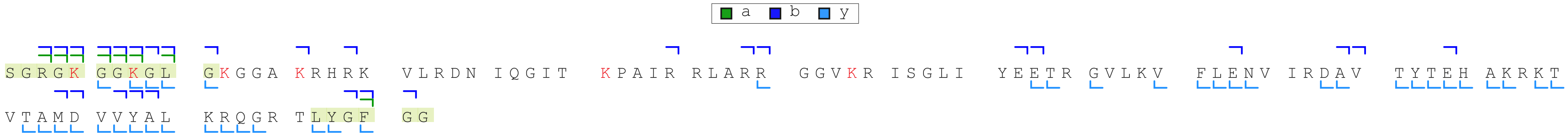

Proteoform:

Index	Protein	SC [%]	SVP [%]	IC [%]	MS Score [a.u.]	Var. Mods	Mono Mass [Da]	Chemical Formula
3	H4Kac	50.5	16.7	39.1	19.7	Ace: K5, K8, K12, K16, K20, K31	11481.4046	C506H849N163O140S

a b y

SGRGK GGKGL GKGGA KRHRK VLRDN IQGIT KPAIR RLARR GGVKR ISGLI YEETR GVLKV FLENV IRDAV TYTEH AKRKT
VTAMD VVYAL KRQGR TLYGF GG

Proteoform:	Index	Protein	SC [%]	SVP [%]	IC [%]	MS Score [a.u.]	Var. Mods	Mono Mass [Da]	Chemical Formula
	4	H4Kac	50.5	16.7	39.1	19.7	Ace: K5, K8, K12, K20, K31, K44	11481.4046	C506H849N163O140S

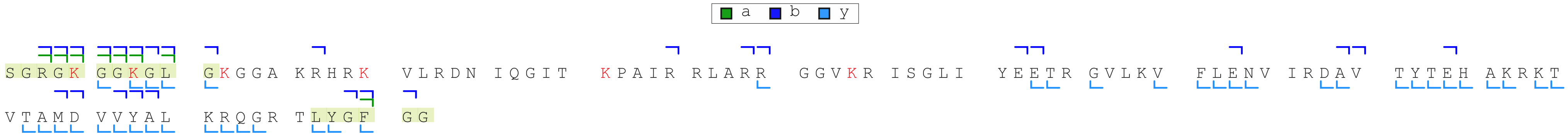

Proteoform:

Index	Protein	SC [%]	SVP [%]	IC [%]	MS Score [a.u.]	Var. Mods	Mono Mass [Da]	Chemical Formula
5	H4Kac	50.5	16.7	39.1	19.7	Ace: K5, K8, K16, K20, K31, K44	11481.4046	C506H849N163O140S

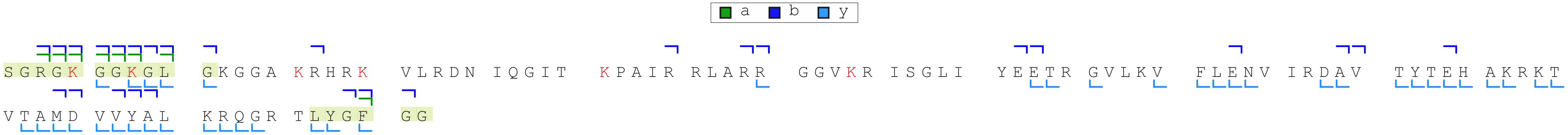

Proteoform:

Index	Protein	SC [%]	SVP [%]	IC [%]	MS Score [a.u.]	Var. Mods	Mono Mass [Da]	Chemical Formula
6	H4Kac	51.5	16.7	36.4	18.8	Ace: K5, K8, K12, K16, K20, K59	11481.4046	C506H849N163O140S

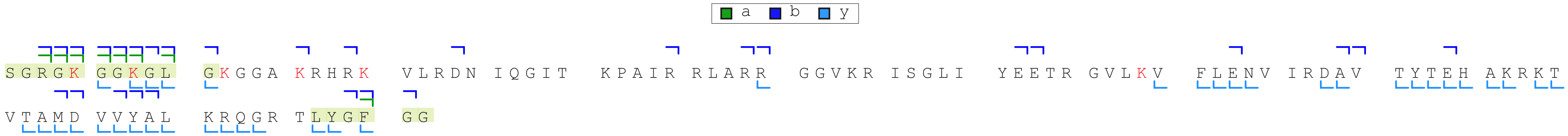

Proteoform:

Index	Protein	SC [%]	SVP [%]	IC [%]	MS Score [a.u.]	Var. Mods	Mono Mass [Da]	Chemical Formula
7	H4Kac	50.5	16.7	36.3	18.3	Ace: K5, K8, K12, K16, K31, K59	11481.4046	C506H849N163O140S

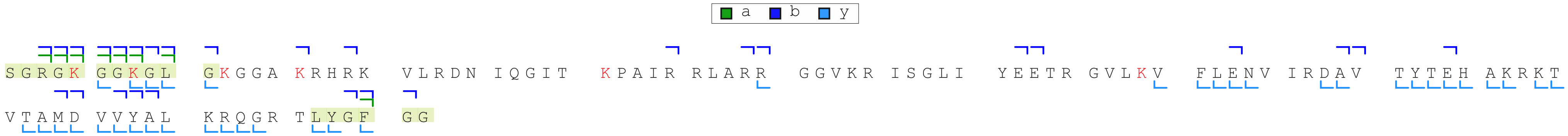

Proteoform:

Index	Protein	SC [%]	SVP [%]	IC [%]	MS Score [a.u.]	Var. Mods	Mono Mass [Da]	Chemical Formula
8	H4Kac	49.5	16.7	36.2	17.9	Ace: K5, K8, K12, K20, K31, K59	11481.4046	C506H849N163O140S

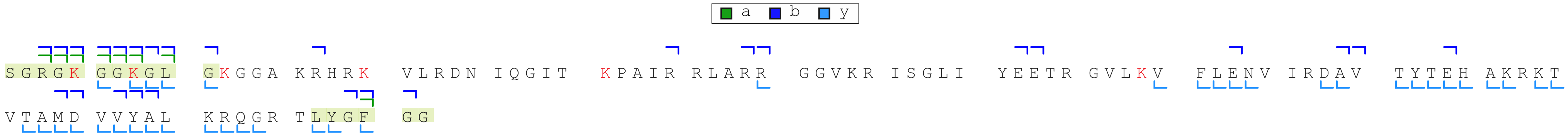

Proteoform:

Index	Protein	SC [%]	SVP [%]	IC [%]	MS Score [a.u.]	Var. Mods	Mono Mass [Da]	Chemical Formula
9	H4Kac	49.5	16.7	36.2	17.9	Ace: K5, K8, K16, K20, K31, K59	11481.4046	C506H849N163O140S

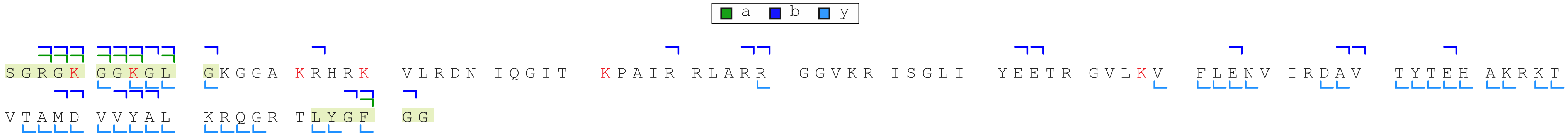

Proteoform:

Index	Protein	SC [%]	SVP [%]	IC [%]	MS Score [a.u.]	Var. Mods	Mono Mass [Da]	Chemical Formula
10	H4Kac	47.5	12.7	36.2	17.2	Ace: K5, K12, K16, K20, K31, K44	11481.4046	$C_{506}H_{849}N_{163}O_{140}S$

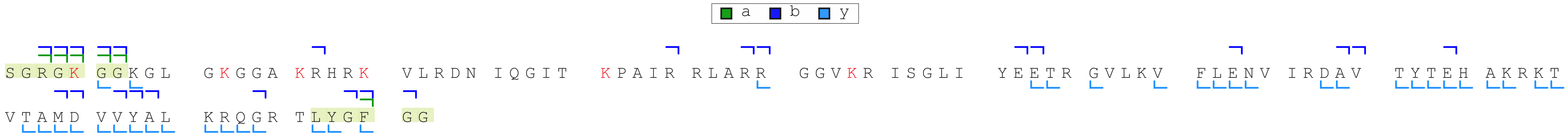

Error Plot

Proteoform:

Index	Protein	SC [%]	SVP [%]	IC [%]	MS Score [a.u.]	Var. Mods	Mono Mass [Da]	Chemical Formula
1	H4Kac	52.5	16.7	39.3	20.6	Ace: K5, K8, K12, K16, K20, K44	11481.4046	C506H849N163O140S

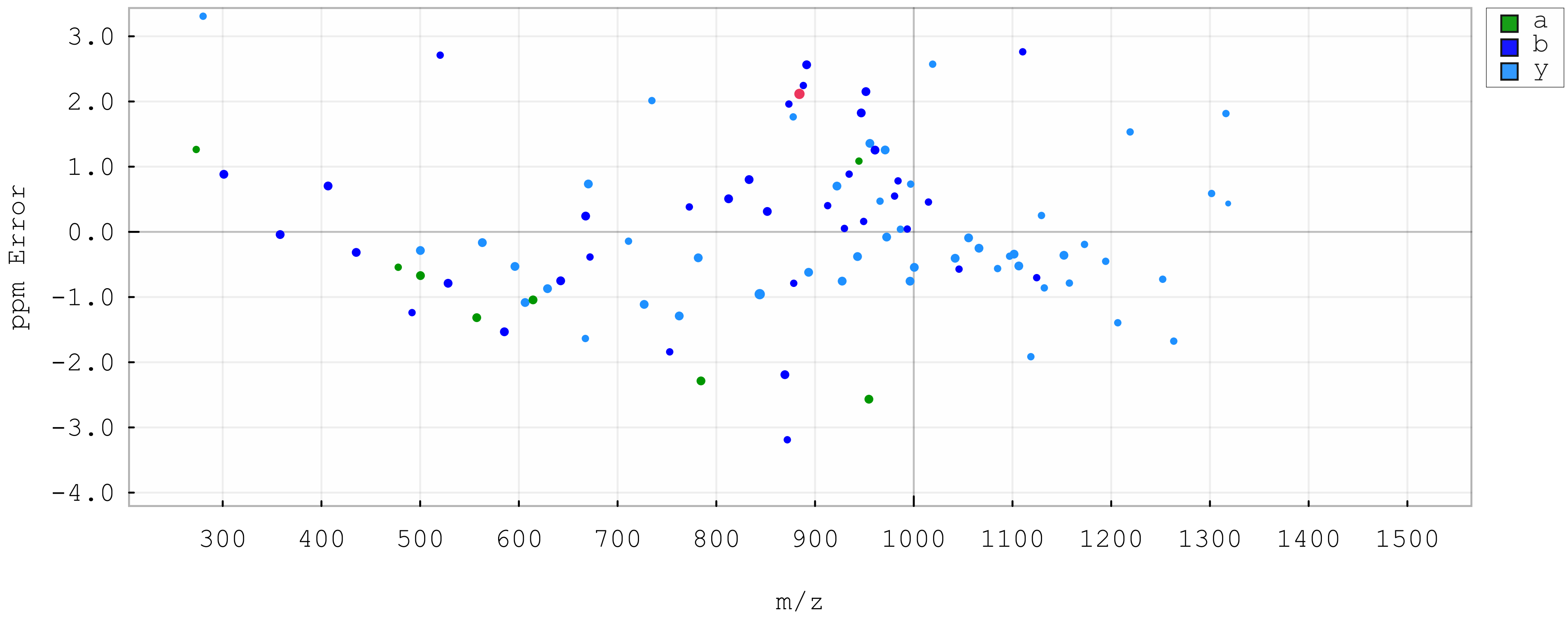

Proteoform:

Index	Protein	SC [%]	SVP [%]	IC [%]	MS Score [a.u.]	Var. Mods	Mono Mass [Da]	Chemical Formula
2	H4Kac	51.5	16.7	39.1	20.2	Ace: K5, K8, K12, K16, K31, K44	11481.4046	C506H849N163O140S

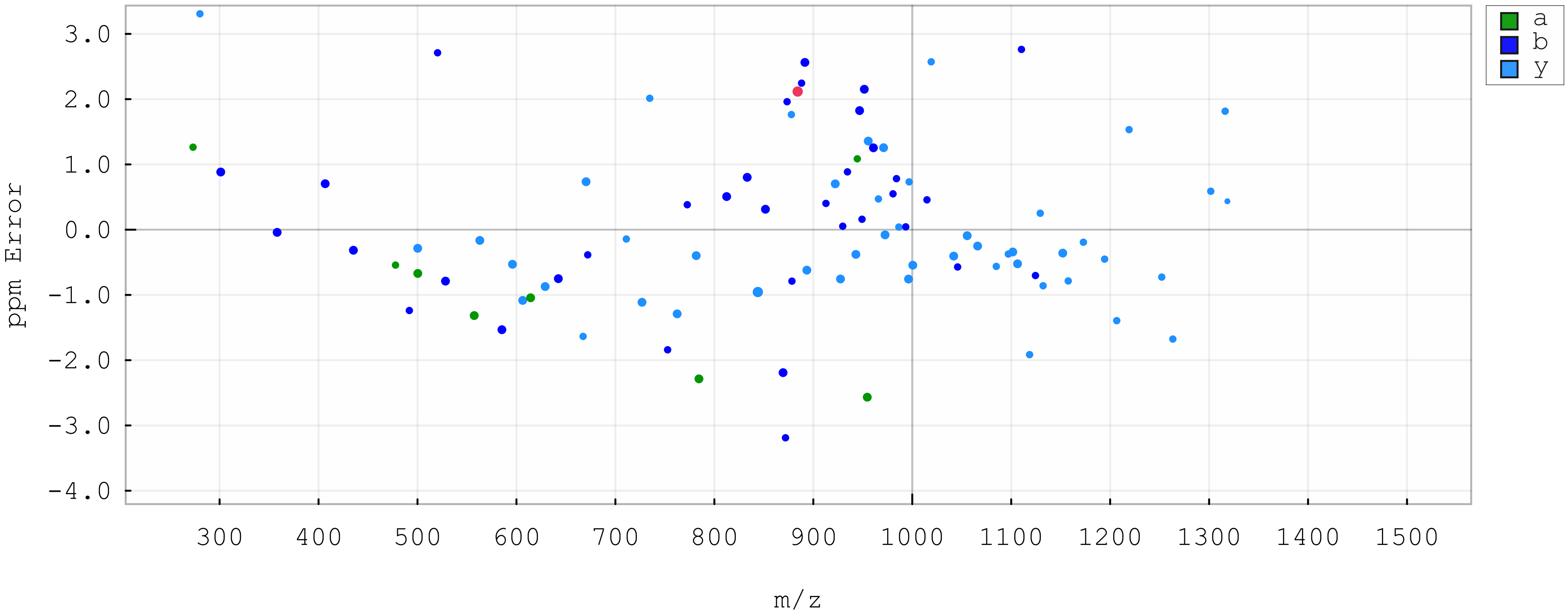

Proteoform:

Index	Protein	SC [%]	SVP [%]	IC [%]	MS Score [a.u.]	Var. Mods	Mono Mass [Da]	Chemical Formula
3	H4Kac	50.5	16.7	39.1	19.7	Ace: K5, K8, K12, K16, K20, K31	11481.4046	C506H849N163O140S

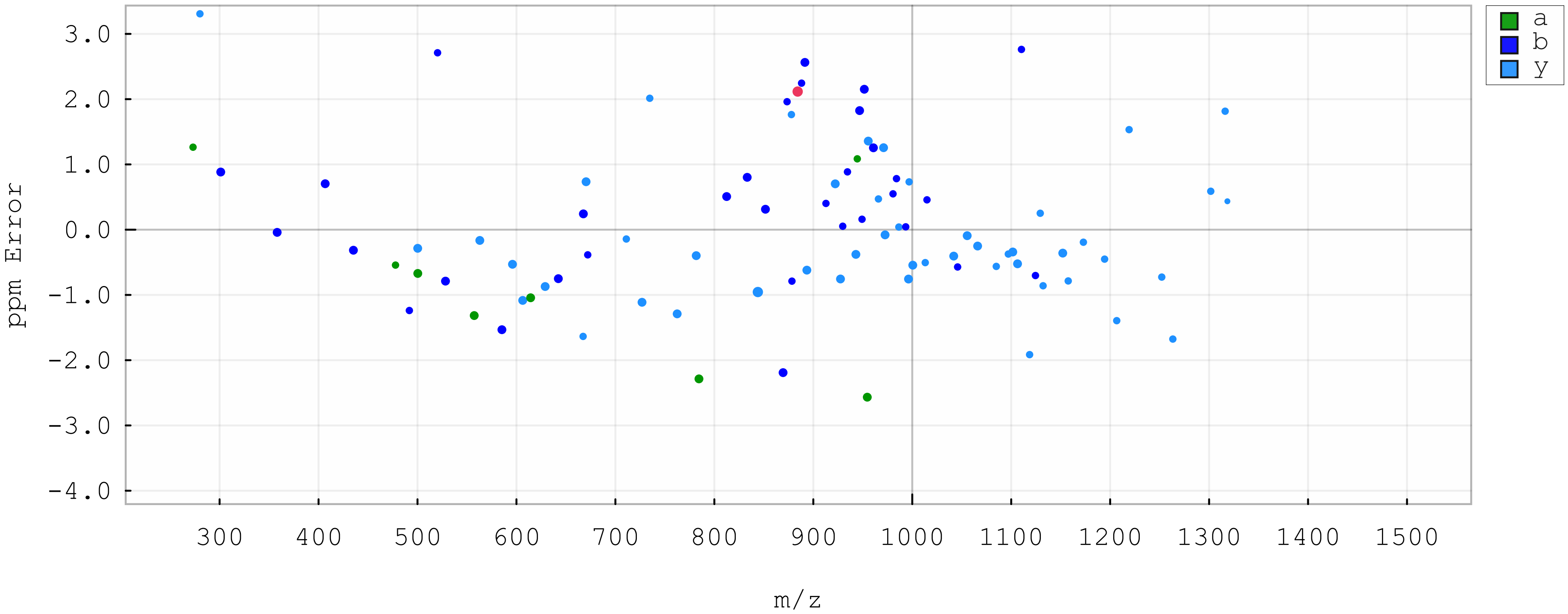

Proteoform:

Index	Protein	SC [%]	SVP [%]	IC [%]	MS Score [a.u.]	Var. Mods	Mono Mass [Da]	Chemical Formula
4	H4Kac	50.5	16.7	39.1	19.7	Ace: K5, K8, K12, K20, K31, K44	11481.4046	C506H849N163O140S

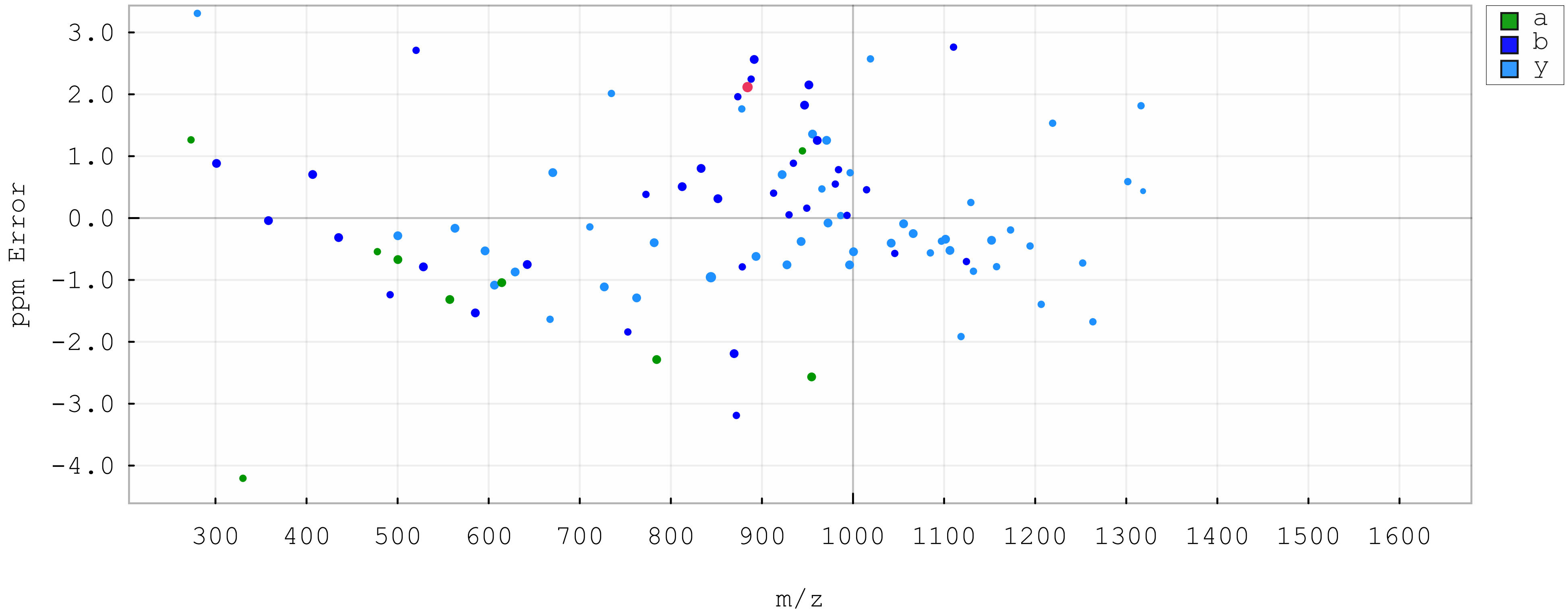

Proteoform:

Index	Protein	SC [%]	SVP [%]	IC [%]	MS Score [a.u.]	Var. Mods	Mono Mass [Da]	Chemical Formula
5	H4Kac	50.5	16.7	39.1	19.7	Ace: K5, K8, K16, K20, K31, K44	11481.4046	C506H849N163O140S

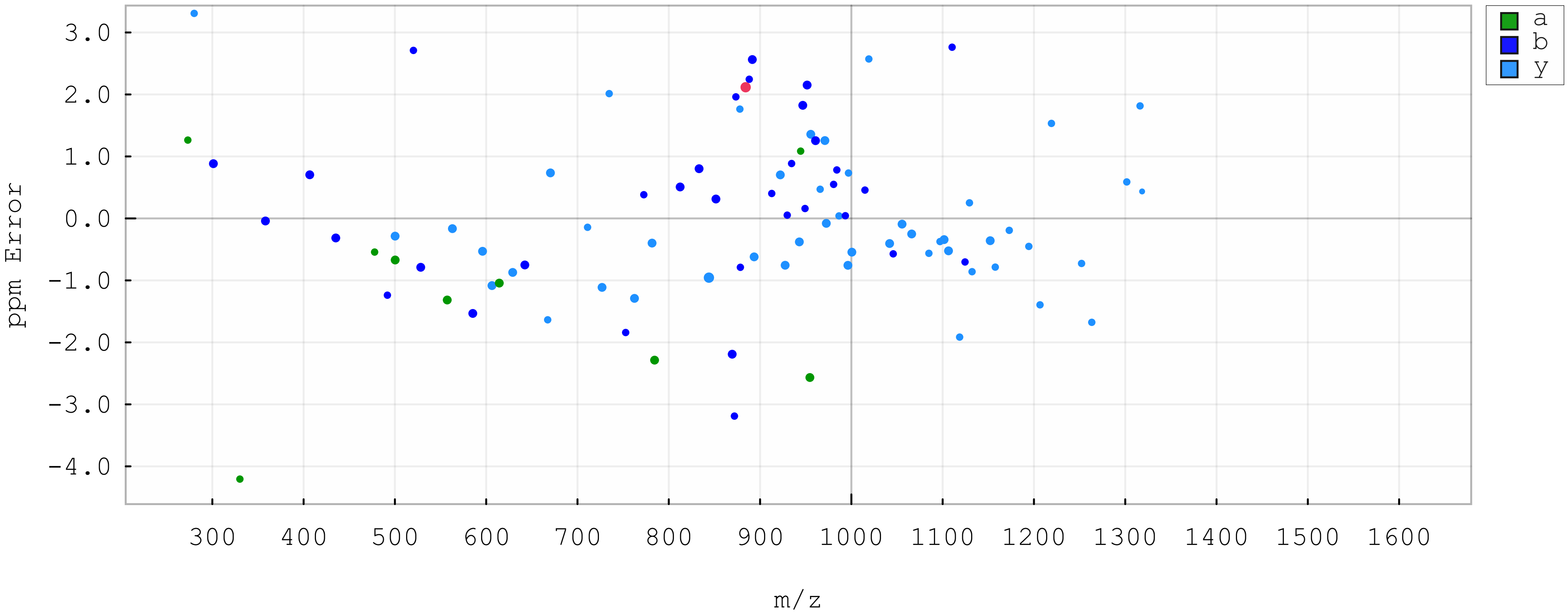

Proteoform:

Index	Protein	SC [%]	SVP [%]	IC [%]	MS Score [a.u.]	Var. Mods	Mono Mass [Da]	Chemical Formula
6	H4Kac	51.5	16.7	36.4	18.8	Ace: K5, K8, K12, K16, K20, K59	11481.4046	C506H849N163O140S

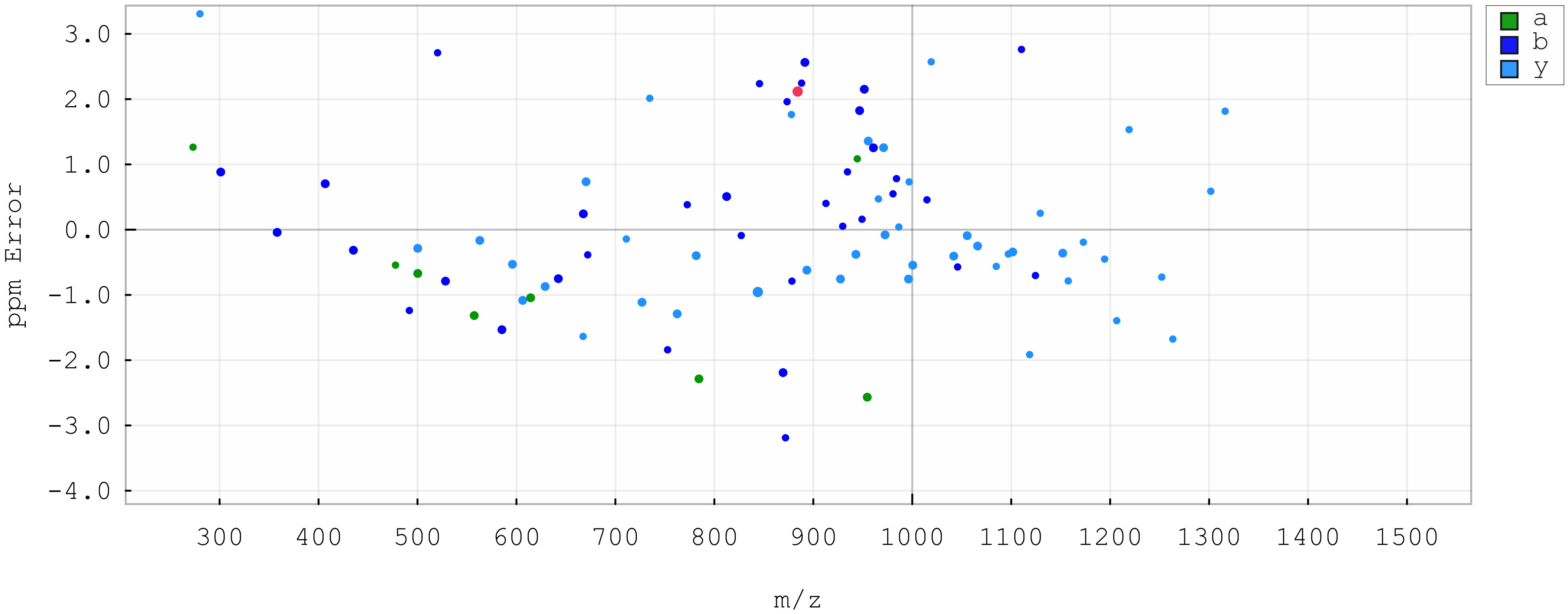

Proteoform:

Index	Protein	SC [%]	SVP [%]	IC [%]	MS Score [a.u.]	Var. Mods	Mono Mass [Da]	Chemical Formula
7	H4Kac	50.5	16.7	36.3	18.3	Ace: K5, K8, K12, K16, K31, K59	11481.4046	C506H849N163O140S

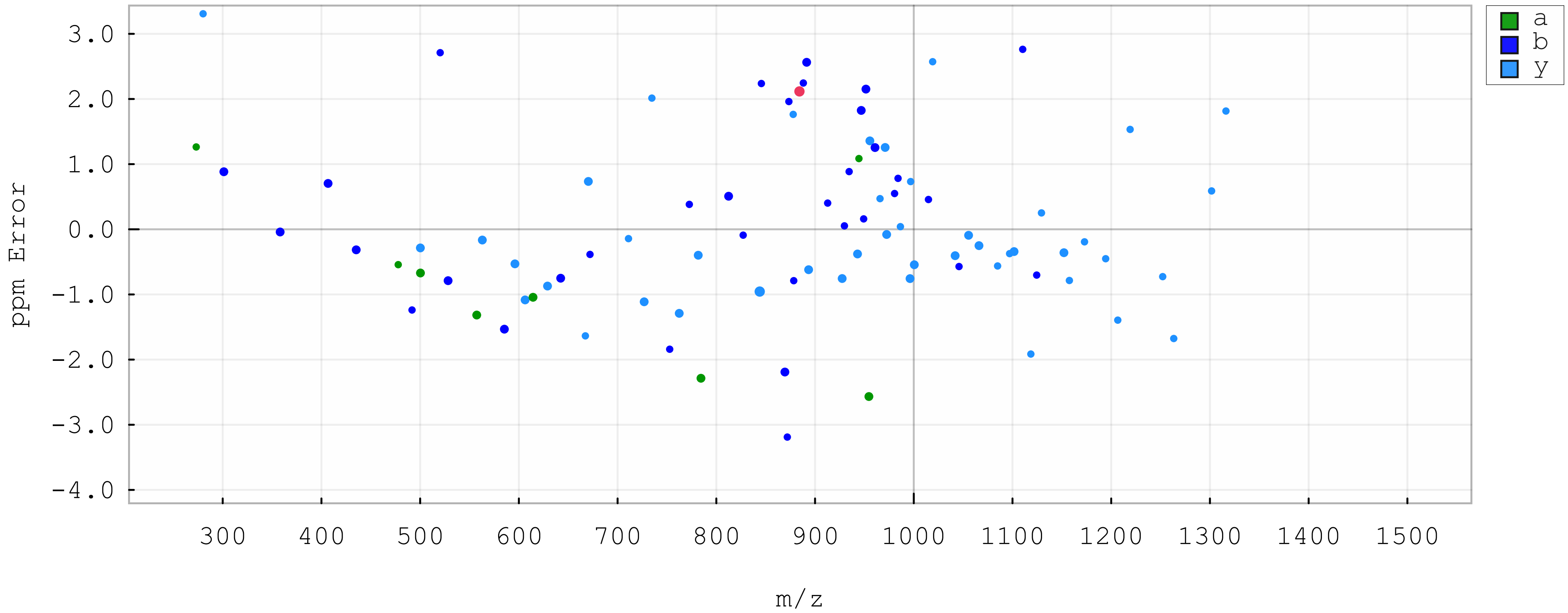

Proteoform:

Index	Protein	SC [%]	SVP [%]	IC [%]	MS Score [a.u.]	Var. Mods	Mono Mass [Da]	Chemical Formula
8	H4Kac	49.5	16.7	36.2	17.9	Ace: K5, K8, K12, K20, K31, K59	11481.4046	C506H849N163O140S

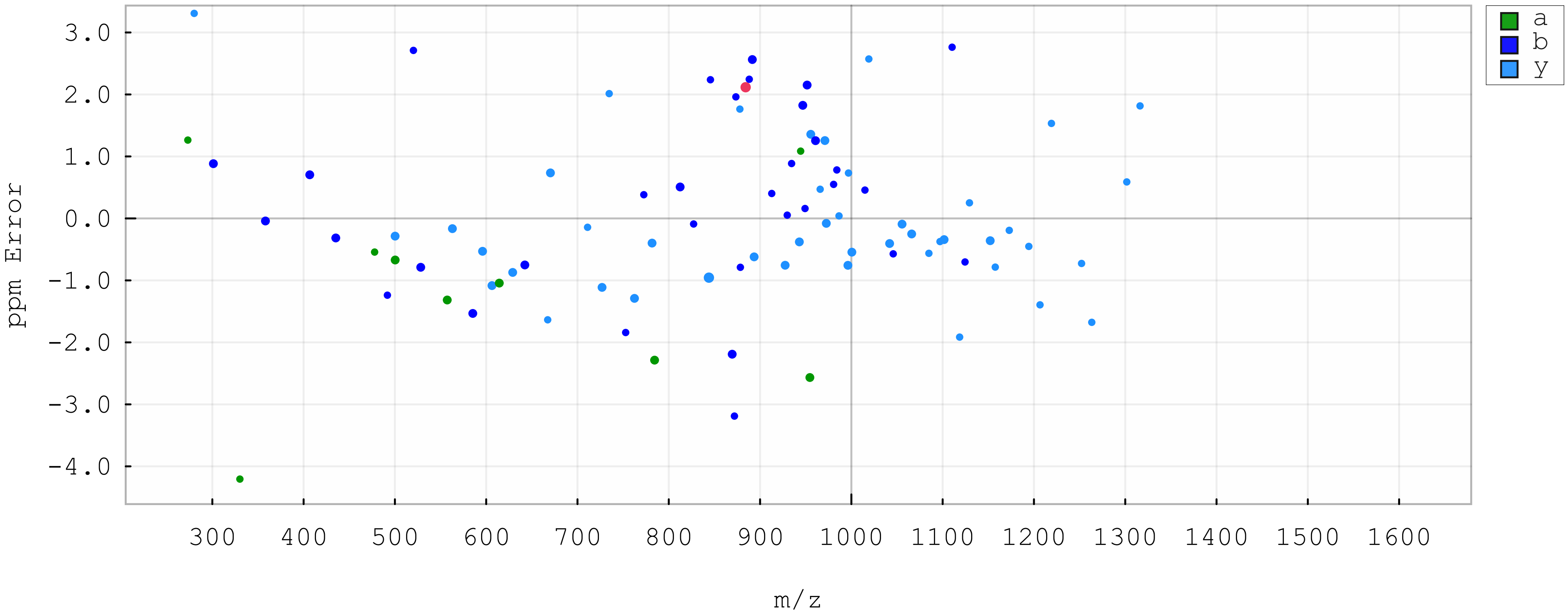

Proteoform:

Index	Protein	SC [%]	SVP [%]	IC [%]	MS Score [a.u.]	Var. Mods	Mono Mass [Da]	Chemical Formula
9	H4Kac	49.5	16.7	36.2	17.9	Ace: K5, K8, K16, K20, K31, K59	11481.4046	C506H849N163O140S

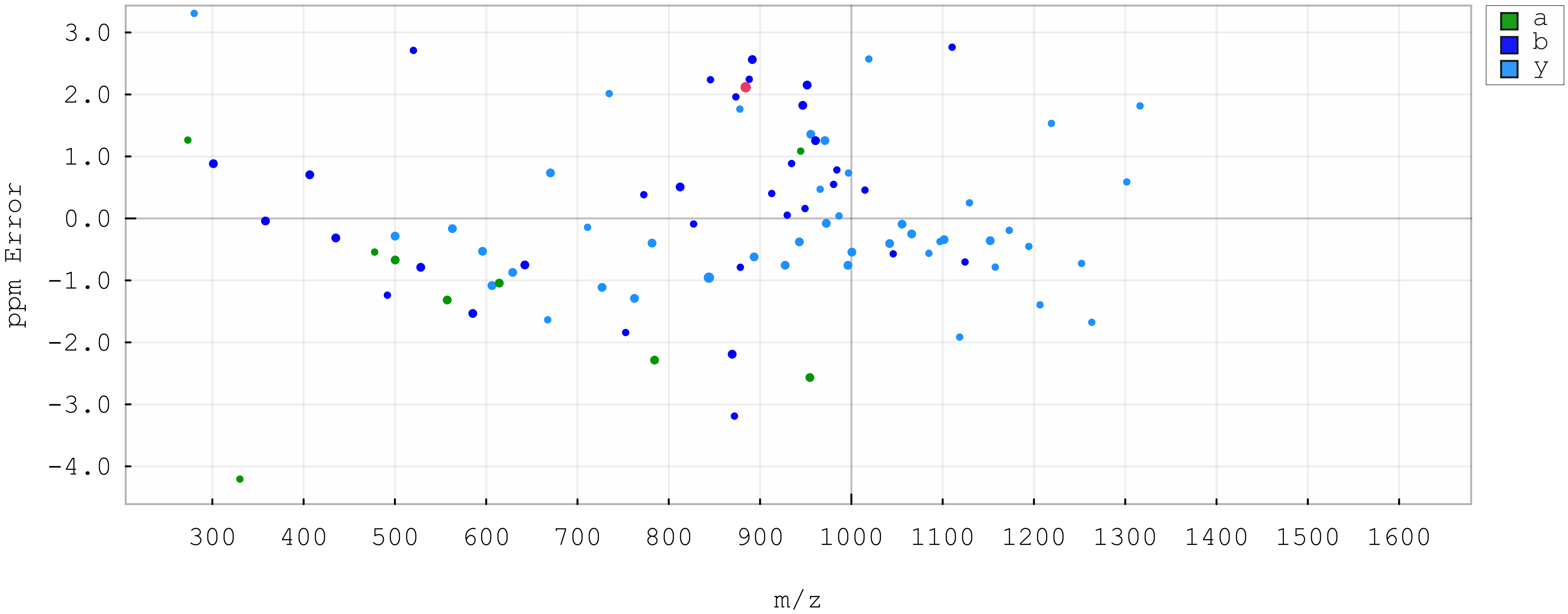

Proteoform:

Index	Protein	SC [%]	SVP [%]	IC [%]	MS Score [a.u.]	Var. Mods	Mono Mass [Da]	Chemical Formula
10	H4Kac	47.5	12.7	36.2	17.2	Ace: K5, K12, K16, K20, K31, K44	11481.4046	C506H849N163O140S

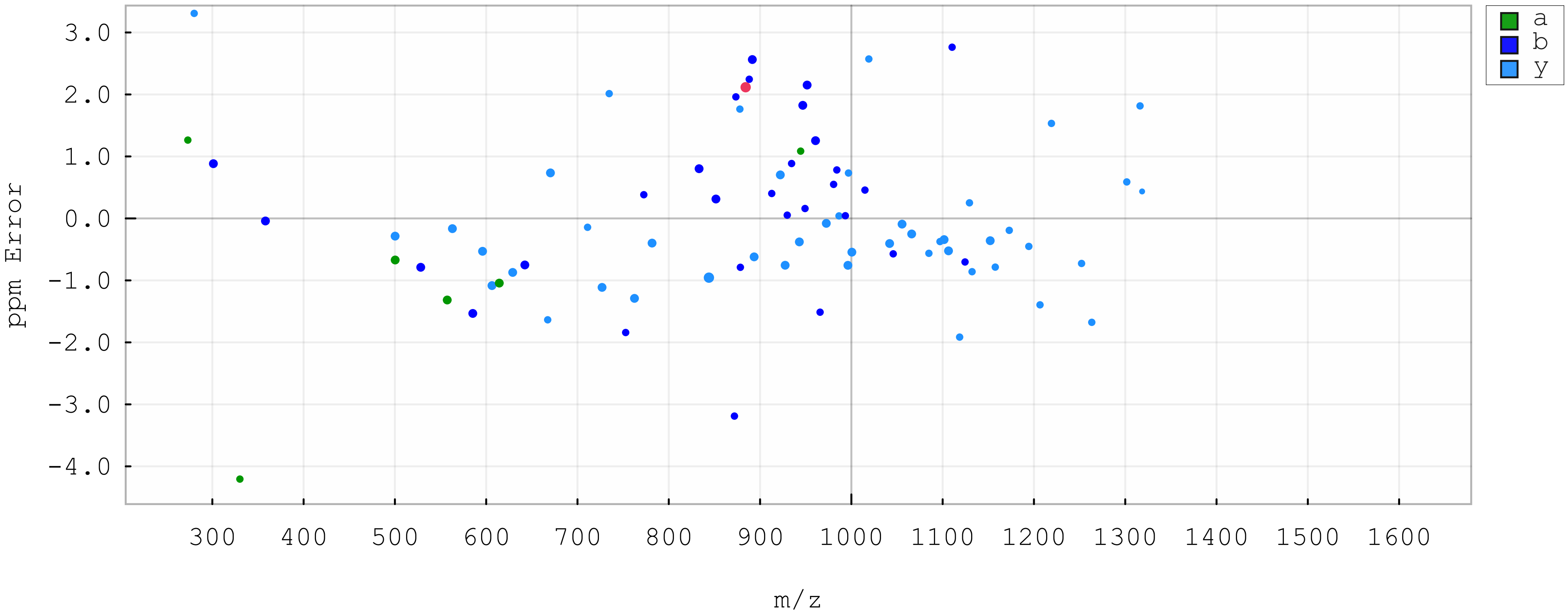

Primary Fragment Plots

Proteoform:

Index	Protein	SC [%]	SVP [%]	IC [%]	MS Score [a.u.]	Var. Mods	Mono Mass [Da]	Chemical Formula
1	H4Kac	52.5	16.7	39.3	20.6	Ace: K5, K8, K12, K16, K20, K44	11481.4046	C506H849N163O140S

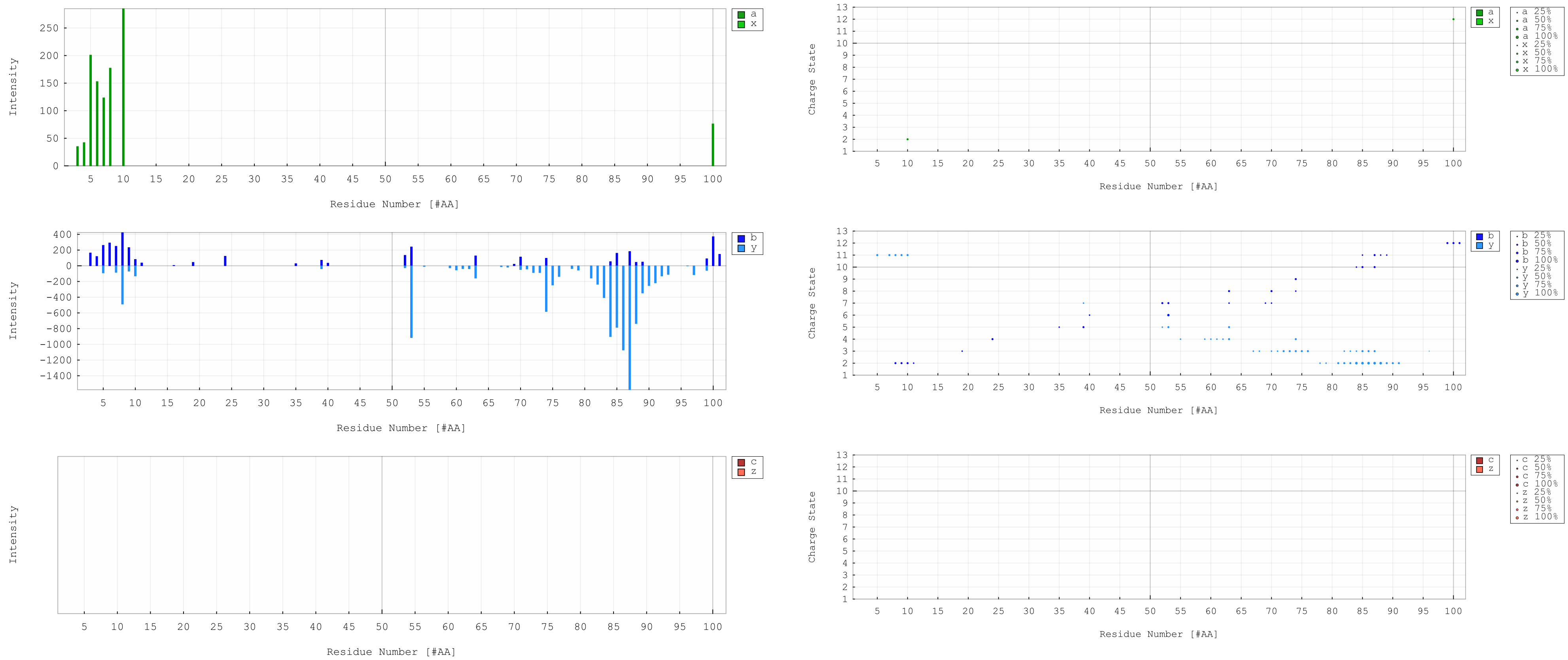

Proteoform:

Index	Protein	SC [%]	SVP [%]	IC [%]	MS Score [a.u.]	Var. Mods	Mono Mass [Da]	Chemical Formula
2	H4Kac	51.5	16.7	39.1	20.2	Ace: K5, K8, K12, K16, K31, K44	11481.4046	C506H849N163O140S

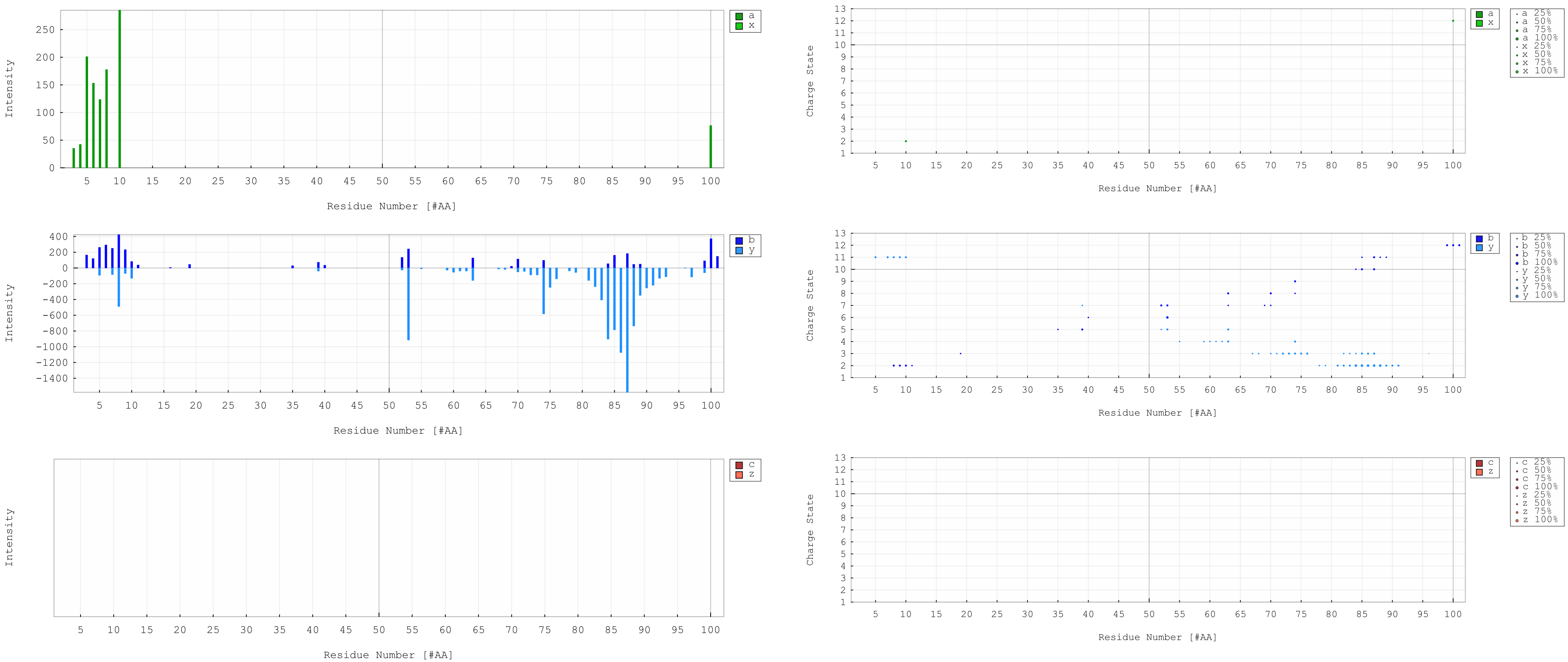

Proteoform:

Index	Protein	SC [%]	SVP [%]	IC [%]	MS Score [a.u.]	Var. Mods	Mono Mass [Da]	Chemical Formula
3	H4Kac	50.5	16.7	39.1	19.7	Ace: K5, K8, K12, K16, K20, K31	11481.4046	C506H849N163O140S

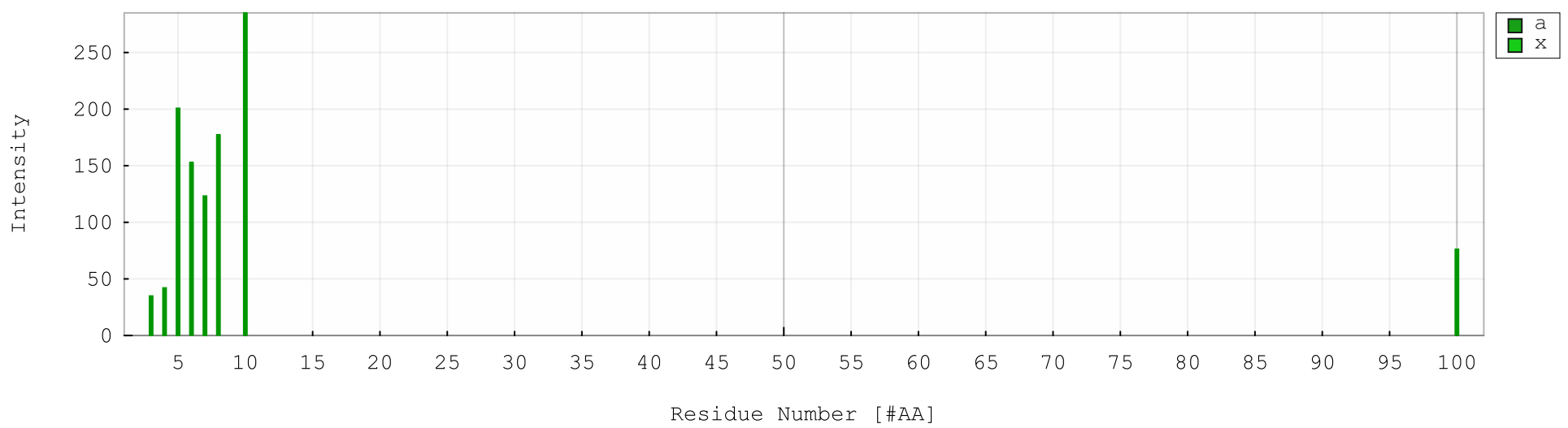

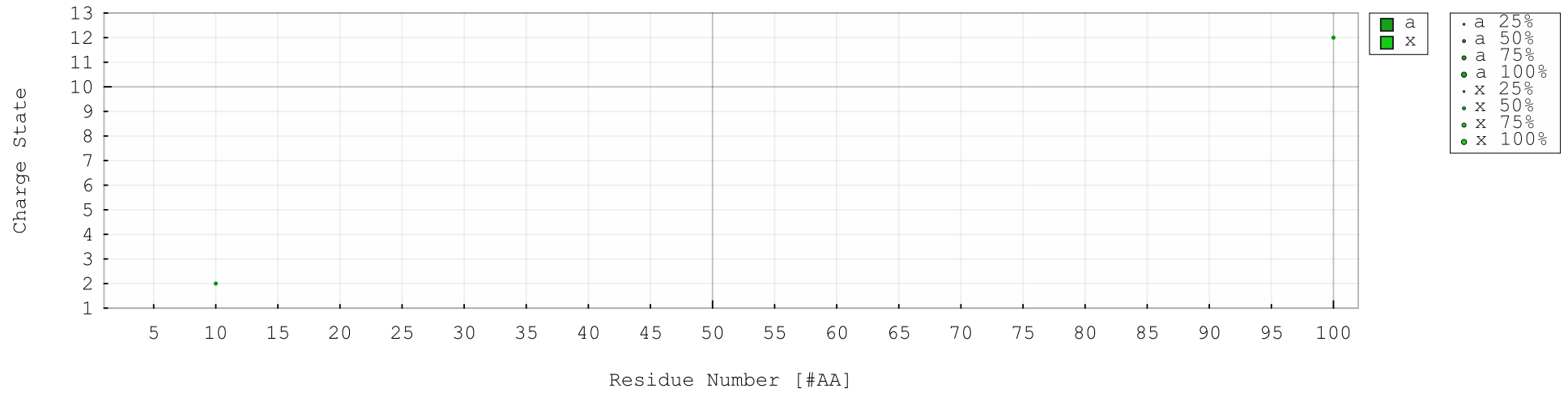

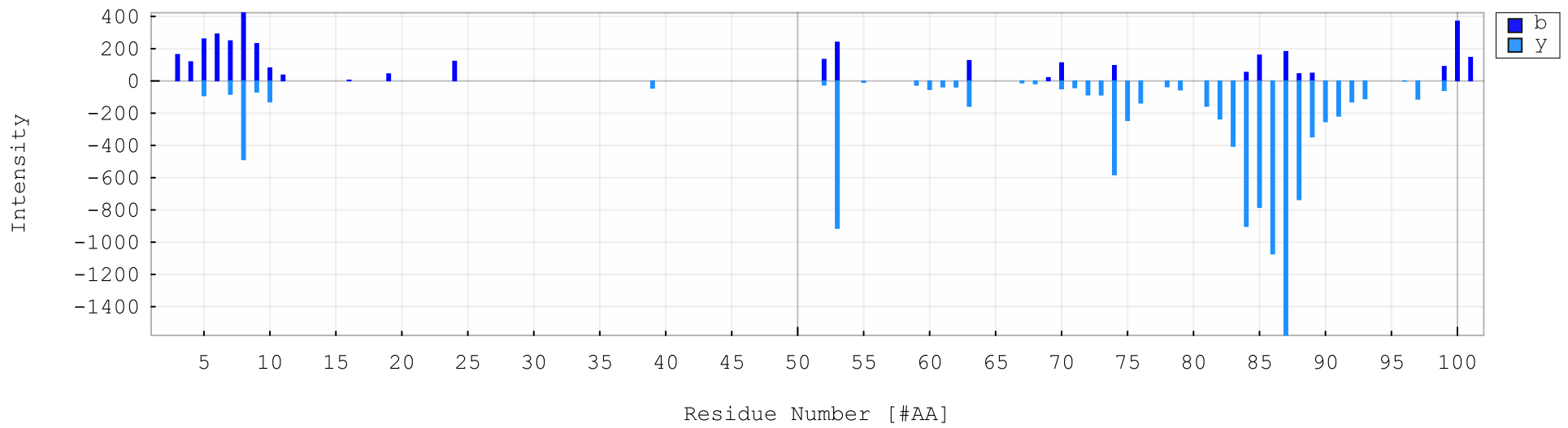

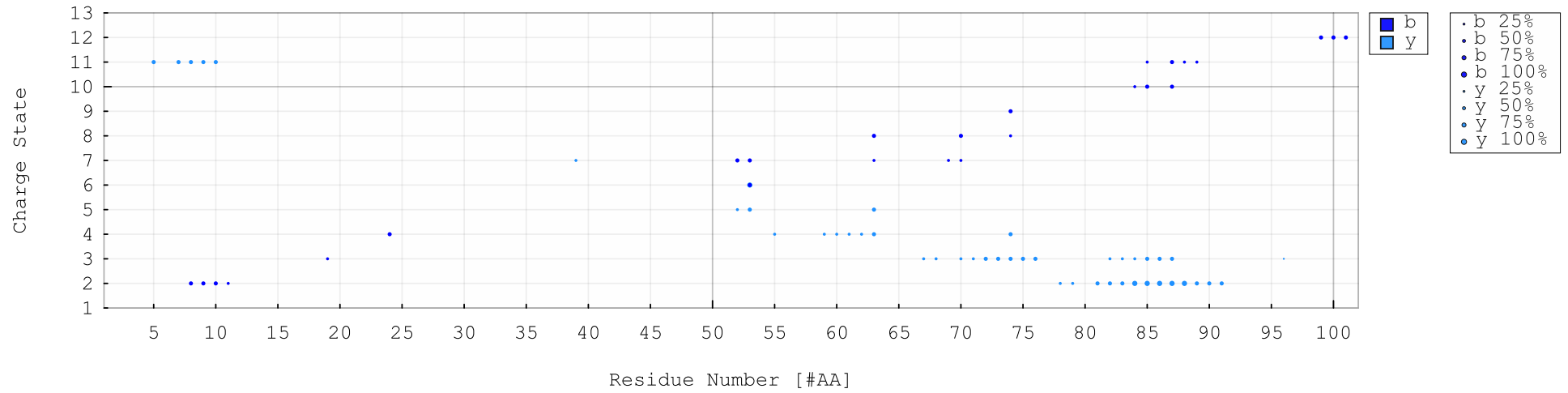

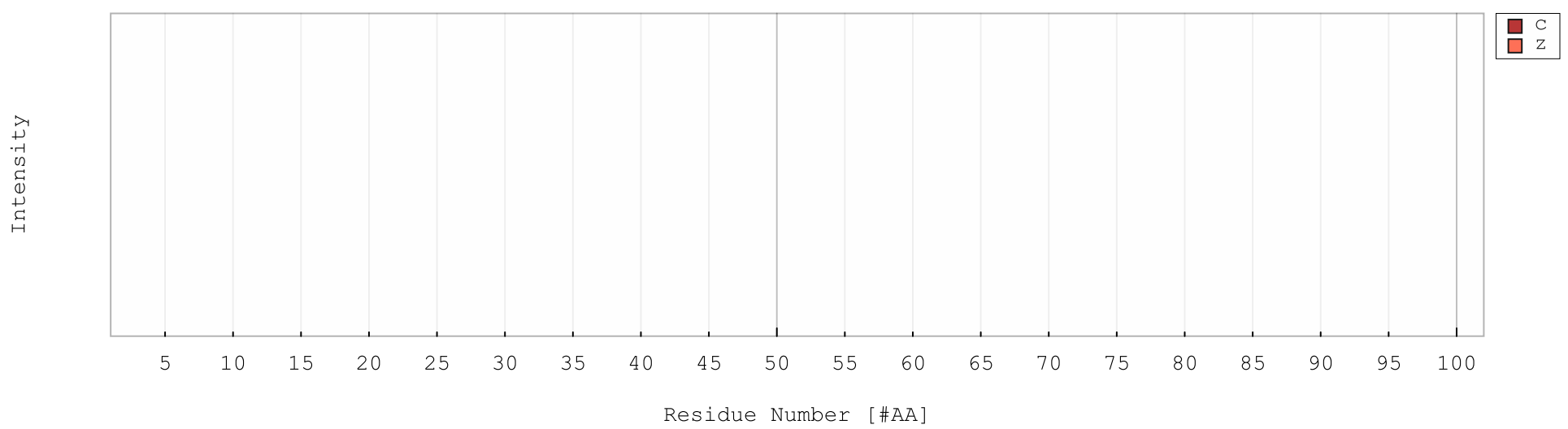

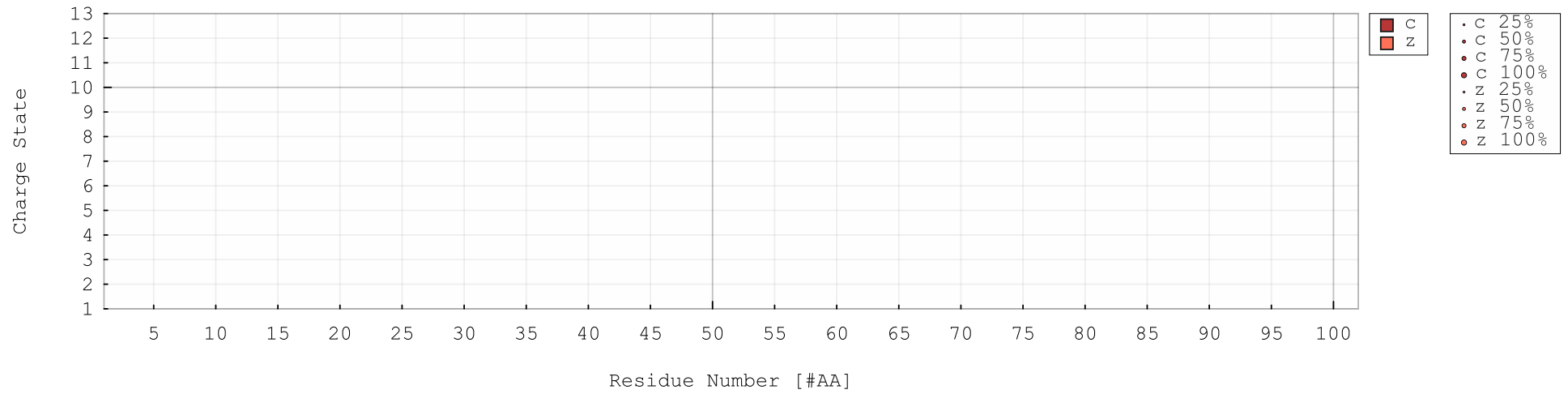

Proteoform:

Index	Protein	SC [%]	SVP [%]	IC [%]	MS Score [a.u.]	Var. Mods	Mono Mass [Da]	Chemical Formula
4	H4Kac	50.5	16.7	39.1	19.7	Ace: K5, K8, K12, K20, K31, K44	11481.4046	C506H849N163O140S

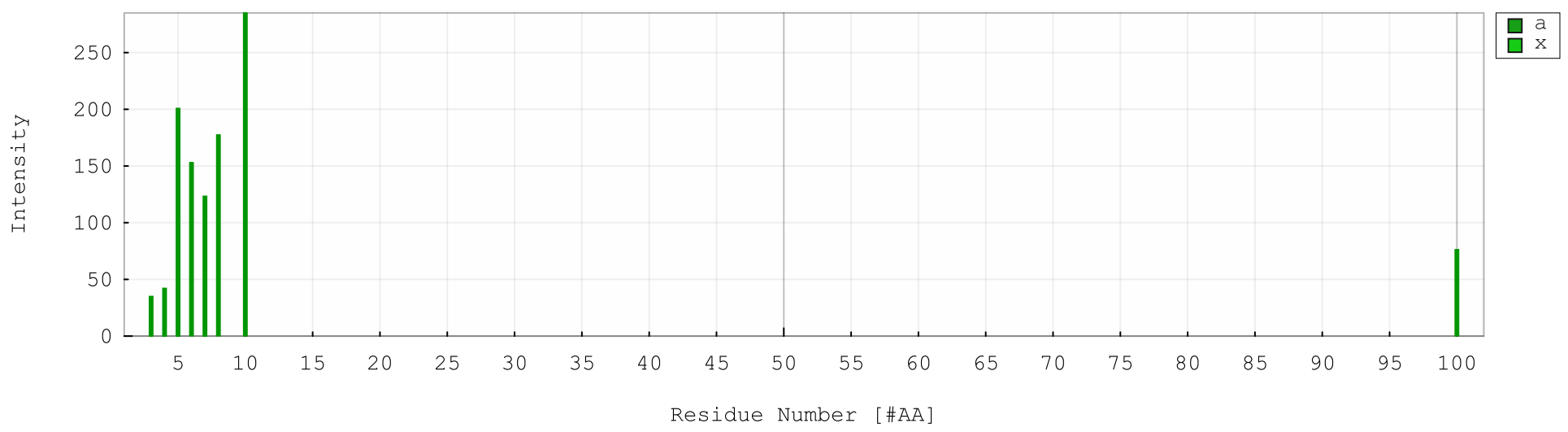

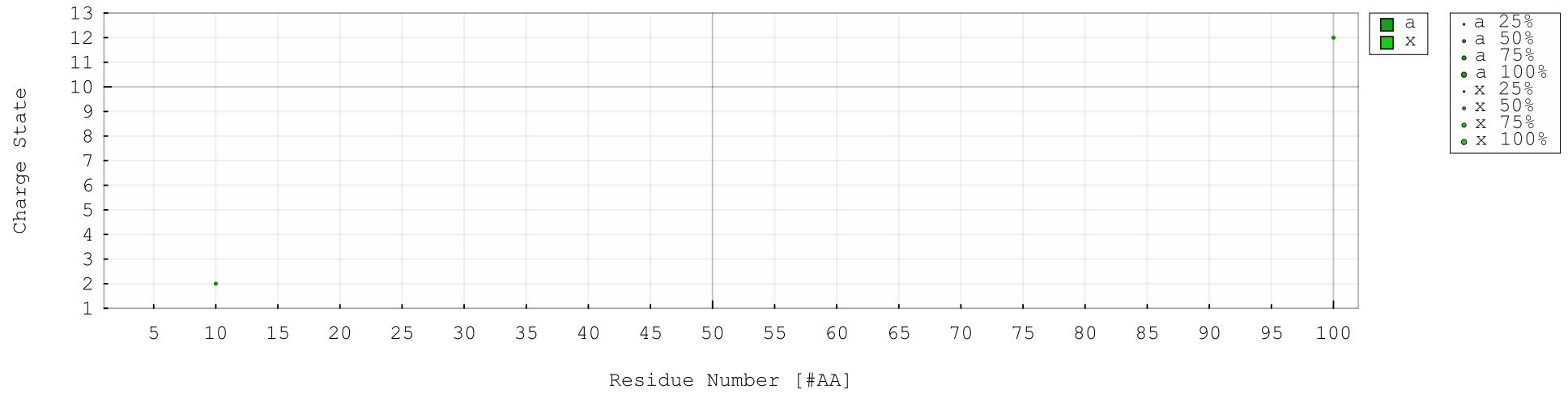

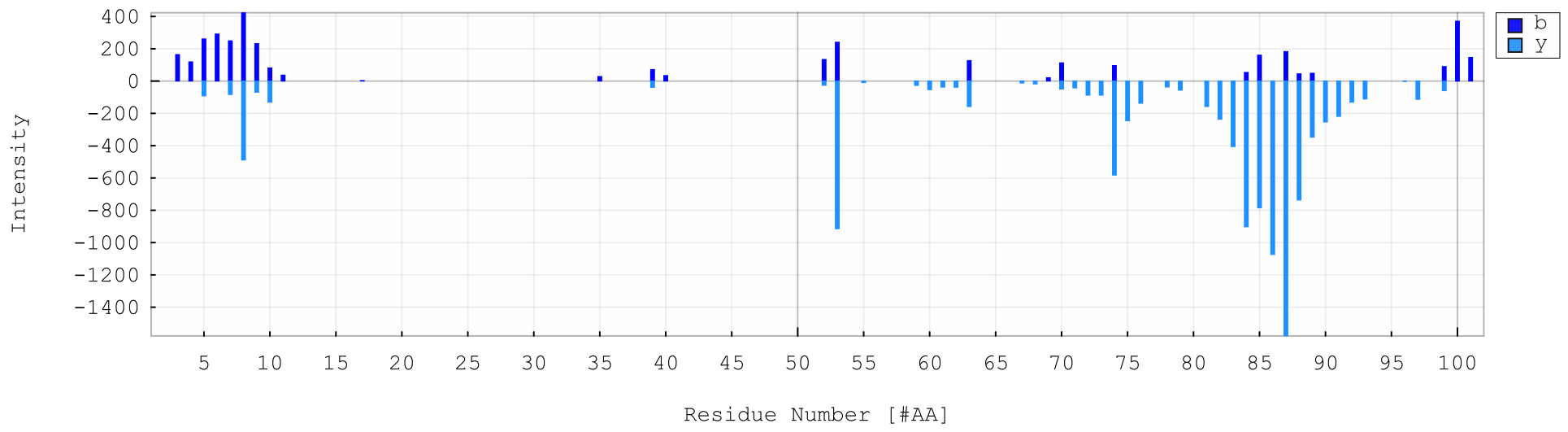

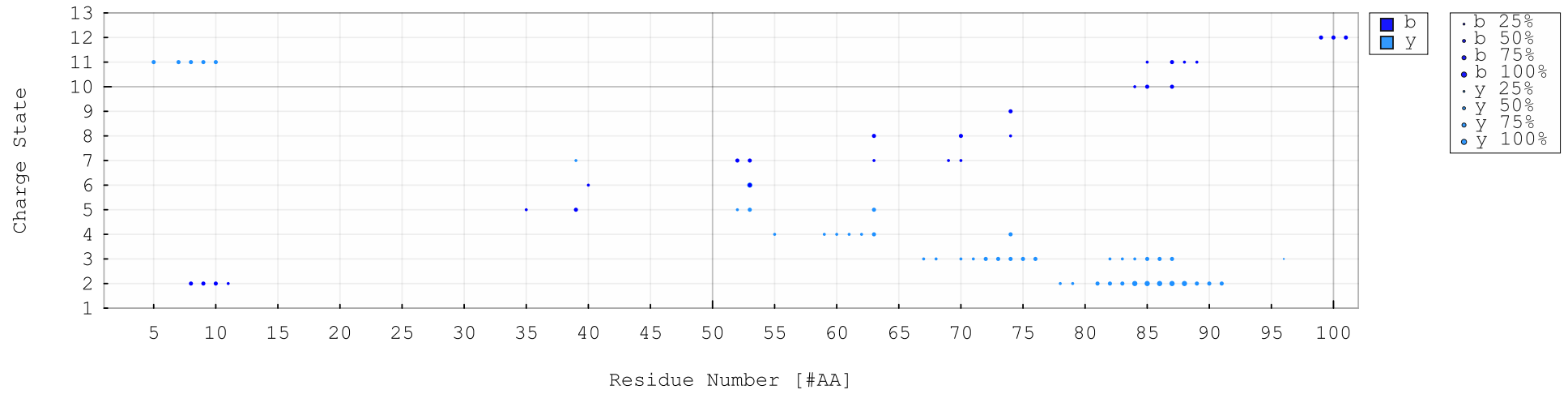

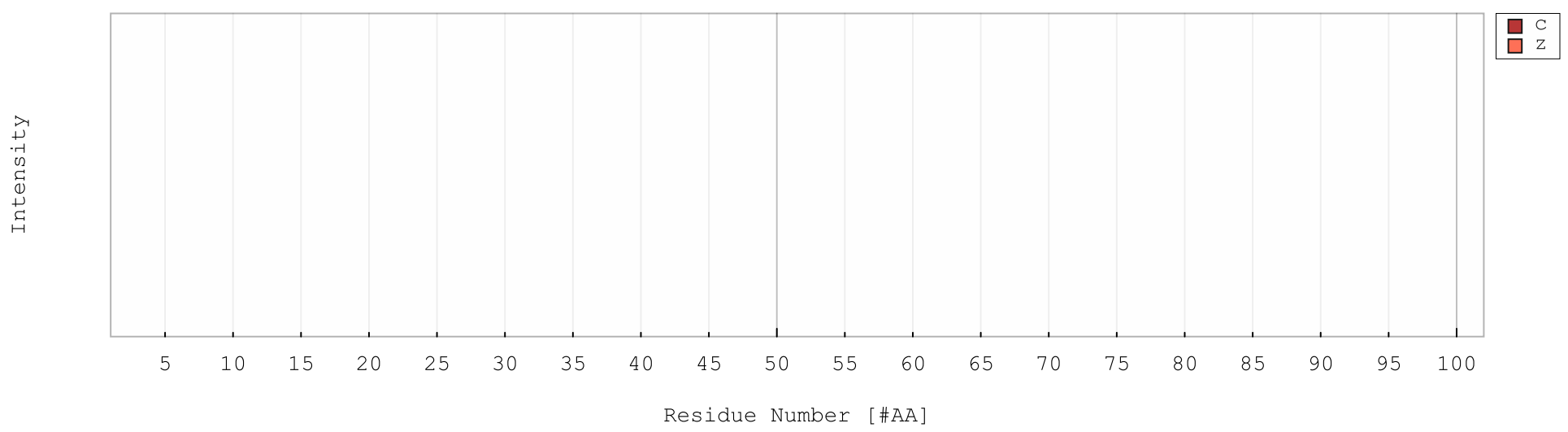

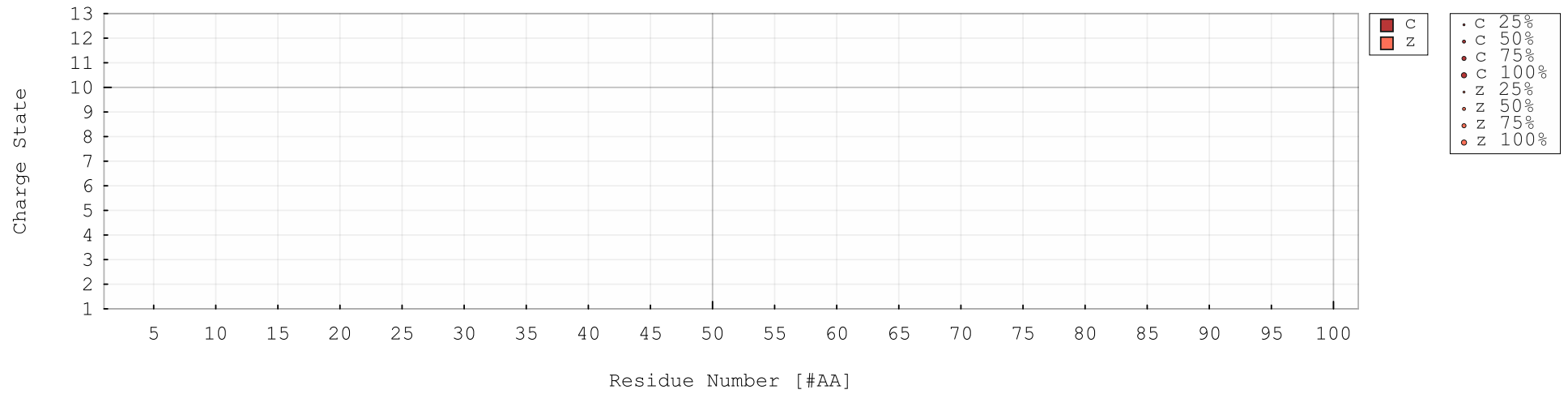

Proteoform:	Index	Protein	SC [%]	SVP [%]	IC [%]	MS Score [a.u.]	Var. Mods	Mono Mass [Da]	Chemical Formula
	5	H4Kac	50.5	16.7	39.1	19.7	Ace: K5, K8, K16, K20, K31, K44	11481.4046	C506H849N163O140S

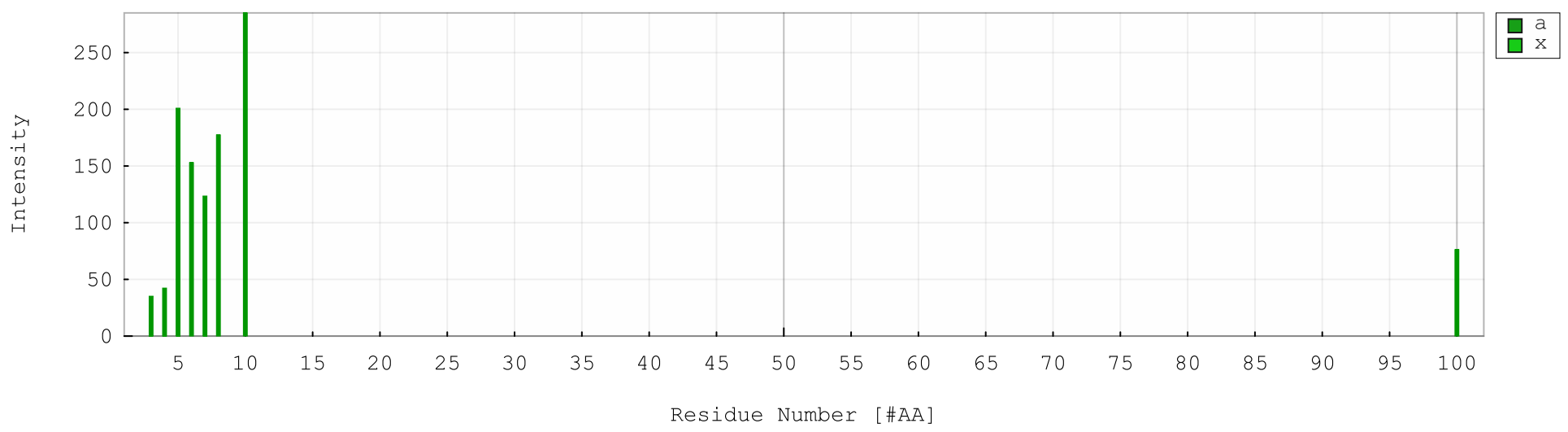

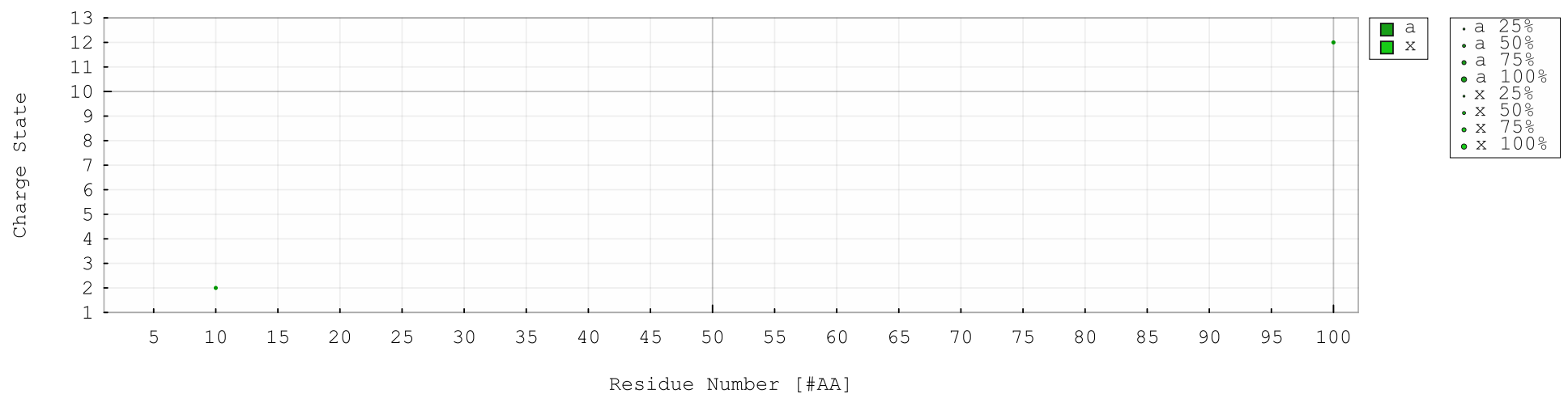

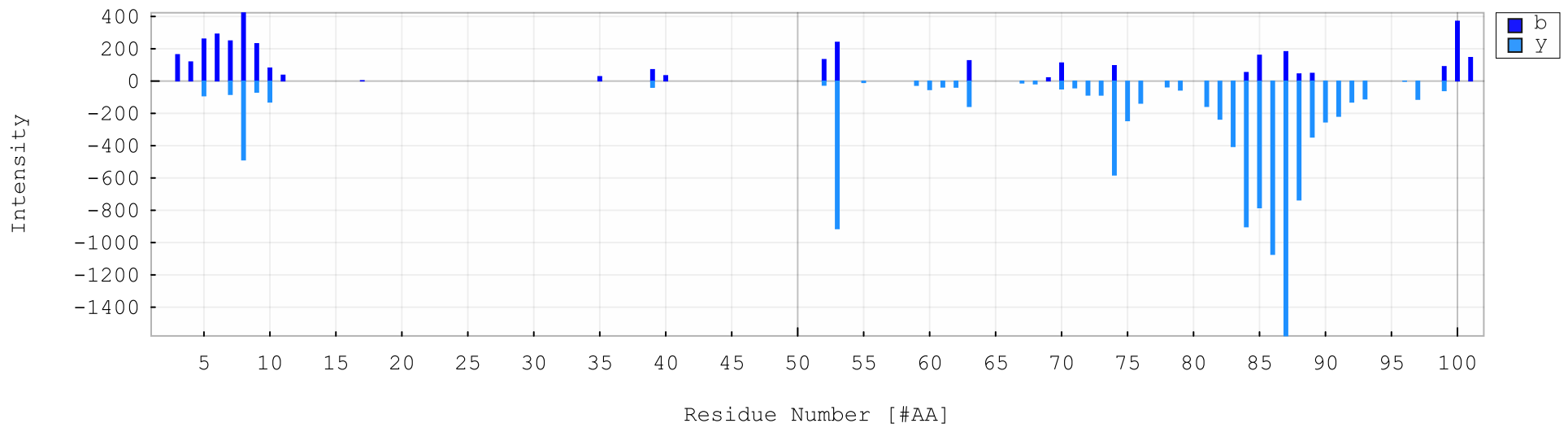

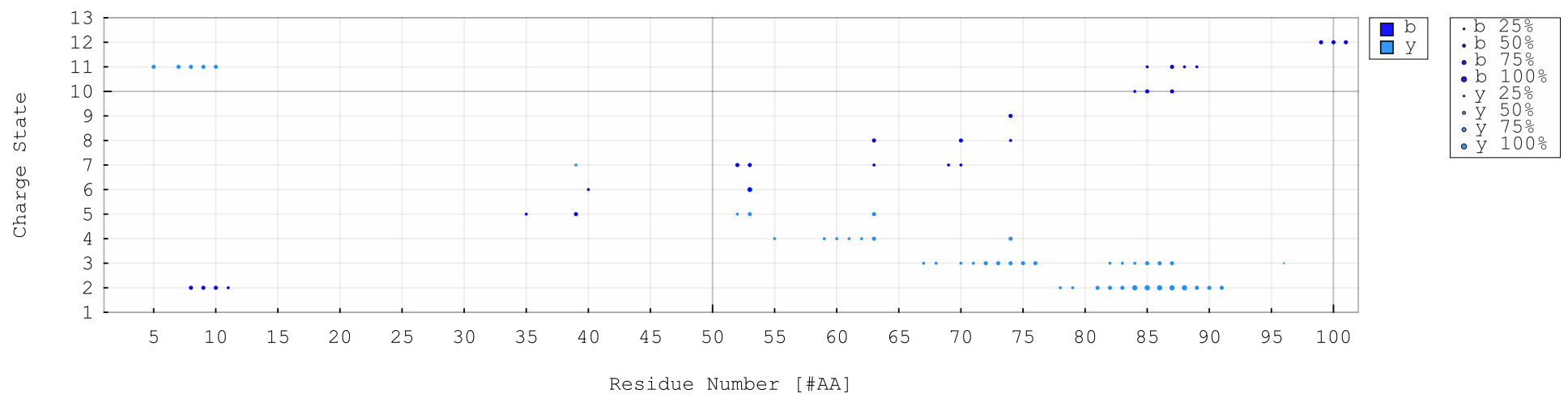

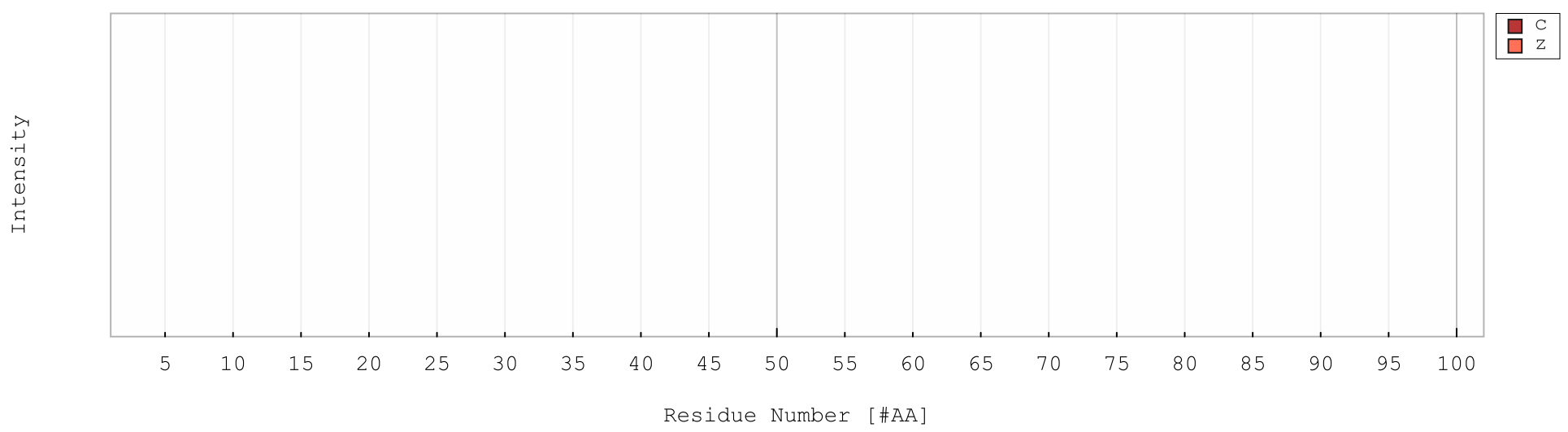

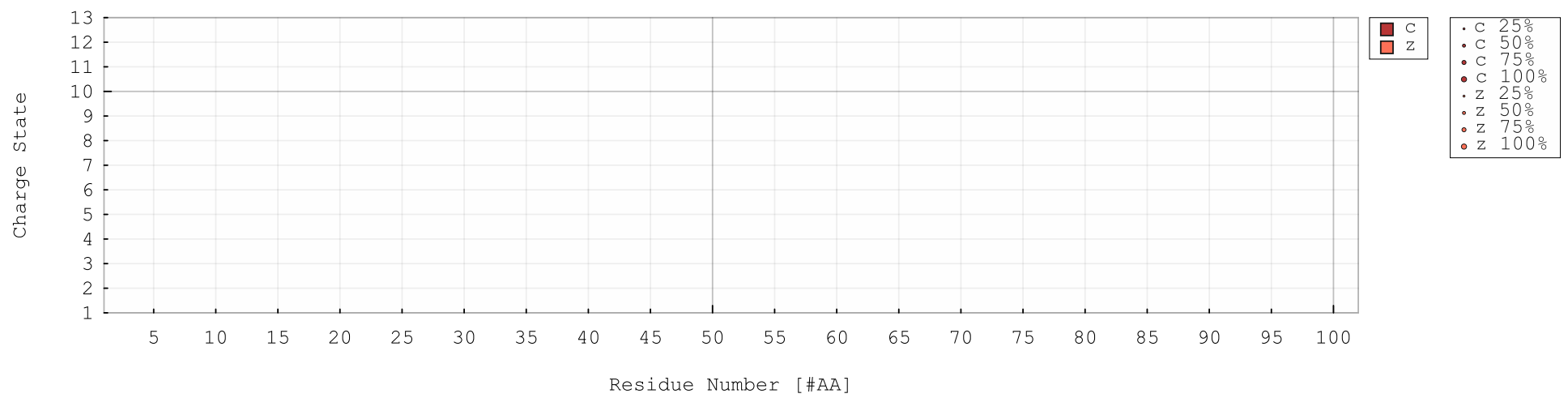

Proteoform:

Index	Protein	SC [%]	SVP [%]	IC [%]	MS Score [a.u.]	Var. Mods	Mono Mass [Da]	Chemical Formula
6	H4Kac	51.5	16.7	36.4	18.8	Ace: K5, K8, K12, K16, K20, K59	11481.4046	C506H849N163O140S

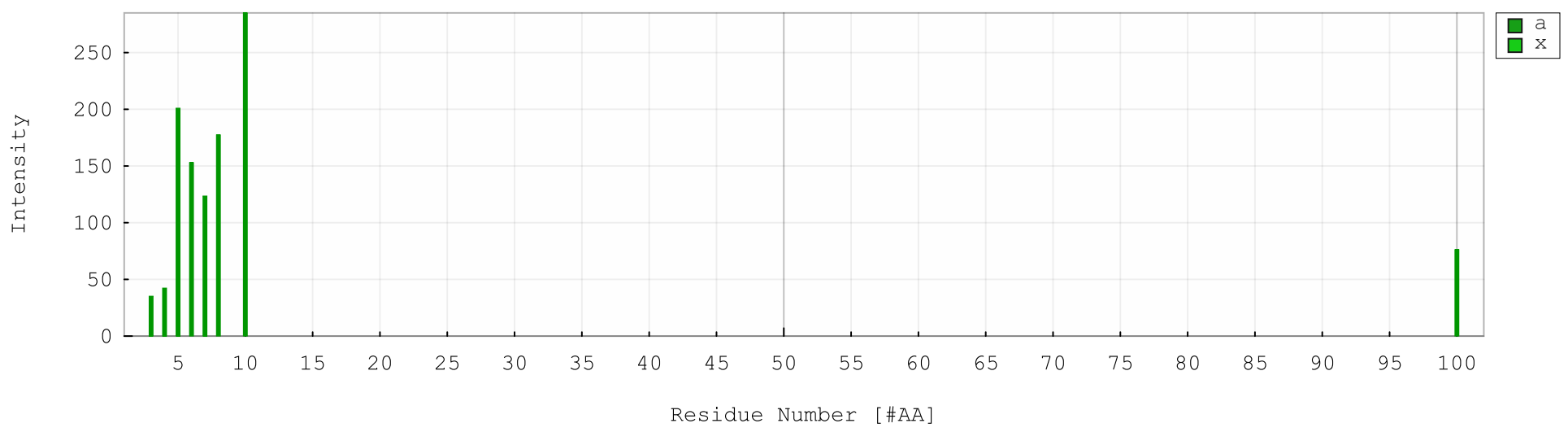

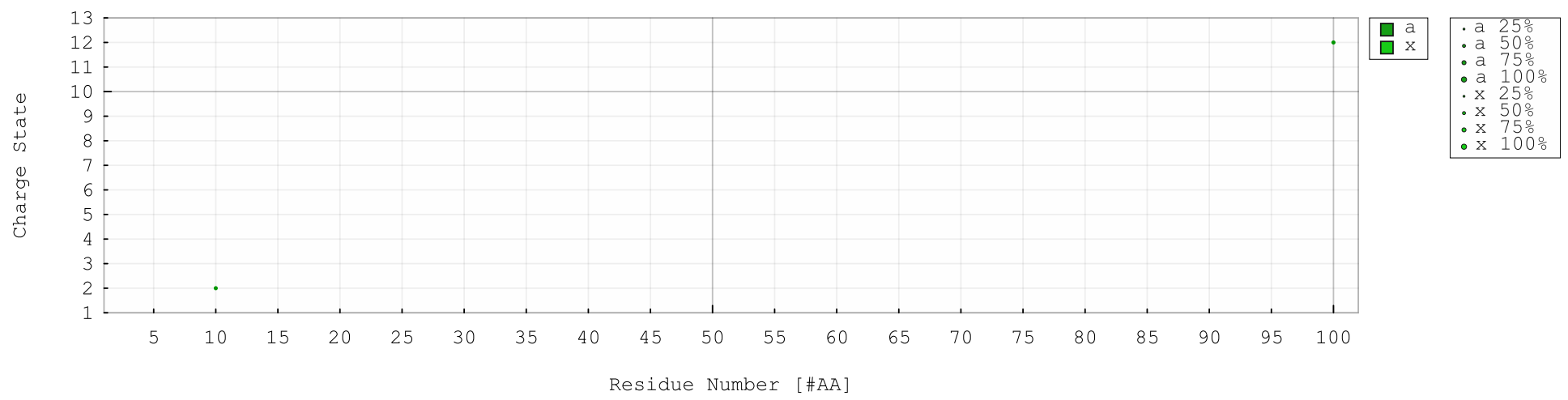

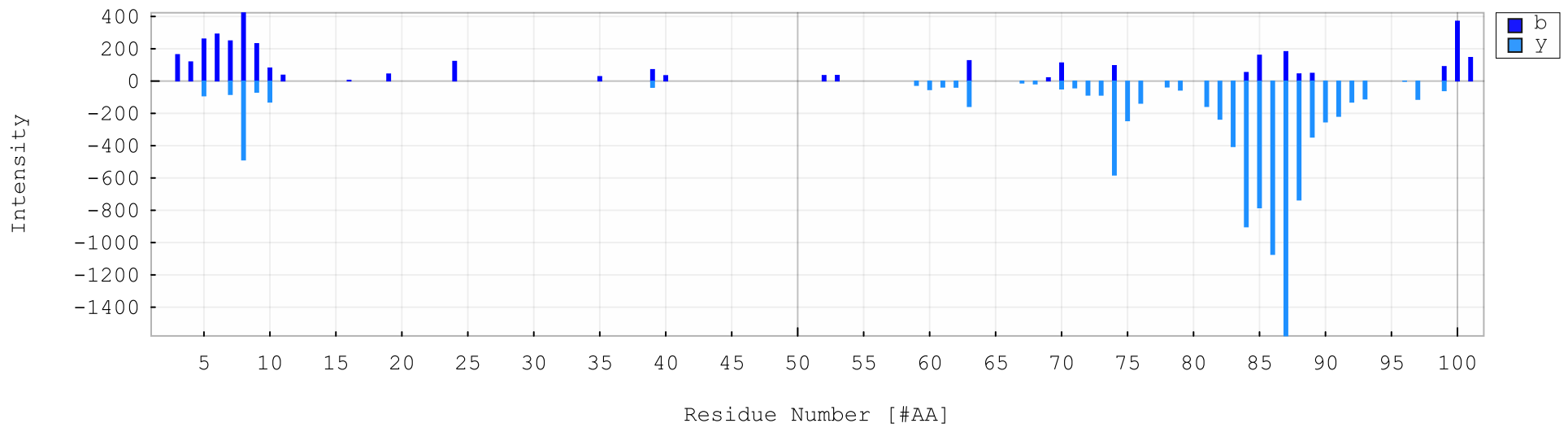

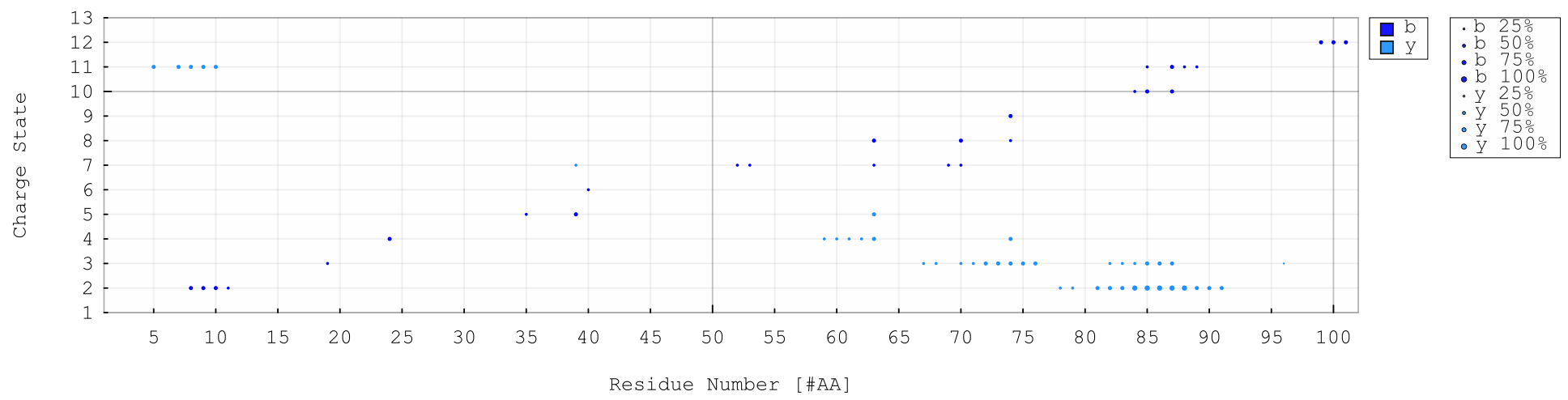

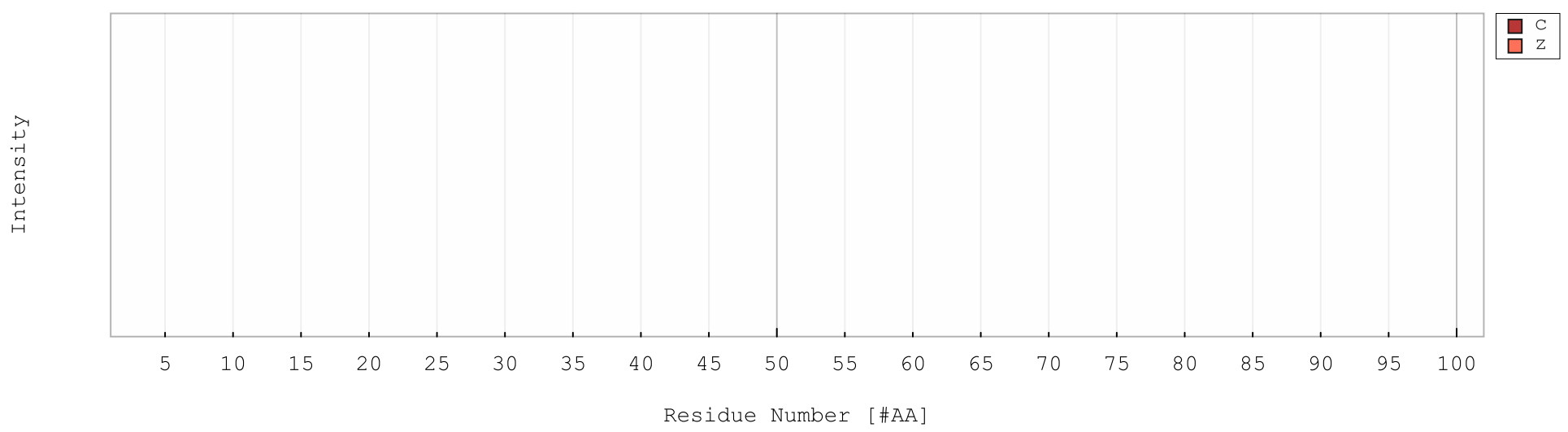

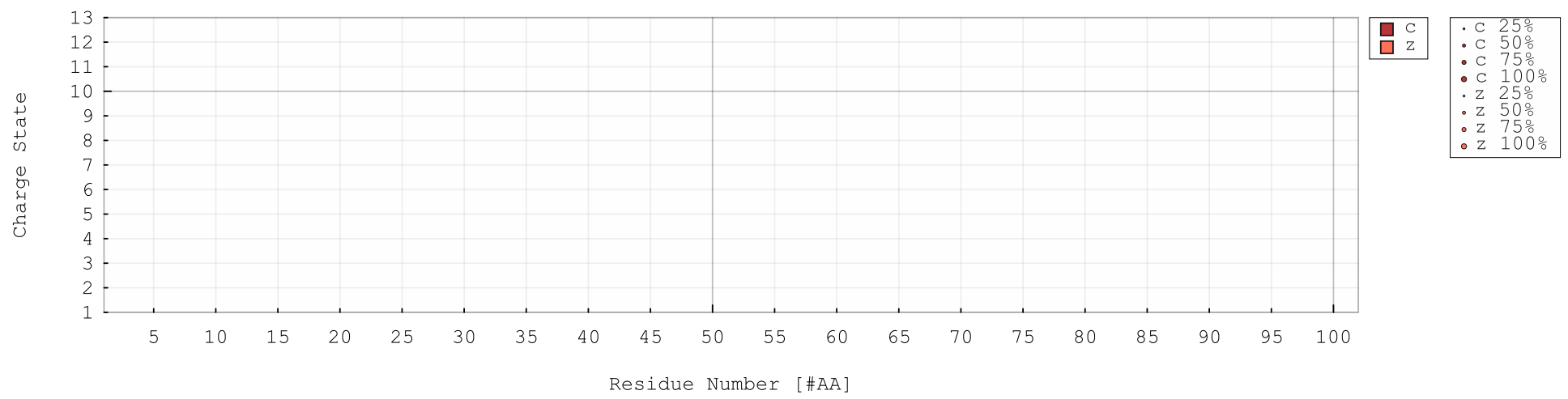

Proteoform:

Index	Protein	SC [%]	SVP [%]	IC [%]	MS Score [a.u.]	Var. Mods	Mono Mass [Da]	Chemical Formula
7	H4Kac	50.5	16.7	36.3	18.3	Ace: K5, K8, K12, K16, K31, K59	11481.4046	C506H849N163O140S

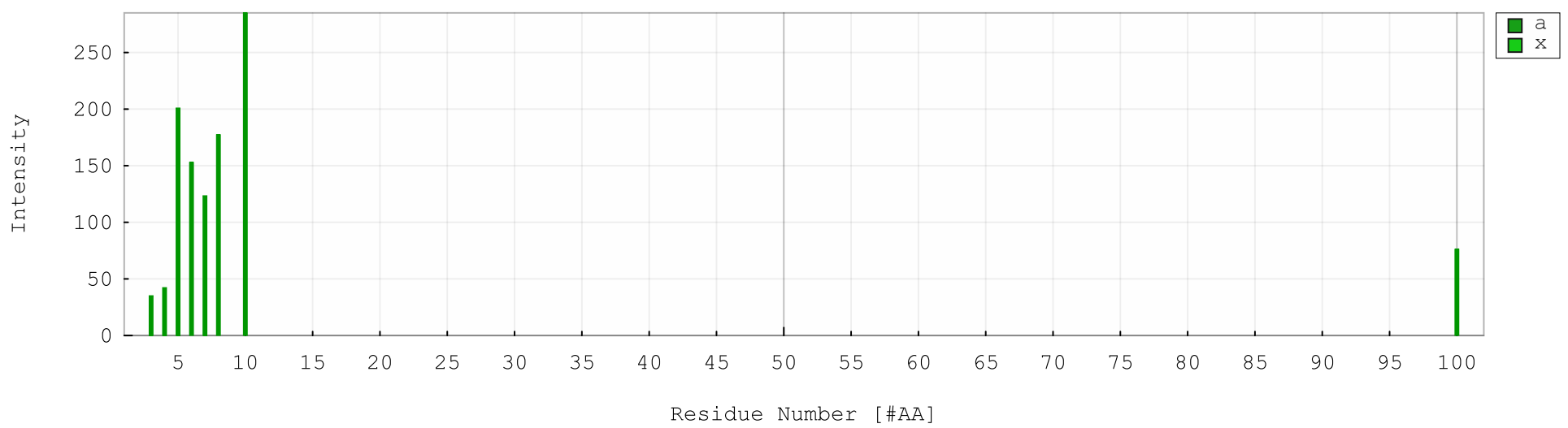

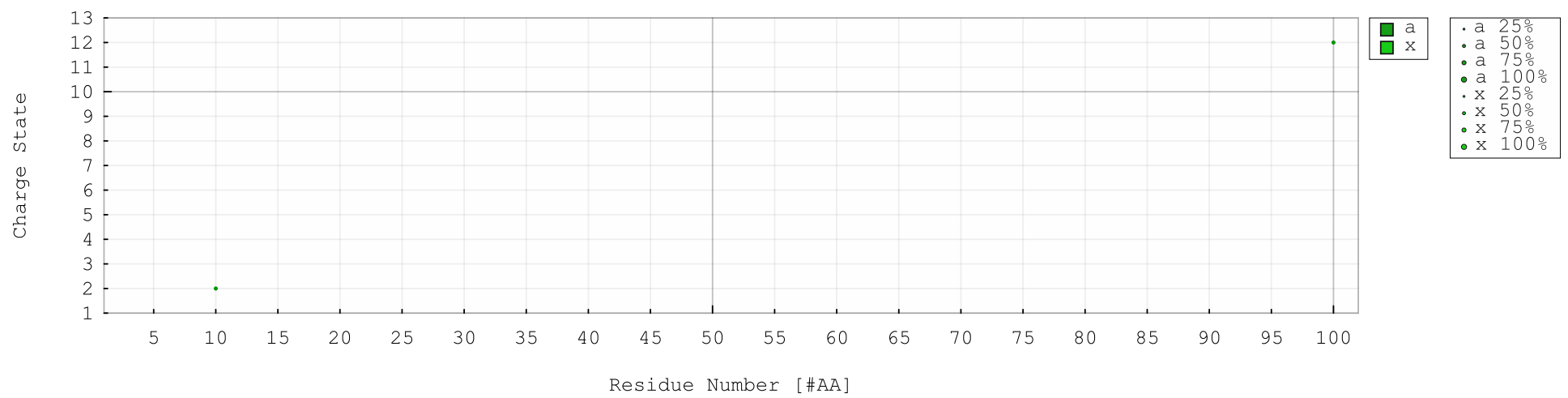

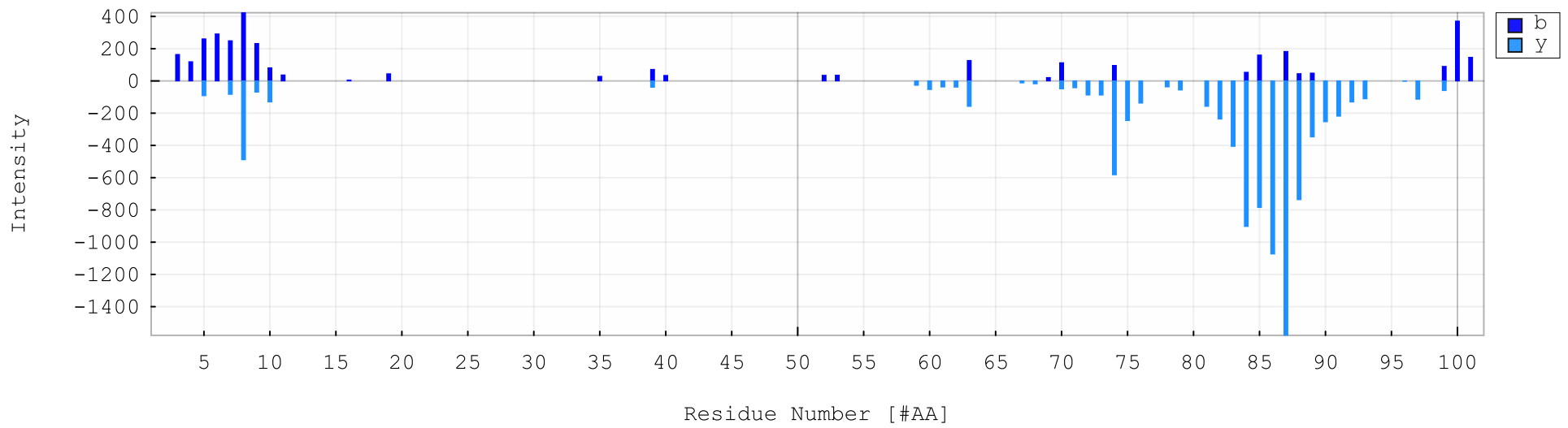

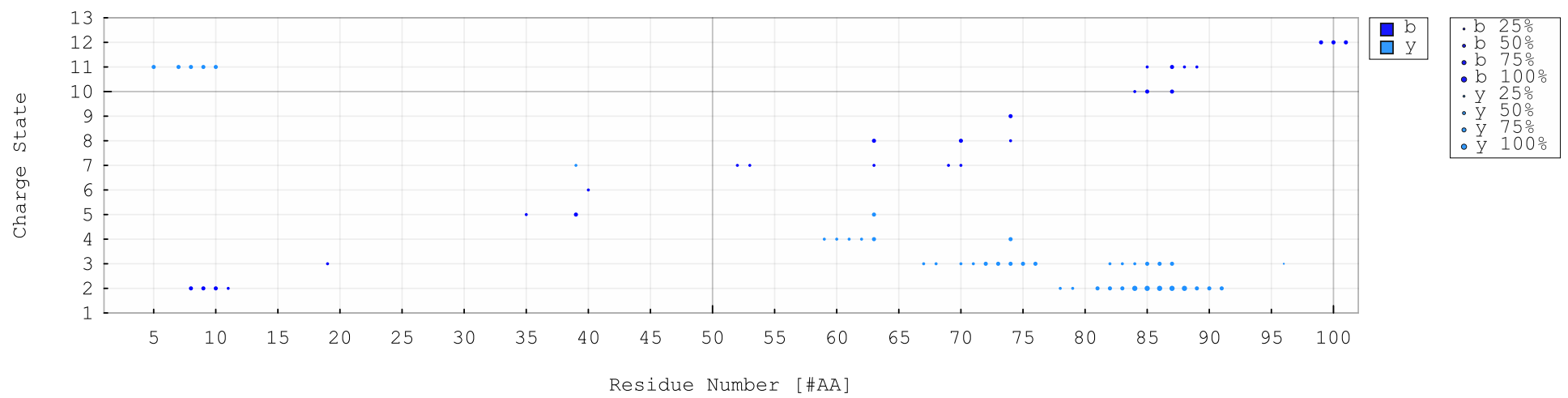

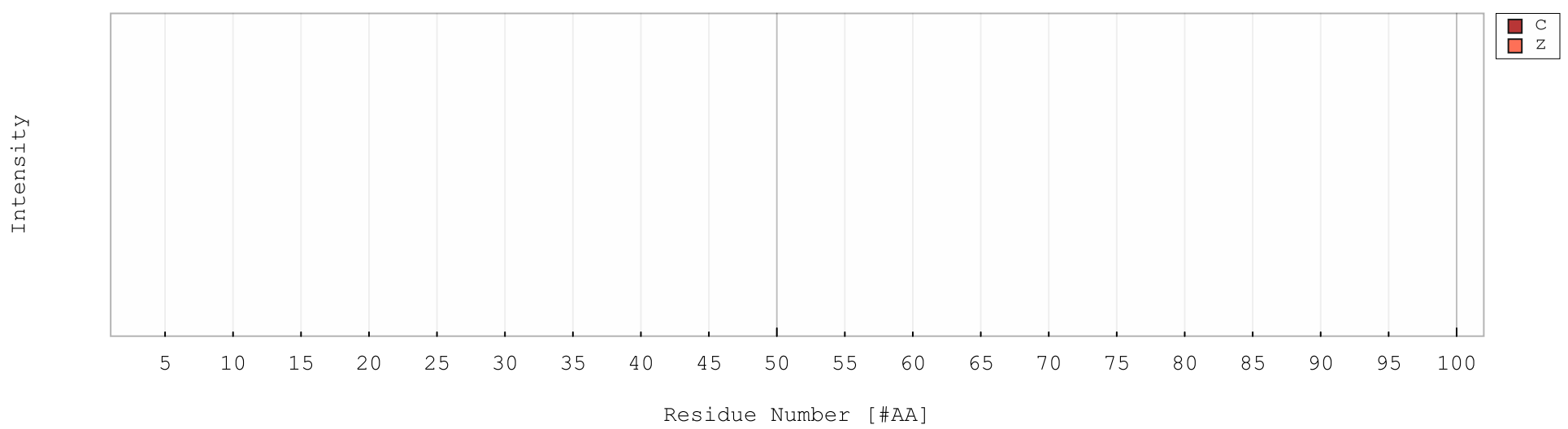

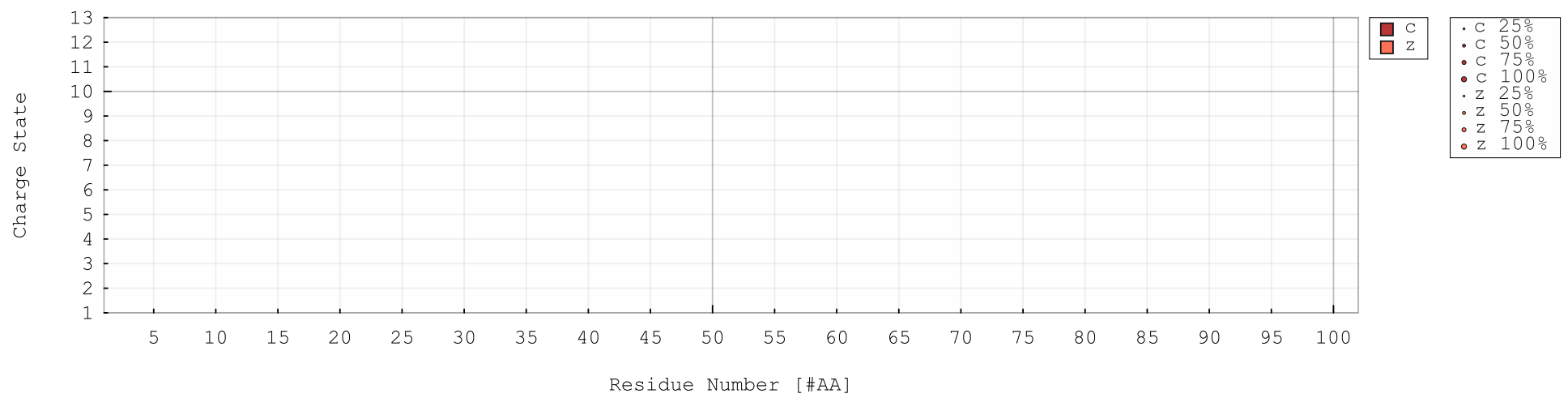

Proteoform:

Index	Protein	SC [%]	SVP [%]	IC [%]	MS Score [a.u.]	Var. Mods	Mono Mass [Da]	Chemical Formula
8	H4Kac	49.5	16.7	36.2	17.9	Ace: K5, K8, K12, K20, K31, K59	11481.4046	C506H849N163O140S

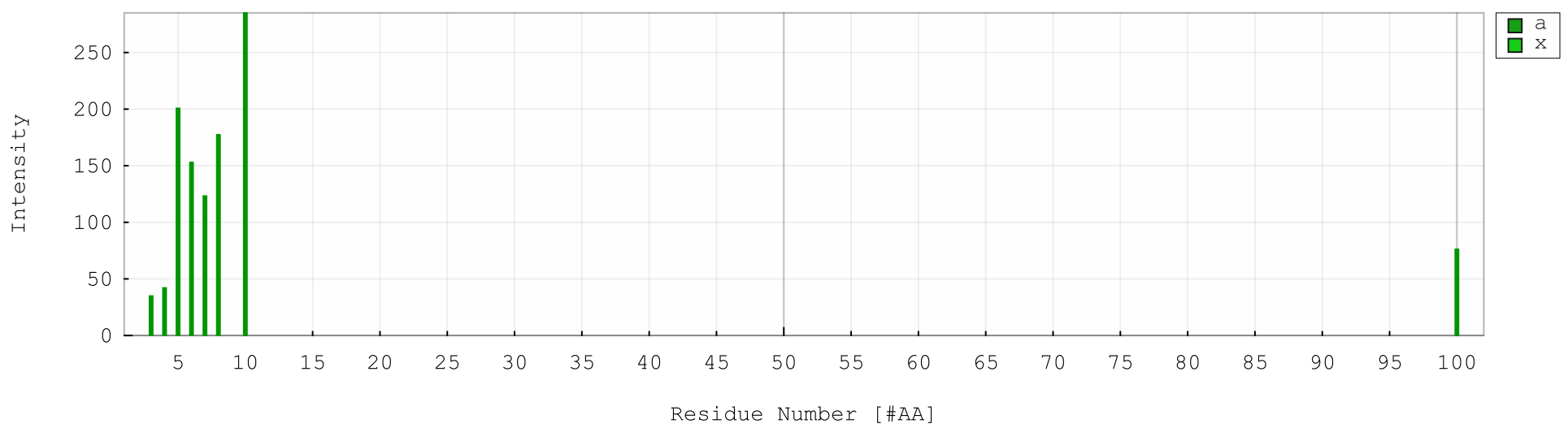

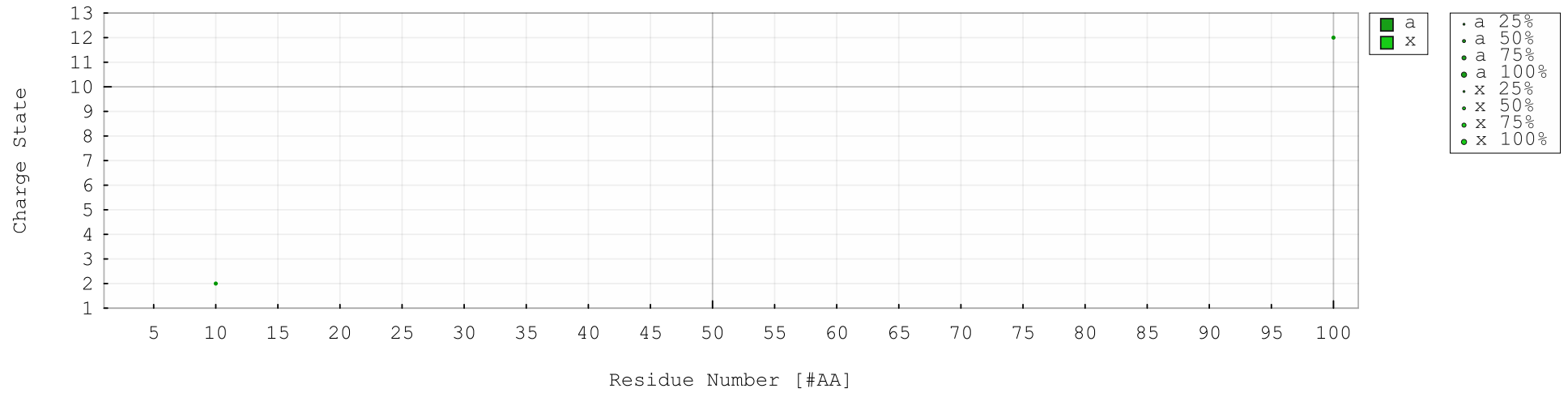

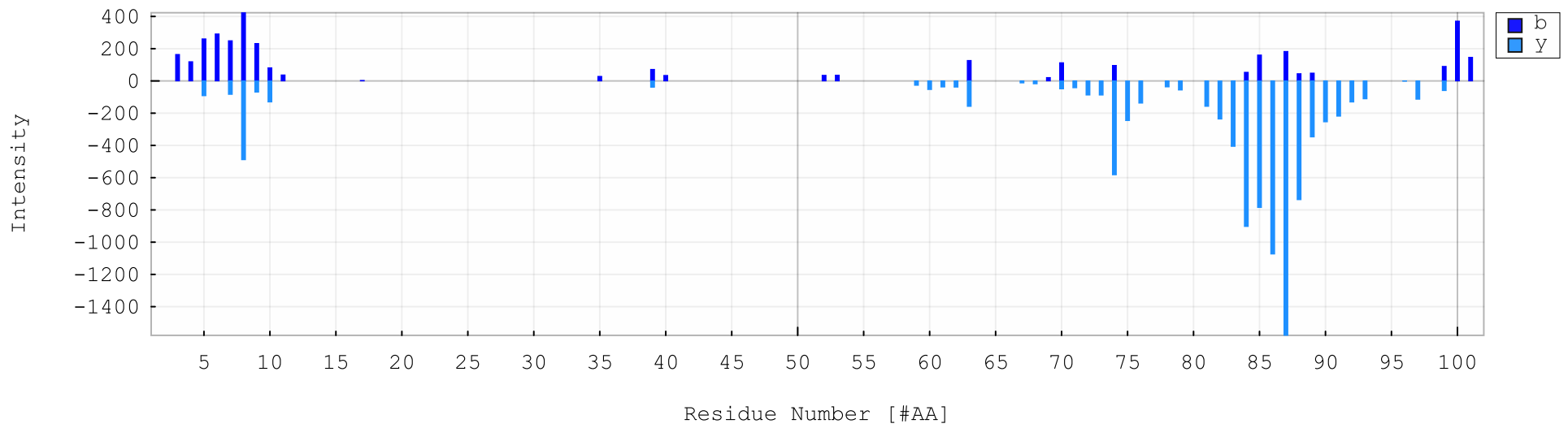

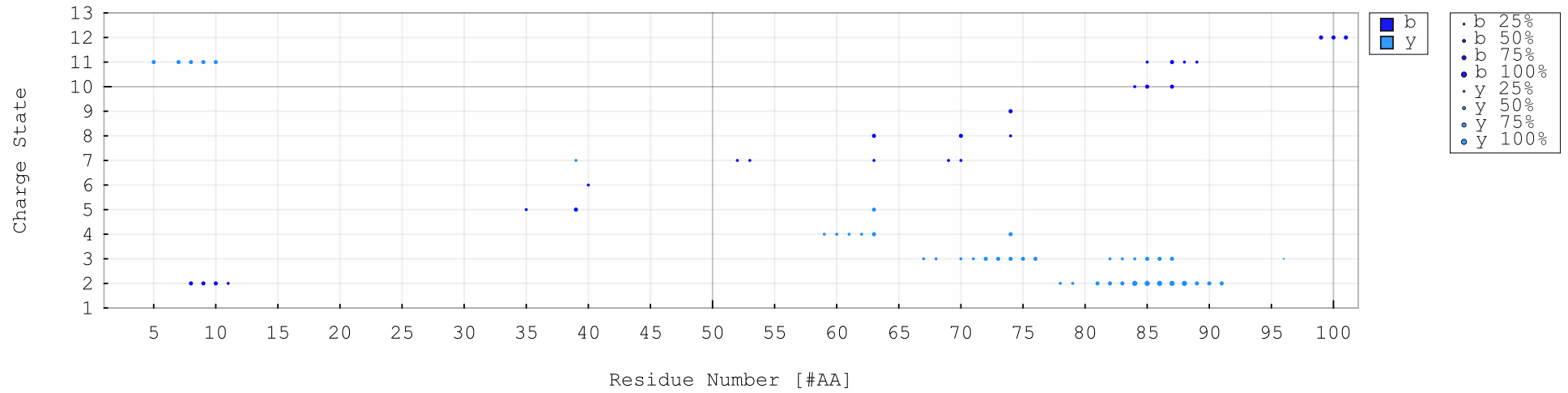

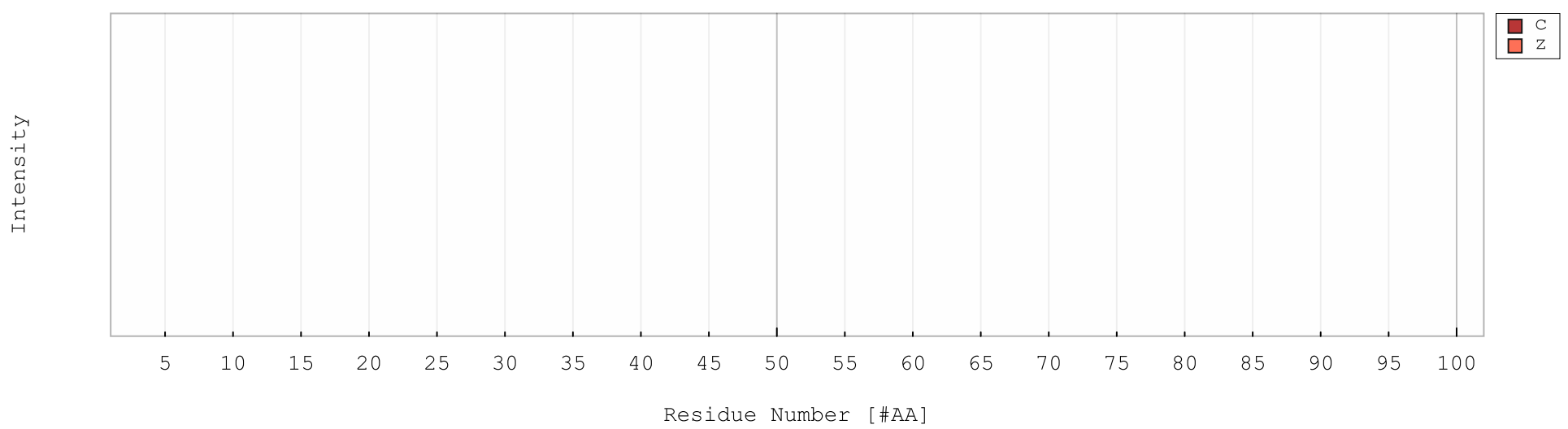

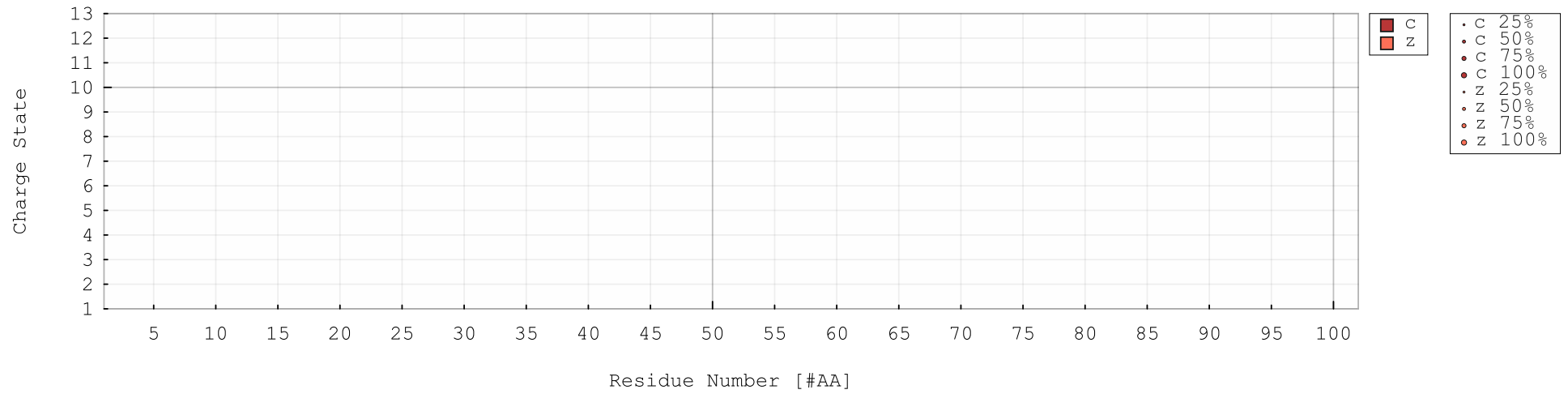

Proteoform:

Index	Protein	SC [%]	SVP [%]	IC [%]	MS Score [a.u.]	Var. Mods	Mono Mass [Da]	Chemical Formula
9	H4Kac	49.5	16.7	36.2	17.9	Ace: K5, K8, K16, K20, K31, K59	11481.4046	C506H849N163O140S

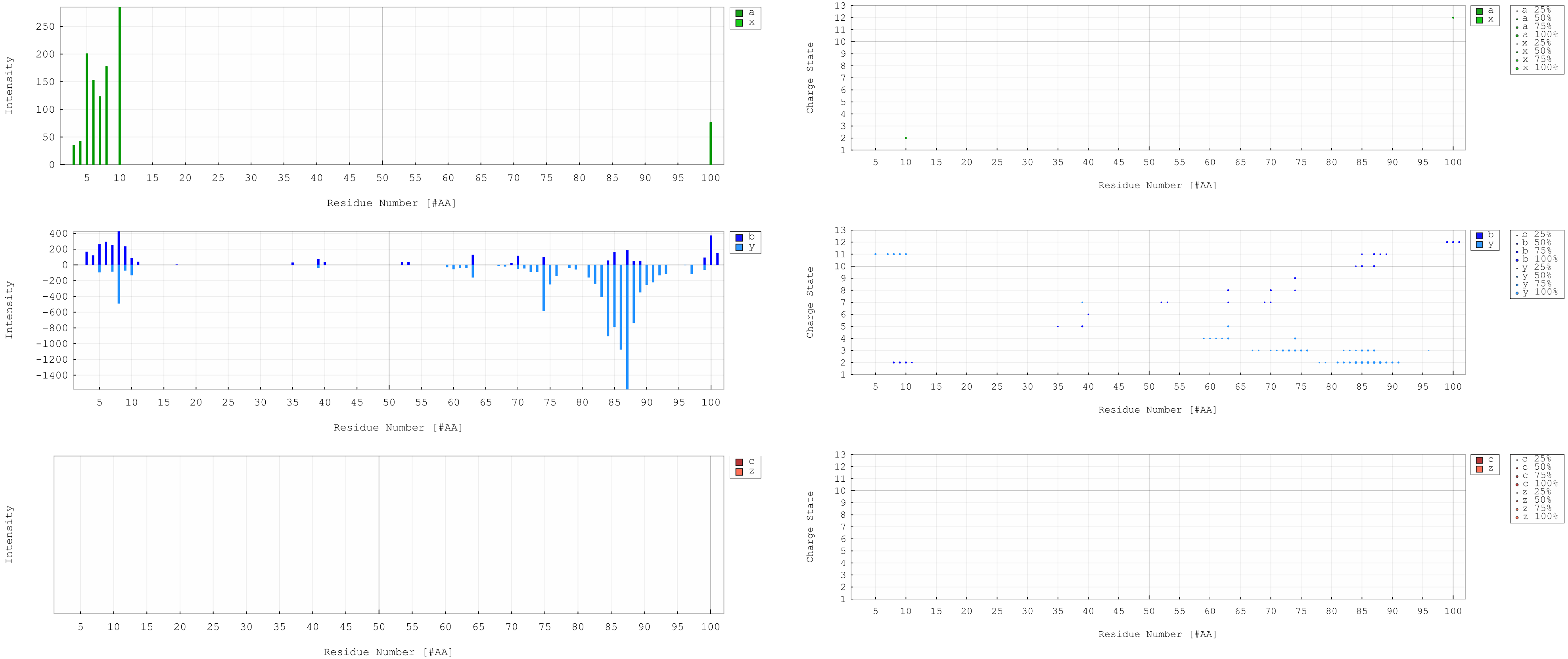

Proteoform:	Index	Protein	SC [%]	SVP [%]	IC [%]	MS Score [a.u.]	Var. Mods	Mono Mass [Da]	Chemical Formula
	10	H4Kac	47.5	12.7	36.2	17.2	Ace: K5, K12, K16, K20, K31, K44	11481.4046	C506H849N163O140S

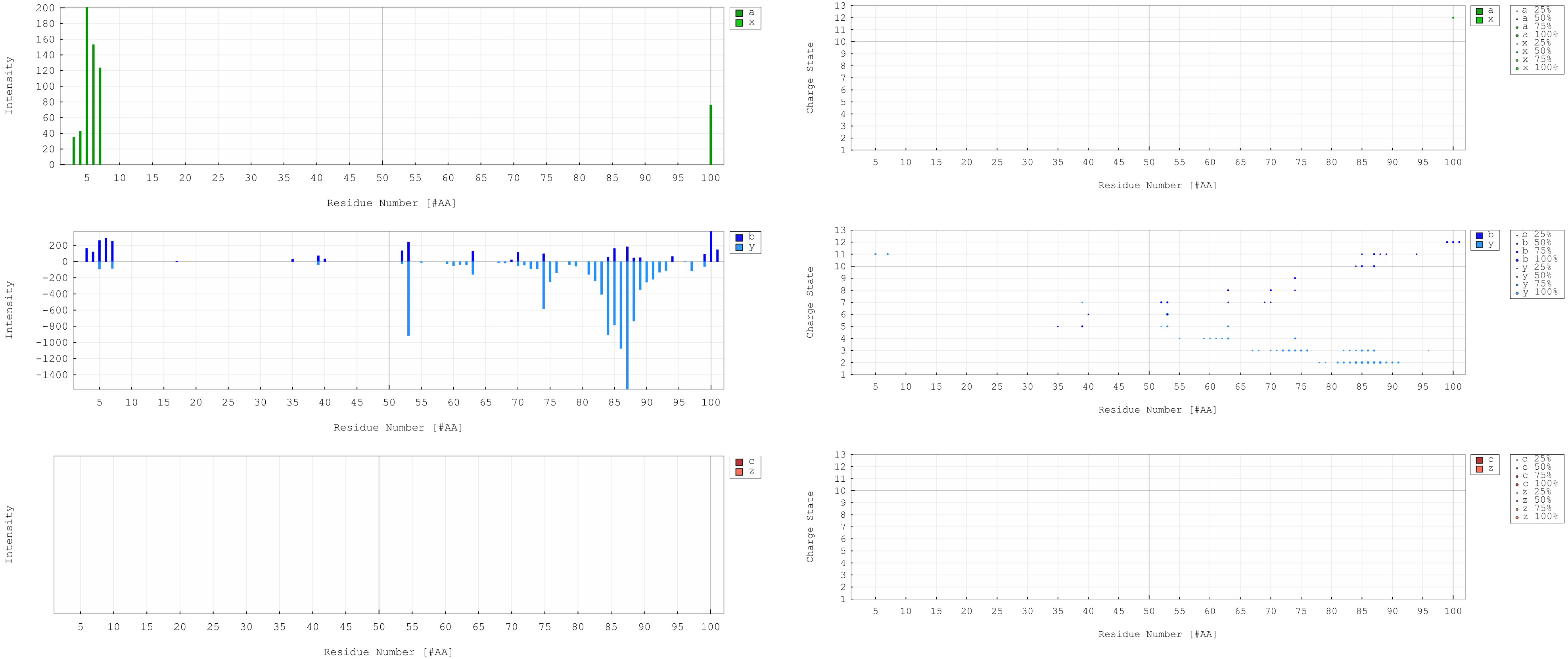

Supplemental Data S4

H4 - p300, 60 min

**Report**	5 of 7
**Experiment**	MS2
**Histone/sample**	H4
**Enzyme**	p300
**Reaction/source**	60 min
**Precursor**	H4Kac7
**Charge state**	13+
**Fragmentation / workflow**	ECD
**Original file**	MS2_H4Kac7_13+_ECD.pdf

Project Information

Project Directory: C:/Users/LAB-ADMIN
Processing date: Tue Apr 28 13:01:31 2026

Sample Info & Protocols

Name: Top-Down Protein Sequencing ExD

Dataset

Imported spectrum file name:	250428 H4_P300-50_60min z13ac7 MS2 ECD_001-487_calibrated.txt
Dataset name:	Information is only available from imported JPEAKS files
Date acquired:	Information is only available from imported JPEAKS files
Date imported:	Information is only available from imported JPEAKS files
Instrument Name:	Information is only available from imported JPEAKS files
Instrument Serial Number:	Information is only available from imported JPEAKS files

Workflow Result Info

Analysis: 250428 H4_P300-50_60min z13ac7 MS2 ECD_001-487_calibrated
Location: C:/Users/LAB-ADMIN
Method: -

Method Information

Name: Unnamed

Spectral Parameters

Mass Error [ppm]: 6
Intensity Deviation [au]: 0.15
Min/Max Charges: 1-13
Experiment type: ExD

a ions	b ions	c ions	x ions	y ions	z ions	satellite	internal	precursor
a	b	c		y	z+1			M
a+1		c+1			z+2			M+-2H

Noise threshold calculation: Simple moving average noise threshold (SMA)
Window size [datapoints]: 600
Noise curve offset: 3.0306919
SVP parameters: terminal gap: 2, internal gap: 1

Proteoforms

(top 10)

Index	Parent Sequence	Chain	Var. Mods	Mono Mass [Da]	Chemical Composition
1	H4Kac	H4KacK5[Ace]K8[Ace]K12[Ace]K16[Ace]K20[Ace]K31[Ace]K44[Ace]	K5, K8, K12, K16, K20, K31, K44	11523.4152	C508H851N163O141S
2	H4Kac	H4KacK5[Ace]K8[Ace]K12[Ace]K16[Ace]K20[Ace]K31[Ace]K59[Ace]	K5, K8, K12, K16, K20, K31, K59	11523.4152	C508H851N163O141S
3	H4Kac	H4KacK5[Ace]K8[Ace]K12[Ace]K16[Ace]K20[Ace]K44[Ace]K59[Ace]	K5, K8, K12, K16, K20, K44, K59	11523.4152	C508H851N163O141S
4	H4Kac	H4KacK5[Ace]K8[Ace]K12[Ace]K16[Ace]K20[Ace]K31[Ace]K77[Ace]	K5, K8, K12, K16, K20, K31, K77	11523.4152	C508H851N163O141S
5	H4Kac	H4KacK5[Ace]K8[Ace]K12[Ace]K16[Ace]K20[Ace]K31[Ace]K79[Ace]	K5, K8, K12, K16, K20, K31, K79	11523.4152	C508H851N163O141S
6	H4Kac	H4KacK5[Ace]K8[Ace]K12[Ace]K16[Ace]K31[Ace]K44[Ace]K59[Ace]	K5, K8, K12, K16, K31, K44, K59	11523.4152	C508H851N163O141S
7	H4Kac	H4KacK5[Ace]K8[Ace]K12[Ace]K20[Ace]K31[Ace]K44[Ace]K59[Ace]	K5, K8, K12, K20, K31, K44, K59	11523.4152	C508H851N163O141S
8	H4Kac	H4KacK5[Ace]K8[Ace]K12[Ace]K16[Ace]K20[Ace]K44[Ace]K77[Ace]	K5, K8, K12, K16, K20, K44, K77	11523.4152	C508H851N163O141S
9	H4Kac	H4KacK5[Ace]K8[Ace]K16[Ace]K20[Ace]K31[Ace]K44[Ace]K59[Ace]	K5, K8, K16, K20, K31, K44, K59	11523.4152	C508H851N163O141S
10	H4Kac	H4KacK5[Ace]K12[Ace]K16[Ace]K20[Ace]K31[Ace]K44[Ace]K59[Ace]	K5, K12, K16, K20, K31, K44, K59	11523.4152	C508H851N163O141S

Results

Proteoforms Table (top 10)

Index	Protein	SC [%]	SVP [%]	IC [%]	MS Score [a.u.]	Var. Mods	Mono Mass [Da]	Chemical Formula
1	H4Kac	87.1	62.7	58.1	50.6	Ace: K5, K8, K12, K16, K20, K31, K44	11523.4152	C508H851N163O141S
2	H4Kac	85.1	54.9	54.0	46.0	Ace: K5, K8, K12, K16, K20, K31, K59	11523.4152	C508H851N163O141S
3	H4Kac	82.2	29.4	48.8	40.1	Ace: K5, K8, K12, K16, K20, K44, K59	11523.4152	C508H851N163O141S
4	H4Kac	80.2	54.9	48.4	38.8	Ace: K5, K8, K12, K16, K20, K31, K77	11523.4152	C508H851N163O141S
5	H4Kac	79.2	54.9	47.8	37.9	Ace: K5, K8, K12, K16, K20, K31, K79	11523.4152	C508H851N163O141S
6	H4Kac	82.2	25.5	43.7	35.9	Ace: K5, K8, K12, K16, K31, K44, K59	11523.4152	C508H851N163O141S
7	H4Kac	80.2	25.5	42.2	33.8	Ace: K5, K8, K12, K20, K31, K44, K59	11523.4152	C508H851N163O141S
8	H4Kac	77.2	29.4	43.2	33.4	Ace: K5, K8, K12, K16, K20, K44, K77	11523.4152	C508H851N163O141S
9	H4Kac	80.2	11.8	41.4	33.2	Ace: K5, K8, K16, K20, K31, K44, K59	11523.4152	C508H851N163O141S
10	H4Kac	80.2	11.8	40.8	32.7	Ace: K5, K12, K16, K20, K31, K44, K59	11523.4152	C508H851N163O141S

Sequence

Name: H4Kac

```
                 10                    20                    30                    40                    50                    60
S G R G K G G K G L   G K G G A K R H R K   V L R D N I Q G I T   K P A I R R L A R R   G G V K R I S G L I   Y E E T R G V L K V
                 70                    80                    90                   100                   110                   120
F L E N V I R D A V   T Y T E H A K R K T   V T A M D V V Y A L   K R Q G R T L Y G F   G G
```

Modifications

Modification	Position	Type
Acetyl	5	variable
Acetyl	8	variable
Acetyl	12	variable
Acetyl	16	variable
Acetyl	20	variable
Acetyl	31	variable
Acetyl	44	variable
Acetyl	59	variable
Acetyl	77	variable
Acetyl	79	variable

Sequence Map

Proteoform:

Index	Protein	SC [%]	SVP [%]	IC [%]	MS Score [a.u.]	Var. Mods	Mono Mass [Da]	Chemical Formula
1	H4Kac	87.1	62.7	58.1	50.6	Ace: K5, K8, K12, K16, K20, K31, K44	11523.4152	C508H851N163O141S

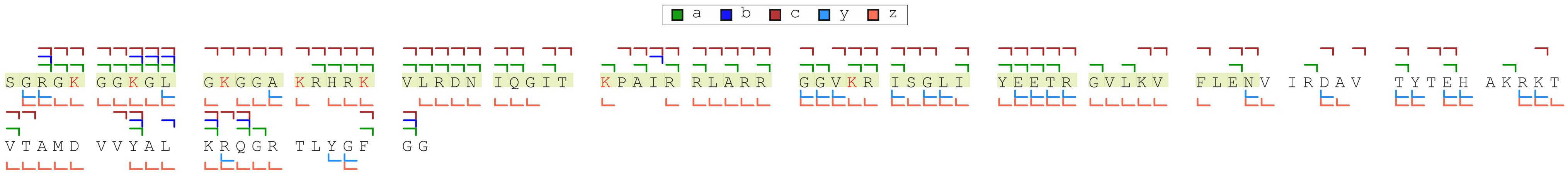

Proteoform:

Index	Protein	SC [%]	SVP [%]	IC [%]	MS Score [a.u.]	Var. Mods	Mono Mass [Da]	Chemical Formula
2	H4Kac	85.1	54.9	54.0	46.0	Ace: K5, K8, K12, K16, K20, K31, K59	11523.4152	C508H851N163O141S

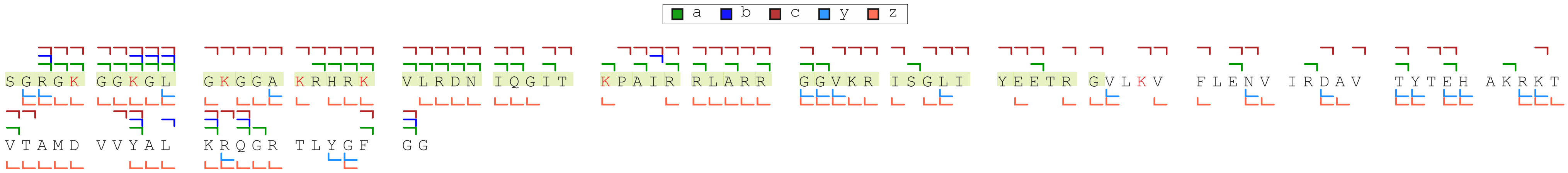

Top-Down Result OmniScape 2025
Sequence Confirmation
250428 H4_P300-50_60min z13ac7 MS2 ECD_001-487_calibrated

Proteoform:

Index	Protein	SC [%]	SVP [%]	IC [%]	MS Score [a.u.]	Var. Mods	Mono Mass [Da]	Chemical Formula
3	H4Kac	82.2	29.4	48.8	40.1	Ace: K5, K8, K12, K16, K20, K44, K59	11523.4152	C508H851N163O141S

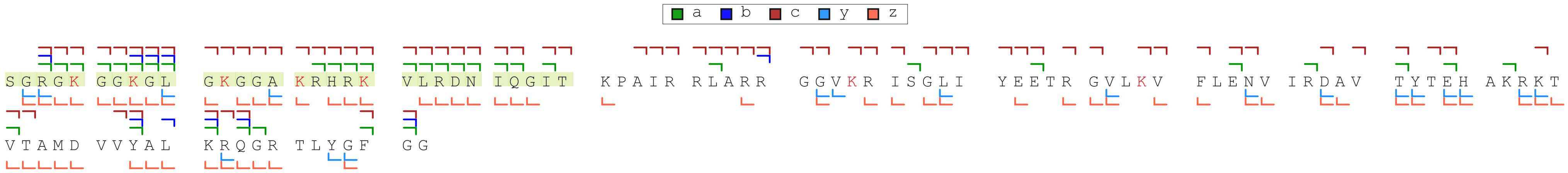

Proteoform:

Index	Protein	SC [%]	SVP [%]	IC [%]	MS Score [a.u.]	Var. Mods	Mono Mass [Da]	Chemical Formula
4	H4Kac	80.2	54.9	48.4	38.8	Ace: K5, K8, K12, K16, K20, K31, K77	11523.4152	C508H851N163O141S

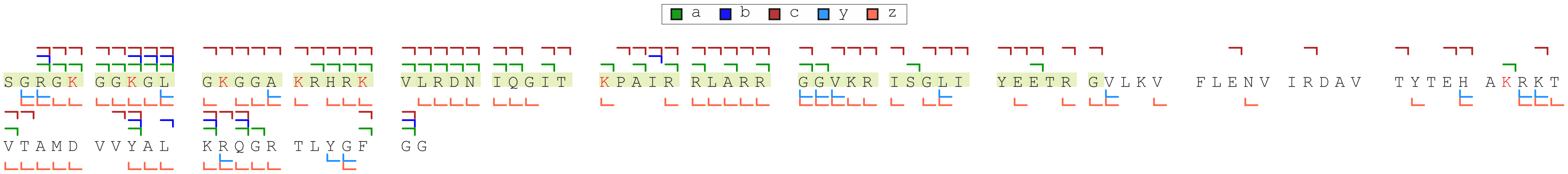

Proteoform:

Index	Protein	SC [%]	SVP [%]	IC [%]	MS Score [a.u.]	Var. Mods	Mono Mass [Da]	Chemical Formula
5	H4Kac	79.2	54.9	47.8	37.9	Ace: K5, K8, K12, K16, K20, K31, K79	11523.4152	C508H851N163O141S

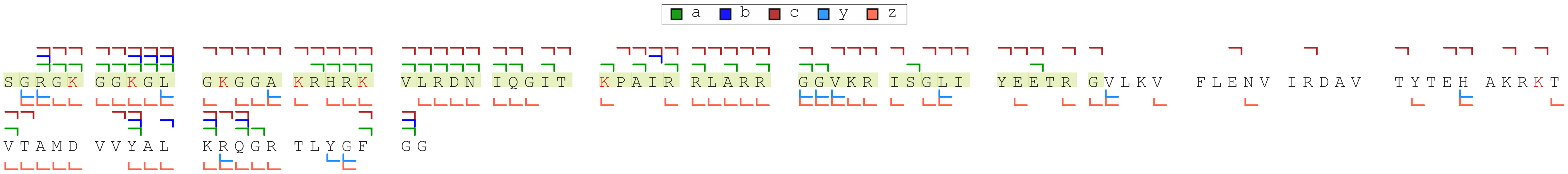

Proteoform:

Index	Protein	SC [%]	SVP [%]	IC [%]	MS Score [a.u.]	Var. Mods	Mono Mass [Da]	Chemical Formula
6	H4Kac	82.2	25.5	43.7	35.9	Ace: K5, K8, K12, K16, K31, K44, K59	11523.4152	C508H851N163O141S

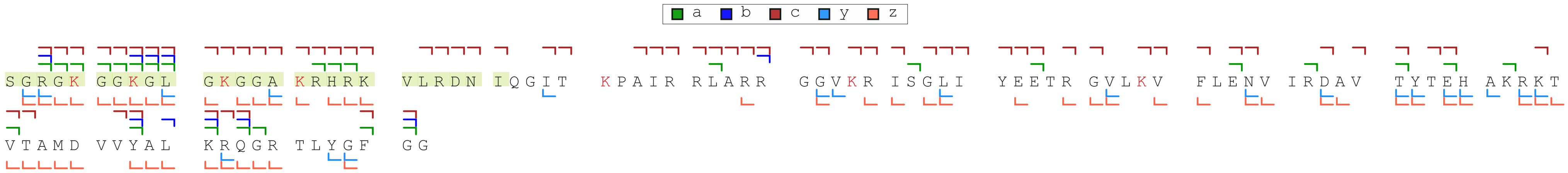

Proteoform:

Index	Protein	SC [%]	SVP [%]	IC [%]	MS Score [a.u.]	Var. Mods	Mono Mass [Da]	Chemical Formula
7	H4Kac	80.2	25.5	42.2	33.8	Ace: K5, K8, K12, K20, K31, K44, K59	11523.4152	C508H851N163O141S

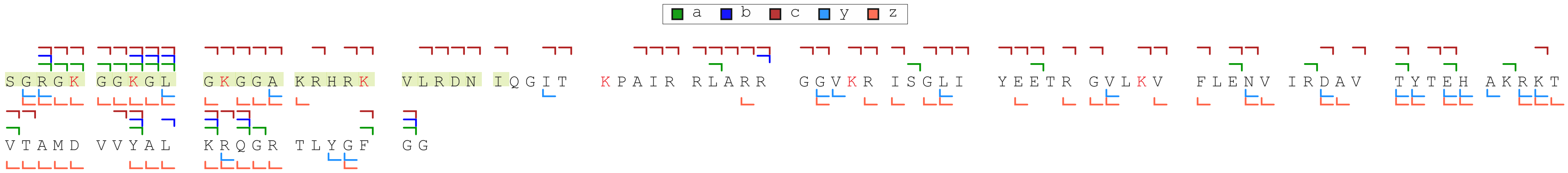

Proteoform:

Index	Protein	SC [%]	SVP [%]	IC [%]	MS Score [a.u.]	Var. Mods	Mono Mass [Da]	Chemical Formula
8	H4Kac	77.2	29.4	43.2	33.4	Ace: K5, K8, K12, K16, K20, K44, K77	11523.4152	C508H851N163O141S

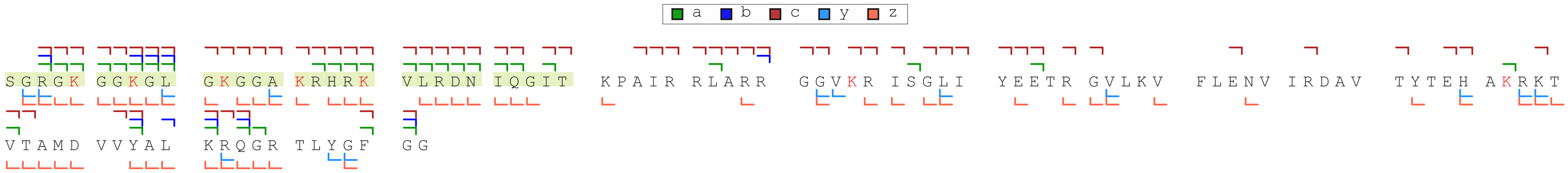

Proteoform:

Index	Protein	SC [%]	SVP [%]	IC [%]	MS Score [a.u.]	Var. Mods	Mono Mass [Da]	Chemical Formula
9	H4Kac	80.2	11.8	41.4	33.2	Ace: K5, K8, K16, K20, K31, K44, K59	11523.4152	C508H851N163O141S

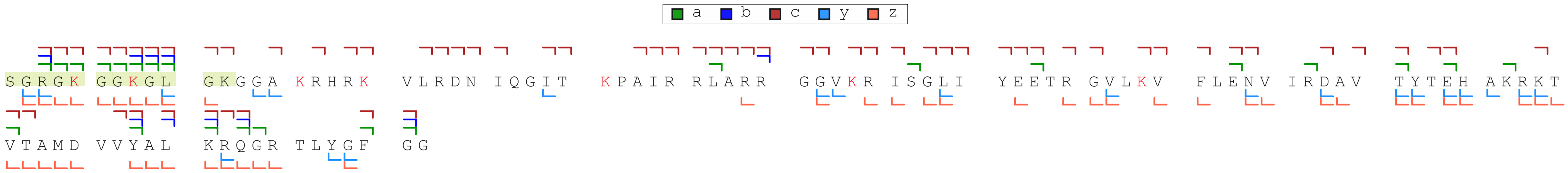

Proteoform:

Index	Protein	SC [%]	SVP [%]	IC [%]	MS Score [a.u.]	Var. Mods	Mono Mass [Da]	Chemical Formula
10	H4Kac	80.2	11.8	40.8	32.7	Ace: K5, K12, K16, K20, K31, K44, K59	11523.4152	C508H851N163O141S

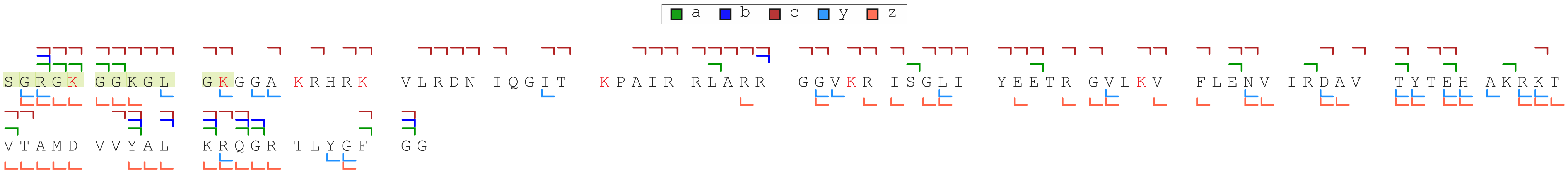

Error Plot

Proteoform:

Index	Protein	SC [%]	SVP [%]	IC [%]	MS Score [a.u.]	Var. Mods	Mono Mass [Da]	Chemical Formula
1	H4Kac	87.1	62.7	58.1	50.6	Ace: K5, K8, K12, K16, K20, K31, K44	11523.4152	C508H851N163O141S

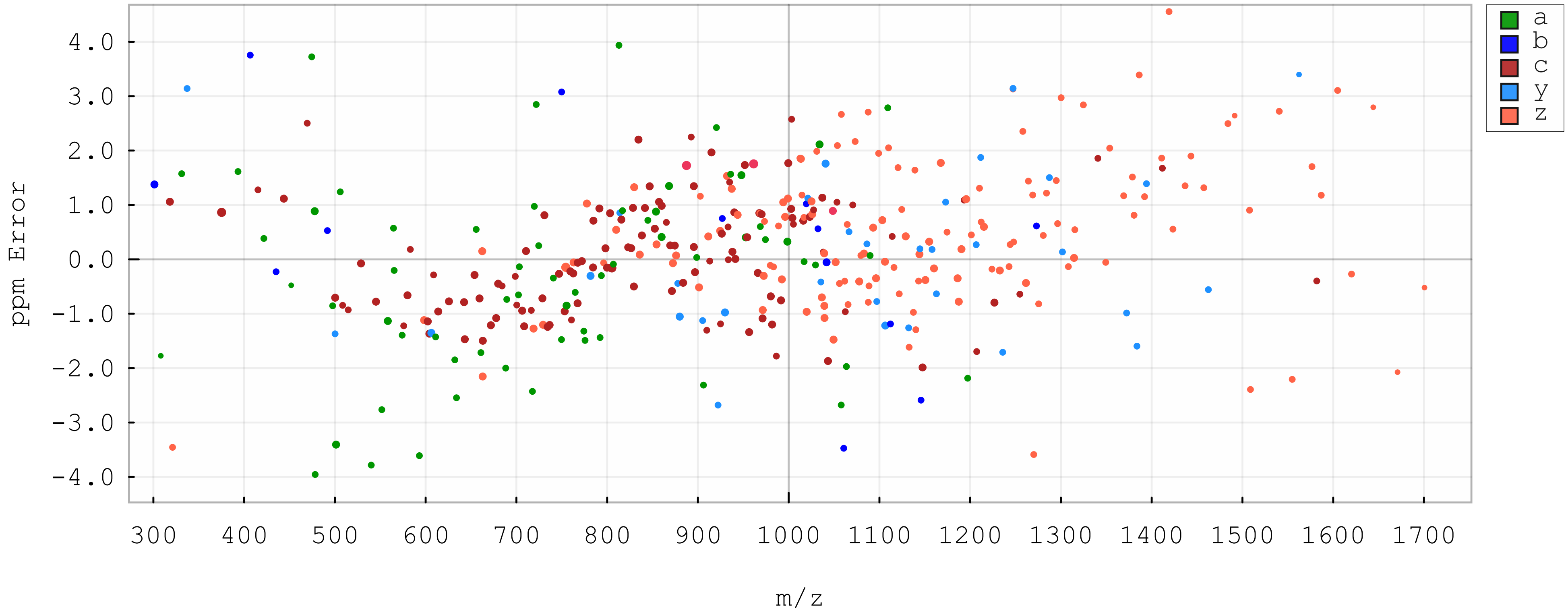

Proteoform:

Index	Protein	SC [%]	SVP [%]	IC [%]	MS Score [a.u.]	Var. Mods	Mono Mass [Da]	Chemical Formula
2	H4Kac	85.1	54.9	54.0	46.0	Ace: K5, K8, K12, K16, K20, K31, K59	11523.4152	C508H851N163O141S

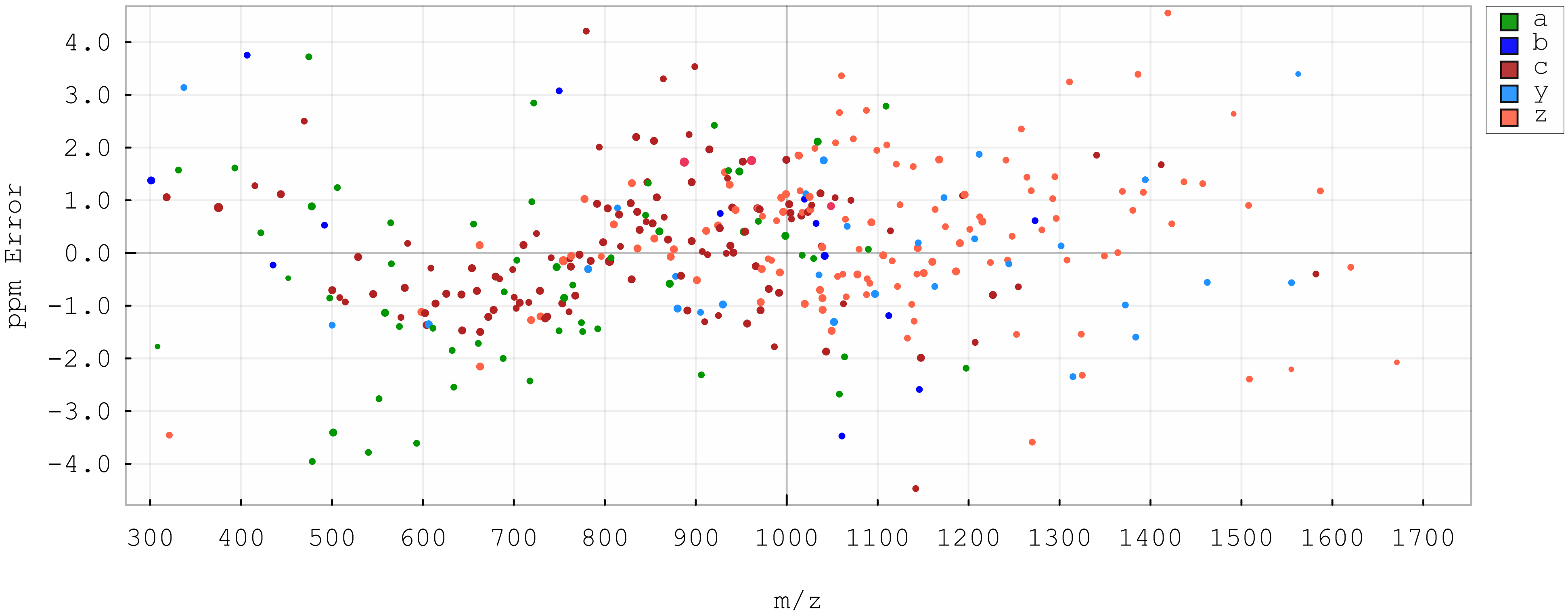

Proteoform:

Index	Protein	SC [%]	SVP [%]	IC [%]	MS Score [a.u.]	Var. Mods	Mono Mass [Da]	Chemical Formula
3	H4Kac	82.2	29.4	48.8	40.1	Ace: K5, K8, K12, K16, K20, K44, K59	11523.4152	C508H851N163O141S

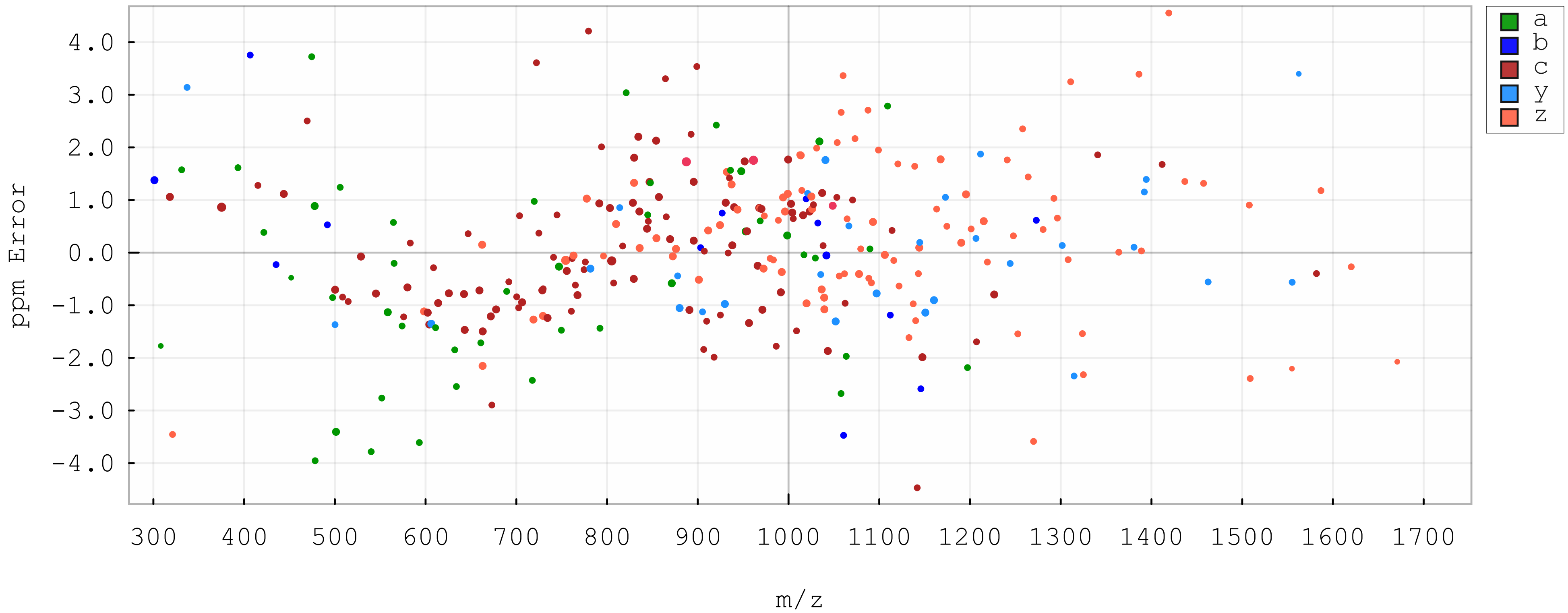

Proteoform:

Index	Protein	SC [%]	SVP [%]	IC [%]	MS Score [a.u.]	Var. Mods	Mono Mass [Da]	Chemical Formula
4	H4Kac	80.2	54.9	48.4	38.8	Ace: K5, K8, K12, K16, K20, K31, K77	11523.4152	C508H851N163O141S

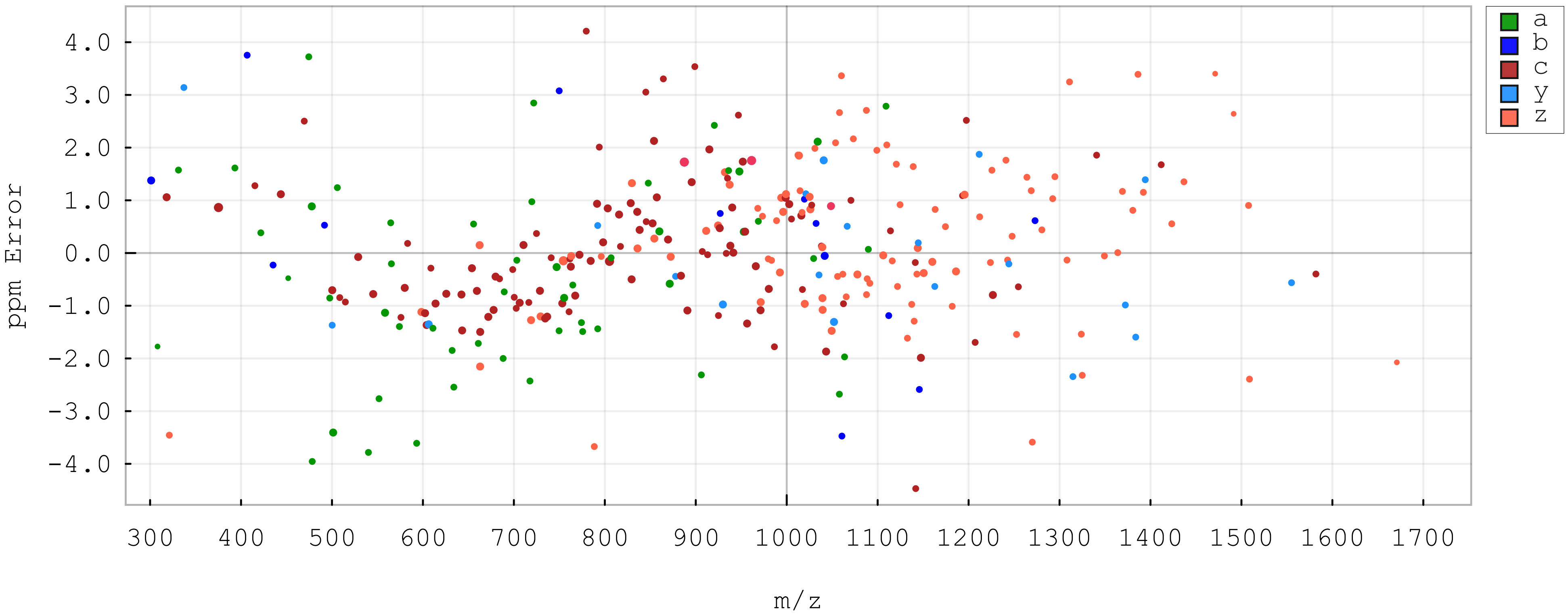

Proteoform:

Index	Protein	SC [%]	SVP [%]	IC [%]	MS Score [a.u.]	Var. Mods	Mono Mass [Da]	Chemical Formula
5	H4Kac	79.2	54.9	47.8	37.9	Ace: K5, K8, K12, K16, K20, K31, K79	11523.4152	C508H851N163O141S

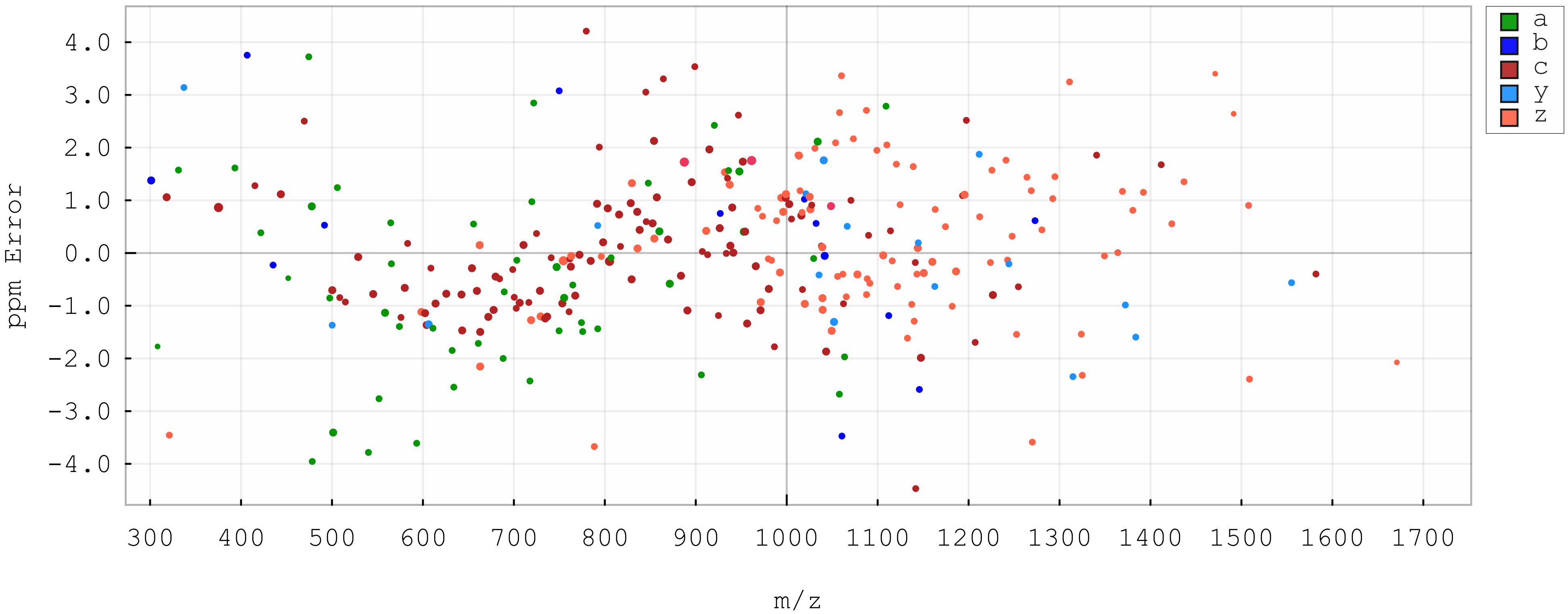

Proteoform:

Index	Protein	SC [%]	SVP [%]	IC [%]	MS Score [a.u.]	Var. Mods	Mono Mass [Da]	Chemical Formula
6	H4Kac	82.2	25.5	43.7	35.9	Ace: K5, K8, K12, K16, K31, K44, K59	11523.4152	C508H851N163O141S

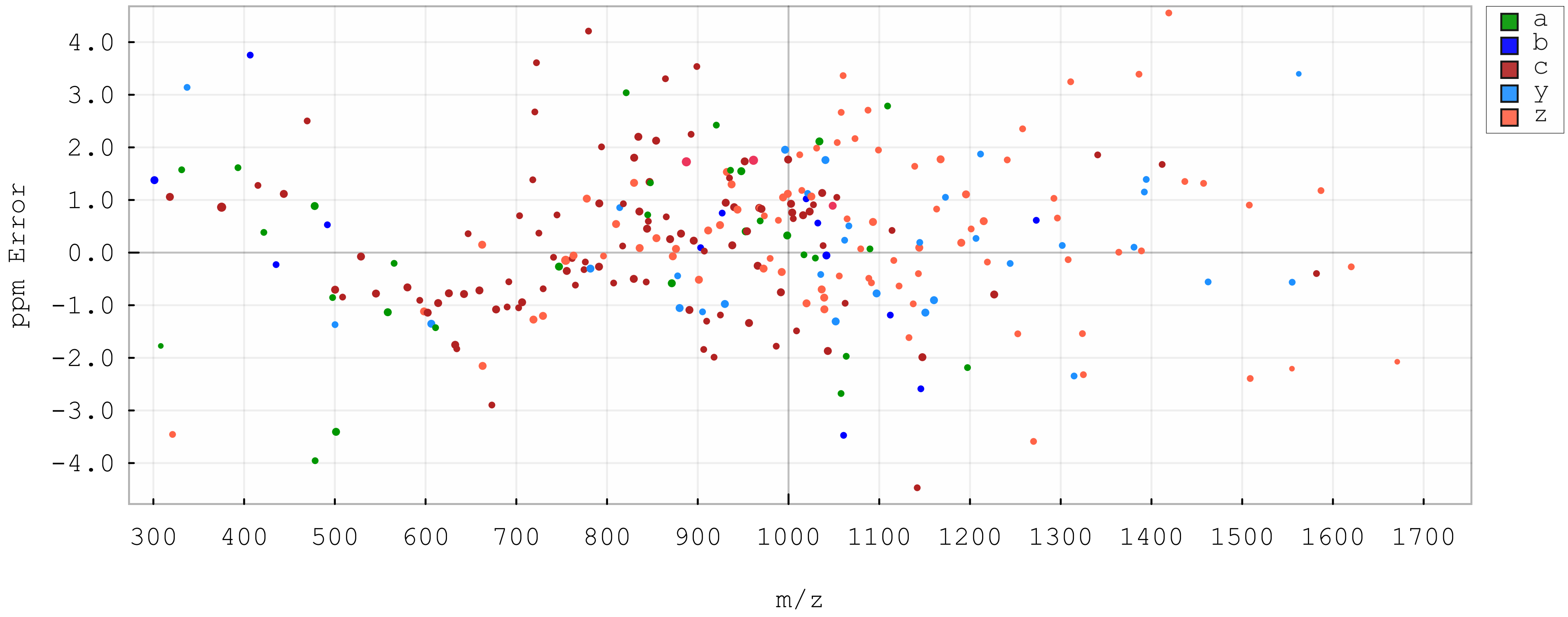

Proteoform:

Index	Protein	SC [%]	SVP [%]	IC [%]	MS Score [a.u.]	Var. Mods	Mono Mass [Da]	Chemical Formula
7	H4Kac	80.2	25.5	42.2	33.8	Ace: K5, K8, K12, K20, K31, K44, K59	11523.4152	C508H851N163O141S

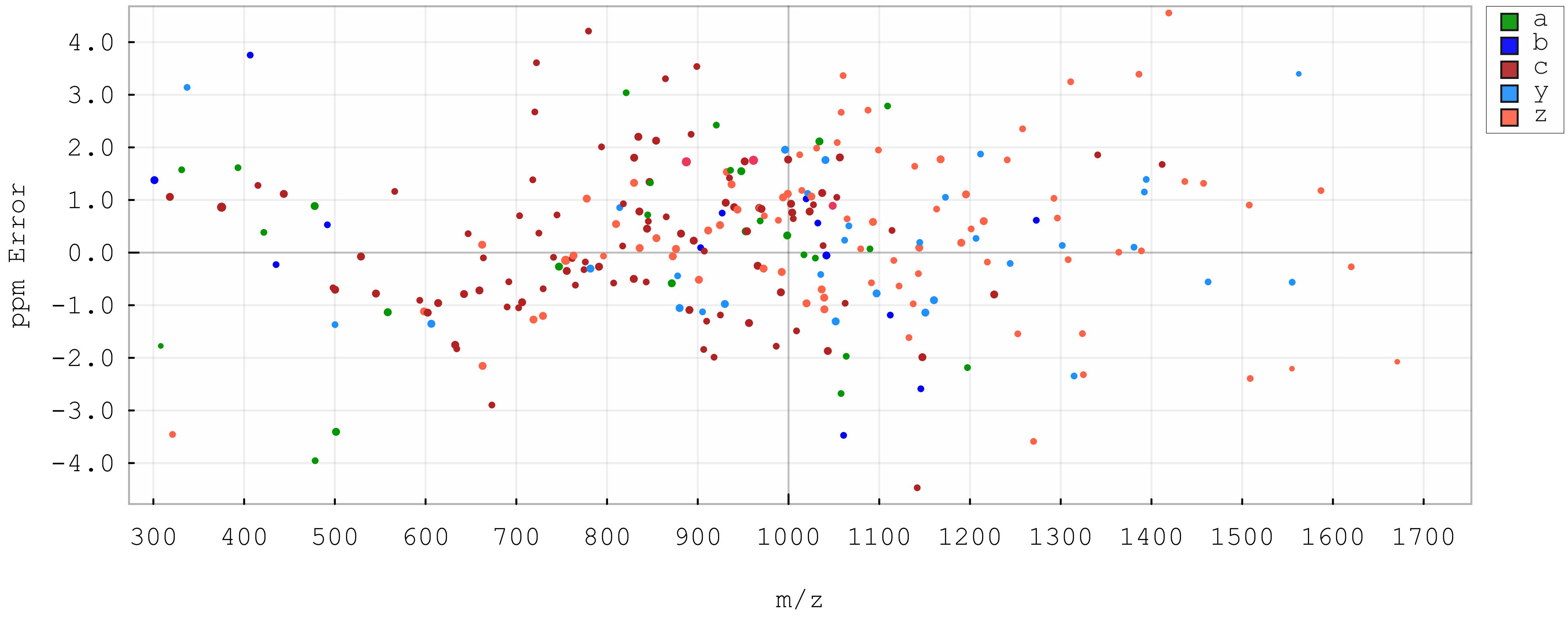

Proteoform:

Index	Protein	SC [%]	SVP [%]	IC [%]	MS Score [a.u.]	Var. Mods	Mono Mass [Da]	Chemical Formula
8	H4Kac	77.2	29.4	43.2	33.4	Ace: K5, K8, K12, K16, K20, K44, K77	11523.4152	C508H851N163O141S

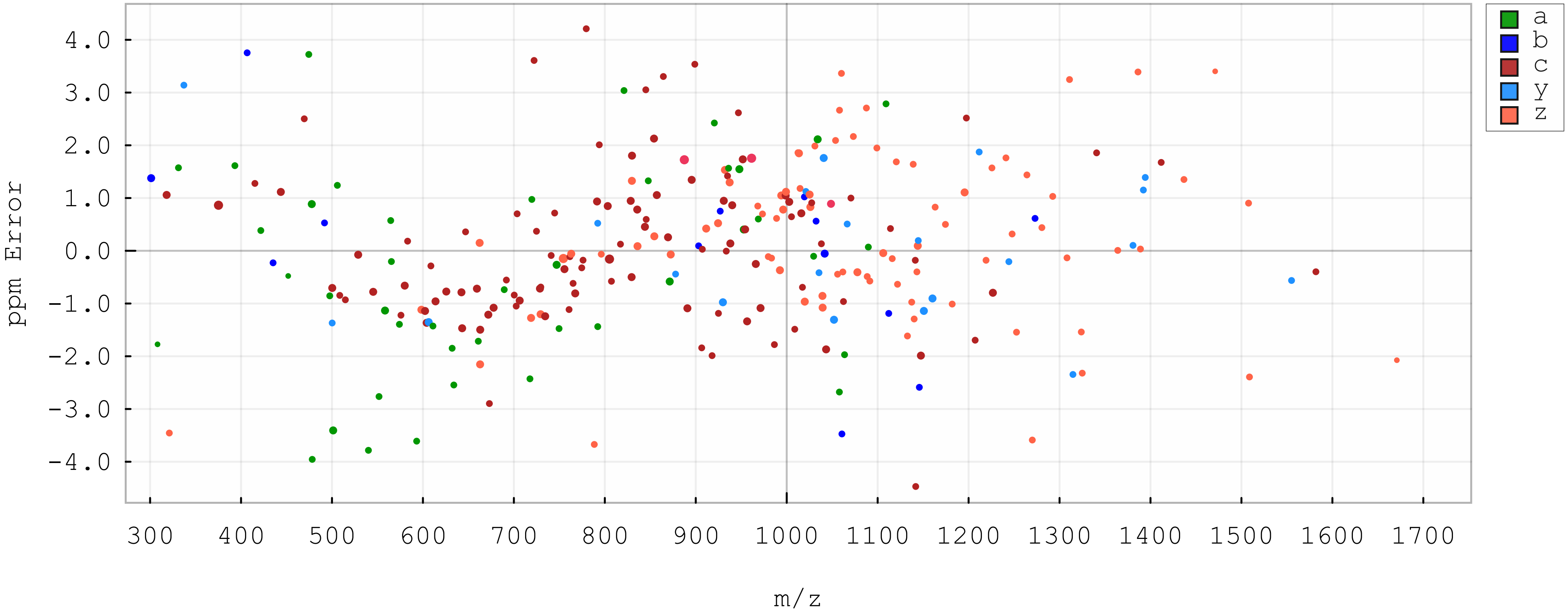

Proteoform:

Index	Protein	SC [%]	SVP [%]	IC [%]	MS Score [a.u.]	Var. Mods	Mono Mass [Da]	Chemical Formula
9	H4Kac	80.2	11.8	41.4	33.2	Ace: K5, K8, K16, K20, K31, K44, K59	11523.4152	C508H851N163O141S

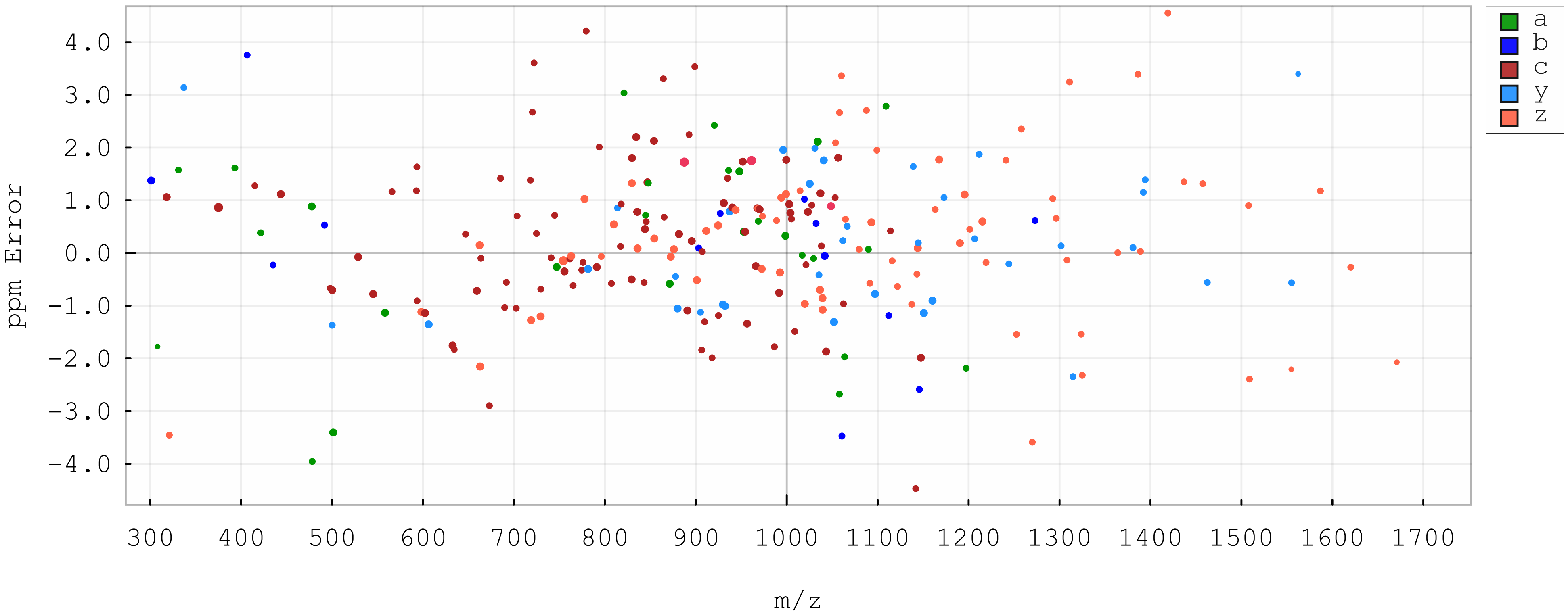

Proteoform:

Index	Protein	SC [%]	SVP [%]	IC [%]	MS Score [a.u.]	Var. Mods	Mono Mass [Da]	Chemical Formula
10	H4Kac	80.2	11.8	40.8	32.7	Ace: K5, K12, K16, K20, K31, K44, K59	11523.4152	C508H851N163O141S

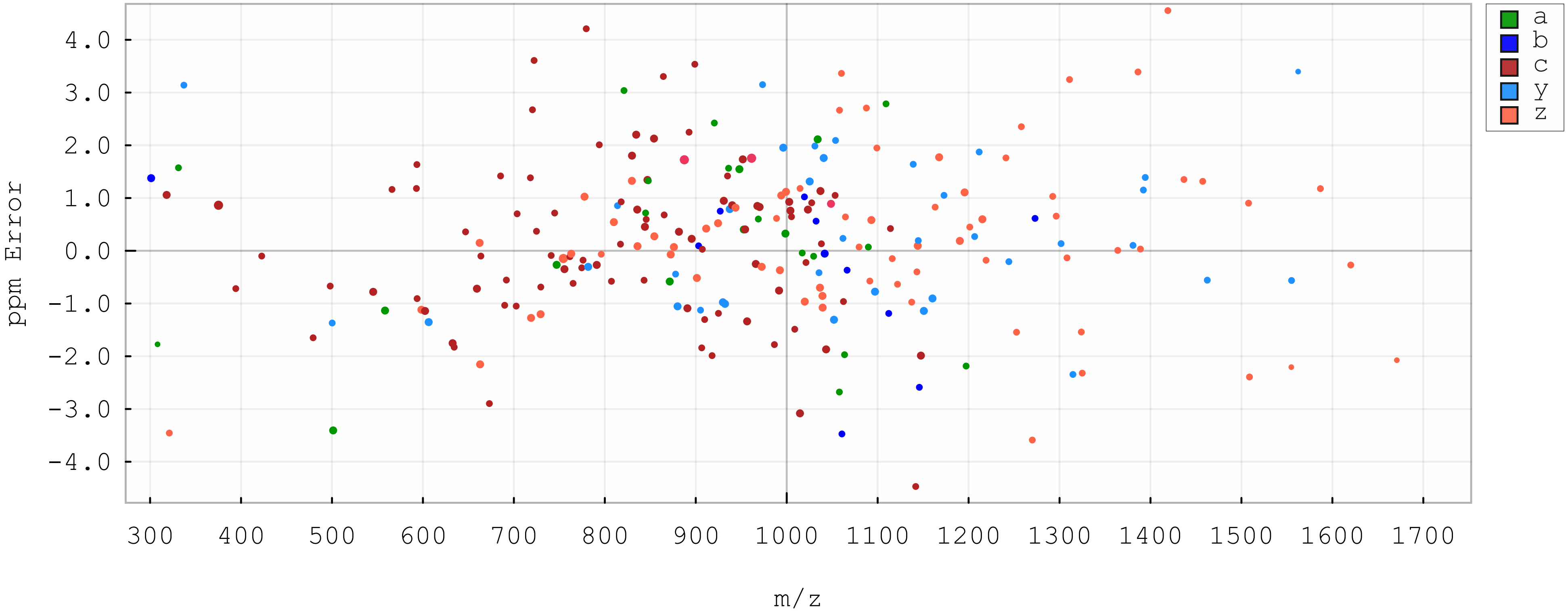

Primary Fragment Plots

Proteoform:

Index	Protein	SC [%]	SVP [%]	IC [%]	MS Score [a.u.]	Var. Mods	Mono Mass [Da]	Chemical Formula
1	H4Kac	87.1	62.7	58.1	50.6	Ace: K5, K8, K12, K16, K20, K31, K44	11523.4152	C508H851N163O141S

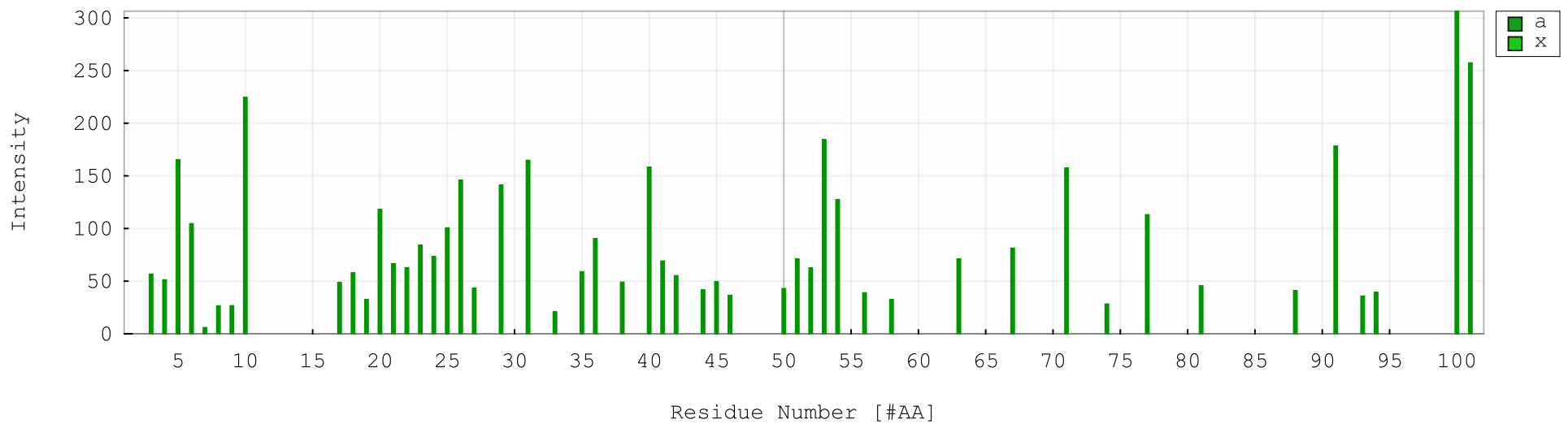

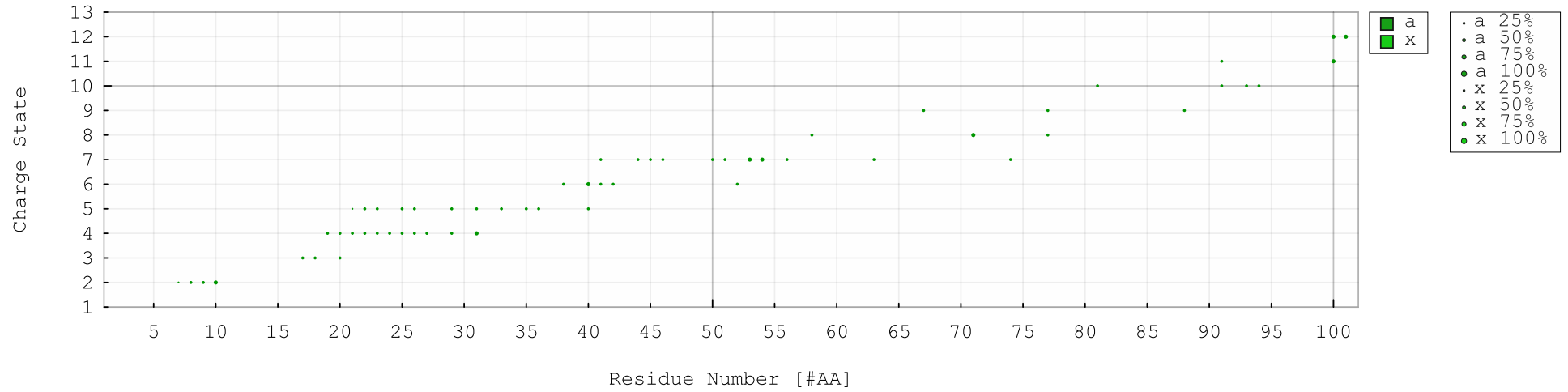

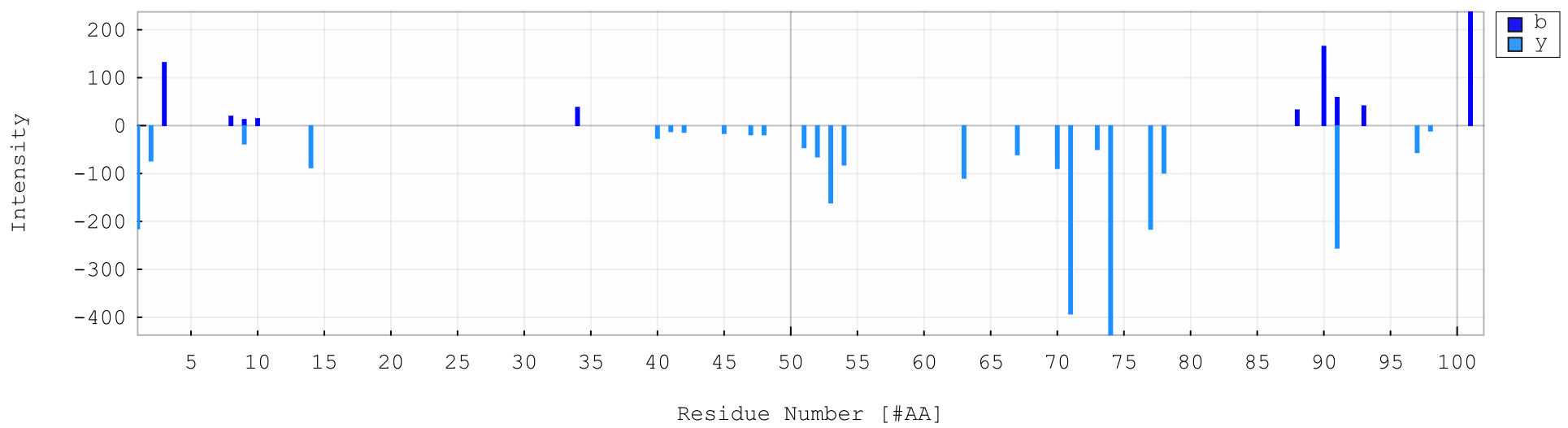

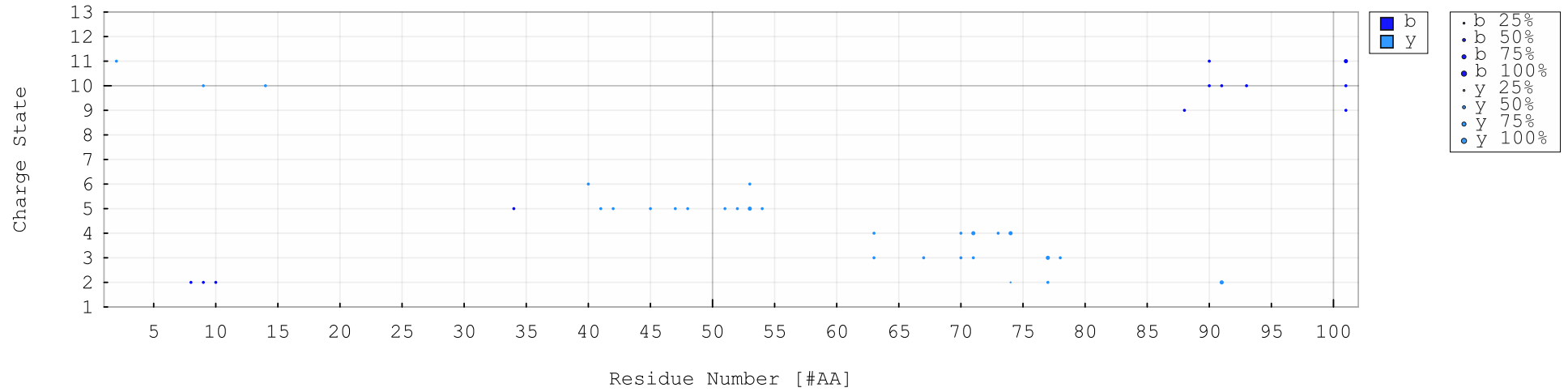

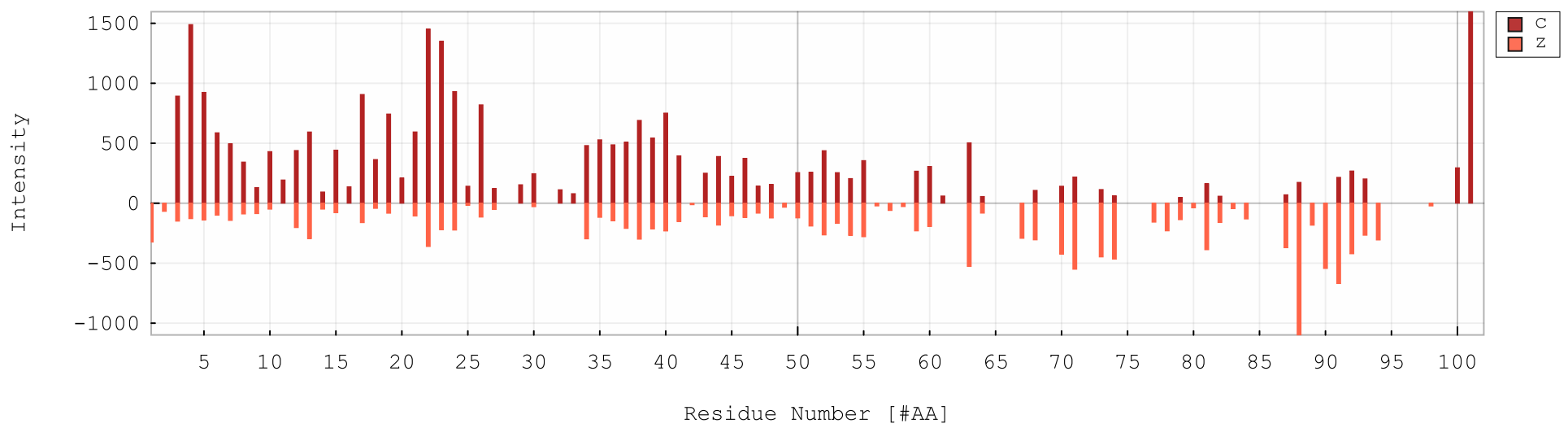

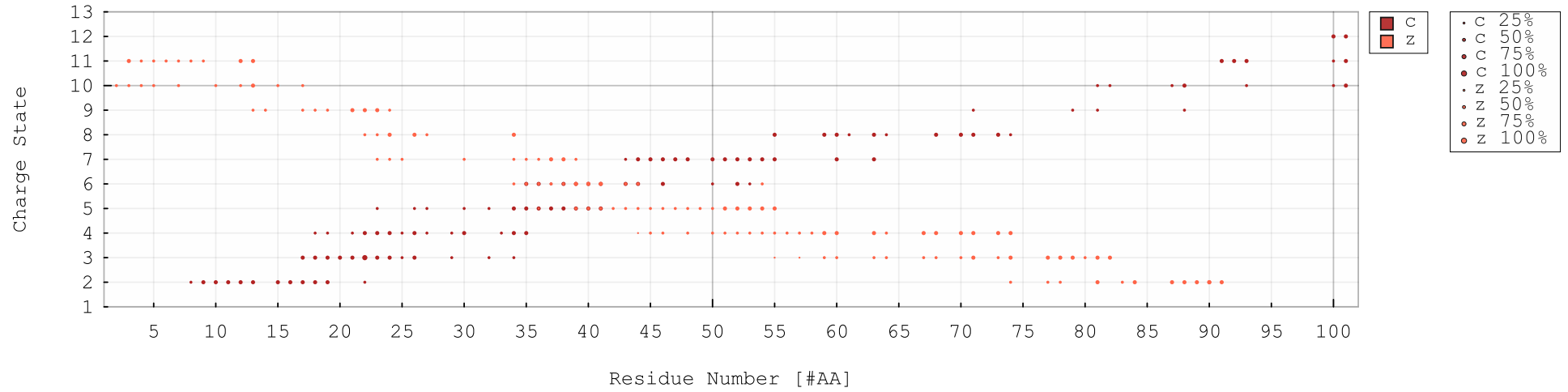

Proteoform:

Index	Protein	SC [%]	SVP [%]	IC [%]	MS Score [a.u.]	Var. Mods	Mono Mass [Da]	Chemical Formula
2	H4Kac	85.1	54.9	54.0	46.0	Ace: K5, K8, K12, K16, K20, K31, K59	11523.4152	C508H851N163O141S

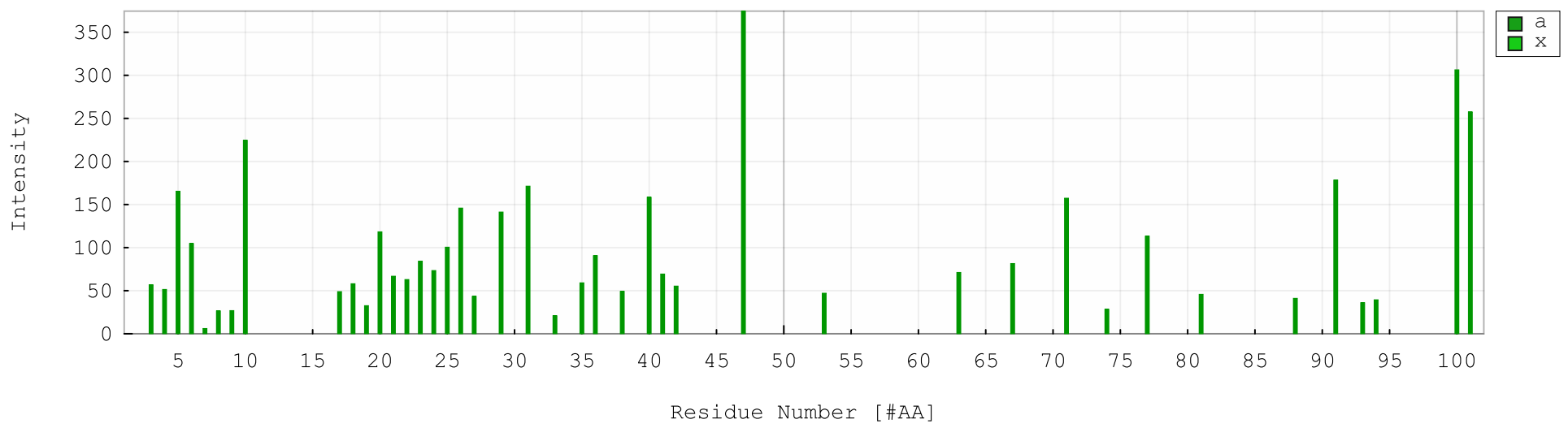

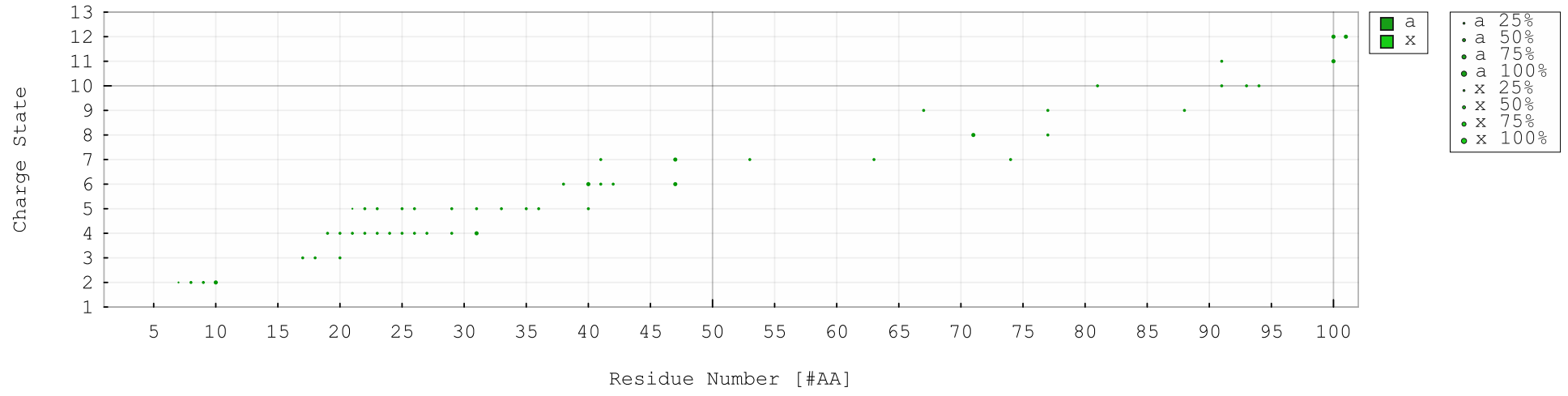

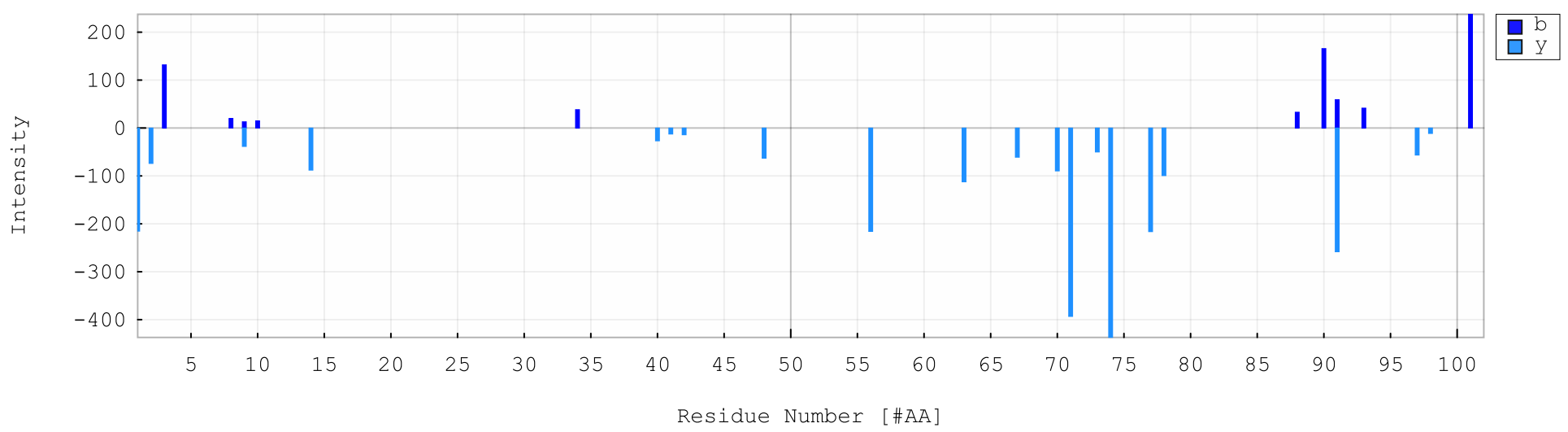

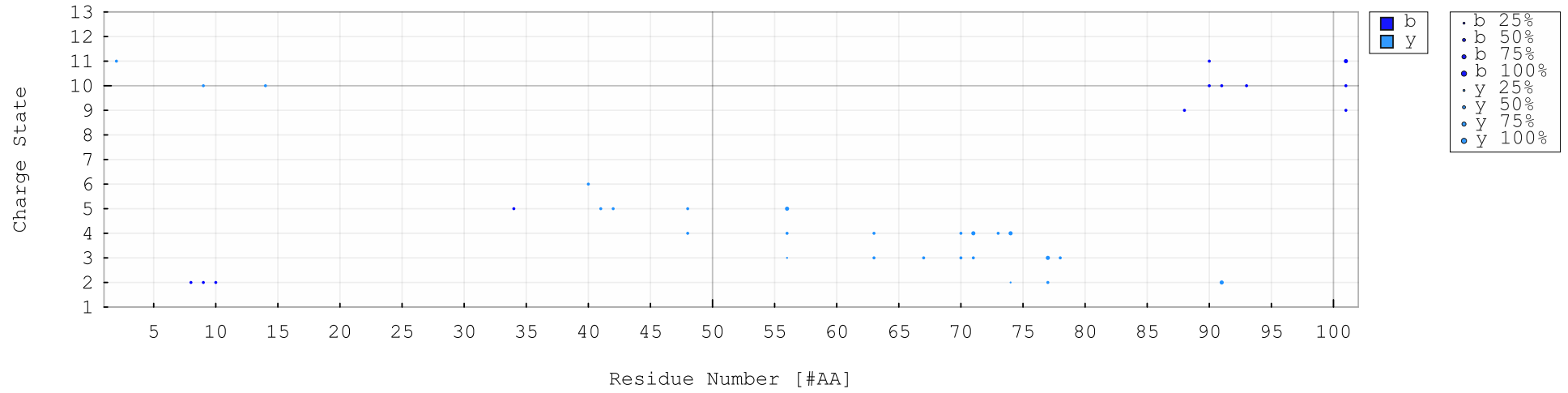

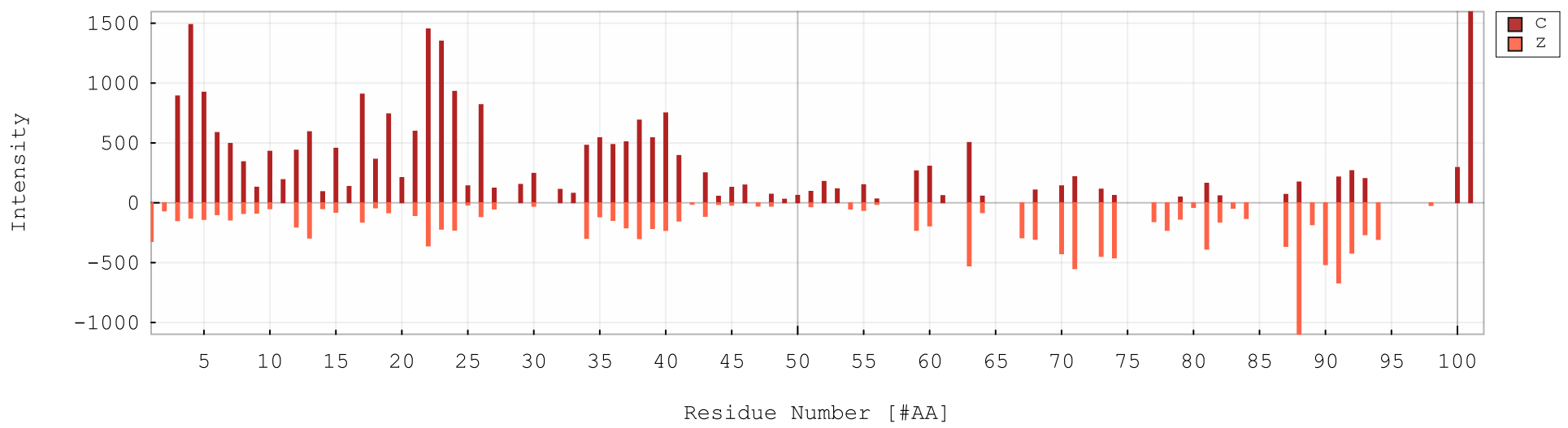

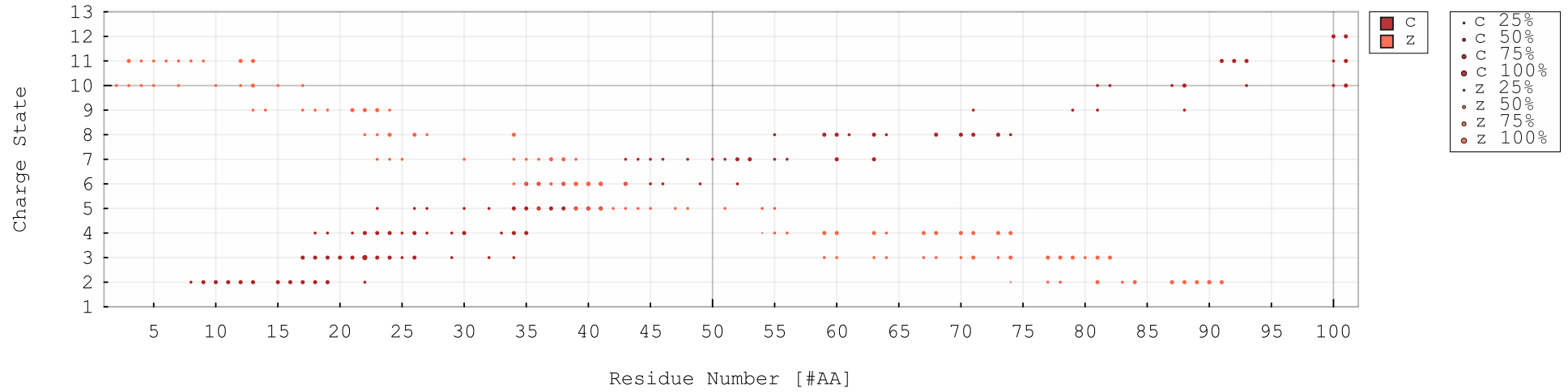

Proteoform:

Index	Protein	SC [%]	SVP [%]	IC [%]	MS Score [a.u.]	Var. Mods	Mono Mass [Da]	Chemical Formula
3	H4Kac	82.2	29.4	48.8	40.1	Ace: K5, K8, K12, K16, K20, K44, K59	11523.4152	C508H851N163O141S

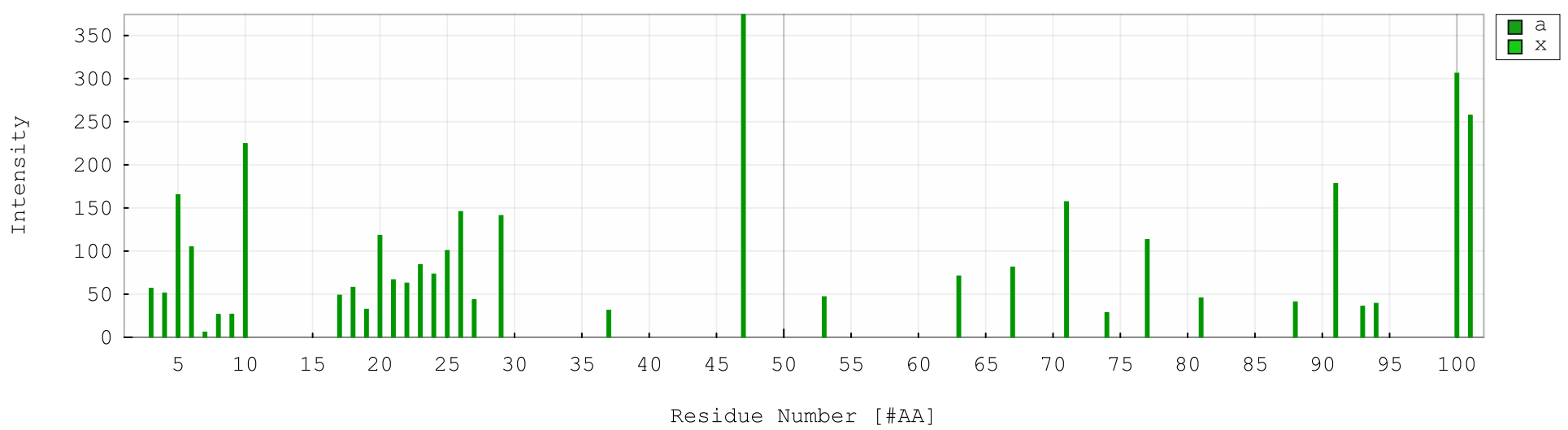

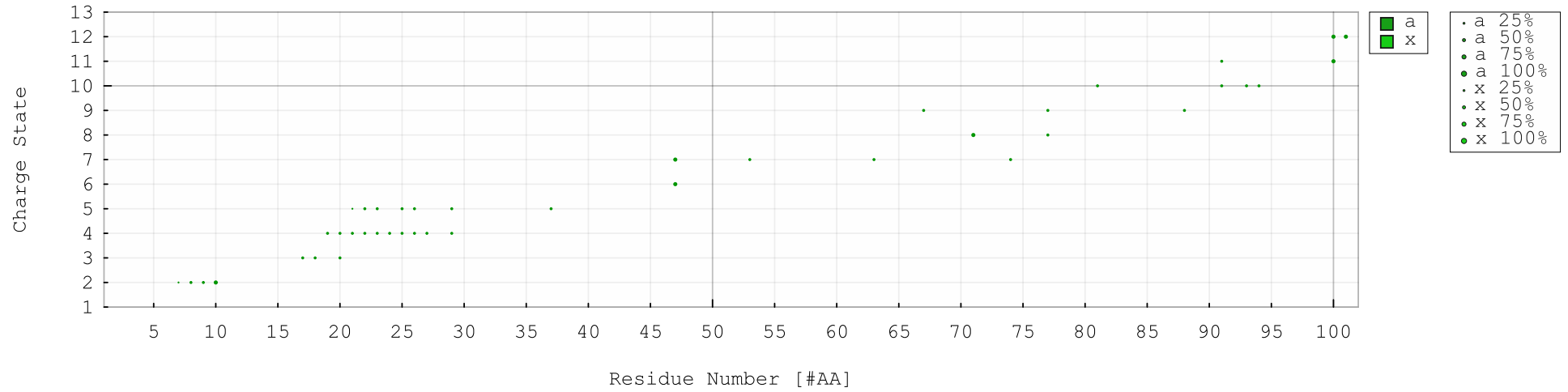

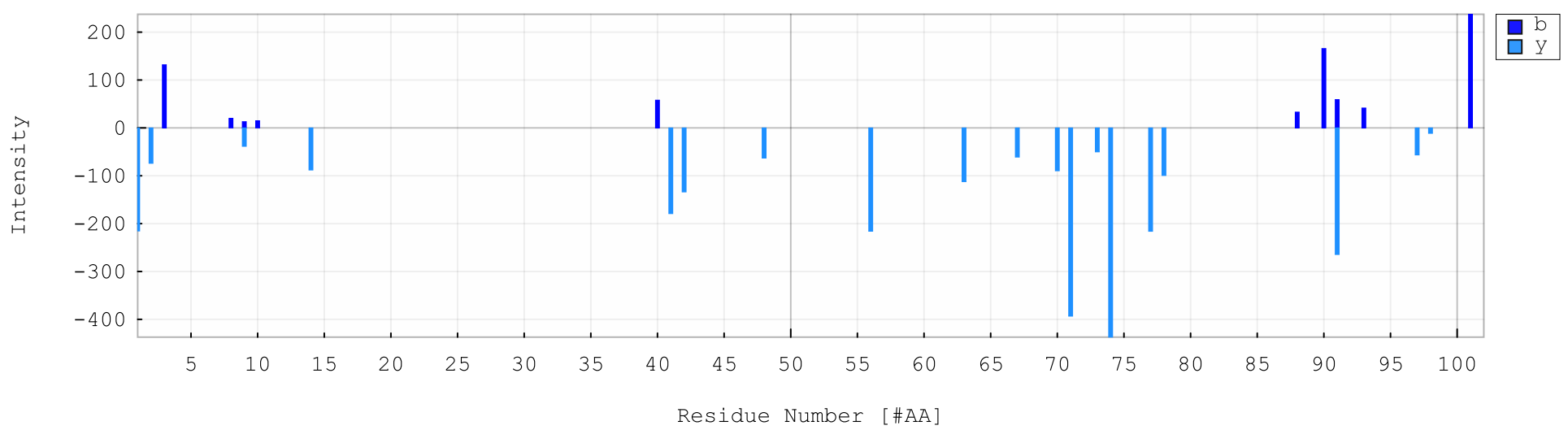

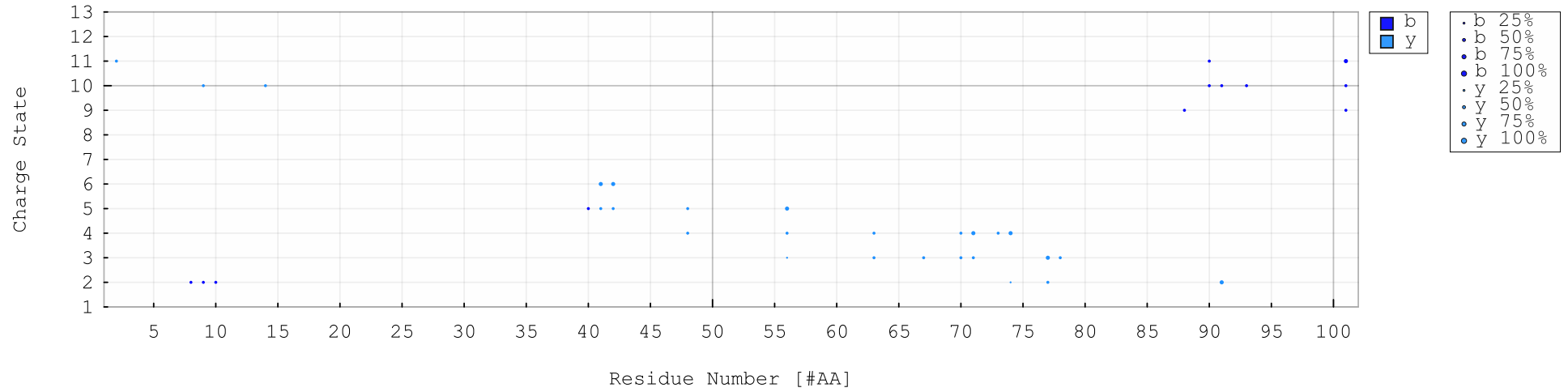

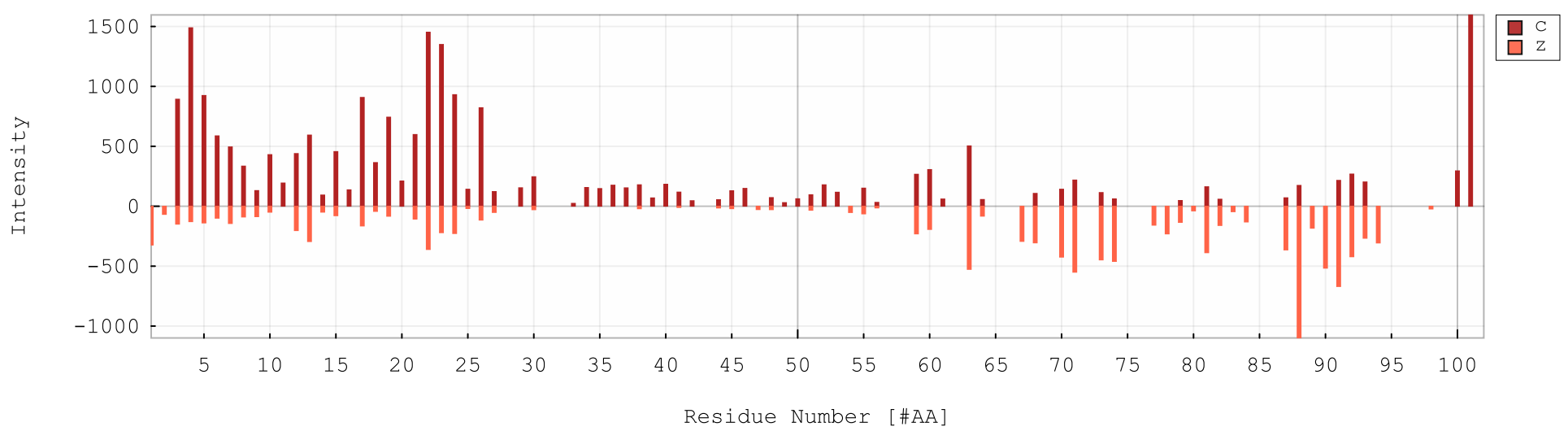

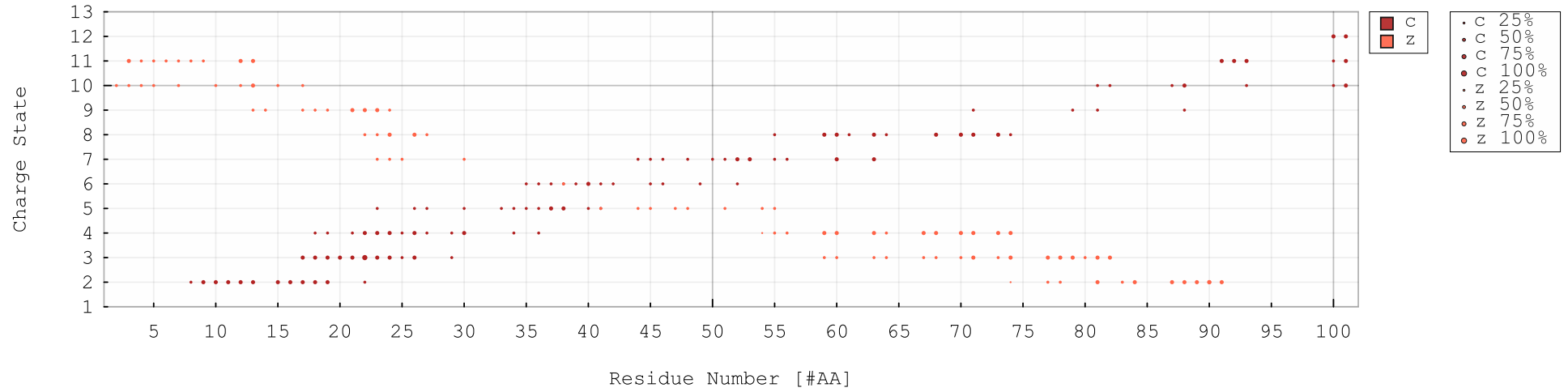

Proteoform:

Index	Protein	SC [%]	SVP [%]	IC [%]	MS Score [a.u.]	Var. Mods	Mono Mass [Da]	Chemical Formula
4	H4Kac	80.2	54.9	48.4	38.8	Ace: K5, K8, K12, K16, K20, K31, K77	11523.4152	C508H851N163O141S

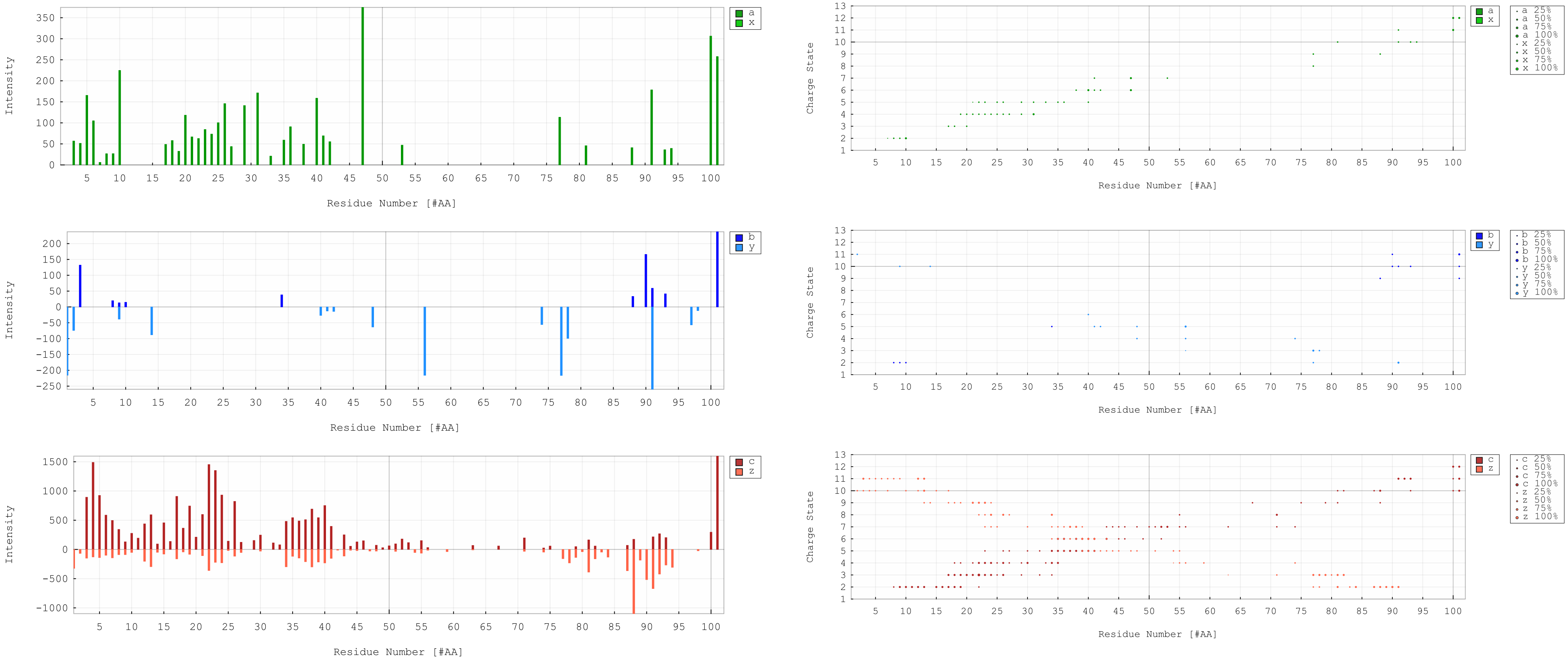

Proteoform:

Index	Protein	SC [%]	SVP [%]	IC [%]	MS Score [a.u.]	Var. Mods	Mono Mass [Da]	Chemical Formula
5	H4Kac	79.2	54.9	47.8	37.9	Ace: K5, K8, K12, K16, K20, K31, K79	11523.4152	$C_{508}H_{851}N_{163}O_{141}S$

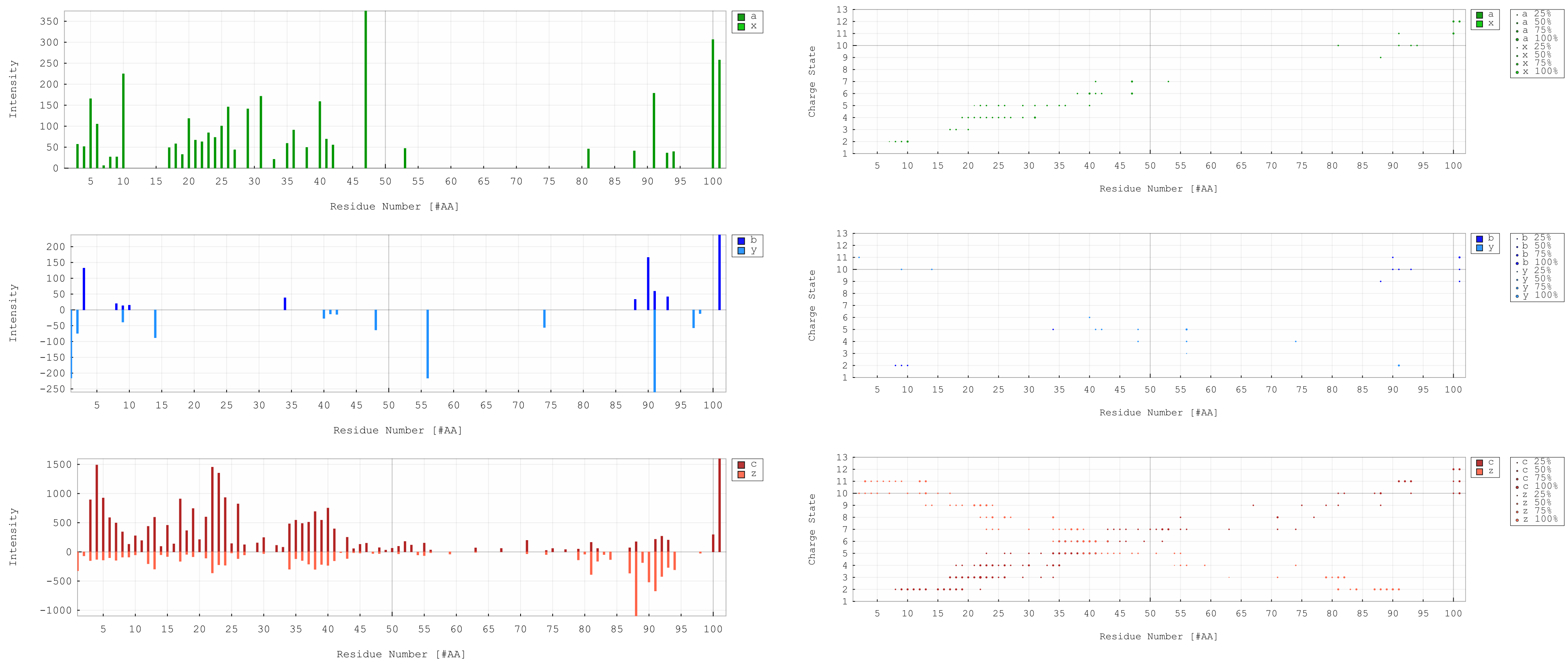

Top-Down Result OmniScape 2025
Sequence Confirmation
250428 H4_P300-50_60min z13ac7 MS2 ECD_001-487_calibrated

Proteoform:

Index	Protein	SC [%]	SVP [%]	IC [%]	MS Score [a.u.]	Var. Mods	Mono Mass [Da]	Chemical Formula
6	H4Kac	82.2	25.5	43.7	35.9	Ace: K5, K8, K12, K16, K31, K44, K59	11523.4152	C508H851N163O141S

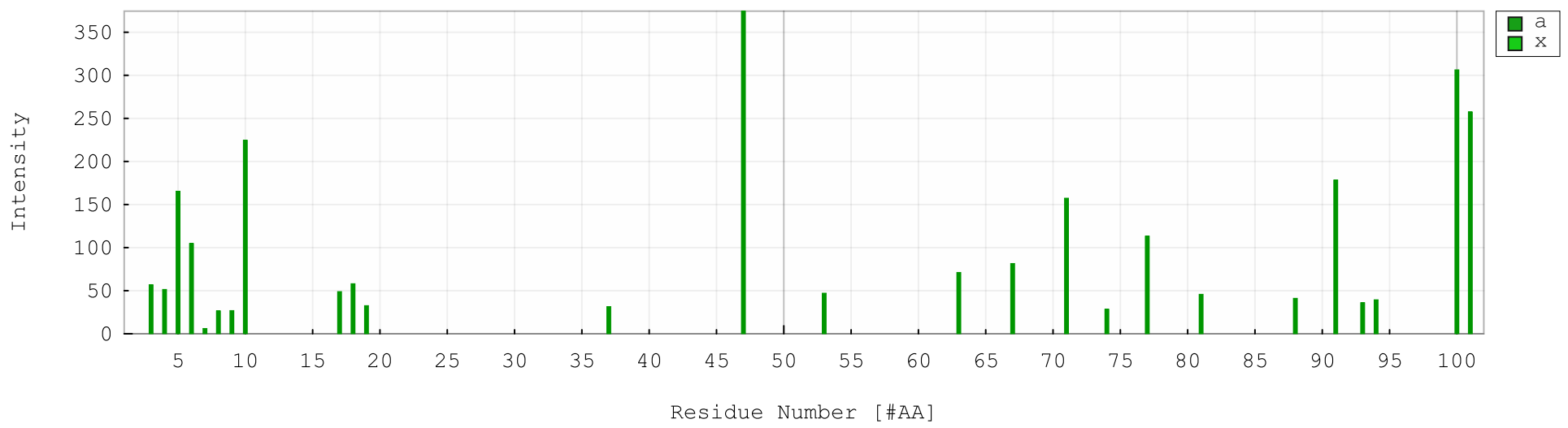

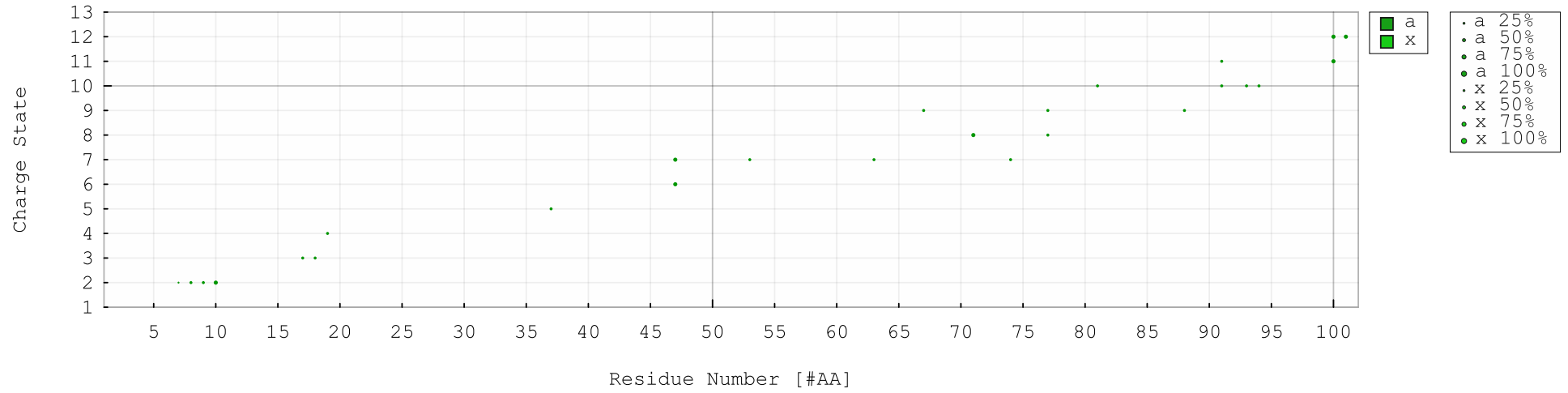

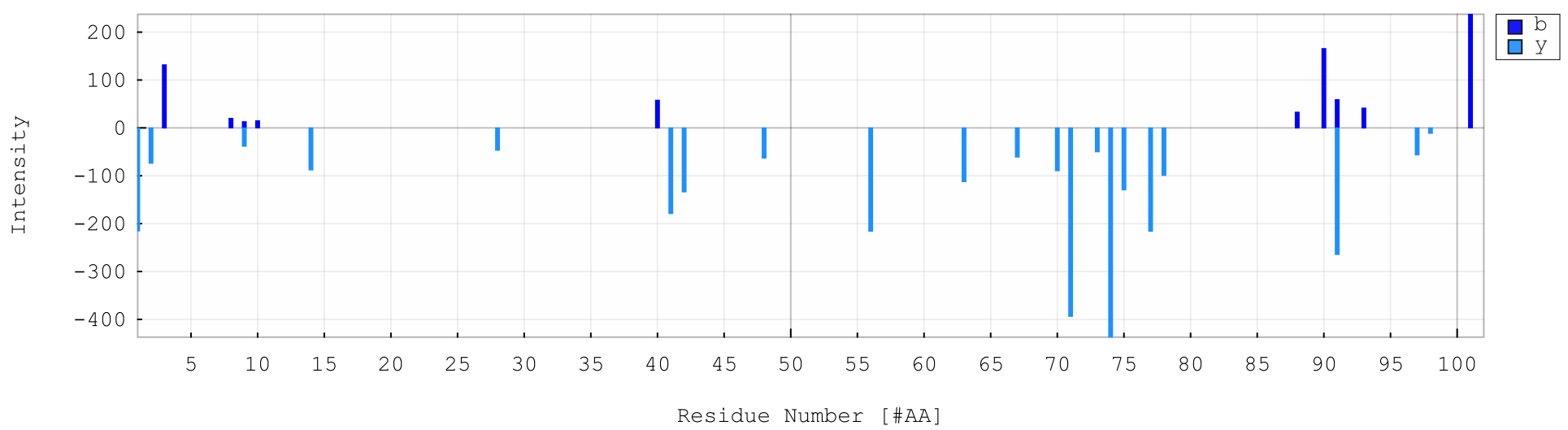

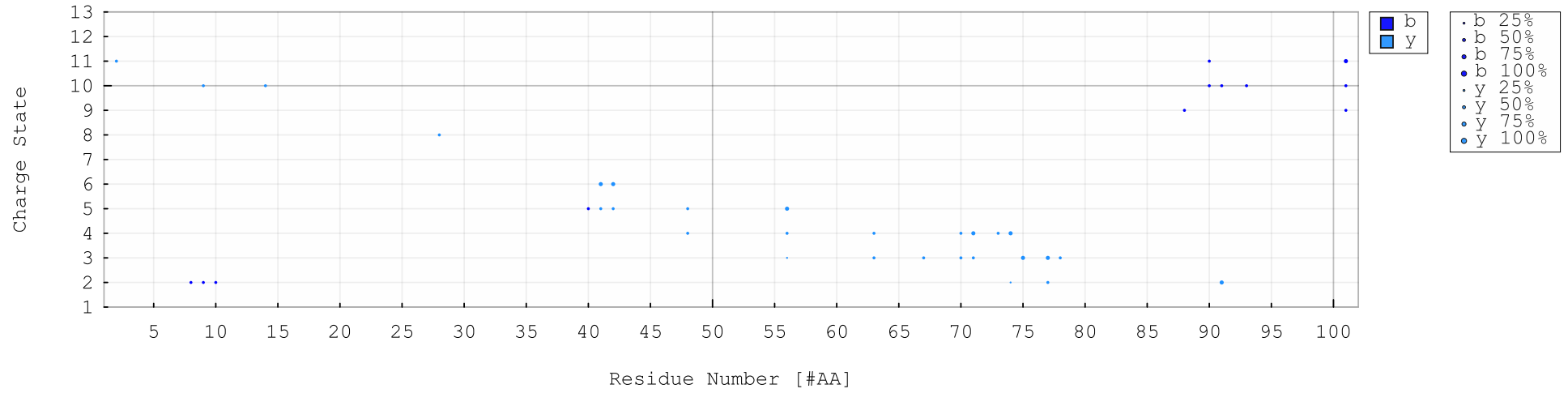

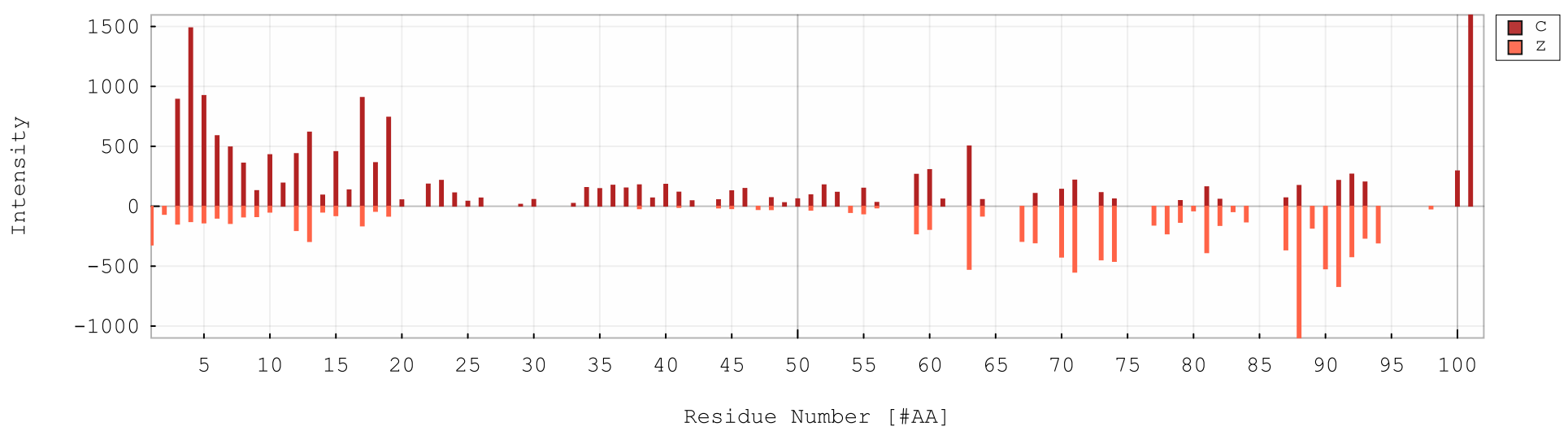

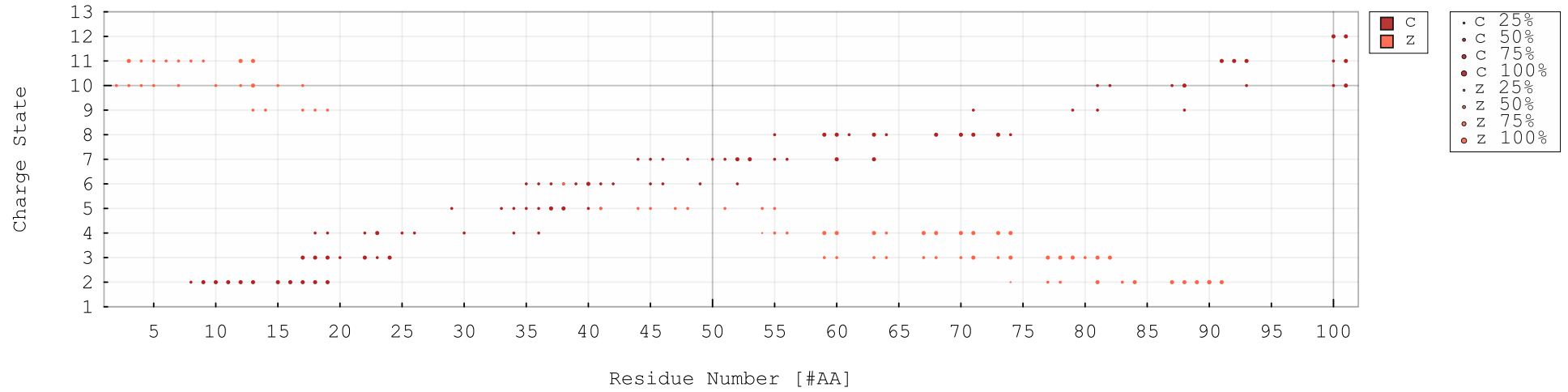

Proteoform:

Index	Protein	SC [%]	SVP [%]	IC [%]	MS Score [a.u.]	Var. Mods	Mono Mass [Da]	Chemical Formula
7	H4Kac	80.2	25.5	42.2	33.8	Ace: K5, K8, K12, K20, K31, K44, K59	11523.4152	C508H851N163O141S

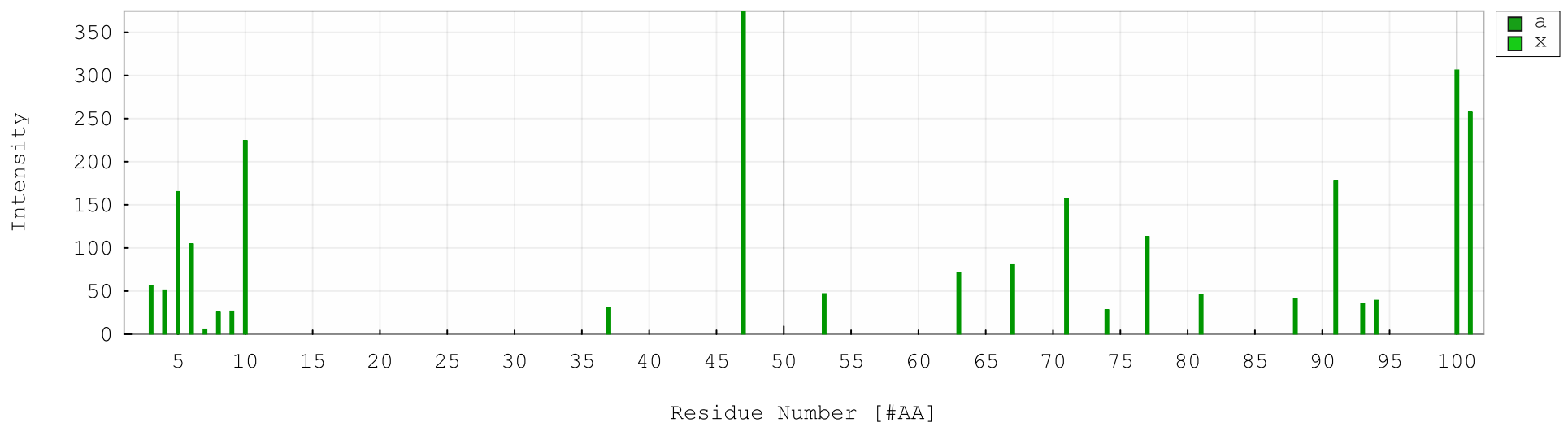

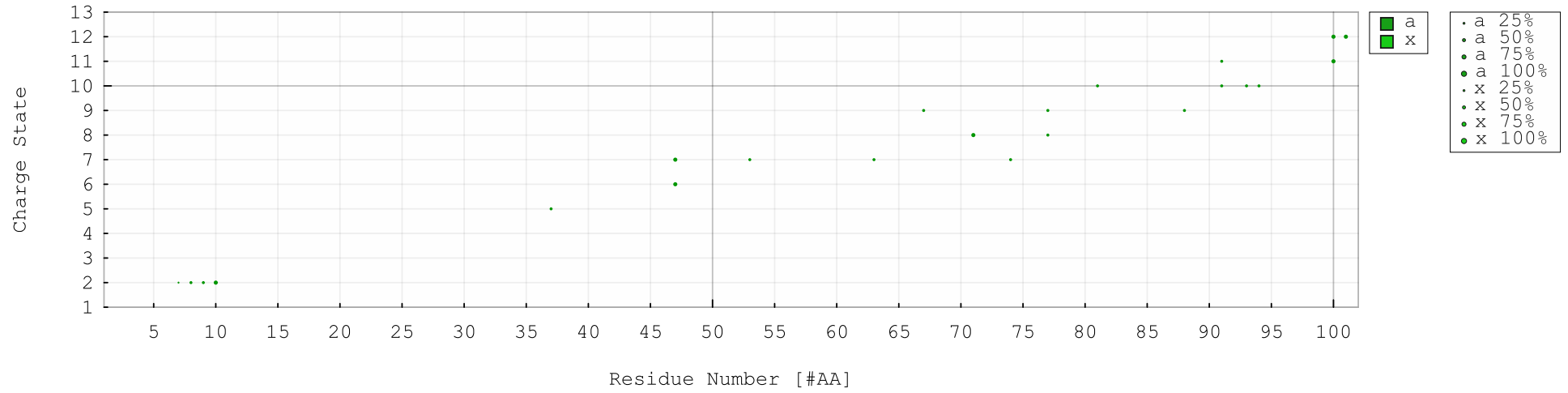

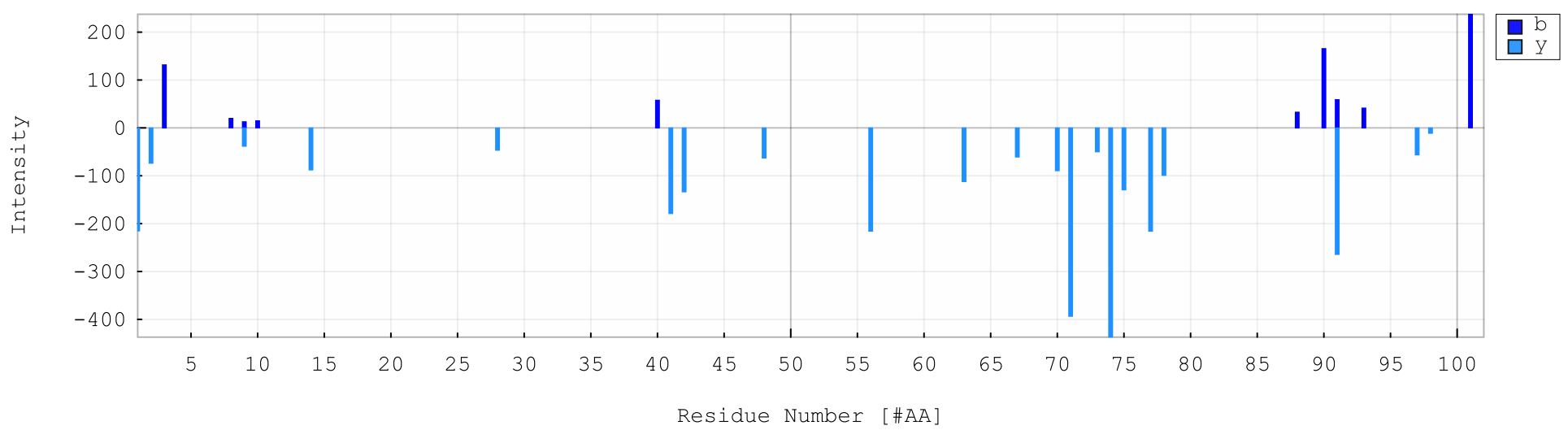

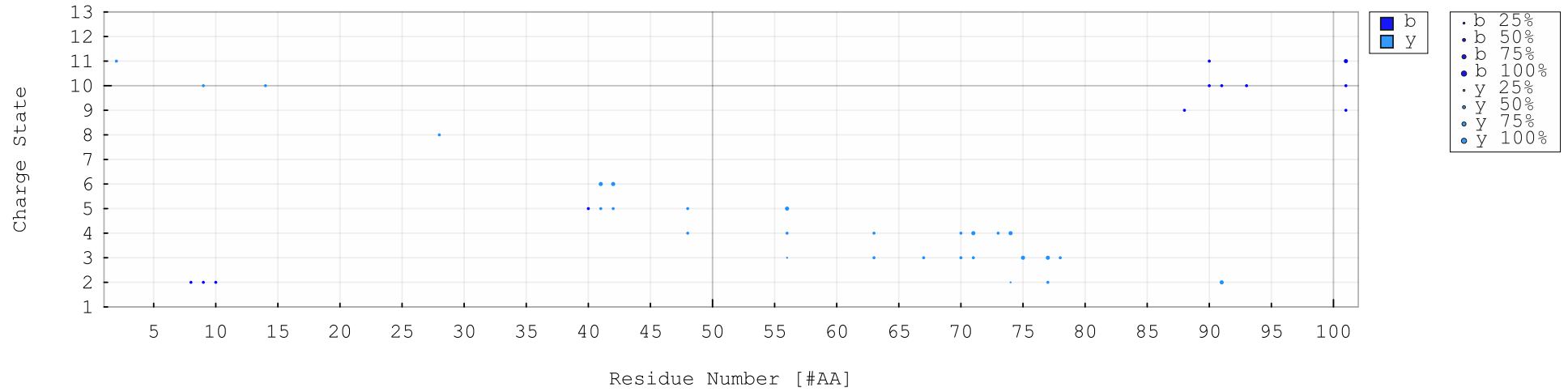

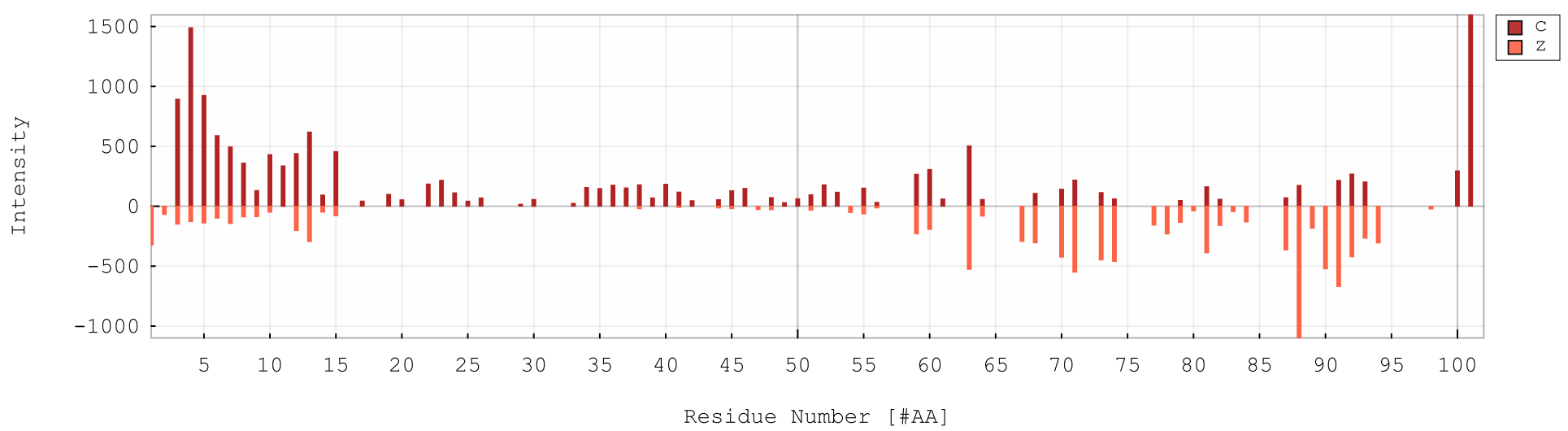

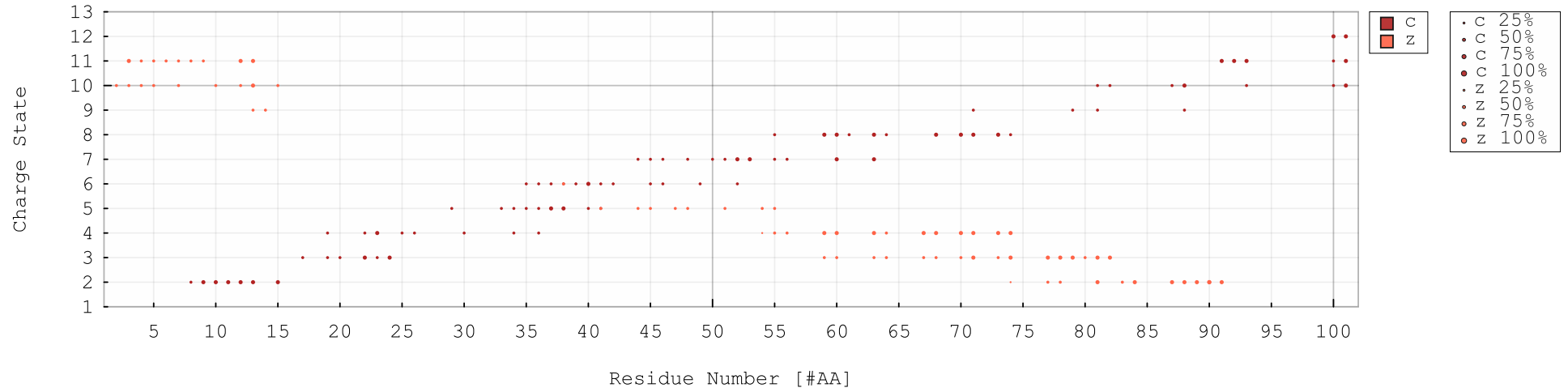

Proteoform:

Index	Protein	SC [%]	SVP [%]	IC [%]	MS Score [a.u.]	Var. Mods	Mono Mass [Da]	Chemical Formula
8	H4Kac	77.2	29.4	43.2	33.4	Ace: K5, K8, K12, K16, K20, K44, K77	11523.4152	C508H851N163O141S

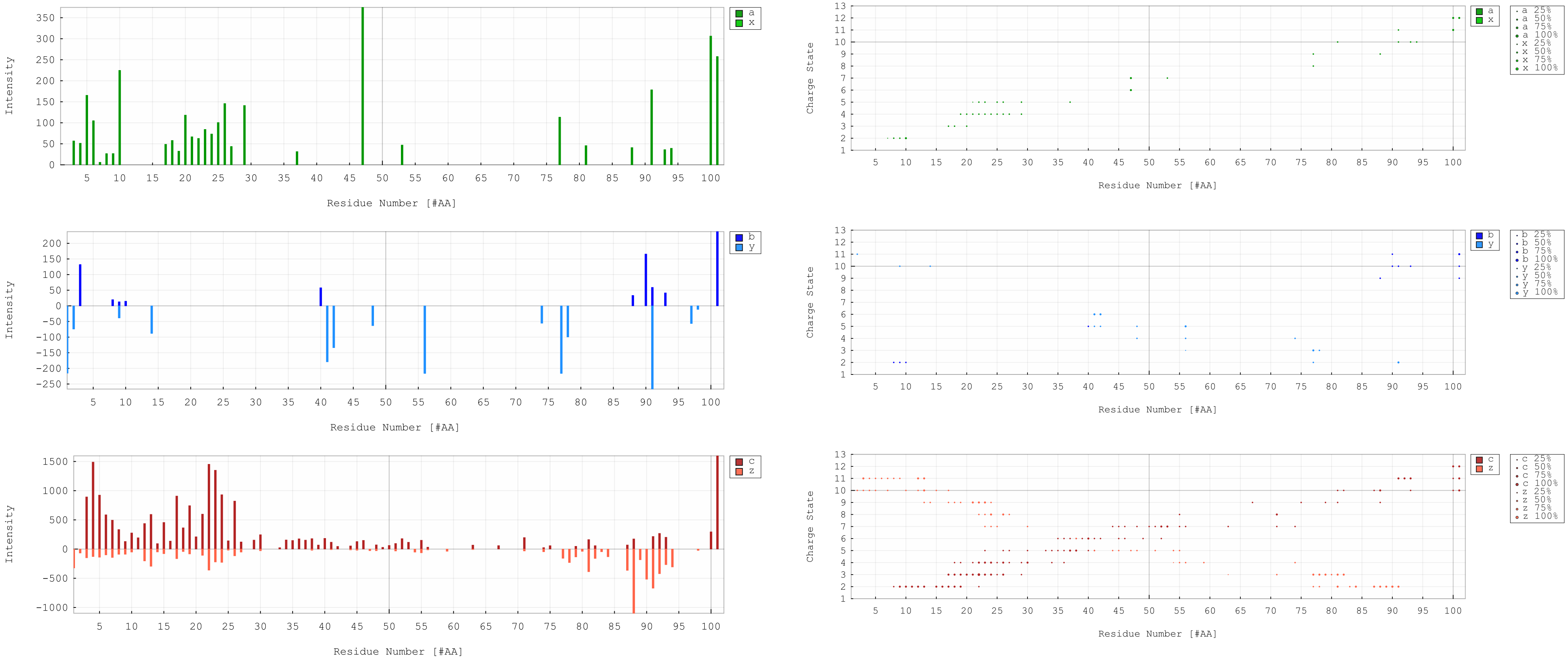

Proteoform:

Index	Protein	SC [%]	SVP [%]	IC [%]	MS Score [a.u.]	Var. Mods	Mono Mass [Da]	Chemical Formula
9	H4Kac	80.2	11.8	41.4	33.2	Ace: K5, K8, K16, K20, K31, K44, K59	11523.4152	C508H851N163O141S

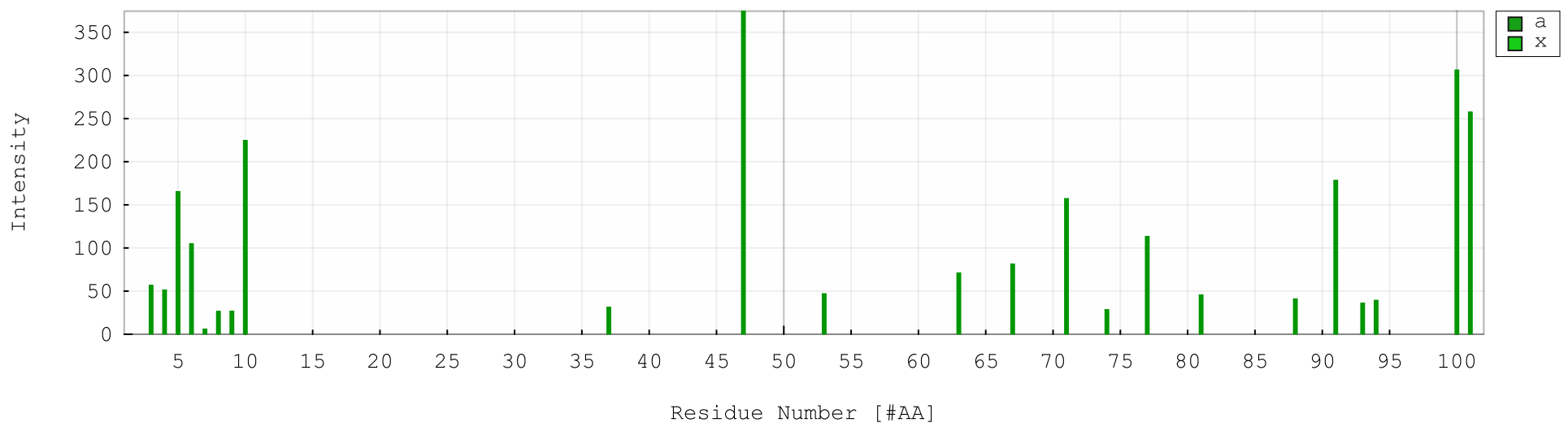

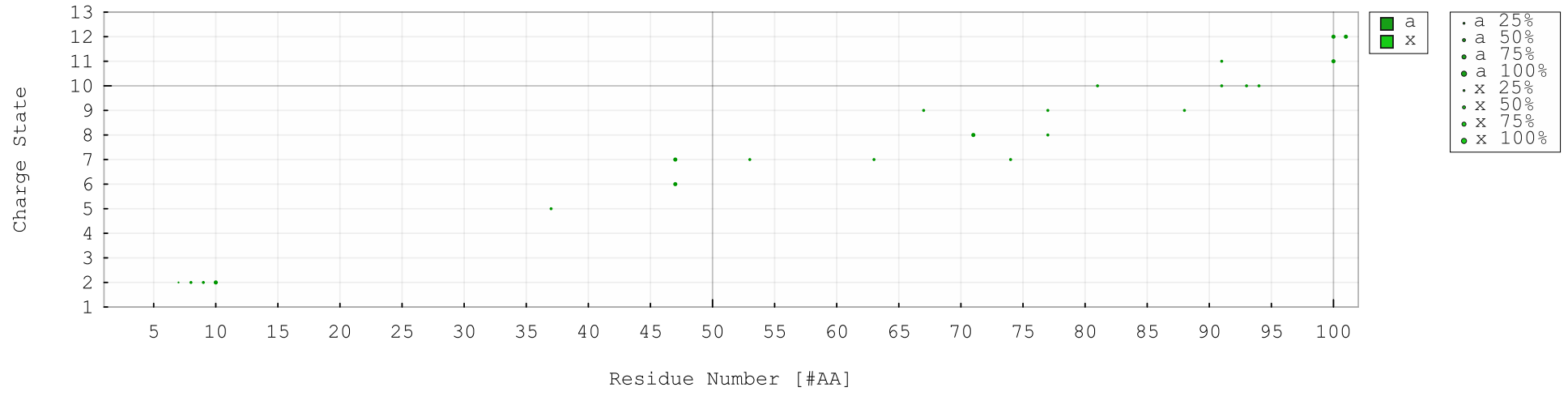

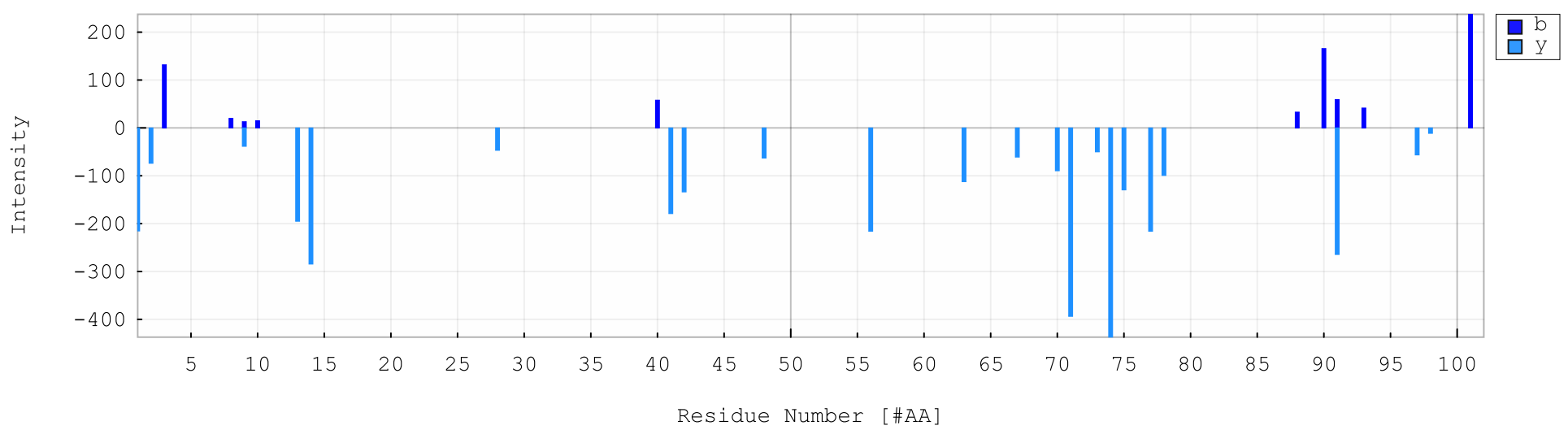

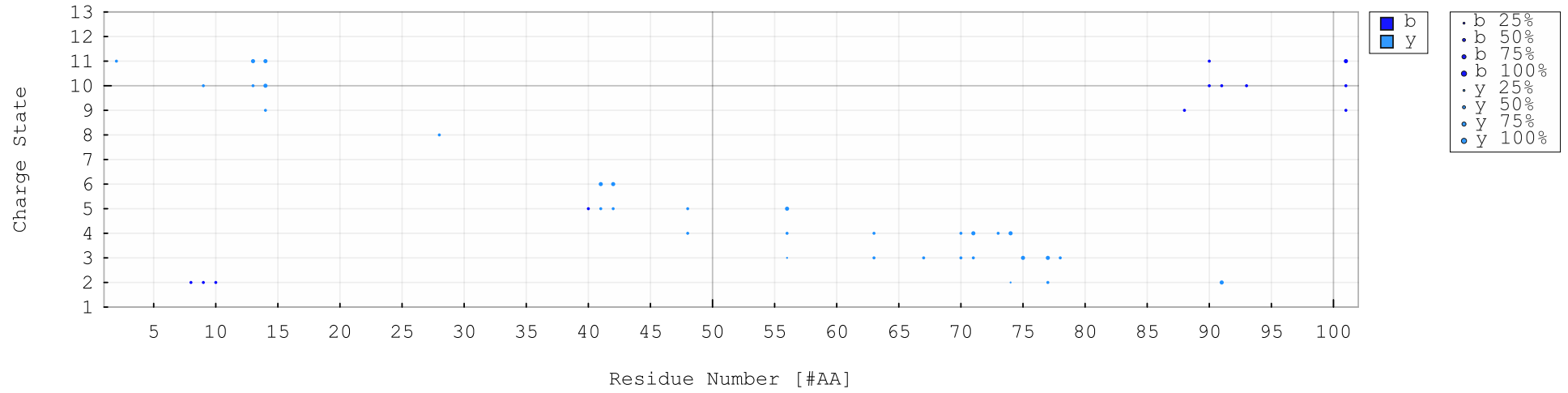

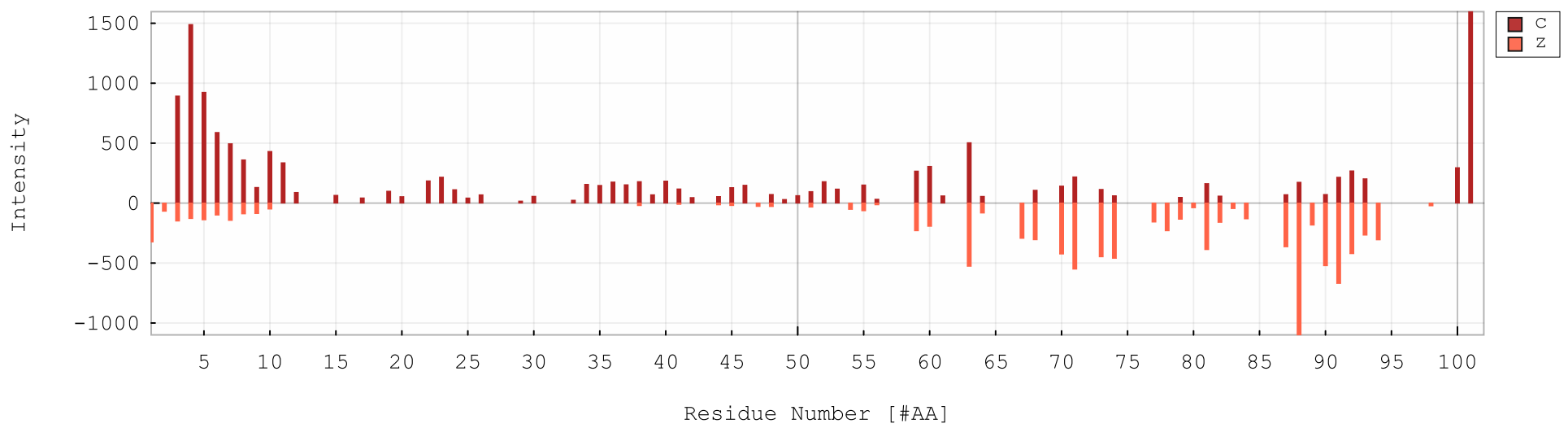

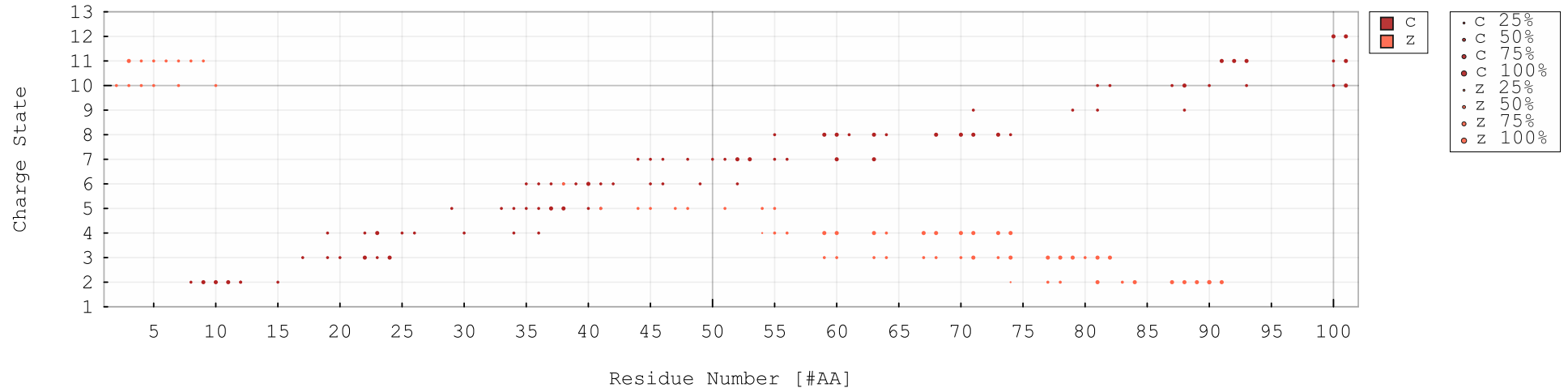

Proteoform:	Index	Protein	SC [%]	SVP [%]	IC [%]	MS Score [a.u.]	Var. Mods	Mono Mass [Da]	Chemical Formula
	10	H4Kac	80.2	11.8	40.8	32.7	Ace: K5, K12, K16, K20, K31, K44, K59	11523.4152	C508H851N163O141S

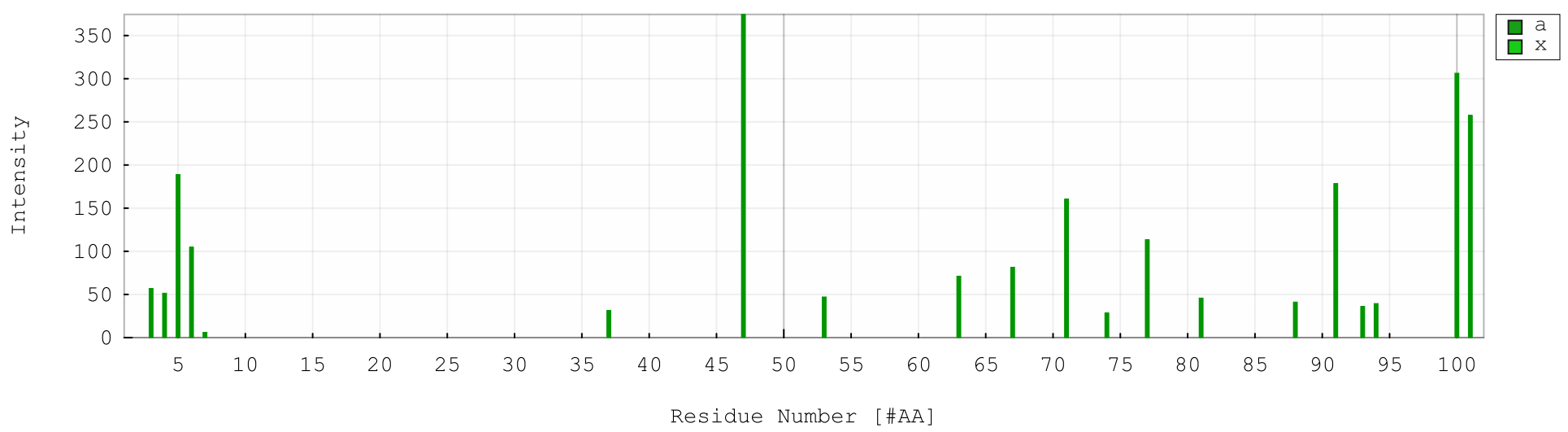

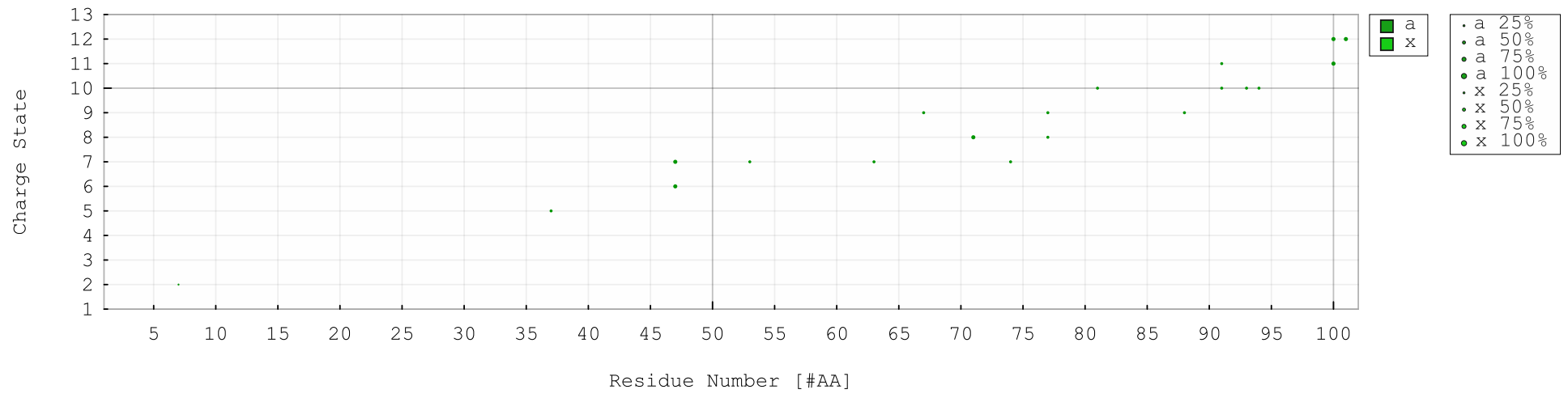

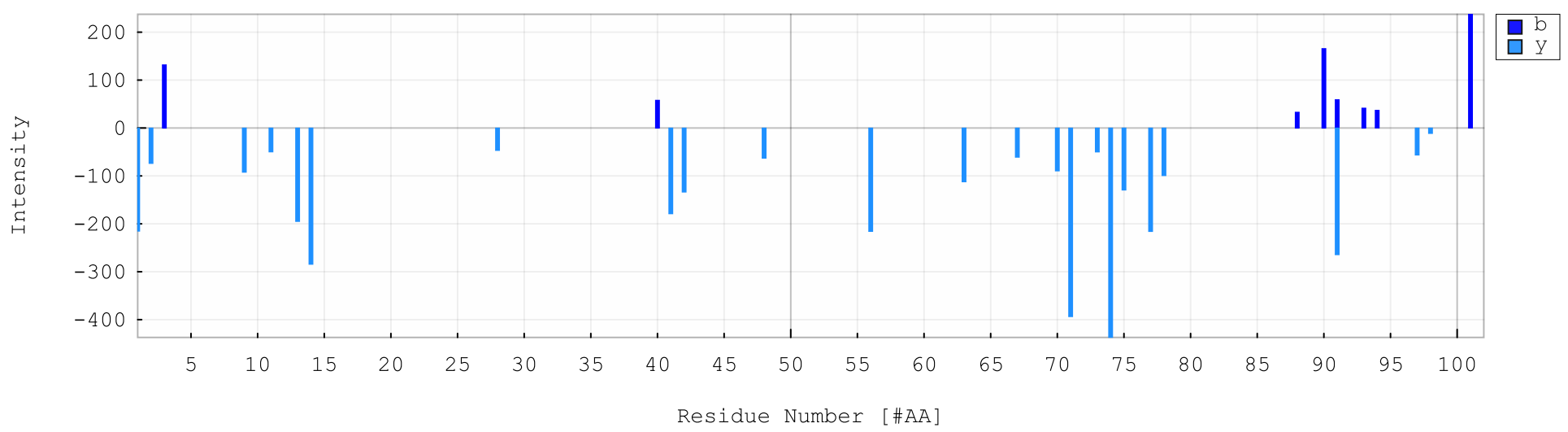

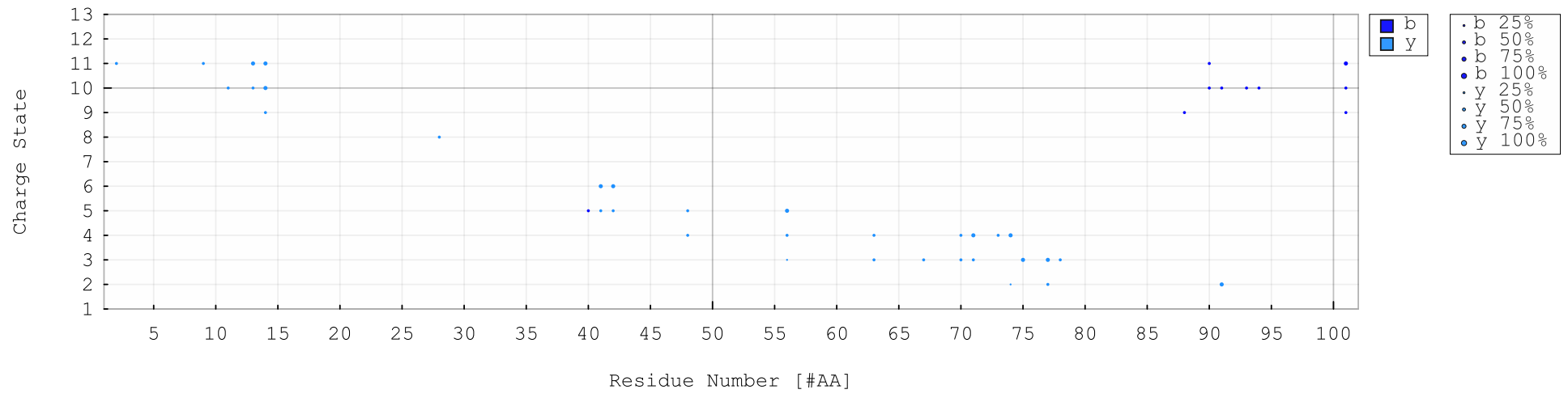

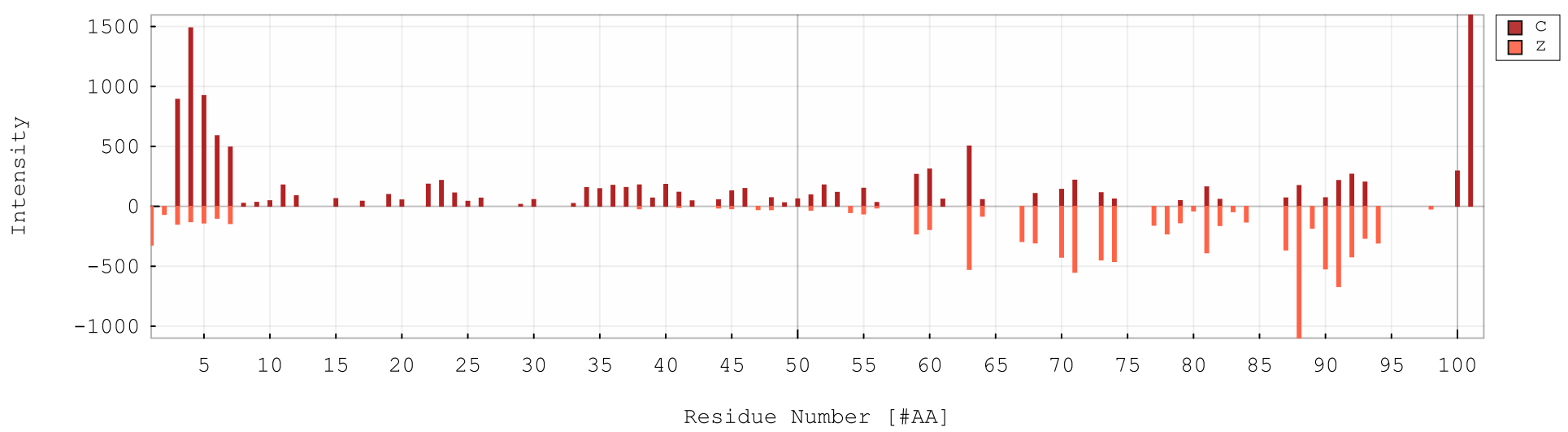

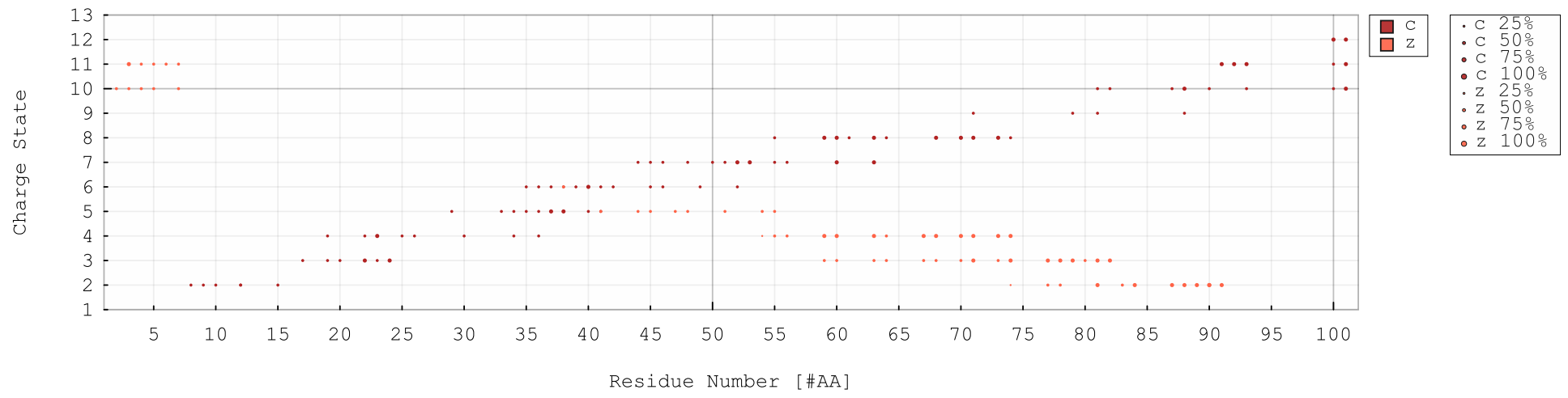

Supplemental Data S4

H4 - p300, 60 min

**Report**	6 of 7
**Experiment**	MS2
**Histone/sample**	H4
**Enzyme**	p300
**Reaction/source**	60 min
**Precursor**	H4Kac7
**Charge state**	13+
**Fragmentation / workflow**	RCID
**Original file**	MS2_H4Kac7_13+_RCID.pdf

Project Information

Project Directory: C:/Users/LAB-ADMIN
Processing date: Tue Apr 28 13:03:32 2026

Sample Info & Protocols

Name: Top-Down Protein Sequencing CID

Dataset

Imported spectrum file name:	250428 H4_P300-50_60min z13ac7 MS2 shCID_001-733_calibrated.txt
Dataset name:	Information is only available from imported JPEAKS files
Date acquired:	Information is only available from imported JPEAKS files
Date imported:	Information is only available from imported JPEAKS files
Instrument Name:	Information is only available from imported JPEAKS files
Instrument Serial Number:	Information is only available from imported JPEAKS files

Workflow Result Info

Analysis: 250428 H4_P300-50_60min z13ac7 MS2 shCID_001-733_calibrated
Location: C:/Users/LAB-ADMIN
Method: -

Method Information

Name: Unnamed

Spectral Parameters

Mass Error [ppm]: 6
Intensity Deviation [au]: 0.15
Min/Max Charges: 1-13
Experiment type: CID

a ions	b ions	c ions	x ions	y ions	z ions	satellite	internal	precursor
a	b			y				M

Noise threshold calculation: Simple moving average noise threshold (SMA)
Window size [datapoints]: 600
Noise curve offset: 1.7928286
SVP parameters: terminal gap: 2, internal gap: 1

Proteoforms

(top 10)

Index	Parent Sequence	Chain	Var. Mods	Mono Mass [Da]	Chemical Composition
1	H4-K4ac	K4acK5[Ace]K8[Ace]K12[Ace]K16[Ace]K20[Ace]K31[Ace]K44[Ace]	K5, K8, K12, K16, K20, K31, K44	11523.4152	C508H851N163O141S
2	H4-K4ac	K4acK5[Ace]K8[Ace]K12[Ace]K16[Ace]K20[Ace]K44[Ace]K59[Ace]	K5, K8, K12, K16, K20, K44, K59	11523.4152	C508H851N163O141S
3	H4-K4ac	K4acK5[Ace]K8[Ace]K12[Ace]K16[Ace]K20[Ace]K31[Ace]K59[Ace]	K5, K8, K12, K16, K20, K31, K59	11523.4152	C508H851N163O141S
4	H4-K4ac	K4acK5[Ace]K8[Ace]K12[Ace]K16[Ace]K31[Ace]K44[Ace]K59[Ace]	K5, K8, K12, K16, K31, K44, K59	11523.4152	C508H851N163O141S
5	H4-K4ac	K4acK5[Ace]K8[Ace]K12[Ace]K20[Ace]K31[Ace]K44[Ace]K59[Ace]	K5, K8, K12, K20, K31, K44, K59	11523.4152	C508H851N163O141S
6	H4-K4ac	K4acK5[Ace]K8[Ace]K16[Ace]K20[Ace]K31[Ace]K44[Ace]K59[Ace]	K5, K8, K16, K20, K31, K44, K59	11523.4152	C508H851N163O141S
7	H4-K4ac	K4acK5[Ace]K8[Ace]K12[Ace]K16[Ace]K20[Ace]K59[Ace]K77[Ace]	K5, K8, K12, K16, K20, K59, K77	11523.4152	C508H851N163O141S
8	H4-K4ac	K4acK5[Ace]K8[Ace]K12[Ace]K16[Ace]K20[Ace]K44[Ace]K77[Ace]	K5, K8, K12, K16, K20, K44, K77	11523.4152	C508H851N163O141S
9	H4-K4ac	K4acK5[Ace]K8[Ace]K12[Ace]K16[Ace]K20[Ace]K31[Ace]K77[Ace]	K5, K8, K12, K16, K20, K31, K77	11523.4152	C508H851N163O141S
10	H4-K4ac	K4acK5[Ace]K8[Ace]K12[Ace]K16[Ace]K20[Ace]K44[Ace]K79[Ace]	K5, K8, K12, K16, K20, K44, K79	11523.4152	C508H851N163O141S

Results

Proteoforms Table (top 10)

Index	Protein	SC [%]	SVP [%]	IC [%]	MS Score [a.u.]	Var. Mods	Mono Mass [Da]	Chemical Formula
1	H4Kac	61.4	23.5	43.4	26.6	Ace: K5, K8, K12, K16, K20, K31, K44	11523.4152	C508H851N163O141S
2	H4Kac	59.4	20.6	39.3	23.4	Ace: K5, K8, K12, K16, K20, K44, K59	11523.4152	C508H851N163O141S
3	H4Kac	58.4	20.6	39.8	23.3	Ace: K5, K8, K12, K16, K20, K31, K59	11523.4152	C508H851N163O141S
4	H4Kac	55.4	20.6	38.8	21.5	Ace: K5, K8, K12, K16, K31, K44, K59	11523.4152	C508H851N163O141S
5	H4Kac	53.5	19.6	38.8	20.7	Ace: K5, K8, K12, K20, K31, K44, K59	11523.4152	C508H851N163O141S
6	H4Kac	50.5	15.7	38.6	19.5	Ace: K5, K8, K16, K20, K31, K44, K59	11523.4152	C508H851N163O141S
7	H4Kac	47.5	23.5	36.5	17.3	Ace: K5, K8, K12, K16, K20, K59, K77	11523.4152	C508H851N163O141S
8	H4Kac	46.5	20.6	36.9	17.2	Ace: K5, K8, K12, K16, K20, K44, K77	11523.4152	C508H851N163O141S
9	H4Kac	45.5	20.6	37.4	17.0	Ace: K5, K8, K12, K16, K20, K31, K77	11523.4152	C508H851N163O141S
10	H4Kac	45.5	20.6	36.9	16.8	Ace: K5, K8, K12, K16, K20, K44, K79	11523.4152	C508H851N163O141S

Sequence

Name: H4Kac

```
                  10                    20                    30                    40                    50                    60
S G R G K G G K G L   G K G G A K R H R K   V L R D N I Q G I T   K P A I R R L A R R   G G V K R I S G L I   Y E E T R G V L K V
                  70                    80                    90                   100                   110                   120
F L E N V I R D A V   T Y T E H A K R K T   V T A M D V V Y A L   K R Q G R T L Y G F   G G
```

Modifications

Modification	Position	Type
Acetyl	5	variable
Acetyl	8	variable
Acetyl	12	variable
Acetyl	16	variable
Acetyl	20	variable
Acetyl	31	variable
Acetyl	44	variable
Acetyl	59	variable
Acetyl	77	variable
Acetyl	79	variable

Sequence Map

Proteoform:

Index	Protein	SC [%]	SVP [%]	IC [%]	MS Score [a.u.]	Var. Mods	Mono Mass [Da]	Chemical Formula
1	H4Kac	61.4	23.5	43.4	26.6	Ace: K5, K8, K12, K16, K20, K31, K44	11523.4152	C508H851N163O141S

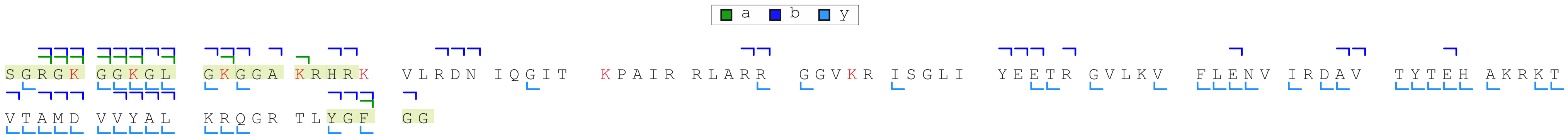

Proteoform:

Index	Protein	SC [%]	SVP [%]	IC [%]	MS Score [a.u.]	Var. Mods	Mono Mass [Da]	Chemical Formula
2	H4Kac	59.4	20.6	39.3	23.4	Ace: K5, K8, K12, K16, K20, K44, K59	11523.4152	C508H851N163O141S

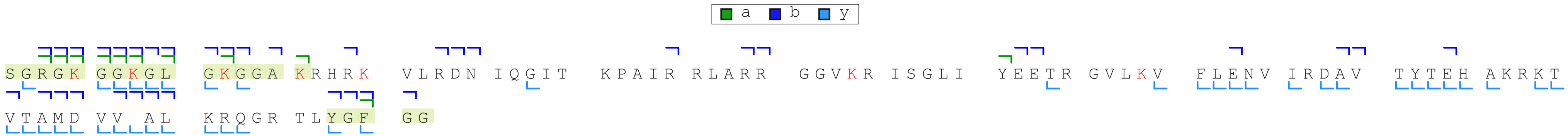

Proteoform:	Index	Protein	SC [%]	SVP [%]	IC [%]	MS Score [a.u.]	Var. Mods	Mono Mass [Da]	Chemical Formula
	3	H4Kac	58.4	20.6	39.8	23.3	Ace: K5, K8, K12, K16, K20, K31, K59	11523.4152	C508H851N163O141S

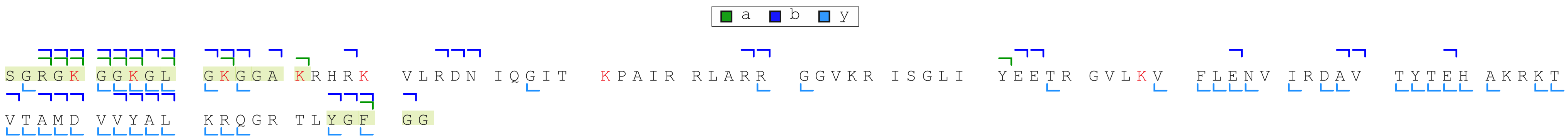

Proteoform:	Index	Protein	SC [%]	SVP [%]	IC [%]	MS Score [a.u.]	Var. Mods	Mono Mass [Da]	Chemical Formula
	4	H4Kac	55.4	20.6	38.8	21.5	Ace: K5, K8, K12, K16, K31, K44, K59	11523.4152	C508H851N163O141S

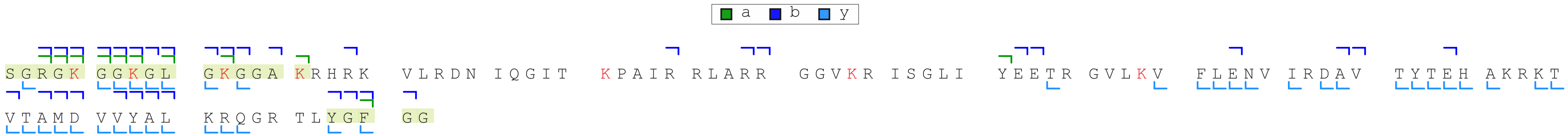

Proteoform:	Index	Protein	SC [%]	SVP [%]	IC [%]	MS Score [a.u.]	Var. Mods	Mono Mass [Da]	Chemical Formula
	5	H4Kac	53.5	19.6	38.8	20.7	Ace: K5, K8, K12, K20, K31, K44, K59	11523.4152	C508H851N163O141S

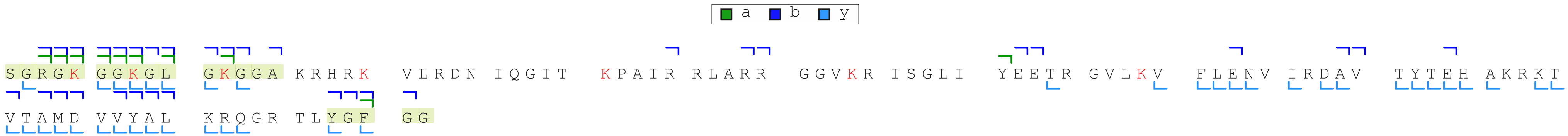

Proteoform:

Index	Protein	SC [%]	SVP [%]	IC [%]	MS Score [a.u.]	Var. Mods	Mono Mass [Da]	Chemical Formula
6	H4Kac	50.5	15.7	38.6	19.5	Ace: K5, K8, K16, K20, K31, K44, K59	11523.4152	C508H851N163O141S

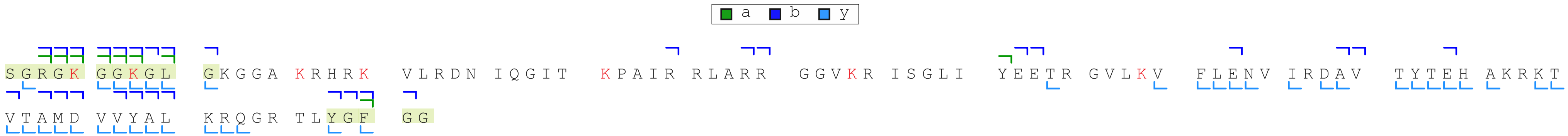

Proteoform:

Index	Protein	SC [%]	SVP [%]	IC [%]	MS Score [a.u.]	Var. Mods	Mono Mass [Da]	Chemical Formula
7	H4Kac	47.5	23.5	36.5	17.3	Ace: K5, K8, K12, K16, K20, K59, K77	11523.4152	C508H851N163O141S

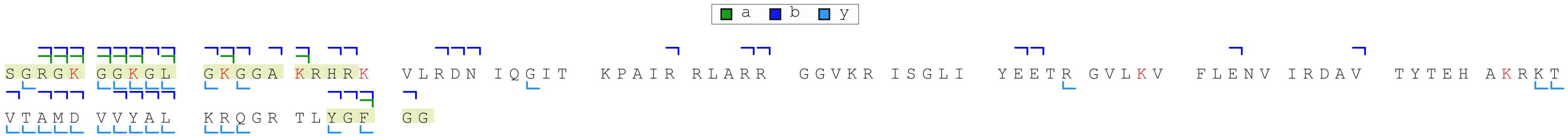

Proteoform:

Index	Protein	SC [%]	SVP [%]	IC [%]	MS Score [a.u.]	Var. Mods	Mono Mass [Da]	Chemical Formula
8	H4Kac	46.5	20.6	36.9	17.2	Ace: K5, K8, K12, K16, K20, K44, K77	11523.4152	C508H851N163O141S

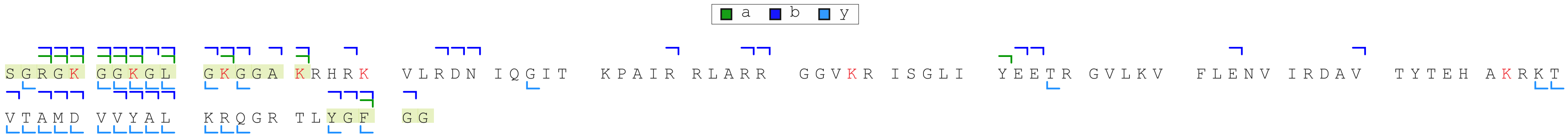

Proteoform:	Index	Protein	SC [%]	SVP [%]	IC [%]	MS Score [a.u.]	Var. Mods	Mono Mass [Da]	Chemical Formula
	9	H4Kac	45.5	20.6	37.4	17.0	Ace: K5, K8, K12, K16, K20, K31, K77	11523.4152	C508H851N163O141S

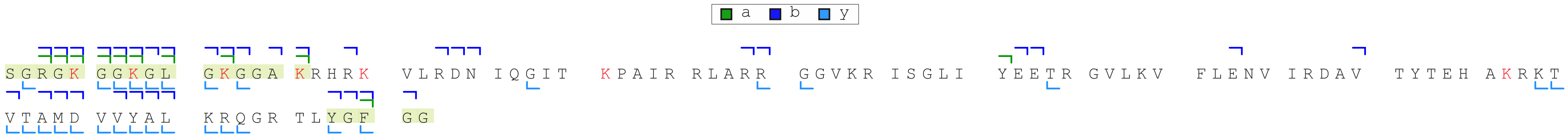

Proteoform:	Index	Protein	SC [%]	SVP [%]	IC [%]	MS Score [a.u.]	Var. Mods	Mono Mass [Da]	Chemical Formula
	10	H4Kac	45.5	20.6	36.9	16.8	Ace: K5, K8, K12, K16, K20, K44, K79	11523.4152	C508H851N163O141S

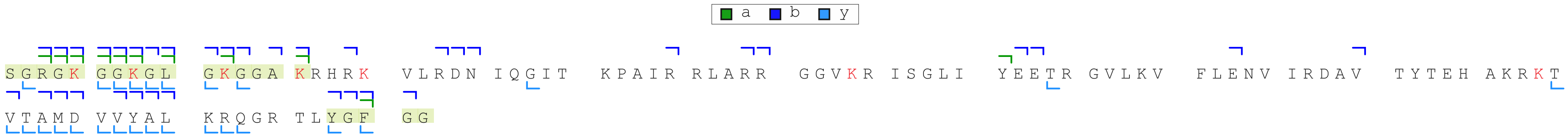

Error Plot

Proteoform:	Index	Protein	SC [%]	SVP [%]	IC [%]	MS Score [a.u.]	Var. Mods	Mono Mass [Da]	Chemical Formula
	1	H4Kac	61.4	23.5	43.4	26.6	Ace: K5, K8, K12, K16, K20, K31, K44	11523.4152	C508H851N163O141S

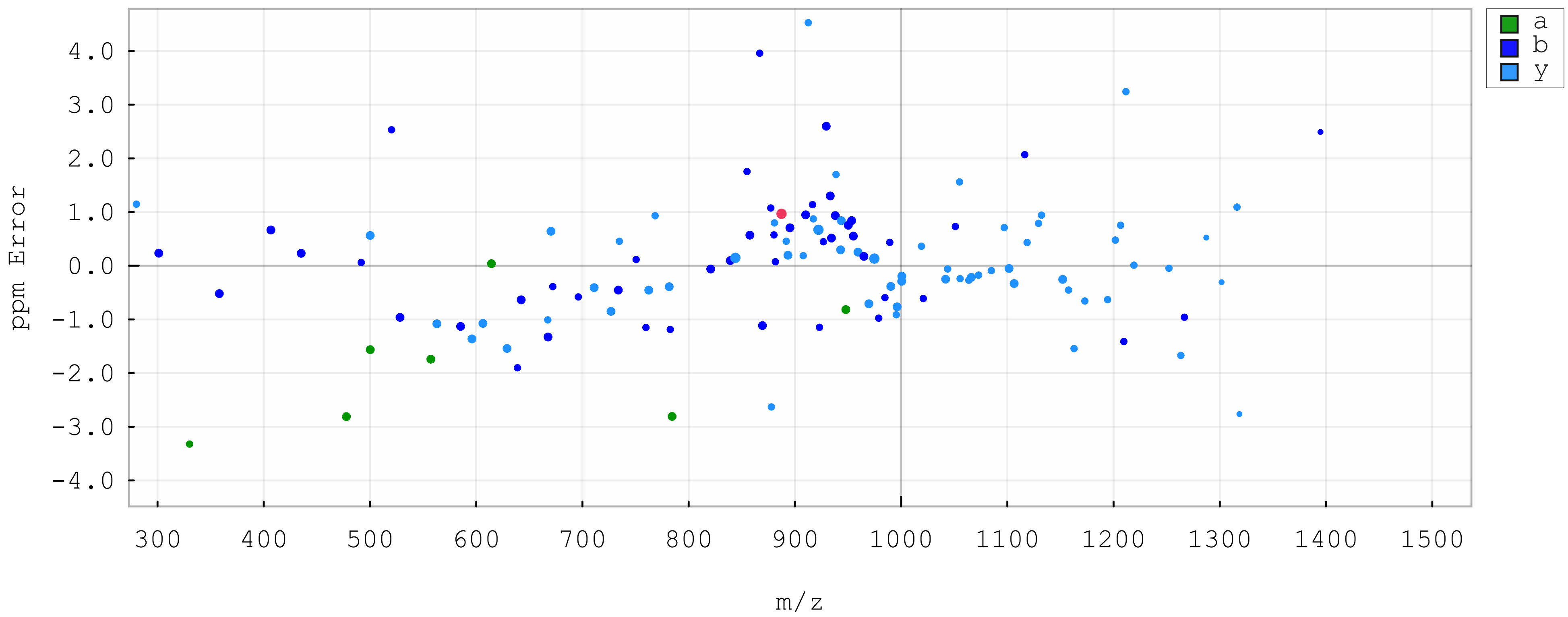

Proteoform:

Index	Protein	SC [%]	SVP [%]	IC [%]	MS Score [a.u.]	Var. Mods	Mono Mass [Da]	Chemical Formula
2	H4Kac	59.4	20.6	39.3	23.4	Ace: K5, K8, K12, K16, K20, K44, K59	11523.4152	C508H851N163O141S

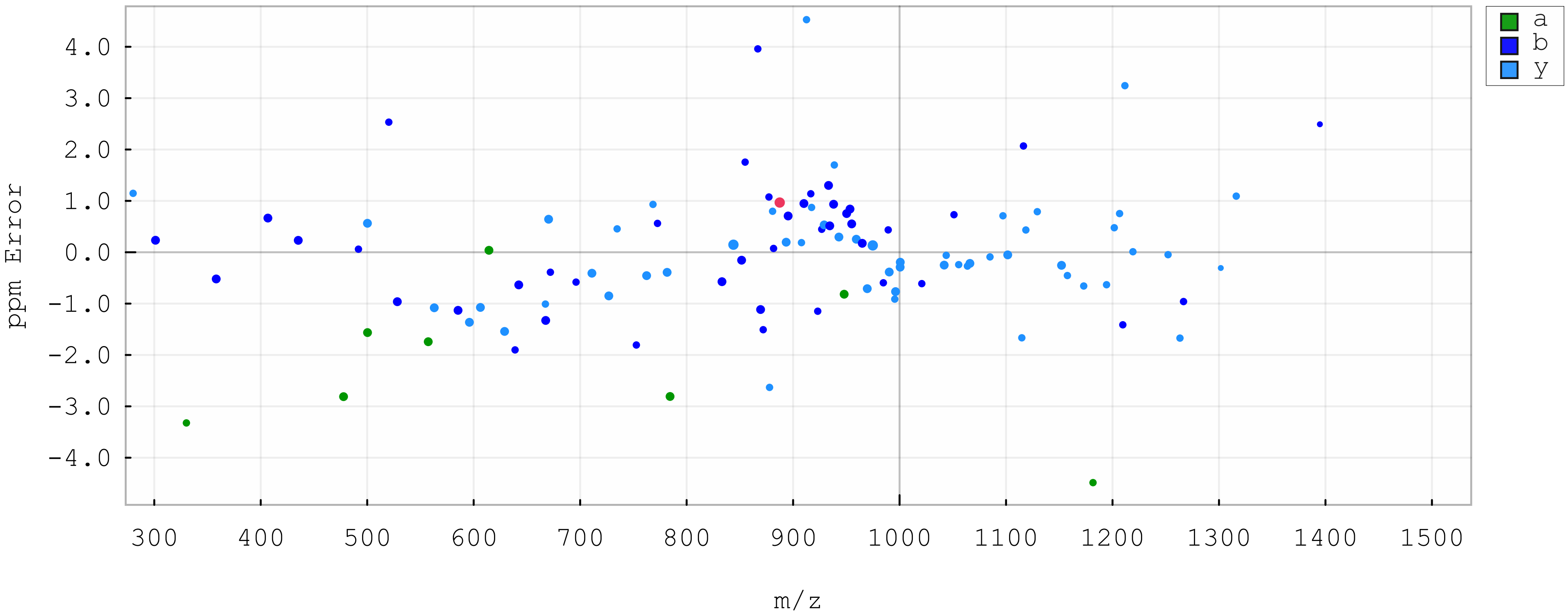

Proteoform:

Index	Protein	SC [%]	SVP [%]	IC [%]	MS Score [a.u.]	Var. Mods	Mono Mass [Da]	Chemical Formula
3	H4Kac	58.4	20.6	39.8	23.3	Ace: K5, K8, K12, K16, K20, K31, K59	11523.4152	C508H851N163O141S

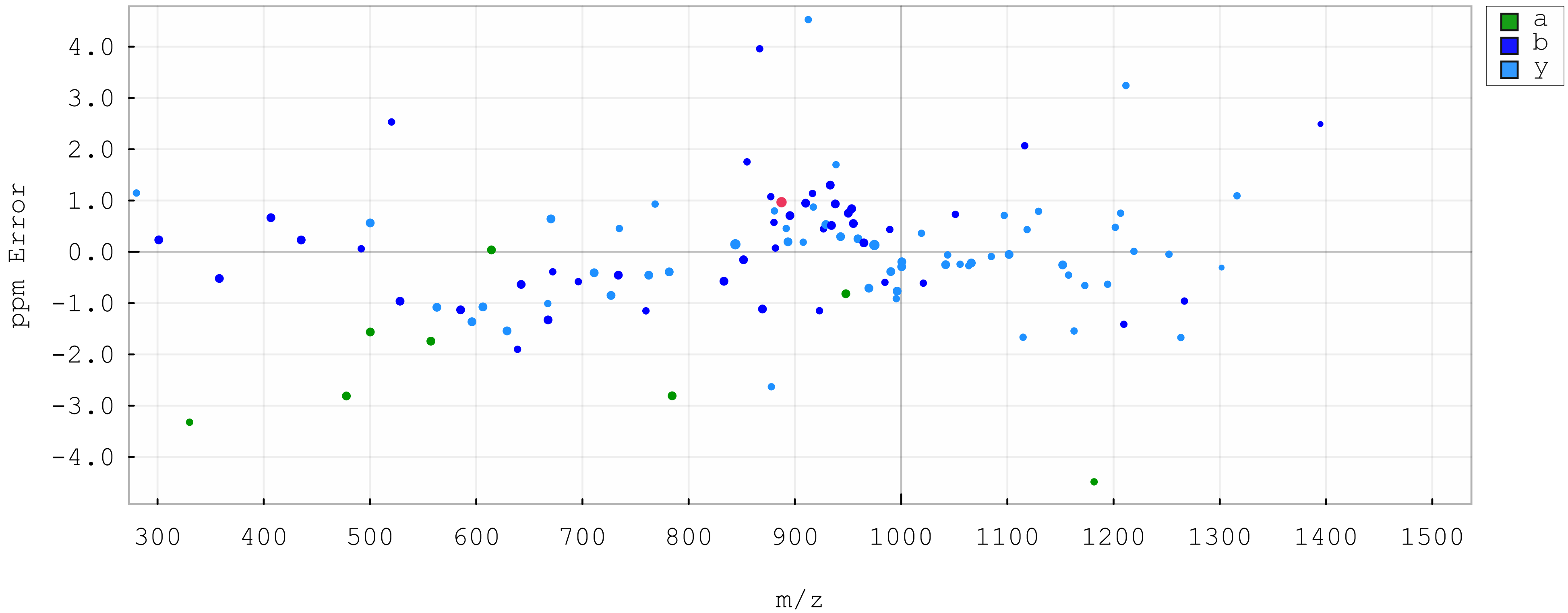

Proteoform:

Index	Protein	SC [%]	SVP [%]	IC [%]	MS Score [a.u.]	Var. Mods	Mono Mass [Da]	Chemical Formula
4	H4Kac	55.4	20.6	38.8	21.5	Ace: K5, K8, K12, K16, K31, K44, K59	11523.4152	C508H851N163O141S

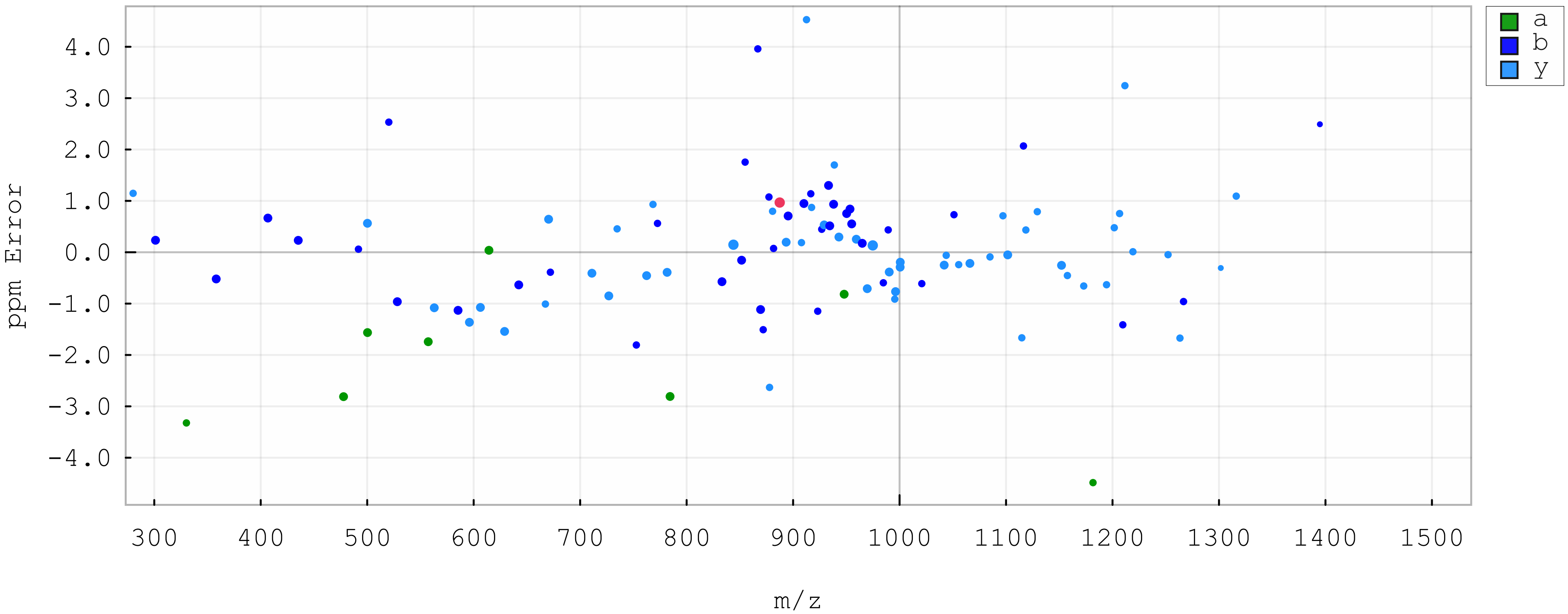

Proteoform:

Index	Protein	SC [%]	SVP [%]	IC [%]	MS Score [a.u.]	Var. Mods	Mono Mass [Da]	Chemical Formula
5	H4Kac	53.5	19.6	38.8	20.7	Ace: K5, K8, K12, K20, K31, K44, K59	11523.4152	C508H851N163O141S

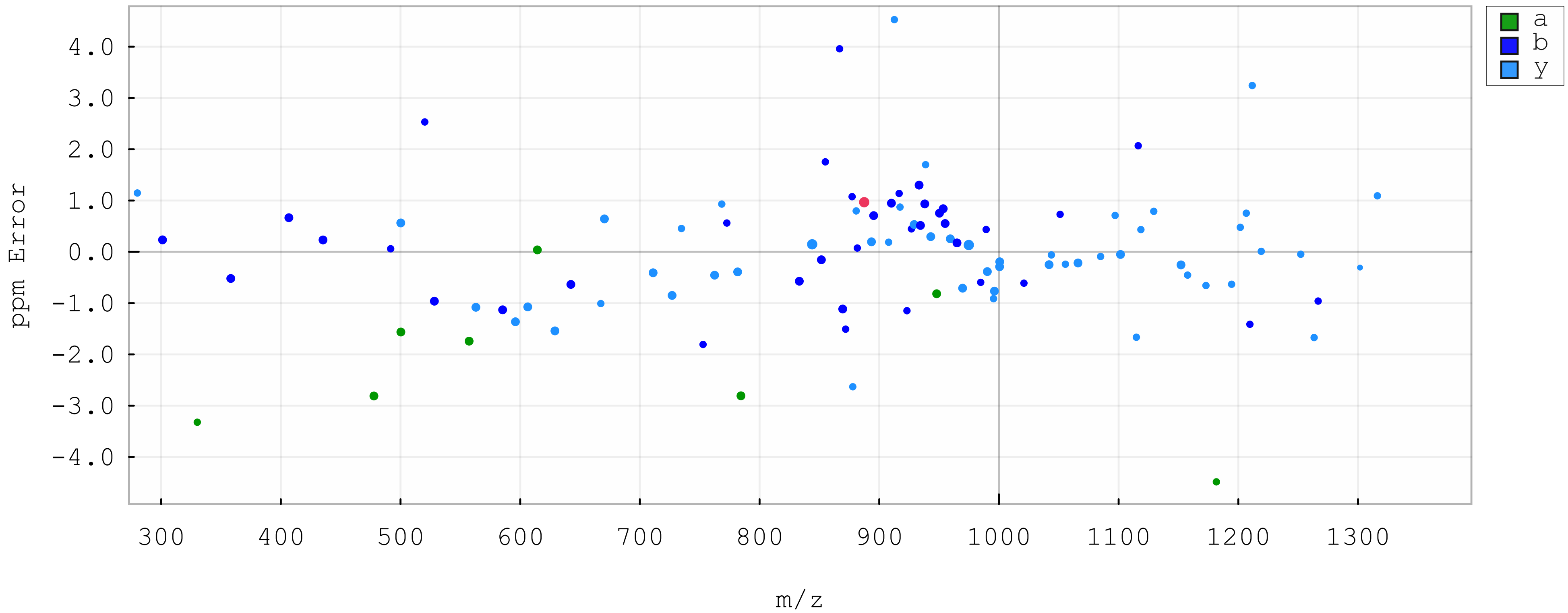

Proteoform:

Index	Protein	SC [%]	SVP [%]	IC [%]	MS Score [a.u.]	Var. Mods	Mono Mass [Da]	Chemical Formula
6	H4Kac	50.5	15.7	38.6	19.5	Ace: K5, K8, K16, K20, K31, K44, K59	11523.4152	C508H851N163O141S

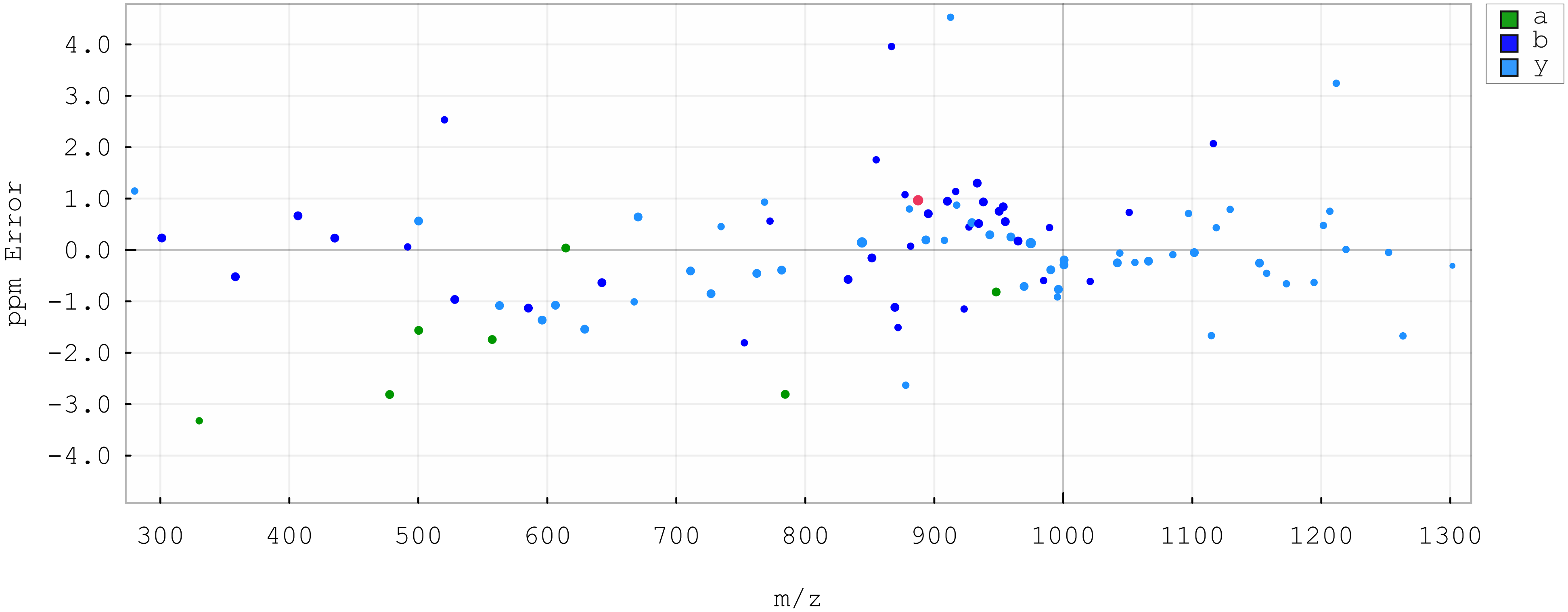

Proteoform:

Index	Protein	SC [%]	SVP [%]	IC [%]	MS Score [a.u.]	Var. Mods	Mono Mass [Da]	Chemical Formula
7	H4Kac	47.5	23.5	36.5	17.3	Ace: K5, K8, K12, K16, K20, K59, K77	11523.4152	C508H851N163O141S

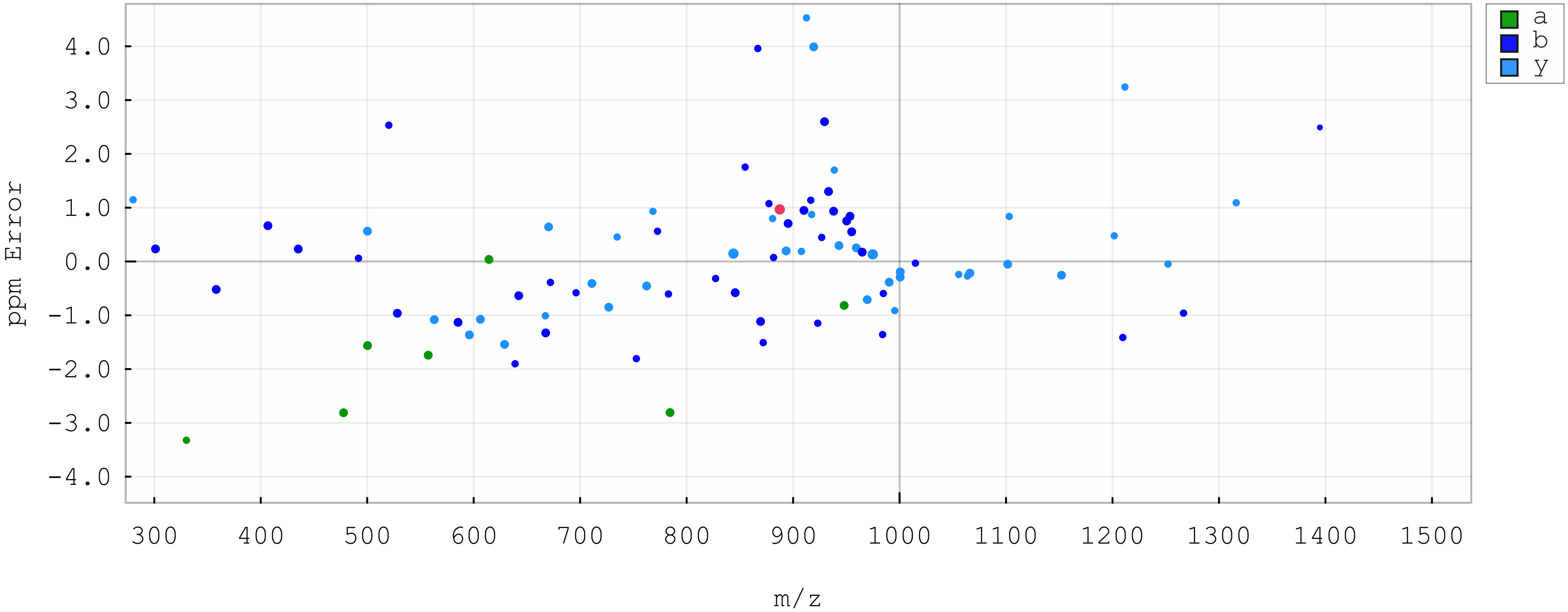

Proteoform:

Index	Protein	SC [%]	SVP [%]	IC [%]	MS Score [a.u.]	Var. Mods	Mono Mass [Da]	Chemical Formula
8	H4Kac	46.5	20.6	36.9	17.2	Ace: K5, K8, K12, K16, K20, K44, K77	11523.4152	C508H851N163O141S

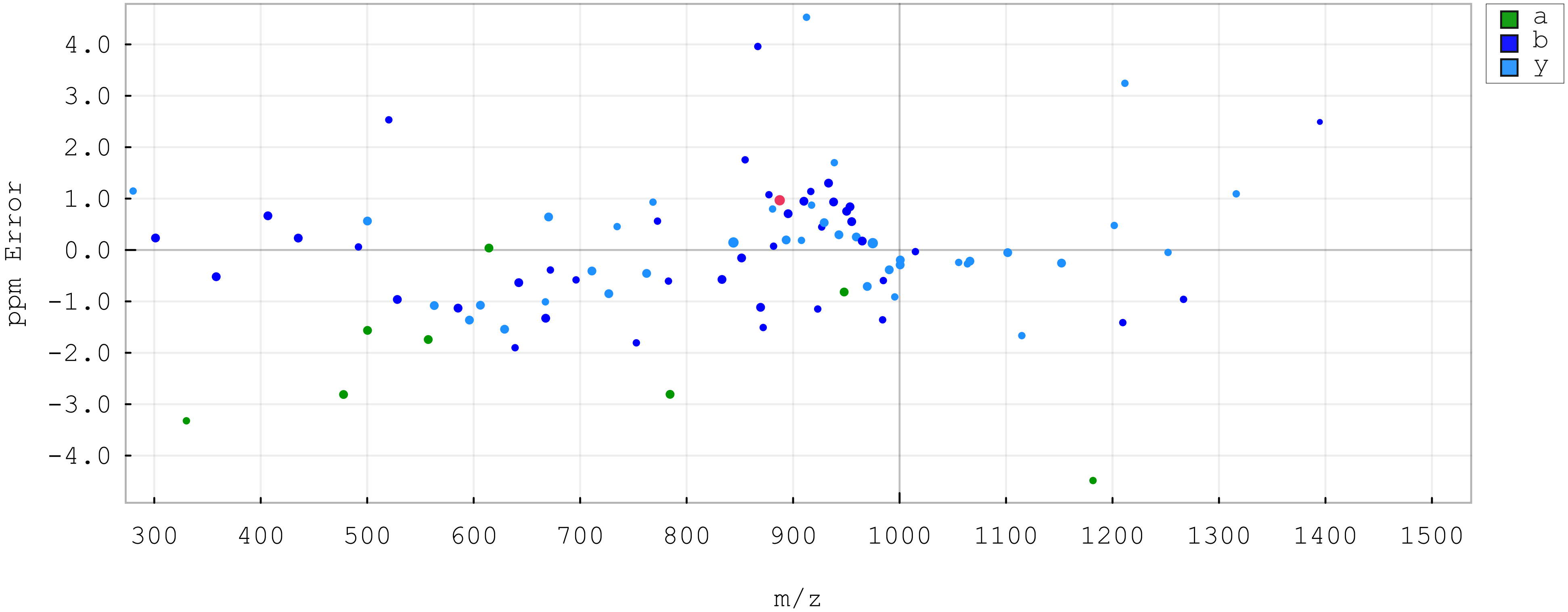

Proteoform:

Index	Protein	SC [%]	SVP [%]	IC [%]	MS Score [a.u.]	Var. Mods	Mono Mass [Da]	Chemical Formula
9	H4Kac	45.5	20.6	37.4	17.0	Ace: K5, K8, K12, K16, K20, K31, K77	11523.4152	C508H851N163O141S

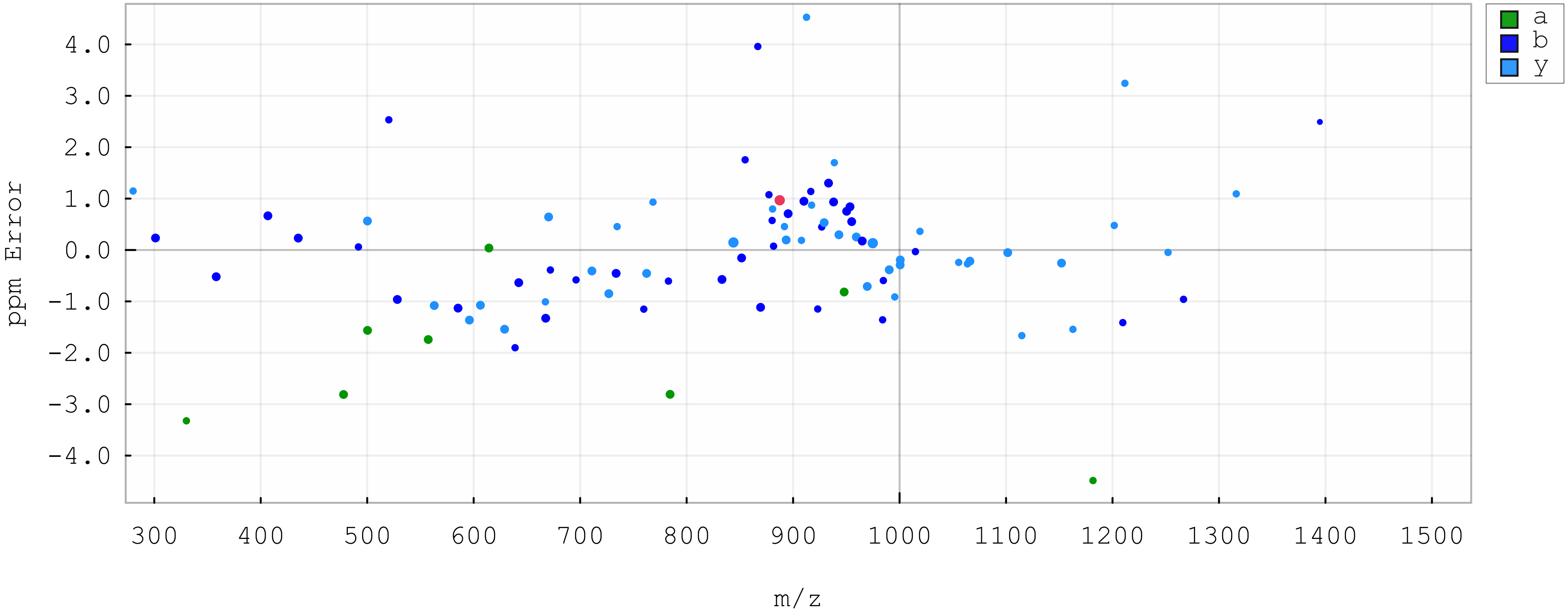

Proteoform:

Index	Protein	SC [%]	SVP [%]	IC [%]	MS Score [a.u.]	Var. Mods	Mono Mass [Da]	Chemical Formula
10	H4Kac	45.5	20.6	36.9	16.8	Ace: K5, K8, K12, K16, K20, K44, K79	11523.4152	C508H851N163O141S

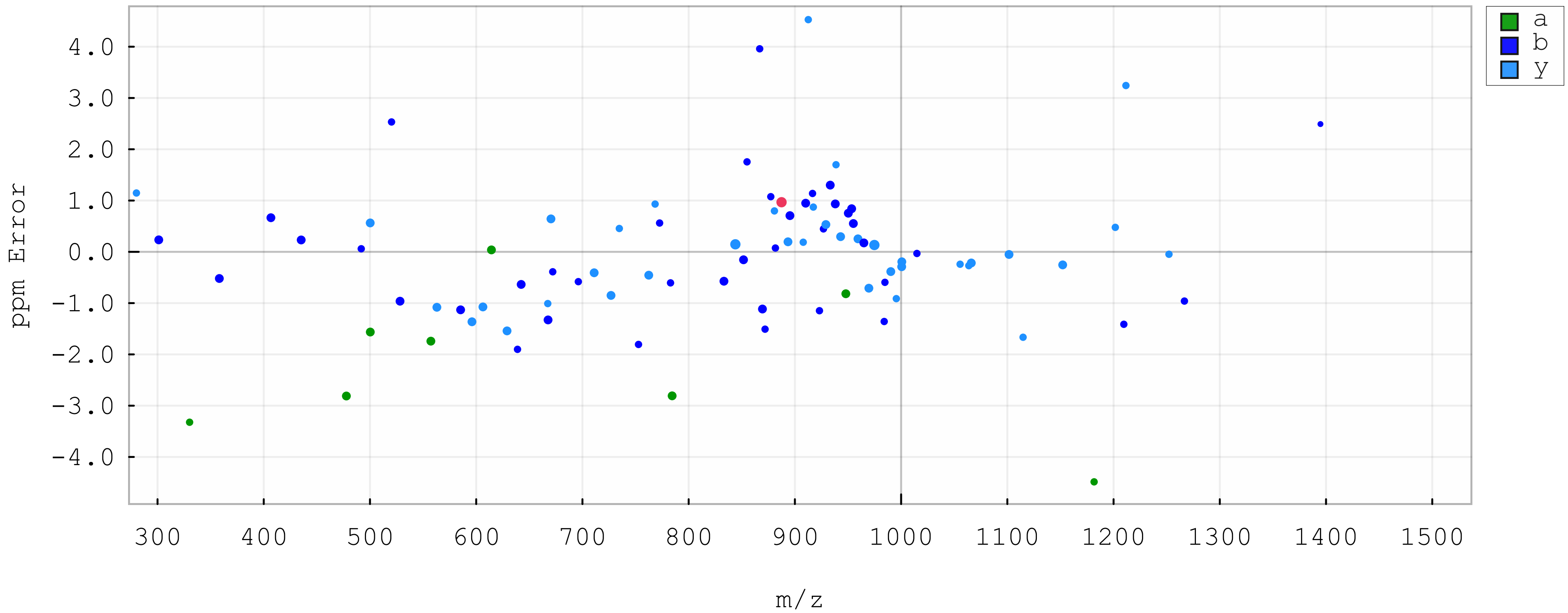

Primary Fragment Plots

Proteoform:

Index	Protein	SC [%]	SVP [%]	IC [%]	MS Score [a.u.]	Var. Mods	Mono Mass [Da]	Chemical Formula
1	H4Kac	61.4	23.5	43.4	26.6	Ace: K5, K8, K12, K16, K20, K31, K44	11523.4152	C508H851N163O141S

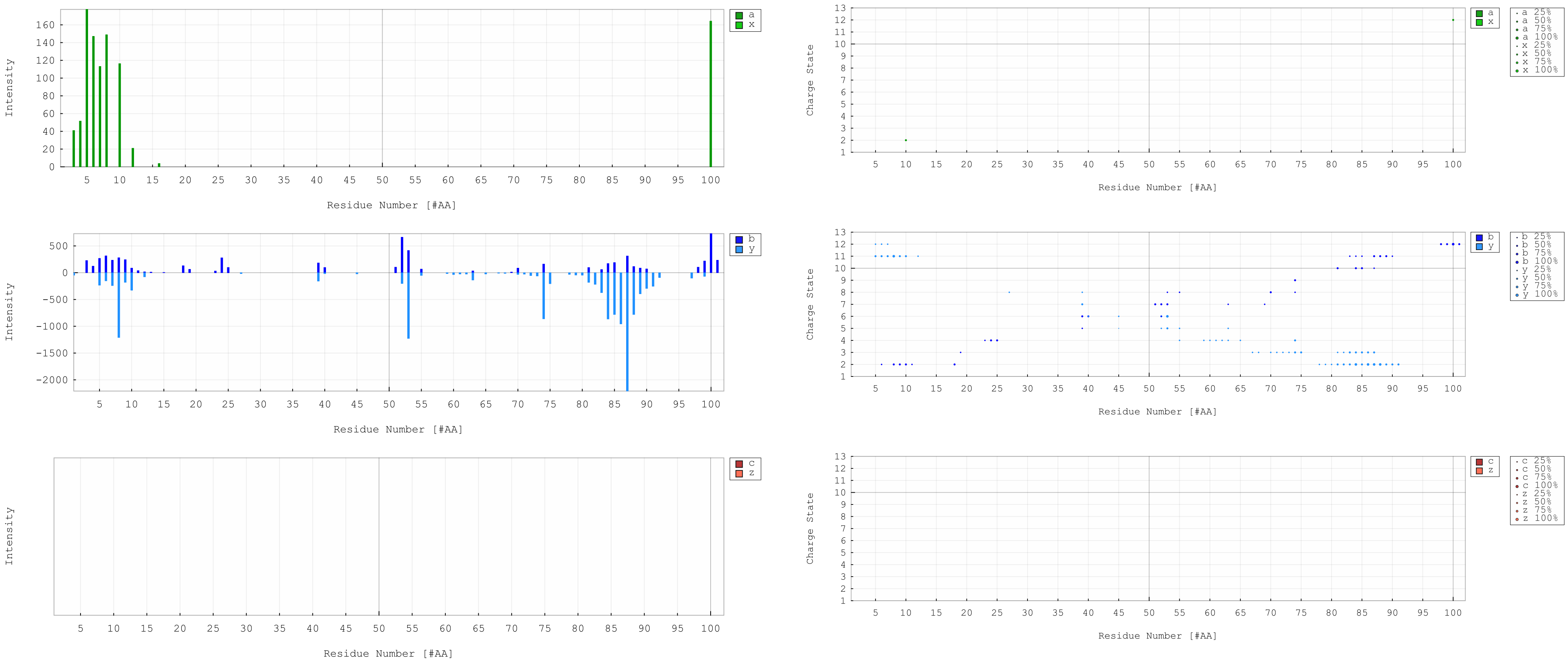

Proteoform:

Index	Protein	SC [%]	SVP [%]	IC [%]	MS Score [a.u.]	Var. Mods	Mono Mass [Da]	Chemical Formula
2	H4Kac	59.4	20.6	39.3	23.4	Ace: K5, K8, K12, K16, K20, K44, K59	11523.4152	$C_{508}H_{851}N_{163}O_{141}S$

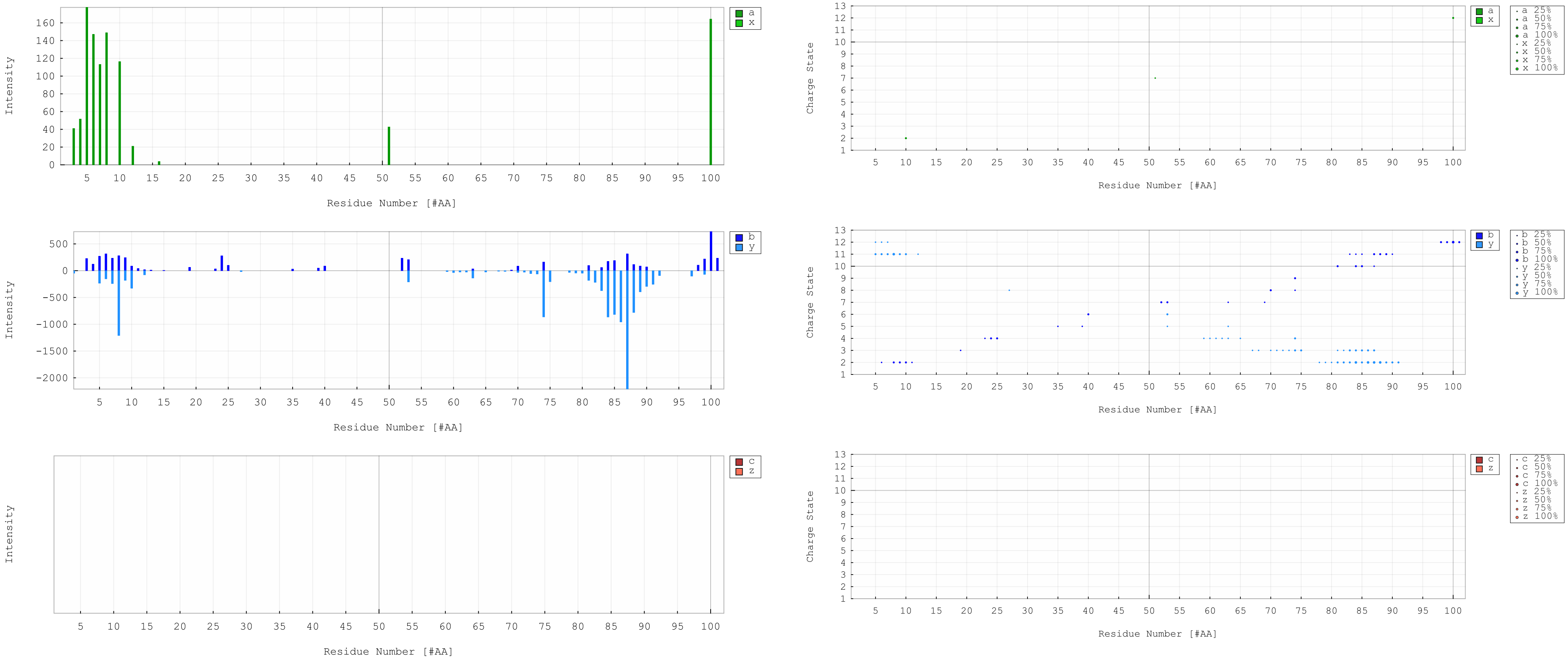

Top-Down Result OmniScape 2025
Sequence Confirmation
250428 H4_P300-50_60min z13ac7 MS2 shCID_001-733_calibrated

Proteoform:

Index	Protein	SC [%]	SVP [%]	IC [%]	MS Score [a.u.]	Var. Mods	Mono Mass [Da]	Chemical Formula
3	H4Kac	58.4	20.6	39.8	23.3	Ace: K5, K8, K12, K16, K20, K31, K59	11523.4152	C508H851N163O141S

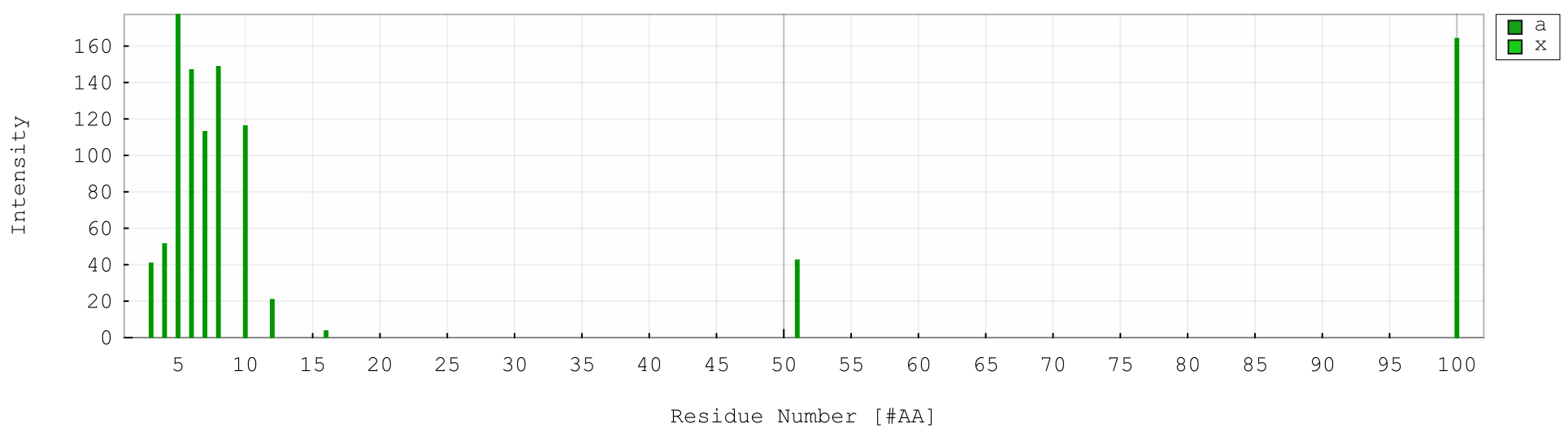

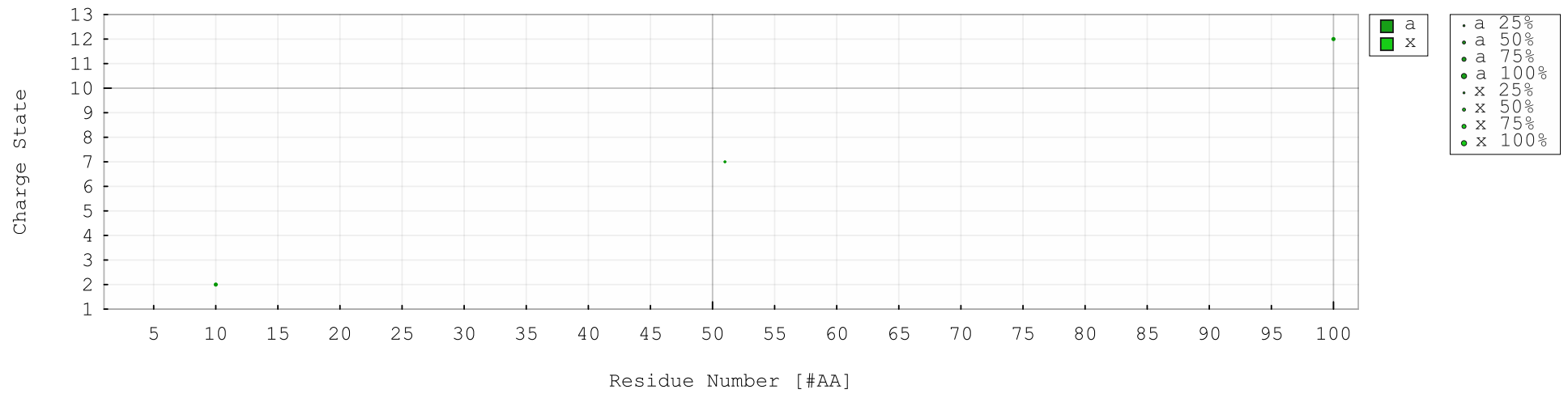

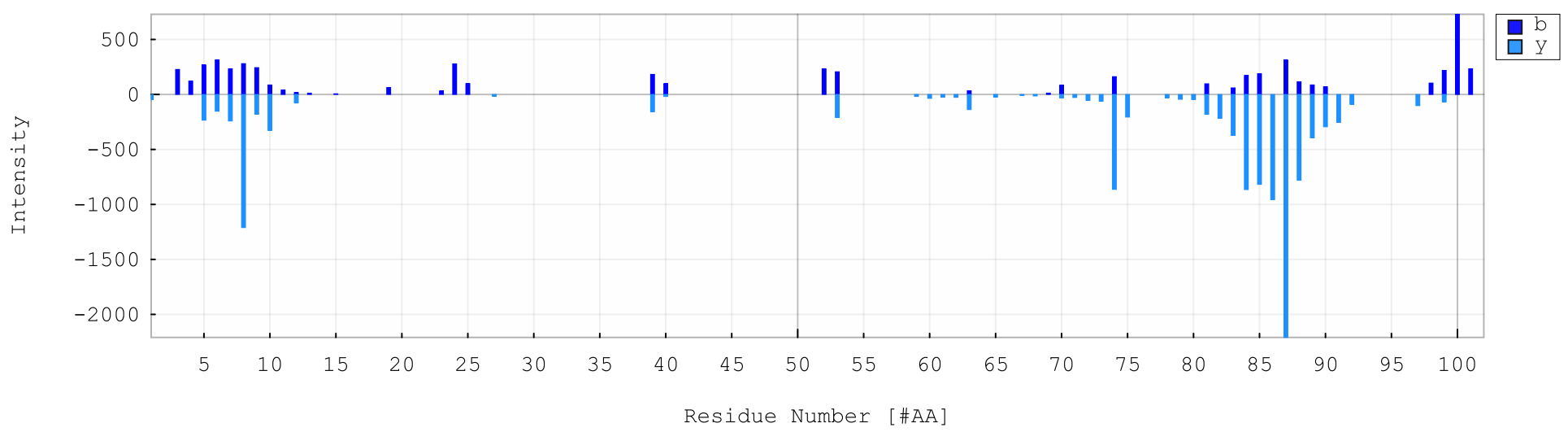

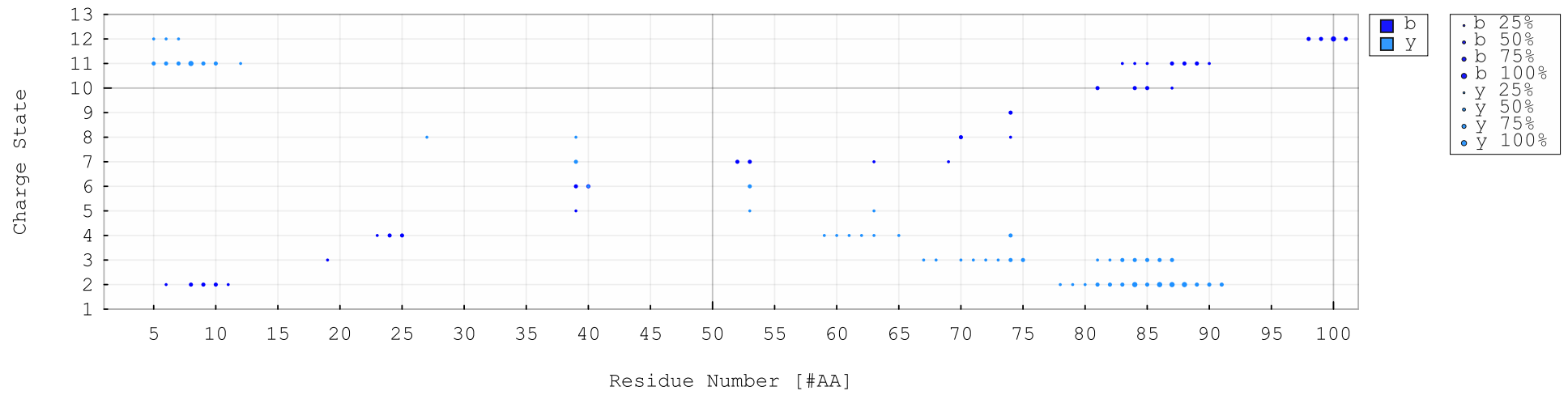

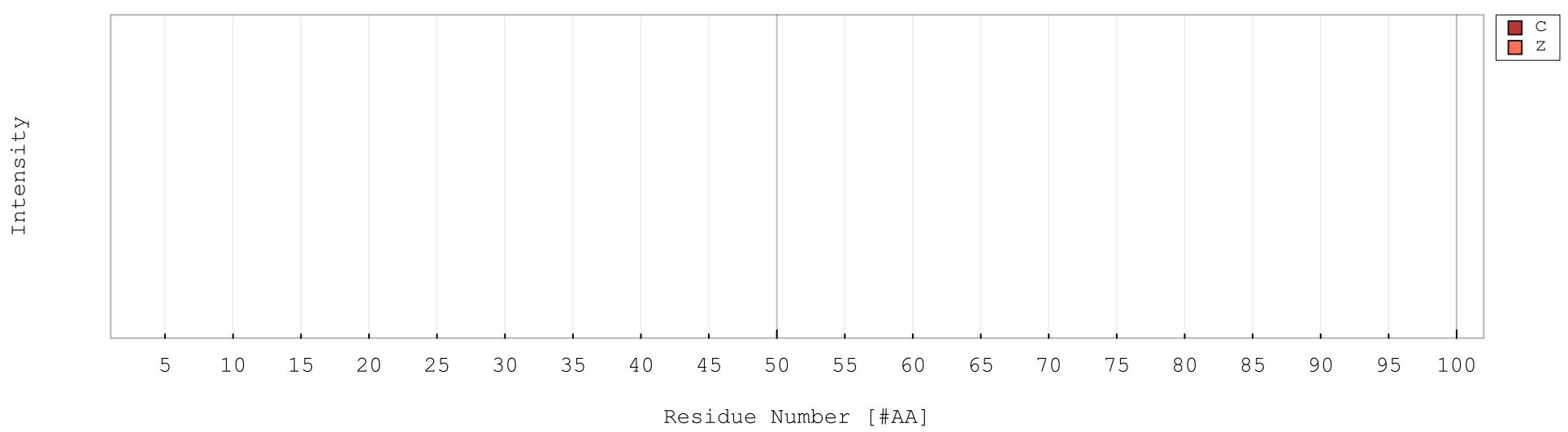

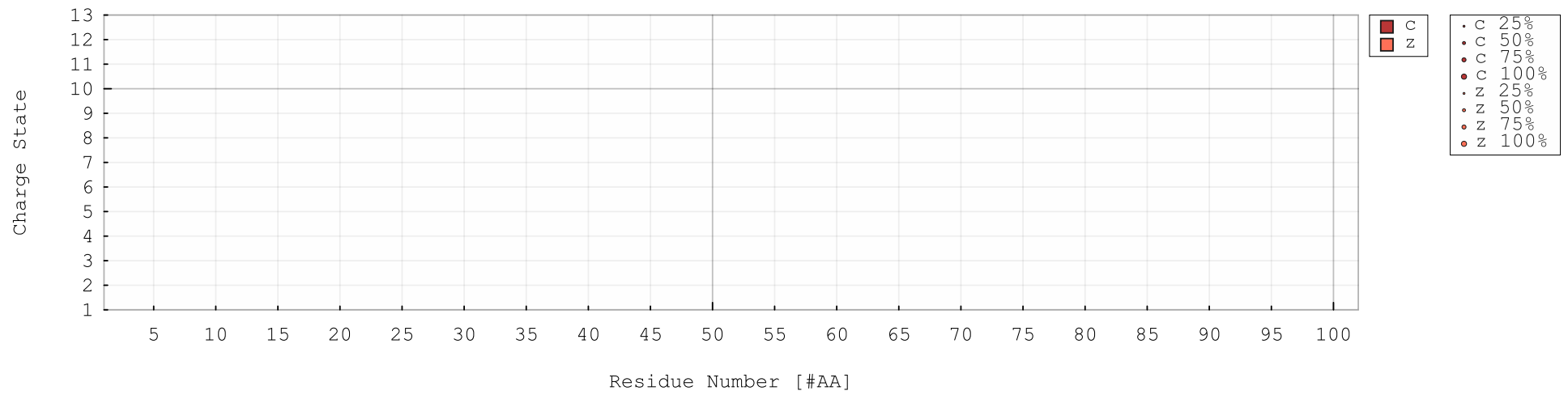

Proteoform:

Index	Protein	SC [%]	SVP [%]	IC [%]	MS Score [a.u.]	Var. Mods	Mono Mass [Da]	Chemical Formula
4	H4Kac	55.4	20.6	38.8	21.5	Ace: K5, K8, K12, K16, K31, K44, K59	11523.4152	C508H851N163O141S

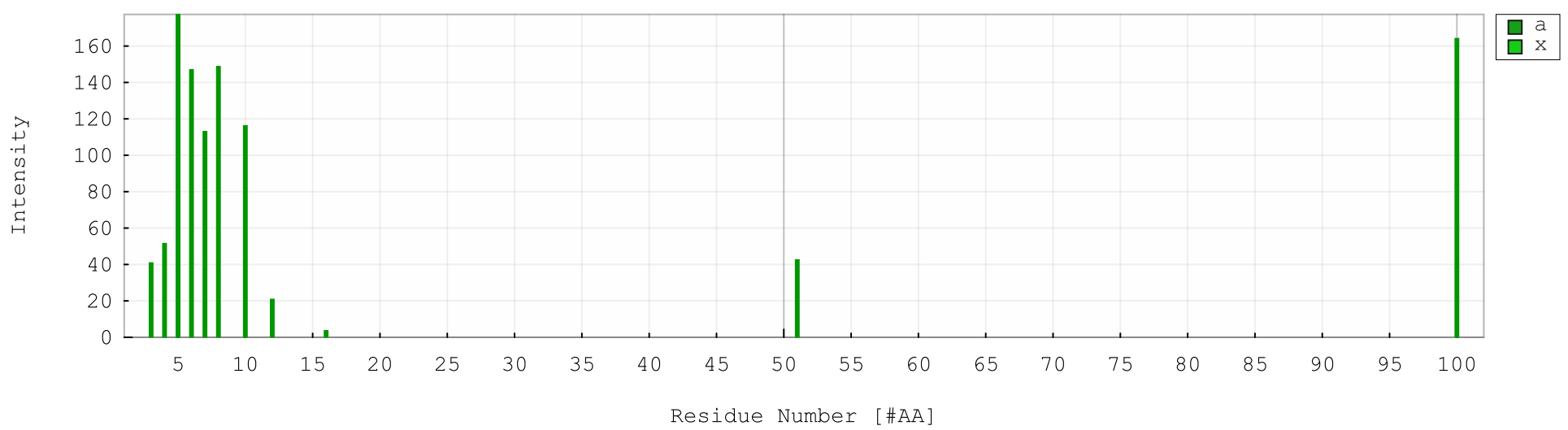

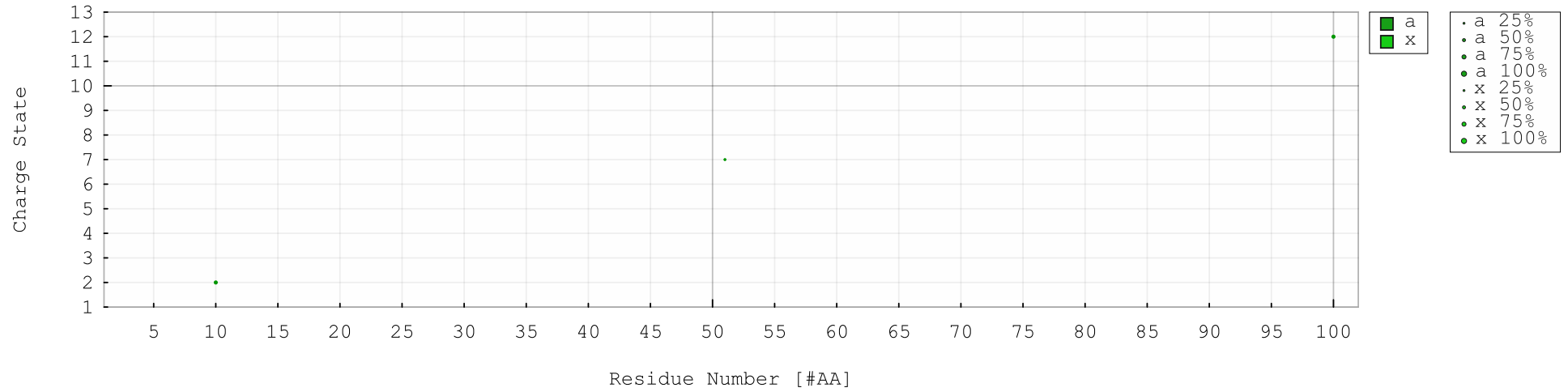

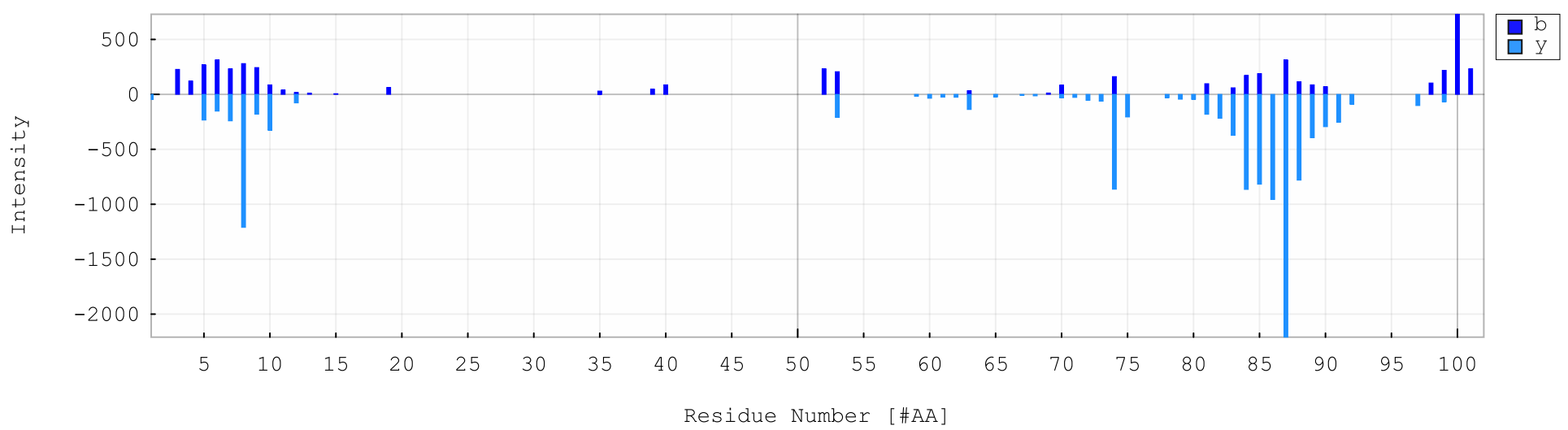

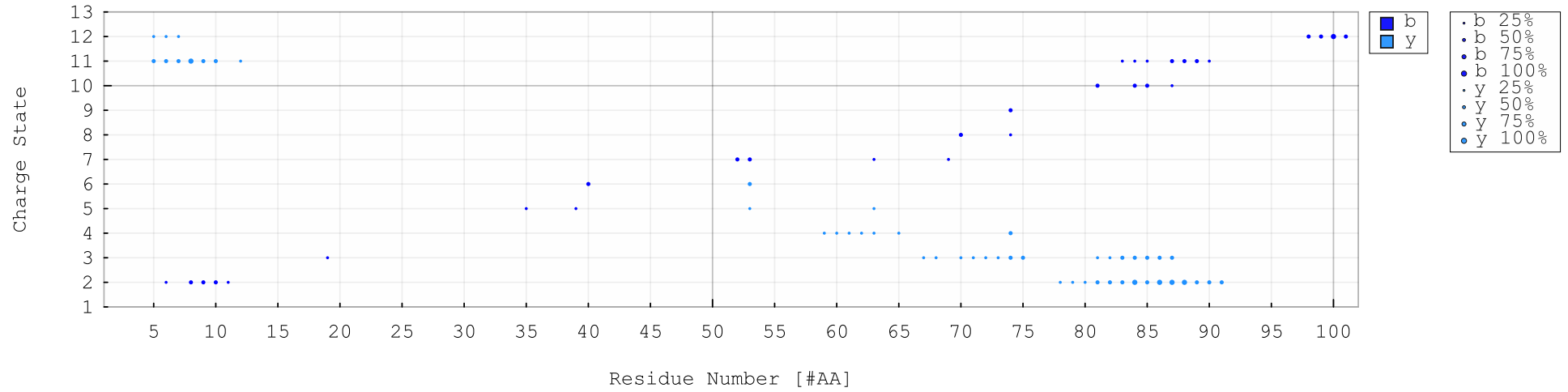

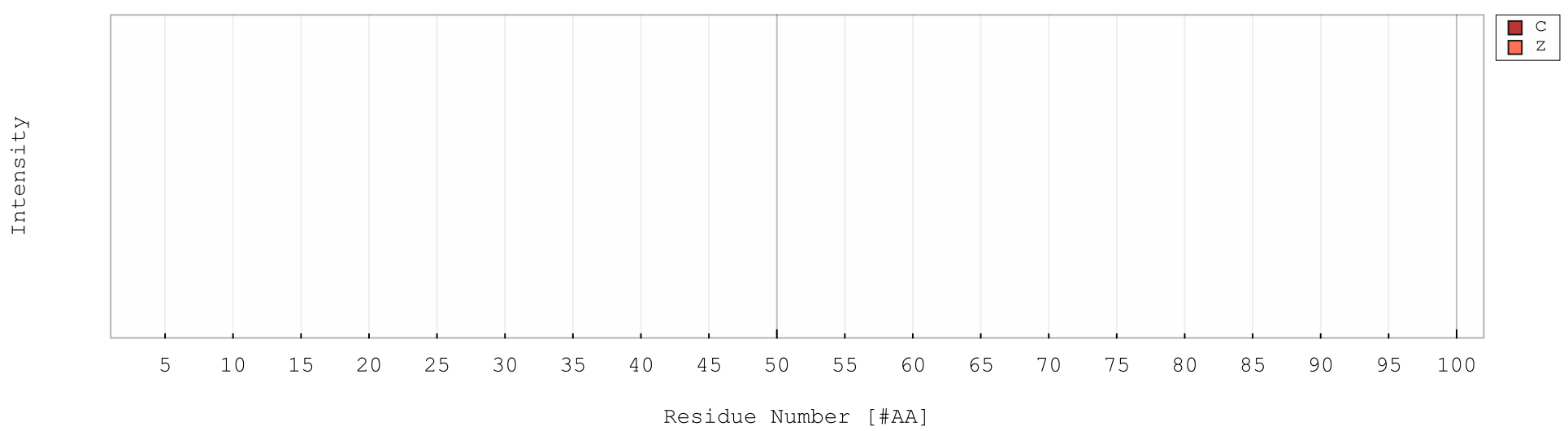

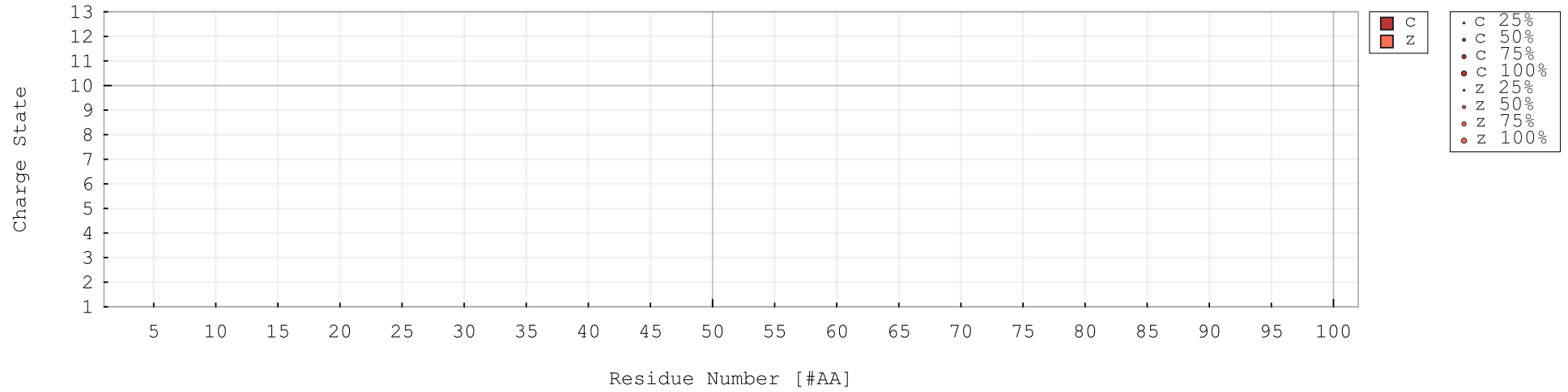

Proteoform:

Index	Protein	SC [%]	SVP [%]	IC [%]	MS Score [a.u.]	Var. Mods	Mono Mass [Da]	Chemical Formula
5	H4Kac	53.5	19.6	38.8	20.7	Ace: K5, K8, K12, K20, K31, K44, K59	11523.4152	C508H851N163O141S

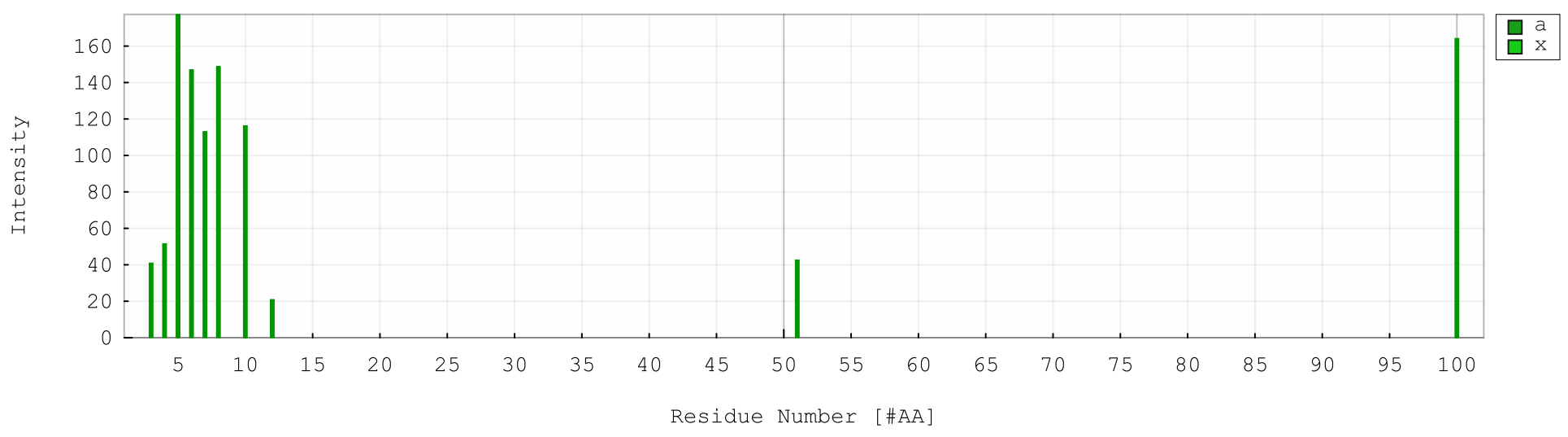

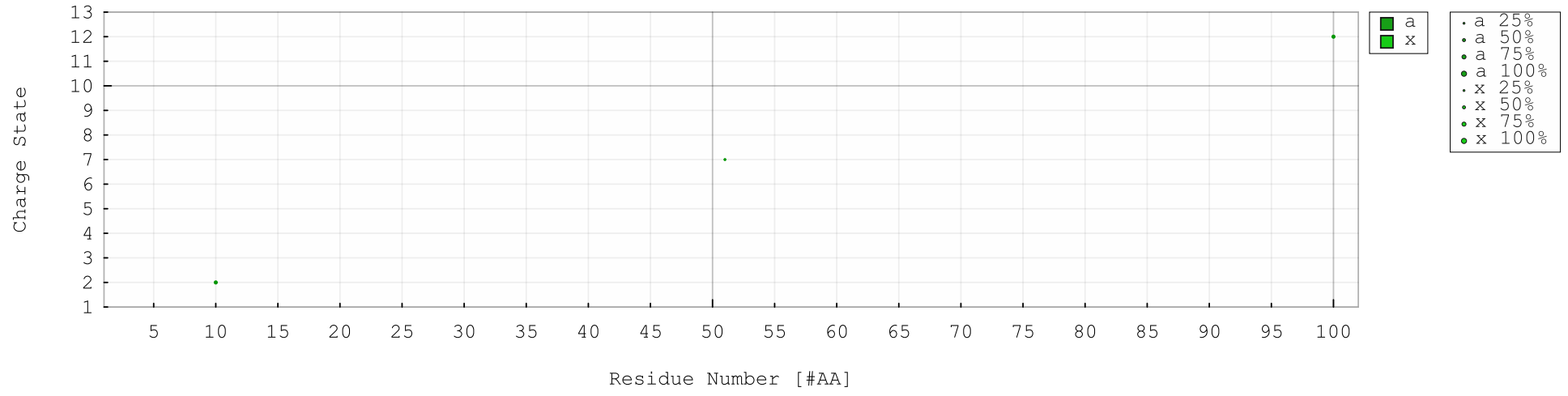

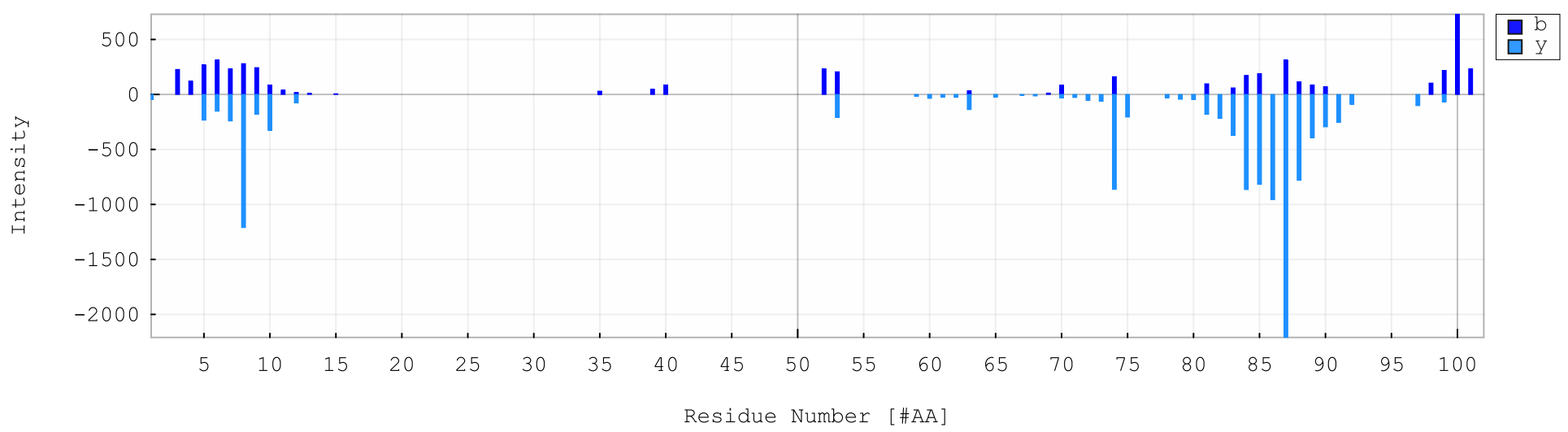

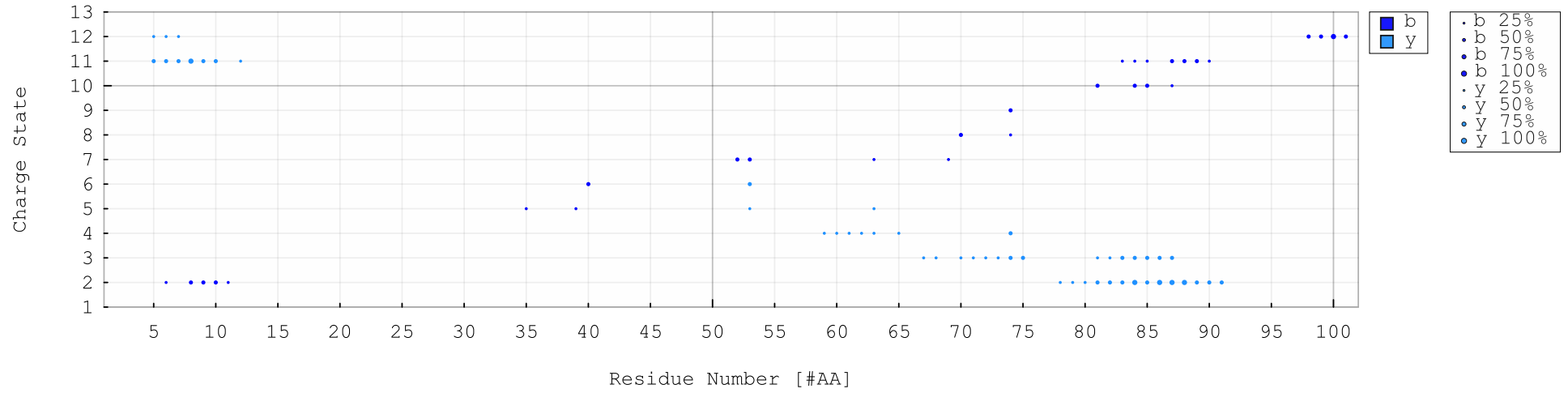

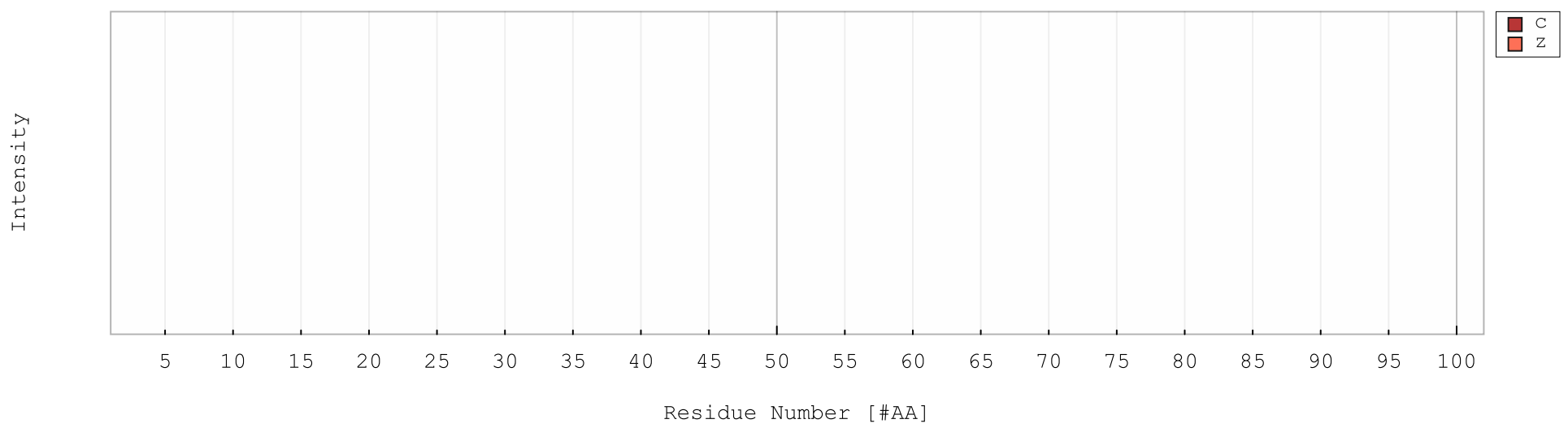

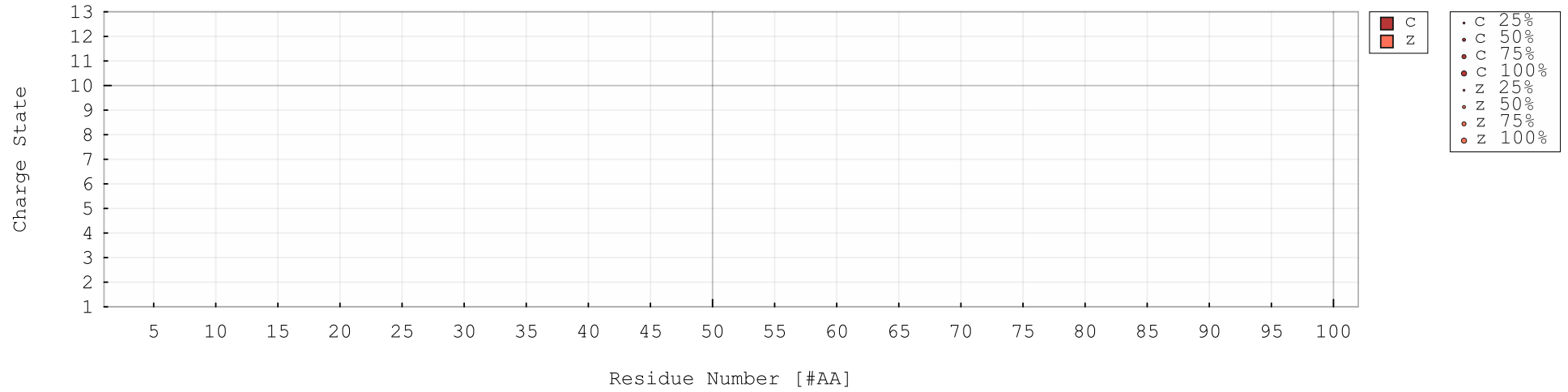

Top-Down Result OmniScape 2025
Sequence Confirmation
250428 H4_P300-50_60min z13ac7 MS2 shCID_001-733_calibrated

Proteoform:

Index	Protein	SC [%]	SVP [%]	IC [%]	MS Score [a.u.]	Var. Mods	Mono Mass [Da]	Chemical Formula
6	H4Kac	50.5	15.7	38.6	19.5	Ace: K5, K8, K16, K20, K31, K44, K59	11523.4152	C508H851N163O141S

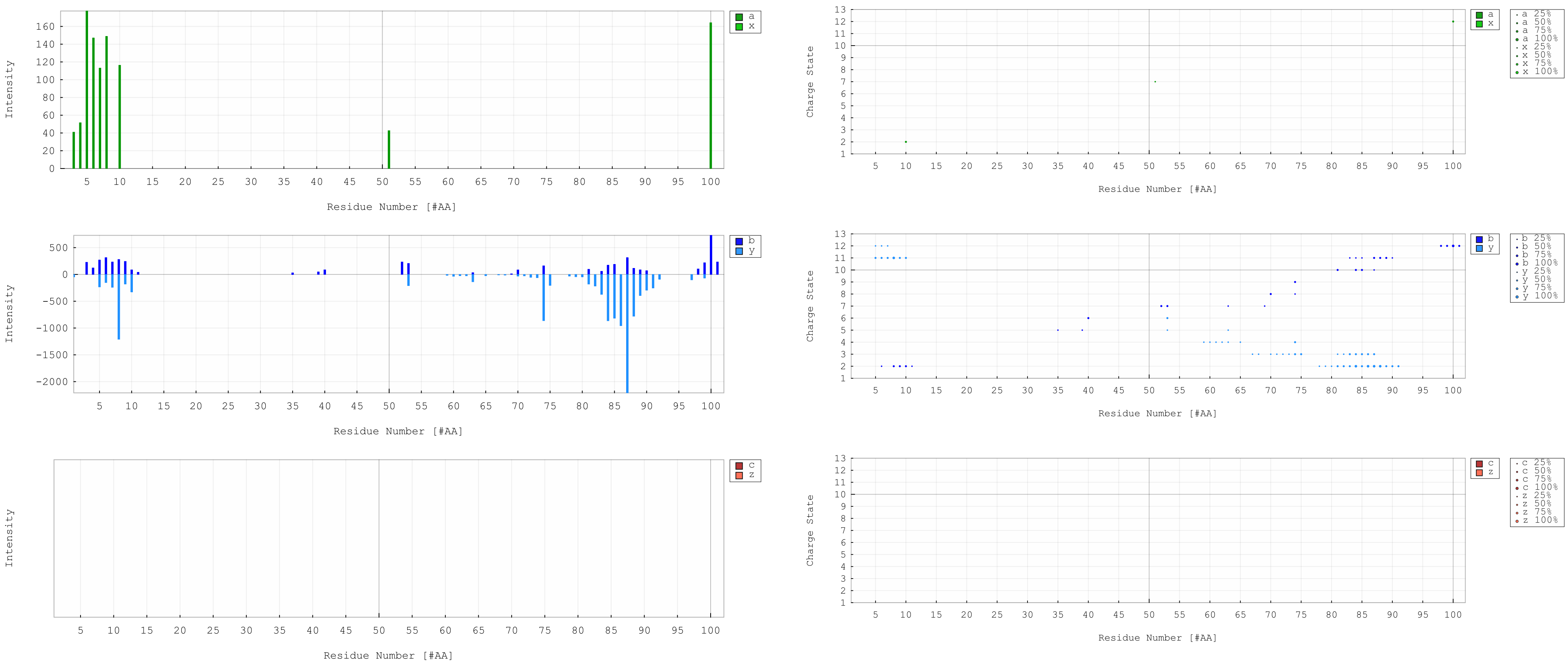

Proteoform:

Index	Protein	SC [%]	SVP [%]	IC [%]	MS Score [a.u.]	Var. Mods	Mono Mass [Da]	Chemical Formula
7	H4Kac	47.5	23.5	36.5	17.3	Ace: K5, K8, K12, K16, K20, K59, K77	11523.4152	C508H851N163O141S

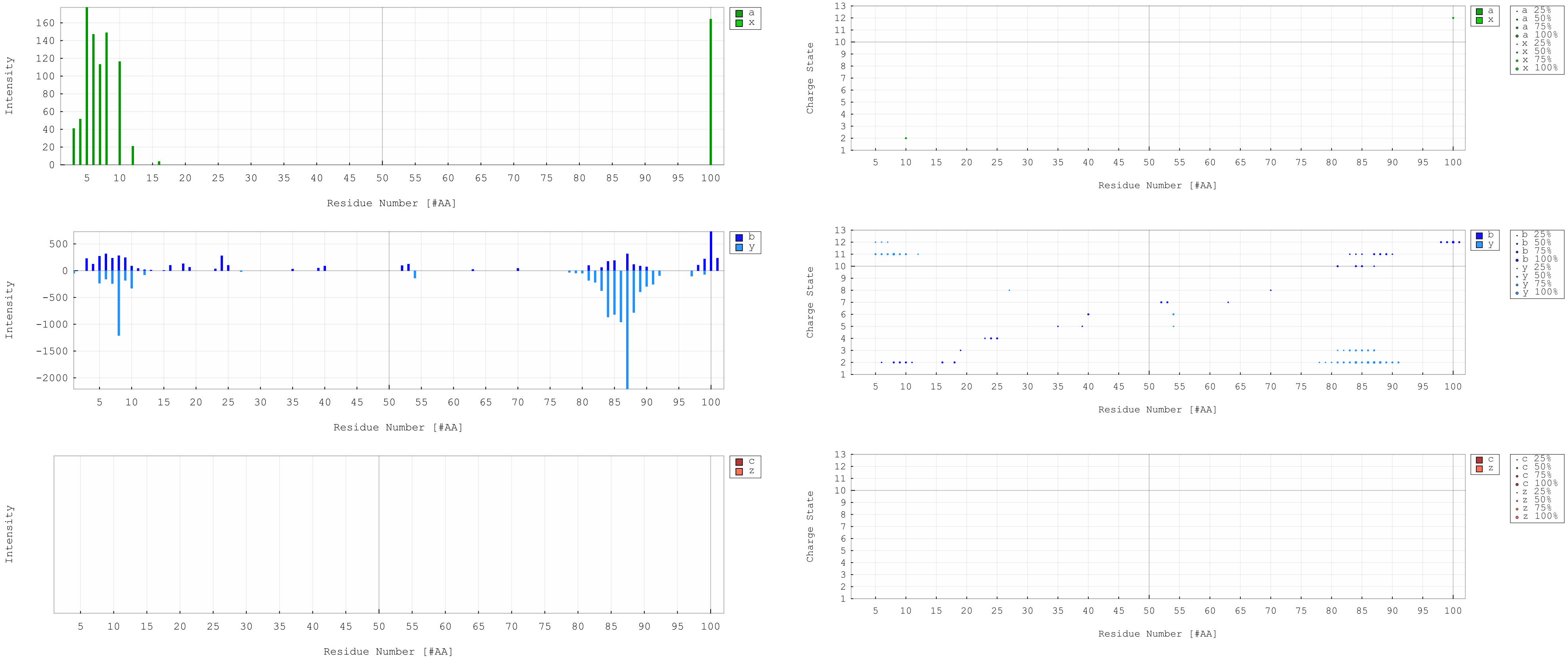

Proteoform:

Index	Protein	SC [%]	SVP [%]	IC [%]	MS Score [a.u.]	Var. Mods	Mono Mass [Da]	Chemical Formula
8	H4Kac	46.5	20.6	36.9	17.2	Ace: K5, K8, K12, K16, K20, K44, K77	11523.4152	C508H851N163O141S

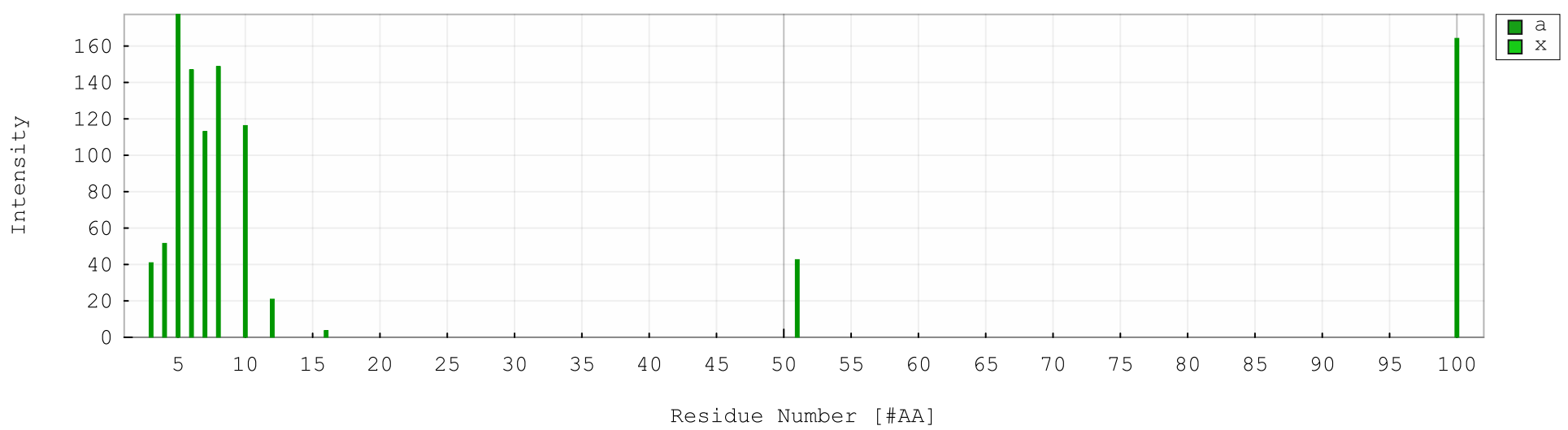

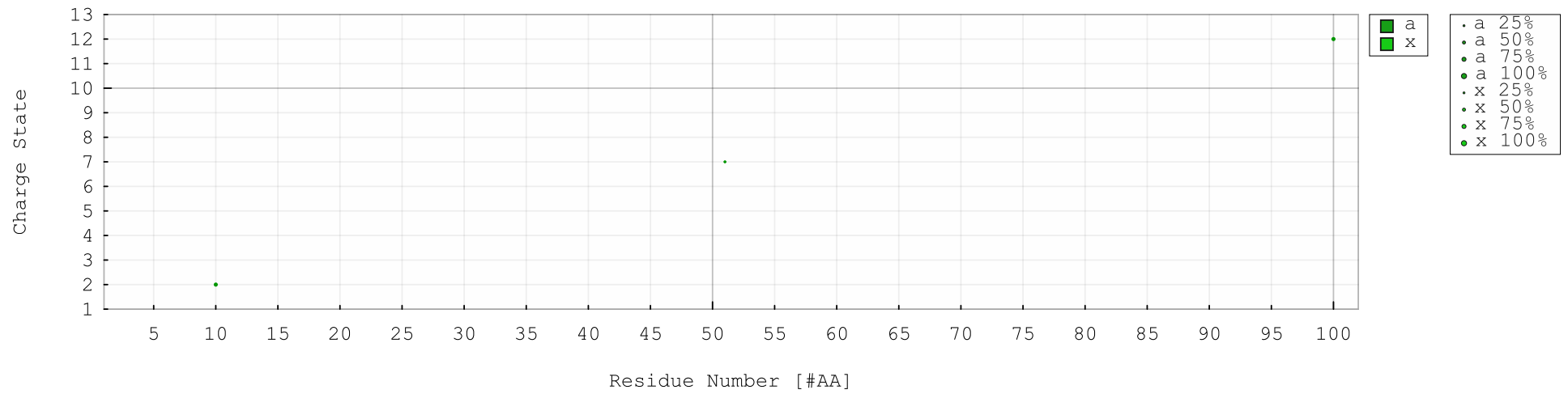

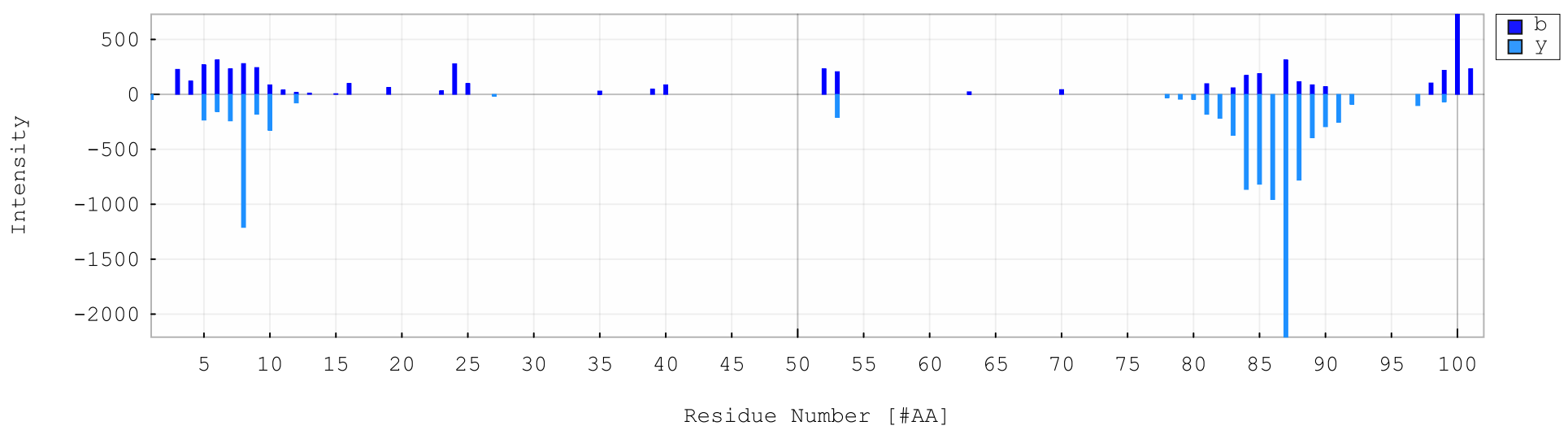

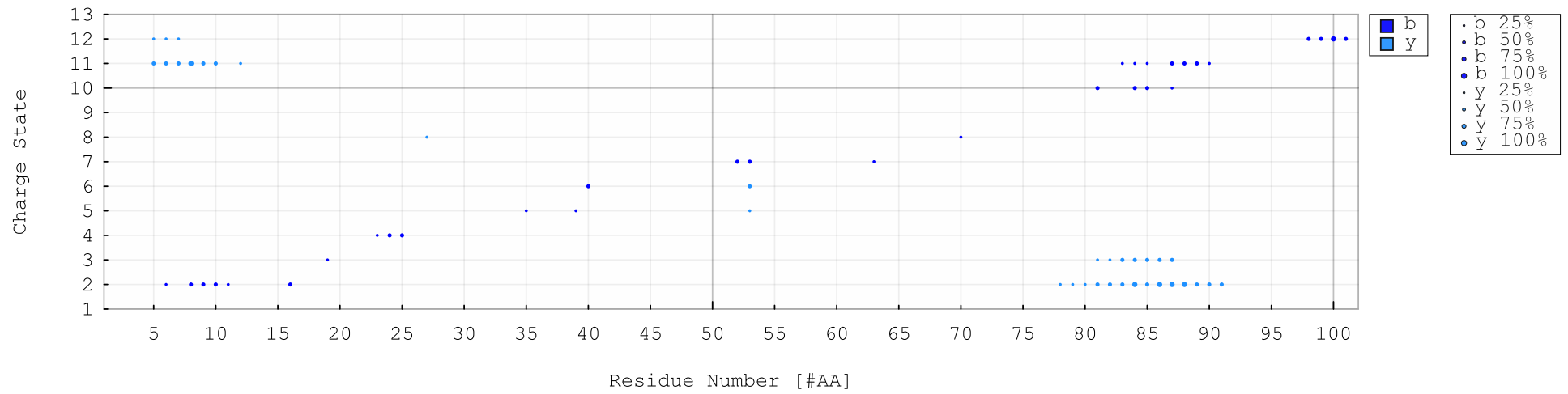

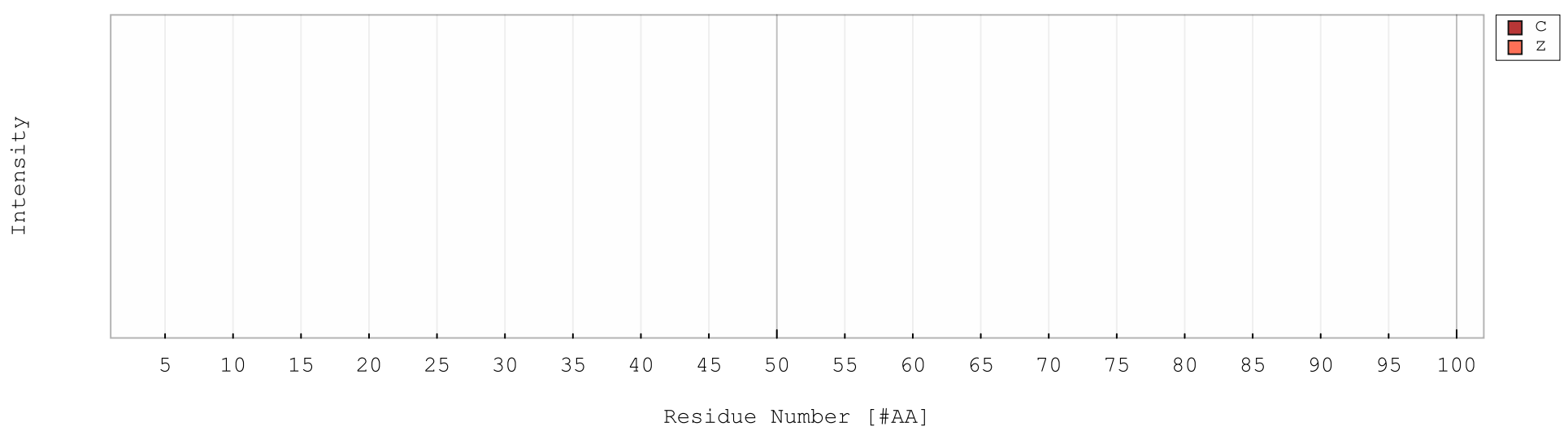

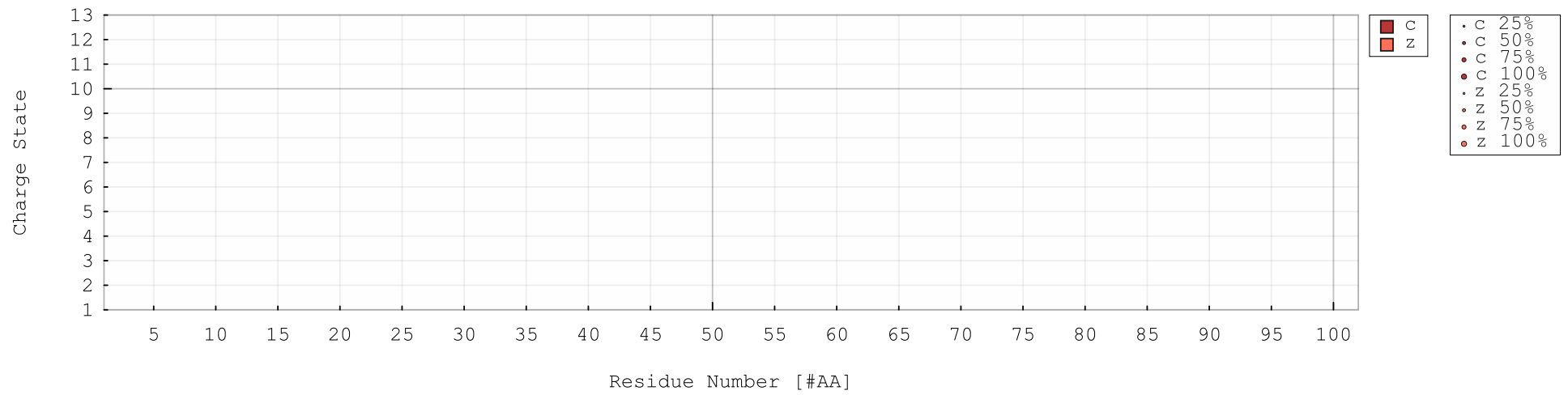

	Index	Protein	SC [%]	SVP [%]	IC [%]	MS Score [a.u.]	Var. Mods	Mono Mass [Da]	Chemical Formula
Proteoform:	9	H4Kac	45.5	20.6	37.4	17.0	Ace: K5, K8, K12, K16, K20, K31, K77	11523.4152	C508H851N163O141S

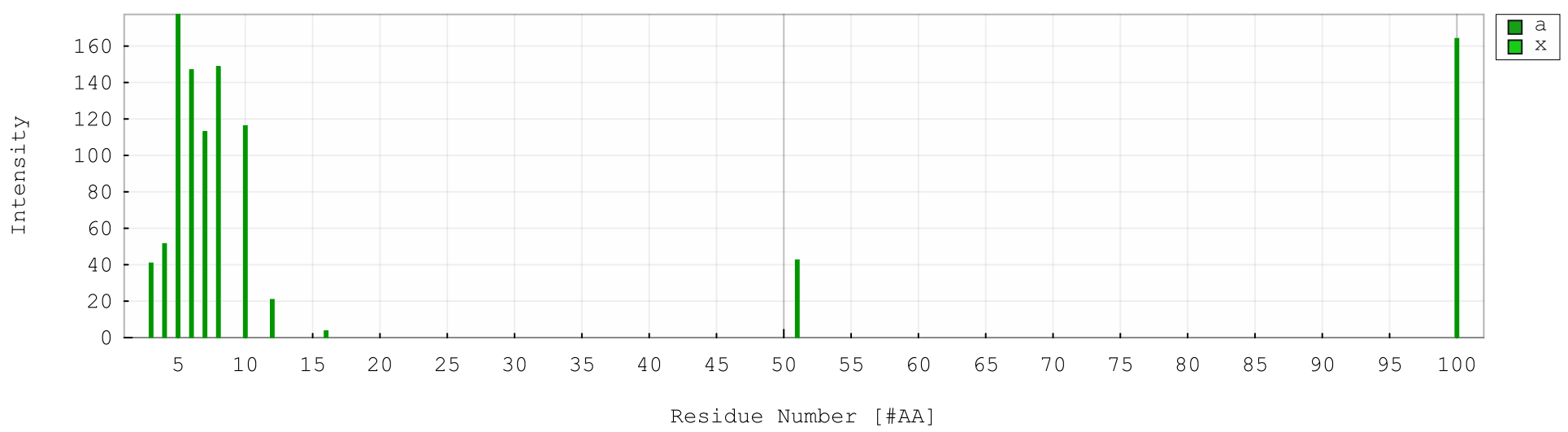

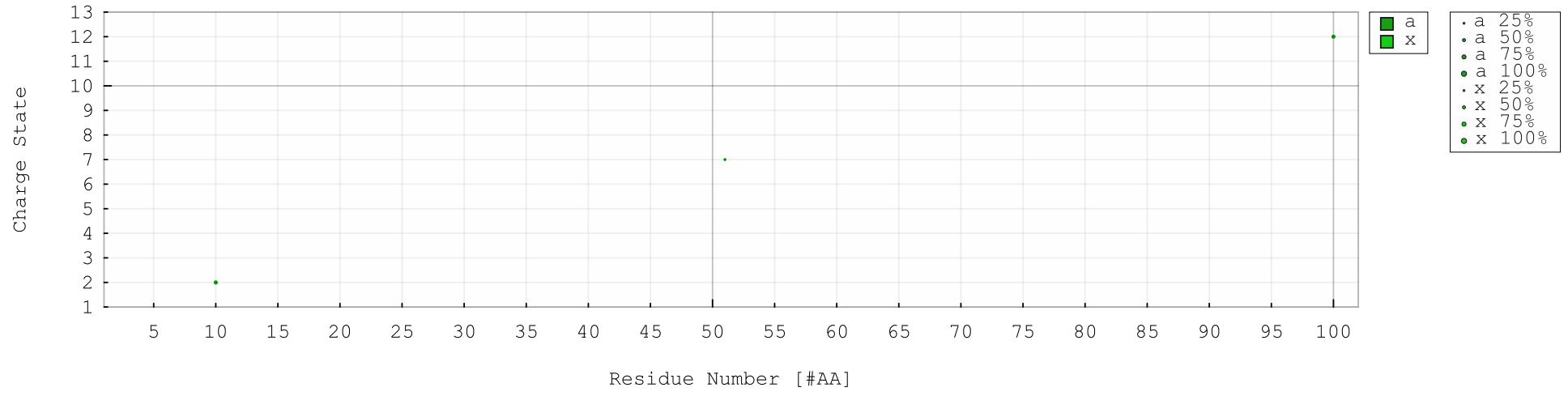

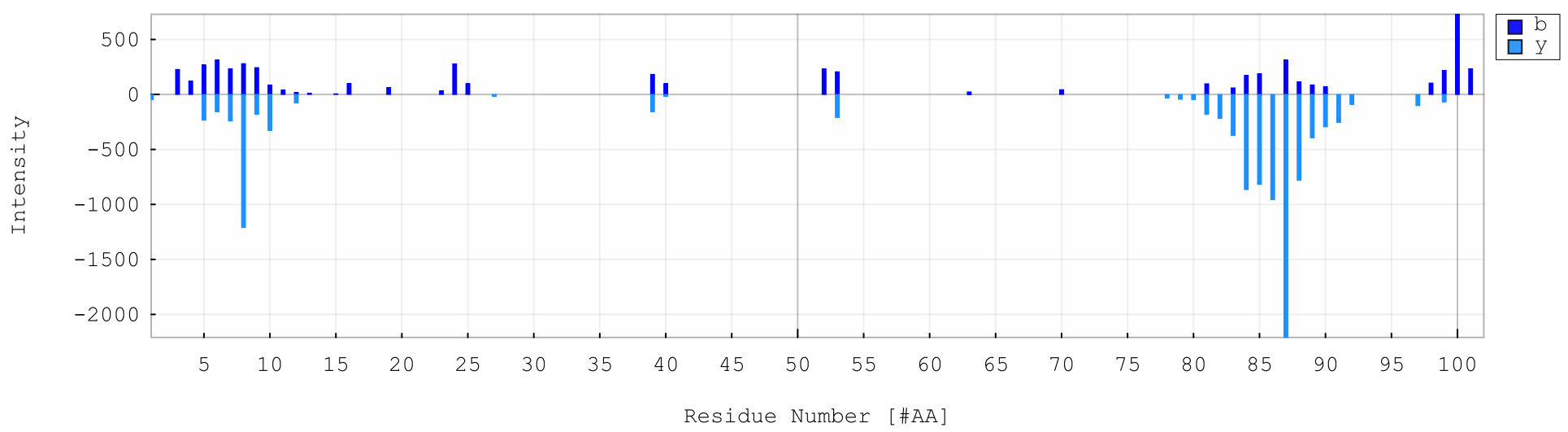

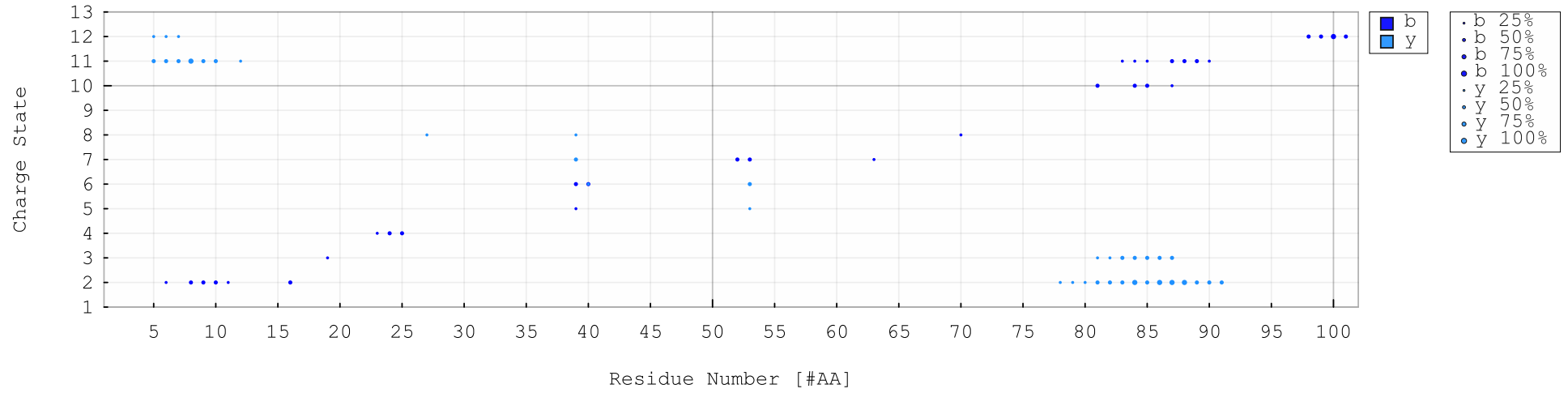

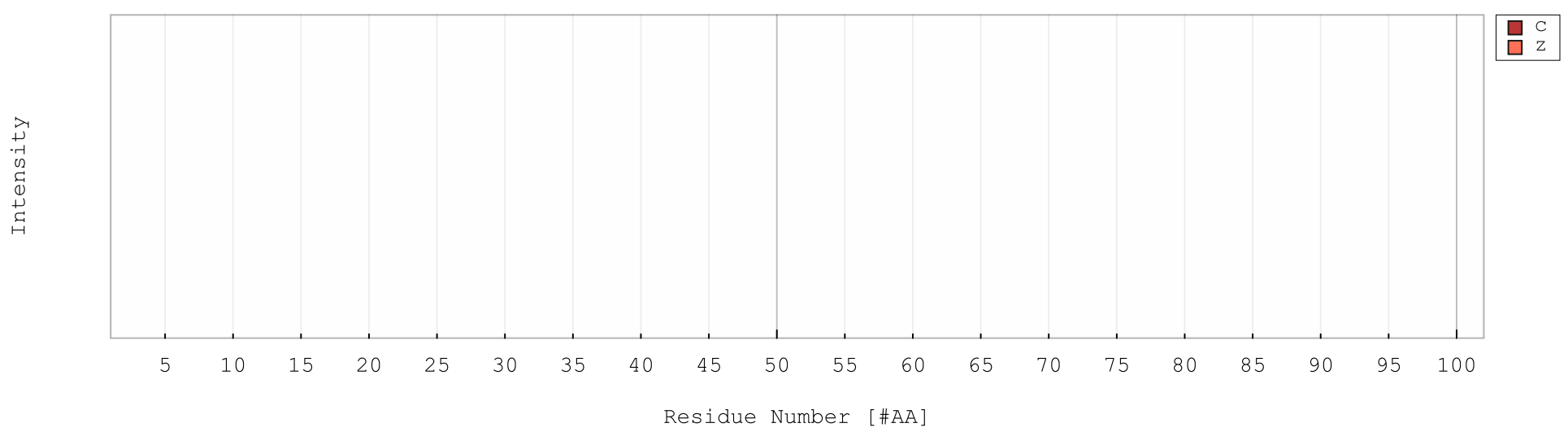

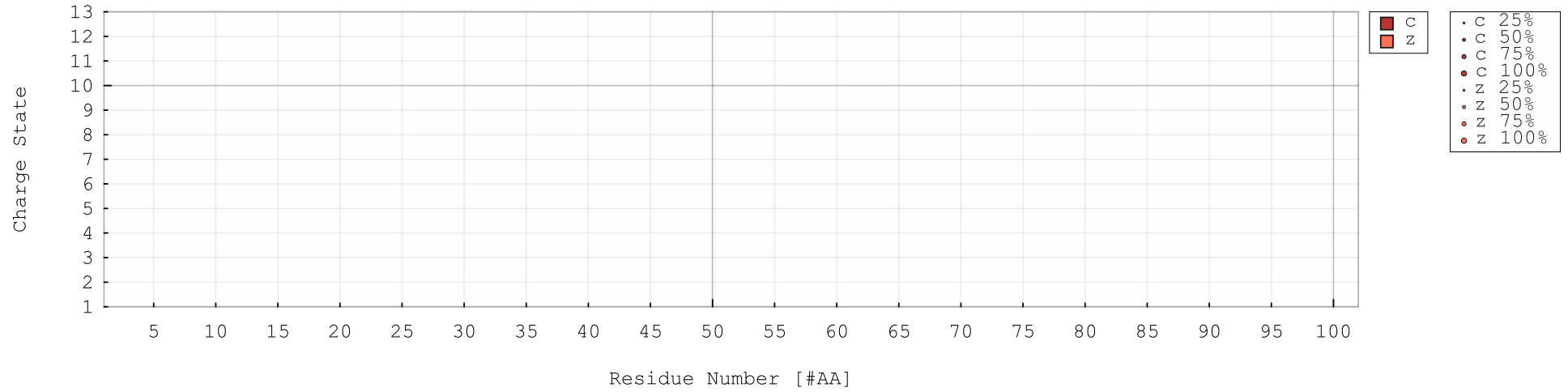

Proteoform:	Index	Protein	SC [%]	SVP [%]	IC [%]	MS Score [a.u.]	Var. Mods	Mono Mass [Da]	Chemical Formula
	10	H4Kac	45.5	20.6	36.9	16.8	Ace: K5, K8, K12, K16, K20, K44, K79	11523.4152	C508H851N163O141S

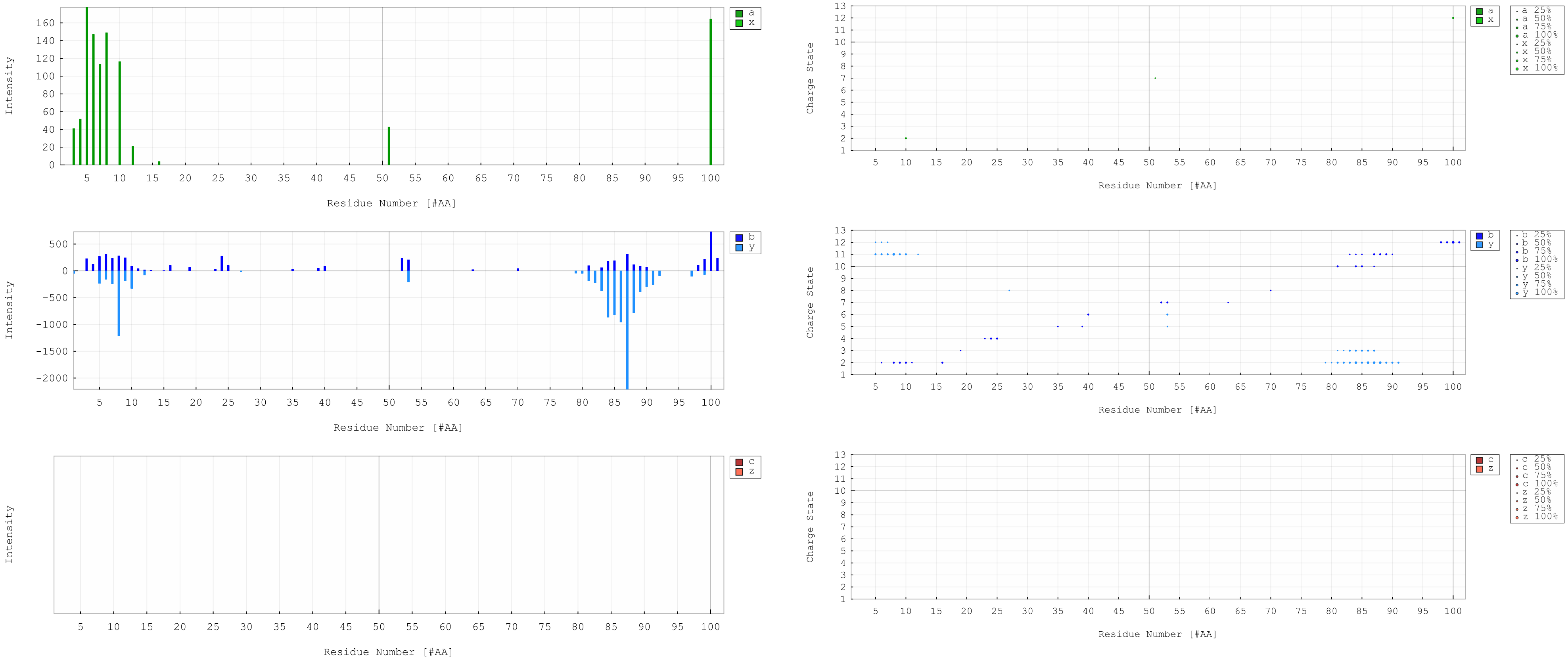

Supplemental Data S4

H4 - p300, 60 min

**Report**	7 of 7
**Experiment**	MS3
**Histone/sample**	H4
**Enzyme**	p300
**Reaction/source**	60 min
**Precursor**	H4Kac6
**Charge state**	13+
**Fragmentation / workflow**	MS2 ECD -> MS3 RCID
**Original file**	MS3_H4Kac6_13+_MS2-ECD_MS3-RCID.pdf

Project Information

Project Directory: C:/Users/LAB-ADMIN
Processing date: Tue Apr 28 13:00:12 2026

Sample Info & Protocols

Name: Top-Down Protein Sequencing ExD

Dataset

Imported spectrum file name:	250430 H4_P300-50_60min z13ac6 MS2 ECD MS3 shCID c22_3+m _2_013-195_calibrated2.txt
Dataset name:	Information is only available from imported JPEAKS files
Date acquired:	Information is only available from imported JPEAKS files
Date imported:	Information is only available from imported JPEAKS files
Instrument Name:	Information is only available from imported JPEAKS files
Instrument Serial Number:	Information is only available from imported JPEAKS files

Workflow Result Info

Analysis: 250430 H4_P300-50_60min z13ac6 MS2 ECD MS3 shCID c22_3+m _2_013-195_calibrated2
Location: C:/Users/LAB-ADMIN
Method: -

Method Information

Name: Unnamed

Spectral Parameters

Mass Error [ppm]: 6
Intensity Deviation [au]: 0.15
Min/Max Charges: 1-13
Experiment type: ExD

a ions	b ions	c ions	x ions	y ions	z ions	satellite	internal	precursor
a	b	c		y	z+1			M
a+1		c+1			z+2			M+-2H

Noise threshold calculation: Simple moving average noise threshold (SMA)
Window size [datapoints]: 600
Noise curve offset: 0.06374714
SVP parameters: terminal gap: 2, internal gap: 1

Proteoforms

(top 1)

Index	Parent Sequence	Chain	Var. Mods	Mono Mass [Da]	Chemical Composition
1	H4[1-22]Kac	H4[1-22]KacK-5[Ace]K8[Ace]K12[Ace]K16[Ace]K20[Ace]L22[Cte]	Ace: K5, K8, K12, K16, K20	2412.3938	C103H181N39O28

Results

Proteoforms Table (top 1)

Index	Protein	SC [%]	SVP [%]	IC [%]	MS Score [a.u.]	Var. Mods	Mono Mass [Da]	Chemical Formula
1	H4[1-22]Kac	95.2	100.0	17.8	17.0	Ace: K5, K8, K12, K16, K20	2412.3938	C103H181N39O28

Sequence

Name: H4[1-22]Kac

10 20
S G R G K G G K G L G K G G A K R H R K V L

Modifications

Modification	Position	Type
Acetyl	5	variable
Acetyl	8	variable
Acetyl	12	variable
Acetyl	16	variable
Acetyl	20	variable
Cterm-OH-to-NH2	22	fixed

Sequence Map

Proteoform:	Index	Protein	SC [%]	SVP [%]	IC [%]	MS Score [a.u.]	Var. Mods	Mono Mass [Da]	Chemical Formula
	1	H4[1-22]Kac	95.2	100.0	17.8	17.0	Ace: K5, K8, K12, K16, K20	2412.3938	C103H181N39O28

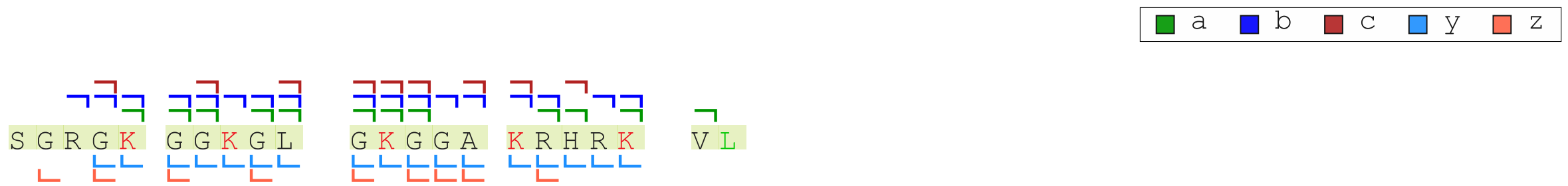

Error Plot

Proteoform:

Index	Protein	SC [%]	SVP [%]	IC [%]	MS Score [a.u.]	Var. Mods	Mono Mass [Da]	Chemical Formula
1	H4[1-22]Kac	95.2	100.0	17.8	17.0	Ace: K5, K8, K12, K16, K20	2412.3938	C103H181N39O28

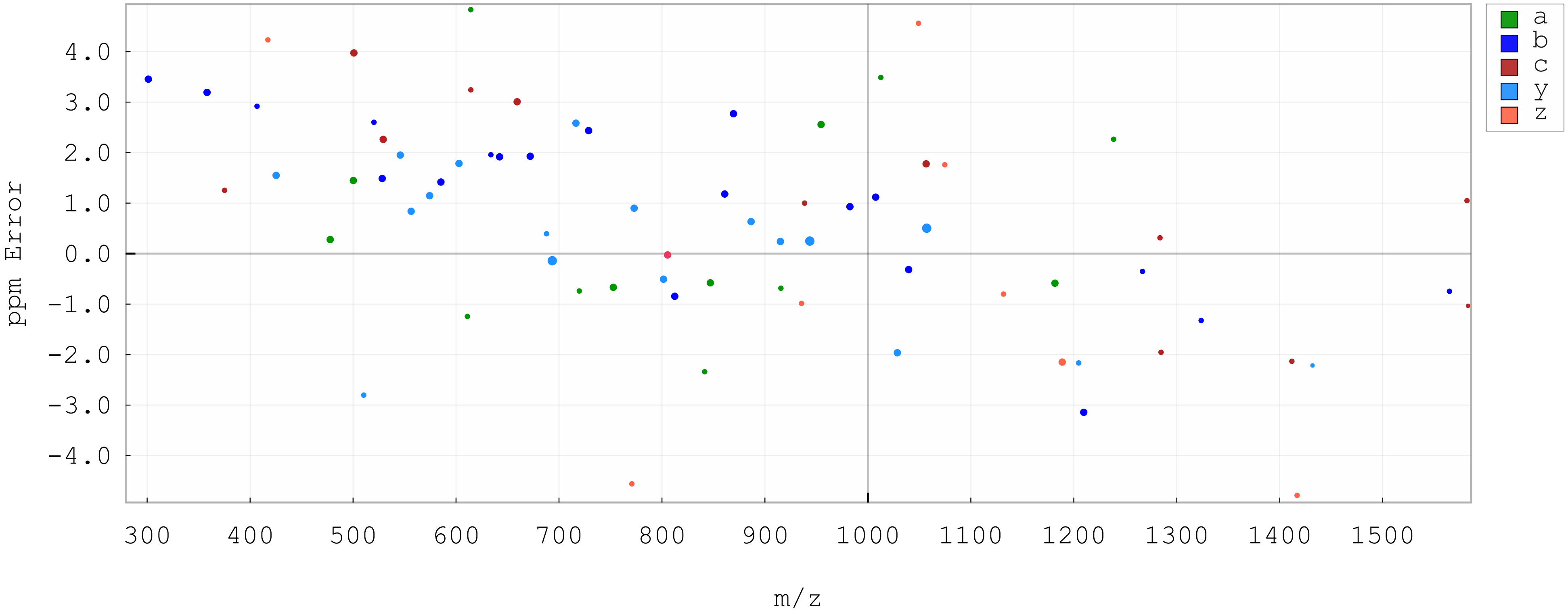

Primary Fragment Plots

Proteoform:

Index	Protein	SC [%]	SVP [%]	IC [%]	MS Score [a.u.]	Var. Mods	Mono Mass [Da]	Chemical Formula
1	H4[1-22]Kac	95.2	100.0	17.8	17.0	Ace: K5, K8, K12, K16, K20	2412.3938	C103H181N39O28

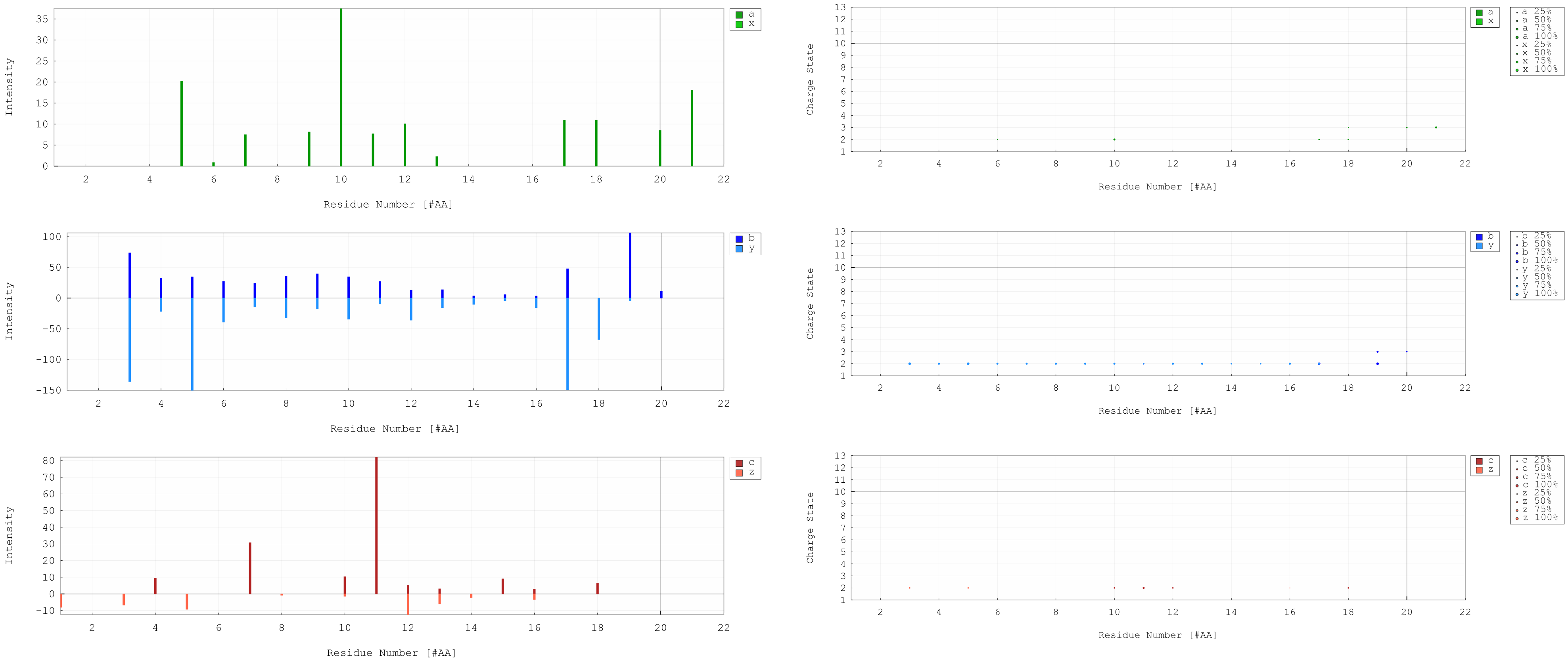

Supplement: Supplementary file 4 [file mmc4.pdf]
