# Supplementary material for: Top-Down Sequencing of Intact Proteoforms Using the timsOmni Mass Spectrometer: Accurate Determination of Co-Occurring Histone Modifications
Source: Mol Cell Proteomics. 2026 Aug 17;25(9):101640. doi: 10.1016/j.mcpro.2026.101640 (PMC13595144; doi:10.1016/j.mcpro.2026.101640)

Supplemental Data S5. H4 - PCAF, 30 min - OmniScape reports

OmniScape reports for mono- and diacetylated H4 generated by PCAF.

**Histone**	H4
**Enzyme**	PCAF
**Reaction/source**	30 min
**Nb of Osc reports**	8

Included reports

#	Experiment	Precursor	Charge	Fragmentation / workflow	File
1	MS2	H4Kac1	13+	ECciD	MS2_H4Kac1_13+_ECciD.pdf
2	MS2	H4Kac1	13+	ECD	MS2_H4Kac1_13+_ECD.pdf
3	MS2	H4Kac1	13+	EID	MS2_H4Kac1_13+_EID.pdf
4	MS2	H4Kac1	13+	RCID	MS2_H4Kac1_13+_RCID.pdf
5	MS2	H4Kac2	13+	ECD	MS2_H4Kac2_13+_ECD.pdf
6	MS2	H4Kac2	13+	RCID	MS2_H4Kac2_13+_RCID.pdf
7	MS3	H4Kac1	13+	MS2 RCID -> MS3 ECD	MS3_H4Kac1_13+_MS2-RCID_MS3-ECD.pdf
8	MS3	H4Kac2	13+	MS2 RCID -> MS3 ECD	MS3_H4Kac2_13+_MS2-RCID_MS3-ECD.pdf

Supplemental Data S5

H4 - PCAF, 30 min

**Report**	1 of 8
**Experiment**	MS2
**Histone/sample**	H4
**Enzyme**	PCAF
**Reaction/source**	30 min
**Precursor**	H4Kac1
**Charge state**	13+
**Fragmentation / workflow**	ECciD
**Original file**	MS2_H4Kac1_13+_ECciD.pdf

Project Information

Project Directory: C:/Users/LAB-ADMIN
Processing date: Tue Apr 28 14:44:29 2026

Sample Info & Protocols

Name: Top-Down Protein Sequencing ExD

Dataset

Imported spectrum file name:	250428 H4-PCAF100-30min z13ac1 MS2 CA-ECD_001-686_calibrated.txt
Dataset name:	Information is only available from imported JPEAKS files
Date acquired:	Information is only available from imported JPEAKS files
Date imported:	Information is only available from imported JPEAKS files
Instrument Name:	Information is only available from imported JPEAKS files
Instrument Serial Number:	Information is only available from imported JPEAKS files

Workflow Result Info

Analysis: 250428 H4-PCAF100-30min z13ac1 MS2 CA-ECD_001-686_calibrated2
Location: C:/Users/LAB-ADMIN
Method: -

Method Information

Name: Unnamed

Spectral Parameters

Mass Error [ppm]: 6
Intensity Deviation [au]: 0.15
Min/Max Charges: 1-13
Experiment type: ExD

a ions	b ions	c ions	x ions	y ions	z ions	satellite	internal	precursor
a	b	c		y	z+1			M
a+1		c+1			z+2			M+-2H

Noise threshold calculation: Simple moving average noise threshold (SMA)
Window size [datapoints]: 600
Noise curve offset: 3.7653408
SVP parameters: terminal gap: 2, internal gap: 1

Proteoforms

(top 10)

Index	Parent Sequence	Chain	Var. Mods	Mono Mass [Da]	Chemical Composition
1	H4Kac	H4KacK8[Ace]	Ace: K8	11271.3518	C496H839N163O135S
2	H4Kac	H4KacK12[Ace]	Ace: K12	11271.3518	C496H839N163O135S
3	H4Kac	H4KacK16[Ace]	Ace: K16	11271.3518	C496H839N163O135S
4	H4Kac	H4KacK5[Ace]	Ace: K5	11271.3518	C496H839N163O135S
5	H4Kac	H4KacK20[Ace]	Ace: K20	11271.3518	C496H839N163O135S
6	H4Kac	H4KacK31[Ace]	Ace: K31	11271.3518	C496H839N163O135S
7	H4Kac	H4KacK44[Ace]	Ace: K44	11271.3518	C496H839N163O135S
8	H4Kac	H4KacK59[Ace]	Ace: K59	11271.3518	C496H839N163O135S
9	H4Kac	H4Kac	None	11229.3412	C494H837N163O134S
10	H4Kac	H4KacK77[Ace]	Ace: K77	11271.3518	C496H839N163O135S

Results

Proteoforms Table (top 10)

Index	Protein	SC [%]	SVP [%]	IC [%]	MS Score [a.u.]	Var. Mods	Mono Mass [Da]	Chemical Formula
1	H4Kac	77.2	35.3	26.6	20.6	Ace: K8	11271.3518	C496H839N163O135S
2	H4Kac	77.2	35.3	25.6	19.8	Ace: K12	11271.3518	C496H839N163O135S
3	H4Kac	76.2	16.7	25.2	19.2	Ace: K16	11271.3518	C496H839N163O135S
4	H4Kac	74.3	8.8	25.0	18.6	Ace: K5	11271.3518	C496H839N163O135S
5	H4Kac	72.3	16.7	23.8	17.2	Ace: K20	11271.3518	C496H839N163O135S
6	H4Kac	63.4	16.7	19.7	12.5	Ace: K31	11271.3518	C496H839N163O135S
7	H4Kac	58.4	16.7	16.9	9.9	Ace: K44	11271.3518	C496H839N163O135S
8	H4Kac	52.5	16.7	13.4	7.1	Ace: K59	11271.3518	C496H839N163O135S
9	H4Kac	59.4	16.7	9.4	5.6	None	11229.3412	C494H837N163O134S
10	H4Kac	44.6	16.7	11.2	5.0	Ace: K77	11271.3518	C496H839N163O135S

Sequence

Name: H4Kac

```
                   10                    20                    30                    40                    50                    60
S G R G K G G K G L   G K G G A K R H R K   V L R D N I Q G I T   K P A I R R L A R R   G G V K R I S G L I   Y E E T R G V L K V
                   70                    80                    90                   100                   110                   120
F L E N V I R D A V   T Y T E H A K R K T   V T A M D V V Y A L   K R Q G R T L Y G F   G G
```

Modifications

Modification	Position	Type
Acetyl	5	variable
Acetyl	8	variable
Acetyl	12	variable
Acetyl	16	variable
Acetyl	20	variable
Acetyl	31	variable
Acetyl	44	variable
Acetyl	59	variable
Acetyl	77	variable
Acetyl	79	variable
Acetyl	91	variable

Sequence Map

Proteoform:

Index	Protein	SC [%]	SVP [%]	IC [%]	MS Score [a.u.]	Var. Mods	Mono Mass [Da]	Chemical Formula
1	H4Kac	77.2	35.3	26.6	20.6	Ace: K8	11271.3518	C496H839N163O135S

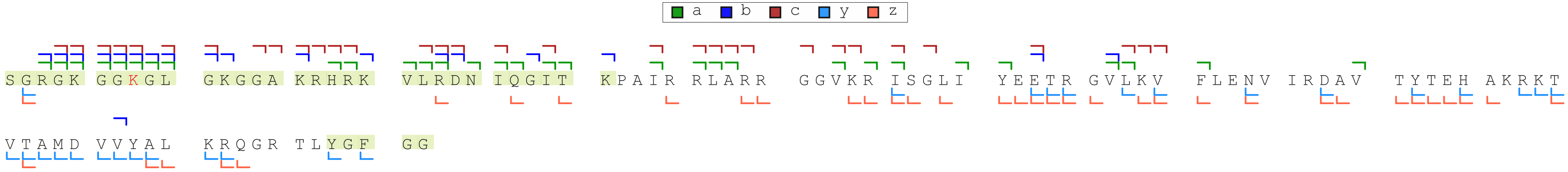

Proteoform:

Index	Protein	SC [%]	SVP [%]	IC [%]	MS Score [a.u.]	Var. Mods	Mono Mass [Da]	Chemical Formula
2	H4Kac	77.2	35.3	25.6	19.8	Ace: K12	11271.3518	C496H839N163O135S

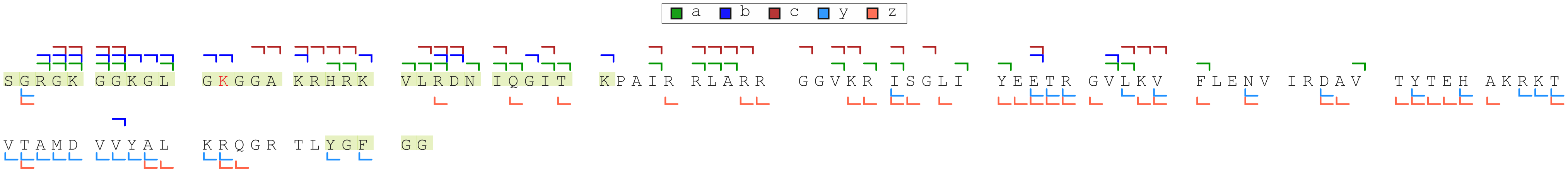

Proteoform:

Index	Protein	SC [%]	SVP [%]	IC [%]	MS Score [a.u.]	Var. Mods	Mono Mass [Da]	Chemical Formula
3	H4Kac	76.2	16.7	25.2	19.2	Ace: K16	11271.3518	C496H839N163O135S

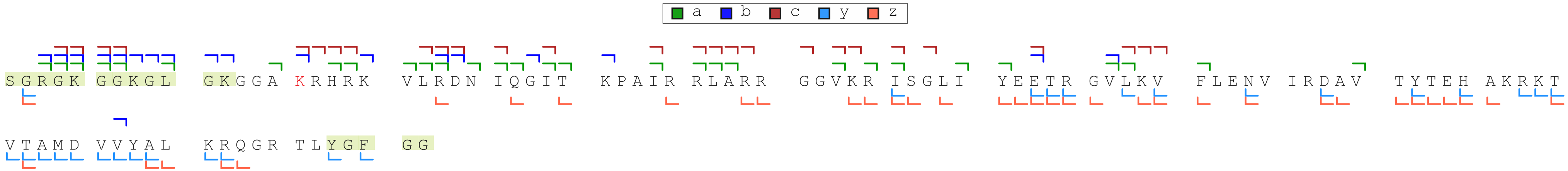

Proteoform:

Index	Protein	SC [%]	SVP [%]	IC [%]	MS Score [a.u.]	Var. Mods	Mono Mass [Da]	Chemical Formula
4	H4Kac	74.3	8.8	25.0	18.6	Ace: K5	11271.3518	C496H839N163O135S

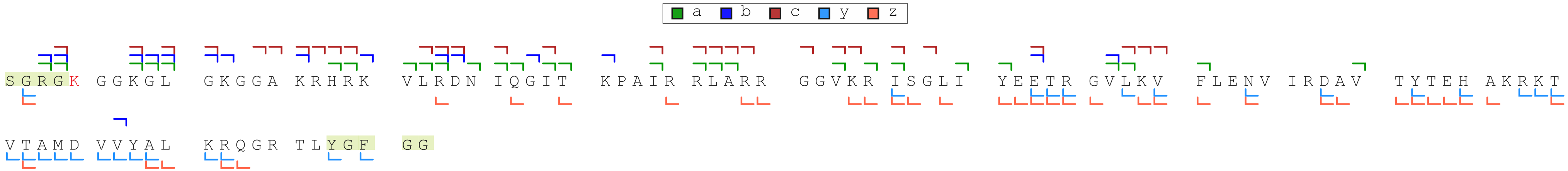

Top-Down Result OmniScape 2025
Sequence Confirmation
250428 H4-PCAF100-30min z13ac1 MS2 CA-ECD_001-686_calibrated2

Proteoform:

Index	Protein	SC [%]	SVP [%]	IC [%]	MS Score [a.u.]	Var. Mods	Mono Mass [Da]	Chemical Formula
5	H4Kac	72.3	16.7	23.8	17.2	Ace: K20	11271.3518	C496H839N163O135S

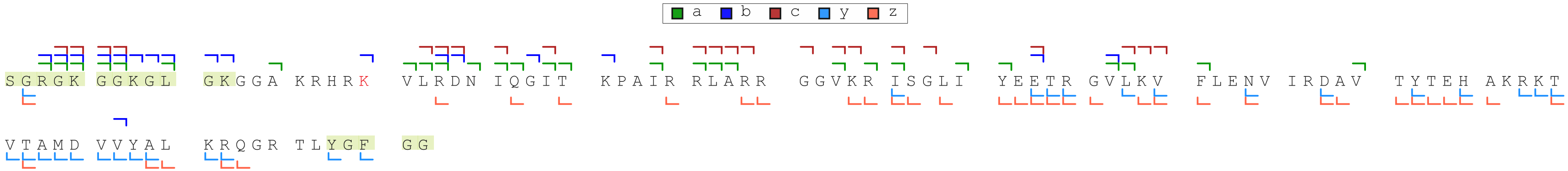

Proteoform:

Index	Protein	SC [%]	SVP [%]	IC [%]	MS Score [a.u.]	Var. Mods	Mono Mass [Da]	Chemical Formula
6	H4Kac	63.4	16.7	19.7	12.5	Ace: K31	11271.3518	C496H839N163O135S

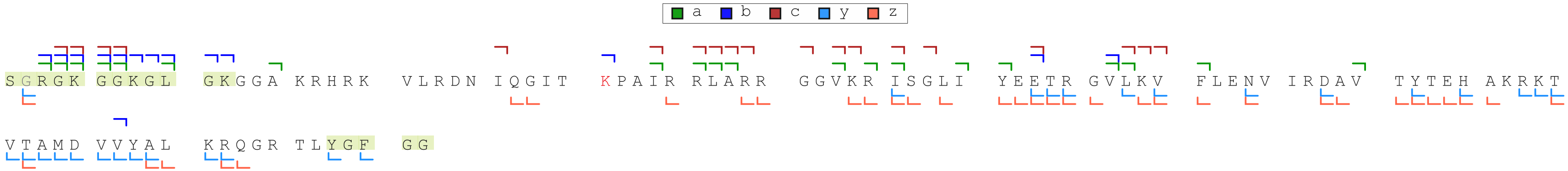

Proteoform:

Index	Protein	SC [%]	SVP [%]	IC [%]	MS Score [a.u.]	Var. Mods	Mono Mass [Da]	Chemical Formula
7	H4Kac	58.4	16.7	16.9	9.9	Ace: K44	11271.3518	C496H839N163O135S

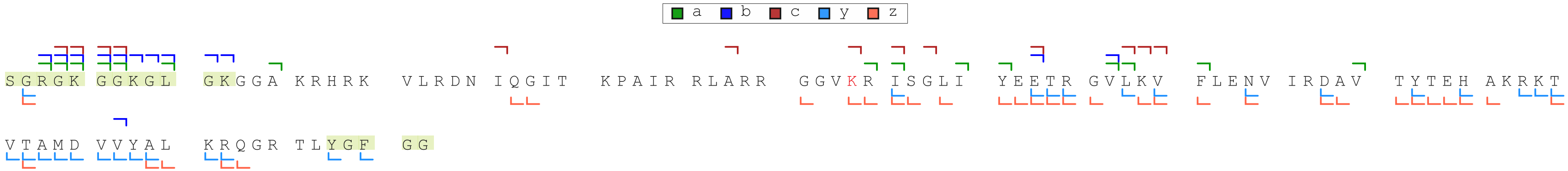

Proteoform:

Index	Protein	SC [%]	SVP [%]	IC [%]	MS Score [a.u.]	Var. Mods	Mono Mass [Da]	Chemical Formula
8	H4Kac	52.5	16.7	13.4	7.1	Ace: K59	11271.3518	C496H839N163O135S

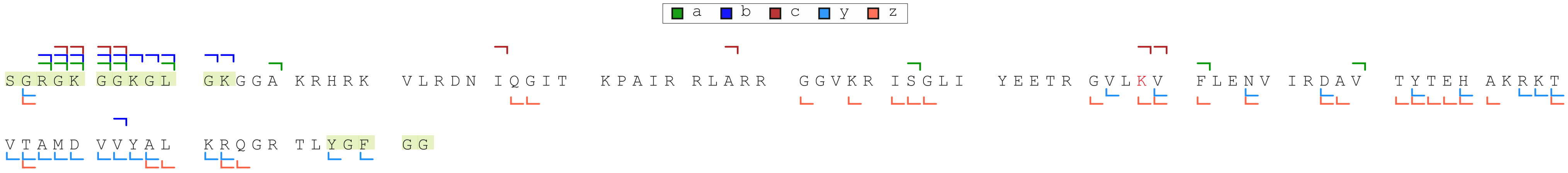

Proteoform:	Index	Protein	SC [%]	SVP [%]	IC [%]	MS Score [a.u.]	Var. Mods	Mono Mass [Da]	Chemical Formula
	9	H4Kac	59.4	16.7	9.4	5.6	None	11229.3412	C494H837N163O134S

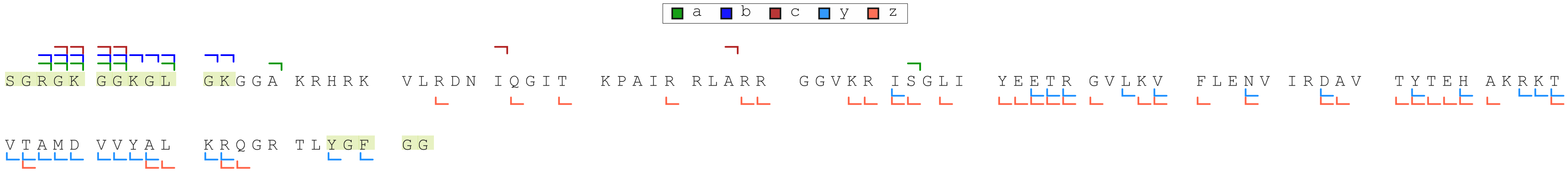

Proteoform:

Index	Protein	SC [%]	SVP [%]	IC [%]	MS Score [a.u.]	Var. Mods	Mono Mass [Da]	Chemical Formula
10	H4Kac	44.6	16.7	11.2	5.0	Ace: K77	11271.3518	C496H839N163O135S

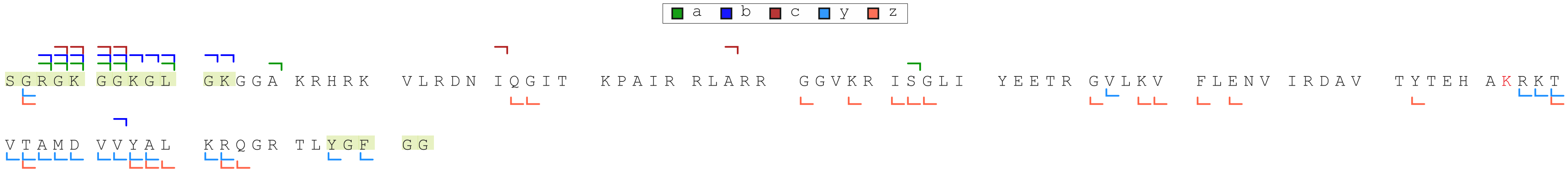

Error Plot

Proteoform:

Index	Protein	SC [%]	SVP [%]	IC [%]	MS Score [a.u.]	Var. Mods	Mono Mass [Da]	Chemical Formula
1	H4Kac	77.2	35.3	26.6	20.6	Ace: K8	11271.3518	C496H839N163O135S

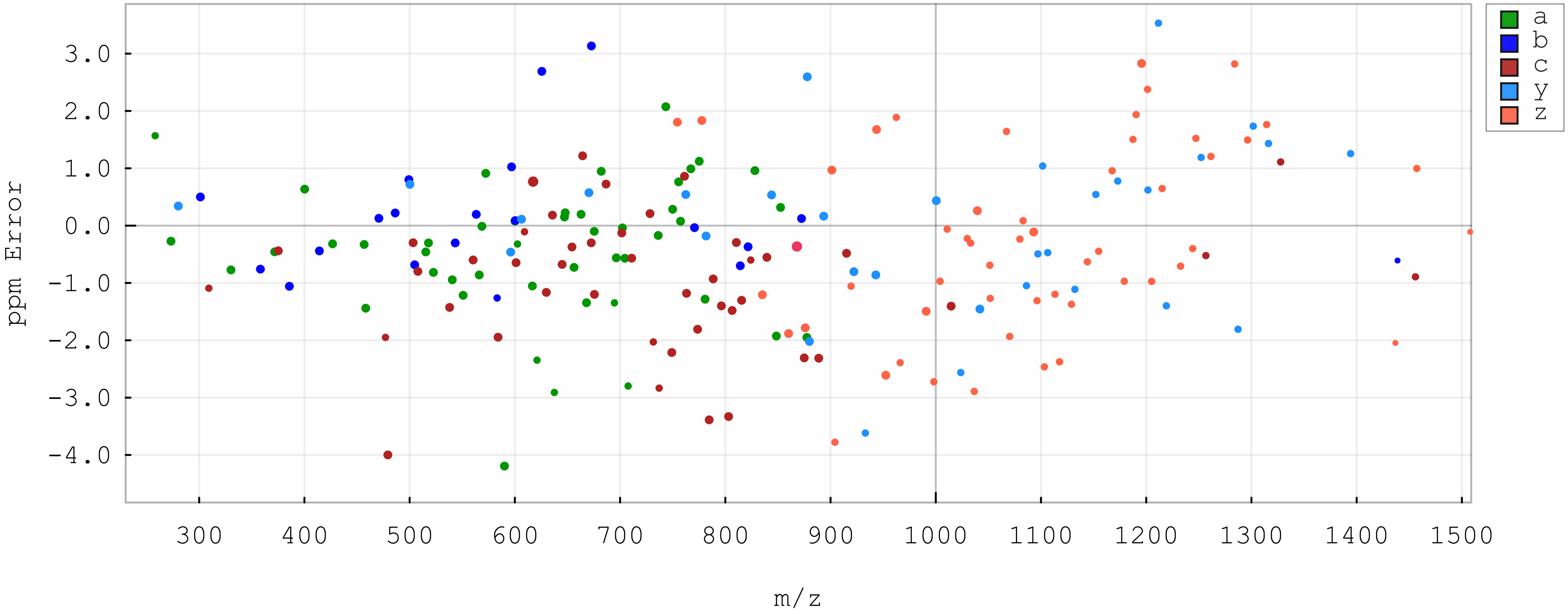

Proteoform:

Index	Protein	SC [%]	SVP [%]	IC [%]	MS Score [a.u.]	Var. Mods	Mono Mass [Da]	Chemical Formula
2	H4Kac	77.2	35.3	25.6	19.8	Ace: K12	11271.3518	$C_{496}H_{839}N_{163}O_{135}S$

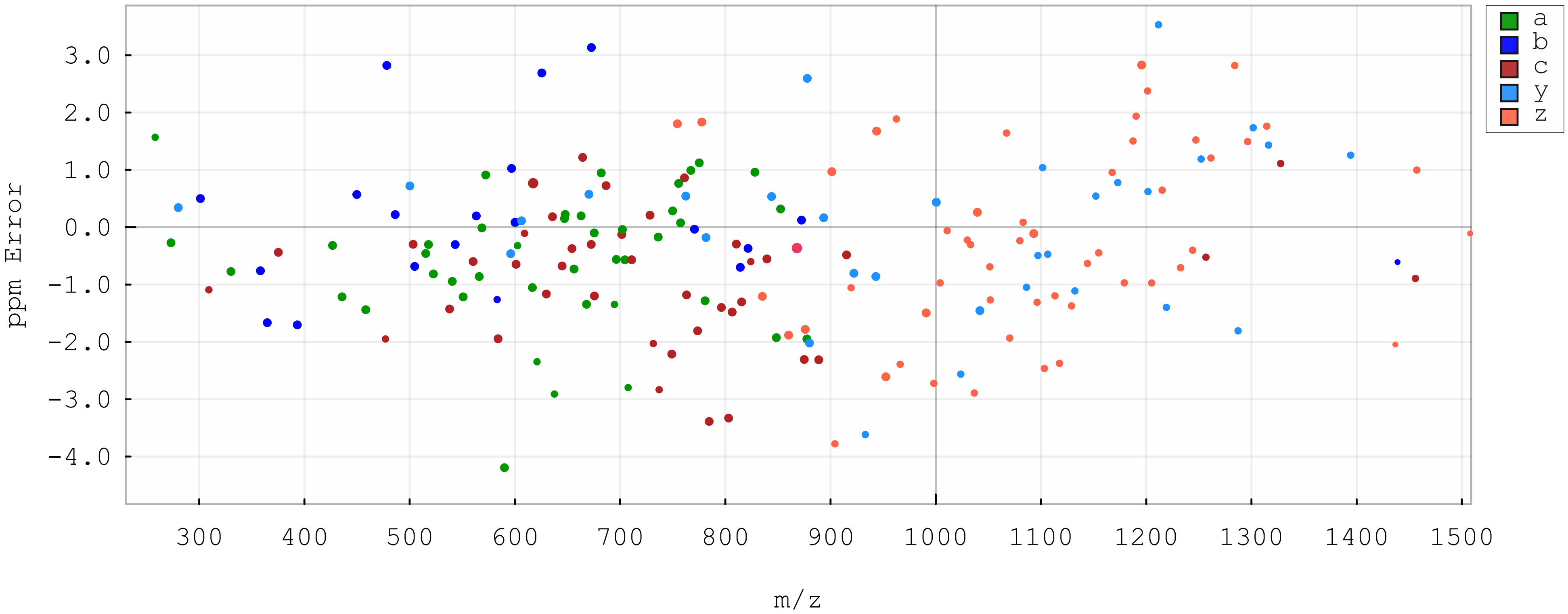

Proteoform:

Index	Protein	SC [%]	SVP [%]	IC [%]	MS Score [a.u.]	Var. Mods	Mono Mass [Da]	Chemical Formula
3	H4Kac	76.2	16.7	25.2	19.2	Ace: K16	11271.3518	C496H839N163O135S

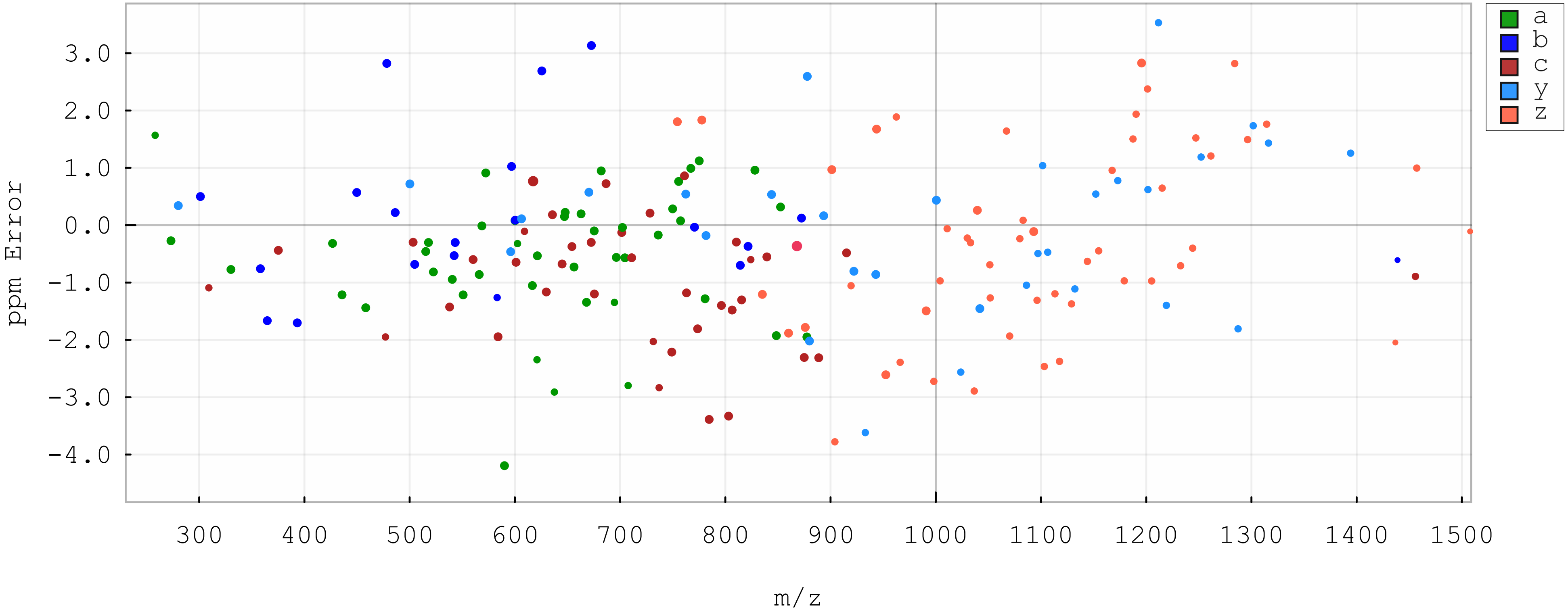

Proteoform:	Index	Protein	SC [%]	SVP [%]	IC [%]	MS Score [a.u.]	Var. Mods	Mono Mass [Da]	Chemical Formula
	4	H4Kac	74.3	8.8	25.0	18.6	Ace: K5	11271.3518	C496H839N163O135S

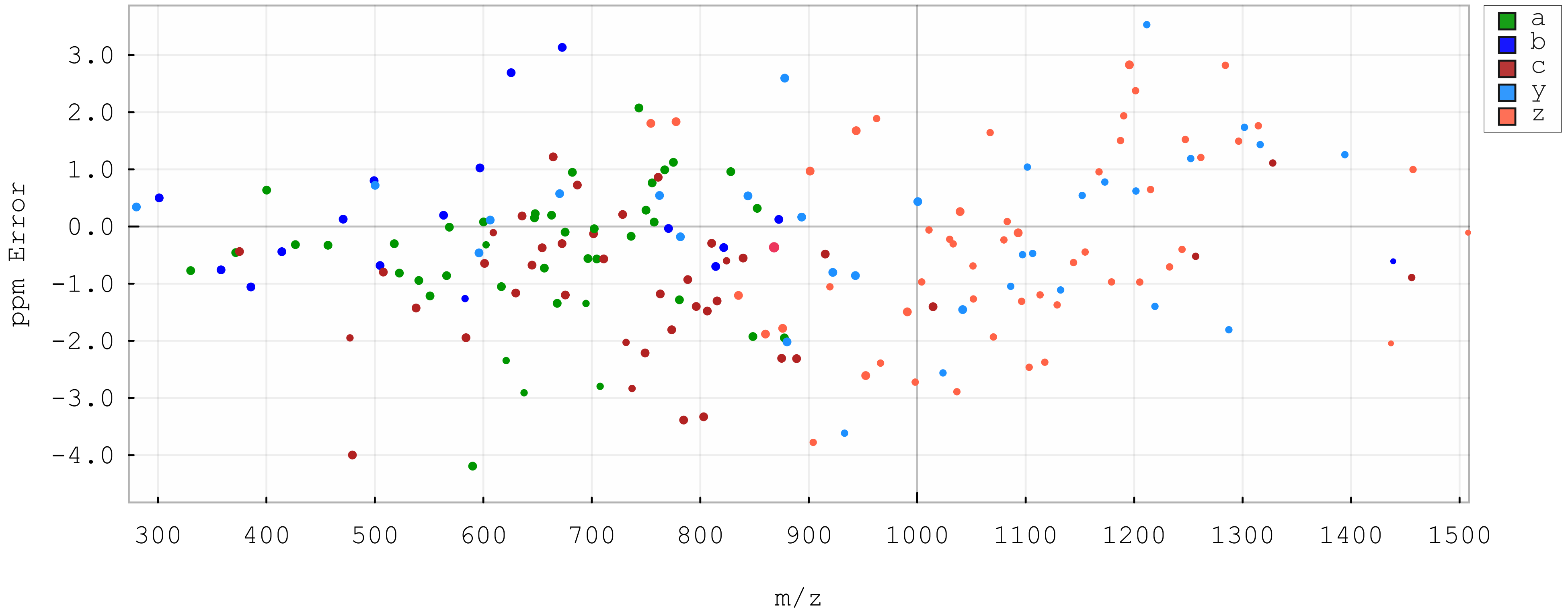

Proteoform:

Index	Protein	SC [%]	SVP [%]	IC [%]	MS Score [a.u.]	Var. Mods	Mono Mass [Da]	Chemical Formula
5	H4Kac	72.3	16.7	23.8	17.2	Ace: K20	11271.3518	C496H839N163O135S

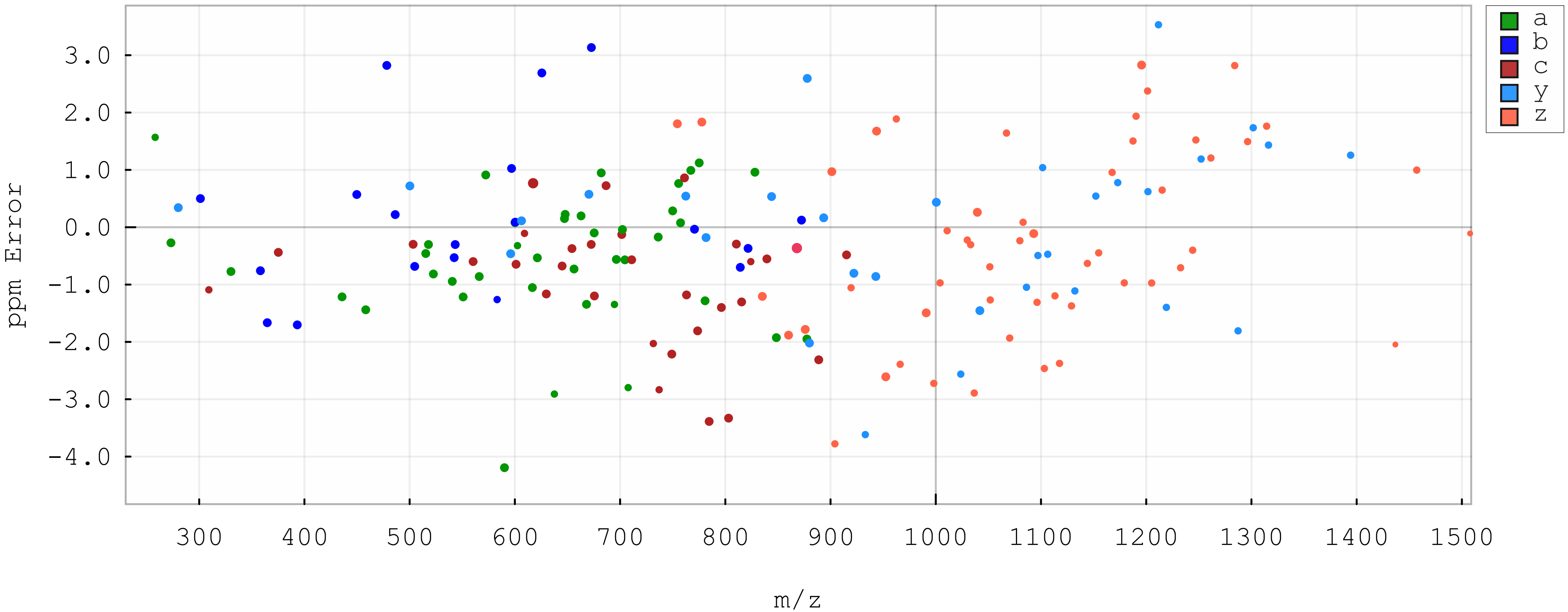

Proteoform:

Index	Protein	SC [%]	SVP [%]	IC [%]	MS Score [a.u.]	Var. Mods	Mono Mass [Da]	Chemical Formula
6	H4Kac	63.4	16.7	19.7	12.5	Ace: K31	11271.3518	C496H839N163O135S

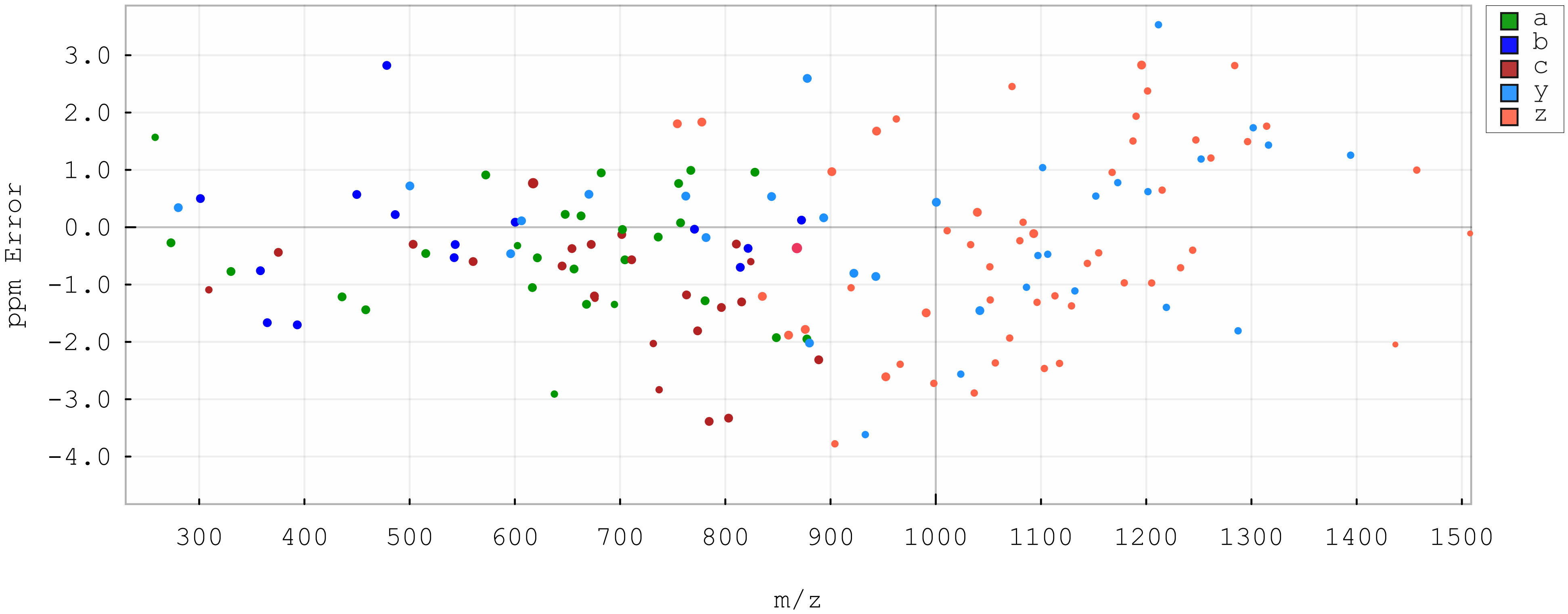

Proteoform:

Index	Protein	SC [%]	SVP [%]	IC [%]	MS Score [a.u.]	Var. Mods	Mono Mass [Da]	Chemical Formula
7	H4Kac	58.4	16.7	16.9	9.9	Ace: K44	11271.3518	C496H839N163O135S

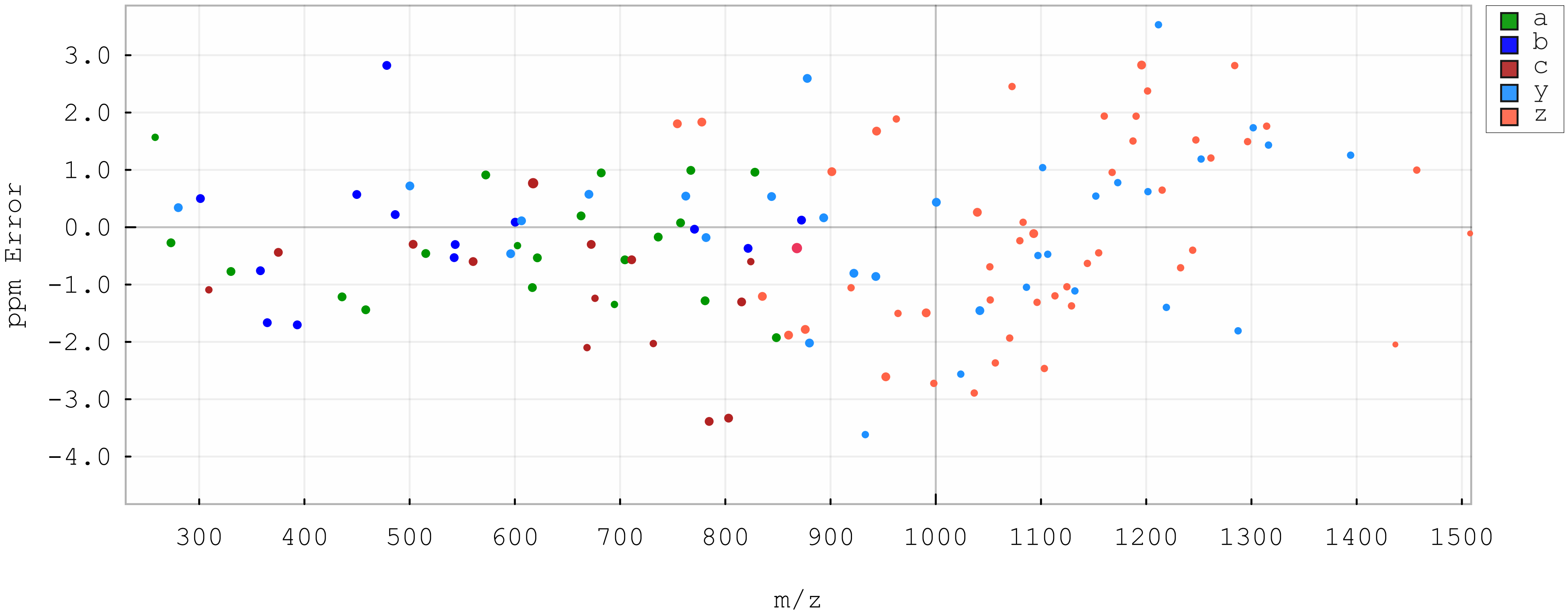

Proteoform:

Index	Protein	SC [%]	SVP [%]	IC [%]	MS Score [a.u.]	Var. Mods	Mono Mass [Da]	Chemical Formula
8	H4Kac	52.5	16.7	13.4	7.1	Ace: K59	11271.3518	C496H839N163O135S

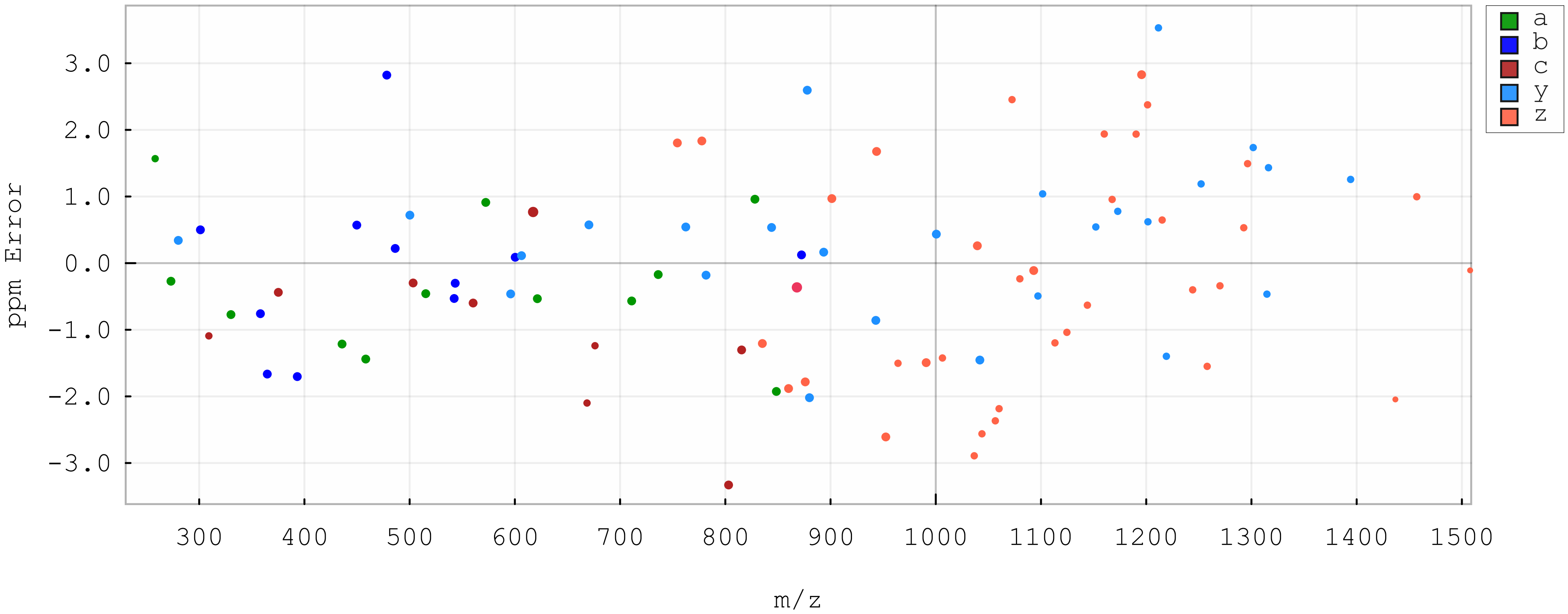

Proteoform:

Index	Protein	SC [%]	SVP [%]	IC [%]	MS Score [a.u.]	Var. Mods	Mono Mass [Da]	Chemical Formula
9	H4Kac	59.4	16.7	9.4	5.6	None	11229.3412	C494H837N163O134S

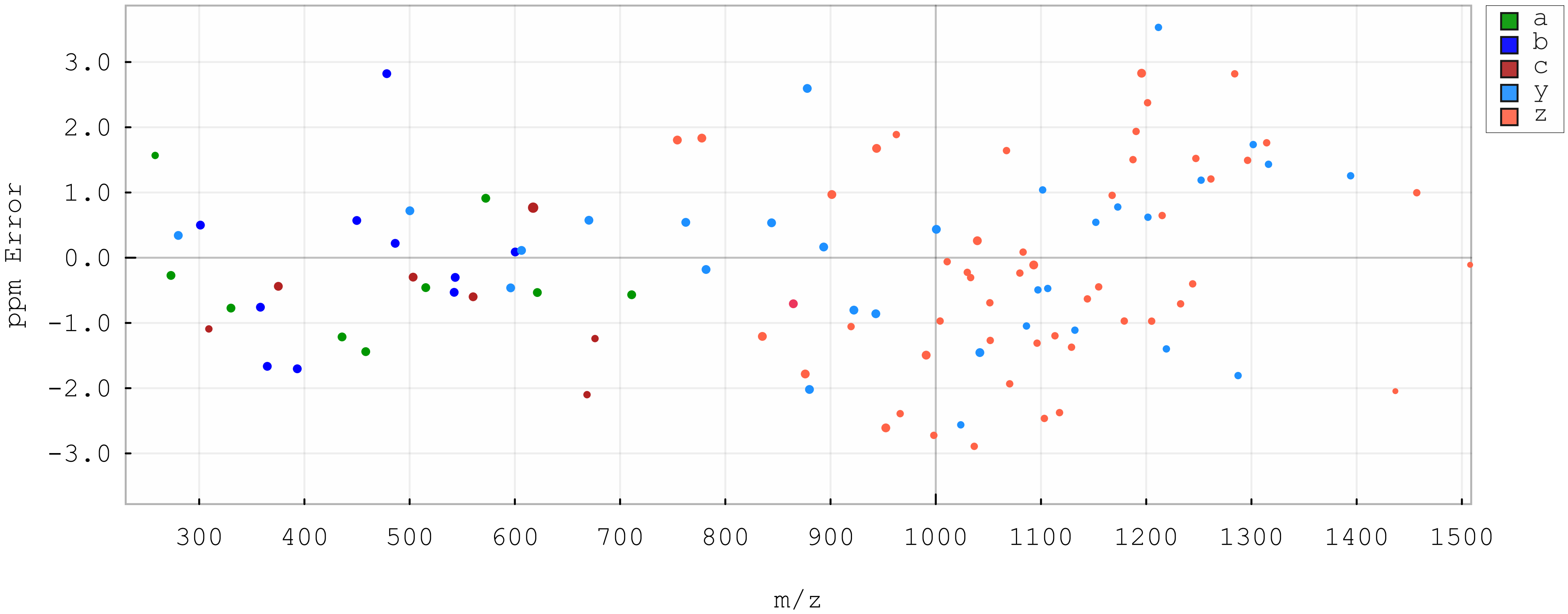

Proteoform:

Index	Protein	SC [%]	SVP [%]	IC [%]	MS Score [a.u.]	Var. Mods	Mono Mass [Da]	Chemical Formula
10	H4Kac	44.6	16.7	11.2	5.0	Ace: K77	11271.3518	C496H839N163O135S

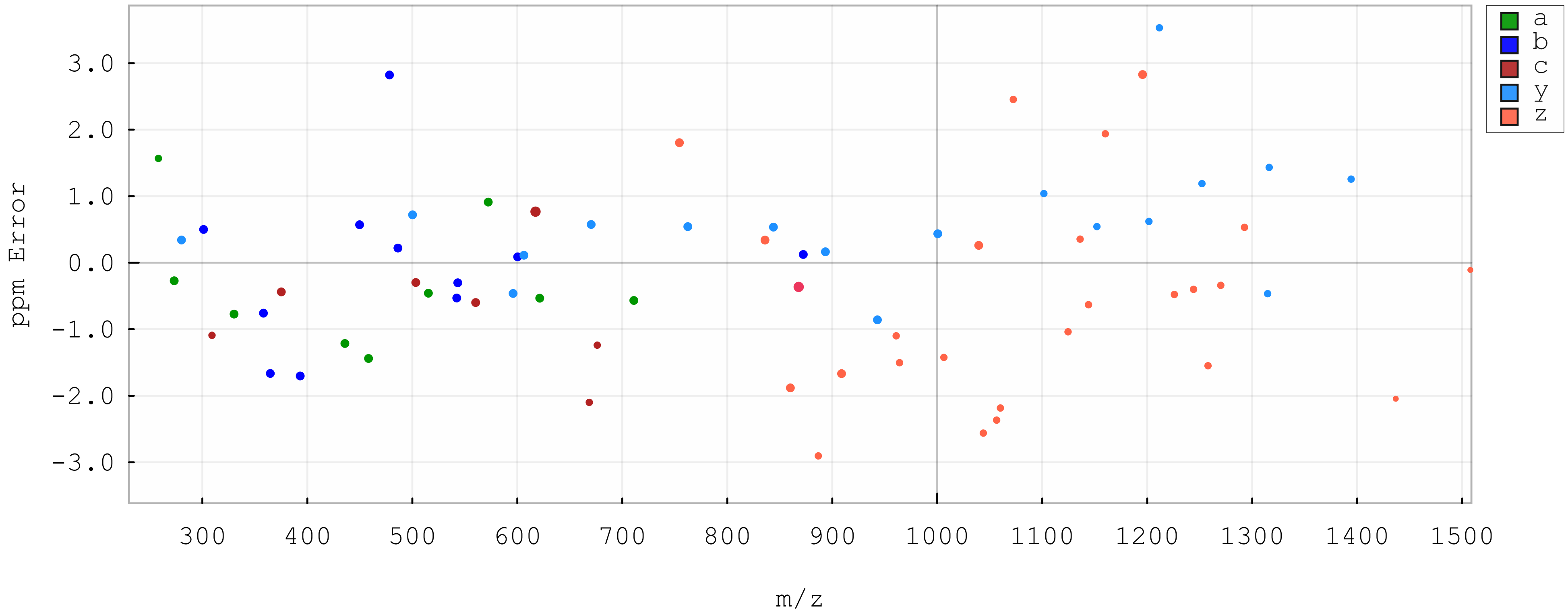

Primary Fragment Plots

Proteoform:

Index	Protein	SC [%]	SVP [%]	IC [%]	MS Score [a.u.]	Var. Mods	Mono Mass [Da]	Chemical Formula
1	H4Kac	77.2	35.3	26.6	20.6	Ace: K8	11271.3518	C496H839N163O135S

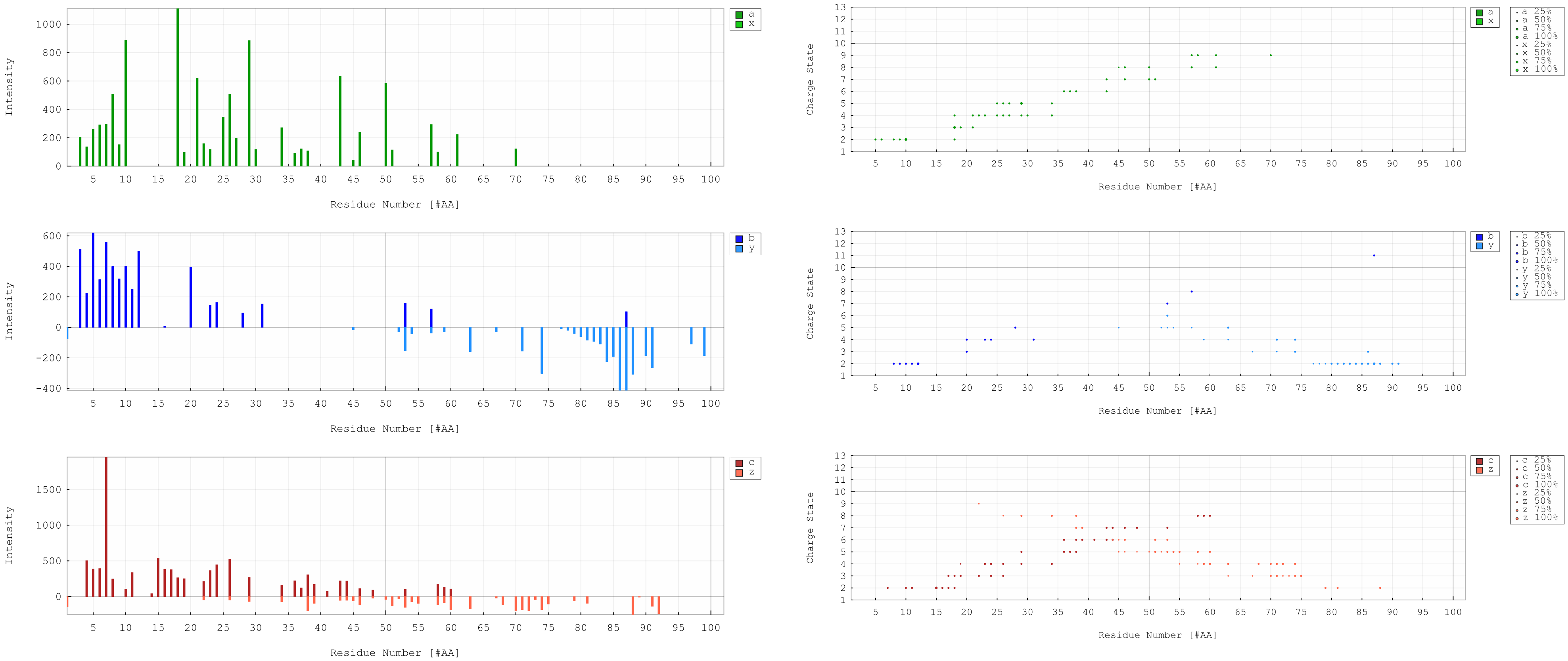

Proteoform:

Index	Protein	SC [%]	SVP [%]	IC [%]	MS Score [a.u.]	Var. Mods	Mono Mass [Da]	Chemical Formula
2	H4Kac	77.2	35.3	25.6	19.8	Ace: K12	11271.3518	C496H839N163O135S

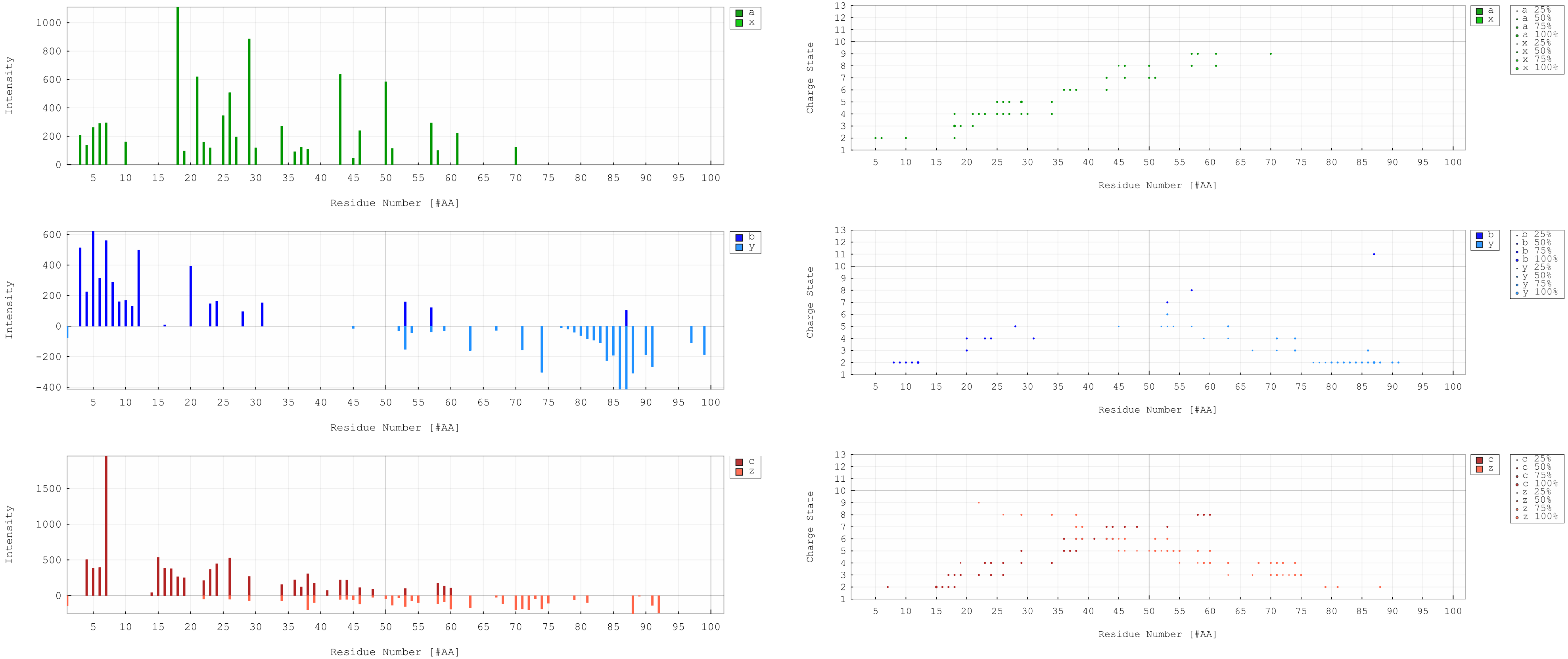

Proteoform:

Index	Protein	SC [%]	SVP [%]	IC [%]	MS Score [a.u.]	Var. Mods	Mono Mass [Da]	Chemical Formula
3	H4Kac	76.2	16.7	25.2	19.2	Ace: K16	11271.3518	C496H839N163O135S

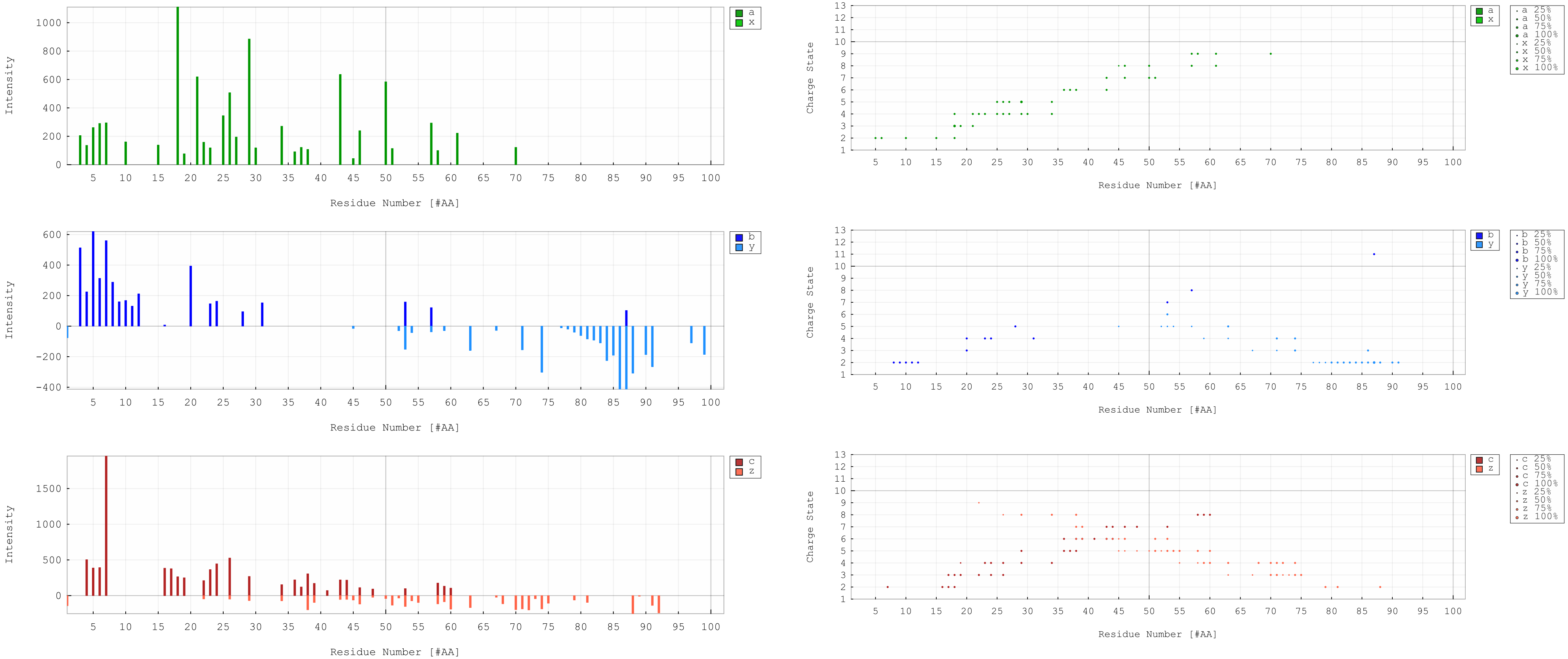

Proteoform:

Index	Protein	SC [%]	SVP [%]	IC [%]	MS Score [a.u.]	Var. Mods	Mono Mass [Da]	Chemical Formula
4	H4Kac	74.3	8.8	25.0	18.6	Ace: K5	11271.3518	C496H839N163O135S

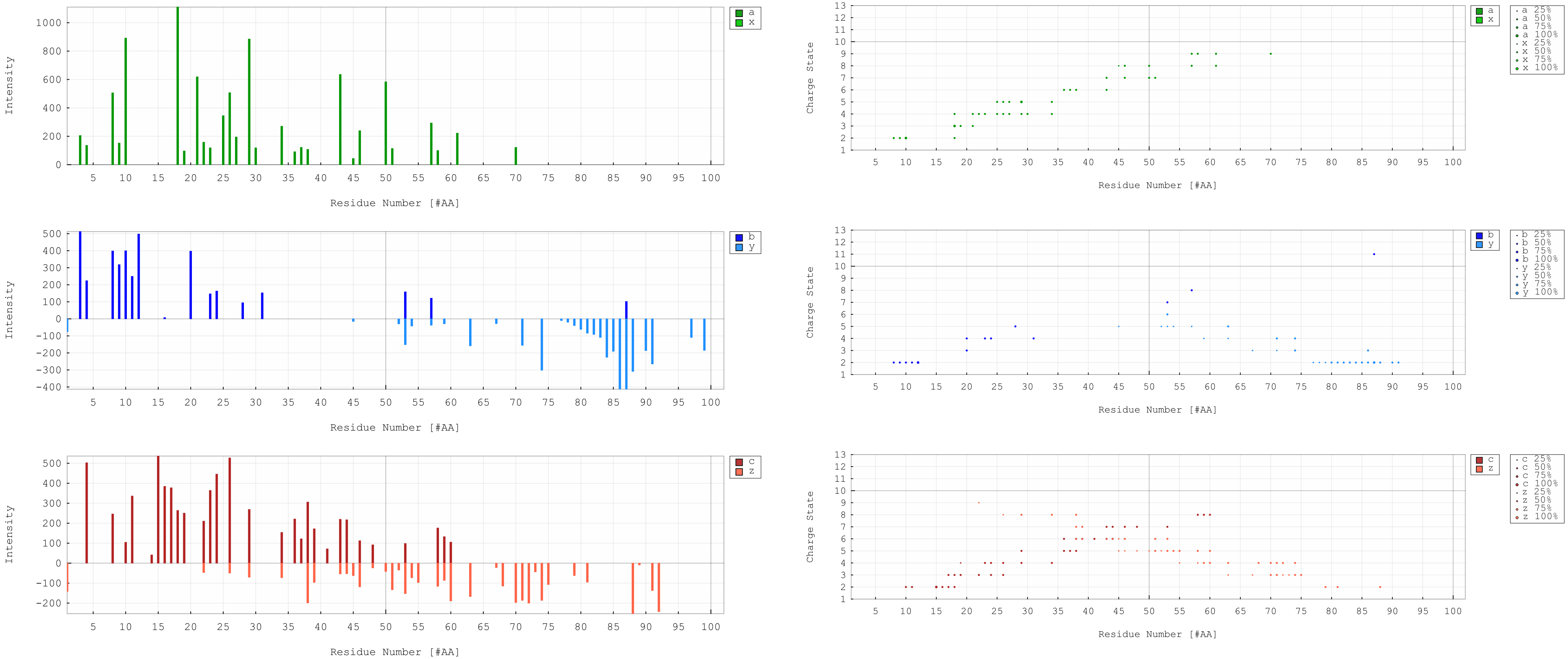

Proteoform:	Index	Protein	SC [%]	SVP [%]	IC [%]	MS Score [a.u.]	Var. Mods	Mono Mass [Da]	Chemical Formula
	5	H4Kac	72.3	16.7	23.8	17.2	Ace: K20	11271.3518	C496H839N163O135S

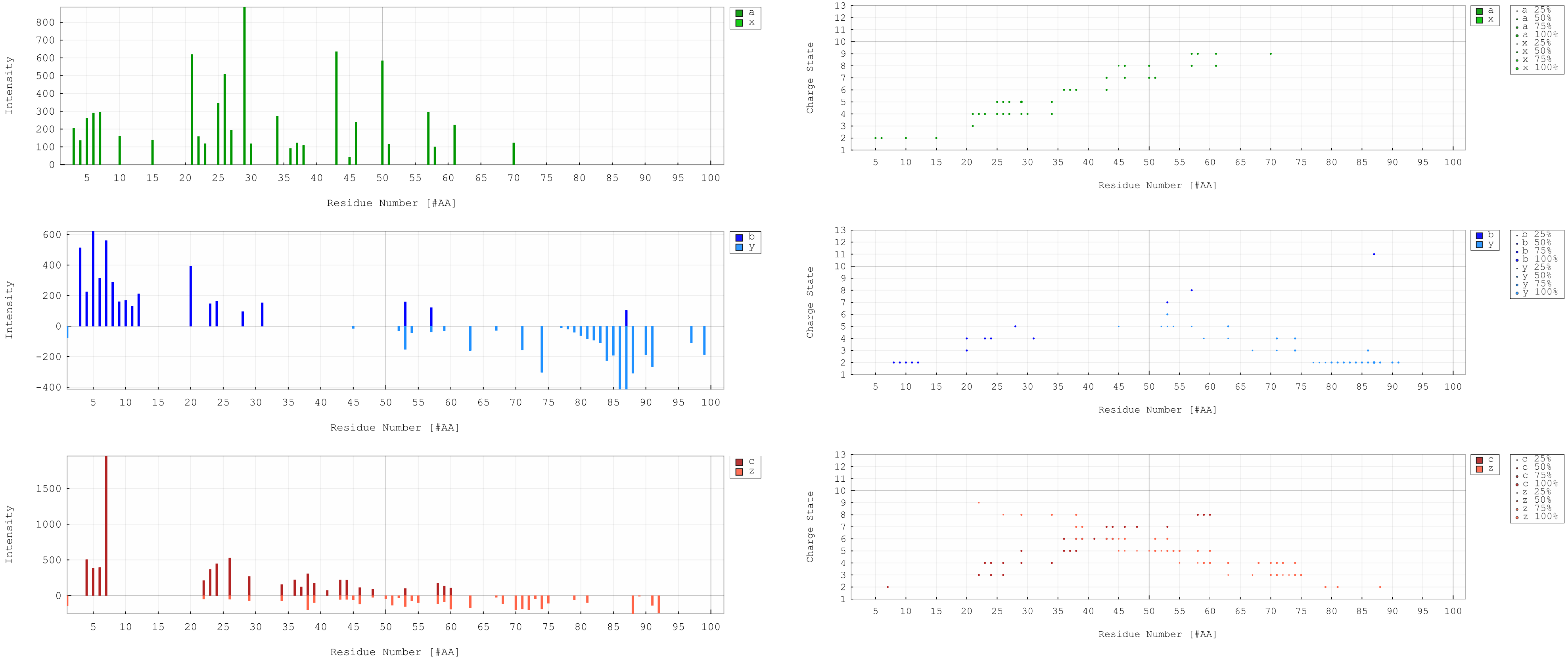

Proteoform:	Index	Protein	SC [%]	SVP [%]	IC [%]	MS Score [a.u.]	Var. Mods	Mono Mass [Da]	Chemical Formula
	6	H4Kac	63.4	16.7	19.7	12.5	Ace: K31	11271.3518	C496H839N163O135S

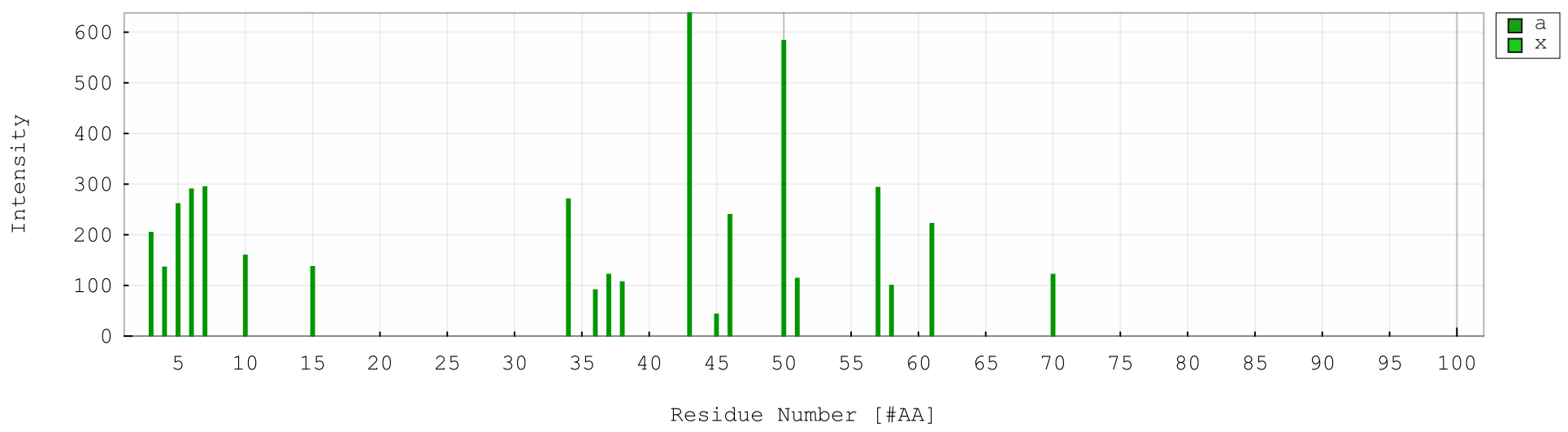

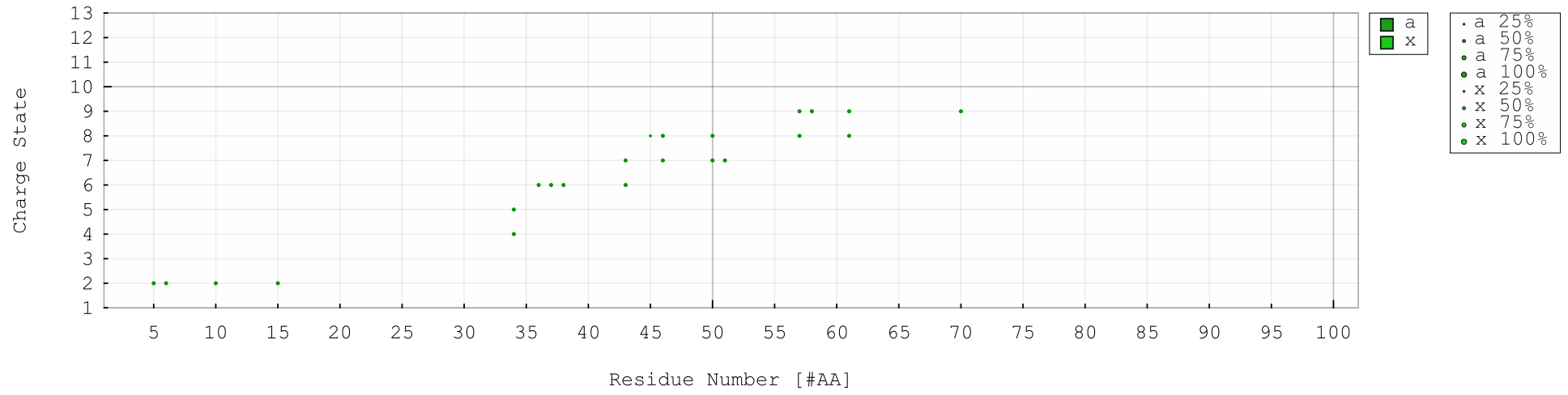

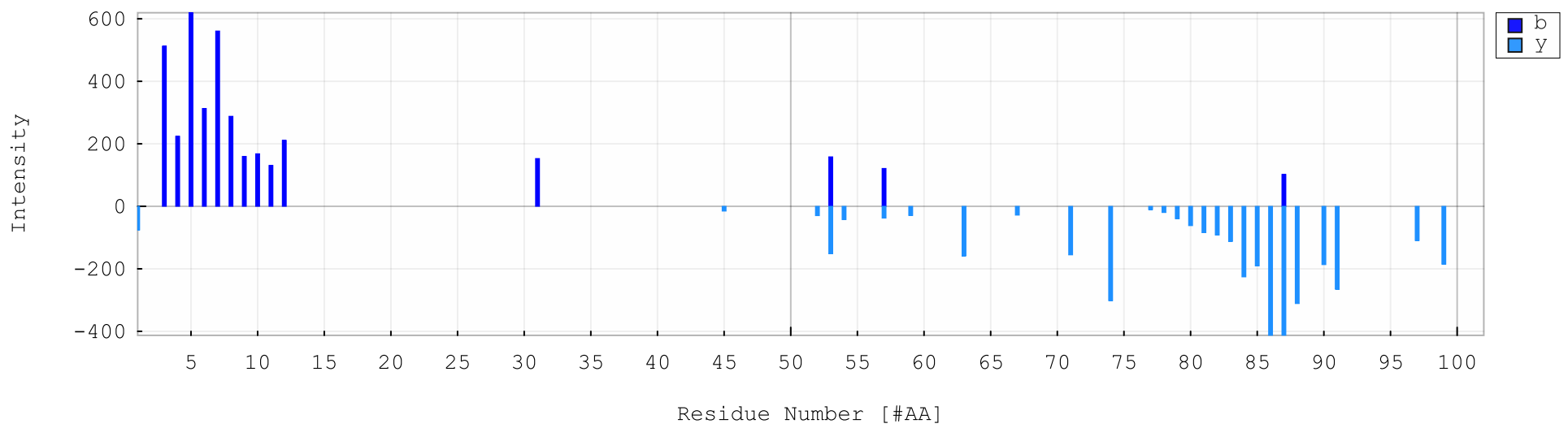

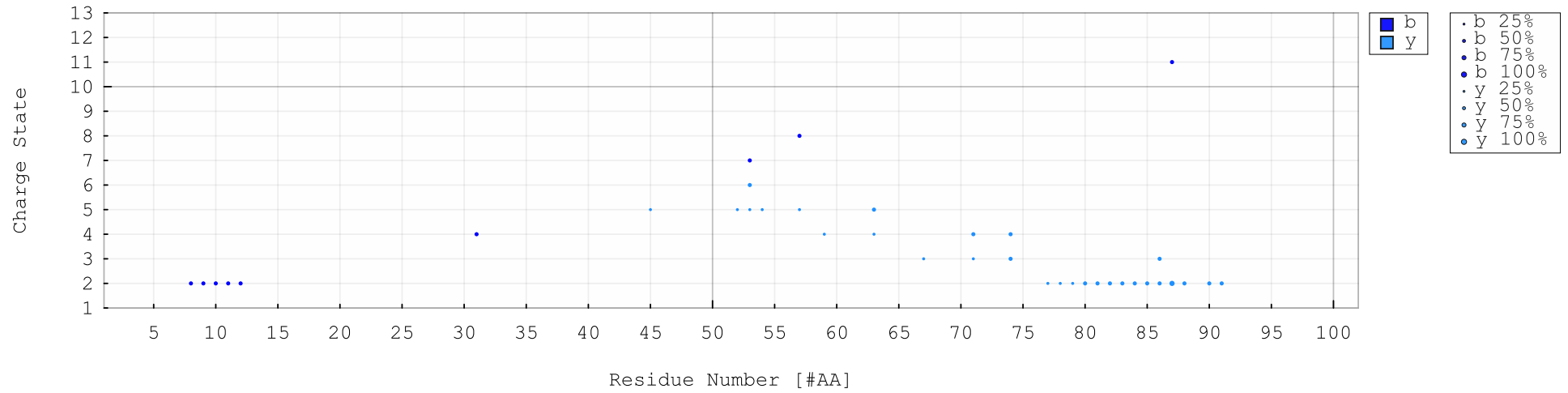

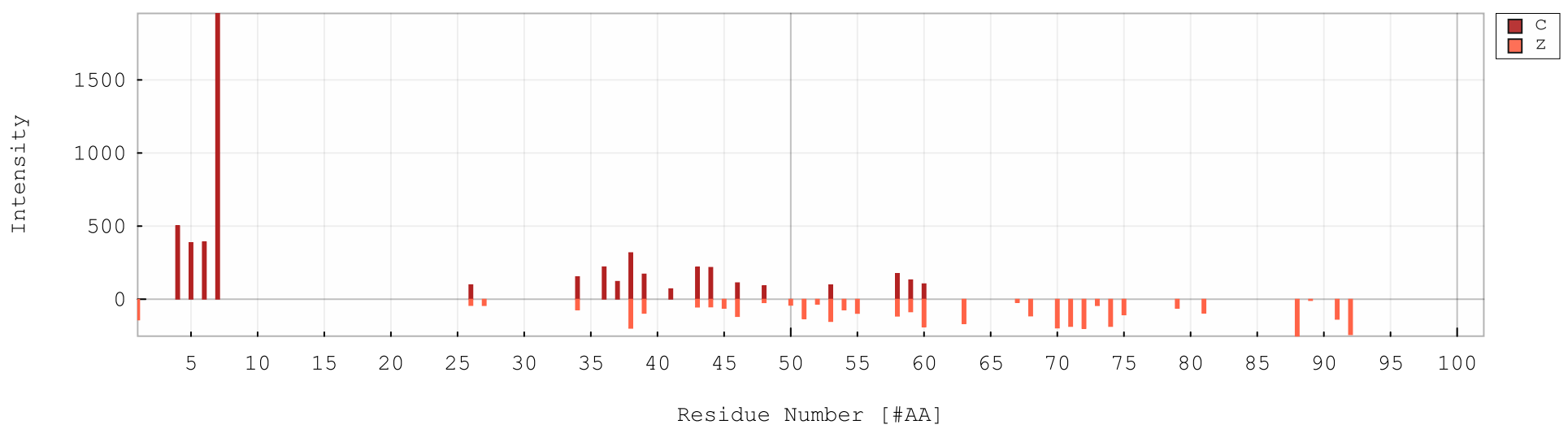

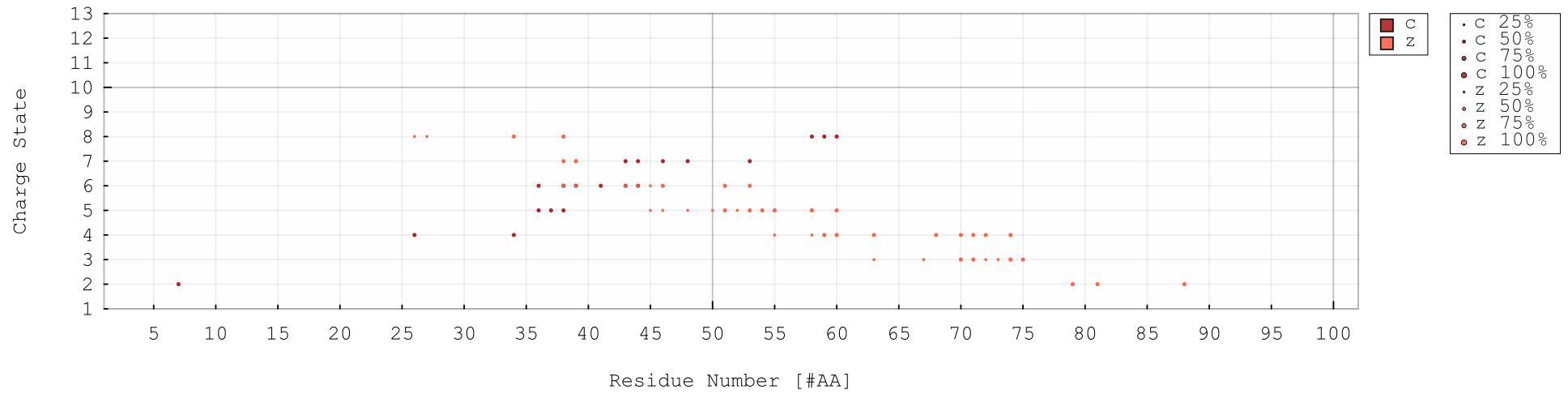

Proteoform:

Index	Protein	SC [%]	SVP [%]	IC [%]	MS Score [a.u.]	Var. Mods	Mono Mass [Da]	Chemical Formula
7	H4Kac	58.4	16.7	16.9	9.9	Ace: K44	11271.3518	C496H839N163O135S

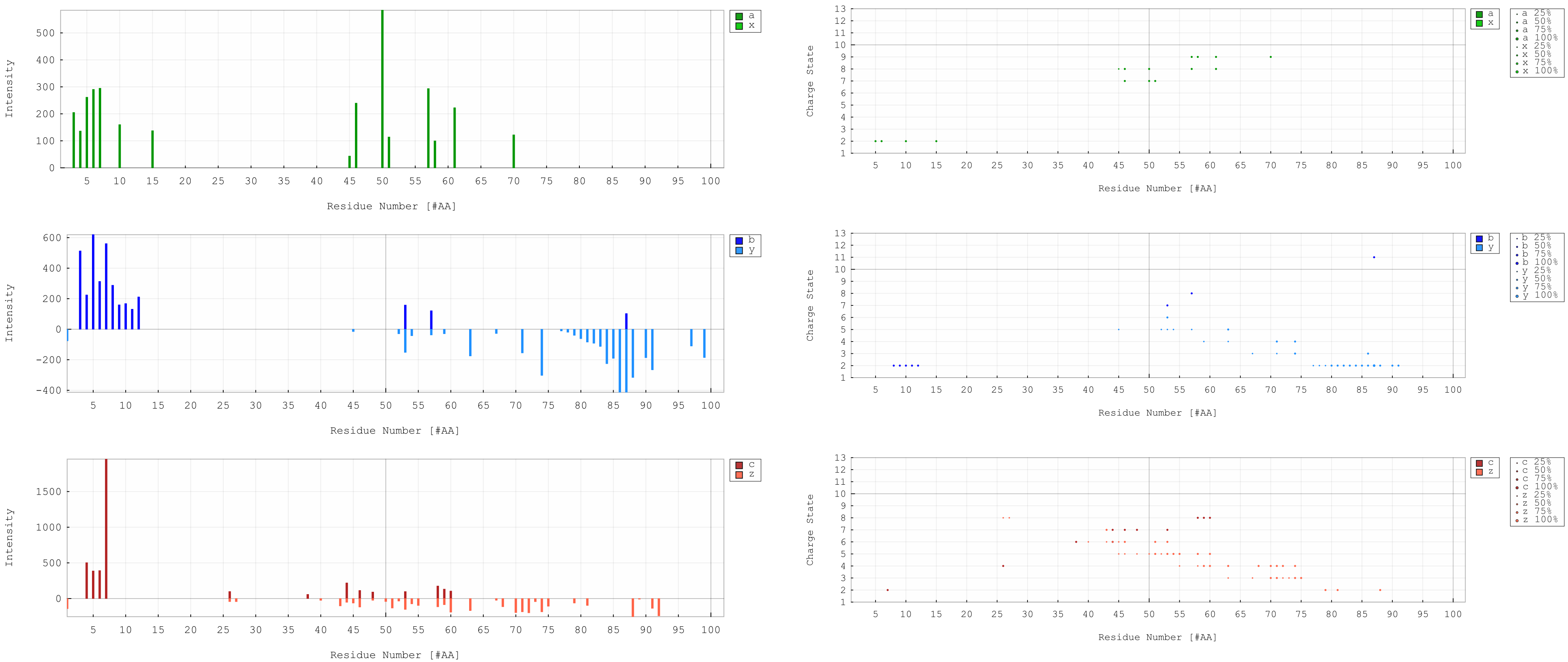

Proteoform:

Index	Protein	SC [%]	SVP [%]	IC [%]	MS Score [a.u.]	Var. Mods	Mono Mass [Da]	Chemical Formula
8	H4Kac	52.5	16.7	13.4	7.1	Ace: K59	11271.3518	C496H839N163O135S

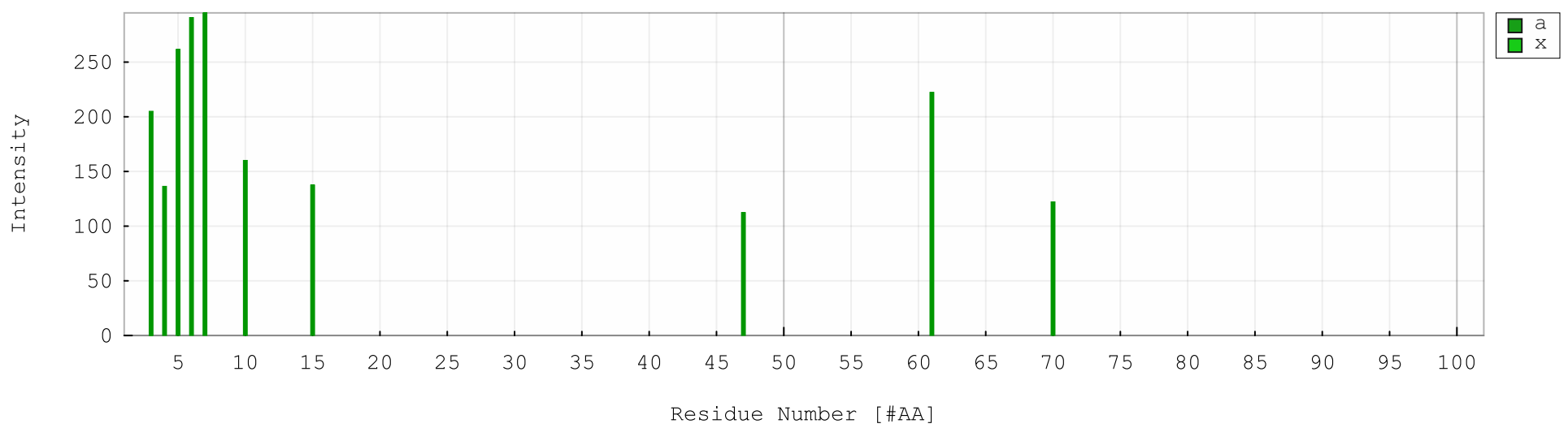

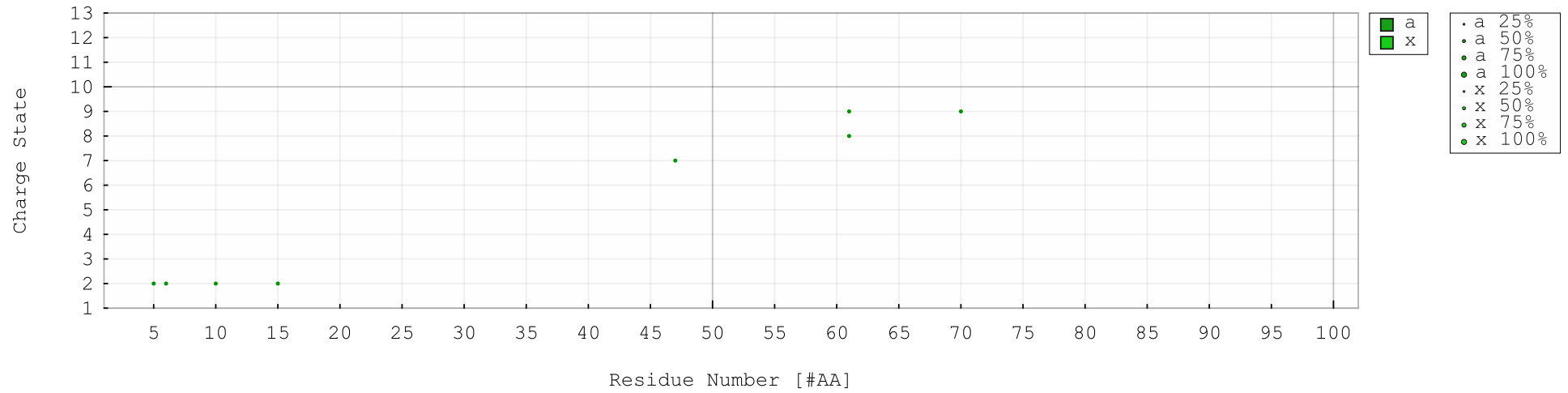

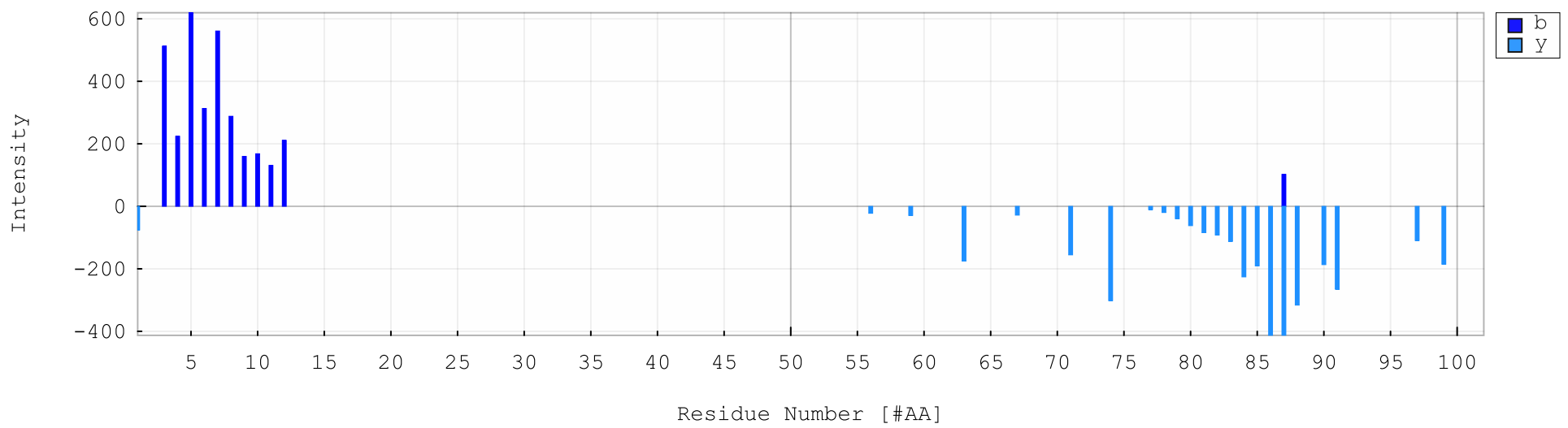

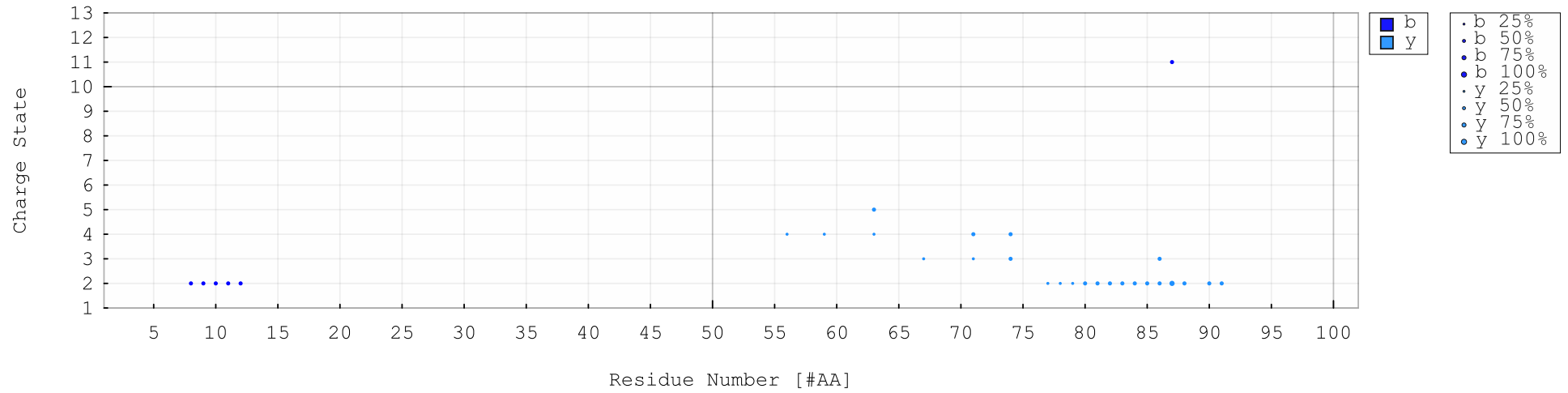

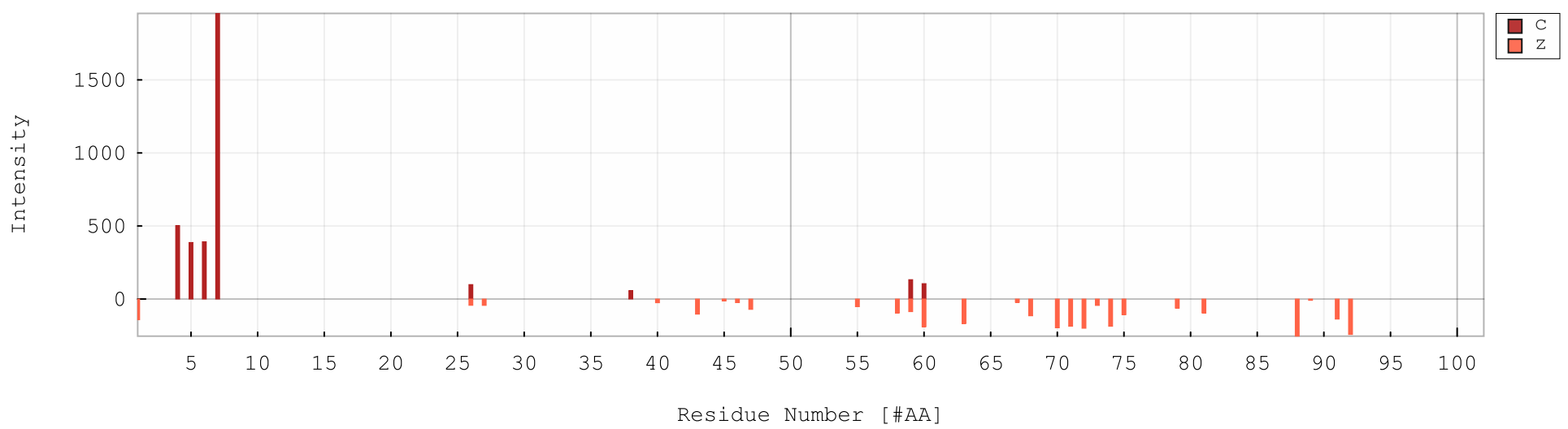

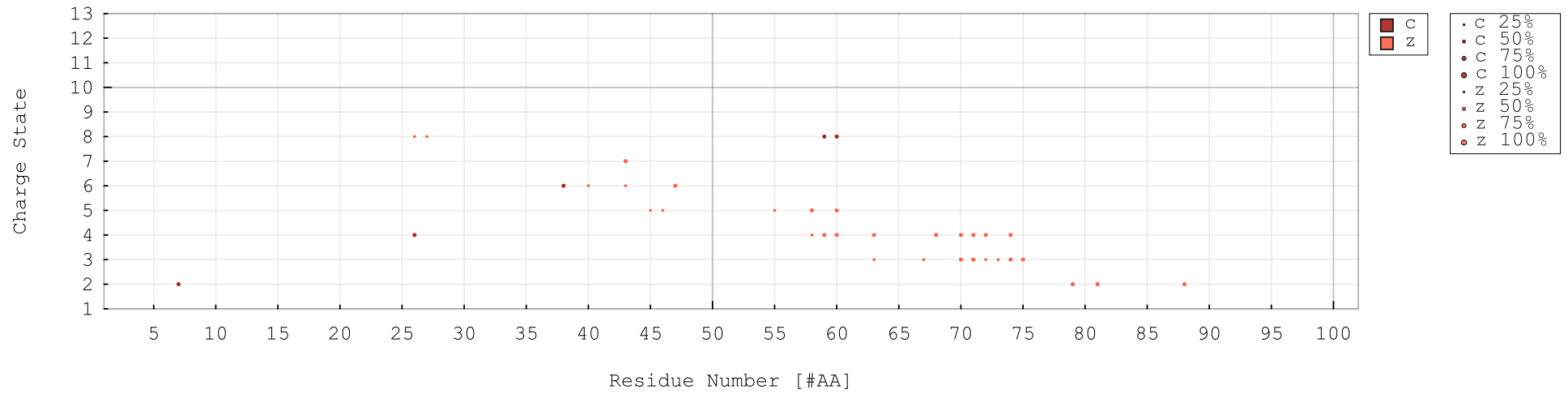

Proteoform:

Index	Protein	SC [%]	SVP [%]	IC [%]	MS Score [a.u.]	Var. Mods	Mono Mass [Da]	Chemical Formula
9	H4Kac	59.4	16.7	9.4	5.6	None	11229.3412	C494H837N163O134S

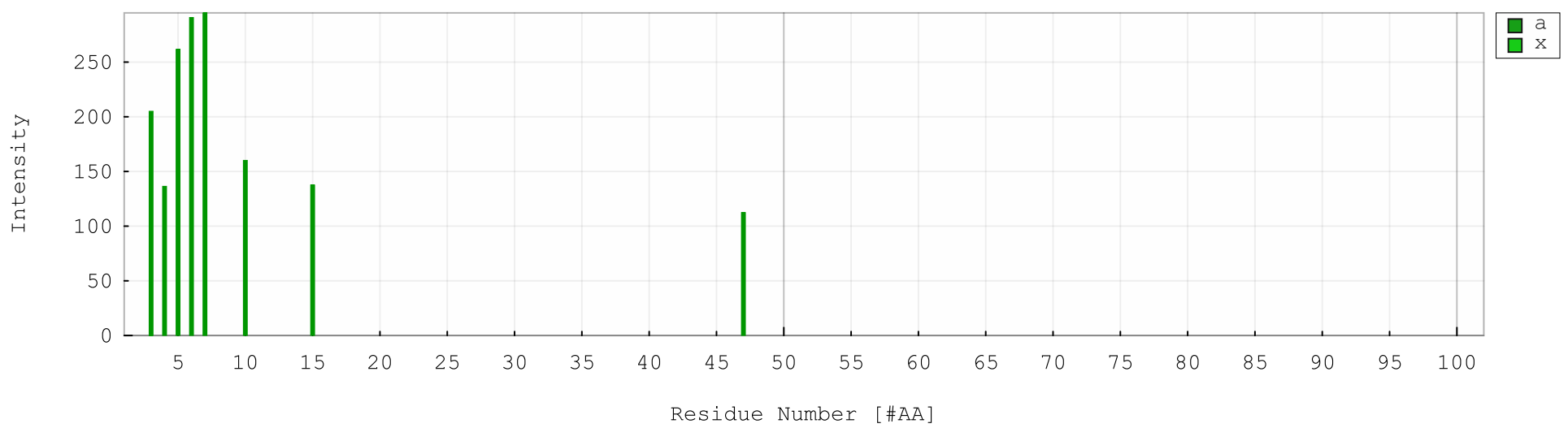

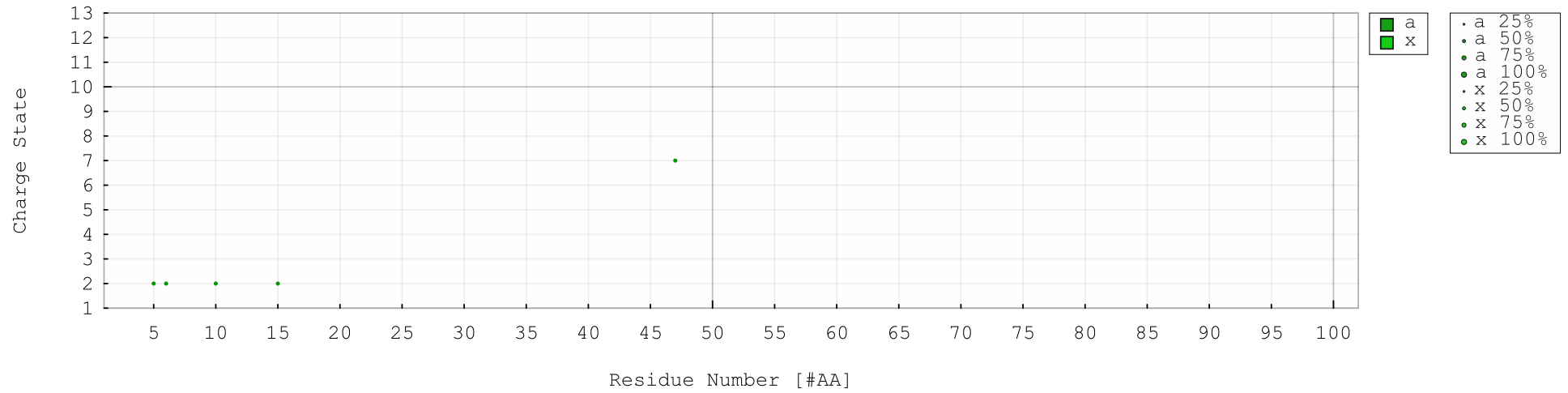

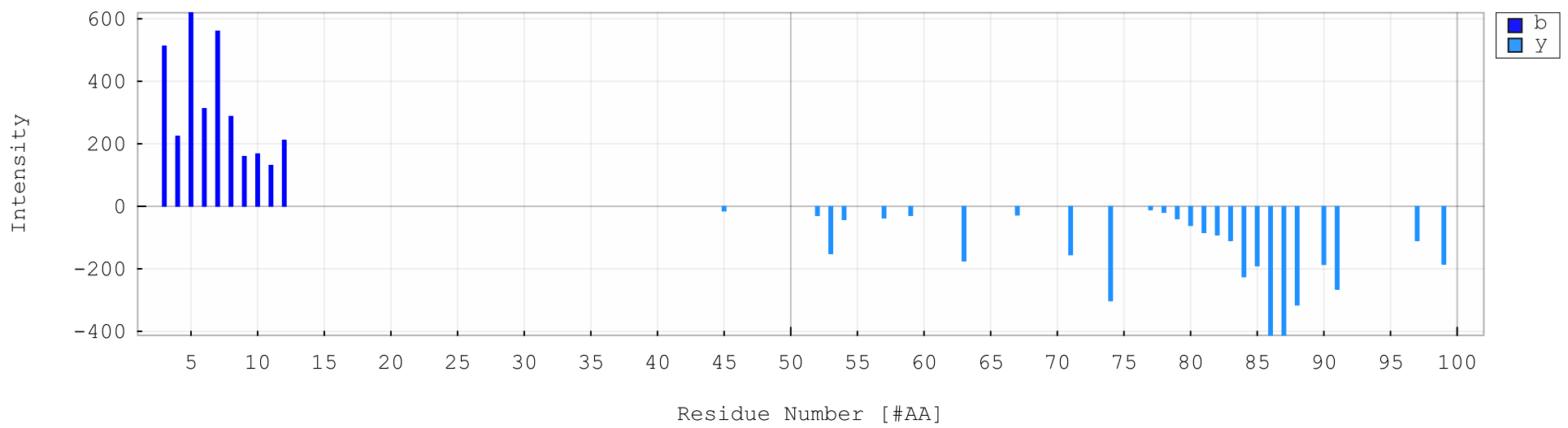

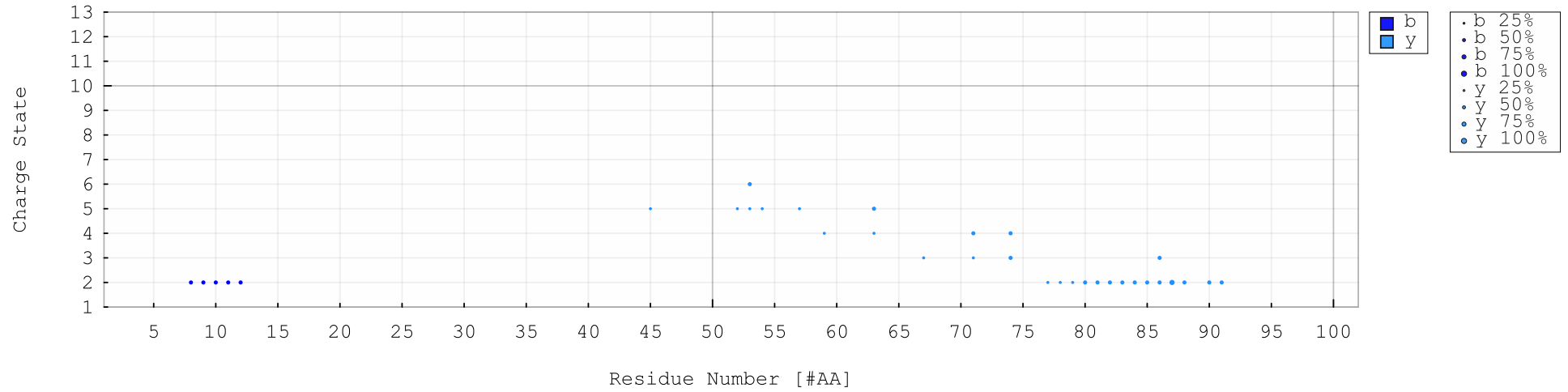

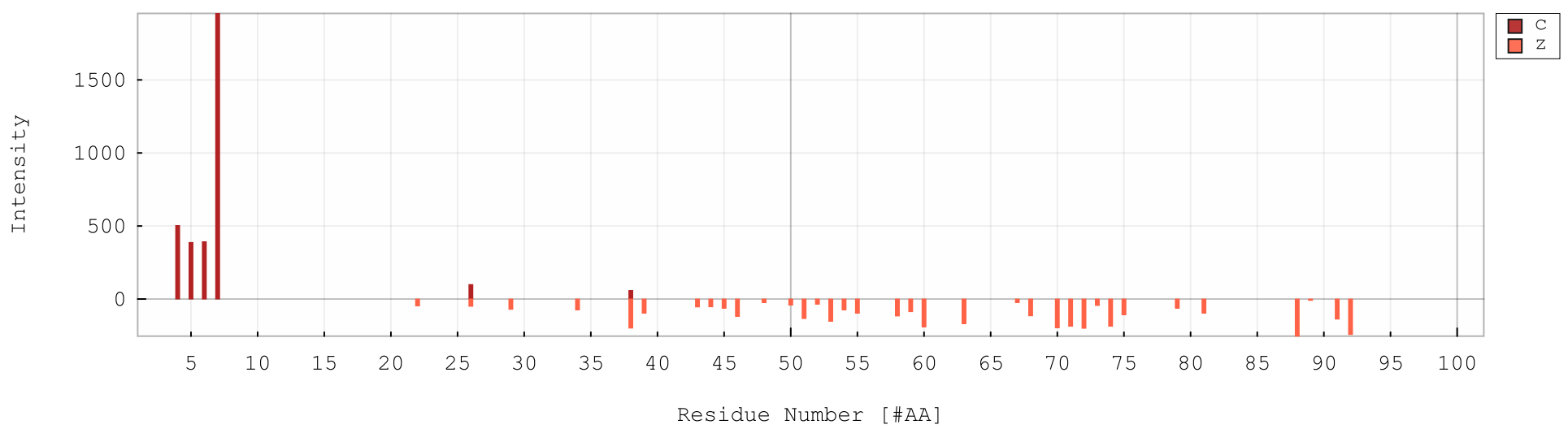

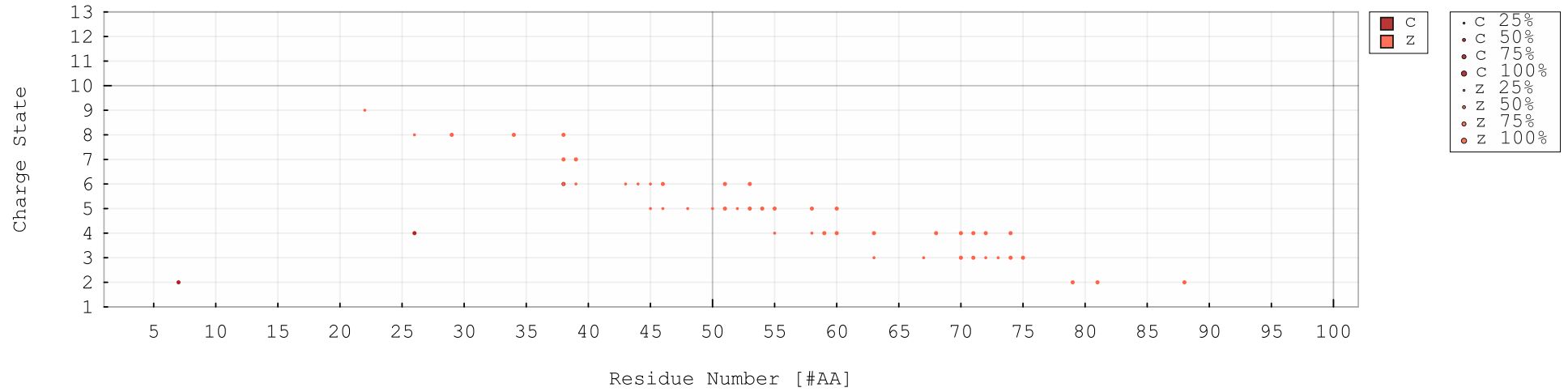

Top-Down Result OmniScape 2025
Sequence Confirmation
250428 H4-PCAF100-30min z13ac1 MS2 CA-ECD_001-686_calibrated2

Proteoform:

Index	Protein	SC [%]	SVP [%]	IC [%]	MS Score [a.u.]	Var. Mods	Mono Mass [Da]	Chemical Formula
10	H4Kac	44.6	16.7	11.2	5.0	Ace: K77	11271.3518	C496H839N163O135S

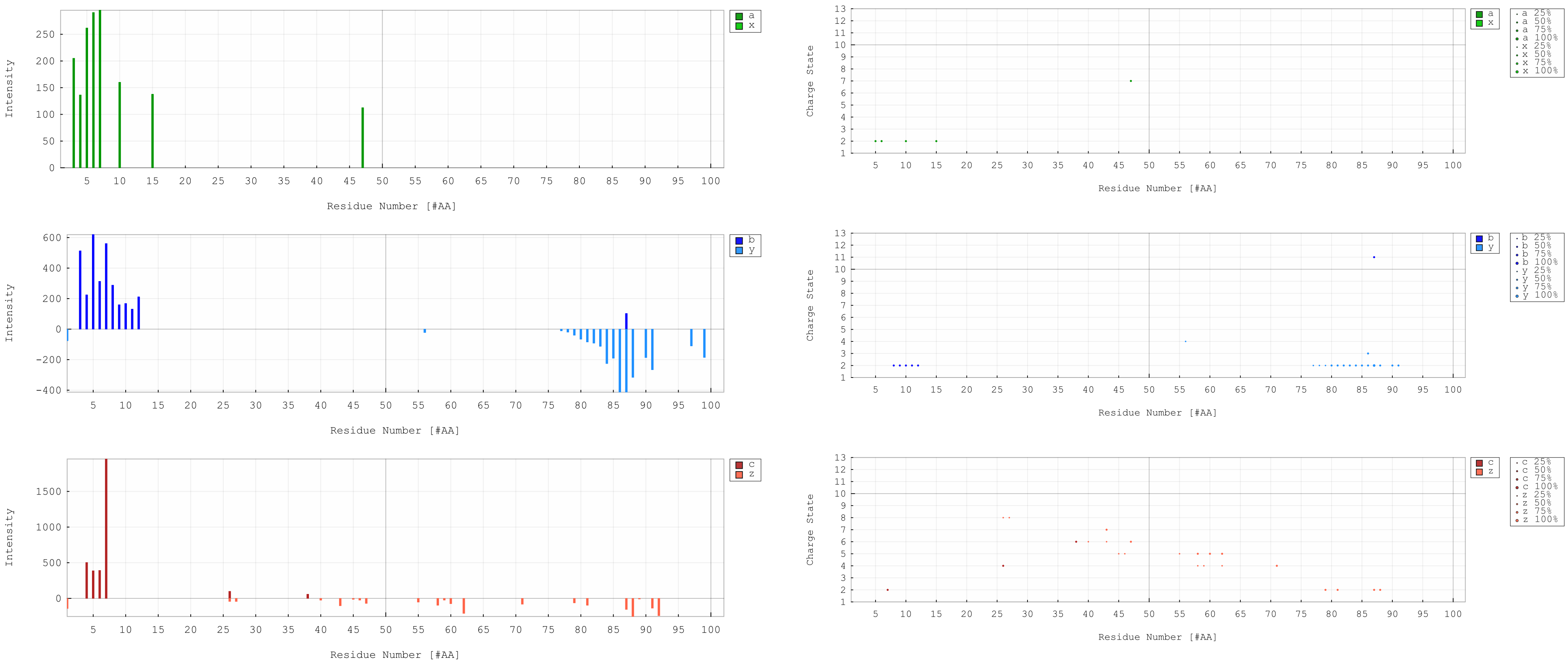

Supplemental Data S5

H4 - PCAF, 30 min

**Report**	2 of 8
**Experiment**	MS2
**Histone/sample**	H4
**Enzyme**	PCAF
**Reaction/source**	30 min
**Precursor**	H4Kac1
**Charge state**	13+
**Fragmentation / workflow**	ECD
**Original file**	MS2_H4Kac1_13+_ECD.pdf

Project Information

Project Directory: C:/Users/LAB-ADMIN
Processing date: Tue Apr 28 14:42:32 2026

Sample Info & Protocols

Name: Top-Down Protein Sequencing ExD

Dataset

Imported spectrum file name:	250428 H4-PCAF100-30min z13ac1 MS2 ECD_001-573_calibrated.txt
Dataset name:	Information is only available from imported JPEAKS files
Date acquired:	Information is only available from imported JPEAKS files
Date imported:	Information is only available from imported JPEAKS files
Instrument Name:	Information is only available from imported JPEAKS files
Instrument Serial Number:	Information is only available from imported JPEAKS files

Workflow Result Info

Analysis: 250428 H4-PCAF100-30min z13ac1 MS2 ECD_001-573_calibrated2
Location: C:/Users/LAB-ADMIN
Method: -

Method Information

Name: Unnamed

Spectral Parameters

Mass Error [ppm]: 6
Intensity Deviation [au]: 0.15
Min/Max Charges: 1-13
Experiment type: ExD

a ions	b ions	c ions	x ions	y ions	z ions	satellite	internal	precursor
a	b	c		y	z+1			M
a+1		c+1			z+2			M+-2H

Noise threshold calculation: Simple moving average noise threshold (SMA)
Window size [datapoints]: 600
Noise curve offset: 4.2683644
SVP parameters: terminal gap: 2, internal gap: 1

Proteoforms

(top 10)

Index	Parent Sequence	Chain	Var. Mods	Mono Mass [Da]	Chemical Composition
1	H4Kac	H4KacK8[Ace]	Ace: K8	11271.3518	C496H839N163O135S
2	H4Kac	H4KacK12[Ace]	Ace: K12	11271.3518	C496H839N163O135S
3	H4Kac	H4KacK16[Ace]	Ace: K16	11271.3518	C496H839N163O135S
4	H4Kac	H4KacK5[Ace]	Ace: K5	11271.3518	C496H839N163O135S
5	H4Kac	H4KacK20[Ace]	Ace: K20	11271.3518	C496H839N163O135S
6	H4Kac	H4KacK31[Ace]	Ace: K31	11271.3518	C496H839N163O135S
7	H4Kac	H4KacK44[Ace]	Ace: K44	11271.3518	C496H839N163O135S
8	H4Kac	H4KacK59[Ace]	Ace: K59	11271.3518	C496H839N163O135S
9	H4Kac	H4KacK77[Ace]	Ace: K77	11271.3518	C496H839N163O135S
10	H4Kac	H4KacK79[Ace]	Ace: K79	11271.3518	C496H839N163O135S

Results

Proteoforms Table (top 10)

Index	Protein	SC [%]	SVP [%]	IC [%]	MS Score [a.u.]	Var. Mods	Mono Mass [Da]	Chemical Formula
1	H4Kac	91.1	97.1	69.1	63.0	Ace: K8	11271.3518	C496H839N163O135S
2	H4Kac	91.1	97.1	68.1	62.0	Ace: K12	11271.3518	C496H839N163O135S
3	H4Kac	91.1	97.1	65.7	59.9	Ace: K16	11271.3518	C496H839N163O135S
4	H4Kac	88.1	38.2	67.8	59.7	Ace: K5	11271.3518	C496H839N163O135S
5	H4Kac	91.1	97.1	63.8	58.1	Ace: K20	11271.3518	C496H839N163O135S
6	H4Kac	89.1	97.1	57.5	51.3	Ace: K31	11271.3518	C496H839N163O135S
7	H4Kac	86.1	62.7	50.1	43.2	Ace: K44	11271.3518	C496H839N163O135S
8	H4Kac	82.2	62.7	42.0	34.5	Ace: K59	11271.3518	C496H839N163O135S
9	H4Kac	75.2	52.9	33.7	25.3	Ace: K77	11271.3518	C496H839N163O135S
10	H4Kac	73.3	51.0	33.2	24.3	Ace: K79	11271.3518	C496H839N163O135S

Sequence

Name: H4Kac

```
         10         20         30         40         50         60
S G R G K G G K G L  G K G G A K R H R K  V L R D N I Q G I T  K P A I R R L A R R  G G V K R I S G L I  Y E E T R G V L K V
         70         80         90        100        110        120
F L E N V I R D A V  T Y T E H A K R K T  V T A M D V V Y A L  K R Q G R T L Y G F  G G
```

Modifications

Modification	Position	Type
Acetyl	5	variable
Acetyl	8	variable
Acetyl	12	variable
Acetyl	16	variable
Acetyl	20	variable
Acetyl	31	variable
Acetyl	44	variable
Acetyl	59	variable
Acetyl	77	variable
Acetyl	79	variable
Acetyl	91	variable

Sequence Map

Proteoform:

Index	Protein	SC [%]	SVP [%]	IC [%]	MS Score [a.u.]	Var. Mods	Mono Mass [Da]	Chemical Formula
1	H4Kac	91.1	97.1	69.1	63.0	Ace: K8	11271.3518	C496H839N163O135S

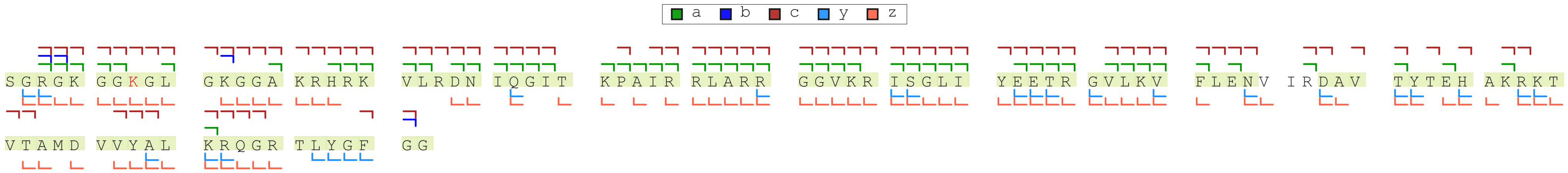

Proteoform:

Index	Protein	SC [%]	SVP [%]	IC [%]	MS Score [a.u.]	Var. Mods	Mono Mass [Da]	Chemical Formula
2	H4Kac	91.1	97.1	68.1	62.0	Ace: K12	11271.3518	C496H839N163O135S

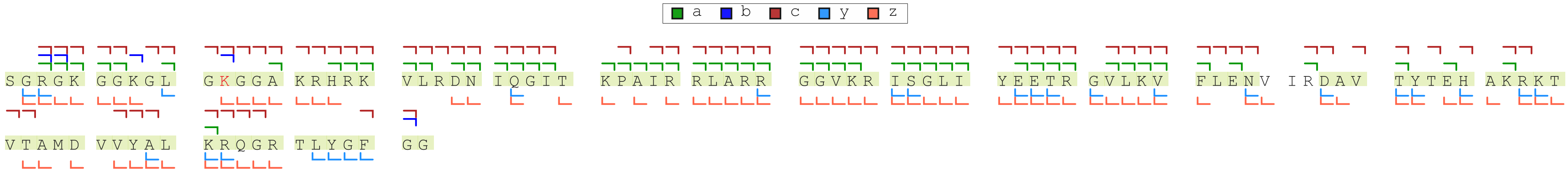

Proteoform:

Index	Protein	SC [%]	SVP [%]	IC [%]	MS Score [a.u.]	Var. Mods	Mono Mass [Da]	Chemical Formula
3	H4Kac	91.1	97.1	65.7	59.9	Ace: K16	11271.3518	C496H839N163O135S

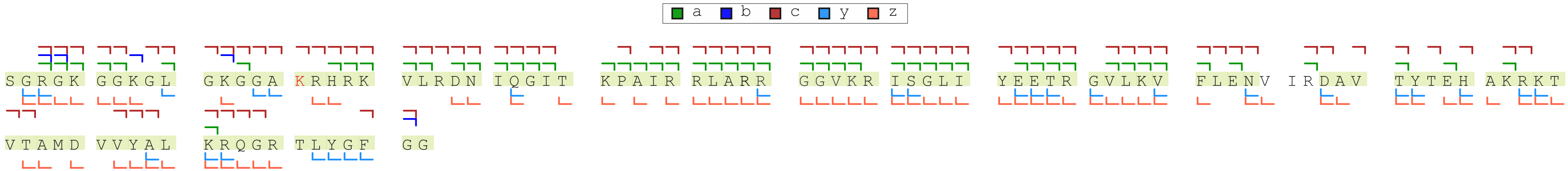

Proteoform:

Index	Protein	SC [%]	SVP [%]	IC [%]	MS Score [a.u.]	Var. Mods	Mono Mass [Da]	Chemical Formula
4	H4Kac	88.1	38.2	67.8	59.7	Ace: K5	11271.3518	C496H839N163O135S

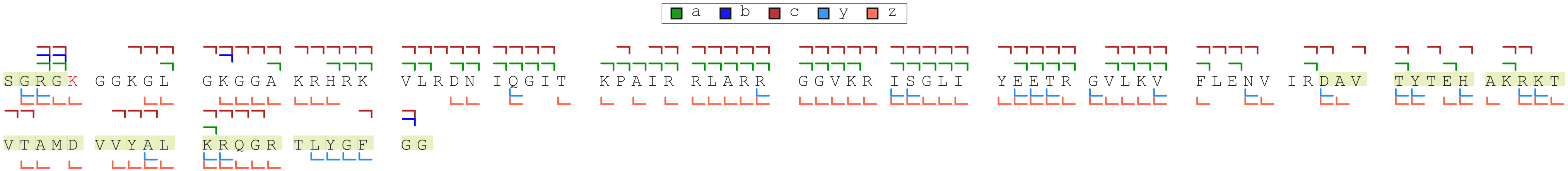

Proteoform:	Index	Protein	SC [%]	SVP [%]	IC [%]	MS Score [a.u.]	Var. Mods	Mono Mass [Da]	Chemical Formula
	5	H4Kac	91.1	97.1	63.8	58.1	Ace: K20	11271.3518	C496H839N163O135S

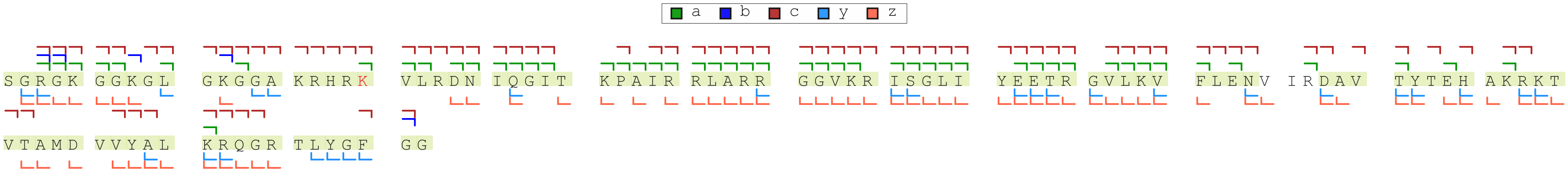

Proteoform:

Index	Protein	SC [%]	SVP [%]	IC [%]	MS Score [a.u.]	Var. Mods	Mono Mass [Da]	Chemical Formula
6	H4Kac	89.1	97.1	57.5	51.3	Ace: K31	11271.3518	C496H839N163O135S

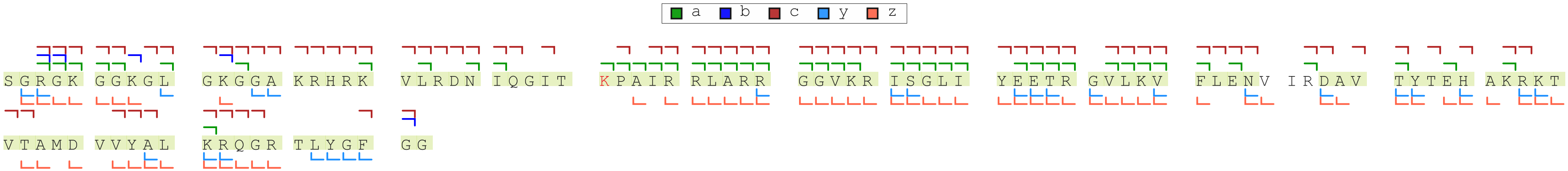

Top-Down Result OmniScape 2025
Sequence Confirmation
250428 H4-PCAF100-30min z13ac1 MS2 ECD_001-573_calibrated2

Proteoform:	Index	Protein	SC [%]	SVP [%]	IC [%]	MS Score [a.u.]	Var. Mods	Mono Mass [Da]	Chemical Formula
	7	H4Kac	86.1	62.7	50.1	43.2	Ace: K44	11271.3518	C496H839N163O135S

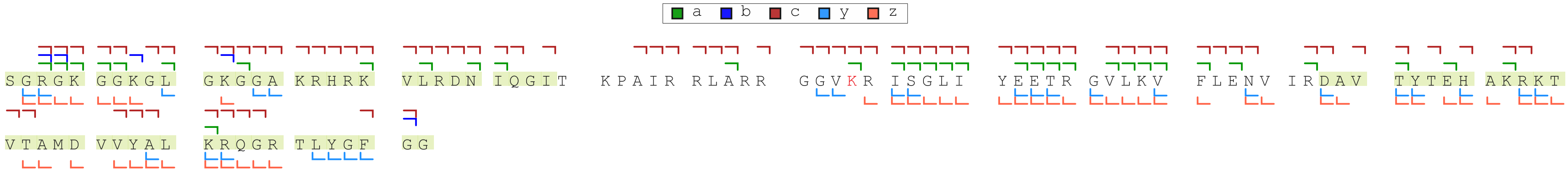

Proteoform:

Index	Protein	SC [%]	SVP [%]	IC [%]	MS Score [a.u.]	Var. Mods	Mono Mass [Da]	Chemical Formula
8	H4Kac	82.2	62.7	42.0	34.5	Ace: K59	11271.3518	C496H839N163O135S

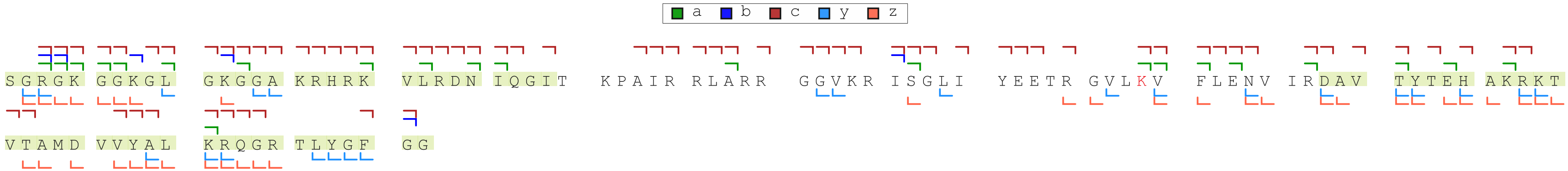

Proteoform:

Index	Protein	SC [%]	SVP [%]	IC [%]	MS Score [a.u.]	Var. Mods	Mono Mass [Da]	Chemical Formula
9	H4Kac	75.2	52.9	33.7	25.3	Ace: K77	11271.3518	C496H839N163O135S

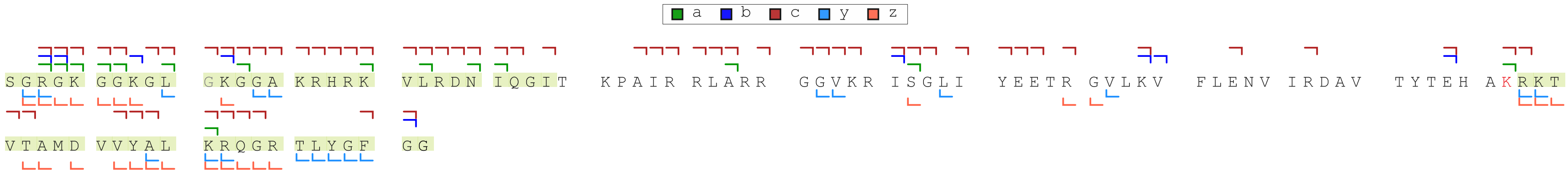

Proteoform:

Index	Protein	SC [%]	SVP [%]	IC [%]	MS Score [a.u.]	Var. Mods	Mono Mass [Da]	Chemical Formula
10	H4Kac	73.3	51.0	33.2	24.3	Ace: K79	11271.3518	C496H839N163O135S

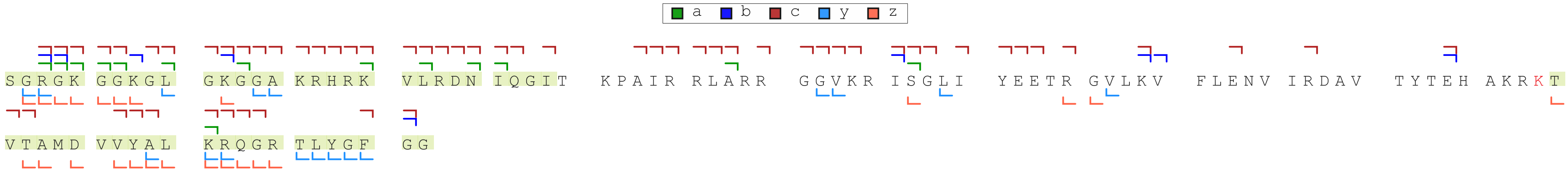

Error Plot

Proteoform:

Index	Protein	SC [%]	SVP [%]	IC [%]	MS Score [a.u.]	Var. Mods	Mono Mass [Da]	Chemical Formula
1	H4Kac	91.1	97.1	69.1	63.0	Ace: K8	11271.3518	C496H839N163O135S

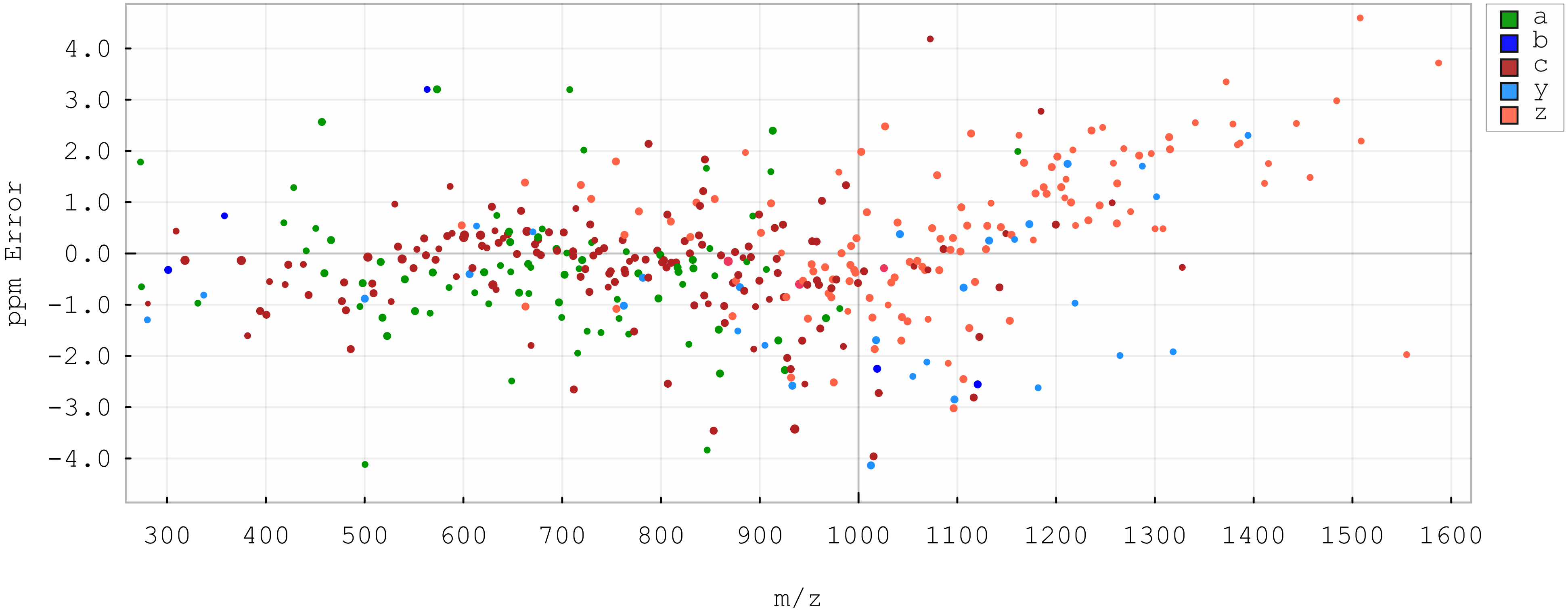

Proteoform:

Index	Protein	SC [%]	SVP [%]	IC [%]	MS Score [a.u.]	Var. Mods	Mono Mass [Da]	Chemical Formula
2	H4Kac	91.1	97.1	68.1	62.0	Ace: K12	11271.3518	C496H839N163O135S

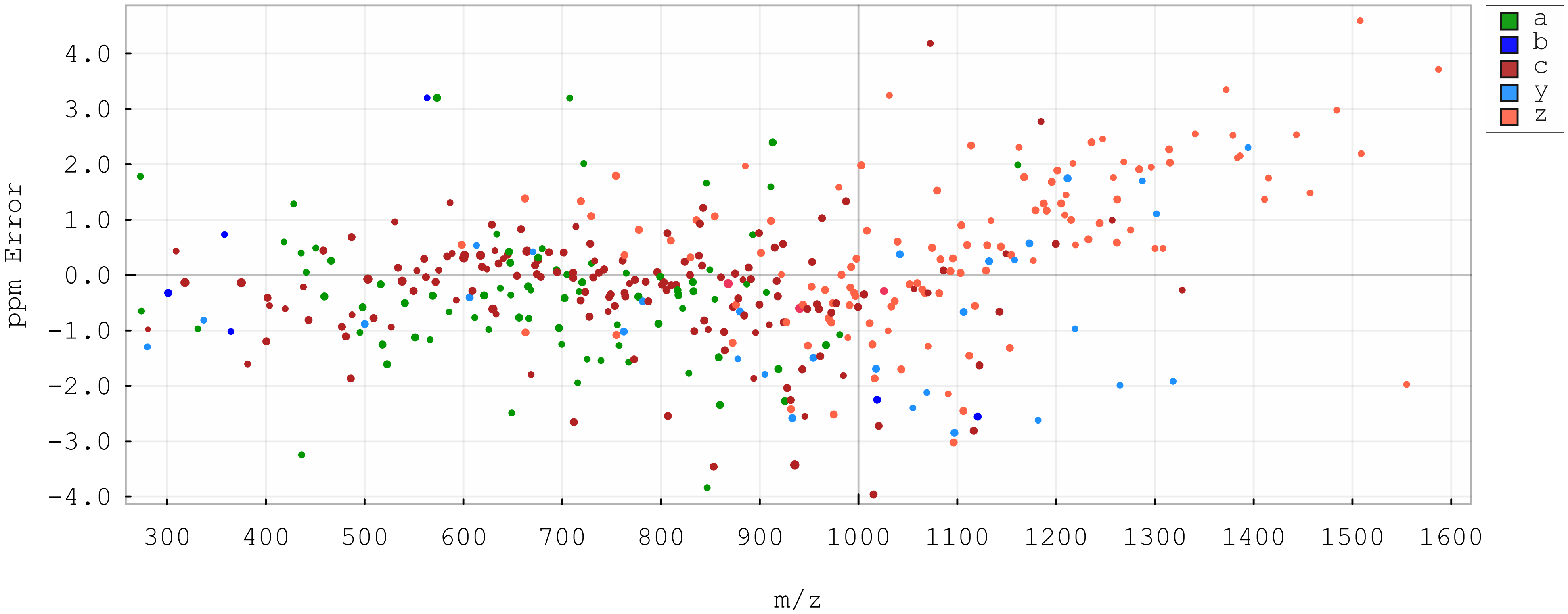

Proteoform:

Index	Protein	SC [%]	SVP [%]	IC [%]	MS Score [a.u.]	Var. Mods	Mono Mass [Da]	Chemical Formula
3	H4Kac	91.1	97.1	65.7	59.9	Ace: K16	11271.3518	C496H839N163O135S

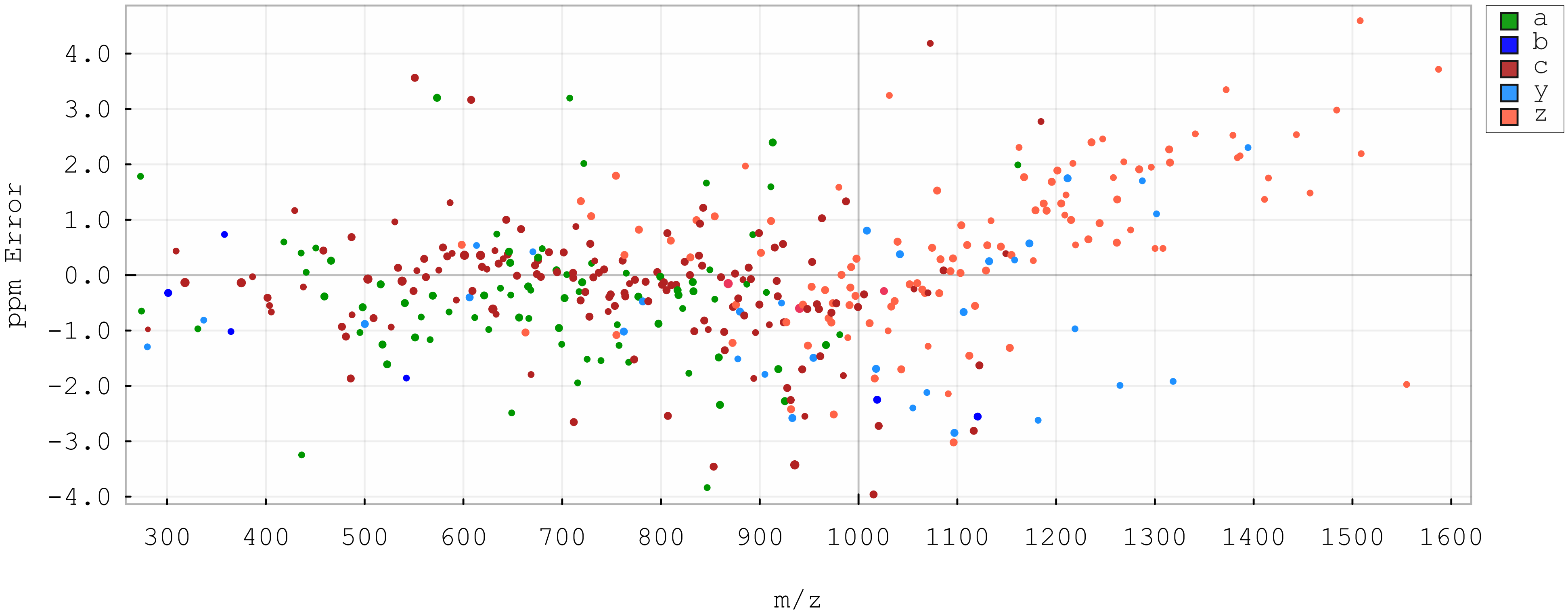

Proteoform:

Index	Protein	SC [%]	SVP [%]	IC [%]	MS Score [a.u.]	Var. Mods	Mono Mass [Da]	Chemical Formula
4	H4Kac	88.1	38.2	67.8	59.7	Ace: K5	11271.3518	C496H839N163O135S

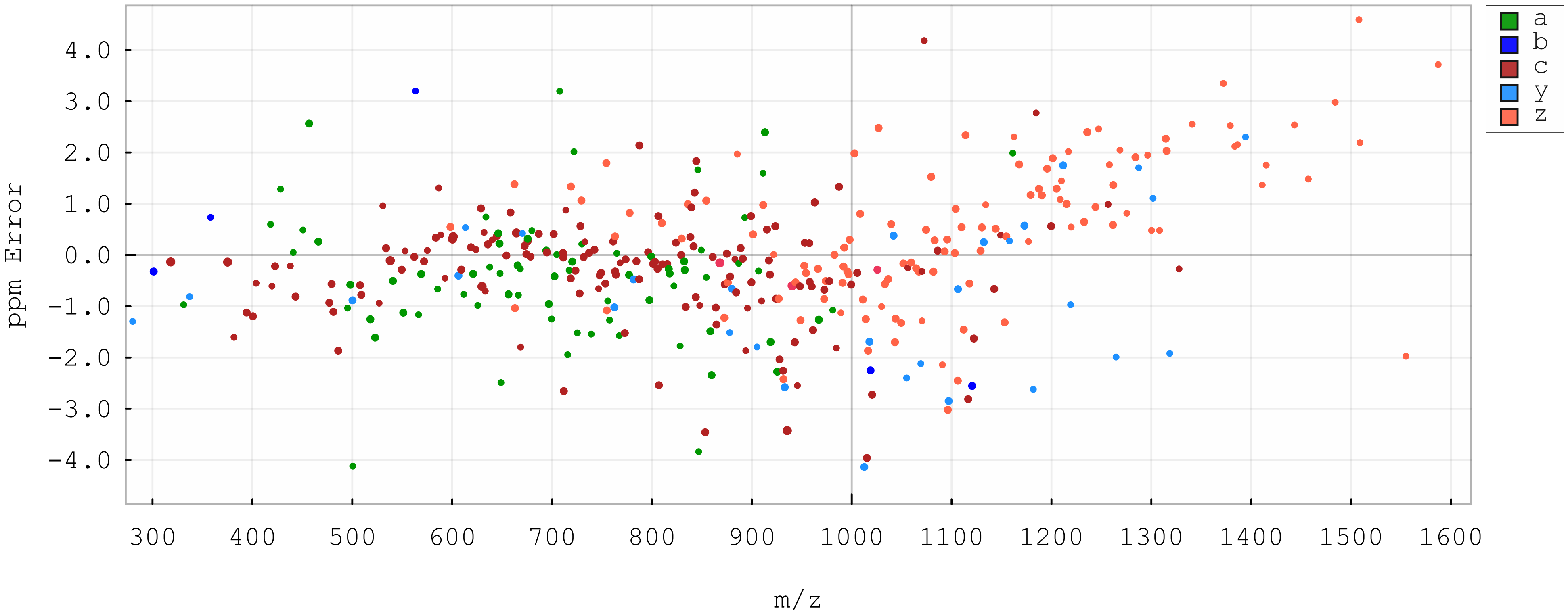

Proteoform:

Index	Protein	SC [%]	SVP [%]	IC [%]	MS Score [a.u.]	Var. Mods	Mono Mass [Da]	Chemical Formula
5	H4Kac	91.1	97.1	63.8	58.1	Ace: K20	11271.3518	C496H839N163O135S

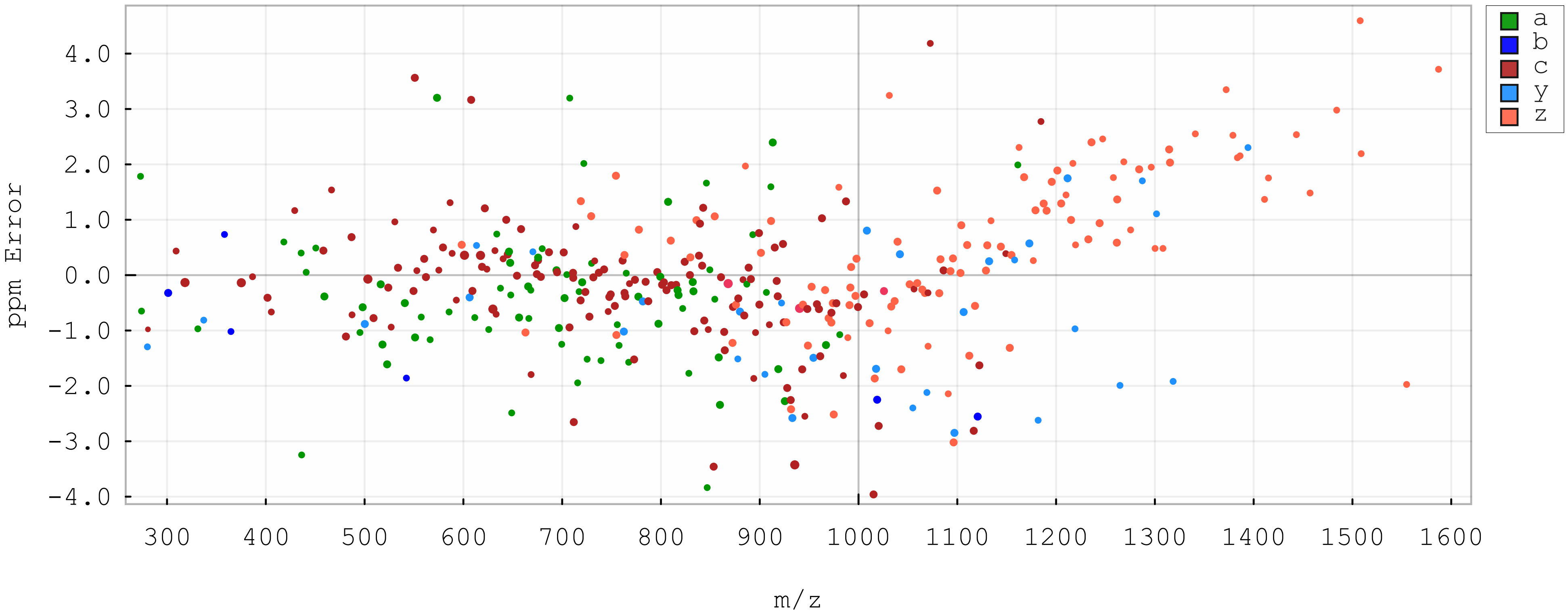

Proteoform:

Index	Protein	SC [%]	SVP [%]	IC [%]	MS Score [a.u.]	Var. Mods	Mono Mass [Da]	Chemical Formula
6	H4Kac	89.1	97.1	57.5	51.3	Ace: K31	11271.3518	C496H839N163O135S

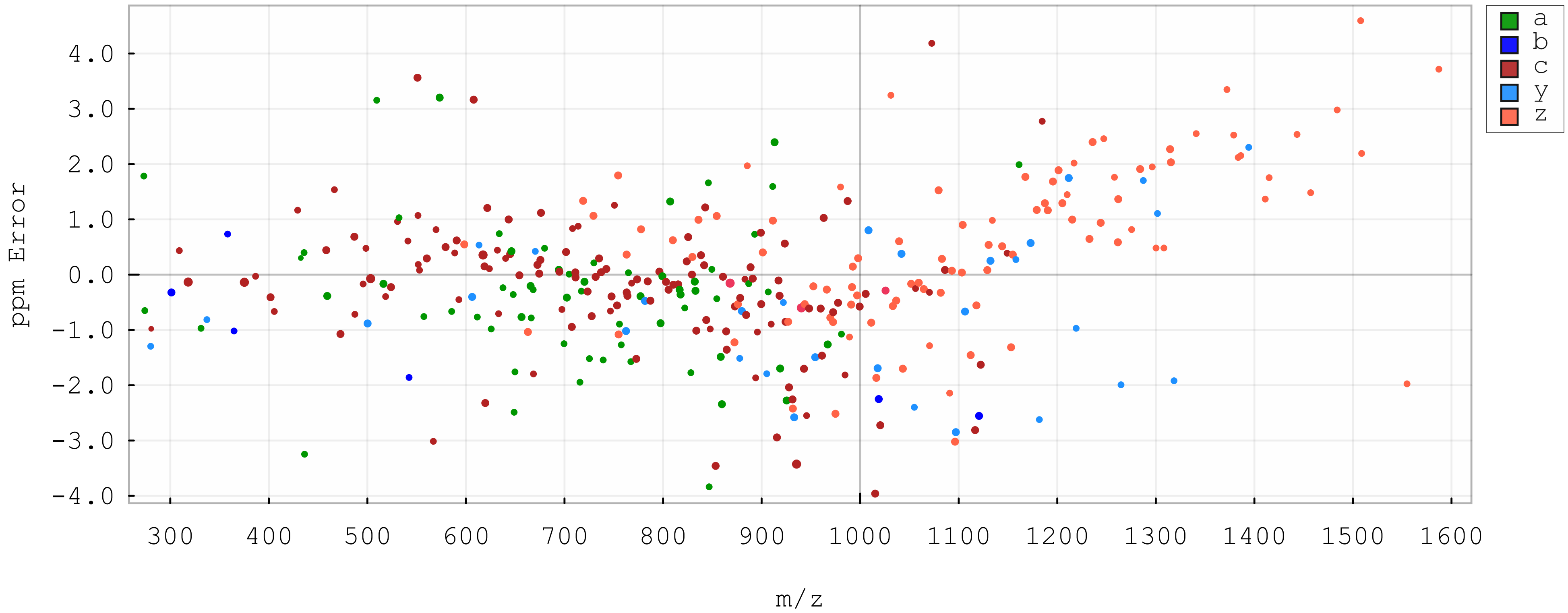

Proteoform:

Index	Protein	SC [%]	SVP [%]	IC [%]	MS Score [a.u.]	Var. Mods	Mono Mass [Da]	Chemical Formula
7	H4Kac	86.1	62.7	50.1	43.2	Ace: K44	11271.3518	C496H839N163O135S

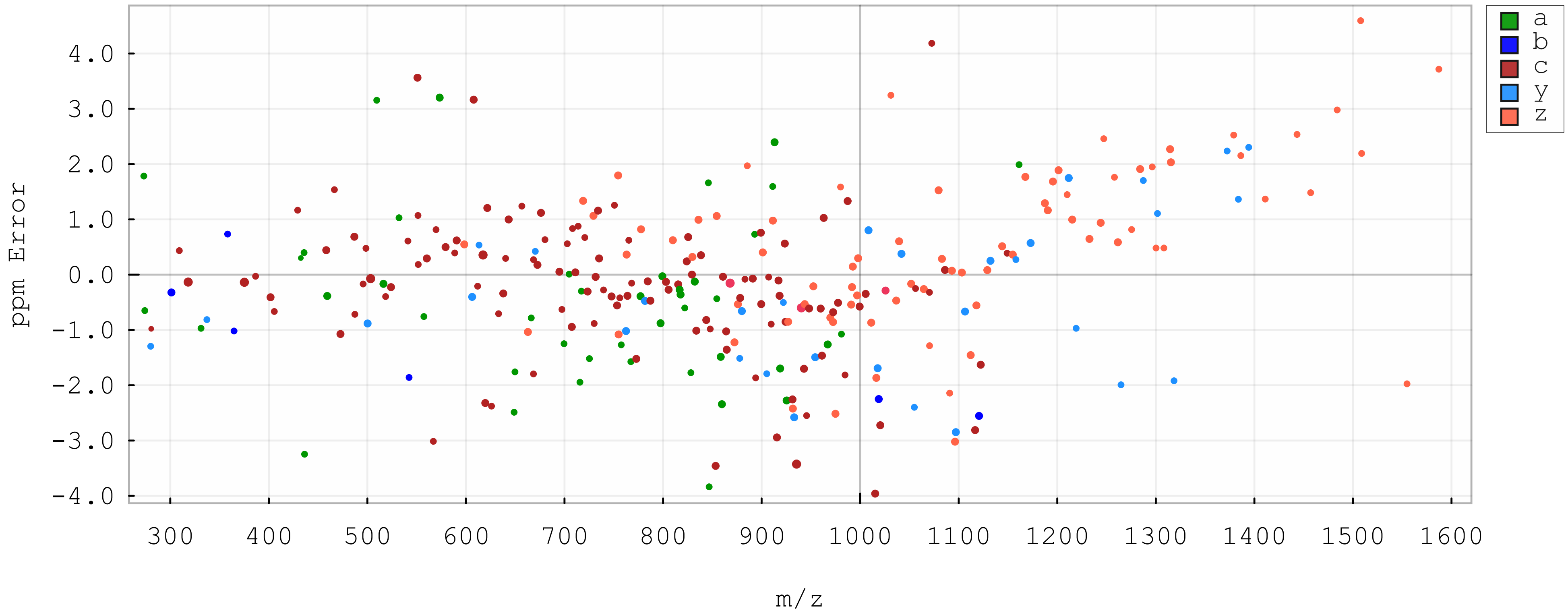

Proteoform:

Index	Protein	SC [%]	SVP [%]	IC [%]	MS Score [a.u.]	Var. Mods	Mono Mass [Da]	Chemical Formula
8	H4Kac	82.2	62.7	42.0	34.5	Ace: K59	11271.3518	C496H839N163O135S

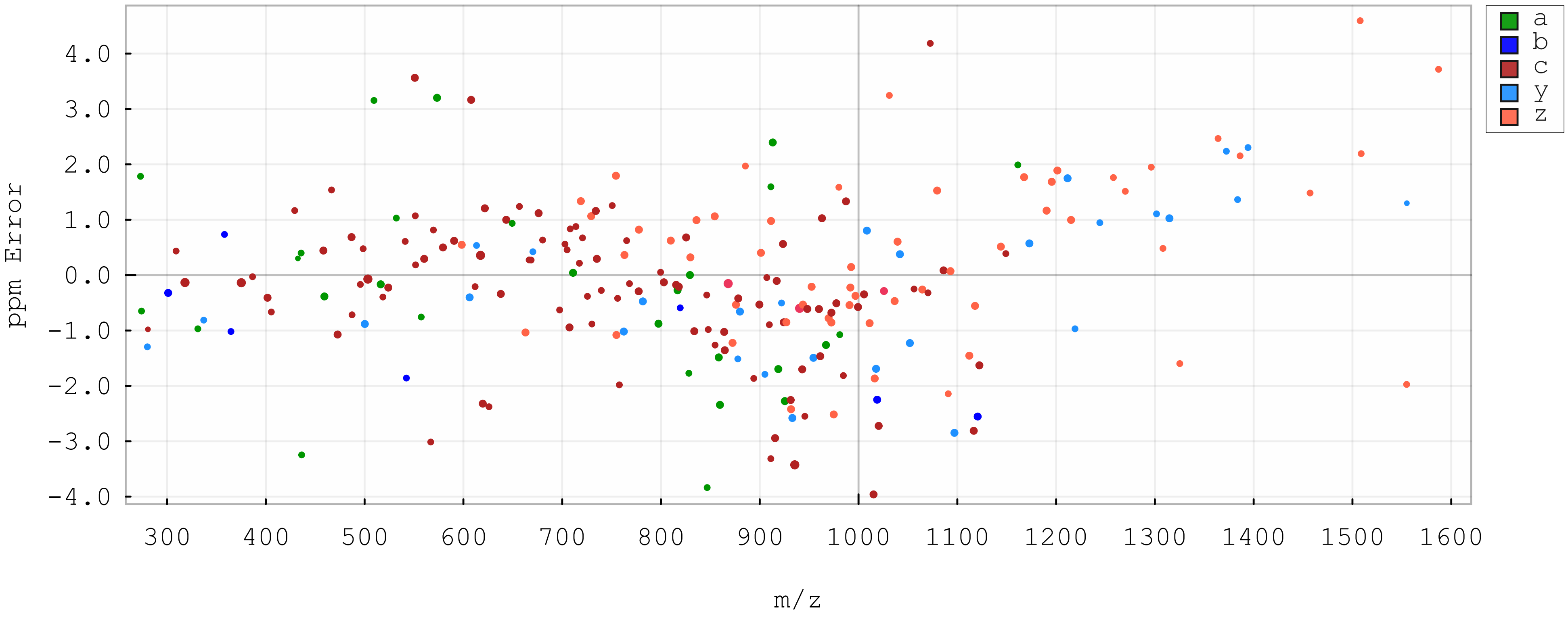

Proteoform:

Index	Protein	SC [%]	SVP [%]	IC [%]	MS Score [a.u.]	Var. Mods	Mono Mass [Da]	Chemical Formula
9	H4Kac	75.2	52.9	33.7	25.3	Ace: K77	11271.3518	C496H839N163O135S

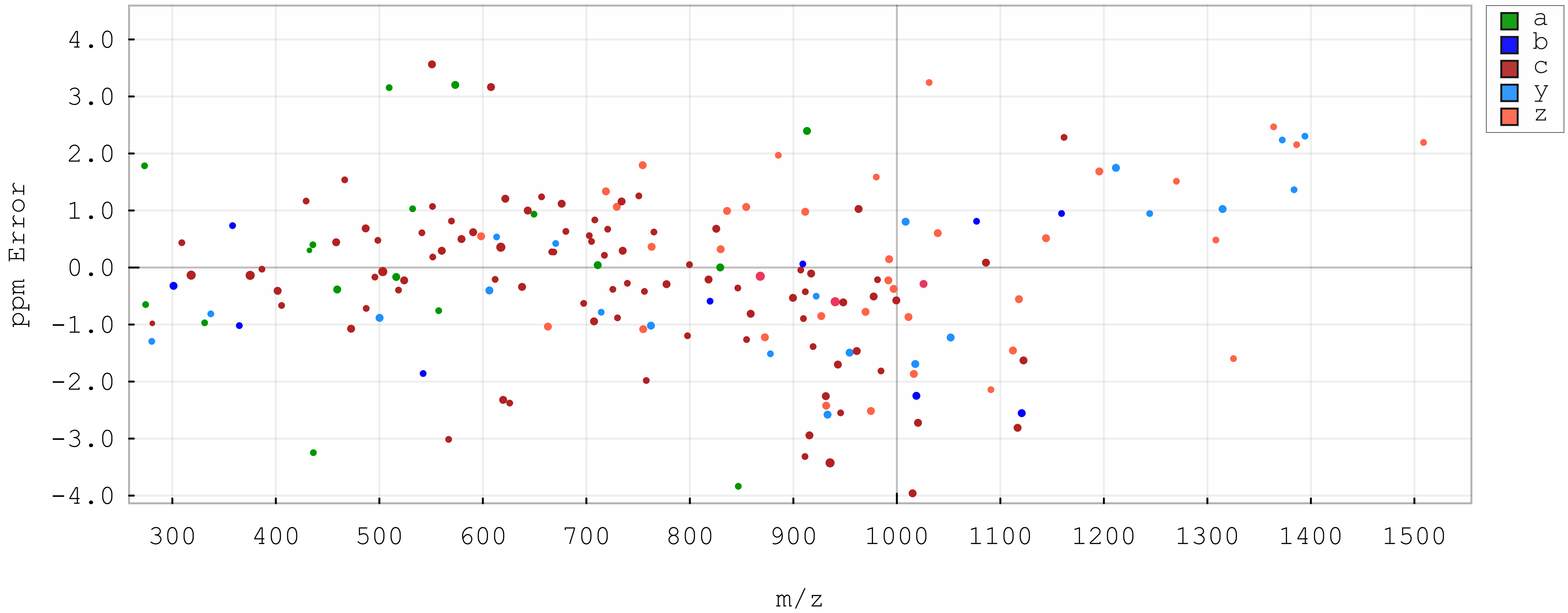

Proteoform:

Index	Protein	SC [%]	SVP [%]	IC [%]	MS Score [a.u.]	Var. Mods	Mono Mass [Da]	Chemical Formula
10	H4Kac	73.3	51.0	33.2	24.3	Ace: K79	11271.3518	C496H839N163O135S

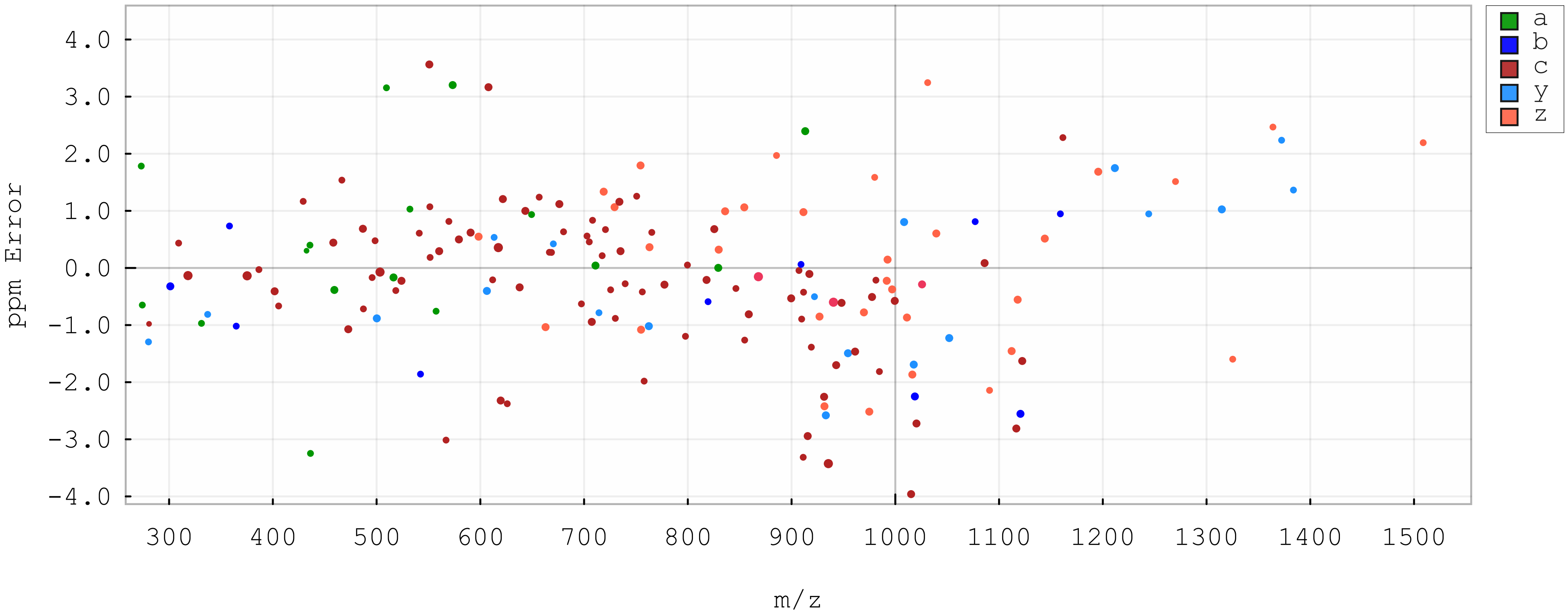

Primary Fragment Plots

Proteoform:

Index	Protein	SC [%]	SVP [%]	IC [%]	MS Score [a.u.]	Var. Mods	Mono Mass [Da]	Chemical Formula
1	H4Kac	91.1	97.1	69.1	63.0	Ace: K8	11271.3518	C496H839N163O135S

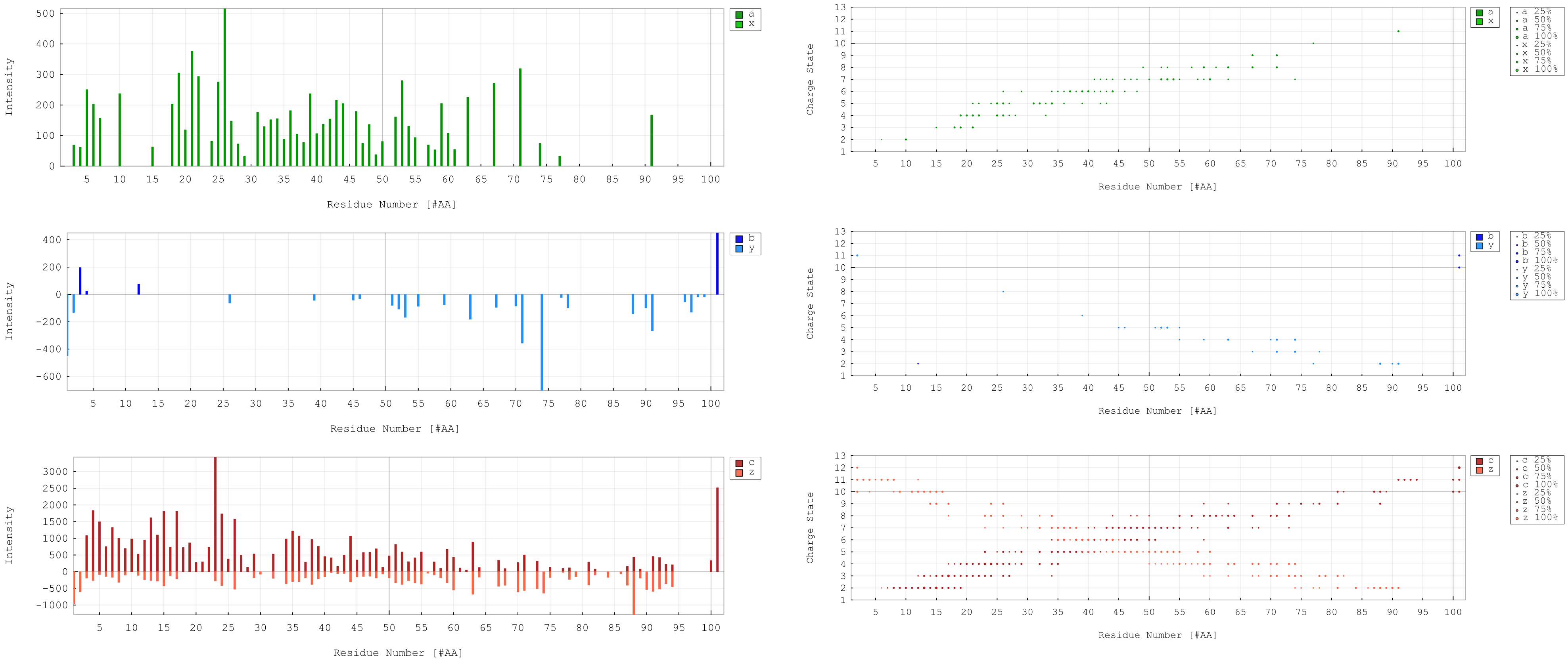

Proteoform:

Index	Protein	SC [%]	SVP [%]	IC [%]	MS Score [a.u.]	Var. Mods	Mono Mass [Da]	Chemical Formula
2	H4Kac	91.1	97.1	68.1	62.0	Ace: K12	11271.3518	C496H839N163O135S

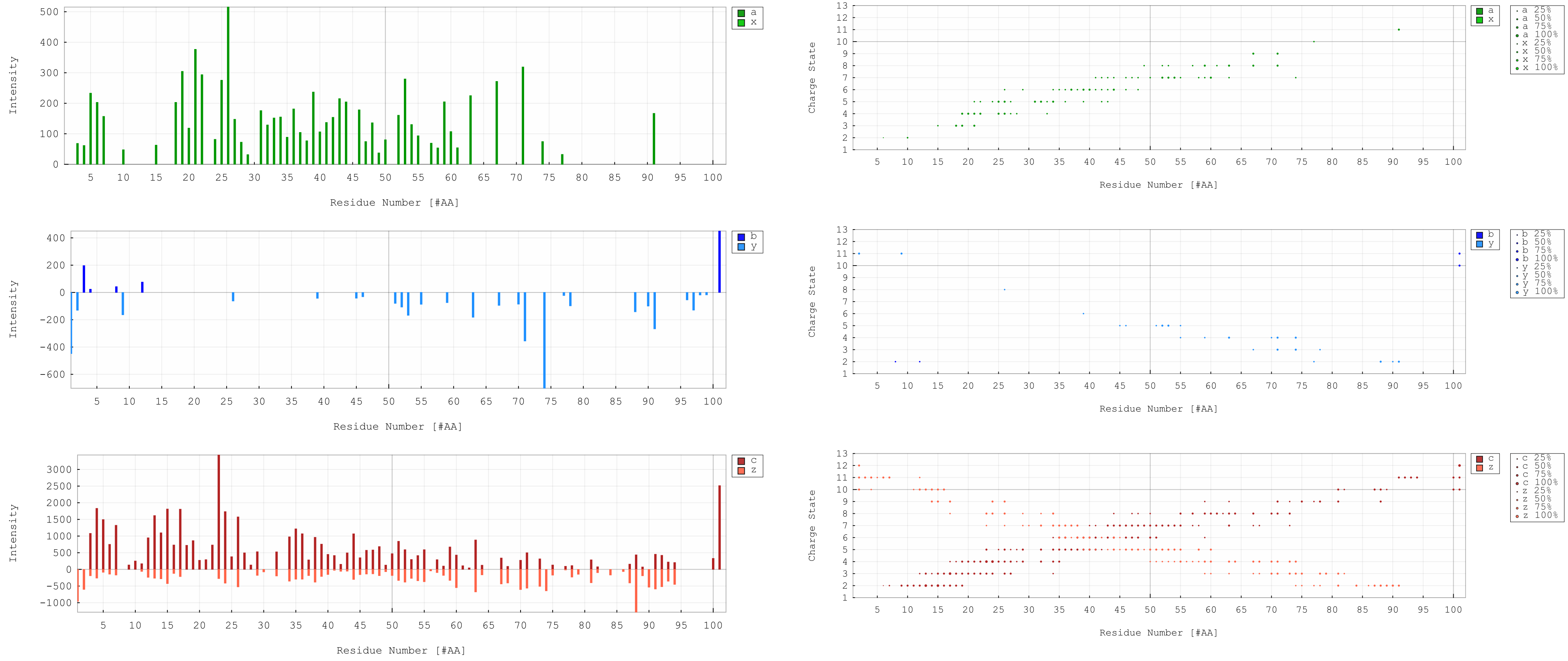

Top-Down Result OmniScape 2025
Sequence Confirmation
250428 H4-PCAF100-30min z13ac1 MS2 ECD_001-573_calibrated2

Proteoform:

Index	Protein	SC [%]	SVP [%]	IC [%]	MS Score [a.u.]	Var. Mods	Mono Mass [Da]	Chemical Formula
3	H4Kac	91.1	97.1	65.7	59.9	Ace: K16	11271.3518	C496H839N163O135S

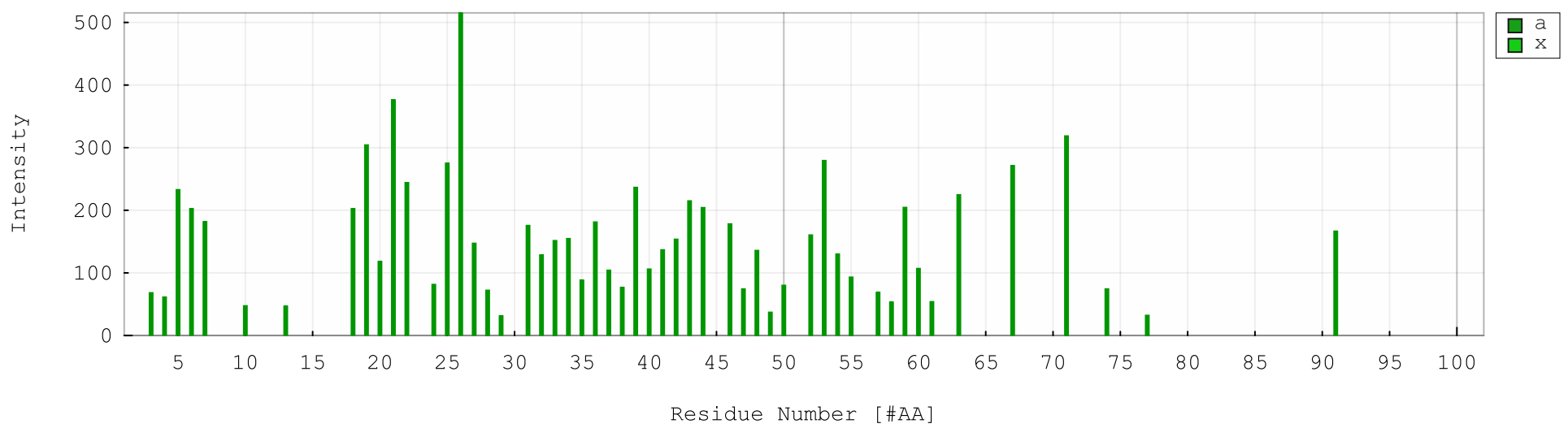

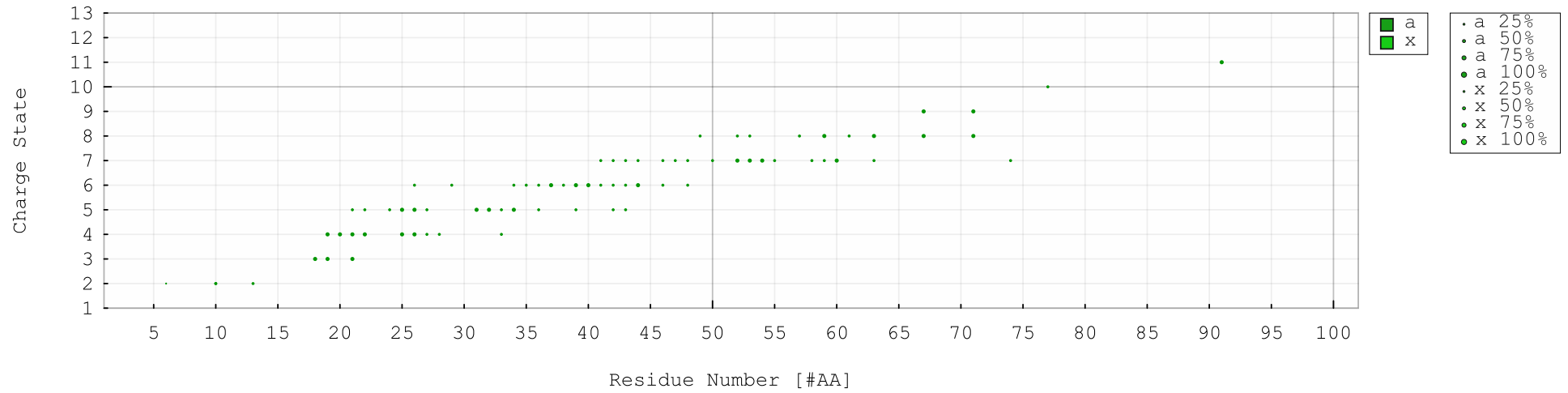

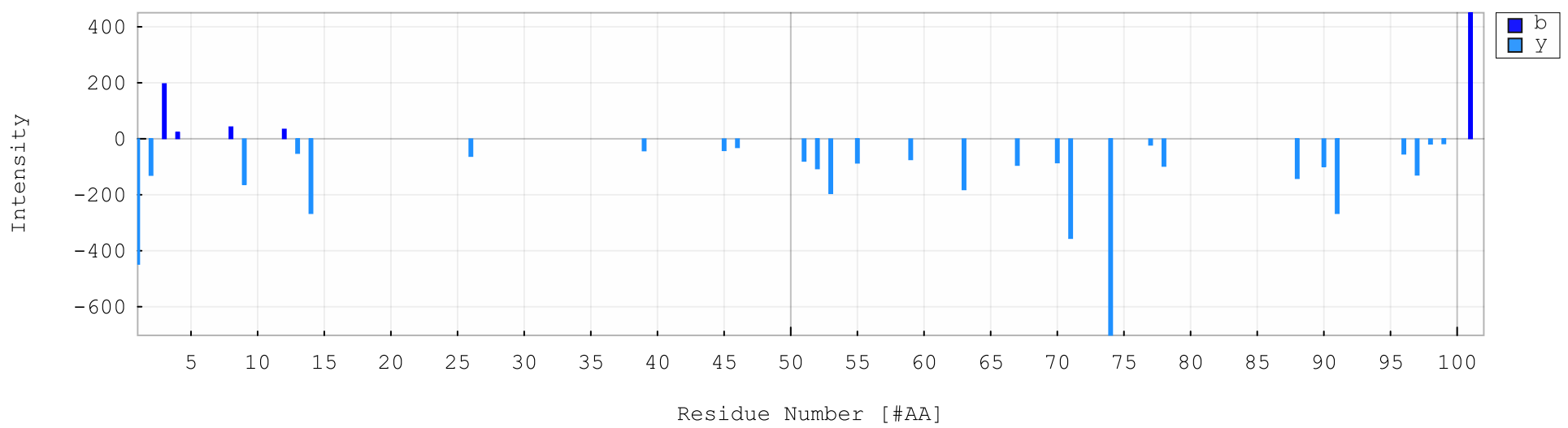

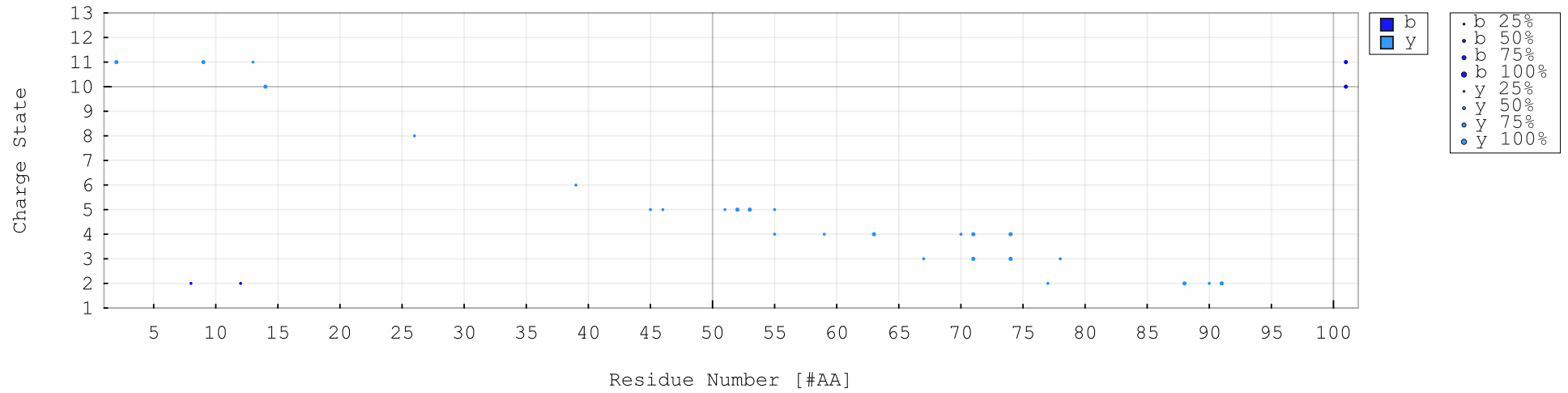

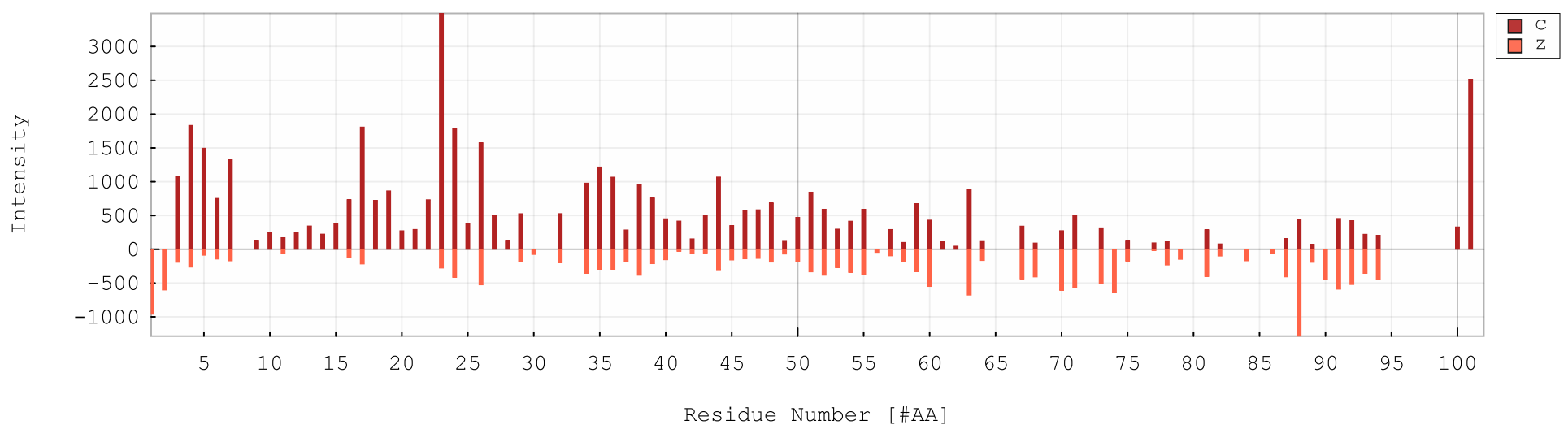

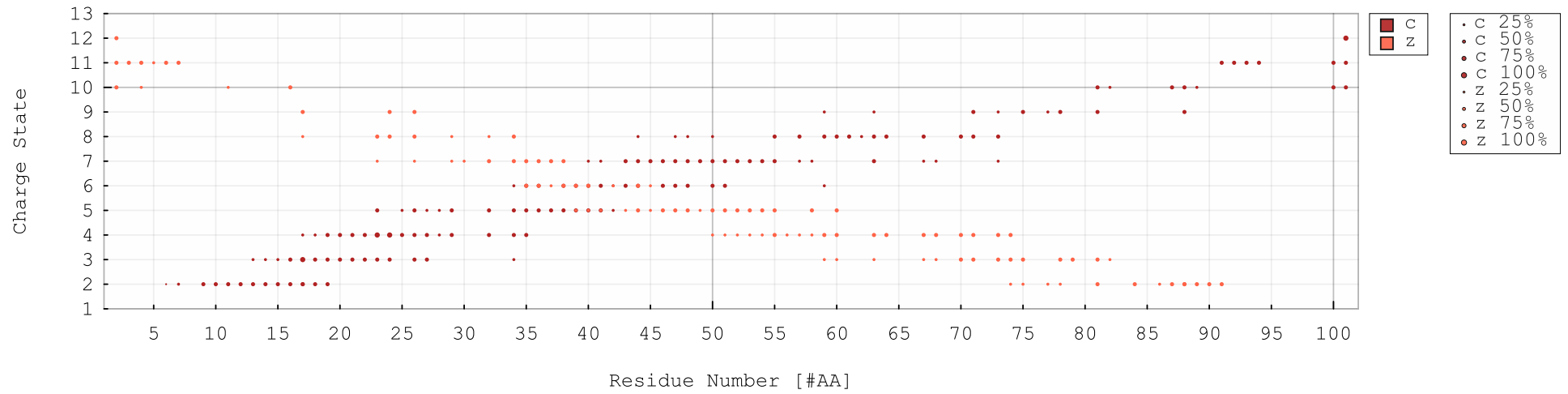

Proteoform:

Index	Protein	SC [%]	SVP [%]	IC [%]	MS Score [a.u.]	Var. Mods	Mono Mass [Da]	Chemical Formula
4	H4Kac	88.1	38.2	67.8	59.7	Ace: K5	11271.3518	C496H839N163O135S

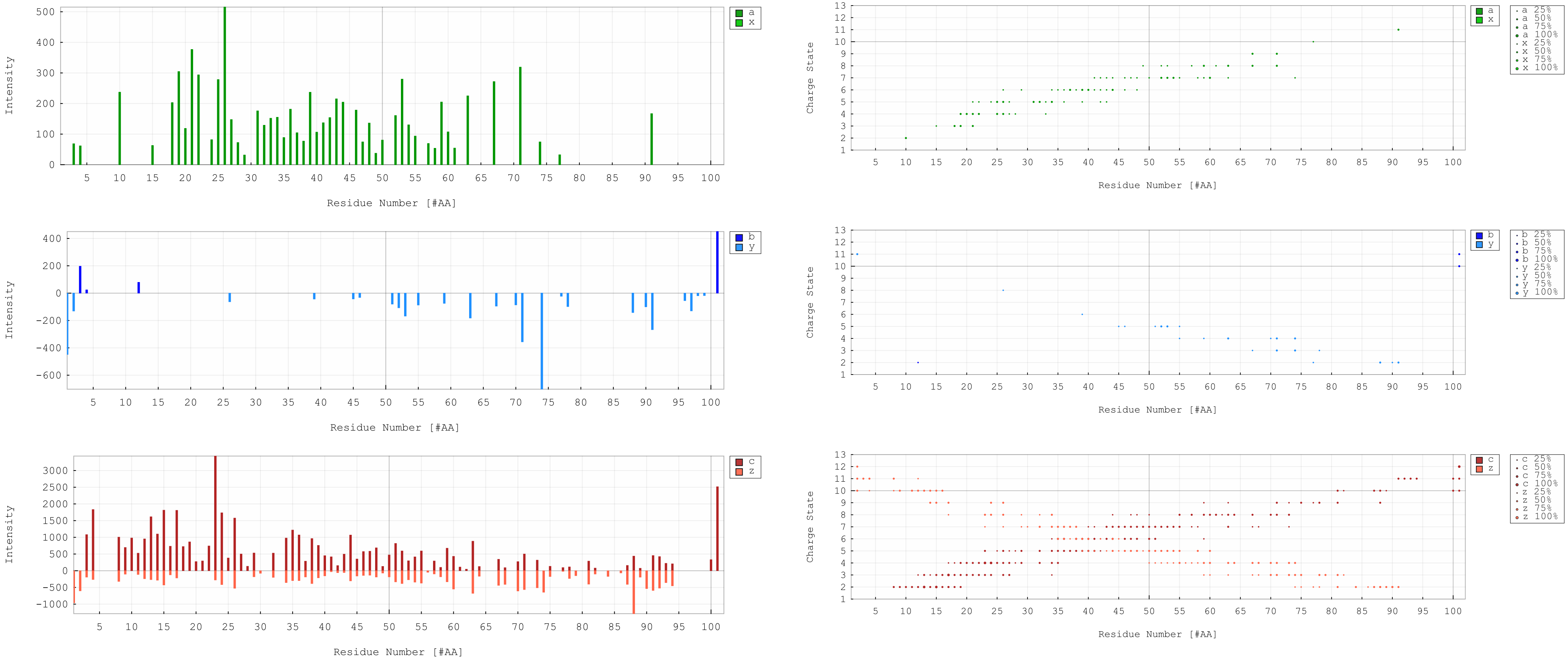

Proteoform:

Index	Protein	SC [%]	SVP [%]	IC [%]	MS Score [a.u.]	Var. Mods	Mono Mass [Da]	Chemical Formula
5	H4Kac	91.1	97.1	63.8	58.1	Ace: K20	11271.3518	C496H839N163O135S

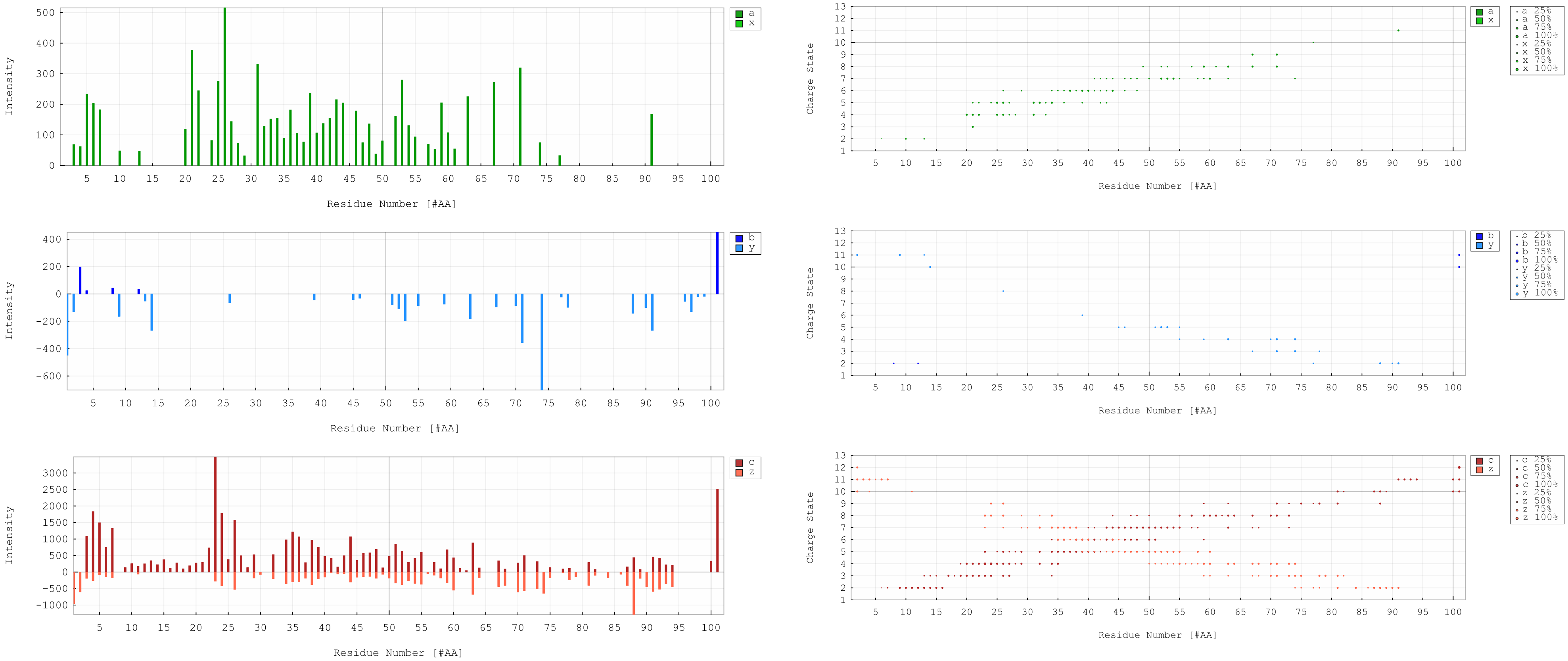

Proteoform:	Index	Protein	SC [%]	SVP [%]	IC [%]	MS Score [a.u.]	Var. Mods	Mono Mass [Da]	Chemical Formula
	6	H4Kac	89.1	97.1	57.5	51.3	Ace: K31	11271.3518	C496H839N163O135S

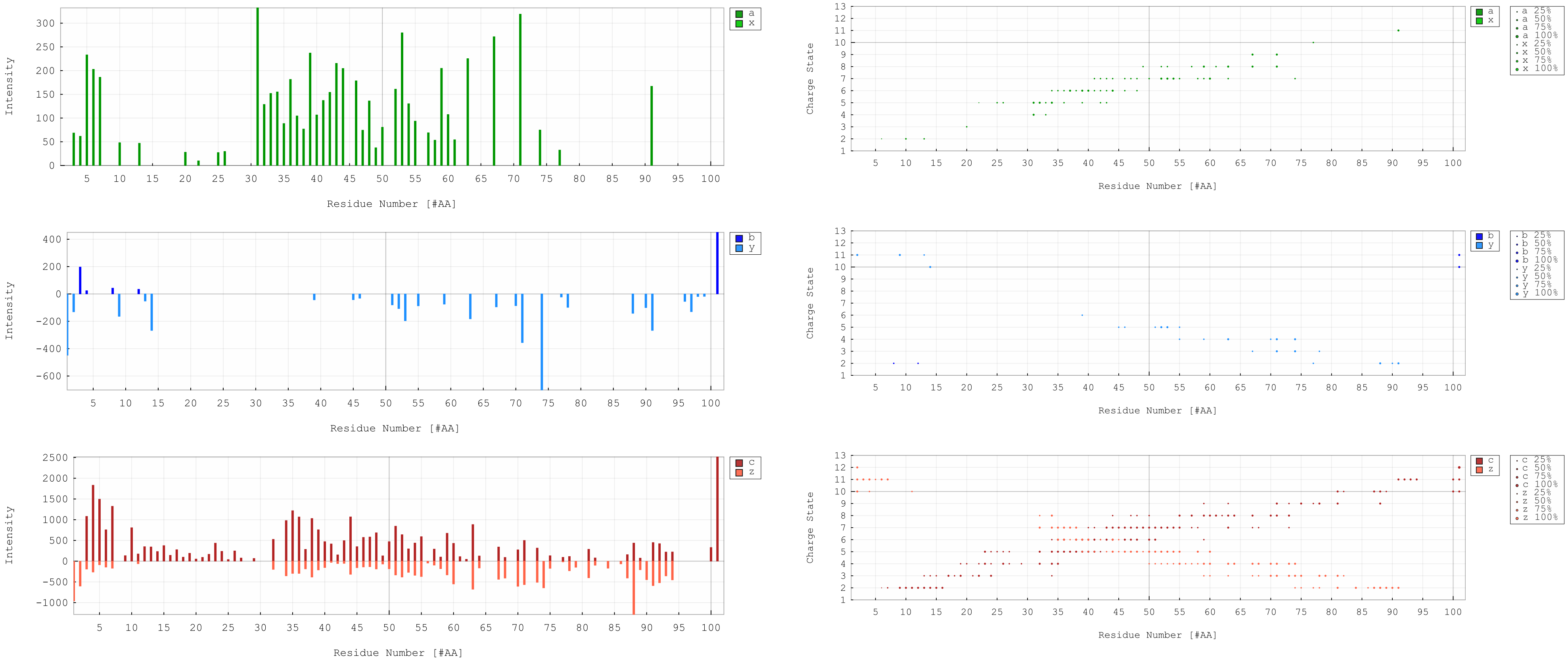

Top-Down Result OmniScape 2025
Sequence Confirmation
250428 H4-PCAF100-30min z13ac1 MS2 ECD_001-573_calibrated2

Proteoform:

Index	Protein	SC [%]	SVP [%]	IC [%]	MS Score [a.u.]	Var. Mods	Mono Mass [Da]	Chemical Formula
7	H4Kac	86.1	62.7	50.1	43.2	Ace: K44	11271.3518	C496H839N163O135S

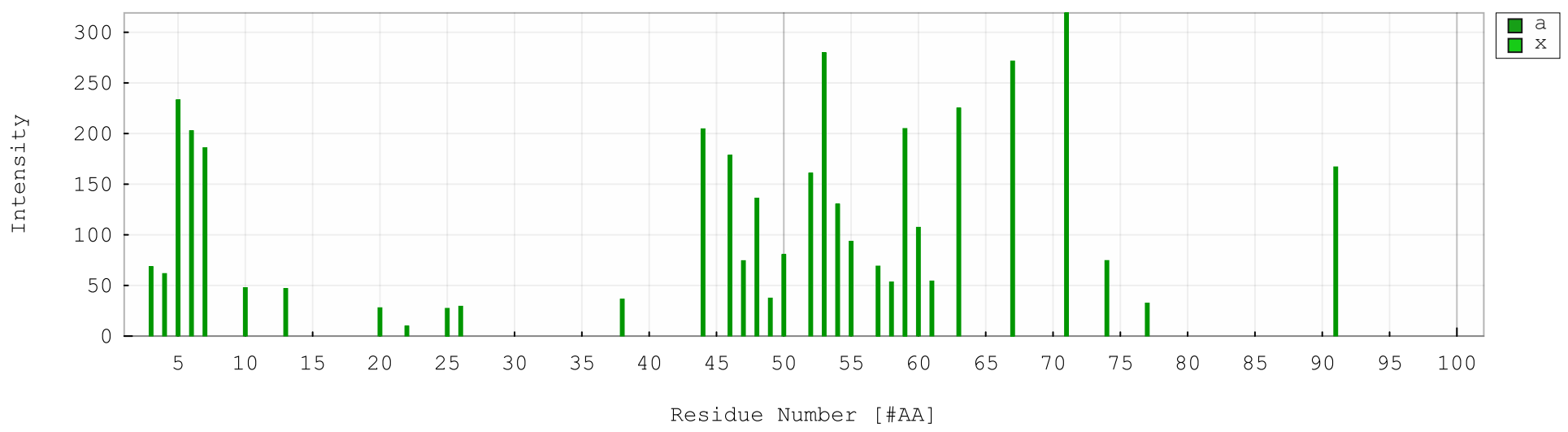

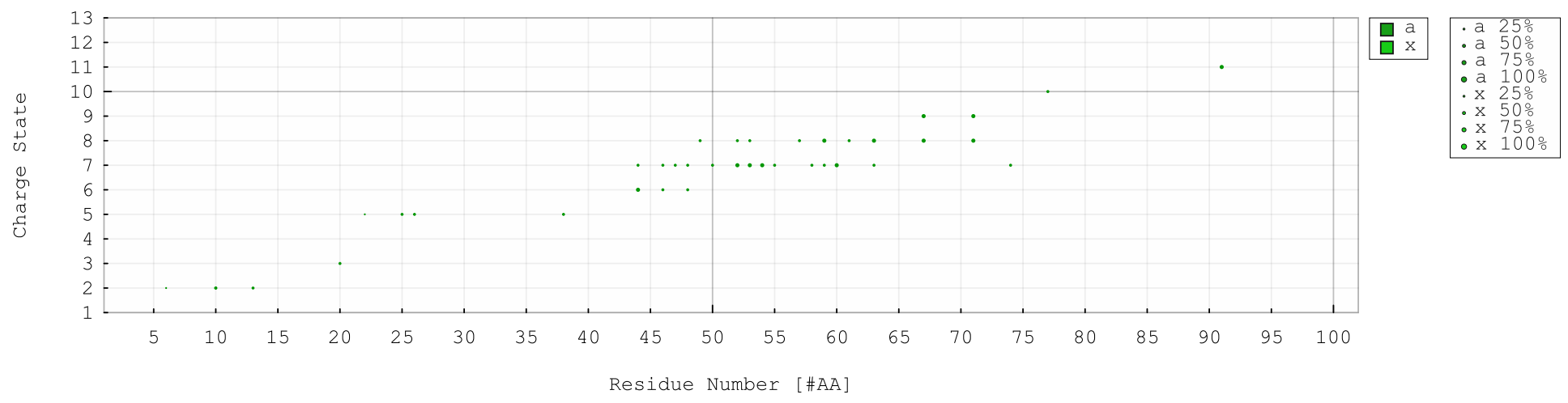

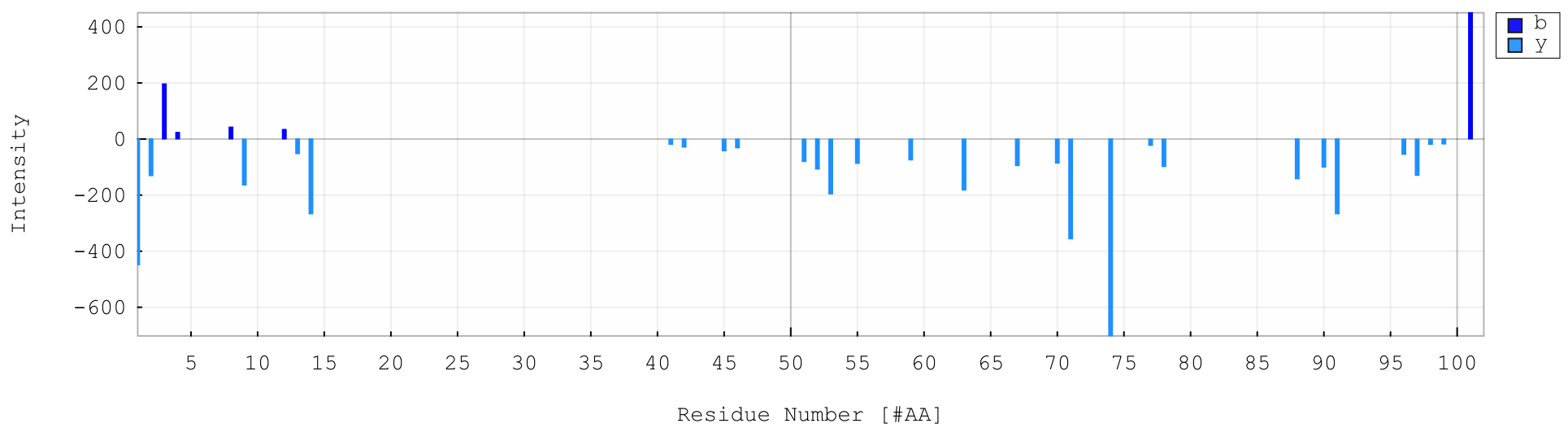

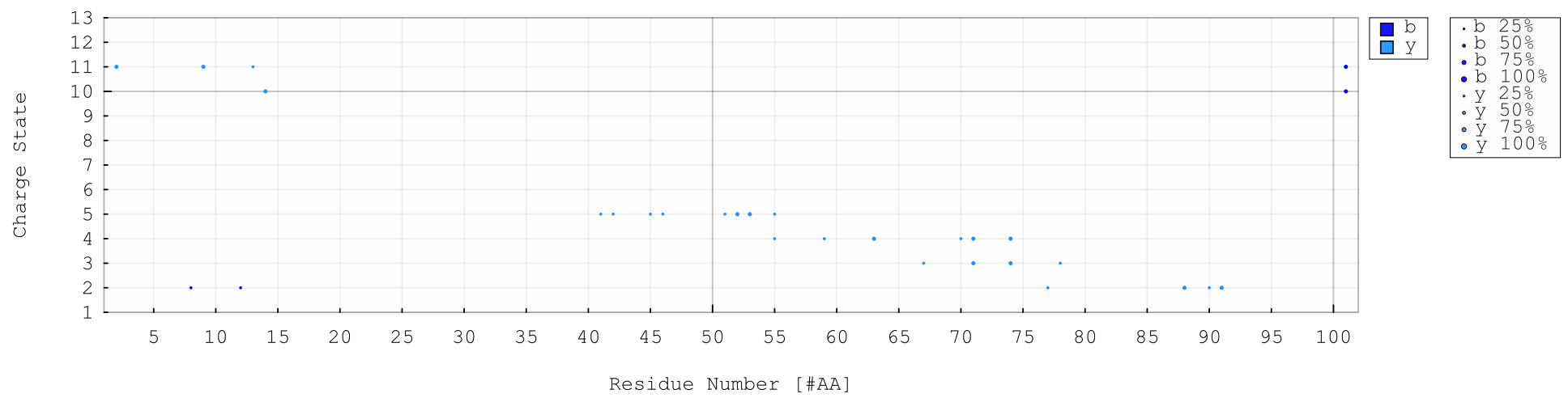

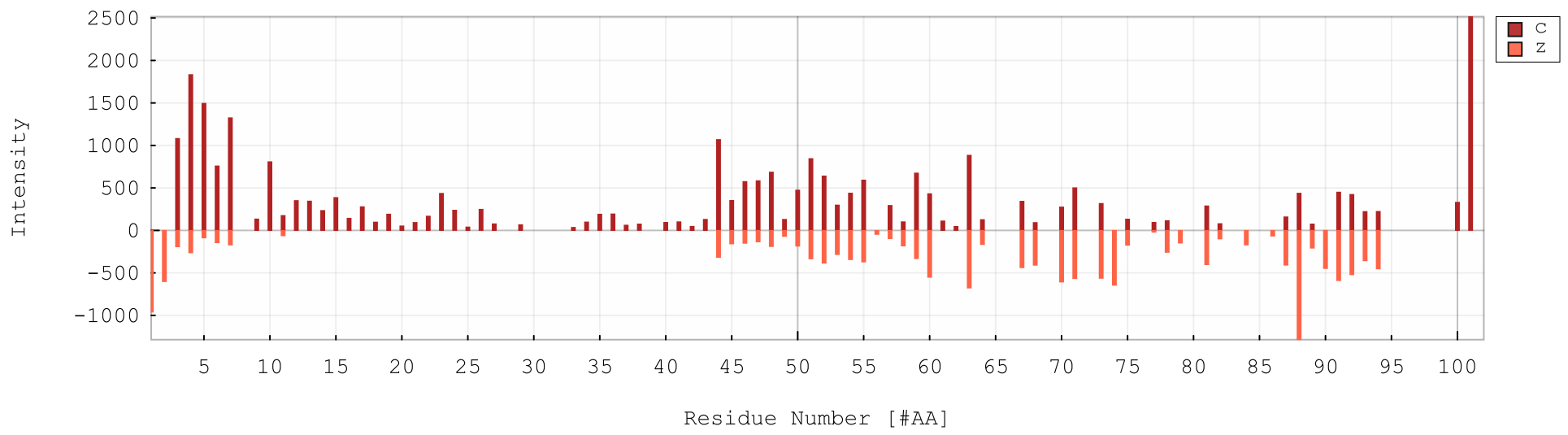

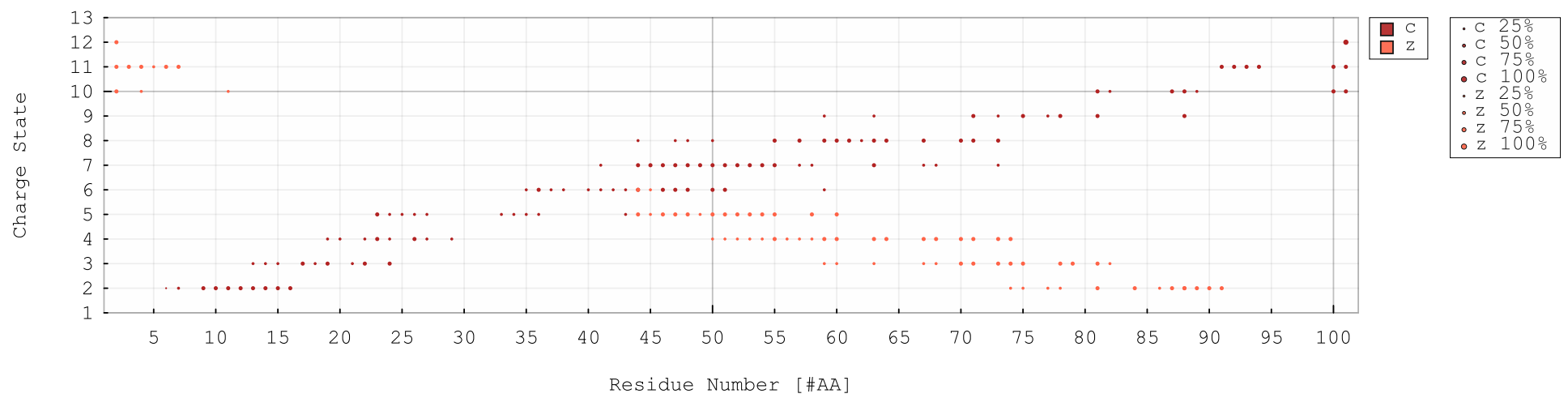

Proteoform:

Index	Protein	SC [%]	SVP [%]	IC [%]	MS Score [a.u.]	Var. Mods	Mono Mass [Da]	Chemical Formula
8	H4Kac	82.2	62.7	42.0	34.5	Ace: K59	11271.3518	C496H839N163O135S

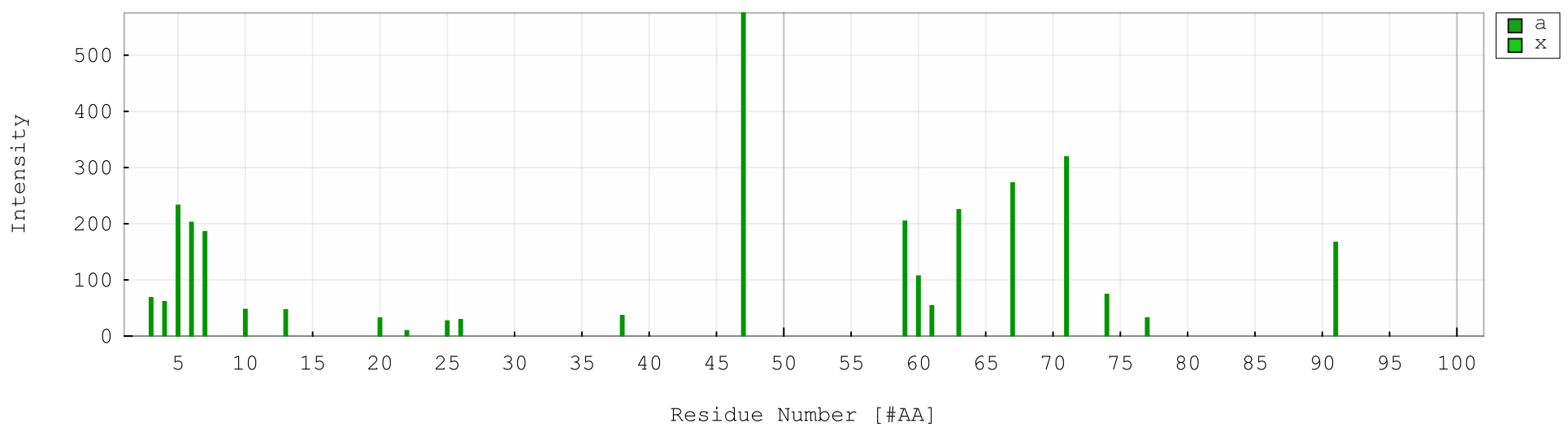

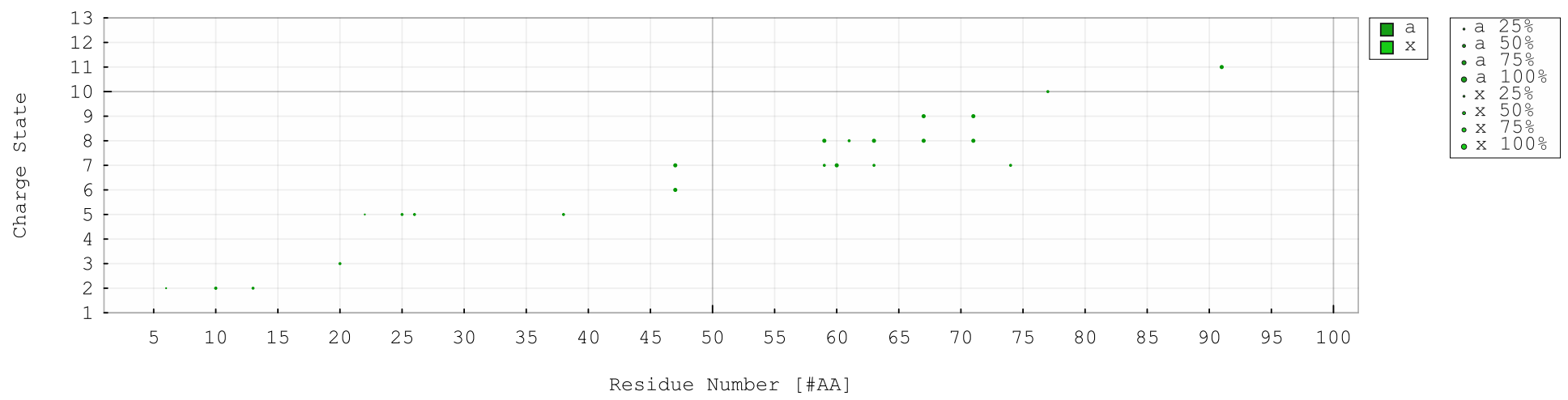

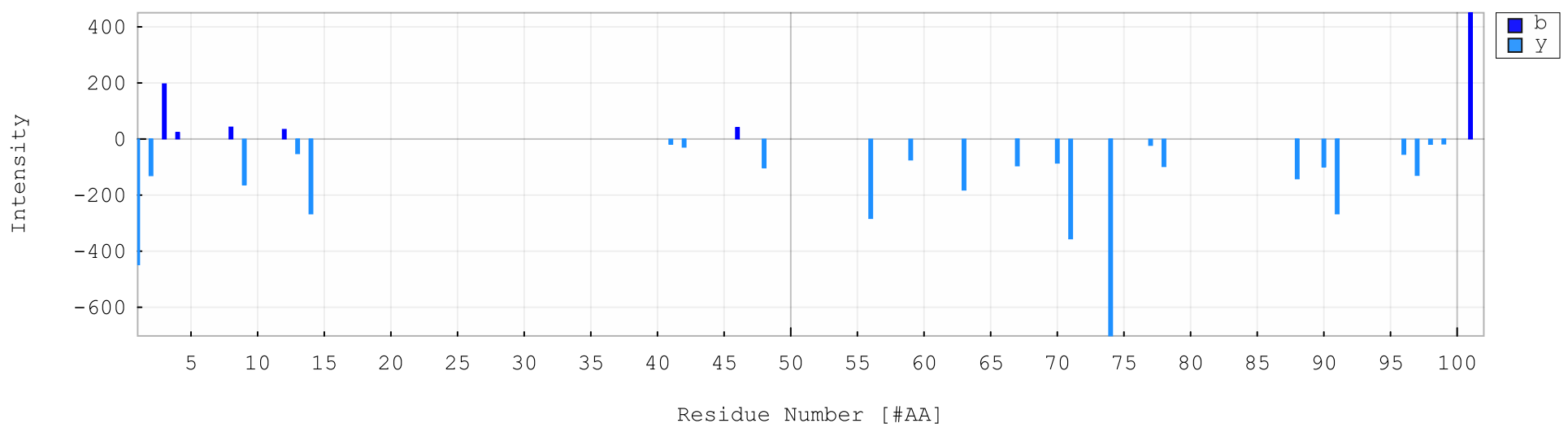

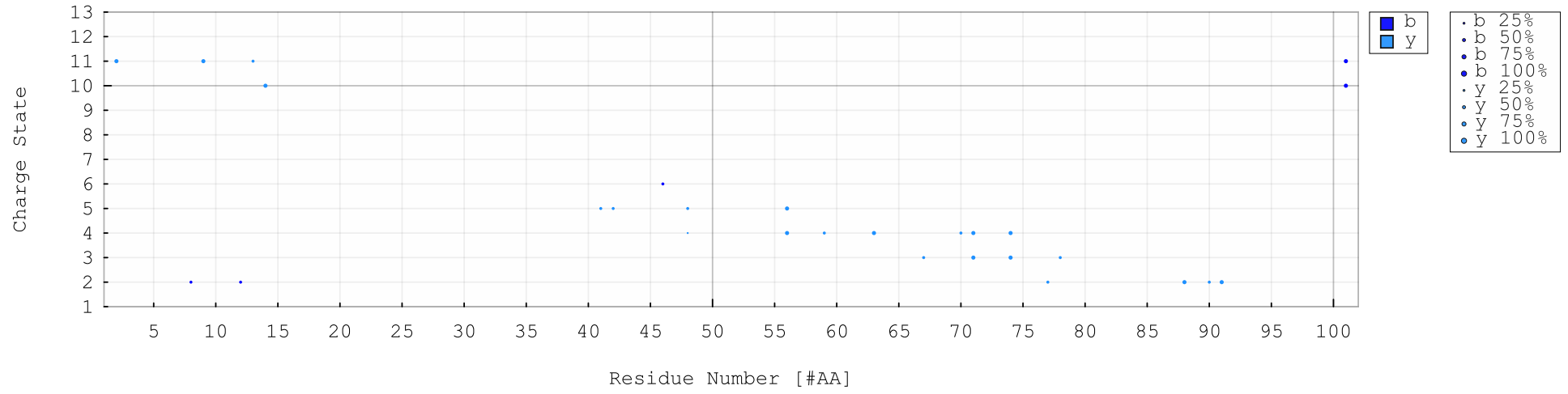

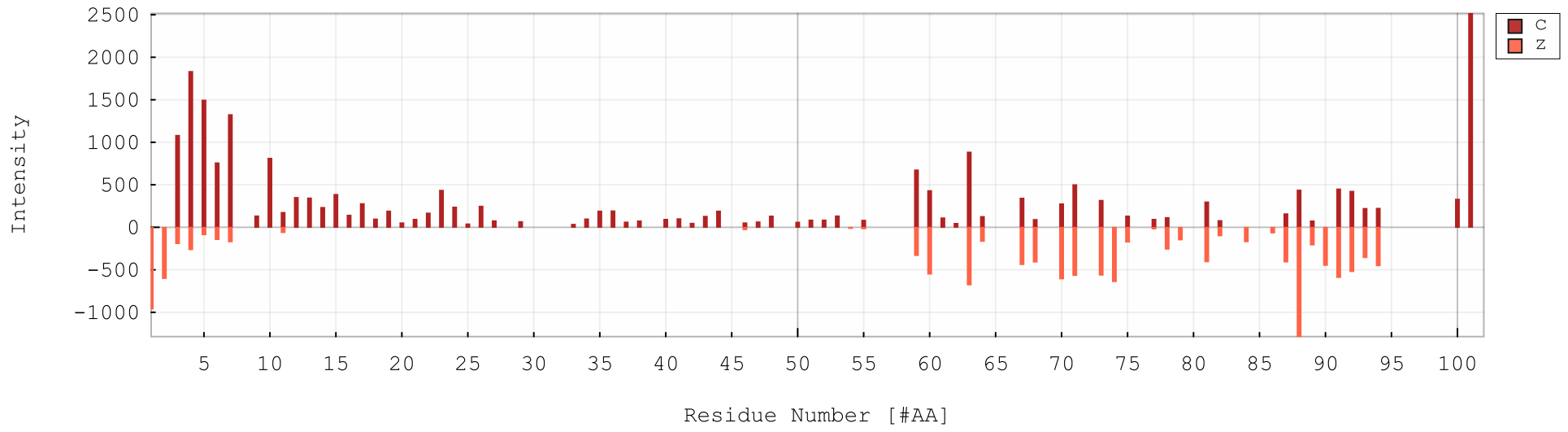

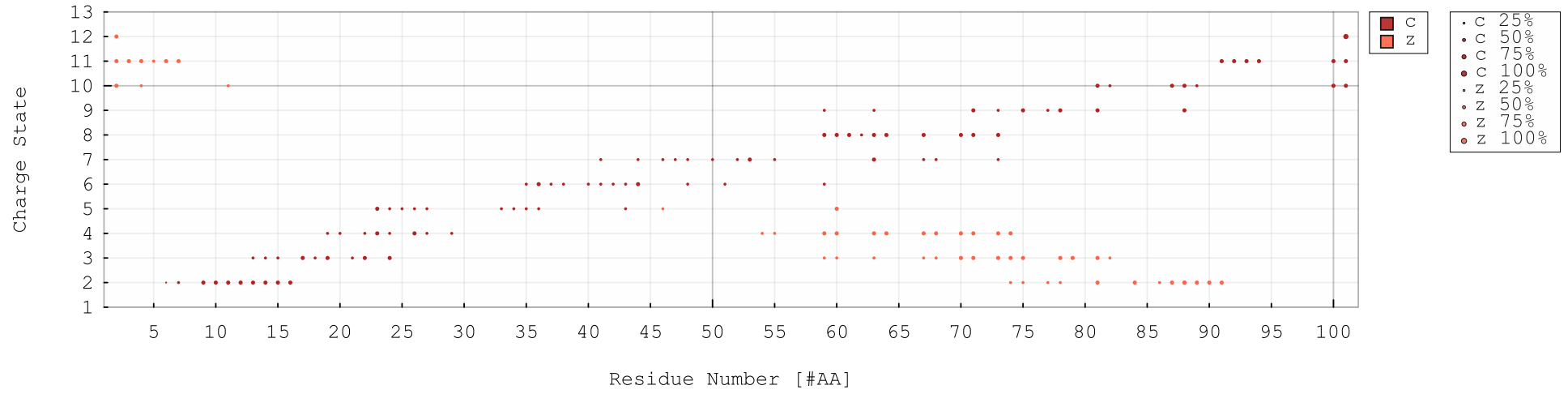

Proteoform:	Index	Protein	SC [%]	SVP [%]	IC [%]	MS Score [a.u.]	Var. Mods	Mono Mass [Da]	Chemical Formula
	9	H4Kac	75.2	52.9	33.7	25.3	Ace: K77	11271.3518	C496H839N163O135S

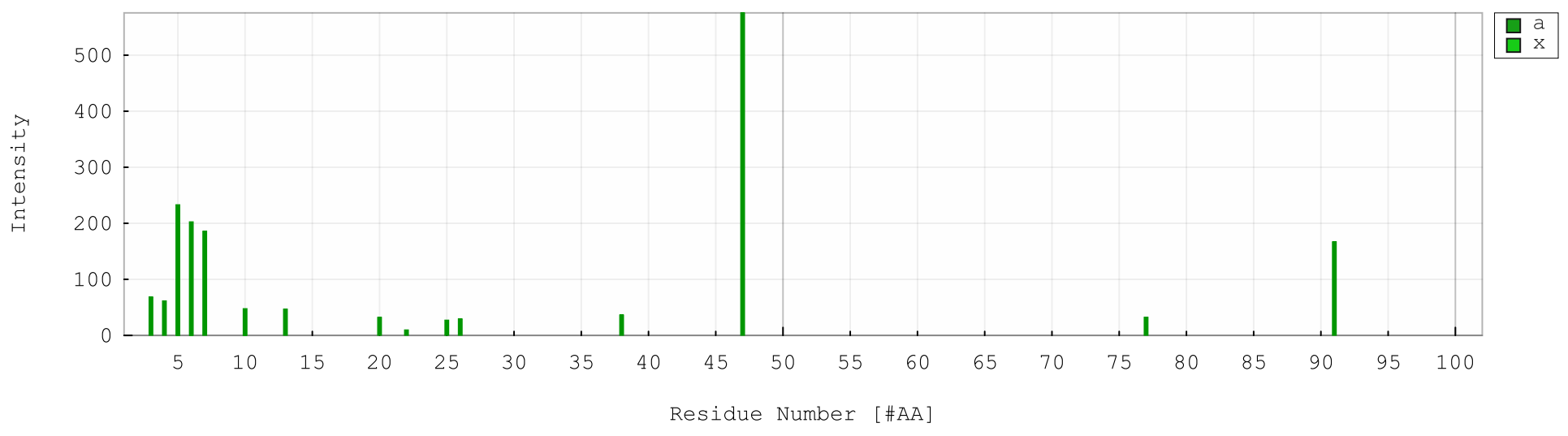

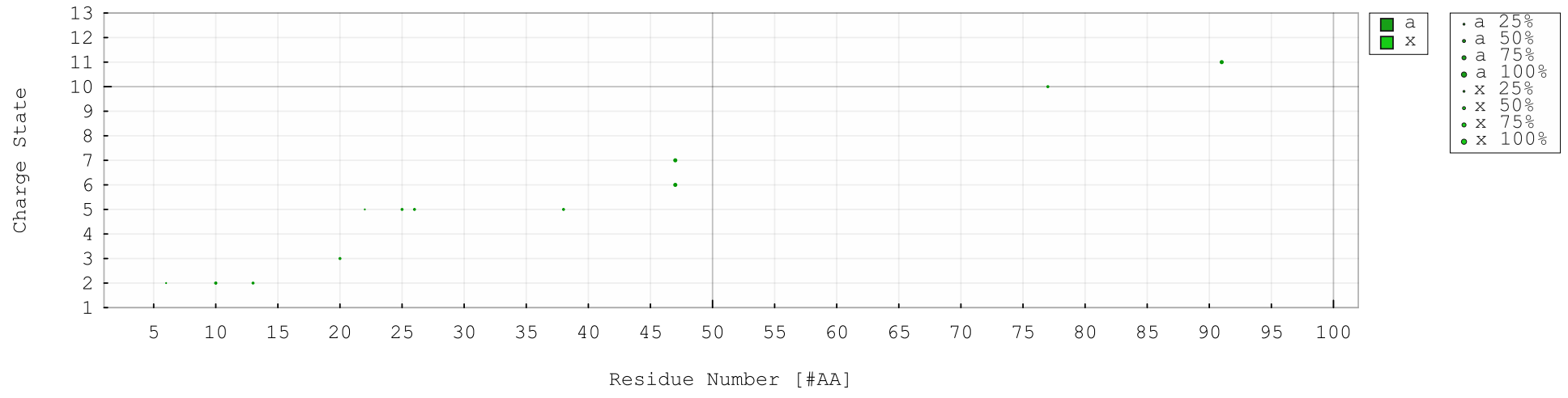

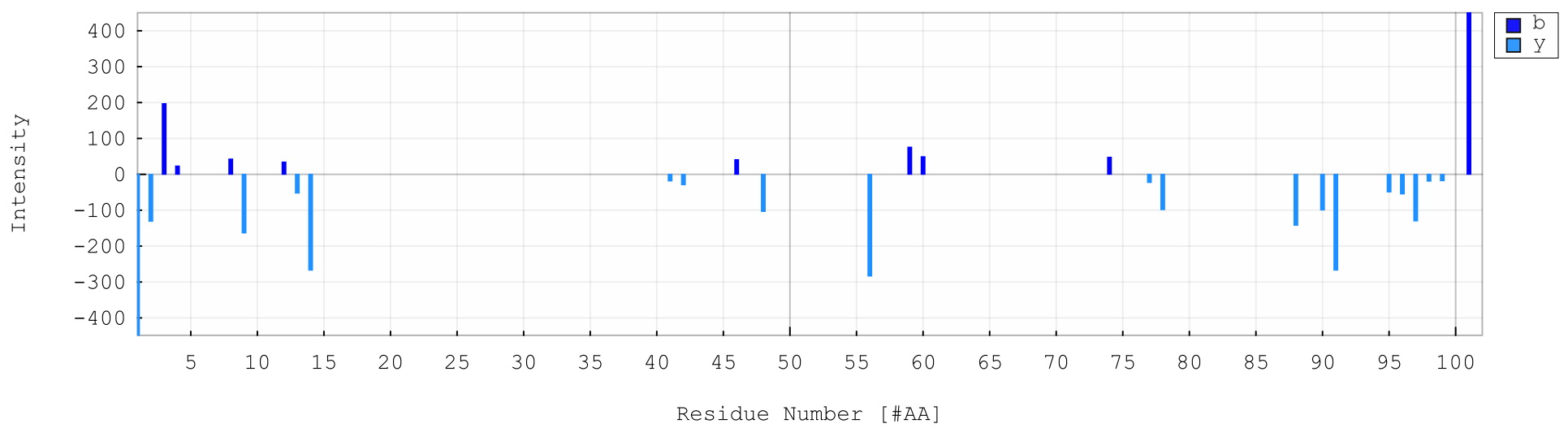

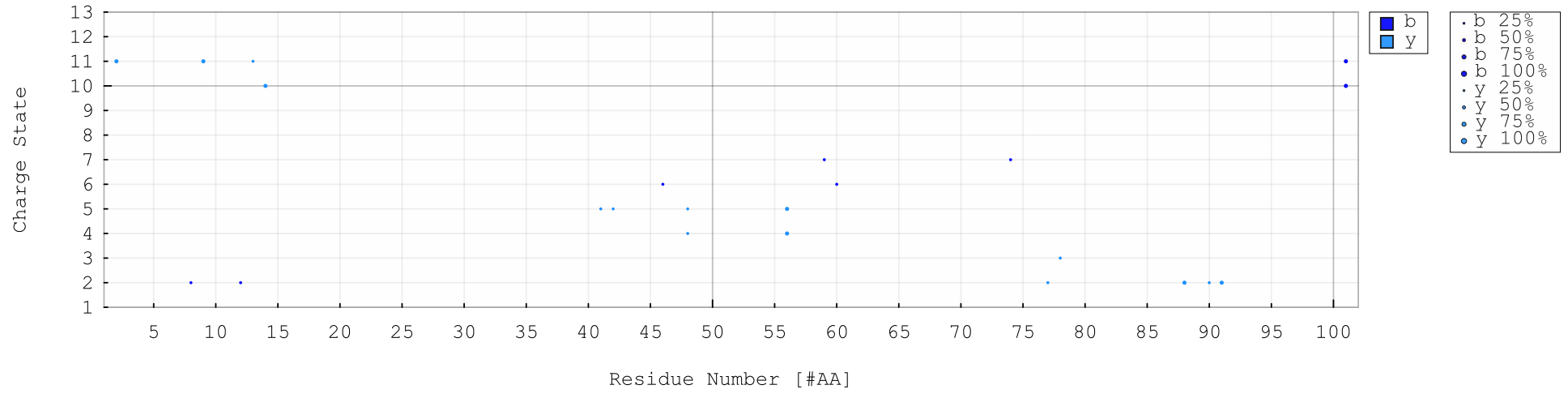

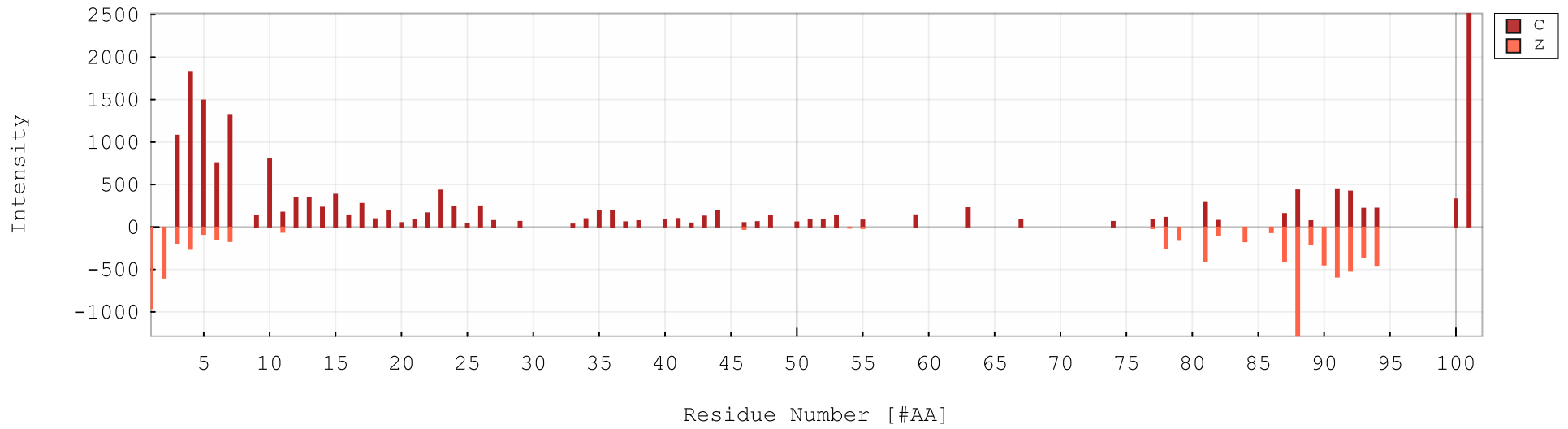

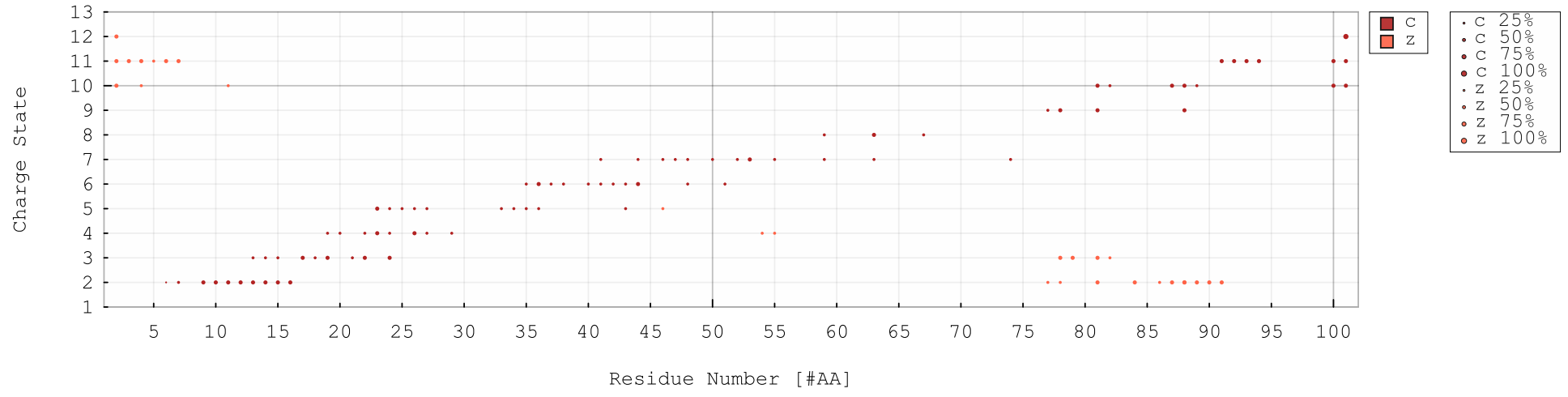

Proteoform:

Index	Protein	SC [%]	SVP [%]	IC [%]	MS Score [a.u.]	Var. Mods	Mono Mass [Da]	Chemical Formula
10	H4Kac	73.3	51.0	33.2	24.3	Ace: K79	11271.3518	C496H839N163O135S

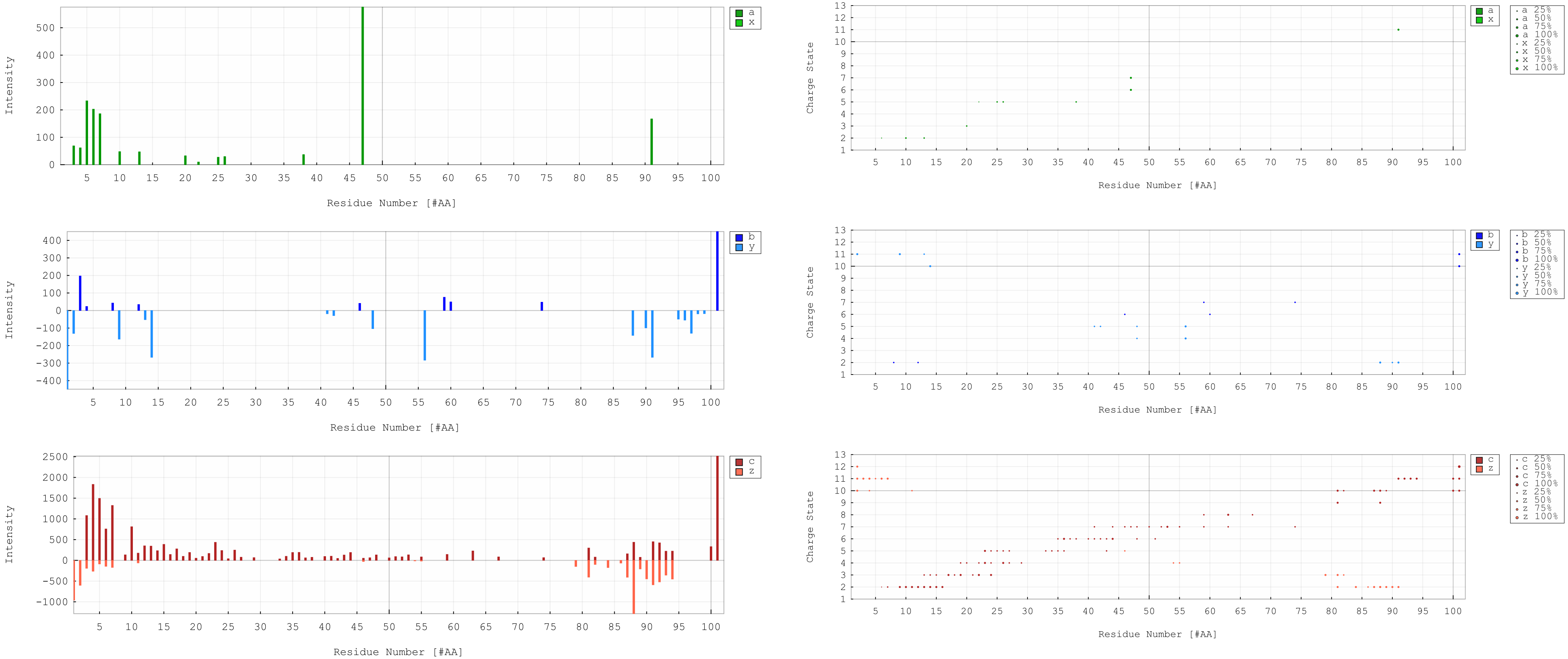

Supplemental Data S5

H4 - PCAF, 30 min

**Report**	3 of 8
**Experiment**	MS2
**Histone/sample**	H4
**Enzyme**	PCAF
**Reaction/source**	30 min
**Precursor**	H4Kac1
**Charge state**	13+
**Fragmentation / workflow**	EID
**Original file**	MS2_H4Kac1_13+_EID.pdf

Project Information

Project Directory: C:/Users/LAB-ADMIN
Processing date: Tue Apr 28 14:43:52 2026

Sample Info & Protocols

Name: Top-Down Protein Sequencing EID

Dataset

Imported spectrum file name:	250428 H4-PCAF100-30min z13ac1 MS2 EID_1-692_calibrated.txt
Dataset name:	Information is only available from imported JPEAKS files
Date acquired:	Information is only available from imported JPEAKS files
Date imported:	Information is only available from imported JPEAKS files
Instrument Name:	Information is only available from imported JPEAKS files
Instrument Serial Number:	Information is only available from imported JPEAKS files

Workflow Result Info

Analysis: 250428 H4-PCAF100-30min z13ac1 MS2 EID_1-692_calibrated2
Location: C:/Users/LAB-ADMIN
Method: -

Method Information

Name: Unnamed

Spectral Parameters

Mass Error [ppm]: 6
Intensity Deviation [au]: 0.15
Min/Max Charges: 1-13
Experiment type: EID

a ions	b ions	c ions	x ions	y ions	z ions	satellite	internal	precursor
a	b	c	x	y	z			M
a+1			x+1	y-1	z+1			M+-1H

Noise threshold calculation: Simple moving average noise threshold (SMA)
Window size [datapoints]: 600
Noise curve offset: 4.562201
SVP parameters: terminal gap: 2, internal gap: 1

Proteoforms

(top 10)

Index	Parent Sequence	Chain	Var. Mods	Mono Mass [Da]	Chemical Composition
1	H4Kac	H4KacK8[Ace]	Ace: K8	11271.3518	C496H839N163O135S
2	H4Kac	H4KacK12[Ace]	Ace: K12	11271.3518	C496H839N163O135S
3	H4Kac	H4KacK5[Ace]	Ace: K5	11271.3518	C496H839N163O135S
4	H4Kac	H4KacK16[Ace]	Ace: K16	11271.3518	C496H839N163O135S
5	H4Kac	H4KacK20[Ace]	Ace: K20	11271.3518	C496H839N163O135S
6	H4Kac	H4KacK31[Ace]	Ace: K31	11271.3518	C496H839N163O135S
7	H4Kac	H4KacK44[Ace]	Ace: K44	11271.3518	C496H839N163O135S
8	H4Kac	H4KacK59[Ace]	Ace: K59	11271.3518	C496H839N163O135S
9	H4Kac	H4Kac	None	11229.3412	C494H837N163O134S
10	H4Kac	H4KacK77[Ace]	Ace: K77	11271.3518	C496H839N163O135S

Results

Proteoforms Table (top 10)

Index	Protein	SC [%]	SVP [%]	IC [%]	MS Score [a.u.]	Var. Mods	Mono Mass [Da]	Chemical Formula
1	H4Kac	89.1	66.7	47.5	42.3	Ace: K8	11271.3518	C496H839N163O135S
2	H4Kac	89.1	66.7	46.2	41.1	Ace: K12	11271.3518	C496H839N163O135S
3	H4Kac	86.1	8.8	46.9	40.4	Ace: K5	11271.3518	C496H839N163O135S
4	H4Kac	88.1	66.7	45.1	39.7	Ace: K16	11271.3518	C496H839N163O135S
5	H4Kac	88.1	66.7	43.4	38.3	Ace: K20	11271.3518	C496H839N163O135S
6	H4Kac	85.1	66.7	33.9	28.9	Ace: K31	11271.3518	C496H839N163O135S
7	H4Kac	76.2	33.3	27.7	21.1	Ace: K44	11271.3518	C496H839N163O135S
8	H4Kac	71.3	33.3	20.9	14.9	Ace: K59	11271.3518	C496H839N163O135S
9	H4Kac	82.2	33.3	14.9	12.2	None	11229.3412	C494H837N163O134S
10	H4Kac	61.4	33.3	16.7	10.2	Ace: K77	11271.3518	C496H839N163O135S

Sequence

Name: H4Kac

```
                  10                    20                    30                    40                    50                    60
S G R G K G G K G L   G K G G A K R H R K   V L R D N I Q G I T   K P A I R R L A R R   G G V K R I S G L I   Y E E T R G V L K V
                  70                    80                    90                   100                   110                   120
F L E N V I R D A V   T Y T E H A K R K T   V T A M D V V Y A L   K R Q G R T L Y G F   G G
```

Modifications

Modification	Position	Type
Acetyl	5	variable
Acetyl	8	variable
Acetyl	12	variable
Acetyl	16	variable
Acetyl	20	variable
Acetyl	31	variable
Acetyl	44	variable
Acetyl	59	variable
Acetyl	77	variable
Acetyl	79	variable
Acetyl	91	variable

Sequence Map

Proteoform:

Index	Protein	SC [%]	SVP [%]	IC [%]	MS Score [a.u.]	Var. Mods	Mono Mass [Da]	Chemical Formula
1	H4Kac	89.1	66.7	47.5	42.3	Ace: K8	11271.3518	C496H839N163O135S

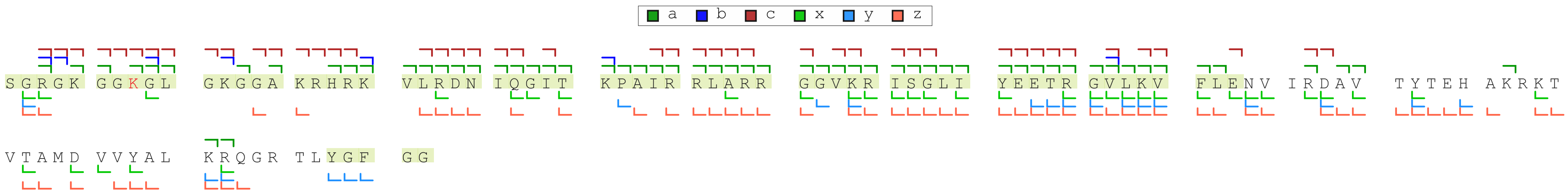

Proteoform:	Index	Protein	SC [%]	SVP [%]	IC [%]	MS Score [a.u.]	Var. Mods	Mono Mass [Da]	Chemical Formula
	2	H4Kac	89.1	66.7	46.2	41.1	Ace: K12	11271.3518	C496H839N163O135S

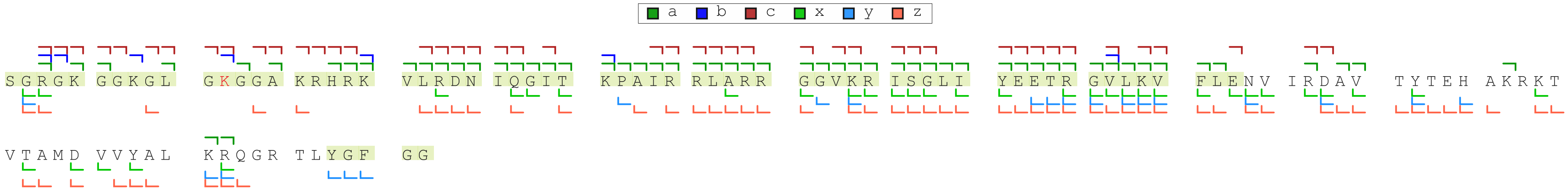

Proteoform:

Index	Protein	SC [%]	SVP [%]	IC [%]	MS Score [a.u.]	Var. Mods	Mono Mass [Da]	Chemical Formula
3	H4Kac	86.1	8.8	46.9	40.4	Ace: K5	11271.3518	C496H839N163O135S

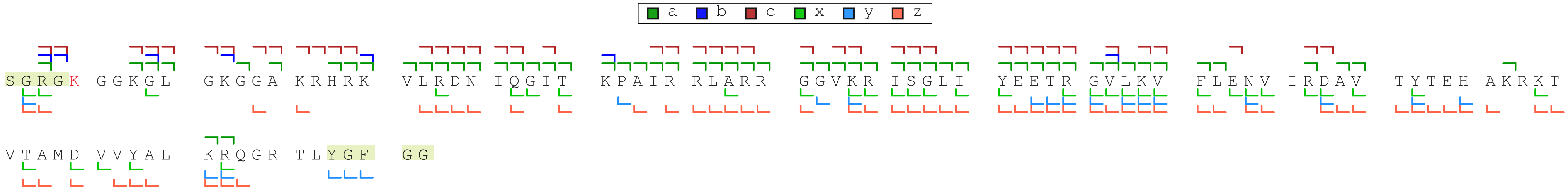

Proteoform:

Index	Protein	SC [%]	SVP [%]	IC [%]	MS Score [a.u.]	Var. Mods	Mono Mass [Da]	Chemical Formula
4	H4Kac	88.1	66.7	45.1	39.7	Ace: K16	11271.3518	C496H839N163O135S

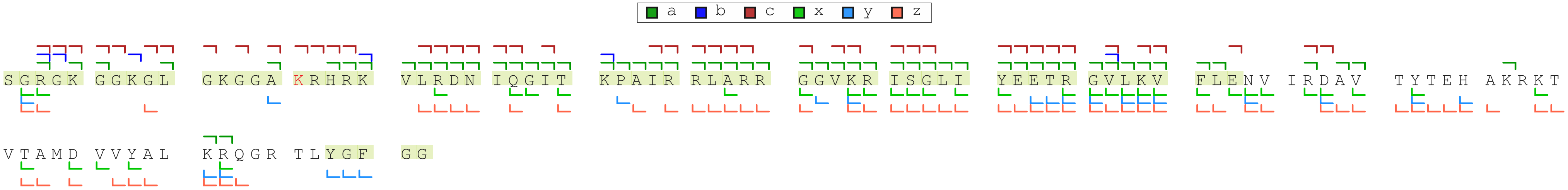

Proteoform:	Index	Protein	SC [%]	SVP [%]	IC [%]	MS Score [a.u.]	Var. Mods	Mono Mass [Da]	Chemical Formula
	5	H4Kac	88.1	66.7	43.4	38.3	Ace: K20	11271.3518	C496H839N163O135S

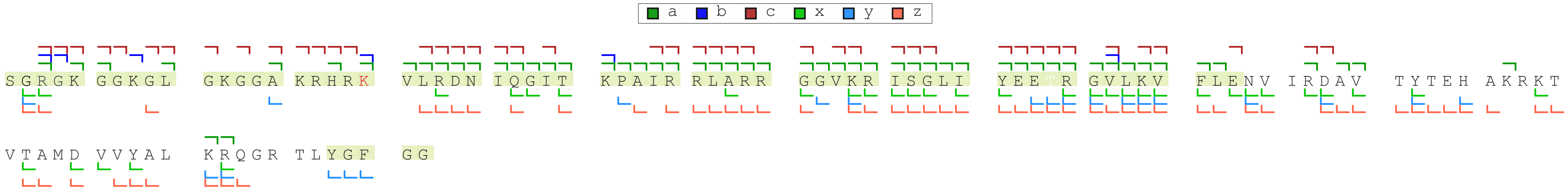

Proteoform:

Index	Protein	SC [%]	SVP [%]	IC [%]	MS Score [a.u.]	Var. Mods	Mono Mass [Da]	Chemical Formula
6	H4Kac	85.1	66.7	33.9	28.9	Ace: K31	11271.3518	C496H839N163O135S

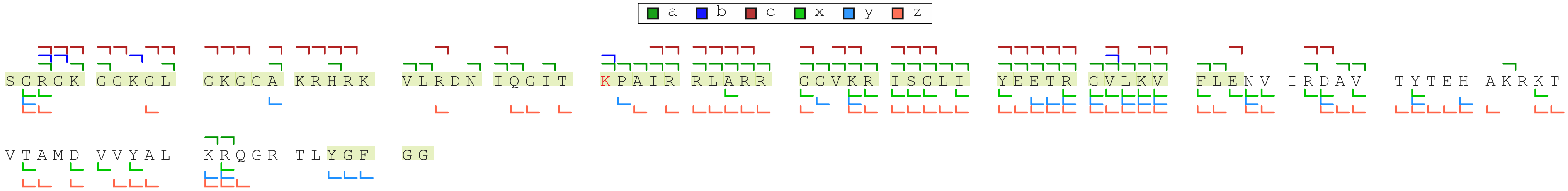

Proteoform:

Index	Protein	SC [%]	SVP [%]	IC [%]	MS Score [a.u.]	Var. Mods	Mono Mass [Da]	Chemical Formula
7	H4Kac	76.2	33.3	27.7	21.1	Ace: K44	11271.3518	C496H839N163O135S

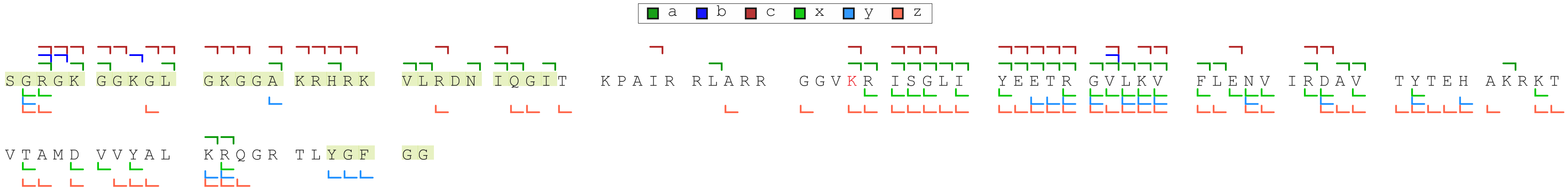

Proteoform:	Index	Protein	SC [%]	SVP [%]	IC [%]	MS Score [a.u.]	Var. Mods	Mono Mass [Da]	Chemical Formula
	8	H4Kac	71.3	33.3	20.9	14.9	Ace: K59	11271.3518	C496H839N163O135S

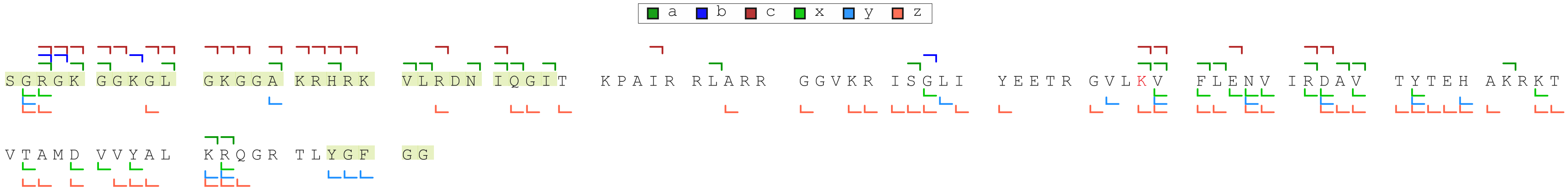

Proteoform:

Index	Protein	SC [%]	SVP [%]	IC [%]	MS Score [a.u.]	Var. Mods	Mono Mass [Da]	Chemical Formula
9	H4Kac	82.2	33.3	14.9	12.2	None	11229.3412	C494H837N163O134S

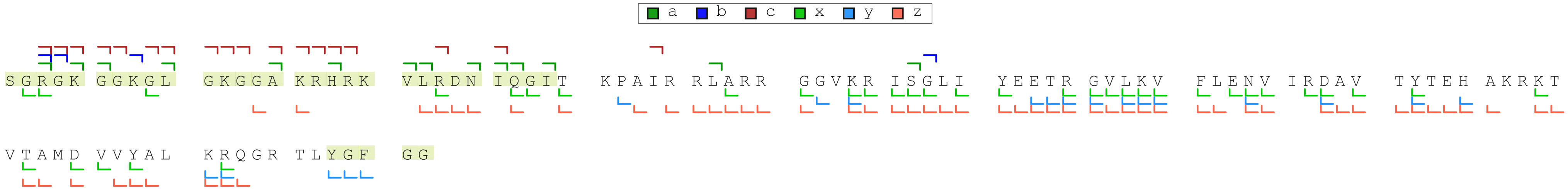

Proteoform:

Index	Protein	SC [%]	SVP [%]	IC [%]	MS Score [a.u.]	Var. Mods	Mono Mass [Da]	Chemical Formula
10	H4Kac	61.4	33.3	16.7	10.2	Ace: K77	11271.3518	C496H839N163O135S

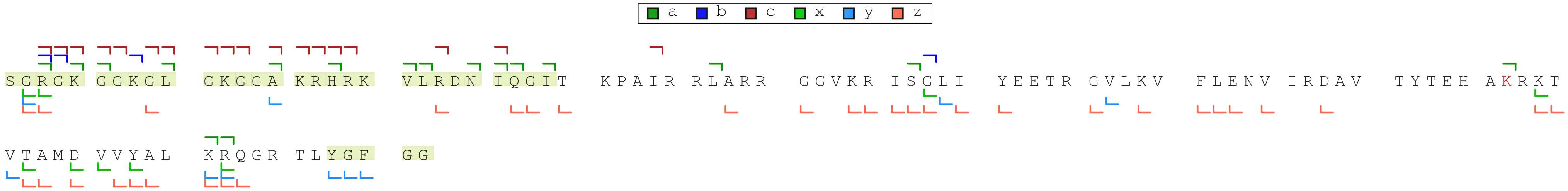

Error Plot

Proteoform:

Index	Protein	SC [%]	SVP [%]	IC [%]	MS Score [a.u.]	Var. Mods	Mono Mass [Da]	Chemical Formula
1	H4Kac	89.1	66.7	47.5	42.3	Ace: K8	11271.3518	C496H839N163O135S

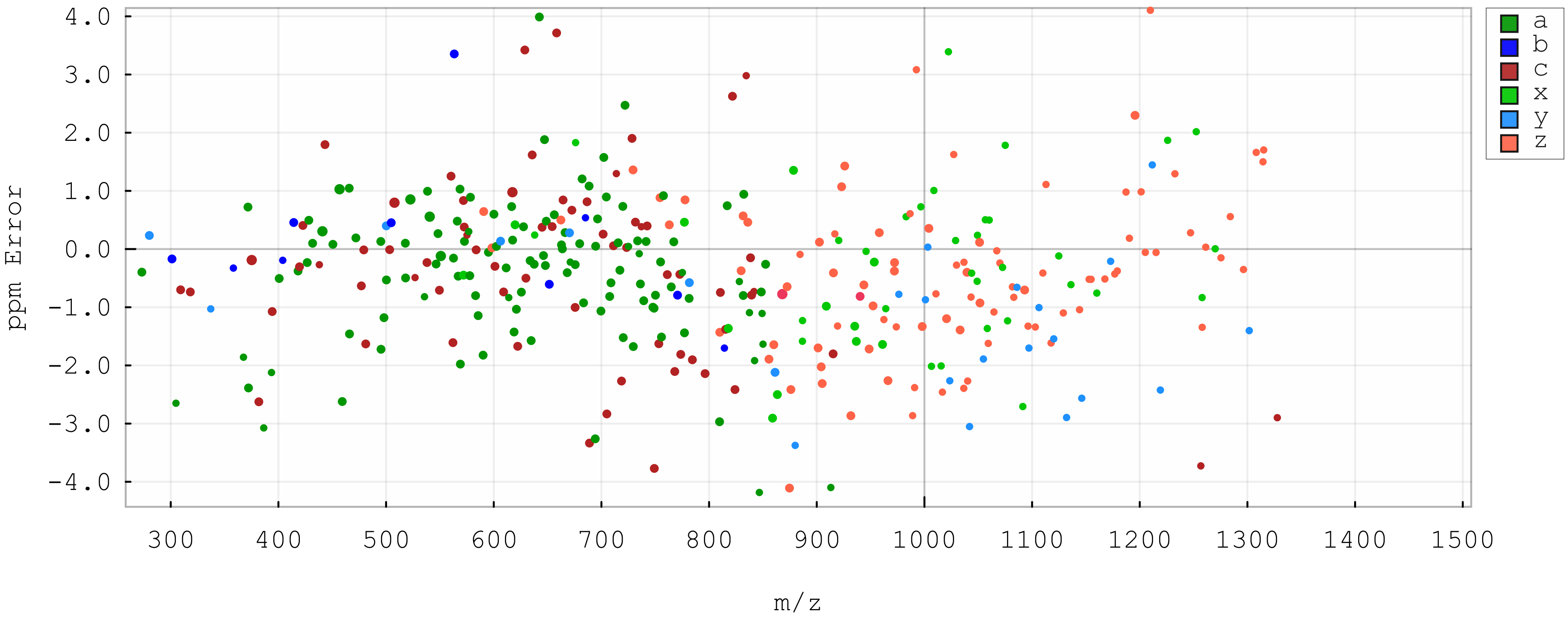

Proteoform:

Index	Protein	SC [%]	SVP [%]	IC [%]	MS Score [a.u.]	Var. Mods	Mono Mass [Da]	Chemical Formula
2	H4Kac	89.1	66.7	46.2	41.1	Ace: K12	11271.3518	C496H839N163O135S

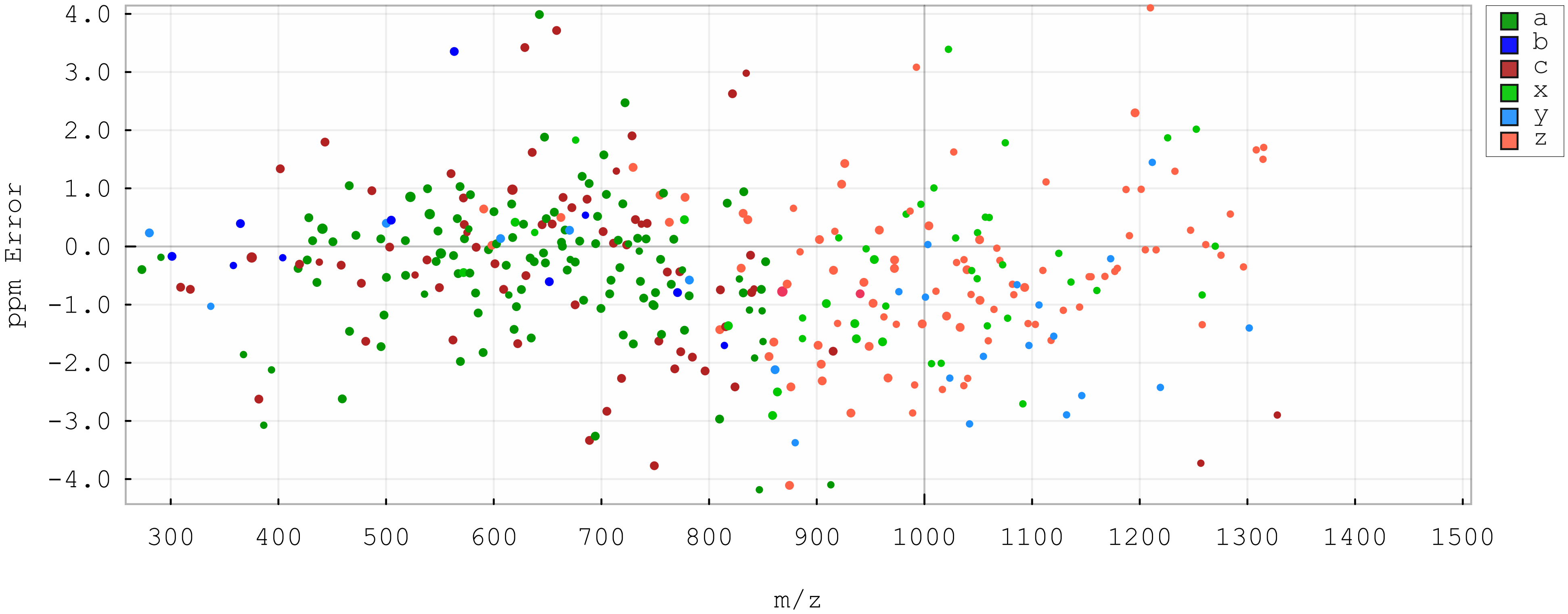

Proteoform:	Index	Protein	SC [%]	SVP [%]	IC [%]	MS Score [a.u.]	Var. Mods	Mono Mass [Da]	Chemical Formula
	3	H4Kac	86.1	8.8	46.9	40.4	Ace: K5	11271.3518	C496H839N163O135S

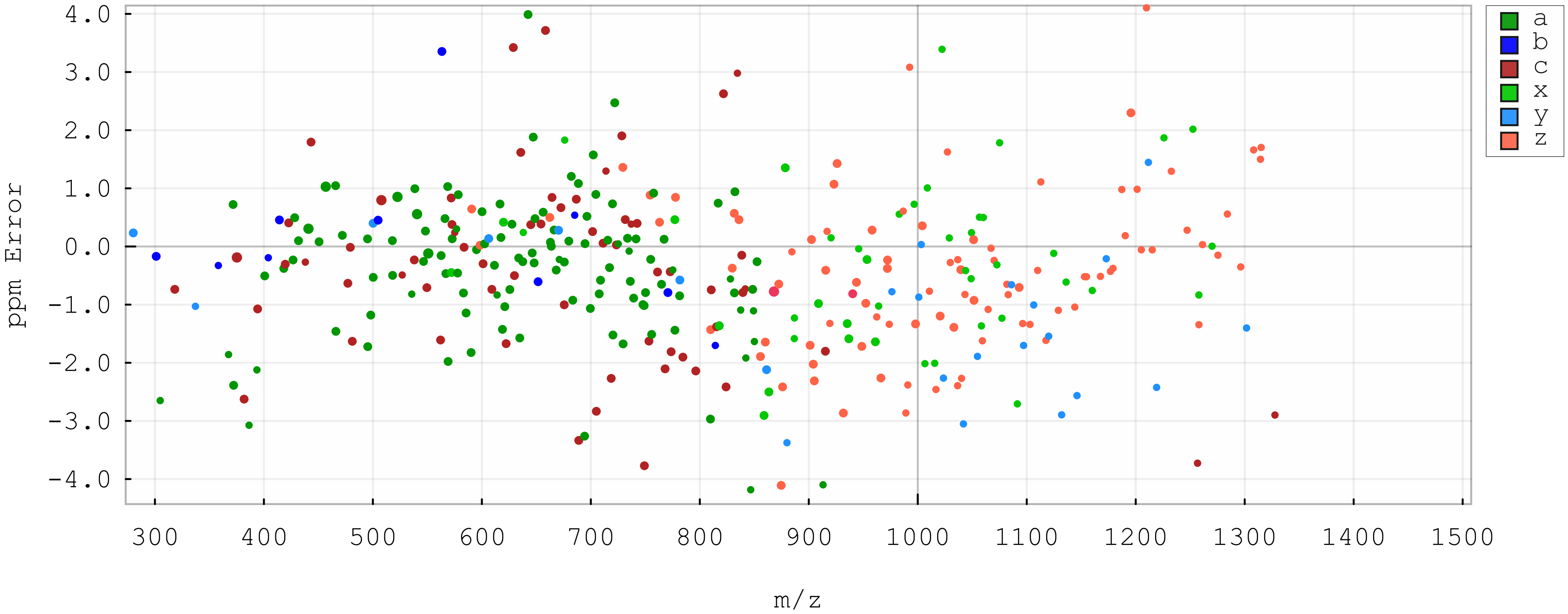

Proteoform:

Index	Protein	SC [%]	SVP [%]	IC [%]	MS Score [a.u.]	Var. Mods	Mono Mass [Da]	Chemical Formula
4	H4Kac	88.1	66.7	45.1	39.7	Ace: K16	11271.3518	C496H839N163O135S

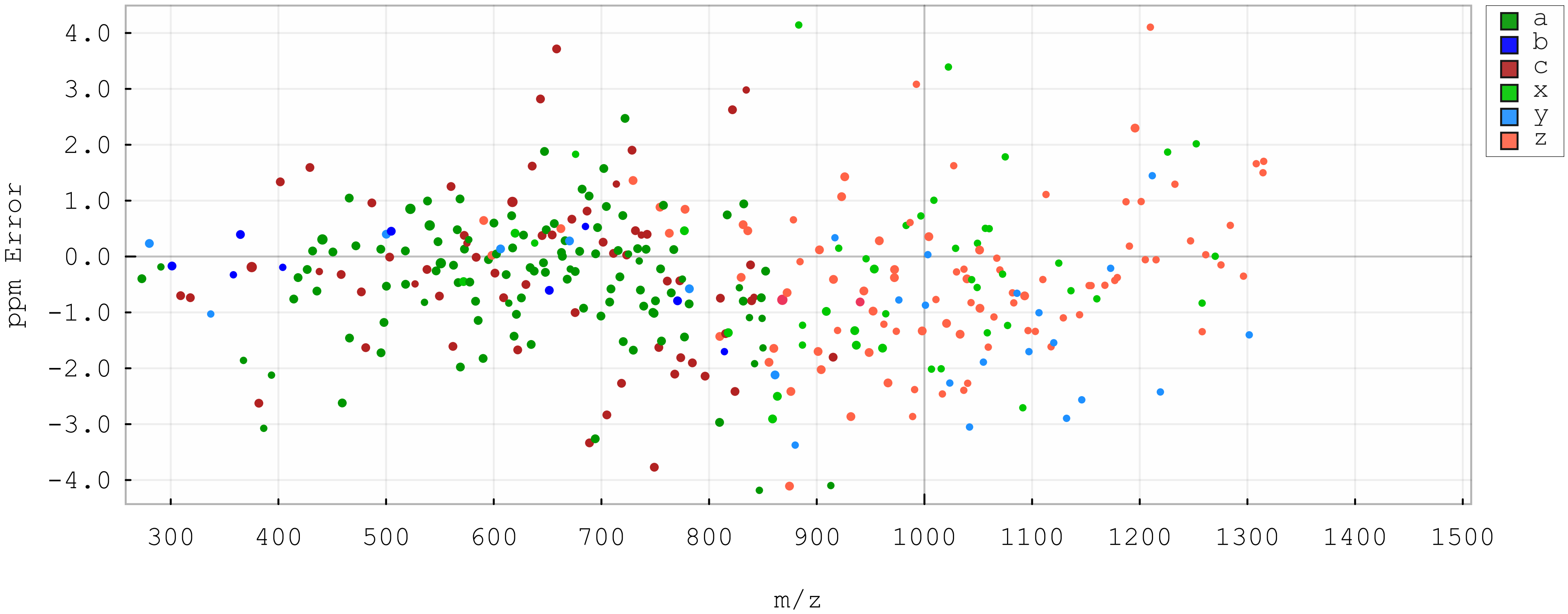

Proteoform:

Index	Protein	SC [%]	SVP [%]	IC [%]	MS Score [a.u.]	Var. Mods	Mono Mass [Da]	Chemical Formula
5	H4Kac	88.1	66.7	43.4	38.3	Ace: K20	11271.3518	C496H839N163O135S

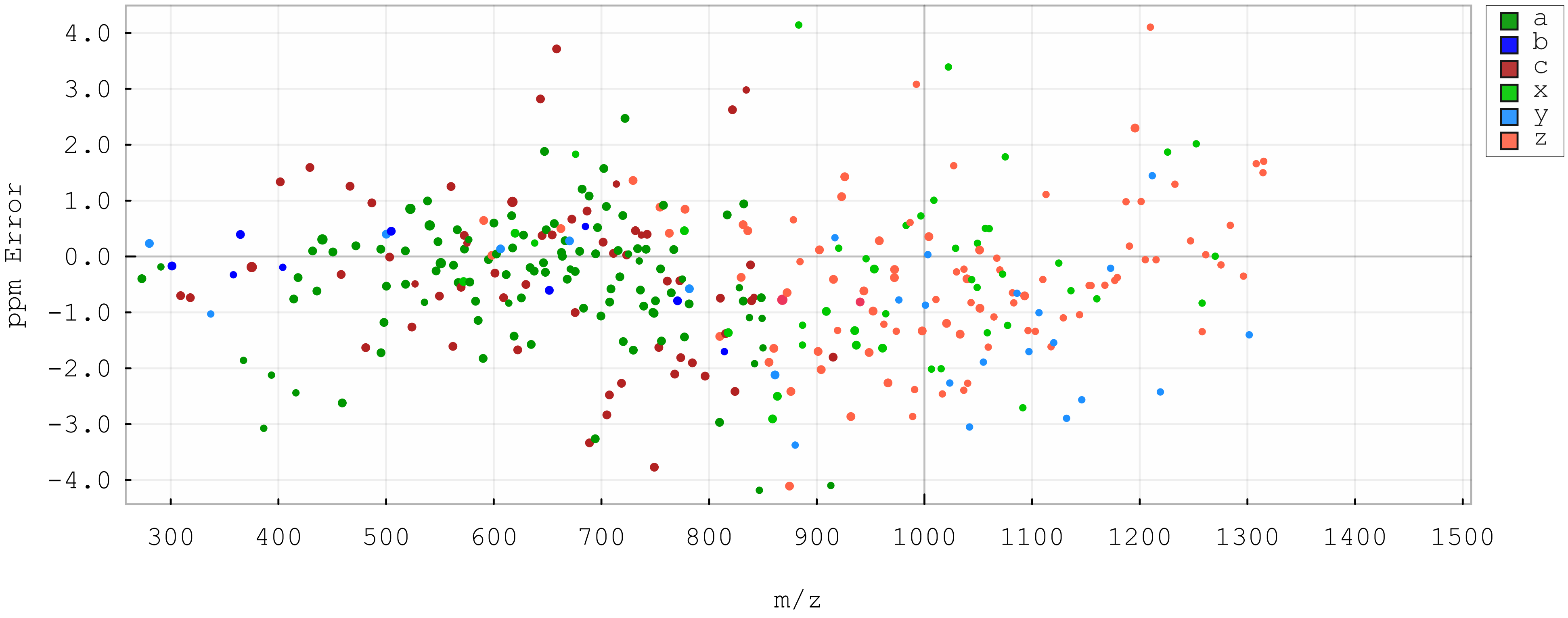

Proteoform:

Index	Protein	SC [%]	SVP [%]	IC [%]	MS Score [a.u.]	Var. Mods	Mono Mass [Da]	Chemical Formula
6	H4Kac	85.1	66.7	33.9	28.9	Ace: K31	11271.3518	C496H839N163O135S

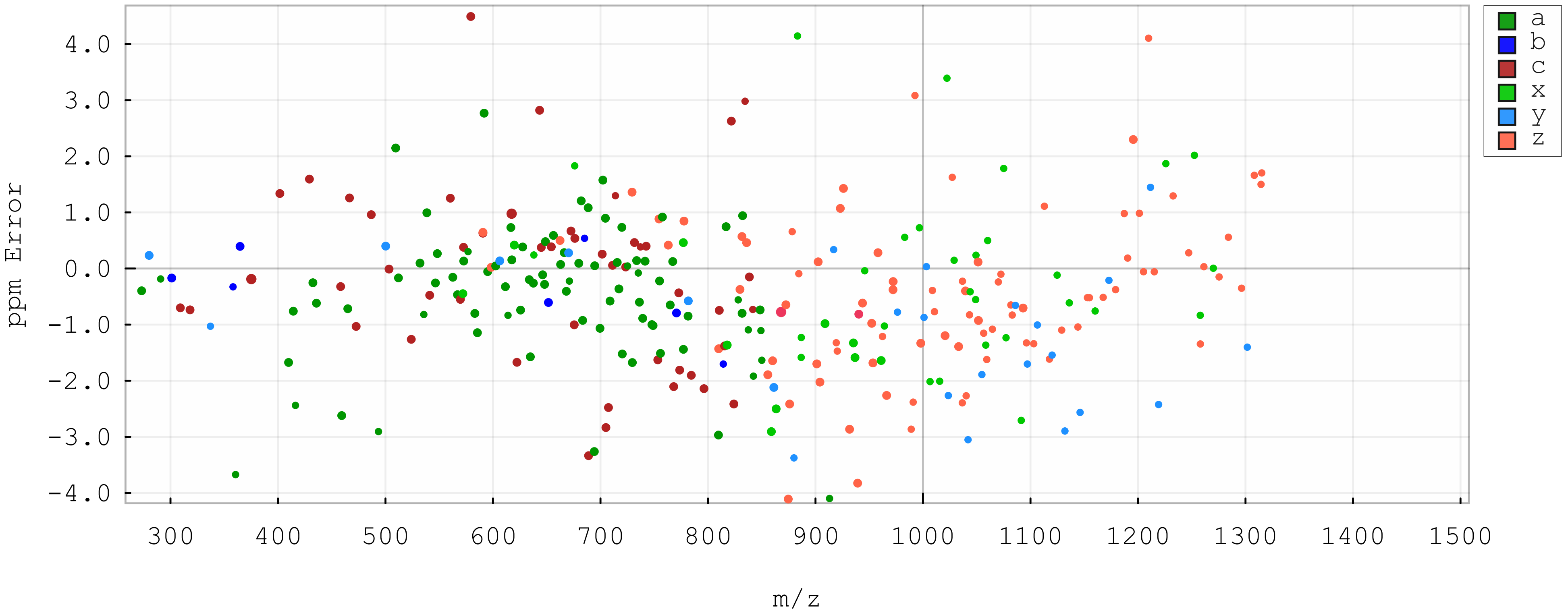

Proteoform:

Index	Protein	SC [%]	SVP [%]	IC [%]	MS Score [a.u.]	Var. Mods	Mono Mass [Da]	Chemical Formula
7	H4Kac	76.2	33.3	27.7	21.1	Ace: K44	11271.3518	$C_{496}H_{839}N_{163}O_{135}S$

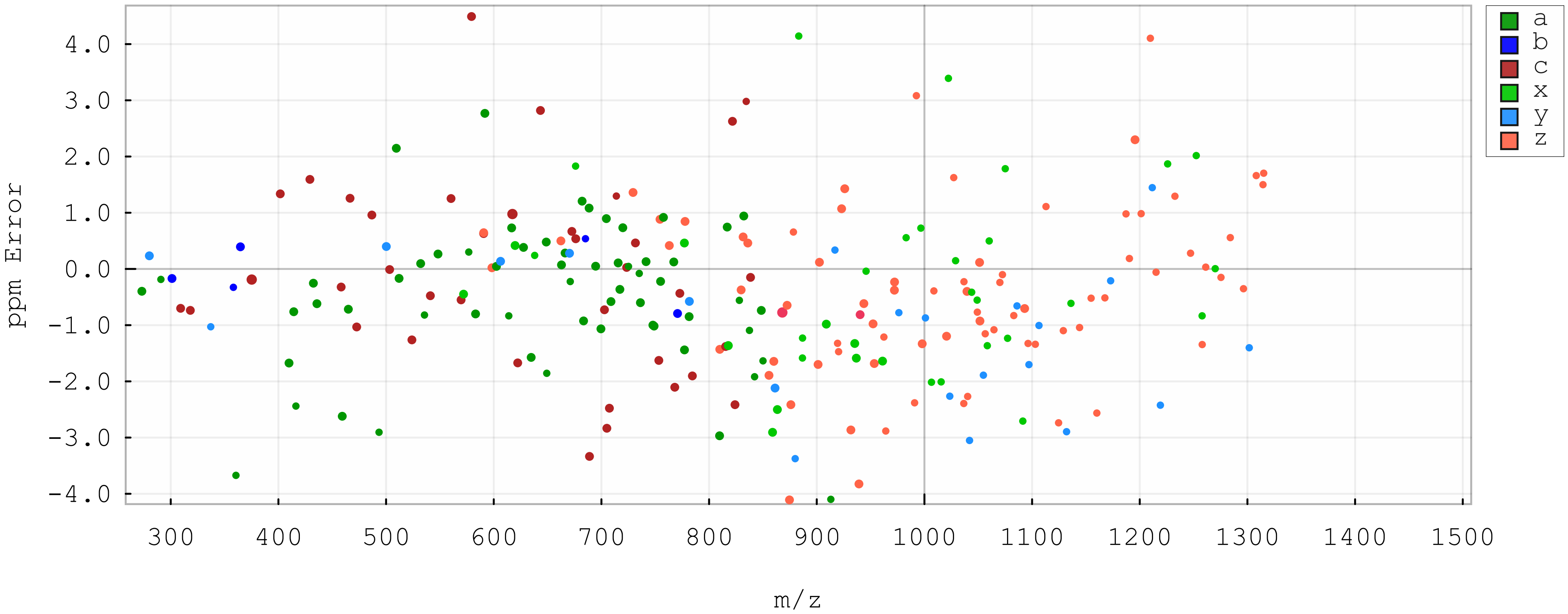

Proteoform:

Index	Protein	SC [%]	SVP [%]	IC [%]	MS Score [a.u.]	Var. Mods	Mono Mass [Da]	Chemical Formula
8	H4Kac	71.3	33.3	20.9	14.9	Ace: K59	11271.3518	C496H839N163O135S

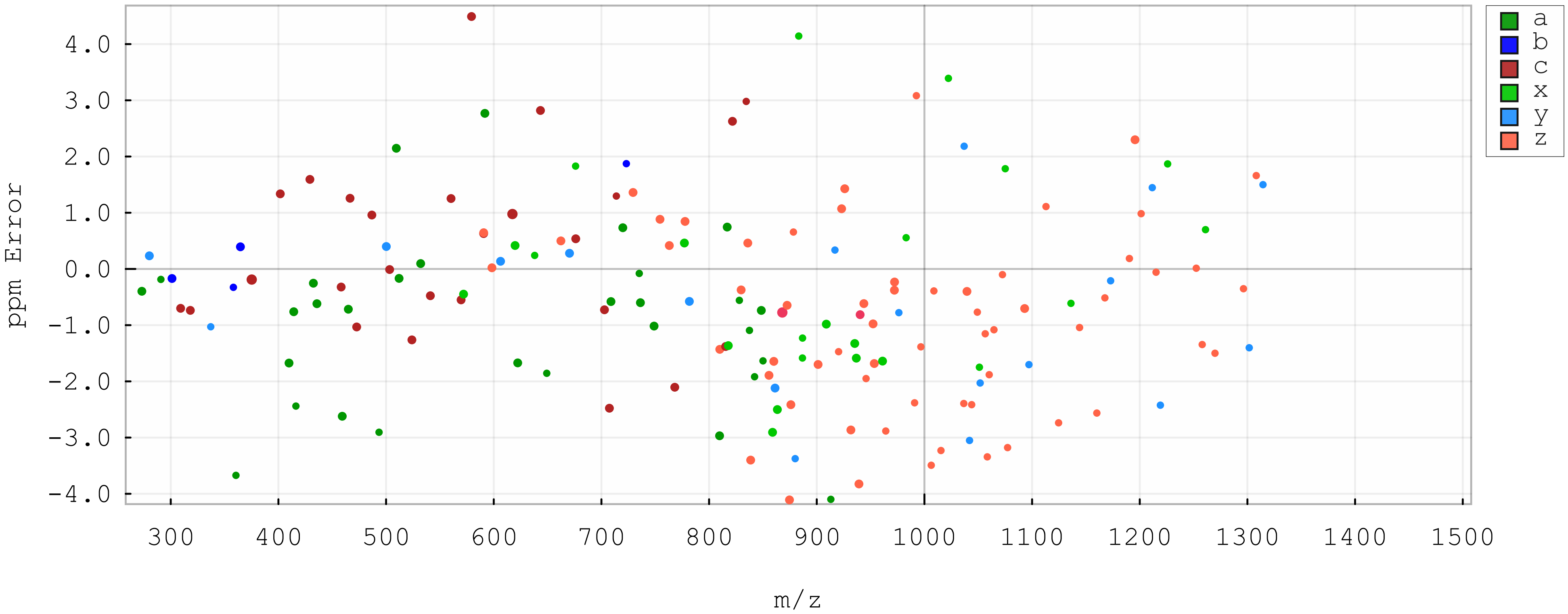

Proteoform:

Index	Protein	SC [%]	SVP [%]	IC [%]	MS Score [a.u.]	Var. Mods	Mono Mass [Da]	Chemical Formula
9	H4Kac	82.2	33.3	14.9	12.2	None	11229.3412	C494H837N163O134S

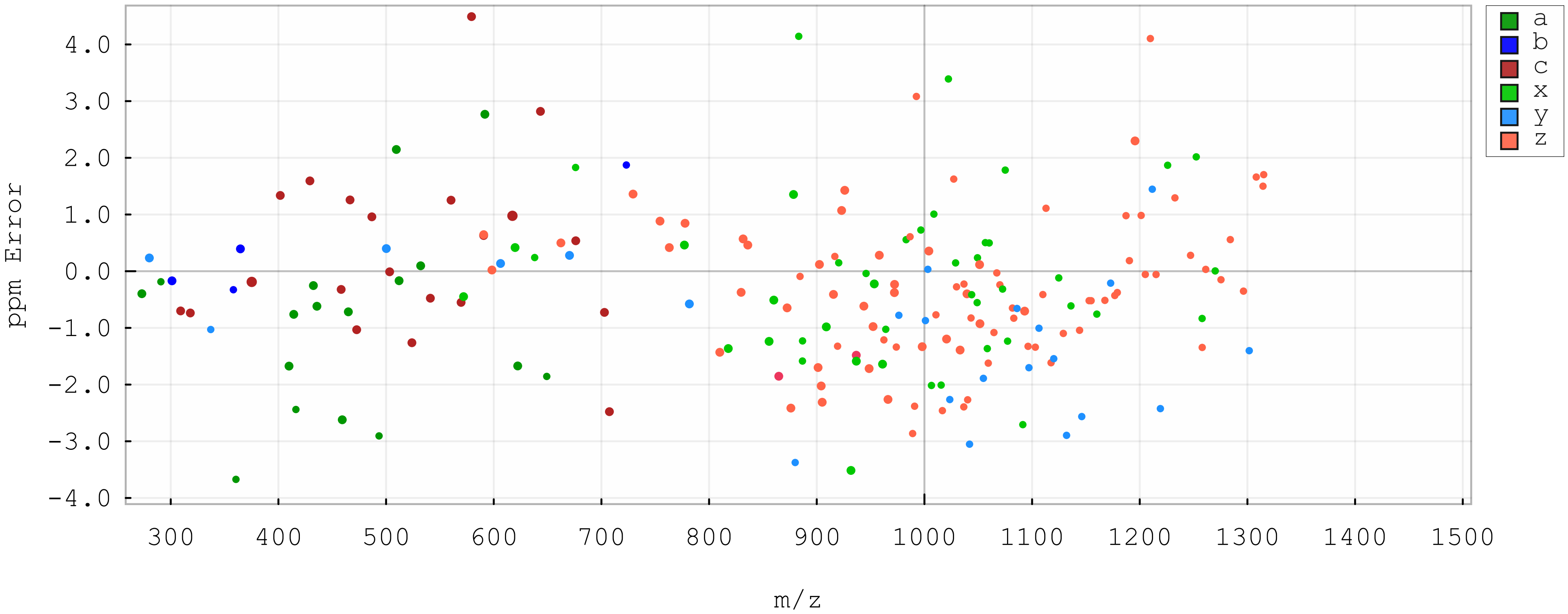

Proteoform:

Index	Protein	SC [%]	SVP [%]	IC [%]	MS Score [a.u.]	Var. Mods	Mono Mass [Da]	Chemical Formula
10	H4Kac	61.4	33.3	16.7	10.2	Ace: K77	11271.3518	C496H839N163O135S

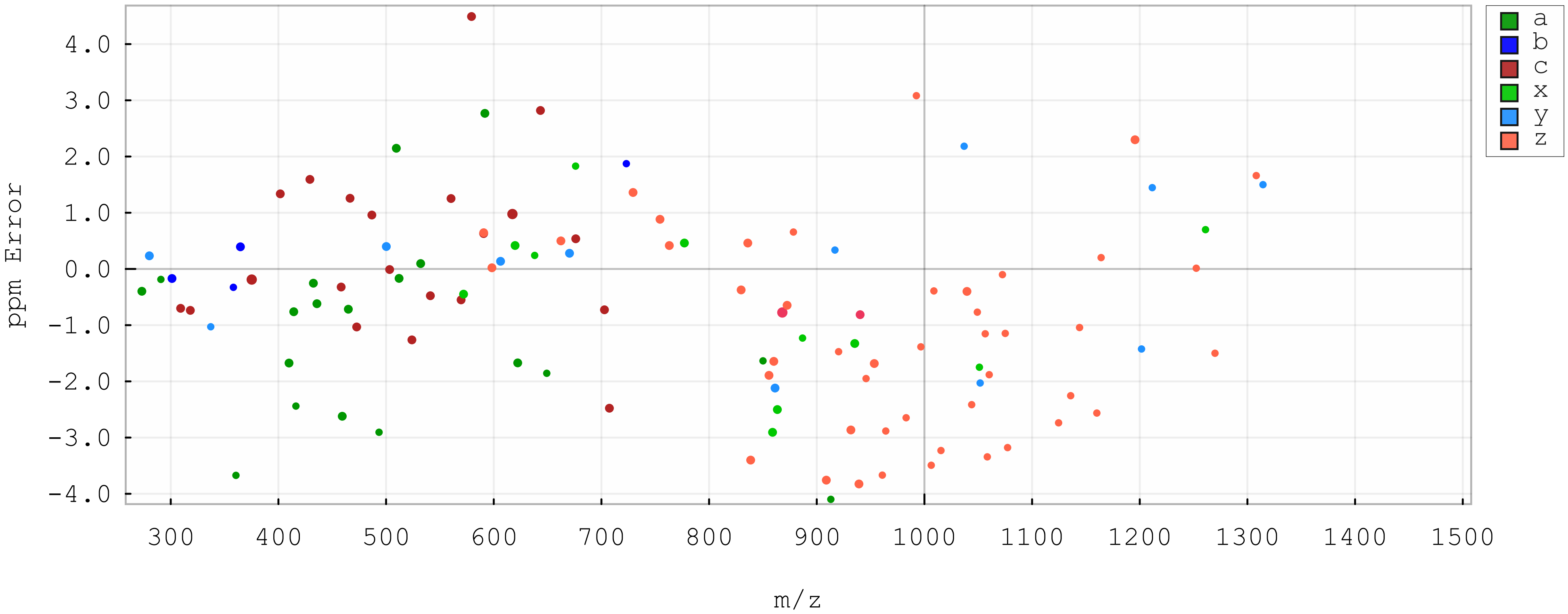

Primary Fragment Plots

Proteoform:

Index	Protein	SC [%]	SVP [%]	IC [%]	MS Score [a.u.]	Var. Mods	Mono Mass [Da]	Chemical Formula
1	H4Kac	89.1	66.7	47.5	42.3	Ace: K8	11271.3518	C496H839N163O135S

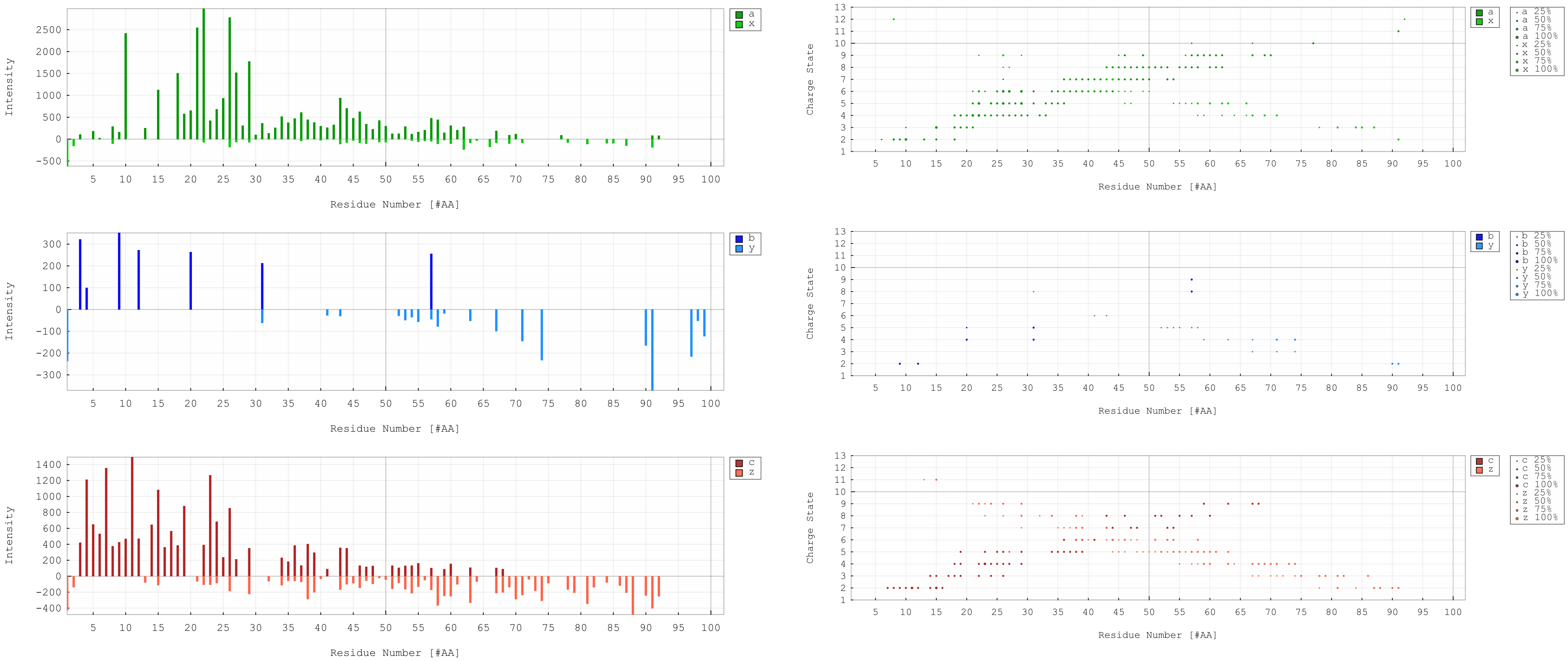

Proteoform:

Index	Protein	SC [%]	SVP [%]	IC [%]	MS Score [a.u.]	Var. Mods	Mono Mass [Da]	Chemical Formula
2	H4Kac	89.1	66.7	46.2	41.1	Ace: K12	11271.3518	C496H839N163O135S

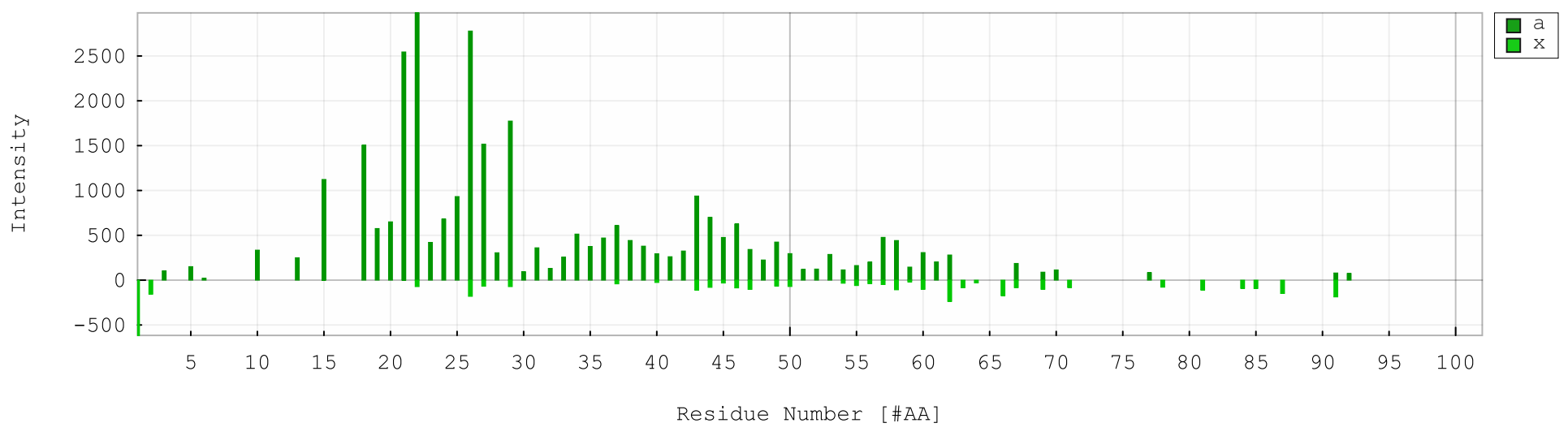

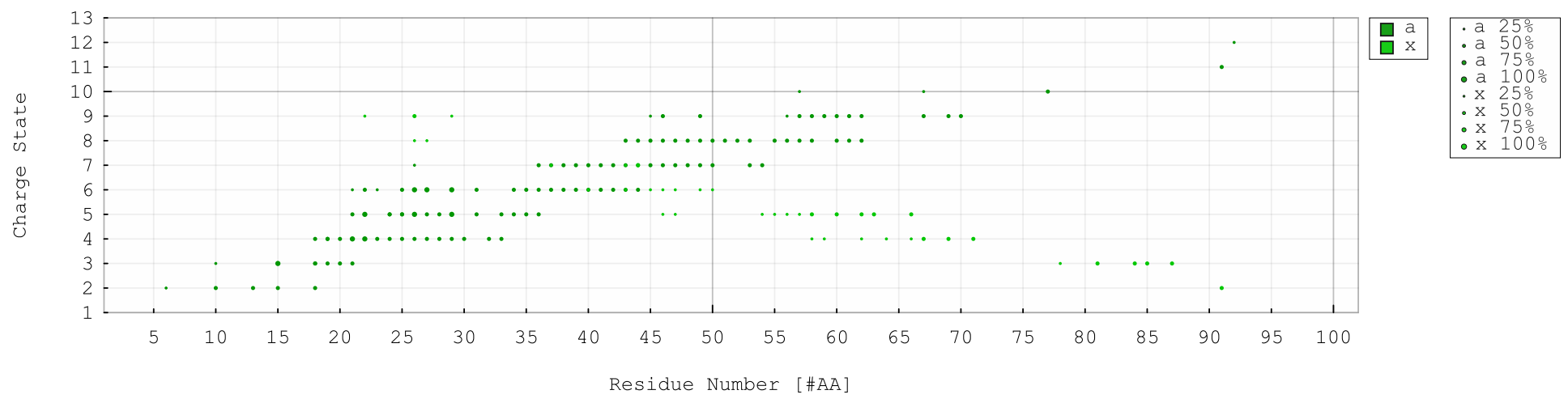

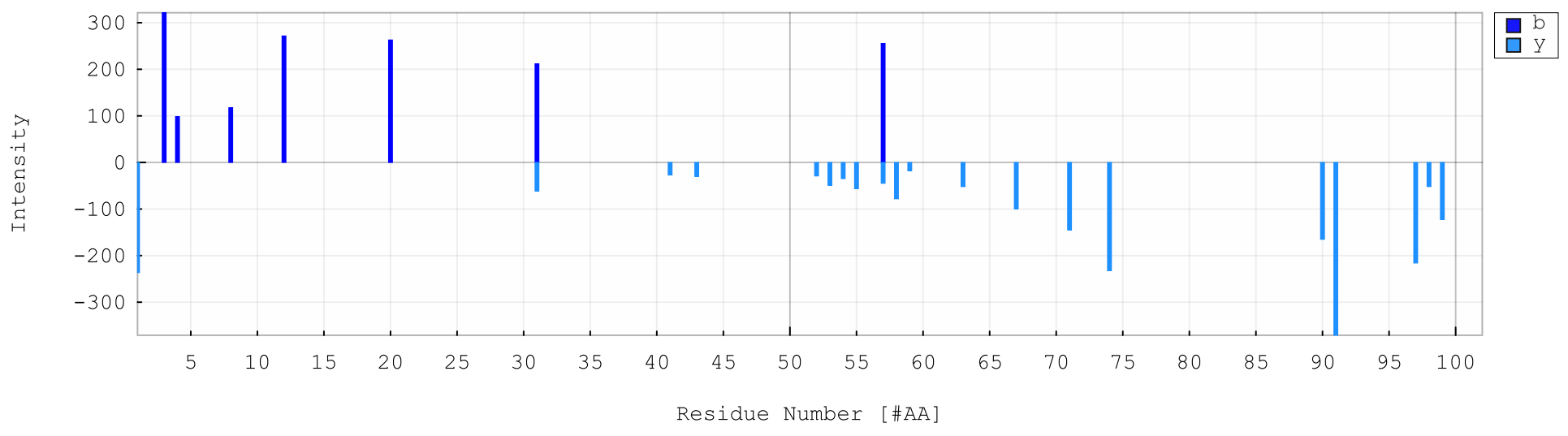

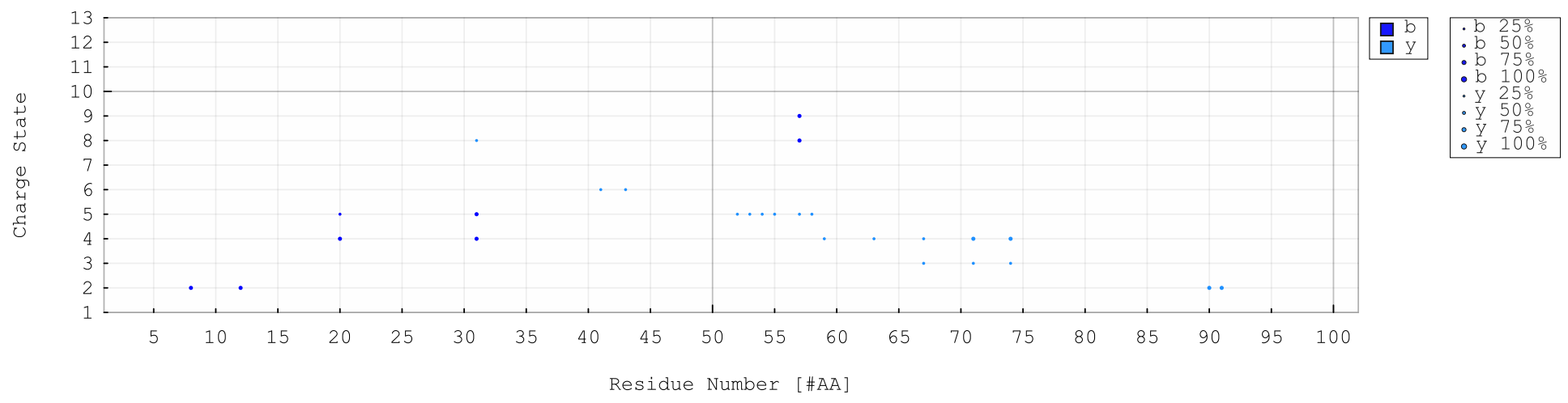

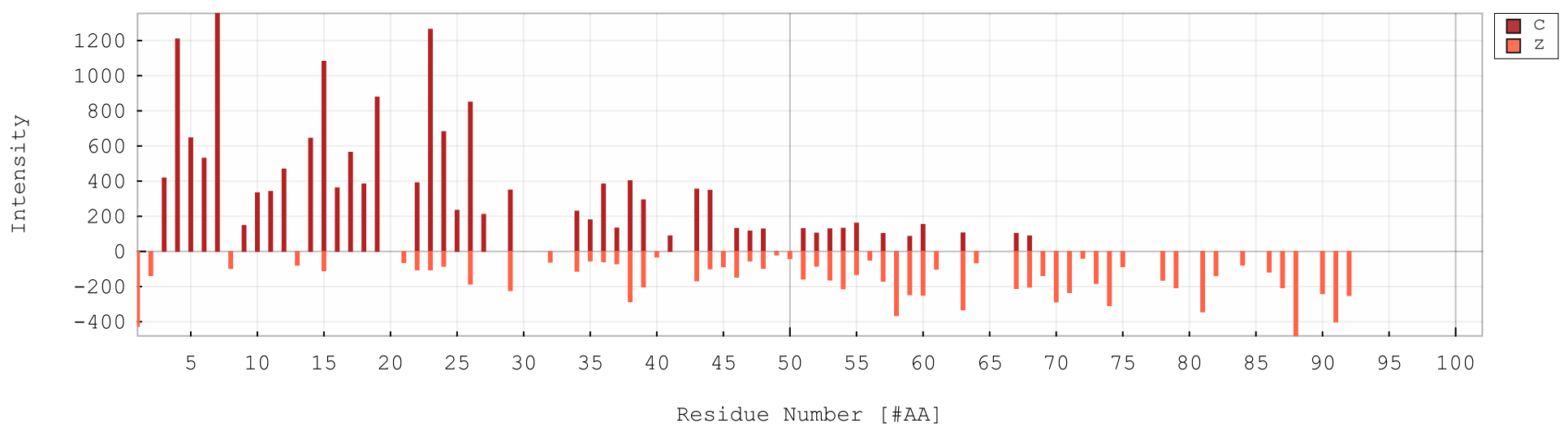

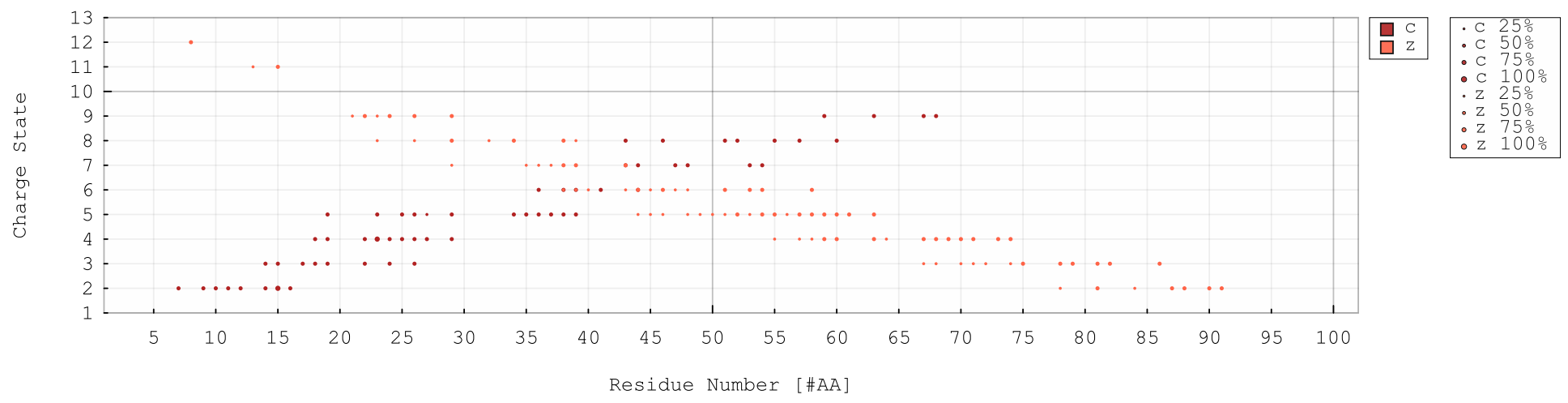

Proteoform:

Index	Protein	SC [%]	SVP [%]	IC [%]	MS Score [a.u.]	Var. Mods	Mono Mass [Da]	Chemical Formula
3	H4Kac	86.1	8.8	46.9	40.4	Ace: K5	11271.3518	C496H839N163O135S

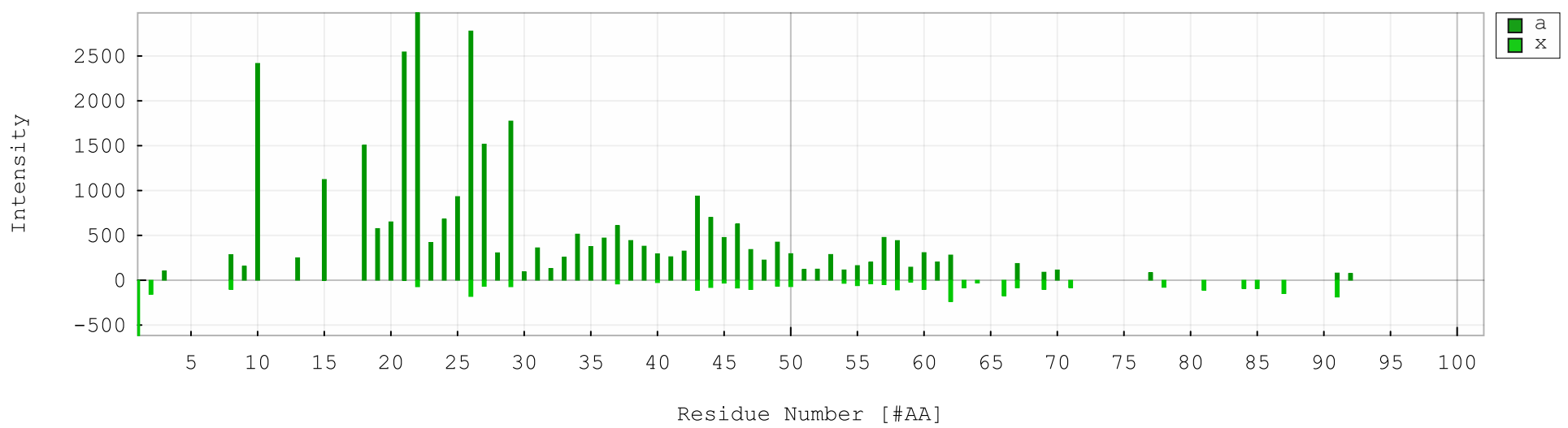

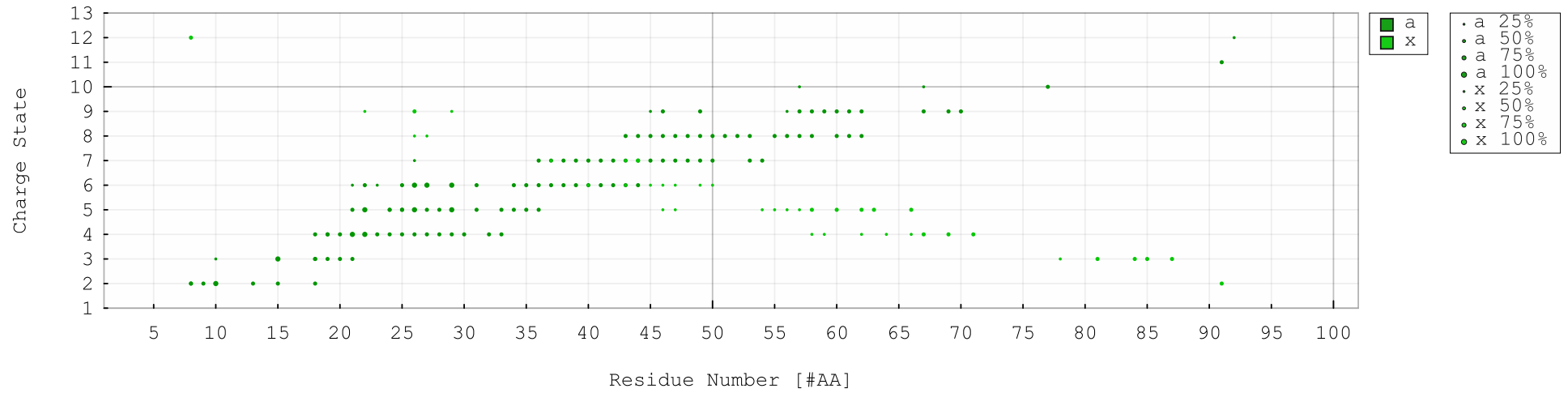

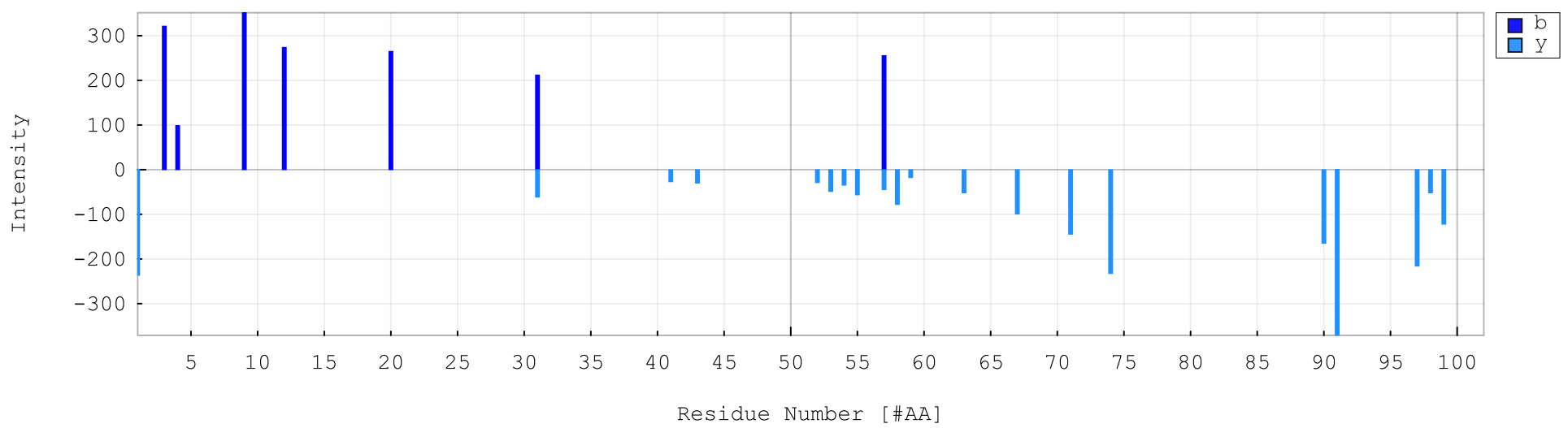

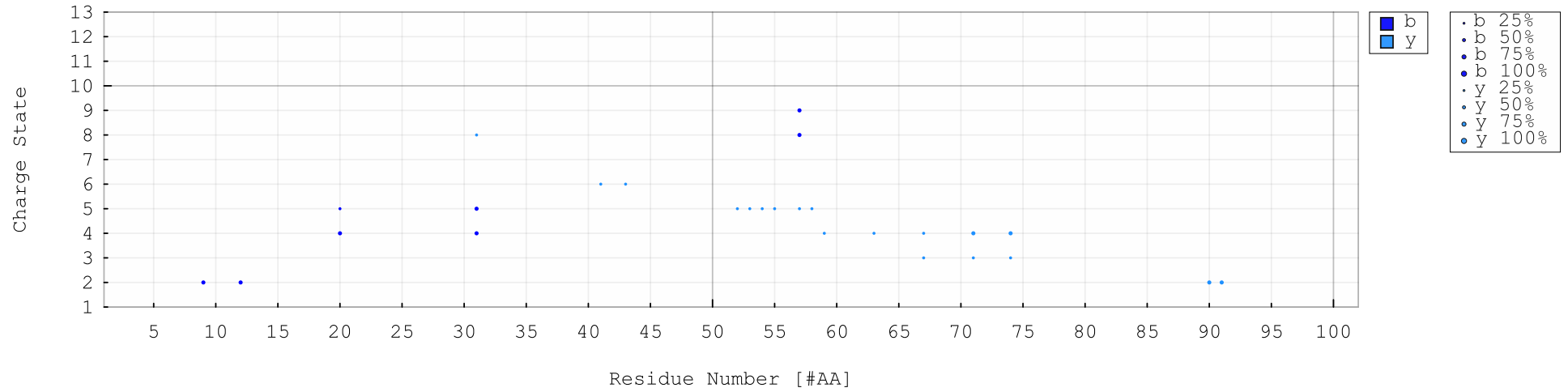

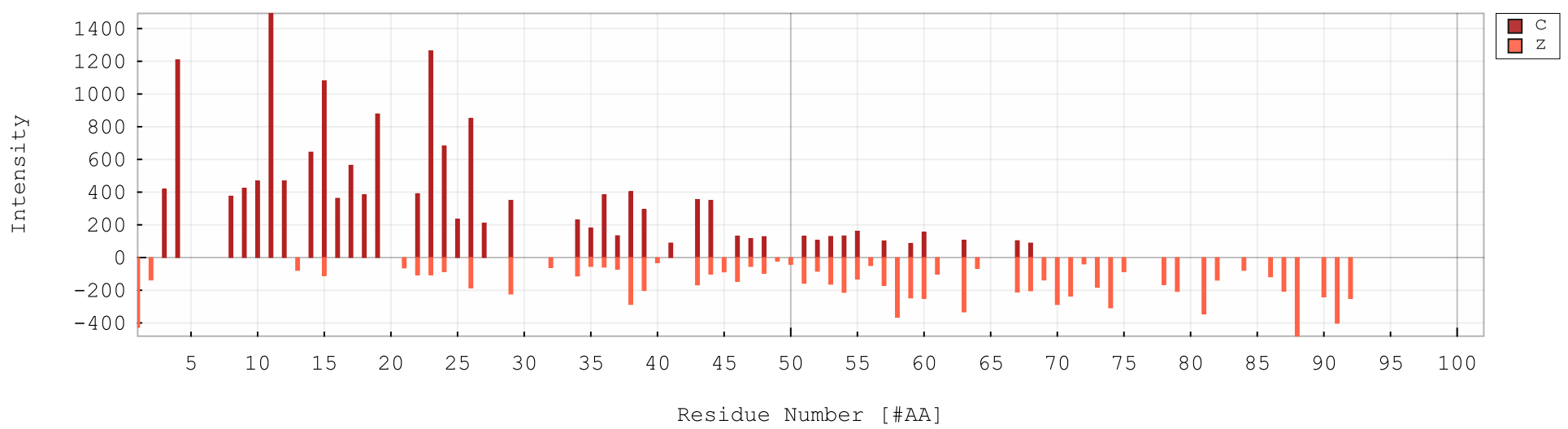

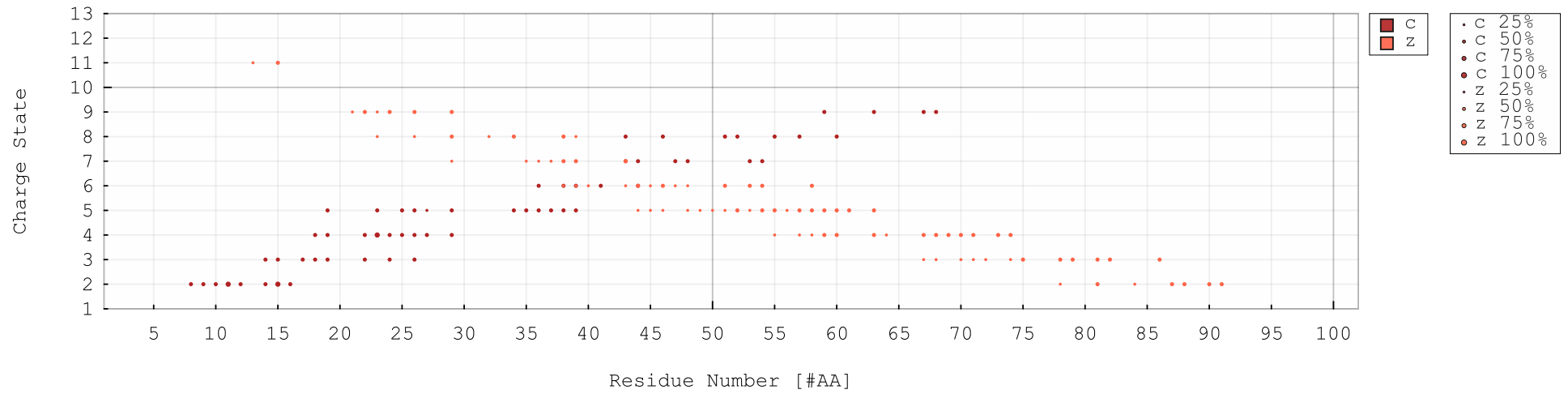

Proteoform:

Index	Protein	SC [%]	SVP [%]	IC [%]	MS Score [a.u.]	Var. Mods	Mono Mass [Da]	Chemical Formula
4	H4Kac	88.1	66.7	45.1	39.7	Ace: K16	11271.3518	C496H839N163O135S

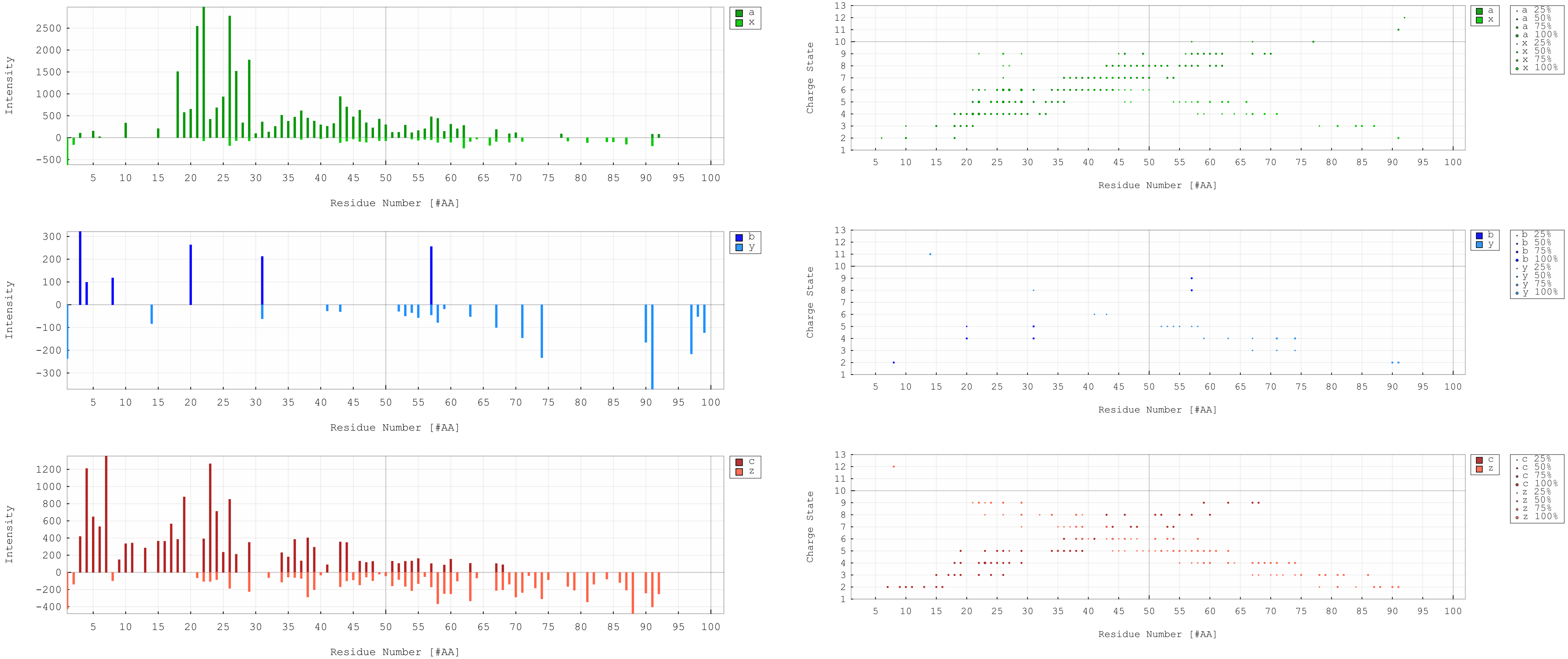

Proteoform:

Index	Protein	SC [%]	SVP [%]	IC [%]	MS Score [a.u.]	Var. Mods	Mono Mass [Da]	Chemical Formula
5	H4Kac	88.1	66.7	43.4	38.3	Ace: K20	11271.3518	C496H839N163O135S

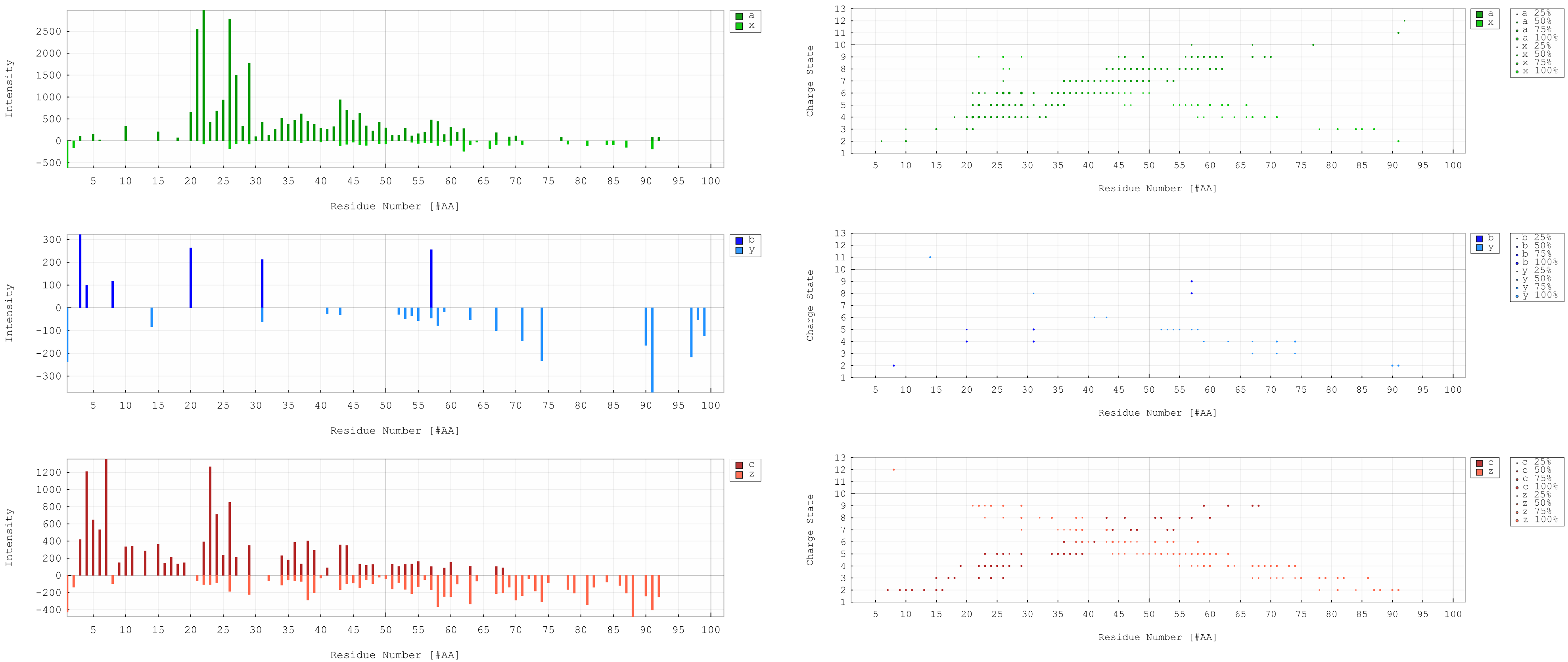

Proteoform:	Index	Protein	SC [%]	SVP [%]	IC [%]	MS Score [a.u.]	Var. Mods	Mono Mass [Da]	Chemical Formula
	6	H4Kac	85.1	66.7	33.9	28.9	Ace: K31	11271.3518	C496H839N163O135S

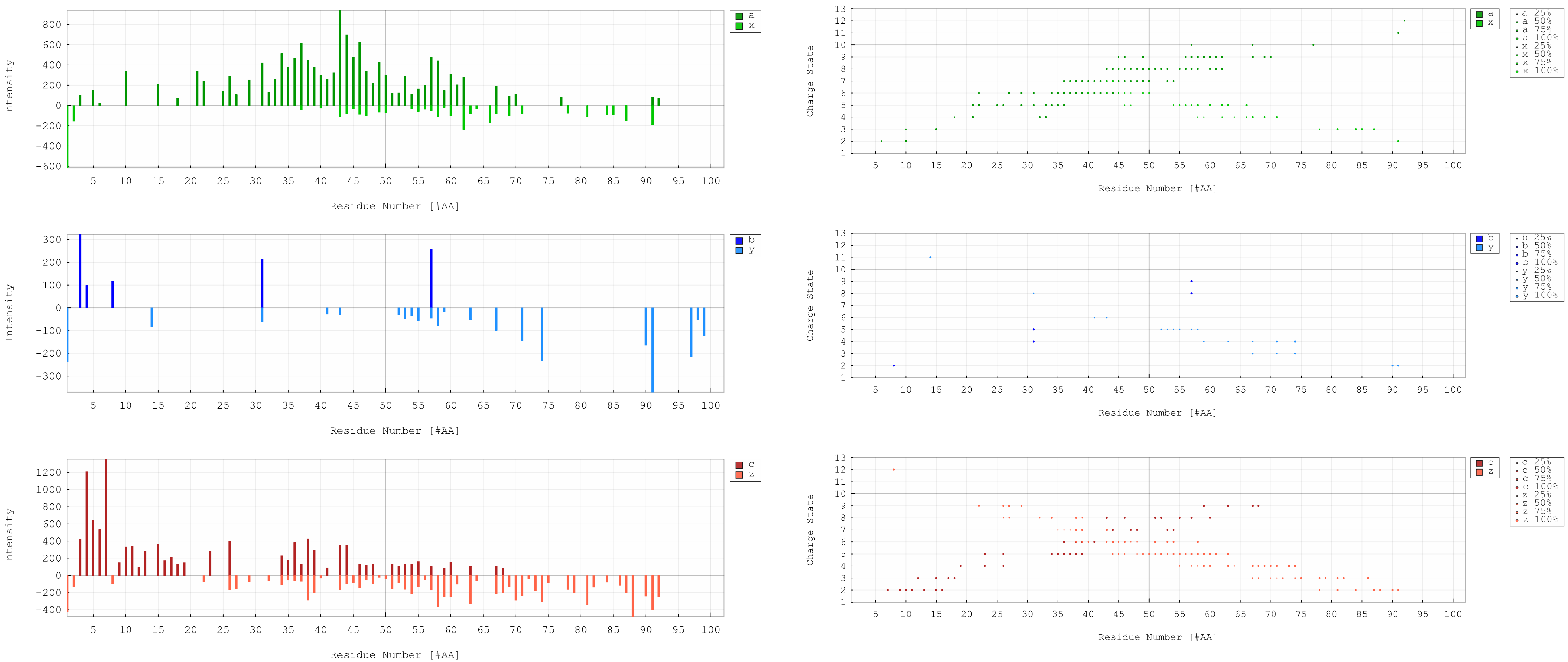

Proteoform:

Index	Protein	SC [%]	SVP [%]	IC [%]	MS Score [a.u.]	Var. Mods	Mono Mass [Da]	Chemical Formula
7	H4Kac	76.2	33.3	27.7	21.1	Ace: K44	11271.3518	C496H839N163O135S

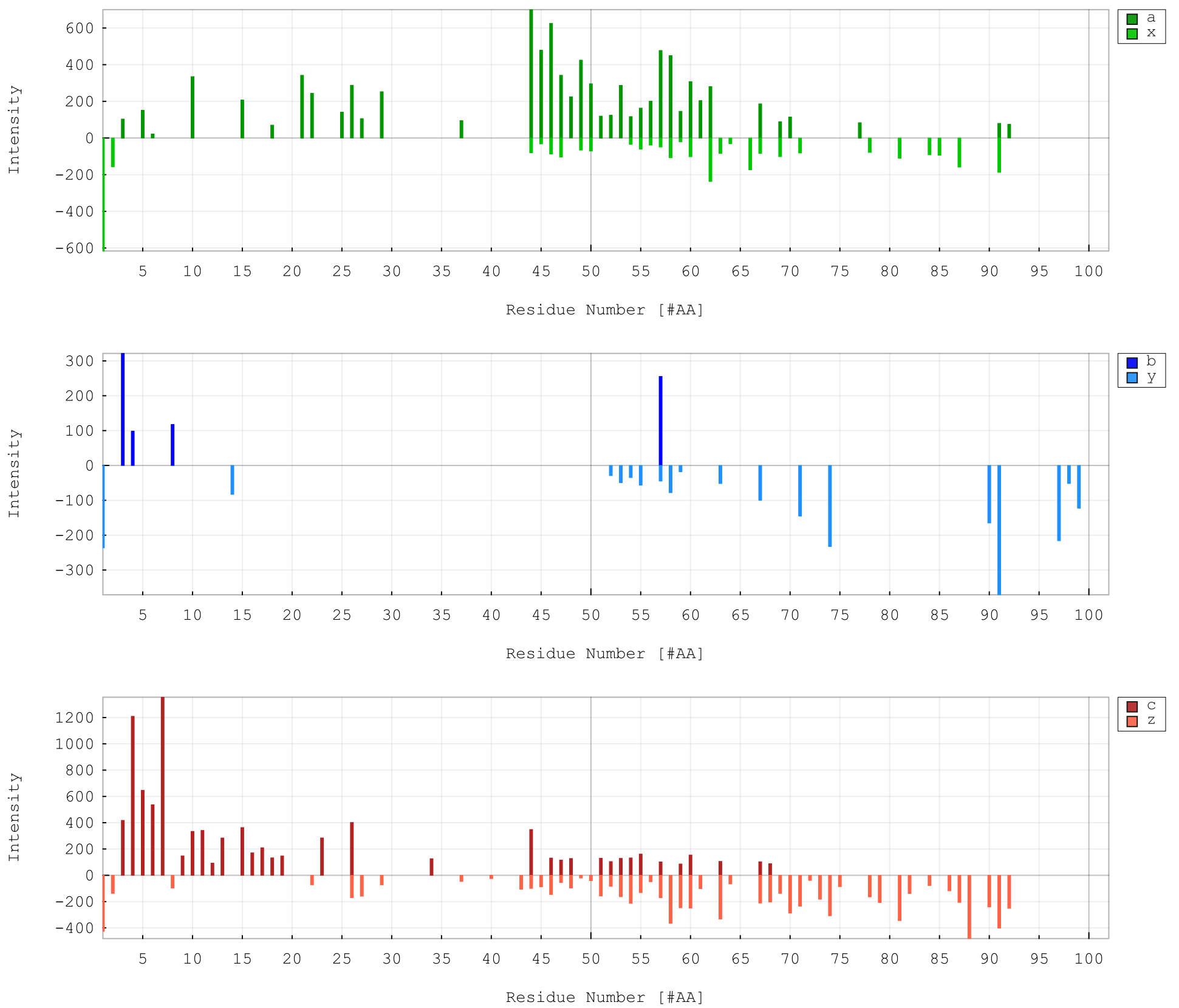

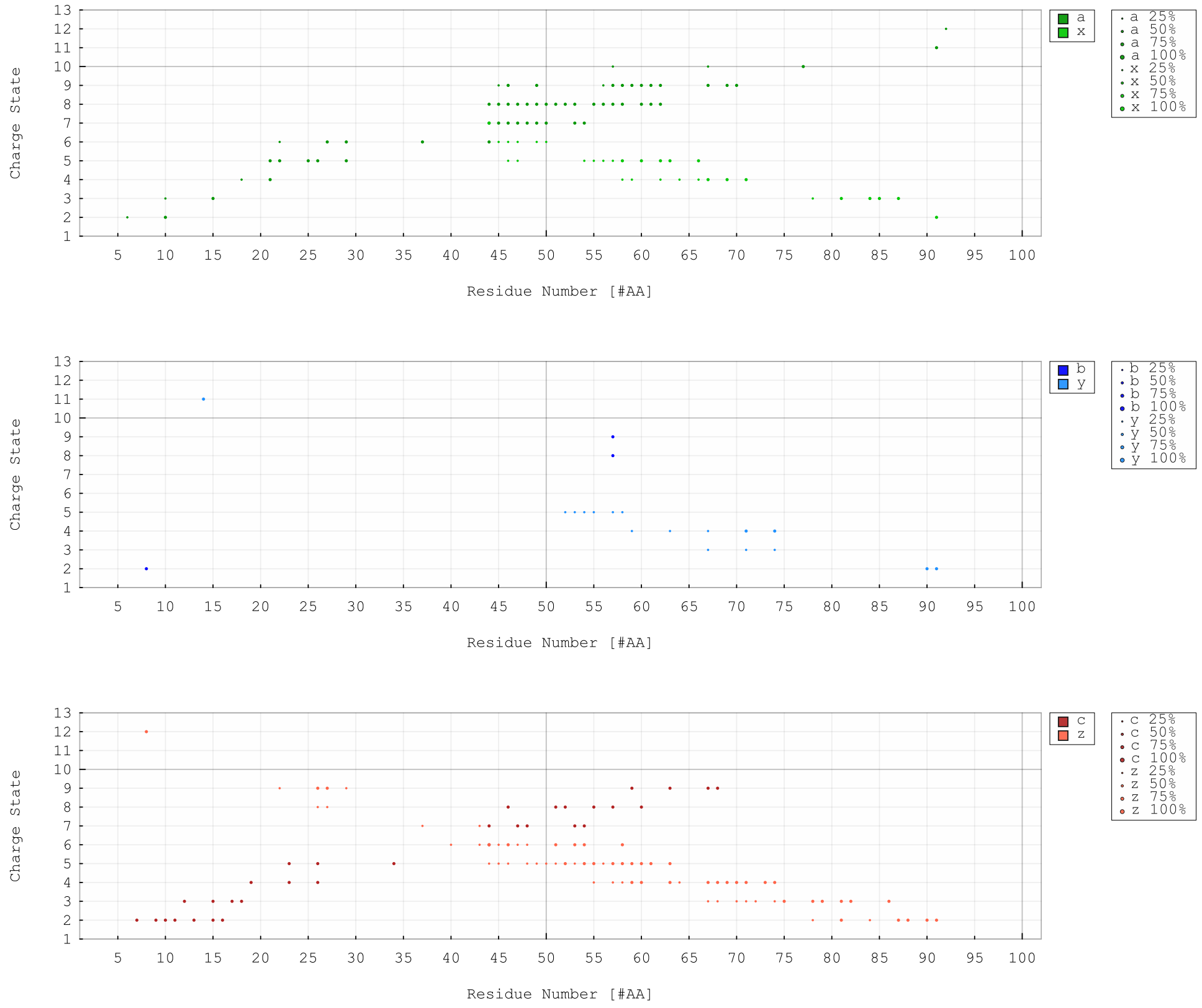

Proteoform:

Index	Protein	SC [%]	SVP [%]	IC [%]	MS Score [a.u.]	Var. Mods	Mono Mass [Da]	Chemical Formula
8	H4Kac	71.3	33.3	20.9	14.9	Ace: K59	11271.3518	C496H839N163O135S

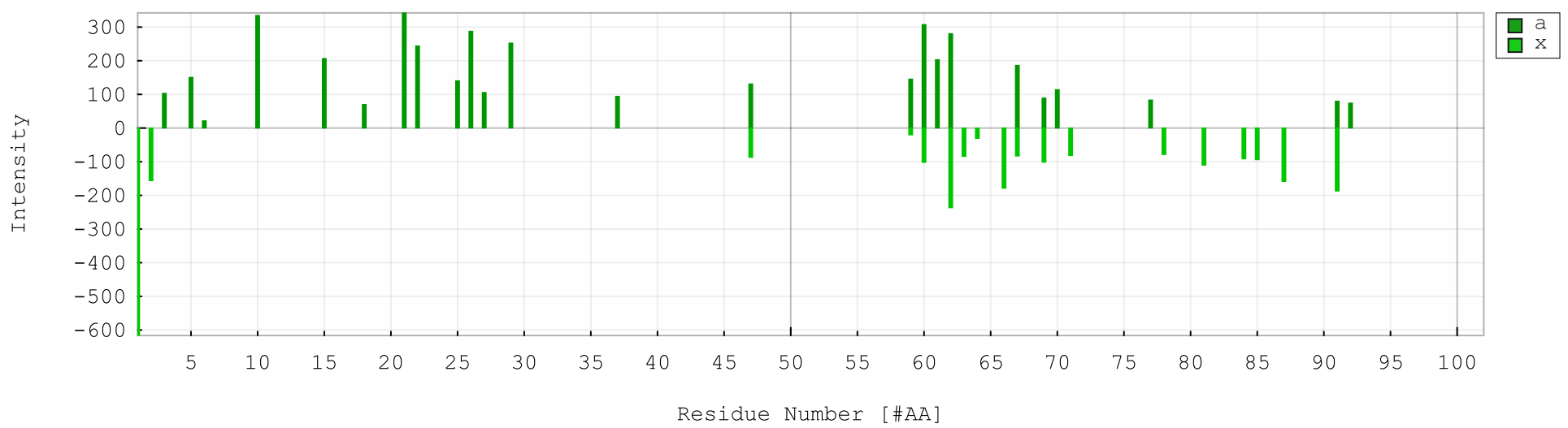

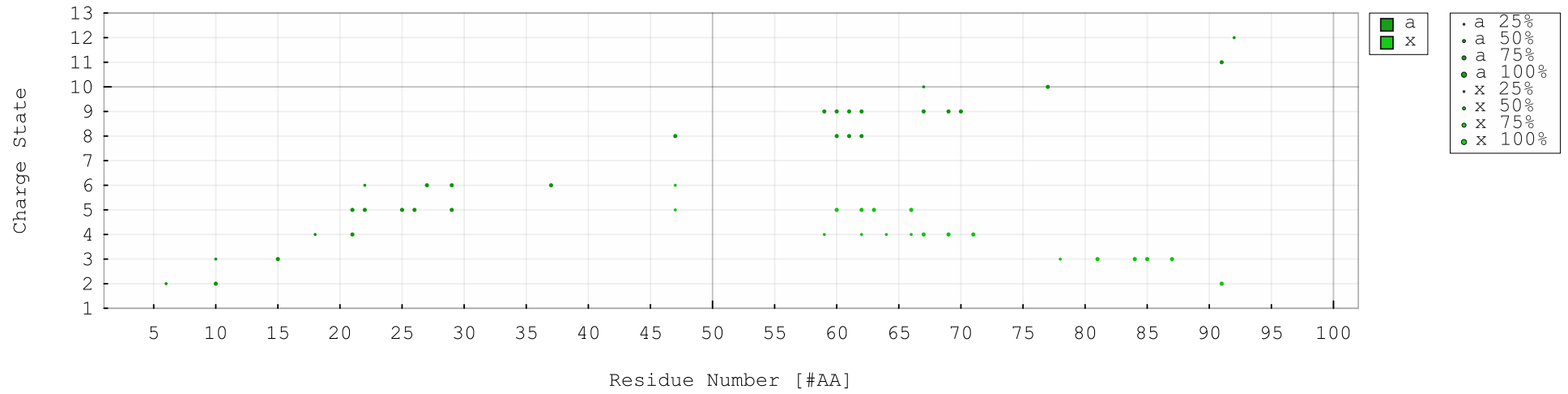

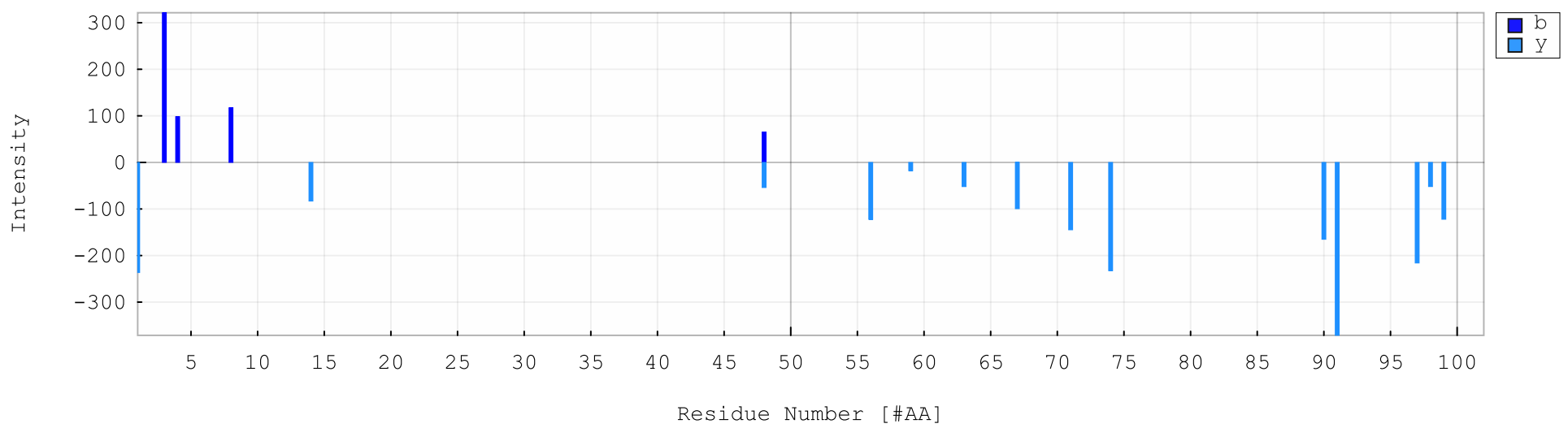

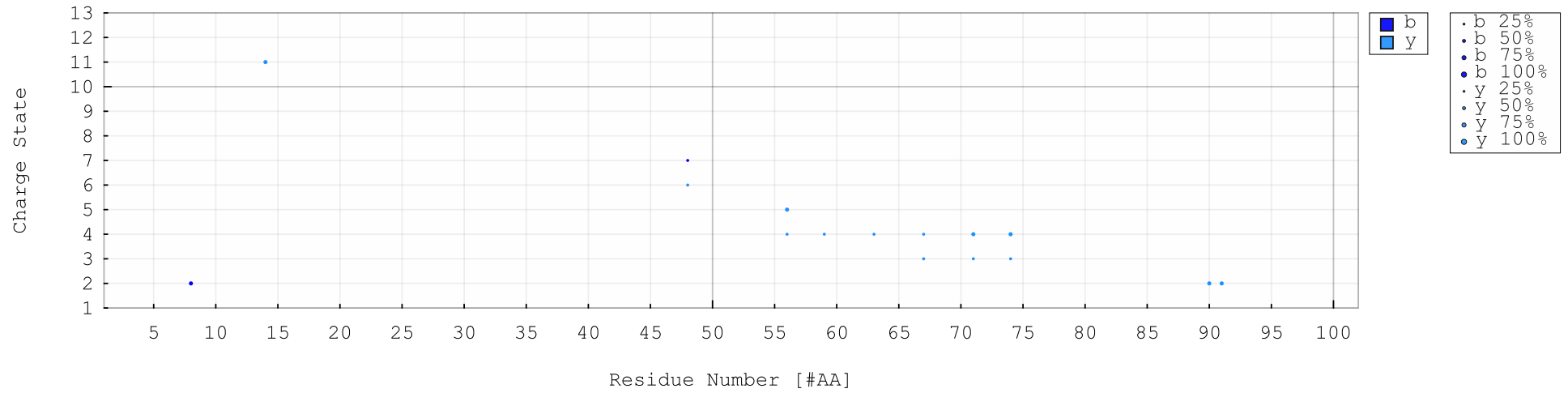

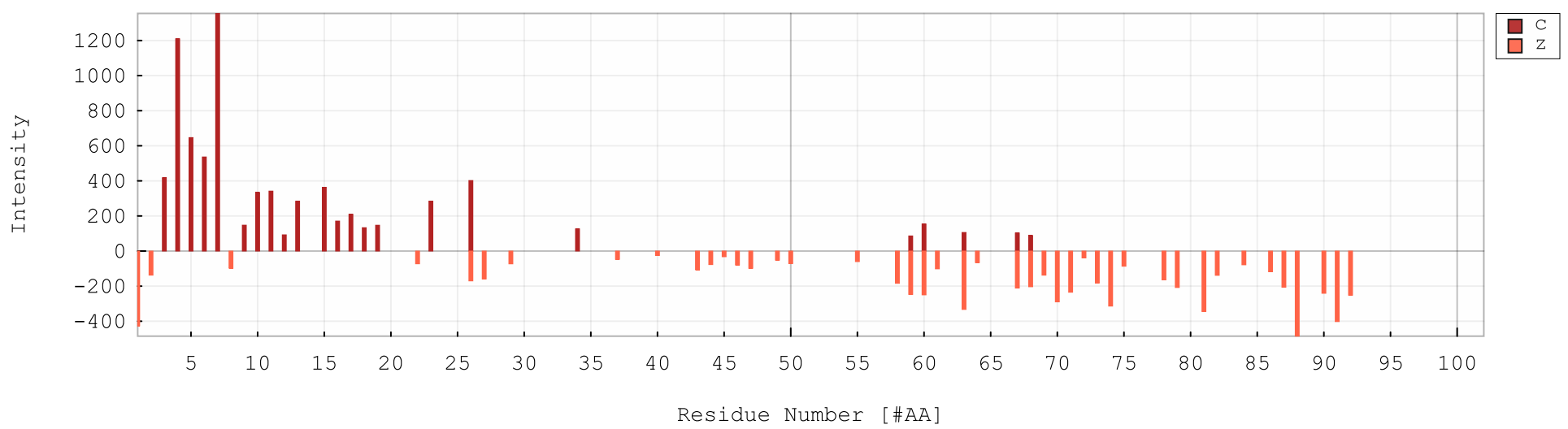

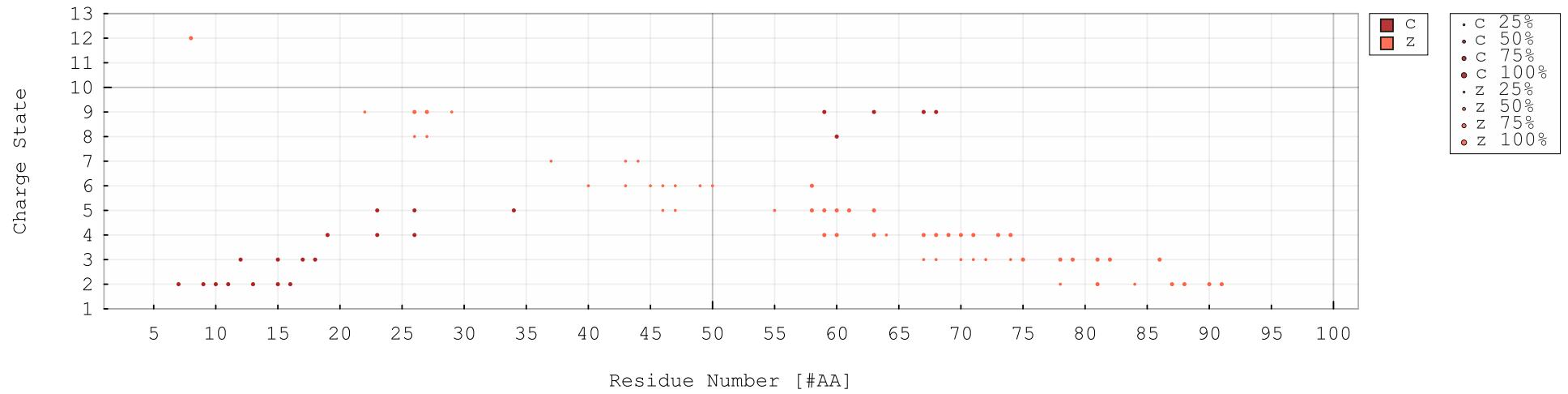

Top-Down Result OmniScape 2025
Sequence Confirmation
250428 H4-PCAF100-30min z13ac1 MS2 EID_1-692_calibrated2

Proteoform:

Index	Protein	SC [%]	SVP [%]	IC [%]	MS Score [a.u.]	Var. Mods	Mono Mass [Da]	Chemical Formula
9	H4Kac	82.2	33.3	14.9	12.2	None	11229.3412	C494H837N163O134S

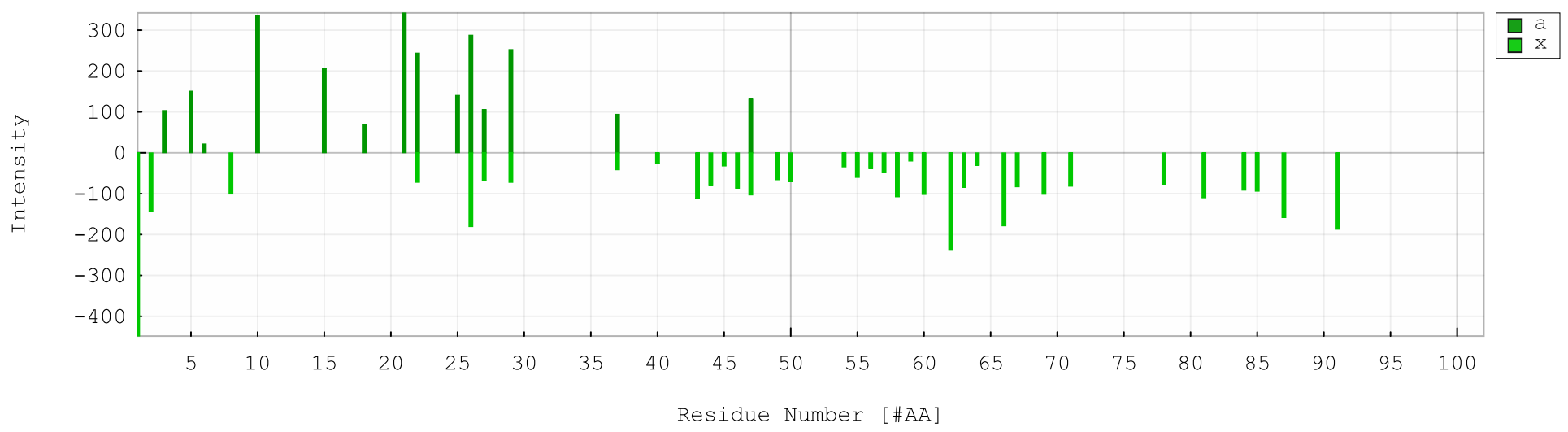

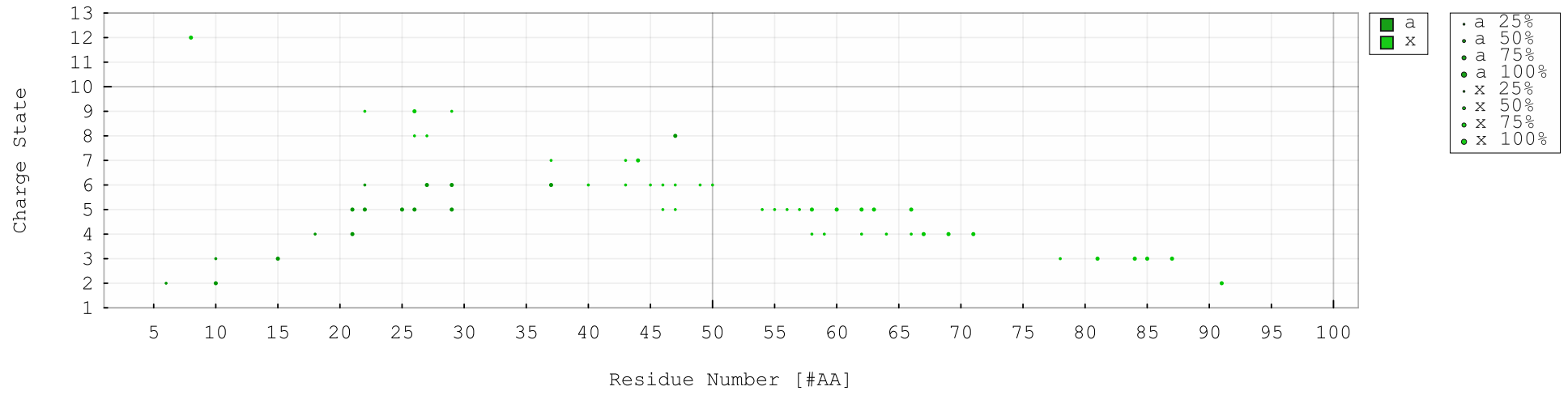

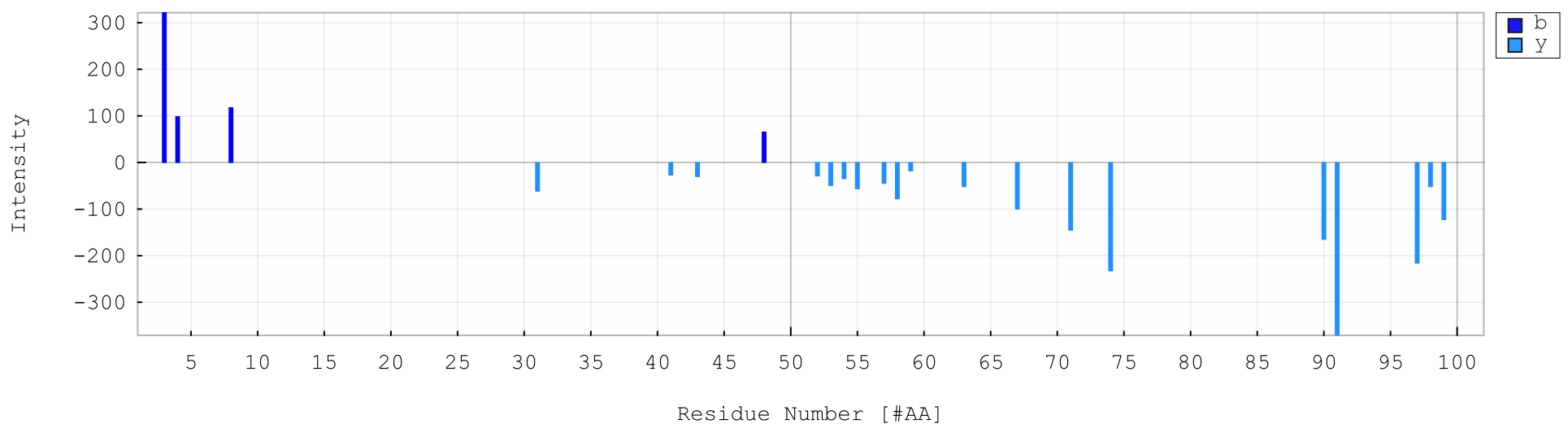

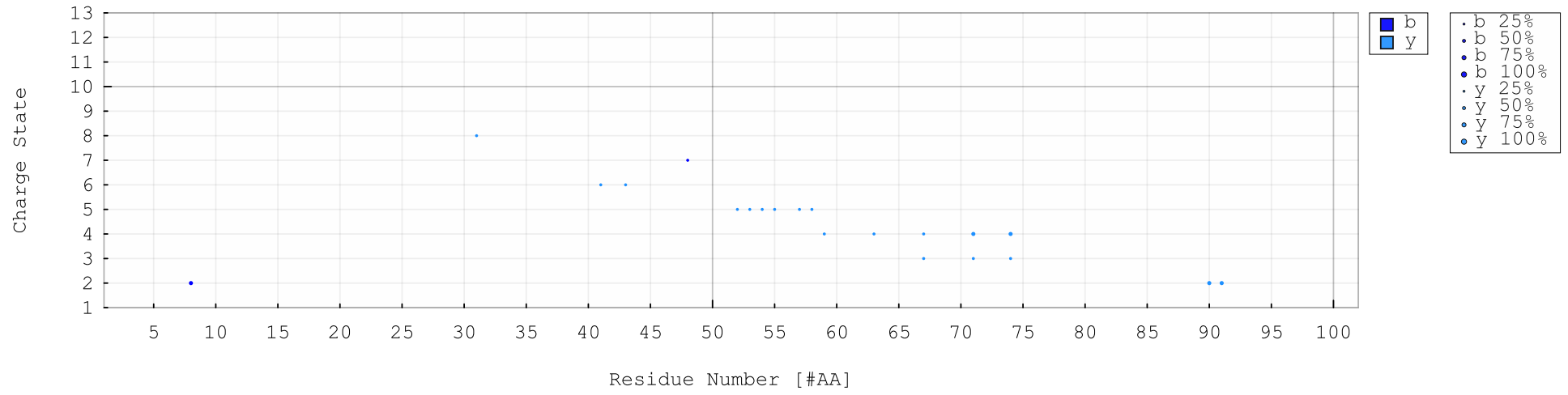

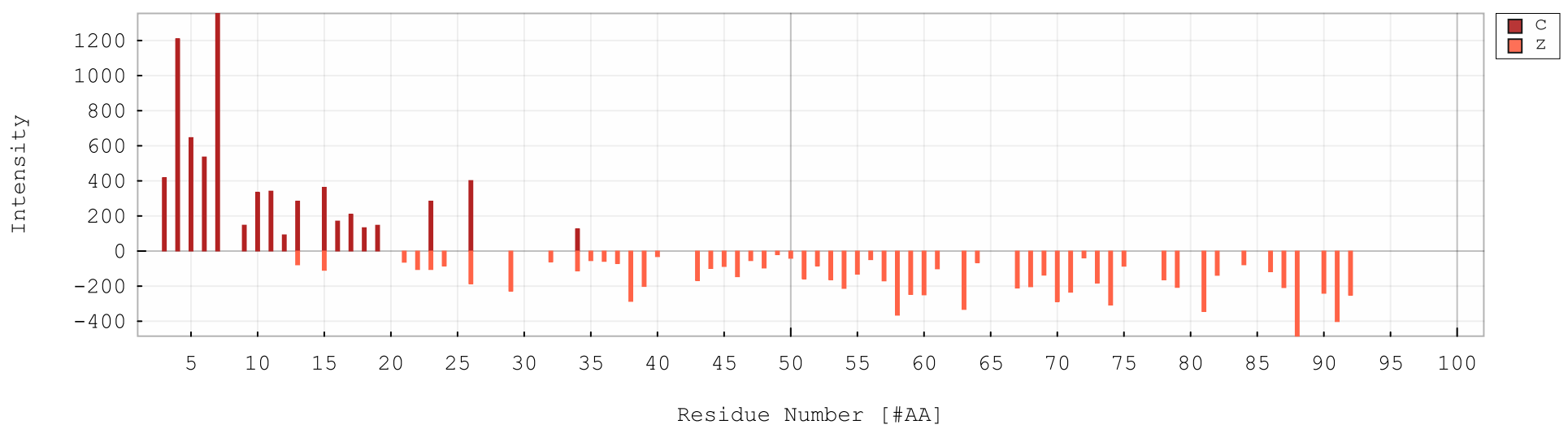

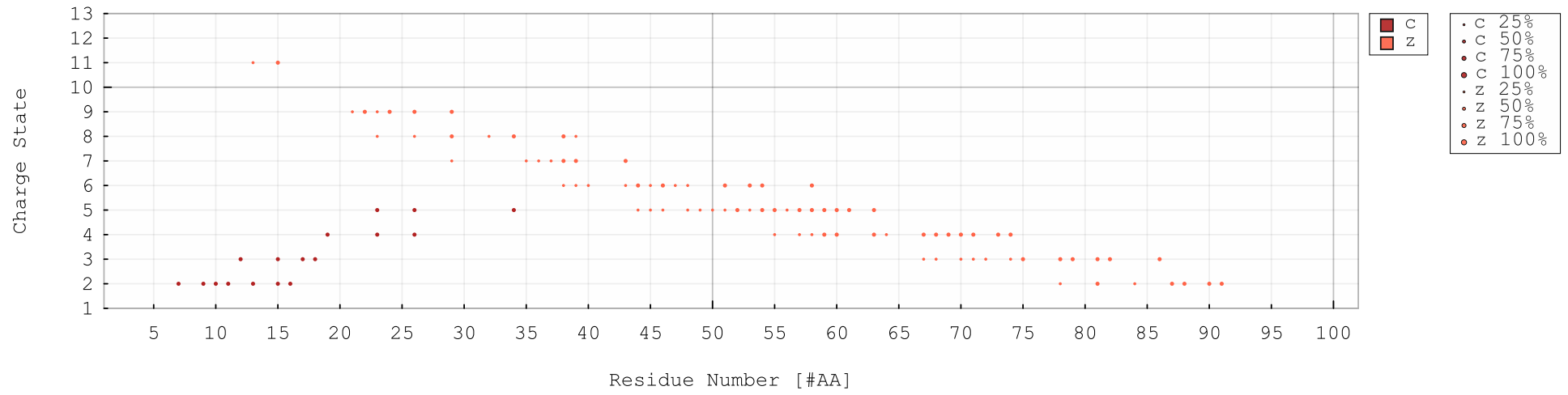

Top-Down Result OmniScape 2025
Sequence Confirmation
250428 H4-PCAF100-30min z13ac1 MS2 EID_1-692_calibrated2

Proteoform:

Index	Protein	SC [%]	SVP [%]	IC [%]	MS Score [a.u.]	Var. Mods	Mono Mass [Da]	Chemical Formula
10	H4Kac	61.4	33.3	16.7	10.2	Ace: K77	11271.3518	C496H839N163O135S

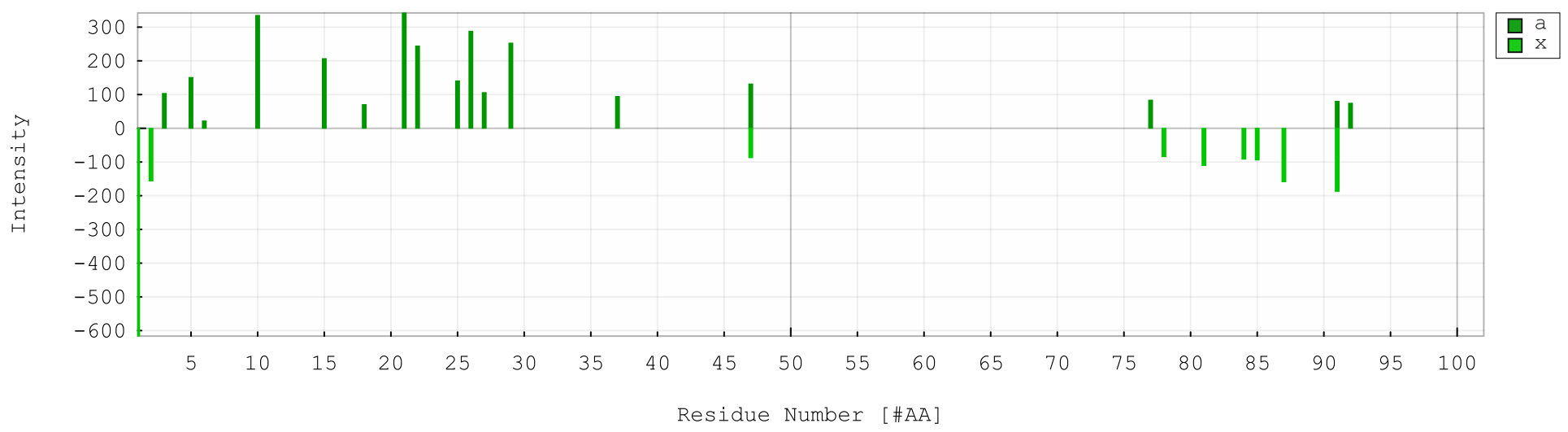

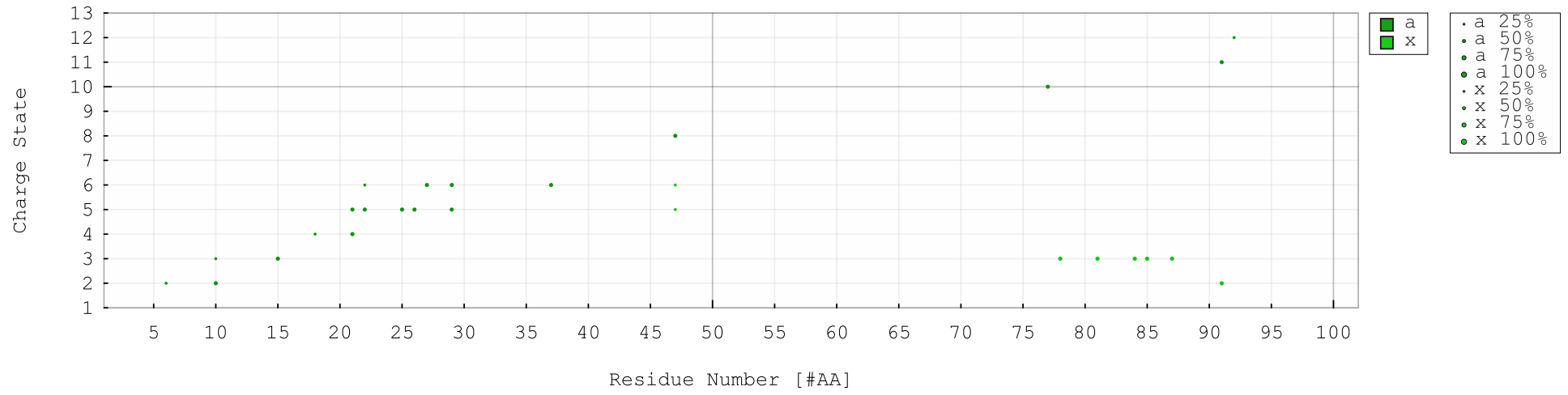

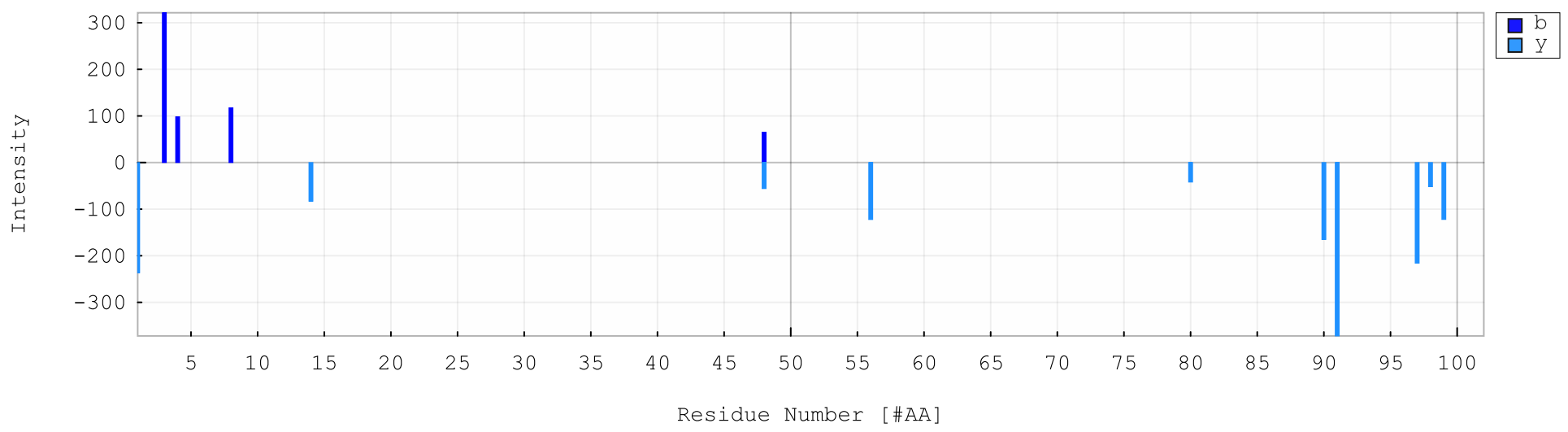

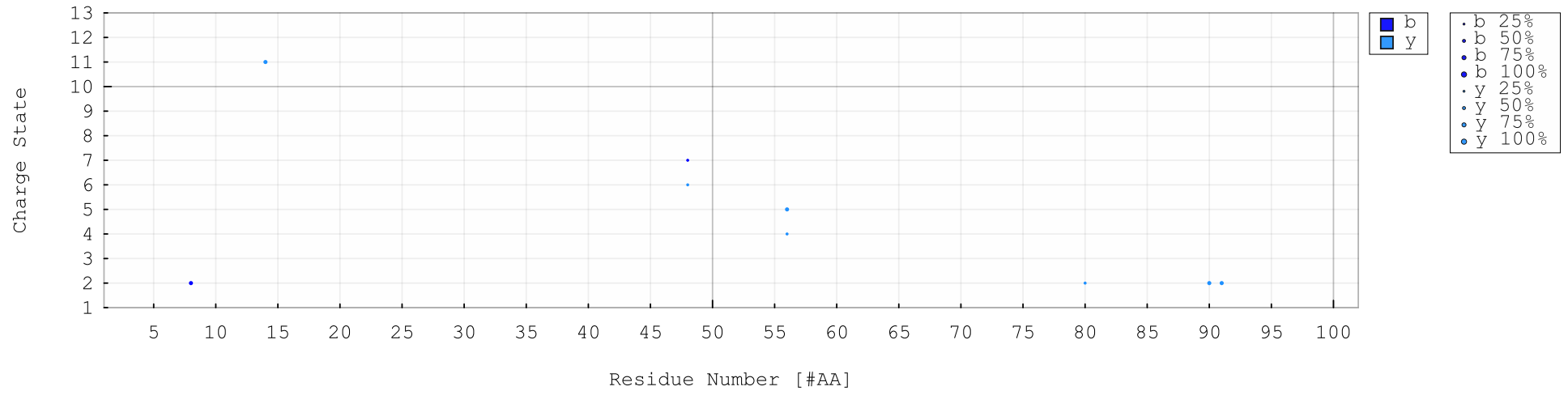

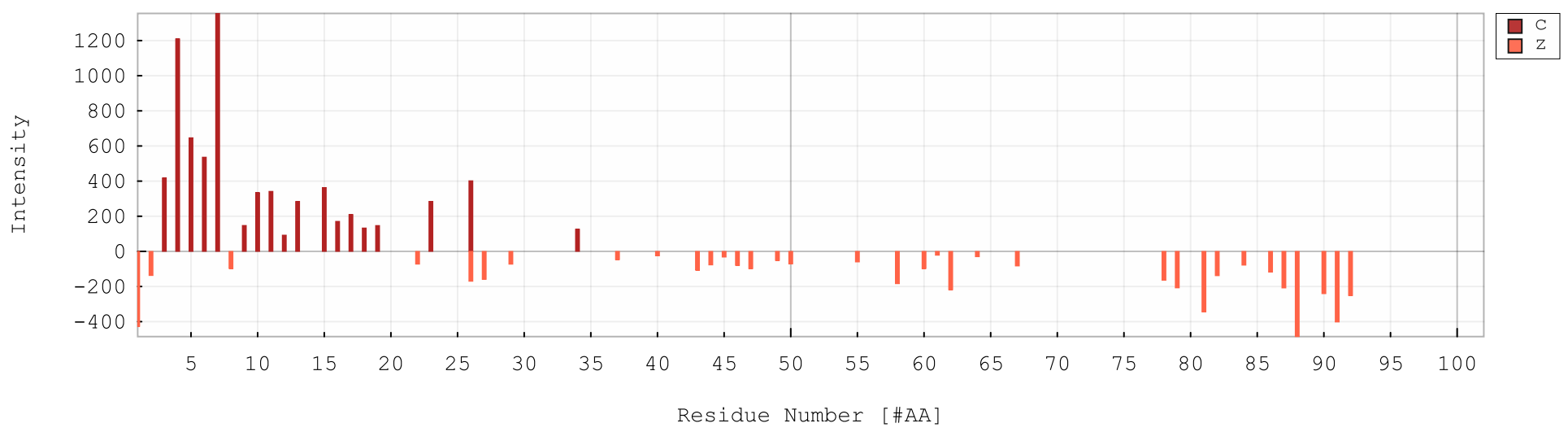

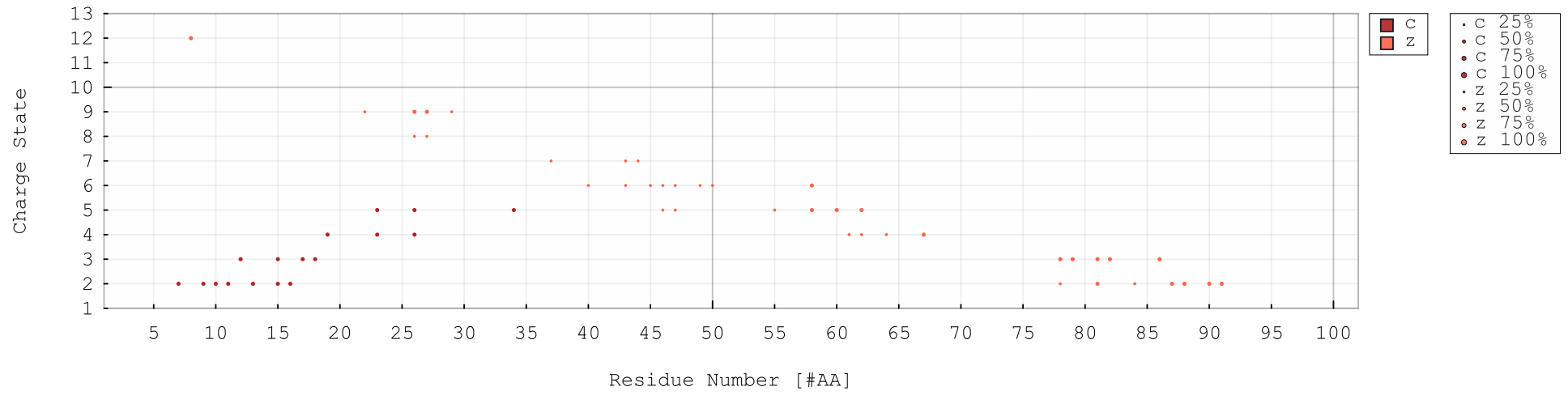

Supplemental Data S5

H4 - PCAF, 30 min

**Report**	4 of 8
**Experiment**	MS2
**Histone/sample**	H4
**Enzyme**	PCAF
**Reaction/source**	30 min
**Precursor**	H4Kac1
**Charge state**	13+
**Fragmentation / workflow**	RCID
**Original file**	MS2_H4Kac1_13+_RCID.pdf

Project Information

Project Directory: C:/Users/LAB-ADMIN
Processing date: Tue Apr 28 14:45:04 2026

Sample Info & Protocols

Name: Top-Down Protein Sequencing CID

Dataset

Imported spectrum file name:	250428 H4-PCAF100-30min z13ac1 MS2 shCID_001-1536_calibrated.txt
Dataset name:	Information is only available from imported JPEAKS files
Date acquired:	Information is only available from imported JPEAKS files
Date imported:	Information is only available from imported JPEAKS files
Instrument Name:	Information is only available from imported JPEAKS files
Instrument Serial Number:	Information is only available from imported JPEAKS files

Workflow Result Info

Analysis: 250428 H4-PCAF100-30min z13ac1 MS2 shCID_001-1536_calibrated2
Location: C:/Users/LAB-ADMIN
Method: -

Method Information

Name: Unnamed

Spectral Parameters

Mass Error [ppm]: 6
Intensity Deviation [au]: 0.15
Min/Max Charges: 1-13
Experiment type: CID

a ions	b ions	c ions	x ions	y ions	z ions	satellite	internal	precursor
a	b			y				M

Noise threshold calculation: Simple moving average noise threshold (SMA)
Window size [datapoints]: 600
Noise curve offset: 1.8430412
SVP parameters: terminal gap: 2, internal gap: 1

Proteoforms

(top 10)

Index	Parent Sequence	Chain	Var. Mods	Mono Mass [Da]	Chemical Composition
1	H4Kac	H4KacK8[Ace]	Ace: K8	11271.3518	C496H839N163O135S
2	H4Kac	H4KacK12[Ace]	Ace: K12	11271.3518	C496H839N163O135S
3	H4Kac	H4KacK5[Ace]	Ace: K5	11271.3518	C496H839N163O135S
4	H4Kac	H4KacK16[Ace]	Ace: K16	11271.3518	C496H839N163O135S
5	H4Kac	H4KacK20[Ace]	Ace: K20	11271.3518	C496H839N163O135S
6	H4Kac	H4KacK31[Ace]	Ace: K31	11271.3518	C496H839N163O135S
7	H4Kac	H4KacK44[Ace]	Ace: K44	11271.3518	C496H839N163O135S
8	H4Kac	H4KacK59[Ace]	Ace: K59	11271.3518	C496H839N163O135S
9	H4Kac	H4Kac	None	11229.3412	C494H837N163O134S
10	H4Kac	H4KacK77[Ace]	Ace: K77	11271.3518	C496H839N163O135S

Results

Proteoforms Table (top 10)

Index	Protein	SC [%]	SVP [%]	IC [%]	MS Score [a.u.]	Var. Mods	Mono Mass [Da]	Chemical Formula
1	H4Kac	58.4	23.5	34.1	19.9	Ace: K8	11271.3518	C496H839N163O135S
2	H4Kac	58.4	23.5	33.3	19.4	Ace: K12	11271.3518	C496H839N163O135S
3	H4Kac	55.4	8.8	33.4	18.5	Ace: K5	11271.3518	C496H839N163O135S
4	H4Kac	56.4	16.7	32.5	18.4	Ace: K16	11271.3518	C496H839N163O135S
5	H4Kac	55.4	16.7	32.4	18.0	Ace: K20	11271.3518	C496H839N163O135S
6	H4Kac	53.5	16.7	32.0	17.1	Ace: K31	11271.3518	C496H839N163O135S
7	H4Kac	52.5	16.7	31.1	16.3	Ace: K44	11271.3518	C496H839N163O135S
8	H4Kac	48.5	16.7	26.6	12.9	Ace: K59	11271.3518	C496H839N163O135S
9	H4Kac	48.5	16.7	18.6	9.0	None	11229.3412	C494H837N163O134S
10	H4Kac	34.7	16.7	22.4	7.8	Ace: K77	11271.3518	C496H839N163O135S

Sequence

Name: H4Kac

```
                  10                    20                    30                    40                    50                    60
S G R G K G G K G L   G K G G A K R H R K   V L R D N I Q G I T   K P A I R R L A R R   G G V K R I S G L I   Y E E T R G V L K V
                  70                    80                    90                   100                   110                   120
F L E N V I R D A V   T Y T E H A K R K T   V T A M D V V Y A L   K R Q G R T L Y G F   G G
```

Modifications

Modification	Position	Type
Acetyl	5	variable
Acetyl	8	variable
Acetyl	12	variable
Acetyl	16	variable
Acetyl	20	variable
Acetyl	31	variable
Acetyl	44	variable
Acetyl	59	variable
Acetyl	77	variable
Acetyl	79	variable
Acetyl	91	variable

Sequence Map

Proteoform:

Index	Protein	SC [%]	SVP [%]	IC [%]	MS Score [a.u.]	Var. Mods	Mono Mass [Da]	Chemical Formula
1	H4Kac	58.4	23.5	34.1	19.9	Ace: K8	11271.3518	C496H839N163O135S

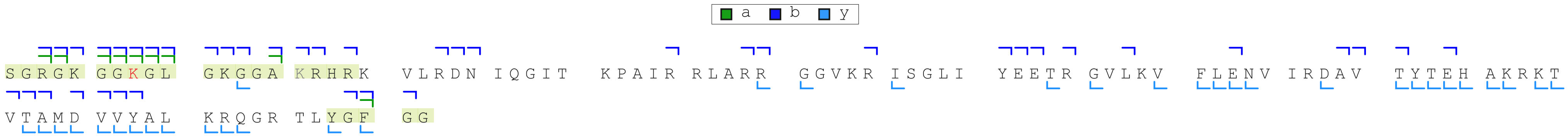

Proteoform:

Index	Protein	SC [%]	SVP [%]	IC [%]	MS Score [a.u.]	Var. Mods	Mono Mass [Da]	Chemical Formula
2	H4Kac	58.4	23.5	33.3	19.4	Ace: K12	11271.3518	C496H839N163O135S

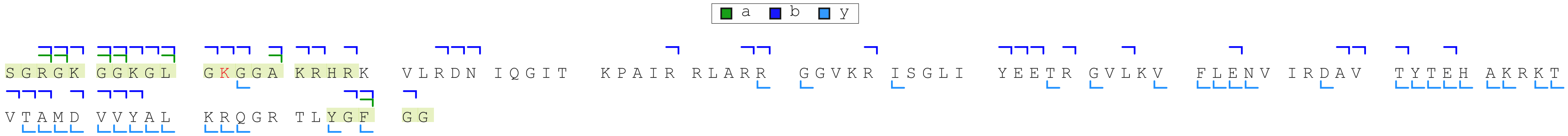

Top-Down Result OmniScape 2025
Sequence Confirmation
250428 H4-PCAF100-30min z13ac1 MS2 shCID_001-1536_calibrated2

Proteoform:

Index	Protein	SC [%]	SVP [%]	IC [%]	MS Score [a.u.]	Var. Mods	Mono Mass [Da]	Chemical Formula
3	H4Kac	55.4	8.8	33.4	18.5	Ace: K5	11271.3518	C496H839N163O135S

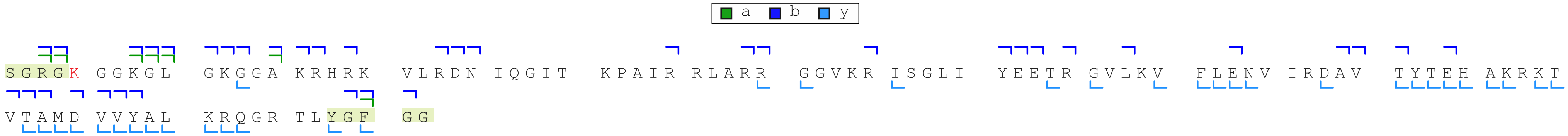

Proteoform:

Index	Protein	SC [%]	SVP [%]	IC [%]	MS Score [a.u.]	Var. Mods	Mono Mass [Da]	Chemical Formula
4	H4Kac	56.4	16.7	32.5	18.4	Ace: K16	11271.3518	C496H839N163O135S

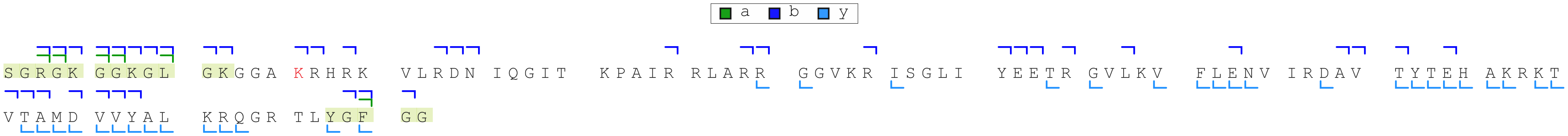

Proteoform:

Index	Protein	SC [%]	SVP [%]	IC [%]	MS Score [a.u.]	Var. Mods	Mono Mass [Da]	Chemical Formula
5	H4Kac	55.4	16.7	32.4	18.0	Ace: K20	11271.3518	C496H839N163O135S

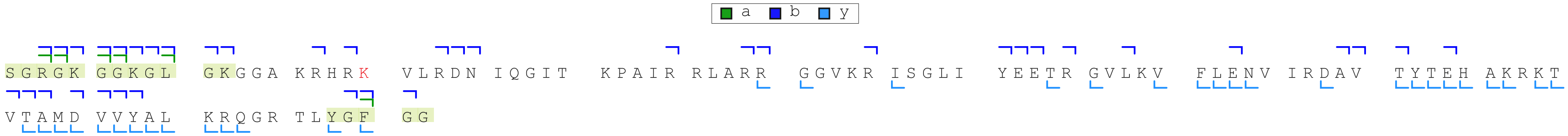

Proteoform:

Index	Protein	SC [%]	SVP [%]	IC [%]	MS Score [a.u.]	Var. Mods	Mono Mass [Da]	Chemical Formula
6	H4Kac	53.5	16.7	32.0	17.1	Ace: K31	11271.3518	C496H839N163O135S

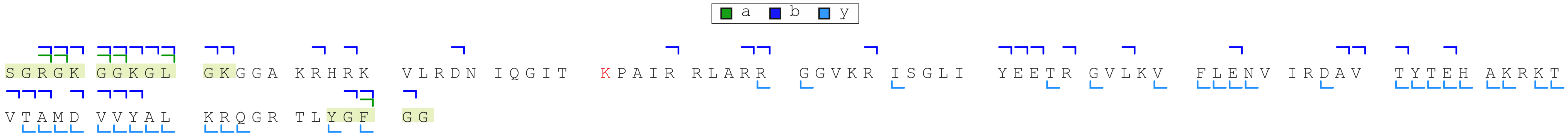

Proteoform:

Index	Protein	SC [%]	SVP [%]	IC [%]	MS Score [a.u.]	Var. Mods	Mono Mass [Da]	Chemical Formula
7	H4Kac	52.5	16.7	31.1	16.3	Ace: K44	11271.3518	C496H839N163O135S

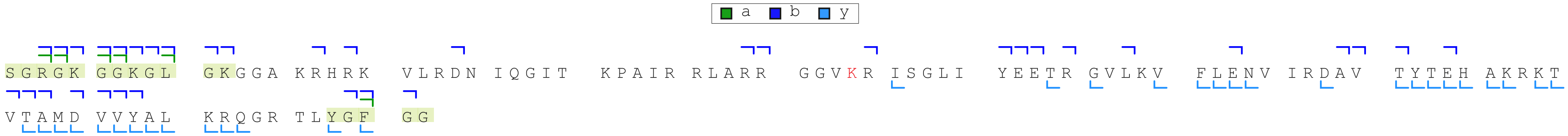

Proteoform:

Index	Protein	SC [%]	SVP [%]	IC [%]	MS Score [a.u.]	Var. Mods	Mono Mass [Da]	Chemical Formula
8	H4Kac	48.5	16.7	26.6	12.9	Ace: K59	11271.3518	C496H839N163O135S

a b y

SGRGK GGKGL GKGGA KRHRK VLRDN IQGIT KPAIR RLARR GGVKR I LI YEETR GVLKV FLENV IRDAV TYTEH AKRKT
VTAMD VVYAL KRQGR TLYGF GG

Proteoform:

Index	Protein	SC [%]	SVP [%]	IC [%]	MS Score [a.u.]	Var. Mods	Mono Mass [Da]	Chemical Formula
9	H4Kac	48.5	16.7	18.6	9.0	None	11229.3412	C494H837N163O134S

a b y

SGRGK GGKGL GKGGA KRHRK VLRDN IQGIT KPAIR RLARR GGVKR ISGLI YEETR GVLKV FLENV IRDAV TYTEH AKRKT
VTAMD VVYAL KRQGR TLYGF GG

Proteoform:

Index	Protein	SC [%]	SVP [%]	IC [%]	MS Score [a.u.]	Var. Mods	Mono Mass [Da]	Chemical Formula
10	H4Kac	34.7	16.7	22.4	7.8	Ace: K77	11271.3518	C496H839N163O135S

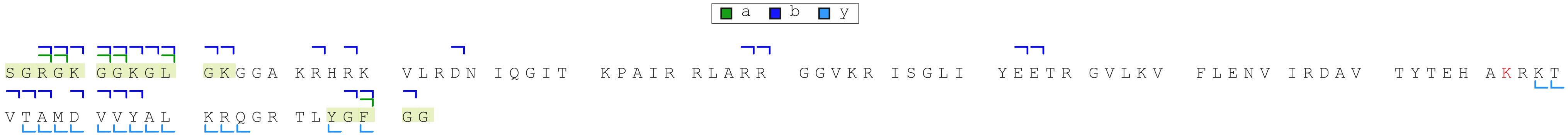

Error Plot

Proteoform:

Index	Protein	SC [%]	SVP [%]	IC [%]	MS Score [a.u.]	Var. Mods	Mono Mass [Da]	Chemical Formula
1	H4Kac	58.4	23.5	34.1	19.9	Ace: K8	11271.3518	C496H839N163O135S

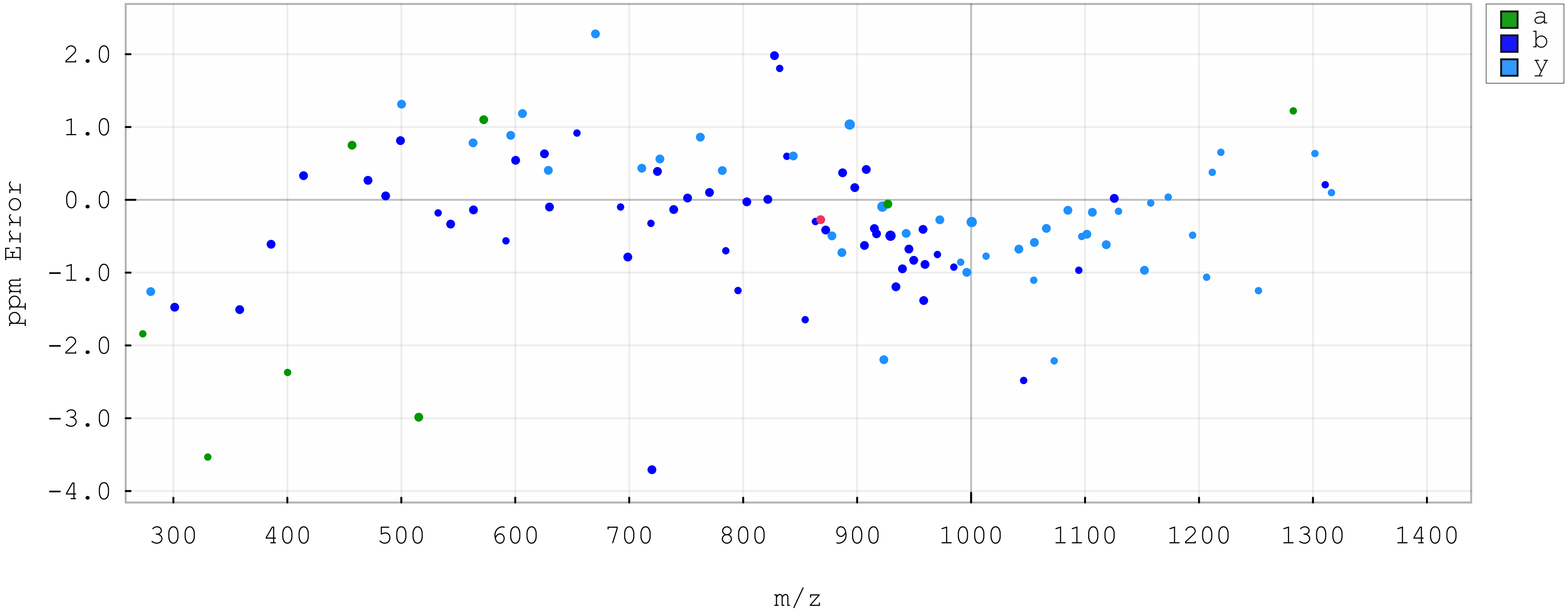

Proteoform:

Index	Protein	SC [%]	SVP [%]	IC [%]	MS Score [a.u.]	Var. Mods	Mono Mass [Da]	Chemical Formula
2	H4Kac	58.4	23.5	33.3	19.4	Ace: K12	11271.3518	C496H839N163O135S

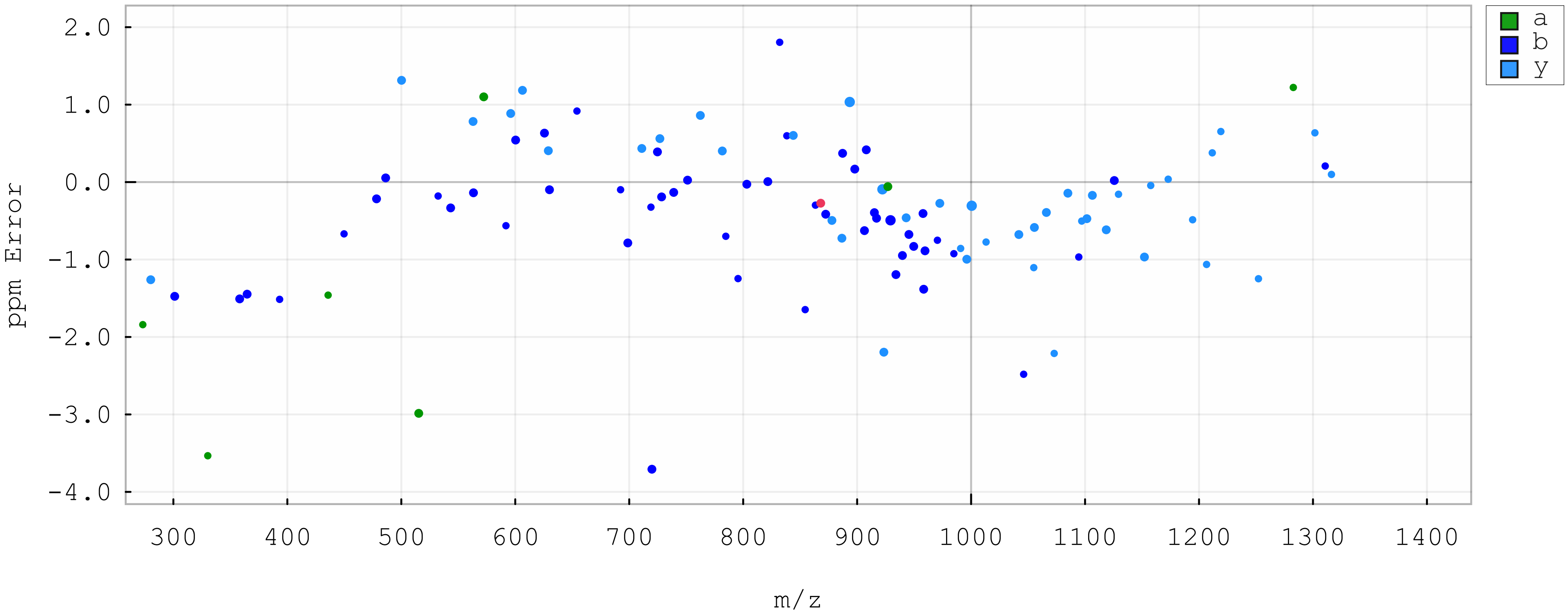

Proteoform:

Index	Protein	SC [%]	SVP [%]	IC [%]	MS Score [a.u.]	Var. Mods	Mono Mass [Da]	Chemical Formula
3	H4Kac	55.4	8.8	33.4	18.5	Ace: K5	11271.3518	C496H839N163O135S

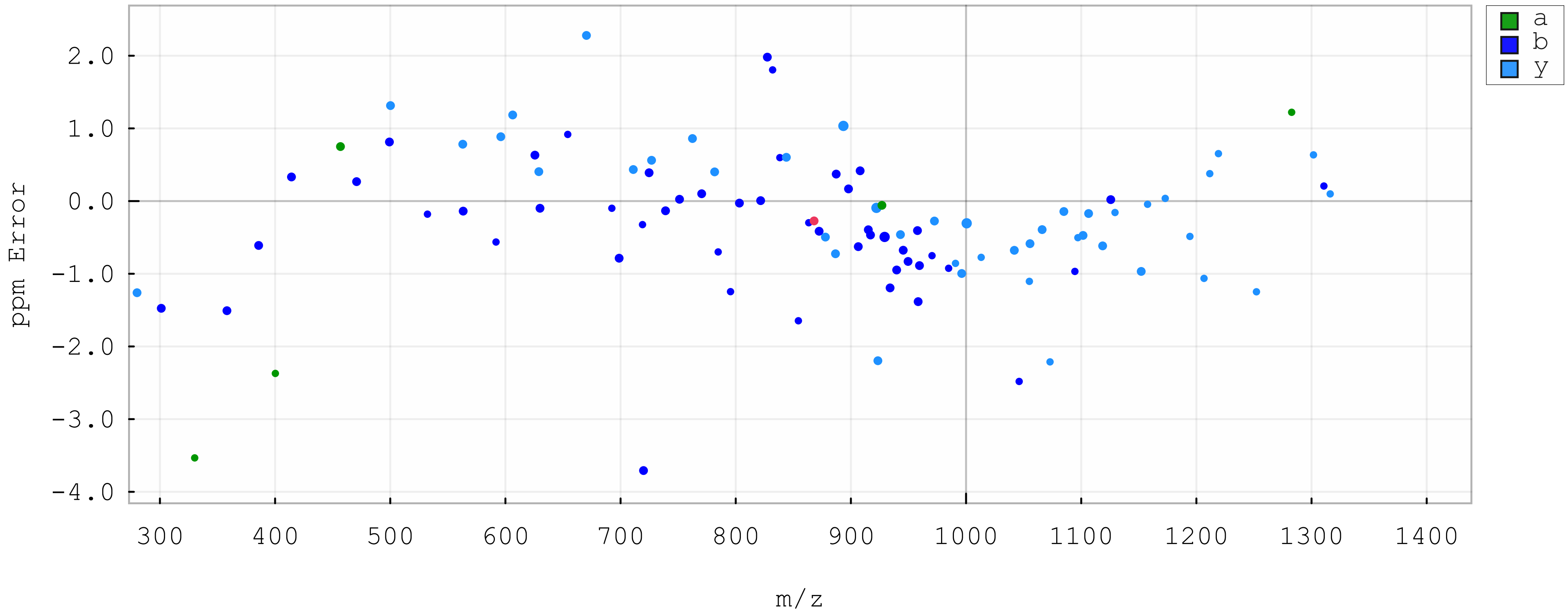

Proteoform:

Index	Protein	SC [%]	SVP [%]	IC [%]	MS Score [a.u.]	Var. Mods	Mono Mass [Da]	Chemical Formula
4	H4Kac	56.4	16.7	32.5	18.4	Ace: K16	11271.3518	C496H839N163O135S

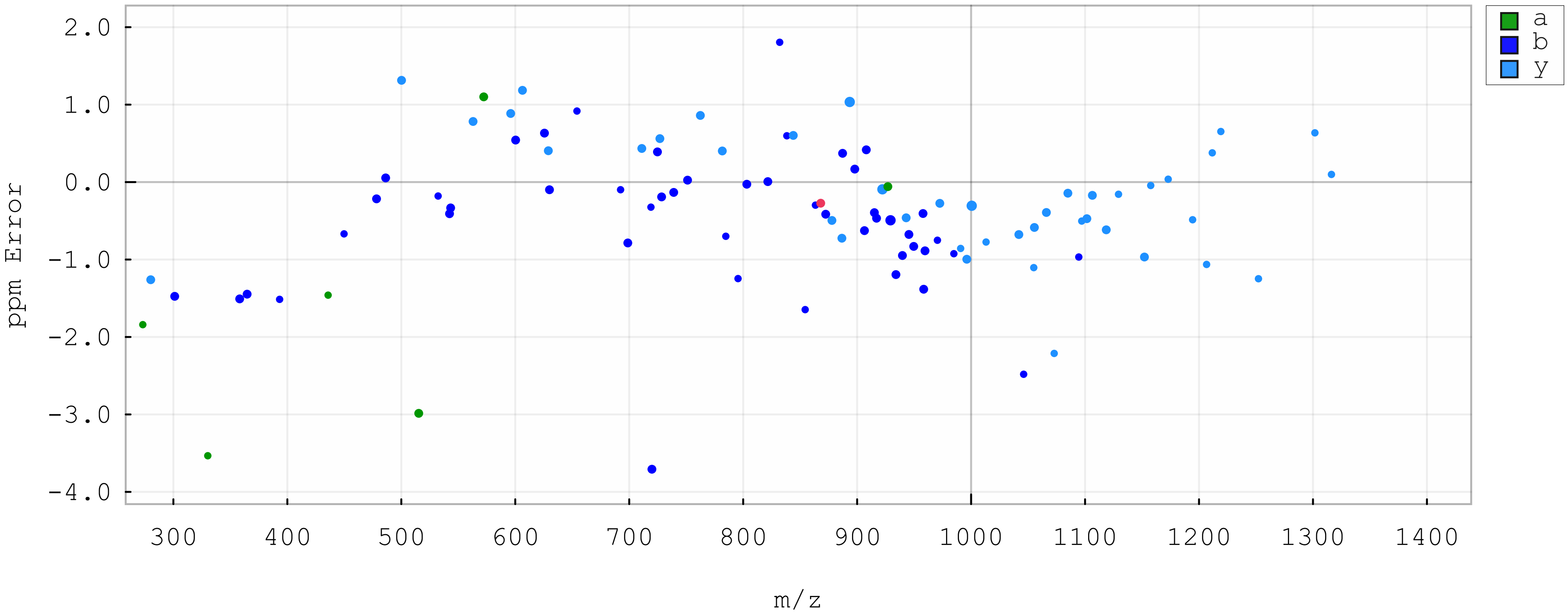

Proteoform:

Index	Protein	SC [%]	SVP [%]	IC [%]	MS Score [a.u.]	Var. Mods	Mono Mass [Da]	Chemical Formula
5	H4Kac	55.4	16.7	32.4	18.0	Ace: K20	11271.3518	C496H839N163O135S

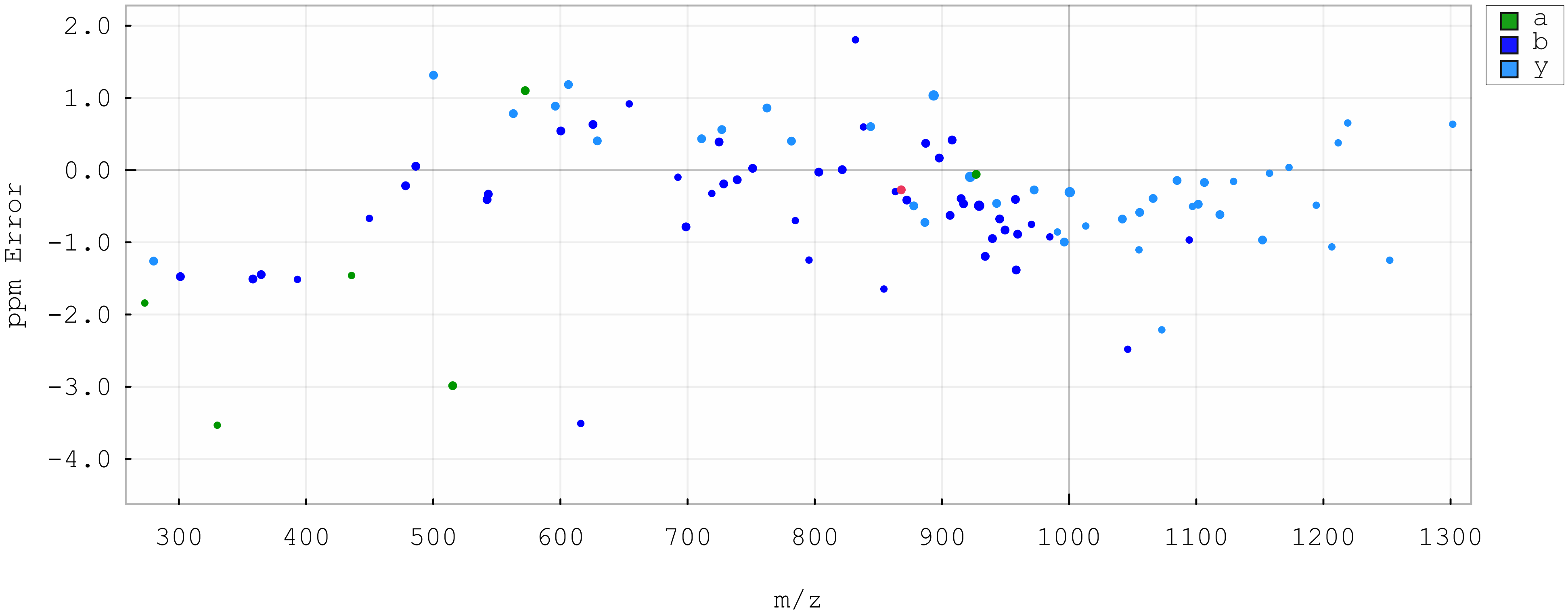

Proteoform:

Index	Protein	SC [%]	SVP [%]	IC [%]	MS Score [a.u.]	Var. Mods	Mono Mass [Da]	Chemical Formula
6	H4Kac	53.5	16.7	32.0	17.1	Ace: K31	11271.3518	C496H839N163O135S

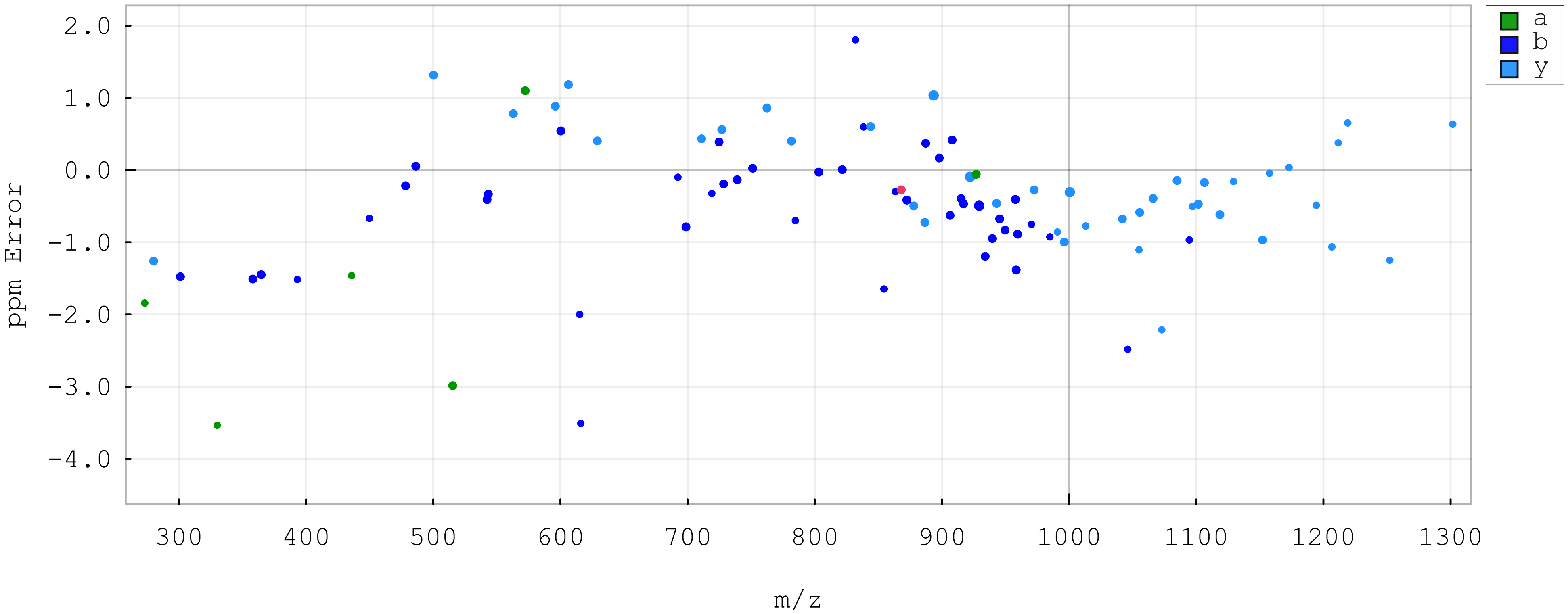

Proteoform:

Index	Protein	SC [%]	SVP [%]	IC [%]	MS Score [a.u.]	Var. Mods	Mono Mass [Da]	Chemical Formula
7	H4Kac	52.5	16.7	31.1	16.3	Ace: K44	11271.3518	C496H839N163O135S

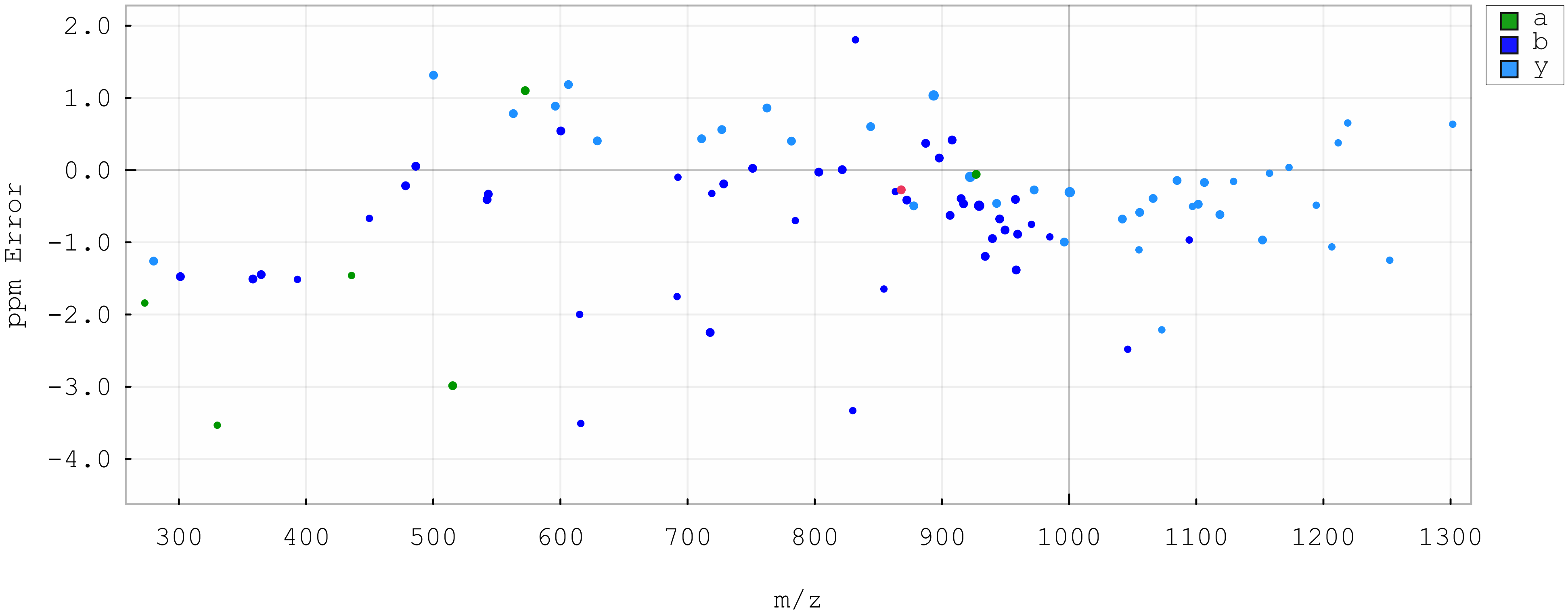

Proteoform:

Index	Protein	SC [%]	SVP [%]	IC [%]	MS Score [a.u.]	Var. Mods	Mono Mass [Da]	Chemical Formula
8	H4Kac	48.5	16.7	26.6	12.9	Ace: K59	11271.3518	C496H839N163O135S

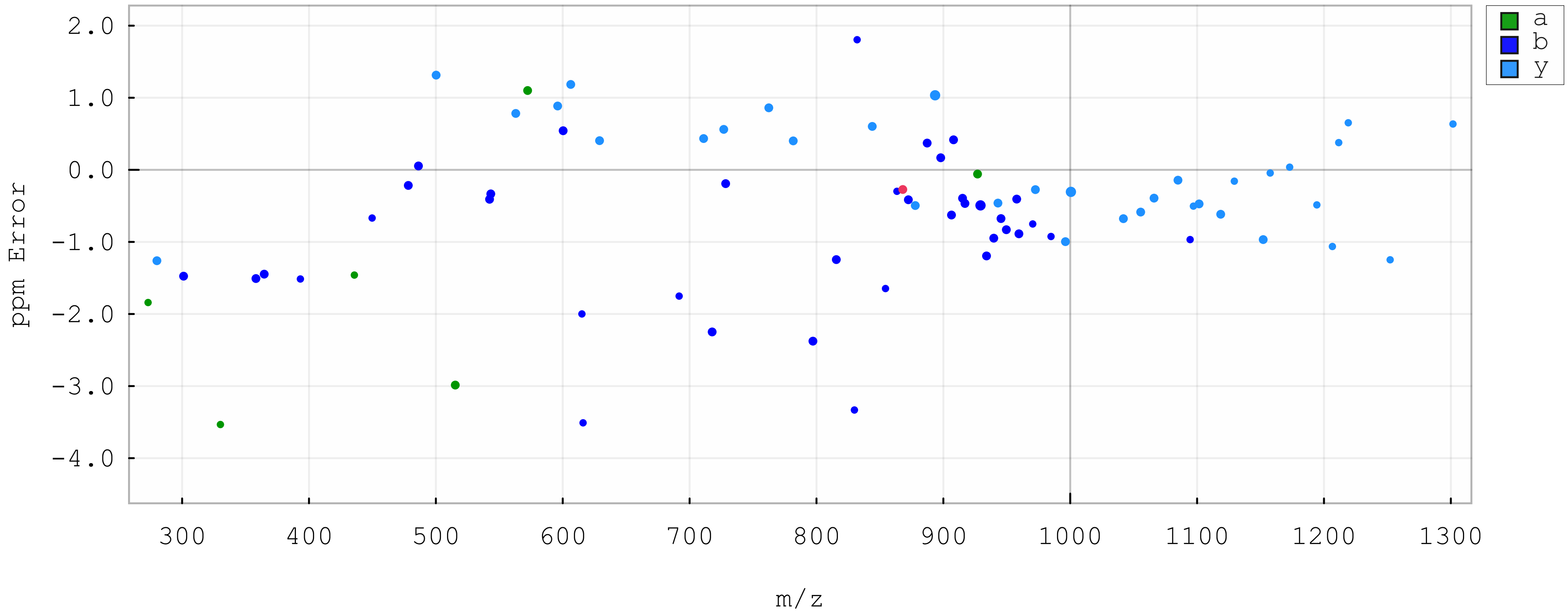

Proteoform:

Index	Protein	SC [%]	SVP [%]	IC [%]	MS Score [a.u.]	Var. Mods	Mono Mass [Da]	Chemical Formula
9	H4Kac	48.5	16.7	18.6	9.0	None	11229.3412	C494H837N163O134S

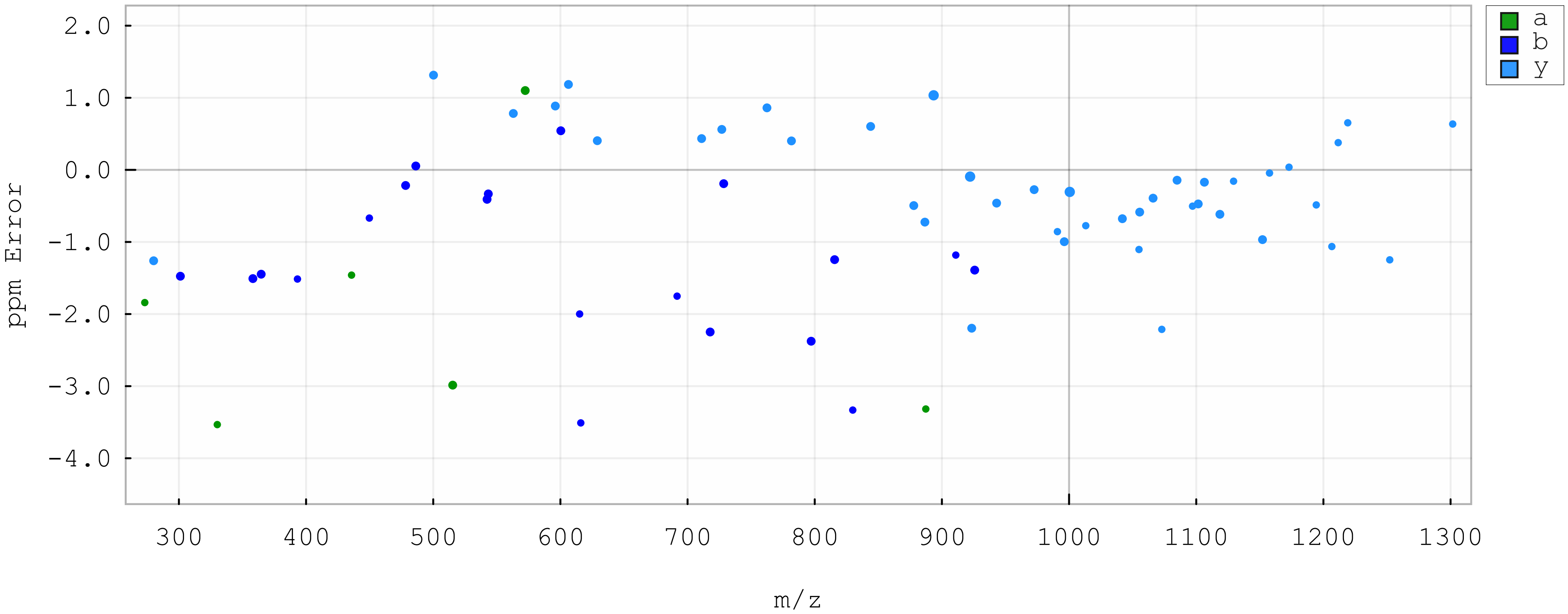

Proteoform:

Index	Protein	SC [%]	SVP [%]	IC [%]	MS Score [a.u.]	Var. Mods	Mono Mass [Da]	Chemical Formula
10	H4Kac	34.7	16.7	22.4	7.8	Ace: K77	11271.3518	C496H839N163O135S

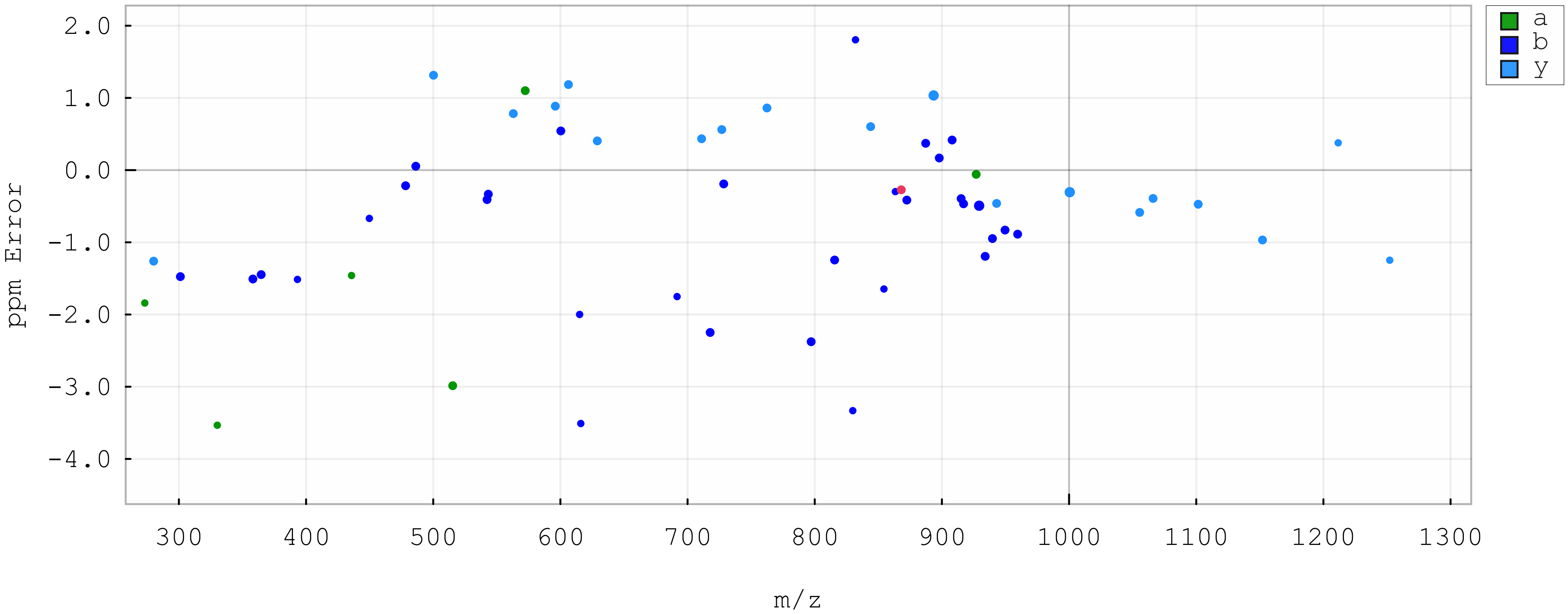

Primary Fragment Plots

Proteoform:

Index	Protein	SC [%]	SVP [%]	IC [%]	MS Score [a.u.]	Var. Mods	Mono Mass [Da]	Chemical Formula
1	H4Kac	58.4	23.5	34.1	19.9	Ace: K8	11271.3518	C496H839N163O135S

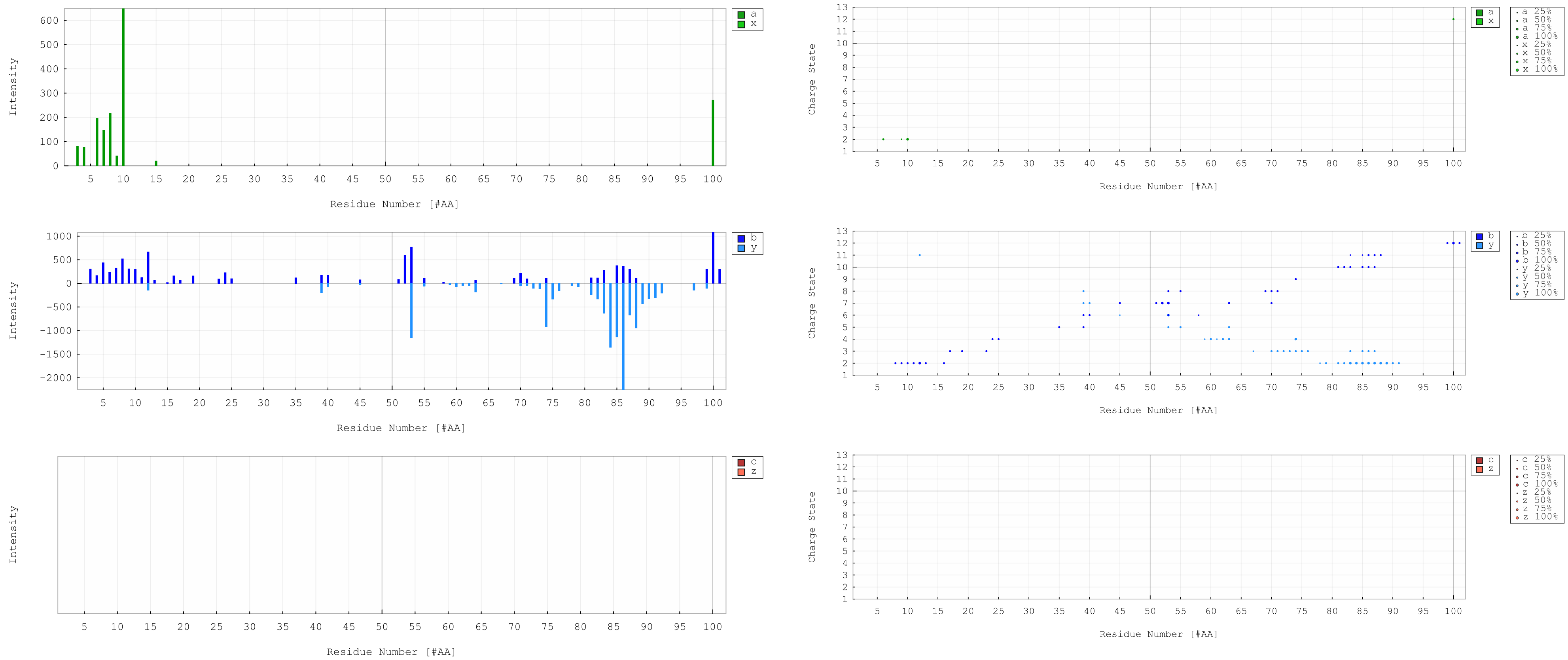

Proteoform:

Index	Protein	SC [%]	SVP [%]	IC [%]	MS Score [a.u.]	Var. Mods	Mono Mass [Da]	Chemical Formula
2	H4Kac	58.4	23.5	33.3	19.4	Ace: K12	11271.3518	C496H839N163O135S

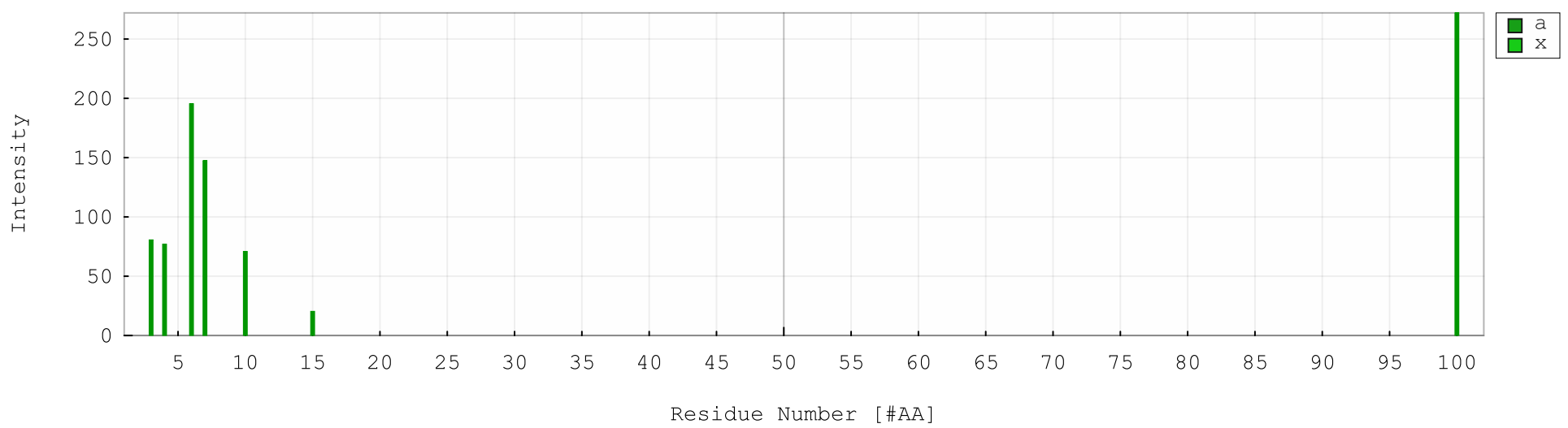

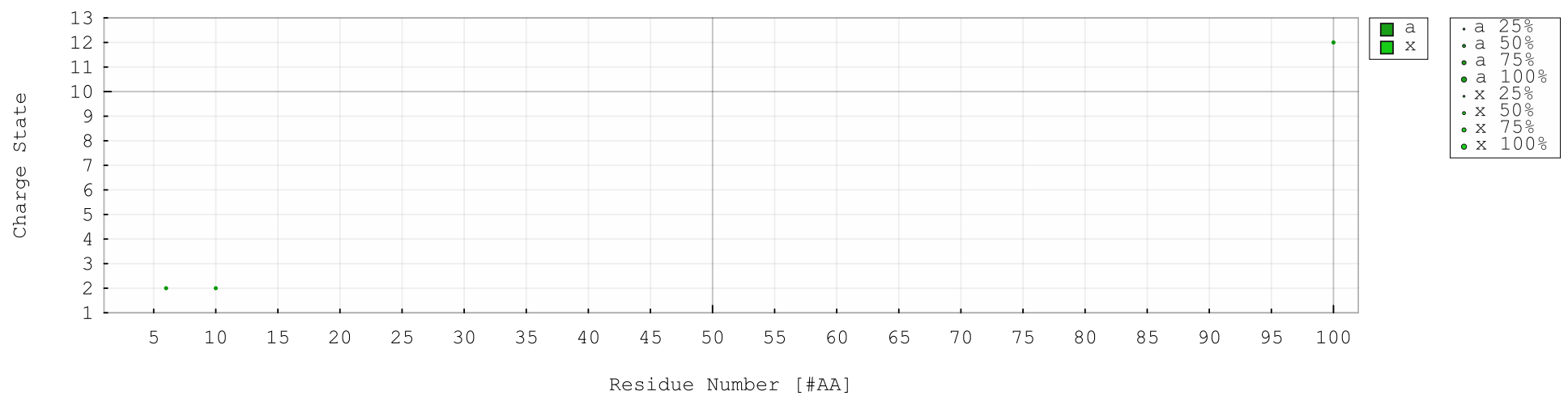

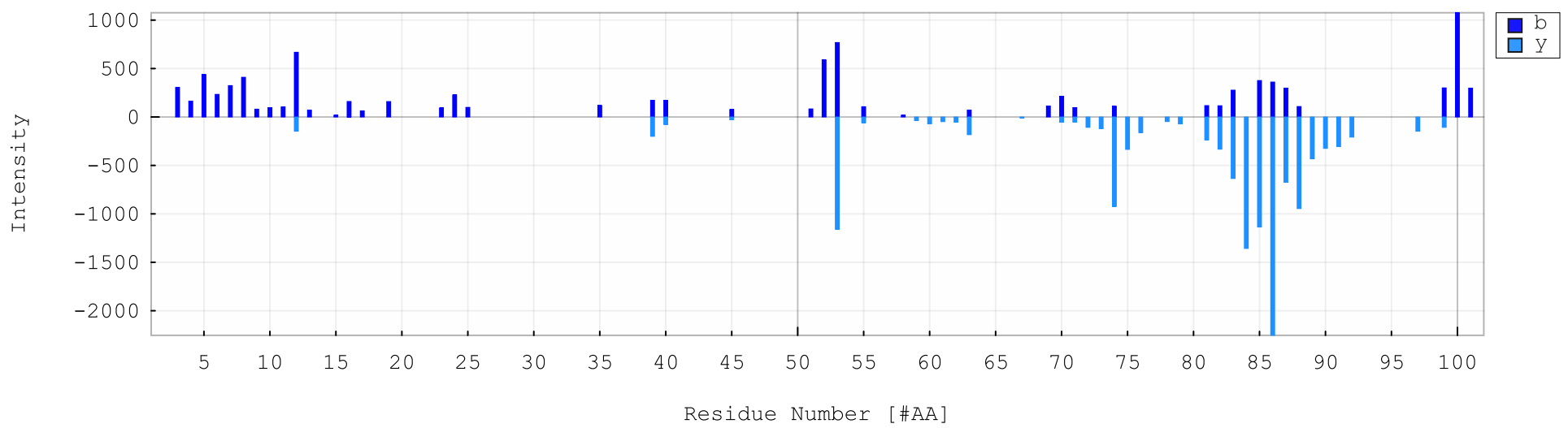

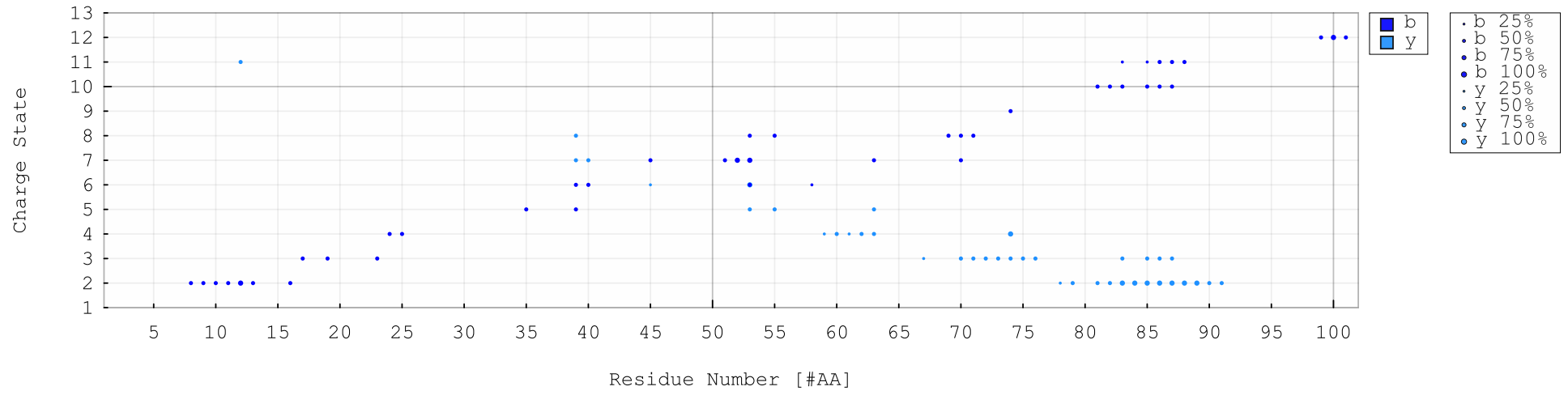

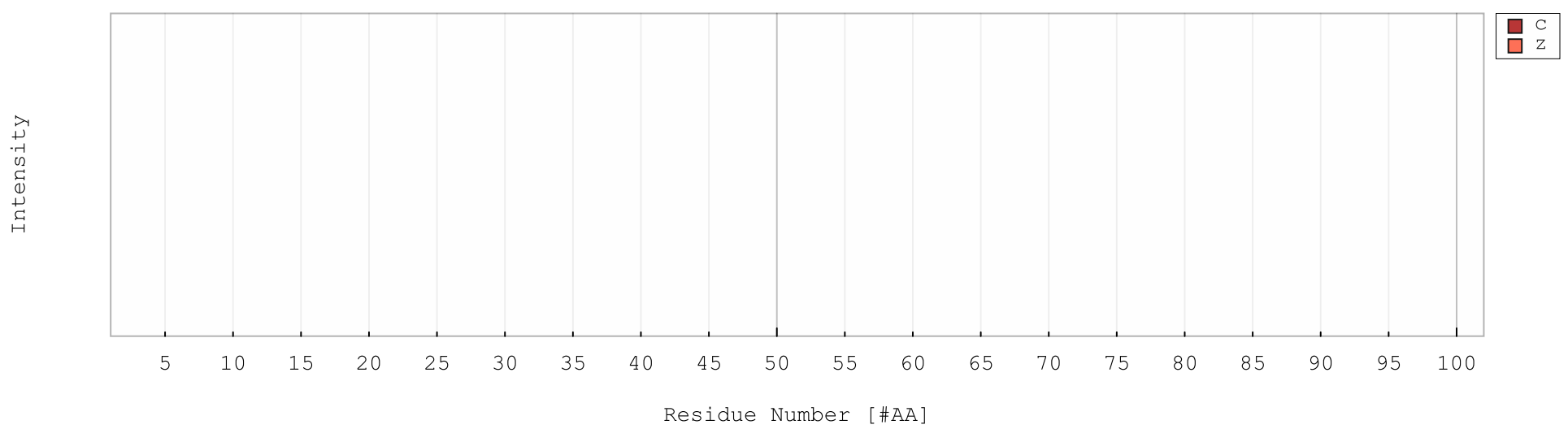

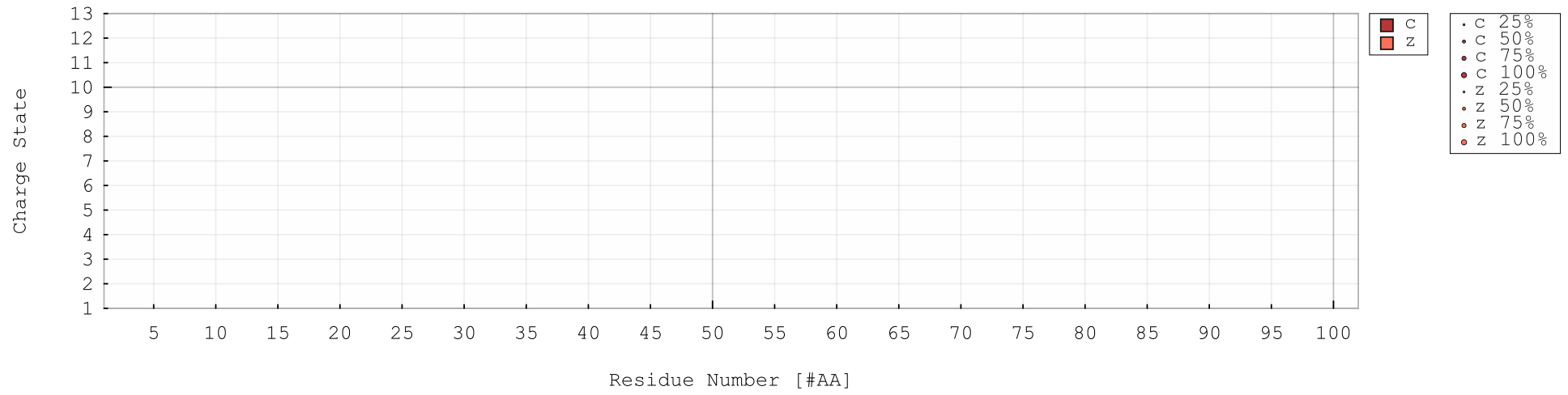

Proteoform:

Index	Protein	SC [%]	SVP [%]	IC [%]	MS Score [a.u.]	Var. Mods	Mono Mass [Da]	Chemical Formula
3	H4Kac	55.4	8.8	33.4	18.5	Ace: K5	11271.3518	C496H839N163O135S

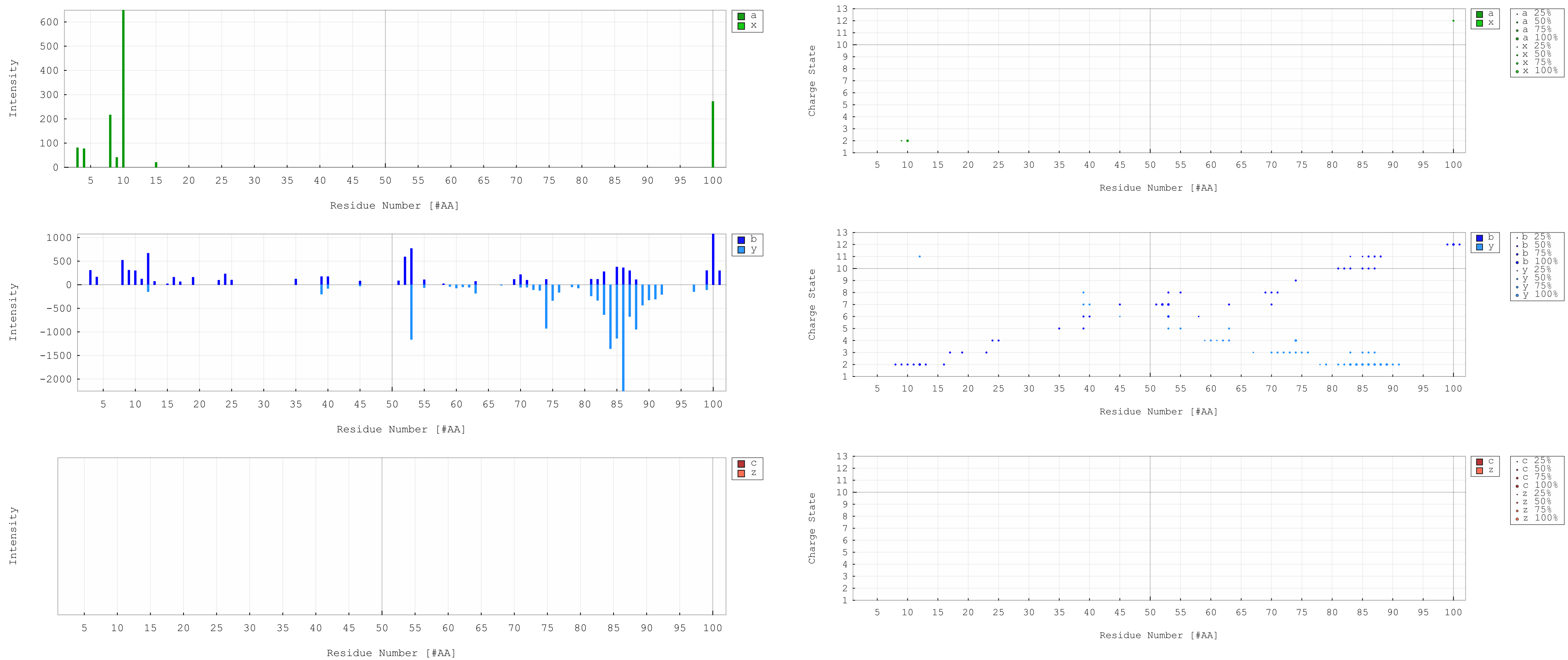

Proteoform:

Index	Protein	SC [%]	SVP [%]	IC [%]	MS Score [a.u.]	Var. Mods	Mono Mass [Da]	Chemical Formula
4	H4Kac	56.4	16.7	32.5	18.4	Ace: K16	11271.3518	C496H839N163O135S

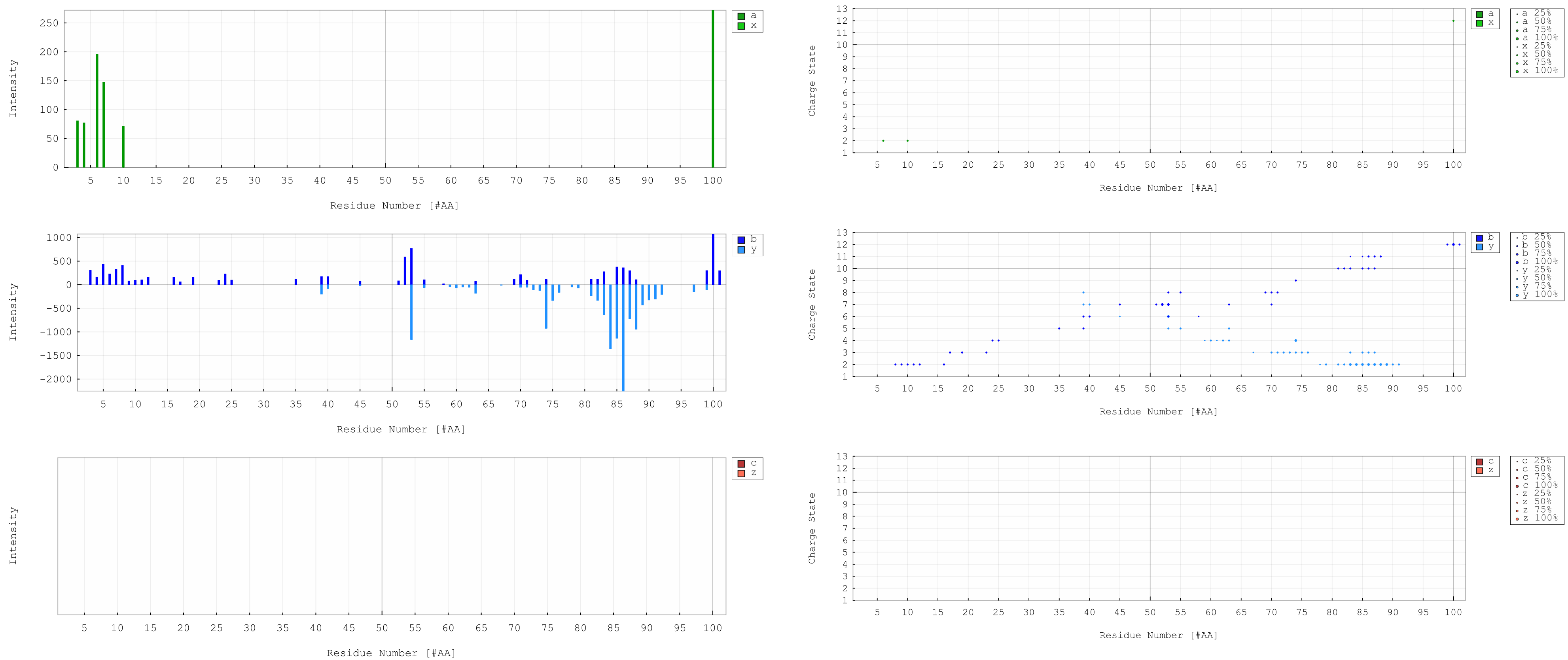

Proteoform:

Index	Protein	SC [%]	SVP [%]	IC [%]	MS Score [a.u.]	Var. Mods	Mono Mass [Da]	Chemical Formula
5	H4Kac	55.4	16.7	32.4	18.0	Ace: K20	11271.3518	C496H839N163O135S

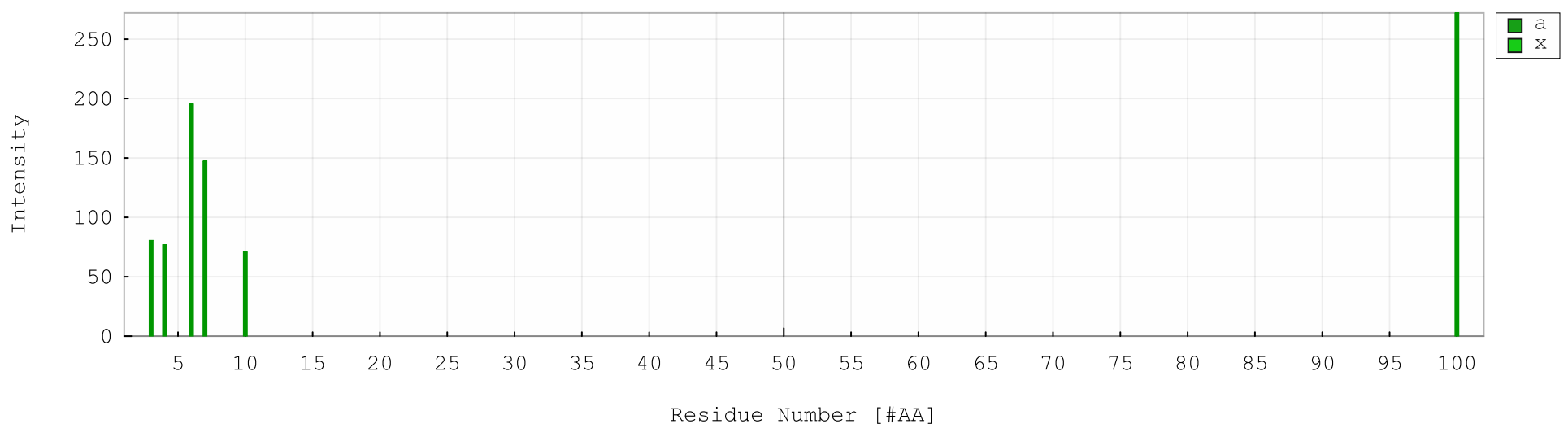

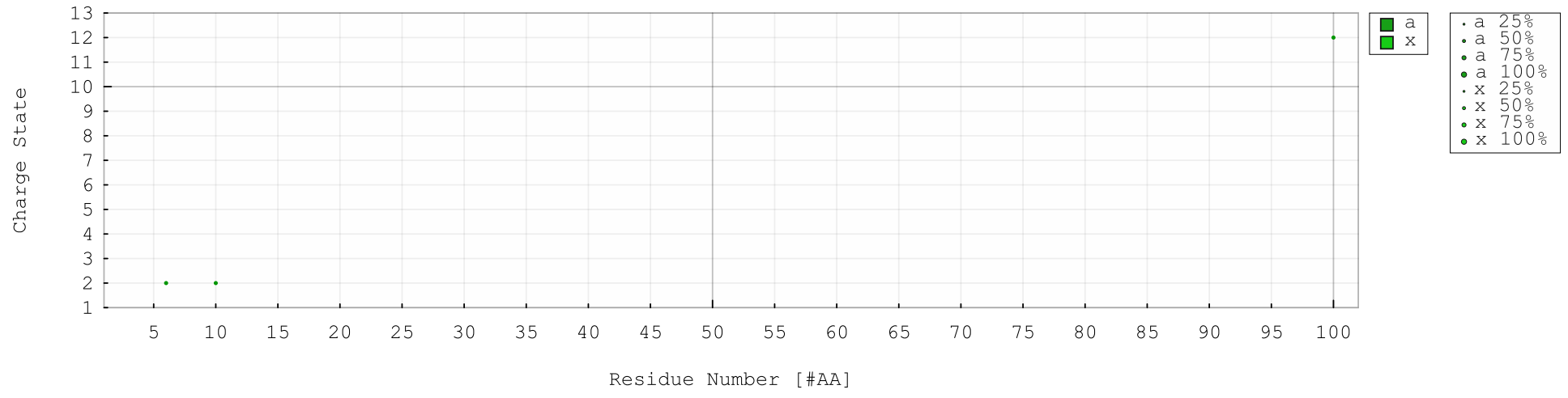

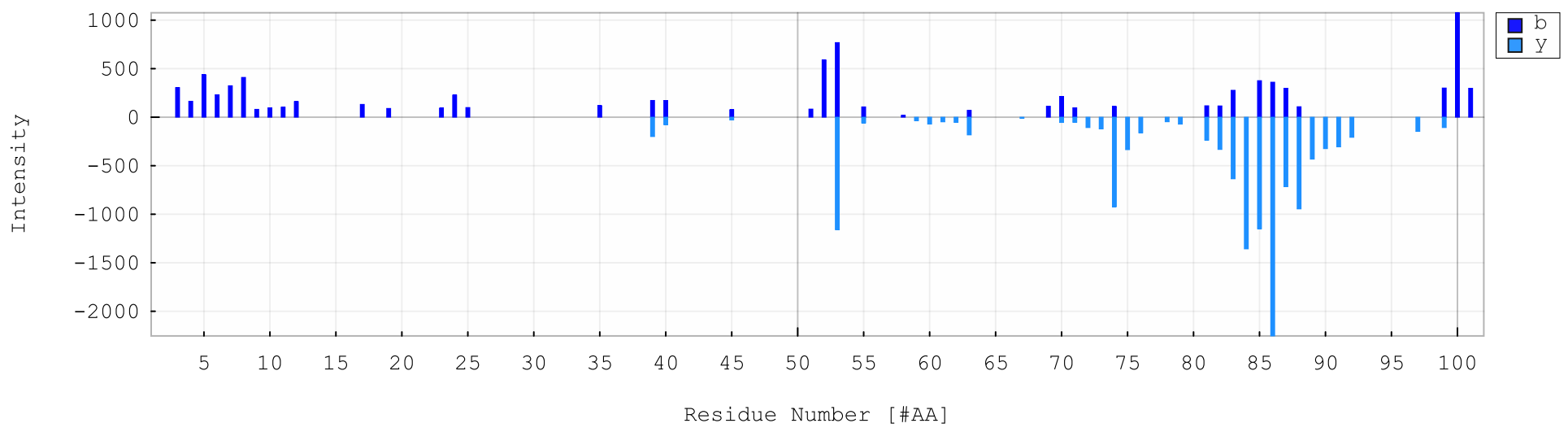

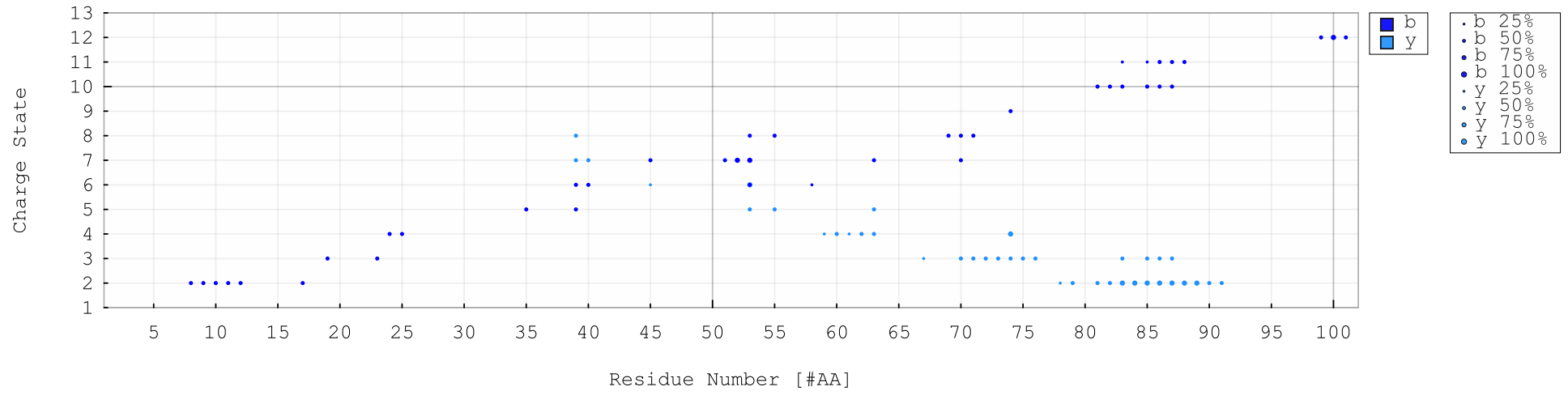

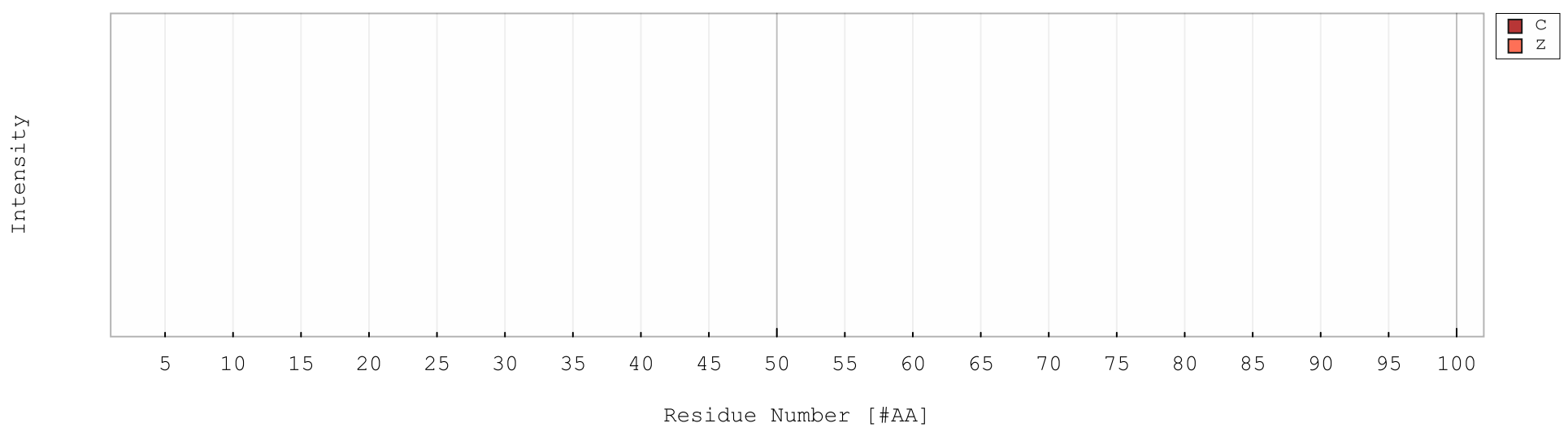

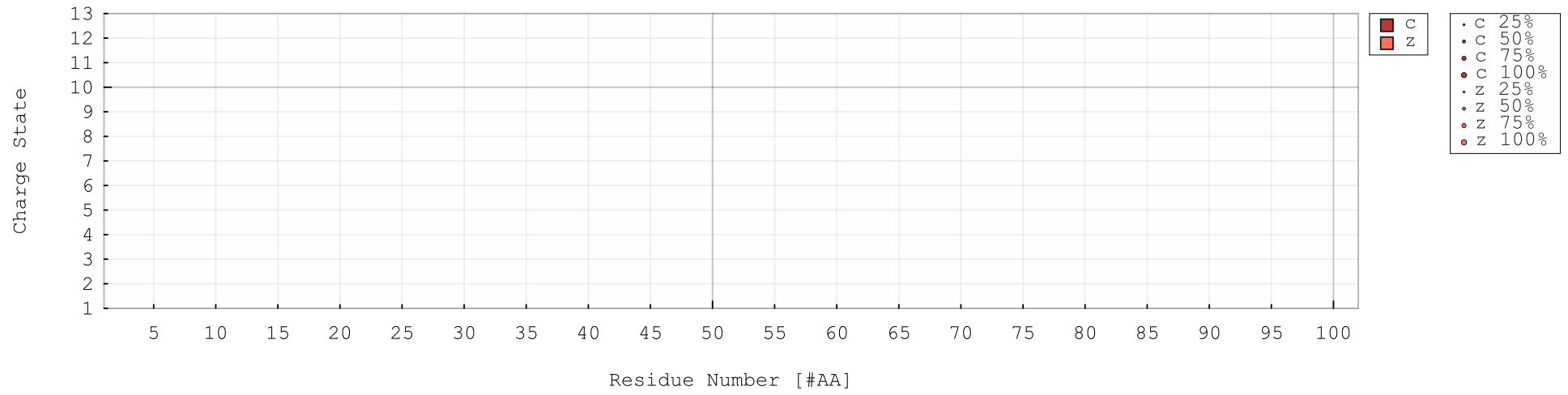

Proteoform:

Index	Protein	SC [%]	SVP [%]	IC [%]	MS Score [a.u.]	Var. Mods	Mono Mass [Da]	Chemical Formula
6	H4Kac	53.5	16.7	32.0	17.1	Ace: K31	11271.3518	C496H839N163O135S

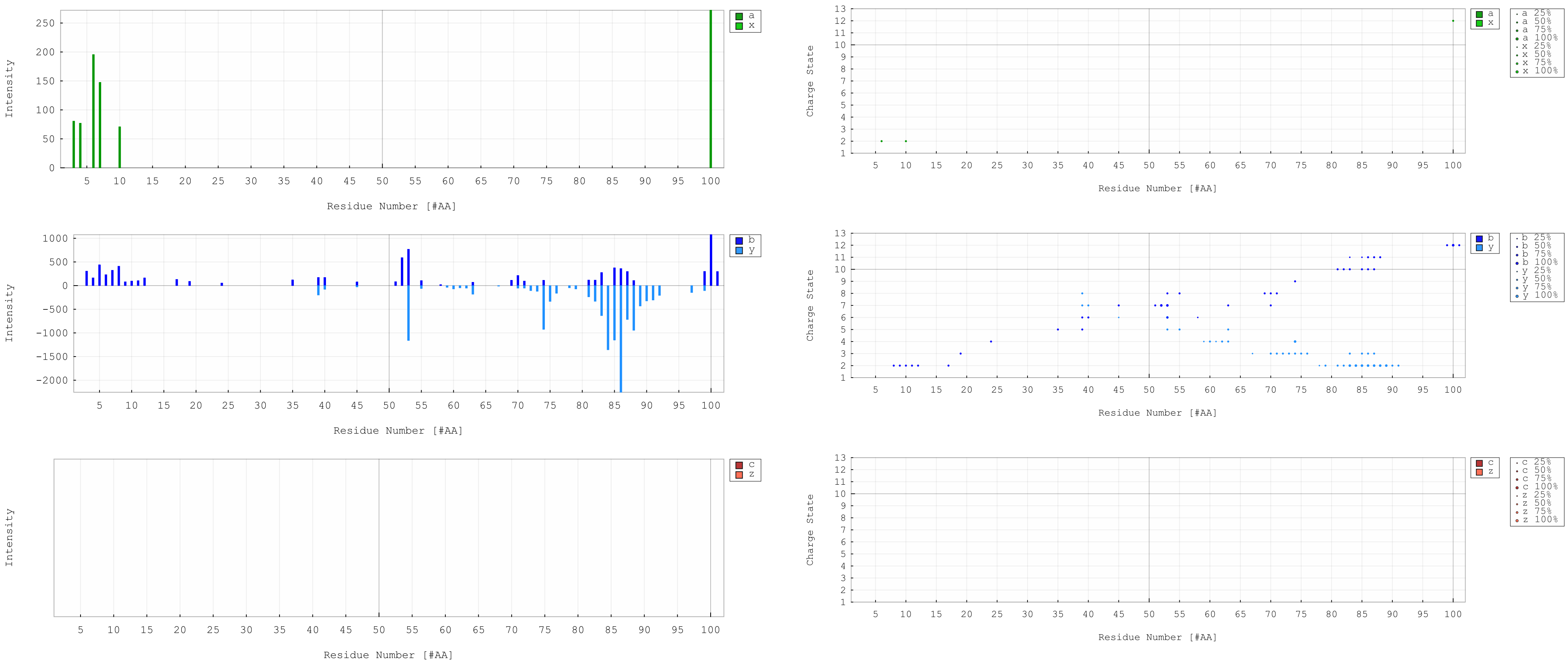

Proteoform:

Index	Protein	SC [%]	SVP [%]	IC [%]	MS Score [a.u.]	Var. Mods	Mono Mass [Da]	Chemical Formula
7	H4Kac	52.5	16.7	31.1	16.3	Ace: K44	11271.3518	C496H839N163O135S

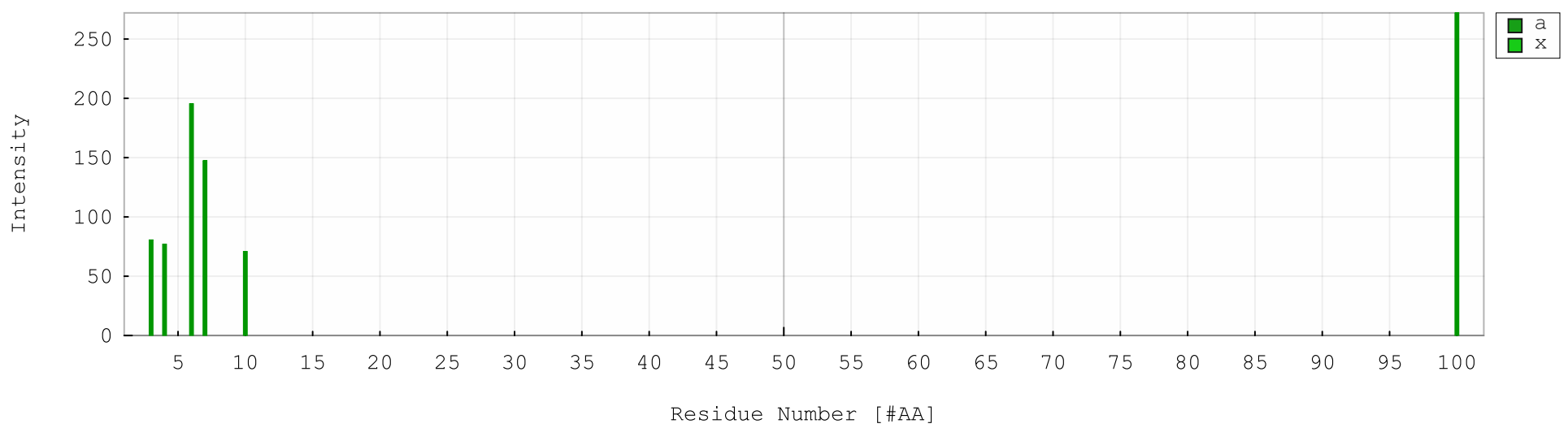

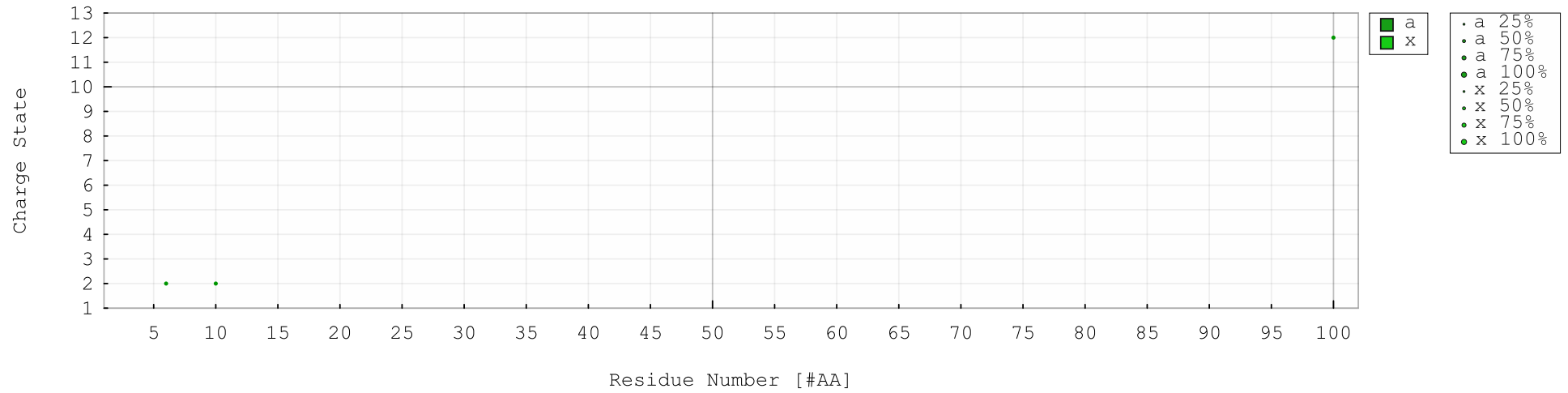

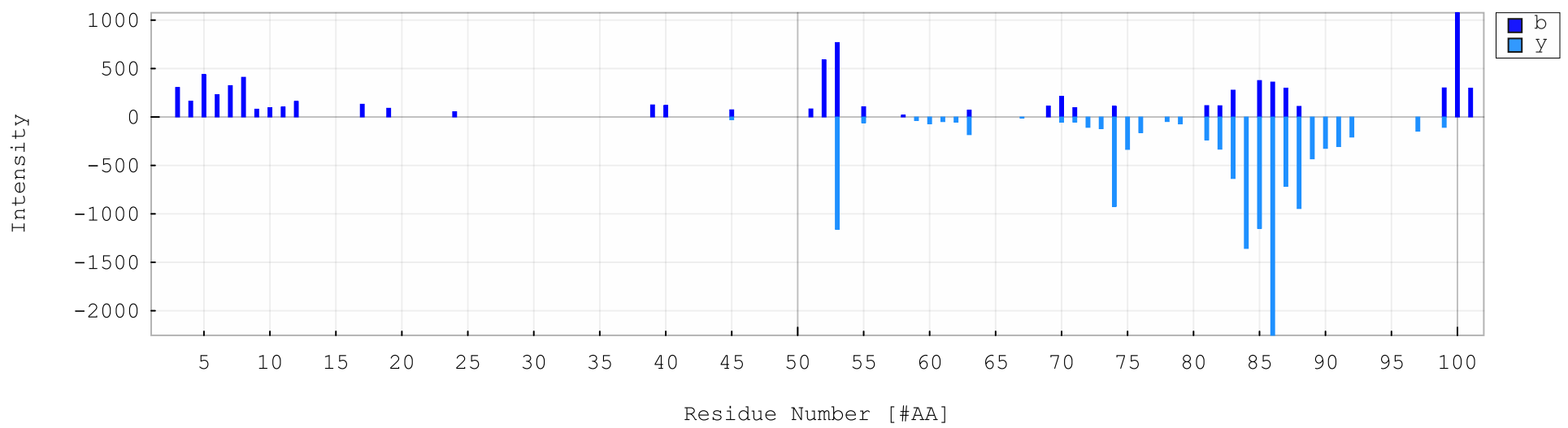

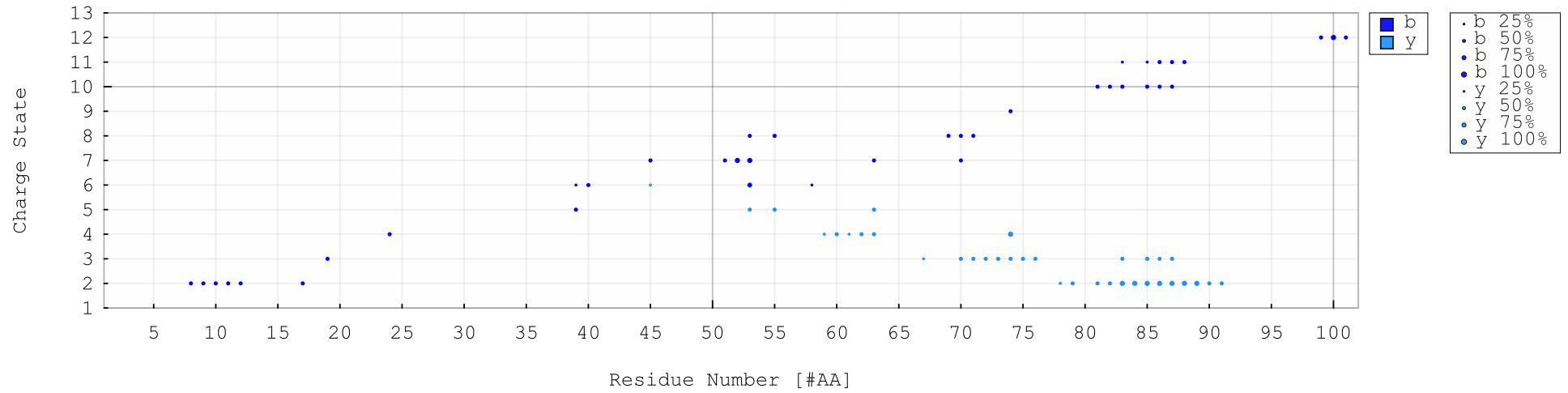

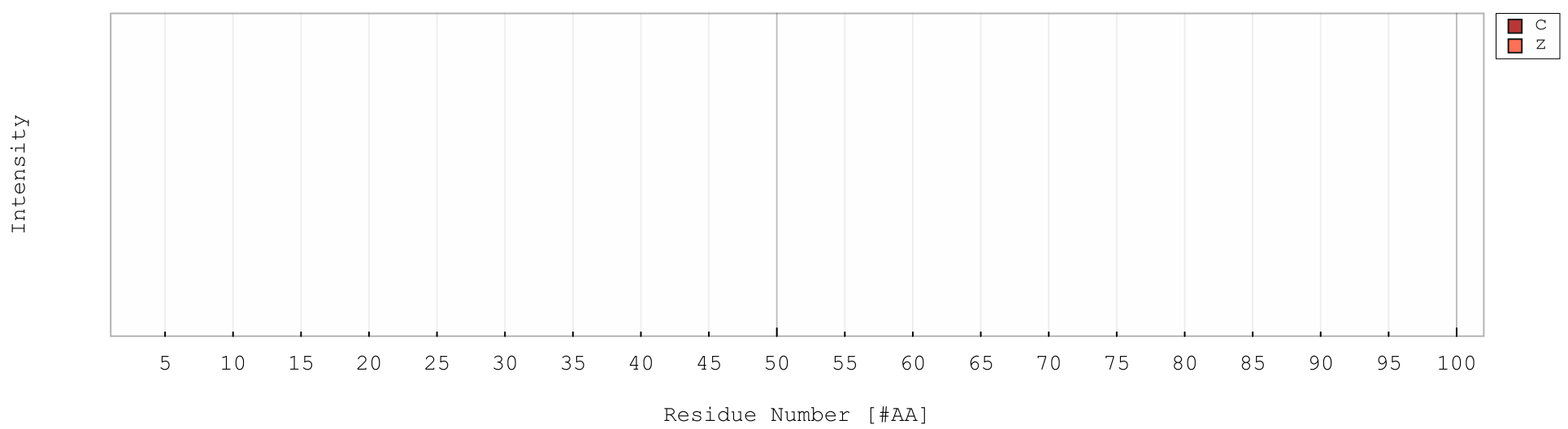

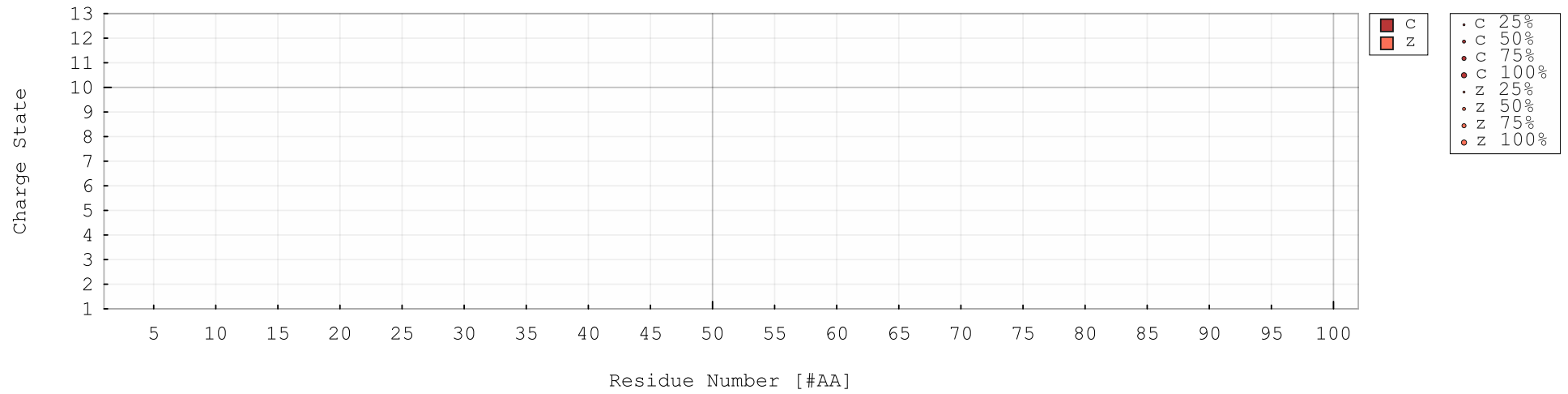

Top-Down Result OmniScape 2025
Sequence Confirmation
250428 H4-PCAF100-30min z13ac1 MS2 shCID_001-1536_calibrated2

Proteoform:	Index	Protein	SC [%]	SVP [%]	IC [%]	MS Score [a.u.]	Var. Mods	Mono Mass [Da]	Chemical Formula
	8	H4Kac	48.5	16.7	26.6	12.9	Ace: K59	11271.3518	C496H839N163O135S

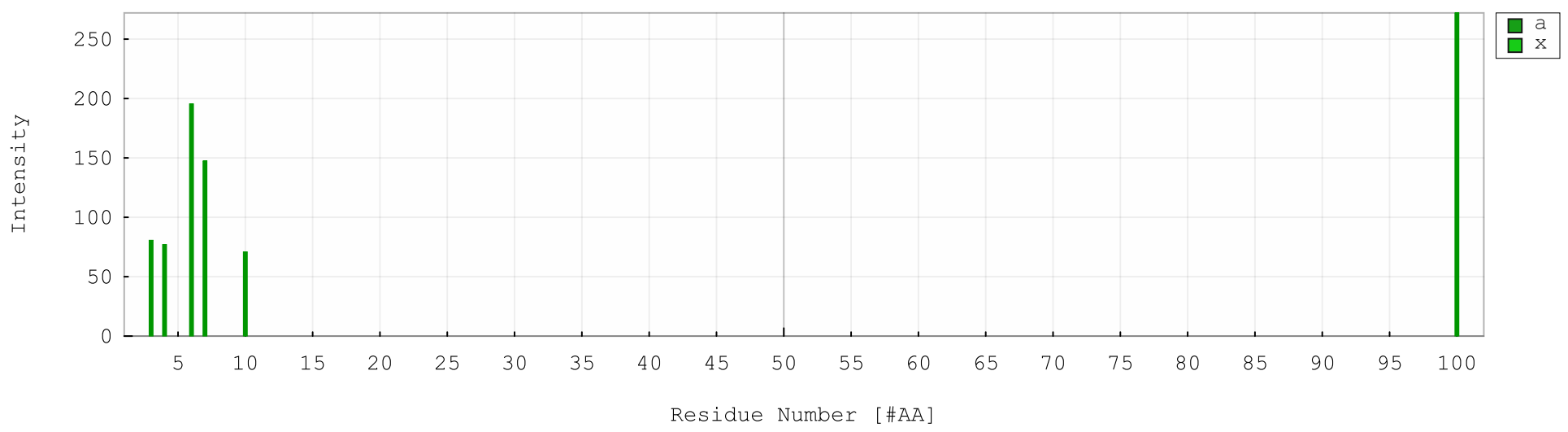

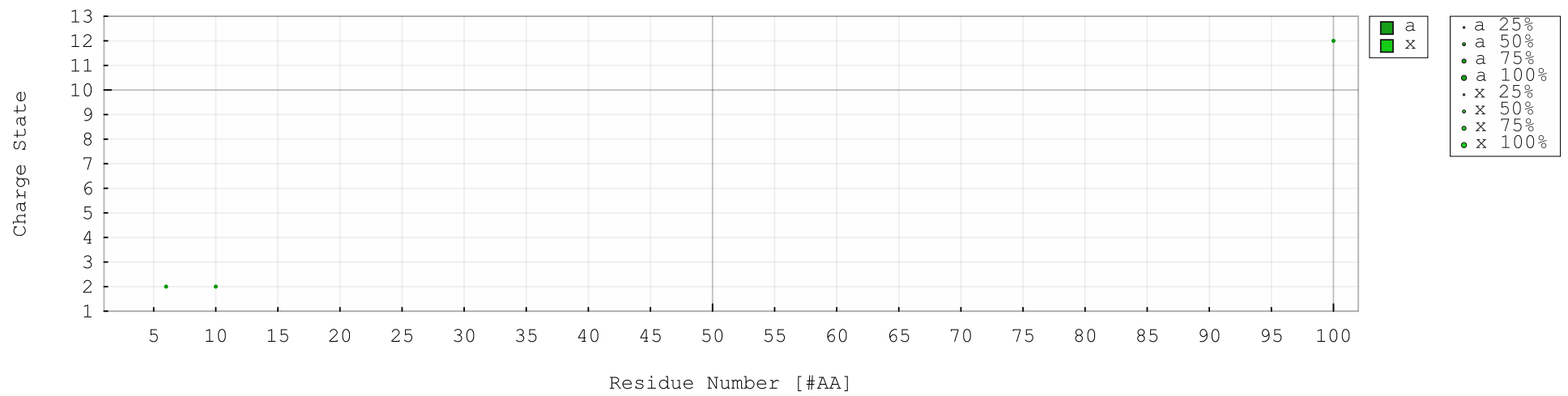

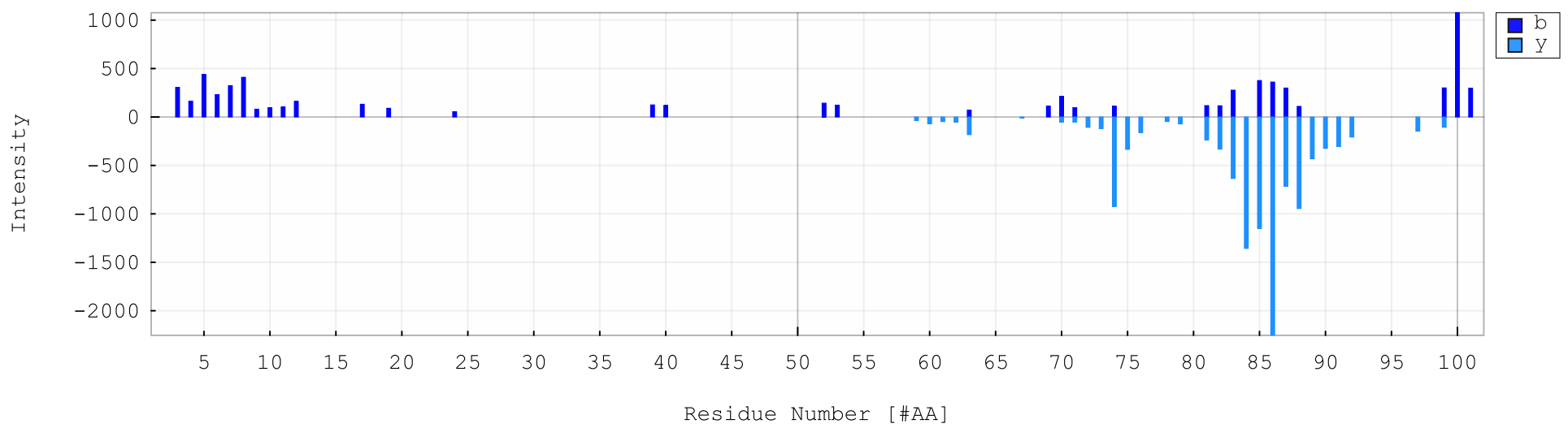

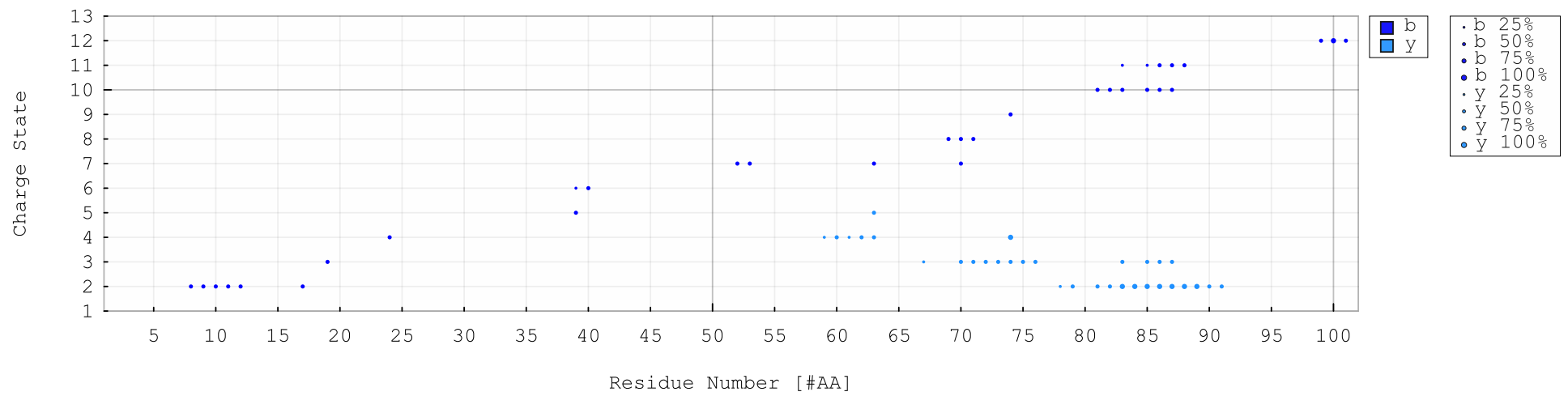

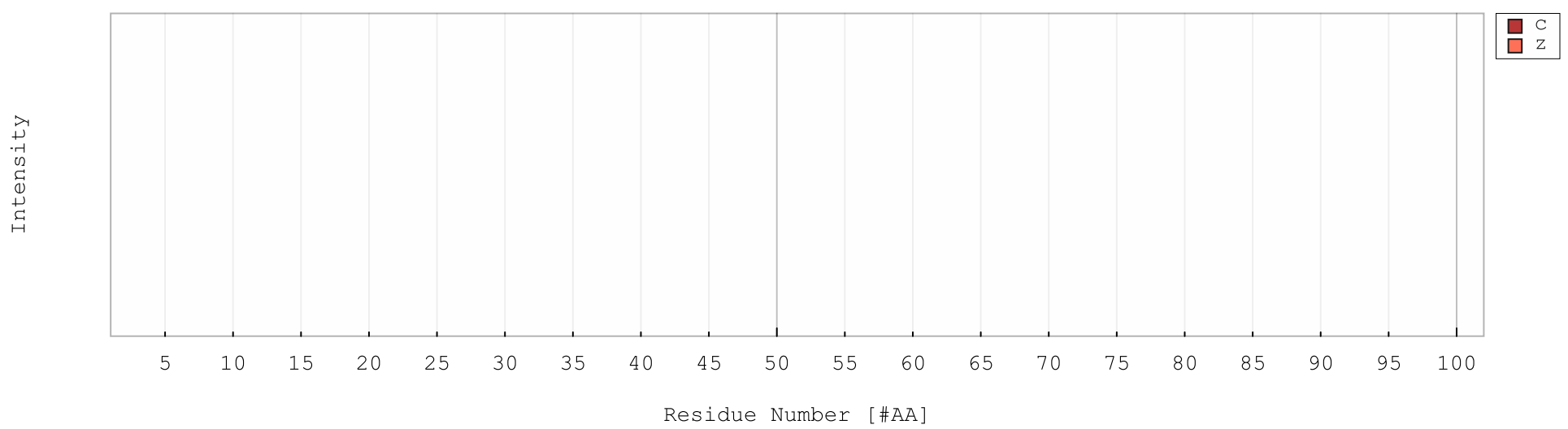

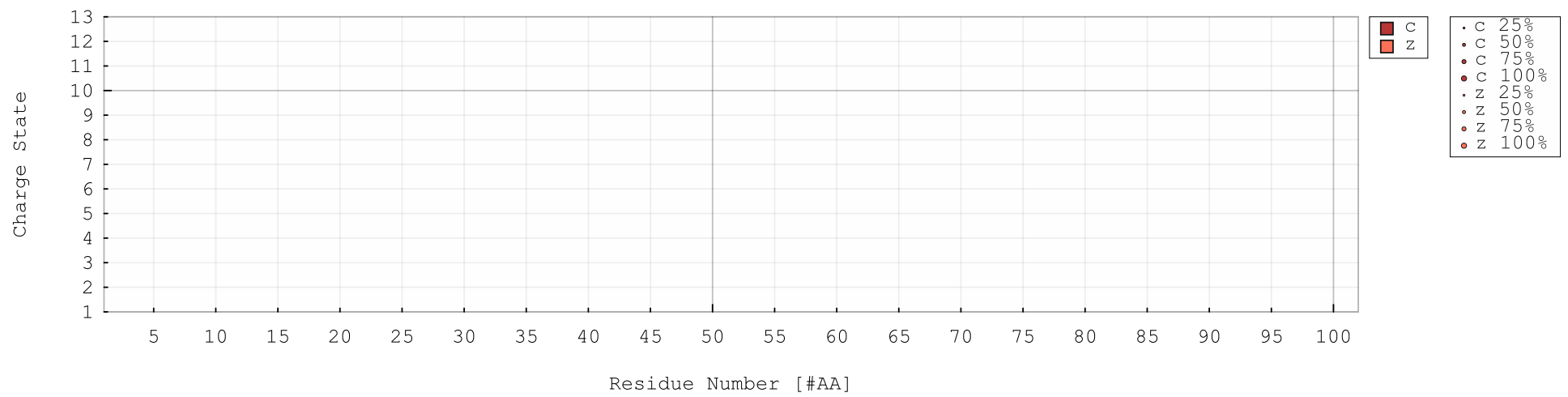

Top-Down Result OmniScape 2025
Sequence Confirmation
250428 H4-PCAF100-30min z13ac1 MS2 shCID_001-1536_calibrated2

Proteoform:

Index	Protein	SC [%]	SVP [%]	IC [%]	MS Score [a.u.]	Var. Mods	Mono Mass [Da]	Chemical Formula
9	H4Kac	48.5	16.7	18.6	9.0	None	11229.3412	C494H837N163O134S

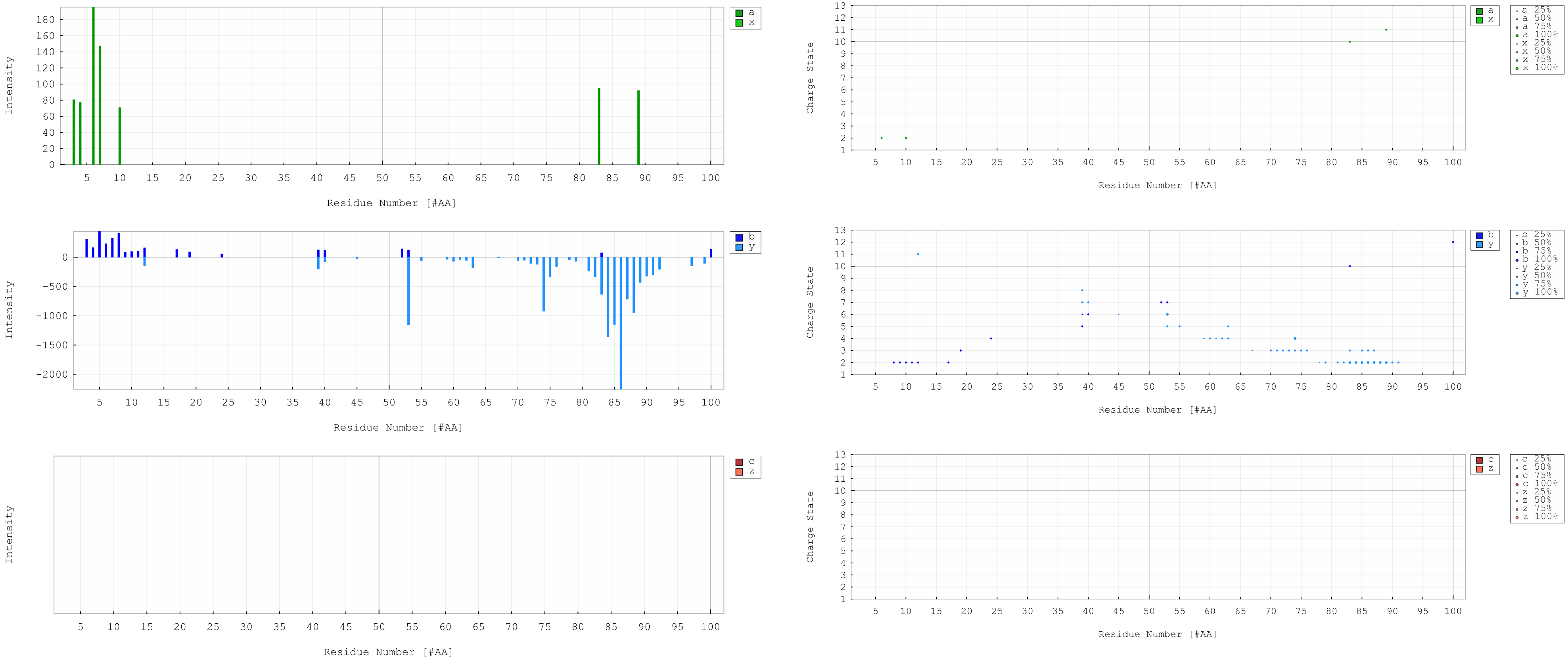

Proteoform:

Index	Protein	SC [%]	SVP [%]	IC [%]	MS Score [a.u.]	Var. Mods	Mono Mass [Da]	Chemical Formula
10	H4Kac	34.7	16.7	22.4	7.8	Ace: K77	11271.3518	C496H839N163O135S

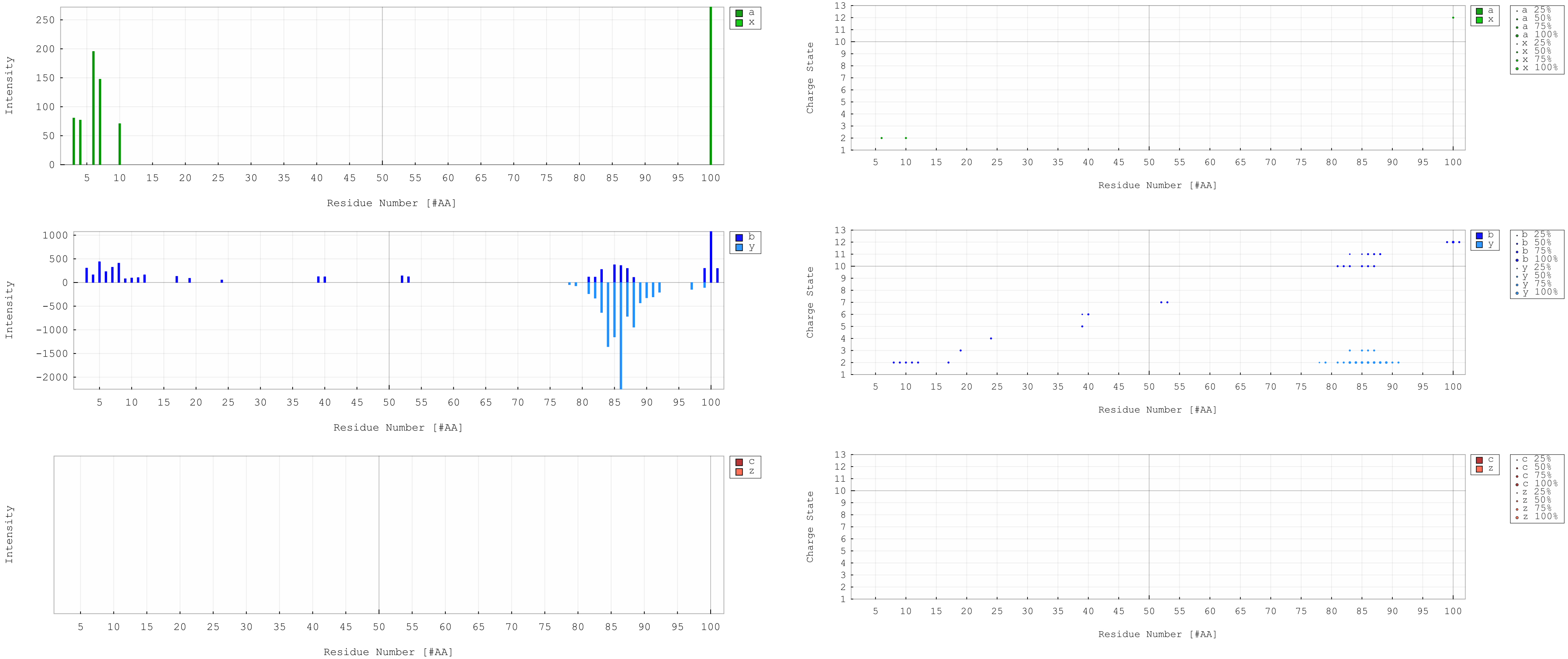

Supplemental Data S5

H4 - PCAF, 30 min

**Report**	5 of 8
**Experiment**	MS2
**Histone/sample**	H4
**Enzyme**	PCAF
**Reaction/source**	30 min
**Precursor**	H4Kac2
**Charge state**	13+
**Fragmentation / workflow**	ECD
**Original file**	MS2_H4Kac2_13+_ECD.pdf

Project Information

Project Directory: C:/Users/LAB-ADMIN
Processing date: Tue Apr 28 14:48:12 2026

Sample Info & Protocols

Name: Top-Down Protein Sequencing ExD

Dataset

Imported spectrum file name:	250428 H4-PCAF100-30min z13ac2 MS2 ECD_1-908_calibrated.txt
Dataset name:	Information is only available from imported JPEAKS files
Date acquired:	Information is only available from imported JPEAKS files
Date imported:	Information is only available from imported JPEAKS files
Instrument Name:	Information is only available from imported JPEAKS files
Instrument Serial Number:	Information is only available from imported JPEAKS files

Workflow Result Info

Analysis: 250428 H4-PCAF100-30min z13ac2 MS2 ECD_1-908_calibrated2
Location: C:/Users/LAB-ADMIN
Method: -

Method Information

Name: Unnamed

Spectral Parameters

Mass Error [ppm]: 6
Intensity Deviation [au]: 0.15
Min/Max Charges: 1-13
Experiment type: ExD

a ions	b ions	c ions	x ions	y ions	z ions	satellite	internal	precursor
a	b	c		y	z+1			M
a+1		c+1			z+2			M+-2H

Noise threshold calculation: Simple moving average noise threshold (SMA)
Window size [datapoints]: 600
Noise curve offset: 2.7606566
SVP parameters: terminal gap: 2, internal gap: 1

Proteoforms
(top 10)

Index	Parent Sequence	Chain	Var. Mods	Mono Mass [Da]	Chemical Composition
1	H4Kac	H4KacK8[Ace]K44[Ace]	Ace: K8, K44	11313.3624	C498H841N163O136S
2	H4Kac	H4KacK12[Ace]K44[Ace]	Ace: K12, K44	11313.3624	C498H841N163O136S
3	H4Kac	H4KacK16[Ace]K44[Ace]	Ace: K16, K44	11313.3624	C498H841N163O136S
4	H4Kac	H4KacK8[Ace]K31[Ace]	Ace: K8, K31	11313.3624	C498H841N163O136S
5	H4Kac	H4KacK5[Ace]K44[Ace]	Ace: K5, K44	11313.3624	C498H841N163O136S
6	H4Kac	H4KacK20[Ace]K44[Ace]	Ace: K20, K44	11313.3624	C498H841N163O136S
7	H4Kac	H4KacK12[Ace]K31[Ace]	Ace: K12, K31	11313.3624	C498H841N163O136S
8	H4Kac	H4KacK8[Ace]K59[Ace]	Ace: K8, K59	11313.3624	C498H841N163O136S
9	H4Kac	H4KacK16[Ace]K31[Ace]	Ace: K16, K31	11313.3624	C498H841N163O136S
10	H4Kac	H4KacK12[Ace]K59[Ace]	Ace: K12, K59	11313.3624	C498H841N163O136S

Results

Proteoforms Table (top 10)

Index	Protein	SC [%]	SVP [%]	IC [%]	MS Score [a.u.]	Var. Mods	Mono Mass [Da]	Chemical Formula
1	H4Kac	85.1	73.5	50.3	42.8	Ace: K8, K44	11313.3624	C498H841N163O136S
2	H4Kac	85.1	73.5	49.7	42.3	Ace: K12, K44	11313.3624	C498H841N163O136S
3	H4Kac	85.1	73.5	48.2	41.0	Ace: K16, K44	11313.3624	C498H841N163O136S
4	H4Kac	84.2	73.5	48.1	40.4	Ace: K8, K31	11313.3624	C498H841N163O136S
5	H4Kac	82.2	18.6	49.1	40.3	Ace: K5, K44	11313.3624	C498H841N163O136S
6	H4Kac	85.1	73.5	47.3	40.3	Ace: K20, K44	11313.3624	C498H841N163O136S
7	H4Kac	84.2	73.5	47.5	40.0	Ace: K12, K31	11313.3624	C498H841N163O136S
8	H4Kac	84.2	73.5	46.2	38.8	Ace: K8, K59	11313.3624	C498H841N163O136S
9	H4Kac	84.2	73.5	46.0	38.7	Ace: K16, K31	11313.3624	C498H841N163O136S
10	H4Kac	84.2	73.5	45.6	38.4	Ace: K12, K59	11313.3624	C498H841N163O136S

Sequence

Name: H4Kac

```
                  10                   20                   30                   40                   50                   60
S G R G K G G K G L  G K G G A K R H R K  V L R D N I Q G I T  K P A I R R L A R R  G G V K R I S G L I  Y E E T R G V L K V
                  70                   80                   90                  100                  110                  120
F L E N V I R D A V  T Y T E H A K R K T  V T A M D V V Y A L  K R Q G R T L Y G F  G G
```

Modifications

Modification	Position	Type
Acetyl	5	variable
Acetyl	8	variable
Acetyl	12	variable
Acetyl	16	variable
Acetyl	20	variable
Acetyl	31	variable
Acetyl	44	variable
Acetyl	59	variable
Acetyl	77	variable
Acetyl	79	variable
Acetyl	91	variable

Sequence Map

Proteoform:

Index	Protein	SC [%]	SVP [%]	IC [%]	MS Score [a.u.]	Var. Mods	Mono Mass [Da]	Chemical Formula
1	H4Kac	85.1	73.5	50.3	42.8	Ace: K8, K44	11313.3624	C498H841N163O136S

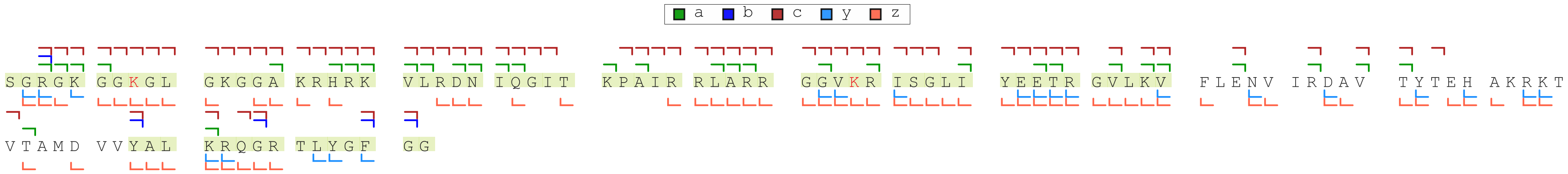

Proteoform:

Index	Protein	SC [%]	SVP [%]	IC [%]	MS Score [a.u.]	Var. Mods	Mono Mass [Da]	Chemical Formula
2	H4Kac	85.1	73.5	49.7	42.3	Ace: K12, K44	11313.3624	C498H841N163O136S

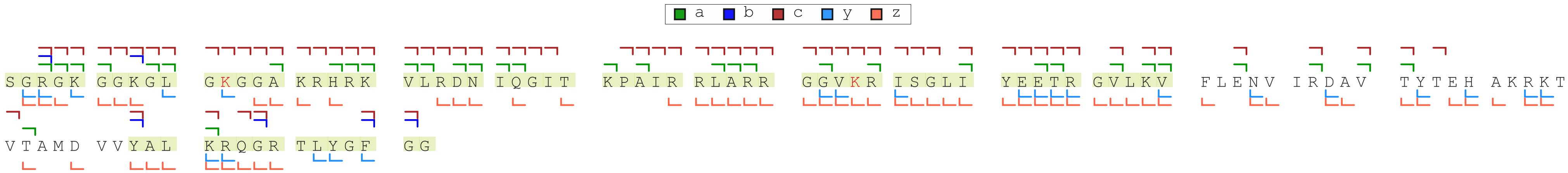

Proteoform:

Index	Protein	SC [%]	SVP [%]	IC [%]	MS Score [a.u.]	Var. Mods	Mono Mass [Da]	Chemical Formula
3	H4Kac	85.1	73.5	48.2	41.0	Ace: K16, K44	11313.3624	C498H841N163O136S

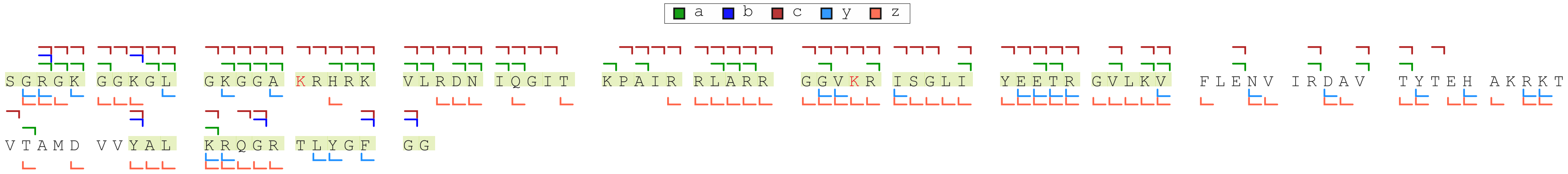

Proteoform:

Index	Protein	SC [%]	SVP [%]	IC [%]	MS Score [a.u.]	Var. Mods	Mono Mass [Da]	Chemical Formula
4	H4Kac	84.2	73.5	48.1	40.4	Ace: K8, K31	11313.3624	C498H841N163O136S

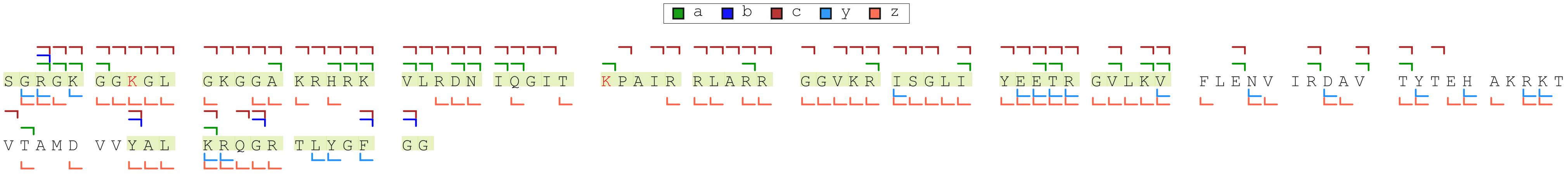

Proteoform:

Index	Protein	SC [%]	SVP [%]	IC [%]	MS Score [a.u.]	Var. Mods	Mono Mass [Da]	Chemical Formula
5	H4Kac	82.2	18.6	49.1	40.3	Ace: K5, K44	11313.3624	C498H841N163O136S

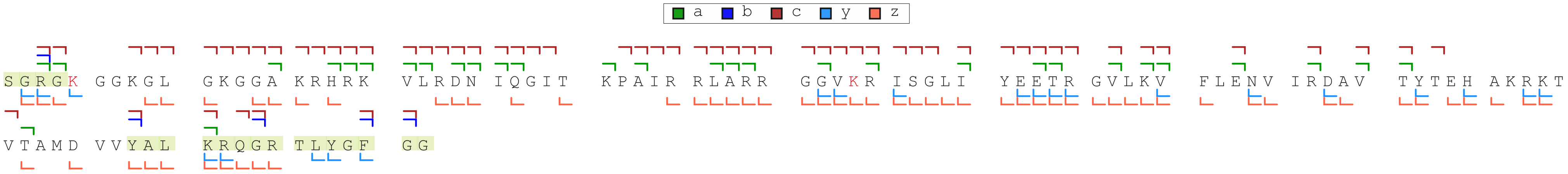

Proteoform:

Index	Protein	SC [%]	SVP [%]	IC [%]	MS Score [a.u.]	Var. Mods	Mono Mass [Da]	Chemical Formula
6	H4Kac	85.1	73.5	47.3	40.3	Ace: K20, K44	11313.3624	C498H841N163O136S

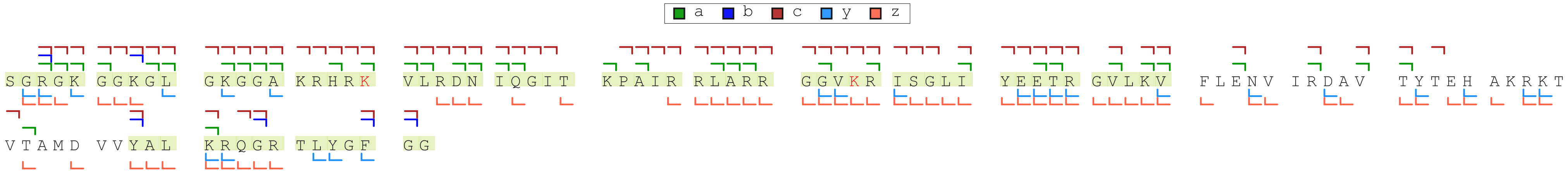

Proteoform:

Index	Protein	SC [%]	SVP [%]	IC [%]	MS Score [a.u.]	Var. Mods	Mono Mass [Da]	Chemical Formula
7	H4Kac	84.2	73.5	47.5	40.0	Ace: K12, K31	11313.3624	C498H841N163O136S

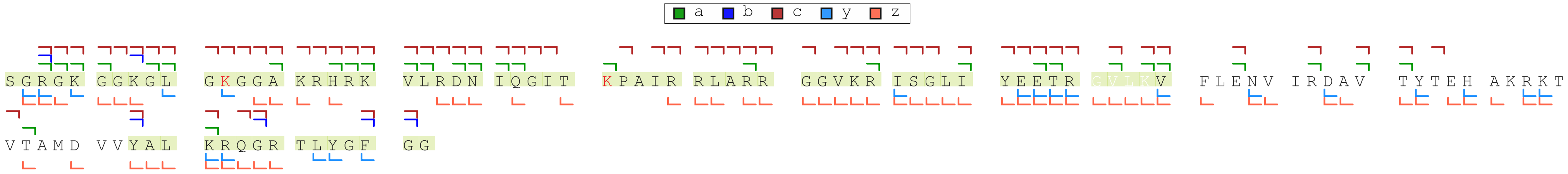

Proteoform:

Index	Protein	SC [%]	SVP [%]	IC [%]	MS Score [a.u.]	Var. Mods	Mono Mass [Da]	Chemical Formula
8	H4Kac	84.2	73.5	46.2	38.8	Ace: K8, K59	11313.3624	C498H841N163O136S

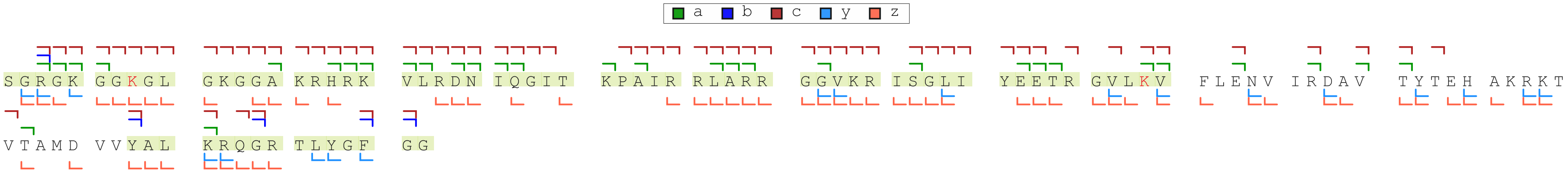

Proteoform:

Index	Protein	SC [%]	SVP [%]	IC [%]	MS Score [a.u.]	Var. Mods	Mono Mass [Da]	Chemical Formula
9	H4Kac	84.2	73.5	46.0	38.7	Ace: K16, K31	11313.3624	C498H841N163O136S

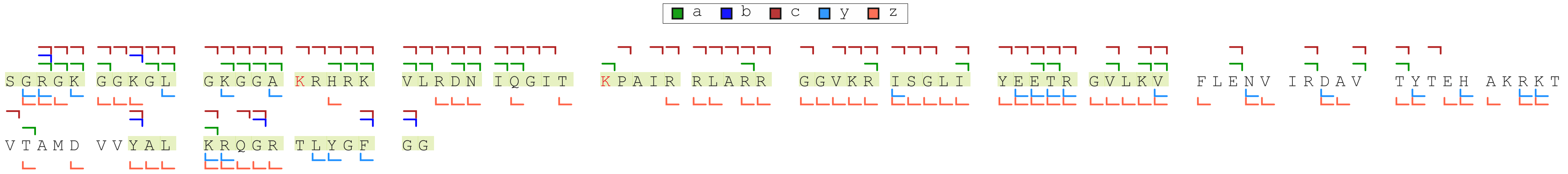

Proteoform:

Index	Protein	SC [%]	SVP [%]	IC [%]	MS Score [a.u.]	Var. Mods	Mono Mass [Da]	Chemical Formula
10	H4Kac	84.2	73.5	45.6	38.4	Ace: K12, K59	11313.3624	C498H841N163O136S

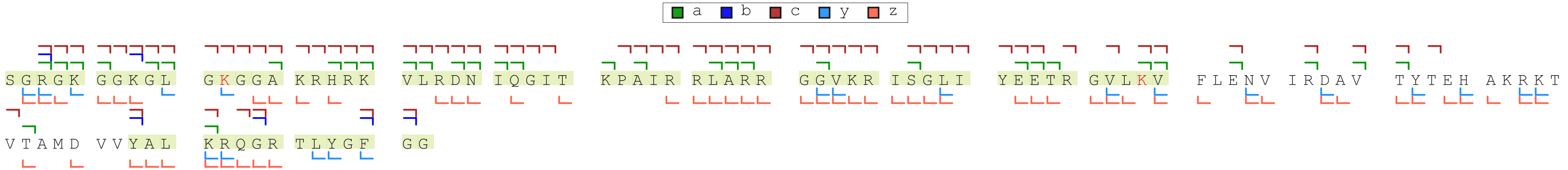

Error Plot

Proteoform:

Index	Protein	SC [%]	SVP [%]	IC [%]	MS Score [a.u.]	Var. Mods	Mono Mass [Da]	Chemical Formula
1	H4Kac	85.1	73.5	50.3	42.8	Ace: K8, K44	11313.3624	C498H841N163O136S

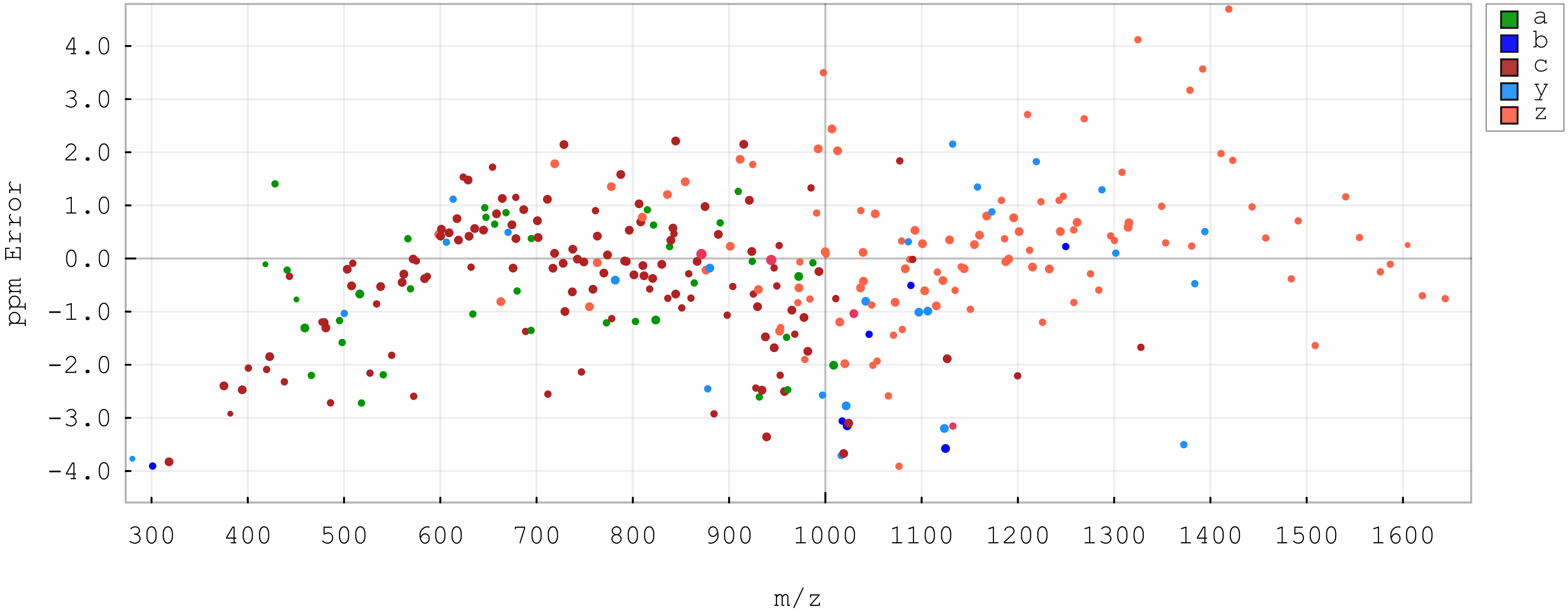

Proteoform:

Index	Protein	SC [%]	SVP [%]	IC [%]	MS Score [a.u.]	Var. Mods	Mono Mass [Da]	Chemical Formula
2	H4Kac	85.1	73.5	49.7	42.3	Ace: K12, K44	11313.3624	C498H841N163O136S

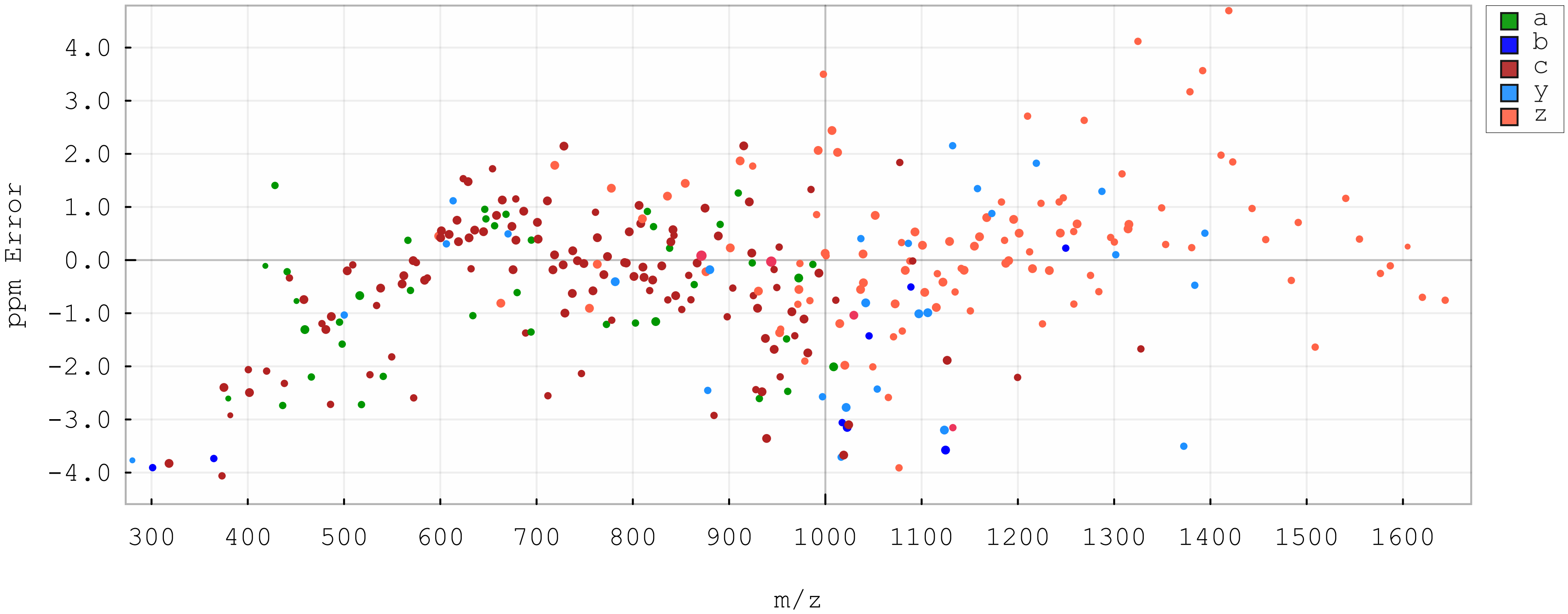

Proteoform:

Index	Protein	SC [%]	SVP [%]	IC [%]	MS Score [a.u.]	Var. Mods	Mono Mass [Da]	Chemical Formula
3	H4Kac	85.1	73.5	48.2	41.0	Ace: K16, K44	11313.3624	C498H841N163O136S

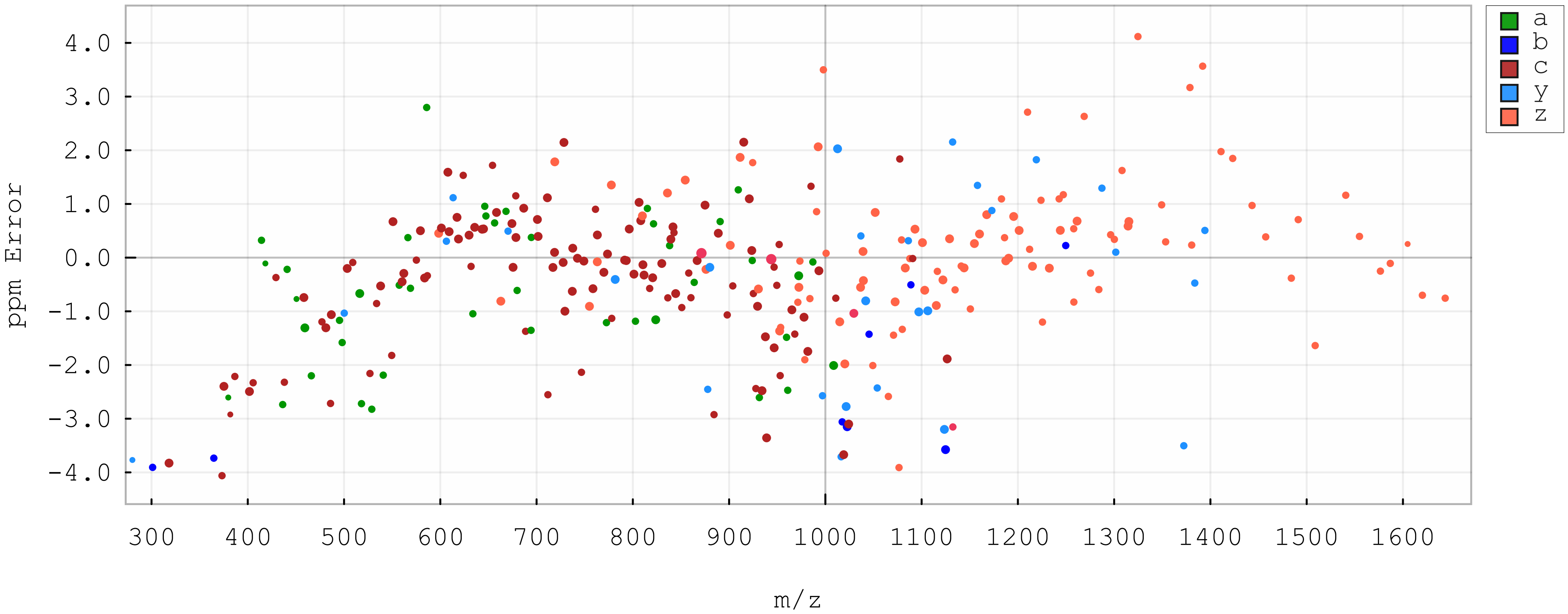

Proteoform:

Index	Protein	SC [%]	SVP [%]	IC [%]	MS Score [a.u.]	Var. Mods	Mono Mass [Da]	Chemical Formula
4	H4Kac	84.2	73.5	48.1	40.4	Ace: K8, K31	11313.3624	$C_{498}H_{841}N_{163}O_{136}S$

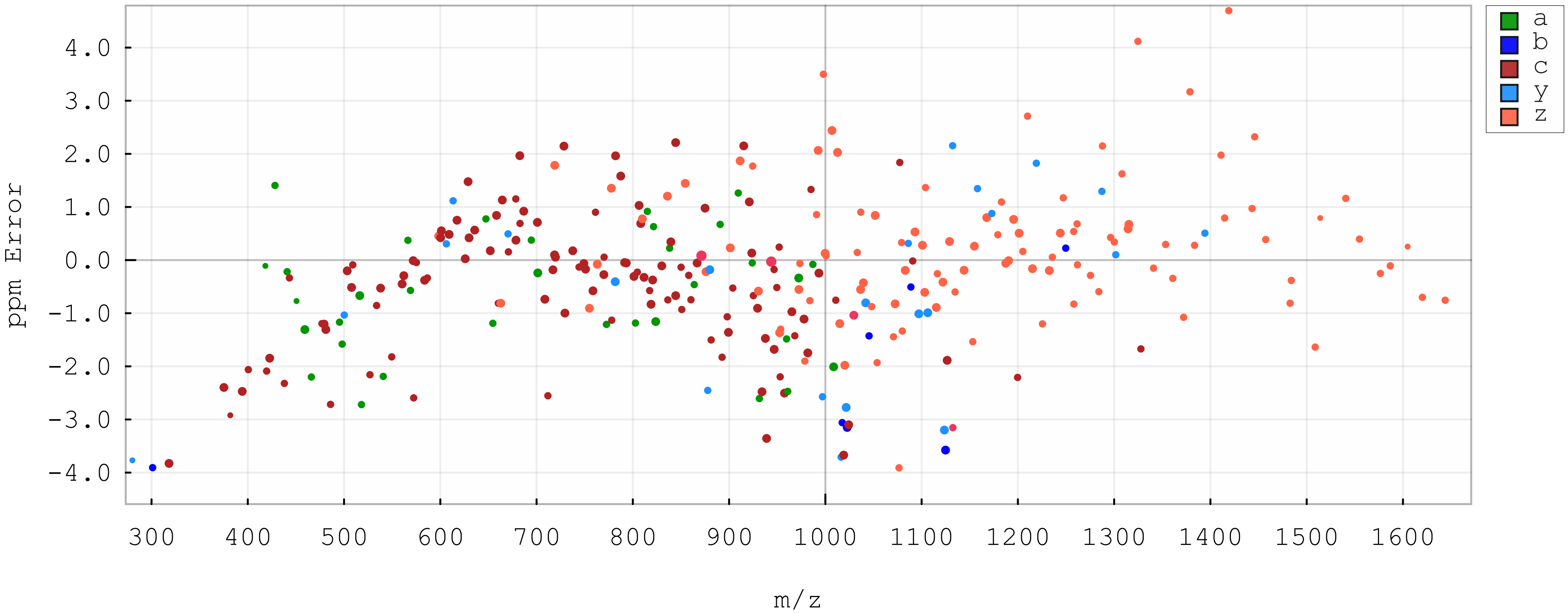

Proteoform:

Index	Protein	SC [%]	SVP [%]	IC [%]	MS Score [a.u.]	Var. Mods	Mono Mass [Da]	Chemical Formula
5	H4Kac	82.2	18.6	49.1	40.3	Ace: K5, K44	11313.3624	C498H841N163O136S

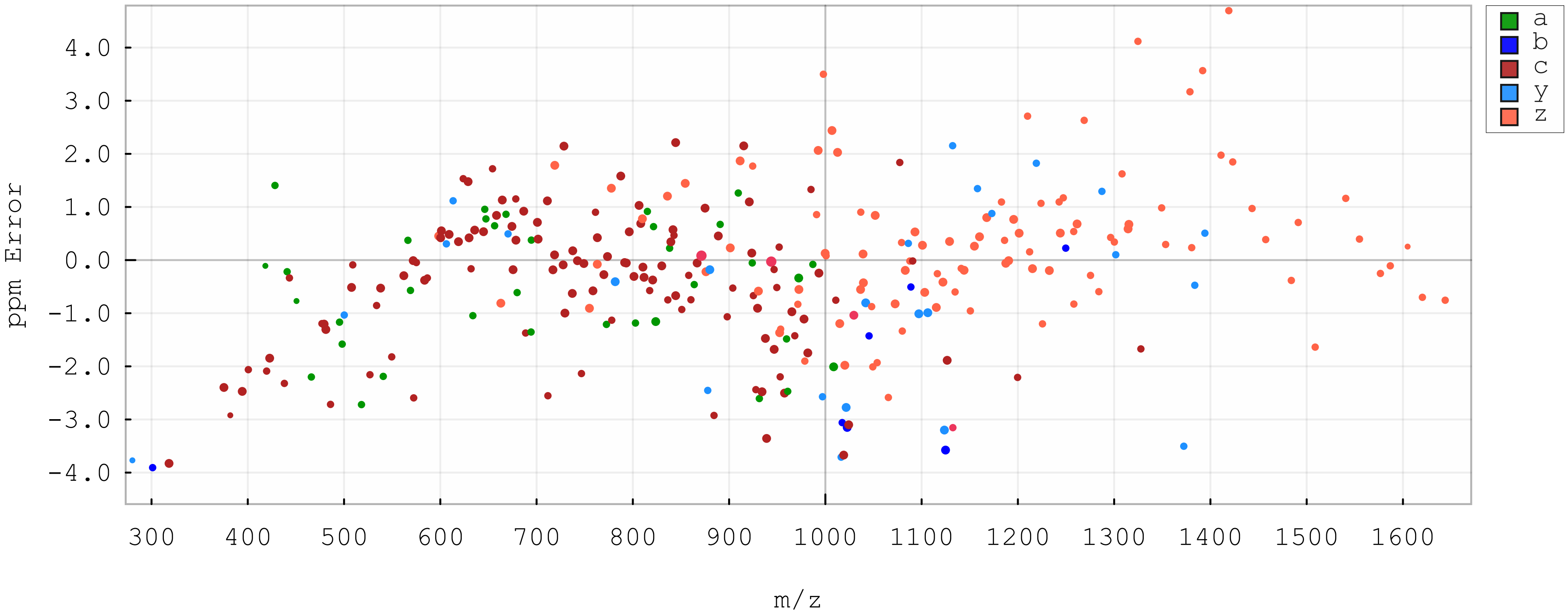

Proteoform:	Index	Protein	SC [%]	SVP [%]	IC [%]	MS Score [a.u.]	Var. Mods	Mono Mass [Da]	Chemical Formula
	6	H4Kac	85.1	73.5	47.3	40.3	Ace: K20, K44	11313.3624	C498H841N163O136S

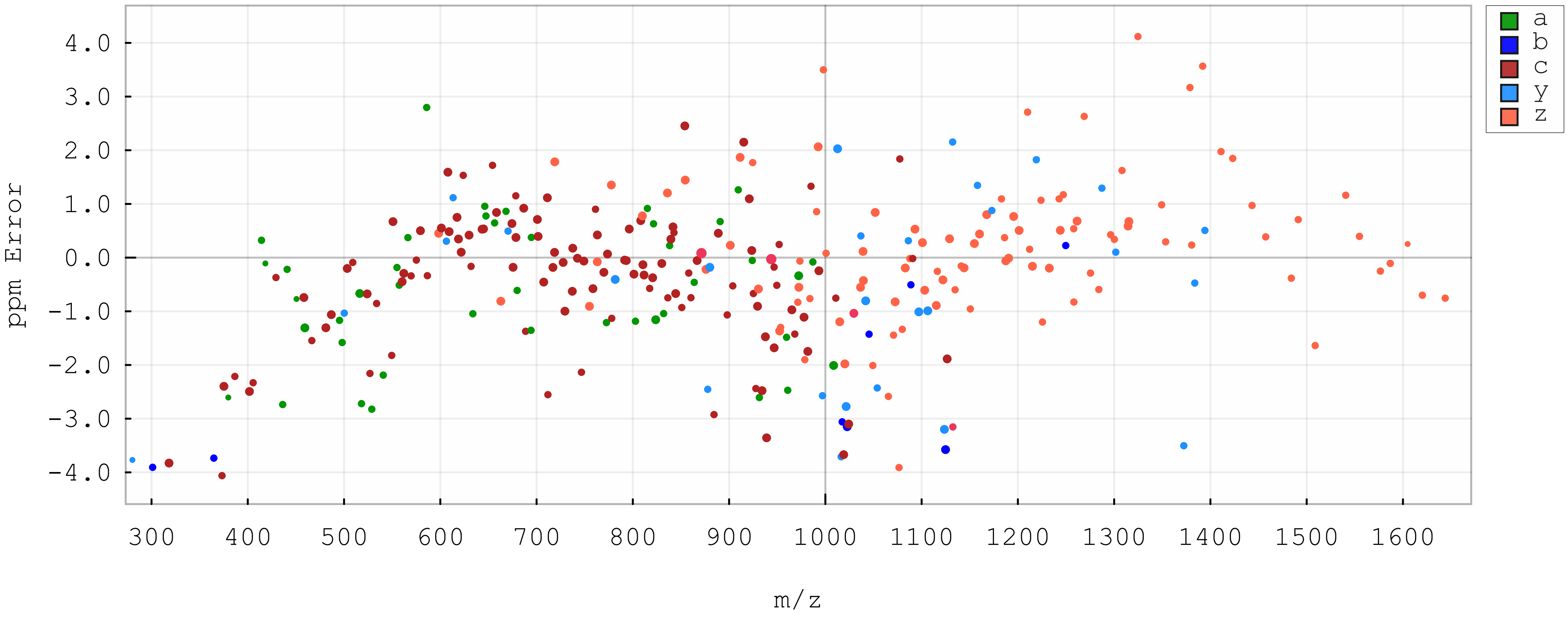

Proteoform:	Index	Protein	SC [%]	SVP [%]	IC [%]	MS Score [a.u.]	Var. Mods	Mono Mass [Da]	Chemical Formula
	7	H4Kac	84.2	73.5	47.5	40.0	Ace: K12, K31	11313.3624	C498H841N163O136S

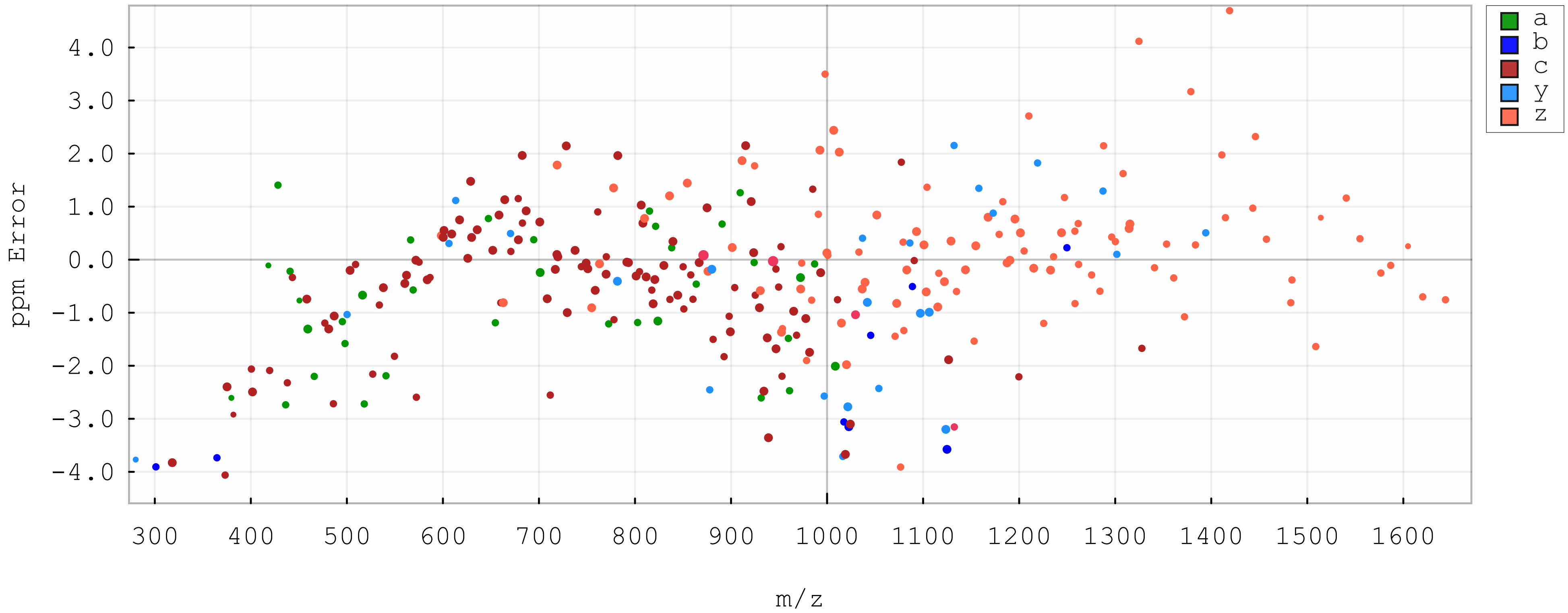

Proteoform:

Index	Protein	SC [%]	SVP [%]	IC [%]	MS Score [a.u.]	Var. Mods	Mono Mass [Da]	Chemical Formula
8	H4Kac	84.2	73.5	46.2	38.8	Ace: K8, K59	11313.3624	C498H841N163O136S

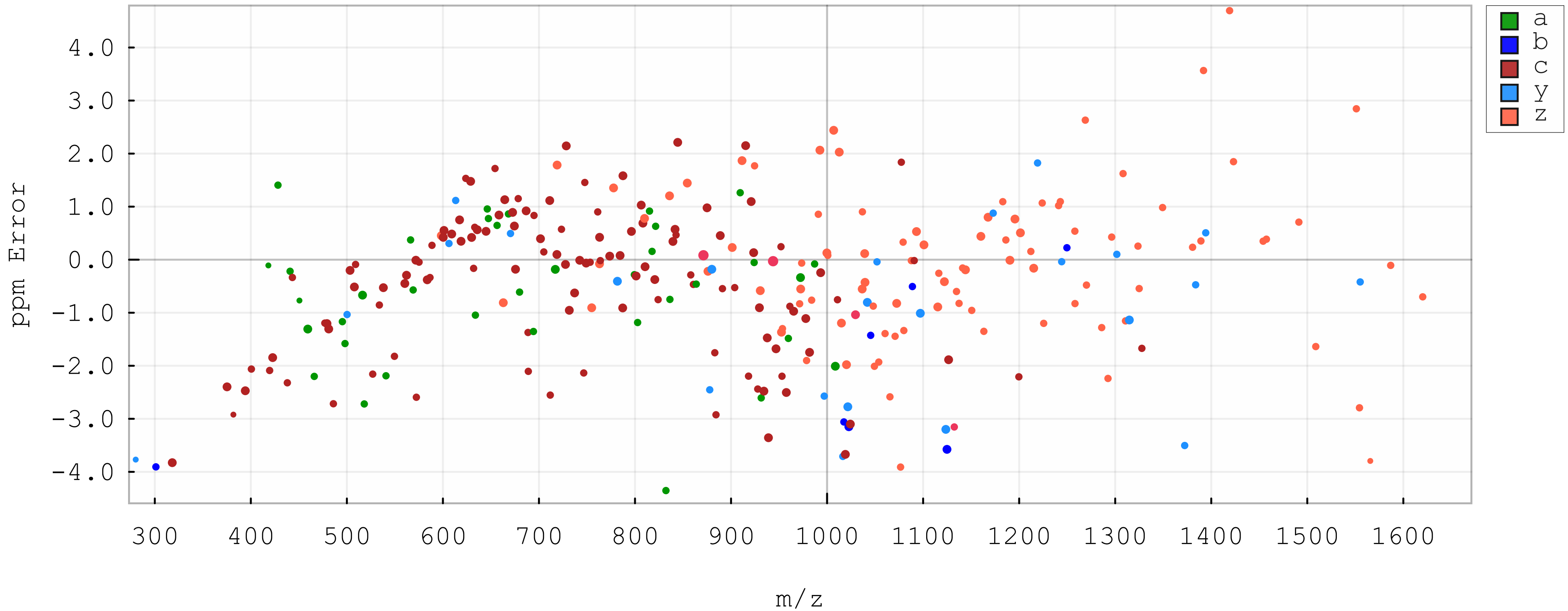

Proteoform:	Index	Protein	SC [%]	SVP [%]	IC [%]	MS Score [a.u.]	Var. Mods	Mono Mass [Da]	Chemical Formula
	9	H4Kac	84.2	73.5	46.0	38.7	Ace: K16, K31	11313.3624	C498H841N163O136S

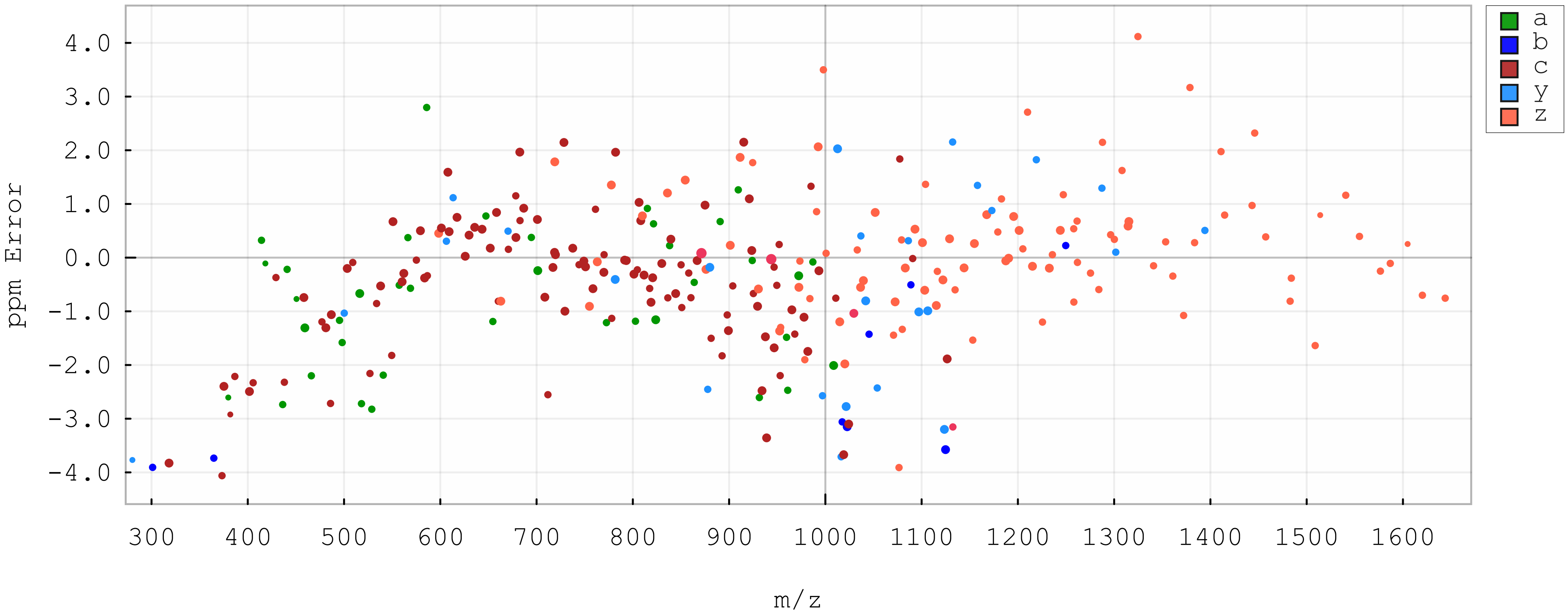

Proteoform:

Index	Protein	SC [%]	SVP [%]	IC [%]	MS Score [a.u.]	Var. Mods	Mono Mass [Da]	Chemical Formula
10	H4Kac	84.2	73.5	45.6	38.4	Ace: K12, K59	11313.3624	C498H841N163O136S

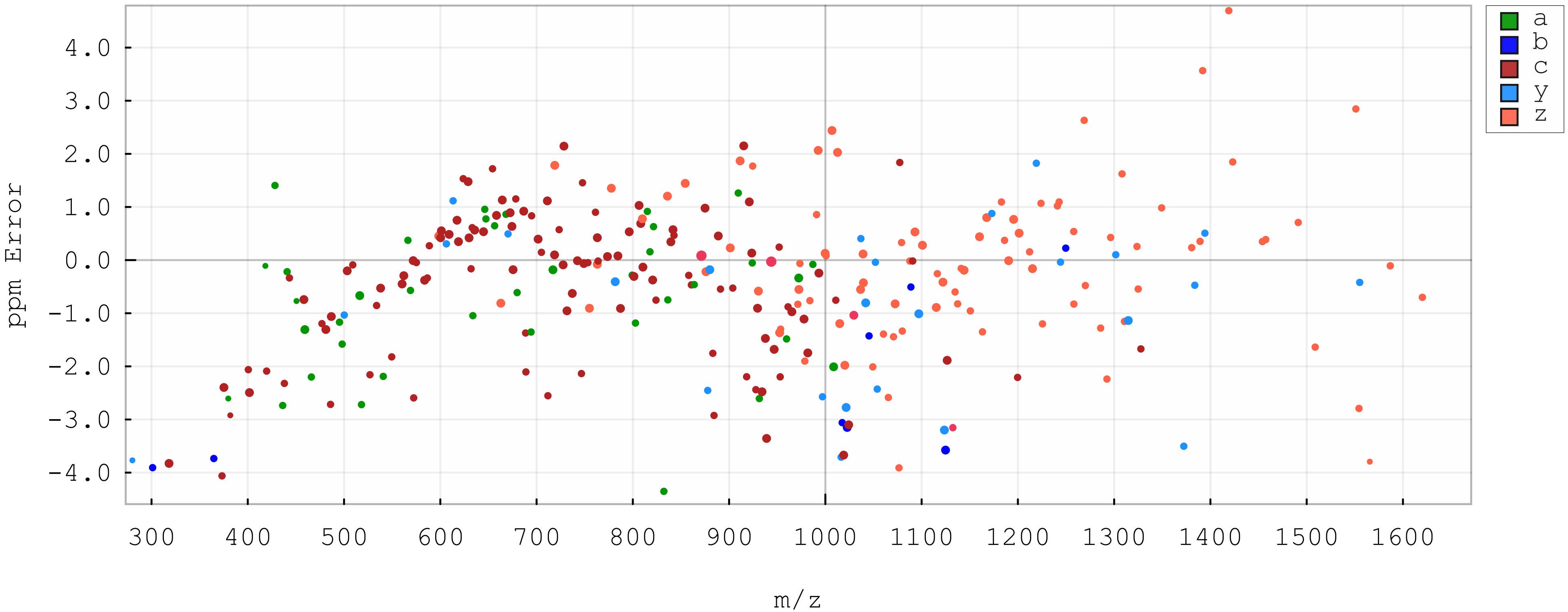

Primary Fragment Plots

Proteoform:

Index	Protein	SC [%]	SVP [%]	IC [%]	MS Score [a.u.]	Var. Mods	Mono Mass [Da]	Chemical Formula
1	H4Kac	85.1	73.5	50.3	42.8	Ace: K8, K44	11313.3624	C498H841N163O136S

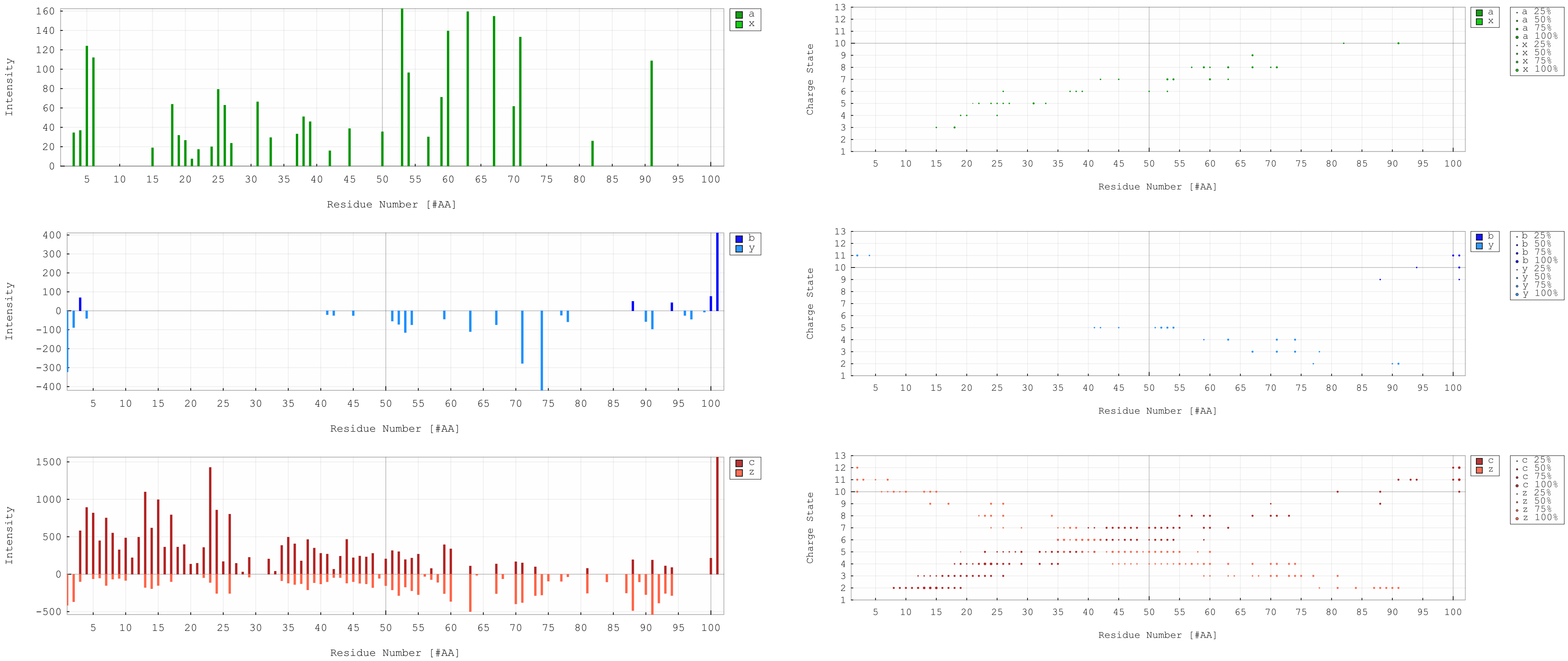

Top-Down Result OmniScape 2025
Sequence Confirmation
250428 H4-PCAF100-30min z13ac2 MS2 ECD_1-908_calibrated2

Proteoform:

Index	Protein	SC [%]	SVP [%]	IC [%]	MS Score [a.u.]	Var. Mods	Mono Mass [Da]	Chemical Formula
2	H4Kac	85.1	73.5	49.7	42.3	Ace: K12, K44	11313.3624	C498H841N163O136S

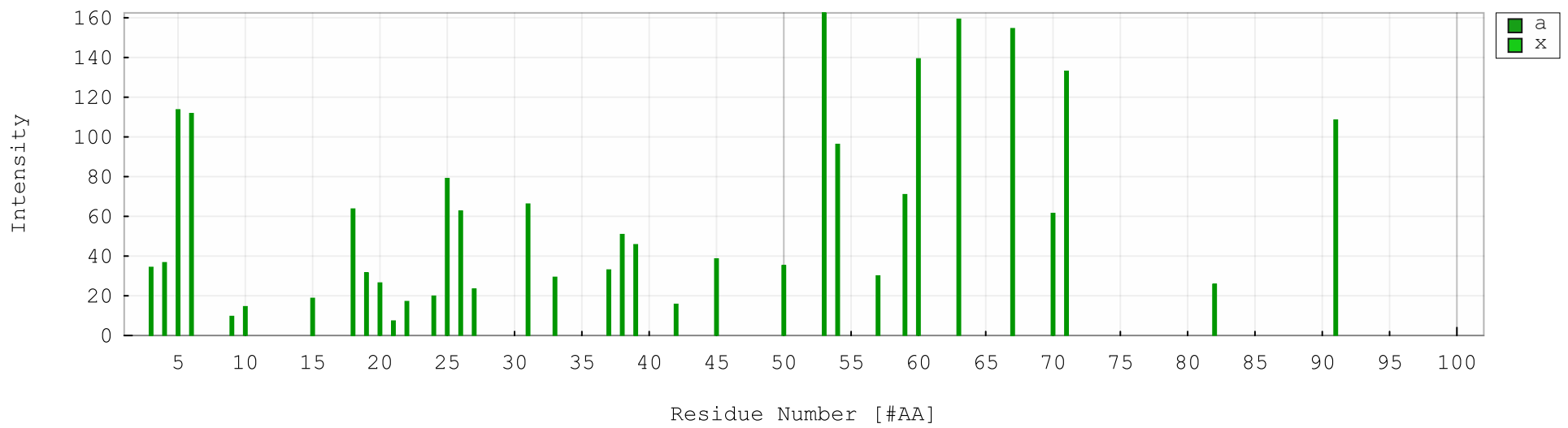

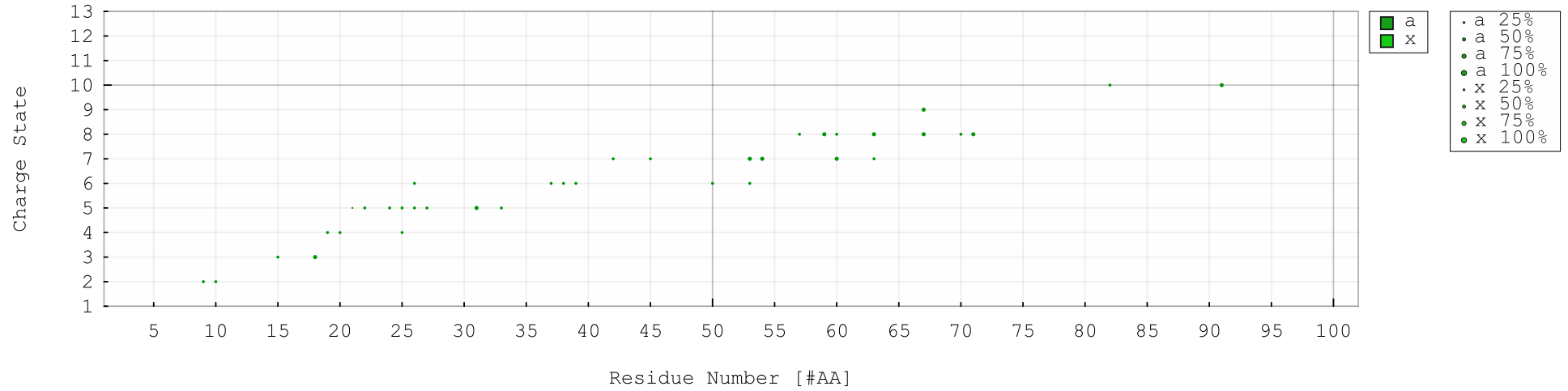

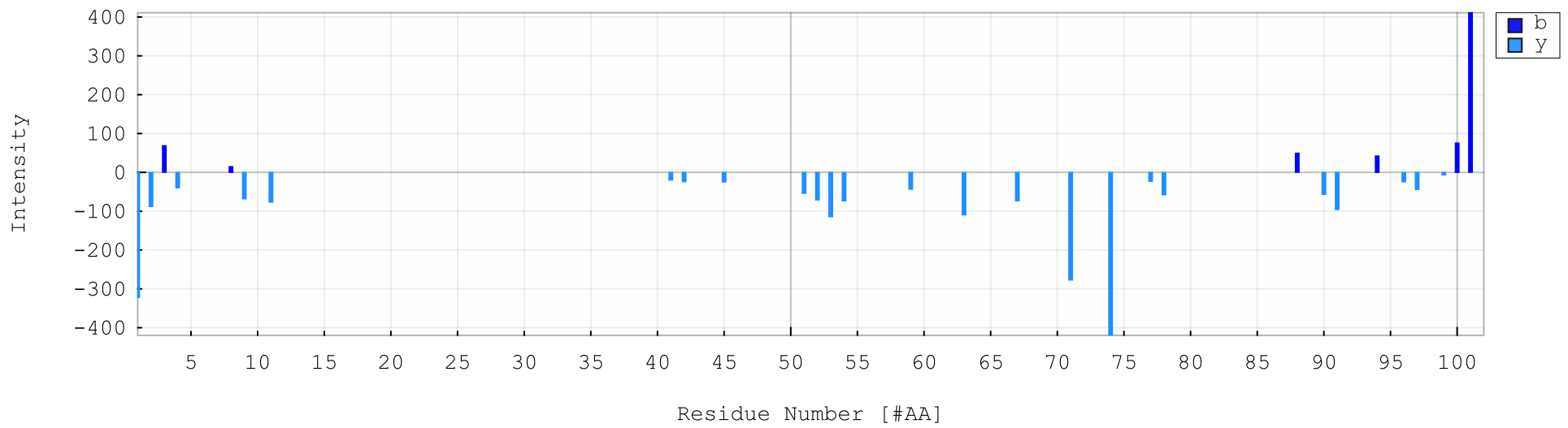

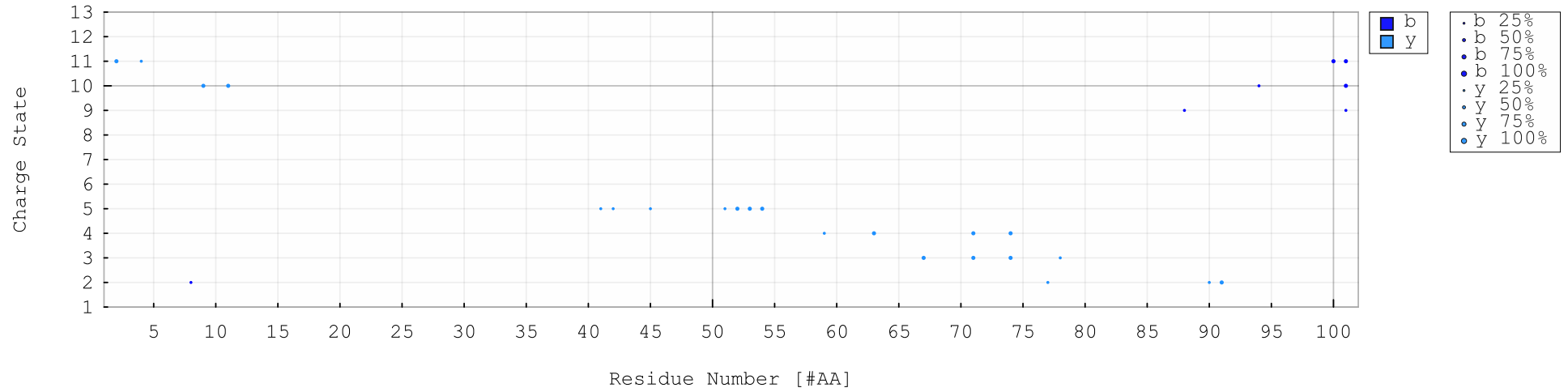

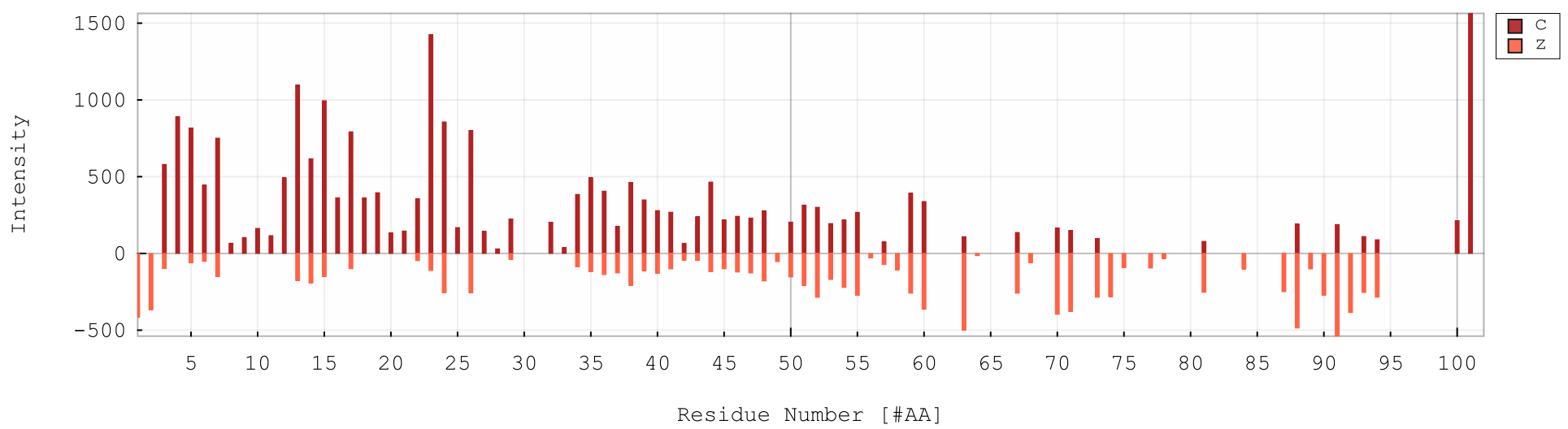

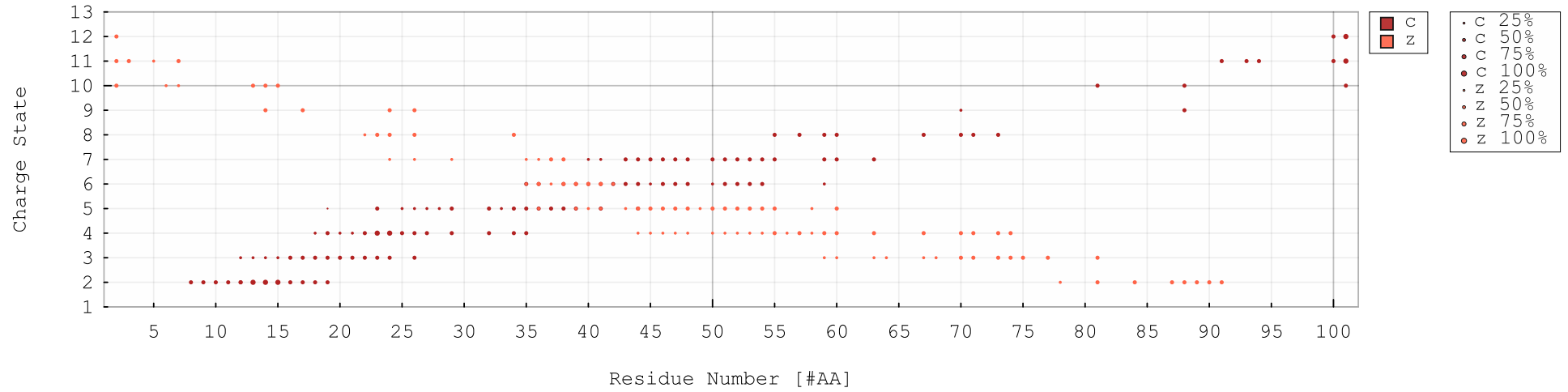

Proteoform:

Index	Protein	SC [%]	SVP [%]	IC [%]	MS Score [a.u.]	Var. Mods	Mono Mass [Da]	Chemical Formula
3	H4Kac	85.1	73.5	48.2	41.0	Ace: K16, K44	11313.3624	C498H841N163O136S

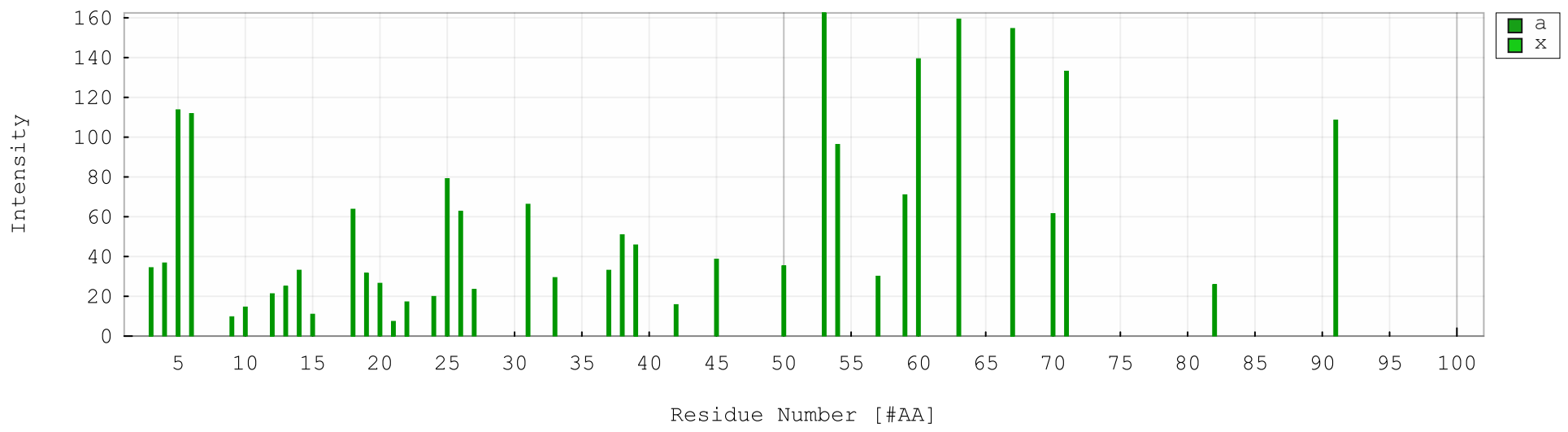

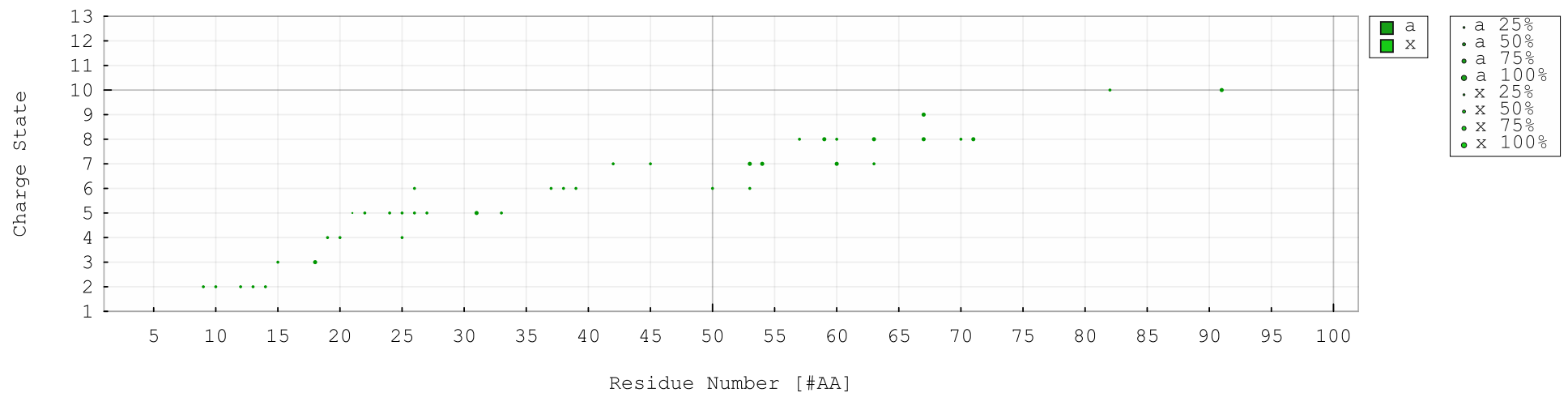

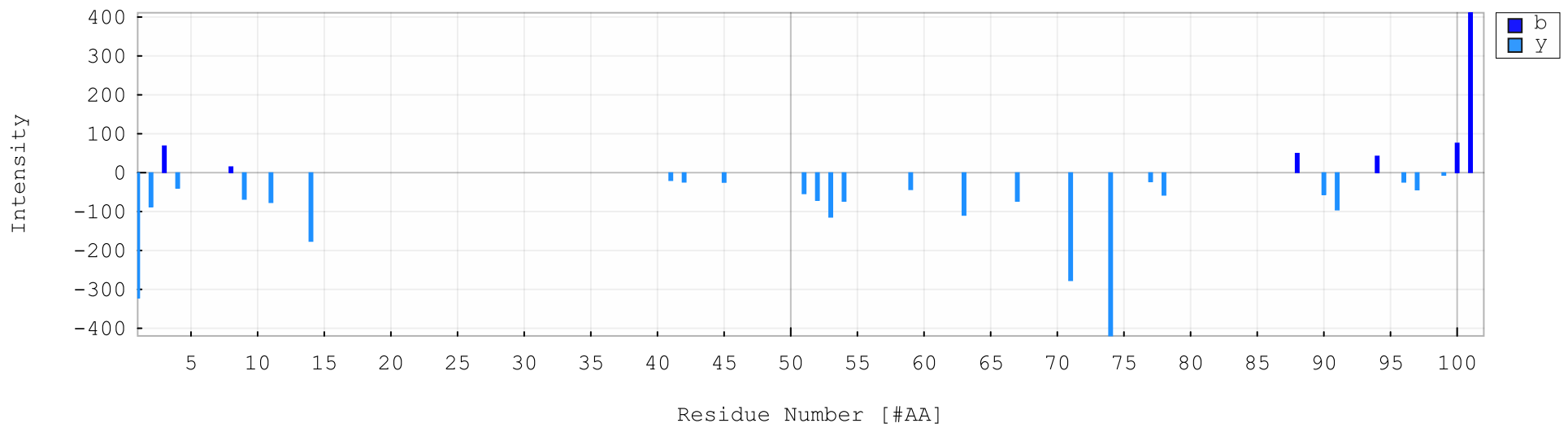

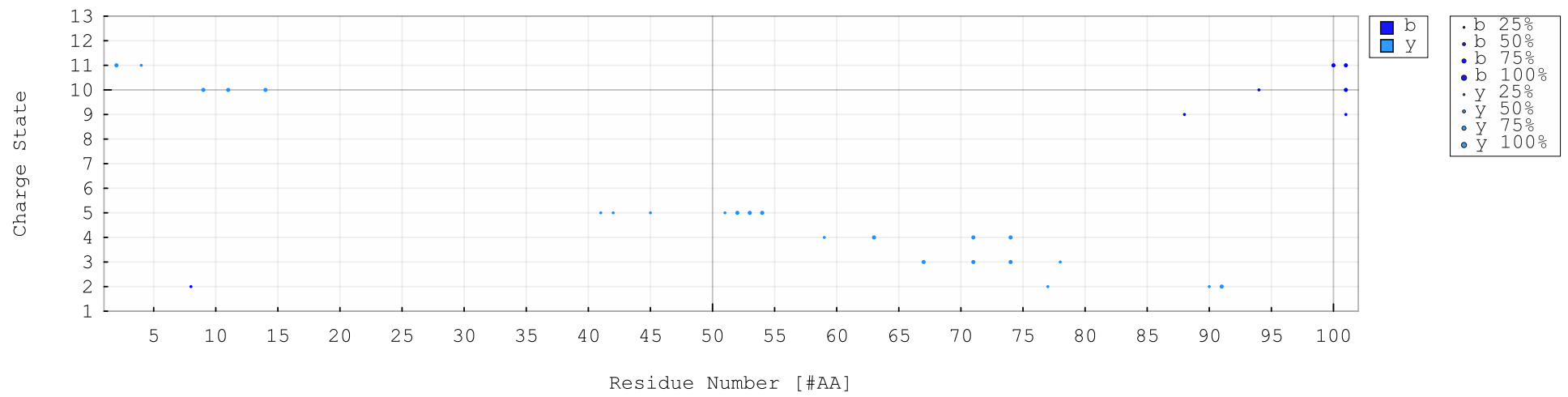

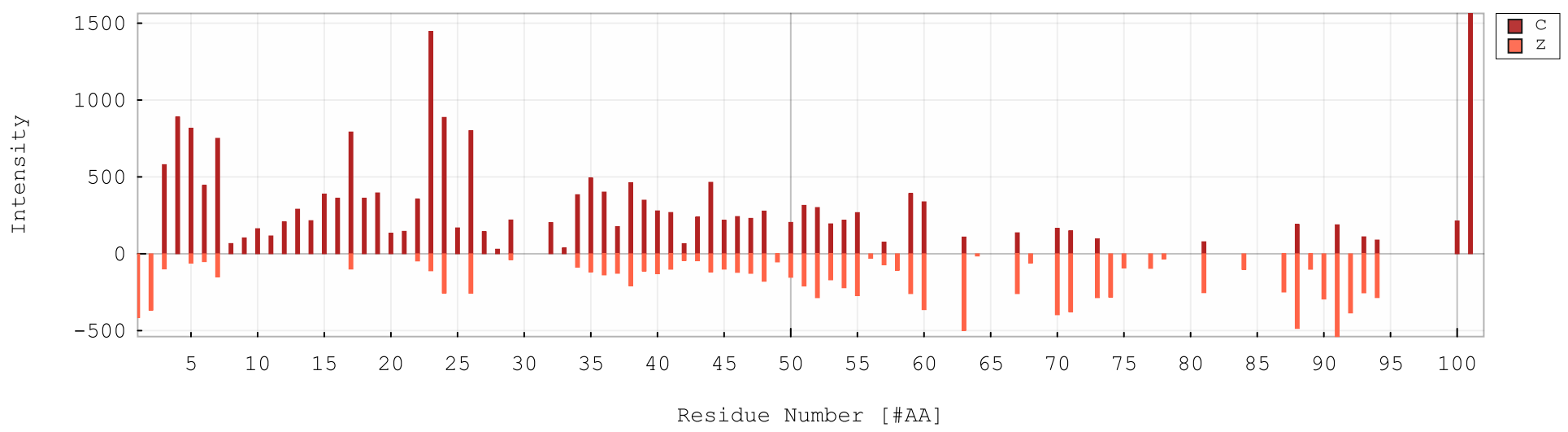

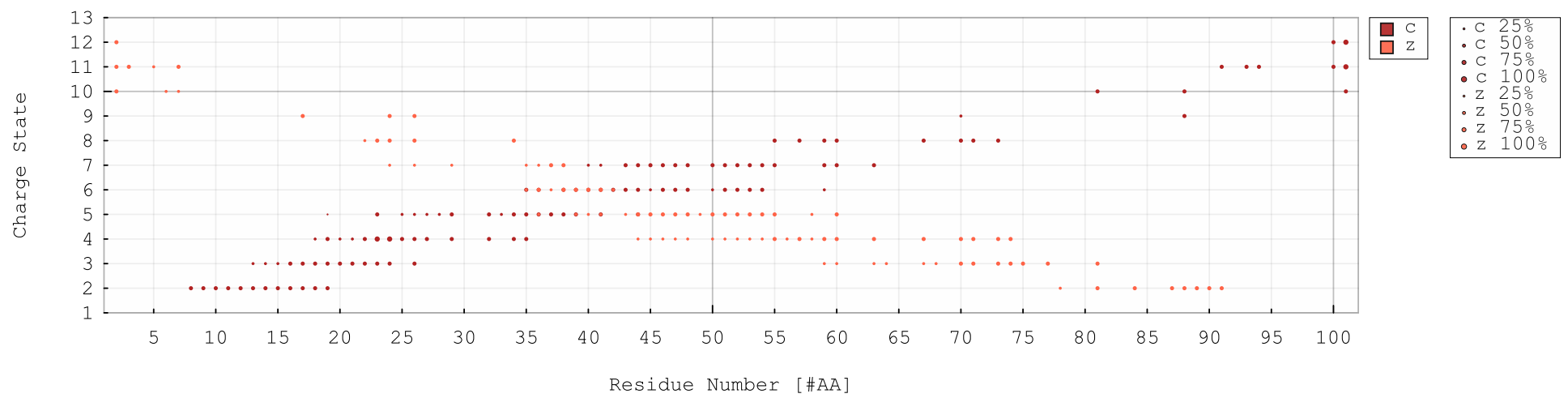

Proteoform:	Index	Protein	SC [%]	SVP [%]	IC [%]	MS Score [a.u.]	Var. Mods	Mono Mass [Da]	Chemical Formula
	4	H4Kac	84.2	73.5	48.1	40.4	Ace: K8, K31	11313.3624	C498H841N163O136S

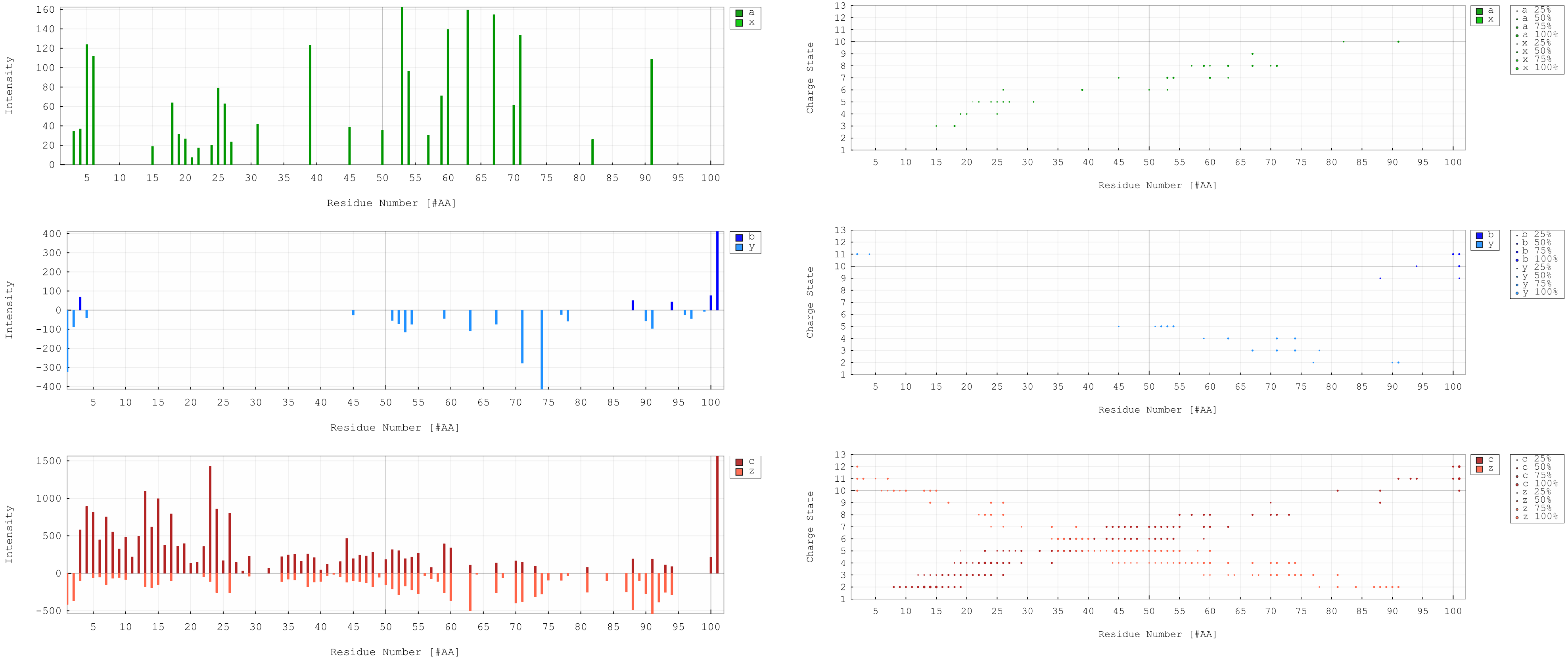

Proteoform:

Index	Protein	SC [%]	SVP [%]	IC [%]	MS Score [a.u.]	Var. Mods	Mono Mass [Da]	Chemical Formula
5	H4Kac	82.2	18.6	49.1	40.3	Ace: K5, K44	11313.3624	C498H841N163O136S

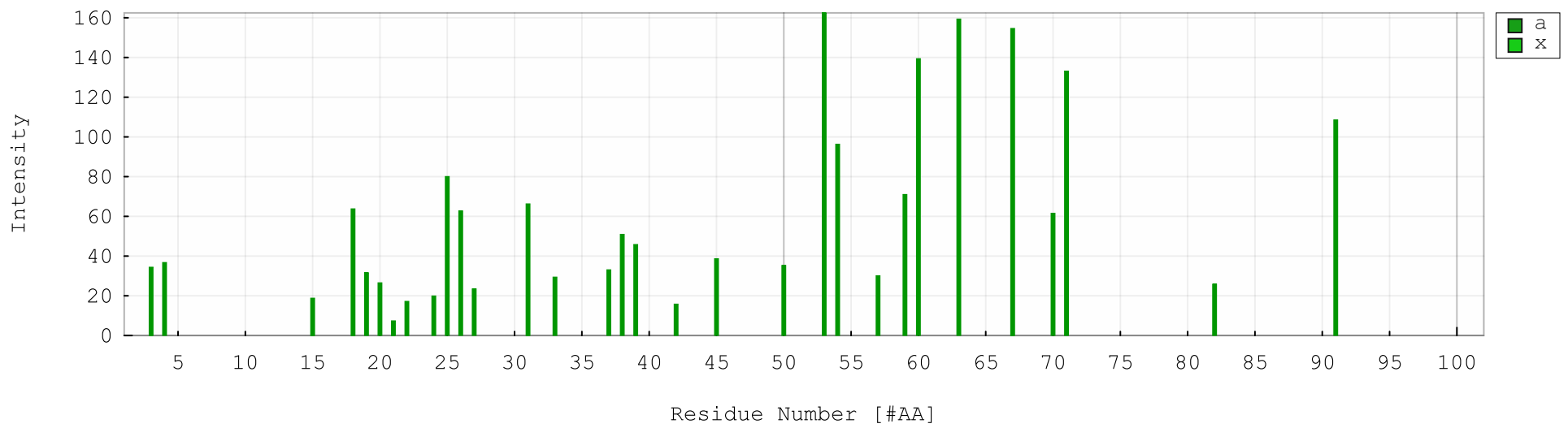

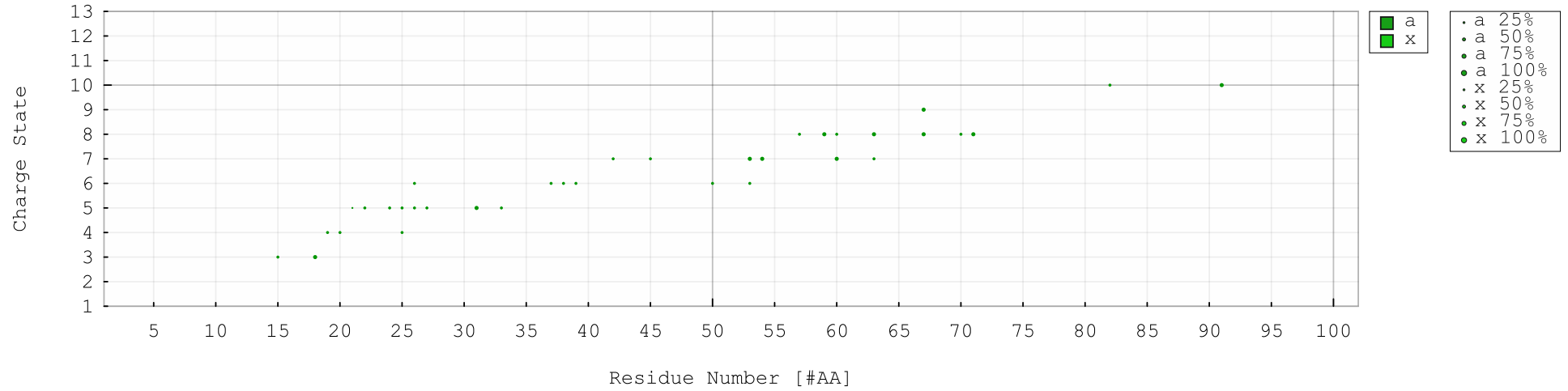

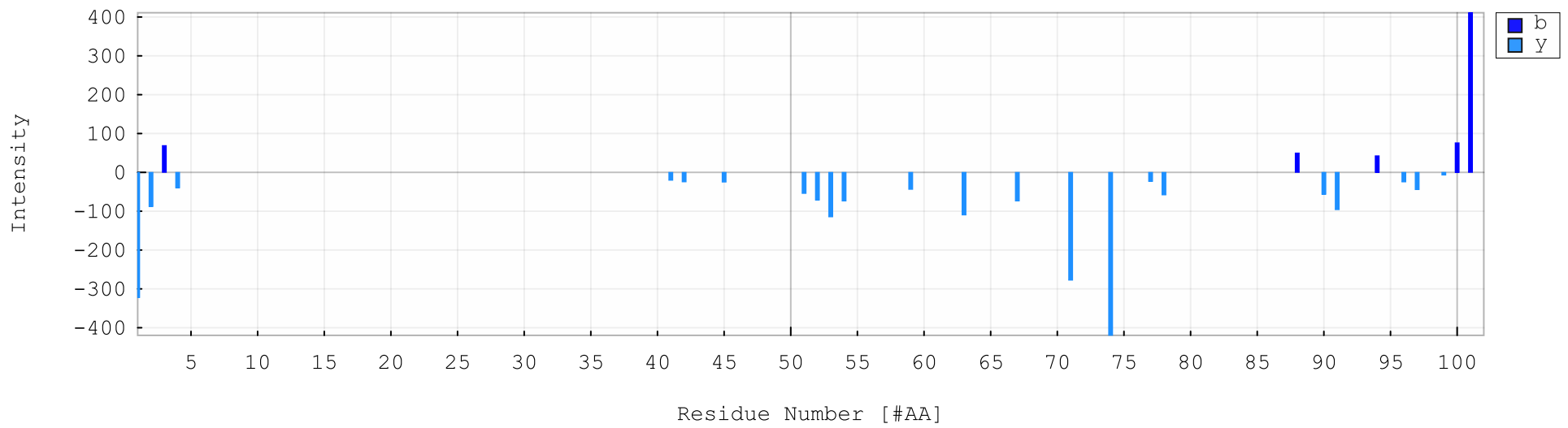

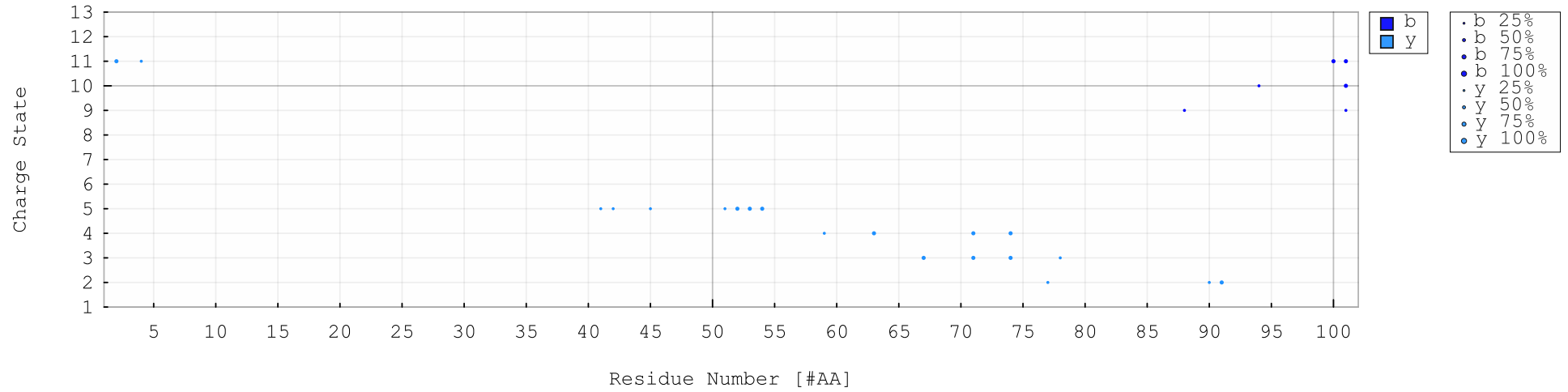

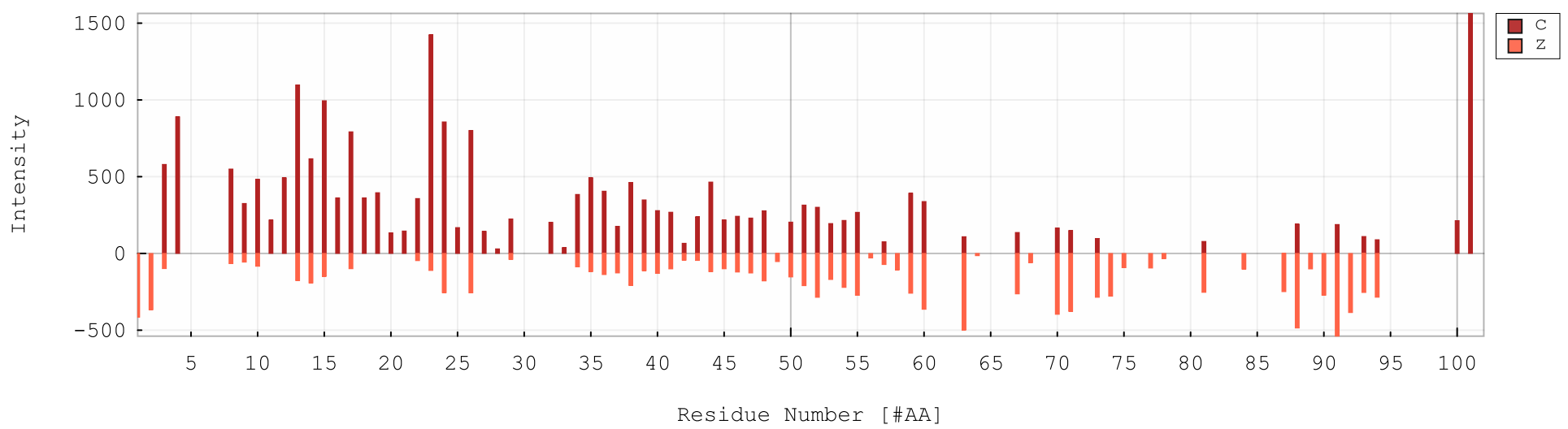

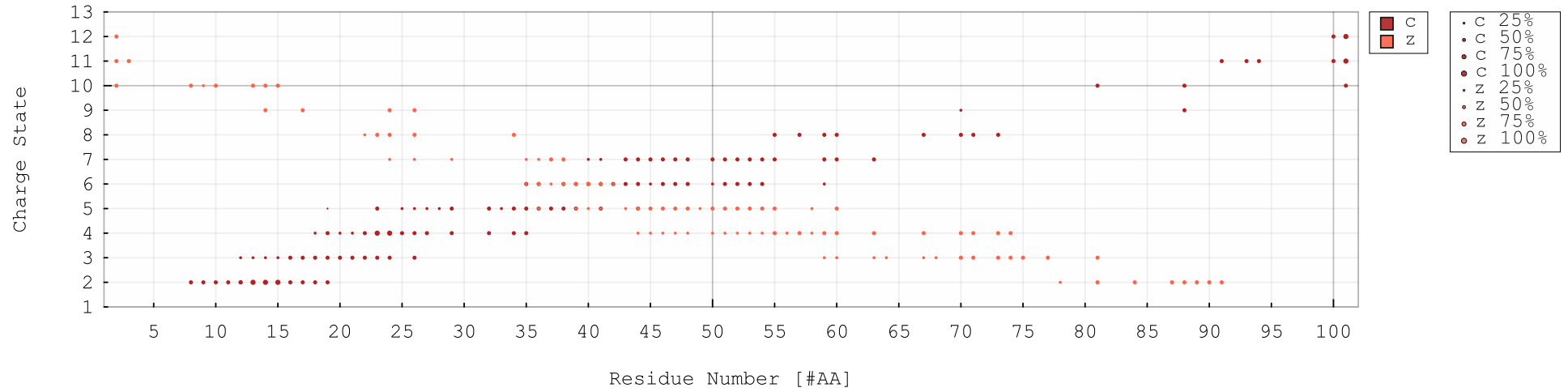

Top-Down Result OmniScape 2025
Sequence Confirmation
250428 H4-PCAF100-30min z13ac2 MS2 ECD_1-908_calibrated2

Proteoform:

Index	Protein	SC [%]	SVP [%]	IC [%]	MS Score [a.u.]	Var. Mods	Mono Mass [Da]	Chemical Formula
6	H4Kac	85.1	73.5	47.3	40.3	Ace: K20, K44	11313.3624	C498H841N163O136S

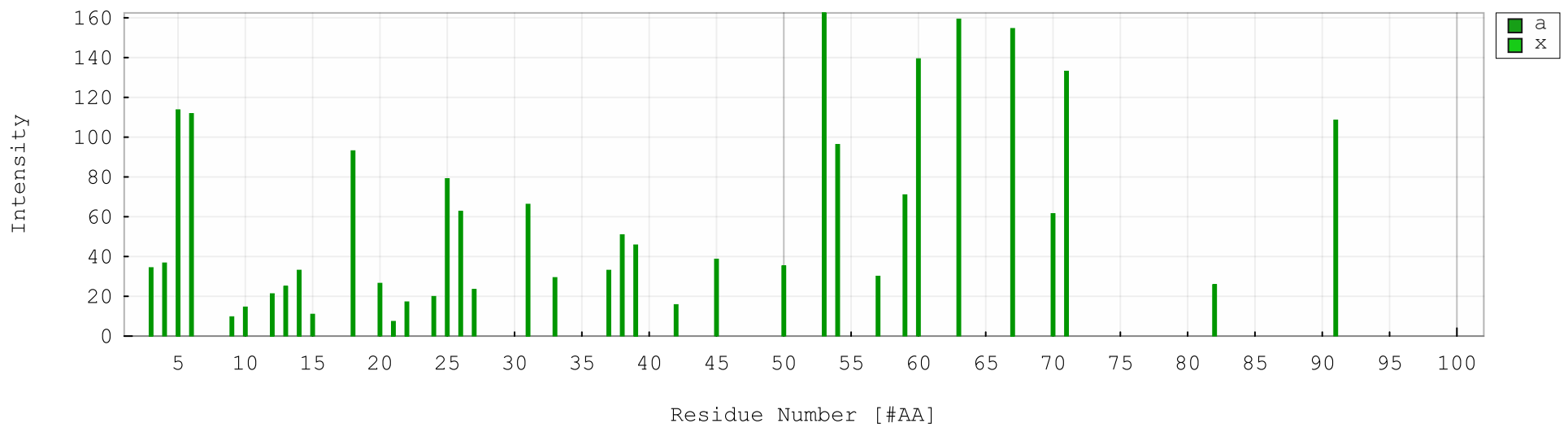

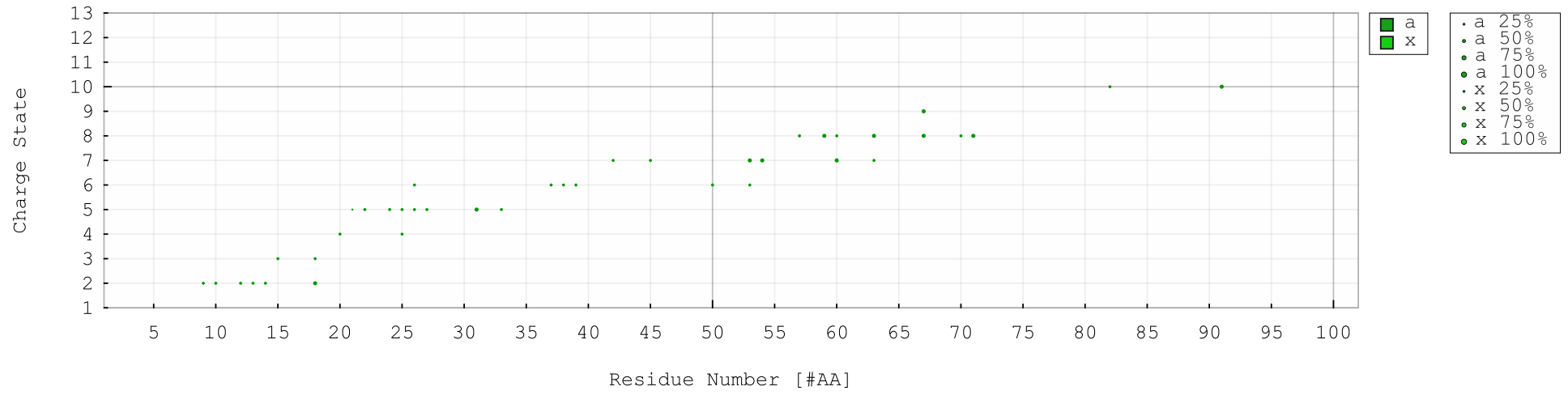

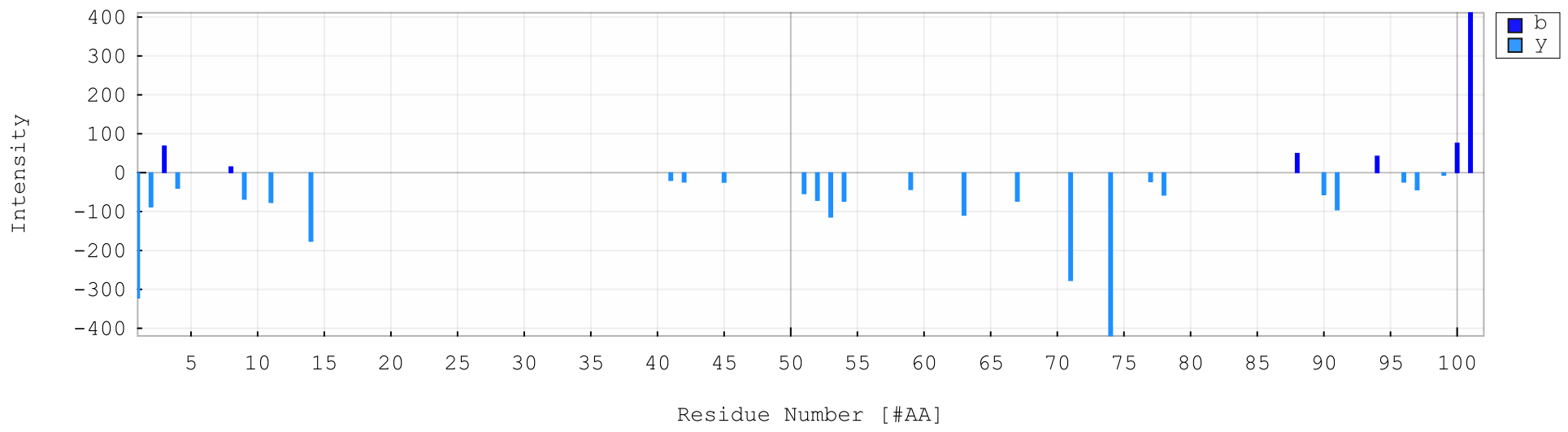

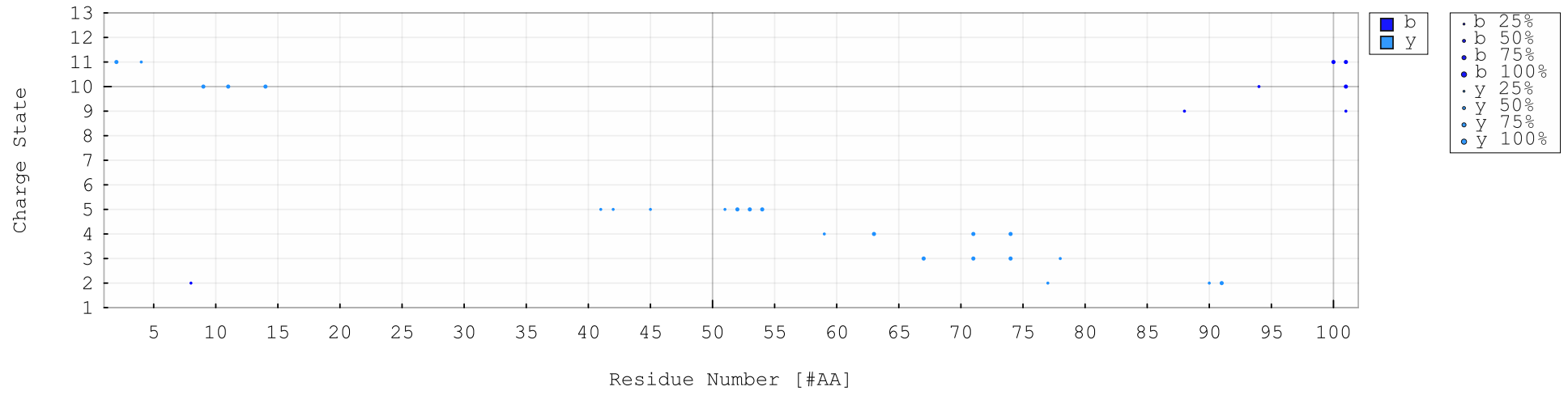

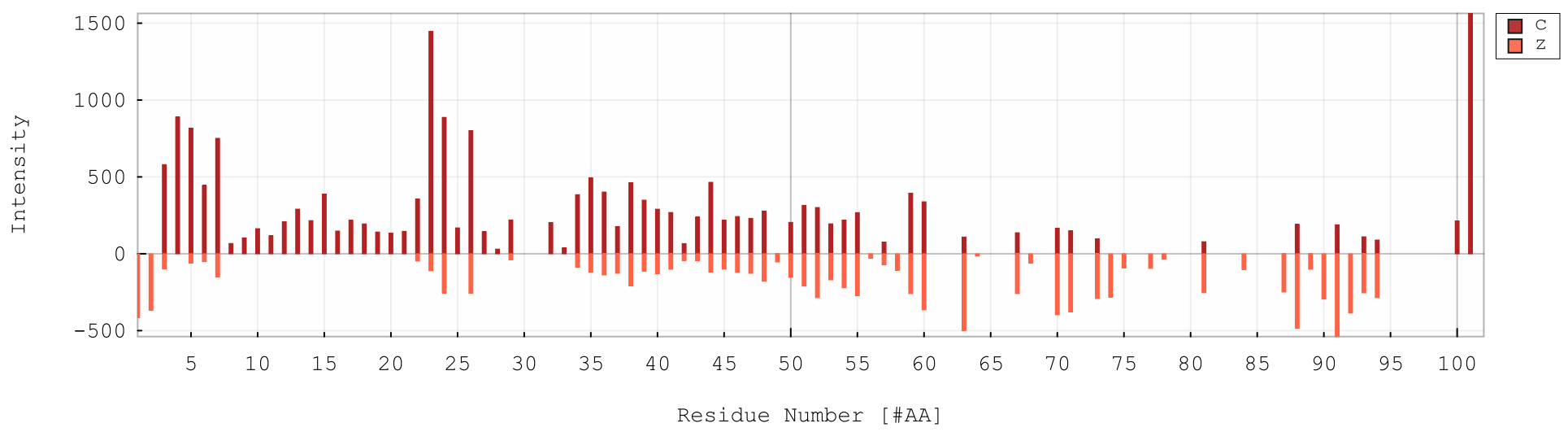

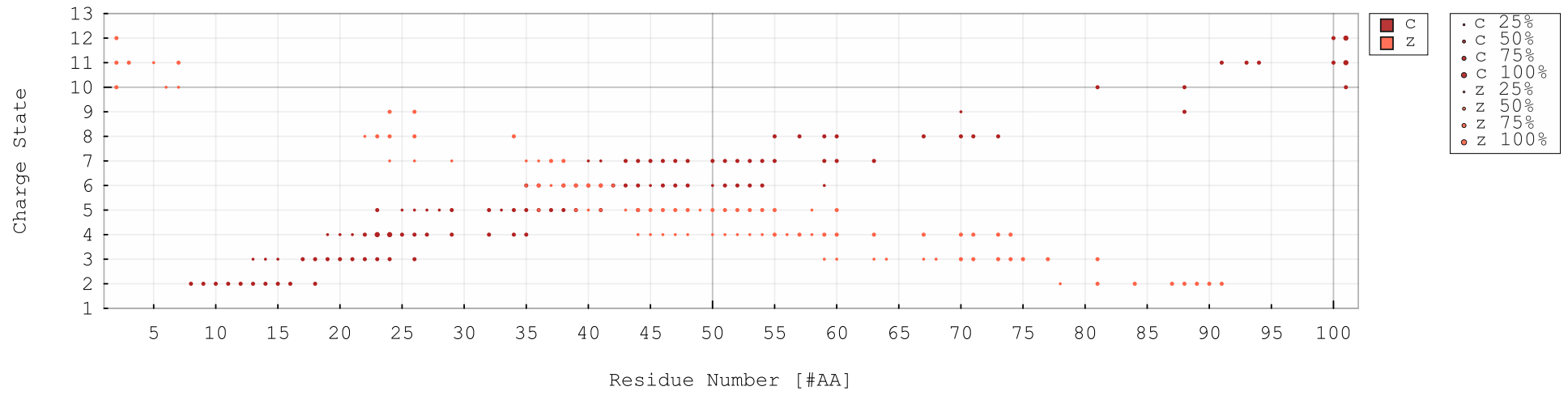

Proteoform:

Index	Protein	SC [%]	SVP [%]	IC [%]	MS Score [a.u.]	Var. Mods	Mono Mass [Da]	Chemical Formula
7	H4Kac	84.2	73.5	47.5	40.0	Ace: K12, K31	11313.3624	C498H841N163O136S

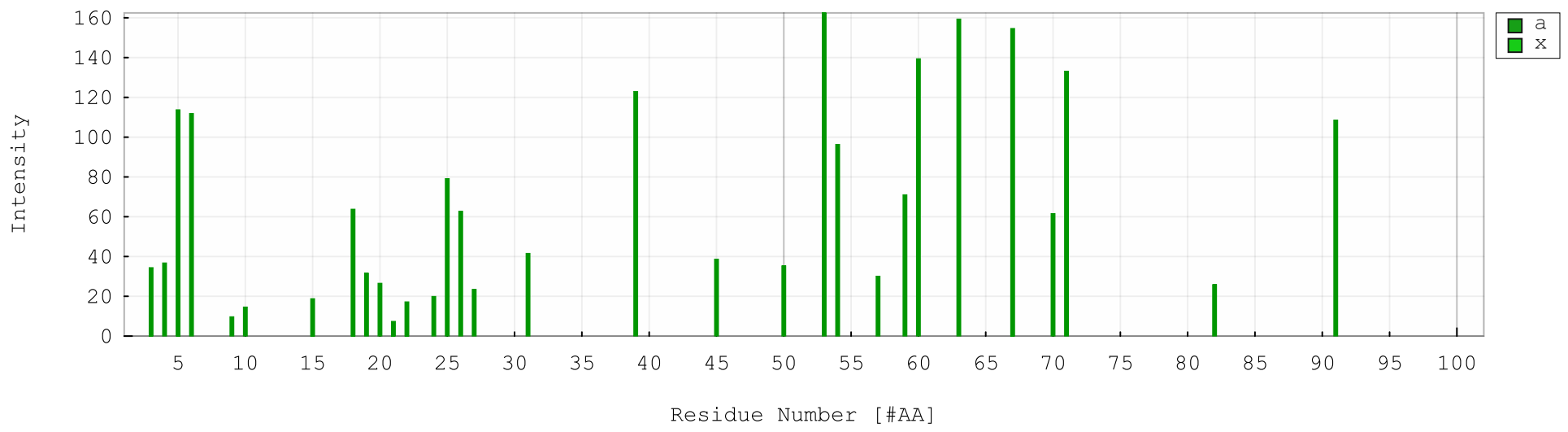

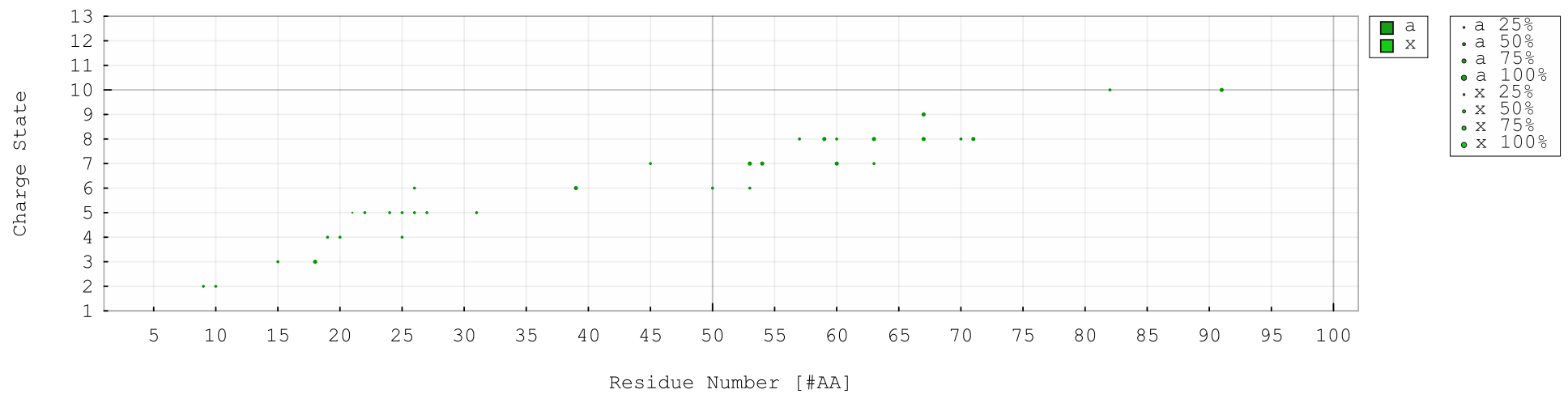

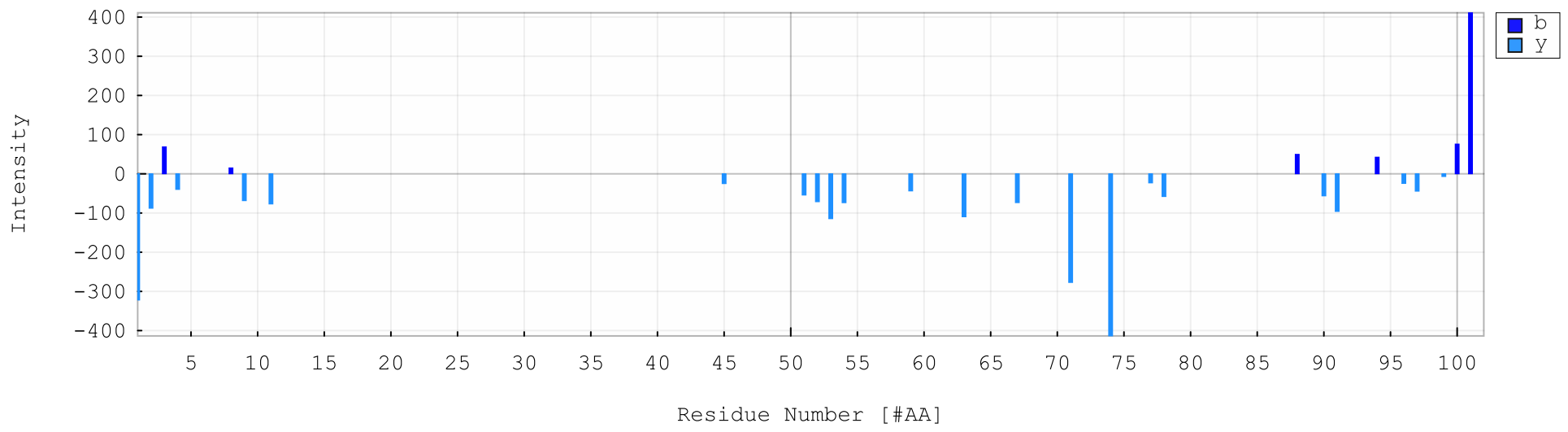

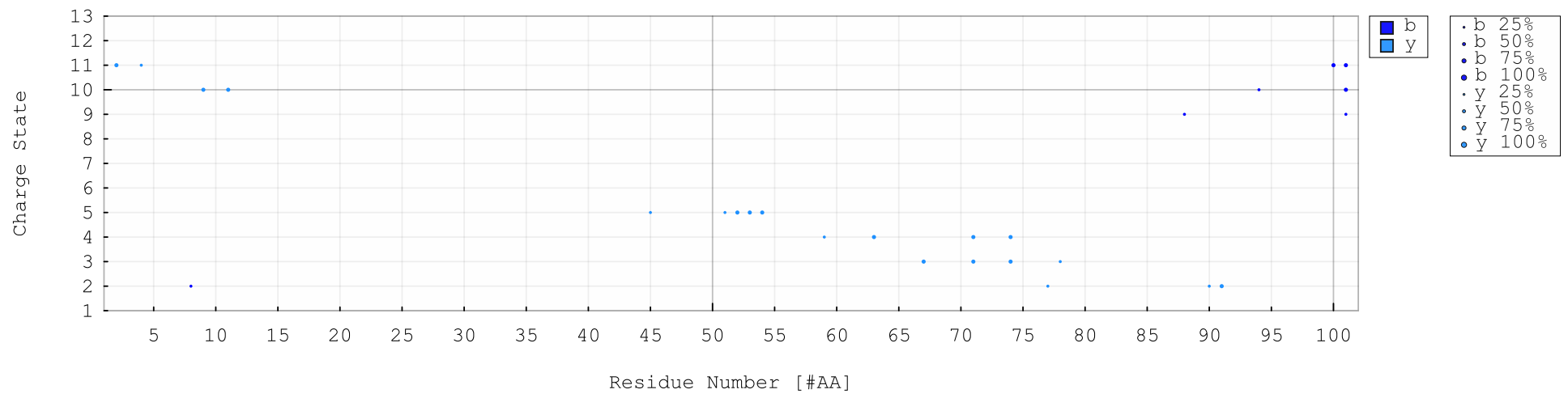

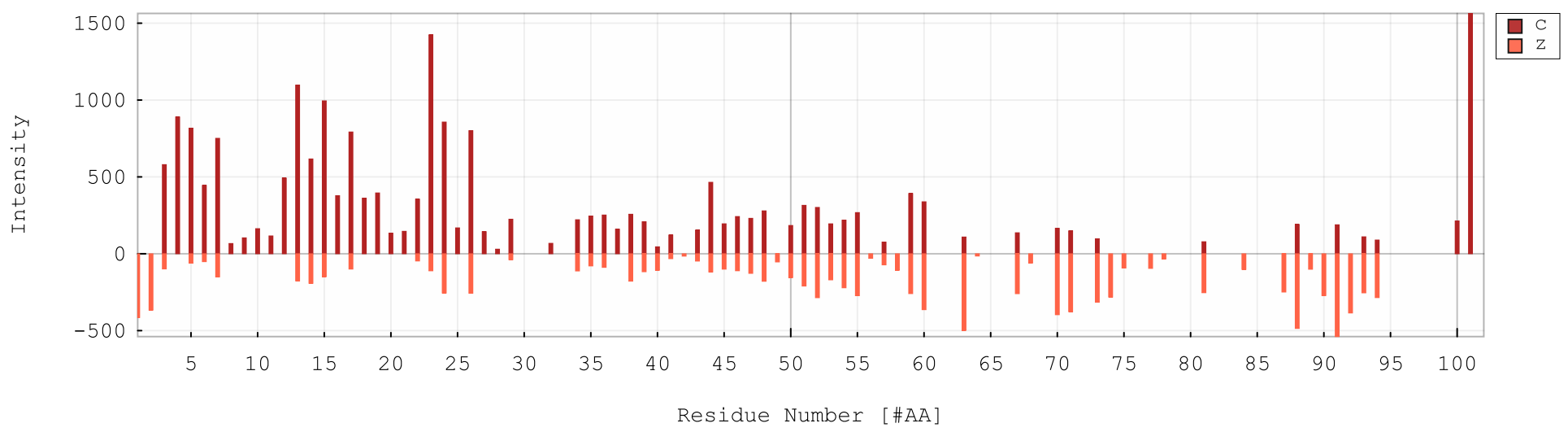

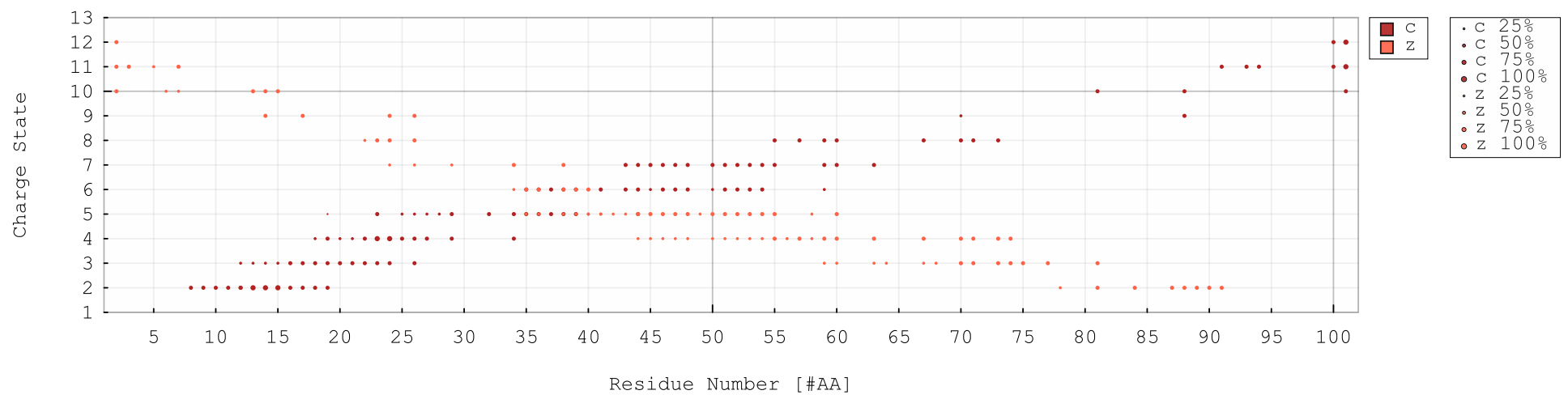

Proteoform:

Index	Protein	SC [%]	SVP [%]	IC [%]	MS Score [a.u.]	Var. Mods	Mono Mass [Da]	Chemical Formula
8	H4Kac	84.2	73.5	46.2	38.8	Ace: K8, K59	11313.3624	C498H841N163O136S

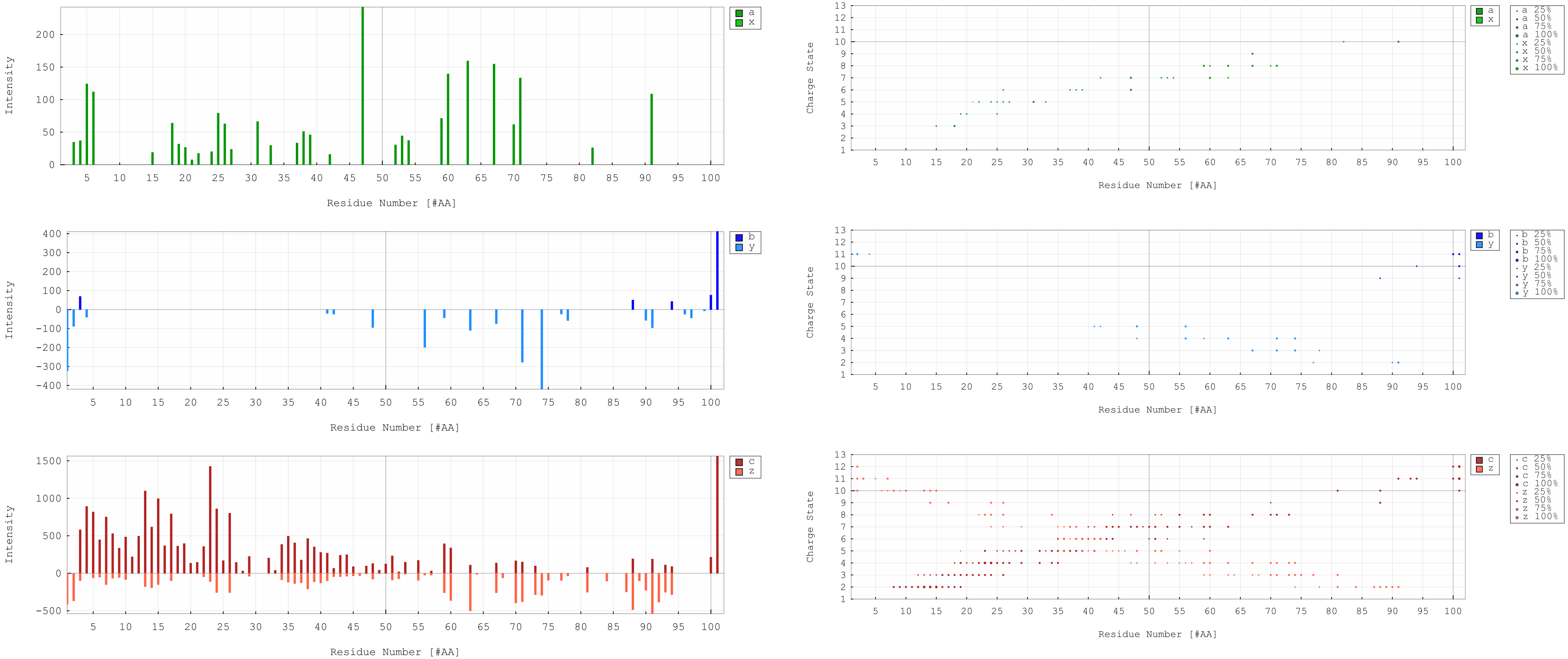

Proteoform:

Index	Protein	SC [%]	SVP [%]	IC [%]	MS Score [a.u.]	Var. Mods	Mono Mass [Da]	Chemical Formula
9	H4Kac	84.2	73.5	46.0	38.7	Ace: K16, K31	11313.3624	C498H841N163O136S

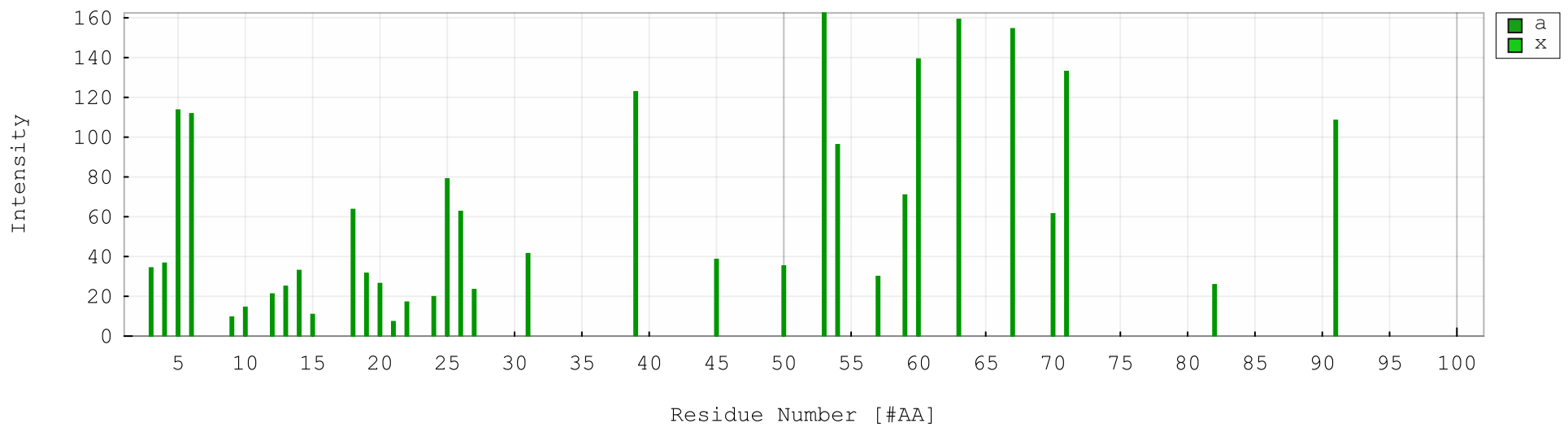

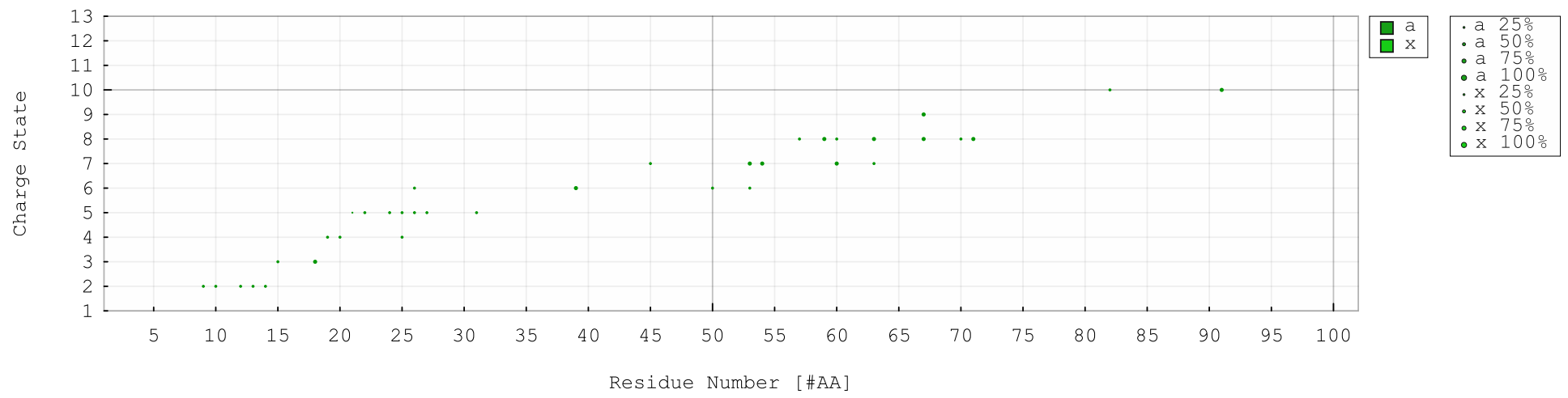

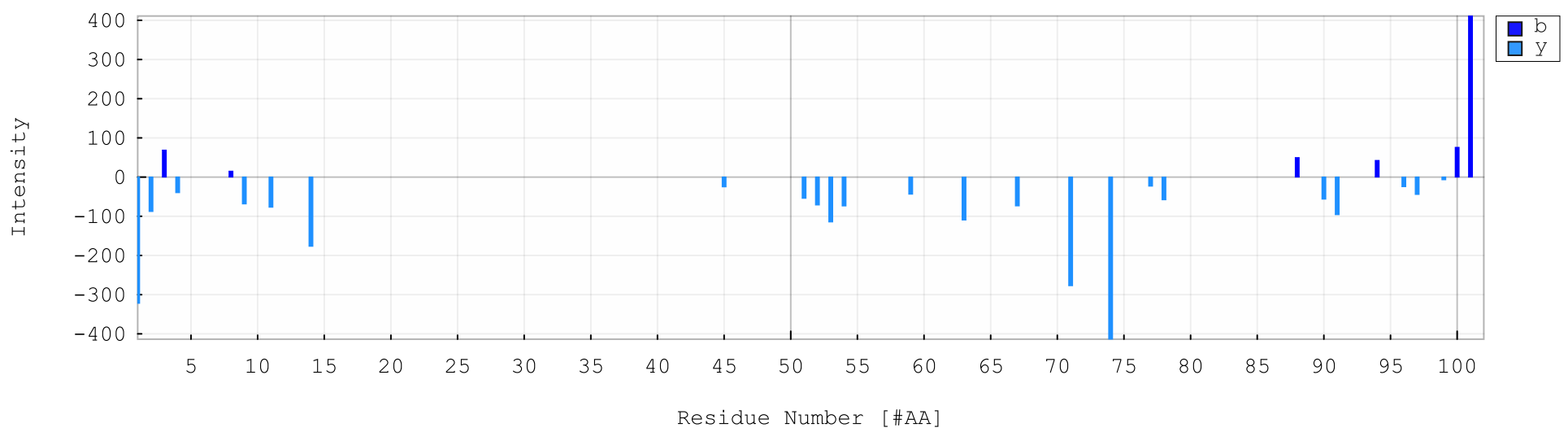

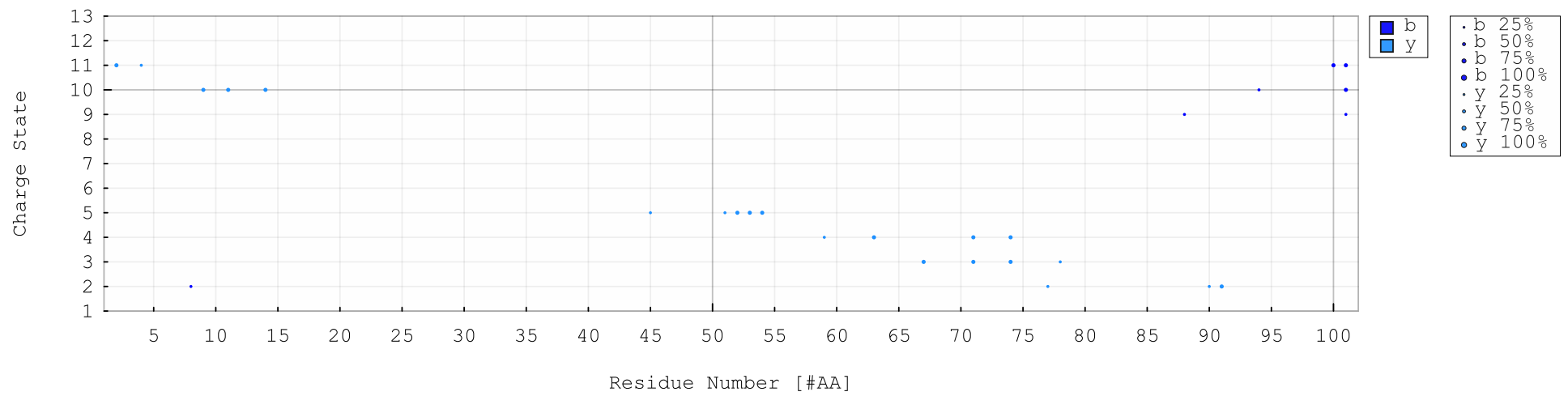

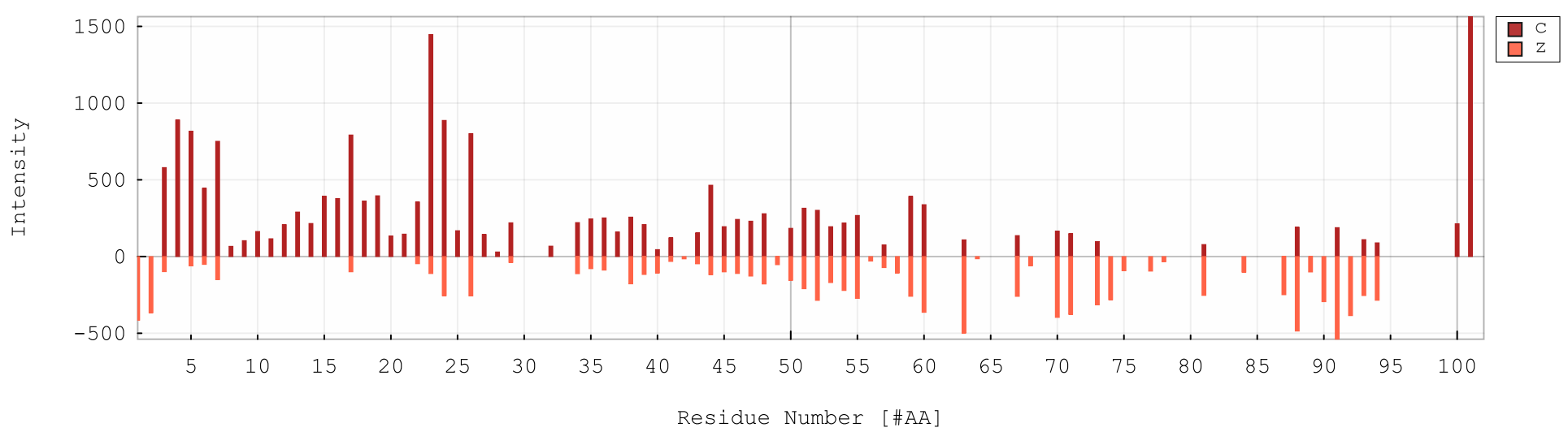

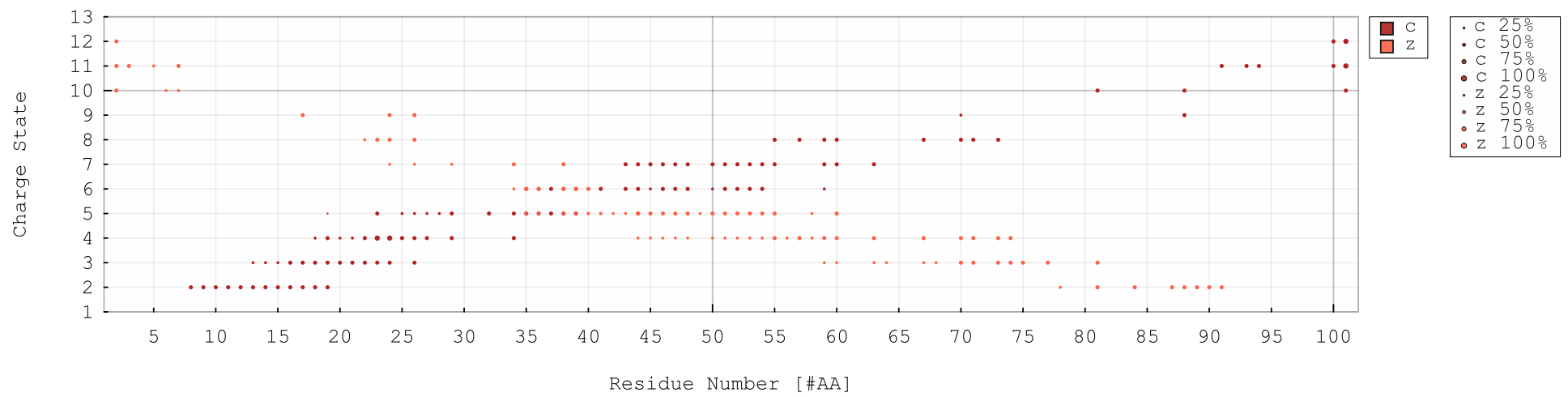

Proteoform:

Index	Protein	SC [%]	SVP [%]	IC [%]	MS Score [a.u.]	Var. Mods	Mono Mass [Da]	Chemical Formula
10	H4Kac	84.2	73.5	45.6	38.4	Ace: K12, K59	11313.3624	C498H841N163O136S

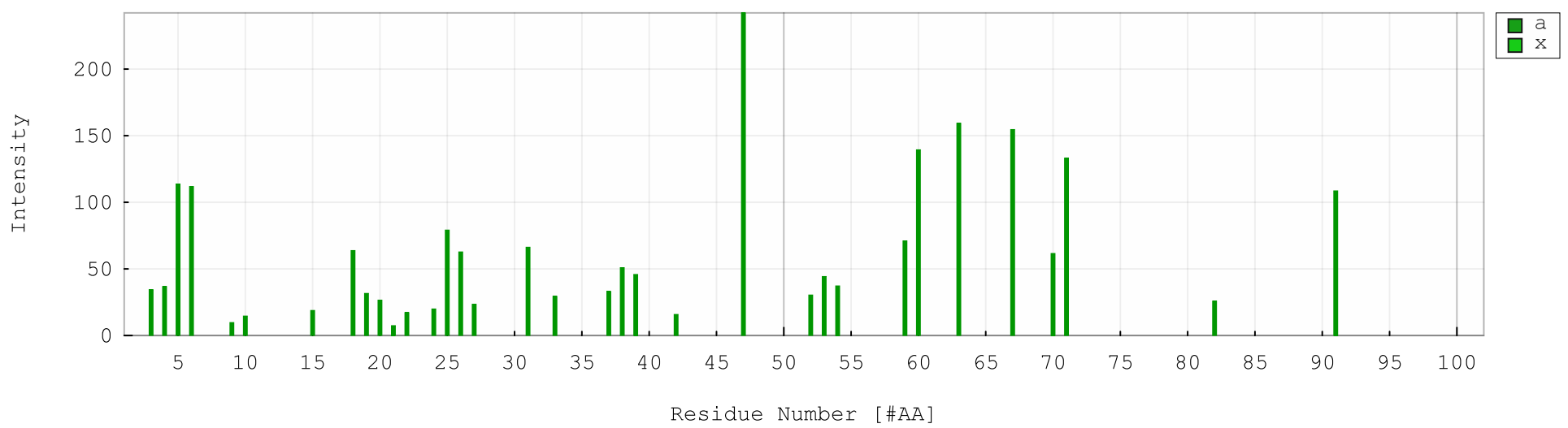

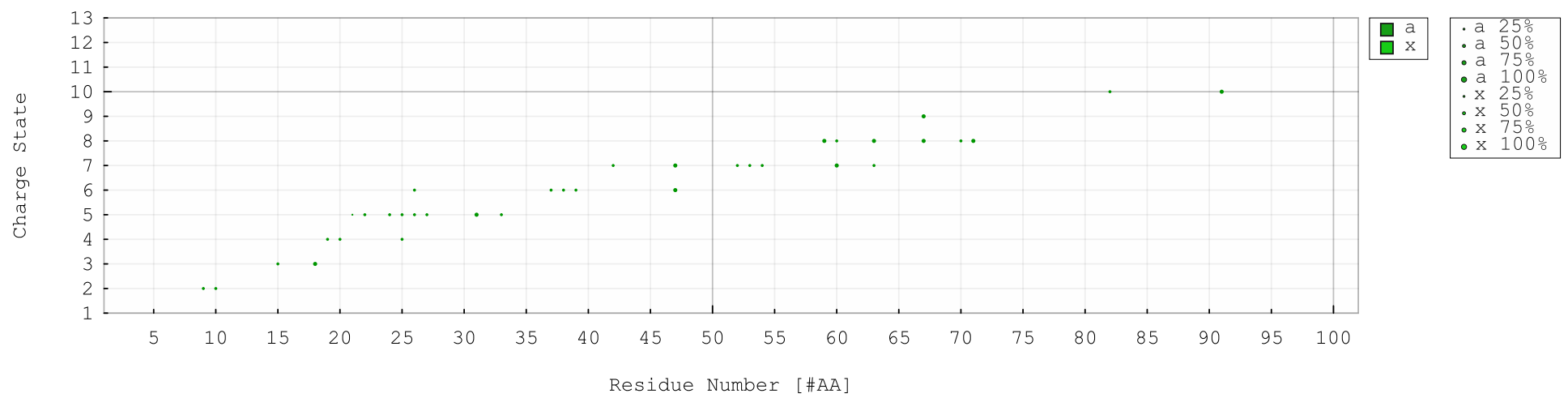

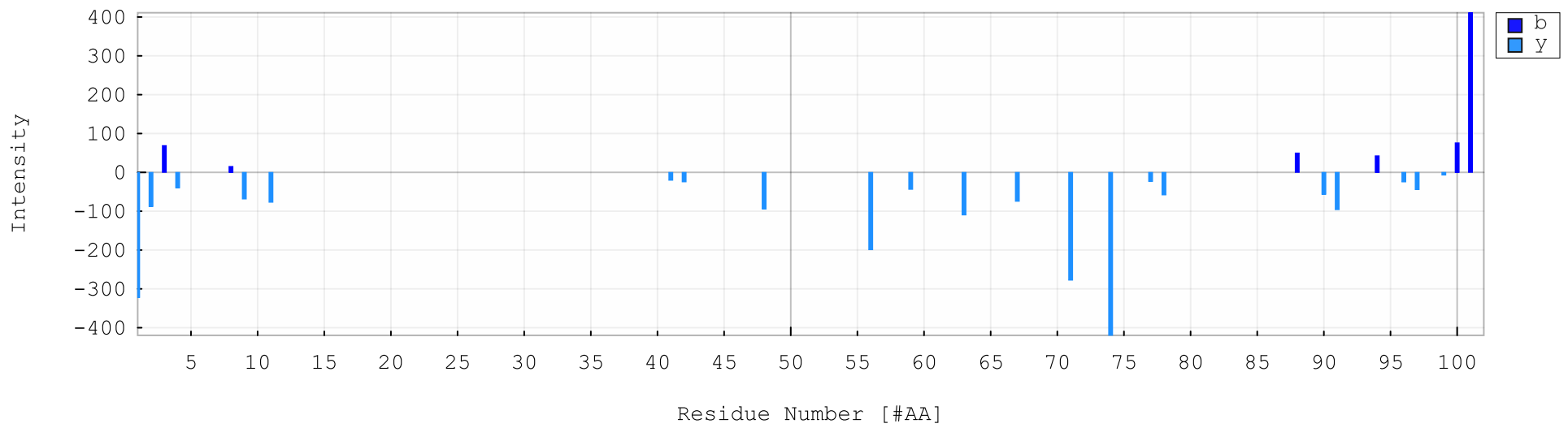

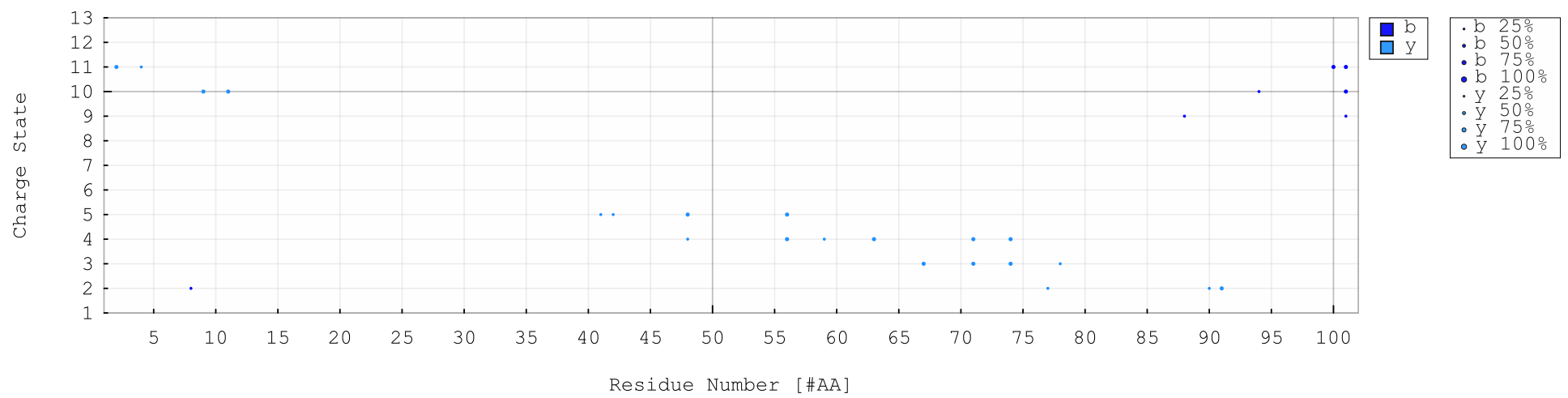

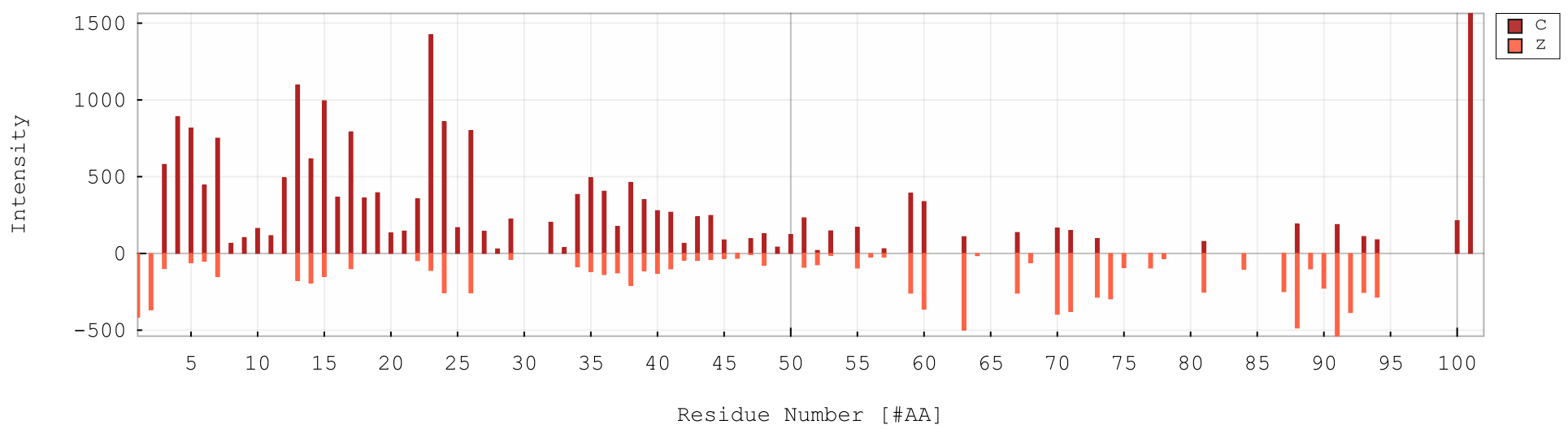

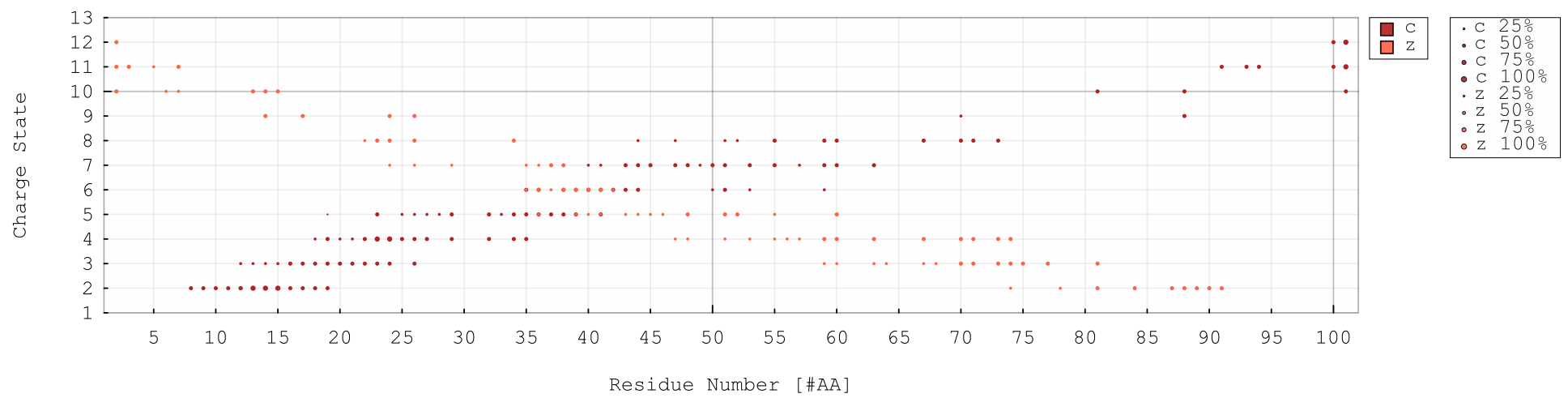

Supplemental Data S5

H4 - PCAF, 30 min

**Report**	6 of 8
**Experiment**	MS2
**Histone/sample**	H4
**Enzyme**	PCAF
**Reaction/source**	30 min
**Precursor**	H4Kac2
**Charge state**	13+
**Fragmentation / workflow**	RCID
**Original file**	MS2_H4Kac2_13+_RCID.pdf

Project Information

Project Directory: C:/Users/LAB-ADMIN
Processing date: Tue Apr 28 14:56:29 2026

Sample Info & Protocols

Name: Top-Down Protein Sequencing CID

Dataset

Imported spectrum file name:	250428 H4-PCAF100-30min z13ac2 MS2 shCID_1-945_calibrated.txt
Dataset name:	Information is only available from imported JPEAKS files
Date acquired:	Information is only available from imported JPEAKS files
Date imported:	Information is only available from imported JPEAKS files
Instrument Name:	Information is only available from imported JPEAKS files
Instrument Serial Number:	Information is only available from imported JPEAKS files

Workflow Result Info

Analysis: 250428 H4-PCAF100-30min z13ac2 MS2 shCID_1-945_calibrated
Location: C:/Users/LAB-ADMIN
Method: -

Method Information

Name: Unnamed

Spectral Parameters

Mass Error [ppm]: 6
Intensity Deviation [au]: 0.15
Min/Max Charges: 1-13
Experiment type: CID

a ions	b ions	c ions	x ions	y ions	z ions	satellite	internal	precursor
a	b			y				M

Noise threshold calculation: Simple moving average noise threshold (SMA)
Window size [datapoints]: 600
Noise curve offset: 1.6955872
SVP parameters: terminal gap: 2, internal gap: 1

Proteoforms

(top 10)

Index	Parent Sequence	Chain	Var. Mods	Mono Mass [Da]	Chemical Composition
1	H4Kac	H4KacK8[Ace]K20[Ace]	Ace: K8, K20	11313.3624	C498H841N163O136S
2	H4Kac	H4KacK12[Ace]K20[Ace]	Ace: K12, K20	11313.3624	C498H841N163O136S
3	H4Kac	H4KacK8[Ace]K16[Ace]	Ace: K8, K16	11313.3624	C498H841N163O136S
4	H4Kac	H4KacK12[Ace]K16[Ace]	Ace: K12, K16	11313.3624	C498H841N163O136S
5	H4Kac	H4KacK5[Ace]K20[Ace]	Ace: K5, K20	11313.3624	C498H841N163O136S
6	H4Kac	H4KacK16[Ace]K20[Ace]	Ace: K16, K20	11313.3624	C498H841N163O136S
7	H4Kac	H4KacK5[Ace]K16[Ace]	Ace: K5, K16	11313.3624	C498H841N163O136S
8	H4Kac	H4KacK8[Ace]K12[Ace]	Ace: K8, K12	11313.3624	C498H841N163O136S
9	H4Kac	H4KacK5[Ace]K12[Ace]	Ace: K5, K12	11313.3624	C498H841N163O136S
10	H4Kac	H4KacK5[Ace]K8[Ace]	Ace: K5, K8	11313.3624	C498H841N163O136S

Results

Proteoforms Table (top 10)

Index	Protein	SC [%]	SVP [%]	IC [%]	MS Score [a.u.]	Var. Mods	Mono Mass [Da]	Chemical Formula
1	H4Kac	60.4	24.5	30.6	18.5	Ace: K8, K20	11313.3624	C498H841N163O136S
2	H4Kac	60.4	24.5	30.0	18.1	Ace: K12, K20	11313.3624	C498H841N163O136S
3	H4Kac	59.4	20.6	30.4	18.1	Ace: K8, K16	11313.3624	C498H841N163O136S
4	H4Kac	59.4	20.6	29.7	17.7	Ace: K12, K16	11313.3624	C498H841N163O136S
5	H4Kac	57.4	9.8	30.0	17.2	Ace: K5, K20	11313.3624	C498H841N163O136S
6	H4Kac	58.4	17.6	29.5	17.2	Ace: K16, K20	11313.3624	C498H841N163O136S
7	H4Kac	56.4	9.8	29.8	16.8	Ace: K5, K16	11313.3624	C498H841N163O136S
8	H4Kac	56.4	16.7	29.8	16.8	Ace: K8, K12	11313.3624	C498H841N163O136S
9	H4Kac	53.5	9.8	29.2	15.6	Ace: K5, K12	11313.3624	C498H841N163O136S
10	H4Kac	49.5	9.8	27.9	13.8	Ace: K5, K8	11313.3624	C498H841N163O136S

Sequence

Name: H4Kac

```
                   10                    20                    30                    40                    50                    60
S G R G K G G K G L   G K G G A K R H R K   V L R D N I Q G I T   K P A I R R L A R R   G G V K R I S G L I   Y E E T R G V L K V
                   70                    80                    90                   100                   110                   120
F L E N V I R D A V   T Y T E H A K R K T   V T A M D V V Y A L   K R Q G R T L Y G F   G G
```

Modifications

Modification	Position	Type
Acetyl	5	variable
Acetyl	8	variable
Acetyl	12	variable
Acetyl	16	variable
Acetyl	20	variable

Sequence Map

Proteoform:	Index	Protein	SC [%]	SVP [%]	IC [%]	MS Score [a.u.]	Var. Mods	Mono Mass [Da]	Chemical Formula
	1	H4Kac	60.4	24.5	30.6	18.5	Ace: K8, K20	11313.3624	C498H841N163O136S

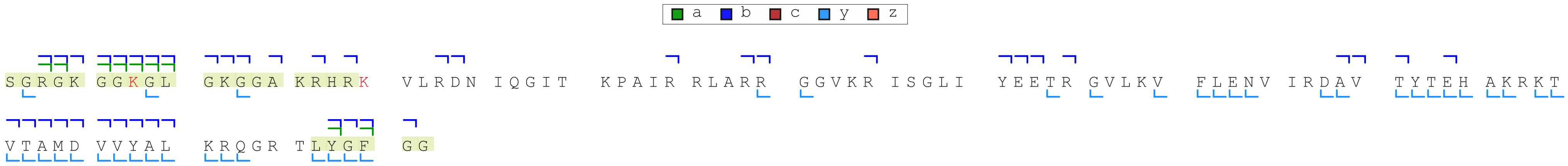

Proteoform:	Index	Protein	SC [%]	SVP [%]	IC [%]	MS Score [a.u.]	Var. Mods	Mono Mass [Da]	Chemical Formula
	2	H4Kac	60.4	24.5	30.0	18.1	Ace: K12, K20	11313.3624	C498H841N163O136S

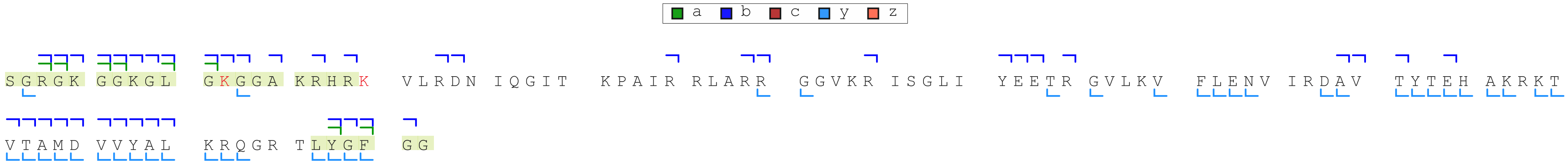

Proteoform:

Index	Protein	SC [%]	SVP [%]	IC [%]	MS Score [a.u.]	Var. Mods	Mono Mass [Da]	Chemical Formula
3	H4Kac	59.4	20.6	30.4	18.1	Ace: K8, K16	11313.3624	C498H841N163O136S

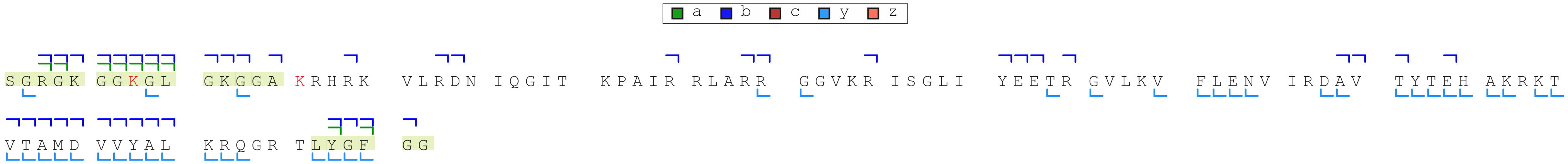

Proteoform:

Index	Protein	SC [%]	SVP [%]	IC [%]	MS Score [a.u.]	Var. Mods	Mono Mass [Da]	Chemical Formula
4	H4Kac	59.4	20.6	29.7	17.7	Ace: K12, K16	11313.3624	C498H841N163O136S

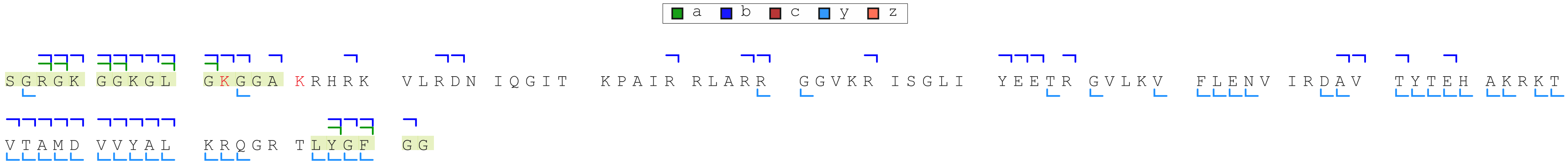

Proteoform:	Index	Protein	SC [%]	SVP [%]	IC [%]	MS Score [a.u.]	Var. Mods	Mono Mass [Da]	Chemical Formula
	5	H4Kac	57.4	9.8	30.0	17.2	Ace: K5, K20	11313.3624	C498H841N163O136S

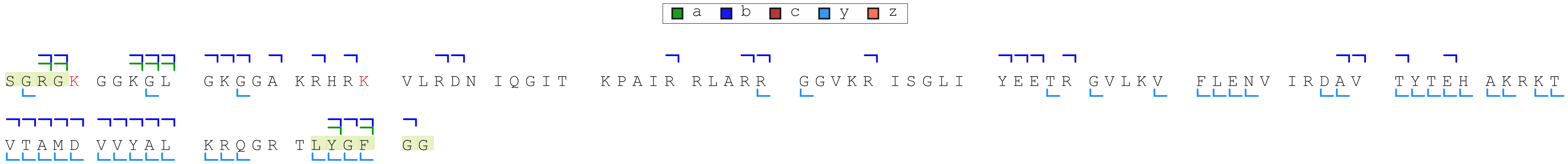

Proteoform:

Index	Protein	SC [%]	SVP [%]	IC [%]	MS Score [a.u.]	Var. Mods	Mono Mass [Da]	Chemical Formula
6	H4Kac	58.4	17.6	29.5	17.2	Ace: K16, K20	11313.3624	C498H841N163O136S

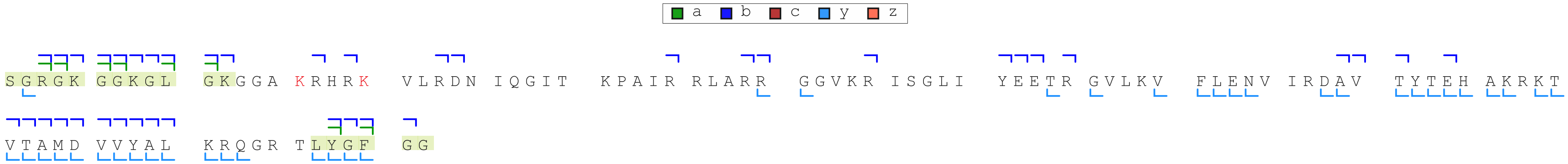

Proteoform:

Index	Protein	SC [%]	SVP [%]	IC [%]	MS Score [a.u.]	Var. Mods	Mono Mass [Da]	Chemical Formula
7	H4Kac	56.4	9.8	29.8	16.8	Ace: K5, K16	11313.3624	C498H841N163O136S

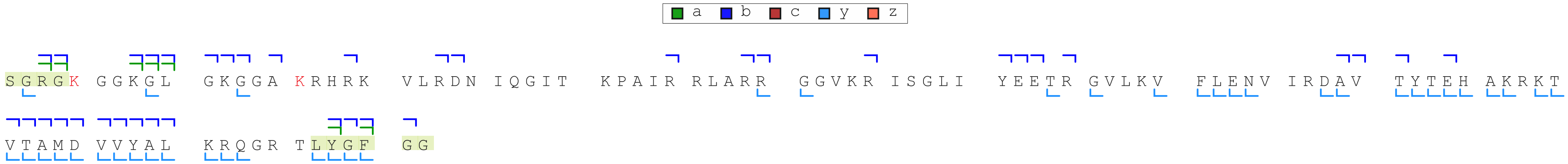

Proteoform:

Index	Protein	SC [%]	SVP [%]	IC [%]	MS Score [a.u.]	Var. Mods	Mono Mass [Da]	Chemical Formula
8	H4Kac	56.4	16.7	29.8	16.8	Ace: K8, K12	11313.3624	C498H841N163O136S

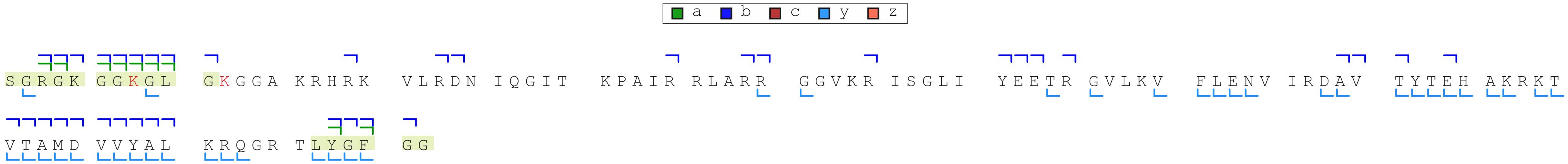

Proteoform:

Index	Protein	SC [%]	SVP [%]	IC [%]	MS Score [a.u.]	Var. Mods	Mono Mass [Da]	Chemical Formula
9	H4Kac	53.5	9.8	29.2	15.6	Ace: K5, K12	11313.3624	C498H841N163O136S

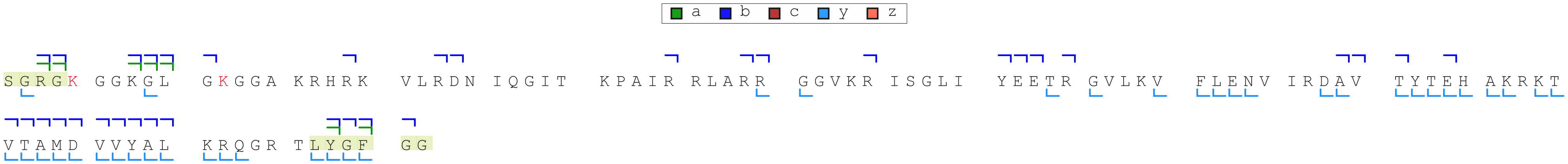

Proteoform:

Index	Protein	SC [%]	SVP [%]	IC [%]	MS Score [a.u.]	Var. Mods	Mono Mass [Da]	Chemical Formula
10	H4Kac	49.5	9.8	27.9	13.8	Ace: K5, K8	11313.3624	C498H841N163O136S

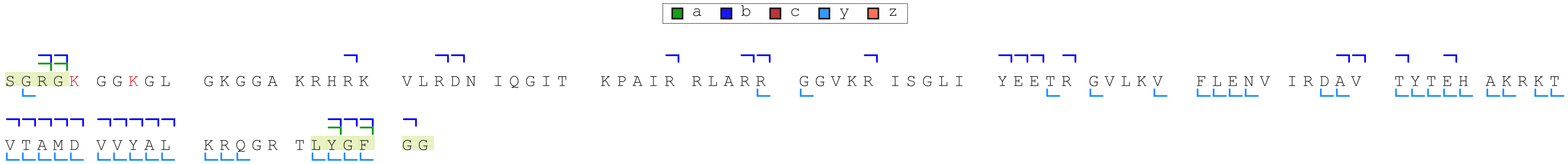

Error Plot

Proteoform:

Index	Protein	SC [%]	SVP [%]	IC [%]	MS Score [a.u.]	Var. Mods	Mono Mass [Da]	Chemical Formula
1	H4Kac	60.4	24.5	30.6	18.5	Ace: K8, K20	11313.3624	C498H841N163O136S

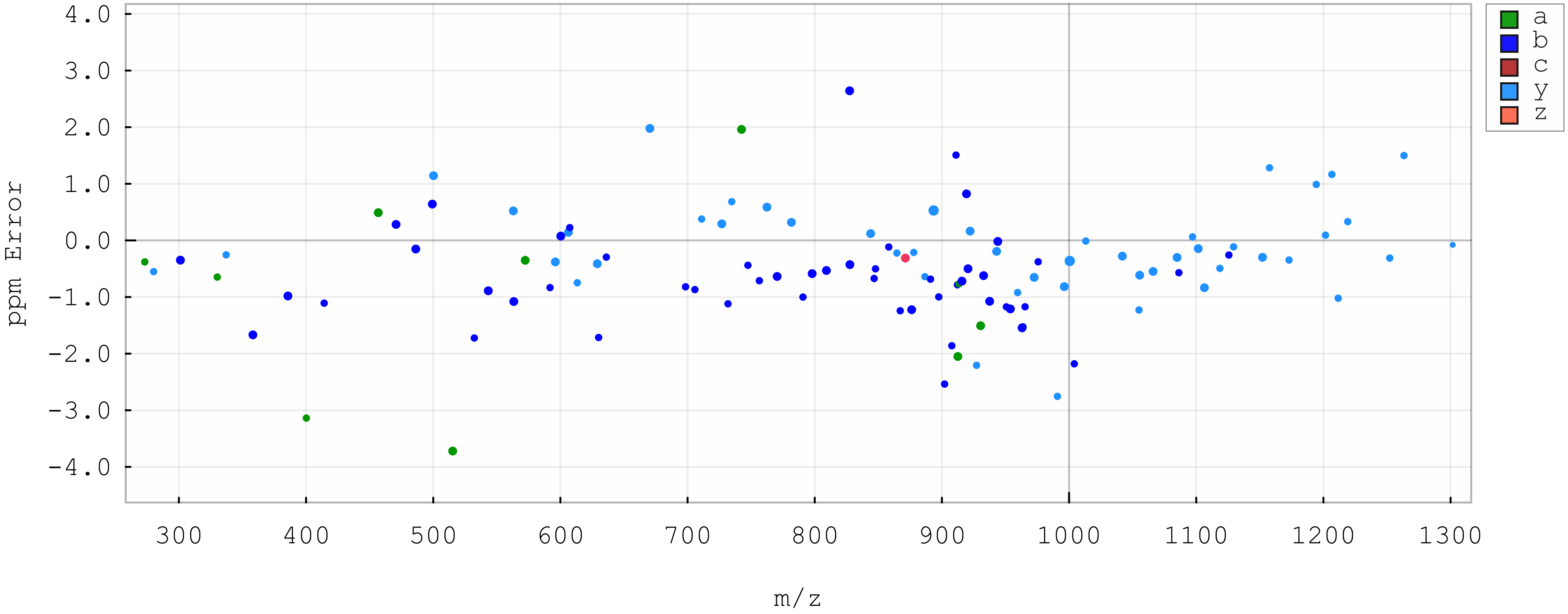

Proteoform:	Index	Protein	SC [%]	SVP [%]	IC [%]	MS Score [a.u.]	Var. Mods	Mono Mass [Da]	Chemical Formula
	2	H4Kac	60.4	24.5	30.0	18.1	Ace: K12, K20	11313.3624	C498H841N163O136S

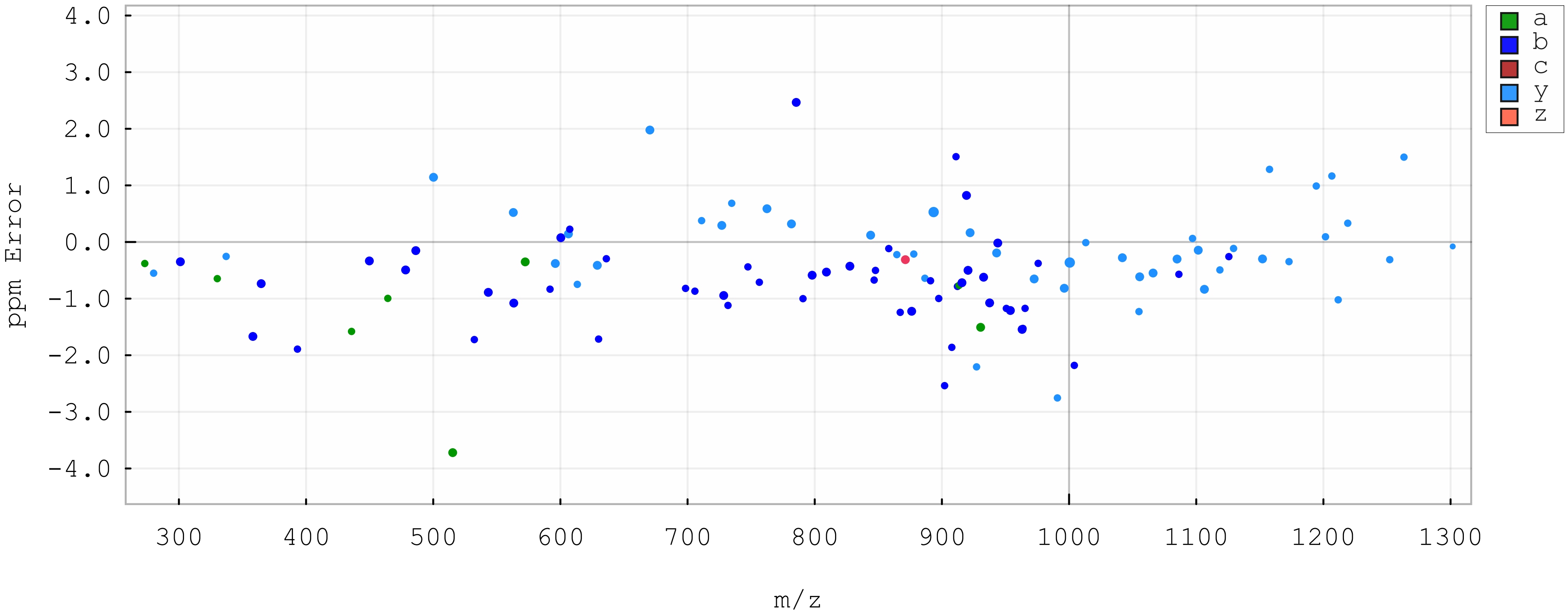

Proteoform:

Index	Protein	SC [%]	SVP [%]	IC [%]	MS Score [a.u.]	Var. Mods	Mono Mass [Da]	Chemical Formula
3	H4Kac	59.4	20.6	30.4	18.1	Ace: K8, K16	11313.3624	C498H841N163O136S

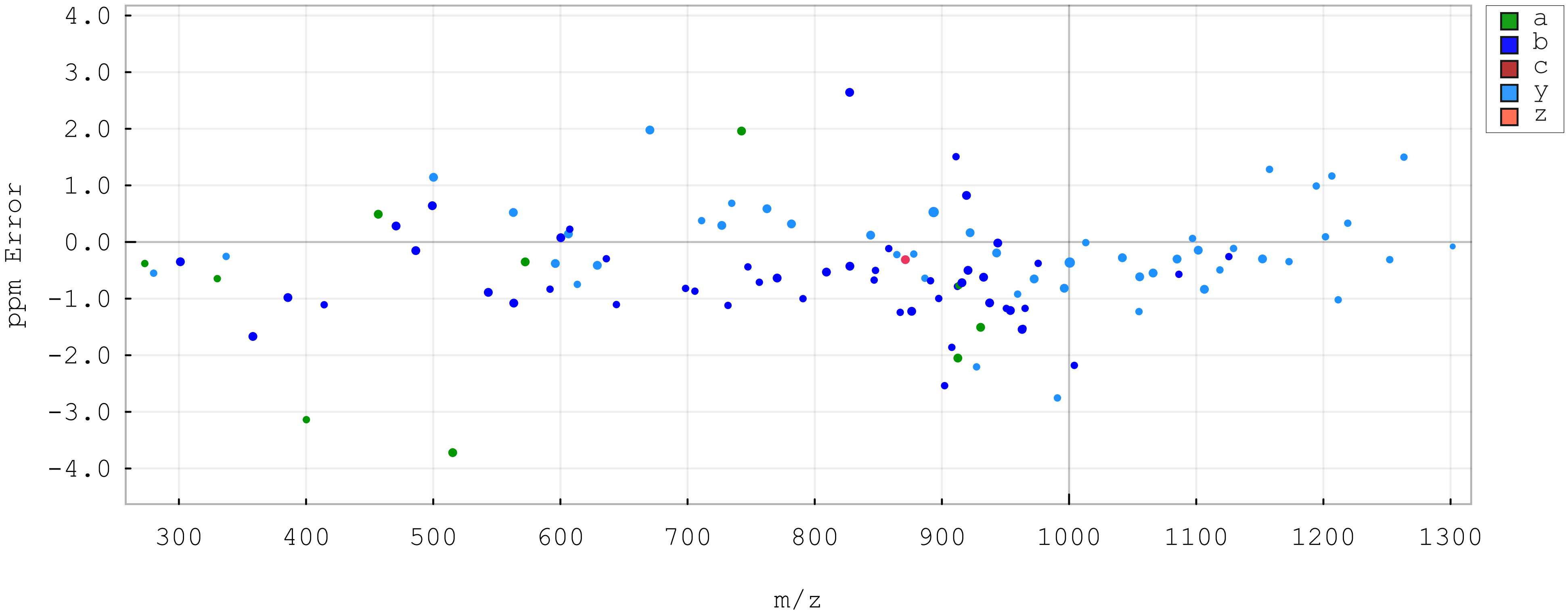

Proteoform:

Index	Protein	SC [%]	SVP [%]	IC [%]	MS Score [a.u.]	Var. Mods	Mono Mass [Da]	Chemical Formula
4	H4Kac	59.4	20.6	29.7	17.7	Ace: K12, K16	11313.3624	C498H841N163O136S

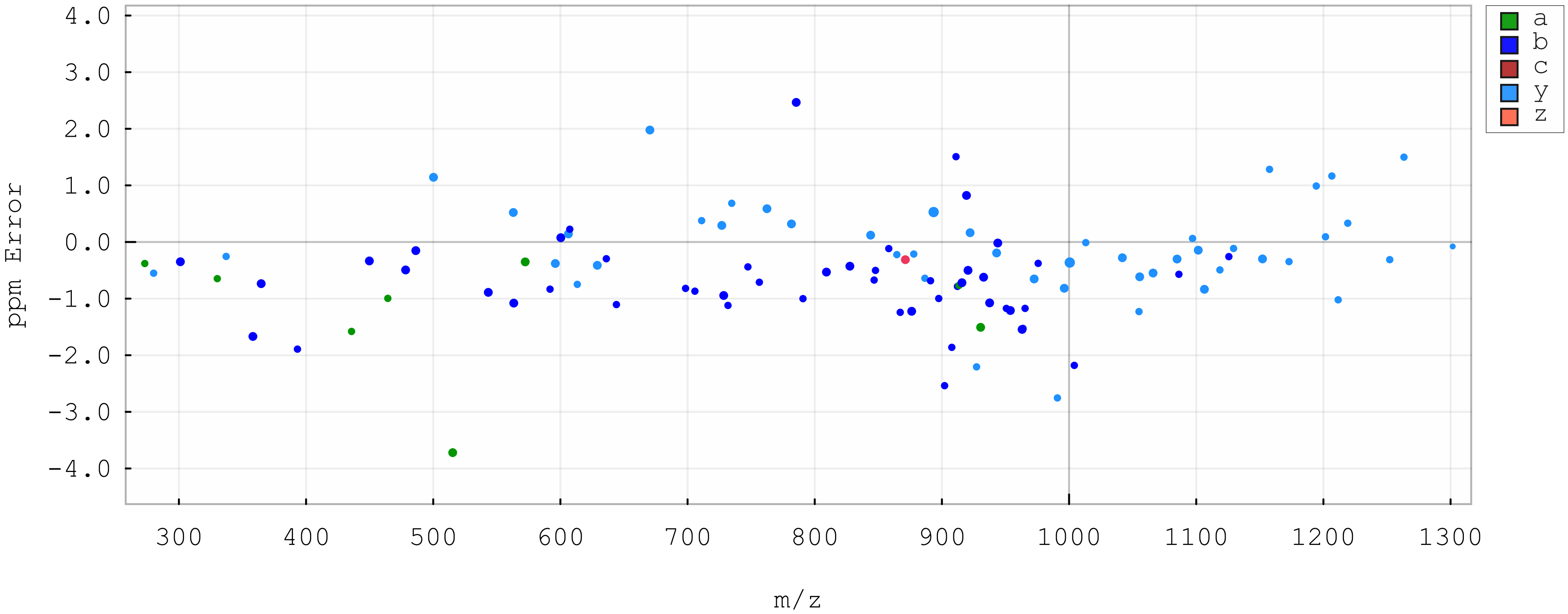

Proteoform:

Index	Protein	SC [%]	SVP [%]	IC [%]	MS Score [a.u.]	Var. Mods	Mono Mass [Da]	Chemical Formula
5	H4Kac	57.4	9.8	30.0	17.2	Ace: K5, K20	11313.3624	C498H841N163O136S

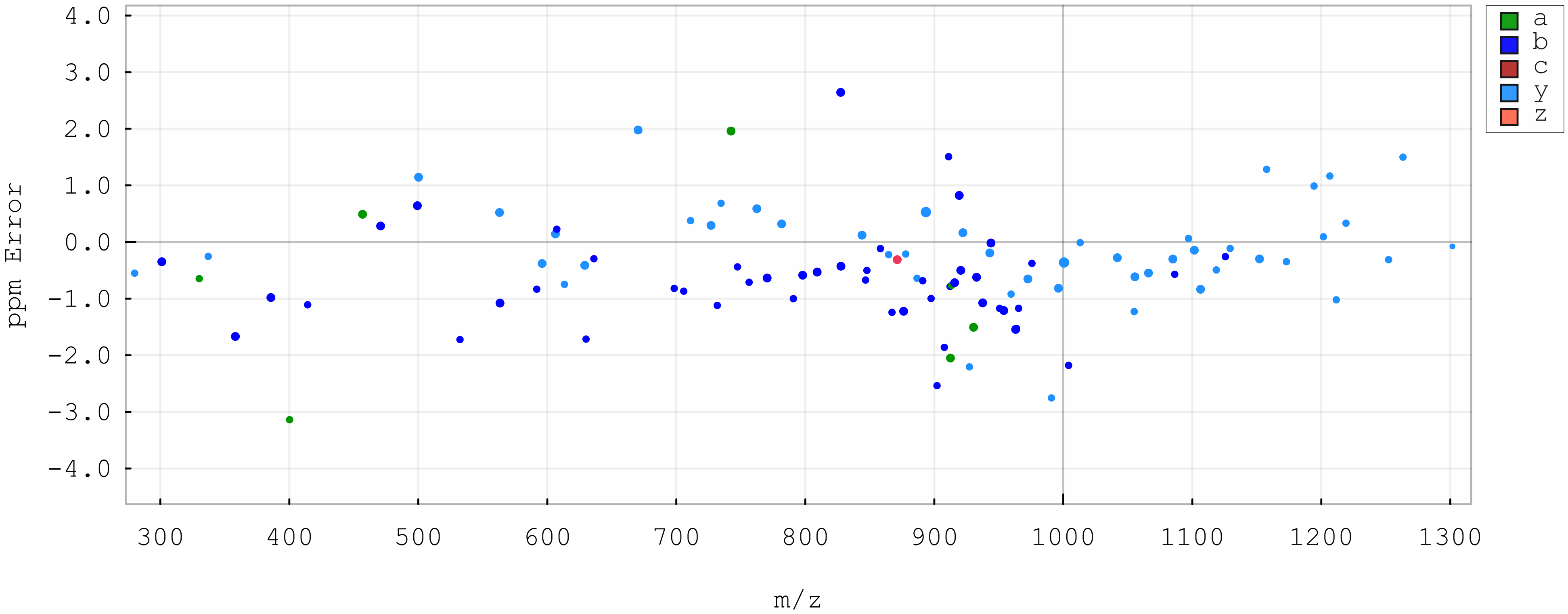

Proteoform:

Index	Protein	SC [%]	SVP [%]	IC [%]	MS Score [a.u.]	Var. Mods	Mono Mass [Da]	Chemical Formula
6	H4Kac	58.4	17.6	29.5	17.2	Ace: K16, K20	11313.3624	C498H841N163O136S

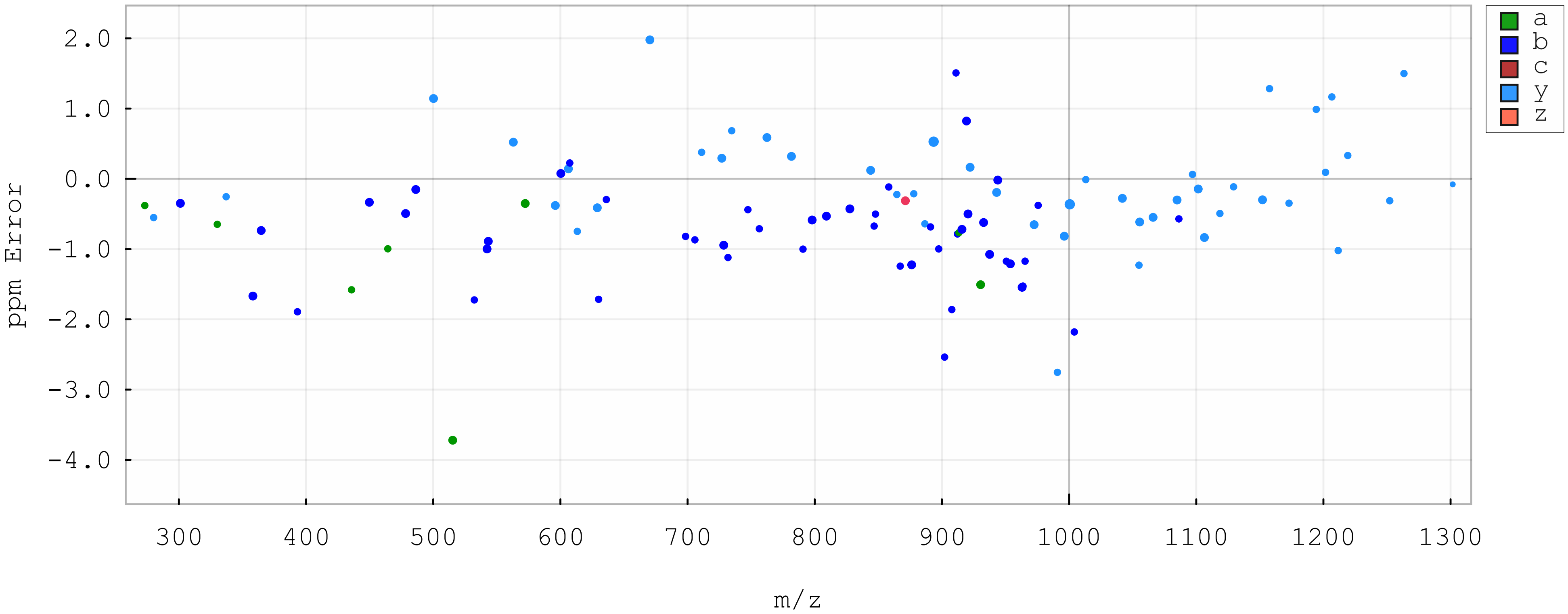

Proteoform:

Index	Protein	SC [%]	SVP [%]	IC [%]	MS Score [a.u.]	Var. Mods	Mono Mass [Da]	Chemical Formula
7	H4Kac	56.4	9.8	29.8	16.8	Ace: K5, K16	11313.3624	C498H841N163O136S

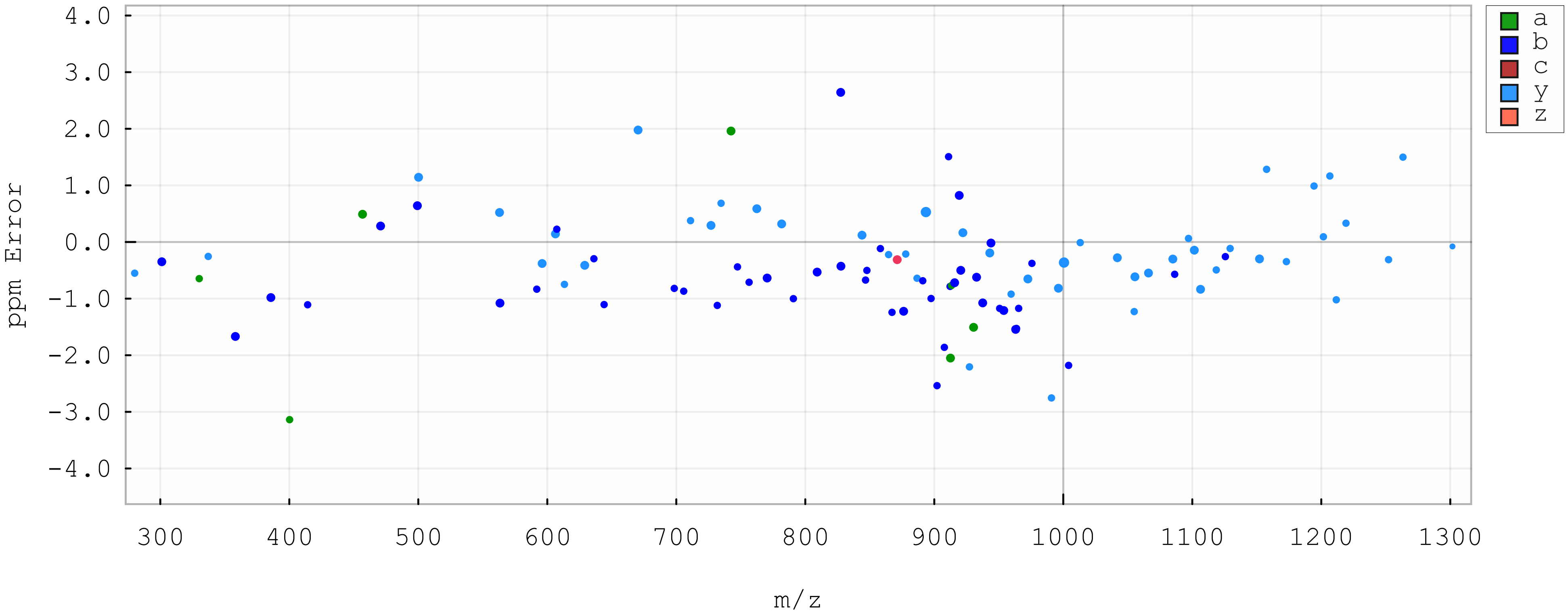

Proteoform:

Index	Protein	SC [%]	SVP [%]	IC [%]	MS Score [a.u.]	Var. Mods	Mono Mass [Da]	Chemical Formula
8	H4Kac	56.4	16.7	29.8	16.8	Ace: K8, K12	11313.3624	C498H841N163O136S

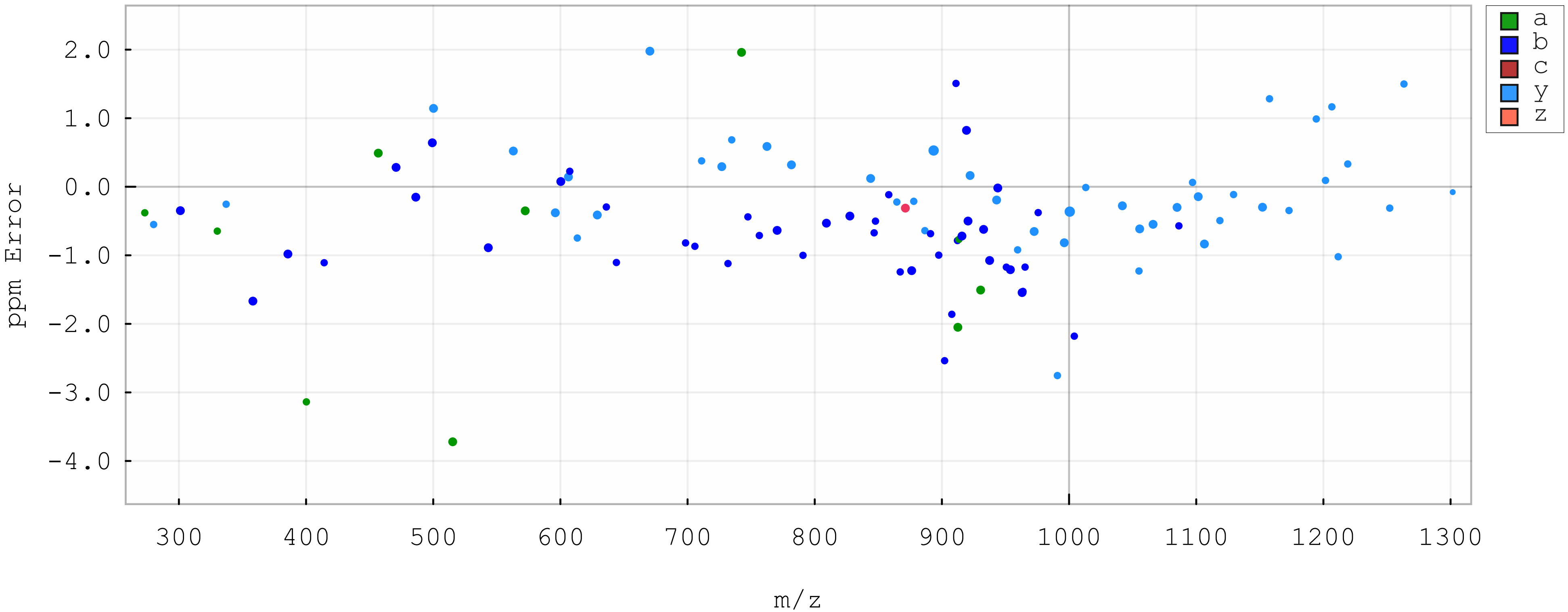

Proteoform:

Index	Protein	SC [%]	SVP [%]	IC [%]	MS Score [a.u.]	Var. Mods	Mono Mass [Da]	Chemical Formula
9	H4Kac	53.5	9.8	29.2	15.6	Ace: K5, K12	11313.3624	C498H841N163O136S

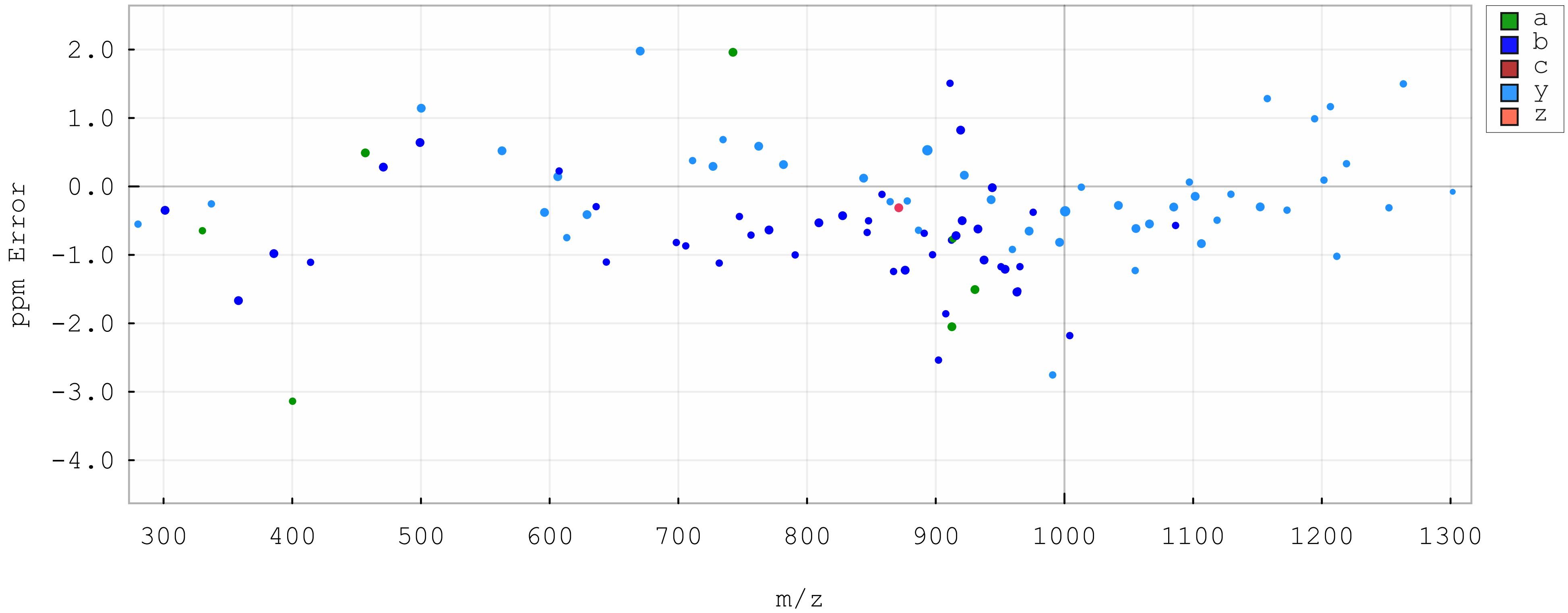

Proteoform:

Index	Protein	SC [%]	SVP [%]	IC [%]	MS Score [a.u.]	Var. Mods	Mono Mass [Da]	Chemical Formula
10	H4Kac	49.5	9.8	27.9	13.8	Ace: K5, K8	11313.3624	C498H841N163O136S

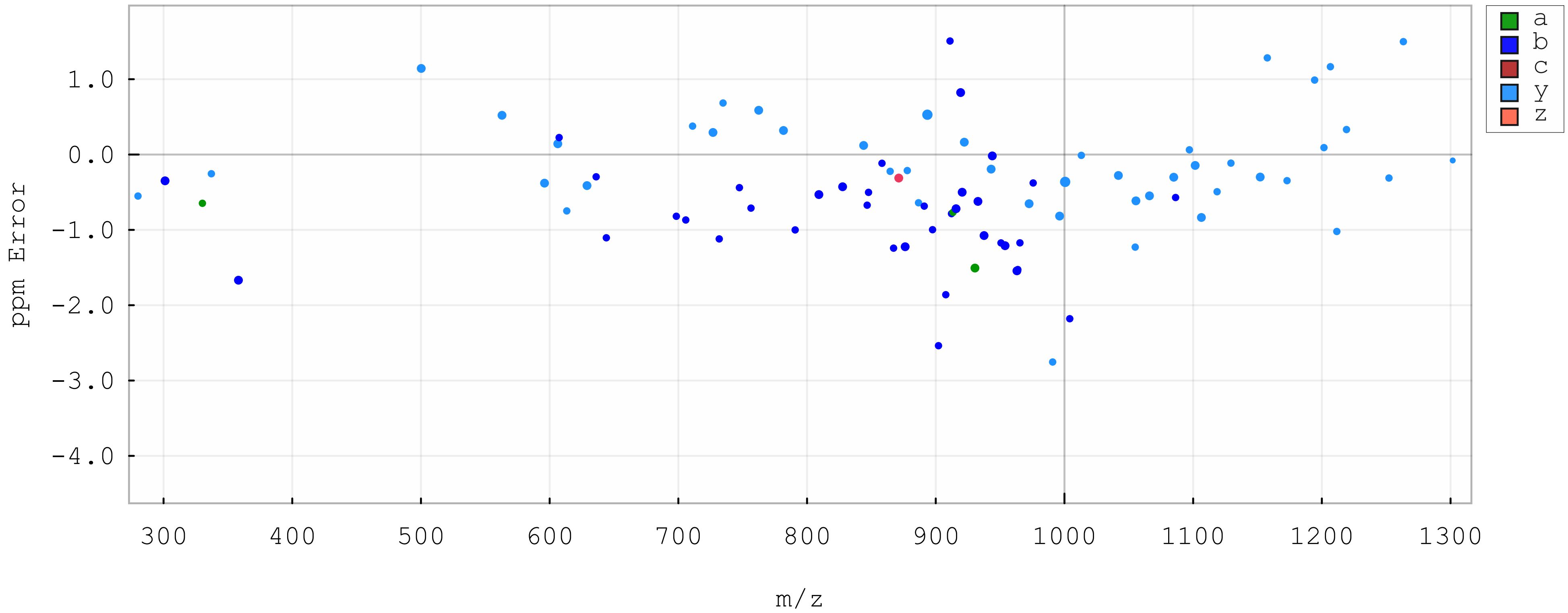

Primary Fragment Plots

Proteoform:

Index	Protein	SC [%]	SVP [%]	IC [%]	MS Score [a.u.]	Var. Mods	Mono Mass [Da]	Chemical Formula
1	H4Kac	60.4	24.5	30.6	18.5	Ace: K8, K20	11313.3624	C498H841N163O136S

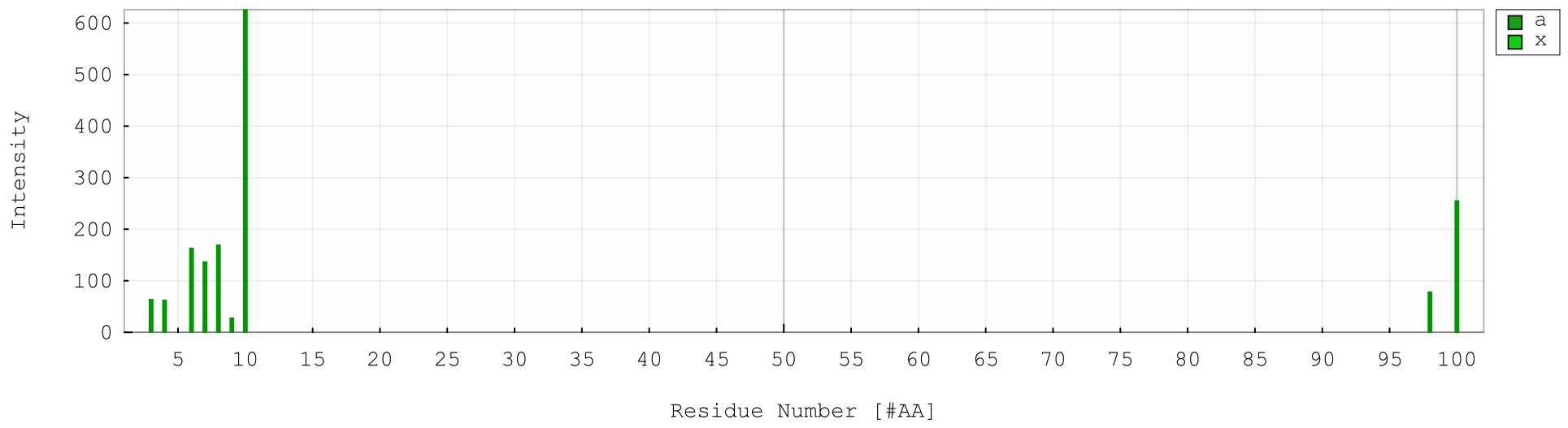

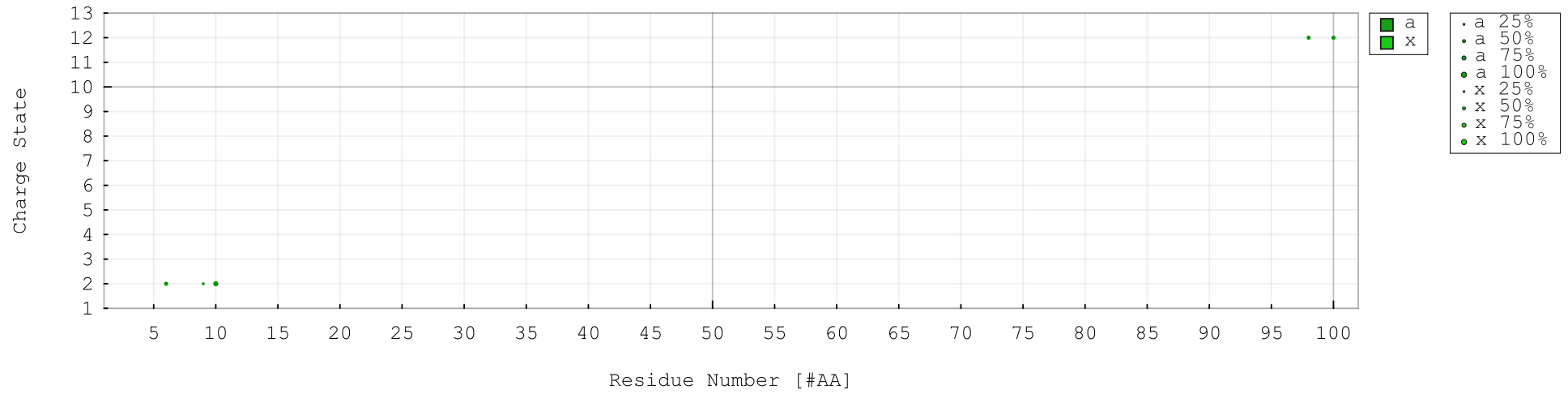

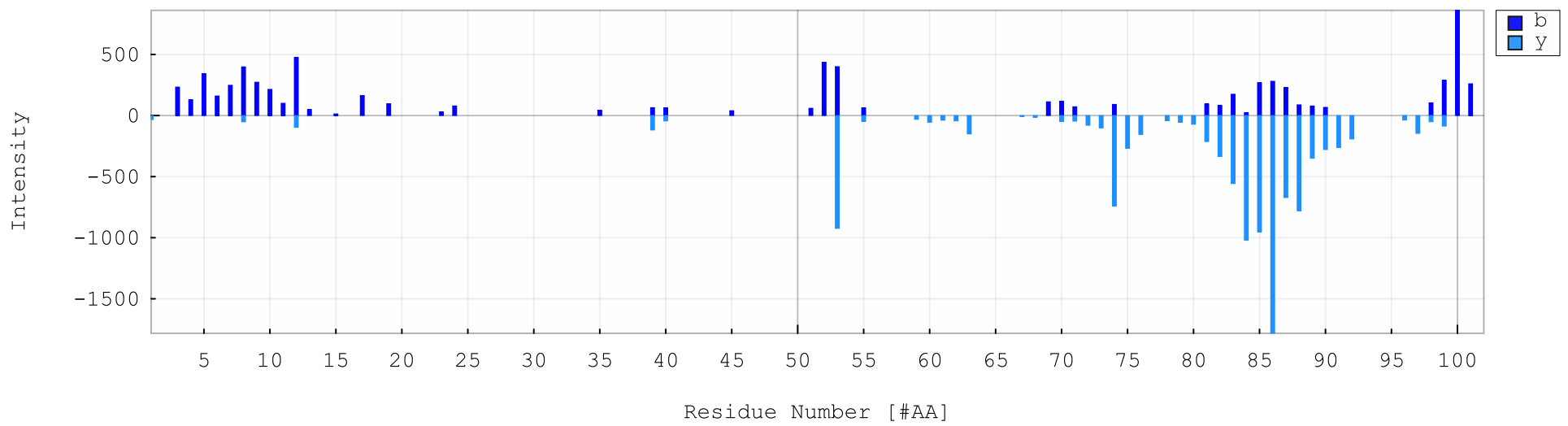

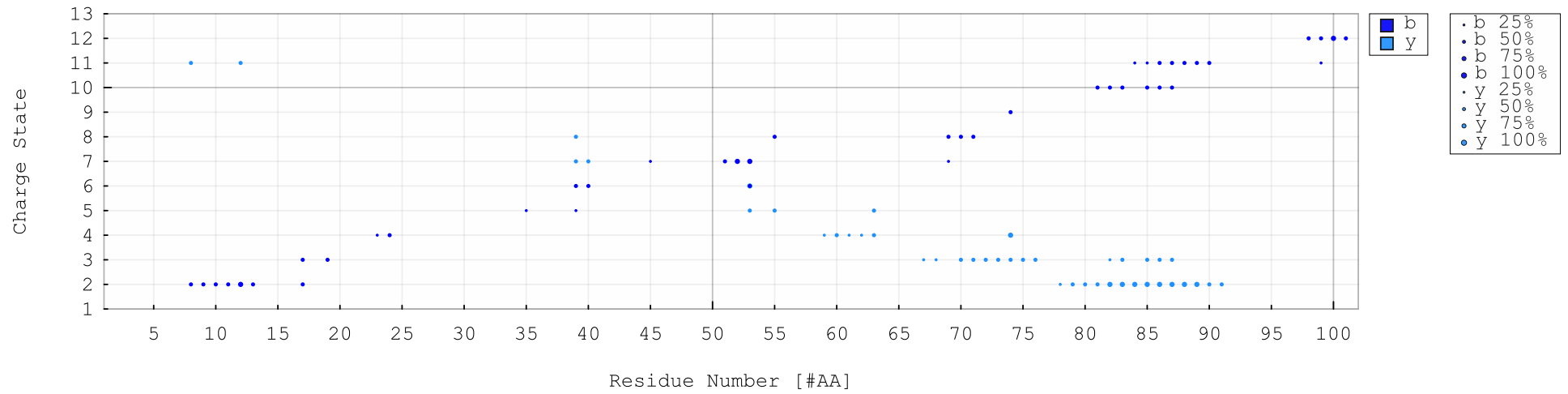

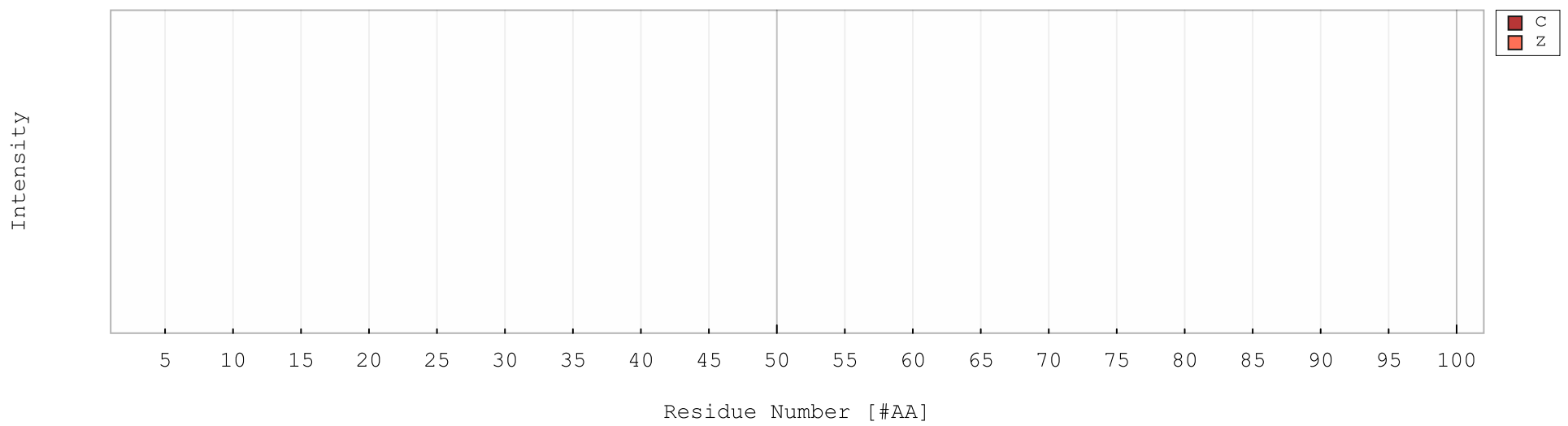

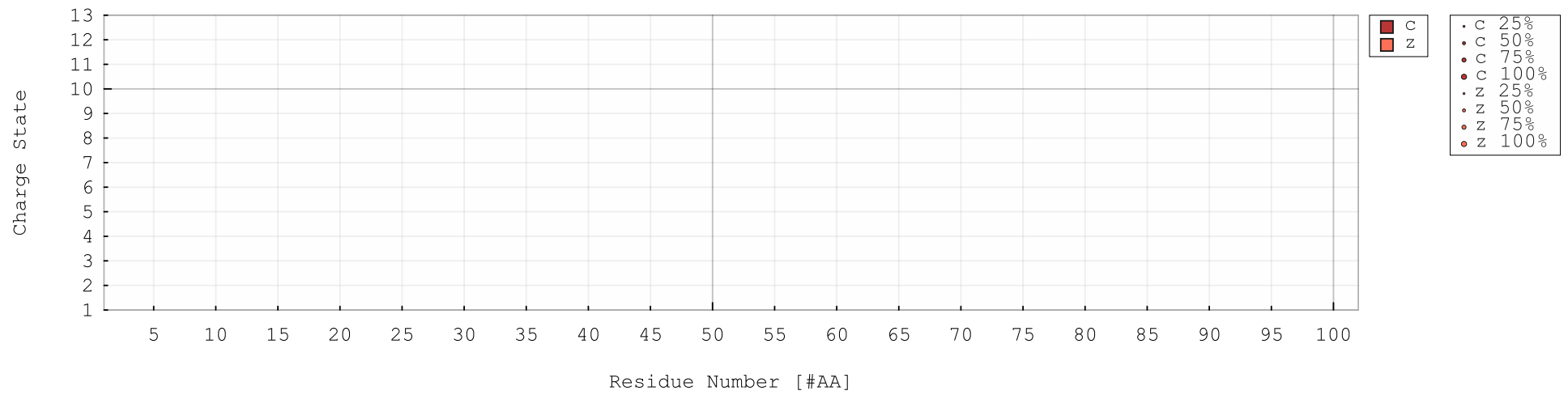

Proteoform:

Index	Protein	SC [%]	SVP [%]	IC [%]	MS Score [a.u.]	Var. Mods	Mono Mass [Da]	Chemical Formula
2	H4Kac	60.4	24.5	30.0	18.1	Ace: K12, K20	11313.3624	C498H841N163O136S

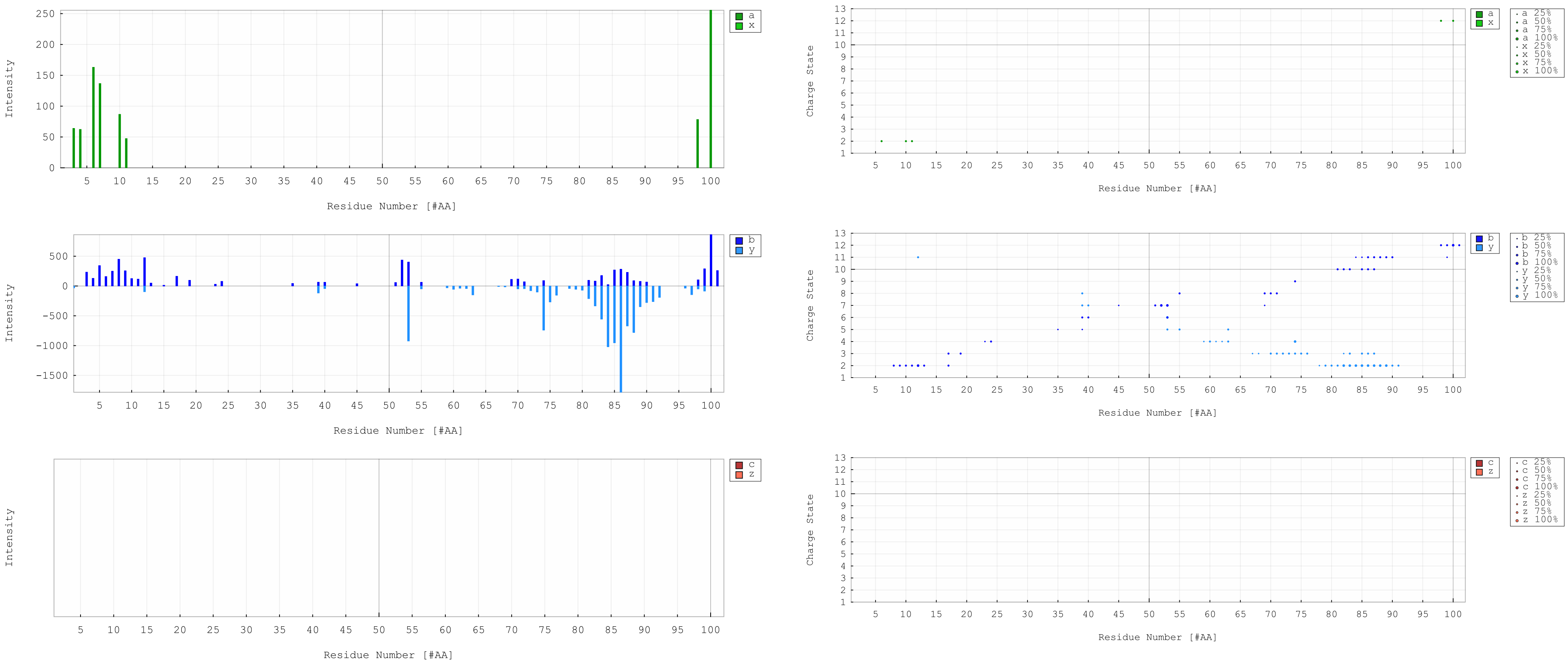

Proteoform:

Index	Protein	SC [%]	SVP [%]	IC [%]	MS Score [a.u.]	Var. Mods	Mono Mass [Da]	Chemical Formula
3	H4Kac	59.4	20.6	30.4	18.1	Ace: K8, K16	11313.3624	C498H841N163O136S

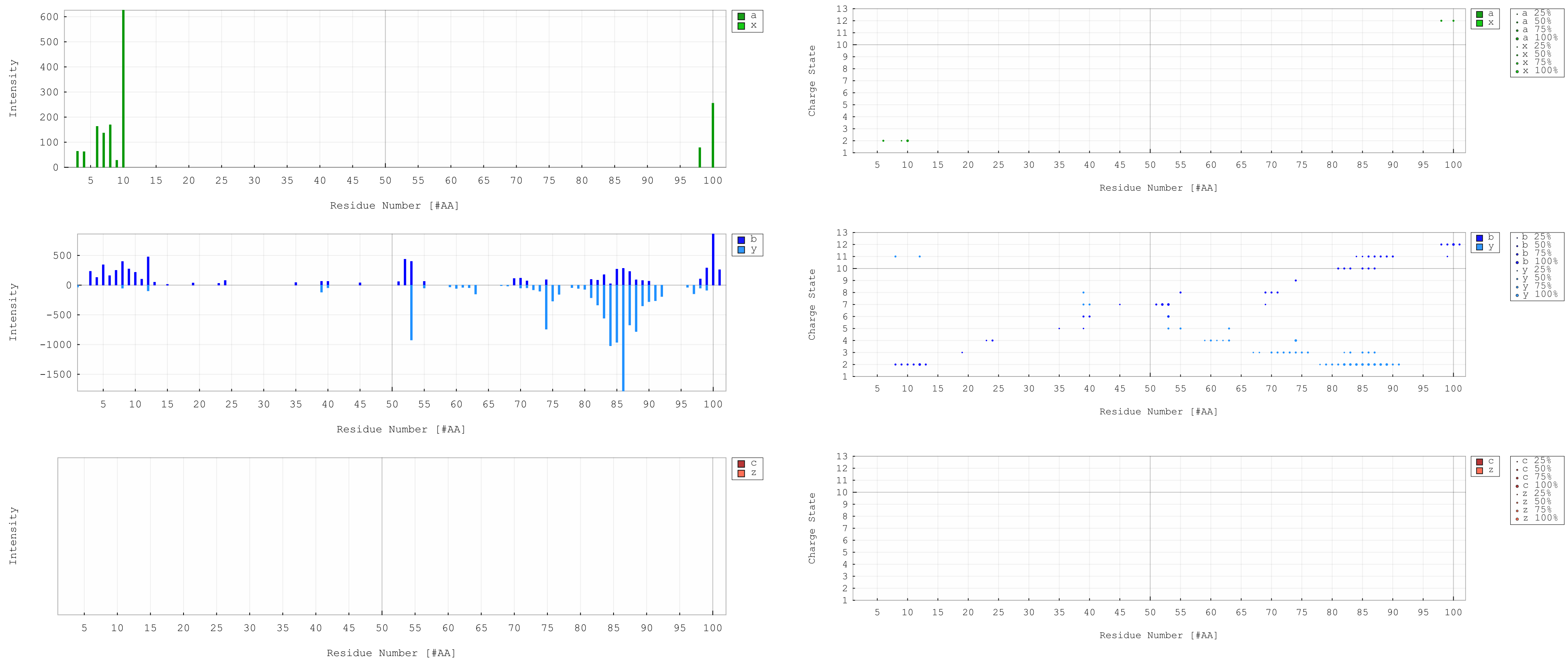

Proteoform:

Index	Protein	SC [%]	SVP [%]	IC [%]	MS Score [a.u.]	Var. Mods	Mono Mass [Da]	Chemical Formula
4	H4Kac	59.4	20.6	29.7	17.7	Ace: K12, K16	11313.3624	C498H841N163O136S

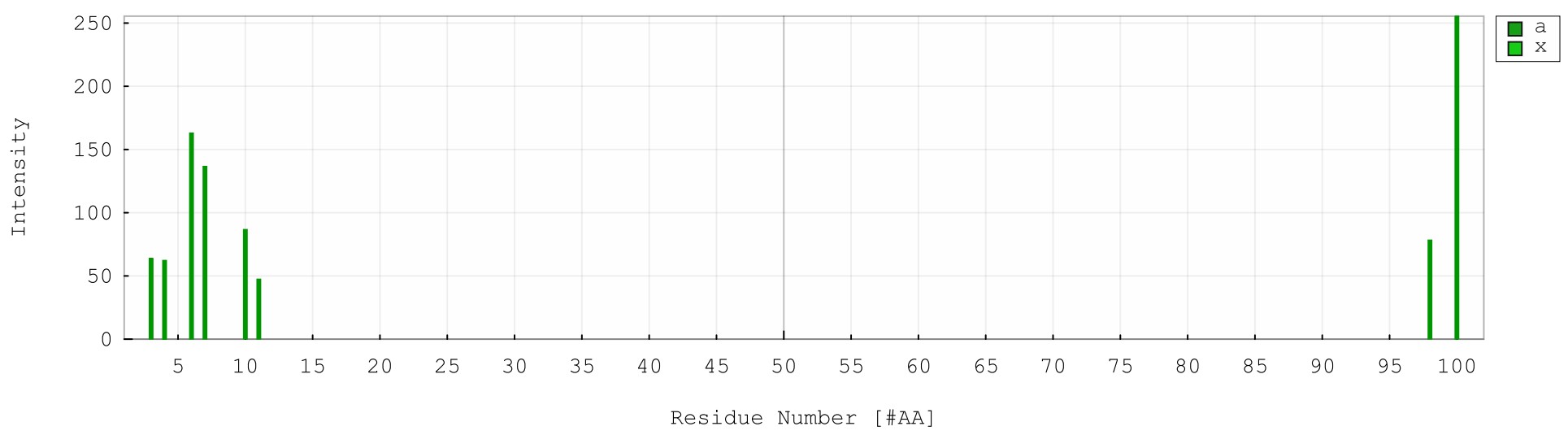

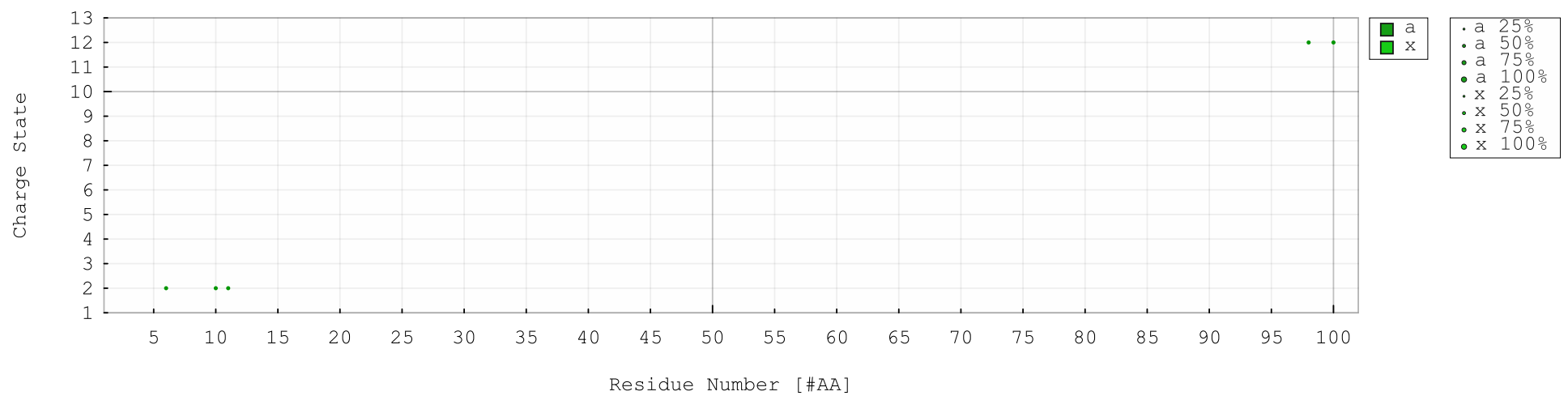

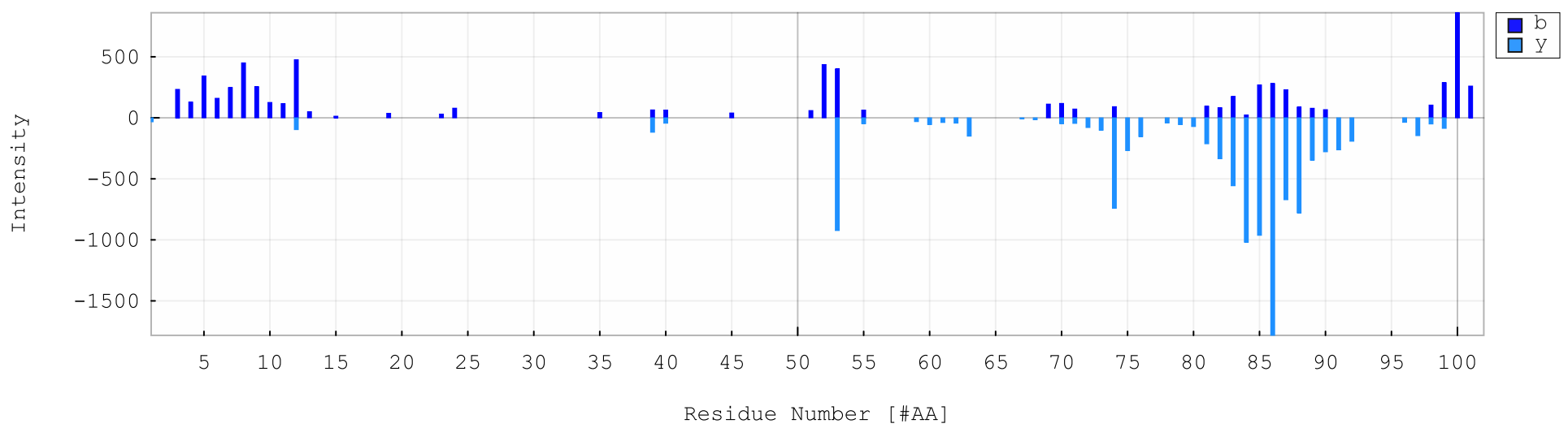

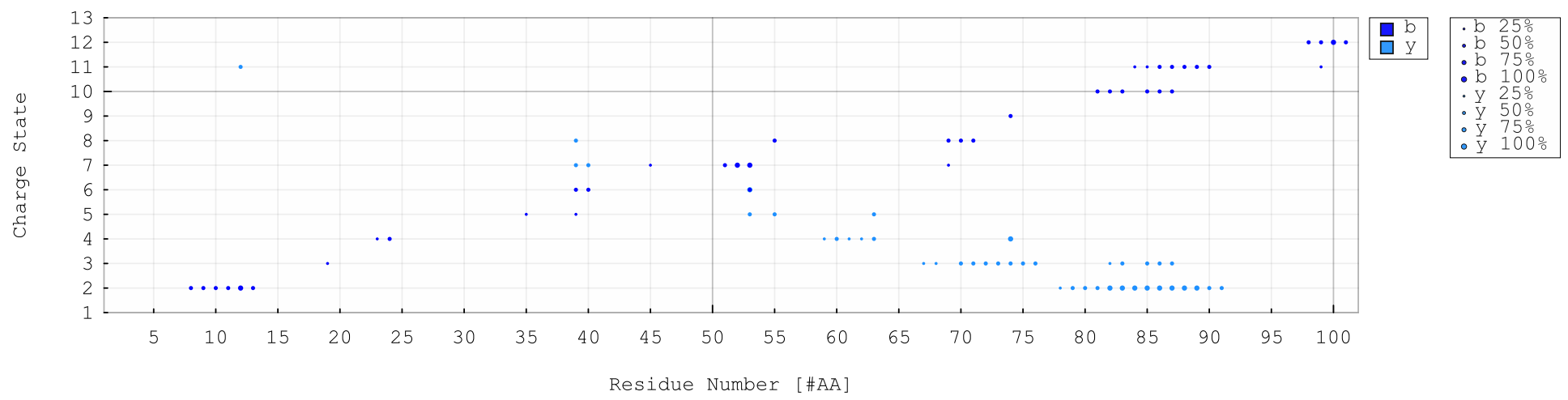

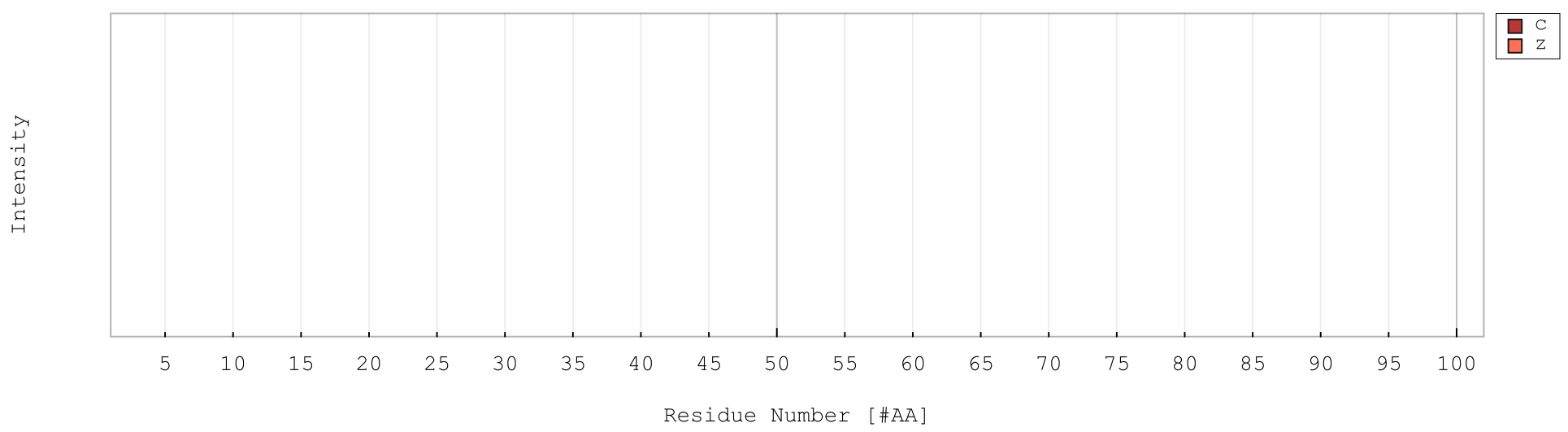

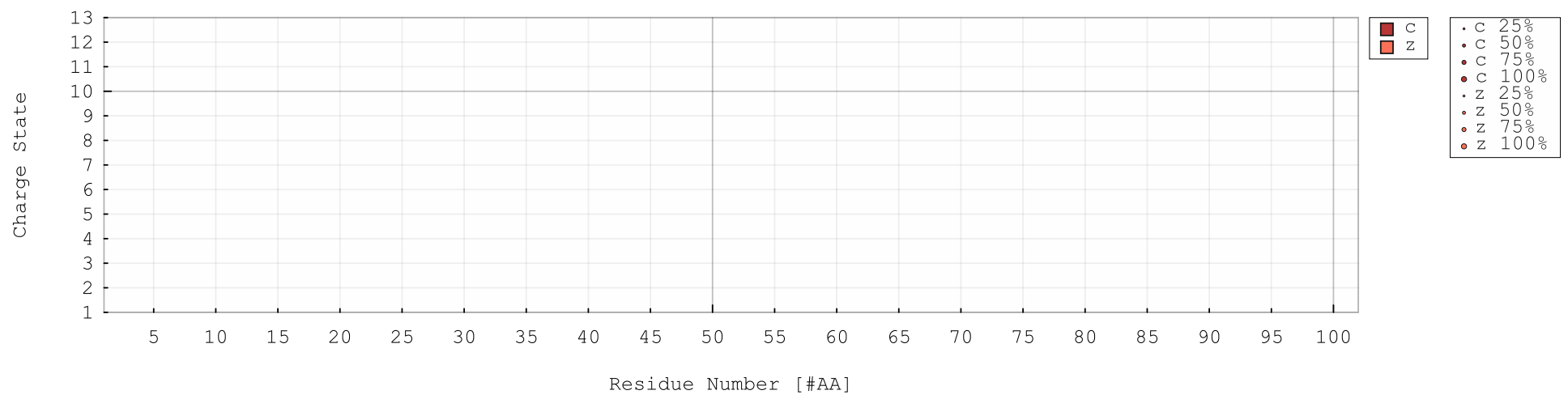

Proteoform:

Index	Protein	SC [%]	SVP [%]	IC [%]	MS Score [a.u.]	Var. Mods	Mono Mass [Da]	Chemical Formula
5	H4Kac	57.4	9.8	30.0	17.2	Ace: K5, K20	11313.3624	C498H841N163O136S

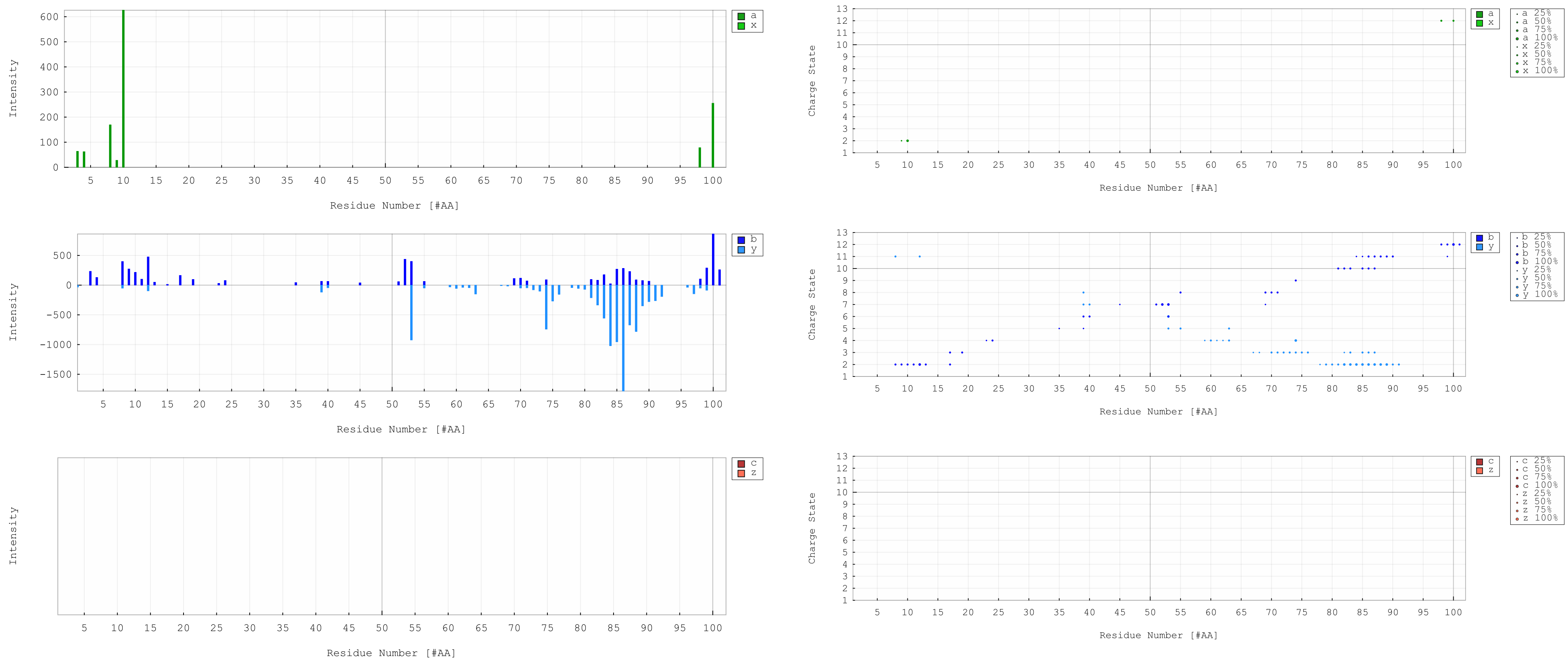

Proteoform:

Index	Protein	SC [%]	SVP [%]	IC [%]	MS Score [a.u.]	Var. Mods	Mono Mass [Da]	Chemical Formula
6	H4Kac	58.4	17.6	29.5	17.2	Ace: K16, K20	11313.3624	C498H841N163O136S

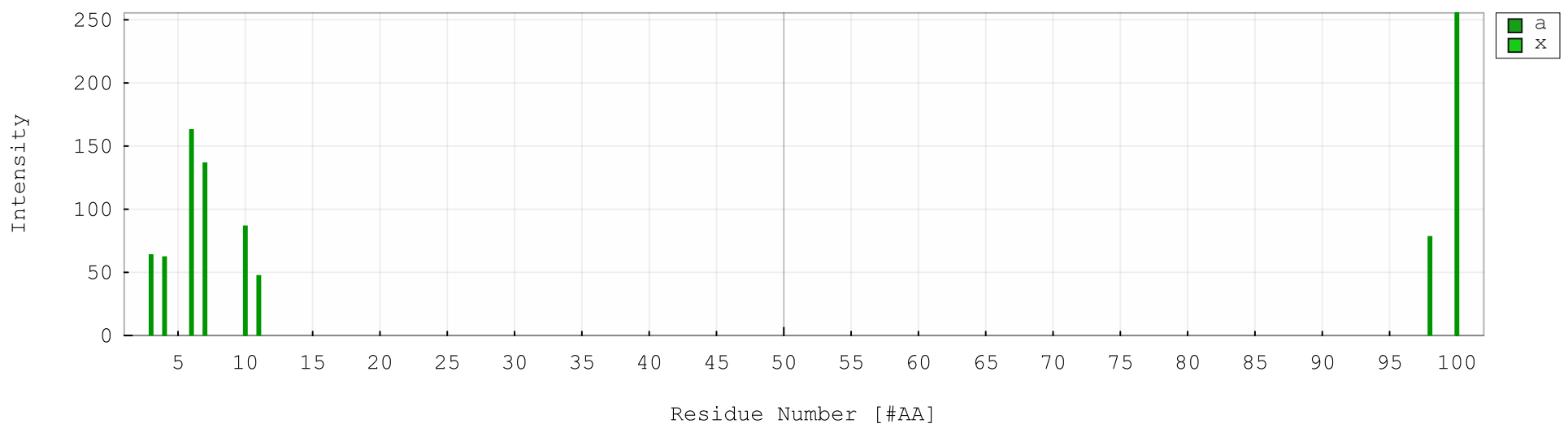

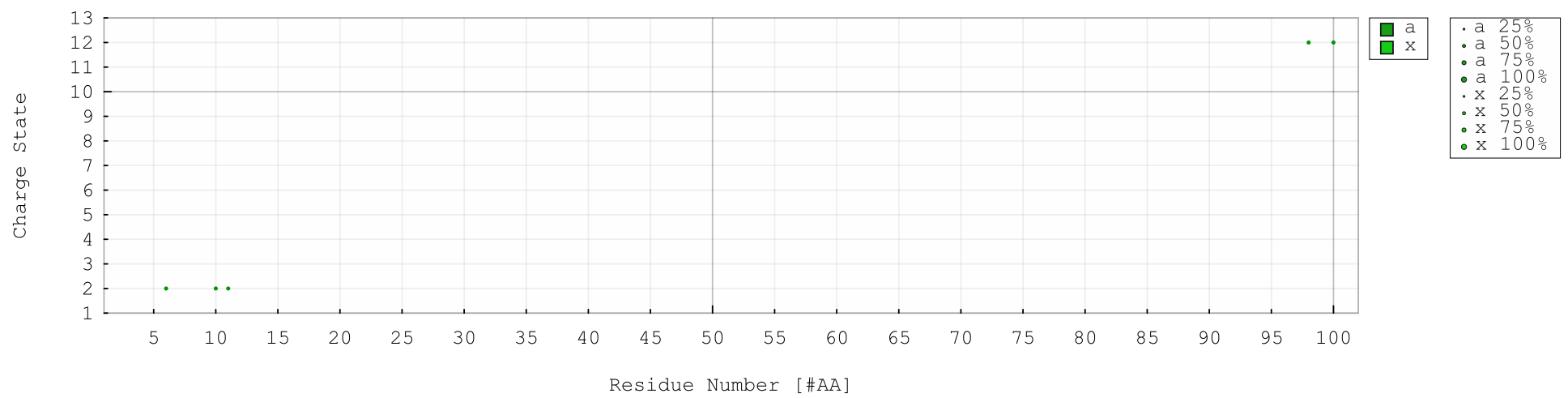

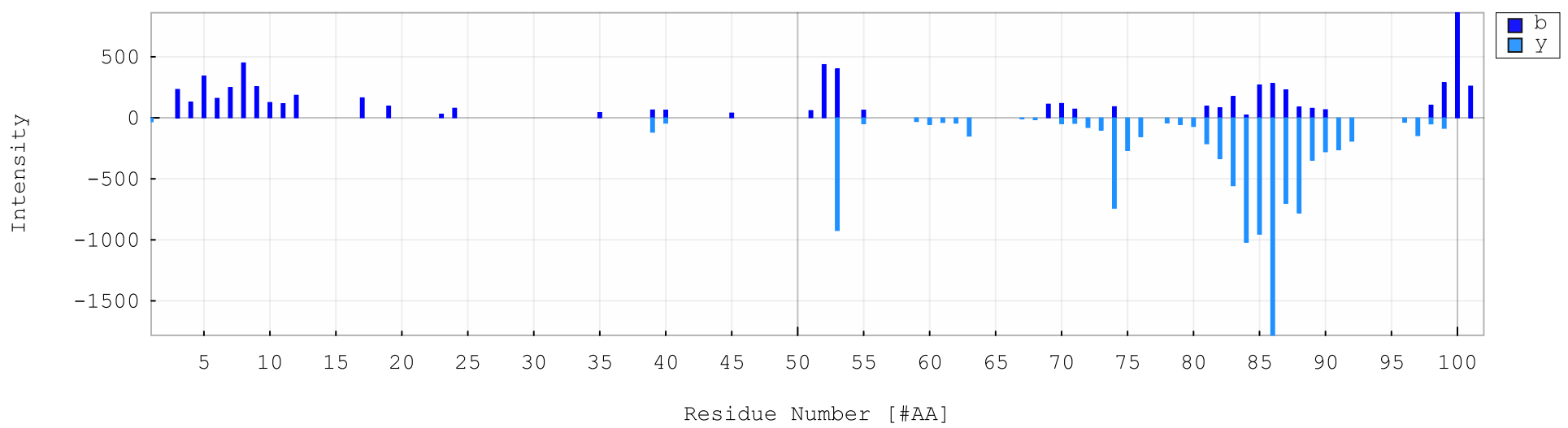

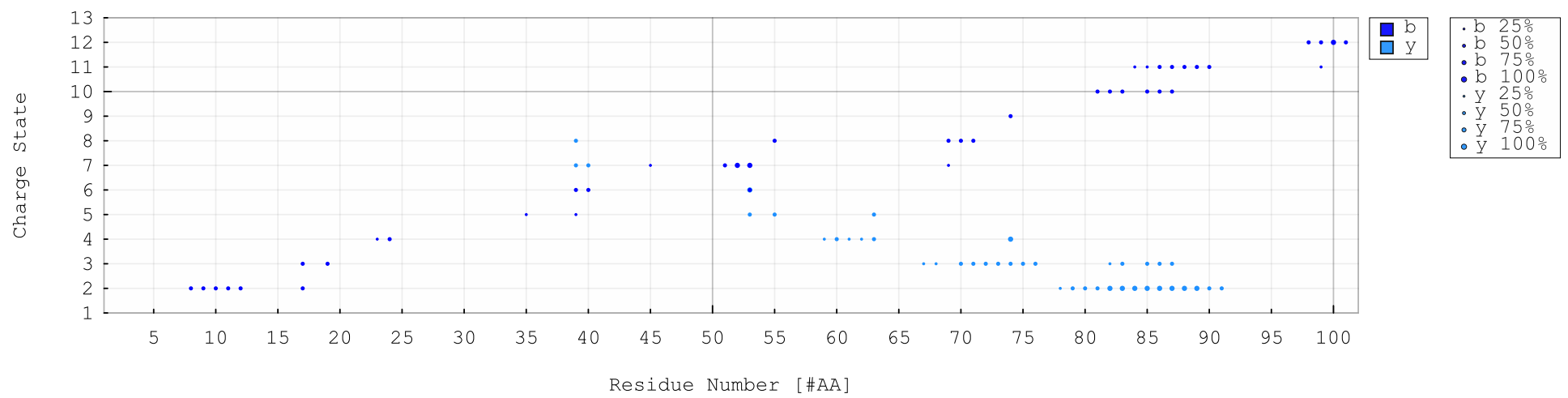

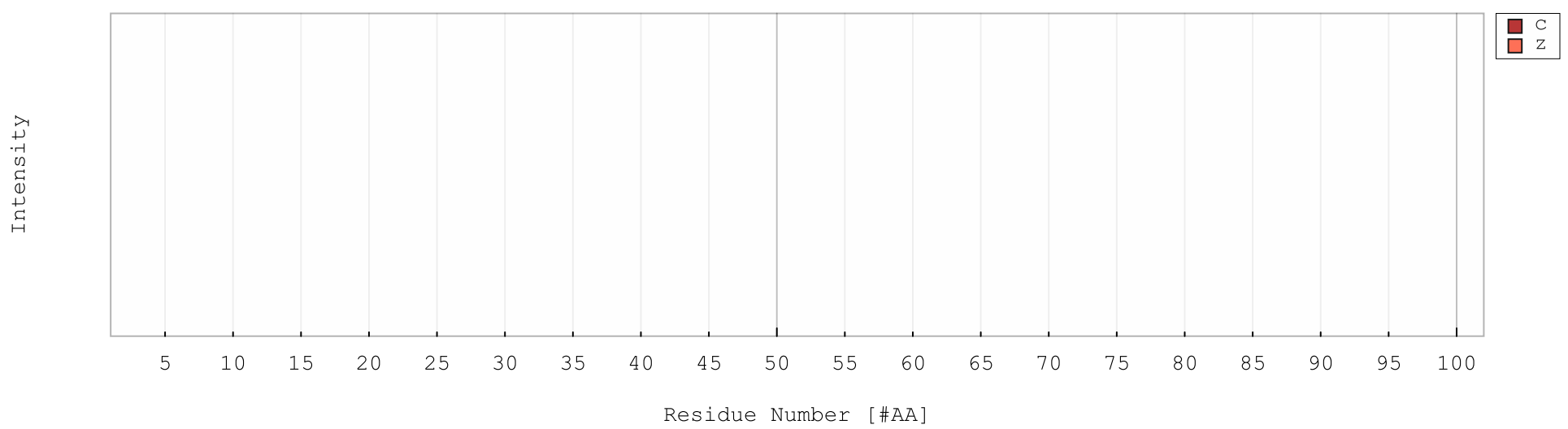

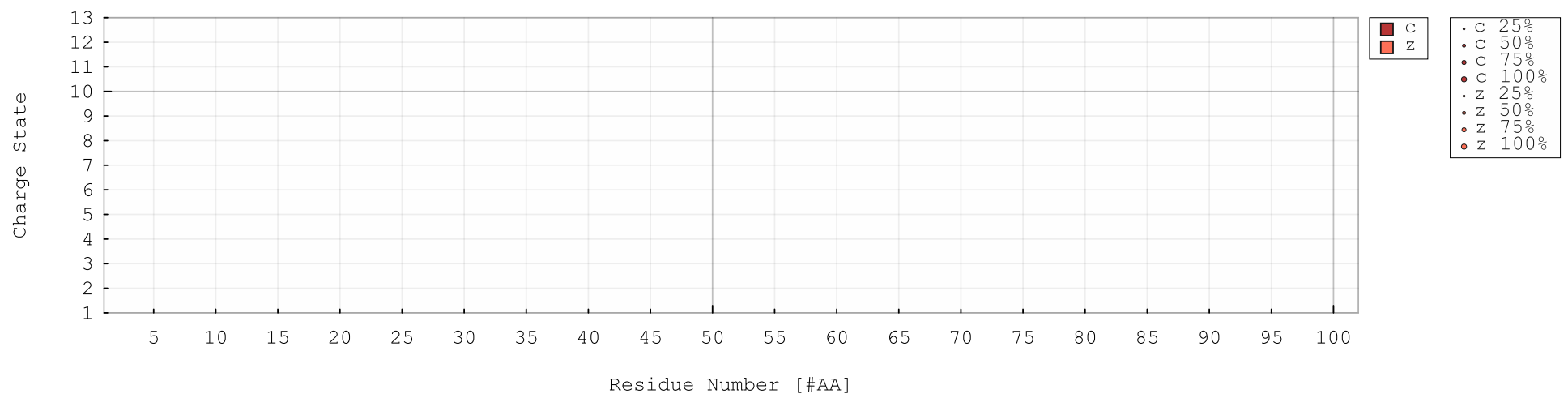

Proteoform:

Index	Protein	SC [%]	SVP [%]	IC [%]	MS Score [a.u.]	Var. Mods	Mono Mass [Da]	Chemical Formula
7	H4Kac	56.4	9.8	29.8	16.8	Ace: K5, K16	11313.3624	C498H841N163O136S

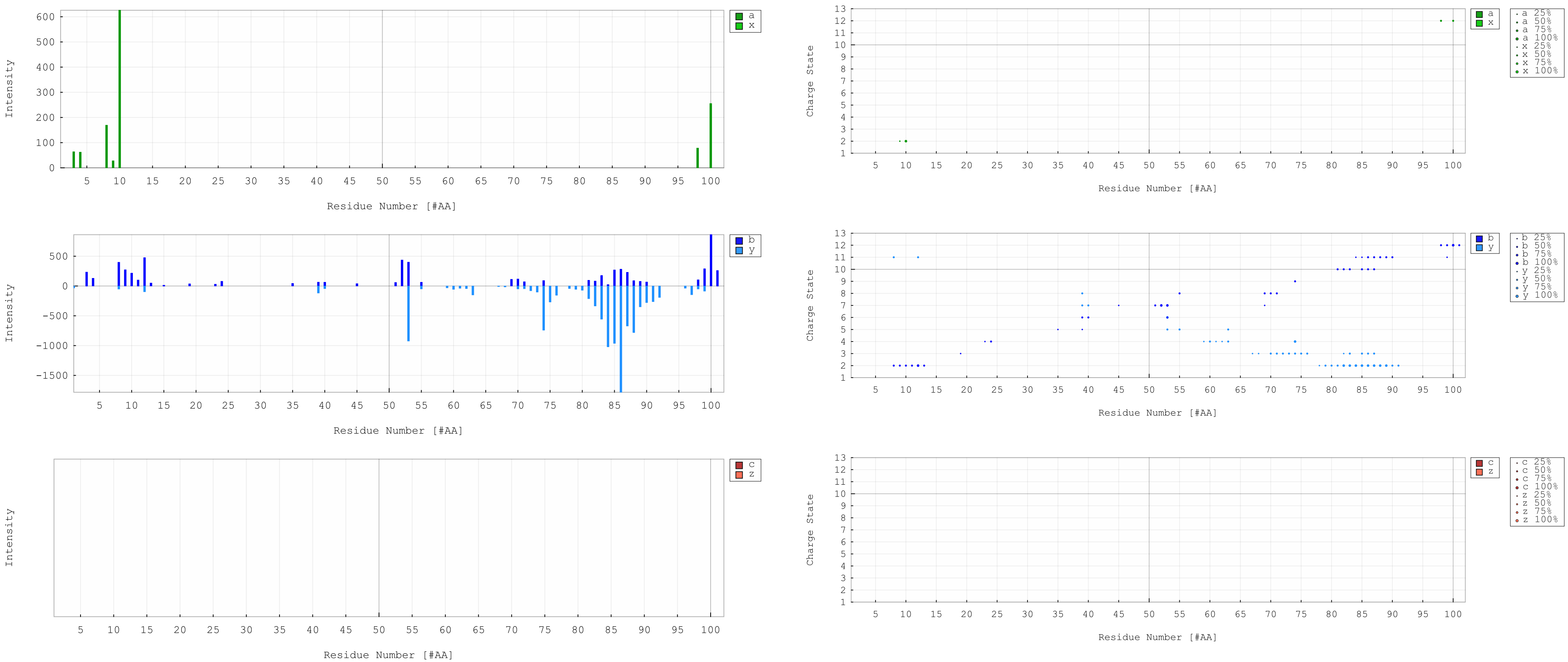

Proteoform:	Index	Protein	SC [%]	SVP [%]	IC [%]	MS Score [a.u.]	Var. Mods	Mono Mass [Da]	Chemical Formula
	8	H4Kac	56.4	16.7	29.8	16.8	Ace: K8, K12	11313.3624	C498H841N163O136S

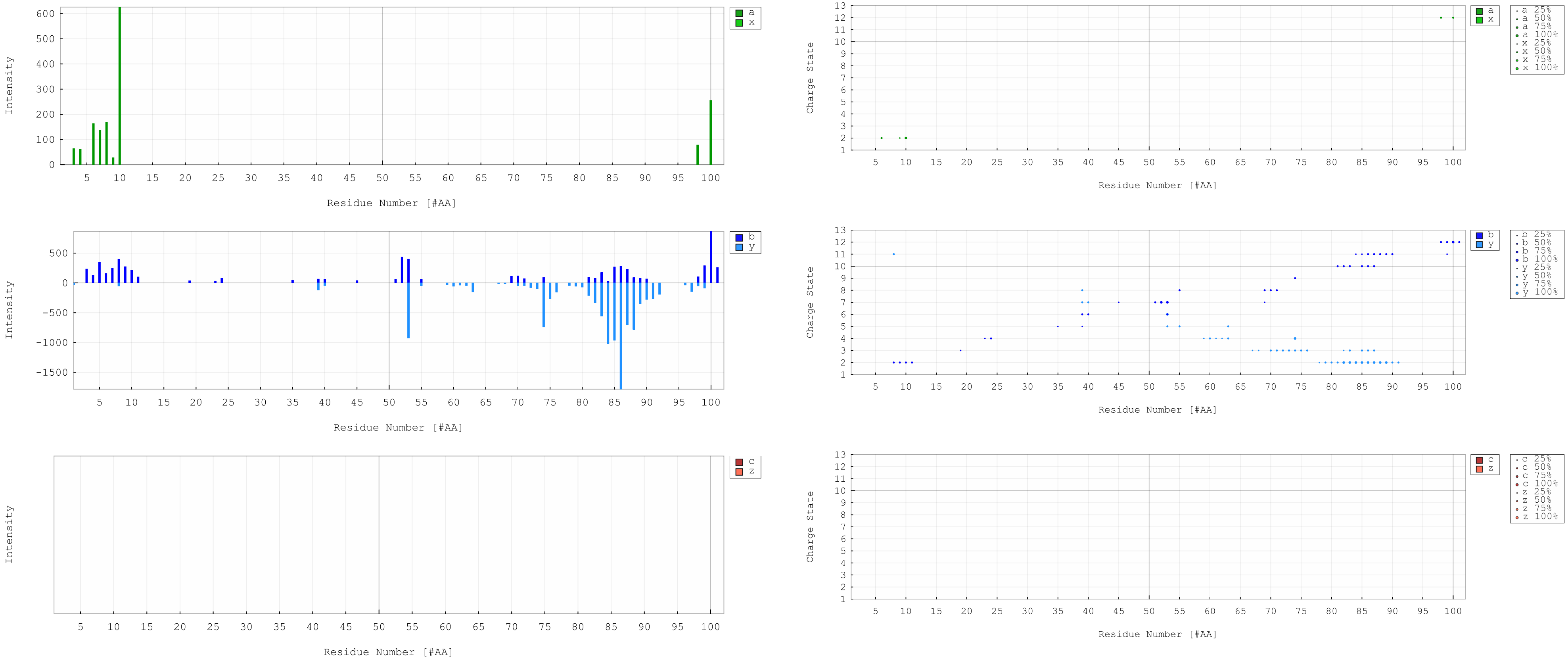

Proteoform:

Index	Protein	SC [%]	SVP [%]	IC [%]	MS Score [a.u.]	Var. Mods	Mono Mass [Da]	Chemical Formula
9	H4Kac	53.5	9.8	29.2	15.6	Ace: K5, K12	11313.3624	C498H841N163O136S

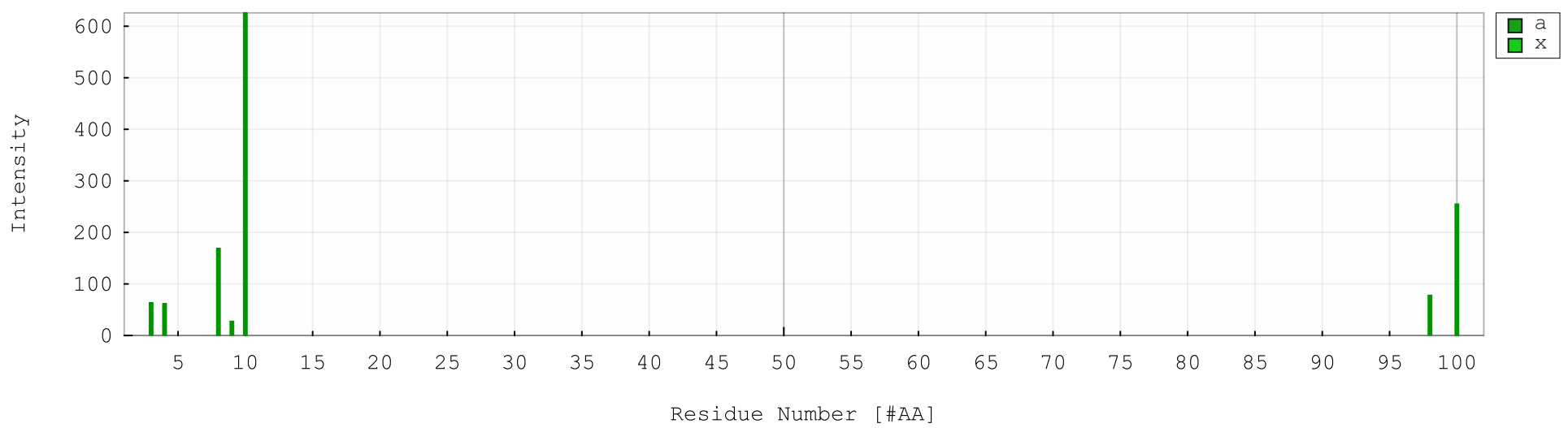

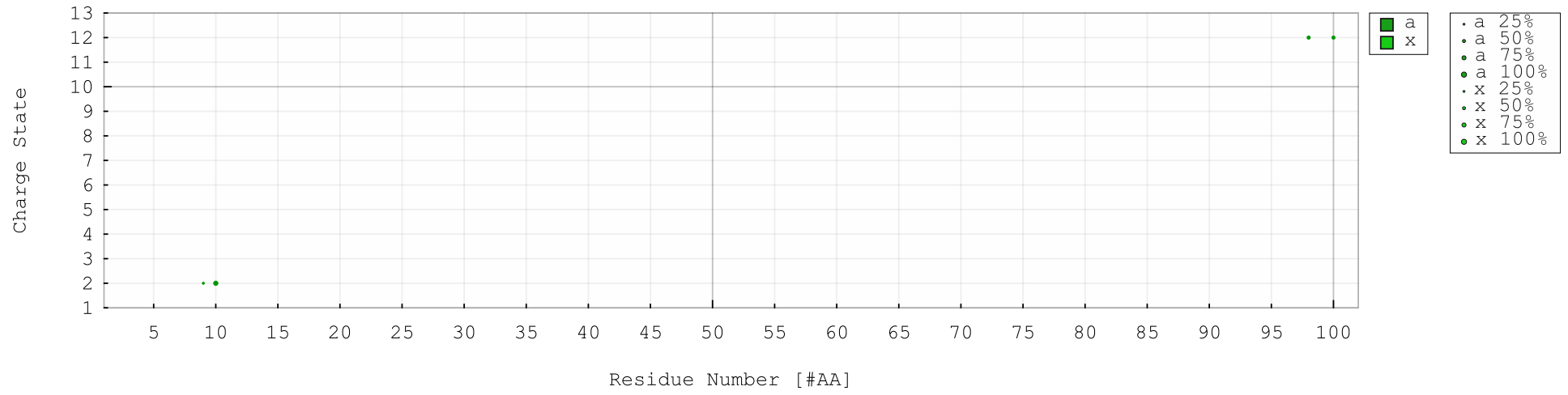

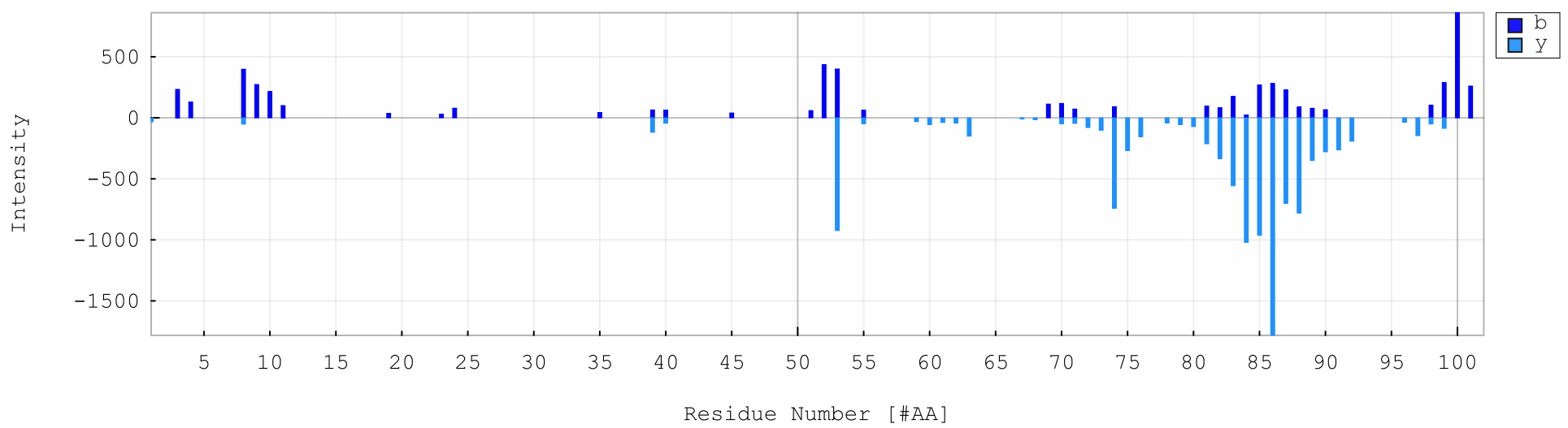

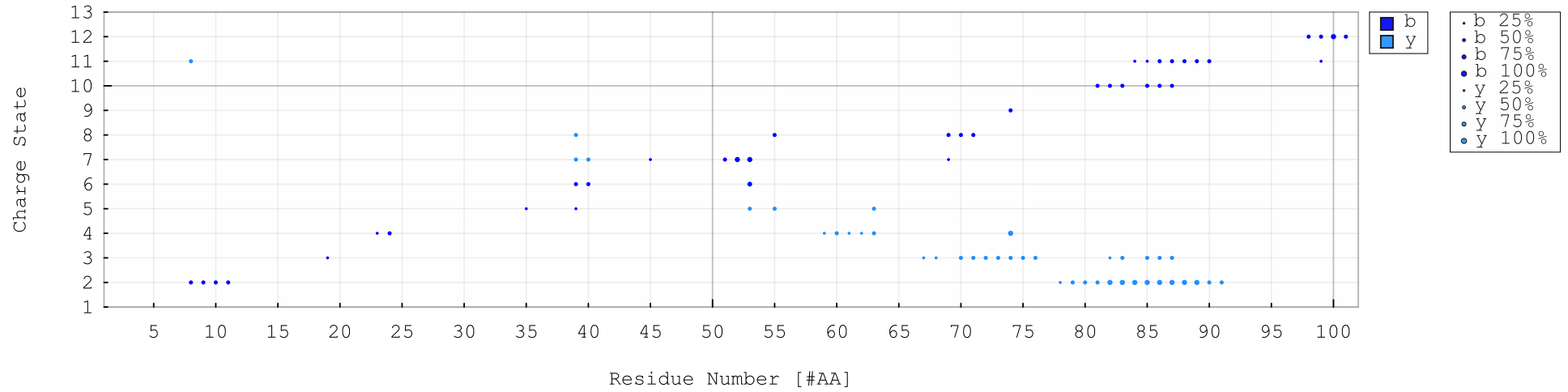

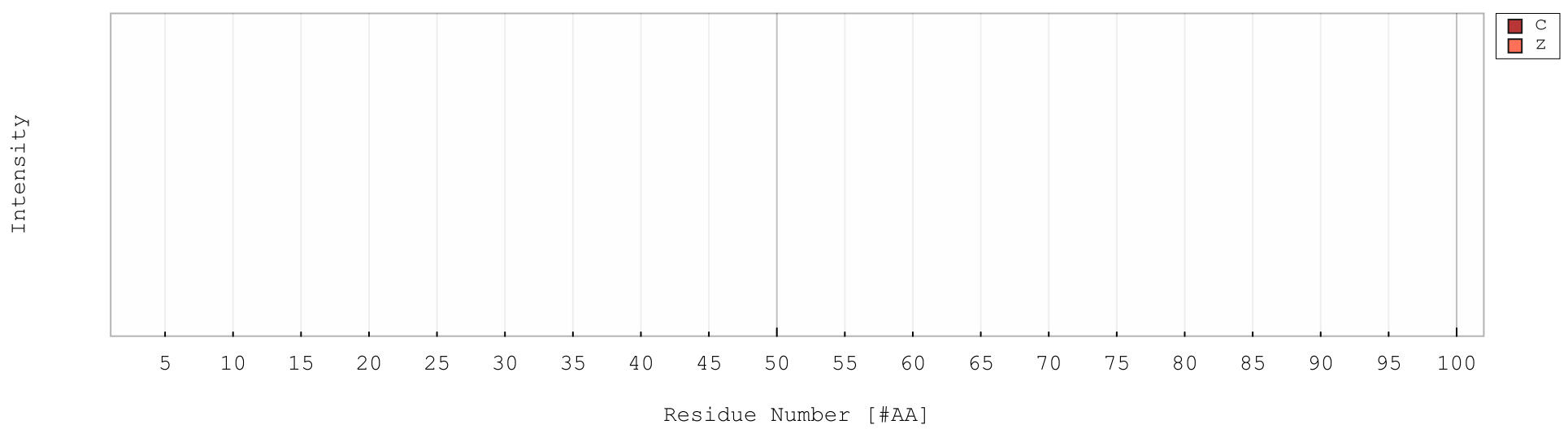

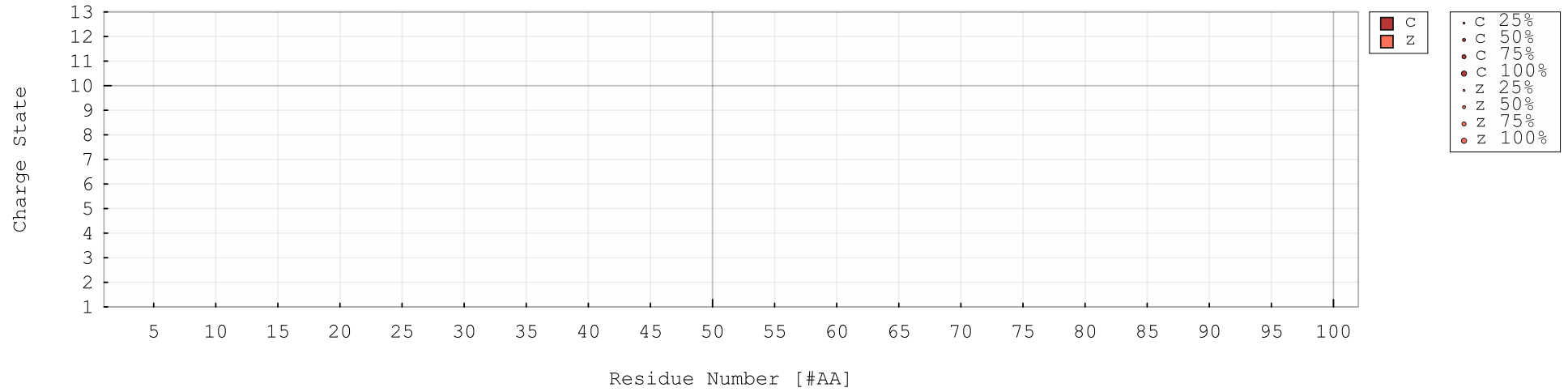

Top-Down Result OmniScape 2025
Sequence Confirmation
250428 H4-PCAF100-30min z13ac2 MS2 shCID_1-945_calibrated

Proteoform:

Index	Protein	SC [%]	SVP [%]	IC [%]	MS Score [a.u.]	Var. Mods	Mono Mass [Da]	Chemical Formula
10	H4Kac	49.5	9.8	27.9	13.8	Ace: K5, K8	11313.3624	C498H841N163O136S

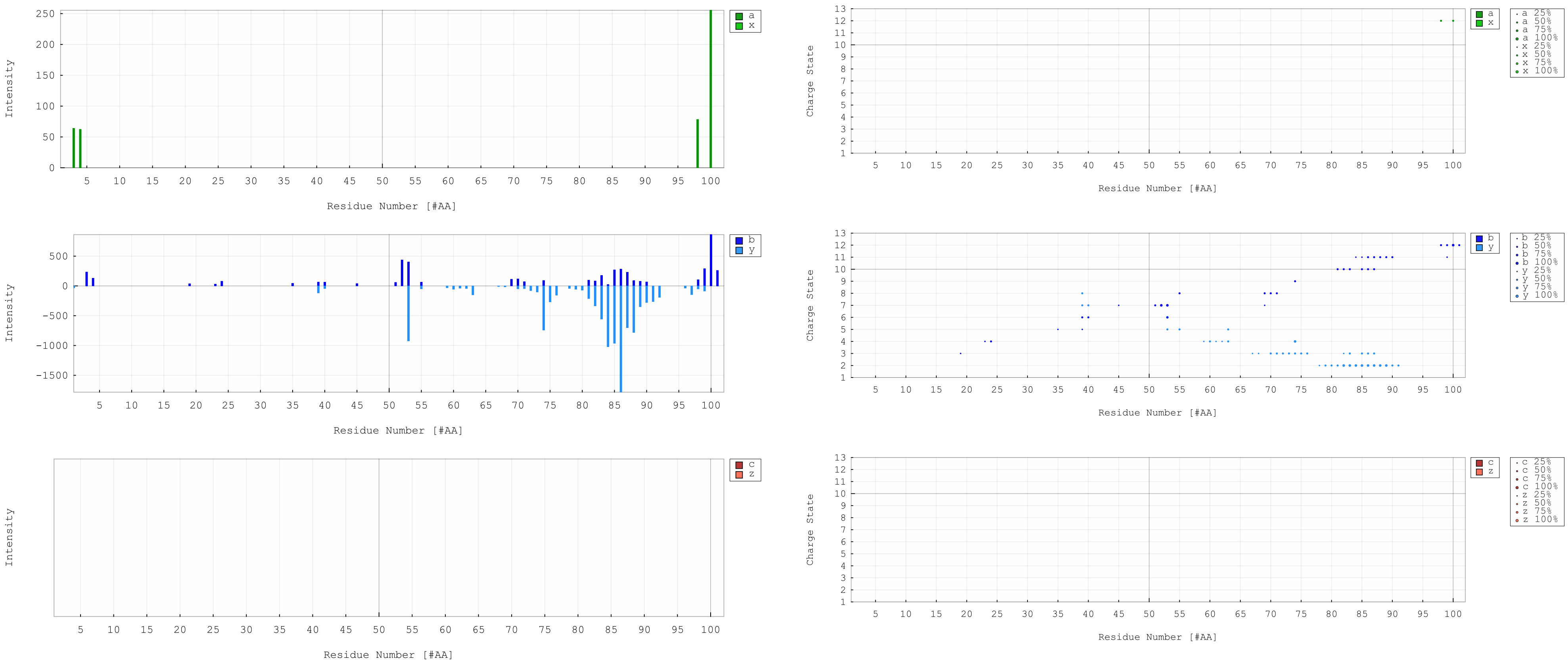

Supplemental Data S5

H4 - PCAF, 30 min

**Report**	7 of 8
**Experiment**	MS3
**Histone/sample**	H4
**Enzyme**	PCAF
**Reaction/source**	30 min
**Precursor**	H4Kac1
**Charge state**	13+
**Fragmentation / workflow**	MS2 RCID -> MS3 ECD
**Original file**	MS3_H4Kac1_13+_MS2-RCID_MS3-ECD.pdf

Project Information

Project Directory: C:/Users/LAB-ADMIN
Processing date: Tue Apr 28 14:47:23 2026

Sample Info & Protocols

Name: Top-Down Protein Sequencing ExD

Dataset

Imported spectrum file name:	250428 H4-PCAF100-30min z13ac1 MS2 shCID ACC MS3 ECD b52ac1_1-360_calibrated_calibrated2.txt
Dataset name:	Information is only available from imported JPEAKS files
Date acquired:	Information is only available from imported JPEAKS files
Date imported:	Information is only available from imported JPEAKS files
Instrument Name:	Information is only available from imported JPEAKS files
Instrument Serial Number:	Information is only available from imported JPEAKS files

Workflow Result Info

Analysis: 250428 H4-PCAF100-30min z13ac1 MS2 shCID ACC MS3 ECD b52ac1_1-360_calibrated4
Location: C:/Users/LAB-ADMIN
Method: -

Method Information

Name: Unnamed

Spectral Parameters

Mass Error [ppm]: 6
Intensity Deviation [au]: 0.1
Min/Max Charges: 1-7
Experiment type: ExD

a ions	b ions	c ions	x ions	y ions	z ions	satellite	internal	precursor
a	b	c		y	z			M
a+1		c+1			z+1			M+-2H
					z+2			

Noise threshold calculation: Simple moving average noise threshold (SMA)
Window size [datapoints]: 600
Noise curve offset: 0.2849536
SVP parameters: terminal gap: 2, internal gap: 1

Proteoforms

(top 7)

Index	Parent Sequence	Chain	Var. Mods	Mono Mass [Da]	Chemical Composition
1	H4[1-52]Kac	H4[1-52]KacK8[Ace]_Cterm[bIon-Cterm]	Ace: K8	5615.3054	C243H428N90O63
2	H4[1-52]Kac	H4[1-52]KacK12[Ace]_Cterm[bIon-Cterm]	Ace: K12	5615.3054	C243H428N90O63
3	H4[1-52]Kac	H4[1-52]KacK16[Ace]_Cterm[bIon-Cterm]	Ace: K16	5615.3054	C243H428N90O63
4	H4[1-52]Kac	H4[1-52]KacK5[Ace]_Cterm[bIon-Cterm]	Ace: K5	5615.3054	C243H428N90O63
5	H4[1-52]Kac	H4[1-52]KacK20[Ace]_Cterm[bIon-Cterm]	Ace: K20	5615.3054	C243H428N90O63
6	H4[1-52]Kac	H4[1-52]KacK31[Ace]_Cterm[bIon-Cterm]	Ace: K31	5615.3054	C243H428N90O63
7	H4[1-52]Kac	H4[1-52]KacK44[Ace]_Cterm[bIon-Cterm]	Ace: K44	5615.3054	C243H428N90O63

Results

Proteoforms Table (top 7)

Index	Protein	SC [%]	SVP [%]	IC [%]	MS Score [a.u.]	Var. Mods	Mono Mass [Da]	Chemical Formula
1	H4[1-52]Kac	82.4	80.8	34.1	28.1	Ace: K8	5615.3054	C243H428N90O63
2	H4[1-52]Kac	78.4	21.2	33.2	26.1	Ace: K12	5615.3054	C243H428N90O63
3	H4[1-52]Kac	82.4	21.2	30.4	25.0	Ace: K16	5615.3054	C243H428N90O63
4	H4[1-52]Kac	78.4	11.5	31.8	24.9	Ace: K5	5615.3054	C243H428N90O63
5	H4[1-52]Kac	80.4	21.2	25.1	20.2	Ace: K20	5615.3054	C243H428N90O63
6	H4[1-52]Kac	78.4	48.1	17.1	13.4	Ace: K31	5615.3054	C243H428N90O63
7	H4[1-52]Kac	64.7	48.1	11.6	7.5	Ace: K44	5615.3054	C243H428N90O63

Sequence

Name: H4[1-52]Kac

10 20 30 40 50 S G R G K G G K G
L G K G G A K R H R K V L R D N I Q G I T K P A I R R L A R R G G V K R I S G L I Y E

Modifications

Modification	Position	Type
bIon-Cterm	C-term	fixed
Acetyl	5	variable
Acetyl	8	variable
Acetyl	12	variable
Acetyl	16	variable
Acetyl	20	variable
Acetyl	31	variable
Acetyl	44	variable

Sequence Map

Proteoform:	Index	Protein	SC [%]	SVP [%]	IC [%]	MS Score [a.u.]	Var. Mods	Mono Mass [Da]	Chemical Formula
	1	H4[1-52]Kac	82.4	80.8	34.1	28.1	Ace: K8	5615.3054	C243H428N90O63

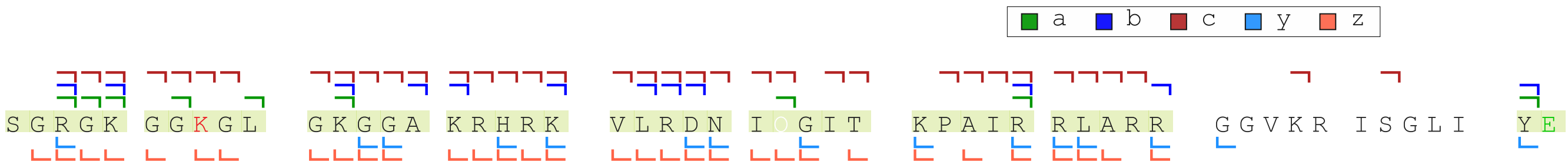

Proteoform:

Index	Protein	SC [%]	SVP [%]	IC [%]	MS Score [a.u.]	Var. Mods	Mono Mass [Da]	Chemical Formula
2	H4[1-52]Kac	78.4	21.2	33.2	26.1	Ace: K12	5615.3054	C243H428N90O63

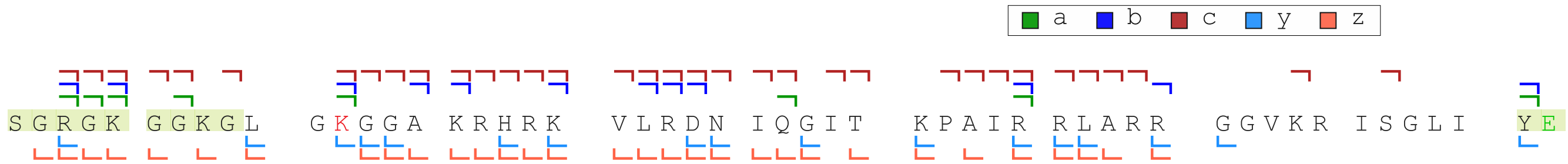

Proteoform:	Index	Protein	SC [%]	SVP [%]	IC [%]	MS Score [a.u.]	Var. Mods	Mono Mass [Da]	Chemical Formula
	3	H4[1-52]Kac	82.4	21.2	30.4	25.0	Ace: K16	5615.3054	C243H428N90O63

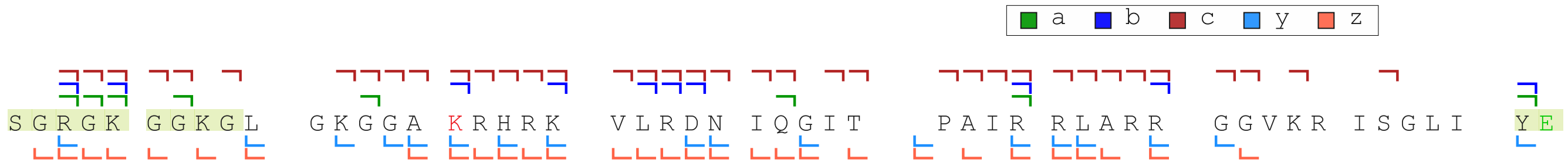

Proteoform:

Index	Protein	SC [%]	SVP [%]	IC [%]	MS Score [a.u.]	Var. Mods	Mono Mass [Da]	Chemical Formula
4	H4[1-52]Kac	78.4	11.5	31.8	24.9	Ace: K5	5615.3054	C243H428N90O63

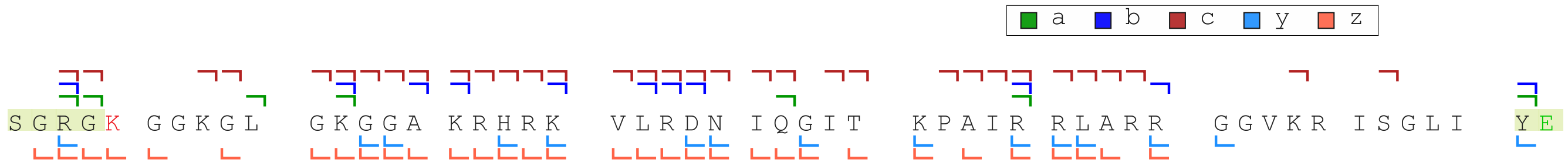

Proteoform:

Index	Protein	SC [%]	SVP [%]	IC [%]	MS Score [a.u.]	Var. Mods	Mono Mass [Da]	Chemical Formula
5	H4[1-52]Kac	80.4	21.2	25.1	20.2	Ace: K20	5615.3054	C243H428N90O63

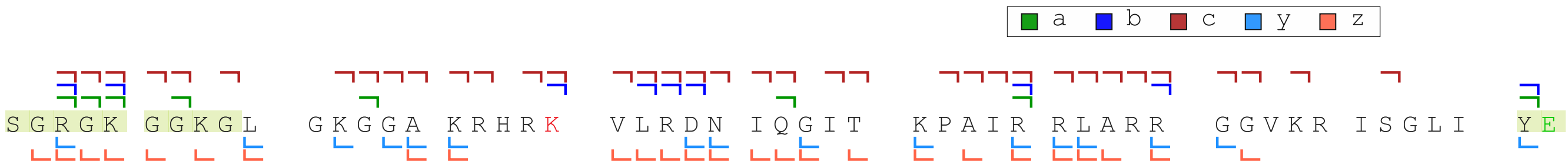

Top-Down Result OmniScape 2025
Sequence Confirmation
250428 H4-PCAF100-30min z13ac1 MS2 shCID ACC MS3 ECD b52ac1_1-360_-calibrated4

Proteoform:

Index	Protein	SC [%]	SVP [%]	IC [%]	MS Score [a.u.]	Var. Mods	Mono Mass [Da]	Chemical Formula
6	H4[1-52]Kac	78.4	48.1	17.1	13.4	Ace: K31	5615.3054	C243H428N90O63

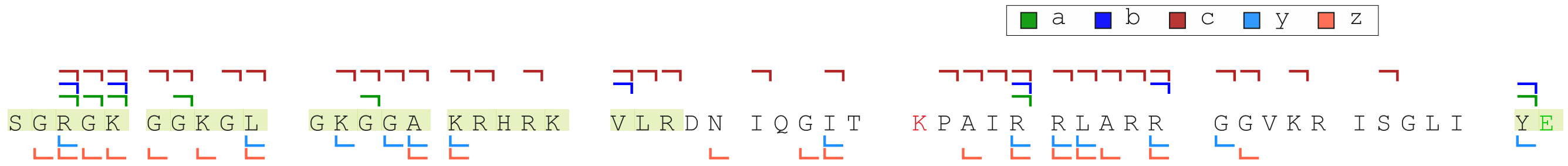

Proteoform:

Index	Protein	SC [%]	SVP [%]	IC [%]	MS Score [a.u.]	Var. Mods	Mono Mass [Da]	Chemical Formula
7	H4[1-52]Kac	64.7	48.1	11.6	7.5	Ace: K44	5615.3054	C243H428N90O63

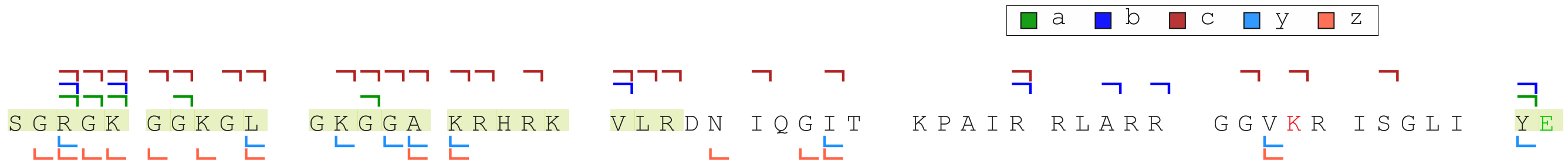

Error Plot

Proteoform:

Index	Protein	SC [%]	SVP [%]	IC [%]	MS Score [a.u.]	Var. Mods	Mono Mass [Da]	Chemical Formula
1	H4[1-52]Kac	82.4	80.8	34.1	28.1	Ace: K8	5615.3054	C243H428N90O63

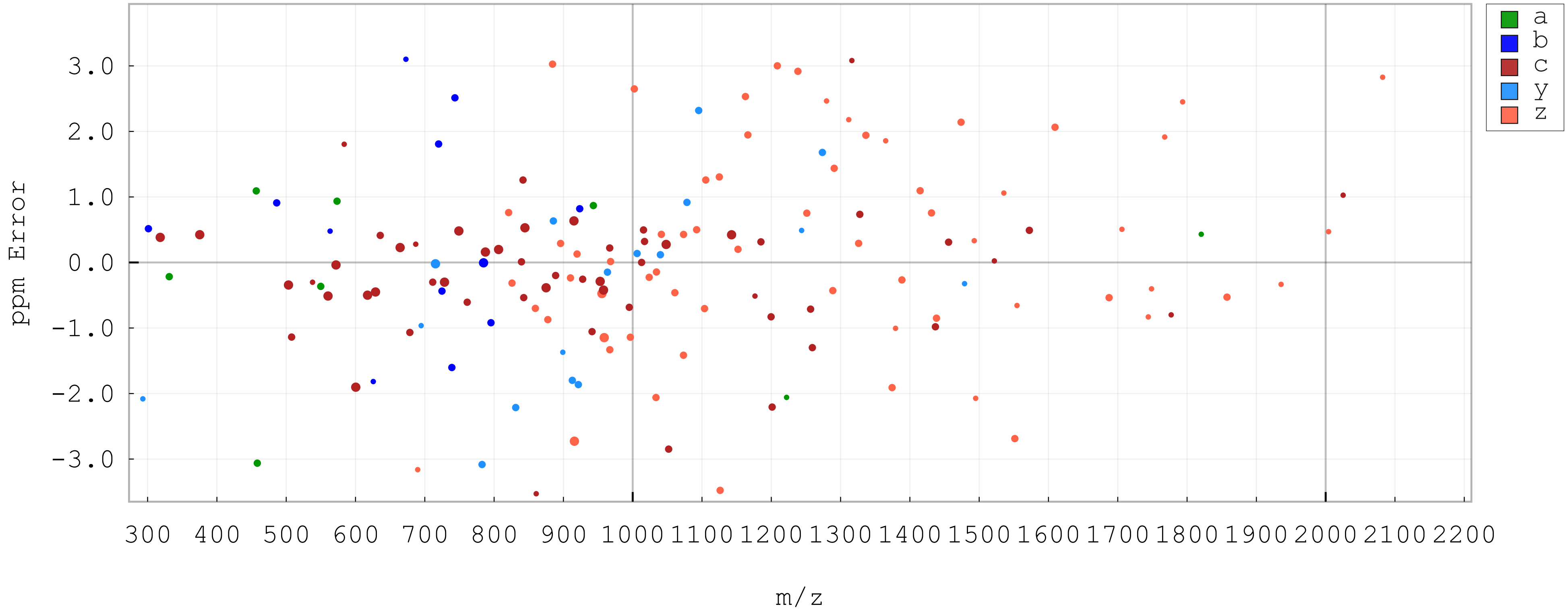

Proteoform:

Index	Protein	SC [%]	SVP [%]	IC [%]	MS Score [a.u.]	Var. Mods	Mono Mass [Da]	Chemical Formula
2	H4[1-52]Kac	78.4	21.2	33.2	26.1	Ace: K12	5615.3054	C243H428N90O63

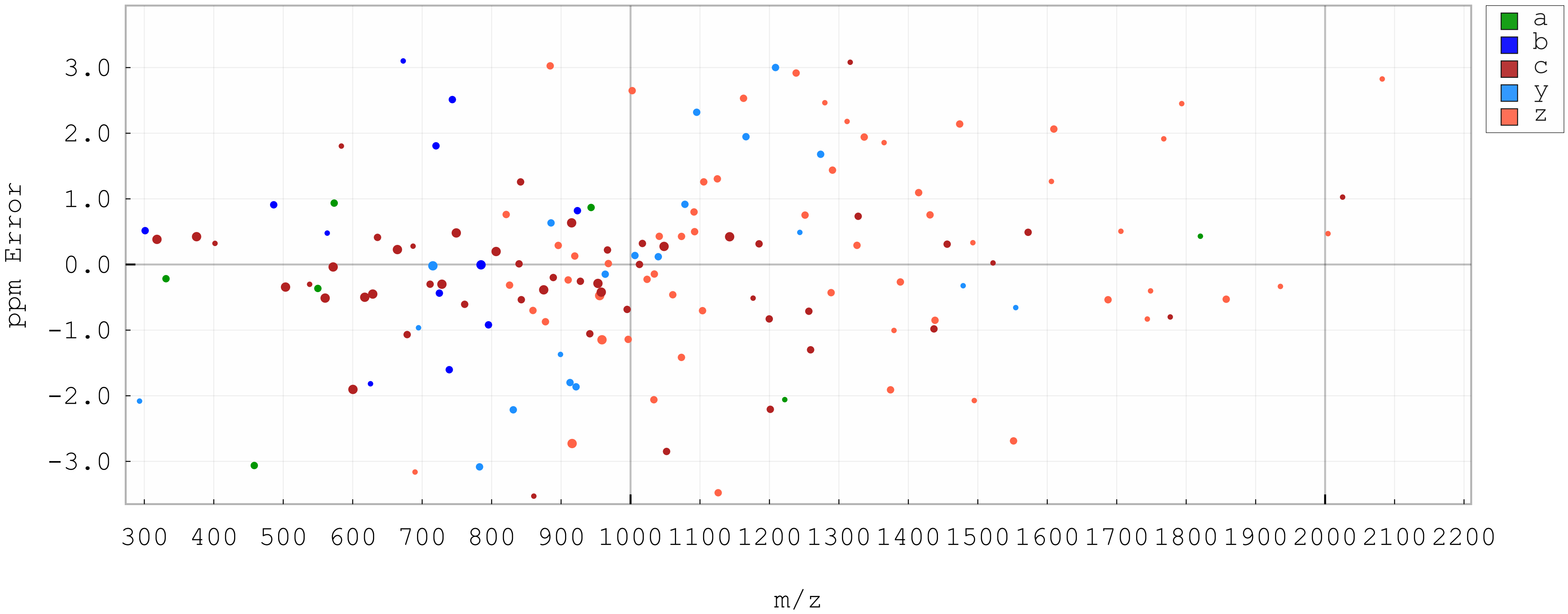

Proteoform:

Index	Protein	SC [%]	SVP [%]	IC [%]	MS Score [a.u.]	Var. Mods	Mono Mass [Da]	Chemical Formula
3	H4[1-52]Kac	82.4	21.2	30.4	25.0	Ace: K16	5615.3054	C243H428N90O63

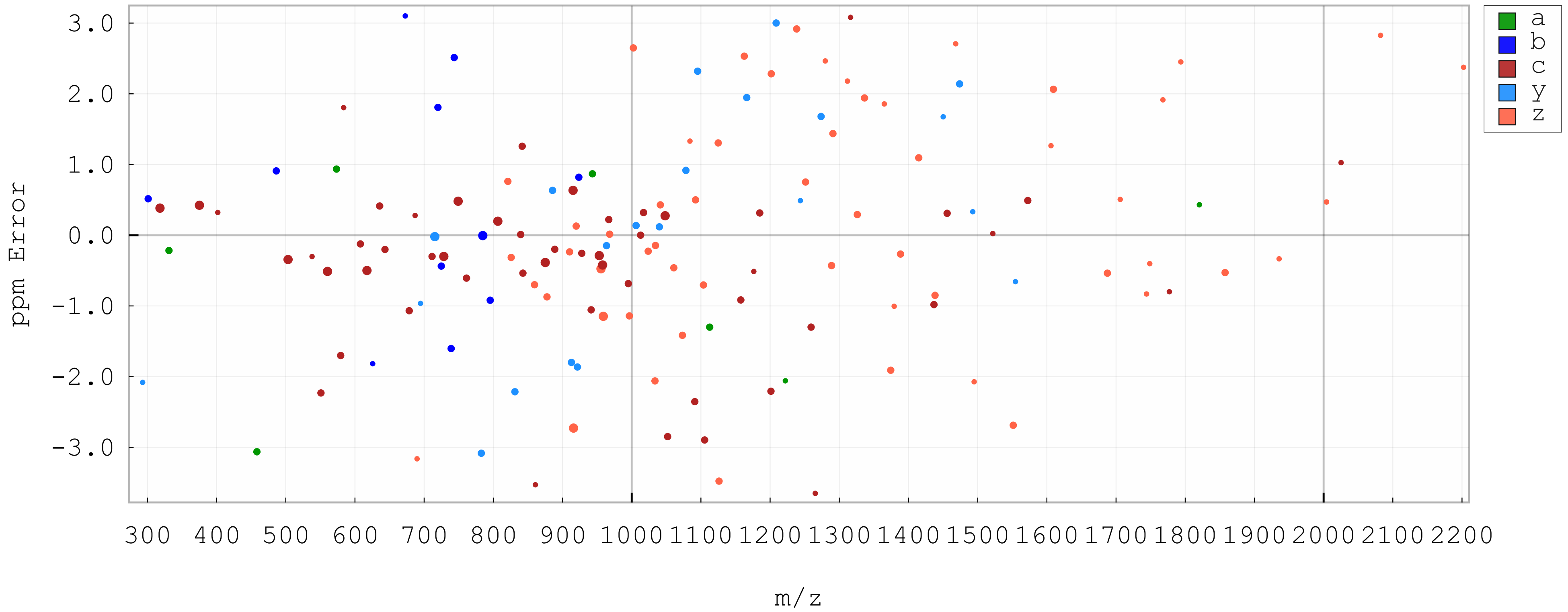

Proteoform:

Index	Protein	SC [%]	SVP [%]	IC [%]	MS Score [a.u.]	Var. Mods	Mono Mass [Da]	Chemical Formula
4	H4[1-52]Kac	78.4	11.5	31.8	24.9	Ace: K5	5615.3054	C243H428N90O63

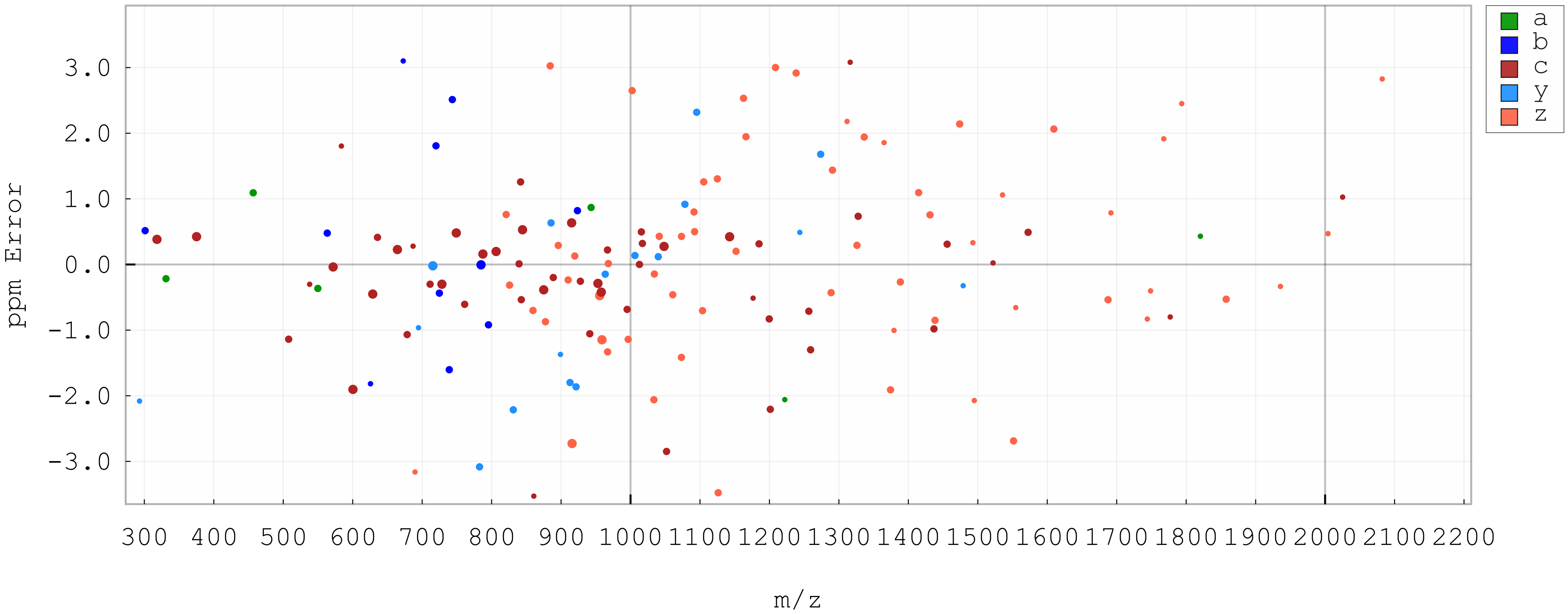

Proteoform:

Index	Protein	SC [%]	SVP [%]	IC [%]	MS Score [a.u.]	Var. Mods	Mono Mass [Da]	Chemical Formula
5	H4[1-52]Kac	80.4	21.2	25.1	20.2	Ace: K20	5615.3054	C243H428N90O63

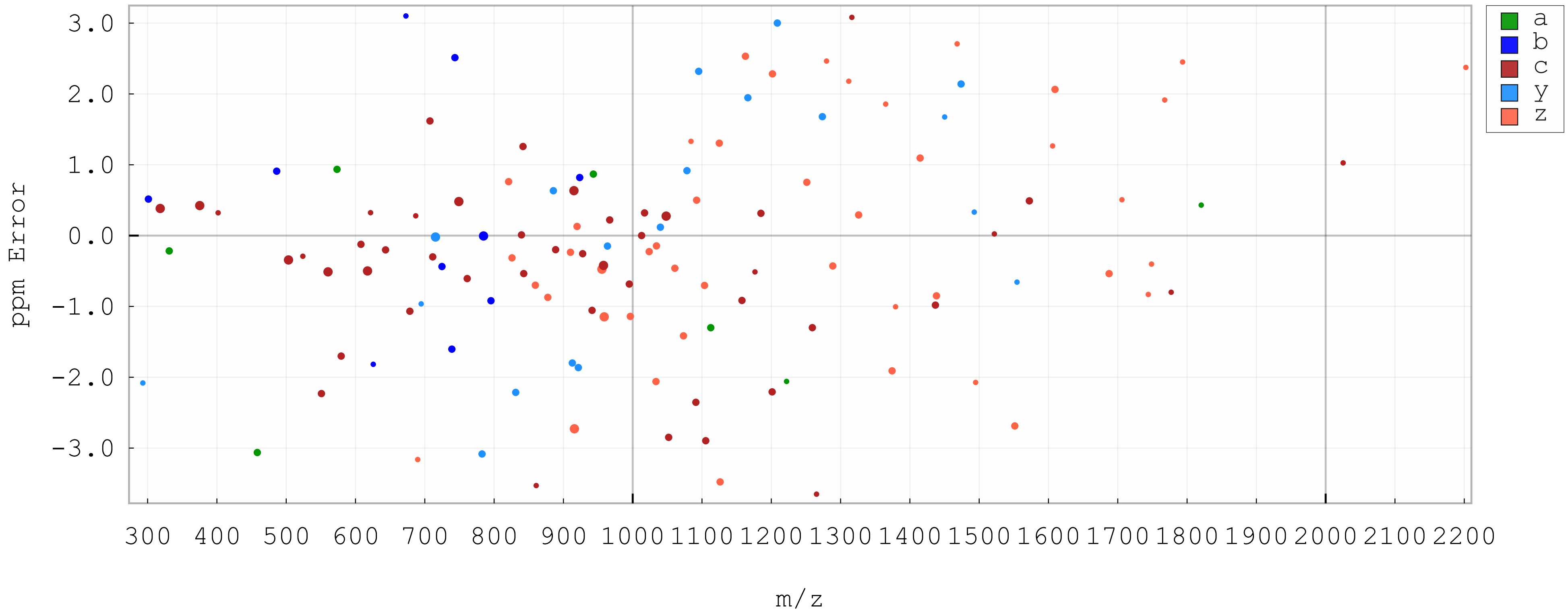

Proteoform:

Index	Protein	SC [%]	SVP [%]	IC [%]	MS Score [a.u.]	Var. Mods	Mono Mass [Da]	Chemical Formula
6	H4[1-52]Kac	78.4	48.1	17.1	13.4	Ace: K31	5615.3054	C243H428N90O63

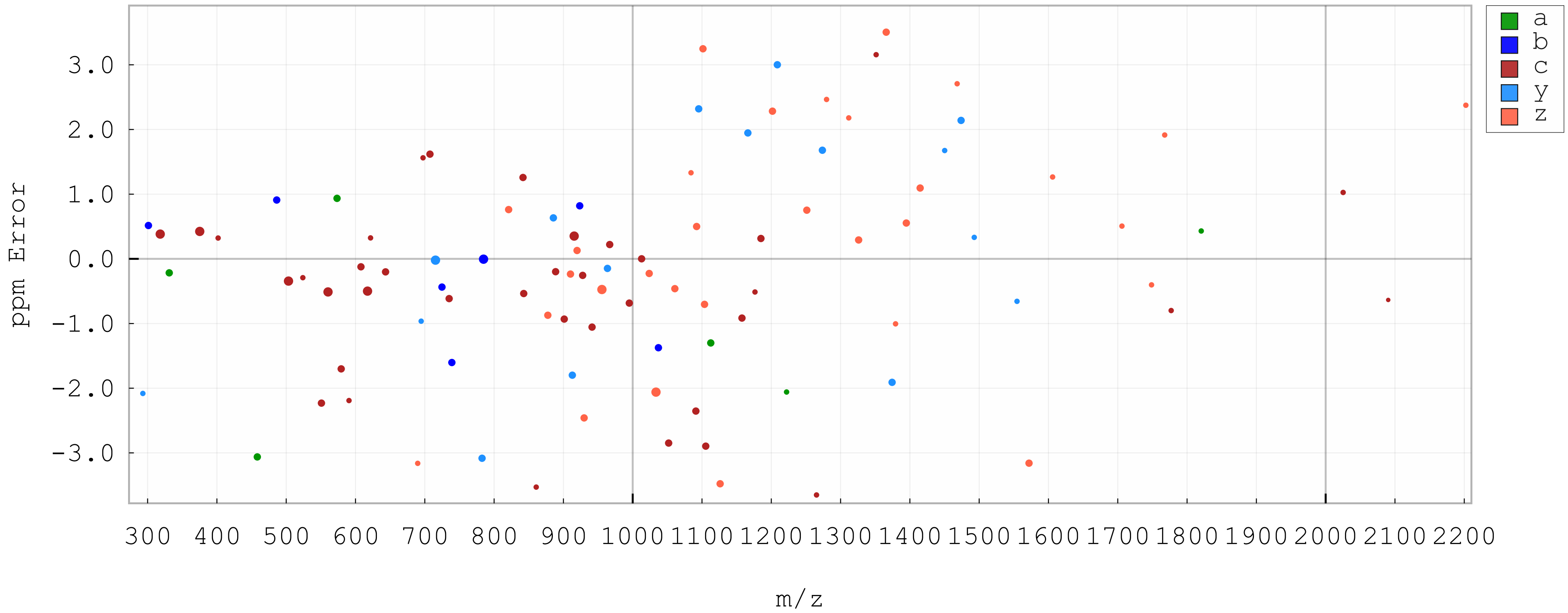

Proteoform:

Index	Protein	SC [%]	SVP [%]	IC [%]	MS Score [a.u.]	Var. Mods	Mono Mass [Da]	Chemical Formula
7	H4[1-52]Kac	64.7	48.1	11.6	7.5	Ace: K44	5615.3054	C243H428N90O63

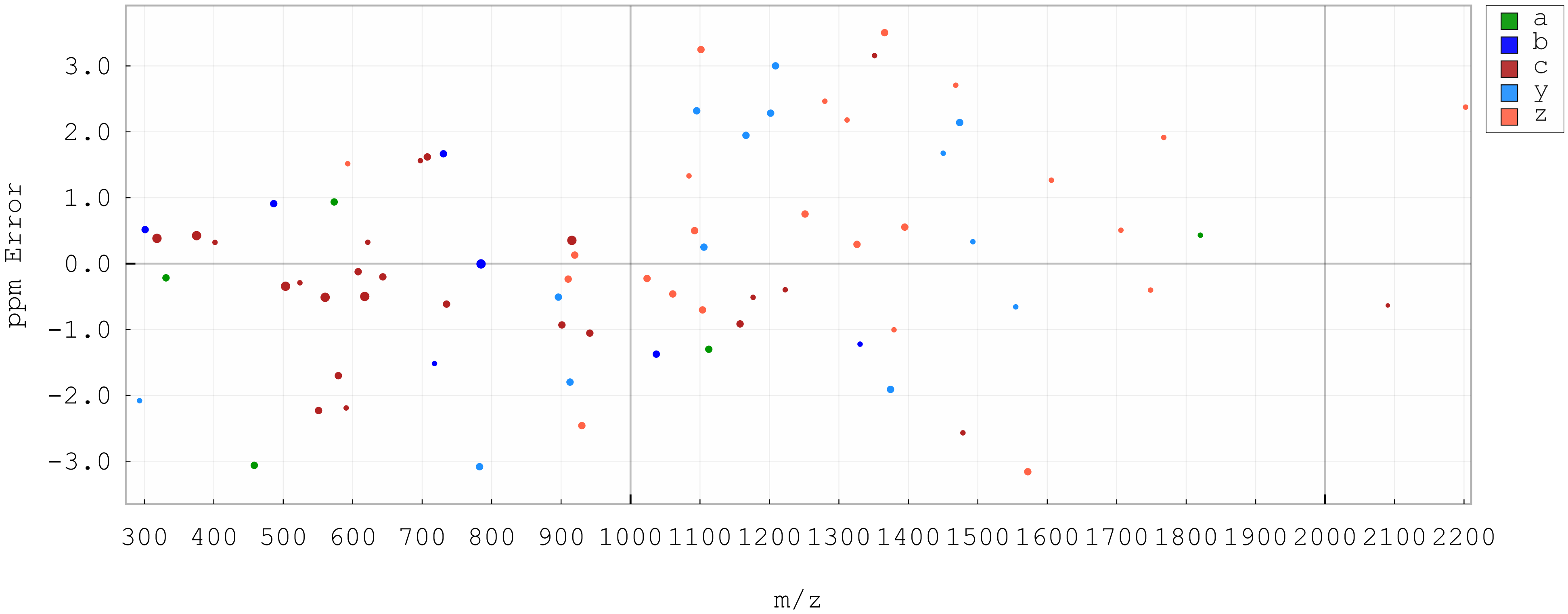

Primary Fragment Plots

Proteoform:

Index	Protein	SC [%]	SVP [%]	IC [%]	MS Score [a.u.]	Var. Mods	Mono Mass [Da]	Chemical Formula
1	H4[1-52]Kac	82.4	80.8	34.1	28.1	Ace: K8	5615.3054	C243H428N90O63

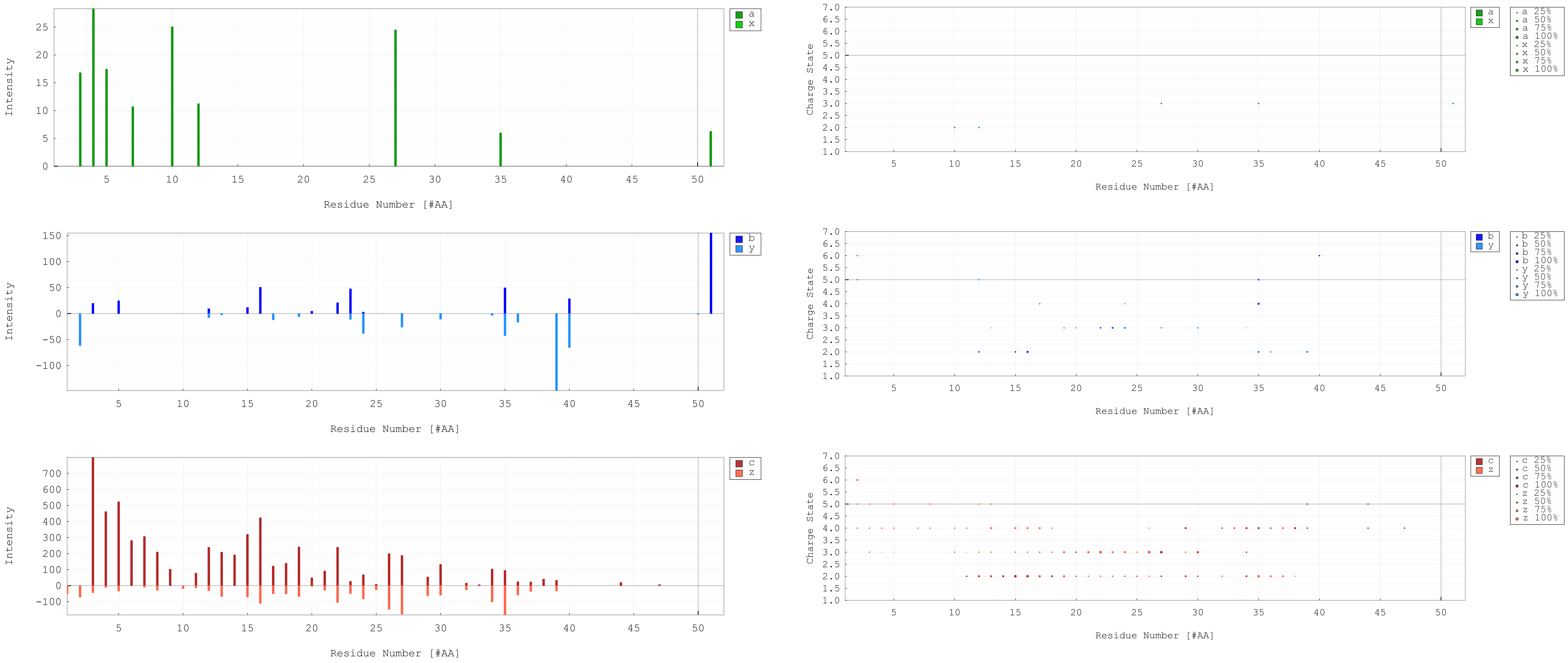

Top-Down Result OmniScape 2025
Sequence Confirmation
250428 H4-PCAF100-30min z13ac1 MS2 shCID ACC MS3 ECD b52ac1_1-360_-calibrated4

Proteoform:

Index	Protein	SC [%]	SVP [%]	IC [%]	MS Score [a.u.]	Var. Mods	Mono Mass [Da]	Chemical Formula
2	H4[1-52]Kac	78.4	21.2	33.2	26.1	Ace: K12	5615.3054	C243H428N90O63

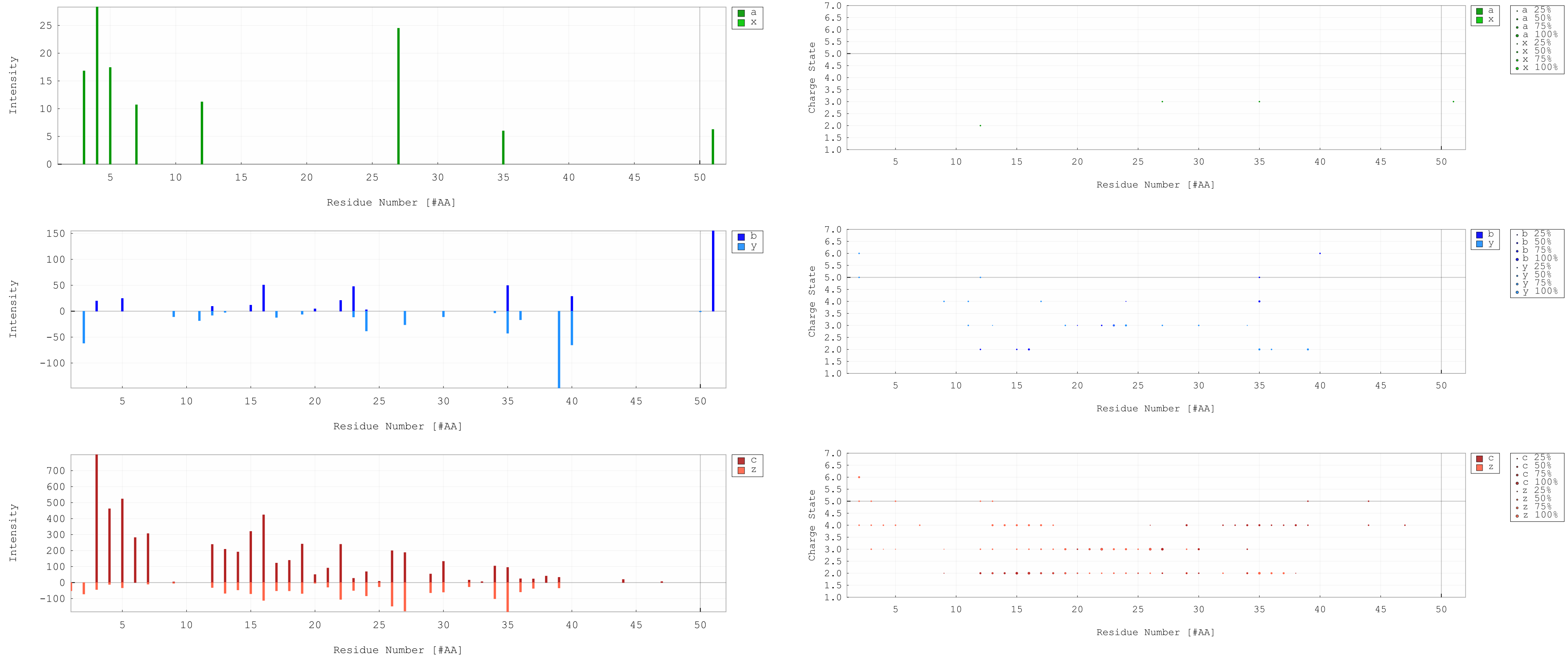

Proteoform:

Index	Protein	SC [%]	SVP [%]	IC [%]	MS Score [a.u.]	Var. Mods	Mono Mass [Da]	Chemical Formula
3	H4[1-52]Kac	82.4	21.2	30.4	25.0	Ace: K16	5615.3054	C243H428N90O63

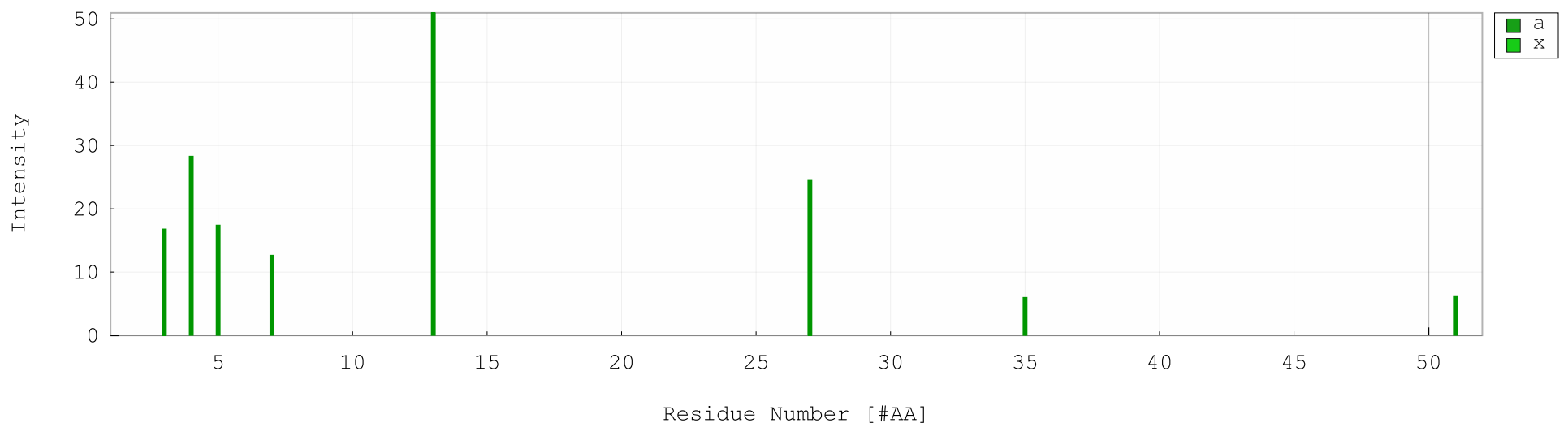

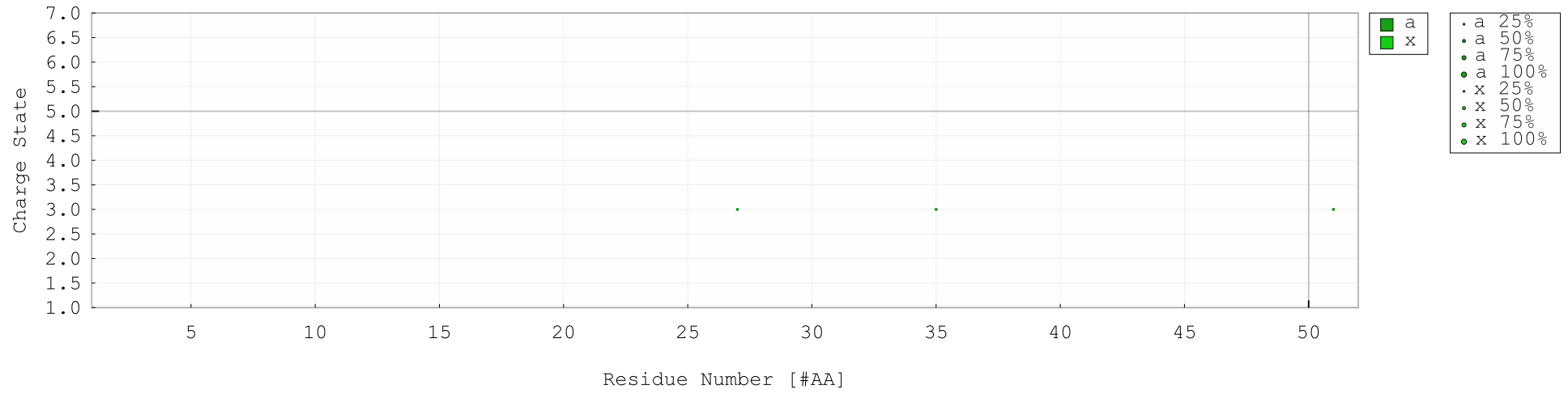

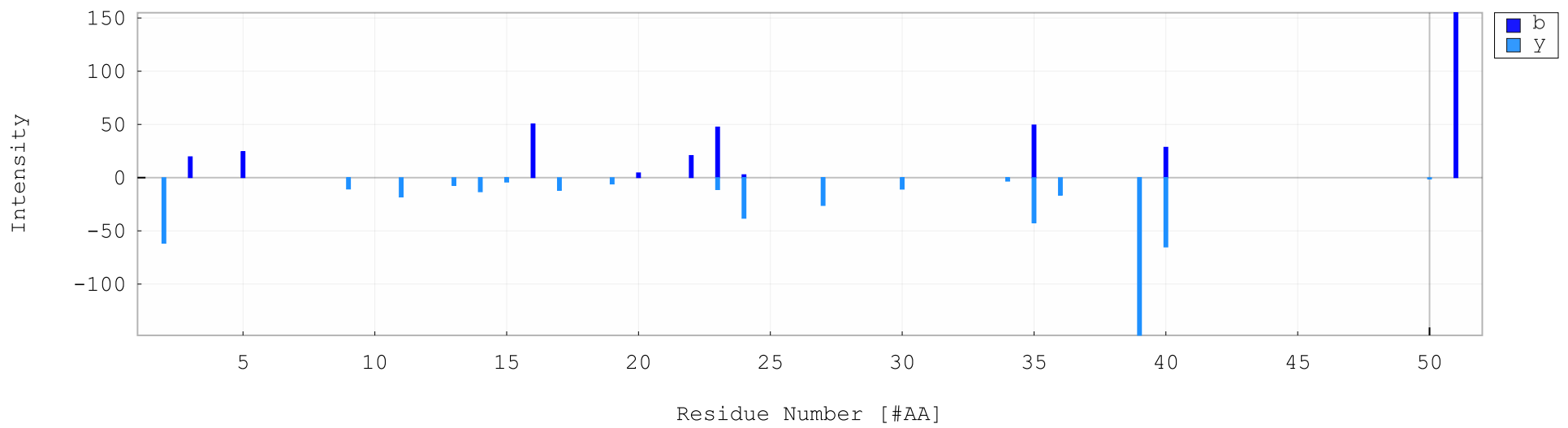

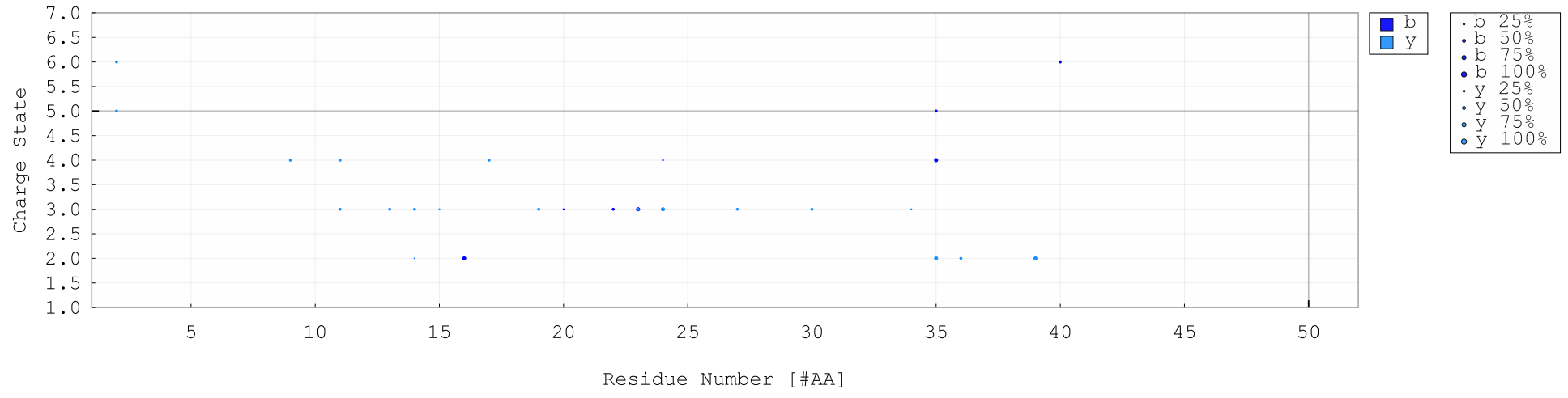

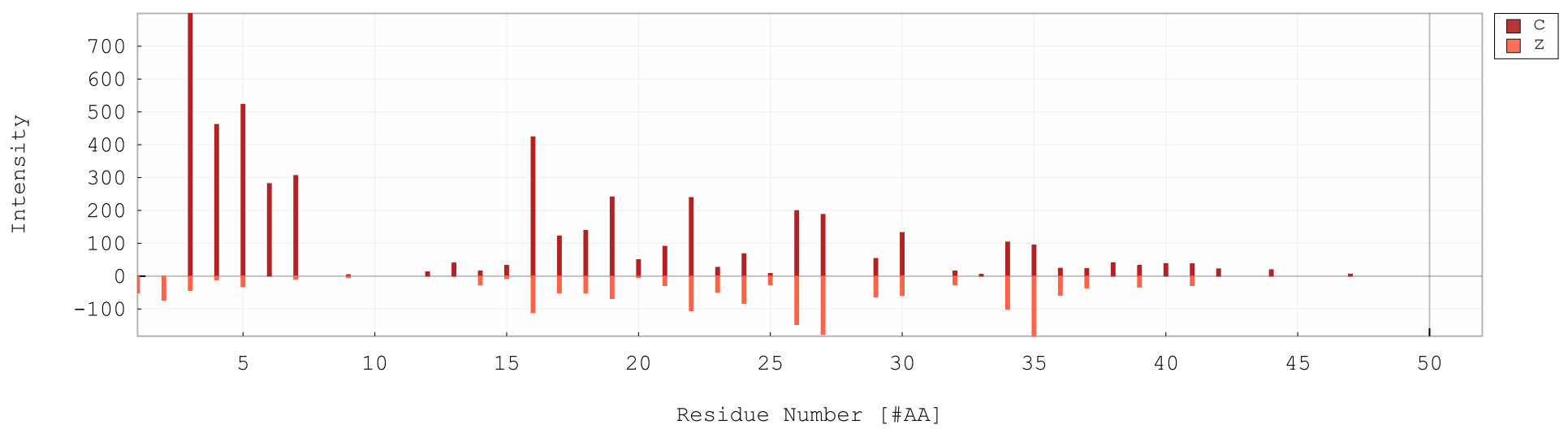

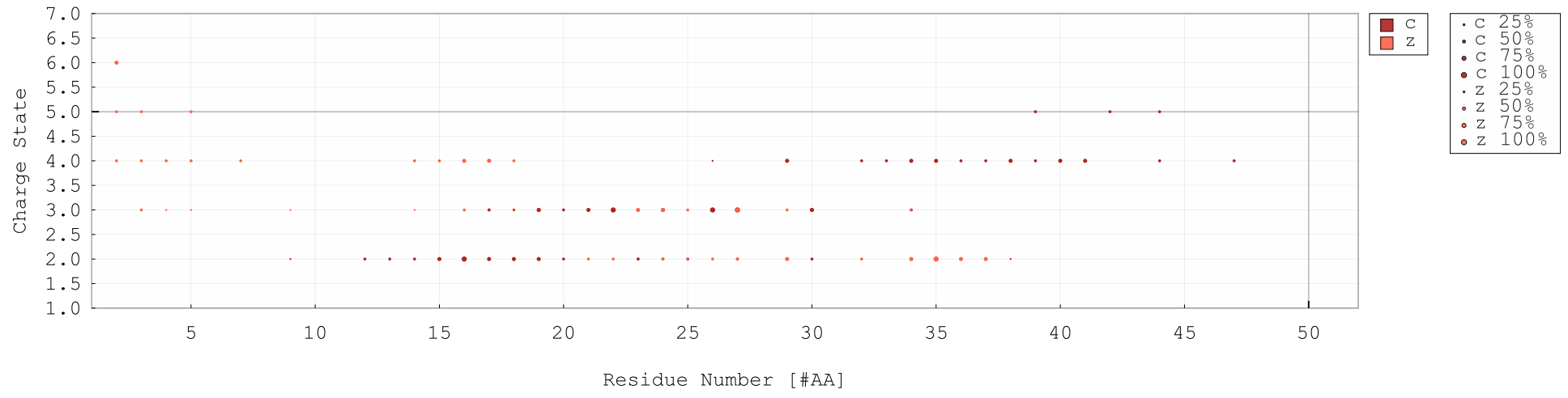

Proteoform:

Index	Protein	SC [%]	SVP [%]	IC [%]	MS Score [a.u.]	Var. Mods	Mono Mass [Da]	Chemical Formula
4	H4[1-52]Kac	78.4	11.5	31.8	24.9	Ace: K5	5615.3054	C243H428N90O63

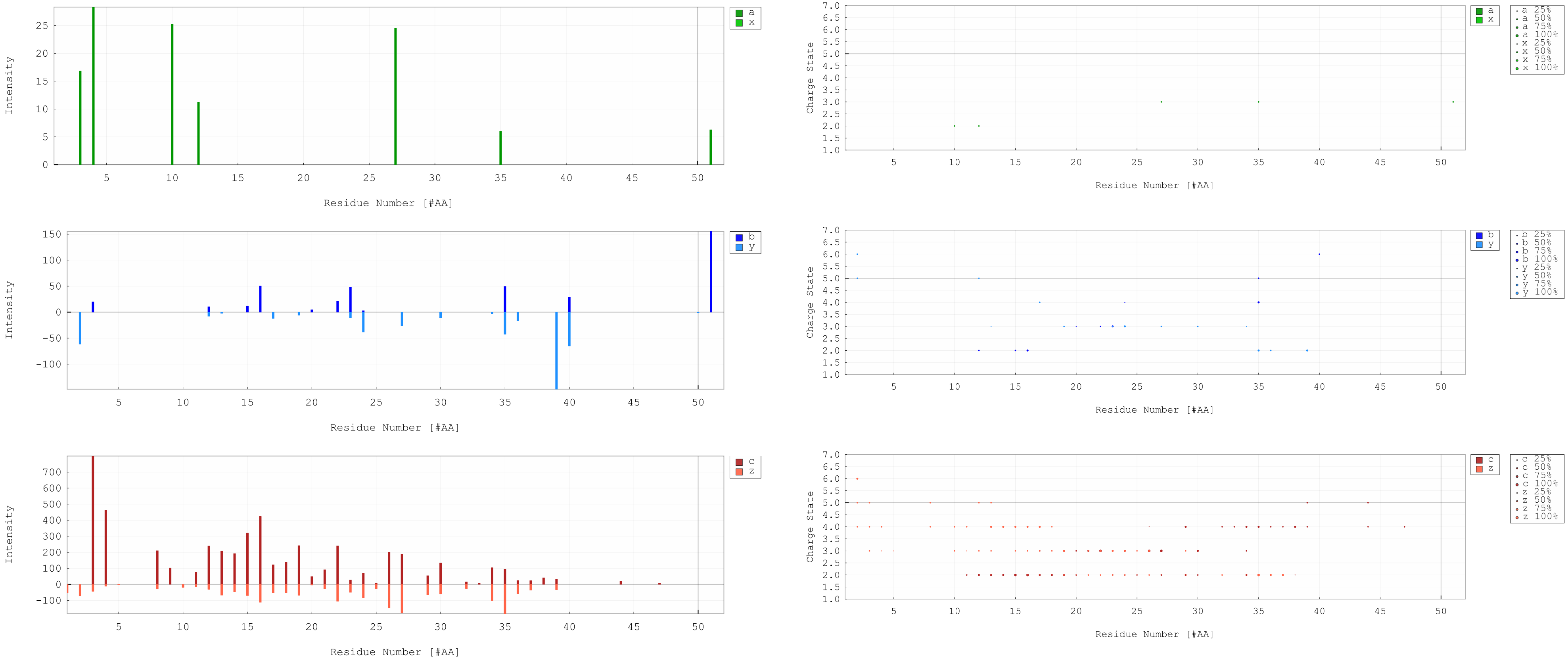

Top-Down Result OmniScape 2025
Sequence Confirmation
250428 H4-PCAF100-30min z13ac1 MS2 shCID ACC MS3 ECD b52ac1_1-360_-calibrated4

Proteoform:

Index	Protein	SC [%]	SVP [%]	IC [%]	MS Score [a.u.]	Var. Mods	Mono Mass [Da]	Chemical Formula
5	H4[1-52]Kac	80.4	21.2	25.1	20.2	Ace: K20	5615.3054	C243H428N90O63

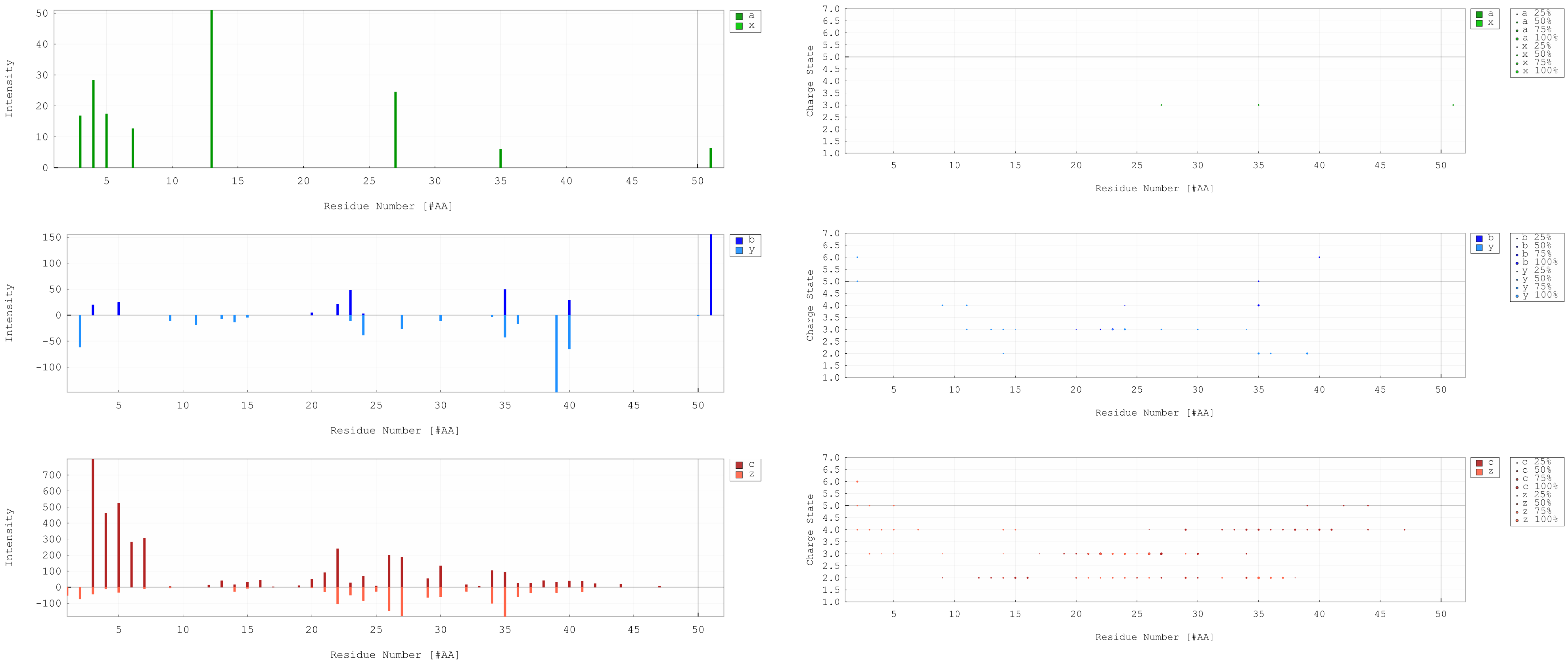

Proteoform:

Index	Protein	SC [%]	SVP [%]	IC [%]	MS Score [a.u.]	Var. Mods	Mono Mass [Da]	Chemical Formula
6	H4[1-52]Kac	78.4	48.1	17.1	13.4	Ace: K31	5615.3054	C243H428N90O63

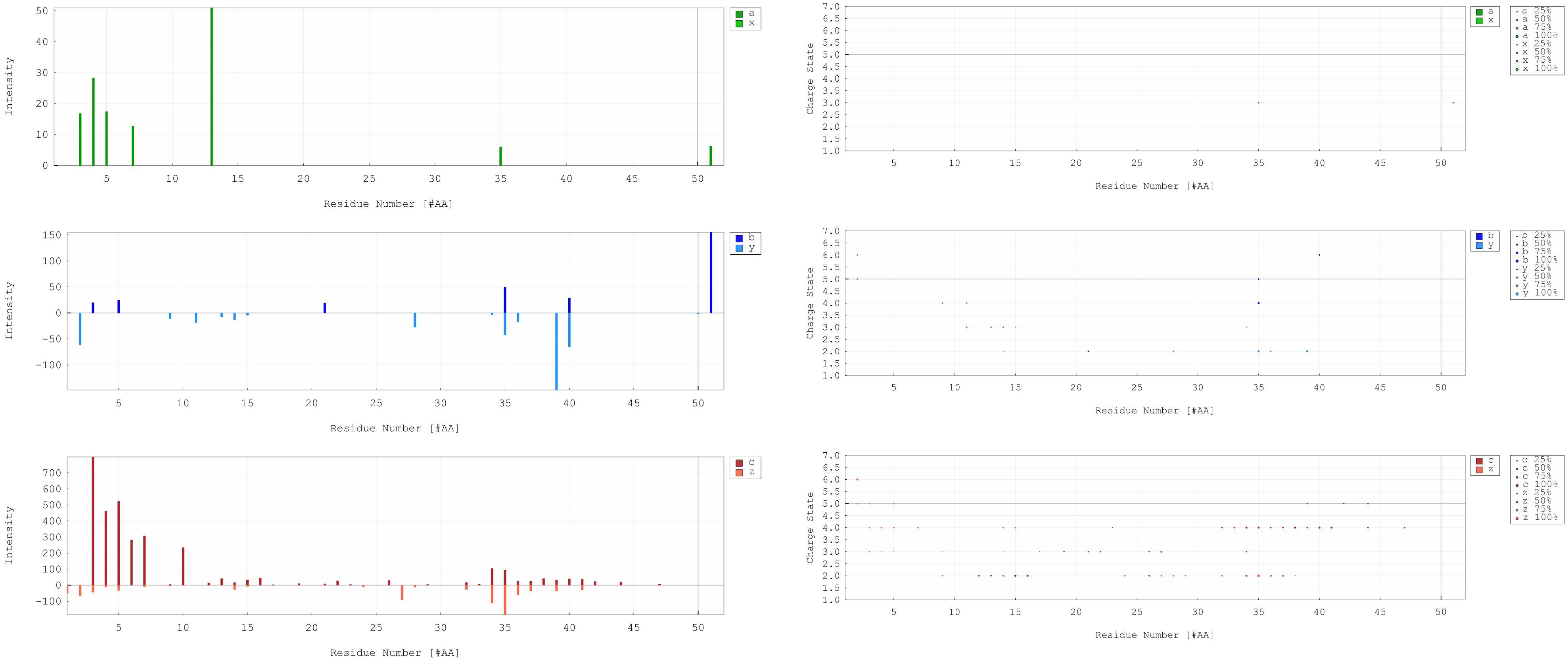

Proteoform:

Index	Protein	SC [%]	SVP [%]	IC [%]	MS Score [a.u.]	Var. Mods	Mono Mass [Da]	Chemical Formula
7	H4[1-52]Kac	64.7	48.1	11.6	7.5	Ace: K44	5615.3054	C243H428N90O63

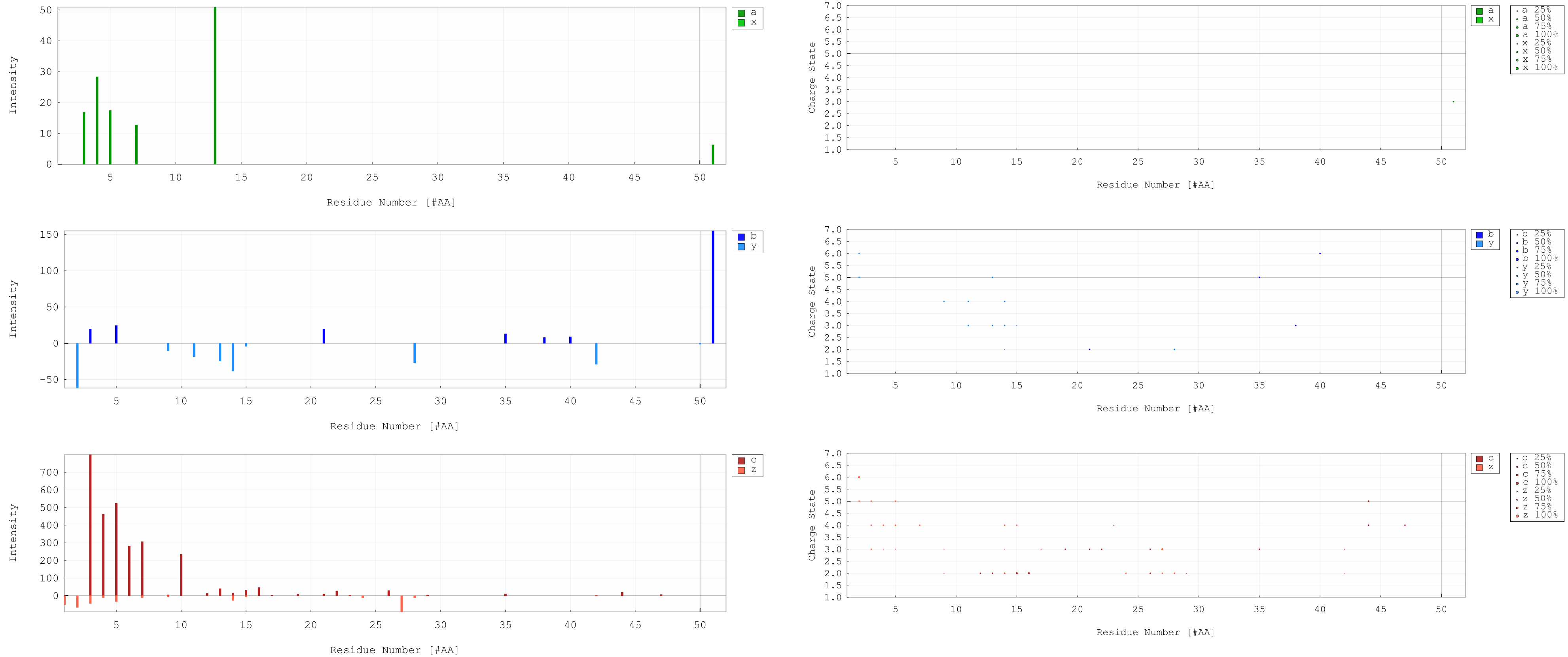

Supplemental Data S5

H4 - PCAF, 30 min

**Report**	8 of 8
**Experiment**	MS3
**Histone/sample**	H4
**Enzyme**	PCAF
**Reaction/source**	30 min
**Precursor**	H4Kac2
**Charge state**	13+
**Fragmentation / workflow**	MS2 RCID -> MS3 ECD
**Original file**	MS3_H4Kac2_13+_MS2-RCID_MS3-ECD.pdf

Project Information

Project Directory: C:/Users/LAB-ADMIN
Processing date: Tue Apr 28 14:57:07 2026

Sample Info & Protocols

Name: Top-Down Protein Sequencing ExD

Dataset

Imported spectrum file name:	250428 H4-PCAF100-30min z13ac2 MS2 shCID ACC MS3 ECD b52ac2_001-225_calibrated2.txt
Dataset name:	Information is only available from imported JPEAKS files
Date acquired:	Information is only available from imported JPEAKS files
Date imported:	Information is only available from imported JPEAKS files
Instrument Name:	Information is only available from imported JPEAKS files
Instrument Serial Number:	Information is only available from imported JPEAKS files

Workflow Result Info

Analysis: 250428 H4-PCAF100-30min z13ac2 MS2 shCID ACC MS3 ECD b52ac2_001-225_calibrated2
Location: C:/Users/LAB-ADMIN
Method: -

Method Information

Name: Unnamed

Spectral Parameters

Mass Error [ppm]: 6
Intensity Deviation [au]: 0.05
Min/Max Charges: 1-7
Experiment type: ExD

a ions	b ions	c ions	x ions	y ions	z ions	satellite	internal	precursor
a	b	c		y	z			M
a+1		c+1			z+1			M+-2H
					z+2			

Noise threshold calculation: Simple moving average noise threshold (SMA)
Window size [datapoints]: 600
Noise curve offset: 0.13890007
SVP parameters: terminal gap: 2, internal gap: 1

Proteoforms

(top 10)

Index	Parent Sequence	Chain	Var. Mods	Mono Mass [Da]	Chemical Composition
1	H4[1-52]Kac	H4[1-52]KacK8[Ace]K31[Ace]_Cterm[bIon-Cterm]	Ace: K8, K31	5657.3160	C245H430N90O64
2	H4[1-52]Kac	H4[1-52]KacK8[Ace]K44[Ace]_Cterm[bIon-Cterm]	Ace: K8, K44	5657.3160	C245H430N90O64
3	H4[1-52]Kac	H4[1-52]KacK12[Ace]K31[Ace]_Cterm[bIon-Cterm]	Ace: K12, K31	5657.3160	C245H430N90O64
4	H4[1-52]Kac	H4[1-52]KacK8[Ace]K20[Ace]_Cterm[bIon-Cterm]	Ace: K8, K20	5657.3160	C245H430N90O64
5	H4[1-52]Kac	H4[1-52]KacK12[Ace]K44[Ace]_Cterm[bIon-Cterm]	Ace: K12, K44	5657.3160	C245H430N90O64
6	H4[1-52]Kac	H4[1-52]KacK8[Ace]K16[Ace]_Cterm[bIon-Cterm]	Ace: K8, K16	5657.3160	C245H430N90O64
7	H4[1-52]Kac	H4[1-52]KacK5[Ace]K31[Ace]_Cterm[bIon-Cterm]	Ace: K5, K31	5657.3160	C245H430N90O64
8	H4[1-52]Kac	H4[1-52]KacK16[Ace]K31[Ace]_Cterm[bIon-Cterm]	Ace: K16, K31	5657.3160	C245H430N90O64
9	H4[1-52]Kac	H4[1-52]KacK12[Ace]K20[Ace]_Cterm[bIon-Cterm]	Ace: K12, K20	5657.3160	C245H430N90O64
10	H4[1-52]Kac	H4[1-52]KacK5[Ace]K44[Ace]_Cterm[bIon-Cterm]	Ace: K5, K44	5657.3160	C245H430N90O64

Results

Proteoforms Table (top 10)

Index	Protein	SC [%]	SVP [%]	IC [%]	MS Score [a.u.]	Var. Mods	Mono Mass [Da]	Chemical Formula
1	H4[1-52]Kac	64.7	44.2	16.2	10.5	Ace: K8, K31	5657.3160	C245H430N90O64
2	H4[1-52]Kac	62.7	44.2	15.7	9.9	Ace: K8, K44	5657.3160	C245H430N90O64
3	H4[1-52]Kac	60.8	13.5	15.6	9.5	Ace: K12, K31	5657.3160	C245H430N90O64
4	H4[1-52]Kac	62.7	50.0	15.2	9.5	Ace: K8, K20	5657.3160	C245H430N90O64
5	H4[1-52]Kac	58.8	13.5	15.1	8.9	Ace: K12, K44	5657.3160	C245H430N90O64
6	H4[1-52]Kac	62.7	50.0	13.9	8.7	Ace: K8, K16	5657.3160	C245H430N90O64
7	H4[1-52]Kac	58.8	7.7	14.6	8.6	Ace: K5, K31	5657.3160	C245H430N90O64
8	H4[1-52]Kac	62.7	13.5	13.7	8.6	Ace: K16, K31	5657.3160	C245H430N90O64
9	H4[1-52]Kac	58.8	13.5	14.6	8.6	Ace: K12, K20	5657.3160	C245H430N90O64
10	H4[1-52]Kac	56.9	7.7	14.1	8.0	Ace: K5, K44	5657.3160	C245H430N90O64

Sequence

Name: H4[1-52]Kac

```
                    10                    20                    30                    40                    50
S G R G K G G K G L G K G G A K R H R K V L R D N I Q G I T K P A I R R L A R R G G V K R I S G L I Y E
```

Modifications

Modification	Position	Type
bIon-Cterm	C-term	fixed
Acetyl	5	variable
Acetyl	8	variable
Acetyl	12	variable
Acetyl	16	variable
Acetyl	20	variable
Acetyl	31	variable
Acetyl	44	variable

Sequence Map

Proteoform:

Index	Protein	SC [%]	SVP [%]	IC [%]	MS Score [a.u.]	Var. Mods	Mono Mass [Da]	Chemical Formula
1	H4[1-52]Kac	64.7	44.2	16.2	10.5	Ace: K8, K31	5657.3160	C245H430N90O64

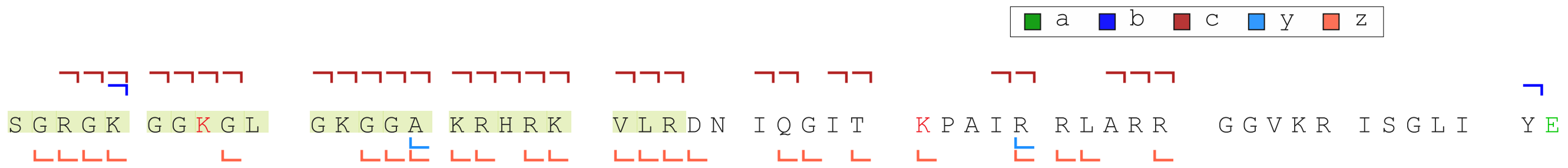

Top-Down Result OmniScape 2025
Sequence Confirmation
250428 H4-PCAF100-30min z13ac2 MS2 shCID ACC MS3 ECD
b52ac2_001-225_calibrated2

Proteoform:

Index	Protein	SC [%]	SVP [%]	IC [%]	MS Score [a.u.]	Var. Mods	Mono Mass [Da]	Chemical Formula
2	H4[1-52]Kac	62.7	44.2	15.7	9.9	Ace: K8, K44	5657.3160	C245H430N90O64

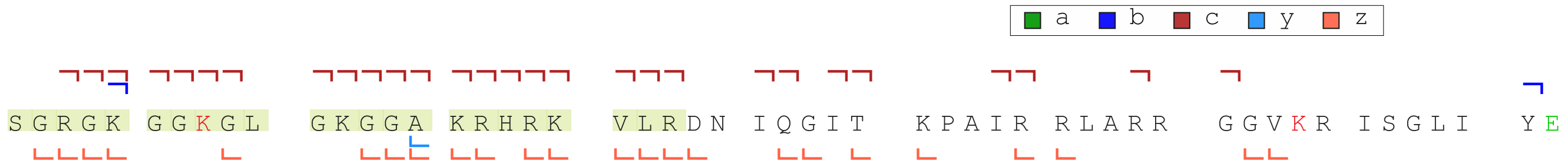

Proteoform:

Index	Protein	SC [%]	SVP [%]	IC [%]	MS Score [a.u.]	Var. Mods	Mono Mass [Da]	Chemical Formula
3	H4[1-52]Kac	60.8	13.5	15.6	9.5	Ace: K12, K31	5657.3160	C245H430N90O64

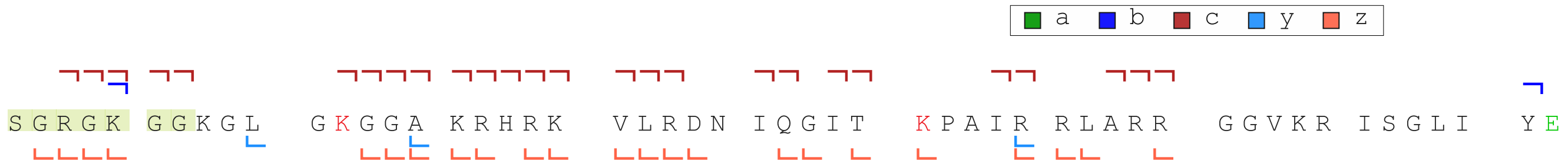

Proteoform:

Index	Protein	SC [%]	SVP [%]	IC [%]	MS Score [a.u.]	Var. Mods	Mono Mass [Da]	Chemical Formula
4	H4[1-52]Kac	62.7	50.0	15.2	9.5	Ace: K8, K20	5657.3160	C245H430N90O64

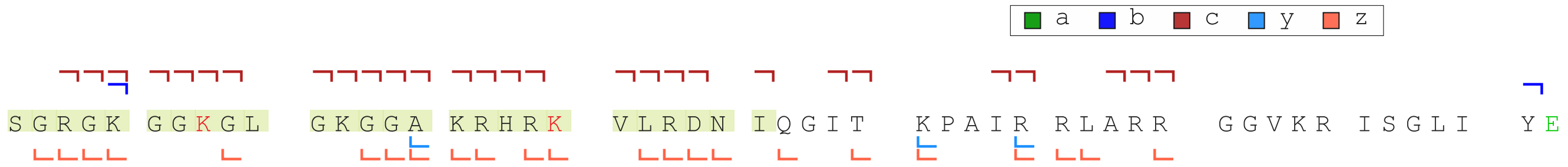

Proteoform:

Index	Protein	SC [%]	SVP [%]	IC [%]	MS Score [a.u.]	Var. Mods	Mono Mass [Da]	Chemical Formula
5	H4[1-52]Kac	58.8	13.5	15.1	8.9	Ace: K12, K44	5657.3160	C245H430N90O64

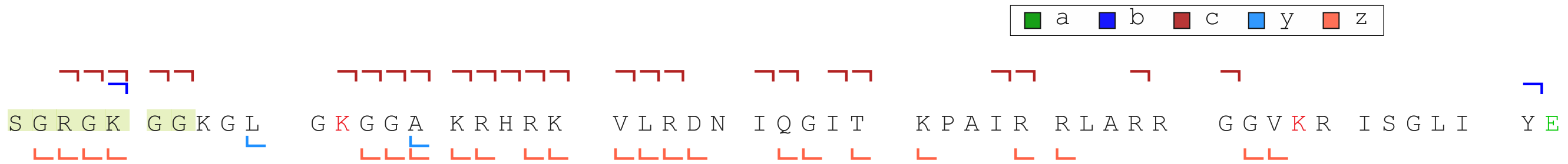

Top-Down Result OmniScape 2025
Sequence Confirmation
250428 H4-PCAF100-30min z13ac2 MS2 shCID ACC MS3 ECD
b52ac2_001-225_calibrated2

Proteoform:

Index	Protein	SC [%]	SVP [%]	IC [%]	MS Score [a.u.]	Var. Mods	Mono Mass [Da]	Chemical Formula
6	H4[1-52]Kac	62.7	50.0	13.9	8.7	Ace: K8, K16	5657.3160	C245H430N90O64

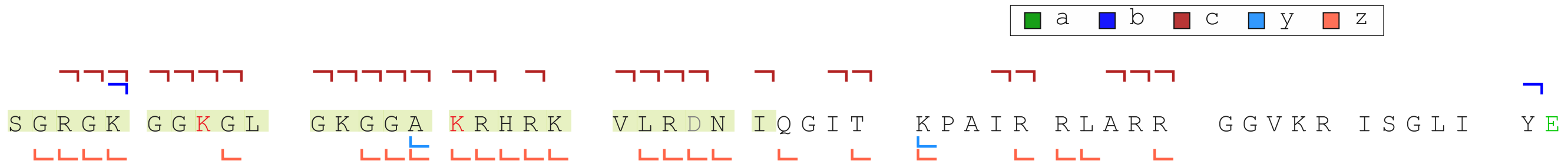

Proteoform:	Index	Protein	SC [%]	SVP [%]	IC [%]	MS Score [a.u.]	Var. Mods	Mono Mass [Da]	Chemical Formula
	7	H4[1-52]Kac	58.8	7.7	14.6	8.6	Ace: K5, K31	5657.3160	C245H430N90O64

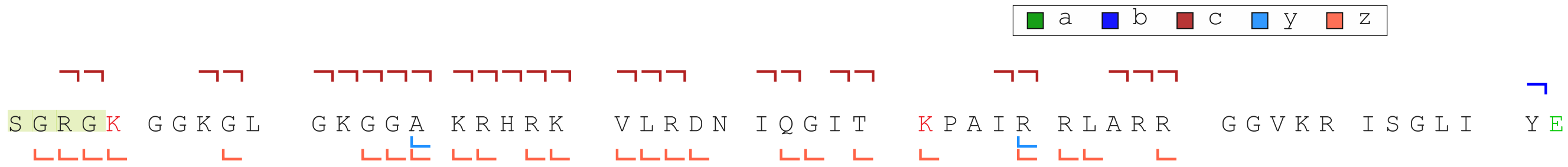

Proteoform:

Index	Protein	SC [%]	SVP [%]	IC [%]	MS Score [a.u.]	Var. Mods	Mono Mass [Da]	Chemical Formula
8	H4[1-52]Kac	62.7	13.5	13.7	8.6	Ace: K16, K31	5657.3160	C245H430N90O64

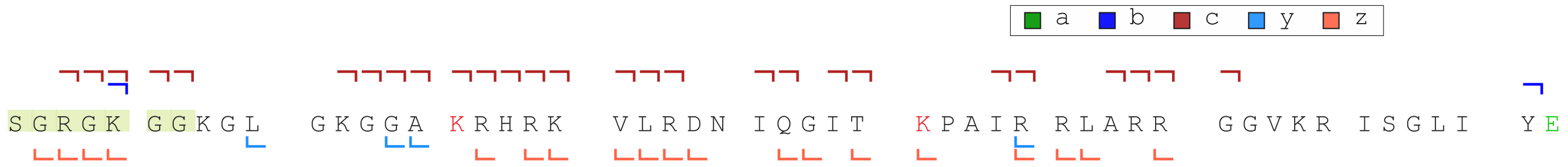

Proteoform:

Index	Protein	SC [%]	SVP [%]	IC [%]	MS Score [a.u.]	Var. Mods	Mono Mass [Da]	Chemical Formula
9	H4[1-52]Kac	58.8	13.5	14.6	8.6	Ace: K12, K20	5657.3160	C245H430N90O64

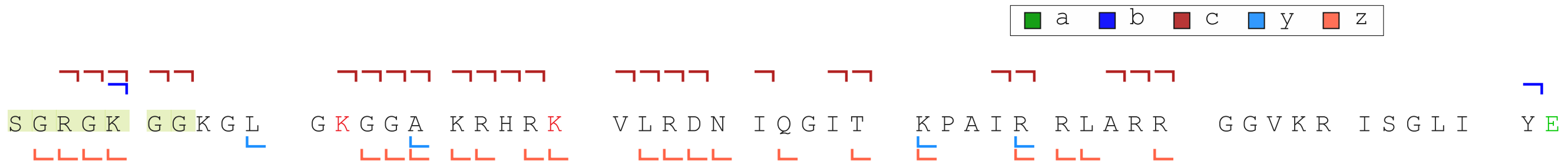

Proteoform:

Index	Protein	SC [%]	SVP [%]	IC [%]	MS Score [a.u.]	Var. Mods	Mono Mass [Da]	Chemical Formula
10	H4[1-52]Kac	56.9	7.7	14.1	8.0	Ace: K5, K44	5657.3160	C245H430N90O64

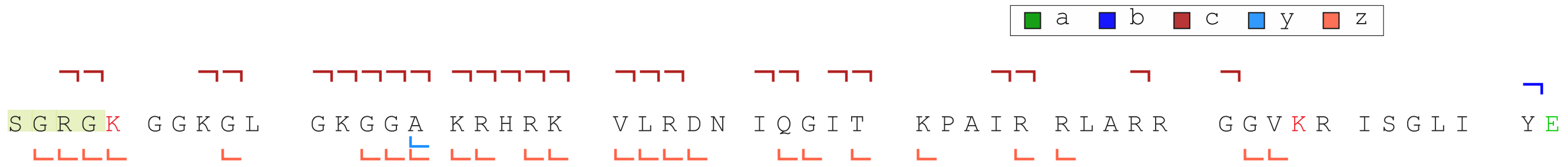

Error Plot

Proteoform:

Index	Protein	SC [%]	SVP [%]	IC [%]	MS Score [a.u.]	Var. Mods	Mono Mass [Da]	Chemical Formula
1	H4[1-52]Kac	64.7	44.2	16.2	10.5	Ace: K8, K31	5657.3160	C245H430N90O64

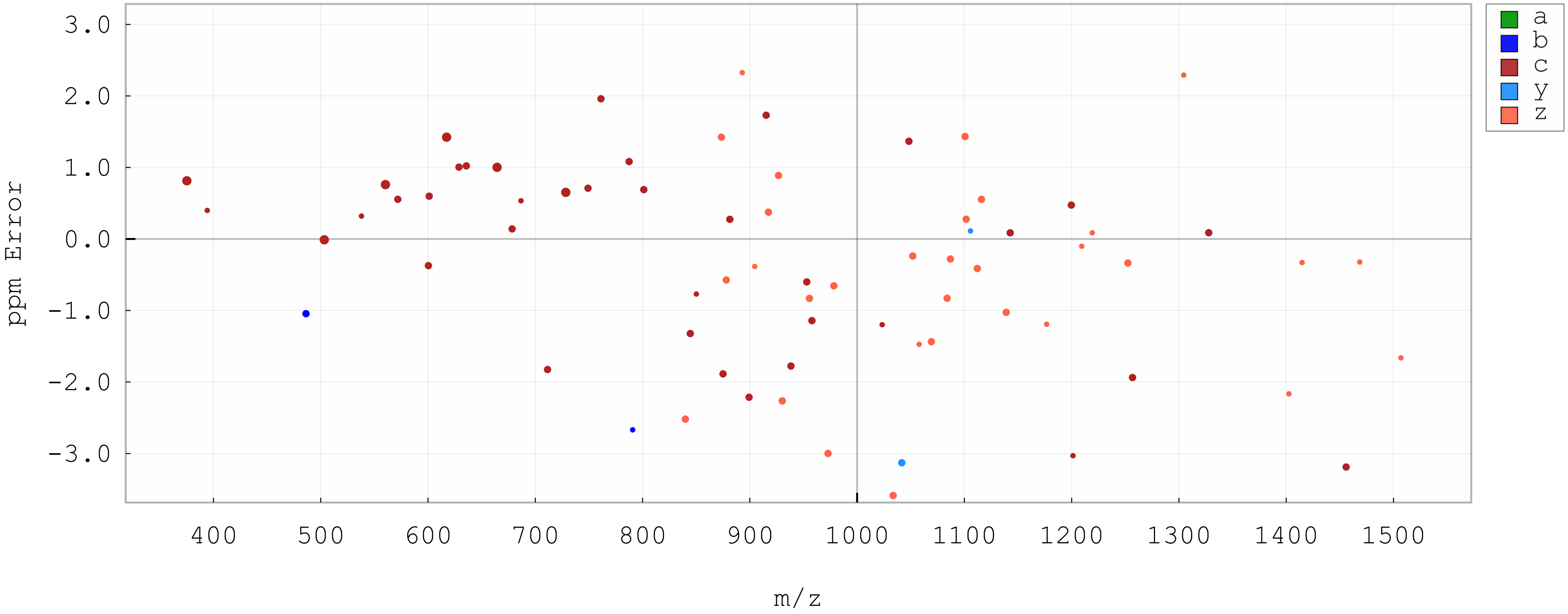

Proteoform:

Index	Protein	SC [%]	SVP [%]	IC [%]	MS Score [a.u.]	Var. Mods	Mono Mass [Da]	Chemical Formula
2	H4[1-52]Kac	62.7	44.2	15.7	9.9	Ace: K8, K44	5657.3160	C245H430N90O64

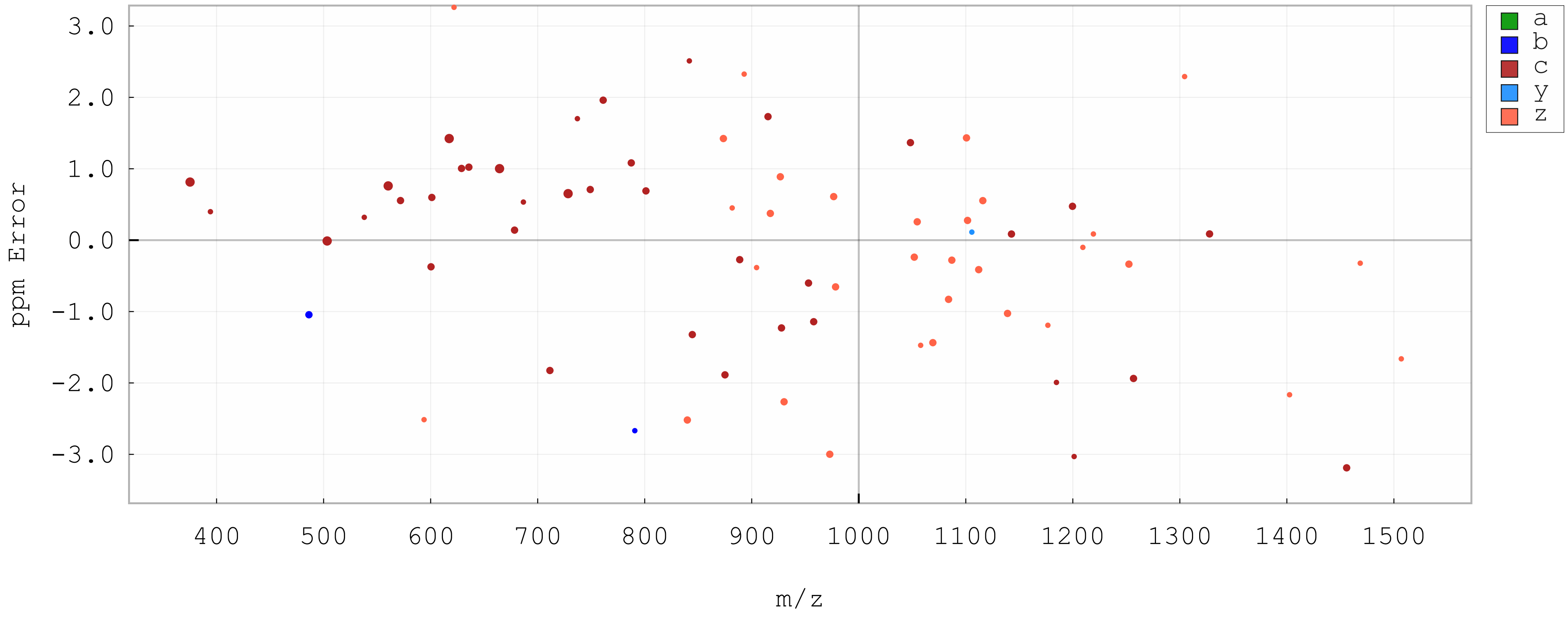

Proteoform:

Index	Protein	SC [%]	SVP [%]	IC [%]	MS Score [a.u.]	Var. Mods	Mono Mass [Da]	Chemical Formula
3	H4[1-52]Kac	60.8	13.5	15.6	9.5	Ace: K12, K31	5657.3160	C245H430N90O64

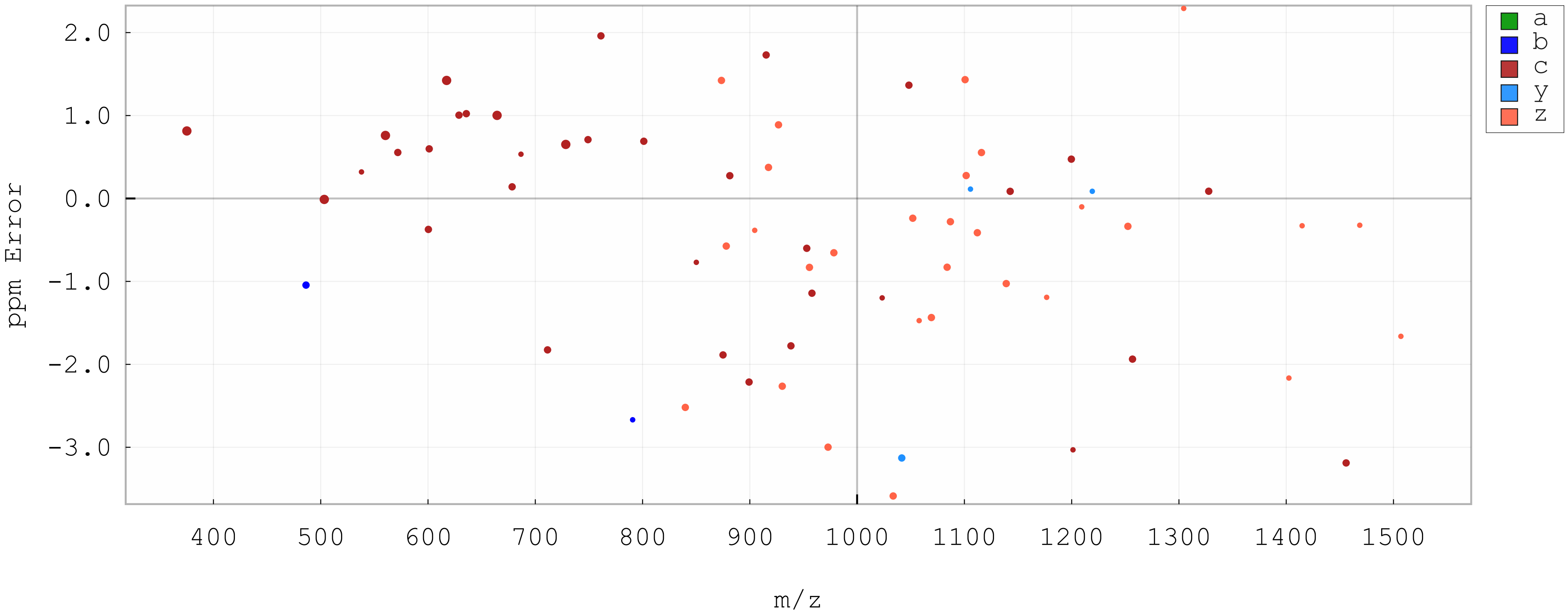

Proteoform:

Index	Protein	SC [%]	SVP [%]	IC [%]	MS Score [a.u.]	Var. Mods	Mono Mass [Da]	Chemical Formula
4	H4[1-52]Kac	62.7	50.0	15.2	9.5	Ace: K8, K20	5657.3160	C245H430N90O64

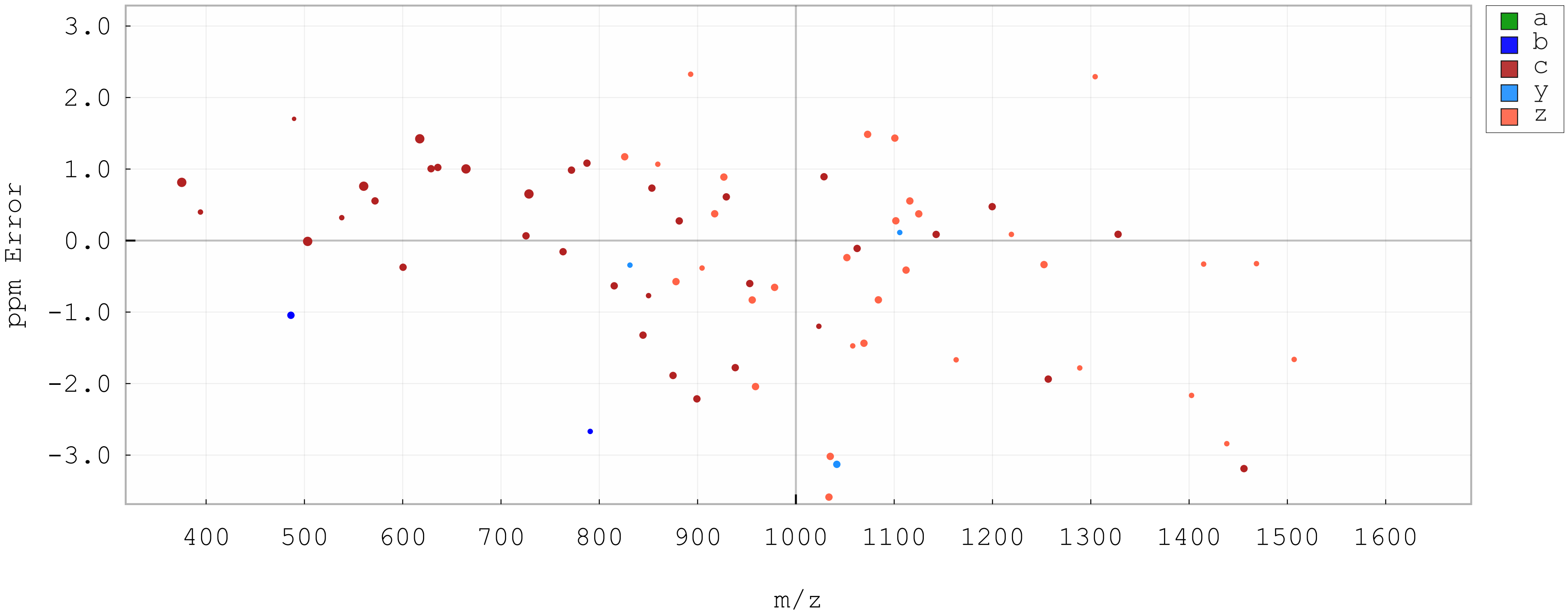

Proteoform:

Index	Protein	SC [%]	SVP [%]	IC [%]	MS Score [a.u.]	Var. Mods	Mono Mass [Da]	Chemical Formula
5	H4[1-52]Kac	58.8	13.5	15.1	8.9	Ace: K12, K44	5657.3160	C245H430N90O64

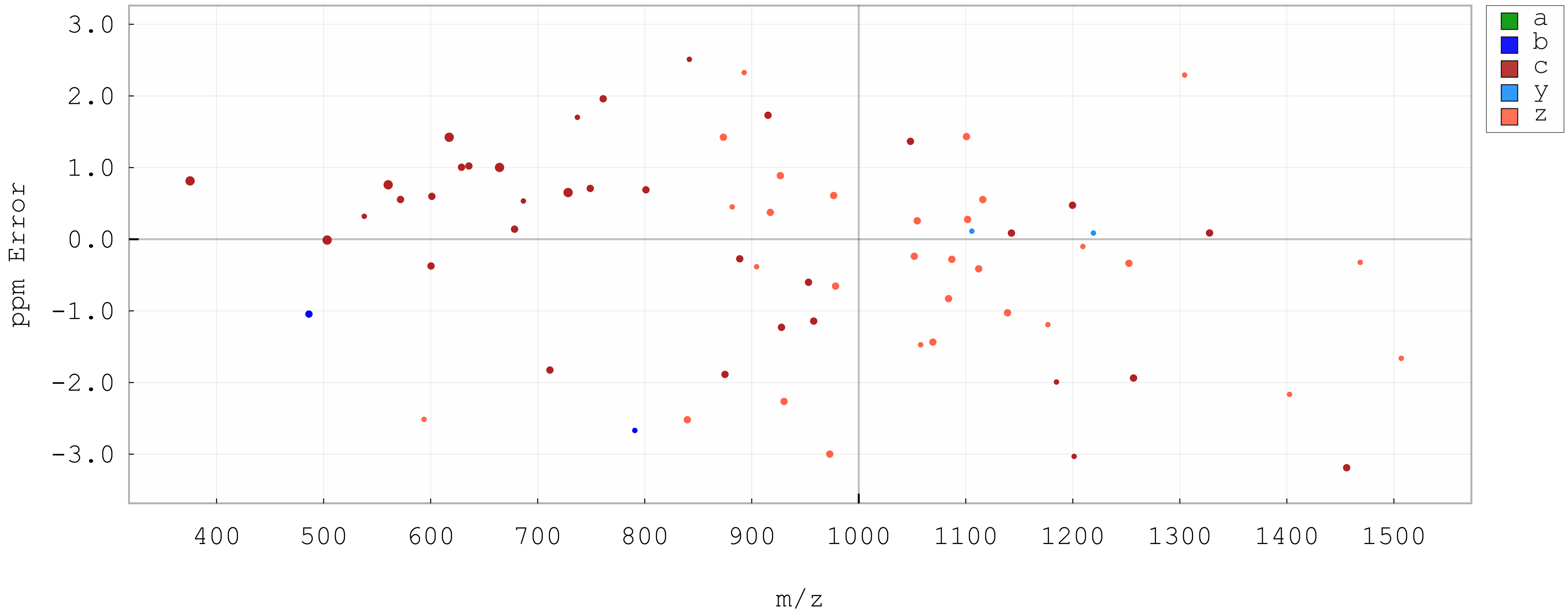

Proteoform:

Index	Protein	SC [%]	SVP [%]	IC [%]	MS Score [a.u.]	Var. Mods	Mono Mass [Da]	Chemical Formula
6	H4[1-52]Kac	62.7	50.0	13.9	8.7	Ace: K8, K16	5657.3160	C245H430N90O64

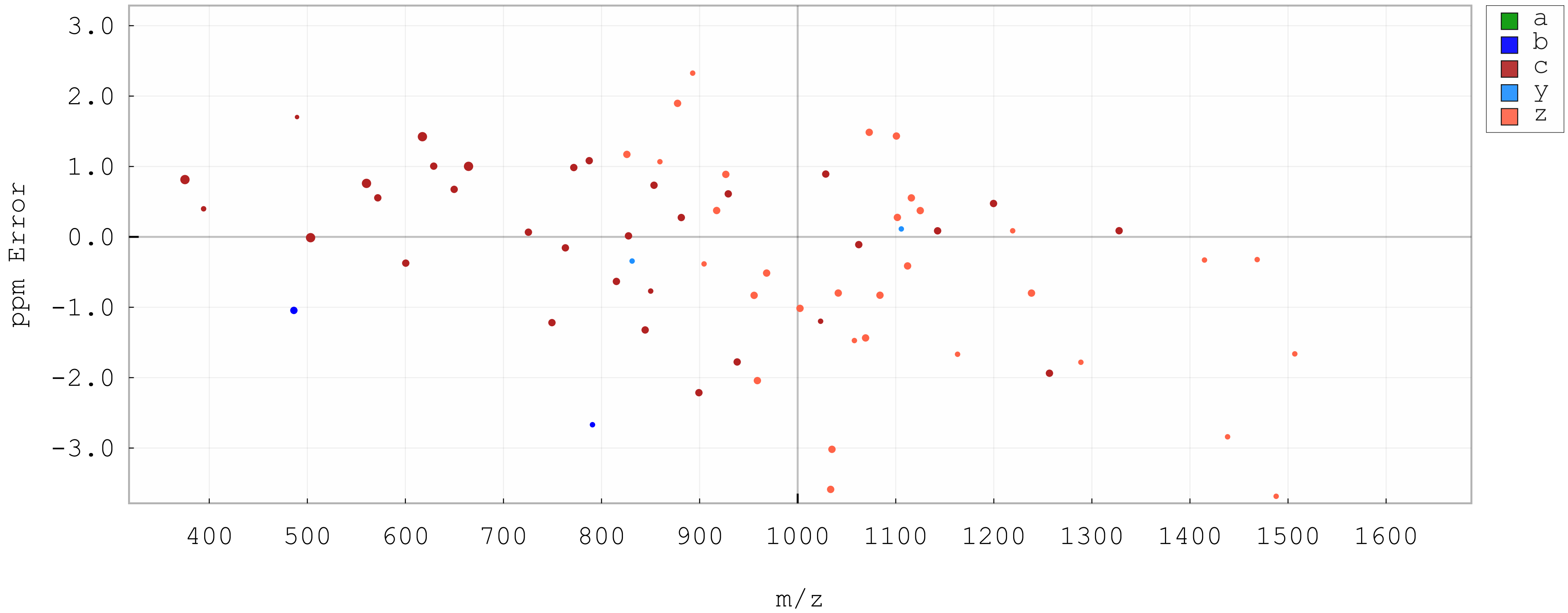

Proteoform:

Index	Protein	SC [%]	SVP [%]	IC [%]	MS Score [a.u.]	Var. Mods	Mono Mass [Da]	Chemical Formula
7	H4[1-52]Kac	58.8	7.7	14.6	8.6	Ace: K5, K31	5657.3160	C245H430N90O64

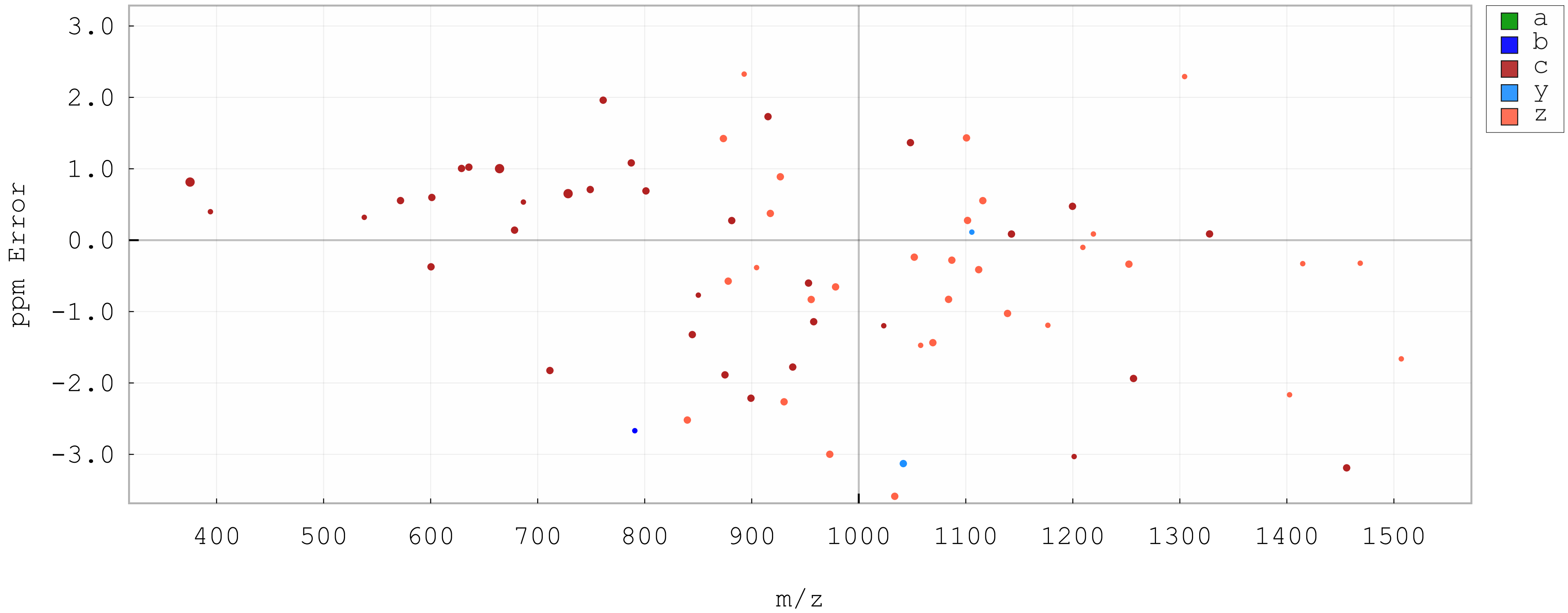

Proteoform:

Index	Protein	SC [%]	SVP [%]	IC [%]	MS Score [a.u.]	Var. Mods	Mono Mass [Da]	Chemical Formula
8	H4[1-52]Kac	62.7	13.5	13.7	8.6	Ace: K16, K31	5657.3160	C245H430N90O64

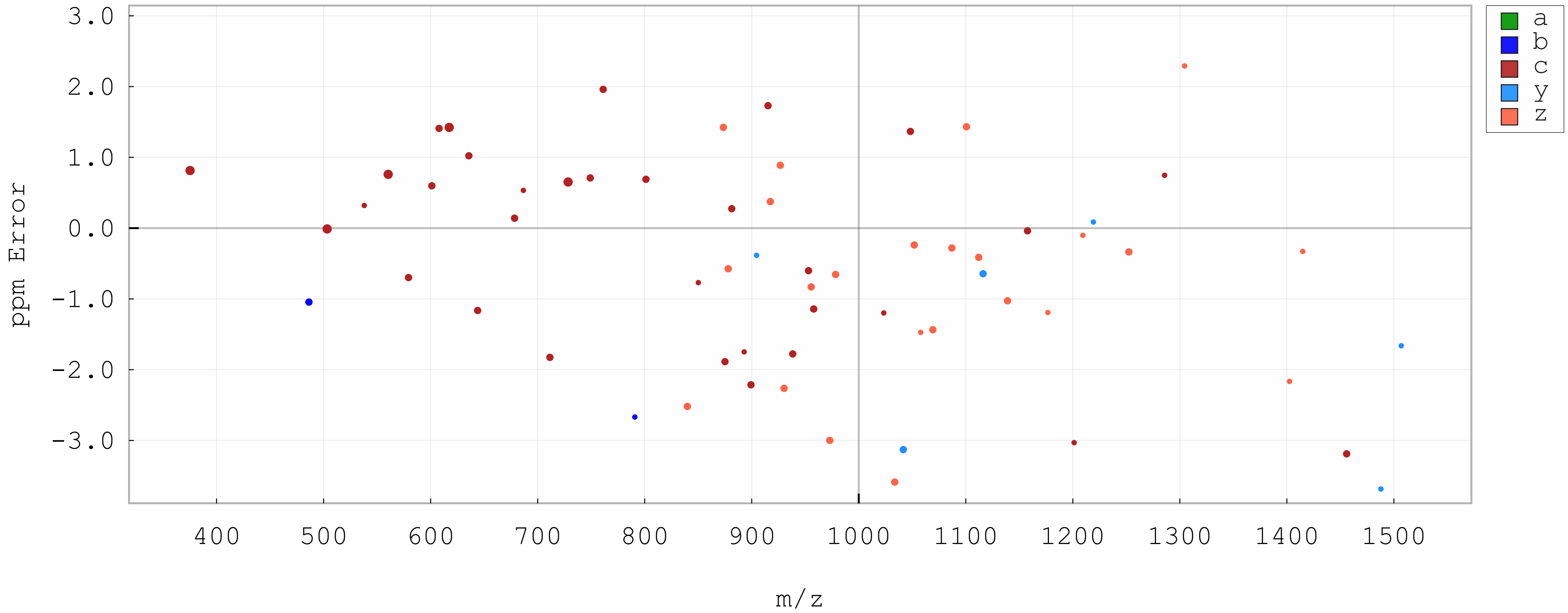

Proteoform:

Index	Protein	SC [%]	SVP [%]	IC [%]	MS Score [a.u.]	Var. Mods	Mono Mass [Da]	Chemical Formula
9	H4[1-52]Kac	58.8	13.5	14.6	8.6	Ace: K12, K20	5657.3160	C245H430N90O64

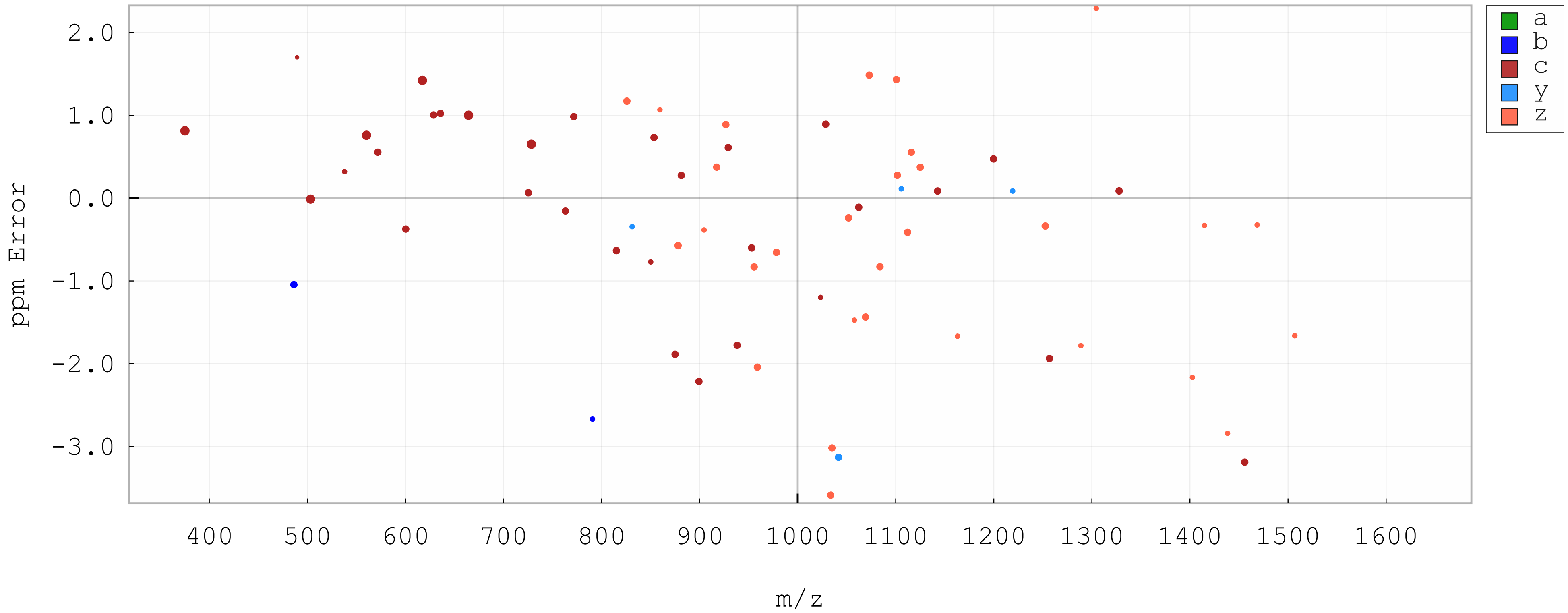

Proteoform:

Index	Protein	SC [%]	SVP [%]	IC [%]	MS Score [a.u.]	Var. Mods	Mono Mass [Da]	Chemical Formula
10	H4[1-52]Kac	56.9	7.7	14.1	8.0	Ace: K5, K44	5657.3160	C245H430N90O64

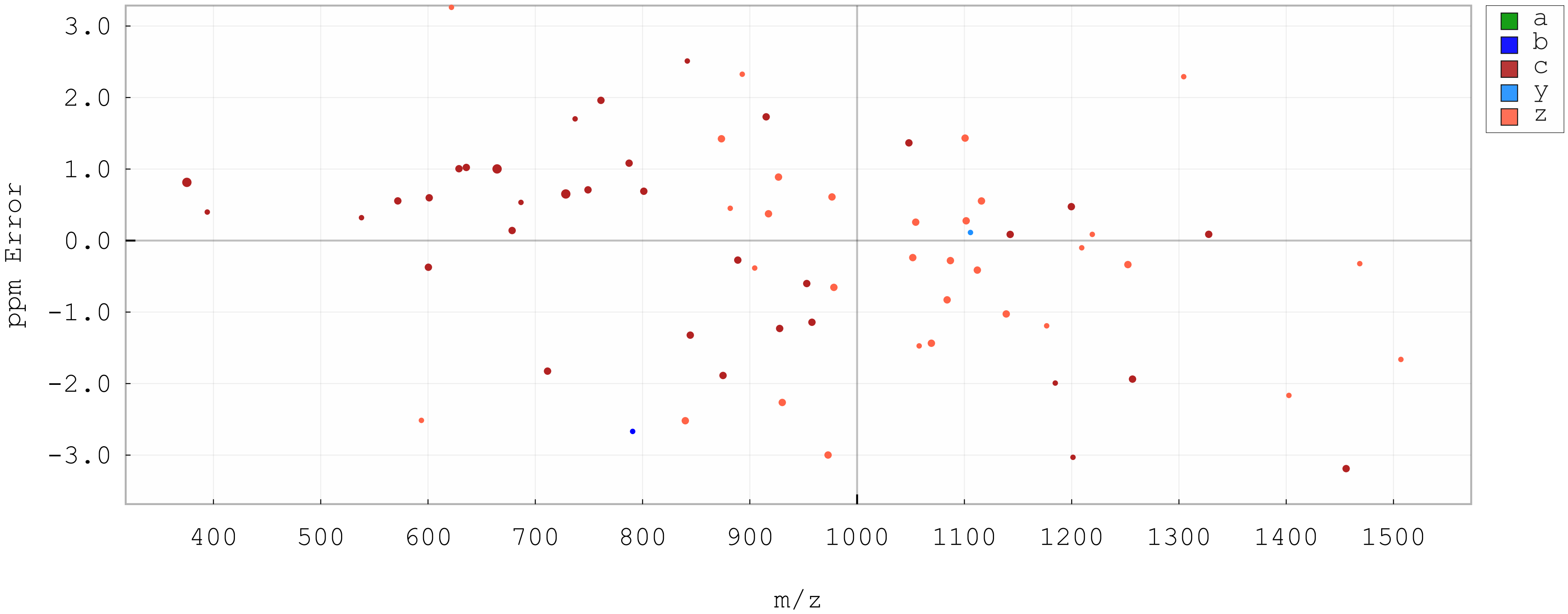

Primary Fragment Plots

Proteoform:

Index	Protein	SC [%]	SVP [%]	IC [%]	MS Score [a.u.]	Var. Mods	Mono Mass [Da]	Chemical Formula
1	H4[1-52]Kac	64.7	44.2	16.2	10.5	Ace: K8, K31	5657.3160	C245H430N90O64

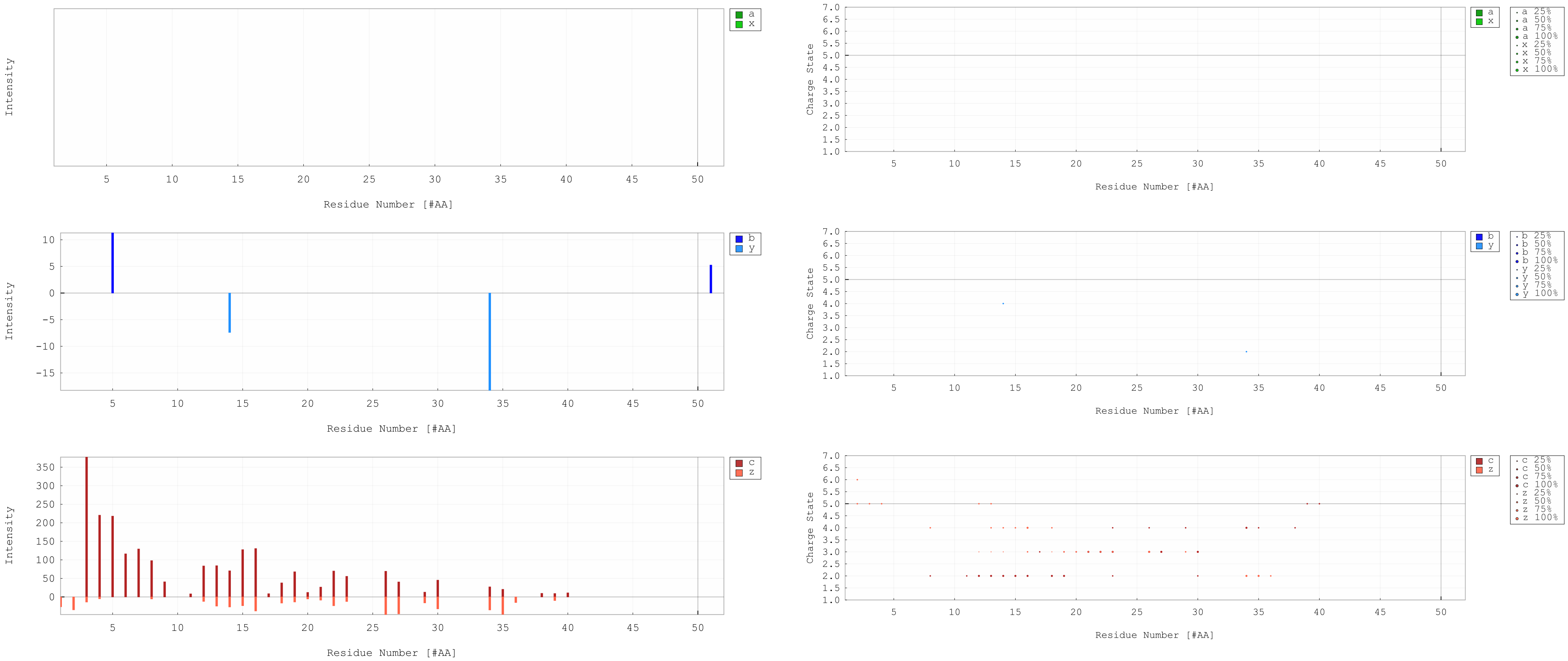

Proteoform:

Index	Protein	SC [%]	SVP [%]	IC [%]	MS Score [a.u.]	Var. Mods	Mono Mass [Da]	Chemical Formula
2	H4[1-52]Kac	62.7	44.2	15.7	9.9	Ace: K8, K44	5657.3160	C245H430N90O64

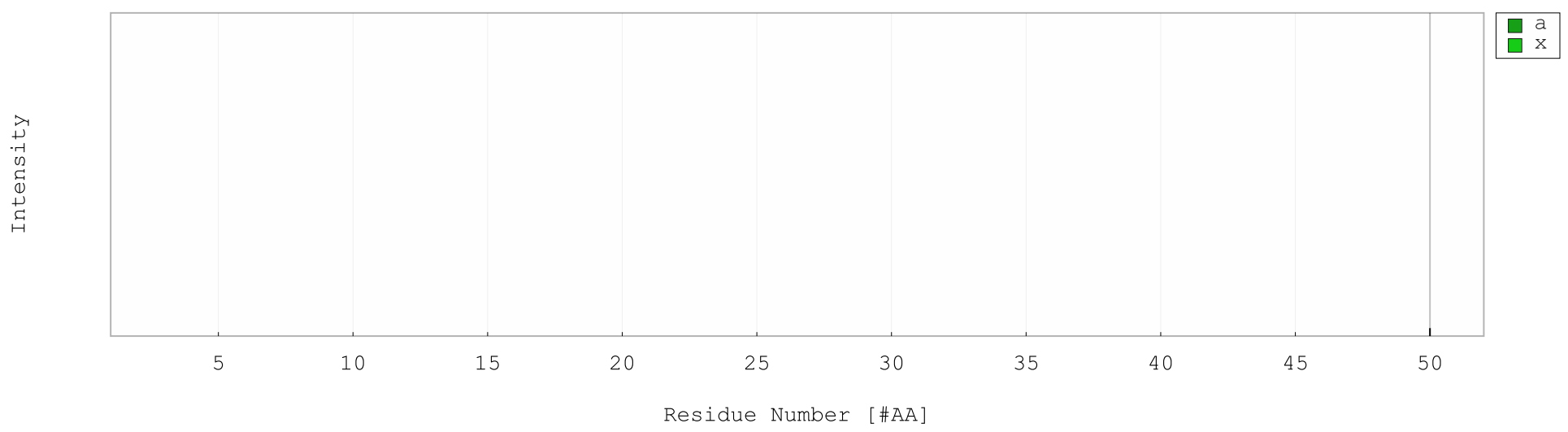

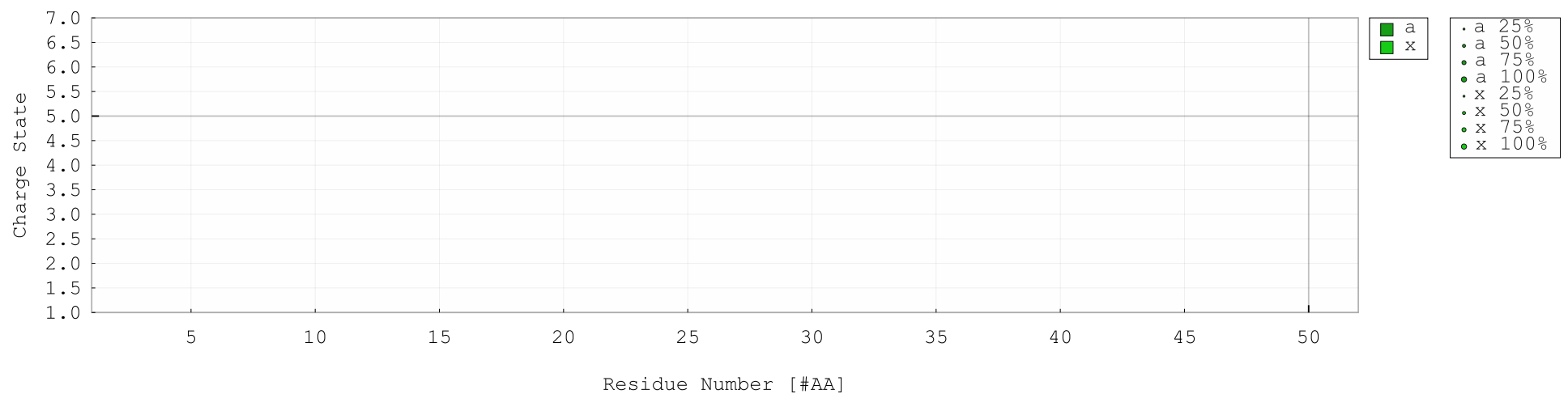

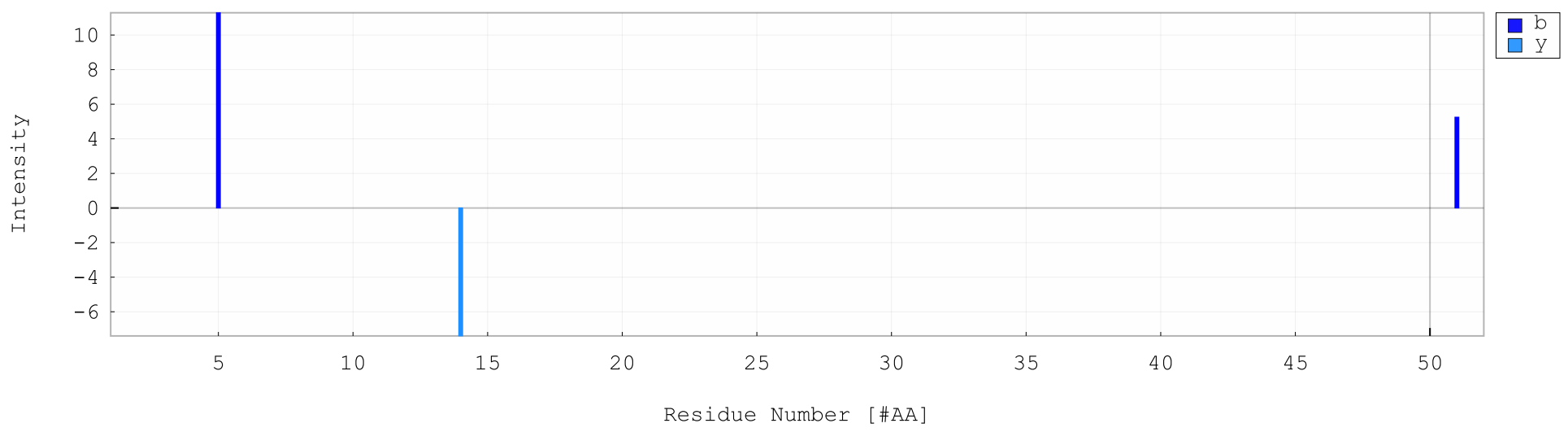

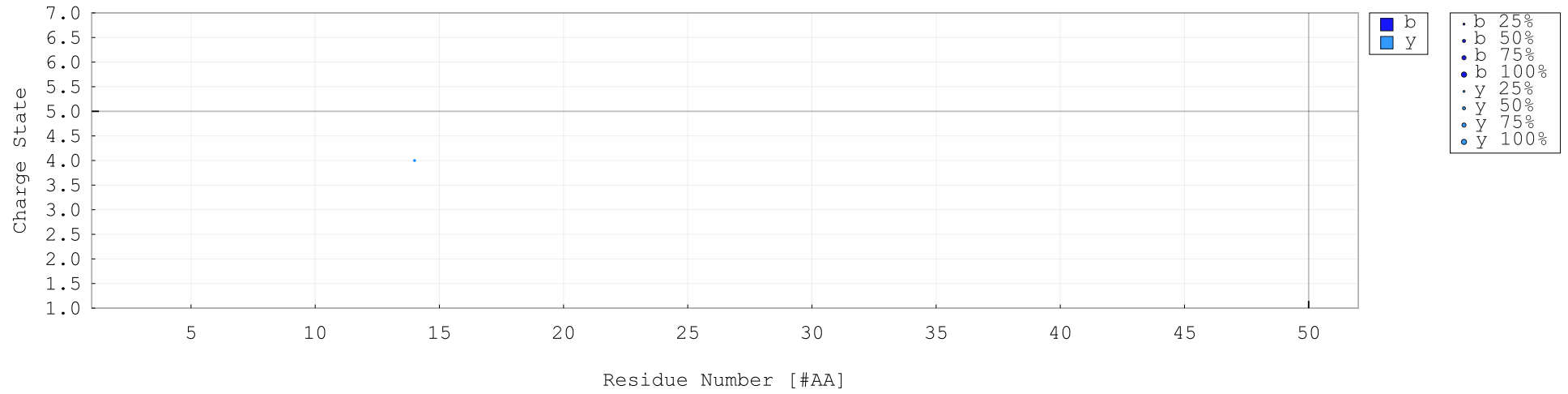

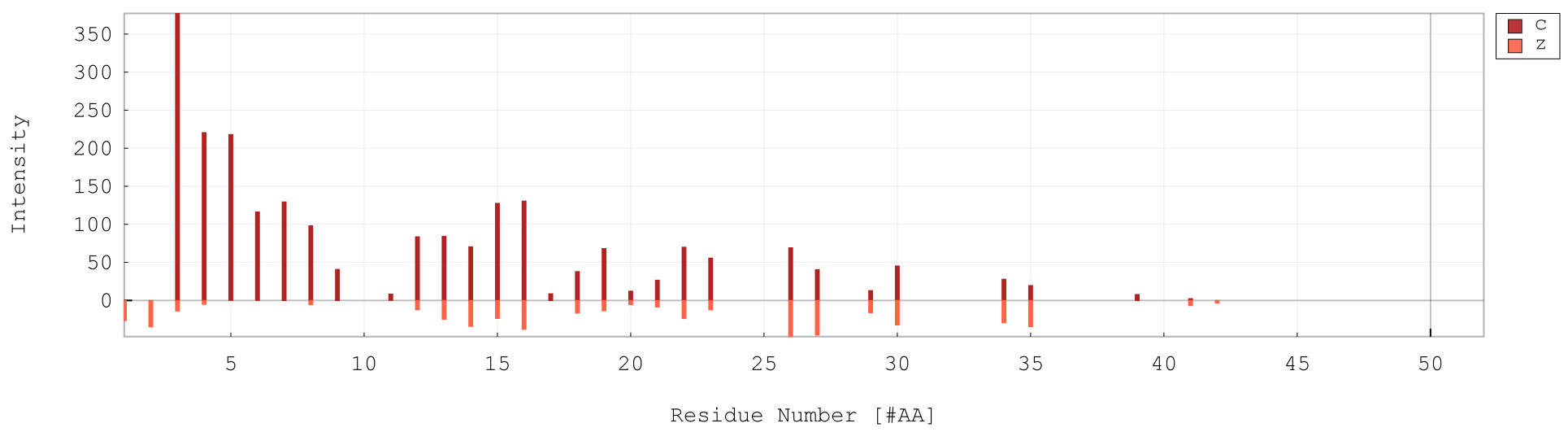

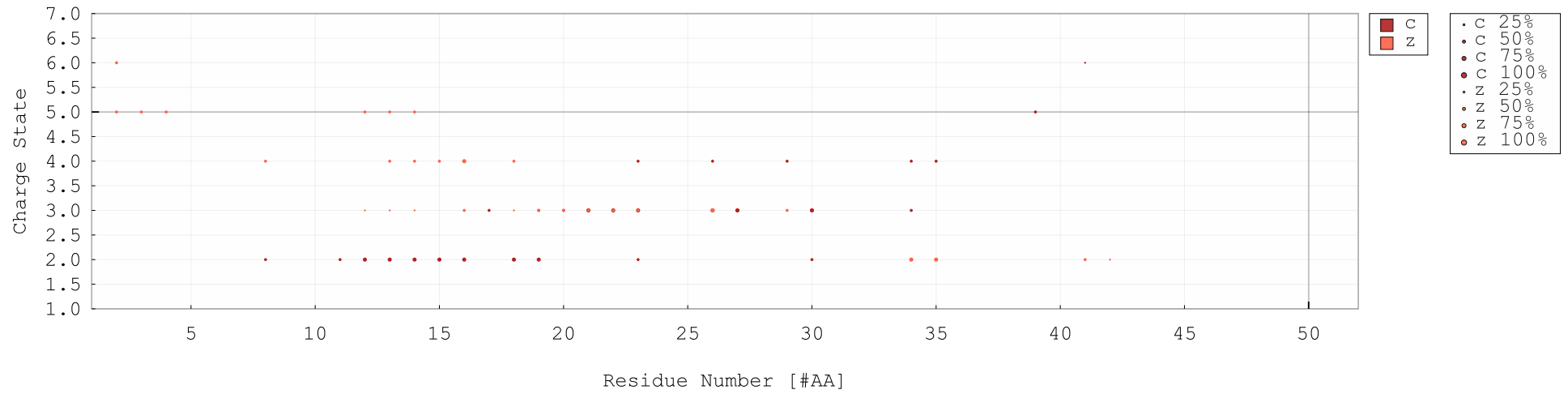

Proteoform:

Index	Protein	SC [%]	SVP [%]	IC [%]	MS Score [a.u.]	Var. Mods	Mono Mass [Da]	Chemical Formula
3	H4[1-52]Kac	60.8	13.5	15.6	9.5	Ace: K12, K31	5657.3160	C245H430N90O64

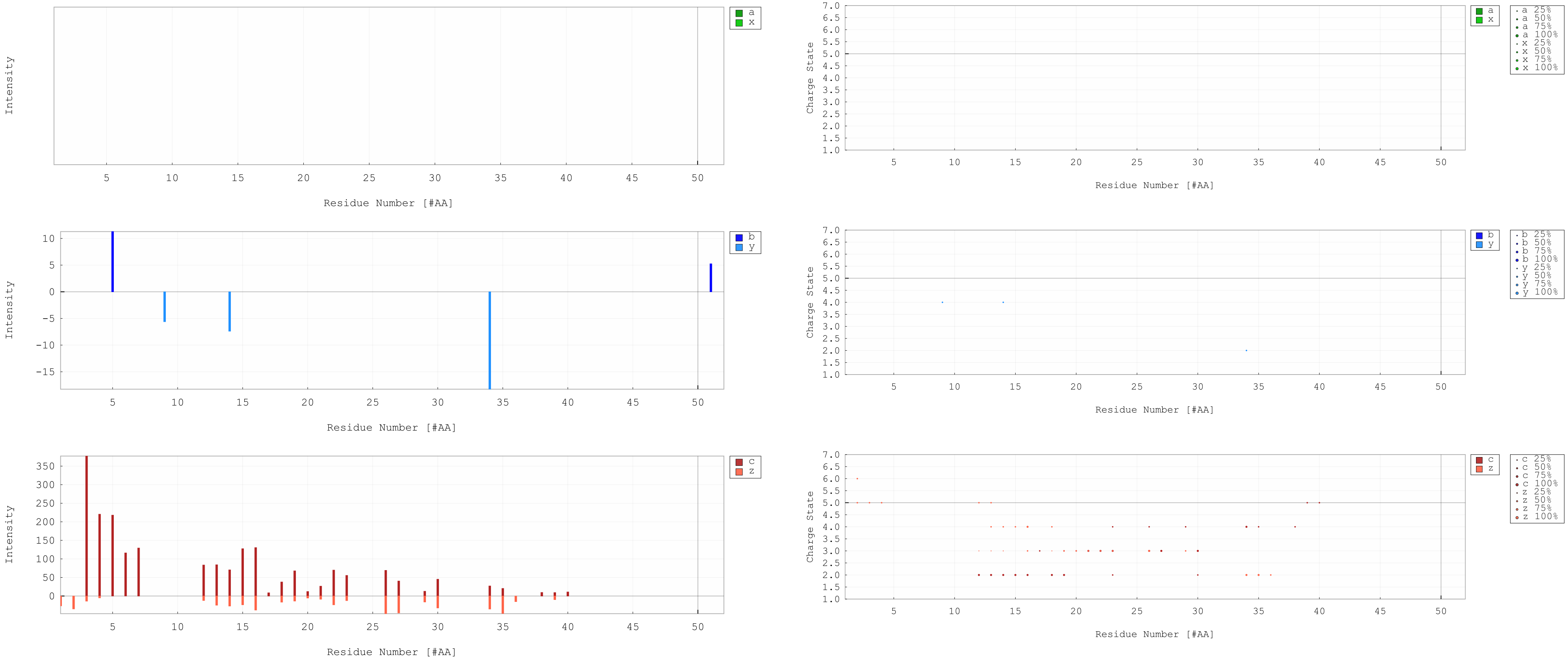

Proteoform:	Index	Protein	SC [%]	SVP [%]	IC [%]	MS Score [a.u.]	Var. Mods	Mono Mass [Da]	Chemical Formula
	4	H4[1-52]Kac	62.7	50.0	15.2	9.5	Ace: K8, K20	5657.3160	C245H430N90O64

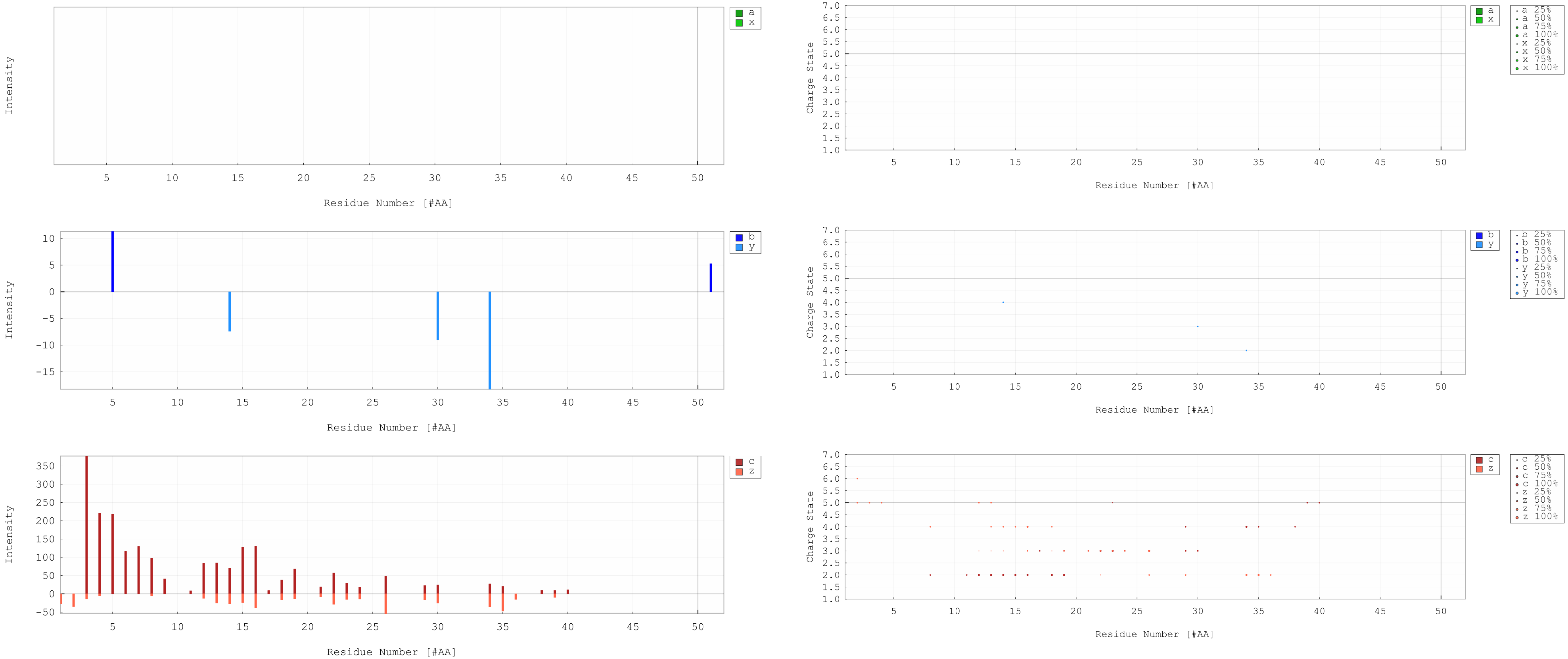

Proteoform:

Index	Protein	SC [%]	SVP [%]	IC [%]	MS Score [a.u.]	Var. Mods	Mono Mass [Da]	Chemical Formula
5	H4[1-52]Kac	58.8	13.5	15.1	8.9	Ace: K12, K44	5657.3160	C245H430N90O64

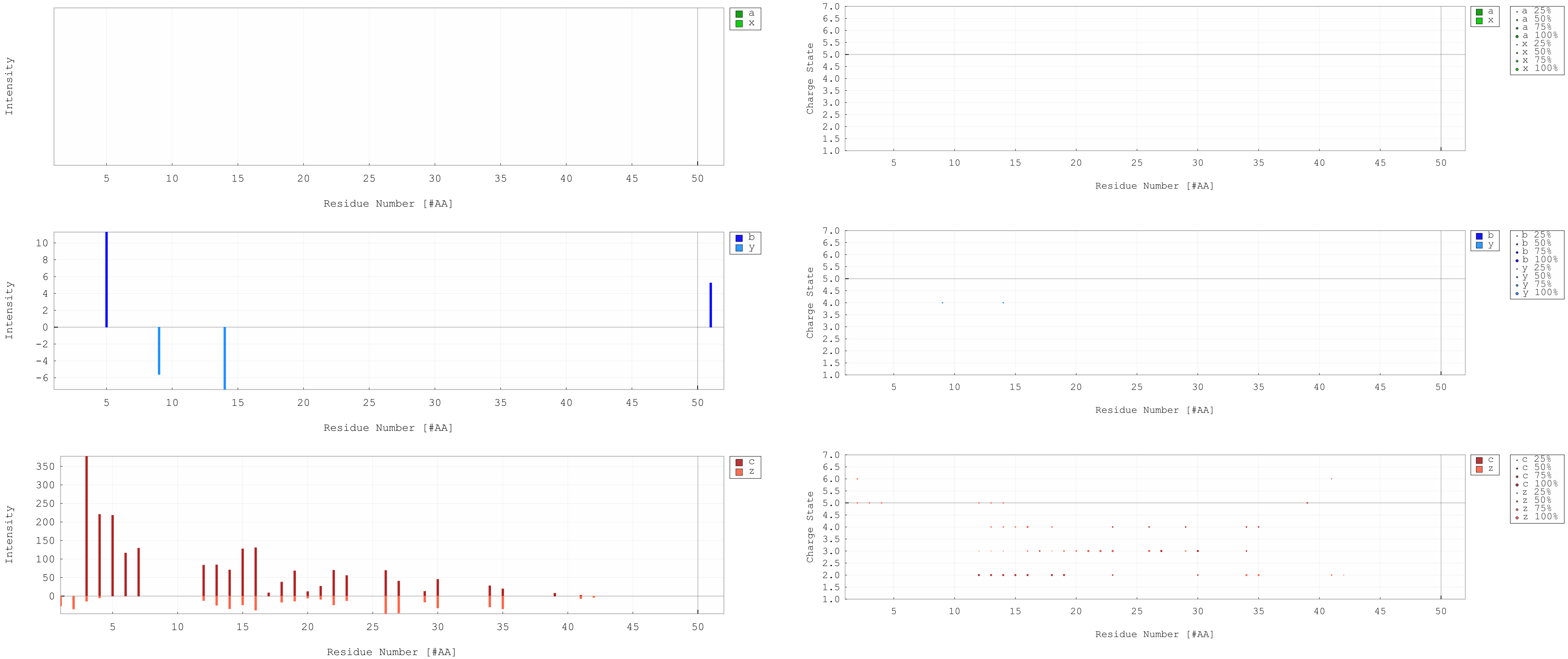

Proteoform:

Index	Protein	SC [%]	SVP [%]	IC [%]	MS Score [a.u.]	Var. Mods	Mono Mass [Da]	Chemical Formula
6	H4[1-52]Kac	62.7	50.0	13.9	8.7	Ace: K8, K16	5657.3160	C245H430N90O64

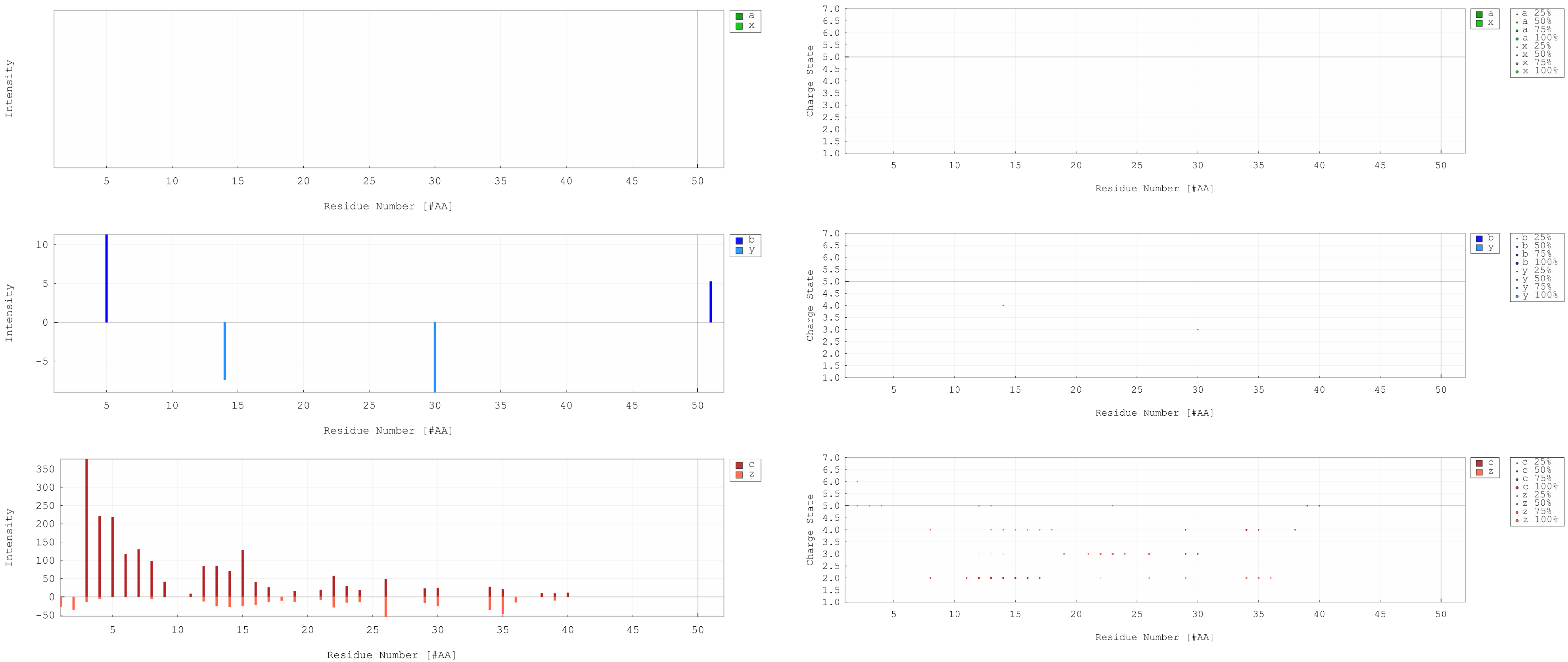

Proteoform:

Index	Protein	SC [%]	SVP [%]	IC [%]	MS Score [a.u.]	Var. Mods	Mono Mass [Da]	Chemical Formula
7	H4[1-52]Kac	58.8	7.7	14.6	8.6	Ace: K5, K31	5657.3160	C245H430N90O64

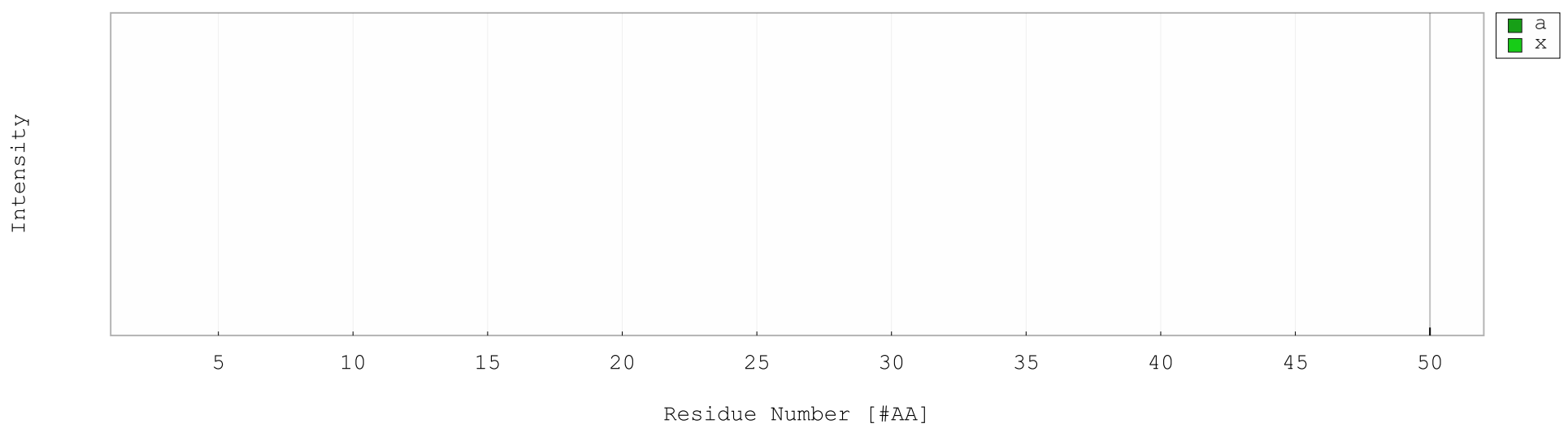

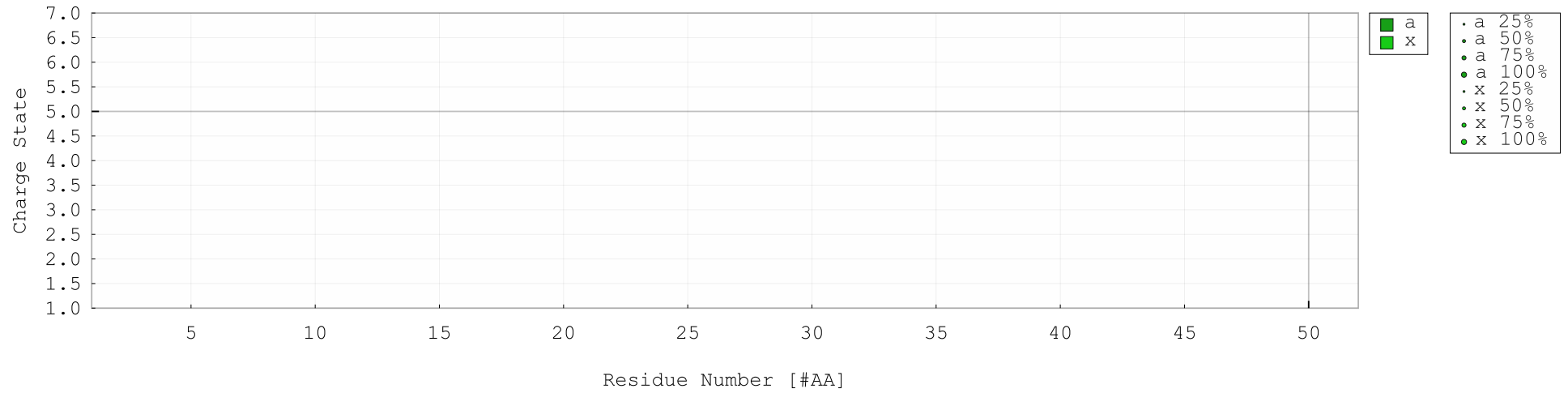

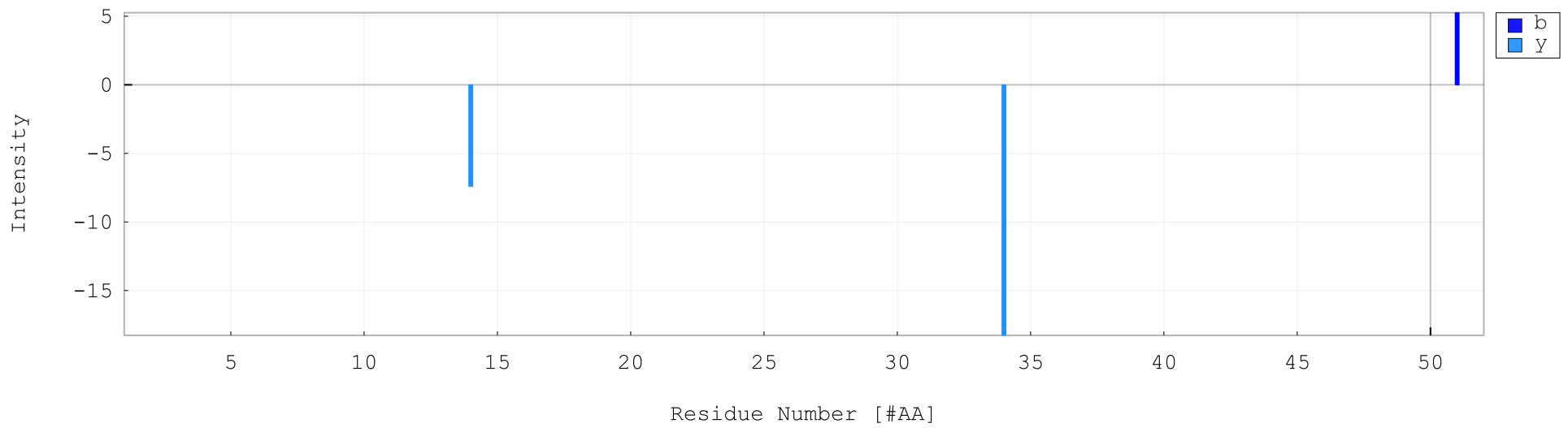

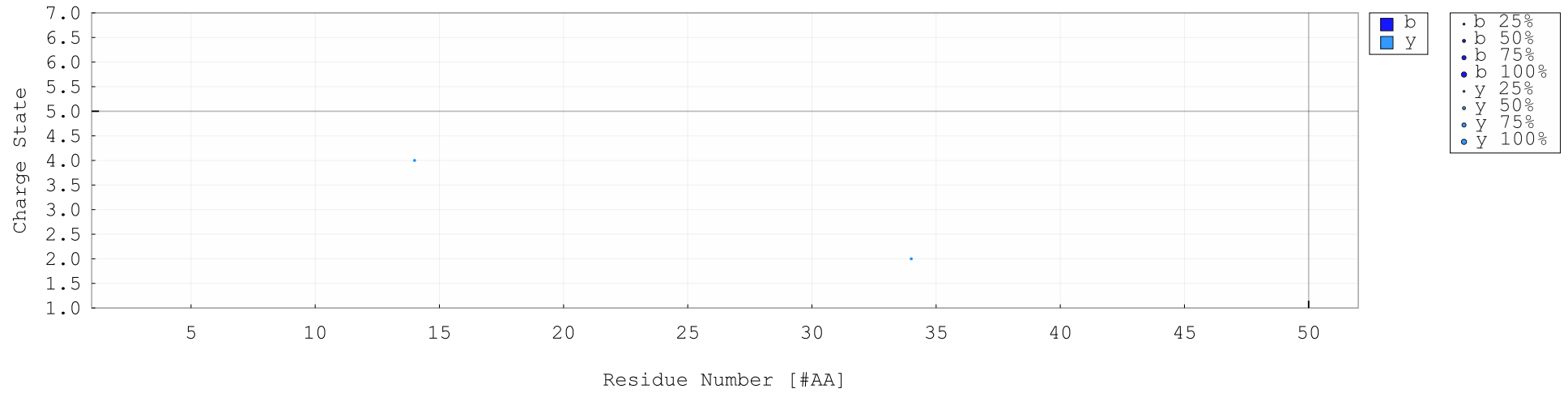

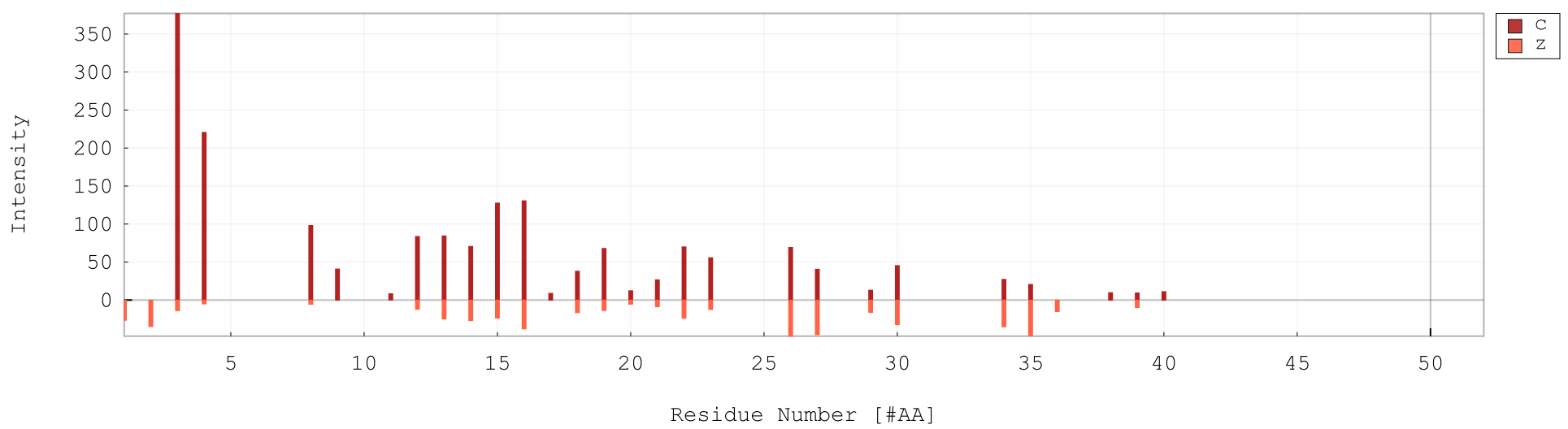

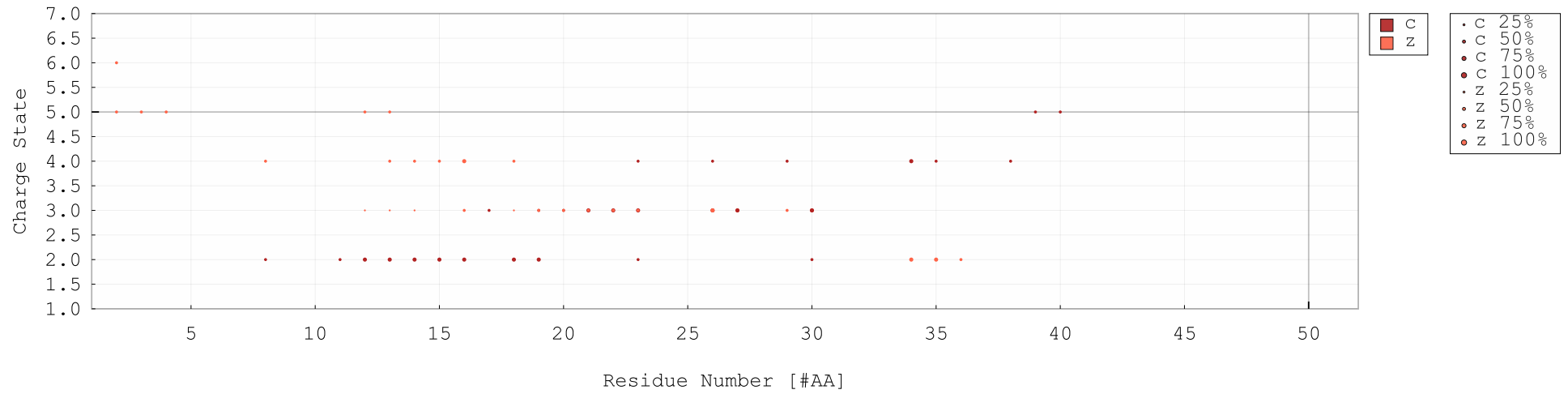

Proteoform:	Index	Protein	SC [%]	SVP [%]	IC [%]	MS Score [a.u.]	Var. Mods	Mono Mass [Da]	Chemical Formula
	8	H4[1-52]Kac	62.7	13.5	13.7	8.6	Ace: K16, K31	5657.3160	C245H430N90O64

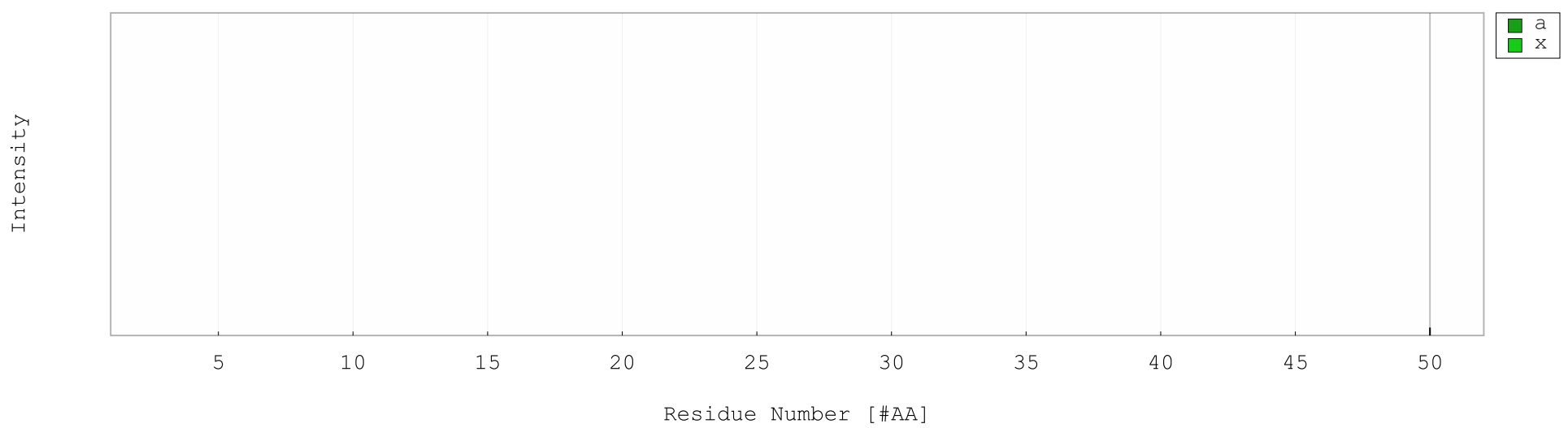

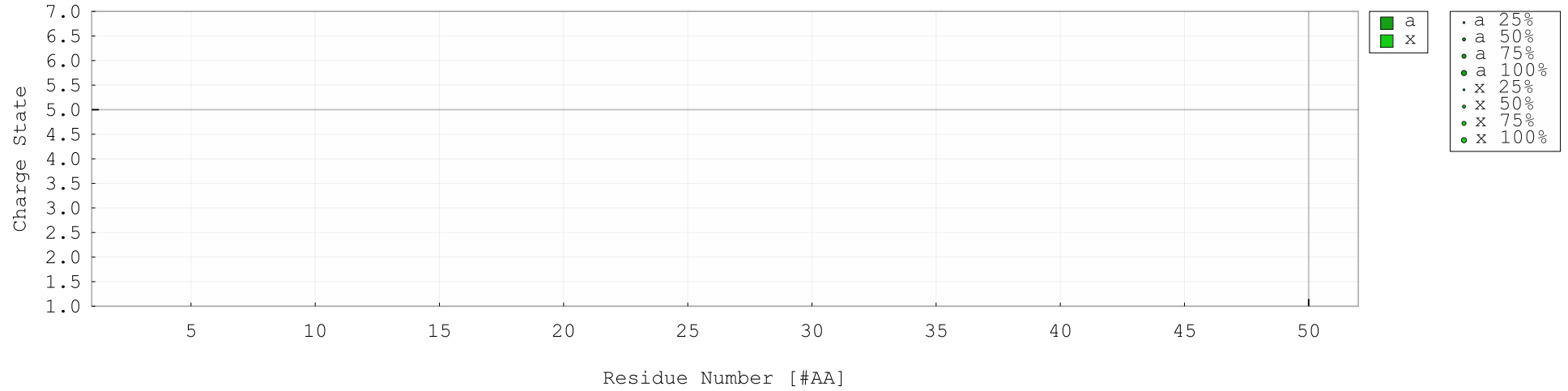

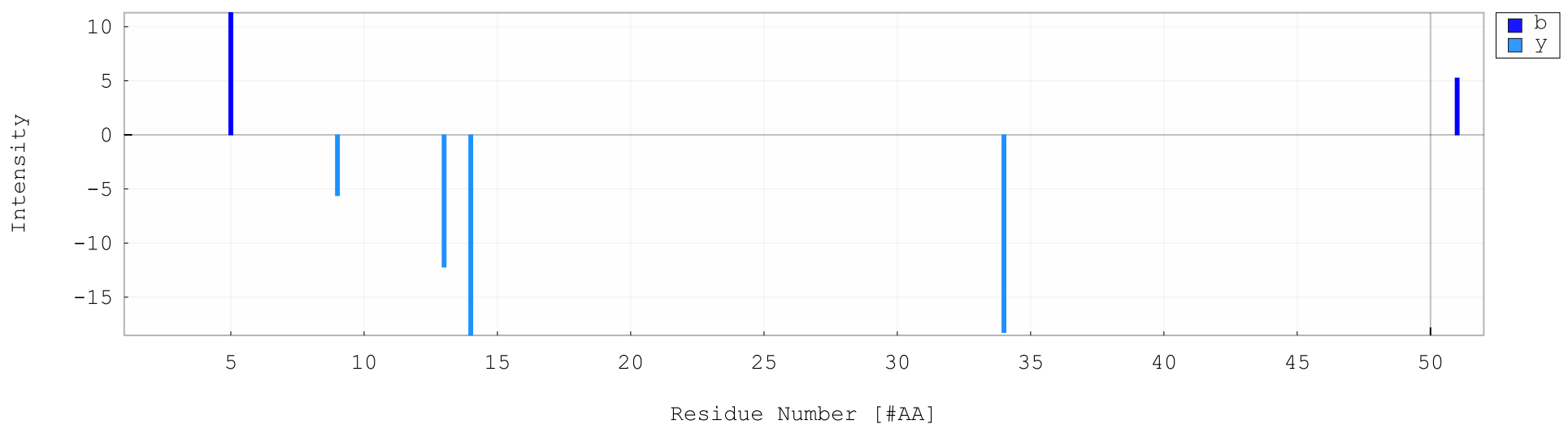

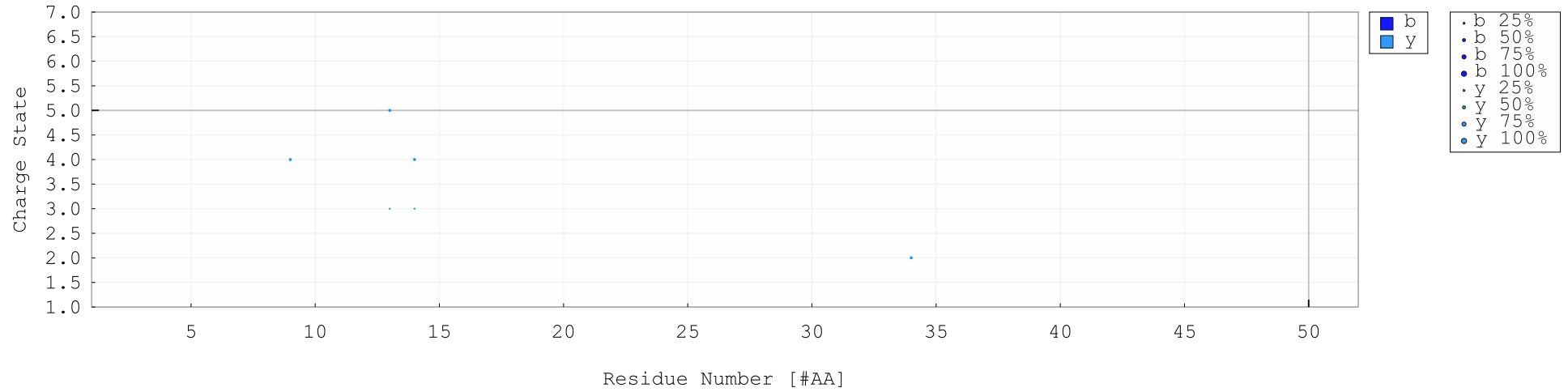

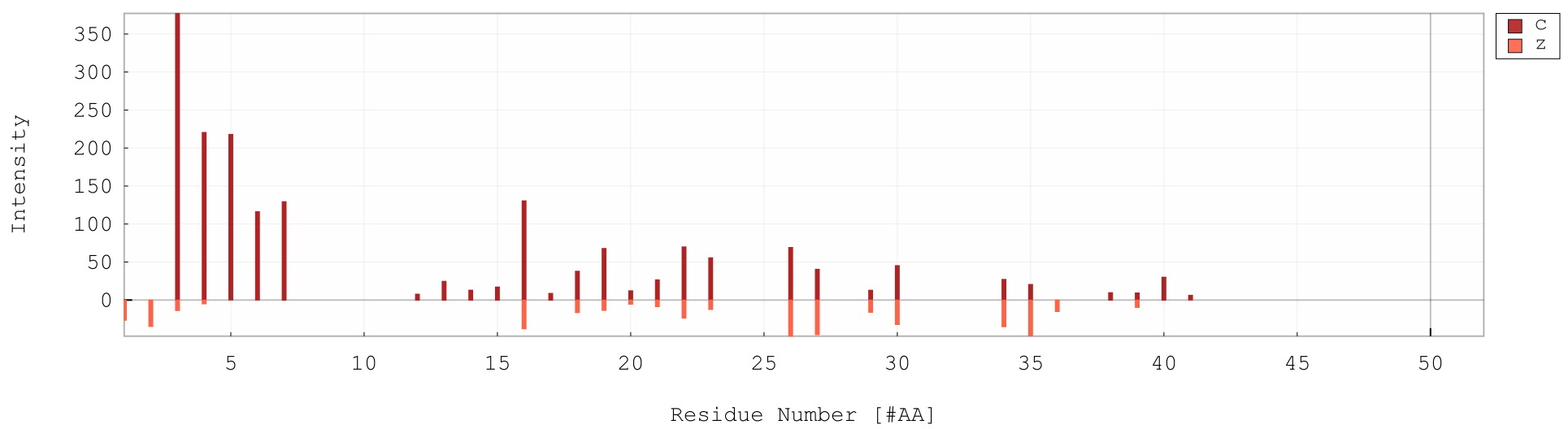

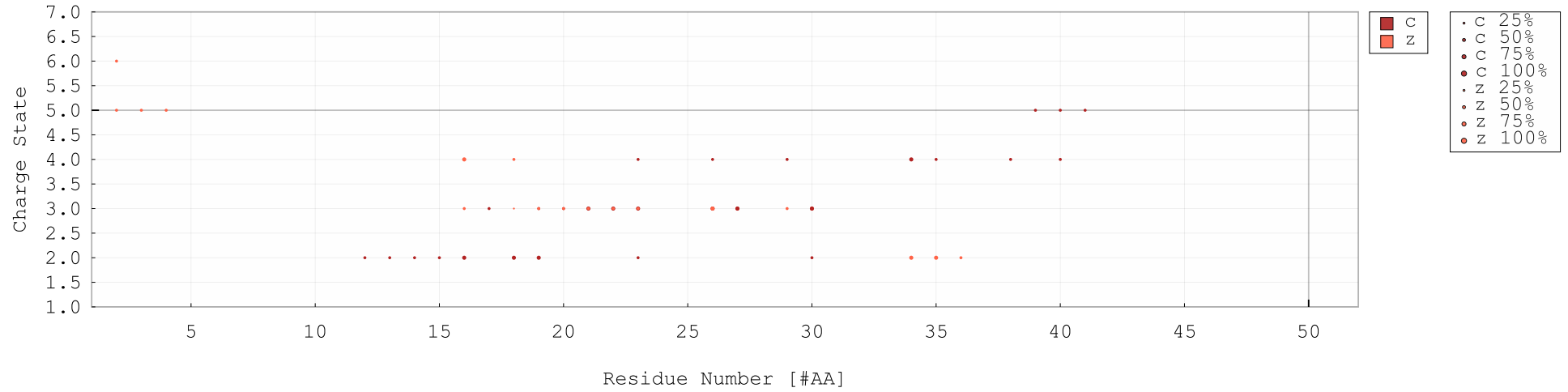

Proteoform:

Index	Protein	SC [%]	SVP [%]	IC [%]	MS Score [a.u.]	Var. Mods	Mono Mass [Da]	Chemical Formula
9	H4[1-52]Kac	58.8	13.5	14.6	8.6	Ace: K12, K20	5657.3160	C245H430N90O64

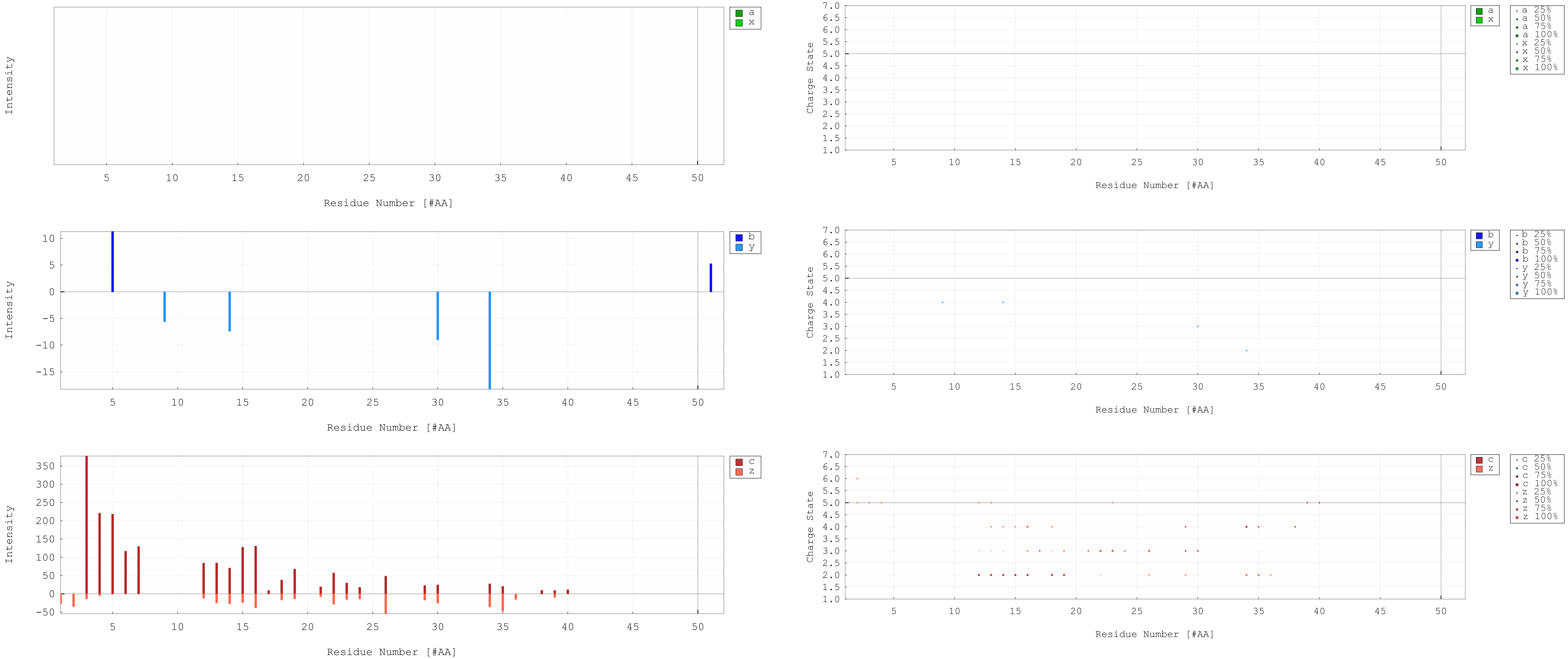

Proteoform:

Index	Protein	SC [%]	SVP [%]	IC [%]	MS Score [a.u.]	Var. Mods	Mono Mass [Da]	Chemical Formula
10	H4[1-52]Kac	56.9	7.7	14.1	8.0	Ace: K5, K44	5657.3160	C245H430N90O64

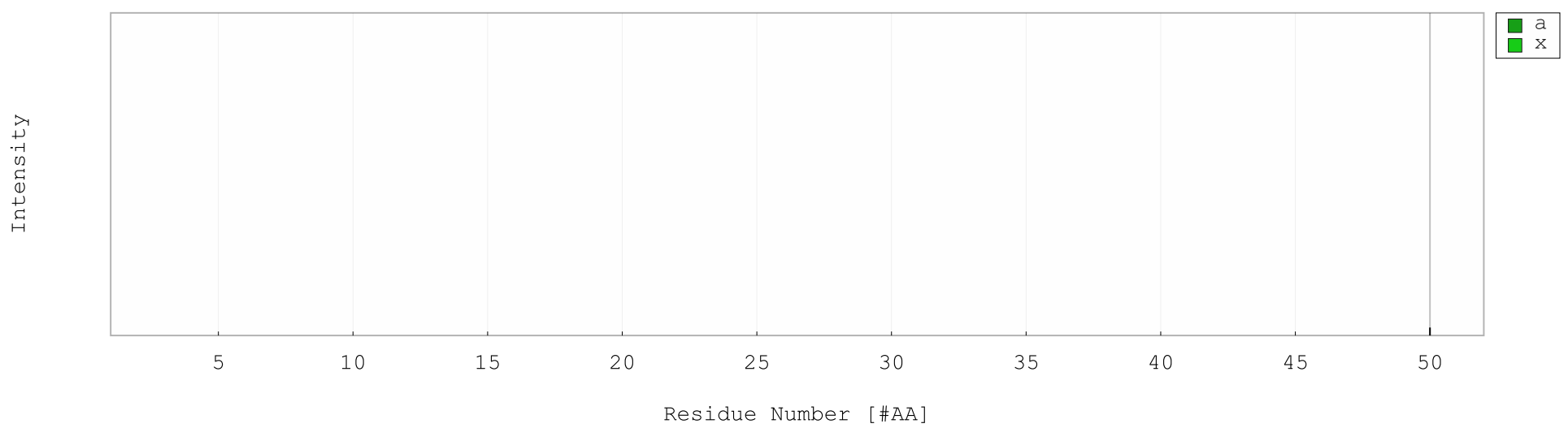

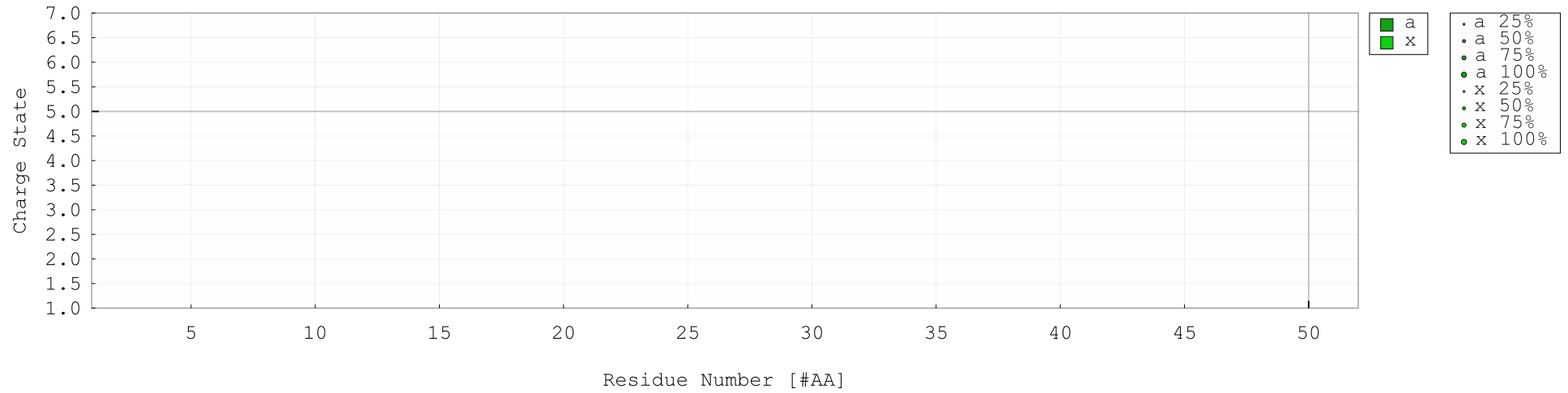

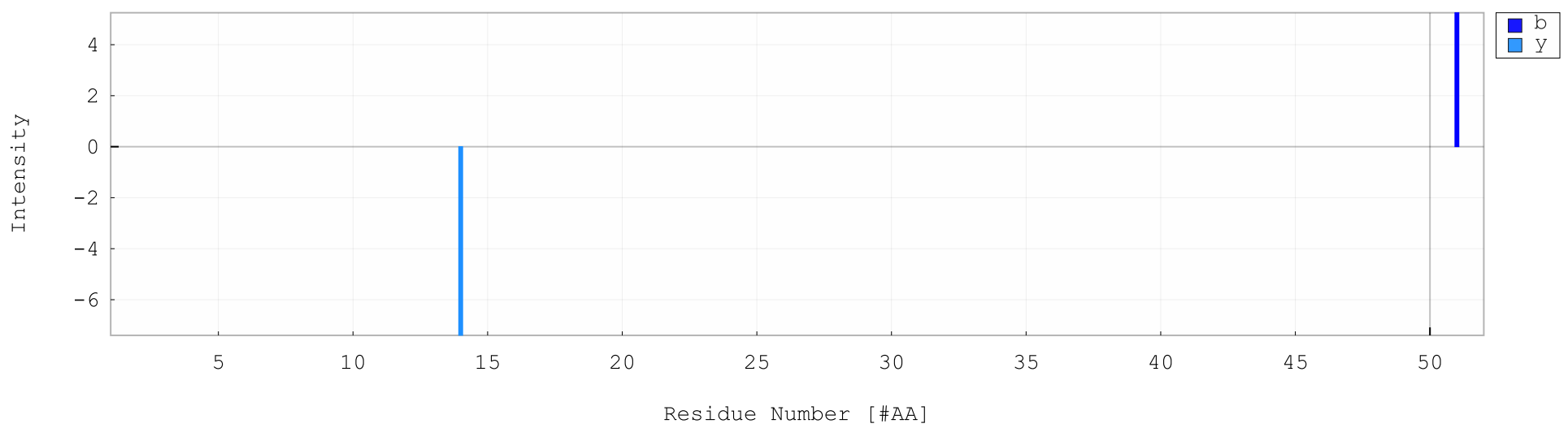

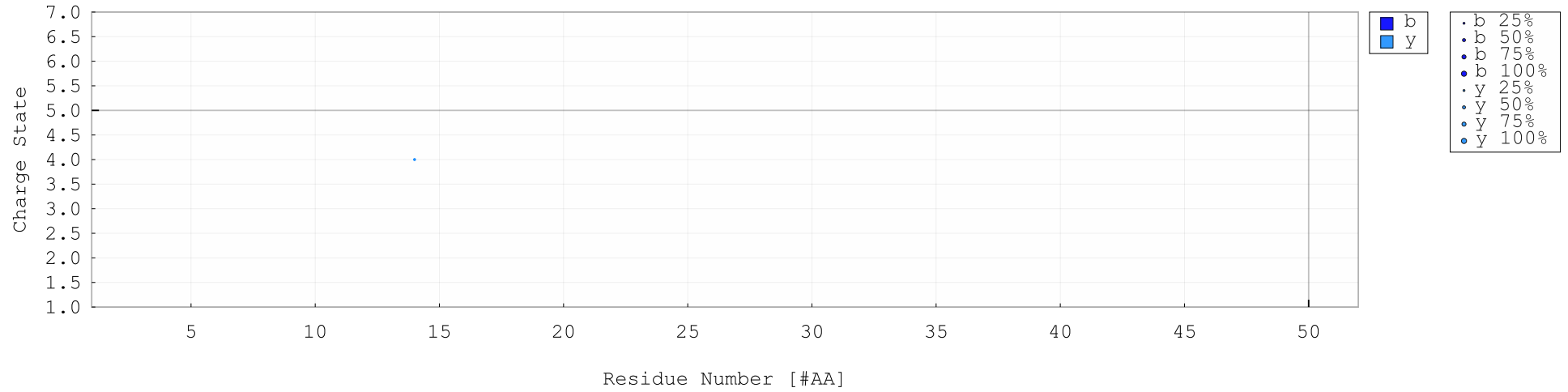

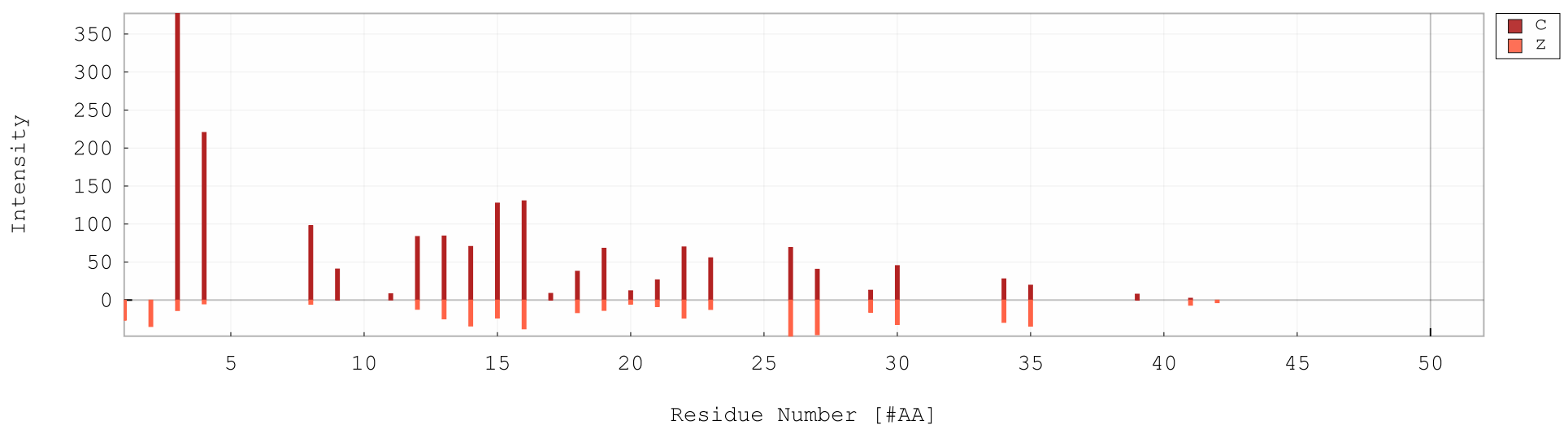

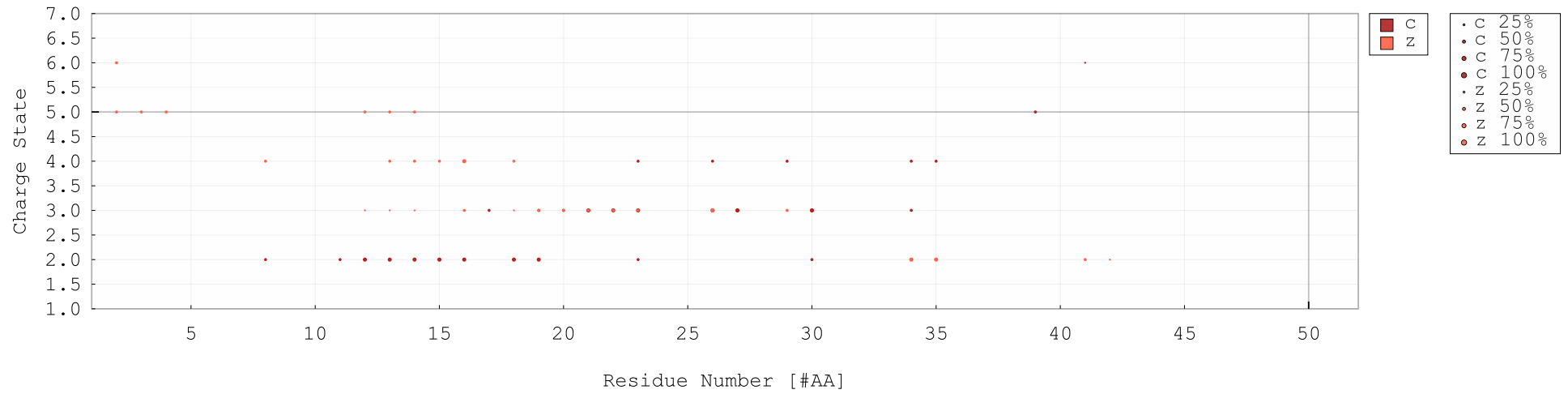

Supplement: Supplementary file 5 [file mmc5.pdf]
